# Genome-wide pathway analysis identifies VEGF pathway association with oral ulceration in systemic lupus erythematosus

**DOI:** 10.1186/s13075-017-1345-6

**Published:** 2017-06-15

**Authors:** Adrià Aterido, Antonio Julià, Patricia Carreira, Ricardo Blanco, José Javier López-Longo, José Javier Pérez Venegas, Àlex Olivé, José Luís Andreu, Maria Ángeles Aguirre-Zamorano, Paloma Vela, Joan M. Nolla, José Luís Marenco-de la Fuente, Antonio Zea, José María Pego, Mercedes Freire, Elvira Díez, María López-Lasanta, Mireia López-Corbeto, Núria Palau, Raül Tortosa, Josep Lluís Gelpí, Devin Absher, Richard M Myers, Antonio Fernández-Nebro, Sara Marsal

**Affiliations:** 1 Rheumatology Research Group, Vall d’Hebron Research Institute, Barcelona, 08035 Spain; 2 Department of Experimental and Health Sciences, Universitat Pompeu Fabra, Barcelona, 08005 Spain; 3 Rheumatology Department, Hospital Universitario 12 de Octubre, Madrid, 28041 Spain; 4 Rheumatology Department, Hospital Universitario Marqués de Valdecilla, Santander, 39008 Spain; 5 Rheumatology Department, Hospital Universitario Gregorio Marañón, Madrid, 28007 Spain; 6 Rheumatology Department, Hospital del SAS de Jerez de la Frontera, Cádiz, 11407 Spain; 7 Rheumatology Department, Hospital Universitari Germans Trias i Pujol, Badalona, 08916 Spain; 8 Rheumatology Department, Hospital Universitario Puerta de Hierro, Madrid, 28222 Spain; 9 Rheumatology Department, Hospital Universitario Reina Sofía, Córdoba, 14004 Spain; 10 Rheumatology Department, Hospital General Universitario de Alicante, Alicante, 03010 Spain; 11 Rheumatology Department, Hospital Universitari de Bellvitge, Barcelona, 08907 Spain; 12 Rheumatology Department, Hospital de Valme, Sevilla, 41014 Spain; 13 Rheumatology Department, Hospital Universitario Ramón y Cajal, 28034 Madrid, Spain; 14 Instituto de Investigación Biomédica de Vigo, Ourense y Pontevedra, 36204 Spain; 15 Rheumatology Department, Hospital Universitario A Coruña, A Coruña, 15006 Spain; 16 Rheumatology Department, Hospital Complejo Asistencial Universitario de León, León, 24001 Spain; 17 Life Sciences, Barcelona Supercomputing Centre, Barcelona, 08034 Spain; 18 HudsonAlpha Institute for Biotechnology, Huntsville, Alabama 35806 USA; 19 Rheumatology Department, Hospital Regional Universitario de Málaga, Málaga, 29010 Spain; 20 Instituto de Investigación Biomédica de Málaga, Universidad de Málaga, Málaga, 29010 Spain

**Keywords:** Genome-wide pathway analysis, Genetics, Systemic lupus erythematosus, Oral ulceration, Clinical phenotypes

## Abstract

**Background:**

Systemic lupus erythematosus (SLE) is a genetically complex rheumatic disease characterized by heterogeneous clinical manifestations of unknown etiology. Recent studies have suggested the existence of a genetic basis for SLE heterogeneity. The objective of the present study was to identify new genetic variation associated with the clinically relevant phenotypes in SLE.

**Methods:**

A two-stage pathway-based approach was used to identify the genetic variation associated with the main clinical phenotypes in SLE. In the discovery stage, 482 SLE patients were genotyped using Illumina Human Quad610 microarrays. Association between 798 reference genetic pathways from the Molecular Signatures Database and 11 SLE phenotypes was tested using the set-based method implemented in PLINK software. Pathways significantly associated after multiple test correction were subsequently tested for replication in an independent cohort of 425 SLE patients. Using an *in silico* approach, we analyzed the functional effects of common SLE therapies on the replicated genetic pathways. The association of known SLE risk variants with the development of the clinical phenotypes was also analyzed.

**Results:**

In the discovery stage, we found a significant association between the vascular endothelial growth factor (VEGF) pathway and oral ulceration (*P* value for false discovery rate (*P*_FDR_) < 0.05), and between the negative regulation signaling pathway of retinoic acid inducible gene-I/melanoma differentiation associated gene 5 and the production of antinuclear antibodies (*P*_FDR_ < 0.05). In the replication stage, we validated the association between the VEGF pathway and oral ulceration. Therapies commonly used to treat mucocutaneous phenotypes in SLE were found to strongly influence VEGF pathway gene expression (*P* = 4.60e-4 to 5.38e-14). Analysis of known SLE risk loci identified a strong association between *PTPN22* and the risk of hematologic disorder and with the development of antinuclear antibodies.

**Conclusions:**

The present study has identified VEGF genetic pathway association with the risk of oral ulceration in SLE. New therapies targeting the VEGF pathway could be more effective in reducing the severity of this phenotype. These findings represent a first step towards the understanding of the genetic basis of phenotype heterogeneity in SLE.

**Electronic supplementary material:**

The online version of this article (doi:10.1186/s13075-017-1345-6) contains supplementary material, which is available to authorized users.

## Background

Systemic lupus erythematosus (SLE) is a disabling multisystem rheumatic disease with substantial epidemiological variation [1]. SLE is characterized by the dysregulation of the immune system and high phenotypical diversity [2]. This phenotypical heterogeneity includes a wide range of clinical manifestations that are exemplified by the current use of multiple clinical phenotypes as criteria to diagnose the disease [3]. So far, however, little is known about the causes of this phenotypic variation. Understanding the molecular mechanisms associated with the pathogenesis of SLE phenotypes could therefore be of high relevance to develop more efficient therapeutic approaches and preventive strategies.

SLE is characterized by a strong genetic component, with a sibling recurrence rate (λ_s_) of 8–29 and estimated heritability of approximately 66% [4]. To date, nine genome-wide association studies (GWAS) of SLE risk have been performed in European and Asian populations [5]. Together these studies have led to the identification of >40 loci associated with SLE susceptibility. Despite this extraordinary success, there is still a lack of understanding of the genetic variation that is relevant for the development of specific phenotypes within the disease. There is evidence, however, that the main clinical phenotypes in SLE aggregate in families [6], suggesting a genetic basis underlying disease heterogeneity.

To date, only a few candidate gene studies have been performed to uncover the genetics of clinical heterogeneity in SLE [7]. These studies have identified immunity-related genes associated with clinically relevant SLE phenotypes [8]. From these, the most significant associations have been detected between renal disorder and genetic variation in the *ITGAM* and *STAT4* genes, which have been also associated with discoid rash and oral ulceration, respectively [9, 10]. Other significant findings include the association between renal disorder and *TNFSF4*, malar rash and *FCGR2A* and hematological disorder and variation in the *IL21* gene. So far, however, the genetic component for most SLE phenotypes has been only partially explained. Therefore, the analysis of genetic variation at a genome-wide scale is needed to identify additional variation associated with SLE clinical heterogeneity.

Complex traits like disease risk or clinical phenotypes have been shown to be caused by multiple genes of small effect size [11]. The identification of these small-effect genes is currently one of the major challenges in the characterization of the genetic background for disease phenotypes [12]. Importantly, single-marker GWAS do not allow the identification of genetic variants with small effect sizes, unless extremely large sample sizes are used [13]. In this common type of GWAS, a large number of markers are tested for association and, consequently, stringent significance thresholds are applied, which makes the identification of small-effect variants very difficult [14]. In addition, single-marker GWAS ignore the joint contribution of multiple genes that act coordinately in the same biological process [15]. The characterization of the genetic basis of many complex traits will therefore require the development of new powerful methods that are able to leverage biological knowledge and efficiently integrate the evidence from multiple loci with moderate to small effect sizes.

Recently, novel statistical methodologies that are able to test genetic risk associations at the pathway level have been developed [16]. Pathway-based approaches test whether sets of functionally related genes are jointly associated with a particular phenotype [17]. This methodology strongly reduces the number of association tests and, therefore, it can substantially increase the power to identify new genetic variation compared to single-marker GWAS [18]. The genome-wide pathway analysis (GWPA) has been recently used to characterize the genetic basis of several complex diseases like cancer [19]. Very recently, using the GWPA approach we have identified new genetic variation associated with psoriasis, an autoimmune disease of the skin [20]. This result confirms the utility of GWPA in the study of the genetics of autoimmune diseases.

To gain a better understanding of the genetic basis underlying phenotype heterogeneity in SLE we have performed, for the first time, a GWAS of clinical phenotypes using the GWPA approach. In this study we have analyzed a discovery cohort of 482 SLE patients of European ancestry to determine the association between 798 reference biological pathways and the main clinical phenotypes of SLE represented that are used as diagnosis criteria. Using an independent cohort of 425 SLE patients from the same ancestry, we have then performed a validation study of the most significant genetic pathways. Based on these results, we have performed an *in silico* validation analysis to evaluate the functional impact of drugs commonly used to treat the associated phenotype. Our findings provide new insights into the biological mechanisms associated with clinical phenotypes of SLE.

## Methods

### Study population

In the discovery stage, a total of 482 SLE patients were recruited. SLE patients were collected from the outpatient clinics of the rheumatology departments of 15 Spanish University Hospitals belonging to the Immune-Mediated Inflammatory Disease (IMID) Consortium [21]. All patients were diagnosed by a rheumatologist. Only those patients with SLE that fulfilled ≥4 of the 1982 revised American College of Rheumatology (ACR) diagnosis criteria were included in the present study [3]. All patients included in this study were >16 years old at the time of sample collection and had >3 years of evolution from the diagnosis date. SLE patients with psoriasis, inflammatory bowel disease (Crohn’s disease or ulcerative colitis) or other rheumatic diseases like rheumatoid arthritis, or multiple sclerosis were excluded from the study. All SLE patients were Caucasian European with all four grandparents born in Spain.

In the validation stage, an independent cohort of 425 SLE patients was used to replicate the genetic pathways that were significantly associated with the SLE phenotypes in the discovery stage. All patients from the validation cohort fulfilled the ACR diagnostic criteria for SLE and were also collected from the IMID Consortium, following the same inclusion and exclusion criteria as for the discovery cohort. All the procedures were followed in compliance with the principles of the Declaration of Helsinki.

The main epidemiological and clinical variables of the discovery and validation cohorts are summarized in Table 1. The distribution of each variable was compared between the discovery and validation cohorts using Fisher’s exact test or Student’s *t* test for categorical and quantitative variables, respectively.

**Table 1:** Main epidemiological and clinical features of the discovery and validation patient cohorts

Clinical and epidemiological variables^a^	Discovery cohort	Validation cohort	*P*
Genetic background	CEU	CEU	-
Number of individuals (*N*)	482	425	-
Gender (% females)	443 (91.91%)	398 (93.65%)	0.37
Average age at onset (m ± SD)	33.50 ± 14.09	32.39 ± 13.32	0.22
Malar rash (*N* ^+^/*N* ^-^)	185/174	183/175	1.00
Discoid rash (*N* ^+^/*N* ^-^)	46/313	49/309	0.74
Photosensitivity (*N* ^+^/*N* ^-^)	191/168	173/185	0.20
Oral ulcers (*N* ^+^/*N* ^-^)	153/206	172/186	0.15
Arthritis (*N* ^+^/*N* ^-^)	275/84	267/91	0.54
Serositis (*N* ^+^/*N* ^-^)	110/249	122/236	0.34
Renal disorder (*N* ^+^/*N* ^-^)	120/239	108/250	0.38
Neurologic disorder (*N* ^+^/*N* ^-^)	25/334	35/293	0.10
Hematologic disorder (*N* ^+^/*N* ^-^)	341/18	337/21	0.63
Immunologic disorder (*N* ^+^/*N* ^-^)	289/70	292/66	0.77
Antinuclear antibodies (*N* ^+^/*N* ^-^)	346/13	347/11	0.84

*Abbreviations*: *CEU* Caucasian European, *M* mean, *N* sample size, *N* ^*+*^ sample size of positive individuals for the clinical variable, *N* ^*-*^ sample size of negative individuals for the clinical variable, *P P* value, *SD* standard deviation

^a^Number of individuals shown in the table represents the total number of patients that were initially recruited for the present study. Conversely, the sample size of positive/negative individuals for the indicated clinical phenotype represents the final number of SLE patients having data on both genotype and phenotype available for association analysis

### SLE phenotypes

The diagnosis of SLE is of major importance to guide both the disease classification and the patient therapy [22]. Given the high phenotypic heterogeneity of SLE, in order to analyze the most relevant clinical manifestations for disease diagnosis we defined the SLE phenotypes according to the established ACR diagnostic criteria for SLE [3]. Consequently, the 11 SLE phenotypes represented by the ACR diagnostic criteria were analyzed using the GWPA approach. These criteria include malar rash, discoid rash, photosensitivity, oral ulcers, arthritis, serositis, renal disorder, neurologic disorder, hematologic disorder, immunologic disorder and antinuclear antibodies. The distribution of each clinical phenotype in the discovery and replication cohorts is shown in Table 1.

### DNA extraction and genome-wide genotyping in the discovery and validation patients

In the discovery stage, the genome-wide genotyping of the 482 SLE patients was performed using the Illumina Quad610 Beadchips (Illumina, San Diego, CA, USA) at the Centro Nacional de Genotipado (CeGen, Madrid, Spain). The genotyping quality control analysis was performed using PLINK software (Additional file 1: Figure S1) [23]. To evaluate the presence of potential population stratification in the SLE patient cohorts, we used the principal component analysis (PCA) implemented in the EIGENSOFT (v4.2) software [24]. Using the first 10 PCs of variation over 10 iterations we identified 14 samples showing an outlier genetic background and were excluded from downstream analysis (Additional file 1: Figure S1). After the quality control analysis, a final dataset of 507,051 single nucleotide polymorphisms (SNPs) and 395 SLE patients was available for the GWPA.

The validation of the two genetic pathways associated with SLE in the discovery stage required the genotyping and analysis of a total 1347 SNPs. Given the large number of variants to be tested and the utility of genome-wide data for accurate genetic ancestry identification, the 425 SLE patients in the validation cohort were genotyped using the same microarray platform. Genotyping for the validation stage was performed at the HudsonAlpha Institute for Biotechnology (Huntsville, AL, USA). The same quality control analysis as in the discovery stage was performed (Additional file 1). A total of 394 SLE patients and all 1347 SNPs from the two genetic pathways passed the quality control and were available for the pathway-based analysis of the validation stage.

### Analysis of association between established SLE risk SNPs and SLE phenotypes

#### Genetic variants associated with SLE risk

The list of established genetic variants (*P* < 5e-8) for SLE risk was obtained from a recent GWAS meta-analysis in a case-control cohort of European ancestry [5]. A total of 43 genetic variants associated with SLE risk were identified and selected for the analysis of association with SLE clinical phenotypes (Additional file 1: Table S6).

#### Imputation of genetic variants associated with SLE risk

From the established autosomal SLE risk SNPs (N = 41 SNPs), the genetic variants that were not directly genotyped by the GWAS Quad610 genotyping array (N = 17 SNPs) were imputed (Additional file 1). Those SNPs that did not pass the stringent imputation quality control filter (N = 1 SNP, information quality metric <0.8) were excluded from the study. Therefore, after excluding two non-autosomal variants and a low-quality imputed SNP, a total of 40 from the initial 43 established SLE risk SNPs were finally available for analysis of association with SLE phenotypes in the discovery cohort. In the validation cohort, the same procedure was followed to obtain the genotypes of the risk variants to be tested for replication.

#### Statistical association analysis

The statistical association analysis between the allele dosage of the established SLE risk SNPs and the SLE clinical phenotypes was performed using the logistic regression model implemented in the SNPTEST v2 software (Oxford, UK) [25]. In this model, the allele dosage was defined as follows:

$$ \mathrm{Allele}\ {\mathrm{Dosage}}_{\mathrm{i}}={\displaystyle \sum_{\mathrm{g}=0}^2} \Pr \left(\mathrm{G}=\mathrm{g}\right)*\mathrm{g} $$

Where *g* represents each genotype of a particular genetic variant *i* and *Pr(G = i)* is the marginal posterior probability obtained by imputation. The allele dosage takes values between 0 and 2. SLE patients without phenotypical data available for the phenotype analyzed were excluded from the association analysis. Finally, the *P* values obtained from the discovery and replication stages were combined using the METAL software [26].

### GWPA

#### GWPA method

The gene-set analysis, also referred to as pathway analysis, is a very powerful methodology to analyze the genetic architecture of complex diseases using GWAS data [27, 28]. An important advantage of this approach is that the hypothesis space is significantly reduced compared to single-marker GWAS. While extremely large number of markers (>500,000 to several millions) are independently tested for association in single-marker GWAS, the number of simultaneous tests is several orders of magnitude lower in pathway-based GWAS (typical range 500–2000 pathways). Consequently, the threshold for significance is much less stringent than the consensus threshold used for single-marker GWAS (*P* < 5e-8) [18, 27]. In addition, pathway-based studies integrate the effects of multiple genetic risk variants that participate in the same biological processes. For these reasons, pathway-based studies can have high statistical power to discover new susceptibility genetic variants provided that they operate within the analyzed pathways.

In the present study, the GWPA was performed using the set-based test implemented in the PLINK software as described previously [20, 23]. Compared to other methods, this set-based test uses genotype data to estimate pathway association instead of *P* values for significance. Importantly, this approach accounts for the linkage disequilibrium between SNPs and therefore avoids an increase in false positive results due to genes with multiple, highly correlated markers. For each pathway, independent SNPs are first identified (linkage disequilibrium of *r* ^2^ < 0.2 here), and from these an average statistic is calculated. Finally, the statistical significance of the pathway is computed using permutation, thereby efficiently correcting by the number of SNPs within the pathway (Additional file 1). As described for the analysis of association with established SLE risk SNPs, those patients with missing data for the phenotype tested for association were excluded from the analysis. In order to account for multiple testing, the false discovery rate (FDR) method was used. The corrected empirical *P* values from the discovery and validation stages were combined using Fisher’s method.

#### Gene set definition

Reference biological pathway annotation databases BioCarta, Kyoto Encyclopedia of Genes and Genomes (KEGG) and Reactome were used for the present study [29]. A total of 217, 186 and 674 curated biological pathways from the Biocarta, KEGG and Reactome databases, respectively, were included, respectively (5th October 2015). Very small uninformative pathways (i.e. <=15 genes) were excluded from the analysis. As described previously, we also excluded large genetic pathways (i.e. >300 genes) [17]. The SNP-gene mapping was performed using the NCBI RefSeq database release 63 (12th October 2015) and an SNP-gene distance window of 20 Kb [30]. The final gene set used for the present GWPA was composed of 211,724 SNPs mapping to 798 different pathways. The list of genetic pathways included in the GWPA is shown in Additional file 1: Table S7.

#### *In silico* analysis of VEGF pathway genes after treatment with topical immunotherapies for cutaneous SLE

In the GWPA, we identified significant genetic association between oral ulceration and the VEGF pathway. The VEGF pathway plays a crucial role in angiogenesis, and there is increasing evidence supporting the implication of this biological process in the pathogenesis of SLE cutaneous phenotypes [31]. These disease phenotypes are commonly treated with steroid and non-steroid topical immunotherapies in SLE [32, 33]. Consequently, we hypothesized that the topical immunotherapies prescribed for cutaneous SLE mediate their therapeutic effect in this tissue through the VEGF pathway and, therefore, should induce significant transcriptional changes in the pathway genes. In order to test this hypothesis, we used transcriptional data from microarray experiments in the NCBI Gene Expression Omnibus microarray database (GEO, https://www.ncbi.nlm.nih.gov/geo/). In this database, we searched for whole genome expression profiling datasets generated from cutaneous/mucocutaneous human samples or cell cultures (5 November 2015). From these, we looked for tissue or cell cultures treated with any of the common steroid and non-steroid topical immunotherapies most widely used in SLE (Additional file 1). We found a total of three datasets analyzing the transcriptional variation after treatment with four common immunotherapies: betamethasone valerate and pimecrolimus (GSE32473), diphencyprone (GSE52360) and imiquimod (GSE68182). The first two transcriptional datasets (i.e. GSE32473 and GSE52360) were obtained from skin biopsies and the latter (GSE68182) from an in vitro study on vaginal mucosal cells (i.e. cell line Vk2/E6E7). For each gene expression dataset, we performed quality control analysis and subsequent normalization on the log2 scale using the quantile normalization method. The analysis of differential expression of the VEGF pathway genes between treated and non-treated samples was performed using Student’s *t* test. The statistical significance of the global perturbation of the VEGF pathway was assessed using the binomial test. All analyses were performed using the R statistical software [34].

## Results

### Phenotypic characterization of the studied cohorts

The epidemiological and phenotypical characteristics of the discovery and replication SLE populations are shown in Table 1. There were no significant differences between the discovery and replication cohorts in the distribution of the epidemiological and phenotypical variables (*P* > 0.05, Table 1).

### Identification of SLE risk genetic variants associated with SLE phenotypes

In the discovery stage, we found that 19 out of the 43 SNPs previously associated with SLE risk were also significantly associated with one or more clinical phenotypes (*P*_Discovery_ < 0.05, Table 2). The association results between each established genetic variant and SLE phenotype are summarized in Additional file 1: Table S1. However, in the independent validation cohort, only the association between *PTPN22* and hematologic disorder (*P*_Replication_ = 0.043_*,*_ *P*_Combined_ = 8.25e-4) and between *PTPN22* and the production of antinuclear antibodies (*P*_Replication_ = 0.028_,_ *P*_Combined_ = 0.001) were significantly replicated. Combining the statistical evidence from the two cohorts, an additional seven loci were found to be associated with SLE phenotypes at the nominal level (*P*_Combined_ < 0.05, Table 2).

**Table 2:** SLE risk SNPs association with clinical phenotypes

SNP	Chr	Pos	Gene	RA	Phenotype	*P*_*D*_	*P*_*V*_	*P*_*C*_	OR (CI, 95%)
**rs2476601** ^a^	**1**	**114377568**	***PTPN22***	**A**	**Hematologic disorder**	**0.0039**	**0.0433**	**5.20e-4**	**9.488 (1.310-68.726)**
					**Antinuclear antibodies**	**0.0146**	**0.0281**	**0.0011**	**0.168 (0.004-0.990)**
rs704840	1	173226195	*TNFSF4*	G	Renal disorder	0.0407	0.9210	0.1290	1.199 (0.941-1.527)
**rs3024505**	**1**	**206939904**	***IL10***	**T**	**Arthritis**	**0.0325**	**0.1540**	**0.0170**	**1.557 (1.091-2.221)**
**rs3768792**	**2**	**213871709**	***IKZF2***	C	Photosensitivity	0.0437	0.3320	0.4580	0.893 (0.674-1.184)
					**Hematologic disorder**	**0.0319**	**0.1880**	**0.0143**	**0.507 (0.301-0.855)**
rs9311676	3	58470351	*ABHD6,PXK*	C	Oral ulcers	0.0338	0.7960	0.9210	0.832 (0.674-1.028)
rs564799	3	159728987	*IL12A*	C	Discoid rash	0.0411	0.1410	0.6853	0.942 (0.674-1.316)
**rs1270942**	**6**	**31918860**	***MHC class III***	**C**	**Arthritis**	**0.0138**	**0.6670**	**0.0407**	**1.530 (1.010-2.346)**
**rs9462027**	**6**	**34797241**	***UHRF1BP1***	**A**	**Malar rash**	**0.0263**	**0.1730**	**0.0137**	**1.333 (1.071-1.660)**
					Oral ulcers	0.0251	0.8950	0.0933	1.210 (0.971-1.508)
rs6932056	6	138242437	*TNFAIP3*	C	Serositis	0.0193	0.8140	0.1364	1.580 (0.898-2.781)
rs4917014	7	50305863	*IKZF1*	T	Oral ulcers	0.0280	0.1550	0.5821	0.944 (0.753-1.184)
rs2663052	10	50069395	*WDFY4*	C	Neurologic disorder	0.0370	0.1590	0.6311	0.963 (0.662-1.401)
rs4948496	10	63805617	*ARID5B*	C	Malar rash	0.0331	0.4580	0.3258	1.116 (0.906-1.375)
					Photosensitivity	0.0202	0.9480	0.0911	1.203 (0.977-1.483)
rs2732549	11	35088399	*CD44*	T	Immunologic disorder	0.0163	0.9910	0.0907	1.277 (0.976-1.670)
**rs7941765**	**11**	**128499000**	***ETS1,FLI1***	**C**	**Arthritis**	**0.0072**	**0.0701**	**0.0015**	**1.470 (1.151-1.877)**
**rs10774625**	**12**	**111910219**	***SH2B3***	**A**	**Serositis**	**0.0207**	**0.0756**	**0.0038**	**0.715 (0.571-0.895)**
					Renal disorder	0.0423	0.6410	0.0773	0.814 (0.650-1.018)
					**Immunologic disorder**	**0.0051**	**0.8190**	**0.0321**	**0.738 (0.566-0.964)**
**rs1059312**	**12**	**129278864**	***SLC15A4***	**C**	**Serositis**	**0.0253**	**0.2370**	**0.0156**	**1.321 (1.053-1.658)**
rs4902562	14	68731458	*RAD51B*	A	Renal disorder	0.0130	0.8670	0.1010	0.834 (0.664-1.047)
rs2941509	17	37921194	*IKZF3*	A	Antinuclear Antibodies	0.0304	0.0413	0.4943	0.676 (0.237-1.930)
rs2304256	19	10475652	*TYK2*	C	Arthritis	0.0298	0.7690	0.1841	0.826 (0.622-1.096)

A total of 19 SLE risk genetic variants were significantly associated with different systemic lupus erythematosus (SLE) phenotypes in the discovery stage. From these, the association between *PTPN22* and hematologic disorder and between *PTPN22* and the production of antinuclear antibodies (^a^) were significantly replicated in the validation cohort. Combining the statistical evidence from the two cohorts, seven additional genetic variants were found to be nominally associated with SLE phenotypes (shown in bold). *Abbreviations*: *SNP* single-nucleotide polymorphism, *Chr* chromosome, *Pos* SNP base pair in build GRCh37/hg19, *RA* disease risk allele, *P*_*D*_ *P* value discovery cohort, *P*_*V*_ *P* value validation cohort, *P*_*C*_ *P* value combined, *OR* odds ratio according to the allele associated with disease risk, *CI* confidence interval (95%)

### Identification of genetic pathways associated with SLE phenotypes

In the GWPA, two genetic pathways were found to be significantly associated with an SLE phenotype after multiple test correction (*P* value for false discovery rate (*P*_FDR_) < 0.05, Table 3). The VEGF pathway was associated with the presence of oral ulcers (*P*_FDR_ = 0.044) and the RIG-I/MDA5 negative regulation signaling pathway was associated with the production of antinuclear antibodies (*P*_FDR_ = 0.016). The results for analysis of association between each genetic pathway and SLE phenotype are shown in Additional file 1: Table S2.

**Table 3:** Genetic pathways associated with the SLE phenotypes in the discovery stage

Pathway	Phenotype	#Genes	SNPs	*P*_*D*_	FDR _D_	*P*_*V*_	FDR _V_	*P*_*C*_
RIG-I/MDA5 negative regulation signaling	Antinuclear antibodies	31	464	2.00e-5	0.016	0.234	0.234	-
VEGF^a^	Oral ulcers	29	883	5.58e-5	0.044	0.013	0.026	2.08e-5

Two genetic pathways were significantly associated with systemic lupus erythematosus (SLE) phenotypes in the discovery stage

*Abbreviations*: *SNP* single-nucleotide polymorphism, *P*_*D*_ *P* value discovery cohort, *FDR*_*D*_ false discovery rate discovery cohort, *P*_*V*_ *P* value validation cohort, *FDR*_*V*_ false discovery rate validation cohort, *P*_*C*_ *P* value combined

^a^The association between the vascular endothelial growth factor (VEGF) pathway and oral ulcers was significantly replicated in the validation cohort

Using the independent validation cohort, the association between the VEGF genetic pathway and oral ulcers in SLE was significantly replicated (*P*_FDR_ = 0.026, Table 3). The details of association between the VEGF pathway and oral ulcers are shown in Additional file 1: Table S3.

### Perturbation of the VEGF genetic pathway by current therapies for cutaneous SLE

**Fig. 1:**
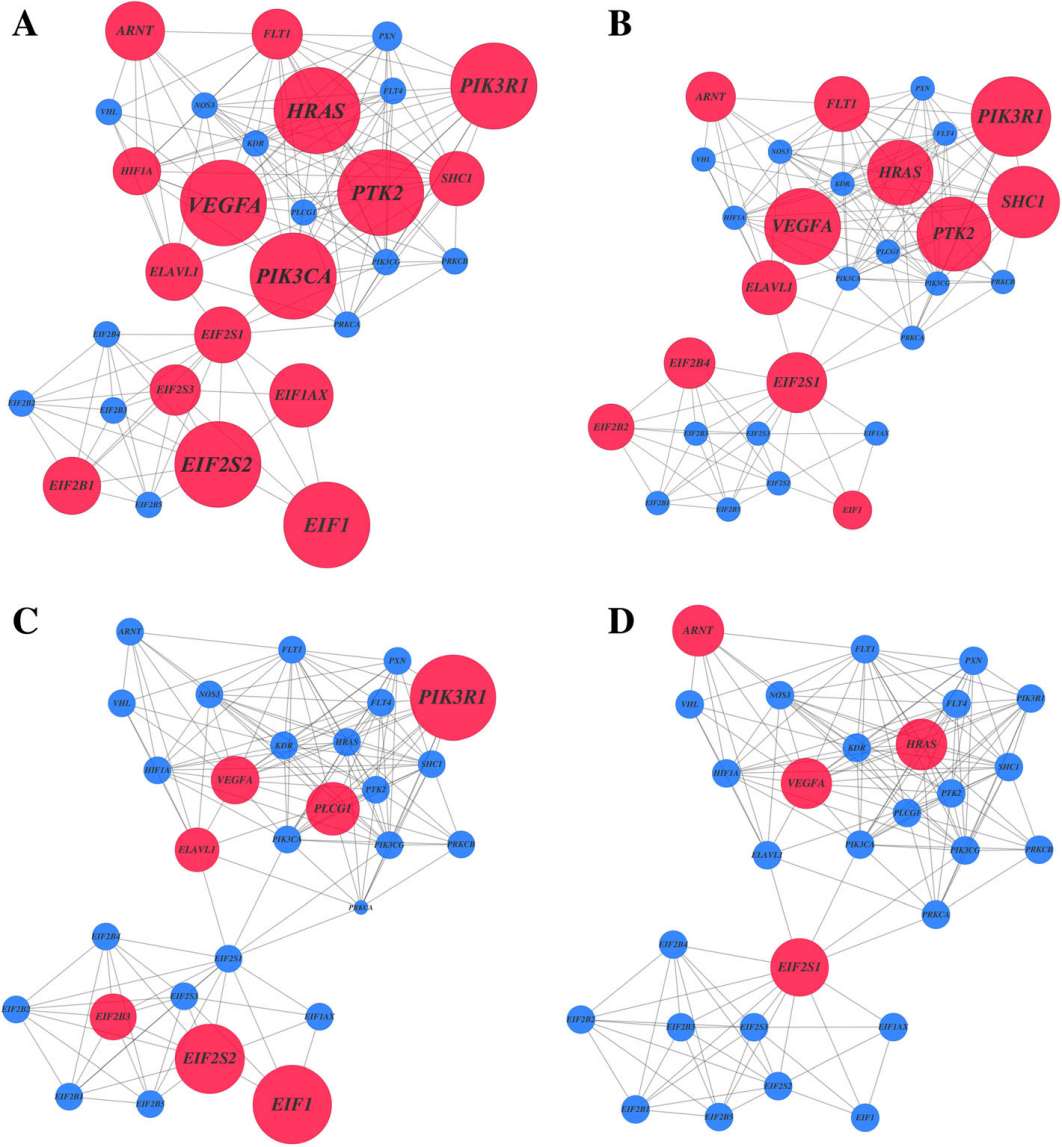
Vascular endothelial growth factor (VEGF) pathway perturbation after topical immunotherapy. Network representation of VEGF genes according to the differential gene expression after treatment with common topical immunotherapies: imiquimod (**a**), betamethasone valerate (**b**), diphencyprone (**c**) and pimecrolimus (**d**). Genes are represented as nodes and are connected by edges according to experimental or computational evidence of interaction between their encoded proteins. The diameter of each node is proportional to the significance of differential expression, with significant genes (*P* < 0.05) in *red* and non-significant genes (*P* ≥ 0.05) in *blue*

Topical immunotherapies are drugs commonly used to treat cutaneous phenotypes of SLE like oral ulceration. Given the observed genetic association between oral ulcers and VEGF pathway, we performed an *in silico* analysis to evaluate the effect of four current topical immunotherapies on the expression of its constituent genes. Using whole genome expression datasets from patients and relevant cell types treated with these therapies, we found that three out of the four analyzed drugs significantly perturb the expression of VEGF pathway genes (Fig. 1, Additional file 1: Table S4). A total of 16, 12 and 7 genes out of the 29 genes from the VEGF pathway were significantly differentially expressed after imiquimod (*P* = 5.38e-14), betamethasone valerate (*P* = 5.69e-9) and diphencyprone (*P* = 4.59e-4) treatment, respectively (Additional file 1: Table S5).

## Discussion

One of the major challenges in the pathogenesis of SLE is to understand the biological mechanisms responsible for its phenotypic heterogeneity. Although single-marker GWAS have successfully identified a large number of genetic variants associated with SLE risk, the genetic basis of SLE phenotypes has so far been analyzed only in a few candidate gene studies. In order to identify new genetic variation, we have here performed the first GWAS on SLE phenotypes using a pathway-based approach. Using a discovery cohort of individuals with European ancestry and a validation cohort with the same ancestry, we have identified and validated the association between VEGF genetic pathway and oral ulcers, a common manifestation of SLE. The results of this study provide new insights into the genetic basis and the biological mechanisms associated with the clinical heterogeneity of SLE.

The VEGF pathway is a network of genes that are involved in the transduction of different intracellular signals and act coordinately to modulate inflammatory and angiogenic processes [35, 36]. The dysregulation of angiogenesis has been described as an important biological mechanism in the pathogenesis of SLE [37]. In particular, there is growing evidence that angiogenesis is also involved in the development of skin manifestations in SLE patients [38, 39]. The serum levels of VEGFA protein itself have been suggested as a useful marker for disease activity monitoring in SLE patients [40]. Importantly, the serum levels of VEGFA have also been found to be significantly elevated in SLE patients with cutaneous manifestations [41]. Despite this clinical evidence, the VEGF pathway had not been previously associated with SLE at the genetic level. Our study provides the first evidence of a genetic association between the VEGF pathway and oral ulceration in SLE.

Oral ulcers are frequently chronic mucocutaneous lesions that affect up to 54% of patients with SLE [42, 43]. This clinical manifestation is characterized by high angiogenic activity and a loss of the epithelial and connective tissue in the oral mucosa [44, 45]. Accordingly, anti-angiogenic therapies like thalidomide have been suggested to promote oral ulcer healing and to control ulcer recurrence [46, 47]. From a clinical perspective, oral ulceration has been associated with an increase in the disease activity and a worse prognosis in SLE [48, 49]. The early detection of oral ulcers is therefore highly valuable as it contributes to an earlier diagnosis of SLE and, consequently, to faster initiation of treatment.

The genetic association identified in this study is consistent with previous evidence from other ulcer-related diseases like Behçet disease (BD), recurrent aphtosus ulceration (RAU) and gastroduodenal ulcers. BD is an inflammatory disorder characterized by an extremely high frequency of oral ulcers (>95%) [50]. Clinical evidence suggests that VEGF cytokine could be directly implicated in the formation of oral ulcers in BD [51]. In RAU, the most common oral mucosal disease, the salivary levels of VEGF have been also associated with oral ulceration [52]. Finally, genetic variation in the *VEGF* gene has been also associated with the risk of developing gastroduodenal ulcers [53]. Evidence from these studies implicates angiogenesis in the pathophysiological development of oral ulcers, at both the genetic and at the functional level. Accordingly, the genes in the VEGF pathway are strong candidates for susceptibility in diseases with a high prevalence of oral ulcers like BD or RAU. Future studies aimed at testing the association between VEGF pathway genes and these diseases are therefore warranted.

Topical steroid and non-steroid immunotherapies have been successfully used for the treatment of the cutaneous manifestations in SLE. However, topical immunotherapies are not exempt from side effects, including skin atrophy or telangiectasias with corticosteroids and the exacerbation of the inflammatory processes with non-steroid immunotherapies like imiquimod [32]. In this study, we have demonstrated that topical steroid and non-steroid immunomodulators significantly affect the expression of the VEGF pathway genes. The results of this *in silico* analysis indicate that the VEGF genetic pathway could be a key mediator of the benefits of topical immunotherapy to reduce oral ulceration. Therefore, our findings suggest that the VEGF pathway is a relevant source of new drug targets for oral ulceration that could allow more specific treatment while reducing the undesirable side effects of current therapies. In order to confirm the VEGF pathway as new source for drug discovery in the treatment of SLE oral ulceration, further prospective studies using oral ulcer samples from SLE patients are clearly needed.

In the present study, we have also identified and validated the association between SLE risk locus *PTPN22* and the production of antinuclear antibodies and with the presence of a hematologic disorder. It is the first time that this coding SNP (rs2476601) has been associated with the development of hematologic disease in SLE. A previous study reported a non-significant trend for association between *PTPN22* and antinuclear antibody positivity [54]. The results from our study provide the evidence to confirm the association between this susceptibility gene and the production of a common autoantibody in SLE. Also, the previously identified association between genetic variation in the *TNFSF4* gene and renal disorder was replicated in the discovery cohort [9]. Conversely, the reported association between renal disorder and *ITGAM* and *STAT4* genes was not replicated. The renal disorder phenotype encompasses different clinical manifestations (e.g. persistent proteinuria or cellular casts) and, therefore, differences in frequencies in any of these sub-phenotypes could have prevented the replication. Additional studies performing specific analysis of association for each renal disease subtype could help to further refine this genetic association. Oral ulceration was also previously found to be associated with variation in *STAT4.* This association was not replicated in the present single-marker analysis. The lack of replication could be explained by the comparatively smaller sample size of our study cohorts and by the small effect size reported for the association (OR ~ 1.12). For example, in the European cohort, *STAT4* association was not statistically significant despite analyzing >4,000 individuals [9]. Finally, after combining both patient cohorts, we also found seven other SLE risk loci - *IL10, IKZF2, MHC class III, UHRF1BP1, ETS1-FLI1, SH2B3* and *SLC15A4 -* to be nominally associated with different phenotypes. These also represent new genetic associations with SLE heterogeneity. Further studies using independent cohorts of phenotypically well-characterized SLE patients like the present one are needed to corroborate these associations.

GWPA represents a new and powerful approach to identify genetic variation associated with complex phenotypes. However, this study has limitations. First, the sample size of the discovery and replication cohorts is moderate compared to recent case-control GWAS and this could have led to missed pathway associations with SLE clinical phenotypes. Second, the statistical power to detect significant associations is lower for those clinical phenotypes with more extreme frequencies (e.g. 7% of SLE patients have a neurologic disorder). Therefore, larger cohorts of well-characterized SLE patients will be needed to identify additional genetic variation associated with clinical phenotypes. Finally, the GWPA methodology also has intrinsic limitations, mainly related to the current knowledge of biological processes and subsequent definition of the pathways [55]. For many human genes, the functional annotation is far from complete, which precludes their mapping to reference biological pathways. Also, genomic variation located far from the transcribed region itself could be relevant for the regulation of the gene expression. Conversely, variants within genes could be influencing the activity of other distant genes or genes in other chromosomes (i.e. trans-regulation). With the increase in knowledge of genomic regulation [56], the mapping of SNPs to their functionally related genes will clearly improve. Consequently, the integration of this knowledge into GWPA will likely increase the power of this approach to identify new pathways associated with human diseases.

## Conclusions

In conclusion, we have performed, for the first time, a genome-wide association analysis to identify genetic risk factors for the main phenotypes in SLE. To do this, we have used a pathway-based analysis approach. Using this approach, we have identified and validated the association between the VEGF genetic pathway and the presence of oral ulcers in SLE. These findings show the existence of a genetic basis underlying SLE heterogeneity that is independent from the genetic component associated with disease risk. The results of the present study could contribute to the development of more efficient therapies to treat cutaneous manifestations of SLE in the near future.

## Additional file

Additional file 1: Supplementary information is available in the online *Supplementary Material* file. (PDF 13965 kb)

## Abbreviations

ACR: American College of Rheumatology
BD: Behçet disease
CEU: Caucasian European
CHR: Chromosome
CI: Confidence interval
*ETS1*: ETS Proto-Oncogene 1 gene
FDR: False discovery rate
FDR_D_: False discovery rate discovery cohort
FDR_V_: False discovery rate validation cohort
*FLI1*: Fli-1 Proto-Oncogene gene
GEO: Gene Expression Omnibus
GWAS: Genome-wide association study
GWPA: Genome-wide pathway analysis
*IKZF2*: Zinc finger DNA binding protein helios, subfamily 1A, 2 gene
*IL10*: Interleukin-10 gene
IMID: Immune-mediated inflammatory disease
KEGG: Kyoto Encyclopedia of Genes and Genomes
M: Mean
*MDA5*: Melanoma differentiation-associated protein 5 gene
MHC: Major histocompatibility complex
N: Sample size
N^-^: Sample size of negative individuals for a particular clinical variable
N^+^: Sample size of positive individuals for a particular clinical variable
OR: Odds ratio
*P*: *P* value
*P*_C_: *P* value combined
*P*_D_: *P* value discovery cohort
POS: single nucleotide polymorphism base pair in build GRCh37/hg19
*PTPN22*: Protein tyrosine phosphatase, non-receptor type 22 gene
*P*_V_: *P* value validation cohort
RA: Disease risk allele
RAU: Recurrent aphtosus ulceration
*RIG-I*: Retinoic acid-inducible gene 1 gene
SD: Standard deviation
*SH2B3*: Lymphocyte-specific adapter protein Lnk 3 gene
*SLC15A4*: Solute carrier family 15 member 4 gene
SLE: Systemic lupus erythematosus
SNP: Single nucleotide polymorphism
*UHRF1BP1*: Ubiquitin-like containing PHD and RING finger domains 1-binding protein 1 gene
VEGF: Vascular endothelial growth factor
λ_s_: Sibling recurrence rate
