# Supplementary material for: Genome-wide pathway analysis identifies VEGF pathway association with oral ulceration in systemic lupus erythematosus
Source: Arthritis Res Ther. 2017 Jun 15;19:138. doi: 10.1186/s13075-017-1345-6 (PMC5471877; doi:10.1186/s13075-017-1345-6)
Supplement: Supplementary file 1 — Supplementary information is available in the online Supplementary Material file. (PDF 13965 kb) [file 13075_2017_1345_MOESM1_ESM.pdf]

SUPPLEMENTARY MATERIAL

INDEX

1. Supplementary Material and Methods **2**

1.1 Study patients 2

1.2 DNA extraction and genome-wide genotyping in the discovery and validation patients 2

1.3 Imputation of genetic variants associated with SLE risk 4

1.4 Pathway-based association analysis 5

1.5 Differential expression analysis of VEGF pathway genes after topical immunotherapies 5

2. Supplementary Tables **7**

2.1 Table S1. Association of SLE risk genes with SLE clinical phenotypes in the discovery cohort 7

2.2 Table S2. Genome-wide pathway associations obtained analyzing the 11 SLE clinical phenotypes in the discovery cohort 8

2.3 Table S3. Association results of the SNPs from the VEGF pathway associated with oral ulcers in SLE 105

2.4 Table S4. Statistical significance of the differential expression analysis performed on the VEGF pathway genes after immunotherapy 106

2.5 Table S5. Statistical significance of the VEGF genetic pathway perturbation after topical immunotherapy 107

2.6 Table S6. Established genetic variants for SLE risk 108

2.7 Table S7. Reference biological pathways selected for the present study 109

3. Supplementary Figures **117**

3.1 Figure S1. Principal components of the SLE discovery cohort 117

4. References **118**

1. Supplementary Material and Methods

1.1 Study patients

In the discovery stage, a total of 482 SLE patients were recruited. SLE patients were collected from the outpatient's clinics of the rheumatology departments of 15 Spanish University Hospitals belonging to the Immune-Mediated Inflammatory Disease (IMID) Consortium [1]. The IMID Consortium is a Spanish network of researchers working on the genetic basis of IMIDs. All SLE patients were diagnosed using the 1982 revised ACR diagnosis criteria [2]. All patients included in this study were >16 years old at the time of sample collection and had >3 years of evolution from the diagnosis date. SLE patients with psoriasis, inflammatory bowel disease (Crohn's disease or ulcerative colitis) or other rheumatic diseases like rheumatoid arthritis, or multiple sclerosis were excluded from the study. All SLE patients were Caucasian European with all four grandparents born in Spain.

In the validation stage, an independent cohort of 425 SLE patients was used to replicate the genetic pathways that were significantly associated with the SLE phenotypes in the discovery stage. All patients from the validation cohort fulfilled the ACR diagnostic criteria for SLE and were also collected from the IMID Consortium, following the same inclusion and exclusion criteria as for the discovery cohort.

1.2 DNA extraction and genome-wide genotyping in the discovery and validation patients

Whole blood samples (5 mL) were collected from all SLE patients from the discovery and replication cohorts. Genomic DNA was then isolated using the Chemagic Magnetic Separation Module I (PerkinElmer, Waltham, MA).

In the discovery stage, the genome-wide genotyping of the 482 SLE patients was performed using the Illumina Quad610 Beadchips (Illumina, San Diego, California, USA) at the Centro Nacional de Genotipado (CeGen, Madrid, Spain). Genotype calling was performed using the GenomeStudio data analysis software v2011.1 (Illumina, San Diego, California, USA). The genotyping quality control analysis was performed using PLINK software [3]. From the 598,258 SNPs available in the Illumina genotyping array, a total of 582,539 autosomal SNPs were selected for the quality control analysis. The SNPs included in the association analysis had a minor allele frequency >0.05 and <5% of missing data (94.17% SNPs). Using an additional cohort of 1,558 healthy controls from the same population, we tested the deviation of the SNPs from the Hardy-Weinberg equilibrium [4]. Those SNPs that were not in Hardy-Weinberg equilibrium in this cohort (0.03% SNPs, P<1e-4) were subsequently removed. To evaluate the presence of potential population stratification in the SLE patient cohorts, we used the principal component analysis (PCA) implemented in EIGENSOFT (v4.2) software [5]. Using the first 10 PCs of variation over 10 iterations we identified 14 samples showing an outlier genetic background and were excluded from downstream analysis. After the quality control analysis, a final data set of 507,051 SNPs and 395 SLE patients was available for the GWPA.

The validation of the two genetic pathways associated in the discovery stage required the genotyping and analysis of a total 1,347 SNPs. Given the relatively large number of variants and the utility of genome-wide data for accurate ancestry outlier identification, the 425 SLE patients of the validation cohort were also genotyped using Illumina Quad610 microarray (Illumina, San Diego, California, USA) at the HudsonAlpha Institute for Biotechnology (Huntsville, Alabama, USA). For the pathway-based analysis, we excluded all non-autosomal SNPs, SNPs with a minor allele frequency

<0.05, >5% of missing data (85.01%) as well as those SNPs that were not in Hardy-Weinberg equilibrium (0.03% SNPs, *P*<1e-4) in a cohort of 1,558 controls from the same population [4]. All 1,347 SNPs from the two genetic pathways associated in the discovery stage passed the quality control. In order to estimate the principal components (PCs) of variation, we analyzed the genome-wide genetic variation using the EIGENSOFT (v4.2) software. Based on the first 10 PCs of variation over 10 iterations, we identified 4 samples showing an outlier genetic background that were subsequently discarded from the pathway-based analysis. A total of 394 SLE patients and all 1,347 SNPs from the two genetic pathways passed the quality control and were available for the pathway-based analysis of the validation stage.

1.3 Imputation of genetic variants associated with SLE risk

From the established autosomal SLE risk SNPs (N=41 SNPs), the genetic variants that were not directly genotyped by the GWAS Quad610 genotyping array (N=17 SNPs) were imputed. SHAPEIT V2-644 (Oxford, UK) software was used to pre-phase the haplotypes of the SNPs that passed the genotyping quality control analysis [6], and these haplotypes were subsequently analyzed using IMPUTE V2 (Oxford, UK) software [7] to impute the missing SLE risk SNPs. The reference genotyping data used for imputation was obtained from the European cohort (N=379 samples) of the latest release of the 1000 Genomes Project (phase 1, version 3) [8]. In the imputation quality control analysis, we excluded those SNPs that had an IMPUTE2 info quality metric < 0.8 (N=1 SNP). After the imputation quality control, a total of 40 SLE risk SNPs were finally available for the association analysis with SLE phenotypes in the discovery cohort. In the validation cohort, the same procedure was followed to obtain the genotypes of the risk variants to be tested for replication.

1.4 Pathway-based association analysis

The set- or pathway-based association analysis implemented in the PLINK software is composed by four steps. In the first step, the raw genotype data is used to compute the linkage disequilibrium (r^2) between pathway SNPs in order to identify truly independent SNPs (r^2<0.2). In the second step, the association between each SNP and SLE clinical phenotype is tested using the allelic $\chi 2$ test. The independent SNPs that are nominally associated with the SLE phenotype are then selected for each genetic pathway. In the third step, the pathway statistic is computed as the average of the $\chi 2$ statistics of the selected SNPs. In the last step, the statistical significance of the pathway association with the SLE phenotype is computed using a permutation-based approach. In the permutation procedure, the SLE phenotype is randomly assigned to the patient cohort and the $\chi 2$ statistic is subsequently computed for each pathway. This analysis is then repeated multiple times (n=1,000,000 permutations). Finally, the empirical P-value of each pathway is computed as the proportion of permuted $\chi 2$ statistics that are higher than the observed $\chi 2$ statistic.

1.5 Differential expression analysis of VEGF pathway genes after topical immunotherapies

Cutaneous and mucocutaneous phenotypes in SLE are treated with a diverse group of immunotherapies. Given the observed genetic association between VEGF and oral ulcers, we hypothesized that the topical immunotherapies commonly prescribed for cutaneous SLE should induce significant transcriptional changes in the VEGF pathway genes. In order to test this hypothesis, we used transcriptional data from microarray experiments at the NCBI Gene Expression Omnibus microarray database (GEO, http://www.ncbi.nim.nih.gov/geo/) [9]. In this database, we searched for whole genome expression profiling datasets from cutaneous/mucocutaneous human samples collected

from non-cancer individuals or cellular cultures (5th November 2015). From these, we looked for tissue or cell cultures treated with any of the common steroid and non-steroid topical immunotherapies most widely used in SLE. The former group was composed by 6 drugs (betamethasone valerate, clobetasol propionate, flucocinolone acetonide, fluocinonide, hydrocortisone butirate and triamcinolone acetonide) [10] and the latter group included a total of 12 drug (ABT-281, anthralin, calcipotriol, diphencyprone, imiquimod, intralesional interferon, pimecrolimus, sirolimus, squartic acid dibutyl ester, tacrolimus, topical interferon and topical zinc) [11]. Given that sample size is one of the major concerns to confidently identify differentially expressed genes between two experimental groups [12], small datasets were excluded from the search. We found a total of three gene expression datasets generated after treatment with four common immunotherapies for cutaneous SLE: betamethasone valerate and pimecrolimus (GSE32473), diphencyprone (GSE52360) and imiquimod (GSE68182). For each gene expression dataset, we performed quality control analysis and subsequent normalization on the log2-scale using the quantile normalization method (Figure S3) [13]. The differential expression analysis for the VEGF pathway genes between treated and non-treated samples was performed using Student's t-test. The statistical significance of the global perturbation of the VEGF pathway was assessed using the Binomial test. All analyses were performed using the R statistical software [14].

2. Supplementary Tables

2.1 Table S1. Association of SLE risk genes with SLE clinical phenotypes in the discovery cohort.

SNP	CHR	POS	GENE	MALAR RASH		DISCOID RASH		PHOTOSENSITIVITY		ORAL ULCERS		ARTHRITIS		SEROSITIS		RENAL DISORDER		NEUROLOGIC DISORDER		HEMATOLOGIC DISORDER		IMMUNOLOGIC DISORDER		ANTINUCLEAR ANTIBODIES	
				P	OR (95% CI)	P	OR (95% CI)	P	OR (95% CI)	P	OR (95% CI)	P	OR (95% CI)	P	OR (95% CI)	P	OR (95% CI)	P	OR (95% CI)	P	OR (95% CI)	P	OR (95% CI)	P	OR (95% CI)
rs2476601	1	114377568	*PTPN22*	5,28E-01	0.86 (0.53-1.37)	9,45E-01	0.97 (0.48-1.97)	5,41E-01	0.86 (0.54-1.38)	9,37E-01	1.02 (0.63-1.64)	2,44E-01	0.7 (0.38-1.28)	3,12E-01	0.77 (0.47-1.26)	6,47E-01	0.89 (0.54-1.45)	4,92E-01	0.73 (0.32-1.69)	3,92E-03	NA (NA-NA)	9,41E-01	0.98 (0.54-1.77)	1,46E-02	NA (NA-NA)
rs1801274	1	161479745	*FCGR2A*	7,99E-01	0.96 (0.72-1.29)	4,49E-01	1.18 (0.75-1.85)	1,69E-01	1.22 (0.91-1.64)	1,25E-01	1.25 (0.93-1.69)	2,86E-01	1.2 (0.85-1.7)	9,09E-01	0.98 (0.71-1.35)	1,51E-01	0.8 (0.59-1.09)	1,57E-01	0.67 (0.38-1.19)	1,66E-01	1.58 (0.81-3.11)	1,37E-01	0.76 (0.52-1.11)	1,62E-01	1.72 (0.78-3.8)
rs704840	1	173226195	*TNFSF4*	1,95E-01	1.23 (0.89-1.7)	1,03E-01	0.67 (0.39-1.13)	4,27E-01	1.13 (0.82-1.57)	3,04E-01	0.85 (0.61-1.18)	9,64E-01	1.01 (0.69-1.48)	6,13E-02	1.37 (0.97-1.93)	4,07E-02	1.4 (1.01-1.96)	1,15E-01	0.59 (0.29-1.21)	8,56E-01	1.07 (0.51-2.26)	2,14E-01	1.29 (0.84-1.96)	5,12E-01	0.77 (0.34-1.75)
rs3024505	1	206939904	*IL10*	1,77E-01	0.77 (0.52-1.14)	6,47E-01	0.87 (0.48-1.6)	3,79E-01	0.85 (0.57-1.25)	1,70E-01	0.77 (0.51-1.14)	3,25E-02	1.68 (1.01-2.81)	1,71E-01	1.32 (0.88-1.99)	7,89E-01	0.95 (0.63-1.43)	3,45E-01	1.4 (0.69-2.81)	8,95E-02	0.52 (0.24-1.1)	3,13E-01	0.79 (0.49-1.26)	4,16E-01	0.68 (0.27-1.73)
rs9782955	1	236039877	*LYST*	9,19E-01	1.02 (0.71-1.46)	2,38E-01	1.4 (0.78-2.52)	7,55E-01	1.06 (0.74-1.51)	8,56E-01	1.03 (0.72-1.49)	5,45E-01	1.14 (0.75-1.72)	8,87E-01	1.03 (0.7-1.52)	8,36E-01	0.96 (0.66-1.4)	5,48E-01	1.25 (0.59-2.62)	7,82E-01	0.89 (0.38-2.07)	9,48E-01	1.01 (0.65-1.59)	8,13E-01	1.12 (0.44-2.83)
rs6740462	2	65667272	*SPRED2*	1,67E-01	0.79 (0.56-1.11)	6,72E-01	1.11 (0.66-1.88)	4,46E-01	0.88 (0.63-1.24)	1,64E-01	0.79 (0.56-1.11)	3,08E-02	1.51 (1.03-2.21)	5,89E-01	0.91 (0.63-1.3)	5,36E-01	1.12 (0.78-1.6)	8,50E-01	1.06 (0.54-2.08)	1,18E-01	1.74 (0.86-3.51)	8,42E-01	1.04 (0.68-1.59)	7,96E-01	1.12 (0.46-2.7)
rs2111485	2	163110536	*IFIH1*	2,68E-01	0.84 (0.62-1.14)	9,42E-01	1.02 (0.64-1.61)	5,67E-01	1.09 (0.81-1.48)	6,64E-01	0.94 (0.69-1.27)	6,35E-01	1.09 (0.76-1.56)	3,45E-01	0.86 (0.62-1.19)	4,89E-01	0.89 (0.65-1.23)	2,87E-01	0.73 (0.41-1.31)	5,51E-01	1.23 (0.62-2.43)	5,30E-01	1.13 (0.77-1.65)	7,93E-01	0.9 (0.39-2.04)
rs11889341	2	191943742	*STAT4*	5,20E-02	0.73 (0.54-1.01)	5,74E-01	1.14 (0.72-1.82)	4,38E-01	0.88 (0.65-1.21)	3,23E-01	0.85 (0.62-1.17)	7,71E-01	0.95 (0.65-1.37)	9,00E-01	0.98 (0.7-1.38)	8,05E-01	0.96 (0.69-1.34)	4,04E-01	0.77 (0.4-1.46)	8,53E-02	1.98 (0.86-4.59)	7,52E-01	1.07 (0.71-1.59)	3,15E-01	1.57 (0.62-3.96)
rs3768792	2	213871709	*IKZF2*	2,76E-01	1.25 (0.84-1.86)	6,03E-01	1.18 (0.63-2.2)	4,37E-02	1.51 (1.02-2.25)	5,44E-01	1.13 (0.76-1.7)	3,66E-01	1.24 (0.79-1.95)	1,96E-01	0.76 (0.5-1.14)	7,97E-01	1.06 (0.69-1.61)	3,78E-01	1.47 (0.61-3.53)	3,19E-02	2.38 (1.14-4.99)	9,75E-01	1.01 (0.61-1.66)	1,71E-01	1.94 (0.8-4.73)
rs9311676	3	58470351	*ABHD6,PXK*	5,59E-01	0.91 (0.68-1.23)	3,22E-01	1.26 (0.81-1.96)	5,60E-01	1.09 (0.81-1.47)	3,38E-02	0.72 (0.53-0.97)	5,30E-01	1.12 (0.79-1.6)	4,40E-01	0.88 (0.64-1.21)	8,06E-01	1.04 (0.76-1.42)	1,10E-01	0.61 (0.33-1.12)	6,02E-01	1.21 (0.61-2.4)	3,01E-01	0.82 (0.56-1.18)	1,06E-01	2.03 (0.85-4.86)
rs564799	3	159728987	*IL12A*	9,22E-01	1.02 (0.73-1.41)	4,11E-02	1.63 (1.03-2.58)	3,81E-01	0.86 (0.62-1.2)	3,30E-01	1.18 (0.85-1.63)	7,39E-01	0.94 (0.64-1.37)	9,52E-01	1.01 (0.71-1.44)	9,46E-01	0.99 (0.7-1.39)	1,30E-01	1.6 (0.88-2.9)	9,22E-01	1.04 (0.49-2.19)	2,13E-01	1.31 (0.85-2.0)	1,09E-01	2.25 (0.76-6.6)
rs10028805	4	102737250	*BANK1*	4,64E-01	0.89 (0.65-1.21)	6,43E-01	1.11 (0.7-1.76)	4,21E-01	0.88 (0.65-1.2)	8,19E-01	1.04 (0.76-1.42)	7,98E-02	1.39 (0.96-2.03)	3,84E-01	1.16 (0.83-1.61)	7,12E-01	1.06 (0.77-1.47)	8,17E-01	1.07 (0.59-1.95)	9,83E-02	1.9 (0.85-4.23)	7,35E-01	0.94 (0.64-1.38)	6,80E-01	1.19 (0.51-2.78)
rs7726414	5	133431834	*TCF7,SKP1*	6,94E-01	1.12 (0.66-1.91)	5,46E-01	1.28 (0.61-2.7)	7,33E-01	0.91 (0.53-1.55)	4,66E-01	1.23 (0.72-2.1)	2,41E-01	1.53 (0.76-3.08)	1,79E-01	1.49 (0.86-2.58)	2,52E-01	1.4 (0.81-2.41)	5,50E-01	0.7 (0.21-2.31)	1,78E-01	3.26 (0.44-24.26)	8,78E-02	0.57 (0.31-1.03)	5,73E-01	0.68 (0.2-2.33)
rs10036748	5	150458146	*TNIP1*	8,21E-01	0.96 (0.69-1.33)	3,97E-01	0.8 (0.48-1.34)	3,94E-01	0.87 (0.63-1.2)	6,60E-01	1.08 (0.78-1.5)	8,72E-01	1.03 (0.7-1.52)	6,05E-01	1.1 (0.77-1.56)	5,20E-01	0.89 (0.63-1.26)	3,08E-01	0.7 (0.35-1.4)	2,99E-01	0.68 (0.34-1.37)	6,05E-01	1.12 (0.74-1.7)	8,83E-01	1.07 (0.44-2.58)
rs2431697	5	159879978	*MIR146A*	5,74E-01	0.92 (0.68-1.24)	2,49E-01	0.76 (0.48-1.22)	6,96E-01	0.94 (0.7-1.27)	7,79E-01	0.96 (0.71-1.3)	2,39E-01	1.24 (0.86-1.78)	9,23E-01	1.02 (0.73-1.41)	3,46E-01	1.16 (0.85-1.6)	8,60E-01	1.05 (0.59-1.9)	2,94E-01	0.7 (0.36-1.36)	9,65E-02	0.73 (0.5-1.06)	4,39E-01	0.73 (0.33-1.61)
rs1270942	6	31918860	*MHC class III*	6,72E-01	0.9 (0.55-1.46)	1,75E-01	1.59 (0.84-3.04)	4,21E-01	1.22 (0.75-1.99)	5,35E-02	1.62 (0.99-2.63)	1,38E-02	2.3 (1.12-4.72)	7,01E-01	0.9 (0.53-1.54)	1,46E-01	0.67 (0.39-1.16)	9,62E-01	0.98 (0.38-2.55)	8,06E-02	4.14 (0.56-30.71)	1,56E-01	0.66 (0.38-1.15)	6,56E-01	1.38 (0.32-5.95)
rs9462027	6	34797241	*UHRF1BP1*	2,63E-02	0.7 (0.51-0.95)	2,63E-01	1.31 (0.83-2.06)	8,90E-01	1.02 (0.75-1.39)	2,51E-02	0.69 (0.5-0.95)	6,25E-01	0.91 (0.63-1.31)	2,45E-01	1.23 (0.88-1.71)	1,14E-01	0.76 (0.54-1.06)	6,06E-01	1.18 (0.65-2.13)	8,41E-01	0.93 (0.46-1.87)	9,55E-01	1.01 (0.69-1.49)	6,79E-01	0.84 (0.37-1.87)
rs6568431	6	106588806	*PRDM1,ATG5*	7,35E-01	1.05 (0.78-1.42)	4,40E-01	1.2 (0.76-1.9)	1,20E-01	1.27 (0.94-1.71)	6,53E-01	0.93 (0.69-1.26)	8,35E-01	0.96 (0.67-1.37)	1,10E-01	0.77 (0.56-1.06)	6,04E-01	1.09 (0.79-1.5)	4,77E-01	0.81 (0.45-1.45)	6,39E-02	0.5 (0.23-1.08)	5,21E-02	0.68 (0.46-1.01)	9,33E-01	0.97 (0.43-2.16)
rs6932056	6	138242437	*TNFAIP3*	2,88E-01	1.48 (0.7-3.13)	4,91E-01	0.66 (0.18-2.39)	4,82E-01	1.3 (0.62-2.73)	4,85E-01	0.77 (0.36-1.64)	4,16E-01	1.45 (0.56-3.8)	1,93E-02	2.37 (1.14-4.95)	6,58E-02	1.97 (0.95-4.1)	5,86E-01	1.43 (0.41-5.01)	6,67E-01	0.72 (0.17-3.13)	1,15E-01	2.37 (0.69-8.12)	1,29E-01	53.54 (0.0-296604.0)
rs849142	7	28185891	*JAZF1*	9,52E-01	0.99 (0.74-1.33)	9,88E-01	1.0 (0.64-1.56)	6,19E-01	1.08 (0.8-1.45)	5,69E-01	1.09 (0.81-1.47)	9,09E-01	1.02 (0.72-1.45)	1,72E-01	1.26 (0.91-1.73)	2,74E-01	0.84 (0.61-1.14)	7,11E-01	1.12 (0.63-2.0)	3,52E-01	0.72 (0.36-1.43)	8,85E-02	0.72 (0.49-1.04)	4,07E-01	0.71 (0.32-1.58)
rs4917014	7	50305863	*IKZF1*	4,08E-01	1.14 (0.83-1.57)	9,54E-01	1.01 (0.63-1.64)	3,10E-01	0.85 (0.62-1.17)	2,80E-02	1.42 (1.03-1.96)	5,00E-01	1.14 (0.77-1.67)	1,22E-01	0.76 (0.53-1.09)	1,28E-01	1.29 (0.92-1.8)	7,13E-01	1.12 (0.6-2.08)	9,11E-01	0.96 (0.46-1.99)	1,01E-01	1.41 (0.92-2.15)	7,40E-01	1.16 (0.48-2.79)
rs10488631	7	128594183	*IRF5*	8,62E-01	0.96 (0.64-1.45)	5,40E-02	0.5 (0.24-1.07)	9,37E-01	0.98 (0.65-1.48)	2,08E-01	0.76 (0.5-1.16)	1,69E-01	1.43 (0.85-2.4)	8,58E-01	0.96 (0.62-1.5)	2,67E-01	1.27 (0.84-1.94)	3,65E-01	1.42 (0.69-2.92)	8,00E-01	1.13 (0.43-2.98)	5,09E-01	1.19 (0.7-2.04)	2,96E-01	0.59 (0.23-1.51)
rs2736340	8	11343973	*BLK*	1,22E-01	1.31 (0.94-1.84)	2,53E-01	0.73 (0.43-1.25)	5,92E-01	0.91 (0.65-1.27)	6,08E-01	1.1 (0.78-1.53)	6,44E-01	1.1 (0.74-1.64)	6,51E-01	1.09 (0.76-1.56)	9,56E-01	1.01 (0.71-1.44)	7,30E-01	0.89 (0.45-1.73)	5,54E-01	0.79 (0.38-1.65)	1,09E-01	1.45 (0.92-2.26)	4,15E-01	1.5 (0.56-4.05)
rs2663052	10	50069395	*WDFY4*	5,38E-01	0.91 (0.68-1.23)	6,61E-01	0.91 (0.58-1.41)	8,24E-01	0.97 (0.72-1.3)	6,70E-01	1.07 (0.79-1.43)	5,63E-01	1.11 (0.78-1.57)	3,59E-01	0.86 (0.63-1.19)	6,98E-01	0.94 (0.69-1.29)	3,70E-02	1.86 (1.01-3.44)	3,89E-01	0.75 (0.38-1.48)	4,46E-01	0.87 (0.6-1.26)	9,59E-01	1.02 (0.47-2.24)
rs4948496	10	63805617	*ARID5B*	3,31E-02	0.73 (0.54-0.97)	1,60E-01	0.73 (0.47-1.13)	2,02E-02	0.7 (0.52-0.95)	6,86E-01	0.94 (0.7-1.26)	6,18E-01	0.91 (0.65-1.3)	6,84E-01	0.94 (0.68-1.29)	2,68E-01	0.84 (0.61-1.14)	8,28E-01	1.07 (0.6-1.9)	9,74E-01	0.99 (0.51-1.94)	5,14E-01	0.88 (0.61-1.28)	5,12E-01	1.3 (0.59-2.86)
rs12802200	11	566936	*IRF7*	4,38E-01	1.15 (0.78-1.7)	1,07E-01	1.52 (0.89-2.59)	2,64E-01	0.81 (0.55-1.2)	7,65E-01	1.06 (0.72-1.56)	8,32E-01	0.96 (0.61-1.5)	9,09E-01	0.98 (0.64-1.49)	7,38E-01	0.94 (0.62-1.41)	1,38E-01	0.55 (0.22-1.37)	9,06E-01	0.95 (0.4-2.28)	7,65E-01	0.93 (0.58-1.51)	4,86E-01	1.45 (0.45-4.65)
rs2732549	11	35088399	*CD44*	8,62E-01	0.97 (0.72-1.31)	4,20E-01	0.83 (0.53-1.29)	8,82E-01	1.02 (0.76-1.38)	3,83E-01	0.87 (0.65-1.18)	1,59E-01	1.29 (0.91-1.83)	5,71E-01	0.91 (0.66-1.25)	6,13E-01	0.92 (0.67-1.26)	4,06E-01	1.29 (0.71-2.35)	7,40E-01	0.89 (0.45-1.77)	1,63E-02	1.59 (1.09-2.3)	6,36E-01	1.21 (0.55-2.66)
rs3794060	11	71187679	*DHCR7,NADSYN1*	6,56E-01	0.93 (0.68-1.27)	2,44E-01	1.33 (0.82-2.17)	9,45E-01	0.99 (0.73-1.35)	9,83E-01	1.0 (0.73-1.37)	4,69E-01	1.15 (0.8-1.65)	3,65E-01	0.86 (0.61-1.19)	9,29E-01	0.99 (0.71-1.37)	5,83E-01	0.84 (0.47-1.53)	8,96E-01	0.95 (0.47-1.94)	5,43E-01	0.88 (0.6-1.31)	2,14E-01	1.67 (0.76-3.67)
rs7941765	11	128499000	*ETS1,FLI1*	4,40E-01	1.12 (0.83-1.5)	7,56E-01	1.07 (0.69-1.67)	8,52E-01	0.97 (0.73-1.31)	4,33E-01	1.12 (0.83-1.51)	7,15E-03	0.63 (0.44-0.89)	8,19E-01	0.96 (0.7-1.33)	3,63E-01	1.15 (0.84-1.57)	8,71E-01	0.95 (0.54-1.7)	9,46E-01	0.98 (0.5-1.91)	5,56E-01	1.11 (0.77-1.61)	6,99E-02	2.05 (0.9-4.66)
rs10774625	12	111910219	*SH2B3*	3,98E-01	0.88 (0.65-1.18)	1,99E-01	1.35 (0.86-2.1)	9,76E-01	1.0 (0.74-1.34)	2,86E-01	0.85 (0.63-1.14)	4,65E-01	1.14 (0.81-1.62)	2,07E-02	0.68 (0.49-0.94)	4,23E-02	0.72 (0.53-0.98)	6,61E-01	1.14 (0.64-2.03)	6,45E-01	0.85 (0.43-1.66)	5,10E-03	0.58 (0.4-0.84)	7,70E-01	1.13 (0.51-2.47)
rs1059312	12	129278864	*SLC15A4*	3,82E-01	1.14 (0.85-1.53)	2,99E-01	1.26 (0.81-1.96)	3,89E-01	0.88 (0.65-1.18)	7,35E-01	0.95 (0.71-1.28)	9,71E-01	0.99 (0.7-1.41)	2,53E-02	0.7 (0.5-0.96)	3,13E-01	0.85 (0.62-1.17)	5,63E-01	0.84 (0.47-1.52)	7,83E-01	1.1 (0.56-2.17)	7,58E-01	0.94 (0.65-1.37)	8,70E-01	1.07 (0.48-2.36)
rs4902562	14	68731458	*RAD51B*	9,88E-01	1.0 (0.74-1.35)	9,89E-01	1.0 (0.63-1.59)	9,32E-02	1.29 (0.95-1.75)	5,86E-01	1.09 (0.8-1.48)	9,23E-02	1.35 (0.95-1.92)	3,74E-01	0.86 (0.62-1.2)	1,30E-02	0.67 (0.49-0.93)	6,70E-01	1.14 (0.62-2.08)	6,56E-01	0.85 (0.42-1.74)	9,09E-01	1.02 (0.7-1.5)	8,15E-01	0.91 (0.4-2.07)
rs2289583	15	75311036	*CSK*	8,61E-01	0.97 (0.72-1.32)	4,80E-01	0.84 (0.53-1.34)	8,72E-01	0.97 (0.72-1.32)	2,99E-01	0.85 (0.62-1.15)	5,80E-01	1.11 (0.77-1.6)	4,86E-01	0.89 (0.64-1.23)	7,05E-01	0.94 (0.68-1.3)	5,43E-02	1.8 (1.01-3.2)	2,01E-01	0.63 (0.32-1.24)	7,27E-01	1.07 (0.73-1.58)	5,65E-01	0.79 (0.36-1.74)
rs9652601	16	11174365	*CIITA,SOCS1*	7,20E-01	0.95 (0.69-1.3)	7,59E-01	1.07 (0.67-1.72)	4,15E-01	1.13 (0.83-1.56)	7,78E-01	1.05 (0.76-1.44)	9,56E-01	1.01 (0.69-1.47)	5,82E-01	1.1 (0.78-1.54)	2,85E-01	0.84 (0.6-1.18)	1,30E-01	0.61 (0.31-1.22)	9,89E-02	1.9 (0.82-4.41)	8,74E-01	0.97 (0.65-1.44)	1,56E-01	1.91 (0.71-5.13)
rs34572943	16	31272353	*ITGAM*	2,74E-01	0.83 (0.59-1.17)	6,15E-01	1.13 (0.68-1.88)	7,57E-01	1.05 (0.75-1.48)	6,73E-01	1.07 (0.76-1.52)	1,11E-01	0.74 (0.5-1.09)	8,85E-02	1.35 (0.94-1.94)	5,68E-01	1.11 (0.77-1.58)	8,30E-01	0.93 (0.47-1.84)	4,27E-01	1.38 (0.59-3.22)	9,21E-01	0.98 (0.64-1.51)	3,94E-01	0.7 (0.3-1.63)
rs11644034	16	85972612	*IRF8*	6,07E-01	1.11 (0.73-1.7)	5,96E-01	0.84 (0.43-1.64)	3,68E-01	0.83 (0.54-1.26)	8,15E-02	0.69 (0.44-1.07)	4,66E-01	0.84 (0.52-1.36)	8,62E-01	0.96 (0.61-1.52)	9,03E-01	0.97 (0.62-1.52)	1,68E-01	0.52 (0.18-1.47)	9,89E-01	1.01 (0.38-2.65)	6,63E-01	1.13 (0.65-1.94)	3,02E-01	1.99 (0.46-8.54)
rs2286672	17	4712617	*PLD2*	1,68E-01	1.47 (0.83-2.59)	4,41E-01	0.71 (0.27-1.83)	7,95E-01	1.07 (0.61-1.88)	1,93E-01	1.43 (0.82-2.5)	1,15E-01	1.76 (0.81-3.81)	2,38E-01	1.4 (0.79-2.5)	5,75E-01	0.85 (0.46-1.56)	8,63E-01	1.09 (0.38-3.17)	6,39E-01	1.38 (0.32-5.9)	8,38E-02	1.98 (0.83-4.74)	4,21E-01	0.6 (0.17-2.06)
rs2941509	17	37921194	*IKZF3*	3,22E-01	1.36 (0.72-2.56)	2,51E-01	1.86 (0.56-6.13)	4,48E-01	1.26 (0.67-2.38)	9,88E-01	1.0 (0.53-1.9)	3,10E-01	0.67 (0.29-1.54)	8,61E-02	1.87 (0.85-4.12)	5,80E-01	1.2 (0.6-2.4)	4,74E-01	0.68 (0.23-1.98)	3,77E-01	0.46 (0.06-3.44)	6,69E-01	0.84 (0.37-1.94)	3,04E-02	NA (NA-NA)
rs2304256	19	10475652	*TYK2*	8,51E-01	0.97 (0.68-1.37)	8,04E-02	0.6 (0.33-1.1)	5,49E-01	0.9 (0.63-1.28)	6,96E-01	0.93 (0.65-1.33)	2,98E-02	0.64 (0.43-0.95)	5,15E-01	0.88 (0.6-1.29)	1,33E-01	1.32 (0.92-1.9)	7,99E-01	0.91 (0.45-1.84)	1,30E-01	0.57 (0.28-1.16)	7,19E-01	1.08 (0.69-1.7)	6,34E-02	0.45 (0.2-1.02)
rs7444	22	21976934	*UBE2L3*	9,35E-01	1.01 (0.71-1.44)	5,70E-01	1.17 (0.7-1.95)	4,00E-01	1.17 (0.82-1.66)	1,12E-01	1.34 (0.94-1.9)	9,98E-01	1.0 (0.66-1.52)	3,10E-01	1.22 (0.84-1.77)	4,41E-01	0.86 (0.59-1.25)	5,60E-01	1.22 (0.63-2.36)	4,63E-01	0.75 (0.35-1.58)	7,99E-01	1.06 (0.68-1.66)	6,78E-01	1.23 (0.46-3.32)

Abbreviations: Chr, chromosome; CI, confidence interval; OR, odds ratio; NA, odds ratio was not estimated due to the presence of zero-cell counts; Position, SNP base pair in build GRCh37/hg19; P, P-value obtained from the association analysis; SNP, Single-Nucleotide Polymorphism.

2.2 Table S2. Genome-wide pathway associations obtained analyzing the 11 SLE clinical phenotypes in the discovery cohort.

SLE PHENOTYPE	PATHWAY	DATABASE	$SNPS_{PATH}$	$SNPS_1$	$SNPS_2$	P	FDR	SNPS
Antinuclear Antibodies	NEGATIVE_REGULATORS_OF_RIG_I_MDA5_SIGNALING	REACTOME	464	9	4	2,00E-05	1,60E-02	rs12599479|rs9936269|rs26538|rs7262903
Antinuclear Antibodies	P75_NTR_RECEPTOR_MEDIATED_SIGNALLING	REACTOME	2669	40	20	2,60E-03	1,00E+00	rs11638112|rs13405741|rs10499305|rs625572|rs1373928|rs7741028|rs6853|rs10872715|rs10496037|rs8030681|rs11072660|rs13383577|rs4289301|rs6019300|rs656709|rs12480096|rs8081687|rs790647|rs10495562|rs8024932
Antinuclear Antibodies	METABOLISM_OF_NON_CODING_RNA	REACTOME	611	21	11	9,20E-03	1,00E+00	rs3026106|rs197438|rs1478785|rs5766533|rs13219815|rs9615025|rs8088313|rs10852553|rs715749|rs2944346|rs1471454
Antinuclear Antibodies	INCRETIN_SYNTHESIS_SECRETION_AND_INACTIVATION	REACTOME	540	23	8	1,14E-02	1,00E+00	rs7713485|rs17696124|rs9883073|rs10903341|rs11135469|rs17153747|rs1573611|rs3741920
Antinuclear Antibodies	TERMINATION_OF_O_GLYCAN_BIOSYNTHESIS	REACTOME	961	45	20	1,63E-02	1,00E+00	rs485141|rs657224|rs4949631|rs7934606|rs12129945|rs11245935|rs6669101|rs12422396|rs387831|rs501426|rs13264573|rs1370714|rs1868435|rs649328|rs10878507|rs6988999|rs1571142|rs2945743|rs28678421|rs341016
Antinuclear Antibodies	NRAGE_SIGNALS_DEATH_THROUGH_JNK	REACTOME	1919	37	20	1,65E-02	1,00E+00	rs13405741|rs10499305|rs625572|rs1373928|rs7741028|rs10872715|rs13383577|rs4289301|rs6019300|rs656709|rs12480096|rs8081687|rs836556|rs4714028|rs4318271|rs1883888|rs10904508|rs8024521|rs179047|rs1875059
Antinuclear Antibodies	ACTIVATION_OF_BH3_ONLY_PROTEINS	REACTOME	325	21	8	1,94E-02	1,00E+00	rs13405741|rs12957347|rs12955104|rs12867405|rs12454840|rs12958785|rs1791330|rs12259267
Antinuclear Antibodies	INTERACTIONS_OF_VPR_WITH_HOST_CELLULAR_PROTEINS	REACTOME	480	20	11	2,18E-02	1,00E+00	rs3026106|rs1478785|rs5766533|rs1151514|rs13219815|rs17201246|rs9615025|rs8088313|rs10852553|rs2944346|rs1471454
Antinuclear Antibodies	REGULATION_OF_GLUCOKINASE_BY_GLUCOKINASE_REGULATORY_PROTEIN	REACTOME	459	16	9	2,20E-02	1,00E+00	rs3026106|rs1478785|rs5766533|rs13219815|rs9615025|rs6505776|rs10852553|rs2944346|rs1471454
Antinuclear Antibodies	CELL_DEATH_SIGNALLING_VIA_NRAGE_NRIF_AND_NADE	REACTOME	2069	37	20	2,45E-02	1,00E+00	rs13405741|rs10499305|rs625572|rs1373928|rs7741028|rs10872715|rs13383577|rs4289301|rs6019300|rs656709|rs12480096|rs8081687|rs836556|rs4714028|rs4318271|rs1883888|rs10904508|rs8024521|rs179047|rs1875059
Antinuclear Antibodies	SIGNALLING_BY_NGF	REACTOME	7869	33	20	2,97E-02	1,00E+00	rs10521466|rs11638112|rs13405741|rs3886478|rs2267737|rs10499305|rs625572|rs902522|rs1373928|rs7741028|rs3799663|rs6853|rs10872715|rs10496037|rs12958400|rs8030681|rs7999458|rs11072660|rs11102314|rs13383577
Antinuclear Antibodies	IL1_SIGNALING	REACTOME	1322	65	20	3,08E-02	1,00E+00	rs12619383|rs6020238|rs6853|rs10485101|rs242389|rs2529737|rs4851547|rs12413938|rs1144171|rs1019033|rs708030|rs2871444|rs17090708|rs6940350|rs1923075|rs331455|rs12216864|rs13217057|rs10498974|rs739559
Antinuclear Antibodies	TRANSPORT_OF_RIBONUCLEOPROTEINS_INTO_THE_HOST_NUCLEUS	REACTOME	452	17	10	3,27E-02	1,00E+00	rs3026106|rs1478785|rs5766533|rs13219815|rs17201246|rs9615025|rs6505776|rs10852553|rs2944346|rs1471454
Antinuclear Antibodies	NUCLEOTIDE_LIKE_PURINERGIC_RECEPTORS	REACTOME	272	8	8	3,78E-02	1,00E+00	rs12139261|rs2800880|rs11235714|rs1920397|rs13094696|rs10857898|rs523907|rs1371101
Antinuclear Antibodies	GLUCOSE_TRANSPORT	REACTOME	689	17	10	3,87E-02	1,00E+00	rs3026106|rs1478785|rs5766533|rs13219815|rs9615025|rs8088313|rs10852553|rs841861|rs2944346|rs1471454
Antinuclear Antibodies	FORMATION_OF_ATP_BY_CHEMIOSMOTIC_COUPLING	REACTOME	77	2	2	4,40E-02	1,00E+00	rs8133991|rs968698
Antinuclear Antibodies	AMINE_LIGAND_BINDING_RECEPTORS	REACTOME	2247	33	20	4,55E-02	1,00E+00	rs6861578|rs297672|rs1072319|rs11706283|rs13185639|rs1578303|rs6994883|rs6429140|rs1936600|rs6885612|rs2115512|rs12119540|rs13190572|rs9526313|rs10485512|rs1471107|rs746622|rs731779|rs9688436|rs6893566
Antinuclear Antibodies	CTLA4_PATHWAY	BIOCARTA	662	32	16	4,80E-02	1,00E+00	rs933984|rs4419220|rs3850890|rs2715267|rs9896052|rs152105|rs1550805|rs4767879|rs7591705|rs6449969|rs1599796|rs9838117|rs1427363|rs35265698|rs1917534|rs16859030
Antinuclear Antibodies	REGULATION_OF_KIT_SIGNALING	REACTOME	587	36	9	4,91E-02	1,00E+00	rs10777125|rs8081512|rs11153318|rs9896052|rs8085725|rs10858795|rs2239167|rs1544241|rs1030982
Antinuclear Antibodies	G_ALPHA1213_SIGNALLING_EVENTS	REACTOME	2855	29	20	5,52E-02	1,00E+00	rs625572|rs10499305|rs1373928|rs7741028|rs297672|rs10872715|rs6994883|rs16947151|rs13383577|rs4289301|rs6019300|rs6807740|rs656709|rs12480096|rs8081687|rs4243583|rs10485512|rs1253704|rs836556|rs4714028
Antinuclear Antibodies	PRIMARY_IMMUNODEFICIENCY	KEGG	742	28	20	5,62E-02	1,00E+00	rs933984|rs4419220|rs3782735|rs7905645|rs179307|rs10507441|rs371927|rs1561144|rs7591705|rs2430682|401754|rs322931|rs1427363|rs3741920|rs11652968|rs4072864|rs11687510|rs1399602|rs11597989|rs2228603
Antinuclear Antibodies	RHO_PATHWAY	BIOCARTA	480	17	9	5,75E-02	1,00E+00	rs10800842|rs11869351|rs1731183|rs17048140|rs465316|rs883751|rs2362941|rs1958913|rs2855726
Antinuclear Antibodies	NEP_NS2_INTERACTS_WITH_THE_CELLULAR_EXPORT_MACHINERY	REACTOME	456	22	12	5,77E-02	1,00E+00	rs3026106|rs1478785|rs5766533|rs17010833|rs13219815|rs4671408|rs9615025|rs8088313|rs10852553|rs2178077|rs2944346|rs1471454
Antinuclear Antibodies	RNA_POL_III_TRANSCRIPTION	REACTOME	861	30	20	6,63E-02	1,00E+00	rs4921702|rs16858688|rs2227301|rs4922152|rs13296301|rs10961534|rs4922157|rs6081157|rs1372349|rs13270944|rs10121140|rs17092536|rs11989385|rs13290272|rs17213362|rs683474|rs6928843|rs2165951|rs7529557|rs520812
Antinuclear Antibodies	PRE_NOTCH_TRANSCRIPTION_AND_TRANSLATION	REACTOME	835	32	14	6,96E-02	1,00E+00	rs17825554|rs9465734|rs11604821|rs9291482|rs12867405|rs566607|rs11621593|rs9295464|rs624797|rs6681035|rs6820015|rs1114312|rs9907255|rs1872568
Antinuclear Antibodies	IL1R_PATHWAY	BIOCARTA	1001	55	20	7,20E-02	1,00E+00	rs12619383|rs6853|rs10485101|rs2069837|rs4851547|rs12199654|rs1144171|rs604045|rs708030|rs1923075|rs10863388|rs7526672|rs331455|rs13217057|rs10498974|rs2415290|rs739559|rs6914621|rs9345117|rs721025
Antinuclear Antibodies	INHIBITION_OF_INSULIN_SECRETION_BY_ADRENALINE_NORADRENALINE	REACTOME	789	36	20	7,27E-02	1,00E+00	rs16947151|rs6807740|rs4243583|rs1253704|rs17360897|rs10228945|rs12075532|rs11715227|rs953672|rs6415964|rs12147761|rs6972935|rs2714645|rs4807284|rs1878247|rs16917244|rs17124529|rs4480744|rs2523189|rs11622145
Antinuclear Antibodies	MCM_PATHWAY	BIOCARTA	234	15	4	7,31E-02	1,00E+00	rs10777125|rs10858795|rs4805487|rs3087474
Antinuclear Antibodies	RIG_I_MDA5_MEDIATED_INDUCTION_OF_IFN_ALPHA_BETA_PATHWAYS	REACTOME	1129	32	20	7,34E-02	1,00E+00	rs12599479|rs2829946|rs13119830|rs466609|rs6850472|rs10029721|rs2207701|rs3014866|rs9936269|rs331455|rs375369|rs3750512|rs13116389|rs2415290|rs793794|rs26538|rs457581|rs1467719|rs17220720|rs793881
Antinuclear Antibodies	DEFENSINS	REACTOME	495	21	9	7,97E-02	1,00E+00	rs3782735|rs6057657|rs2271585|rs1324528|rs1348181|rs17163745|rs1342137|rs6046492|rs3741920
Antinuclear Antibodies	PROPANOATE_METABOLISM	KEGG	1219	38	20	8,02E-02	1,00E+00	rs17070285|rs406303|rs4331688|rs2132302|rs2017571|rs9534734|rs4489814|rs1881717|rs11636141|rs357749|rs7558379|rs12274651|rs6547525|rs837322|rs2306787|rs2273684|rs2902374|rs4646772|rs7602538|rs10973734
Antinuclear Antibodies	INNATE_IMMUNE_SYSTEM	REACTOME	6278	33	20	8,14E-02	1,00E+00	rs12599479|rs6020238|rs3886478|rs16896849|rs2829946|rs3782735|rs499205|rs3799663|rs6853|rs6057657|rs10485101|rs13119830|rs242389|rs12220324|rs12571782|rs2529737|rs971075|rs11876920|rs1143664|rs466609
Antinuclear Antibodies	PGC1A_PATHWAY	BIOCARTA	959	41	17	8,28E-02	1,00E+00	rs3774909|rs2236891|rs7665102|rs6448226|rs306090|rs2274316|rs7694484|rs4699014|rs2055226|rs873592|rs3746259|rs650356|rs216113|rs2236893|rs1011398|rs4240286|rs1554668
Antinuclear Antibodies	ABCA_TRANSPORTERS_IN_LIPID_HOMEOSTASIS	REACTOME	464	19	7	8,47E-02	1,00E+00	rs7419736|rs10877173|rs10179921|rs2286419|rs150926|rs956133|rs737453
Antinuclear Antibodies	ADP_SIGNALLING_THROUGH_P2RY1	REACTOME	863	41	20	8,47E-02	1,00E+00	rs230624|rs16947151|rs6807740|rs4243583|rs1253704|rs6683451|rs12199654|rs12075532|rs11715227|rs11583351|rs12147761|rs6972935|rs612355|rs4807284|rs17124529|rs13094696|rs16826789|rs4480744|rs11622145|rs11880582
Antinuclear Antibodies	SYNTHESIS_SECRETION_AND_INACTIVATION_OF_GLP1	REACTOME	363	15	5	8,66E-02	1,00E+00	rs17696124|rs9883073|rs11135469|rs1573611|rs3741920
Antinuclear Antibodies	TRANSPORT_OF_MATURE_MRNA_DERIVED_FROM_AN_INTRONLESS_TRANSCRIPT	REACTOME	524	30	14	8,84E-02	1,00E+00	rs3026106|rs1478785|rs5766533|rs10495562|rs13219815|rs633742|rs4798|rs9615025|rs6505776|rs10852553|rs6599528|rs715749|rs2944346|rs1471454
Antinuclear Antibodies	PROXIMAL_TUBULE_BICARBONATE_RECLAMATION	KEGG	458	16	8	8,86E-02	1,00E+00	rs11685570|rs1200138|rs7302925|rs10919062|rs2296063|rs11265346|rs10244884|rs17710985
Antinuclear Antibodies	AMI_PATHWAY	BIOCARTA	574	38	16	8,92E-02	1,00E+00	rs1247571|rs4536647|rs9521666|rs903352|rs10804016|rs2047076|rs595325|rs13137121|rs7322495|rs10492497|rs7580146|rs9288147|rs9457997|rs3823055|rs616008|rs4619602
Antinuclear Antibodies	CYTOKINE_SIGNALING_IN_IMMUNE_SYSTEM	REACTOME	6819	50	20	9,02E-02	1,00E+00	rs12619383|rs3026106|rs6020238|rs367503|rs2352946|rs468440|rs17375044|rs6853|rs1478785|rs7905645|rs7665102|rs7999458|rs10485101|rs13119830|rs242389|rs4900218|rs5766533|rs1227631|rs2529737|rs2069837
Antinuclear Antibodies	LIGAND_GATED_ION_CHANNEL_TRANSPORT	REACTOME	1051	53	20	9,11E-02	1,00E+00	rs4906888|rs28671569|rs1863455|rs426783|rs208147|rs488447|rs1863464|rs7179956|rs981347|rs4906892|rs12441474|rs1006679|rs4283207|rs10015366|rs1863459|rs12647358|rs170020|rs8040901|rs4517759|rs13176858
Antinuclear Antibodies	GLUCAGON_SIGNALING_IN_METABOLIC_REGULATION	REACTOME	1044	26	20	9,35E-02	1,00E+00	rs902522|rs6807740|rs1030098|rs1404912|rs4243583|rs1253704|rs17360897|rs2287778|rs12075532|rs11715227|rs6501512|rs2302784|rs953672|rs12147761|rs13155334|rs6972935|rs4807284|rs7703532|rs17124529|rs883751
Antinuclear Antibodies	NITROGEN_METABOLISM	KEGG	587	30	14	9,39E-02	1,00E+00	rs11685570|rs1024898|rs6540114|rs10932334|rs6748782|rs1402028|rs7302925|rs2202044|rs580789|rs759688|rs7173285|rs2296063|rs10494266|rs8023341
Antinuclear Antibodies	GABA_RECEPTOR_ACTIVATION	REACTOME	2705	30	20	9,44E-02	1,00E+00	rs4906888|rs28671569|rs902522|rs2591164|rs1863455|rs16947151|rs948215|rs1843622|rs208147|rs488447|rs1404912|rs4243583|rs1253704|rs17360897|rs10228945|rs2019463|rs2287778|rs2275404|rs12075532|rs1863464
Antinuclear Antibodies	ACTIVATION_OF_CHAPERONE_GENES_BY_XBP1S	REACTOME	761	39	20	9,47E-02	1,00E+00	rs9841006|rs499205|rs1406961|rs1030098|rs12145743|rs2302784|rs848189|rs2717229|rs1838105|rs1391962|rs9810770|rs2969891|rs11708761|rs9302083|rs7649928|rs478309|rs1865536|rs4247202|rs10895987|rs10421782
Antinuclear Antibodies	ACTIVATED_AMPK_STIMULATES_FATTY_ACID_OXIDATION_IN_MUSCLE	REACTOME	421	20	13	9,76E-02	1,00E+00	rs1881628|rs17762352|rs12540943|rs7795096|rs1001684|rs4595037|rs2430682|rs13431868|rs10169231|rs7328100|rs4726084|rs11971243|rs7778077
Antinuclear Antibodies	CACAM_PATHWAY	BIOCARTA	401	20	10	9,94E-02	1,00E+00	rs2236891|rs7665102|rs9898774|rs306090|rs2055226|rs873592|rs1011398|rs2236893|rs4240286|rs1554668
Antinuclear Antibodies	REGULATION_OF_INSULIN_SECRETION_BY_GLUCAGON_LIKE_PEPTIDE1	REACTOME	1388	23	20	1,00E-01	1,00E+00	rs7600722|rs11102314|rs3820852|rs16947151|rs6807740|rs1030098|rs4243583|rs1253704|rs3769309|rs17360897|rs10879852|rs12075532|rs11715227|rs6501512|rs2302784|rs953672|rs10495676|rs12147761|rs7303557|rs6972935
Antinuclear Antibodies	SIGNAL_AMPLIFICATION	REACTOME	1023	40	20	1,02E-01	1,00E+00	rs230624|rs16947151|rs6807740|rs4243583|rs1253704|rs10228945|rs6683451|rs12199654|rs12075532|rs11715227|rs11583351|rs12147761|rs6972935|rs2714645|rs612355|rs4807284|rs17124529|rs13094696|rs16826789|rs4480744
Antinuclear Antibodies	SPHINGOLIPID_DE_NOVO_BIOSYNTHESIS	REACTOME	1020	35	20	1,10E-01	1,00E+00	rs17752182|rs678194|rs3011777|rs12081589|rs1493130|rs346811|rs17333779|rs723506|rs1466447|rs2041120|rs17785945|rs10493211|rs4592839|rs12817211|rs10220397|rs10826161|rs10041669|rs1307813|rs12648528|rs11705811
Antinuclear Antibodies	EIF_PATHWAY	BIOCARTA	158	7	5	1,13E-01	1,00E+00	rs6059703|rs4751691|rs8059570|rs2293603|rs1555322
Antinuclear Antibodies	O_LINKED_GLYCOSYLATION_OF_MUCINS	REACTOME	2402	43	20	1,14E-01	1,00E+00	rs485141|rs657224|rs4949631|rs13035081|rs4846948|rs6095638|rs7934606|rs12129945|rs11245935|rs6669101|rs12699009|rs10256029|rs12422396|rs387831|rs501426|rs4726132|rs10477100|rs13264573|rs1370714|rs10213212
Antinuclear Antibodies	ACTIVATION_OF_THE_MRNA_UPON_BINDING_OF_THE_CAP_BINDING_COMPLEX_AND_EI T_BINDING_TO_43S	REACTOME	780	45	20	1,15E-01	1,00E+00	rs6059703|rs1039153|rs7902136|rs2175376|rs4751691|rs1953910|rs17121120|rs1548597|rs7826090|rs11002569|rs2951967|rs11733964|rs11002552|rs1953912|rs26528|rs1956099|rs10839957|rs10895987|rs12676238|rs2293603
Antinuclear Antibodies	ENDOSOMAL_SORTING_COMPLEX_REQUIRED_FOR_TRANSPORT_ESCRT	REACTOME	318	19	7	1,15E-01	1,00E+00	rs12430144|rs4537255|rs2132302|rs4664531|rs16830728|rs7816946|rs13258200
Antinuclear Antibodies	CELL_CYCLE	KEGG	2581	40	20	1,16E-01	1,00E+00	rs766295|rs12078447|rs9886419|rs9465734|rs7812998|rs1558461|rs1027692|rs17057833|rs1561928|rs11604821|rs9562187|rs6836883|rs10820827|rs6432018|rs4350299|rs12867405|rs848189|rs1426511|rs604045|rs7015841
Antinuclear Antibodies	PAR1_PATHWAY	BIOCARTA	1941	33	20	1,17E-01	1,00E+00	rs13279485|rs10800842|rs625572|rs13269070|rs10485101|rs9515380|rs8081512|rs1404912|rs169143|rs10228945|rs2047076|rs1731183|rs2206422|rs1144171|rs198178|rs11136430|rs10485723|rs2714645|rs1923075|rs708925
Antinuclear Antibodies	NFKB_PATHWAY	BIOCARTA	870	70	20	1,17E-01	1,00E+00	rs12619383|rs6853|rs10485101|rs4851547|rs1144171|rs1923075|rs4483225|rs331455|rs13217057|rs10498974|rs2415290|rs6914621|rs10513330|rs9345117|rs721025|rs1061624|rs12191179|rs12289836|rs7745771|rs10872893
Antinuclear Antibodies	THE_ROLE_OF_NEF_IN_HIV1_REPLICATION_AND_DISEASE_PATHOGENESIS	REACTOME	936	50	20	1,24E-01	1,00E+00	rs13272985|rs3782735|rs17646824|rs951453|rs836556|rs1316638|rs17559604|rs1357287|rs261072|rs11153318|rs1420320|rs2541094|rs4385221|rs1611493|rs30079|rs17170799|rs1477953|rs724489|rs17738444|rs3741920
Antinuclear Antibodies	WNT_PATHWAY	BIOCARTA	1363	39	20	1,24E-01	1,00E+00	rs7028704|rs9886419|rs10485101|rs7812998|rs1561928|rs11604821|rs11175617|rs9883073|rs1250090|rs1144171|rs1426511|rs1923075|rs17296588|rs204680|rs13217057|rs10498974|rs6037819|rs815846|rs6914621|rs9345117
Antinuclear Antibodies	RNA_POL_III_TRANSCRIPTION_INITIATION_FROM_TYPE_3_PROMOTER	REACTOME	501	30	20	1,31E-01	1,00E+00	rs4921702|rs16858688|rs2227301|rs4922152|rs4922157|rs6081157|rs1372349|rs13270944|rs17092536|rs11989385|rs6928843|rs7529557|rs13277342|rs6081150|rs3848006|rs2684456|rs10918673|rs9635238|rs2293603|rs1917534
Antinuclear Antibodies	APOPTOSIS	KEGG	1727	68	20	1,31E-01	1,00E+00	rs12619383|rs12765241|rs6853|rs622175|rs7830593|rs1030098|rs4851547|rs7694484|rs9314261|rs16845834|rs6501512|rs2302784|rs708030|rs1550805|rs2100143|rs883751|rs3750512|rs4699014|rs6449969|rs2415290
Antinuclear Antibodies	UNFOLDED_PROTEIN_RESPONSE	REACTOME	1176	32	20	1,32E-01	1,00E+00	rs9841006|rs499205|rs1406961|rs708272|rs1030098|rs1000174|rs12145743|rs2302784|rs848189|rs10863981|rs2717229|rs13332704|rs1838105|rs679840|rs1391962|rs9810770|rs2969891|rs3795837|rs11860327|rs11708761
Antinuclear Antibodies	GLUCAGON_TYPE_LIGAND_RECEPTORS	REACTOME	786	30	20	1,33E-01	1,00E+00	rs2267737|rs16947151|rs6807740|rs4243583|rs1253704|rs12075532|rs11715227|rs12147761|rs4798777|rs6972935|rs4807284|rs17124529|rs311334|rs4480744|rs11622145|rs11880582|rs12668955|rs6945601|rs4723037|rs4797684
Antinuclear Antibodies	GLYCOSPHINGOLIPID_BIOSYNTHESIS_LACTO_AND_NEOLACTO_SERIES	KEGG	670	55	17	1,35E-01	1,00E+00	rs595341|rs597697|rs12207089|rs608475|rs7928391|rs2230906|rs240543|rs9391221|rs1225999|rs4599653|rs2298475|rs2230279|rs4818059|rs12870|rs3798745|rs722248|rs6433006
Antinuclear Antibodies	ANTIVIRAL_MECHANISM_BY_IFN_STIMULATED_GENES	REACTOME	1077	43	20	1,35E-01	1,00E+00	rs3026106|rs367503|rs468440|rs1478785|rs5766533|rs13029532|rs2085021|rs13219815|rs17201246|rs310244|rs8034917|rs17296210|rs2259091|rs9615025|rs6505776|rs468811|rs9876561|rs10852553|rs1551319|rs1451763
Antinuclear Antibodies	CLASS_B_2_SECRETIN_FAMILY_RECEPTORS	REACTOME	2257	26	20	1,35E-01	1,00E+00	rs7218567|rs2732794|rs2267737|rs16947151|rs1219615|rs6807740|rs8074816|rs4243583|rs1253704|rs10843091|rs7976725|rs1477031|rs1108386|rs10493754|rs11715227|rs12147761|rs4798777|rs6972935|rs3895916|rs4807284
Antinuclear Antibodies	VOLTAGE_GATED_POTASSIUM_CHANNELS	REACTOME	2491	38	20	1,37E-01	1,00E+00	rs4233891|rs3757105|rs2469515|rs631037|rs7600722|rs9865301|rs2283169|rs7820372|rs12760791|rs9693916|rs1316784|rs10774293|rs10879852|rs1587014|rs326751|rs7106909|rs10495676|rs569724|rs349335|rs12072776
Antinuclear Antibodies	POTASSIUM_CHANNELS	REACTOME	4940	41	20	1,38E-01	1,00E+00	rs4233891|rs3757105|rs2469515|rs2591164|rs2855790|rs631037|rs7600722|rs9817294|rs9865301|rs2283169|rs36960|rs1972058|rs16947151|rs1843622|rs7820372|rs12411068|rs1039128|rs12760791|rs904068|rs9693916
Antinuclear Antibodies	FAS_PATHWAY	BIOCARTA	980	51	20	1,38E-01	1,00E+00	rs3789587|rs12765241|rs10485101|rs2529737|rs1403101|rs1144171|rs1923075|rs12216864|rs13217057|rs10498974|rs6914621|rs9345117|rs721025|rs311646|rs12191179|rs4934433|rs4353229|rs6681035|rs8178179|rs7841459
Antinuclear Antibodies	TRAFFICKING_OF_GLUR2_CONTAINING_AMPA_RECEPTORS	REACTOME	721	35	13	1,40E-01	1,00E+00	rs1422893|rs4685165|rs8081512|rs169143|rs7730730|rs10030915|rs198178|rs10515699|rs3785396|rs2012859|rs4754133|rs1904949|rs9925433
Antinuclear Antibodies	COSTIMULATION_BY_THE_CD28_FAMILY	REACTOME	1479	52	20	1,41E-01	1,00E+00	rs933984|rs4419220|rs3782735|rs836556|rs3850890|rs1403101|rs2715267|rs11153318|rs12413938|rs9896052|rs3117230|rs4802906|rs1550805|rs2239167|rs10105118|rs9469357|rs10494270|rs7591705|rs6449969|rs2267423
Antinuclear Antibodies	RNA_POL_III_TRANSCRIPTION_TERMINATION	REACTOME	680	28	20	1,41E-01	1,00E+00	rs4921702|rs2227301|rs4922152|rs13296301|rs10961534|rs4922157|rs6081157|rs1372349|rs13270944|rs10121140|rs17092536|rs11989385|rs13290272|rs17213362|rs683474|rs2165951|rs13277342|rs6081150|rs3848006|rs2684456
Antinuclear Antibodies	MYOSIN_PATHWAY	BIOCARTA	1253	31	20	1,41E-01	1,00E+00	rs13279485|rs10800842|rs625572|rs13269070|rs9515380|rs8081512|rs169143|rs1731183|rs2206422|rs198178|rs11136430|rs10485723|rs708925|rs4242549|rs3785396|rs13262708|rs10229817|rs831168|rs2362941|rs2745755
Antinuclear Antibodies	IL17_PATHWAY	BIOCARTA	316	21	9	1,45E-01	1,00E+00	rs10777125|rs3782735|rs2069837|rs10858795|rs3741920|rs797999|rs1917534|rs16859030|rs7739687
Antinuclear Antibodies	THROMBIN_SIGNALLING_THROUGH_PROTEINASE_ACTIVATED_RECEPTORS_PARS	REACTOME	690	35	20	1,46E-01	1,00E+00	rs16947151|rs877711|rs6807740|rs4243583|rs1253704|rs2047076|rs12075532|rs11715227|rs12147761|rs6972935|rs4807284|rs17124529|rs4480744|rs11622145|rs11880582|rs658573|rs10229817|rs931647|rs8100484|rs208354
Antinuclear Antibodies	MAPK_SIGNALING_PATHWAY	KEGG	9833	39	20	1,47E-01	1,00E+00	rs12619383|rs10804900|rs3821709|rs3886478|rs12765241|rs1106865|rs6452735|rs9810230|rs4238022|rs38553|rs1924210|rs11970312|rs9886419|rs6806686|rs10485101|rs230624|rs7812998|rs11102314|rs10920115|rs9736587
Antinuclear Antibodies	G_PROTEIN_ACTIVATION	REACTOME	703	38	20	1,48E-01	1,00E+00	rs16947151|rs6807740|rs4243583|rs1253704|rs10228945|rs9306383|rs12075532|rs11715227|rs12147761|rs6972935|rs2714645|rs4807284|rs17124529|rs4480744|rs2523189|rs11622145|rs11880582|rs13232987|rs931647|rs8100484
Antinuclear Antibodies	CARM_ER_PATHWAY	BIOCARTA	1617	33	20	1,48E-01	1,00E+00	rs3774909|rs16852052|rs6448226|rs4484585|rs2904530|rs11604821|rs4852054|rs9636384|rs3791428|rs9981655|rs6959501|rs10878450|rs12579363|rs801521|rs352066|rs2229840|rs11883623|rs1882175|rs6928843|rs701038
Antinuclear Antibodies	SYNTHESIS_OF_GLYCOSYLPHOSPHATIDYLINOSITOL_GPI	REACTOME	485	18	8	1,49E-01	1,00E+00	rs281215|rs17164861|rs2275404|rs7210224|rs11809878|rs2273595|rs17418632|rs1135945
Antinuclear Antibodies	NEF_MEDIATES_DOWN_MODULATION_OF_CELL_SURFACE_RECEPTORS_BY_RECRUITIN N_ADAPTERS	REACTOME	366	22	8	1,49E-01	1,00E+00	rs13272985|rs3782735|rs951453|rs1611493|rs1477953|rs3741920|rs5763140|rs3745245
Antinuclear Antibodies	CYSTEINE_AND_METHIONINE_METABOLISM	KEGG	982	47	20	1,50E-01	1,00E+00	rs2028900|rs4788547|rs731727|rs2132302|rs10068900|rs1881717|rs566509|rs11636141|rs158850|rs2202044|rs12274651|rs978114|rs2306787|rs297961|rs580789|rs1834058|rs1205357|rs2039585|rs3793932|rs13338289
Antinuclear Antibodies	3_UTR_MEDIATED_TRANSLATIONAL_REGULATION	REACTOME	1235	46	20	1,55E-01	1,00E+00	rs6059703|rs1039153|rs7902136|rs7176919|rs2175376|rs12602594|rs4751691|rs1953910|rs17121120|rs1548597|rs10065570|rs7826090|rs11002569|rs4777170|rs2951967|rs6895379|rs11733964|rs2301723|rs13325754|rs11002552

Antinuclear Antibodies	INTRINSIC_PATHWAY_FOR_APOPTOSIS	REACTOME	532	34	15	1,56E-01	1,00E+00	rs13405741|rs12957347|rs12955104|rs12867405|rs2239923|rs12454840|rs1439124|rs39416|rs4353229|rs12870|rs2040558|rs12958785|rs1791330|rs12259267|rs10268780
Antinuclear Antibodies	AMYLOIDS	REACTOME	972	29	17	1,58E-01	1,00E+00	rs2829946|rs11177603|rs12203803|rs466609|rs2290501|rs12562819|rs2794502|rs6882087|rs17205146|rs12524841|rs375369|rs457581|rs871443|rs4654986|rs9358533|rs9356821|rs10917070
Antinuclear Antibodies	CK1_PATHWAY	BIOCARTA	759	33	20	1,61E-01	1,00E+00	rs13185639|rs6885612|rs1030098|rs13190572|rs1471107|rs2866250|rs2206422|rs6893566|rs6501512|rs2302784|rs10485723|rs708925|rs883751|rs4699014|rs2303924|rs7445117|rs2745755|rs6055995|rs8123677|rs6056252
Antinuclear Antibodies	NO1_PATHWAY	BIOCARTA	1482	45	20	1,65E-01	1,00E+00	rs2618681|rs12737847|rs4262039|rs10841528|rs7069974|rs10740411|rs10925415|rs12032218|rs1030098|rs11045301|rs6501512|rs2302784|rs4606345|rs10762562|rs400330|rs7907343|rs11249743|rs1194491|rs883751|rs2055226
Antinuclear Antibodies	SIGNALING_BY_NOTCH1	REACTOME	3563	25	20	1,66E-01	1,00E+00	rs17825554|rs7028704|rs11790630|rs9886419|rs7812998|rs4484585|rs767087|rs877711|rs877860|rs1561928|rs4852054|rs1838343|rs9291482|rs10495562|rs9636384|rs4903104|rs209948|rs3791428|rs6959501|rs1426511
Antinuclear Antibodies	WNT_SIGNALING_PATHWAY	KEGG	5611	33	20	1,67E-01	1,00E+00	rs7218567|rs853275|rs11181613|rs2938004|rs2107003|rs9886419|rs7665102|rs10485101|rs7812998|rs1219615|rs17833716|rs1561928|rs11604821|rs1407101|rs8081512|rs7212246|rs7145301|rs11175617|rs9883073|rs8006609
Antinuclear Antibodies	ENERGY_DEPENDENT_REGULATION_OF_MTOR_BY_LKB1_AMPK	REACTOME	461	21	14	1,67E-01	1,00E+00	rs1881628|rs17762352|rs12540943|rs7795096|rs1001684|rs4595037|rs13431868|rs10169231|rs7328100|rs4726084|rs11971243|rs9930956|rs7778077|rs2589149
Antinuclear Antibodies	HEMATOPOIETIC_CELL_LINEAGE	KEGG	1963	76	20	1,69E-01	1,00E+00	rs12619383|rs10777125|rs3782735|rs17375044|rs2335525|rs1227631|rs2069837|rs13253242|rs4851547|rs6440961|rs1019033|rs353625|rs10173487|rs2871444|rs10858795|rs2182913|rs193276|rs163567|rs6861460|rs7547373
Antinuclear Antibodies	INTERFERON_SIGNALING	REACTOME	3448	38	20	1,70E-01	1,00E+00	rs3026106|rs367503|rs2352946|rs468440|rs17375044|rs1478785|rs7665102|rs13119830|rs4900218|rs5766533|rs13029532|rs2038171|rs6850472|rs10029721|rs608914|rs353625|rs3117230|rs2085021|rs13219815|rs161130
Antinuclear Antibodies	SPHINGOLIPID_METABOLISM	KEGG	1101	40	20	1,71E-01	1,00E+00	rs17752182|rs2167239|rs3011777|rs12081589|rs1493130|rs346811|rs10121681|rs11707667|rs17785945|rs10493211|rs4592839|rs7855135|rs10220397|rs9626899|rs11624852|rs10826161|rs10041669|rs12648528|rs12258132|rs2983364
Antinuclear Antibodies	SPRY_PATHWAY	BIOCARTA	910	50	19	1,72E-01	1,00E+00	rs253447|rs6452735|rs1114275|rs10879175|rs6563216|rs9545290|rs9896052|rs1215930|rs300574|rs2465810|rs10061008|rs13139048|rs9318729|rs11178323|rs6563245|rs2584028|rs919594|rs17073080|rs3097907
Antinuclear Antibodies	SYNTHESIS_AND_INTERCONVERSION_OF_NUCLEOTIDE_DI_AND_TRIPHOSPHATES	REACTOME	306	20	10	1,73E-01	1,00E+00	rs2815307|rs2978663|rs2387094|rs4243969|rs913987|rs4135192|rs10040697|rs2776|rs7546131|rs7512897
Antinuclear Antibodies	POST_TRANSLATIONAL_MODIFICATION_SYNTHESIS_OF_GPI_ANCHORED_PROTEINS	REACTOME	586	20	9	1,74E-01	1,00E+00	rs281215|rs17164861|rs2275404|rs6088520|rs7210224|rs11809878|rs2273595|rs17418632|rs1135945
Antinuclear Antibodies	NA_CL_DEPENDENT_NEUROTRANSMITTER_TRANSPORTERS	REACTOME	718	34	20	1,76E-01	1,00E+00	rs2276433|rs12125560|rs4685165|rs10088489|rs492315|rs4640842|rs16842|rs488192|rs7727745|rs9820724|rs4923345|rs720625|rs973188|rs10493116|rs953115|rs11919568|rs17298481|rs9861586|rs999969|rs1497025
Antinuclear Antibodies	REGULATION_OF_WATER_BALANCE_BY_RENAL_AQUAPORINS	REACTOME	1393	24	20	1,76E-01	1,00E+00	rs902522|rs16947151|rs6807740|rs1030098|rs1404912|rs4243583|rs1253704|rs17360897|rs8181443|rs2287778|rs10493754|rs11715227|rs6501512|rs2302784|rs953672|rs12147761|rs13155334|rs6972935|rs4807284|rs7703532
Antinuclear Antibodies	RNA_POL_III_CHAIN_ELONGATION	REACTOME	364	22	15	1,76E-01	1,00E+00	rs4921702|rs2227301|rs4922152|rs4922157|rs6081157|rs1372349|rs13270944|rs17092536|rs11989385|rs13277342|rs6081150|rs3848006|rs2684456|rs2293603|rs871403
Antinuclear Antibodies	SIGNALING_BY_NOTCH	REACTOME	4553	30	20	1,76E-01	1,00E+00	rs17825554|rs7028704|rs11790630|rs9886419|rs9465734|rs7812998|rs4484585|rs767087|rs877711|rs6780684|rs877860|rs1561928|rs11604821|rs4852054|rs1838343|rs10898931|rs11851062|rs9291482|rs10495562|rs9636384
Antinuclear Antibodies	BETA_DEFENSINS	REACTOME	333	15	5	1,76E-01	1,00E+00	rs6057657|rs1324528|rs1348181|rs1342137|rs6046492
Antinuclear Antibodies	MAPK_PATHWAY	BIOCARTA	2690	32	20	1,77E-01	1,00E+00	rs10804900|rs3886478|rs11970312|rs9886419|rs10485101|rs7812998|rs1561928|rs2529737|rs2274316|rs13029532|rs836556|rs2005292|rs731571|rs1403101|rs885126|rs12199654|rs12413938|rs1144171|rs602837|rs9896052
Antinuclear Antibodies	INTERFERON_ALPHA_BETA_SIGNALING	REACTOME	1477	33	20	1,78E-01	1,00E+00	rs367503|rs2352946|rs468440|rs13119830|rs4900218|rs13029532|rs2038171|rs6850472|rs10029721|rs2239167|rs276947|rs1611493|rs13116389|rs310244|rs490531|rs793794|401754|rs468811|rs2246010|rs793881
Antinuclear Antibodies	APC_CDC20_MEDIATED_DEGRADATION_OF_NEK2A	REACTOME	396	46	15	1,79E-01	1,00E+00	rs766295|rs1027692|rs9562187|rs6836883|rs10514915|rs1106950|rs11734845|rs11679897|rs10018121|rs6856643|rs9876561|rs7683090|rs1509000|rs2089053|rs10032586
Antinuclear Antibodies	KINESINS	REACTOME	566	36	18	1,79E-01	1,00E+00	rs994626|rs4301693|rs1579282|rs1890981|rs6869991|rs7142555|rs961548|rs12256435|rs2896489|rs1833868|rs2887830|rs1464898|rs6580153|rs7603456|rs11867476|rs6583830|rs1967243|rs351291
Antinuclear Antibodies	CELL_ADHESION_MOLECULES_CAMS	KEGG	8813	38	20	1,80E-01	1,00E+00	rs933984|rs6764017|rs7124686|rs4419220|rs7241357|rs647241|rs3782735|rs2826418|rs8133991|rs12744678|rs567595|rs173840|rs2887480|rs10934067|rs229451|rs11240308|rs8123347|rs17836166|rs1215|rs1838343
Antinuclear Antibodies	PPARA_ACTIVATES_GENE_EXPRESSION	REACTOME	3675	29	20	1,82E-01	1,00E+00	rs3774909|rs11225161|rs10499305|rs11615846|rs7741028|rs4589495|rs10872715|rs6448226|rs706762|rs6467259|rs722420|rs3789823|rs11610288|rs11611796|rs2305913|rs10056261|rs6440618|rs12785137|rs4702326|rs9395247
Antinuclear Antibodies	MYD88_MAL_CASCADE_INITIATED_ON_PLASMA_MEMBRANE	REACTOME	2527	39	20	1,83E-01	1,00E+00	rs6020238|rs3886478|rs2829946|rs499205|rs3799663|rs6853|rs10485101|rs242389|rs2529737|rs466609|rs2005292|rs12199654|rs1144171|rs708030|rs17090708|rs6940350|rs1923075|rs3014866|rs4483225|rs4802906
Antinuclear Antibodies	GLYCOSAMINOGLYCAN_BIOSYNTHESIS_HEPARAN_SULFATE	KEGG	2273	51	20	1,84E-01	1,00E+00	rs2865155|rs11223958|rs4467961|rs11729868|rs9584410|rs1606724|rs1866430|rs17486477|rs7929855|rs2187467|rs7165082|rs10457277|rs12991269|rs913427|rs7334014|rs2098266|rs6675798|rs13333993|rs150344|rs11562932
Antinuclear Antibodies	INTRINSIC_PATHWAY	REACTOME	264	19	8	1,85E-01	1,00E+00	rs2074437|rs216872|rs9998003|rs216321|rs13133050|rs11063954|rs917857|rs584337
Antinuclear Antibodies	TRANSLATION	REACTOME	1803	49	20	1,89E-01	1,00E+00	rs6059703|rs1039153|rs499205|rs7902136|rs1504632|rs2175376|rs12602594|rs4743335|rs4751691|rs1953910|rs4743347|rs17121120|rs1548597|rs10065570|rs7826090|rs11002569|rs896946|rs4777170|rs2951967|rs6895379
Antinuclear Antibodies	INHIBITION_OF_THE_PROTEOLYTIC_ACTIVITY_OF_APC_C_REQUIRED_FOR_THE_ONSET_	REACTOME	365	46	15	1,89E-01	1,00E+00	rs766295|rs1027692|rs9562187|rs6836883|rs10514915|rs1106950|rs11734845|rs11679897|rs13135303|rs6856643|rs9876561|rs7683090|rs1509000|rs10032586|rs2089053
	TOTIC_SPINDLE_CHECKPOINT_COMPONENTS							
Antinuclear Antibodies	GLYCOLYSIS	REACTOME	558	34	16	1,90E-01	1,00E+00	rs10903939|rs11251480|rs7084464|rs4802906|rs2239167|rs7092772|rs10105118|rs2068455|rs2516617|rs7074424|rs11251694|rs11251588|rs12220754|rs7089499|rs10903959|rs1116003
Antinuclear Antibodies	TRAF6_MEDIATED_INDUCTION_OF_NFKB_AND_MAP_KINASES_UPON_TLR7_8_OR_9_ACT	REACTOME	2239	36	20	1,92E-01	1,00E+00	rs6020238|rs3886478|rs2829946|rs11970312|rs6853|rs10485101|rs242389|rs2529737|rs466609|rs2005292|rs12199654|rs1144171|rs708030|rs17090708|rs6940350|rs1923075|rs3014866|rs4802906|rs7822830|rs331455
Antinuclear Antibodies	REGULATION_OF_GENE_EXPRESSION_IN_BETA_CELLS	REACTOME	716	27	12	1,92E-01	1,00E+00	rs2943547|rs9549096|rs1390088|rs9576967|rs1343882|rs9886592|rs17008412|rs7329423|rs4264821|rs4812823|rs16939060|rs2922769
Antinuclear Antibodies	G_BETA_GAMMA_SIGNALLING_THROUGH_PLC_BETA	REACTOME	624	33	20	1,94E-01	1,00E+00	rs16947151|rs6807740|rs4243583|rs1253704|rs12075532|rs11715227|rs2206422|rs12147761|rs6972935|rs10485723|rs4807284|rs708925|rs17124529|rs4480744|rs11622145|rs11880582|rs2745755|rs6055995|rs8123677|rs931647
Antinuclear Antibodies	ADP_SIGNALLING_THROUGH_P2RY12	REACTOME	438	38	20	1,96E-01	1,00E+00	rs16947151|rs6807740|rs4243583|rs1253704|rs10228945|rs12075532|rs11715227|rs12147761|rs6972935|rs2714645|rs4807284|rs17124529|rs4480744|rs2523189|rs11622145|rs11880582|rs13232987|rs931647|rs8100484|rs3741920
Antinuclear Antibodies	PEROXISOME	KEGG	2047	32	20	1,96E-01	1,00E+00	rs10877173|rs7918164|rs6853|rs6077242|rs406303|rs8139802|rs395538|rs11605276|rs2073211|rs2305913|rs12435671|rs6140362|rs17341221|rs6108011|rs10483096|rs7005777|rs10199038|rs3734087|rs4148073|rs11130651
Antinuclear Antibodies	INHIBITION_OF_VOLTAGE_GATED_CA2_CHANNELS_VIA_GBETA_GAMMA_SUBUNITS	REACTOME	1082	31	20	1,99E-01	1,00E+00	rs2591164|rs16947151|rs948215|rs1843622|rs4243583|rs1253704|rs13415851|rs2275404|rs10493754|rs12147761|rs4807284|rs17124529|rs12482075|rs9898685|rs774505|rs1514236|rs11622145|rs11880582|rs2835908|rs8129867
Antinuclear Antibodies	TAK1_ACTIVATES_NFKB_BY_PHOSPHORYLATION_AND_ACTIVATION_OF_IKKS_COMPLE	REACTOME	777	25	20	2,00E-01	1,00E+00	rs2829946|rs10485101|rs466609|rs1144171|rs708030|rs6940350|rs1923075|rs3014866|rs331455|rs12216864|rs375369|rs13217057|rs10498974|rs2415290|rs6914621|rs457581|rs9345117|rs721025|rs311646|rs12191179
Antinuclear Antibodies	SMAD2_SMAD3_SMAD4_HETEROTRIMER_REGULATES_TRANSCRIPTION	REACTOME	715	30	20	2,00E-01	1,00E+00	rs9886419|rs7812998|rs722420|rs1561928|rs12867405|rs6440618|rs17787978|rs1426511|rs6803863|rs85440|rs727929|rs1333050|rs4939659|rs372334|rs12901499|rs1468934|rs12915039|rs4681521|rs9512215|rs12869996
Antinuclear Antibodies	PRE_NOTCH_EXPRESSION_AND_PROCESSING	REACTOME	1434	44	20	2,02E-01	1,00E+00	rs17825554|rs9465734|rs6780684|rs11604821|rs10898931|rs11851062|rs9291482|rs229798|rs12867405|rs4904087|rs9915261|rs566607|rs11621593|rs10140642|rs2372239|rs4788102|rs2293370|rs9295464|rs624797|rs17642827
Antinuclear Antibodies	EXTRACELLULAR_MATRIX_ORGANIZATION	REACTOME	3783	29	20	2,02E-01	1,00E+00	rs7092269|rs12668754|rs1490742|rs1247571|rs12751561|rs890813|rs4536647|rs4747097|rs4085170|rs1878510|rs9521666|rs1487798|rs967119|rs4490127|rs903352|rs756258|rs738791|rs10999501|rs12792912|rs16893529
Antinuclear Antibodies	BOTULINUM_NEUROTOXICITY	REACTOME	522	35	20	2,05E-01	1,00E+00	rs10800842|rs12409084|rs17702579|rs2695872|rs7220482|rs7137937|rs6026402|rs12369479|rs10861133|rs2206161|rs1983773|rs2278637|rs2113557|rs6503224|rs2362941|rs218454|rs362570|rs11087010|rs363012|rs10773810
Antinuclear Antibodies	MPR_PATHWAY	BIOCARTA	624	33	17	2,09E-01	1,00E+00	rs1030098|rs12438754|rs1404912|rs10228945|rs6501512|rs2302784|rs17048140|rs465316|rs2714645|rs2983460|rs4809258|rs883751|rs2523189|rs2471226|rs13232987|rs6062662|rs12148317
Antinuclear Antibodies	SPLICEOSOME	KEGG	1685	45	20	2,11E-01	1,00E+00	rs17825554|rs6081864|rs622175|rs12441204|rs3743121|rs1151514|rs12457331|rs200177|rs10140923|rs6062340|rs3857113|rs11621593|rs8033479|rs11845708|rs9381336|rs8006767|rs3826425|rs734570|rs11721883|rs4435501
Antinuclear Antibodies	GABA_B_RECEPTOR_ACTIVATION	REACTOME	1863	26	20	2,13E-01	1,00E+00	rs902522|rs2591164|rs16947151|rs948215|rs1843622|rs1404912|rs4243583|rs1253704|rs17360897|rs10228945|rs13415851|rs2287778|rs2275404|rs12075532|rs953672|rs12147761|rs13155334|rs2714645|rs4807284|rs7703532
Antinuclear Antibodies	P53_SIGNALING_PATHWAY	KEGG	1897	36	20	2,16E-01	1,00E+00	rs12765241|rs13159031|rs10515857|rs7830593|rs12957347|rs11604821|rs2387094|rs10820827|rs12955104|rs10742717|rs10931598|rs12454840|rs4664688|rs626481|rs2166504|rs9962338|rs6760597|rs7015841|rs4807284|rs10509534
Antinuclear Antibodies	DEGRADATION_OF_THE_EXTRACELLULAR_MATRIX	REACTOME	580	48	19	2,18E-01	1,00E+00	rs1247571|rs4085170|rs1487798|rs738791|rs12792912|rs10514392|rs1353739|rs2425019|rs17163745|rs2659122|rs3933239|rs656759|rs2397446|rs2664368|rs9457997|rs3823055|rs1555322|rs1420228|rs4786370
Antinuclear Antibodies	INTEGRATION_OF_ENERGY_METABOLISM	REACTOME	4501	24	20	2,18E-01	1,00E+00	rs1881628|rs7713485|rs902522|rs9372365|rs7600722|rs1578180|rs12540943|rs17696124|rs6429140|rs11102314|rs3820852|rs16947151|rs6807740|rs8081512|rs2017571|rs1030098|rs12119540|rs1404912|rs4243583|rs9883073
Antinuclear Antibodies	FRUCTOSE_AND_MANNOSE_METABOLISM	KEGG	984	40	20	2,19E-01	1,00E+00	rs9391926|rs10903939|rs11251480|rs7084464|rs12203614|rs4129219|rs7768483|rs7092772|rs2068455|rs2516617|rs7074424|rs11242726|rs11251694|rs9640883|rs11251588|rs12220754|rs973986|rs7089499|rs10903959|rs234915
Antinuclear Antibodies	HOST_INTERACTIONS_OF_HIV_FACTORS	REACTOME	2270	45	20	2,22E-01	1,00E+00	rs3026106|rs13272985|rs3782735|rs1478785|rs17646824|rs5766533|rs1151514|rs951453|rs836556|rs1316638|rs17559604|rs1403101|rs261072|rs11153318|rs17010833|rs1420320|rs2541094|rs13219815|rs443678|rs4385221
Antinuclear Antibodies	ACTIVATED_TLR4_SIGNALLING	REACTOME	2676	39	20	2,23E-01	1,00E+00	rs6020238|rs3886478|rs2829946|rs499205|rs11970312|rs6853|rs10485101|rs242389|rs2529737|rs466609|rs2005292|rs12199654|rs1144171|rs708030|rs17090708|rs6940350|rs1923075|rs3014866|rs4483225|rs4802906
Antinuclear Antibodies	RNA_POL_III_TRANSCRIPTION_INITIATION_FROM_TYPE_2_PROMOTER	REACTOME	417	25	17	2,23E-01	1,00E+00	rs4921702|rs2227301|rs4922152|rs4922157|rs6081157|rs1372349|rs13270944|rs17092536|rs11989385|rs6928843|rs520812|rs13277342|rs6081150|rs3848006|rs2684456|rs2293603|rs871403
Antinuclear Antibodies	MUSCLE_CONTRACTION	REACTOME	1023	42	20	2,24E-01	1,00E+00	rs12765241|rs955760|rs7895441|rs201603|rs947486|rs8060975|rs220076|rs17456435|rs10982444|rs2819369|rs7481661|rs1106950|rs2055226|rs943347|rs2278998|rs12707180|rs6467557|rs6861460|rs3818540|rs3738281
Antinuclear Antibodies	MICRORNA_MIRNA_BIOGENESIS	REACTOME	227	17	10	2,24E-01	1,00E+00	rs550353|rs2227301|rs6869442|rs576070|rs443678|rs7141416|rs633742|rs604965|rs2178077|rs2293603
Antinuclear Antibodies	AQUAPORIN_MEDIATED_TRANSPORT	REACTOME	1510	23	20	2,25E-01	1,00E+00	rs902522|rs16947151|rs6807740|rs1030098|rs1404912|rs4243583|rs1253704|rs17360897|rs8181443|rs2287778|rs12075532|rs11715227|rs6501512|rs7302925|rs2302784|rs953672|rs12147761|rs13155334|rs6972935|rs4807284
Antinuclear Antibodies	SIGNALING_BY_CONSTITUTIVELY_ACTIVE_EGFR	REACTOME	503	15	5	2,28E-01	1,00E+00	rs1397529|rs9896052|rs1550805|rs6449969|rs9838117
Antinuclear Antibodies	G_PROTEIN_BETA_GAMMA_SIGNALLING	REACTOME	798	32	20	2,30E-01	1,00E+00	rs16947151|rs6807740|rs4243583|rs1253704|rs10493754|rs11715227|rs2206422|rs12147761|rs6972935|rs10485723|rs4807284|rs708925|rs17124529|rs4480744|rs7804859|rs11622145|rs11880582|rs17153527|rs2745755|rs6055995
Antinuclear Antibodies	TRAFFICKING_OF_AMPA_RECEPTORS	REACTOME	1132	45	20	2,31E-01	1,00E+00	rs1422893|rs7665102|rs720863|rs4685165|rs8081512|rs169143|rs7730730|rs9343330|rs10030915|rs198178|rs4806674|rs1474976|rs13189947|rs11654578|rs873592|rs3755771|rs745686|rs3755768|rs4916598|rs2189290
Antinuclear Antibodies	INTESTINAL_IMMUNE_NETWORK_FOR_IGA_PRODUCTION	KEGG	1392	43	20	2,32E-01	1,00E+00	rs933984|rs4419220|rs2069837|rs2284191|rs3850890|rs2715267|rs2395300|rs3117230|rs2229135|rs7668305|rs9469357|rs7371043|rs11813145|rs7591705|rs3097646|rs1599796|rs10191360|rs17488134|rs441576|rs8179181
Antinuclear Antibodies	INTRINSIC_PATHWAY	BIOCARTA	603	34	14	2,34E-01	1,00E+00	rs4536647|rs9521666|rs903352|rs9998003|rs2047076|rs595325|rs13137121|rs7322495|rs10492497|rs13133050|rs9287095|rs616008|rs12755775|rs4619602
Antinuclear Antibodies	IRON_UPTAKE_AND_TRANSPORT	REACTOME	733	35	20	2,35E-01	1,00E+00	rs2725252|rs7853552|rs13272985|rs11230882|rs2166504|rs16861582|rs1390943|rs16870060|rs6895379|rs7646203|rs4148155|rs8177190|rs1477953|rs1800007|rs10032109|rs296917|rs1481017|rs4927870|rs1390938|rs2458284
Antinuclear Antibodies	SMOOTH_MUSCLE_CONTRACTION	REACTOME	658	43	19	2,35E-01	1,00E+00	rs12765241|rs955760|rs7895441|rs8060975|rs220076|rs17456435|rs2819369|rs2055226|rs943347|rs12707180|rs6467557|rs6861460|rs3818540|rs4934433|rs3800702|rs11673699|rs2272555|rs2820312|rs2119685
Antinuclear Antibodies	SIGNALING_BY_RHO_GTPASES	REACTOME	4877	29	20	2,35E-01	1,00E+00	rs625572|rs10499305|rs1373928|rs7741028|rs3742324|rs10872715|rs4902208|rs2017328|rs11947791|rs13383577|rs11714363|rs6531770|rs1145157|rs4289301|rs6019300|rs11175219|rs673377|rs656709|rs1822861|rs12480096
Antinuclear Antibodies	METABOLISM_OF_CARBOHYDRATES	REACTOME	9072	24	20	2,37E-01	1,00E+00	rs3026106|rs2865155|rs16895527|rs7552867|rs1980779|rs9309906|rs11223958|rs10903939|rs17375044|rs4467961|rs11251480|rs7084464|rs1478785|rs4535751|rs11729868|rs10861272|rs9584410|rs1606724|rs879162|rs1866430
Antinuclear Antibodies	INWARDLY_RECTIFYING_K_CHANNELS	REACTOME	1317	40	20	2,40E-01	1,00E+00	rs2591164|rs2855790|rs16947151|rs1843622|rs4243583|rs1253704|rs1882459|rs2275404|rs12075532|rs12147761|rs4807284|rs17124529|rs12482075|rs9898685|rs774505|rs1514236|rs11622145|rs11880582|rs2835908|rs8129867
Antinuclear Antibodies	INFLAMMASOMES	REACTOME	468	22	10	2,40E-01	1,00E+00	rs2829946|rs466609|rs11072638|rs375369|rs1792634|rs17178663|rs457581|rs4135192|rs2776|rs12958785
Antinuclear Antibodies	ERK5_PATHWAY	BIOCARTA	595	22	11	2,42E-01	1,00E+00	rs3886478|rs2274316|rs9896052|rs1550805|rs6449969|rs7804859|rs9838117|rs17153527|rs3746259|rs4730197|rs17153654
Antinuclear Antibodies	INSULIN_SIGNALING_PATHWAY	KEGG	3744	43	20	2,44E-01	1,00E+00	rs3774909|rs1881628|rs955760|rs4436949|rs12540943|rs7999458|rs6448226|rs10841528|rs9549096|rs7895441|rs2017571|rs4603757|rs1030098|rs4129219|rs2038171|rs7258382|rs11045301|rs885126|rs9576967|rs6501512
Antinuclear Antibodies	FORMATION_OF_THE_TERNARY_COMPLEX_AND_SUBSEQUENTLY_THE_43S_COMPLEX	REACTOME	677	41	20	2,45E-01	1,00E+00	rs6059703|rs7902136|rs2175376|rs4751691|rs1953910|rs17121120|rs1548597|rs7826090|rs11002569|rs11733964|rs11002552|rs1953912|rs26528|rs1956099|rs10839957|rs10895987|rs12676238|rs2140386|rs1720261|rs2352907
Antinuclear Antibodies	RAP1_SIGNALLING	REACTOME	835	37	20	2,49E-01	1,00E+00	rs11102314|rs3820852|rs7069974|rs10740411|rs3769309|rs9901827|rs10762562|rs12124283|rs7907343|rs1194491|rs883751|rs3767132|rs1546924|rs3818942|rs2128699|rs7306680|rs211085|rs12135009|rs4734512|rs4734497
Antinuclear Antibodies	NEUROACTIVE_LIGAND_RECEPTOR_INTERACTION	KEGG	13469	31	20	2,50E-01	1,00E+00	rs12139261|rs4906888|rs1806201|rs17047183|rs571312|rs1247571|rs2267737|rs28671569|rs6861578|rs297672|rs9819978|rs1863455|rs7676645|rs1578180|rs11706283|rs1422893|rs13185639|rs1578303|rs6994883|rs17613247
Antinuclear Antibodies	PENTOSE_PHOSPHATE_PATHWAY	KEGG	616	37	16	2,53E-01	1,00E+00	rs10903939|rs11251480|rs7084464|rs4129219|rs2068455|rs7074424|rs11251694|rs11251588|rs6855439|rs2306181|rs4234960|rs12220754|rs6531592|rs7089499|rs10903959|rs17301901
Antinuclear Antibodies	NCAM1_INTERACTIONS	REACTOME	1896	34	20	2,54E-01	1,00E+00	rs12668754|rs9327283|rs10920115|rs4536647|rs1895464|rs9521666|rs4490127|rs903352|rs10787635|rs2279447|rs9898731|rs346852|rs677152|rs9288163|rs6415964|rs595325|rs4663712|rs3794621|rs161130|rs1455777
Antinuclear Antibodies	NOTCH1_INTRACELLULAR_DOMAIN_REGULATES_TRANSCRIPTION	REACTOME	2852	28	20	2,57E-01	1,00E+00	rs17825554|rs7028704|rs11790630|rs9886419|rs7812998|rs4484585|rs1561928|rs4852054|rs9291482|rs9636384|rs3791428|rs6959501|rs1426511|rs566607|rs8182084|rs11621593|rs801521|rs352066|rs17296588|rs2229840
Antinuclear Antibodies	P38MAPK_PATHWAY	BIOCARTA	1904	36	20	2,59E-01	1,00E+00	rs9886419|rs10485101|rs230624|rs7812998|rs1561928|rs2529737|rs2274316|rs13029532|rs836556|rs731571|rs885126|rs6683451|rs12199654|rs1144171|rs9896052|rs11583351|rs1426511|rs604045|rs612355|rs1923075
Antinuclear Antibodies	PYRUVATE_METABOLISM_AND_CITRIC_ACID_TCA_CYCLE	REACTOME	905	39	20	2,60E-01	1,00E+00	rs17070285|rs4331688|rs9534734|rs2028548|rs1830426|rs7558379|rs2978169|rs4239020|rs3134010|rs6547525|rs2902374|rs7602538|rs6471412|rs836624|rs2012859|rs9534844|rs11893686|rs7600094|rs7713458|rs2970358
Antinuclear Antibodies	REGULATION_OF_ACTIN_CYTOSKELETON	KEGG	7089	26	20	2,62E-01	1,00E+00	rs17718875|rs10499305|rs2974254|rs12869090|rs7741028|rs12909256|rs1924210|rs1578180|rs10872715|rs11869351|rs12357398|rs6429140|rs9307949|rs9515380|rs4448606|rs9736587|rs1326086|rs9805351|rs3129678|rs656709
Antinuclear Antibodies	ACTIVATED_NOTCH1_TRANSMITS_SIGNAL_TO_THE_NUCLEUS	REACTOME	719	36	18	2,63E-01	1,00E+00	rs767087|rs877711|rs877860|rs1838343|rs10495562|rs4903104|rs209948|rs1474817|rs7763013|rs11177624|rs17660210|rs658573|rs10494266|rs17581619|rs4905994|rs1022790|rs1427281|rs1040923
Antinuclear Antibodies	CHREBP2_PATHWAY	BIOCARTA	1059	36	20	2,63E-01	1,00E+00	rs1881628|rs12540943|rs2017571|rs1030098|rs6432018|rs1480159|rs6501512|rs2302784|rs13434196|rs2976282|rs4802906|rs10105118|rs883751|rs7795096|rs1001684|rs4595037|rs10929592|rs4502283|rs11160664|rs4734512
Antinuclear Antibodies	CD28_DEPENDENT_PI3K_AKT_SIGNALING	REACTOME	572	20	9	2,64E-01	1,00E+00	rs3850890|rs2715267|rs11153318|rs12413938|rs1550805|rs10494270|rs6449969|rs1599796|rs9838117
Antinuclear Antibodies	DEADENYLATION_OF_MRNA	REACTOME	331	24	11	2,64E-01	1,00E+00	rs1039153|rs11228985|rs1000174|rs10732882|rs2951967|rs10498060|rs11178058|rs10853869|rs2293603|rs4782165|rs1533079
Antinuclear Antibodies	NOD1_2_SIGNALING_PATHWAY	REACTOME	881	53	20	2,64E-01	1,00E+00	rs6020238|rs10485101|rs12199654|rs1144171|rs708030|rs6940350|rs1923075|rs331455|rs12216864|rs13217057|rs10498974|rs739559|rs6914621|rs9345117|rs721025|rs311646|rs6012846|rs12191179|rs7841459|rs7745771
Antinuclear Antibodies	TRANSCRIPTIONAL_REGULATION_OF_WHITE_ADIPOCYTE_DIFFERENTIATION	REACTOME	2719	30	20	2,65E-01	1,00E+00	rs3774909|rs11615846|rs6473282|rs6448226|rs706762|rs1327620|rs3743466|rs1110060|rs1036519|rs10520809|rs3789823|rs11611796|rs11610288|rs1411355|rs12916220|rs10056261|rs4702326|rs964330|rs1834210|rs7865653
Antinuclear Antibodies	CA_DEPENDENT_EVENTS	REACTOME	1487	42	20	2,65E-01	1,00E+00	rs902522|rs230624|rs306090|rs8081512|rs1030098|rs1404912|rs17360897|rs2287778|rs6683451|rs6501512|rs2302784|rs953672|rs11583351|rs13155334|rs612355|rs7703532|rs883751|rs17649232|rs16826789|rs2055226
Antinuclear Antibodies	SIGNALING_BY_HIPPO	REACTOME	627	31	20	2,66E-01	1,00E+00	rs11225161|rs722420|rs244896|rs6440618|rs904534|rs17787978|rs478243|rs6803863|rs6694744|rs1763321|rs6875678|rs2842258|rs7330095|rs13169010|rs4976608|rs4681521|rs7110355|rs7994886|rs785422|rs10102594
Antinuclear Antibodies	AMINO_ACID_SYNTHESIS_AND_INTERCONVERSION_TRANSAMINATION	REACTOME	530	30	12	2,67E-01	1,00E+00	rs11685570|rs501819|rs1757947|rs7302925|rs7027964|rs10122711|rs2296063|rs9927205|rs3793932|rs2183838|rs13338289|rs11137607
Antinuclear Antibodies	CDC42RAC_PATHWAY	BIOCARTA	435	12	6	2,67E-01	1,00E+00	rs836556|rs17048140|rs465316|rs1550805|rs6449969|rs9838117
Antinuclear Antibodies	DEADENYLATION_DEPENDENT_MRNA_DECAY	REACTOME	622	30	13	2,67E-01	1,00E+00	rs1039153|rs11228985|rs1000174|rs2096914|rs10732882|rs2951967|rs10498060|rs11178058|rs10853869|rs2733537|rs2293603|rs4782165|rs1533079
Antinuclear Antibodies	REGULATORY_RNA_PATHWAYS	REACTOME	345	18	11	2,68E-01	1,00E+00	rs550353|rs2227301|rs6869442|rs576070|rs443678|rs7141416|rs633742|rs604965|rs2178077|rs2293603|rs9907255
Antinuclear Antibodies	REGULATION_OF_IFNA_SIGNALING	REACTOME	339	12	5	2,68E-01	1,00E+00	rs13029532|rs2038171|rs2239167|rs310244|rs2834115
Antinuclear Antibodies	INSULIN_RECEPTOR_RECYCLING	REACTOME	492	22	13	2,68E-01	1,00E+00	rs7853552|rs13272985|rs7258382|rs17254521|rs1390943|rs16870060|rs6895379|rs7646203|rs1477953|rs296917|rs1390938|rs2458284|rs2453997
Antinuclear Antibodies	TRANSPORT_OF_MATURE_TRANSCRIPT_TO_CYTOPLASM	REACTOME	693	36	18	2,69E-01	1,00E+00	rs3026106|rs1478785|rs5766533|rs10495562|rs13219815|rs633742|rs4798|rs13038619|rs9615025|rs8088313|rs8013586|rs10852553|rs706130|rs150926|rs6599528|rs715749|rs2944346|rs1471454

Antinuclear Antibodies	SIGNALING_BY_ILS	REACTOME	2996	60	20	2,69E-01	1,00E+00	rs12619383|rs6020238|rs6853|rs7905645|rs10485101|rs242389|rs1227631|rs2529737|rs2069837|rs13253242|rs13029532|rs4851547|rs11153318|rs12413938|rs1144171|rs1019033|rs9896052|rs708030|rs2871444|rs17090708
Antinuclear Antibodies	PKB_MEDIATED_EVENTS	REACTOME	583	23	16	2,69E-01	1,00E+00	rs1881628|rs17762352|rs12540943|rs7795096|rs1001684|rs4595037|rs8059570|rs13431868|rs10169231|rs7328100|rs4726084|rs11971243|rs9930956|rs7778077|rs2293603|rs2589149
Antinuclear Antibodies	COLLAGEN_FORMATION	REACTOME	3203	27	20	2,71E-01	1,00E+00	rs7092269|rs12668754|rs1490742|rs12751561|rs890813|rs4536647|rs4747097|rs1878510|rs9521666|rs967119|rs4490127|rs903352|rs756258|rs10999501|rs16893529|rs17388474|rs677152|rs9288163|rs1687410|rs1297941
Antinuclear Antibodies	NTHI_PATHWAY	BIOCARTA	876	38	20	2,71E-01	1,00E+00	rs6853|rs10485101|rs11746792|rs7653262|rs12199654|rs4868204|rs1144171|rs1923075|rs478061|rs13217057|rs10498974|rs2415290|rs739559|rs6914621|rs9345117|rs721025|rs12191179|rs372334|rs12901499|rs12915039
Antinuclear Antibodies	NOTCH_SIGNALING_PATHWAY	KEGG	1387	47	20	2,75E-01	1,00E+00	rs17825554|rs2938004|rs767087|rs10495562|rs9291482|rs4903104|rs1250090|rs209948|rs566607|rs1474817|rs11621593|rs352066|rs2229840|rs7763013|rs11929034|rs701038|rs11891208|rs906304|rs718949|rs10083154
Antinuclear Antibodies	G_BETA_GAMMA_SIGNALLING_THROUGH_PI3KGAMMA	REACTOME	487	35	20	2,76E-01	1,00E+00	rs16947151|rs6807740|rs4243583|rs1253704|rs12075532|rs11715227|rs12147761|rs6972935|rs4807284|rs17124529|rs4480744|rs7804859|rs11622145|rs11880582|rs17153527|rs4730197|rs931647|rs8100484|rs3741920|rs12944446
Antinuclear Antibodies	SPHINGOLIPID_METABOLISM	REACTOME	1944	37	20	2,76E-01	1,00E+00	rs17752182|rs6448180|rs678194|rs2167239|rs3011777|rs12081589|rs1493130|rs346811|rs17333779|rs723506|rs10121681|rs11707667|rs11714594|rs1466447|rs2041120|rs17785945|rs873592|rs2194051|rs10493211|rs4592839
Antinuclear Antibodies	SULFUR_AMINO_ACID_METABOLISM	REACTOME	930	37	20	2,76E-01	1,00E+00	rs2028900|rs731727|rs17636594|rs10068900|rs17062147|rs158850|rs2202044|rs677012|rs4774770|rs978114|rs297961|rs7758711|rs580789|rs1484197|rs10153006|rs809942|rs1205357|rs2039585|rs3793932|rs12148892
Antinuclear Antibodies	HS_GAG_BIOSYNTHESIS	REACTOME	3154	40	20	2,77E-01	1,00E+00	rs2865155|rs1980779|rs4467961|rs11729868|rs9584410|rs1606724|rs879162|rs1866430|rs12853685|rs17486477|rs205148|rs8055371|rs7165082|rs10457277|rs12991269|rs1445253|rs2290501|rs1924384|rs913427|rs7334014
Antinuclear Antibodies	SIGNALING_BY_PDGF	REACTOME	4427	36	20	2,77E-01	1,00E+00	rs12668754|rs1247571|rs6452735|rs902522|rs2974254|rs10490696|rs12958400|rs9307949|rs4536647|rs306090|rs9549096|rs9521666|rs8081512|rs4490127|rs903352|rs1030098|rs1404912|rs13029532|rs17360897|rs2287778
Antinuclear Antibodies	PHENYLALANINE_METABOLISM	KEGG	442	34	11	2,79E-01	1,00E+00	rs4788547|rs2000466|rs2070460|rs5760102|rs10745952|rs12085083|rs1834058|rs12802641|rs3793932|rs13338289|rs4822461
Antinuclear Antibodies	ETS_PATHWAY	BIOCARTA	682	30	17	2,84E-01	1,00E+00	rs197438|rs10790928|rs6689823|rs2005292|rs7108562|rs352066|rs9787850|rs2229840|rs701038|rs906304|rs7547373|rs12294634|rs4936059|rs333968|rs773878|rs6681035|rs12318072
Antinuclear Antibodies	PRE_NOTCH_PROCESSING_IN_GOLGI	REACTOME	691	23	11	2,85E-01	1,00E+00	rs10898931|rs11851062|rs229798|rs4904087|rs9915261|rs10140642|rs2372239|rs4788102|rs17642827|rs1114312|rs12894167
Antinuclear Antibodies	CHOLESTEROL_BIOSYNTHESIS	REACTOME	259	18	8	2,86E-01	1,00E+00	rs6671536|rs1792331|rs3812656|rs4420519|rs4841600|rs1792225|rs4819211|rs2684874
Antinuclear Antibodies	ION_CHANNEL_TRANSPORT	REACTOME	2741	29	20	2,86E-01	1,00E+00	rs4906888|rs28671569|rs1863455|rs7358822|rs4921297|rs422598|rs29658|rs486535|rs208147|rs1200138|rs488447|rs13316275|rs35673516|rs1863464|rs9915261|rs10919062|rs7179956|rs416330|rs9511405|rs981347
Antinuclear Antibodies	SEMA4D_INDUCED_CELL_MIGRATION_AND_GROWTH_CONE_COLLAPSE	REACTOME	433	33	12	2,86E-01	1,00E+00	rs6559349|rs2269529|rs836556|rs9410475|rs8060975|rs220076|rs1571534|rs2040532|rs11670669|rs2855726|rs2272555|rs6531240
Antinuclear Antibodies	STATHMIN_PATHWAY	BIOCARTA	456	24	12	2,87E-01	1,00E+00	rs7665102|rs306090|rs1030098|rs6501512|rs2302784|rs883751|rs873592|rs4240286|rs1554668|rs797999|rs1917534|rs16859030
Antinuclear Antibodies	GRAFT_VERSUS_HOST_DISEASE	KEGG	1446	41	20	2,89E-01	1,00E+00	rs10772294|rs12765241|rs2069837|rs2284191|rs3850890|rs2715267|rs2395300|rs3117230|rs1611493|rs9469357|rs3097646|rs1599796|rs3014996|rs4934433|rs2246010|rs4747064|rs441576|rs35265698|rs2523554|rs2154254
Antinuclear Antibodies	PTDINS_PATHWAY	BIOCARTA	1058	52	20	2,90E-01	1,00E+00	rs10903939|rs11251480|rs7084464|rs767087|rs3824561|rs836556|rs2510257|rs209948|rs4441060|rs1474817|rs2068455|rs7074424|rs2595199|rs11251694|rs11251588|rs4952809|rs12220754|rs7089499|rs4952779|rs10903959
Antinuclear Antibodies	PYK2_PATHWAY	BIOCARTA	752	33	13	2,92E-01	1,00E+00	rs8081512|rs2529737|rs169143|rs836556|rs12199654|rs9896052|rs198178|rs2055226|rs2163177|rs3785396|rs6681035|rs12259267|rs9925433
Antinuclear Antibodies	PPARA_PATHWAY	BIOCARTA	2445	32	20	2,93E-01	1,00E+00	rs3774909|rs9886419|rs7812998|rs6448226|rs11746792|rs1561928|rs8081512|rs1030098|rs6553052|rs12498345|rs169143|rs1471921|rs2305913|rs4868204|rs6501512|rs2822946|rs2302784|rs1426511|rs198178|rs10463193
Antinuclear Antibodies	APC_C_CDC20_MEDIATED_DEGRADATION_OF_MITOTIC_PROTEINS	REACTOME	1094	42	20	2,93E-01	1,00E+00	rs766295|rs1027692|rs17057833|rs9562187|rs6836883|rs10514915|rs11845708|rs7187327|rs12447239|rs698856|rs1106950|rs425594|rs11922435|401754|rs2431097|rs13207114|rs11734845|rs3787573|rs11679897|rs10018121
Antinuclear Antibodies	NUCLEOTIDE_BINDING_DOMAIN_LEUCINE_RICH_REPEAT_CONTAINING_RECEPTOR_NLRYS	REACTOME	1337	31	20	2,94E-01	1,00E+00	rs6020238|rs2829946|rs10485101|rs466609|rs12199654|rs1144171|rs11072638|rs708030|rs6940350|rs1923075|rs331455|rs12216864|rs375369|rs1792634|rs13217057|rs10498974|rs17178663|rs739559|rs6914621|rs457581
Antinuclear Antibodies	FATTY_ACID_TRIACYLGLYCEROL_AND_KETONE_BODY_METABOLISM	REACTOME	5526	30	20	2,97E-01	1,00E+00	rs3774909|rs1881628|rs11225161|rs10499305|rs11615846|rs7741028|rs4589495|rs10872715|rs12540943|rs6448226|rs706762|rs6467259|rs722420|rs2017571|rs17009821|rs3789823|rs11610288|rs11611796|rs9458157|rs2305913
Antinuclear Antibodies	TOLL_PATHWAY	BIOCARTA	1147	31	20	2,99E-01	1,00E+00	rs499205|rs6853|rs10485101|rs2529737|rs2005292|rs12199654|rs1144171|rs6940350|rs1923075|rs4483225|rs331455|rs13217057|rs10498974|rs2415290|rs1053079|rs739559|rs6914621|rs9345117|rs10513330|rs721025
Antinuclear Antibodies	MELANOGENESIS	KEGG	4077	32	20	2,99E-01	1,00E+00	rs7218567|rs6059709|rs10777125|rs902522|rs7665102|rs1219615|rs1407101|rs288496|rs8081512|rs7212246|rs1404912|rs9883073|rs169143|rs17360897|rs10228945|rs2327162|rs2287778|rs7623679|rs1477031|rs1108386
Antinuclear Antibodies	NETRIN1_SIGNALING	REACTOME	3710	32	20	3,03E-01	1,00E+00	rs4791330|rs12286714|rs1917072|rs10520737|rs10502934|rs12357398|rs4868489|rs4448606|rs10866630|rs4777919|rs7435615|rs1950045|rs10830183|rs836556|rs6555831|rs11153318|rs4791812|rs7164857|rs12584914|rs4357599
Antinuclear Antibodies	CREB_PATHWAY	BIOCARTA	969	40	20	3,05E-01	1,00E+00	rs7665102|rs8081512|rs1030098|rs1404912|rs169143|rs836556|rs12199654|rs6501512|rs2302784|rs9896052|rs198178|rs1550805|rs883751|rs6449969|rs873592|rs2471226|rs9838117|rs3785396|rs4240286|rs1554668
Antinuclear Antibodies	AMINE_COMPOUND_SLC_TRANSPORTERS	REACTOME	1298	36	20	3,06E-01	1,00E+00	rs2276433|rs12125560|rs4685165|rs6673947|rs10088489|rs492315|rs4640842|rs16842|rs12349416|rs488192|rs7727745|rs9820724|rs4923345|rs17097955|rs720625|rs4949673|rs973188|rs2844456|rs1892465|rs10493116
Antinuclear Antibodies	NEURONAL_SYSTEM	REACTOME	13378	32	20	3,09E-01	1,00E+00	rs4906888|rs1806201|rs4233891|rs3757105|rs10800842|rs28671569|rs2469515|rs4869685|rs902522|rs9810230|rs2591164|rs2847381|rs11685570|rs631037|rs7600722|rs3799663|rs1863455|rs1422893|rs9817294|rs9865301
Antinuclear Antibodies	TOLL_RECEPTOR_CASCADES	REACTOME	3778	38	20	3,10E-01	1,00E+00	rs6020238|rs3886478|rs16896849|rs2829946|rs499205|rs3799663|rs6853|rs10485101|rs242389|rs2529737|rs11876920|rs466609|rs2005292|rs12199654|rs10445097|rs1144171|rs203170|rs4441060|rs708030|rs17090708
Antinuclear Antibodies	PROSTACYCLIN_SIGNALLING_THROUGH_PROSTACYCLIN_RECEPTOR	REACTOME	353	35	19	3,11E-01	1,00E+00	rs16947151|rs6807740|rs4243583|rs1253704|rs10493754|rs11715227|rs12147761|rs6972935|rs4807284|rs17124529|rs4480744|rs11622145|rs11880582|rs931647|rs8100484|rs3741920|rs10414848|rs1246159|rs7612445
Antinuclear Antibodies	REGULATION_OF_INSULIN_SECRETION	REACTOME	3741	24	20	3,14E-01	1,00E+00	rs7713485|rs9372365|rs7600722|rs1072319|rs17696124|rs6429140|rs11102314|rs3820852|rs16947151|rs6807740|rs8081512|rs1030098|rs12119540|rs4243583|rs9883073|rs1253704|rs3769309|rs17360897|rs10228945|rs10879852
Antinuclear Antibodies	SHC_RELATED_EVENTS	REACTOME	302	16	6	3,14E-01	1,00E+00	rs7258382|rs9896052|rs17254521|rs944482|rs2392794|rs1125467
Antinuclear Antibodies	FORMATION_OF_FIBRIN_CLOT_CLOTTING_CASCADE	REACTOME	703	35	20	3,18E-01	1,00E+00	rs2074437|rs216872|rs6910246|rs10804016|rs9998003|rs13137121|rs5978|rs7580146|rs6048526|rs216321|rs749005|rs9288147|rs11757860|rs220988|rs12021681|rs9328355|rs13133050|rs11063954|rs917857|rs9287095
Antinuclear Antibodies	MEMBRANE_TRAFFICKING	REACTOME	2276	31	20	3,19E-01	1,00E+00	rs11576760|rs11230882|rs12430144|rs877711|rs3731828|rs4537255|rs1406961|rs2132302|rs720863|rs951453|rs1886176|rs17781348|rs3798188|rs9343330|rs4794873|rs2022345|rs16933086|rs16869484|rs1065206|rs1336625
Antinuclear Antibodies	ARAP_PATHWAY	BIOCARTA	292	19	9	3,20E-01	1,00E+00	rs1406961|rs13238780|rs1065206|rs11887079|rs940243|rs2175337|rs2709580|rs4798|rs9611895
Antinuclear Antibodies	PYRUVATE_METABOLISM	REACTOME	209	12	7	3,21E-01	1,00E+00	rs1830426|rs2978169|rs4239020|rs3134010|rs6471412|rs836624|rs2012859
Antinuclear Antibodies	HEPARAN_SULFATE_HEPARIN_HS_GAG_METABOLISM	REACTOME	3879	35	20	3,22E-01	1,00E+00	rs2865155|rs1980779|rs11223958|rs4467961|rs11729868|rs9584410|rs1606724|rs879162|rs1866430|rs12853685|rs17486477|rs205148|rs7929855|rs2187467|rs8055371|rs7165082|rs10457277|rs12991269|rs2591454|rs1445253
Antinuclear Antibodies	HYPERTROPHIC_CARDIOMYOPATHY_HCM	KEGG	4149	33	20	3,22E-01	1,00E+00	rs1881628|rs2618681|rs9810230|rs17053465|rs38553|rs12737847|rs12540943|rs10920115|rs11715062|rs10925415|rs13430329|rs12032218|rs10486963|rs10190488|rs12152349|rs2069837|rs10045068|rs2251788|rs947486|rs658071
Antinuclear Antibodies	SIGNALING_BY_FGFR1_MUTANTS	REACTOME	1243	37	20	3,24E-01	1,00E+00	rs1924210|rs10878951|rs9736587|rs3129678|rs6465836|rs13029532|rs950549|rs6884797|rs9896052|rs3129599|rs249729|rs1550805|rs8000805|rs10808119|rs2031282|rs6449969|rs9506777|rs11177527|rs12672026|rs1078363
Antinuclear Antibodies	INTEGRIN_ALPHAIIB_BETA3_SIGNALING	REACTOME	779	41	20	3,27E-01	1,00E+00	rs11102314|rs3820852|rs216872|rs2038171|rs3769309|rs1404772|rs9896052|rs1537741|rs10821495|rs13137121|rs1546924|rs216321|rs7306680|rs2991216|rs10932619|rs11063954|rs917857|rs11015149|rs2290889|rs1250034
Antinuclear Antibodies	DOWNSTREAM_TCR_SIGNALING	REACTOME	1573	29	20	3,28E-01	1,00E+00	rs6020238|rs11767716|rs3782735|rs10485101|rs13080704|rs1144171|rs2064478|rs6940350|rs1923075|rs1550805|rs331455|rs12216864|rs4357599|rs7904311|rs9469357|rs10509534|rs13217057|rs10498974|rs6449969|rs2415290
Antinuclear Antibodies	GLYCOSYLPHOSPHATIDYLINOSITOL_GPI_ANCHOR_BIOSYNTHESIS	KEGG	318	12	7	3,29E-01	1,00E+00	rs17164861|rs2275404|rs6088520|rs7210224|rs11809878|rs2273595|rs1135945
Antinuclear Antibodies	SYNTHESIS_OF_PC	REACTOME	669	36	18	3,29E-01	1,00E+00	rs10508915|rs16947151|rs6673947|rs3906767|rs12349416|rs10183830|rs1917806|rs17097955|rs7567684|rs4949673|rs2844456|rs1892465|rs10209151|rs11165213|rs1109859|rs2278952|rs11892871|rs630505
Antinuclear Antibodies	TRANSCRIPTIONAL_ACTIVITY_OF_SMAD2_SMAD3_SMAD4_HETEROTRIMER	REACTOME	967	28	20	3,31E-01	1,00E+00	rs9886419|rs7812998|rs722420|rs1561928|rs12867405|rs6440618|rs17787978|rs1426511|rs6803863|rs85440|rs2229840|rs727929|rs1333050|rs701038|rs906304|rs4939659|rs372334|rs12901499|rs1468934|rs12915039
Antinuclear Antibodies	MITOTIC_PROMETAPHASE	REACTOME	1814	43	20	3,33E-01	1,00E+00	rs766295|rs994626|rs1478785|rs1558461|rs1027692|rs4350299|rs9303909|rs12953815|rs17010833|rs4802906|rs10105118|rs3027247|rs12456560|rs4671408|rs1833868|rs3800933|rs11582225|rs1464898|rs4758670|rs8531
Antinuclear Antibodies	PKA_MEDIATED_PHOSPHORYLATION_OF_CREB	REACTOME	767	40	20	3,34E-01	1,00E+00	rs902522|rs1030098|rs1404912|rs17360897|rs2287778|rs6501512|rs2302784|rs953672|rs13155334|rs7703532|rs883751|rs2471226|rs438900|rs6878196|rs6770805|rs4702473|rs4701780|rs10421422|rs13168690|rs7206142
Antinuclear Antibodies	COMPLEMENT_AND_COAGULATION_CASCADES	KEGG	1742	35	20	3,36E-01	1,00E+00	rs1247571|rs216872|rs6910246|rs12220324|rs12571782|rs10804016|rs9998003|rs971075|rs1143664|rs9661996|rs2047076|rs4592582|rs1389623|rs2182913|rs13137121|rs5978|rs11003466|rs10754199|rs3750512|rs1450665
Antinuclear Antibodies	DESTABILIZATION_OF_MRNA_BY_TRISTETRAPROLIN_TTP	REACTOME	179	1	1	3,45E-01	1,00E+00	rs16973403
Antinuclear Antibodies	PROTEIN_EXPORT	KEGG	750	24	12	3,45E-01	1,00E+00	rs499205|rs4743335|rs4743347|rs3114815|rs10272579|rs1358434|rs12891079|rs2050042|rs6962665|rs7805129|rs478309|rs9881405
Antinuclear Antibodies	TNFR2_PATHWAY	BIOCARTA	414	27	12	3,45E-01	1,00E+00	rs1538660|rs11746792|rs4868204|rs3750512|rs2415290|rs1061624|rs4978374|rs17220720|rs10979569|rs12289836|rs10872893|rs3138045
Antinuclear Antibodies	COMPLEMENT_CASCADE	REACTOME	752	43	20	3,46E-01	1,00E+00	rs12220324|rs12571782|rs971075|rs1143664|rs9661996|rs1389623|rs2182913|rs1903946|rs10754199|rs3750512|rs2292727|rs7962629|rs706470|rs34562262|rs13156109|rs10740487|rs10473234|rs624298|rs10762771|rs7083840
Antinuclear Antibodies	HIV_LIFE_CYCLE	REACTOME	1529	32	20	3,46E-01	1,00E+00	rs3026106|rs3782735|rs1478785|rs2132302|rs5766533|rs1151514|rs2239923|rs17010833|rs3786238|rs13219815|rs10732882|rs443678|rs7371043|rs174532|rs4671408|rs1053079|rs633742|rs6928843|rs9615025|rs604965
Antinuclear Antibodies	VIBRIO_CHOLERAE_INFECTION	KEGG	1518	37	20	3,46E-01	1,00E+00	rs7853552|rs13272985|rs2283169|rs7934606|rs8081512|rs11245935|rs4743335|rs169143|rs13238780|rs10445097|rs4743347|rs198178|rs1390943|rs940243|rs883751|rs16870060|rs6895379|rs7945327|rs6980032|rs7805129
Antinuclear Antibodies	NOS1_PATHWAY	BIOCARTA	1133	46	20	3,47E-01	1,00E+00	rs1806201|rs8081512|rs1030098|rs169143|rs2866250|rs4332745|rs6501512|rs2302784|rs2872715|rs198178|rs883751|rs4699014|rs8060068|rs2055226|rs1074042|rs12934132|rs10744029|rs3785396|rs12811583|rs3782221
Antinuclear Antibodies	GROWTH_HORMONE_RECEPTOR_SIGNALING	REACTOME	773	36	20	3,47E-01	1,00E+00	rs7999458|rs10495562|rs13029532|rs2038171|rs12203803|rs7187776|rs17205146|rs12524841|rs4675077|rs2239167|rs2001303|rs2251692|rs11069810|rs2274471|rs13392647|rs2894595|rs10941583|rs7727306|rs9358533|rs9356821
Antinuclear Antibodies	APC_C_CDC20_MEDIATED_DEGRADATION_OF_CYCLIN_B	REACTOME	224	22	8	3,51E-01	1,00E+00	rs9562187|rs6836883|rs10514915|rs1106950|rs11734845|rs11679897|rs9876561|rs10032586
Antinuclear Antibodies	INFLUENZA_LIFE_CYCLE	REACTOME	1671	42	20	3,54E-01	1,00E+00	rs3026106|rs7902136|rs1478785|rs1504632|rs12602594|rs5766533|rs1953910|rs17121120|rs17010833|rs16933086|rs10065570|rs13219815|rs7329817|rs11002569|rs17201246|rs4777170|rs4671408|rs633742|rs6895379|rs11733964
Antinuclear Antibodies	TRANSPORT_OF_INORGANIC_CATIONS_ANIONS_AND_AMINO_ACIDS_OLIGOPEPTIDES	REACTOME	4051	41	20	3,54E-01	1,00E+00	rs13274956|rs4869685|rs1468788|rs7657271|rs12000690|rs9857832|rs1327409|rs2188565|rs4685165|rs13430329|rs4640842|rs12625137|rs4602539|rs11670126|rs17305387|rs1979073|rs13300397|rs488192|rs9289660|rs6436196
Antinuclear Antibodies	NEUROTRANSMITTER_RECEPTOR_BINDING_AND_DOWNSTREAM_TRANSMISSION_IN_THL	REACTOME	6592	32	20	3,56E-01	1,00E+00	rs4906888|rs1806201|rs28671569|rs902522|rs2591164|rs11970312|rs1863455|rs1422893|rs7665102|rs16947151|rs948215|rs1843622|rs9898774|rs306090|rs6807740|rs9447579|rs4685165|rs8081512|rs208147|rs488447
Antinuclear Antibodies	PHOSPHORYLATION_OF_THE_APC_C	REACTOME	219	22	8	3,58E-01	1,00E+00	rs9562187|rs6836883|rs10514915|rs1106950|rs11734845|rs11679897|rs9876561|rs10032586
Antinuclear Antibodies	THROMBOXANE_SIGNALLING_THROUGH_TP_RECEPTOR	REACTOME	492	35	20	3,61E-01	1,00E+00	rs16947151|rs6807740|rs4243583|rs1253704|rs10493754|rs11715227|rs12147761|rs6972935|rs4807284|rs17124529|rs4480744|rs11622145|rs11880582|rs931647|rs8100484|rs3741920|rs10414848|rs1246159|rs7612445|rs10869954
Antinuclear Antibodies	APOPTOSIS	REACTOME	3298	44	20	3,68E-01	1,00E+00	rs13405741|rs12765241|rs1543805|rs7830593|rs12957347|rs10502934|rs11141915|rs12955104|rs1572968|rs9883073|rs1357287|rs1953042|rs12867405|rs2239923|rs16845834|rs12454840|rs10140923|rs17576470|rs336629|rs1930061
Antinuclear Antibodies	ALANINE_ASPARTATE_AND_GLUTAMATE_METABOLISM	KEGG	841	42	19	3,69E-01	1,00E+00	rs11685570|rs2371034|rs4439928|rs2073211|rs10932334|rs6748782|rs7302925|rs10199038|rs759688|rs4538195|rs2296063|rs9927205|rs925102|rs914983|rs10901125|rs3793932|rs1641107|rs13338289|rs3006009
Antinuclear Antibodies	MCALPAIN_PATHWAY	BIOCARTA	543	11	8	3,70E-01	1,00E+00	rs1030098|rs6501512|rs2302784|rs4767886|rs883751|rs6861460|rs625750|rs12146170
Antinuclear Antibodies	MYOGENESIS	REACTOME	1747	41	20	3,70E-01	1,00E+00	rs7241357|rs557831|rs8123347|rs2431016|rs2274316|rs9883073|rs485409|rs6124072|rs4925295|rs3740912|rs12199654|rs10750322|rs1939890|rs4925207|rs17595731|rs1201914|rs11705763|rs739559|rs4482379|rs2700204
Antinuclear Antibodies	POST_TRANSLATIONAL_PROTEIN_MODIFICATION	REACTOME	6008	45	20	3,71E-01	1,00E+00	rs485141|rs657224|rs4949631|rs13035081|rs11152012|rs281215|rs17164861|rs4846948|rs2028900|rs13072457|rs5742858|rs6095638|rs7934606|rs12129945|rs11245935|rs6669101|rs807270|rs12699009|rs10233708|rs12422396
Antinuclear Antibodies	CONVERSION_FROM_APC_C_CDC20_TO_APC_C_CDH1_IN_LATE_ANAPHASE	REACTOME	226	22	8	3,72E-01	1,00E+00	rs9562187|rs6836883|rs10514915|rs1106950|rs11734845|rs11679897|rs9876561|rs10032586
Antinuclear Antibodies	CSK_PATHWAY	BIOCARTA	554	17	14	3,75E-01	1,00E+00	rs3782735|rs1030098|rs1404912|rs6501512|rs2302784|rs1561144|rs883751|rs322931|rs2471226|rs35265698|rs3741920|rs1917534|rs11687510|rs16859030
Antinuclear Antibodies	SLC_MEDIATED_TRANSMEMBRANE_TRANSPORT	REACTOME	8774	50	20	3,75E-01	1,00E+00	rs13274956|rs3026106|rs11174735|rs4869685|rs1468790|rs1478785|rs2276433|rs7657271|rs12000690|rs12125560|rs9857832|rs1327409|rs2188565|rs4685165|rs13430329|rs2000466|rs5766533|rs11776475|rs1139266|rs6673947
Antinuclear Antibodies	VEGF_PATHWAY	BIOCARTA	883	30	20	3,77E-01	1,00E+00	rs6059703|rs4262039|rs8081512|rs169143|rs708030|rs198178|rs400330|rs1550805|rs11249743|rs4767886|rs6449969|rs10517342|rs7804859|rs9838117|rs3785396|rs17153527|rs17086609|rs882951|rs4730197|rs2088402
Antinuclear Antibodies	BLADDER_CANCER	KEGG	1222	38	20	3,80E-01	1,00E+00	rs9886419|rs9465734|rs11141915|rs7812998|rs1561928|rs11604821|rs1953042|rs1426511|rs1930061|rs309770|rs11141911|rs3933239|rs4878066|rs2397446|rs12207548|rs9295464|rs624797|rs1468934|rs3820028|rs1872568
Antinuclear Antibodies	IL6_PATHWAY	BIOCARTA	401	27	11	3,80E-01	1,00E+00	rs2069837|rs2005292|rs9896052|rs6125931|rs204680|rs4767879|rs310244|rs6037819|rs2274471|rs6681035|rs161700
Antinuclear Antibodies	INSULIN_SYNTHESIS_AND_PROCESSING	REACTOME	1033	32	20	3,82E-01	1,00E+00	rs4301693|rs16999102|rs1504610|rs11135469|rs10517365|rs4691213|rs10017098|rs9504016|rs1211554|rs2269012|rs2444043|rs2206161|rs934818|rs2887830|rs4901750|rs2278637|rs6075207|rs362570|rs6131920|rs12040671
Antinuclear Antibodies	CITRATE_CYCLE_TCA_CYCLE	KEGG	1195	54	20	3,83E-01	1,00E+00	rs17070285|rs4331688|rs10813415|rs9534734|rs400426|rs2028548|rs7558379|rs288877|rs6547525|rs10516988|rs2902374|rs7602538|rs10970969|rs9534844|rs10970427|rs11893686|rs7600094|rs955358|rs1946741|rs2970358
Antinuclear Antibodies	MTA3_PATHWAY	BIOCARTA	333	10	4	3,83E-01	1,00E+00	rs6091084|rs2292963|rs2374432|rs6472114
Antinuclear Antibodies	ARGININE_AND_PROLINE_METABOLISM	KEGG	991	43	20	3,84E-01	1,00E+00	rs11685570|rs9671414|rs2371034|rs4526224|rs501819|rs10932334|rs6748782|rs7302925|rs4646772|rs4623951|rs759688|rs10973734|rs17742907|rs2296063|rs1834058|rs2408700|rs914983|rs10901125|rs12811583|rs3793932
Antinuclear Antibodies	PLATELET_AGGREGATION_PLUG_FORMATION	REACTOME	942	42	20	3,88E-01	1,00E+00	rs11102314|rs3820852|rs216872|rs2038171|rs3769309|rs1404772|rs9896052|rs1537741|rs10821495|rs1878247|rs13137121|rs1546924|rs216321|rs7306680|rs2991216|rs10932619|rs11063954|rs917857|rs11015149|rs2290889
Antinuclear Antibodies	PLATELET_CALCIUM_HOMEOSTASIS	REACTOME	1156	35	20	3,90E-01	1,00E+00	rs12286714|rs13430329|rs13316275|rs9915261|rs2060822|rs7303557|rs10490817|rs718314|rs11685090|rs4788102|rs6810046|rs7623572|rs799320|rs2194435|rs10842720|rs7586183|rs684119|rs42307|rs12474136|rs1499772
Antinuclear Antibodies	RAS_ACTIVATION_UOPN_CA2_INFUX_THROUGH_NMDA_RECEPTOR	REACTOME	1033	34	15	3,95E-01	1,00E+00	rs1806201|rs7665102|rs4332745|rs2872715|rs8024521|rs8060068|rs2055226|rs873592|rs1074042|rs12934132|rs10744029|rs729650|rs4240286|rs1554668|rs2160517
Antinuclear Antibodies	EARLY_PHASE_OF_HIV_LIFE_CYCLE	REACTOME	270	15	9	3,96E-01	1,00E+00	rs3782735|rs1151514|rs7371043|rs174532|rs828704|rs10191360|rs1805388|rs301282|rs3741920
Antinuclear Antibodies	GLYCOLYSIS_GLUCONEOGENESIS	KEGG	1391	41	20	3,96E-01	1,00E+00	rs10903939|rs11251480|rs7084464|rs2132302|rs4129219|rs1881717|rs11636141|rs12274651|rs2306787|rs2273684|rs10516988|rs2239167|rs4646772|rs2068455|rs7074424|rs10973734|rs1614972|rs11251694|rs11251588|rs6855439
Antinuclear Antibodies	MITOTIC_G2_G2_M_PHASES	REACTOME	1389	30	20	4,00E-01	1,00E+00	rs1333924|rs9465734|rs1990639|rs11933720|rs10984865|rs945246|rs17010833|rs741339|rs7295385|rs371164|rs1554160|rs4802906|rs3764694|rs4671408|rs870777|rs12517345|rs2111027|rs9295464|rs9576040|rs6505776
Antinuclear Antibodies	INSULIN_PATHWAY	BIOCARTA	725	36	20	4,00E-01	1,00E+00	rs11745726|rs2005292|rs7258382|rs9896052|rs17254521|rs4675077|rs1550805|rs2001303|rs204680|rs4767879|rs6449969|rs2251692|rs6037819|rs7804859|rs2894595|rs13392647|rs9838117|rs17153527|rs6681035|rs4730197
Antinuclear Antibodies	GOLGI_ASSOCIATED_VESICLE_BIOGENESIS	REACTOME	1057	31	20	4,00E-01	1,00E+00	rs11230882|rs877711|rs3731828|rs951453|rs17781348|rs3798188|rs4794873|rs2022345|rs16933086|rs419588|rs14976|rs9347573|rs26538|rs12376522|rs4704470|rs2278637|rs11938266|rs658573|rs913199|rs875462
Antinuclear Antibodies	TPO_PATHWAY	BIOCARTA	814	43	20	4,00E-01	1,00E+00	rs6452735|rs8081512|rs13029532|rs169143|rs2005292|rs9896052|rs198178|rs1550805|rs204680|rs6449969|rs6037819|rs7804859|rs2274471|rs9838117|rs3785396|rs17153527|rs6681035|rs4730197|rs9925433|rs17153654
Antinuclear Antibodies	CALCINEURIN_PATHWAY	BIOCARTA	895	38	15	4,02E-01	1,00E+00	rs9372365|rs8081512|rs169143|rs2866250|rs7148384|rs198178|rs954620|rs4699014|rs2055226|rs12207548|rs761685|rs3785396|rs614569|rs9925433|rs9462209
Antinuclear Antibodies	T_CELL_RECEPTOR_SIGNALING_PATHWAY	KEGG	3546	35	20	4,04E-01	1,00E+00	rs933984|rs4419220|rs11767716|rs3782735|rs10490696|rs10485101|rs656709|rs3824561|rs2005292|rs1357287|rs7694484|rs11153318|rs12199654|rs2510257|rs12413938|rs7148384|rs1144171|rs9896052|rs152105|rs11706098
Antinuclear Antibodies	G0_AND_EARLY_G1	REACTOME	258	12	8	4,05E-01	1,00E+00	rs12867405|rs2298330|rs285164|rs7015841|rs4805487|rs9576040|rs13052269|rs704874

Antinuclear Antibodies	GLUTATHIONE_METABOLISM	KEGG	683	36	20	4,06E-01	1,00E+00	rs2978663|rs9296691|rs2387094|rs316156|rs10456664|rs140289|rs13124794|rs2273684|rs4243969|rs7758711|rs1038121|rs17355469|rs2224198|rs5751902|rs809942|rs396216|rs2970358|rs8192013|rs1892714|rs11757000
Antinuclear Antibodies	PDGF_PATHWAY	BIOCARTA	1021	39	20	4,08E-01	1,00E+00	rs6452735|rs8081512|rs2529737|rs13029532|rs169143|rs2005292|rs9896052|rs198178|rs1550805|rs204680|rs310244|rs6449969|rs6037819|rs7804859|rs9838117|rs2598483|rs3785396|rs16833215|rs17153527|rs6681035
Antinuclear Antibodies	MEF2D_PATHWAY	BIOCARTA	684	38	13	4,08E-01	1,00E+00	rs8081512|rs2274316|rs169143|rs7694484|rs198178|rs352066|rs4699014|rs2055226|rs3788368|rs761685|rs3785396|rs614569|rs9925433
Antinuclear Antibodies	BIOSYNTHESIS_OF_UNSATURATED_FATTY_ACIDS	KEGG	463	22	13	4,08E-01	1,00E+00	rs6853|rs2305913|rs13435303|rs209489|rs12498468|rs12499413|rs174532|rs4665830|rs6838244|rs7680893|rs705648|rs10508533|rs934150
Antinuclear Antibodies	CYTOSOLIC_TRNA_AMINOACYLATION	REACTOME	312	10	6	4,09E-01	1,00E+00	rs244896|rs933181|rs3733977|rs12968882|rs12123041|rs3768976
Antinuclear Antibodies	PEROXISOMAL_LIPID_METABOLISM	REACTOME	435	16	10	4,13E-01	1,00E+00	rs6853|rs11605276|rs2305913|rs11130651|rs10219485|rs174745|rs10500797|rs12300735|rs1507534|rs7625947
Antinuclear Antibodies	INITIAL_TRIGGERING_OF_COMPLEMENT	REACTOME	434	34	12	4,16E-01	1,00E+00	rs12220324|rs12571782|rs1143664|rs1389623|rs1903946|rs7962629|rs2844456|rs10740487|rs10762771|rs7083840|rs4935400|rs1107187
Antinuclear Antibodies	EIF4_PATHWAY	BIOCARTA	890	35	20	4,18E-01	1,00E+00	rs1039153|rs8081512|rs169143|rs885126|rs12199654|rs198178|rs4675077|rs1550805|rs2001303|rs10509534|rs6449969|rs2251692|rs2951967|rs13392647|rs2894595|rs9838117|rs3785396|rs10941583|rs10788582|rs945559
Antinuclear Antibodies	CARDIAC_MUSCLE_CONTRACTION	KEGG	3169	38	20	4,19E-01	1,00E+00	rs2618681|rs9810230|rs38553|rs12737847|rs10920115|rs11715062|rs10925415|rs13430329|rs12032218|rs10486963|rs1200138|rs12152349|rs2251788|rs947485|rs658071|rs38544|rs6415964|rs10919062|rs10425332|rs3773603
Antinuclear Antibodies	CELL_SURFACE_INTERACTIONS_AT_THE_VASCULAR_WALL	REACTOME	2650	34	20	4,19E-01	1,00E+00	rs7124686|rs12668754|rs8133991|rs7657271|rs709505|rs12988898|rs1200138|rs4602539|rs3753306|rs11153318|rs11670126|rs682386|rs1404772|rs9896052|rs2824206|rs10919062|rs4239020|rs2824065|rs1550805|rs2239167
Antinuclear Antibodies	TRANSMISSION_ACROSS_CHEMICAL_SYNAPSES	REACTOME	9089	31	20	4,20E-01	1,00E+00	rs4906888|rs1806201|rs28671569|rs4869685|rs902522|rs9810230|rs2591164|rs11685570|rs3799663|rs1863455|rs1422893|rs7665102|rs10508915|rs16947151|rs948215|rs1843622|rs9898774|rs306090|rs6807740|rs1535501
Antinuclear Antibodies	SEMA4D_IN_SEMAPHORIN_SIGNALING	REACTOME	597	38	15	4,21E-01	1,00E+00	rs6559349|rs2269529|rs836556|rs9410475|rs8060975|rs220076|rs1571534|rs1561144|rs2040532|rs322931|rs2402118|rs11670669|rs2855726|rs2272555|rs6531240
Antinuclear Antibodies	RNA_POL_I_RNA_POL_III_AND_MITOCHONDRIAL_TRANSCRIPTION	REACTOME	1830	28	20	4,23E-01	1,00E+00	rs4921702|rs16858688|rs2227301|rs4922152|rs13296301|rs10961534|rs4922157|rs6081157|rs1372349|rs13270944|rs10121140|rs17092536|rs11989385|rs13290272|rs17213362|rs683474|rs10951129|rs6928843|rs2844456|rs10924771
Antinuclear Antibodies	SELENOAMINO_ACID_METABOLISM	KEGG	742	42	19	4,24E-01	1,00E+00	rs2028900|rs10068900|rs566509|rs158850|rs2202044|rs2290536|rs12443981|rs978114|rs277884|rs297961|rs580789|rs1038121|rs6119535|rs5751902|rs1205357|rs520002|rs17602403|rs4512145|rs165064
Antinuclear Antibodies	G1_PHASE	REACTOME	625	33	20	4,26E-01	1,00E+00	rs12078447|rs9465734|rs11604821|rs12867405|rs2976282|rs4802906|rs10105118|rs1333050|rs12207548|rs9295464|rs7315599|rs624797|rs2237572|rs3820028|rs7781436|rs1872568|rs3745245|rs1116003|rs2932777|rs9462209
Antinuclear Antibodies	GLEEVEC_PATHWAY	BIOCARTA	954	35	20	4,26E-01	1,00E+00	rs9886419|rs7812998|rs1561928|rs2529737|rs13029532|rs2005292|rs9896052|rs1426511|rs1550805|rs6449969|rs7804859|rs2274471|rs9838117|rs1468934|rs17153527|rs6681035|rs5751636|rs3788358|rs4730197|rs131702
Antinuclear Antibodies	IGF1R_PATHWAY	BIOCARTA	735	35	19	4,26E-01	1,00E+00	rs1030098|rs1404912|rs7168213|rs6501512|rs2302784|rs9896052|rs4675077|rs7181975|rs1550805|rs2001303|rs883751|rs6449969|rs2251692|rs13392647|rs2894595|rs2471226|rs8038056|rs9838117|rs12901358
Antinuclear Antibodies	GLYOXYLATE_AND_DICARBOXYLATE_METABOLISM	KEGG	704	51	18	4,28E-01	1,00E+00	rs6077242|rs10813415|rs400426|rs6140362|rs6108011|rs288877|rs8011839|rs10970969|rs745686|rs17023457|rs765567|rs942382|rs10923799|rs10970427|rs11789963|rs6086075|rs955358|rs6055253
Antinuclear Antibodies	PML_PATHWAY	BIOCARTA	384	22	14	4,29E-01	1,00E+00	rs12765241|rs12477596|rs824939|rs17342653|rs1678192|rs1061624|rs1835432|rs4934433|rs1430659|rs992454|rs2154254|rs2105436|rs1926189|rs3754941
Antinuclear Antibodies	NCAM_SIGNALING_FOR_NEURITE_OUT_GROWTH	REACTOME	2480	32	20	4,30E-01	1,00E+00	rs12668754|rs9327283|rs10920115|rs4536647|rs1895464|rs9521666|rs4490127|rs903352|rs12593435|rs10787635|rs2279447|rs11153318|rs9898731|rs346852|rs677152|rs9896052|rs9288163|rs6415964|rs595325|rs4663712
Antinuclear Antibodies	NDKDYNAMIN_PATHWAY	BIOCARTA	548	17	7	4,38E-01	1,00E+00	rs7694484|rs2475556|rs750997|rs4699014|rs2055226|rs10806791|rs10178199
Antinuclear Antibodies	GLYCOSAMINOGLYCAN_METABOLISM	REACTOME	6096	29	20	4,40E-01	1,00E+00	rs2865155|rs16895527|rs1980779|rs11223958|rs17375044|rs4467961|rs4535751|rs11729868|rs10861272|rs9584410|rs1606724|rs879162|rs1866430|rs6095638|rs12853685|rs17486477|rs831423|rs205148|rs7929855|rs2187467
Antinuclear Antibodies	LATE_PHASE_OF_HIV_LIFE_CYCLE	REACTOME	1259	35	20	4,40E-01	1,00E+00	rs3026106|rs1478785|rs2132302|rs5766533|rs2239923|rs17010833|rs3786238|rs13219815|rs10732882|rs443678|rs4671408|rs1053079|rs633742|rs6928843|rs9615025|rs604965|rs8088313|rs399491|rs10852553|rs2178077
Antinuclear Antibodies	CITRIC_ACID_CYCLE_TCA_CYCLE	REACTOME	675	40	17	4,40E-01	1,00E+00	rs17070285|rs4331688|rs9534734|rs2028548|rs7558379|rs6547525|rs2902374|rs7602538|rs9534844|rs11893686|rs7600094|rs7713458|rs2970358|rs7611269|rs1409488|rs1597039|rs7629728
Antinuclear Antibodies	TRAF6_MEDIATED_IRF7_ACTIVATION	REACTOME	433	24	13	4,40E-01	1,00E+00	rs13119830|rs6850472|rs10029721|rs2207701|rs331455|rs3750512|rs13116389|rs793794|rs17220720|rs793881|rs1342852|rs17686080|rs7267297
Antinuclear Antibodies	MET_PATHWAY	BIOCARTA	1415	32	20	4,40E-01	1,00E+00	rs11745726|rs12357398|rs11102314|rs4448606|rs1397529|rs10830183|rs2005292|rs9896052|rs1550805|rs4767886|rs4767879|rs10901096|rs10509534|rs11245214|rs6449969|rs1546924|rs2163177|rs7804859|rs2909580|rs4751323
Antinuclear Antibodies	IGF1_PATHWAY	BIOCARTA	813	36	20	4,42E-01	1,00E+00	rs11745726|rs2005292|rs11632057|rs9896052|rs4675077|rs7181975|rs1550805|rs2001303|rs204680|rs4767879|rs6449969|rs2251692|rs6037819|rs7804859|rs2894595|rs13392647|rs8038056|rs9838117|rs17153527|rs6681035
Antinuclear Antibodies	CDMAC_PATHWAY	BIOCARTA	1018	30	20	4,43E-01	1,00E+00	rs9886419|rs7812998|rs1561928|rs8081512|rs169143|rs2005292|rs2206422|rs1426511|rs198178|rs10485723|rs708925|rs2415290|rs3785396|rs1468934|rs2745755|rs6681035|rs12289836|rs6055995|rs8123677|rs6599711
Antinuclear Antibodies	GAP_JUNCTION_TRAFFICKING	REACTOME	576	39	20	4,43E-01	1,00E+00	rs11576760|rs720863|rs1886176|rs9343330|rs16933086|rs16869484|rs1065206|rs1336625|rs3849768|rs7321901|rs2078750|rs9292741|rs12187127|rs9509119|rs7764196|rs4957233|rs8064669|rs9450659|rs4596408|rs7213960
Antinuclear Antibodies	GLYCOSAMINOGLYCAN_BIOSYNTHESIS_CHONDROITIN_SULFATE	KEGG	1372	29	20	4,46E-01	1,00E+00	rs11223958|rs4535751|rs10861272|rs7929855|rs2187467|rs3789823|rs17079451|rs2979874|rs2172350|rs10934779|rs6675798|rs8058274|rs17407149|rs11862371|rs7926027|rs4936233|rs12189648|rs11772554|rs10161421|rs4937880
Antinuclear Antibodies	SEMAPHORIN_INTERACTIONS	REACTOME	2884	40	20	4,47E-01	1,00E+00	rs12579612|rs281215|rs999426|rs6702300|rs9645353|rs407266|rs2106673|rs7829347|rs6559349|rs864106|rs2269529|rs836556|rs10827245|rs1403101|rs17260912|rs9410475|rs8060975|rs11153318|rs3774876|rs11594539
Antinuclear Antibodies	LOSS_OF_NLP_FROM_MITOTIC_CENTROSOMES	REACTOME	1100	43	20	4,47E-01	1,00E+00	rs1333924|rs1990639|rs11933720|rs10984865|rs945246|rs741339|rs7295385|rs371164|rs1554160|rs4802906|rs3764694|rs870777|rs12517345|rs2111027|rs6505776|rs3889570|rs4836798|rs10860122|rs4865047|rs3844450
Antinuclear Antibodies	CHONDROITIN_SULFATE_BIOSYNTHESIS	REACTOME	846	37	20	4,48E-01	1,00E+00	rs4535751|rs10861272|rs3789823|rs2591454|rs2979874|rs2172350|rs937618|rs951781|rs10934779|rs9967331|rs17407149|rs11772554|rs10161421|rs10074448|rs2238675|rs7077321|rs17621618|rs2081807|rs4921656|rs6858978
Antinuclear Antibodies	ACTIVATED_POINT_MUTANTS_OF_FGFR2	REACTOME	873	30	20	4,49E-01	1,00E+00	rs1924210|rs9736587|rs3129678|rs950549|rs6884797|rs3129599|rs249729|rs4751828|rs12592277|rs8000805|rs9506777|rs2420932|rs1078363|rs152523|rs17287322|rs4770259|rs10477181|rs9510129|rs9509983|rs249923
Antinuclear Antibodies	GPCR_PATHWAY	BIOCARTA	1102	41	20	4,52E-01	1,00E+00	rs8081512|rs1030098|rs1404912|rs169143|rs2005292|rs10228945|rs2866250|rs7148384|rs6501512|rs2302784|rs198178|rs2714645|rs883751|rs4699014|rs2055226|rs2523189|rs761685|rs2471226|rs3785396|rs6681035
Antinuclear Antibodies	BASIGIN_INTERACTIONS	REACTOME	595	33	18	4,53E-01	1,00E+00	rs7657271|rs1200138|rs4602539|rs11670126|rs10919062|rs4239020|rs748680|rs7657780|rs10434201|rs910795|rs12891079|rs2012859|rs6850624|rs2056275|rs12879346|rs13108353|rs7693577|rs12146170
Antinuclear Antibodies	TRANS_GOLGI_NETWORK_VESICLE_BUDDING	REACTOME	1106	31	20	4,53E-01	1,00E+00	rs11230882|rs877711|rs3731828|rs951453|rs17781348|rs3798188|rs4794873|rs2022345|rs16933086|rs419588|rs14976|rs9347573|rs26538|rs12376522|rs7730112|rs2278637|rs11938266|rs658573|rs913199|rs875462
Antinuclear Antibodies	ACTIVATION_OF_GENES_BY_ATF4	REACTOME	316	14	8	4,55E-01	1,00E+00	rs708272|rs1000174|rs10863981|rs679840|rs3795837|rs2884515|rs4782165|rs1533079
Antinuclear Antibodies	TRANSFERRIN_ENDOCYTOSIS_AND_RECYCLING	REACTOME	513	20	14	4,55E-01	1,00E+00	rs7853552|rs13272985|rs2166504|rs1390943|rs16870060|rs6895379|rs7646203|rs8177190|rs1477953|rs296917|rs4927870|rs1390938|rs2458284|rs2453997
Antinuclear Antibodies	INFLAM_PATHWAY	BIOCARTA	747	31	20	4,55E-01	1,00E+00	rs3782735|rs1227631|rs2069837|rs13253242|rs11921898|rs604045|rs1498737|rs10863388|rs7668305|rs7526672|rs7547373|rs3014996|rs333968|rs7610160|rs16906063|rs8179181|rs1603059|rs35265698|rs3741920|rs6509940
Antinuclear Antibodies	IL10_PATHWAY	BIOCARTA	288	21	9	4,56E-01	1,00E+00	rs2069837|rs10892201|rs13029532|rs310244|rs2598483|rs16833215|rs652107|rs268691|rs16833239
Antinuclear Antibodies	CGMP_EFFECTS	REACTOME	1520	48	20	4,56E-01	1,00E+00	rs11729954|rs9817294|rs1972058|rs10841528|rs7069974|rs10740411|rs1358786|rs11045301|rs10762562|rs7907343|rs1194491|rs17649232|rs6534152|rs211085|rs314140|rs6922908|rs9838117|rs1432515|rs7299937|rs7762369
Antinuclear Antibodies	NONSENSE_MEDIATED_DECAY_ENHANCED_BY_THE_EXON_JUNCTION_COMPLEX	REACTOME	1261	49	20	4,61E-01	1,00E+00	rs1039153|rs7902136|rs7176919|rs12602594|rs9562187|rs1953910|rs17121120|rs2976282|rs10065570|rs346524|rs4802906|rs11002569|rs4777170|rs2951967|rs6895379|rs11733964|rs4780769|rs2301723|rs13325754|rs11002552
Antinuclear Antibodies	PERK_REGULATED_GENE_EXPRESSION	REACTOME	334	14	8	4,61E-01	1,00E+00	rs708272|rs1000174|rs10863981|rs679840|rs3795837|rs2884515|rs8051249|rs1533079
Antinuclear Antibodies	IL2RB_PATHWAY	BIOCARTA	1296	31	20	4,63E-01	1,00E+00	rs12765241|rs9886419|rs7812998|rs1561928|rs2005292|rs9896052|rs1426511|rs10821495|rs4675077|rs1550805|rs2239167|rs2001303|rs310244|rs6449969|rs2251692|rs7804859|rs13392647|rs2894595|rs2991216|rs9838117
Antinuclear Antibodies	EPITHELIAL_CELL_SIGNALING_IN_HELICOBACTER_PYLORI_INFECTION	KEGG	1535	33	20	4,63E-01	1,00E+00	rs7853552|rs13272985|rs8133991|rs10953942|rs2529737|rs10495562|rs836556|rs12199654|rs682386|rs10445097|rs1390943|rs4767879|rs16870060|rs2415290|rs6879217|rs6895379|rs7646203|rs12803146|rs1196471|rs1477953
Antinuclear Antibodies	ERK_PATHWAY	BIOCARTA	1025	37	17	4,63E-01	1,00E+00	rs9886419|rs7812998|rs1561928|rs10879175|rs885126|rs7168213|rs9896052|rs1426511|rs7181975|rs2798464|rs8038056|rs1468934|rs2465810|rs12136101|rs12146170|rs12901358|rs751437
Antinuclear Antibodies	FACTORS_INVOLVED_IN_MEGAKARYOCYTE_DEVELOPMENT_AND_PLATELET_PRODUCT	REACTOME	3549	24	20	4,64E-01	1,00E+00	rs994626|rs4301693|rs753536|rs1579282|rs12357398|rs13119830|rs4448606|rs17646824|rs1890981|rs584922|rs1030098|rs10830183|rs6869991|rs836556|rs1316638|rs17559604|rs6850472|rs10029721|rs261072|rs10903341
Antinuclear Antibodies	NEGATIVE_REGULATION_OF_FGFR_SIGNALING	REACTOME	1536	39	20	4,66E-01	1,00E+00	rs1924210|rs9736587|rs1114275|rs3129678|rs6563216|rs885126|rs950549|rs9545290|rs6884797|rs9896052|rs3129599|rs1215930|rs249729|rs4751828|rs12592277|rs948992|rs4802906|rs8000805|rs10105118|rs9506777
Antinuclear Antibodies	ARF_PATHWAY	BIOCARTA	733	32	20	4,66E-01	1,00E+00	rs9886419|rs7812998|rs1561928|rs2227301|rs836556|rs1426511|rs1550805|rs6449969|rs2189000|rs7804859|rs9838117|rs1468934|rs17153527|rs878745|rs3848006|rs4730197|rs2583836|rs1056209|rs17153654|rs751437
Antinuclear Antibodies	ACTIVATION_OF_NMDA_RECEPTOR_UPON_GLUTAMATE_BINDING_AND_POSTSYNAPTIC	REACTOME	2117	46	20	4,67E-01	1,00E+00	rs1806201|rs3799663|rs1422893|rs7665102|rs9898774|rs306090|rs1404912|rs7730730|rs4332745|rs10030915|rs2872715|rs8024521|rs10515699|rs8060068|rs2055226|rs873592|rs1074042|rs378373|rs3799584|rs12934132
Antinuclear Antibodies	GALACTOSE_METABOLISM	KEGG	759	44	19	4,68E-01	1,00E+00	rs10903939|rs11251480|rs7084464|rs2078024|rs11707667|rs2068455|rs7074424|rs11251694|rs9640883|rs11251588|rs6855439|rs871443|rs12220754|rs16962243|rs6531592|rs7089499|rs10903959|rs16973008|rs17301901
Antinuclear Antibodies	SIGNALING_BY_FGFR_MUTANTS	REACTOME	1657	37	20	4,69E-01	1,00E+00	rs1924210|rs10878951|rs9736587|rs3129678|rs6465836|rs1397529|rs13029532|rs950549|rs6884797|rs9896052|rs3129599|rs249729|rs4751828|rs12592277|rs1550805|rs8000805|rs10808119|rs2031282|rs6449969|rs9506777
Antinuclear Antibodies	ADHERENS_JUNCTION	KEGG	4520	45	20	4,69E-01	1,00E+00	rs6764017|rs955760|rs647241|rs11869351|rs567595|rs10485101|rs10934067|rs10879175|rs7895441|rs17020744|rs2974154|rs12961323|rs9883073|rs2038171|rs6091084|rs836556|rs7258382|rs17231134|rs2862944|rs11632057
Antinuclear Antibodies	RIP_MEDIATED_NFKB_ACTIVATION_VIA_DAI	REACTOME	328	22	15	4,70E-01	1,00E+00	rs2829946|rs466609|rs7148384|rs3014866|rs375369|rs2415290|rs457581|rs1467719|rs5743312|rs1519309|rs12289836|rs10872893|rs2388548|rs3138045|rs17074691
Antinuclear Antibodies	EPO_PATHWAY	BIOCARTA	255	16	8	4,70E-01	1,00E+00	rs2005292|rs9896052|rs2239167|rs204680|rs6037819|rs2274471|rs6681035|rs12259267
Antinuclear Antibodies	G_ALPHA_Z_SIGNALLING_EVENTS	REACTOME	2191	28	20	4,72E-01	1,00E+00	rs902522|rs16947151|rs6807740|rs8081512|rs968539|rs1404912|rs4243583|rs1253704|rs169143|rs17360897|rs10228945|rs9306383|rs2287778|rs10493754|rs11715227|rs953672|rs12147761|rs13155334|rs6972935|rs198178
Antinuclear Antibodies	CTCF_PATHWAY	BIOCARTA	1035	31	20	4,72E-01	1,00E+00	rs9886419|rs7812998|rs9968396|rs1561928|rs7653262|rs1426511|rs604045|rs478061|rs10863388|rs1550805|rs7526672|rs10509534|rs6449969|rs7804859|rs9838117|rs1468934|rs17153527|rs6672952|rs10788582|rs945559
Antinuclear Antibodies	GLYCINE_SERINE_AND_THREONINE_METABOLISM	KEGG	564	30	15	4,73E-01	1,00E+00	rs2510257|rs1757947|rs2202044|rs7027964|rs10199038|rs10122711|rs4740848|rs4623951|rs580789|rs4538195|rs11999442|rs7030760|rs2183838|rs916620|rs11137607
Antinuclear Antibodies	GAP_JUNCTION	KEGG	4388	36	20	4,74E-01	1,00E+00	rs3886478|rs902522|rs2974254|rs13185639|rs1578303|rs9307949|rs17041308|rs6885612|rs7069974|rs1407101|rs10740411|rs8081512|rs13190572|rs1404912|rs9526313|rs169143|rs1471107|rs17360897|rs10228945|rs2327162
Antinuclear Antibodies	ACTINY_PATHWAY	BIOCARTA	290	18	8	4,74E-01	1,00E+00	rs836556|rs17048140|rs465316|rs1756472|rs3791247|rs932734|rs924483|rs10164916
Antinuclear Antibodies	BIOPEPTIDES_PATHWAY	BIOCARTA	1081	51	20	4,75E-01	1,00E+00	rs7665102|rs8081512|rs13029532|rs169143|rs10228945|rs11153318|rs12199654|rs9896052|rs198178|rs2714645|rs2303924|rs2055226|rs873592|rs2163177|rs2274471|rs2523189|rs2598483|rs3785396|rs16833215|rs13232987
Antinuclear Antibodies	COMP_PATHWAY	BIOCARTA	628	47	20	4,75E-01	1,00E+00	rs12220324|rs12571782|rs971075|rs1143664|rs9661996|rs1389623|rs11003466|rs2292727|rs7962629|rs706470|rs2844456|rs13156109|rs10740487|rs10473234|rs624298|rs10762771|rs7083840|rs4935400|rs1107187|rs11953280
Antinuclear Antibodies	BIOSYNTHESIS_OF_THE_N_GLYCAN_PRECURSOR_DOLICHOL_LIPID_LINKED_OLIGOSACC RANSFER_TO_A_NASCENT_PROTEIN	REACTOME	355	23	7	4,76E-01	1,00E+00	rs13072457|rs12755096|rs12423422|rs12304768|rs10844762|rs484747|rs11165281
Antinuclear Antibodies	PLC_BETA_MEDIATED_EVENTS	REACTOME	2313	31	20	4,76E-01	1,00E+00	rs902522|rs230624|rs306090|rs1407101|rs8081512|rs1030098|rs1404912|rs17360897|rs10228945|rs2327162|rs9306383|rs2287778|rs6683451|rs2206422|rs6501512|rs2302784|rs953672|rs11583351|rs7303557|rs13155334
Antinuclear Antibodies	VIP_PATHWAY	BIOCARTA	1000	36	20	4,78E-01	1,00E+00	rs9886419|rs7812998|rs1561928|rs1030098|rs2866250|rs6501512|rs2302784|rs1426511|rs883751|rs4699014|rs311334|rs2055226|rs2415290|rs7905229|rs761685|rs1468934|rs12289836|rs10872893|rs3138045|rs311326
Antinuclear Antibodies	TCR_SIGNALING	REACTOME	2040	36	20	4,80E-01	1,00E+00	rs6020238|rs11767716|rs3782735|rs10485101|rs1190976|rs1403101|rs13080704|rs1144171|rs152105|rs2064478|rs6940350|rs1923075|rs1550805|rs1561144|rs331455|rs12216864|rs4357599|rs7904311|rs9469357|rs10509534
Antinuclear Antibodies	CTLA4_INHIBITORY_SIGNALING	REACTOME	447	22	9	4,81E-01	1,00E+00	rs3850890|rs2715267|rs11153318|rs4802906|rs10105118|rs1599796|rs1544241|rs11160664|rs1116003
Antinuclear Antibodies	FGFR_LIGAND_BINDING_AND_ACTIVATION	REACTOME	984	27	20	4,82E-01	1,00E+00	rs1924210|rs9736587|rs3129678|rs950549|rs6884797|rs3129599|rs249729|rs4751828|rs12592277|rs948992|rs8000805|rs9506777|rs2420932|rs4522197|rs1078363|rs152523|rs376618|rs17287322|rs4770259|rs10477181
Antinuclear Antibodies	LATENT_INFECTION_OF_HOMO_SAPIENS_WITH_MYCOBACTERIUM_TUBERCULOSIS	REACTOME	589	22	14	4,83E-01	1,00E+00	rs7853552|rs13272985|rs1390943|rs4420519|rs16870060|rs6895379|rs7646203|rs12811583|rs1477953|rs296917|rs3782221|rs1390938|rs2458284|rs2453997
Antinuclear Antibodies	TEL_PATHWAY	BIOCARTA	978	52	18	4,85E-01	1,00E+00	rs9886419|rs7812998|rs1561928|rs8081512|rs6601327|rs7168213|rs1426511|rs7181975|rs1993596|rs12679892|rs8038056|rs828704|rs1468934|rs11787063|rs12958785|rs12901358|rs7837054|rs751437
Antinuclear Antibodies	BCR_PATHWAY	BIOCARTA	1053	44	20	4,86E-01	1,00E+00	rs7905645|rs8081512|rs169143|rs836556|rs2005292|rs7694484|rs12199654|rs7148384|rs9896052|rs198178|rs10821495|rs4699014|rs2055226|rs8102571|rs2991216|rs761685|rs3785396|rs6681035|rs10424498|rs2290889
Antinuclear Antibodies	HIV_INFECTION	REACTOME	3237	43	20	4,87E-01	1,00E+00	rs3026106|rs13272985|rs3782735|rs1478785|rs2132302|rs17646824|rs5766533|rs1151514|rs951453|rs836556|rs1316638|rs17559604|rs1357287|rs2239923|rs261072|rs11153318|rs17010833|rs1420320|rs2541094|rs3786238
Antinuclear Antibodies	REGULATION_OF_MITOTIC_CELL_CYCLE	REACTOME	1318	37	20	4,88E-01	1,00E+00	rs766295|rs1027692|rs17057833|rs9562187|rs6836883|rs10514915|rs11845708|rs7187327|rs12447239|rs3027247|rs698856|rs1106950|rs425594|rs11922435|401754|rs2431097|rs13207114|rs11734845|rs3787573|rs11679897
Antinuclear Antibodies	BAD_PATHWAY	BIOCARTA	927	35	20	4,90E-01	1,00E+00	rs10777125|rs1030098|rs1404912|rs11632057|rs6501512|rs2302784|rs10858795|rs7181975|rs1550805|rs883751|rs6449969|rs7804859|rs2471226|rs8038056|rs9838117|rs17153527|rs4730197|rs12958785|rs12901358|rs17153654
Antinuclear Antibodies	PLATELET_HOMEOSTASIS	REACTOME	4080	42	20	4,91E-01	1,00E+00	rs12286714|rs11729954|rs9817294|rs230624|rs1972058|rs10841528|rs16947151|rs6807740|rs7069974|rs10740411|rs13430329|rs4243583|rs1253704|rs1358786|rs11045301|rs13316275|rs6683451|rs12199654|rs12075532|rs11715227
Antinuclear Antibodies	DIABETES_PATHWAYS	REACTOME	3395	28	20	4,92E-01	1,00E+00	rs9841006|rs1247571|rs499205|rs4301693|rs1406961|rs708272|rs16999102|rs1030098|rs1504610|rs1000174|rs1411355|rs7865534|rs12145743|rs11135469|rs7523465|rs10517365|rs2302784|rs848189|rs4691213|rs10017098
Antinuclear Antibodies	SIGNALING_BY_INSULIN_RECEPTOR	REACTOME	3432	35	20	4,94E-01	1,00E+00	rs1881628|rs7853552|rs13272985|rs1924210|rs17762352|rs12540943|rs7999458|rs9736587|rs3129678|rs1397529|rs11876920|rs7258382|rs950549|rs6884797|rs9896052|rs3129599|rs203170|rs249729|rs4751828|rs12592277
Antinuclear Antibodies	DORSO_VENTRAL_AXIS_FORMATION	KEGG	1149	37	20	4,95E-01	1,00E+00	rs10845433|rs7496727|rs10790928|rs2213176|rs7312361|rs7963072|rs9896052|rs7134204|rs10926234|rs7108562|rs9787850|rs2008864|rs12096679|rs4759659|rs12124187|rs12294634|rs4936059|rs4397403|rs7314811|rs1114312
Antinuclear Antibodies	EGF_PATHWAY	BIOCARTA	1048	42	20	4,96E-01	1,00E+00	rs6452735|rs8081512|rs2529737|rs13029532|rs169143|rs2005292|rs9896052|rs198178|rs1550805|rs204680|rs310244|rs6449969|rs6037819|rs9838117|rs2598483|rs3785396|rs16833215|rs6681035|rs16833239|rs12259267
Antinuclear Antibodies	ADIPOCYTOKINE_SIGNALING_PATHWAY	KEGG	2106	27	20	4,96E-01	1,00E+00	rs3774909|rs1881628|rs12540943|rs7999458|rs6448226|rs9898774|rs213213|rs11612383|rs4675077|rs2001303|rs156283|rs4357599|rs3750512|rs7904311|rs4767879|rs7795096|rs1001684|rs2251692|rs11069810|rs2415290
Antinuclear Antibodies	INTERFERON_GAMMA_SIGNALING	REACTOME	2043	29	20	4,97E-01	1,00E+00	rs2352946|rs17375044|rs7665102|rs13119830|rs13029532|rs2038171|rs6850472|rs10029721|rs608914|rs353625|rs3117230|rs161130|rs2239167|rs276947|rs1611493|rs9469357|rs193276|rs13116389|rs310244|rs873592
Antinuclear Antibodies	SMALL_CELL_LUNG_CANCER	KEGG	3747	35	20	5,01E-01	1,00E+00	rs2056777|rs9877140|rs9886419|rs9465734|rs2236891|rs7812998|rs4536647|rs1561928|rs11604821|rs9521666|rs903352|rs2365491|rs213213|rs1404772|rs17668326|rs595325|rs7373003|rs10173487|rs1286762|rs1426511
Antinuclear Antibodies	HDAC_PATHWAY	BIOCARTA	1231	33	20	5,01E-01	1,00E+00	rs3886478|rs2236891|rs2274316|rs2866250|rs7258382|rs11632057|rs12199654|rs17254521|rs7181975|rs1550805|rs4699014|rs6449969|rs2055226|rs739559|rs7804859|rs3788368|rs761685|rs8038056|rs9838117|rs17153527
Antinuclear Antibodies	PROTEASOME_PATHWAY	BIOCARTA	280	8	4	5,03E-01	1,00E+00	rs2719921|rs11845708|rs11922435|rs13207114
Antinuclear Antibodies	SHC_MEDIATED_CASCADE	REACTOME	1070	31	20	5,06E-01	1,00E+00	rs1924210|rs9736587|rs3129678|rs950549|rs6884797|rs9896052|rs3129599|rs249729|rs4751828|rs12592277|rs948992|rs8000805|rs9506777|rs2420932|rs4522197|rs1078363|rs152523|rs376618|rs17287322|rs4770259
Antinuclear Antibodies	CELLCYCLE_PATHWAY	BIOCARTA	459	26	15	5,06E-01	1,00E+00	rs12078447|rs11604821|rs12867405|rs4805487|rs1333050|rs12207548|rs7315599|rs9576040|rs624797|rs2237572|rs7781436|rs1872568|rs1465701|rs2932777|rs9462209
Antinuclear Antibodies	IGF1MTOR_PATHWAY	BIOCARTA	582	16	13	5,07E-01	1,00E+00	rs6059703|rs7168213|rs7181975|rs1550805|rs10509534|rs6449969|rs8038056|rs9838117|rs10788582|rs945559|rs12901358|rs334550|rs7935699
Antinuclear Antibodies	TNFR1_PATHWAY	BIOCARTA	862	41	20	5,07E-01	1,00E+00	rs10485101|rs2529737|rs1357287|rs1144171|rs11614624|rs1923075|rs12817202|rs10859598|rs4761528|rs3750512|rs13217057|rs10498974|rs2697920|rs6914621|rs9345117|rs721025|rs12191179|rs6681035|rs8178179|rs7745771
Antinuclear Antibodies	SYSTEMIC_LUPUS_ERYTHEMATOSUS	KEGG	2453	35	20	5,07E-01	1,00E+00	rs1806201|rs6698806|rs2284191|rs971075|rs1143664|rs9661996|rs3850890|rs2715267|rs4332745|rs2395300|rs2872715|rs2064478|rs1389623|rs3750512|rs2292727|rs7962629|rs9469357|rs8060068|rs706470|rs1074042
Antinuclear Antibodies	TRIF_MEDIATED_TLR3_SIGNALING	REACTOME	2075	27	20	5,07E-01	1,00E+00	rs3886478|rs2829946|rs3799663|rs10485101|rs2529737|rs466609|rs2005292|rs12199654|rs7148384|rs1144171|rs708030|rs6940350|rs1923075|rs3014866|rs4802906|rs7822830|rs331455|rs12216864|rs375369|rs10105118

Antinuclear Antibodies	GLUCOSE_METABOLISM	REACTOME	1554	33	20	5,09E-01	1,00E+00	rs9309906|rs10903939|rs11251480|rs7084464|rs4129219|rs13095735|rs4802906|rs8052416|rs2239167|rs7092772|rs10105118|rs883751|rs2068455|rs11918537|rs2516617|rs7074424|rs2055226|rs13245850|rs11251694|rs11251588
Antinuclear Antibodies	G_ALPHA_S_SIGNALLING_EVENTS	REACTOME	5019	37	20	5,12E-01	1,00E+00	rs571312|rs2267737|rs902522|rs13185639|rs1936600|rs10841528|rs16947151|rs7747433|rs202451|rs6885612|rs6807740|rs2115512|rs9974032|rs11682325|rs7579411|rs13190572|rs1404912|rs4243583|rs1253704|rs2327672
Antinuclear Antibodies	INTEGRATION_OF_PROVIRUS	REACTOME	122	7	4	5,14E-01	1,00E+00	rs1151514|rs828704|rs1805388|rs301282
Antinuclear Antibodies	BASAL_TRANSCRIPTION_FACTORS	KEGG	386	16	8	5,15E-01	1,00E+00	rs17397852|rs2140551|rs633742|rs6928843|rs6469842|rs399491|rs7524919|rs2978263
Antinuclear Antibodies	RECRUITMENT_OF_MITOTIC_CENTROSOME_PROTEINS_AND_COMPLEXES	REACTOME	1177	43	20	5,15E-01	1,00E+00	rs1333924|rs1990639|rs11933720|rs10984865|rs945246|rs741339|rs7295385|rs371164|rs1399960|rs4802906|rs3764694|rs870777|rs12517345|rs2111027|rs6505776|rs3889570|rs4836798|rs10860122|rs3813739|rs4865047
Antinuclear Antibodies	SIGNALLING_TO_RAS	REACTOME	468	22	11	5,16E-01	1,00E+00	rs6977082|rs12199654|rs9896052|rs1811208|rs944482|rs2392794|rs2519090|rs2798464|rs1125467|rs12136101|rs10496561
Antinuclear Antibodies	ARMS_MEDIATED_ACTIVATION	REACTOME	393	7	7	5,16E-01	1,00E+00	rs11102314|rs13008971|rs10901096|rs1546924|rs2798464|rs12136101|rs869382
Antinuclear Antibodies	LYSOSOME_VESICLE_BIOGENESIS	REACTOME	420	24	14	5,18E-01	1,00E+00	rs877711|rs3731828|rs951453|rs16933086|rs419588|rs14976|rs12376522|rs2278637|rs658573|rs913199|rs1254901|rs956323|rs5763140|rs1465701
Antinuclear Antibodies	PROGESTERONE_MEDIATED_OOCYTE_MATURATION	KEGG	2535	38	20	5,20E-01	1,00E+00	rs902522|rs11970312|rs7496727|rs10841528|rs1027692|rs9562187|rs6836883|rs1404912|rs17360897|rs10228945|rs2287778|rs11045301|rs7168213|rs12199654|rs953672|rs13155334|rs2714645|rs10514915|rs7703532|rs7181975
Antinuclear Antibodies	PI3K_CASCADE	REACTOME	2702	33	20	5,21E-01	1,00E+00	rs1881628|rs1924210|rs17762352|rs12540943|rs7999458|rs9736587|rs3129678|rs1397529|rs11876920|rs7258382|rs950549|rs6884797|rs9896052|rs3129599|rs203170|rs249729|rs4751828|rs12592277|rs948992|rs17254521
Antinuclear Antibodies	ECM_RECEPTOR_INTERACTION	KEGG	3602	33	20	5,22E-01	1,00E+00	rs12668754|rs17594552|rs17375044|rs2236891|rs12751561|rs709505|rs4536647|rs216872|rs1878510|rs9521666|rs4490127|rs362698|rs903352|rs4932543|rs2290501|rs1404772|rs353625|rs9288163|rs595325|rs362780
Antinuclear Antibodies	LEISHMANIA_INFECTION	KEGG	2265	29	20	5,23E-01	1,00E+00	rs6698806|rs6853|rs10485101|rs13029532|rs2284191|rs169143|rs2005292|rs11921898|rs608914|rs12199654|rs2395300|rs1144171|rs604045|rs3117230|rs198178|rs6940350|rs1389623|rs1498737|rs1923075|rs4483225
Antinuclear Antibodies	PROTEOLYTIC_CLEAVAGE_OF_SNARE_COMPLEX_PROTEINS	REACTOME	382	28	17	5,27E-01	1,00E+00	rs17702579|rs2695872|rs7220482|rs7137937|rs6026402|rs12369479|rs2206161|rs1983773|rs2278637|rs12952153|rs6503224|rs218454|rs362570|rs11087010|rs363012|rs10773810|rs6133821
Antinuclear Antibodies	CD28_CO_STIMULATION	REACTOME	797	46	18	5,28E-01	1,00E+00	rs836556|rs3850890|rs1403101|rs2715267|rs11153318|rs12413938|rs9896052|rs1550805|rs10494270|rs6449969|rs2267423|rs8102571|rs1599796|rs9838117|rs1544241|rs10424498|rs2617822|rs137966
Antinuclear Antibodies	ACETYLCHOLINE_BINDING_AND_DOWNSTREAM_EVENTS	REACTOME	242	17	8	5,35E-01	1,00E+00	rs12900519|rs11637923|rs12904458|rs7167632|rs2163177|rs10026108|rs1550097|rs3828246
Antinuclear Antibodies	SIGNALING_BY_ROBO_RECEPTOR	REACTOME	1900	44	20	5,36E-01	1,00E+00	rs1917072|rs10490696|rs1145157|rs11175219|rs1190976|rs7435615|rs836556|rs1403101|rs6672395|rs12639497|rs3911489|rs2028510|rs7577243|rs1531640|rs1344075|rs12485454|rs4578998|rs10938796|rs17819911|rs7650723
Antinuclear Antibodies	SNARE_INTERACTIONS_IN_VESICULAR_TRANSPORT	KEGG	828	46	20	5,36E-01	1,00E+00	rs3731828|rs17702579|rs2695872|rs7220482|rs7137937|rs6026402|rs12369479|rs1838105|rs2206161|rs1983773|rs14976|rs7903017|rs2278637|rs12952153|rs6503224|rs29784|rs218454|rs1254901|rs362570|rs11087010
Antinuclear Antibodies	CYTOSOLIC_DNA_SENSING_PATHWAY	KEGG	620	21	13	5,38E-01	1,00E+00	rs2227301|rs2069837|rs2210463|rs7148384|rs6081157|rs2415290|rs1330383|rs6081150|rs3848006|rs12289836|rs10872893|rs3138045|rs7267297
Antinuclear Antibodies	SHC1_EVENTS_IN_ERBB4_SIGNALING	REACTOME	1072	36	18	5,39E-01	1,00E+00	rs987320|rs3756675|rs4458837|rs4373483|rs9896052|rs2439312|rs6712652|rs7818821|rs2439281|rs6879217|rs4236723|rs7819063|rs1481762|rs7425448|rs4733263|rs10503913|rs1481758|rs2975498
Antinuclear Antibodies	ACTIVATED_TAK1_MEDIATES_P38_MAPK_ACTIVATION	REACTOME	649	51	20	5,40E-01	1,00E+00	rs10485101|rs12199654|rs1144171|rs708030|rs6940350|rs1923075|rs331455|rs12216864|rs13217057|rs10498974|rs739559|rs6914621|rs9345117|rs721025|rs311646|rs12191179|rs7841459|rs7745771|rs817565|rs7745444
Antinuclear Antibodies	DILATED_CARDIOMYOPATHY	KEGG	4636	30	20	5,42E-01	1,00E+00	rs2618681|rs902522|rs9810230|rs17053465|rs38553|rs12737847|rs10920115|rs11715062|rs10925415|rs13430329|rs12032218|rs10486963|rs10190488|rs12152349|rs1404912|rs10045068|rs2251788|rs17360897|rs2287778|rs947485
Antinuclear Antibodies	STARCH_AND_SUCROSE_METABOLISM	KEGG	2178	37	20	5,44E-01	1,00E+00	rs7552867|rs9309906|rs6448180|rs7619524|rs9493105|rs6661612|rs9493133|rs12057334|rs13095735|rs3193507|rs11918537|rs7617253|rs718137|rs6855439|rs1999480|rs7768480|rs11892031|rs9290264|rs4148280|rs6531592
Antinuclear Antibodies	MAP_KINASE_ACTIVATION_IN_TLR_CASCADE	REACTOME	1697	27	20	5,45E-01	1,00E+00	rs3886478|rs11970312|rs10485101|rs2529737|rs2005292|rs12199654|rs1144171|rs708030|rs6940350|rs1923075|rs4802906|rs7822830|rs331455|rs12216864|rs10105118|rs11787332|rs13217057|rs10498974|rs378373|rs3799584
Antinuclear Antibodies	TRAF6_MEDIATED_NFKB_ACTIVATION	REACTOME	351	25	15	5,46E-01	1,00E+00	rs2829946|rs466609|rs3014866|rs331455|rs375369|rs3750512|rs2415290|rs457581|rs1467719|rs12289836|rs10872893|rs2388548|rs3138045|rs7267297|rs17074691
Antinuclear Antibodies	GSK3_PATHWAY	BIOCARTA	1055	42	20	5,47E-01	1,00E+00	rs499205|rs6853|rs11604821|rs9883073|rs10228945|rs2714645|rs4483225|rs1550805|rs6449969|rs1053079|rs10513330|rs2523189|rs9838117|rs478309|rs624797|rs10856989|rs7764196|rs12289836|rs13232987|rs1609566
Antinuclear Antibodies	INOSITOL_PHOSPHATE_METABOLISM	KEGG	2355	31	20	5,51E-01	1,00E+00	rs928807|rs6481369|rs1967328|rs1407101|rs17593321|rs11922130|rs11876920|rs663591|rs2327162|rs4394764|rs2239923|rs10445097|rs2206422|rs7897517|rs203170|rs2475556|rs6834625|rs10485723|rs750997|rs708925
Antinuclear Antibodies	A_TETRASACCHARIDE_LINKER_SEQUENCE_IS_REQUIRED_FOR_GAG_SYNTHESIS	REACTOME	1731	36	20	5,51E-01	1,00E+00	rs1980779|rs11223958|rs12853685|rs7929855|rs2187467|rs2591454|rs1445253|rs2290501|rs1924384|rs937618|rs2637451|rs951781|rs7577243|rs6675798|rs1459433|rs7926027|rs7007840|rs4936233|rs2590509|rs4937880
Antinuclear Antibodies	PANTOTHENATE_AND_COA_BIOSYNTHESIS	KEGG	611	25	14	5,54E-01	1,00E+00	rs158898|rs17664707|rs9493105|rs9493133|rs2294757|rs9437663|rs12209262|rs738814|rs7768480|rs11047702|rs16928217|rs12044858|rs6569765|rs7267297
Antinuclear Antibodies	FATTY_ACYL_COA_BIOSYNTHESIS	REACTOME	404	19	10	5,55E-01	1,00E+00	rs2017571|rs13435303|rs209489|rs12498468|rs12499413|rs4502283|rs6838244|rs7680893|rs10498146|rs934150
Antinuclear Antibodies	ALPHA_LINOLENIC_ACID_METABOLISM	KEGG	571	41	14	5,60E-01	1,00E+00	rs230624|rs2305913|rs6683451|rs11583351|rs612355|rs16826789|rs631134|rs622450|rs2278964|rs7546956|rs4466603|rs776691|rs174576|rs10732980
Antinuclear Antibodies	SIGNALING_BY_FGFR1_FUSION_MUTANTS	REACTOME	639	39	19	5,61E-01	1,00E+00	rs10878951|rs6465836|rs13029532|rs9896052|rs1550805|rs10808119|rs2031282|rs6449969|rs11177527|rs12672026|rs9838117|rs1880345|rs7327207|rs11762037|rs5751636|rs1098528|rs3788358|rs404284|rs131702
Antinuclear Antibodies	PYRUVATE_METABOLISM	KEGG	908	39	20	5,66E-01	1,00E+00	rs2132302|rs2017571|rs1881717|rs11636141|rs12274651|rs2306787|rs2273684|rs12448432|rs10516988|rs4646772|rs594361|rs10973734|rs2430682|rs9640883|rs1781735|rs765567|rs2408700|rs498981|rs11789963|rs1946741
Antinuclear Antibodies	JNK_C_JUN_KINASES_PHOSPHORYLATION_AND_ACTIVATION_MEDIATED_BY_ACTIVATI	REACTOME	728	48	20	5,67E-01	1,00E+00	rs10485101|rs2529737|rs1144171|rs708030|rs6940350|rs1923075|rs331455|rs12216864|rs13217057|rs10498974|rs6914621|rs9345117|rs721025|rs311646|rs12191179|rs4403040|rs7841459|rs7745771|rs7745444|rs12259267
Antinuclear Antibodies	SIGNALLING_TO_ERKS	REACTOME	794	31	17	5,67E-01	1,00E+00	rs11102314|rs6977082|rs12199654|rs9896052|rs1811208|rs13008971|rs944482|rs2392794|rs10901096|rs1546924|rs2519090|rs2798464|rs515533|rs1125467|rs12136101|rs10496561|rs869382
Antinuclear Antibodies	MHC_CLASS_II_ANTIGEN_PRESENTATION	REACTOME	2226	25	20	5,70E-01	1,00E+00	rs994626|rs4301693|rs1579282|rs625061|rs1890981|rs951453|rs2284191|rs6869991|rs2395300|rs16933086|rs2064478|rs7142555|rs1554160|rs2292963|rs961548|rs9469357|rs12256435|rs1010651|rs1488540|rs2896489
Antinuclear Antibodies	OOCYTE_MEIOSIS	KEGG	3114	35	20	5,70E-01	1,00E+00	rs902522|rs3799663|rs7665102|rs7496727|rs17041308|rs1027692|rs17057833|rs9562187|rs6836883|rs1404912|rs6432018|rs17360897|rs4350299|rs7694484|rs2287778|rs11632057|rs3805018|rs953672|rs7303557|rs13155334
Antinuclear Antibodies	ERK_MAPK_TARGETS	REACTOME	704	27	19	5,72E-01	1,00E+00	rs3886478|rs3799663|rs12199654|rs4802906|rs7822830|rs10105118|rs11787332|rs378373|rs3799584|rs2071941|rs770082|rs3799613|rs16899429|rs909768|rs3799627|rs583759|rs707768|rs1116003|rs2341797
Antinuclear Antibodies	RECYCLING_PATHWAY_OF_L1	REACTOME	874	30	20	5,72E-01	1,00E+00	rs3799663|rs1579282|rs7829347|rs6869991|rs4903104|rs16933086|rs961548|rs378373|rs3799584|rs12376522|rs6580153|rs2071941|rs3799613|rs16899429|rs909768|rs3799627|rs956323|rs1784653|rs351291|rs583759
Antinuclear Antibodies	CCR3_PATHWAY	BIOCARTA	939	29	16	5,73E-01	1,00E+00	rs10800842|rs8081512|rs169143|rs2206422|rs198178|rs10485723|rs708925|rs3785396|rs10160860|rs2362941|rs2745755|rs6055995|rs8123677|rs2855726|rs6056252|rs9925433
Antinuclear Antibodies	SHH_PATHWAY	BIOCARTA	679	24	16	5,73E-01	1,00E+00	rs12155027|rs1030098|rs7805291|rs2298330|rs6501512|rs2302784|rs883751|rs12667722|rs7860361|rs13052269|rs4849909|rs17172040|rs28536742|rs12609590|rs6750309|rs334550
Antinuclear Antibodies	B_CELL_RECEPTOR_SIGNALING_PATHWAY	KEGG	2278	33	20	5,74E-01	1,00E+00	rs11767716|rs4436949|rs7905645|rs12571935|rs656709|rs4603757|rs3824561|rs169143|rs836556|rs2005292|rs7694484|rs2510257|rs10445097|rs7148384|rs9896052|rs198178|rs10821495|rs2182913|rs1550805|rs2239167
Antinuclear Antibodies	YAP1_AND_WWTR1_TAZ_STIMULATED_GENE_EXPRESSION	REACTOME	1047	30	20	5,74E-01	1,00E+00	rs10501995|rs722420|rs3789823|rs6440618|rs12562819|rs12526196|rs17787978|rs450110|rs6803863|rs9493149|rs9968207|rs1999562|rs6018631|rs6120708|rs4681521|rs2164349|rs7110355|rs2235734|rs4857747|rs4857702
Antinuclear Antibodies	REGULATION_OF_AUTOPHAGY	KEGG	933	23	12	5,75E-01	1,00E+00	rs1215|rs2050249|rs11876920|rs203170|rs1001684|rs26538|rs307118|rs4243272|rs3014996|rs1374642|rs11208061|rs1465701
Antinuclear Antibodies	PIP3_ACTIVATES_AKT_SIGNALING	REACTOME	587	26	14	5,76E-01	1,00E+00	rs12958400|rs9549096|rs9576967|rs7329423|rs10494270|rs10509534|rs12207548|rs665821|rs9480865|rs10788582|rs945559|rs9930956|rs541425|rs9462209
Antinuclear Antibodies	BRANCHED_CHAIN_AMINO_ACID_CATABOLISM	REACTOME	507	21	7	5,77E-01	1,00E+00	rs534800|rs9361660|rs11047702|rs16928217|rs947223|rs9350868|rs4604577
Antinuclear Antibodies	ASTHMA	KEGG	770	26	9	5,78E-01	1,00E+00	rs2284191|rs2395300|rs3117230|rs9469357|rs2276039|rs10792095|rs3097646|rs441576|rs35265698
Antinuclear Antibodies	CCR5_PATHWAY	BIOCARTA	696	28	15	5,78E-01	1,00E+00	rs8081512|rs169143|rs2005292|rs12199654|rs198178|rs7371043|rs11813145|rs2055226|rs2163177|rs3785396|rs10191360|rs6681035|rs17488134|rs12259267|rs9925433
Antinuclear Antibodies	OXYGEN_DEPENDENT_PROLINE_HYDROXYLATION_OF_HYPOXIA_INDUCIBLE_FACTOR_	REACTOME	313	14	11	5,79E-01	1,00E+00	rs11010056|rs7542797|rs708030|rs2891268|rs1000531|rs1053079|rs10198379|rs11629133|rs1680694|rs4982138|rs17496856
Antinuclear Antibodies	SIGNALING_BY_TGF_BETA_RECEPTOR_COMPLEX	REACTOME	1605	24	20	5,82E-01	1,00E+00	rs9886419|rs7812998|rs722420|rs1561928|rs12867405|rs17231134|rs7653262|rs6440618|rs17010833|rs17787978|rs1426511|rs6025738|rs6803863|rs85440|rs478061|rs2229840|rs4671408|rs11009607|rs727929|rs1333050
Antinuclear Antibodies	PROLONGED_ERK_ACTIVATION_EVENTS	REACTOME	429	11	8	5,83E-01	1,00E+00	rs11102314|rs13008971|rs10901096|rs1546924|rs2798464|rs515533|rs12136101|rs869382
Antinuclear Antibodies	AMINO_SUGAR_AND_NUCLEOTIDE_SUGAR_METABOLISM	KEGG	956	35	20	5,83E-01	1,00E+00	rs9391926|rs10738958|rs12203614|rs10814369|rs2078024|rs7768483|rs10019381|rs1476295|rs10857871|rs11242726|rs6855439|rs4852246|rs871443|rs7442192|rs16962243|rs137132|rs6531592|rs6115227|rs11124097|rs820857
Antinuclear Antibodies	PORPHYRIN_AND_CHLOROPHYLL_METABOLISM	KEGG	1163	25	17	5,85E-01	1,00E+00	rs11230882|rs7216331|rs7857932|rs16861582|rs11078220|rs10401042|rs12123041|rs11892031|rs1558120|rs16948995|rs4148280|rs4895324|rs12188286|rs1800007|rs7214862|rs10521258|rs268691
Antinuclear Antibodies	CYTOKINE_PATHWAY	BIOCARTA	491	12	8	5,86E-01	1,00E+00	rs2069837|rs11921898|rs1498737|rs7668305|rs3014996|rs7610160|rs7739687|rs918519
Antinuclear Antibodies	ANTIGEN_PROCESSING_AND_PRESENTATION	KEGG	1754	52	20	5,86E-01	1,00E+00	rs10772294|rs3782735|rs2284191|rs2395300|rs10507441|rs3117230|rs1611493|rs9469357|rs1010651|rs4616886|rs4841600|401754|rs3097646|rs2246010|rs441576|rs35265698|rs3741920|rs2523554|rs4072864|rs12310310
Antinuclear Antibodies	N_GLYCAN_ANTENNAE_ELONGATION_IN_THE_MEDIAL_TRANS_GOLGI	REACTOME	962	19	15	5,88E-01	1,00E+00	rs11152012|rs6095638|rs2279447|rs13035520|rs1455777|rs1490776|rs12424060|rs359327|rs4141587|rs1352323|rs10508527|rs999925|rs3784732|rs6707591|rs7607690
Antinuclear Antibodies	POST_NMDA_RECEPTOR_ACTIVATION_EVENTS	REACTOME	1753	39	20	5,91E-01	1,00E+00	rs1806201|rs11970312|rs7665102|rs9898774|rs306090|rs1404912|rs4332745|rs2872715|rs8024521|rs8060068|rs2055226|rs873592|rs1074042|rs378373|rs3799584|rs12934132|rs2471226|rs10744029|rs729650|rs2071941
Antinuclear Antibodies	AMINO_ACID_TRANSPORT_ACROSS_THE_PLASMA_MEMBRANE	REACTOME	1025	32	20	5,91E-01	1,00E+00	rs7657271|rs4685165|rs4640842|rs4602539|rs11670126|rs488192|rs7727745|rs4296412|rs748680|rs7657780|rs720625|rs10434201|rs910795|rs12891079|rs11919568|rs6850624|rs2056275|rs9861586|rs999969|rs12879346
Antinuclear Antibodies	TYPE_I_DIABETES_MELLITUS	KEGG	1975	38	20	5,92E-01	1,00E+00	rs12765241|rs2284191|rs4439928|rs3807811|rs3850890|rs11921898|rs2715267|rs2395300|rs4691213|rs10017098|rs2064478|rs1498737|rs2110333|rs1611493|rs934818|rs9469357|rs1242775|rs3097646|rs1599796|rs2008341
Antinuclear Antibodies	AXON_GUIDANCE	KEGG	9506	34	20	5,93E-01	1,00E+00	rs4791330|rs12579612|rs12109609|rs11745726|rs1917072|rs281215|rs10490696|rs999426|rs13200697|rs10502934|rs10423119|rs4868489|rs13383577|rs10464035|rs10866630|rs11922528|rs1145157|rs11175219|rs6702300|rs9645353
Antinuclear Antibodies	NFKB_AND_MAP_KINASES_ACTIVATION_MEDIATED_BY_TLR4_SIGNALING_REPERTOIRE	REACTOME	2248	28	20	5,94E-01	1,00E+00	rs3886478|rs2829946|rs11970312|rs10485101|rs2529737|rs466609|rs2005292|rs12199654|rs1144171|rs708030|rs6940350|rs1923075|rs3014866|rs4483225|rs4802906|rs7822830|rs331455|rs12216864|rs375369|rs10105118
Antinuclear Antibodies	SYNTHESIS_OF_PA	REACTOME	1057	45	20	5,95E-01	1,00E+00	rs230624|rs17009821|rs9458157|rs6683451|rs11922502|rs11583351|rs3764899|rs612355|rs11195843|rs1880540|rs16826789|rs13098743|rs631134|rs11195662|rs622450|rs829608|rs9825310|rs4819329|rs7546956|rs2803594
Antinuclear Antibodies	LONG_TERM_POTENTIATION	KEGG	3620	41	20	5,95E-01	1,00E+00	rs1806201|rs3799663|rs1422893|rs7665102|rs11102314|rs17041308|rs306090|rs1407101|rs8081512|rs1404912|rs169143|rs7730730|rs2327162|rs7694484|rs4332745|rs3805018|rs2206422|rs10030915|rs2872715|rs7303557
Antinuclear Antibodies	ALLOGRAFT_REJECTION	KEGG	1510	41	20	5,95E-01	1,00E+00	rs12765241|rs2284191|rs3850890|rs11921898|rs2715267|rs2395300|rs2064478|rs1498737|rs1611493|rs9469357|rs3097646|rs1599796|rs3014996|rs4934433|rs2246010|rs4747064|rs7610160|rs441576|rs35265698|rs2523554
Antinuclear Antibodies	ACTIVATION_OF_KAINATE_RECEPTORS_UPON_GLUTAMATE_BINDING	REACTOME	1577	26	20	5,99E-01	1,00E+00	rs16947151|rs6807740|rs4243583|rs2211779|rs1253704|rs12075532|rs11715227|rs2206422|rs12147761|rs6972935|rs10485723|rs10892616|rs4807284|rs4363614|rs708925|rs645156|rs17124529|rs2510877|rs9485557|rs4291239
Antinuclear Antibodies	ACYL_CHAIN_REMODELLING_OF_PG	REACTOME	571	34	12	6,00E-01	1,00E+00	rs230624|rs6683451|rs11583351|rs612355|rs16826789|rs631134|rs622450|rs7546956|rs4466603|rs1712429|rs776691|rs10732980
Antinuclear Antibodies	NUCLEAR_EVENTS_KINASE_AND_TRANSCRIPTION_FACTOR_ACTIVATION	REACTOME	766	27	19	6,03E-01	1,00E+00	rs3886478|rs11970312|rs12199654|rs4802906|rs7822830|rs10105118|rs11787332|rs378373|rs3799584|rs2071941|rs770082|rs3799613|rs16899429|rs909768|rs3799627|rs583759|rs707768|rs1116003|rs2341797
Antinuclear Antibodies	NON_SMALL_CELL_LUNG_CANCER	KEGG	2585	38	20	6,03E-01	1,00E+00	rs2056777|rs9877140|rs9465734|rs11604821|rs8081512|rs169143|rs2365491|rs213213|rs10445097|rs17668326|rs9896052|rs7373003|rs1286762|rs198178|rs12493462|rs6787664|rs6776486|rs1550805|rs213392|rs1286665
Antinuclear Antibodies	GAP_JUNCTION_ASSEMBLY	REACTOME	330	14	11	6,05E-01	1,00E+00	rs11576760|rs1886176|rs1336625|rs7321901|rs9509119|rs7764196|rs8064669|rs9450659|rs7213960|rs1978809|rs7319745
Antinuclear Antibodies	TOLL_LIKE_RECEPTOR_SIGNALING_PATHWAY	KEGG	2339	30	20	6,06E-01	1,00E+00	rs499205|rs6853|rs10485101|rs2529737|rs2069837|rs13029532|rs836556|rs2005292|rs3850890|rs11921898|rs2715267|rs12199654|rs12413938|rs1144171|rs6940350|rs1498737|rs1923075|rs4483225|rs4693923|rs1550805
Antinuclear Antibodies	SPPA_PATHWAY	BIOCARTA	1428	43	20	6,07E-01	1,00E+00	rs230624|rs8081512|rs169143|rs10228945|rs2047076|rs6683451|rs2206422|rs11583351|rs41717|rs198178|rs10485723|rs2714645|rs612355|rs10821495|rs708925|rs16826789|rs2523189|rs6861460|rs2991216|rs3785396
Antinuclear Antibodies	PPAR_SIGNALING_PATHWAY	KEGG	1324	51	20	6,07E-01	1,00E+00	rs955760|rs6473282|rs6853|rs7895441|rs1110060|rs2305913|rs213213|rs4975057|rs17456435|rs11130651|rs943347|rs12609870|rs2046446|rs3818540|rs7620635|rs10954289|rs4810476|rs7859229|rs10498146|rs7625947
Antinuclear Antibodies	DAG_AND_IP3_SIGNALING	REACTOME	1644	36	20	6,08E-01	1,00E+00	rs902522|rs306090|rs8081512|rs1030098|rs1404912|rs17360897|rs2287778|rs6501512|rs2302784|rs953672|rs7303557|rs13155334|rs718314|rs7703532|rs883751|rs17649232|rs2055226|rs2595199|rs10842720|rs4952809
Antinuclear Antibodies	FRS2_MEDIATED_CASCADE	REACTOME	1192	31	20	6,09E-01	1,00E+00	rs1924210|rs9736587|rs3129678|rs950549|rs6884797|rs9896052|rs3129599|rs249729|rs4751828|rs12592277|rs948992|rs8000805|rs9506777|rs2420932|rs4522197|rs1078363|rs152523|rs376618|rs17287322|rs4770259
Antinuclear Antibodies	ONE_CARBON_POOL_BY_FOLATE	KEGG	441	8	6	6,10E-01	1,00E+00	rs1404772|rs4673965|rs8011839|rs745686|rs942382|rs4819211
Antinuclear Antibodies	REGULATION_OF_HYPOXIA_INDUCIBLE_FACTOR_HIF_BY_OXYGEN	REACTOME	443	14	11	6,13E-01	1,00E+00	rs11010056|rs7542797|rs708030|rs2891268|rs1000531|rs1053079|rs10198379|rs11629133|rs1680694|rs4982138|rs17496856
Antinuclear Antibodies	TRANSPORT_OF_GLUCOSE_AND_OTHER_SUGARS_BILE_SALTS_AND_ORGANIC_ACIDS_M<br>NE_COMPOUNDS	REACTOME	3234	53	20	6,17E-01	1,00E+00	rs11174735|rs2276433|rs12125560|rs4685165|rs2000466|rs11776475|rs6673947|rs10088489|rs492315|rs2073211|rs4640842|rs274088|rs213213|rs2070460|rs16842|rs12349416|rs4239020|rs488192|rs16861582|rs7727745
Antinuclear Antibodies	ETHER_LIPID_METABOLISM	KEGG	1041	53	20	6,20E-01	1,00E+00	rs17139|rs230624|rs12081589|rs6683451|rs11583351|rs3764899|rs612355|rs16826789|rs10493211|rs13098743|rs631134|rs622450|rs10041669|rs2278964|rs7546956|rs747820|rs6552765|rs4466603|rs776691|rs10732980
Antinuclear Antibodies	UBIQUITIN_MEDIATED_PROTEOLYSIS	KEGG	3227	36	20	6,21E-01	1,00E+00	rs875785|rs10072359|rs9347562|rs447921|rs10490113|rs11010056|rs13195756|rs9562187|rs6836883|rs4984803|rs708030|rs4940693|rs11706098|rs2719921|rs10514915|rs9355359|rs2047597|rs6675798|rs331455|rs930567
Antinuclear Antibodies	ALDOSTERONE_REGULATED_SODIUM_REABSORPTION	KEGG	1794	36	20	6,22E-01	1,00E+00	rs2847381|rs7999458|rs9493844|rs8081512|rs1200138|rs7688347|rs169143|rs6837298|rs7258382|rs10919062|rs4940693|rs198178|rs17484678|rs17254521|rs2200984|rs4675077|rs1550805|rs2001303|rs6449969|rs2251692
Antinuclear Antibodies	DESTABILIZATION_OF_MRNA_BY_KSRP	REACTOME	205	9	6	6,22E-01	1,00E+00	rs1000174|rs12199654|rs4734512|rs4734497|rs4782165|rs1533079
Antinuclear Antibodies	INTEGRIN_CELL_SURFACE_INTERACTIONS	REACTOME	2614	32	20	6,22E-01	1,00E+00	rs12668754|rs8133991|rs11102314|rs3820852|rs4536647|rs216872|rs9521666|rs903352|rs2038171|rs3769309|rs682386|rs1404772|rs9896052|rs595325|rs9388700|rs1537741|rs4693923|rs10821495|rs13137121|rs10510697
Antinuclear Antibodies	VIRAL_MYOCARDITIS	KEGG	2750	36	20	6,24E-01	1,00E+00	rs17053465|rs7630352|rs11604821|rs10045068|rs2269529|rs2284191|rs836556|rs3850890|rs6672395|rs201603|rs2715267|rs8060975|rs11153318|rs2395300|rs2824206|rs9388700|rs3117230|rs2824065|rs4434365|rs2273684
Antinuclear Antibodies	PREFOLDIN_MEDIATED_TRANSFER_OF_SUBSTRATE_TO_CCT_TRIC	REACTOME	288	10	6	6,28E-01	1,00E+00	rs292117|rs371164|rs3892375|rs710764|rs3844450|rs6865121
Antinuclear Antibodies	DOWNSTREAM_SIGNAL_TRANSDUCTION	REACTOME	3083	34	20	6,30E-01	1,00E+00	rs11745726|rs902522|rs10490696|rs12958400|rs306090|rs9549096|rs8081512|rs1030098|rs1404912|rs13029532|rs17360897|rs2287778|rs9576967|rs6501512|rs2302784|rs953672|rs9896052|rs7303557|rs13155334|rs718314
Antinuclear Antibodies	PI_3K_CASCADE	REACTOME	1868	35	20	6,30E-01	1,00E+00	rs1924210|rs12958400|rs9736587|rs3129678|rs9549096|rs1397529|rs9576967|rs950549|rs6884797|rs9896052|rs3129599|rs249729|rs4751828|rs12592277|rs7329423|rs948992|rs1550805|rs8000805|rs10494270|rs10509534
Antinuclear Antibodies	METABOLISM_OF_NUCLEOTIDES	REACTOME	1559	34	20	6,31E-01	1,00E+00	rs2815307|rs2978663|rs4789145|rs819864|rs2387094|rs595890|rs1404772|rs371927|rs653241|rs9437663|rs4243969|rs4673965|rs913987|rs951409|rs738814|rs4135192|rs10040697|rs4543167|rs2776|rs1001179
Antinuclear Antibodies	TGFB_PATHWAY	BIOCARTA	817	40	20	6,32E-01	1,00E+00	rs10485101|rs7653262|rs1144171|rs604045|rs1923075|rs478061|rs10863388|rs7526672|rs13217057|rs10498974|rs6914621|rs9345117|rs721025|rs12191179|rs4939659|rs372334|rs12901499|rs12915039|rs7745771|rs8179181
Antinuclear Antibodies	APOPTOTIC_EXECUTION_PHASE	REACTOME	1211	38	20	6,34E-01	1,00E+00	rs1543805|rs1572968|rs9883073|rs1357287|rs10140923|rs17576470|rs336629|rs9959147|rs11845708|rs4357599|rs7904311|rs17201246|rs8006767|rs4915501|rs17398435|rs998733|rs1467719|rs3810006|rs2757632|rs4353229
Antinuclear Antibodies	CYTOKINE_CYTOKINE_RECEPTOR_INTERACTION	KEGG	6425	53	20	6,34E-01	1,00E+00	rs12619383|rs12765241|rs10777125|rs2974254|rs7830593|rs7572399|rs4262039|rs3805558|rs9307949|rs2154948|rs1227631|rs2069837|rs13253242|rs10892201|rs12203803|rs13044457|rs4851547|rs12466337|rs4662532|rs11921898
Antinuclear Antibodies	APOPTOTIC_CLEAVAGE_OF_CELLULAR_PROTEINS	REACTOME	1052	37	20	6,38E-01	1,00E+00	rs1543805|rs1572968|rs9883073|rs10140923|rs17576470|rs336629|rs9959147|rs11845708|rs4357599|rs7904311|rs8006767|rs4915501|rs998733|rs17398435|rs3810006|rs2757632|rs4353229|rs1556003|rs785422|rs2031221
Antinuclear Antibodies	PENTOSE_AND_GLUCURONATE_INTERCONVERSIONS	KEGG	874	11	6	6,39E-01	1,00E+00	rs6490551|rs9640883|rs11892031|rs4148280|rs169044|rs9550639

Antinuclear Antibodies	INSULIN_RECEPTOR_SIGNALLING_CASCADE	REACTOME	3006	33	20	6,41E-01	1,00E+00	rs1881628|rs1924210|rs17762352|rs12540943|rs7999458|rs9736587|rs3129678|rs1397529|rs11876920|rs7258382|rs950549|rs6884797|rs9896052|rs3129599|rs203170|rs249729|rs4751828|rs12592277|rs948992|rs17254521
Antinuclear Antibodies	SIGNALING_BY_SCF_KIT	REACTOME	1989	45	20	6,41E-01	1,00E+00	rs10777125|rs12958400|rs2106673|rs9549096|rs8081512|rs13029532|rs836556|rs11153318|rs9576967|rs9896052|rs7329423|rs8085725|rs10858795|rs1550805|rs2239167|rs17564471|rs2715129|rs10494270|rs10509534|rs3933239
Antinuclear Antibodies	AXON_GUIDANCE	REACTOME	13131	32	20	6,42E-01	1,00E+00	rs4791330|rs12286714|rs12668754|rs12579612|rs2469515|rs1917072|rs9327283|rs3799663|rs281215|rs10490696|rs2938818|rs10520737|rs1579282|rs10502934|rs12357398|rs4868489|rs10920115|rs4948420|rs4448606|rs10866630
Antinuclear Antibodies	PI3K_EVENTS_IN_ERBB2_SIGNALING	REACTOME	1886	37	20	6,45E-01	1,00E+00	rs987320|rs12958400|rs9549096|rs1397529|rs3756675|rs4458837|rs9576967|rs4373483|rs9896052|rs2439312|rs6712652|rs7329423|rs7818821|rs1550805|rs10494270|rs10509534|rs2439281|rs6449969|rs6879217|rs4236723
Antinuclear Antibodies	TCR_PATHWAY	BIOCARTA	1517	46	20	6,45E-01	1,00E+00	rs11745726|rs8081512|rs2529737|rs169143|rs836556|rs2005292|rs7694484|rs11153318|rs7148384|rs9896052|rs198178|rs1550805|rs4699014|rs6449969|rs2055226|rs2415290|rs12737056|rs7804859|rs8102571|rs761685
Antinuclear Antibodies	PYRIMIDINE_METABOLISM	REACTOME	792	17	10	6,46E-01	1,00E+00	rs4789145|rs595890|rs653241|rs9437663|rs738814|rs4543167|rs16853627|rs12044858|rs3887873|rs7524799
Antinuclear Antibodies	CTNNB1_PHOSPHORYLATION_CASCADE	REACTOME	316	12	6	6,46E-01	1,00E+00	rs9883073|rs4802906|rs10105118|rs11160664|rs1116003|rs7712431
Antinuclear Antibodies	IL22BP_PATHWAY	BIOCARTA	296	22	10	6,47E-01	1,00E+00	rs10892201|rs13029532|rs310244|rs2274471|rs3934861|rs2598483|rs16833215|rs652107|rs16833239|rs276567
Antinuclear Antibodies	APC_C_CDH1_MEDIATED_DEGRADATION_OF_CDC20_AND_OTHER_APC_C_CDH1_TARGE TE_MITOSIS_EARLY_G1	REACTOME	959	38	20	6,47E-01	1,00E+00	rs17057833|rs9562187|rs6836883|rs10514915|rs11845708|rs7187327|rs12447239|rs3027247|rs698856|rs1106950|rs425594|rs11922435|401754|rs2431097|rs13207114|rs11734845|rs6032895|rs11679897|rs8531|rs2107459
Antinuclear Antibodies	SIGNALING_BY_NODAL	REACTOME	632	28	15	6,48E-01	1,00E+00	rs7168655|rs12466337|rs4662532|rs10961689|rs6729501|rs1562253|rs7566998|rs4939659|rs372334|rs12901499|rs12915039|rs4965897|rs12476865|rs9480865|rs16827454
Antinuclear Antibodies	VASCULAR_SMOOTH_MUSCLE_CONTRACTION	KEGG	5845	34	20	6,49E-01	1,00E+00	rs12765241|rs10800842|rs902522|rs297672|rs9817294|rs6994883|rs230624|rs1972058|rs10920115|rs17041308|rs7069974|rs1407101|rs10740411|rs8081512|rs1404912|rs10485512|rs169143|rs17360897|rs2327162|rs2670128
Antinuclear Antibodies	NITRIC_OXIDE_STIMULATES_GUANYLATE_CYCLASE	REACTOME	1895	47	20	6,50E-01	1,00E+00	rs11729954|rs9817294|rs1972058|rs10841528|rs7069974|rs10740411|rs1358786|rs11045301|rs10762562|rs12364403|rs7907343|rs1194491|rs17649232|rs3806777|rs6534152|rs211085|rs314140|rs6922908|rs9838117|rs1432515
Antinuclear Antibodies	PURINE_METABOLISM	REACTOME	581	24	17	6,51E-01	1,00E+00	rs4789145|rs819864|rs595890|rs1404772|rs371927|rs653241|rs4673965|rs951409|rs1001179|rs13418515|rs16885|rs4832609|rs10840415|rs7100550|rs3006009|rs2871427|rs13379232
Antinuclear Antibodies	GRB2_EVENTS_IN_ERBB2_SIGNALING	REACTOME	1202	36	18	6,52E-01	1,00E+00	rs987320|rs3756675|rs4458837|rs4373483|rs9896052|rs2439312|rs6712652|rs7818821|rs2439281|rs6879217|rs4236723|rs7819063|rs1481762|rs7425448|rs4733263|rs10503913|rs1481758|rs2919384
Antinuclear Antibodies	TYROSINE_METABOLISM	KEGG	1169	45	20	6,52E-01	1,00E+00	rs4788547|rs2000466|rs2070460|rs8042782|rs5760102|rs4672866|rs2290536|rs10738258|rs277884|rs1614972|rs1834058|rs12238357|rs12802641|rs9678469|rs11789947|rs3793932|rs1126673|rs1826909|rs10830228|rs13338289
Antinuclear Antibodies	KERATAN_SULFATE_BIOSYNTHESIS	REACTOME	674	24	13	6,52E-01	1,00E+00	rs6095638|rs3743398|rs13264573|rs10800925|rs1868435|rs6988999|rs1424013|rs2945743|rs4141587|rs10110481|rs2084512|rs12870|rs4755327
Antinuclear Antibodies	ION_TRANSPORT_BY_P_TYPE_ATPASES	REACTOME	1690	26	20	6,52E-01	1,00E+00	rs7358822|rs4921297|rs422598|rs486535|rs1200138|rs13316275|rs35673516|rs9915261|rs10919062|rs416330|rs9511405|rs10490817|rs4432122|rs17416101|rs10459725|rs1933199|rs4788102|rs2414009|rs6810046|rs11265346
Antinuclear Antibodies	MAPK_TARGETS_NUCLEAR_EVENTS_MEDIATED_BY_MAP_KINASES	REACTOME	980	29	20	6,55E-01	1,00E+00	rs3886478|rs3799663|rs2005292|rs12199654|rs4802906|rs7822830|rs10105118|rs11787332|rs378373|rs3799584|rs2071941|rs770082|rs6681035|rs4403040|rs3799613|rs16899429|rs909768|rs3799627|rs583759|rs12259267
Antinuclear Antibodies	GH_PATHWAY	BIOCARTA	963	32	20	6,56E-01	1,00E+00	rs8081512|rs169143|rs7258382|rs9896052|rs198178|rs17254521|rs4675077|rs1550805|rs2239167|rs2001303|rs6449969|rs2251692|rs7804859|rs2274471|rs2894595|rs13392647|rs9838117|rs3785396|rs17153527|rs10941583
Antinuclear Antibodies	SRP_DEPENDENT_COTRANSLATIONAL_PROTEIN_TARGETING_TO_MEMBRANE	REACTOME	1378	45	20	6,63E-01	1,00E+00	rs499205|rs7902136|rs7176919|rs12602594|rs4743335|rs1953910|rs4743347|rs17121120|rs10065570|rs11002569|rs4777170|rs6895379|rs11733964|rs7805129|rs478309|rs2301723|rs13325754|rs11002552|rs1953912|rs1956099
Antinuclear Antibodies	GLUTATHIONE_CONJUGATION	REACTOME	407	20	15	6,65E-01	1,00E+00	rs9296691|rs316156|rs10456664|rs13124794|rs2273684|rs7758711|rs17355469|rs2207951|rs5751902|rs809942|rs396216|rs8192013|rs3822045|rs17817077|rs17089368
Antinuclear Antibodies	CHONDROITIN_SULFATE_DERMATAN_SULFATE_METABOLISM	REACTOME	2784	29	20	6,65E-01	1,00E+00	rs1980779|rs11223958|rs4535751|rs10861272|rs12853685|rs7929855|rs2187467|rs3789823|rs2591454|rs1445253|rs17079451|rs2290501|rs2979874|rs1924384|rs2172350|rs937618|rs2637451|rs951781|rs7577243|rs10934779
Antinuclear Antibodies	PI3K_EVENTS_IN_ERBB4_SIGNALING	REACTOME	1709	35	20	6,68E-01	1,00E+00	rs987320|rs12958400|rs9549096|rs3756675|rs4458837|rs9576967|rs4373483|rs2439312|rs6712652|rs7329423|rs7818821|rs1550805|rs10494270|rs10509534|rs2439281|rs6449969|rs6879217|rs4236723|rs12207548|rs9838117
Antinuclear Antibodies	NKT_PATHWAY	BIOCARTA	638	24	19	6,70E-01	1,00E+00	rs3782735|rs11921898|rs608914|rs604045|rs1498737|rs10863388|rs7526672|rs7371043|rs1921622|rs3014996|rs10191360|rs7610160|rs3761041|rs393540|rs8179181|rs13431828|rs1603059|rs3741920|rs918519
Antinuclear Antibodies	JAK_STAT_SIGNALING_PATHWAY	KEGG	4346	33	20	6,70E-01	1,00E+00	rs253447|rs9886419|rs7812998|rs2043111|rs1114275|rs1561928|rs11604821|rs7597061|rs1227631|rs2631661|rs2069837|rs13253242|rs10892201|rs13029532|rs12203803|rs6563216|rs11921898|rs608914|rs9545290|rs6419221
Antinuclear Antibodies	INTERACTION_BETWEEN_L1_AND_ANKYRINS	REACTOME	970	53	20	6,71E-01	1,00E+00	rs2469515|rs4948420|rs12593435|rs13255835|rs12355908|rs12461158|rs11997827|rs170679|rs10930162|rs1904398|rs2469520|rs6989203|rs2469643|rs9299440|rs2058194|rs3785568|rs2613843|rs4968681|rs6573568|rs1437824
Antinuclear Antibodies	G1_S_SPECIFIC_TRANSCRIPTION	REACTOME	194	9	5	6,71E-01	1,00E+00	rs12867405|rs4805487|rs9576040|rs3087474|rs2871029
Antinuclear Antibodies	CHRONIC_MYELOID_LEUKEMIA	KEGG	2538	37	20	6,73E-01	1,00E+00	rs2938004|rs9886419|rs9465734|rs7812998|rs1561928|rs11604821|rs13049777|rs7653262|rs884421|rs3863105|rs9896052|rs2834652|rs1426511|rs604045|rs11706098|rs8031813|rs352066|rs478061|rs10863388|rs1550805
Antinuclear Antibodies	ACYL_CHAIN_REMODELLING_OF_PC	REACTOME	693	36	14	6,76E-01	1,00E+00	rs230624|rs6683451|rs11583351|rs612355|rs13008971|rs16826789|rs631134|rs622450|rs2386983|rs7546956|rs4466603|rs1712429|rs776691|rs10732980
Antinuclear Antibodies	ANTIGEN_PROCESSING_UBIQUITINATION_PROTEASOME_DEGRADATION	REACTOME	4788	39	20	6,77E-01	1,00E+00	rs875785|rs6020238|rs2608578|rs12145857|rs9347562|rs11253250|rs447921|rs11010056|rs13195756|rs9562187|rs6836883|rs12466600|rs10493118|rs567962|rs3088095|rs12147541|rs708030|rs11706098|rs2719921|rs10514915
Antinuclear Antibodies	DARPP_32_EVENTS	REACTOME	879	35	20	6,79E-01	1,00E+00	rs1030098|rs7694484|rs6501512|rs2302784|rs10514862|rs35305|rs4802906|rs13176940|rs10105118|rs883751|rs4699014|rs2303924|rs2055226|rs7522367|rs12060491|rs7517992|rs153968|rs10939840|rs10421422|rs1116003
Antinuclear Antibodies	CREB_PHOSPHORYLATION_THROUGH_THE_ACTIVATION_OF_RAS	REACTOME	1349	42	20	6,80E-01	1,00E+00	rs1806201|rs11970312|rs7665102|rs4332745|rs2872715|rs8024521|rs8060068|rs2055226|rs873592|rs1074042|rs378373|rs3799584|rs12934132|rs10744029|rs729650|rs2071941|rs4240286|rs1554668|rs3799613|rs16899429
Antinuclear Antibodies	GASTRIN_CREB_SIGNALLING_PATHWAY_VIA_PKC_AND_MAPK	REACTOME	8122	30	20	6,80E-01	1,00E+00	rs12286714|rs3886478|rs2829946|rs1373928|rs297672|rs11970312|rs1072319|rs1578303|rs6994883|rs17696124|rs6429140|rs16947151|rs4289301|rs6807740|rs1407101|rs11209971|rs8081512|rs11235714|rs12119540|rs4243583
Antinuclear Antibodies	LAIR_PATHWAY	BIOCARTA	479	21	13	6,80E-01	1,00E+00	rs2069837|rs971075|rs3753306|rs1389623|rs1450665|rs13156109|rs10473234|rs3176874|rs4607021|rs9287095|rs170962|rs12146170|rs12755775
Antinuclear Antibodies	STEROID_HORMONES	REACTOME	693	40	19	6,81E-01	1,00E+00	rs1990842|rs12591359|rs7682870|rs831022|rs4233828|rs1010651|rs12441382|rs34562262|rs9640883|rs4844486|rs2300697|rs2271461|rs17270248|rs2271463|rs4616447|rs17703982|rs12555788|rs12586718|rs166050
Antinuclear Antibodies	RAS_PATHWAY	BIOCARTA	557	20	13	6,81E-01	1,00E+00	rs6977082|rs836556|rs1811208|rs1550805|rs6449969|rs13098743|rs7804859|rs2519090|rs9838117|rs17153527|rs12289836|rs4730197|rs17153654
Antinuclear Antibodies	RNA_POLYMERASE	KEGG	351	32	9	6,84E-01	1,00E+00	rs2227301|rs6081157|rs1887455|rs633742|rs604965|rs6081150|rs3848006|rs2286405|rs2293603
Antinuclear Antibodies	FC_GAMMA_R_MEDIATED_PHAGOCYTOSIS	KEGG	3795	35	20	6,85E-01	1,00E+00	rs6698806|rs9372365|rs4436949|rs230624|rs12081589|rs656709|rs17646824|rs346811|rs8081512|rs4603757|rs3824561|rs169143|rs836556|rs1316638|rs17559604|rs261072|rs6683451|rs2510257|rs10445097|rs11583351
Antinuclear Antibodies	HIVNEF_PATHWAY	BIOCARTA	1008	35	20	6,85E-01	1,00E+00	rs12765241|rs6452735|rs1357287|rs11614624|rs12817202|rs10859598|rs4761528|rs3750512|rs2415290|rs1061624|rs1439124|rs4934433|rs39416|rs4353229|rs8178179|rs12289836|rs4363056|rs10872893|rs12958785|rs2154254
Antinuclear Antibodies	HEDGEHOG_SIGNALING_PATHWAY	KEGG	2151	36	20	6,85E-01	1,00E+00	rs7218567|rs1990842|rs12155027|rs8074816|rs7805291|rs13044457|rs9392915|rs831022|rs1358776|rs4233828|rs1579036|rs12054856|rs6107891|rs6099433|rs2225265|rs17635121|rs883751|rs2041120|rs8005983|rs9475399
Antinuclear Antibodies	MAL_PATHWAY	BIOCARTA	225	6	3	6,86E-01	1,00E+00	rs836556|rs2855726|rs12259267
Antinuclear Antibodies	PHOSPHOLIPASE_C_MEDIATED_CASCADE	REACTOME	2628	24	20	6,89E-01	1,00E+00	rs902522|rs1924210|rs9736587|rs306090|rs3129678|rs8081512|rs1030098|rs1404912|rs17360897|rs2287778|rs950549|rs6501512|rs6884797|rs2302784|rs953672|rs3129599|rs249729|rs4751828|rs7303557|rs13155334
Antinuclear Antibodies	MITOTIC_G1_G1_S_PHASES	REACTOME	2477	30	20	6,91E-01	1,00E+00	rs12078447|rs9886419|rs9465734|rs7812998|rs1561928|rs11604821|rs3786136|rs12867405|rs512604|rs2298330|rs285164|rs1426511|rs2976282|rs7015841|rs4802906|rs11845708|rs7187327|rs10105118|rs12447239|rs741048
Antinuclear Antibodies	KERATAN_SULFATE_KERATIN_METABOLISM	REACTOME	737	29	15	6,96E-01	1,00E+00	rs6095638|rs3743398|rs11707667|rs13264573|rs10800925|rs1868435|rs6988999|rs1424013|rs2945743|rs4141587|rs10110481|rs2084512|rs12870|rs820857|rs4755327
Antinuclear Antibodies	EDG1_PATHWAY	BIOCARTA	1432	37	20	6,99E-01	1,00E+00	rs346811|rs8081512|rs10804342|rs1404912|rs169143|rs836556|rs10228945|rs2206422|rs198178|rs10485723|rs2714645|rs708925|rs1550805|rs6449969|rs7804859|rs2523189|rs2471226|rs9838117|rs3785396|rs17153527
Antinuclear Antibodies	MELANOMA	KEGG	2894	32	20	7,02E-01	1,00E+00	rs2974254|rs1924210|rs9465734|rs9307949|rs9736587|rs3129678|rs11604821|rs288496|rs7623679|rs7168213|rs1532684|rs950549|rs17423437|rs6884797|rs3129599|rs249729|rs12592277|rs948992|rs7181975|rs1550805
Antinuclear Antibodies	NOD_LIKE_RECEPTOR_SIGNALING_PATHWAY	KEGG	1406	26	20	7,02E-01	1,00E+00	rs10485101|rs2069837|rs6561682|rs12199654|rs1144171|rs11072638|rs6940350|rs10065570|rs1923075|rs159289|rs331455|rs12216864|rs13217057|rs10498974|rs17178663|rs2415290|rs6914621|rs9345117|rs721025|rs311646
Antinuclear Antibodies	PHOSPHORYLATION_OF_CD3_AND_TCR_ZETA_CHAINS	REACTOME	521	24	11	7,04E-01	1,00E+00	rs3782735|rs2064478|rs1561144|rs9469357|rs322931|rs441576|rs7824819|rs35265698|rs3741920|rs1917534|rs16859030
Antinuclear Antibodies	MITOTIC_M_M_G1_PHASES	REACTOME	3176	41	20	7,05E-01	1,00E+00	rs766295|rs994626|rs9465734|rs1478785|rs1558461|rs1027692|rs3786136|rs4350299|rs512604|rs9303909|rs12953815|rs17010833|rs4802906|rs11845708|rs7187327|rs12447239|rs10105118|rs3027247|rs12456560|rs741048
Antinuclear Antibodies	RESPONSE_TO_ELEVATED_PLATELET_CYTOSOLIC_CA2_	REACTOME	2301	32	20	7,05E-01	1,00E+00	rs1247571|rs2829946|rs216872|rs6910246|rs8081512|rs169143|rs466609|rs3753306|rs1404772|rs17189568|rs604045|rs198178|rs4592582|rs10863388|rs309770|rs13137121|rs5978|rs7526672|rs375369|rs1318557
Antinuclear Antibodies	FCER1_PATHWAY	BIOCARTA	1564	52	20	7,05E-01	1,00E+00	rs230624|rs2529737|rs169143|rs2005292|rs1403101|rs7694484|rs6683451|rs7148384|rs9896052|rs11583351|rs198178|rs612355|rs10821495|rs1550805|rs4699014|rs16826789|rs6449969|rs2055226|rs7804859|rs8102571
Antinuclear Antibodies	NUCLEAR_SIGNALING_BY_ERBB4	REACTOME	2231	25	20	7,07E-01	1,00E+00	rs987320|rs11225161|rs10495562|rs12203803|rs3756675|rs4458837|rs7187776|rs4373483|rs2439312|rs10521025|rs6940350|rs6712652|rs8055815|rs17205146|rs12524841|rs7818821|rs16948286|rs4508428|rs11813145|rs2439281
Antinuclear Antibodies	NEUROTRANSMITTER_RELEASE_CYCLE	REACTOME	1400	31	20	7,08E-01	1,00E+00	rs11685570|rs10508915|rs4439928|rs492315|rs7302925|rs133940|rs488192|rs2815717|rs10861133|rs2206161|rs9820724|rs1917806|rs12528795|rs2781562|rs973188|rs2278637|rs1641107|rs5998714|rs6931045|rs362570
Antinuclear Antibodies	METABOLISM_OF_STEROID_HORMONES_AND_VITAMINS_A_AND_D	REACTOME	745	41	20	7,08E-01	1,00E+00	rs1990842|rs12591359|rs7682870|rs831022|rs4233828|rs1010651|rs12441859|rs34562262|rs9640883|rs4844486|rs2300697|rs2271461|rs17270248|rs2271463|rs4616447|rs4889294|rs17703982|rs11647597|rs12555788|rs12586718
Antinuclear Antibodies	PLATELET_SENSITIZATION_BY_LDL	REACTOME	676	42	19	7,10E-01	1,00E+00	rs230624|rs6683451|rs12199654|rs11583351|rs612355|rs4802906|rs2239167|rs2231863|rs10105118|rs16826789|rs13396400|rs12474685|rs11160664|rs4466603|rs550619|rs1288497|rs1116003|rs328539|rs10732980
Antinuclear Antibodies	ANTIGEN_ACTIVATES_B_CELL_RECEPTOR_LEADING_TO_GENERATION_OF_SECOND_ME	REACTOME	1299	35	20	7,10E-01	1,00E+00	rs7905645|rs12571935|rs11153318|rs10445097|rs9896052|rs2060822|rs11706098|rs7303557|rs718314|rs10821495|rs1550805|rs930567|rs6449969|rs2055226|rs10842720|rs8102571|rs2470697|rs2991216|rs4240896|rs684119
Antinuclear Antibodies	ASSOCIATION_OF_TRIC_CCT_WITH_TARGET_PROTEINS_DURING_BIOSYNTHESIS	REACTOME	359	10	8	7,11E-01	1,00E+00	rs346811|rs9383320|rs3750512|rs12701264|rs710764|rs6584453|rs10985014|rs6865121
Antinuclear Antibodies	PHASE1_FUNCTIONALIZATION_OF_COMPOUNDS	REACTOME	1515	43	20	7,11E-01	1,00E+00	rs7918164|rs6052369|rs12591359|rs41717|rs8187950|rs6116189|rs2273684|rs8187890|rs17564210|rs2002700|rs1614972|rs12441859|rs2182859|rs34562262|rs4808358|rs4288415|rs7256675|rs17703982|rs1126673|rs1826909
Antinuclear Antibodies	NEPHRIN_INTERACTIONS	REACTOME	1617	38	20	7,12E-01	1,00E+00	rs10490696|rs7950175|rs6963699|rs10229458|rs2075014|rs11153318|rs2714700|rs11220521|rs10261468|rs1550805|rs13207896|rs7775178|rs9649304|rs12705567|rs6449969|rs6955922|rs1919262|rs6590202|rs7109913|rs7793860
Antinuclear Antibodies	PROCESSING_OF_CAPPED_INTRON_CONTAINING_PRE_MRNA	REACTOME	1547	37	20	7,15E-01	1,00E+00	rs3026106|rs606119|rs1478785|rs5766533|rs10495562|rs6062340|rs13219815|rs6827036|rs3809425|rs3826425|rs734570|rs633742|rs4435501|rs4798|rs1534680|rs1881697|rs13038619|rs9615025|rs604965|rs8088313
Antinuclear Antibodies	GNRH_SIGNALING_PATHWAY	KEGG	3992	32	20	7,15E-01	1,00E+00	rs3886478|rs902522|rs7665102|rs230624|rs10920115|rs17041308|rs1407101|rs8081512|rs2529737|rs1404912|rs169143|rs17360897|rs2327162|rs2287778|rs658071|rs6683451|rs12199654|rs3805018|rs2206422|rs602837
Antinuclear Antibodies	CHEMICAL_PATHWAY	BIOCARTA	585	25	13	7,16E-01	1,00E+00	rs8081512|rs13029532|rs169143|rs198178|rs4767886|rs1439124|rs3785396|rs39416|rs4353229|rs12958785|rs874583|rs10268780|rs9925433
Antinuclear Antibodies	NEUROTROPHIN_SIGNALING_PATHWAY	KEGG	3689	41	20	7,17E-01	1,00E+00	rs3886478|rs4238022|rs11970312|rs1560977|rs7665102|rs7999458|rs11102314|rs306090|rs1397529|rs6432018|rs836556|rs12199654|rs7187776|rs10445097|rs9896052|rs260415|rs708030|rs8031813|rs4675077|rs13008971
Antinuclear Antibodies	PD1_SIGNALING	REACTOME	398	24	11	7,17E-01	1,00E+00	rs3782735|rs3117230|rs2239167|rs9469357|rs441576|rs7024586|rs35265698|rs3741920|rs1917534|rs16859030|rs7865645
Antinuclear Antibodies	LEUKOCYTE_TRANSENDOTHELIAL_MIGRATION	KEGG	4793	28	20	7,19E-01	1,00E+00	rs7124686|rs8133991|rs183718|rs11102314|rs3820852|rs6531770|rs656709|rs8081512|rs17020744|rs1215|rs2974154|rs3824561|rs11643524|rs9883073|rs7776133|rs169143|rs836556|rs3769309|rs10228945|rs2862944
Antinuclear Antibodies	NGF_SIGNALLING_VIA_TRKA_FROM_THE_PLASMA_MEMBRANE	REACTOME	4847	31	20	7,20E-01	1,00E+00	rs3886478|rs2267737|rs902522|rs11970312|rs12958400|rs7999458|rs11102314|rs306090|rs6977082|rs9549096|rs8081512|rs1030098|rs1404912|rs17360897|rs2287778|rs12199654|rs9576967|rs6501512|rs2302784|rs953672
Antinuclear Antibodies	NKCELLS_PATHWAY	BIOCARTA	678	35	20	7,20E-01	1,00E+00	rs10772294|rs836556|rs10821495|rs1550805|rs2239167|rs1611493|rs6449969|rs2163177|rs8102571|rs2991216|rs9838117|rs10424498|rs252299|rs2290889|rs12146170|rs2617822|rs8053800|rs12310310|rs2733840|rs7874712
Antinuclear Antibodies	GLYCOSPHINGOLIPID_METABOLISM	REACTOME	924	40	20	7,21E-01	1,00E+00	rs6448180|rs2167239|rs10121681|rs11707667|rs11714594|rs873592|rs2194051|rs7855135|rs9626899|rs918167|rs11624852|rs718137|rs12258132|rs1688416|rs2903808|rs7671785|rs6863289|rs1638720|rs820857|rs13128386
Antinuclear Antibodies	RAC1_PATHWAY	BIOCARTA	763	29	18	7,22E-01	1,00E+00	rs10800842|rs836556|rs1048121|rs1550805|rs6449969|rs2303924|rs13098743|rs7804859|rs8102571|rs9838117|rs17153527|rs2362941|rs10424498|rs4730197|rs2617822|rs2855726|rs27111|rs17153654
Antinuclear Antibodies	FANCONI_ANEMIA_PATHWAY	REACTOME	461	10	6	7,23E-01	1,00E+00	rs10490113|rs1230|rs4325734|rs6734368|rs11571805|rs144848
Antinuclear Antibodies	PI3K_AKT_ACTIVATION	REACTOME	1161	33	20	7,24E-01	1,00E+00	rs12958400|rs7999458|rs9549096|rs9576967|rs7329423|rs4675077|rs1550805|rs2001303|rs10494270|rs10509534|rs6449969|rs2251692|rs11069810|rs2798464|rs12207548|rs13392647|rs2894595|rs9838117|rs665821|rs9480865
Antinuclear Antibodies	VALINE_LEUCINE_AND_ISOLEUCINE_DEGRADATION	KEGG	1149	44	20	7,25E-01	1,00E+00	rs534800|rs6853|rs11082773|rs4672866|rs357749|rs9361660|rs837322|rs4646772|rs10973734|rs2408700|rs4665830|rs1641107|rs11047702|rs16928217|rs695983|rs7620635|rs947223|rs9350868|rs4604577|rs13290293
Antinuclear Antibodies	CIRCADIAN_REPRESSION_OF_EXPRESSION_BY_REV_ERBA	REACTOME	1358	35	20	7,25E-01	1,00E+00	rs3774909|rs4589495|rs6448226|rs3789823|rs450110|rs17270181|rs1567804|rs9968207|rs17204910|rs6018631|rs6120708|rs1370429|rs339969|rs2062091|rs17024869|rs2164349|rs650356|rs216113|rs12909890|rs7176740
Antinuclear Antibodies	OTHER_GLYCAN_DEGRADATION	KEGG	648	41	15	7,26E-01	1,00E+00	rs4862816|rs17801853|rs11707667|rs2614635|rs1379411|rs1563591|rs1632770|rs6830655|rs6552245|rs3907136|rs17068194|rs10019475|rs2681338|rs4690557|rs820857
Antinuclear Antibodies	PEPTIDE_CHAIN_ELONGATION	REACTOME	947	42	20	7,26E-01	1,00E+00	rs7902136|rs1504632|rs12602594|rs1953910|rs17121120|rs10065570|rs11002569|rs4777170|rs6895379|rs11733964|rs2301723|rs13325754|rs11002552|rs1953912|rs1956099|rs10895987|rs1569498|rs8179823|rs2140386|rs1720261
Antinuclear Antibodies	PHASE_II_CONJUGATION	REACTOME	2059	34	20	7,27E-01	1,00E+00	rs2028900|rs9296691|rs138055|rs10068900|rs17062147|rs158850|rs316156|rs10456664|rs13124794|rs978114|rs2273684|rs297961|rs7758711|rs17355469|rs2224198|rs4646267|rs5751902|rs809942|rs11892031|rs1205357
Antinuclear Antibodies	CHEMOKINE_SIGNALING_PATHWAY	KEGG	5407	23	20	7,27E-01	1,00E+00	rs10499305|rs902522|rs7741028|rs10872715|rs11102314|rs16947151|rs877711|rs6019300|rs6807740|rs8141065|rs1407101|rs656709|rs12480096|rs17646824|rs3824561|rs1404912|rs4243583|rs13029532|rs1253704|rs169143
Antinuclear Antibodies	NO2IL12_PATHWAY	BIOCARTA	461	21	16	7,27E-01	1,00E+00	rs3782735|rs13029532|rs11921898|rs1498737|rs2274471|rs3014996|rs16833215|rs7610160|rs3761041|rs393540|rs16833239|rs3741920|rs797999|rs1917534|rs16859030|rs918519
Antinuclear Antibodies	IL7_PATHWAY	BIOCARTA	699	34	14	7,28E-01	1,00E+00	rs1227631|rs13253242|rs11153318|rs1550805|rs310244|rs6449969|rs2163177|rs7804859|rs9838117|rs17153527|rs4730197|rs16906063|rs12958785|rs17153654
Antinuclear Antibodies	OPIOID_SIGNALLING	REACTOME	3594	32	20	7,28E-01	1,00E+00	rs902522|rs230624|rs16947151|rs306090|rs6807740|rs1407101|rs8081512|rs1030098|rs1404912|rs4243583|rs1253704|rs17360897|rs10228945|rs2327162|rs9306383|rs6532915|rs2287778|rs6683451|rs10493754|rs11715227
Antinuclear Antibodies	REGULATION_OF_BETA_CELL_DEVELOPMENT	REACTOME	1288	42	20	7,29E-01	1,00E+00	rs2943547|rs9549096|rs1337728|rs9291482|rs1390088|rs9576967|rs1343882|rs9886592|rs2247328|rs17008412|rs7329423|rs8025430|rs874511|rs4264821|rs7209835|rs3094509|rs8100003|rs2001726|rs4812823|rs16939060
Antinuclear Antibodies	AMYOTROPHIC_LATERAL_SCLEROSIS_ALS	KEGG	1813	49	20	7,29E-01	1,00E+00	rs1806201|rs1422893|rs836556|rs7730730|rs2866250|rs4332745|rs12199654|rs10030915|rs2872715|rs7590550|rs13189947|rs4699014|rs8060068|rs1074042|rs739559|rs12934132|rs1061624|rs10744029|rs1439124|rs12811583
Antinuclear Antibodies	STRIATED_MUSCLE_CONTRACTION	REACTOME	453	26	12	7,31E-01	1,00E+00	rs201603|rs947485|rs10982444|rs7481661|rs1106950|rs2278998|rs3738281|rs174745|rs4743112|rs6538998|rs11673699|rs1405712
Antinuclear Antibodies	ABC_FAMILY_PROTEINS_MEDIATED_TRANSPORT	REACTOME	1753	36	20	7,31E-01	1,00E+00	rs7419736|rs10877173|rs2214102|rs9898731|rs17189568|rs2108258|rs10179921|rs35595|rs2286419|rs8075890|rs1800136|rs4148549|rs4147952|rs1550646|rs150926|rs956133|rs737453|rs1925850|rs2238475|200096
Antinuclear Antibodies	ARRHYTHMOGENIC_RIGHT_VENTRICULAR_CARDIOMYOPATHY_ARVC	KEGG	5871	35	20	7,31E-01	1,00E+00	rs2618681|rs9810230|rs17053465|rs7241357|rs38553|rs12737847|rs10920115|rs11715062|rs10925415|rs13430329|rs12032218|rs10486963|rs17020744|rs2974154|rs12152349|rs10045068|rs9883073|rs485409|rs2251788|rs2862944
Antinuclear Antibodies	FATTY_ACID_METABOLISM	KEGG	776	33	16	7,32E-01	1,00E+00	rs6853|rs11082773|rs2305913|rs4646772|rs10973734|rs1614972|rs12609870|rs2408700|rs4142082|rs4665830|rs1126673|rs1826909|rs7620635|rs13290293|rs558275|rs10498146
Antinuclear Antibodies	ACTIVATION_OF_NF_KAPPAB_IN_B_CELLS	REACTOME	1396	29	20	7,33E-01	1,00E+00	rs11767716|rs10485101|rs169143|rs1144171|rs198178|rs1923075|rs11845708|rs7187327|rs12447239|rs13217057|rs10498974|rs698856|rs2415290|rs425594|rs11922435|rs6914621|401754|rs9345117|rs721025|rs1182137
Antinuclear Antibodies	RORA_ACTIVATES_CIRCADIAN_EXPRESSION	REACTOME	1377	34	20	7,37E-01	1,00E+00	rs3774909|rs4589495|rs6448226|rs3789823|rs450110|rs17270181|rs1567804|rs9968207|rs17204910|rs6018631|rs6088590|rs1370429|rs339969|rs2062091|rs17024869|rs2164349|rs650356|rs216113|rs12909890|rs7176740
Antinuclear Antibodies	TID_PATHWAY	BIOCARTA	479	13	12	7,37E-01	1,00E+00	rs2418588|rs608914|rs16934382|rs2415290|rs2274471|rs17137037|rs1061624|rs3014996|rs12289836|rs10872893|rs7103780|rs3138045
Antinuclear Antibodies	THYROID_CANCER	KEGG	1104	37	20	7,38E-01	1,00E+00	rs9886419|rs7812998|rs1561928|rs11604821|rs9883073|rs213213|rs1426511|rs6719501|rs7895307|rs10821612|rs624797|rs1468934|rs1362943|rs10856989|rs12573128|rs2148887|rs11196203|rs1872568|rs7859229|rs1626088
Antinuclear Antibodies	ABC_TRANSPORTERS	KEGG	2684	37	20	7,42E-01	1,00E+00	rs2725252|rs7419736|rs10877173|rs2214102|rs9898731|rs17189568|rs2108258|rs10259451|rs4148073|rs10179921|rs35595|rs2286419|rs2303924|rs8075890|401754|rs6980032|rs4148155|rs7469197|rs4148549|rs2472510

Antinuclear Antibodies	HOMOLOGOUS_RECOMBINATION_REPAIR_OF_REPLICATION_INDEPENDENT_DOUBLE_ST	REACTOME	376	16	8	7,47E-01	1,00E+00	rs12453935|rs3786136|rs741048|rs11762146|rs11571805|rs2508678|rs17545935|rs144848
Antinuclear Antibodies	BASAL_CELL_CARCINOMA	KEGG	2166	33	20	7,50E-01	1,00E+00	rs7218567|rs1219615|rs12155027|rs7212246|rs7805291|rs9883073|rs13044457|rs1477031|rs1108386|rs1350171|rs6107891|rs12998820|rs11010252|rs2225265|rs17635121|rs1472513|rs8005983|rs6719501|rs12667722|rs7860361
Antinuclear Antibodies	MRNA_PROCESSING	REACTOME	1942	37	20	7,50E-01	1,00E+00	rs3026106|rs606119|rs1478785|rs5766533|rs10431307|rs10495562|rs7979377|rs6062340|rs13219815|rs6827036|rs3809425|rs3826425|rs734570|rs633742|rs13183577|rs4435501|rs4798|rs1534680|rs1881697|rs13038619
Antinuclear Antibodies	E2F_MEDIATED_REGULATION_OF_DNA_REPLICATION	REACTOME	452	15	10	7,51E-01	1,00E+00	rs12867405|rs512604|rs4802906|rs10105118|rs4805487|rs9576040|rs3087474|rs1860652|rs1116003|rs2871029
Antinuclear Antibodies	PROTEIN_FOLDING	REACTOME	675	20	16	7,51E-01	1,00E+00	rs292117|rs346811|rs9383320|rs371164|rs7554192|rs11759402|rs3750512|rs3892375|rs1443096|rs12701264|rs710764|rs6584453|rs10985014|rs3844450|rs6865121|rs8074277
Antinuclear Antibodies	POST_CHAPERONIN_TUBULIN_FOLDING_PATHWAY	REACTOME	254	6	6	7,52E-01	1,00E+00	rs371164|rs7554192|rs11759402|rs1443096|rs3844450|rs8074277
Antinuclear Antibodies	PARKINSONS_DISEASE	KEGG	2833	35	20	7,52E-01	1,00E+00	rs875785|rs8133991|rs9347562|rs13195756|rs10088489|rs1411451|rs2201144|rs11326|rs16842|rs968698|rs10425332|rs3822362|rs11669029|rs9355359|rs6675798|rs6757526|rs10406627|rs7738279|rs7251331|rs10068191
Antinuclear Antibodies	PTM_GAMMA_CARBOXYLATION_HYPUSINE_FORMATION_AND_ARYLSULFATASE_ACTIV	REACTOME	377	18	12	7,53E-01	1,00E+00	rs2028900|rs11714594|rs873592|rs2194051|rs10936665|rs918167|rs1688416|rs7671785|rs6863289|rs13128386|rs12486780|rs754567
Antinuclear Antibodies	NATURAL_KILLER_CELL_MEDIATED_CYTOTOXICITY	KEGG	3607	43	20	7,55E-01	1,00E+00	rs10772294|rs12765241|rs7830593|rs656709|rs8081512|rs3824561|rs169143|rs836556|rs7694484|rs9314261|rs16845834|rs608914|rs11153318|rs2510257|rs10445097|rs7148384|rs9896052|rs198178|rs8031813|rs12181819
Antinuclear Antibodies	MTOR_PATHWAY	BIOCARTA	509	11	10	7,55E-01	1,00E+00	rs885126|rs4751691|rs1550805|rs10509534|rs6449969|rs9838117|rs10788582|rs945559|rs9930956|rs2293603
Antinuclear Antibodies	CARDIACEGF_PATHWAY	BIOCARTA	1289	39	20	7,55E-01	1,00E+00	rs9886419|rs7812998|rs1561928|rs8081512|rs169143|rs1870510|rs2005292|rs1426511|rs198178|rs9422812|rs3785396|rs1468934|rs6681035|rs12289836|rs1278302|rs4681446|rs9462724|rs812196|rs9925433|rs751437
Antinuclear Antibodies	UCALPAIN_PATHWAY	BIOCARTA	426	9	6	7,58E-01	1,00E+00	rs836556|rs4767886|rs6861460|rs625750|rs4902681|rs12146170
Antinuclear Antibodies	PLATELET_ACTIVATION_SIGNALING_AND_AGGREGATION	REACTOME	6965	26	20	7,59E-01	1,00E+00	rs12286714|rs12668754|rs247571|rs2829946|rs230624|rs11102314|rs3820852|rs16947151|rs877711|rs6807740|rs216872|rs656709|rs6910246|rs8081512|rs4243583|rs9533044|rs1253704|rs2038171|rs169143|rs836556
Antinuclear Antibodies	AMINO_ACID_AND_OLIGOPEPTIDE_SLC_TRANSPORTERS	REACTOME	1707	30	20	7,59E-01	1,00E+00	rs4869685|rs7657271|rs4685165|rs4640842|rs4602539|rs11670126|rs1979073|rs488192|rs7727745|rs4296412|rs748680|rs7657780|rs720625|rs10434201|rs1552336|rs910795|rs1881998|rs12891079|rs10847697|rs4772118
Antinuclear Antibodies	ELONGATION_ARREST_AND_RECOVERY	REACTOME	246	9	7	7,59E-01	1,00E+00	rs3786238|rs10732882|rs1053079|rs633742|rs604965|rs4799085|rs2293603
Antinuclear Antibodies	EGFR_DOWNREGULATION	REACTOME	1037	38	16	7,62E-01	1,00E+00	rs1114275|rs6563216|rs9545290|rs9896052|rs1215930|rs16933086|rs4664531|rs16830728|rs12376522|rs300574|rs13139048|rs9318729|rs956323|rs6563245|rs17073080|rs3097907
Antinuclear Antibodies	DESTABILIZATION_OF_MRNA_BY_AUF1_HNRNP_D0	REACTOME	811	37	18	7,62E-01	1,00E+00	rs1039153|rs11845708|rs7187327|rs6827036|rs12447239|rs698856|rs425594|rs2951967|rs11922435|401754|rs13207114|rs3787573|rs2107459|rs11732320|rs577357|rs2293603|rs992507|rs4973377
Antinuclear Antibodies	TRIGLYCERIDE_BIOSYNTHESIS	REACTOME	1182	31	20	7,65E-01	1,00E+00	rs2017571|rs17009821|rs9458157|rs13435303|rs3906767|rs11922502|rs209489|rs12498468|rs10183830|rs11195843|rs1880540|rs12499413|rs7567684|rs11195662|rs10209151|rs829608|rs9825310|rs4502283|rs4819329|rs6838244
Antinuclear Antibodies	ADHERENS_JUNCTIONS_INTERACTIONS	REACTOME	5496	33	20	7,66E-01	1,00E+00	rs6764017|rs7241357|rs183718|rs12496792|rs10934067|rs12494510|rs8123347|rs7730479|rs12446828|rs11643524|rs9883073|rs904750|rs9859918|rs485409|rs6124072|rs4925295|rs2937524|rs10511074|rs17299353|rs16896657
Antinuclear Antibodies	LINOLEIC_ACID_METABOLISM	KEGG	716	41	14	7,67E-01	1,00E+00	rs230624|rs6683451|rs11583351|rs612355|rs16826789|rs631134|rs622450|rs2278964|rs7546956|rs4466603|rs776691|rs2470890|rs12125747|rs10732980
Antinuclear Antibodies	PYRIMIDINE_METABOLISM	KEGG	1900	34	20	7,67E-01	1,00E+00	rs4668507|rs4789145|rs2227301|rs2387094|rs595890|rs12604|rs6081157|rs10919062|rs11699316|rs653241|rs6557674|rs9437663|rs11188513|rs4243969|rs1887455|rs2278998|rs633742|rs738814|rs4815390|rs1870131
Antinuclear Antibodies	GPVI_MEDIATED_ACTIVATION_CASCADE	REACTOME	1181	30	20	7,67E-01	1,00E+00	rs12668754|rs656709|rs836556|rs11153318|rs10445097|rs10821495|rs1550805|rs6449969|rs7804859|rs7537605|rs8102571|rs2991216|rs9838117|rs17153527|rs315796|rs2125749|rs2504469|rs10424498|rs11716652|rs4730197
Antinuclear Antibodies	METABOLISM_OF_AMINO_ACIDS_AND_DERIVATIVES	REACTOME	5246	39	20	7,68E-01	1,00E+00	rs7918164|rs11685570|rs534800|rs6077242|rs2028900|rs7572399|rs6052369|rs4788547|rs9671414|rs731727|rs2371034|rs17636594|rs11685496|rs4526224|rs501819|rs10068900|rs10932334|rs6140362|rs566509|rs6748782
Antinuclear Antibodies	G2_PATHWAY	BIOCARTA	354	16	9	7,69E-01	1,00E+00	rs6432018|rs2983460|rs4809258|rs12207548|rs10929592|rs878179|rs6062662|rs1465701|rs9462209
Antinuclear Antibodies	TIE2_SIGNALING	REACTOME	790	17	11	7,69E-01	1,00E+00	rs12988898|rs9896052|rs1550805|rs6449969|rs3923113|rs413825|rs9838117|rs530365|rs6469131|rs11716652|rs10967750
Antinuclear Antibodies	SYNTHESIS_SECRETION_AND_DEACYLATION_OF_GHRELIN	REACTOME	624	33	12	7,71E-01	1,00E+00	rs1411355|rs11135469|rs12638016|rs10124038|rs10979023|rs2140483|rs1880143|rs9854178|rs4978670|rs1360257|rs10513618|rs10759237
Antinuclear Antibodies	ENDOCYTOSIS	KEGG	6484	26	20	7,72E-01	1,00E+00	rs3026106|rs987320|rs3785784|rs12430144|rs11947791|rs4262039|rs877711|rs673377|rs8141065|rs4537255|rs1406961|rs11203999|rs2132302|rs1896198|rs6531424|rs1822676|rs3756675|rs7595184|rs2047076|rs8181443
Antinuclear Antibodies	PRION_DISEASES	KEGG	1585	41	20	7,73E-01	1,00E+00	rs2826418|rs229451|rs2069837|rs971075|rs9661996|rs7509723|rs11153318|rs1783280|rs161130|rs883751|rs3750512|rs2292727|rs706470|rs7282990|rs8134936|rs13156109|rs12279261|rs2827709|rs10473234|rs624298
Antinuclear Antibodies	CELL_CYCLE_CHECKPOINTS	REACTOME	1961	39	20	7,74E-01	1,00E+00	rs766295|rs11558461|rs1027692|rs9562187|rs6836883|rs3786136|rs7015841|rs10514915|rs11845708|rs7187327|rs12447239|rs741048|rs2393374|rs698856|rs1106950|rs425594|rs4805487|rs11922435|rs3800933|401754
Antinuclear Antibodies	STEROID_BIOSYNTHESIS	KEGG	247	15	7	7,75E-01	1,00E+00	rs1792331|rs4841600|rs1792225|rs4819211|rs7531307|rs2492778|rs2684874
Antinuclear Antibodies	G_ALPHA_I_SIGNALLING_EVENTS	REACTOME	6453	33	20	7,75E-01	1,00E+00	rs12139261|rs2829946|rs6861578|rs902522|rs11706283|rs299826|rs16947151|rs2800880|rs6807740|rs6690854|rs1404912|rs4243583|rs1253704|rs17360897|rs466609|rs10228945|rs746622|rs9306383|rs2287778|rs1920397
Antinuclear Antibodies	SIGNALING_BY_BMP	REACTOME	918	40	20	7,75E-01	1,00E+00	rs9968396|rs4075546|rs13044457|rs12466337|rs4662532|rs10961689|rs6107891|rs2225265|rs6729501|rs7566998|rs232657|rs1147695|rs6816834|rs12476865|rs235757|rs6140076|rs4776831|rs7560426|rs4278364|rs1992600
Antinuclear Antibodies	PATHOGENIC_ESCHERICHIA_COLI_INFECTION	KEGG	1256	44	20	7,75E-01	1,00E+00	rs10490696|rs8081512|rs9883073|rs6432018|rs361540|rs11153318|rs4483225|rs371164|rs6580921|rs17427173|rs1053079|rs10513330|rs11253141|rs10929592|rs4734512|rs4734497|rs778584|rs17247633|rs1392125|rs12622971
Antinuclear Antibodies	GENERATION_OF_SECOND_MESSENGER_MOLECULES	REACTOME	616	41	19	7,76E-01	1,00E+00	rs3782735|rs1190976|rs1403101|rs152105|rs2064478|rs9469357|rs2267423|rs315796|rs441576|rs252299|rs35265698|rs3741920|rs137966|rs8053800|rs1917534|rs11687510|rs16859030|rs1190993|rs8045689
Antinuclear Antibodies	LYSOSOME	KEGG	2448	42	20	7,76E-01	1,00E+00	rs13272985|rs1478785|rs2167239|rs4862816|rs7496727|rs951453|rs3798188|rs12114861|rs17801853|rs11707667|rs16933086|rs1065206|rs6557674|rs3795438|rs11714594|rs2292963|rs2614635|rs11652474|rs1010651|rs1379411
Antinuclear Antibodies	NICOTINATE_AND_NICOTINAMIDE_METABOLISM	KEGG	963	29	20	7,77E-01	1,00E+00	rs4789145|rs595890|rs9493105|rs17341221|rs4672866|rs9493133|rs653241|rs3734087|rs1922502|rs792225|rs7768480|rs9813586|rs16849307|rs179883|rs7713458|rs4832609|rs6683452|rs6569765|rs4703261|rs1288869
Antinuclear Antibodies	REGULATION_OF_MRNA_STABILITY_BY_PROTEINS_THAT_BIND_AU_RICH_ELEMENTS	REACTOME	1372	43	20	7,78E-01	1,00E+00	rs1039153|rs8081512|rs1000174|rs12199654|rs17010833|rs16973403|rs11845708|rs7187327|rs6827036|rs12447239|rs4671408|rs698856|rs425594|rs2951967|rs11922435|401754|rs13207114|rs6032895|rs4734512|rs2107459
Antinuclear Antibodies	RNA_DEGRADATION	KEGG	1123	32	20	7,78E-01	1,00E+00	rs12520016|rs1000174|rs111930|rs7716228|rs1472169|rs12102745|rs2239167|rs3809425|rs10498060|rs11178058|rs2270048|rs10853869|rs905067|rs2733537|rs17034884|rs234592|rs4782165|rs744675|rs17214407|rs1533079
Antinuclear Antibodies	AUTOIMMUNE_THYROID_DISEASE	KEGG	1646	45	20	7,79E-01	1,00E+00	rs12765241|rs2284191|rs3850890|rs2715267|rs2395300|rs2064478|rs1611493|rs9469357|rs3097646|rs1599796|rs9678469|rs4934433|rs2246010|rs4747064|rs441576|rs35265698|rs3783941|rs2523554|rs2154254|rs7157900
Antinuclear Antibodies	NFAT_PATHWAY	BIOCARTA	1626	34	20	7,80E-01	1,00E+00	rs2236891|rs306090|rs1030098|rs7694484|rs12199654|rs10903341|rs7148384|rs6501512|rs12562819|rs2302784|rs11089441|rs17153747|rs1550805|rs883751|rs4699014|rs6449969|rs2055226|rs6879217|rs7804859|rs9304671
Antinuclear Antibodies	INFLUENZA_VIRAL_RNA_TRANSCRIPTION_AND_REPLICATION	REACTOME	1153	38	20	7,80E-01	1,00E+00	rs7902136|rs7176919|rs12602594|rs1953910|rs17121120|rs10065570|rs7329817|rs11002569|rs4777170|rs633742|rs6895379|rs11733964|rs9302083|rs2301723|rs13325754|rs604965|rs11002552|rs1953912|rs1956099|rs9556844
Antinuclear Antibodies	REGULATION_OF_APOPTOSIS	REACTOME	1432	34	20	7,80E-01	1,00E+00	rs10502934|rs11141915|rs1357287|rs1953042|rs1930061|rs11845708|rs7187327|rs12447239|rs1114191|rs4746080|rs4556886|rs698856|rs425594|rs11922435|rs1504508|rs4878066|401754|rs11660123|rs7243527|rs13207114
Antinuclear Antibodies	CLASS_I_MHC_MEDIATED_ANTIGEN_PROCESSING_PRESENTATION	REACTOME	5895	41	20	7,81E-01	1,00E+00	rs875785|rs6020238|rs2608578|rs12145857|rs9347562|rs11253250|rs447921|rs1010056|rs13195756|rs9562187|rs6836883|rs4743335|rs12466600|rs10493118|rs567962|rs3088095|rs4743347|rs10050860|rs12147541|rs708030
Antinuclear Antibodies	HUNTINGTONS_DISEASE	KEGG	4311	30	20	7,81E-01	1,00E+00	rs3774909|rs1806201|rs8133991|rs6448226|rs17041308|rs6467259|rs1407101|rs438657|rs2327162|rs1411451|rs3805018|rs2206422|rs968698|rs13241028|rs16933086|rs1179605|rs1882095|rs10425332|rs3800602|rs1065206
Antinuclear Antibodies	CALCIUM_SIGNALING_PATHWAY	KEGG	9275	26	20	7,81E-01	1,00E+00	rs987320|rs2618681|rs902522|rs297672|rs928807|rs1072319|rs12737847|rs13185639|rs7665102|rs1578303|rs6994883|rs6429140|rs1936600|rs10920115|rs17041308|rs306090|rs1950650|rs2115512|rs1407101|rs10925415
Antinuclear Antibodies	TGF_BETA_RECEPTOR_SIGNALING_IN_EMT_EPITHELIAL_TO_MESENCHYMAL_TRANSITI(	REACTOME	429	16	10	7,81E-01	1,00E+00	rs17231134|rs7653262|rs478061|rs1009607|rs6481924|rs7462455|rs2337360|rs8179181|rs11576837|rs8110645
Antinuclear Antibodies	CYCLIN_E_ASSOCIATED_EVENTS_DURING_G1_S_TRANSITION_	REACTOME	1212	36	20	7,82E-01	1,00E+00	rs9886419|rs7812998|rs1561928|rs1426511|rs7015841|rs11845708|rs7187327|rs12447239|rs425594|rs4805487|rs11922435|401754|rs13207114|rs12207548|rs3787573|rs9576040|rs1468934|rs2107459|rs577357|rs992507
Antinuclear Antibodies	METAL_ION_SLC_TRANSPORTERS	REACTOME	653	24	12	7,82E-01	1,00E+00	rs11776475|rs213213|rs16861582|rs6829701|rs4981345|rs13266634|rs7299019|rs2133509|rs2293963|rs12040671|rs13436110|rs2213028
Antinuclear Antibodies	O_GLYCAN_BIOSYNTHESIS	KEGG	1579	38	20	7,82E-01	1,00E+00	rs13035081|rs4846948|rs6095638|rs12699009|rs10233708|rs4726132|rs10477100|rs13264573|rs10213212|rs1868435|rs6988999|rs1860351|rs1646613|rs4719095|rs12658319|rs2288100|rs2945743|rs4141587|rs4585627|rs1495288
Antinuclear Antibodies	EICOSANOID_LIGAND_BINDING_RECEPTORS	REACTOME	507	7	5	7,85E-01	1,00E+00	rs10941508|rs350089|rs3766333|rs931647|rs12123324
Antinuclear Antibodies	RIBOFLAVIN_METABOLISM	KEGG	295	14	7	7,86E-01	1,00E+00	rs9493105|rs9493133|rs7768480|rs10830228|rs973986|rs7258535|rs6569765
Antinuclear Antibodies	DRUG_METABOLISM_CYTOCHROME_P450	KEGG	1661	40	20	7,92E-01	1,00E+00	rs9296691|rs316156|rs4672866|rs10456664|rs140289|rs13124794|rs10912887|rs17564210|rs1614972|rs17355469|rs2207951|rs12802641|rs11892031|rs7256675|rs1126673|rs4148280|rs1826909|rs16864125|rs396216|rs8192013
Antinuclear Antibodies	DOWNREGULATION_OF_TGF_BETA_RECEPTOR_SIGNALING	REACTOME	521	19	12	7,93E-01	1,00E+00	rs7653262|rs17010833|rs6025738|rs478061|rs4671408|rs4939659|rs372334|rs12901499|rs12915039|rs8179181|rs128116|rs13044132
Antinuclear Antibodies	TCA_CYCLE_AND_RESPIRATORY_ELECTRON_TRANSPORT	REACTOME	2599	27	20	7,94E-01	1,00E+00	rs8133991|rs17070285|rs4331688|rs9534734|rs2028548|rs1411451|rs653263|rs1830426|rs7558379|rs968698|rs10425332|rs2978169|rs4239020|rs3134010|rs3822362|rs11669029|rs6547525|rs2902374|rs10406627|rs7602538
Antinuclear Antibodies	SYNTHESIS_OF_PIPS_AT_THE_GOLGI_MEMBRANE	REACTOME	788	16	7	7,96E-01	1,00E+00	rs11876920|rs203170|rs307118|rs4243272|rs10160860|rs1374642|rs130635
Antinuclear Antibodies	SIGNALING_BY_FGFR_IN_DISEASE	REACTOME	4545	34	20	7,97E-01	1,00E+00	rs902522|rs1924210|rs10878951|rs12958400|rs9736587|rs1853300|rs306090|rs3129678|rs6465836|rs9549096|rs8081512|rs1397529|rs1030098|rs1404912|rs13029532|rs17360897|rs6563216|rs2287778|rs885126|rs9576967
Antinuclear Antibodies	G_ALPHA_Q_SIGNALLING_EVENTS	REACTOME	7428	29	20	7,97E-01	1,00E+00	rs12286714|rs2829946|rs1373928|rs297672|rs1578180|rs1578303|rs6994883|rs17696124|rs6429140|rs16947151|rs4289301|rs6807740|rs1407101|rs12099717|rs11235714|rs12119540|rs4243583|rs9526313|rs10485512|rs9533044
Antinuclear Antibodies	RESOLUTION_OF_AP_SITES_VIA_THE_MULTIPLE_NUCLEOTIDE_PATCH_REPLACEMENT_I	REACTOME	192	9	6	7,98E-01	1,00E+00	rs2029166|rs174532|rs2278998|rs1405655|rs9930956|rs1872635
Antinuclear Antibodies	ERBB_SIGNALING_PATHWAY	KEGG	4135	33	20	7,98E-01	1,00E+00	rs987320|rs9886419|rs10490696|rs7665102|rs7812998|rs1561928|rs8081512|rs1397529|rs2529737|rs169143|rs3756675|rs1403101|rs4458837|rs6672395|rs10884065|rs10445097|rs4373483|rs9896052|rs2439312|rs2111654
Antinuclear Antibodies	TIGHT_JUNCTION_INTERACTIONS	REACTOME	715	30	19	8,03E-01	1,00E+00	rs1215|rs7776133|rs17231134|rs2072168|rs9561869|rs2000546|rs1009607|rs7427173|rs9561879|rs6785483|rs4244991|rs17462455|rs2063492|rs1954436|rs4598521|rs3844450|rs11576837|rs962320|rs2871029
Antinuclear Antibodies	MISMATCH_REPAIR	KEGG	576	28	17	8,04E-01	1,00E+00	rs3786136|rs6753949|rs6544990|rs741048|rs2393374|rs3136247|rs10514805|rs2278998|rs2306597|rs11762146|rs3136337|rs1832265|rs13178429|rs7328146|rs1405655|rs9540647|rs17545935
Antinuclear Antibodies	TGF_BETA_RECEPTOR_SIGNALING_ACTIVATES_SMADS	REACTOME	563	19	12	8,05E-01	1,00E+00	rs7653262|rs17010833|rs6025738|rs478061|rs4671408|rs4939659|rs372334|rs12901499|rs12915039|rs8179181|rs128116|rs13044132
Antinuclear Antibodies	RACCYCD_PATHWAY	BIOCARTA	551	28	17	8,06E-01	1,00E+00	rs11604821|rs836556|rs12867405|rs1550805|rs6449969|rs2415290|rs4805487|rs12207548|rs9838117|rs624797|rs2237572|rs12289836|rs7781436|rs10872893|rs1872568|rs3138045|rs9462209
Antinuclear Antibodies	VEGF_SIGNALING_PATHWAY	KEGG	2111	43	20	8,06E-01	1,00E+00	rs230624|rs4262039|rs346811|rs8081512|rs169143|rs836556|rs6532915|rs6683451|rs12199654|rs10445097|rs7148384|rs11583351|rs198178|rs612355|rs1550805|rs2392794|rs4767886|rs4699014|rs16826789|rs6449969
Antinuclear Antibodies	RIBOSOME	KEGG	1118	41	20	8,06E-01	1,00E+00	rs7902136|rs7176919|rs12602594|rs1953910|rs17121120|rs10065570|rs11002569|rs4777170|rs6895379|rs11733964|rs2301723|rs13325754|rs11002552|rs1953912|rs1956099|rs10895987|rs1569498|rs953978|rs8179823|rs2140386
Antinuclear Antibodies	MATURITY_ONSET_DIABETES_OF_THE_YOUNG	KEGG	992	38	19	8,07E-01	1,00E+00	rs2943547|rs1337728|rs1390088|rs1343882|rs9886592|rs2247328|rs17008412|rs8025430|rs874511|rs4264821|rs7209835|rs3094509|rs2001726|rs746508|rs4812823|rs16939060|rs7529495|rs9839009|rs2941433
Antinuclear Antibodies	FC_EPSILON_RI_SIGNALING_PATHWAY	KEGG	2758	44	20	8,07E-01	1,00E+00	rs4436949|rs230624|rs656709|rs8081512|rs4603757|rs2529737|rs3824561|rs169143|rs836556|rs6683451|rs11153318|rs12199654|rs2510257|rs10445097|rs9896052|rs11583351|rs198178|rs612355|rs10821495|rs1550805
Antinuclear Antibodies	LAGGING_STRAND_SYNTHESIS	REACTOME	550	19	13	8,08E-01	1,00E+00	rs3786136|rs512604|rs741048|rs2393374|rs174532|rs2278998|rs11762146|rs1832265|rs7328146|rs1405655|rs9540647|rs1860652|rs17545935
Antinuclear Antibodies	FMLP_PATHWAY	BIOCARTA	1148	38	20	8,10E-01	1,00E+00	rs2236891|rs836556|rs2866250|rs12199654|rs206422|rs7148384|rs10485723|rs708925|rs4699014|rs2055226|rs2415290|rs739559|rs761685|rs10160860|rs2745755|rs12289836|rs6055995|rs8123677|rs1011398|rs2236893
Antinuclear Antibodies	DOWNSTREAM_SIGNALING_OF_ACTIVATED_FGFR	REACTOME	3636	29	20	8,10E-01	1,00E+00	rs902522|rs1924210|rs12958400|rs9736587|rs306090|rs3129678|rs9549096|rs8081512|rs1397529|rs1030098|rs1404912|rs17360897|rs2287778|rs9576967|rs950549|rs6501512|rs6884797|rs2302784|rs953672|rs9896052
Antinuclear Antibodies	ALK_PATHWAY	BIOCARTA	1932	30	20	8,11E-01	1,00E+00	rs10485101|rs9968396|rs9883073|rs4075546|rs13044457|rs7653262|rs10903341|rs1144171|rs12562819|rs604045|rs358776|rs1923075|rs17153747|rs6107891|rs478061|rs10863388|rs7526672|rs6099433|rs2225265|rs17635121
Antinuclear Antibodies	HER2_PATHWAY	BIOCARTA	1326	33	20	8,15E-01	1,00E+00	rs987320|rs2904530|rs2069837|rs9896052|rs10878450|rs12579363|rs6712652|rs1550805|rs1882175|rs6449969|rs7804859|rs9838117|rs1168312|rs17153527|rs7425448|rs4730197|rs10748053|rs161700|rs1160676|rs4283063
Antinuclear Antibodies	AT1R_PATHWAY	BIOCARTA	1224	39	16	8,15E-01	1,00E+00	rs8081512|rs2529737|rs2274316|rs169143|rs836556|rs9896052|rs198178|rs2055226|rs2163177|rs3785396|rs6681035|rs3746259|rs4681446|rs12259267|rs812196|rs9925433
Antinuclear Antibodies	TH1TH2_PATHWAY	BIOCARTA	535	16	13	8,16E-01	1,00E+00	rs11921898|rs2715267|rs608914|rs1498737|rs1921622|rs3014996|rs7610160|rs3761041|rs393540|rs1431828|rs35265698|rs6602392|rs918519
Antinuclear Antibodies	STRESS_PATHWAY	BIOCARTA	633	30	17	8,17E-01	1,00E+00	rs2529737|rs12199654|rs11614624|rs12817202|rs10859598|rs4761528|rs3750512|rs2415290|rs739559|rs17220720|rs6681035|rs12289836|rs12451722|rs10872893|rs3138045|rs12259267|rs12818362
Antinuclear Antibodies	DNA_REPLICATION	REACTOME	3548	41	20	8,18E-01	1,00E+00	rs766295|rs994626|rs9465734|rs1478785|rs1558461|rs1027692|rs3786136|rs4350299|rs12604|rs9303909|rs12953815|rs17010833|rs4802906|rs11845708|rs7187327|rs3764694|rs10105118|rs12447239|rs3027247|rs12456560
Antinuclear Antibodies	BASE_EXCISION_REPAIR	REACTOME	209	9	6	8,19E-01	1,00E+00	rs2029166|rs174532|rs2278998|rs1405655|rs9930956|rs1872635
Antinuclear Antibodies	PHOSPHATIDYLINOSITOL_SIGNALING_SYSTEM	KEGG	3813	24	20	8,20E-01	1,00E+00	rs4436949|rs928807|rs17041308|rs1967328|rs1407101|rs8081512|rs17593321|rs4603757|rs11922130|rs9533044|rs11876920|rs169143|rs663591|rs2327162|rs4394764|rs2239923|rs2358073|rs3805018|rs10445097|rs2206422
Antinuclear Antibodies	TRNA_AMINOACYLATION	REACTOME	795	21	14	8,21E-01	1,00E+00	rs244896|rs959086|rs933181|rs3733977|rs12968882|rs2123041|rs2645303|rs4959338|rs10452026|rs9405818|rs854221|rs7135097|rs3768976|rs6768533
Antinuclear Antibodies	RIG_I_LIKE_RECEPTOR_SIGNALING_PATHWAY	KEGG	1361	24	20	8,21E-01	1,00E+00	rs10485101|rs11921898|rs12199654|rs1144171|rs2207701|rs1498737|rs1923075|rs331455|rs3750512|rs13217057|rs10498974|rs7026552|rs2415290|rs26538|rs6914621|rs9345117|rs721025|rs4246868|rs12191179|rs17220720
Antinuclear Antibodies	TRANSCRIPTION	REACTOME	2708	27	20	8,22E-01	1,00E+00	rs4921702|rs16858688|rs2227301|rs10495562|rs4922152|rs13296301|rs10961534|rs4922157|rs6081157|rs3786238|rs1372349|rs10732882|rs13270944|rs10121140|rs17092536|rs11989385|rs13290272|rs3809425|rs17213362|rs683474
Antinuclear Antibodies	LONG_TERM_DEPRESSION	KEGG	3926	54	20	8,22E-01	1,00E+00	rs7676645|rs1422893|rs230624|rs17041308|rs7069974|rs1407101|rs10740411|rs1385405|rs8081512|rs169143|rs7730730|rs10228945|rs2327162|rs9306383|rs168213|rs6683451|rs3805018|rs2206422|rs10030915|rs1583351
Antinuclear Antibodies	SIGNALING_BY_ERBB2	REACTOME	3847	29	20	8,22E-01	1,00E+00	rs987320|rs902522|rs12958400|rs306090|rs9549096|rs8081512|rs1397529|rs1030098|rs1404912|rs17360897|rs3756675|rs4458837|rs2287778|rs11153318|rs9576967|rs6501512|rs4373483|rs2302784|rs953672|rs9896052
Antinuclear Antibodies	CELL_CELL_COMMUNICATION	REACTOME	9725	29	20	8,22E-01	1,00E+00	rs4791330|rs6764017|rs7241357|rs10490696|rs2236891|rs183718|rs10502934|rs12496792|rs10934067|rs709505|rs12494510|rs8123347|rs7950175|rs7730479|rs1215|rs1254160|rs12446828|rs11643524|rs6963699|rs10229458
Antinuclear Antibodies	INTEGRIN_PATHWAY	BIOCARTA	1027	36	19	8,24E-01	1,00E+00	rs10800842|rs11102314|rs11153318|rs9896052|rs4767886|rs10901096|rs1546924|rs6861460|rs625750|rs2362941|rs6681035|rs3791906|rs5751636|rs3788358|rs4902681|rs131702|rs12146170|rs12259267|rs869382
Antinuclear Antibodies	BETA_ALANINE_METABOLISM	KEGG	711	20	15	8,25E-01	1,00E+00	rs406303|rs4439928|rs9437663|rs4646772|rs10973734|rs738814|rs2408700|rs4665830|rs1641107|rs7620635|rs12044858|rs13290293|rs17817077|rs12957330|rs17089368
Antinuclear Antibodies	OXIDATIVE_PHOSPHORYLATION	KEGG	2486	34	20	8,25E-01	1,00E+00	rs7853552|rs13272985|rs8133991|rs7216331|rs959086|rs1411451|rs968698|rs9511405|rs10425332|rs4147774|rs11669029|rs1390943|rs6757526|rs10406627|rs7251331|rs16870060|rs11078220|rs10401042|rs12699165|rs6895379
Antinuclear Antibodies	HS_GAG_DEGRADATION	REACTOME	1386	42	20	8,28E-01	1,00E+00	rs1980779|rs12853685|rs1445253|rs2290501|rs1924384|rs11707667|rs2637451|rs7577243|rs1459433|rs7007840|rs2590509|rs3897502|rs10492501|rs996463|rs7586891|rs11724804|rs9523229|rs4773741|rs10492625|rs4654986
Antinuclear Antibodies	METABOLISM_OF_XENOBIOTICS_BY_CYTOCHROME_P450	KEGG	1674	35	20	8,28E-01	1,00E+00	rs9296691|rs11252845|rs316156|rs10456664|rs140289|rs13124794|rs10904449|rs1614972|rs17355469|rs2207951|rs12802641|rs11892031|rs7256675|rs1126673|rs4148280|rs1826909|rs4525119|rs396216|rs8192013|rs1892714
Antinuclear Antibodies	IL2_PATHWAY	BIOCARTA	452	25	13	8,29E-01	1,00E+00	rs2005292|rs9896052|rs10821495|rs204680|rs10244|rs6037819|rs2991216|rs6681035|rs228947|rs2290889|rs6602392|rs12259267|rs7874712
Antinuclear Antibodies	REGULATION_OF_SIGNALING_BY_CBL	REACTOME	703	33	19	8,29E-01	1,00E+00	rs7905645|rs11153318|rs9896052|rs10821495|rs1550805|rs10901096|rs6449969|rs8102571|rs2991216|rs4240896|rs9838117|rs1544241|rs10424498|rs11716652|rs2290889|rs2617822|rs11597989|rs869382|rs7874712
Antinuclear Antibodies	REGULATION_OF_INSULIN_LIKE_GROWTH_FACTOR_IGF_ACTIVITY_BY_INSULIN_LIKE_G	REACTOME	570	30	20	8,29E-01	1,00E+00	rs1247571|rs7865534|rs7523465|rs12379210|rs2659122|rs2397446|rs788723|rs7874262|rs9457997|rs2372866|rs6951912|rs1349323|rs7979974|rs3823055|rs4507975|rs1486137|rs1420228|rs4278081|rs10982987|rs1998499

	DING_PROTEINS_IGFBPS								
Antinuclear Antibodies	ENOS_ACTIVATION_AND_REGULATION	REACTOME	336	13	5	8,31E-01	1,00E+00	rs10961649|rs2055226|rs4142081|rs10961558|rs980720
Antinuclear Antibodies	GAB1_SIGNALOSOME	REACTOME	1077	41	20	8,31E-01	1,00E+00	rs12958400|rs9549096|rs1397529|rs9576967|rs9896052|rs7329423|rs1550805|rs10494270|rs10509534|rs6449969|rs12207548|rs9838117|rs665821|rs9480865|rs10788582|rs945559|rs9930956|rs7824819|rs541425|rs9462209
Antinuclear Antibodies	GLUCONEOGENESIS	REACTOME	490	15	9	8,31E-01	1,00E+00	rs4129219|rs2239167|rs883751|rs13245850|rs9927205|rs3793932|rs13338289|rs10421422|rs6716901
Antinuclear Antibodies	GLYCEROPHOSPHOLIPID_METABOLISM	KEGG	2549	38	20	8,32E-01	1,00E+00	rs10508915|rs230624|rs16947151|rs12081589|rs9533044|rs17009821|rs9458157|rs6683451|rs2358073|rs903927|rs974130|rs11922502|rs11583351|rs3764899|rs726297|rs182034|rs612355|rs1917806|rs13008971|rs11195843
Antinuclear Antibodies	AUTODEGRADATION_OF_CDH1_BY_CDH1_APC_C	REACTOME	857	43	20	8,33E-01	1,00E+00	rs9562187|rs6836883|rs10514915|rs11845708|rs7187327|rs12447239|rs1106950|rs425594|rs11922435|401754|rs13207114|rs11734845|rs3787573|rs11679897|rs2107459|rs9876561|rs577357|rs992507|rs10032586|rs4973377
Antinuclear Antibodies	BMAL1_CLOCK_NPAS2_ACTIVATES_CIRCADIAN_EXPRESSION	REACTOME	1728	33	20	8,36E-01	1,00E+00	rs3774909|rs4589495|rs6448226|rs3789823|rs3824872|rs450110|rs17270181|rs1567804|rs9968207|rs11113179|rs2422840|rs17204910|rs2278637|rs6018631|rs6120708|rs1370429|rs179883|rs339969|rs4912911|rs2062091
Antinuclear Antibodies	PURINE_METABOLISM	KEGG	5442	31	20	8,38E-01	1,00E+00	rs2198511|rs902522|rs11729954|rs9877140|rs10841528|rs7747433|rs4789145|rs2227301|rs819864|rs2387094|rs1404912|rs2327672|rs1358786|rs17360897|rs595890|rs2365491|rs9493105|rs2287778|rs11045301|rs512604
Antinuclear Antibodies	ACYL_CHAIN_REMODELLING_OF_PE	REACTOME	696	42	19	8,41E-01	1,00E+00	rs230624|rs6683451|rs11583351|rs612355|rs13008971|rs16826789|rs631134|rs622450|rs717208|rs2386983|rs2457345|rs2472756|rs7546956|rs625964|rs4466603|rs1712429|rs776691|rs943571|rs10732980
Antinuclear Antibodies	CELL_CELL_JUNCTION_ORGANIZATION	REACTOME	6211	33	20	8,45E-01	1,00E+00	rs6764017|rs7241357|rs183718|rs12496792|rs10934067|rs12494510|rs8123347|rs7730479|rs1215|rs12446828|rs11643524|rs9883073|rs904750|rs7776133|rs9859918|rs485409|rs6124072|rs4925295|rs2937524|rs17231134
Antinuclear Antibodies	SIGNALING_BY_FGFR	REACTOME	4154	32	20	8,47E-01	1,00E+00	rs902522|rs1924210|rs12958400|rs9736587|rs1853300|rs306090|rs3129678|rs9549096|rs8081512|rs1397529|rs1030098|rs1404912|rs17360897|rs6563216|rs2287778|rs885126|rs9576967|rs950549|rs6501512|rs9545290
Antinuclear Antibodies	BUTANOATE_METABOLISM	KEGG	763	17	12	8,47E-01	1,00E+00	rs4439928|rs9858256|rs10516988|rs4646772|rs10973734|rs2408700|rs4665830|rs1641107|rs7620635|rs1946741|rs13290293|rs558275
Antinuclear Antibodies	FOCAL_ADHESION	KEGG	7733	23	20	8,49E-01	1,00E+00	rs12668754|rs2974254|rs12591920|rs12902258|rs2236891|rs12357398|rs4262039|rs11102314|rs12751561|rs9307949|rs4448606|rs4536647|rs216872|rs1878510|rs11604821|rs656709|rs9521666|rs8081512|rs4490127|rs362698
Antinuclear Antibodies	P53_PATHWAY	BIOCARTA	347	16	8	8,49E-01	1,00E+00	rs11604821|rs4805487|rs12207548|rs1439124|rs624797|rs12958785|rs1872568|rs9462209
Antinuclear Antibodies	BIOLOGICAL_OXIDATIONS	REACTOME	3568	35	20	8,55E-01	1,00E+00	rs7918164|rs2028900|rs6052369|rs9296691|rs138055|rs12591359|rs10068900|rs566509|rs158850|rs316156|rs10456664|rs13124794|rs41717|rs8187950|rs6116189|rs978114|rs2273684|rs8187890|rs297961|rs7758711
Antinuclear Antibodies	EFFECTS_OF_PIP2_HYDROLYSIS	REACTOME	1651	32	20	8,55E-01	1,00E+00	rs12286714|rs9533044|rs836556|rs2358073|rs7303557|rs726297|rs182034|rs718314|rs4357599|rs2852786|rs7904311|rs2116531|rs2595199|rs10842720|rs4952809|rs11620516|rs2336408|rs684119|rs1499772|rs11224851
Antinuclear Antibodies	CELL_JUNCTION_ORGANIZATION	REACTOME	6781	33	20	8,56E-01	1,00E+00	rs6764017|rs7241357|rs2236891|rs173840|rs12496792|rs10934067|rs12494510|rs8123347|rs7730479|rs1215|rs11254160|rs12446828|rs11643524|rs9883073|rs904750|rs7776133|rs9859918|rs485409|rs6124072|rs4925295
Antinuclear Antibodies	PHOSPHOLIPID_METABOLISM	REACTOME	6226	40	20	8,59E-01	1,00E+00	rs17752182|rs6448180|rs678194|rs2167239|rs10508915|rs230624|rs3011777|rs16947151|rs12081589|rs1493130|rs346811|rs7291756|rs6673947|rs11876920|rs17009821|rs9458157|rs6683451|rs17333779|rs731395|rs723506
Antinuclear Antibodies	DC_PATHWAY	BIOCARTA	573	15	9	8,60E-01	1,00E+00	rs11921898|rs1498737|rs4483225|rs10513330|rs3014996|rs7610160|rs7406439|rs797999|rs918519
Antinuclear Antibodies	ABORTIVE_ELONGATION_OF_HIV1_TRANSCRIPT_IN_THE_ABSENCE_OF_TAT	REACTOME	195	8	6	8,61E-01	1,00E+00	rs3786238|rs633742|rs604965|rs4799085|rs715749|rs2293603
Antinuclear Antibodies	PROCESSING_OF_CAPPED_INTRONLESS_PRE_MRNA	REACTOME	274	19	9	8,64E-01	1,00E+00	rs10495562|rs3809425|rs13183577|rs4798|rs6599528|rs715749|rs234592|rs17214407|rs6064389
Antinuclear Antibodies	GLUCURONIDATION	REACTOME	656	3	2	8,64E-01	1,00E+00	rs11892031|rs4148280
Antinuclear Antibodies	SIGNAL_TRANSDUCTION_BY_L1	REACTOME	1310	34	20	8,67E-01	1,00E+00	rs11970312|rs836556|rs10827245|rs11594539|rs16933086|rs161130|rs10510697|rs204680|rs6037819|rs378373|rs3799584|rs11009356|rs6550492|rs12376522|rs12279261|rs2071941|rs1447460|rs3799613|rs16899429|rs909768
Antinuclear Antibodies	METABOLISM_OF_MRNA	REACTOME	3017	46	20	8,67E-01	1,00E+00	rs1039153|rs7902136|rs1504632|rs12602594|rs11228985|rs9562187|rs8081512|rs1000174|rs1111930|rs12199654|rs1953910|rs17121120|rs17010833|rs2976282|rs10065570|rs346524|rs10732882|rs4802906|rs16973403|rs11002569
Antinuclear Antibodies	RNA_POL_I_PROMOTER_OPENING	REACTOME	501	2	2	8,67E-01	1,00E+00	rs871443|rs9303601
Antinuclear Antibodies	EXTENSION_OF_TELOMERES	REACTOME	618	21	14	8,68E-01	1,00E+00	rs3786136|rs512604|rs741048|rs2393374|rs174532|rs2278998|rs11762146|rs1832265|rs7328146|rs1405655|rs9540647|rs4968207|rs1860652|rs17545935
Antinuclear Antibodies	CXCR4_PATHWAY	BIOCARTA	1099	39	20	8,68E-01	1,00E+00	rs8081512|rs169143|rs10228945|rs198178|rs2714645|rs1550805|rs4767886|rs7371043|rs11813145|rs6449969|rs2163177|rs2523189|rs9838117|rs3785396|rs10191360|rs10160860|rs17488134|rs12289836|rs13232987|rs9925433
Antinuclear Antibodies	NGF_PATHWAY	BIOCARTA	566	24	15	8,70E-01	1,00E+00	rs2005292|rs9896052|rs1550805|rs204680|rs6449969|rs6037819|rs7804859|rs2798464|rs9838117|rs17153527|rs6681035|rs4730197|rs12136101|rs12259267|rs17153654
Antinuclear Antibodies	G1_PATHWAY	BIOCARTA	611	32	20	8,70E-01	1,00E+00	rs11604821|rs12867405|rs604045|rs10863388|rs7526672|rs4805487|rs1333050|rs12207548|rs372334|rs12901499|rs9576040|rs624797|rs2237572|rs12915039|rs8179181|rs7781436|rs1603059|rs1872568|rs334550|rs2583836
Antinuclear Antibodies	GLYCEROPHOSPHOLIPID_BIOSYNTHESIS	REACTOME	2483	40	20	8,71E-01	1,00E+00	rs10508915|rs230624|rs16947151|rs6673947|rs17009821|rs9458157|rs6683451|rs731395|rs903927|rs974130|rs12349416|rs11922502|rs11583351|rs3764899|rs612355|rs10183830|rs1917806|rs13008971|rs17097955|rs11195843
Antinuclear Antibodies	DOWNSTREAM_SIGNALING_EVENTS_OF_B_CELL_RECEPTOR_BCR	REACTOME	2127	33	20	8,72E-01	1,00E+00	rs11767716|rs12958400|rs10485101|rs9549096|rs169143|rs9576967|rs1144171|rs198178|rs1923075|rs7329423|rs11845708|rs7187327|rs12447239|rs10494270|rs10509534|rs13217057|rs10498974|rs698856|rs2415290|rs425594
Antinuclear Antibodies	ASSEMBLY_OF_THE_PRE_REPLICATIVE_COMPLEX	REACTOME	911	29	17	8,74E-01	1,00E+00	rs9465734|rs11845708|rs7187327|rs12447239|rs698856|rs425594|rs11922435|401754|rs13207114|rs6032895|rs9295464|rs2107459|rs3087474|rs3820028|rs577357|rs992507|rs4973377
Antinuclear Antibodies	GLYCOGEN_BREAKDOWN_GLYCOGENOLYSIS	REACTOME	246	13	7	8,74E-01	1,00E+00	rs8052416|rs2055226|rs6855439|rs3772573|rs6531592|rs594249|rs17301901
Antinuclear Antibodies	DEPOSITION_OF_NEW_CENPA_CONTAINING_NUCLEOSOMES_AT_THE_CENTROMERE	REACTOME	637	15	10	8,75E-01	1,00E+00	rs8134439|rs11562980|rs2833736|rs1976101|rs2833752|rs372543|rs10492892|rs704874|rs1957173|rs7606893
Antinuclear Antibodies	MEIOTIC_SYNAPSIS	REACTOME	1031	30	16	8,78E-01	1,00E+00	rs4984803|rs4350299|rs214952|rs11913200|rs2258974|rs4645434|rs909914|rs12201313|rs4902264|rs2813494|rs360589|rs695983|rs10144225|rs3738593|rs2485664|rs9397084
Antinuclear Antibodies	AKT_PATHWAY	BIOCARTA	682	28	16	8,79E-01	1,00E+00	rs9549096|rs9576967|rs7329423|rs1550805|rs6449969|rs2415290|rs7804859|rs9838117|rs17153527|rs10941583|rs9480865|rs12289836|rs4730197|rs10872893|rs3138045|rs17153654
Antinuclear Antibodies	DNA_STRAND_ELONGATION	REACTOME	672	22	16	8,81E-01	1,00E+00	rs3786136|rs512604|rs3764694|rs741048|rs2393374|rs174532|rs2278998|rs6990366|rs11762146|rs1832265|rs7328146|rs1405655|rs9540647|rs1860652|rs17545935|rs2871029
Antinuclear Antibodies	COLORECTAL_CANCER	KEGG	2539	32	20	8,82E-01	1,00E+00	rs9886419|rs10502934|rs7812998|rs1561928|rs11604821|rs9883073|rs836556|rs2005292|rs7653262|rs1426511|rs604045|rs6753949|rs6544990|rs478061|rs10863388|rs1550805|rs7526672|rs4556886|rs6449969|rs3136247
Antinuclear Antibodies	TRANSPORT_OF_VITAMINS_NUCLEOSIDES_AND_RELATED_MOLECULES	REACTOME	1154	34	20	8,84E-01	1,00E+00	rs1139266|rs9357436|rs6885006|rs4854776|rs7719773|rs12582527|rs1515766|rs4980346|rs6496836|rs2422840|rs1643033|rs3803390|rs7627477|rs10841644|rs2179845|rs4149023|rs6870697|rs17598180|rs17185623|rs6062840
Antinuclear Antibodies	ALZHEIMERS_DISEASE	KEGG	5029	29	20	8,84E-01	1,00E+00	rs1806201|rs12765241|rs2829946|rs10920115|rs17041308|rs2776341|rs1407101|rs10495562|rs466609|rs2327162|rs1411451|rs7694484|rs658071|rs4332745|rs3805018|rs2206422|rs1072869|rs6440961|rs9915261|rs2872715
Antinuclear Antibodies	RELA_PATHWAY	BIOCARTA	208	7	6	8,85E-01	1,00E+00	rs331455|rs2415290|rs1061624|rs12289836|rs10872893|rs3138045
Antinuclear Antibodies	PTEN_PATHWAY	BIOCARTA	544	17	9	8,89E-01	1,00E+00	rs9896052|rs1550805|rs10509534|rs6449969|rs9838117|rs9480865|rs10788582|rs945559|rs12146170
Antinuclear Antibodies	IL_3_5_AND_GM_CSF_SIGNALING	REACTOME	1134	32	20	8,90E-01	1,00E+00	rs7905645|rs11153318|rs9896052|rs10821495|rs1550805|rs2239167|rs10901096|rs310244|rs6449969|rs163567|rs2274471|rs8102571|rs2991216|rs4240896|rs9838117|rs1544241|rs4734512|rs4734497|rs228947|rs10424498
Antinuclear Antibodies	MRNA_SPLICING_MINOR_PATHWAY	REACTOME	383	14	8	8,92E-01	1,00E+00	rs6062340|rs3826425|rs633742|rs4435501|rs13038619|rs604965|rs715749|rs2293603
Antinuclear Antibodies	ARACHIDONIC_ACID_METABOLISM	KEGG	1356	45	20	8,93E-01	1,00E+00	rs230624|rs6683451|rs11583351|rs312462|rs41717|rs612355|rs312460|rs1038121|rs16826789|rs9911427|rs631134|rs4808358|rs622450|rs6119535|rs5751902|rs2278964|rs7256675|rs7546956|rs4525119|rs500670
Antinuclear Antibodies	XENOBIOTICS	REACTOME	231	5	2	8,93E-01	1,00E+00	rs7256675|rs2470890
Antinuclear Antibodies	ACTIVATION_OF_THE_PRE_REPLICATIVE_COMPLEX	REACTOME	565	12	9	8,94E-01	1,00E+00	rs3786136|rs512604|rs741048|rs11762146|rs3087474|rs1860652|rs768255|rs17545935|rs2871029
Antinuclear Antibodies	SYNTHESIS_OF_PIPS_AT_THE_PLASMA_MEMBRANE	REACTOME	1050	33	20	8,94E-01	1,00E+00	rs7291756|rs2475556|rs6834625|rs750997|rs1550805|rs883751|rs3775616|rs10509534|rs6449969|rs11013140|rs7804859|rs10806791|rs4240896|rs9838117|rs17153527|rs10160860|rs10788582|rs945559|rs11716652|rs4730197
Antinuclear Antibodies	ANTIGEN_PRESENTATION_FOLDING_ASSEMBLY_AND_PEPTIDE_LOADING_OF_CLASS_I_M	REACTOME	830	18	7	8,96E-01	1,00E+00	rs10050860|rs1611493|rs1559085|401754|rs2246010|rs2523554|rs10824031
Antinuclear Antibodies	DOWNREGULATION_OF_SMAD2_3_SMAD4_TRANSCRIPTIONAL_ACTIVITY	REACTOME	453	14	11	8,96E-01	1,00E+00	rs85440|rs2229840|rs727929|rs701038|rs906304|rs4939659|rs372334|rs12901499|rs12915039|rs874583|rs12318072
Antinuclear Antibodies	SIGNALING_BY_ERBB4	REACTOME	3434	31	20	8,97E-01	1,00E+00	rs987320|rs11225161|rs12958400|rs9549096|rs10495562|rs12203803|rs3756675|rs4458837|rs9576967|rs7187776|rs4373483|rs9896052|rs2439312|rs10521025|rs6940350|rs6712652|rs7329423|rs8055815|rs17205146|rs12524841
Antinuclear Antibodies	GLYCOSAMINOGLYCAN_DEGRADATION	KEGG	709	33	14	8,98E-01	1,00E+00	rs1866430|rs2098266|rs11707667|rs6502282|rs3897502|rs1859906|rs12603445|rs4239128|rs11724804|rs6863289|rs16947829|rs820848|rs9303080|rs754567
Antinuclear Antibodies	IL_RECEPTOR_SHC_SIGNALING	REACTOME	624	25	13	8,98E-01	1,00E+00	rs9896052|rs1550805|rs2239167|rs310244|rs6449969|rs163567|rs2274471|rs4240896|rs9838117|rs228947|rs11716652|rs6602392|rs7935699
Antinuclear Antibodies	MITOCHONDRIAL_TRNA_AMINOACYLATION	REACTOME	500	11	8	8,99E-01	1,00E+00	rs959086|rs2645303|rs4959338|rs10452026|rs9405818|rs854221|rs7135097|rs6768533
Antinuclear Antibodies	STEROID_HORMONE_BIOSYNTHESIS	KEGG	1491	42	20	8,99E-01	1,00E+00	rs11252845|rs12591359|rs213213|rs7682870|rs10904449|rs2002700|rs12441859|rs34562262|rs4844486|rs2300697|rs4288415|rs11892031|rs17703982|rs12056107|rs4148280|rs4525119|rs2589998|rs4559587|rs12555788|rs2684874
Antinuclear Antibodies	CIRCADIAN_CLOCK	REACTOME	2075	34	20	9,00E-01	1,00E+00	rs3774909|rs4589495|rs6448226|rs2274316|rs3789823|rs3824872|rs450110|rs17270181|rs1567804|rs11113179|rs9968207|rs2422840|rs17204910|rs2278637|rs6018631|rs6088590|rs1370429|rs179883|rs339969|rs4912911
Antinuclear Antibodies	GLIOMA	KEGG	2070	37	20	9,02E-01	1,00E+00	rs9465734|rs7665102|rs11604821|rs8081512|rs169143|rs7168213|rs10445097|rs9896052|rs198178|rs8031813|rs7181975|rs1550805|rs944482|rs2392794|rs10509534|rs6449969|rs2055226|rs873592|rs7804859|rs12207548
Antinuclear Antibodies	BCELLSURVIVAL_PATHWAY	BIOCARTA	500	14	11	9,02E-01	1,00E+00	rs2005292|rs1550805|rs6449969|rs7804859|rs6861460|rs9838117|rs17153527|rs4353229|rs4730197|rs12146170|rs17153654
Antinuclear Antibodies	KERATINOCYTE_PATHWAY	BIOCARTA	1952	31	20	9,03E-01	1,00E+00	rs12765241|rs10790928|rs8081512|rs2529737|rs169143|rs2005292|rs12199654|rs198178|rs7108562|rs9787850|rs4357599|rs3750512|rs7904311|rs2415290|rs739559|rs2595199|rs1061624|rs4952809|rs3785396|rs12294634
Antinuclear Antibodies	MITOCHONDRIAL_PROTEIN_IMPORT	REACTOME	609	20	16	9,04E-01	1,00E+00	rs6461670|rs4143897|rs7716228|rs2819369|rs10068191|rs13245850|rs2362866|rs1130635|rs2197801|rs10262258|rs10826223|rs744675|rs6716901|rs4689604|rs868179|rs2885631
Antinuclear Antibodies	FORMATION_OF_TUBULIN_FOLDING_INTERMEDIATES_BY_CCT_TRIC	REACTOME	227	6	4	9,04E-01	1,00E+00	rs371164|rs710764|rs3844450|rs6865121
Antinuclear Antibodies	GATA3_PATHWAY	BIOCARTA	898	21	15	9,04E-01	1,00E+00	rs1030098|rs12199654|rs6501512|rs2302784|rs1444788|rs883751|rs1415882|rs761685|rs1324880|rs10514476|rs173645|rs12443665|rs955921|rs12261421|rs614569
Antinuclear Antibodies	G1_S_TRANSITION	REACTOME	1941	29	20	9,04E-01	1,00E+00	rs9886419|rs7812998|rs1561928|rs3786136|rs12867405|rs512604|rs1426511|rs7015841|rs4802906|rs11845708|rs7187327|rs10105118|rs12447239|rs741048|rs425594|rs4805487|rs11922435|401754|rs13207114|rs12207548
Antinuclear Antibodies	DNA_REPLICATION	KEGG	740	21	15	9,04E-01	1,00E+00	rs3786136|rs512604|rs741048|rs2393374|rs174532|rs2278998|rs2306597|rs11762146|rs1832265|rs7328146|rs1405655|rs9540647|rs1860652|rs6591188|rs17545935
Antinuclear Antibodies	TGF_BETA_SIGNALING_PATHWAY	KEGG	3535	26	20	9,05E-01	1,00E+00	rs9886419|rs7572399|rs7812998|rs9968396|rs159480|rs1561928|rs4075546|rs13044457|rs12466337|rs12867405|rs4662532|rs9392915|rs7653262|rs6987276|rs1601667|rs238630|rs6706968|rs10946347|rs1426511|rs604045
Antinuclear Antibodies	SIGNALING_BY_EGFR_IN_CANCER	REACTOME	4030	32	20	9,08E-01	1,00E+00	rs902522|rs12958400|rs1853300|rs306090|rs9549096|rs8081512|rs1397529|rs1030098|rs1404912|rs10495562|rs17360897|rs1870510|rs6563216|rs2287778|rs9576967|rs12633819|rs6501512|rs9545290|rs2302784|rs953672
Antinuclear Antibodies	ASCORBATE_AND_ALDARATE_METABOLISM	KEGG	874	12	7	9,09E-01	1,00E+00	rs4646772|rs10973734|rs2408700|rs11892031|rs4148280|rs9616854|rs13290293
Antinuclear Antibodies	N_GLYCAN_BIOSYNTHESIS	KEGG	1792	34	20	9,10E-01	1,00E+00	rs13072457|rs5742858|rs2106673|rs12603183|rs807270|rs13035520|rs11621456|rs1490776|rs11203713|rs12755096|rs12946266|rs7642575|rs12423422|rs6989485|rs12304768|rs10844762|rs2720602|rs999925|rs484747|rs12881142
Antinuclear Antibodies	AMINOACYL_TRNA_BIOSYNTHESIS	KEGG	731	19	13	9,11E-01	1,00E+00	rs244896|rs933181|rs3733977|rs12968882|rs12123041|rs2645303|rs4959338|rs10452026|rs9405818|rs854221|rs7135097|rs3768976|rs6768533
Antinuclear Antibodies	TFF_PATHWAY	BIOCARTA	697	30	14	9,11E-01	1,00E+00	rs9883073|rs9896052|rs1550805|rs6449969|rs7804859|rs9838117|rs1439124|rs17153527|rs39416|rs10941583|rs4730197|rs12146170|rs10268780|rs17153654
Antinuclear Antibodies	RENAL_CELL_CARCINOMA	KEGG	2164	34	20	9,11E-01	1,00E+00	rs11102314|rs10790928|rs11010056|rs1397529|rs836556|rs1357287|rs9896052|rs7542797|rs604045|rs708030|rs2891268|rs7108562|rs10863388|rs1550805|rs9787850|rs309770|rs7526672|rs4767879|rs10901096|rs1000531
Antinuclear Antibodies	HCMV_PATHWAY	BIOCARTA	547	20	11	9,12E-01	1,00E+00	rs12199654|rs1550805|rs6449969|rs739559|rs7804859|rs9838117|rs17153527|rs12289836|rs4730197|rs817565|rs17153654
Antinuclear Antibodies	GABA_SYNTHESIS_RELEASE_REUPTAKE_AND_DEGRADATION	REACTOME	746	29	19	9,12E-01	1,00E+00	rs4439928|rs488192|rs2815717|rs10861133|rs2206161|rs9820724|rs12528795|rs2781562|rs973188|rs2278637|rs1641107|rs6931045|rs362570|rs9350450|rs11087010|rs9446655|rs363012|rs6133821|rs1245769
Antinuclear Antibodies	LYSINE_DEGRADATION	KEGG	952	33	19	9,12E-01	1,00E+00	rs10767617|rs2072168|rs6885410|rs2268075|rs7644640|rs4646772|rs3125785|rs10973734|rs2844456|rs2269879|rs2408700|rs12490099|rs4665830|rs3088050|rs12459350|rs7620635|rs2602248|rs13290293|rs1870073
Antinuclear Antibodies	TRANSPORT_TO_THE_GOLGI_AND_SUBSEQUENT_MODIFICATION	REACTOME	1557	34	20	9,13E-01	1,00E+00	rs11152012|rs6095638|rs807270|rs2279447|rs13203167|rs13035520|rs9363217|rs1455777|rs1490776|rs12424060|rs359327|rs4141587|rs1352323|rs10508527|rs222547|rs999925|rs6932267|rs3784732|rs10498989|rs6707591
Antinuclear Antibodies	CERAMIDE_PATHWAY	BIOCARTA	425	8	8	9,15E-01	1,00E+00	rs2529737|rs3750512|rs2130070|rs39416|rs12289836|rs12958785|rs12259267|rs10268780
Antinuclear Antibodies	S_PHASE	REACTOME	2076	28	20	9,15E-01	1,00E+00	rs9886419|rs7812998|rs1561928|rs11604821|rs3786136|rs512604|rs1426511|rs7015841|rs11845708|rs7187327|rs12447239|rs3764694|rs741048|rs2393374|rs174532|rs698856|rs425594|rs4805487|rs11922435|rs2278998
Antinuclear Antibodies	NUCLEAR_RECEPTOR_TRANSCRIPTION_PATHWAY	REACTOME	2750	31	20	9,16E-01	1,00E+00	rs9819978|rs4589495|rs2943547|rs1886124|rs1337728|rs7688347|rs6837298|rs213213|rs1556695|rs577787|rs2247328|rs17484678|rs7157428|rs17270181|rs9843745|rs10463193|rs2200984|rs1568400|rs3749376|rs1570500
Antinuclear Antibodies	VIF_MEDIATED_DEGRADATION_OF_APOBEC3G	REACTOME	738	23	14	9,17E-01	1,00E+00	rs11845708|rs7187327|rs12447239|rs698856|rs425594|rs11922435|rs1053079|401754|rs13207114|rs3787573|rs2107459|rs577357|rs992507|rs4973377
Antinuclear Antibodies	DOUBLE_STRAND_BREAK_REPAIR	REACTOME	506	21	12	9,17E-01	1,00E+00	rs12453935|rs3786136|rs741048|rs11762146|rs828704|rs1805388|rs8178179|rs301282|rs11571805|rs2508678|rs17545935|rs144848
Antinuclear Antibodies	IMMUNOREGULATORY_INTERACTIONS_BETWEEN_A_LYMPHOID_AND_A_NON_LYMPHO	REACTOME	1325	38	20	9,17E-01	1,00E+00	rs10772294|rs1749313|rs2824206|rs1389623|rs2824065|rs1611493|rs6488285|rs272422|rs2823997|rs202873|rs2246010|rs11759611|rs3176874|rs4607021|rs8105340|rs9305793|rs170962|rs12146170|rs2523554|rs4968681
Antinuclear Antibodies	IL12_PATHWAY	BIOCARTA	589	31	20	9,17E-01	1,00E+00	rs13029532|rs11921898|rs12199654|rs1498737|rs1921622|rs739559|rs2274471|rs3014996|rs16833215|rs7610160|rs6681035|rs3761041|rs393540|rs12451722|rs13431828|rs16833239|rs1917534|rs16859030|rs12259267|rs918519
Antinuclear Antibodies	TRYPTOPHAN_METABOLISM	KEGG	953	35	18	9,17E-01	1,00E+00	rs11685496|rs4672866|rs10169957|rs11808201|rs975533|rs4646772|rs10973734|rs1487276|rs2645303|rs2408700|rs4665830|rs1001179|rs7620635|rs13290293|rs1472080|rs9307938|rs2470890|rs10227423
Antinuclear Antibodies	PANCREATIC_CANCER	KEGG	1920	29	20	9,19E-01	1,00E+00	rs9465734|rs11604821|rs6977082|rs13029532|rs836556|rs7653262|rs604045|rs1811208|rs478061|rs10863388|rs309770|rs1550805|rs7526672|rs310244|rs6449969|rs13098743|rs7804859|rs2519090|rs9295464|rs4939659
Antinuclear Antibodies	CDK_MEDIATED_PHOSPHORYLATION_AND_REMOVAL_OF_CDC6	REACTOME	698	22	13	9,20E-01	1,00E+00	rs11845708|rs7187327|rs12447239|rs698856|rs425594|rs11922435|401754|rs13207114|rs3787573|rs2107459|rs577357|rs992507|rs4973377
Antinuclear Antibodies	ENDOMETRIAL_CANCER	KEGG	3028	53	20	9,21E-01	1,00E+00	rs9886419|rs7812998|rs1561928|rs11604821|rs17020744|rs2974154|rs9883073|rs2862944|rs7605056|rs1432175|rs9896052|rs671772|rs1426511|rs6480244|rs1550805|rs17017611|rs10509534|rs13015589|rs6449969|rs6719501
Antinuclear Antibodies	FORMATION_OF_RNA_POL_II_ELONGATION_COMPLEX_	REACTOME	401	11	9	9,22E-01	1,00E+00	rs3786238|rs10732882|rs1053079|rs633742|rs604965|rs4799085|rs715749|rs2293603|rs2932777
Antinuclear Antibodies	L1CAM_INTERACTIONS	REACTOME	3914	38	20	9,24E-01	1,00E+00	rs2469515|rs11970312|rs1579282|rs4948420|rs7829347|rs1838343|rs12593435|rs6869991|rs836556|rs10827245|rs13255835|rs4903104|rs11594539|rs2729137|rs16933086|rs12355908|rs12461158|rs161130|rs10185592|rs11997827
Antinuclear Antibodies	P53_INDEPENDENT_G1_S_DNA_DAMAGE_CHECKPOINT	REACTOME	729	22	13	9,24E-01	1,00E+00	rs11845708|rs7187327|rs12447239|rs698856|rs425594|rs11922435|401754|rs13207114|rs3787573|rs2107459|rs577357|rs992507|rs4973377
Antinuclear Antibodies	41BB_PATHWAY	BIOCARTA	341	12	11	9,31E-01	1,00E+00	rs12199654|rs3750512|rs2415290|rs3014996|rs6681035|rs12289836|rs4363056|rs10139934|rs10872893|rs3138045|rs12259267
Antinuclear Antibodies	LIPID_DIGESTION_MOBILIZATION_AND_TRANSPORT	REACTOME	1389	30	20	9,31E-01	1,00E+00	rs6473282|rs1532624|rs1110060|rs2290501|rs10179921|rs883751|rs4708|rs11632627|rs2271461|rs13396400|rs2271463|rs7469197|rs2336408|rs12474685|rs633695|rs2472510|rs9457997|rs7449650|rs6441791|rs4654986
Antinuclear Antibodies	AUTODEGRADATION_OF_THE_E3_UBIQUITIN_LIGASE_COP1	REACTOME	766	22	13	9,32E-01	1,00E+00	rs11845708|rs7187327|rs12447239|rs698856|rs425594|rs11922435|401754|rs13207114|rs3787573|rs2107459|rs577357|rs992507|rs4973377

Antinuclear Antibodies	SIGNALING_BY_THE_B_CELL_RECEPTOR_BCR	REACTOME	3426	37	20	9,32E-01	1,00E+00	rs11767716|rs7905645|rs12958400|rs10485101|rs12571935|rs9549096|rs169143|rs11153318|rs9576967|rs10445097|rs1144171|rs9896052|rs2060822|rs11706098|rs7303557|rs198178|rs1923075|rs718314|rs7329423|rs10821495
Antinuclear Antibodies	RNA_POL_I_TRANSCRIPTION_TERMINATION	REACTOME	335	11	8	9,34E-01	1,00E+00	rs2227301|rs9303601|rs3848006|rs3759978|rs2293603|rs417271|rs2932777|rs35455442
Antinuclear Antibodies	RENIN_ANGIOTENSIN_SYSTEM	KEGG	467	8	5	9,34E-01	1,00E+00	rs6440961|rs2331406|rs2903808|rs4681446|rs812196
Antinuclear Antibodies	M_G1_TRANSITION	REACTOME	1315	34	20	9,35E-01	1,00E+00	rs9465734|rs3786136|rs512604|rs11845708|rs7187327|rs12447239|rs741048|rs698856|rs425594|rs11922435|401754|rs13207114|rs3787573|rs9295464|rs11762146|rs2107459|rs3087474|rs3820028|rs577357|rs1860652
Antinuclear Antibodies	MTOR_SIGNALING_PATHWAY	KEGG	1290	27	20	9,36E-01	1,00E+00	rs3799663|rs17762352|rs309770|rs1550805|rs1001684|rs6449969|rs378373|rs3799584|rs7804859|rs17296210|rs4240896|rs13431868|rs9838117|rs2071941|rs10169231|rs17153527|rs7328100|rs11716652|rs9930956|rs4730197
Antinuclear Antibodies	TELOMERE_MAINTENANCE	REACTOME	1133	21	14	9,37E-01	1,00E+00	rs3786136|rs512604|rs741048|rs2393374|rs174532|rs2278998|rs11762146|rs1832265|rs7328146|rs1405655|rs9540647|rs4968207|rs1860652|rs17545935
Antinuclear Antibodies	ACUTE_MYELOID_LEUKEMIA	KEGG	1892	37	20	9,39E-01	1,00E+00	rs9886419|rs7812998|rs1561928|rs11604821|rs13049777|rs9896052|rs2834652|rs1426511|rs1550805|rs10891629|rs6449969|rs6719501|rs2697920|rs7804859|rs7895307|rs4240896|rs9838117|rs485550|rs9576040|rs624797
Antinuclear Antibodies	CROSS_PRESENTATION_OF_SOLUBLE_EXOGENOUS_ANTIGENS_ENDOSOMES	REACTOME	713	23	14	9,41E-01	1,00E+00	rs11845708|rs7187327|rs12447239|rs698856|rs425594|rs11922435|401754|rs13207114|rs6032895|rs2107459|rs2080390|rs577357|rs992507|rs4973377
Antinuclear Antibodies	P53_DEPENDENT_G1_DNA_DAMAGE_RESPONSE	REACTOME	838	28	17	9,41E-01	1,00E+00	rs7015841|rs11845708|rs7187327|rs12447239|rs698856|rs425594|rs4805487|rs11922435|401754|rs13207114|rs12207548|rs3787573|rs2107459|rs577357|rs992507|rs4973377|rs9462209
Antinuclear Antibodies	TOB1_PATHWAY	BIOCARTA	652	22	16	9,41E-01	1,00E+00	rs7653262|rs604045|rs478061|rs10863388|rs7526672|rs372334|rs12901499|rs3014996|rs12915039|rs4278789|rs6672952|rs8179181|rs1603059|rs6602392|rs1917534|rs16859030
Antinuclear Antibodies	BASE_EXCISION_REPAIR	KEGG	500	20	14	9,42E-01	1,00E+00	rs7693247|rs17153747|rs2029166|rs174532|rs2278998|rs2430682|rs1467719|rs1405655|rs9930956|rs2580065|rs2388548|rs874583|rs1872635|rs17074691
Antinuclear Antibodies	ER_PHAGOSOME_PATHWAY	REACTOME	1416	46	19	9,42E-01	1,00E+00	rs4743335|rs4743347|rs11845708|rs7187327|rs12447239|rs1611493|rs698856|rs425594|rs11922435|401754|rs13207114|rs3787573|rs7805129|rs2246010|rs2107459|rs577357|rs2523554|rs992507|rs4973377
Antinuclear Antibodies	P53HYPOXIA_PATHWAY	BIOCARTA	517	14	11	9,43E-01	1,00E+00	rs2214102|rs3786136|rs12207548|rs788723|rs6951912|rs4640402|rs1486137|rs12259267|rs4278081|rs7712431|rs9462209
Antinuclear Antibodies	MRNA_3_END_PROCESSING	REACTOME	320	24	12	9,44E-01	1,00E+00	rs10495562|rs3809425|rs4798|rs13038619|rs8013586|rs706130|rs150926|rs6599528|rs715749|rs234592|rs17214407|rs6064389
Antinuclear Antibodies	ECM_PATHWAY	BIOCARTA	753	20	12	9,44E-01	1,00E+00	rs11153318|rs1550805|rs4767886|rs6449969|rs7804859|rs6861460|rs9838117|rs17153527|rs4730197|rs1958913|rs12146170|rs17153654
Antinuclear Antibodies	PROSTATE_CANCER	KEGG	2870	42	20	9,45E-01	1,00E+00	rs2974254|rs9465734|rs9307949|rs11604821|rs9549096|rs9883073|rs11632057|rs9576967|rs17423437|rs9896052|rs4751828|rs7015841|rs7329423|rs11981754|rs7181975|rs1550805|rs2659122|rs10509534|rs6449969|rs2415290
Antinuclear Antibodies	REGULATION_OF_ORNITHINE_DECARBOXYLASE_ODC	REACTOME	773	23	14	9,45E-01	1,00E+00	rs11845708|rs7187327|rs12447239|rs698856|rs425594|rs11922435|401754|rs13207114|rs3787573|rs2107459|rs11633399|rs577357|rs992507|rs4973377
Antinuclear Antibodies	RNA_POL_I_TRANSCRIPTION_INITIATION	REACTOME	427	12	9	9,45E-01	1,00E+00	rs2227301|rs2844456|rs9303601|rs3848006|rs3759978|rs2293603|rs417271|rs2932777|rs35455442
Antinuclear Antibodies	PRIMARY_BILE_ACID_BIOSYNTHESIS	KEGG	439	12	9	9,46E-01	1,00E+00	rs11130651|rs10904449|rs2002700|rs4288415|rs12056107|rs2589998|rs12192544|rs2841027|rs7625947
Antinuclear Antibodies	METABOLISM_OF_VITAMINS_AND_COFACTORS	REACTOME	1119	34	20	9,47E-01	1,00E+00	rs158898|rs17664707|rs9493105|rs10947842|rs9493133|rs8011839|rs3004068|rs1792225|rs745686|rs4502283|rs7768480|rs11848862|rs9813586|rs179883|rs16849307|rs1024908|rs137132|rs841861|rs373542|rs6683452
Antinuclear Antibodies	PROTEASOME	KEGG	821	25	15	9,47E-01	1,00E+00	rs11845708|rs7187327|rs12447239|rs698856|rs425594|rs11922435|401754|rs13207114|rs3787573|rs3014996|rs1404508|rs2107459|rs577357|rs992507|rs4973377
Antinuclear Antibodies	MRNA_SPLICING	REACTOME	1057	32	20	9,48E-01	1,00E+00	rs606119|rs10495562|rs6062340|rs6827036|rs3809425|rs3826425|rs734570|rs633742|rs4435501|rs4798|rs1534680|rs1881697|rs13038619|rs604965|rs8013586|rs706130|rs150926|rs6599528|rs11732320|rs10817476
Antinuclear Antibodies	DEATH_PATHWAY	BIOCARTA	505	26	19	9,48E-01	1,00E+00	rs7830593|rs16845834|rs2100143|rs3750512|rs2415290|rs1439124|rs39416|rs3007421|rs4353229|rs12289836|rs11778166|rs10872893|rs12958785|rs2270418|rs3138045|rs3738593|rs2485664|rs337475|rs10268780
Antinuclear Antibodies	CDT1_ASSOCIATION_WITH_THE_CDC6_ORC_ORIGIN_COMPLEX	REACTOME	793	24	14	9,48E-01	1,00E+00	rs11845708|rs7187327|rs12447239|rs698856|rs425594|rs11922435|401754|rs13207114|rs3787573|rs2107459|rs3087474|rs577357|rs992507|rs4973377
Antinuclear Antibodies	CHEMOKINE_RECEPTORS_BIND_CHEMOKINES	REACTOME	726	13	9	9,50E-01	1,00E+00	rs1875266|rs7371043|rs11813145|rs2037596|rs10191360|rs223848|rs17488134|rs9890937|rs2173755
Antinuclear Antibodies	SCFSKP2_MEDIATED_DEGRADATION_OF_P27_P21	REACTOME	813	28	17	9,51E-01	1,00E+00	rs7015841|rs11845708|rs7187327|rs12447239|rs425594|rs4805487|rs11922435|401754|rs13207114|rs12207548|rs6032895|rs9576040|rs2107459|rs577357|rs992507|rs4973377|rs9462209
Antinuclear Antibodies	SCF_BETA_TRCP_MEDIATED_DEGRADATION_OF_EMI1	REACTOME	793	25	14	9,51E-01	1,00E+00	rs11845708|rs7187327|rs12447239|rs698856|rs425594|rs11922435|401754|rs13207114|rs6032895|rs2107459|rs3127244|rs577357|rs992507|rs4973377
Antinuclear Antibodies	CHROMOSOME_MAINTENANCE	REACTOME	1949	28	20	9,54E-01	1,00E+00	rs3786136|rs4984803|rs4350299|rs512604|rs8134439|rs214952|rs11562980|rs11913200|rs762224|rs2258974|rs741048|rs2393374|rs174532|rs4645434|rs2278998|rs909914|rs12201313|rs1976101|rs11762146|rs4902264
Antinuclear Antibodies	FORMATION_OF_THE_HIV1_EARLY_ELONGATION_COMPLEX	REACTOME	329	9	7	9,54E-01	1,00E+00	rs3786238|rs633742|rs604965|rs4799085|rs715749|rs2293603|rs2932777
Antinuclear Antibodies	AGR_PATHWAY	BIOCARTA	1981	37	20	9,54E-01	1,00E+00	rs836556|rs3756675|rs1357287|rs4458837|rs10884065|rs4373483|rs2439312|rs9388700|rs10883934|rs4401940|rs7818821|rs4767886|rs2439281|rs933106|rs4236723|rs12204892|rs10884989|rs12528747|rs6861460|rs7819063
Antinuclear Antibodies	GLYCEROLIPID_METABOLISM	KEGG	1872	30	20	9,58E-01	1,00E+00	rs12081589|rs9533044|rs17009821|rs9458157|rs2358073|rs4975057|rs726297|rs182034|rs13008971|rs4646772|rs11195843|rs1880540|rs10973734|rs2116531|rs10493211|rs11632627|rs11195662|rs9965251|rs717208|rs9640883
Antinuclear Antibodies	CLEAVAGE_OF_GROWING_TRANSCRIPT_IN_THE_TERMINATION_REGION_	REACTOME	435	25	13	9,60E-01	1,00E+00	rs10495562|rs3809425|rs13183577|rs4798|rs13038619|rs8013586|rs706130|rs150926|rs6599528|rs715749|rs234592|rs17214407|rs6064389
Antinuclear Antibodies	BILE_ACID_AND_BILE_SALT_METABOLISM	REACTOME	1176	35	20	9,60E-01	1,00E+00	rs9898731|rs16963144|rs9519173|rs11130651|rs4149126|rs10904449|rs2002700|rs1515766|rs4288415|rs1014030|rs9514193|rs174745|rs10841644|rs12056107|rs8013586|rs4149023|rs2589998|rs9558159|rs12192544|rs2841027
Antinuclear Antibodies	ANTIGEN_PROCESSING_CROSS_PRESENTATION	REACTOME	1601	41	20	9,63E-01	1,00E+00	rs4743335|rs4743347|rs11845708|rs7187327|rs12447239|rs4420519|rs1611493|rs698856|rs425594|rs11922435|401754|rs13207114|rs6032895|rs7805129|rs2246010|rs2107459|rs10954289|rs2080390|rs577357|rs2523554
Antinuclear Antibodies	RETINOL_METABOLISM	KEGG	1512	43	20	9,66E-01	1,00E+00	rs12435671|rs8187950|rs8187890|rs725957|rs2067062|rs1614972|rs2038118|rs2182859|rs17270248|rs11892031|rs7256675|rs4889294|rs1126673|rs4148280|rs1826909|rs11647597|rs9309462|rs12887581|rs2470890|rs12062307
Antinuclear Antibodies	ACH_PATHWAY	BIOCARTA	602	19	12	9,67E-01	1,00E+00	rs4401940|rs1550805|rs6449969|rs2163177|rs7804859|rs9838117|rs17153527|rs9480865|rs4730197|rs1550097|rs3828246|rs17153654
Antinuclear Antibodies	RESPIRATORY_ELECTRON_TRANSPORT_ATP_SYNTHESIS_BY_CHEMIOSMOTIC_COUPLIN TION_BY_UNCOUPLING_PROTEINS_	REACTOME	1741	37	20	9,69E-01	1,00E+00	rs8133991|rs1411451|rs653263|rs968698|rs10425332|rs4147774|rs11669029|rs10406627|rs7251331|rs7250000|rs12699165|rs10409325|rs6871722|rs12540223|rs39416|rs632663|rs11559290|rs10221449|rs1316932|rs778584
Antinuclear Antibodies	RNA_POL_I_TRANSCRIPTION	REACTOME	937	14	11	9,70E-01	1,00E+00	rs2227301|rs10951129|rs2844456|rs871443|rs9303601|rs3848006|rs3759978|rs2293603|rs417271|rs2932777|rs35455442
Antinuclear Antibodies	SIGNALING_BY_WNT	REACTOME	1071	36	20	9,70E-01	1,00E+00	rs9883073|rs4802906|rs11845708|rs7187327|rs12447239|rs10105118|rs698856|rs425594|rs11922435|401754|rs13207114|rs6032895|rs11160664|rs2107459|rs3127244|rs577357|rs992507|rs4973377|rs1116003|rs7712431
Antinuclear Antibodies	ASPARAGINE_N_LINKED_GLYCOSYLATION	REACTOME	2760	29	20	9,70E-01	1,00E+00	rs11152012|rs13072457|rs5742858|rs6095638|rs807270|rs2279447|rs13203167|rs13035520|rs11621456|rs9363217|rs1455777|rs1391962|rs1490776|rs11203713|rs12755096|rs12424060|rs11708761|rs359327|rs4141587|rs12423422
Antinuclear Antibodies	GCR_PATHWAY	BIOCARTA	736	16	12	9,71E-01	1,00E+00	rs12562819|rs1550805|rs6449969|rs2055226|rs7804859|rs9838117|rs17153527|rs4912911|rs12289836|rs4730197|rs12521436|rs17153654
Antinuclear Antibodies	ACTIVATION_OF_ATR_IN_RESPONSE_TO_REPLICATION_STRESS	REACTOME	653	15	11	9,71E-01	1,00E+00	rs3786136|rs741048|rs2393374|rs11762146|rs1832265|rs7328146|rs3087474|rs9540647|rs768255|rs17545935|rs2871029
Antinuclear Antibodies	IL_2_SIGNALING	REACTOME	936	33	18	9,72E-01	1,00E+00	rs9896052|rs10821495|rs1550805|rs2239167|rs310244|rs6449969|rs163567|rs2163177|rs2274471|rs2991216|rs4240896|rs9838117|rs228947|rs11716652|rs2290889|rs6602392|rs7935699|rs7874712
Antinuclear Antibodies	MEIOTIC_RECOMBINATION	REACTOME	993	10	8	9,72E-01	1,00E+00	rs3786136|rs741048|rs11762146|rs871443|rs11571805|rs2508678|rs17545935|rs144848
Antinuclear Antibodies	VASOPRESSIN_REGULATED_WATER_REABSORPTION	KEGG	1100	32	20	9,74E-01	1,00E+00	rs625061|rs953672|rs9825747|rs11981754|rs1554160|rs883751|rs1488540|rs313393|rs2422840|rs7121257|rs41307|rs2278637|rs438900|rs6968464|rs6499096|rs2958757|rs10421422|rs16874653|rs628426|rs7206142
Antinuclear Antibodies	SYNTHESIS_OF_BILE_ACIDS_AND_BILE_SALTS	REACTOME	546	15	11	9,76E-01	1,00E+00	rs11130651|rs10904449|rs2002700|rs4288415|rs174745|rs12056107|rs2589998|rs12192544|rs2841027|rs4148791|rs7625947
Antinuclear Antibodies	G2_M_CHECKPOINTS	REACTOME	710	15	11	9,78E-01	1,00E+00	rs3786136|rs741048|rs2393374|rs11762146|rs1832265|rs7328146|rs3087474|rs9540647|rs768255|rs17545935|rs2871029
Antinuclear Antibodies	RNA_POL_II_PRE_TRANSCRIPTION_EVENTS	REACTOME	532	19	13	9,78E-01	1,00E+00	rs3786238|rs10732882|rs1053079|rs633742|rs6928843|rs604965|rs399491|rs4799085|rs715749|rs2293603|rs2932777|rs7524919|rs2978263
Antinuclear Antibodies	FORMATION_OF_INCISION_COMPLEX_IN_GG_NER	REACTOME	545	13	10	9,79E-01	1,00E+00	rs3786136|rs741048|rs11762146|rs11615|rs9651726|rs2733537|rs2036958|rs1794337|rs17545935|rs2932777
Antinuclear Antibodies	MEIOSIS	REACTOME	1674	31	20	9,79E-01	1,00E+00	rs3786136|rs4984803|rs4350299|rs214952|rs11913200|rs2258974|rs741048|rs4645434|rs909914|rs12201313|rs11762146|rs871443|rs4902264|rs2813494|rs360589|rs695983|rs10144225|rs11571805|rs2508678|rs3738593
Antinuclear Antibodies	CHYLOMICRON_MEDIATED_LIPID_TRANSPORT	REACTOME	435	15	10	9,80E-01	1,00E+00	rs2290501|rs11632627|rs13396400|rs12474685|rs633695|rs4654986|rs550619|rs7162855|rs10917070|rs328539
Antinuclear Antibodies	PI_METABOLISM	REACTOME	1808	36	20	9,82E-01	1,00E+00	rs7291756|rs11876920|rs203170|rs2475556|rs6834625|rs750997|rs1550805|rs883751|rs10509534|rs3775616|rs6449969|rs11013140|rs7804859|rs10806791|rs4240896|rs9838117|rs307118|rs4243272|rs17153527|rs10160860
Antinuclear Antibodies	ORC1_REMOVAL_FROM_CHROMATIN	REACTOME	930	27	17	9,82E-01	1,00E+00	rs11845708|rs7187327|rs12447239|rs698856|rs425594|rs11922435|401754|rs13207114|rs12207548|rs6032895|rs9576040|rs2107459|rs3087474|rs577357|rs992507|rs4973377|rs9462209
Antinuclear Antibodies	TYPE_II_DIABETES_MELLITUS	KEGG	2053	25	20	9,82E-01	1,00E+00	rs7999458|rs175338|rs7258382|rs658071|rs9898731|rs9886592|rs12408625|rs1860094|rs17254521|rs4675077|rs1550805|rs2001303|rs6449969|rs2251692|rs11069810|rs1460118|rs7804859|rs2359453|rs2595199|rs882193
Antinuclear Antibodies	HISTIDINE_METABOLISM	KEGG	654	25	15	9,83E-01	1,00E+00	rs2290536|rs277888|rs4646772|rs10973734|rs925102|rs2408700|rs12802641|rs1455996|rs4819211|rs13290293|rs17817077|rs10496793|rs12957330|rs13023679|rs17089368
Antinuclear Antibodies	SYNTHESIS_OF_DNA	REACTOME	1545	27	20	9,83E-01	1,00E+00	rs3786136|rs512604|rs11845708|rs7187327|rs12447239|rs3764694|rs741048|rs2393374|rs174532|rs698856|rs425594|rs11922435|rs2278998|401754|rs13207114|rs12207548|rs6032895|rs6990366|rs11762146|rs9576040
Antinuclear Antibodies	PEPTIDE_LIGAND_BINDING_RECEPTORS	REACTOME	5053	33	20	9,84E-01	1,00E+00	rs571312|rs2829946|rs17696124|rs202451|rs11209971|rs9974032|rs6690854|rs4729366|rs16999138|rs466609|rs2047076|rs12209443|rs1875266|rs2427430|rs499062|rs12527223|rs6533102|rs1389623|rs10412841|rs9321852
Antinuclear Antibodies	TIGHT_JUNCTION	KEGG	6242	29	20	9,85E-01	1,00E+00	rs7630352|rs8133991|rs678194|rs2227301|rs1864509|rs8081512|rs17020744|rs1215|rs2974154|rs6963699|rs10229458|rs2269529|rs9883073|rs7776133|rs169143|rs1480159|rs10228945|rs2075014|rs201603|rs17231134
Antinuclear Antibodies	TRANSCRIPTION_COUPLED_NER_TC_NER	REACTOME	834	22	16	9,86E-01	1,00E+00	rs3786136|rs741048|rs2393374|rs2278998|rs633742|rs11762146|rs604965|rs1832265|rs7328146|rs1405655|rs11615|rs9540647|rs1794337|rs2293603|rs17545935|rs2932777
Antinuclear Antibodies	CYTOCHROME_P450_ARRANGED_BY_SUBSTRATE_TYPE	REACTOME	1163	42	17	9,87E-01	1,00E+00	rs12591359|rs41717|rs2002700|rs12441859|rs2182859|rs34562262|rs4808358|rs4288415|rs7256675|rs17703982|rs500670|rs9309462|rs12192544|rs2470890|rs576119|rs12125747|rs3810427
Antinuclear Antibodies	NUCLEOTIDE_EXCISION_REPAIR	KEGG	914	24	18	9,87E-01	1,00E+00	rs3786136|rs9356399|rs741048|rs2393374|rs2278998|rs2306597|rs11762146|rs1832265|rs7328146|rs1405655|rs11615|rs9651726|rs9540647|rs2733537|rs2036958|rs1794337|rs17545935|rs2932777
Antinuclear Antibodies	FORMATION_OF_TRANSCRIPTION_COUPLED_NER_TC_NER_REPAIR_COMPLEX	REACTOME	389	7	6	9,87E-01	1,00E+00	rs633742|rs604965|rs11615|rs1794337|rs2293603|rs2932777
Antinuclear Antibodies	HOMOLOGOUS_RECOMBINATION	KEGG	730	20	15	9,87E-01	1,00E+00	rs3786136|rs741048|rs11158757|rs861547|rs2278998|rs11762146|rs1404508|rs1405655|rs1956534|rs11571805|rs2508678|rs2331780|rs17545935|rs2930961|rs144848
Antinuclear Antibodies	RNA_POL_II_TRANSCRIPTION_PRE_INITIATION_AND_PROMOTER_OPENING	REACTOME	383	13	8	9,88E-01	1,00E+00	rs633742|rs6928843|rs604965|rs399491|rs2293603|rs2932777|rs7524919|rs2978263
Antinuclear Antibodies	RNA_POL_II_TRANSCRIPTION	REACTOME	1050	37	20	9,91E-01	1,00E+00	rs10495562|rs3786238|rs10732882|rs3809425|rs1053079|rs633742|rs13183577|rs6928843|rs4798|rs13038619|rs604965|rs399491|rs8013586|rs706130|rs150926|rs6599528|rs4799085|rs715749|rs2293603|rs6454694
Antinuclear Antibodies	ATRBRCA_PATHWAY	BIOCARTA	238	3	3	9,92E-01	1,00E+00	rs11571805|rs2508678|rs144848
Antinuclear Antibodies	DRUG_METABOLISM_OTHER_ENZYMES	KEGG	1609	23	14	9,92E-01	1,00E+00	rs819864|rs9437663|rs738814|rs4646267|rs11892031|rs4148280|rs13277723|rs16853627|rs12044858|rs13418515|rs8362|rs3887873|rs7524799|rs2271025
Antinuclear Antibodies	MITOCHONDRIA_PATHWAY	BIOCARTA	317	9	6	9,93E-01	1,00E+00	rs1439124|rs39416|rs4353229|rs12870|rs12958785|rs10268780
Antinuclear Antibodies	GLOBAL_GENOMIC_NER_GG_NER	REACTOME	823	22	16	9,93E-01	1,00E+00	rs3786136|rs741048|rs2393374|rs2278998|rs11762146|rs1832265|rs7328146|rs1405655|rs11615|rs9651726|rs9540647|rs2733537|rs715749|rs1794337|rs17545935|rs2932777
Antinuclear Antibodies	MRNA_CAPPING	REACTOME	388	7	6	9,94E-01	1,00E+00	rs633742|rs604965|rs715749|rs2293603|rs6454694|rs2932777
Antinuclear Antibodies	LIPOPROTEIN_METABOLISM	REACTOME	1037	29	20	9,96E-01	1,00E+00	rs1532624|rs2290501|rs4708|rs11632627|rs2271461|rs13396400|rs2271463|rs7469197|rs12474685|rs633695|rs2472510|rs9457997|rs7449650|rs4654986|rs4810476|rs550619|rs7162855|rs2482424|rs10917070|rs328539
Antinuclear Antibodies	NUCLEOTIDE_EXCISION_REPAIR	REACTOME	956	26	19	9,96E-01	1,00E+00	rs3786136|rs741048|rs2393374|rs2278998|rs633742|rs11762146|rs604965|rs1832265|rs7328146|rs1405655|rs11615|rs9651726|rs9540647|rs2733537|rs715749|rs1794337|rs2293603|rs17545935|rs2932777
Antinuclear Antibodies	RESPIRATORY_ELECTRON_TRANSPORT	REACTOME	1631	40	20	9,97E-01	1,00E+00	rs1411451|rs10425332|rs3822362|rs11669029|rs10406627|rs7251331|rs7250000|rs12699165|rs10409325|rs6871722|rs12540223|rs39416|rs632663|rs11559290|rs10221449|rs1316932|rs778584|rs7180462|rs6510333|rs12985735
Antinuclear Antibodies	DNA_REPAIR	REACTOME	2111	36	20	9,97E-01	1,00E+00	rs12241573|rs10490113|rs12453935|rs3786136|rs2029166|rs3824759|rs4751077|rs741048|rs2393374|rs174532|rs2278998|rs633742|rs1230|rs11762146|rs4325734|rs604965|rs828704|rs1832265|rs7328146|rs1805388
Antinuclear Antibodies	ATM_PATHWAY	BIOCARTA	397	12	12	9,97E-01	1,00E+00	rs2415290|rs12207548|rs12731705|rs6681035|rs12289836|rs10872893|rs2508678|rs3138045|rs2583836|rs12259267|rs1056209|rs9462209
Antinuclear Antibodies	TASTE_TRANSDUCTION	KEGG	1100	23	20	9,98E-01	1,00E+00	rs953672|rs11261073|rs4701850|rs883751|rs17649232|rs7712262|rs4926242|rs1432515|rs684119|rs11054080|rs12984970|rs16015|rs11882861|rs11739224|rs11745136|rs3741920|rs2949739|rs319276|rs9461937|rs10421422
Antinuclear Antibodies	CASPASE_PATHWAY	BIOCARTA	327	11	9	9,98E-01	1,00E+00	rs1439124|rs39416|rs4747064|rs4353229|rs566275|rs874583|rs3738593|rs2485664|rs10268780
Antinuclear Antibodies	DESTABILIZATION_OF_MRNA_BY_BRF1	REACTOME	204	0	0	1,00E+00	1,00E+00	NA
Antinuclear Antibodies	PACKAGING_OF_TELOMERE_ENDS	REACTOME	515	0	0	1,00E+00	1,00E+00	NA
Arthritis	CK1_PATHWAY	BIOCARTA	759	35	20	2,20E-03	7,09E-01	rs7125415|rs2302234|rs2734838|rs888298|rs2251238|rs12129768|rs4130345|rs2514227|rs7719360|rs17064682|rs4936270|rs2207317|rs6039211|rs6118219|rs7703433|rs10485723|rs2223538|rs4373974|rs265993|rs583720
Arthritis	MPR_PATHWAY	BIOCARTA	624	36	20	3,30E-03	7,09E-01	rs17153079|rs2302234|rs17162190|rs998235|rs888298|rs2724113|rs12129768|rs6062662|rs2015810|rs1554465|rs9244|rs1534131|rs885244|rs2005219|rs1002487|rs1535211|rs182524|rs583720|rs7531583|rs7077462
Arthritis	G_ALPHA_I_SIGNALLING_EVENTS	REACTOME	6453	42	20	3,40E-03	7,09E-01	rs7125415|rs17153079|rs1179766|rs10501367|rs17272364|rs1509849|rs384067|rs2734838|rs2422669|rs10980811|rs11850274|rs4596491|rs12044675|rs1023262|rs9353528|rs3107373|rs2724113|rs4908127|rs4130345|rs2498323
Arthritis	VIP_PATHWAY	BIOCARTA	1000	31	20	3,80E-03	7,09E-01	rs2302234|rs4733828|rs4872533|rs759648|rs888298|rs9792218|rs2251238|rs832574|rs12129768|rs1499368|rs10869979|rs3793228|rs230530|rs2542417|rs4404878|rs6560613|rs1740005|rs177820|rs10505528|rs6470648
Arthritis	IGF1R_PATHWAY	BIOCARTA	735	28	20	4,70E-03	7,09E-01	rs10515086|rs2302234|rs17162190|rs998235|rs888298|rs12129768|rs9672254|rs2229765|rs17237251|rs2494738|rs1554465|rs2302975|rs7169544|rs422437|rs1534131|rs885244|rs2005219|rs10498208|rs12652661|rs1002487
Arthritis	PEPTIDE_LIGAND_BINDING_RECEPTORS	REACTOME	5053	37	20	6,50E-03	7,09E-01	rs980545|rs1179766|rs10501367|rs17272364|rs1419791|rs2422669|rs9471843|rs11850274|rs4596491|rs646749|rs11962370|rs9381141|rs12672801|rs1023262|rs9318516|rs9389870|rs7679849|rs17764941|rs12812470|rs9394696
Arthritis	DEADENYLATION_OF_MRNA	REACTOME	331	34	9	7,20E-03	7,09E-01	rs11228985|rs1239898|rs12639287|rs3125804|rs7300901|rs2453176|rs6893735|rs7403900|rs3105433
Arthritis	GLEEVEC_PATHWAY	BIOCARTA	954	26	20	7,30E-03	7,09E-01	rs10515086|rs4733828|rs2726550|rs759648|rs9792218|rs832574|rs1499368|rs4899554|rs10974873|rs17237251|rs2494738|rs2542417|rs4404878|rs11887698|rs2302975|rs10505528|rs422437|rs2005219|rs12652661|rs6470648
Arthritis	WNT_PATHWAY	BIOCARTA	1363	31	20	8,00E-03	7,09E-01	rs4733828|rs2894401|rs4510937|rs10867720|rs759648|rs1076465|rs584846|rs9792218|rs1999090|rs1499368|rs739141|rs1498792|rs10485408|rs815865|rs389673|rs864631|rs10455526|rs2655638|rs2542417|rs4404878
Arthritis	BAD_PATHWAY	BIOCARTA	927	29	20	9,10E-03	7,11E-01	rs10515086|rs2302234|rs17162190|rs998235|rs888298|rs12129768|rs735563|rs9672254|rs2229765|rs17237251|rs2494738|rs1554465|rs2302975|rs12454712|rs7169544|rs422437|rs1534131|rs885244|rs2005219|rs8096471
Arthritis	SYNTHESIS_OF_PA	REACTOME	1057	35	20	9,80E-03	7,11E-01	rs4478182|rs1093733|rs3757031|rs10752974|rs11589805|rs10004130|rs910721|rs2017802|rs10912025|rs1339082|rs3132941|rs230632|rs1567102|rs360402|rs800719|rs4128634|rs3782323|rs11573253|rs495183|rs1938512
Arthritis	DEADENYLATION_DEPENDENT_MRNA_DECAY	REACTOME	622	46	15	1,32E-02	8,53E-01	rs11228985|rs1239898|rs12639287|rs3125804|rs7300901|rs2229032|rs10419232|rs369052|rs2453176|rs11062040|rs6893735|rs4382556|rs7403900|rs3105433|rs1871856

Arthritis	CREB_PATHWAY	BIOCARTA	969	27	20	1,39E-02	8,53E-01	rs10515086|rs2302234|rs17162190|rs998235|rs888298|rs12129768|rs1029471|rs10147042|rs17237251|rs2494738|rs1554465|rs2238493|rs2302975|rs6533710|rs422437|rs1534131|rs885244|rs2005219|rs851010|rs12652661
Arthritis	CXCR4_PATHWAY	BIOCARTA	1099	39	20	1,63E-02	9,27E-01	rs10515086|rs17153079|rs10505811|rs1023262|rs577684|rs2724113|rs10869979|rs306966|rs230530|rs17237251|rs10204606|rs2238493|rs9244|rs2302975|rs6560613|rs422437|rs2005219|rs12652661|rs4440020|rs1016269
Arthritis	ARF_PATHWAY	BIOCARTA	733	27	20	1,75E-02	9,27E-01	rs10515086|rs4733828|rs759648|rs9792218|rs1499368|rs9512817|rs1535565|rs17237251|rs11149485|rs2542417|rs4404878|rs2302975|rs10505528|rs422437|rs12652661|rs6470648|rs849376|rs1561928|rs1106841|rs7721613
Arthritis	ETS_PATHWAY	BIOCARTA	682	26	20	1,92E-02	9,27E-01	rs12418638|rs2209358|rs12146687|rs3020829|rs2836815|rs2836656|rs4899554|rs991707|rs2836816|rs10893818|rs701078|rs6590343|rs12421164|rs4937316|rs7122859|rs4245079|rs7126621|rs6874642|rs7101705|rs1110904
Arthritis	PPARA_PATHWAY	BIOCARTA	2445	35	20	2,20E-02	9,27E-01	rs10515086|rs2302234|rs4733828|rs6784193|rs297|rs759648|rs4697435|rs888298|rs9792218|rs2823183|rs2057034|rs12129768|rs2282529|rs1499368|rs1397829|rs701078|rs4817754|rs7689213|rs2823209|rs17237251
Arthritis	IL_RECEPTOR_SHC_SIGNALING	REACTOME	624	45	20	2,34E-02	9,27E-01	rs10515086|rs310209|rs228942|rs7373276|rs163577|rs3761049|rs735563|rs3856848|rs10974873|rs785512|rs17237251|rs2302975|rs11914132|rs422437|rs7508025|rs12652661|rs12129709|rs2292572|rs11575812|rs693293
Arthritis	ACETYLCHOLINE_BINDING_AND_DOWNSTREAM_EVENTS	REACTOME	242	10	3	2,54E-02	9,27E-01	rs4417522|rs1514254|rs891398
Arthritis	NO1_PATHWAY	BIOCARTA	1482	30	20	3,00E-02	9,27E-01	rs2302234|rs16835926|rs11850274|rs1194491|rs888298|rs12129768|rs12505096|rs7077665|rs12058655|rs11045087|rs1464459|rs2805434|rs10083148|rs10507386|rs6700222|rs1959053|rs17080484|rs2494738|rs10822867|rs1740005
Arthritis	PRE_NOTCH_PROCESSING_IN_GOLGI	REACTOME	691	37	16	3,16E-02	9,27E-01	rs12944845|rs3761441|rs4078830|rs1652583|rs1959011|rs3134942|rs1007637|rs1652593|rs12274970|rs2906142|rs1887735|rs17677652|rs17514846|rs958937|rs9323731|rs12323801
Arthritis	CD28_DEPENDENT_PI3K_AKT_SIGNALING	REACTOME	572	29	11	3,34E-02	9,27E-01	rs10515086|rs6084337|rs785512|rs17237251|rs2494738|rs2302975|rs422437|rs12652661|rs2670289|rs7721613|rs16829957
Arthritis	SMAD2_SMAD3_SMAD4_HETEROTRIMER_REGULATES_TRANSCRIPTION	REACTOME	715	24	20	3,34E-02	9,27E-01	rs4733828|rs759648|rs2227631|rs9792218|rs1499368|rs7234567|rs266339|rs2337143|rs2542417|rs4404878|rs11874742|rs4776344|rs10505528|rs920660|rs6470648|rs6016004|rs2383208|rs744910|rs998910|rs6912892
Arthritis	IL_3_5_AND_GM_CSF_SIGNALING	REACTOME	1134	37	20	3,37E-02	9,27E-01	rs10515086|rs2510139|rs310209|rs11188666|rs12129768|rs228942|rs7373276|rs163577|rs3761049|rs735563|rs3856848|rs10974873|rs10901096|rs785512|rs17237251|rs2302975|rs11914132|rs422437|rs7508025|rs12652661
Arthritis	GSK3_PATHWAY	BIOCARTA	1055	32	20	3,40E-02	9,27E-01	rs10515086|rs17153079|rs1076465|rs10983934|rs10739508|rs389673|rs12555480|rs230530|rs17237251|rs2494738|rs1554973|rs9244|rs2302975|rs7740467|rs10759930|rs422437|rs7703433|rs12503104|rs6478330|rs694428
Arthritis	STATHMIN_PATHWAY	BIOCARTA	456	27	15	3,49E-02	9,27E-01	rs2302234|rs2949654|rs888298|rs12129768|rs1029471|rs6533710|rs704859|rs1618879|rs2072439|rs1551565|rs583720|rs7077462|rs13133676|rs6501512|rs2056626
Arthritis	IL2RB_PATHWAY	BIOCARTA	1296	32	20	3,58E-02	9,27E-01	rs10515086|rs4733828|rs759648|rs2510152|rs9792218|rs310209|rs1499368|rs228942|rs3761049|rs4899554|rs17237251|rs2494738|rs2542417|rs4404878|rs2302975|rs11914132|rs12454712|rs10505528|rs422437|rs2005219
Arthritis	NFKB_PATHWAY	BIOCARTA	870	61	20	3,60E-02	9,27E-01	rs3917265|rs1999090|rs832574|rs2287047|rs10983934|rs739141|rs10739508|rs10485408|rs12555480|rs10455526|rs230530|rs2655638|rs1554973|rs2048874|rs10759930|rs1556091|rs9353794|rs6478330|rs694428|rs1061624
Arthritis	ERK_PATHWAY	BIOCARTA	1025	30	20	3,88E-02	9,27E-01	rs4733828|rs17162190|rs759648|rs998235|rs9792218|rs2724113|rs1499368|rs12026714|rs10147042|rs9672254|rs2229765|rs3863373|rs2542417|rs4404878|rs880183|rs7169544|rs10505528|rs1468727|rs7703433|rs2005219
Arthritis	CYCLIN_E_ASSOCIATED_EVENTS_DURING_G1_S_TRANSITION_	REACTOME	1212	34	20	3,94E-02	9,27E-01	rs6564984|rs4733828|rs759648|rs9792218|rs3815844|rs6575121|rs1499368|rs2280162|rs2550461|rs10818964|rs2284704|rs2542417|rs4404878|rs7404213|rs10271133|rs10505528|rs6142963|rs228271|rs6470648|rs13160147
Arthritis	OPIOID_SIGNALLING	REACTOME	3594	39	20	4,35E-02	9,27E-01	rs17153079|rs2302234|rs1509849|rs384067|rs2422669|rs12457181|rs888298|rs10752974|rs16889129|rs2724113|rs2251238|rs12129768|rs11048665|rs11615994|rs4979032|rs10912025|rs6056526|rs1339082|rs230632|rs7955100
Arthritis	CARDIACEGF_PATHWAY	BIOCARTA	1289	33	20	4,44E-02	9,27E-01	rs4733828|rs9471843|rs759648|rs9381141|rs9792218|rs9318516|rs1499368|rs9394696|rs9574197|rs4899554|rs1278302|rs230530|rs4962318|rs2542417|rs4404878|rs2238493|rs10505528|rs388488|rs1468727|rs7097346
Arthritis	TRANSCRIPTIONAL_ACTIVITY_OF_SMAD2_SMAD3_SMAD4_HETEROTRIMER	REACTOME	967	23	20	4,97E-02	9,27E-01	rs4733828|rs759648|rs2227631|rs9792218|rs1499368|rs7234567|rs266339|rs2337143|rs701078|rs1989138|rs2542417|rs4404878|rs1991403|rs627205|rs11874742|rs556765|rs4776344|rs10505528|rs11582563|rs920660
Arthritis	ECM_PATHWAY	BIOCARTA	753	41	15	5,13E-02	9,27E-01	rs10515086|rs1958918|rs4509829|rs3784166|rs306966|rs17237251|rs2302975|rs422437|rs2005219|rs12652661|rs849376|rs7721613|rs238242|rs1550955|rs4981146
Arthritis	RECYCLING_PATHWAY_OF_L1	REACTOME	874	25	14	5,19E-02	9,27E-01	rs1295217|rs17162190|rs998235|rs961548|rs845641|rs6580153|rs11919795|rs10147042|rs882551|rs1002487|rs4710090|rs3808734|rs874277|rs2224957
Arthritis	CDC42RAC_PATHWAY	BIOCARTA	435	19	8	5,21E-02	9,27E-01	rs10515086|rs17237251|rs2302975|rs422437|rs12652661|rs2501279|rs6809452|rs7721613
Arthritis	AMINO_ACID_SYNTHESIS_AND_INTERCONVERSION_TRANSAMINATION	REACTOME	530	40	15	5,22E-02	9,27E-01	rs1923939|rs863944|rs10911063|rs1082214|rs11245507|rs10867272|rs7870042|rs11649592|rs4529531|rs10486010|rs867376|rs549042|rs7858423|rs2275272|rs11137861
Arthritis	GLUCAGON_SIGNALING_IN_METABOLIC_REGULATION	REACTOME	1044	40	20	5,31E-02	9,27E-01	rs2302234|rs1509849|rs384067|rs888298|rs2724113|rs12129768|rs4979032|rs10498440|rs13190668|rs1509844|rs4807284|rs1554465|rs448852|rs1369450|rs1534131|rs885244|rs1396980|rs583720|rs291353|rs7531583
Arthritis	ERK5_PATHWAY	BIOCARTA	595	30	16	5,35E-02	9,27E-01	rs10515086|rs17162190|rs998235|rs2119945|rs17237251|rs2494738|rs2302975|rs422437|rs2005219|rs12652661|rs1002487|rs325383|rs849376|rs4661229|rs7721613|rs2235366
Arthritis	NEUROACTIVE_LIGAND_RECEPTOR_INTERACTION	KEGG	13469	35	20	5,47E-02	9,27E-01	rs7125415|rs980545|rs10501367|rs17272364|rs712775|rs12944845|rs9485742|rs4632486|rs4417522|rs2734838|rs10980811|rs2975758|rs11850274|rs4596491|rs12044675|rs34647055|rs646749|rs11962370|rs17455628|rs724224
Arthritis	GLYCEROPHOSPHOLIPID_METABOLISM	KEGG	2549	30	20	5,54E-02	9,27E-01	rs4478182|rs9834264|rs11541600|rs1093733|rs3757031|rs10103493|rs10752974|rs4637750|rs11589805|rs10004130|rs910721|rs4668600|rs2017802|rs10912025|rs1339082|rs3132941|rs6788054|rs230632|rs1567102|rs360402
Arthritis	METABOLISM_OF_MRNA	REACTOME	3017	37	20	5,76E-02	9,27E-01	rs11228985|rs7636810|rs6564984|rs284541|rs1239898|rs12639287|rs1149754|rs3815844|rs6575121|rs1239900|rs2280162|rs2550461|rs7300901|rs4554825|rs2229032|rs760782|rs10818964|rs7153386|rs6503113|rs2291782
Arthritis	RAS_PATHWAY	BIOCARTA	557	47	17	5,77E-02	9,27E-01	rs10515086|rs626713|rs360402|rs2073923|rs230530|rs17237251|rs2494738|rs4128634|rs2302975|rs422437|rs2005219|rs12652661|rs2501279|rs13116385|rs849376|rs7721613|rs7662536
Arthritis	SIGNAL_AMPLIFICATION	REACTOME	1023	40	20	5,83E-02	9,27E-01	rs17153079|rs10752974|rs2724113|rs4979032|rs10869979|rs10912025|rs1339082|rs308039|rs230632|rs10498440|rs11652805|rs4807284|rs9244|rs6560613|rs1938512|rs10911946|rs851010|rs4806942|rs9380546|rs182524
Arthritis	GPCR_PATHWAY	BIOCARTA	1102	36	20	5,85E-02	9,27E-01	rs17153079|rs2302234|rs888298|rs2724113|rs2251238|rs12129768|rs10869979|rs4899554|rs1554465|rs2238493|rs9244|rs6560613|rs1740005|rs177820|rs1534131|rs885244|rs2005219|rs7233024|rs182524|rs12431766
Arthritis	MTOR_PATHWAY	BIOCARTA	509	25	12	5,89E-02	9,27E-01	rs10515086|rs12639287|rs17237251|rs2494738|rs2302975|rs422437|rs7703433|rs12652661|rs739500|rs7721613|rs7094771|rs2735352
Arthritis	NEPHRIN_INTERACTIONS	REACTOME	1617	37	20	5,90E-02	9,27E-01	rs1523843|rs10515086|rs952316|rs10245351|rs13231956|rs493725|rs4671952|rs3807689|rs11880466|rs2074646|rs583795|rs2065198|rs17237251|rs619208|rs2302975|rs422437|rs354232|rs2689775|rs12652661|rs10248125
Arthritis	PLC_BETA_MEDIATED_EVENTS	REACTOME	2313	34	20	5,97E-02	9,27E-01	rs17153079|rs2302234|rs1509849|rs384067|rs888298|rs10752974|rs12129768|rs11048665|rs11615994|rs10912025|rs6056526|rs1339082|rs230632|rs7955100|rs4259245|rs13190668|rs1509844|rs2207317|rs6039211|rs1554465
Arthritis	IL_2_SIGNALING	REACTOME	936	38	20	6,11E-02	9,27E-01	rs10515086|rs310209|rs228942|rs7373276|rs163577|rs3761049|rs735563|rs3856848|rs10974873|rs785512|rs17237251|rs2302975|rs11914132|rs12038990|rs422437|rs2005219|rs7508025|rs12652661|rs6876|rs12129709
Arthritis	G_ALPHA_Q_SIGNALLING_EVENTS	REACTOME	7428	29	20	6,13E-02	9,27E-01	rs10515086|rs980545|rs3924457|rs1419791|rs9834264|rs9471843|rs10980811|rs11850274|rs4596491|rs11962370|rs9381141|rs12672801|rs17455628|rs9318516|rs9389870|rs2724113|rs1843593|rs11048665|rs11779546|rs9526301
Arthritis	ABC_TRANSPORTERS	KEGG	2684	53	20	6,24E-02	9,27E-01	rs17388072|rs12700229|rs7330196|rs1881078|rs557491|rs4472462|rs11185630|rs3755160|rs2487031|rs10233470|rs10223929|rs2071469|rs574741|rs17686852|rs1025447|rs7566779|rs4148483|rs11979757|rs11077414|rs882992
Arthritis	PROXIMAL_TUBULE_BICARBONATE_RECLAMATION	KEGG	458	34	16	6,45E-02	9,27E-01	rs4839066|rs9421580|rs1082214|rs12544346|rs4938440|rs17368402|rs16846419|rs16831518|rs10488782|rs11216567|rs954319|rs6092495|rs6694434|rs10504814|rs850610|rs987390
Arthritis	GAP_JUNCTION	KEGG	4388	44	20	6,64E-02	9,27E-01	rs7125415|rs17153079|rs1509849|rs384067|rs2734838|rs10980811|rs1194491|rs2705462|rs12129768|rs11048665|rs9526301|rs11615994|rs4130345|rs7077665|rs10869979|rs6056526|rs2514227|rs7676376|rs308039|rs7719360
Arthritis	GASTRIN_CREB_SIGNALLING_PATHWAY_VIA_PKC_AND_MAPK	REACTOME	8122	29	20	6,69E-02	9,27E-01	rs10515086|rs980545|rs3924457|rs1419791|rs9834264|rs9471843|rs10980811|rs11850274|rs4596491|rs17162190|rs11962370|rs9381141|rs12672801|rs998235|rs17455628|rs9318516|rs9389870|rs2724113|rs1843593|rs11048665
Arthritis	IL17_PATHWAY	BIOCARTA	316	10	8	7,26E-02	9,27E-01	rs2949654|rs13208597|rs1892275|rs704859|rs10484879|rs1618879|rs1974226|rs2056626
Arthritis	PKA_MEDIATED_PHOSPHORYLATION_OF_CREB	REACTOME	767	41	20	7,31E-02	9,27E-01	rs2302234|rs1509849|rs384067|rs888298|rs12129768|rs13190668|rs1509844|rs1554465|rs448852|rs1369450|rs1534131|rs885244|rs1396980|rs583720|rs263232|rs7844964|rs4263480|rs6501512|rs6988340|rs6864771
Arthritis	SIGNALING_BY_THE_B_CELL_RECEPTOR_BCR	REACTOME	3426	35	20	7,43E-02	9,27E-01	rs10515086|rs2647394|rs6564984|rs2755209|rs13014054|rs2510152|rs573323|rs3815844|rs1999090|rs6575121|rs11188666|rs11048665|rs6084337|rs11615994|rs2280162|rs2550461|rs6461856|rs10818964|rs10485408|rs7955100
Arthritis	TEL_PATHWAY	BIOCARTA	978	29	20	7,66E-02	9,27E-01	rs4733828|rs759648|rs7829843|rs9792218|rs1499368|rs10503384|rs7567258|rs9672254|rs2229765|rs2494738|rs2542417|rs4404878|rs12454712|rs7169544|rs10505528|rs1468727|rs7703433|rs17730505|rs10101132|rs8096471
Arthritis	GLYCEROPHOSPHOLIPID_BIOSYNTHESIS	REACTOME	2483	30	20	7,72E-02	9,27E-01	rs4478182|rs11541600|rs1093733|rs3757031|rs10103493|rs10752974|rs4637750|rs11589805|rs10004130|rs2295514|rs910721|rs4668600|rs2017802|rs10912025|rs7419959|rs1339082|rs3132941|rs230632|rs1567102|rs360402
Arthritis	GLUTATHIONE_METABOLISM	KEGG	683	33	12	7,73E-02	9,27E-01	rs10735234|rs2132847|rs3763009|rs1167174|rs7687822|rs3934860|rs707143|rs5751901|rs2042235|rs1356553|rs12646059|rs6496603
Arthritis	APOPTOSIS	KEGG	1727	49	20	7,93E-02	9,27E-01	rs10515086|rs2302234|rs3917265|rs888298|rs2251238|rs12129768|rs2287047|rs12026714|rs1921082|rs735563|rs3844279|rs785512|rs230530|rs17237251|rs2494738|rs2302975|rs2048874|rs12454712|rs3773953|rs422437
Arthritis	REGULATION_OF_INSULIN_SECRETION_BY_GLUCAGON_LIKE_PEPTIDE1	REACTOME	1388	40	20	8,38E-02	9,27E-01	rs2302234|rs1509849|rs988896|rs888298|rs2724113|rs12129768|rs11048665|rs11615994|rs4979032|rs7955100|rs10498440|rs4259245|rs1509844|rs4807284|rs448852|rs1369450|rs2345517|rs1396980|rs1536041|rs583720
Arthritis	IL1_SIGNALING	REACTOME	1322	51	20	8,41E-02	9,27E-01	rs3917265|rs2726550|rs1999090|rs12987393|rs2287047|rs748197|rs739141|rs1921082|rs10485408|rs5751022|rs3844279|rs10455526|rs2698537|rs2655638|rs1240073|rs2048874|rs218921|rs3773953|rs10271133|rs1189133
Arthritis	G2_PATHWAY	BIOCARTA	354	20	13	8,45E-02	9,27E-01	rs17162190|rs998235|rs2229032|rs6062662|rs12069919|rs12459759|rs2015810|rs1002487|rs1535211|rs1799949|rs6432018|rs7077462|rs5758211
Arthritis	ASSEMBLY_OF_THE_PRE_REPLICATIVE_COMPLEX	REACTOME	911	44	20	8,45E-02	9,27E-01	rs6564984|rs3815844|rs6575121|rs2280162|rs9350245|rs2550461|rs3820716|rs10818964|rs2075995|rs510862|rs7404213|rs6142963|rs228271|rs9450763|rs4134943|rs8063430|rs3819716|rs13332962|rs12453759|rs971963
Arthritis	AQUAPORIN_MEDIATED_TRANSPORT	REACTOME	1510	37	20	8,51E-02	9,27E-01	rs2302234|rs1509849|rs384067|rs888298|rs2724113|rs12129768|rs467323|rs1082214|rs4979032|rs10498440|rs11631950|rs13190668|rs1509844|rs4807284|rs1554465|rs448852|rs1369450|rs1534131|rs885244|rs1396980
Arthritis	CSK_PATHWAY	BIOCARTA	554	35	19	8,72E-02	9,27E-01	rs2302234|rs2949654|rs888298|rs2724113|rs12129768|rs1554465|rs704859|rs1534131|rs885244|rs1618879|rs3135353|rs9268832|rs583720|rs7531583|rs2858870|rs8046065|rs6501512|rs129988|rs2056626
Arthritis	ACTIVATION_OF_NF_KAPPAB_IN_B_CELLS	REACTOME	1396	35	20	9,13E-02	9,27E-01	rs2647394|rs6564984|rs3815844|rs1999090|rs6575121|rs2280162|rs2550461|rs6461856|rs10818964|rs10485408|rs10455526|rs2655638|rs2238493|rs7404213|rs10271133|rs1182136|rs1556091|rs6142963|rs228271|rs9353794
Arthritis	MCALPAIN_PATHWAY	BIOCARTA	543	33	14	9,15E-02	9,27E-01	rs2302234|rs4509829|rs888298|rs12129768|rs306966|rs1468727|rs2005219|rs583720|rs3796944|rs6501512|rs1550955|rs6974790|rs759159|rs4925479
Arthritis	ACYL_CHAIN_REMODELLING_OF_PE	REACTOME	696	34	20	9,27E-02	9,27E-01	rs1093733|rs10752974|rs11589805|rs4668600|rs10912025|rs1339082|rs230632|rs1567102|rs4712469|rs800719|rs11573253|rs1938512|rs2287541|rs10911946|rs4712480|rs3788428|rs9729852|rs12045689|rs2872821|rs3810322
Arthritis	CA_DEPENDENT_EVENTS	REACTOME	1487	26	20	9,80E-02	9,27E-01	rs2302234|rs1509849|rs384067|rs888298|rs10752974|rs12129768|rs10912025|rs1339082|rs230632|rs13190668|rs1509844|rs1554465|rs1740005|rs448852|rs1153138|rs1369450|rs1534131|rs1249950|rs1938512|rs885244
Arthritis	ACTIVATED_NOTCH1_TRANSMITS_SIGNAL_TO_THE_NUCLEUS	REACTOME	719	40	20	9,81E-02	9,27E-01	rs4906007|rs7155649|rs6108727|rs11678000|rs6104663|rs2293806|rs1938593|rs1474817|rs17189227|rs280372|rs2068143|rs13383306|rs13392868|rs2273608|rs2396644|rs1861617|rs10149661|rs2871921|rs2239282|rs6074170
Arthritis	REGULATION_OF_SIGNALING_BY_CBL	REACTOME	703	38	16	9,84E-02	9,27E-01	rs10515086|rs2510139|rs11188666|rs10901096|rs785512|rs17237251|rs2302975|rs422437|rs12652661|rs8095469|rs3012739|rs290255|rs2660489|rs693293|rs12608960|rs7721613
Arthritis	DOWNSTREAM_TCR_SIGNALING	REACTOME	1573	34	20	1,00E-01	9,27E-01	rs10515086|rs2647394|rs2949654|rs1999090|rs9277334|rs6461856|rs975167|rs10485408|rs10455526|rs17237251|rs9469357|rs2655638|rs1240073|rs2302975|rs704859|rs218921|rs1182136|rs1556091|rs422437|rs2439909
Arthritis	NFAT_PATHWAY	BIOCARTA	1626	28	20	1,01E-01	9,27E-01	rs10515086|rs2302234|rs9471843|rs9381141|rs888298|rs2251238|rs12129768|rs9394696|rs11250154|rs17237251|rs2494738|rs12924903|rs2302975|rs1740005|rs177820|rs4695885|rs11038977|rs422437|rs12458|rs2005219
Arthritis	G_PROTEIN_ACTIVATION	REACTOME	703	41	20	1,02E-01	9,27E-01	rs17153079|rs2422669|rs2724113|rs4979032|rs10498440|rs10485058|rs4807284|rs1319339|rs9244|rs6045733|rs6035331|rs13338253|rs182524|rs291353|rs7531583|rs12147761|rs3788341|rs304732|rs4731190|rs8053370
Arthritis	SIGNALING_BY_FGFR1_FUSION_MUTANTS	REACTOME	639	30	15	1,02E-01	9,27E-01	rs10515086|rs2313626|rs1734729|rs17237251|rs11887698|rs2302975|rs422437|rs12652661|rs7315446|rs407366|rs12168342|rs2312968|rs2292572|rs7721613|rs2235366
Arthritis	GLYCEROLIPID_METABOLISM	KEGG	1872	32	20	1,03E-01	9,27E-01	rs4478182|rs9834264|rs297|rs3757031|rs10004130|rs910721|rs2152078|rs4668600|rs2017802|rs3132941|rs6788054|rs1038378|rs1525079|rs4712469|rs4823164|rs7865905|rs12703402|rs12133338|rs2278102|rs10507505
Arthritis	HCMV_PATHWAY	BIOCARTA	547	35	15	1,05E-01	9,27E-01	rs10515086|rs832574|rs230530|rs17237251|rs2494738|rs2302975|rs422437|rs2005219|rs851010|rs12652661|rs2237093|rs13116385|rs849376|rs7721613|rs7662536
Arthritis	AUTODEGRADATION_OF_THE_E3_UBIQUITIN_LIGASE_COP1	REACTOME	766	45	20	1,05E-01	9,27E-01	rs6564984|rs10494494|rs3815844|rs6575121|rs2280162|rs2550461|rs10818964|rs7404213|rs6142963|rs228271|rs8063430|rs3819716|rs6665385|rs13332962|rs12453759|rs971963|rs9904424|rs584184|rs10753110|rs8004624
Arthritis	EDG1_PATHWAY	BIOCARTA	1432	28	20	1,07E-01	9,27E-01	rs10515086|rs17153079|rs12044675|rs2724113|rs4908127|rs306966|rs17237251|rs12407294|rs2207317|rs2494738|rs6039211|rs1554465|rs2238493|rs9244|rs2302975|rs422437|rs1534131|rs6118219|rs10485723|rs885244
Arthritis	ACUTE_MYELOID_LEUKEMIA	KEGG	1892	24	20	1,07E-01	9,27E-01	rs10515086|rs4733828|rs2894401|rs759648|rs2834772|rs9792218|rs1499368|rs7277670|rs11884056|rs2242747|rs785512|rs2834950|rs230530|rs17237251|rs2494738|rs7988301|rs2542417|rs4404878|rs238896|rs2302975
Arthritis	PROGESTERONE_MEDIATED_OOCYTE_MATURATION	KEGG	2535	31	20	1,08E-01	9,27E-01	rs10515086|rs17153079|rs1509849|rs384067|rs17162190|rs998235|rs12129768|rs11045087|rs12139487|rs10083148|rs845641|rs785512|rs13190668|rs9672254|rs2229765|rs17237251|rs1509844|rs3898028|rs2494738|rs1554465
Arthritis	PACKAGING_OF_TELOMERE_ENDS	REACTOME	515	71	11	1,08E-01	9,27E-01	rs1427575|rs742047|rs9972635|rs12928076|rs7745603|rs639373|rs12196939|rs6499137|rs1593756|rs199750|rs6456688
Arthritis	CTCF_PATHWAY	BIOCARTA	1035	25	20	1,10E-01	9,27E-01	rs10515086|rs4733828|rs759648|rs9792218|rs1499368|rs17237251|rs4522809|rs11625323|rs2542417|rs4404878|rs2302975|rs10505528|rs422437|rs7703433|rs4955159|rs12652661|rs6470648|rs849376|rs1561928|rs2029828
Arthritis	P38MAPK_PATHWAY	BIOCARTA	1904	29	20	1,12E-01	9,27E-01	rs4733828|rs2726550|rs759648|rs10752974|rs9792218|rs1999090|rs832574|rs1499368|rs10912025|rs1339082|rs17326052|rs10485408|rs230632|rs10455526|rs10147042|rs2119945|rs11625323|rs2655638|rs2542417|rs4404878
Arthritis	CDT1_ASSOCIATION_WITH_THE_CDC6_ORC_ORIGIN_COMPLEX	REACTOME	793	46	20	1,12E-01	9,27E-01	rs6564984|rs3815844|rs6575121|rs2280162|rs2550461|rs3820716|rs10818964|rs510862|rs7404213|rs6142963|rs228271|rs9450763|rs8063430|rs3819716|rs13332962|rs12453759|rs971963|rs9904424|rs584184|rs565211
Arthritis	AUTODEGRADATION_OF_CDH1_BY_CDH1_APC_C	REACTOME	857	43	20	1,13E-01	9,27E-01	rs6564984|rs4858552|rs3815844|rs6575121|rs2280162|rs2550461|rs10818964|rs3898028|rs7404213|rs6142963|rs228271|rs8063430|rs3819716|rs12497066|rs13332962|rs12453759|rs971963|rs9904424|rs584184|rs12623041
Arthritis	LINOLEIC_ACID_METABOLISM	KEGG	716	39	20	1,14E-01	9,27E-01	rs1093733|rs10752974|rs11589805|rs10912025|rs1339082|rs230632|rs1567102|rs7081491|rs800719|rs7496336|rs11573253|rs1938512|rs11188059|rs10911946|rs3788428|rs11812285|rs9729852|rs12045689|rs2872821|rs7530853
Arthritis	REGULATION_OF_WATER_BALANCE_BY_RENAL_AQUAPORINS	REACTOME	1393	42	20	1,14E-01	9,27E-01	rs2302234|rs1509849|rs384067|rs888298|rs2724113|rs12129768|rs467323|rs4979032|rs10498440|rs13190668|rs1509844|rs4807284|rs1554465|rs448852|rs1369450|rs1534131|rs885244|rs1396980|rs583720|rs291353
Arthritis	BCELLSURVIVAL_PATHWAY	BIOCARTA	500	24	13	1,14E-01	9,27E-01	rs10515086|rs4899554|rs17237251|rs10419232|rs2494738|rs2302975|rs422437|rs12652661|rs1976955|rs849376|rs2019978|rs7721613|rs7149441
Arthritis	ALPHA_LINOLENIC_ACID_METABOLISM	KEGG	571	36	18	1,17E-01	9,27E-01	rs1093733|rs10752974|rs11589805|rs10912025|rs1339082|rs230632|rs1567102|rs800719|rs7496336|rs11573253|rs1938512|rs10911946|rs3788428|rs9729852|rs12045689|rs2872821|rs7530853|rs7551550
Arthritis	SIGNALING_BY_ILS	REACTOME	2996	46	20	1,18E-01	9,27E-01	rs10515086|rs3917265|rs2726550|rs2510139|rs310209|rs1999090|rs11188666|rs12129768|rs12987393|rs228942|rs2287047|rs748197|rs7373276|rs163577|rs739141|rs3761049|rs1921082|rs10485408|rs735563|rs3856848
Arthritis	ETHER_LIPID_METABOLISM	KEGG	1041	38	20	1,20E-01	9,27E-01	rs1093733|rs10752974|rs2289887|rs11589805|rs10912025|rs1339082|rs230632|rs1567102|rs360402|rs800719|rs12133338|rs1217957|rs4128634|rs7496336|rs11573253|rs6844506|rs2404716|rs1938512|rs10911946|rs3788428
Arthritis	BUTANOATE_METABOLISM	KEGG	763	41	18	1,24E-01	9,27E-01	rs6784193|rs4765026|rs6856710|rs7614173|rs876848|rs1865806|rs2306619|rs10846824|rs7865905|rs4439928|rs4836272|rs8061692|rs7700981|rs9930187|rs10013515|rs11047649|rs13177937|rs1923431
Arthritis	COMPLEMENT_AND_COAGULATION_CASCADES	KEGG	1742	58	20	1,24E-01	9,27E-01	rs1179766|rs881376|rs11850274|rs7772062|rs2227631|rs434602|rs437179|rs1303|rs2749531|rs4957381|rs1959053|rs3024422|rs835223|rs4422662|rs11038977|rs6597197|rs945039|rs2270151|rs926788|rs10063791
Arthritis	IL1R_PATHWAY	BIOCARTA	1001	60	20	1,26E-01	9,27E-01	rs3917265|rs1999090|rs832574|rs2287047|rs739141|rs1921082|rs10485408|rs3844279|rs10455526|rs230530|rs11625323|rs2655638|rs2048874|rs3773953|rs1556091|rs9353794|rs402330|rs851010|rs7771959|rs2811650
Arthritis	DORSO_VENTRAL_AXIS_FORMATION	KEGG	1149	26	20	1,26E-01	9,27E-01	rs10732562|rs12418638|rs12146687|rs2836815|rs3134942|rs2836656|rs2836816|rs10893818|rs9959145|rs6590343|rs12421164|rs9662100|rs4937316|rs7122859|rs17841176|rs4245079|rs7126621|rs1468727|rs2005219|rs7101705
Arthritis	INHIBITION_OF_THE_PROTEOLYTIC_ACTIVITY_OF_APC_C_REQUIRED_FOR_THE_ONSET_[illegible]<br>TOTIC_SPINDLE_CHECKPOINT_COMPONENTS	REACTOME	365	11	7	1,26E-01	9,27E-01	rs4858552|rs12243192|rs3898028|rs12497066|rs12623041|rs3814026|rs1996182
Arthritis	GLUCONEOGENESIS	REACTOME	490	22	8	1,28E-01	9,27E-01	rs863944|rs12129768|rs10993282|rs11649592|rs583720|rs6092495|rs238228|rs6591226
Arthritis	ENOS_ACTIVATION_AND_REGULATION	REACTOME	336	21	9	1,29E-01	9,27E-01	rs551037|rs1106726|rs2494738|rs1740005|rs10516652|rs4694006|rs10278782|rs1402840|rs12431766
Arthritis	GLYCOSPHINGOLIPID_BIOSYNTHESIS_LACTO_AND_NEOLACTO_SERIES	KEGG	670	37	17	1,29E-01	9,27E-01	rs1544136|rs1336954|rs4840141|rs3761049|rs10499007|rs6745253|rs4434479|rs659115|rs4233366|rs629027|rs3756948|rs2717263|rs2239385|rs16854131|rs10841952|rs2302600|rs6605
Arthritis	ACYL_CHAIN_REMODELLING_OF_PC	REACTOME	693	34	20	1,31E-01	9,27E-01	rs1093733|rs10752974|rs11589805|rs4668600|rs10912025|rs1339082|rs230632|rs1567102|rs800719|rs11573253|rs1938512|rs2287541|rs10911946|rs3788428|rs243840|rs9729852|rs12045689|rs10475030|rs2872821|rs3810322

Arthritis	GLUTATHIONE_CONJUGATION	REACTOME	407	15	6	1,31E-01	9,27E-01	rs2132847|rs4970774|rs7687822|rs5751901|rs1356553|rs12646059
Arthritis	SCF_BETA_TRCP_MEDIATED_DEGRADATION_OF_EMI1	REACTOME	793	43	20	1,36E-01	9,27E-01	rs6564984|rs3815844|rs6575121|rs2280162|rs2550461|rs10818964|rs7756899|rs9371645|rs7404213|rs10271133|rs6142963|rs228271|rs8063430|rs3819716|rs13332962|rs12453759|rs971963|rs9904424|rs584184|rs8004624
Arthritis	MAPK_PATHWAY	BIOCARTA	2690	31	20	1,36E-01	9,27E-01	rs10424773|rs4733828|rs2726550|rs17162190|rs759648|rs998235|rs9792218|rs1999090|rs832574|rs1499368|rs1370480|rs17326052|rs4899554|rs10485408|rs845641|rs10455526|rs10147042|rs2119945|rs230530|rs11625323
Arthritis	NGF_SIGNALLING_VIA_TRKA_FROM_THE_PLASMA_MEMBRANE	REACTOME	4847	34	20	1,38E-01	9,27E-01	rs10515086|rs2302234|rs1509849|rs384067|rs626713|rs17162190|rs2755209|rs1662695|rs998235|rs888298|rs7021722|rs2083828|rs4821822|rs12129768|rs11048665|rs6084337|rs11615994|rs12026714|rs845641|rs7955100
Arthritis	JAK_STAT_SIGNALING_PATHWAY	KEGG	4346	29	20	1,38E-01	9,27E-01	rs10515086|rs9318729|rs4733828|rs1145965|rs759648|rs11744747|rs2510139|rs9792218|rs310209|rs2243176|rs10811561|rs1499368|rs228942|rs975405|rs254441|rs7373276|rs163577|rs11749831|rs3761049|rs12040948
Arthritis	SELENOAMINO_ACID_METABOLISM	KEGG	742	42	20	1,38E-01	9,27E-01	rs357181|rs7448803|rs9314048|rs1414397|rs11135315|rs234784|rs17610788|rs11209658|rs5751901|rs257685|rs720938|rs203219|rs2964917|rs17114050|rs6879291|rs2028900|rs2341510|rs11701664|rs11949489|rs11760275
Arthritis	3_UTR_MEDIATED_TRANSLATIONAL_REGULATION	REACTOME	1235	37	20	1,39E-01	9,27E-01	rs7636810|rs284541|rs12639287|rs1149754|rs2316692|rs10509398|rs7757405|rs8109578|rs6503113|rs2291782|rs36621|rs8076790|rs10824609|rs2053984|rs5756219|rs12484030|rs2574693|rs7508025|rs2004997|rs10467685
Arthritis	ALANINE_ASPARTATE_AND_GLUTAMATE_METABOLISM	KEGG	841	44	20	1,39E-01	9,27E-01	rs9421580|rs863944|rs10911063|rs1082214|rs876848|rs612380|rs1865806|rs4439928|rs3006009|rs11649592|rs7717823|rs12730102|rs10486010|rs1967559|rs186471|rs2058925|rs10901268|rs2306084|rs3758938|rs7355583
Arthritis	ARACHIDONIC_ACID_METABOLISM	KEGG	1356	36	20	1,42E-01	9,27E-01	rs7694491|rs1093733|rs10752974|rs11589805|rs3763009|rs10912025|rs1339082|rs230632|rs1567102|rs7081491|rs800719|rs707143|rs7496336|rs5751901|rs6012696|rs11573253|rs17418275|rs1938512|rs11188059|rs10911946
Arthritis	RNA_POL_III_TRANSCRIPTION_INITIATION_FROM_TYPE_3_PROMOTER	REACTOME	501	19	12	1,44E-01	9,27E-01	rs1075|rs9512817|rs2173115|rs217689|rs1535565|rs11149485|rs12541335|rs1497021|rs510395|rs1532445|rs1106841|rs2559658
Arthritis	P53_DEPENDENT_G1_DNA_DAMAGE_RESPONSE	REACTOME	838	45	20	1,44E-01	9,27E-01	rs6564984|rs10494494|rs3815844|rs6575121|rs2280162|rs2550461|rs10818964|rs7404213|rs6142963|rs228271|rs8063430|rs3819716|rs2487144|rs13332962|rs12453759|rs971963|rs9904424|rs584184|rs10753110|rs8004624
Arthritis	FANCONI_ANEMIA_PATHWAY	REACTOME	461	25	13	1,46E-01	9,27E-01	rs2215849|rs2894401|rs2229032|rs414000|rs1541546|rs226981|rs1799949|rs11571805|rs11125757|rs1344459|rs1207952|rs11693731|rs8106959
Arthritis	PHENYLALANINE_METABOLISM	KEGG	442	15	6	1,47E-01	9,27E-01	rs863944|rs4328106|rs11649592|rs9608216|rs7312593|rs2279124
Arthritis	RESPIRATORY_ELECTRON_TRANSPORT_ATP_SYNTHESIS_BY_CHEMIOSMOTIC_COUPLIN TION_BY_UNCOUPLING_PROTEINS_	REACTOME	1741	26	20	1,47E-01	9,27E-01	rs222289|rs10226904|rs3753362|rs999918|rs3900417|rs10423721|rs1626521|rs766549|rs17476583|rs9293478|rs2834287|rs7254601|rs414000|rs11957871|rs7255086|rs7247484|rs1581512|rs1264901|rs4634927|rs6470250
Arthritis	DNA_REPAIR	REACTOME	2111	38	20	1,47E-01	9,27E-01	rs34180180|rs2215849|rs2894401|rs1762421|rs7901425|rs1323806|rs7310681|rs4426221|rs2229032|rs12139487|rs414000|rs639373|rs7567258|rs2284704|rs2097289|rs3218801|rs1075496|rs1541549|rs7086428|rs1925089
Arthritis	ER_PHAGOSOME_PATHWAY	REACTOME	1416	41	20	1,49E-01	9,27E-01	rs6564984|rs2253907|rs3815844|rs6575121|rs2280162|rs2550461|rs10818964|rs2071469|rs2596574|rs12260965|rs7404213|rs6142963|rs228271|rs2071554|rs8063430|rs3819716|rs12267330|rs429511|rs2394185|rs13332962
Arthritis	FACTORS_INVOLVED_IN_MEGAKARYOCYTE_DEVELOPMENT_AND_PLATELET_PRODUCT	REACTOME	3549	34	20	1,49E-01	9,27E-01	rs10282181|rs2302234|rs958934|rs1295217|rs888298|rs2295281|rs11815834|rs4454616|rs10952167|rs12129768|rs2860228|rs11255764|rs927221|rs1049618|rs6557837|rs961548|rs4902628|rs200968|rs911263|rs10106386
Arthritis	GABA_SYNTHESIS_RELEASE_REUPTAKE_AND_DEGRADATION	REACTOME	746	23	15	1,54E-01	9,27E-01	rs2815727|rs2950798|rs999448|rs876848|rs1865806|rs4439928|rs9871974|rs6931045|rs17046119|rs6071120|rs1728803|rs1682821|rs17518345|rs1934124|rs829473
Arthritis	CYSTEINE_AND_METHIONINE_METABOLISM	KEGG	982	55	20	1,54E-01	9,27E-01	rs863944|rs357181|rs7448803|rs9314048|rs11135315|rs234784|rs17610788|rs9481119|rs11209658|rs257685|rs720938|rs11649592|rs11254408|rs2964917|rs6879291|rs2028900|rs1977420|rs4921028|rs11701664|rs173635
Arthritis	G1_S_TRANSITION	REACTOME	1941	25	20	1,56E-01	9,27E-01	rs6564984|rs4733828|rs759648|rs9792218|rs3815844|rs6575121|rs1499368|rs2280162|rs2550461|rs3820716|rs10818964|rs510862|rs7756899|rs2284704|rs3218801|rs4936661|rs2542417|rs4404878|rs1541549|rs9371645
Arthritis	PLATELET_CALCIUM_HOMEOSTASIS	REACTOME	1156	36	20	1,57E-01	9,27E-01	rs3924457|rs12944845|rs11048665|rs11615994|rs9858042|rs7955100|rs1007637|rs4259245|rs929708|rs12475881|rs758642|rs13430329|rs6442178|rs184477|rs11686356|rs1536041|rs13155995|rs1865395|rs12579456|rs1994474
Arthritis	GLYCOGEN_BREAKDOWN_GLYCOGENOLYSIS	REACTOME	246	28	7	1,60E-01	9,27E-01	rs3753491|rs816411|rs8004788|rs1740005|rs12431766|rs929959|rs6844466
Arthritis	APC_C_CDC20_MEDIATED_DEGRADATION_OF_MITOTIC_PROTEINS	REACTOME	1094	40	20	1,61E-01	9,27E-01	rs6564984|rs4858552|rs12243192|rs3815844|rs6575121|rs2280162|rs2550461|rs10818964|rs3898028|rs7404213|rs6142963|rs228271|rs8063430|rs3819716|rs12497066|rs13332962|rs12453759|rs971963|rs9904424|rs584184
Arthritis	DOWNSTREAM_SIGNALING_EVENTS_OF_B_CELL_RECEPTOR_BCR	REACTOME	2127	39	20	1,63E-01	9,27E-01	rs2647394|rs6564984|rs2755209|rs13014054|rs3815844|rs1999090|rs6575121|rs6084337|rs2280162|rs2550461|rs6461856|rs10818964|rs10485408|rs10455526|rs2494738|rs2655638|rs13408995|rs2238493|rs12038990|rs7404213
Arthritis	VIF_MEDIATED_DEGRADATION_OF_APOBEC3G	REACTOME	738	41	20	1,63E-01	9,27E-01	rs6564984|rs3815844|rs6575121|rs2280162|rs2550461|rs10818964|rs5751022|rs7404213|rs6142963|rs228271|rs8063430|rs3819716|rs13332962|rs12453759|rs971963|rs9904424|rs584184|rs8004624|rs4664417|rs7200210
Arthritis	PLATELET_ACTIVATION_SIGNALING_AND_AGGREGATION	REACTOME	6965	39	20	1,66E-01	9,27E-01	rs10515086|rs17153079|rs3924457|rs9834264|rs7330196|rs881376|rs7772062|rs2227631|rs434602|rs10752974|rs17661774|rs12445650|rs3107373|rs2724113|rs11048665|rs11615994|rs4979032|rs10869979|rs10912025|rs1339082
Arthritis	CHRONIC_MYELOID_LEUKEMIA	KEGG	2538	29	20	1,67E-01	9,27E-01	rs10515086|rs4733828|rs759648|rs2834772|rs2510152|rs9792218|rs12633242|rs1499368|rs7277670|rs9350245|rs266339|rs721128|rs2242747|rs2075995|rs2715252|rs785512|rs2834950|rs12632728|rs1498444|rs230530
Arthritis	SIGNALING_BY_EGFR_IN_CANCER	REACTOME	4030	35	20	1,68E-01	9,27E-01	rs10515086|rs9318729|rs2302234|rs1509849|rs384067|rs2755209|rs888298|rs2510152|rs12129768|rs11048665|rs6084337|rs11615994|rs1278302|rs7955100|rs1379194|rs300550|rs11919795|rs4833920|rs4259245|rs13190668
Arthritis	BLADDER_CANCER	KEGG	1222	31	20	1,69E-01	9,27E-01	rs4733828|rs1816594|rs759648|rs9792218|rs1499368|rs9350245|rs2075995|rs10746803|rs10147042|rs2542417|rs4404878|rs17301608|rs2889977|rs12038990|rs10505528|rs1051442|rs17475756|rs1468727|rs2005219|rs6470648
Arthritis	TRANSLATION	REACTOME	1803	33	20	1,69E-01	9,27E-01	rs7636810|rs284541|rs12631297|rs12639287|rs1149754|rs2316692|rs10099003|rs4554825|rs7757405|rs2715609|rs8109578|rs6503113|rs2291782|rs12541647|rs16974510|rs12260965|rs36621|rs8076790|rs10824609|rs2053984
Arthritis	RNA_POL_III_TRANSCRIPTION_INITIATION_FROM_TYPE_2_PROMOTER	REACTOME	417	18	11	1,69E-01	9,27E-01	rs1075|rs9512817|rs2173115|rs1535565|rs11149485|rs12541335|rs1497021|rs510395|rs1532445|rs1106841|rs2559658
Arthritis	SIGNALING_BY_TGF_BETA_RECEPTOR_COMPLEX	REACTOME	1605	26	20	1,72E-01	9,27E-01	rs4733828|rs759648|rs2227631|rs9792218|rs1499368|rs7975139|rs7234567|rs1331683|rs9972635|rs266339|rs2337143|rs701078|rs1989138|rs4522809|rs2542417|rs4404878|rs1991403|rs627205|rs11874742|rs556765
Arthritis	TRIGLYCERIDE_BIOSYNTHESIS	REACTOME	1182	32	20	1,72E-01	9,27E-01	rs4478182|rs149293|rs3757031|rs10004130|rs910721|rs2017802|rs7419959|rs3132941|rs2419627|rs3745013|rs3782323|rs6065338|rs12692461|rs1834452|rs4669835|rs10197259|rs34129552|rs2017571|rs9861404|rs10495589
Arthritis	BETA_ALANINE_METABOLISM	KEGG	711	37	14	1,74E-01	9,27E-01	rs6784193|rs876848|rs1865806|rs2280010|rs2306619|rs7865905|rs4439928|rs4836272|rs507170|rs12568335|rs7700981|rs7534754|rs8136239|rs1923431
Arthritis	APC_C_CDH1_MEDIATED_DEGRADATION_OF_CDC20_AND_OTHER_APC_C_CDH1_TARGE TE_MITOSIS_EARLY_G1	REACTOME	959	40	20	1,74E-01	9,27E-01	rs6564984|rs4858552|rs3815844|rs6575121|rs2280162|rs2550461|rs10818964|rs3898028|rs7404213|rs6142963|rs228271|rs13160147|rs8063430|rs3819716|rs12497066|rs13332962|rs12453759|rs971963|rs9904424|rs584184
Arthritis	ACH_PATHWAY	BIOCARTA	602	26	13	1,76E-01	9,27E-01	rs10515086|rs306966|rs17237251|rs2494738|rs2302975|rs422437|rs12652661|rs849376|rs484754|rs2322606|rs891398|rs7721613|rs1550955
Arthritis	PRE_NOTCH_EXPRESSION_AND_PROCESSING	REACTOME	1434	35	20	1,78E-01	9,27E-01	rs12944845|rs7943506|rs3761441|rs4078830|rs1652583|rs17005603|rs1959010|rs3134942|rs9350245|rs176943|rs1007637|rs1652593|rs12274970|rs6817684|rs2906142|rs11021494|rs4134943|rs6771941|rs1887735|rs17677652
Arthritis	REGULATION_OF_ORNITHINE_DECARBOXYLASE_ODC	REACTOME	773	41	20	1,78E-01	9,27E-01	rs6564984|rs3815844|rs6575121|rs2280162|rs2550461|rs10818964|rs2965749|rs7404213|rs6142963|rs228271|rs8063430|rs3819716|rs13332962|rs12453759|rs971963|rs9904424|rs584184|rs8004624|rs4664417|rs7200210
Arthritis	G_ALPHA_S_SIGNALLING_EVENTS	REACTOME	5019	44	20	1,79E-01	9,27E-01	rs17153079|rs1419791|rs1509849|rs384067|rs1562782|rs34647055|rs646749|rs12672801|rs1344925|rs16889129|rs2724113|rs17764941|rs17304935|rs4979032|rs3793228|rs11045087|rs612238|rs6755901|rs3761049|rs10083148
Arthritis	APC_CDC20_MEDIATED_DEGRADATION_OF_NEK2A	REACTOME	396	12	8	1,80E-01	9,27E-01	rs4858552|rs12243192|rs3898028|rs12497066|rs1773356|rs12623041|rs3814026|rs1996182
Arthritis	ANTIGEN_ACTIVATES_B_CELL_RECEPTOR_LEADING_TO_GENERATION_OF_SECOND_MES	REACTOME	1299	42	20	1,80E-01	9,27E-01	rs10515086|rs2510139|rs573323|rs11188666|rs11048665|rs11615994|rs7955100|rs2715252|rs4259245|rs2921256|rs17237251|rs2736360|rs2302975|rs1740005|rs422437|rs12652661|rs9937676|rs1992055|rs1536041|rs12431766
Arthritis	RAP1_SIGNALLING	REACTOME	835	28	19	1,81E-01	9,27E-01	rs12943009|rs1194491|rs6502555|rs12129768|rs7077665|rs11653675|rs10822867|rs11652683|rs12256137|rs2317462|rs4077990|rs12259464|rs11001056|rs6876|rs583720|rs17549153|rs7359618|rs7222829|rs1373497
Arthritis	AMINE_LIGAND_BINDING_RECEPTORS	REACTOME	2247	37	20	1,81E-01	9,27E-01	rs7125415|rs2734838|rs34647055|rs17455628|rs3107373|rs11779546|rs9526301|rs4130345|rs2514227|rs2875512|rs7719360|rs12090480|rs17064682|rs4936270|rs9526282|rs12498102|rs12759541|rs10194776|rs2296973|rs9526257
Arthritis	RENIN_ANGIOTENSIN_SYSTEM	KEGG	467	31	13	1,81E-01	9,27E-01	rs9289775|rs6440971|rs3737656|rs1981846|rs388488|rs6510701|rs1601383|rs989692|rs9653951|rs9821034|rs878893|rs6496603|rs4353
Arthritis	ADP_SIGNALLING_THROUGH_P2RY12	REACTOME	438	35	15	1,82E-01	9,27E-01	rs17153079|rs2724113|rs4979032|rs10498440|rs4807284|rs9244|rs182524|rs291353|rs7531583|rs12147761|rs16863356|rs304732|rs4731190|rs10276215|rs2965208
Arthritis	ADP_SIGNALLING_THROUGH_P2RY1	REACTOME	863	42	20	1,83E-01	9,27E-01	rs10752974|rs2724113|rs4979032|rs10869979|rs10912025|rs1339082|rs308039|rs230632|rs10498440|rs4807284|rs6560613|rs1938512|rs10911946|rs851010|rs2237093|rs3746070|rs291353|rs9729852|rs7531583|rs12147761
Arthritis	SIGNALING_BY_WNT	REACTOME	1071	45	20	1,86E-01	9,27E-01	rs6564984|rs3815844|rs6575121|rs972984|rs2280162|rs2550461|rs10818964|rs389673|rs4936661|rs7404213|rs10271133|rs7703433|rs6142963|rs228271|rs8063430|rs1116003|rs10490823|rs3819716|rs13332962|rs12453759
Arthritis	RNA_POL_III_CHAIN_ELONGATION	REACTOME	364	18	11	1,86E-01	9,27E-01	rs1075|rs9512817|rs2173115|rs1535565|rs11149485|rs12541335|rs1497021|rs510395|rs1532445|rs1106841|rs2559658
Arthritis	INTEGRATION_OF_PROVIRUS	REACTOME	122	6	4	1,89E-01	9,27E-01	rs4426221|rs7567258|rs1872066|rs9514816
Arthritis	PHOSPHORYLATION_OF_CD3_AND_TCR_ZETA_CHAINS	REACTOME	521	17	9	1,89E-01	9,27E-01	rs2949654|rs2301226|rs9469357|rs704859|rs1618879|rs6473257|rs2858870|rs3130192|rs2056626
Arthritis	RHO_PATHWAY	BIOCARTA	480	27	10	1,89E-01	9,27E-01	rs1958918|rs4509829|rs3784166|rs11038977|rs7515836|rs2855726|rs6809452|rs238242|rs3767397|rs4981146
Arthritis	RESPIRATORY_ELECTRON_TRANSPORT	REACTOME	1631	28	20	1,90E-01	9,27E-01	rs222289|rs10226904|rs3753362|rs999918|rs3900417|rs10423721|rs766549|rs17476583|rs9293478|rs7254601|rs414000|rs11957871|rs7255086|rs7247484|rs1581512|rs4634927|rs6470250|rs2871775|rs2402111|rs1136224
Arthritis	ATRBRCA_PATHWAY	BIOCARTA	238	13	6	1,91E-01	9,27E-01	rs2894401|rs2229032|rs1799949|rs11571805|rs1207952|rs27162
Arthritis	ARGININE_AND_PROLINE_METABOLISM	KEGG	991	41	20	1,91E-01	9,27E-01	rs1923939|rs863944|rs10911063|rs1082214|rs612380|rs11245507|rs1038378|rs4767520|rs7865905|rs11671781|rs9481119|rs9658570|rs11649592|rs4836272|rs186471|rs2058925|rs2016108|rs10901268|rs549042|rs7700981
Arthritis	LYSOSOME_VESICLE_BIOGENESIS	REACTOME	420	14	10	1,91E-01	9,27E-01	rs6733550|rs849749|rs11048060|rs11962800|rs737883|rs2232739|rs1045895|rs3808734|rs2224957|rs2267138
Arthritis	PEPTIDE_CHAIN_ELONGATION	REACTOME	947	35	20	1,93E-01	9,27E-01	rs7636810|rs284541|rs1149754|rs4554825|rs760782|rs6503113|rs2291782|rs36621|rs8076790|rs10824609|rs2053984|rs12484030|rs2574693|rs7508025|rs2004997|rs10467685|rs10197539|rs7902136|rs10762770|rs14398
Arthritis	PI_METABOLISM	REACTOME	1808	41	20	1,93E-01	9,27E-01	rs10515086|rs1024355|rs10505811|rs577684|rs1476592|rs1130635|rs849749|rs7771000|rs3775616|rs785512|rs7234952|rs17237251|rs2302975|rs422437|rs228271|rs965976|rs12357384|rs875858|rs12652661|rs987380
Arthritis	GATA3_PATHWAY	BIOCARTA	898	46	20	1,96E-01	9,27E-01	rs2302234|rs958934|rs888298|rs4454616|rs12129768|rs11255764|rs177820|rs11150222|rs4749844|rs851010|rs4307960|rs16951505|rs9380546|rs7233024|rs9804388|rs583720|rs2243248|rs4799062|rs1242987|rs6501512
Arthritis	RNA_DEGRADATION	KEGG	1123	63	20	1,96E-01	9,27E-01	rs11853191|rs1239898|rs3125804|rs3809425|rs437179|rs7300901|rs2229032|rs16969711|rs4859879|rs10419232|rs369052|rs2453176|rs7592215|rs11062040|rs7857803|rs1432295|rs6893735|rs4382556|rs13163920|rs238228
Arthritis	PRION_DISEASES	KEGG	1585	26	20	2,00E-01	9,27E-01	rs2826356|rs1179766|rs12129768|rs17554245|rs2826861|rs11700900|rs1899949|rs2826586|rs660361|rs2246589|rs835223|rs2826343|rs2827107|rs2048874|rs2407581|rs4937993|rs994781|rs2827076|rs2827037|rs2005219
Arthritis	ACTIVATION_OF_CHAPERONE_GENES_BY_XBP1S	REACTOME	761	36	20	2,00E-01	9,27E-01	rs848189|rs888298|rs6442982|rs1864427|rs1836581|rs2715609|rs4143282|rs13332660|rs6785659|rs4686432|rs1880666|rs2005219|rs402330|rs2444218|rs2176246|rs1869241|rs11051241|rs2237813|rs2285346|rs12004
Arthritis	RAC1_PATHWAY	BIOCARTA	763	51	19	2,00E-01	9,27E-01	rs10515086|rs4766522|rs832574|rs360402|rs17237251|rs4128634|rs2302975|rs17303923|rs422437|rs2855726|rs12652661|rs164351|rs339959|rs849376|rs2660489|rs12608960|rs7721613|rs3886691|rs3767397
Arthritis	SULFUR_AMINO_ACID_METABOLISM	REACTOME	930	52	20	2,01E-01	9,27E-01	rs357181|rs7448803|rs355396|rs9314048|rs11135315|rs234784|rs17610788|rs11209658|rs257685|rs720938|rs2272300|rs2964917|rs6879291|rs10153006|rs2028900|rs1915190|rs1977420|rs4921028|rs1356553|rs11701664
Arthritis	ORC1_REMOVAL_FROM_CHROMATIN	REACTOME	930	46	20	2,02E-01	9,27E-01	rs6564984|rs3815844|rs6575121|rs2280162|rs2550461|rs3820716|rs10818964|rs510862|rs7404213|rs6142963|rs228271|rs9450763|rs8063430|rs3819716|rs13332962|rs12453759|rs971963|rs9904424|rs584184|rs565211
Arthritis	PD1_SIGNALING	REACTOME	398	18	9	2,02E-01	9,27E-01	rs2949654|rs2301226|rs9469357|rs704859|rs1618879|rs1360238|rs2858870|rs3130192|rs2056626
Arthritis	SIGNALING_BY_NOTCH	REACTOME	4553	23	20	2,02E-01	9,27E-01	rs12944845|rs4733828|rs7943506|rs4510937|rs3761441|rs10867720|rs759648|rs584846|rs4078830|rs9792218|rs1652583|rs4906007|rs7155649|rs17005603|rs1959010|rs1499368|rs6108727|rs3213615|rs11678000|rs6104663
Arthritis	CACAM_PATHWAY	BIOCARTA	401	22	11	2,03E-01	9,27E-01	rs7314454|rs1029471|rs10849860|rs6533710|rs1740005|rs758642|rs2072439|rs1551565|rs12431766|rs13133676|rs6809452
Arthritis	TOLL_PATHWAY	BIOCARTA	1147	37	20	2,03E-01	9,27E-01	rs2726550|rs1999090|rs832574|rs10983934|rs739141|rs10739508|rs4899554|rs10485408|rs12555480|rs10455526|rs230530|rs2655638|rs1554973|rs10759930|rs1556091|rs9353794|rs6478330|rs851010|rs694428|rs7691384
Arthritis	PHOSPHOLIPID_METABOLISM	REACTOME	6226	28	20	2,05E-01	9,27E-01	rs10515086|rs4478182|rs1024355|rs1806328|rs10505811|rs11541600|rs1093733|rs3757031|rs10103493|rs10752974|rs10820938|rs577684|rs1476592|rs4637750|rs11589805|rs11683765|rs1130635|rs10004130|rs849749|rs2295514
Arthritis	ERBB_SIGNALING_PATHWAY	KEGG	4135	32	20	2,07E-01	9,27E-01	rs10515086|rs17101139|rs4733828|rs7079919|rs2726550|rs17406681|rs759648|rs2510152|rs9792218|rs1499368|rs1029471|rs7562947|rs4673677|rs306966|rs11191432|rs2715252|rs2423441|rs785512|rs2921256|rs17237251
Arthritis	PAR1_PATHWAY	BIOCARTA	1941	32	20	2,09E-01	9,27E-01	rs10515086|rs17153079|rs7007884|rs1999090|rs2724113|rs10869979|rs10485408|rs10455526|rs9555756|rs17237251|rs11652805|rs2207317|rs2655638|rs6039211|rs1554465|rs2238493|rs4242546|rs9244|rs2302975|rs6560613
Arthritis	ANTIGEN_PROCESSING_CROSS_PRESENTATION	REACTOME	1601	38	20	2,10E-01	9,27E-01	rs6564984|rs2253907|rs3815844|rs6575121|rs2280162|rs2550461|rs10818964|rs1981846|rs2071469|rs2596574|rs12260965|rs7404213|rs6142963|rs228271|rs2071554|rs8063430|rs3819716|rs12267330|rs429511|rs2394185
Arthritis	MEIOTIC_SYNAPSIS	REACTOME	1031	68	20	2,10E-01	9,27E-01	rs1427575|rs742047|rs9972635|rs2229032|rs12928076|rs1051644|rs7745603|rs639373|rs12196939|rs9384022|rs633891|rs6499137|rs959573|rs1256063|rs1799949|rs12110479|rs1593756|rs199750|rs4821818|rs17639579
Arthritis	CDK_MEDIATED_PHOSPHORYLATION_AND_REMOVAL_OF_CDC6	REACTOME	698	41	20	2,11E-01	9,27E-01	rs6564984|rs3815844|rs6575121|rs2280162|rs2550461|rs10818964|rs7404213|rs6142963|rs228271|rs8063430|rs3819716|rs13332962|rs12453759|rs971963|rs9904424|rs584184|rs8004624|rs4664417|rs7200210|rs8842
Arthritis	CHONDROITIN_SULFATE_DERMATAN_SULFATE_METABOLISM	REACTOME	2784	36	20	2,12E-01	9,27E-01	rs16943314|rs12756105|rs2119297|rs4454830|rs16943623|rs9523322|rs4307360|rs10955078|rs7727897|rs1041883|rs10808350|rs9589298|rs342711|rs9516073|rs1289459|rs2374409|rs2589204|rs971656|rs12408228|rs3939465
Arthritis	SIGNALING_BY_FGFR_IN_DISEASE	REACTOME	4545	35	20	2,13E-01	9,27E-01	rs10515086|rs9318729|rs2302234|rs1509849|rs384067|rs2755209|rs888298|rs2510139|rs2313626|rs12129768|rs11048665|rs6084337|rs11615994|rs1734729|rs7674434|rs7955100|rs4259245|rs13190668|rs8002487|rs17237251
Arthritis	CCR3_PATHWAY	BIOCARTA	939	40	20	2,13E-01	9,27E-01	rs10505811|rs4509829|rs577684|rs2724113|rs10869979|rs306966|rs2207317|rs6039211|rs2238493|rs6560613|rs6118219|rs10485723|rs2005219|rs2855726|rs2223538|rs4471960|rs2076409|rs9913908|rs7531583|rs198182
Arthritis	SRP_DEPENDENT_COTRANSLATIONAL_PROTEIN_TARGETING_TO_MEMBRANE	REACTOME	1378	32	20	2,14E-01	9,27E-01	rs7636810|rs284541|rs12631297|rs1149754|rs10509398|rs760782|rs2715609|rs6503113|rs2291782|rs16974510|rs12260965|rs36621|rs8076790|rs10824609|rs2053984|rs7659414|rs6584|rs1880666|rs12484030|rs2574693
Arthritis	DAG_AND_IP3_SIGNALING	REACTOME	1644	30	20	2,16E-01	9,27E-01	rs2302234|rs1509849|rs384067|rs888298|rs12129768|rs11048665|rs11615994|rs7955100|rs4259245|rs13190668|rs1509844|rs1554465|rs1740005|rs448852|rs1153138|rs1369450|rs1534131|rs1249950|rs885244|rs935656
Arthritis	COLORECTAL_CANCER	KEGG	2539	24	20	2,16E-01	9,27E-01	rs10515086|rs2348238|rs4733828|rs626713|rs759648|rs9792218|rs1499368|rs266339|rs4899554|rs11884056|rs389673|rs9840663|rs785512|rs2073923|rs17237251|rs2494738|rs4522809|rs11625323|rs2542417|rs4404878
Arthritis	CROSS_PRESENTATION_OF_SOLUBLE_EXOGENOUS_ANTIGENS_ENDOSOMES	REACTOME	713	41	20	2,17E-01	9,27E-01	rs6564984|rs3815844|rs6575121|rs2280162|rs2550461|rs10818964|rs7404213|rs6142963|rs228271|rs8063430|rs3819716|rs13332962|rs12453759|rs971963|rs9904424|rs584184|rs8004624|rs4664417|rs7200210|rs8842
Arthritis	INFLUENZA_VIRAL_RNA_TRANSCRIPTION_AND_REPLICATION	REACTOME	1153	39	20	2,17E-01	9,27E-01	rs7636810|rs284541|rs1149754|rs7993717|rs4554825|rs760782|rs4772030|rs6503113|rs2291782|rs36621|rs8076790|rs10824609|rs2053984|rs12484030|rs2574693|rs7508025|rs12870730|rs2004997|rs10467685|rs10197539
Arthritis	A_TETRASACCHARIDE_LINKER_SEQUENCE_IS_REQUIRED_FOR_GAG_SYNTHESIS	REACTOME	1731	34	20	2,18E-01	9,27E-01	rs12756105|rs2119297|rs4454830|rs9523322|rs10955078|rs7727897|rs10808350|rs9589298|rs342711|rs9516073|rs1289459|rs2589204|rs12408228|rs3939465|rs7007840|rs7991374|rs9523299|rs9522712|rs9351798|rs9523512
Arthritis	SCFSKP2_MEDIATED_DEGRADATION_OF_P27_P21	REACTOME	813	42	20	2,19E-01	9,27E-01	rs6564984|rs3815844|rs6575121|rs2280162|rs2550461|rs10818964|rs7404213|rs10271133|rs6142963|rs228271|rs13160147|rs8063430|rs3819716|rs13332962|rs12453759|rs971963|rs9904424|rs584184|rs33671|rs8004624
Arthritis	REGULATION_OF_MITOTIC_CELL_CYCLE	REACTOME	1318	40	20	2,20E-01	9,27E-01	rs6564984|rs4858552|rs12243192|rs3815844|rs6575121|rs2280162|rs2550461|rs10818964|rs7756899|rs3898028|rs9371645|rs7404213|rs10271133|rs6142963|rs228271|rs13160147|rs8063430|rs3819716|rs12497066|rs13332962
Arthritis	P53_INDEPENDENT_G1_S_DNA_DAMAGE_CHECKPOINT	REACTOME	729	41	20	2,20E-01	9,27E-01	rs6564984|rs3815844|rs6575121|rs2280162|rs2550461|rs10818964|rs7404213|rs6142963|rs228271|rs8063430|rs3819716|rs13332962|rs12453759|rs971963|rs9904424|rs584184|rs8004624|rs4664417|rs7200210|rs8842
Arthritis	S_PHASE	REACTOME	2076	26	20	2,21E-01	9,27E-01	rs6564984|rs4733828|rs759648|rs9792218|rs3815844|rs6575121|rs1499368|rs2280162|rs2550461|rs3820716|rs10818964|rs510862|rs2284704|rs3218801|rs2542417|rs4404878|rs1541549|rs1925089|rs7404213|rs10271133
Arthritis	RNA_POL_I_PROMOTER_OPENING	REACTOME	501	77	7	2,26E-01	9,42E-01	rs742047|rs7745603|rs639373|rs12196939|rs2005219|rs199750|rs6456688
Arthritis	TCR_SIGNALING	REACTOME	2040	34	20	2,28E-01	9,43E-01	rs10515086|rs2647394|rs2949654|rs1999090|rs2301226|rs137991|rs6461856|rs975167|rs10485408|rs10455526|rs17237251|rs9469357|rs2655638|rs1240073|rs2302975|rs704859|rs218921|rs1182136|rs1556091|rs422437
Arthritis	SIGNALING_BY_FGFR	REACTOME	4154	35	20	2,30E-01	9,43E-01	rs10515086|rs9318729|rs2302234|rs1509849|rs384067|rs2755209|rs888298|rs2510139|rs12129768|rs11048665|rs6084337|rs11615994|rs7674434|rs7955100|rs4259245|rs13190668|rs8002487|rs17237251|rs1509844|rs9591501

Arthritis	ACYL_CHAIN_REMODELLING_OF_PG	REACTOME	571	35	20	2,30E-01	9,43E-01	rs1093733|rs10752974|rs1158980|rs10912025|rs1339082|rs230632|rs1567102|rs800719|rs11573253|rs1938512|rs10911946|rs3788428|rs9729852|rs12045689|rs17221851|rs10475030|rs2872821|rs11630961|rs7530853|rs7551550
Arthritis	MITOCHONDRIAL_TRNA_AMINOACYLATION	REACTOME	500	28	11	2,33E-01	9,43E-01	rs4959341|rs6932040|rs1891222|rs17563587|rs9450740|rs13144832|rs6764923|rs2341510|rs9405818|rs7744190|rs6926774
Arthritis	SIGNALING_BY_CONSTITUTIVELY_ACTIVE_EGFR	REACTOME	503	31	15	2,33E-01	9,43E-01	rs10515086|rs2510139|rs17237251|rs2302975|rs12038990|rs422437|rs1468727|rs12652661|rs10842514|rs3796944|rs7721613|rs2235366|rs6974790|rs759159|rs2168043
Arthritis	GABA_B_RECEPTOR_ACTIVATION	REACTOME	1863	32	20	2,34E-01	9,43E-01	rs17153079|rs1509849|rs384067|rs12457181|rs2835909|rs2724113|rs4979032|rs10498440|rs13190668|rs1509844|rs2836034|rs4807284|rs1554465|rs9244|rs448852|rs1369450|rs1534131|rs885244|rs9907685|rs2226741
Arthritis	PHASE_II_CONJUGATION	REACTOME	2059	39	20	2,36E-01	9,43E-01	rs357181|rs7448803|rs2132847|rs9314048|rs6586711|rs4970774|rs1135315|rs6586734|rs737866|rs7687822|rs16970280|rs17610788|rs13254048|rs17563587|rs13332660|rs11203956|rs5751901|rs257685|rs720938|rs2005219
Arthritis	OXIDATIVE_PHOSPHORYLATION	KEGG	2486	27	20	2,37E-01	9,43E-01	rs222289|rs10226904|rs17152064|rs3753362|rs2001515|rs999918|rs3900417|rs10423721|rs4669621|rs766549|rs17476583|rs9293478|rs2834287|rs7254601|rs414000|rs11957871|rs9291289|rs13144832|rs1497021|rs16919915
Arthritis	THROMBOXANE_SIGNALLING_THROUGH_TP_RECEPTOR	REACTOME	492	38	15	2,39E-01	9,43E-01	rs2724113|rs4979032|rs10869979|rs308039|rs10498440|rs11652805|rs4807284|rs6560613|rs4806942|rs3746070|rs291353|rs7531583|rs12147761|rs2296781|rs2965208
Arthritis	FCER1_PATHWAY	BIOCARTA	1564	28	20	2,42E-01	9,43E-01	rs10515086|rs2726550|rs10752974|rs2251238|rs832574|rs10912025|rs1339082|rs4899554|rs230632|rs17237251|rs2238493|rs2302975|rs1740005|rs177820|rs422437|rs1938512|rs2005219|rs10911946|rs12652661|rs2501873
Arthritis	DESTABILIZATION_OF_MRNA_BY_AUF1_HNRNP_D0	REACTOME	811	41	20	2,43E-01	9,43E-01	rs6564984|rs3815844|rs6575121|rs2280162|rs2550461|rs10818964|rs7404213|rs6142963|rs228271|rs8063430|rs3819716|rs2011786|rs13332962|rs12453759|rs971963|rs9904424|rs584184|rs2908201|rs8004624|rs4664417
Arthritis	CARDIAC_MUSCLE_CONTRACTION	KEGG	3169	30	20	2,43E-01	9,43E-01	rs4839066|rs16835926|rs4509829|rs1868513|rs3753362|rs6785307|rs9878916|rs9311552|rs3900417|rs2238517|rs10423721|rs12058655|rs1464459|rs4938440|rs17476583|rs2805434|rs9293478|rs1405331|rs7254601|rs6700222
Arthritis	CD28_CO_STIMULATION	REACTOME	797	37	17	2,43E-01	9,43E-01	rs10515086|rs137991|rs6084337|rs785512|rs17237251|rs2494738|rs2302975|rs422437|rs12652661|rs2501279|rs8095469|rs2660489|rs2670289|rs12608960|rs7721613|rs16829957|rs12166465
Arthritis	LYSINE_DEGRADATION	KEGG	952	45	20	2,44E-01	9,43E-01	rs6784193|rs7643465|rs11029795|rs12445568|rs12487322|rs1038378|rs3803915|rs7865905|rs6850897|rs4836272|rs519417|rs2592980|rs1913185|rs7700981|rs6885410|rs2077807|rs10835129|rs7692005|rs10835128|rs6797159
Arthritis	CDMAC_PATHWAY	BIOCARTA	1018	24	20	2,45E-01	9,43E-01	rs4733828|rs759648|rs9792218|rs1499368|rs4899554|rs230530|rs2207317|rs2542417|rs4404878|rs6039211|rs2238493|rs10505528|rs6118219|rs10485723|rs2005219|rs2223538|rs6470648|rs6599711|rs4471960|rs13116385
Arthritis	M_G1_TRANSITION	REACTOME	1315	35	20	2,46E-01	9,43E-01	rs6564984|rs3815844|rs6575121|rs2280162|rs9350245|rs2550461|rs3820716|rs10818964|rs2075995|rs510862|rs3218801|rs1541549|rs7404213|rs6142963|rs228271|rs6463716|rs9450763|rs4134943|rs8063430|rs3819716
Arthritis	NON_SMALL_CELL_LUNG_CANCER	KEGG	2585	29	20	2,50E-01	9,43E-01	rs10515086|rs4302350|rs13097687|rs6806748|rs1994908|rs1821942|rs6788821|rs9809773|rs9350245|rs2205361|rs4679627|rs13077354|rs7562947|rs7634772|rs2075995|rs721207|rs1569355|rs2856029|rs785512|rs17237251
Arthritis	MELANOGENESIS	KEGG	4077	32	20	2,51E-01	9,43E-01	rs17153079|rs1509849|rs384067|rs9471843|rs9381141|rs1076465|rs9318516|rs937643|rs12129768|rs1029471|rs10869979|rs3121310|rs9394696|rs6056526|rs11614438|rs765015|rs9574197|rs765543|rs2371933|rs11884056
Arthritis	SMALL_CELL_LUNG_CANCER	KEGG	3747	22	20	2,52E-01	9,43E-01	rs10515086|rs4733828|rs4302350|rs759648|rs9792218|rs13097687|rs6806748|rs1994908|rs1499368|rs1821942|rs6788821|rs9809773|rs9350245|rs2205361|rs869936|rs4679627|rs13077354|rs7634772|rs306966|rs630943
Arthritis	RNA_POL_I_TRANSCRIPTION_TERMINATION	REACTOME	335	14	10	2,55E-01	9,43E-01	rs1056616|rs9512817|rs1535565|rs2284704|rs11149485|rs17011628|rs10514603|rs10200632|rs1106841|rs16902819
Arthritis	INHIBITION_OF_INSULIN_SECRETION_BY_ADRENALINE_NORADRENALINE	REACTOME	789	39	20	2,55E-01	9,43E-01	rs17153079|rs3107373|rs2724113|rs4979032|rs10498440|rs4807284|rs1277748|rs9244|rs12416052|rs13338253|rs11014144|rs182524|rs12220858|rs291353|rs7531583|rs12147761|rs304732|rs4731190|rs11644870|rs10276215
Arthritis	GCR_PATHWAY	BIOCARTA	736	28	20	2,56E-01	9,43E-01	rs10515086|rs2724113|rs230530|rs17237251|rs2494738|rs17601663|rs2302975|rs1740005|rs422437|rs12652661|rs12555078|rs4581120|rs17350396|rs17341964|rs12431766|rs10781146|rs12562952|rs13116385|rs849376|rs7531583
Arthritis	SYNTHESIS_OF_PIPS_AT_THE_GOLGI_MEMBRANE	REACTOME	788	38	17	2,57E-01	9,43E-01	rs10505811|rs577684|rs1130635|rs849749|rs7771000|rs7234952|rs965976|rs875858|rs987380|rs4146444|rs9956023|rs6568603|rs9961683|rs1865288|rs13209604|rs12957142|rs518345
Arthritis	LAIR_PATHWAY	BIOCARTA	479	16	10	2,58E-01	9,43E-01	rs1179766|rs1899949|rs3783621|rs2048874|rs1822824|rs751138|rs7027797|rs155110|rs2070947|rs17201075
Arthritis	COSTIMULATION_BY_THE_CD28_FAMILY	REACTOME	1479	38	20	2,58E-01	9,43E-01	rs10515086|rs2949654|rs2301226|rs972984|rs137991|rs6084337|rs785512|rs17237251|rs9469357|rs2494738|rs4936661|rs2302975|rs704859|rs2838527|rs422437|rs7703433|rs1618879|rs12652661|rs2501279|rs1360238
Arthritis	METABOLISM_OF_AMINO_ACIDS_AND_DERIVATIVES	REACTOME	5246	43	20	2,59E-01	9,43E-01	rs6564984|rs4724065|rs1923939|rs863944|rs357181|rs7448803|rs355396|rs11029795|rs3815844|rs6575121|rs12894797|rs9314048|rs7149154|rs10911063|rs1082214|rs2280162|rs12211979|rs2550461|rs612380|rs9352866
Arthritis	AKT_PATHWAY	BIOCARTA	682	45	19	2,59E-01	9,43E-01	rs10515086|rs2755209|rs230530|rs17237251|rs2494738|rs2302975|rs422437|rs7703433|rs12652661|rs12115174|rs4435111|rs11739840|rs1782804|rs13116385|rs849376|rs2951787|rs484754|rs7721613|rs7662536
Arthritis	PROTEASOME	KEGG	821	41	20	2,60E-01	9,43E-01	rs6564984|rs3815844|rs6575121|rs2280162|rs2550461|rs10818964|rs7404213|rs6142963|rs228271|rs8063430|rs3819716|rs13332962|rs12453759|rs971963|rs9904424|rs584184|rs8004624|rs4664417|rs7200210|rs6955026
Arthritis	HS_GAG_DEGRADATION	REACTOME	1386	35	20	2,60E-01	9,43E-01	rs12756105|rs4454830|rs9523322|rs10955078|rs10808350|rs9589298|rs342711|rs9516073|rs2589204|rs12408228|rs3939465|rs7007840|rs7991374|rs9523299|rs9522712|rs9523512|rs1335328|rs7988839|rs16947827|rs10748760
Arthritis	SIGNALING_BY_NOTCH1	REACTOME	3563	22	20	2,63E-01	9,46E-01	rs4733828|rs7943506|rs4510937|rs10867721|rs759648|rs584846|rs9792218|rs4906007|rs7155649|rs17005603|rs1499368|rs610872|rs3213615|rs11678000|rs6104663|rs2293806|rs1872618|rs1938593|rs176943|rs6543534
Arthritis	PROSTACYCLIN_SIGNALLING_THROUGH_PROSTACYCLIN_RECEPTOR	REACTOME	353	19	8	2,63E-01	9,46E-01	rs2724113|rs4979032|rs10498440|rs4807284|rs291353|rs7531583|rs12147761|rs2965208
Arthritis	CELL_ADHESION_MOLECULES_CAMS	KEGG	8813	29	20	2,68E-01	9,51E-01	rs2826356|rs10487920|rs2972100|rs12756105|rs2205210|rs2253907|rs13230925|rs12432833|rs12421648|rs10273151|rs2107128|rs1914779|rs9955719|rs7931895|rs12969349|rs2301226|rs777709|rs2160919|rs17554245|rs10255503
Arthritis	RIBOSOME	KEGG	1118	36	20	2,68E-01	9,51E-01	rs7636810|rs284541|rs1149754|rs4554825|rs7757405|rs6503113|rs2291782|rs36621|rs8076790|rs10824609|rs2053984|rs12484030|rs2574693|rs7508025|rs12485248|rs2004997|rs10467685|rs10197539|rs7902136|rs10762770
Arthritis	CELL_CELL_COMMUNICATION	REACTOME	9725	47	20	2,69E-01	9,51E-01	rs1523843|rs10515086|rs952316|rs1903014|rs2195316|rs2210281|rs7123151|rs4791330|rs7732120|rs12421648|rs10245351|rs139077|rs1437236|rs7931895|rs4395067|rs7201194|rs13231956|rs6124082|rs6779752|rs233560
Arthritis	EARLY_PHASE_OF_HIV_LIFE_CYCLE	REACTOME	270	18	7	2,69E-01	9,51E-01	rs4426221|rs7567258|rs10204606|rs1872066|rs9514816|rs4440020|rs1016269
Arthritis	DOWNSTREAM_SIGNAL_TRANSDUCTION	REACTOME	3083	32	20	2,71E-01	9,51E-01	rs10515086|rs2302234|rs1509849|rs384067|rs2755209|rs888298|rs12129768|rs11048665|rs6084337|rs11615994|rs7955100|rs10901096|rs4259245|rs13190668|rs17237251|rs1509844|rs2494738|rs1554465|rs11887698|rs2302975
Arthritis	CALCIUM_SIGNALING_PATHWAY	KEGG	9275	31	20	2,73E-01	9,51E-01	rs980545|rs1509849|rs12944845|rs6674511|rs384067|rs16835926|rs4417522|rs17406681|rs11850274|rs34647055|rs12457181|rs17455628|rs9318516|rs665197|rs1533939|rs2251238|rs12129768|rs11048665|rs11779546|rs9526301
Arthritis	IMMUNOREGULATORY_INTERACTIONS_BETWEEN_A_LYMPHOID_AND_A_NON_LYMPHOI	REACTOME	1325	23	20	2,73E-01	9,51E-01	rs11574599|rs4320015|rs2253907|rs2949654|rs2270238|rs11880466|rs2956754|rs3783621|rs12604328|rs704859|rs2824327|rs12626798|rs12720356|rs1618879|rs2643075|rs9937676|rs2272300|rs2824280|rs4141178|rs1340976
Arthritis	INTEGRATION_OF_ENERGY_METABOLISM	REACTOME	4501	34	20	2,75E-01	9,56E-01	rs17153079|rs2302234|rs1509849|rs384067|rs988896|rs888298|rs3107373|rs4725424|rs2724113|rs12129768|rs11048665|rs11615994|rs4979032|rs10869979|rs3132941|rs308039|rs389673|rs953221|rs7955100|rs10498440
Arthritis	JNK_C_JUN_KINASES_PHOSPHORYLATION_AND_ACTIVATION_MEDIATED_BY_ACTIVATE	REACTOME	728	41	20	2,78E-01	9,59E-01	rs2726550|rs1999090|rs739141|rs10485408|rs3844278|rs10455526|rs2655638|rs1240073|rs218921|rs1556091|rs402330|rs9353794|rs7771959|rs2811650|rs311636|rs9362733|rs2322376|rs9353872|rs17521993|rs4961116
Arthritis	MITOTIC_G1_G1_S_PHASES	REACTOME	2477	26	20	2,79E-01	9,59E-01	rs6564984|rs4733828|rs759648|rs9792218|rs3815844|rs6575121|rs1499368|rs2280162|rs9350245|rs2550461|rs3820716|rs10818964|rs2075995|rs10862|rs4817863|rs7756899|rs2284704|rs10255859|rs3218801|rs4936661
Arthritis	G_ALPHA_Z_SIGNALLING_EVENTS	REACTOME	2191	35	20	2,82E-01	9,59E-01	rs17153079|rs1509849|rs384067|rs3107373|rs2724113|rs3910736|rs4979032|rs17083833|rs975167|rs10498440|rs13190668|rs1509844|rs4807284|rs1957895|rs1554465|rs2238493|rs9244|rs448852|rs1369450
Arthritis	PDGF_PATHWAY	BIOCARTA	1021	39	20	2,86E-01	9,59E-01	rs10515086|rs2726550|rs310209|rs832574|rs4899554|rs17237251|rs11887698|rs2238493|rs2302975|rs422437|rs2005219|rs12652661|rs12129709|rs849376|rs9913908|rs198182|rs2322376|rs7721613|rs7149441|rs1998762
Arthritis	SNARE_INTERACTIONS_IN_VESICULAR_TRANSPORT	KEGG	828	41	20	2,86E-01	9,59E-01	rs10760706|rs12445568|rs6733550|rs16982131|rs6070531|rs9837294|rs16958408|rs1487608|rs7463863|rs11759401|rs7962097|rs2232739|rs2072439|rs9893164|rs12740112|rs7220482|rs17741348|rs486409|rs3286|rs17518345
Arthritis	SYNTHESIS_OF_PIPS_AT_THE_PLASMA_MEMBRANE	REACTOME	1050	41	20	2,89E-01	9,59E-01	rs10515086|rs10505811|rs577684|rs1476592|rs3775616|rs785512|rs17237251|rs2302975|rs422437|rs228271|rs12357384|rs12652661|rs3788428|rs849376|rs975136|rs693293|rs518345|rs7721613|rs7094771|rs10470165
Arthritis	EGF_PATHWAY	BIOCARTA	1048	40	20	2,89E-01	9,59E-01	rs10515086|rs2726550|rs310209|rs832574|rs4899554|rs17237251|rs11887698|rs2238493|rs2302975|rs422437|rs1468727|rs2005219|rs12652661|rs12129709|rs9913908|rs198182|rs2322376|rs3796944|rs7721613|rs7149441
Arthritis	FORMATION_OF_ATP_BY_CHEMIOSMOTIC_COUPLING	REACTOME	77	4	3	2,91E-01	9,59E-01	rs2834287|rs1264901|rs12359107
Arthritis	POST_NMDA_RECEPTOR_ACTIVATION_EVENTS	REACTOME	1753	30	20	2,92E-01	9,59E-01	rs1509849|rs17162190|rs998235|rs6557801|rs216073|rs12129768|rs1029471|rs1379271|rs845641|rs16970502|rs172677|rs1509844|rs7259683|rs1554465|rs6533710|rs1740005|rs448852|rs1369450|rs1534131|rs758642
Arthritis	REGULATION_OF_KIT_SIGNALING	REACTOME	587	19	13	2,92E-01	9,59E-01	rs713130|rs2510139|rs1992344|rs4891843|rs8095469|rs4891846|rs9913908|rs3020827|rs218273|rs1948607|rs904141|rs2168043|rs12150089
Arthritis	RNA_POL_I_TRANSCRIPTION	REACTOME	937	67	20	2,92E-01	9,59E-01	rs1056616|rs742047|rs9512817|rs7745603|rs639373|rs12196939|rs1535565|rs2284704|rs11149485|rs2005219|rs519417|rs17011628|rs6771941|rs10514603|rs4253126|rs10200632|rs199750|rs1106841|rs2068477|rs16902819
Arthritis	NOTCH1_INTRACELLULAR_DOMAIN_REGULATES_TRANSCRIPTION	REACTOME	2852	28	20	2,95E-01	9,59E-01	rs4733828|rs7943506|rs4510937|rs10867721|rs759648|rs584846|rs9792218|rs17005603|rs1499368|rs3213615|rs1872618|rs176943|rs6543534|rs815865|rs2286003|rs5751022|rs864631|rs701078|rs9850957|rs2415078
Arthritis	CHREBP2_PATHWAY	BIOCARTA	1059	34	20	2,97E-01	9,59E-01	rs2302234|rs888298|rs4725424|rs12129768|rs972984|rs953221|rs4936661|rs11156470|rs6978142|rs875864|rs7703433|rs4894270|rs2017571|rs6876|rs758985|rs10872828|rs1116003|rs4897759|rs11760502|rs583720
Arthritis	PLATELET_HOMEOSTASIS	REACTOME	4080	35	20	2,97E-01	9,59E-01	rs3924457|rs12944845|rs1194491|rs10936997|rs2705462|rs13449253|rs10752974|rs2724113|rs11048665|rs972984|rs11615994|rs4979032|rs7077665|rs9817294|rs10912025|rs11045087|rs9858042|rs1339082|rs10083148|rs230632
Arthritis	DARPP_32_EVENTS	REACTOME	879	38	20	2,97E-01	9,59E-01	rs2302234|rs888298|rs16889129|rs2251238|rs12129768|rs4936661|rs1740005|rs7703433|rs10035950|rs1116003|rs4700325|rs12431766|rs583720|rs1439|rs6877743|rs7539471|rs17031191|rs6501512|rs986400|rs783037
Arthritis	PTM_GAMMA_CARBOXYLATION_HYPUSINE_FORMATION_AND_ARYLSULFATASE_ACTIV	REACTOME	377	27	13	2,99E-01	9,59E-01	rs7294|rs722572|rs6893444|rs6533743|rs13178105|rs10126|rs5896|rs11710876|rs7641926|rs2028900|rs17514846|rs8178610|rs8066279
Arthritis	SPHINGOLIPID_DE_NOVO_BIOSYNTHESIS	REACTOME	1020	29	20	3,01E-01	9,59E-01	rs1806328|rs10820938|rs2299920|rs12659791|rs964987|rs8008553|rs13391857|rs12133338|rs369861|rs918957|rs7251590|rs6481273|rs17544336|rs2404716|rs718545|rs17808500|rs1956461|rs2242507|rs1402840|rs11844637
Arthritis	PTEN_PATHWAY	BIOCARTA	544	28	15	3,02E-01	9,59E-01	rs10515086|rs306966|rs17237251|rs2494738|rs2302975|rs422437|rs2005219|rs12652661|rs739500|rs484754|rs7721613|rs1550955|rs7094771|rs2168043|rs2735352
Arthritis	GLYOXYLATE_AND_DICARBOXYLATE_METABOLISM	KEGG	704	42	17	3,08E-01	9,59E-01	rs12342878|rs11752779|rs10120052|rs17186108|rs1265327|rs7863381|rs6055239|rs11758761|rs6557111|rs175853|rs6140342|rs6923669|rs7024400|rs556808|rs6905272|rs7857257|rs12195019
Arthritis	DESTABILIZATION_OF_MRNA_BY_TRISTETRAPROLIN_TTP	REACTOME	179	7	3	3,08E-01	9,59E-01	rs2229032|rs6876|rs4382556
Arthritis	GNRH_SIGNALING_PATHWAY	KEGG	3992	29	20	3,09E-01	9,59E-01	rs1509849|rs384067|rs2726550|rs1816594|rs1093733|rs10752974|rs11589805|rs832574|rs1843593|rs12129768|rs11048665|rs1029471|rs11615994|rs972072|rs10869979|rs10912025|rs6056526|rs1339082|rs7688857|rs308039
Arthritis	TAK1_ACTIVATES_NFKB_BY_PHOSPHORYLATION_AND_ACTIVATION_OF_IKKS_COMPLE	REACTOME	777	40	20	3,14E-01	9,59E-01	rs1999090|rs3134942|rs739141|rs10485408|rs3844279|rs10455526|rs2655638|rs1240073|rs218921|rs1556091|rs402330|rs9353794|rs34129552|rs469632|rs7771959|rs2811650|rs12115174|rs9551925|rs311636|rs9362733
Arthritis	CTLA4_PATHWAY	BIOCARTA	662	37	19	3,15E-01	9,59E-01	rs10515086|rs2949654|rs17237251|rs2302975|rs704859|rs2838527|rs422437|rs1618879|rs12652661|rs3135353|rs9268832|rs2858870|rs2670289|rs11575812|rs7721613|rs16829957|rs1559931|rs2056626|rs231793
Arthritis	THYROID_CANCER	KEGG	1104	25	20	3,16E-01	9,59E-01	rs4733828|rs759648|rs9792218|rs1499368|rs11884056|rs389673|rs920964|rs2542417|rs4404878|rs6719501|rs12038990|rs10505528|rs12503104|rs2005219|rs2854028|rs6470648|rs10490823|rs2938395|rs11196146|rs592412
Arthritis	REGULATION_OF_INSULIN_SECRETION	REACTOME	3741	32	20	3,16E-01	9,59E-01	rs17153079|rs2302234|rs1509849|rs988896|rs888298|rs3107373|rs2724113|rs12129768|rs11048665|rs11615994|rs4979032|rs10869979|rs308039|rs389673|rs7955100|rs10498440|rs11250154|rs12090480|rs4259245|rs16974510
Arthritis	TERMINATION_OF_O_GLYCAN_BIOSYNTHESIS	REACTOME	961	43	20	3,17E-01	9,59E-01	rs13282872|rs2547067|rs1566250|rs7524886|rs2978023|rs6781|rs4897731|rs7827061|rs676269|rs1554324|rs7820063|rs4897728|rs3911575|rs17116558|rs2071174|rs12732852|rs6766574|rs4421636|rs8059245|rs6689933
Arthritis	SIGNALLING_BY_NGF	REACTOME	7869	31	20	3,18E-01	9,59E-01	rs10515086|rs2302234|rs1509849|rs384067|rs907400|rs626713|rs17162190|rs753290|rs2755209|rs1662695|rs998235|rs888298|rs7021722|rs2083828|rs4821822|rs12129768|rs11048665|rs1052116|rs11683765|rs6084337
Arthritis	SIGNALING_BY_SCF_KIT	REACTOME	1989	40	20	3,19E-01	9,59E-01	rs713130|rs10515086|rs2755209|rs2510139|rs137991|rs6084337|rs10974873|rs785512|rs17237251|rs2494738|rs11952637|rs11887698|rs2302975|rs12038990|rs422437|rs2005219|rs12652661|rs1992344|rs6876|rs4891843
Arthritis	SHH_PATHWAY	BIOCARTA	679	29	20	3,20E-01	9,59E-01	rs2302234|rs888298|rs12129768|rs3779173|rs4817863|rs1191355|rs4848608|rs3801210|rs1233556|rs10234024|rs583720|rs10275082|rs13298230|rs6460004|rs17193211|rs3857649|rs1636874|rs12711539|rs6438549|rs718939
Arthritis	TGFB_PATHWAY	BIOCARTA	817	31	20	3,21E-01	9,59E-01	rs1999090|rs266339|rs739141|rs10485408|rs2337143|rs10455526|rs4522809|rs11625323|rs2655638|rs4776344|rs1556091|rs4955159|rs2005219|rs9353794|rs920660|rs649676|rs7771959|rs2811650|rs744910|rs9362733
Arthritis	RACCYCD_PATHWAY	BIOCARTA	551	34	17	3,22E-01	9,59E-01	rs10515086|rs230530|rs17237251|rs2494738|rs10255859|rs2302975|rs422437|rs2005219|rs12652661|rs12115174|rs592412|rs13116385|rs9577581|rs7721613|rs1605729|rs7662536|rs7318844
Arthritis	TFF_PATHWAY	BIOCARTA	697	35	18	3,23E-01	9,59E-01	rs10515086|rs306966|rs389673|rs17237251|rs2494738|rs2302975|rs422437|rs1468727|rs2005219|rs12652661|rs10490823|rs11739840|rs849376|rs7721613|rs1550955|rs6974790|rs759159|rs2168043
Arthritis	DESTABILIZATION_OF_MRNA_BY_BRF1	REACTOME	204	11	7	3,24E-01	9,59E-01	rs2229032|rs7153386|rs2494738|rs11629182|rs6876|rs4382556|rs194770
Arthritis	GLUCOSE_METABOLISM	REACTOME	1554	43	20	3,26E-01	9,59E-01	rs3753491|rs863944|rs12129768|rs2516614|rs9817863|rs17018889|rs11127721|rs975488|rs3814587|rs4936661|rs10993282|rs1487111|rs764189|rs816411|rs8004788|rs1740005|rs7624990|rs1477694|rs2054780|rs11649592
Arthritis	SIGNALING_BY_ERBB2	REACTOME	3847	33	20	3,26E-01	9,59E-01	rs10515086|rs2302234|rs1509849|rs384067|rs17406681|rs2755209|rs888298|rs12129768|rs11048665|rs6084337|rs11615994|rs4673677|rs7955100|rs4259245|rs13190668|rs17237251|rs1509844|rs4622647|rs2494738|rs6725181
Arthritis	NGF_PATHWAY	BIOCARTA	566	28	16	3,26E-01	9,59E-01	rs10515086|rs12026714|rs4899554|rs17237251|rs2302975|rs422437|rs2005219|rs12652661|rs12090216|rs849376|rs7721613|rs7149441|rs1998762|rs2235366|rs2168043|rs2017750
Arthritis	KINESINS	REACTOME	566	14	9	3,28E-01	9,59E-01	rs1295217|rs961548|rs6580153|rs882551|rs3805941|rs4347670|rs4906434|rs6504889|rs3130276
Arthritis	MYOSIN_PATHWAY	BIOCARTA	1253	35	20	3,28E-01	9,59E-01	rs1958918|rs7007884|rs4509829|rs2724113|rs3784166|rs10869979|rs9555756|rs11652805|rs2207317|rs6039211|rs2238493|rs4242546|rs6560613|rs6118219|rs7515836|rs10485723|rs4938800|rs2287699|rs2223538|rs4773341
Arthritis	ERK_MAPK_TARGETS	REACTOME	704	35	19	3,28E-01	9,59E-01	rs17162190|rs998235|rs845641|rs10147042|rs2119945|rs4936661|rs7703433|rs2229105|rs10858840|rs851010|rs1002487|rs4710090|rs874277|rs325383|rs1116003|rs9380546|rs704061|rs2301682|rs10421191
Arthritis	BIOSYNTHESIS_OF_THE_N_GLYCAN_PRECURSOR_DOLICHOL_LIPID_LINKED_OLIGOSACC RANSFER_TO_A_NASCENT_PROTEIN	REACTOME	355	26	12	3,29E-01	9,59E-01	rs1865806|rs6478803|rs639373|rs2426227|rs1722705|rs7350509|rs2025631|rs4915899|rs6650384|rs9925239|rs11208199|rs1915393
Arthritis	ECM_RECEPTOR_INTERACTION	KEGG	3602	27	20	3,30E-01	9,59E-01	rs1360290|rs12756105|rs2021832|rs4756195|rs10273151|rs2107128|rs4841920|rs4341231|rs1437236|rs10955078|rs9277912|rs353639|rs1271449|rs10808350|rs869936|rs9346596|rs630943|rs2589204|rs879904|rs622486
Arthritis	ARAP_PATHWAY	BIOCARTA	292	12	9	3,31E-01	9,59E-01	rs2267364|rs849749|rs8141057|rs2793422|rs1115535|rs1997885|rs5756581|rs12004|rs2273102
Arthritis	NOTCH_SIGNALING_PATHWAY	KEGG	1387	40	20	3,31E-01	9,59E-01	rs7943506|rs3761441|rs17005603|rs608727|rs6104663|rs3134942|rs2293806|rs1938593|rs176943|rs1474817|rs17189227|rs701078|rs11919795|rs2250994|rs6817684|rs2906142|rs11021494|rs6771941|rs6822207|rs1110904
Arthritis	CELL_CYCLE	KEGG	2581	27	20	3,32E-01	9,59E-01	rs4733828|rs10820827|rs759648|rs848189|rs9792218|rs12243192|rs1499368|rs9350245|rs2229032|rs266339|rs3820716|rs12139487|rs1109998|rs2075995|rs12069919|rs5751022|rs3898028|rs4807284|rs10255859|rs11625323
Arthritis	IL7_PATHWAY	BIOCARTA	699	39	19	3,33E-01	9,59E-01	rs10515086|rs310209|rs3761049|rs17237251|rs2302975|rs2454712|rs422437|rs7508025|rs8096471|rs12652661|rs12129709|rs849376|rs2322606|rs891398|rs7721613|rs8046065|rs2302600|rs129988|rs5758211
Arthritis	CELL_CYCLE_CHECKPOINTS	REACTOME	1961	34	20	3,33E-01	9,59E-01	rs6564984|rs4858552|rs10494494|rs12243192|rs3815844|rs6575121|rs2280162|rs2550461|rs2229032|rs3820716|rs10818964|rs3898028|rs1541549|rs1925089|rs7404213|rs6142963|rs228271|rs6463716|rs9540055|rs2290822
Arthritis	PLATELET_SENSITIZATION_BY_LDL	REACTOME	676	36	19	3,35E-01	9,59E-01	rs10752974|rs972984|rs10912025|rs1339082|rs230632|rs4936661|rs312027|rs7703433|rs1938512|rs10911946|rs17042892|rs851010|rs1116003|rs9380546|rs9729852|rs7587610|rs328539|rs2285346|rs10421191
Arthritis	EIF_PATHWAY	BIOCARTA	158	7	5	3,37E-01	9,59E-01	rs12639287|rs4783446|rs8059561|rs3170368|rs7154496
Arthritis	BIOLOGICAL_OXIDATIONS	REACTOME	3568	45	20	3,40E-01	9,59E-01	rs4813547|rs12720462|rs357181|rs7448803|rs338593|rs2132847|rs9314048|rs6586711|rs305968|rs4970774|rs8187890|rs1135315|rs6586734|rs6139335|rs737866|rs7687822|rs7081491|rs16970280|rs17610788|rs13254048
Arthritis	IGF1MTOR_PATHWAY	BIOCARTA	582	41	18	3,41E-01	9,59E-01	rs10515086|rs9672254|rs2229765|rs17237251|rs2494738|rs2302975|rs7169544|rs422437|rs7703433|rs12652661|rs7136446|rs739500|rs8037855|rs10048024|rs6438549|rs7721613|rs7094771|rs2735352
Arthritis	MRNA_3_END_PROCESSING	REACTOME	320	15	11	3,42E-01	9,59E-01	rs3809425|rs4788605|rs234784|rs1943344|rs2240769|rs485152|rs2402111|rs11604322|rs11701664|rs6573908|rs2805767
Arthritis	HER2_PATHWAY	BIOCARTA	1326	31	20	3,47E-01	9,59E-01	rs10515086|rs17406681|rs12821519|rs4673677|rs17237251|rs4622647|rs6725181|rs2302975|rs11176203|rs422437|rs1468727|rs6706010|rs10506495|rs2005219|rs6722322|rs12652661|rs7971370|rs2219872|rs7598440|rs849376
Arthritis	TYPE_I_DIABETES_MELLITUS	KEGG	1975	36	20	3,47E-01	9,59E-01	rs2253907|rs6952172|rs10017098|rs9277334|rs4328874|rs876848|rs7144673|rs9276711|rs2071469|rs4493656|rs2421059|rs1263560|rs4439928|rs2596574|rs9469357|rs221300|rs2048874|rs2284191|rs3130141|rs1264456
Arthritis	SIGNALING_BY_PDGF	REACTOME	4427	31	20	3,49E-01	9,59E-01	rs10515086|rs2302234|rs1509849|rs384067|rs2755209|rs4841920|rs888298|rs4341231|rs12129768|rs11048665|rs6084337|rs11615994|rs9346596|rs7676376|rs630943|rs7955100|rs13419630|rs10901096|rs622486|rs4259245

Arthritis	NOS1_PATHWAY	BIOCARTA	1133	35	20	3,49E-01	9,59E-01	rs2302234|rs888298|rs2160732|rs2251238|rs12129768|rs4767520|rs172677|rs2238493|rs9658570|rs1740005|rs316375|rs1065832|rs10492142|rs11074659|rs1362816|rs12431766|rs583720|rs10505778|rs9925203|rs9913908
Arthritis	FORMATION_OF_FIBRIN_CLOT_CLOTTING_CASCADE	REACTOME	703	38	20	3,49E-01	9,59E-01	rs881376|rs7772062|rs434602|rs3024422|rs4422662|rs5896|rs6597197|rs11869909|rs2074437|rs2270151|rs6489695|rs1951626|rs1318041|rs10874870|rs2457996|rs8178610|rs12317523|rs12369177|rs10900986|rs10179730
Arthritis	FAS_PATHWAY	BIOCARTA	980	34	20	3,51E-01	9,59E-01	rs2726550|rs1999090|rs832574|rs1051644|rs10485408|rs10455526|rs2655638|rs1240073|rs218921|rs1556091|rs9353794|rs1937332|rs7771959|rs2811650|rs959573|rs10931934|rs1976955|rs311636|rs9362733|rs2019978
Arthritis	FORMATION_OF_TUBULIN_FOLDING_INTERMEDIATES_BY_CCT_TRIC	REACTOME	227	12	5	3,52E-01	9,59E-01	rs502930|rs4129373|rs10735819|rs6490620|rs7328603
Arthritis	INSULIN_SIGNALING_PATHWAY	KEGG	3744	35	20	3,53E-01	9,59E-01	rs10515086|rs2302234|rs13278594|rs4697435|rs2755209|rs888298|rs2510139|rs4725424|rs12129768|rs7975139|rs11045087|rs7088546|rs19334|rs1397829|rs10083148|rs953221|rs525428|rs2715252|rs10901096|rs785512
Arthritis	SYNTHESIS_AND_INTERCONVERSION_OF_NUCLEOTIDE_DI_AND_TRIPHOSPHATES	REACTOME	306	25	8	3,53E-01	9,59E-01	rs2602930|rs17380592|rs2313602|rs1840246|rs12144794|rs1049927|rs2215290|rs17100521
Arthritis	SYSTEMIC_LUPUS_ERYTHEMATOSUS	KEGG	2453	81	20	3,54E-01	9,59E-01	rs1179766|rs2160732|rs9277334|rs10836031|rs742047|rs437179|rs7745603|rs1899949|rs9276711|rs2071469|rs3867132|rs639373|rs12196939|rs3798106|rs172677|rs835223|rs9469357|rs2284191|rs316375|rs1065832
Arthritis	TCR_PATHWAY	BIOCARTA	1517	30	20	3,55E-01	9,59E-01	rs10515086|rs2726550|rs2949654|rs2251238|rs832574|rs4899554|rs230530|rs17237251|rs2238493|rs2302975|rs1740005|rs704859|rs177820|rs422437|rs1618879|rs2005219|rs12652661|rs11150675|rs7233024|rs12431766
Arthritis	TGF_BETA_SIGNALING_PATHWAY	KEGG	3535	25	20	3,57E-01	9,59E-01	rs4724065|rs4733828|rs6677615|rs13037653|rs759648|rs9792218|rs186659|rs1033872|rs1499368|rs527929|rs1276408|rs266339|rs3820716|rs9346596|rs13038229|rs5751022|rs2337143|rs11962800|rs11169953|rs6728934
Arthritis	STRIATED_MUSCLE_CONTRACTION	REACTOME	453	23	10	3,58E-01	9,59E-01	rs4509829|rs864302|rs4792010|rs12882128|rs4576532|rs8014568|rs10752073|rs1050255|rs3729704|rs1536950
Arthritis	RESOLUTION_OF_AP_SITES_VIA_THE_MULTIPLE_NUCLEOTIDE_PATCH_REPLACEMENT_P	REACTOME	192	4	3	3,58E-01	9,59E-01	rs7310681|rs6107581|rs6809452
Arthritis	BASE_EXCISION_REPAIR	REACTOME	209	4	3	3,59E-01	9,59E-01	rs7310681|rs6107581|rs6809452
Arthritis	FATTY_ACID_METABOLISM	KEGG	776	41	16	3,62E-01	9,59E-01	rs6784193|rs4605929|rs1038378|rs12609870|rs7865905|rs1265303|rs3745469|rs2419627|rs4836272|rs869013|rs11736582|rs7700981|rs7534754|rs9307238|rs6832271|rs1923431
Arthritis	SPPA_PATHWAY	BIOCARTA	1428	32	20	3,63E-01	9,59E-01	rs17153079|rs10752974|rs2724113|rs10912025|rs1339082|rs306966|rs230632|rs2207317|rs6039211|rs2238493|rs9244|rs5896|rs6118219|rs10485723|rs1938512|rs2005219|rs10911946|rs2223538|rs10063791|rs182524
Arthritis	FORMATION_OF_THE_TERNARY_COMPLEX_AND_SUBSEQUENTLY_THE_43S_COMPLEX	REACTOME	677	29	16	3,64E-01	9,59E-01	rs1149754|rs2316692|rs10509398|rs8109578|rs36621|rs10824609|rs2053984|rs5756219|rs2574693|rs7902136|rs10762770|rs14398|rs1975804|rs7920205|rs4758037|rs1149750
Arthritis	LONG_TERM_DEPRESSION	KEGG	3926	32	20	3,66E-01	9,59E-01	rs17153079|rs34164|rs1093733|rs1194491|rs2705462|rs10752974|rs11589805|rs11048665|rs11615994|rs7077665|rs6532359|rs10869979|rs10912025|rs6056526|rs1339082|rs308039|rs17420541|rs230632|rs1567102|rs7943420
Arthritis	CYTOKINE_SIGNALING_IN_IMMUNE_SYSTEM	REACTOME	6819	37	20	3,68E-01	9,59E-01	rs10515086|rs3917265|rs2726550|rs4858552|rs1145965|rs2253907|rs4756195|rs12639287|rs2510152|rs310209|rs1999090|rs11188666|rs9277334|rs12129768|rs12987393|rs228942|rs1029471|rs2287047|rs353639|rs748197
Arthritis	ALDOSTERONE_REGULATED_SODIUM_REABSORPTION	KEGG	1794	39	20	3,75E-01	9,59E-01	rs4839066|rs10515086|rs1743940|rs2200984|rs4938440|rs6837298|rs785512|rs7986346|rs336209|rs17237251|rs7674926|rs17368402|rs2238493|rs2302975|rs422437|rs2005219|rs6566955|rs10498208|rs12652661|rs11152059
Arthritis	VEGF_SIGNALING_PATHWAY	KEGG	2111	33	20	3,75E-01	9,59E-01	rs10515086|rs1093733|rs10752974|rs11589805|rs2251238|rs12505096|rs10912025|rs1339082|rs306966|rs230632|rs1567102|rs785512|rs800719|rs17237251|rs2494738|rs2238493|rs7496336|rs2302975|rs177820|rs12038990
Arthritis	CARM_ER_PATHWAY	BIOCARTA	1617	33	20	3,76E-01	9,59E-01	rs12821519|rs6701290|rs4697435|rs2823183|rs3213615|rs1872618|rs6543534|rs1397829|rs7808621|rs701078|rs4817754|rs11124179|rs7811991|rs2823209|rs10257046|rs10219488|rs10506495|rs10177901|rs12659397|rs7971370
Arthritis	CERAMIDE_PATHWAY	BIOCARTA	425	24	13	3,78E-01	9,59E-01	rs2726550|rs832574|rs230530|rs12454712|rs2005219|rs8096471|rs10931934|rs2270941|rs13116385|rs2322376|rs13402616|rs7662536|rs2017750
Arthritis	TRANS_GOLGI_NETWORK_VESICLE_BUDDING	REACTOME	1106	37	20	3,78E-01	9,59E-01	rs6733550|rs12445650|rs13120989|rs849749|rs4963442|rs11048060|rs10957967|rs355419|rs2072824|rs11962800|rs9944192|rs4963446|rs737883|rs2232739|rs6080886|rs1045895|rs3808734|rs2231754|rs9990333|rs2224957
Arthritis	NUCLEAR_EVENTS_KINASE_AND_TRANSCRIPTION_FACTOR_ACTIVATION	REACTOME	766	35	19	3,78E-01	9,59E-01	rs17162190|rs998235|rs845641|rs10147042|rs2119945|rs4936661|rs7703433|rs2005219|rs7972776|rs851010|rs1002487|rs4710090|rs874277|rs325383|rs1116003|rs9380546|rs704061|rs2301682|rs10421191
Arthritis	TCA_CYCLE_AND_RESPIRATORY_ELECTRON_TRANSPORT	REACTOME	2599	25	20	3,80E-01	9,59E-01	rs222289|rs10226904|rs3753362|rs999918|rs3900417|rs10423721|rs1626521|rs766549|rs17476583|rs9293478|rs2834287|rs12459759|rs7254601|rs414000|rs3934860|rs1366819|rs11957871|rs7255086|rs7247484|rs1581512
Arthritis	NEUROTRANSMITTER_RELEASE_CYCLE	REACTOME	1400	38	20	3,81E-01	9,59E-01	rs2815727|rs2950798|rs999448|rs1082214|rs2378089|rs876848|rs1865806|rs1288338|rs3766781|rs137500|rs4439928|rs13014176|rs9871974|rs5754189|rs6931045|rs17046094|rs10974530|rs6071120|rs1728803|rs1682821
Arthritis	SPRY_PATHWAY	BIOCARTA	910	42	20	3,81E-01	9,59E-01	rs9318729|rs2510152|rs254441|rs11745862|rs300550|rs4833920|rs8002487|rs1468727|rs2005219|rs9545330|rs632547|rs9565708|rs7443175|rs4105088|rs348029|rs17226465|rs9545836|rs3796944|rs2329172|rs9545295
Arthritis	BIOPEPTIDES_PATHWAY	BIOCARTA	1081	39	20	3,84E-01	9,59E-01	rs17153079|rs2724113|rs1029471|rs308039|rs10974873|rs11887698|rs2238493|rs9244|rs6533710|rs1740005|rs5896|rs2005219|rs851010|rs2072439|rs9380546|rs182524|rs12431766|rs9913908|rs7531583|rs198182
Arthritis	NEGATIVE_REGULATORS_OF_RIG_I_MDA5_SIGNALING	REACTOME	464	21	13	3,84E-01	9,59E-01	rs9938543|rs1815811|rs4784754|rs7670291|rs7259683|rs6052147|rs1549669|rs150895|rs2717533|rs10863389|rs4946730|rs9783665|rs3862833
Arthritis	NITROGEN_METABOLISM	KEGG	587	32	17	3,88E-01	9,59E-01	rs1923939|rs10911063|rs1082214|rs12544346|rs12708477|rs11209658|rs10486010|rs3829305|rs186471|rs2058925|rs4501567|rs7841820|rs13257749|rs569688|rs987390|rs10108007|rs12561944
Arthritis	NOD1_2_SIGNALING_PATHWAY	REACTOME	881	35	20	3,89E-01	9,59E-01	rs1999090|rs666723|rs739141|rs10485408|rs3844278|rs10455526|rs2655638|rs1240073|rs218921|rs1556091|rs402330|rs9353794|rs851010|rs7771959|rs2811650|rs12115174|rs9380546|rs10931934|rs311636|rs9362733
Arthritis	TELOMERE_MAINTENANCE	REACTOME	1133	65	20	3,90E-01	9,59E-01	rs1427575|rs742047|rs9972635|rs12928076|rs7745603|rs639373|rs12196939|rs3218801|rs1541549|rs1925089|rs6463716|rs9540055|rs6499137|rs5030740|rs1593756|rs616953|rs6107581|rs17466949|rs199750|rs9539838
Arthritis	HUNTINGTONS_DISEASE	KEGG	4311	31	20	3,91E-01	9,59E-01	rs222289|rs2076384|rs10226904|rs4697435|rs3753362|rs4821822|rs2160732|rs999918|rs3900417|rs10423721|rs2842992|rs10869979|rs766549|rs6056526|rs2545886|rs17476583|rs1397829|rs9293478|rs111171|rs2834287
Arthritis	GH_PATHWAY	BIOCARTA	963	30	20	3,92E-01	9,59E-01	rs10515086|rs17162190|rs998235|rs10974873|rs17237251|rs2238493|rs2302975|rs422437|rs2005219|rs10498208|rs12652661|rs1002487|rs11739840|rs849376|rs9913908|rs198182|rs1560251|rs7721613|rs7258382|rs2274471
Arthritis	MITOTIC_M_M_G1_PHASES	REACTOME	3176	28	20	3,95E-01	9,59E-01	rs6564984|rs11653144|rs12243192|rs3815844|rs6575121|rs972984|rs7975139|rs2280162|rs9350245|rs2550461|rs1614490|rs3820716|rs10818964|rs617621|rs1975305|rs2075995|rs4387213|rs525428|rs510862|rs7756899
Arthritis	STARCH_AND_SUCROSE_METABOLISM	KEGG	2178	42	20	4,00E-01	9,59E-01	rs3753491|rs2722639|rs11185438|rs9817863|rs1330403|rs17018889|rs11127721|rs525428|rs11185381|rs7552867|rs975488|rs17014930|rs1880924|rs1487111|rs2670854|rs8004788|rs7624990|rs1477694|rs6543374|rs894391
Arthritis	OOCYTE_MEIOSIS	KEGG	3114	27	20	4,05E-01	9,59E-01	rs1509849|rs384067|rs17162190|rs998235|rs577684|rs2251238|rs12129768|rs11048665|rs972984|rs1029471|rs7975139|rs11615994|rs617621|rs12139487|rs845641|rs5751022|rs7955100|rs4259245|rs13190668|rs7756899
Arthritis	NEUROTRANSMITTER_RECEPTOR_BINDING_AND_DOWNSTREAM_TRANSMISSION_IN_THE L	REACTOME	6592	33	20	4,06E-01	9,59E-01	rs17153079|rs1509849|rs9485742|rs384067|rs4417522|rs17162190|rs12457181|rs998235|rs7002363|rs2160732|rs2835909|rs2724113|rs12129768|rs6937943|rs2387333|rs1029471|rs4979032|rs2238517|rs1379271|rs9499025
Arthritis	XENOBIOTICS	REACTOME	231	21	6	4,07E-01	9,59E-01	rs305968|rs7081491|rs11188059|rs17726963|rs11812285|rs11879413
Arthritis	CLASS_B_2_SECRETIN_FAMILY_RECEPTORS	REACTOME	2257	31	20	4,07E-01	9,59E-01	rs8107904|rs1562782|rs16979900|rs1076465|rs2724113|rs937643|rs4979032|rs3793228|rs3121310|rs11614438|rs765543|rs2371933|rs467439|rs10498440|rs6732651|rs2473242|rs6462246|rs4807284|rs351359|rs937642
Arthritis	VEGF_PATHWAY	BIOCARTA	883	31	20	4,08E-01	9,59E-01	rs10515086|rs12498317|rs306966|rs10507386|rs3844279|rs17080484|rs17237251|rs2238493|rs2302975|rs422437|rs12652661|rs849376|rs9913908|rs4714696|rs198182|rs7982283|rs6855099|rs9508029|rs7721613|rs9513085
Arthritis	NO2IL12_PATHWAY	BIOCARTA	461	20	14	4,08E-01	9,59E-01	rs2949654|rs10974873|rs4493656|rs2421059|rs704859|rs382410|rs12720356|rs1618879|rs755004|rs11741918|rs2304237|rs2274471|rs2056626|rs426132
Arthritis	SIGNALING_BY_ERBB4	REACTOME	3434	43	20	4,08E-01	9,59E-01	rs10515086|rs17406681|rs4624193|rs1995549|rs2755209|rs1023262|rs9922434|rs6084337|rs3744473|rs7500549|rs7206663|rs4673677|rs5751022|rs10974873|rs1991406|rs1207774|rs2068143|rs7744633|rs17237251|rs4622647
Arthritis	SPLICEOSOME	KEGG	1685	31	20	4,09E-01	9,59E-01	rs10061188|rs6733550|rs6044228|rs9908594|rs4788605|rs2163773|rs176943|rs234784|rs1928630|rs1533303|rs8065368|rs7585767|rs10419232|rs369052|rs1075496|rs2074733|rs11718099|rs4697051|rs17573579|rs2240769
Arthritis	REGULATION_OF_MRNA_STABILITY_BY_PROTEINS_THAT_BIND_AU_RICH_ELEMENTS	REACTOME	1372	39	20	4,10E-01	9,59E-01	rs6564984|rs3815844|rs6575121|rs2280162|rs2550461|rs2229032|rs10818964|rs7153386|rs2494738|rs11244329|rs7404213|rs11629182|rs6142963|rs228271|rs851010|rs6876|rs8063430|rs3819716|rs9380546|rs2011786
Arthritis	APC_C_CDC20_MEDIATED_DEGRADATION_OF_CYCLIN_B	REACTOME	224	11	7	4,11E-01	9,59E-01	rs4858552|rs3898028|rs12497066|rs7077462|rs12623041|rs3814026|rs1996182
Arthritis	PHOSPHORYLATION_OF_THE_APC_C	REACTOME	219	11	7	4,13E-01	9,59E-01	rs4858552|rs3898028|rs12497066|rs7077462|rs12623041|rs3814026|rs1996182
Arthritis	RNA_POL_III_TRANSCRIPTION_TERMINATION	REACTOME	680	33	18	4,13E-01	9,59E-01	rs1075|rs9512817|rs958957|rs2173115|rs1535565|rs11149485|rs12541335|rs1497021|rs1923352|rs510395|rs10961346|rs1011531|rs303723|rs1532445|rs7873382|rs1106841|rs12339356|rs2559658
Arthritis	CHONDROITIN_SULFATE_BIOSYNTHESIS	REACTOME	846	48	20	4,15E-01	9,59E-01	rs16943314|rs16943623|rs4307360|rs2374409|rs10503653|rs17514963|rs6972852|rs4148944|rs12956990|rs10075474|rs4616261|rs11112057|rs7720425|rs9422277|rs2228603|rs4747229|rs17704048|rs9987322|rs1519134|rs11593516
Arthritis	IL22BP_PATHWAY	BIOCARTA	296	20	11	4,15E-01	9,59E-01	rs310209|rs3761049|rs10974873|rs11887698|rs12720356|rs7508025|rs12129709|rs7552086|rs2302600|rs2304237|rs2274471
Arthritis	NOD_LIKE_RECEPTOR_SIGNALING_PATHWAY	KEGG	1406	33	20	4,15E-01	9,59E-01	rs1233650|rs16981882|rs1999090|rs17178663|rs739141|rs10485408|rs10455526|rs2291782|rs230530|rs9913432|rs2655638|rs1240073|rs218921|rs1556091|rs2005219|rs9353794|rs1362765|rs851010|rs9912659|rs7771959
Arthritis	ACTIVATION_OF_KAINATE_RECEPTORS_UPON_GLUTAMATE_BINDING	REACTOME	1577	27	20	4,19E-01	9,59E-01	rs9485742|rs2724113|rs6937943|rs2387333|rs4979032|rs9499025|rs10498440|rs11264003|rs7012061|rs2207317|rs4807284|rs7517274|rs6039211|rs12135742|rs7765952|rs7644219|rs1024217|rs1740005|rs537958|rs9377426
Arthritis	NKCELLS_PATHWAY	BIOCARTA	678	33	17	4,20E-01	9,59E-01	rs10515086|rs2270238|rs17237251|rs2302975|rs422437|rs2005219|rs12652661|rs11150675|rs429511|rs290255|rs2322606|rs2660489|rs891398|rs12608960|rs7721613|rs9260759|rs252308
Arthritis	CONVERSION_FROM_APC_C_CDC20_TO_APC_C_CDH1_IN_LATE_ANAPHASE	REACTOME	226	11	7	4,20E-01	9,59E-01	rs4858552|rs3898028|rs12497066|rs2270694|rs12623041|rs3814026|rs1996182
Arthritis	ACTIVATION_OF_THE_MRNA_UPON_BINDING_OF_THE_CAP_BINDING_COMPLEX_AND_EIF T_BINDING_TO_43S	REACTOME	780	32	19	4,20E-01	9,59E-01	rs12639287|rs1149754|rs2316692|rs4554825|rs8109578|rs36621|rs10824609|rs2053984|rs5756219|rs2574693|rs7902136|rs10762770|rs1636462|rs14398|rs1975804|rs7920205|rs4758037|rs1149750|rs3105433
Arthritis	REGULATION_OF_IFNA_SIGNALING	REACTOME	339	51	8	4,21E-01	9,59E-01	rs310209|rs1330320|rs11887698|rs12720356|rs12129709|rs637949|rs2304237|rs6091153
Arthritis	ION_TRANSPORT_BY_P_TYPE_ATPASES	REACTOME	1690	37	20	4,21E-01	9,59E-01	rs4839066|rs12944845|rs4906762|rs9858042|rs4938440|rs12960272|rs11939228|rs2700865|rs1007637|rs17368402|rs929708|rs4799017|rs8053211|rs4906745|rs2575484|rs1872380|rs4906654|rs8025577|rs943055|rs869339
Arthritis	NONSENSE_MEDIATED_DECAY_ENHANCED_BY_THE_EXON_JUNCTION_COMPLEX	REACTOME	1261	35	20	4,21E-01	9,59E-01	rs7636810|rs284541|rs1149754|rs4554825|rs760782|rs6503113|rs2291782|rs36621|rs8076790|rs10824609|rs2053984|rs7703433|rs12484030|rs2574693|rs7508025|rs2004997|rs10467685|rs10197539|rs7902136|rs10762770
Arthritis	GRAFT_VERSUS_HOST_DISEASE	KEGG	1446	39	20	4,23E-01	9,59E-01	rs2253907|rs2301226|rs2270238|rs7144673|rs3948793|rs2071469|rs2596574|rs9469357|rs2048874|rs2284191|rs3130141|rs1264456|rs2071554|rs1937332|rs3819716|rs7758736|rs9268832|rs2394185|rs206762|rs453779
Arthritis	CREB_PHOSPHORYLATION_THROUGH_THE_ACTIVATION_OF_RAS	REACTOME	1349	35	20	4,24E-01	9,59E-01	rs17162190|rs998235|rs6557801|rs2160732|rs1029471|rs1379271|rs845641|rs16970502|rs172677|rs7259683|rs6533710|rs1740005|rs316375|rs1373967|rs1065832|rs1002487|rs2072439|rs4710090|rs874277|rs10492142
Arthritis	TRANSCRIPTIONAL_REGULATION_OF_WHITE_ADIPOCYTE_DIFFERENTIATION	REACTOME	2719	31	20	4,24E-01	9,59E-01	rs9487444|rs297|rs1880143|rs4697435|rs929779|rs1886449|rs2195003|rs10831104|rs7180301|rs933910|rs1991147|rs168405|rs7982719|rs1397829|rs10947994|rs13342054|rs701078|rs10816540|rs934782|rs9850957
Arthritis	ONE_CARBON_POOL_BY_FOLATE	KEGG	441	21	12	4,24E-01	9,59E-01	rs11752779|rs713160|rs1265327|rs3737964|rs11758761|rs6557111|rs175853|rs10934761|rs6923669|rs556808|rs6905272|rs12195019
Arthritis	CLEAVAGE_OF_GROWING_TRANSCRIPT_IN_THE_TERMINATION_REGION_	REACTOME	435	16	12	4,25E-01	9,59E-01	rs3809425|rs4788605|rs234784|rs1943344|rs2240769|rs485152|rs2402111|rs11604322|rs11701664|rs8136239|rs6573908|rs2805767
Arthritis	COMP_PATHWAY	BIOCARTA	628	44	15	4,26E-01	9,59E-01	rs1179766|rs437179|rs4957381|rs835223|rs12218966|rs2204758|rs2384167|rs1822824|rs3105782|rs751138|rs2248678|rs7027797|rs11714564|rs17201075|rs1917186
Arthritis	GENERATION_OF_SECOND_MESSENGER_MOLECULES	REACTOME	616	24	14	4,27E-01	9,59E-01	rs2949654|rs9277334|rs137991|rs9469357|rs704859|rs1618879|rs11150675|rs2858870|rs12166465|rs252308|rs3130192|rs2056626|rs2235366|rs2840945
Arthritis	TIGHT_JUNCTION_INTERACTIONS	REACTOME	715	31	18	4,28E-01	9,59E-01	rs17123151|rs1331683|rs9972635|rs9561897|rs12867610|rs2835406|rs2886235|rs11009607|rs16935300|rs7643214|rs6481924|rs12495548|rs1108371|rs4934671|rs2835402|rs13056098|rs17744842|rs876644
Arthritis	MITOCHONDRIAL_PROTEIN_IMPORT	REACTOME	609	27	18	4,28E-01	9,59E-01	rs1130635|rs989925|rs7566988|rs6433589|rs10910536|rs2718794|rs1513887|rs10164810|rs6586356|rs2802928|rs247459|rs11583023|rs6850094|rs1057358|rs3899437|rs7449072|rs4744826|rs970797
Arthritis	RNA_POL_I_TRANSCRIPTION_INITIATION	REACTOME	427	19	14	4,28E-01	9,59E-01	rs1056616|rs9512817|rs1535565|rs2284704|rs11149485|rs519417|rs17011628|rs6771941|rs10514603|rs4253126|rs10200632|rs1106841|rs2068477|rs16902819
Arthritis	INTERFERON_GAMMA_SIGNALING	REACTOME	2043	36	20	4,30E-01	9,59E-01	rs1145965|rs2253907|rs4756195|rs310209|rs9277334|rs1029471|rs353639|rs10974873|rs660361|rs2596574|rs12607710|rs9469357|rs10521318|rs7259683|rs11887698|rs17375044|rs3783621|rs6533710|rs4937993|rs1459665
Arthritis	ACTIVATED_TAK1_MEDIATES_P38_MAPK_ACTIVATION	REACTOME	649	41	20	4,31E-01	9,59E-01	rs1999090|rs739141|rs10485408|rs3844279|rs10455526|rs2655638|rs1240073|rs218921|rs1556091|rs402330|rs9353794|rs851010|rs7771959|rs2811650|rs2237093|rs311636|rs9362733|rs9353872|rs17521993|rs4961116
Arthritis	NUCLEAR_SIGNALING_BY_ERBB4	REACTOME	2231	41	20	4,31E-01	9,59E-01	rs17406681|rs4624193|rs1995549|rs1023262|rs9922434|rs9908594|rs7500549|rs7206663|rs4673677|rs5751022|rs10974873|rs1207774|rs2068143|rs7744633|rs4622647|rs6725181|rs7002832|rs10271133|rs9940608|rs10492908
Arthritis	EFFECTS_OF_PIP2_HYDROLYSIS	REACTOME	1651	30	20	4,31E-01	9,59E-01	rs3924457|rs9834264|rs11048665|rs11615994|rs975167|rs6788054|rs7955100|rs1525079|rs4259245|rs1957895|rs2278102|rs10507505|rs3819863|rs2358025|rs2439909|rs6467709|rs935656|rs6783586|rs3800631|rs1536041
Arthritis	G_BETA_GAMMA_SIGNALLING_THROUGH_PI3KGAMMA	REACTOME	487	22	11	4,33E-01	9,59E-01	rs2724113|rs4979032|rs10498440|rs2494738|rs4807284|rs849376|rs291353|rs7531583|rs12147761|rs714407|rs2965208
Arthritis	DOWNSTREAM_SIGNALING_OF_ACTIVATED_FGFR	REACTOME	3636	31	20	4,33E-01	9,59E-01	rs10515086|rs2302234|rs1509849|rs384067|rs2755209|rs888298|rs12129768|rs11048665|rs6084337|rs11615994|rs7674434|rs7955100|rs4259245|rs13190668|rs17237251|rs1509844|rs9591501|rs2494738|rs2288696|rs6490668
Arthritis	ENDOMETRIAL_CANCER	KEGG	3028	25	20	4,33E-01	9,59E-01	rs10515086|rs4733828|rs759648|rs9792218|rs1541947|rs671802|rs1499368|rs7605223|rs4402182|rs11884056|rs1901335|rs389673|rs785512|rs17237251|rs2494738|rs2542417|rs4404878|rs1511196|rs12611756|rs11599789
Arthritis	NEF_MEDIATES_DOWN_MODULATION_OF_CELL_SURFACE_RECEPTORS_BY_RECRUITING N_ADAPTERS	REACTOME	366	10	7	4,34E-01	9,59E-01	rs849749|rs11919795|rs16919915|rs737883|rs429511|rs9260759|rs2267138
Arthritis	MTOR_SIGNALING_PATHWAY	KEGG	1290	32	20	4,35E-01	9,59E-01	rs10515086|rs17162190|rs998235|rs845641|rs785512|rs4868684|rs17237251|rs2494738|rs2302975|rs2589142|rs422437|rs2005219|rs12652661|rs1002487|rs4710090|rs874277|rs2333526|rs2289762|rs416203|rs7136446
Arthritis	BILE_ACID_AND_BILE_SALT_METABOLISM	REACTOME	1176	46	20	4,35E-01	9,59E-01	rs17292284|rs9300791|rs12445568|rs3755160|rs279942|rs12657454|rs2228468|rs11253048|rs10841798|rs12059934|rs1274744|rs479682|rs6491730|rs2254636|rs3916932|rs16856261|rs10509564|rs7997209|rs2465079|rs2560718
Arthritis	INSULIN_SYNTHESIS_AND_PROCESSING	REACTOME	1033	45	20	4,35E-01	9,59E-01	rs2269010|rs10017098|rs4328874|rs7792010|rs13175238|rs6080670|rs9993582|rs2899048|rs12530513|rs11971531|rs11736419|rs4396776|rs3792747|rs2451819|rs2322291|rs9504898|rs1211554|rs8024439|rs12641893|rs16999051
Arthritis	GAP_JUNCTION_TRAFFICKING	REACTOME	576	31	18	4,39E-01	9,60E-01	rs2359106|rs17745948|rs1011038|rs11919795|rs835223|rs2313602|rs7740467|rs11855809|rs4924132|rs9343330|rs9315439|rs8064669|rs17707738|rs1572371|rs1086602|rs690824|rs7164838|rs6925183
Arthritis	MAP_KINASE_ACTIVATION_IN_TLR_CASCADE	REACTOME	1697	31	20	4,45E-01	9,60E-01	rs2726550|rs17162190|rs998235|rs1999090|rs739141|rs4899554|rs10485408|rs845641|rs3844278|rs10455526|rs10147042|rs2119945|rs4936661|rs2655638|rs1240073|rs218921|rs1556091|rs7703433|rs2005219|rs7972776
Arthritis	PANCREATIC_CANCER	KEGG	1920	33	20	4,45E-01	9,60E-01	rs10515086|rs626713|rs310209|rs9350245|rs266339|rs7562947|rs2075995|rs360402|rs785512|rs2073923|rs230530|rs17237251|rs2494738|rs10255859|rs4522809|rs11625323|rs11887698|rs4128634|rs2302975|rs4776344
Arthritis	MEIOSIS	REACTOME	1674	60	20	4,45E-01	9,60E-01	rs1427575|rs742047|rs9972635|rs2229032|rs12928076|rs1051644|rs7745603|rs639373|rs12196939|rs7547056|rs10520896|rs1541549|rs9384022|rs3101017|rs633891|rs6463716|rs6499137|rs2029778|rs959573|rs1256063
Arthritis	MISMATCH_REPAIR	KEGG	576	21	14	4,50E-01	9,60E-01	rs2348238|rs1635516|rs1541549|rs1925089|rs6463716|rs9540055|rs5030740|rs616953|rs6107581|rs17466949|rs3136245|rs9539838|rs9539836|rs12540373
Arthritis	LIPID_DIGESTION_MOBILIZATION_AND_TRANSPORT	REACTOME	1389	50	20	4,53E-01	9,60E-01	rs881376|rs297|rs12129768|rs7975139|rs2487031|rs7897550|rs1025447|rs991811|rs312027|rs12541335|rs882992|rs2502805|rs2269318|rs4148102|rs17042892|rs10278782|rs10904831|rs2420287|rs11685669|rs1054135
Arthritis	UBIQUITIN_MEDIATED_PROTEOLYSIS	KEGG	3227	39	20	4,53E-01	9,60E-01	rs1406257|rs2215849|rs4858552|rs1048447|rs10494494|rs2510152|rs832574|rs2365999|rs7773233|rs16826424|rs2291916|rs12139487|rs12459759|rs5751022|rs3844279|rs2715252|rs925861|rs1991406|rs11242213|rs12995534
Arthritis	ALK_PATHWAY	BIOCARTA	1932	38	20	4,56E-01	9,60E-01	rs13037653|rs1076465|rs4509829|rs4422194|rs1999090|rs186659|rs1276408|rs13038229|rs10485408|rs389673|rs11250154|rs10455526|rs4522809|rs11625323|rs2655638|rs235772|rs9475399|rs1556091|rs2149639|rs4955159
Arthritis	HS_GAG_BIOSYNTHESIS	REACTOME	3154	34	20	4,56E-01	9,60E-01	rs12756105|rs2635205|rs4454830|rs9523322|rs16974808|rs10955078|rs8055955|rs7583856|rs4438310|rs11867655|rs10808350|rs17862059|rs9589298|rs482700|rs342711|rs9516073|rs9937575|rs2589204|rs7674443|rs11990095
Arthritis	TNFR1_PATHWAY	BIOCARTA	862	37	20	4,56E-01	9,60E-01	rs2726550|rs1999090|rs832574|rs1051644|rs10485408|rs10455526|rs2655638|rs1556091|rs9353794|rs7771959|rs2811650|rs959573|rs10931934|rs1976955|rs9362733|rs1051006|rs2019978|rs2322376|rs13402616|rs9353872
Arthritis	GABA_RECEPTOR_ACTIVATION	REACTOME	2705	30	20	4,57E-01	9,60E-01	rs17153079|rs1509849|rs384067|rs12457181|rs2835909|rs2724113|rs4979032|rs1593609|rs10519568|rs8023912|rs10498440|rs10431855|rs13190668|rs1509844|rs2836034|rs4807284|rs1554465|rs9244|rs4906883|rs448852

Arthritis	ACTIVATION_OF_NMDA_RECEPTOR_UPON_GLUTAMATE_BINDING_AND_POSTSYNAPTIC_	REACTOME	2117	30	20	4,59E-01	9,60E-01	rs1509849|rs17162190|rs998235|rs7002363|rs2160732|rs12129768|rs1029471|rs1379271|rs845641|rs16970502|rs172677|rs1509844|rs7259683|rs1554465|rs6533710|rs1740005|rs448852|rs1369450|rs1534131|rs758642
Arthritis	EXTRACELLULAR_MATRIX_ORGANIZATION	REACTOME	3783	41	20	4,61E-01	9,60E-01	rs12080986|rs9927989|rs7643465|rs1816594|rs4841920|rs4341231|rs4259003|rs17632760|rs7714843|rs1985690|rs12487322|rs11775756|rs9277912|rs536435|rs4736285|rs6577977|rs1054480|rs630943|rs1380582|rs13260549
Arthritis	CGMP_EFFECTS	REACTOME	1520	26	20	4,62E-01	9,60E-01	rs1194491|rs10936997|rs1344925|rs7077665|rs9817294|rs11045087|rs10083148|rs6781173|rs10822867|rs7621366|rs17226220|rs1153138|rs1009708|rs12256137|rs9290640|rs10516652|rs4910170|rs1249950|rs4694006|rs578654
Arthritis	VOLTAGE_GATED_POTASSIUM_CHANNELS	REACTOME	2491	29	20	4,62E-01	9,60E-01	rs11826873|rs988896|rs6089908|rs13260906|rs12631297|rs4129625|rs13207257|rs12080989|rs999420|rs9343015|rs1501569|rs179435|rs10176051|rs1823506|rs9442835|rs4565432|rs10136789|rs13285989|rs11119565|rs10955548
Arthritis	LEUKOCYTE_TRANSENDOTHELIAL_MIGRATION	KEGG	4793	38	20	4,62E-01	9,60E-01	rs10515086|rs17153079|rs1816594|rs1958918|rs1023262|rs4509829|rs1541947|rs671802|rs777709|rs233560|rs661820|rs3784166|rs7605223|rs4503419|rs306966|rs1901335|rs389673|rs9561897|rs3867132|rs785512
Arthritis	RESPONSE_TO_ELEVATED_PLATELET_CYTOSOLIC_CA2_	REACTOME	2301	39	20	4,63E-01	9,60E-01	rs7330196|rs881376|rs7772062|rs2227631|rs434602|rs17661774|rs12445650|rs1303|rs2749531|rs8177190|rs3024422|rs4148483|rs11625323|rs4422662|rs2238493|rs1740005|rs1051442|rs6597197|rs2270151|rs2582367
Arthritis	BETA_DEFENSINS	REACTOME	333	12	7	4,64E-01	9,60E-01	rs9644758|rs11362|rs2738173|rs2223526|rs6046492|rs2131283|rs6139074
Arthritis	INTRINSIC_PATHWAY	REACTOME	264	24	10	4,65E-01	9,60E-01	rs881376|rs5896|rs11869909|rs2074437|rs2270151|rs6489695|rs12317523|rs12369177|rs238242|rs1800378
Arthritis	BASIGIN_INTERACTIONS	REACTOME	595	22	15	4,66E-01	9,60E-01	rs10145863|rs9308208|rs12459759|rs12644755|rs11734488|rs12644806|rs10278782|rs1376088|rs12609177|rs4843718|rs12290253|rs1884544|rs6572731|rs17206198|rs299133
Arthritis	CHEMOKINE_SIGNALING_PATHWAY	KEGG	5407	35	20	4,66E-01	9,60E-01	rs10515086|rs17153079|rs1509849|rs384067|rs1233650|rs1023262|rs917526|rs11532670|rs2724113|rs12129768|rs4979032|rs1331683|rs6056526|rs3761049|rs306966|rs10974873|rs10498440|rs785512|rs9649643|rs13190668
Arthritis	NITRIC_OXIDE_STIMULATES_GUANYLATE_CYCLASE	REACTOME	1895	27	20	4,67E-01	9,60E-01	rs1194491|rs10936997|rs2705462|rs1344925|rs7077665|rs9817294|rs11045087|rs10083148|rs7943420|rs4767520|rs2014174|rs6781173|rs10822867|rs7621366|rs9658570|rs17226220|rs1153138|rs1009708|rs12256137|rs9290640
Arthritis	FMLP_PATHWAY	BIOCARTA	1148	34	20	4,68E-01	9,60E-01	rs10505811|rs577684|rs2724113|rs2251238|rs832574|rs230530|rs2207317|rs6039211|rs1740005|rs177820|rs6118219|rs10485723|rs2005219|rs851010|rs2223538|rs2237093|rs7233024|rs12431766|rs3746070|rs4471960
Arthritis	EIF4_PATHWAY	BIOCARTA	890	33	20	4,71E-01	9,60E-01	rs10515086|rs12639287|rs17237251|rs2494738|rs2238493|rs2302975|rs422437|rs2005219|rs851010|rs10498208|rs12652661|rs9380546|rs11739840|rs739500|rs9913908|rs198182|rs1560251|rs7721613|rs7094771|rs3105433
Arthritis	GAP_JUNCTION_ASSEMBLY	REACTOME	330	19	15	4,71E-01	9,60E-01	rs2359106|rs17745948|rs1011038|rs2313602|rs7740467|rs11855809|rs4924132|rs9315439|rs8064669|rs17707738|rs1572371|rs1086602|rs690824|rs7164838|rs6925183
Arthritis	ABCA_TRANSPORTERS_IN_LIPID_HOMEOSTASIS	REACTOME	464	43	11	4,72E-01	9,60E-01	rs12941297|rs1025447|rs7566779|rs11077414|rs9302891|rs4148102|rs641382|rs11685669|rs737453|rs4148123|rs6605
Arthritis	DOWNREGULATION_OF_SMAD2_3_SMAD4_TRANSCRIPTIONAL_ACTIVITY	REACTOME	453	29	20	4,73E-01	9,60E-01	rs7234567|rs266339|rs701078|rs1989138|rs1991403|rs627205|rs11874742|rs556765|rs4776344|rs11582563|rs6016004|rs2173049|rs150895|rs1110904|rs11057706|rs744910|rs948604|rs3782267|rs4955588|rs2293514
Arthritis	VIBRIO_CHOLERAE_INFECTION	KEGG	1518	30	20	4,75E-01	9,60E-01	rs384067|rs11826873|rs12129768|rs10952789|rs849749|rs2275431|rs4669621|rs179435|rs12260965|rs2238493|rs1497021|rs16919915|rs899130|rs6977910|rs2188198|rs2966431|rs2071174|rs721950|rs2004997|rs12267330
Arthritis	ABC_FAMILY_PROTEINS_MEDIATED_TRANSPORT	REACTOME	1753	61	20	4,75E-01	9,60E-01	rs12700229|rs7330196|rs12941297|rs10233470|rs574741|rs1025447|rs7566779|rs4148483|rs11077414|rs9302891|rs4148102|rs641382|rs11685669|rs1476485|rs446924|rs7990574|rs9590190|rs3758953|rs4147839|rs737453
Arthritis	ALLOGRAFT_REJECTION	KEGG	1510	37	20	4,75E-01	9,60E-01	rs2253907|rs2301226|rs7144673|rs9276711|rs2071469|rs4493656|rs2421059|rs2596574|rs9469357|rs2284191|rs3130141|rs1264456|rs2071554|rs1937332|rs755004|rs3819716|rs7758736|rs9268832|rs2394185|rs206762
Arthritis	PREFOLDIN_MEDIATED_TRANSFER_OF_SUBSTRATE_TO_CCT_TRIC	REACTOME	288	16	7	4,76E-01	9,60E-01	rs502930|rs4129373|rs10735819|rs2966431|rs6490620|rs14398|rs7328603
Arthritis	O_GLYCAN_BIOSYNTHESIS	KEGG	1579	31	20	4,76E-01	9,60E-01	rs13282872|rs1942237|rs7680255|rs2653831|rs6740075|rs2978023|rs4897731|rs6463634|rs7827061|rs751645|rs11935600|rs7026938|rs7571016|rs1554324|rs7820063|rs10161277|rs17058946|rs4897728|rs4761874|rs3776996
Arthritis	KERATAN_SULFATE_BIOSYNTHESIS	REACTOME	674	35	20	4,77E-01	9,60E-01	rs13282872|rs3761049|rs2978023|rs1667255|rs4897731|rs7827061|rs1554324|rs7820063|rs4897728|rs12964617|rs944949|rs896344|rs8024016|rs4233366|rs1126823|rs17572584|rs2717263|rs8059245|rs2302600|rs4897729
Arthritis	MYD88_MAL_CASCADE_INITIATED_ON_PLASMA_MEMBRANE	REACTOME	2527	34	20	4,77E-01	9,60E-01	rs2726550|rs17162190|rs998235|rs1999090|rs832574|rs12987393|rs10983934|rs3134942|rs739141|rs10739508|rs4899554|rs1921082|rs10485408|rs845641|rs12555480|rs3844278|rs10455526|rs10147042|rs2119945|rs2698537
Arthritis	NETRIN1_SIGNALING	REACTOME	3710	42	20	4,80E-01	9,60E-01	rs3924457|rs4238475|rs4791330|rs4399429|rs6497081|rs1480689|rs12653501|rs11815834|rs17709306|rs2817683|rs975167|rs10520729|rs8030422|rs306966|rs1465522|rs10765093|rs1549910|rs17206556|rs1237775|rs166225
Arthritis	TRAF6_MEDIATED_NFKB_ACTIVATION	REACTOME	351	26	10	4,80E-01	9,60E-01	rs832574|rs3134942|rs6052147|rs34129552|rs469632|rs12115174|rs9551925|rs3014866|rs17074629|rs3014885
Arthritis	CALCINEURIN_PATHWAY	BIOCARTA	895	50	20	4,81E-01	9,60E-01	rs1809119|rs2251238|rs867458|rs10869979|rs12988452|rs7565375|rs2238493|rs6560613|rs1740005|rs177820|rs7761143|rs3845743|rs7233024|rs12431766|rs9913908|rs198182|rs2615519|rs4799062|rs17031191|rs1078633
Arthritis	REGULATION_OF_APOPTOSIS	REACTOME	1432	34	20	4,81E-01	9,60E-01	rs6564984|rs3815844|rs6575121|rs2280162|rs2550461|rs10818964|rs9840663|rs10746803|rs2889977|rs7404213|rs1237775|rs17475756|rs6142963|rs228271|rs9964201|rs8063430|rs17748116|rs3819716|rs7243444|rs6417121
Arthritis	ANTIGEN_PROCESSING_UBIQUITINATION_PROTEASOME_DEGRADATION	REACTOME	4788	29	20	4,83E-01	9,62E-01	rs1406257|rs6564984|rs4858552|rs1048447|rs3815844|rs6575121|rs3753362|rs8028608|rs10498691|rs10836031|rs1483867|rs6084337|rs2365999|rs2280162|rs2550461|rs12105524|rs9477982|rs7773233|rs4971821|rs10818964
Arthritis	REGULATION_OF_INSULIN_LIKE_GROWTH_FACTOR_IGF_ACTIVITY_BY_INSULIN_LIKE_G<br>DING_PROTEINS_IGFBPS	REACTOME	570	39	20	4,87E-01	9,63E-01	rs1816594|rs10275333|rs16849826|rs12694392|rs17301608|rs5896|rs10229987|rs11859163|rs8047464|rs10759829|rs243840|rs2465836|rs12290253|rs879965|rs1782632|rs2035567|rs11640811|rs10913158|rs7974876|rs1053605
Arthritis	THROMBIN_SIGNALLING_THROUGH_PROTEINASE_ACTIVATED_RECEPTORS_PARS	REACTOME	690	49	20	4,88E-01	9,63E-01	rs2724113|rs4979032|rs10869979|rs308039|rs10498440|rs11652805|rs4807284|rs6560613|rs11038977|rs2005219|rs10063791|rs3746070|rs291353|rs7531583|rs12147761|rs208353|rs2296781|rs250736|rs1393098|rs2965208
Arthritis	CTNNB1_PHOSPHORYLATION_CASCADE	REACTOME	316	21	9	4,89E-01	9,63E-01	rs972984|rs389673|rs4936661|rs7703433|rs1116003|rs10490823|rs11189119|rs2285346|rs10421191
Arthritis	PROCESSING_OF_CAPPED_INTRONLESS_PRE_MRNA	REACTOME	274	10	6	4,89E-01	9,63E-01	rs3809425|rs1943344|rs2402111|rs11604322|rs8136239|rs2805767
Arthritis	LEISHMANIA_INFECTION	KEGG	2265	37	20	4,90E-01	9,64E-01	rs1145965|rs310209|rs1999090|rs9277334|rs10983934|rs739141|rs10739508|rs4899554|rs10485408|rs12555480|rs3948793|rs2071469|rs10974873|rs4493656|rs2421059|rs10455526|rs230530|rs9469357|rs11625323|rs2655638
Arthritis	ALZHEIMERS_DISEASE	KEGG	5029	35	20	4,99E-01	9,71E-01	rs222289|rs12944845|rs10226904|rs297|rs6440971|rs3753362|rs2160732|rs2251238|rs11048665|rs10888621|rs11615994|rs6517656|rs3900417|rs10423721|rs10869979|rs766549|rs6056526|rs17476583|rs7167430|rs9293478
Arthritis	CHYLOMICRON_MEDIATED_LIPID_TRANSPORT	REACTOME	435	27	10	4,99E-01	9,71E-01	rs297|rs991811|rs312027|rs2502805|rs17042892|rs1136224|rs7587610|rs328539|rs1534650|rs10516449
Arthritis	BIOSYNTHESIS_OF_UNSATURATED_FATTY_ACIDS	KEGG	463	12	7	5,00E-01	9,71E-01	rs1393492|rs7567258|rs2054780|rs10413616|rs17448411|rs7686686|rs3978768
Arthritis	TRAF6_MEDIATED_INDUCTION_OF_NFKB_AND_MAP_KINASES_UPON_TLR7_8_OR_9_ACTI	REACTOME	2239	36	20	5,00E-01	9,71E-01	rs2726550|rs17162190|rs998235|rs1999090|rs832574|rs12987393|rs3134942|rs739141|rs4899554|rs10485408|rs845641|rs3844278|rs10455526|rs10147042|rs2119945|rs2698537|rs4936661|rs2655638|rs1240073|rs218921
Arthritis	METAL_ION_SLC_TRANSPORTERS	REACTOME	653	29	15	5,03E-01	9,71E-01	rs1849067|rs151377|rs13175238|rs16861650|rs457490|rs2451819|rs2854028|rs1472487|rs10505292|rs1793704|rs10171891|rs10049581|rs10931697|rs9438392|rs12074420
Arthritis	G_ALPHA1213_SIGNALLING_EVENTS	REACTOME	2855	27	20	5,05E-01	9,71E-01	rs10515086|rs1753290|rs17455628|rs2724113|rs11052116|rs11683765|rs11779546|rs4979032|rs1379271|rs4573339|rs9380633|rs2834287|rs16970502|rs10498440|rs785512|rs9861401|rs17237251|rs338513|rs11652805|rs2409415
Arthritis	KERATAN_SULFATE_KERATIN_METABOLISM	REACTOME	737	34	20	5,07E-01	9,71E-01	rs13282872|rs3761049|rs2978023|rs1667255|rs4897731|rs7827061|rs1554324|rs7820063|rs4897728|rs12964617|rs944949|rs896344|rs8024016|rs4233366|rs1126823|rs17572584|rs2717263|rs8059245|rs4438612|rs2302600
Arthritis	TIE2_SIGNALING	REACTOME	790	33	20	5,07E-01	9,71E-01	rs10515086|rs1014898|rs17237251|rs2302975|rs13274462|rs12038990|rs422437|rs12652661|rs481294|rs16876241|rs10842514|rs10967753|rs1334811|rs13279908|rs4276711|rs693293|rs2922861|rs7721613|rs3818282|rs2168043
Arthritis	B_CELL_RECEPTOR_SIGNALING_PATHWAY	KEGG	2278	32	20	5,07E-01	9,71E-01	rs10515086|rs2647394|rs13014054|rs573323|rs11188666|rs2251238|rs6461856|rs4899554|rs785512|rs230530|rs17237251|rs2494738|rs13408995|rs2238493|rs2302975|rs177820|rs12038990|rs1182136|rs422437|rs2005219
Arthritis	SYNTHESIS_SECRETION_AND_DEACYLATION_OF_GHRELIN	REACTOME	624	18	12	5,10E-01	9,71E-01	rs1880143|rs10816540|rs16974510|rs12682930|rs7659414|rs3792747|rs1972384|rs10476552|rs4979515|rs11133152|rs10017353|rs269853
Arthritis	TOLL_LIKE_RECEPTOR_SIGNALING_PATHWAY	KEGG	2339	31	20	5,10E-01	9,71E-01	rs10515086|rs2726550|rs1999090|rs10983934|rs739141|rs10739508|rs4899554|rs10485408|rs12555480|rs4493656|rs2421059|rs785512|rs10455526|rs1330320|rs230530|rs17237251|rs2494738|rs2655638|rs1554973|rs7259683
Arthritis	TID_PATHWAY	BIOCARTA	479	25	14	5,11E-01	9,71E-01	rs1145965|rs10974873|rs5030280|rs2400842|rs230530|rs1061624|rs12115174|rs13116385|rs932503|rs3758953|rs2807897|rs2274471|rs7662536|rs7552664
Arthritis	COLLAGEN_FORMATION	REACTOME	3203	38	20	5,12E-01	9,71E-01	rs12080986|rs7643465|rs4841920|rs4341231|rs4259003|rs17632760|rs7714843|rs1985690|rs12487322|rs11775756|rs9277912|rs536435|rs4736285|rs6577977|rs1054480|rs630943|rs1380582|rs13419630|rs622486|rs1563389
Arthritis	UNFOLDED_PROTEIN_RESPONSE	REACTOME	1176	35	20	5,12E-01	9,71E-01	rs848189|rs888298|rs6442982|rs1864427|rs1836581|rs6926079|rs2715609|rs4143282|rs13332660|rs6785659|rs4686432|rs1880666|rs2005219|rs402330|rs2444218|rs2176246|rs1869241|rs10486010|rs11051241|rs2237813
Arthritis	FRUCTOSE_AND_MANNOSE_METABOLISM	KEGG	984	31	20	5,13E-01	9,71E-01	rs1024355|rs2516614|rs1865806|rs2012025|rs742556|rs3814587|rs10993282|rs764189|rs11752769|rs2054780|rs3800046|rs11599872|rs12753370|rs2814811|rs17632013|rs9392354|rs1475411|rs1053903|rs943|rs3829185
Arthritis	CHOLESTEROL_BIOSYNTHESIS	REACTOME	259	9	6	5,18E-01	9,71E-01	rs3761739|rs6592055|rs2274941|rs17488814|rs3794624|rs676584
Arthritis	RNA_POL_III_TRANSCRIPTION	REACTOME	861	34	19	5,23E-01	9,71E-01	rs1075|rs9512817|rs958957|rs2173115|rs217689|rs1535565|rs11149485|rs12541335|rs1497021|rs1923352|rs510395|rs10961346|rs1011531|rs303723|rs1532445|rs7873382|rs1106841|rs12339356|rs2559658
Arthritis	INSULIN_PATHWAY	BIOCARTA	725	29	16	5,24E-01	9,71E-01	rs10515086|rs4899554|rs17237251|rs2302975|rs422437|rs2005219|rs10498208|rs12652661|rs849376|rs1560251|rs7721613|rs7258382|rs7149441|rs1998762|rs2168043|rs2017750
Arthritis	SIGNALLING_TO_RAS	REACTOME	468	26	14	5,24E-01	9,71E-01	rs626713|rs12026714|rs2073923|rs12038990|rs2005219|rs851010|rs6876|rs12090216|rs9380546|rs10842514|rs7077462|rs4661229|rs944485|rs2168043
Arthritis	PYRUVATE_METABOLISM	KEGG	908	37	20	5,25E-01	9,71E-01	rs4813547|rs6856710|rs989925|rs2306619|rs9806826|rs1877908|rs1727505|rs7865905|rs7729671|rs4836272|rs2017571|rs10898483|rs7700981|rs7940587|rs6092495|rs10013515|rs12448432|rs6591226|rs1938930|rs13426329
Arthritis	STRESS_PATHWAY	BIOCARTA	633	25	12	5,25E-01	9,71E-01	rs2726550|rs832574|rs230530|rs851010|rs12115174|rs9380546|rs13116385|rs2322376|rs7568498|rs7662536|rs1998762|rs2017750
Arthritis	NFKB_AND_MAP_KINASES_ACTIVATION_MEDIATED_BY_TLR4_SIGNALING_REPERTOIRE	REACTOME	2248	32	20	5,26E-01	9,71E-01	rs2726550|rs17162190|rs998235|rs1999090|rs10983934|rs3134942|rs739141|rs10739508|rs4899554|rs10485408|rs845641|rs12555480|rs3844278|rs10455526|rs10147042|rs2119945|rs4936661|rs2655638|rs1554973|rs1240073
Arthritis	INFLAMMASOMES	REACTOME	468	21	11	5,27E-01	9,71E-01	rs17178663|rs9913432|rs12454712|rs1049927|rs1362765|rs9912659|rs8096471|rs469632|rs4073612|rs1468635|rs17646330
Arthritis	ANTIGEN_PROCESSING_AND_PRESENTATION	KEGG	1754	65	20	5,27E-01	9,71E-01	rs2253907|rs2301226|rs2270238|rs6926079|rs3948793|rs2071469|rs1330319|rs2596574|rs9469357|rs2284191|rs3130141|rs1264456|rs2071554|rs2031812|rs3819716|rs7758736|rs9268832|rs2394185|rs206762|rs637949
Arthritis	RNA_POL_I_RNA_POL_III_AND_MITOCHONDRIAL_TRANSCRIPTION	REACTOME	1830	50	20	5,29E-01	9,71E-01	rs1075|rs1056616|rs742047|rs9512817|rs7745603|rs639373|rs958957|rs2173115|rs217689|rs12196939|rs1535565|rs2284704|rs11149485|rs12541335|rs1497021|rs1923352|rs510395|rs2005219|rs10961346|rs519417
Arthritis	TASTE_TRANSDUCTION	KEGG	1100	40	20	5,29E-01	9,71E-01	rs1509849|rs12129768|rs6488326|rs3741845|rs1014070|rs4259245|rs1509844|rs765128|rs448852|rs6488361|rs4499352|rs1369450|rs1396980|rs1536041|rs16035|rs8055868|rs583720|rs7531583|rs263232|rs7844964
Arthritis	IGF1_PATHWAY	BIOCARTA	813	38	20	5,30E-01	9,71E-01	rs10515086|rs4899554|rs9672254|rs2229765|rs17237251|rs2302975|rs7169544|rs422437|rs2005219|rs10498208|rs12652661|rs7136446|rs8037855|rs849376|rs1560251|rs10048024|rs7721613|rs7149441|rs1998762|rs2168043
Arthritis	PPAR_SIGNALING_PATHWAY	KEGG	1324	45	20	5,32E-01	9,71E-01	rs2894401|rs6784193|rs297|rs1393492|rs7088546|rs12609870|rs12657454|rs3745469|rs2419627|rs2228468|rs11188349|rs2502805|rs2854028|rs12059934|rs1054135|rs1136224|rs6595870|rs2938395|rs11736582|rs1051006
Arthritis	ASSOCIATION_OF_TRIC_CCT_WITH_TARGET_PROTEINS_DURING_BIOSYNTHESIS	REACTOME	359	14	9	5,33E-01	9,71E-01	rs13247369|rs502930|rs12139487|rs1115535|rs2876472|rs1559405|rs12197692|rs2273102|rs7270022
Arthritis	ACTIVATED_AMPK_STIMULATES_FATTY_ACID_OXIDATION_IN_MUSCLE	REACTOME	421	17	8	5,34E-01	9,71E-01	rs4725424|rs953221|rs6978142|rs11760502|rs11736582|rs12188129|rs4528168|rs6832271
Arthritis	SYNTHESIS_OF_DNA	REACTOME	1545	35	20	5,35E-01	9,71E-01	rs6564984|rs3815844|rs6575121|rs2280162|rs2550461|rs3820716|rs10818964|rs510862|rs3218801|rs1541549|rs1925089|rs7404213|rs6142963|rs228271|rs6463716|rs9540055|rs9450763|rs8063430|rs3819716|rs5030740
Arthritis	DEPOSITION_OF_NEW_CENPA_CONTAINING_NUCLEOSOMES_AT_THE_CENTROMERE	REACTOME	637	65	13	5,36E-01	9,71E-01	rs742047|rs7745603|rs639373|rs12196939|rs3737147|rs653844|rs226981|rs944949|rs16835131|rs16894005|rs199750|rs1980705|rs6456668
Arthritis	HEPARAN_SULFATE_HEPARIN_HS_GAG_METABOLISM	REACTOME	3879	24	20	5,37E-01	9,71E-01	rs12756105|rs2119297|rs2635205|rs4454830|rs9523322|rs16974808|rs10955078|rs8055955|rs7727897|rs7583856|rs4438310|rs11867655|rs10808350|rs17862059|rs9589298|rs482700|rs342711|rs9516073|rs1289459|rs9937575
Arthritis	AMINOACYL_TRNA_BIOSYNTHESIS	KEGG	731	30	14	5,39E-01	9,71E-01	rs4959341|rs6932040|rs1891222|rs17563587|rs9450740|rs3101017|rs6764923|rs2341510|rs9405818|rs4758462|rs895639|rs7744190|rs925210|rs6926774
Arthritis	AUTOIMMUNE_THYROID_DISEASE	KEGG	1646	62	20	5,39E-01	9,71E-01	rs2253907|rs2301226|rs7144673|rs3948793|rs1514684|rs2071469|rs1330320|rs2596574|rs9469357|rs2284191|rs3130141|rs1264456|rs2071554|rs1937332|rs3819716|rs7758736|rs9268832|rs2394185|rs206762|rs637949
Arthritis	PROPANOATE_METABOLISM	KEGG	1219	54	19	5,42E-01	9,71E-01	rs4813547|rs6784193|rs1458868|rs1865806|rs2306619|rs10484429|rs7865905|rs1366819|rs4836272|rs2017571|rs7647085|rs9534842|rs10862246|rs7700981|rs7534754|rs12485670|rs7335993|rs7208613|rs1923431
Arthritis	SIGNALING_BY_RHO_GTPASES	REACTOME	4877	41	20	5,42E-01	9,71E-01	rs4912610|rs6823854|rs881376|rs11101371|rs1753290|rs400052|rs17057836|rs11052116|rs11683765|rs6716352|rs6006880|rs1035712|rs6891143|rs2072255|rs1379271|rs9598051|rs4573339|rs17097481|rs1379935|rs9380633
Arthritis	DNA_REPLICATION	REACTOME	3548	28	20	5,42E-01	9,71E-01	rs6564984|rs11653144|rs12243192|rs3815844|rs6575121|rs972984|rs7975139|rs2280162|rs9350245|rs2550461|rs1614490|rs3820716|rs10818964|rs617621|rs1975305|rs2075995|rs4387213|rs525428|rs510862|rs7756899
Arthritis	ACTIVATED_TLR4_SIGNALLING	REACTOME	2676	34	20	5,45E-01	9,71E-01	rs2726550|rs17162190|rs998235|rs1999090|rs832574|rs12987393|rs10983934|rs3134942|rs739141|rs10739508|rs4899554|rs1921082|rs10485408|rs845641|rs12555480|rs3844278|rs10455526|rs10147042|rs2119945|rs2698537
Arthritis	POST_CHAPERONIN_TUBULIN_FOLDING_PATHWAY	REACTOME	254	16	8	5,45E-01	9,71E-01	rs354616|rs4129373|rs354599|rs10735819|rs6490620|rs6884279|rs2112549|rs7328603
Arthritis	PI3K_AKT_ACTIVATION	REACTOME	1161	35	20	5,46E-01	9,71E-01	rs10515086|rs2755209|rs6084337|rs12026714|rs7986346|rs336209|rs17237251|rs2494738|rs2302975|rs422437|rs10498208|rs12652661|rs12090216|rs8000641|rs4435111|rs1782804|rs2951787|rs484754|rs1560251|rs4661229
Arthritis	MAPK_TARGETS_NUCLEAR_EVENTS_MEDIATED_BY_MAP_KINASES	REACTOME	980	34	20	5,47E-01	9,71E-01	rs17162190|rs998235|rs4899554|rs845641|rs10147042|rs2119945|rs4936661|rs7703433|rs2005219|rs7972776|rs851010|rs1002487|rs4710090|rs874277|rs325383|rs1116003|rs2237093|rs704061|rs2301682|rs7149441
Arthritis	PHASE1_FUNCTIONALIZATION_OF_COMPOUNDS	REACTOME	1515	51	20	5,48E-01	9,71E-01	rs4813547|rs12720462|rs338593|rs305968|rs8187890|rs6139335|rs7081491|rs2228468|rs6012696|rs11188059|rs12459140|rs11124634|rs17726963|rs4144915|rs6583859|rs11812285|rs2585422|rs4736319|rs8105815|rs4852846
Arthritis	GLYCOSYLPHOSPHATIDYLINOSITOL_GPI_ANCHOR_BIOSYNTHESIS	KEGG	318	16	11	5,49E-01	9,71E-01	rs6478803|rs11162293|rs763158|rs1772253|rs17014179|rs2306595|rs525624|rs191827|rs2717533|rs6104159|rs2038491
Arthritis	RIG_I_LIKE_RECEPTOR_SIGNALING_PATHWAY	KEGG	1361	63	20	5,50E-01	9,71E-01	rs1999090|rs832574|rs10811561|rs1815811|rs10485408|rs4493656|rs2421059|rs10455526|rs1330319|rs230530|rs2655638|rs7259683|rs1556091|rs6052147|rs9353794|rs851010|rs755004|rs7771959|rs2811650|rs12115174
Arthritis	INFLUENZA_LIFE_CYCLE	REACTOME	1671	40	20	5,54E-01	9,71E-01	rs7636810|rs284541|rs1149754|rs7993717|rs10509398|rs7757405|rs4772030|rs6503113|rs2291782|rs36621|rs11244329|rs8076790|rs10824609|rs2053984|rs12484030|rs2574693|rs7508025|rs12870730|rs6829581|rs2004997
Arthritis	NTHI_PATHWAY	BIOCARTA	876	31	20	5,55E-01	9,71E-01	rs1999090|rs266339|rs10485408|rs10455526|rs230530|rs4522809|rs2655638|rs4776344|rs1556091|rs4955159|rs9353794|rs851010|rs7771959|rs2811650|rs12115174|rs2237093|rs17341964|rs744910|rs13116385|rs9362733
Arthritis	AMINO_SUGAR_AND_NUCLEOTIDE_SUGAR_METABOLISM	KEGG	956	32	20	5,58E-01	9,71E-01	rs7580273|rs137126|rs1865806|rs2012025|rs2267452|rs742556|rs11752769|rs4323713|rs6543374|rs2025631|rs3800046|rs894391|rs12753370|rs888352|rs2814811|rs758985|rs9392354|rs1475411|rs17501218|rs943
Arthritis	ION_CHANNEL_TRANSPORT	REACTOME	2741	32	20	5,58E-01	9,71E-01	rs4839066|rs12944845|rs6852066|rs4695937|rs4906762|rs9858042|rs4938440|rs12960272|rs1593609|rs923677|rs11939228|rs4691391|rs10519568|rs8023912|rs2700865|rs10431855|rs1007637|rs17368402|rs929708|rs4799017
Arthritis	SIGNAL_TRANSDUCTION_BY_L1	REACTOME	1310	28	20	5,59E-01	9,71E-01	rs17162190|rs998235|rs10827245|rs845641|rs879904|rs11919795|rs10147042|rs660361|rs2288696|rs4937993|rs994781|rs3117582|rs11819443|rs1468727|rs2005219|rs2212020|rs1002487|rs4710090|rs3808734|rs874277
Arthritis	CLASS_I_MHC_MEDIATED_ANTIGEN_PROCESSING_PRESENTATION	REACTOME	5895	29	20	5,59E-01	9,71E-01	rs1406257|rs6564984|rs4858552|rs2253907|rs1048447|rs3815844|rs6575121|rs3753362|rs8028608|rs10498691|rs10836031|rs1483867|rs6084337|rs2365999|rs2280162|rs2550461|rs12105524|rs7773233|rs9477982|rs4971821
Arthritis	NKT_PATHWAY	BIOCARTA	638	33	16	5,62E-01	9,71E-01	rs1145965|rs4493656|rs2421059|rs11625323|rs10204606|rs382410|rs2283563|rs755004|rs4440020|rs1016269|rs11741918|rs604045|rs11575812|rs2243248|rs2807897|rs426132
Arthritis	PRIMARY_BILE_ACID_BIOSYNTHESIS	KEGG	439	24	9	5,62E-01	9,71E-01	rs12445568|rs2228468|rs11253048|rs12059934|rs2254636|rs10509564|rs2465079|rs2560718|rs6467729
Arthritis	GOLGI_ASSOCIATED_VESICLE_BIOGENESIS	REACTOME	1057	37	20	5,63E-01	9,71E-01	rs6733550|rs13120989|rs849749|rs4963442|rs10957967|rs355419|rs2072824|rs11962800|rs9944192|rs4963446|rs737883|rs2232739|rs6080886|rs1045895|rs3808734|rs2231754|rs9990333|rs2224957|rs688359|rs2267138
Arthritis	CASPASE_PATHWAY	BIOCARTA	327	20	10	5,64E-01	9,71E-01	rs666723|rs7144673|rs1051644|rs959573|rs10931934|rs1976955|rs2019978|rs7949293|rs13402616|rs1805410
Arthritis	ASTHMA	KEGG	770	49	20	5,65E-01	9,71E-01	rs9277334|rs547110|rs9276711|rs2071469|rs9469357|rs2284191|rs12602891|rs10144126|rs2501873|rs2071554|rs3135353|rs1136224|rs3819716|rs9268832|rs206762|rs453779|rs2858870|rs3021094|rs2243248|rs3024505
Arthritis	P53HYPOXIA_PATHWAY	BIOCARTA	517	23	15	5,67E-01	9,71E-01	rs10275333|rs6750100|rs2965749|rs12069919|rs2494738|rs10229987|rs5030740|rs2270941|rs13426368|rs879965|rs11762916|rs6503222|rs5758211|rs4128846|rs2017750
Arthritis	PARKINSONS_DISEASE	KEGG	2833	24	20	5,67E-01	9,71E-01	rs222289|rs10226904|rs3753362|rs999918|rs3900417|rs10423721|rs766549|rs7773233|rs17476583|rs9293478|rs2834287|rs7254601|rs414000|rs13258461|rs6987649|rs6918198|rs11957871|rs9291289|rs7255086|rs7247484

Arthritis	MRNA_SPLICING	REACTOME	1057	28	20	5,68E-01	9,71E-01	rs6044228|rs3809425|rs9908594|rs4788605|rs234784|rs1533303|rs8065368|rs7585767|rs1943344|rs2074733|rs17573579|rs2240769|rs485152|rs6111235|rs16865262|rs4894239|rs2402111|rs11604322|rs2011786|rs1782345
Arthritis	DRUG_METABOLISM_CYTOCHROME_P450	KEGG	1661	64	18	5,72E-01	9,71E-01	rs12720462|rs10735234|rs2132847|rs7687822|rs7081491|rs305985|rs7343168|rs11188059|rs2348023|rs17726963|rs11812285|rs4609955|rs12646059|rs11568318|rs9307238|rs34187424|rs2279124|rs11879413
Arthritis	GLYCOSAMINOGLYCAN_METABOLISM	REACTOME	6096	26	20	5,72E-01	9,71E-01	rs16943314|rs12756105|rs13282872|rs2119297|rs4756195|rs2635205|rs4454830|rs16943623|rs9523322|rs4307360|rs16974808|rs10955078|rs8055955|rs353639|rs7727897|rs1041883|rs7583856|rs4438310|rs11867655|rs10808350
Arthritis	LIPOPROTEIN_METABOLISM	REACTOME	1037	52	20	5,72E-01	9,71E-01	rs881376|rs297|rs2487031|rs7897550|rs991811|rs312027|rs12541335|rs882992|rs2502805|rs2269318|rs4148102|rs17042892|rs10904831|rs1136224|rs7043894|rs7587610|rs328539|rs1534650|rs7200210|rs12579740
Arthritis	ENERGY_DEPENDENT_REGULATION_OF_MTOR_BY_LKB1_AMPK	REACTOME	461	19	10	5,73E-01	9,71E-01	rs4725424|rs953221|rs2589142|rs6978142|rs11760502|rs2289762|rs12188129|rs999977|rs4528168|rs9908195
Arthritis	GLYCOSAMINOGLYCAN_BIOSYNTHESIS_CHONDROITIN_SULFATE	KEGG	1372	40	20	5,73E-01	9,71E-01	rs2119297|rs4307360|rs7727897|rs1041883|rs1289459|rs2374409|rs971656|rs10503653|rs3749894|rs17514963|rs11860576|rs6972852|rs4148944|rs9351798|rs10791360|rs10075474|rs11112057|rs4238646|rs7720425|rs2500537
Arthritis	MCM_PATHWAY	BIOCARTA	234	13	6	5,73E-01	9,71E-01	rs3820716|rs510862|rs9450763|rs565211|rs2307325|rs13213285
Arthritis	IL10_PATHWAY	BIOCARTA	288	18	8	5,76E-01	9,73E-01	rs310209|rs11887698|rs2048874|rs12129709|rs1181574|rs3021094|rs3024505|rs12075255
Arthritis	TRANSMISSION_ACROSS_CHEMICAL_SYNAPSES	REACTOME	9089	29	20	5,78E-01	9,74E-01	rs17153079|rs1509849|rs9485742|rs384067|rs4417522|rs17162190|rs2815727|rs12457181|rs998235|rs1868513|rs2950798|rs6557801|rs4637750|rs2160732|rs2835909|rs6785307|rs2724113|rs9878916|rs12129768|rs999448
Arthritis	FORMATION_OF_INCISION_COMPLEX_IN_GG_NER	REACTOME	545	35	12	5,80E-01	9,74E-01	rs1323806|rs2284704|rs2097289|rs1541549|rs6463716|rs2808673|rs5030740|rs9554903|rs10492771|rs17466949|rs16902819|rs12540373
Arthritis	IL6_PATHWAY	BIOCARTA	401	24	14	5,81E-01	9,74E-01	rs310209|rs3761049|rs4899554|rs10974873|rs12720356|rs2005219|rs7508025|rs12129709|rs2304237|rs2302600|rs2274471|rs7149441|rs1998762|rs2168043
Arthritis	RNA_POLYMERASE	KEGG	351	13	10	5,82E-01	9,74E-01	rs9512817|rs1535565|rs11149485|rs12541335|rs9261290|rs1150743|rs6761|rs1106841|rs1131384|rs2559658
Arthritis	PKB_MEDIATED_EVENTS	REACTOME	583	23	12	5,82E-01	9,74E-01	rs4725424|rs953221|rs4783446|rs2589142|rs6978142|rs11760502|rs8059561|rs2289762|rs12188129|rs999977|rs4528168|rs9908195
Arthritis	P53_SIGNALING_PATHWAY	KEGG	1897	28	20	5,84E-01	9,74E-01	rs10820827|rs10275333|rs2227631|rs10494494|rs17781459|rs2229032|rs1109998|rs12069919|rs1369272|rs4807284|rs10255859|rs4035233|rs9381100|rs9286378|rs2890109|rs1051442|rs4121859|rs6975917|rs9950483|rs9696753
Arthritis	KERATINOCYTE_PATHWAY	BIOCARTA	1952	24	20	5,84E-01	9,74E-01	rs2726550|rs12418638|rs12146687|rs832574|rs2836815|rs975167|rs2836656|rs4899554|rs2836816|rs10893818|rs230530|rs6590343|rs12421164|rs3757639|rs1957895|rs2238493|rs4937316|rs7122859|rs4245079|rs12454712
Arthritis	RAS_ACTIVATION_UOPN_CA2_INFUX_THROUGH_NMDA_RECEPTOR	REACTOME	1033	36	20	5,87E-01	9,74E-01	rs7002363|rs2160732|rs1029471|rs1379271|rs16970502|rs172677|rs6533710|rs1740005|rs316375|rs1823024|rs1065832|rs2072439|rs10492142|rs11074659|rs696879|rs1362816|rs12431766|rs10505778|rs9925203|rs8064140
Arthritis	PIP3_ACTIVATES_AKT_SIGNALING	REACTOME	587	22	10	5,88E-01	9,74E-01	rs2755209|rs6084337|rs2494738|rs4435111|rs1782804|rs2951787|rs484754|rs523478|rs7094771|rs2735352
Arthritis	TH1TH2_PATHWAY	BIOCARTA	535	28	14	5,89E-01	9,74E-01	rs1145965|rs4493656|rs2421059|rs382410|rs2283563|rs3135353|rs755004|rs9268832|rs2858870|rs11741918|rs11575812|rs2243248|rs2807897|rs426132
Arthritis	MEMBRANE_TRAFFICKING	REACTOME	2276	28	20	5,90E-01	9,74E-01	rs2359106|rs6733550|rs12445650|rs17745948|rs13120989|rs849749|rs1011038|rs2415508|rs4963442|rs11048060|rs6854328|rs10957967|rs355419|rs2072824|rs11962800|rs11919795|rs835223|rs7740467|rs9944192|rs11730728
Arthritis	AXON_GUIDANCE	KEGG	9506	29	20	5,91E-01	9,74E-01	rs17153079|rs2942544|rs881758|rs4791330|rs3799792|rs3109384|rs6551454|rs17819911|rs1480689|rs400052|rs1023262|rs12914000|rs12653501|rs7338655|rs1381571|rs12354155|rs2251238|rs11683765|rs164294|rs7781126
Arthritis	ANTIGEN_PRESENTATION_FOLDING_ASSEMBLY_AND_PEPTIDE_LOADING_OF_CLASS_I_M	REACTOME	830	52	18	5,96E-01	9,80E-01	rs2253907|rs2415508|rs6854328|rs2071469|rs2596574|rs11730728|rs2071554|rs3819716|rs429511|rs2394185|rs983694|rs993675|rs17677598|rs1634747|rs7383287|rs9260759|rs28367729|rs241429
Arthritis	HEMATOPOIETIC_CELL_LINEAGE	KEGG	1963	61	20	6,00E-01	9,81E-01	rs3917265|rs4756195|rs6440971|rs3020829|rs2287047|rs353639|rs7373276|rs163577|rs3856848|rs1514684|rs7988301|rs17375044|rs2048874|rs11230335|rs2283563|rs9937676|rs6874642|rs3135353|rs11869835|rs2310169
Arthritis	PHOSPHOLIPASE_C_MEDIATED_CASCADE	REACTOME	2628	27	20	6,04E-01	9,81E-01	rs2302234|rs1509849|rs384067|rs888298|rs12129768|rs11048665|rs11615994|rs7674434|rs7955100|rs4259245|rs13190668|rs1509844|rs9591501|rs2288696|rs6490668|rs1554465|rs10503587|rs1740005|rs448852|rs1153138
Arthritis	PI3K_EVENTS_IN_ERBB4_SIGNALING	REACTOME	1709	31	20	6,06E-01	9,81E-01	rs10515086|rs17406681|rs2755209|rs6084337|rs4673677|rs17237251|rs4622647|rs2494738|rs6725181|rs2302975|rs7002832|rs422437|rs6706010|rs6722322|rs12652661|rs12676976|rs7598440|rs4435111|rs1782804|rs2976527
Arthritis	METABOLISM_OF_XENOBIOTICS_BY_CYTOCHROME_P450	KEGG	1674	66	19	6,06E-01	9,81E-01	rs10735234|rs338593|rs2132847|rs305968|rs7687822|rs7081491|rs11253048|rs11188059|rs11124634|rs17726963|rs2270941|rs11812285|rs12646059|rs11568318|rs8105815|rs9307238|rs4149226|rs34187424|rs2279124
Arthritis	THE_ROLE_OF_NEF_IN_HIV1_REPLICATION_AND_DISEASE_PATHOGENESIS	REACTOME	936	29	17	6,09E-01	9,81E-01	rs2949654|rs11532670|rs849749|rs11919795|rs704859|rs16919915|rs737883|rs1618879|rs7729998|rs429511|rs4723596|rs6890504|rs6876868|rs9260759|rs2267138|rs2056626|rs261060
Arthritis	TPO_PATHWAY	BIOCARTA	814	29	20	6,11E-01	9,81E-01	rs10515086|rs4899554|rs10974873|rs17237251|rs11887698|rs2238493|rs2302975|rs422437|rs2005219|rs12652661|rs849376|rs9913908|rs198182|rs7721613|rs2274471|rs7149441|rs1998762|rs2235366|rs2168043|rs12150089
Arthritis	ARMS_MEDIATED_ACTIVATION	REACTOME	393	13	10	6,13E-01	9,81E-01	rs12026714|rs10901096|rs12038990|rs2005219|rs6876|rs12090216|rs3012739|rs10842514|rs7077462|rs4661229
Arthritis	MYOGENESIS	REACTOME	1747	33	20	6,17E-01	9,81E-01	rs732779|rs3898276|rs6124082|rs12797894|rs3087949|rs12606601|rs884938|rs389673|rs6071574|rs10485470|rs1939890|rs2119945|rs808950|rs6071562|rs1305277|rs9849714|rs1895080|rs851010|rs9304511|rs6089247
Arthritis	HOST_INTERACTIONS_OF_HIV_FACTORS	REACTOME	2270	27	20	6,17E-01	9,81E-01	rs6564984|rs2949654|rs3815844|rs6575121|rs11532670|rs849749|rs2280162|rs2550461|rs10818964|rs5751022|rs11919795|rs11244329|rs704859|rs16919915|rs7404213|rs6142963|rs737883|rs228271|rs1618879|rs7729998
Arthritis	AMYLOIDS	REACTOME	972	104	19	6,17E-01	9,81E-01	rs742047|rs7745603|rs1667255|rs639373|rs12196939|rs1207774|rs7744633|rs1709276|rs469632|rs10841798|rs17350396|rs13204006|rs12562952|rs1935005|rs199750|rs196034|rs6850389|rs6456688|rs2092058
Arthritis	O_LINKED_GLYCOSYLATION_OF_MUCINS	REACTOME	2402	30	20	6,17E-01	9,81E-01	rs13282872|rs1942237|rs7680255|rs2653831|rs2547067|rs1566250|rs6740075|rs7524886|rs3761049|rs2978023|rs6781|rs4897731|rs6463634|rs7827061|rs676269|rs751645|rs11935600|rs7026938|rs7571016|rs1554324
Arthritis	TRIF_MEDIATED_TLR3_SIGNALING	REACTOME	2075	33	20	6,18E-01	9,81E-01	rs2726550|rs17162190|rs998235|rs1999090|rs3134942|rs739141|rs4899554|rs10485408|rs845641|rs3844279|rs10455526|rs10147042|rs2119945|rs4936661|rs2655638|rs7259683|rs1240073|rs218921|rs1556091|rs7703433
Arthritis	TRYPTOPHAN_METABOLISM	KEGG	953	50	20	6,18E-01	9,81E-01	rs6784193|rs1038378|rs6725631|rs1891222|rs7865905|rs6850897|rs12294809|rs4276678|rs1487275|rs4836272|rs11124634|rs2348023|rs255165|rs11032695|rs7838116|rs7700981|rs930420|rs10227423|rs7692005|rs7846217
Arthritis	CYTOKINE_CYTOKINE_RECEPTOR_INTERACTION	KEGG	6425	35	20	6,19E-01	9,81E-01	rs3917265|rs4724065|rs1145965|rs13037653|rs1233650|rs11744747|rs1023262|rs917526|rs2243176|rs186659|rs3020829|rs10811561|rs228942|rs13208597|rs975405|rs12505096|rs2287047|rs1276408|rs7373276|rs3820716
Arthritis	NUCLEOTIDE_BINDING_DOMAIN_LEUCINE_RICH_REPEAT_CONTAINING_RECEPTOR_NLR YS	REACTOME	1337	30	20	6,21E-01	9,81E-01	rs1999090|rs17178663|rs666723|rs739141|rs10485408|rs3844278|rs10455526|rs9913432|rs2655638|rs1240073|rs12454712|rs218921|rs1556091|rs1049927|rs9353794|rs402330|rs1362765|rs851010|rs9912659|rs8096471
Arthritis	DIABETES_PATHWAYS	REACTOME	3395	35	20	6,21E-01	9,81E-01	rs1816594|rs1880143|rs10275333|rs848189|rs888298|rs2269010|rs10017098|rs6442982|rs16849826|rs1864427|rs4328874|rs12694392|rs1836581|rs6926079|rs7792010|rs13175238|rs10816540|rs2715609|rs16974510|rs4143282
Arthritis	MAPK_SIGNALING_PATHWAY	KEGG	9833	30	20	6,21E-01	9,81E-01	rs3917265|rs6674511|rs4733828|rs10820827|rs2726550|rs11653144|rs1093733|rs17162190|rs759648|rs13014054|rs1662695|rs998235|rs10752974|rs9792218|rs7021722|rs1999090|rs1868513|rs2083828|rs12633242|rs6785307
Arthritis	TRNA_AMINOACYLATION	REACTOME	795	37	17	6,22E-01	9,81E-01	rs4959341|rs6932040|rs1891222|rs17563587|rs9450740|rs13144832|rs3101017|rs10999211|rs6764923|rs2341510|rs9405818|rs10458221|rs4758462|rs895639|rs7744190|rs925210|rs6926774
Arthritis	MEIOTIC_RECOMBINATION	REACTOME	993	99	20	6,23E-01	9,81E-01	rs742047|rs7745603|rs639373|rs12196939|rs7547056|rs10520896|rs1541549|rs3101017|rs6463716|rs2029778|rs1799949|rs10805751|rs5030740|rs11571805|rs299247|rs1207952|rs17466949|rs199750|rs6456688|rs12540373
Arthritis	HDAC_PATHWAY	BIOCARTA	1231	36	20	6,24E-01	9,81E-01	rs10515086|rs2251238|rs2119945|rs9672254|rs2229765|rs17237251|rs2494738|rs2302975|rs1740005|rs177820|rs7169544|rs422437|rs851010|rs12652661|rs325383|rs2237093|rs7233024|rs12431766|rs7136446|rs8037855
Arthritis	FATTY_ACID_TRIACYLGLYCEROL_AND_KETONE_BODY_METABOLISM	REACTOME	5526	29	20	6,25E-01	9,81E-01	rs4478182|rs3741496|rs9487444|rs149293|rs3757031|rs4697435|rs4725424|rs7614173|rs2195003|rs12425122|rs10004130|rs6084337|rs1458868|rs910721|rs10831104|rs6750100|rs933910|rs2017802|rs3761739|rs2487031
Arthritis	MITOTIC_PROMETAPHASE	REACTOME	1814	38	20	6,26E-01	9,81E-01	rs11653144|rs12243192|rs972984|rs7975139|rs1614490|rs617621|rs1975305|rs4387213|rs525428|rs9913432|rs4936661|rs7556434|rs7703433|rs3737147|rs12119063|rs944949|rs12475700|rs10510504|rs1116003|rs4076557
Arthritis	DILATED_CARDIOMYOPATHY	KEGG	4636	31	20	6,27E-01	9,81E-01	rs1509849|rs384067|rs16835926|rs10273151|rs2107128|rs4509829|rs1868513|rs6785307|rs9878916|rs12129768|rs9311552|rs2238517|rs12058655|rs1464459|rs2805434|rs9510351|rs1405331|rs6700222|rs879904|rs13190668
Arthritis	IL2_PATHWAY	BIOCARTA	452	35	16	6,28E-01	9,81E-01	rs310209|rs228942|rs3761049|rs4899554|rs11914132|rs2005219|rs7508025|rs12129709|rs290255|rs11575812|rs2302600|rs7149441|rs1998762|rs2235329|rs2168043|rs2017750
Arthritis	BASE_EXCISION_REPAIR	KEGG	500	19	13	6,29E-01	9,81E-01	rs1826121|rs7310681|rs7995747|rs3218801|rs12458|rs7686012|rs9551925|rs6107581|rs323881|rs6809452|rs17074629|rs1805410|rs16939904
Arthritis	PEROXISOMAL_LIPID_METABOLISM	REACTOME	435	42	14	6,29E-01	9,81E-01	rs11614129|rs966099|rs889927|rs11049951|rs1372316|rs495183|rs802098|rs802036|rs12059934|rs11604748|rs2560718|rs31653|rs6130946|rs2555491
Arthritis	T_CELL_RECEPTOR_SIGNALING_PATHWAY	KEGG	3546	30	20	6,34E-01	9,88E-01	rs10515086|rs2647394|rs2949654|rs2510139|rs1999090|rs2251238|rs137991|rs6461856|rs975167|rs4899554|rs10485408|rs2715252|rs2423441|rs785512|rs10455526|rs230530|rs2921253|rs17237251|rs2494738|rs2655638
Arthritis	SIGNALING_BY_FGFR1_MUTANTS	REACTOME	1243	25	20	6,36E-01	9,88E-01	rs10515086|rs2313626|rs1734729|rs17237251|rs2288696|rs6490668|rs11887698|rs10503587|rs2302975|rs249923|rs422437|rs263931|rs7105655|rs12652661|rs7315446|rs407366|rs12168342|rs2312968|rs9510021|rs6490721
Arthritis	CYTOSOLIC_DNA_SENSING_PATHWAY	KEGG	620	64	18	6,36E-01	9,88E-01	rs9512817|rs1342326|rs1535565|rs1330319|rs230530|rs11149485|rs7259683|rs12541335|rs6052147|rs12115174|rs637949|rs3733236|rs13116385|rs10863389|rs1106841|rs13181561|rs7662536|rs2559658
Arthritis	IRON_UPTAKE_AND_TRANSPORT	REACTOME	733	27	18	6,38E-01	9,88E-01	rs4669621|rs4963442|rs8177190|rs2249572|rs16861650|rs1497021|rs16919915|rs4963446|rs6977910|rs721950|rs2004997|rs3816954|rs12742531|rs9990333|rs577866|rs10503675|rs4732330|rs2513873
Arthritis	IL12_PATHWAY	BIOCARTA	589	27	18	6,45E-01	9,94E-01	rs2949654|rs10974873|rs4493656|rs2421059|rs704859|rs382410|rs12720356|rs1618879|rs851010|rs755004|rs9380546|rs11741918|rs2304237|rs2274471|rs2056626|rs1998762|rs426132|rs2017750
Arthritis	VASCULAR_SMOOTH_MUSCLE_CONTRACTION	KEGG	5845	26	20	6,46E-01	9,94E-01	rs1509849|rs384067|rs1093733|rs1194491|rs10936997|rs2705462|rs17455628|rs10752974|rs11589805|rs12129768|rs2673474|rs11048665|rs11779546|rs7975139|rs11615994|rs7077665|rs9817294|rs10869979|rs10912025|rs6056526
Arthritis	INTERACTION_BETWEEN_L1_AND_ANKYRINS	REACTOME	970	22	15	6,46E-01	9,94E-01	rs6089908|rs4671952|rs11814752|rs393569|rs13426300|rs1439791|rs354232|rs907136|rs1432546|rs857724|rs1672460|rs12469667|rs7087489|rs10761445|rs10994398
Arthritis	SMOOTH_MUSCLE_CONTRACTION	REACTOME	658	23	13	6,47E-01	9,94E-01	rs7088546|rs1441734|rs216166|rs2218989|rs1740005|rs11188349|rs6467563|rs11583023|rs12431766|rs17168150|rs1050255|rs7811|rs7097467
Arthritis	FC_EPSILON_RI_SIGNALING_PATHWAY	KEGG	2758	34	20	6,48E-01	9,94E-01	rs10515086|rs2726550|rs1093733|rs10752974|rs11589805|rs10912025|rs1339082|rs547110|rs230632|rs1567102|rs785512|rs800719|rs17237251|rs2494738|rs2238493|rs7496336|rs2302975|rs12038990|rs11573253|rs422437
Arthritis	EGFR_DOWNREGULATION	REACTOME	1037	39	20	6,51E-01	9,95E-01	rs9318729|rs2510152|rs300550|rs11919795|rs4833920|rs8002487|rs1468727|rs9545330|rs2501279|rs3808734|rs9565708|rs348029|rs9545836|rs3796944|rs2224957|rs2329172|rs9545295|rs17424600|rs6974790|rs759159
Arthritis	G1_PHASE	REACTOME	625	33	20	6,51E-01	9,95E-01	rs9350245|rs2075995|rs2284704|rs10255859|rs4936661|rs9381100|rs10271133|rs7703433|rs12822144|rs2383208|rs13160147|rs4134943|rs1116003|rs11063059|rs592412|rs4554318|rs33671|rs243538|rs9577581|rs16902819
Arthritis	SYNTHESIS_OF_BILE_ACIDS_AND_BILE_SALTS	REACTOME	546	52	14	6,52E-01	9,95E-01	rs12445568|rs3755160|rs2228468|rs11253048|rs12059934|rs479682|rs2254636|rs16856261|rs10509564|rs2465079|rs2560718|rs6130946|rs6467729|rs2555491
Arthritis	ACTIVATION_OF_BH3_ONLY_PROTEINS	REACTOME	325	17	13	6,53E-01	9,95E-01	rs17781459|rs2494738|rs12454712|rs9950483|rs8065435|rs8096471|rs6876|rs17772748|rs7240884|rs11660783|rs9577581|rs12456032|rs2017750
Arthritis	STEROID_HORMONES	KEGG	693	22	11	6,55E-01	9,95E-01	rs529439|rs7897550|rs4622471|rs10904831|rs853988|rs2585422|rs7029101|rs4736319|rs2181874|rs12907723|rs1062033
Arthritis	G_BETA_GAMMA_SIGNALLING_THROUGH_PLC_BETA	REACTOME	624	33	18	6,56E-01	9,95E-01	rs2724113|rs4979032|rs10498440|rs2207317|rs4807284|rs6039211|rs6118219|rs10485723|rs2223538|rs4471960|rs291353|rs2076409|rs7531583|rs12147761|rs11087808|rs6039206|rs227134|rs2965208
Arthritis	TRANSPORT_OF_VITAMINS_NUCLEOSIDES_AND_RELATED_MOLECULES	REACTOME	1154	44	20	6,57E-01	9,95E-01	rs4627290|rs953888|rs7616492|rs12593834|rs11853372|rs11720811|rs883431|rs3818057|rs10501421|rs2190748|rs12912997|rs13093879|rs10841798|rs8025438|rs6595870|rs1706767|rs2290272|rs12910476|rs11140525|rs12097741
Arthritis	VALINE_LEUCINE_AND_ISOLEUCINE_DEGRADATION	KEGG	1149	53	20	6,60E-01	9,97E-01	rs6784193|rs1458868|rs9352866|rs1865806|rs1038378|rs10484429|rs7865905|rs4836272|rs10447552|rs11762112|rs2348023|rs13197064|rs869013|rs10991781|rs11166413|rs2047554|rs7700981|rs7534754|rs284662|rs17369983
Arthritis	COMPLEMENT_CASCADE	REACTOME	752	46	19	6,62E-01	9,98E-01	rs1179766|rs437179|rs4957381|rs835223|rs11265265|rs12218966|rs2204758|rs2384167|rs1822824|rs3105782|rs751138|rs8178610|rs2248678|rs7027797|rs11714564|rs1060287|rs17201075|rs1917186|rs2842754
Arthritis	FATTY_ACYL_COA_BIOSYNTHESIS	REACTOME	404	16	8	6,67E-01	9,99E-01	rs149293|rs2419627|rs2017571|rs11736582|rs10413616|rs6832271|rs7208613|rs7686686
Arthritis	CYTOKINE_PATHWAY	BIOCARTA	491	25	15	6,67E-01	9,99E-01	rs13208597|rs4493656|rs2421059|rs1892275|rs2048874|rs10484879|rs1974226|rs755004|rs11741918|rs3021094|rs11575812|rs2243248|rs3024505|rs12075255|rs12907723
Arthritis	POTASSIUM_CHANNELS	REACTOME	4940	29	20	6,68E-01	9,99E-01	rs11826873|rs988896|rs6089908|rs13260906|rs10936997|rs12631297|rs4129625|rs2471093|rs13207257|rs2835909|rs2724113|rs12080989|rs999420|rs4979032|rs9817294|rs9343015|rs9727842|rs10498440|rs1501569|rs179435
Arthritis	LIGAND_GATED_ION_CHANNEL_TRANSPORT	REACTOME	1051	40	20	6,69E-01	9,99E-01	rs6852066|rs4695937|rs1593609|rs923677|rs4691391|rs10519568|rs8023912|rs10431855|rs4906883|rs745850|rs4695945|rs3777522|rs834396|rs1432132|rs10043074|rs3777514|rs17648940|rs4432228|rs7167756|rs9359845
Arthritis	PRE_NOTCH_TRANSCRIPTION_AND_TRANSLATION	REACTOME	835	36	20	6,74E-01	9,99E-01	rs7943506|rs17005603|rs3134942|rs9350245|rs176943|rs6817684|rs11021494|rs4134943|rs6771941|rs6822207|rs592412|rs4692386|rs12646059|rs13119079|rs9577581|rs2068477|rs8046065|rs948164|rs7934164|rs129988
Arthritis	PROTEOLYTIC_CLEAVAGE_OF_SNARE_COMPLEX_PROTEINS	REACTOME	382	36	19	6,74E-01	9,99E-01	rs12445568|rs16982131|rs6070531|rs16958408|rs1487608|rs11759401|rs7962097|rs9893164|rs12740112|rs7220482|rs17741348|rs486409|rs17518345|rs6503206|rs879687|rs6912580|rs2398526|rs9388996|rs8071380
Arthritis	NEURONAL_SYSTEM	REACTOME	13378	28	20	6,78E-01	9,99E-01	rs17153079|rs1509849|rs9485742|rs384067|rs4417522|rs11826873|rs988896|rs17162190|rs6089908|rs13260906|rs2815727|rs4838857|rs10936997|rs12445568|rs12457181|rs12631297|rs998235|rs4129625|rs2471093|rs5771086
Arthritis	BASAL_TRANSCRIPTION_FACTORS	KEGG	386	6	5	6,78E-01	9,99E-01	rs6755901|rs12040438|rs11677350|rs2295625|rs10813843
Arthritis	RENAL_CELL_CARCINOMA	KEGG	2164	25	20	6,80E-01	9,99E-01	rs10515086|rs12418638|rs12146687|rs7562947|rs10893818|rs5751022|rs3844278|rs2423441|rs10901096|rs785512|rs17237251|rs6590343|rs12421164|rs2494738|rs11625323|rs4937316|rs7122859|rs2302975|rs4245079|rs12038990
Arthritis	PI3K_EVENTS_IN_ERBB2_SIGNALING	REACTOME	1886	31	20	6,85E-01	9,99E-01	rs10515086|rs17406681|rs2755209|rs6084337|rs4673677|rs17237251|rs4622647|rs2494738|rs6725181|rs2302975|rs7002832|rs422437|rs1468727|rs6706010|rs6722322|rs12652661|rs12676976|rs7598440|rs4435111|rs1782804
Arthritis	DEFENSINS	REACTOME	495	16	9	6,85E-01	9,99E-01	rs9644758|rs11362|rs2738173|rs2223526|rs6046492|rs2131283|rs2981398|rs3888293|rs6139074
Arthritis	TOB1_PATHWAY	BIOCARTA	652	25	20	6,86E-01	9,99E-01	rs2949654|rs11079937|rs266339|rs4522809|rs11625323|rs704859|rs4776344|rs4955159|rs1618879|rs744910|rs2029828|rs604045|rs11575812|rs2243248|rs11129399|rs6768081|rs9876727|rs12915039|rs2056626|rs11129414
Arthritis	PURINE_METABOLISM	KEGG	581	34	20	6,86E-01	9,99E-01	rs510862|rs11753050|rs3006009|rs10936022|rs12294809|rs12038990|rs4744663|rs6584|rs7932321|rs3903689|rs12730102|rs2871427|rs11032695|rs10869134|rs2306084|rs565211|rs185925|rs7924176|rs4910146|rs17264901
Arthritis	INTERFERON_SIGNALING	REACTOME	3448	40	20	6,87E-01	9,99E-01	rs4858552|rs1145965|rs2253907|rs4756195|rs12639287|rs310209|rs9277334|rs1029471|rs353639|rs2167977|rs7215005|rs10974873|rs1991406|rs660361|rs1330320|rs2596574|rs12607710|rs9469357|rs10521318|rs7259683
Arthritis	SPHINGOLIPID_METABOLISM	KEGG	1101	34	20	6,87E-01	9,99E-01	rs10820938|rs11683765|rs1870653|rs2299920|rs1667255|rs12133338|rs1369861|rs6481273|rs4074469|rs2404716|rs13386477|rs12964617|rs11722041|rs17635854|rs7387252|rs10981150|rs4438612|rs7531629|rs12035812|rs12635761
Arthritis	NA_CL_DEPENDENT_NEUROTRANSMITTER_TRANSPORTERS	REACTOME	718	20	17	6,89E-01	9,99E-01	rs2950798|rs999448|rs4269967|rs13258461|rs9871974|rs10161479|rs10446439|rs17183671|rs1728803|rs1682821|rs363227|rs11116502|rs3773162|rs2947146|rs11116469|rs6794801|rs10503670
Arthritis	RIG_I_MDA5_MEDIATED_INDUCTION_OF_IFN_ALPHA_BETA_PATHWAYS	REACTOME	1129	66	20	6,91E-01	9,99E-01	rs9938543|rs832574|rs1815811|rs3134942|rs4784754|rs1330320|rs7670291|rs7259683|rs17715481|rs6052147|rs34129552|rs1549669|rs469632|rs12115174|rs1105207|rs10931934|rs150895|rs9551925|rs637949|rs793899
Arthritis	AMINO_ACID_AND_OLIGOPEPTIDE_SLC_TRANSPORTERS	REACTOME	1707	40	20	6,91E-01	9,99E-01	rs1289392|rs10145863|rs3782999|rs2712623|rs10434201|rs1288338|rs3766781|rs12644755|rs4269967|rs11734488|rs17306529|rs9792165|rs17632120|rs10974530|rs7508025|rs10161479|rs7978366|rs10446439|rs17183671|rs180427
Arthritis	PEROXISOME	KEGG	2047	34	20	6,93E-01	9,99E-01	rs6784193|rs7839056|rs4605929|rs2842992|rs17186108|rs11614129|rs1821901|rs966099|rs3934860|rs7567258|rs1265303|rs12294809|rs4708838|rs889927|rs11049951|rs2419627|rs17727473|rs1372316|rs495183|rs6055239
Arthritis	PHOSPHATIDYLINOSITOL_SIGNALING_SYSTEM	KEGG	3813	29	20	6,93E-01	9,99E-01	rs10515086|rs9834264|rs10505811|rs577684|rs1476592|rs11048665|rs1130635|rs11615994|rs6056526|rs6788054|rs3775616|rs7955100|rs785512|rs1525079|rs4259245|rs2239921|rs11130036|rs7234952|rs17237251|rs2207317
Arthritis	PROTEIN_FOLDING	REACTOME	675	34	19	6,94E-01	9,99E-01	rs13247369|rs502930|rs12139487|rs354616|rs4129373|rs354599|rs10735819|rs2966431|rs6490620|rs6884279|rs1115535|rs2112549|rs14398|rs2876472|rs1559405|rs7328603|rs12197692|rs2273102|rs7270022
Arthritis	DEGRADATION_OF_THE_EXTRACELLULAR_MATRIX	REACTOME	580	38	20	6,94E-01	9,99E-01	rs9927989|rs1816594|rs13260549|rs17301608|rs10090518|rs11859163|rs7945819|rs17514846|rs243840|rs2465836|rs1922314|rs12290253|rs1782632|rs2889529|rs7004307|rs11640811|rs738792|rs1053605|rs17722347|rs1964986
Arthritis	MITOCHONDRIA_PATHWAY	BIOCARTA	317	9	8	6,96E-01	9,99E-01	rs6003071|rs12454712|rs8096471|rs10931934|rs2270941|rs1976955|rs2019978|rs13402616
Arthritis	MHC_CLASS_II_ANTIGEN_PRESENTATION	REACTOME	2226	32	20	7,02E-01	9,99E-01	rs1295217|rs2301226|rs849749|rs961548|rs2415508|rs1259952|rs6854328|rs9276711|rs2071469|rs6580153|rs13103662|rs11919795|rs4690859|rs1025447|rs9469357|rs2284191|rs11730728|rs882551|rs8065435|rs737883
Arthritis	N_GLYCAN_ANTENNAE_ELONGATION_IN_THE_MEDIAL_TRANS_GOLGI	REACTOME	962	50	19	7,03E-01	9,99E-01	rs1147443|rs187462|rs839136|rs1667255|rs7701590|rs10863128|rs11630088|rs7148147|rs12912184|rs12964617|rs10066593|rs4902379|rs6766574|rs4233366|rs2717263|rs16830064|rs2318305|rs1257193|rs12311449
Arthritis	GAB1_SIGNALOSOME	REACTOME	1077	43	20	7,04E-01	9,99E-01	rs10515086|rs2755209|rs6084337|rs17237251|rs2494738|rs2302975|rs422437|rs1468727|rs6473257|rs12652661|rs4435111|rs1782804|rs2951787|rs484754|rs3796944|rs523478|rs7721613|rs7094771|rs6974790|rs759159

Arthritis	METABOLISM_OF_CARBOHYDRATES	REACTOME	9072	39	20	7,09E-01	9,99E-01	rs3753491|rs16943314|rs12756105|rs13282872|rs863944|rs2119297|rs4756195|rs2635205|rs4454830|rs16943623|rs9523322|rs4307360|rs12129768|rs16974808|rs10955078|rs8055955|rs2722639|rs11185438|rs2516614|rs353639
Arthritis	P53_PATHWAY	BIOCARTA	347	11	9	7,14E-01	9,99E-01	rs12069919|rs137500|rs12454712|rs8096471|rs592412|rs2270941|rs6107581|rs11605729|rs17318844
Arthritis	SYNTHESIS_OF_GLYCOSYLPHOSPHATIDYLINOSITOL_GPI	REACTOME	485	25	11	7,15E-01	9,99E-01	rs12914000|rs6478803|rs763158|rs2306595|rs525624|rs1912640|rs1025145|rs281317|rs2038491|rs1993336|rs7170708
Arthritis	ANTIVIRAL_MECHANISM_BY_IFN_STIMULATED_GENES	REACTOME	1077	40	20	7,16E-01	9,99E-01	rs4858552|rs12639287|rs310209|rs2167977|rs1991406|rs7259683|rs11887698|rs11244329|rs2005219|rs6829581|rs6768994|rs12129709|rs3026106|rs12497066|rs2717533|rs6763238|rs1438019|rs12604538|rs12149031|rs2094881
Arthritis	BOTULINUM_NEUROTOXICITY	REACTOME	522	37	20	7,16E-01	9,99E-01	rs12445568|rs16982131|rs6070531|rs16958408|rs1487608|rs11759401|rs17046094|rs7962097|rs9893164|rs12740112|rs7220482|rs17741348|rs486409|rs17518345|rs6503206|rs879687|rs6912580|rs2398526|rs9388996|rs8071380
Arthritis	NUCLEOTIDE_LIKE_PURINERGIC_RECEPTORS	REACTOME	272	11	7	7,17E-01	9,99E-01	rs8109578|rs1790063|rs6668383|rs1220996|rs2510881|rs16863356|rs2247867
Arthritis	N_GLYCAN_BIOSYNTHESIS	KEGG	1792	44	20	7,17E-01	9,99E-01	rs1869676|rs17115614|rs1147443|rs187462|rs6478803|rs2720611|rs639373|rs2720644|rs2426227|rs7701590|rs6933285|rs2042993|rs1421247|rs1063588|rs17122705|rs7350509|rs7148147|rs1429088|rs4915899|rs10066593
Arthritis	NCAM1_INTERACTIONS	REACTOME	1896	28	20	7,19E-01	9,99E-01	rs4841920|rs4341231|rs2631926|rs2072277|rs1054645|rs999012|rs11993990|rs630943|rs13419630|rs622486|rs660361|rs12022389|rs1277748|rs9583467|rs11630088|rs4937993|rs9521623|rs1459665|rs4739284|rs12686426
Arthritis	DC_PATHWAY	BIOCARTA	573	38	20	7,19E-01	9,99E-01	rs10983934|rs10739508|rs12555480|rs4493656|rs2421059|rs1554973|rs10759930|rs6478330|rs694428|rs755004|rs1514762|rs11741918|rs3021094|rs2243248|rs175119|rs3024505|rs6496603|rs12075255|rs2780242|rs3826656
Arthritis	DESTABILIZATION_OF_MRNA_BY_KSRP	REACTOME	205	9	5	7,22E-01	9,99E-01	rs2494738|rs851010|rs2237093|rs4382556|rs7403900
Arthritis	OXYGEN_DEPENDENT_PROLINE_HYDROXYLATION_OF_HYPOXIA_INDUCIBLE_FACTOR_	REACTOME	313	13	9	7,23E-01	9,99E-01	rs5751022|rs3844279|rs4953347|rs2881321|rs150895|rs797351|rs1000531|rs1809461|rs17102132
Arthritis	MUSCLE_CONTRACTION	REACTOME	1023	38	20	7,23E-01	9,99E-01	rs4509829|rs7088546|rs1441734|rs864302|rs216166|rs2218989|rs4792010|rs12882128|rs1740005|rs11188349|rs4576532|rs6467563|rs8014568|rs11583023|rs10752073|rs12431766|rs17168150|rs1050255|rs3729704|rs1536950
Arthritis	INTRINSIC_PATHWAY_FOR_APOPTOSIS	REACTOME	532	29	20	7,24E-01	9,99E-01	rs17781459|rs7144673|rs2239921|rs2494738|rs12454712|rs9950483|rs8065435|rs8096471|rs6876|rs17772748|rs10931934|rs7240884|rs2270941|rs1976955|rs2019978|rs11660783|rs13402616|rs9577581|rs12456032|rs2017750
Arthritis	AMINE_COMPOUND_SLC_TRANSPORTERS	REACTOME	1298	26	20	7,26E-01	9,99E-01	rs2950798|rs999448|rs2378089|rs2298720|rs4269967|rs13258461|rs13014176|rs9871974|rs1942574|rs11590799|rs10161479|rs10446439|rs17183671|rs1728803|rs1682821|rs2802112|rs12117457|rs363227|rs1385956|rs11116502
Arthritis	CHROMOSOME_MAINTENANCE	REACTOME	1949	51	20	7,26E-01	9,99E-01	rs1427575|rs742047|rs9972635|rs2229032|rs12928076|rs1051644|rs7745603|rs639373|rs12196939|rs3218801|rs1541549|rs9384022|rs1925089|rs633891|rs6463716|rs3737147|rs653844|rs9540055|rs6499137|rs226981
Arthritis	RIBOFLAVIN_METABOLISM	KEGG	295	18	11	7,27E-01	9,99E-01	rs1024355|rs17632013|rs946904|rs2659063|rs3766510|rs1053903|rs897811|rs12129752|rs4921744|rs1942492|rs884431
Arthritis	TGF_BETA_RECEPTOR_SIGNALING_ACTIVATES_SMADS	REACTOME	563	29	20	7,30E-01	9,99E-01	rs7975139|rs266339|rs2337143|rs4522809|rs1991403|rs4776344|rs4955159|rs920660|rs649676|rs611251|rs967990|rs17514846|rs744910|rs948604|rs2029828|rs11129399|rs6768081|rs9876727|rs12915039|rs9822117
Arthritis	APOPTOTIC_CLEAVAGE_OF_CELLULAR_PROTEINS	REACTOME	1052	40	20	7,30E-01	9,99E-01	rs6772402|rs2275431|rs2757596|rs975167|rs1051644|rs306966|rs389673|rs10484326|rs2439909|rs959573|rs10490823|rs10752073|rs10931934|rs1976955|rs2019978|rs13402616|rs2258689|rs1550955|rs4744826|rs4479095
Arthritis	PYK2_PATHWAY	BIOCARTA	752	48	20	7,30E-01	9,99E-01	rs2726550|rs832574|rs10869979|rs2238493|rs6560613|rs1740005|rs2005219|rs851010|rs9380546|rs12431766|rs9913908|rs198182|rs2322606|rs2322376|rs891398|rs1998762|rs2235366|rs2168043|rs12150089|rs2017750
Arthritis	CCR5_PATHWAY	BIOCARTA	696	53	20	7,34E-01	9,99E-01	rs1023262|rs10869979|rs4899554|rs10204606|rs2238493|rs6560613|rs1740005|rs851010|rs4440020|rs1016269|rs9380546|rs12431766|rs9913908|rs198182|rs2322606|rs891398|rs7149441|rs1998762|rs2235366|rs12150089
Arthritis	DOUBLE_STRAND_BREAK_REPAIR	REACTOME	506	26	17	7,36E-01	9,99E-01	rs4426221|rs639373|rs7567258|rs1075496|rs1541549|rs1872066|rs9514816|rs6463716|rs1799949|rs5030740|rs11571805|rs1207952|rs17466949|rs12453935|rs9262143|rs11614523|rs12540373
Arthritis	HISTIDINE_METABOLISM	KEGG	654	23	11	7,37E-01	9,99E-01	rs10496793|rs1038378|rs7865905|rs4836272|rs7424403|rs3829305|rs7700981|rs3758938|rs1455996|rs2279124|rs1923431
Arthritis	UCALPAIN_PATHWAY	BIOCARTA	426	9	6	7,41E-01	9,99E-01	rs306966|rs3867132|rs11869835|rs2073307|rs1550955|rs4925479
Arthritis	METABOLISM_OF_STEROID_HORMONES_AND_VITAMINS_A_AND_D	REACTOME	745	26	14	7,41E-01	9,99E-01	rs529439|rs7897550|rs4622471|rs201823|rs10904831|rs2420287|rs853988|rs2585422|rs7029101|rs4736319|rs7188923|rs2181874|rs12907723|rs1062033
Arthritis	SIGNALING_BY_ROBO_RECEPTOR	REACTOME	1900	43	20	7,43E-01	9,99E-01	rs17819911|rs1480689|rs400052|rs11718389|rs1975305|rs2675188|rs7630397|rs1465522|rs2423441|rs1441956|rs751503|rs13099223|rs2140645|rs10513359|rs4680967|rs13066384|rs162871|rs2501279|rs2171958|rs10171349
Arthritis	NDKDYNAMIN_PATHWAY	BIOCARTA	548	27	15	7,44E-01	9,99E-01	rs2251238|rs929629|rs11919795|rs10263645|rs1740005|rs10265891|rs2215290|rs12431766|rs10267034|rs17031191|rs10470165|rs13240006|rs4146043|rs10171785|rs543293
Arthritis	LONG_TERM_POTENTIATION	KEGG	3620	30	20	7,45E-01	9,99E-01	rs1509849|rs17162190|rs998235|rs2160732|rs2251238|rs12129768|rs11048665|rs1029471|rs7975139|rs11615994|rs10869979|rs6056526|rs845641|rs7955100|rs4259245|rs172677|rs11130036|rs1509844|rs2207317|rs6039211
Arthritis	TRANSFERRIN_ENDOCYTOSIS_AND_RECYCLING	REACTOME	513	15	13	7,47E-01	9,99E-01	rs4669621|rs8177190|rs1497021|rs16919915|rs6977910|rs721950|rs2004997|rs12742531|rs9990333|rs577866|rs10503675|rs4732330|rs2513873
Arthritis	MAL_PATHWAY	BIOCARTA	225	12	8	7,47E-01	9,99E-01	rs832574|rs2005219|rs2855726|rs2501279|rs2254779|rs7310409|rs2017750|rs4925479
Arthritis	CELL_SURFACE_INTERACTIONS_AT_THE_VASCULAR_WALL	REACTOME	2650	31	20	7,51E-01	9,99E-01	rs10515086|rs4320015|rs10145863|rs1437236|rs1014898|rs661820|rs9308208|rs12459759|rs12644755|rs11734488|rs17237251|rs312027|rs2302975|rs13274462|rs2824327|rs12038990|rs5896|rs17306529|rs422437|rs9804672
Arthritis	NUCLEAR_RECEPTOR_TRANSCRIPTION_PATHWAY	REACTOME	2750	29	20	7,52E-01	9,99E-01	rs7636810|rs4632486|rs2894401|rs1393385|rs218289|rs2200984|rs2683540|rs4742771|rs6837298|rs7674926|rs782918|rs4470447|rs8012921|rs12173906|rs10519068|rs2044756|rs2502805|rs11143958|rs17082499|rs16918155
Arthritis	INTESTINAL_IMMUNE_NETWORK_FOR_IGA_PRODUCTION	KEGG	1392	44	20	7,52E-01	9,99E-01	rs1023262|rs9277334|rs9276711|rs2071469|rs8177585|rs9469357|rs10204606|rs2284191|rs2838527|rs2391645|rs2000059|rs2272300|rs2071554|rs3135353|rs4440020|rs1016269|rs3819716|rs9268832|rs206762|rs453779
Arthritis	ADHERENS_JUNCTIONS_INTERACTIONS	REACTOME	5496	43	20	7,53E-01	9,99E-01	rs1903014|rs2195316|rs7732120|rs12421648|rs7931895|rs4395067|rs7201194|rs6124082|rs6779752|rs233560|rs11217357|rs997101|rs12606601|rs4867023|rs13153654|rs4403186|rs2963711|rs11215438|rs4482907|rs884938
Arthritis	TRANSPORT_TO_THE_GOLGI_AND_SUBSEQUENT_MODIFICATION	REACTOME	1557	40	20	7,53E-01	9,99E-01	rs2613547|rs1147443|rs187462|rs839136|rs2415508|rs6854328|rs1667255|rs7701590|rs6933285|rs9494677|rs10863128|rs11630088|rs11730728|rs7148147|rs12912184|rs12964617|rs10066593|rs4902379|rs6766574|rs983694
Arthritis	VASOPRESSIN_REGULATED_WATER_REABSORPTION	KEGG	1100	31	18	7,53E-01	9,99E-01	rs384067|rs12445650|rs12129768|rs467323|rs1025447|rs352500|rs8065435|rs11606012|rs352494|rs583720|rs7800004|rs2929358|rs889870|rs2299269|rs217513|rs664310|rs1017416|rs1013900
Arthritis	G_PROTEIN_BETA_GAMMA_SIGNALLING	REACTOME	798	35	20	7,53E-01	9,99E-01	rs2724113|rs4979032|rs10498440|rs2207317|rs2494738|rs4807284|rs6039211|rs6118219|rs10485723|rs2223538|rs4471960|rs849376|rs291353|rs2076409|rs7531583|rs12147761|rs11087808|rs6039206|rs714407|rs227134
Arthritis	GLYCINE_SERINE_AND_THREONINE_METABOLISM	KEGG	564	19	13	7,54E-01	9,99E-01	rs234784|rs10867272|rs11209658|rs7870042|rs7627178|rs7717823|rs4529531|rs867376|rs7858423|rs11701664|rs11137861|rs7355583|rs12561944
Arthritis	DOWNREGULATION_OF_TGF_BETA_RECEPTOR_SIGNALING	REACTOME	521	30	20	7,54E-01	9,99E-01	rs7975139|rs266339|rs2337143|rs4522809|rs1991403|rs4776344|rs4955159|rs920660|rs649676|rs611251|rs967990|rs744910|rs948604|rs2029828|rs11129399|rs6768081|rs9876727|rs12915039|rs11129414|rs12906930
Arthritis	LATENT_INFECTION_OF_HOMO_SAPIENS_WITH_MYCOBACTERIUM_TUBERCULOSIS	REACTOME	589	18	14	7,55E-01	9,99E-01	rs4669621|rs4767520|rs9658570|rs1497021|rs16919915|rs6977910|rs721950|rs2004997|rs12742531|rs741997|rs3794624|rs10503675|rs4732330|rs2513873
Arthritis	AGR_PATHWAY	BIOCARTA	1981	51	20	7,56E-01	9,99E-01	rs17101139|rs7079919|rs10457763|rs666199|rs306966|rs11191432|rs2423441|rs7766787|rs9285505|rs9376865|rs7002832|rs1468727|rs2005219|rs2501279|rs12676976|rs2976527|rs6039524|rs7084937|rs2237248|rs12570337
Arthritis	POST_TRANSLATIONAL_MODIFICATION_SYNTHESIS_OF_GPI_ANCHORED_PROTEINS	REACTOME	586	30	16	7,57E-01	9,99E-01	rs12914000|rs6478803|rs11162293|rs763158|rs2426227|rs2306595|rs525624|rs191827|rs1912640|rs1025145|rs281311|rs6104159|rs2038491|rs1993336|rs7170708|rs2286960
Arthritis	PGC1A_PATHWAY	BIOCARTA	959	36	20	7,58E-01	9,99E-01	rs4697435|rs2251238|rs1029471|rs1397829|rs2119945|rs6533710|rs1740005|rs2072439|rs325383|rs1551565|rs12431766|rs12501032|rs11728280|rs16873852|rs13133676|rs6809452|rs17613848|rs2970873|rs887522|rs17031191
Arthritis	INTRINSIC_PATHWAY	BIOCARTA	603	25	15	7,59E-01	9,99E-01	rs630943|rs13419630|rs622486|rs9583467|rs9521623|rs11038977|rs10063791|rs1951626|rs13382950|rs4773200|rs912946|rs8178610|rs7981946|rs250736|rs1373745
Arthritis	PYRUVATE_METABOLISM	REACTOME	209	6	5	7,59E-01	9,99E-01	rs12459759|rs739500|rs2956114|rs3133978|rs2978129
Arthritis	CTLA4_INHIBITORY_SIGNALING	REACTOME	447	21	11	7,59E-01	9,99E-01	rs972984|rs2494738|rs4936661|rs7703433|rs1116003|rs8095469|rs2670289|rs2285346|rs16829957|rs231793|rs10421191
Arthritis	CELL_CELL_JUNCTION_ORGANIZATION	REACTOME	6211	39	20	7,61E-01	9,99E-01	rs1903014|rs2195316|rs17123151|rs7732120|rs12421648|rs7931895|rs4395067|rs7201194|rs6124082|rs6779752|rs233560|rs11217357|rs997101|rs1331683|rs9972635|rs12606601|rs4867023|rs13153654|rs4403186|rs2963711
Arthritis	CHEMOKINE_RECEPTORS_BIND_CHEMOKINES	REACTOME	726	39	15	7,63E-01	9,99E-01	rs1023262|rs10204606|rs7619161|rs10489216|rs7651347|rs886434|rs4440020|rs1016269|rs72301|rs3733236|rs17416271|rs9288627|rs2457996|rs6849878|rs2277618
Arthritis	AMINO_ACID_TRANSPORT_ACROSS_THE_PLASMA_MEMBRANE	REACTOME	1025	30	20	7,64E-01	9,99E-01	rs10145863|rs9308208|rs12644755|rs4269967|rs11734488|rs17306529|rs9792165|rs17632120|rs10161479|rs10446439|rs17183671|rs18042 7|rs1376088|rs12609177|rs10880923|rs4843718|rs3742058|rs11116502|rs2293067|rs3773162
Arthritis	CELL_DEATH_SIGNALLING_VIA_NRAGE_NRIF_AND_NADE	REACTOME	2069	26	20	7,66E-01	9,99E-01	rs1753290|rs11052116|rs11683765|rs748197|rs1379271|rs4573339|rs12026714|rs9380633|rs2834287|rs16970502|rs9861401|rs2068143|rs338513|rs2409415|rs549590|rs7539762|rs16835687|rs17303923|rs1016088|rs7515836
Arthritis	ASPARAGINE_N_LINKED_GLYCOSYLATION	REACTOME	2760	34	20	7,67E-01	9,99E-01	rs1869676|rs17115614|rs2613547|rs6442982|rs1864427|rs1147443|rs187462|rs1865806|rs1836581|rs839136|rs2415508|rs6478803|rs6854328|rs1667255|rs2720611|rs639373|rs2720644|rs2426227|rs7701590|rs6933285
Arthritis	PPARA_ACTIVATES_GENE_EXPRESSION	REACTOME	3675	32	20	7,67E-01	9,99E-01	rs3741496|rs9487444|rs4697435|rs2195003|rs12425122|rs6084337|rs10831104|rs6750100|rs933910|rs3761739|rs2487031|rs168405|rs2304731|rs7757405|rs6926079|rs1397829|rs10947994|rs13342054|rs9850957|rs7728127
Arthritis	NRAGE_SIGNALS_DEATH_THROUGH_JNK	REACTOME	1919	26	20	7,68E-01	9,99E-01	rs1753290|rs11052116|rs11683765|rs1379271|rs4573339|rs12026714|rs9380633|rs2834287|rs16970502|rs9861401|rs338513|rs2409415|rs549590|rs7539762|rs16835687|rs17303923|rs1016088|rs7515836|rs4635166|rs11909747
Arthritis	INTERFERON_ALPHA_BETA_SIGNALING	REACTOME	1477	49	20	7,69E-01	9,99E-01	rs2253907|rs310209|rs7215005|rs1330319|rs2596574|rs10521318|rs7259683|rs11887698|rs8076790|rs12930771|rs4905164|rs17715481|rs731707|rs12720356|rs12129709|rs3819716|rs1105207|rs429511|rs2394185|rs637949
Arthritis	SPHINGOLIPID_METABOLISM	REACTOME	1944	30	20	7,74E-01	9,99E-01	rs1806328|rs10820938|rs11683765|rs2299920|rs12659791|rs964987|rs8008553|rs13391857|rs12133338|rs1369861|rs6893444|rs6533743|rs918957|rs7251590|rs6481273|rs13178105|rs4074469|rs17544336|rs11710876|rs2404716
Arthritis	SYNTHESIS_OF_PC	REACTOME	669	37	20	7,75E-01	9,99E-01	rs4637750|rs7419959|rs3745013|rs608881|rs6065338|rs12692461|rs11590799|rs4669835|rs10197259|rs10495589|rs2802112|rs4925048|rs12117457|rs624177|rs422084|rs915012|rs642926|rs665575|rs6976053|rs1045075
Arthritis	ASCORBATE_AND_ALDARATE_METABOLISM	KEGG	874	20	7	7,77E-01	9,99E-01	rs2306619|rs7865905|rs4836272|rs7700981|rs11568318|rs34187424|rs1923431
Arthritis	AT1R_PATHWAY	BIOCARTA	1224	46	20	7,79E-01	9,99E-01	rs2726550|rs832574|rs10869979|rs306966|rs2119945|rs2238493|rs6560613|rs1740005|rs388488|rs1468727|rs2005219|rs325383|rs12431766|rs9821034|rs9913908|rs198182|rs2322606|rs2322376|rs891398|rs1550955
Arthritis	APOPTOSIS	REACTOME	3298	31	20	7,79E-01	9,99E-01	rs6564984|rs3815844|rs6575121|rs6772402|rs17781459|rs2280162|rs2275431|rs2550461|rs10818964|rs2757596|rs7144673|rs975167|rs1051644|rs306966|rs389673|rs9840663|rs10746803|rs2239921|rs2494738|rs10484326
Arthritis	ENDOCYTOSIS	KEGG	6484	37	20	7,82E-01	9,99E-01	rs4912610|rs6823854|rs17406681|rs2253907|rs223138|rs2510152|rs1476592|rs911713|rs3020829|rs6753831|rs228942|rs7522616|rs1035712|rs6891143|rs12498317|rs1397312|rs1331683|rs9972635|rs17097481|rs4673677
Arthritis	ADIPOCYTOKINE_SIGNALING_PATHWAY	KEGG	2106	23	20	7,84E-01	9,99E-01	rs4697435|rs7314454|rs4725424|rs10849860|rs2245657|rs975167|rs1397829|rs953221|rs525428|rs12609870|rs10974873|rs7986346|rs336209|rs230530|rs2494738|rs2670854|rs3745469|rs2419627|rs2186245|rs6978142
Arthritis	NEUROTROPHIN_SIGNALING_PATHWAY	KEGG	3689	29	20	7,85E-01	9,99E-01	rs10515086|rs17162190|rs1662695|rs998235|rs7021722|rs2083828|rs832574|rs1029471|rs12026714|rs1921082|rs845641|rs3844278|rs10901096|rs785512|rs10147042|rs7986346|rs336209|rs230530|rs17237251|rs2494738
Arthritis	MRNA_SPLICING_MINOR_PATHWAY	REACTOME	383	20	12	7,86E-01	9,99E-01	rs9910260|rs8065368|rs17573579|rs2240769|rs16865262|rs7235319|rs1134231|rs8067833|rs6761|rs8136239|rs1131384|rs2805767
Arthritis	PURINE_METABOLISM	KEGG	5442	26	20	7,87E-01	9,99E-01	rs1509849|rs384067|rs2705462|rs1344925|rs13097687|rs16889129|rs6806748|rs1821942|rs6788821|rs1336472|rs17304935|rs10951303|rs3820539|rs12672267|rs2205361|rs9512817|rs11045087|rs4679627|rs2602930|rs6138531
Arthritis	PYRIMIDINE_METABOLISM	REACTOME	792	26	15	7,88E-01	9,99E-01	rs2280010|rs1424241|rs11753050|rs4241760|rs7717823|rs3903689|rs507170|rs1160638|rs12562047|rs12568335|rs2814472|rs8136239|rs17264901|rs17664900|rs13007683
Arthritis	RECRUITMENT_OF_MITOTIC_CENTROSOME_PROTEINS_AND_COMPLEXES	REACTOME	1177	30	20	7,88E-01	9,99E-01	rs9868985|rs1975305|rs437707|rs523975|rs7325678|rs413666|rs3095329|rs3858659|rs17742788|rs11701361|rs17635854|rs2299603|rs4911507|rs4752932|rs1333924|rs1773356|rs10818415|rs7077462|rs1544285|rs7320567
Arthritis	INITIAL_TRIGGERING_OF_COMPLEMENT	REACTOME	434	23	9	7,89E-01	9,99E-01	rs437179|rs11265265|rs12218966|rs2204758|rs2384167|rs3105782|rs2248678|rs11714564|rs1917186
Arthritis	PROTEASOME_PATHWAY	BIOCARTA	280	21	13	7,90E-01	9,99E-01	rs2280162|rs10818964|rs6142963|rs228271|rs12453759|rs12595506|rs9904424|rs584184|rs8004624|rs4664417|rs2906041|rs2286670|rs1962040
Arthritis	P75_NTR_RECEPTOR_MEDIATED_SIGNALLING	REACTOME	2669	28	20	7,90E-01	9,99E-01	rs907400|rs1753290|rs11052116|rs11683765|rs748197|rs1379271|rs4573339|rs12026714|rs9380633|rs2834287|rs16970502|rs9861401|rs2068143|rs338513|rs2409415|rs4078063|rs1240073|rs549590|rs7539762|rs16835687
Arthritis	SYNTHESIS_SECRETION_AND_INACTIVATION_OF_GLP1	REACTOME	363	20	12	7,90E-01	9,99E-01	rs389673|rs16974510|rs7659414|rs641568|rs3792747|rs1614778|rs10490823|rs10476552|rs11133152|rs12462800|rs10017353|rs269853
Arthritis	TGF_BETA_RECEPTOR_SIGNALING_IN_EMT_EPITHELIAL_TO_MESENCHYMAL_TRANSITI	REACTOME	429	24	14	7,93E-01	9,99E-01	rs1331683|rs9972635|rs4522809|rs4955159|rs11009607|rs16935300|rs4244991|rs4934671|rs2029828|rs11129399|rs6768081|rs9876727|rs11129414|rs876644
Arthritis	CITRATE_CYCLE_TCA_CYCLE	KEGG	1195	37	19	7,94E-01	9,99E-01	rs12342878|rs6856710|rs10120052|rs3934860|rs1366819|rs7863381|rs4634927|rs7647085|rs2871775|rs9534842|rs11630013|rs6427588|rs7024400|rs6092495|rs10013515|rs6591226|rs12485670|rs2994985|rs7857257
Arthritis	PATHOGENIC_ESCHERICHIA_COLI_INFECTION	KEGG	1256	34	20	7,96E-01	9,99E-01	rs10983934|rs3845862|rs10739508|rs389673|rs12555480|rs1554973|rs3095329|rs4129373|rs10759930|rs10735819|rs6478330|rs694428|rs7477292|rs2966431|rs6490620|rs2501279|rs10171349|rs10490823|rs1514762|rs6432018
Arthritis	APOPTOTIC_EXECUTION_PHASE	REACTOME	1211	41	20	7,99E-01	9,99E-01	rs6772402|rs2275431|rs2757596|rs975167|rs1051644|rs306966|rs389673|rs10484326|rs13194781|rs2439909|rs959573|rs10490823|rs10752073|rs10931934|rs9551925|rs1976955|rs2019978|rs199750|rs17074629|rs13402616
Arthritis	GLYCOLYSIS	REACTOME	558	27	14	8,01E-01	9,99E-01	rs2516614|rs3814587|rs4936661|rs764189|rs2054780|rs7703433|rs11599872|rs1116003|rs10508244|rs238228|rs1060287|rs10903963|rs10421191|rs2388268
Arthritis	GLYCOSAMINOGLYCAN_BIOSYNTHESIS_HEPARAN_SULFATE	KEGG	2273	40	20	8,02E-01	9,99E-01	rs2119297|rs2635205|rs16974808|rs7727897|rs7583856|rs11867655|rs17862059|rs482700|rs1289459|rs7674443|rs11990095|rs7840309|rs11860576|rs12503813|rs9351798|rs6448633|rs10791361|rs7210506|rs1454870|rs9995245
Arthritis	HIV_INFECTION	REACTOME	3237	26	20	8,04E-01	9,99E-01	rs6564984|rs2949654|rs3815844|rs6575121|rs11532670|rs849749|rs2280162|rs2550461|rs4426221|rs10818964|rs5751022|rs11919795|rs10906842|rs7567258|rs2239921|rs2284704|rs10204606|rs11244329|rs704859|rs16919915
Arthritis	MITOTIC_G2_G2_M_PHASES	REACTOME	1389	32	20	8,05E-01	9,99E-01	rs9868985|rs9350245|rs1975305|rs437707|rs523975|rs7325678|rs413666|rs2284704|rs3095329|rs3858659|rs17742788|rs11701361|rs17635854|rs2299603|rs4134943|rs4911507|rs4076557|rs1333924|rs1773356|rs10818415
Arthritis	HOMOLOGOUS_RECOMBINATION	KEGG	730	34	20	8,05E-01	9,99E-01	rs927221|rs4902628|rs911263|rs7796764|rs2236184|rs1541549|rs6463716|rs12142240|rs6573851|rs5030740|rs11571805|rs714973|rs1207952|rs17466949|rs11623543|rs12896969|rs761951|rs11614523|rs6955026|rs12540373
Arthritis	CELL_JUNCTION_ORGANIZATION	REACTOME	6781	40	20	8,05E-01	9,99E-01	rs1903014|rs2195316|rs17123151|rs7732120|rs12421648|rs139077|rs7931895|rs4395067|rs7201194|rs6124082|rs6779752|rs233560|rs11217357|rs997101|rs1331683|rs9972635|rs12606601|rs6006693|rs4867023|rs13153654
Arthritis	PTDINS_PATHWAY	BIOCARTA	1058	29	17	8,05E-01	9,99E-01	rs849749|rs6108727|rs6104663|rs1474817|rs17189227|rs11919795|rs3814587|rs2494738|rs935656|rs2929358|rs11125034|rs10508244|rs6438549|rs6074170|rs10903963|rs2235366|rs2388268
Arthritis	INSULIN_RECEPTOR_RECYCLING	REACTOME	492	12	11	8,08E-01	9,99E-01	rs4669621|rs1497021|rs16919915|rs6977910|rs721950|rs2004997|rs12742531|rs10503675|rs7258382|rs4732330|rs2513873
Arthritis	GLUCAGON_TYPE_LIGAND_RECEPTORS	REACTOME	786	47	20	8,10E-01	9,99E-01	rs2724113|rs4979032|rs3793228|rs10498440|rs6462246|rs4807284|rs1869527|rs1812572|rs6748349|rs1558477|rs291353|rs4723037|rs7531583|rs12147761|rs6942576|rs789030|rs2730260|rs6979985|rs9792050|rs311346
Arthritis	SHC1_EVENTS_IN_ERBB4_SIGNALING	REACTOME	1072	32	20	8,10E-01	9,99E-01	rs17406681|rs4673677|rs4622647|rs6725181|rs7002832|rs12038990|rs6706010|rs2005219|rs6722322|rs6876|rs12676976|rs7598440|rs2976527|rs10842514|rs13021324|rs10173674|rs7077462|rs7594456|rs4673633|rs2371706
Arthritis	TNFR2_PATHWAY	BIOCARTA	414	17	10	8,11E-01	9,99E-01	rs832574|rs230530|rs1061624|rs12115174|rs13116385|rs17715219|rs9783665|rs7568498|rs7662536|rs7552664
Arthritis	TRANSCRIPTION	REACTOME	2708	46	20	8,12E-01	9,99E-01	rs1075|rs1056616|rs3809425|rs742047|rs4788605|rs9512817|rs234784|rs7745603|rs639373|rs958957|rs2173115|rs217689|rs12196939|rs1535565|rs1943344|rs2284704|rs11149485|rs12541335|rs1497021|rs1923352
Arthritis	PROLONGED_ERK_ACTIVATION_EVENTS	REACTOME	429	16	13	8,13E-01	9,99E-01	rs12026714|rs10901096|rs12038990|rs2005219|rs6876|rs12090216|rs3012739|rs10842514|rs7077462|rs10878989|rs4661229|rs17813377|rs2235366
Arthritis	G1_S_SPECIFIC_TRANSCRIPTION	REACTOME	194	13	10	8,14E-01	9,99E-01	rs510862|rs7756899|rs9371645|rs6107581|rs7077462|rs565211|rs9577581|rs2073758|rs13056098|rs629310
Arthritis	SIGNALLING_TO_ERKS	REACTOME	794	33	20	8,15E-01	9,99E-01	rs626713|rs12026714|rs10901096|rs2073923|rs12038990|rs2005219|rs851010|rs6876|rs12090216|rs1878679|rs2237093|rs3012739|rs10842514|rs7077462|rs10878989|rs4661229|rs17813377|rs612939|rs2235366|rs944485
Arthritis	INCRETIN_SYNTHESIS_SECRETION_AND_INACTIVATION	REACTOME	540	29	16	8,16E-01	9,99E-01	rs389673|rs11250154|rs16974510|rs7659414|rs12458|rs641568|rs3792747|rs1614778|rs10490823|rs1842242|rs10476552|rs11133152|rs12462800|rs10017353|rs7442627|rs269853
Arthritis	OTHER_GLYCAN_DEGRADATION	KEGG	648	23	15	8,18E-01	9,99E-01	rs11683765|rs228614|rs17831521|rs2250361|rs13386477|rs2301796|rs2715518|rs2271100|rs7440877|rs6833962|rs4438612|rs6552396|rs7655585|rs12635761|rs4861781
Arthritis	FOCAL_ADHESION	KEGG	7733	32	20	8,19E-01	9,99E-01	rs10515086|rs1360290|rs2021832|rs1958918|rs10273151|rs2107128|rs139077|rs4841920|rs4766522|rs4341231|rs1476592|rs11815834|rs7975139|rs12498317|rs9277912|rs1271449|rs3784166|rs6006693|rs869936|rs9346596
Arthritis	NUCLEOTIDE_EXCISION_REPAIR	KEGG	914	42	20	8,21E-01	9,99E-01	rs1323806|rs5751022|rs2284704|rs2097289|rs3218801|rs1541549|rs1925089|rs6463716|rs2808673|rs9540055|rs5030740|rs9554903|rs616953|rs10492771|rs6107581|rs17466949|rs4253126|rs9539838|rs16902819|rs9539836

Arthritis	MET_PATHWAY	BIOCARTA	1415	28	20	8,21E-01	9,99E-01	rs10515086|rs11815834|rs4899554|rs306966|rs10901096|rs10765093|rs17237251|rs2302975|rs422437|rs2005219|rs12652661|rs11017973|rs3012739|rs849376|rs7474861|rs873185|rs2322606|rs891398|rs7721613|rs4293044
Arthritis	NCAM_SIGNALING_FOR_NEURITE_OUT_GROWTH	REACTOME	2480	27	20	8,21E-01	9,99E-01	rs4841920|rs4341231|rs2631926|rs2072277|rs1054645|rs4671952|rs999012|rs393569|rs11993990|rs306966|rs630943|rs13419630|rs10147042|rs622486|rs660361|rs12022389|rs2288696|rs1277748|rs9583467|rs11630088
Arthritis	GLOBAL_GENOMIC_NER_GG_NER	REACTOME	823	45	19	8,23E-01	9,99E-01	rs1323806|rs2284704|rs2097289|rs3218801|rs1541549|rs1925089|rs6463716|rs2808673|rs9540055|rs5030740|rs9554903|rs616953|rs10492771|rs6107581|rs17466949|rs9539838|rs16902819|rs9539836|rs12540373
Arthritis	GLIOMA	KEGG	2070	32	20	8,23E-01	9,99E-01	rs10515086|rs1029471|rs9350245|rs7562947|rs2075995|rs785512|rs9672254|rs2229765|rs17237251|rs2494738|rs10255859|rs2238493|rs2302975|rs6533710|rs1740005|rs1007662|rs12038990|rs7169544|rs422437|rs1468727
Arthritis	HYPERTROPHIC_CARDIOMYOPATHY_HCM	KEGG	4149	31	20	8,25E-01	9,99E-01	rs16835926|rs10273151|rs2107128|rs4509829|rs1868513|rs4725424|rs6785307|rs9878916|rs9311552|rs2238517|rs12058655|rs1464459|rs2805434|rs9510351|rs953221|rs1405331|rs6700222|rs879904|rs1851048|rs4787985
Arthritis	RIP_MEDIATED_NFKB_ACTIVATION_VIA_DAI	REACTOME	328	26	11	8,31E-01	9,99E-01	rs3134942|rs7691384|rs34129552|rs469632|rs12115174|rs9551925|rs3014866|rs11938703|rs17074629|rs4608848|rs3014885
Arthritis	WNT_SIGNALING_PATHWAY	KEGG	5611	25	20	8,33E-01	9,99E-01	rs4733828|rs2894401|rs759648|rs1076465|rs9792218|rs1999090|rs2251238|rs937643|rs12129768|rs7695009|rs972984|rs1499368|rs2719355|rs1029471|rs3121310|rs6056526|rs266339|rs11614438|rs765543|rs1561662
Arthritis	AMI_PATHWAY	BIOCARTA	574	36	19	8,34E-01	9,99E-01	rs630943|rs13419630|rs622486|rs9583467|rs9521623|rs5896|rs10063791|rs4889673|rs1951626|rs13382950|rs2465836|rs4773200|rs912946|rs1782632|rs8178610|rs7981946|rs10179730|rs250736|rs1373745
Arthritis	REGULATION_OF_GENE_EXPRESSION_IN_BETA_CELLS	REACTOME	716	23	13	8,35E-01	9,99E-01	rs2755209|rs2494738|rs1337906|rs1614778|rs10841798|rs758985|rs2977330|rs4435111|rs1782804|rs2951787|rs736824|rs2254779|rs7310409
Arthritis	TIGHT_JUNCTION	KEGG	6242	44	20	8,36E-01	9,99E-01	rs1523843|rs17123151|rs10245351|rs17372614|rs4766522|rs1541947|rs671802|rs13231956|rs777709|rs2275431|rs661820|rs1331683|rs9972635|rs3845862|rs1563371|rs7605223|rs4503419|rs975167|rs7792010|rs1901335
Arthritis	REGULATION_OF_AUTOPHAGY	KEGG	933	67	16	8,43E-01	9,99E-01	rs12134779|rs9832407|rs1330319|rs7234952|rs965976|rs2004837|rs987380|rs637949|rs4146444|rs9956023|rs9961683|rs12188129|rs1865288|rs12957142|rs4946730|rs3862833
Arthritis	41BB_PATHWAY	BIOCARTA	341	22	12	8,44E-01	9,99E-01	rs832574|rs230530|rs851010|rs12115174|rs9380546|rs13116385|rs11575812|rs2243248|rs3783408|rs7662536|rs1998762|rs2017750
Arthritis	METABOLISM_OF_NUCLEOTIDES	REACTOME	1559	26	20	8,44E-01	9,99E-01	rs2280010|rs2602930|rs510862|rs1424241|rs17380592|rs11753050|rs4241760|rs3006009|rs10936022|rs2313602|rs12294809|rs12038990|rs1840246|rs4744663|rs6584|rs12144794|rs1049927|rs7717823|rs7932321|rs3903689
Arthritis	GPVI_MEDIATED_ACTIVATION_CASCADE	REACTOME	1181	30	20	8,48E-01	9,99E-01	rs10515086|rs785512|rs17237251|rs2494738|rs342056|rs2302975|rs422437|rs12652661|rs2501873|rs2501279|rs11150675|rs1136224|rs1887630|rs849376|rs290255|rs12043814|rs2289083|rs4889426|rs2660489|rs693293
Arthritis	PORPHYRIN_AND_CHLOROPHYLL_METABOLISM	KEGG	1163	37	18	8,51E-01	9,99E-01	rs4963442|rs639373|rs9872119|rs10519667|rs2194082|rs16861650|rs4963446|rs10521264|rs4233366|rs6797163|rs1181574|rs4919393|rs10037268|rs11568318|rs746590|rs16936474|rs34187424|rs10070906
Arthritis	CHEMICAL_PATHWAY	BIOCARTA	585	16	14	8,52E-01	9,99E-01	rs306966|rs2494738|rs11887698|rs2238493|rs12454712|rs8096471|rs2270941|rs1976955|rs9913908|rs198182|rs2019978|rs1550955|rs1805410|rs12150089
Arthritis	STEROID_HORMONE_BIOSYNTHESIS	KEGG	1491	33	12	8,53E-01	9,99E-01	rs529439|rs737866|rs11253048|rs11124634|rs2854028|rs2465079|rs7029101|rs4736319|rs11568318|rs34187424|rs6467729|rs1062033
Arthritis	DRUG_METABOLISM_OTHER_ENZYMES	KEGG	1609	45	20	8,55E-01	9,99E-01	rs6586711|rs2280010|rs6586734|rs305985|rs13254048|rs10936022|rs11203956|rs7343168|rs507170|rs1160638|rs12562047|rs12568335|rs1495750|rs2814472|rs4609955|rs185925|rs8136239|rs11568318|rs34187424|rs11879413
Arthritis	GRB2_EVENTS_IN_ERBB2_SIGNALING	REACTOME	1202	31	20	8,57E-01	9,99E-01	rs17406681|rs4673677|rs4622647|rs6725181|rs7002832|rs12038990|rs1468727|rs6706010|rs2005219|rs6722322|rs6876|rs12676976|rs7598440|rs2976527|rs10842514|rs13021324|rs10173674|rs7077462|rs7594456|rs4673633
Arthritis	SIGNALING_BY_BMP	REACTOME	918	36	20	8,58E-01	9,99E-01	rs4659995|rs3820716|rs2337143|rs1373651|rs1991403|rs235772|rs2149639|rs2522528|rs920660|rs2173049|rs150895|rs12128976|rs1147720|rs4776846|rs1392392|rs1020522|rs8122397|rs9631455|rs11867294|rs6085775
Arthritis	STEROID_BIOSYNTHESIS	KEGG	247	9	7	8,59E-01	9,99E-01	rs6592055|rs2274941|rs2254636|rs17488814|rs676584|rs7974876|rs2493117
Arthritis	AXON_GUIDANCE	REACTOME	13131	22	20	8,59E-01	9,99E-01	rs3924457|rs4238475|rs881758|rs4791330|rs4399429|rs6497081|rs1295217|rs17819911|rs17162190|rs6089908|rs2160327|rs1480689|rs400052|rs4841920|rs998235|rs4341231|rs12914000|rs1476592|rs12653501|rs11815834
Arthritis	TYPE_II_DIABETES_MELLITUS	KEGG	2053	31	20	8,60E-01	9,99E-01	rs10515086|rs6674511|rs6693236|rs2609485|rs785512|rs7986346|rs336209|rs17237251|rs765128|rs2302975|rs4499352|rs422437|rs2005219|rs10498208|rs935656|rs12652661|rs11130377|rs199955|rs758985|rs16035
Arthritis	NEGATIVE_REGULATION_OF_FGFR_SIGNALING	REACTOME	1536	31	20	8,61E-01	9,99E-01	rs9318729|rs2510152|rs7674434|rs8002487|rs9591501|rs2288696|rs6490668|rs10503587|rs249923|rs7703433|rs263931|rs2005219|rs9545330|rs7105655|rs1116003|rs17320222|rs9565708|rs9510021|rs6490721|rs9578453
Arthritis	LOSS_OF_NLP_FROM_MITOTIC_CENTROSOMES	REACTOME	1100	32	20	8,62E-01	9,99E-01	rs9868985|rs1975305|rs437707|rs523975|rs413666|rs3095329|rs3858659|rs11701361|rs17635854|rs2299603|rs4911507|rs4752932|rs1333924|rs1773356|rs10818415|rs7077462|rs4917968|rs1333904|rs10421191|rs6538791
Arthritis	G0_AND_EARLY_G1	REACTOME	258	9	8	8,62E-01	9,99E-01	rs4817863|rs231216|rs6107581|rs17193211|rs16835131|rs7077462|rs2871921|rs9577581
Arthritis	HOMOLOGOUS_RECOMBINATION_REPAIR_OF_REPLICATION_INDEPENDENT_DOUBLE_ST	REACTOME	376	20	13	8,65E-01	9,99E-01	rs639373|rs1075496|rs1541549|rs6463716|rs1799949|rs5030740|rs11571805|rs1207952|rs17466949|rs12453935|rs9262143|rs11614523|rs12540373
Arthritis	SIGNALING_BY_FGFR_MUTANTS	REACTOME	1657	25	20	8,65E-01	9,99E-01	rs10515086|rs2313626|rs1734729|rs17237251|rs2288696|rs6490668|rs11887698|rs10503587|rs2302975|rs12038990|rs249923|rs422437|rs263931|rs7105655|rs12652661|rs7315446|rs407366|rs17320222|rs12168342|rs2312968
Arthritis	CITRIC_ACID_CYCLE_TCA_CYCLE	REACTOME	675	15	10	8,66E-01	9,99E-01	rs3934860|rs1366819|rs4634927|rs7647085|rs2871775|rs9534842|rs11630013|rs6427588|rs12485670|rs2994985
Arthritis	RETINOL_METABOLISM	KEGG	1512	37	20	8,67E-01	9,99E-01	rs8187890|rs7081491|rs305985|rs2287611|rs201823|rs7343168|rs9749171|rs2038118|rs11188059|rs17726963|rs4144915|rs6583859|rs12435671|rs11812285|rs4609955|rs7188923|rs11568318|rs4852846|rs11626721|rs9307238
Arthritis	E2F_MEDIATED_REGULATION_OF_DNA_REPLICATION	REACTOME	452	30	18	8,67E-01	9,99E-01	rs3820716|rs510862|rs7756899|rs4936661|rs9371645|rs7703433|rs9450763|rs1116003|rs6107581|rs7077462|rs565211|rs9577581|rs2073758|rs236164|rs13056098|rs10421191|rs13213285|rs629310
Arthritis	TOLL_RECEPTOR_CASCADES	REACTOME	3778	34	20	8,69E-01	9,99E-01	rs2726550|rs17162190|rs998235|rs1999090|rs832574|rs12987393|rs10983934|rs3134942|rs739141|rs10739508|rs4899554|rs1921082|rs10485408|rs845641|rs12555480|rs1532122|rs3844279|rs10455526|rs10147042|rs6908267
Arthritis	BCR_PATHWAY	BIOCARTA	1053	32	20	8,71E-01	9,99E-01	rs11188666|rs2251238|rs832574|rs4899554|rs2238493|rs1740005|rs177820|rs2005219|rs851010|rs2237093|rs7233024|rs12431766|rs290255|rs9913908|rs198182|rs2660489|rs12608960|rs4799062|rs17031191|rs1078633
Arthritis	ATM_PATHWAY	BIOCARTA	397	13	8	8,71E-01	9,99E-01	rs12069919|rs230530|rs1799949|rs13116385|rs299247|rs7662536|rs1998762|rs2017750
Arthritis	L1CAM_INTERACTIONS	REACTOME	3914	23	20	8,72E-01	9,99E-01	rs1295217|rs17162190|rs6089908|rs2160327|rs998235|rs4671952|rs11814752|rs961548|rs13076532|rs10827245|rs393569|rs6782375|rs845641|rs1666559|rs6580153|rs13426300|rs280372|rs879904|rs11919795|rs10147042
Arthritis	TYROSINE_METABOLISM	KEGG	1169	52	16	8,74E-01	9,99E-01	rs863944|rs3743422|rs737866|rs1514684|rs1948080|rs11649592|rs2348023|rs1446271|rs9608216|rs9307238|rs1942492|rs4285563|rs1416572|rs2279124|rs10171105|rs12348188
Arthritis	FORMATION_OF_TRANSCRIPTION_COUPLED_NER_TC_NER_REPAIR_COMPLEX	REACTOME	389	23	8	8,75E-01	9,99E-01	rs2284704|rs2097289|rs9554903|rs10492771|rs4253126|rs6761|rs16902819|rs1131384
Arthritis	INTEGRIN_CELL_SURFACE_INTERACTIONS	REACTOME	2614	26	20	8,77E-01	9,99E-01	rs1360290|rs10273151|rs2107128|rs661820|rs1271449|rs3809805|rs306966|rs630943|rs12459759|rs879904|rs13419630|rs622486|rs10982536|rs2494738|rs9583467|rs3783621|rs9521623|rs1051442|rs9804672|rs2270151
Arthritis	GLYCOLYSIS_GLUCONEOGENESIS	KEGG	1391	36	20	8,78E-01	9,99E-01	rs4813547|rs6856710|rs2152078|rs1038378|rs525428|rs3814587|rs7865905|rs10993282|rs2670854|rs4836272|rs12475700|rs758985|rs7700981|rs943|rs3829185|rs7349412|rs10508244|rs6092495|rs10013515|rs238228
Arthritis	HEDGEHOG_SIGNALING_PATHWAY	KEGG	2151	28	20	8,79E-01	9,99E-01	rs13037653|rs186659|rs12129768|rs1276408|rs3121310|rs13038229|rs2371933|rs3779173|rs11962800|rs2473242|rs7251590|rs235772|rs351359|rs11191355|rs9475399|rs4848608|rs2149639|rs3823038|rs3801210|rs4943461
Arthritis	MICRORNA_MIRNA_BIOGENESIS	REACTOME	227	12	9	8,84E-01	9,99E-01	rs11624081|rs1105567|rs1096699|rs11620631|rs6761|rs1131384|rs10149095|rs10140275|rs8010588
Arthritis	SEMA4D_INDUCED_CELL_MIGRATION_AND_GROWTH_CONE_COLLAPSE	REACTOME	433	25	15	8,85E-01	9,99E-01	rs216166|rs342056|rs788330|rs7515836|rs4938800|rs2855726|rs2501279|rs2290822|rs2289083|rs9410321|rs3817003|rs4252627|rs10908892|rs11217861|rs7811
Arthritis	PROTEIN_EXPORT	KEGG	750	43	19	8,87E-01	9,99E-01	rs2715609|rs16974510|rs12260965|rs7659414|rs6584|rs1880666|rs13224529|rs6978512|rs1525500|rs12591842|rs12267330|rs13247393|rs2416771|rs11133152|rs7806611|rs10017353|rs438824|rs6572731|rs2280673
Arthritis	PI_3K_CASCADE	REACTOME	1868	32	20	8,87E-01	9,99E-01	rs10515086|rs2755209|rs6084337|rs7674434|rs17237251|rs9591501|rs2494738|rs2288696|rs6490668|rs10503587|rs2302975|rs249923|rs422437|rs263931|rs7105655|rs12652661|rs17320222|rs4435111|rs1782804|rs9510021
Arthritis	PROCESSING_OF_CAPPED_INTRON_CONTAINING_PRE_MRNA	REACTOME	1547	29	20	8,87E-01	9,99E-01	rs6044228|rs3809425|rs9908594|rs4788605|rs234784|rs1533303|rs8065368|rs7585767|rs1943344|rs11244329|rs2074733|rs17573579|rs2240769|rs485152|rs6111235|rs16865262|rs6532208|rs4894239|rs2402111|rs6768994
Arthritis	G1_PATHWAY	BIOCARTA	611	25	18	8,87E-01	9,99E-01	rs2229032|rs266339|rs10255859|rs11625323|rs4776344|rs2383208|rs13160147|rs592412|rs744910|rs33671|rs7077462|rs604045|rs9577581|rs6438549|rs11605729|rs17318844|rs12915039|rs12906930
Arthritis	CYTOCHROME_P450_ARRANGED_BY_SUBSTRATE_TYPE	REACTOME	1163	48	20	8,89E-01	9,99E-01	rs338593|rs305968|rs7081491|rs2228468|rs6012696|rs11188059|rs12459140|rs11124634|rs17726963|rs4144915|rs6583859|rs11812285|rs2585422|rs4736319|rs8105815|rs4852846|rs2181874|rs1062033|rs11879413|rs4918430
Arthritis	MTA3_PATHWAY	BIOCARTA	333	16	8	8,93E-01	9,99E-01	rs10735819|rs12056536|rs6490620|rs10194712|rs7328603|rs930420|rs2908201|rs1709183
Arthritis	NATURAL_KILLER_CELL_MEDIATED_CYTOTOXICITY	KEGG	3607	51	20	8,93E-01	9,99E-01	rs10515086|rs2844521|rs1145965|rs2949654|rs2251238|rs2270238|rs7144673|rs11880466|rs785512|rs1330320|rs17237251|rs2238493|rs2302975|rs704859|rs177820|rs1007662|rs12038990|rs422437|rs2848713|rs2523454
Arthritis	SEMA4D_IN_SEMAPHORIN_SIGNALING	REACTOME	597	28	17	8,93E-01	9,99E-01	rs216166|rs342056|rs7259683|rs788330|rs7515836|rs4938800|rs2855726|rs2501279|rs17289513|rs2290822|rs2289083|rs9410321|rs3817003|rs4252627|rs10908892|rs11217861|rs7811
Arthritis	POST_TRANSLATIONAL_PROTEIN_MODIFICATION	REACTOME	6008	24	20	8,93E-01	9,99E-01	rs13282872|rs1942237|rs7294|rs7680255|rs12914000|rs2653831|rs2547067|rs1869676|rs17115614|rs1566250|rs6740075|rs2613547|rs6442982|rs7524886|rs1864427|rs1147443|rs187462|rs1865806|rs1836581|rs839136
Arthritis	METABOLISM_OF_VITAMINS_AND_COFACTORS	REACTOME	1119	25	20	8,93E-01	9,99E-01	rs137126|rs719917|rs2267452|rs3737964|rs2426227|rs16960059|rs222682|rs17754686|rs6052147|rs7789066|rs12146097|rs2106303|rs7781839|rs4683428|rs7531583|rs897811|rs17153112|rs228578|rs9299496|rs12025180
Arthritis	ACTIVATION_OF_GENES_BY_ATF4	REACTOME	316	13	6	8,94E-01	9,99E-01	rs6926079|rs10486011|rs4382556|rs10918342|rs7403900|rs10456499
Arthritis	SIGNALING_BY_HIPPO	REACTOME	627	22	13	8,94E-01	9,99E-01	rs2275431|rs16897076|rs12545656|rs6876|rs1698976|rs1976955|rs17551539|rs2019978|rs6783790|rs6897282|rs9682157|rs4744826|rs6796705
Arthritis	EICOSANOID_LIGAND_BINDING_RECEPTORS	REACTOME	507	12	9	8,95E-01	9,99E-01	rs350031|rs2287699|rs4806942|rs6893430|rs11804627|rs6683555|rs930420|rs8005977|rs6893565
Arthritis	CYTOSOLIC_TRNA_AMINOACYLATION	REACTOME	312	9	6	8,96E-01	9,99E-01	rs3101017|rs10999211|rs10458221|rs4758462|rs895639|rs925210
Arthritis	DNA_REPLICATION	KEGG	740	20	14	8,98E-01	9,99E-01	rs4804208|rs3218801|rs1541549|rs1925089|rs6463716|rs9540055|rs5030740|rs616953|rs6107581|rs17466949|rs9539838|rs2307325|rs9539836|rs12540373
Arthritis	RELA_PATHWAY	BIOCARTA	208	15	10	9,01E-01	9,99E-01	rs230530|rs1061624|rs12659397|rs12115174|rs13116385|rs8046065|rs129988|rs7662536|rs5758211|rs7552664
Arthritis	INNATE_IMMUNE_SYSTEM	REACTOME	6278	31	20	9,01E-01	9,99E-01	rs1179766|rs2726550|rs17162190|rs9938543|rs998235|rs1999090|rs832574|rs12987393|rs17178663|rs437179|rs9644758|rs1815811|rs10983934|rs4784754|rs666723|rs1049618|rs739141|rs10739508|rs4899554|rs1921082
Arthritis	PERK_REGULATED_GENE_EXPRESSION	REACTOME	334	13	6	9,03E-01	9,99E-01	rs6926079|rs10486011|rs4382556|rs10918342|rs7403900|rs10456499
Arthritis	TRAFFICKING_OF_AMPA_RECEPTORS	REACTOME	1132	26	20	9,03E-01	9,99E-01	rs1029471|rs2238517|rs11919795|rs4787985|rs7644219|rs1024217|rs2238493|rs6533710|rs2072439|rs9343330|rs3803815|rs11860503|rs1461234|rs9913908|rs11074618|rs198182|rs8103771|rs13133676|rs3773162|rs4821503
Arthritis	DEATH_PATHWAY	BIOCARTA	505	14	9	9,04E-01	9,99E-01	rs230530|rs12454712|rs8096471|rs10931934|rs1976955|rs13116385|rs2019978|rs13402616|rs7662536
Arthritis	NUCLEOTIDE_EXCISION_REPAIR	REACTOME	956	41	20	9,04E-01	9,99E-01	rs1323806|rs2284704|rs2097289|rs3218801|rs1541549|rs1925089|rs6463716|rs2808673|rs9540055|rs5030740|rs9554903|rs616953|rs10492771|rs6107581|rs17466949|rs4253126|rs9539838|rs6761|rs16902819|rs1131384
Arthritis	PYRIMIDINE_METABOLISM	KEGG	1900	32	20	9,06E-01	9,99E-01	rs9512817|rs2280010|rs6138531|rs306196|rs737866|rs2076559|rs1424241|rs1535565|rs11753050|rs4241760|rs11149485|rs3218801|rs2274821|rs12541335|rs4582902|rs9261290|rs3903689|rs507170|rs1160638|rs2215290
Arthritis	EPO_PATHWAY	BIOCARTA	255	11	10	9,07E-01	9,99E-01	rs4899554|rs10974873|rs2005219|rs2274471|rs7149441|rs1998762|rs2235366|rs506597|rs2168043|rs2017750
Arthritis	GROWTH_HORMONE_RECEPTOR_SIGNALING	REACTOME	773	32	19	9,15E-01	9,99E-01	rs10974873|rs7986346|rs1207774|rs7744633|rs336209|rs11887698|rs2005219|rs10498208|rs13204006|rs8000641|rs11739840|rs1935005|rs1560251|rs6492218|rs196034|rs2274471|rs6091153|rs2092058|rs11891922
Arthritis	ACTIVATION_OF_ATR_IN_RESPONSE_TO_REPLICATION_STRESS	REACTOME	653	27	20	9,15E-01	9,99E-01	rs2229032|rs3820716|rs1541549|rs1925089|rs6463716|rs9540055|rs2290822|rs9450763|rs10906320|rs5030740|rs616953|rs17466949|rs9539838|rs2307325|rs11258248|rs505892|rs2073758|rs9539836|rs236164|rs13056098
Arthritis	TRAF6_MEDIATED_IRF7_ACTIVATION	REACTOME	433	51	12	9,15E-01	9,99E-01	rs1330320|rs7259683|rs17715481|rs6052147|rs1105207|rs637949|rs793899|rs10863389|rs8046065|rs129988|rs7568498|rs5758211
Arthritis	INOSITOL_PHOSPHATE_METABOLISM	KEGG	2355	37	20	9,17E-01	9,99E-01	rs10505811|rs665197|rs1476592|rs1130635|rs6056526|rs3775616|rs2239921|rs7234952|rs2207317|rs6039211|rs969489|rs13044386|rs6118219|rs10485723|rs228271|rs965976|rs12357384|rs2223538|rs987380|rs2212042
Arthritis	GLYCOSAMINOGLYCAN_DEGRADATION	KEGG	709	22	14	9,17E-01	9,99E-01	rs11867655|rs510862|rs6893444|rs13178105|rs7210506|rs10748760|rs7226075|rs2322973|rs565211|rs4438612|rs8080564|rs17565276|rs12635761|rs1954294
Arthritis	VIRAL_MYOCARDITIS	KEGG	2750	33	20	9,19E-01	9,99E-01	rs4320015|rs2253907|rs9277334|rs9510351|rs3948793|rs2071469|rs2074876|rs2596574|rs216166|rs9469357|rs4792010|rs788330|rs12882128|rs2284191|rs2824327|rs4704876|rs6556055|rs12626798|rs2643075|rs17665136
Arthritis	BASAL_CELL_CARCINOMA	KEGG	2166	27	20	9,20E-01	9,99E-01	rs1076465|rs937643|rs3121310|rs11614438|rs765543|rs2371933|rs11884056|rs389673|rs3779173|rs11919795|rs2473242|rs235772|rs351359|rs937642|rs11191355|rs6719501|rs4848608|rs315644|rs2149639|rs12503104
Arthritis	EXTENSION_OF_TELOMERES	REACTOME	618	17	12	9,20E-01	9,99E-01	rs3218801|rs1541549|rs1925089|rs6463716|rs9540055|rs5030740|rs616953|rs6107581|rs17466949|rs9539838|rs9539836|rs12540373
Arthritis	TRANSPORT_OF_INORGANIC_CATIONS_ANIONS_AND_AMINO_ACIDS_OLIGOPEPTIDES	REACTOME	4051	35	20	9,21E-01	9,99E-01	rs1289392|rs10145863|rs6706564|rs9873087|rs1539683|rs3782999|rs12000690|rs2712623|rs10434201|rs1288338|rs1468788|rs3766781|rs12644755|rs4269967|rs4904903|rs10498067|rs11734488|rs6987649|rs12636395|rs12475881
Arthritis	INHIBITION_OF_VOLTAGE_GATED_CA2_CHANNELS_VIA_GBETA_GAMMA_SUBUNITS	REACTOME	1082	33	20	9,24E-01	9,99E-01	rs2835909|rs2724113|rs4979032|rs10498440|rs2836034|rs4807284|rs9907685|rs2226741|rs7212176|rs6590359|rs291353|rs7531583|rs12147761|rs1005316|rs3787835|rs2835845|rs1859962|rs2121089|rs4937381|rs2965208
Arthritis	SEMAPHORIN_INTERACTIONS	REACTOME	2884	32	20	9,25E-01	9,99E-01	rs881758|rs12914000|rs1476592|rs7781126|rs637746|rs10827245|rs2185383|rs4702617|rs7302972|rs17259861|rs11880466|rs153745|rs216166|rs2478816|rs1494825|rs342056|rs7259683|rs788330|rs6778210|rs12739500
Arthritis	G2_M_CHECKPOINTS	REACTOME	710	27	20	9,25E-01	9,99E-01	rs2229032|rs3820716|rs1541549|rs1925089|rs6463716|rs9540055|rs2290822|rs9450763|rs10906320|rs5030740|rs616953|rs7077462|rs17466949|rs9539838|rs2307325|rs11258248|rs505892|rs2073758|rs9539836|rs236164
Arthritis	SHC_RELATED_EVENTS	REACTOME	302	8	8	9,25E-01	9,99E-01	rs12038990|rs2005219|rs6876|rs10842514|rs7077462|rs7258382|rs944485|rs2168043
Arthritis	PROSTATE_CANCER	KEGG	2870	35	20	9,27E-01	9,99E-01	rs10515086|rs2755209|rs9350245|rs7676376|rs7562947|rs11884056|rs2075995|rs389673|rs785512|rs9672254|rs2229765|rs230530|rs17237251|rs2494738|rs2288696|rs352500|rs2302975|rs6719501|rs12454712|rs12038990
Arthritis	BRANCHED_CHAIN_AMINO_ACID_CATABOLISM	REACTOME	507	23	12	9,28E-01	9,99E-01	rs9352866|rs10447552|rs11762112|rs11752339|rs10991781|rs11166413|rs2047554|rs284662|rs17369983|rs17454070|rs10842402|rs7967945
Arthritis	TRANSPORT_OF_GLUCOSE_AND_OTHER_SUGARS_BILE_SALTS_AND_ORGANIC_ACIDS_M NE_COMPOUNDS	REACTOME	3234	27	20	9,28E-01	9,99E-01	rs1849067|rs2950798|rs999448|rs151377|rs2378089|rs2298720|rs13175238|rs684690|rs4758562|rs4269967|rs218676|rs13258461|rs11173480|rs13014176|rs9871974|rs16861650|rs1942574|rs7485002|rs11590799|rs218669
Arthritis	INFLAM_PATHWAY	BIOCARTA	747	29	16	9,32E-01	9,99E-01	rs4493656|rs2421059|rs11625323|rs2048874|rs3135353|rs755004|rs9268832|rs2858870|rs11741918|rs604045|rs3021094|rs11575812|rs333966|rs2243248|rs3024505|rs12075255
Arthritis	ACTIVATION_OF_THE_PRE_REPLICATIVE_COMPLEX	REACTOME	565	29	18	9,32E-01	9,99E-01	rs3820716|rs510862|rs3218801|rs1541549|rs6463716|rs9450763|rs10906320|rs5030740|rs17466949|rs565211|rs2307325|rs11258248|rs505892|rs2073758|rs236164|rs13056098|rs13213285|rs12540373
Arthritis	REGULATION_OF_HYPOXIA_INDUCIBLE_FACTOR_HIF_BY_OXYGEN	REACTOME	443	19	13	9,33E-01	9,99E-01	rs5751022|rs3844279|rs4953347|rs2881321|rs150895|rs797351|rs4714696|rs1000531|rs8046065|rs129988|rs1809461|rs506597|rs17102132
Arthritis	FC_GAMMA_R_MEDIATED_PHAGOCYTOSIS	KEGG	3795	28	20	9,34E-01	9,99E-01	rs10515086|rs10752974|rs1476592|rs911713|rs7522616|rs10912025|rs929629|rs1339082|rs1180398|rs230632|rs360402|rs785512|rs800719|rs12133338|rs17237251|rs2494738|rs2238493|rs10263645|rs4128634|rs7496336
Arthritis	ARRHYTHMOGENIC_RIGHT_VENTRICULAR_CARDIOMYOPATHY_ARVC	KEGG	5871	24	20	9,35E-01	9,99E-01	rs16835926|rs10273151|rs2107128|rs1541947|rs671802|rs1868513|rs6785307|rs9878916|rs9311552|rs2238517|rs12058655|rs1464459|rs12606601|rs7605223|rs4402182|rs2757596|rs2805434|rs11884056|rs1901335|rs389673
Arthritis	LAGGING_STRAND_SYNTHESIS	REACTOME	550	14	11	9,38E-01	9,99E-01	rs1541549|rs1925089|rs6463716|rs9540055|rs5030740|rs616953|rs6107581|rs17466949|rs9539838|rs9539836|rs12540373
Arthritis	PYRUVATE_METABOLISM_AND_CITRIC_ACID_TCA_CYCLE	REACTOME	905	21	15	9,39E-01	9,99E-01	rs12459759|rs3934860|rs1366819|rs4634927|rs7647085|rs2871775|rs9534842|rs739500|rs11630013|rs6427588|rs2956114|rs3133978|rs2978129|rs12485670|rs2994985
Arthritis	AMYOTROPHIC_LATERAL_SCLEROSIS_ALS	KEGG	1813	33	20	9,41E-01	9,99E-01	rs7002363|rs2160732|rs2251238|rs4767520|rs172677|rs3219153|rs12294809|rs9658570|rs12454712|rs2502805|rs316375|rs1065832|rs851010|rs8096471|rs1061624|rs10492142|rs1136224|rs11074659|rs9979630|rs11032695
Arthritis	RNA_POL_II_TRANSCRIPTION_PRE_INITIATION_AND_PROMOTER_OPENING	REACTOME	383	8	4	9,41E-01	9,99E-01	rs2284704|rs6761|rs16902819|rs1131384
Arthritis	YAP1_AND_WWTR1_TAZ_STIMULATED_GENE_EXPRESSION	REACTOME	1047	33	20	9,42E-01	9,99E-01	rs2304731|rs760782|rs9850957|rs13072667|rs7651271|rs1883636|rs2288481|rs1953148|rs16828012|rs6771941|rs10504474|rs16944293|rs17350396|rs1490047|rs12562952|rs12678139|rs6783790|rs2316972|rs12155521|rs2068477

Arthritis	GLUCURONIDATION	REACTOME	656	9	2	9,42E-01	9,99E-01	rs12097741|rs11568318
Arthritis	PRIMARY_IMMUNODEFICIENCY	KEGG	742	32	19	9,44E-01	9,99E-01	rs11188666|rs3761049|rs2071469|rs7508025|rs9937676|rs2071554|rs2031812|rs3819716|rs2759303|rs2228603|rs7383287|rs1559931|rs2302600|rs9612371|rs4273077|rs3752008|rs3818716|rs16939904|rs241429
Arthritis	NICOTINATE_AND_NICOTINAMIDE_METABOLISM	KEGG	963	30	18	9,45E-01	9,99E-01	rs719917|rs1821901|rs11753050|rs17155896|rs7789066|rs3903689|rs12146097|rs2348023|rs2106303|rs4683428|rs7531583|rs17341747|rs17153112|rs1289265|rs17264901|rs1153996|rs17155849|rs11973260
Arthritis	PI3K_CASCADE	REACTOME	2702	25	20	9,46E-01	9,99E-01	rs10515086|rs4725424|rs6084337|rs7674434|rs953221|rs7986346|rs4783446|rs7234952|rs336209|rs17237251|rs9591501|rs2288696|rs6490668|rs10503587|rs2302975|rs2589142|rs6978142|rs249923|rs422437|rs263931
Arthritis	GLYCOSPHINGOLIPID_METABOLISM	REACTOME	924	30	20	9,46E-01	9,99E-01	rs11683765|rs6893444|rs6533743|rs13178105|rs4074469|rs11710876|rs13386477|rs17635854|rs7641926|rs7387252|rs762571|rs4697350|rs10981150|rs7678820|rs8066279|rs4438612|rs1032147|rs12635761|rs943154|rs10498611
Arthritis	SIGNALING_BY_NODAL	REACTOME	632	31	16	9,49E-01	9,99E-01	rs266339|rs3820716|rs4965877|rs4776344|rs17514846|rs744910|rs484754|rs1106887|rs948604|rs1020522|rs10915896|rs12915039|rs6598456|rs2121212|rs4662263|rs12906930
Arthritis	REGULATORY_RNA_PATHWAYS	REACTOME	345	14	10	9,54E-01	9,99E-01	rs11624081|rs1105567|rs1096699|rs11620631|rs6761|rs1131384|rs2613514|rs10149095|rs10140275|rs8010588
Arthritis	INWARDLY_RECTIFYING_K_CHANNELS	REACTOME	1317	32	20	9,55E-01	9,99E-01	rs2835909|rs2724113|rs4979032|rs10498440|rs2836034|rs4807284|rs9907685|rs2226741|rs7212176|rs6590359|rs291353|rs7531583|rs12147761|rs1005316|rs3758953|rs3787835|rs2835845|rs1859962|rs7117162|rs2121089
Arthritis	MRNA_PROCESSING	REACTOME	1942	30	20	9,55E-01	9,99E-01	rs6044228|rs3809425|rs9910260|rs4788605|rs234784|rs1533303|rs8065368|rs7585767|rs1943344|rs2284704|rs11244329|rs2074733|rs17573579|rs2240769|rs485152|rs6111235|rs16865262|rs6829581|rs4894239|rs2402111
Arthritis	LYSOSOME	KEGG	2448	28	20	9,56E-01	9,99E-01	rs3759745|rs228614|rs3132941|rs11048060|rs355419|rs13103662|rs3803915|rs510862|rs17831521|rs12927924|rs4690859|rs919214|rs6893444|rs13178105|rs9944192|rs16919915|rs6977910|rs11710876|rs737883|rs894251
Arthritis	CELLCYCLE_PATHWAY	BIOCARTA	459	20	13	9,57E-01	9,99E-01	rs10255859|rs9381100|rs12822144|rs2383208|rs11063059|rs592412|rs4554318|rs7077462|rs9577581|rs16902819|rs11607976|rs17318844|rs10849024
Arthritis	ACTINY_PATHWAY	BIOCARTA	290	4	4	9,60E-01	9,99E-01	rs6142963|rs6809452|rs4661229|rs4925479
Arthritis	MELANOMA	KEGG	2894	32	20	9,61E-01	9,99E-01	rs10515086|rs9350245|rs765015|rs7676376|rs2075995|rs1336703|rs785512|rs9585824|rs7986923|rs9672254|rs2229765|rs17237251|rs2494738|rs10255859|rs2288696|rs6490668|rs1540657|rs10503587|rs2302975|rs12038990
Arthritis	RNA_POL_II_TRANSCRIPTION	REACTOME	1050	28	19	9,61E-01	9,99E-01	rs3809425|rs4788605|rs234784|rs1943344|rs2284704|rs2240769|rs485152|rs2402111|rs11604322|rs4796995|rs17503919|rs11701664|rs6761|rs8136239|rs16902819|rs1325006|rs1131384|rs6573908|rs2805767
Arthritis	TRANSCRIPTION_COUPLED_NER_TC_NER	REACTOME	834	40	20	9,64E-01	9,99E-01	rs2284704|rs2097289|rs3218801|rs1541549|rs1925089|rs6463716|rs9540055|rs5030740|rs9554903|rs616953|rs10492771|rs6107581|rs17466949|rs4253126|rs9539838|rs6761|rs16902819|rs1131384|rs9539836|rs12540373
Arthritis	EPITHELIAL_CELL_SIGNALING_IN_HELICOBACTER_PYLORI_INFECTION	KEGG	1535	29	20	9,64E-01	9,99E-01	rs2726550|rs661820|rs4669621|rs230530|rs1497021|rs16919915|rs6977910|rs9804672|rs1468727|rs851010|rs2501279|rs721950|rs2004997|rs12115174|rs2237093|rs542939|rs12742531|rs1976955|rs13116385|rs2019978
Arthritis	FORMATION_OF_THE_HIV1_EARLY_ELONGATION_COMPLEX	REACTOME	329	9	5	9,64E-01	9,99E-01	rs2284704|rs6761|rs16902819|rs1131384|rs2805767
Arthritis	ADHERENS_JUNCTION	KEGG	4520	30	20	9,65E-01	9,99E-01	rs9955719|rs1999090|rs1541947|rs671802|rs12969349|rs11217357|rs1331683|rs7605223|rs266339|rs7088546|rs4503419|rs10163905|rs11884056|rs10485408|rs1901335|rs389673|rs874536|rs3867132|rs10455526|rs9672254
Arthritis	MEF2D_PATHWAY	BIOCARTA	684	22	15	9,65E-01	9,99E-01	rs2251238|rs2238493|rs1740005|rs177820|rs7233024|rs12431766|rs9913908|rs198182|rs4799062|rs17031191|rs1078633|rs5758211|rs12605781|rs12150089|rs6067805
Arthritis	MRNA_CAPPING	REACTOME	388	13	8	9,67E-01	9,99E-01	rs2284704|rs4796995|rs17503919|rs6761|rs16902819|rs1325006|rs1131384|rs2805767
Arthritis	TRANSPORT_OF_MATURE_TRANSCRIPT_TO_CYTOPLASM	REACTOME	693	22	20	9,68E-01	9,99E-01	rs4788605|rs234784|rs11244329|rs2240769|rs485152|rs6532208|rs2402111|rs6768994|rs3026106|rs11701664|rs6763238|rs1438019|rs12604538|rs12149031|rs10232205|rs11097233|rs9615022|rs6573908|rs10197130|rs11683501
Arthritis	PANTOTHENATE_AND_COA_BIOSYNTHESIS	KEGG	611	19	11	9,69E-01	9,99E-01	rs2280010|rs6052147|rs507170|rs12568335|rs2047554|rs8136239|rs12025180|rs17369983|rs11185762|rs10842402|rs7967945
Arthritis	SHC_MEDIATED_CASCADE	REACTOME	1070	26	20	9,73E-01	9,99E-01	rs7674434|rs9591501|rs2288696|rs6490668|rs10503587|rs12038990|rs249923|rs263931|rs7105655|rs17320222|rs9510021|rs10842514|rs6490721|rs9578453|rs3135763|rs465174|rs17431894|rs6884797|rs9552472|rs34019
Arthritis	HIVNEF_PATHWAY	BIOCARTA	1008	34	20	9,73E-01	9,99E-01	rs832574|rs1051644|rs306966|rs230530|rs12454712|rs8096471|rs1061624|rs1937332|rs959573|rs10931934|rs1976955|rs13116385|rs2019978|rs13402616|rs2282851|rs1550955|rs7662536|rs1805410|rs7097467|rs7552664
Arthritis	FGFR_LIGAND_BINDING_AND_ACTIVATION	REACTOME	984	26	20	9,74E-01	9,99E-01	rs7674434|rs9591501|rs2288696|rs6490668|rs10503587|rs249923|rs263931|rs7105655|rs17320222|rs9510021|rs6490721|rs9578453|rs3135763|rs465174|rs17431894|rs6884797|rs9552472|rs34019|rs3787011|rs11203812
Arthritis	GALACTOSE_METABOLISM	KEGG	759	30	16	9,75E-01	9,99E-01	rs525428|rs3814587|rs1880924|rs2670854|rs12475700|rs758985|rs943|rs3829185|rs7349412|rs10508244|rs4438612|rs929959|rs12635761|rs10903963|rs6844466|rs2388268
Arthritis	INSULIN_RECEPTOR_SIGNALLING_CASCADE	REACTOME	3006	25	20	9,75E-01	9,99E-01	rs10515086|rs4725424|rs6084337|rs7674434|rs953221|rs7986346|rs4783446|rs7234952|rs336209|rs17237251|rs9591501|rs2288696|rs6490668|rs10503587|rs2302975|rs2589142|rs6978142|rs12038990|rs249923|rs422437
Arthritis	BMAL1_CLOCK_NPAS2_ACTIVATES_CIRCADIAN_EXPRESSION	REACTOME	1728	28	20	9,77E-01	9,99E-01	rs4697435|rs2227631|rs719917|rs1397829|rs9850957|rs782918|rs17041758|rs13072667|rs10519068|rs7651271|rs7789066|rs12659397|rs16828012|rs13260060|rs1490047|rs17341964|rs2106303|rs12501032|rs11634318|rs10851690
Arthritis	FORMATION_OF_RNA_POL_II_ELONGATION_COMPLEX_	REACTOME	401	9	5	9,77E-01	9,99E-01	rs2284704|rs6761|rs16902819|rs1131384|rs2805767
Arthritis	FRS2_MEDIATED_CASCADE	REACTOME	1192	25	20	9,79E-01	9,99E-01	rs7674434|rs9591501|rs2288696|rs6490668|rs10503587|rs12038990|rs249923|rs263931|rs2005219|rs7105655|rs6876|rs17320222|rs9510021|rs10842514|rs7318923|rs9578453|rs3135763|rs465174|rs17431894|rs7077462
Arthritis	PENTOSE_PHOSPHATE_PATHWAY	KEGG	616	13	9	9,79E-01	9,99E-01	rs3814587|rs10993282|rs1843217|rs10508244|rs12306431|rs929959|rs10903963|rs6844466|rs2388268
Arthritis	GLUCOSE_TRANSPORT	REACTOME	689	30	20	9,83E-01	9,99E-01	rs525428|rs2670854|rs11244329|rs6532208|rs12475700|rs758985|rs6768994|rs3026106|rs943|rs3829185|rs7349412|rs6763238|rs1438019|rs12604538|rs12149031|rs11210751|rs10232205|rs11097233|rs9615022|rs11609211
Arthritis	CIRCADIAN_REPRESSION_OF_EXPRESSION_BY_REV_ERBA	REACTOME	1358	31	20	9,85E-01	9,99E-01	rs4697435|rs1397829|rs9850957|rs782918|rs13072667|rs10519068|rs7651271|rs12659397|rs16828012|rs10504474|rs1490047|rs12501032|rs11634318|rs10851690|rs11728280|rs11630018|rs7699745|rs12678139|rs2316972|rs17613848
Arthritis	PLATELET_AGGREGATION_PLUG_FORMATION	REACTOME	942	28	20	9,85E-01	9,99E-01	rs3107373|rs306966|rs2494738|rs5896|rs2270151|rs11869835|rs6489695|rs12220858|rs290255|rs7085145|rs12369177|rs12317523|rs12772391|rs238242|rs1550955|rs850737|rs6091153|rs16854526|rs2168043|rs1800378
Arthritis	SIGNALING_BY_INSULIN_RECEPTOR	REACTOME	3432	23	20	9,86E-01	9,99E-01	rs10515086|rs4725424|rs6084337|rs4669621|rs7674434|rs953221|rs7986346|rs4783446|rs7234952|rs336209|rs17237251|rs9591501|rs2288696|rs6490668|rs10503587|rs2302975|rs1497021|rs16919915|rs2589142|rs6978142
Arthritis	INTEGRIN_PATHWAY	BIOCARTA	1027	29	18	9,86E-01	9,99E-01	rs306966|rs3867132|rs10901096|rs3791936|rs2005219|rs934039|rs10278782|rs2059409|rs12168342|rs3012739|rs11686435|rs2073307|rs1550955|rs1890875|rs1998762|rs2168043|rs2017750|rs4925479
Arthritis	RORA_ACTIVATES_CIRCADIAN_EXPRESSION	REACTOME	1377	31	20	9,86E-01	9,99E-01	rs4697435|rs1397829|rs9850957|rs782918|rs13072667|rs10519068|rs7651271|rs12659397|rs16828012|rs13260060|rs1490047|rs12501032|rs11634318|rs10851690|rs11728280|rs11630018|rs7699745|rs12678139|rs2316972|rs17613848
Arthritis	REGULATION_OF_ACTIN_CYTOSKELETON	KEGG	7089	29	20	9,89E-01	9,99E-01	rs10515086|rs11850274|rs10273151|rs2107128|rs4509829|rs1476592|rs11815834|rs7975139|rs7985340|rs7676376|rs4573339|rs1180398|rs3809805|rs1592108|rs306966|rs1336703|rs3867132|rs879904|rs2423441|rs785512
Arthritis	REGULATION_OF_GLUCOKINASE_BY_GLUCOKINASE_REGULATORY_PROTEIN	REACTOME	459	19	14	9,89E-01	9,99E-01	rs11244329|rs6532208|rs758985|rs6768994|rs3026106|rs6763238|rs1438019|rs12604538|rs12149031|rs10232205|rs11097233|rs9615022|rs10197130|rs11683501
Arthritis	ELONGATION_ARREST_AND_RECOVERY	REACTOME	246	3	2	9,90E-01	9,99E-01	rs6761|rs1131384
Arthritis	ENDOSOMAL_SORTING_COMPLEX_REQUIRED_FOR_TRANSPORT_ESCRT	REACTOME	318	5	5	9,90E-01	9,99E-01	rs1053903|rs2747566|rs4921744|rs11781369|rs3820706
Arthritis	ABORTIVE_ELONGATION_OF_HIV1_TRANSCRIPT_IN_THE_ABSENCE_OF_TAT	REACTOME	195	4	3	9,91E-01	9,99E-01	rs6761|rs1131384|rs2805767
Arthritis	RNA_POL_II_PRE_TRANSCRIPTION_EVENTS	REACTOME	532	9	5	9,91E-01	9,99E-01	rs2284704|rs6761|rs16902819|rs1131384|rs2805767
Arthritis	NEP_NS2_INTERACTS_WITH_THE_CELLULAR_EXPORT_MACHINERY	REACTOME	456	18	13	9,92E-01	9,99E-01	rs11244329|rs6532208|rs6768994|rs3026106|rs6763238|rs1438019|rs12604538|rs12149031|rs10232205|rs11097233|rs9615022|rs10197130|rs11683501
Arthritis	TRANSPORT_OF_RIBONUCLEOPROTEINS_INTO_THE_HOST_NUCLEUS	REACTOME	452	18	13	9,92E-01	9,99E-01	rs11244329|rs6532208|rs6768994|rs3026106|rs6763238|rs1438019|rs12604538|rs12149031|rs10232205|rs11097233|rs9615022|rs10197130|rs11683501
Arthritis	SLC_MEDIATED_TRANSMEMBRANE_TRANSPORT	REACTOME	8774	31	20	9,92E-01	9,99E-01	rs1289392|rs1849067|rs4627290|rs10145863|rs2950798|rs6706564|rs999448|rs9873087|rs1539683|rs151377|rs953888|rs2378089|rs3782999|rs12000690|rs2712623|rs2298720|rs9308208|rs1288338|rs1468790|rs3766781
Arthritis	DNA_STRAND_ELONGATION	REACTOME	672	19	15	9,93E-01	9,99E-01	rs1541549|rs1925089|rs6463716|rs9540055|rs5030740|rs616953|rs6107581|rs17466949|rs9539838|rs2307325|rs2073758|rs9539836|rs236164|rs13056098|rs12540373
Arthritis	INTERACTIONS_OF_VPR_WITH_HOST_CELLULAR_PROTEINS	REACTOME	480	19	14	9,93E-01	9,99E-01	rs11244329|rs6829581|rs6768994|rs3026106|rs6850094|rs6763238|rs1438019|rs12604538|rs12149031|rs10232205|rs11097233|rs9615022|rs10197130|rs11683501
Arthritis	PENTOSE_AND_GLUCURONATE_INTERCONVERSIONS	KEGG	874	12	4	9,93E-01	9,99E-01	rs2270941|rs9509138|rs11568318|rs34187424
Arthritis	CIRCADIAN_CLOCK	REACTOME	2075	25	20	9,94E-01	9,99E-01	rs4697435|rs2227631|rs719917|rs1397829|rs9850957|rs782918|rs17041758|rs13072667|rs10519068|rs10271133|rs7651271|rs7789066|rs12659397|rs16828012|rs13260060|rs1490047|rs17341964|rs2106303|rs12501032|rs11634318
Arthritis	MATURITY_ONSET_DIABETES_OF_THE_YOUNG	KEGG	992	27	17	9,94E-01	9,99E-01	rs7799010|rs1337906|rs1614778|rs10841798|rs12027362|rs758985|rs2977330|rs6493610|rs4290087|rs4278686|rs2651505|rs10998853|rs736824|rs746508|rs2254779|rs11799695|rs7310409
Arthritis	TRANSPORT_OF_MATURE_MRNA_DERIVED_FROM_AN_INTRONLESS_TRANSCRIPT	REACTOME	524	20	15	9,95E-01	9,99E-01	rs11244329|rs6829581|rs2402111|rs6768994|rs3026106|rs6763238|rs1438019|rs12604538|rs12149031|rs10232205|rs11097233|rs9615022|rs10197130|rs11683501|rs2805767
Arthritis	PML_PATHWAY	BIOCARTA	384	14	10	9,95E-01	9,99E-01	rs1061624|rs1937332|rs1078555|rs11259989|rs8046065|rs2239839|rs129988|rs7097467|rs6754024|rs7552664
Arthritis	HIV_LIFE_CYCLE	REACTOME	1529	32	20	9,95E-01	9,99E-01	rs4426221|rs10906842|rs7567258|rs2239921|rs2284704|rs10204606|rs11244329|rs1872066|rs9514816|rs6829581|rs4440020|rs6768994|rs1016269|rs4796995|rs3026106|rs17503919|rs1053903|rs4921744|rs6763238|rs6761
Arthritis	INTEGRIN_ALPHAIIB_BETA3_SIGNALING	REACTOME	779	22	16	9,95E-01	9,99E-01	rs306966|rs2494738|rs2270151|rs11869835|rs6489695|rs290255|rs7085145|rs12369177|rs12317523|rs12772391|rs1550955|rs850737|rs6091153|rs16854526|rs2168043|rs1800378
Arthritis	TRAFFICKING_OF_GLUR2_CONTAINING_AMPA_RECEPTORS	REACTOME	721	13	12	9,95E-01	9,99E-01	rs11919795|rs2238493|rs1461234|rs9913908|rs198182|rs8103771|rs3773162|rs9324750|rs688053|rs12150089|rs6794801|rs17588123
Arthritis	ACTIVATED_POINT_MUTANTS_OF_FGFR2	REACTOME	873	26	20	9,96E-01	9,99E-01	rs6490668|rs10503587|rs249923|rs263931|rs7105655|rs17320222|rs9510021|rs7318923|rs9578453|rs3135763|rs465174|rs6884797|rs9552472|rs34019|rs3787011|rs11203812|rs10467553|rs10160812|rs891978|rs823422
Arthritis	REGULATION_OF_BETA_CELL_DEVELOPMENT	REACTOME	1288	37	20	9,97E-01	9,99E-01	rs2755209|rs2494738|rs1337906|rs1614778|rs10841798|rs12027362|rs758985|rs2977330|rs17320222|rs4435111|rs6493610|rs1782804|rs4692386|rs4290087|rs2951787|rs4278686|rs2651505|rs10998853|rs736824|rs2254779
Arthritis	METABOLISM_OF_NON_CODING_RNA	REACTOME	611	23	18	9,99E-01	9,99E-01	rs11244329|rs1264901|rs6532208|rs6768994|rs3026106|rs2316972|rs6763238|rs12155521|rs8136239|rs1438019|rs12604538|rs12149031|rs10232205|rs11097233|rs9615022|rs10197130|rs11683501|rs2805767
Arthritis	LATE_PHASE_OF_HIV_LIFE_CYCLE	REACTOME	1259	27	20	9,99E-01	9,99E-01	rs10906842|rs2239921|rs2284704|rs11244329|rs6829581|rs6768994|rs4796995|rs3026106|rs17503919|rs1053903|rs4921744|rs6763238|rs6761|rs1438019|rs11781369|rs12604538|rs12149031|rs16902819|rs1325006|rs10232205
Photosensitivity	SYNTHESIS_SECRETION_AND_DEACYLATION_OF_GHRELIN	REACTOME	624	50	17	4,00E-04	3,19E-01	rs4978629|rs7639015|rs1096215|rs7034018|rs2070776|rs1545163|rs10733565|rs16974510|rs10816539|rs7429483|rs4979515|rs2049311|rs6805120|rs3020619|rs4979065|rs6976|rs11920160
Photosensitivity	PYRIMIDINE_METABOLISM	KEGG	1900	47	20	3,50E-03	7,86E-01	rs12516561|rs10017797|rs12401542|rs12127542|rs2559658|rs885838|rs528455|rs139873|rs12023630|rs822567|rs11165798|rs730283|rs6759948|rs2235116|rs8063431|rs4789848|rs3088264|rs7112576|rs4819850|rs3782093
Photosensitivity	BIOSYNTHESIS_OF_UNSATURATED_FATTY_ACIDS	KEGG	463	46	14	4,00E-03	7,86E-01	rs9030|rs2072114|rs11737840|rs11254654|rs6813255|rs7482316|rs2720320|rs2239621|rs7893486|rs2303842|rs174534|rs1135889|rs6835929|rs11734609
Photosensitivity	DIABETES_PATHWAYS	REACTOME	3395	40	20	5,10E-03	7,86E-01	rs4978629|rs7571971|rs7639015|rs8049837|rs1096215|rs362623|rs9864104|rs7034018|rs11243152|rs2172680|rs883541|rs2070776|rs4548895|rs1545163|rs2493047|rs10733565|rs16974510|rs1275509|rs17443293|rs10816539
Photosensitivity	BETA_ALANINE_METABOLISM	KEGG	711	79	20	5,80E-03	7,86E-01	rs1487302|rs12127542|rs528455|rs3006845|rs1555481|rs2887|rs12023630|rs4878808|rs822567|rs11165798|rs7865905|rs290821|rs1330581|rs12044858|rs12467996|rs7506051|rs7577|rs7590991|rs4835913|rs11165754
Photosensitivity	BLADDER_CANCER	KEGG	1222	39	20	6,30E-03	7,86E-01	rs1030609|rs743682|rs721412|rs10796820|rs6909633|rs11568993|rs12512817|rs3740643|rs2236947|rs943084|rs3124238|rs2005219|rs13060691|rs17746482|rs1048691|rs1144396|rs9368179|rs911361|rs1432442|rs984654
Photosensitivity	METABOLISM_OF_XENOBIOTICS_BY_CYTOCHROME_P450	KEGG	1674	126	20	6,90E-03	7,86E-01	rs11192001|rs8192772|rs1913264|rs9423399|rs4148329|rs6741148|rs4147536|rs3902902|rs1937881|rs10744116|rs8192712|rs9418982|rs6761246|rs2361502|rs2246709|rs2860975|rs308328|rs1027473|rs7074522|rs6591256
Photosensitivity	PML_PATHWAY	BIOCARTA	384	13	6	8,30E-03	8,28E-01	rs17339080|rs1061631|rs17200733|rs12477596|rs10167683|rs7552664
Photosensitivity	FATTY_ACYL_COA_BIOSYNTHESIS	REACTOME	404	31	11	1,00E-02	8,46E-01	rs9030|rs11737840|rs17674015|rs6813255|rs2395926|rs247011|rs10024579|rs9905601|rs6835929|rs6832271|rs10182865
Photosensitivity	MITOCHONDRIAL_TRNA_AMINOACYLATION	REACTOME	500	23	9	1,06E-02	8,46E-01	rs4945300|rs12730226|rs191833|rs17140468|rs7766094|rs2645303|rs4771704|rs6926631|rs2432749
Photosensitivity	RNA_POL_III_CHAIN_ELONGATION	REACTOME	364	13	9	1,44E-02	9,44E-01	rs2559658|rs11785348|rs885838|rs139873|rs3088264|rs11989385|rs13280649|rs2227301|rs6586938
Photosensitivity	PHOSPHORYLATION_OF_CD3_AND_TCR_ZETA_CHAINS	REACTOME	521	16	7	1,55E-02	9,44E-01	rs3916765|rs2060581|rs10918693|rs858490|rs9275582|rs1003815|rs7523940
Photosensitivity	POST_CHAPERONIN_TUBULIN_FOLDING_PATHWAY	REACTOME	254	10	3	1,84E-02	9,44E-01	rs9687161|rs9507572|rs11954532
Photosensitivity	BIOLOGICAL_OXIDATIONS	REACTOME	3568	76	20	1,92E-02	9,44E-01	rs11192001|rs8192772|rs10487665|rs8049837|rs1913264|rs8109054|rs4148329|rs4548895|rs6741148|rs10059065|rs2672487|rs4147536|rs11214959|rs294588|rs2277119|rs10744116|rs10167564|rs8192712|rs9418982|rs6761246
Photosensitivity	CTNNB1_PHOSPHORYLATION_CASCADE	REACTOME	316	23	5	1,97E-02	9,44E-01	rs351408|rs8004556|rs442683|rs6599127|rs1056348
Photosensitivity	PYRIMIDINE_METABOLISM	REACTOME	792	72	20	2,10E-02	9,44E-01	rs10017797|rs1487302|rs12127542|rs528455|rs12023630|rs1507069|rs11165798|rs6759948|rs290821|rs12044858|rs9362203|rs16899972|rs16853627|rs1275522|rs2464974|rs17282057|rs10521023|rs11165754|rs11587873|rs6004171
Photosensitivity	RNA_POL_III_TRANSCRIPTION_INITIATION_FROM_TYPE_3_PROMOTER	REACTOME	501	17	12	2,16E-02	9,44E-01	rs2559658|rs11785348|rs885838|rs139873|rs12418104|rs7148769|rs3088264|rs11989385|rs13280649|rs2227301|rs6586938|rs4255740
Photosensitivity	DRUG_METABOLISM_CYTOCHROME_P450	KEGG	1661	138	20	2,23E-02	9,44E-01	rs11192001|rs8192772|rs1913264|rs4148329|rs4147536|rs3902902|rs1986415|rs10744116|rs2348024|rs8192712|rs9418982|rs6761246|rs2361502|rs2246709|rs2860975|rs308328|rs1027473|rs6591256|rs11572172|rs2243822
Photosensitivity	PERK_REGULATED_GENE_EXPRESSION	REACTOME	334	18	8	2,36E-02	9,44E-01	rs7571971|rs11189224|rs33556|rs3764261|rs9543118|rs1613070|rs12666800|rs11642944
Photosensitivity	ASPARAGINE_N_LINKED_GLYCOSYLATION	REACTOME	2760	41	20	2,53E-02	9,44E-01	rs1487984|rs10108742|rs1667271|rs13262159|rs227867|rs2101809|rs10093209|rs7233900|rs12762685|rs9325770|rs3811250|rs12036045|rs2581830|rs12426170|rs1275509|rs1257180|rs2117642|rs3751467|rs12891257|rs2130177
Photosensitivity	RNA_POLYMERASE	KEGG	351	14	9	2,62E-02	9,44E-01	rs2559658|rs885838|rs139873|rs730283|rs3088264|rs7112576|rs1625691|rs1561330|rs2227301
Photosensitivity	METABOLISM_OF_VITAMINS_AND_COFACTORS	REACTOME	1119	38	20	2,62E-02	9,44E-01	rs11192001|rs3826608|rs7636269|rs17170428|rs13231383|rs11210751|rs2501920|rs9845929|rs2838969|rs9974754|rs2423067|rs1256107|rs4726741|rs3134295|rs6777158|rs4845881|rs2180448|rs1776967|rs16967576|rs9493133
Photosensitivity	GRB2_EVENTS_IN_ERBB2_SIGNALING	REACTOME	1202	39	20	2,78E-02	9,44E-01	rs721412|rs264336|rs2888104|rs11568993|rs16849142|rs17329875|rs6435692|rs4452759|rs7817546|rs2005219|rs13060691|rs13260061|rs960824|rs17746482|rs6827871|rs4236723|rs1432442|rs984654|rs10171977|rs904453
Photosensitivity	UNFOLDED_PROTEIN_RESPONSE	REACTOME	1176	37	20	2,90E-02	9,44E-01	rs7571971|rs8049837|rs11243152|rs2172680|rs883541|rs4548895|rs1275509|rs17443293|rs6785659|rs11189224|rs9842582|rs33556|rs7427632|rs3764261|rs1836581|rs9543118|rs3825073|rs1211975|rs231395|rs1613070
Photosensitivity	NUCLEOTIDE_LIKE_PURINERGIC_RECEPTORS	REACTOME	272	15	5	3,12E-02	9,44E-01	rs11687066|rs13419955|rs2236624|rs9875152|rs6809526
Photosensitivity	PHASE_II_CONJUGATION	REACTOME	2059	111	20	3,13E-02	9,44E-01	rs11192001|rs8049837|rs1913264|rs8109054|rs4148329|rs4548895|rs10059065|rs2672487|rs11214959|rs294588|rs10744116|rs10167564|rs6761246|rs2544800|rs7738142|rs9569|rs17287615|rs2361502|rs11958300|rs7577
Photosensitivity	N_GLYCAN_BIOSYNTHESIS	KEGG	1792	40	20	3,24E-02	9,44E-01	rs10108742|rs13262159|rs227867|rs11073961|rs2101809|rs10093209|rs9325770|rs3811250|rs12036045|rs2581830|rs1873588|rs1257180|rs3751467|rs12891257|rs6444192|rs7429360|rs13017917|rs12589188|rs3004318|rs17793809
Photosensitivity	METABOLISM_OF_CARBOHYDRATES	REACTOME	9072	31	20	3,34E-02	9,44E-01	rs723988|rs11184173|rs9515779|rs8049837|rs4870775|rs9913031|rs1667271|rs6793913|rs335124|rs2317400|rs13100173|rs9999101|rs12195809|rs16851527|rs3800794|rs9320488|rs7205890|rs3811755|rs7233900|rs1880924
Photosensitivity	ENDOMETRIAL_CANCER	KEGG	3028	43	20	3,45E-02	9,44E-01	rs1030609|rs6480245|rs721412|rs10796820|rs4433471|rs10822936|rs11568993|rs3795994|rs7906982|rs13429335|rs7898559|rs3740643|rs4918785|rs2862499|rs11685301|rs2005219|rs4074716|rs13388538|rs2673825|rs13060691
Photosensitivity	PRE_NOTCH_TRANSCRIPTION_AND_TRANSLATION	REACTOME	835	49	20	3,55E-02	9,44E-01	rs10796820|rs6909633|rs13129525|rs4753743|rs7188960|rs6816542|rs1030211|rs3740643|rs11724190|rs10017961|rs8080480|rs9368179|rs911361|rs1075617|rs2788865|rs1812402|rs17051003|rs1464434|rs4525961|rs11128940
Photosensitivity	RNA_POL_III_TRANSCRIPTION_INITIATION_FROM_TYPE_2_PROMOTER	REACTOME	417	16	11	3,77E-02	9,51E-01	rs2559658|rs11785348|rs885838|rs139873|rs3088264|rs2583793|rs11989385|rs13280649|rs2227301|rs6586938|rs8270
Photosensitivity	NKT_PATHWAY	BIOCARTA	638	35	18	3,82E-02	9,51E-01	rs2228428|rs583911|rs17047703|rs2243300|rs3864277|rs4143832|rs2421057|rs12215787|rs17811014|rs17283264|rs10206753|rs1932971|rs4921508|rs4334868|rs9972960|rs40401|rs17187469|rs9402884

Photosensitivity	PROPANOATE_METABOLISM	KEGG	1219	57	20	4,01E-02	9,51E-01	rs9585457|rs3741049|rs891274|rs7572995|rs767504|rs1555481|rs4754298|rs12577790|rs4878808|rs9534842|rs9567895|rs10849968|rs7865905|rs1359393|rs12485908|rs12328118|rs6050291|rs7590991|rs6458651|rs4835913
Photosensitivity	PANTOTHENATE_AND_COA_BIOSYNTHESIS	KEGG	611	62	16	4,18E-02	9,51E-01	rs1487302|rs12127542|rs528455|rs12023630|rs822567|rs11165798|rs2029986|rs290821|rs12044858|rs9493133|rs11165754|rs11047681|rs11587873|rs6004171|rs3950612|rs11582179
Photosensitivity	PD1_SIGNALING	REACTOME	398	15	7	4,24E-02	9,51E-01	rs3916765|rs10918693|rs16923201|rs858490|rs9275582|rs16923198|rs1003815
Photosensitivity	TRANSPORT_OF_VITAMINS_NUCLEOSIDES_AND_RELATED_MOLECULES	REACTOME	1154	50	20	4,30E-02	9,51E-01	rs2252035|rs16928705|rs4854786|rs7636169|rs7027670|rs9357436|rs4944074|rs709370|rs7865947|rs7629416|rs7106761|rs4854785|rs7181178|rs2270059|rs1932649|rs6480507|rs7174549|rs6783575|rs12824715|rs17596814
Photosensitivity	FGFR_LIGAND_BINDING_AND_ACTIVATION	REACTOME	984	26	20	4,41E-02	9,51E-01	rs743682|rs666336|rs17487009|rs250201|rs10886918|rs9580274|rs883355|rs13250792|rs3129601|rs465174|rs3176292|rs10437827|rs6859263|rs10160812|rs250092|rs7982293|rs4575197|rs9634328|rs2608819|rs7670903
Photosensitivity	GLYCOSAMINOGLYCAN_METABOLISM	REACTOME	6096	30	20	5,03E-02	9,60E-01	rs723988|rs9515779|rs4870775|rs9913031|rs1667271|rs6793913|rs13100173|rs9999101|rs12195809|rs16851527|rs7786151|rs9320488|rs7205890|rs3811755|rs7233900|rs7995233|rs2303076|rs1606724|rs10100391|rs6680621
Photosensitivity	ENDOCYTOSIS	KEGG	6484	31	20	5,24E-02	9,60E-01	rs13137847|rs11085070|rs743682|rs721412|rs6854185|rs2888104|rs7023913|rs7544627|rs2717719|rs11568993|rs12251307|rs17086820|rs9963622|rs13182713|rs16849142|rs10461355|rs9260759|rs1546713|rs10886918|rs17329875
Photosensitivity	KERATAN_SULFATE_KERATIN_METABOLISM	REACTOME	737	44	20	5,31E-02	9,60E-01	rs1667271|rs6793913|rs16851527|rs7233900|rs7628381|rs11900673|rs7115683|rs7173652|rs9858362|rs6697045|rs4971224|rs10081532|rs13275988|rs278364|rs10859103|rs11073810|rs2945744|rs2595361|rs1372288|rs17201864
Photosensitivity	FRS2_MEDIATED_CASCADE	REACTOME	1192	26	20	5,55E-02	9,60E-01	rs743682|rs666336|rs17487009|rs250201|rs10886918|rs9580274|rs2005219|rs13060691|rs883355|rs13250792|rs3129601|rs465174|rs3176292|rs10437827|rs6859263|rs10160812|rs250092|rs7982293|rs4575197|rs1432442
Photosensitivity	MAPK_SIGNALING_PATHWAY	KEGG	9833	36	20	5,60E-02	9,60E-01	rs7871111|rs4748472|rs2299168|rs1030608|rs743682|rs721412|rs666336|rs2239094|rs17487009|rs1798619|rs963065|rs11568993|rs6705101|rs7174521|rs42050|rs10912414|rs630426|rs250201|rs17047703|rs4756788
Photosensitivity	NO2IL12_PATHWAY	BIOCARTA	461	22	13	5,73E-02	9,60E-01	rs7601754|rs583911|rs10918693|rs11080358|rs858490|rs2421057|rs17811014|rs4921508|rs2531882|rs4334868|rs1003815|rs1517352|rs2297518
Photosensitivity	SHC_MEDIATED_CASCADE	REACTOME	1070	26	20	5,77E-02	9,60E-01	rs743682|rs666336|rs17487009|rs250201|rs10886918|rs9580274|rs883355|rs13250792|rs3129601|rs465174|rs3176292|rs10437827|rs6859263|rs10160812|rs250092|rs7982293|rs4575197|rs10171977|rs9634328|rs2608819
Photosensitivity	BASAL_CELL_CARCINOMA	KEGG	2166	32	20	5,78E-02	9,60E-01	rs12516561|rs7571877|rs544704|rs7640326|rs4945061|rs288746|rs17389792|rs3781730|rs4716923|rs4918785|rs11685301|rs11060782|rs10261329|rs3823734|rs12698335|rs442683|rs232651|rs1922081|rs1402292|rs6599127
Photosensitivity	ACTIVATION_OF_CHAPERONE_GENES_BY_XBP1S	REACTOME	761	39	20	5,82E-02	9,60E-01	rs8049837|rs11243152|rs883541|rs4548895|rs1275509|rs17443293|rs6785659|rs9842582|rs7427632|rs1836581|rs3825073|rs1211975|rs231395|rs11217129|rs6762892|rs1429997|rs1350386|rs2275075|rs17043569|rs918167
Photosensitivity	ACTIVATION_OF_KAINATE_RECEPTORS_UPON_GLUTAMATE_BINDING	REACTOME	1577	44	20	5,84E-02	9,60E-01	rs7007251|rs12364414|rs466767|rs10107483|rs513472|rs6589846|rs9377381|rs6656317|rs215811|rs17414857|rs7830150|rs12215960|rs2832484|rs218422|rs11157863|rs6055990|rs2719795|rs8115925|rs9880331|rs6992873
Photosensitivity	MEIOSIS	REACTOME	1674	68	20	6,09E-02	9,60E-01	rs3915766|rs1366452|rs2244012|rs1883848|rs1126432|rs3213191|rs9366694|rs884241|rs3778279|rs992276|rs323368|rs2030419|rs2256191|rs1048691|rs1801516|rs2508678|rs129128|rs7357092|rs12534920|rs4646536
Photosensitivity	WNT_SIGNALING_PATHWAY	KEGG	5611	34	20	6,17E-02	9,60E-01	rs12516561|rs1030608|rs7571877|rs959176|rs10796820|rs544704|rs7640326|rs351408|rs4945061|rs963065|rs9864104|rs2946998|rs7016063|rs9576193|rs2241604|rs2461489|rs3781730|rs17031191|rs3740643|rs873417
Photosensitivity	CARDIACEGF_PATHWAY	BIOCARTA	1289	47	20	6,34E-02	9,60E-01	rs1030609|rs721412|rs12873185|rs1878404|rs11568993|rs3908461|rs873417|rs4884075|rs4962318|rs17746482|rs1324792|rs4381631|rs4411531|rs3729908|rs984654|rs6999335|rs758658|rs1812402|rs10956433|rs4140770
Photosensitivity	RNA_POL_III_TRANSCRIPTION_TERMINATION	REACTOME	680	39	18	6,37E-02	9,60E-01	rs2559658|rs11785348|rs885838|rs10491776|rs139873|rs1408314|rs10756504|rs3088264|rs4668168|rs4740560|rs11989385|rs13280649|rs1322990|rs10961347|rs8192986|rs7027532|rs2227301|rs6586938
Photosensitivity	METABOLISM_OF_NUCLEOTIDES	REACTOME	1559	58	20	6,43E-02	9,60E-01	rs10017797|rs12401542|rs12127542|rs528455|rs1126432|rs2269340|rs12023630|rs1507069|rs11165798|rs6759948|rs1042427|rs7545638|rs11162326|rs3760469|rs290821|rs12044858|rs2071017|rs7125888|rs4952085|rs3817657
Photosensitivity	CELLCYCLE_PATHWAY	BIOCARTA	459	33	12	6,49E-02	9,60E-01	rs10796820|rs997669|rs3740643|rs1049612|rs6797587|rs1048691|rs11667347|rs4646536|rs10273030|rs2417223|rs649392|rs1561734
Photosensitivity	MELANOMA	KEGG	2894	35	20	6,60E-02	9,60E-01	rs5745752|rs721412|rs666336|rs10796820|rs17487009|rs6909633|rs11568993|rs12280614|rs250201|rs12531099|rs1546713|rs9580274|rs3740643|rs7653308|rs2005219|rs2684794|rs2673825|rs13060691|rs17526983|rs883355
Photosensitivity	NITRIC_OXIDE_STIMULATES_GUANYLATE_CYCLASE	REACTOME	1895	45	20	6,66E-02	9,60E-01	rs6443589|rs13115024|rs602159|rs2573083|rs32574|rs16927077|rs1025598|rs4754175|rs1783138|rs11203213|rs11080358|rs17106293|rs10823217|rs11042916|rs4243390|rs4834779|rs10497482|rs10497599|rs11178236|rs1940386
Photosensitivity	GAP_JUNCTION_TRAFFICKING	REACTOME	576	59	19	6,98E-02	9,60E-01	rs7023913|rs13182713|rs1436908|rs8064669|rs6929363|rs2432216|rs3003608|rs12338625|rs1919870|rs3805787|rs4804528|rs7703742|rs1546281|rs12127091|rs7164838|rs3812042|rs843339|rs11154022|rs17745948
Photosensitivity	MCALPAIN_PATHWAY	BIOCARTA	543	36	16	7,18E-02	9,60E-01	rs721412|rs11568993|rs883541|rs10940270|rs2005219|rs17746482|rs870992|rs600135|rs984654|rs4140770|rs2876980|rs918167|rs4745514|rs1254403|rs1015793|rs12535482
Photosensitivity	RESPONSE_TO_ELEVATED_PLATELET_CYTOSOLIC_CA2_	REACTOME	2301	36	20	7,23E-02	9,60E-01	rs5745752|rs721412|rs8049837|rs2511989|rs1742932|rs11568993|rs17047703|rs12531099|rs12512817|rs13062355|rs6796803|rs873417|rs943084|rs11629242|rs8132767|rs11702267|rs4381631|rs4411531|rs3729908|rs879180
Photosensitivity	FOCAL_ADHESION	KEGG	7733	45	20	7,51E-02	9,60E-01	rs1630986|rs5745752|rs2483852|rs721412|rs17312104|rs2245617|rs2861315|rs12654563|rs10796820|rs1798619|rs11568993|rs17086820|rs7174521|rs9333118|rs12280614|rs747574|rs10461355|rs12531099|rs1546713|rs12512817
Photosensitivity	SPLICEOSOME	KEGG	1685	32	20	7,55E-02	9,60E-01	rs4683295|rs12243448|rs12251307|rs2665840|rs10197258|rs9462232|rs215749|rs2588882|rs2270048|rs2305797|rs7652849|rs12937256|rs1140409|rs3857111|rs17200733|rs12406805|rs236346|rs4646296|rs8111911|rs4807225
Photosensitivity	ACTIVATED_POINT_MUTANTS_OF_FGFR2	REACTOME	873	28	20	7,72E-02	9,60E-01	rs666336|rs17487009|rs250201|rs10886918|rs9580274|rs883355|rs13250792|rs3129601|rs465174|rs3176292|rs10437827|rs6859263|rs10160812|rs250092|rs7982293|rs4575197|rs9634328|rs11986428|rs10070885|rs7703260
Photosensitivity	DRUG_METABOLISM_OTHER_ENZYMES	KEGG	1609	139	20	7,75E-02	9,60E-01	rs12401542|rs12127542|rs528455|rs4148329|rs12023630|rs1507069|rs11165798|rs6759948|rs12927815|rs6761246|rs290821|rs12044858|rs2361502|rs2246709|rs1833182|rs4952085|rs2859778|rs16853627|rs6756897|rs9297056
Photosensitivity	STARCH_AND_SUCROSE_METABOLISM	KEGG	2178	96	20	8,38E-02	9,60E-01	rs11184173|rs2317400|rs11236101|rs4148329|rs1880924|rs7535245|rs7534879|rs12064556|rs2269260|rs12030805|rs620281|rs11184300|rs12798453|rs9864435|rs6761246|rs13080791|rs7797360|rs2361502|rs9493133|rs217463
Photosensitivity	PURINE_METABOLISM	KEGG	5442	27	20	8,40E-02	9,60E-01	rs12516561|rs13115024|rs9868156|rs602159|rs2573083|rs2559658|rs1457145|rs4764112|rs885838|rs215697|rs1606119|rs32574|rs1126432|rs139873|rs1025598|rs2672487|rs4754175|rs4736746|rs1783138|rs11203213
Photosensitivity	PROXIMAL_TUBULE_BICARBONATE_RECLAMATION	KEGG	458	7	6	8,56E-02	9,60E-01	rs973608|rs2236941|rs17688758|rs2064393|rs1465216|rs3885388
Photosensitivity	AMINOACYL_TRNA_BIOSYNTHESIS	KEGG	731	33	14	8,66E-02	9,60E-01	rs4945300|rs12730226|rs759243|rs191833|rs17140468|rs7766094|rs12961130|rs2645303|rs707937|rs4771704|rs6926631|rs268739|rs12022181|rs2432749
Photosensitivity	DESTABILIZATION_OF_MRNA_BY_TRISTETRAPROLIN_TTP	REACTOME	179	13	4	8,94E-02	9,60E-01	rs13082092|rs11189224|rs33556|rs9543118
Photosensitivity	PLATELET_HOMEOSTASIS	REACTOME	4080	39	20	9,13E-02	9,60E-01	rs6443589|rs13115024|rs7120938|rs602159|rs2573083|rs13064158|rs351408|rs12048929|rs10430939|rs32574|rs1866901|rs16927077|rs1025598|rs7586183|rs4754175|rs1783138|rs12143542|rs11203213|rs11080358|rs17106293
Photosensitivity	IL12_PATHWAY	BIOCARTA	589	30	14	9,27E-02	9,60E-01	rs7601754|rs583911|rs10918693|rs858490|rs2421057|rs17811014|rs10204137|rs11924144|rs2716121|rs4921508|rs4334868|rs1003815|rs1517352|rs9380546
Photosensitivity	AUTOIMMUNE_THYROID_DISEASE	KEGG	1646	53	20	9,35E-02	9,60E-01	rs4903967|rs3916765|rs2268467|rs2371462|rs2243300|rs9260759|rs2268477|rs2110695|rs4143832|rs2284734|rs68600|rs9275582|rs6074024|rs9264219|rs624035|rs4297024|rs9261950|rs9378229|rs12075255|rs206775
Photosensitivity	GLUTATHIONE_CONJUGATION	REACTOME	407	34	12	9,46E-02	9,60E-01	rs11192001|rs1913264|rs10744116|rs7738142|rs7577|rs1027473|rs6591256|rs11101992|rs4149195|rs6955931|rs6088659|rs9463826
Photosensitivity	NDKDYNAMIN_PATHWAY	BIOCARTA	548	46	20	9,68E-02	9,60E-01	rs7023913|rs963065|rs2461489|rs17031191|rs2851045|rs3003608|rs6852347|rs12338625|rs10499602|rs3760469|rs10265223|rs2866250|rs11727778|rs6462856|rs999613|rs843339|rs873270|rs2267808|rs2254562|rs6659310
Photosensitivity	CALCINEURIN_PATHWAY	BIOCARTA	895	41	20	9,72E-02	9,60E-01	rs13396925|rs963065|rs16862130|rs2461489|rs17031191|rs873417|rs2851045|rs6852347|rs4381631|rs4411531|rs3729908|rs758658|rs907956|rs7694484|rs2028889|rs11727778|rs4811158|rs7193148|rs11725047|rs4239343
Photosensitivity	G1_PHASE	REACTOME	625	37	14	9,72E-02	9,60E-01	rs10796820|rs6909633|rs3740643|rs1049612|rs1048691|rs9368179|rs911361|rs1056348|rs4646536|rs10273030|rs2417223|rs1825183|rs649392|rs1561734
Photosensitivity	SIGNALING_BY_FGFR_MUTANTS	REACTOME	1657	26	20	1,01E-01	9,60E-01	rs743682|rs666336|rs17487009|rs13231090|rs250201|rs10886918|rs9580274|rs10899496|rs6467779|rs883355|rs13250792|rs3129601|rs465174|rs3176292|rs10437827|rs6859263|rs10160812|rs706733|rs706724|rs11048778
Photosensitivity	RNA_POL_III_TRANSCRIPTION	REACTOME	861	36	20	1,04E-01	9,60E-01	rs2559658|rs11785348|rs885838|rs10491776|rs139873|rs1408314|rs10756504|rs12418104|rs7148769|rs3088264|rs4668168|rs302917|rs4740560|rs11989385|rs13280649|rs1322990|rs10961347|rs8192986|rs7027532|rs2227301
Photosensitivity	GLYOXYLATE_AND_DICARBOXYLATE_METABOLISM	KEGG	704	45	20	1,04E-01	9,60E-01	rs2045616|rs941425|rs10813821|rs992710|rs838534|rs7260781|rs6476361|rs1256107|rs2840246|rs2839947|rs6910267|rs519861|rs6900628|rs6086074|rs7042042|rs309453|rs471579|rs6055065|rs4242684|rs13201018
Photosensitivity	GLYCOGEN_BREAKDOWN_GLYCOGENOLYSIS	REACTOME	246	8	5	1,04E-01	9,60E-01	rs7535245|rs2269260|rs620281|rs217463|rs855314
Photosensitivity	TRYPTOPHAN_METABOLISM	KEGG	953	31	20	1,06E-01	9,60E-01	rs3741049|rs6741148|rs12730226|rs351676|rs1555481|rs4754298|rs12577790|rs4878808|rs1986415|rs7846217|rs7865905|rs2348024|rs12138459|rs5019656|rs1472080|rs407867|rs2645303|rs11238133|rs10227423|rs2129575
Photosensitivity	RESOLUTION_OF_AP_SITES_VIA_THE_MULTIPLE_NUCLEOTIDE_PATCH_REPLACEMENT_F	REACTOME	192	9	5	1,06E-01	9,60E-01	rs174537|rs2275008|rs1713460|rs10861152|rs11236183
Photosensitivity	SHC1_EVENTS_IN_ERBB4_SIGNALING	REACTOME	1072	39	20	1,06E-01	9,60E-01	rs264336|rs2888104|rs16849142|rs17329875|rs6435692|rs4452759|rs7817546|rs2005219|rs13060691|rs13260061|rs960824|rs6827871|rs4236723|rs1432442|rs10171977|rs904453|rs16879142|rs12620896|rs2876980|rs1357141
Photosensitivity	SYNTHESIS_OF_PC	REACTOME	669	23	9	1,07E-01	9,60E-01	rs12141345|rs2577254|rs859068|rs7022410|rs182411|rs1979021|rs4838541|rs13404506|rs11690125
Photosensitivity	TRIGLYCERIDE_BIOSYNTHESIS	REACTOME	1182	45	20	1,10E-01	9,60E-01	rs9030|rs11737840|rs17674015|rs3737358|rs1488|rs6559175|rs6813255|rs1421050|rs2577254|rs2838464|rs2962061|rs2395926|rs2060780|rs11987150|rs3788094|rs17028984|rs1352436|rs3913795|rs247011|rs6762236
Photosensitivity	SEMA4D_INDUCED_CELL_MIGRATION_AND_GROWTH_CONE_COLLAPSE	REACTOME	433	21	11	1,12E-01	9,60E-01	rs2988067|rs12364414|rs1359647|rs962199|rs10512197|rs833480|rs10835147|rs4666390|rs2075511|rs1005570|rs1094561
Photosensitivity	FATTY_ACID_TRIACYLGLYCEROL_AND_KETONE_BODY_METABOLISM	REACTOME	5526	48	20	1,12E-01	9,60E-01	rs9030|rs11737840|rs9585457|rs17674015|rs6579757|rs3741049|rs307176|rs10774816|rs174546|rs3800794|rs6829223|rs2203801|rs32574|rs4148329|rs3737358|rs1488|rs4917342|rs4754298|rs6559175|rs6942490
Photosensitivity	PROTEIN_FOLDING	REACTOME	675	27	12	1,12E-01	9,60E-01	rs9687161|rs17714754|rs7571143|rs6912751|rs40428|rs4716213|rs760849|rs9507572|rs11954532|rs7558608|rs2189659|rs4235379
Photosensitivity	CHROMOSOME_MAINTENANCE	REACTOME	1949	54	20	1,13E-01	9,60E-01	rs3915766|rs7146101|rs174546|rs1266294|rs1883848|rs1126432|rs372543|rs11070327|rs9366694|rs884241|rs3778279|rs2256191|rs3004318|rs3782093|rs129128|rs7357092|rs676114|rs7043114|rs12534920|rs3214004
Photosensitivity	CYTOKINE_CYTOKINE_RECEPTOR_INTERACTION	KEGG	6425	32	20	1,18E-01	9,60E-01	rs17389324|rs5745752|rs2228428|rs721412|rs2008018|rs12654563|rs12130311|rs11568993|rs12251307|rs17086820|rs583911|rs6180|rs17047703|rs2243300|rs10461355|rs12531099|rs8193036|rs12512817|rs7321414|rs6973108
Photosensitivity	CYTOCHROME_P450_ARRANGED_BY_SUBSTRATE_TYPE	REACTOME	1163	48	20	1,18E-01	9,60E-01	rs8192772|rs10487665|rs6741148|rs2277119|rs4803415|rs9418982|rs1048691|rs6068799|rs576119|rs2246709|rs17523880|rs3732860|rs4646491|rs7907528|rs2291617|rs2860975|rs2289105|rs10882168|rs11572172|rs11596645
Photosensitivity	MET_PATHWAY	BIOCARTA	1415	43	20	1,20E-01	9,60E-01	rs5745752|rs2483852|rs12531099|rs4766987|rs2302456|rs10940270|rs10830099|rs2005219|rs2673825|rs13060691|rs17526983|rs6090585|rs706733|rs706724|rs870992|rs11792737|rs3012738|rs2112166|rs1432442|rs10171977
Photosensitivity	SEMA4D_IN_SEMAPHORIN_SIGNALING	REACTOME	597	26	15	1,21E-01	9,60E-01	rs2988067|rs12364414|rs2060581|rs17526983|rs1359647|rs962199|rs10512197|rs833480|rs10835147|rs41741|rs7523940|rs4666390|rs2075511|rs1005570|rs1094561
Photosensitivity	MEIOTIC_RECOMBINATION	REACTOME	993	87	20	1,21E-01	9,60E-01	rs1366452|rs2244012|rs3213191|rs9366694|rs3778279|rs992276|rs323368|rs2030419|rs1048691|rs1801516|rs2508678|rs129128|rs7357092|rs4646536|rs7728406|rs10484434|rs707937|rs9379818|rs3857546|rs1362599
Photosensitivity	EICOSANOID_LIGAND_BINDING_RECEPTORS	REACTOME	507	17	7	1,21E-01	9,60E-01	rs11687066|rs13419955|rs4455605|rs1323556|rs2744902|rs3181384|rs2241362
Photosensitivity	REGULATORY_RNA_PATHWAYS	REACTOME	345	19	13	1,21E-01	9,60E-01	rs885838|rs139873|rs7188960|rs1030211|rs8080480|rs7112576|rs1075617|rs1625691|rs8012576|rs17409275|rs2227301|rs2161006|rs7160403
Photosensitivity	STATHMIN_PATHWAY	BIOCARTA	456	20	11	1,22E-01	9,60E-01	rs883541|rs10918693|rs759947|rs3822295|rs858490|rs17463958|rs7684832|rs891944|rs918167|rs1003815|rs4745514
Photosensitivity	CYTOKINE_PATHWAY	BIOCARTA	491	37	17	1,26E-01	9,60E-01	rs583911|rs3848180|rs2243300|rs8193036|rs4143832|rs2421057|rs17200733|rs17811014|rs1912124|rs25887|rs4921508|rs12075255|rs4334868|rs8193040|rs4722164|rs40401|rs16882131
Photosensitivity	SIGNALING_BY_FGFR1_MUTANTS	REACTOME	1243	37	20	1,26E-01	9,60E-01	rs666336|rs17487009|rs13231090|rs250201|rs9580274|rs10899496|rs6467779|rs883355|rs13250792|rs3129601|rs465174|rs3176292|rs10437827|rs706733|rs706724|rs11048778|rs250092|rs7982293|rs9634328|rs11986428
Photosensitivity	ABORTIVE_ELONGATION_OF_HIV1_TRANSCRIPT_IN_THE_ABSENCE_OF_TAT	REACTOME	195	8	7	1,26E-01	9,60E-01	rs885838|rs139873|rs7112576|rs1625691|rs1613070|rs4799085|rs585811
Photosensitivity	DORSO_VENTRAL_AXIS_FORMATION	KEGG	1149	35	20	1,28E-01	9,60E-01	rs2124724|rs496485|rs7120183|rs2239175|rs11221383|rs2005219|rs7937445|rs10743964|rs17746482|rs1944850|rs2008864|rs11221335|rs2723808|rs1107764|rs11221388|rs1432442|rs984654|rs566326|rs7122859|rs10171977
Photosensitivity	LINOLEIC_ACID_METABOLISM	KEGG	716	65	20	1,31E-01	9,60E-01	rs8192772|rs12048929|rs12143542|rs7176475|rs10798089|rs9418982|rs1800627|rs2246709|rs12439430|rs17764723|rs330712|rs2430119|rs9729852|rs7172971|rs4144116|rs2860975|rs11572172|rs1859690|rs4820315|rs10911933
Photosensitivity	MYOGENESIS	REACTOME	1747	27	20	1,33E-01	9,60E-01	rs17090050|rs1201981|rs17007262|rs6015839|rs4445973|rs2426913|rs6061779|rs6142828|rs4950914|rs16943951|rs944248|rs944261|rs17135097|rs12479463|rs442683|rs17089925|rs16943810|rs1017809|rs6599127|rs2551447
Photosensitivity	TIGHT_JUNCTION	KEGG	6242	30	20	1,33E-01	9,60E-01	rs17809941|rs6480245|rs9969775|rs918285|rs4433471|rs10457562|rs1524967|rs1088672|rs10822936|rs628877|rs3795994|rs10499837|rs7906982|rs13429335|rs7855204|rs4756788|rs11669442|rs7898559|rs1342653|rs6950691
Photosensitivity	ARAP_PATHWAY	BIOCARTA	292	26	13	1,34E-01	9,60E-01	rs2239822|rs7215100|rs6501243|rs11574849|rs8081973|rs7703742|rs10240988|rs933241|rs2744570|rs12771352|rs3768331|rs1894599|rs9611895
Photosensitivity	ARRHYTHMOGENIC_RIGHT_VENTRICULAR_CARDIOMYOPATHY_ARVC	KEGG	5871	38	20	1,35E-01	9,60E-01	rs4748472|rs2299168|rs6480245|rs12876542|rs4433471|rs2239094|rs10822936|rs9333118|rs3795994|rs42050|rs7906982|rs13429335|rs1866901|rs2107118|rs4445973|rs4532407|rs7898559|rs7586183|rs140040|rs16943951
Photosensitivity	RNA_POL_I_RNA_POL_III_AND_MITOCHONDRIAL_TRANSCRIPTION	REACTOME	1830	41	20	1,35E-01	9,60E-01	rs2559658|rs11785348|rs885838|rs10491776|rs139873|rs1408314|rs9366694|rs10756504|rs2005219|rs3778279|rs16867175|rs12418104|rs4253042|rs7148769|rs4253126|rs3088264|rs1381257|rs4668168|rs302917|rs12026838
Photosensitivity	GLUTATHIONE_METABOLISM	KEGG	683	38	13	1,38E-01	9,60E-01	rs11192001|rs1913264|rs10744116|rs7738142|rs3817657|rs1027473|rs6591256|rs11101992|rs2042235|rs4149195|rs6955931|rs6088659|rs9463826
Photosensitivity	TOB1_PATHWAY	BIOCARTA	652	21	16	1,39E-01	9,60E-01	rs12251307|rs4658265|rs17047703|rs2243300|rs10918693|rs858490|rs2770191|rs10493860|rs1932971|rs12244380|rs1003815|rs9866764|rs266363|rs17187469|rs7163945|rs1887054
Photosensitivity	HEDGEHOG_SIGNALING_PATHWAY	KEGG	2151	33	20	1,40E-01	9,60E-01	rs12516561|rs544704|rs7640326|rs4945061|rs9576193|rs288746|rs17389792|rs3781730|rs4716923|rs2475249|rs10261329|rs3823734|rs12698335|rs6099454|rs232651|rs1922081|rs1402292|rs7796435|rs6085938|rs1125413
Photosensitivity	MEF2D_PATHWAY	BIOCARTA	684	43	19	1,41E-01	9,60E-01	rs963065|rs2499611|rs2461489|rs17031191|rs873417|rs2851045|rs6852347|rs4381631|rs4411531|rs3729908|rs738809|rs758658|rs7694484|rs11727778|rs4811158|rs7193148|rs11725047|rs4239343|rs8090692
Photosensitivity	CITRIC_ACID_CYCLE_TCA_CYCLE	REACTOME	675	52	16	1,43E-01	9,60E-01	rs891274|rs7572995|rs1079396|rs767504|rs9534842|rs9567895|rs1359393|rs12485908|rs2341694|rs12328118|rs9814396|rs1486837|rs2158839|rs10247064|rs12427929|rs11674417
Photosensitivity	ALPHA_LINOLENIC_ACID_METABOLISM	KEGG	571	46	18	1,43E-01	9,60E-01	rs2072114|rs12048929|rs12143542|rs7176475|rs10798089|rs7482316|rs1800627|rs12439430|rs330712|rs2430119|rs9729852|rs7172971|rs4144116|rs1135889|rs3788535|rs10911933|rs1487492|rs16827441
Photosensitivity	NEGATIVE_REGULATION_OF_FGFR_SIGNALING	REACTOME	1536	29	20	1,43E-01	9,60E-01	rs743682|rs666336|rs17487009|rs250201|rs10886918|rs9580274|rs2005219|rs534870|rs883355|rs13250792|rs3129601|rs465174|rs3176292|rs10437827|rs6090585|rs6859263|rs10160812|rs1215447|rs250092|rs7982293
Photosensitivity	PACKAGING_OF_TELOMERE_ENDS	REACTOME	515	53	12	1,44E-01	9,60E-01	rs3915766|rs1126432|rs9366694|rs3778279|rs129128|rs12534920|rs3214004|rs10484434|rs1427575|rs9379818|rs3857546|rs1362599
Photosensitivity	SPRY_PATHWAY	BIOCARTA	910	47	20	1,45E-01	9,60E-01	rs721412|rs11568993|rs986345|rs2005219|rs13060691|rs534870|rs17746482|rs3097920|rs6090585|rs1215447|rs2112166|rs1432442|rs984654|rs10171977|rs904453|rs4140770|rs2876980|rs4502102|rs2794235|rs7984951
Photosensitivity	MPR_PATHWAY	BIOCARTA	624	34	20	1,46E-01	9,60E-01	rs883541|rs912239|rs6062356|rs3912541|rs1393849|rs10501976|rs1404912|rs2005219|rs6090585|rs727735|rs2886612|rs2876980|rs1525660|rs7556894|rs918167|rs576479|rs2081066|rs4745514|rs10238438|rs11224629
Photosensitivity	GLYCOSPHINGOLIPID_BIOSYNTHESIS_LACTO_AND_NEOLACTO_SERIES	KEGG	670	44	18	1,47E-01	9,60E-01	rs7742857|rs504117|rs281380|rs7628381|rs9322615|rs11900673|rs611542|rs11673407|rs1653385|rs281407|rs9789488|rs278364|rs658234|rs4553380|rs7582020|rs4144272|rs12482962|rs10841999
Photosensitivity	G1_PATHWAY	BIOCARTA	611	35	16	1,49E-01	9,60E-01	rs10796820|rs997669|rs17047703|rs3740643|rs6797587|rs1048691|rs1801516|rs11667347|rs1932971|rs4646536|rs10273030|rs2417223|rs649392|rs266363|rs7163945|rs228591
Photosensitivity	EGF_PATHWAY	BIOCARTA	1048	30	20	1,49E-01	9,60E-01	rs721412|rs7601754|rs11568993|rs873417|rs2005219|rs13060691|rs17746482|rs706733|rs706724|rs4381631|rs4411531|rs2112166|rs3729908|rs1432442|rs984654|rs10171977|rs758658|rs2716121|rs904453|rs4140770
Photosensitivity	PYRUVATE_METABOLISM_AND_CITRIC_ACID_TCA_CYCLE	REACTOME	905	51	20	1,49E-01	9,60E-01	rs891274|rs7572995|rs1079396|rs7001334|rs767504|rs9534842|rs9567895|rs1359393|rs847682|rs12485908|rs2341694|rs10264649|rs12328118|rs7566884|rs9811074|rs4447777|rs1486837|rs2158839|rs12675850|rs10247064
Photosensitivity	VALINE_LEUCINE_AND_ISOLEUCINE_DEGRADATION	KEGG	1149	39	20	1,51E-01	9,60E-01	rs9585457|rs3741049|rs12337100|rs1555481|rs4754298|rs12577790|rs4878808|rs10849785|rs1986415|rs7865905|rs2348024|rs2029986|rs10755397|rs2239621|rs7590991|rs4305722|rs10485285|rs17454070|rs4835913|rs2783224
Photosensitivity	BUTANOATE_METABOLISM	KEGG	763	33	20	1,51E-01	9,60E-01	rs3741049|rs3006845|rs1555481|rs4754298|rs12577790|rs4878808|rs10849785|rs7865905|rs2744601|rs1330581|rs12467996|rs4765026|rs2567352|rs4699509|rs2301770|rs9811074|rs7685363|rs1946741|rs4835913|rs558275
Photosensitivity	XENOBIOTICS	REACTOME	231	36	8	1,56E-01	9,60E-01	rs8192772|rs8192712|rs9418982|rs2246709|rs2860975|rs11572172|rs1859690|rs1709081
Photosensitivity	BASAL_TRANSCRIPTION_FACTORS	KEGG	386	21	11	1,58E-01	9,60E-01	rs1953738|rs3800453|rs4717903|rs10491310|rs9989245|rs1613070|rs7766094|rs7109662|rs12878503|rs6062014|rs2273006
Photosensitivity	NOS1_PATHWAY	BIOCARTA	1133	40	20	1,59E-01	9,60E-01	rs963065|rs883541|rs7190913|rs2461489|rs17031191|rs873417|rs2851045|rs4780550|rs2872715|rs6852347|rs17550532|rs2284406|rs4381631|rs4411531|rs3729908|rs1900718|rs7202071|rs758658|rs2866250|rs11727778

Photosensitivity	PROSTATE_CANCER	KEGG	2870	37	20	1,65E-01	9,60E-01	rs721412|rs10796820|rs6909633|rs12666636|rs11568993|rs997669|rs12280614|rs160343|rs2324528|rs1546713|rs10886918|rs1029897|rs3740643|rs4918785|rs17276467|rs11685301|rs2005219|rs2684794|rs2673825|rs13060691
Photosensitivity	ELONGATION_ARREST_AND_RECOVERY	REACTOME	246	10	9	1,66E-01	9,60E-01	rs885838|rs139873|rs6981243|rs7112576|rs1625691|rs1613070|rs4799085|rs17211813|rs585811
Photosensitivity	PI3K_EVENTS_IN_ERBB2_SIGNALING	REACTOME	1886	35	20	1,67E-01	9,60E-01	rs721412|rs264336|rs2888104|rs11568993|rs16849142|rs2324528|rs17329875|rs6435692|rs4452759|rs7817546|rs2673825|rs13260061|rs960824|rs17746482|rs706733|rs6827871|rs706724|rs12626158|rs4236723|rs984654
Photosensitivity	KERATAN_SULFATE_BIOSYNTHESIS	REACTOME	674	45	20	1,68E-01	9,60E-01	rs1667271|rs16851527|rs7233900|rs7628381|rs11900673|rs7115683|rs7173652|rs6697045|rs4971224|rs10081532|rs13275988|rs278364|rs10859103|rs11073810|rs2945744|rs2595361|rs1372288|rs17201864|rs1518893|rs4149495
Photosensitivity	BIOSYNTHESIS_OF_THE_N_GLYCAN_PRECURSOR_DOLICHOL_LIPID_LINKED_OLIGOSACC RANSFER_TO_A_NASCENT_PROTEIN	REACTOME	355	20	6	1,70E-01	9,60E-01	rs12036045|rs2581830|rs3004318|rs2850308|rs4847372|rs3749237
Photosensitivity	P53_DEPENDENT_G1_DNA_DAMAGE_RESPONSE	REACTOME	838	33	20	1,71E-01	9,60E-01	rs16827840|rs997669|rs9904424|rs4802351|rs743272|rs9673361|rs4148876|rs1801516|rs1957107|rs12292911|rs11667347|rs4664414|rs1973619|rs2550461|rs1217|rs8063430|rs10484565|rs2417223|rs2286670|rs12453325
Photosensitivity	SYNTHESIS_SECRETION_AND_INACTIVATION_OF_GLP1	REACTOME	363	12	6	1,71E-01	9,60E-01	rs16974510|rs442683|rs6599127|rs1806180|rs9551421|rs11177
Photosensitivity	CSK_PATHWAY	BIOCARTA	554	23	14	1,71E-01	9,60E-01	rs2060581|rs883541|rs10918693|rs2395175|rs1404912|rs858490|rs6736735|rs1525660|rs918167|rs28530648|rs1003815|rs4745514|rs7523940|rs3129882
Photosensitivity	TGF_BETA_SIGNALING_PATHWAY	KEGG	3535	34	20	1,72E-01	9,60E-01	rs17389324|rs1630986|rs1030608|rs7171416|rs1545550|rs6443969|rs885838|rs17047703|rs2247792|rs6973108|rs4849900|rs2005219|rs2475249|rs7771384|rs6120815|rs340595|rs6099454|rs7212632|rs7229153|rs232651
Photosensitivity	G1_S_SPECIFIC_TRANSCRIPTION	REACTOME	194	16	5	1,72E-01	9,60E-01	rs997669|rs6797587|rs11667347|rs837763|rs555812
Photosensitivity	CHRONIC_MYELOID_LEUKEMIA	KEGG	2538	34	20	1,75E-01	9,60E-01	rs1030609|rs10796820|rs6909633|rs2834662|rs2499611|rs2946998|rs17047703|rs4766987|rs7638569|rs10899496|rs3740643|rs1511603|rs2302456|rs2005219|rs13060691|rs764967|rs722813|rs2834670|rs706733|rs6060563
Photosensitivity	INFLAM_PATHWAY	BIOCARTA	747	36	20	1,76E-01	9,60E-01	rs583911|rs17047703|rs2243300|rs2395175|rs3864277|rs4143832|rs2421057|rs17200733|rs17811014|rs1999713|rs1932971|rs7000123|rs4921508|rs12075255|rs4334868|rs4722164|rs28530648|rs1441438|rs40401|rs3129882
Photosensitivity	DEPOSITION_OF_NEW_CENPA_CONTAINING_NUCLEOSOMES_AT_THE_CENTROMERE	REACTOME	637	61	18	1,76E-01	9,60E-01	rs7146101|rs1266294|rs372543|rs11070327|rs9366694|rs3778279|rs3004318|rs129128|rs7043114|rs7453050|rs7161938|rs10484434|rs9379818|rs2099505|rs3857546|rs4267943|rs1362599|rs8011302
Photosensitivity	FATTY_ACID_METABOLISM	KEGG	776	34	20	1,77E-01	9,60E-01	rs17674015|rs3741049|rs1555481|rs4754298|rs12577790|rs4147536|rs4878808|rs10849785|rs7865905|rs2395926|rs2239621|rs247011|rs4835913|rs10024579|rs1135889|rs558275|rs3803695|rs11791018|rs6832271|rs10182865
Photosensitivity	TH1TH2_PATHWAY	BIOCARTA	535	34	17	1,81E-01	9,60E-01	rs12251307|rs583911|rs2243300|rs2395175|rs2421057|rs12215787|rs17811014|rs10204137|rs6131015|rs4921508|rs12244380|rs4334868|rs28530648|rs17187469|rs3129882|rs1569723|rs9402884
Photosensitivity	MRNA_SPLICING_MINOR_PATHWAY	REACTOME	383	17	12	1,81E-01	9,60E-01	rs885838|rs139873|rs9914884|rs7112576|rs1625691|rs1613070|rs17629022|rs10859963|rs16987990|rs626785|rs6030839|rs10494363
Photosensitivity	PYRUVATE_METABOLISM	KEGG	908	27	20	1,82E-01	9,60E-01	rs3741049|rs1555481|rs4754298|rs12577790|rs4878808|rs7112309|rs964092|rs10849968|rs7865905|rs2840246|rs8047464|rs1052176|rs6458065|rs1877908|rs843748|rs4699509|rs6050291|rs2064393|rs9811074|rs3763142
Photosensitivity	MTA3_PATHWAY	BIOCARTA	333	15	10	1,84E-01	9,60E-01	rs8049837|rs4983411|rs1047920|rs16939099|rs1298954|rs9507572|rs3020368|rs8179838|rs16938992|rs11155813
Photosensitivity	CK1_PATHWAY	BIOCARTA	759	27	20	1,85E-01	9,60E-01	rs963065|rs883541|rs17031191|rs2851045|rs6852347|rs17806303|rs6055990|rs265993|rs2719795|rs6893566|rs8115925|rs2223538|rs7123697|rs2866250|rs6140619|rs11727778|rs4938019|rs879606|rs1422919|rs12216492
Photosensitivity	TRNA_AMINOACYLATION	REACTOME	795	31	14	1,85E-01	9,60E-01	rs4945300|rs12730226|rs191833|rs17140468|rs7766094|rs12961130|rs2645303|rs2302330|rs707937|rs4771704|rs6926631|rs268739|rs12022181|rs2432749
Photosensitivity	THYROID_CANCER	KEGG	1104	35	20	1,86E-01	9,60E-01	rs1030608|rs10796820|rs4917342|rs3740643|rs4918785|rs11685301|rs2005219|rs442683|rs6599127|rs914852|rs157869|rs2120825|rs6722568|rs1152005|rs1432442|rs10520405|rs10492292|rs4918805|rs10956433|rs17178655
Photosensitivity	MEIOTIC_SYNAPSIS	REACTOME	1031	59	20	1,86E-01	9,60E-01	rs3915766|rs1883848|rs1126432|rs9366694|rs884241|rs3778279|rs2256191|rs129128|rs12534920|rs3214004|rs2275075|rs10484434|rs7738912|rs1427575|rs9379818|rs2036847|rs4645434|rs9371583|rs3857546|rs2178009
Photosensitivity	DESTABILIZATION_OF_MRNA_BY_BRF1	REACTOME	204	14	5	1,89E-01	9,60E-01	rs13082092|rs11189224|rs33556|rs9543118|rs2498804
Photosensitivity	CARDIAC_MUSCLE_CONTRACTION	KEGG	3169	26	20	1,91E-01	9,60E-01	rs4748472|rs2299168|rs973608|rs2239094|rs42050|rs1500147|rs1866901|rs2107118|rs7586183|rs140040|rs2238012|rs2799686|rs2733954|rs9888368|rs2239062|rs10486972|rs2238043|rs1812144|rs11642949|rs6120890
Photosensitivity	INTRINSIC_PATHWAY	BIOCARTA	603	23	13	1,92E-01	9,60E-01	rs2511989|rs13062355|rs6796803|rs6722598|rs16975259|rs3769641|rs13028299|rs6727477|rs10513803|rs5030102|rs492560|rs6739977|rs4253236
Photosensitivity	UCALPAIN_PATHWAY	BIOCARTA	426	12	6	1,92E-01	9,60E-01	rs10940270|rs6090585|rs870992|rs600135|rs17106421|rs1742501
Photosensitivity	HS_GAG_DEGRADATION	REACTOME	1386	34	20	1,93E-01	9,60E-01	rs9515779|rs6793913|rs7995233|rs6680621|rs2881925|rs9589052|rs7562354|rs4812893|rs8000454|rs4773614|rs12405481|rs1445252|rs9858362|rs12507011|rs6680835|rs9301668|rs7340397|rs12050059|rs2589204|rs7319605
Photosensitivity	ARACHIDONIC_ACID_METABOLISM	KEGG	1356	51	20	1,94E-01	9,60E-01	rs8192772|rs10487665|rs10912414|rs12143542|rs2816|rs7176475|rs10798089|rs8192712|rs9418982|rs576119|rs1800627|rs12439430|rs17764723|rs330712|rs2430119|rs9729852|rs7172971|rs2801904|rs4144116|rs2860975
Photosensitivity	E2F_MEDIATED_REGULATION_OF_DNA_REPLICATION	REACTOME	452	22	8	1,94E-01	9,60E-01	rs997669|rs6797587|rs3782093|rs1056348|rs11667347|rs676114|rs837763|rs555812
Photosensitivity	ASSOCIATION_OF_TRIC_CCT_WITH_TARGET_PROTEINS_DURING_BIOSYNTHESIS	REACTOME	359	16	8	1,95E-01	9,60E-01	rs17714754|rs7571143|rs6912751|rs40428|rs4716213|rs760849|rs7558608|rs4235379
Photosensitivity	EARLY_PHASE_OF_HIV_LIFE_CYCLE	REACTOME	270	14	5	1,95E-01	9,60E-01	rs174546|rs9587505|rs1193693|rs1408906|rs301287
Photosensitivity	N_GLYCAN_ANTENNAE_ELONGATION_IN_THE_MEDIAL_TRANS_GOLGI	REACTOME	962	34	20	1,95E-01	9,60E-01	rs1487984|rs1667271|rs7233900|rs12762685|rs1257180|rs2130177|rs6444192|rs13017917|rs12589188|rs11117162|rs7967976|rs7597227|rs11675416|rs17776120|rs10873193|rs2595361|rs863395|rs17754044|rs10735253|rs4686823
Photosensitivity	INFLUENZA_VIRAL_RNA_TRANSCRIPTION_AND_REPLICATION	REACTOME	1153	33	20	1,98E-01	9,60E-01	rs7993566|rs885838|rs5995711|rs7210156|rs139873|rs12903497|rs11623678|rs6927367|rs2816697|rs7329817|rs7112576|rs3825073|rs1625691|rs1211975|rs1613070|rs11217129|rs12624027|rs7670903|rs219493|rs1452404
Photosensitivity	SHH_PATHWAY	BIOCARTA	679	37	20	1,99E-01	9,60E-01	rs883541|rs288746|rs17389792|rs4716923|rs10261329|rs3823734|rs12698335|rs1922081|rs7796435|rs1125413|rs1889616|rs10256288|rs4716979|rs707485|rs2363923|rs4716945|rs775806|rs918167|rs4745514|rs2835709
Photosensitivity	LYSINE_DEGRADATION	KEGG	952	29	20	2,00E-01	9,60E-01	rs2602248|rs1982510|rs3741049|rs1555481|rs4754298|rs12577790|rs4878808|rs7390989|rs7865905|rs12631730|rs2459139|rs1523074|rs1447011|rs4835913|rs2242259|rs1556488|rs578641|rs7907802|rs10520183|rs11791018
Photosensitivity	BRANCHED_CHAIN_AMINO_ACID_CATABOLISM	REACTOME	507	30	13	2,00E-01	9,60E-01	rs3741049|rs12337100|rs4754298|rs12577790|rs2029986|rs10755397|rs7590991|rs10485285|rs17454070|rs1502762|rs2158839|rs11047681|rs3950612
Photosensitivity	PRE_NOTCH_EXPRESSION_AND_PROCESSING	REACTOME	1434	42	20	2,00E-01	9,60E-01	rs10796820|rs6909633|rs13129525|rs4753743|rs7188960|rs6816542|rs1030211|rs3740643|rs7628381|rs11724190|rs10017961|rs8080480|rs17116491|rs9368179|rs911361|rs1075617|rs2788865|rs17117189|rs1812402|rs12323801
Photosensitivity	P53_PATHWAY	BIOCARTA	347	29	12	2,01E-01	9,60E-01	rs10796820|rs997669|rs3740643|rs1048691|rs1801516|rs11667347|rs4646536|rs12458289|rs649392|rs228591|rs4319556|rs2236719
Photosensitivity	CHONDROITIN_SULFATE_DERMATAN_SULFATE_METABOLISM	REACTOME	2784	32	20	2,02E-01	9,60E-01	rs723988|rs9515779|rs13100173|rs12195809|rs3800794|rs7995233|rs10100391|rs6680621|rs2881925|rs9589052|rs10458997|rs1342761|rs7562354|rs707576|rs8000454|rs2449393|rs13180395|rs4773614|rs17593178|rs12405481
Photosensitivity	DEADENYLATION_DEPENDENT_MRNA_DECAY	REACTOME	622	37	20	2,03E-01	9,60E-01	rs3812278|rs215749|rs13082092|rs581045|rs11189224|rs3105429|rs6948145|rs33556|rs10784820|rs9543118|rs6772823|rs9857753|rs1524246|rs10892279|rs6462370|rs17305345|rs1388794|rs9881693|rs4641872|rs11642944
Photosensitivity	CITRATE_CYCLE_TCA_CYCLE	KEGG	1195	50	20	2,03E-01	9,60E-01	rs891274|rs7572995|rs1079396|rs10813821|rs767504|rs992710|rs9534842|rs9567895|rs6476361|rs1359393|rs12485908|rs2341694|rs12328118|rs4699509|rs2064393|rs7042042|rs9811074|rs7685363|rs1946741|rs4242684
Photosensitivity	GLUCONEOGENESIS	REACTOME	490	15	11	2,04E-01	9,60E-01	rs8049837|rs6499969|rs11189224|rs8057892|rs2490286|rs2064393|rs8062792|rs16961113|rs4745514|rs678363|rs12925511
Photosensitivity	ASSEMBLY_OF_THE_PRE_REPLICATIVE_COMPLEX	REACTOME	911	31	20	2,05E-01	9,60E-01	rs16827840|rs6909633|rs9904424|rs4802351|rs743272|rs9673361|rs4148876|rs1957107|rs12292911|rs9368179|rs911361|rs4664414|rs1973619|rs2550461|rs837763|rs1217|rs555812|rs8063430|rs2071749|rs10484565
Photosensitivity	VEGF_PATHWAY	BIOCARTA	883	37	20	2,07E-01	9,60E-01	rs12654563|rs17086820|rs10461355|rs873417|rs943084|rs11133360|rs706733|rs7671214|rs706724|rs4381631|rs4411531|rs3729908|rs3774208|rs6059703|rs758658|rs33344|rs3829387|rs6806377|rs10515068|rs6842830
Photosensitivity	NUCLEAR_SIGNALING_BY_ERBB4	REACTOME	2231	36	20	2,08E-01	9,60E-01	rs2008018|rs264336|rs2888104|rs8046010|rs6180|rs3169733|rs16849142|rs17329875|rs6435692|rs10501976|rs4452759|rs7817546|rs12937256|rs13331974|rs13260061|rs960824|rs6827871|rs2221449|rs17797430|rs4236723
Photosensitivity	PANCREATIC_CANCER	KEGG	1920	43	20	2,09E-01	9,60E-01	rs721412|rs10796820|rs6909633|rs11568993|rs17047703|rs7227801|rs12512817|rs3740643|rs943084|rs2005219|rs13060691|rs17746482|rs706733|rs6060563|rs12454987|rs706724|rs1048691|rs9368179|rs911361|rs1432442
Photosensitivity	NOTCH_SIGNALING_PATHWAY	KEGG	1387	38	20	2,10E-01	9,60E-01	rs2701623|rs13129525|rs2499611|rs4753743|rs2946998|rs6816542|rs2604882|rs9997981|rs8105009|rs10017961|rs10793994|rs6074170|rs2423511|rs4813046|rs177387|rs6040334|rs1732803|rs2788865|rs17189227|rs17051003
Photosensitivity	MRNA_CAPPING	REACTOME	388	12	9	2,14E-01	9,60E-01	rs885838|rs139873|rs7112576|rs1625691|rs1613070|rs7766094|rs1618536|rs1561734|rs1143407
Photosensitivity	M_G1_TRANSITION	REACTOME	1315	33	20	2,14E-01	9,60E-01	rs16827840|rs10874743|rs6909633|rs9904424|rs4802351|rs6693314|rs743272|rs9673361|rs4148876|rs10906320|rs3782093|rs1957107|rs12292911|rs9368179|rs911361|rs10493851|rs4664414|rs1973619|rs2550461|rs7357092
Photosensitivity	PHASE1_FUNCTIONALIZATION_OF_COMPOUNDS	REACTOME	1515	51	20	2,15E-01	9,60E-01	rs8192772|rs10487665|rs6741148|rs4147536|rs2277119|rs4803415|rs9418982|rs1048691|rs6068799|rs576119|rs722921|rs6116201|rs2246709|rs17523880|rs945772|rs3732860|rs4646491|rs7907528|rs2291617|rs2095639
Photosensitivity	DARPP_32_EVENTS	REACTOME	879	41	20	2,15E-01	9,60E-01	rs1457145|rs963065|rs883541|rs17031191|rs2851045|rs6860907|rs6852347|rs4384209|rs1056348|rs13178509|rs2866250|rs11727778|rs13186687|rs879606|rs16889495|rs36066827|rs918167|rs294474|rs945969|rs11725047
Photosensitivity	GLYCOSAMINOGLYCAN_BIOSYNTHESIS_HEPARAN_SULFATE	KEGG	2273	33	20	2,16E-01	9,60E-01	rs9913031|rs9999101|rs12195809|rs9320488|rs7205890|rs3811755|rs1606724|rs6825825|rs17443293|rs10458997|rs1342761|rs10034047|rs7220560|rs6820268|rs7197164|rs11945500|rs598240|rs12148317|rs7660418|rs2199998
Photosensitivity	GAP_JUNCTION_ASSEMBLY	REACTOME	330	24	9	2,16E-01	9,60E-01	rs1436908|rs8064669|rs6929363|rs1919870|rs3805787|rs12127091|rs7164838|rs11154022|rs17745948
Photosensitivity	TID_PATHWAY	BIOCARTA	479	9	8	2,16E-01	9,60E-01	rs2279664|rs1716543|rs1061631|rs12215787|rs17200733|rs6500596|rs7552664|rs9402884
Photosensitivity	NON_SMALL_CELL_LUNG_CANCER	KEGG	2585	41	20	2,16E-01	9,60E-01	rs721412|rs9868156|rs10796820|rs6909633|rs11568993|rs1656463|rs4917342|rs3740643|rs873417|rs2236947|rs10866044|rs13071758|rs1604003|rs2005219|rs7616062|rs13060691|rs17746482|rs213392|rs706733|rs2629899
Photosensitivity	DC_PATHWAY	BIOCARTA	573	31	16	2,18E-01	9,60E-01	rs583911|rs2243300|rs3864277|rs4143832|rs2421057|rs17811014|rs10818151|rs6074024|rs10983791|rs4921508|rs11863903|rs12075255|rs4334868|rs40401|rs10983917|rs1569723
Photosensitivity	CLASS_B_2_SECRETIN_FAMILY_RECEPTORS	REACTOME	2257	32	20	2,18E-01	9,60E-01	rs7571877|rs544704|rs7640326|rs4945061|rs288746|rs7794503|rs10502294|rs3781730|rs4716923|rs376620|rs17159371|rs10224073|rs11060782|rs7652849|rs4807916|rs17810148|rs12698335|rs1922081|rs11157863|rs1402292
Photosensitivity	MITOTIC_G1_G1_S_PHASES	REACTOME	2477	46	20	2,20E-01	9,61E-01	rs1030608|rs16827840|rs10874743|rs10796820|rs6909633|rs997669|rs9904424|rs3740643|rs4802351|rs1049612|rs6693314|rs743272|rs7687399|rs9673361|rs4148876|rs6797587|rs1048691|rs10906320|rs3782093|rs1957107
Photosensitivity	HEPARAN_SULFATE_HEPARIN_HS_GAG_METABOLISM	REACTOME	3879	32	20	2,21E-01	9,61E-01	rs9515779|rs9913031|rs6793913|rs9999101|rs12195809|rs9320488|rs7205890|rs3811755|rs7995233|rs1606724|rs6680621|rs2881925|rs9589052|rs6825825|rs10458997|rs1342761|rs7562354|rs10034047|rs707576|rs7220560
Photosensitivity	P53_INDEPENDENT_G1_S_DNA_DAMAGE_CHECKPOINT	REACTOME	729	24	20	2,22E-01	9,61E-01	rs16827840|rs9904424|rs4802351|rs743272|rs9673361|rs4148876|rs6797587|rs1801516|rs1957107|rs12292911|rs4664414|rs1973619|rs2550461|rs1217|rs8063430|rs10484565|rs2286670|rs12453325|rs1877354|rs540723
Photosensitivity	VIF_MEDIATED_DEGRADATION_OF_APOBEC3G	REACTOME	738	32	20	2,26E-01	9,67E-01	rs16827840|rs9904424|rs7949655|rs4802351|rs743272|rs9673361|rs4148876|rs1957107|rs12292911|rs4664414|rs1973619|rs2550461|rs1217|rs12807544|rs8063430|rs10484565|rs2286670|rs12453325|rs1877354|401754
Photosensitivity	SIGNALING_BY_WNT	REACTOME	1071	38	20	2,26E-01	9,67E-01	rs16827840|rs351408|rs9904424|rs4802351|rs8004556|rs743272|rs442683|rs9673361|rs4148876|rs6599127|rs1957107|rs12292911|rs1056348|rs4664414|rs1973619|rs2550461|rs1217|rs8063430|rs10484565|rs2286670
Photosensitivity	SCFSKP2_MEDIATED_DEGRADATION_OF_P27_P21	REACTOME	813	33	20	2,27E-01	9,67E-01	rs16827840|rs997669|rs9904424|rs4802351|rs743272|rs9673361|rs4148876|rs1957107|rs12292911|rs11667347|rs4664414|rs1973619|rs2550461|rs1217|rs8063430|rs10484565|rs2417223|rs2286670|rs12453325|rs1877354
Photosensitivity	REGULATION_OF_APOPTOSIS	REACTOME	1432	29	20	2,28E-01	9,67E-01	rs16827840|rs7234019|rs1798619|rs9904424|rs4400714|rs4802351|rs3124238|rs743272|rs9673361|rs4148876|rs2277257|rs1957107|rs12292911|rs12962203|rs12501768|rs2879414|rs4664414|rs1973619|rs2550461|rs729823
Photosensitivity	SEMAPHORIN_INTERACTIONS	REACTOME	2884	32	20	2,32E-01	9,68E-01	rs2498315|rs473500|rs12364414|rs17140205|rs1798619|rs2060581|rs2560635|rs2964652|rs589869|rs4391668|rs842009|rs10940270|rs2242499|rs7183098|rs797831|rs10740870|rs17526983|rs17806303|rs4404490|rs1359647
Photosensitivity	TRANSPORT_TO_THE_GOLGI_AND_SUBSEQUENT_MODIFICATION	REACTOME	1557	30	20	2,33E-01	9,68E-01	rs1487984|rs1667271|rs7233900|rs12762685|rs1275509|rs1257180|rs2130177|rs6444192|rs13017917|rs12589188|rs2162400|rs11730732|rs9452812|rs7148403|rs7754906|rs4806|rs6931728|rs1872183|rs11887744|rs11117162
Photosensitivity	AXON_GUIDANCE	KEGG	9506	37	20	2,34E-01	9,68E-01	rs10423119|rs36041|rs2988067|rs1464463|rs7234019|rs473500|rs12364414|rs13100173|rs17140205|rs575590|rs7894232|rs1798619|rs963065|rs11035695|rs11242599|rs4857260|rs6862713|rs4696738|rs4400714|rs9998674
Photosensitivity	PGC1A_PATHWAY	BIOCARTA	959	43	20	2,35E-01	9,68E-01	rs963065|rs6829223|rs2461489|rs17031191|rs2851045|rs759947|rs3822295|rs6852347|rs4469064|rs4361373|rs3815076|rs17463958|rs135549|rs2866250|rs7684832|rs6817487|rs891944|rs11727778|rs1283614|rs12655172
Photosensitivity	CDK_MEDIATED_PHOSPHORYLATION_AND_REMOVAL_OF_CDC6	REACTOME	698	27	18	2,35E-01	9,68E-01	rs16827840|rs9904424|rs4802351|rs743272|rs9673361|rs4148876|rs1957107|rs12292911|rs4664414|rs1973619|rs2550461|rs1217|rs8063430|rs10484565|rs2286670|rs12453325|rs1877354|401754
Photosensitivity	RACCYCD_PATHWAY	BIOCARTA	551	44	20	2,36E-01	9,68E-01	rs10796820|rs997669|rs3740643|rs2005219|rs13060691|rs706733|rs706724|rs1048691|rs11667347|rs904453|rs4646536|rs2876980|rs33344|rs10273030|rs2417223|rs649392|rs10515068|rs1423236|rs3815701|rs10054692
Photosensitivity	41BB_PATHWAY	BIOCARTA	341	12	5	2,37E-01	9,68E-01	rs2243300|rs1982235|rs2716121|rs832540|rs2237093
Photosensitivity	CROSS_PRESENTATION_OF_SOLUBLE_EXOGENOUS_ANTIGENS_ENDOSOMES	REACTOME	713	27	18	2,38E-01	9,68E-01	rs16827840|rs9904424|rs4802351|rs743272|rs9673361|rs4148876|rs1957107|rs12292911|rs4664414|rs1973619|rs2550461|rs1217|rs8063430|rs10484565|rs2286670|rs12453325|rs1877354|401754
Photosensitivity	RENAL_CELL_CARCINOMA	KEGG	2164	28	20	2,44E-01	9,88E-01	rs5745752|rs1798619|rs7120183|rs17047703|rs12531099|rs12512817|rs11210751|rs11221383|rs4766987|rs2302456|rs943084|rs2005219|rs13060691|rs7937445|rs17526983|rs6039576|rs1944850|rs1867788|rs706733|rs11221332
Photosensitivity	POST_TRANSLATIONAL_PROTEIN_MODIFICATION	REACTOME	6008	39	20	2,46E-01	9,90E-01	rs1487984|rs10108742|rs1667271|rs13262159|rs227867|rs2101809|rs590666|rs10093209|rs7233900|rs12536849|rs6976905|rs11729278|rs12762685|rs1202640|rs9325770|rs6560519|rs3811250|rs2305965|rs12036045|rs3809816
Photosensitivity	SIGNALING_BY_ERBB4	REACTOME	3434	29	20	2,48E-01	9,91E-01	rs721412|rs2008018|rs264336|rs2888104|rs11568993|rs8046010|rs6180|rs3169733|rs16849142|rs2324528|rs17329875|rs6435692|rs10501976|rs4452759|rs7817546|rs2005219|rs12937256|rs13331974|rs2673825|rs13060691
Photosensitivity	PLATELET_CALCIUM_HOMEOSTASIS	REACTOME	1156	69	20	2,51E-01	9,91E-01	rs7120938|rs13064158|rs10430939|rs1866901|rs7586183|rs4684045|rs12313273|rs1317122|rs7961587|rs495435|rs4481994|rs7951076|rs3862986|rs2272294|rs6806666|rs6799104|rs1938985|rs830131|rs7159779|rs6758359
Photosensitivity	NETRIN1_SIGNALING	REACTOME	3710	39	20	2,52E-01	9,91E-01	rs2483852|rs7234019|rs17651767|rs29452|rs7894232|rs6862713|rs3866217|rs4400714|rs9998674|rs6416579|rs353189|rs1384862|rs12054167|rs1807245|rs152342|rs10018142|rs8041800|rs2938767|rs6850501|rs10830099
Photosensitivity	PROTEASOME_PATHWAY	BIOCARTA	280	14	10	2,52E-01	9,91E-01	rs9904424|rs4802351|rs7429360|rs1957107|rs12292911|rs4664414|rs2526025|rs2714732|rs2286670|rs12453325
Photosensitivity	INTERFERON_GAMMA_SIGNALING	REACTOME	2043	63	20	2,53E-01	9,91E-01	rs7946527|rs599683|rs3916765|rs10922552|rs12747584|rs9260759|rs2252405|rs10922613|rs2228615|rs3822295|rs12732279|rs743272|rs11115|rs12215787|rs16951524|rs4421571|rs591042|rs9275595|rs729396|rs1169307
Photosensitivity	GPCR_PATHWAY	BIOCARTA	1102	38	20	2,55E-01	9,91E-01	rs963065|rs883541|rs2461489|rs17031191|rs873417|rs1404912|rs2851045|rs2005219|rs13060691|rs6852347|rs4381631|rs4411531|rs3729908|rs1432442|rs758658|rs2886612|rs2866250|rs1812402|rs904453|rs11727778
Photosensitivity	KERATINOCYTE_PATHWAY	BIOCARTA	1952	32	20	2,55E-01	9,91E-01	rs721412|rs1088672|rs628877|rs11568993|rs7120183|rs11221383|rs353189|rs873417|rs2005219|rs13060691|rs7937445|rs1061631|rs17746482|rs1944850|rs1124787|rs17200733|rs6740453|rs11221335|rs4381631|rs1020445
Photosensitivity	GH_PATHWAY	BIOCARTA	963	29	20	2,57E-01	9,95E-01	rs6180|rs4804366|rs2070776|rs873417|rs2005219|rs13060691|rs706733|rs706724|rs4381631|rs4411531|rs1169307|rs3729908|rs1432442|rs10171977|rs758658|rs3020619|rs2254779|rs904453|rs2876980|rs33344
Photosensitivity	BCR_PATHWAY	BIOCARTA	1053	35	20	2,59E-01	9,96E-01	rs963065|rs2070776|rs2461489|rs17031191|rs873417|rs2851045|rs2005219|rs13060691|rs6852347|rs4381631|rs4411531|rs3729908|rs1432442|rs10171977|rs758658|rs3020619|rs2866250|rs2716121|rs904453|rs11727778
Photosensitivity	CELL_CYCLE	KEGG	2581	50	20	2,61E-01	9,96E-01	rs1030609|rs10874743|rs10796820|rs6909633|rs9374432|rs997669|rs17047703|rs3740643|rs1647253|rs1049612|rs6693314|rs3170660|rs3105453|rs478859|rs6577168|rs13130657|rs6797587|rs1048691|rs1801516|rs239560
Photosensitivity	PI_3K_CASCADE	REACTOME	1868	25	20	2,61E-01	9,96E-01	rs743682|rs666336|rs17487009|rs250201|rs2324528|rs10886918|rs9580274|rs2673825|rs883355|rs13250792|rs3129601|rs465174|rs3176292|rs10437827|rs6859263|rs10160812|rs706733|rs706724|rs250092|rs7982293
Photosensitivity	MELANOGENESIS	KEGG	4077	27	20	2,62E-01	9,96E-01	rs12516561|rs7571877|rs544704|rs7640326|rs4945061|rs12873185|rs3908461|rs3781730|rs4736746|rs873417|rs4918785|rs17276467|rs1404912|rs11685301|rs11060782|rs10745478|rs2005219|rs4884075|rs13060691|rs3822295
Photosensitivity	CYCLIN_E_ASSOCIATED_EVENTS_DURING_G1_S_TRANSITION_	REACTOME	1212	40	20	2,64E-01	9,97E-01	rs1030608|rs16827840|rs997669|rs9904424|rs4802351|rs743272|rs9673361|rs4148876|rs6797587|rs1957107|rs12292911|rs11667347|rs992241|rs4664414|rs1973619|rs2550461|rs10956433|rs1217|rs8063430|rs10484565
Photosensitivity	FORMATION_OF_FIBRIN_CLOT_CLOTTING_CASCADE	REACTOME	703	35	20	2,69E-01	9,99E-01	rs2511989|rs1742932|rs1245480|rs13062355|rs6796803|rs1778193|rs1520481|rs226372|rs584337|rs13217372|rs2274393|rs10513803|rs5030102|rs3024409|rs11243069|rs7107322|rs1429638|rs969936|rs6048526|rs2086297
Photosensitivity	DESTABILIZATION_OF_MRNA_BY_AUF1_HNRNP_D0	REACTOME	811	28	20	2,81E-01	9,99E-01	rs16827840|rs9904424|rs4802351|rs3105429|rs743272|rs9673361|rs4148876|rs1957107|rs12292911|rs4664414|rs1973619|rs10019349|rs2550461|rs1217|rs8063430|rs10484565|rs2286670|rs12453325|rs1877354|rs7004284
Photosensitivity	JAK_STAT_SIGNALING_PATHWAY	KEGG	4346	37	20	2,82E-01	9,99E-01	rs1522772|rs1030609|rs2008018|rs10796820|rs7601754|rs12251307|rs583911|rs6180|rs2243300|rs986345|rs4766987|rs3864277|rs3740643|rs997406|rs1049612|rs4143832|rs534870|rs12324479|rs2421057|rs722813
Photosensitivity	HS_GAG_BIOSYNTHESIS	REACTOME	3154	35	20	2,82E-01	9,99E-01	rs9515779|rs9913031|rs9999101|rs9320488|rs7205890|rs3811755|rs7995233|rs1606724|rs6680621|rs2881925|rs9589052|rs6825825|rs7562354|rs10034047|rs6032142|rs7220560|rs8000454|rs6820268|rs4773614|rs11945500

Photosensitivity	RNA_POL_II_TRANSCRIPTION_PRE_INITIATION_AND_PROMOTER_OPENING	REACTOME	383	17	14	2,83E-01	9,99E-01	rs885838|rs139873|rs3800453|rs7112576|rs1625691|rs1613070|rs7766094|rs7109662|rs12878503|rs6062014|rs2273006|rs1618536|rs1561734|rs1143407
Photosensitivity	ATRBRCA_PATHWAY	BIOCARTA	238	24	11	2,83E-01	9,99E-01	rs2244012|rs10472945|rs6927367|rs1801516|rs2508678|rs10833766|rs7113416|rs17032295|rs540723|rs228591|rs799906
Photosensitivity	CHONDROITIN_SULFATE_BIOSYNTHESIS	REACTOME	846	36	20	2,83E-01	9,99E-01	rs723988|rs3800794|rs10100391|rs13180395|rs17593178|rs12521344|rs2680406|rs4886728|rs1519143|rs951781|rs11247267|rs10497386|rs2394847|rs1445780|rs2290673|rs962066|rs3731918|rs151251|rs1107613|rs4808931
Photosensitivity	A_TETRASACCHARIDE_LINKER_SEQUENCE_IS_REQUIRED_FOR_GAG_SYNTHESIS	REACTOME	1731	36	20	2,84E-01	9,99E-01	rs9515779|rs12195809|rs7995233|rs6680621|rs2881925|rs9589052|rs10458997|rs1342761|rs7562354|rs4812893|rs8000454|rs13180395|rs4773614|rs12405481|rs1445252|rs4571176|rs4886728|rs6680835|rs11223878|rs9301668
Photosensitivity	G_ALPHA_S_SIGNALLING_EVENTS	REACTOME	5019	27	20	2,88E-01	9,99E-01	rs4903967|rs12412993|rs2573083|rs1457145|rs4971851|rs1886957|rs2268467|rs1606119|rs2371462|rs17037898|rs1025598|rs11727674|rs7794503|rs1553474|rs10502294|rs4736746|rs17159371|rs1404912|rs2268477|rs10224073
Photosensitivity	DNA_REPAIR	REACTOME	2111	35	20	2,88E-01	9,99E-01	rs174537|rs885838|rs2244012|rs1464251|rs139873|rs7258303|rs2275008|rs7590342|rs914573|rs10978769|rs9587505|rs6926662|rs7158356|rs2228529|rs7085203|rs1167998|rs4253126|rs6981243|rs6743916|rs7112576
Photosensitivity	ACYL_CHAIN_REMODELLING_OF_PE	REACTOME	696	38	16	2,91E-01	9,99E-01	rs10912414|rs1410119|rs12143542|rs1356410|rs10798089|rs1668547|rs1800627|rs330712|rs2430119|rs9729852|rs992893|rs4144116|rs4820315|rs10911933|rs1487492|rs16827441
Photosensitivity	AUTODEGRADATION_OF_THE_E3_UBIQUITIN_LIGASE_COP1	REACTOME	766	26	20	2,91E-01	9,99E-01	rs16827840|rs9904424|rs4802351|rs743272|rs9673361|rs4148876|rs1801516|rs1957107|rs12292911|rs4664414|rs1973619|rs2550461|rs1217|rs8063430|rs10484565|rs2286670|rs12453325|rs704832|rs1877354|rs228591
Photosensitivity	SIGNALING_BY_NOTCH	REACTOME	4553	39	20	2,97E-01	9,99E-01	rs1030608|rs2701623|rs10796820|rs7850433|rs6909633|rs13129525|rs2499611|rs4753743|rs6756261|rs4851975|rs7188960|rs468141|rs6816542|rs7177654|rs1030211|rs1887719|rs3740643|rs7628381|rs9997981|rs2520361
Photosensitivity	MICRORNA_MIRNA_BIOGENESIS	REACTOME	227	11	9	3,01E-01	9,99E-01	rs885838|rs139873|rs7112576|rs1625691|rs8012576|rs17409275|rs2227301|rs2161006|rs7160403
Photosensitivity	NCAM1_INTERACTIONS	REACTOME	1896	50	20	3,02E-01	9,99E-01	rs4748472|rs1487984|rs2059198|rs7946527|rs8115602|rs1426182|rs7782469|rs6722598|rs1459591|rs10930910|rs13216351|rs1570939|rs591042|rs16975259|rs3769641|rs3763045|rs13028299|rs1409202|rs6727477|rs10740993
Photosensitivity	G0_AND_EARLY_G1	REACTOME	258	21	7	3,09E-01	9,99E-01	rs997669|rs7687399|rs6797587|rs11667347|rs2871921|rs2835709|rs2835760
Photosensitivity	OTHER_GLYCAN_DEGRADATION	KEGG	648	11	8	3,09E-01	9,99E-01	rs6793913|rs9858362|rs3180018|rs17716609|rs10520433|rs12502245|rs7440877|rs1454218
Photosensitivity	SYNTHESIS_OF_PA	REACTOME	1057	34	20	3,10E-01	9,99E-01	rs10912417|rs3737358|rs1488|rs12143542|rs6559175|rs1421050|rs10798089|rs1668547|rs2838464|rs2962061|rs1800627|rs2060780|rs330712|rs11987150|rs2430119|rs9729852|rs3788094|rs4144116|rs17028984|rs1352436
Photosensitivity	ADHERENS_JUNCTION	KEGG	4520	34	20	3,10E-01	9,99E-01	rs6480245|rs4433471|rs10822936|rs11872382|rs3795994|rs10163905|rs4804366|rs7906982|rs13429335|rs1571131|rs7986566|rs17367018|rs1546713|rs16974215|rs1007369|rs7898559|rs558034|rs4918785|rs2862499|rs2675585
Photosensitivity	SPHINGOLIPID_METABOLISM	KEGG	1101	39	16	3,11E-01	9,99E-01	rs1667271|rs6793913|rs7233900|rs3786327|rs10981168|rs5770953|rs9858362|rs3180018|rs927047|rs7604173|rs1541296|rs12136817|rs7874985|rs2595361|rs895344|rs1548984
Photosensitivity	AMYLOIDS	REACTOME	972	61	20	3,14E-01	9,99E-01	rs3775461|rs1812923|rs9366694|rs3778279|rs8132767|rs11702267|rs13070740|rs11966463|rs6532183|rs129128|rs2424577|rs11024600|rs7744710|rs2280214|rs10484434|rs7739889|rs9379818|rs7751843|rs3857546|rs2595361
Photosensitivity	AUTODEGRADATION_OF_CDH1_BY_CDH1_APC_C	REACTOME	857	25	20	3,16E-01	9,99E-01	rs16827840|rs9904424|rs1647253|rs4802351|rs743272|rs9673361|rs4148876|rs1957107|rs12292911|rs4664414|rs1973619|rs2550461|rs1217|rs8063430|rs1994174|rs10484565|rs2286670|rs11687619|rs12647998|rs12453325
Photosensitivity	CTLA4_INHIBITORY_SIGNALING	REACTOME	447	26	9	3,21E-01	9,99E-01	rs351408|rs12435569|rs6090585|rs1056348|rs624035|rs3730050|rs7834615|rs6438537|rs2498804
Photosensitivity	INFLUENZA_LIFE_CYCLE	REACTOME	1671	34	20	3,21E-01	9,99E-01	rs7993566|rs885838|rs5995711|rs7210156|rs139873|rs12903497|rs11623678|rs10183020|rs6927367|rs7622028|rs2816697|rs7329817|rs2038743|rs7770657|rs7112576|rs3825073|rs2328136|rs1625691|rs1209621|rs1613070
Photosensitivity	HYPERTROPHIC_CARDIOMYOPATHY_HCM	KEGG	4149	35	20	3,21E-01	9,99E-01	rs4748472|rs2299168|rs12876542|rs2239094|rs9333118|rs42050|rs17047703|rs1866901|rs2107118|rs3922737|rs7586183|rs140040|rs2238012|rs2799686|rs10940270|rs7778077|rs4725424|rs9888368|rs2239062|rs10486972
Photosensitivity	EIF_PATHWAY	BIOCARTA	158	4	2	3,22E-01	9,99E-01	rs10787899|rs6059703
Photosensitivity	IMMUNOREGULATORY_INTERACTIONS_BETWEEN_A_LYMPHOID_AND_A_NON_LYMPHO	REACTOME	1325	42	20	3,22E-01	9,99E-01	rs3745264|rs1493101|rs12747584|rs9260759|rs10918693|rs2643075|rs2228615|rs858490|rs11115|rs4806798|rs11246067|rs2824271|rs6074024|rs9264219|rs1298954|rs9637987|rs4818317|rs9378229|rs2289495|rs3176861
Photosensitivity	REGULATION_OF_ACTIN_CYTOSKELETON	KEGG	7089	42	20	3,24E-01	9,99E-01	rs2483852|rs743682|rs721412|rs666336|rs12364414|rs1594513|rs10761189|rs17487009|rs1798619|rs11568993|rs9333118|rs12280614|rs250201|rs4756788|rs16974215|rs345306|rs10886918|rs9580274|rs2302456|rs10940270
Photosensitivity	IGF1R_PATHWAY	BIOCARTA	735	38	20	3,25E-01	9,99E-01	rs883541|rs1546713|rs1404912|rs2005219|rs2684794|rs13060691|rs706733|rs706724|rs7162314|rs1432442|rs10171977|rs904453|rs2876980|rs1525660|rs6518758|rs33344|rs7169544|rs918167|rs10515068|rs1423236
Photosensitivity	REGULATION_OF_MRNA_STABILITY_BY_PROTEINS_THAT_BIND_AU_RICH_ELEMENTS	REACTOME	1372	25	20	3,28E-01	9,99E-01	rs16827840|rs9904424|rs873417|rs4802351|rs13082092|rs11189224|rs3105429|rs3105453|rs743272|rs9673361|rs33556|rs4148876|rs4381631|rs4411531|rs1957107|rs9543118|rs12292911|rs1613070|rs4664414|rs758658
Photosensitivity	BASE_EXCISION_REPAIR	REACTOME	209	13	9	3,28E-01	9,99E-01	rs174546|rs2275008|rs3744357|rs1713460|rs3135998|rs10861152|rs3213403|rs11236183|rs3213255
Photosensitivity	TRANSCRIPTIONAL_REGULATION_OF_WHITE_ADIPOCYTE_DIFFERENTIATION	REACTOME	2719	33	20	3,28E-01	9,99E-01	rs4978629|rs307176|rs10774816|rs3800794|rs6829223|rs7034018|rs16973991|rs17056109|rs4917342|rs10733565|rs10816539|rs4979515|rs16975547|rs7715522|rs17200733|rs17090102|rs11067630|rs1048691|rs4469064|rs949745
Photosensitivity	ACTIVATION_OF_ATR_IN_RESPONSE_TO_REPLICATION_STRESS	REACTOME	653	24	16	3,30E-01	9,99E-01	rs10874743|rs10472945|rs6693314|rs6797587|rs10906320|rs10493851|rs7357092|rs4520495|rs11258248|rs2071749|rs1949009|rs1359389|rs1629511|rs7328146|rs2071744|rs540723
Photosensitivity	ACTIVATION_OF_THE_PRE_REPLICATIVE_COMPLEX	REACTOME	565	23	14	3,31E-01	9,99E-01	rs10874743|rs6693314|rs10906320|rs3782093|rs10493851|rs7357092|rs676114|rs837763|rs4520495|rs555812|rs11258248|rs2071749|rs1949009|rs2071744
Photosensitivity	GLIOMA	KEGG	2070	36	20	3,32E-01	9,99E-01	rs721412|rs10796820|rs6909633|rs11568993|rs1546713|rs3740643|rs873417|rs2005219|rs2684794|rs2673825|rs13060691|rs3822295|rs17746482|rs706733|rs17746897|rs706724|rs1048691|rs4381631|rs4411531|rs4775789
Photosensitivity	REGULATION_OF_ORNITHINE_DECARBOXYLASE_ODC	REACTOME	773	28	19	3,33E-01	9,99E-01	rs16827840|rs9904424|rs4802351|rs743272|rs9673361|rs4148876|rs1957107|rs12292911|rs4664414|rs1973619|rs2550461|rs1217|rs8063430|rs10484565|rs2286670|rs7841955|rs12453325|rs1877354|401754
Photosensitivity	MEMBRANE_TRAFFICKING	REACTOME	2276	31	20	3,34E-01	9,99E-01	rs7023913|rs7544627|rs5014235|rs13182713|rs1436908|rs8064669|rs904115|rs11574849|rs6917198|rs6929363|rs1275509|rs2432216|rs12126748|rs3003608|rs13196275|rs12338625|rs10042243|rs6090585|rs1919870|rs2074797
Photosensitivity	ETS_PATHWAY	BIOCARTA	682	39	20	3,34E-01	9,99E-01	rs2499611|rs7120183|rs11221383|rs7937445|rs1944850|rs11221332|rs1107764|rs11221388|rs3815076|rs7122859|rs1999713|rs1812402|rs2836747|rs12793676|rs2088229|rs7130878|rs3782280|rs6590281|rs1242991|rs1882832
Photosensitivity	AMINO_ACID_AND_OLIGOPEPTIDE_SLC_TRANSPORTERS	REACTOME	1707	23	20	3,35E-01	9,99E-01	rs8105903|rs10974591|rs12490575|rs2455280|rs2886790|rs2507821|rs3820201|rs2588233|rs3887266|rs2236941|rs1892250|rs2671444|rs876985|rs12327843|rs3498|rs2398438|rs6546120|rs7983644|rs4646214|rs10506269
Photosensitivity	CACAM_PATHWAY	BIOCARTA	401	13	7	3,36E-01	9,99E-01	rs759947|rs3822295|rs17463958|rs1558294|rs7684832|rs891944|rs4844851
Photosensitivity	S_PHASE	REACTOME	2076	44	20	3,39E-01	9,99E-01	rs1030608|rs16827840|rs10796820|rs174537|rs997669|rs9904424|rs3740643|rs4802351|rs743272|rs9673361|rs4148876|rs6797587|rs1048691|rs3782093|rs1957107|rs12292911|rs11667347|rs6999335|rs4664414|rs11987150
Photosensitivity	TFF_PATHWAY	BIOCARTA	697	30	20	3,40E-01	9,99E-01	rs6180|rs2070776|rs2005219|rs17746482|rs442683|rs706733|rs706724|rs6599127|rs984654|rs10171977|rs3020619|rs6461845|rs4140770|rs2876980|rs33344|rs10515068|rs39454|rs1423236|rs3815701|rs10054692
Photosensitivity	ERK_PATHWAY	BIOCARTA	1025	28	20	3,42E-01	9,99E-01	rs1030609|rs1546713|rs2005219|rs2684794|rs13060691|rs17746482|rs6090585|rs17746897|rs7162314|rs1444043|rs1432442|rs195204|rs984654|rs1294383|rs10171977|rs6999335|rs12436076|rs904453|rs10956433|rs4140770
Photosensitivity	CGMP_EFFECTS	REACTOME	1520	51	20	3,42E-01	9,99E-01	rs6443589|rs2573083|rs32574|rs16927077|rs1025598|rs11203213|rs10823217|rs11042916|rs4243390|rs4834779|rs10497482|rs10497599|rs11178236|rs11045359|rs1544863|rs13089659|rs12255651|rs4611248|rs1492119|rs7922491
Photosensitivity	PHOSPHOLIPASE_C_MEDIATED_CASCADE	REACTOME	2628	25	20	3,44E-01	9,99E-01	rs743682|rs666336|rs17487009|rs628877|rs883541|rs250201|rs10886918|rs9580274|rs4736746|rs873417|rs1404912|rs759947|rs883355|rs13250792|rs3129601|rs465174|rs3176292|rs10437827|rs6859263|rs1124787
Photosensitivity	LAIR_PATHWAY	BIOCARTA	479	28	15	3,47E-01	9,99E-01	rs12747584|rs6796803|rs2228615|rs1440046|rs11115|rs17200733|rs1376177|rs10513803|rs3176861|rs1008761|rs5030102|rs4722164|rs3093032|rs896117|rs8264
Photosensitivity	SPPA_PATHWAY	BIOCARTA	1428	30	20	3,49E-01	9,99E-01	rs10487665|rs10912414|rs873417|rs12143542|rs10940270|rs2005219|rs10798089|rs13060691|rs6090585|rs6055990|rs4381631|rs870992|rs4411531|rs2719795|rs8115925|rs3729908|rs1432442|rs330712|rs2223538|rs758658
Photosensitivity	CDT1_ASSOCIATION_WITH_THE_CDC6_ORC_ORIGIN_COMPLEX	REACTOME	793	28	20	3,50E-01	9,99E-01	rs16827840|rs9904424|rs4802351|rs743272|rs9673361|rs4148876|rs1957107|rs12292911|rs4664414|rs1973619|rs2550461|rs837763|rs1217|rs555812|rs8063430|rs10484565|rs2286670|rs12453325|rs1877354|401754
Photosensitivity	ECM_RECEPTOR_INTERACTION	KEGG	3602	42	20	3,50E-01	9,99E-01	rs1630986|rs17312104|rs2245617|rs2861315|rs709505|rs9333118|rs747574|rs2303076|rs6680621|rs10487172|rs2881925|rs10940270|rs707576|rs7782469|rs1440046|rs2701270|rs11688011|rs11789200|rs12405481|rs6685612
Photosensitivity	TELOMERE_MAINTENANCE	REACTOME	1133	62	20	3,51E-01	9,99E-01	rs3915766|rs174546|rs1126432|rs9366694|rs3778279|rs3782093|rs129128|rs7357092|rs676114|rs12534920|rs3214004|rs10484434|rs1427575|rs9379818|rs1359389|rs1629511|rs11236183|rs3857546|rs7328146|rs1362599
Photosensitivity	GENERATION_OF_SECOND_MESSENGER_MOLECULES	REACTOME	616	33	15	3,51E-01	9,99E-01	rs1798619|rs3916765|rs10918693|rs858490|rs9275595|rs10799312|rs6736735|rs2084385|rs7704354|rs11799692|rs1003815|rs10915840|rs444556|rs1190986|rs6555894
Photosensitivity	HOMOLOGOUS_RECOMBINATION_REPAIR_OF_REPLICATION_INDEPENDENT_DOUBLE_ST	REACTOME	376	15	7	3,53E-01	9,99E-01	rs2244012|rs1801516|rs2508678|rs7357092|rs10849605|rs228591|rs799906
Photosensitivity	GLYCOSAMINOGLYCAN_DEGRADATION	KEGG	709	35	20	3,56E-01	9,99E-01	rs9913031|rs6793913|rs13100173|rs7220560|rs598240|rs9858362|rs2199998|rs12507011|rs10053925|rs555812|rs10025120|rs2098026|rs17818103|rs447998|rs6584238|rs1283|rs709607|rs7226014|rs36929|rs6453415
Photosensitivity	ACTIVATION_OF_GENES_BY_ATF4	REACTOME	316	15	7	3,56E-01	9,99E-01	rs11189224|rs33556|rs3764261|rs9543118|rs1613070|rs12666800|rs11642944
Photosensitivity	ER_PHAGOSOME_PATHWAY	REACTOME	1416	27	20	3,57E-01	9,99E-01	rs16827840|rs9904424|rs9260759|rs4802351|rs743272|rs9673361|rs6952793|rs4148876|rs1957107|rs12292911|rs9264219|rs162202|rs4664414|rs1973619|rs2550461|rs1217|rs10988774|rs9378229|rs8063430|rs10484565
Photosensitivity	NEPHRIN_INTERACTIONS	REACTOME	1617	49	20	3,57E-01	9,99E-01	rs17809941|rs918285|rs10499837|rs16974215|rs6950691|rs10252126|rs573946|rs493725|rs7789885|rs706733|rs706724|rs7809986|rs1799027|rs2068338|rs519514|rs2589949|rs2708507|rs17106421|rs1742501|rs12474808
Photosensitivity	SCF_BETA_TRCP_MEDIATED_DEGRADATION_OF_EMI1	REACTOME	793	28	19	3,58E-01	9,99E-01	rs16827840|rs9904424|rs4802351|rs743272|rs9673361|rs4148876|rs1957107|rs12292911|rs4664414|rs1973619|rs2550461|rs1217|rs8063430|rs10484565|rs2286670|rs12453325|rs1877354|rs633384|401754
Photosensitivity	SHC_RELATED_EVENTS	REACTOME	302	23	11	3,59E-01	9,99E-01	rs4804366|rs2005219|rs13060691|rs1432442|rs10171977|rs904453|rs2876980|rs10771184|rs2115386|rs2914559|rs4255740
Photosensitivity	BAD_PATHWAY	BIOCARTA	927	34	20	3,60E-01	9,99E-01	rs883541|rs1546713|rs1404912|rs10777109|rs2005219|rs2684794|rs706733|rs6060563|rs706724|rs17811365|rs2726648|rs7162314|rs25887|rs2876980|rs218282|rs7310517|rs1525660|rs12458289|rs9606893|rs33344
Photosensitivity	ALLOGRAFT_REJECTION	KEGG	1510	54	20	3,60E-01	9,99E-01	rs3916765|rs583911|rs2243300|rs9260759|rs4143832|rs2421057|rs68600|rs17200733|rs17811014|rs9275595|rs6074024|rs9264219|rs624035|rs9261950|rs4921508|rs9378229|rs12075255|rs206768|rs4334868|rs10484565
Photosensitivity	PROTEASOME	KEGG	821	27	20	3,62E-01	9,99E-01	rs16827840|rs9904424|rs4802351|rs743272|rs9673361|rs4148876|rs1957107|rs12292911|rs4664414|rs1973619|rs2550461|rs7320128|rs1217|rs8063430|rs10484565|rs2286670|rs12453325|rs1582175|rs1877354|401754
Photosensitivity	NEUROTRANSMITTER_RELEASE_CYCLE	REACTOME	1400	41	20	3,62E-01	9,99E-01	rs10974591|rs9682960|rs362623|rs3006845|rs10778102|rs2886790|rs5998705|rs3820201|rs1710879|rs2744601|rs1330581|rs12467996|rs13289148|rs363251|rs12461006|rs13215369|rs1905752|rs11710097|rs4077707|rs2930142
Photosensitivity	FORMATION_OF_TRANSCRIPTION_COUPLED_NER_TC_NER_REPAIR_COMPLEX	REACTOME	389	25	14	3,64E-01	9,99E-01	rs885838|rs139873|rs4253042|rs4253126|rs6981243|rs7112576|rs1625691|rs7766094|rs2025448|rs1618536|rs1561734|rs1143407|rs794078|rs794084
Photosensitivity	FORMATION_OF_THE_HIV1_EARLY_ELONGATION_COMPLEX	REACTOME	329	14	11	3,65E-01	9,99E-01	rs885838|rs139873|rs7112576|rs1625691|rs1613070|rs7766094|rs4799085|rs585811|rs1618536|rs1561734|rs1143407
Photosensitivity	SIGNALING_BY_RHO_GTPASES	REACTOME	4877	39	20	3,65E-01	9,99E-01	rs13137847|rs12364414|rs10761189|rs6854185|rs7174521|rs4055938|rs13163604|rs463717|rs246660|rs7227801|rs6781236|rs6894946|rs12778761|rs345306|rs4810783|rs9825026|rs10483764|rs4273488|rs12993643|rs733675
Photosensitivity	IL_2_SIGNALING	REACTOME	936	45	20	3,67E-01	9,99E-01	rs12251307|rs3864277|rs10899496|rs4143832|rs2005219|rs13060691|rs706733|rs706724|rs17811365|rs1432442|rs10171977|rs4240910|rs2292979|rs904453|rs2876980|rs12244380|rs10771184|rs2278278|rs273266|rs33344
Photosensitivity	PLATELET_ACTIVATION_SIGNALING_AND_AGGREGATION	REACTOME	6965	32	20	3,67E-01	9,99E-01	rs5745752|rs721412|rs8049837|rs2511989|rs1742932|rs1088672|rs885838|rs628877|rs10487781|rs4732260|rs11568993|rs10912417|rs17047703|rs12531099|rs12512817|rs345306|rs353189|rs13062355|rs6796803|rs3778818
Photosensitivity	ALK_PATHWAY	BIOCARTA	1932	23	20	3,70E-01	9,99E-01	rs7171416|rs6443969|rs885838|rs4658265|rs17047703|rs1982235|rs10072402|rs2475249|rs2770191|rs6099454|rs442683|rs7212632|rs232651|rs12505085|rs6556066|rs6599127|rs6884482|rs6085938|rs991880|rs6107902
Photosensitivity	AXON_GUIDANCE	REACTOME	13131	38	20	3,70E-01	9,99E-01	rs4748472|rs1487984|rs2483852|rs2498315|rs7234019|rs17651767|rs473500|rs12364414|rs2059198|rs17140205|rs7946527|rs7023913|rs29452|rs7894232|rs1798619|rs2060581|rs6862713|rs17333297|rs2070776|rs3866217
Photosensitivity	ACETYLCHOLINE_BINDING_AND_DOWNSTREAM_EVENTS	REACTOME	242	10	6	3,72E-01	9,99E-01	rs2946542|rs1567883|rs11071503|rs4618346|rs4779939|rs1514254
Photosensitivity	MRNA_SPLICING	REACTOME	1057	34	20	3,75E-01	9,99E-01	rs885838|rs10197258|rs139873|rs2588882|rs2305797|rs9914884|rs2526388|rs7112576|rs17704641|rs4646296|rs1798177|rs6433636|rs1625691|rs1613070|rs17629022|rs1398969|rs10019349|rs4238961|rs2074732|rs3745216
Photosensitivity	PI3K_CASCADE	REACTOME	2702	29	20	3,75E-01	9,99E-01	rs743682|rs666336|rs17487009|rs4804366|rs250201|rs10886918|rs9580274|rs1941590|rs2440455|rs2809244|rs7778077|rs4725424|rs4595861|rs883355|rs13250792|rs3129601|rs4725435|rs465174|rs3176292|rs10437827
Photosensitivity	INCRETIN_SYNTHESIS_SECRETION_AND_INACTIVATION	REACTOME	540	17	9	3,76E-01	9,99E-01	rs16974510|rs442683|rs6599127|rs1806180|rs6990313|rs9551421|rs2898292|rs11177|rs6867918
Photosensitivity	PTM_GAMMA_CARBOXYLATION_HYPUSINE_FORMATION_AND_ARYLSULFATASE_ACTIV	REACTOME	377	26	13	3,78E-01	9,99E-01	rs11729278|rs3809816|rs13062355|rs5770953|rs2121397|rs891944|rs10020382|rs10053925|rs918167|rs17818103|rs9307398|rs447998|rs6453415
Photosensitivity	TYPE_I_DIABETES_MELLITUS	KEGG	1975	52	20	3,80E-01	9,99E-01	rs12698214|rs3916765|rs583911|rs3006845|rs9260759|rs1638742|rs4909242|rs7783909|rs2421057|rs68600|rs17200733|rs17811014|rs9275595|rs3807843|rs1330581|rs12467996|rs9264219|rs624035|rs221213|rs2056449
Photosensitivity	VIP_PATHWAY	BIOCARTA	1000	48	20	3,82E-01	9,99E-01	rs1030609|rs963065|rs883541|rs2461489|rs17031191|rs2851045|rs6852347|rs7823327|rs992241|rs7694484|rs10956433|rs11727778|rs4811158|rs10740099|rs16902654|rs918167|rs4733581|rs633596|rs6997983|rs11725047
Photosensitivity	COSTIMULATION_BY_THE_CD28_FAMILY	REACTOME	1479	40	20	3,82E-01	9,99E-01	rs351408|rs1798619|rs3916765|rs10918693|rs12435569|rs16923201|rs858490|rs10206295|rs6090585|rs706733|rs706724|rs2838522|rs9275595|rs2234167|rs2838523|rs1056348|rs12626158|rs624035|rs3109032|rs4301703
Photosensitivity	FORMATION_OF_RNA_POL_II_ELONGATION_COMPLEX_	REACTOME	401	16	13	3,85E-01	9,99E-01	rs885838|rs139873|rs6981243|rs7112576|rs1625691|rs1613070|rs7766094|rs4799085|rs17211813|rs585811|rs1618536|rs1561734|rs1143407
Photosensitivity	PLATELET_SENSITIZATION_BY_LDL	REACTOME	676	51	18	3,88E-01	9,99E-01	rs351408|rs10912414|rs12143542|rs10798089|rs8004556|rs1607581|rs1056348|rs330712|rs1288480|rs2430119|rs9729852|rs4144116|rs13398007|rs10911933|rs1487492|rs2237093|rs16827441|rs4908343
Photosensitivity	CTCF_PATHWAY	BIOCARTA	1035	33	20	3,91E-01	9,99E-01	rs1030609|rs2070776|rs4658265|rs17047703|rs2673825|rs2770191|rs706733|rs12505085|rs706724|rs10493860|rs6999335|rs3020619|rs1932971|rs10956433|rs33344|rs2417223|rs16902654|rs10515068|rs4733581|rs6997983
Photosensitivity	CELL_CYCLE_CHECKPOINTS	REACTOME	1961	35	20	3,92E-01	9,99E-01	rs16827840|rs10874743|rs10472945|rs997669|rs9904424|rs1647253|rs4802351|rs6693314|rs13130657|rs743272|rs9673361|rs4148876|rs6797587|rs10906320|rs1801516|rs1957107|rs12292911|rs10493851|rs3996325|rs11592827
Photosensitivity	O_GLYCAN_BIOSYNTHESIS	KEGG	1579	47	20	3,94E-01	9,99E-01	rs590666|rs12536849|rs6976905|rs1202640|rs6560519|rs2305965|rs1465231|rs12477460|rs11247000|rs1481373|rs7686995|rs719430|rs10060240|rs890797|rs10081532|rs6860445|rs6760219|rs836022|rs13275988|rs12479422
Photosensitivity	RNA_POL_I_TRANSCRIPTION	REACTOME	937	59	20	3,96E-01	9,99E-01	rs885838|rs9366694|rs2005219|rs3778279|rs16867175|rs2228529|rs4253126|rs1381257|rs129128|rs7766094|rs10484434|rs11128940|rs2025448|rs9379818|rs2357266|rs11676697|rs1618536|rs1561734|rs3857546|rs1561330
Photosensitivity	IL17_PATHWAY	BIOCARTA	316	17	12	3,99E-01	9,99E-01	rs8193036|rs10918693|rs10745478|rs858490|rs25887|rs7310517|rs8193040|rs4722164|rs1003815|rs40401|rs938486|rs16882131
Photosensitivity	MCM_PATHWAY	BIOCARTA	234	24	9	3,99E-01	9,99E-01	rs997669|rs10777109|rs11667347|rs837763|rs555812|rs7310517|rs2071749|rs2417223|rs2071744
Photosensitivity	G_PROTEIN_ACTIVATION	REACTOME	703	26	14	4,01E-01	9,99E-01	rs1937600|rs2269340|rs2236941|rs11157863|rs13196610|rs12174208|rs501163|rs2886612|rs2301230|rs7152036|rs584845|rs8104096|rs10238438|rs11810173
Photosensitivity	PPAR_SIGNALING_PATHWAY	KEGG	1324	38	20	4,02E-01	9,99E-01	rs2072114|rs17674015|rs1571131|rs4917342|rs7482316|rs914852|rs10882615|rs6740107|rs1144396|rs10846765|rs157869|rs2395926|rs2120825|rs1152005|rs2239621|rs6818701|rs3732860|rs549062|rs135549|rs7109662
Photosensitivity	ATM_PATHWAY	BIOCARTA	397	19	7	4,05E-01	9,99E-01	rs2244012|rs1801516|rs2508678|rs1812402|rs540723|rs228591|rs799906
Photosensitivity	LYSOSOME_VESICLE_BIOGENESIS	REACTOME	420	19	8	4,06E-01	9,99E-01	rs7544627|rs14976|rs4804528|rs7740227|rs7548844|rs3768331|rs10399621|rs2077852
Photosensitivity	SPHINGOLIPID_DE_NOVO_BIOSYNTHESIS	REACTOME	1020	39	18	4,06E-01	9,99E-01	rs225829|rs6793037|rs10444692|rs3786327|rs10189241|rs977122|rs1998355|rs492369|rs927047|rs1466447|rs1020603|rs7604173|rs1541296|rs12136817|rs12147361|rs12589992|rs7874985|rs895344
Photosensitivity	RIBOFLAVIN_METABOLISM	KEGG	295	4	3	4,07E-01	9,99E-01	rs2501920|rs9493133|rs885239
Photosensitivity	RNA_DEGRADATION	KEGG	1123	30	20	4,08E-01	9,99E-01	rs3812278|rs215749|rs2270048|rs13082092|rs581045|rs10266401|rs11189224|rs6948145|rs33556|rs10784820|rs9543118|rs8111911|rs4807225|rs6772823|rs4476170|rs2047321|rs9857753|rs1524246|rs4784049|rs10892279
Photosensitivity	RNA_POL_II_PRE_TRANSCRIPTION_EVENTS	REACTOME	532	21	18	4,09E-01	9,99E-01	rs885838|rs139873|rs3800453|rs6981243|rs7112576|rs1625691|rs1613070|rs7766094|rs7109662|rs12878503|rs6062014|rs2273006|rs4799085|rs17211813|rs585811|rs1618536|rs1561734|rs1143407
Photosensitivity	PENTOSE_PHOSPHATE_PATHWAY	KEGG	616	33	20	4,09E-01	9,99E-01	rs8049837|rs335124|rs7535245|rs2269260|rs2306569|rs3895063|rs10903843|rs217463|rs11246300|rs11818509|rs2256817|rs10794929|rs2454829|rs1556632|rs10903939|rs678363|rs2306676|rs855314|rs2900367|rs2306181
Photosensitivity	CDMAC_PATHWAY	BIOCARTA	1018	28	20	4,09E-01	9,99E-01	rs1030608|rs873417|rs2005219|rs13060691|rs17200733|rs6055990|rs4381631|rs4411531|rs2719795|rs8115925|rs3729908|rs1432442|rs2223538|rs10492292|rs758658|rs1812402|rs904453|rs10956433|rs6140619|rs2876980
Photosensitivity	FANCONI_ANEMIA_PATHWAY	REACTOME	461	28	13	4,10E-01	9,99E-01	rs7258303|rs7590342|rs6926662|rs1167998|rs6743916|rs1801516|rs226981|rs10833766|rs6545712|rs7113416|rs17032295|rs228591|rs799906

Photosensitivity	TPO_PATHWAY	BIOCARTA	814	38	20	4,10E-01	9,99E-01	rs885838|rs873417|rs2005219|rs13060691|rs706733|rs706724|rs4381631|rs4411531|rs2112166|rs3729908|rs1432442|rs10171977|rs758658|rs2716121|rs904453|rs33344|rs10515068|rs6452735|rs7193148|rs1423236
Photosensitivity	G1_S_TRANSITION	REACTOME	1941	44	20	4,12E-01	9,99E-01	rs1030609|rs16827840|rs10874743|rs997669|rs9904424|rs4802351|rs6693314|rs743272|rs9673361|rs4148876|rs6797587|rs10906320|rs3782093|rs1957107|rs12292911|rs1056348|rs10493851|rs11667347|rs6999335|rs4664414
Photosensitivity	SIGNALING_BY_NOTCH1	REACTOME	3563	38	20	4,12E-01	9,99E-01	rs1030608|rs2701623|rs7850433|rs13129525|rs9374432|rs4753743|rs6756261|rs4851975|rs468141|rs6816542|rs7177654|rs1887719|rs9997981|rs2520361|rs278899|rs7593722|rs1427282|rs10017961|rs10780472|rs3791406
Photosensitivity	ACYL_CHAIN_REMODELLING_OF_PG	REACTOME	571	38	18	4,15E-01	9,99E-01	rs12048929|rs12143542|rs1356410|rs10798089|rs1668547|rs2962061|rs1663171|rs1800627|rs330712|rs2430119|rs9729852|rs1884642|rs4144116|rs10911933|rs1487492|rs4975554|rs16827441|rs36982
Photosensitivity	CYTOSOLIC_DNA_SENSING_PATHWAY	KEGG	620	23	13	4,15E-01	9,99E-01	rs2559658|rs10813821|rs9426832|rs3088264|rs2064393|rs7042042|rs12229321|rs4722164|rs742724|rs9972960|rs2227301|rs3181384|rs669260
Photosensitivity	INTESTINAL_IMMUNE_NETWORK_FOR_IGA_PRODUCTION	KEGG	1392	51	20	4,15E-01	9,99E-01	rs3916765|rs2243300|rs7321414|rs4143832|rs17160128|rs10206295|rs1440046|rs2229135|rs68600|rs2838522|rs12943647|rs3850997|rs9275582|rs2838523|rs6074024|rs624035|rs11671930|rs12075255|rs206768|rs6002551
Photosensitivity	FCER1_PATHWAY	BIOCARTA	1564	45	20	4,17E-01	9,99E-01	rs1798619|rs963065|rs10912414|rs2461489|rs17031191|rs12143542|rs2851045|rs2005219|rs10798089|rs13060691|rs6852347|rs706733|rs706724|rs3729908|rs1432442|rs330712|rs10171977|rs2430119|rs9729852|rs6532915
Photosensitivity	TCA_CYCLE_AND_RESPIRATORY_ELECTRON_TRANSPORT	REACTOME	2599	44	20	4,18E-01	9,99E-01	rs891274|rs7572995|rs1079396|rs7001334|rs1500147|rs4854053|rs767504|rs9775695|rs4147750|rs714621|rs9534842|rs9567895|rs2733954|rs1812144|rs1359393|rs10971025|rs1345638|rs17321133|rs7795088|rs11669029
Photosensitivity	CONVERSION_FROM_APC_C_CDC20_TO_APC_C_CDH1_IN_LATE_ANAPHASE	REACTOME	226	11	7	4,27E-01	9,99E-01	rs1647253|rs478859|rs6577168|rs1994174|rs11687619|rs12647998|rs2270694
Photosensitivity	SIGNALING_BY_ROBO_RECEPTOR	REACTOME	1900	35	20	4,27E-01	9,99E-01	rs1798619|rs6781236|rs9825026|rs12054167|rs1807245|rs10018142|rs2029206|rs4716146|rs6850501|rs7562354|rs1154398|rs4697130|rs10865573|rs6516498|rs990309|rs1977298|rs267131|rs7622781|rs12480230|rs1542568
Photosensitivity	DILATED_CARDIOMYOPATHY	KEGG	4636	35	20	4,28E-01	9,99E-01	rs4748472|rs2299168|rs12876542|rs2239094|rs9333118|rs42050|rs17047703|rs1866901|rs2107118|rs4532407|rs7586183|rs140040|rs4736746|rs2238012|rs1404912|rs2799686|rs10940270|rs9888368|rs2239062|rs10486972
Photosensitivity	AMINE_COMPOUND_SLC_TRANSPORTERS	REACTOME	1298	32	20	4,29E-01	9,99E-01	rs12141345|rs9682960|rs12490575|rs859068|rs7022410|rs7131598|rs1972873|rs1710879|rs182411|rs363251|rs6796254|rs720625|rs1905752|rs2531748|rs3785155|rs923073|rs1979021|rs3760586|rs6763180|rs11710097
Photosensitivity	INSULIN_RECEPTOR_SIGNALLING_CASCADE	REACTOME	3006	29	20	4,29E-01	9,99E-01	rs743682|rs666336|rs17487009|rs4804366|rs250201|rs10886918|rs9580274|rs1941590|rs2440455|rs2809244|rs2302456|rs7778077|rs4725424|rs2005219|rs4595861|rs13060691|rs883355|rs13250792|rs3129601|rs4725435
Photosensitivity	NCAM_SIGNALING_FOR_NEURITE_OUT_GROWTH	REACTOME	2480	35	20	4,30E-01	9,99E-01	rs4748472|rs1487984|rs2059198|rs7946527|rs8115602|rs2005219|rs1426182|rs13060691|rs7782469|rs6090585|rs6722598|rs1459591|rs10930910|rs13216351|rs591042|rs1570939|rs16975259|rs1165838|rs3769641|rs3763045
Photosensitivity	MITOCHONDRIA_PATHWAY	BIOCARTA	317	20	12	4,33E-01	9,99E-01	rs7953290|rs3212153|rs6060563|rs12501768|rs1800627|rs6461845|rs11899004|rs12458289|rs39454|rs4319556|rs2236719|rs12673033
Photosensitivity	G2_M_CHECKPOINTS	REACTOME	710	26	18	4,34E-01	9,99E-01	rs10874743|rs10472945|rs6693314|rs6797587|rs10906320|rs1801516|rs10493851|rs7357092|rs4520495|rs11258248|rs2071749|rs1949009|rs1359389|rs1629511|rs7328146|rs2071744|rs540723|rs228591
Photosensitivity	EXTRACELLULAR_MATRIX_ORGANIZATION	REACTOME	3783	36	20	4,34E-01	9,99E-01	rs262065|rs10091563|rs1507263|rs2036984|rs625820|rs2214375|rs747574|rs7213204|rs1010698|rs9307326|rs10090518|rs7844188|rs2194860|rs2808787|rs7782469|rs7089663|rs10952049|rs6722598|rs13216351|rs4736297
Photosensitivity	PI3K_EVENTS_IN_ERBB4_SIGNALING	REACTOME	1709	38	20	4,35E-01	9,99E-01	rs264336|rs2888104|rs16849142|rs2324528|rs17329875|rs6435692|rs4452759|rs7817546|rs2673825|rs13260061|rs960824|rs706733|rs6827871|rs706724|rs12626158|rs4236723|rs3730050|rs16879142|rs12620896|rs11664248
Photosensitivity	ASCORBATE_AND_ALDARATE_METABOLISM	KEGG	874	164	12	4,36E-01	9,99E-01	rs4148329|rs1555481|rs4878808|rs7865905|rs6761246|rs2361502|rs4835913|rs2608822|rs376109|rs11791018|rs6830865|rs2687964
Photosensitivity	COLLAGEN_FORMATION	REACTOME	3203	35	20	4,37E-01	9,99E-01	rs262065|rs10091563|rs1507263|rs2036984|rs625820|rs2214375|rs747574|rs9307326|rs7844188|rs2194860|rs2808787|rs7782469|rs7089663|rs10952049|rs6722598|rs13216351|rs4736297|rs9342782|rs16975259|rs7743423
Photosensitivity	STEROID_HORMONE_BIOSYNTHESIS	KEGG	1491	127	20	4,38E-01	9,99E-01	rs8109054|rs9423399|rs4148329|rs6741148|rs1937881|rs6761246|rs2544800|rs4702379|rs2361502|rs2246709|rs17523880|rs2289105|rs7074522|rs2050308|rs6415602|rs619824|rs2277603|rs9423584|rs1859690|rs376109
Photosensitivity	P75_NTR_RECEPTOR_MEDIATED_SIGNALLING	REACTOME	2669	30	20	4,38E-01	9,99E-01	rs12364414|rs10761189|rs4296917|rs7174521|rs13377154|rs10504873|rs4350297|rs10509783|rs345306|rs4360891|rs4810783|rs754743|rs7218427|rs9371867|rs1398028|rs7761427|rs790640|rs6692050|rs1332684|rs7027092
Photosensitivity	APC_C_CDH1_MEDIATED_DEGRADATION_OF_CDC20_AND_OTHER_APC_C_CDH1_TARGET<br>TE_MITOSIS_EARLY_G1	REACTOME	959	24	20	4,40E-01	9,99E-01	rs16827840|rs9904424|rs1647253|rs4802351|rs743272|rs9673361|rs4148876|rs1957107|rs12292911|rs4664414|rs1973619|rs2550461|rs1217|rs7500128|rs8063430|rs1994174|rs10484565|rs2286670|rs11687619|rs12647998
Photosensitivity	APC_C_CDC20_MEDIATED_DEGRADATION_OF_MITOTIC_PROTEINS	REACTOME	1094	24	20	4,41E-01	9,99E-01	rs16827840|rs9904424|rs1647253|rs4802351|rs13130657|rs743272|rs9673361|rs4148876|rs1957107|rs12292911|rs11592827|rs4664414|rs1973619|rs2550461|rs1217|rs8063430|rs1994174|rs10484565|rs2286670|rs11687619
Photosensitivity	TEL_PATHWAY	BIOCARTA	978	41	20	4,42E-01	9,99E-01	rs1030609|rs1546713|rs873417|rs2684794|rs17746482|rs1713434|rs4381631|rs4411531|rs7162314|rs984654|rs6999335|rs758658|rs10956433|rs4140770|rs3093926|rs10771184|rs12458289|rs7169544|rs16902654|rs4733581
Photosensitivity	GABA_SYNTHESIS_RELEASE_REUPTAKE_AND_DEGRADATION	REACTOME	746	39	20	4,44E-01	9,99E-01	rs9682960|rs362623|rs3006845|rs10778102|rs1710879|rs2744601|rs1330581|rs12467996|rs13215369|rs1905752|rs11710097|rs2930142|rs1402611|rs9446432|rs9826914|rs1795994|rs2492970|rs9990174|rs6090020|rs2126624
Photosensitivity	RAP1_SIGNALLING	REACTOME	835	32	20	4,48E-01	9,99E-01	rs829370|rs1557087|rs6502555|rs1530378|rs10823217|rs13060691|rs3105453|rs8065651|rs904453|rs12255651|rs4790372|rs7922491|rs1922130|rs17706710|rs7902949|rs735176|rs12252380|rs3829170|rs4745514|rs3133539
Photosensitivity	FORMATION_OF_THE_TERNARY_COMPLEX_AND_SUBSEQUENTLY_THE_43S_COMPLEX	REACTOME	677	23	15	4,52E-01	9,99E-01	rs11986063|rs11623678|rs2816697|rs7006843|rs10787899|rs3825073|rs6059703|rs1211975|rs11217129|rs2278498|rs13248744|rs4979938|rs10090331|rs2049974|rs11604561
Photosensitivity	RNA_POL_I_PROMOTER_OPENING	REACTOME	501	47	10	4,54E-01	9,99E-01	rs9366694|rs2005219|rs3778279|rs1381257|rs129128|rs10484434|rs9379818|rs3857546|rs1362599|rs4283294
Photosensitivity	INTRINSIC_PATHWAY	REACTOME	264	19	10	4,57E-01	9,99E-01	rs2511989|rs6796803|rs226372|rs584337|rs10513803|rs5030102|rs7107322|rs969936|rs2086297|rs4253236
Photosensitivity	NOTCH1_INTRACELLULAR_DOMAIN_REGULATES_TRANSCRIPTION	REACTOME	2852	43	20	4,58E-01	9,99E-01	rs1030609|rs7850433|rs13129525|rs4296917|rs4753743|rs6756261|rs4851975|rs468141|rs6816542|rs7177654|rs1887719|rs9997981|rs2520361|rs7593722|rs10017961|rs10780472|rs3791406|rs2046380|rs1444547|rs12005290
Photosensitivity	TYROSINE_METABOLISM	KEGG	1169	46	20	4,60E-01	9,99E-01	rs13100173|rs6499969|rs4147536|rs3902902|rs1986415|rs2065367|rs2348024|rs958842|rs8057892|rs7043787|rs983121|rs2490286|rs1003784|rs16971853|rs308328|rs12683218|rs11238133|rs8062792|rs10809674|rs1999377
Photosensitivity	FORMATION_OF_INCISION_COMPLEX_IN_GG_NER	REACTOME	545	15	8	4,60E-01	9,99E-01	rs1464251|rs10978769|rs7357092|rs7766094|rs1618536|rs1561734|rs1143407|rs6477562
Photosensitivity	NICOTINATE_AND_NICOTINAMIDE_METABOLISM	KEGG	963	41	18	4,61E-01	9,99E-01	rs4519903|rs7636269|rs10154999|rs11214959|rs1986415|rs2348024|rs6777158|rs9493133|rs9362203|rs1713460|rs2165613|rs1922495|rs2243822|rs10464000|rs7720125|rs10498268|rs2704976|rs7226068
Photosensitivity	GATA3_PATHWAY	BIOCARTA	898	49	20	4,61E-01	9,99E-01	rs883541|rs2243300|rs4307960|rs7896001|rs4143832|rs404694|rs8060741|rs1243974|rs2646388|rs13329842|rs4749775|rs4888977|rs4811158|rs1415879|rs918167|rs2348182|rs4745514|rs4239343|rs8044109|rs7206380
Photosensitivity	PDGF_PATHWAY	BIOCARTA	1021	36	20	4,62E-01	9,99E-01	rs7601754|rs873417|rs2005219|rs13060691|rs706733|rs17746897|rs706724|rs4381631|rs4411531|rs2112166|rs3729908|rs1444043|rs1432442|rs10171977|rs758658|rs1812402|rs904453|rs8065164|rs33344|rs167769
Photosensitivity	MITOTIC_M_M_G1_PHASES	REACTOME	3176	36	20	4,62E-01	9,99E-01	rs16827840|rs10874743|rs351408|rs6909633|rs6507992|rs9904424|rs12757772|rs2028455|rs4802351|rs6693314|rs12452736|rs996482|rs8004556|rs6769686|rs13130657|rs743272|rs7129085|rs9673361|rs259871|rs4148876
Photosensitivity	ORC1_REMOVAL_FROM_CHROMATIN	REACTOME	930	24	20	4,62E-01	9,99E-01	rs16827840|rs9904424|rs4802351|rs743272|rs9673361|rs4148876|rs1957107|rs12292911|rs4664414|rs1973619|rs2550461|rs837763|rs1217|rs555812|rs8063430|rs2071749|rs10484565|rs2417223|rs2286670|rs12453325
Photosensitivity	PARKINSONS_DISEASE	KEGG	2833	36	20	4,63E-01	9,99E-01	rs10519706|rs3775461|rs1812923|rs1500147|rs4854053|rs9775695|rs4147750|rs714621|rs1982235|rs11326|rs13195186|rs2733954|rs4412175|rs2017089|rs1812144|rs6446298|rs6120890|rs4461665|rs9456772|rs10971025
Photosensitivity	TRANSPORT_OF_RIBONUCLEOPROTEINS_INTO_THE_HOST_NUCLEUS	REACTOME	452	24	11	4,64E-01	9,99E-01	rs10183020|rs7628051|rs2038743|rs7770657|rs2328136|rs10871221|rs3026106|rs4684126|rs6961391|rs6467593|rs3115761
Photosensitivity	GSK3_PATHWAY	BIOCARTA	1055	44	20	4,67E-01	9,99E-01	rs10796820|rs3740643|rs442683|rs4592915|rs2232571|rs706733|rs1919870|rs10818151|rs706724|rs3805787|rs6599127|rs7843776|rs2465153|rs2239621|rs2886612|rs10983791|rs33344|rs649392|rs10515068|rs1423236
Photosensitivity	CARM_ER_PATHWAY	BIOCARTA	1617	37	20	4,69E-01	9,99E-01	rs10796820|rs2499611|rs6756261|rs6829223|rs4851975|rs468141|rs3740643|rs2520361|rs7593722|rs3791406|rs4469064|rs4361373|rs3791601|rs3815076|rs1613070|rs6817487|rs1283614|rs12878503|rs12655172|rs10474293
Photosensitivity	ION_TRANSPORT_BY_P_TYPE_ATPASES	REACTOME	1690	49	20	4,70E-01	9,99E-01	rs973608|rs13064158|rs500951|rs1998834|rs3011500|rs4121751|rs4684045|rs11640901|rs12456346|rs1317122|rs12148810|rs1403588|rs495435|rs2019535|rs1764646|rs13146994|rs17082351|rs6067931|rs9511834|rs2575527
Photosensitivity	GLYCEROPHOSPHOLIPID_BIOSYNTHESIS	REACTOME	2483	33	20	4,71E-01	9,99E-01	rs12141345|rs10912414|rs1410119|rs3737358|rs6076792|rs1488|rs6133219|rs12143542|rs6559175|rs1356410|rs1421050|rs2577254|rs859068|rs7022410|rs10798089|rs260879|rs11046741|rs10505887|rs1668547|rs11046500
Photosensitivity	INTERACTIONS_OF_VPR_WITH_HOST_CELLULAR_PROTEINS	REACTOME	480	24	11	4,71E-01	9,99E-01	rs10183020|rs7628051|rs2038743|rs7770657|rs2328136|rs10871221|rs3026106|rs4684126|rs6961391|rs6467593|rs3115761
Photosensitivity	CTLA4_PATHWAY	BIOCARTA	662	29	20	4,72E-01	9,99E-01	rs10918693|rs2395175|rs4766987|rs858490|rs10206295|rs706733|rs706724|rs2838522|rs2838523|rs624035|rs33344|rs10515068|rs28530648|rs1003815|rs7562030|rs13407072|rs1423236|rs3815701|rs10054692|rs17187469
Photosensitivity	TRANSMISSION_ACROSS_CHEMICAL_SYNAPSES	REACTOME	9089	25	20	4,73E-01	9,99E-01	rs4748472|rs7007251|rs10974591|rs9682960|rs12364414|rs872091|rs362623|rs466767|rs10107483|rs7174521|rs513472|rs2455280|rs3006845|rs10986577|rs7190913|rs6589846|rs9377381|rs2107118|rs10778102|rs11656925
Photosensitivity	MAL_PATHWAY	BIOCARTA	225	16	10	4,74E-01	9,99E-01	rs2005219|rs13060691|rs1169307|rs1432442|rs6580191|rs2254779|rs904453|rs2876980|rs832540|rs4255740
Photosensitivity	LAGGING_STRAND_SYNTHESIS	REACTOME	550	12	8	4,75E-01	9,99E-01	rs174537|rs3782093|rs7357092|rs676114|rs1359389|rs1629511|rs11236183|rs7328146
Photosensitivity	EFFECTS_OF_PIP2_HYDROLYSIS	REACTOME	1651	37	20	4,76E-01	9,99E-01	rs1088672|rs628877|rs10487781|rs4732260|rs353189|rs3778818|rs36873|rs13073829|rs1124787|rs6740453|rs7961587|rs1020445|rs729396|rs4481994|rs11684301|rs161338|rs11924144|rs17033965|rs6720975|rs2026
Photosensitivity	TRANSCRIPTION	REACTOME	2708	35	20	4,76E-01	9,99E-01	rs2559658|rs11785348|rs885838|rs10491776|rs139873|rs3800453|rs1408314|rs9366694|rs10756504|rs2005219|rs3778279|rs16867175|rs12418104|rs2228529|rs7148769|rs4253126|rs6981243|rs3088264|rs7112576|rs1381257
Photosensitivity	ASTHMA	KEGG	770	63	17	4,78E-01	9,99E-01	rs3916765|rs2243300|rs4143832|rs68600|rs17200733|rs9275595|rs6065925|rs25887|rs12075255|rs206768|rs10484565|rs9500927|rs40401|rs7767167|rs6074022|rs2427832|rs206762
Photosensitivity	TRANSPORT_OF_MATURE_MRNA_DERIVED_FROM_AN_INTRONLESS_TRANSCRIPT	REACTOME	524	24	11	4,78E-01	9,99E-01	rs10183020|rs7628051|rs2038743|rs7770657|rs2328136|rs10871221|rs3026106|rs4684126|rs6961391|rs6467593|rs3115761
Photosensitivity	DNA_REPLICATION	REACTOME	3548	38	20	4,80E-01	9,99E-01	rs16827840|rs10874743|rs351408|rs174537|rs6909633|rs6507992|rs9904424|rs12757772|rs2028455|rs4802351|rs6693314|rs12452736|rs996482|rs8004556|rs6769686|rs13130657|rs743272|rs7129085|rs9673361|rs259871
Photosensitivity	IL10_PATHWAY	BIOCARTA	288	9	9	4,82E-01	9,99E-01	rs7601754|rs17200733|rs2247526|rs12075255|rs4722164|rs2071749|rs167769|rs1517352|rs10892204
Photosensitivity	REGULATION_OF_KIT_SIGNALING	REACTOME	587	26	15	4,83E-01	9,99E-01	rs873417|rs10745478|rs17809666|rs6090585|rs4381631|rs4411531|rs2726648|rs10171977|rs758658|rs218282|rs7310517|rs7834615|rs4406072|rs7244774|rs3986302
Photosensitivity	PRION_DISEASES	KEGG	1585	36	20	4,84E-01	9,99E-01	rs7946527|rs2826881|rs8115602|rs9981283|rs382094|rs2827432|rs2005219|rs11088869|rs11953280|rs198095|rs591042|rs2827709|rs2826727|rs1432442|rs2826912|rs2827875|rs8134910|rs1448369|rs2828104|rs10489623
Photosensitivity	AMINO_ACID_TRANSPORT_ACROSS_THE_PLASMA_MEMBRANE	REACTOME	1025	30	20	4,92E-01	9,99E-01	rs8105903|rs12490575|rs2507821|rs2588233|rs2236941|rs876985|rs3498|rs6546120|rs10506269|rs17244832|rs12828089|rs6796254|rs720625|rs7670671|rs12894506|rs2531748|rs478085|rs28479223|rs4768689|rs3851117
Photosensitivity	EXTENSION_OF_TELOMERES	REACTOME	618	12	8	4,92E-01	9,99E-01	rs174537|rs3782093|rs7357092|rs676114|rs1359389|rs1629511|rs11236183|rs7328146
Photosensitivity	GLYCOSAMINOGLYCAN_BIOSYNTHESIS_CHONDROITIN_SULFATE	KEGG	1372	33	20	4,93E-01	9,99E-01	rs723988|rs12195809|rs3800794|rs10100391|rs10458997|rs1342761|rs2449393|rs7197164|rs17593178|rs12521344|rs11223878|rs11247267|rs11647711|rs1478748|rs2394847|rs7186245|rs3731918|rs1463462|rs4937902|rs4780677
Photosensitivity	HDAC_PATHWAY	BIOCARTA	1231	41	20	4,96E-01	9,99E-01	rs963065|rs4804366|rs1546713|rs2461489|rs17031191|rs2851045|rs2684794|rs6852347|rs706733|rs706724|rs7162314|rs3815076|rs738809|rs7694484|rs11727778|rs1283614|rs4811158|rs12655172|rs10474293|rs9606893
Photosensitivity	SIGNALING_BY_ERBB2	REACTOME	3847	28	20	4,97E-01	9,99E-01	rs721412|rs264336|rs2888104|rs628877|rs11568993|rs883541|rs16849142|rs2324528|rs17329875|rs6435692|rs7949655|rs4736746|rs873417|rs1404912|rs759947|rs4452759|rs7817546|rs2005219|rs2673825|rs13060691
Photosensitivity	VEGF_SIGNALING_PATHWAY	KEGG	2111	36	20	4,98E-01	9,99E-01	rs963065|rs12048929|rs10461355|rs2461489|rs17031191|rs873417|rs12143542|rs2851045|rs943084|rs2005219|rs7176475|rs10798089|rs13060691|rs6852347|rs11133360|rs6090585|rs706733|rs7671214|rs706724|rs4381631
Photosensitivity	GLUCURONIDATION	REACTOME	656	132	6	4,99E-01	9,99E-01	rs4148329|rs6761246|rs2361502|rs2608822|rs6830865|rs2687964
Photosensitivity	PHENYLALANINE_METABOLISM	KEGG	442	37	16	4,99E-01	9,99E-01	rs13100173|rs6499969|rs3902902|rs8057892|rs2490286|rs6539055|rs1003784|rs308328|rs11238133|rs10507149|rs8062792|rs2242259|rs16961113|rs17634958|rs12925511|rs1283
Photosensitivity	ERBB_SIGNALING_PATHWAY	KEGG	4135	29	20	5,00E-01	9,99E-01	rs1030609|rs721412|rs264336|rs2888104|rs1798619|rs11568993|rs16849142|rs17329875|rs6435692|rs873417|rs2302456|rs4452759|rs7817546|rs2005219|rs13060691|rs3822295|rs13260061|rs1414867|rs10748994|rs722813
Photosensitivity	REGULATION_OF_GENE_EXPRESSION_IN_BETA_CELLS	REACTOME	716	30	20	5,00E-01	9,99E-01	rs2324528|rs11669442|rs1462436|rs2424476|rs6093978|rs9512906|rs1806180|rs1052176|rs1169307|rs10409222|rs2254779|rs3730050|rs4435111|rs7981979|rs6075965|rs8000689|rs1203951|rs9512928|rs4941961|rs4943805
Photosensitivity	BIOPEPTIDES_PATHWAY	BIOCARTA	1081	25	20	5,01E-01	9,99E-01	rs7601754|rs6796803|rs873417|rs2005219|rs13060691|rs3822295|rs4381631|rs4411531|rs3729908|rs1432442|rs10171977|rs758658|rs2292979|rs2886612|rs7684832|rs904453|rs891944|rs2876980|rs10513803|rs5030102
Photosensitivity	ION_CHANNEL_TRANSPORT	REACTOME	2741	32	20	5,01E-01	9,99E-01	rs973608|rs13064158|rs13116864|rs500951|rs1998834|rs3011500|rs4121751|rs2715583|rs164369|rs1012549|rs12440234|rs4684045|rs11640901|rs12456346|rs1317122|rs12148810|rs980982|rs1403588|rs495435|rs209350
Photosensitivity	ARMS_MEDIATED_ACTIVATION	REACTOME	393	27	15	5,02E-01	9,99E-01	rs2302456|rs2005219|rs13060691|rs11792737|rs3012738|rs1432442|rs195204|rs1294383|rs904453|rs2876980|rs10771184|rs3012719|rs869382|rs4255740|rs4626883
Photosensitivity	PROLONGED_ERK_ACTIVATION_EVENTS	REACTOME	429	27	15	5,05E-01	9,99E-01	rs2302456|rs2005219|rs13060691|rs11792737|rs3012738|rs1432442|rs195204|rs1294383|rs904453|rs2876980|rs10771184|rs3012719|rs869382|rs4255740|rs4626883
Photosensitivity	SIGNALING_BY_CONSTITUTIVELY_ACTIVE_EGFR	REACTOME	503	47	19	5,05E-01	9,99E-01	rs721412|rs11568993|rs17746482|rs706733|rs706724|rs984654|rs10171977|rs4140770|rs10771184|rs33344|rs10515068|rs1423236|rs3815701|rs10054692|rs1015793|rs11953134|rs12535482|rs4976129|rs1490819
Photosensitivity	RETINOL_METABOLISM	KEGG	1512	106	20	5,06E-01	9,99E-01	rs4148329|rs747574|rs4147536|rs4926354|rs4803415|rs6761246|rs722921|rs2361502|rs2246709|rs7521256|rs4926339|rs7907528|rs2860975|rs10882168|rs11572172|rs1867049|rs11596645|rs17382342|rs7167150|rs8187890
Photosensitivity	ACUTE_MYELOID_LEUKEMIA	KEGG	1892	32	20	5,08E-01	9,99E-01	rs1030608|rs10796820|rs2834662|rs3740643|rs4918785|rs11685301|rs2005219|rs1597301|rs13060691|rs764967|rs2834670|rs706733|rs706724|rs2726648|rs402311|rs6722568|rs1432442|rs10520405|rs10171977|rs6999335
Photosensitivity	GLYCEROLIPID_METABOLISM	KEGG	1872	34	20	5,09E-01	9,99E-01	rs10487781|rs4732260|rs1410119|rs3737358|rs1488|rs3778818|rs1555481|rs880887|rs4878808|rs1421050|rs17821232|rs36873|rs13073829|rs7865905|rs2838464|rs12254574|rs11987150|rs161338|rs11924144|rs992893
Photosensitivity	NRAGE_SIGNALS_DEATH_THROUGH_JNK	REACTOME	1919	25	20	5,10E-01	9,99E-01	rs12364414|rs10761189|rs7174521|rs345306|rs4810783|rs754743|rs7218427|rs9371867|rs1398028|rs7761427|rs6692050|rs1332684|rs7027092|rs1359647|rs926693|rs4778857|rs2882936|rs4817403|rs2289321|rs2440056
Photosensitivity	SYNTHESIS_OF_PIPS_AT_THE_GOLGI_MEMBRANE	REACTOME	788	30	14	5,13E-01	9,99E-01	rs12490575|rs1941590|rs2440455|rs4595861|rs1374670|rs1339959|rs3768331|rs752216|rs9945673|rs7671799|rs2451078|rs4919128|rs11604561|rs10853477
Photosensitivity	HIV_INFECTION	REACTOME	3237	34	20	5,14E-01	9,99E-01	rs16827840|rs174537|rs885838|rs1798619|rs139873|rs9904424|rs9260759|rs3800453|rs10918693|rs7949655|rs4802351|rs10183020|rs9587505|rs7622028|rs858490|rs2038743|rs743272|rs259916|rs9673361|rs4148876
Photosensitivity	FMLP_PATHWAY	BIOCARTA	1148	39	20	5,15E-01	9,99E-01	rs963065|rs2461489|rs17031191|rs2851045|rs2005219|rs13060691|rs6852347|rs1374670|rs6055990|rs2719795|rs8115925|rs1432442|rs2223538|rs7694484|rs904453|rs6140619|rs11727778|rs4811158|rs2876980|rs11725047
Photosensitivity	TRANSLATION	REACTOME	1803	32	20	5,15E-01	9,99E-01	rs11243152|rs5995711|rs7210156|rs11986063|rs12903497|rs11623678|rs16974510|rs6927367|rs3105429|rs3793371|rs2816697|rs7006843|rs7429360|rs6952793|rs10787899|rs3825073|rs162202|rs6059703|rs1211975|rs11217129
Photosensitivity	3_UTR_MEDIATED_TRANSLATIONAL_REGULATION	REACTOME	1235	37	20	5,15E-01	9,99E-01	rs5995711|rs7210156|rs11986063|rs12903497|rs11623678|rs6927367|rs3105429|rs2816697|rs7006843|rs10787899|rs3825073|rs6059703|rs1211975|rs11217129|rs12624027|rs7670903|rs219493|rs1452404|rs1452396|rs2278498
Photosensitivity	IL_RECEPTOR_SHC_SIGNALING	REACTOME	624	49	20	5,16E-01	9,99E-01	rs12251307|rs3864277|rs10899496|rs4143832|rs706733|rs706724|rs17811365|rs10171977|rs4240910|rs12244380|rs273266|rs33344|rs3218295|rs10515068|rs1423236|rs3815701|rs10054692|rs40401|rs11953134|rs4976129
Photosensitivity	SLC_MEDIATED_TRANSMEMBRANE_TRANSPORT	REACTOME	8774	35	20	5,17E-01	9,99E-01	rs2252035|rs16928705|rs8105903|rs12141345|rs10974591|rs9682960|rs12490575|rs4854786|rs2455280|rs7722287|rs1866901|rs11210751|rs7586183|rs270607|rs11810507|rs10183020|rs2886790|rs720436|rs859068|rs7636169
Photosensitivity	IGF1_PATHWAY	BIOCARTA	813	40	20	5,17E-01	9,99E-01	rs1546713|rs4766987|rs2005219|rs2684794|rs13060691|rs706733|rs706724|rs2112166|rs7162314|rs1432442|rs10171977|rs2716121|rs904453|rs33344|rs7169544|rs10515068|rs6452735|rs1423236|rs3815701|rs30394
Photosensitivity	ECM_PATHWAY	BIOCARTA	753	34	20	5,17E-01	9,99E-01	rs10940270|rs2005219|rs8009215|rs13060691|rs6090585|rs17386030|rs706733|rs706724|rs870992|rs1432442|rs6580191|rs904453|rs2876980|rs33344|rs1295907|rs10515068|rs1423236|rs3815701|rs1254403|rs10054692
Photosensitivity	ANTIGEN_PROCESSING_CROSS_PRESENTATION	REACTOME	1601	27	20	5,20E-01	9,99E-01	rs16827840|rs9904424|rs9260759|rs4802351|rs743272|rs9673361|rs6952793|rs4148876|rs1957107|rs12292911|rs9264219|rs162202|rs4664414|rs1973619|rs2550461|rs1217|rs10988774|rs9378229|rs8063430|rs10484565
Photosensitivity	INSULIN_RECEPTOR_RECYCLING	REACTOME	492	18	11	5,20E-01	9,99E-01	rs4804366|rs2515210|rs17200733|rs871516|rs16919915|rs17749106|rs2115386|rs2914559|rs13238736|rs8846|rs7836003
Photosensitivity	EGFR_DOWNREGULATION	REACTOME	1037	56	20	5,21E-01	9,99E-01	rs721412|rs11568993|rs986345|rs534870|rs17746482|rs3097920|rs1215447|rs984654|rs4140770|rs4502102|rs10795505|rs843339|rs2794235|rs7984951|rs2357291|rs17567|rs17491036|rs1982837|rs9574595|rs11604757
Photosensitivity	EIF4_PATHWAY	BIOCARTA	890	29	20	5,21E-01	9,99E-01	rs6180|rs873417|rs3105429|rs2005219|rs2673825|rs706733|rs706724|rs4381631|rs4411531|rs3729908|rs847682|rs758658|rs2876980|rs33344|rs10515068|rs7193148|rs1423236|rs3815701|rs10054692|rs11687971
Photosensitivity	DOUBLE_STRAND_BREAK_REPAIR	REACTOME	506	27	13	5,22E-01	9,99E-01	rs2244012|rs9587505|rs7158356|rs1801516|rs2508678|rs7357092|rs10849605|rs1193693|rs1408906|rs301287|rs7003908|rs228591|rs799906
Photosensitivity	VASCULAR_SMOOTH_MUSCLE_CONTRACTION	KEGG	5845	33	20	5,22E-01	9,99E-01	rs6443589|rs13115024|rs602159|rs12364414|rs2239094|rs1088672|rs628877|rs1878404|rs12048929|rs16927077|rs6948564|rs4754175|rs353189|rs17426222|rs4736746|rs873417|rs1783138|rs12143542|rs2238012|rs1404912

Photosensitivity	TIGHT_JUNCTION_INTERACTIONS	REACTOME	715	21	11	5,23E-01	9,99E-01	rs7528744|rs13080849|rs4934517|rs3822964|rs928844|rs1425113|rs4819804|rs1331681|rs1617306|rs2297221|rs13051127
Photosensitivity	INHIBITION_OF_THE_PROTEOLYTIC_ACTIVITY_OF_APC_C_REQUIRED_FOR_THE_ONSET_ TOTIC_SPINDLE_CHECKPOINT_COMPONENTS	REACTOME	365	10	7	5,25E-01	9,99E-01	rs1647253|rs13130657|rs11592827|rs1994174|rs11687619|rs12647998|rs3759839
Photosensitivity	IL2_PATHWAY	BIOCARTA	452	27	14	5,26E-01	9,99E-01	rs12251307|rs2005219|rs13060691|rs1432442|rs10171977|rs2716121|rs904453|rs12244380|rs2278278|rs1993703|rs3218295|rs6037986|rs4255740|rs1882832
Photosensitivity	DEADENYLATION_OF_MRNA	REACTOME	331	25	11	5,29E-01	9,99E-01	rs3812278|rs3105429|rs6948145|rs10784820|rs9857753|rs1524246|rs1388794|rs11642944|rs2453176|rs7004284|rs10516145
Photosensitivity	SIGNALING_BY_INSULIN_RECEPTOR	REACTOME	3432	29	20	5,30E-01	9,99E-01	rs743682|rs666336|rs17487009|rs4804366|rs250201|rs2515210|rs10886918|rs9580274|rs8087566|rs2440455|rs2809244|rs2302456|rs7778077|rs4725424|rs2005219|rs4595861|rs13060691|rs883355|rs13250792|rs3129601
Photosensitivity	APC_C_CDC20_MEDIATED_DEGRADATION_OF_CYCLIN_B	REACTOME	224	6	4	5,33E-01	9,99E-01	rs1647253|rs1994174|rs11687619|rs7683982
Photosensitivity	INTERFERON_SIGNALING	REACTOME	3448	54	20	5,33E-01	9,99E-01	rs7946527|rs599683|rs3916765|rs10922552|rs11638243|rs12747584|rs9260759|rs2252405|rs10813821|rs10922613|rs10183020|rs9426832|rs2005219|rs2228615|rs3822295|rs7973701|rs7628051|rs12732279|rs2038743|rs743272
Photosensitivity	HER2_PATHWAY	BIOCARTA	1326	42	20	5,33E-01	9,99E-01	rs2888104|rs16849142|rs17329875|rs6435692|rs2005219|rs13060691|rs960824|rs17746482|rs706733|rs706724|rs1432442|rs984654|rs10171977|rs904453|rs4140770|rs12620896|rs2876980|rs1357141|rs924822|rs3020368
Photosensitivity	AMYOTROPHIC_LATERAL_SCLEROSIS_ALS	KEGG	1813	39	20	5,33E-01	9,99E-01	rs963065|rs7190913|rs2461489|rs17031191|rs2110739|rs10065813|rs2851045|rs4780550|rs2872715|rs6852347|rs1061631|rs17550532|rs17200733|rs6060563|rs2284406|rs1900718|rs12501768|rs7202071|rs6461845|rs7694484
Photosensitivity	SYNTHESIS_AND_INTERCONVERSION_OF_NUCLEOTIDE_DI_AND_TRIPHOSPHATES	REACTOME	306	16	6	5,34E-01	9,99E-01	rs7545638|rs11162326|rs3760469|rs3817657|rs4135223|rs6690084
Photosensitivity	ADHERENS_JUNCTIONS_INTERACTIONS	REACTOME	5496	34	20	5,34E-01	9,99E-01	rs7943417|rs1369918|rs4457095|rs10058333|rs1201981|rs1559303|rs11645947|rs13332623|rs1366492|rs6015839|rs4411488|rs4445973|rs2426913|rs7202575|rs1007369|rs11215438|rs6061779|rs1582924|rs6142828|rs16899078
Photosensitivity	DESTABILIZATION_OF_MRNA_BY_KSRP	REACTOME	205	19	9	5,34E-01	9,99E-01	rs11189224|rs3105453|rs33556|rs9543118|rs1613070|rs3133539|rs11642944|rs9380546|rs2498804
Photosensitivity	TCR_PATHWAY	BIOCARTA	1517	34	20	5,36E-01	9,99E-01	rs963065|rs10918693|rs2461489|rs17031191|rs873417|rs2851045|rs2005219|rs13060691|rs6852347|rs858490|rs706733|rs706724|rs4381631|rs4411531|rs2112166|rs3729908|rs1432442|rs10171977|rs758658|rs7694484
Photosensitivity	NFAT_PATHWAY	BIOCARTA	1626	38	20	5,37E-01	9,99E-01	rs963065|rs883541|rs2461489|rs17031191|rs10072402|rs2851045|rs759947|rs2005219|rs13060691|rs6852347|rs283427|rs706733|rs12945671|rs6556066|rs706724|rs6884482|rs6990313|rs1432442|rs17463958|rs2866250
Photosensitivity	FAS_PATHWAY	BIOCARTA	980	33	20	5,38E-01	9,99E-01	rs1798619|rs3212153|rs10504873|rs884241|rs7841459|rs991880|rs12501768|rs1800627|rs7930377|rs1812402|rs12216864|rs12191736|rs11899004|rs2275075|rs8065164|rs2084385|rs437932|rs2036847|rs7742254|rs1782449
Photosensitivity	KINESINS	REACTOME	566	17	12	5,39E-01	9,99E-01	rs7148495|rs12452736|rs10137053|rs3805941|rs2099095|rs10490798|rs2062400|rs351291|rs9890710|rs1525704|rs1618536|rs961548
Photosensitivity	REGULATION_OF_MITOTIC_CELL_CYCLE	REACTOME	1318	26	20	5,39E-01	9,99E-01	rs16827840|rs9904424|rs1647253|rs4802351|rs599215|rs6577168|rs13130657|rs743272|rs9673361|rs4148876|rs1957107|rs12292911|rs11592827|rs4664414|rs1973619|rs2550461|rs1217|rs7500128|rs8063430|rs1994174
Photosensitivity	SIGNALING_BY_FGFR1_FUSION_MUTANTS	REACTOME	639	44	15	5,39E-01	9,99E-01	rs13231090|rs10899496|rs6467779|rs706733|rs706724|rs11048778|rs12311257|rs33344|rs10515068|rs1423236|rs3815701|rs10054692|rs11953134|rs4976129|rs1490819
Photosensitivity	GLYCOLYSIS_GLUCONEOGENESIS	KEGG	1391	36	20	5,40E-01	9,99E-01	rs8049837|rs7535245|rs1555481|rs2269260|rs4147536|rs11189224|rs3902902|rs4878808|rs10234790|rs7865905|rs1052176|rs10903843|rs217463|rs4699509|rs6050291|rs2064393|rs308328|rs9811074|rs7685363|rs1946741
Photosensitivity	CREB_PATHWAY	BIOCARTA	969	34	20	5,41E-01	9,99E-01	rs883541|rs873417|rs1404912|rs2005219|rs3822295|rs706733|rs706724|rs4381631|rs4411531|rs3729908|rs10171977|rs12436076|rs758658|rs7684832|rs891944|rs2876980|rs1525660|rs33344|rs918167|rs10515068
Photosensitivity	PROTEOLYTIC_CLEAVAGE_OF_SNARE_COMPLEX_PROTEINS	REACTOME	382	12	7	5,41E-01	9,99E-01	rs362623|rs11657426|rs729463|rs11654567|rs6570608|rs11658644|rs6503203
Photosensitivity	EPO_PATHWAY	BIOCARTA	255	21	10	5,43E-01	9,99E-01	rs2005219|rs13060691|rs4804164|rs1432442|rs10171977|rs1812402|rs904453|rs6037986|rs4255740|rs1882832
Photosensitivity	RAS_ACTIVATION_UOPN_CA2_INFUX_THROUGH_NMDA_RECEPTOR	REACTOME	1033	38	19	5,44E-01	9,99E-01	rs7174521|rs7190913|rs4780550|rs2872715|rs3822295|rs17550532|rs4778857|rs2284406|rs1900718|rs7202071|rs7684832|rs891944|rs1379271|rs10045942|rs12102827|rs6498771|rs4310702|rs11055486|rs12449282
Photosensitivity	CERAMIDE_PATHWAY	BIOCARTA	425	25	17	5,47E-01	9,99E-01	rs4386969|rs2005219|rs13060691|rs1432442|rs7930377|rs6461845|rs904453|rs11899004|rs2876980|rs8065164|rs12458289|rs39454|rs832540|rs4255740|rs7818823|rs2236719|rs12673033
Photosensitivity	ACYL_CHAIN_REMODELLING_OF_PC	REACTOME	693	36	17	5,47E-01	9,99E-01	rs10912414|rs12143542|rs1356410|rs10798089|rs1668547|rs2962061|rs1800627|rs330712|rs2430119|rs9729852|rs4144116|rs4820315|rs10911933|rs1487492|rs16827441|rs4975554|rs36982
Photosensitivity	GAB1_SIGNALOSOME	REACTOME	1077	34	20	5,50E-01	9,99E-01	rs721412|rs11568993|rs2324528|rs2673825|rs17746482|rs6090585|rs706733|rs706724|rs12626158|rs984654|rs4140770|rs3730050|rs11664248|rs4435111|rs33344|rs2417223|rs10494270|rs10515068|rs8000689|rs2145068
Photosensitivity	REGULATION_OF_GLUCOKINASE_BY_GLUCOKINASE_REGULATORY_PROTEIN	REACTOME	459	25	12	5,52E-01	9,99E-01	rs10183020|rs7628051|rs2038743|rs7770657|rs2328136|rs10871221|rs3026106|rs4684126|rs6961391|rs6467593|rs1260326|rs3115761
Photosensitivity	ACTIVATION_OF_THE_MRNA_UPON_BINDING_OF_THE_CAP_BINDING_COMPLEX_AND_EI T_BINDING_TO_43S	REACTOME	780	26	17	5,53E-01	9,99E-01	rs11986063|rs11623678|rs3105429|rs2816697|rs7006843|rs10787899|rs3825073|rs6059703|rs1209621|rs11217129|rs2278498|rs13248744|rs4979938|rs10090331|rs2049974|rs7004284|rs11604561
Photosensitivity	CASPASE_PATHWAY	BIOCARTA	327	16	11	5,53E-01	9,99E-01	rs3212153|rs884241|rs12501768|rs1800627|rs6461845|rs11899004|rs2275075|rs2036847|rs39454|rs4319556|rs12673033
Photosensitivity	SMOOTH_MUSCLE_CONTRACTION	REACTOME	658	33	12	5,54E-01	9,99E-01	rs1571131|rs6948564|rs10940270|rs870992|rs10882615|rs549062|rs1254403|rs2414810|rs10188058|rs2469763|rs2075511|rs12091348
Photosensitivity	ALZHEIMERS_DISEASE	KEGG	5029	30	20	5,54E-01	9,99E-01	rs7571971|rs2239094|rs3775461|rs963065|rs1812923|rs1500147|rs2172680|rs7190913|rs4854053|rs9775695|rs2461489|rs4147750|rs17031191|rs714621|rs1982235|rs2238012|rs2851045|rs2005219|rs4780550|rs2872715
Photosensitivity	APOPTOTIC_EXECUTION_PHASE	REACTOME	1211	34	20	5,55E-01	9,99E-01	rs1798619|rs3212153|rs353189|rs9951341|rs884241|rs442683|rs6599127|rs729396|rs12501768|rs1449079|rs1800627|rs231395|rs36032521|rs7992176|rs10502568|rs7327256|rs12216125|rs11899004|rs2275075|rs7321613
Photosensitivity	NEP_NS2_INTERACTS_WITH_THE_CELLULAR_EXPORT_MACHINERY	REACTOME	456	25	12	5,57E-01	9,99E-01	rs10183020|rs7628051|rs2038743|rs7770657|rs2328136|rs10871221|rs3026106|rs4684126|rs6961391|rs6467593|rs6545886|rs3115761
Photosensitivity	CCR3_PATHWAY	BIOCARTA	939	29	20	5,58E-01	9,99E-01	rs873417|rs2005219|rs13060691|rs1374670|rs6055990|rs4381631|rs4411531|rs2719795|rs907698|rs8115925|rs3729908|rs1432442|rs2223538|rs758658|rs904453|rs6140619|rs2876980|rs7193148|rs4816085|rs4255740
Photosensitivity	METABOLISM_OF_MRNA	REACTOME	3017	28	20	5,58E-01	9,99E-01	rs16827840|rs5995711|rs7210156|rs3812278|rs9904424|rs12903497|rs215749|rs11623678|rs873417|rs4802351|rs13082092|rs6926662|rs581045|rs11189224|rs3105429|rs3105453|rs2816697|rs7079622|rs743272|rs6948145
Photosensitivity	COMPLEMENT_AND_COAGULATION_CASCADES	KEGG	1742	26	20	5,59E-01	9,99E-01	rs2511989|rs1742932|rs1245480|rs13062355|rs6796803|rs1778193|rs4610335|rs11953280|rs346044|rs11629242|rs1409432|rs17662737|rs10824840|rs8077638|rs10489623|rs1520481|rs226372|rs13217372|rs2274393|rs3812042
Photosensitivity	GLYCEROPHOSPHOLIPID_METABOLISM	KEGG	2549	34	20	5,61E-01	9,99E-01	rs10487781|rs4732260|rs10912414|rs1410119|rs3737358|rs6076792|rs1488|rs3778818|rs12143542|rs1421050|rs7176475|rs10798089|rs260879|rs36873|rs11046741|rs13073829|rs10505887|rs7599290|rs11046500|rs2838464
Photosensitivity	G_ALPHA_I_SIGNALLING_EVENTS	REACTOME	6453	27	20	5,65E-01	9,99E-01	rs11687066|rs2228428|rs2112508|rs12567677|rs872091|rs10514964|rs1937600|rs10986577|rs2183249|rs1034992|rs11143600|rs1341463|rs8110105|rs6701418|rs6796803|rs7853418|rs4736746|rs10818955|rs1404912|rs9985440
Photosensitivity	NUCLEOTIDE_EXCISION_REPAIR	REACTOME	956	33	20	5,66E-01	9,99E-01	rs885838|rs1464251|rs139873|rs10978769|rs2228529|rs4253126|rs6981243|rs7112576|rs1625691|rs7357092|rs7766094|rs2025448|rs1359389|rs1629511|rs11236183|rs1618536|rs1561734|rs7328146|rs1143407|rs6477562
Photosensitivity	HISTIDINE_METABOLISM	KEGG	654	48	19	5,67E-01	9,99E-01	rs1973077|rs1555481|rs2887|rs3902902|rs4878808|rs7865905|rs7506051|rs1025607|rs7577|rs12626746|rs308328|rs11238133|rs1493282|rs4835913|rs9981981|rs11791018|rs17634958|rs3829305|rs1980983
Photosensitivity	FORMATION_OF_TUBULIN_FOLDING_INTERMEDIATES_BY_CCT_TRIC	REACTOME	227	5	4	5,69E-01	9,99E-01	rs7571143|rs40428|rs9507572|rs7558608
Photosensitivity	TRAFFICKING_OF_AMPA_RECEPTORS	REACTOME	1132	26	20	5,71E-01	9,99E-01	rs2107118|rs1945975|rs140040|rs873417|rs10065813|rs3822295|rs11642949|rs4381631|rs4411531|rs9880331|rs3729908|rs3755768|rs758658|rs7684832|rs891944|rs9876485|rs1438946|rs843339|rs1938958|rs12152961
Photosensitivity	ACTIVATED_NOTCH1_TRANSMITS_SIGNAL_TO_THE_NUCLEUS	REACTOME	719	43	20	5,74E-01	9,99E-01	rs2701623|rs278899|rs1427282|rs6074170|rs2423511|rs4813046|rs177387|rs6040334|rs1732803|rs17189227|rs1051007|rs4973189|rs2871921|rs973322|rs11614358|rs694335|rs7605496|rs745338|rs12372413|rs12128690
Photosensitivity	AMINO_ACID_SYNTHESIS_AND_INTERCONVERSION_TRANSAMINATION	REACTOME	530	24	10	5,74E-01	9,99E-01	rs6499969|rs10512079|rs8057892|rs10125637|rs2490286|rs8062792|rs2275273|rs16961113|rs7900964|rs12925511
Photosensitivity	DNA_REPLICATION	KEGG	740	21	13	5,74E-01	9,99E-01	rs174546|rs7336417|rs2408291|rs3782093|rs7357092|rs676114|rs2071749|rs1359389|rs1629511|rs11236183|rs7328146|rs2071744|rs9535565
Photosensitivity	GALACTOSE_METABOLISM	KEGG	759	22	17	5,74E-01	9,99E-01	rs6793913|rs1880924|rs7535245|rs2269260|rs9858362|rs7797360|rs10903843|rs217463|rs11818509|rs2256817|rs10794929|rs3003514|rs1556632|rs10903939|rs855314|rs2388595|rs3771763
Photosensitivity	SRP_DEPENDENT_COTRANSLATIONAL_PROTEIN_TARGETING_TO_MEMBRANE	REACTOME	1378	34	20	5,75E-01	9,99E-01	rs11243152|rs5995711|rs7210156|rs12903497|rs11623678|rs16974510|rs6927367|rs2816697|rs7429360|rs6952793|rs3825073|rs162202|rs1211975|rs11217129|rs12624027|rs7670903|rs219493|rs10988774|rs1452404|rs1452396
Photosensitivity	MYOSIN_PATHWAY	BIOCARTA	1253	30	20	5,75E-01	9,99E-01	rs12364414|rs4532638|rs873417|rs8009215|rs17386030|rs1359647|rs6055990|rs4381631|rs4411531|rs2719795|rs907698|rs8115925|rs11717281|rs3729908|rs2223538|rs758658|rs11655004|rs6140619|rs942648|rs1295907
Photosensitivity	INSULIN_PATHWAY	BIOCARTA	725	36	20	5,75E-01	9,99E-01	rs4804366|rs4766987|rs2005219|rs13060691|rs706733|rs706724|rs2112166|rs1432442|rs10171977|rs2716121|rs904453|rs33344|rs2115386|rs2914559|rs10515068|rs6452735|rs1423236|rs3815701|rs10054692|rs30394
Photosensitivity	BASIGIN_INTERACTIONS	REACTOME	595	29	15	5,81E-01	9,99E-01	rs973608|rs2507821|rs876985|rs1144396|rs17244832|rs7670671|rs2268875|rs28479223|rs762349|rs11568429|rs2912464|rs12644755|rs3885388|rs3746028|rs8008775
Photosensitivity	PROCESSING_OF_CAPPED_INTRON_CONTAINING_PRE_MRNA	REACTOME	1547	35	20	5,81E-01	9,99E-01	rs885838|rs10197258|rs139873|rs2588882|rs10183020|rs2305797|rs12937256|rs7628051|rs2038743|rs2526388|rs7770657|rs7112576|rs17704641|rs4646296|rs1798177|rs2328136|rs6433636|rs1625691|rs1613070|rs17629022
Photosensitivity	MHC_CLASS_II_ANTIGEN_PRESENTATION	REACTOME	2226	36	20	5,82E-01	9,99E-01	rs7023913|rs3916765|rs7148495|rs1471910|rs4800180|rs6979207|rs3003608|rs12452736|rs6745653|rs12338625|rs2237591|rs68600|rs10137053|rs2162400|rs11730732|rs2099095|rs3805941|rs7126344|rs9275582|rs4804528
Photosensitivity	RELA_PATHWAY	BIOCARTA	208	5	5	5,84E-01	9,99E-01	rs1061631|rs17200733|rs7930377|rs7552664|rs5030437
Photosensitivity	INHIBITION_OF_INSULIN_SECRETION_BY_ADRENALINE_NORADRENALINE	REACTOME	789	33	18	5,84E-01	9,99E-01	rs4748472|rs2236941|rs11157863|rs1570939|rs12261976|rs1409202|rs2886612|rs10740993|rs7152036|rs17683046|rs11014358|rs584845|rs2138551|rs8104096|rs10238438|rs11810173|rs4748444|rs2203615
Photosensitivity	HOST_INTERACTIONS_OF_HIV_FACTORS	REACTOME	2270	31	20	5,86E-01	9,99E-01	rs16827840|rs1798619|rs9904424|rs9260759|rs10918693|rs7949655|rs4802351|rs10183020|rs7622028|rs858490|rs2038743|rs743272|rs259916|rs9673361|rs4148876|rs7770657|rs2111035|rs1957107|rs12292911|rs2328136
Photosensitivity	SPHINGOLIPID_METABOLISM	KEGG	1944	31	20	5,88E-01	9,99E-01	rs225829|rs6793913|rs6793037|rs10444692|rs11729278|rs3786327|rs10189241|rs10981168|rs5770953|rs977122|rs1998355|rs9858362|rs3180018|rs492369|rs927047|rs891944|rs1466447|rs1020603|rs10020382|rs10053925
Photosensitivity	DOWNSTREAM_SIGNALING_OF_ACTIVATED_FGFR	REACTOME	3636	25	20	5,89E-01	9,99E-01	rs743682|rs666336|rs17487009|rs628877|rs883541|rs250201|rs2324528|rs10886918|rs9580274|rs4736746|rs873417|rs1404912|rs759947|rs2005219|rs2673825|rs13060691|rs883355|rs13250792|rs3129601|rs465174
Photosensitivity	PPARA_PATHWAY	BIOCARTA	2445	30	20	5,90E-01	9,99E-01	rs1030608|rs6553019|rs6829223|rs883541|rs12757772|rs4917342|rs873417|rs11080358|rs7703021|rs2005219|rs4243461|rs17200733|rs706733|rs706724|rs4381631|rs4469064|rs914852|rs4411531|rs4361373|rs3729908
Photosensitivity	ABCA_TRANSPORTERS_IN_LIPID_HOMEOSTASIS	REACTOME	464	17	8	5,91E-01	9,99E-01	rs12615195|rs737453|rs3792009|rs8072153|rs3752237|rs7586353|rs225436|rs9302893
Photosensitivity	APC_CDC20_MEDIATED_DEGRADATION_OF_NEK2A	REACTOME	396	11	8	5,92E-01	9,99E-01	rs1647253|rs13130657|rs11592827|rs3795833|rs1994174|rs11687619|rs7683982|rs3759839
Photosensitivity	STEROID_BIOSYNTHESIS	KEGG	247	18	10	5,93E-01	9,99E-01	rs1048691|rs2254670|rs885561|rs3825073|rs1211975|rs2291617|rs604778|rs7922269|rs12678891|rs3735810
Photosensitivity	METABOLISM_OF_NON_CODING_RNA	REACTOME	611	30	14	5,93E-01	9,99E-01	rs10183020|rs7628051|rs2038743|rs11623624|rs7770657|rs2328136|rs10871221|rs3026106|rs10859963|rs4684126|rs6961391|rs6467593|rs3115761|rs7726729
Photosensitivity	CHREBP2_PATHWAY	BIOCARTA	1059	39	20	5,94E-01	9,99E-01	rs351408|rs883541|rs7778077|rs4725424|rs12435569|rs3105453|rs4725435|rs6851340|rs1052176|rs1056348|rs4705117|rs2727564|rs10889011|rs7788160|rs6490267|rs9606893|rs715891|rs6965834|rs1881624|rs918167
Photosensitivity	FC_GAMMA_R_MEDIATED_PHAGOCYTOSIS	KEGG	3795	30	20	5,95E-01	9,99E-01	rs17651767|rs7023913|rs29452|rs628877|rs2060581|rs10912414|rs3866217|rs345306|rs10899496|rs873417|rs12143542|rs152342|rs2302456|rs1356410|rs3003608|rs2005219|rs7176475|rs10798089|rs13060691|rs12338625
Photosensitivity	PHOSPHORYLATION_OF_THE_APC_C	REACTOME	219	7	5	5,96E-01	9,99E-01	rs1647253|rs7500128|rs1994174|rs11687619|rs7683982
Photosensitivity	IL6_PATHWAY	BIOCARTA	401	23	12	5,98E-01	9,99E-01	rs4766987|rs2005219|rs13060691|rs1432442|rs10171977|rs1812402|rs904453|rs4722164|rs6037986|rs4255740|rs1882832|rs739787
Photosensitivity	IL_3_5_AND_GM_CSF_SIGNALING	REACTOME	1134	41	20	5,99E-01	9,99E-01	rs12251307|rs3864277|rs10899496|rs2302456|rs4143832|rs3105453|rs706733|rs706724|rs11792737|rs3012738|rs17811365|rs10171977|rs4240910|rs12244380|rs2278278|rs273266|rs33344|rs3012719|rs1993703|rs3218295
Photosensitivity	HEMATOPOIETIC_CELL_LINEAGE	KEGG	1963	31	20	6,00E-01	9,99E-01	rs885838|rs12251307|rs2243300|rs2395175|rs3864277|rs10940270|rs10777109|rs4143832|rs1423096|rs1440046|rs2701270|rs17200733|rs11688011|rs870992|rs8108707|rs10166912|rs2726648|rs11263909|rs3789938|rs1999713
Photosensitivity	INTEGRATION_OF_PROVIRUS	REACTOME	122	9	4	6,00E-01	9,99E-01	rs9587505|rs1193693|rs1408906|rs301287
Photosensitivity	GLUCOSE_TRANSPORT	REACTOME	689	31	16	6,00E-01	9,99E-01	rs11210751|rs10183020|rs7628051|rs2038743|rs7770657|rs2328136|rs3815076|rs11217129|rs10871221|rs3026106|rs4684126|rs6961391|rs6467593|rs1260326|rs3115761|rs3771763
Photosensitivity	ANTIVIRAL_MECHANISM_BY_IFN_STIMULATED_GENES	REACTOME	1077	34	20	6,00E-01	9,99E-01	rs10813821|rs10183020|rs2005219|rs7973701|rs7622028|rs2038743|rs6446298|rs6445954|rs7770657|rs11616903|rs11540270|rs2328136|rs1912401|rs10871221|rs3026106|rs7042042|rs17790482|rs9944218|rs4684126|rs7629936
Photosensitivity	NEUROACTIVE_LIGAND_RECEPTOR_INTERACTION	KEGG	13469	33	20	6,01E-01	9,99E-01	rs4903967|rs2112508|rs2008018|rs12364414|rs872091|rs1594513|rs4686174|rs4971851|rs466767|rs13116864|rs12873185|rs1878404|rs10514964|rs1886957|rs2268467|rs1937600|rs3908461|rs6180|rs823920|rs10986577
Photosensitivity	HOMOLOGOUS_RECOMBINATION	KEGG	730	28	13	6,01E-01	9,99E-01	rs2244012|rs3213191|rs2244814|rs2508678|rs7357092|rs6464262|rs4899244|rs10849605|rs17783214|rs11236183|rs3760410|rs5028394|rs17106012
Photosensitivity	AGR_PATHWAY	BIOCARTA	1981	31	20	6,02E-01	9,99E-01	rs264336|rs1798619|rs10940270|rs4452759|rs7817546|rs2005219|rs2507821|rs13260061|rs1414867|rs10748994|rs17746482|rs6039576|rs6090585|rs2237247|rs870992|rs12480230|rs1336299|rs12292911|rs544978|rs4236723
Photosensitivity	RNA_POL_I_TRANSCRIPTION_INITIATION	REACTOME	427	27	14	6,04E-01	9,99E-01	rs885838|rs16867175|rs4253042|rs4253126|rs7766094|rs11128940|rs2025448|rs2357266|rs11676697|rs1618536|rs1561734|rs1561330|rs2227301|rs1143407
Photosensitivity	CELL_DEATH_SIGNALLING_VIA_NRAGE_NRIF_AND_NADE	REACTOME	2069	25	20	6,04E-01	9,99E-01	rs12364414|rs10761189|rs7174521|rs345306|rs4810783|rs754743|rs7218427|rs9371867|rs1398028|rs7761427|rs6692050|rs1332684|rs7027092|rs1359647|rs926693|rs4778857|rs2882936|rs3004318|rs4817403|rs2289321
Photosensitivity	G2_PATHWAY	BIOCARTA	354	22	12	6,05E-01	9,99E-01	rs912239|rs6062356|rs6797587|rs1801516|rs7500128|rs6518758|rs576479|rs1968648|rs540723|rs7003908|rs228591|rs799906
Photosensitivity	REGULATION_OF_BETA_CELL_DEVELOPMENT	REACTOME	1288	32	20	6,08E-01	9,99E-01	rs6816542|rs2324528|rs11669442|rs3790826|rs1462436|rs2424476|rs6859263|rs6093978|rs9512906|rs1806180|rs189800|rs2440335|rs1052176|rs1169307|rs2788865|rs10409222|rs2254779|rs3730050|rs4525961|rs4435111
Photosensitivity	LIGAND_GATED_ION_CHANNEL_TRANSPORT	REACTOME	1051	24	20	6,12E-01	9,99E-01	rs13116864|rs2715583|rs164369|rs1012549|rs12440234|rs980982|rs209350|rs10024014|rs6879622|rs1367959|rs735983|rs17462431|rs17738087|rs4572353|rs1467257|rs10476364|rs6881515|rs11758041|rs6606866|rs3922665
Photosensitivity	INHIBITION_OF_VOLTAGE_GATED_CA2_CHANNELS_VIA_GBETA_GAMMA_SUBUNITS	REACTOME	1082	37	20	6,13E-01	9,99E-01	rs872091|rs10986577|rs11656925|rs10818955|rs17780119|rs7218368|rs11157863|rs180054|rs2835933|rs9903422|rs2779572|rs7152036|rs8075236|rs529269|rs10210535|rs2779524|rs3106651|rs584845|rs4793513|rs8104096
Photosensitivity	SNARE_INTERACTIONS_IN_VESICULAR_TRANSPORT	KEGG	828	37	20	6,13E-01	9,99E-01	rs362623|rs4729105|rs6583640|rs14976|rs2014613|rs11657426|rs8107108|rs3923878|rs751262|rs11987321|rs7917657|rs870142|rs7814958|rs12267930|rs729463|rs10157510|rs11654567|rs9313618|rs9644545|rs6570608
Photosensitivity	METABOLISM_OF_AMINO_ACIDS_AND_DERIVATIVES	REACTOME	5246	31	20	6,15E-01	9,99E-01	rs17389324|rs16827840|rs3741049|rs12337100|rs10143550|rs1918498|rs9904424|rs1154121|rs941425|rs10059065|rs884678|rs6973108|rs1409150|rs351676|rs6499969|rs848692|rs4802351|rs4754298|rs4849900|rs12577790
Photosensitivity	CA_DEPENDENT_EVENTS	REACTOME	1487	34	20	6,16E-01	9,99E-01	rs10912414|rs883541|rs4736746|rs873417|rs12143542|rs1404912|rs759947|rs10798089|rs4381631|rs10497599|rs4411531|rs729396|rs17463958|rs330712|rs4268746|rs758658|rs2430119|rs9729852|rs4144116|rs13166852
Photosensitivity	ONE_CARBON_POOL_BY_FOLATE	KEGG	441	33	16	6,20E-01	9,99E-01	rs2045616|rs1256107|rs2839947|rs6910267|rs4845881|rs519861|rs6900628|rs12626746|rs13201018|rs3772078|rs3821466|rs561882|rs9981981|rs1530637|rs1980983|rs895988
Photosensitivity	RIG_I_MDA5_MEDIATED_INDUCTION_OF_IFN_ALPHA_BETA_PATHWAYS	REACTOME	1129	26	20	6,21E-01	9,99E-01	rs2252405|rs10813821|rs11574849|rs1548992|rs891140|rs6446298|rs8132767|rs11702267|rs11540270|rs10490561|rs4975032|rs4946730|rs7930377|rs7992176|rs7327256|rs11899004|rs11024600|rs12586226|rs7042042|rs12229321
Photosensitivity	RHO_PATHWAY	BIOCARTA	480	26	16	6,22E-01	9,99E-01	rs3912541|rs8009215|rs6090585|rs17386030|rs1359647|rs727735|rs907698|rs6580191|rs7556894|rs1295907|rs4745514|rs1254403|rs1958916|rs1094561|rs10258757|rs4969391
Photosensitivity	NA_CL_DEPENDENT_NEUROTRANSMITTER_TRANSPORTERS	REACTOME	718	22	18	6,24E-01	9,99E-01	rs9682960|rs12490575|rs7131598|rs1710879|rs363251|rs6796254|rs720625|rs1905752|rs2531748|rs3785155|rs6763180|rs11710097|rs2930142|rs9826914|rs11747247|rs894747|rs9990174|rs13257289
Photosensitivity	ANTIGEN_PROCESSING_UBIQUITINATION_PROTEASOME_DEGRADATION	REACTOME	4788	27	20	6,25E-01	9,99E-01	rs4915953|rs16827840|rs17011585|rs6735360|rs17258702|rs12621817|rs903231|rs9904424|rs7949655|rs11073790|rs1647253|rs4802351|rs13195186|rs696500|rs7973701|rs350769|rs6758984|rs4412175|rs705571|rs722813
Photosensitivity	BOTULINUM_NEUROTOXICITY	REACTOME	522	25	12	6,26E-01	9,99E-01	rs362623|rs10778102|rs11657426|rs907698|rs729463|rs11654567|rs1402611|rs6570608|rs11658644|rs1795994|rs2126624|rs6503203
Photosensitivity	RIBOSOME	KEGG	1118	51	20	6,26E-01	9,99E-01	rs5995711|rs7210156|rs12903497|rs11623678|rs12911537|rs6926662|rs2816697|rs3825073|rs1209621|rs11217129|rs12624027|rs7670903|rs219493|rs1452404|rs1452396|rs2278498|rs2729812|rs17800095|rs4979938|rs4255740

Photosensitivity	RNA_POL_I_TRANSCRIPTION_TERMINATION	REACTOME	335	14	10	6,27E-01	9,99E-01	rs885838|rs16867175|rs7766094|rs2357266|rs11676697|rs1618536|rs1561734|rs1561330|rs2227301|rs1143407
Photosensitivity	TRANS_GOLGI_NETWORK_VESICLE_BUDDING	REACTOME	1106	47	20	6,29E-01	9,99E-01	rs7544627|rs5014235|rs11574849|rs6917198|rs12126748|rs13196275|rs10042243|rs14976|rs4804528|rs7740227|rs9996742|rs4715996|rs16972294|rs7548844|rs6864833|rs12771352|rs10069818|rs1460040|rs3768331|rs17138406
Photosensitivity	NO1_PATHWAY	BIOCARTA	1482	38	20	6,29E-01	9,99E-01	rs12654563|rs17086820|rs883541|rs10461355|rs6796803|rs943084|rs10823217|rs2507821|rs11133360|rs7671214|rs1416798|rs544978|rs11045359|rs12255651|rs10513803|rs478085|rs2819770|rs5030102|rs1492119|rs2618663
Photosensitivity	NEGATIVE_REGULATORS_OF_RIG_I_MDA5_SIGNALING	REACTOME	464	21	17	6,31E-01	9,99E-01	rs10813821|rs1548992|rs891140|rs6446298|rs11540270|rs4975032|rs4946730|rs12586226|rs7042042|rs12229321|rs1994174|rs7629936|rs2081066|rs627346|rs9938543|rs6568433|rs669260
Photosensitivity	DOWNSTREAM_TCR_SIGNALING	REACTOME	1573	31	20	6,31E-01	9,99E-01	rs3916765|rs10504873|rs10918693|rs353189|rs2673825|rs7973701|rs858490|rs706733|rs7841459|rs706724|rs991880|rs6420554|rs9275582|rs10804473|rs1049679|rs10214840|rs6461856|rs12216864|rs12191736|rs273266
Photosensitivity	VIRAL_MYOCARDITIS	KEGG	2750	54	20	6,32E-01	9,99E-01	rs12876542|rs10796820|rs3916765|rs1493101|rs9260759|rs3922737|rs3740643|rs2643075|rs2228615|rs68600|rs11115|rs12945671|rs9317095|rs9275582|rs2824271|rs6065925|rs9264219|rs12501768|rs3904322|rs624035
Photosensitivity	BASE_EXCISION_REPAIR	KEGG	500	24	17	6,33E-01	9,99E-01	rs174537|rs2275008|rs3828486|rs3744357|rs7992176|rs7327256|rs1713460|rs3135998|rs1889363|rs10861152|rs3093926|rs3213403|rs17064538|rs11236183|rs9684452|rs1467719|rs3213255
Photosensitivity	HIVNEF_PATHWAY	BIOCARTA	1008	29	20	6,33E-01	9,99E-01	rs1798619|rs3212153|rs884241|rs11107199|rs1061631|rs17200733|rs729396|rs2112166|rs12501768|rs11107202|rs1800627|rs7930377|rs6461845|rs11899004|rs2275075|rs2084385|rs12458289|rs2036847|rs6452735|rs39454
Photosensitivity	ENDOSOMAL_SORTING_COMPLEX_REQUIRED_FOR_TRANSPORT_ESCRT	REACTOME	318	10	6	6,36E-01	9,99E-01	rs904115|rs7836184|rs10795505|rs2357291|rs12052202|rs2064505
Photosensitivity	THE_ROLE_OF_NEF_IN_HIV1_REPLICATION_AND_DISEASE_PATHOGENESIS	REACTOME	936	31	18	6,37E-01	9,99E-01	rs1798619|rs9260759|rs10918693|rs858490|rs259916|rs2111035|rs4723645|rs2084385|rs16919915|rs843339|rs2724002|rs1647791|rs17646250|rs3768331|rs1003815|rs12518125|rs17187469|rs11604757
Photosensitivity	AMINE_LIGAND_BINDING_RECEPTORS	REACTOME	2247	33	20	6,40E-01	9,99E-01	rs2112508|rs1594513|rs10514964|rs11727674|rs17426222|rs4497195|rs2507821|rs16892078|rs9353399|rs8038887|rs6139415|rs12261976|rs6810358|rs265993|rs13436303|rs10802816|rs2880695|rs6893566|rs544978|rs4345419
Photosensitivity	VASOPRESSIN_REGULATED_WATER_REABSORPTION	KEGG	1100	30	20	6,42E-01	9,99E-01	rs12666636|rs160343|rs1029897|rs17276467|rs6979207|rs2237591|rs10486593|rs10486588|rs10233653|rs7126344|rs2237360|rs6948572|rs16874525|rs2299278|rs2238431|rs352493|rs3792009|rs42714|rs1910553|rs2099505
Photosensitivity	TRAFFICKING_OF_GLUR2_CONTAINING_AMPA_RECEPTORS	REACTOME	721	20	15	6,43E-01	9,99E-01	rs1945975|rs873417|rs10065813|rs4381631|rs4411531|rs3729908|rs758658|rs1438946|rs843339|rs1938958|rs12152961|rs7193148|rs11604757|rs10502051|rs9883238
Photosensitivity	SIGNALLING_BY_NGF	REACTOME	7869	31	20	6,44E-01	9,99E-01	rs7871111|rs12364414|rs10761189|rs7023913|rs628877|rs4296917|rs7174521|rs630426|rs883541|rs13377154|rs13288396|rs1401875|rs2324528|rs10504873|rs4350297|rs10509783|rs2130617|rs345306|rs4360891|rs10502294
Photosensitivity	APOPTOTIC_CLEAVAGE_OF_CELLULAR_PROTEINS	REACTOME	1052	35	20	6,45E-01	9,99E-01	rs3212153|rs353189|rs9951341|rs884241|rs442683|rs6599127|rs729396|rs12501768|rs1449079|rs1800627|rs231395|rs36032521|rs10502568|rs11899004|rs2275075|rs7321613|rs6785093|rs2036847|rs3778337|rs12143423
Photosensitivity	HIV_LIFE_CYCLE	REACTOME	1529	32	20	6,46E-01	9,99E-01	rs174537|rs885838|rs139873|rs3800453|rs10183020|rs9587505|rs7628051|rs2038743|rs7770657|rs6981243|rs7112576|rs2328136|rs1625691|rs1613070|rs10871221|rs7766094|rs3026106|rs7109662|rs12878503|rs6062014
Photosensitivity	PPARA_ACTIVATES_GENE_EXPRESSION	REACTOME	3675	47	20	6,48E-01	9,99E-01	rs6579757|rs307176|rs10774816|rs174546|rs3800794|rs723394|rs2203801|rs32574|rs4148329|rs4917342|rs6942490|rs6927367|rs10519096|rs7115063|rs9371867|rs17797652|rs6761246|rs2882936|rs11067630|rs4469064
Photosensitivity	CHEMOKINE_RECEPTORS_BIND_CHEMOKINES	REACTOME	726	26	20	6,48E-01	9,99E-01	rs2228428|rs17160128|rs497916|rs17283264|rs10184090|rs10429892|rs1051007|rs9993375|rs10491118|rs11671930|rs11574914|rs10972195|rs12409982|rs6456156|rs12360225|rs9972960|rs1429638|rs2236938|rs11217072|rs1737168
Photosensitivity	MITOCHONDRIAL_PROTEIN_IMPORT	REACTOME	609	28	14	6,50E-01	9,99E-01	rs2673969|rs17578228|rs10264536|rs976973|rs2802998|rs4693596|rs17178655|rs17409808|rs4082843|rs7042509|rs2270104|rs10265548|rs3810622|rs2674002
Photosensitivity	LEUKOCYTE_TRANSENDOTHELIAL_MIGRATION	KEGG	4793	44	20	6,50E-01	9,99E-01	rs6480245|rs4433471|rs10822936|rs1559303|rs3795994|rs12747584|rs7906982|rs13429335|rs6829390|rs345306|rs7898559|rs4766987|rs873417|rs13080849|rs2862499|rs4074716|rs13388538|rs2228615|rs8009215|rs9414948
Photosensitivity	NGF_SIGNALLING_VIA_TRKA_FROM_THE_PLASMA_MEMBRANE	REACTOME	4847	29	20	6,52E-01	9,99E-01	rs7871111|rs7023913|rs628877|rs630426|rs883541|rs1401875|rs2324528|rs2130617|rs10502294|rs4736746|rs873417|rs1012421|rs2302456|rs1404912|rs759947|rs3003608|rs2005219|rs2673825|rs13060691|rs12338625
Photosensitivity	INTERACTION_BETWEEN_L1_AND_ANKYRINS	REACTOME	970	30	20	6,52E-01	9,99E-01	rs17333297|rs2070776|rs10994214|rs10099843|rs1720344|rs1165838|rs2241538|rs2355254|rs9832586|rs2288358|rs857725|rs2883727|rs12357972|rs1616|rs10103618|rs2267887|rs2393603|rs7823044|rs16904685|rs4948410
Photosensitivity	RESPIRATORY_ELECTRON_TRANSPORT	REACTOME	1631	40	20	6,54E-01	9,99E-01	rs1500147|rs4854053|rs9775695|rs4147750|rs714621|rs2733954|rs1812144|rs10971025|rs1345638|rs17321133|rs7795088|rs11669029|rs13277184|rs10487881|rs10116346|rs6461845|rs567697|rs6510333|rs1840819|rs254741
Photosensitivity	MITOTIC_PROMETAPHASE	REACTOME	1814	46	20	6,55E-01	9,99E-01	rs351408|rs6507992|rs12757772|rs2028455|rs12452736|rs996482|rs8004556|rs6769686|rs13130657|rs7129085|rs259871|rs3004318|rs1056348|rs3996325|rs11592827|rs7043114|rs1713676|rs2345029|rs2580347|rs7500128
Photosensitivity	GROWTH_HORMONE_RECEPTOR_SIGNALING	REACTOME	773	24	20	6,59E-01	9,99E-01	rs2008018|rs6180|rs2005219|rs2221449|rs11966463|rs3020619|rs7491948|rs1023168|rs7744710|rs2876980|rs7221341|rs9915291|rs7739889|rs7751843|rs10521023|rs1604422|rs9905081|rs11687971|rs2251692|rs7834615
Photosensitivity	TRAF6_MEDIATED_NFKB_ACTIVATION	REACTOME	351	21	16	6,59E-01	9,99E-01	rs10813821|rs11574849|rs8132767|rs11702267|rs11540270|rs7992176|rs7327256|rs11024600|rs7042042|rs2282295|rs832540|rs2839362|rs1467719|rs669260|rs5030437|rs400603
Photosensitivity	TCR_SIGNALING	REACTOME	2040	32	20	6,62E-01	9,99E-01	rs1798619|rs3916765|rs2060581|rs10918693|rs10504873|rs353189|rs2673825|rs7973701|rs858490|rs706733|rs7841459|rs706724|rs991880|rs6420554|rs9275582|rs10804473|rs1049679|rs10214840|rs6461856|rs12216864
Photosensitivity	CYTOKINE_SIGNALING_IN_IMMUNE_SYSTEM	REACTOME	6819	44	20	6,62E-01	9,99E-01	rs5745752|rs2008018|rs7946527|rs599683|rs3916765|rs12251307|rs10922552|rs11638243|rs6180|rs12747584|rs12531099|rs9260759|rs2252405|rs10813821|rs10504873|rs10922613|rs3864277|rs10899496|rs11574849|rs10183020
Photosensitivity	CXCR4_PATHWAY	BIOCARTA	1099	31	20	6,66E-01	9,99E-01	rs873417|rs2302456|rs2005219|rs13060691|rs1374670|rs706733|rs706724|rs4381631|rs4411531|rs3729908|rs1432442|rs758658|rs2292979|rs2886612|rs904453|rs2876980|rs33344|rs12360225|rs10515068|rs7193148
Photosensitivity	CELL_CELL_COMMUNICATION	REACTOME	9725	33	20	6,67E-01	9,99E-01	rs17809941|rs7234019|rs709505|rs918285|rs7943417|rs1369918|rs4457095|rs10058333|rs1201981|rs1559303|rs11645947|rs10499837|rs13332623|rs1998777|rs1366492|rs2884345|rs6015839|rs4411488|rs16974215|rs4445973
Photosensitivity	APOPTOSIS	REACTOME	3298	33	20	6,68E-01	9,99E-01	rs16827840|rs7234019|rs1798619|rs7953290|rs9904424|rs3212153|rs4400714|rs353189|rs4802351|rs9951341|rs884241|rs3124238|rs743272|rs442683|rs1398028|rs17200733|rs9673361|rs6060563|rs4148876|rs6599127
Photosensitivity	HUNTINGTONS_DISEASE	KEGG	4311	35	20	6,68E-01	9,99E-01	rs1953738|rs885838|rs12666636|rs2499611|rs723394|rs1500147|rs139873|rs160343|rs4854053|rs9775695|rs4147750|rs1029897|rs714621|rs1982235|rs6942490|rs17276467|rs7694687|rs2733954|rs1812144|rs6120890
Photosensitivity	RECYCLING_PATHWAY_OF_L1	REACTOME	874	35	19	6,68E-01	9,99E-01	rs7023913|rs3003608|rs12338625|rs6090585|rs6456115|rs2099095|rs4804528|rs177387|rs12436076|rs2062400|rs2876980|rs843339|rs351291|rs550840|rs6918886|rs11604757|rs2077852|rs7752989|rs961548
Photosensitivity	WNT_PATHWAY	BIOCARTA	1363	46	20	6,70E-01	9,99E-01	rs1030609|rs959176|rs10796820|rs3740643|rs442683|rs10780472|rs6599127|rs991880|rs2465153|rs6999335|rs10956433|rs12191736|rs815847|rs437932|rs16902654|rs649392|rs4733581|rs6997983|rs7742254|rs17101044
Photosensitivity	YAP1_AND_WWTR1_TAZ_STIMULATED_GENE_EXPRESSION	REACTOME	1047	49	20	6,70E-01	9,99E-01	rs3800794|rs6941971|rs4917342|rs6927367|rs7115063|rs1928532|rs914852|rs2046380|rs877988|rs7636407|rs135549|rs6440618|rs6773879|rs659555|rs10948238|rs6917644|rs11128940|rs3782836|rs1882110|rs1545255
Photosensitivity	ACTINY_PATHWAY	BIOCARTA	290	12	7	6,71E-01	9,99E-01	rs3912541|rs727735|rs7982250|rs7556894|rs2474293|rs10258757|rs2133814
Photosensitivity	PEPTIDE_CHAIN_ELONGATION	REACTOME	947	43	20	6,72E-01	9,99E-01	rs5995711|rs7210156|rs12903497|rs11623678|rs6926662|rs2816697|rs3825073|rs1211975|rs11217129|rs12624027|rs7670903|rs219493|rs1452404|rs1452396|rs2278498|rs17800095|rs4979938|rs4255740|rs2134927|rs4789636
Photosensitivity	LATE_PHASE_OF_HIV_LIFE_CYCLE	REACTOME	1259	28	20	6,73E-01	9,99E-01	rs885838|rs139873|rs3800453|rs10183020|rs7622028|rs2038743|rs7770657|rs6981243|rs7112576|rs2328136|rs1625691|rs1613070|rs10871221|rs7766094|rs3026106|rs7109662|rs12878503|rs6062014|rs4684126|rs2273006
Photosensitivity	CHEMICAL_PATHWAY	BIOCARTA	585	30	19	6,75E-01	9,99E-01	rs3212153|rs873417|rs6060563|rs4381631|rs1801516|rs4411531|rs3729908|rs12501768|rs1800627|rs758658|rs6461845|rs12458289|rs7193148|rs39454|rs228591|rs4319556|rs2236719|rs2498804|rs12673033
Photosensitivity	SIGNALING_BY_FGFR_IN_DISEASE	REACTOME	4545	26	20	6,80E-01	9,99E-01	rs743682|rs666336|rs17487009|rs13231090|rs628877|rs883541|rs250201|rs2324528|rs10886918|rs9580274|rs10899496|rs4736746|rs873417|rs1404912|rs759947|rs2005219|rs2673825|rs13060691|rs6467779|rs534870
Photosensitivity	NEURONAL_SYSTEM	REACTOME	13378	28	20	6,80E-01	9,99E-01	rs4748472|rs7007251|rs6443589|rs870031|rs10974591|rs9682960|rs12364414|rs872091|rs362623|rs466767|rs10107483|rs7174521|rs513472|rs2455280|rs3006845|rs10986577|rs7190913|rs6589846|rs9377381|rs2107118
Photosensitivity	SMALL_CELL_LUNG_CANCER	KEGG	3747	45	20	6,82E-01	9,99E-01	rs1030608|rs9868156|rs10796820|rs6909633|rs997669|rs1656463|rs4917342|rs3740643|rs10866044|rs13071758|rs11080358|rs1604003|rs7616062|rs2673825|rs2701270|rs213392|rs706733|rs16951524|rs11688011|rs6060563
Photosensitivity	CELL_CELL_JUNCTION_ORGANIZATION	REACTOME	6211	34	20	6,85E-01	9,99E-01	rs7943417|rs1369918|rs4457095|rs10058333|rs1201981|rs1559303|rs11645947|rs13332623|rs1366492|rs6015839|rs4411488|rs4445973|rs2426913|rs7202575|rs1007369|rs11215438|rs6061779|rs1582924|rs6142828|rs16899078
Photosensitivity	SIGNALLING_TO_ERKS	REACTOME	794	34	20	6,85E-01	9,99E-01	rs2302456|rs2005219|rs13060691|rs6090585|rs11792737|rs3012738|rs625658|rs1432442|rs195204|rs1294383|rs10171977|rs904453|rs10468964|rs2876980|rs10771184|rs3012719|rs869382|rs4255740|rs4626883|rs9380546
Photosensitivity	PYK2_PATHWAY	BIOCARTA	752	34	20	6,88E-01	9,99E-01	rs873417|rs2005219|rs13060691|rs6090585|rs4381631|rs4411531|rs3729908|rs1432442|rs10171977|rs758658|rs2292979|rs2716121|rs904453|rs2876980|rs8065164|rs7193148|rs832540|rs4255740|rs2237093|rs10869985
Photosensitivity	GOLGI_ASSOCIATED_VESICLE_BIOGENESIS	REACTOME	1057	47	20	6,88E-01	9,99E-01	rs7544627|rs5014235|rs6917198|rs12126748|rs13196275|rs10042243|rs14976|rs4804528|rs7740227|rs9996742|rs4715996|rs16972294|rs6588140|rs6864833|rs10069818|rs1460040|rs3768331|rs17138406|rs6080886|rs355425
Photosensitivity	MATURITY_ONSET_DIABETES_OF_THE_YOUNG	KEGG	992	30	20	6,89E-01	9,99E-01	rs11669442|rs3790826|rs1462436|rs2424476|rs6093978|rs9512906|rs1806180|rs189800|rs2440335|rs1052176|rs1169307|rs10409222|rs2254779|rs7981979|rs6075965|rs724767|rs731573|rs2821367|rs17626423|rs1203951
Photosensitivity	SIGNALLING_TO_RAS	REACTOME	468	26	14	6,91E-01	9,99E-01	rs2005219|rs13060691|rs6090585|rs1432442|rs195204|rs1294383|rs10171977|rs904453|rs2876980|rs10771184|rs4255740|rs4626883|rs2237093|rs12701767
Photosensitivity	AMI_PATHWAY	BIOCARTA	574	25	12	6,92E-01	9,99E-01	rs13062355|rs6722598|rs16975259|rs3769641|rs13028299|rs6727477|rs1520481|rs2064712|rs492560|rs3823055|rs6935921|rs6739977
Photosensitivity	CREB_PHOSPHORYLATION_THROUGH_THE_ACTIVATION_OF_RAS	REACTOME	1349	34	20	6,93E-01	9,99E-01	rs7174521|rs7190913|rs4780550|rs2872715|rs13060691|rs3822295|rs17550532|rs4778857|rs2284406|rs6456115|rs1900718|rs7202071|rs7684832|rs904453|rs891944|rs1379271|rs10045942|rs2876980|rs12102827|rs6498771
Photosensitivity	TRAF6_MEDIATED_IRF7_ACTIVATION	REACTOME	433	17	12	6,93E-01	9,99E-01	rs2252405|rs10813821|rs11540270|rs10490561|rs7042042|rs12229321|rs2797507|rs4326049|rs1882364|rs12470772|rs669260|rs5030437
Photosensitivity	SIGNALING_BY_BMP	REACTOME	918	33	20	6,94E-01	9,99E-01	rs7171416|rs7212632|rs7229153|rs232651|rs12505085|rs6085938|rs6107902|rs2439368|rs17206172|rs6038619|rs15985|rs1048070|rs1392392|rs1994174|rs263526|rs2042555|rs733877|rs10926304|rs12456328|rs4662515
Photosensitivity	ABC_FAMILY_PROTEINS_MEDIATED_TRANSPORT	REACTOME	1753	34	20	6,96E-01	9,99E-01	rs12615195|rs7805271|rs4147837|rs4147830|rs1858947|rs2297636|rs483904|rs927204|rs2993589|rs737453|rs35596|rs557026|rs17189299|rs8187843|rs2235046|rs1467257|rs4781699|rs3792009|rs1914361|rs8072153
Photosensitivity	CELL_ADHESION_MOLECULES_CAMS	KEGG	8813	31	20	7,01E-01	9,99E-01	rs963314|rs7943417|rs7946527|rs7808274|rs3916765|rs3745264|rs11872382|rs1201981|rs1559303|rs9333118|rs10163905|rs2060581|rs12747584|rs17333297|rs2826881|rs6015839|rs9260759|rs4445973|rs2426913|rs11677389
Photosensitivity	O_LINKED_GLYCOSYLATION_OF_MUCINS	REACTOME	2402	47	20	7,02E-01	9,99E-01	rs590666|rs12536849|rs6976905|rs1202640|rs6560519|rs2305965|rs1465231|rs11900673|rs12477460|rs11247000|rs1481373|rs338047|rs6444192|rs7480563|rs6667721|rs10060240|rs890797|rs289686|rs10081532|rs6860445
Photosensitivity	APOPTOSIS	KEGG	1727	34	20	7,03E-01	9,99E-01	rs963065|rs883541|rs3212153|rs2461489|rs17031191|rs2851045|rs6852347|rs17200733|rs706733|rs6060563|rs706724|rs1801516|rs600135|rs17811365|rs12501768|rs195204|rs1294383|rs1800627|rs2239621|rs25887
Photosensitivity	POST_NMDA_RECEPTOR_ACTIVATION_EVENTS	REACTOME	1753	27	20	7,04E-01	9,99E-01	rs7174521|rs7190913|rs4736746|rs1404912|rs759947|rs4780550|rs2872715|rs13060691|rs3822295|rs17550532|rs4778857|rs2284406|rs6456115|rs1900718|rs17463958|rs1558294|rs7202071|rs7684832|rs904453|rs891944
Photosensitivity	ANTIGEN_ACTIVATES_B_CELL_RECEPTOR_LEADING_TO_GENERATION_OF_SECOND_ME	REACTOME	1299	44	20	7,05E-01	9,99E-01	rs7120938|rs10430939|rs2070776|rs722813|rs12313273|rs706733|rs7961587|rs706724|rs4580153|rs10171977|rs4240910|rs3020619|rs7951076|rs2448074|rs4919048|rs2278278|rs33344|rs1993703|rs10515068|rs1423236
Photosensitivity	NITROGEN_METABOLISM	KEGG	587	31	14	7,06E-01	9,99E-01	rs7845438|rs11071746|rs3765966|rs11863558|rs1025607|rs6748782|rs4650044|rs12091891|rs2780749|rs1532423|rs745338|rs939341|rs3829305|rs6424389
Photosensitivity	METABOLISM_OF_STEROID_HORMONES_AND_VITAMINS_A_AND_D	REACTOME	745	32	20	7,09E-01	9,99E-01	rs3848180|rs6439858|rs17431426|rs1048691|rs780825|rs6068799|rs4622471|rs4702379|rs17523880|rs2291617|rs2289105|rs209967|rs4667591|rs12991585|rs619824|rs927650|rs2277603|rs13325144|rs6127112|rs8041933
Photosensitivity	IL22BP_PATHWAY	BIOCARTA	296	10	9	7,09E-01	9,99E-01	rs7601754|rs7221341|rs9915291|rs10484796|rs167769|rs1517352|rs9905081|rs10892204|rs4969168
Photosensitivity	RAS_PATHWAY	BIOCARTA	557	47	20	7,11E-01	9,99E-01	rs7227801|rs2005219|rs13060691|rs706733|rs6060563|rs12454987|rs706724|rs1432442|rs904453|rs33344|rs328994|rs10515068|rs1423236|rs3815701|rs10054692|rs1813100|rs4255740|rs11953134|rs4976129|rs1388122
Photosensitivity	NEF_MEDIATES_DOWN_MODULATION_OF_CELL_SURFACE_RECEPTORS_BY_RECRUITIN N_ADAPTERS	REACTOME	366	10	6	7,13E-01	9,99E-01	rs9260759|rs16919915|rs843339|rs3768331|rs17187469|rs11604757
Photosensitivity	INTERFERON_ALPHA_BETA_SIGNALING	REACTOME	1477	34	20	7,15E-01	9,99E-01	rs4656092|rs11638243|rs9260759|rs2252405|rs9426832|rs743272|rs11246067|rs4148876|rs1169307|rs9264219|rs305095|rs276947|rs4436753|rs7221341|rs9378229|rs9915291|rs4500694|rs9933359|rs10484565|rs12662501
Photosensitivity	NONSENSE_MEDIATED_DECAY_ENHANCED_BY_THE_EXON_JUNCTION_COMPLEX	REACTOME	1261	44	20	7,18E-01	9,99E-01	rs5995711|rs7210156|rs12903497|rs11623678|rs6926662|rs3105429|rs2816697|rs7079622|rs3825073|rs1056348|rs1209621|rs11217129|rs12624027|rs7670903|rs219493|rs1452404|rs1452396|rs2278498|rs3803809|rs1825183
Photosensitivity	RNA_POL_II_TRANSCRIPTION	REACTOME	1050	21	20	7,19E-01	9,99E-01	rs885838|rs139873|rs3800453|rs6981243|rs7112576|rs17704641|rs4646296|rs1798177|rs1625691|rs1613070|rs7766094|rs7109662|rs10859963|rs12878503|rs16987990|rs13183577|rs6030839|rs6062014|rs2273006|rs4799085
Photosensitivity	UBIQUITIN_MEDIATED_PROTEOLYSIS	KEGG	3227	26	20	7,19E-01	9,99E-01	rs4915953|rs17011585|rs17258702|rs7590342|rs7949655|rs11073790|rs1647253|rs7735274|rs13195186|rs696500|rs7973701|rs6758984|rs4412175|rs705571|rs722813|rs2017089|rs6446298|rs16951524|rs9456772|rs7633774
Photosensitivity	PENTOSE_AND_GLUCURONATE_INTERCONVERSIONS	KEGG	874	161	11	7,20E-01	9,99E-01	rs4148329|rs6761246|rs2361502|rs1093685|rs2183207|rs9315648|rs2608822|rs376109|rs6830865|rs709607|rs2687964
Photosensitivity	G_ALPHA_Z_SIGNALLING_EVENTS	REACTOME	2191	27	20	7,21E-01	9,99E-01	rs1088672|rs628877|rs1154029|rs353189|rs4736746|rs873417|rs1404912|rs2790090|rs9285537|rs1124787|rs2236941|rs7762618|rs6740453|rs11157863|rs6981243|rs12261976|rs4381631|rs1020445|rs4411531|rs729396
Photosensitivity	INWARDLY_RECTIFYING_K_CHANNELS	REACTOME	1317	36	20	7,22E-01	9,99E-01	rs872091|rs10986577|rs11656925|rs10818955|rs17780119|rs7218368|rs11157863|rs180054|rs2835933|rs9903422|rs2779572|rs7152036|rs8075236|rs529269|rs10210535|rs2779524|rs3106651|rs584845|rs4793513|rs4148661
Photosensitivity	TYPE_II_DIABETES_MELLITUS	KEGG	2053	21	20	7,23E-01	9,99E-01	rs2239094|rs628877|rs4804366|rs2238012|rs2005219|rs7525299|rs9888368|rs2239062|rs2238043|rs1993430|rs1124787|rs17200733|rs706733|rs6740453|rs706724|rs1020445|rs9512906|rs729396|rs1052176|rs11666813
Photosensitivity	SYSTEMIC_LUPUS_ERYTHEMATOSUS	KEGG	2453	56	20	7,24E-01	9,99E-01	rs3916765|rs7190913|rs9366694|rs3778279|rs4780550|rs2872715|rs11953280|rs16927253|rs68600|rs17550532|rs17200733|rs2284406|rs9275582|rs4668168|rs6065925|rs1900718|rs624035|rs129128|rs7202071|rs10489623
Photosensitivity	AT1R_PATHWAY	BIOCARTA	1224	24	20	7,25E-01	9,99E-01	rs873417|rs1982235|rs2005219|rs13060691|rs17746482|rs6090585|rs4381631|rs4411531|rs3729908|rs1432442|rs984654|rs10171977|rs758658|rs2292979|rs1812402|rs904453|rs4140770|rs1283614|rs2876980|rs12655172
Photosensitivity	LATENT_INFECTION_OF_HOMO_SAPIENS_WITH_MYCOBACTERIUM_TUBERCULOSIS	REACTOME	589	21	13	7,25E-01	9,99E-01	rs2515210|rs11080358|rs17200733|rs13070740|rs871516|rs2531882|rs16919915|rs17749106|rs2297518|rs13238736|rs2139634|rs8846|rs7836003
Photosensitivity	NEUROTRANSMITTER_RECEPTOR_BINDING_AND_DOWNSTREAM_TRANSMISSION_IN_TH L	REACTOME	6592	25	20	7,27E-01	9,99E-01	rs7007251|rs12364414|rs872091|rs466767|rs10107483|rs7174521|rs513472|rs10986577|rs7190913|rs6589846|rs9377381|rs2107118|rs11656925|rs2715583|rs164369|rs2946542|rs1945975|rs140040|rs4736746|rs873417
Photosensitivity	RESPIRATORY_ELECTRON_TRANSPORT_ATP_SYNTHESIS_BY_CHEMIOSMOTIC_COUPLIN TION_BY_UNCOUPLING_PROTEINS_	REACTOME	1741	40	20	7,27E-01	9,99E-01	rs1500147|rs4854053|rs9775695|rs4147750|rs714621|rs2733954|rs1812144|rs10971025|rs1345638|rs17321133|rs7795088|rs11669029|rs13277184|rs10487881|rs6818701|rs10116346|rs6461845|rs567697|rs6510333|rs1840819
Photosensitivity	SIGNALING_BY_FGFR	REACTOME	4154	25	20	7,29E-01	9,99E-01	rs743682|rs666336|rs17487009|rs628877|rs883541|rs250201|rs2324528|rs10886918|rs9580274|rs4736746|rs873417|rs1404912|rs759947|rs2005219|rs2673825|rs13060691|rs534870|rs883355|rs13250792|rs3129601
Photosensitivity	SYNTHESIS_OF_DNA	REACTOME	1545	27	20	7,30E-01	9,99E-01	rs16827840|rs174546|rs9904424|rs4802351|rs743272|rs9673361|rs4148876|rs3782093|rs1957107|rs12292911|rs4664414|rs11987150|rs1973619|rs2550461|rs7357092|rs676114|rs837763|rs1217|rs555812|rs8063430
Photosensitivity	PROTEIN_EXPORT	KEGG	750	41	14	7,30E-01	9,99E-01	rs16974510|rs37646|rs7785319|rs6952793|rs10282292|rs162202|rs1528034|rs10988774|rs11568429|rs1358024|rs10953655|rs11177|rs10487300|rs7806148
Photosensitivity	BILE_ACID_AND_BILE_SALT_METABOLISM	REACTOME	1176	37	20	7,31E-01	9,99E-01	rs9423399|rs696910|rs4772523|rs2277119|rs2254670|rs4646296|rs3732860|rs9514217|rs2801527|rs9519033|rs7922269|rs1336596|rs13434020|rs9554926|rs1925978|rs12824715|rs7492089|rs2228468|rs3756462|rs16856261
Photosensitivity	ABC_TRANSPORTERS	KEGG	2684	31	20	7,31E-01	9,99E-01	rs12615195|rs7805271|rs4540359|rs4147837|rs4147830|rs6946623|rs333937|rs4148876|rs1858947|rs2297636|rs2740484|rs554931|rs927204|rs2993589|rs10248040|rs737453|rs35596|rs557026|rs17189299|rs6977474
Photosensitivity	ACTIVATION_OF_NMDA_RECEPTOR_UPON_GLUTAMATE_BINDING_AND_POSTSYNAPTIC_	REACTOME	2117	26	20	7,32E-01	9,99E-01	rs7174521|rs7190913|rs1945975|rs4736746|rs1404912|rs10065813|rs759947|rs4780550|rs2872715|rs13060691|rs3822295|rs17550532|rs4778857|rs2284406|rs6456115|rs1900718|rs17463958|rs1558294|rs7202071|rs7684832
Photosensitivity	PHOSPHOLIPID_METABOLISM	REACTOME	6226	30	20	7,33E-01	9,99E-01	rs225829|rs6793913|rs12141345|rs12490575|rs6793037|rs10912414|rs10444692|rs11729278|rs1410119|rs3786327|rs3737358|rs6076792|rs1488|rs6133219|rs1941590|rs2440455|rs12143542|rs6559175|rs1356410|rs10189241
Photosensitivity	SIGNAL_AMPLIFICATION	REACTOME	1023	36	20	7,33E-01	9,99E-01	rs10912414|rs12143542|rs10798089|rs6090585|rs2236941|rs11157863|rs330712|rs501163|rs2430119|rs2886612|rs2301230|rs9729852|rs11655004|rs4144116|rs7152036|rs9875152|rs584845|rs6809526|rs8104096|rs10238438
Photosensitivity	GLEEVEC_PATHWAY	BIOCARTA	954	38	20	7,34E-01	9,99E-01	rs1030608|rs2005219|rs13060691|rs706733|rs706724|rs1432442|rs10171977|rs992241|rs2716121|rs904453|rs10956433|rs8065164|rs33344|rs16902654|rs10515068|rs4733581|rs6997983|rs1423236|rs3815701|rs10054692
Photosensitivity	TRANSCRIPTION_COUPLED_NER_TC_NER	REACTOME	834	30	19	7,34E-01	9,99E-01	rs885838|rs139873|rs4253042|rs4253126|rs6981243|rs7112576|rs1625691|rs7357092|rs7766094|rs2025448|rs1359389|rs1629511|rs11236183|rs1618536|rs1561734|rs7328146|rs1143407|rs794078|rs794084
Photosensitivity	TNFR1_PATHWAY	BIOCARTA	862	32	20	7,34E-01	9,99E-01	rs1798619|rs884241|rs11107199|rs17200733|rs991880|rs12501768|rs11107202|rs7930377|rs1812402|rs12191736|rs11899004|rs2275075|rs8065164|rs2084385|rs437932|rs2036847|rs7742254|rs1782449|rs832540|rs7003908
Photosensitivity	INFLAMMASOMES	REACTOME	468	43	16	7,37E-01	9,99E-01	rs591874|rs8132767|rs6060563|rs11702267|rs882937|rs1718119|rs6685474|rs4135223|rs2716932|rs12458289|rs11653896|rs917686|rs8079727|rs9806416|rs2236719|rs400603
Photosensitivity	OOCYTE_MEIOSIS	KEGG	3114	34	20	7,37E-01	9,99E-01	rs351408|rs963065|rs997669|rs1546713|rs2461489|rs17031191|rs1647253|rs4736746|rs10501976|rs1404912|rs2851045|rs2005219|rs2684794|rs996482|rs3822295|rs8004556|rs6852347|rs3105453|rs6769686|rs13130657

Photosensitivity	CELL_JUNCTION_ORGANIZATION	REACTOME	6781	33	20	7,38E-01	9,99E-01	rs7943417|rs1369918|rs4457095|rs10058333|rs1201981|rs1559303|rs11645947|rs13332623|rs1998777|rs1366492|rs2884345|rs6015839|rs4411488|rs4445973|rs2426913|rs7202575|rs1007369|rs9614362|rs11215438|rs6061779
Photosensitivity	ADIPOCYTOKINE_SIGNALING_PATHWAY	KEGG	2106	36	20	7,40E-01	9,99E-01	rs17674015|rs6829223|rs11210751|rs4917342|rs4766987|rs353189|rs7778077|rs4725424|rs1061631|rs4725435|rs10849968|rs17200733|rs12533743|rs4469064|rs914852|rs157869|rs4361373|rs2395926|rs1558294|rs2727564
Photosensitivity	EDG1_PATHWAY	BIOCARTA	1432	25	20	7,42E-01	9,99E-01	rs6701418|rs873417|rs1404912|rs2005219|rs6090585|rs706733|rs17746897|rs706724|rs6055990|rs11164184|rs4381631|rs4411531|rs2719795|rs8115925|rs3729908|rs1444043|rs2223538|rs758658|rs2886612|rs10437031
Photosensitivity	MRNA_PROCESSING	REACTOME	1942	33	20	7,42E-01	9,99E-01	rs885838|rs10197258|rs139873|rs2588882|rs10183020|rs2305797|rs9426832|rs12937256|rs7622028|rs2038743|rs2526388|rs7770657|rs7112576|rs17704641|rs4646296|rs1798177|rs2328136|rs6433636|rs1625691|rs1613070
Photosensitivity	GLUCOSE_METABOLISM	REACTOME	1554	30	20	7,43E-01	9,99E-01	rs8049837|rs7535245|rs6499969|rs2269260|rs11189224|rs620281|rs9864435|rs8057892|rs13080791|rs1052176|rs1056348|rs2267844|rs2490286|rs10903843|rs217463|rs3750667|rs2064393|rs10514738|rs943117|rs8062792
Photosensitivity	COLORECTAL_CANCER	KEGG	2539	34	20	7,43E-01	9,99E-01	rs1030609|rs7234019|rs10796820|rs17047703|rs3740643|rs4918785|rs11685301|rs2005219|rs13060691|rs442683|rs706733|rs706724|rs6599127|rs12962203|rs6722568|rs12501768|rs1432442|rs2879414|rs10520405|rs992241
Photosensitivity	GLUCAGON_TYPE_LIGAND_RECEPTORS	REACTOME	786	35	18	7,46E-01	9,99E-01	rs7794503|rs10502294|rs10224073|rs17810148|rs11157863|rs9470963|rs3786122|rs2345589|rs1862009|rs7152036|rs7785534|rs7218549|rs584845|rs633596|rs8104096|rs1468687|rs11810173|rs9966172
Photosensitivity	CLASS_I_MHC_MEDIATED_ANTIGEN_PROCESSING_PRESENTATION	REACTOME	5895	27	20	7,47E-01	9,99E-01	rs4915953|rs16827840|rs17011585|rs6735360|rs17258702|rs12621817|rs903231|rs9904424|rs9260759|rs7949655|rs11073790|rs1647253|rs4802351|rs13195186|rs696500|rs7973701|rs350769|rs6758984|rs4412175|rs705571
Photosensitivity	ETHER_LIPID_METABOLISM	KEGG	1041	48	20	7,48E-01	9,99E-01	rs10912417|rs1348850|rs12143542|rs7176475|rs10798089|rs2962061|rs1800627|rs12439430|rs330712|rs2430119|rs9729852|rs7172971|rs4144116|rs12136817|rs2286670|rs12952976|rs895344|rs4820315|rs10911933|rs1487492
Photosensitivity	DAG_AND_IP3_SIGNALING	REACTOME	1644	25	20	7,50E-01	9,99E-01	rs628877|rs883541|rs4736746|rs873417|rs1404912|rs759947|rs1124787|rs6740453|rs7961587|rs4381631|rs10497599|rs1020445|rs4411531|rs729396|rs17463958|rs4268746|rs11684301|rs758658|rs17033965|rs6720975
Photosensitivity	PEPTIDE_LIGAND_BINDING_RECEPTORS	REACTOME	5053	28	20	7,51E-01	9,99E-01	rs2228428|rs12412993|rs12873185|rs1878404|rs1886957|rs1937600|rs3908461|rs11143600|rs2810585|rs6796803|rs7853418|rs4751433|rs6048417|rs4884075|rs17160128|rs10890041|rs2069662|rs2268496|rs10878001|rs10512014
Photosensitivity	SMAD2_SMAD3_SMAD4_HETEROTRIMER_REGULATES_TRANSCRIPTION	REACTOME	715	26	15	7,52E-01	9,99E-01	rs1030608|rs7229153|rs6999335|rs6440618|rs10956433|rs12585793|rs4305170|rs16902654|rs4733581|rs6997983|rs266363|rs12456328|rs4798085|rs7163945|rs9954608
Photosensitivity	FACTORS_INVOLVED_IN_MEGAKARYOCYTE_DEVELOPMENT_AND_PLATELET_PRODUCT	REACTOME	3549	52	20	7,55E-01	9,99E-01	rs2483852|rs4296917|rs4369779|rs9483803|rs883541|rs2252405|rs7007134|rs7148495|rs7896001|rs974208|rs10765118|rs12452736|rs11715034|rs4741823|rs3801778|rs353087|rs259916|rs6935954|rs10137053|rs925031
Photosensitivity	ENERGY_DEPENDENT_REGULATION_OF_MTOR_BY_LKB1_AMPK	REACTOME	461	29	20	7,55E-01	9,99E-01	rs2809244|rs7778077|rs4725424|rs4725435|rs12943041|rs2727564|rs12948054|rs10889011|rs7788160|rs6490267|rs2289762|rs13409720|rs6965834|rs1881624|rs10901219|rs4901977|rs4435442|rs7221948|rs721575|rs7502124
Photosensitivity	TAK1_ACTIVATES_NFKB_BY_PHOSPHORYLATION_AND_ACTIVATION_OF_IKKS_COMPLE	REACTOME	777	27	20	7,56E-01	9,99E-01	rs10504873|rs11574849|rs8132767|rs7841459|rs11702267|rs991880|rs10214840|rs7992176|rs7327256|rs12216864|rs12191736|rs11024600|rs437932|rs2282295|rs7742254|rs1782449|rs2839362|rs6955931|rs4141907|rs1467719
Photosensitivity	PKB_MEDIATED_EVENTS	REACTOME	583	29	20	7,57E-01	9,99E-01	rs2809244|rs7778077|rs4725424|rs4725435|rs12943041|rs2727564|rs12948054|rs10889011|rs7788160|rs3730050|rs6490267|rs2289762|rs13409720|rs6965834|rs1881624|rs10901219|rs4901977|rs4435442|rs7221948|rs721575
Photosensitivity	RIP_MEDIATED_NFKB_ACTIVATION_VIA_DAI	REACTOME	328	14	13	7,58E-01	9,99E-01	rs11574849|rs8132767|rs11702267|rs7992176|rs7327256|rs11024600|rs2064393|rs2282295|rs742724|rs2839362|rs3181384|rs1467719|rs400603
Photosensitivity	PLATELET_AGGREGATION_PLUG_FORMATION	REACTOME	942	21	14	7,59E-01	9,99E-01	rs885838|rs2302456|rs703009|rs6090585|rs12261976|rs10171977|rs786870|rs2278278|rs1993703|rs2138551|rs17706710|rs10520094|rs2498804|rs2203615
Photosensitivity	PEROXISOMAL_LIPID_METABOLISM	REACTOME	435	18	9	7,63E-01	9,99E-01	rs1348850|rs1911597|rs2239621|rs13434020|rs3756462|rs12747412|rs1135889|rs10219485|rs384346
Photosensitivity	G_ALPHA_Q_SIGNALLING_EVENTS	REACTOME	7428	27	20	7,64E-01	9,99E-01	rs11687066|rs12412993|rs1594513|rs12873185|rs1088672|rs628877|rs1878404|rs10487781|rs4732260|rs3908461|rs11143600|rs2810585|rs353189|rs6796803|rs7853418|rs3778818|rs17426222|rs9985440|rs4751433|rs4497195
Photosensitivity	GLYCOSPHINGOLIPID_METABOLISM	REACTOME	924	34	19	7,64E-01	9,99E-01	rs6793913|rs11729278|rs10981168|rs5770953|rs9858362|rs3180018|rs891944|rs10020382|rs10053925|rs375396|rs1843485|rs918167|rs2854992|rs4697371|rs17818103|rs9307398|rs447998|rs1548984|rs6453415
Photosensitivity	OPIOID_SIGNALLING	REACTOME	3594	34	20	7,66E-01	9,99E-01	rs1457145|rs963065|rs1937600|rs10912414|rs883541|rs17031191|rs4736746|rs873417|rs12143542|rs1404912|rs2851045|rs2269340|rs7230634|rs759947|rs6860907|rs10798089|rs6852347|rs2236941|rs7961587|rs11157863
Photosensitivity	PREFOLDIN_MEDIATED_TRANSFER_OF_SUBSTRATE_TO_CCT_TRIC	REACTOME	288	6	5	7,67E-01	9,99E-01	rs7571143|rs40428|rs9507572|rs7558608|rs2189659
Photosensitivity	ACTIVATION_OF_NF_KAPPAB_IN_B_CELLS	REACTOME	1396	24	20	7,67E-01	9,99E-01	rs16827840|rs9904424|rs4802351|rs743272|rs9673361|rs4148876|rs991880|rs6420554|rs1957107|rs12292911|rs3729908|rs4664414|rs6461856|rs1973619|rs2550461|rs12191736|rs1217|rs12713430|rs8063430|rs10484565
Photosensitivity	THROMBIN_SIGNALLING_THROUGH_PROTEINASE_ACTIVATED_RECEPTORS_PARS	REACTOME	690	16	13	7,69E-01	9,99E-01	rs2005219|rs2069662|rs6090585|rs11157863|rs11655004|rs1051007|rs7152036|rs2876980|rs584845|rs10229817|rs8104096|rs11810173|rs10869985
Photosensitivity	INTEGRIN_PATHWAY	BIOCARTA	1027	32	20	7,70E-01	9,99E-01	rs10940270|rs2005219|rs13060691|rs6090585|rs870992|rs11792737|rs600135|rs3012738|rs907698|rs1432442|rs10171977|rs2716121|rs904453|rs17106421|rs2876980|rs1742501|rs3791938|rs3012719|rs11684943|rs869382
Photosensitivity	SELENOAMINO_ACID_METABOLISM	KEGG	742	31	15	7,71E-01	9,99E-01	rs10059065|rs2672487|rs294588|rs9569|rs17287615|rs11958300|rs11203172|rs4650044|rs1363071|rs2780749|rs9429768|rs4583905|rs729352|rs3734969|rs6424389
Photosensitivity	GABA_B_RECEPTOR_ACTIVATION	REACTOME	1863	32	20	7,74E-01	9,99E-01	rs872091|rs10986577|rs11656925|rs4736746|rs10818955|rs1404912|rs7230634|rs17780119|rs2236941|rs7218368|rs11157863|rs180054|rs624549|rs2835933|rs9903422|rs4268746|rs501163|rs2779572|rs2886612|rs2301230
Photosensitivity	ERK_MAPK_TARGETS	REACTOME	704	38	18	7,75E-01	9,99E-01	rs1401875|rs1012421|rs2005219|rs6456115|rs1056348|rs12436076|rs11105194|rs1283614|rs2876980|rs12655172|rs10474293|rs4732986|rs7305095|rs6918886|rs7752989|rs1366594|rs7821268|rs9380546
Photosensitivity	COMPLEMENT_CASCADE	REACTOME	752	31	17	7,76E-01	9,99E-01	rs13062355|rs4610335|rs11953280|rs17662737|rs10824840|rs10489623|rs3812042|rs1376177|rs1008761|rs12492885|rs11588887|rs587585|rs6690215|rs6540433|rs10824842|rs896117|rs8264
Photosensitivity	LEISHMANIA_INFECTION	KEGG	2265	41	20	7,76E-01	9,99E-01	rs3916765|rs583911|rs17047703|rs2243300|rs11080358|rs2005219|rs2421057|rs1440046|rs68600|rs12215787|rs17200733|rs17811014|rs10818151|rs991880|rs9275582|rs3729908|rs2239621|rs10214840|rs1932971|rs1812402
Photosensitivity	GCR_PATHWAY	BIOCARTA	736	39	17	7,78E-01	9,99E-01	rs11143600|rs7853418|rs17209216|rs10512014|rs706733|rs706724|rs4912652|rs33344|rs10515068|rs1423236|rs3815701|rs10054692|rs11953134|rs4976129|rs1388122|rs2498804|rs11959113
Photosensitivity	FC_EPSILON_RI_SIGNALING_PATHWAY	KEGG	2758	30	20	7,79E-01	9,99E-01	rs628877|rs10912414|rs2243300|rs345306|rs3864277|rs10899496|rs873417|rs12143542|rs4143832|rs2005219|rs7176475|rs10798089|rs13060691|rs1124787|rs17200733|rs706733|rs6692050|rs1332684|rs7580869|rs6740453
Photosensitivity	B_CELL_RECEPTOR_SIGNALING_PATHWAY	KEGG	2278	34	20	7,80E-01	9,99E-01	rs963065|rs6705101|rs2070776|rs2461489|rs345306|rs17031191|rs2851045|rs2005219|rs13060691|rs6852347|rs706733|rs6692050|rs1332684|rs7580869|rs11246067|rs706724|rs6811327|rs6420554|rs16763|rs3729908
Photosensitivity	INTEGRIN_ALPHAIIB_BETA3_SIGNALING	REACTOME	779	13	10	7,82E-01	9,99E-01	rs2302456|rs703009|rs6090585|rs10171977|rs786870|rs2278278|rs1993703|rs17706710|rs10520094|rs2498804
Photosensitivity	CDC42RAC_PATHWAY	BIOCARTA	435	30	16	7,83E-01	9,99E-01	rs3912541|rs706733|rs17746897|rs706724|rs727735|rs1444043|rs7556894|rs33344|rs10515068|rs1423236|rs3815701|rs10054692|rs11953134|rs4976129|rs1490819|rs10258757
Photosensitivity	PRE_NOTCH_PROCESSING_IN_GOLGI	REACTOME	691	14	9	7,85E-01	9,99E-01	rs7628381|rs17116491|rs17117189|rs12323801|rs278364|rs1242558|rs17604893|rs17111840|rs1036570
Photosensitivity	AKT_PATHWAY	BIOCARTA	682	36	20	7,85E-01	9,99E-01	rs6180|rs2070776|rs2324528|rs706733|rs706724|rs3020619|rs4435111|rs9606893|rs33344|rs10515068|rs8000689|rs1423236|rs3815701|rs10054692|rs4941961|rs4943805|rs11953134|rs4976129|rs1490819|rs2498804
Photosensitivity	ALANINE_ASPARTATE_AND_GLUTAMATE_METABOLISM	KEGG	841	32	17	7,89E-01	9,99E-01	rs3006845|rs6499969|rs8057892|rs2744601|rs1330581|rs12467996|rs4538195|rs2490286|rs6748782|rs1493282|rs16899972|rs8062792|rs1275522|rs16961113|rs10521023|rs12925511|rs2498804
Photosensitivity	BMAL1_CLOCK_NPAS2_ACTIVATES_CIRCADIAN_EXPRESSION	REACTOME	1728	30	20	7,90E-01	9,99E-01	rs3800794|rs6829223|rs4917342|rs6858138|rs11048994|rs17209216|rs10519096|rs7610825|rs4469064|rs914852|rs3789327|rs4912652|rs2046380|rs940224|rs4361373|rs4600866|rs6740234|rs877988|rs7636407|rs12502678
Photosensitivity	SIGNALING_BY_ILS	REACTOME	2996	27	20	7,91E-01	9,99E-01	rs5745752|rs12251307|rs12531099|rs10504873|rs3864277|rs10899496|rs11574849|rs2302456|rs4143832|rs2005219|rs13060691|rs3105453|rs7973701|rs706733|rs7841459|rs706724|rs991880|rs11792737|rs3012738|rs17811365
Photosensitivity	PYRUVATE_METABOLISM	REACTOME	209	10	6	7,93E-01	9,99E-01	rs847682|rs10264649|rs7566884|rs9811074|rs2158839|rs12151618
Photosensitivity	TRANSPORT_OF_MATURE_TRANSCRIPT_TO_CYTOPLASM	REACTOME	693	30	17	7,93E-01	9,99E-01	rs10183020|rs7628051|rs2038743|rs7770657|rs4646296|rs2328136|rs10871221|rs3026106|rs16987990|rs6030839|rs4684126|rs2282763|rs6961391|rs3803695|rs6467593|rs1888523|rs3115761
Photosensitivity	OXIDATIVE_PHOSPHORYLATION	KEGG	2486	40	20	7,96E-01	9,99E-01	rs1998834|rs1500147|rs2515210|rs4854053|rs9775695|rs4147750|rs714621|rs1982235|rs2733954|rs1812144|rs6120890|rs17200733|rs10971025|rs1345638|rs7795088|rs11669029|rs13277184|rs10487881|rs871516|rs10116346
Photosensitivity	GLYCOLYSIS	REACTOME	558	29	18	7,98E-01	9,99E-01	rs8049837|rs11189224|rs1052176|rs1056348|rs2267844|rs10903843|rs3750667|rs943117|rs11818509|rs2256817|rs10794929|rs3003514|rs4747886|rs1556632|rs10903939|rs7096344|rs7092772|rs2388595
Photosensitivity	ANTIGEN_PRESENTATION_FOLDING_ASSEMBLY_AND_PEPTIDE_LOADING_OF_CLASS_I_M	REACTOME	830	32	16	8,01E-01	9,99E-01	rs9260759|rs4148876|rs2162400|rs11730732|rs7148403|rs9264219|rs1350386|rs9378229|rs1876125|rs10484565|rs12662501|rs9461688|rs17040388|rs988419|401754|rs1736956
Photosensitivity	P53_SIGNALING_PATHWAY	KEGG	1897	39	20	8,02E-01	9,99E-01	rs10796820|rs997669|rs1940269|rs2303076|rs3740643|rs1049612|rs2673825|rs3170660|rs6583634|rs1048691|rs2347343|rs1801516|rs12501768|rs11667347|rs10491802|rs6461845|rs788778|rs4646536|rs11899004|rs1429554
Photosensitivity	ADP_SIGNALLING_THROUGH_P2RY12	REACTOME	438	13	11	8,04E-01	9,99E-01	rs2236941|rs11157863|rs501163|rs2886612|rs2301230|rs7152036|rs9875152|rs584845|rs8104096|rs10238438|rs11810173
Photosensitivity	CIRCADIAN_REPRESSION_OF_EXPRESSION_BY_REV_ERBA	REACTOME	1358	34	20	8,05E-01	9,99E-01	rs3800794|rs6829223|rs4917342|rs10519096|rs4469064|rs914852|rs3789327|rs2046380|rs940224|rs4361373|rs6740234|rs877988|rs9818162|rs135549|rs6817487|rs17303530|rs3112256|rs6773879|rs782933|rs10741613
Photosensitivity	NUCLEAR_RECEPTOR_TRANSCRIPTION_PATHWAY	REACTOME	2750	36	20	8,05E-01	9,99E-01	rs577787|rs1414529|rs17611421|rs12757772|rs4917342|rs11117644|rs3790826|rs10501976|rs1462436|rs7703021|rs17209216|rs11143958|rs7157192|rs10519096|rs6093978|rs946465|rs10177924|rs914852|rs4912652|rs940224
Photosensitivity	IL2RB_PATHWAY	BIOCARTA	1296	37	20	8,06E-01	9,99E-01	rs1030608|rs12251307|rs2005219|rs13060691|rs706733|rs6060563|rs706724|rs10171977|rs992241|rs904453|rs10956433|rs2876980|rs12244380|rs7221341|rs9915291|rs2278278|rs12458289|rs33344|rs1993703|rs16902654
Photosensitivity	TRANSPORT_OF_INORGANIC_CATIONS_ANIONS_AND_AMINO_ACIDS_OLIGOPEPTIDES	REACTOME	4051	25	20	8,07E-01	9,99E-01	rs8105903|rs10974591|rs12490575|rs2455280|rs7722287|rs1866901|rs7586183|rs2886790|rs720436|rs2507821|rs3820201|rs12434284|rs957910|rs2588233|rs3887266|rs2236941|rs7154465|rs1892250|rs2671444|rs876985
Photosensitivity	IGF1MTOR_PATHWAY	BIOCARTA	582	37	20	8,07E-01	9,99E-01	rs1546713|rs2684794|rs2673825|rs706733|rs706724|rs7162314|rs847682|rs6059703|rs33344|rs7169544|rs6806377|rs10515068|rs1423236|rs3815701|rs10054692|rs4912474|rs11953134|rs4976129|rs1388122|rs2498804
Photosensitivity	NUCLEAR_EVENTS_KINASE_AND_TRANSCRIPTION_FACTOR_ACTIVATION	REACTOME	766	38	18	8,08E-01	9,99E-01	rs1401875|rs1012421|rs2005219|rs6456115|rs1056348|rs12436076|rs11105194|rs1283614|rs2876980|rs12655172|rs10474293|rs4732986|rs7305095|rs6918886|rs7752989|rs1366594|rs7821268|rs2237093
Photosensitivity	TRANSFERRIN_ENDOCYTOSIS_AND_RECYCLING	REACTOME	513	15	10	8,10E-01	9,99E-01	rs2515210|rs17200733|rs871516|rs16919915|rs17749106|rs7250572|rs1358024|rs13238736|rs8846|rs7836003
Photosensitivity	STEROID_HORMONES	REACTOME	693	33	20	8,10E-01	9,99E-01	rs3848180|rs17431426|rs1048691|rs780825|rs6068799|rs4622471|rs4702379|rs17523880|rs2291617|rs2289105|rs209967|rs4667591|rs12991585|rs619824|rs927650|rs2277603|rs6127112|rs8041933|rs2241190|rs439046
Photosensitivity	GAP_JUNCTION	KEGG	4388	30	20	8,13E-01	9,99E-01	rs721412|rs13115024|rs602159|rs11568993|rs12280614|rs1436908|rs4754175|rs4736746|rs873417|rs1783138|rs1404912|rs2005219|rs17106293|rs10823217|rs13060691|rs17746482|rs1872902|rs6090585|rs17746897|rs2236941
Photosensitivity	RORA_ACTIVATES_CIRCADIAN_EXPRESSION	REACTOME	1377	34	20	8,14E-01	9,99E-01	rs3800794|rs6829223|rs4917342|rs10519096|rs4469064|rs914852|rs3789327|rs2046380|rs940224|rs4361373|rs6740234|rs877988|rs7636407|rs135549|rs6817487|rs17303530|rs3112256|rs6773879|rs782933|rs10741613
Photosensitivity	PURINE_METABOLISM	REACTOME	581	25	20	8,15E-01	9,99E-01	rs1126432|rs2269340|rs1042427|rs2071017|rs7125888|rs4952085|rs9362203|rs1713460|rs3802917|rs555812|rs860762|rs6756897|rs10511997|rs4909926|rs7029217|rs3772078|rs6714794|rs10498268|rs10840415|rs10179712
Photosensitivity	POST_TRANSLATIONAL_MODIFICATION_SYNTHESIS_OF_GPI_ANCHORED_PROTEINS	REACTOME	586	10	8	8,15E-01	9,99E-01	rs346044|rs8039143|rs1028307|rs17249299|rs477077|rs17069296|rs17069572|rs281282
Photosensitivity	NUCLEOTIDE_BINDING_DOMAIN_LEUCINE_RICH_REPEAT_CONTAINING_RECEPTOR_NLR<br>YS	REACTOME	1337	34	20	8,16E-01	9,99E-01	rs10504873|rs1548992|rs591874|rs7973701|rs8132767|rs6060563|rs7841459|rs11702267|rs991880|rs882937|rs1049679|rs1718119|rs10214840|rs6685474|rs4135223|rs12216864|rs12191736|rs11899004|rs2716932|rs12458289
Photosensitivity	TRANSPORT_OF_GLUCOSE_AND_OTHER_SUGARS_BILE_SALTS_AND_ORGANIC_ACIDS_M<br>NE_COMPOUNDS	REACTOME	3234	53	20	8,16E-01	9,99E-01	rs12141345|rs9682960|rs12490575|rs11210751|rs270607|rs11810507|rs859068|rs7047481|rs7131598|rs1972873|rs2143044|rs2665355|rs1710879|rs6581351|rs9621386|rs182411|rs12500086|rs3091470|rs10497704|rs886285
Photosensitivity	GASTRIN_CREB_SIGNALLING_PATHWAY_VIA_PKC_AND_MAPK	REACTOME	8122	28	20	8,16E-01	9,99E-01	rs11687066|rs12412993|rs1594513|rs12873185|rs1088672|rs628877|rs1878404|rs10487781|rs4732260|rs3908461|rs11143600|rs2810585|rs1010698|rs353189|rs6796803|rs7853418|rs3778818|rs17426222|rs873417|rs9985440
Photosensitivity	ARGININE_AND_PROLINE_METABOLISM	KEGG	991	32	20	8,19E-01	9,99E-01	rs2089855|rs6499969|rs1555481|rs11080358|rs4878808|rs7865905|rs8057892|rs1814288|rs2490286|rs6748782|rs2531882|rs8062792|rs156031|rs2275273|rs4835913|rs16961113|rs2297518|rs11791018|rs7900964|rs12925511
Photosensitivity	CELL_SURFACE_INTERACTIONS_AT_THE_VASCULAR_WALL	REACTOME	2650	38	20	8,25E-01	9,99E-01	rs973608|rs709505|rs1493101|rs3787565|rs2756900|rs13062355|rs2643075|rs2507821|rs6132106|rs7782469|rs1536913|rs6090585|rs706733|rs706724|rs876985|rs1144396|rs2824271|rs1607581|rs10171977|rs17244832
Photosensitivity	LONG_TERM_POTENTIATION	KEGG	3620	25	20	8,25E-01	9,99E-01	rs2239094|rs963065|rs7190913|rs2461489|rs17031191|rs4736746|rs873417|rs2238012|rs1404912|rs2851045|rs10065813|rs759947|rs2005219|rs4780550|rs2872715|rs13060691|rs9888368|rs3822295|rs6852347|rs2239062
Photosensitivity	ACTIVATED_AMPK_STIMULATES_FATTY_ACID_OXIDATION_IN_MUSCLE	REACTOME	421	25	18	8,27E-01	9,99E-01	rs7778077|rs4725424|rs4725435|rs10849968|rs2727564|rs10889011|rs7788160|rs6490267|rs13409720|rs6965834|rs10024579|rs1881624|rs4435442|rs6832271|rs721575|rs10849926|rs2746342|rs3764640
Photosensitivity	LONG_TERM_DEPRESSION	KEGG	3926	36	20	8,28E-01	9,99E-01	rs13115024|rs602159|rs12048929|rs12645274|rs1546713|rs4754175|rs873417|rs1783138|rs12143542|rs10065813|rs2005219|rs17106293|rs7176475|rs2684794|rs10823217|rs10798089|rs13060691|rs2236941|rs7961587|rs6055990
Photosensitivity	T_CELL_RECEPTOR_SIGNALING_PATHWAY	KEGG	3546	34	20	8,29E-01	9,99E-01	rs1798619|rs963065|rs2060581|rs2243300|rs10918693|rs2461489|rs345306|rs3864277|rs353189|rs17031191|rs2851045|rs4143832|rs2005219|rs13060691|rs6852347|rs858490|rs722813|rs10206295|rs6516498|rs17200733
Photosensitivity	CYSTEINE_AND_METHIONINE_METABOLISM	KEGG	982	30	20	8,32E-01	9,99E-01	rs10059065|rs6499969|rs294588|rs6546045|rs8057892|rs10811629|rs17287615|rs2490286|rs11958300|rs11203172|rs934615|rs4650044|rs8062792|rs1363071|rs9750503|rs2780749|rs16961113|rs9429768|rs4583905|rs10904907
Photosensitivity	CHYLOMICRON_MEDIATED_LIPID_TRANSPORT	REACTOME	435	13	9	8,35E-01	9,99E-01	rs2881925|rs17821232|rs1607581|rs1816879|rs13398007|rs6083|rs3760627|rs10413089|rs2228671
Photosensitivity	DNA_STRAND_ELONGATION	REACTOME	672	17	13	8,35E-01	9,99E-01	rs174546|rs3782093|rs11987150|rs7357092|rs676114|rs2071749|rs1359389|rs1629511|rs11236183|rs7328146|rs6990366|rs2071744|rs7819949
Photosensitivity	RIG_I_LIKE_RECEPTOR_SIGNALING_PATHWAY	KEGG	1361	30	20	8,36E-01	9,99E-01	rs583911|rs10813821|rs1548992|rs2421057|rs17200733|rs17811014|rs11540270|rs991880|rs10490561|rs4946730|rs7930377|rs12191736|rs11899004|rs12586226|rs4921508|rs7042042|rs12229321|rs4334868|rs437932|rs2081066
Photosensitivity	NGF_PATHWAY	BIOCARTA	566	38	20	8,40E-01	9,99E-01	rs2005219|rs13060691|rs706733|rs706724|rs1432442|rs195204|rs1294383|rs10171977|rs1812402|rs904453|rs33344|rs10515068|rs1423236|rs3815701|rs10054692|rs6037986|rs2671683|rs4255740|rs1882832|rs11953134
Photosensitivity	INTRINSIC_PATHWAY_FOR_APOPTOSIS	REACTOME	532	29	18	8,40E-01	9,99E-01	rs7953290|rs1398028|rs6060563|rs2289321|rs12501768|rs6461845|rs11899004|rs11858141|rs12458289|rs9953959|rs11630670|rs39454|rs10515981|rs11681263|rs4319556|rs2236719|rs2498804|rs12673033
Photosensitivity	REGULATION_OF_INSULIN_SECRETION	REACTOME	3741	28	20	8,41E-01	9,99E-01	rs4748472|rs1594513|rs362623|rs883541|rs16974215|rs11210751|rs16974510|rs4736746|rs873417|rs4497195|rs442683|rs2236941|rs7961587|rs11157863|rs9470963|rs6599127|rs1570939|rs6055990|rs12261976|rs4381631
Photosensitivity	SIGNALING_BY_HIPPO	REACTOME	627	25	12	8,43E-01	9,99E-01	rs1499395|rs11949188|rs12501768|rs11121648|rs6440618|rs1498730|rs10077631|rs9552348|rs1422422|rs9682157|rs7555293|rs2922068
Photosensitivity	RAC1_PATHWAY	BIOCARTA	763	40	20	8,45E-01	9,99E-01	rs7227801|rs17806303|rs706733|rs17746897|rs16955157|rs706724|rs907698|rs1444043|rs33344|rs328994|rs10515068|rs1423236|rs3815701|rs10054692|rs1254403|rs832540|rs1813100|rs7248911|rs11953134|rs4976129
Photosensitivity	P38MAPK_PATHWAY	BIOCARTA	1904	38	20	8,45E-01	9,99E-01	rs1030608|rs10912414|rs17047703|rs1982235|rs12143542|rs10798089|rs2699027|rs991880|rs330712|rs992241|rs12436076|rs2430119|rs1932971|rs760166|rs9729852|rs10956433|rs4144116|rs12191736|rs1283614|rs12655172
Photosensitivity	MTOR_PATHWAY	BIOCARTA	509	31	16	8,45E-01	9,99E-01	rs2809244|rs2673825|rs706733|rs706724|rs10787899|rs847682|rs33344|rs10515068|rs10901219|rs1423236|rs3815701|rs10054692|rs11953134|rs4976129|rs1490819|rs2498804
Photosensitivity	PTDINS_PATHWAY	BIOCARTA	1058	33	20	8,45E-01	9,99E-01	rs628877|rs1124787|rs6740453|rs6074170|rs1020445|rs16763|rs2423511|rs4813046|rs6040334|rs11684301|rs17189227|rs10903843|rs17033965|rs6720975|rs667420|rs843339|rs11888552|rs3768331|rs11818509|rs2256817
Photosensitivity	STRESS_PATHWAY	BIOCARTA	633	19	10	8,45E-01	9,99E-01	rs11107199|rs17200733|rs10490561|rs11107202|rs1812402|rs8065164|rs832540|rs1882364|rs12470772|rs9380546
Photosensitivity	COMP_PATHWAY	BIOCARTA	628	22	12	8,47E-01	9,99E-01	rs11953280|rs17662737|rs10824840|rs10489623|rs3812042|rs1376177|rs1008761|rs12492885|rs587585|rs10824842|rs896117|rs8264
Photosensitivity	NEUROTROPHIN_SIGNALING_PATHWAY	KEGG	3689	32	20	8,48E-01	9,99E-01	rs7871111|rs630426|rs10520656|rs10504873|rs2130617|rs4766987|rs2302456|rs759947|rs2005219|rs13060691|rs3822295|rs3105453|rs2013566|rs706733|rs7841459|rs706724|rs6456115|rs11792737|rs3012738|rs729396
Photosensitivity	GABA_RECEPTOR_ACTIVATION	REACTOME	2705	28	20	8,50E-01	9,99E-01	rs872091|rs10986577|rs11656925|rs2715583|rs164369|rs4736746|rs10818955|rs1404912|rs1012549|rs7230634|rs12440234|rs17780119|rs2236941|rs7218368|rs11157863|rs980982|rs209350|rs180054|rs624549|rs2835933
Photosensitivity	NUCLEOTIDE_EXCISION_REPAIR	KEGG	914	30	15	8,52E-01	9,99E-01	rs1464251|rs10978769|rs4253042|rs4253126|rs7357092|rs7766094|rs2025448|rs1359389|rs1629511|rs11236183|rs1618536|rs1561734|rs7328146|rs1143407|rs6477562
Photosensitivity	SULFUR_AMINO_ACID_METABOLISM	REACTOME	930	35	20	8,53E-01	9,99E-01	rs1918498|rs10059065|rs294588|rs7738142|rs10811629|rs17287615|rs2490286|rs11958300|rs11203172|rs7577|rs4650044|rs3213403|rs1363071|rs9750503|rs2780749|rs4583905|rs729352|rs12438405|rs2293429|rs6424389
Photosensitivity	CHOLESTEROL_BIOSYNTHESIS	REACTOME	259	14	10	8,54E-01	9,99E-01	rs1052176|rs3825073|rs1211975|rs6426075|rs604778|rs12678891|rs11076696|rs1891805|rs3735810|rs2287182
Photosensitivity	SIGNALING_BY_THE_B_CELL_RECEPTOR_BCR	REACTOME	3426	37	20	8,54E-01	9,99E-01	rs7120938|rs16827840|rs6705101|rs10430939|rs2070776|rs9904424|rs2324528|rs4802351|rs2673825|rs722813|rs12313273|rs743272|rs706733|rs9673361|rs7961587|rs4148876|rs706724|rs991880|rs6420554|rs1957107
Photosensitivity	ADP_SIGNALLING_THROUGH_P2RY1	REACTOME	863	38	19	8,55E-01	9,99E-01	rs12048929|rs12143542|rs10798089|rs6090585|rs11157863|rs330712|rs2430119|rs9729852|rs4144116|rs7152036|rs584845|rs2061584|rs8104096|rs11810173|rs10911933|rs1487492|rs9380546|rs16827441|rs10869985

Photosensitivity	PRIMARY_BILE_ACID_BIOSYNTHESIS	KEGG	439	33	18	8,55E-01	9,99E-01	rs9423399|rs2277119|rs2254670|rs3732860|rs2801527|rs7922269|rs13434020|rs2228468|rs3756462|rs12747412|rs2228467|rs2050308|rs6415602|rs9423584|rs960891|rs384346|rs2431049|rs9472810
Photosensitivity	MAPK_PATHWAY	BIOCARTA	2690	35	20	8,56E-01	9,99E-01	rs1030609|rs1798619|rs17047703|rs1370480|rs1488|rs1982235|rs7228129|rs2214890|rs2005219|rs13060691|rs2699027|rs6456115|rs991880|rs10851507|rs1432442|rs2338887|rs992241|rs12436076|rs6803944|rs1932971
Photosensitivity	ANTIGEN_PROCESSING_AND_PRESENTATION	KEGG	1754	82	20	8,59E-01	9,99E-01	rs3916765|rs9260759|rs743272|rs68600|rs17200733|rs9275595|rs9264219|rs815017|rs9261950|rs9378229|rs206768|rs273266|rs10484565|rs12662501|rs9461688|rs9500927|rs11208754|rs7767167|rs938486|rs3735810
Photosensitivity	SIGNALING_BY_SCF_KIT	REACTOME	1989	33	20	8,60E-01	9,99E-01	rs2324528|rs10899496|rs873417|rs10745478|rs7183098|rs2005219|rs17809666|rs2673825|rs13060691|rs6090585|rs706733|rs706724|rs4381631|rs4411531|rs2726648|rs12626158|rs1432442|rs10171977|rs758658|rs904453
Photosensitivity	GLOBAL_GENOMIC_NER_GG_NER	REACTOME	823	19	12	8,61E-01	9,99E-01	rs1464251|rs10978769|rs7357092|rs7766094|rs1359389|rs1629511|rs11236183|rs1618536|rs1561734|rs7328146|rs1143407|rs6477562
Photosensitivity	REGULATION_OF_AUTOPHAGY	KEGG	933	35	16	8,63E-01	9,99E-01	rs6657501|rs1941590|rs2440455|rs4595861|rs4946730|rs10889011|rs3845563|rs2780882|rs4254156|rs752216|rs9945673|rs10803002|rs11208082|rs2746342|rs6568433|rs10853477
Photosensitivity	MAPK_TARGETS_NUCLEAR_EVENTS_MEDIATED_BY_MAP_KINASES	REACTOME	980	40	20	8,64E-01	9,99E-01	rs1401875|rs1982235|rs1012421|rs2005219|rs6456115|rs1056348|rs12436076|rs1009619|rs1812402|rs1283614|rs2876980|rs12655172|rs10474293|rs4732986|rs7305095|rs6918886|rs1882832|rs7752989|rs1366594|rs4640664
Photosensitivity	G_BETA_GAMMA_SIGNALLING_THROUGH_PLC_BETA	REACTOME	624	13	12	8,64E-01	9,99E-01	rs11157863|rs6055990|rs2719795|rs8115925|rs2223538|rs6140619|rs7152036|rs584845|rs8104096|rs4816085|rs11810173|rs936213
Photosensitivity	PLC_BETA_MEDIATED_EVENTS	REACTOME	2313	34	20	8,66E-01	9,99E-01	rs12048929|rs883541|rs4736746|rs873417|rs12143542|rs1404912|rs2269340|rs759947|rs10798089|rs2236941|rs7961587|rs6055990|rs4381631|rs10497599|rs4411531|rs2719795|rs729396|rs8115925|rs17463958|rs330712
Photosensitivity	INITIAL_TRIGGERING_OF_COMPLEMENT	REACTOME	434	7	6	8,66E-01	9,99E-01	rs17662737|rs10824840|rs12492885|rs1588887|rs587585|rs10824842
Photosensitivity	NOD1_2_SIGNALING_PATHWAY	REACTOME	881	28	19	8,67E-01	9,99E-01	rs10504873|rs1548992|rs7973701|rs7841459|rs991880|rs1049679|rs10214840|rs12216864|rs12191736|rs11899004|rs17790482|rs437932|rs7742254|rs1782449|rs6955931|rs4141907|rs145768|rs9380546|rs5030437
Photosensitivity	G_ALPHA1213_SIGNALLING_EVENTS	REACTOME	2855	23	20	8,68E-01	9,99E-01	rs12364414|rs10761189|rs7174521|rs345306|rs4810783|rs17426222|rs754743|rs7218427|rs9371867|rs7761427|rs706733|rs6692050|rs1332684|rs7027092|rs1359647|rs926693|rs4778857|rs11157863|rs2882936|rs706724
Photosensitivity	REGULATION_OF_HYPOXIA_INDUCIBLE_FACTOR_HIF_BY_OXYGEN	REACTOME	443	13	10	8,69E-01	9,99E-01	rs943084|rs1867788|rs3774208|rs1447563|rs1994174|rs1881124|rs3736329|rs11688133|rs900360|rs9357421
Photosensitivity	TIE2_SIGNALING	REACTOME	790	46	19	8,69E-01	9,99E-01	rs3787565|rs2756900|rs1536913|rs706733|rs706724|rs10171977|rs10771184|rs273266|rs33344|rs10515068|rs1423236|rs3815701|rs10054692|rs7035592|rs11775442|rs11953134|rs7820162|rs4976129|rs1490819
Photosensitivity	GRAFT_VERSUS_HOST_DISEASE	KEGG	1446	84	20	8,70E-01	9,99E-01	rs3916765|rs9260759|rs68600|rs17200733|rs9275595|rs9264219|rs624035|rs9261950|rs9378229|rs206768|rs4722164|rs10484565|rs12662501|rs9461688|rs9500927|rs17187469|rs7767167|rs2537763|rs6438537|rs206762
Photosensitivity	CCR5_PATHWAY	BIOCARTA	696	23	13	8,71E-01	9,99E-01	rs873417|rs4381631|rs4411531|rs3729908|rs758658|rs2292979|rs2716121|rs12360225|rs9972960|rs7193148|rs1882832|rs2237093|rs10869985
Photosensitivity	PTEN_PATHWAY	BIOCARTA	544	30	18	8,72E-01	9,99E-01	rs2005219|rs2673825|rs706733|rs706724|rs847682|rs10171977|rs7109662|rs2876980|rs33344|rs2417223|rs10515068|rs1423236|rs3815701|rs10054692|rs11953134|rs4976129|rs1388122|rs2498804
Photosensitivity	DEATH_PATHWAY	BIOCARTA	505	18	14	8,74E-01	9,99E-01	rs3212153|rs12501768|rs1800627|rs7930377|rs6461845|rs11899004|rs2275075|rs12458289|rs365238|rs39454|rs231983|rs4319556|rs2236719|rs12673033
Photosensitivity	CD28_CO_STIMULATION	REACTOME	797	34	20	8,75E-01	9,99E-01	rs1798619|rs6090585|rs706733|rs706724|rs12626158|rs624035|rs4301703|rs3730050|rs2084385|rs273266|rs33344|rs10494270|rs10515068|rs2145068|rs1423236|rs3815701|rs10054692|rs17187469|rs7834615|rs7248911
Photosensitivity	DOWNSTREAM_SIGNALING_EVENTS_OF_B_CELL_RECEPTOR_BCR	REACTOME	2127	28	20	8,75E-01	9,99E-01	rs16827840|rs6705101|rs9904424|rs2324528|rs4802351|rs2673825|rs743272|rs9673361|rs1448876|rs991880|rs6420554|rs1957107|rs12292911|rs12626158|rs3729908|rs4664414|rs6461856|rs1973619|rs2550461|rs12191736
Photosensitivity	PAR1_PATHWAY	BIOCARTA	1941	26	20	8,76E-01	9,99E-01	rs12364414|rs4532638|rs873417|rs1404912|rs706733|rs1359647|rs706724|rs6055990|rs4381631|rs991880|rs4411531|rs2719795|rs907698|rs8115925|rs11717281|rs3729908|rs2223538|rs758658|rs2292979|rs2886612
Photosensitivity	ENOS_ACTIVATION_AND_REGULATION	REACTOME	336	10	7	8,77E-01	9,99E-01	rs4804528|rs6749871|rs10810175|rs10516652|rs7604402|rs13284733|rs2498804
Photosensitivity	PEROXISOME	KEGG	2047	46	20	8,80E-01	9,99E-01	rs17674015|rs4519903|rs941425|rs1348850|rs11080358|rs838534|rs7260781|rs767445|rs2395926|rs4538195|rs2239621|rs1039857|rs4952085|rs6086074|rs2165613|rs2303842|rs2531882|rs11587145|rs13434020|rs247011
Photosensitivity	INTEGRIN_CELL_SURFACE_INTERACTIONS	REACTOME	2614	42	20	8,81E-01	9,99E-01	rs657635|rs3745264|rs9333118|rs12747584|rs2302456|rs10940270|rs2228615|rs7782469|rs703009|rs11115|rs6090585|rs11789200|rs6722598|rs16975259|rs870992|rs11637293|rs4237337|rs3769641|rs10516798|rs13028299
Photosensitivity	DEFENSINS	REACTOME	495	22	9	8,81E-01	9,99E-01	rs2738133|rs717064|rs6923691|rs2702938|rs1126287|rs6456156|rs5001275|rs7823808|rs9994111
Photosensitivity	TGF_BETA_RECEPTOR_SIGNALING_IN_EMT_EPITHELIAL_TO_MESENCHYMAL_TRANSITIO	REACTOME	429	12	5	8,82E-01	9,99E-01	rs4934517|rs1331681|rs1617306|rs2297221|rs9866764
Photosensitivity	OXYGEN_DEPENDENT_PROLINE_HYDROXYLATION_OF_HYPOXIA_INDUCIBLE_FACTOR_A	REACTOME	313	11	8	8,84E-01	9,99E-01	rs1867788|rs3774208|rs1447563|rs1994174|rs1881124|rs3736329|rs11688133|rs900360
Photosensitivity	INTEGRATION_OF_ENERGY_METABOLISM	REACTOME	4501	28	20	8,85E-01	9,99E-01	rs4748472|rs1594513|rs362623|rs883541|rs16974215|rs11210751|rs16974510|rs4736746|rs873417|rs1404912|rs7778077|rs2306569|rs4497195|rs4725424|rs4725435|rs10849968|rs442683|rs2236941|rs7961587|rs11157863
Photosensitivity	CIRCADIAN_CLOCK	REACTOME	2075	29	20	8,85E-01	9,99E-01	rs3800794|rs723393|rs4917342|rs1982235|rs6858138|rs11048994|rs17209216|rs10519096|rs7610825|rs4469064|rs914852|rs3789327|rs4912652|rs2046380|rs940224|rs4361373|rs4600866|rs6740234|rs877988|rs7636407
Photosensitivity	SIGNALING_BY_EGFR_IN_CANCER	REACTOME	4030	22	20	8,91E-01	9,99E-01	rs721412|rs628877|rs11568993|rs883541|rs2324528|rs986345|rs4736746|rs873417|rs1404912|rs759947|rs2005219|rs2673825|rs1427282|rs13060691|rs4962318|rs534870|rs17746482|rs3097920|rs6090585|rs1124787
Photosensitivity	P53HYPOXIA_PATHWAY	BIOCARTA	517	14	11	8,94E-01	9,99E-01	rs2203801|rs1801516|rs788778|rs2235046|rs4851802|rs3803809|rs17502828|rs12994383|rs228591|rs2084779|rs2498804
Photosensitivity	FORMATION_OF_ATP_BY_CHEMIOSMOTIC_COUPLING	REACTOME	77	2	2	8,95E-01	9,99E-01	rs1044228|rs2834287
Photosensitivity	PROSTACYCLIN_SIGNALLING_THROUGH_PROSTACYCLIN_RECEPTOR	REACTOME	353	6	5	8,97E-01	9,99E-01	rs11157863|rs7152036|rs584845|rs8104096|rs11810173
Photosensitivity	PIP3_ACTIVATES_AKT_SIGNALING	REACTOME	587	17	14	8,98E-01	9,99E-01	rs2324528|rs2673825|rs12626158|rs3730050|rs11664248|rs4435111|rs2417223|rs10494270|rs8000689|rs2145068|rs4941961|rs4943805|rs2498804|rs10760398
Photosensitivity	TNFR2_PATHWAY	BIOCARTA	414	14	10	8,99E-01	9,99E-01	rs1061631|rs17200733|rs10490561|rs12586226|rs4978760|rs2112661|rs832540|rs1882364|rs12470772|rs7552664
Photosensitivity	DEGRADATION_OF_THE_EXTRACELLULAR_MATRIX	REACTOME	580	27	20	9,01E-01	9,99E-01	rs7213204|rs595840|rs10090518|rs2454347|rs12924764|rs2664368|rs2064712|rs4789921|rs1034375|rs17297226|rs9667864|rs1382104|rs7947031|rs6469206|rs3823055|rs1276289|rs4754876|rs6935921|rs4406450|rs4253236
Photosensitivity	ARF_PATHWAY	BIOCARTA	733	40	19	9,01E-01	9,99E-01	rs1030609|rs706733|rs706724|rs992241|rs10956433|rs33344|rs16902654|rs10515068|rs4733581|rs6997983|rs1423236|rs3815701|rs10054692|rs1561330|rs2227301|rs9889520|rs11953134|rs4976129|rs1490819
Photosensitivity	PROCESSING_OF_CAPPED_INTRONLESS_PRE_MRNA	REACTOME	274	13	10	9,01E-01	9,99E-01	rs17704641|rs1798177|rs10859963|rs13183577|rs17054866|rs2282763|rs4653186|rs4982753|rs7936604|rs2025366
Photosensitivity	PRIMARY_IMMUNODEFICIENCY	KEGG	742	30	20	9,03E-01	9,99E-01	rs2060581|rs10206295|rs4148876|rs12943647|rs6065925|rs815017|rs6736735|rs6002551|rs10484565|rs1567697|rs5759855|rs7562030|rs2256817|rs13407072|rs7907802|rs7523940|rs4822423|rs938486|rs6074022|rs401754
Photosensitivity	TGFB_PATHWAY	BIOCARTA	817	26	18	9,03E-01	9,99E-01	rs17047703|rs2005219|rs7229153|rs991880|rs288706|rs1432442|rs1932971|rs12191736|rs437932|rs9866764|rs266363|rs7742254|rs12456328|rs1782449|rs7163945|rs4255740|rs4141907|rs145768
Photosensitivity	SYNTHESIS_OF_BILE_ACIDS_AND_BILE_SALTS	REACTOME	546	35	20	9,04E-01	9,99E-01	rs9423399|rs2277119|rs2254670|rs3732860|rs2801527|rs7922269|rs13434020|rs2228468|rs3756462|rs16856261|rs12747412|rs2228467|rs2050308|rs6415602|rs2287618|rs9423584|rs960891|rs384346|rs2431049|rs9472810
Photosensitivity	NATURAL_KILLER_CELL_MEDIATED_CYTOTOXICITY	KEGG	3607	31	20	9,06E-01	9,99E-01	rs963065|rs9260759|rs10918693|rs2461489|rs345306|rs4766987|rs3864277|rs17031191|rs873417|rs2851045|rs2005219|rs2228615|rs13060691|rs6852347|rs858490|rs901662|rs11115|rs12215787|rs17200733|rs706733
Photosensitivity	POTASSIUM_CHANNELS	REACTOME	4940	33	20	9,08E-01	9,99E-01	rs6443589|rs870031|rs872091|rs10986577|rs305691|rs11656925|rs2545457|rs12234043|rs10818955|rs3816044|rs12433739|rs2012618|rs2680344|rs6671309|rs2237888|rs12753051|rs10099843|rs1014665|rs2255512|rs17780119
Photosensitivity	MRNA_3_END_PROCESSING	REACTOME	320	11	10	9,12E-01	9,99E-01	rs17704641|rs4646296|rs1798177|rs16987990|rs6030839|rs3803695|rs4982753|rs1888523|rs7936604|rs2025366
Photosensitivity	MTOR_SIGNALING_PATHWAY	KEGG	1290	33	20	9,13E-01	9,99E-01	rs12512817|rs2809244|rs943084|rs2005219|rs706733|rs706724|rs6456115|rs4240910|rs12943041|rs12948054|rs10889011|rs3730050|rs2876980|rs10520358|rs273266|rs2289762|rs33344|rs13409720|rs10515068|rs10901219
Photosensitivity	BETA_DEFENSINS	REACTOME	333	18	7	9,14E-01	9,99E-01	rs717064|rs6923691|rs1126287|rs6456156|rs5001275|rs7461062|rs9994111
Photosensitivity	NKCELLS_PATHWAY	BIOCARTA	678	35	19	9,17E-01	9,99E-01	rs9260759|rs2005219|rs706733|rs706724|rs1432442|rs2292979|rs2278278|rs33344|rs1993703|rs10515068|rs1423236|rs3815701|rs10054692|rs4255740|rs7248911|rs11953134|rs4976129|rs2537763|rs1490819
Photosensitivity	GLYCOSYLPHOSPHATIDYLINOSITOL_GPI_ANCHOR_BIOSYNTHESIS	KEGG	318	5	5	9,17E-01	9,99E-01	rs1028307|rs17249299|rs2744570|rs17069296|rs17069752
Photosensitivity	ACTIVATION_OF_BH3_ONLY_PROTEINS	REACTOME	325	13	10	9,17E-01	9,99E-01	rs1398028|rs2289321|rs11858141|rs12458289|rs9953959|rs11630670|rs10515981|rs11681263|rs2236719|rs2498804
Photosensitivity	GLYCINE_SERINE_AND_THREONINE_METABOLISM	KEGG	564	29	16	9,18E-01	9,99E-01	rs10512079|rs10125637|rs2073815|rs4538195|rs2282159|rs667420|rs11203172|rs4650044|rs16899972|rs10815450|rs2651236|rs10521023|rs2158839|rs2285177|rs6424389|rs893363
Photosensitivity	INSULIN_SYNTHESIS_AND_PROCESSING	REACTOME	1033	29	17	9,21E-01	9,99E-01	rs362623|rs2493047|rs852099|rs2536799|rs3809540|rs4976008|rs2412693|rs2493046|rs17587610|rs203745|rs4532481|rs16998899|rs908024|rs1955161|rs8042411|rs11845376|rs7144018
Photosensitivity	SIGNAL_TRANSDUCTION_BY_L1	REACTOME	1310	44	20	9,21E-01	9,99E-01	rs7946527|rs2005219|rs10740870|rs17746482|rs2242445|rs591042|rs6456115|rs1432442|rs984654|rs12436076|rs4140770|rs2876980|rs843339|rs550840|rs2804875|rs6037986|rs6918886|rs605843|rs2804503|rs11604757
Photosensitivity	VIBRIO_CHOLERAE_INFECTION	KEGG	1518	30	20	9,21E-01	9,99E-01	rs2515210|rs873417|rs2012618|rs2237888|rs7480563|rs17200733|rs6952793|rs4381631|rs4411531|rs231349|rs3729908|rs871516|rs162202|rs4580153|rs758658|rs10988774|rs16919915|rs17749106|rs2238431|rs3768331
Photosensitivity	ACTIVATED_TAK1_MEDIATES_P38_MAPK_ACTIVATION	REACTOME	649	22	14	9,24E-01	9,99E-01	rs10504873|rs7841459|rs991880|rs10214840|rs12216864|rs12191736|rs437932|rs7742254|rs1782449|rs6955931|rs4141907|rs145768|rs9380546|rs5030437
Photosensitivity	SIGNALING_BY_NODAL	REACTOME	632	23	13	9,25E-01	9,99E-01	rs3806702|rs2074797|rs2084148|rs10195713|rs11247300|rs1048070|rs2042555|rs266363|rs10933439|rs2859593|rs7163945|rs4662515|rs8027819
Photosensitivity	METAL_ION_SLC_TRANSPORTERS	REACTOME	653	8	5	9,27E-01	9,99E-01	rs10497704|rs4976008|rs7623663|rs9653564|rs17032126
Photosensitivity	BCELLSURVIVAL_PATHWAY	BIOCARTA	500	37	19	9,32E-01	9,99E-01	rs10940270|rs7571143|rs706733|rs706724|rs870992|rs12501768|rs33344|rs10515068|rs1109627|rs1423236|rs3815701|rs10054692|rs1882832|rs11953134|rs7558608|rs4976129|rs17304039|rs1388122|rs2498804
Photosensitivity	DOWNREGULATION_OF_TGF_BETA_RECEPTOR_SIGNALING	REACTOME	521	15	10	9,33E-01	9,99E-01	rs7229153|rs17272673|rs1288706|rs728506|rs4955316|rs266363|rs12456328|rs6070233|rs7163945|rs6545886
Photosensitivity	IRON_UPTAKE_AND_TRANSPORT	REACTOME	733	23	16	9,33E-01	9,99E-01	rs2515210|rs17200733|rs2674490|rs10497704|rs871516|rs16919915|rs17749106|rs2071749|rs7623663|rs7250572|rs1358024|rs259874|rs13238736|rs8846|rs7836003|rs11639998
Photosensitivity	MAP_KINASE_ACTIVATION_IN_TLR_CASCADE	REACTOME	1697	33	20	9,35E-01	9,99E-01	rs1401875|rs10504873|rs1982235|rs1012421|rs2005219|rs7841459|rs6456115|rs991880|rs1056348|rs1432442|rs10214840|rs12436076|rs1105194|rs1812402|rs12216864|rs12191736|rs1283614|rs2876980|rs12655172|rs10474293
Photosensitivity	SYNTHESIS_OF_GLYCOSYLPHOSPHATIDYLINOSITOL_GPI	REACTOME	485	8	6	9,35E-01	9,99E-01	rs8039143|rs17249299|rs47077|rs17069296|rs17069752|rs281282
Photosensitivity	JNK_C_JUN_KINASES_PHOSPHORYLATION_AND_ACTIVATION_MEDIATED_BY_ACTIVATE	REACTOME	728	22	15	9,35E-01	9,99E-01	rs10504873|rs7841459|rs991880|rs10214840|rs12216864|rs12191736|rs8065164|rs437932|rs7742254|rs1782449|rs6955931|rs4141907|rs4640664|rs145768|rs5030437
Photosensitivity	PI_METABOLISM	REACTOME	1808	38	20	9,37E-01	9,99E-01	rs12490575|rs1941590|rs2440455|rs4595861|rs2673825|rs1374670|rs706733|rs706724|rs1339959|rs7291756|rs2667100|rs4240910|rs999613|rs2283877|rs273266|rs33344|rs12453325|rs3768331|rs2254562|rs10515068
Photosensitivity	CALCIUM_SIGNALING_PATHWAY	KEGG	9275	27	20	9,37E-01	9,99E-01	rs2239094|rs1594513|rs13064158|rs2888104|rs12873185|rs1878404|rs963065|rs3908461|rs215697|rs7190913|rs1866901|rs16849142|rs12568960|rs17276674|rs2461489|rs17329875|rs6435692|rs7586183|rs2946542|rs17031191
Photosensitivity	PKA_MEDIATED_PHOSPHORYLATION_OF_CREB	REACTOME	767	34	20	9,38E-01	9,99E-01	rs883541|rs4736746|rs1404912|rs4268746|rs13166852|rs10063916|rs1525660|rs2238431|rs4371986|rs1023095|rs918167|rs2099505|rs72001|rs4745514|rs7196832|rs3181384|rs10062299|rs3892156|rs4401545|rs6470863
Photosensitivity	REGULATION_OF_SIGNALING_BY_CBL	REACTOME	703	34	20	9,39E-01	9,99E-01	rs2302456|rs706733|rs706724|rs1792737|rs3012738|rs4240910|rs2278278|rs273266|rs33344|rs3012719|rs1993703|rs10515068|rs1423236|rs3815701|rs10054692|rs7834615|rs869382|rs7248911|rs11953134|rs4976129
Photosensitivity	TGF_BETA_RECEPTOR_SIGNALING_ACTIVATES_SMADS	REACTOME	563	15	10	9,39E-01	9,99E-01	rs7229153|rs17272673|rs1288706|rs728506|rs4955316|rs266363|rs12456328|rs6070233|rs7163945|rs6545886
Photosensitivity	DOWNSTREAM_SIGNAL_TRANSDUCTION	REACTOME	3083	22	20	9,40E-01	9,99E-01	rs628877|rs883541|rs2324528|rs4736746|rs873417|rs2302456|rs1404912|rs759947|rs2005219|rs2673825|rs13060691|rs6090585|rs1124787|rs706733|rs17746897|rs6740453|rs7961587|rs706724|rs4381631|rs10497599
Photosensitivity	G_PROTEIN_BETA_GAMMA_SIGNALLING	REACTOME	798	15	14	9,42E-01	9,99E-01	rs11157863|rs6055990|rs2719795|rs8115925|rs2223538|rs6140619|rs3730050|rs7152036|rs584845|rs8104096|rs4816085|rs11810173|rs936213|rs2498804
Photosensitivity	L1CAM_INTERACTIONS	REACTOME	3914	37	20	9,42E-01	9,99E-01	rs7946527|rs7023913|rs17333297|rs2070776|rs2727997|rs10940270|rs3003608|rs17037203|rs2005219|rs278899|rs10994214|rs1990708|rs10740870|rs12338625|rs10099843|rs17746482|rs6090585|rs2242445|rs591042|rs6456115
Photosensitivity	RENIN_ANGIOTENSIN_SYSTEM	KEGG	467	6	5	9,45E-01	9,99E-01	rs2561193|rs6676670|rs5705|rs3789662|rs243978
Photosensitivity	REGULATION_OF_INSULIN_LIKE_GROWTH_FACTOR_IGF_ACTIVITY_BY_INSULIN_LIKE_GROWTH_FACTOR_BINDING_PROTEINS_IGFBPS	REACTOME	570	14	11	9,46E-01	9,99E-01	rs8047464|rs1144396|rs12924764|rs788778|rs12666800|rs2064712|rs720683|rs17502828|rs1989775|rs3823055|rs6935921
Photosensitivity	CYTOSOLIC_TRNA_AMINOACYLATION	REACTOME	312	8	5	9,46E-01	9,99E-01	rs12961130|rs2302330|rs707937|rs268739|rs12022181
Photosensitivity	ACH_PATHWAY	BIOCARTA	602	31	16	9,49E-01	9,99E-01	rs6090585|rs706733|rs706724|rs12292911|rs2292979|rs16915435|rs9606893|rs33344|rs10515068|rs1423236|rs3815701|rs10054692|rs11953134|rs4976129|rs1388122|rs2498804
Photosensitivity	MUSCLE_CONTRACTION	REACTOME	1023	43	19	9,50E-01	9,99E-01	rs1571131|rs6948564|rs2799686|rs10940270|rs870992|rs10882615|rs549062|rs907611|rs7213488|rs1254403|rs2414810|rs4576532|rs10188058|rs2469763|rs2075511|rs12091348|rs3759614|rs10778125|rs4673510
Photosensitivity	NOD_LIKE_RECEPTOR_SIGNALING_PATHWAY	KEGG	1406	30	20	9,50E-01	9,99E-01	rs10504873|rs2005219|rs6591116|rs475097|rs17200733|rs7841459|rs991880|rs10214840|rs6685474|rs12216864|rs12191736|rs11899004|rs2876980|rs2716932|rs11653896|rs4722164|rs437932|rs917686|rs8079727|rs1429638
Photosensitivity	CLEAVAGE_OF_GROWING_TRANSCRIPT_IN_THE_TERMINATION_REGION_	REACTOME	435	18	15	9,54E-01	9,99E-01	rs17704641|rs4646296|rs1798177|rs10859963|rs16987990|rs13183577|rs6030839|rs17054866|rs2282763|rs4653186|rs3803695|rs4982753|rs1888523|rs7936604|rs2025366
Photosensitivity	PORPHYRIN_AND_CHLOROPHYLL_METABOLISM	KEGG	1163	174	20	9,55E-01	9,99E-01	rs4148329|rs2170643|rs6761246|rs2361502|rs1871705|rs12961130|rs11954404|rs2071749|rs10061417|rs7623663|rs12022181|rs4979243|rs259874|rs376109|rs6830865|rs7922546|rs709607|rs2287182|rs818702|rs11639998
Photosensitivity	THROMBOXANE_SIGNALLING_THROUGH_TP_RECEPTOR	REACTOME	492	9	7	9,56E-01	9,99E-01	rs11157863|rs11655004|rs7152036|rs584845|rs8104096|rs11810173|rs10869985
Photosensitivity	SIGNALING_BY_PDGF	REACTOME	4427	27	20	9,57E-01	9,99E-01	rs1630986|rs628877|rs12280614|rs883541|rs2324528|rs4736746|rs873417|rs1404912|rs2302456|rs759947|rs2005219|rs2673825|rs13060691|rs7782469|rs1872902|rs6090585|rs1124787|rs706733|rs17746897|rs6740453
Photosensitivity	GPVI_MEDIATED_ACTIVATION_CASCADE	REACTOME	1181	34	20	9,59E-01	9,99E-01	rs345306|rs7782469|rs706733|rs6692050|rs1332684|rs706724|rs962199|rs4580153|rs3730050|rs10835147|rs2278278|rs273266|rs33344|rs12039907|rs1993703|rs10515068|rs1423236|rs3815701|rs10054692|rs12716928
Photosensitivity	TRAF6_MEDIATED_INDUCTION_OF_NFKB_AND_MAP_KINASES_UPON_TLR7_8_OR_9_ACT	REACTOME	2239	28	20	9,59E-01	9,99E-01	rs1401875|rs10504873|rs11574849|rs1982235|rs1012421|rs2005219|rs7973701|rs8132767|rs7841459|rs11702267|rs6456115|rs991880|rs1056348|rs1432442|rs1049679|rs2239621|rs10214840|rs12436076|rs7992176|rs11105194
Photosensitivity	GLUCAGON_SIGNALING_IN_METABOLIC_REGULATION	REACTOME	1044	33	20	9,59E-01	9,99E-01	rs883541|rs4736746|rs1404912|rs11157863|rs4268746|rs13166852|rs10063916|rs7152036|rs1525660|rs2238431|rs4371986|rs1023095|rs918167|rs584845|rs2099505|rs72001|rs4745514|rs8104096|rs7196832|rs3181384
Photosensitivity	G_BETA_GAMMA_SIGNALLING_THROUGH_PI3KGAMMA	REACTOME	487	8	7	9,60E-01	9,99E-01	rs11157863|rs3730050|rs7152036|rs584845|rs8104096|rs11810173|rs2498804
Photosensitivity	TRIF_MEDIATED_TLR3_SIGNALING	REACTOME	2075	30	20	9,61E-01	9,99E-01	rs1401875|rs10504873|rs11574849|rs1982235|rs1012421|rs2005219|rs8132767|rs7841459|rs11702267|rs6456115|rs991880|rs1056348|rs1432442|rs10214840|rs12436076|rs7992176|rs11105194|rs7327256|rs2716121|rs12216864
Photosensitivity	ALDOSTERONE_REGULATED_SODIUM_REABSORPTION	KEGG	1794	33	20	9,61E-01	9,99E-01	rs973608|rs4804366|rs873417|rs2005219|rs3170660|rs706733|rs706724|rs4381631|rs4411531|rs3729908|rs4240910|rs758658|rs7491948|rs1023168|rs2876980|rs10771184|rs273266|rs33344|rs12374234|rs2115386
Photosensitivity	NFKB_PATHWAY	BIOCARTA	870	22	17	9,62E-01	9,99E-01	rs1061631|rs17200733|rs10818151|rs991880|rs2239621|rs7930377|rs10983791|rs12191736|rs437932|rs7742254|rs1782449|rs10983917|rs832540|rs4141907|rs7552664|rs145768|rs5030437
Photosensitivity	TERMINATION_OF_O_GLYCAN_BIOSYNTHESIS	REACTOME	961	37	20	9,64E-01	9,99E-01	rs6444192|rs7480563|rs6667721|rs289686|rs10081532|rs17128554|rs2114567|rs13275988|rs3822163|rs665178|rs6685141|rs17776120|rs6593523|rs844518|rs4949715|rs2945744|rs7103978|rs4686823|rs11604757|rs3744050
Photosensitivity	TRANSCRIPTIONAL_ACTIVITY_OF_SMAD2_SMAD3_SMAD4_HETEROTRIMER	REACTOME	967	30	20	9,64E-01	9,99E-01	rs1030609|rs7229153|rs10492292|rs6440618|rs10956433|rs12585793|rs2088229|rs4305170|rs1994174|rs3782280|rs16902654|rs263526|rs1242991|rs4733581|rs6997983|rs266363|rs12456328|rs4798085|rs4901977|rs7163945
Photosensitivity	PI3K_AKT_ACTIVATION	REACTOME	1161	28	20	9,64E-01	9,99E-01	rs2324528|rs2673825|rs706733|rs706724|rs12626158|rs195204|rs1294383|rs7491948|rs3730050|rs1023168|rs1664248|rs4435111|rs273266|rs33344|rs2417223|rs10494270|rs10515068|rs8000689|rs2145068|rs1423236
Photosensitivity	NFKB_AND_MAP_KINASES_ACTIVATION_MEDIATED_BY_TLR4_SIGNALING_REPERTOIRE	REACTOME	2248	29	20	9,64E-01	9,99E-01	rs1401875|rs10504873|rs11574849|rs1982235|rs1012421|rs2005219|rs8132767|rs10818151|rs7841459|rs11702267|rs6456115|rs991880|rs7843776|rs1056348|rs1432442|rs10214840|rs12436076|rs7992176|rs1009619|rs7327256

Photosensitivity	MITOTIC_G2_G2_M_PHASES	REACTOME	1389	34	20	9,65E-01	9,99E-01	rs6909633|rs10927020|rs9550174|rs6797587|rs7320567|rs9368179|rs911361|rs1056348|rs1475663|rs2416745|rs2580347|rs7500128|rs3795833|rs7101374|rs2847255|rs12952976|rs1968648|rs1561734|rs10954269|rs6545886
Photosensitivity	SYNTHESIS_OF_PIPS_AT_THE_PLASMA_MEMBRANE	REACTOME	1050	31	20	9,66E-01	9,99E-01	rs2673825|rs1374670|rs706733|rs706724|rs7291756|rs2667100|rs4240910|rs999613|rs2283877|rs273266|rs33344|rs12453325|rs2254562|rs10515068|rs1423236|rs4745514|rs3815701|rs10054692|rs17438150|rs7671799
Photosensitivity	LIPOPROTEIN_METABOLISM	REACTOME	1037	32	20	9,66E-01	9,99E-01	rs2881925|rs17821232|rs17431426|rs9355296|rs780825|rs3764261|rs1607581|rs2740484|rs226372|rs1816879|rs13398007|rs6083|rs969936|rs2066716|rs3760627|rs225436|rs7838257|rs10413089|rs2228671|rs11254275
Photosensitivity	LIPID_DIGESTION_MOBILIZATION_AND_TRANSPORT	REACTOME	1389	26	20	9,67E-01	9,99E-01	rs2881925|rs880887|rs17821232|rs17431426|rs9355296|rs780825|rs4682900|rs3764261|rs1607581|rs2740484|rs226372|rs3792009|rs13100991|rs1816879|rs13398007|rs4745514|rs6083|rs969936|rs2066716|rs7612822
Photosensitivity	PROGESTERONE_MEDIATED_OOCYTE_MATURATION	KEGG	2535	26	20	9,67E-01	9,99E-01	rs1546713|rs1647253|rs4736746|rs10501976|rs1404912|rs2005219|rs2684794|rs13060691|rs13130657|rs706733|rs2236941|rs6797587|rs706724|rs6456115|rs11227147|rs7162314|rs1432442|rs11045359|rs4268746|rs4240910
Photosensitivity	REGULATION_OF_IFNA_SIGNALING	REACTOME	339	4	4	9,68E-01	9,99E-01	rs7221341|rs9915291|rs9905081|rs4969168
Photosensitivity	ERK5_PATHWAY	BIOCARTA	595	32	17	9,68E-01	9,99E-01	rs2005219|rs706733|rs706724|rs1283614|rs2876980|rs12655172|rs10474293|rs33344|rs10515068|rs1423236|rs3815701|rs10054692|rs1366594|rs11953134|rs4976129|rs1388122|rs2498804
Photosensitivity	EPITHELIAL_CELL_SIGNALING_IN_HELICOBACTER_PYLORI_INFECTION	KEGG	1535	37	20	9,69E-01	9,99E-01	rs2515210|rs4766987|rs1427282|rs17526983|rs17746482|rs6090585|rs17200733|rs871516|rs12501768|rs984654|rs4580153|rs2716121|rs4140770|rs8065164|rs16919915|rs17749106|rs1019221|rs1011692|rs11807930|rs41741
Photosensitivity	PATHOGENIC_ESCHERICHIA_COLI_INFECTION	KEGG	1256	28	20	9,69E-01	9,99E-01	rs873417|rs3105453|rs442683|rs10818151|rs6599127|rs4381631|rs4411531|rs7843776|rs758658|rs10983791|rs9507572|rs12474808|rs464901|rs11695567|rs468789|rs3133539|rs1909530|rs10983917|rs336508|rs11884955
Photosensitivity	AMINO_SUGAR_AND_NUCLEOTIDE_SUGAR_METABOLISM	KEGG	956	31	20	9,72E-01	9,99E-01	rs7535245|rs2269260|rs12508162|rs3800070|rs226463|rs217463|rs17501218|rs3823271|rs1500488|rs3823261|rs6672661|rs4959663|rs3749237|rs6695928|rs2608822|rs7601269|rs17592080|rs9503031|rs2687964|rs855314
Photosensitivity	HCMV_PATHWAY	BIOCARTA	547	32	17	9,72E-01	9,99E-01	rs2005219|rs706733|rs706724|rs1432442|rs2876980|rs33344|rs10515068|rs1423236|rs3815701|rs10054692|rs832540|rs4255740|rs11953134|rs4976129|rs1388122|rs9380546|rs2498804
Photosensitivity	TOLL_RECEPTOR_CASCADES	REACTOME	3778	28	20	9,73E-01	9,99E-01	rs7023913|rs1401875|rs10504873|rs1941590|rs11574849|rs2440455|rs1982235|rs1012421|rs3003608|rs2005219|rs4595861|rs7973701|rs12338625|rs4592915|rs2232571|rs353087|rs8132767|rs10818151|rs7841459|rs11702267
Photosensitivity	IL1_SIGNALING	REACTOME	1322	28	20	9,73E-01	9,99E-01	rs10504873|rs7973701|rs7841459|rs991880|rs1432442|rs1049679|rs2239621|rs10214840|rs4301703|rs12216864|rs12191736|rs8065164|rs1152494|rs17790482|rs2310173|rs437932|rs736939|rs7742254|rs1782449|rs633384
Photosensitivity	FRUCTOSE_AND_MANNOSE_METABOLISM	KEGG	984	31	20	9,74E-01	9,99E-01	rs8049837|rs3800070|rs2267844|rs226463|rs10903843|rs3750667|rs3823271|rs943117|rs3823261|rs11818509|rs4959663|rs2256817|rs10794929|rs3749237|rs2454829|rs4747886|rs1556632|rs10903939|rs678363|rs17592080
Photosensitivity	MYD88_MAL_CASCADE_INITIATED_ON_PLASMA_MEMBRANE	REACTOME	2527	28	20	9,77E-01	9,99E-01	rs1401875|rs10504873|rs11574849|rs1982235|rs1012421|rs2005219|rs7973701|rs8132767|rs10818151|rs7841459|rs11702267|rs6456115|rs991880|rs7843776|rs1056348|rs1432442|rs1049679|rs2239621|rs10214840|rs12436076
Photosensitivity	INSULIN_SIGNALING_PATHWAY	KEGG	3744	30	20	9,80E-01	9,99E-01	rs723393|rs4804366|rs883541|rs1571131|rs2324528|rs2809244|rs2302456|rs7778077|rs4725424|rs2005219|rs13060691|rs722813|rs4725435|rs10849968|rs706733|rs7580869|rs706724|rs4469064|rs11792737|rs10882615
Photosensitivity	CD28_DEPENDENT_PI3K_AKT_SIGNALING	REACTOME	572	34	20	9,80E-01	9,99E-01	rs706733|rs706724|rs12626158|rs624035|rs4301703|rs3730050|rs273266|rs33344|rs10494270|rs10515068|rs2145068|rs1423236|rs3815701|rs10054692|rs17187469|rs11953134|rs4976129|rs1490819|rs6438537|rs2498804
Photosensitivity	GNRH_SIGNALING_PATHWAY	KEGG	3992	26	20	9,80E-01	9,99E-01	rs2239094|rs10912414|rs1488|rs4736746|rs873417|rs12143542|rs2238012|rs1404912|rs2005219|rs7176475|rs10798089|rs13060691|rs9888368|rs3822295|rs2239062|rs2238043|rs17746482|rs6090585|rs7961587|rs6055990
Photosensitivity	STRIATED_MUSCLE_CONTRACTION	REACTOME	453	12	9	9,81E-01	9,99E-01	rs2799686|rs907611|rs7213488|rs2414810|rs4576532|rs12091348|rs3759614|rs10778125|rs4673510
Photosensitivity	NTHI_PATHWAY	BIOCARTA	876	28	16	9,83E-01	9,99E-01	rs17209216|rs17200733|rs991880|rs4912652|rs2239621|rs12191736|rs437932|rs9866764|rs266363|rs7742254|rs1782449|rs2112661|rs7163945|rs4141907|rs1145768|rs2237093
Photosensitivity	PHOSPHATIDYLINOSITOL_SIGNALING_SYSTEM	KEGG	3813	28	20	9,84E-01	9,99E-01	rs10487781|rs4732260|rs6076792|rs3778818|rs8087566|rs873417|rs2440455|rs4595861|rs2673825|rs1374670|rs36873|rs13073829|rs17593321|rs706733|rs7580869|rs7961587|rs706724|rs6055990|rs4381631|rs4411531
Photosensitivity	REGULATION_OF_INSULIN_SECRETION_BY_GLUCAGON_LIKE_PEPTIDE1	REACTOME	1388	33	19	9,84E-01	9,99E-01	rs883541|rs16974215|rs4736746|rs7961587|rs11157863|rs9470963|rs506958|rs2589949|rs7152036|rs4371986|rs1023095|rs918167|rs584845|rs17706710|rs4745514|rs8104096|rs11810173|rs6470863|rs1379966
Photosensitivity	AQUAPORIN_MEDIATED_TRANSPORT	REACTOME	1510	30	20	9,85E-01	9,99E-01	rs883541|rs4736746|rs1194610|rs9945925|rs1404912|rs11157863|rs4268746|rs13166852|rs10063916|rs7152036|rs1525660|rs2238431|rs4371986|rs1023095|rs1465216|rs918167|rs584845|rs938508|rs2099505|rs72001
Photosensitivity	TASTE_TRANSDUCTION	KEGG	1100	32	20	9,87E-01	9,99E-01	rs4736746|rs1993430|rs6686865|rs10497599|rs11666813|rs2499711|rs506958|rs8113506|rs4926278|rs4371986|rs1023095|rs584845|rs6897599|rs4745514|rs12447134|rs3181384|rs936213|rs4701849|rs2495973|rs6470863
Photosensitivity	REGULATION_OF_WATER_BALANCE_BY_RENAL_AQUAPORINS	REACTOME	1393	31	20	9,87E-01	9,99E-01	rs883541|rs4736746|rs9945925|rs1404912|rs11157863|rs4268746|rs13166852|rs10063916|rs7152036|rs1525660|rs2238431|rs4371986|rs1023095|rs1465216|rs918167|rs584845|rs2099505|rs72001|rs4745514|rs8104096
Photosensitivity	TOLL_LIKE_RECEPTOR_SIGNALING_PATHWAY	KEGG	2339	28	20	9,88E-01	9,99E-01	rs583911|rs2005219|rs2421057|rs4592915|rs2232571|rs17200733|rs706733|rs17811014|rs10818151|rs706724|rs991880|rs7843776|rs6065925|rs10516798|rs1432442|rs2239621|rs624035|rs10214840|rs7930377|rs4240910
Photosensitivity	ACTIVATED_TLR4_SIGNALLING	REACTOME	2676	28	20	9,88E-01	9,99E-01	rs1401875|rs10504873|rs11574849|rs1982235|rs1012421|rs2005219|rs7973701|rs8132767|rs10818151|rs7841459|rs11702267|rs6456115|rs991880|rs7843776|rs1056348|rs1432442|rs1049679|rs2239621|rs10214840|rs12436076
Photosensitivity	LYSOSOME	KEGG	2448	28	20	9,89E-01	9,99E-01	rs6793913|rs13100173|rs5014235|rs12694622|rs5770953|rs2235116|rs2254670|rs9858362|rs3180018|rs7703742|rs885561|rs11902634|rs17716609|rs10520433|rs2340252|rs649529|rs7930237|rs10504983|rs10053925|rs555812
Photosensitivity	IL1R_PATHWAY	BIOCARTA	1001	30	18	9,89E-01	9,99E-01	rs17047703|rs17200733|rs991880|rs2239621|rs1932971|rs2716121|rs12191736|rs4722164|rs437932|rs7742254|rs1782449|rs9877268|rs11465990|rs832540|rs4141907|rs1145768|rs2237093|rs5030437
Photosensitivity	IL7_PATHWAY	BIOCARTA	699	28	16	9,90E-01	9,99E-01	rs706733|rs706724|rs2292979|rs7000123|rs12458289|rs33344|rs11567697|rs10515068|rs1441438|rs1423236|rs3815701|rs10054692|rs11953134|rs4976129|rs2236719|rs1388122
Photosensitivity	INOSITOL_PHOSPHATE_METABOLISM	KEGG	2355	31	20	9,92E-01	9,99E-01	rs8087566|rs2440455|rs4595861|rs2673825|rs1374670|rs17593321|rs6055990|rs2719795|rs1339959|rs7630340|rs8115925|rs2667100|rs4580153|rs2223538|rs4240910|rs7043114|rs6140619|rs4797615|rs909270|rs708777
Photosensitivity	MISMATCH_REPAIR	KEGG	576	8	8	9,92E-01	9,99E-01	rs7357092|rs12112229|rs1863332|rs1359389|rs1629511|rs11236183|rs7328146|rs1047840
Photosensitivity	RECRUITMENT_OF_MITOTIC_CENTROSOME_PROTEINS_AND_COMPLEXES	REACTOME	1177	25	19	9,95E-01	9,99E-01	rs10927020|rs9550174|rs7320567|rs1056348|rs1475663|rs2416745|rs2580347|rs7500128|rs3795833|rs7101374|rs2847255|rs12952976|rs10954269|rs2580367|rs1558729|rs4837752|rs3747941|rs4793039|rs7968231
Photosensitivity	DOWNREGULATION_OF_SMAD2_3_SMAD4_TRANSCRIPTIONAL_ACTIVITY	REACTOME	453	11	11	9,95E-01	9,99E-01	rs2088229|rs4305170|rs1994174|rs3782280|rs263526|rs1242991|rs266363|rs4798085|rs4901977|rs7163945|rs9954608
Photosensitivity	SIGNALING_BY_TGF_BETA_RECEPTOR_COMPLEX	REACTOME	1605	32	20	9,96E-01	9,99E-01	rs1030608|rs4934517|rs7229153|rs17272673|rs1288706|rs992241|rs6440618|rs10956433|rs12585793|rs728506|rs2088229|rs4305170|rs1994174|rs3782280|rs1331681|rs1617306|rs16902654|rs263526|rs11807930|rs1242991
Photosensitivity	VOLTAGE_GATED_POTASSIUM_CHANNELS	REACTOME	2491	26	20	9,96E-01	9,99E-01	rs305691|rs2012618|rs2237888|rs12753051|rs10099843|rs1014665|rs2255512|rs987996|rs7957246|rs11030613|rs231349|rs6453613|rs9847518|rs498033|rs10451557|rs506958|rs3767943|rs1012105|rs746080|rs4143555
Photosensitivity	LOSS_OF_NLP_FROM_MITOTIC_CENTROSOMES	REACTOME	1100	22	16	9,97E-01	9,99E-01	rs10927020|rs1056348|rs1475663|rs2416745|rs2580347|rs7500128|rs3795833|rs7101374|rs2847255|rs12952976|rs10954269|rs2580367|rs1558729|rs4837752|rs4793039|rs7968231
Photosensitivity	INNATE_IMMUNE_SYSTEM	REACTOME	6278	28	20	9,97E-01	9,99E-01	rs7023913|rs1401875|rs2252405|rs10813821|rs10504873|rs13062355|rs1941590|rs11574849|rs2440455|rs1982235|rs1012421|rs1548992|rs4610335|rs3003608|rs2005219|rs591874|rs11953280|rs4595861|rs7973701|rs12338625
Photosensitivity	TOLL_PATHWAY	BIOCARTA	1147	36	20	9,97E-01	9,99E-01	rs10818151|rs991880|rs7843776|rs2239621|rs10214840|rs135549|rs2716121|rs10983791|rs12191736|rs8065164|rs437932|rs7742254|rs1909530|rs1782449|rs10983917|rs832540|rs1882832|rs4141907|rs1145768|rs2237093
Photosensitivity	CHEMOKINE_SIGNALING_PATHWAY	KEGG	5407	22	20	1,00E+00	1,00E+00	rs2228428|rs345306|rs4810783|rs4736746|rs1404912|rs2302456|rs6440041|rs2005219|rs17160128|rs13060691|rs4934517|rs754743|rs9371867|rs497916|rs706733|rs6692050|rs259916|rs2236941|rs1332684|rs926693
Oral Ulcers	VEGF_PATHWAY	BIOCARTA	883	55	20	5,58E-05	4,45E-02	rs2373929|rs17709898|rs743507|rs10940145|rs7396875|rs7726943|rs10461535|rs227913|rs2372183|rs4976028|rs904132|rs11133360|rs9892886|rs11860367|rs10515068|rs7719763|rs8051034|rs863818|rs10515059|rs6504424
Oral Ulcers	VEGF_SIGNALING_PATHWAY	KEGG	2111	38	20	6,00E-04	1,92E-01	rs2373929|rs17709898|rs6852347|rs743507|rs10940145|rs7396875|rs7726943|rs16897175|rs227913|rs2372183|rs354263|rs6096351|rs4976028|rs13434873|rs7518498|rs904132|rs7542020|rs11133360|rs9892886|rs11860367
Oral Ulcers	NEPHRIN_INTERACTIONS	REACTOME	1617	46	20	9,00E-04	1,92E-01	rs10940145|rs809192|rs3807671|rs7726943|rs7775178|rs1918930|rs16897175|rs3807663|rs483644|rs3769504|rs13239642|rs2372183|rs2510705|rs3754806|rs4976028|rs7789660|rs6967983|rs4851835|rs3779325|rs663268
Oral Ulcers	GCR_PATHWAY	BIOCARTA	736	53	20	1,20E-03	1,92E-01	rs2373929|rs743507|rs10940145|rs7726943|rs10461535|rs2372183|rs4976028|rs4694868|rs1352514|rs10515068|rs1297783|rs7719763|rs931640|rs11143705|rs863818|rs11794852|rs10515059|rs230530|rs10515621|rs17640419
Oral Ulcers	HDAC_PATHWAY	BIOCARTA	1231	41	20	1,20E-03	1,92E-01	rs770466|rs6852347|rs10940145|rs7726943|rs10461535|rs7169544|rs1019731|rs2372183|rs6096351|rs4976028|rs13434873|rs1422189|rs2267172|rs2851045|rs10515068|rs1297783|rs7719763|rs931640|rs6214|rs863818
Oral Ulcers	ANTIGEN_PRESENTATION_FOLDING_ASSEMBLY_AND_PEPTIDE_LOADING_OF_CLASS_I_M	REACTOME	830	66	10	2,30E-03	2,63E-01	rs2853923|rs11730732|rs9348863|rs17108797|rs1634776|rs3775836|rs4148876|rs2523580|rs2162400|rs7745906
Oral Ulcers	CHEMOKINE_SIGNALING_PATHWAY	KEGG	5407	35	20	2,80E-03	2,63E-01	rs852350|rs701724|rs263241|rs10940145|rs10489469|rs7396875|rs1541821|rs7726943|rs6018981|rs12334868|rs16897175|rs1779344|rs16904360|rs4710181|rs952784|rs12600516|rs2256156|rs266087|rs2372183|rs2833264
Oral Ulcers	REGULATION_OF_SIGNALING_BY_CBL	REACTOME	703	51	20	3,00E-03	2,63E-01	rs10940145|rs809192|rs7726943|rs10461535|rs952784|rs2372183|rs290240|rs4976028|rs182832|rs2660481|rs10515068|rs17718517|rs7719763|rs3746188|rs863818|rs1551746|rs10120669|rs7834615|rs2660489|rs10515059
Oral Ulcers	NITRIC_OXIDE_STIMULATES_GUANYLATE_CYCLASE	REACTOME	1895	30	20	3,10E-03	2,63E-01	rs2373929|rs743507|rs13419755|rs7964046|rs7762369|rs10823962|rs11690740|rs1840572|rs7893773|rs12619532|rs6759996|rs988146|rs10034450|rs2251273|rs13064262|rs12495203|rs4691787|rs17033553|rs2128650|rs1896610
Oral Ulcers	IL_3_5_AND_GM_CSF_SIGNALING	REACTOME	1134	42	20	3,30E-03	2,63E-01	rs10940145|rs7396875|rs809192|rs7726943|rs16897175|rs952784|rs2372183|rs290240|rs4976028|rs182832|rs1153465|rs310209|rs2660481|rs163546|rs628542|rs10515068|rs17718517|rs17606924|rs7719763|rs706779
Oral Ulcers	TCR_PATHWAY	BIOCARTA	1517	37	20	4,30E-03	2,89E-01	rs6852347|rs10940145|rs7396875|rs809192|rs7726943|rs16897175|rs11683207|rs227913|rs2372183|rs6096351|rs4976028|rs13434873|rs502514|rs2660481|rs9892886|rs11860367|rs2851045|rs10515068|rs17718517|rs1297783
Oral Ulcers	SYNTHESIS_SECRETION_AND_INACTIVATION_OF_GLP1	REACTOME	363	15	7	4,60E-03	2,89E-01	rs721355|rs2239512|rs686699|rs10074809|rs874627|rs10835826|rs4731437
Oral Ulcers	GPVI_MEDIATED_ACTIVATION_CASCADE	REACTOME	1181	43	20	4,70E-03	2,89E-01	rs10940145|rs809192|rs7726943|rs16897175|rs952784|rs810687|rs2372183|rs290240|rs4976028|rs1029999|rs182832|rs1332684|rs17020294|rs421587|rs2660481|rs7543435|rs10515068|rs17718517|rs4888197|rs7719763
Oral Ulcers	NFAT_PATHWAY	BIOCARTA	1626	38	20	5,70E-03	3,14E-01	rs770466|rs6852347|rs10940145|rs7396875|rs7726943|rs10461535|rs1019731|rs2372183|rs6096351|rs4976028|rs13434873|rs1422189|rs2851045|rs10057083|rs10515068|rs1297783|rs6771638|rs12114452|rs1292035|rs7719763
Oral Ulcers	IL_RECEPTOR_SHC_SIGNALING	REACTOME	624	51	20	5,90E-03	3,14E-01	rs10940145|rs7396875|rs7726943|rs16897175|rs2372183|rs4976028|rs1153465|rs310209|rs163546|rs628542|rs10515068|rs17606924|rs7719763|rs706779|rs3746188|rs863818|rs10515059|rs7409343|rs163577|rs5756391
Oral Ulcers	ERK5_PATHWAY	BIOCARTA	595	50	20	7,50E-03	3,46E-01	rs770466|rs10940145|rs7396875|rs7726943|rs16897175|rs2372183|rs4976028|rs1422189|rs10515068|rs7719763|rs863818|rs10515059|rs1888861|rs11633249|rs12442313|rs2699905|rs1490791|rs7379303|rs7645550|rs412458
Oral Ulcers	FCER1_PATHWAY	BIOCARTA	1564	37	20	7,70E-03	3,46E-01	rs6852347|rs10940145|rs7396875|rs7726943|rs16897175|rs952784|rs2372183|rs354263|rs6096351|rs290240|rs4976028|rs13434873|rs182832|rs2660481|rs11860367|rs2851045|rs926324|rs10515068|rs17718517|rs1297783
Oral Ulcers	CREB_PATHWAY	BIOCARTA	969	61	20	8,00E-03	3,46E-01	rs10940145|rs7396875|rs7726943|rs10461535|rs919741|rs3822295|rs227913|rs2372183|rs4976028|rs2285703|rs9892886|rs11860367|rs10515068|rs7719763|rs6533711|rs8051034|rs863818|rs7524898|rs872077|rs10515059
Oral Ulcers	SIGNALLING_BY_NGF	REACTOME	7869	32	20	8,50E-03	3,46E-01	rs852350|rs770466|rs701724|rs263241|rs10940145|rs10489469|rs7396875|rs1541821|rs7726943|rs13311977|rs6018981|rs10869736|rs12334868|rs16897175|rs16904360|rs2667783|rs2256156|rs1351726|rs7841459|rs227913
Oral Ulcers	CTLA4_PATHWAY	BIOCARTA	662	65	20	9,30E-03	3,46E-01	rs10940145|rs7726943|rs10461535|rs1344638|rs2372183|rs4976028|rs3135363|rs30117|rs502514|rs10515068|rs13003194|rs7719763|rs7192|rs863818|rs9368726|rs2949659|rs10515059|rs10195135|rs9983407|rs2699905
Oral Ulcers	ENOS_ACTIVATION_AND_REGULATION	REACTOME	336	13	8	9,60E-03	3,46E-01	rs2373929|rs743507|rs1297783|rs931640|rs688242|rs3779258|rs904160|rs7259810
Oral Ulcers	IGF1MTOR_PATHWAY	BIOCARTA	582	47	20	9,90E-03	3,46E-01	rs10940145|rs7726943|rs16897175|rs7169544|rs1019731|rs2372183|rs4976028|rs628542|rs10515068|rs6771638|rs1292035|rs7719763|rs6214|rs863818|rs10515059|rs2239953|rs6605631|rs7070956|rs1546713|rs2230392
Oral Ulcers	CD28_CO_STIMULATION	REACTOME	797	54	20	1,02E-02	3,46E-01	rs10940145|rs809192|rs7726943|rs10461535|rs952784|rs2372183|rs4976028|rs182832|rs2660481|rs10515068|rs17718517|rs7719763|rs3746188|rs8139544|rs863818|rs7834615|rs2660489|rs10515059|rs17813240|rs2689420
Oral Ulcers	INCRETIN_SYNTHESIS_SECRETION_AND_INACTIVATION	REACTOME	540	30	13	1,04E-02	3,46E-01	rs4131677|rs721355|rs2239512|rs686699|rs10074809|rs17236135|rs12114452|rs11785555|rs874627|rs10835826|rs4731437|rs2068701|rs2256241
Oral Ulcers	SIGNALING_BY_ROBO_RECEPTOR	REACTOME	1900	49	20	1,09E-02	3,48E-01	rs7431017|rs219862|rs10511054|rs10889117|rs3769504|rs1350136|rs4459934|rs267131|rs3754806|rs936213|rs9810156|rs11244164|rs196532|rs3828334|rs3813982|rs11715608|rs4851835|rs1490311|rs6039529|rs6808931
Oral Ulcers	LATENT_INFECTION_OF_HOMO_SAPIENS_WITH_MYCOBACTERIUM_TUBERCULOSIS	REACTOME	589	38	20	1,16E-02	3,53E-01	rs2373929|rs743507|rs1871093|rs5750326|rs2346412|rs1006049|rs17078876|rs2734574|rs6983249|rs2004997|rs7579208|rs13192469|rs3814043|rs1018088|rs9828099|rs4646812|rs921968|rs498193|rs2139634|rs2072712
Oral Ulcers	CXCR4_PATHWAY	BIOCARTA	1099	53	20	1,23E-02	3,53E-01	rs10940145|rs7396875|rs7726943|rs16897175|rs1779344|rs227913|rs266087|rs2372183|rs4976028|rs9892886|rs11860367|rs1816971|rs10515068|rs11688530|rs7719763|rs8051034|rs863818|rs11145635|rs2781548|rs10515059
Oral Ulcers	IL_2_SIGNALING	REACTOME	936	48	20	1,24E-02	3,53E-01	rs10940145|rs7396875|rs7726943|rs10461535|rs2372183|rs290240|rs4976028|rs1153465|rs310209|rs163546|rs628542|rs10515068|rs17606924|rs7719763|rs706779|rs3746188|rs863818|rs1551746|rs10120669|rs10515059
Oral Ulcers	IGF1_PATHWAY	BIOCARTA	813	48	20	1,34E-02	3,57E-01	rs10940145|rs7396875|rs7726943|rs16897175|rs7169544|rs1019731|rs2372183|rs4976028|rs1922529|rs205898|rs10515068|rs7719763|rs6214|rs863818|rs10515059|rs4326648|rs1546713|rs2699905|rs1490791|rs7379303
Oral Ulcers	ECM_PATHWAY	BIOCARTA	753	51	20	1,46E-02	3,57E-01	rs10940145|rs7396875|rs809192|rs7726943|rs10461535|rs2372183|rs4976028|rs10515068|rs7719763|rs6887205|rs11009157|rs820336|rs863818|rs4530474|rs10515059|rs2239206|rs12340264|rs238238|rs2153875|rs3763630
Oral Ulcers	TIE2_SIGNALING	REACTOME	790	54	20	1,49E-02	3,57E-01	rs10940145|rs7396875|rs7726943|rs2022950|rs10461535|rs2372183|rs4976028|rs10515068|rs2782434|rs11895467|rs7719763|rs3746188|rs863818|rs6469131|rs6039132|rs10515059|rs1283651|rs7568547|rs899425|rs2699905
Oral Ulcers	IGF1R_PATHWAY	BIOCARTA	735	47	20	1,50E-02	3,57E-01	rs10940145|rs7396875|rs7726943|rs16897175|rs7169544|rs2372183|rs4976028|rs1922529|rs2267172|rs10515068|rs7719763|rs863818|rs7524898|rs872077|rs10515059|rs7526798|rs3729877|rs1546713|rs316689|rs2699905
Oral Ulcers	TFF_PATHWAY	BIOCARTA	697	44	20	1,51E-02	3,57E-01	rs10940145|rs7396875|rs7726943|rs10461535|rs2372183|rs4976028|rs39425|rs17673576|rs10515068|rs7719763|rs11009157|rs863818|rs2288713|rs2877260|rs2955234|rs10515059|rs12153009|rs13157622|rs4292454|rs11976696
Oral Ulcers	TPO_PATHWAY	BIOCARTA	814	61	20	1,52E-02	3,57E-01	rs10940145|rs7396875|rs7726943|rs16897175|rs227913|rs2372183|rs4976028|rs205898|rs9892886|rs11860367|rs10515068|rs7719763|rs8051034|rs863818|rs10515059|rs6504424|rs8078231|rs4326648|rs9927848|rs11646540
Oral Ulcers	GH_PATHWAY	BIOCARTA	963	55	20	1,68E-02	3,83E-01	rs10940145|rs7396875|rs7726943|rs16897175|rs227913|rs2372183|rs2254779|rs4976028|rs1922529|rs9892886|rs11860367|rs10515068|rs7719763|rs8051034|rs863818|rs2955234|rs10515059|rs6504424|rs12153009|rs13157622
Oral Ulcers	PROGESTERONE_MEDIATED_OOCYTE_MATURATION	KEGG	2535	40	20	1,84E-02	3,93E-01	rs263241|rs10940145|rs7964046|rs1541821|rs7726943|rs12334868|rs16897175|rs7169544|rs16904360|rs2256156|rs1019731|rs2372183|rs4976028|rs72001|rs7566051|rs8033106|rs2575676|rs2058166|rs378373|rs1896610
Oral Ulcers	NKCELLS_PATHWAY	BIOCARTA	678	57	20	1,95E-02	3,93E-01	rs10940145|rs7726943|rs10461535|rs2372183|rs290240|rs4976028|rs2660481|rs10515068|rs17718517|rs2733840|rs7719763|rs11009157|rs863818|rs1551746|rs10120669|rs2660489|rs10515059|rs2153875|rs169724|rs2689420
Oral Ulcers	PGC1A_PATHWAY	BIOCARTA	959	40	20	1,95E-02	3,93E-01	rs770466|rs6852347|rs1555208|rs919741|rs3822295|rs13434873|rs1422189|rs2285703|rs2267172|rs2851045|rs1297783|rs2946385|rs931640|rs6533711|rs1800206|rs6819019|rs16874509|rs873592|rs135557|rs2946390
Oral Ulcers	EGF_PATHWAY	BIOCARTA	1048	50	20	2,03E-02	3,93E-01	rs10940145|rs7396875|rs7726943|rs10461535|rs227913|rs2372183|rs4976028|rs205898|rs310209|rs9892886|rs11860367|rs17673576|rs10515068|rs7719763|rs2721854|rs8051034|rs863818|rs7601754|rs2877260|rs2529718
Oral Ulcers	RENAL_CELL_CARCINOMA	KEGG	2164	52	20	2,06E-02	3,93E-01	rs10940145|rs10489469|rs219862|rs7396875|rs7726943|rs2965012|rs10461535|rs4690785|rs2333512|rs2372183|rs4976028|rs2341694|rs936213|rs508594|rs7552931|rs12532699|rs6039529|rs10515068|rs5758168|rs13053242
Oral Ulcers	IL2RB_PATHWAY	BIOCARTA	1296	39	20	2,10E-02	3,93E-01	rs10940145|rs7396875|rs7726943|rs16897175|rs9635726|rs9658702|rs2372183|rs290240|rs4976028|rs1922529|rs310209|rs8074003|rs11580042|rs1982673|rs10515068|rs16893188|rs1292035|rs7910825|rs7719763|rs706779
Oral Ulcers	IL7_PATHWAY	BIOCARTA	699	58	20	2,11E-02	3,93E-01	rs10940145|rs809192|rs7726943|rs16897175|rs2372183|rs4976028|rs310209|rs1982673|rs10515068|rs13053242|rs7719763|rs863818|rs10515059|rs2887501|rs2156192|rs7409343|rs2046136|rs7230970|rs2717548|rs2699905
Oral Ulcers	PDGF_PATHWAY	BIOCARTA	1021	53	20	2,12E-02	3,93E-01	rs10940145|rs7396875|rs7726943|rs16897175|rs227913|rs2372183|rs4976028|rs205898|rs310209|rs9892886|rs11860367|rs10515068|rs7719763|rs2721854|rs8051034|rs863818|rs7601754|rs2529718|rs10515059|rs6504424
Oral Ulcers	EIF4_PATHWAY	BIOCARTA	890	53	20	2,18E-02	3,95E-01	rs10940145|rs7726943|rs16897175|rs227913|rs2372183|rs4976028|rs1922529|rs3843552|rs17460250|rs9892886|rs11860367|rs10515068|rs1292035|rs7719763|rs8051034|rs863818|rs10515059|rs6504424|rs2239953|rs12153009
Oral Ulcers	MAL_PATHWAY	BIOCARTA	225	5	4	2,36E-02	4,19E-01	rs7396875|rs2254779|rs1002063|rs2689420
Oral Ulcers	RAS_PATHWAY	BIOCARTA	557	49	20	2,47E-02	4,28E-01	rs10940145|rs7396875|rs7726943|rs13311977|rs10461535|rs2372183|rs4976028|rs10515068|rs7719763|rs863818|rs10515059|rs230530|rs83615|rs6060627|rs2689420|rs6951760|rs2699905|rs1490791|rs7379303|rs7645550
Oral Ulcers	SYNTHESIS_OF_PIPS_AT_THE_PLASMA_MEMBRANE	REACTOME	1050	46	20	2,60E-02	4,30E-01	rs10940145|rs7726943|rs16897175|rs2372183|rs4976028|rs3775601|rs6796883|rs17391840|rs2283877|rs2667100|rs13119090|rs1816971|rs628542|rs10515068|rs7719763|rs3746188|rs863818|rs951850|rs613229|rs872077
Oral Ulcers	NATURAL_KILLER_CELL_MEDIATED_CYTOTOXICITY	KEGG	3607	31	20	2,62E-02	4,30E-01	rs2853923|rs6852347|rs10940145|rs7396875|rs809192|rs7726943|rs16897175|rs11683207|rs227913|rs9658702|rs2372183|rs6096351|rs10866819|rs290240|rs4976028|rs13434873|rs4647693|rs9266629|rs1332684|rs9357122
Oral Ulcers	EDG1_PATHWAY	BIOCARTA	1432	46	20	2,65E-02	4,30E-01	rs10940145|rs7726943|rs16897175|rs227913|rs2372183|rs12409786|rs4976028|rs16824774|rs6577244|rs227142|rs373983|rs17605348|rs11658221|rs1983967|rs12407294|rs9892886|rs11860367|rs10515068|rs12407211|rs13021389
Oral Ulcers	SIGNALING_BY_CONSTITUTIVELY_ACTIVE_EGFR	REACTOME	503	54	20	2,70E-02	4,30E-01	rs10940145|rs7396875|rs7726943|rs16897175|rs4690785|rs2372183|rs4976028|rs17673576|rs10515068|rs7719763|rs863818|rs2877260|rs10515059|rs11976696|rs2699905|rs1490791|rs7379303|rs7645550|rs3815701|rs2510139
Oral Ulcers	CELL_SURFACE_INTERACTIONS_AT_THE_VASCULAR_WALL	REACTOME	2650	36	20	2,76E-02	4,30E-01	rs10940145|rs7396875|rs809192|rs7726943|rs2022950|rs10461535|rs1176387|rs2680182|rs952784|rs12524876|rs1516640|rs810687|rs2372183|rs4976028|rs1029999|rs182832|rs12755775|rs17605348|rs421587|rs11658221
Oral Ulcers	AKT_PATHWAY	BIOCARTA	682	47	20	2,89E-02	4,30E-01	rs10940145|rs7726943|rs16897175|rs2372183|rs4976028|rs9576991|rs2267172|rs11580042|rs10515068|rs7719763|rs9566508|rs17254382|rs863818|rs2955234|rs10515059|rs12153009|rs230530|rs13157622|rs4292454|rs17446614

Oral Ulcers	NO1_PATHWAY	BIOCARTA	1482	35	20	2,89E-02	4,30E-01	rs2373929|rs17709898|rs743507|rs7964046|rs10823962|rs2167568|rs7893773|rs904132|rs11133360|rs1896610|rs1297783|rs16925106|rs1030460|rs1002063|rs10490975|rs211085|rs931640|rs7898516|rs1409052|rs6671391
Oral Ulcers	ABC_TRANSPORTERS	KEGG	2684	37	20	2,91E-02	4,30E-01	rs2373929|rs743507|rs215066|rs2274410|rs4721914|rs2074315|rs1858941|rs6461509|rs7805271|rs7802291|rs1331924|rs2740484|rs9656611|rs10877201|rs2027401|rs2303924|rs3789375|rs1989886|rs600880|rs4148045
Oral Ulcers	B_CELL_RECEPTOR_SIGNALING_PATHWAY	KEGG	2278	35	20	3,18E-02	4,35E-01	rs6852347|rs10940145|rs7396875|rs7726943|rs10461535|rs2239512|rs952784|rs2372183|rs6096351|rs290240|rs4976028|rs13434873|rs182832|rs1332684|rs17020294|rs13353326|rs10951010|rs2660481|rs11860367|rs2851045
Oral Ulcers	TOB1_PATHWAY	BIOCARTA	652	50	16	3,36E-02	4,35E-01	rs12906930|rs2965012|rs11631839|rs6680614|rs4658265|rs7552931|rs502514|rs706779|rs202654|rs12958604|rs2949659|rs266366|rs2243290|rs8110090|rs4803455|rs16859085
Oral Ulcers	BCR_PATHWAY	BIOCARTA	1053	40	20	3,39E-02	4,35E-01	rs6852347|rs7396875|rs952784|rs227913|rs6096351|rs290240|rs13434873|rs182832|rs2660481|rs9892886|rs11860367|rs2851045|rs17718517|rs1297783|rs931640|rs8051034|rs7236492|rs1551746|rs10120669|rs7834615
Oral Ulcers	COSTIMULATION_BY_THE_CD28_FAMILY	REACTOME	1479	42	20	3,41E-02	4,35E-01	rs10940145|rs809192|rs7726943|rs16897175|rs952784|rs1344638|rs2372183|rs3997854|rs4976028|rs182832|rs502514|rs2660481|rs1741143|rs10515068|rs17718517|rs13003194|rs7719763|rs3746188|rs8139544|rs874627
Oral Ulcers	ARF_PATHWAY	BIOCARTA	733	54	20	3,48E-02	4,35E-01	rs10940145|rs7726943|rs10461535|rs2372183|rs4976028|rs11244164|rs6924483|rs10515068|rs16893188|rs7719763|rs10092081|rs719667|rs863818|rs492553|rs11792889|rs1535565|rs10515059|rs2049824|rs311572|rs3789724
Oral Ulcers	ACH_PATHWAY	BIOCARTA	602	53	19	3,52E-02	4,35E-01	rs10940145|rs7726943|rs16897175|rs10980573|rs2372183|rs4976028|rs2267172|rs11580042|rs10515068|rs7719763|rs863818|rs10980551|rs10515059|rs2699905|rs1490791|rs7379303|rs7645550|rs3815701|rs16897819
Oral Ulcers	INTEGRIN_CELL_SURFACE_INTERACTIONS	REACTOME	2614	45	20	3,55E-02	4,35E-01	rs6974002|rs10489469|rs1074801|rs11657731|rs668842|rs6854563|rs1075188|rs9301436|rs810687|rs290240|rs6973683|rs10991|rs1029999|rs6942446|rs10178458|rs17605348|rs508594|rs421587|rs11658221|rs4764521
Oral Ulcers	DILATED_CARDIOMYOPATHY	KEGG	4636	25	20	3,63E-02	4,35E-01	rs6974002|rs263241|rs1541821|rs628258|rs3774609|rs2965012|rs12334868|rs2108639|rs697928|rs9507041|rs16904360|rs479623|rs1075188|rs2283551|rs2283984|rs1536723|rs2256156|rs2282755|rs1019731|rs762898
Oral Ulcers	CGMP_EFFECTS	REACTOME	1520	35	20	3,64E-02	4,35E-01	rs13419755|rs7964046|rs7762369|rs10823962|rs11690740|rs7893773|rs12619532|rs6759996|rs988146|rs10034450|rs2251273|rs13064262|rs12495203|rs1896610|rs1430145|rs10497484|rs3008008|rs756576|rs4275567|rs1030460
Oral Ulcers	RAC1_PATHWAY	BIOCARTA	763	49	20	3,67E-02	4,35E-01	rs10940145|rs7726943|rs16897175|rs2372183|rs4976028|rs2660481|rs10515068|rs7719763|rs17718517|rs1292035|rs7719763|rs820336|rs863818|rs4530474|rs2660489|rs10515059|rs1559715|rs83615|rs2689420
Oral Ulcers	SYNTHESIS_OF_PIPS_AT_THE_GOLGI_MEMBRANE	REACTOME	788	15	7	3,72E-02	4,35E-01	rs1941593|rs12964783|rs1816971|rs7505711|rs7194558|rs9953631|rs1461710
Oral Ulcers	ANTIGEN_ACTIVATES_B_CELL_RECEPTOR_LEADING_TO_GENERATION_OF_SECOND_MES	REACTOME	1299	44	20	3,77E-02	4,35E-01	rs10940145|rs809192|rs7726943|rs16897175|rs952784|rs2372183|rs290240|rs4976028|rs182832|rs2660481|rs2399106|rs10515068|rs17718517|rs1297783|rs4888197|rs7719763|rs4888191|rs931640|rs863818|rs1551746
Oral Ulcers	NDKDYNAMIN_PATHWAY	BIOCARTA	548	39	20	3,79E-02	4,35E-01	rs6852347|rs934826|rs1866236|rs2276575|rs13434873|rs6713701|rs2851045|rs10499605|rs1297783|rs931640|rs2118458|rs3003610|rs495942|rs17489570|rs744373|rs2240853|rs904160|rs2534578|rs7259810|rs12701650
Oral Ulcers	TASTE_TRANSDUCTION	KEGG	1100	32	20	3,80E-02	4,35E-01	rs263241|rs4926290|rs12334868|rs16904360|rs17199565|rs936213|rs2495971|rs12536735|rs4920552|rs4908932|rs1430145|rs2495973|rs7204560|rs874627|rs3935570|rs17221445|rs4666589|rs7524898|rs872077|rs152745
Oral Ulcers	NGF_SIGNALLING_VIA_TRKA_FROM_THE_PLASMA_MEMBRANE	REACTOME	4847	37	20	3,80E-02	4,35E-01	rs770466|rs263241|rs10940145|rs10489469|rs7396875|rs1541821|rs7726943|rs13311977|rs12334868|rs16897175|rs16904360|rs2256156|rs227913|rs2372183|rs281508|rs4976028|rs1922529|rs1422189|rs72001|rs9576991
Oral Ulcers	NGF_PATHWAY	BIOCARTA	566	48	19	3,82E-02	4,35E-01	rs10940145|rs7396875|rs7726943|rs10461535|rs2372183|rs4976028|rs205898|rs10515068|rs7719763|rs863818|rs10515059|rs4326648|rs2699905|rs1490791|rs7379303|rs8021524|rs7645550|rs3815701|rs16897819
Oral Ulcers	EPO_PATHWAY	BIOCARTA	255	6	4	3,88E-02	4,36E-01	rs7396875|rs205898|rs4326648|rs8021524
Oral Ulcers	PLATELET_HOMEOSTASIS	REACTOME	4080	30	20	3,97E-02	4,40E-01	rs2373929|rs743507|rs13419755|rs7964046|rs13064158|rs7762369|rs10823962|rs11690740|rs26797|rs6590911|rs354263|rs6442178|rs10895184|rs1840572|rs7893773|rs768710|rs12619532|rs6759996|rs988146|rs10034450
Oral Ulcers	T_CELL_RECEPTOR_SIGNALING_PATHWAY	KEGG	3546	38	20	4,17E-02	4,50E-01	rs6852347|rs10940145|rs21986|rs7396875|rs809192|rs7726943|rs10461535|rs3769504|rs1344638|rs11683207|rs2372183|rs3754806|rs6096351|rs4976028|rs936213|rs13434873|rs711272|rs1332684|rs30117|rs17020294
Oral Ulcers	DOWNSTREAM_TCR_SIGNALING	REACTOME	1573	39	20	4,34E-02	4,50E-01	rs10940145|rs7726943|rs10461535|rs1351726|rs7841459|rs2372183|rs3997854|rs4976028|rs711272|rs4507261|rs10745|rs10508312|rs4613794|rs10951010|rs502514|rs10515068|rs10097279|rs11107008|rs7719763|rs12205556
Oral Ulcers	SIGNALING_BY_SCF_KIT	REACTOME	1989	48	20	4,37E-02	4,50E-01	rs10940145|rs7396875|rs809192|rs7726943|rs17809666|rs16897175|rs952784|rs227913|rs2372183|rs4976028|rs182832|rs9576991|rs1968228|rs2660481|rs9892886|rs10515068|rs17718517|rs34322|rs7719763|rs9566508
Oral Ulcers	ARRHYTHMOGENIC_RIGHT_VENTRICULAR_CARDIOMYOPATHY_ARVC	KEGG	5871	42	20	4,38E-02	4,50E-01	rs6974002|rs10844435|rs10844440|rs7072330|rs628258|rs13191992|rs3774609|rs2108639|rs697928|rs9507041|rs1075188|rs2283551|rs2283984|rs1536723|rs2282755|rs762898|rs6973683|rs768710|rs12631566|rs10049492
Oral Ulcers	PROLONGED_ERK_ACTIVATION_EVENTS	REACTOME	429	24	8	4,42E-02	4,50E-01	rs10489469|rs7396875|rs508594|rs1888861|rs716851|rs10173234|rs943851|rs3012750
Oral Ulcers	CALCIUM_SIGNALING_PATHWAY	KEGG	9275	27	20	4,45E-02	4,50E-01	rs2373929|rs6852347|rs743507|rs263241|rs4926290|rs753724|rs13064158|rs1541821|rs6090338|rs1317242|rs9922023|rs3774609|rs12334868|rs1328684|rs6306|rs890707|rs4074889|rs6804671|rs7689418|rs16904360
Oral Ulcers	RNA_POL_III_TRANSCRIPTION	REACTOME	861	30	20	4,45E-02	4,50E-01	rs6081172|rs6928833|rs10810056|rs12375629|rs1372348|rs35076|rs6924483|rs579096|rs13302756|rs10961346|rs1853195|rs7021837|rs7355118|rs7148769|rs10122910|rs137831|rs492553|rs393543|rs1535565|rs2072102
Oral Ulcers	CLASS_I_MHC_MEDIATED_ANTIGEN_PROCESSING_PRESENTATION	REACTOME	5895	45	20	4,57E-02	4,53E-01	rs4072410|rs2853923|rs11730732|rs1626645|rs1894752|rs2294183|rs6545358|rs12533310|rs3749316|rs328349|rs10490578|rs5750326|rs2644494|rs3873381|rs399672|rs12519126|rs952901|rs1549487|rs17108797|rs1002941
Oral Ulcers	BAD_PATHWAY	BIOCARTA	927	43	20	4,63E-02	4,53E-01	rs10940145|rs7726943|rs16897175|rs7169544|rs1019731|rs2372183|rs4976028|rs2267172|rs1982673|rs10515068|rs7719763|rs6214|rs863818|rs7524898|rs872077|rs10515059|rs2156192|rs7526798|rs3729877|rs2046136
Oral Ulcers	DOWNSTREAM_SIGNAL_TRANSDUCTION	REACTOME	3083	43	20	4,68E-02	4,53E-01	rs263241|rs10940145|rs7396875|rs1541821|rs7726943|rs12334868|rs16897175|rs16904360|rs3769504|rs2256156|rs227913|rs2372183|rs281508|rs3754806|rs4976028|rs72001|rs9576991|rs7566051|rs4851835|rs9892886
Oral Ulcers	PKA_MEDIATED_PHOSPHORYLATION_OF_CREB	REACTOME	767	36	20	4,81E-02	4,53E-01	rs263241|rs1541821|rs12334868|rs16904360|rs2256156|rs72001|rs7566051|rs2058166|rs1593933|rs3892156|rs2601787|rs1993716|rs17221445|rs7524898|rs872077|rs7725101|rs7526798|rs3729877|rs3338087|rs2239310
Oral Ulcers	LYSOSOME	KEGG	2448	37	20	4,85E-02	4,53E-01	rs1642272|rs4704458|rs26529|rs10889147|rs7607927|rs3850615|rs748630|rs1871093|rs1897759|rs13100173|rs1434039|rs4747195|rs373983|rs2183933|rs1019367|rs1759269|rs1904432|rs11956308|rs7908969|rs10009214
Oral Ulcers	INSULIN_PATHWAY	BIOCARTA	725	47	20	4,86E-02	4,53E-01	rs10940145|rs7396875|rs7726943|rs10461535|rs2372183|rs4976028|rs1922529|rs205898|rs10515068|rs7719763|rs863818|rs10515059|rs4326648|rs4804366|rs2699905|rs1490791|rs7379303|rs8021524|rs7645550|rs11066323
Oral Ulcers	CTCF_PATHWAY	BIOCARTA	1035	52	20	4,88E-02	4,53E-01	rs10940145|rs7726943|rs2965012|rs16897175|rs2372183|rs6680614|rs4976028|rs4658265|rs6692647|rs10515068|rs16893188|rs34322|rs1292035|rs7719763|rs10092081|rs863818|rs12958604|rs10515059|rs7070956|rs2049824
Oral Ulcers	PANCREATIC_CANCER	KEGG	1920	51	20	4,99E-02	4,56E-01	rs10940145|rs12906930|rs7726943|rs13311977|rs2965012|rs11631839|rs10461535|rs2333512|rs2372183|rs4976028|rs310209|rs6692647|rs2575676|rs3764351|rs17673576|rs10515068|rs7719763|rs3746188|rs3749791|rs863818
Oral Ulcers	DOWNREGULATION_OF_SMAD2_3_SMAD4_TRANSCRIPTIONAL_ACTIVITY	REACTOME	453	44	17	5,05E-02	4,56E-01	rs12906930|rs10773086|rs11631839|rs7512482|rs838511|rs12426133|rs7225993|rs12958604|rs937341|rs11874742|rs266366|rs9961435|rs263526|rs1242991|rs16955746|rs9635893|rs906304
Oral Ulcers	ARMS_MEDIATED_ACTIVATION	REACTOME	393	24	8	5,09E-02	4,56E-01	rs10489469|rs7396875|rs508594|rs1888861|rs716851|rs10173234|rs943851|rs3012750
Oral Ulcers	REGULATION_OF_KIT_SIGNALING	REACTOME	587	45	14	5,33E-02	4,64E-01	rs809192|rs17809666|rs952784|rs227913|rs182832|rs9892886|rs7834615|rs6504424|rs1077156|rs17813240|rs8078231|rs2510152|rs1004420|rs2237259
Oral Ulcers	GLIOMA	KEGG	2070	41	20	5,36E-02	4,64E-01	rs10940145|rs7396875|rs7726943|rs10461535|rs7169544|rs4074889|rs919741|rs3822295|rs227913|rs1019731|rs2372183|rs4976028|rs2285703|rs9892886|rs11860367|rs17673576|rs10515068|rs1297783|rs10519194|rs4888197
Oral Ulcers	PROTEIN_EXPORT	KEGG	750	22	8	5,38E-02	4,64E-01	rs721355|rs2940385|rs1528036|rs2050042|rs1354991|rs3829406|rs12433985|rs1220783
Oral Ulcers	G_ALPHA1213_SIGNALLING_EVENTS	REACTOME	2855	39	20	5,43E-02	4,64E-01	rs852350|rs701724|rs10940145|rs7726943|rs6018981|rs16897175|rs2291049|rs2372183|rs2833264|rs4976028|rs1332684|rs17020294|rs2660481|rs7543435|rs10515068|rs2833470|rs17718517|rs1086250|rs11130558|rs7719763
Oral Ulcers	SIGNALING_BY_FGFR1_MUTANTS	REACTOME	1243	45	20	5,47E-02	4,64E-01	rs10940145|rs7726943|rs10461535|rs11167777|rs2372183|rs4976028|rs12542904|rs17487009|rs9510050|rs152442|rs12523624|rs12020729|rs10515068|rs733895|rs750535|rs365836|rs9366078|rs263932|rs2290755|rs7719763
Oral Ulcers	DEFENSINS	REACTOME	495	46	11	5,55E-02	4,66E-01	rs11776120|rs4710181|rs717064|rs2367486|rs6057657|rs9349534|rs874627|rs10867025|rs2180584|rs1555679|rs4300027
Oral Ulcers	SIGNALING_BY_FGFR_MUTANTS	REACTOME	1657	39	20	5,95E-02	4,91E-01	rs10940145|rs7396875|rs7726943|rs16897175|rs4690785|rs11167777|rs2372183|rs4976028|rs12542904|rs17487009|rs9510050|rs152442|rs12523624|rs12020729|rs11199823|rs10515068|rs733895|rs750535|rs10788173|rs365836
Oral Ulcers	CD28_DEPENDENT_PI3K_AKT_SIGNALING	REACTOME	572	48	19	6,08E-02	4,91E-01	rs10940145|rs809192|rs7726943|rs16897175|rs2372183|rs4976028|rs10515068|rs7719763|rs3746188|rs863818|rs10515059|rs2699905|rs1490791|rs7379303|rs7645550|rs3815701|rs1004420|rs16897819|rs2237259
Oral Ulcers	SYNTHESIS_AND_INTERCONVERSION_OF_NUCLEOTIDE_DI_AND_TRIPHOSPHATES	REACTOME	306	12	7	6,08E-02	4,91E-01	rs2853229|rs2278092|rs17100521|rs3779647|rs2304891|rs743961|rs2030598
Oral Ulcers	RACCYCD_PATHWAY	BIOCARTA	551	46	20	6,09E-02	4,91E-01	rs10940145|rs7396875|rs7726943|rs16897175|rs2372183|rs4976028|rs10515068|rs34322|rs7719763|rs863818|rs10515059|rs230530|rs1321311|rs2689420|rs2699905|rs1490791|rs7379303|rs7645550|rs3818411|rs3815701
Oral Ulcers	INSULIN_SYNTHESIS_AND_PROCESSING	REACTOME	1033	55	20	6,31E-02	4,98E-01	rs10485567|rs10483681|rs9299670|rs7272324|rs7662154|rs753129|rs6044586|rs7143894|rs4976178|rs852107|rs10074809|rs2286746|rs12106074|rs1845376|rs17687250|rs1459506|rs7906224|rs12206548|rs2206161|rs6136105
Oral Ulcers	HCMV_PATHWAY	BIOCARTA	547	44	17	6,32E-02	4,98E-01	rs10940145|rs7726943|rs16897175|rs2372183|rs4976028|rs10515068|rs2364598|rs7719763|rs863818|rs10515059|rs230530|rs2699905|rs1490791|rs7379303|rs7645550|rs3815701|rs16897819
Oral Ulcers	NEGATIVE_REGULATORS_OF_RIG_I_MDA5_SIGNALING	REACTOME	464	25	15	6,37E-02	4,98E-01	rs7699006|rs9909750|rs26529|rs3813982|rs2144825|rs1244837|rs10783580|rs11868917|rs668033|rs728868|rs7198642|rs12878111|rs5743291|rs3247831|rs16948923
Oral Ulcers	LONG_TERM_POTENTIATION	KEGG	3620	27	20	6,47E-02	5,01E-01	rs6852347|rs263241|rs10489469|rs7396875|rs9922023|rs12334868|rs4074889|rs6804671|rs16904360|rs919741|rs3822295|rs227913|rs936213|rs13434873|rs227142|rs508594|rs2285703|rs2119802|rs11055671|rs3805021
Oral Ulcers	ALK_PATHWAY	BIOCARTA	1932	24	20	6,53E-02	5,01E-01	rs770466|rs10933441|rs2965012|rs1858577|rs2254779|rs6680614|rs1422189|rs711272|rs1026487|rs4658265|rs2469109|rs1005351|rs6692647|rs4613794|rs924311|rs2540590|rs6771638|rs12114452|rs2685127|rs17126825
Oral Ulcers	RNA_POL_III_TRANSCRIPTION_INITIATION_FROM_TYPE_2_PROMOTER	REACTOME	417	22	12	6,73E-02	5,11E-01	rs6081172|rs6928833|rs1372348|rs6924483|rs579096|rs137831|rs492553|rs393543|rs1535565|rs2072102|rs729360|rs10089021
Oral Ulcers	APOPTOSIS	KEGG	1727	38	20	7,01E-02	5,23E-01	rs6852347|rs10940145|rs7726943|rs10461535|rs9658702|rs2372183|rs10866819|rs4976028|rs13434873|rs4647693|rs39425|rs11580042|rs2851045|rs1982673|rs10515068|rs560978|rs7910825|rs7719763|rs1926189|rs3746188
Oral Ulcers	CDT1_ASSOCIATION_WITH_THE_CDC6_ORC_ORIGIN_COMPLEX	REACTOME	793	54	17	7,06E-02	5,23E-01	rs2294183|rs194825|rs328349|rs7808273|rs8066176|rs8009527|rs4710834|rs3820667|rs4148876|rs2584624|rs1045288|rs3859192|rs1543947|rs1023238|rs653079|rs4980335|rs2597189
Oral Ulcers	IL6_PATHWAY	BIOCARTA	401	22	9	7,08E-02	5,23E-01	rs7396875|rs7801617|rs205898|rs310209|rs12535797|rs7409343|rs4326648|rs8021524|rs11066323
Oral Ulcers	TCR_SIGNALING	REACTOME	2040	39	20	7,39E-02	5,29E-01	rs10940145|rs7726943|rs10461535|rs11683207|rs1351726|rs7841459|rs2372183|rs3997854|rs4976028|rs711272|rs4507261|rs30117|rs895275|rs510745|rs10508312|rs4613794|rs10951010|rs502514|rs10515068|rs7523940
Oral Ulcers	CHRONIC_MYELOID_LEUKEMIA	KEGG	2538	50	20	7,46E-02	5,29E-01	rs10940145|rs12906930|rs7396875|rs7726943|rs12632728|rs2965012|rs11631839|rs16897175|rs2372183|rs4976028|rs11244164|rs8126845|rs6692647|rs900030|rs17643974|rs2399106|rs10515068|rs10519194|rs16893188|rs2252585
Oral Ulcers	DEPOSITION_OF_NEW_CENPA_CONTAINING_NUCLEOSOMES_AT_THE_CENTROMERE	REACTOME	637	24	8	7,48E-02	5,29E-01	rs2051186|rs8132833|rs9302109|rs1980705|rs10514815|rs9379780|rs17690723|rs7755833
Oral Ulcers	G_ALPHA_S_SIGNALLING_EVENTS	REACTOME	5019	27	20	7,48E-02	5,29E-01	rs263241|rs13419755|rs7964046|rs13227318|rs1541821|rs12334868|rs7762369|rs890707|rs11690740|rs16904360|rs17611047|rs4305747|rs1078523|rs2256156|rs12411535|rs859253|rs324978|rs1320301|rs1992139|rs12619532
Oral Ulcers	RNA_POL_III_TRANSCRIPTION_INITIATION_FROM_TYPE_3_PROMOTER	REACTOME	501	32	16	7,64E-02	5,29E-01	rs6081172|rs6928833|rs12375629|rs1372348|rs6924483|rs579096|rs7355118|rs7148769|rs137831|rs492553|rs1535565|rs2072102|rs3810213|rs16858688|rs729360|rs10089021
Oral Ulcers	MET_PATHWAY	BIOCARTA	1415	63	20	7,72E-02	5,29E-01	rs10940145|rs10489469|rs7396875|rs7726943|rs10461535|rs4690785|rs2372183|rs4976028|rs508594|rs2532699|rs10515068|rs7719763|rs1002063|rs6887205|rs11009157|rs863818|rs10515059|rs10273273|rs2239206|rs7070956
Oral Ulcers	REGULATION_OF_ACTIN_CYTOSKELETON	KEGG	7089	40	20	7,77E-02	5,29E-01	rs6974002|rs701724|rs10940145|rs9846559|rs219862|rs9538396|rs7396875|rs7726943|rs1953128|rs11657731|rs9557798|rs10461535|rs6854563|rs1075188|rs11167777|rs9528021|rs1525910|rs7996198|rs2372183|rs2833264
Oral Ulcers	ANTIVIRAL_MECHANISM_BY_IFN_STIMULATED_GENES	REACTOME	1077	28	20	7,81E-02	5,29E-01	rs7699006|rs9909750|rs4611262|rs8127870|rs10852553|rs3851366|rs310209|rs2094881|rs12024682|rs839241|rs11107008|rs4684126|rs4869498|rs1718483|rs1616903|rs11150846|rs12472874|rs1407439|rs1868917|rs2383477
Oral Ulcers	CHONDROITIN_SULFATE_BIOSYNTHESIS	REACTOME	846	43	20	7,82E-02	5,29E-01	rs2339331|rs219233|rs2433591|rs4886456|rs2591454|rs7966281|rs7812088|rs33598|rs765940|rs7071860|rs608923|rs8089993|rs6983139|rs1607483|rs7972201|rs781677|rs11247267|rs11112149|rs2559640|rs9967331
Oral Ulcers	OOCYTE_MEIOSIS	KEGG	3114	25	20	8,02E-02	5,29E-01	rs6852347|rs263241|rs1541821|rs12334868|rs7169544|rs4074889|rs6804671|rs16904360|rs919741|rs3822295|rs2256156|rs1019731|rs13434873|rs72001|rs2025258|rs2285703|rs2119802|rs136569|rs3805021|rs2267172
Oral Ulcers	PTEN_PATHWAY	BIOCARTA	544	47	20	8,15E-02	5,29E-01	rs10940145|rs7726943|rs16897175|rs2372183|rs4976028|rs11580042|rs10515068|rs34322|rs7719763|rs1009157|rs863818|rs10515059|rs2239953|rs7070956|rs2153875|rs2230392|rs2699905|rs1490791|rs7379303|rs7645550
Oral Ulcers	SIGNALING_BY_PDGF	REACTOME	4427	33	20	8,15E-02	5,29E-01	rs263241|rs10940145|rs3811159|rs7396875|rs1541821|rs7726943|rs12334868|rs16897175|rs16904360|rs3769504|rs2256156|rs227913|rs9301436|rs810687|rs2372183|rs281508|rs3754806|rs4976028|rs1029999|rs72001
Oral Ulcers	REGULATION_OF_INSULIN_SECRETION	REACTOME	3741	35	20	8,19E-02	5,29E-01	rs263241|rs4926290|rs10489469|rs4131677|rs721355|rs12334868|rs2239512|rs686699|rs16904360|rs7272324|rs2241952|rs227913|rs2074315|rs936213|rs227142|rs10869950|rs1162211|rs508594|rs10925809|rs1144430
Oral Ulcers	REGULATION_OF_AUTOPHAGY	KEGG	933	32	16	8,21E-02	5,29E-01	rs10512488|rs1941593|rs26529|rs6696559|rs12964783|rs1001684|rs7505711|rs3818637|rs9953631|rs4977686|rs17316576|rs1461710|rs7630417|rs3732817|rs8075157|rs3792826
Oral Ulcers	AMINO_ACID_AND_OLIGOPEPTIDE_SLC_TRANSPORTERS	REACTOME	1707	40	20	8,21E-02	5,29E-01	rs1366632|rs1023681|rs2562582|rs11741316|rs9883639|rs1320301|rs1980943|rs867422|rs1384319|rs10833748|rs9036|rs11206093|rs17244832|rs7983644|rs12644755|rs3760670|rs7583682|rs828933|rs12543594|rs17279465
Oral Ulcers	ACETYLCHOLINE_BINDING_AND_DOWNSTREAM_EVENTS	REACTOME	242	13	4	8,24E-02	5,29E-01	rs2167568|rs8032156|rs10026108|rs4861065
Oral Ulcers	ELONGATION_ARREST_AND_RECOVERY	REACTOME	246	8	6	8,29E-02	5,29E-01	rs4487686|rs2257205|rs1131384|rs2072102|rs1613070|rs8102597
Oral Ulcers	TRANSMISSION_ACROSS_CHEMICAL_SYNAPSES	REACTOME	9089	33	20	8,76E-02	5,44E-01	rs1264866|rs1366632|rs263241|rs1023681|rs4926290|rs7396875|rs1541821|rs11654455|rs9322668|rs9922023|rs2562582|rs12334868|rs16837945|rs697928|rs11741316|rs16904360|rs1232222|rs919741|rs10063828|rs7549591
Oral Ulcers	SYNTHESIS_SECRETION_AND_DEACYLATION_OF_GHRELIN	REACTOME	624	43	15	8,91E-02	5,44E-01	rs721355|rs12351531|rs7864185|rs1051236|rs4979136|rs10074809|rs4979065|rs7037931|rs1707709|rs2955234|rs10816540|rs4731437|rs10217338|rs1440678|rs7653493
Oral Ulcers	PAR1_PATHWAY	BIOCARTA	1941	40	20	8,92E-02	5,44E-01	rs10940145|rs6981540|rs7726943|rs16897175|rs1861954|rs227913|rs2372183|rs4976028|rs711272|rs227142|rs4613794|rs9892886|rs11860367|rs17830077|rs4773326|rs10515068|rs12600950|rs3767423|rs4876265|rs11130558
Oral Ulcers	PYRUVATE_METABOLISM	KEGG	908	35	20	8,95E-02	5,44E-01	rs1019635|rs7105863|rs11983987|rs10973340|rs10516988|rs3741049|rs1885490|rs1945146|rs788953|rs2181139|rs6050291|rs6037013|rs2048022|rs7047872|rs1646744|rs2057374|rs674965|rs2408700|rs10516985|rs15677
Oral Ulcers	SIGNALING_BY_ERBB2	REACTOME	3847	41	20	9,05E-02	5,44E-01	rs263241|rs10940145|rs7396875|rs1541821|rs809192|rs7726943|rs12334868|rs10461535|rs16904360|rs4690785|rs2256156|rs227913|rs2372183|rs281508|rs4976028|rs72001|rs9576991|rs16847152|rs7566051|rs9892886
Oral Ulcers	SIGNALING_BY_FGFR1_FUSION_MUTANTS	REACTOME	639	52	20	9,13E-02	5,44E-01	rs10940145|rs7726943|rs16897175|rs2372183|rs4976028|rs10515068|rs365836|rs9366078|rs7719763|rs863818|rs10784765|rs1087818|rs10515059|rs5759636|rs2529232|rs2699905|rs2017342|rs6581864|rs1490791|rs7379303
Oral Ulcers	SIGNALING_BY_RHO_GTPASES	REACTOME	4877	36	20	9,14E-02	5,44E-01	rs852350|rs2778665|rs6894946|rs701724|rs6018981|rs1350136|rs4859319|rs2291049|rs2833264|rs7511739|rs258800|rs1332684|rs17157602|rs3813982|rs2778652|rs7093726|rs17020294|rs11943039|rs10026556|rs1715608
Oral Ulcers	DOWNSTREAM_SIGNALING_OF_ACTIVATED_FGFR	REACTOME	3636	36	20	9,17E-02	5,44E-01	rs263241|rs10940145|rs7396875|rs1541821|rs7726943|rs12334868|rs10461535|rs16904360|rs4690785|rs11167777|rs2256156|rs227913|rs2372183|rs281508|rs4976028|rs72001|rs12542904|rs17487009|rs9576991|rs9510050
Oral Ulcers	BCELLSURVIVAL_PATHWAY	BIOCARTA	500	52	20	9,21E-02	5,44E-01	rs10940145|rs7726943|rs10461535|rs2372183|rs4976028|rs4647693|rs10515068|rs7719763|rs6887205|rs11009157|rs863818|rs10515059|rs2153875|rs2699905|rs1490791|rs7379303|rs8021524|rs7645550|rs3815701|rs16897819
Oral Ulcers	PI_METABOLISM	REACTOME	1808	43	20	9,21E-02	5,44E-01	rs10940145|rs1941593|rs7726943|rs10461535|rs2372183|rs4976028|rs3775601|rs6796883|rs17391840|rs2283877|rs2667100|rs12964783|rs13119090|rs1816971|rs628542|rs10515068|rs1429871|rs7719763|rs3746188|rs7194558
Oral Ulcers	LATE_PHASE_OF_HIV_LIFE_CYCLE	REACTOME	1259	29	20	9,31E-02	5,46E-01	rs4611262|rs6928833|rs6508425|rs4487686|rs2257205|rs10852553|rs2756382|rs1131384|rs3734261|rs12024682|rs6142711|rs4150454|rs16902819|rs595158|rs10500836|rs4684126|rs11756329|rs4869498|rs2235852|rs11752001
Oral Ulcers	ABORTIVE_ELONGATION_OF_HIV1_TRANSCRIPT_IN_THE_ABSENCE_OF_TAT	REACTOME	195	8	6	9,57E-02	5,50E-01	rs4487686|rs2257205|rs1131384|rs2072102|rs1613070|rs8102597
Oral Ulcers	RNA_POL_II_PRE_TRANSCRIPTION_EVENTS	REACTOME	532	39	18	9,69E-02	5,50E-01	rs6928833|rs6508425|rs4487686|rs2257205|rs1131384|rs3734261|rs6142711|rs4150454|rs16902819|rs2072102|rs1613070|rs7142641|rs2532929|rs4151374|rs4150667|rs2020892|rs8102597|rs1471420
Oral Ulcers	DIABETES_PATHWAYS	REACTOME	3395	42	20	9,77E-02	5,50E-01	rs9284892|rs10485567|rs10483681|rs721355|rs9299670|rs4661681|rs7272324|rs12351531|rs1714748|rs7864185|rs198972|rs7216531|rs8061066|rs7662154|rs753129|rs6044586|rs7143894|rs10512367|rs4976178|rs4979136
Oral Ulcers	CDC42RAC_PATHWAY	BIOCARTA	435	51	20	9,80E-02	5,50E-01	rs10940145|rs7726943|rs16897175|rs268852|rs2372183|rs4976028|rs10515068|rs7719763|rs863818|rs2287064|rs688242|rs10515059|rs3779258|rs3549591|rs2689420|rs2699905|rs1490791|rs7379303|rs7645550|rs3815701
Oral Ulcers	FC_EPSILON_RI_SIGNALING_PATHWAY	KEGG	2758	38	20	9,87E-02	5,50E-01	rs10940145|rs7396875|rs809192|rs7726943|rs16897175|rs952784|rs227913|rs2372183|rs281508|rs354263|rs290240|rs4976028|rs182832|rs1332684|rs7542020|rs17020294|rs2575676|rs2660481|rs9892886|rs11860367
Oral Ulcers	INTEGRATION_OF_ENERGY_METABOLISM	REACTOME	4501	32	20	9,98E-02	5,50E-01	rs263241|rs4926290|rs10489469|rs4131677|rs1541821|rs721355|rs12334868|rs2239512|rs686699|rs16904360|rs7272324|rs2241952|rs2256156|rs227913|rs2074315|rs936213|rs72001|rs227142|rs10869950|rs1162211
Oral Ulcers	FOCAL_ADHESION	KEGG	7733	40	20	1,00E-01	5,50E-01	rs6974002|rs17709898|rs10940145|rs10489469|rs219862|rs3811159|rs7396875|rs809192|rs7726943|rs10461535|rs6494330|rs7169544|rs6854563|rs1075188|rs2333512|rs227913|rs9301436|rs810687|rs1019731|rs2372183
Oral Ulcers	TYPE_II_DIABETES_MELLITUS	KEGG	2053	58	20	1,01E-01	5,50E-01	rs10940145|rs4926290|rs7726943|rs3774609|rs10461535|rs2074315|rs2372183|rs281508|rs4976028|rs1922529|rs10049492|rs7294534|rs10161181|rs2575676|rs8074003|rs923273|rs10515068|rs9588044|rs4953334|rs7719763

Oral Ulcers	HYPERTROPHIC_CARDIOMYOPATHY_HCM	KEGG	4149	30	20	1,02E-01	5,50E-01	rs6974002|rs628258|rs3774609|rs2965012|rs2108639|rs697928|rs9507041|rs479623|rs1075188|rs2283551|rs2283984|rs1536723|rs2282755|rs1019731|rs762898|rs7801617|rs6973683|rs768710|rs12631566|rs2393550
Oral Ulcers	P75_NTR_RECEPTOR_MEDIATED_SIGNALLING	REACTOME	2669	24	20	1,02E-01	5,50E-01	rs852350|rs701724|rs6018981|rs2667783|rs1351726|rs7841459|rs2291049|rs2833264|rs4647693|rs1332684|rs17020294|rs12900004|rs2660481|rs7543435|rs10097279|rs2833470|rs17718517|rs726430|rs2269757|rs11086250
Oral Ulcers	CHONDROITIN_SULFATE_DERMATAN_SULFATE_METABOLISM	REACTOME	2784	34	20	1,03E-01	5,50E-01	rs4949320|rs2182542|rs11751562|rs2339331|rs219233|rs13100173|rs2433591|rs4886456|rs2591454|rs12408228|rs7966281|rs7943044|rs3828334|rs11753710|rs7812088|rs33598|rs765940|rs4707862|rs7071860|rs608923
Oral Ulcers	HER2_PATHWAY	BIOCARTA	1326	43	20	1,03E-01	5,50E-01	rs10940145|rs7396875|rs7726943|rs10461535|rs2372183|rs4976028|rs7801617|rs16847152|rs11895509|rs17673576|rs10515068|rs13053242|rs12990694|rs7719763|rs12535797|rs863818|rs10932383|rs2877260|rs2888104|rs10515059
Oral Ulcers	MELANOMA	KEGG	2894	39	20	1,03E-01	5,50E-01	rs10940145|rs9846559|rs7396875|rs7726943|rs9557798|rs16897175|rs7169544|rs11167777|rs1019731|rs1525910|rs2372183|rs4976028|rs12542904|rs17487009|rs9510050|rs152442|rs12523624|rs12532699|rs7947946|rs12020729
Oral Ulcers	CA_DEPENDENT_EVENTS	REACTOME	1487	44	20	1,03E-01	5,50E-01	rs263241|rs1541821|rs12334868|rs16904360|rs2256156|rs227913|rs354263|rs72001|rs7566051|rs9892886|rs2058166|rs926324|rs1430145|rs1297783|rs13356161|rs3892156|rs2601787|rs931640|rs2383623|rs1993716
Oral Ulcers	GLUTATHIONE_METABOLISM	KEGG	683	28	20	1,04E-01	5,51E-01	rs6088659|rs2853229|rs2278092|rs4260|rs9972327|rs2262377|rs4149186|rs3779647|rs8024587|rs1027472|rs2024092|rs7896809|rs2304891|rs4241932|rs6458861|rs17608022|rs2275984|rs7593466|rs6734788|rs10874814
Oral Ulcers	PURINE_METABOLISM	KEGG	5442	27	20	1,07E-01	5,60E-01	rs263241|rs13419755|rs7964046|rs4679640|rs1541821|rs6081172|rs12334868|rs7762369|rs11690740|rs16904360|rs940296|rs2853229|rs9493105|rs2256156|rs4487686|rs1324299|rs1840572|rs1131384|rs12619532|rs7544426
Oral Ulcers	RESPONSE_TO_ELEVATED_PLATELET_CYTOSOLIC_CA2_	REACTOME	2301	38	20	1,08E-01	5,62E-01	rs1074801|rs2965012|rs3758919|rs10889117|rs2680182|rs2274410|rs2333512|rs2288810|rs227913|rs1799852|rs4747195|rs12755775|rs17605348|rs6692647|rs11658221|rs12532699|rs4764521|rs9892886|rs11860367|rs7908969
Oral Ulcers	DOWNREGULATION_OF_TGF_BETA_RECEPTOR_SIGNALING	REACTOME	521	40	17	1,09E-01	5,63E-01	rs12906930|rs518822|rs427278|rs11631839|rs6015091|rs7225993|rs4802517|rs266366|rs3736242|rs9961435|rs8110090|rs4803455|rs16955746|rs842796|rs17414380|rs9635893|rs4810108
Oral Ulcers	GAB1_SIGNALOSOME	REACTOME	1077	56	20	1,09E-01	5,63E-01	rs10940145|rs7726943|rs16897175|rs4690785|rs2372183|rs4976028|rs9576991|rs10957993|rs17673576|rs10515068|rs34322|rs7719763|rs9566508|rs17254382|rs863818|rs2877260|rs10515059|rs7070956|rs11976696|rs1321311
Oral Ulcers	SPHINGOLIPID_METABOLISM	KEGG	1101	48	20	1,12E-01	5,75E-01	rs1642272|rs4834403|rs17221476|rs9626899|rs1667255|rs373983|rs6574916|rs9326038|rs1667271|rs7536157|rs11647934|rs42931|rs1572057|rs7917488|rs8022732|rs3115673|rs4438612|rs12650546|rs8018273|rs6842323
Oral Ulcers	PLATELET_CALCIUM_HOMEOSTASIS	REACTOME	1156	46	20	1,15E-01	5,83E-01	rs13064158|rs26797|rs6590911|rs6442178|rs10895184|rs768710|rs11917518|rs4622889|rs2373788|rs13100251|rs9842059|rs12472352|rs725027|rs1230960|rs12650123|rs2304891|rs1392171|rs808933|rs1861266|rs4713646
Oral Ulcers	STEROID_BIOSYNTHESIS	KEGG	247	9	5	1,18E-01	5,92E-01	rs2183933|rs1043180|rs7315995|rs6586179|rs12081312
Oral Ulcers	ACTIVATION_OF_KAINATE_RECEPTORS_UPON_GLUTAMATE_BINDING	REACTOME	1577	59	20	1,20E-01	5,92E-01	rs1264866|rs9322668|rs1232222|rs7549591|rs4935752|rs7768094|rs936213|rs1160752|rs227142|rs949298|rs1297783|rs1933992|rs7007251|rs9499109|rs3767087|rs6086543|rs931640|rs874627|rs10892629|rs9322604
Oral Ulcers	MTOR_PATHWAY	BIOCARTA	509	47	20	1,20E-01	5,92E-01	rs10940145|rs7726943|rs16897175|rs2372183|rs4976028|rs10515068|rs1292035|rs7719763|rs863818|rs10515059|rs2239953|rs6605631|rs7070956|rs7874234|rs2230392|rs10438740|rs2699905|rs1490791|rs7379303|rs7645550
Oral Ulcers	MTOR_SIGNALING_PATHWAY	KEGG	1290	44	20	1,20E-01	5,92E-01	rs10940145|rs7726943|rs16897175|rs2333512|rs1019731|rs2372183|rs4976028|rs378373|rs1001684|rs10515068|rs721575|rs1292035|rs7719763|rs3746188|rs491700|rs6214|rs863818|rs12498670|rs4889863|rs613229
Oral Ulcers	FAS_PATHWAY	BIOCARTA	980	32	20	1,21E-01	5,92E-01	rs1351726|rs7841459|rs9658702|rs7003908|rs4647693|rs711272|rs4613794|rs11580042|rs2193360|rs10097279|rs7910825|rs12205556|rs2721854|rs1926189|rs12191179|rs4807269|rs2154254|rs2529718|rs9345126|rs921
Oral Ulcers	SIGNALLING_TO_RAS	REACTOME	468	12	8	1,21E-01	5,92E-01	rs7396875|rs13311977|rs2076139|rs1888861|rs4919890|rs12462766|rs6951760|rs2073925
Oral Ulcers	SIGNALING_BY_EGFR_IN_CANCER	REACTOME	4030	42	20	1,24E-01	5,97E-01	rs263241|rs10940145|rs7396875|rs1541821|rs7726943|rs12334868|rs16897175|rs16904360|rs4690785|rs2256156|rs227913|rs2372183|rs281508|rs12251014|rs4976028|rs1022373|rs72001|rs9576991|rs895275|rs17753395
Oral Ulcers	CALCINEURIN_PATHWAY	BIOCARTA	895	41	20	1,24E-01	5,97E-01	rs6852347|rs227913|rs6096351|rs13434873|rs9892886|rs11860367|rs2851045|rs2364598|rs1297783|rs12223|rs17233751|rs931640|rs8051034|rs7236492|rs11145635|rs6504424|rs7565375|rs3780305|rs16862115|rs8078231
Oral Ulcers	SIGNALING_BY_ILS	REACTOME	2996	35	20	1,25E-01	5,97E-01	rs10940145|rs7396875|rs809192|rs7726943|rs16897175|rs952784|rs1351726|rs7841459|rs2372183|rs290240|rs4976028|rs7801617|rs711272|rs182832|rs1153465|rs310209|rs4613794|rs2555454|rs12532699|rs2660481
Oral Ulcers	NEUROTROPHIN_SIGNALING_PATHWAY	KEGG	3689	42	20	1,26E-01	5,97E-01	rs10940145|rs10489469|rs7396875|rs7726943|rs16897175|rs4074889|rs3850615|rs919741|rs4690785|rs3822295|rs1351726|rs7841459|rs2372183|rs4976028|rs1922529|rs11244164|rs508594|rs2285703|rs12797855|rs2267172
Oral Ulcers	INTEGRIN_ALPHAIIB_BETA3_SIGNALING	REACTOME	779	57	20	1,26E-01	5,97E-01	rs10489469|rs767513|rs2680182|rs290240|rs17605348|rs508594|rs11658221|rs4764521|rs11698821|rs1551746|rs708382|rs10120669|rs7588661|rs2074532|rs1317763|rs2081406|rs17706710|rs169724|rs3763630|rs2118773
Oral Ulcers	GLEEVEC_PATHWAY	BIOCARTA	954	47	20	1,31E-01	6,10E-01	rs10940145|rs7396875|rs7726943|rs16897175|rs2372183|rs4976028|rs10515068|rs16893188|rs7719763|rs2721854|rs10092081|rs863818|rs2529718|rs10515059|rs2049824|rs4326648|rs5759636|rs2699905|rs2017342|rs1490791
Oral Ulcers	NOTCH1_INTRACELLULAR_DOMAIN_REGULATES_TRANSCRIPTION	REACTOME	2852	35	20	1,31E-01	6,10E-01	rs4877524|rs4852054|rs10773086|rs2457400|rs1995243|rs1328413|rs4739987|rs10272709|rs838511|rs273473|rs1483023|rs304987|rs4687504|rs17801563|rs1332879|rs13291796|rs12426133|rs9855959|rs874754|rs1328406
Oral Ulcers	RNA_POL_III_TRANSCRIPTION_TERMINATION	REACTOME	680	26	19	1,31E-01	6,10E-01	rs6081172|rs10810056|rs1372348|rs535076|rs6924483|rs579096|rs13302756|rs10961346|rs1853195|rs7021837|rs10122910|rs137831|rs492553|rs1535565|rs2072102|rs3920568|rs729360|rs10810124|rs10089021
Oral Ulcers	GABA_B_RECEPTOR_ACTIVATION	REACTOME	1863	34	20	1,32E-01	6,13E-01	rs263241|rs1541821|rs11654455|rs12334868|rs16837945|rs16904360|rs2155548|rs2256156|rs72001|rs2898334|rs7566051|rs2058166|rs2192990|rs4937390|rs2835991|rs1593933|rs3892156|rs2601787|rs8066734|rs874627
Oral Ulcers	ION_TRANSPORT_BY_P_TYPE_ATPASES	REACTOME	1690	38	20	1,33E-01	6,13E-01	rs1041052|rs13064158|rs4770728|rs1403478|rs306401|rs26797|rs9313857|rs6442178|rs1380754|rs11917518|rs4622889|rs9551233|rs2414007|rs13100251|rs4906864|rs9842059|rs8041124|rs725027|rs6851867|rs13185341
Oral Ulcers	NEUROTRANSMITTER_RECEPTOR_BINDING_AND_DOWNSTREAM_TRANSMISSION_IN_THE<br>L	REACTOME	6592	28	20	1,36E-01	6,21E-01	rs1264866|rs263241|rs7396875|rs1541821|rs11654455|rs9322668|rs9922023|rs12334868|rs16837945|rs16904360|rs1232222|rs919741|rs10063828|rs7549591|rs2283551|rs2167568|rs2155548|rs2283984|rs4935752|rs3822295
Oral Ulcers	MEF2D_PATHWAY	BIOCARTA	684	46	20	1,39E-01	6,31E-01	rs6852347|rs227913|rs6096351|rs13434873|rs9892886|rs11860367|rs2851045|rs1297783|rs13053242|rs931640|rs8051034|rs7236492|rs6504424|rs8078231|rs422674|rs660749|rs9927848|rs11646540|rs904160|rs7259810
Oral Ulcers	ECM_RECEPTOR_INTERACTION	KEGG	3602	29	20	1,39E-01	6,31E-01	rs6974002|rs4949320|rs3811159|rs8038073|rs16854563|rs1075188|rs9301436|rs810687|rs6973683|rs510991|rs1029999|rs6942446|rs12408228|rs2298928|rs17605348|rs10487182|rs10264893|rs421587|rs11658221|rs10489329
Oral Ulcers	RNA_POL_III_CHAIN_ELONGATION	REACTOME	364	15	10	1,43E-01	6,46E-01	rs6081172|rs1372348|rs6924483|rs579096|rs137831|rs492553|rs1535565|rs2072102|rs729360|rs10089021
Oral Ulcers	MEMBRANE_TRAFFICKING	REACTOME	2276	41	20	1,45E-01	6,50E-01	rs11730732|rs4704458|rs9438040|rs26529|rs3850615|rs1078523|rs6988243|rs3849774|rs4653061|rs17108797|rs506233|rs1904432|rs11956308|rs2231997|rs153060|rs7712189|rs999095|rs595158|rs4484406|rs10500836
Oral Ulcers	ARGININE_AND_PROLINE_METABOLISM	KEGG	991	41	20	1,49E-01	6,60E-01	rs2373929|rs743507|rs6703674|rs10911044|rs2540627|rs1885490|rs9937648|rs2181139|rs8045775|rs9493000|rs6748782|rs914977|rs10794004|rs11592789|rs2057374|rs3918347|rs2408700|rs10915896|rs2302909|rs11684145
Oral Ulcers	RNA_POL_I_RNA_POL_III_AND_MITOCHONDRIAL_TRANSCRIPTION	REACTOME	1830	31	20	1,50E-01	6,60E-01	rs6081172|rs6928833|rs10810056|rs12375629|rs11646787|rs1372348|rs659445|rs535076|rs6924483|rs579096|rs13302756|rs10961346|rs3008598|rs1853195|rs7021837|rs4150454|rs16902819|rs7355118|rs7148769|rs10122910
Oral Ulcers	BETA_DEFENSINS	REACTOME	333	25	6	1,51E-01	6,60E-01	rs4710181|rs717064|rs6057657|rs9349534|rs2180584|rs1555679
Oral Ulcers	INTERFERON_SIGNALING	REACTOME	3448	30	20	1,52E-01	6,60E-01	rs2853923|rs7699006|rs9909750|rs4611262|rs919741|rs3822295|rs8127870|rs3997854|rs7196849|rs10852553|rs9357122|rs3851366|rs2025258|rs266549|rs310209|rs2285703|rs2094881|rs6540245|rs305059|rs12024682
Oral Ulcers	CYTOKINE_SIGNALING_IN_IMMUNE_SYSTEM	REACTOME	6819	32	20	1,52E-01	6,60E-01	rs2853923|rs7699006|rs10940145|rs9909750|rs7396875|rs809192|rs7726943|rs4611262|rs10461535|rs919741|rs952784|rs3822295|rs8127870|rs1351726|rs7841459|rs1207774|rs2372183|rs3997854|rs7196849|rs10852553
Oral Ulcers	MCM_PATHWAY	BIOCARTA	234	23	8	1,52E-01	6,60E-01	rs194825|rs7808273|rs4645742|rs34322|rs133416|rs2071744|rs2307344|rs4645752
Oral Ulcers	ABC_FAMILY_PROTEINS_MEDIATED_TRANSPORT	REACTOME	1753	43	20	1,55E-01	6,69E-01	rs215066|rs2274410|rs4721914|rs2074315|rs1858941|rs6461509|rs7805271|rs7802291|rs10877201|rs3789375|rs1989886|rs4148045|rs17205859|rs10512523|rs1191231|rs6977876|rs736528|rs4148330|rs1889547|rs671872
Oral Ulcers	FACTORS_INVOLVED_IN_MEGAKARYOCYTE_DEVELOPMENT_AND_PLATELET_PRODUCT	REACTOME	3549	64	20	1,58E-01	6,69E-01	rs13095652|rs7252293|rs13250684|rs3801796|rs9299670|rs12587001|rs617467|rs17064262|rs16873564|rs1579282|rs11244164|rs10757845|rs6557837|rs4543516|rs2608988|rs1244157|rs4444637|rs2303924|rs13053242|rs439786
Oral Ulcers	CELL_DEATH_SIGNALLING_VIA_NRAGE_NRIF_AND_NADE	REACTOME	2069	25	20	1,59E-01	6,69E-01	rs852350|rs701724|rs6018981|rs2291049|rs2833264|rs4647693|rs1332684|rs17020294|rs2660481|rs7543435|rs2833470|rs17718517|rs726430|rs11086250|rs11130558|rs6066683|rs1995303|rs4653544|rs7218427|rs4790152
Oral Ulcers	SIGNALING_BY_FGFR	REACTOME	4154	36	20	1,59E-01	6,69E-01	rs263241|rs10940145|rs7396875|rs1541821|rs7726943|rs12334868|rs10461535|rs16904360|rs4690785|rs11167777|rs2256156|rs227913|rs2372183|rs281508|rs4976028|rs72001|rs12542904|rs17487009|rs9576991|rs9510050
Oral Ulcers	NRAGE_SIGNALS_DEATH_THROUGH_JNK	REACTOME	1919	26	20	1,60E-01	6,69E-01	rs852350|rs701724|rs6018981|rs2291049|rs2833264|rs1332684|rs17020294|rs2660481|rs7543435|rs2833470|rs17718517|rs726430|rs11086250|rs11130558|rs6066683|rs1995303|rs4653544|rs7218427|rs13048019|rs6018970
Oral Ulcers	TYROSINE_METABOLISM	KEGG	1169	33	20	1,61E-01	6,69E-01	rs10499133|rs13100173|rs388483|rs10831551|rs10960455|rs727299|rs10960488|rs2242259|rs13075694|rs9937648|rs2026326|rs3764351|rs8045775|rs17210001|rs2232248|rs5993891|rs10809666|rs2348024|rs7806863|rs4646812
Oral Ulcers	RNA_POLYMERASE	KEGG	351	21	14	1,61E-01	6,69E-01	rs6081172|rs4487686|rs1131384|rs7712322|rs6924483|rs719667|rs137831|rs492553|rs1535565|rs2072102|rs311572|rs3789724|rs2303801|rs4878155
Oral Ulcers	BIOSYNTHESIS_OF_THE_N_GLYCAN_PRECURSOR_DOLICHOL_LIPID_LINKED_OLIGOSACC<br>RANSFER_TO_A_NASCENT_PROTEIN	REACTOME	355	15	6	1,62E-01	6,69E-01	rs902417|rs1525902|rs9611613|rs3749791|rs6867398|rs6792345
Oral Ulcers	PROTEASOME_PATHWAY	BIOCARTA	280	24	10	1,62E-01	6,69E-01	rs2294183|rs8066176|rs2811491|rs8009527|rs4710834|rs3820667|rs11543947|rs11023238|rs653079|rs2597189
Oral Ulcers	ORC1_REMOVAL_FROM_CHROMATIN	REACTOME	930	50	20	1,63E-01	6,69E-01	rs2294183|rs194825|rs328349|rs7808273|rs8066176|rs8009527|rs4645742|rs34322|rs4710834|rs3820667|rs4148876|rs2665833|rs1045288|rs133416|rs3859192|rs11543947|rs2071744|rs11023238|rs1321311|rs653079
Oral Ulcers	GLUCAGON_SIGNALING_IN_METABOLIC_REGULATION	REACTOME	1044	34	20	1,64E-01	6,69E-01	rs263241|rs1541821|rs12334868|rs16904360|rs2256156|rs72001|rs7566051|rs2058166|rs13356161|rs3892156|rs2601787|rs874627|rs1993716|rs17221445|rs7524898|rs872077|rs7725101|rs7526798|rs3729877|rs13338087
Oral Ulcers	BIOPEPTIDES_PATHWAY	BIOCARTA	1081	50	20	1,65E-01	6,70E-01	rs7396875|rs809192|rs919741|rs3822295|rs227913|rs2285703|rs9892886|rs11860367|rs2303924|rs1297783|rs931640|rs6533711|rs8051034|rs820336|rs7601754|rs4530474|rs6504424|rs8078231|rs873592|rs9927848
Oral Ulcers	PROTEOLYTIC_CLEAVAGE_OF_SNARE_COMPLEX_PROTEINS	REACTOME	382	21	13	1,66E-01	6,74E-01	rs127433|rs7272324|rs11654567|rs3813356|rs1549333|rs2788942|rs2206161|rs7970029|rs2168856|rs2304911|rs2695872|rs505431|rs362599
Oral Ulcers	GSK3_PATHWAY	BIOCARTA	1055	47	20	1,68E-01	6,75E-01	rs10940145|rs7726943|rs10461535|rs2372183|rs4976028|rs2540590|rs10515068|rs2334659|rs6771638|rs2685127|rs7719763|rs1429529|rs863818|rs1935624|rs10782213|rs10515059|rs230530|rs5743942|rs12197250|rs3845785
Oral Ulcers	ASPARAGINE_N_LINKED_GLYCOSYLATION	REACTOME	2760	35	20	1,68E-01	6,75E-01	rs9284892|rs11730732|rs902417|rs7143906|rs3759917|rs11714748|rs1667255|rs11998428|rs8037921|rs1525902|rs636757|rs1319090|rs17108797|rs2410283|rs12426170|rs7633722|rs4493419|rs12131285|rs2811491|rs11629679
Oral Ulcers	RNA_POL_II_TRANSCRIPTION_PRE_INITIATION_AND_PROMOTER_OPENING	REACTOME	383	38	17	1,70E-01	6,75E-01	rs6928833|rs6508425|rs4487686|rs1131384|rs3734261|rs6142711|rs4150454|rs16902819|rs2072102|rs1613070|rs4899786|rs2532929|rs4151374|rs4150667|rs2020892|rs8102597|rs1471420
Oral Ulcers	YAP1_AND_WWTR1_TAZ_STIMULATED_GENE_EXPRESSION	REACTOME	1047	30	20	1,70E-01	6,75E-01	rs4759430|rs1995243|rs1555208|rs1920558|rs219233|rs7812088|rs4757060|rs12200211|rs9296459|rs4113925|rs6789040|rs1800206|rs12787239|rs2047564|rs17410829|rs11655550|rs12312282|rs11067162|rs1889155|rs6794849
Oral Ulcers	TRANSCRIPTIONAL_ACTIVITY_OF_SMAD2_SMAD3_SMAD4_HETEROTRIMER	REACTOME	967	38	20	1,71E-01	6,77E-01	rs12906930|rs10773086|rs11631839|rs7512482|rs838511|rs12426133|rs7225993|rs2364598|rs16893188|rs9579015|rs10092081|rs12958604|rs1011970|rs937341|rs11874742|rs266366|rs3736242|rs9961435|rs2049824|rs1537377
Oral Ulcers	TRANSFERRIN_ENDOCYTOSIS_AND_RECYCLING	REACTOME	513	35	15	1,73E-01	6,78E-01	rs1551907|rs2346412|rs1799852|rs1006049|rs2734574|rs6983249|rs2004997|rs7579208|rs13192469|rs3814043|rs1018088|rs9828099|rs4646812|rs498193|rs1390943
Oral Ulcers	INTERFERON_ALPHA_BETA_SIGNALING	REACTOME	1477	38	20	1,73E-01	6,78E-01	rs2853923|rs8127870|rs7196849|rs9357122|rs2025258|rs490531|rs310209|rs2094881|rs6540245|rs305059|rs8074003|rs12521868|rs11247653|rs1634776|rs11698821|rs17389541|rs4148876|rs2871398|rs766908|rs2523580
Oral Ulcers	GLYCOSAMINOGLYCAN_METABOLISM	REACTOME	6096	29	20	1,74E-01	6,78E-01	rs1554324|rs4949320|rs2182542|rs11751562|rs3749862|rs2339331|rs7664795|rs6984293|rs219233|rs4073229|rs13100173|rs2433591|rs10470840|rs4886456|rs1667255|rs12680336|rs4438820|rs2591454|rs2098026|rs1886634
Oral Ulcers	PI_3K_CASCADE	REACTOME	1868	41	20	1,78E-01	6,78E-01	rs10940145|rs7726943|rs16897175|rs4690785|rs11167777|rs2372183|rs4976028|rs12542904|rs17487009|rs9576991|rs9510050|rs152442|rs12523624|rs12020729|rs11199823|rs10515068|rs733895|rs750535|rs10788173|rs263932
Oral Ulcers	PURINE_METABOLISM	REACTOME	581	21	10	1,78E-01	6,78E-01	rs940296|rs7544426|rs6816128|rs3826543|rs2071017|rs1653249|rs12797274|rs510619|rs13150967|rs11001078
Oral Ulcers	PYRUVATE_METABOLISM_AND_CITRIC_ACID_TCA_CYCLE	REACTOME	905	30	20	1,81E-01	6,78E-01	rs11983987|rs2341694|rs9972327|rs12681770|rs738140|rs3133976|rs2885856|rs1918094|rs8024587|rs11982165|rs12155014|rs4146945|rs17085|rs137831|rs11072732|rs12652118|rs2239953|rs2267366|rs7647085|rs1886446
Oral Ulcers	ASSEMBLY_OF_THE_PRE_REPLICATIVE_COMPLEX	REACTOME	911	50	20	1,82E-01	6,78E-01	rs2294183|rs194825|rs328349|rs7808273|rs8066176|rs8009527|rs4645742|rs4710834|rs3820667|rs4148876|rs2665833|rs1045288|rs133416|rs3859192|rs11543947|rs2071744|rs11023238|rs653079|rs2307344|rs4980335
Oral Ulcers	CARM_ER_PATHWAY	BIOCARTA	1617	39	20	1,82E-01	6,78E-01	rs770466|rs4852054|rs10773086|rs6928833|rs10272709|rs838511|rs1422189|rs12426133|rs1726614|rs4150454|rs13053242|rs2946385|rs11680658|rs6819019|rs4661661|rs2076139|rs937341|rs11655550|rs7135224|rs1613070
Oral Ulcers	P53_SIGNALING_PATHWAY	KEGG	1897	29	20	1,83E-01	6,78E-01	rs4621371|rs2853229|rs2155548|rs929461|rs872611|rs9658702|rs1019731|rs10866819|rs13186063|rs4647693|rs39425|rs7597597|rs11968166|rs16937996|rs9948303|rs10872027|rs9960342|rs2053273|rs7589528|rs7910825
Oral Ulcers	MELANOGENESIS	KEGG	4077	23	20	1,83E-01	6,78E-01	rs263241|rs7396875|rs1541821|rs12334868|rs4074889|rs16904360|rs2796806|rs6943342|rs919741|rs2241952|rs3822295|rs2256156|rs227913|rs4098686|rs936213|rs6732834|rs10960455|rs72001|rs227142|rs4665287
Oral Ulcers	SHC_RELATED_EVENTS	REACTOME	302	5	4	1,84E-01	6,78E-01	rs7396875|rs4919890|rs4804366|rs12462766
Oral Ulcers	FORMATION_OF_FIBRIN_CLOT_CLOTTING_CASCADE	REACTOME	703	34	20	1,84E-01	6,78E-01	rs1074801|rs3758919|rs1861309|rs11233361|rs12755775|rs2086297|rs12725867|rs4764521|rs11165206|rs2069916|rs2729376|rs16846520|rs3026237|rs3024377|rs11683986|rs12644965|rs238238|rs4959377|rs762484|rs13133050
Oral Ulcers	AXON_GUIDANCE	KEGG	9506	38	20	1,85E-01	6,78E-01	rs7431017|rs1486757|rs6852347|rs352602|rs219862|rs17140205|rs4957661|rs7396875|rs809192|rs10511054|rs6898594|rs1779344|rs1598915|rs3769504|rs1350136|rs4459934|rs267131|rs1511279|rs4085398|rs17086899
Oral Ulcers	SIGNALING_BY_TGF_BETA_RECEPTOR_COMPLEX	REACTOME	1605	45	20	1,85E-01	6,78E-01	rs12906930|rs518822|rs10773086|rs427278|rs11631839|rs7512482|rs838511|rs6015091|rs12426133|rs7225993|rs2364598|rs743102|rs16893188|rs9579015|rs10092081|rs12958604|rs1011970|rs937341|rs1854363|rs4802517
Oral Ulcers	ACTIVATED_POINT_MUTANTS_OF_FGFR2	REACTOME	873	27	20	1,88E-01	6,78E-01	rs11167777|rs12542904|rs17487009|rs9510050|rs152442|rs12523624|rs12020729|rs11199823|rs733895|rs750535|rs10788173|rs263932|rs11263615|rs4980694|rs2290755|rs11776272|rs951904|rs7981220|rs9506843|rs11745375
Oral Ulcers	VIRAL_MYOCARDITIS	KEGG	2750	54	20	1,88E-01	6,78E-01	rs2853923|rs809192|rs9507041|rs6088659|rs1536723|rs3997854|rs4647693|rs9317036|rs3135363|rs11244164|rs3873381|rs244982|rs39425|rs1050162|rs4958418|rs3746821|rs1490311|rs7754832|rs1634776|rs7192
Oral Ulcers	GNRH_SIGNALING_PATHWAY	KEGG	3992	29	20	1,89E-01	6,78E-01	rs263241|rs7396875|rs1541821|rs3774609|rs12334868|rs4074889|rs6804671|rs16904360|rs919741|rs3822295|rs2256156|rs227913|rs354263|rs936213|rs10049492|rs72001|rs227142|rs7542020|rs2285703|rs2119802
Oral Ulcers	CROSS_PRESENTATION_OF_SOLUBLE_EXOGENOUS_ANTIGENS_ENDOSOMES	REACTOME	713	39	15	1,89E-01	6,78E-01	rs2294183|rs328349|rs8066176|rs8009527|rs4710834|rs3820667|rs4148876|rs2584624|rs1045288|rs3859192|rs11543947|rs11023238|rs653079|rs4980335|rs2597189
Oral Ulcers	CDK_MEDIATED_PHOSPHORYLATION_AND_REMOVAL_OF_CDC6	REACTOME	698	39	15	1,89E-01	6,78E-01	rs2294183|rs328349|rs8066176|rs8009527|rs4710834|rs3820667|rs4148876|rs2665833|rs1045288|rs3859192|rs11543947|rs11023238|rs653079|rs4980335|rs2597189
Oral Ulcers	GRAFT_VERSUS_HOST_DISEASE	KEGG	1446	79	20	1,90E-01	6,78E-01	rs2853923|rs9658702|rs3997854|rs7801617|rs3135363|rs9348862|rs11580042|rs7910825|rs1634776|rs1926189|rs12535797|rs7192|rs2523580|rs9368726|rs376892|rs2154254|rs13192469|rs7745906|rs11672983|rs9275582
Oral Ulcers	INTRINSIC_PATHWAY	REACTOME	264	19	9	1,92E-01	6,78E-01	rs3758919|rs11233361|rs2086297|rs4764521|rs2729376|rs3026237|rs238238|rs13133050|rs3822058
Oral Ulcers	HOMOLOGOUS_RECOMBINATION_REPAIR_OF_REPLICATION_INDEPENDENT_DOUBLE_ST	REACTOME	376	19	6	1,92E-01	6,78E-01	rs1805812|rs2697675|rs1467464|rs6489769|rs1801516|rs7222799
Oral Ulcers	A_TETRASACCHARIDE_LINKER_SEQUENCE_IS_REQUIRED_FOR_GAG_SYNTHESIS	REACTOME	1731	28	20	1,94E-01	6,78E-01	rs4949320|rs2182542|rs4886456|rs2591454|rs12408228|rs7966281|rs7943044|rs3828334|rs33598|rs4707862|rs11070048|rs1382498|rs2748331|rs753353|rs2682185|rs931932|rs4773567|rs9555997|rs1944886|rs7981707
Oral Ulcers	GLYCOSPHINGOLIPID_METABOLISM	REACTOME	924	41	20	1,96E-01	6,78E-01	rs1642272|rs17221476|rs9626899|rs4747195|rs373983|rs6574916|rs7908969|rs10008543|rs11647934|rs42931|rs4697371|rs8022732|rs3115673|rs4438612|rs10805890|rs7678820|rs873592|rs8018273|rs3763630|rs2163315
Oral Ulcers	ACTIVATION_OF_CHAPERONE_GENES_BY_XBP1S	REACTOME	761	33	20	1,97E-01	6,78E-01	rs9284892|rs4661681|rs11714748|rs7633722|rs3753067|rs4493419|rs4425237|rs17365477|rs390200|rs3846158|rs9853582|rs2667469|rs10843894|rs2842363|rs3763630|rs560577|rs240940|rs164662|rs2730324|rs6806768
Oral Ulcers	METABOLISM_OF_STEROID_HORMONES_AND_VITAMINS_A_AND_D	REACTOME	745	42	20	1,97E-01	6,78E-01	rs11254339|rs276167|rs17522553|rs4889298|rs2043833|rs4665287|rs6564862|rs4668123|rs7788953|rs4667591|rs1646744|rs3753519|rs12333245|rs222014|rs1242105|rs4778636|rs1993116|rs206834|rs6847603|rs2486758
Oral Ulcers	UNFOLDED_PROTEIN_RESPONSE	REACTOME	1176	32	20	1,99E-01	6,78E-01	rs9284892|rs4661681|rs11714748|rs7216531|rs8061066|rs7633722|rs3753067|rs4493419|rs2722191|rs4425237|rs17365477|rs2075755|rs390200|rs3846158|rs2217332|rs2152092|rs9853582|rs2518054|rs2667469|rs10843894
Oral Ulcers	INTESTINAL_IMMUNE_NETWORK_FOR_IGA_PRODUCTION	KEGG	1392	47	20	2,01E-01	6,78E-01	rs1779344|rs1344638|rs5758511|rs266087|rs3997854|rs7801617|rs3135363|rs6510799|rs3746821|rs12922317|rs6835391|rs11679328|rs13003194|rs12535797|rs7192|rs9368726|rs376892|rs2781548|rs12511035|rs12638201
Oral Ulcers	ERK_MAPK_TARGETS	REACTOME	704	21	13	2,01E-01	6,78E-01	rs770466|rs1422189|rs378373|rs491700|rs12830764|rs2076139|rs2223405|rs388372|rs12541220|rs11633249|rs12442313|rs661311|rs4936661
Oral Ulcers	SIGNALING_BY_THE_B_CELL_RECEPTOR_BCR	REACTOME	3426	39	20	2,02E-01	6,78E-01	rs10940145|rs2294183|rs7396875|rs809192|rs7726943|rs10461535|rs328349|rs952784|rs2372183|rs290240|rs4976028|rs711272|rs182832|rs9576991|rs4613794|rs8066176|rs10951010|rs2660481|rs11860367|rs2399106
Oral Ulcers	PLC_BETA_MEDIATED_EVENTS	REACTOME	2313	38	20	2,03E-01	6,78E-01	rs263241|rs1541821|rs12334868|rs16904360|rs2241952|rs2256156|rs227913|rs354263|rs936213|rs72001|rs227142|rs7566051|rs9892886|rs2058166|rs2269340|rs926324|rs1430145|rs899236|rs1297783|rs13356161
Oral Ulcers	PML_PATHWAY	BIOCARTA	384	21	14	2,03E-01	6,78E-01	rs7396875|rs9658702|rs13398147|rs11580042|rs7910825|rs1926189|rs6736007|rs2059755|rs2154254|rs13192469|rs5746014|rs2288356|rs11681644|rs4886844

<table>
<tr><th></th><th></th><th></th><th></th><th></th><th></th><th></th><th></th><th></th></tr>
<tr><td>Oral Ulcers</td><td>HIV_LIFE_CYCLE</td><td>REACTOME</td><td>1529</td><td>28</td><td>20</td><td>2,03E-01</td><td>6,78E-01</td><td>rs4611262|rs6928833|rs6508426|rs4487686|rs2257205|rs10852553|rs2756382|rs1131384|rs3734261|rs12024682|rs6142711|rs4150454|rs11688530|rs16902819|rs595158|rs10500836|rs4684126|rs11756329|rs4869498|rs874627</td></tr>
<tr><td>Oral Ulcers</td><td>PHOSPHATIDYLINOSITOL_SIGNALING_SYSTEM</td><td>KEGG</td><td>3813</td><td>37</td><td>20</td><td>2,03E-01</td><td>6,78E-01</td><td>rs10940145|rs753724|rs1941593|rs7726943|rs10461535|rs4074889|rs6804671|rs1470352|rs227913|rs2372183|rs4976028|rs936213|rs3775601|rs6467706|rs227142|rs161338|rs838709|rs17391840|rs2119802|rs2283877</td></tr>
<tr><td>Oral Ulcers</td><td>SHC_MEDIATED_CASCADE</td><td>REACTOME</td><td>1070</td><td>25</td><td>20</td><td>2,03E-01</td><td>6,78E-01</td><td>rs7396875|rs11167777|rs12542904|rs17487009|rs9510050|rs152442|rs12523624|rs12020729|rs11199823|rs733895|rs750535|rs10788173|rs263932|rs244731|rs11263615|rs4980694|rs2290755|rs11776272|rs951904|rs7981220</td></tr>
<tr><td>Oral Ulcers</td><td>TRANSCRIPTION</td><td>REACTOME</td><td>2708</td><td>32</td><td>20</td><td>2,04E-01</td><td>6,78E-01</td><td>rs6081172|rs6928833|rs10810056|rs6508425|rs12375629|rs4487686|rs11646787|rs2257205|rs2756382|rs1372348|rs659445|rs1131384|rs3734261|rs535076|rs6924483|rs579096|rs13302756|rs4648873|rs10961346|rs3008598</td></tr>
<tr><td>Oral Ulcers</td><td>POST_NMDA_RECEPTOR_ACTIVATION_EVENTS</td><td>REACTOME</td><td>1753</td><td>41</td><td>20</td><td>2,05E-01</td><td>6,78E-01</td><td>rs263241|rs7396875|rs9922023|rs12334868|rs16904360|rs919741|rs3822295|rs2285703|rs11055671|rs7566051|rs378373|rs1297783|rs10492130|rs931640|rs6533711|rs491700|rs17221445|rs7524898|rs2223405|rs388372</td></tr>
<tr><td>Oral Ulcers</td><td>DAG_AND_IP3_SIGNALING</td><td>REACTOME</td><td>1644</td><td>44</td><td>20</td><td>2,05E-01</td><td>6,78E-01</td><td>rs263241|rs1541821|rs12334868|rs16904360|rs2256156|rs227913|rs281508|rs72001|rs7566051|rs9892886|rs2058166|rs1430145|rs1297783|rs4953334|rs13356161|rs3892156|rs2601787|rs931640|rs1993716|rs4952779</td></tr>
<tr><td>Oral Ulcers</td><td>TERMINATION_OF_O_GLYCAN_BIOSYNTHESIS</td><td>REACTOME</td><td>961</td><td>54</td><td>20</td><td>2,06E-01</td><td>6,78E-01</td><td>rs1554324|rs12680336|rs3791039|rs410076|rs11882828|rs4435373|rs3862899|rs12495023|rs517036|rs741900|rs10878626|rs1401346|rs8100230|rs2877822|rs12064337|rs10751635|rs12091356|rs2910368|rs647890|rs2922464</td></tr>
<tr><td>Oral Ulcers</td><td>IRON_UPTAKE_AND_TRANSPORT</td><td>REACTOME</td><td>733</td><td>37</td><td>20</td><td>2,08E-01</td><td>6,78E-01</td><td>rs1871093|rs2346412|rs1799852|rs1399959|rs1006049|rs4645742|rs2734574|rs6983249|rs9307048|rs2004997|rs133416|rs7579208|rs13192469|rs3814043|rs1018088|rs9828099|rs4646812|rs17554|rs498193|rs11674142</td></tr>
<tr><td>Oral Ulcers</td><td>SIGNALING_BY_INSULIN_RECEPTOR</td><td>REACTOME</td><td>3432</td><td>37</td><td>20</td><td>2,09E-01</td><td>6,78E-01</td><td>rs10940145|rs7396875|rs1941593|rs7726943|rs10461535|rs4690785|rs1871093|rs11167777|rs2346412|rs2372183|rs4976028|rs1922529|rs2393550|rs1006049|rs12542904|rs17487009|rs9510050|rs152442|rs12964783|rs12523624</td></tr>
<tr><td>Oral Ulcers</td><td>KERATAN_SULFATE_BIOSYNTHESIS</td><td>REACTOME</td><td>674</td><td>41</td><td>18</td><td>2,09E-01</td><td>6,78E-01</td><td>rs1554324|rs1667255|rs12680336|rs3791039|rs741900|rs1667271|rs1401346|rs11038507|rs4756006|rs7646486|rs7409343|rs2922464|rs7019909|rs1702956|rs13282872|rs17195011|rs10800919|rs7854765</td></tr>
<tr><td>Oral Ulcers</td><td>SIGNALLING_TO_ERKS</td><td>REACTOME</td><td>794</td><td>32</td><td>14</td><td>2,10E-01</td><td>6,78E-01</td><td>rs10489469|rs7396875|rs13311977|rs508594|rs2076139|rs1888861|rs4919890|rs6716851|rs10173234|rs12462766|rs6951760|rs943851|rs3012750|rs2073925</td></tr>
<tr><td>Oral Ulcers</td><td>COLORECTAL_CANCER</td><td>KEGG</td><td>2539</td><td>47</td><td>20</td><td>2,11E-01</td><td>6,78E-01</td><td>rs10940145|rs12906930|rs7726943|rs2965012|rs11631839|rs16897175|rs2372183|rs4976028|rs4647693|rs6732834|rs39425|rs6692647|rs1373179|rs2575676|rs1982673|rs10515068|rs6771638|rs16893188|rs2685127|rs7719763</td></tr>
<tr><td>Oral Ulcers</td><td>AMI_PATHWAY</td><td>BIOCARTA</td><td>574</td><td>31</td><td>15</td><td>2,12E-01</td><td>6,78E-01</td><td>rs767513|rs1861309|rs9301436|rs6436669|rs3783101|rs2396463|rs17588591|rs2069916|rs4503663|rs16846520|rs13021572|rs11683986|rs7991229|rs6727477|rs7325855</td></tr>
<tr><td>Oral Ulcers</td><td>NON_SMALL_CELL_LUNG_CANCER</td><td>KEGG</td><td>2585</td><td>38</td><td>20</td><td>2,13E-01</td><td>6,78E-01</td><td>rs10940145|rs4679640|rs7396875|rs7726943|rs16897175|rs227913|rs2372183|rs4976028|rs2049847|rs1562525|rs9311748|rs6779755|rs1385812|rs9892886|rs1910236|rs11860367|rs3764351|rs2236947|rs17673576|rs10515068</td></tr>
<tr><td>Oral Ulcers</td><td>PLATELET_AGGREGATION_PLUG_FORMATION</td><td>REACTOME</td><td>942</td><td>53</td><td>20</td><td>2,14E-01</td><td>6,78E-01</td><td>rs10489469|rs1074801|rs16854563|rs290240|rs17605348|rs508594|rs11658221|rs4764521|rs11698821|rs1551746|rs708382|rs10120669|rs7588661|rs2074532|rs1317763|rs2081406|rs238238|rs17706710|rs169724|rs3763630</td></tr>
<tr><td>Oral Ulcers</td><td>MICRORNA_MIRNA_BIOGENESIS</td><td>REACTOME</td><td>227</td><td>16</td><td>9</td><td>2,17E-01</td><td>6,78E-01</td><td>rs4487686|rs1131384|rs7752811|rs2282265|rs1110386|rs1954417|rs2072102|rs7717331|rs7720494</td></tr>
<tr><td>Oral Ulcers</td><td>CARDIAC_MUSCLE_CONTRACTION</td><td>KEGG</td><td>3169</td><td>35</td><td>20</td><td>2,18E-01</td><td>6,78E-01</td><td>rs628258|rs3774609|rs11665724|rs2108639|rs697928|rs479623|rs2283551|rs2283984|rs2282755|rs762898|rs768710|rs12631566|rs10049492|rs916288|rs17620912|rs16946204|rs9880354|rs6414573|rs7294534|rs9855109</td></tr>
<tr><td>Oral Ulcers</td><td>SELENOAMINO_ACID_METABOLISM</td><td>KEGG</td><td>742</td><td>33</td><td>20</td><td>2,18E-01</td><td>6,78E-01</td><td>rs10931791|rs10499133|rs11592638|rs388483|rs7444620|rs12651454|rs2651244|rs13075694|rs2232248|rs3124455|rs2726675|rs7522196|rs2275984|rs7806863|rs1526897|rs7719164|rs9491563|rs17131359|rs165064|rs2228224</td></tr>
<tr><td>Oral Ulcers</td><td>ANTIGEN_PROCESSING_CROSS_PRESENTATION</td><td>REACTOME</td><td>1601</td><td>70</td><td>20</td><td>2,19E-01</td><td>6,78E-01</td><td>rs2853923|rs2294183|rs328349|rs5750326|rs9348862|rs8066176|rs8009527|rs4710834|rs3820667|rs1634776|rs4148876|rs2523580|rs2665833|rs1045288|rs3859192|rs1527479|rs1220783|rs11543947|rs28397299|rs11023238</td></tr>
<tr><td>Oral Ulcers</td><td>TGF_BETA_RECEPTOR_SIGNALING_ACTIVATES_SMADS</td><td>REACTOME</td><td>563</td><td>48</td><td>20</td><td>2,19E-01</td><td>6,78E-01</td><td>rs12906930|rs518822|rs427278|rs11631839|rs6015091|rs7225993|rs12958604|rs4802517|rs266366|rs3736242|rs9961435|rs8110090|rs2532100|rs4803455|rs16955746|rs842796|rs6041970|rs17414380|rs9635893|rs4810108</td></tr>
<tr><td>Oral Ulcers</td><td>NCAM_SIGNALING_FOR_NEURITE_OUT_GROWTH</td><td>REACTOME</td><td>2480</td><td>29</td><td>20</td><td>2,19E-01</td><td>6,78E-01</td><td>rs3811159|rs7396875|rs809192|rs2295522|rs4803342|rs9301436|rs810687|rs3759917|rs4984637|rs1029999|rs6436669|rs8037921|rs2610983|rs421587|rs10098621|rs11629679|rs3783101|rs10745384|rs6090117|rs625087</td></tr>
<tr><td>Oral Ulcers</td><td>IL2_PATHWAY</td><td>BIOCARTA</td><td>452</td><td>35</td><td>13</td><td>2,19E-01</td><td>6,78E-01</td><td>rs7396875|rs290240|rs205898|rs310209|rs706779|rs1551746|rs10120669|rs7409343|rs4326648|rs169724|rs3218295|rs8021524|rs1004420</td></tr>
<tr><td>Oral Ulcers</td><td>ACUTE_MYELOID_LEUKEMIA</td><td>KEGG</td><td>1892</td><td>42</td><td>20</td><td>2,19E-01</td><td>6,78E-01</td><td>rs10940145|rs7396875|rs7726943|rs10956814|rs10461535|rs2372183|rs4976028|rs6732834|rs8126845|rs11079013|rs485550|rs10515068|rs16893188|rs2252585|rs1292035|rs7719763|rs3746188|rs10092081|rs863818|rs613229</td></tr>
<tr><td>Oral Ulcers</td><td>RNA_POL_II_TRANSCRIPTION</td><td>REACTOME</td><td>1050</td><td>35</td><td>20</td><td>2,20E-01</td><td>6,78E-01</td><td>rs6928833|rs6508426|rs4487686|rs2257205|rs2756382|rs1131384|rs3734261|rs4648873|rs6142711|rs4150454|rs16902819|rs11756329|rs16987990|rs11752001|rs2072102|rs1613070|rs6103330|rs2288055|rs9351162|rs4899786</td></tr>
<tr><td>Oral Ulcers</td><td>SIGNALING_BY_FGFR_IN_DISEASE</td><td>REACTOME</td><td>4545</td><td>36</td><td>20</td><td>2,20E-01</td><td>6,78E-01</td><td>rs263241|rs10940145|rs7396875|rs1541821|rs7726943|rs12334868|rs10461535|rs16904360|rs4690785|rs11167777|rs2256156|rs227913|rs2372183|rs281508|rs4976028|rs72001|rs12542904|rs17487009|rs9576991|rs9510050</td></tr>
<tr><td>Oral Ulcers</td><td>ANTIGEN_PROCESSING_UBIQUITINATION_PROTEASOME_DEGRADATION</td><td>REACTOME</td><td>4788</td><td>45</td><td>20</td><td>2,22E-01</td><td>6,81E-01</td><td>rs4072410|rs1626645|rs1894752|rs2294183|rs6545358|rs12533310|rs3749316|rs328349|rs10490578|rs2644494|rs399672|rs12519126|rs952901|rs1549487|rs1002941|rs1376679|rs371513|rs8066176|rs502514|rs10112123</td></tr>
<tr><td>Oral Ulcers</td><td>ER_PHAGOSOME_PATHWAY</td><td>REACTOME</td><td>1416</td><td>80</td><td>20</td><td>2,23E-01</td><td>6,81E-01</td><td>rs2853923|rs2294183|rs328349|rs9348862|rs8066176|rs8009527|rs4710834|rs3820667|rs1634776|rs4148876|rs2523580|rs2584624|rs1045288|rs3859192|rs1220783|rs11543947|rs7745906|rs11023238|rs653079|rs4980335</td></tr>
<tr><td>Oral Ulcers</td><td>TGFB_PATHWAY</td><td>BIOCARTA</td><td>817</td><td>45</td><td>20</td><td>2,24E-01</td><td>6,83E-01</td><td>rs12906930|rs2965012|rs11631839|rs711272|rs7552931|rs4613794|rs13053242|rs12205556|rs12191179|rs12958604|rs9345126|rs266366|rs3736242|rs9961435|rs9345117|rs538723|rs1884839|rs8110090|rs10431924|rs4783689</td></tr>
<tr><td>Oral Ulcers</td><td>PI3K_AKT_ACTIVATION</td><td>REACTOME</td><td>1161</td><td>50</td><td>20</td><td>2,26E-01</td><td>6,86E-01</td><td>rs10940145|rs7726943|rs10461535|rs2372183|rs4976028|rs1922529|rs9576991|rs10515068|rs9588044|rs34322|rs7719763|rs9566508|rs3746188|rs17254382|rs863818|rs10515059|rs1888861|rs7070956|rs1321311|rs17446614</td></tr>
<tr><td>Oral Ulcers</td><td>GABA_RECEPTOR_ACTIVATION</td><td>REACTOME</td><td>2705</td><td>48</td><td>20</td><td>2,27E-01</td><td>6,86E-01</td><td>rs263241|rs1541821|rs11654455|rs12334868|rs16837945|rs16904360|rs10063828|rs2155548|rs2256156|rs7672100|rs72001|rs2898334|rs11949658|rs7566051|rs1035750|rs2058166|rs2192990|rs1477310|rs4937390|rs2835991</td></tr>
<tr><td>Oral Ulcers</td><td>ALDOSTERONE_REGULATED_SODIUM_REABSORPTION</td><td>KEGG</td><td>1794</td><td>44</td><td>20</td><td>2,28E-01</td><td>6,87E-01</td><td>rs10940145|rs7726943|rs10461535|rs227913|rs1019731|rs2372183|rs17199565|rs4976028|rs1922529|rs1763510|rs9892886|rs11860367|rs10515068|rs9588044|rs4940646|rs2243492|rs7719763|rs7204560|rs3746188|rs8051034</td></tr>
<tr><td>Oral Ulcers</td><td>G1_PATHWAY</td><td>BIOCARTA</td><td>611</td><td>47</td><td>20</td><td>2,30E-01</td><td>6,91E-01</td><td>rs12906930|rs2965012|rs11631839|rs11244164|rs7552931|rs6771638|rs34322|rs11792889|rs12958604|rs1011970|rs266366|rs1537377|rs1801516|rs8110090|rs2251403|rs1321311|rs4803455|rs6797587|rs17279275|rs2229032</td></tr>
<tr><td>Oral Ulcers</td><td>AT1R_PATHWAY</td><td>BIOCARTA</td><td>1224</td><td>36</td><td>20</td><td>2,36E-01</td><td>7,01E-01</td><td>rs770466|rs7396875|rs227913|rs1422189|rs9892886|rs11860367|rs17673576|rs1297783|rs3863998|rs2721854|rs931640|rs8051034|rs11145635|rs2877260|rs2529718|rs6504424|rs1456680|rs6440546|rs3780305|rs8078231</td></tr>
<tr><td>Oral Ulcers</td><td>BIOSYNTHESIS_OF_UNSATURATED_FATTY_ACIDS</td><td>KEGG</td><td>463</td><td>19</td><td>14</td><td>2,36E-01</td><td>7,01E-01</td><td>rs17621311|rs7679085|rs7684686|rs209510|rs7674113|rs934153|rs7252966|rs10452184|rs3789512|rs4926125|rs6533493|rs3109650|rs244016|rs174534</td></tr>
<tr><td>Oral Ulcers</td><td>SCF_BETA_TRCP_MEDIATED_DEGRADATION_OF_EMI1</td><td>REACTOME</td><td>793</td><td>39</td><td>15</td><td>2,37E-01</td><td>7,01E-01</td><td>rs2294183|rs328349|rs8066176|rs8009527|rs4710834|rs3820667|rs4148876|rs2665833|rs1045288|rs3859192|rs11543947|rs11023238|rs653079|rs4980335|rs2597189</td></tr>
<tr><td>Oral Ulcers</td><td>INSULIN_RECEPTOR_SIGNALLING_CASCADE</td><td>REACTOME</td><td>3006</td><td>38</td><td>20</td><td>2,37E-01</td><td>7,01E-01</td><td>rs10940145|rs7396875|rs1941593|rs7726943|rs16897175|rs4690785|rs11167777|rs2372183|rs4976028|rs1922529|rs2393550|rs12542904|rs17487009|rs9510050|rs152442|rs12964783|rs12523624|rs12020729|rs11199823|rs1001684</td></tr>
<tr><td>Oral Ulcers</td><td>BASAL_TRANSCRIPTION_FACTORS</td><td>KEGG</td><td>386</td><td>25</td><td>13</td><td>2,41E-01</td><td>7,11E-01</td><td>rs6928833|rs6508425|rs3734261|rs6142711|rs6956055|rs10075302|rs1613070|rs4899786|rs2532929|rs4150667|rs10813843|rs8102597|rs1471420</td></tr>
<tr><td>Oral Ulcers</td><td>NUCLEAR_EVENTS_KINASE_AND_TRANSCRIPTION_FACTOR_ACTIVATION</td><td>REACTOME</td><td>766</td><td>21</td><td>13</td><td>2,44E-01</td><td>7,13E-01</td><td>rs770466|rs1422189|rs378373|rs491700|rs12830764|rs2076139|rs2223405|rs388372|rs12541220|rs11633249|rs12442313|rs661311|rs4936661</td></tr>
<tr><td>Oral Ulcers</td><td>CHOLESTEROL_BIOSYNTHESIS</td><td>REACTOME</td><td>259</td><td>2</td><td>2</td><td>2,45E-01</td><td>7,13E-01</td><td>rs1043180|rs12081312</td></tr>
<tr><td>Oral Ulcers</td><td>GLUCOSE_TRANSPORT</td><td>REACTOME</td><td>689</td><td>20</td><td>15</td><td>2,46E-01</td><td>7,13E-01</td><td>rs4611262|rs8067341|rs10852553|rs12024682|rs923273|rs4684126|rs4869498|rs2171385|rs12472874|rs2383477|rs2670854|rs580438|rs1972947|rs3115752|rs13054435</td></tr>
<tr><td>Oral Ulcers</td><td>NOS1_PATHWAY</td><td>BIOCARTA</td><td>1133</td><td>41</td><td>20</td><td>2,50E-01</td><td>7,13E-01</td><td>rs6852347|rs9922023|rs227913|rs13434873|rs11055671|rs9892886|rs11860367|rs2851045|rs1297783|rs390200|rs10492130|rs931640|rs8051034|rs7524898|rs872077|rs6504424|rs7526798|rs3729877|rs8078231|rs7972433</td></tr>
<tr><td>Oral Ulcers</td><td>TRANSPORT_OF_MATURE_MRNA_DERIVED_FROM_AN_INTRONLESS_TRANSCRIPT</td><td>REACTOME</td><td>524</td><td>12</td><td>11</td><td>2,51E-01</td><td>7,13E-01</td><td>rs4611262|rs10852553|rs12024682|rs4684126|rs4869498|rs12472874|rs2383477|rs580438|rs1972947|rs3115752|rs13054435</td></tr>
<tr><td>Oral Ulcers</td><td>GPCR_PATHWAY</td><td>BIOCARTA</td><td>1102</td><td>38</td><td>20</td><td>2,51E-01</td><td>7,13E-01</td><td>rs6852347|rs7396875|rs227913|rs6096351|rs13434873|rs9892886|rs11860367|rs2851045|rs1297783|rs931640|rs8051034|rs7236492|rs11145635|rs7524898|rs872077|rs6504424|rs3780305|rs7526798|rs3729877|rs8078231</td></tr>
<tr><td>Oral Ulcers</td><td>NEP_NS2_INTERACTS_WITH_THE_CELLULAR_EXPORT_MACHINERY</td><td>REACTOME</td><td>456</td><td>12</td><td>11</td><td>2,52E-01</td><td>7,13E-01</td><td>rs4611262|rs10852553|rs12024682|rs4684126|rs4869498|rs12472874|rs2383477|rs580438|rs1972947|rs3115752|rs13054435</td></tr>
<tr><td>Oral Ulcers</td><td>INTERACTIONS_OF_VPR_WITH_HOST_CELLULAR_PROTEINS</td><td>REACTOME</td><td>480</td><td>12</td><td>11</td><td>2,53E-01</td><td>7,13E-01</td><td>rs4611262|rs10852553|rs12024682|rs4684126|rs4869498|rs12472874|rs2383477|rs580438|rs1972947|rs3115752|rs13054435</td></tr>
<tr><td>Oral Ulcers</td><td>HS_GAG_BIOSYNTHESIS</td><td>REACTOME</td><td>3154</td><td>33</td><td>20</td><td>2,53E-01</td><td>7,13E-01</td><td>rs4949320|rs2182542|rs3749862|rs7664795|rs4073229|rs10470840|rs4438820|rs2098026|rs1886634|rs12408228|rs1013120|rs17665827|rs3828334|rs9372417|rs11098419|rs3811755|rs11648676|rs12952904|rs11070048|rs10955842</td></tr>
<tr><td>Oral Ulcers</td><td>CELL_CYCLE</td><td>KEGG</td><td>2581</td><td>41</td><td>20</td><td>2,55E-01</td><td>7,13E-01</td><td>rs12906930|rs194825|rs2965012|rs11631839|rs4661681|rs929461|rs872611|rs7003908|rs7808273|rs11244164|rs9289642|rs6692647|rs136569|rs2267172|rs11968166|rs5758168|rs4645742|rs6771638|rs13053242|rs16893188</td></tr>
<tr><td>Oral Ulcers</td><td>OTHER_GLYCAN_DEGRADATION</td><td>KEGG</td><td>648</td><td>35</td><td>17</td><td>2,56E-01</td><td>7,13E-01</td><td>rs9484764|rs1897759|rs1434039|rs10009214|rs10520400|rs2611031|rs1454212|rs2168040|rs3115673|rs4438612|rs10805890|rs11937114|rs6552275|rs7656870|rs13107405|rs1379410|rs12504394</td></tr>
<tr><td>Oral Ulcers</td><td>REGULATORY_RNA_PATHWAYS</td><td>REACTOME</td><td>345</td><td>27</td><td>13</td><td>2,56E-01</td><td>7,13E-01</td><td>rs4487686|rs1131384|rs4411562|rs7752811|rs2282265|rs1110386|rs9897312|rs1954417|rs2072102|rs7717331|rs10521128|rs9611286|rs7720494</td></tr>
<tr><td>Oral Ulcers</td><td>AUTODEGRADATION_OF_THE_E3_UBIQUITIN_LIGASE_COP1</td><td>REACTOME</td><td>766</td><td>40</td><td>16</td><td>2,57E-01</td><td>7,13E-01</td><td>rs2294183|rs328349|rs8066176|rs8009527|rs4710834|rs3820667|rs4148876|rs2665833|rs1045288|rs3859192|rs11543947|rs11023238|rs1801516|rs653079|rs4980335|rs2597189</td></tr>
<tr><td>Oral Ulcers</td><td>PPARA_PATHWAY</td><td>BIOCARTA</td><td>2445</td><td>38</td><td>20</td><td>2,57E-01</td><td>7,13E-01</td><td>rs10940145|rs7726943|rs10773086|rs16897175|rs1555208|rs838511|rs227913|rs2372183|rs4976028|rs7581571|rs7518498|rs2112662|rs12426133|rs9892886|rs11860367|rs10515068|rs13149400|rs2364598|rs12038031|rs13053242</td></tr>
<tr><td>Oral Ulcers</td><td>METABOLISM_OF_CARBOHYDRATES</td><td>REACTOME</td><td>9072</td><td>26</td><td>20</td><td>2,57E-01</td><td>7,13E-01</td><td>rs1554324|rs4949320|rs2182542|rs11751562|rs3749862|rs2339331|rs4611262|rs4880592|rs7664795|rs7924157|rs11983987|rs6984293|rs219233|rs8067341|rs1327257|rs4073229|rs1539234|rs13100173|rs2433591|rs10470840</td></tr>
<tr><td>Oral Ulcers</td><td>TRANSPORT_OF_RIBONUCLEOPROTEINS_INTO_THE_HOST_NUCLEUS</td><td>REACTOME</td><td>452</td><td>12</td><td>11</td><td>2,57E-01</td><td>7,13E-01</td><td>rs4611262|rs10852553|rs12024682|rs4684126|rs4869498|rs12472874|rs2383477|rs580438|rs1972947|rs3115752|rs13054435</td></tr>
<tr><td>Oral Ulcers</td><td>TRANSPORT_OF_INORGANIC_CATIONS_ANIONS_AND_AMINO_ACIDS_OLIGOPEPTIDES</td><td>REACTOME</td><td>4051</td><td>46</td><td>20</td><td>2,58E-01</td><td>7,13E-01</td><td>rs1366632|rs1023681|rs6112470|rs2562582|rs7611368|rs11741316|rs9883639|rs1320301|rs768710|rs1980943|rs660526|rs867422|rs1327410|rs1384319|rs6046170|rs10833748|rs9036|rs6575242|rs12586368|rs12625137</td></tr>
<tr><td>Oral Ulcers</td><td>REGULATION_OF_GLUCOKINASE_BY_GLUCOKINASE_REGULATORY_PROTEIN</td><td>REACTOME</td><td>459</td><td>12</td><td>11</td><td>2,59E-01</td><td>7,13E-01</td><td>rs4611262|rs10852553|rs12024682|rs4684126|rs4869498|rs12472874|rs2383477|rs580438|rs1972947|rs3115752|rs13054435</td></tr>
<tr><td>Oral Ulcers</td><td>REGULATION_OF_WATER_BALANCE_BY_RENAL_AQUAPORINS</td><td>REACTOME</td><td>1393</td><td>33</td><td>20</td><td>2,60E-01</td><td>7,13E-01</td><td>rs263241|rs1541821|rs12334868|rs16904360|rs2256156|rs72001|rs1017414|rs7566051|rs2058166|rs13356161|rs10510036|rs3892156|rs2601787|rs2414891|rs874627|rs1993716|rs951388|rs17221445|rs7524898|rs872077</td></tr>
<tr><td>Oral Ulcers</td><td>PI3K_CASCADE</td><td>REACTOME</td><td>2702</td><td>38</td><td>20</td><td>2,60E-01</td><td>7,13E-01</td><td>rs10940145|rs1941593|rs7726943|rs10461535|rs4690785|rs11167777|rs2372183|rs4976028|rs1922529|rs2393550|rs12542904|rs17487009|rs9510050|rs152442|rs12964783|rs12523624|rs12020729|rs11199823|rs1001684|rs10515068</td></tr>
<tr><td>Oral Ulcers</td><td>DESTABILIZATION_OF_MRNA_BY_AUF1_HNRNP_D0</td><td>REACTOME</td><td>811</td><td>39</td><td>20</td><td>2,62E-01</td><td>7,15E-01</td><td>rs2294183|rs328349|rs3843552|rs17460250|rs8066176|rs8009527|rs4710834|rs3820667|rs4148876|rs2584624|rs1045288|rs3859192|rs11218954|rs2227956|rs3115673|rs11543947|rs11023238|rs653079|rs4980335|rs2597189</td></tr>
<tr><td>Oral Ulcers</td><td>GAP_JUNCTION</td><td>KEGG</td><td>4388</td><td>24</td><td>20</td><td>2,63E-01</td><td>7,15E-01</td><td>rs263241|rs7396875|rs1541821|rs12334868|rs1328684|rs6306|rs10823962|rs6804671|rs16904360|rs2256156|rs227913|rs936213|rs1840572|rs7893773|rs4978964|rs72001|rs227142|rs973908|rs1008157|rs2119802</td></tr>
<tr><td>Oral Ulcers</td><td>CITRATE_CYCLE_TCA_CYCLE</td><td>KEGG</td><td>1195</td><td>37</td><td>20</td><td>2,64E-01</td><td>7,15E-01</td><td>rs1019635|rs11983987|rs2341694|rs10516988|rs9972327|rs738140|rs2885856|rs1918094|rs8024587|rs2048022|rs12155014|rs4146945|rs17085|rs137831|rs11072732|rs10516985|rs7647085|rs1886446|rs7593466|rs6734788</td></tr>
<tr><td>Oral Ulcers</td><td>TCA_CYCLE_AND_RESPIRATORY_ELECTRON_TRANSPORT</td><td>REACTOME</td><td>2599</td><td>30</td><td>20</td><td>2,65E-01</td><td>7,15E-01</td><td>rs11631423|rs11665724|rs7689418|rs17163678|rs11983987|rs4975854|rs2341694|rs2240760|rs17620912|rs9972327|rs39425|rs12681770|rs11956638|rs10952211|rs7904875|rs6722509|rs738140|rs3133976|rs2885856|rs1918094</td></tr>
<tr><td>Oral Ulcers</td><td>CTLA4_INHIBITORY_SIGNALING</td><td>REACTOME</td><td>447</td><td>22</td><td>11</td><td>2,65E-01</td><td>7,15E-01</td><td>rs809192|rs952784|rs182832|rs1741143|rs7834615|rs17813240|rs12541220|rs231793|rs1004420|rs1998038|rs4936661</td></tr>
<tr><td>Oral Ulcers</td><td>PROTEASOME</td><td>KEGG</td><td>821</td><td>41</td><td>16</td><td>2,66E-01</td><td>7,15E-01</td><td>rs2294183|rs328349|rs8066176|rs8009527|rs4710834|rs3820667|rs4148876|rs2665833|rs1045288|rs3859192|rs7320128|rs11543947|rs11023238|rs653079|rs4980335|rs2597189</td></tr>
<tr><td>Oral Ulcers</td><td>ACTIVATED_TAK1_MEDIATES_P38_MAPK_ACTIVATION</td><td>REACTOME</td><td>649</td><td>25</td><td>15</td><td>2,68E-01</td><td>7,15E-01</td><td>rs1351726|rs7841459|rs711272|rs4613794|rs10097279|rs12205556|rs12191179|rs2076139|rs9345126|rs9345117|rs538723|rs559052|rs39502|rs1884839|rs5743291</td></tr>
<tr><td>Oral Ulcers</td><td>ERBB_SIGNALING_PATHWAY</td><td>KEGG</td><td>4135</td><td>45</td><td>20</td><td>2,71E-01</td><td>7,15E-01</td><td>rs10940145|rs219862|rs7396875|rs7726943|rs16897175|rs919741|rs3769504|rs4690785|rs3822295|rs227913|rs2372183|rs3754806|rs4976028|rs936213|rs11244164|rs2285703|rs16847152|rs4851835|rs2575676|rs9892886</td></tr>
<tr><td>Oral Ulcers</td><td>SPHINGOLIPID_METABOLISM</td><td>REACTOME</td><td>1944</td><td>35</td><td>20</td><td>2,71E-01</td><td>7,15E-01</td><td>rs1642272|rs225890|rs17221476|rs9837945|rs9626899|rs10497340|rs4747195|rs373983|rs1019367|rs9326038|rs7908969|rs29214|rs2165406|rs7536157|rs10008543|rs7148794|rs10142295|rs11647934|rs1191566|rs42931</td></tr>
<tr><td>Oral Ulcers</td><td>FGFR_LIGAND_BINDING_AND_ACTIVATION</td><td>REACTOME</td><td>984</td><td>25</td><td>20</td><td>2,72E-01</td><td>7,15E-01</td><td>rs11167777|rs12542904|rs17487009|rs9510050|rs152442|rs12523624|rs12020729|rs11199823|rs733895|rs750535|rs10788173|rs263932|rs244731|rs11263615|rs4980694|rs2290755|rs11776272|rs951904|rs7981220|rs9506843</td></tr>
<tr><td>Oral Ulcers</td><td>PHOSPHOLIPID_METABOLISM</td><td>REACTOME</td><td>6226</td><td>34</td><td>20</td><td>2,72E-01</td><td>7,15E-01</td><td>rs1642272|rs10940145|rs1941593|rs7726943|rs225890|rs10461535|rs17221476|rs9837945|rs9626899|rs11892871|rs1470352|rs2372183|rs7930593|rs354263|rs4976028|rs10497340|rs11670988|rs17561950|rs4813754|rs6133248</td></tr>
<tr><td>Oral Ulcers</td><td>CELL_CELL_COMMUNICATION</td><td>REACTOME</td><td>9725</td><td>35</td><td>20</td><td>2,73E-01</td><td>7,15E-01</td><td>rs10940145|rs2587374|rs1703047|rs1975673|rs422384|rs809192|rs3807671|rs7726943|rs12921373|rs7775178|rs1918930|rs16897175|rs3807663|rs483644|rs1462307|rs3769504|rs4782685|rs1304648|rs13239642|rs2372183</td></tr>
<tr><td>Oral Ulcers</td><td>PROSTATE_CANCER</td><td>KEGG</td><td>2870</td><td>36</td><td>20</td><td>2,73E-01</td><td>7,15E-01</td><td>rs10940145|rs7396875|rs7726943|rs10461535|rs7169544|rs6943342|rs12539927|rs1019731|rs2372183|rs4976028|rs198972|rs6732834|rs9576991|rs7947946|rs7794304|rs11199823|rs2722191|rs3764351|rs1982673|rs17673576</td></tr>
<tr><td>Oral Ulcers</td><td>MAPK_SIGNALING_PATHWAY</td><td>KEGG</td><td>9833</td><td>31</td><td>20</td><td>2,73E-01</td><td>7,15E-01</td><td>rs770466|rs6852347|rs4926290|rs10489469|rs9846559|rs7396875|rs628258|rs3774609|rs12632728|rs2965012|rs9557798|rs2108639|rs697928|rs2283551|rs2283984|rs11167777|rs929461|rs872611|rs227913|rs12410731</td></tr>
<tr><td>Oral Ulcers</td><td>CACAM_PATHWAY</td><td>BIOCARTA</td><td>401</td><td>27</td><td>11</td><td>2,77E-01</td><td>7,23E-01</td><td>rs919741|rs3822295|rs2285703|rs1297783|rs931640|rs6533711|rs873592|rs1644512|rs904160|rs10009286|rs7259810</td></tr>
<tr><td>Oral Ulcers</td><td>SIGNALING_BY_NOTCH1</td><td>REACTOME</td><td>3563</td><td>31</td><td>20</td><td>2,78E-01</td><td>7,23E-01</td><td>rs4877524|rs4852054|rs10773086|rs2457400|rs1995243|rs1328413|rs4739987|rs10272709|rs838511|rs273473|rs1483023|rs304987|rs4687504|rs17801563|rs1332879|rs506233|rs13291796|rs12426133|rs9855959|rs874754</td></tr>
<tr><td>Oral Ulcers</td><td>TRAFFICKING_OF_AMPA_RECEPTORS</td><td>REACTOME</td><td>1132</td><td>60</td><td>20</td><td>2,79E-01</td><td>7,23E-01</td><td>rs919741|rs2283551|rs2283984|rs3822295|rs227913|rs2285703|rs9036|rs9892886|rs11860367|rs2022174|rs2267357|rs6533711|rs8051034|rs8111479|rs2238517|rs6504424|rs2267366|rs8078231|rs873592|rs9622429</td></tr>
<tr><td>Oral Ulcers</td><td>OPIOID_SIGNALLING</td><td>REACTOME</td><td>3594</td><td>29</td><td>20</td><td>2,80E-01</td><td>7,23E-01</td><td>rs6852347|rs263241|rs1541821|rs12334868|rs16904360|rs2241952|rs2256156|rs227913|rs354263|rs936213|rs13434873|rs72001|rs227142|rs10491353|rs4665287|rs7566051|rs9892886|rs2058166|rs2269340|rs2851045</td></tr>
<tr><td>Oral Ulcers</td><td>INTRINSIC_PATHWAY</td><td>BIOCARTA</td><td>603</td><td>35</td><td>19</td><td>2,86E-01</td><td>7,35E-01</td><td>rs767513|rs3758919|rs9301436|rs6436669|rs12755775|rs3783101|rs2396463|rs17588591|rs2069916|rs2729376|rs4503663|rs16846520|rs13021572|rs11683986|rs7991229|rs6727477|rs13133050|rs3822058|rs7325855</td></tr>
<tr><td>Oral Ulcers</td><td>MITOCHONDRIAL_TRNA_AMINOACYLATION</td><td>REACTOME</td><td>500</td><td>27</td><td>15</td><td>2,87E-01</td><td>7,37E-01</td><td>rs9369476|rs10931791|rs17023223|rs11243014|rs38494|rs7763309|rs2726486|rs2713829|rs2532929|rs2270003|rs3600|rs3131921|rs12730226|rs797145|rs2647242</td></tr>
<tr><td>Oral Ulcers</td><td>TRANSPORT_TO_THE_GOLGI_AND_SUBSEQUENT_MODIFICATION</td><td>REACTOME</td><td>1557</td><td>39</td><td>20</td><td>2,89E-01</td><td>7,37E-01</td><td>rs11730732|rs7143906|rs3759917|rs1667255|rs8037921|rs636757|rs1319090|rs17108797|rs11629679|rs4284481|rs12495023|rs1667271|rs17102896|rs6917694|rs3775836|rs4902433|rs3769693|rs11684754|rs1499570|rs9489810</td></tr>
<tr><td>Oral Ulcers</td><td>FRS2_MEDIATED_CASCADE</td><td>REACTOME</td><td>1192</td><td>25</td><td>20</td><td>2,89E-01</td><td>7,37E-01</td><td>rs7396875|rs11167777|rs12542904|rs17487009|rs9510050|rs152442|rs12523624|rs12020729|rs11199823|rs733895|rs750535|rs10788173|rs263932|rs244731|rs11263615|rs4980694|rs2290755|rs11776272|rs951904|rs7981220</td></tr>
<tr><td>Oral Ulcers</td><td>SNARE_INTERACTIONS_IN_VESICULAR_TRANSPORT</td><td>KEGG</td><td>828</td><td>29</td><td>20</td><td>2,92E-01</td><td>7,38E-01</td><td>rs41524|rs127433|rs7272324|rs11654567|rs3813356|rs1549333|rs173878|rs2788942|rs7917657|rs1408817|rs697673|rs511744|rs12547538|rs12359794|rs2206161|rs7970029|rs4918779|rs10215958|rs4980335|rs2168856</td></tr>
<tr><td>Oral Ulcers</td><td>REGULATION_OF_GENE_EXPRESSION_IN_BETA_CELLS</td><td>REACTOME</td><td>716</td><td>33</td><td>14</td><td>2,92E-01</td><td>7,38E-01</td><td>rs686699|rs2254779|rs752402|rs9576991|rs199812|rs9566508|rs17254382|rs10835826|rs2178603|rs10516713|rs17446614|rs1950130|rs1751853|rs2425640</td></tr>
<tr><td>Oral Ulcers</td><td>ALLOGRAFT_REJECTION</td><td>KEGG</td><td>1510</td><td>92</td><td>20</td><td>2,92E-01</td><td>7,38E-01</td><td>rs2853923|rs9658702|rs3997854|rs3135363|rs3873381|rs3746821|rs11580042|rs7910825|rs1634776|rs1926189|rs7192|rs2523580|rs9368726|rs376892|rs2154254|rs13192469|rs7745906|rs2243290|rs9275582|rs7643177</td></tr>
<tr><td>Oral Ulcers</td><td>HEPARAN_SULFATE_HEPARIN_HS_GAG_METABOLISM</td><td>REACTOME</td><td>3879</td><td>28</td><td>20</td><td>2,93E-01</td><td>7,38E-01</td><td>rs4949320|rs2182542|rs3749862|rs7664795|rs4073229|rs10470840|rs4886456|rs4438820|rs2591454|rs2098026|rs1886634|rs12408228|rs7966281|rs1013120|rs17665827|rs7943044|rs3828334|rs4917833|rs33598|rs9372417</td></tr>
<tr><td>Oral Ulcers</td><td>CASPASE_PATHWAY</td><td>BIOCARTA</td><td>327</td><td>18</td><td>8</td><td>2,97E-01</td><td>7,44E-01</td><td>rs4647693|rs39425|rs2193360|rs4807269|rs2288713|rs921|rs12765134|rs13402616</td></tr>
<tr><td>Oral Ulcers</td><td>ETS_PATHWAY</td><td>BIOCARTA</td><td>682</td><td>32</td><td>18</td><td>2,98E-01</td><td>7,44E-01</td><td>rs7396875|rs10773086|rs2836786|rs838511|rs12426133|rs2237085|rs7924735|rs937341|rs7189819|rs12122011|rs4326648|rs736813|rs197414|rs3746882|rs8021524|rs1242991|rs4245079|rs906304</td></tr>
<tr><td>Oral Ulcers</td><td>FORMATION_OF_RNA_POL_II_ELONGATION_COMPLEX_</td><td>REACTOME</td><td>401</td><td>24</td><td>12</td><td>3,00E-01</td><td>7,44E-01</td><td>rs4487686|rs2257205|rs1131384|rs4150454|rs16902819|rs2072102|rs1613070|rs2532929|rs4151374|rs4150667|rs2020892|rs8102597</td></tr>
<tr><td>Oral Ulcers</td><td>PYRIMIDINE_METABOLISM</td><td>KEGG</td><td>1900</td><td>37</td><td>20</td><td>3,00E-01</td><td>7,44E-01</td><td>rs6081172|rs6707705|rs17471640|rs2853229|rs4487686|rs1131384|rs6816128|rs7712322|rs4669069|rs6924483|rs11165845|rs716604|rs1405655|rs439786|rs7552825|rs6759948|rs17210001|rs3181115|rs1346793|rs719667</td></tr>
<tr><td>Oral Ulcers</td><td>GLYCOSAMINOGLYCAN_DEGRADATION</td><td>KEGG</td><td>709</td><td>38</td><td>20</td><td>3,00E-01</td><td>7,44E-01</td><td>rs13100173|rs2098026|rs1013120|rs17665827|rs4917833|rs10883109|rs12952904|rs7205379|rs4559283|rs10883150|rs764428|rs11975640|rs4438612|rs10805890|rs643084|rs551674|rs12572945|rs17565276|rs25413|rs4792347</td></tr>
<tr><td>Oral Ulcers</td><td>TRAF6_MEDIATED_IRF7_ACTIVATION</td><td>REACTOME</td><td>433</td><td>23</td><td>12</td><td>3,02E-01</td><td>7,46E-01</td><td>rs9909750|rs3813982|rs13053242|rs12521868|rs2871398|rs766908|rs4977686|rs11868917|rs793801|rs3775545|rs11132242|rs10964832</td></tr>
<tr><td>Oral Ulcers</td><td>TRANSPORT_OF_VITAMINS_NUCLEOSIDES_AND_RELATED_MOLECULES</td><td>REACTOME</td><td>1154</td><td>39</td><td>20</td><td>3,03E-01</td><td>7,47E-01</td><td>rs1417210|rs3195701|rs1246264|rs4980358|rs3758773|rs4002407|rs7116044|rs1362985|rs2313152|rs4149023|rs1885277|rs6595861|rs7340717|rs852550|rs4301004|rs1422443|rs3787537|rs9804732|rs1932652|rs10868155</td></tr>
<tr><td>Oral Ulcers</td><td>PARKINSONS_DISEASE</td><td>KEGG</td><td>2833</td><td>30</td><td>20</td><td>3,04E-01</td><td>7,47E-01</td><td>rs5748403|rs11665724|rs7689418|rs17163678|rs4975854|rs4647693|rs10831551|rs363333|rs2240760|rs17620912|rs952901|rs39425|rs9605004|rs3775256|rs371513|rs11956638|rs769293|rs10952211|rs7904875|rs6722509</td></tr>
<tr><td>Oral Ulcers</td><td>STRIATED_MUSCLE_CONTRACTION</td><td>REACTOME</td><td>453</td><td>20</td><td>10</td><td>3,08E-01</td><td>7,49E-01</td><td>rs399672|rs17605348|rs16946204|rs3764351|rs4764808|rs6671391|rs907606|rs3816551|rs17192160|rs12408988</td></tr>
</table>

Oral Ulcers	GLYCOSAMINOGLYCAN_BIOSYNTHESIS_CHONDROITIN_SULFATE	KEGG	1372	29	20	3,10E-01	7,49E-01	rs11751562|rs219233|rs7943044|rs11753710|rs4564548|rs4344725|rs7812088|rs4707862|rs7071860|rs1382498|rs2748331|rs753353|rs2682185|rs931932|rs8058794|rs6983139|rs1944886|rs9377158|rs3017976|rs1939726
Oral Ulcers	NUCLEOTIDE_LIKE_PURINERGIC_RECEPTORS	REACTOME	272	16	7	3,10E-01	7,49E-01	rs859253|rs2364813|rs11687066|rs2800881|rs7542294|rs9903817|rs2250509
Oral Ulcers	NEURONAL_SYSTEM	REACTOME	13378	31	20	3,11E-01	7,49E-01	rs1264866|rs1366632|rs263241|rs1023681|rs4926290|rs7396875|rs1541821|rs11654455|rs9322668|rs9922023|rs2562582|rs10835507|rs12334868|rs1489635|rs7938219|rs16837945|rs697928|rs11741316|rs16904360|rs127433
Oral Ulcers	KERATAN_SULFATE_KERATIN_METABOLISM	REACTOME	737	43	20	3,12E-01	7,49E-01	rs1554324|rs1667255|rs12680336|rs3791039|rs741900|rs1667271|rs1401346|rs11038507|rs4756006|rs7646486|rs4438612|rs10805890|rs7409343|rs2922464|rs7019909|rs1702956|rs13282872|rs17195011|rs10800919|rs7854765
Oral Ulcers	IL10_PATHWAY	BIOCARTA	288	25	12	3,12E-01	7,49E-01	rs4803342|rs7801617|rs310209|rs4645742|rs12535797|rs133416|rs7601754|rs13192469|rs2307344|rs17121510|rs4645752|rs13029532
Oral Ulcers	FORMATION_OF_THE_HIV1_EARLY_ELONGATION_COMPLEX	REACTOME	329	24	12	3,13E-01	7,49E-01	rs4487686|rs2257205|rs1131384|rs4150454|rs16902819|rs2072102|rs1613070|rs2532929|rs4151374|rs4150667|rs2020892|rs8102597
Oral Ulcers	ATM_PATHWAY	BIOCARTA	397	35	11	3,14E-01	7,49E-01	rs1805812|rs2697675|rs11244164|rs11792889|rs12966840|rs540723|rs230530|rs4326648|rs1801516|rs2251403|rs1321311
Oral Ulcers	AUTOIMMUNE_THYROID_DISEASE	KEGG	1646	81	20	3,14E-01	7,49E-01	rs2853923|rs9658702|rs3997854|rs3135363|rs9357122|rs3746821|rs11580042|rs7910825|rs1634776|rs1926189|rs7192|rs2523580|rs4977686|rs9368726|rs376892|rs2154254|rs28397299|rs949832|rs2243290|rs9275582
Oral Ulcers	SIGNALING_BY_BMP	REACTOME	918	45	20	3,15E-01	7,49E-01	rs7512482|rs11858577|rs2700262|rs2469109|rs1005351|rs924311|rs7225993|rs1438868|rs1499916|rs4630700|rs1733324|rs7349350|rs12958604|rs2216930|rs3736242|rs6430191|rs879081|rs263526|rs893912|rs4659986
Oral Ulcers	SIGNALING_BY_NOTCH	REACTOME	4553	32	20	3,17E-01	7,51E-01	rs4877524|rs4852054|rs10773086|rs2457400|rs12589158|rs1995243|rs1328413|rs4739987|rs10272709|rs838511|rs273473|rs12588669|rs1483023|rs4411562|rs304987|rs3791039|rs3130304|rs4687504|rs17801563|rs1154827
Oral Ulcers	CIRCADIAN_CLOCK	REACTOME	2075	28	20	3,18E-01	7,51E-01	rs770466|rs7167741|rs1995243|rs1555208|rs219233|rs11071587|rs2278722|rs1422189|rs1472782|rs7812088|rs8042149|rs10162630|rs13053242|rs17024814|rs2946385|rs6789040|rs6809156|rs1800206|rs6819019|rs2047564
Oral Ulcers	RESPIRATORY_ELECTRON_TRANSPORT_ATP_SYNTHESIS_BY_CHEMIOSMOTIC_COUPLIN<br>TION_BY_UNCOUPLING_PROTEINS_	REACTOME	1741	37	20	3,20E-01	7,51E-01	rs11631423|rs11665724|rs7689418|rs17163678|rs4975854|rs2240760|rs17620912|rs39425|rs11956638|rs10952211|rs7904875|rs6722509|rs2971117|rs11255374|rs10424816|rs12965084|rs2061781|rs688958|rs1031609|rs4646812
Oral Ulcers	INTERFERON_GAMMA_SIGNALING	REACTOME	2043	43	20	3,20E-01	7,51E-01	rs2853923|rs919741|rs3822295|rs3997854|rs7196849|rs9348863|rs2025258|rs310209|rs2285703|rs6540245|rs305059|rs8074003|rs8052106|rs12521868|rs625087|rs1634776|rs11698821|rs17389541|rs6533711|rs2871398
Oral Ulcers	TRYPTOPHAN_METABOLISM	KEGG	953	27	20	3,21E-01	7,51E-01	rs1932440|rs3741049|rs17023223|rs1885490|rs13027051|rs2181139|rs172424|rs6719977|rs3774051|rs12155014|rs2293682|rs2878125|rs10196846|rs2057374|rs2348024|rs12797274|rs2408700|rs15677|rs11124907|rs352929
Oral Ulcers	N_GLYCAN_ANTENNAE_ELONGATION_IN_THE_MEDIAL_TRANS_GOLGI	REACTOME	962	39	20	3,21E-01	7,51E-01	rs7143906|rs3759917|rs1667255|rs8037921|rs636757|rs11629679|rs4284481|rs12495023|rs1667271|rs17102896|rs4902433|rs3769693|rs11684754|rs1386775|rs2318310|rs3748896|rs8087849|rs12962777|rs745775|rs11158590
Oral Ulcers	DNA_REPAIR	REACTOME	2111	22	20	3,22E-01	7,51E-01	rs7086428|rs4487686|rs3793910|rs1805812|rs1861415|rs7003908|rs2697675|rs1131384|rs2239359|rs1861145|rs4439380|rs942592|rs2722191|rs1711655|rs12571913|rs1762430|rs13029039|rs1405655|rs9299131|rs4150454
Oral Ulcers	PI3K_EVENTS_IN_ERBB2_SIGNALING	REACTOME	1886	42	20	3,25E-01	7,51E-01	rs10940145|rs7726943|rs10461535|rs4690785|rs2372183|rs4976028|rs9576991|rs16847152|rs3764351|rs11895509|rs16999922|rs17673576|rs10515068|rs17719687|rs12990694|rs34322|rs7719763|rs9566508|rs896577|rs17254382
Oral Ulcers	DARPP_32_EVENTS	REACTOME	879	32	20	3,25E-01	7,51E-01	rs6852347|rs13434873|rs10491353|rs2851045|rs2303924|rs1297783|rs931640|rs539322|rs16889921|rs12453682|rs294482|rs7524898|rs872077|rs6449471|rs37691|rs7526798|rs3729877|rs17780860|rs316689|rs1078368
Oral Ulcers	INSULIN_RECEPTOR_RECYCLING	REACTOME	492	35	15	3,26E-01	7,51E-01	rs1871093|rs2346412|rs1006049|rs2734574|rs6983249|rs2004997|rs7579208|rs13192469|rs3814043|rs1018088|rs9828099|rs4646812|rs498193|rs4804366|rs1390943
Oral Ulcers	GLYOXYLATE_AND_DICARBOXYLATE_METABOLISM	KEGG	704	32	17	3,28E-01	7,51E-01	rs7752450|rs11983987|rs10973340|rs738140|rs7047872|rs4816009|rs137831|rs7660423|rs175862|rs11627525|rs309455|rs2223534|rs7742684|rs942382|rs2768659|rs10970972|rs1555179
Oral Ulcers	RESPIRATORY_ELECTRON_TRANSPORT	REACTOME	1631	40	20	3,28E-01	7,51E-01	rs11631423|rs11665724|rs7689418|rs17163678|rs4975854|rs2240760|rs17620912|rs39425|rs11956638|rs10952211|rs6722509|rs2971117|rs10424816|rs12965084|rs2061781|rs688958|rs1031609|rs4646812|rs7560797|rs2284087
Oral Ulcers	METABOLISM_OF_NUCLEOTIDES	REACTOME	1559	40	20	3,28E-01	7,51E-01	rs6707705|rs17471640|rs940296|rs2853229|rs2278092|rs7544426|rs6816128|rs17100521|rs11165845|rs3826543|rs7552825|rs3779647|rs6759948|rs2071017|rs1653249|rs2304891|rs743961|rs10747488|rs17246034|rs2853129
Oral Ulcers	CCR5_PATHWAY	BIOCARTA	696	42	20	3,29E-01	7,51E-01	rs1779344|rs227913|rs266087|rs9892886|rs11860367|rs1297783|rs11688530|rs931640|rs8051034|rs11145635|rs2781548|rs6504424|rs3780305|rs8078231|rs4245843|rs4326648|rs4954592|rs1855531|rs9927848|rs11646540
Oral Ulcers	KINESINS	REACTOME	566	19	11	3,30E-01	7,51E-01	rs9299670|rs12587001|rs1579282|rs2286746|rs8082530|rs7156395|rs2377473|rs988435|rs7642843|rs11713101|rs1775725
Oral Ulcers	ION_CHANNEL_TRANSPORT	REACTOME	2741	51	20	3,31E-01	7,52E-01	rs1041052|rs13064158|rs10063828|rs4770728|rs1403478|rs306401|rs26797|rs9313857|rs6442178|rs1380754|rs7672100|rs11949658|rs11917518|rs1035750|rs4622889|rs9551233|rs2414007|rs13100251|rs4906864|rs154111
Oral Ulcers	NUCLEAR_RECEPTOR_TRANSCRIPTION_PATHWAY	REACTOME	2750	36	20	3,33E-01	7,53E-01	rs6800549|rs2664339|rs7167741|rs1555208|rs11117791|rs11071587|rs9850879|rs2683530|rs17783432|rs1562737|rs7635612|rs1849|rs6665426|rs7567451|rs1405655|rs8042149|rs10162630|rs6771638|rs487359|rs3750420
Oral Ulcers	RAP1_SIGNALLING	REACTOME	835	46	20	3,36E-01	7,53E-01	rs10489469|rs10823962|rs7893773|rs508594|rs2317467|rs4275567|rs10490975|rs211085|rs7898516|rs3767132|rs10822117|rs7524898|rs872077|rs4935216|rs10490969|rs2074532|rs7526798|rs4734507|rs2081406|rs4086289
Oral Ulcers	AQUAPORIN_MEDIATED_TRANSPORT	REACTOME	1510	33	20	3,36E-01	7,53E-01	rs263241|rs1541821|rs12334868|rs16904360|rs2256156|rs72001|rs1017414|rs7566051|rs2058166|rs13356161|rs10510036|rs3892156|rs2601787|rs2414891|rs874627|rs1993716|rs951388|rs17221445|rs7524898|rs872077
Oral Ulcers	SIGNALING_BY_NODAL	REACTOME	632	52	20	3,37E-01	7,53E-01	rs12906930|rs11631839|rs2643358|rs1438868|rs1499916|rs4630700|rs3784491|rs7349350|rs12958604|rs2216930|rs266366|rs10915896|rs11713702|rs9961435|rs6430191|rs3827494|rs10514714|rs10799329|rs2532100|rs1520494
Oral Ulcers	JNK_C_JUN_KINASES_PHOSPHORYLATION_AND_ACTIVATION_MEDIATED_BY_ACTIVATE	REACTOME	728	34	20	3,38E-01	7,53E-01	rs1351726|rs7841459|rs711272|rs4613794|rs2575676|rs10097279|rs12205556|rs2721854|rs3749791|rs12191179|rs2529718|rs9345126|rs730688|rs9345117|rs3810213|rs538723|rs2869442|rs556335|rs39502|rs1884839
Oral Ulcers	PHOSPHOLIPASE_C_MEDIATED_CASCADE	REACTOME	2628	32	20	3,38E-01	7,53E-01	rs263241|rs1541821|rs12334868|rs16904360|rs11167777|rs2256156|rs227913|rs281508|rs72001|rs12542904|rs17487009|rs9510050|rs152442|rs7566051|rs12523624|rs9892886|rs12020729|rs2058166|rs11199823|rs733895
Oral Ulcers	PYRUVATE_METABOLISM	REACTOME	209	17	6	3,38E-01	7,53E-01	rs12681770|rs3133976|rs11982165|rs2239953|rs2267366|rs2230392
Oral Ulcers	PLATELET_ACTIVATION_SIGNALING_AND_AGGREGATION	REACTOME	6965	36	20	3,39E-01	7,53E-01	rs10940145|rs10489469|rs809192|rs7726943|rs1074801|rs2965012|rs16897175|rs3758919|rs10889117|rs2680182|rs2274410|rs952784|rs2333512|rs2288810|rs6590911|rs227913|rs810687|rs2372183|rs281508|rs1799852
Oral Ulcers	CITRIC_ACID_CYCLE_TCA_CYCLE	REACTOME	675	32	19	3,40E-01	7,53E-01	rs11983987|rs2341694|rs9972327|rs738140|rs2885856|rs1918094|rs8024587|rs12155014|rs4146945|rs17085|rs137831|rs11072732|rs12652118|rs7647085|rs1886446|rs1326481|rs11894798|rs6451722|rs2422821
Oral Ulcers	TRNA_AMINOACYLATION	REACTOME	795	30	20	3,41E-01	7,53E-01	rs9369476|rs10931791|rs10914625|rs17023223|rs3197232|rs11243014|rs38494|rs685653|rs7763309|rs2726486|rs2227956|rs480092|rs2713829|rs3101017|rs2532929|rs2270003|rs3600|rs4401711|rs2876124|rs12730226
Oral Ulcers	VIF_MEDIATED_DEGRADATION_OF_APOBEC3G	REACTOME	738	45	19	3,42E-01	7,53E-01	rs2294183|rs328349|rs8066176|rs8009527|rs5758168|rs4710834|rs3820667|rs4148876|rs2584624|rs1045288|rs3859192|rs15677|rs11543947|rs926914|rs11023238|rs653079|rs4980335|rs2597189|rs12807544
Oral Ulcers	ACTIVATION_OF_NMDA_RECEPTOR_UPON_GLUTAMATE_BINDING_AND_POSTSYNAPTIC_	REACTOME	2117	41	20	3,43E-01	7,53E-01	rs263241|rs7396875|rs9922023|rs12334868|rs16904360|rs919741|rs3822295|rs2285703|rs11055671|rs7566051|rs378373|rs1297783|rs10492130|rs931640|rs6533711|rs491700|rs17221445|rs7524898|rs2223405|rs388372
Oral Ulcers	P53_INDEPENDENT_G1_S_DNA_DAMAGE_CHECKPOINT	REACTOME	729	42	18	3,44E-01	7,54E-01	rs2294183|rs328349|rs8066176|rs8009527|rs4710834|rs3820667|rs4148876|rs2584624|rs1045288|rs3859192|rs540723|rs11543947|rs11023238|rs1801516|rs653079|rs4980335|rs2597189|rs6797587
Oral Ulcers	REGULATION_OF_MRNA_STABILITY_BY_PROTEINS_THAT_BIND_AU_RICH_ELEMENTS	REACTOME	1372	43	20	3,46E-01	7,55E-01	rs2294183|rs10942495|rs328349|rs227913|rs3843552|rs17460250|rs8066176|rs9892886|rs8009527|rs2075755|rs4710834|rs3820667|rs4148876|rs2665833|rs2152092|rs1045288|rs3859192|rs2076139|rs6792874|rs11218954
Oral Ulcers	GROWTH_HORMONE_RECEPTOR_SIGNALING	REACTOME	773	34	20	3,47E-01	7,55E-01	rs952784|rs1207774|rs1922529|rs182832|rs11759745|rs8074003|rs9588044|rs404220|rs11698821|rs13212670|rs2955234|rs7834615|rs12153009|rs11751260|rs17813240|rs13157622|rs4292454|rs9350403|rs9301405|rs1576166
Oral Ulcers	SIGNALING_BY_HIPPO	REACTOME	627	17	10	3,48E-01	7,55E-01	rs4647693|rs9767555|rs7271519|rs390200|rs6031944|rs4790152|rs2400273|rs11748886|rs2062784|rs1719336
Oral Ulcers	PTDINS_PATHWAY	BIOCARTA	1058	41	20	3,48E-01	7,55E-01	rs4880592|rs7924157|rs952784|rs17020888|rs281508|rs182832|rs1931865|rs6771638|rs1292035|rs4953334|rs1887319|rs3829916|rs4952779|rs11251481|rs7834615|rs3768751|rs17813240|rs1028846|rs4436465|rs4074080
Oral Ulcers	AMINOACYL_TRNA_BIOSYNTHESIS	KEGG	731	31	20	3,50E-01	7,56E-01	rs9369476|rs10931791|rs10914625|rs17023223|rs3197232|rs11243014|rs38494|rs685653|rs7763309|rs2227956|rs480092|rs3101017|rs7085513|rs2532929|rs2270003|rs3600|rs12730226|rs797145|rs707937|rs2228224
Oral Ulcers	FRUCTOSE_AND_MANNOSE_METABOLISM	KEGG	984	34	20	3,52E-01	7,59E-01	rs4880592|rs7924157|rs1539234|rs226468|rs17592080|rs7788953|rs923273|rs1931865|rs9611613|rs2171385|rs3829916|rs12210306|rs11251481|rs1646744|rs3778533|rs2569860|rs600130|rs3750671|rs10761339|rs7817826
Oral Ulcers	ENDOSOMAL_SORTING_COMPLEX_REQUIRED_FOR_TRANSPORT_ESCRT	REACTOME	318	16	8	3,54E-01	7,60E-01	rs1078523|rs153060|rs595158|rs10500836|rs7836184|rs1488360|rs11230611|rs12714198
Oral Ulcers	SPRY_PATHWAY	BIOCARTA	910	40	20	3,55E-01	7,60E-01	rs7396875|rs1022373|rs17753395|rs2123381|rs7981942|rs9601762|rs17673576|rs6563230|rs11618221|rs9574902|rs2877260|rs1333411|rs17814416|rs2908528|rs13131772|rs1036253|rs9307531|rs2503378|rs9318814|rs11976696
Oral Ulcers	HS_GAG_DEGRADATION	REACTOME	1386	39	20	3,57E-01	7,64E-01	rs4949320|rs2182542|rs12408228|rs3828334|rs4917833|rs10883109|rs11070048|rs10883150|rs4773567|rs9555997|rs7981707|rs1836911|rs8000454|rs659897|rs9301667|rs2057524|rs7988257|rs7998675|rs4438612|rs11616669
Oral Ulcers	WNT_SIGNALING_PATHWAY	KEGG	5611	28	20	3,61E-01	7,71E-01	rs6684209|rs6852347|rs6843675|rs12906930|rs11631839|rs1995243|rs2796806|rs919741|rs3822295|rs227913|rs6096351|rs4098686|rs936213|rs13434873|rs711272|rs205898|rs2860529|rs6732834|rs227142|rs2285703
Oral Ulcers	CIRCADIAN_REPRESSION_OF_EXPRESSION_BY_REV_ERBA	REACTOME	1358	34	20	3,64E-01	7,74E-01	rs7167741|rs1995243|rs1555208|rs219233|rs11071587|rs2278722|rs7812088|rs8042149|rs10162630|rs13053242|rs17024814|rs2946385|rs6789040|rs1800206|rs6819019|rs2047564|rs11655550|rs1889155|rs16874509|rs6794849
Oral Ulcers	DESTABILIZATION_OF_MRNA_BY_TRISTETRAPROLIN_TTP	REACTOME	179	11	5	3,65E-01	7,75E-01	rs10942495|rs2152092|rs6792874|rs31726|rs2229032
Oral Ulcers	AUTODEGRADATION_OF_CDH1_BY_CDH1_APC_C	REACTOME	857	40	20	3,66E-01	7,75E-01	rs2294183|rs328349|rs399672|rs8066176|rs8009527|rs6736093|rs4710834|rs3820667|rs4148876|rs6841355|rs2665833|rs1045288|rs3859192|rs11543947|rs2061185|rs11023238|rs653079|rs4980335|rs2597189|rs12485431
Oral Ulcers	GLYCINE_SERINE_AND_THREONINE_METABOLISM	KEGG	564	27	14	3,69E-01	7,75E-01	rs1025690|rs2651244|rs666930|rs12449313|rs3124455|rs17246034|rs7522196|rs9687411|rs3918347|rs2273026|rs17131359|rs216195|rs2431332|rs7849881
Oral Ulcers	POST_CHAPERONIN_TUBULIN_FOLDING_PATHWAY	REACTOME	254	9	5	3,70E-01	7,75E-01	rs2291688|rs7589023|rs2270461|rs3745571|rs3212358
Oral Ulcers	LONG_TERM_DEPRESSION	KEGG	3926	24	20	3,71E-01	7,75E-01	rs4926290|rs7396875|rs7169544|rs10823962|rs6804671|rs952784|rs2241952|rs227913|rs1019731|rs354263|rs936213|rs1840572|rs7893773|rs182832|rs227142|rs7542020|rs2119802|rs3805021|rs4691787|rs17033553
Oral Ulcers	MYOSIN_PATHWAY	BIOCARTA	1253	36	20	3,71E-01	7,75E-01	rs6981540|rs1861954|rs227913|rs227142|rs9892886|rs11860367|rs17830077|rs4773326|rs12600950|rs3767423|rs4876265|rs11130558|rs6086543|rs8051034|rs820336|rs4530474|rs11145635|rs6086402|rs6504424|rs3780305
Oral Ulcers	BOTULINUM_NEUROTOXICITY	REACTOME	522	25	17	3,71E-01	7,75E-01	rs127433|rs7272324|rs11654567|rs3813356|rs1549333|rs2788942|rs1795994|rs11114112|rs2206161|rs7970029|rs2168856|rs2304911|rs7967341|rs2695872|rs505431|rs1381885|rs362599
Oral Ulcers	RORA_ACTIVATES_CIRCADIAN_EXPRESSION	REACTOME	1377	34	20	3,75E-01	7,80E-01	rs7167741|rs1995243|rs1555208|rs219233|rs11071587|rs2278722|rs7812088|rs8042149|rs10162630|rs13053242|rs17024814|rs2946385|rs6789040|rs1800206|rs6819019|rs2047564|rs11655550|rs1889155|rs6794849|rs16874509
Oral Ulcers	G_ALPHA_Z_SIGNALLING_EVENTS	REACTOME	2191	38	20	3,76E-01	7,80E-01	rs263241|rs1541821|rs12334868|rs16904360|rs2256156|rs227913|rs281508|rs72001|rs510745|rs10508312|rs7566051|rs9892886|rs11860367|rs2058166|rs4953334|rs1593933|rs3892156|rs2601787|rs8051034|rs874627
Oral Ulcers	ALZHEIMERS_DISEASE	KEGG	5029	34	20	3,78E-01	7,84E-01	rs6852347|rs1317242|rs9922023|rs3774609|rs11665724|rs4074889|rs6804671|rs17163678|rs4975854|rs11630176|rs9658702|rs936213|rs13434873|rs4647693|rs10049492|rs7216531|rs2240760|rs17620912|rs227142|rs39425
Oral Ulcers	PI3K_EVENTS_IN_ERBB4_SIGNALING	REACTOME	1709	44	20	3,80E-01	7,85E-01	rs10940145|rs7726943|rs16897175|rs2372183|rs4976028|rs9576991|rs16847152|rs11895509|rs16999923|rs10515068|rs17719687|rs12990694|rs34322|rs7719763|rs9566508|rs896577|rs17254382|rs863818|rs10932383|rs2888104
Oral Ulcers	PHOSPHORYLATION_OF_THE_APC_C	REACTOME	219	7	5	3,81E-01	7,85E-01	rs399672|rs6736093|rs6841355|rs2061185|rs12485431
Oral Ulcers	APC_C_CDC20_MEDIATED_DEGRADATION_OF_CYCLIN_B	REACTOME	224	7	5	3,82E-01	7,85E-01	rs399672|rs6736093|rs6841355|rs2061185|rs12485431
Oral Ulcers	IMMUNOREGULATORY_INTERACTIONS_BETWEEN_A_LYMPHOID_AND_A_NON_LYMPHO	REACTOME	1325	50	20	3,84E-01	7,85E-01	rs2853923|rs3873381|rs721336|rs13353326|rs3746821|rs7773464|rs502514|rs10910843|rs2733840|rs11009157|rs1634776|rs11569429|rs10432228|rs7255954|rs2523580|rs2279623|rs2949659|rs2363863|rs11673632|rs28397299
Oral Ulcers	CONVERSION_FROM_APC_C_CDC20_TO_APC_C_CDH1_IN_LATE_ANAPHASE	REACTOME	226	7	5	3,84E-01	7,85E-01	rs399672|rs6736093|rs6841355|rs2061185|rs12485431
Oral Ulcers	INNATE_IMMUNE_SYSTEM	REACTOME	6278	35	20	3,85E-01	7,85E-01	rs770466|rs11776120|rs7699006|rs9909750|rs1941593|rs26529|rs4710181|rs17020888|rs1351726|rs7841459|rs919308|rs1422189|rs711272|rs659445|rs896117|rs9988620|rs3813982|rs3738448|rs4613794|rs2555454
Oral Ulcers	ONE_CARBON_POOL_BY_FOLATE	KEGG	441	17	13	3,86E-01	7,85E-01	rs7752450|rs12442814|rs4646745|rs12449313|rs7660423|rs4646706|rs175862|rs11627525|rs2273026|rs7742684|rs942382|rs1555179|rs12404124
Oral Ulcers	GALACTOSE_METABOLISM	KEGG	759	31	20	3,86E-01	7,85E-01	rs4880592|rs7924157|rs8067341|rs10265585|rs7788953|rs923273|rs1931865|rs2171385|rs3829916|rs1880924|rs11251481|rs11150846|rs1646744|rs4438612|rs4436465|rs919005|rs2670854|rs7019909|rs1702956|rs2838544
Oral Ulcers	THROMBIN_SIGNALLING_THROUGH_PROTEINASE_ACTIVATED_RECEPTORS_PARS	REACTOME	690	31	13	3,94E-01	7,97E-01	rs10869950|rs1162211|rs11948805|rs1144430|rs506233|rs874627|rs11145635|rs11880198|rs11145517|rs12938589|rs208358|rs6429146|rs12610831
Oral Ulcers	MUSCLE_CONTRACTION	REACTOME	1023	36	20	3,94E-01	7,97E-01	rs9658702|rs399672|rs2347897|rs1050162|rs17605348|rs16946204|rs3764351|rs4764808|rs1297783|rs7910825|rs6887205|rs931640|rs820336|rs3800702|rs1653249|rs725926|rs4530474|rs6671391|rs216163|rs907606
Oral Ulcers	GLYCOLYSIS_GLUCONEOGENESIS	KEGG	1391	31	20	3,99E-01	8,04E-01	rs1019635|rs4880592|rs7924157|rs8067341|rs10516988|rs1885490|rs923273|rs1931865|rs2181139|rs9691221|rs6050291|rs6037013|rs2048022|rs2171385|rs3829916|rs7534552|rs11251481|rs2057374|rs600130|rs2408700
Oral Ulcers	REGULATION_OF_ORNITHINE_DECARBOXYLASE_ODC	REACTOME	773	45	19	4,02E-01	8,08E-01	rs2294183|rs328349|rs8066176|rs8009527|rs4710834|rs3820667|rs4148876|rs2665833|rs1045288|rs2917682|rs3859192|rs2513892|rs11543947|rs11023238|rs757233|rs653079|rs4980335|rs6991188|rs2597189
Oral Ulcers	UBIQUITIN_MEDIATED_PROTEOLYSIS	KEGG	3227	32	20	4,04E-01	8,10E-01	rs4072410|rs1894752|rs12533310|rs2088747|rs2853229|rs1861415|rs2644494|rs399672|rs1861145|rs952901|rs371513|rs502514|rs10112123|rs8074003|rs7225993|rs2399106|rs9355387|rs13029039|rs6490100|rs5758168
Oral Ulcers	GLYCOSAMINOGLYCAN_BIOSYNTHESIS_HEPARAN_SULFATE	KEGG	2273	29	20	4,06E-01	8,13E-01	rs3749862|rs7664795|rs10470840|rs4438820|rs2098026|rs1886634|rs1013120|rs17665827|rs7943044|rs4564548|rs4344725|rs9372417|rs11098419|rs3811755|rs12952904|rs4707862|rs1382498|rs10955842|rs2748331|rs753353
Oral Ulcers	INFLAM_PATHWAY	BIOCARTA	747	53	20	4,09E-01	8,13E-01	rs2965012|rs7801617|rs3135363|rs6692647|rs6835391|rs1058511|rs12535797|rs7192|rs874627|rs9368726|rs13192469|rs12511035|rs17506083|rs2243290|rs2717548|rs9916158|rs8110090|rs7643177|rs4694636|rs4803455
Oral Ulcers	NTHI_PATHWAY	BIOCARTA	876	45	20	4,09E-01	8,13E-01	rs12906930|rs11631839|rs711272|rs4613794|rs2112662|rs13053242|rs12205556|rs12191179|rs12958604|rs2076139|rs13192469|rs9345126|rs230530|rs266366|rs9345117|rs538723|rs12517663|rs1884839|rs1143634|rs4694636
Oral Ulcers	CYTOSOLIC_DNA_SENSING_PATHWAY	KEGG	620	33	20	4,09E-01	8,13E-01	rs6081172|rs7801617|rs7712322|rs3813982|rs6924483|rs2890704|rs12535797|rs137831|rs492553|rs4977686|rs1535565|rs230530|rs742724|rs6128102|rs1143634|rs2303801|rs8118279|rs10964832|rs3818411|rs4981508
Oral Ulcers	TRANSCRIPTIONAL_REGULATION_OF_WHITE_ADIPOCYTE_DIFFERENTIATION	REACTOME	2719	29	20	4,11E-01	8,13E-01	rs7170179|rs10773086|rs1995243|rs1555208|rs219233|rs12351531|rs838511|rs8031224|rs780885|rs7864185|rs7812088|rs17176425|rs11968166|rs10512367|rs4979136|rs12426133|rs10520789|rs7138692|rs4979065|rs244647
Oral Ulcers	TRAF6_MEDIATED_NFKB_ACTIVATION	REACTOME	351	10	9	4,15E-01	8,16E-01	rs9909750|rs11024603|rs452987|rs11868917|rs11603089|rs17074679|rs9550565|rs3130283|rs3818411
Oral Ulcers	RIG_I_MDA5_MEDIATED_INDUCTION_OF_IFN_ALPHA_BETA_PATHWAYS	REACTOME	1129	32	20	4,16E-01	8,16E-01	rs7699006|rs9909750|rs26529|rs3813982|rs13053242|rs12521868|rs2144825|rs11024603|rs2871398|rs766908|rs12448377|rs452987|rs4977686|rs10783580|rs11868917|rs668033|rs555745|rs11603089|rs17074679|rs9550565
Oral Ulcers	P38MAPK_PATHWAY	BIOCARTA	1904	30	20	4,16E-01	8,16E-01	rs770466|rs7396875|rs2965012|rs354263|rs1422189|rs711272|rs6692647|rs4613794|rs926324|rs16893188|rs12205556|rs2721854|rs10092081|rs2383623|rs12191179|rs4902371|rs12315146|rs2529718|rs9345126|rs2049824
Oral Ulcers	APC_C_CDH1_MEDIATED_DEGRADATION_OF_CDC20_AND_OTHER_APC_C_CDH1_TARGE<br>TE_MITOSIS_EARLY_G1	REACTOME	959	40	20	4,17E-01	8,16E-01	rs2294183|rs328349|rs399672|rs8066176|rs8009527|rs3826543|rs6736093|rs4710834|rs3820667|rs4148876|rs6841355|rs2665833|rs1045288|rs3859192|rs11543947|rs2061185|rs11023238|rs653079|rs4980335|rs2597189
Oral Ulcers	NOTCH_SIGNALING_PATHWAY	KEGG	1387	26	20	4,17E-01	8,16E-01	rs10773086|rs838511|rs17255455|rs3130304|rs4687504|rs11145770|rs900030|rs17643974|rs12426133|rs9855959|rs2293797|rs13053242|rs2701623|rs569977|rs390200|rs9423033|rs4972661|rs10933711|rs1887319|rs17287454
Oral Ulcers	ACTINY_PATHWAY	BIOCARTA	290	20	12	4,18E-01	8,16E-01	rs268852|rs924483|rs2504786|rs1002063|rs560873|rs2287064|rs688242|rs1888861|rs3779258|rs2689420|rs12466364|rs3769676
Oral Ulcers	TRANSPORT_OF_MATURE_TRANSCRIPT_TO_CYTOPLASM	REACTOME	693	18	15	4,23E-01	8,22E-01	rs4611262|rs10852553|rs12024682|rs4648873|rs4684126|rs4869498|rs16987990|rs12472874|rs2383477|rs6103330|rs580438|rs1972947|rs9916811|rs3115752|rs13054435
Oral Ulcers	TGF_BETA_SIGNALING_PATHWAY	KEGG	3535	32	20	4,24E-01	8,22E-01	rs10933441|rs12906930|rs2965012|rs11631839|rs11858577|rs1825443|rs16883016|rs1047033|rs7562511|rs4235993|rs7966281|rs267840|rs1026487|rs2469109|rs1005351|rs7552931|rs924311|rs7225993|rs1438868|rs1499916
Oral Ulcers	INTEGRIN_PATHWAY	BIOCARTA	1027	42	20	4,25E-01	8,22E-01	rs10489469|rs7396875|rs809192|rs3791978|rs508594|rs3767423|rs750132|rs1002063|rs6887205|rs11009157|rs2239206|rs4326648|rs5759636|rs2153875|rs3763630|rs735986|rs943851|rs2017342|rs1195962|rs3012750
Oral Ulcers	ACTIVATION_OF_THE_PRE_REPLICATIVE_COMPLEX	REACTOME	565	31	12	4,26E-01	8,22E-01	rs194825|rs7808273|rs4645742|rs1467464|rs133416|rs13447454|rs5748218|rs2071744|rs2307344|rs4645752|rs9349915|rs1539604
Oral Ulcers	SMAD2_SMAD3_SMAD4_HETEROTRIMER_REGULATES_TRANSCRIPTION	REACTOME	715	36	17	4,29E-01	8,22E-01	rs12906930|rs11631839|rs2364598|rs16893188|rs9579015|rs10092081|rs12958604|rs1011970|rs11874742|rs266366|rs3736242|rs9961435|rs2049824|rs1537377|rs16955746|rs9635893|rs4733792
Oral Ulcers	AMYLOIDS	REACTOME	972	22	15	4,29E-01	8,22E-01	rs1207774|rs1667255|rs17078876|rs11759745|rs6882087|rs11024603|rs452987|rs13212670|rs11751260|rs11603089|rs9379780|rs2139634|rs12340264|rs34719083|rs9350403
Oral Ulcers	PEROXISOME	KEGG	2047	26	20	4,30E-01	8,22E-01	rs9687490|rs13054099|rs17485390|rs12146602|rs2842994|rs9972327|rs1342645|rs2565050|rs5992985|rs10024579|rs10877201|rs12054736|rs1882567|rs1127745|rs934153|rs8024587|rs3774051|rs4148045|rs6852527|rs17562893

Oral Ulcers	NEF_MEDIATES_DOWN_MODULATION_OF_CELL_SURFACE_RECEPTORS_BY_RECRUITINC N_ADAPTERS	REACTOME	366	7	4	4,30E-01	8,22E-01	rs1551907|rs874627|rs11604757|rs1004420
Oral Ulcers	P53_DEPENDENT_G1_DNA_DAMAGE_RESPONSE	REACTOME	838	44	19	4,31E-01	8,22E-01	rs2294183|rs328349|rs8066176|rs8009527|rs34322|rs4710834|rs3820667|rs4148876|rs2584624|rs1045288|rs3859192|rs11543947|rs11023238|rs1801516|rs1321311|rs6989591|rs653079|rs4980335|rs2597189
Oral Ulcers	PENTOSE_PHOSPHATE_PATHWAY	KEGG	616	35	20	4,32E-01	8,22E-01	rs4880592|rs7924157|rs10489436|rs2032539|rs1931865|rs6963707|rs3829916|rs11251481|rs600130|rs10761339|rs12699949|rs10902218|rs335124|rs4436465|rs7633966|rs919005|rs7804628|rs2838544|rs945142|rs422732
Oral Ulcers	TYPE_I_DIABETES_MELLITUS	KEGG	1975	61	20	4,33E-01	8,22E-01	rs2853923|rs9658702|rs3997854|rs1076434|rs3135363|rs9348862|rs7662154|rs11580042|rs7589023|rs7910825|rs1634776|rs1926189|rs7192|rs3807840|rs2523580|rs9368726|rs376892|rs2154254|rs13192469|rs17687250
Oral Ulcers	CK1_PATHWAY	BIOCARTA	759	28	20	4,34E-01	8,22E-01	rs6852347|rs13434873|rs227142|rs2851045|rs2303924|rs6589386|rs6086543|rs12453682|rs7524898|rs872077|rs6086402|rs7526798|rs6118062|rs3729877|rs2179798|rs316689|rs4816089|rs7755078|rs1983635|rs6055748
Oral Ulcers	SHH_PATHWAY	BIOCARTA	679	29	20	4,36E-01	8,25E-01	rs3779173|rs12464700|rs9986932|rs2299146|rs1991769|rs6771638|rs6961071|rs6709236|rs7524898|rs872077|rs10278993|rs11762523|rs7526798|rs3729877|rs10949798|rs17440799|rs1568836|rs12698335|rs316689|rs2001595
Oral Ulcers	TH1TH2_PATHWAY	BIOCARTA	535	32	8	4,37E-01	8,25E-01	rs3135363|rs3746821|rs706779|rs7192|rs9368726|rs2243290|rs7643177|rs1041973
Oral Ulcers	MITOTIC_PROMETAPHASE	REACTOME	1814	25	20	4,44E-01	8,32E-01	rs1934497|rs4611262|rs4804147|rs5758511|rs11603446|rs6507992|rs17007652|rs1741143|rs17614161|rs3826543|rs2885987|rs1934520|rs2459975|rs6550498|rs1980705|rs12489804|rs1062492|rs6696295|rs4758695|rs10514815
Oral Ulcers	METABOLISM_OF_NON_CODING_RNA	REACTOME	611	15	13	4,45E-01	8,32E-01	rs4611262|rs10852553|rs12024682|rs4684126|rs4869498|rs12472874|rs2383477|rs197414|rs580438|rs1972947|rs2847117|rs3115752|rs13054435
Oral Ulcers	FMLP_PATHWAY	BIOCARTA	1148	26	20	4,48E-01	8,32E-01	rs6852347|rs7396875|rs6096351|rs13434873|rs227142|rs1816971|rs2851045|rs1297783|rs6086543|rs931640|rs7236492|rs11880198|rs6086402|rs230530|rs6118062|rs2179798|rs660749|rs4816089|rs2689420|rs904160
Oral Ulcers	RIBOFLAVIN_METABOLISM	KEGG	295	22	10	4,50E-01	8,32E-01	rs9493105|rs3766505|rs6569759|rs7766061|rs11988|rs7817826|rs946904|rs973986|rs2434449|rs2811750
Oral Ulcers	E2F_MEDIATED_REGULATION_OF_DNA_REPLICATION	REACTOME	452	21	8	4,50E-01	8,32E-01	rs194825|rs7808273|rs5748218|rs12541220|rs6797587|rs9349915|rs1539604|rs4936661
Oral Ulcers	THROMBOXANE_SIGNALLING_THROUGH_TP_RECEPTOR	REACTOME	492	23	11	4,51E-01	8,32E-01	rs10869950|rs1162211|rs1144430|rs874627|rs11145635|rs3786989|rs11880198|rs11145517|rs12938589|rs6429146|rs12610831
Oral Ulcers	PATHOGENIC_ESCHERICHIA_COLI_INFECTION	KEGG	1256	57	20	4,51E-01	8,32E-01	rs809192|rs3769504|rs227913|rs3754806|rs11244164|rs4851835|rs9892886|rs7589023|rs10490697|rs11009157|rs1935624|rs11792889|rs10495567|rs2270461|rs688242|rs6504424|rs17427173|rs3745571|rs8078231|rs4734507
Oral Ulcers	CHEMOKINE_RECEPTORS_BIND_CHEMOKINES	REACTOME	726	39	20	4,51E-01	8,32E-01	rs1779344|rs4710181|rs266087|rs7604823|rs6840660|rs11679328|rs2268539|rs2781548|rs9872121|rs12638201|rs17283264|rs4245843|rs4954592|rs12644965|rs1855531|rs1860264|rs3732378|rs4694636|rs2037596|rs3760386
Oral Ulcers	PEPTIDE_LIGAND_BINDING_RECEPTORS	REACTOME	5053	28	20	4,52E-01	8,32E-01	rs961552|rs13227318|rs6090338|rs13152353|rs1779344|rs17611047|rs4710181|rs16087|rs266087|rs324978|rs6076798|rs7604823|rs16920907|rs2503097|rs4930591|rs6566045|rs1425903|rs1878404|rs884697|rs4665287
Oral Ulcers	BUTANOATE_METABOLISM	KEGG	763	41	20	4,54E-01	8,32E-01	rs1019635|rs10516988|rs2170624|rs3741049|rs1885490|rs2181139|rs3774051|rs2048022|rs11058042|rs2057374|rs2408700|rs10516985|rs15677|rs1273422|rs2313427|rs1514974|rs10776672|rs1923431|rs4235426|rs1161111
Oral Ulcers	GLUTATHIONE_CONJUGATION	REACTOME	407	29	15	4,54E-01	8,32E-01	rs6088659|rs4260|rs2262377|rs4149186|rs1027472|rs7896809|rs4241932|rs6458861|rs17608022|rs2275984|rs10874814|rs6060124|rs2160512|rs2608654|rs4863687
Oral Ulcers	TRIGLYCERIDE_BIOSYNTHESIS	REACTOME	1182	29	20	4,55E-01	8,32E-01	rs11892871|rs17621311|rs7684686|rs209510|rs2046731|rs10024579|rs10520097|rs2804616|rs2936523|rs2936517|rs2135284|rs6559176|rs6852527|rs11080994|rs1554336|rs7252966|rs442873|rs3913795|rs13160153|rs10452184
Oral Ulcers	SIGNALING_BY_ERBB4	REACTOME	3434	37	20	4,56E-01	8,32E-01	rs10940145|rs7396875|rs7726943|rs16897175|rs1779344|rs17629022|rs266087|rs1207774|rs2372183|rs4976028|rs4887997|rs9576991|rs16847152|rs11759745|rs3751832|rs10112123|rs11895509|rs16999922|rs17673576|rs10515068
Oral Ulcers	LYSINE_DEGRADATION	KEGG	952	32	20	4,56E-01	8,32E-01	rs659445|rs2268075|rs2242259|rs3741049|rs1885490|rs10835128|rs2181139|rs1058511|rs3774051|rs12155014|rs2293682|rs12226573|rs918459|rs2057374|rs2408700|rs15677|rs939293|rs4865483|rs28660993|rs10776672
Oral Ulcers	ATRBRCA_PATHWAY	BIOCARTA	238	15	7	4,56E-01	8,32E-01	rs1805812|rs2697675|rs540723|rs7707427|rs1801516|rs2229032|rs10833776
Oral Ulcers	GLYCEROPHOSPHOLIPID_BIOSYNTHESIS	REACTOME	2483	27	20	4,58E-01	8,32E-01	rs11892871|rs1470352|rs7930593|rs354263|rs11670988|rs17561950|rs4813754|rs6133248|rs659445|rs17494100|rs4803329|rs6038092|rs2046731|rs10520097|rs1712426|rs11997392|rs10842078|rs2804616|rs8074003|rs2936523
Oral Ulcers	SMOOTH_MUSCLE_CONTRACTION	REACTOME	658	37	20	4,59E-01	8,32E-01	rs9658702|rs2347897|rs1050162|rs16946204|rs1297783|rs7910825|rs6887205|rs931640|rs820336|rs3800702|rs1653249|rs725926|rs4530474|rs216163|rs1326935|rs3763630|rs1017813|rs904160|rs1015350|rs7259810
Oral Ulcers	STATHMIN_PATHWAY	BIOCARTA	456	30	16	4,61E-01	8,32E-01	rs919741|rs3822295|rs2285703|rs502514|rs6533711|rs2949659|rs7524898|rs872077|rs7526798|rs3729877|rs873592|rs316689|rs1644512|rs797999|rs10009286|rs16859085
Oral Ulcers	FORMATION_OF_TRANSCRIPTION_COUPLED_NER_TC_NER_REPAIR_COMPLEX	REACTOME	389	26	11	4,61E-01	8,32E-01	rs4487686|rs1131384|rs4150454|rs16902819|rs12446158|rs2072102|rs2532929|rs4151374|rs4150667|rs2020892|rs158570
Oral Ulcers	M_G1_TRANSITION	REACTOME	1315	50	20	4,65E-01	8,37E-01	rs2294183|rs194825|rs328349|rs7808273|rs8066176|rs8009527|rs4645742|rs4710834|rs3820667|rs4148876|rs1467464|rs2584624|rs1045288|rs133416|rs3859192|rs11543947|rs13447454|rs5748218|rs2071744|rs11023238
Oral Ulcers	RAS_ACTIVATION_UOPN_CA2_INFUX_THROUGH_NMDA_RECEPTOR	REACTOME	1033	40	20	4,66E-01	8,37E-01	rs7396875|rs9922023|rs919741|rs3822295|rs2285703|rs11055671|rs1297783|rs10492130|rs931640|rs6533711|rs873592|rs7972433|rs12817623|rs1833161|rs1074368|rs1071502|rs9925203|rs904160|rs2870112|rs7710039
Oral Ulcers	CCR3_PATHWAY	BIOCARTA	939	40	20	4,71E-01	8,40E-01	rs7396875|rs227913|rs227142|rs9892886|rs11860367|rs1816971|rs3767423|rs6086543|rs8051034|rs11145635|rs6086402|rs6504424|rs3780305|rs6118062|rs8078231|rs2179798|rs9927848|rs11646540|rs4816089|rs17192160
Oral Ulcers	SCFSKP2_MEDIATED_DEGRADATION_OF_P27_P21	REACTOME	813	44	19	4,71E-01	8,40E-01	rs2294183|rs328349|rs8066176|rs8009527|rs34322|rs4710834|rs3820667|rs4148876|rs2584624|rs1045288|rs3859192|rs11543947|rs11023238|rs1321311|rs6989591|rs653079|rs4980335|rs2597189|rs17279275
Oral Ulcers	PANTOTHENATE_AND_COA_BIOSYNTHESIS	KEGG	611	60	20	4,72E-01	8,40E-01	rs17471640|rs9493105|rs11165845|rs6942306|rs6569759|rs837642|rs7766061|rs7552825|rs12218194|rs10747488|rs2853129|rs1034215|rs2029986|rs4415625|rs9483495|rs17100610|rs11165784|rs2840805|rs10808389|rs1577897
Oral Ulcers	ARAP_PATHWAY	BIOCARTA	292	11	9	4,73E-01	8,40E-01	rs933241|rs1467079|rs11956308|rs5758941|rs7284821|rs12710561|rs12724113|rs4656256|rs4796825
Oral Ulcers	SYNTHESIS_OF_DNA	REACTOME	1545	46	20	4,74E-01	8,40E-01	rs2294183|rs194825|rs328349|rs7808273|rs8066176|rs942592|rs8009527|rs1405655|rs4645742|rs34322|rs4710834|rs3820667|rs4148876|rs1467464|rs2584624|rs1045288|rs133416|rs3859192|rs690705|rs11543947
Oral Ulcers	PYK2_PATHWAY	BIOCARTA	752	43	19	4,75E-01	8,40E-01	rs7396875|rs227913|rs9892886|rs11860367|rs1297783|rs2721854|rs931640|rs8051034|rs11145635|rs2529718|rs6504424|rs3780305|rs8078231|rs4326648|rs9927848|rs11646540|rs2689420|rs904160|rs7259810
Oral Ulcers	ANTIGEN_PROCESSING_AND_PRESENTATION	KEGG	1754	84	20	4,76E-01	8,40E-01	rs2853923|rs3997854|rs3135363|rs3873381|rs2733840|rs1634776|rs7192|rs874627|rs2523580|rs4977686|rs9368726|rs376892|rs13192469|rs11218954|rs2227956|rs3115673|rs7745906|rs11672983|rs7322307|rs1242105
Oral Ulcers	MRNA_CAPPING	REACTOME	388	33	17	4,76E-01	8,40E-01	rs4487686|rs2756382|rs1131384|rs4150454|rs16902819|rs11756329|rs11752001|rs2072102|rs1613070|rs9351162|rs2532929|rs2610763|rs4151374|rs4150667|rs2020892|rs8102597|rs9451102
Oral Ulcers	IL1R_PATHWAY	BIOCARTA	1001	28	20	4,77E-01	8,40E-01	rs2965012|rs7801617|rs711272|rs7552931|rs4613794|rs419598|rs2334659|rs12205556|rs12535797|rs12191179|rs1152918|rs2088169|rs13192469|rs17766515|rs9345126|rs230530|rs5743942|rs9345117|rs4326648|rs538723
Oral Ulcers	PHASE_II_CONJUGATION	REACTOME	2059	30	20	4,78E-01	8,40E-01	rs1938725|rs6088659|rs11592638|rs4260|rs7444620|rs12651454|rs1112310|rs2262377|rs4149186|rs17210001|rs1885277|rs5993891|rs1027472|rs7896809|rs2726675|rs4241932|rs6458861|rs17608022|rs2275984|rs2201554
Oral Ulcers	DEATH_PATHWAY	BIOCARTA	505	18	13	4,81E-01	8,43E-01	rs10866819|rs4647693|rs39425|rs1982673|rs2288713|rs230530|rs2156192|rs555745|rs2046136|rs7230970|rs10438740|rs13402616|rs3818411
Oral Ulcers	NOD_LIKE_RECEPTOR_SIGNALING_PATHWAY	KEGG	1406	34	20	4,82E-01	8,43E-01	rs1351726|rs7841459|rs7801617|rs711272|rs9988620|rs3738448|rs4613794|rs2575676|rs2722191|rs10097279|rs12205556|rs10065570|rs44267|rs12535797|rs3749791|rs12191179|rs2076139|rs13192469|rs9345126|rs186493
Oral Ulcers	HIV_INFECTION	REACTOME	3237	35	20	4,86E-01	8,46E-01	rs2294183|rs809192|rs4611262|rs328349|rs6508425|rs1551907|rs4487686|rs2257205|rs10852553|rs2756382|rs1131384|rs3734261|rs8066176|rs12024682|rs6142711|rs8009527|rs13245967|rs5758168|rs4150454|rs1420426
Oral Ulcers	OXIDATIVE_PHOSPHORYLATION	KEGG	2486	35	20	4,88E-01	8,46E-01	rs11665724|rs17163678|rs4975854|rs4770728|rs1871093|rs2346412|rs2240760|rs17620912|rs1006049|rs11245177|rs11956638|rs10952211|rs7904875|rs6722509|rs7903954|rs2734574|rs1058511|rs2971117|rs10901761|rs11255374
Oral Ulcers	MRNA_SPLICING_MINOR_PATHWAY	REACTOME	383	21	15	4,88E-01	8,46E-01	rs17629022|rs4487686|rs1131384|rs16987990|rs788018|rs2227956|rs2072102|rs1613070|rs480092|rs6103330|rs11661922|rs2847117|rs9916811|rs8102597|rs132793
Oral Ulcers	PRIMARY_IMMUNODEFICIENCY	KEGG	742	26	19	4,89E-01	8,46E-01	rs1344638|rs11683207|rs5758511|rs3746821|rs7523940|rs599779|rs13003194|rs4148876|rs874627|rs10195135|rs7409343|rs7322307|rs10919619|rs17002022|rs10753848|rs2838544|rs16939904|rs1004420|rs6002551
Oral Ulcers	PEROXISOMAL_LIPID_METABOLISM	REACTOME	435	28	17	4,90E-01	8,46E-01	rs13054099|rs17485390|rs12146602|rs1127745|rs257977|rs3213945|rs250412|rs246966|rs7593466|rs6734788|rs138328|rs11022984|rs8116218|rs7162180|rs1480627|rs10437066|rs854151
Oral Ulcers	HOMOLOGOUS_RECOMBINATION	KEGG	730	29	13	4,94E-01	8,46E-01	rs1805812|rs2697675|rs5756895|rs1405655|rs12449313|rs1467464|rs5750488|rs6489769|rs3816551|rs419893|rs5750432|rs7939226|rs2294914
Oral Ulcers	RNA_POL_I_TRANSCRIPTION_INITIATION	REACTOME	427	42	19	4,96E-01	8,46E-01	rs11646787|rs659445|rs6924483|rs3008598|rs4150454|rs16902819|rs719667|rs492553|rs8048412|rs1535565|rs11714203|rs2532929|rs10171594|rs311572|rs4151374|rs3789724|rs4150667|rs2020892|rs4669463
Oral Ulcers	HUNTINGTONS_DISEASE	KEGG	4311	28	20	4,97E-01	8,46E-01	rs6928833|rs11665724|rs6804671|rs7689418|rs17163678|rs6508426|rs6943342|rs4975854|rs12539927|rs4487686|rs2842994|rs936213|rs4647693|rs1131384|rs2240760|rs17620912|rs227142|rs39425|rs2119802|rs11055671
Oral Ulcers	MITOTIC_M_M_G1_PHASES	REACTOME	3176	31	20	4,97E-01	8,46E-01	rs2294183|rs194825|rs1934497|rs4611262|rs328349|rs4804147|rs5758511|rs7808273|rs11603446|rs8066176|rs6507992|rs17007652|rs1741143|rs17614161|rs8009527|rs4645742|rs3826543|rs2885987|rs1934520|rs2459975
Oral Ulcers	NCAM1_INTERACTIONS	REACTOME	1896	31	20	4,98E-01	8,46E-01	rs3811159|rs9301436|rs810687|rs3759917|rs4984637|rs1029999|rs10178458|rs8037921|rs2610983|rs421587|rs10098621|rs11629679|rs3783101|rs10745384|rs6090117|rs625087|rs2072277|rs12693523|rs2396463|rs11197567
Oral Ulcers	ENDOMETRIAL_CANCER	KEGG	3028	40	20	4,98E-01	8,46E-01	rs10940145|rs7072330|rs7396875|rs7726943|rs10461535|rs2372183|rs4976028|rs6732834|rs12464541|rs3764351|rs17673576|rs10515068|rs7922708|rs10996746|rs17017349|rs6771638|rs16893188|rs2685127|rs4381268|rs7719763
Oral Ulcers	NA_CL_DEPENDENT_NEUROTRANSMITTER_TRANSPORTERS	REACTOME	718	39	18	4,99E-01	8,46E-01	rs9883639|rs363333|rs1384319|rs9036|rs1728803|rs2282661|rs6885505|rs17279465|rs13064472|rs13081924|rs9818982|rs2042449|rs3776084|rs7977721|rs10458887|rs4685158|rs13058781|rs11116408
Oral Ulcers	TRANSPORT_OF_GLUCOSE_AND_OTHER_SUGARS_BILE_SALTS_AND_ORGANIC_ACIDS_M NE_COMPOUNDS	REACTOME	3234	32	20	5,00E-01	8,46E-01	rs7583382|rs17122678|rs9883639|rs1327257|rs1421184|rs218669|rs4890568|rs1128127|rs659445|rs363333|rs16837576|rs6650226|rs1384319|rs10819283|rs9036|rs7971218|rs4976178|rs7530862|rs13103835|rs1728803
Oral Ulcers	DC_PATHWAY	BIOCARTA	573	19	10	5,00E-01	8,46E-01	rs1697531|rs3746821|rs595158|rs1935624|rs2243290|rs7643177|rs10739508|rs797999|rs247008|rs10964832
Oral Ulcers	G_PROTEIN_ACTIVATION	REACTOME	703	17	10	5,02E-01	8,46E-01	rs2241952|rs4665287|rs2269340|rs899236|rs874627|rs510619|rs10226374|rs899227|rs6429146|rs7738233
Oral Ulcers	AMINE_COMPOUND_SLC_TRANSPORTERS	REACTOME	1298	47	20	5,03E-01	8,46E-01	rs9883639|rs1421183|rs4890568|rs659445|rs363333|rs1384319|rs9036|rs1728803|rs2282661|rs6885505|rs900967|rs2705363|rs17279465|rs13064472|rs13081924|rs1385337|rs9818982|rs2042449|rs3776084|rs7977721
Oral Ulcers	DEGRADATION_OF_THE_EXTRACELLULAR_MATRIX	REACTOME	580	33	20	5,03E-01	8,46E-01	rs198972|rs3744787|rs17251|rs2664368|rs2367486|rs2616487|rs198979|rs16883803|rs243840|rs1345319|rs7004307|rs5759997|rs7834603|rs11225394|rs17241017|rs1558666|rs1586475|rs7115014|rs13090|rs2532100
Oral Ulcers	ACYL_CHAIN_REMODELLING_OF_PG	REACTOME	571	32	15	5,03E-01	8,46E-01	rs354263|rs17494100|rs1712426|rs926324|rs2034521|rs666511|rs2383623|rs442873|rs525380|rs13160153|rs4406174|rs7162607|rs492738|rs12130951|rs4434808
Oral Ulcers	MATURITY_ONSET_DIABETES_OF_THE_YOUNG	KEGG	992	31	20	5,04E-01	8,46E-01	rs686699|rs2254779|rs752402|rs4687504|rs6665426|rs9855959|rs2639676|rs199812|rs487359|rs12041297|rs10933711|rs10835826|rs17709930|rs2178603|rs10516713|rs1490752|rs1899745|rs4147452|rs6768674|rs4794756
Oral Ulcers	G_ALPHA_I_SIGNALLING_EVENTS	REACTOME	6453	28	20	5,08E-01	8,46E-01	rs961552|rs263241|rs1541821|rs12334868|rs2039141|rs13152353|rs1779344|rs16904360|rs4710181|rs16087|rs2256156|rs266087|rs12409786|rs7604823|rs16920907|rs4978964|rs2503097|rs9730423|rs4930591|rs12191325
Oral Ulcers	PHOSPHORYLATION_OF_CD3_AND_TCR_ZETA_CHAINS	REACTOME	521	29	13	5,08E-01	8,46E-01	rs3997854|rs895275|rs502514|rs7523940|rs599779|rs874627|rs2949659|rs1964995|rs10919619|rs10753848|rs16859085|rs1004420|rs3130192
Oral Ulcers	CHEMICAL_PATHWAY	BIOCARTA	585	45	20	5,08E-01	8,46E-01	rs227913|rs4647693|rs39425|rs9892886|rs11860367|rs1982673|rs8051034|rs2288713|rs6504424|rs2239206|rs2156192|rs8078231|rs2046136|rs7230970|rs6060627|rs1801516|rs9927848|rs11646540|rs3763630|rs13029532
Oral Ulcers	VASOPRESSIN_REGULATED_WATER_REABSORPTION	KEGG	1100	37	20	5,08E-01	8,46E-01	rs6943342|rs12539927|rs2256156|rs1017414|rs7566051|rs7794304|rs2058166|rs2193360|rs10437653|rs2601787|rs2414891|rs6979207|rs7524898|rs872077|rs921|rs7526798|rs13338087|rs177580|rs42733|rs2239310
Oral Ulcers	AMINE_LIGAND_BINDING_RECEPTORS	REACTOME	2247	38	20	5,11E-01	8,47E-01	rs1328684|rs6306|rs890707|rs4305747|rs12411535|rs12191325|rs7809318|rs10038844|rs973908|rs10925809|rs9389015|rs168333|rs2702313|rs2005187|rs4569679|rs6787134|rs6589386|rs17025198|rs1034258|rs4732874
Oral Ulcers	LIPOPROTEIN_METABOLISM	REACTOME	1037	31	20	5,12E-01	8,47E-01	rs11254339|rs4804147|rs1331924|rs2740484|rs2027401|rs7163052|rs7819541|rs600880|rs7761293|rs838893|rs838920|rs9919066|rs2518054|rs4148083|rs2482430|rs4149295|rs914189|rs881395|rs3026237|rs34719083
Oral Ulcers	PRIMARY_BILE_ACID_BIOSYNTHESIS	KEGG	439	14	8	5,12E-01	8,47E-01	rs1931669|rs1127745|rs639434|rs257977|rs250412|rs246966|rs10437066|rs17380969
Oral Ulcers	LEUKOCYTE_TRANSENDOTHELIAL_MIGRATION	KEGG	4793	41	20	5,12E-01	8,47E-01	rs10940145|rs10489469|rs7072330|rs7726943|rs10461535|rs1779344|rs227913|rs5750326|rs266087|rs2372183|rs4976028|rs1332684|rs30117|rs508594|rs17020294|rs12464541|rs777710|rs12678166|rs2660481|rs9892886
Oral Ulcers	VASCULAR_SMOOTH_MUSCLE_CONTRACTION	KEGG	5845	29	20	5,15E-01	8,47E-01	rs263241|rs1541821|rs3774609|rs12334868|rs10823962|rs4074889|rs6804671|rs16904360|rs1078523|rs2256156|rs227913|rs9658702|rs859253|rs281508|rs354263|rs936213|rs1840572|rs7893773|rs10049492|rs72001
Oral Ulcers	REGULATION_OF_APOPTOSIS	REACTOME	1432	38	20	5,15E-01	8,47E-01	rs2294183|rs328349|rs3128501|rs4647693|rs11141915|rs1373179|rs8066176|rs8009527|rs4710834|rs3820667|rs2940520|rs4148876|rs2665833|rs1045288|rs3859192|rs17477673|rs11543947|rs10868558|rs7169989|rs11023238
Oral Ulcers	CTNNB1_PHOSPHORYLATION_CASCADE	REACTOME	316	7	5	5,17E-01	8,49E-01	rs10057083|rs1741143|rs2685127|rs12541220|rs4936661
Oral Ulcers	DOUBLE_STRAND_BREAK_REPAIR	REACTOME	506	26	12	5,19E-01	8,49E-01	rs1805812|rs7003908|rs2697675|rs1467464|rs6489769|rs1105451|rs1801516|rs2731858|rs1478486|rs7222799|rs132765|rs17674880
Oral Ulcers	CELL_ADHESION_MOLECULES_CAMS	KEGG	8813	29	20	5,22E-01	8,49E-01	rs6974002|rs2853923|rs4949320|rs11713106|rs1176387|rs7278532|rs2239512|rs1462307|rs1344638|rs1075188|rs6697344|rs6753833|rs3997854|rs10500175|rs6973683|rs2221513|rs6942446|rs3135363|rs2591454|rs9348863
Oral Ulcers	TAK1_ACTIVATES_NFKB_BY_PHOSPHORYLATION_AND_ACTIVATION_OF_IKKS_COMPLE	REACTOME	777	30	20	5,22E-01	8,49E-01	rs1351726|rs7841459|rs711272|rs4613794|rs10097279|rs12205556|rs11024603|rs452987|rs12191179|rs9345126|rs9345117|rs11603089|rs538723|rs494340|rs39502|rs17074679|rs1884839|rs9550565|rs5743291|rs3130283
Oral Ulcers	INSULIN_SIGNALING_PATHWAY	KEGG	3744	36	20	5,24E-01	8,49E-01	rs10940145|rs7964046|rs7396875|rs7726943|rs10461535|rs4074889|rs8067341|rs2372183|rs4976028|rs1922529|rs2393550|rs9576991|rs2575676|rs8074003|rs2399106|rs1896610|rs923273|rs1001684|rs10515068|rs1297783
Oral Ulcers	MPR_PATHWAY	BIOCARTA	624	28	17	5,25E-01	8,49E-01	rs7396875|rs10889117|rs268852|rs1002063|rs2287064|rs7524898|rs872077|rs7526798|rs3729877|rs12805701|rs316689|rs10226374|rs12155410|rs660541|rs7778387|rs12112953|rs885244
Oral Ulcers	LAIR_PATHWAY	BIOCARTA	479	32	16	5,26E-01	8,49E-01	rs7801617|rs896117|rs12755775|rs11009157|rs11569429|rs12535797|rs2279623|rs3917744|rs13192469|rs2329591|rs2153875|rs12469449|rs3782522|rs4694636|rs9976299|rs2455314
Oral Ulcers	MITOCHONDRIA_PATHWAY	BIOCARTA	317	19	13	5,28E-01	8,49E-01	rs4647693|rs39425|rs7953290|rs1982673|rs2288713|rs2156192|rs375555|rs2046136|rs7230970|rs6060627|rs9461882|rs13402616|rs210134
Oral Ulcers	IL22BP_PATHWAY	BIOCARTA	296	18	8	5,28E-01	8,49E-01	rs310209|rs8074003|rs10484798|rs7601754|rs17066096|rs7409343|rs17121510|rs13029532
Oral Ulcers	VIP_PATHWAY	BIOCARTA	1000	28	20	5,29E-01	8,49E-01	rs6852347|rs6096351|rs13434873|rs2851045|rs1297783|rs16893188|rs10092081|rs931640|rs7236492|rs11145635|rs7524898|rs872077|rs476886|rs230530|rs3780305|rs7526798|rs3729877|rs2049824|rs660749|rs316689
Oral Ulcers	GAP_JUNCTION_ASSEMBLY	REACTOME	330	17	11	5,29E-01	8,49E-01	rs11240147|rs4653061|rs999095|rs6593803|rs10782213|rs12197250|rs7328044|rs11154022|rs4706352|rs728447|rs550963
Oral Ulcers	MYOGENESIS	REACTOME	1747	41	20	5,34E-01	8,49E-01	rs770466|rs1603218|rs1422189|rs11244164|rs1479340|rs2236415|rs6121666|rs9920504|rs6071447|rs6071360|rs6128983|rs1468966|rs7264432|rs1401191|rs11792889|rs8087860|rs2076139|rs6129093|rs6124083|rs6142852
Oral Ulcers	GENERATION_OF_SECOND_MESSENGER_MOLECULES	REACTOME	616	35	16	5,34E-01	8,49E-01	rs11683207|rs3997854|rs30117|rs502514|rs427829|rs8139544|rs874627|rs2949659|rs1964995|rs171847|rs10512702|rs3804250|rs9611234|rs16859085|rs1004420|rs3130192
Oral Ulcers	GOLGI_ASSOCIATED_VESICLE_BIOGENESIS	REACTOME	1057	33	20	5,35E-01	8,49E-01	rs4704458|rs26529|rs3850615|rs6988243|rs506233|rs1904432|rs4484406|rs365936|rs11218954|rs6080887|rs495942|rs10515171|rs837036|rs1555422|rs11987678|rs16972294|rs760306|rs355421|rs9456870|rs913859
Oral Ulcers	TNFR1_PATHWAY	BIOCARTA	862	32	20	5,36E-01	8,49E-01	rs7003908|rs4647693|rs711272|rs4613794|rs2193360|rs12205556|rs2721854|rs12191179|rs4807269|rs13192469|rs4761529|rs2529718|rs9345126|rs921|rs12297002|rs733751|rs9345117|rs555745|rs4326648|rs538723
Oral Ulcers	PRE_NOTCH_PROCESSING_IN_GOLGI	REACTOME	691	44	20	5,37E-01	8,49E-01	rs12589158|rs12588669|rs3791039|rs3130304|rs1154827|rs10484135|rs4904040|rs12437161|rs8011971|rs4899822|rs10498559|rs10141409|rs7019909|rs1888363|rs1702956|rs765614|rs2532100|rs3130320|rs10484160|rs8003146
Oral Ulcers	REGULATION_OF_BETA_CELL_DEVELOPMENT	REACTOME	1288	28	20	5,38E-01	8,49E-01	rs686699|rs2254779|rs752402|rs4687504|rs9576991|rs6665426|rs9855959|rs2639676|rs199812|rs958425|rs6046818|rs9566508|rs487359|rs17254382|rs12041297|rs10933711|rs10835826|rs17709930|rs2178603|rs10516713
Oral Ulcers	ENDOCYTOSIS	KEGG	6484	35	20	5,38E-01	8,49E-01	rs2853923|rs17127410|rs17709898|rs7396875|rs7169544|rs4804147|rs17020888|rs1078523|rs4859319|rs605832|rs6768921|rs11249638|rs2465880|rs3849774|rs9348863|rs904132|rs17391840|rs3768969|rs1467079|rs11943039
Oral Ulcers	SLC_MEDIATED_TRANSMEMBRANE_TRANSPORT	REACTOME	8774	39	20	5,39E-01	8,49E-01	rs1366632|rs1023681|rs6112470|rs2562582|rs4611262|rs7611368|rs7583382|rs1417210|rs11741316|rs17122678|rs3195701|rs9883639|rs8067341|rs1327257|rs1421183|rs1320301|rs218669|rs10852553|rs4890568|rs1128127
Oral Ulcers	SMALL_CELL_LUNG_CANCER	KEGG	3747	39	20	5,39E-01	8,49E-01	rs10940145|rs4679640|rs7726943|rs16897175|rs2680182|rs9301436|rs2372183|rs4976028|rs510991|rs2298928|rs39425|rs7518498|rs2049847|rs1562525|rs9311748|rs6779755|rs1385812|rs1910236|rs677136|rs1982673
Oral Ulcers	PHENYLALANINE_METABOLISM	KEGG	442	21	15	5,39E-01	8,49E-01	rs13100173|rs12301933|rs2242259|rs1539259|rs9937648|rs8045775|rs7977571|rs4646812|rs11649592|rs5760093|rs1498688|rs10521054|rs8075157|rs4313593|rs3923499

Oral Ulcers	FC_GAMMA_R_MEDIATED_PHAGOCYTOSIS	KEGG	3795	36	20	5,40E-01	8,49E-01	rs10940145|rs7726943|rs16897175|rs952784|rs227913|rs2372183|rs281508|rs354263|rs290240|rs4976028|rs924483|rs182832|rs1332684|rs17391840|rs1467079|rs17020294|rs1712426|rs9326038|rs2660481|rs9892886
Oral Ulcers	RNA_POL_I_TRANSCRIPTION	REACTOME	937	34	20	5,43E-01	8,52E-01	rs11646787|rs659445|rs6924483|rs3008598|rs4150454|rs16902819|rs719667|rs492553|rs8048412|rs1535565|rs10951129|rs11714203|rs2532929|rs9379780|rs10171594|rs311572|rs4151374|rs3789724|rs4150667|rs1730265
Oral Ulcers	PORPHYRIN_AND_CHLOROPHYLL_METABOLISM	KEGG	1163	74	17	5,44E-01	8,52E-01	rs4803342|rs7238897|rs1112310|rs1531348|rs4645742|rs4895337|rs133416|rs4348243|rs1156684|rs376109|rs1105879|rs8077302|rs760306|rs2307344|rs4148298|rs4645752|rs4919393
Oral Ulcers	TRANS_GOLGI_NETWORK_VESICLE_BUDDING	REACTOME	1106	33	20	5,46E-01	8,52E-01	rs4704458|rs26529|rs3850615|rs6988243|rs506233|rs1904432|rs4484406|rs2293682|rs365936|rs11218954|rs6080887|rs495942|rs10515171|rs837036|rs1555422|rs11987678|rs16972294|rs760306|rs355421|rs9456870
Oral Ulcers	CARDIACEGF_PATHWAY	BIOCARTA	1289	39	20	5,47E-01	8,52E-01	rs7396875|rs227913|rs12251014|rs1878404|rs1972228|rs9892886|rs11860367|rs17673576|rs16893188|rs3863998|rs10128229|rs10092081|rs8051034|rs2877260|rs6504424|rs1456680|rs6440546|rs230530|rs8078231|rs2049824
Oral Ulcers	MAP_KINASE_ACTIVATION_IN_TLR_CASCADE	REACTOME	1697	26	20	5,47E-01	8,52E-01	rs770466|rs1351726|rs7841459|rs1422189|rs711272|rs4613794|rs2575676|rs378373|rs10097279|rs12205556|rs2721854|rs491700|rs3749791|rs12830764|rs12191179|rs2076139|rs2529718|rs2223405|rs9345126|rs388372
Oral Ulcers	TRAFFICKING_OF_GLUR2_CONTAINING_AMPA_RECEPTORS	REACTOME	721	33	13	5,48E-01	8,52E-01	rs227913|rs9036|rs9892886|rs11860367|rs8051034|rs6504424|rs2267366|rs8078231|rs2973139|rs9310449|rs9927848|rs11646540|rs11604757
Oral Ulcers	CYTOKINE_CYTOKINE_RECEPTOR_INTERACTION	KEGG	6425	28	20	5,49E-01	8,52E-01	rs17709898|rs10933441|rs1025690|rs2965012|rs1779344|rs4710181|rs12600516|rs2333512|rs5758511|rs9658702|rs266087|rs1207774|rs9357530|rs10866819|rs7801617|rs7604823|rs7562511|rs1234313|rs1153465|rs4560819
Oral Ulcers	REGULATION_OF_IFNA_SIGNALING	REACTOME	339	24	7	5,50E-01	8,52E-01	rs310209|rs8074003|rs11698821|rs4977686|rs2834126|rs10964832|rs13029532
Oral Ulcers	ADP_SIGNALLING_THROUGH_P2RY1	REACTOME	863	33	14	5,51E-01	8,52E-01	rs354263|rs10869950|rs1162211|rs1144430|rs926324|rs2383623|rs874627|rs11145635|rs11880198|rs11145517|rs6429146|rs12610831|rs12130951|rs4434808
Oral Ulcers	TOLL_LIKE_RECEPTOR_SIGNALING_PATHWAY	KEGG	2339	41	20	5,53E-01	8,53E-01	rs10940145|rs7726943|rs10461535|rs2372183|rs4976028|rs7801617|rs711272|rs3813982|rs4613794|rs3746821|rs2575676|rs10515068|rs2334659|rs2144825|rs7719763|rs12205556|rs2721854|rs12535797|rs3746188|rs3749791
Oral Ulcers	INOSITOL_PHOSPHATE_METABOLISM	KEGG	2355	29	20	5,55E-01	8,53E-01	rs753724|rs1941593|rs7899724|rs936213|rs3775601|rs227142|rs17391840|rs2283877|rs2667100|rs12964783|rs13119090|rs1816971|rs628542|rs7505711|rs4888197|rs912261|rs12267364|rs4888191|rs6086543|rs9953631
Oral Ulcers	RNA_POL_I_TRANSCRIPTION_TERMINATION	REACTOME	335	36	17	5,55E-01	8,53E-01	rs11646787|rs6924483|rs3008598|rs4150454|rs16902819|rs719667|rs492553|rs8048412|rs1535565|rs2532929|rs10171594|rs311572|rs4151374|rs3789724|rs4150667|rs2020892|rs4669463
Oral Ulcers	PD1_SIGNALING	REACTOME	398	19	8	5,56E-01	8,53E-01	rs3997854|rs502514|rs874627|rs2949659|rs1964995|rs16859085|rs1004420|rs3130192
Oral Ulcers	IL1_SIGNALING	REACTOME	1322	23	20	5,59E-01	8,54E-01	rs1351726|rs7841459|rs711272|rs4613794|rs2555454|rs419598|rs10097279|rs5758168|rs2334659|rs721575|rs11107008|rs12205556|rs2721854|rs12191179|rs1152918|rs2088169|rs12892005|rs2529718|rs17766515|rs9345126
Oral Ulcers	ACTIVATION_OF_NF_KAPPAB_IN_B_CELLS	REACTOME	1396	35	20	5,60E-01	8,54E-01	rs2294183|rs328349|rs711272|rs4613794|rs8066176|rs10951010|rs11860367|rs8009527|rs4710834|rs3820667|rs12205556|rs4148876|rs8051034|rs2584624|rs12191179|rs1045288|rs3859192|rs9345126|rs12700653|rs11543947
Oral Ulcers	GLYCOLYSIS	REACTOME	558	30	17	5,60E-01	8,54E-01	rs4880592|rs7924157|rs1539234|rs1931865|rs3829916|rs7534552|rs11251481|rs3750671|rs4436465|rs238238|rs12541220|rs2838544|rs945142|rs422732|rs13293668|rs3816699|rs4936661
Oral Ulcers	HIVNEF_PATHWAY	BIOCARTA	1008	29	20	5,62E-01	8,55E-01	rs9658702|rs7003908|rs4647693|rs39425|rs11580042|rs2193360|rs1982673|rs7910825|rs1926189|rs4807269|rs2288713|rs2154254|rs13192469|rs4761529|rs5746014|rs921|rs230530|rs12297002|rs733751|rs2156192
Oral Ulcers	BMAL1_CLOCK_NPAS2_ACTIVATES_CIRCADIAN_EXPRESSION	REACTOME	1728	34	20	5,65E-01	8,58E-01	rs7167741|rs1995243|rs1555208|rs219233|rs11071587|rs2278722|rs1472782|rs7812088|rs8042149|rs10162630|rs13053242|rs17024814|rs2946385|rs6789040|rs6809156|rs1800206|rs6819019|rs2047564|rs11655550|rs1889155
Oral Ulcers	GLYCOSYLPHOSPHATIDYLINOSITOL_GPI_ANCHOR_BIOSYNTHESIS	KEGG	318	8	5	5,66E-01	8,59E-01	rs2285173|rs2306595|rs7763|rs10518440|rs6776064
Oral Ulcers	SPHINGOLIPID_DE_NOVO_BIOSYNTHESIS	REACTOME	1020	35	18	5,69E-01	8,61E-01	rs225890|rs9837945|rs10497340|rs9326038|rs29214|rs2165406|rs7536157|rs7148794|rs10142295|rs1191566|rs1572057|rs7917488|rs17114642|rs4668089|rs12954327|rs16981947|rs7157767|rs12336689
Oral Ulcers	CREB_PHOSPHORYLATION_THROUGH_THE_ACTIVATION_OF_RAS	REACTOME	1349	41	20	5,70E-01	8,61E-01	rs7396875|rs9922023|rs919741|rs3822295|rs2285703|rs11055671|rs378373|rs1297783|rs10492130|rs931640|rs6533711|rs491700|rs2223405|rs388372|rs873592|rs7972433|rs12817623|rs1833161|rs1074368|rs1071502
Oral Ulcers	GLUCOSE_METABOLISM	REACTOME	1554	37	20	5,71E-01	8,61E-01	rs4880592|rs7924157|rs11983987|rs1539234|rs1461612|rs2639256|rs9937648|rs1297783|rs1931865|rs8045775|rs7615371|rs931640|rs6778614|rs3829916|rs7534552|rs11251481|rs7524898|rs872077|rs600130|rs3750671
Oral Ulcers	O_GLYCAN_BIOSYNTHESIS	KEGG	1579	28	20	5,74E-01	8,65E-01	rs1554324|rs836022|rs12680336|rs2141911|rs4621175|rs10902513|rs10777177|rs4925524|rs13102584|rs741900|rs4766276|rs7783170|rs1401346|rs13728|rs1547140|rs10849124|rs11722206|rs7592907|rs7674455|rs16841278
Oral Ulcers	IL17_PATHWAY	BIOCARTA	316	21	14	5,77E-01	8,66E-01	rs1176387|rs7801617|rs502514|rs12535797|rs874627|rs2949659|rs1937146|rs9916158|rs4694636|rs797999|rs17640030|rs2275913|rs16859085|rs10802189
Oral Ulcers	GAP_JUNCTION_TRAFFICKING	REACTOME	576	40	20	5,78E-01	8,67E-01	rs11240147|rs3849774|rs4653061|rs11956308|rs1994882|rs999095|rs6593803|rs7721839|rs10782213|rs3003610|rs10515171|rs16869234|rs12197250|rs7328044|rs2078750|rs11154022|rs4706352|rs728447|rs700221|rs550963
Oral Ulcers	DEADENYLATION_OF_MRNA	REACTOME	331	9	7	5,80E-01	8,67E-01	rs3843552|rs17460250|rs10424816|rs11150846|rs16963758|rs10438740|rs11957360
Oral Ulcers	STEROID_HORMONES	REACTOME	693	38	20	5,81E-01	8,67E-01	rs11254339|rs17522553|rs2043833|rs4665287|rs4668123|rs7788953|rs4667591|rs1646744|rs3753519|rs12333245|rs222014|rs1242105|rs4778636|rs1993116|rs206834|rs6847603|rs2486758|rs17431426|rs11254284|rs251128
Oral Ulcers	APOPTOSIS	REACTOME	3298	30	20	5,81E-01	8,67E-01	rs2294183|rs13191992|rs328349|rs3128501|rs9658702|rs10140923|rs10866819|rs4647693|rs2155948|rs11141915|rs39425|rs7953290|rs510745|rs1373179|rs10508312|rs8066176|rs9948303|rs11580042|rs1982673|rs8009527
Oral Ulcers	MITOCHONDRIAL_PROTEIN_IMPORT	REACTOME	609	38	20	5,82E-01	8,67E-01	rs4644138|rs2969363|rs738140|rs12471146|rs7531062|rs3796908|rs137831|rs352336|rs11881243|rs3757446|rs3810213|rs6434930|rs4685064|rs2969344|rs2122013|rs439401|rs12151388|rs962273|rs10826223|rs17541818
Oral Ulcers	APC_C_CDC20_MEDIATED_DEGRADATION_OF_MITOTIC_PROTEINS	REACTOME	1094	40	20	5,85E-01	8,67E-01	rs2294183|rs328349|rs399672|rs8066176|rs8009527|rs6736093|rs4710834|rs3820667|rs4148876|rs6841355|rs2584624|rs1045288|rs3859192|rs11543947|rs2061185|rs11023238|rs6856440|rs653079|rs4980335|rs2031894
Oral Ulcers	DRUG_METABOLISM_OTHER_ENZYMES	KEGG	1609	71	20	5,88E-01	8,67E-01	rs6707705|rs17471640|rs11165845|rs1112310|rs1833182|rs7552825|rs6759948|rs10747488|rs2853129|rs305985|rs817365|rs1420566|rs1974204|rs2201554|rs376109|rs2842936|rs7247903|rs1105879|rs2282010|rs12599795
Oral Ulcers	APOPTOTIC_CLEAVAGE_OF_CELLULAR_PROTEINS	REACTOME	1052	38	20	5,88E-01	8,67E-01	rs13191992|rs10140923|rs4647693|rs2155948|rs510745|rs10508312|rs11136336|rs9951341|rs336629|rs9392904|rs2076802|rs2387399|rs661891|rs12340264|rs12570830|rs1719336|rs10431924|rs4783689|rs1442723|rs242557
Oral Ulcers	VIBRIO_CHOLERAE_INFECTION	KEGG	1518	39	20	5,89E-01	8,67E-01	rs1551907|rs2256156|rs227913|rs2346412|rs1006049|rs7566051|rs9892886|rs11860367|rs2058166|rs4888197|rs2734574|rs163171|rs2601787|rs4888191|rs6983249|rs8051034|rs2004997|rs7579208|rs13192469|rs3814043
Oral Ulcers	FATTY_ACYL_COA_BIOSYNTHESIS	REACTOME	404	21	16	5,89E-01	8,67E-01	rs17621311|rs7684686|rs209510|rs10024579|rs6852527|rs1554336|rs7252966|rs10452184|rs4926125|rs6533493|rs244016|rs6828840|rs4862439|rs7726875|rs1633399|rs4862451
Oral Ulcers	ADHERENS_JUNCTIONS_INTERACTIONS	REACTOME	5496	31	20	5,90E-01	8,67E-01	rs2587374|rs1703047|rs1975673|rs422384|rs12921373|rs1462307|rs4782685|rs1304648|rs7355953|rs4573711|rs6549064|rs7498814|rs750146|rs2042441|rs12639225|rs17566483|rs4949141|rs16957844|rs12293402|rs2236415
Oral Ulcers	HOST_INTERACTIONS_OF_HIV_FACTORS	REACTOME	2270	30	20	5,91E-01	8,67E-01	rs2294183|rs809192|rs4611262|rs328349|rs1551907|rs10852553|rs8066176|rs12024682|rs8009527|rs13245967|rs5758168|rs1420426|rs4710834|rs3820667|rs2700986|rs4684126|rs17670092|rs12656935|rs4869498|rs4148876
Oral Ulcers	INFLUENZA_LIFE_CYCLE	REACTOME	1671	31	20	5,91E-01	8,67E-01	rs4611262|rs4487686|rs2278722|rs10852553|rs1131384|rs634760|rs4792792|rs13353326|rs633161|rs12024682|rs10467685|rs1426075|rs10065570|rs4684126|rs7331943|rs4869498|rs4777186|rs1458332|rs5003866|rs12472874
Oral Ulcers	FANCONI_ANEMIA_PATHWAY	REACTOME	461	30	14	5,94E-01	8,70E-01	rs1861415|rs2239359|rs1861145|rs4439380|rs13029039|rs7588962|rs4399710|rs1801516|rs6758100|rs13399274|rs2229032|rs10833776|rs16966142|rs10196564
Oral Ulcers	POST_TRANSLATIONAL_PROTEIN_MODIFICATION	REACTOME	6008	27	20	5,97E-01	8,72E-01	rs1554324|rs9284892|rs11730732|rs902417|rs7143906|rs3759917|rs11714748|rs1667255|rs11998428|rs836022|rs12680336|rs2141911|rs3791039|rs8037921|rs1525902|rs636757|rs1319090|rs17108797|rs410076|rs2410283
Oral Ulcers	SPPA_PATHWAY	BIOCARTA	1428	49	20	6,00E-01	8,74E-01	rs7396875|rs227913|rs354263|rs290240|rs227142|rs9892886|rs11860367|rs926324|rs6887205|rs11009157|rs6086543|rs8051034|rs2383623|rs1551746|rs10120669|rs6086402|rs6504424|rs6118062|rs8078231|rs2179798
Oral Ulcers	INFLUENZA_VIRAL_RNA_TRANSCRIPTION_AND_REPLICATION	REACTOME	1153	36	20	6,00E-01	8,74E-01	rs4487686|rs2278722|rs1131384|rs634760|rs4792792|rs13353326|rs8001181|rs10467685|rs1426075|rs10065570|rs7331943|rs4777186|rs1458332|rs5003866|rs2072102|rs1613070|rs8076790|rs7502891|rs4583476|rs2280401
Oral Ulcers	SIGNALING_BY_WNT	REACTOME	1071	43	20	6,05E-01	8,78E-01	rs2294183|rs328349|rs8066176|rs10057083|rs1741143|rs8009527|rs2685127|rs4710834|rs3820667|rs4148876|rs2584624|rs1045288|rs3859192|rs11543947|rs11023238|rs12541220|rs653079|rs4980335|rs2597189|rs4936661
Oral Ulcers	CSK_PATHWAY	BIOCARTA	554	43	20	6,06E-01	8,78E-01	rs11683207|rs3135363|rs502514|rs7523940|rs599779|rs7192|rs874627|rs9368726|rs2949659|rs7524898|rs872077|rs7526798|rs3729877|rs10919619|rs316689|rs12155410|rs10753848|rs7778387|rs16859085|rs12112953
Oral Ulcers	FORMATION_OF_TUBULIN_FOLDING_INTERMEDIATES_BY_CCT_TRIC	REACTOME	227	10	6	6,06E-01	8,78E-01	rs7589023|rs11133604|rs2270461|rs3745571|rs3212358|rs2832157
Oral Ulcers	G2_PATHWAY	BIOCARTA	354	15	12	6,12E-01	8,83E-01	rs7003908|rs2267172|rs13053242|rs7928738|rs10495567|rs540723|rs12481679|rs1801516|rs1321311|rs6797587|rs9287712|rs2229032
Oral Ulcers	EICOSANOID_LIGAND_BINDING_RECEPTORS	REACTOME	507	24	12	6,13E-01	8,83E-01	rs1992139|rs2352037|rs1776503|rs11687066|rs6719977|rs3786989|rs2257772|rs17831706|rs2548145|rs602383|rs4546432|rs17253026
Oral Ulcers	DNA_REPLICATION	REACTOME	3548	29	20	6,13E-01	8,83E-01	rs2294183|rs194825|rs1934497|rs4611262|rs328349|rs4804147|rs5758511|rs7808273|rs11603446|rs8066176|rs6507992|rs942592|rs17007652|rs1741143|rs17614161|rs8009527|rs1405655|rs4645742|rs3826543|rs2885987
Oral Ulcers	RIG_I_LIKE_RECEPTOR_SIGNALING_PATHWAY	KEGG	1361	37	20	6,17E-01	8,87E-01	rs9909750|rs26529|rs711272|rs3813982|rs4613794|rs2575676|rs2144825|rs12205556|rs3749791|rs12191179|rs4977686|rs2076139|rs13192469|rs11868917|rs9345126|rs668033|rs730688|rs230530|rs9345117|rs555745
Oral Ulcers	ASCORBATE_AND_ALDARATE_METABOLISM	KEGG	874	67	9	6,19E-01	8,88E-01	rs1112310|rs1885490|rs2181139|rs2057374|rs2408700|rs376109|rs1105879|rs1923431|rs4148298
Oral Ulcers	TEL_PATHWAY	BIOCARTA	978	47	20	6,21E-01	8,88E-01	rs7169544|rs227913|rs1022258|rs9892886|rs1713423|rs1982673|rs17673576|rs16893188|rs10092081|rs2877260|rs6504424|rs2156192|rs8078231|rs2049824|rs2046136|rs7230970|rs11976696|rs1546713|rs132793|rs2871864
Oral Ulcers	CYCLIN_E_ASSOCIATED_EVENTS_DURING_G1_S_TRANSITION_	REACTOME	1212	39	20	6,22E-01	8,88E-01	rs2294183|rs328349|rs8066176|rs8009527|rs16893188|rs16902819|rs7928738|rs34322|rs4710834|rs3820667|rs10092081|rs4148876|rs2665833|rs1045288|rs4902371|rs3859192|rs11543947|rs2049824|rs11023238|rs4151374
Oral Ulcers	NICOTINATE_AND_NICOTINAMIDE_METABOLISM	KEGG	963	40	19	6,23E-01	8,88E-01	rs9687490|rs9493105|rs12054736|rs6569759|rs1882567|rs7766061|rs17562893|rs974244|rs9790066|rs11242506|rs12652118|rs1922503|rs2348024|rs4235646|rs950233|rs1345621|rs12656536|rs6451722|rs9834257
Oral Ulcers	NOD1_2_SIGNALING_PATHWAY	REACTOME	881	32	20	6,23E-01	8,88E-01	rs1351726|rs7841459|rs711272|rs4613794|rs10097279|rs11107008|rs12205556|rs12191179|rs2076139|rs9345126|rs9345117|rs12597|rs538723|rs556335|rs39502|rs1884839|rs728868|rs5743291|rs16948923|rs13402616
Oral Ulcers	G_BETA_GAMMA_SIGNALLING_THROUGH_PLC_BETA	REACTOME	624	14	10	6,28E-01	8,90E-01	rs936213|rs227142|rs6086543|rs874627|rs6086402|rs6118062|rs2179798|rs4816089|rs6429146|rs6055748
Oral Ulcers	RIBOSOME	KEGG	1118	46	20	6,28E-01	8,90E-01	rs1669796|rs2278722|rs4506807|rs4792792|rs13353326|rs10467685|rs8009062|rs11856823|rs10065570|rs4777186|rs1458332|rs5003866|rs12594729|rs8076790|rs7502891|rs4583476|rs2280401|rs3796934|rs11002444|rs7210156
Oral Ulcers	PYRIMIDINE_METABOLISM	REACTOME	792	62	20	6,28E-01	8,90E-01	rs6707705|rs17471640|rs6816128|rs11165845|rs7552825|rs6759948|rs10747488|rs17246034|rs2853129|rs9687411|rs1034215|rs2282010|rs714190|rs4415625|rs17100610|rs11165784|rs4657493|rs10238679|rs10808389|rs1577897
Oral Ulcers	INTRINSIC_PATHWAY_FOR_APOPTOSIS	REACTOME	532	30	19	6,31E-01	8,90E-01	rs4647693|rs39425|rs7953290|rs9948303|rs1982673|rs726430|rs2288713|rs12456032|rs1439287|rs2156192|rs375555|rs1398030|rs2046136|rs7230970|rs6060627|rs9461882|rs13402616|rs8085349|rs210134
Oral Ulcers	HEDGEHOG_SIGNALING_PATHWAY	KEGG	2151	24	20	6,33E-01	8,90E-01	rs3779173|rs1927081|rs12464700|rs153880|rs267840|rs1026487|rs1005351|rs9986932|rs1392224|rs2299146|rs8069437|rs4668123|rs10057083|rs1991769|rs4667591|rs6771638|rs267184|rs17126825|rs6871469|rs6961071
Oral Ulcers	NEGATIVE_REGULATION_OF_FGFR_SIGNALING	REACTOME	1536	25	20	6,34E-01	8,90E-01	rs11167777|rs12542904|rs17487009|rs9510050|rs152442|rs12523624|rs12020729|rs11199823|rs7981942|rs9601762|rs733895|rs750535|rs10788173|rs263932|rs6563230|rs244731|rs11263615|rs4980694|rs2290755|rs11776272
Oral Ulcers	XENOBIOTICS	REACTOME	231	8	4	6,35E-01	8,90E-01	rs7921420|rs305985|rs7247903|rs915908
Oral Ulcers	AMINO_ACID_SYNTHESIS_AND_INTERCONVERSION_TRANSAMINATION	REACTOME	530	28	14	6,37E-01	8,90E-01	rs6703674|rs10911044|rs666930|rs9937648|rs8045775|rs10794004|rs11592789|rs3771311|rs11649592|rs2985439|rs10521054|rs7849881|rs12098522|rs7790127
Oral Ulcers	CELL_CYCLE_CHECKPOINTS	REACTOME	1961	41	20	6,38E-01	8,90E-01	rs2294183|rs194825|rs328349|rs7808273|rs399672|rs8066176|rs942592|rs8009527|rs4645742|rs6736093|rs7928738|rs34322|rs4710834|rs3820667|rs1062492|rs4148876|rs6841355|rs1467464|rs2584624|rs1045288
Oral Ulcers	MHC_CLASS_II_ANTIGEN_PRESENTATION	REACTOME	2226	43	20	6,41E-01	8,90E-01	rs11730732|rs9299670|rs12587001|rs9843124|rs3997854|rs1579282|rs17108797|rs5027023|rs6508259|rs4634140|rs2286746|rs3775836|rs6979207|rs2878125|rs376892|rs3003610|rs2162400|rs1964995|rs10515171|rs8082530
Oral Ulcers	BLADDER_CANCER	KEGG	1222	37	20	6,42E-01	8,90E-01	rs7396875|rs2333512|rs3128501|rs11141915|rs3764351|rs2236947|rs17673576|rs16893188|rs10092081|rs243840|rs1345319|rs12498670|rs2877260|rs17477673|rs11850328|rs943080|rs2049824|rs10868558|rs7169989|rs11976696
Oral Ulcers	STARCH_AND_SUCROSE_METABOLISM	KEGG	2178	54	20	6,44E-01	8,90E-01	rs8067341|rs9493105|rs1461612|rs9841414|rs4536829|rs1112310|rs2639256|rs10265585|rs12064556|rs6569759|rs923273|rs7766061|rs6778614|rs2171385|rs4697371|rs1880924|rs11150846|rs7536956|rs376109|rs7678820
Oral Ulcers	ACTIVATION_OF_BH3_ONLY_PROTEINS	REACTOME	325	15	10	6,44E-01	8,90E-01	rs9948303|rs1982673|rs726430|rs12456032|rs1439287|rs2156192|rs1398030|rs2046136|rs7230970|rs8085349
Oral Ulcers	AGR_PATHWAY	BIOCARTA	1981	40	20	6,44E-01	8,90E-01	rs6570626|rs219862|rs2167568|rs10980573|rs936213|rs6039529|rs12663692|rs17673576|rs2364598|rs17719687|rs1002063|rs6887205|rs7754832|rs11009157|rs3924461|rs10980551|rs2877260|rs6926573|rs1529712|rs10954863
Oral Ulcers	ADHERENS_JUNCTION	KEGG	4520	34	20	6,45E-01	8,90E-01	rs12906930|rs7072330|rs809192|rs11631839|rs7169544|rs1462307|rs924483|rs711272|rs205898|rs6020218|rs6732834|rs4130485|rs12639225|rs1503185|rs1968228|rs1992373|rs12464541|rs4613794|rs12293402|rs3764351
Oral Ulcers	CDMAC_PATHWAY	BIOCARTA	1018	37	20	6,45E-01	8,90E-01	rs7396875|rs227913|rs227142|rs9892886|rs11860367|rs16893188|rs10092081|rs6086543|rs8051034|rs13192469|rs6086402|rs6504424|rs230530|rs6118062|rs8078231|rs2049824|rs4326648|rs2179798|rs9927848|rs11646540
Oral Ulcers	PRION_DISEASES	KEGG	1585	30	20	6,45E-01	8,90E-01	rs809192|rs7278532|rs7801617|rs896117|rs11145770|rs2827541|rs2826665|rs2827484|rs17600315|rs625087|rs2827156|rs12535797|rs1783396|rs2226441|rs2826877|rs7524898|rs2329591|rs872077|rs2227956|rs480092
Oral Ulcers	MAPK_TARGETS_NUCLEAR_EVENTS_MEDIATED_BY_MAP_KINASES	REACTOME	980	37	19	6,46E-01	8,90E-01	rs770466|rs1422189|rs2575676|rs378373|rs491700|rs3749791|rs12830764|rs2076139|rs2223405|rs388372|rs730688|rs4326648|rs2904103|rs12541220|rs11633249|rs12442313|rs8021524|rs661311|rs4936661
Oral Ulcers	NITROGEN_METABOLISM	KEGG	587	44	18	6,47E-01	8,90E-01	rs6688840|rs6703674|rs10911044|rs2651244|rs6748782|rs3124455|rs3765964|rs7522196|rs2302909|rs290558|rs11684145|rs3771311|rs10957133|rs2985439|rs17131359|rs7790127|rs9288423|rs1825000
Oral Ulcers	FATTY_ACID_METABOLISM	KEGG	776	29	20	6,47E-01	8,90E-01	rs10024579|rs3741049|rs1885490|rs2181139|rs390200|rs3774051|rs6852527|rs1887574|rs2293682|rs1554336|rs2057374|rs2408700|rs15677|rs515324|rs1146581|rs2075633|rs1693439|rs10776672|rs1923431|rs6828840
Oral Ulcers	EPITHELIAL_CELL_SIGNALING_IN_HELICOBACTER_PYLORI_INFECTION	KEGG	1535	34	20	6,49E-01	8,91E-01	rs952784|rs1871093|rs2346412|rs4647693|rs182832|rs1006049|rs2575676|rs17673576|rs4888197|rs2734574|rs2721854|rs4888191|rs6983249|rs3749791|rs11979908|rs2004997|rs2877260|rs2076139|rs7579208|rs13192469
Oral Ulcers	HEMATOPOIETIC_CELL_LINEAGE	KEGG	1963	26	20	6,51E-01	8,91E-01	rs1697531|rs2239512|rs7801617|rs3135363|rs1153465|rs17605348|rs11658221|rs502514|rs163546|rs6857303|rs1058511|rs595158|rs17606924|rs6887205|rs706779|rs12535797|rs7192|rs874627|rs2237085|rs12409675
Oral Ulcers	PACKAGING_OF_TELOMERE_ENDS	REACTOME	515	6	4	6,52E-01	8,91E-01	rs153060|rs12538587|rs9379780|rs792637
Oral Ulcers	CERAMIDE_PATHWAY	BIOCARTA	425	11	10	6,52E-01	8,91E-01	rs39425|rs1982673|rs2721854|rs2529718|rs230530|rs2156192|rs555745|rs2046136|rs7230970|rs13402616
Oral Ulcers	GLYCEROPHOSPHOLIPID_METABOLISM	KEGG	2549	29	20	6,54E-01	8,92E-01	rs1470352|rs7930593|rs354263|rs17561950|rs17494100|rs6467706|rs161338|rs838709|rs7542020|rs6038092|rs2046731|rs7811862|rs9326038|rs11997392|rs10842078|rs2804616|rs8074003|rs7788248|rs926324|rs1045075
Oral Ulcers	ALPHA_LINOLENIC_ACID_METABOLISM	KEGG	571	24	10	6,58E-01	8,94E-01	rs354263|rs7542020|rs926324|rs2383623|rs525380|rs132985|rs492738|rs2278964|rs12130951|rs4463623
Oral Ulcers	INHIBITION_OF_THE_PROTEOLYTIC_ACTIVITY_OF_APC_C_REQUIRED_FOR_THE_ONSET_[illegible] TOTIC_SPINDLE_CHECKPOINT_COMPONENTS	REACTOME	365	12	7	6,60E-01	8,94E-01	rs399672|rs6736093|rs6841355|rs2061185|rs6856440|rs2031894|rs12485431
Oral Ulcers	APC_CDC20_MEDIATED_DEGRADATION_OF_NEK2A	REACTOME	396	12	7	6,63E-01	8,94E-01	rs399672|rs6736093|rs6841355|rs2061185|rs16997627|rs2031894|rs12485431
Oral Ulcers	MEIOTIC_RECOMBINATION	REACTOME	993	40	14	6,63E-01	8,94E-01	rs1805812|rs2697675|rs12449313|rs138465|rs1467464|rs12966840|rs11743379|rs3131379|rs28366162|rs17312807|rs9379780|rs1801516|rs2294914|rs805292
Oral Ulcers	SEMA4D_INDUCED_CELL_MIGRATION_AND_GROWTH_CONE_COLLAPSE	REACTOME	433	16	9	6,63E-01	8,94E-01	rs4405007|rs1050162|rs3764351|rs216163|rs2284037|rs2689420|rs822570|rs216164|rs2269529
Oral Ulcers	INFLAMMASOMES	REACTOME	468	20	13	6,65E-01	8,94E-01	rs9988620|rs3738448|rs1982673|rs452987|rs186493|rs3751143|rs2156192|rs2046136|rs7230970|rs17525809|rs7312642|rs6060627|rs12565738
Oral Ulcers	JAK_STAT_SIGNALING_PATHWAY	KEGG	4346	41	20	6,66E-01	8,94E-01	rs2860850|rs10940145|rs7726943|rs16897175|rs1207774|rs2372183|rs4976028|rs7801617|rs1022373|rs1542994|rs1153465|rs2025258|rs310209|rs17753395|rs11968166|rs13032509|rs2123381|rs11759745|rs12228989|rs8074003
Oral Ulcers	METAL_ION_SLC_TRANSPORTERS	REACTOME	653	31	20	6,66E-01	8,94E-01	rs7583382|rs16837576|rs4976178|rs7530862|rs6822371|rs2165993|rs1603942|rs10184058|rs2771354|rs6702177|rs10505292|rs921968|rs9429875|rs7608598|rs1123109|rs6449986|rs7034425|rs11627628|rs1695715|rs7546852
Oral Ulcers	BRANCHED_CHAIN_AMINO_ACID_CATABOLISM	REACTOME	507	16	10	6,67E-01	8,94E-01	rs3741049|rs837642|rs10991869|rs1454465|rs6479333|rs15677|rs11083620|rs723587|rs2029986|rs12807544
Oral Ulcers	HISTIDINE_METABOLISM	KEGG	654	27	17	6,67E-01	8,94E-01	rs10499133|rs388483|rs1885490|rs13075694|rs2181139|rs2232248|rs2057374|rs2408700|rs7806863|rs4646812|rs9491563|rs1923431|rs8075157|rs4313593|rs10519263|rs3923499|rs854151
Oral Ulcers	FORMATION_OF_ATP_BY_CHEMIOSMOTIC_COUPLING	REACTOME	77	6	6	6,68E-01	8,94E-01	rs7904875|rs11255374|rs2829890|rs3891250|rs962273|rs17541818
Oral Ulcers	G2_M_CHECKPOINTS	REACTOME	710	43	20	6,69E-01	8,94E-01	rs194825|rs7808273|rs942592|rs4645742|rs7928738|rs1467464|rs133416|rs690705|rs540723|rs1543551|rs13447454|rs5748218|rs2071744|rs7707427|rs1801516|rs2307344|rs4943189|rs4645752|rs6797587|rs2229032

Oral Ulcers	ARACHIDONIC_ACID_METABOLISM	KEGG	1356	35	20	6,71E-01	8,94E-01	rs8133052|rs354263|rs7518498|rs7542020|rs2565050|rs10033662|rs926324|rs12038031|rs2383623|rs7921420|rs2024092|rs8066922|rs7215288|rs16931419|rs4646530|rs2275984|rs2816|rs7857431|rs525380|rs915908
Oral Ulcers	REGULATION_OF_INSULIN_SECRETION_BY_GLUCAGON_LIKE_PEPTIDE1	REACTOME	1388	37	20	6,72E-01	8,94E-01	rs263241|rs10489469|rs12334868|rs16904360|rs508594|rs16984239|rs874627|rs17221445|rs7524898|rs872077|rs6758546|rs2074532|rs7526798|rs3729877|rs4713646|rs6019816|rs2081406|rs3742609|rs11048665|rs17706710
Oral Ulcers	MAPK_PATHWAY	BIOCARTA	2690	22	20	6,73E-01	8,94E-01	rs770466|rs7396875|rs2965012|rs1422189|rs711272|rs4940003|rs6692647|rs4613794|rs2575676|rs378373|rs2364598|rs4150454|rs16893188|rs721575|rs1292035|rs12205556|rs2721854|rs4940011|rs10092081|rs491700
Oral Ulcers	INWARDLY_RECTIFYING_K_CHANNELS	REACTOME	1317	46	20	6,78E-01	8,94E-01	rs11654455|rs16837945|rs2155548|rs2074315|rs2898334|rs2192990|rs4937390|rs2835991|rs8066734|rs874627|rs4462751|rs10512532|rs2835955|rs874690|rs2835910|rs8128418|rs858027|rs2846700|rs6429146|rs2808542
Oral Ulcers	EFFECTS_OF_PIP2_HYDROLYSIS	REACTOME	1651	34	20	6,78E-01	8,94E-01	rs6590911|rs281508|rs10895184|rs6467706|rs161338|rs838709|rs510745|rs10508312|rs7811862|rs7788248|rs4953334|rs1230960|rs9968130|rs12650123|rs1392171|rs4952779|rs11868917|rs567384|rs3768751|rs4407400
Oral Ulcers	DESTABILIZATION_OF_MRNA_BY_KSRP	REACTOME	205	7	6	6,79E-01	8,94E-01	rs2075755|rs2152092|rs2076139|rs4734507|rs6468757|rs16963758
Oral Ulcers	MCALPAIN_PATHWAY	BIOCARTA	543	33	20	6,80E-01	8,94E-01	rs7396875|rs17673576|rs1002063|rs6887205|rs11009157|rs820336|rs4530474|rs2877260|rs7524898|rs872077|rs2239206|rs7526798|rs3729877|rs11976696|rs2153875|rs316689|rs3763630|rs1195962|rs17192160|rs2216058
Oral Ulcers	CELLCYCLE_PATHWAY	BIOCARTA	459	12	9	6,80E-01	8,94E-01	rs11968166|rs16902819|rs34322|rs4130023|rs1011970|rs1537377|rs1321311|rs4445045|rs6797587
Oral Ulcers	PPARA_ACTIVATES_GENE_EXPRESSION	REACTOME	3675	28	20	6,81E-01	8,94E-01	rs4759430|rs7167741|rs1995243|rs1555208|rs219233|rs11071587|rs2278722|rs9357530|rs780885|rs1331924|rs2740484|rs7581571|rs7812088|rs1043180|rs4757060|rs10024579|rs17176425|rs1112310|rs12200211|rs2027401
Oral Ulcers	ACTIVATION_OF_ATR_IN_RESPONSE_TO_REPLICATION_STRESS	REACTOME	653	43	19	6,82E-01	8,94E-01	rs194825|rs7808273|rs942592|rs4645742|rs1467464|rs133416|rs690705|rs540723|rs1543551|rs13447454|rs5748218|rs2071744|rs7707427|rs2307344|rs4943189|rs4645752|rs6797587|rs2229032|rs2393374
Oral Ulcers	KERATINOCYTE_PATHWAY	BIOCARTA	1952	35	20	6,83E-01	8,94E-01	rs7396875|rs2836786|rs227913|rs9658702|rs281508|rs510745|rs10508312|rs9892886|rs11860367|rs11580042|rs1982673|rs17673576|rs2364598|rs4953334|rs7910825|rs2721854|rs1926189|rs8051034|rs11221077|rs2877260
Oral Ulcers	N_GLYCAN_BIOSYNTHESIS	KEGG	1792	31	20	6,84E-01	8,94E-01	rs902417|rs7143906|rs11998428|rs1525902|rs636757|rs1319090|rs2410283|rs2811491|rs1873588|rs12495023|rs352766|rs7832401|rs17102896|rs6917694|rs4902433|rs3769693|rs11157596|rs11684754|rs227003|rs1499570
Oral Ulcers	EGFR_DOWNREGULATION	REACTOME	1037	44	20	6,84E-01	8,94E-01	rs1022373|rs17753395|rs2123381|rs7981942|rs9601762|rs17673576|rs6563230|rs11618221|rs9574902|rs2877260|rs1333411|rs13131772|rs1036253|rs9307531|rs2503378|rs10515171|rs9318814|rs1555422|rs11976696|rs11841981
Oral Ulcers	GLYCOSPHINGOLIPID_BIOSYNTHESIS_LACTO_AND_NEOLACTO_SERIES	KEGG	670	28	11	6,85E-01	8,94E-01	rs17135399|rs3791039|rs646723|rs11673407|rs9322615|rs673086|rs7409343|rs7019909|rs1702956|rs1225999|rs7854765
Oral Ulcers	O_LINKED_GLYCOSYLATION_OF_MUCINS	REACTOME	2402	38	20	6,90E-01	9,00E-01	rs1554324|rs836022|rs12680336|rs2141911|rs3791039|rs410076|rs11882828|rs4621175|rs4435373|rs10902513|rs3862899|rs4925524|rs12495023|rs517036|rs13102584|rs741900|rs4766276|rs7783170|rs10878661|rs1401346
Oral Ulcers	COLLAGEN_FORMATION	REACTOME	3203	35	20	6,93E-01	9,03E-01	rs3811159|rs2763341|rs17040061|rs17786760|rs9301436|rs810687|rs1029999|rs805693|rs2581625|rs1002993|rs10178458|rs421587|rs1982850|rs891718|rs9464379|rs2707973|rs6946256|rs1995126|rs10759686|rs3783101
Oral Ulcers	PENTOSE_AND_GLUCURONATE_INTERCONVERSIONS	KEGG	874	67	10	6,97E-01	9,05E-01	rs1112310|rs7788953|rs2070487|rs151618|rs1646744|rs376109|rs1105879|rs4148298|rs868022|rs4616468
Oral Ulcers	TRIF_MEDIATED_TLR3_SIGNALING	REACTOME	2075	24	20	6,98E-01	9,06E-01	rs770466|rs1351726|rs7841459|rs1422189|rs711272|rs3813982|rs4613794|rs2575676|rs378373|rs10097279|rs12205556|rs11024603|rs2721854|rs491700|rs3749791|rs12830764|rs452987|rs12191179|rs2076139|rs2529718
Oral Ulcers	WNT_PATHWAY	BIOCARTA	1363	27	20	7,02E-01	9,10E-01	rs711272|rs205898|rs1332879|rs900030|rs4613794|rs2540590|rs874754|rs10057083|rs6771638|rs16893188|rs2685127|rs12205556|rs3739581|rs10092081|rs1429529|rs12191179|rs12958604|rs9345126|rs7866986|rs815865
Oral Ulcers	BETA_ALANINE_METABOLISM	KEGG	711	51	20	7,05E-01	9,12E-01	rs17471640|rs11165845|rs1885490|rs2181139|rs7552825|rs3774051|rs10747488|rs2853129|rs2057374|rs2408700|rs1974204|rs1146581|rs1273422|rs2313427|rs10776672|rs1923431|rs4415625|rs17100610|rs11165784|rs10808389
Oral Ulcers	NKT_PATHWAY	BIOCARTA	638	20	13	7,11E-01	9,14E-01	rs2965012|rs7552931|rs11688530|rs874627|rs17283264|rs4245843|rs2243290|rs4954592|rs8110090|rs7643177|rs1041973|rs4803455|rs247008
Oral Ulcers	LINOLEIC_ACID_METABOLISM	KEGG	716	27	12	7,11E-01	9,14E-01	rs354263|rs7542020|rs926324|rs2383623|rs7921420|rs525380|rs915908|rs132985|rs492738|rs2278964|rs12130951|rs4463623
Oral Ulcers	GRB2_EVENTS_IN_ERBB2_SIGNALING	REACTOME	1202	34	20	7,12E-01	9,14E-01	rs7396875|rs16847152|rs3764351|rs11895509|rs16999923|rs17673576|rs17719687|rs12990694|rs896577|rs10932383|rs2877260|rs2888104|rs16999874|rs10954863|rs10164675|rs4859417|rs6745717|rs7425448|rs11976696|rs6739390
Oral Ulcers	INHIBITION_OF_VOLTAGE_GATED_CA2_CHANNELS_VIA_GBETA_GAMMA_SUBUNITS	REACTOME	1082	37	20	7,12E-01	9,14E-01	rs11654455|rs16837945|rs2155548|rs2898334|rs2192990|rs4937390|rs2835991|rs8066734|rs874627|rs4462751|rs10512532|rs2835955|rs874690|rs2835910|rs8128418|rs858027|rs2846700|rs6429146|rs2808542|rs978708
Oral Ulcers	GLUCURONIDATION	REACTOME	656	48	5	7,12E-01	9,14E-01	rs1112310|rs1885277|rs1105879|rs4148298|rs4616468
Oral Ulcers	PREFOLDIN_MEDIATED_TRANSFER_OF_SUBSTRATE_TO_CCT_TRIC	REACTOME	288	11	7	7,14E-01	9,14E-01	rs7589023|rs11133604|rs2270461|rs3745571|rs6867870|rs3212358|rs2832157
Oral Ulcers	NUCLEOTIDE_BINDING_DOMAIN_LEUCINE_RICH_REPEAT_CONTAINING_RECEPTOR_NLR YS	REACTOME	1337	30	20	7,18E-01	9,18E-01	rs1351726|rs7841459|rs711272|rs12745508|rs3738448|rs4613794|rs1982673|rs10097279|rs11107008|rs12205556|rs452987|rs12191179|rs2076139|rs9345126|rs186493|rs3751143|rs2156192|rs9345117|rs538723|rs12597
Oral Ulcers	GASTRIN_CREB_SIGNALLING_PATHWAY_VIA_PKC_AND_MAPK	REACTOME	8122	32	20	7,19E-01	9,18E-01	rs10940145|rs13227318|rs7396875|rs6090338|rs7726943|rs1328684|rs16897175|rs6306|rs2239512|rs6590911|rs227913|rs2372183|rs281508|rs324978|rs4976028|rs6076798|rs936213|rs10895184|rs4978964|rs2503097
Oral Ulcers	INTERACTION_BETWEEN_L1_AND_ANKYRINS	REACTOME	970	26	20	7,25E-01	9,20E-01	rs2295522|rs4803342|rs3847171|rs7896287|rs10509119|rs10505589|rs170680|rs9894841|rs4245585|rs12333431|rs10956645|rs1684895|rs10994493|rs12357972|rs444583|rs13032976|rs2155854|rs3782478|rs4948385|rs11672523
Oral Ulcers	CYTOSOLIC_TRNA_AMINOACYLATION	REACTOME	312	13	11	7,27E-01	9,20E-01	rs10914625|rs3197232|rs38494|rs685653|rs2227956|rs480092|rs3101017|rs4401711|rs2876124|rs707937|rs2228224
Oral Ulcers	APOPTOTIC_EXECUTION_PHASE	REACTOME	1211	37	20	7,28E-01	9,20E-01	rs13191992|rs10140923|rs4647693|rs2155948|rs510745|rs10508312|rs11136336|rs9951341|rs336629|rs9392904|rs2076802|rs2387399|rs661891|rs17074679|rs12340264|rs9550565|rs12570830|rs1719336|rs3600|rs10431924
Oral Ulcers	BASAL_CELL_CARCINOMA	KEGG	2166	26	20	7,28E-01	9,20E-01	rs3779173|rs2796806|rs4098686|rs12464700|rs6732834|rs1026487|rs1005351|rs9986932|rs1392224|rs2299146|rs8069437|rs2540590|rs1991769|rs6771638|rs2685127|rs17126825|rs6961071|rs390200|rs1160047|rs6709236
Oral Ulcers	LIPID_DIGESTION_MOBILIZATION_AND_TRANSPORT	REACTOME	1389	28	20	7,28E-01	9,20E-01	rs11254339|rs4804147|rs1331924|rs2740484|rs2027401|rs7163052|rs7819541|rs600880|rs7761293|rs838893|rs838920|rs880887|rs7524898|rs872077|rs9919066|rs567384|rs2518054|rs4148083|rs2482430|rs7526798
Oral Ulcers	CELL_CELL_JUNCTION_ORGANIZATION	REACTOME	6211	31	20	7,28E-01	9,20E-01	rs2587374|rs1703047|rs1975673|rs422384|rs12921373|rs1462307|rs4782685|rs1304648|rs7355953|rs4573711|rs6549064|rs7498814|rs750146|rs2042441|rs12639225|rs17566483|rs4949141|rs16957844|rs12293402|rs2236415
Oral Ulcers	ACYL_CHAIN_REMODELLING_OF_PE	REACTOME	696	34	17	7,30E-01	9,20E-01	rs354263|rs1712426|rs926324|rs2034521|rs666511|rs9358309|rs2383623|rs442873|rs525380|rs2472756|rs6716851|rs132985|rs1366444|rs7162607|rs492738|rs12130951|rs4434808
Oral Ulcers	COMPLEMENT_AND_COAGULATION_CASCADES	KEGG	1742	47	20	7,30E-01	9,20E-01	rs1074801|rs3758919|rs1861309|rs896117|rs659445|rs12755775|rs12725867|rs4764521|rs698090|rs11569429|rs10801555|rs2279623|rs11165201|rs2329591|rs2069916|rs12333245|rs2729376|rs16846520|rs626457|rs2891447
Oral Ulcers	RELA_PATHWAY	BIOCARTA	208	9	6	7,33E-01	9,22E-01	rs13053242|rs13192469|rs5746014|rs230530|rs555745|rs3818411
Oral Ulcers	G_ALPHA_Q_SIGNALLING_EVENTS	REACTOME	7428	33	20	7,34E-01	9,22E-01	rs10940145|rs13227318|rs6090338|rs7726943|rs1328684|rs10461535|rs6306|rs2239512|rs6590911|rs2372183|rs281508|rs324978|rs4976028|rs6076798|rs936213|rs10895184|rs4978964|rs2503097|rs9730423|rs6467706
Oral Ulcers	CLASS_B_2_SECRETIN_FAMILY_RECEPTORS	REACTOME	2257	30	20	7,35E-01	9,22E-01	rs252552|rs9917042|rs2796806|rs1078523|rs1320301|rs4098686|rs8081471|rs1392224|rs8069437|rs1198823|rs2540590|rs4988494|rs252568|rs724450|rs10771385|rs6961071|rs11766609|rs1160047|rs1429529|rs874627
Oral Ulcers	G_PROTEIN_BETA_GAMMA_SIGNALLING	REACTOME	798	16	11	7,37E-01	9,22E-01	rs936213|rs227142|rs6086543|rs874627|rs613229|rs6086402|rs6118062|rs2179798|rs4816089|rs6429146|rs6055748
Oral Ulcers	DOWNSTREAM_SIGNALING_EVENTS_OF_B_CELL_RECEPTOR_BCR	REACTOME	2127	31	20	7,41E-01	9,22E-01	rs2294183|rs7396875|rs328349|rs711272|rs9576991|rs4613794|rs8066176|rs10951010|rs11860367|rs8009527|rs34322|rs4710834|rs3820667|rs12205556|rs9566508|rs17254382|rs4148876|rs8051034|rs2665833|rs12191179
Oral Ulcers	ABCA_TRANSPORTERS_IN_LIPID_HOMEOSTASIS	REACTOME	464	41	13	7,43E-01	9,22E-01	rs10877201|rs4148045|rs10512523|rs736528|rs671872|rs2072102|rs4141177|rs4148083|rs914189|rs881395|rs4952689|rs10493872|rs4245794
Oral Ulcers	RNA_DEGRADATION	KEGG	1123	44	20	7,43E-01	9,22E-01	rs964450|rs1503236|rs17135399|rs437179|rs1921592|rs8111911|rs1346793|rs2152092|rs10424816|rs7534552|rs9628|rs2227956|rs202841|rs480092|rs10814518|rs17034385|rs10198826|rs7497342|rs238238|rs16963758
Oral Ulcers	S_PHASE	REACTOME	2076	42	20	7,44E-01	9,22E-01	rs2294183|rs194825|rs328349|rs7808273|rs8066176|rs942592|rs8009527|rs1405655|rs4645742|rs16893188|rs16902819|rs7928738|rs34322|rs4710834|rs3820667|rs10092081|rs4148876|rs1467464|rs2584624|rs1045288
Oral Ulcers	ASTHMA	KEGG	770	53	15	7,45E-01	9,22E-01	rs3997854|rs3135363|rs3746821|rs7192|rs9368726|rs376892|rs13192469|rs2243290|rs9275595|rs7152062|rs10797094|rs129653|rs25895|rs7758736|rs3130192
Oral Ulcers	MTA3_PATHWAY	BIOCARTA	333	17	11	7,48E-01	9,22E-01	rs6020218|rs2374432|rs7589023|rs10415156|rs7761846|rs6020170|rs10431924|rs1284300|rs4783689|rs3020375|rs17679678
Oral Ulcers	SYNTHESIS_OF_PA	REACTOME	1057	33	20	7,48E-01	9,22E-01	rs354263|rs2046731|rs2804616|rs2936523|rs926324|rs2936517|rs8030796|rs2135284|rs6559176|rs2383623|rs442873|rs3913795|rs525380|rs13160153|rs4819361|rs3134603|rs83615|rs836178|rs7908446|rs4406174
Oral Ulcers	G1_S_TRANSITION	REACTOME	1941	42	20	7,48E-01	9,22E-01	rs2294183|rs194825|rs328349|rs7808273|rs8066176|rs8009527|rs4645742|rs16893188|rs16902819|rs7928738|rs34322|rs4710834|rs3820667|rs10092081|rs4148876|rs1467464|rs2665833|rs1045288|rs4902371|rs133416
Oral Ulcers	SYNTHESIS_OF_PC	REACTOME	669	22	15	7,49E-01	9,22E-01	rs11892871|rs659445|rs10520097|rs1045075|rs11080994|rs4461819|rs4669784|rs12340765|rs10431453|rs3810947|rs12141345|rs10820780|rs884534|rs13013930|rs7567183
Oral Ulcers	SYSTEMIC_LUPUS_ERYTHEMATOSUS	KEGG	2453	55	20	7,50E-01	9,22E-01	rs9922023|rs3997854|rs3135363|rs659445|rs896117|rs1549487|rs1376679|rs11055671|rs3746821|rs2271696|rs10492130|rs11569429|rs2157338|rs2279623|rs9368726|rs376892|rs13192469|rs2329591|rs12333245|rs9268644
Oral Ulcers	TRAF6_MEDIATED_INDUCTION_OF_NFKB_AND_MAP_KINASES_UPON_TLR7_8_OR_9_ACTI	REACTOME	2239	22	20	7,50E-01	9,22E-01	rs770466|rs1351726|rs7841459|rs1422189|rs711272|rs4613794|rs2555454|rs2575676|rs378373|rs10097279|rs11107008|rs12205556|rs11024603|rs2721854|rs491700|rs3749791|rs12830764|rs452987|rs12191179|rs12892005
Oral Ulcers	RESOLUTION_OF_AP_SITES_VIA_THE_MULTIPLE_NUCLEOTIDE_PATCH_REPLACEMENT_P	REACTOME	192	9	6	7,51E-01	9,22E-01	rs2722191|rs1405655|rs3816551|rs4135048|rs7939226|rs174534
Oral Ulcers	REGULATION_OF_MITOTIC_CELL_CYCLE	REACTOME	1318	41	20	7,51E-01	9,22E-01	rs2294183|rs328349|rs399672|rs8066176|rs8009527|rs3826543|rs6736093|rs4710834|rs3820667|rs4148876|rs6841355|rs2584624|rs1045288|rs3859192|rs11543947|rs2061185|rs11023238|rs11936705|rs653079|rs4980335
Oral Ulcers	TNFR2_PATHWAY	BIOCARTA	414	11	8	7,53E-01	9,23E-01	rs2112662|rs2144825|rs13192469|rs5746014|rs230530|rs12517663|rs12878111|rs3818411
Oral Ulcers	PPAR_SIGNALING_PATHWAY	KEGG	1324	23	20	7,60E-01	9,30E-01	rs1555208|rs7679085|rs7581571|rs7674113|rs13126170|rs10024579|rs1127745|rs3774051|rs6852527|rs6595861|rs1554336|rs1800206|rs725926|rs10518221|rs1527479|rs1889155|rs1372299|rs7701539|rs11988|rs1422443
Oral Ulcers	AMINO_ACID_TRANSPORT_ACROSS_THE_PLASMA_MEMBRANE	REACTOME	1025	33	20	7,61E-01	9,30E-01	rs9883639|rs1384319|rs9036|rs17244832|rs12644755|rs7583682|rs12543594|rs17279465|rs3829406|rs13081924|rs12433985|rs1376088|rs10506265|rs10211524|rs2465602|rs13177627|rs6818743|rs9818982|rs7977721|rs4685158
Oral Ulcers	EXTRACELLULAR_MATRIX_ORGANIZATION	REACTOME	3783	32	20	7,64E-01	9,32E-01	rs3811159|rs2763341|rs17040061|rs17786760|rs9301436|rs810687|rs1029999|rs198972|rs805693|rs2581625|rs1002993|rs6436669|rs3744787|rs421587|rs17251|rs1982850|rs2664368|rs891718|rs9464379|rs2707973
Oral Ulcers	BASE_EXCISION_REPAIR	REACTOME	209	10	7	7,66E-01	9,33E-01	rs2722191|rs1405655|rs2854501|rs3816551|rs4135048|rs7939226|rs174534
Oral Ulcers	SEMA4D_IN_SEMAPHORIN_SIGNALING	REACTOME	597	23	14	7,68E-01	9,34E-01	rs4405007|rs1050162|rs3764351|rs7523940|rs599779|rs216163|rs10919619|rs2284037|rs2689420|rs11083855|rs822570|rs10753848|rs216164|rs2269529
Oral Ulcers	SHC1_EVENTS_IN_ERBB4_SIGNALING	REACTOME	1072	37	20	7,70E-01	9,36E-01	rs7396875|rs16847152|rs11895509|rs16999923|rs17719687|rs12990694|rs896577|rs10932383|rs2888104|rs16999874|rs10954863|rs10164675|rs4859417|rs6745717|rs7425448|rs6739390|rs10185729|rs10503920|rs13008370|rs1370618
Oral Ulcers	SPLICEOSOME	KEGG	1685	31	20	7,73E-01	9,37E-01	rs4143678|rs17629022|rs10140923|rs200177|rs13236851|rs4683295|rs1140409|rs9462015|rs2734574|rs8111911|rs7564183|rs9789521|rs202654|rs896064|rs1463558|rs16987990|rs7764770|rs10424816|rs4683441|rs11150846
Oral Ulcers	LEISHMANIA_INFECTION	KEGG	2265	35	20	7,74E-01	9,37E-01	rs2965012|rs5750326|rs3997854|rs711272|rs3135363|rs7518498|rs310209|rs7552931|rs4613794|rs11860367|rs12038031|rs12205556|rs11009157|rs11569429|rs8051034|rs7192|rs1935624|rs12191179|rs11584662|rs2279623
Oral Ulcers	TOLL_RECEPTOR_CASCADES	REACTOME	3778	25	20	7,76E-01	9,37E-01	rs770466|rs1941593|rs17020888|rs1351726|rs7841459|rs919308|rs1422189|rs711272|rs3813982|rs4613794|rs2555454|rs12964783|rs2575676|rs378373|rs2722191|rs10097279|rs1429871|rs4888197|rs32019|rs2144825
Oral Ulcers	GLUCONEOGENESIS	REACTOME	490	17	16	7,77E-01	9,37E-01	rs11983987|rs9937648|rs8045775|rs7534552|rs7524898|rs872077|rs600130|rs10761339|rs7526798|rs11649592|rs238238|rs316689|rs10521054|rs422732|rs13293668|rs1633399
Oral Ulcers	DEADENYLATION_DEPENDENT_MRNA_DECAY	REACTOME	622	21	15	7,82E-01	9,37E-01	rs17135399|rs3843552|rs17460250|rs2152092|rs10424816|rs11150846|rs9628|rs2227956|rs480092|rs7497342|rs16963758|rs10438740|rs2241521|rs11957360|rs2229032
Oral Ulcers	CELL_JUNCTION_ORGANIZATION	REACTOME	6781	30	20	7,83E-01	9,37E-01	rs2587374|rs1703047|rs1975673|rs422384|rs12921373|rs1462307|rs4782685|rs1304648|rs7355953|rs805693|rs4573711|rs2298928|rs1983609|rs6549064|rs7498814|rs750146|rs826699|rs2042441|rs12639225|rs17566483
Oral Ulcers	NUCLEOTIDE_EXCISION_REPAIR	REACTOME	956	40	20	7,85E-01	9,37E-01	rs4487686|rs1131384|rs942592|rs1405655|rs9299131|rs4150454|rs16902819|rs1467464|rs12446158|rs690705|rs2072102|rs11988|rs1543551|rs2532929|rs4151374|rs4150667|rs3816551|rs2020892|rs7939226|rs4943189
Oral Ulcers	BIOLOGICAL_OXIDATIONS	REACTOME	3568	26	20	7,86E-01	9,37E-01	rs1938725|rs6088659|rs17522553|rs1324299|rs4260|rs7444620|rs7518498|rs4665287|rs12651454|rs1112310|rs2262377|rs4149186|rs12038031|rs6050291|rs639434|rs6037013|rs17210001|rs1885277|rs5993891|rs1027472
Oral Ulcers	MEIOSIS	REACTOME	1674	52	20	7,86E-01	9,37E-01	rs1805812|rs2141150|rs2697675|rs2025258|rs136569|rs153060|rs12449313|rs138465|rs12173486|rs1467464|rs2178009|rs12966840|rs11743379|rs12538587|rs2092602|rs3101017|rs28366162|rs17312807|rs9379780|rs9322379
Oral Ulcers	PROPANOATE_METABOLISM	KEGG	1219	29	20	7,87E-01	9,37E-01	rs3741049|rs1885490|rs2885856|rs2181139|rs6050291|rs1918094|rs6037013|rs3774051|rs4146945|rs2490128|rs17085|rs2057374|rs2408700|rs15677|rs7647085|rs1886446|rs1146581|rs4772302|rs1273422|rs2313427
Oral Ulcers	ADIPOCYTOKINE_SIGNALING_PATHWAY	KEGG	2106	27	20	7,89E-01	9,37E-01	rs1555208|rs16087|rs8067341|rs1922529|rs2393550|rs4665287|rs510745|rs10508312|rs10024579|rs2575676|rs8074003|rs1001684|rs9588044|rs2946385|rs6852527|rs3749791|rs1554336|rs1800206|rs6819019|rs1527479
Oral Ulcers	THE_ROLE_OF_NEF_IN_HIV1_REPLICATION_AND_DISEASE_PATHOGENESIS	REACTOME	936	34	18	7,90E-01	9,37E-01	rs809192|rs1871093|rs13245967|rs1420426|rs2700986|rs17670092|rs12656935|rs874627|rs2949659|rs10488019|rs2689420|rs7797162|rs11604757|rs16859085|rs1004420|rs17644822|rs4723612|rs2237259
Oral Ulcers	UCALPAIN_PATHWAY	BIOCARTA	426	23	13	7,91E-01	9,37E-01	rs17605348|rs11658221|rs1002063|rs6887205|rs11009157|rs2239206|rs2153875|rs3763630|rs735986|rs2689420|rs1195962|rs2292867|rs2216058
Oral Ulcers	GLYCOGEN_BREAKDOWN_GLYCOGENOLYSIS	REACTOME	246	12	8	7,91E-01	9,37E-01	rs1297783|rs7615371|rs931640|rs919005|rs11710943|rs904160|rs746748|rs7259810
Oral Ulcers	STEROID_HORMONE_BIOSYNTHESIS	KEGG	1491	72	14	7,92E-01	9,37E-01	rs17522553|rs1931669|rs1112310|rs17210001|rs5993891|rs10196846|rs3753519|rs12333245|rs376109|rs1105879|rs206834|rs6847603|rs4148298|rs2486758
Oral Ulcers	CHROMOSOME_MAINTENANCE	REACTOME	1949	51	20	7,93E-01	9,37E-01	rs2051186|rs8132833|rs2141150|rs2025258|rs136569|rs942592|rs153060|rs1405655|rs9302109|rs1980705|rs12173486|rs1467464|rs2178009|rs10514815|rs690705|rs12538587|rs2092602|rs1543551|rs9379780|rs9322379
Oral Ulcers	RETINOL_METABOLISM	KEGG	1512	95	19	7,93E-01	9,37E-01	rs276167|rs4889298|rs6564862|rs1112310|rs7921420|rs305985|rs1673130|rs376109|rs1042026|rs10153788|rs7247903|rs1693439|rs1105879|rs8187889|rs6541030|rs1927473|rs1644731|rs4148298|rs2892805
Oral Ulcers	INITIAL_TRIGGERING_OF_COMPLEMENT	REACTOME	434	36	12	7,94E-01	9,37E-01	rs659445|rs698090|rs11569429|rs2279623|rs12333245|rs2891447|rs2384013|rs13090|rs16861729|rs2384124|rs13064994|rs12571712
Oral Ulcers	NEUROACTIVE_LIGAND_RECEPTOR_INTERACTION	KEGG	13469	26	20	7,94E-01	9,37E-01	rs6800549|rs17752444|rs961552|rs6090338|rs9322668|rs9922023|rs1328684|rs13152353|rs6306|rs890707|rs17611047|rs4305747|rs1232222|rs10063828|rs7549591|rs2167568|rs1595043|rs4935752|rs9850879|rs2683530
Oral Ulcers	MEIOTIC_SYNAPSIS	REACTOME	1031	41	17	7,96E-01	9,38E-01	rs2141150|rs2025258|rs136569|rs153060|rs12173486|rs2178009|rs12538587|rs2092602|rs9379780|rs9322379|rs2813510|rs792637|rs11715257|rs10431453|rs9371581|rs2229032|rs34301839
Oral Ulcers	LOSS_OF_NLP_FROM_MITOTIC_CENTROSOMES	REACTOME	1100	33	20	7,97E-01	9,38E-01	rs3811741|rs1558729|rs1156214|rs12331742|rs17692377|rs7589023|rs9366078|rs4790152|rs6538797|rs2193009|rs3745571|rs3729877|rs3764694|rs2416739|rs2024490|rs12517345|rs12651631|rs1399960|rs983679|rs11611613
Oral Ulcers	RENIN_ANGIOTENSIN_SYSTEM	KEGG	467	9	6	8,03E-01	9,44E-01	rs3863998|rs9653951|rs1456680|rs6440546|rs9825391|rs1466097
Oral Ulcers	FORMATION_OF_INCISION_COMPLEX_IN_GG_NER	REACTOME	545	30	11	8,04E-01	9,44E-01	rs9299131|rs4150454|rs16902819|rs1467464|rs12446158|rs11988|rs2532929|rs4151374|rs4150667|rs2020892|rs4978688
Oral Ulcers	RHO_PATHWAY	BIOCARTA	480	32	18	8,09E-01	9,45E-01	rs268852|rs17391840|rs3767423|rs820336|rs4530474|rs4513276|rs872077|rs10125301|rs12340264|rs238238|rs4969254|rs713253|rs4076037|rs3763630|rs822570|rs17192160|rs2741853|rs4745451
Oral Ulcers	NFKB_AND_MAP_KINASES_ACTIVATION_MEDIATED_BY_TLR4_SIGNALING_REPERTOIRE	REACTOME	2248	26	20	8,10E-01	9,45E-01	rs770466|rs1351726|rs7841459|rs1422189|rs711272|rs4613794|rs2575676|rs378373|rs10097279|rs12205556|rs11024603|rs2721854|rs491700|rs3749791|rs12830764|rs452987|rs1935624|rs12191179|rs2076139|rs2529718
Oral Ulcers	TRANSCRIPTION_COUPLED_NER_TC_NER	REACTOME	834	44	20	8,11E-01	9,45E-01	rs4487686|rs1131384|rs942592|rs1405655|rs4150454|rs16902819|rs1467464|rs12446158|rs690705|rs2072102|rs1543551|rs2532929|rs4151374|rs4150667|rs3816551|rs2020892|rs7939226|rs4943189|rs158570|rs2393374
Oral Ulcers	RECRUITMENT_OF_MITOTIC_CENTROSOME_PROTEINS_AND_COMPLEXES	REACTOME	1177	32	20	8,13E-01	9,45E-01	rs3811741|rs1558729|rs1156214|rs12331742|rs17692377|rs7589023|rs9366078|rs8043036|rs4790152|rs6538797|rs2193009|rs3745571|rs3729877|rs3764694|rs2416739|rs2024490|rs12517345|rs4792930|rs12651631|rs1554160
Oral Ulcers	BASIGIN_INTERACTIONS	REACTOME	595	24	16	8,15E-01	9,45E-01	rs17244832|rs12644755|rs2269757|rs11009157|rs1882694|rs3829406|rs12433985|rs1376088|rs2267366|rs1033805|rs6818743|rs847693|rs2153875|rs2230392|rs7694744|rs882126
Oral Ulcers	ACYL_CHAIN_REMODELLING_OF_PC	REACTOME	693	38	19	8,18E-01	9,45E-01	rs354263|rs1712426|rs926324|rs2034521|rs666511|rs243840|rs2383623|rs442873|rs525380|rs13160153|rs6716851|rs132985|rs1366444|rs1558666|rs4406174|rs7162607|rs492738|rs12130951|rs4463623
Oral Ulcers	ACTIVATED_AMPK_STIMULATES_FATTY_ACID_OXIDATION_IN_MUSCLE	REACTOME	421	23	17	8,19E-01	9,45E-01	rs2393550|rs10024579|rs1001684|rs721575|rs6852527|rs1554336|rs6950343|rs4726070|rs6593732|rs7788961|rs6828840|rs13384784|rs1881624|rs4862439|rs2268400|rs3792826|rs4862451
Oral Ulcers	PRE_NOTCH_EXPRESSION_AND_PROCESSING	REACTOME	1434	45	20	8,19E-01	9,45E-01	rs12589158|rs12588669|rs4411562|rs3791039|rs3130304|rs1154827|rs10484135|rs13053242|rs569977|rs17287454|rs4904040|rs9897312|rs12437161|rs17117189|rs4899822|rs11714203|rs6682678|rs4326648|rs10521128|rs10498559

Oral Ulcers	SYNTHESIS_OF_BILE_ACIDS_AND_BILE_SALTS	REACTOME	546	19	11	8,19E-01	9,45E-01	rs1931669|rs1127745|rs639434|rs257977|rs250412|rs246966|rs8116218|rs7162180|rs10437066|rs17380969|rs854151
Oral Ulcers	LIGAND_GATED_ION_CHANNEL_TRANSPORT	REACTOME	1051	52	20	8,21E-01	9,45E-01	rs10063828|rs7672100|rs11949658|rs1035750|rs154111|rs1477310|rs8040103|rs7179575|rs745013|rs6606866|rs208136|rs4778147|rs6497213|rs4912521|rs1491411|rs6881515|rs13176858|rs9444675|rs7129190|rs2297391
Oral Ulcers	CYTOCHROME_P450_ARRANGED_BY_SUBSTRATE_TYPE	REACTOME	1163	28	15	8,22E-01	9,45E-01	rs17522553|rs4665287|rs639434|rs7921420|rs4646530|rs305985|rs10196846|rs12333245|rs11085969|rs10153788|rs7247903|rs915908|rs1927473|rs1993116|rs2486758
Oral Ulcers	AXON_GUIDANCE	REACTOME	13131	30	20	8,23E-01	9,45E-01	rs7431017|rs219862|rs3811159|rs17140205|rs7396875|rs809192|rs10511054|rs2295522|rs11713106|rs10889117|rs4803342|rs6898594|rs3769504|rs1350136|rs4459934|rs267131|rs6590911|rs9301436|rs810687|rs3754806
Oral Ulcers	BILE_ACID_AND_BILE_SALT_METABOLISM	REACTOME	1176	41	20	8,23E-01	9,45E-01	rs1931669|rs4149023|rs1127745|rs9519052|rs9554926|rs639434|rs1336596|rs257977|rs250412|rs246966|rs2575139|rs852550|rs7701539|rs2575153|rs7707569|rs9804732|rs1274749|rs8116218|rs11568563|rs7328957
Oral Ulcers	G_BETA_GAMMA_SIGNALLING_THROUGH_PI3KGAMMA	REACTOME	487	4	3	8,23E-01	9,45E-01	rs874627|rs613229|rs6429146
Oral Ulcers	PROCESSING_OF_CAPPED_INTRON_CONTAINING_PRE_MRNA	REACTOME	1547	23	20	8,24E-01	9,45E-01	rs4611262|rs17629022|rs4487686|rs10852553|rs1131384|rs12024682|rs4648873|rs7564183|rs4684126|rs4869498|rs202654|rs16987990|rs10783580|rs12472874|rs788018|rs2383477|rs2227956|rs2072102|rs1613070|rs480092
Oral Ulcers	FATTY_ACID_TRIACYLGLYCEROL_AND_KETONE_BODY_METABOLISM	REACTOME	5526	24	20	8,26E-01	9,45E-01	rs10773086|rs4759430|rs7167741|rs1995243|rs1555208|rs11892871|rs219233|rs11071587|rs838511|rs17621311|rs2278722|rs9357530|rs780885|rs7684686|rs1331924|rs2740484|rs7581571|rs209510|rs7812088|rs1043180
Oral Ulcers	SYNTHESIS_OF_GLYCOSYLPHOSPHATIDYLINOSITOL_GPI	REACTOME	485	12	7	8,27E-01	9,45E-01	rs2285173|rs2306595|rs7763|rs8031322|rs1025145|rs6776064|rs957566
Oral Ulcers	AMYOTROPHIC_LATERAL_SCLEROSIS_ALS	KEGG	1813	28	20	8,28E-01	9,45E-01	rs6852347|rs9922023|rs13434873|rs4647693|rs39425|rs11055671|rs2851045|rs1982673|rs10492130|rs2288713|rs2076139|rs13192469|rs5746014|rs12797274|rs2156192|rs2046136|rs7230970|rs1319621|rs2973139|rs12817623
Oral Ulcers	TOLL_PATHWAY	BIOCARTA	1147	30	20	8,29E-01	9,45E-01	rs1555208|rs711272|rs4613794|rs2334659|rs12205556|rs2721854|rs1935624|rs12191179|rs1800206|rs8108561|rs2529718|rs11725282|rs9345126|rs230530|rs5743942|rs9345117|rs4326648|rs538723|rs559052|rs1884839
Oral Ulcers	CHREBP2_PATHWAY	BIOCARTA	1059	30	20	8,33E-01	9,45E-01	rs2393550|rs4897759|rs2267172|rs1741143|rs16838740|rs1001684|rs6889043|rs9419702|rs4790152|rs10495567|rs6950343|rs7524898|rs872077|rs4726070|rs4527882|rs7526798|rs3729877|rs4734507|rs6468757|rs6593732
Oral Ulcers	POTASSIUM_CHANNELS	REACTOME	4940	25	20	8,33E-01	9,45E-01	rs11654455|rs10835507|rs1489635|rs7938219|rs16837945|rs2155548|rs2074315|rs3826046|rs17260690|rs2898334|rs701229|rs2790372|rs10228248|rs13064262|rs12495203|rs10495897|rs11978545|rs3847171|rs1890843|rs4496877
Oral Ulcers	MRNA_PROCESSING	REACTOME	1942	23	20	8,33E-01	9,45E-01	rs4611262|rs17629022|rs4487686|rs10852553|rs2756382|rs1131384|rs12024682|rs4648873|rs4150454|rs16902819|rs7564183|rs4684126|rs11756329|rs4869498|rs202654|rs16987990|rs10783580|rs11752001|rs12472874|rs788018
Oral Ulcers	NFKB_PATHWAY	BIOCARTA	870	25	16	8,33E-01	9,45E-01	rs711272|rs4613794|rs12205556|rs1935624|rs12191179|rs13192469|rs17766515|rs5746014|rs9345126|rs230530|rs9345117|rs555745|rs538723|rs1884839|rs10739508|rs3818411
Oral Ulcers	DORSO_VENTRAL_AXIS_FORMATION	KEGG	1149	39	20	8,36E-01	9,45E-01	rs2836786|rs3130304|rs11145770|rs1982530|rs8033106|rs9806913|rs2051526|rs17673576|rs11221077|rs2877260|rs1037666|rs2961557|rs879081|rs11976696|rs736813|rs7310017|rs3746882|rs783528|rs3130320|rs4245079
Oral Ulcers	LYSOSOME_VESICLE_BIOGENESIS	REACTOME	420	10	8	8,39E-01	9,45E-01	rs506233|rs2293682|rs365936|rs11218954|rs10515171|rs1555422|rs2304911|rs3808755
Oral Ulcers	METABOLISM_OF_XENOBIOTICS_BY_CYTOCHROME_P450	KEGG	1674	69	20	8,39E-01	9,45E-01	rs1931669|rs1112310|rs2262377|rs4149186|rs1027472|rs7921420|rs7896809|rs4241932|rs305985|rs6458861|rs10196846|rs17608022|rs4646812|rs376109|rs1042026|rs1693439|rs1105879|rs915908|rs3766934|rs4148298
Oral Ulcers	ALANINE_ASPARTATE_AND_GLUTAMATE_METABOLISM	KEGG	841	40	20	8,39E-01	9,45E-01	rs6703674|rs10911044|rs9937648|rs8045775|rs6748782|rs914977|rs3749791|rs17246034|rs13030665|rs9687411|rs6867398|rs2302909|rs11684145|rs3771311|rs1273422|rs11649592|rs2313427|rs13150967|rs2985439|rs10521054
Oral Ulcers	SIGNAL_AMPLIFICATION	REACTOME	1023	40	18	8,39E-01	9,45E-01	rs354263|rs10869950|rs1162211|rs1144430|rs926324|rs2383623|rs874627|rs11145635|rs3786989|rs11880198|rs11145517|rs12938589|rs510619|rs10226374|rs6429146|rs12610831|rs12130951|rs4434808
Oral Ulcers	ACTIVATED_TLR4_SIGNALLING	REACTOME	2676	22	20	8,40E-01	9,45E-01	rs770466|rs1351726|rs7841459|rs1422189|rs711272|rs3813982|rs4613794|rs2555454|rs2575676|rs378373|rs10097279|rs2144825|rs11107008|rs12205556|rs11024603|rs2721854|rs491700|rs3749791|rs12830764|rs452987
Oral Ulcers	NUCLEAR_SIGNALING_BY_ERBB4	REACTOME	2231	24	20	8,41E-01	9,46E-01	rs1779344|rs17629022|rs266087|rs1207774|rs4887997|rs16847152|rs11759745|rs3751832|rs11895509|rs16999923|rs5758168|rs17719687|rs12990694|rs404220|rs896577|rs4887950|rs377651|rs10932383|rs17724641|rs8045079
Oral Ulcers	MYD88_MAL_CASCADE_INITIATED_ON_PLASMA_MEMBRANE	REACTOME	2527	22	20	8,44E-01	9,47E-01	rs770466|rs1351726|rs7841459|rs1422189|rs711272|rs4613794|rs2555454|rs2575676|rs378373|rs10097279|rs11107008|rs12205556|rs11024603|rs2721854|rs491700|rs3749791|rs12830764|rs452987|rs1935624|rs12191179
Oral Ulcers	REGULATION_OF_INSULIN_LIKE_GROWTH_FACTOR_IGF_ACTIVITY_BY_INSULIN_LIKE_GR DING_PROTEINS_IGFBPS	REACTOME	570	24	16	8,45E-01	9,47E-01	rs198972|rs7315995|rs940794|rs243840|rs1345319|rs11691253|rs12379469|rs10435873|rs17241017|rs1558666|rs720683|rs879965|rs7865455|rs1462282|rs10275903|rs7479065
Oral Ulcers	IL12_PATHWAY	BIOCARTA	589	17	9	8,46E-01	9,47E-01	rs6780296|rs502514|rs7601754|rs2949659|rs4326648|rs7643177|rs1041973|rs16859085|rs13029532
Oral Ulcers	VOLTAGE_GATED_POTASSIUM_CHANNELS	REACTOME	2491	33	20	8,53E-01	9,53E-01	rs10835507|rs1489635|rs7938219|rs17260690|rs2790372|rs10228248|rs10495897|rs11978545|rs3847171|rs1890843|rs4496877|rs16984239|rs6672114|rs750574|rs163171|rs10505589|rs17256687|rs3111229|rs8048412|rs2389736
Oral Ulcers	REGULATION_OF_HYPOXIA_INDUCIBLE_FACTOR_HIF_BY_OXYGEN	REACTOME	443	12	8	8,55E-01	9,53E-01	rs5758168|rs13053242|rs1431054|rs917948|rs943080|rs7092999|rs17256317|rs1447562
Oral Ulcers	MISMATCH_REPAIR	KEGG	576	24	14	8,61E-01	9,53E-01	rs2228006|rs942592|rs1405655|rs1467464|rs690705|rs6753949|rs1543551|rs9287256|rs3816551|rs3796517|rs7939226|rs4943189|rs3136337|rs2393374
Oral Ulcers	GLYCEROLIPID_METABOLISM	KEGG	1872	31	20	8,61E-01	9,53E-01	rs6467706|rs161338|rs838709|rs2046731|rs7811862|rs9326038|rs2804616|rs7788248|rs1885490|rs7788953|rs7163052|rs2181139|rs2135284|rs7536157|rs9358309|rs9968130|rs10518221|rs1572057|rs880887|rs1646744
Oral Ulcers	CHYLOMICRON_MEDIATED_LIPID_TRANSPORT	REACTOME	435	13	9	8,64E-01	9,53E-01	rs4804147|rs7163052|rs12750012|rs635141|rs439401|rs10797094|rs8102273|rs2194562|rs717966
Oral Ulcers	SEMAPHORIN_INTERACTIONS	REACTOME	2884	23	20	8,64E-01	9,53E-01	rs17140205|rs809192|rs4405007|rs7759295|rs153641|rs6949364|rs696964|rs1050162|rs11945109|rs268487|rs3764351|rs7523940|rs599779|rs2303924|rs17296526|rs2785262|rs12508543|rs6887205|rs4844687|rs11009157
Oral Ulcers	PEPTIDE_CHAIN_ELONGATION	REACTOME	947	40	20	8,65E-01	9,53E-01	rs2278722|rs4792792|rs13353326|rs10467685|rs11856823|rs10065570|rs4777186|rs1458332|rs5003866|rs8076790|rs7502891|rs4583476|rs2280401|rs3796934|rs11002444|rs7210156|rs12886774|rs31726|rs12602594|rs7636810
Oral Ulcers	PHASE1_FUNCTIONALIZATION_OF_COMPOUNDS	REACTOME	1515	31	20	8,66E-01	9,53E-01	rs17522553|rs7518498|rs4665287|rs12038031|rs6050291|rs639434|rs6037013|rs7921420|rs11584662|rs4646530|rs305985|rs10196846|rs12333245|rs11085969|rs2075633|rs10153788|rs7247903|rs1693439|rs915908|rs8187889
Oral Ulcers	OXYGEN_DEPENDENT_PROLINE_HYDROXYLATION_OF_HYPOXIA_INDUCIBLE_FACTOR_/	REACTOME	313	6	6	8,66E-01	9,53E-01	rs5758168|rs1431054|rs917948|rs926914|rs17256317|rs1447562
Oral Ulcers	METABOLISM_OF_MRNA	REACTOME	3017	37	20	8,67E-01	9,53E-01	rs2294183|rs10942495|rs328349|rs227913|rs2278722|rs17135399|rs4792792|rs3843552|rs17460250|rs13353326|rs8066176|rs9892886|rs10467685|rs8009527|rs1426075|rs2075755|rs4710834|rs3820667|rs10065570|rs4148876
Oral Ulcers	TIGHT_JUNCTION	KEGG	6242	27	20	8,67E-01	9,53E-01	rs7072330|rs7396875|rs3807671|rs1918930|rs3807663|rs6088659|rs227913|rs13239642|rs281508|rs9875970|rs1990433|rs205898|rs1050162|rs510745|rs4897759|rs12464541|rs10508312|rs6967983|rs6924483|rs777710
Oral Ulcers	ETHER_LIPID_METABOLISM	KEGG	1041	41	20	8,68E-01	9,53E-01	rs354263|rs7542020|rs9326038|rs926324|rs7536157|rs243840|rs2383623|rs11132224|rs3213945|rs1572057|rs442873|rs525380|rs13160153|rs132985|rs83615|rs1558666|rs4406174|rs7162607|rs10431453|rs492738
Oral Ulcers	NEUROTRANSMITTER_RELEASE_CYCLE	REACTOME	1400	29	20	8,69E-01	9,53E-01	rs7272324|rs1320301|rs1980943|rs363333|rs11206093|rs305494|rs1728803|rs3760670|rs13064472|rs9446432|rs11218954|rs1795994|rs817365|rs11114112|rs8138938|rs3771311|rs1273422|rs2313427|rs2206161|rs9341360
Oral Ulcers	MITOTIC_G2_G2_M_PHASES	REACTOME	1389	34	20	8,72E-01	9,53E-01	rs3811741|rs1558729|rs1156214|rs12331742|rs17692377|rs7589023|rs9366078|rs16902819|rs7928738|rs8043036|rs4790152|rs6538797|rs12481679|rs2193009|rs3745571|rs3729877|rs3764694|rs2416739|rs4151374|rs2024490
Oral Ulcers	PROSTACYCLIN_SIGNALLING_THROUGH_PROSTACYCLIN_RECEPTOR	REACTOME	353	2	2	8,72E-01	9,53E-01	rs874627|rs6429146
Oral Ulcers	MRNA_SPLICING	REACTOME	1057	27	20	8,73E-01	9,53E-01	rs17629022|rs4487686|rs1131384|rs4648873|rs7564183|rs202654|rs16987990|rs10783580|rs788018|rs2227956|rs2072102|rs1613070|rs480092|rs6103330|rs6105651|rs2288055|rs6080397|rs11661922|rs11905250|rs2920706
Oral Ulcers	SIGNAL_TRANSDUCTION_BY_L1	REACTOME	1310	31	20	8,73E-01	9,53E-01	rs205898|rs17605348|rs11658221|rs378373|rs17673576|rs17296526|rs2785262|rs625087|rs11009157|rs491700|rs805262|rs2877260|rs708382|rs2223405|rs388372|rs10515171|rs7952158|rs1555422|rs11976696|rs2065364
Oral Ulcers	DESTABILIZATION_OF_MRNA_BY_BRF1	REACTOME	204	5	4	8,74E-01	9,53E-01	rs2152092|rs6792874|rs6573856|rs2229032
Oral Ulcers	PIP3_ACTIVATES_AKT_SIGNALING	REACTOME	587	15	10	8,74E-01	9,53E-01	rs9576991|rs34322|rs9566508|rs17254382|rs7070956|rs1321311|rs17446614|rs7232459|rs1751853|rs3818411
Oral Ulcers	3_UTR_MEDIATED_TRANSLATIONAL_REGULATION	REACTOME	1235	39	20	8,79E-01	9,57E-01	rs2278722|rs4792792|rs3843552|rs17460250|rs13353326|rs10467685|rs11856823|rs10065570|rs4777186|rs1458332|rs5003866|rs3808593|rs8076790|rs7502891|rs4583476|rs2280401|rs153107|rs4559682|rs2556560|rs3796934
Oral Ulcers	COMP_PATHWAY	BIOCARTA	628	55	18	8,83E-01	9,59E-01	rs896117|rs659445|rs698090|rs11569429|rs2279623|rs2329591|rs12333245|rs2891447|rs6699859|rs2384013|rs13090|rs16861729|rs2384124|rs13064994|rs700221|rs2597738|rs12571712|rs1885002
Oral Ulcers	METABOLISM_OF_AMINO_ACIDS_AND_DERIVATIVES	REACTOME	5246	29	20	8,84E-01	9,60E-01	rs1932440|rs2294183|rs328349|rs1949575|rs13197942|rs10973340|rs7562511|rs10831551|rs7444620|rs6703674|rs2268075|rs4665287|rs2564848|rs10911044|rs12301933|rs2651244|rs2242259|rs8066176|rs2540627|rs3741049
Oral Ulcers	VALINE_LEUCINE_AND_ISOLEUCINE_DEGRADATION	KEGG	1149	27	20	8,86E-01	9,60E-01	rs3741049|rs1885490|rs837642|rs10991869|rs2181139|rs3774051|rs2490128|rs1454465|rs6479333|rs2057374|rs2348024|rs2408700|rs15677|rs515324|rs1146581|rs11083620|rs723587|rs4772302|rs1273422|rs2313427
Oral Ulcers	RECYCLING_PATHWAY_OF_L1	REACTOME	874	26	14	8,86E-01	9,60E-01	rs1579282|rs378373|rs2293797|rs491700|rs2223405|rs3003610|rs388372|rs10515171|rs1555422|rs12590918|rs11213366|rs988435|rs11604757|rs3808755
Oral Ulcers	INHIBITION_OF_INSULIN_SECRETION_BY_ADRENALINE_NORADRENALINE	REACTOME	789	15	9	8,91E-01	9,61E-01	rs2241952|rs899236|rs874627|rs2068356|rs10226374|rs899227|rs10787318|rs6429146|rs2489214
Oral Ulcers	SRP_DEPENDENT_COTRANSLATIONAL_PROTEIN_TARGETING_TO_MEMBRANE	REACTOME	1378	40	20	8,93E-01	9,61E-01	rs721355|rs2278722|rs4792792|rs13353326|rs2811491|rs10467685|rs11856823|rs10065570|rs4777186|rs1458332|rs5003866|rs12631297|rs1220783|rs8076790|rs7502891|rs4583476|rs2280401|rs3796934|rs2842363|rs11002444
Oral Ulcers	COMPLEMENT_CASCADE	REACTOME	752	47	20	8,93E-01	9,61E-01	rs896117|rs659445|rs698090|rs11569429|rs10801555|rs2279623|rs2329591|rs12333245|rs626457|rs2891447|rs17006738|rs6699859|rs2384013|rs13090|rs16861729|rs2384124|rs13064994|rs700221|rs2455314|rs12571712
Oral Ulcers	NO2IL12_PATHWAY	BIOCARTA	461	12	8	8,94E-01	9,61E-01	rs502514|rs874627|rs7601754|rs2949659|rs7643177|rs797999|rs16859085|rs13029532
Oral Ulcers	NUCLEOTIDE_EXCISION_REPAIR	KEGG	914	38	20	8,94E-01	9,61E-01	rs942592|rs1405655|rs5758168|rs9299131|rs4150454|rs16902819|rs1467464|rs12446158|rs690705|rs10116799|rs11988|rs1543551|rs2532929|rs926914|rs4151374|rs4150667|rs3816551|rs2020892|rs3796517|rs7939226
Oral Ulcers	DRUG_METABOLISM_CYTOCHROME_P450	KEGG	1661	71	20	8,97E-01	9,63E-01	rs1112310|rs2262377|rs4149186|rs1027472|rs7921420|rs7896809|rs4241932|rs305985|rs6458861|rs17608022|rs2348024|rs4646812|rs376109|rs2075633|rs7247903|rs1693439|rs1105879|rs915908|rs950233|rs2076320
Oral Ulcers	ERK_PATHWAY	BIOCARTA	1025	30	20	9,02E-01	9,66E-01	rs7396875|rs7169544|rs17673576|rs16893188|rs11009157|rs10092081|rs2877260|rs17814416|rs10414066|rs2049824|rs2203232|rs1559715|rs11976696|rs1546713|rs2153875|rs11178364|rs9325190|rs2871864|rs11178478|rs17084392
Oral Ulcers	NONSENSE_MEDIATED_DECAY_ENHANCED_BY_THE_EXON_JUNCTION_COMPLEX	REACTOME	1261	40	20	9,02E-01	9,66E-01	rs2278722|rs4792792|rs3843552|rs17460250|rs13353326|rs10467685|rs1426075|rs10065570|rs4777186|rs1458332|rs5003866|rs11150846|rs8076790|rs7502891|rs4527882|rs4583476|rs2280401|rs3796934|rs11002444|rs7210156
Oral Ulcers	ASSOCIATION_OF_TRIC_CCT_WITH_TARGET_PROTEINS_DURING_BIOSYNTHESIS	REACTOME	359	17	13	9,07E-01	9,68E-01	rs9383320|rs11133604|rs202841|rs17361782|rs6926745|rs6856727|rs17170180|rs4720087|rs10517564|rs2832157|rs2422821|rs6856075|rs2714804
Oral Ulcers	DNA_REPLICATION	KEGG	740	29	20	9,07E-01	9,68E-01	rs751539|rs942592|rs1405655|rs4645742|rs6561611|rs1467464|rs133416|rs690705|rs10116799|rs4583476|rs1543551|rs2071744|rs1999043|rs3816551|rs2307344|rs3796517|rs7939226|rs4943189|rs4645752|rs174534
Oral Ulcers	MITOTIC_G1_G1_S_PHASES	REACTOME	2477	42	20	9,09E-01	9,68E-01	rs2294183|rs194825|rs328349|rs7808273|rs11968166|rs8066176|rs8009527|rs4645742|rs16893188|rs16902819|rs7928738|rs34322|rs4710834|rs3820667|rs10092081|rs4148876|rs1467464|rs4130023|rs2665833|rs1045288
Oral Ulcers	PROTEIN_FOLDING	REACTOME	675	27	19	9,09E-01	9,68E-01	rs2291688|rs9383320|rs7589023|rs11133604|rs2270461|rs202841|rs17361782|rs6926745|rs3745571|rs6856727|rs17170180|rs6867870|rs4720087|rs3212358|rs10517564|rs2832157|rs2422821|rs6856075|rs2714804
Oral Ulcers	STRESS_PATHWAY	BIOCARTA	633	12	9	9,11E-01	9,69E-01	rs2721854|rs13192469|rs4761529|rs2529718|rs230530|rs12297002|rs733751|rs4326648|rs3818411
Oral Ulcers	ACTIVATION_OF_GENES_BY_ATF4	REACTOME	316	10	9	9,12E-01	9,69E-01	rs2075755|rs2217332|rs2152092|rs2518054|rs11581556|rs16963758|rs4694636|rs7790127|rs10275903
Oral Ulcers	INTEGRATION_OF_PROVIRUS	REACTOME	122	6	5	9,13E-01	9,69E-01	rs1105451|rs2731858|rs1478486|rs132793|rs17674880
Oral Ulcers	CYTOKINE_PATHWAY	BIOCARTA	491	29	14	9,15E-01	9,69E-01	rs7801617|rs6835391|rs12535797|rs13192469|rs12511035|rs2243290|rs9357726|rs4778636|rs7643177|rs4694636|rs25895|rs17640030|rs2275913|rs10964832
Oral Ulcers	ENERGY_DEPENDENT_REGULATION_OF_MTOR_BY_LKB1_AMPK	REACTOME	461	26	17	9,16E-01	9,69E-01	rs2393550|rs1001684|rs721575|rs4889863|rs6950343|rs12943041|rs4726070|rs7874234|rs17713451|rs6593732|rs7788961|rs4969266|rs13384784|rs1881624|rs1062935|rs3792826|rs2809244
Oral Ulcers	CYSTEINE_AND_METHIONINE_METABOLISM	KEGG	982	21	15	9,18E-01	9,69E-01	rs7444620|rs2651244|rs9937648|rs8045775|rs3124455|rs7522196|rs11951539|rs1526897|rs11649592|rs7719164|rs2132302|rs10521054|rs17131359|rs10965163|rs165064
Oral Ulcers	PERK_REGULATED_GENE_EXPRESSION	REACTOME	334	10	9	9,19E-01	9,69E-01	rs2075755|rs2217332|rs2152092|rs2518054|rs11581556|rs16963758|rs4694636|rs7790127|rs10275903
Oral Ulcers	RNA_POL_I_PROMOTER_OPENING	REACTOME	501	3	2	9,21E-01	9,69E-01	rs9379780|rs1259813
Oral Ulcers	BASE_EXCISION_REPAIR	KEGG	500	19	15	9,24E-01	9,69E-01	rs1043180|rs2722191|rs1405655|rs10116799|rs2854501|rs4769346|rs17074679|rs9550565|rs10520376|rs3816551|rs4135048|rs7939226|rs9580991|rs174534|rs16939904
Oral Ulcers	GATA3_PATHWAY	BIOCARTA	898	37	19	9,25E-01	9,69E-01	rs6096351|rs4404082|rs1244157|rs8046585|rs7236492|rs7524898|rs872077|rs9783189|rs12932040|rs17483588|rs7526798|rs3729877|rs2243290|rs660749|rs316689|rs578268|rs17469061|rs1862721|rs3859314
Oral Ulcers	THYROID_CANCER	KEGG	1104	24	17	9,26E-01	9,69E-01	rs7396875|rs6732834|rs12024682|rs16893188|rs10864912|rs10092081|rs2383477|rs1889155|rs1888861|rs6719501|rs2049824|rs10431924|rs4783689|rs3115752|rs1028629|rs7867728|rs4733792
Oral Ulcers	ACTIVATION_OF_THE_MRNA_UPON_BINDING_OF_THE_CAP_BINDING_COMPLEX_AND_EIF T_BINDING_TO_43S	REACTOME	780	21	17	9,27E-01	9,69E-01	rs3843552|rs17460250|rs13353326|rs3808593|rs6605631|rs4583476|rs2280401|rs153107|rs4559682|rs2556560|rs11002444|rs222999|rs7844203|rs12886774|rs31726|rs10438740|rs1145170
Oral Ulcers	G1_PHASE	REACTOME	625	20	15	9,28E-01	9,69E-01	rs11968166|rs16902819|rs34322|rs4130023|rs1011970|rs7189819|rs4527882|rs1537377|rs4151374|rs1321311|rs12541220|rs4445045|rs2020892|rs17279275|rs4936661
Oral Ulcers	SULFUR_AMINO_ACID_METABOLISM	REACTOME	930	35	18	9,28E-01	9,69E-01	rs1949575|rs7444620|rs2651244|rs3124455|rs6493163|rs7522196|rs12101736|rs11951539|rs2670811|rs3123808|rs7719164|rs10874814|rs12908274|rs17131359|rs2289578|rs2733250|rs10965163|rs165064
Oral Ulcers	GLOBAL_GENOMIC_NER_GG_NER	REACTOME	823	43	19	9,29E-01	9,69E-01	rs942592|rs1405655|rs9299131|rs4150454|rs16902819|rs1467464|rs12446158|rs690705|rs11988|rs1543551|rs2532929|rs4151374|rs4150667|rs3816551|rs2020892|rs7939226|rs4943189|rs4978688|rs2393374
Oral Ulcers	P53HYPOXIA_PATHWAY	BIOCARTA	517	19	13	9,30E-01	9,69E-01	rs10057083|rs13053242|rs2917682|rs12720066|rs2227956|rs480092|rs2889450|rs1801516|rs1321311|rs6750100|rs879965|rs17256317|rs1462282
Oral Ulcers	POST_TRANSLATIONAL_MODIFICATION_SYNTHESIS_OF_GPI_ANCHORED_PROTEINS	REACTOME	586	15	10	9,31E-01	9,69E-01	rs2285173|rs2306595|rs7763|rs10518440|rs8031322|rs1025145|rs6776064|rs2286960|rs4803646|rs957566
Oral Ulcers	TID_PATHWAY	BIOCARTA	479	11	10	9,32E-01	9,69E-01	rs763583|rs13192469|rs5746014|rs2227956|rs480092|rs230530|rs2279664|rs2235107|rs7103780|rs2400842
Oral Ulcers	EARLY_PHASE_OF_HIV_LIFE_CYCLE	REACTOME	270	13	10	9,35E-01	9,70E-01	rs11688530|rs874627|rs4245843|rs1105451|rs4954592|rs2731858|rs1478486|rs132765|rs174534|rs17674880
Oral Ulcers	PKB_MEDIATED_EVENTS	REACTOME	583	29	20	9,36E-01	9,70E-01	rs2393550|rs1001684|rs721575|rs1292035|rs4889863|rs6950343|rs12943041|rs4726070|rs6605631|rs7874234|rs17713451|rs6593732|rs7788961|rs4969266|rs1993116|rs13384784|rs1881624|rs1062935|rs3792826|rs2809244
Oral Ulcers	MRNA_3_END_PROCESSING	REACTOME	320	14	10	9,36E-01	9,70E-01	rs4648873|rs16987990|rs6103330|rs2288055|rs11905250|rs2920706|rs7936604|rs9916811|rs2474871|rs4982753
Oral Ulcers	PRE_NOTCH_TRANSCRIPTION_AND_TRANSLATION	REACTOME	835	32	16	9,39E-01	9,70E-01	rs4411562|rs3130304|rs13053242|rs569977|rs17287454|rs9897312|rs11714203|rs6682678|rs4326648|rs10521128|rs9611286|rs3130320|rs3796627|rs514907|rs4863687|rs1978763
Oral Ulcers	PLATELET_SENSITIZATION_BY_LDL	REACTOME	676	22	12	9,39E-01	9,70E-01	rs354263|rs926324|rs1741143|rs2383623|rs12541220|rs635141|rs2194562|rs12130951|rs717966|rs4463623|rs1288502|rs4936661
Oral Ulcers	AMINO_SUGAR_AND_NUCLEOTIDE_SUGAR_METABOLISM	KEGG	956	32	20	9,41E-01	9,70E-01	rs226468|rs17592080|rs923273|rs9611613|rs3749791|rs2171385|rs12210306|rs13030665|rs3778533|rs2569860|rs6115227|rs6867398|rs10805890|rs137126|rs919005|rs2569859|rs2886117|rs1861854|rs10494133|rs422732
Oral Ulcers	P53_PATHWAY	BIOCARTA	347	14	10	9,42E-01	9,70E-01	rs1982673|rs2288713|rs2156192|rs2046136|rs7230970|rs1801516|rs1321311|rs130274|rs135152|rs5754285
Oral Ulcers	TRANSLATION	REACTOME	1803	35	20	9,42E-01	9,70E-01	rs721355|rs2278722|rs4792792|rs3843552|rs17460250|rs13353326|rs2811491|rs10467685|rs1426075|rs10065570|rs4777186|rs1458332|rs5003866|rs3808593|rs12631297|rs1220783|rs8076790|rs7502891|rs6605631|rs4583476
Oral Ulcers	GABA_SYNTHESIS_RELEASE_REUPTAKE_AND_DEGRADATION	REACTOME	746	21	17	9,43E-01	9,70E-01	rs7272324|rs1728803|rs13064472|rs9446432|rs11218954|rs1795994|rs817365|rs11114112|rs1273422|rs2313427|rs2206161|rs9341360|rs2304911|rs7967341|rs2754042|rs1381885|rs362599
Oral Ulcers	TIGHT_JUNCTION_INTERACTIONS	REACTOME	715	25	15	9,48E-01	9,74E-01	rs743102|rs6942609|rs8111177|rs10229749|rs1854363|rs7642120|rs12866697|rs17427173|rs11590812|rs2835406|rs2835358|rs1348564|rs988140|rs1095687|rs7068747
Oral Ulcers	G1_S_SPECIFIC_TRANSCRIPTION	REACTOME	194	2	2	9,49E-01	9,74E-01	rs5748218|rs6797587

<table>
<tr><td>Oral Ulcers</td><td>EIF_PATHWAY</td><td>BIOCARTA</td><td>158</td><td>2</td><td>2</td><td>9,53E-01</td><td>9,75E-01</td><td>rs10438740|rs2403144</td></tr>
<tr><td>Oral Ulcers</td><td>G0_AND_EARLY_G1</td><td>REACTOME</td><td>258</td><td>3</td><td>3</td><td>9,53E-01</td><td>9,75E-01</td><td>rs7189819|rs6989591|rs6797587</td></tr>
<tr><td>Oral Ulcers</td><td>GLUCAGON_TYPE_LIGAND_RECEPTORS</td><td>REACTOME</td><td>786</td><td>23</td><td>15</td><td>9,56E-01</td><td>9,76E-01</td><td>rs8081471|rs4988494|rs11766609|rs874627|rs4797684|rs1085998|rs476886|rs342543|rs1941673|rs17810148|rs2918939|rs6429146|rs6503264|rs218527|rs11682382</td></tr>
<tr><td>Oral Ulcers</td><td>RIP_MEDIATED_NFKB_ACTIVATION_VIA_DAI</td><td>REACTOME</td><td>328</td><td>13</td><td>12</td><td>9,61E-01</td><td>9,79E-01</td><td>rs11024603|rs452987|rs11725282|rs11603089|rs17074679|rs9550565|rs742724|rs6128102|rs3130283|rs8118279|rs3818411|rs4981508</td></tr>
<tr><td>Oral Ulcers</td><td>L1CAM_INTERACTIONS</td><td>REACTOME</td><td>3914</td><td>26</td><td>20</td><td>9,61E-01</td><td>9,79E-01</td><td>rs2295522|rs11713106|rs4803342|rs510991|rs1579282|rs205898|rs12637447|rs17605348|rs11658221|rs3847171|rs378373|rs2293797|rs677136|rs443052|rs17673576|rs4686213|rs17296526|rs2065551|rs4627775|rs2785262</td></tr>
<tr><td>Oral Ulcers</td><td>PTM_GAMMA_CARBOXYLATION_HYPUSINE_FORMATION_AND_ARYLSULFATASE_ACTIV</td><td>REACTOME</td><td>377</td><td>16</td><td>7</td><td>9,65E-01</td><td>9,79E-01</td><td>rs10008543|rs2069916|rs873592|rs11683986|rs25413|rs2532100|rs11934601</td></tr>
<tr><td>Oral Ulcers</td><td>CLEAVAGE_OF_GROWING_TRANSCRIPT_IN_THE_TERMINATION_REGION_</td><td>REACTOME</td><td>435</td><td>14</td><td>10</td><td>9,65E-01</td><td>9,79E-01</td><td>rs4648873|rs16987990|rs6103330|rs2288055|rs11905250|rs2920706|rs7936604|rs9916811|rs2474871|rs4982753</td></tr>
<tr><td>Oral Ulcers</td><td>41BB_PATHWAY</td><td>BIOCARTA</td><td>341</td><td>5</td><td>4</td><td>9,65E-01</td><td>9,79E-01</td><td>rs230530|rs4326648|rs2243290|rs3818411</td></tr>
<tr><td>Oral Ulcers</td><td>FORMATION_OF_THE_TERNARY_COMPLEX_AND_SUBSEQUENTLY_THE_43S_COMPLEX</td><td>REACTOME</td><td>677</td><td>15</td><td>12</td><td>9,65E-01</td><td>9,79E-01</td><td>rs13353326|rs3808593|rs4583476|rs2280401|rs153107|rs4559682|rs2556560|rs11002444|rs7844203|rs12886774|rs31726|rs1145170</td></tr>
<tr><td>Oral Ulcers</td><td>ACTIVATED_NOTCH1_TRANSMITS_SIGNAL_TO_THE_NUCLEUS</td><td>REACTOME</td><td>719</td><td>22</td><td>16</td><td>9,70E-01</td><td>9,82E-01</td><td>rs506233|rs2293797|rs2701623|rs1887319|rs1028846|rs11177623|rs12724113|rs1918953|rs2959523|rs12590918|rs1888578|rs4656256|rs6077977|rs12966680|rs1028479|rs2091918</td></tr>
<tr><td>Oral Ulcers</td><td>NETRIN1_SIGNALING</td><td>REACTOME</td><td>3710</td><td>27</td><td>20</td><td>9,71E-01</td><td>9,82E-01</td><td>rs809192|rs6898594|rs6590911|rs10895184|rs11134531|rs510745|rs1479340|rs1373179|rs10508312|rs2817701|rs9920504|rs11931784|rs265042|rs11134558|rs17614174|rs860537|rs12649350|rs2168802|rs4868223|rs6774541</td></tr>
<tr><td>Oral Ulcers</td><td>LAGGING_STRAND_SYNTHESIS</td><td>REACTOME</td><td>550</td><td>22</td><td>13</td><td>9,76E-01</td><td>9,86E-01</td><td>rs942592|rs1405655|rs1467464|rs690705|rs1543551|rs3816551|rs7939226|rs4943189|rs174534|rs9349915|rs1539604|rs3758626|rs2393374</td></tr>
<tr><td>Oral Ulcers</td><td>ADP_SIGNALLING_THROUGH_P2RY12</td><td>REACTOME</td><td>438</td><td>4</td><td>4</td><td>9,77E-01</td><td>9,86E-01</td><td>rs874627|rs510619|rs10226374|rs6429146</td></tr>
<tr><td>Oral Ulcers</td><td>TGF_BETA_RECEPTOR_SIGNALING_IN_EMT_EPITHELIAL_TO_MESENCHYMAL_TRANSITIC</td><td>REACTOME</td><td>429</td><td>9</td><td>7</td><td>9,79E-01</td><td>9,87E-01</td><td>rs743102|rs1854363|rs8110090|rs1095687|rs4803455|rs6041970|rs7068747</td></tr>
<tr><td>Oral Ulcers</td><td>DNA_STRAND_ELONGATION</td><td>REACTOME</td><td>672</td><td>30</td><td>20</td><td>9,83E-01</td><td>9,89E-01</td><td>rs942592|rs1405655|rs4645742|rs1467464|rs133416|rs690705|rs1543551|rs5748218|rs2071744|rs3764694|rs3816551|rs2307344|rs7939226|rs4943189|rs4645752|rs174534|rs9349915|rs1539604|rs3758626|rs2393374</td></tr>
<tr><td>Oral Ulcers</td><td>EXTENSION_OF_TELOMERES</td><td>REACTOME</td><td>618</td><td>22</td><td>13</td><td>9,86E-01</td><td>9,90E-01</td><td>rs942592|rs1405655|rs1467464|rs690705|rs1543551|rs3816551|rs7939226|rs4943189|rs174534|rs9349915|rs1539604|rs3758626|rs2393374</td></tr>
<tr><td>Oral Ulcers</td><td>METABOLISM_OF_VITAMINS_AND_COFACTORS</td><td>REACTOME</td><td>1119</td><td>34</td><td>20</td><td>9,86E-01</td><td>9,90E-01</td><td>rs9493105|rs6569759|rs3783438|rs12449313|rs12218194|rs7896809|rs9790066|rs8129445|rs3133810|rs11627525|rs2273026|rs137126|rs1788571|rs2027993|rs568388|rs1715367|rs11696527|rs4726739|rs3758626|rs4755142</td></tr>
<tr><td>Oral Ulcers</td><td>PROCESSING_OF_CAPPED_INTRONLESS_PRE_MRNA</td><td>REACTOME</td><td>274</td><td>8</td><td>6</td><td>9,90E-01</td><td>9,91E-01</td><td>rs2288055|rs11905250|rs2920706|rs7936604|rs2474871|rs4982753</td></tr>
<tr><td>Oral Ulcers</td><td>TELOMERE_MAINTENANCE</td><td>REACTOME</td><td>1133</td><td>28</td><td>17</td><td>9,90E-01</td><td>9,91E-01</td><td>rs942592|rs153060|rs1405655|rs1467464|rs690705|rs12538587|rs1543551|rs9379780|rs792637|rs3816551|rs7939226|rs4943189|rs174534|rs9349915|rs1539604|rs3758626|rs2393374</td></tr>
<tr><td>Oral Ulcers</td><td>PROXIMAL_TUBULE_BICARBONATE_RECLAMATION</td><td>KEGG</td><td>458</td><td>20</td><td>10</td><td>9,99E-01</td><td>9,99E-01</td><td>rs1453445|rs1033805|rs3771311|rs6555357|rs882126|rs6694434|rs7551983|rs10518087|rs11585375|rs16845804</td></tr>
<tr><td>Discoid Rash</td><td>GLYCOSAMINOGLYCAN_BIOSYNTHESIS_HEPARAN_SULFATE</td><td>KEGG</td><td>2273</td><td>59</td><td>20</td><td>4,10E-03</td><td>9,96E-01</td><td>rs4662862|rs8058794|rs4338508|rs2445904|rs3115775|rs6825790|rs2635297|rs2119299|rs2191068|rs11644271|rs240940|rs10002047|rs1506793|rs897005|rs1874238|rs13024819|rs7113440|rs960438|rs7987510|rs6933665</td></tr>
<tr><td>Discoid Rash</td><td>PRION_DISEASES</td><td>KEGG</td><td>1585</td><td>34</td><td>20</td><td>4,90E-03</td><td>9,96E-01</td><td>rs1783391|rs11088869|rs17115184|rs2827641|rs2826836|rs2205502|rs1586488|rs4815715|rs957575|rs2826622|rs9975242|rs2826727|rs978815|rs17042407|rs11701180|rs2826213|rs1940735|rs7282990|rs2826841|rs2827312</td></tr>
<tr><td>Discoid Rash</td><td>HUNTINGTONS_DISEASE</td><td>KEGG</td><td>4311</td><td>36</td><td>20</td><td>6,10E-03</td><td>9,96E-01</td><td>rs10835201|rs1151996|rs304051|rs7149317|rs1511451|rs6108160|rs3851422|rs17203281|rs728633|rs2036713|rs1544470|rs901849|rs4731630|rs12065184|rs2839604|rs4920706|rs218979|rs4474804|rs12102923|rs2280231</td></tr>
<tr><td>Discoid Rash</td><td>THE_ROLE_OF_NEF_IN_HIV1_REPLICATION_AND_DISEASE_PATHOGENESIS</td><td>REACTOME</td><td>936</td><td>30</td><td>20</td><td>6,50E-03</td><td>9,96E-01</td><td>rs2546507|rs2338809|rs2717952|rs1882070|rs2392492|rs12657308|rs1882078|rs4723673|rs401618|rs7203231|rs372332|rs11783925|rs2046252|rs6121337|rs6462777|rs1182091|rs1882086|rs11075904|rs4723645|rs2707212</td></tr>
<tr><td>Discoid Rash</td><td>SIGNALING_BY_HIPPO</td><td>REACTOME</td><td>627</td><td>47</td><td>20</td><td>1,09E-02</td><td>9,96E-01</td><td>rs4779683|rs6862868|rs6130713|rs3019294|rs2144649|rs1357386|rs10907200|rs17429690|rs12916064|rs965938|rs2033367|rs17551608|rs9439603|rs2287704|rs10492947|rs2267858|rs7616812|rs1538579|rs16897076|rs1765719</td></tr>
<tr><td>Discoid Rash</td><td>CALCIUM_SIGNALING_PATHWAY</td><td>KEGG</td><td>9275</td><td>42</td><td>20</td><td>1,32E-02</td><td>9,96E-01</td><td>rs997559|rs1422909|rs304051|rs10488600|rs4681446|rs1064448|rs6108160|rs703745|rs233966|rs2638362|rs4868513|rs7574462|rs4864510|rs11133313|rs1897104|rs13278849|rs6670616|rs13429751|rs732050|rs7820461</td></tr>
<tr><td>Discoid Rash</td><td>SULFUR_AMINO_ACID_METABOLISM</td><td>REACTOME</td><td>930</td><td>37</td><td>20</td><td>1,68E-02</td><td>9,96E-01</td><td>rs1466202|rs2651237|rs11740544|rs12075963|rs10493474|rs2651236|rs11953266|rs4920037|rs11083711|rs7161825|rs756208|rs10874814|rs1414073|rs8039174|rs1484197|rs575425|rs13328108|rs2886722|rs6826022|rs11209643</td></tr>
<tr><td>Discoid Rash</td><td>NITROGEN_METABOLISM</td><td>KEGG</td><td>587</td><td>34</td><td>20</td><td>1,71E-02</td><td>9,96E-01</td><td>rs17024981|rs2651237|rs12075963|rs10493474|rs2136703|rs2780749|rs17762822|rs1229564|rs1339374|rs6540114|rs2274327|rs10876891|rs7465573|rs1414073|rs1921915|rs12091891|rs4567871|rs1912445|rs4338916|rs11209643</td></tr>
<tr><td>Discoid Rash</td><td>CHEMOKINE_SIGNALING_PATHWAY</td><td>KEGG</td><td>5407</td><td>46</td><td>20</td><td>2,27E-02</td><td>9,96E-01</td><td>rs2546507|rs12602195|rs1064448|rs16834223|rs2833293|rs6108160|rs4976132|rs11137533|rs2338809|rs11767121|rs2717952|rs1490801|rs1882070|rs7820461|rs2392492|rs12657308|rs2159917|rs1877563|rs1327489|rs16834018</td></tr>
<tr><td>Discoid Rash</td><td>PYRUVATE_METABOLISM_AND_CITRIC_ACID_TCA_CYCLE</td><td>REACTOME</td><td>905</td><td>44</td><td>20</td><td>2,50E-02</td><td>9,96E-01</td><td>rs6789203|rs1539624|rs10264649|rs1326481|rs1978566|rs12065184|rs9534724|rs4585672|rs12693008|rs6467993|rs4832076|rs854093|rs7337764|rs9611598|rs2144575|rs10247064|rs977103|rs2025776|rs10186197|rs710887</td></tr>
<tr><td>Discoid Rash</td><td>SIGNALLING_TO_RAS</td><td>REACTOME</td><td>468</td><td>32</td><td>8</td><td>2,52E-02</td><td>9,96E-01</td><td>rs11137533|rs6130713|rs17540656|rs1808416|rs10494878|rs2859131|rs4963872|rs7364173</td></tr>
<tr><td>Discoid Rash</td><td>CDC42RAC_PATHWAY</td><td>BIOCARTA</td><td>435</td><td>33</td><td>14</td><td>2,59E-02</td><td>9,96E-01</td><td>rs4976132|rs11767121|rs4864510|rs1490801|rs11133313|rs3448|rs4976028|rs10073996|rs1008163|rs2501276|rs7719763|rs10070379|rs10940145|rs4131890</td></tr>
<tr><td>Discoid Rash</td><td>DOWNREGULATION_OF_SMAD2_3_SMAD4_TRANSCRIPTIONAL_ACTIVITY</td><td>REACTOME</td><td>453</td><td>29</td><td>14</td><td>2,80E-02</td><td>9,96E-01</td><td>rs1992215|rs11608551|rs12102171|rs11661651|rs12607345|rs238135|rs838875|rs2314664|rs4305170|rs4368250|rs2173049|rs837948|rs4776862|rs7359174</td></tr>
<tr><td>Discoid Rash</td><td>RENIN_ANGIOTENSIN_SYSTEM</td><td>KEGG</td><td>467</td><td>58</td><td>12</td><td>2,85E-02</td><td>9,96E-01</td><td>rs4681446|rs2638362|rs7646258|rs4329|rs2331406|rs188019|rs394643|rs4951309|rs8076157|rs7647324|rs12909844|rs7611664</td></tr>
<tr><td>Discoid Rash</td><td>GABA_SYNTHESIS_RELEASE_REUPTAKE_AND_DEGRADATION</td><td>REACTOME</td><td>746</td><td>38</td><td>20</td><td>2,96E-02</td><td>9,96E-01</td><td>rs2789603|rs10455248|rs2496531|rs362988|rs6077718|rs892601|rs1710887|rs4083221|rs6755814|rs6133821|rs2950798|rs6924133|rs6077680|rs12930556|rs350658|rs761177|rs9442769|rs1273422|rs17518345|rs2697135</td></tr>
<tr><td>Discoid Rash</td><td>CK1_PATHWAY</td><td>BIOCARTA</td><td>759</td><td>25</td><td>16</td><td>3,11E-02</td><td>9,96E-01</td><td>rs1422909|rs6108160|rs703745|rs4868513|rs2179984|rs6118062|rs6140561|rs6086525|rs6039189|rs6055889|rs4478191|rs7212835|rs4896857|rs13224682|rs9755490|rs17529477</td></tr>
<tr><td>Discoid Rash</td><td>CTNNB1_PHOSPHORYLATION_CASCADE</td><td>REACTOME</td><td>316</td><td>15</td><td>5</td><td>3,13E-02</td><td>9,96E-01</td><td>rs11189119|rs8003274|rs3916990|rs440804|rs4355511</td></tr>
<tr><td>Discoid Rash</td><td>METABOLISM_OF_AMINO_ACIDS_AND_DERIVATIVES</td><td>REACTOME</td><td>5246</td><td>30</td><td>20</td><td>3,13E-02</td><td>9,96E-01</td><td>rs1466202|rs17024981|rs1215985|rs1581676|rs9342101|rs2651237|rs2293576|rs4746076|rs11740544|rs7856089|rs13210742|rs12075963|rs17533359|rs6719977|rs10493474|rs6539460|rs2136703|rs4963801|rs6637|rs12529764</td></tr>
<tr><td>Discoid Rash</td><td>HOST_INTERACTIONS_OF_HIV_FACTORS</td><td>REACTOME</td><td>2270</td><td>33</td><td>20</td><td>3,42E-02</td><td>9,96E-01</td><td>rs2546507|rs2228375|rs590945|rs2338809|rs2293576|rs2717952|rs1882070|rs2392492|rs12657308|rs1882078|rs4723673|rs667707|rs328331|rs401618|rs217797|rs7203231|rs372332|rs4740420|rs11783925|rs2046252</td></tr>
<tr><td>Discoid Rash</td><td>SHC_RELATED_EVENTS</td><td>REACTOME</td><td>302</td><td>25</td><td>6</td><td>3,89E-02</td><td>9,96E-01</td><td>rs11137533|rs6130713|rs7507911|rs1808416|rs4804103|rs4963872</td></tr>
<tr><td>Discoid Rash</td><td>UBIQUITIN_MEDIATED_PROTEOLYSIS</td><td>KEGG</td><td>3227</td><td>41</td><td>20</td><td>3,90E-02</td><td>9,96E-01</td><td>rs9430244|rs1018326|rs13195756|rs590945|rs4467482|rs2173461|rs825218|rs7642557|rs10933849|rs4804505|rs7255657|rs2189713|rs3810168|rs2903698|rs1445059|rs9653414|rs667707|rs7781554|rs6784447|rs11217175</td></tr>
<tr><td>Discoid Rash</td><td>ERK5_PATHWAY</td><td>BIOCARTA</td><td>595</td><td>43</td><td>20</td><td>3,99E-02</td><td>9,96E-01</td><td>rs4976132|rs918418|rs1490801|rs700591|rs7175248|rs6860708|rs11633249|rs2664537|rs2254137|rs4976028|rs10073996|rs8041078|rs247587|rs11576136|rs1002487|rs11958574|rs6598858|rs11892543|rs7719763|rs10070379</td></tr>
<tr><td>Discoid Rash</td><td>NEUROTROPHIN_SIGNALING_PATHWAY</td><td>KEGG</td><td>3689</td><td>33</td><td>20</td><td>4,32E-02</td><td>9,96E-01</td><td>rs10835201|rs1577452|rs4976132|rs11137533|rs3765692|rs728633|rs7138947|rs1490801|rs6130713|rs10492093|rs17540656|rs17088180|rs10483453|rs16898963|rs6496466|rs3739804|rs2241694|rs6922031|rs3019260|rs17103274</td></tr>
<tr><td>Discoid Rash</td><td>TID_PATHWAY</td><td>BIOCARTA</td><td>479</td><td>19</td><td>11</td><td>4,52E-02</td><td>9,96E-01</td><td>rs2834219|rs1327489|rs10483453|rs8130924|rs17103274|rs496888|rs3782165|rs3747581|rs2029812|rs986475|rs7969498</td></tr>
<tr><td>Discoid Rash</td><td>SIGNALING_BY_FGFR1_MUTANTS</td><td>REACTOME</td><td>1243</td><td>31</td><td>20</td><td>4,59E-02</td><td>9,96E-01</td><td>rs17072071|rs4976132|rs10047718|rs1490801|rs12056820|rs950549|rs9552595|rs2290755|rs34000|rs11167777|rs2031283|rs6490668|rs410187|rs2664537|rs4976028|rs10073996|rs2070715|rs7327237|rs2476509|rs750535</td></tr>
<tr><td>Discoid Rash</td><td>GLUTATHIONE_CONJUGATION</td><td>REACTOME</td><td>407</td><td>13</td><td>9</td><td>4,89E-02</td><td>9,96E-01</td><td>rs1466202|rs12646059|rs34511626|rs10874814|rs316156|rs7355084|rs6462210|rs2132847|rs1356553</td></tr>
<tr><td>Discoid Rash</td><td>ACTIVATED_POINT_MUTANTS_OF_FGFR2</td><td>REACTOME</td><td>873</td><td>26</td><td>20</td><td>4,95E-02</td><td>9,96E-01</td><td>rs17072071|rs10047718|rs12056820|rs950549|rs9552595|rs2290755|rs34000|rs11167777|rs17102287|rs6490668|rs6882257|rs10419120|rs2070715|rs7327237|rs2476509|rs750535|rs4616067|rs12542904|rs9509984|rs7329697</td></tr>
<tr><td>Discoid Rash</td><td>GAP_JUNCTION</td><td>KEGG</td><td>4388</td><td>41</td><td>20</td><td>5,18E-02</td><td>9,96E-01</td><td>rs1422909|rs304051|rs4779683|rs1064448|rs6108160|rs703745|rs4868513|rs4864510|rs11133313|rs7820461|rs2479982|rs901849|rs10517655|rs1357386|rs467504|rs3785383|rs1476946|rs10058673|rs12916064|rs4382132</td></tr>
<tr><td>Discoid Rash</td><td>HEDGEHOG_SIGNALING_PATHWAY</td><td>KEGG</td><td>2151</td><td>40</td><td>20</td><td>5,22E-02</td><td>9,96E-01</td><td>rs2744738|rs590945|rs4724083|rs2744728|rs652673|rs7517829|rs932659|rs6709236|rs17360053|rs7542242|rs913069|rs6054474|rs6077047|rs667707|rs2363923|rs6946781|rs12344289|rs17476911|rs7741360|rs1958636</td></tr>
<tr><td>Discoid Rash</td><td>DESTABILIZATION_OF_MRNA_BY_BRF1</td><td>REACTOME</td><td>204</td><td>13</td><td>5</td><td>5,75E-02</td><td>9,96E-01</td><td>rs6130713|rs1545229|rs6783810|rs11189224|rs10494878</td></tr>
<tr><td>Discoid Rash</td><td>DESTABILIZATION_OF_MRNA_BY_KSRP</td><td>REACTOME</td><td>205</td><td>5</td><td>3</td><td>5,77E-02</td><td>9,96E-01</td><td>rs1545229|rs3019260|rs11189224</td></tr>
<tr><td>Discoid Rash</td><td>PGC1A_PATHWAY</td><td>BIOCARTA</td><td>959</td><td>37</td><td>20</td><td>5,90E-02</td><td>9,96E-01</td><td>rs1511451|rs918418|rs700591|rs2036713|rs7175248|rs6860708|rs2241694|rs11633249|rs135549|rs2356932|rs7656250|rs1995917|rs1011973|rs1644512|rs1159176|rs12150519|rs8041078|rs247587|rs16873852|rs11958574</td></tr>
<tr><td>Discoid Rash</td><td>ARGININE_AND_PROLINE_METABOLISM</td><td>KEGG</td><td>991</td><td>35</td><td>20</td><td>6,04E-02</td><td>9,96E-01</td><td>rs1215985|rs627354|rs6539460|rs156025|rs11788224|rs816296|rs9324063|rs9604911|rs4836277|rs7031579|rs11080358|rs2650163|rs1142530|rs14868|rs10876891|rs2270823|rs9935162|rs1921915|rs4567871|rs818167</td></tr>
<tr><td>Discoid Rash</td><td>INSULIN_RECEPTOR_SIGNALLING_CASCADE</td><td>REACTOME</td><td>3006</td><td>39</td><td>20</td><td>6,06E-02</td><td>9,96E-01</td><td>rs17072071|rs1577452|rs4976132|rs10047718|rs11137533|rs1490801|rs12056820|rs6130713|rs950549|rs2848780|rs9552595|rs4947475|rs7438406|rs6970522|rs2290755|rs34000|rs9568203|rs11167777|rs7185040|rs7599150</td></tr>
<tr><td>Discoid Rash</td><td>SMAD2_SMAD3_SMAD4_HETEROTRIMER_REGULATES_TRANSCRIPTION</td><td>REACTOME</td><td>715</td><td>37</td><td>20</td><td>6,23E-02</td><td>9,96E-01</td><td>rs1992215|rs998910|rs2648888|rs12102171|rs17199521|rs11661651|rs12547643|rs12607345|rs4733859|rs238135|rs2314664|rs4305170|rs4368250|rs2896452|rs10505507|rs4776862|rs7616812|rs6985681|rs6987802|rs2874714</td></tr>
<tr><td>Discoid Rash</td><td>ACTINY_PATHWAY</td><td>BIOCARTA</td><td>290</td><td>6</td><td>4</td><td>6,86E-02</td><td>9,96E-01</td><td>rs11767121|rs560873|rs9400356|rs12660791</td></tr>
<tr><td>Discoid Rash</td><td>BRANCHED_CHAIN_AMINO_ACID_CATABOLISM</td><td>REACTOME</td><td>507</td><td>44</td><td>10</td><td>7,41E-02</td><td>9,96E-01</td><td>rs7856089|rs4963801|rs12529764|rs9343977|rs10842402|rs4585672|rs12322525|rs976145|rs9443804|rs10505945</td></tr>
<tr><td>Discoid Rash</td><td>HIV_INFECTION</td><td>REACTOME</td><td>3237</td><td>40</td><td>20</td><td>7,43E-02</td><td>9,96E-01</td><td>rs2546507|rs16834223|rs2228375|rs590945|rs2338809|rs2293576|rs2717952|rs1882070|rs2392492|rs12657308|rs16834018|rs1882078|rs4723673|rs667707|rs328331|rs401618|rs217797|rs2279083|rs6740091|rs7203231</td></tr>
<tr><td>Discoid Rash</td><td>LIPID_DIGESTION_MOBILIZATION_AND_TRANSPORT</td><td>REACTOME</td><td>1389</td><td>39</td><td>20</td><td>7,48E-02</td><td>9,96E-01</td><td>rs8133852|rs1110060|rs1519491|rs2176472|rs157580|rs12597002|rs879667|rs7677213|rs6494005|rs12370375|rs2297409|rs4665639|rs6598863|rs17743079|rs874546|rs4299376|rs7544500|rs11611433|rs2336408|rs1503777</td></tr>
<tr><td>Discoid Rash</td><td>TRANSCRIPTIONAL_ACTIVITY_OF_SMAD2_SMAD3_SMAD4_HETEROTRIMER</td><td>REACTOME</td><td>967</td><td>30</td><td>20</td><td>7,57E-02</td><td>9,96E-01</td><td>rs1992215|rs11608551|rs998910|rs1499367|rs12102171|rs17199521|rs11661651|rs12547643|rs12607345|rs4733859|rs238135|rs838875|rs2314664|rs4305170|rs4368250|rs2896452|rs10505507|rs2173049|rs837948|rs4776862</td></tr>
<tr><td>Discoid Rash</td><td>TIGHT_JUNCTION_INTERACTIONS</td><td>REACTOME</td><td>715</td><td>30</td><td>18</td><td>7,60E-02</td><td>9,96E-01</td><td>rs2140825|rs6766201|rs7546890|rs7528744|rs3774023|rs1705007|rs11929772|rs1215|rs2835365|rs3773770|rs1523270|rs7516332|rs481781|rs9556476|rs10255195|rs6685551|rs2786551|rs2492450</td></tr>
<tr><td>Discoid Rash</td><td>MYOGENESIS</td><td>REACTOME</td><td>1747</td><td>39</td><td>20</td><td>7,73E-02</td><td>9,96E-01</td><td>rs918418|rs1420965|rs2588478|rs700591|rs944248|rs12962729|rs2068606|rs2113445|rs1785765|rs7175248|rs6860708|rs11633249|rs12052006|rs12955716|rs17269733|rs440804|rs7236100|rs2245976|rs764699|rs7176908</td></tr>
<tr><td>Discoid Rash</td><td>THYROID_CANCER</td><td>KEGG</td><td>1104</td><td>39</td><td>20</td><td>7,78E-02</td><td>9,96E-01</td><td>rs1151996|rs6719501|rs11884056|rs1499367|rs10864912|rs10749127|rs17199521|rs12547643|rs4733859|rs440804|rs11725638|rs11675205|rs7642041|rs1271546|rs10505507|rs6851442|rs17431892|rs1930788|rs4918801|rs10510419</td></tr>
<tr><td>Discoid Rash</td><td>AMINE_LIGAND_BINDING_RECEPTORS</td><td>REACTOME</td><td>2247</td><td>35</td><td>20</td><td>7,84E-02</td><td>9,96E-01</td><td>rs1422909|rs10488600|rs703745|rs4868513|rs13278849|rs732050|rs2479982|rs10488602|rs13171967|rs613479|rs10806386|rs324677|rs1364402|rs11760092|rs10944288|rs9526276|rs6993912|rs2800143|rs10060486|rs10748546</td></tr>
<tr><td>Discoid Rash</td><td>EARLY_PHASE_OF_HIV_LIFE_CYCLE</td><td>REACTOME</td><td>270</td><td>28</td><td>9</td><td>7,93E-02</td><td>9,96E-01</td><td>rs16834223|rs16834018|rs6740091|rs1325382|rs11687599|rs2707212|rs4802436|rs4724321|rs4954580</td></tr>
<tr><td>Discoid Rash</td><td>SIGNALING_BY_INSULIN_RECEPTOR</td><td>REACTOME</td><td>3432</td><td>32</td><td>20</td><td>8,24E-02</td><td>9,96E-01</td><td>rs17072071|rs1577452|rs4976132|rs10047718|rs11137533|rs1490801|rs12056820|rs6130713|rs950549|rs2848780|rs9552595|rs4947475|rs7438406|rs6970522|rs2041689|rs2290755|rs34000|rs9568203|rs10982300|rs11167777</td></tr>
<tr><td>Discoid Rash</td><td>BASAL_CELL_CARCINOMA</td><td>KEGG</td><td>2166</td><td>37</td><td>20</td><td>8,33E-02</td><td>9,96E-01</td><td>rs2744738|rs4724083|rs2744728|rs652673|rs7517829|rs6709236|rs17360053|rs6719501|rs11884056|rs7542242|rs10749127|rs6054474|rs6077047|rs3916990|rs2363923|rs440804|rs11725638|rs11675205|rs6946781|rs2131858</td></tr>
<tr><td>Discoid Rash</td><td>FGFR_LIGAND_BINDING_AND_ACTIVATION</td><td>REACTOME</td><td>984</td><td>26</td><td>20</td><td>8,40E-02</td><td>9,96E-01</td><td>rs17072071|rs10047718|rs12056820|rs950549|rs9552595|rs2290755|rs34000|rs11167777|rs17102287|rs6490668|rs6882257|rs10419120|rs2070715|rs7327237|rs2476509|rs750535|rs4616067|rs12542904|rs392490|rs9509984</td></tr>
<tr><td>Discoid Rash</td><td>AT1R_PATHWAY</td><td>BIOCARTA</td><td>1224</td><td>53</td><td>20</td><td>8,41E-02</td><td>9,96E-01</td><td>rs4681446|rs2638362|rs918418|rs700591|rs7175248|rs6557991|rs6860708|rs3785383|rs11633249|rs4433842|rs392715|rs4700008|rs2811876|rs8041078|rs188019|rs8051531|rs247587|rs11958574|rs11646540|rs17753569</td></tr>
<tr><td>Discoid Rash</td><td>PRE_NOTCH_PROCESSING_IN_GOLGI</td><td>REACTOME</td><td>691</td><td>57</td><td>20</td><td>8,45E-02</td><td>9,96E-01</td><td>rs10149996|rs1036047|rs10484153|rs1152349|rs12895242|rs2256905|rs1628691|rs2372200|rs799103|rs1379244|rs7151516|rs12881723|rs204990|rs11624992|rs876287|rs1343687|rs7153719|rs1777696|rs12433856|rs10132156</td></tr>
<tr><td>Discoid Rash</td><td>TRANSCRIPTIONAL_REGULATION_OF_WHITE_ADIPOCYTE_DIFFERENTIATION</td><td>REACTOME</td><td>2719</td><td>35</td><td>20</td><td>8,58E-02</td><td>9,96E-01</td><td>rs1151996|rs16973982|rs1511451|rs1110060|rs177421|rs2036713|rs33194|rs11608551|rs998910|rs7026534|rs11999806|rs12438895|rs737479|rs1316379|rs10520797|rs791595|rs7167270|rs4921252|rs838875|rs12593344</td></tr>
<tr><td>Discoid Rash</td><td>CELL_ADHESION_MOLECULES_CAMS</td><td>KEGG</td><td>8813</td><td>26</td><td>20</td><td>8,93E-02</td><td>9,96E-01</td><td>rs3094691|rs1783391|rs11088869|rs2767326|rs6766201|rs17115184|rs2827641|rs2826836|rs4726774|rs1420965|rs7564976|rs2205502|rs7546890|rs1586488|rs944248|rs2212020|rs12962729|rs197721|rs231726|rs6759316</td></tr>
<tr><td>Discoid Rash</td><td>SNARE_INTERACTIONS_IN_VESICULAR_TRANSPORT</td><td>KEGG</td><td>828</td><td>29</td><td>20</td><td>9,00E-02</td><td>9,96E-01</td><td>rs751262|rs362988|rs6077718|rs6585186|rs7953586|rs17808583|rs11733672|rs838958|rs4239107|rs6133821|rs2331902|rs7460879|rs6583640|rs6077680|rs1815239|rs3822904|rs7556644|rs728827|rs4917329|rs218449</td></tr>
<tr><td>Discoid Rash</td><td>GLYCINE_SERINE_AND_THREONINE_METABOLISM</td><td>KEGG</td><td>564</td><td>45</td><td>20</td><td>9,01E-02</td><td>9,96E-01</td><td>rs2651237|rs12075963|rs10493474|rs6539460|rs672413|rs2780749|rs4920037|rs1142530|rs4585672|rs11137606|rs1414073|rs10514153|rs809359|rs944514|rs11209643|rs17131320|rs586199|rs4740848|rs7868916|rs129934</td></tr>
<tr><td>Discoid Rash</td><td>ARRHYTHMOGENIC_RIGHT_VENTRICULAR_CARDIOMYOPATHY_ARVC</td><td>KEGG</td><td>5871</td><td>32</td><td>20</td><td>9,62E-02</td><td>9,96E-01</td><td>rs12213754|rs12356797|rs1583609|rs4990414|rs1420965|rs7564976|rs6942458|rs2326761|rs7074696|rs2212020|rs12962729|rs197721|rs12707506|rs6759316|rs2068606|rs6719501|rs4821506|rs11884056|rs10509237|rs11012843</td></tr>
<tr><td>Discoid Rash</td><td>RACCYCD_PATHWAY</td><td>BIOCARTA</td><td>551</td><td>23</td><td>12</td><td>9,63E-02</td><td>9,96E-01</td><td>rs4976132|rs1490801|rs10483453|rs17103274|rs3448|rs4976028|rs10073996|rs7719763|rs10070379|rs10940145|rs2374589|rs4131890</td></tr>
<tr><td>Discoid Rash</td><td>ARMS_MEDIATED_ACTIVATION</td><td>REACTOME</td><td>393</td><td>11</td><td>5</td><td>9,71E-02</td><td>9,96E-01</td><td>rs6130713|rs17540656|rs10793894|rs4963872|rs2296950</td></tr>
<tr><td>Discoid Rash</td><td>GLYCOSAMINOGLYCAN_BIOSYNTHESIS_CHONDROITIN_SULFATE</td><td>KEGG</td><td>1372</td><td>46</td><td>20</td><td>9,92E-02</td><td>9,96E-01</td><td>rs8058794|rs7976582|rs10520079|rs2128333|rs9390633|rs10161421|rs2119299|rs11644271|rs519604|rs7113440|rs960438|rs6933665|rs7451498|rs1891523|rs7929855|rs17480734|rs11224035|rs13215358|rs12598653|rs10100391</td></tr>
<tr><td>Discoid Rash</td><td>INTEGRIN_CELL_SURFACE_INTERACTIONS</td><td>REACTOME</td><td>2614</td><td>33</td><td>20</td><td>9,99E-02</td><td>9,96E-01</td><td>rs12213754|rs4729129|rs7546890|rs2326761|rs2212020|rs197721|rs11223702|rs11694554|rs679668|rs3095208|rs1858822|rs1351140|rs10464587|rs7328731|rs10451555|rs6722598|rs3917016|rs1981316|rs631428|rs17364665</td></tr>
<tr><td>Discoid Rash</td><td>NEPHRIN_INTERACTIONS</td><td>REACTOME</td><td>1617</td><td>40</td><td>20</td><td>1,00E-01</td><td>9,96E-01</td><td>rs4976132|rs11767121|rs17031463|rs13308578|rs1490801|rs12668317|rs2441686|rs17106413|rs1475035|rs16831|rs2376977|rs17150562|rs9641392|rs1108906|rs4851831|rs798343|rs11685896|rs4976028|rs10073996|rs7605126</td></tr>
<tr><td>Discoid Rash</td><td>HS_GAG_BIOSYNTHESIS</td><td>REACTOME</td><td>3154</td><td>52</td><td>20</td><td>1,01E-01</td><td>9,96E-01</td><td>rs4662862|rs4338508|rs2468133|rs3115775|rs6825790|rs4303490|rs2635297|rs2191068|rs10002047|rs7990210|rs1506793|rs897005|rs1572342|rs4144545|rs1874238|rs13024819|rs7993144|rs7984041|rs2439525|rs7987510</td></tr>
<tr><td>Discoid Rash</td><td>GLUCURONIDATION</td><td>REACTOME</td><td>656</td><td>33</td><td>4</td><td>1,03E-01</td><td>9,96E-01</td><td>rs10028494|rs1458226|rs294755|rs6848997</td></tr>
<tr><td>Discoid Rash</td><td>JAK_STAT_SIGNALING_PATHWAY</td><td>KEGG</td><td>4346</td><td>26</td><td>20</td><td>1,03E-01</td><td>9,96E-01</td><td>rs2834219|rs4976132|rs1417718|rs13131772|rs1673459|rs3217810|rs724055|rs1490801|rs7642557|rs12720279|rs7626849|rs12466948|rs10933849|rs1278150|rs2648888|rs17262993|rs11884872|rs8177732|rs7717673|rs2138992</td></tr>
<tr><td>Discoid Rash</td><td>PROLONGED_ERK_ACTIVATION_EVENTS</td><td>REACTOME</td><td>429</td><td>13</td><td>6</td><td>1,04E-01</td><td>9,96E-01</td><td>rs6130713|rs17540656|rs10793894|rs2664537|rs4963872|rs2296950</td></tr>
<tr><td>Discoid Rash</td><td>FACTORS_INVOLVED_IN_MEGAKARYOCYTE_DEVELOPMENT_AND_PLATELET_PRODUCT</td><td>REACTOME</td><td>3549</td><td>28</td><td>20</td><td>1,04E-01</td><td>9,96E-01</td><td>rs2546507|rs2664904|rs2338809|rs1521780|rs2331780|rs12104926|rs9517623|rs1521774|rs963917|rs4759348|rs12657308|rs1149907|rs1327489|rs396567|rs2396190|rs10905533|rs12248916|rs11814342|rs277317|rs1060561</td></tr>
<tr><td>Discoid Rash</td><td>L1CAM_INTERACTIONS</td><td>REACTOME</td><td>3914</td><td>29</td><td>20</td><td>1,05E-01</td><td>9,96E-01</td><td>rs6941522|rs17652604|rs17115184|rs7916174|rs3846912|rs2393610|rs2212020|rs197721|rs1612989|rs679668|rs12420766|rs13076532|rs1351140|rs16898963|rs7023913|rs11672523|rs277317|rs10509119|rs9693071|rs6777489</td></tr>
<tr><td>Discoid Rash</td><td>CXCR4_PATHWAY</td><td>BIOCARTA</td><td>1099</td><td>48</td><td>20</td><td>1,07E-01</td><td>9,96E-01</td><td>rs16834223|rs4976132|rs1490801|rs16834018|rs6557991|rs3785383|rs6740091|rs4433842|rs12706724|rs11687599|rs392715|rs7971352|rs2664537|rs4976028|rs10073996|rs537885|rs8051531|rs266078|rs11646540|rs17753569</td></tr>
<tr><td>Discoid Rash</td><td>DESTABILIZATION_OF_MRNA_BY_TRISTETRAPROLIN_TTP</td><td>REACTOME</td><td>179</td><td>14</td><td>6</td><td>1,10E-01</td><td>9,96E-01</td><td>rs6130713|rs1545229|rs6783810|rs11189224|rs10494878|rs2277743</td></tr>
<tr><td>Discoid Rash</td><td>FRS2_MEDIATED_CASCADE</td><td>REACTOME</td><td>1192</td><td>28</td><td>20</td><td>1,10E-01</td><td>9,96E-01</td><td>rs17072071|rs10047718|rs12056820|rs6130713|rs950549|rs9552595|rs2290755|rs34000|rs11167777|rs17102287|rs6490668|rs6882257|rs10419120|rs2070715|rs7327237|rs2476509|rs750535|rs4616067|rs12542904|rs392490</td></tr>
<tr><td>Discoid Rash</td><td>NEUROTRANSMITTER_RELEASE_CYCLE</td><td>REACTOME</td><td>1400</td><td>34</td><td>20</td><td>1,10E-01</td><td>9,96E-01</td><td>rs2789603|rs10455248|rs2250128|rs362988|rs6077718|rs305522|rs7616006|rs5998523|rs892601|rs1710887|rs4083221|rs6755814|rs6133821|rs137309|rs2950798|rs2471583|rs10876891|rs6924133|rs8178992|rs2618406</td></tr>
<tr><td>Discoid Rash</td><td>LIPOPROTEIN_METABOLISM</td><td>REACTOME</td><td>1037</td><td>43</td><td>20</td><td>1,11E-01</td><td>9,96E-01</td><td>rs8133852|rs1519491|rs2176472|rs157580|rs12597002|rs7677213|rs6494005|rs10846722|rs2297409|rs4665639|rs6598863|rs7544500|rs11611433|rs1503777|rs4654986|rs17042892|rs3924843|rs394643|rs838878|rs9644636</td></tr>
<tr><td>Discoid Rash</td><td>TIGHT_JUNCTION</td><td>KEGG</td><td>6242</td><td>39</td><td>20</td><td>1,15E-01</td><td>9,96E-01</td><td>rs2140825|rs1456721|rs4779683|rs6766201|rs12356797|rs4990414|rs13308578|rs7546890|rs11710619|rs7074696|rs12668317|rs7349397|rs11223702|rs10822661|rs10996198|rs6800292|rs873418|rs1879039|rs9973085|rs2204204</td></tr>
</table>

Discoid Rash	INTESTINAL_IMMUNE_NETWORK_FOR_IGA_PRODUCTION	KEGG	1392	44	20	1,15E-01	9,96E-01	rs16834223|rs7564976|rs9559300|rs8177732|rs4280293|rs3096866|rs3759333|rs16834018|rs3104404|rs6740091|rs9276284|rs11687599|rs16939964|rs743479|rs4683148|rs6710279|rs1224174|rs266078|rs28383344|rs10242595
Discoid Rash	SHC_MEDIATED_CASCADE	REACTOME	1070	26	20	1,16E-01	9,96E-01	rs17072071|rs10047718|rs12056820|rs950549|rs9552595|rs2290755|rs34000|rs11167777|rs17102287|rs6490668|rs6882257|rs10419120|rs2070715|rs7327237|rs2476509|rs750535|rs4616067|rs12542904|rs392490|rs9509984
Discoid Rash	BASAL_TRANSCRIPTION_FACTORS	KEGG	386	14	6	1,18E-01	9,96E-01	rs7149317|rs757575|rs2296085|rs2140551|rs12469348|rs7735993
Discoid Rash	AUTOIMMUNE_THYROID_DISEASE	KEGG	1646	66	20	1,20E-01	9,96E-01	rs3094691|rs9342101|rs231726|rs7755852|rs3779965|rs1611350|rs3104404|rs3130473|rs9276284|rs4977686|rs11591298|rs2245548|rs4927594|rs762084|rs12111032|rs9295986|rs28383344|rs13191519|rs1736956|rs3129882
Discoid Rash	DOWNSTREAM_SIGNALING_OF_ACTIVATED_FGFR	REACTOME	3636	36	20	1,20E-01	9,96E-01	rs17072071|rs1064448|rs4976132|rs10047718|rs1490801|rs1897104|rs12056820|rs13429751|rs6130713|rs950549|rs7820461|rs7349397|rs9552595|rs873418|rs2204204|rs2344662|rs2290755|rs34000|rs11167777|rs10058673
Discoid Rash	NUCLEAR_EVENTS_KINASE_AND_TRANSCRIPTION_FACTOR_ACTIVATION	REACTOME	766	36	20	1,21E-01	9,96E-01	rs918418|rs700591|rs13281383|rs16898963|rs7175248|rs6860708|rs11633249|rs7823751|rs2254137|rs10494878|rs8041078|rs247587|rs11576136|rs1002487|rs11958574|rs6598858|rs11892543|rs7312093|rs3799658|rs16899429
Discoid Rash	CLASS_I_MHC_MEDIATED_ANTIGEN_PROCESSING_PRESENTATION	REACTOME	5895	35	20	1,23E-01	9,96E-01	rs3094691|rs1018326|rs13195756|rs590945|rs2293576|rs4467482|rs2173461|rs825218|rs2057566|rs7642557|rs7755852|rs10933849|rs4804505|rs7602023|rs593234|rs7255657|rs2189713|rs3810168|rs1611350|rs12198323
Discoid Rash	PI_3K_CASCADE	REACTOME	1868	31	20	1,24E-01	9,96E-01	rs17072071|rs4976132|rs10047718|rs1490801|rs12056820|rs950549|rs9552595|rs873418|rs2290755|rs34000|rs11167777|rs7322396|rs7981045|rs7185040|rs4943769|rs9817|rs17102287|rs6490668|rs6882257|rs2254137
Discoid Rash	PTM_GAMMA_CARBOXYLATION_HYPUSINE_FORMATION_AND_ARYLSULFATASE_ACTIV	REACTOME	377	15	8	1,25E-01	9,96E-01	rs6866961|rs7216490|rs672413|rs12150519|rs2270982|rs25413|rs766183|rs17818103
Discoid Rash	DOWNREGULATION_OF_TGF_BETA_RECEPTOR_SIGNALING	REACTOME	521	35	17	1,27E-01	9,96E-01	rs1992215|rs6128143|rs12102171|rs1417416|rs11661651|rs7781554|rs927171|rs17743079|rs2314664|rs4776862|rs6099847|rs10819641|rs1019855|rs7632716|rs10801165|rs7359174|rs7345275
Discoid Rash	TGF_BETA_RECEPTOR_SIGNALING_ACTIVATES_SMADS	REACTOME	563	41	18	1,29E-01	9,96E-01	rs1992215|rs6128143|rs12102171|rs1417416|rs11661651|rs7781554|rs927171|rs17743079|rs2314664|rs4368250|rs4776862|rs6099847|rs10819641|rs1019855|rs7632716|rs710140|rs7345275|rs7359174
Discoid Rash	PHOSPHOLIPASE_C_MEDIATED_CASCADE	REACTOME	2628	33	20	1,30E-01	9,96E-01	rs17072071|rs1064448|rs10047718|rs1897104|rs12056820|rs13429751|rs950549|rs7820461|rs7349397|rs9552595|rs2204204|rs2344662|rs2290755|rs34000|rs11167777|rs10058673|rs17649232|rs4382132|rs10475376|rs4433842
Discoid Rash	IMMUNOREGULATORY_INTERACTIONS_BETWEEN_A_LYMPHOID_AND_A_NON_LYMPHO	REACTOME	1325	36	20	1,30E-01	9,96E-01	rs3094691|rs4806802|rs7755852|rs157580|rs9937676|rs1611350|rs8101605|rs4283898|rs3130473|rs3917016|rs4320015|rs2776068|rs235370|rs377702|rs208898|rs12111032|rs11880466|rs938213|rs281414|rs9295986
Discoid Rash	WNT_PATHWAY	BIOCARTA	1363	29	20	1,34E-01	9,96E-01	rs590945|rs11189119|rs1043395|rs1499367|rs2335855|rs17199521|rs1845451|rs667707|rs12547643|rs4733859|rs7747353|rs3916990|rs440804|rs1572003|rs11725638|rs7856994|rs1888855|rs4368250|rs4713859|rs10505507
Discoid Rash	CARM_ER_PATHWAY	BIOCARTA	1617	44	20	1,37E-01	9,96E-01	rs1511451|rs918418|rs17103105|rs700591|rs2036713|rs11608551|rs17150238|rs851982|rs6860708|rs799917|rs11176420|rs838875|rs12228293|rs17081685|rs7656250|rs9905578|rs6977642|rs1995917|rs11124176|rs1159176
Discoid Rash	SIGNALING_BY_FGFR_MUTANTS	REACTOME	1657	31	20	1,38E-01	9,96E-01	rs17072071|rs4976132|rs10047718|rs1490801|rs12056820|rs950549|rs9552595|rs2290755|rs34000|rs11167777|rs2031283|rs17102287|rs6490668|rs410187|rs2664537|rs6882257|rs4976028|rs10419120|rs10073996|rs2070715
Discoid Rash	AXON_GUIDANCE	KEGG	9506	38	20	1,38E-01	9,96E-01	rs11036094|rs16834223|rs1429743|rs6935980|rs3797980|rs258014|rs744834|rs10499888|rs4282339|rs2140459|rs7916174|rs17031463|rs6057009|rs2172860|rs10491393|rs2636785|rs346462|rs4732523|rs622996|rs11936149
Discoid Rash	PERK_REGULATED_GENE_EXPRESSION	REACTOME	334	16	8	1,40E-01	9,96E-01	rs2136703|rs1545229|rs17019438|rs1229564|rs11189224|rs1912445|rs10263979|rs1496496
Discoid Rash	NEP_NS2_INTERACTS_WITH_THE_CELLULAR_EXPORT_MACHINERY	REACTOME	456	20	10	1,40E-01	9,96E-01	rs2228375|rs217797|rs4740420|rs11177355|rs2018928|rs2016266|rs7782920|rs10271506|rs13431263|rs857587
Discoid Rash	PI3K_CASCADE	REACTOME	2702	34	20	1,42E-01	9,96E-01	rs17072071|rs1577452|rs4976132|rs10047718|rs1490801|rs12056820|rs950549|rs2848780|rs9552595|rs7438406|rs6970522|rs2290755|rs34000|rs9568203|rs11167777|rs7185040|rs7599150|rs17102287|rs2727528|rs336230
Discoid Rash	ION_TRANSPORT_BY_P_TYPE_ATPASES	REACTOME	1690	48	20	1,43E-01	9,96E-01	rs997559|rs4695247|rs6799104|rs7162581|rs6491069|rs938076|rs2341140|rs17637194|rs4384980|rs13336802|rs306401|rs9511834|rs8112614|rs4907563|rs11600216|rs1345101|rs4121751|rs2430890|rs11661180|rs4580096
Discoid Rash	WNT_SIGNALING_PATHWAY	KEGG	5611	32	20	1,44E-01	9,96E-01	rs2744738|rs4763782|rs590945|rs6108160|rs2744728|rs16870629|rs652673|rs1992215|rs3217810|rs7517829|rs6462795|rs11189119|rs12769610|rs11181634|rs17360053|rs6719501|rs11884056|rs1499367|rs7542242|rs10749127
Discoid Rash	TRANSPORT_OF_RIBONUCLEOPROTEINS_INTO_THE_HOST_NUCLEUS	REACTOME	452	20	10	1,45E-01	9,96E-01	rs2228375|rs217797|rs4740420|rs11177355|rs2018928|rs2016266|rs7782920|rs10271506|rs13431263|rs857587
Discoid Rash	G_ALPHA_S_SIGNALLING_EVENTS	REACTOME	5019	41	20	1,45E-01	9,96E-01	rs1422909|rs12418204|rs1064448|rs703745|rs4868513|rs9342101|rs1897104|rs8093607|rs8112063|rs13429751|rs7820461|rs6988640|rs1607987|rs2268657|rs2159917|rs7522367|rs244570|rs931646|rs10058673|rs17649232
Discoid Rash	TCA_CYCLE_AND_RESPIRATORY_ELECTRON_TRANSPORT	REACTOME	2599	39	20	1,45E-01	9,96E-01	rs6789203|rs1539624|rs10264649|rs3851422|rs1326481|rs1978566|rs12065184|rs2839604|rs4920706|rs218979|rs4474804|rs2280231|rs527216|rs4854073|rs9534724|rs12586783|rs1142530|rs4719793|rs4585672|rs10061728
Discoid Rash	RAS_PATHWAY	BIOCARTA	557	33	12	1,46E-01	9,96E-01	rs4976132|rs1490801|rs2124147|rs3448|rs4976028|rs10073996|rs2501276|rs7719763|rs10070379|rs10940145|rs13075194|rs4131890
Discoid Rash	ACTIVATION_OF_GENES_BY_ATF4	REACTOME	316	16	8	1,47E-01	9,96E-01	rs2136703|rs1545229|rs17019438|rs1229564|rs11189224|rs1912445|rs10263979|rs1496496
Discoid Rash	SELENOAMINO_ACID_METABOLISM	KEGG	742	42	20	1,47E-01	9,96E-01	rs2651237|rs11740544|rs12075963|rs10493474|rs2651236|rs11953266|rs4920037|rs2282367|rs1414073|rs17552854|rs17430359|rs13328108|rs2886722|rs11209643|rs17131320|rs6569455|rs10752294|rs10521140|rs297951|rs2755427
Discoid Rash	REGULATION_OF_GLUCOKINASE_BY_GLUCOKINASE_REGULATORY_PROTEIN	REACTOME	459	20	10	1,47E-01	9,96E-01	rs2228375|rs217797|rs4740420|rs11177355|rs2018928|rs2016266|rs7782920|rs10271506|rs13431263|rs857587
Discoid Rash	PATHOGENIC_ESCHERICHIA_COLI_INFECTION	KEGG	1256	28	20	1,49E-01	9,96E-01	rs11767121|rs17326309|rs17031463|rs10186875|rs467504|rs1928298|rs3019260|rs2376977|rs3774023|rs440804|rs4433842|rs3448|rs4851831|rs4604517|rs1827153|rs10875943|rs11685896|rs7605126|rs12003046|rs1994350
Discoid Rash	INTERFERON_GAMMA_SIGNALING	REACTOME	2043	45	20	1,49E-01	9,96E-01	rs3094691|rs2834219|rs17115184|rs7755852|rs1611350|rs1327489|rs8130924|rs10498252|rs11865882|rs2241694|rs3104404|rs1940735|rs3130473|rs9276284|rs3917016|rs3848468|rs1893217|rs1011973|rs1532471|rs4646982
Discoid Rash	ANTIGEN_PROCESSING_UBIQUITINATION_PROTEASOME_DEGRADATION	REACTOME	4788	36	20	1,51E-01	9,96E-01	rs1018326|rs13195756|rs590945|rs2293576|rs4467482|rs2173461|rs825227|rs2057566|rs7642557|rs10933849|rs4804505|rs7602023|rs593234|rs7255657|rs2189713|rs3810168|rs12198323|rs2903698|rs1445059|rs12155423
Discoid Rash	TASTE_TRANSDUCTION	KEGG	1100	42	20	1,52E-01	9,96E-01	rs799942|rs1897104|rs13429751|rs7820461|rs1120559|rs10772380|rs3759333|rs6467221|rs17649232|rs237485|rs6092704|rs10783282|rs3935570|rs965808|rs884423|rs2426154|rs10424440|rs802952|rs2499689|rs4565471
Discoid Rash	BIOSYNTHESIS_OF_UNSATURATED_FATTY_ACIDS	KEGG	463	17	8	1,55E-01	9,96E-01	rs209512|rs2243816|rs7656124|rs3798719|rs2075863|rs17448411|rs12804822|rs10452184
Discoid Rash	CLASS_B_2_SECRETIN_FAMILY_RECEPTORS	REACTOME	2257	42	20	1,55E-01	9,96E-01	rs2744738|rs2744728|rs2641600|rs652673|rs7517829|rs17360053|rs6988640|rs1607987|rs2268657|rs7542242|rs2159917|rs931646|rs4807988|rs2363923|rs11810173|rs6092704|rs6946781|rs2131858|rs1219626|rs10228431
Discoid Rash	ERK_MAPK_TARGETS	REACTOME	704	36	20	1,57E-01	9,96E-01	rs918418|rs700591|rs13281383|rs16898963|rs7175248|rs6860708|rs11633249|rs7823751|rs8041078|rs247587|rs11576136|rs1002487|rs11958574|rs6598858|rs7312093|rs3799658|rs16899429|rs6905447|rs7156757|rs17555326
Discoid Rash	SIGNALING_BY_TGF_BETA_RECEPTOR_COMPLEX	REACTOME	1605	25	20	1,57E-01	9,96E-01	rs1992215|rs7546890|rs11608551|rs998910|rs6128143|rs1499367|rs12102171|rs1417416|rs17199521|rs11661651|rs12547643|rs7781554|rs12607345|rs4733859|rs927171|rs17743079|rs3448|rs238135|rs838875|rs2314664
Discoid Rash	LYSINE_DEGRADATION	KEGG	952	44	20	1,58E-01	9,96E-01	rs1581676|rs2587940|rs11710718|rs11788224|rs9568203|rs4836277|rs11029795|rs7922341|rs11725638|rs3818299|rs4245924|rs1160604|rs12634033|rs4597027|rs7630694|rs10247064|rs11725821|rs710887|rs2810716|rs7253181
Discoid Rash	PYRUVATE_METABOLISM	REACTOME	209	33	9	1,59E-01	9,96E-01	rs1539624|rs10264649|rs4585672|rs12693008|rs854093|rs2025776|rs2076371|rs875511|rs12274651
Discoid Rash	CHRONIC_MYELOID_LEUKEMIA	KEGG	2538	35	20	1,61E-01	9,96E-01	rs4976132|rs11137533|rs1992215|rs1490801|rs7642557|rs10933849|rs1511592|rs1499367|rs13324208|rs12102171|rs10483453|rs1445059|rs2335855|rs17199521|rs17047703|rs12547643|rs2834684|rs17103274|rs4733859|rs6784447
Discoid Rash	CELL_SURFACE_INTERACTIONS_AT_THE_VASCULAR_WALL	REACTOME	2650	42	20	1,62E-01	9,96E-01	rs2767326|rs4976132|rs4729129|rs1539624|rs7546890|rs1490801|rs1519491|rs2176472|rs11223702|rs1283663|rs1858822|rs3829406|rs10464587|rs4665639|rs1544241|rs4667731|rs8120756|rs4431992|rs1439502|rs4320015
Discoid Rash	CCR5_PATHWAY	BIOCARTA	696	41	20	1,63E-01	9,96E-01	rs16834223|rs16834018|rs6557991|rs3785383|rs6740091|rs4433842|rs11687599|rs392715|rs2811876|rs2664537|rs8051531|rs266078|rs8013053|rs11646540|rs17753569|rs17811655|rs1634523|rs2741343|rs17488134|rs17786782
Discoid Rash	GLYCOSAMINOGLYCAN_METABOLISM	REACTOME	6096	43	20	1,63E-01	9,96E-01	rs4662862|rs4338508|rs2468133|rs3115775|rs7976582|rs6825790|rs10520079|rs11038519|rs2128333|rs9390633|rs6866961|rs10161421|rs4303490|rs7301863|rs2635297|rs672413|rs2119299|rs2191068|rs10002047|rs7990210
Discoid Rash	DOWNSTREAM_SIGNAL_TRANSDUCTION	REACTOME	3083	40	20	1,65E-01	9,96E-01	rs1064448|rs4976132|rs17031463|rs4864510|rs1490801|rs11133313|rs1897104|rs13429751|rs6130713|rs7820461|rs7349397|rs873418|rs2204204|rs2344662|rs10058673|rs17649232|rs2376977|rs7322396|rs4382132|rs7981045
Discoid Rash	SMALL_CELL_LUNG_CANCER	KEGG	3747	34	20	1,66E-01	9,96E-01	rs12213754|rs4976132|rs1490801|rs1483831|rs1447981|rs2326761|rs6759316|rs6445205|rs1499367|rs7623479|rs679668|rs1947152|rs873418|rs10483453|rs17199521|rs7640419|rs212063|rs7328731|rs12547643|rs11080358
Discoid Rash	CYTOKINE_SIGNALING_IN_IMMUNE_SYSTEM	REACTOME	6819	32	20	1,66E-01	9,96E-01	rs3094691|rs2834219|rs2228375|rs590945|rs1577452|rs4976132|rs17115184|rs1417718|rs1490801|rs486907|rs6130713|rs12720279|rs1043395|rs7755852|rs1278150|rs17262993|rs7438406|rs1611350|rs1327489|rs1532
Discoid Rash	EDG1_PATHWAY	BIOCARTA	1432	37	20	1,66E-01	9,96E-01	rs6108160|rs4976132|rs4864510|rs1490801|rs11133313|rs3785383|rs4433842|rs12706724|rs1388452|rs3448|rs2179984|rs1589880|rs392715|rs11164184|rs6118062|rs6140561|rs12061503|rs4976028|rs10073996|rs2056131
Discoid Rash	TRANSPORT_OF_MATURE_MRNA_DERIVED_FROM_AN_INTRONLESS_TRANSCRIPT	REACTOME	524	25	14	1,67E-01	9,96E-01	rs2228375|rs7438406|rs217797|rs4740420|rs12586783|rs11177355|rs2018928|rs2016266|rs7782920|rs10271506|rs10007831|rs13431263|rs11887079|rs857587
Discoid Rash	CITRIC_ACID_CYCLE_TCA_CYCLE	REACTOME	675	41	17	1,71E-01	9,96E-01	rs6789203|rs1326481|rs12065184|rs9534724|rs4585672|rs6467993|rs4832076|rs7337764|rs9611598|rs10247064|rs977103|rs10186197|rs710887|rs1536002|rs2970358|rs11919650|rs1274500
Discoid Rash	METABOLISM_OF_NON_CODING_RNA	REACTOME	611	25	12	1,71E-01	9,96E-01	rs2228375|rs217797|rs4740420|rs2295682|rs11177355|rs2018928|rs3786509|rs2016266|rs7782920|rs10271506|rs13431263|rs857587
Discoid Rash	DILATED_CARDIOMYOPATHY	KEGG	4636	29	20	1,72E-01	9,96E-01	rs12213754|rs1064448|rs7564976|rs6942458|rs2326761|rs2212020|rs197721|rs7820461|rs12707506|rs6759316|rs4821506|rs11012843|rs2050140|rs2239016|rs7637998|rs3095208|rs12475881|rs1351140|rs1108385|rs17047703
Discoid Rash	RAC1_PATHWAY	BIOCARTA	763	51	20	1,73E-01	9,96E-01	rs4976132|rs4864510|rs1490801|rs11133313|rs2124147|rs4700008|rs935865|rs4807101|rs4976028|rs10073996|rs1008163|rs10800842|rs7719763|rs7258040|rs10070379|rs10940145|rs7212835|rs13100237|rs13075194|rs12610223
Discoid Rash	NUCLEAR_SIGNALING_BY_ERBB4	REACTOME	2231	33	20	1,76E-01	9,96E-01	rs590945|rs1417718|rs7574462|rs16947307|rs17262993|rs4236723|rs1396702|rs1327489|rs7658842|rs851982|rs1481624|rs9356797|rs924870|rs667707|rs16847102|rs17762822|rs16847122|rs17737362|rs17081685|rs10503899
Discoid Rash	SIGNALING_BY_PDGF	REACTOME	4427	38	20	1,78E-01	9,96E-01	rs1064448|rs4976132|rs4729129|rs17031463|rs4864510|rs1490801|rs11133313|rs1897104|rs9455013|rs13429751|rs6130713|rs7820461|rs7349397|rs11694554|rs10517655|rs873418|rs2204204|rs1858822|rs2344662|rs9406337
Discoid Rash	CYSTEINE_AND_METHIONINE_METABOLISM	KEGG	982	38	20	1,78E-01	9,96E-01	rs2651237|rs11740544|rs12075963|rs10493474|rs2780749|rs11953266|rs4920037|rs11636141|rs2282367|rs9935162|rs1414073|rs17552854|rs7194392|rs575425|rs13328108|rs2886722|rs6826022|rs11209643|rs17131320|rs2025776
Discoid Rash	REGULATION_OF_ACTIN_CYTOSKELETON	KEGG	7089	29	20	1,79E-01	9,96E-01	rs10488600|rs17072071|rs2833293|rs4976132|rs10047718|rs10488226|rs11767121|rs7564976|rs6057009|rs4864510|rs1490801|rs11133313|rs12056820|rs2212020|rs197721|rs950549|rs6759316|rs17725737|rs945032|rs9552595
Discoid Rash	PI3K_AKT_ACTIVATION	REACTOME	1161	36	20	1,81E-01	9,96E-01	rs1577452|rs4976132|rs1490801|rs17540656|rs873418|rs7322396|rs7981045|rs7185040|rs4943769|rs3448|rs9817|rs7599150|rs336230|rs2254137|rs4976028|rs10073996|rs17188448|rs719737|rs11618989|rs769412
Discoid Rash	IL_2_SIGNALING	REACTOME	936	39	20	1,81E-01	9,96E-01	rs4976132|rs1490801|rs6130713|rs1278150|rs1327489|rs6557991|rs7072398|rs12722589|rs36686|rs4976028|rs10073996|rs7899538|rs4744512|rs1755991|rs2450132|rs4749882|rs1585461|rs2741343|rs693293|rs7719763
Discoid Rash	VIBRIO_CHOLERAE_INFECTION	KEGG	1518	36	20	1,82E-01	9,96E-01	rs4779683|rs4930007|rs10055964|rs2283214|rs2041689|rs1357386|rs10982300|rs2283194|rs3785383|rs12267330|rs12916064|rs4433842|rs11783925|rs6092704|rs392715|rs11769043|rs2664537|rs4255520|rs6464929|rs8051531
Discoid Rash	PEPTIDE_LIGAND_BINDING_RECEPTORS	REACTOME	5053	50	20	1,82E-01	9,96E-01	rs10486008|rs4681446|rs16834223|rs233966|rs2638362|rs13410673|rs8093607|rs1829651|rs7871222|rs12489843|rs721608|rs945032|rs4692540|rs3796974|rs6960789|rs1877563|rs906895|rs17520|rs16834018|rs2106987
Discoid Rash	NKT_PATHWAY	BIOCARTA	638	44	20	1,83E-01	9,96E-01	rs2834219|rs16834223|rs16834018|rs1532|rs17047703|rs6740091|rs11687599|rs2707212|rs1833754|rs12029576|rs1634523|rs2268626|rs7250425|rs918519|rs1801275|rs485499|rs2305740|rs924080|rs2373154|rs2965015
Discoid Rash	ENDOMETRIAL_CANCER	KEGG	3028	46	20	1,86E-01	9,96E-01	rs12356797|rs4976132|rs4990414|rs1490801|rs7074696|rs6719501|rs11884056|rs2648888|rs10822661|rs10996198|rs10749127|rs873418|rs17199521|rs7576479|rs1864548|rs6480209|rs12547643|rs4733859|rs3916990|rs440804
Discoid Rash	BIOLOGICAL_OXIDATIONS	REACTOME	3568	33	20	1,87E-01	9,96E-01	rs1466202|rs10028494|rs11740544|rs10306202|rs2284205|rs17466747|rs163253|rs7598552|rs16989226|rs9886369|rs11953266|rs11773597|rs12646059|rs6583859|rs13332660|rs34511626|rs2297812|rs10493270|rs756208|rs2131463
Discoid Rash	SIGNALLING_TO_ERKS	REACTOME	794	37	12	1,88E-01	9,96E-01	rs11137533|rs6130713|rs17540656|rs10793894|rs2664537|rs1808416|rs10494878|rs2859131|rs525291|rs4963872|rs7364173|rs2296950
Discoid Rash	FC_GAMMA_R_MEDIATED_PHAGOCYTOSIS	KEGG	3795	36	20	1,89E-01	9,96E-01	rs2546507|rs4976132|rs2338809|rs10488226|rs11767121|rs1490801|rs7349397|rs11153413|rs560873|rs12657308|rs3922668|rs2204204|rs2124147|rs2344662|rs10114577|rs11761049|rs10494605|rs7023913|rs659732|rs4463623
Discoid Rash	MAPK_TARGETS_NUCLEAR_EVENTS_MEDIATED_BY_MAP_KINASES	REACTOME	980	32	20	1,89E-01	9,96E-01	rs918418|rs700591|rs13281383|rs16898963|rs7175248|rs6860708|rs11633249|rs7823751|rs2811876|rs2254137|rs10494878|rs8041078|rs6854208|rs247587|rs11576136|rs8013053|rs3775167|rs1002487|rs11958574|rs6598858
Discoid Rash	GROWTH_HORMONE_RECEPTOR_SIGNALING	REACTOME	773	31	20	1,89E-01	9,96E-01	rs1577452|rs1417718|rs17262993|rs1327489|rs9356797|rs7599150|rs336230|rs196034|rs452335|rs7139113|rs17188448|rs11970435|rs6512654|rs719737|rs3782415|rs11618989|rs336209|rs7705676|rs2092058|rs1414320
Discoid Rash	CARDIACEGF_PATHWAY	BIOCARTA	1289	50	20	1,90E-01	9,96E-01	rs4681446|rs2638362|rs1499367|rs748289|rs17199521|rs3785383|rs12547643|rs4733859|rs12098279|rs4433842|rs3448|rs392715|rs4639051|rs2811876|rs2664537|rs10505507|rs188019|rs8051531|rs8013053|rs11646540
Discoid Rash	REGULATION_OF_INSULIN_LIKE_GROWTH_FACTOR_IGF_ACTIVITY_BY_INSULIN_LIKE_G<br>DING_PROTEINS_IGFBPS	REACTOME	570	36	18	1,90E-01	9,96E-01	rs10743111|rs2294654|rs6478233|rs4341514|rs7033487|rs879966|rs4431992|rs7945189|rs10932667|rs519806|rs11643163|rs1496496|rs6728171|rs4234019|rs1420228|rs17302884|rs17561224|rs11764490
Discoid Rash	NGF_SIGNALLING_VIA_TRKA_FROM_THE_PLASMA_MEMBRANE	REACTOME	4847	37	20	1,94E-01	9,96E-01	rs1064448|rs1577452|rs4976132|rs11137533|rs918418|rs1490801|rs1897104|rs700591|rs13429751|rs6130713|rs13281383|rs7820461|rs7349397|rs17540656|rs17088180|rs873418|rs2204204|rs2344662|rs16898963|rs3739804
Discoid Rash	HEPARAN_SULFATE_HEPARIN_HS_GAG_METABOLISM	REACTOME	3879	52	20	1,96E-01	9,96E-01	rs4662862|rs4338508|rs2445904|rs3115775|rs6825790|rs4303490|rs2635297|rs2119299|rs2191068|rs10002047|rs7990210|rs1506793|rs897005|rs1572342|rs4144545|rs1874238|rs13024819|rs7993144|rs7984041|rs2439525
Discoid Rash	ERK_PATHWAY	BIOCARTA	1025	35	20	1,96E-01	9,96E-01	rs12580224|rs4864510|rs11133313|rs17540656|rs1499367|rs17199521|rs12547643|rs4733859|rs6092704|rs10505507|rs1317636|rs10506598|rs965808|rs11576136|rs1008163|rs1002487|rs1187095|rs7166565|rs10794486|rs6598858
Discoid Rash	SIGNALING_BY_FGFR	REACTOME	4154	36	20	1,98E-01	9,96E-01	rs17072071|rs1064448|rs4976132|rs10047718|rs1490801|rs1897104|rs12056820|rs13429751|rs6130713|rs950549|rs7820461|rs7349397|rs9552595|rs873418|rs2204204|rs2344662|rs2290755|rs34000|rs11167777|rs10058673
Discoid Rash	HYPERTROPHIC_CARDIOMYOPATHY_HCM	KEGG	4149	26	20	2,00E-01	9,96E-01	rs12213754|rs7564976|rs6942458|rs2326761|rs2212020|rs197721|rs12707506|rs6759316|rs4821506|rs11012843|rs2050140|rs2239016|rs4329|rs7637998|rs6970522|rs3095208|rs12475881|rs1351140|rs1108385|rs17047703
Discoid Rash	TRNA_AMINOACYLATION	REACTOME	795	45	20	2,00E-01	9,96E-01	rs2713834|rs3765144|rs4699172|rs1316319|rs2503838|rs603392|rs707938|rs6450986|rs7751261|rs2326613|rs1441042|rs4409225|rs6779394|rs6926774|rs6676647|rs2295085|rs2064108|rs959086|rs585362|rs3136646
Discoid Rash	PRE_NOTCH_EXPRESSION_AND_PROCESSING	REACTOME	1434	41	20	2,02E-01	9,96E-01	rs10149996|rs1036047|rs10484153|rs1152349|rs6483495|rs10519523|rs12895242|rs6414283|rs2256905|rs1628691|rs2372200|rs799103|rs1379244|rs12646059|rs2811876|rs2888183|rs12417347|rs12881723|rs204990|rs11624992
Discoid Rash	VIRAL_MYOCARDITIS	KEGG	2750	47	20	2,03E-01	9,96E-01	rs12213754|rs3094691|rs1456721|rs2326761|rs7755852|rs1611350|rs3104404|rs965938|rs3130473|rs9276284|rs4704795|rs4719793|rs3866330|rs4320015|rs2776068|rs3745511|rs235370|rs208898|rs12111032|rs12003046
Discoid Rash	PHASE_II_CONJUGATION	REACTOME	2059	37	20	2,10E-01	9,96E-01	rs1466202|rs10028494|rs11740544|rs163253|rs7598552|rs11953266|rs12646059|rs13332660|rs34511626|rs756208|rs10874814|rs1458226|rs163269|rs316156|rs17430359|rs7355084|rs294755|rs13328108|rs2886722|rs8109050
Discoid Rash	NKCELLS_PATHWAY	BIOCARTA	678	42	20	2,10E-01	9,96E-01	rs4976132|rs1490801|rs3922668|rs6557991|rs401618|rs4807101|rs4976028|rs252308|rs10073996|rs4744512|rs1755991|rs1187095|rs1585461|rs2394250|rs2741343|rs166152|rs6457109|rs7719763|rs7258040|rs10070379
Discoid Rash	CDMAC_PATHWAY	BIOCARTA	1018	32	20	2,11E-01	9,96E-01	rs6108160|rs2648888|rs10483453|rs17199521|rs3785383|rs12547643|rs17103274|rs4733859|rs4433842|rs2179984|rs392715|rs2811876|rs6118062|rs6140561|rs10505507|rs8051531|rs8013053|rs11646540|rs6086525|rs17753569
Discoid Rash	MELANOMA	KEGG	2894	33	20	2,14E-01	9,96E-01	rs17072071|rs4976132|rs10047718|rs4864510|rs1490801|rs11133313|rs12056820|rs950549|rs9552595|rs10517655|rs873418|rs2290755|rs34000|rs5745752|rs11167777|rs879772|rs9817|rs4021|rs6490668|rs6882257
Discoid Rash	INSULIN_SIGNALING_PATHWAY	KEGG	3744	39	20	2,14E-01	9,96E-01	rs2140825|rs1511451|rs1577452|rs4976132|rs11137533|rs1490801|rs2036713|rs7642557|rs10933849|rs8081659|rs7438406|rs6970522|rs1445059|rs2418001|rs6784447|rs11217175|rs7322396|rs7981045|rs7630432|rs7185040
Discoid Rash	CA_DEPENDENT_EVENTS	REACTOME	1487	42	20	2,18E-01	9,96E-01	rs1064448|rs1897104|rs13429751|rs7820461|rs10494605|rs659732|rs4463623|rs10058673|rs17649232|rs4382132|rs10475376|rs4433842|rs17826588|rs1589880|rs1474797|rs10783282|rs1980444|rs1644512|rs2254137|rs1148757
Discoid Rash	INTERFERON_SIGNALING	REACTOME	3448	33	20	2,18E-01	9,96E-01	rs3094691|rs2834219|rs2228375|rs17115184|rs486907|rs12720279|rs7755852|rs7438406|rs1611350|rs1327489|rs1532|rs10498252|rs11865882|rs2241694|rs3104404|rs217797|rs2407705|rs1940709|rs4740420|rs3130473
Discoid Rash	ION_CHANNEL_TRANSPORT	REACTOME	2741	46	20	2,19E-01	9,96E-01	rs997559|rs4695247|rs745013|rs6799104|rs7162581|rs6491069|rs938076|rs10515829|rs2341140|rs17637194|rs4238490|rs10937160|rs4384980|rs12221649|rs13336802|rs306401|rs9511834|rs8112614|rs4572353|rs4907563
Discoid Rash	GABA_B_RECEPTOR_ACTIVATION	REACTOME	1863	36	20	2,19E-01	9,96E-01	rs1064448|rs2808539|rs17779442|rs7820461|rs2190550|rs2159917|rs3025646|rs8094168|rs931646|rs10058673|rs4382132|rs1540011|rs8081911|rs11810173|rs10475376|rs12706724|rs17826588|rs1589880|rs13000959|rs10783282
Discoid Rash	PDGF_PATHWAY	BIOCARTA	1021	48	20	2,22E-01	9,96E-01	rs4976132|rs4864510|rs1490801|rs11133313|rs3785383|rs4433842|rs392715|rs4700008|rs2811876|rs2664537|rs4976028|rs10073996|rs8051531|rs1008163|rs8013053|rs11646540|rs17753569|rs17811655|rs7719763|rs198182
Discoid Rash	EICOSANOID_LIGAND_BINDING_RECEPTORS	REACTOME	507	41	10	2,24E-01	9,96E-01	rs6670616|rs6719977|rs931646|rs1334997|rs2744907|rs520171|rs1020360|rs1390376|rs2278583|rs1254600
Discoid Rash	AKT_PATHWAY	BIOCARTA	682	30	17	2,27E-01	9,96E-01	rs4976132|rs1490801|rs10483453|rs17103274|rs7322396|rs7981045|rs4943769|rs4976028|rs10073996|rs11621560|rs7719763|rs10070379|rs10940145|rs1268160|rs4131890|rs10492682|rs9549123
Discoid Rash	INTERACTIONS_OF_VPR_WITH_HOST_CELLULAR_PROTEINS	REACTOME	480	21	11	2,27E-01	9,96E-01	rs2228375|rs217797|rs4740420|rs11177355|rs2018928|rs2016266|rs7782920|rs10271506|rs13431263|rs857587|rs11734260
Discoid Rash	BUTANOATE_METABOLISM	KEGG	763	32	20	2,28E-01	9,96E-01	rs2301770|rs11788224|rs4836277|rs13064360|rs12368128|rs6755814|rs11725638|rs2273828|rs4883216|rs4956145|rs12930556|rs28703192|rs163269|rs2144575|rs13098723|rs1898251|rs1273422|rs2810716|rs2275591|rs6532592

Discoid Rash	ARF_PATHWAY	BIOCARTA	733	43	20	2,30E-01	9,96E-01	rs4976132|rs1490801|rs1499367|rs17199521|rs12547643|rs4733859|rs9817|rs10505507|rs4976028|rs10073996|rs12003046|rs6734172|rs769412|rs7594007|rs7719763|rs6985681|rs10070379|rs10940145|rs6987802|rs10225279
Discoid Rash	CYTOSOLIC_TRNA_AMINOACYLATION	REACTOME	312	24	9	2,30E-01	9,96E-01	rs3765144|rs1316319|rs603392|rs707938|rs6450986|rs6779394|rs585362|rs10798918|rs13192727
Discoid Rash	NGF_PATHWAY	BIOCARTA	566	31	16	2,31E-01	9,96E-01	rs4976132|rs1490801|rs17540656|rs2811876|rs2664537|rs1317636|rs4976028|rs10073996|rs8013053|rs7719763|rs10070379|rs6677589|rs17183482|rs10940145|rs11207306|rs4131890
Discoid Rash	ASCORBATE_AND_ALDARATE_METABOLISM	KEGG	874	42	10	2,32E-01	9,96E-01	rs10028494|rs11788224|rs4836277|rs1458226|rs294755|rs6848997|rs2810716|rs293408|rs730870|rs480245
Discoid Rash	ER_PHAGOSOME_PATHWAY	REACTOME	1416	58	20	2,33E-01	9,96E-01	rs3094691|rs2293576|rs7755852|rs1611350|rs328331|rs12267330|rs3130473|rs2314664|rs12599594|rs371500|rs13394402|rs12111032|rs9295986|rs13191519|rs1736956|rs2844575|rs9266018|rs6457109|rs12463483|rs3731973
Discoid Rash	PLATELET_HOMEOSTASIS	REACTOME	4080	49	20	2,33E-01	9,96E-01	rs997559|rs12418204|rs1897104|rs9844737|rs1519491|rs13429751|rs2176472|rs6799104|rs2159917|rs8003274|rs12475881|rs816296|rs10494605|rs931646|rs659732|rs4434808|rs4665639|rs11080358|rs17649232|rs2650163
Discoid Rash	IL17_PATHWAY	BIOCARTA	316	15	9	2,33E-01	9,96E-01	rs2767326|rs10948680|rs10484882|rs2707212|rs10242595|rs10161145|rs2995094|rs10950917|rs870875
Discoid Rash	NCAM1_INTERACTIONS	REACTOME	1896	36	20	2,37E-01	9,96E-01	rs4729129|rs17115184|rs9455013|rs11694554|rs4815715|rs11012843|rs1858822|rs10464587|rs2240220|rs434222|rs10889285|rs7328731|rs10451555|rs6722598|rs1940735|rs1981316|rs631428|rs7069531|rs1459574|rs652462
Discoid Rash	SIGNALING_BY_ERBB4	REACTOME	3434	31	20	2,37E-01	9,96E-01	rs590945|rs4976132|rs1417718|rs7574462|rs1490801|rs6130713|rs16947307|rs17262993|rs4236723|rs873418|rs1396702|rs1327489|rs7658842|rs851982|rs1481624|rs9356797|rs924870|rs667707|rs16847102|rs17762822
Discoid Rash	ADHERENS_JUNCTION	KEGG	4520	38	20	2,41E-01	9,96E-01	rs4779683|rs12356797|rs11767121|rs4990414|rs1992215|rs7074696|rs157580|rs16938992|rs6719501|rs11884056|rs560873|rs10509237|rs4682242|rs619957|rs10996198|rs10749127|rs7735422|rs12102171|rs1357386|rs613113
Discoid Rash	NATURAL_KILLER_CELL_MEDIATED_CYTOTOXICITY	KEGG	3607	46	20	2,41E-01	9,96E-01	rs3094691|rs2834219|rs6919586|rs4976132|rs11137533|rs1490801|rs7755852|rs3922668|rs8130924|rs10114577|rs9267404|rs6557991|rs3785383|rs401618|rs965938|rs4283898|rs3130473|rs4433842|rs1007662|rs392715
Discoid Rash	ARACHIDONIC_ACID_METABOLISM	KEGG	1356	35	20	2,41E-01	9,96E-01	rs17024981|rs10306202|rs2284205|rs10494605|rs659732|rs4463623|rs4792203|rs7017417|rs3448|rs1474797|rs1712432|rs10493270|rs1980444|rs2131463|rs1148757|rs9911427|rs6697553|rs3787016|rs915907|rs878400
Discoid Rash	SPPA_PATHWAY	BIOCARTA	1428	36	20	2,42E-01	9,96E-01	rs6108160|rs10306202|rs2284205|rs1351140|rs10494605|rs659732|rs4434808|rs3785383|rs4433842|rs12706724|rs2179984|rs392715|rs1474797|rs6118062|rs6140561|rs1980444|rs1148757|rs4744512|rs8051531|rs1755991
Discoid Rash	HER2_PATHWAY	BIOCARTA	1326	52	20	2,42E-01	9,96E-01	rs4976132|rs7574462|rs17103105|rs1490801|rs851982|rs16847102|rs16847122|rs11176420|rs12228293|rs17081685|rs12623434|rs4976028|rs12580112|rs10073996|rs1465828|rs6435604|rs6435670|rs10242595|rs6435689|rs2813554
Discoid Rash	ABCA_TRANSPORTERS_IN_LIPID_HOMEOSTASIS	REACTOME	464	16	6	2,46E-01	9,96E-01	rs8133852|rs4299376|rs323066|rs4148217|rs12468887|rs1025447
Discoid Rash	IL_RECEPTOR_SHC_SIGNALING	REACTOME	624	33	19	2,48E-01	9,96E-01	rs4976132|rs1490801|rs1278150|rs1327489|rs7072398|rs12722589|rs36686|rs4976028|rs7899538|rs10073996|rs2450132|rs4749882|rs693293|rs7719763|rs10070379|rs10940145|rs2290608|rs4131890|rs739719
Discoid Rash	NICOTINATE_AND_NICOTINAMIDE_METABOLISM	KEGG	963	58	20	2,48E-01	9,96E-01	rs34229|rs12493507|rs1428878|rs946173|rs2247016|rs249393|rs13190660|rs8072869|rs11707887|rs242253|rs7701167|rs11754555|rs11828|rs7636269|rs10935348|rs12516214|rs675220|rs4789145|rs295464|rs6935458
Discoid Rash	PTEN_PATHWAY	BIOCARTA	544	29	13	2,49E-01	9,96E-01	rs4976132|rs1490801|rs873418|rs4976028|rs10073996|rs1187095|rs9927309|rs7719763|rs875511|rs10070379|rs10940145|rs1268160|rs4131890
Discoid Rash	SIGNALING_BY_CONSTITUTIVELY_ACTIVE_EGFR	REACTOME	503	28	14	2,50E-01	9,96E-01	rs4976132|rs1490801|rs11217175|rs2314664|rs2664537|rs4976028|rs10073996|rs11621560|rs7719763|rs10070379|rs10940145|rs4963872|rs2740764|rs4131890
Discoid Rash	INFLUENZA_VIRAL_RNA_TRANSCRIPTION_AND_REPLICATION	REACTOME	1153	33	20	2,54E-01	9,96E-01	rs285055|rs2958522|rs12896325|rs10509398|rs7255657|rs3810168|rs4558175|rs2816764|rs2314664|rs3764536|rs7331868|rs2001558|rs3787016|rs11621560|rs9394299|rs633161|rs4803238|rs634760|rs36631|rs7331770
Discoid Rash	SIGNALLING_BY_NGF	REACTOME	7869	31	20	2,56E-01	9,96E-01	rs2140825|rs1064448|rs2833293|rs1577452|rs4976132|rs11137533|rs918418|rs6797559|rs1490801|rs1897104|rs700591|rs13429751|rs6130713|rs13281383|rs7820461|rs7349397|rs17540656|rs9647067|rs17088180|rs4918129
Discoid Rash	MTOR_PATHWAY	BIOCARTA	509	30	16	2,57E-01	9,96E-01	rs4976132|rs1490801|rs7438406|rs873418|rs7185040|rs4976028|rs10073996|rs7719763|rs10007831|rs875511|rs10070379|rs10940145|rs494469|rs2519757|rs4131890|rs10747658
Discoid Rash	INHIBITION_OF_VOLTAGE_GATED_CA2_CHANNELS_VIA_GBETA_GAMMA_SUBUNITS	REACTOME	1082	29	20	2,60E-01	9,96E-01	rs2808539|rs17779442|rs2190550|rs2159917|rs3025646|rs931646|rs1540011|rs8081911|rs11810173|rs13000959|rs2154552|rs529269|rs1321649|rs6478676|rs6517442|rs17178083|rs1860316|rs8069222|rs11601431|rs10512554
Discoid Rash	EIF4_PATHWAY	BIOCARTA	890	38	20	2,60E-01	9,96E-01	rs4976132|rs1490801|rs7438406|rs873418|rs3785383|rs4433842|rs392715|rs7599150|rs4976028|rs10073996|rs17188448|rs8051531|rs11646540|rs17753569|rs17811655|rs7719763|rs10007831|rs198182|rs875511|rs10070379
Discoid Rash	GLUCOSE_TRANSPORT	REACTOME	689	33	15	2,61E-01	9,96E-01	rs2228375|rs2297972|rs217797|rs4740420|rs11177355|rs2018928|rs12150519|rs1000251|rs2016266|rs17387775|rs741811|rs7782920|rs10271506|rs13431263|rs857587
Discoid Rash	INFLUENZA_LIFE_CYCLE	REACTOME	1671	38	20	2,61E-01	9,96E-01	rs2228375|rs285055|rs2958522|rs12896325|rs4554825|rs7255657|rs3810168|rs4558175|rs2816764|rs217797|rs4740420|rs2314664|rs11177355|rs2018928|rs3764536|rs7331868|rs2001558|rs3787016|rs11621560|rs9394299
Discoid Rash	RIBOSOME	KEGG	1118	32	20	2,62E-01	9,96E-01	rs2958522|rs12896325|rs12594750|rs4554825|rs7255657|rs3810168|rs4558175|rs2816764|rs2314664|rs3764536|rs527015|rs1462395|rs2001558|rs9394299|rs4803238|rs36631|rs4738315|rs2012243|rs8016600|rs2277743
Discoid Rash	IL22BP_PATHWAY	BIOCARTA	296	10	7	2,63E-01	9,96E-01	rs12720279|rs1327489|rs36686|rs281414|rs7310731|rs7572482|rs12100005
Discoid Rash	LEUKOCYTE_TRANSENDOTHELIAL_MIGRATION	KEGG	4793	46	20	2,63E-01	9,96E-01	rs6766201|rs16834223|rs12356797|rs4976132|rs4990414|rs7564976|rs7546890|rs1490801|rs7074696|rs11223702|rs10509237|rs10996198|rs16834018|rs10114577|rs6557991|rs7576479|rs17106413|rs1864548|rs3785383|rs6480209
Discoid Rash	ANTIGEN_ACTIVATES_B_CELL_RECEPTOR_LEADING_TO_GENERATION_OF_SECOND_ME	REACTOME	1299	37	20	2,66E-01	9,96E-01	rs4976132|rs1490801|rs7642557|rs10933849|rs9937676|rs1445059|rs6784447|rs11217175|rs7630432|rs3827867|rs2664537|rs11595566|rs4807101|rs4976028|rs10073996|rs4744512|rs4142696|rs1755991|rs1585461|rs7911684
Discoid Rash	SIGNALING_BY_FGFR_IN_DISEASE	REACTOME	4545	36	20	2,67E-01	9,96E-01	rs17072071|rs1064448|rs4976132|rs10047718|rs1490801|rs1897104|rs12056820|rs13429751|rs6130713|rs950549|rs7820461|rs7349397|rs9552595|rs873418|rs2204204|rs2344662|rs2290755|rs34000|rs11167777|rs10058673
Discoid Rash	MATURITY_ONSET_DIABETES_OF_THE_YOUNG	KEGG	992	34	20	2,69E-01	9,96E-01	rs17626394|rs719714|rs1429447|rs6788164|rs265451|rs8024399|rs10504605|rs2926615|rs9512906|rs1562147|rs1572579|rs6017335|rs7272694|rs947591|rs1875343|rs7822381|rs4687500|rs6817184|rs1806180|rs17192831
Discoid Rash	IL_3_5_AND_GM_CSF_SIGNALING	REACTOME	1134	29	20	2,71E-01	9,96E-01	rs4976132|rs1490801|rs1278150|rs1327489|rs3019260|rs1544241|rs11217175|rs10793894|rs6121337|rs7072398|rs12722589|rs4807101|rs36686|rs4976028|rs10073996|rs7899538|rs4744512|rs1755991|rs2450132|rs4749882
Discoid Rash	CTCF_PATHWAY	BIOCARTA	1035	46	20	2,74E-01	9,96E-01	rs4976132|rs1490801|rs2648888|rs873418|rs17199521|rs17047703|rs12547643|rs4733859|rs9817|rs4368250|rs10505507|rs4976028|rs10073996|rs12029576|rs2058194|rs769412|rs2268626|rs10819641|rs1019855|rs7719763
Discoid Rash	NEGATIVE_REGULATION_OF_FGFR_SIGNALING	REACTOME	1536	27	20	2,75E-01	9,96E-01	rs17072071|rs10047718|rs12056820|rs950549|rs9552595|rs2290755|rs34000|rs11167777|rs11217175|rs2314664|rs7328506|rs17102287|rs6490668|rs6882257|rs10419120|rs2070715|rs7327237|rs2476509|rs750535|rs4616067
Discoid Rash	GRAFT_VERSUS_HOST_DISEASE	KEGG	1446	63	20	2,76E-01	9,96E-01	rs3094691|rs7755852|rs1611350|rs17042407|rs3104404|rs3130473|rs9276284|rs11591298|rs2245548|rs762084|rs12111032|rs9295986|rs28383344|rs10242595|rs13191519|rs1736956|rs3129882|rs1937332|rs2844575|rs9266018
Discoid Rash	NEUROACTIVE_LIGAND_RECEPTOR_INTERACTION	KEGG	13469	30	20	2,76E-01	9,96E-01	rs1422909|rs4753598|rs10488600|rs4681446|rs703745|rs233966|rs2638362|rs7912496|rs4868513|rs7635518|rs9342101|rs1417718|rs2808539|rs745013|rs13278849|rs17779442|rs8093607|rs6670616|rs1829651|rs8112063
Discoid Rash	NO2IL12_PATHWAY	BIOCARTA	461	20	15	2,78E-01	9,96E-01	rs2767326|rs12720279|rs1327489|rs11080358|rs2707212|rs1833754|rs281414|rs7572482|rs7250425|rs2995094|rs918519|rs485499|rs2305740|rs924080|rs870875
Discoid Rash	GSK3_PATHWAY	BIOCARTA	1055	40	20	2,79E-01	9,96E-01	rs4976132|rs17326309|rs1490801|rs1360094|rs3916990|rs440804|rs12706724|rs11725638|rs4604517|rs1827153|rs6851442|rs4976028|rs10073996|rs4355511|rs1891442|rs13232987|rs1468242|rs1035153|rs1819328|rs7719763
Discoid Rash	P38MAPK_PATHWAY	BIOCARTA	1904	29	20	2,80E-01	9,96E-01	rs918418|rs700591|rs1499367|rs17199521|rs7175248|rs6860708|rs17047703|rs10494605|rs1845451|rs659732|rs4434808|rs6922031|rs11633249|rs12547643|rs4733859|rs7747353|rs13115100|rs1474797|rs4700008|rs294514
Discoid Rash	DEGRADATION_OF_THE_EXTRACELLULAR_MATRIX	REACTOME	580	35	20	2,81E-01	9,96E-01	rs3025066|rs10132585|rs1276284|rs615098|rs278897|rs9995366|rs11225401|rs4431992|rs1386763|rs7945189|rs10090518|rs6469604|rs519806|rs11643163|rs743257|rs4253331|rs9927309|rs1555322|rs12677791|rs1420228
Discoid Rash	PLATELET_CALCIUM_HOMEOSTASIS	REACTOME	1156	47	20	2,81E-01	9,96E-01	rs997559|rs6799104|rs12475881|rs9309056|rs12459087|rs1425614|rs2430890|rs4142696|rs984707|rs380975|rs403630|rs709641|rs17032984|rs11048447|rs13079701|rs11048495|rs4952447|rs374153|rs11048657|rs10842797
Discoid Rash	TFF_PATHWAY	BIOCARTA	697	26	14	2,82E-01	9,96E-01	rs4976132|rs1490801|rs440804|rs3448|rs4719793|rs4976028|rs10073996|rs1187095|rs7719763|rs10070379|rs10940145|rs2740764|rs4131890|rs1439123
Discoid Rash	KERATAN_SULFATE_BIOSYNTHESIS	REACTOME	674	32	14	2,82E-01	9,96E-01	rs11038519|rs6485628|rs7844620|rs6981912|rs1079022|rs1868431|rs3740702|rs2595371|rs36686|rs7543148|rs6714411|rs1868430|rs2978056|rs7848472
Discoid Rash	PI3K_EVENTS_IN_ERBB4_SIGNALING	REACTOME	1709	32	20	2,83E-01	9,96E-01	rs4976132|rs7574462|rs1490801|rs4236723|rs873418|rs7658842|rs1481624|rs16847102|rs16847122|rs7322396|rs7981045|rs7185040|rs4943769|rs9817|rs10503899|rs12623434|rs2254137|rs4976028|rs10073996|rs1465828
Discoid Rash	ECM_RECEPTOR_INTERACTION	KEGG	3602	31	20	2,84E-01	9,96E-01	rs12213754|rs4729129|rs7564976|rs2326761|rs2212020|rs197721|rs6759316|rs679668|rs3095208|rs1858822|rs1351140|rs16848353|rs9406337|rs10464587|rs7328731|rs747574|rs4298437|rs8027117|rs17182135|rs1981316
Discoid Rash	ACTIVATION_OF_BH3_ONLY_PROTEINS	REACTOME	325	16	6	2,85E-01	9,96E-01	rs6130713|rs1877330|rs8098717|rs954954|rs7242402|rs2849380
Discoid Rash	PYRUVATE_METABOLISM	KEGG	908	31	20	2,85E-01	9,96E-01	rs3752556|rs2736655|rs3763142|rs11788224|rs4836277|rs985773|rs11636141|rs4585672|rs2273828|rs6467993|rs843726|rs10774641|rs2268387|rs17147932|rs722158|rs17138930|rs1898251|rs674965|rs2810716|rs2512994
Discoid Rash	ACUTE_MYELOID_LEUKEMIA	KEGG	1892	33	20	2,86E-01	9,96E-01	rs4976132|rs1490801|rs6719501|rs7322302|rs11884056|rs1499367|rs10749127|rs10838702|rs17199521|rs12547643|rs2834684|rs4733859|rs1990966|rs11725638|rs11675205|rs2242724|rs681200|rs4713859|rs12290032|rs10505507
Discoid Rash	METABOLISM_OF_CARBOHYDRATES	REACTOME	9072	40	20	2,87E-01	9,96E-01	rs4662862|rs4338508|rs2468133|rs2228375|rs3115775|rs7976582|rs6825790|rs7092772|rs10520079|rs2243816|rs11038519|rs12638582|rs2128333|rs9390633|rs6866961|rs1932001|rs10161421|rs4303490|rs7301863|rs2635297
Discoid Rash	GH_PATHWAY	BIOCARTA	963	34	20	2,89E-01	9,96E-01	rs4976132|rs1490801|rs1327489|rs3785383|rs4433842|rs392715|rs7599150|rs7507911|rs2664537|rs12150519|rs4976028|rs10073996|rs17188448|rs8051531|rs11576136|rs1002487|rs11646540|rs17753569|rs17811655|rs6598858
Discoid Rash	CTLA4_INHIBITORY_SIGNALING	REACTOME	447	22	7	2,89E-01	9,96E-01	rs231726|rs8003274|rs1544241|rs6506537|rs10497873|rs2612102|rs610902
Discoid Rash	PPARA_PATHWAY	BIOCARTA	2445	36	20	2,91E-01	9,96E-01	rs1511451|rs4976132|rs1490801|rs2036713|rs12198642|rs11608551|rs2648888|rs1979570|rs10483453|rs17199521|rs9376452|rs3785383|rs12547643|rs11080358|rs17103274|rs4733859|rs4433842|rs392715|rs838875|rs135549
Discoid Rash	ENOS_ACTIVATION_AND_REGULATION	REACTOME	336	6	5	2,92E-01	9,96E-01	rs11767121|rs11621560|rs4804528|rs10961558|rs7595027
Discoid Rash	G_BETA_GAMMA_SIGNALLING_THROUGH_PLC_BETA	REACTOME	624	23	15	2,92E-01	9,96E-01	rs6108160|rs2159917|rs931646|rs11810173|rs2179984|rs6118062|rs6140561|rs6086525|rs765044|rs6039189|rs6055889|rs2873409|rs6955457|rs12905386|rs17755118
Discoid Rash	IGF1MTOR_PATHWAY	BIOCARTA	582	30	16	2,92E-01	9,96E-01	rs4976132|rs1490801|rs7438406|rs873418|rs4976028|rs10073996|rs7166565|rs10794486|rs7719763|rs10007831|rs875511|rs10070379|rs10940145|rs2684777|rs2684790|rs4131890
Discoid Rash	SYNTHESIS_OF_PA	REACTOME	1057	34	20	2,96E-01	9,96E-01	rs6559175|rs2124147|rs10494605|rs659732|rs11127284|rs4434808|rs2341422|rs1474797|rs2912024|rs1559746|rs4955238|rs1980444|rs2838464|rs17028998|rs11099616|rs1148757|rs204990|rs6870197|rs6697553|rs776698
Discoid Rash	TPO_PATHWAY	BIOCARTA	814	37	20	2,96E-01	9,96E-01	rs4976132|rs1490801|rs1327489|rs3785383|rs4433842|rs392715|rs2811876|rs2664537|rs4976028|rs10073996|rs8051531|rs8013053|rs11646540|rs17753569|rs17811655|rs7719763|rs198182|rs10070379|rs6677589|rs17183482
Discoid Rash	GLYCOSPHINGOLIPID_METABOLISM	REACTOME	924	41	20	2,97E-01	9,96E-01	rs7145896|rs11722340|rs6866961|rs17123551|rs672413|rs1413219|rs4904376|rs6008944|rs10508925|rs17820573|rs25413|rs12050431|rs17561000|rs394643|rs766183|rs2008644|rs8015076|rs6586688|rs1617580|rs1642272
Discoid Rash	INSULIN_SYNTHESIS_AND_PROCESSING	REACTOME	1033	34	20	2,98E-01	9,96E-01	rs362988|rs6077718|rs2317222|rs1024175|rs17377483|rs9392155|rs6133821|rs9505014|rs6080616|rs12210050|rs2269044|rs6077680|rs6957026|rs211258|rs6075164|rs12537119|rs11754408|rs17518345|rs9328416|rs35243
Discoid Rash	DAG_AND_IP3_SIGNALING	REACTOME	1644	47	20	2,99E-01	9,96E-01	rs1064448|rs1897104|rs13429751|rs7820461|rs7349397|rs2204204|rs2344662|rs10058673|rs17649232|rs4382132|rs10475376|rs4433842|rs17826588|rs1589880|rs2664537|rs10783282|rs1644512|rs2254137|rs4701770|rs4142696
Discoid Rash	VALINE_LEUCINE_AND_ISOLEUCINE_DEGRADATION	KEGG	1149	59	20	3,00E-01	9,96E-01	rs7856089|rs4963801|rs12529764|rs11788224|rs9343977|rs4836277|rs2247016|rs7335993|rs11725638|rs10842402|rs4585672|rs2273828|rs6851442|rs7136392|rs12930556|rs976145|rs1273422|rs2810716|rs730870|rs480245
Discoid Rash	ALPHA_LINOLENIC_ACID_METABOLISM	KEGG	571	21	11	3,00E-01	9,96E-01	rs10494605|rs659732|rs4434808|rs1474797|rs1712432|rs1980444|rs1148757|rs6697553|rs776691|rs16827441|rs16829110
Discoid Rash	METABOLISM_OF_STEROID_HORMONES_AND_VITAMINS_A_AND_D	REACTOME	745	33	19	3,03E-01	9,96E-01	rs2031321|rs9342101|rs846906|rs879667|rs222014|rs6837549|rs2476923|rs2277603|rs10210408|rs4809958|rs2856448|rs1998615|rs1458928|rs2271463|rs10082248|rs3890520|rs439046|rs1010651|rs2762926
Discoid Rash	PROTEOLYTIC_CLEAVAGE_OF_SNARE_COMPLEX_PROTEINS	REACTOME	382	27	17	3,03E-01	9,96E-01	rs362988|rs6077718|rs7953586|rs17808583|rs4239107|rs6133821|rs2331902|rs6077680|rs3822904|rs218449|rs17518345|rs11870073|rs10848194|rs4896703|rs6108463|rs2632601|rs25680
Discoid Rash	GLEEVEC_PATHWAY	BIOCARTA	954	45	20	3,05E-01	9,96E-01	rs4976132|rs1490801|rs2648888|rs1327489|rs17199521|rs12547643|rs4733859|rs4700008|rs2811876|rs10505507|rs4976028|rs10073996|rs8013053|rs7719763|rs6985681|rs10070379|rs6677589|rs17183482|rs10940145|rs9914858
Discoid Rash	GLUCAGON_SIGNALING_IN_METABOLIC_REGULATION	REACTOME	1044	38	20	3,06E-01	9,96E-01	rs1064448|rs7820461|rs2159917|rs931646|rs10058673|rs4382132|rs11810173|rs10475376|rs6092704|rs17826588|rs1589880|rs10783282|rs4701770|rs965808|rs884423|rs6806529|rs4289537|rs7196832|rs765044|rs4724400
Discoid Rash	MAPK_SIGNALING_PATHWAY	KEGG	9833	25	20	3,07E-01	9,96E-01	rs10835201|rs17072071|rs10047718|rs3934648|rs918418|rs12580224|rs728633|rs7138947|rs4864510|rs11133313|rs6942458|rs12056820|rs700591|rs879221|rs950549|rs13281383|rs10492093|rs12707506|rs4846349|rs1043395
Discoid Rash	COLLAGEN_FORMATION	REACTOME	3203	37	20	3,07E-01	9,96E-01	rs4729129|rs1200562|rs2290519|rs4440614|rs13094190|rs4909444|rs11694554|rs4746912|rs9289822|rs2318340|rs11710718|rs1858822|rs10464587|rs12074848|rs10889285|rs7328731|rs1400240|rs747574|rs10451555|rs9324493
Discoid Rash	G2_PATHWAY	BIOCARTA	354	27	13	3,09E-01	9,96E-01	rs6062356|rs879221|rs2983452|rs799917|rs9817|rs11576136|rs1002487|rs6598858|rs769412|rs7928738|rs699235|rs11585171|rs4035540
Discoid Rash	GLUTATHIONE_METABOLISM	KEGG	683	26	20	3,09E-01	9,96E-01	rs1466202|rs6637|rs12646059|rs3448|rs2274760|rs10505018|rs10874814|rs818167|rs3787016|rs316156|rs7355084|rs2978662|rs6462210|rs2970358|rs10521140|rs1265143|rs11686084|rs3746162|rs2132847|rs3779647
Discoid Rash	TRAF6_MEDIATED_NFKB_ACTIVATION	REACTOME	351	18	10	3,10E-01	9,96E-01	rs9900686|rs10483453|rs17103274|rs4700008|rs204990|rs9550565|rs2839343|rs3136646|rs1056890|rs16886445
Discoid Rash	TOLL_PATHWAY	BIOCARTA	1147	33	20	3,10E-01	9,96E-01	rs17326309|rs1043395|rs10483453|rs1845451|rs1360094|rs17103274|rs7747353|rs956239|rs4700008|rs135549|rs16939964|rs4604517|rs2811876|rs1827153|rs559052|rs100043|rs10485405|rs8013053|rs1891442|rs565665
Discoid Rash	ALANINE_ASPARTATE_AND_GLUTAMATE_METABOLISM	KEGG	841	35	20	3,12E-01	9,96E-01	rs1215985|rs2136703|rs2509713|rs7031579|rs1229564|rs6755814|rs3006009|rs10876891|rs9935162|rs1921915|rs12930556|rs4567871|rs1912445|rs2282219|rs7194392|rs4338916|rs17493602|rs10263979|rs1273422|rs7874077
Discoid Rash	PROPANOATE_METABOLISM	KEGG	1219	38	20	3,13E-01	9,96E-01	rs6789203|rs1326481|rs11788224|rs4836277|rs7335993|rs9534724|rs11636141|rs3818299|rs4832076|rs10774641|rs12930556|rs2268387|rs7337764|rs17138930|rs977103|rs10186197|rs1273422|rs2810716|rs1536002|rs4813547
Discoid Rash	HEMATOPOIETIC_CELL_LINEAGE	KEGG	1963	31	20	3,13E-01	9,96E-01	rs2767326|rs7564976|rs6759316|rs7322302|rs1278150|rs9937676|rs3810168|rs1351140|rs175133|rs216124|rs17042407|rs2275475|rs7072398|rs4927594|rs8102574|rs8108707|rs12722589|rs13120102|rs2707212|rs7899538
Discoid Rash	BCELLSURVIVAL_PATHWAY	BIOCARTA	500	27	16	3,14E-01	9,96E-01	rs4976132|rs1490801|rs1351140|rs965938|rs4976028|rs10073996|rs8013053|rs1187095|rs4808776|rs7719763|rs10070379|rs17183482|rs10940145|rs4131890|rs2126953|rs10940277
Discoid Rash	INTERACTION_BETWEEN_L1_AND_ANKYRINS	REACTOME	970	35	20	3,14E-01	9,96E-01	rs17652604|rs2393610|rs11672523|rs10509119|rs9693071|rs10497292|rs658624|rs10956658|rs11158561|rs7594117|rs7373157|rs7831492|rs10876210|rs2058194|rs521184|rs393569|rs2038442|rs17656487|rs10509123|rs17155074
Discoid Rash	TGF_BETA_RECEPTOR_SIGNALING_IN_EMT_EPITHELIAL_TO_MESENCHYMAL_TRANSITI	REACTOME	429	14	10	3,14E-01	9,96E-01	rs7546890|rs7781554|rs3448|rs2314664|rs1705007|rs10819641|rs1019855|rs7632716|rs2786551|rs2492450
Discoid Rash	SIGNALING_BY_WNT	REACTOME	1071	40	20	3,17E-01	9,96E-01	rs590945|rs2293576|rs11189119|rs8003274|rs667707|rs328331|rs3916990|rs440804|rs2314664|rs12599594|rs371500|rs13394402|rs4355511|rs17760556|rs12463483|rs3731973|rs7212835|rs3751497|rs8005252|rs6966980
Discoid Rash	CELLCYCLE_PATHWAY	BIOCARTA	459	4	4	3,18E-01	9,96E-01	rs3217810|rs10849006|rs2374589|rs3217827
Discoid Rash	PKA_MEDIATED_PHOSPHORYLATION_OF_CREB	REACTOME	767	43	20	3,21E-01	9,96E-01	rs1064448|rs7820461|rs10058673|rs4382132|rs10475376|rs17826588|rs1589880|rs10783282|rs2254137|rs4701770|rs884423|rs6806529|rs4289537|rs7196832|rs4724400|rs11892543|rs9840967|rs4565471|rs7461923|rs13224682
Discoid Rash	ATM_PATHWAY	BIOCARTA	397	33	18	3,23E-01	9,96E-01	rs3765692|rs879221|rs17772583|rs10483453|rs17103274|rs799917|rs9817|rs2811876|rs12003046|rs769412|rs6677589|rs699235|rs11207306|rs11585171|rs535801|rs3765731|rs739719|rs4035540
Discoid Rash	ACTIVATION_OF_THE_MRNA_UPON_BINDING_OF_THE_CAP_BINDING_COMPLEX_AND_EI T_BINDING_TO_43S	REACTOME	780	33	20	3,23E-01	9,96E-01	rs12896325|rs8112063|rs10509398|rs7438406|rs4558175|rs2816764|rs3764536|rs222999|rs2001558|rs4803238|rs36631|rs8016600|rs2277743|rs7894696|rs2574693|rs226206|rs11604561|rs4758038|rs4845360|rs2278498
Discoid Rash	GPVI_MEDIATED_ACTIVATION_CASCADE	REACTOME	1181	36	20	3,23E-01	9,96E-01	rs4976132|rs4729129|rs1490801|rs3922668|rs1858822|rs10464587|rs3448|rs17020055|rs4807101|rs4976028|rs252308|rs10073996|rs4744512|rs4531312|rs1755991|rs1219769|rs1585461|rs5756573|rs166152|rs2501276
Discoid Rash	PEPTIDE_CHAIN_ELONGATION	REACTOME	947	32	20	3,25E-01	9,96E-01	rs2958522|rs12896325|rs10509398|rs7255657|rs3810168|rs4558175|rs2816764|rs2314664|rs3764536|rs2001558|rs9394299|rs4803238|rs36631|rs4738315|rs2012243|rs8016600|rs2277743|rs7894696|rs2574693|rs8179823
Discoid Rash	ACH_PATHWAY	BIOCARTA	602	34	15	3,25E-01	9,96E-01	rs4976132|rs1490801|rs6557991|rs12292911|rs4976028|rs10073996|rs2741343|rs7719763|rs10070379|rs10940145|rs10980549|rs1268160|rs540171|rs7018577|rs4131890
Discoid Rash	AGR_PATHWAY	BIOCARTA	1981	30	20	3,27E-01	9,96E-01	rs12213754|rs6057009|rs2326761|rs4236723|rs1111807|rs1351140|rs1481624|rs12292911|rs3866330|rs935865|rs2811876|rs10503899|rs2992776|rs11697698|rs956429|rs13045381|rs12267783|rs1187095|rs2292354|rs6056969
Discoid Rash	SIGNAL_TRANSDUCTION_BY_L1	REACTOME	1310	25	20	3,28E-01	9,96E-01	rs17115184|rs7916174|rs2212020|rs197721|rs16898963|rs372332|rs1940735|rs4623845|rs1549599|rs2804502|rs12782894|rs4646982|rs2056131|rs2302518|rs3851570|rs12151442|rs4507864|rs11576136|rs1002487|rs1187095
Discoid Rash	STEROID_HORMONES	REACTOME	693	32	18	3,29E-01	9,96E-01	rs2031321|rs9342101|rs846906|rs222014|rs6837549|rs2476923|rs2277603|rs10210408|rs4809958|rs2856448|rs1998615|rs1458928|rs2271463|rs10082248|rs3890520|rs439046|rs1010651|rs2762926
Discoid Rash	3_UTR_MEDIATED_TRANSLATIONAL_REGULATION	REACTOME	1235	31	20	3,30E-01	9,96E-01	rs2958522|rs12896325|rs8112063|rs4554825|rs7255657|rs7438406|rs3810168|rs4558175|rs2816764|rs2314664|rs3764536|rs222999|rs2001558|rs9394299|rs4803238|rs36631|rs4738315|rs2012243|rs8016600|rs2277743

Discoid Rash	G1_PATHWAY	BIOCARTA	611	25	12	3,30E-01	9,96E-01	rs1992215|rs12102171|rs17047703|rs4368250|rs4776862|rs12003046|rs12029576|rs13160147|rs2268626|rs2374589|rs7359174|rs2965015
Discoid Rash	CREB_PATHWAY	BIOCARTA	969	32	20	3,30E-01	9,96E-01	rs4976132|rs1490801|rs2241694|rs3785383|rs4433842|rs6092704|rs1589880|rs392715|rs1011973|rs2254137|rs4976028|rs10073996|rs8051531|rs965808|rs11576136|rs1002487|rs11646540|rs17753569|rs17811655|rs6598858
Discoid Rash	LATENT_INFECTION_OF_HOMO_SAPIENS_WITH_MYCOBACTERIUM_TUBERCULOSIS	REACTOME	589	18	15	3,30E-01	9,96E-01	rs2041689|rs816296|rs10982300|rs11080358|rs2650163|rs11783925|rs11769043|rs2682826|rs12670097|rs4683236|rs4732330|rs10954211|rs4979427|rs2454039|rs3856465
Discoid Rash	REGULATION_OF_BETA_CELL_DEVELOPMENT	REACTOME	1288	34	20	3,32E-01	9,96E-01	rs17626394|rs719714|rs1429447|rs6788164|rs265451|rs8024399|rs958425|rs10504605|rs7322396|rs7981045|rs4943769|rs2926615|rs9512906|rs6035613|rs1562147|rs1572579|rs6017335|rs7272694|rs1875343|rs7822381
Discoid Rash	TYPE_I_DIABETES_MELLITUS	KEGG	1975	48	20	3,32E-01	9,96E-01	rs3094691|rs7755852|rs10234067|rs870020|rs9654713|rs1611350|rs17042407|rs3104404|rs881468|rs3130473|rs6755814|rs9276284|rs11591298|rs2245548|rs762084|rs12111032|rs1833754|rs9295986|rs12670364|rs28383344
Discoid Rash	REGULATION_OF_SIGNALING_BY_CBL	REACTOME	703	34	20	3,35E-01	9,96E-01	rs4976132|rs1490801|rs1544241|rs11217175|rs10793894|rs6121337|rs4807101|rs4976028|rs10073996|rs4744512|rs1755991|rs1585461|rs7911684|rs693293|rs7719763|rs6506537|rs7258040|rs10070379|rs10940145|rs12610223
Discoid Rash	G_ALPHA_Q_SIGNALLING_EVENTS	REACTOME	7428	45	20	3,36E-01	9,96E-01	rs10486008|rs4681446|rs6108160|rs4976132|rs233966|rs2638362|rs6797559|rs1490801|rs13278849|rs13410673|rs7871222|rs8112063|rs2479982|rs7349397|rs945032|rs4692540|rs3796974|rs13171967|rs2159917|rs7810652
Discoid Rash	ANTIGEN_PROCESSING_CROSS_PRESENTATION	REACTOME	1601	56	20	3,36E-01	9,96E-01	rs3094691|rs2293576|rs7755852|rs1611350|rs328331|rs12267330|rs3130473|rs2314664|rs12599594|rs371500|rs13394402|rs12111032|rs12151442|rs9295986|rs13191519|rs1736956|rs2844575|rs9266018|rs6457109|rs2075221
Discoid Rash	HDAC_PATHWAY	BIOCARTA	1231	37	20	3,36E-01	9,96E-01	rs4976132|rs918418|rs1490801|rs700591|rs7175248|rs6860708|rs11633249|rs2356932|rs7507911|rs9608247|rs4976028|rs10073996|rs8041078|rs100043|rs247587|rs11958574|rs7166565|rs10794486|rs4478191|rs4844477
Discoid Rash	TRYPTOPHAN_METABOLISM	KEGG	953	51	20	3,38E-01	9,96E-01	rs1581676|rs6719977|rs11788224|rs4836277|rs13400749|rs2247016|rs11725638|rs9808531|rs13020622|rs2273828|rs6851442|rs2206716|rs6676647|rs10247064|rs710887|rs2810716|rs1800440|rs7699981|rs13275833|rs11685496
Discoid Rash	VOLTAGE_GATED_POTASSIUM_CHANNELS	REACTOME	2491	34	20	3,39E-01	9,96E-01	rs1564372|rs17652604|rs4930007|rs2471637|rs4254605|rs2283214|rs500677|rs4727899|rs2365669|rs8013313|rs2283194|rs9693071|rs10863856|rs13426554|rs326736|rs709688|rs10956658|rs237485|rs1155593|rs2793476
Discoid Rash	CHYLOMICRON_MEDIATED_LIPID_TRANSPORT	REACTOME	435	42	20	3,39E-01	9,96E-01	rs1519491|rs2176472|rs157580|rs7677213|rs6494005|rs4665639|rs6598863|rs7544500|rs1503777|rs4654986|rs17042892|rs3924843|rs9644636|rs1968687|rs264|rs16940372|rs6714773|rs908679|rs2228671|rs7588415
Discoid Rash	GLYCOLYSIS	REACTOME	558	19	13	3,40E-01	9,96E-01	rs7092772|rs2243816|rs1932001|rs12240387|rs10508243|rs6682376|rs7071157|rs9664019|rs884289|rs2075863|rs9664762|rs10905856|rs12357621
Discoid Rash	TGFB_PATHWAY	BIOCARTA	817	38	20	3,41E-01	9,96E-01	rs1992215|rs1043395|rs12102171|rs17047703|rs11661651|rs1845451|rs7747353|rs4368250|rs4776862|rs4355511|rs12029576|rs10485405|rs2268626|rs10819641|rs565665|rs1019855|rs7632716|rs1114328|rs284509|rs7359174
Discoid Rash	ACTIVATION_OF_THE_PRE_REPLICATIVE_COMPLEX	REACTOME	565	20	11	3,43E-01	9,96E-01	rs7916990|rs1535010|rs495026|rs9396464|rs10508455|rs10146912|rs2891652|rs10948689|rs3782093|rs747232|rs11258231
Discoid Rash	CITRATE_CYCLE_TCA_CYCLE	KEGG	1195	37	20	3,46E-01	9,96E-01	rs6789203|rs1326481|rs12065184|rs9534724|rs12238815|rs9905601|rs4585672|rs6467993|rs4832076|rs17147932|rs7337764|rs9611598|rs10247064|rs1898251|rs977103|rs10186197|rs710887|rs6532592|rs1536002|rs2970358
Discoid Rash	ACTIVATED_NOTCH1_TRANSMITS_SIGNAL_TO_THE_NUCLEUS	REACTOME	719	33	19	3,47E-01	9,96E-01	rs2179688|rs10139403|rs4923889|rs6732014|rs17762822|rs2314664|rs9905578|rs3787051|rs6040055|rs1888578|rs6721342|rs13394622|rs6133987|rs6108803|rs10916066|rs4710755|rs173003|rs2011781|rs12590932
Discoid Rash	NCAM_SIGNALING_FOR_NEURITE_OUT_GROWTH	REACTOME	2480	36	20	3,50E-01	9,96E-01	rs4729129|rs17115184|rs9455013|rs6130713|rs11694554|rs4815715|rs11012843|rs1858822|rs10464587|rs2240220|rs11672523|rs434222|rs10889285|rs7328731|rs10451555|rs6722598|rs1940735|rs1981316|rs11158561|rs631428
Discoid Rash	LEISHMANIA_INFECTION	KEGG	2265	34	20	3,50E-01	9,96E-01	rs2834219|rs7564976|rs1043395|rs1327489|rs10483453|rs1532|rs17047703|rs1845451|rs17042407|rs1360094|rs3785383|rs3104404|rs11080358|rs17103274|rs7747353|rs9276284|rs392715|rs4604517|rs2811876|rs235370
Discoid Rash	NFKB_PATHWAY	BIOCARTA	870	37	20	3,52E-01	9,96E-01	rs1043395|rs10483453|rs1845451|rs17042407|rs1928298|rs17103274|rs7747353|rs4700008|rs16939964|rs4604517|rs496888|rs10485405|rs13035227|rs1891442|rs1545092|rs565665|rs1819328|rs1114328|rs10984022|rs7216796
Discoid Rash	ANTIGEN_PROCESSING_AND_PRESENTATION	KEGG	1754	65	20	3,53E-01	9,96E-01	rs3094691|rs7755852|rs1611350|rs3104404|rs709688|rs3130473|rs9276284|rs4977686|rs11208830|rs2254137|rs2707212|rs4781016|rs12111032|rs9295986|rs11621560|rs28383344|rs13191519|rs1736956|rs3129882|rs2844575
Discoid Rash	GABA_RECEPTOR_ACTIVATION	REACTOME	2705	30	20	3,55E-01	9,96E-01	rs1064448|rs2808539|rs745013|rs17779442|rs7820461|rs2190550|rs2159917|rs10515829|rs3025646|rs8094168|rs4238490|rs931646|rs10058673|rs4382132|rs1540011|rs8081911|rs11810173|rs10475376|rs12706724|rs17826588
Discoid Rash	CD28_DEPENDENT_PI3K_AKT_SIGNALING	REACTOME	572	23	12	3,61E-01	9,96E-01	rs4976132|rs1490801|rs16939964|rs4976028|rs10073996|rs7719763|rs10070379|rs7216796|rs10940145|rs2489863|rs4131890|rs610902
Discoid Rash	INWARDLY_RECTIFYING_K_CHANNELS	REACTOME	1317	29	20	3,61E-01	9,96E-01	rs2808539|rs17779442|rs2190550|rs2159917|rs3025646|rs931646|rs1540011|rs8081911|rs11810173|rs13000959|rs2154552|rs529269|rs1321649|rs6478676|rs6517442|rs17178083|rs1860316|rs8069222|rs11601431|rs10512554
Discoid Rash	P75_NTR_RECEPTOR_MEDIATED_SIGNALLING	REACTOME	2669	32	20	3,61E-01	9,96E-01	rs2140825|rs2833293|rs6797559|rs17540656|rs9647067|rs4918129|rs538957|rs10483453|rs4598601|rs1007429|rs17762822|rs17103274|rs965938|rs11678490|rs3448|rs11812127|rs580004|rs2314664|rs7908373|rs1877330
Discoid Rash	TRAFFICKING_OF_AMPA_RECEPTORS	REACTOME	1132	29	20	3,63E-01	9,96E-01	rs4821506|rs2050140|rs17036351|rs2241694|rs3785383|rs372332|rs4433842|rs392715|rs9817|rs1461234|rs17036576|rs4502882|rs2283556|rs1011973|rs6453850|rs11746889|rs1940964|rs2302518|rs8051531|rs1000014
Discoid Rash	EPITHELIAL_CELL_SIGNALING_IN_HELICOBACTER_PYLORI_INFECTION	KEGG	1535	37	20	3,64E-01	9,96E-01	rs4779683|rs7546890|rs11223702|rs2041689|rs10483453|rs1357386|rs10982300|rs12916064|rs17103274|rs965938|rs11783925|rs16939964|rs11769043|rs2811876|rs2664537|rs12647506|rs3775167|rs12670097|rs2859129|rs4732330
Discoid Rash	LINOLEIC_ACID_METABOLISM	KEGG	716	26	15	3,64E-01	9,96E-01	rs10494605|rs659732|rs4434808|rs11773597|rs1474797|rs1712432|rs10493270|rs1980444|rs1148757|rs6697553|rs915907|rs776691|rs16827441|rs11572172|rs16829110
Discoid Rash	41BB_PATHWAY	BIOCARTA	341	19	9	3,66E-01	9,96E-01	rs10483453|rs6922031|rs17103274|rs4700008|rs2811876|rs17654215|rs6677589|rs11207306|rs16886445
Discoid Rash	KERATAN_SULFATE_KERATIN_METABOLISM	REACTOME	737	33	15	3,67E-01	9,96E-01	rs11038519|rs6485628|rs7844620|rs6981912|rs1079022|rs1868431|rs3740702|rs2595371|rs36686|rs7543148|rs6714411|rs17561000|rs1868430|rs2978056|rs7848472
Discoid Rash	PLATELET_SENSITIZATION_BY_LDL	REACTOME	676	48	20	3,68E-01	9,96E-01	rs1519491|rs2176472|rs8003274|rs10494605|rs659732|rs4434808|rs4665639|rs1474797|rs1980444|rs1148757|rs6697553|rs17042892|rs16827441|rs4908343|rs6714773|rs908679|rs7588415|rs679899|rs16829110|rs17564408
Discoid Rash	REGULATION_OF_IFNA_SIGNALING	REACTOME	339	29	6	3,68E-01	9,96E-01	rs12720279|rs4977686|rs281414|rs6512654|rs4977682|rs1810503
Discoid Rash	POST_CHAPERONIN_TUBULIN_FOLDING_PATHWAY	REACTOME	254	1	1	3,68E-01	9,96E-01	rs10875943
Discoid Rash	TRANSPORT_OF_MATURE_TRANSCRIPT_TO_CYTOPLASM	REACTOME	693	31	17	3,71E-01	9,96E-01	rs2228375|rs7438406|rs217797|rs4740420|rs12586783|rs11177355|rs2018928|rs11249163|rs2016266|rs7782920|rs10271506|rs10007831|rs11249167|rs13431263|rs11887079|rs857587|rs4947058
Discoid Rash	MELANOGENESIS	KEGG	4077	37	20	3,72E-01	9,96E-01	rs2744738|rs1064448|rs6108160|rs2744728|rs652673|rs7517829|rs7820461|rs17360053|rs6719501|rs11884056|rs7542242|rs10749127|rs2241694|rs3785383|rs10058673|rs10738290|rs4382132|rs7863513|rs440804|rs10475376
Discoid Rash	G_BETA_GAMMA_SIGNALLING_THROUGH_PI3KGAMMA	REACTOME	487	11	8	3,72E-01	9,96E-01	rs2159917|rs931646|rs11810173|rs3448|rs765044|rs2873409|rs6955457|rs17755118
Discoid Rash	FOCAL_ADHESION	KEGG	7733	32	20	3,73E-01	9,96E-01	rs12213754|rs4976132|rs11137533|rs4729129|rs7564976|rs6057009|rs4864510|rs3217810|rs1490801|rs11133313|rs2326761|rs2212020|rs197721|rs6759316|rs679668|rs10517655|rs873418|rs3095208|rs1858822|rs1351140
Discoid Rash	AMI_PATHWAY	BIOCARTA	574	32	13	3,76E-01	9,96E-01	rs11694554|rs7328731|rs10451555|rs6722598|rs1981316|rs631428|rs595325|rs4255644|rs9521585|rs2141831|rs4773155|rs9521666|rs6723547
Discoid Rash	PI3K_EVENTS_IN_ERBB2_SIGNALING	REACTOME	1886	32	20	3,76E-01	9,96E-01	rs4976132|rs7574462|rs1490801|rs4236723|rs873418|rs7658842|rs1481624|rs16847102|rs16847122|rs7322396|rs7981045|rs7185040|rs4943769|rs9817|rs10503899|rs12623434|rs2254137|rs4976028|rs10073996|rs1465828
Discoid Rash	NFKB_AND_MAP_KINASES_ACTIVATION_MEDIATED_BY_TLR4_SIGNALING_REPERTOIRE	REACTOME	2248	26	20	3,77E-01	9,96E-01	rs918418|rs17326309|rs700591|rs13281383|rs1043395|rs10483453|rs16898963|rs7175248|rs6860708|rs1845451|rs1928298|rs11633249|rs17103274|rs7747353|rs7823751|rs956239|rs2314664|rs4604517|rs2052834|rs2811876
Discoid Rash	RELA_PATHWAY	BIOCARTA	208	6	5	3,79E-01	9,96E-01	rs10483453|rs17103274|rs496888|rs11741808|rs986475
Discoid Rash	FRUCTOSE_AND_MANNOSE_METABOLISM	KEGG	984	46	20	3,80E-01	9,96E-01	rs7092772|rs9391934|rs2243816|rs1004779|rs7983595|rs10163323|rs600130|rs12240387|rs548348|rs10508243|rs1000251|rs9405506|rs6909392|rs7071157|rs12343547|rs9664019|rs884289|rs2075863|rs3800064|rs234953
Discoid Rash	SIGNALING_BY_ERBB2	REACTOME	3847	37	20	3,82E-01	9,96E-01	rs1064448|rs4976132|rs7574462|rs1490801|rs1897104|rs13429751|rs6130713|rs7820461|rs7349397|rs4236723|rs873418|rs2204204|rs2344662|rs7658842|rs1481624|rs16847102|rs1544241|rs10058673|rs17649232|rs16847122
Discoid Rash	BOTULINUM_NEUROTOXICITY	REACTOME	522	34	20	3,82E-01	9,96E-01	rs362988|rs6077718|rs7953586|rs17808583|rs4083221|rs4239107|rs6133821|rs2331902|rs6077680|rs3822904|rs218449|rs17518345|rs11870073|rs10848194|rs10800842|rs4896703|rs6108463|rs2632601|rs25680|rs10778256
Discoid Rash	ALLOGRAFT_REJECTION	KEGG	1510	61	20	3,84E-01	9,96E-01	rs3094691|rs7755852|rs1611350|rs3104404|rs3130473|rs9276284|rs11591298|rs2245548|rs762084|rs12111032|rs1833754|rs9295986|rs28383344|rs13191519|rs1736956|rs3129882|rs1937332|rs2844575|rs9266018|rs6457109
Discoid Rash	VEGF_PATHWAY	BIOCARTA	883	39	20	3,85E-01	9,96E-01	rs4976132|rs1490801|rs3785383|rs4433842|rs307805|rs392715|rs722503|rs7324547|rs2664537|rs4976028|rs10073996|rs8051531|rs11646540|rs17753569|rs17811655|rs4767884|rs7719763|rs198182|rs10070379|rs10940145
Discoid Rash	PROSTACYCLIN_SIGNALLING_THROUGH_PROSTACYCLIN_RECEPTOR	REACTOME	353	13	9	3,86E-01	9,96E-01	rs2159917|rs931646|rs11810173|rs6092704|rs965808|rs765044|rs2873409|rs6955457|rs17755118
Discoid Rash	MTA3_PATHWAY	BIOCARTA	333	29	12	3,88E-01	9,96E-01	rs16938992|rs6719977|rs851982|rs17081685|rs10875943|rs2813554|rs7450824|rs2485209|rs2504070|rs2278583|rs7252741|rs3020418
Discoid Rash	PAR1_PATHWAY	BIOCARTA	1941	36	20	3,89E-01	9,96E-01	rs6108160|rs4976132|rs1490801|rs8069620|rs6557991|rs1845451|rs3785383|rs7747353|rs4433842|rs12706724|rs2179984|rs3448|rs1589880|rs392715|rs9588385|rs17288929|rs6118062|rs6140561|rs6792241|rs4976028
Discoid Rash	COLORECTAL_CANCER	KEGG	2539	32	20	3,89E-01	9,96E-01	rs4976132|rs1992215|rs1490801|rs6719501|rs11884056|rs2648888|rs10749127|rs12102171|rs12327358|rs17199521|rs17047703|rs11661651|rs9950125|rs12547643|rs4733859|rs965938|rs11875845|rs3916990|rs440804|rs11725638
Discoid Rash	INTERFERON_ALPHA_BETA_SIGNALING	REACTOME	1477	57	20	3,89E-01	9,96E-01	rs3094691|rs486907|rs12720279|rs7755852|rs1611350|rs11865882|rs3130473|rs4977686|rs1532471|rs12886562|rs12111032|rs281414|rs9295986|rs793801|rs13191519|rs1736956|rs6512654|rs4079379|rs9430018|rs2844575
Discoid Rash	ARAP_PATHWAY	BIOCARTA	292	14	7	3,90E-01	9,96E-01	rs5756581|rs738532|rs2666219|rs7703742|rs1890060|rs933241|rs11887079
Discoid Rash	G_PROTEIN_BETA_GAMMA_SIGNALLING	REACTOME	798	24	16	3,91E-01	9,96E-01	rs6108160|rs2159917|rs931646|rs11810173|rs3448|rs2179984|rs6118062|rs6140561|rs6086525|rs765044|rs6039189|rs6055889|rs2873409|rs6955457|rs12905386|rs17755118
Discoid Rash	IL2RB_PATHWAY	BIOCARTA	1296	30	20	3,92E-01	9,96E-01	rs4976132|rs1490801|rs2648888|rs17199521|rs12547643|rs4733859|rs11217175|rs7599150|rs11591298|rs2245548|rs7072398|rs10505507|rs12722589|rs36686|rs4976028|rs954954|rs10073996|rs7899538|rs17188448|rs4744512
Discoid Rash	SHC1_EVENTS_IN_ERBB4_SIGNALING	REACTOME	1072	61	20	3,94E-01	9,96E-01	rs7574462|rs6130713|rs4236723|rs7658842|rs1481624|rs16847102|rs16847122|rs10503899|rs12623434|rs1465828|rs6435604|rs6435670|rs6435689|rs2976532|rs16846100|rs12162287|rs10185729|rs1836729|rs10105518|rs4963872
Discoid Rash	CCR3_PATHWAY	BIOCARTA	939	27	20	3,97E-01	9,96E-01	rs6108160|rs3785383|rs4433842|rs6092704|rs3448|rs2179984|rs392715|rs7971352|rs6118062|rs6140561|rs537885|rs8051531|rs965808|rs11646540|rs6086525|rs17753569|rs17811655|rs6039189|rs6055889|rs3815341
Discoid Rash	SIGNALING_BY_FGFR1_FUSION_MUTANTS	REACTOME	639	29	17	3,98E-01	9,96E-01	rs4976132|rs1490801|rs2031283|rs410187|rs2664537|rs4976028|rs10073996|rs12305434|rs2450132|rs912410|rs11765661|rs7719763|rs10070379|rs7324038|rs10940145|rs4131890|rs13231090
Discoid Rash	INTEGRATION_OF_PROVIRUS	REACTOME	122	6	2	4,00E-01	9,96E-01	rs1325382|rs4724321
Discoid Rash	SRP_DEPENDENT_COTRANSLATIONAL_PROTEIN_TARGETING_TO_MEMBRANE	REACTOME	1378	32	20	4,09E-01	9,96E-01	rs2958522|rs12896325|rs4254605|rs4554825|rs7255657|rs3810168|rs4558175|rs12267330|rs2816764|rs1551368|rs1358024|rs2314664|rs3764536|rs2001558|rs9394299|rs4803238|rs36631|rs4738315|rs2012243|rs8016600
Discoid Rash	VASCULAR_SMOOTH_MUSCLE_CONTRACTION	KEGG	5845	49	20	4,09E-01	9,96E-01	rs304051|rs4681446|rs1064448|rs6108160|rs2638362|rs158205|rs13278849|rs9844737|rs7820461|rs7349397|rs901849|rs13171967|rs2239016|rs2204204|rs2344662|rs1108385|rs10494605|rs931646|rs659732|rs4434808
Discoid Rash	ACYL_CHAIN_REMODELLING_OF_PG	REACTOME	571	27	14	4,09E-01	9,96E-01	rs10494605|rs659732|rs4434808|rs6680326|rs1474797|rs1980444|rs1148757|rs6870197|rs6697553|rs776698|rs776691|rs16827441|rs666511|rs16829110
Discoid Rash	PENTOSE_AND_GLUCURONATE_INTERCONVERSIONS	KEGG	874	45	10	4,10E-01	9,96E-01	rs10028494|rs9315608|rs1458226|rs294755|rs6848997|rs2270941|rs17475847|rs293408|rs4770035|rs7336556
Discoid Rash	GASTRIN_CREB_SIGNALLING_PATHWAY_VIA_PKC_AND_MAPK	REACTOME	8122	45	20	4,10E-01	9,96E-01	rs10486008|rs4681446|rs6108160|rs4976132|rs233966|rs2638362|rs6797559|rs1490801|rs13278849|rs13410673|rs7871222|rs8112063|rs6130713|rs2479982|rs7349397|rs945032|rs4692540|rs3796974|rs13171967|rs2159917
Discoid Rash	NEGATIVE_REGULATORS_OF_RIG_I_MDA5_SIGNALING	REACTOME	464	16	11	4,12E-01	9,96E-01	rs9900686|rs4785452|rs11217175|rs2314664|rs12130290|rs730554|rs1545092|rs1539243|rs12229321|rs4906269|rs10782267
Discoid Rash	MICRORNA_MIRNA_BIOGENESIS	REACTOME	227	27	10	4,12E-01	9,96E-01	rs7160403|rs11089328|rs12186785|rs12878043|rs10071110|rs17408227|rs3787016|rs12897280|rs3746162|rs11746052
Discoid Rash	SIGNALING_BY_ILS	REACTOME	2996	27	20	4,12E-01	9,96E-01	rs590945|rs4976132|rs1490801|rs6130713|rs12720279|rs1043395|rs1278150|rs1327489|rs6557991|rs5745752|rs1845451|rs667707|rs329499|rs17042407|rs3019260|rs1544241|rs7747353|rs2698537|rs11217175|rs10200259
Discoid Rash	HCMV_PATHWAY	BIOCARTA	547	36	15	4,13E-01	9,96E-01	rs4976132|rs1490801|rs4700008|rs2254137|rs4976028|rs10073996|rs100043|rs11892543|rs7719763|rs10070379|rs10940145|rs2715815|rs17202235|rs4131890|rs16886445
Discoid Rash	GLUCONEOGENESIS	REACTOME	490	42	11	4,14E-01	9,96E-01	rs1932001|rs600130|rs548348|rs6467993|rs9935162|rs7196146|rs17147932|rs6725388|rs6682376|rs12343547|rs13912
Discoid Rash	ETS_PATHWAY	BIOCARTA	682	34	20	4,14E-01	9,96E-01	rs11608551|rs1477577|rs946577|rs2303091|rs216124|rs3746882|rs838875|rs2811876|rs837948|rs8013053|rs500207|rs2836722|rs12417930|rs13188584|rs2836774|rs6677589|rs17183482|rs919751|rs948254|rs11207306
Discoid Rash	NEF_MEDIATES_DOWN_MODULATION_OF_CELL_SURFACE_RECEPTORS_BY_RECRUITIN<br>N_ADAPTERS	REACTOME	366	21	11	4,17E-01	9,96E-01	rs401618|rs7203231|rs372332|rs11783925|rs11075904|rs2707212|rs2302518|rs2394250|rs312181|rs6457109|rs9259650
Discoid Rash	EXTRACELLULAR_MATRIX_ORGANIZATION	REACTOME	3783	38	20	4,17E-01	9,96E-01	rs4729129|rs1200562|rs2290519|rs4440614|rs13094190|rs4909444|rs11694554|rs4746912|rs9289822|rs2318340|rs11710718|rs3025066|rs1858822|rs10132585|rs10464587|rs12074848|rs2846707|rs10889285|rs7328731|rs615098
Discoid Rash	ACTIVATION_OF_NF_KAPPAB_IN_B_CELLS	REACTOME	1396	31	20	4,18E-01	9,96E-01	rs590945|rs2293576|rs10483453|rs1845451|rs667707|rs328331|rs3785383|rs17103274|rs7747353|rs392715|rs2314664|rs12599594|rs371500|rs13394402|rs8051531|rs10485405|rs11646540|rs17753569|rs17811655|rs565665
Discoid Rash	GLYCOSAMINOGLYCAN_DEGRADATION	KEGG	709	22	11	4,19E-01	9,96E-01	rs6866961|rs672413|rs3826061|rs10254660|rs8080388|rs25413|rs7214164|rs17561000|rs8082455|rs1860124|rs17818103
Discoid Rash	FORMATION_OF_THE_TERNARY_COMPLEX_AND_SUBSEQUENTLY_THE_43S_COMPLEX	REACTOME	677	35	20	4,23E-01	9,96E-01	rs12896325|rs8112063|rs4554825|rs4558175|rs2816764|rs3764536|rs2001558|rs4803238|rs36631|rs8016600|rs2277743|rs7894696|rs2574693|rs226206|rs11604561|rs4758038|rs2278498|rs4845360|rs4807928|rs494469
Discoid Rash	ERBB_SIGNALING_PATHWAY	KEGG	4135	40	20	4,24E-01	9,96E-01	rs4976132|rs11137533|rs7574462|rs17031463|rs6057009|rs1490801|rs7642557|rs10933849|rs1499367|rs4236723|rs7658842|rs1481624|rs1445059|rs17199521|rs11681491|rs16847102|rs2241694|rs3785383|rs12547643|rs4733859
Discoid Rash	TRAFFICKING_OF_GLUR2_CONTAINING_AMPA_RECEPTORS	REACTOME	721	34	20	4,24E-01	9,96E-01	rs17036351|rs3785383|rs372332|rs4433842|rs392715|rs1461234|rs17036576|rs4502882|rs11746889|rs1940964|rs2302518|rs8051531|rs11953799|rs11646540|rs12417624|rs17753569|rs17811655|rs312181|rs2076371|rs198182
Discoid Rash	CYTOKINE_CYTOKINE_RECEPTOR_INTERACTION	KEGG	6425	32	20	4,29E-01	9,96E-01	rs2834219|rs12602195|rs16834223|rs1417718|rs10489273|rs4864510|rs724055|rs11133313|rs7626849|rs7322302|rs1278150|rs9559300|rs17262993|rs8177732|rs913069|rs7717673|rs10517655|rs2138992|rs6054474|rs4280293
Discoid Rash	PLC_BETA_MEDIATED_EVENTS	REACTOME	2313	39	20	4,30E-01	9,96E-01	rs1064448|rs6108160|rs1897104|rs13429751|rs7820461|rs10494605|rs659732|rs4434808|rs10058673|rs17649232|rs4382132|rs10475376|rs4433842|rs12706724|rs17826588|rs2179984|rs1589880|rs1474797|rs6118062|rs10783282
Discoid Rash	PROTEASOME_PATHWAY	BIOCARTA	280	14	10	4,32E-01	9,96E-01	rs2293576|rs2189713|rs371500|rs757439|rs12463483|rs1977035|rs7212835|rs3751497|rs8005252|rs12903486
Discoid Rash	PPAR_SIGNALING_PATHWAY	KEGG	1324	34	20	4,33E-01	9,96E-01	rs1151996|rs1110060|rs3851422|rs7742826|rs12985511|rs2031766|rs4431992|rs135549|rs4713859|rs7945189|rs10846765|rs11039149|rs1930788|rs247011|rs854151|rs10510419|rs519806|rs11666579|rs2286814|rs876873
Discoid Rash	ENDOCYTOSIS	KEGG	6484	37	20	4,34E-01	9,96E-01	rs2140825|rs3094691|rs16834223|rs1027803|rs7574462|rs4864510|rs11133313|rs7642557|rs7755852|rs10933849|rs2432216|rs7216490|rs6998100|rs178844|rs11709925|rs1611350|rs2124147|rs659396|rs16834018|rs2267568
Discoid Rash	GCR_PATHWAY	BIOCARTA	736	38	19	4,34E-01	9,96E-01	rs4976132|rs1490801|rs7871222|rs6092704|rs2039979|rs4976028|rs10073996|rs11959113|rs965808|rs11621560|rs7034411|rs7719763|rs10070379|rs2351791|rs10940145|rs4912652|rs7595027|rs4131890|rs4744678
Discoid Rash	TCR_PATHWAY	BIOCARTA	1517	36	20	4,36E-01	9,96E-01	rs4976132|rs3934648|rs1490801|rs3922668|rs10483453|rs3785383|rs17103274|rs4433842|rs392715|rs4700008|rs2811876|rs2664537|rs4807101|rs4976028|rs252308|rs10073996|rs8051531|rs12069950|rs8013053|rs11646540
Discoid Rash	HOMOLOGOUS_RECOMBINATION_REPAIR_OF_REPLICATION_INDEPENDENT_DOUBLE_ST	REACTOME	376	16	6	4,42E-01	9,96E-01	rs17772583|rs799917|rs4802436|rs1784304|rs535801|rs739719
Discoid Rash	TRANSLATION	REACTOME	1803	31	20	4,44E-01	9,96E-01	rs2958522|rs12896325|rs8112063|rs4254605|rs4554825|rs7255657|rs7438406|rs3810168|rs4558175|rs12267330|rs2816764|rs1551368|rs1358024|rs2314664|rs3764536|rs222999|rs2001558|rs9394299|rs4803238|rs36631
Discoid Rash	CTLA4_PATHWAY	BIOCARTA	662	40	20	4,45E-01	9,96E-01	rs4976132|rs1490801|rs231726|rs743479|rs4976028|rs10073996|rs6710279|rs12716251|rs2858870|rs3129882|rs13420393|rs7719763|rs10497873|rs10070379|rs2995094|rs11571312|rs25856|rs10940145|rs11066323|rs11066320
Discoid Rash	TYPE_II_DIABETES_MELLITUS	KEGG	2053	34	20	4,46E-01	9,96E-01	rs1577452|rs4976132|rs1490801|rs7349397|rs2239016|rs1120559|rs2204204|rs2344662|rs1108385|rs2240220|rs11062222|rs7599150|rs1006564|rs336230|rs7507911|rs9512906|rs12150519|rs1000251|rs4976028|rs10073996
Discoid Rash	INTRINSIC_PATHWAY_FOR_APOPTOSIS	REACTOME	532	22	12	4,48E-01	9,96E-01	rs6130713|rs965938|rs1877330|rs4719793|rs8098717|rs954954|rs762084|rs2285427|rs2270941|rs7242402|rs2849380|rs1439123
Discoid Rash	PROSTATE_CANCER	KEGG	2870	30	20	4,50E-01	9,96E-01	rs4976132|rs4864510|rs1490801|rs11133313|rs6719501|rs1544470|rs11884056|rs10517655|rs10749127|rs873418|rs10483453|rs11681491|rs749145|rs17103274|rs7322396|rs7981045|rs440804|rs11725638|rs4943769|rs11675205
Discoid Rash	MAP_KINASE_ACTIVATION_IN_TLR_CASCADE	REACTOME	1697	31	20	4,53E-01	9,96E-01	rs918418|rs700591|rs13281383|rs1043395|rs16898963|rs7175248|rs6860708|rs1845451|rs11633249|rs7747353|rs7823751|rs2811876|rs2254137|rs10494878|rs559052|rs8041078|rs6854208|rs100043|rs247587|rs11576136
Discoid Rash	NTHI_PATHWAY	BIOCARTA	876	35	20	4,54E-01	9,96E-01	rs1992215|rs12102171|rs10483453|rs1845451|rs17103274|rs7747353|rs16939964|rs4368250|rs4776862|rs100043|rs10485405|rs2112661|rs11746792|rs4849123|rs10819641|rs565665|rs1019855|rs7632716|rs1114328|rs7216796

Discoid Rash	GLIOMA	KEGG	2070	46	20	4,55E-01	9,96E-01	rs4976132|rs11137533|rs4864510|rs1490801|rs11133313|rs873418|rs11681491|rs2241694|rs3785383|rs4433842|rs1007662|rs392715|rs9817|rs2664537|rs1808416|rs1011973|rs4976028|rs10073996|rs11253227|rs8051531
Discoid Rash	MYD88_MAL_CASCADE_INITIATED_ON_PLASMA_MEMBRANE	REACTOME	2527	24	20	4,57E-01	9,96E-01	rs918418|rs17326309|rs700591|rs13281383|rs1043395|rs10483453|rs16898963|rs7175248|rs6860708|rs1845451|rs329499|rs1360094|rs11633249|rs17103274|rs7747353|rs2698537|rs7823751|rs2314664|rs4700008|rs10200259
Discoid Rash	EPO_PATHWAY	BIOCARTA	255	12	8	4,58E-01	9,96E-01	rs1327489|rs2811876|rs2664537|rs8108707|rs8013053|rs6677589|rs17183482|rs11207306
Discoid Rash	STEROID_HORMONE_BIOSYNTHESIS	KEGG	1491	56	20	4,59E-01	9,96E-01	rs2031321|rs10028494|rs846906|rs9886369|rs11773597|rs1458226|rs2476923|rs7405231|rs2277603|rs294755|rs8109050|rs6848997|rs1800440|rs2856448|rs1458928|rs293408|rs10082248|rs386084|rs10490621|rs17210001
Discoid Rash	SPHINGOLIPID_METABOLISM	KEGG	1101	32	20	4,60E-01	9,96E-01	rs7145896|rs17533359|rs17123551|rs1413219|rs4904376|rs4441771|rs1493130|rs6008944|rs10508925|rs2131463|rs2595371|rs11005872|rs17820573|rs6588591|rs1020382|rs1160677|rs12050431|rs4912291|rs2008644|rs8015076
Discoid Rash	AQUAPORIN_MEDIATED_TRANSPORT	REACTOME	1510	41	20	4,60E-01	9,96E-01	rs1064448|rs7820461|rs2159917|rs17821056|rs774033|rs931646|rs10058673|rs4382132|rs11810173|rs10475376|rs6092704|rs17826588|rs1589880|rs8023564|rs10783282|rs10876891|rs4701770|rs965808|rs884423|rs6806529
Discoid Rash	SEMAPHORIN_INTERACTIONS	REACTOME	2884	32	20	4,65E-01	9,96E-01	rs3797980|rs258014|rs7916174|rs7680750|rs4732523|rs622996|rs6467966|rs6869550|rs7734835|rs1351140|rs199169|rs1805964|rs1539168|rs1499585|rs4623845|rs797821|rs3448|rs671434|rs1700569|rs3745511
Discoid Rash	G_ALPHA_I_SIGNALLING_EVENTS	REACTOME	6453	44	20	4,67E-01	9,96E-01	rs10488600|rs1064448|rs16834223|rs2808539|rs1329543|rs17779442|rs6670616|rs1829651|rs7871222|rs732050|rs12491596|rs7820461|rs721608|rs6719977|rs945032|rs10488602|rs2159917|rs3025646|rs16834018|rs9818671
Discoid Rash	CHEMOKINE_RECEPTORS_BIND_CHEMOKINES	REACTOME	726	46	19	4,70E-01	9,96E-01	rs16834223|rs1877563|rs16834018|rs6740091|rs1072755|rs11687599|rs266078|rs3093012|rs1634523|rs17488134|rs3815341|rs2419114|rs871144|rs2772548|rs8073784|rs6744322|rs10900022|rs3093010|rs4954580
Discoid Rash	PANTOTHENATE_AND_COA_BIOSYNTHESIS	KEGG	611	54	19	4,71E-01	9,96E-01	rs3756975|rs3793357|rs4963801|rs1625579|rs10842402|rs12205095|rs822607|rs290826|rs12322525|rs11754555|rs2811219|rs12401542|rs34085458|rs11804383|rs10493899|rs6935458|rs7010772|rs11587873|rs10505945
Discoid Rash	IL7_PATHWAY	BIOCARTA	699	37	17	4,71E-01	9,96E-01	rs4976132|rs1490801|rs6557991|rs36686|rs4976028|rs954954|rs10073996|rs16906063|rs2741343|rs7719763|rs446791|rs10070379|rs10940145|rs4739109|rs7242402|rs4131890|rs2849380
Discoid Rash	FANCONI_ANEMIA_PATHWAY	REACTOME	461	30	11	4,71E-01	9,96E-01	rs356669|rs799917|rs2314664|rs4713859|rs12747317|rs3787051|rs11896571|rs107068|rs6758100|rs2192463|rs16912093
Discoid Rash	STATHMIN_PATHWAY	BIOCARTA	456	19	13	4,74E-01	9,96E-01	rs2767326|rs2241694|rs1011973|rs1644512|rs2859129|rs4446309|rs2158196|rs2995094|rs7660775|rs10491334|rs13224682|rs9755490|rs870875
Discoid Rash	KINESINS	REACTOME	566	17	9	4,75E-01	9,96E-01	rs277317|rs260373|rs4656204|rs2331880|rs211258|rs12451623|rs11167733|rs12933548|rs6141285
Discoid Rash	CELL_CYCLE	KEGG	2581	28	20	4,75E-01	9,96E-01	rs1992215|rs3217810|rs879221|rs6130713|rs1499367|rs12528948|rs12102171|rs2031970|rs17199521|rs17047703|rs9653414|rs11661651|rs10510126|rs1535010|rs3019260|rs12547643|rs495026|rs4733859|rs6852016|rs9817
Discoid Rash	MET_PATHWAY	BIOCARTA	1415	41	20	4,75E-01	9,96E-01	rs4976132|rs1490801|rs873418|rs1351140|rs6557991|rs5745752|rs11814342|rs1060561|rs10793894|rs2811876|rs4448606|rs4976028|rs10073996|rs11816982|rs7092134|rs3740546|rs2483847|rs8013053|rs1187095|rs10242168
Discoid Rash	SIGNALING_BY_SCF_KIT	REACTOME	1989	31	20	4,78E-01	9,96E-01	rs4976132|rs1490801|rs6130713|rs4947475|rs873418|rs7735422|rs1327489|rs1544241|rs11217175|rs7322396|rs7981045|rs7185040|rs4433842|rs4943769|rs9817|rs10774623|rs2254137|rs4807101|rs13120102|rs4976028
Discoid Rash	DOUBLE_STRAND_BREAK_REPAIR	REACTOME	506	23	8	4,79E-01	9,96E-01	rs17772583|rs799917|rs1325382|rs4802436|rs1784304|rs7147381|rs535801|rs739719
Discoid Rash	CIRCADIAN_CLOCK	REACTOME	2075	32	20	4,79E-01	9,96E-01	rs590945|rs1511451|rs918418|rs700591|rs2036713|rs16912392|rs6860708|rs667707|rs11634318|rs17237521|rs2314664|rs135549|rs11706165|rs7656250|rs1995917|rs2254137|rs1159176|rs6778657|rs247587|rs16873852
Discoid Rash	DC_PATHWAY	BIOCARTA	573	24	14	4,81E-01	9,96E-01	rs2767326|rs175133|rs1928298|rs4604517|rs1833754|rs1891442|rs1327804|rs918519|rs10984022|rs485499|rs3024498|rs1569723|rs739719|rs12909844
Discoid Rash	ASSOCIATION_OF_TRIC_CCT_WITH_TARGET_PROTEINS_DURING_BIOSYNTHESIS	REACTOME	359	27	10	4,84E-01	9,96E-01	rs17714754|rs3901642|rs3818299|rs4712311|rs6923237|rs6912751|rs12203190|rs3852166|rs4497932|rs7655280
Discoid Rash	G_ALPHA1213_SIGNALLING_EVENTS	REACTOME	2855	38	20	4,84E-01	9,96E-01	rs2833293|rs4976132|rs6797559|rs1490801|rs13278849|rs9647067|rs13171967|rs2159917|rs538957|rs931646|rs1007429|rs11678490|rs11810173|rs3448|rs580004|rs10223823|rs6993912|rs10060486|rs1392912|rs17288929
Discoid Rash	OOCYTE_MEIOSIS	KEGG	3114	34	20	4,85E-01	9,96E-01	rs304051|rs1064448|rs590945|rs6130713|rs7820461|rs901849|rs8003274|rs16898963|rs9653414|rs667707|rs2241694|rs3019260|rs10058673|rs4382132|rs10475376|rs17743079|rs17826588|rs1589880|rs6852016|rs17737362
Discoid Rash	RESPIRATORY_ELECTRON_TRANSPORT_ATP_SYNTHESIS_BY_CHEMIOSMOTIC_COUPLIN<br>TION_BY_UNCOUPLING_PROTEINS_	REACTOME	1741	36	20	4,86E-01	9,96E-01	rs3851422|rs12065184|rs2839604|rs4920706|rs218979|rs4474804|rs2280231|rs527216|rs4854073|rs12586783|rs1142530|rs4719793|rs10061728|rs4853975|rs3776150|rs1434238|rs1500147|rs10181293|rs17528637|rs10231717
Discoid Rash	BASIGIN_INTERACTIONS	REACTOME	595	29	16	4,86E-01	9,96E-01	rs1539624|rs3829406|rs4431992|rs1439502|rs7945189|rs298467|rs12879346|rs1187095|rs519806|rs4724321|rs2301628|rs2076371|rs2065134|rs8013531|rs875511|rs1200136
Discoid Rash	CYTOSOLIC_DNA_SENSING_PATHWAY	KEGG	620	54	18	4,86E-01	9,96E-01	rs10815388|rs10975463|rs7712322|rs10483453|rs17103274|rs4977686|rs10242595|rs12130290|rs4849123|rs1634523|rs1539243|rs3136646|rs12229321|rs10950917|rs9311428|rs2243506|rs10124609|rs4623937
Discoid Rash	TRANSFERRIN_ENDOCYTOSIS_AND_RECYCLING	REACTOME	513	14	13	4,88E-01	9,96E-01	rs2041689|rs10982300|rs8177191|rs11783925|rs1358024|rs11769043|rs12670097|rs4732330|rs10954211|rs4979427|rs2454039|rs3856465|rs2166504
Discoid Rash	AXON_GUIDANCE	REACTOME	13131	29	20	4,89E-01	9,96E-01	rs6941522|rs17652604|rs3797980|rs4729129|rs258014|rs17115184|rs4282339|rs11767121|rs7916174|rs17031463|rs6057009|rs6458997|rs9455013|rs2393610|rs2212020|rs197721|rs2636785|rs6130713|rs7680750|rs4732523
Discoid Rash	RECYCLING_PATHWAY_OF_L1	REACTOME	874	33	20	4,91E-01	9,96E-01	rs12420766|rs16898963|rs7023913|rs277317|rs633505|rs372332|rs260373|rs2302518|rs1994350|rs4507864|rs11576136|rs1002487|rs3824389|rs6598858|rs312181|rs4804528|rs11167733|rs4490948|rs7825468|rs3003608
Discoid Rash	OTHER_GLYCAN_DEGRADATION	KEGG	648	28	14	4,95E-01	9,96E-01	rs13146175|rs7691749|rs10019475|rs7657084|rs3860630|rs7212486|rs1903568|rs13136851|rs17561000|rs1380000|rs9484764|rs400763|rs569945|rs2666052
Discoid Rash	MPR_PATHWAY	BIOCARTA	624	35	19	4,97E-01	9,96E-01	rs6062356|rs2983452|rs12706724|rs6092704|rs1589880|rs17737362|rs601618|rs965808|rs1910381|rs11576136|rs1002487|rs730554|rs159525|rs13232987|rs6598858|rs4724400|rs1468242|rs13224682|rs9755490
Discoid Rash	POTASSIUM_CHANNELS	REACTOME	4940	31	20	4,98E-01	9,96E-01	rs1564372|rs17652604|rs2808539|rs4930007|rs17779442|rs9844737|rs2471637|rs4254605|rs2190550|rs2841616|rs2283214|rs500677|rs2159917|rs7738011|rs4727899|rs3025646|rs4426906|rs2365669|rs8013313|rs1218603
Discoid Rash	TH1TH2_PATHWAY	BIOCARTA	535	24	16	4,99E-01	9,96E-01	rs2834219|rs1532|rs7072398|rs12722589|rs7899538|rs1833754|rs4749882|rs2858870|rs3129882|rs7250425|rs918519|rs1801275|rs485499|rs2305740|rs924080|rs1569723
Discoid Rash	INTRINSIC_PATHWAY	BIOCARTA	603	46	18	4,99E-01	9,96E-01	rs11694554|rs7328731|rs10451555|rs6722598|rs9995366|rs1981316|rs631428|rs595325|rs4255644|rs970741|rs4253331|rs4686799|rs3758919|rs9521585|rs2141831|rs4773155|rs9521666|rs6723547
Discoid Rash	SIGNALING_BY_THE_B_CELL_RECEPTOR_BCR	REACTOME	3426	28	20	5,00E-01	9,96E-01	rs590945|rs4976132|rs2293576|rs1490801|rs7642557|rs10933849|rs9937676|rs873418|rs10483453|rs1445059|rs1551486|rs1845451|rs667707|rs328331|rs3785383|rs17103274|rs6784447|rs7747353|rs11217175|rs7322396
Discoid Rash	PEROXISOME	KEGG	2047	56	20	5,03E-01	9,96E-01	rs34229|rs500800|rs6901445|rs6539460|rs1428878|rs467504|rs4641034|rs11080358|rs2423222|rs249393|rs13190660|rs2564114|rs7017417|rs1510121|rs7788404|rs2842989|rs7957619|rs7701167|rs2206716|rs10865088
Discoid Rash	GNRH_SIGNALING_PATHWAY	KEGG	3992	45	20	5,05E-01	9,96E-01	rs304051|rs1064448|rs6108160|rs9342101|rs7820461|rs901849|rs2239016|rs2124147|rs1108385|rs10132585|rs6557991|rs10494605|rs659732|rs4434808|rs2241694|rs3785383|rs4709487|rs10058673|rs4382132|rs11062222
Discoid Rash	PHASE1_FUNCTIONALIZATION_OF_COMPOUNDS	REACTOME	1515	36	20	5,06E-01	9,96E-01	rs10306202|rs2284205|rs17466747|rs16989226|rs9886369|rs11773597|rs6583859|rs2297812|rs10493270|rs2131463|rs2307492|rs11143438|rs2277603|rs915907|rs1662037|rs6095541|rs4809958|rs6546738|rs1800440|rs2856448
Discoid Rash	APOPTOTIC_CLEAVAGE_OF_CELLULAR_PROTEINS	REACTOME	1052	36	20	5,06E-01	9,96E-01	rs4779683|rs1357386|rs12916064|rs16848195|rs965938|rs440804|rs2298614|rs9517302|rs10904923|rs4355511|rs6503454|rs3775068|rs832152|rs6777221|rs1538579|rs1941184|rs6939137|rs6778301|rs3738593|rs12905328
Discoid Rash	P53_SIGNALING_PATHWAY	KEGG	1897	24	20	5,06E-01	9,96E-01	rs3765692|rs3217810|rs2963087|rs879221|rs873418|rs2903698|rs2031970|rs10497135|rs965938|rs879966|rs7185040|rs9817|rs11591298|rs4719793|rs2245548|rs8098717|rs7564934|rs10505018|rs901478|rs3758813
Discoid Rash	IL1R_PATHWAY	BIOCARTA	1001	32	20	5,07E-01	9,96E-01	rs1043395|rs10483453|rs17047703|rs1845451|rs17042407|rs17103274|rs7747353|rs4700008|rs16939964|rs2811876|rs100043|rs12029576|rs10485405|rs10242595|rs13035227|rs2268626|rs565665|rs6442159|rs1114328|rs6677589
Discoid Rash	APC_C_CDC20_MEDIATED_DEGRADATION_OF_CYCLIN_B	REACTOME	224	17	7	5,07E-01	9,96E-01	rs9653414|rs6852016|rs2314664|rs12480887|rs16941635|rs9867568|rs17775614
Discoid Rash	HIV_LIFE_CYCLE	REACTOME	1529	42	20	5,08E-01	9,96E-01	rs16834223|rs2228375|rs16834018|rs217797|rs2279083|rs6740091|rs4740420|rs1325382|rs11687599|rs2314664|rs964888|rs11177355|rs10896604|rs2018928|rs2707212|rs4802436|rs443678|rs2296085|rs3787016|rs2016266
Discoid Rash	RETINOL_METABOLISM	KEGG	1512	57	20	5,10E-01	9,96E-01	rs10028494|rs17466747|rs747574|rs11773597|rs6583859|rs12402628|rs1458226|rs11143438|rs294755|rs10889769|rs4738315|rs11085554|rs6848997|rs1662037|rs6546738|rs293408|rs11572172|rs17134356|rs707265|rs11592783
Discoid Rash	APC_CDC20_MEDIATED_DEGRADATION_OF_NEK2A	REACTOME	396	25	11	5,12E-01	9,96E-01	rs9653414|rs10510126|rs10494941|rs6852016|rs2314664|rs12480887|rs16941635|rs13130657|rs11248466|rs9867568|rs17775614
Discoid Rash	ACTIVATED_TLR4_SIGNALLING	REACTOME	2676	24	20	5,12E-01	9,96E-01	rs918418|rs17326309|rs700591|rs13281383|rs1043395|rs10483453|rs16898963|rs7175248|rs6860708|rs1845451|rs329499|rs1928298|rs11633249|rs17103274|rs7747353|rs2698537|rs7823751|rs956239|rs2314664|rs4700008
Discoid Rash	SIGNALING_BY_RHO_GTPASES	REACTOME	4877	32	20	5,14E-01	9,96E-01	rs6531866|rs12995474|rs2833293|rs6797559|rs11189119|rs2873688|rs9647067|rs17471022|rs538957|rs7768656|rs10451237|rs6823403|rs2972028|rs2077152|rs11655654|rs12065219|rs1007429|rs4570995|rs1553985|rs11678490
Discoid Rash	FORMATION_OF_FIBRIN_CLOT_CLOTTING_CASCADE	REACTOME	703	41	19	5,15E-01	9,96E-01	rs12758064|rs1058242|rs1050783|rs8120756|rs9995366|rs1482875|rs1245480|rs1902946|rs7766109|rs12198109|rs11611433|rs17141697|rs970741|rs4253331|rs4686799|rs3758919|rs3799558|rs11243078|rs714408
Discoid Rash	DOWNSTREAM_SIGNALING_EVENTS_OF_B_CELL_RECEPTOR_BCR	REACTOME	2127	24	20	5,15E-01	9,96E-01	rs590945|rs2293576|rs873418|rs10483453|rs1551486|rs1845451|rs667707|rs328331|rs3785383|rs17103274|rs7747353|rs7322396|rs7981045|rs7185040|rs4943769|rs17013034|rs392715|rs9817|rs2314664|rs12599594
Discoid Rash	LAIR_PATHWAY	BIOCARTA	479	24	12	5,16E-01	9,96E-01	rs7564976|rs17042407|rs3917016|rs235370|rs10242595|rs1187095|rs4686799|rs2292727|rs10950917|rs3181088|rs986475|rs2277984
Discoid Rash	TRAF6_MEDIATED_INDUCTION_OF_NFKB_AND_MAP_KINASES_UPON_TLR7_8_OR_9_ACT	REACTOME	2239	27	20	5,17E-01	9,96E-01	rs918418|rs700591|rs13281383|rs1043395|rs10483453|rs16898963|rs7175248|rs6860708|rs1845451|rs329499|rs11633249|rs17103274|rs7747353|rs2698537|rs7823751|rs2314664|rs4700008|rs10200259|rs2811876|rs2254137
Discoid Rash	NUCLEAR_RECEPTOR_TRANSCRIPTION_PATHWAY	REACTOME	2750	39	20	5,19E-01	9,96E-01	rs1151996|rs12253611|rs4414112|rs8006145|rs2576261|rs851982|rs265451|rs16839941|rs884799|rs12437118|rs11634318|rs10504605|rs17237521|rs12745466|rs1867653|rs17737362|rs135549|rs4713859|rs17081685|rs11117760
Discoid Rash	BIOSYNTHESIS_OF_THE_N_GLYCAN_PRECURSOR_DOLICHOL_LIPID_LINKED_OLIGOSACC<br>RANSFER_TO_A_NASCENT_PROTEIN	REACTOME	355	15	9	5,19E-01	9,96E-01	rs659758|rs10163323|rs7533303|rs6657738|rs1784304|rs4943435|rs12424735|rs9924614|rs3009577
Discoid Rash	GRB2_EVENTS_IN_ERBB2_SIGNALING	REACTOME	1202	61	20	5,20E-01	9,96E-01	rs7574462|rs6130713|rs4236723|rs7658842|rs1481624|rs16847102|rs16847122|rs10503899|rs12623434|rs1465828|rs6435604|rs6435670|rs6435689|rs2976532|rs16846100|rs12162287|rs10185729|rs1836729|rs10105518|rs4963872
Discoid Rash	SCF_BETA_TRCP_MEDIATED_DEGRADATION_OF_EMI1	REACTOME	793	31	19	5,21E-01	9,96E-01	rs590945|rs2293576|rs667707|rs328331|rs2314664|rs12599594|rs371500|rs13394402|rs17760556|rs12463483|rs3731973|rs7212835|rs3751497|rs8005252|rs6966980|rs3819715|rs2550461|rs241440|rs2298265
Discoid Rash	G_ALPHA_Z_SIGNALLING_EVENTS	REACTOME	2191	33	20	5,22E-01	9,96E-01	rs1064448|rs7820461|rs7349397|rs2159917|rs2204204|rs2344662|rs2661303|rs931646|rs3785383|rs10058673|rs4382132|rs11810173|rs10475376|rs4433842|rs12706724|rs11783925|rs6092704|rs17826588|rs1589880|rs392715
Discoid Rash	ADIPOCYTOKINE_SIGNALING_PATHWAY	KEGG	2106	36	20	5,23E-01	9,96E-01	rs1511451|rs1577452|rs2036713|rs6970522|rs1327489|rs10483453|rs1137101|rs2390961|rs17103274|rs2297972|rs791595|rs2245657|rs7599150|rs135549|rs17369837|rs2727528|rs336230|rs10848568|rs7656250|rs1995917
Discoid Rash	ADP_SIGNALLING_THROUGH_P2RY12	REACTOME	438	24	11	5,25E-01	9,96E-01	rs2159917|rs931646|rs11810173|rs12706724|rs6440739|rs13232987|rs765044|rs1468242|rs2873409|rs6955457|rs17755118
Discoid Rash	FORMATION_OF_INCISION_COMPLEX_IN_GG_NER	REACTOME	545	4	4	5,31E-01	9,96E-01	rs12336762|rs1746837|rs11039149|rs12004883
Discoid Rash	SYNTHESIS_OF_PIPS_AT_THE_PLASMA_MEMBRANE	REACTOME	1050	39	20	5,32E-01	9,96E-01	rs4976132|rs1490801|rs745719|rs873418|rs7983595|rs2218314|rs7971352|rs4976028|rs10073996|rs537885|rs8138677|rs693293|rs11604561|rs7719763|rs10070379|rs10940145|rs11652315|rs10470165|rs11044233|rs4131890
Discoid Rash	TRIGLYCERIDE_BIOSYNTHESIS	REACTOME	1182	28	20	5,32E-01	9,96E-01	rs209512|rs6559175|rs11127284|rs6072343|rs2341422|rs2912024|rs1559746|rs9905601|rs4955238|rs2838464|rs17028998|rs11099616|rs7656124|rs204990|rs3798719|rs6870197|rs247011|rs17138930|rs2792751|rs876873
Discoid Rash	TRIF_MEDIATED_TLR3_SIGNALING	REACTOME	2075	30	20	5,35E-01	9,96E-01	rs918418|rs700591|rs13281383|rs1043395|rs10483453|rs16898963|rs7175248|rs6860708|rs1845451|rs11633249|rs17103274|rs7747353|rs7823751|rs956239|rs2314664|rs2811876|rs2254137|rs10494878|rs556335|rs8041078
Discoid Rash	IL1_SIGNALING	REACTOME	1322	29	20	5,36E-01	9,96E-01	rs590945|rs1043395|rs1845451|rs667707|rs17042407|rs329499|rs7747353|rs2698537|rs10200259|rs2303677|rs556335|rs100043|rs2054492|rs10485405|rs13035227|rs17790482|rs150615|rs565665|rs17760556|rs6442159
Discoid Rash	ANTIGEN_PRESENTATION_FOLDING_ASSEMBLY_AND_PEPTIDE_LOADING_OF_CLASS_I_M	REACTOME	830	67	20	5,37E-01	9,96E-01	rs3094691|rs7755852|rs1611350|rs3130473|rs12111032|rs9295986|rs469783|rs13191519|rs1736956|rs709641|rs2844575|rs9266018|rs6457109|rs1634783|rs7494073|rs9259650|rs2517938|rs2523966|rs4959039|rs3819715
Discoid Rash	CASPASE_PATHWAY	BIOCARTA	327	5	5	5,37E-01	9,96E-01	rs965938|rs4719793|rs762084|rs3738593|rs1439123
Discoid Rash	INSULIN_RECEPTOR_RECYCLING	REACTOME	492	14	12	5,39E-01	9,96E-01	rs2041689|rs10982300|rs11783925|rs11769043|rs7507911|rs12670097|rs4732330|rs10954211|rs4979427|rs2454039|rs3856465|rs4804103
Discoid Rash	SIGNALING_BY_EGFR_IN_CANCER	REACTOME	4030	37	20	5,39E-01	9,96E-01	rs1064448|rs4976132|rs13131772|rs1490801|rs1897104|rs13429751|rs6130713|rs7820461|rs7349397|rs873418|rs2204204|rs2344662|rs748289|rs10058673|rs12098279|rs17649232|rs11217175|rs7322396|rs4382132|rs7981045
Discoid Rash	RESPIRATORY_ELECTRON_TRANSPORT	REACTOME	1631	36	20	5,40E-01	9,96E-01	rs12065184|rs2839604|rs4920706|rs218979|rs4474804|rs2280231|rs527216|rs4854073|rs12586783|rs1142530|rs4719793|rs10061728|rs4853975|rs3776150|rs1434238|rs1500147|rs10181293|rs17528637|rs10231717|rs2267552
Discoid Rash	SPHINGOLIPID_METABOLISM	REACTOME	1944	35	20	5,45E-01	9,96E-01	rs7145896|rs11722340|rs17533359|rs1191571|rs6866961|rs17123551|rs672413|rs3787146|rs1413219|rs4904376|rs1493130|rs6008944|rs10508925|rs17571979|rs2131463|rs11005872|rs12605289|rs17820573|rs1002667|rs6015258
Discoid Rash	PROTEIN_FOLDING	REACTOME	675	29	12	5,49E-01	9,96E-01	rs17714754|rs3901642|rs2273828|rs10875943|rs2272313|rs4712311|rs6923237|rs6912751|rs12203190|rs3852166|rs1359761|rs7655280
Discoid Rash	RIP_MEDIATED_NFKB_ACTIVATION_VIA_DAI	REACTOME	328	12	10	5,51E-01	9,96E-01	rs10483453|rs17103274|rs956239|rs204990|rs9550565|rs2839343|rs3136646|rs1056890|rs11721827|rs4807643
Discoid Rash	ATRBRCA_PATHWAY	BIOCARTA	238	28	10	5,52E-01	9,96E-01	rs17772583|rs356669|rs799917|rs4713859|rs2686830|rs9311428|rs16912093|rs535801|rs739719|rs4035540
Discoid Rash	ECM_PATHWAY	BIOCARTA	753	29	17	5,55E-01	9,96E-01	rs4976132|rs1490801|rs1351140|rs3448|rs4976028|rs10073996|rs1187095|rs4767884|rs7719763|rs10070379|rs10940145|rs11748064|rs13100237|rs12050150|rs4131890|rs2126953|rs10940277
Discoid Rash	DEFENSINS	REACTOME	495	30	11	5,56E-01	9,96E-01	rs2980937|rs9463574|rs7461956|rs2707212|rs3093012|rs6061162|rs2702889|rs1348181|rs6032887|rs8124562|rs3093010
Discoid Rash	PHOSPHOLIPID_METABOLISM	REACTOME	6226	37	20	5,58E-01	9,96E-01	rs2087179|rs7145896|rs4976132|rs2292645|rs11722340|rs1490801|rs6559175|rs4823104|rs17533359|rs1191571|rs2848780|rs6866961|rs17123551|rs672413|rs745719|rs873418|rs7983595|rs2124147|rs10494605|rs659732
Discoid Rash	ACYL_CHAIN_REMODELLING_OF_PC	REACTOME	693	30	16	5,60E-01	9,96E-01	rs10494605|rs659732|rs4463623|rs7929406|rs1474797|rs1980444|rs1148757|rs6870197|rs6697553|rs10750999|rs776698|rs776691|rs9973936|rs16827441|rs666511|rs16829110
Discoid Rash	REGULATION_OF_GENE_EXPRESSION_IN_BETA_CELLS	REACTOME	716	30	20	5,70E-01	9,96E-01	rs1429447|rs10504605|rs7322396|rs7981045|rs4943769|rs2926615|rs9512906|rs1562147|rs1572579|rs6017335|rs7272694|rs7822381|rs6817184|rs1806180|rs17192831|rs3212208|rs2272668|rs10000976|rs17008412|rs2977327
Discoid Rash	PRIMARY_IMMUNODEFICIENCY	KEGG	742	27	20	5,70E-01	9,96E-01	rs9937676|rs4280293|rs3096866|rs7922341|rs17615641|rs36686|rs2707212|rs6710279|rs4781016|rs7911684|rs7916726|rs7288250|rs13420393|rs4851462|rs13374545|rs4822422|rs1898240|rs9603093|rs11571312|rs2057637
Discoid Rash	MAPK_PATHWAY	BIOCARTA	2690	27	20	5,71E-01	9,96E-01	rs918418|rs700591|rs2648888|rs6543094|rs10483453|rs16898963|rs17199521|rs7175248|rs6860708|rs17047703|rs1845451|rs6922031|rs4709487|rs11633249|rs12547643|rs17103274|rs4733859|rs7747353|rs1990966|rs13115100
Discoid Rash	BETA_DEFENSINS	REACTOME	333	24	8	5,71E-01	9,96E-01	rs2980937|rs9463574|rs3093012|rs6061162|rs1348181|rs6032887|rs6109606|rs3093010
Discoid Rash	NON_SMALL_CELL_LUNG_CANCER	KEGG	2585	46	20	5,73E-01	9,96E-01	rs4976132|rs1490801|rs1483831|rs1447981|rs6445205|rs7623479|rs1947152|rs2144649|rs11681491|rs7640419|rs212063|rs3785383|rs10510826|rs4433842|rs392715|rs2664537|rs12631631|rs4976028|rs10073996|rs12638276
Discoid Rash	SIGNALING_BY_NOTCH	REACTOME	4553	23	20	5,74E-01	9,96E-01	rs10149996|rs2179688|rs1036047|rs1470833|rs11608551|rs10484153|rs17150238|rs998910|rs1152349|rs10139403|rs2648888|rs6483495|rs4923889|rs10519523|rs12895242|rs6732014|rs2378384|rs7859384|rs6788164|rs2807305
Discoid Rash	DEADENYLATION_DEPENDENT_MRNA_DECAY	REACTOME	622	14	12	5,75E-01	9,96E-01	rs7438406|rs1545229|rs6783810|rs11189224|rs10896604|rs11129533|rs11602501|rs2361696|rs17694584|rs10007831|rs10747658|rs17810558
Discoid Rash	NDKDYNAMIN_PATHWAY	BIOCARTA	548	18	11	5,77E-01	9,96E-01	rs11761049|rs7023913|rs13031703|rs6947865|rs2302518|rs17014923|rs4478191|rs3003608|rs17497544|rs10470165|rs7595027
Discoid Rash	IGF1R_PATHWAY	BIOCARTA	735	35	20	5,79E-01	9,96E-01	rs4976132|rs1490801|rs1589880|rs7599150|rs4976028|rs10073996|rs17188448|rs11576136|rs1002487|rs7166565|rs10794486|rs6598858|rs4724400|rs7719763|rs10070379|rs10940145|rs13224682|rs9755490|rs2684777|rs2684790
Discoid Rash	METABOLISM_OF_VITAMINS_AND_COFACTORS	REACTOME	1119	36	20	5,80E-01	9,96E-01	rs17780226|rs12493507|rs946173|rs8091374|rs2297972|rs8123436|rs11707887|rs8003379|rs41684|rs11828|rs3008831|rs3133803|rs7636269|rs10935348|rs9948558|rs17387775|rs7253363|rs675220|rs3783438|rs12023117
Discoid Rash	RNA_POL_III_TRANSCRIPTION_TERMINATION	REACTOME	680	45	20	5,80E-01	9,96E-01	rs2224553|rs7873382|rs575660|rs2078639|rs17420770|rs1813529|rs10961329|rs13302756|rs3787016|rs1532445|rs10961439|rs10961483|rs17092361|rs10961347|rs17603298|rs1385309|rs1323348|rs2243506|rs3746162|rs10117199
Discoid Rash	PREFOLDIN_MEDIATED_TRANSFER_OF_SUBSTRATE_TO_CCT_TRIC	REACTOME	288	10	4	5,81E-01	9,96E-01	rs3818299|rs10875943|rs2272313|rs3852166
Discoid Rash	ALDOSTERONE_REGULATED_SODIUM_REABSORPTION	KEGG	1794	33	20	5,81E-01	9,96E-01	rs1577452|rs4976132|rs1490801|rs846906|rs3759333|rs3785383|rs11154765|rs4433842|rs392715|rs7599150|rs7772299|rs336230|rs9402592|rs7507911|rs11600216|rs4976028|rs10073996|rs17188448|rs868840|rs8051531
Discoid Rash	EGF_PATHWAY	BIOCARTA	1048	41	20	5,84E-01	9,96E-01	rs4976132|rs1490801|rs3785383|rs4433842|rs392715|rs4700008|rs2811876|rs2664537|rs4976028|rs10073996|rs8051531|rs8013053|rs11646540|rs17753569|rs17811655|rs7719763|rs198182|rs10070379|rs6677589|rs7572482

Discoid Rash	HISTIDINE_METABOLISM	KEGG	654	25	18	5,88E-01	9,96E-01	rs17024981|rs11788224|rs4836277|rs2509713|rs12605520|rs6430767|rs854151|rs16837500|rs2810716|rs6569455|rs730870|rs480245|rs1968736|rs11768267|rs6756311|rs3809524|rs1465936|rs16840472
Discoid Rash	NUCLEOTIDE_LIKE_PURINERGIC_RECEPTORS	REACTOME	272	16	10	5,91E-01	9,96E-01	rs8112063|rs11235714|rs3814728|rs7208308|rs6440739|rs12026765|rs2364815|rs1042295|rs4382936|rs10898906
Discoid Rash	G_PROTEIN_ACTIVATION	REACTOME	703	36	15	5,91E-01	9,96E-01	rs2159917|rs931646|rs3935975|rs11810173|rs12706724|rs652051|rs11966376|rs13232987|rs875602|rs765044|rs1468242|rs2873409|rs6955457|rs8121495|rs17755118
Discoid Rash	TIE2_SIGNALING	REACTOME	790	42	20	5,92E-01	9,96E-01	rs4976132|rs1490801|rs1283663|rs4667731|rs10967753|rs4976028|rs10073996|rs1888087|rs1283682|rs693293|rs7719763|rs10070379|rs13272391|rs2897911|rs10940145|rs6086286|rs13027527|rs4963872|rs4305876|rs4131890
Discoid Rash	PIP3_ACTIVATES_AKT_SIGNALING	REACTOME	587	21	14	5,92E-01	9,96E-01	rs873418|rs7322396|rs7981045|rs7185040|rs4943769|rs9817|rs2254137|rs769412|rs11892543|rs1268160|rs665821|rs17202235|rs10492682|rs9549123
Discoid Rash	SIGNALING_BY_ROBO_RECEPTOR	REACTOME	1900	32	20	5,93E-01	9,96E-01	rs17031463|rs6057009|rs6797725|rs10154970|rs7612183|rs12634677|rs1843786|rs9882126|rs2376977|rs6785747|rs4851831|rs11697698|rs1480689|rs7641197|rs9830459|rs776136|rs569230|rs11685896|rs13045381|rs7605126
Discoid Rash	INSULIN_PATHWAY	BIOCARTA	725	36	20	5,96E-01	9,96E-01	rs4976132|rs1490801|rs7599150|rs2811876|rs7507911|rs12150519|rs4976028|rs10073996|rs17188448|rs8013053|rs7719763|rs10070379|rs6677589|rs17183482|rs10940145|rs11066323|rs4804103|rs11207306|rs11066320|rs4131890
Discoid Rash	MYOSIN_PATHWAY	BIOCARTA	1253	29	20	5,96E-01	9,96E-01	rs6108160|rs8069620|rs3785383|rs12463361|rs4433842|rs2179984|rs392715|rs9588385|rs17288929|rs6118062|rs6140561|rs6792241|rs8051531|rs11646540|rs6086525|rs17753569|rs17811655|rs3779704|rs6039189|rs6055889
Discoid Rash	PHOSPHORYLATION_OF_THE_APC_C	REACTOME	219	15	6	5,96E-01	9,96E-01	rs9653414|rs6852016|rs12480887|rs16941635|rs9867568|rs17775614
Discoid Rash	REGULATORY_RNA_PATHWAYS	REACTOME	345	29	12	5,96E-01	9,96E-01	rs7160403|rs11089328|rs12186785|rs12878043|rs10071110|rs17408227|rs3787016|rs875188|rs12897280|rs3746162|rs11746052|rs9907255
Discoid Rash	DIABETES_PATHWAYS	REACTOME	3395	28	20	5,97E-01	9,96E-01	rs10743111|rs362988|rs6077718|rs2317222|rs11656072|rs2136703|rs2294654|rs6478233|rs11999806|rs240940|rs4341514|rs1024175|rs17377483|rs7033487|rs1545229|rs4689388|rs9392155|rs17019438|rs4931434|rs791595
Discoid Rash	TERMINATION_OF_O_GLYCAN_BIOSYNTHESIS	REACTOME	961	47	20	5,97E-01	9,96E-01	rs2289241|rs2404839|rs620381|rs1862458|rs7844620|rs6981912|rs10467147|rs1868431|rs10804566|rs1325287|rs10493583|rs10493582|rs7548049|rs2933346|rs4768301|rs1868430|rs10018283|rs12054151|rs2259419|rs2978056
Discoid Rash	GLYOXYLATE_AND_DICARBOXYLATE_METABOLISM	KEGG	704	33	17	6,00E-01	9,96E-01	rs1555179|rs2346892|rs517766|rs2073066|rs2423222|rs12238815|rs6467993|rs8003379|rs9611598|rs2423322|rs1508310|rs6086028|rs13037510|rs12201472|rs7739248|rs11754661|rs1274500
Discoid Rash	NONSENSE_MEDIATED_DECAY_ENHANCED_BY_THE_EXON_JUNCTION_COMPLEX	REACTOME	1261	33	20	6,01E-01	9,96E-01	rs2958522|rs12896325|rs10509398|rs7255657|rs3810168|rs4558175|rs2816764|rs2314664|rs3764536|rs11784209|rs2001558|rs9394299|rs4803238|rs36631|rs4738315|rs2012243|rs8016600|rs2277743|rs7894696|rs2574693
Discoid Rash	MITOCHONDRIAL_TRNA_AMINOACYLATION	REACTOME	500	39	20	6,02E-01	9,96E-01	rs2713834|rs4699172|rs2503838|rs7751261|rs2326613|rs1441042|rs4409225|rs6779394|rs6926774|rs6676647|rs2295085|rs2064108|rs959086|rs3136646|rs2224391|rs9381309|rs9392684|rs9927961|rs9405272|rs10899556
Discoid Rash	ADP_SIGNALLING_THROUGH_P2RY1	REACTOME	863	31	18	6,02E-01	9,96E-01	rs2159917|rs10494605|rs931646|rs659732|rs4463623|rs11810173|rs1474797|rs1980444|rs1148757|rs6697553|rs765044|rs310686|rs17786782|rs16827441|rs2873409|rs16829110|rs6955457|rs17755118
Discoid Rash	XENOBIOTICS	REACTOME	231	5	4	6,06E-01	9,96E-01	rs11773597|rs915907|rs11572172|rs707265
Discoid Rash	DEADENYLATION_OF_MRNA	REACTOME	331	8	7	6,07E-01	9,96E-01	rs7438406|rs10896604|rs11129533|rs11602501|rs2361696|rs10007831|rs10747658
Discoid Rash	DOWNSTREAM_TCR_SIGNALING	REACTOME	1573	45	20	6,07E-01	9,96E-01	rs4976132|rs1490801|rs873418|rs10483453|rs1845451|rs3104404|rs17103274|rs7747353|rs9276284|rs2707212|rs4976028|rs10904923|rs10073996|rs559052|rs10485405|rs28383344|rs17790482|rs17289265|rs150615|rs565665
Discoid Rash	FORMATION_OF_TUBULIN_FOLDING_INTERMEDIATES_BY_CCT_TRIC	REACTOME	227	9	3	6,08E-01	9,96E-01	rs3818299|rs10875943|rs3852166
Discoid Rash	ACYL_CHAIN_REMODELLING_OF_PE	REACTOME	696	30	17	6,09E-01	9,96E-01	rs10494605|rs659732|rs4434808|rs7929406|rs1474797|rs1980444|rs1148757|rs7766133|rs6697553|rs10750999|rs776698|rs776691|rs9973936|rs16827441|rs2472760|rs666511|rs16829110
Discoid Rash	AMINO_ACID_SYNTHESIS_AND_INTERCONVERSION_TRANSAMINATION	REACTOME	530	21	13	6,11E-01	9,96E-01	rs2136703|rs1229564|rs11137606|rs10876891|rs9935162|rs1921915|rs1912445|rs7194392|rs944514|rs10263979|rs7868916|rs6559400|rs7902179
Discoid Rash	DEATH_PATHWAY	BIOCARTA	505	22	17	6,14E-01	9,96E-01	rs10483453|rs17103274|rs965938|rs365238|rs4719793|rs16939964|rs9868605|rs954954|rs3850170|rs11997323|rs3738593|rs7216796|rs7242402|rs2849380|rs1439123|rs2109681|rs9872592
Discoid Rash	PORPHYRIN_AND_CHLOROPHYLL_METABOLISM	KEGG	1163	58	20	6,14E-01	9,96E-01	rs10028494|rs11870213|rs10521258|rs7957619|rs1458226|rs699512|rs10070906|rs1784304|rs16861637|rs9686533|rs294755|rs1560550|rs3853397|rs6502324|rs818704|rs6848997|rs293408|rs13095262|rs9927961|rs867131
Discoid Rash	NOTCH_SIGNALING_PATHWAY	KEGG	1387	29	20	6,16E-01	9,96E-01	rs2179688|rs11608551|rs6483495|rs4923889|rs10519523|rs6788164|rs2335855|rs12646059|rs838875|rs7086797|rs12417347|rs837948|rs3787051|rs204990|rs6040055|rs3781436|rs4687500|rs6133987|rs9855415|rs495494
Discoid Rash	REGULATION_OF_WATER_BALANCE_BY_RENAL_AQUAPORINS	REACTOME	1393	38	20	6,16E-01	9,96E-01	rs1064448|rs7820461|rs2159917|rs931646|rs10058673|rs4382132|rs11810173|rs10475376|rs6092704|rs17826588|rs1589880|rs10783282|rs4701770|rs965808|rs884423|rs6806529|rs4289537|rs7196832|rs765044|rs7069291
Discoid Rash	GLYCEROPHOSPHOLIPID_BIOSYNTHESIS	REACTOME	2483	38	20	6,17E-01	9,96E-01	rs2087179|rs2292645|rs6559175|rs4823104|rs2124147|rs10494605|rs659732|rs11127284|rs4463623|rs6680326|rs6072343|rs2341422|rs7929406|rs1474797|rs2912024|rs1559746|rs11670988|rs11046741|rs4955238|rs1980444
Discoid Rash	RNA_POLYMERASE	KEGG	351	21	9	6,17E-01	9,96E-01	rs1245371|rs7712322|rs3787016|rs6734172|rs7594007|rs2243506|rs3746162|rs4711207|rs754950
Discoid Rash	PROTEIN_EXPORT	KEGG	750	28	16	6,18E-01	9,96E-01	rs3829406|rs7811230|rs12267330|rs1551368|rs10245119|rs1358024|rs10953645|rs1008453|rs1528035|rs37642|rs2065134|rs8013531|rs6946131|rs2333452|rs11156868|rs11536344
Discoid Rash	VIP_PATHWAY	BIOCARTA	1000	37	20	6,22E-01	9,96E-01	rs2648888|rs13275868|rs10483453|rs17199521|rs12547643|rs17103274|rs4733859|rs4700008|rs2664537|rs10505507|rs17674754|rs1124425|rs1410489|rs4478191|rs6985681|rs17786782|rs6987802|rs13224682|rs9755490|rs7595027
Discoid Rash	RAP1_SIGNALLING	REACTOME	835	32	20	6,22E-01	9,96E-01	rs6130713|rs4077990|rs3019260|rs12051711|rs11000160|rs10747521|rs12442869|rs293314|rs2038916|rs11073339|rs11695696|rs8065180|rs293290|rs9904506|rs2860493|rs1937683|rs10508942|rs6480375|rs2454566|rs1904050
Discoid Rash	BETA_ALANINE_METABOLISM	KEGG	711	40	20	6,23E-01	9,96E-01	rs3793357|rs11788224|rs4836277|rs1625579|rs6755814|rs822607|rs290826|rs12930556|rs12605520|rs2786529|rs12401542|rs1273422|rs2810716|rs34085458|rs11804383|rs10493899|rs730870|rs480245|rs7010772|rs11587873
Discoid Rash	DRUG_METABOLISM_OTHER_ENZYMES	KEGG	1609	52	20	6,25E-01	9,96E-01	rs10028494|rs3793357|rs12026896|rs11773597|rs1625579|rs822607|rs290826|rs1458226|rs10865088|rs294755|rs2811219|rs1487302|rs3808830|rs6848997|rs768364|rs34085458|rs11804383|rs10799655|rs293408|rs10493899
Discoid Rash	CYTOCHROME_P450_ARRANGED_BY_SUBSTRATE_TYPE	REACTOME	1163	41	20	6,25E-01	9,96E-01	rs2284205|rs17466747|rs9886369|rs11773597|rs6583859|rs2297812|rs10493270|rs2131463|rs2277603|rs915907|rs6095541|rs4809958|rs6546738|rs1800440|rs2856448|rs1458928|rs11572172|rs10490621|rs707265|rs11592783
Discoid Rash	CELL_DEATH_SIGNALLING_VIA_NRAGE_NRIF_AND_NADE	REACTOME	2069	29	20	6,26E-01	9,96E-01	rs2833293|rs6797559|rs17540656|rs9647067|rs538957|rs1007429|rs17762822|rs965938|rs11678490|rs580004|rs2314664|rs1877330|rs10223823|rs1392912|rs12124096|rs17288929|rs17020055|rs1317636|rs4807101|rs2833264
Discoid Rash	REGULATION_OF_KIT_SIGNALING	REACTOME	587	20	11	6,27E-01	9,96E-01	rs1544241|rs11217175|rs4433842|rs10774623|rs13120102|rs2711414|rs9959750|rs7226990|rs6506537|rs10161145|rs2612102
Discoid Rash	NA_CL_DEPENDENT_NEUROTRANSMITTER_TRANSPORTERS	REACTOME	718	27	14	6,29E-01	9,96E-01	rs10833369|rs2241694|rs892601|rs1710887|rs2950798|rs3779672|rs350658|rs10466424|rs3776511|rs4106010|rs2697135|rs2697159|rs1710885|rs4684213
Discoid Rash	CELL_CELL_COMMUNICATION	REACTOME	9725	33	20	6,29E-01	9,96E-01	rs2140825|rs6766201|rs4976132|rs11767121|rs1420965|rs17031463|rs13308578|rs7546890|rs1490801|rs944248|rs12962729|rs10892148|rs12668317|rs2441686|rs2068606|rs157580|rs1892918|rs4682242|rs2136129|rs7201194
Discoid Rash	ACTIVATED_AMPK_STIMULATES_FATTY_ACID_OXIDATION_IN_MUSCLE	REACTOME	421	23	13	6,29E-01	9,96E-01	rs6970522|rs9568203|rs2727528|rs11878692|rs10774641|rs2746342|rs2268387|rs10278273|rs1348316|rs6743951|rs4974079|rs12540943|rs2466155
Discoid Rash	AMINOACYL_TRNA_BIOSYNTHESIS	KEGG	731	34	20	6,31E-01	9,96E-01	rs3765144|rs2503838|rs707938|rs6450986|rs7751261|rs2326613|rs1441042|rs4409225|rs6779394|rs6926774|rs6676647|rs2295085|rs2064108|rs585362|rs3136646|rs2282293|rs2224391|rs9381309|rs9392684|rs9927961
Discoid Rash	PEROXISOMAL_LIPID_METABOLISM	REACTOME	435	11	7	6,32E-01	9,96E-01	rs500800|rs7788404|rs854151|rs994414|rs17544148|rs3802577|rs7651276
Discoid Rash	ACETYLCHOLINE_BINDING_AND_DOWNSTREAM_EVENTS	REACTOME	242	12	8	6,33E-01	9,96E-01	rs4435224|rs7017417|rs935865|rs10519712|rs2741343|rs12904458|rs4072398|rs11071503
Discoid Rash	IRON_UPTAKE_AND_TRANSPORT	REACTOME	733	16	15	6,33E-01	9,96E-01	rs2041689|rs10982300|rs8177191|rs11783925|rs1358024|rs11769043|rs16861637|rs12670097|rs4732330|rs10954211|rs4979427|rs2454039|rs3856465|rs13095262|rs2166504
Discoid Rash	GAB1_SIGNALOSOME	REACTOME	1077	37	20	6,33E-01	9,96E-01	rs4976132|rs1490801|rs873418|rs7322396|rs7981045|rs7185040|rs4943769|rs9817|rs2254137|rs4976028|rs10073996|rs769412|rs11892543|rs7719763|rs7012200|rs10070379|rs10940145|rs1268160|rs665821|rs17202235
Discoid Rash	BASE_EXCISION_REPAIR	KEGG	500	27	17	6,34E-01	9,96E-01	rs3805169|rs10009807|rs9580991|rs10883663|rs4802436|rs6834946|rs10146912|rs9550565|rs2153608|rs1439349|rs11236181|rs3730463|rs2135615|rs4840583|rs6856498|rs1395480|rs1673041
Discoid Rash	AMINO_SUGAR_AND_NUCLEOTIDE_SUGAR_METABOLISM	KEGG	956	34	18	6,35E-01	9,96E-01	rs9391934|rs1011848|rs1004779|rs10841923|rs17501218|rs10163323|rs1000251|rs9405506|rs6909392|rs12736865|rs163918|rs17561000|rs6843723|rs217463|rs3800064|rs234953|rs10517428|rs2812365
Discoid Rash	COSTIMULATION_BY_THE_CD28_FAMILY	REACTOME	1479	53	20	6,38E-01	9,96E-01	rs4976132|rs1490801|rs231726|rs8003274|rs3104404|rs1544241|rs9276284|rs16939964|rs743479|rs4807101|rs2707212|rs4976028|rs10073996|rs6710279|rs28383344|rs2282055|rs13420393|rs2501276|rs7719763|rs6506537
Discoid Rash	DRUG_METABOLISM_CYTOCHROME_P450	KEGG	1661	53	20	6,39E-01	9,96E-01	rs10028494|rs6637|rs2247016|rs11773597|rs12646059|rs4147618|rs1458226|rs2307492|rs316156|rs915907|rs7355084|rs294755|rs6848997|rs1662037|rs1348316|rs293408|rs4916273|rs11572172|rs2132847|rs707265
Discoid Rash	INHIBITION_OF_THE_PROTEOLYTIC_ACTIVITY_OF_APC_C_REQUIRED_FOR_THE_ONSET_ TOTIC_SPINDLE_CHECKPOINT_COMPONENTS	REACTOME	365	21	9	6,41E-01	9,96E-01	rs9653414|rs10510126|rs6852016|rs12480887|rs16941635|rs13130657|rs11248466|rs9867568|rs17775614
Discoid Rash	VASOPRESSIN_REGULATED_WATER_REABSORPTION	KEGG	1100	42	20	6,41E-01	9,96E-01	rs3912622|rs42065|rs1544470|rs6979207|rs11712712|rs6092704|rs10783282|rs2254137|rs10495907|rs965808|rs3857801|rs7196832|rs11264680|rs11892543|rs3763040|rs10280608|rs17202235|rs2237590|rs6782149|rs660751
Discoid Rash	FCER1_PATHWAY	BIOCARTA	1564	35	20	6,43E-01	9,96E-01	rs4976132|rs1490801|rs10494605|rs659732|rs4463623|rs3785383|rs392715|rs1474797|rs4700008|rs2811876|rs2664537|rs1980444|rs4807101|rs4976028|rs10073996|rs1148757|rs4744512|rs8051531|rs1755991|rs6697553
Discoid Rash	INTRINSIC_PATHWAY	REACTOME	264	7	5	6,43E-01	9,96E-01	rs9995366|rs11611433|rs4253331|rs4686799|rs3758919
Discoid Rash	ONE_CARBON_POOL_BY_FOLATE	KEGG	441	27	15	6,44E-01	9,96E-01	rs1555179|rs2346892|rs517766|rs2073066|rs9984077|rs8003379|rs4646745|rs10165919|rs4646750|rs9966612|rs12201472|rs7739248|rs2273026|rs11754661|rs2847154
Discoid Rash	ACTIVATION_OF_ATR_IN_RESPONSE_TO_REPLICATION_STRESS	REACTOME	653	26	15	6,44E-01	9,96E-01	rs7916990|rs1535010|rs495026|rs2321744|rs10508455|rs7324754|rs614123|rs2891652|rs2686830|rs10948689|rs9311428|rs9598129|rs1753477|rs11258231|rs1328667
Discoid Rash	NOS1_PATHWAY	BIOCARTA	1133	27	20	6,45E-01	9,96E-01	rs17203281|rs11074683|rs816296|rs3785383|rs2650163|rs1805510|rs4433842|rs392715|rs9921476|rs8051531|rs12820037|rs2682826|rs11646540|rs17753569|rs17811655|rs4478191|rs198182|rs10444986|rs8064140|rs1548853
Discoid Rash	REGULATION_OF_MITOTIC_CELL_CYCLE	REACTOME	1318	35	20	6,46E-01	9,96E-01	rs590945|rs2293576|rs9653414|rs10510126|rs667707|rs328331|rs6852016|rs2314664|rs12599594|rs371500|rs13394402|rs16979884|rs12480887|rs13160147|rs16941635|rs17760556|rs12463483|rs13130657|rs3731973|rs7212835
Discoid Rash	OPIOID_SIGNALLING	REACTOME	3594	35	20	6,46E-01	9,96E-01	rs1064448|rs6108160|rs1897104|rs13429751|rs7820461|rs2159917|rs7522367|rs8094168|rs244570|rs10494605|rs931646|rs659732|rs3935975|rs4463623|rs10058673|rs17649232|rs4382132|rs11810173|rs10475376|rs4433842
Discoid Rash	SIGNAL_AMPLIFICATION	REACTOME	1023	41	20	6,46E-01	9,96E-01	rs2159917|rs10494605|rs931646|rs659732|rs4463623|rs11810173|rs12706724|rs1474797|rs1980444|rs1148757|rs6697553|rs6440739|rs13232987|rs765044|rs11652805|rs1468242|rs310686|rs17786782|rs16827441|rs11655004
Discoid Rash	HIVNEF_PATHWAY	BIOCARTA	1008	36	20	6,48E-01	9,96E-01	rs10483453|rs17194635|rs6922031|rs17103274|rs965938|rs9817|rs4700008|rs11591298|rs4719793|rs16939964|rs2245548|rs954954|rs496888|rs6777221|rs1937332|rs769412|rs10859589|rs3738593|rs17654215|rs10916066
Discoid Rash	ANTIVIRAL_MECHANISM_BY_IFN_STIMULATED_GENES	REACTOME	1077	32	20	6,50E-01	9,96E-01	rs2228375|rs7438406|rs217797|rs2407705|rs4740420|rs2314664|rs3935428|rs11177355|rs2664537|rs2018928|rs2016266|rs730554|rs17790482|rs7612407|rs7782920|rs10271506|rs2361696|rs13415733|rs10007831|rs11107018
Discoid Rash	MEIOTIC_SYNAPSIS	REACTOME	1031	41	18	6,53E-01	9,96E-01	rs910831|rs8006145|rs799917|rs9397084|rs718527|rs17298882|rs1784304|rs9383964|rs1416388|rs472594|rs2813554|rs11935024|rs3738593|rs17082796|rs747680|rs12663707|rs7898064|rs964732
Discoid Rash	STRIATED_MUSCLE_CONTRACTION	REACTOME	453	3	3	6,55E-01	9,96E-01	rs2089910|rs11652772|rs178638
Discoid Rash	AMYLOIDS	REACTOME	972	29	19	6,55E-01	9,96E-01	rs1417718|rs17262993|rs9356797|rs196034|rs11023392|rs1784304|rs11970435|rs7544500|rs4654986|rs4683236|rs4694010|rs4932450|rs11935024|rs2092058|rs9368521|rs12520800|rs12202475|rs3857059|rs10841798
Discoid Rash	LYSOSOME	KEGG	2448	34	20	6,55E-01	9,96E-01	rs7145896|rs13146175|rs6866961|rs17123551|rs12051345|rs7691749|rs672413|rs10937820|rs3826061|rs10019475|rs7203231|rs7657084|rs4904376|rs11783925|rs882294|rs2294605|rs11769043|rs11075904|rs7703742|rs11585511
Discoid Rash	PRIMARY_BILE_ACID_BIOSYNTHESIS	KEGG	439	5	4	6,57E-01	9,96E-01	rs9886369|rs1317361|rs2251633|rs7651276
Discoid Rash	PROCESSING_OF_CAPPED_INTRON_CONTAINING_PRE_MRNA	REACTOME	1547	28	20	6,57E-01	9,96E-01	rs2228375|rs6105651|rs6034593|rs7438406|rs217797|rs7148594|rs4740420|rs12586783|rs1783983|rs4814550|rs11177355|rs7147840|rs2018928|rs11684592|rs11249163|rs6433636|rs16979884|rs3787016|rs2016266|rs9611598
Discoid Rash	ENERGY_DEPENDENT_REGULATION_OF_MTOR_BY_LKB1_AMPK	REACTOME	461	23	13	6,58E-01	9,96E-01	rs6970522|rs9568203|rs7185040|rs2727528|rs11878692|rs2746342|rs10278273|rs1348316|rs6743951|rs12540943|rs2519757|rs2271612|rs2466155
Discoid Rash	INHIBITION_OF_INSULIN_SECRETION_BY_ADRENALINE_NORADRENALINE	REACTOME	789	44	20	6,59E-01	9,96E-01	rs11012843|rs2159917|rs931646|rs11810173|rs12706724|rs7069531|rs10783282|rs10509944|rs4748444|rs6806529|rs7895467|rs13232987|rs765044|rs9840967|rs1468242|rs9420082|rs2873409|rs1331328|rs6955457|rs17755118
Discoid Rash	NRAGE_SIGNALS_DEATH_THROUGH_JNK	REACTOME	1919	30	20	6,62E-01	9,96E-01	rs2833293|rs6797559|rs17540656|rs9647067|rs538957|rs1007429|rs11678490|rs580004|rs1877330|rs10223823|rs1392912|rs17288929|rs17020055|rs1317636|rs4807101|rs2833264|rs6792241|rs10844235|rs4531312|rs1808339
Discoid Rash	APOPTOTIC_EXECUTION_PHASE	REACTOME	1211	36	20	6,67E-01	9,96E-01	rs4779683|rs1357386|rs12916064|rs16848195|rs965938|rs440804|rs2298614|rs9517302|rs10904923|rs4355511|rs6503454|rs3775068|rs9550565|rs832152|rs6777221|rs1538579|rs1941184|rs6939137|rs6778301|rs3738593
Discoid Rash	SIGNALING_BY_NOTCH1	REACTOME	3563	26	20	6,72E-01	9,96E-01	rs2179688|rs1470833|rs11608551|rs17150238|rs998910|rs10139403|rs2648888|rs6483495|rs4923889|rs10519523|rs6732014|rs12336218|rs7859384|rs6788164|rs2807305|rs17199521|rs12547643|rs17762822|rs4733859|rs1572003
Discoid Rash	T_CELL_RECEPTOR_SIGNALING_PATHWAY	KEGG	3546	36	20	6,72E-01	9,96E-01	rs4976132|rs17031463|rs6057009|rs1490801|rs231726|rs7642557|rs10933849|rs3922668|rs10483453|rs1445059|rs10114577|rs1845451|rs17103274|rs6784447|rs7747353|rs11217175|rs2376977|rs7630432|rs3448|rs4851831
Discoid Rash	REGULATION_OF_AUTOPHAGY	KEGG	933	43	20	6,72E-01	9,96E-01	rs2848780|rs3818637|rs4658565|rs881939|rs4977686|rs6657471|rs4512683|rs28477715|rs324321|rs1215|rs11665535|rs2746342|rs10853472|rs2150031|rs1496814|rs675566|rs11808494|rs4977682|rs367745|rs2156480
Discoid Rash	STARCH_AND_SUCROSE_METABOLISM	KEGG	2178	52	20	6,74E-01	9,96E-01	rs10028494|rs11722340|rs627354|rs12638582|rs2418001|rs17445697|rs11046104|rs1458226|rs1000251|rs11754555|rs17014930|rs958515|rs294755|rs1871126|rs5024722|rs6848997|rs6583142|rs6796340|rs2218935|rs217463
Discoid Rash	TNFR2_PATHWAY	BIOCARTA	414	28	14	6,75E-01	9,96E-01	rs10483453|rs17103274|rs4700008|rs16939964|rs496888|rs2112661|rs1267034|rs11746792|rs1545092|rs4906269|rs7216796|rs986475|rs10782267|rs16886445
Discoid Rash	CELL_CELL_JUNCTION_ORGANIZATION	REACTOME	6211	34	20	6,75E-01	9,96E-01	rs2140825|rs6766201|rs1420965|rs7546890|rs944248|rs12962729|rs2068606|rs157580|rs4682242|rs2136129|rs7201194|rs6897759|rs1117513|rs1785765|rs2408106|rs976968|rs17385192|rs7528744|rs12596363|rs9293168
Discoid Rash	MHC_CLASS_II_ANTIGEN_PRESENTATION	REACTOME	2226	42	20	6,76E-01	9,96E-01	rs3912622|rs42065|rs6979207|rs2120663|rs7023913|rs277317|rs3104404|rs7203231|rs372332|rs260373|rs9276284|rs4656204|rs6508259|rs11075904|rs2331880|rs211258|rs2302518|rs10495907|rs7865066|rs28383344
Discoid Rash	PKB_MEDIATED_EVENTS	REACTOME	583	26	16	6,76E-01	9,96E-01	rs7438406|rs6970522|rs9568203|rs7185040|rs2727528|rs11878692|rs2746342|rs10278273|rs1348316|rs10007831|rs1009276|rs12540943|rs2519757|rs2271612|rs2466155|rs10747658
Discoid Rash	MAL_PATHWAY	BIOCARTA	225	12	5	6,77E-01	9,96E-01	rs3448|rs4700008|rs2501276|rs11748064|rs16886445
Discoid Rash	TOB1_PATHWAY	BIOCARTA	652	35	20	6,77E-01	9,96E-01	rs1992215|rs12102171|rs17047703|rs4368250|rs7072398|rs12722589|rs7899538|rs4776862|rs12029576|rs4749882|rs2268626|rs10819641|rs1019855|rs7632716|rs2995094|rs7359174|rs12404952|rs870875|rs4278789|rs2965015
Discoid Rash	IL2_PATHWAY	BIOCARTA	452	17	13	6,79E-01	9,96E-01	rs7072398|rs2811876|rs12722589|rs36686|rs7899538|rs4744512|rs1755991|rs8013053|rs4749882|rs1585461|rs6677589|rs17183482|rs11207306
Discoid Rash	P53HYPOXIA_PATHWAY	BIOCARTA	517	21	15	6,79E-01	9,96E-01	rs879221|rs879966|rs9817|rs689452|rs12994383|rs1224540|rs11621560|rs769412|rs1496496|rs2270941|rs3136646|rs10483745|rs699235|rs11585171|rs11764490
Discoid Rash	PML_PATHWAY	BIOCARTA	384	20	12	6,79E-01	9,96E-01	rs1007119|rs10498252|rs11591298|rs2245548|rs496888|rs1937332|rs7582427|rs2507|rs7920305|rs986475|rs7599810|rs17354508
Discoid Rash	IL6_PATHWAY	BIOCARTA	401	19	15	6,81E-01	9,96E-01	rs12720279|rs1327489|rs2811876|rs36686|rs281414|rs10242595|rs8013053|rs6677589|rs17183482|rs10950917|rs11066323|rs7730168|rs11207306|rs11066320|rs10908835
Discoid Rash	ALK_PATHWAY	BIOCARTA	1932	41	20	6,81E-01	9,96E-01	rs918418|rs700591|rs913069|rs6054474|rs16842228|rs6860708|rs17047703|rs6077047|rs1845451|rs7747353|rs3916990|rs440804|rs4368250|rs1958636|rs247587|rs4355511|rs12029576|rs10485405|rs11958574|rs4075546
Discoid Rash	REGULATION_OF_INSULIN_SECRETION_BY_GLUCAGON_LIKE_PEPTIDE1	REACTOME	1388	36	20	6,82E-01	9,96E-01	rs7820461|rs2471637|rs2268657|rs2159917|rs931646|rs13426554|rs11810173|rs237485|rs6092704|rs10783282|rs10747521|rs4142696|rs965808|rs11695696|rs884423|rs6806529|rs2426154|rs17114754|rs765044|rs2446329
Discoid Rash	PHOSPHATIDYLINOSITOL_SIGNALING_SYSTEM	KEGG	3813	42	20	6,82E-01	9,96E-01	rs304051|rs6108160|rs4976132|rs1490801|rs2848780|rs901849|rs7810652|rs745719|rs873418|rs1223577|rs2278214|rs3785383|rs1858608|rs4433842|rs2179984|rs392715|rs2218314|rs7971352|rs1859733|rs7785682
Discoid Rash	NITRIC_OXIDE_STIMULATES_GUANYLATE_CYCLASE	REACTOME	1895	36	20	6,84E-01	9,96E-01	rs12418204|rs1897104|rs9844737|rs13429751|rs816296|rs11080358|rs17649232|rs2650163|rs11000160|rs10034450|rs470922|rs314157|rs293314|rs2038916|rs2682826|rs293290|rs3778248|rs6536096|rs10770645|rs10175364
Discoid Rash	ETHER_LIPID_METABOLISM	KEGG	1041	40	20	6,86E-01	9,96E-01	rs2124147|rs10494605|rs659732|rs4434808|rs10505371|rs1474797|rs1712432|rs16892827|rs1980444|rs1148757|rs6870197|rs6588591|rs6697553|rs1020382|rs867092|rs4912291|rs776691|rs16827441|rs3005888|rs13075194
Discoid Rash	REGULATION_OF_MRNA_STABILITY_BY_PROTEINS_THAT_BIND_AU_RICH_ELEMENTS	REACTOME	1372	39	20	6,89E-01	9,96E-01	rs2293576|rs6130713|rs1545229|rs328331|rs3019260|rs4740420|rs4433842|rs6783810|rs2314664|rs11189224|rs12599594|rs371500|rs10494878|rs13394402|rs6777221|rs2277743|rs12463483|rs7660665|rs3731973|rs7212835
Discoid Rash	EIF_PATHWAY	BIOCARTA	158	6	6	6,89E-01	9,96E-01	rs7438406|rs10007831|rs1555322|rs959083|rs494469|rs10426955

Discoid Rash	EGFR_DOWNREGULATION	REACTOME	1037	29	20	6,89E-01	9,96E-01
Discoid Rash	HOMOLOGOUS_RECOMBINATION	KEGG	730	24	14	6,90E-01	9,96E-01
Discoid Rash	UNFOLDED_PROTEIN_RESPONSE	REACTOME	1176	32	20	6,91E-01	9,96E-01
Discoid Rash	MTOR_SIGNALING_PATHWAY	KEGG	1290	39	20	6,92E-01	9,96E-01
Discoid Rash	CGMP_EFFECTS	REACTOME	1520	44	20	6,95E-01	9,96E-01
Discoid Rash	RNA_POL_III_TRANSCRIPTION	REACTOME	861	45	20	6,95E-01	9,96E-01
Discoid Rash	TAK1_ACTIVATES_NFKB_BY_PHOSPHORYLATION_AND_ACTIVATION_OF_IKKS_COMPLE	REACTOME	777	33	20	6,99E-01	9,96E-01
Discoid Rash	BAD_PATHWAY	BIOCARTA	927	37	20	7,01E-01	9,96E-01
Discoid Rash	GLUCAGON_TYPE_LIGAND_RECEPTORS	REACTOME	786	29	20	7,04E-01	9,96E-01
Discoid Rash	CD28_CO_STIMULATION	REACTOME	797	31	19	7,05E-01	9,96E-01
Discoid Rash	SYNTHESIS_OF_BILE_ACIDS_AND_BILE_SALTS	REACTOME	546	6	5	7,06E-01	9,96E-01
Discoid Rash	TRAF6_MEDIATED_IRF7_ACTIVATION	REACTOME	433	34	10	7,07E-01	9,96E-01
Discoid Rash	SIGNALING_BY_NODAL	REACTOME	632	30	17	7,09E-01	9,96E-01
Discoid Rash	SIGNALING_BY_BMP	REACTOME	918	35	15	7,12E-01	9,96E-01
Discoid Rash	FATTY_ACYL_COA_BIOSYNTHESIS	REACTOME	404	26	14	7,13E-01	9,96E-01
Discoid Rash	CONVERSION_FROM_APC_C_CDC20_TO_APC_C_CDH1_IN_LATE_ANAPHASE	REACTOME	226	17	7	7,13E-01	9,96E-01
Discoid Rash	GATA3_PATHWAY	BIOCARTA	898	41	20	7,13E-01	9,96E-01
Discoid Rash	APOPTOSIS	KEGG	1727	33	20	7,14E-01	9,96E-01
Discoid Rash	ACTIVATED_TAK1_MEDIATES_P38_MAPK_ACTIVATION	REACTOME	649	32	16	7,15E-01	9,96E-01
Discoid Rash	M_G1_TRANSITION	REACTOME	1315	34	20	7,15E-01	9,96E-01
Discoid Rash	METABOLISM_OF_MRNA	REACTOME	3017	31	20	7,15E-01	9,96E-01
Discoid Rash	FATTY_ACID_TRIACYLGLYCEROL_AND_KETONE_BODY_METABOLISM	REACTOME	5526	32	20	7,16E-01	9,96E-01
Discoid Rash	NOD_LIKE_RECEPTOR_SIGNALING_PATHWAY	KEGG	1406	37	20	7,16E-01	9,96E-01
Discoid Rash	OXIDATIVE_PHOSPHORYLATION	KEGG	2486	35	20	7,17E-01	9,96E-01
Discoid Rash	STRESS_PATHWAY	BIOCARTA	633	30	17	7,17E-01	9,96E-01
Discoid Rash	REGULATION_OF_APOPTOSIS	REACTOME	1432	42	20	7,18E-01	9,96E-01
Discoid Rash	CHONDROITIN_SULFATE_BIOSYNTHESIS	REACTOME	846	24	18	7,19E-01	9,96E-01
Discoid Rash	IGF1_PATHWAY	BIOCARTA	813	34	20	7,19E-01	9,96E-01
Discoid Rash	TGF_BETA_SIGNALING_PATHWAY	KEGG	3535	35	20	7,19E-01	9,96E-01
Discoid Rash	PURINE_METABOLISM	KEGG	5442	32	20	7,22E-01	9,96E-01
Discoid Rash	GLUCOSE_METABOLISM	REACTOME	1554	44	20	7,23E-01	9,96E-01
Discoid Rash	UCALPAIN_PATHWAY	BIOCARTA	426	26	16	7,24E-01	9,96E-01
Discoid Rash	CHONDROITIN_SULFATE_DERMATAN_SULFATE_METABOLISM	REACTOME	2784	35	20	7,27E-01	9,96E-01
Discoid Rash	METABOLISM_OF_XENOBIOTICS_BY_CYTOCHROME_P450	KEGG	1674	53	20	7,28E-01	9,96E-01
Discoid Rash	COMPLEMENT_AND_COAGULATION_CASCADES	KEGG	1742	28	20	7,32E-01	9,96E-01
Discoid Rash	TOLL_LIKE_RECEPTOR_SIGNALING_PATHWAY	KEGG	2339	34	20	7,34E-01	9,96E-01
Discoid Rash	BIOPEPTIDES_PATHWAY	BIOCARTA	1081	32	20	7,34E-01	9,96E-01
Discoid Rash	NOTCH1_INTRACELLULAR_DOMAIN_REGULATES_TRANSCRIPTION	REACTOME	2852	25	20	7,34E-01	9,96E-01
Discoid Rash	SPHINGOLIPID_DE_NOVO_BIOSYNTHESIS	REACTOME	1020	33	20	7,37E-01	9,96E-01
Discoid Rash	MCM_PATHWAY	BIOCARTA	234	6	5	7,41E-01	9,96E-01
Discoid Rash	RIBOFLAVIN_METABOLISM	KEGG	295	24	10	7,45E-01	9,96E-01
Discoid Rash	INITIAL_TRIGGERING_OF_COMPLEMENT	REACTOME	434	19	8	7,47E-01	9,96E-01
Discoid Rash	CALCINEURIN_PATHWAY	BIOCARTA	895	33	20	7,47E-01	9,96E-01
Discoid Rash	SYNTHESIS_SECRETION_AND_INACTIVATION_OF_GLP1	REACTOME	363	13	10	7,47E-01	9,96E-01
Discoid Rash	THROMBOXANE_SIGNALLING_THROUGH_TP_RECEPTOR	REACTOME	492	15	11	7,49E-01	9,96E-01
Discoid Rash	PROGESTERONE_MEDIATED_OOCYTE_MATURATION	KEGG	2535	45	20	7,54E-01	9,96E-01
Discoid Rash	TCR_SIGNALING	REACTOME	2040	37	20	7,57E-01	9,96E-01
Discoid Rash	PTDINS_PATHWAY	BIOCARTA	1058	34	19	7,58E-01	9,96E-01
Discoid Rash	CDK_MEDIATED_PHOSPHORYLATION_AND_REMOVAL_OF_CDC6	REACTOME	698	24	15	7,59E-01	9,96E-01
Discoid Rash	SHH_PATHWAY	BIOCARTA	679	20	15	7,60E-01	9,96E-01
Discoid Rash	GLYCOSPHINGOLIPID_BIOSYNTHESIS_LACTO_AND_NEOLACTO_SERIES	KEGG	670	14	13	7,60E-01	9,96E-01
Discoid Rash	MITOTIC_PROMETAPHASE	REACTOME	1814	37	20	7,62E-01	9,96E-01
Discoid Rash	CYCLIN_E_ASSOCIATED_EVENTS_DURING_G1_S_TRANSITION_	REACTOME	1212	36	20	7,66E-01	9,96E-01
Discoid Rash	PARKINSONS_DISEASE	KEGG	2833	31	20	7,67E-01	9,96E-01
Discoid Rash	AMYOTROPHIC_LATERAL_SCLEROSIS_ALS	KEGG	1813	25	20	7,67E-01	9,96E-01
Discoid Rash	PD1_SIGNALING	REACTOME	398	20	10	7,67E-01	9,96E-01
Discoid Rash	MEIOSIS	REACTOME	1674	39	20	7,67E-01	9,96E-01
Discoid Rash	GENERATION_OF_SECOND_MESSENGER_MOLECULES	REACTOME	616	34	18	7,69E-01	9,96E-01
Discoid Rash	NFAT_PATHWAY	BIOCARTA	1626	40	20	7,69E-01	9,96E-01
Discoid Rash	TOLL_RECEPTOR_CASCADES	REACTOME	3778	25	20	7,70E-01	9,96E-01
Discoid Rash	VIF_MEDIATED_DEGRADATION_OF_APOBEC3G	REACTOME	738	24	15	7,71E-01	9,96E-01
Discoid Rash	TRANSPORT_OF_VITAMINS_NUCLEOSIDES_AND_RELATED_MOLECULES	REACTOME	1154	38	20	7,71E-01	9,96E-01
Discoid Rash	LONG_TERM_POTENTIATION	KEGG	3620	33	20	7,74E-01	9,96E-01
Discoid Rash	MITOTIC_M_M_G1_PHASES	REACTOME	3176	36	20	7,75E-01	9,96E-01
Discoid Rash	INTEGRATION_OF_ENERGY_METABOLISM	REACTOME	4501	34	20	7,81E-01	9,96E-01
Discoid Rash	MRNA_PROCESSING	REACTOME	1942	28	20	7,81E-01	9,96E-01
Discoid Rash	PYK2_PATHWAY	BIOCARTA	752	40	20	7,82E-01	9,96E-01
Discoid Rash	CYTOKINE_PATHWAY	BIOCARTA	491	21	14	7,83E-01	9,96E-01
Discoid Rash	RESPONSE_TO_ELEVATED_PLATELET_CYTOSOLIC_CA2_	REACTOME	2301	36	20	7,83E-01	9,96E-01
Discoid Rash	IL10_PATHWAY	BIOCARTA	288	11	7	7,84E-01	9,96E-01
Discoid Rash	CELL_JUNCTION_ORGANIZATION	REACTOME	6781	34	20	7,84E-01	9,96E-01
Discoid Rash	MEIOTIC_RECOMBINATION	REACTOME	993	33	16	7,84E-01	9,96E-01
Discoid Rash	PPARA_ACTIVATES_GENE_EXPRESSION	REACTOME	3675	34	20	7,85E-01	9,96E-01
Discoid Rash	CACAM_PATHWAY	BIOCARTA	401	21	13	7,89E-01	9,96E-01
Discoid Rash	MRNA_CAPPING	REACTOME	388	9	7	7,90E-01	9,96E-01
Discoid Rash	LIGAND_GATED_ION_CHANNEL_TRANSPORT	REACTOME	1051	32	20	7,93E-01	9,96E-01
Discoid Rash	CHREBP2_PATHWAY	BIOCARTA	1059	52	20	7,93E-01	9,96E-01
Discoid Rash	P53_PATHWAY	BIOCARTA	347	14	10	7,93E-01	9,96E-01
Discoid Rash	FORMATION_OF_TRANSCRIPTION_COUPLED_NER_TC_NER_REPAIR_COMPLEX	REACTOME	389	9	5	7,96E-01	9,96E-01
Discoid Rash	REGULATION_OF_INSULIN_SECRETION	REACTOME	3741	32	20	7,97E-01	9,96E-01
Discoid Rash	AUTODEGRADATION_OF_THE_E3_UBIQUITIN_LIGASE_COP1	REACTOME	766	25	16	7,97E-01	9,96E-01
Discoid Rash	TRANSMISSION_ACROSS_CHEMICAL_SYNAPSES	REACTOME	9089	26	20	7,97E-01	9,96E-01
Discoid Rash	G1_S_TRANSITION	REACTOME	1941	39	20	7,97E-01	9,96E-01
Discoid Rash	THROMBIN_SIGNALLING_THROUGH_PROTEINASE_ACTIVATED_RECEPTORS_PARS	REACTOME	690	17	13	7,99E-01	9,96E-01
Discoid Rash	APC_C_CDH1_MEDIATED_DEGRADATION_OF_CDC20_AND_OTHER_APC_C_CDH1_TARGE TE_MITOSIS_EARLY_G1	REACTOME	959	35	20	8,00E-01	9,96E-01
Discoid Rash	ENDOSOMAL_SORTING_COMPLEX_REQUIRED_FOR_TRANSPORT_ESCRT	REACTOME	318	12	8	8,02E-01	9,96E-01
Discoid Rash	PI_METABOLISM	REACTOME	1808	38	20	8,03E-01	9,96E-01
Discoid Rash	MITOCHONDRIA_PATHWAY	BIOCARTA	317	9	7	8,03E-01	9,96E-01
Discoid Rash	PYRIMIDINE_METABOLISM	REACTOME	792	44	20	8,04E-01	9,96E-01
Discoid Rash	JNK_C_JUN_KINASES_PHOSPHORYLATION_AND_ACTIVATION_MEDIATED_BY_ACTIVATI	REACTOME	728	37	17	8,05E-01	9,96E-01
Discoid Rash	KERATINOCYTE_PATHWAY	BIOCARTA	1952	30	20	8,09E-01	9,96E-01

EGFR_DOWNREGULATION	rs13131772|rs11217175|rs372332|rs2314664|rs7328506|rs1993191|rs2302518|rs3113392|rs7865066|rs518569|rs1334384|rs3824389|rs2184818|rs6512102|rs2501276|rs312181|rs4490948|rs34815730|rs12982931|rs1028594
HOMOLOGOUS_RECOMBINATION	rs2331780|rs963917|rs17772583|rs12896969|rs7141573|rs7208872|rs11236181|rs535801|rs4899246|rs10241838|rs1673041|rs10134446|rs927221|rs739719
UNFOLDED_PROTEIN_RESPONSE	rs2136703|rs240940|rs1545229|rs4689388|rs17019438|rs4931434|rs1229564|rs1358024|rs13332660|rs11189224|rs13429247|rs11869535|rs1912445|rs40957|rs17660888|rs3775068|rs741811|rs6762892|rs9924434|rs10263979
MTOR_SIGNALING_PATHWAY	rs4976132|rs1490801|rs7438406|rs16898963|rs9568203|rs7185040|rs11878692|rs4976028|rs10073996|rs11576136|rs2746342|rs7668750|rs1002487|rs7898235|rs6598858|rs13148526|rs693293|rs873350|rs7719763|rs10007831
CGMP_EFFECTS	rs12418204|rs1897104|rs9844737|rs13429751|rs17649232|rs11000160|rs10034450|rs470922|rs314157|rs293314|rs2038916|rs293290|rs3778248|rs10770645|rs10175364|rs1937683|rs7485410|rs6534152|rs10508942|rs7591292
RNA_POL_III_TRANSCRIPTION	rs2224553|rs7873382|rs575660|rs2078639|rs17420770|rs1813529|rs10961329|rs13302756|rs3787016|rs1532445|rs10961439|rs10961483|rs17092361|rs10961347|rs17603298|rs1385309|rs2995094|rs1323348|rs2243506|rs3746162
TAK1_ACTIVATES_NFKB_BY_PHOSPHORYLATION_AND_ACTIVATION_OF_IKKS_COMPLE	rs1043395|rs10483453|rs1845451|rs17103274|rs7747353|rs559052|rs204990|rs10485405|rs9550565|rs2839343|rs150615|rs565665|rs3136646|rs6442159|rs5743369|rs1114328|rs1056890|rs284509|rs8057341|rs6961625
BAD_PATHWAY	rs4976132|rs1490801|rs1589880|rs13120102|rs4976028|rs954954|rs10073996|rs11576136|rs1002487|rs7166565|rs10794486|rs6598858|rs4724400|rs2270941|rs7719763|rs10161354|rs10070379|rs10940145|rs13224682|rs9755490
GLUCAGON_TYPE_LIGAND_RECEPTORS	rs1607987|rs2268657|rs2159917|rs931646|rs11810173|rs6092704|rs4797604|rs965808|rs1124425|rs1410489|rs9945019|rs765044|rs926674|rs17810148|rs2000600|rs8091765|rs2873409|rs578512|rs7233940|rs1880081
CD28_CO_STIMULATION	rs4976132|rs1490801|rs1544241|rs16939964|rs4807101|rs4976028|rs10073996|rs2501276|rs7719763|rs6506537|rs7258040|rs10070379|rs7216796|rs10940145|rs12610223|rs2612102|rs2489863|rs4131890|rs610902
SYNTHESIS_OF_BILE_ACIDS_AND_BILE_SALTS	rs9886369|rs1317361|rs854151|rs2251633|rs7651276
TRAF6_MEDIATED_IRF7_ACTIVATION	rs9900686|rs4977686|rs793801|rs1267034|rs12130290|rs1539243|rs12229321|rs10124609|rs11733679|rs4623937
SIGNALING_BY_NODAL	rs1992215|rs12102171|rs11661651|rs14868|rs4368250|rs4776862|rs11855154|rs11680214|rs8042430|rs6598483|rs1268160|rs10799329|rs7359174|rs11853956|rs6598477|rs17512976|rs3784485
SIGNALING_BY_BMP	rs6054474|rs6077047|rs7781554|rs1259320|rs4368250|rs2173049|rs6701024|rs7546054|rs4075546|rs11680214|rs2423163|rs12759793|rs6085817|rs12625876|rs6691111
FATTY_ACYL_COA_BIOSYNTHESIS	rs209512|rs9905601|rs7656124|rs3798719|rs247011|rs17138930|rs876873|rs6760807|rs2084182|rs4244350|rs12804822|rs1858074|rs149293|rs10452184
CONVERSION_FROM_APC_C_CDC20_TO_APC_C_CDH1_IN_LATE_ANAPHASE	rs9653414|rs6852016|rs12480887|rs16941635|rs9867568|rs17775614|rs599215
GATA3_PATHWAY	rs2664904|rs1149907|rs10905533|rs12248916|rs827405|rs7101305|rs7900250|rs827618|rs12325586|rs7067548|rs4747817|rs13224682|rs9755490|rs7078148|rs7921734|rs434645|rs11150198|rs4749641|rs1243974|rs1416560
APOPTOSIS	rs4976132|rs1490801|rs17540656|rs10483453|rs17042407|rs17103274|rs965938|rs11591298|rs365238|rs4719793|rs16939964|rs2245548|rs9868605|rs4976028|rs954954|rs10073996|rs3850170|rs11997323|rs13035227|rs1937332
ACTIVATED_TAK1_MEDIATES_P38_MAPK_ACTIVATION	rs1043395|rs1845451|rs7747353|rs10494878|rs559052|rs100043|rs10485405|rs150615|rs565665|rs6442159|rs5743369|rs1114328|rs284509|rs2715815|rs8057341|rs6961625
M_G1_TRANSITION	rs7916990|rs2293576|rs1535010|rs328331|rs495026|rs2314664|rs12599594|rs6904263|rs371500|rs13394402|rs9396464|rs10508455|rs10146912|rs2891652|rs10948689|rs12463483|rs2817739|rs4134966|rs3782093|rs3731973
METABOLISM_OF_MRNA	rs2958522|rs2293576|rs12896325|rs6130713|rs4554825|rs7255657|rs7438406|rs3810168|rs1545229|rs328331|rs4558175|rs3019260|rs2816764|rs4740420|rs4433842|rs6783810|rs2314664|rs11189224|rs12599594|rs10896604
FATTY_ACID_TRIACYLGLYCEROL_AND_KETONE_BODY_METABOLISM	rs1151996|rs209512|rs1511451|rs177421|rs2036713|rs6559175|rs11608551|rs998910|rs4731630|rs7026534|rs6970522|rs2303091|rs16912392|rs737479|rs2297409|rs11127284|rs6072343|rs11634318|rs13064360|rs7335993
NOD_LIKE_RECEPTOR_SIGNALING_PATHWAY	rs12602195|rs1043395|rs10483453|rs1845451|rs17103274|rs7747353|rs10500300|rs556335|rs6854208|rs396146|rs10485405|rs11621560|rs10242595|rs3775167|rs2859129|rs4849123|rs150615|rs1545092|rs565665|rs3136646
OXIDATIVE_PHOSPHORYLATION	rs2713834|rs12065184|rs2839604|rs2041689|rs4920706|rs218979|rs4474804|rs4699172|rs10982300|rs2280231|rs527216|rs4854073|rs11870213|rs12586783|rs1142530|rs11783925|rs8112614|rs10061728|rs4853975|rs11769043
STRESS_PATHWAY	rs10483453|rs17194635|rs17103274|rs4700008|rs16939964|rs2811876|rs100043|rs1267034|rs10859589|rs6677589|rs7216796|rs9914858|rs11207306|rs2715815|rs7314748|rs986475|rs16886445
REGULATION_OF_APOPTOSIS	rs2293576|rs12327358|rs10999739|rs328331|rs9950125|rs965938|rs11875845|rs2314664|rs1373762|rs1927976|rs12599594|rs371500|rs13394402|rs12962203|rs4940235|rs9956761|rs2274606|rs12463483|rs3731973|rs7212835
CHONDROITIN_SULFATE_BIOSYNTHESIS	rs7976582|rs10520079|rs10161421|rs7240679|rs17480734|rs10100391|rs10478926|rs4438389|rs1352033|rs1975752|rs9954485|rs11593516|rs7960741|rs4929810|rs2130398|rs10502494|rs2410611|rs8098615
IGF1_PATHWAY	rs4976132|rs1490801|rs7599150|rs2811876|rs4976028|rs10073996|rs17188448|rs8013053|rs7166565|rs10794486|rs7719763|rs10070379|rs6677589|rs17183482|rs10940145|rs11066323|rs11207306|rs2684777|rs11066320|rs2684790
TGF_BETA_SIGNALING_PATHWAY	rs17397603|rs1992215|rs932659|rs1499367|rs913069|rs6054474|rs16842228|rs12102171|rs9406337|rs9358259|rs17199521|rs17768143|rs17047703|rs6077047|rs11661651|rs2543090|rs12547643|rs7781554|rs4733859|rs893222
PURINE_METABOLISM	rs12418204|rs1064448|rs1897104|rs1447981|rs13429751|rs7820461|rs4804505|rs6445205|rs1245371|rs7623479|rs1947152|rs7522367|rs203777|rs7712322|rs7640419|rs244570|rs212063|rs10058673|rs12267330|rs17649232
GLUCOSE_METABOLISM	rs7092772|rs2243816|rs12638582|rs1932001|rs2418001|rs600130|rs12240387|rs548348|rs10508243|rs6467993|rs11046104|rs9935162|rs1011450|rs7196146|rs17147932|rs6725388|rs6682376|rs1871126|rs7071157|rs12343547
UCALPAIN_PATHWAY	rs1351140|rs17106413|rs1475035|rs3448|rs2056131|rs2268969|rs1994350|rs1187095|rs11652772|rs4767884|rs3867132|rs1950712|rs12586937|rs2126953|rs901363|rs10940277
CHONDROITIN_SULFATE_DERMATAN_SULFATE_METABOLISM	rs7976582|rs10520079|rs2128333|rs9390633|rs6866961|rs10161421|rs672413|rs2119299|rs7990210|rs1572342|rs4144545|rs519604|rs7993144|rs7337223|rs7240679|rs2439525|rs7113440|rs6933665|rs7451498|rs1891523
METABOLISM_OF_XENOBIOTICS_BY_CYTOCHROME_P450	rs10028494|rs6637|rs11773597|rs12646059|rs34511626|rs1009668|rs1458226|rs316156|rs915907|rs7355084|rs294755|rs6848997|rs1662037|rs2270941|rs1800440|rs293408|rs11572172|rs10490621|rs2132847|rs1051740
COMPLEMENT_AND_COAGULATION_CASCADES	rs945032|rs12758064|rs1058242|rs11160174|rs4504976|rs7146347|rs1050783|rs8120756|rs9995366|rs1482875|rs1245480|rs4251854|rs2749527|rs12492885|rs4900312|rs10762788|rs1902946|rs7766109|rs12198109|rs941755
TOLL_LIKE_RECEPTOR_SIGNALING_PATHWAY	rs4976132|rs17326309|rs1490801|rs1043395|rs10483453|rs1845451|rs1928298|rs17103274|rs7747353|rs956239|rs4977686|rs4604517|rs2052834|rs2811876|rs1827153|rs4976028|rs10073996|rs559052|rs12647506|rs100043
BIOPEPTIDES_PATHWAY	rs1327489|rs6557991|rs2241694|rs3785383|rs4433842|rs12706724|rs392715|rs2664537|rs1011973|rs8051531|rs6503454|rs11646540|rs17753569|rs17811655|rs13232987|rs2741343|rs4446309|rs4686799|rs2158196|rs1468242
NOTCH1_INTRACELLULAR_DOMAIN_REGULATES_TRANSCRIPTION	rs1470833|rs11608551|rs17150238|rs998910|rs2648888|rs6483495|rs10519523|rs12336218|rs7859384|rs6788164|rs2807305|rs17199521|rs12547643|rs4733859|rs1572003|rs12646059|rs7856994|rs838875|rs2314664|rs1888855
SPHINGOLIPID_DE_NOVO_BIOSYNTHESIS	rs17533359|rs1191571|rs3787146|rs1493130|rs17571979|rs2131463|rs11005872|rs12605289|rs1002667|rs6015258|rs6588591|rs2927714|rs1440985|rs12587100|rs2165406|rs1020382|rs1160677|rs9831488|rs4912291|rs3005888
MCM_PATHWAY	rs1535010|rs2891652|rs10948689|rs10161145|rs747232
RIBOFLAVIN_METABOLISM	rs7983595|rs17191474|rs11754555|rs11039149|rs9866280|rs7253363|rs7545497|rs6935458|rs1003299|rs1042602
INITIAL_TRIGGERING_OF_COMPLEMENT	rs4504976|rs12492885|rs10762788|rs1917186|rs10824854|rs2856448|rs1962474|rs2277984
CALCINEURIN_PATHWAY	rs10191574|rs11153413|rs3785383|rs4433842|rs392715|rs9320442|rs2664537|rs8051531|rs11646540|rs17753569|rs17811655|rs4478191|rs17786782|rs198182|rs7755236|rs11675772|rs12598|rs796495|rs198190|rs7595027
SYNTHESIS_SECRETION_AND_INACTIVATION_OF_GLP1	rs791595|rs440804|rs1551368|rs4731416|rs1806180|rs6467165|rs791600|rs2333452|rs6882366|rs11536344
THROMBOXANE_SIGNALLING_THROUGH_TP_RECEPTOR	rs2159917|rs931646|rs11810173|rs765044|rs11652805|rs310686|rs17786782|rs11655004|rs2873409|rs6955457|rs17755118
PROGESTERONE_MEDIATED_OOCYTE_MATURATION	rs1064448|rs4976132|rs1490801|rs7820461|rs16898963|rs9653414|rs10058673|rs4382132|rs10475376|rs12706724|rs17826588|rs1589880|rs6852016|rs17737362|rs10783282|rs901478|rs4976028|rs601618|rs10073996|rs12647506
TCR_SIGNALING	rs4976132|rs1490801|rs3922668|rs873418|rs10483453|rs1845451|rs3104404|rs17103274|rs7747353|rs9276284|rs2664537|rs2707212|rs4976028|rs252308|rs10904923|rs10073996|rs559052|rs10485405|rs28383344|rs12716251
PTDINS_PATHWAY	rs2179688|rs7349397|rs2204204|rs2344662|rs10114577|rs12240387|rs2664537|rs10508243|rs2302518|rs6040055|rs6133987|rs6108803|rs9664019|rs884289|rs7559522|rs935672|rs506134|rs2519106|rs12357621
CDK_MEDIATED_PHOSPHORYLATION_AND_REMOVAL_OF_CDC6	rs2293576|rs328331|rs2314664|rs12599594|rs371500|rs13394402|rs12463483|rs3731973|rs7212835|rs3751497|rs8005252|rs3819715|rs2550461|rs241440|rs2298265
SHH_PATHWAY	rs4724083|rs6709236|rs2363923|rs12667722|rs4716990|rs3801228|rs7811368|rs288761|rs2354471|rs3823734|rs13224682|rs9755490|rs1922086|rs2835753|rs1992902
GLYCOSPHINGOLIPID_BIOSYNTHESIS_LACTO_AND_NEOLACTO_SERIES	rs4818064|rs17646831|rs4021|rs7754801|rs36686|rs496862|rs10754051|rs759584|rs4599653|rs6714411|rs13410278|rs6570428|rs2418049
MITOTIC_PROMETAPHASE	rs4265409|rs2028455|rs8003274|rs3001083|rs4804149|rs10510126|rs8096557|rs12124096|rs11177355|rs9812706|rs2331880|rs2791152|rs17298882|rs3010110|rs2462456|rs9867712|rs709641|rs12451623|rs867092|rs714447
CYCLIN_E_ASSOCIATED_EVENTS_DURING_G1_S_TRANSITION_	rs2293576|rs1499367|rs17199521|rs328331|rs12547643|rs4733859|rs2314664|rs12599594|rs10505507|rs371500|rs13394402|rs13160147|rs7928738|rs6985681|rs12463483|rs1383595|rs6987802|rs7212835|rs3751497|rs8005252
PARKINSONS_DISEASE	rs13195756|rs12065184|rs2404839|rs2839604|rs4920706|rs218979|rs4474804|rs2280231|rs527216|rs4854073|rs965938|rs4709619|rs12586783|rs9355359|rs1142530|rs13211741|rs5993761|rs4719793|rs10061728|rs10467147
AMYOTROPHIC_LATERAL_SCLEROSIS_ALS	rs157580|rs11074683|rs816296|rs17036351|rs6922031|rs965938|rs2650163|rs1805510|rs3448|rs1461234|rs17036576|rs4719793|rs4502882|rs9921476|rs10875943|rs11746889|rs954954|rs496888|rs100043|rs11953799
PD1_SIGNALING	rs3104404|rs9276284|rs2707212|rs28383344|rs2282055|rs444821|rs4237162|rs2995094|rs3808847|rs870875
MEIOSIS	rs910831|rs8006145|rs17772583|rs16892616|rs799917|rs707938|rs9397084|rs7728406|rs718527|rs17298882|rs1784304|rs9383964|rs1416388|rs472594|rs7208872|rs2813554|rs3774332|rs11935024|rs3738593|rs6127999
GENERATION_OF_SECOND_MESSENGER_MOLECULES	rs3922668|rs3104404|rs9276284|rs2664537|rs2707212|rs252308|rs28383344|rs12716251|rs16845098|rs166152|rs4851462|rs315751|rs444821|rs2995094|rs25856|rs12434820|rs696743|rs870875
NFAT_PATHWAY	rs4976132|rs918418|rs1490801|rs700591|rs283433|rs6860708|rs2356932|rs1644512|rs4976028|rs10073996|rs11913168|rs247587|rs11958574|rs9608854|rs17615558|rs4478191|rs4844477|rs7719763|rs10070379|rs10940145
TOLL_RECEPTOR_CASCADES	rs918418|rs17326309|rs700591|rs13281383|rs1043395|rs2848780|rs10483453|rs16898963|rs7175248|rs6860708|rs1845451|rs7023913|rs329499|rs1360094|rs11633249|rs17103274|rs7747353|rs2698537|rs7823751|rs956239
VIF_MEDIATED_DEGRADATION_OF_APOBEC3G	rs2293576|rs328331|rs2314664|rs12599594|rs371500|rs13394402|rs12463483|rs3731973|rs7212835|rs3751497|rs8005252|rs3819715|rs2550461|rs241440|rs2298265
TRANSPORT_OF_VITAMINS_NUCLEOSIDES_AND_RELATED_MOLECULES	rs6496888|rs17536553|rs12985511|rs4984325|rs17425371|rs12554805|rs1530634|rs2061714|rs11045834|rs4762785|rs2249998|rs11666579|rs2286814|rs2295085|rs6010817|rs10501421|rs7181019|rs387352|rs8040847|rs1545470
LONG_TERM_POTENTIATION	rs304051|rs6108160|rs7820461|rs901849|rs11074683|rs2239016|rs1108385|rs16898963|rs17036351|rs2241694|rs3785383|rs1805510|rs11062222|rs4433842|rs17743079|rs1892879|rs2179984|rs1589880|rs392715|rs1461234
MITOTIC_M_M_G1_PHASES	rs7916990|rs2293576|rs4265409|rs2028455|rs8003274|rs3001083|rs4804149|rs10510126|rs1535010|rs328331|rs8096557|rs495026|rs2314664|rs12124096|rs11177355|rs9812706|rs12599594|rs6904263|rs2331880|rs371500
INTEGRATION_OF_ENERGY_METABOLISM	rs1064448|rs6108160|rs362988|rs7820461|rs6077718|rs11153413|rs2471637|rs11012843|rs2268657|rs2159917|rs1120559|rs6970522|rs931646|rs10058673|rs13426554|rs2297972|rs4382132|rs791595|rs11810173|rs440804
MRNA_PROCESSING	rs2228375|rs6105651|rs6034593|rs7438406|rs217797|rs2279083|rs7148594|rs4740420|rs12586783|rs964888|rs1783983|rs4814550|rs11177355|rs7147840|rs2018928|rs11684592|rs11249163|rs6433636|rs16979884|rs3787016
PYK2_PATHWAY	rs6557991|rs3785383|rs4433842|rs392715|rs4700008|rs2811876|rs2664537|rs8051531|rs11646540|rs17753569|rs17811655|rs2741343|rs9927309|rs17786782|rs198182|rs6677589|rs9914858|rs11207306|rs198190|rs7595027
CYTOKINE_PATHWAY	rs7717673|rs17042407|rs10948680|rs10484882|rs1833754|rs10242595|rs918519|rs10950917|rs1912124|rs10030060|rs485499|rs3024498|rs986475|rs739719
RESPONSE_TO_ELEVATED_PLATELET_CYTOSOLIC_CA2_	rs873705|rs17525472|rs1058242|rs17047703|rs11160174|rs4148482|rs5745752|rs9898|rs17106413|rs3785383|rs1050783|rs17530191|rs1475035|rs8177191|rs4433842|rs14868|rs392715|rs1358024|rs1482875|rs2749527
IL10_PATHWAY	rs17042407|rs699512|rs10242595|rs7572482|rs10950917|rs3024498|rs986475
CELL_JUNCTION_ORGANIZATION	rs2140825|rs6766201|rs1420965|rs7546890|rs944248|rs12962729|rs2068606|rs157580|rs4682242|rs2136129|rs7201194|rs6897759|rs1117513|rs1785765|rs2408106|rs976968|rs17385192|rs7528744|rs12596363|rs9293168
MEIOTIC_RECOMBINATION	rs17772583|rs16892616|rs799917|rs707938|rs7728406|rs1784304|rs472594|rs9916423|rs3774332|rs11935024|rs6127999|rs9368521|rs6452162|rs2973606|rs535801|rs739719
PPARA_ACTIVATES_GENE_EXPRESSION	rs1151996|rs1511451|rs177421|rs2036713|rs998910|rs4731630|rs7026534|rs16912392|rs737479|rs2297409|rs11634318|rs17237521|rs12985511|rs135549|rs7788404|rs10850626|rs1897164|rs7656250|rs8102574|rs1995917
CACAM_PATHWAY	rs2241694|rs2356932|rs1011973|rs1644512|rs2254137|rs4446309|rs2158196|rs11892543|rs4844477|rs7660775|rs10491334|rs17202235|rs7595027
MRNA_CAPPING	rs2279083|rs964888|rs3787016|rs4803238|rs3746162|rs6901395|rs9362529
LIGAND_GATED_ION_CHANNEL_TRANSPORT	rs745013|rs10515829|rs4238490|rs10937160|rs12221649|rs4572353|rs1994313|rs2148174|rs6606888|rs4554269|rs159926|rs4132750|rs17116687|rs4076021|rs17539361|rs592403|rs12441474|rs2880691|rs6846164|rs3843498
CHREBP2_PATHWAY	rs6130713|rs6970522|rs8003274|rs4689407|rs3019260|rs1480157|rs2727528|rs11784209|rs6775793|rs2746342|rs10278273|rs160970|rs6814782|rs17138930|rs1348316|rs1401547|rs13224682|rs9755490|rs12540943|rs11135929
P53_PATHWAY	rs879221|rs9817|rs954954|rs769412|rs2270941|rs699235|rs11585171|rs7242402|rs2849380|rs1439123
FORMATION_OF_TRANSCRIPTION_COUPLED_NER_TC_NER_REPAIR_COMPLEX	rs4253042|rs3787016|rs7722373|rs7081472|rs3746162
REGULATION_OF_INSULIN_SECRETION	rs6108160|rs362988|rs7820461|rs6077718|rs11153413|rs2471637|rs11012843|rs2268657|rs2159917|rs1120559|rs931646|rs13426554|rs2297972|rs791595|rs11810173|rs440804|rs4433842|rs237485|rs12706724|rs6092704
AUTODEGRADATION_OF_THE_E3_UBIQUITIN_LIGASE_COP1	rs2293576|rs328331|rs2314664|rs12599594|rs371500|rs13394402|rs12041226|rs12463483|rs3731973|rs7212835|rs3751497|rs8005252|rs3819715|rs2550461|rs241440|rs2298265
TRANSMISSION_ACROSS_CHEMICAL_SYNAPSES	rs2789603|rs1064448|rs6108160|rs2808539|rs745013|rs17779442|rs10455248|rs2496531|rs362988|rs7820461|rs6077718|rs9292637|rs305522|rs4821506|rs2190550|rs11012843|rs11074683|rs2050140|rs2159917|rs10515829
G1_S_TRANSITION	rs7916990|rs2293576|rs1499367|rs17199521|rs1535010|rs328331|rs12547643|rs495026|rs4733859|rs2314664|rs12599594|rs10505507|rs371500|rs13394402|rs9396464|rs10508455|rs10146912|rs13160147|rs2891652|rs7928738
THROMBIN_SIGNALLING_THROUGH_PROTEINASE_ACTIVATED_RECEPTORS_PARS	rs2159917|rs931646|rs11810173|rs9905578|rs765044|rs773902|rs11652805|rs310686|rs17786782|rs11655004|rs2873409|rs6955457|rs17755118
APC_C_CDH1_MEDIATED_DEGRADATION_OF_CDC20_AND_OTHER_APC_C_CDH1_TARGE TE_MITOSIS_EARLY_G1	rs2293576|rs9653414|rs328331|rs6852016|rs2314664|rs12599594|rs371500|rs13394402|rs16979884|rs12480887|rs13160147|rs16941635|rs12463483|rs3731973|rs7212835|rs10485805|rs3751497|rs7727115|rs8005252|rs9867568
ENDOSOMAL_SORTING_COMPLEX_REQUIRED_FOR_TRANSPORT_ESCRT	rs2314664|rs1387551|rs7816946|rs11997323|rs34815730|rs522073|rs2378026|rs2242268
PI_METABOLISM	rs4976132|rs1490801|rs2848780|rs745719|rs873418|rs7983595|rs2218314|rs7971352|rs4976028|rs10073996|rs537885|rs324321|rs11665535|rs10853472|rs8138677|rs693293|rs11604561|rs7719763|rs10070379|rs1496814
MITOCHONDRIA_PATHWAY	rs965938|rs4719793|rs954954|rs2270941|rs7242402|rs2849380|rs1439123
PYRIMIDINE_METABOLISM	rs3793357|rs12026896|rs1625579|rs8072869|rs822607|rs242253|rs290826|rs2786529|rs12401542|rs2282011|rs768364|rs34085458|rs11804383|rs10799655|rs10493899|rs13023991|rs2349106|rs4789145|rs9966612|rs7010772
JNK_C_JUN_KINASES_PHOSPHORYLATION_AND_ACTIVATION_MEDIATED_BY_ACTIVATI	rs1043395|rs1845451|rs7747353|rs556335|rs12647506|rs10485405|rs3775167|rs150615|rs565665|rs6442159|rs5743369|rs7438872|rs1114328|rs9914858|rs284509|rs8057341|rs6961625
KERATINOCYTE_PATHWAY	rs7349397|rs2204204|rs1477577|rs2344662|rs10483453|rs6922031|rs3785383|rs17103274|rs1990966|rs4433842|rs3746882|rs392715|rs4700008|rs11591298|rs16939964|rs2245548|rs2811876|rs954954|rs10904923|rs496888

Discoid Rash	GAP_JUNCTION_TRAFFICKING	REACTOME	576	24	15	8,10E-01	9,96E-01	rs2432216|rs7023913|rs1886176|rs6453850|rs7703742|rs1857208|rs12862093|rs4804528|rs7174852|rs3003608|rs10870680|rs9552089|rs7984378|rs3845481|rs10080593
Discoid Rash	ABORTIVE_ELONGATION_OF_HIV1_TRANSCRIPT_IN_THE_ABSENCE_OF_TAT	REACTOME	195	4	3	8,11E-01	9,96E-01	rs3787016|rs4803238|rs3746162
Discoid Rash	PHOSPHORYLATION_OF_CD3_AND_TCR_ZETA_CHAINS	REACTOME	521	20	10	8,12E-01	9,96E-01	rs3104404|rs9276284|rs2707212|rs28383344|rs13374545|rs7012200|rs1898240|rs444821|rs2995094|rs870875
Discoid Rash	FMLP_PATHWAY	BIOCARTA	1148	29	20	8,12E-01	9,96E-01	rs6108160|rs10483453|rs17103274|rs2179984|rs4700008|rs7971352|rs2356932|rs6118062|rs6140561|rs100043|rs537885|rs6086525|rs6039189|rs6055889|rs4478191|rs310686|rs4844477|rs11044233|rs2715815|rs12598
Discoid Rash	AUTODEGRADATION_OF_CDH1_BY_CDH1_APC_C	REACTOME	857	38	20	8,14E-01	9,96E-01	rs2293576|rs9653414|rs328331|rs6852016|rs2314664|rs12599594|rs371500|rs13394402|rs12480887|rs16941635|rs12463483|rs7212835|rs3751497|rs8005252|rs9867568|rs17775614|rs3819715|rs2550461|rs241440|rs2298265
Discoid Rash	G2_M_CHECKPOINTS	REACTOME	710	32	19	8,15E-01	9,96E-01	rs7916990|rs1535010|rs495026|rs901478|rs2321744|rs10508455|rs7324754|rs614123|rs2891652|rs2686830|rs7928738|rs10948689|rs9311428|rs9598129|rs1753477|rs3191402|rs11258231|rs1328667|rs4035540
Discoid Rash	FC_EPSILON_RI_SIGNALING_PATHWAY	KEGG	2758	39	20	8,16E-01	9,96E-01	rs4976132|rs1490801|rs7349397|rs3922668|rs2204204|rs2344662|rs10114577|rs10494605|rs659732|rs4434808|rs3785383|rs4433842|rs392715|rs1474797|rs1712432|rs12693008|rs2664537|rs1980444|rs17020055|rs4807101
Discoid Rash	INCRETIN_SYNTHESIS_SECRETION_AND_INACTIVATION	REACTOME	540	16	12	8,18E-01	9,96E-01	rs791595|rs440804|rs1551368|rs4371706|rs4731416|rs1806180|rs6467165|rs791600|rs2333452|rs4840583|rs6882366|rs11536344
Discoid Rash	RIG_I_MDA5_MEDIATED_INDUCTION_OF_IFN_ALPHA_BETA_PATHWAYS	REACTOME	1129	34	20	8,18E-01	9,96E-01	rs9900686|rs10483453|rs4785452|rs17103274|rs11217175|rs4977686|rs2314664|rs4700008|rs204990|rs793801|rs1267034|rs12130290|rs730554|rs9550565|rs2839347|rs1545092|rs1539243|rs3136646|rs12229321|rs4906269
Discoid Rash	ACTIVATION_OF_KAINATE_RECEPTORS_UPON_GLUTAMATE_BINDING	REACTOME	1577	29	20	8,18E-01	9,96E-01	rs6108160|rs2159917|rs931646|rs2252359|rs218428|rs9485630|rs11810173|rs2179984|rs6118062|rs6140561|rs954765|rs7121527|rs17623275|rs6086525|rs7771431|rs12206631|rs2832458|rs765044|rs6039189|rs6055889
Discoid Rash	P53_DEPENDENT_G1_DNA_DAMAGE_RESPONSE	REACTOME	838	28	18	8,19E-01	9,96E-01	rs2293576|rs328331|rs9817|rs2314664|rs12599594|rs371500|rs13394402|rs769412|rs12041226|rs12463483|rs3731973|rs7212835|rs3751497|rs8005252|rs3819715|rs2550461|rs241440|rs2298265
Discoid Rash	PANCREATIC_CANCER	KEGG	1920	50	20	8,19E-01	9,96E-01	rs4976132|rs1992215|rs1490801|rs12102171|rs2124147|rs11681491|rs17047703|rs11661651|rs4368250|rs4976028|rs10073996|rs4776862|rs6854208|rs12029576|rs4690476|rs3775167|rs5756573|rs13148526|rs2501276|rs2268626
Discoid Rash	ASSEMBLY_OF_THE_PRE_REPLICATIVE_COMPLEX	REACTOME	911	27	20	8,21E-01	9,96E-01	rs2293576|rs1535010|rs328331|rs2314664|rs12599594|rs6904263|rs371500|rs13394402|rs2891652|rs10948689|rs12463483|rs2817739|rs4134966|rs3731973|rs7212835|rs3751497|rs8005252|rs746720|rs747232|rs3819715
Discoid Rash	APC_C_CDC20_MEDIATED_DEGRADATION_OF_MITOTIC_PROTEINS	REACTOME	1094	36	20	8,26E-01	9,96E-01	rs2293576|rs9653414|rs10510126|rs328331|rs6852016|rs2314664|rs12599594|rs371500|rs13394402|rs12480887|rs16941635|rs12463483|rs13130657|rs3731973|rs7212835|rs3751497|rs7727115|rs8005252|rs11248466|rs9867568
Discoid Rash	P53_INDEPENDENT_G1_S_DNA_DAMAGE_CHECKPOINT	REACTOME	729	25	16	8,26E-01	9,96E-01	rs2293576|rs328331|rs2314664|rs12599594|rs371500|rs13394402|rs12463483|rs3731973|rs7212835|rs3751497|rs8005252|rs3819715|rs2550461|rs241440|rs4035540|rs2298265
Discoid Rash	AMINO_ACID_TRANSPORT_ACROSS_THE_PLASMA_MEMBRANE	REACTOME	1025	25	14	8,27E-01	9,96E-01	rs3829406|rs1348573|rs4790232|rs1439502|rs298467|rs11836940|rs4482114|rs12879346|rs12373586|rs9792165|rs2065134|rs8013531|rs6738590|rs4684213
Discoid Rash	ACTIVATION_OF_CHAPERONE_GENES_BY_XBP1S	REACTOME	761	32	20	8,31E-01	9,96E-01	rs240940|rs4689388|rs4931434|rs1358024|rs13332660|rs2884289|rs40957|rs17660888|rs3775068|rs741811|rs6762892|rs11243152|rs3738593|rs12933548|rs6774516|rs4684672|rs7635047|rs4669632|rs10095449|rs560577
Discoid Rash	CARDIAC_MUSCLE_CONTRACTION	KEGG	3169	27	20	8,31E-01	9,96E-01	rs6942458|rs12707506|rs4821506|rs11012843|rs2050140|rs2239016|rs7637998|rs4920706|rs12475881|rs1108385|rs4474804|rs6796475|rs9309056|rs11062222|rs7618850|rs10061728|rs1006564|rs1034784|rs7069531|rs11600216
Discoid Rash	IL12_PATHWAY	BIOCARTA	589	24	18	8,31E-01	9,96E-01	rs12720279|rs1327489|rs2811876|rs9818739|rs100043|rs1833754|rs281414|rs7572482|rs6677589|rs7250425|rs2995094|rs918519|rs485499|rs2305740|rs11207306|rs2715815|rs924080|rs870875
Discoid Rash	CELL_CYCLE_CHECKPOINTS	REACTOME	1961	37	20	8,31E-01	9,96E-01	rs7916990|rs2293576|rs9653414|rs10510126|rs1535010|rs328331|rs495026|rs6852016|rs9817|rs2314664|rs12599594|rs901478|rs371500|rs13394402|rs2321744|rs10508455|rs7324754|rs12480887|rs614123|rs2891652
Discoid Rash	NOD1_2_SIGNALING_PATHWAY	REACTOME	881	35	20	8,32E-01	9,96E-01	rs1043395|rs4785452|rs1845451|rs7747353|rs556335|rs100043|rs10485405|rs17790482|rs2859131|rs150615|rs1545092|rs565665|rs6442159|rs5743369|rs1114328|rs284509|rs11107025|rs2715815|rs7364173|rs10782267
Discoid Rash	MITOTIC_G1_G1_S_PHASES	REACTOME	2477	38	20	8,33E-01	9,96E-01	rs7916990|rs2293576|rs3217810|rs2648888|rs17199521|rs1535010|rs328331|rs12547643|rs495026|rs4733859|rs2314664|rs12599594|rs2896452|rs10505507|rs11784209|rs371500|rs13394402|rs9396464|rs10508455|rs10146912
Discoid Rash	ALZHEIMERS_DISEASE	KEGG	5029	37	20	8,35E-01	9,96E-01	rs304051|rs6108160|rs1072869|rs157580|rs901849|rs12065184|rs11074683|rs2239016|rs2839604|rs4920706|rs1108385|rs218979|rs4474804|rs816296|rs2280231|rs527216|rs4854073|rs965938|rs2650163|rs1805510
Discoid Rash	LATE_PHASE_OF_HIV_LIFE_CYCLE	REACTOME	1259	34	20	8,36E-01	9,96E-01	rs2228375|rs217797|rs2279083|rs4740420|rs2314664|rs964888|rs11177355|rs10896604|rs2018928|rs443678|rs2296085|rs3787016|rs2016266|rs4803238|rs2285427|rs7782920|rs10271506|rs7251067|rs522073|rs2874714
Discoid Rash	ELONGATION_ARREST_AND_RECOVERY	REACTOME	246	8	6	8,38E-01	9,96E-01	rs10896604|rs3787016|rs4803238|rs7251067|rs2874714|rs3746162
Discoid Rash	CHEMICAL_PATHWAY	BIOCARTA	585	27	19	8,41E-01	9,96E-01	rs3785383|rs965938|rs4433842|rs392715|rs4719793|rs954954|rs8051531|rs11646540|rs17753569|rs17811655|rs4767884|rs2270941|rs198182|rs198190|rs7242402|rs3785378|rs2849380|rs1439123|rs198178
Discoid Rash	GLYCEROPHOSPHOLIPID_METABOLISM	KEGG	2549	38	20	8,41E-01	9,96E-01	rs2087179|rs2292645|rs7810652|rs2124147|rs10494605|rs659732|rs11127284|rs4434808|rs6680326|rs2341422|rs1474797|rs1712432|rs1859733|rs7785682|rs11046741|rs4955238|rs1980444|rs10278266|rs2838464|rs17028998
Discoid Rash	FATTY_ACID_METABOLISM	KEGG	776	34	19	8,43E-01	9,96E-01	rs6901445|rs11788224|rs4836277|rs11725638|rs3818299|rs4245924|rs247011|rs876873|rs1662037|rs2810716|rs6760807|rs2084182|rs730870|rs480245|rs10415880|rs696337|rs1858074|rs6935622|rs6692958
Discoid Rash	AMINE_COMPOUND_SLC_TRANSPORTERS	REACTOME	1298	39	20	8,43E-01	9,96E-01	rs10833369|rs2241694|rs892601|rs1710887|rs12954053|rs3737475|rs1942574|rs2950798|rs1972873|rs3779672|rs350658|rs2282382|rs10820785|rs6593584|rs10466424|rs3776511|rs4106010|rs2697135|rs150311|rs12604591
Discoid Rash	RESOLUTION_OF_AP_SITES_VIA_THE_MULTIPLE_NUCLEOTIDE_PATCH_REPLACEMENT_F	REACTOME	192	5	4	8,43E-01	9,96E-01	rs4802436|rs2153608|rs11236181|rs1673041
Discoid Rash	PACKAGING_OF_TELOMERE_ENDS	REACTOME	515	8	4	8,44E-01	9,96E-01	rs1784304|rs472594|rs11935024|rs9368521
Discoid Rash	PROTEASOME	KEGG	821	31	17	8,45E-01	9,96E-01	rs2293576|rs328331|rs17086849|rs12599594|rs371500|rs13394402|rs12463483|rs4573787|rs3731973|rs7212835|rs3751497|rs8005252|rs10241838|rs3819715|rs2550461|rs241440|rs2298265
Discoid Rash	NETRIN1_SIGNALING	REACTOME	3710	34	20	8,46E-01	9,96E-01	rs4282339|rs11767121|rs2636785|rs4790589|rs10154970|rs2938769|rs7612183|rs12327358|rs10999739|rs11814342|rs1060561|rs9950125|rs1843786|rs9882126|rs7181913|rs2817675|rs11875845|rs2220105|rs4917753|rs7717558
Discoid Rash	CIRCADIAN_REPRESSION_OF_EXPRESSION_BY_REV_ERBA	REACTOME	1358	33	20	8,47E-01	9,96E-01	rs1511451|rs2036713|rs16912392|rs11634318|rs17237521|rs135549|rs7656250|rs1995917|rs1159176|rs6778657|rs16873852|rs552305|rs4851390|rs1930788|rs17046434|rs8029174|rs4809636|rs9850957|rs11741808|rs11857108
Discoid Rash	N_GLYCAN_BIOSYNTHESIS	KEGG	1792	37	20	8,51E-01	9,96E-01	rs2410274|rs7597227|rs659758|rs10496721|rs11896729|rs7533303|rs6430476|rs909715|rs187798|rs1036979|rs9829340|rs6657738|rs11652334|rs1784304|rs17611658|rs11625540|rs4943435|rs10483776|rs9401188|rs12424735
Discoid Rash	INOSITOL_PHOSPHATE_METABOLISM	KEGG	2355	33	20	8,52E-01	9,96E-01	rs6108160|rs2848780|rs745719|rs873418|rs1223577|rs2278214|rs1858608|rs2179984|rs7899961|rs7072607|rs2218314|rs7971352|rs6118062|rs2664537|rs6140561|rs10747068|rs2955006|rs537885|rs324321|rs11665535
Discoid Rash	POST_TRANSLATIONAL_MODIFICATION_SYNTHESIS_OF_GPI_ANCHORED_PROTEINS	REACTOME	586	9	7	8,54E-01	9,96E-01	rs671434|rs4251854|rs4144003|rs10497796|rs4774479|rs1594496|rs1573402
Discoid Rash	G1_PHASE	REACTOME	625	17	13	8,55E-01	9,96E-01	rs3217810|rs2314664|rs2896452|rs11784209|rs13160147|rs10849006|rs4134966|rs2374589|rs3217827|rs11135929|rs1048527|rs746720|rs6966980
Discoid Rash	NEUROTRANSMITTER_RECEPTOR_BINDING_AND_DOWNSTREAM_TRANSMISSION_IN_TH L	REACTOME	6592	31	20	8,57E-01	9,96E-01	rs1064448|rs6108160|rs2808539|rs745013|rs17779442|rs7820461|rs4821506|rs2190550|rs11074683|rs2050140|rs2159917|rs10515829|rs538957|rs3025646|rs8094168|rs16898963|rs4238490|rs17036351|rs931646|rs2241694
Discoid Rash	RIG_I_LIKE_RECEPTOR_SIGNALING_PATHWAY	KEGG	1361	37	20	8,57E-01	9,96E-01	rs10483453|rs4785452|rs1845451|rs17103274|rs7747353|rs11217175|rs4977686|rs4700008|rs12647506|rs1833754|rs10485405|rs1267034|rs12130290|rs3775167|rs730554|rs10757189|rs2859131|rs565665|rs1539243|rs3136646
Discoid Rash	RORA_ACTIVATES_CIRCADIAN_EXPRESSION	REACTOME	1377	33	20	8,58E-01	9,96E-01	rs1511451|rs2036713|rs16912392|rs11634318|rs17237521|rs135549|rs7656250|rs1995917|rs1159176|rs6778657|rs16873852|rs552305|rs4851390|rs1930788|rs17046434|rs8029174|rs4809636|rs9850957|rs11741808|rs11857108
Discoid Rash	SCFSKP2_MEDIATED_DEGRADATION_OF_P27_P21	REACTOME	813	25	16	8,59E-01	9,96E-01	rs2293576|rs328331|rs2314664|rs12599594|rs371500|rs13394402|rs13160147|rs12463483|rs7212835|rs3751497|rs8005252|rs6966980|rs3819715|rs2550461|rs241440|rs2298265
Discoid Rash	INFLAM_PATHWAY	BIOCARTA	747	26	20	8,61E-01	9,96E-01	rs3810168|rs17047703|rs17042407|rs2707212|rs16906063|rs1833754|rs12029576|rs10242595|rs2858870|rs3129882|rs2268626|rs17700376|rs918519|rs10950917|rs10030060|rs485499|rs3024498|rs4739109|rs986475|rs17025223
Discoid Rash	CDT1_ASSOCIATION_WITH_THE_CDC6_ORC_ORIGIN_COMPLEX	REACTOME	793	28	19	8,61E-01	9,96E-01	rs2293576|rs328331|rs2314664|rs12599594|rs6904263|rs371500|rs13394402|rs2891652|rs12463483|rs2817739|rs3731973|rs7212835|rs3751497|rs8005252|rs747232|rs3819715|rs2550461|rs241440|rs2298265
Discoid Rash	BASE_EXCISION_REPAIR	REACTOME	209	5	4	8,64E-01	9,96E-01	rs4802436|rs2153608|rs11236181|rs1673041
Discoid Rash	PROCESSING_OF_CAPPED_INTRONLESS_PRE_MRNA	REACTOME	274	10	8	8,64E-01	9,96E-01	rs12586783|rs1783983|rs16979884|rs1570558|rs11887079|rs6127737|rs735408|rs4653185
Discoid Rash	FORMATION_OF_THE_HIV1_EARLY_ELONGATION_COMPLEX	REACTOME	329	4	3	8,69E-01	9,96E-01	rs3787016|rs4803238|rs3746162
Discoid Rash	REGULATION_OF_ORNITHINE_DECARBOXYLASE_ODC	REACTOME	773	31	20	8,69E-01	9,96E-01	rs2293576|rs328331|rs10955290|rs12599594|rs689452|rs371500|rs13394402|rs818167|rs2523177|rs12463483|rs3731973|rs7212835|rs11686084|rs3751497|rs8005252|rs3819715|rs2550461|rs13018228|rs241440|rs2298265
Discoid Rash	CROSS_PRESENTATION_OF_SOLUBLE_EXOGENOUS_ANTIGENS_ENDOSOMES	REACTOME	713	25	16	8,70E-01	9,96E-01	rs2293576|rs328331|rs12599594|rs371500|rs13394402|rs2075221|rs12463483|rs3731973|rs7212835|rs2014055|rs3751497|rs8005252|rs3819715|rs2550461|rs241440|rs2298265
Discoid Rash	SMOOTH_MUSCLE_CONTRACTION	REACTOME	658	17	11	8,70E-01	9,96E-01	rs1351140|rs2031766|rs9787358|rs7800283|rs16944640|rs7920305|rs13100237|rs11599166|rs7595027|rs2126953|rs10940277
Discoid Rash	ACTIVATION_OF_NMDA_RECEPTOR_UPON_GLUTAMATE_BINDING_AND_POSTSYNAPTIC	REACTOME	2117	36	20	8,72E-01	9,96E-01	rs7820461|rs11074683|rs538957|rs16898963|rs17036351|rs2241694|rs1805510|rs1589880|rs580004|rs1461234|rs17036576|rs4502882|rs9921476|rs1011973|rs1644512|rs2254137|rs11746889|rs1940964|rs11953799|rs884423
Discoid Rash	B_CELL_RECEPTOR_SIGNALING_PATHWAY	KEGG	2278	29	20	8,72E-01	9,96E-01	rs4976132|rs1490801|rs9937676|rs10483453|rs10114577|rs1551486|rs3785383|rs17103274|rs3827867|rs17013034|rs392715|rs1365492|rs2811876|rs689452|rs11595566|rs17020055|rs4807101|rs2866168|rs4976028|rs10073996
Discoid Rash	TNFR1_PATHWAY	BIOCARTA	862	37	19	8,73E-01	9,96E-01	rs17194635|rs1845451|rs965938|rs7747353|rs4700008|rs2811876|rs11039149|rs10485405|rs10859589|rs565665|rs3738593|rs1114328|rs6677589|rs9914858|rs284509|rs11207306|rs7314748|rs986475|rs16886445
Discoid Rash	NEURONAL_SYSTEM	REACTOME	13378	29	20	8,74E-01	9,96E-01	rs2789603|rs1064448|rs1564372|rs17652604|rs6108160|rs2808539|rs745013|rs4930007|rs17779442|rs10455248|rs2250128|rs362988|rs9844737|rs7820461|rs6077718|rs9292637|rs305522|rs2471637|rs4254605|rs4821506
Discoid Rash	GPCR_PATHWAY	BIOCARTA	1102	33	20	8,74E-01	9,96E-01	rs3785383|rs4433842|rs12706724|rs6092704|rs1589880|rs392715|rs2811876|rs2664537|rs2254137|rs8051531|rs965808|rs8013053|rs11646540|rs17753569|rs17811655|rs13232987|rs4724400|rs4478191|rs11892543|rs1468242
Discoid Rash	LONG_TERM_DEPRESSION	KEGG	3926	33	20	8,75E-01	9,96E-01	rs304051|rs6108160|rs6988640|rs901849|rs1120559|rs816296|rs17036351|rs10494605|rs659732|rs4463623|rs3785383|rs2650163|rs4433842|rs12706724|rs6092704|rs1892879|rs2179984|rs392715|rs1474797|rs1712432
Discoid Rash	DNA_REPLICATION	REACTOME	3548	36	20	8,75E-01	9,96E-01	rs7916990|rs2293576|rs4265409|rs2028455|rs8003274|rs3001083|rs4804149|rs10510126|rs1535010|rs328331|rs8096557|rs495026|rs2314664|rs12124096|rs11177355|rs9812706|rs12599594|rs6904263|rs2331880|rs371500
Discoid Rash	RNA_POL_I_TRANSCRIPTION_INITIATION	REACTOME	427	13	6	8,76E-01	9,96E-01	rs4253042|rs6734172|rs7081472|rs7594007|rs10184938|rs11128940
Discoid Rash	RECRUITMENT_OF_MITOTIC_CENTROSOME_PROTEINS_AND_COMPLEXES	REACTOME	1177	33	20	8,77E-01	9,96E-01	rs10519309|rs3008333|rs7870808|rs10927002|rs10494941|rs6538797|rs2297453|rs11621560|rs11701058|rs17742788|rs867092|rs2490400|rs6606792|rs10745776|rs2024490|rs5750597|rs13224682|rs12371968|rs2580367|rs5771242
Discoid Rash	RNA_POL_III_CHAIN_ELONGATION	REACTOME	364	12	8	8,77E-01	9,96E-01	rs17420770|rs3787016|rs1532445|rs17092361|rs17603298|rs1385309|rs2243506|rs3746162
Discoid Rash	APOPTOSIS	REACTOME	3298	32	20	8,77E-01	9,96E-01	rs4779683|rs2293576|rs6130713|rs12327358|rs10999739|rs1357386|rs328331|rs9950125|rs12916064|rs16848195|rs965938|rs11875845|rs440804|rs2314664|rs1373762|rs1877330|rs11591298|rs365238|rs4719793|rs1927976
Discoid Rash	RNA_POL_I_PROMOTER_OPENING	REACTOME	501	9	5	8,77E-01	9,96E-01	rs1784304|rs11660673|rs11935024|rs7238305|rs9368521
Discoid Rash	PRE_NOTCH_TRANSCRIPTION_AND_TRANSLATION	REACTOME	835	24	16	8,78E-01	9,96E-01	rs6483495|rs10519523|rs12646059|rs2811876|rs12417347|rs204990|rs495494|rs2604931|rs4134966|rs6677589|rs11128940|rs875188|rs11207306|rs11021485|rs746720|rs9907255
Discoid Rash	TRANSPORT_OF_GLUCOSE_AND_OTHER_SUGARS_BILE_SALTS_AND_ORGANIC_ACIDS_M NE_COMPOUNDS	REACTOME	3234	46	20	8,78E-01	9,96E-01	rs10819284|rs1539624|rs1464258|rs10833369|rs13189284|rs2241694|rs10208012|rs892601|rs1710887|rs12954053|rs2297972|rs2276299|rs3737475|rs11074656|rs449095|rs1942574|rs2950798|rs1972873|rs12150519|rs3779672
Discoid Rash	G0_AND_EARLY_G1	REACTOME	258	3	3	8,79E-01	9,96E-01	rs2896452|rs173003|rs2835753
Discoid Rash	MRNA_3_END_PROCESSING	REACTOME	320	15	10	8,80E-01	9,96E-01	rs12586783|rs1783983|rs11249163|rs16979884|rs1570558|rs11249167|rs11887079|rs6127737|rs4947051|rs735408
Discoid Rash	LYSOSOME_VESICLE_BIOGENESIS	REACTOME	420	23	12	8,81E-01	9,96E-01	rs7203231|rs11075904|rs4507864|rs3824389|rs2377674|rs4883201|rs4804528|rs4490948|rs1028594|rs492960|rs1555422|rs1254901
Discoid Rash	HS_GAG_DEGRADATION	REACTOME	1386	32	20	8,82E-01	9,96E-01	rs7990210|rs1572342|rs4144545|rs7993144|rs7984041|rs2439525|rs7327328|rs7999475|rs9516076|rs7544500|rs2352838|rs1932299|rs10492615|rs1980779|rs12620119|rs4654986|rs1016797|rs3924843|rs9523709|rs1459433
Discoid Rash	RNA_POL_II_TRANSCRIPTION_PRE_INITIATION_AND_PROMOTER_OPENING	REACTOME	383	3	3	8,83E-01	9,96E-01	rs2296085|rs3787016|rs3746162
Discoid Rash	CREB_PHOSPHORYLATION_THROUGH_THE_ACTIVATION_OF_RAS	REACTOME	1349	29	20	8,84E-01	9,96E-01	rs11074683|rs538957|rs16898963|rs2241694|rs1805510|rs580004|rs9921476|rs1011973|rs2254137|rs11576136|rs12820037|rs1002487|rs4704710|rs6598858|rs4446309|rs2158196|rs11892543|rs3799658|rs16899429|rs10444986
Discoid Rash	RENAL_CELL_CARCINOMA	KEGG	2164	36	20	8,86E-01	9,96E-01	rs4976132|rs6057009|rs1490801|rs1477577|rs11681491|rs17047703|rs5745752|rs2297972|rs10793894|rs2811876|rs11697698|rs4976028|rs11072922|rs10073996|rs13045381|rs12029576|rs7668750|rs17387775|rs10242168|rs500207
Discoid Rash	TEL_PATHWAY	BIOCARTA	978	37	20	8,86E-01	9,96E-01	rs2648888|rs17199521|rs12547643|rs4733859|rs4433842|rs10505507|rs954954|rs11621560|rs11776114|rs7166565|rs10794486|rs12544019|rs6985681|rs11249961|rs6987802|rs2684777|rs4963872|rs2740764|rs2684790|rs7242402
Discoid Rash	RAS_ACTIVATION_UOPN_CA2_INFUX_THROUGH_NMDA_RECEPTOR	REACTOME	1033	33	20	8,86E-01	9,96E-01	rs11074683|rs538957|rs2241694|rs1805510|rs580004|rs9921476|rs1011973|rs12820037|rs4704710|rs4446309|rs2158196|rs10444986|rs7660775|rs8064140|rs1548853|rs12924552|rs16966016|rs12308275|rs9922023|rs7595027
Discoid Rash	FAS_PATHWAY	BIOCARTA	980	40	20	8,87E-01	9,96E-01	rs1845451|rs965938|rs7747353|rs4700008|rs11591298|rs2245548|rs2811876|rs10485405|rs3827730|rs1937332|rs150615|rs565665|rs3738593|rs1114328|rs6677589|rs9914858|rs7920305|rs284509|rs11207306|rs16886445
Discoid Rash	FORMATION_OF_RNA_POL_II_ELONGATION_COMPLEX_	REACTOME	401	8	6	8,89E-01	9,96E-01	rs10896604|rs3787016|rs4803238|rs7251067|rs2874714|rs3746162
Discoid Rash	MEF2D_PATHWAY	BIOCARTA	684	20	14	8,90E-01	9,96E-01	rs3785383|rs4433842|rs392715|rs9608247|rs8051531|rs11646540|rs17753569|rs17811655|rs4478191|rs198182|rs198190|rs7595027|rs3785378|rs198178
Discoid Rash	VEGF_SIGNALING_PATHWAY	KEGG	2111	35	20	8,90E-01	9,96E-01	rs4976132|rs1490801|rs10494605|rs659732|rs4434808|rs3785383|rs4433842|rs392715|rs1474797|rs1712432|rs2664537|rs1808416|rs689452|rs1980444|rs10494878|rs4976028|rs10073996|rs1148757|rs8051531|rs6697553
Discoid Rash	CERAMIDE_PATHWAY	BIOCARTA	425	18	10	8,91E-01	9,96E-01	rs4700008|rs4719793|rs954954|rs17820573|rs17208515|rs2270941|rs9914858|rs7242402|rs2849380|rs16886445
Discoid Rash	SYNTHESIS_OF_PC	REACTOME	669	14	12	8,91E-01	9,96E-01	rs6072343|rs8178992|rs4916425|rs11101191|rs2282382|rs10820785|rs6593584|rs4798055|rs10803744|rs12705099|rs3793790|rs7861505
Discoid Rash	ORC1_REMOVAL_FROM_CHROMATIN	REACTOME	930	28	19	8,92E-01	9,96E-01	rs2293576|rs1535010|rs328331|rs2314664|rs12599594|rs371500|rs13394402|rs2891652|rs10948689|rs12463483|rs3731973|rs7212835|rs3751497|rs8005252|rs747232|rs3819715|rs2550461|rs241440|rs2298265
Discoid Rash	SEMA4D_INDUCED_CELL_MIGRATION_AND_GROWTH_CONE_COLLAPSE	REACTOME	433	11	10	8,92E-01	9,96E-01	rs3448|rs3745511|rs16944640|rs5756573|rs2012928|rs2501276|rs7470318|rs1055639|rs959058|rs136176
Discoid Rash	INTEGRIN_PATHWAY	BIOCARTA	1027	28	20	8,95E-01	9,96E-01	rs1351140|rs17106413|rs1475035|rs3828280|rs3448|rs10793894|rs2811876|rs2268969|rs1187095|rs2052928|rs890051|rs4767884|rs3867132|rs9927309|rs10800842|rs6677589|rs1549733|rs11207306|rs1950712|rs3791884
Discoid Rash	SYNTHESIS_OF_GLYCOSYLPHOSPHATIDYLINOSITOL_GPI	REACTOME	485	7	5	8,96E-01	9,96E-01	rs671434|rs4144003|rs4774479|rs1594496|rs1573402
Discoid Rash	FORMATION_OF_ATP_BY_CHEMIOSMOTIC_COUPLING	REACTOME	77	1	1	8,97E-01	9,96E-01	rs7108613
Discoid Rash	RNA_POL_III_TRANSCRIPTION_INITIATION_FROM_TYPE_2_PROMOTER	REACTOME	417	12	8	8,97E-01	9,96E-01	rs17420770|rs3787016|rs1532445|rs17092361|rs17603298|rs1385309|rs2243506|rs3746162
Discoid Rash	LOSS_OF_NLP_FROM_MITOTIC_CENTROSOMES	REACTOME	1100	31	18	8,99E-01	9,96E-01	rs10519309|rs7870808|rs10927002|rs10494941|rs6538797|rs2297453|rs11621560|rs11701058|rs867092|rs2490400|rs10745776|rs2024490|rs5750597|rs13224682|rs12371968|rs2580367|rs6718864|rs13749
Discoid Rash	SYNTHESIS_SECRETION_AND_DEACYLATION_OF_GHRELIN	REACTOME	624	18	15	8,99E-01	9,96E-01	rs11999806|rs791595|rs1551368|rs4731416|rs1836458|rs10816554|rs6467165|rs791600|rs3914191|rs4979264|rs2333452|rs7766818|rs1410821|rs6882366|rs11536344
Discoid Rash	MITOCHONDRIAL_PROTEIN_IMPORT	REACTOME	609	35	18	8,99E-01	9,96E-01	rs157580|rs16844196|rs9938257|rs2294928|rs10800799|rs6725388|rs9611598|rs10910536|rs1963268|rs6689634|rs1448539|rs6840620|rs4145875|rs6586361|rs11734260|rs1274500|rs9287215|rs12696105
Discoid Rash	POST_NMDA_RECEPTOR_ACTIVATION_EVENTS	REACTOME	1753	35	20	8,99E-01	9,96E-01	rs7820461|rs11074683|rs538957|rs16898963|rs2241694|rs1805510|rs1589880|rs580004|rs9921476|rs1011973|rs1644512|rs2254137|rs884423|rs11576136|rs12820037|rs1002487|rs4704710|rs6598858|rs4446309|rs4724400
Discoid Rash	ABC_FAMILY_PROTEINS_MEDIATED_TRANSPORT	REACTOME	1753	52	20	9,00E-01	9,96E-01	rs8133852|rs873705|rs4148482|rs17143109|rs11165042|rs2277624|rs4299376|rs152033|rs10266338|rs1729770|rs4912471|rs323066|rs4148217|rs7990574|rs12468887|rs1927496|rs9302064|rs4148490|rs1025447|rs2355017

Discoid Rash	ASTHMA	KEGG	770	30	15	9,01E-01	9,96E-01	rs7717673|rs3104404|rs9276284|rs28383344|rs10853004|rs3129882|rs3815341|rs444821|rs8073784|rs3024498|rs1569723|rs986475|rs3819715|rs241440|rs739719
Discoid Rash	BCR_PATHWAY	BIOCARTA	1053	32	20	9,02E-01	9,96E-01	rs3785383|rs4433842|rs392715|rs4700008|rs2811876|rs2664537|rs4807101|rs4744512|rs8051531|rs1755991|rs8013053|rs1585461|rs11646540|rs17753569|rs17811655|rs7911684|rs2058194|rs4478191|rs7258040|rs198182
Discoid Rash	A_TETRASACCHARIDE_LINKER_SEQUENCE_IS_REQUIRED_FOR_GAG_SYNTHESIS	REACTOME	1731	29	20	9,02E-01	9,96E-01	rs2119299|rs7990210|rs1572342|rs4144545|rs7993144|rs7337223|rs2439525|rs7113440|rs6933665|rs1891523|rs7327328|rs7999475|rs9523670|rs7929855|rs11224035|rs7544500|rs2352838|rs1932299|rs10492615|rs1980779
Discoid Rash	BLADDER_CANCER	KEGG	1222	37	20	9,03E-01	9,96E-01	rs1499367|rs17199521|rs12547643|rs4733859|rs9817|rs4431992|rs1927976|rs7945189|rs10505507|rs4690476|rs519806|rs11643163|rs13148526|rs769412|rs2274606|rs873350|rs6985681|rs4134966|rs4690458|rs6987802
Discoid Rash	PURINE_METABOLISM	REACTOME	581	24	15	9,03E-01	9,96E-01	rs3006009|rs3448|rs9984077|rs2327951|rs2206716|rs10865088|rs5025470|rs10165919|rs10781076|rs11076720|rs2057637|rs819848|rs4789145|rs244073|rs1685748
Discoid Rash	N_GLYCAN_ANTENNAE_ELONGATION_IN_THE_MEDIAL_TRANS_GOLGI	REACTOME	962	35	20	9,05E-01	9,96E-01	rs7597227|rs10496721|rs11104430|rs1516085|rs6430476|rs1036979|rs2595371|rs11625540|rs10483776|rs4569915|rs1519304|rs4429522|rs7701590|rs11852344|rs12054151|rs1147447|rs1965183|rs2318308|rs1455549|rs11241027
Discoid Rash	DESTABILIZATION_OF_MRNA_BY_AUF1_HNRNP_D0	REACTOME	811	28	18	9,05E-01	9,96E-01	rs2293576|rs328331|rs2314664|rs12599594|rs371500|rs13394402|rs12463483|rs7660665|rs3731973|rs7212835|rs10516668|rs3751497|rs8005252|rs2101680|rs3819715|rs2550461|rs241440|rs2298265
Discoid Rash	MRNA_SPLICING	REACTOME	1057	25	20	9,05E-01	9,96E-01	rs6105651|rs6034593|rs12586783|rs1783983|rs4814550|rs7147840|rs11684592|rs11249163|rs6433636|rs16979884|rs3787016|rs9611598|rs7253181|rs1570558|rs788008|rs7660665|rs11249167|rs11887079|rs10516668|rs11205277
Discoid Rash	RNA_POL_II_PRE_TRANSCRIPTION_EVENTS	REACTOME	532	9	7	9,07E-01	9,96E-01	rs10896604|rs2296085|rs3787016|rs4803238|rs7251067|rs2874714|rs3746162
Discoid Rash	MEMBRANE_TRAFFICKING	REACTOME	2276	42	20	9,08E-01	9,96E-01	rs4779683|rs2432216|rs1357386|rs10935320|rs7023913|rs13165062|rs12916064|rs7203231|rs2314664|rs1886176|rs11075904|rs6453850|rs7703742|rs1387551|rs7816946|rs6917198|rs1857208|rs11997323|rs7865066|rs1890060
Discoid Rash	DEPOSITION_OF_NEW_CENPA_CONTAINING_NUCLEOSOMES_AT_THE_CENTROMERE	REACTOME	637	27	14	9,08E-01	9,96E-01	rs10872992|rs12124096|rs924233|rs981951|rs1784304|rs8127350|rs11935024|rs714447|rs12663707|rs7606893|rs11562980|rs6588044|rs7848472|rs8010307
Discoid Rash	CSK_PATHWAY	BIOCARTA	554	19	14	9,09E-01	9,96E-01	rs6092704|rs1589880|rs2707212|rs965808|rs2858870|rs3129882|rs4851462|rs4724400|rs13374545|rs1898240|rs2995094|rs13224682|rs9755490|rs870875
Discoid Rash	RNA_DEGRADATION	KEGG	1123	32	20	9,10E-01	9,96E-01	rs1545229|rs17479462|rs6783810|rs7580140|rs11189224|rs12153180|rs13243520|rs2317847|rs7601401|rs713195|rs905067|rs11129533|rs6682376|rs2906802|rs1570558|rs17694584|rs12921771|rs918760|rs735408|rs1359761
Discoid Rash	SPLICEOSOME	KEGG	1685	25	20	9,13E-01	9,96E-01	rs6105651|rs6034593|rs12517249|rs4750000|rs4686709|rs706844|rs963058|rs4814550|rs7147840|rs11684592|rs6433636|rs227944|rs10884953|rs3132454|rs2339648|rs17127304|rs9611598|rs9267431|rs7253181|rs2361696
Discoid Rash	NO1_PATHWAY	BIOCARTA	1482	28	20	9,14E-01	9,96E-01	rs12418204|rs945032|rs7146347|rs307805|rs722503|rs11000160|rs935865|rs7324547|rs293314|rs4900312|rs2038916|rs11621560|rs293290|rs4686799|rs10770645|rs1937683|rs7485410|rs2069605|rs12058655|rs10508942
Discoid Rash	TYROSINE_METABOLISM	KEGG	1169	37	20	9,18E-01	9,96E-01	rs6637|rs2247016|rs10738290|rs7863513|rs4927594|rs17191474|rs9935162|rs7111341|rs11787776|rs7194392|rs2114716|rs1662037|rs6569455|rs10491749|rs11768267|rs2075508|rs129934|rs11855351|rs17210001|rs12292212
Discoid Rash	ADHERENS_JUNCTIONS_INTERACTIONS	REACTOME	5496	32	20	9,20E-01	9,96E-01	rs1420965|rs944248|rs12962729|rs2068606|rs157580|rs4682242|rs2136129|rs7201194|rs6897759|rs1117513|rs1785765|rs2408106|rs976968|rs17385192|rs12596363|rs9293168|rs555255|rs6437962|rs17118023|rs6452328
Discoid Rash	COMP_PATHWAY	BIOCARTA	628	25	12	9,21E-01	9,96E-01	rs4504976|rs12492885|rs10762788|rs3742088|rs1917186|rs10824854|rs2856448|rs1450665|rs1962474|rs17384711|rs17468519|rs2277984
Discoid Rash	GLYCEROLIPID_METABOLISM	KEGG	1872	27	20	9,23E-01	9,96E-01	rs4823104|rs7810652|rs11788224|rs6494005|rs4836277|rs11127284|rs12604221|rs1859733|rs7785682|rs874546|rs10278266|rs2838464|rs11099616|rs738491|rs204990|rs7766133|rs6588591|rs2336408|rs1020382|rs17799355
Discoid Rash	GLYCOSYLPHOSPHATIDYLINOSITOL_GPI_ANCHOR_BIOSYNTHESIS	KEGG	318	4	3	9,24E-01	9,96E-01	rs4144003|rs10497796|rs1573402
Discoid Rash	CHROMOSOME_MAINTENANCE	REACTOME	1949	39	20	9,31E-01	9,96E-01	rs910831|rs8006145|rs799917|rs10872992|rs9397084|rs12124096|rs924233|rs718527|rs981951|rs9396464|rs4802436|rs17298882|rs1784304|rs2321744|rs9383964|rs1416388|rs472594|rs7324754|rs8127350|rs10146912
Discoid Rash	ABC_TRANSPORTERS	KEGG	2684	54	20	9,32E-01	9,96E-01	rs8133852|rs873705|rs4148482|rs6949736|rs2297409|rs17143109|rs7788404|rs11165042|rs4606009|rs2277624|rs4299376|rs152033|rs4360243|rs10266338|rs1729770|rs1674850|rs7778411|rs2872247|rs323066|rs4148217
Discoid Rash	GLYCOLYSIS_GLUCONEOGENESIS	KEGG	1391	37	20	9,33E-01	9,96E-01	rs1932001|rs11788224|rs4836277|rs600130|rs12240387|rs11636141|rs548348|rs4585672|rs10508243|rs1000251|rs6682376|rs1898251|rs1662037|rs2810716|rs6532592|rs12343547|rs6733848|rs217463|rs9664019|rs4813547
Discoid Rash	MITOTIC_G2_G2_M_PHASES	REACTOME	1389	30	20	9,37E-01	9,96E-01	rs10519309|rs3008333|rs7870808|rs10927002|rs10494941|rs6538797|rs2297453|rs901478|rs11621560|rs11701058|rs17742788|rs867092|rs2490400|rs6606792|rs7928738|rs10745776|rs2024490|rs5750597|rs4134966|rs13224682
Discoid Rash	RNA_POL_I_RNA_POL_III_AND_MITOCHONDRIAL_TRANSCRIPTION	REACTOME	1830	46	20	9,41E-01	9,96E-01	rs2224553|rs7873382|rs575660|rs4253042|rs2078639|rs17420770|rs1813529|rs10419120|rs10961329|rs1784304|rs13302756|rs3787016|rs1532445|rs10961439|rs10924774|rs10961483|rs6734172|rs17092361|rs11660673|rs7081472
Discoid Rash	METABOLISM_OF_NUCLEOTIDES	REACTOME	1559	31	20	9,41E-01	9,96E-01	rs3793357|rs12026896|rs1625579|rs3006009|rs8072869|rs3448|rs822607|rs10505018|rs242253|rs290826|rs9984077|rs2327951|rs2206716|rs10865088|rs5025470|rs2811219|rs12401542|rs3808830|rs768364|rs10165919
Discoid Rash	OXYGEN_DEPENDENT_PROLINE_HYDROXYLATION_OF_HYPOXIA_INDUCIBLE_FACTOR_	REACTOME	313	18	14	9,42E-01	9,96E-01	rs10153462|rs2314664|rs881547|rs11665853|rs12897122|rs2000015|rs10403826|rs12882238|rs10483745|rs17801488|rs4982145|rs1680730|rs1867786|rs17745230
Discoid Rash	DNA_STRAND_ELONGATION	REACTOME	672	19	13	9,44E-01	9,96E-01	rs1535010|rs9396464|rs4802436|rs2321744|rs7324754|rs614123|rs10948689|rs11236181|rs3782093|rs9598129|rs1753477|rs1673041|rs1328667
Discoid Rash	BMAL1_CLOCK_NPAS2_ACTIVATES_CIRCADIAN_EXPRESSION	REACTOME	1728	30	20	9,44E-01	9,96E-01	rs1511451|rs2036713|rs16912392|rs11634318|rs17237521|rs135549|rs11706165|rs7656250|rs1995917|rs1159176|rs6778657|rs7699745|rs552305|rs4851390|rs1930788|rs17046434|rs11130232|rs7684118|rs10462023|rs2100840
Discoid Rash	PENTOSE_PHOSPHATE_PATHWAY	KEGG	616	32	17	9,44E-01	9,96E-01	rs600130|rs12240387|rs548348|rs9850440|rs10508243|rs11100502|rs1009940|rs4465231|rs12343547|rs217463|rs9664019|rs2242301|rs884289|rs10517428|rs2268169|rs2375592|rs12357621
Discoid Rash	SPRY_PATHWAY	BIOCARTA	910	30	20	9,46E-01	9,96E-01	rs13131772|rs11217175|rs7328506|rs1993191|rs10506598|rs3113392|rs518569|rs1334384|rs2184818|rs1867005|rs9574614|rs2329177|rs2740764|rs9545837|rs1198963|rs3853477|rs942233|rs1004988|rs981698|rs1577186
Discoid Rash	CLEAVAGE_OF_GROWING_TRANSCRIPT_IN_THE_TERMINATION_REGION_	REACTOME	435	16	11	9,48E-01	9,96E-01	rs12586783|rs1783983|rs11249163|rs16979884|rs1570558|rs11249167|rs11887079|rs6127737|rs4947058|rs735408|rs4653185
Discoid Rash	PROXIMAL_TUBULE_BICARBONATE_RECLAMATION	KEGG	458	16	12	9,48E-01	9,96E-01	rs11600216|rs10876891|rs1921915|rs10908753|rs1046995|rs345187|rs11805919|rs8107107|rs1200136|rs13912|rs443177|rs3761685
Discoid Rash	EFFECTS_OF_PIP2_HYDROLYSIS	REACTOME	1651	35	20	9,48E-01	9,96E-01	rs7349397|rs7810652|rs2204204|rs2344662|rs1859733|rs7785682|rs874546|rs10278266|rs12442869|rs10904923|rs4142696|rs11073339|rs2336408|rs12883769|rs6777221|rs198448|rs11681604|rs11048447|rs4902049|rs2241873
Discoid Rash	RNA_POL_I_TRANSCRIPTION_TERMINATION	REACTOME	335	5	3	9,48E-01	9,96E-01	rs6734172|rs7594007|rs10184938
Discoid Rash	DORSO_VENTRAL_AXIS_FORMATION	KEGG	1149	41	20	9,48E-01	9,96E-01	rs12723298|rs1477577|rs3746882|rs7311189|rs1797648|rs2855711|rs204990|rs17524639|rs500207|rs2710304|rs2836722|rs12417930|rs2144575|rs11054365|rs783523|rs12759793|rs2836774|rs10505762|rs948254|rs3124594
Discoid Rash	PYRIMIDINE_METABOLISM	KEGG	1900	39	20	9,50E-01	9,96E-01	rs1245371|rs3793357|rs7712322|rs12026896|rs1625579|rs8072869|rs4594412|rs822607|rs10505018|rs242253|rs290826|rs7601401|rs9396464|rs751394|rs13383761|rs3787016|rs10146912|rs2786529|rs6734172|rs1487302
Discoid Rash	REGULATION_OF_HYPOXIA_INDUCIBLE_FACTOR_HIF_BY_OXYGEN	REACTOME	443	19	15	9,50E-01	9,96E-01	rs10153462|rs1339374|rs2314664|rs881547|rs11665853|rs12897122|rs2000015|rs10403826|rs12882238|rs10483745|rs17801488|rs4982145|rs1680730|rs1867786|rs17745230
Discoid Rash	SYNTHESIS_AND_INTERCONVERSION_OF_NUCLEOTIDE_DI_AND_TRIPHOSPHATES	REACTOME	306	5	5	9,51E-01	9,96E-01	rs10505018|rs2978662|rs1265143|rs3779647|rs10914641
Discoid Rash	CHOLESTEROL_BIOSYNTHESIS	REACTOME	259	4	4	9,52E-01	9,96E-01	rs7957619|rs4840583|rs6426075|rs9685465
Discoid Rash	S_PHASE	REACTOME	2076	34	20	9,53E-01	9,96E-01	rs2293576|rs1499367|rs17199521|rs1535010|rs328331|rs12547643|rs4733859|rs2314664|rs12599594|rs10505507|rs371500|rs13394402|rs9396464|rs4802436|rs2321744|rs7324754|rs10146912|rs614123|rs13160147|rs2891652
Discoid Rash	TRANSPORT_TO_THE_GOLGI_AND_SUBSEQUENT_MODIFICATION	REACTOME	1557	33	20	9,55E-01	9,96E-01	rs7597227|rs10496721|rs11104430|rs11896729|rs6430476|rs909715|rs187798|rs1036979|rs2595371|rs4706457|rs11625540|rs13205436|rs10483776|rs709641|rs9401188|rs4569915|rs1342660|rs1519304|rs4429522|rs9498441
Discoid Rash	BILE_ACID_AND_BILE_SALT_METABOLISM	REACTOME	1176	29	14	9,57E-01	9,96E-01	rs2806744|rs9886369|rs1317361|rs2277624|rs11045834|rs854151|rs4762785|rs2260808|rs2251633|rs12430125|rs1730650|rs3803258|rs10841798|rs7651276
Discoid Rash	INTEGRIN_ALPHAIIB_BETA3_SIGNALING	REACTOME	779	25	19	9,58E-01	9,96E-01	rs10747521|rs12442869|rs4744512|rs2056131|rs1537741|rs11073339|rs1755991|rs11695696|rs6512654|rs1585461|rs11652772|rs2860493|rs9927309|rs2050447|rs11895413|rs6482566|rs2194717|rs13410779|rs1810503
Discoid Rash	DNA_REPLICATION	KEGG	740	20	14	9,59E-01	9,96E-01	rs1535010|rs9396464|rs4802436|rs2321744|rs7324754|rs10146912|rs614123|rs10948689|rs11236181|rs3782093|rs9598129|rs1753477|rs1673041|rs1328667
Discoid Rash	RNA_POL_III_TRANSCRIPTION_INITIATION_FROM_TYPE_3_PROMOTER	REACTOME	501	14	10	9,62E-01	9,96E-01	rs17420770|rs3787016|rs1532445|rs17092361|rs17603298|rs1385309|rs2995094|rs2243506|rs3746162|rs870875
Discoid Rash	GLYCOGEN_BREAKDOWN_GLYCOGENOLYSIS	REACTOME	246	7	5	9,62E-01	9,96E-01	rs1011450|rs217463|rs4681471|rs10517428|rs7595027
Discoid Rash	GLOBAL_GENOMIC_NER_GG_NER	REACTOME	823	18	14	9,62E-01	9,96E-01	rs12336762|rs1746837|rs4802436|rs2321744|rs11039149|rs7324754|rs10146912|rs614123|rs11236181|rs9598129|rs1753477|rs12004883|rs1673041|rs1328667
Discoid Rash	NUCLEOTIDE_EXCISION_REPAIR	KEGG	914	25	17	9,63E-01	9,96E-01	rs2228529|rs12336762|rs1746837|rs4802436|rs2321744|rs11039149|rs7324754|rs10146912|rs614123|rs7722373|rs7081472|rs11236181|rs9598129|rs1753477|rs12004883|rs1673041|rs1328667
Discoid Rash	O_LINKED_GLYCOSYLATION_OF_MUCINS	REACTOME	2402	38	20	9,64E-01	9,96E-01	rs2289241|rs2404839|rs17323530|rs620381|rs1862458|rs7844620|rs6731612|rs6981912|rs6494120|rs11662739|rs10467147|rs1868431|rs10804566|rs1325287|rs36686|rs10020087|rs10493583|rs1386583|rs2203890|rs1950148
Discoid Rash	SEMA4D_IN_SEMAPHORIN_SIGNALING	REACTOME	597	20	16	9,65E-01	9,96E-01	rs3448|rs3745511|rs16944640|rs17659879|rs5756573|rs2012928|rs2501276|rs13374545|rs7470318|rs41752|rs1055639|rs1898240|rs11083855|rs311372|rs959058|rs136176
Discoid Rash	SYSTEMIC_LUPUS_ERYTHEMATOSUS	KEGG	2453	34	20	9,65E-01	9,96E-01	rs11074683|rs17106413|rs3104404|rs7914787|rs1475035|rs1805510|rs9276284|rs9921476|rs1784304|rs2268969|rs2052731|rs3742088|rs11051642|rs28383344|rs12820037|rs472594|rs10771875|rs10823484|rs3129882|rs12817263
Discoid Rash	GAP_JUNCTION_ASSEMBLY	REACTOME	330	9	9	9,66E-01	9,96E-01	rs1886176|rs1857208|rs12862093|rs7174852|rs10870680|rs9552089|rs7984378|rs3845481|rs10080593
Discoid Rash	MUSCLE_CONTRACTION	REACTOME	1023	20	14	9,67E-01	9,96E-01	rs1351140|rs2031766|rs2089910|rs9787358|rs7800283|rs16944640|rs11652772|rs178638|rs7920305|rs13100237|rs11599166|rs7595027|rs2126953|rs10940277
Discoid Rash	SYNTHESIS_OF_PIPS_AT_THE_GOLGI_MEMBRANE	REACTOME	788	19	16	9,67E-01	9,96E-01	rs2848780|rs7971352|rs537885|rs324321|rs11665535|rs10853472|rs11604561|rs1496814|rs675566|rs11044233|rs2156480|rs8089834|rs635956|rs11082270|rs4771142|rs2298265
Discoid Rash	DNA_REPAIR	REACTOME	2111	38	20	9,67E-01	9,96E-01	rs4264079|rs17772583|rs356669|rs799917|rs7092494|rs1325382|rs2228529|rs12336762|rs2314664|rs4713859|rs1746837|rs2259409|rs12747317|rs4802436|rs3787051|rs1784304|rs2321744|rs11039149|rs6687381|rs3787016
Discoid Rash	COMPLEMENT_CASCADE	REACTOME	752	24	12	9,67E-01	9,96E-01	rs4504976|rs12492885|rs10762788|rs1917186|rs10824854|rs2856448|rs1450665|rs1782468|rs1962474|rs17384711|rs17468519|rs2277984
Discoid Rash	RNA_POL_I_TRANSCRIPTION	REACTOME	937	22	11	9,68E-01	9,96E-01	rs2228529|rs1784304|rs6734172|rs11660673|rs7081472|rs7594007|rs11935024|rs10184938|rs11128940|rs7238305|rs12663707
Discoid Rash	PLATELET_AGGREGATION_PLUG_FORMATION	REACTOME	942	28	20	9,68E-01	9,96E-01	rs10747521|rs12442869|rs10509944|rs4744512|rs2056131|rs1537741|rs11073339|rs1755991|rs11695696|rs6512654|rs1585461|rs11652772|rs2860493|rs9927309|rs2050447|rs11895413|rs9420082|rs6482566|rs2194717|rs13410779
Discoid Rash	NUCLEOTIDE_BINDING_DOMAIN_LEUCINE_RICH_REPEAT_CONTAINING_RECEPTOR_NLR YS	REACTOME	1337	31	20	9,69E-01	9,96E-01	rs1043395|rs4785452|rs1845451|rs7747353|rs954954|rs559052|rs396146|rs100043|rs10485405|rs17790482|rs2859129|rs150615|rs1545092|rs565665|rs6442159|rs11653896|rs5743369|rs1114328|rs284509|rs11107025
Discoid Rash	LAGGING_STRAND_SYNTHESIS	REACTOME	550	17	11	9,69E-01	9,96E-01	rs9396464|rs4802436|rs2321744|rs7324754|rs614123|rs11236181|rs3782093|rs9598129|rs1753477|rs1673041|rs1328667
Discoid Rash	YAP1_AND_WWTR1_TAZ_STIMULATED_GENE_EXPRESSION	REACTOME	1047	35	20	9,69E-01	9,96E-01	rs4767235|rs135549|rs8102574|rs4714861|rs6778657|rs552305|rs1930788|rs9394299|rs7616812|rs17046434|rs11067009|rs1560001|rs11128940|rs1025256|rs4809636|rs9850957|rs7638140|rs11022509|rs233909|rs1865604
Discoid Rash	DARPP_32_EVENTS	REACTOME	879	34	18	9,72E-01	9,96E-01	rs7522367|rs244570|rs10065699|rs1824154|rs36066827|rs4478191|rs378869|rs11208844|rs7728286|rs27723|rs13224682|rs9755490|rs1457145|rs7595027|rs2271882|rs6896121|rs783062|rs918583
Discoid Rash	SYNTHESIS_OF_DNA	REACTOME	1545	29	20	9,72E-01	9,96E-01	rs2293576|rs1535010|rs328331|rs2314664|rs12599594|rs371500|rs13394402|rs9396464|rs4802436|rs2321744|rs7324754|rs10146912|rs614123|rs2891652|rs10948689|rs12463483|rs11236181|rs3782093|rs3731973|rs7212835
Discoid Rash	INNATE_IMMUNE_SYSTEM	REACTOME	6278	25	20	9,75E-01	9,96E-01	rs918418|rs17326309|rs700591|rs13281383|rs1043395|rs2848780|rs9900686|rs2980937|rs10483453|rs16898963|rs9463574|rs4785452|rs7175248|rs6860708|rs1845451|rs7023913|rs4504976|rs329499|rs1360094|rs11633249
Discoid Rash	MCALPAIN_PATHWAY	BIOCARTA	543	16	11	9,76E-01	9,96E-01	rs1351140|rs1994350|rs1187095|rs4767884|rs13100237|rs13224682|rs9755490|rs2740764|rs2126953|rs901363|rs10940277
Discoid Rash	RHO_PATHWAY	BIOCARTA	480	7	6	9,77E-01	9,96E-01	rs3448|rs10800842|rs11748064|rs13100237|rs12050150|rs11664
Discoid Rash	NUCLEOTIDE_EXCISION_REPAIR	REACTOME	956	27	19	9,77E-01	9,96E-01	rs2228529|rs12336762|rs1746837|rs4802436|rs2321744|rs11039149|rs3787016|rs7324754|rs10146912|rs614123|rs7722373|rs7081472|rs11236181|rs9598129|rs3746162|rs1753477|rs12004883|rs1673041|rs1328667
Discoid Rash	AMINO_ACID_AND_OLIGOPEPTIDE_SLC_TRANSPORTERS	REACTOME	1707	27	20	9,82E-01	9,96E-01	rs9292637|rs3829406|rs11026529|rs1348573|rs4790232|rs1439502|rs298467|rs4482114|rs11836940|rs12879346|rs12373586|rs10860593|rs2183472|rs2956574|rs9792165|rs6895433|rs2967055|rs2065134|rs1288367|rs8013531
Discoid Rash	METAL_ION_SLC_TRANSPORTERS	REACTOME	653	28	16	9,83E-01	9,96E-01	rs10208012|rs449095|rs16861637|rs16889547|rs11264680|rs6469681|rs35243|rs13095262|rs233812|rs10190518|rs1793705|rs1995222|rs1440235|rs421446|rs3852133|rs10981696
Discoid Rash	EXTENSION_OF_TELOMERES	REACTOME	618	19	13	9,83E-01	9,96E-01	rs9396464|rs4802436|rs2321744|rs7324754|rs10146912|rs614123|rs11236181|rs3782093|rs9598129|rs1753477|rs1673041|rs3744258|rs1328667
Discoid Rash	MRNA_SPLICING_MINOR_PATHWAY	REACTOME	383	4	4	9,84E-01	9,96E-01	rs3787016|rs788008|rs11205277|rs3746162
Discoid Rash	ASPARAGINE_N_LINKED_GLYCOSYLATION	REACTOME	2760	37	20	9,86E-01	9,96E-01	rs2410274|rs7597227|rs659758|rs10496721|rs11104430|rs11896729|rs10163323|rs7533303|rs6430476|rs909715|rs187798|rs1036979|rs6657738|rs2595371|rs4706457|rs1784304|rs17611658|rs11625540|rs4943435|rs13205436
Discoid Rash	TRANSCRIPTION_COUPLED_NER_TC_NER	REACTOME	834	23	15	9,86E-01	9,96E-01	rs2228529|rs4802436|rs2321744|rs3787016|rs7324754|rs10146912|rs614123|rs7722373|rs7081472|rs11236181|rs9598129|rs3746162|rs1753477|rs1673041|rs1328667
Discoid Rash	PLATELET_ACTIVATION_SIGNALING_AND_AGGREGATION	REACTOME	6965	29	20	9,86E-01	9,96E-01	rs4976132|rs4729129|rs1490801|rs7349397|rs873705|rs2159917|rs3922668|rs7810652|rs2204204|rs1858822|rs17525472|rs1058242|rs2344662|rs10464587|rs17047703|rs11160174|rs4148482|rs10494605|rs5745752|rs931646
Discoid Rash	TRANS_GOLGI_NETWORK_VESICLE_BUDDING	REACTOME	1106	32	20	9,89E-01	9,96E-01	rs13165062|rs7203231|rs11075904|rs6917198|rs7865066|rs1890060|rs3824389|rs2377674|rs10755299|rs4883201|rs4804528|rs11604561|rs4490948|rs7162209|rs1028594|rs1047631|rs2072824|rs492960|rs1555422|rs17271418
Discoid Rash	O_GLYCAN_BIOSYNTHESIS	KEGG	1579	33	20	9,89E-01	9,96E-01	rs17323530|rs7844620|rs6731612|rs6981912|rs6494120|rs11662739|rs1868431|rs10212715|rs1386583|rs2203890|rs1950148|rs10520248|rs4799402|rs4719118|rs12479422|rs2161393|rs6750051|rs7808818|rs1868430|rs1894008
Discoid Rash	RNA_POL_II_TRANSCRIPTION	REACTOME	1050	28	20	9,89E-01	9,96E-01	rs2279083|rs12586783|rs964888|rs1783983|rs10896604|rs11249163|rs16979884|rs2296085|rs3787016|rs4803238|rs1570558|rs7251067|rs11249167|rs2874714|rs11887079|rs3746162|rs6127737|rs6901395|rs4947058|rs735408
Discoid Rash	SLC_MEDIATED_TRANSMEMBRANE_TRANSPORT	REACTOME	8774	40	20	9,90E-01	9,96E-01	rs2228375|rs10819284|rs1539624|rs10055964|rs6496888|rs4977343|rs9292637|rs1464258|rs3278|rs10833369|rs12475881|rs13189284|rs3829406|rs17536553|rs11026529|rs2241694|rs10208012|rs892601|rs1710887|rs12954053
Discoid Rash	INFLAMMASOMES	REACTOME	468	10	7	9,90E-01	9,96E-01	rs954954|rs396146|rs11653896|rs9908117|rs7242402|rs2159635|rs2849380
Discoid Rash	PHENYLALANINE_METABOLISM	KEGG	442	9	8	9,91E-01	9,96E-01	rs9935162|rs7194392|rs17542122|rs2172873|rs11768267|rs10860900|rs3809524|rs1465936
Discoid Rash	E2F_MEDIATED_REGULATION_OF_DNA_REPLICATION	REACTOME	452	8	6	9,92E-01	9,96E-01	rs9396464|rs2891652|rs3782093|rs9966612|rs747232|rs2847154
Discoid Rash	GALACTOSE_METABOLISM	KEGG	759	13	11	9,93E-01	9,96E-01	rs12240387|rs10508243|rs1000251|rs5024722|rs217463|rs9664019|rs884289|rs10517428|rs715041|rs2812365|rs12357621
Discoid Rash	G1_S_SPECIFIC_TRANSCRIPTION	REACTOME	194	3	3	9,93E-01	9,96E-01	rs9966612|rs747232|rs2847154
Discoid Rash	TRANSCRIPTION	REACTOME	2708	38	20	9,93E-01	9,96E-01	rs2224553|rs7873382|rs575660|rs2279083|rs12586783|rs2228529|rs2078639|rs17420770|rs964888|rs1783983|rs10896604|rs1813529|rs11249163|rs10419120|rs10961329|rs1784304|rs13302756|rs16979884|rs2296085|rs3787016
Discoid Rash	TRANSPORT_OF_INORGANIC_CATIONS_ANIONS_AND_AMINO_ACIDS_OLIGOPEPTIDES	REACTOME	4051	30	20	9,93E-01	9,96E-01	rs10055964|rs4977343|rs9292637|rs3278|rs12475881|rs3829406|rs11026529|rs9309056|rs12459087|rs1425614|rs1348573|rs4790232|rs9074|rs1439502|rs6466182|rs298467|rs4482114|rs11836940|rs12879346|rs11712608
Discoid Rash	STEROID_BIOSYNTHESIS	KEGG	247	2	2	9,93E-01	9,96E-01	rs4840583|rs9685465
Discoid Rash	MISMATCH_REPAIR	KEGG	576	18	12	9,94E-01	9,96E-01	rs4802436|rs2321744|rs7324754|rs614123|rs3774332|rs11236181|rs9598129|rs2526697|rs1753477|rs1673041|rs3136337|rs1328667
Discoid Rash	GOLGI_ASSOCIATED_VESICLE_BIOGENESIS	REACTOME	1057	32	20	9,94E-01	9,96E-01	rs13165062|rs7203231|rs11075904|rs6917198|rs4507864|rs3824389|rs10755299|rs4804528|rs11604561|rs4490948|rs7162209|rs1028594|rs1047631|rs2072824|rs492960|rs1555422|rs17271418|rs1990890|rs1997679|rs1254901
Discoid Rash	TELOMERE_MAINTENANCE	REACTOME	1133	27	17	9,95E-01	9,96E-01	rs9396464|rs4802436|rs1784304|rs2321744|rs472594|rs7324754|rs10146912|rs614123|rs11935024|rs11236181|rs3782093|rs9598129|rs1753477|rs10484400|rs1673041|rs3744258|rs1328667
Discoid Rash	POST_TRANSLATIONAL_PROTEIN_MODIFICATION	REACTOME	6008	36	20	9,96E-01	9,96E-01	rs2410274|rs2289241|rs7597227|rs6866961|rs7216490|rs2404839|rs672413|rs17323530|rs620381|rs1862458|rs7844620|rs659758|rs10496721|rs6731612|rs11104430|rs6981912|rs1516085|rs10163323|rs7533303|rs6494120

Malar Rash	G_ALPHA_I_SIGNALLING_EVENTS	REACTOME	6453	31	20	3,20E-03	9,17E-01	rs6579927|rs11720405|rs7637803|rs10956564|rs9395320|rs12452228|rs4629584|rs4517239|rs12215494|rs2039141|rs11687066|rs17451303|rs198311|rs4683189|rs9410438|rs16891982|rs16904360|rs13148043|rs917427|rs1213352
Malar Rash	BIOSYNTHESIS_OF_UNSATURATED_FATTY_ACIDS	KEGG	463	23	10	8,50E-03	9,17E-01	rs11737840|rs11190483|rs4698832|rs6447868|rs17448763|rs12108641|rs10452184|rs6533493|rs6501847|rs1980360
Malar Rash	COLORECTAL_CANCER	KEGG	2539	32	20	1,80E-02	9,17E-01	rs406334|rs8092360|rs2711008|rs4976123|rs2035256|rs266371|rs9962572|rs16950543|rs4410871|rs12543549|rs4987721|rs11561771|rs8004059|rs9961435|rs36047858|rs11766675|rs6719501|rs880026|rs1636808|rs7173609
Malar Rash	O_GLYCAN_BIOSYNTHESIS	KEGG	1579	35	20	1,87E-02	9,17E-01	rs2736862|rs4717586|rs6976905|rs6860445|rs28757712|rs2364445|rs556586|rs10068322|rs2332211|rs768425|rs17626419|rs4958735|rs4535614|rs6580074|rs11167680|rs2241430|rs7137092|rs1317198|rs1638201|rs10902513
Malar Rash	REGULATION_OF_INSULIN_SECRETION_BY_GLUCAGON_LIKE_PEPTIDE1	REACTOME	1388	46	20	1,90E-02	9,17E-01	rs11720405|rs4972394|rs10956564|rs883541|rs16904360|rs7303990|rs10170651|rs2294451|rs4964005|rs7972342|rs4898725|rs12147761|rs891488|rs6067085|rs2344498|rs11658925|rs472159|rs11621038|rs3934729|rs1861932
Malar Rash	ASSOCIATION_OF_TRIC_CCT_WITH_TARGET_PROTEINS_DURING_BIOSYNTHESIS	REACTOME	359	17	8	2,71E-02	9,17E-01	rs7690973|rs6856075|rs11732516|rs6090921|rs6500380|rs4716195|rs2054758|rs6051076
Malar Rash	SELENOAMINO_ACID_METABOLISM	KEGG	742	26	20	2,74E-02	9,17E-01	rs12142041|rs17552854|rs11135284|rs7798172|rs13178865|rs173792|rs10521140|rs9804151|rs294588|rs6088638|rs7447133|rs7723455|rs17360177|rs12188891|rs17359728|rs4868845|rs17348939|rs4583905|rs2027745|rs234742
Malar Rash	CHRONIC_MYELOID_LEUKEMIA	KEGG	2538	42	20	2,76E-02	9,17E-01	rs9682469|rs4976123|rs4435197|rs7183535|rs266371|rs9410438|rs2834874|rs16950543|rs4410871|rs12543549|rs1152699|rs11918821|rs1543478|rs36047858|rs11766675|rs6938550|rs1636808|rs7173609|rs2720662|rs4074785
Malar Rash	FRUCTOSE_AND_MANNOSE_METABOLISM	KEGG	984	39	20	2,78E-02	9,17E-01	rs756567|rs3823279|rs1708414|rs3800128|rs6560821|rs9392322|rs4747994|rs1227938|rs9503159|rs7815340|rs4747886|rs1545970|rs12527698|rs17687453|rs12189843|rs1557591|rs13225343|rs1769259|rs10794989|rs10823343
Malar Rash	SMAD2_SMAD3_SMAD4_HETEROTRIMER_REGULATES_TRANSCRIPTION	REACTOME	715	37	20	2,97E-02	9,17E-01	rs266371|rs16950543|rs4410871|rs12543549|rs9961435|rs36047858|rs7173609|rs2720662|rs266312|rs3217992|rs6577060|rs11916830|rs9507713|rs7181907|rs944695|rs7163945|rs1011970|rs6930645|rs4130120|rs1547705
Malar Rash	STATHMIN_PATHWAY	BIOCARTA	456	10	6	3,16E-02	9,17E-01	rs883541|rs11098195|rs11658925|rs2767326|rs11938486|rs7213247
Malar Rash	NFAT_PATHWAY	BIOCARTA	1626	47	20	3,54E-02	9,17E-01	rs883541|rs4976123|rs10520259|rs11250154|rs4811174|rs4618461|rs12945671|rs7824704|rs6041884|rs754505|rs815808|rs11766675|rs12646107|rs10223241|rs1636808|rs11658925|rs6500552|rs187571|rs4867697|rs703559
Malar Rash	CGMP_EFFECTS	REACTOME	1520	48	20	3,58E-02	9,17E-01	rs1544863|rs10497482|rs833141|rs575669|rs2573088|rs12196394|rs4243390|rs569647|rs221725|rs7629595|rs10841414|rs4910164|rs12829100|rs10505864|rs634651|rs10751211|rs6931483|rs11929676|rs907851|rs6586341
Malar Rash	PHOSPHORYLATION_OF_CD3_AND_TCR_ZETA_CHAINS	REACTOME	521	26	7	3,76E-02	9,17E-01	rs3767742|rs7756516|rs2281390|rs9277052|rs9277535|rs4404873|rs1932435
Malar Rash	DEADENYLATION_DEPENDENT_MRNA_DECAY	REACTOME	622	36	20	3,88E-02	9,17E-01	rs6764079|rs968053|rs4428160|rs13072044|rs1025814|rs16898766|rs3108908|rs11642944|rs581045|rs4761281|rs4765876|rs17028238|rs6495119|rs12630964|rs6958241|rs2165944|rs3741436|rs12532231|rs10876394|rs12599053
Malar Rash	IL2RB_PATHWAY	BIOCARTA	1296	38	20	3,90E-02	9,17E-01	rs12409333|rs8092360|rs4976123|rs4410871|rs12543549|rs11914132|rs4987721|rs12244380|rs8004059|rs11766675|rs1636808|rs7073236|rs2720662|rs17489214|rs1993703|rs310245|rs706725|rs1760118|rs42154|rs1323653
Malar Rash	VIRAL_MYOCARDITIS	KEGG	2750	38	20	3,99E-02	9,17E-01	rs2290600|rs2711008|rs201603|rs9306310|rs178637|rs7756516|rs788338|rs12945671|rs2060986|rs885945|rs6060124|rs17752721|rs4272873|rs11561771|rs265371|rs2517938|rs2857106|rs9510465|rs9578562|rs4812997
Malar Rash	PRION_DISEASES	KEGG	1585	53	20	4,08E-02	9,17E-01	rs10854450|rs7282326|rs9984570|rs2826327|rs725745|rs198095|rs2826409|rs2827451|rs2827634|rs2826377|rs4957473|rs13047152|rs10473234|rs2827034|rs2483675|rs742720|rs7510476|rs2826965|rs2031749|rs2828214
Malar Rash	PHOSPHOLIPID_METABOLISM	REACTOME	6226	33	20	4,25E-02	9,17E-01	rs11582551|rs1968930|rs1037182|rs2618956|rs7749480|rs1993224|rs4976123|rs2662126|rs977122|rs13427229|rs12542460|rs2701632|rs1907108|rs17114593|rs600415|rs11876701|rs2286489|rs4763508|rs974309|rs7099450
Malar Rash	PD1_SIGNALING	REACTOME	398	23	6	4,46E-02	9,17E-01	rs10807113|rs822349|rs2281390|rs9277052|rs7025291|rs9277535
Malar Rash	GLYCOLYSIS	REACTOME	558	21	10	4,48E-02	9,17E-01	rs756567|rs6560821|rs4747994|rs4747886|rs11881878|rs11084823|rs10794989|rs2388251|rs11251481|rs2388566
Malar Rash	TFF_PATHWAY	BIOCARTA	697	41	20	4,56E-02	9,17E-01	rs2711008|rs4976123|rs4947491|rs11561771|rs11766675|rs7776830|rs880026|rs1636808|rs17172446|rs12537364|rs706725|rs42154|rs7708673|rs6599127|rs2526406|rs249479|rs7840147|rs9863665|rs3020619|rs7219
Malar Rash	DEPOSITION_OF_NEW_CENPA_CONTAINING_NUCLEOSOMES_AT_THE_CENTROMERE	REACTOME	637	29	9	4,71E-02	9,17E-01	rs2833690|rs9381786|rs9461204|rs4267943|rs9302109|rs2099505|rs7776065|rs2014547|rs9358913
Malar Rash	GASTRIN_CREB_SIGNALLING_PATHWAY_VIA_PKC_AND_MAPK	REACTOME	8122	38	20	4,90E-02	9,17E-01	rs6579927|rs11720405|rs11198846|rs3799630|rs4976123|rs4947491|rs8126112|rs4629584|rs12215494|rs6789224|rs11687066|rs17451303|rs11248060|rs2075355|rs237851|rs9533044|rs7303990|rs1235695|rs16903375|rs2996011
Malar Rash	ERK_MAPK_TARGETS	REACTOME	704	51	17	4,93E-02	9,17E-01	rs3799630|rs8029326|rs4618461|rs10223241|rs7183836|rs16899333|rs3799631|rs11105194|rs11881878|rs12436076|rs4709117|rs8030728|rs187571|rs6918384|rs9366043|rs9348131|rs770466
Malar Rash	RECYCLING_PATHWAY_OF_L1	REACTOME	874	45	20	5,00E-02	9,17E-01	rs7702812|rs3799630|rs1422834|rs17182398|rs11213366|rs10787735|rs16899333|rs3799631|rs4804528|rs8013402|rs7023913|rs12436076|rs4709117|rs12338625|rs646289|rs4076950|rs6018027|rs11167725|rs6918384|rs4425006
Malar Rash	SIGNALING_BY_BMP	REACTOME	918	57	20	5,13E-02	9,17E-01	rs7171416|rs15985|rs1515263|rs13044457|rs6054517|rs719563|rs1489152|rs13099637|rs11654392|rs36047858|rs1561902|rs6430191|rs2258224|rs6759721|rs4459683|rs4662505|rs1076352|rs10000023|rs368007|rs11807703
Malar Rash	O_LINKED_GLYCOSYLATION_OF_MUCINS	REACTOME	2402	35	20	5,19E-02	9,17E-01	rs2736862|rs12422396|rs4717586|rs6976905|rs6860445|rs28757712|rs17000836|rs2364445|rs4686823|rs556586|rs10068322|rs2332211|rs768425|rs17626419|rs4958735|rs4535614|rs6580074|rs11167680|rs2241430|rs7137092
Malar Rash	TIGHT_JUNCTION	KEGG	6242	30	20	5,52E-02	9,17E-01	rs3807741|rs12960187|rs10872828|rs2290600|rs201603|rs9306310|rs17018319|rs178637|rs12472674|rs2135732|rs788338|rs12945671|rs2060986|rs17416597|rs6060124|rs4689424|rs28705165|rs714104|rs12866697|rs7257613
Malar Rash	SPHINGOLIPID_METABOLISM	REACTOME	1944	37	20	5,59E-02	9,17E-01	rs1968930|rs1037182|rs2618956|rs1993224|rs2662126|rs977122|rs17114593|rs7099450|rs9285606|rs2842085|rs5770953|rs11627212|rs13128386|rs12323862|rs10826013|rs11850756|rs10138733|rs8016092|rs11684431|rs10758320
Malar Rash	CYSTEINE_AND_METHIONINE_METABOLISM	KEGG	982	27	20	5,65E-02	9,17E-01	rs12142041|rs17552854|rs11677670|rs11135284|rs13178865|rs173792|rs9804151|rs4345650|rs294588|rs7447133|rs7723455|rs11887120|rs17360177|rs12188891|rs17359728|rs4868845|rs17348939|rs2298316|rs4583905|rs2027745
Malar Rash	GLYCOSAMINOGLYCAN_BIOSYNTHESIS_CHONDROITIN_SULFATE	KEGG	1372	42	20	5,70E-02	9,17E-01	rs4780677|rs718356|rs6455394|rs4780667|rs2682185|rs4965800|rs11224169|rs2975419|rs2449393|rs11223842|rs12933411|rs1540675|rs4794136|rs6586829|rs1545970|rs12375275|rs4921662|rs17769571|rs2500513|rs7743868
Malar Rash	EICOSANOID_LIGAND_BINDING_RECEPTORS	REACTOME	507	18	7	5,73E-02	9,17E-01	rs11687066|rs2244507|rs640044|rs990466|rs12651829|rs1811337|rs1395157
Malar Rash	BASAL_TRANSCRIPTION_FACTORS	KEGG	386	20	6	6,01E-02	9,17E-01	rs10208754|rs12587011|rs4953611|rs7017279|rs7982845|rs348334
Malar Rash	EIF_PATHWAY	BIOCARTA	158	9	5	6,04E-02	9,17E-01	rs968053|rs737204|rs6503653|rs17028238|rs2165944
Malar Rash	PROPANOATE_METABOLISM	KEGG	1219	57	20	6,07E-02	9,17E-01	rs7647085|rs962800|rs9381786|rs10973770|rs6791324|rs4550189|rs4267943|rs10210248|rs1490265|rs8049513|rs6088638|rs17425310|rs1326466|rs7990266|rs12874460|rs7993336|rs2268403|rs4646770|rs1921057|rs993736
Malar Rash	CTCF_PATHWAY	BIOCARTA	1035	43	20	6,36E-02	9,17E-01	rs4976123|rs10493860|rs4410871|rs12543549|rs1887054|rs1489152|rs2075751|rs36047858|rs11766675|rs1636808|rs2720662|rs4074785|rs3785568|rs3217992|rs706725|rs42154|rs7708673|rs4130120|rs1030102|rs3773650
Malar Rash	NITRIC_OXIDE_STIMULATES_GUANYLATE_CYCLASE	REACTOME	1895	46	20	6,37E-02	9,17E-01	rs1544863|rs10497482|rs833141|rs575669|rs2573088|rs12196394|rs4243390|rs901779|rs569647|rs221725|rs7629595|rs10841414|rs4910164|rs12829100|rs10505864|rs634651|rs10751211|rs6931483|rs6789945|rs11212022
Malar Rash	HIVNEF_PATHWAY	BIOCARTA	1008	43	20	6,63E-02	9,17E-01	rs8092360|rs2711008|rs10047541|rs4804833|rs1543478|rs4987721|rs11561771|rs4837820|rs4761528|rs15614|rs4807269|rs12537364|rs7922090|rs1997703|rs696|rs8083946|rs2155458|rs13402616|rs2526406|rs7840147
Malar Rash	POST_TRANSLATIONAL_PROTEIN_MODIFICATION	REACTOME	6008	42	20	6,63E-02	9,17E-01	rs2410361|rs1968930|rs2736862|rs12422396|rs4717586|rs6976905|rs6860445|rs2662126|rs2720630|rs718780|rs1947110|rs7156225|rs12431858|rs28757712|rs1295391|rs17000836|rs2364445|rs9401188|rs17108549|rs4686823
Malar Rash	UBIQUITIN_MEDIATED_PROTEOLYSIS	KEGG	3227	38	20	6,69E-02	9,17E-01	rs13093068|rs7690973|rs7639174|rs6856075|rs735842|rs11039149|rs4683620|rs10155|rs11732516|rs12714750|rs8182037|rs4550189|rs10183291|rs4804505|rs4479617|rs4441427|rs11071240|rs17223858|rs2173461|rs17752673
Malar Rash	PYRUVATE_METABOLISM	KEGG	908	58	20	6,90E-02	9,17E-01	rs7216|rs1708414|rs10973770|rs2158839|rs4550189|rs662361|rs1781738|rs12287767|rs1946741|rs6088638|rs10267797|rs1474758|rs843720|rs602498|rs13225343|rs7119426|rs2268403|rs4646770|rs10942262|rs7122406
Malar Rash	BAD_PATHWAY	BIOCARTA	927	32	20	7,28E-02	9,17E-01	rs8092360|rs883541|rs4976123|rs2684789|rs4987721|rs11766675|rs2684790|rs1636808|rs2684802|rs11658925|rs703559|rs7166558|rs2075937|rs706725|rs42154|rs8083946|rs7708673|rs218237|rs249479|rs131849
Malar Rash	TOB1_PATHWAY	BIOCARTA	652	40	20	7,52E-02	9,17E-01	rs266371|rs10493860|rs16950543|rs1887054|rs12244380|rs36047858|rs7073236|rs7173609|rs266312|rs10878721|rs7181907|rs7163945|rs1323653|rs791589|rs3773650|rs7169592|rs2243290|rs8109167|rs2278545|rs2205181
Malar Rash	DORSO_VENTRAL_AXIS_FORMATION	KEGG	1149	41	20	7,56E-02	9,17E-01	rs4947491|rs10772508|rs7111926|rs15985|rs8023333|rs2267644|rs7936180|rs10743964|rs7776830|rs5751957|rs17172446|rs7130878|rs10926254|rs2124724|rs565536|rs621922|rs436388|rs11221390|rs1940973|rs12421034
Malar Rash	IL7_PATHWAY	BIOCARTA	699	45	20	7,58E-02	9,17E-01	rs12409333|rs8092360|rs4976123|rs4987721|rs11766675|rs1636808|rs6500552|rs310245|rs706725|rs6985210|rs42154|rs8083946|rs7708673|rs249479|rs7000615|rs4739147|rs310199|rs11076786|rs129988|rs4131890
Malar Rash	ALK_PATHWAY	BIOCARTA	1932	35	20	7,84E-02	9,17E-01	rs7171416|rs11250154|rs10493860|rs4618461|rs7824704|rs13044457|rs1887054|rs6054517|rs719563|rs1489152|rs11654392|rs9345044|rs2820350|rs36047858|rs2811650|rs10223241|rs3823039|rs880026|rs1561902|rs2475252
Malar Rash	NEPHRIN_INTERACTIONS	REACTOME	1617	52	20	7,85E-02	9,17E-01	rs3807741|rs4976123|rs4715005|rs11220608|rs12610011|rs1485781|rs4711874|rs7129859|rs12799000|rs4935966|rs7787597|rs6967983|rs4730427|rs2868865|rs12705416|rs706725|rs10893560|rs7708673|rs37852|rs3779329
Malar Rash	PTM_GAMMA_CARBOXYLATION_HYPUSINE_FORMATION_AND_ARYLSULFATASE_ACTIV	REACTOME	377	41	14	7,97E-02	9,17E-01	rs1968930|rs2662126|rs3760999|rs5770953|rs6755028|rs13128386|rs9848608|rs555212|rs10187424|rs234687|rs6453423|rs9898599|rs134774|rs6878810
Malar Rash	ARAP_PATHWAY	BIOCARTA	292	23	12	7,98E-02	9,17E-01	rs3761793|rs4821628|rs5750402|rs10169262|rs793683|rs8139798|rs1894599|rs1041894|rs6090921|rs2269593|rs12771352|rs1510796
Malar Rash	L1CAM_INTERACTIONS	REACTOME	3914	34	20	8,02E-02	9,17E-01	rs7702812|rs1864773|rs3799630|rs536173|rs4947491|rs412970|rs9861063|rs9878958|rs1422834|rs6777408|rs2804877|rs17182398|rs6471070|rs1990708|rs12955222|rs11213366|rs2031002|rs7776830|rs572614|rs10787735
Malar Rash	FACTORS_INVOLVED_IN_MEGAKARYOCYTE_DEVELOPMENT_AND_PLATELET_PRODUCT	REACTOME	3549	38	20	8,15E-02	9,17E-01	rs7702812|rs11720405|rs883541|rs875078|rs11250154|rs478882|rs947006|rs1016984|rs7824704|rs13116389|rs1422834|rs10829597|rs16866450|rs8076424|rs10104574|rs1331191|rs12251654|rs17181086|rs1538512|rs6476904
Malar Rash	REGULATION_OF_INSULIN_SECRETION	REACTOME	3741	35	20	8,40E-02	9,17E-01	rs11720405|rs4972394|rs10956564|rs883541|rs8126112|rs11250154|rs16904360|rs1108656|rs7824704|rs7303990|rs12710651|rs17703233|rs2294451|rs891700|rs4964005|rs1688123|rs6133869|rs7972342|rs156005|rs4898725
Malar Rash	APOPTOSIS	KEGG	1727	37	20	8,50E-02	9,17E-01	rs8092360|rs2711008|rs883541|rs4976123|rs394558|rs4851541|rs1543478|rs4987721|rs11561771|rs11766675|rs1636808|rs1177614|rs12635900|rs12537364|rs11658925|rs7922090|rs696|rs2075937|rs706725|rs12546238
Malar Rash	SIGNALING_BY_NODAL	REACTOME	632	45	20	8,59E-02	9,17E-01	rs266371|rs16950543|rs1515263|rs9961435|rs36047858|rs7174882|rs7173609|rs266312|rs6430191|rs7181907|rs2258224|rs6759721|rs4459683|rs7163945|rs4662505|rs7169592|rs17069632|rs2238658|rs2278545|rs10195713
Malar Rash	BUTANOATE_METABOLISM	KEGG	763	61	17	8,75E-02	9,17E-01	rs7216|rs3761793|rs10773148|rs10973770|rs4550189|rs7405412|rs1946741|rs807513|rs4646770|rs12920708|rs1273376|rs7045845|rs17025751|rs1370004|rs2306619|rs4878805|rs4728329
Malar Rash	ANTIGEN_PROCESSING_UBIQUITINATION_PROTEASOME_DEGRADATION	REACTOME	4788	28	20	8,84E-02	9,17E-01	rs13093068|rs7690973|rs12292911|rs12405914|rs7639174|rs6856075|rs8063430|rs6437789|rs11732516|rs12714750|rs8182037|rs10957025|rs4550189|rs11125482|rs573264|rs10183291|rs4804505|rs2131786|rs6115830|rs764885
Malar Rash	HDAC_PATHWAY	BIOCARTA	1231	52	20	8,94E-02	9,17E-01	rs4976123|rs8029326|rs4811174|rs4618461|rs2684789|rs754505|rs815808|rs11766675|rs10223241|rs2684790|rs2189756|rs1636808|rs7183836|rs2684802|rs1750304|rs8030728|rs187571|rs703559|rs7166558|rs706725
Malar Rash	AMYOTROPHIC_LATERAL_SCLEROSIS_ALS	KEGG	1813	35	20	9,01E-02	9,17E-01	rs8092360|rs2711008|rs937210|rs7826050|rs2098469|rs918168|rs4987721|rs507879|rs4958596|rs11561771|rs17181086|rs1868289|rs2189756|rs17313372|rs7298664|rs17830392|rs12537364|rs220593|rs16957102|rs17339754
Malar Rash	TRANSCRIPTIONAL_ACTIVITY_OF_SMAD2_SMAD3_SMAD4_HETEROTRIMER	REACTOME	967	37	20	9,17E-02	9,17E-01	rs266371|rs16950543|rs4410871|rs12543549|rs9961435|rs36047858|rs12580394|rs7173609|rs2720662|rs266312|rs3217992|rs6577060|rs11916830|rs9507713|rs7181907|rs944695|rs7163945|rs1011970|rs6930645|rs4130120
Malar Rash	G_ALPHA_Q_SIGNALLING_EVENTS	REACTOME	7428	37	20	9,37E-02	9,17E-01	rs6579927|rs10937003|rs11198846|rs4976123|rs8126112|rs4629584|rs12215494|rs6789224|rs11687066|rs17451303|rs11248060|rs2075355|rs237851|rs9533044|rs7303990|rs1235695|rs16903375|rs2996011|rs714104|rs2256331
Malar Rash	G_PROTEIN_BETA_GAMMA_SIGNALLING	REACTOME	798	24	20	9,42E-02	9,17E-01	rs10937003|rs8126112|rs2294451|rs11766675|rs4898725|rs1636808|rs12147761|rs195770|rs6140629|rs13045064|rs11621038|rs6133573|rs926505|rs6077420|rs42154|rs7141727|rs887030|rs2179440|rs2745772|rs2287208
Malar Rash	ALZHEIMERS_DISEASE	KEGG	5029	33	20	9,53E-02	9,17E-01	rs9829238|rs4605535|rs4854053|rs2711008|rs8126112|rs1860094|rs12593288|rs766850|rs709318|rs10153042|rs7303990|rs16863205|rs11203280|rs2098469|rs918168|rs2256331|rs1878300|rs11561771|rs886898|rs4964005
Malar Rash	CITRIC_ACID_CYCLE_TCA_CYCLE	REACTOME	675	39	17	9,75E-02	9,17E-01	rs7647085|rs11203280|rs2158839|rs6791324|rs10210248|rs1490265|rs10267797|rs17425310|rs1326466|rs7990266|rs7993336|rs993736|rs10247064|rs8035724|rs12652118|rs9678502|rs6051076
Malar Rash	CASPASE_PATHWAY	BIOCARTA	327	28	12	9,88E-02	9,17E-01	rs2711008|rs10047541|rs507879|rs11561771|rs4807269|rs12537364|rs3758562|rs13402616|rs2526406|rs3812062|rs4646018|rs7315397
Malar Rash	NUCLEOTIDE_LIKE_PURINERGIC_RECEPTORS	REACTOME	272	31	13	9,93E-02	9,17E-01	rs7637803|rs11687066|rs7208308|rs2244507|rs738813|rs2247867|rs1466684|rs1874142|rs10920569|rs10935907|rs16863356|rs1978867|rs2250509
Malar Rash	HEDGEHOG_SIGNALING_PATHWAY	KEGG	2151	36	20	1,01E-01	9,17E-01	rs10463762|rs17270248|rs12988804|rs2544371|rs737681|rs2035901|rs13044457|rs6054517|rs719563|rs7869597|rs189727|rs288746|rs13014164|rs10046817|rs3823039|rs7388704|rs2744718|rs1887439|rs2883154|rs2475252
Malar Rash	TEL_PATHWAY	BIOCARTA	978	34	20	1,02E-01	9,17E-01	rs8092360|rs4947491|rs4410871|rs12543549|rs2684789|rs4987721|rs7776830|rs2684790|rs2720662|rs17172446|rs12549064|rs2684802|rs7166558|rs8083946|rs4130120|rs1030102|rs737063|rs7169544|rs4965436|rs12547643
Malar Rash	ACTIVATION_OF_BH3_ONLY_PROTEINS	REACTOME	325	13	8	1,02E-01	9,17E-01	rs8092360|rs4987721|rs6577060|rs2156223|rs8083946|rs12458289|rs6567322|rs2236719
Malar Rash	CITRATE_CYCLE_TCA_CYCLE	KEGG	1195	44	20	1,03E-01	9,17E-01	rs7647085|rs10758115|rs11203280|rs2158839|rs6791324|rs10210248|rs1490265|rs372957|rs1946741|rs10267797|rs17425310|rs1474758|rs1326466|rs7990266|rs7874815|rs7993336|rs7119426|rs993736|rs10247064|rs8035724
Malar Rash	PROTEASOME_PATHWAY	BIOCARTA	280	9	6	1,03E-01	9,17E-01	rs12292911|rs7224730|rs4790706|rs6142963|rs6017596|rs2011713
Malar Rash	MAP_KINASE_ACTIVATION_IN_TLR_CASCADE	REACTOME	1697	36	20	1,04E-01	9,17E-01	rs3799630|rs8029326|rs394558|rs4618461|rs4804833|rs8004059|rs1546745|rs9345044|rs2820350|rs2811650|rs10223241|rs2189756|rs7183836|rs4256810|rs1177614|rs16899333|rs3799631|rs11105194|rs11881878|rs12436076
Malar Rash	STEROID_HORMONES	REACTOME	693	27	20	1,06E-01	9,17E-01	rs17270248|rs12988804|rs2544371|rs1708414|rs5297|rs12602084|rs13014164|rs1801239|rs13225343|rs2302694|rs2075248|rs11939173|rs274657|rs2296241|rs7788953|rs7174997|rs7546652|rs1352844|rs1242094|rs7922682
Malar Rash	CLASS_I_MHC_MEDIATED_ANTIGEN_PROCESSING_PRESENTATION	REACTOME	5895	25	20	1,06E-01	9,17E-01	rs13093068|rs7690973|rs12292911|rs12405914|rs7639174|rs6856075|rs8063430|rs7156225|rs6437789|rs885945|rs11732516|rs12714750|rs8182037|rs10957025|rs4550189|rs11125482|rs573264|rs17108549|rs10183291|rs4804505
Malar Rash	INTRINSIC_PATHWAY_FOR_APOPTOSIS	REACTOME	532	30	16	1,07E-01	9,17E-01	rs8092360|rs2711008|rs4987721|rs11561771|rs6577060|rs12537364|rs2156223|rs8083946|rs7953290|rs13402616|rs2526406|rs1052571|rs919699|rs12458289|rs6567322|rs2236719
Malar Rash	PHOSPHOLIPASE_C_MEDIATED_CASCADE	REACTOME	2628	40	20	1,07E-01	9,17E-01	rs3117915|rs10956564|rs883541|rs833141|rs16904360|rs7303990|rs6891913|rs11593080|rs17612068|rs714104|rs7257613|rs4964005|rs815808|rs9578440|rs7972342|rs12619351|rs17234079|rs3129601|rs3129596|rs13135439
Malar Rash	CK1_PATHWAY	BIOCARTA	759	40	20	1,09E-01	9,17E-01	rs7445117|rs883541|rs8126112|rs7937641|rs251925|rs6140629|rs13045064|rs11658925|rs10896172|rs10736466|rs6133573|rs926505|rs6077420|rs2179440|rs2745772|rs1549221|rs2206423|rs7213247|rs10516128|rs362848
Malar Rash	NUCLEAR_EVENTS_KINASE_AND_TRANSCRIPTION_FACTOR_ACTIVATION	REACTOME	766	50	20	1,09E-01	9,17E-01	rs3799630|rs8029326|rs4618461|rs10223241|rs7183836|rs4256810|rs16899333|rs3799631|rs11105194|rs11881878|rs12436076|rs4709117|rs8030728|rs187571|rs12329315|rs6918384|rs4675672|rs9366043|rs9348131|rs770466
Malar Rash	DOWNREGULATION_OF_SMAD2_3_SMAD4_TRANSCRIPTIONAL_ACTIVITY	REACTOME	453	32	15	1,09E-01	9,17E-01	rs266371|rs16950543|rs9961435|rs36047858|rs12580394|rs7173609|rs266312|rs7181907|rs7163945|rs837493|rs7169592|rs2278545|rs12327024|rs6494629|rs837462
Malar Rash	TRANSPORT_OF_MATURE_TRANSCRIPT_TO_CYTOPLASM	REACTOME	693	32	20	1,12E-01	9,17E-01	rs9310419|rs968053|rs5766404|rs987607|rs10169262|rs12191724|rs9615022|rs2274913|rs17028238|rs13258200|rs723553|rs2282763|rs2165944|rs2027745|rs4740419|rs12473402|rs4274569|rs9297042|rs7284581|rs4759082
Malar Rash	NEUROTROPHIN_SIGNALING_PATHWAY	KEGG	3689	32	20	1,12E-01	9,17E-01	rs8092360|rs3799630|rs4976123|rs10849275|rs4435197|rs7183535|rs394558|rs13427229|rs9410438|rs10047541|rs4804833|rs1543478|rs4987721|rs1546745|rs815808|rs11766675|rs4734507|rs11098195|rs9588044|rs1636808
Malar Rash	DOWNSTREAM_SIGNALING_OF_ACTIVATED_FGFR	REACTOME	3636	32	20	1,12E-01	9,17E-01	rs3117915|rs10956564|rs883541|rs4976123|rs2721051|rs833141|rs16904360|rs7303990|rs6891913|rs11593080|rs17612068|rs714104|rs7257613|rs4964005|rs815808|rs6115830|rs9578440|rs7972342|rs12619351|rs17234079
Malar Rash	GLYCOSPHINGOLIPID_METABOLISM	REACTOME	924	52	20	1,13E-01	9,17E-01	rs1968930|rs1037182|rs2618956|rs2662126|rs5770953|rs13128386|rs8016092|rs11684431|rs10758320|rs9848608|rs820844|rs1555556|rs10823876|rs439863|rs7508|rs234687|rs6453423|rs9898599|rs7658635|rs11624137
Malar Rash	METABOLISM_OF_STEROID_HORMONES_AND_VITAMINS_A_AND_D	REACTOME	745	27	20	1,14E-01	9,17E-01	rs17270248|rs12988804|rs2544371|rs1708414|rs5297|rs12602084|rs13014164|rs1801239|rs13225343|rs2302694|rs2075248|rs11939173|rs2766581|rs274657|rs2296241|rs7788953|rs7174997|rs201823|rs7546652|rs6439858
Malar Rash	GABA_SYNTHESIS_RELEASE_REUPTAKE_AND_DEGRADATION	REACTOME	746	40	20	1,15E-01	9,17E-01	rs3761793|rs1400155|rs9682960|rs13206801|rs6133869|rs11128537|rs3900078|rs2250474|rs10485738|rs1462923|rs2017903|rs216250|rs9442725|rs807513|rs10744557|rs4234504|rs1273376|rs8891|rs362560|rs542736
Malar Rash	INHIBITION_OF_THE_PROTEOLYTIC_ACTIVITY_OF_APC_C_REQUIRED_FOR_THE_ONSET_ TOTIC_SPINDLE_CHECKPOINT_COMPONENTS	REACTOME	365	22	8	1,17E-01	9,17E-01	rs11248424|rs7678743|rs7894765|rs9990802|rs2004845|rs11592827|rs17754530|rs11593744
Malar Rash	CSK_PATHWAY	BIOCARTA	554	18	9	1,18E-01	9,17E-01	rs883541|rs3767742|rs11658925|rs6500552|rs13041735|rs7213247|rs11076786|rs1932435|rs129988
Malar Rash	GNRH_SIGNALING_PATHWAY	KEGG	3992	37	20	1,18E-01	9,17E-01	rs11582551|rs4605535|rs10956564|rs4947491|rs8126112|rs1860094|rs16904360|rs2701632|rs709318|rs7303990|rs4804833|rs886898|rs4964005|rs1688123|rs4820944|rs815808|rs7776830|rs3774589|rs7972342|rs11098195
Malar Rash	TGFB_PATHWAY	BIOCARTA	817	34	20	1,19E-01	9,17E-01	rs266371|rs16950543|rs7202333|rs9961435|rs9345044|rs2820350|rs36047858|rs2811650|rs7173609|rs266312|rs7181907|rs6500552|rs2501657|rs7163945|rs3773650|rs9342261|rs7169592|rs8061932|rs8109167|rs2278545
Malar Rash	NEUROTRANSMITTER_RECEPTOR_BINDING_AND_DOWNSTREAM_TRANSMISSION_IN_TH L	REACTOME	6592	34	20	1,19E-01	9,17E-01	rs10937003|rs10956564|rs4793521|rs3799630|rs8126112|rs12523641|rs1984334|rs4517239|rs6517438|rs937210|rs16904360|rs926543|rs1396514|rs9861063|rs6558000|rs10183636|rs7403139|rs2098469|rs1385902|rs918168

Malar Rash	TID_PATHWAY	BIOCARTA	479	14	10	1,20E-01	9,17E-01	rs9376268|rs17703233|rs1543478|rs9808753|rs10878721|rs1571221|rs696|rs1327474|rs11968400|rs10483453
Malar Rash	PROTEIN_FOLDING	REACTOME	675	21	11	1,21E-01	9,17E-01	rs7690973|rs6856075|rs11732516|rs6929344|rs6090921|rs6500380|rs4716195|rs11080571|rs354599|rs2054758|rs6051076
Malar Rash	PLATELET_HOMEOSTASIS	REACTOME	4080	39	20	1,23E-01	9,17E-01	rs1544863|rs10937003|rs10497482|rs833141|rs575669|rs2573088|rs12196394|rs4243390|rs901779|rs7303990|rs17025772|rs42792|rs2294451|rs569647|rs221725|rs4964005|rs2192724|rs715305|rs7972342|rs7629595
Malar Rash	ACYL_CHAIN_REMODELLING_OF_PE	REACTOME	696	34	19	1,24E-01	9,17E-01	rs11582551|rs7749480|rs13427229|rs2701632|rs600415|rs4820944|rs156631|rs1487492|rs1327658|rs6680812|rs955835|rs650392|rs12564176|rs591393|rs4821738|rs11677|rs1356410|rs818968|rs10912119
Malar Rash	PANCREATIC_CANCER	KEGG	1920	40	20	1,26E-01	9,17E-01	rs12409333|rs4976123|rs4947491|rs266371|rs16950543|rs9961435|rs36047858|rs11766675|rs7776830|rs6938550|rs1636808|rs7173609|rs4074785|rs266312|rs3217992|rs11568993|rs17172446|rs7631698|rs8139798|rs4894512
Malar Rash	GLYCOSAMINOGLYCAN_BIOSYNTHESIS_HEPARAN_SULFATE	KEGG	2273	47	20	1,27E-01	9,17E-01	rs4780677|rs718356|rs6455394|rs4945522|rs4780667|rs2682185|rs731096|rs11224169|rs2245104|rs12746227|rs7984995|rs241179|rs7926191|rs12933411|rs9890916|rs1540675|rs10010085|rs4794136|rs1994958|rs9913031
Malar Rash	ABCA_TRANSPORTERS_IN_LIPID_HOMEOSTASIS	REACTOME	464	14	8	1,28E-01	9,17E-01	rs1038390|rs12452482|rs3886119|rs10493872|rs225406|rs2172133|rs17031664|rs12615195
Malar Rash	TAK1_ACTIVATES_NFKB_BY_PHOSPHORYLATION_AND_ACTIVATION_OF_IKKS_COMPLE	REACTOME	777	40	20	1,32E-01	9,17E-01	rs394558|rs1543478|rs2256331|rs1546745|rs9345044|rs2820350|rs2811650|rs1177614|rs9506325|rs531486|rs2829946|rs7922090|rs467778|rs696|rs2501657|rs311636|rs9342261|rs2830012|rs9611169|rs12215452
Malar Rash	NOTCH1_INTRACELLULAR_DOMAIN_REGULATES_TRANSCRIPTION	REACTOME	2852	29	20	1,34E-01	9,17E-01	rs7690973|rs13245206|rs6856075|rs10152562|rs6444767|rs4410871|rs10513747|rs12543549|rs2920946|rs11732516|rs1877209|rs1995243|rs4310569|rs2051920|rs669669|rs12580394|rs3739809|rs12911091|rs6804202|rs2720662
Malar Rash	AMINO_ACID_TRANSPORT_ACROSS_THE_PLASMA_MEMBRANE	REACTOME	1025	38	20	1,35E-01	9,17E-01	rs3783260|rs2531747|rs2286489|rs7329421|rs2720546|rs17588888|rs3759608|rs10132368|rs2729384|rs216250|rs6766142|rs17244832|rs999969|rs882953|rs1380412|rs752503|rs10862903|rs10744557|rs2729385|rs3811188
Malar Rash	ANTIVIRAL_MECHANISM_BY_IFN_STIMULATED_GENES	REACTOME	1077	42	20	1,37E-01	9,17E-01	rs12409333|rs9310419|rs968053|rs4284952|rs5766404|rs987607|rs9615022|rs10495915|rs11071240|rs4677039|rs17028238|rs723553|rs310245|rs9398444|rs3024912|rs2165944|rs4740419|rs4585264|rs6802890|rs11684526
Malar Rash	ERK5_PATHWAY	BIOCARTA	595	56	20	1,38E-01	9,17E-01	rs4976123|rs8029326|rs4618461|rs11766675|rs10223241|rs1636808|rs7183836|rs1750304|rs8030728|rs187571|rs706725|rs42154|rs7708673|rs249479|rs12329315|rs4675672|rs7219|rs4131890|rs770466|rs7804859
Malar Rash	TGF_BETA_RECEPTOR_SIGNALING_ACTIVATES_SMADS	REACTOME	563	42	20	1,39E-01	9,17E-01	rs266371|rs16950543|rs6041884|rs9961435|rs36047858|rs7173609|rs266312|rs928163|rs1855469|rs7181907|rs10896172|rs7163945|rs3773650|rs9309664|rs8124053|rs7169592|rs8109167|rs1474880|rs2278545|rs6494629
Malar Rash	VASCULAR_SMOOTH_MUSCLE_CONTRACTION	KEGG	5845	36	20	1,41E-01	9,17E-01	rs1544863|rs11582551|rs4605535|rs10956564|rs8126112|rs1860094|rs16904360|rs2701632|rs709318|rs901779|rs7303990|rs1235695|rs7208308|rs10185142|rs2075751|rs714104|rs31684|rs7257613|rs886898|rs4964005
Malar Rash	CHEMOKINE_SIGNALING_PATHWAY	KEGG	5407	35	20	1,43E-01	9,17E-01	rs11720405|rs10956564|rs11198846|rs4976123|rs8126112|rs4435197|rs7183535|rs4683189|rs16763|rs9410438|rs9371859|rs16904360|rs10197003|rs1543478|rs2294451|rs7095121|rs7093673|rs16832740|rs2882936|rs11766675
Malar Rash	ACTIVATED_POINT_MUTANTS_OF_FGFR2	REACTOME	873	29	20	1,43E-01	9,17E-01	rs3117915|rs6891913|rs11593080|rs17612068|rs9578440|rs17234079|rs3129601|rs3129596|rs11776272|rs4769189|rs477516|rs7989744|rs17004857|rs9634328|rs1501588|rs4866891|rs1458038|rs4489674|rs3135763|rs9580274
Malar Rash	BRANCHED_CHAIN_AMINO_ACID_CATABOLISM	REACTOME	507	29	10	1,44E-01	9,17E-01	rs9294177|rs2158839|rs4550189|rs9352825|rs10267797|rs277973|rs13220420|rs11969591|rs3122126|rs12579934
Malar Rash	INHIBITION_OF_VOLTAGE_GATED_CA2_CHANNELS_VIA_GBETA_GAMMA_SUBUNITS	REACTOME	1082	49	20	1,45E-01	9,17E-01	rs4793521|rs4517239|rs6517438|rs1396514|rs1385902|rs17763873|rs17763595|rs4898725|rs12147761|rs2109051|rs11621038|rs7943324|rs1167767|rs2367005|rs3129073|rs7141727|rs887030|rs6501411|rs1073572|rs2190550
Malar Rash	RNA_DEGRADATION	KEGG	1123	32	20	1,46E-01	9,17E-01	rs6764079|rs7568378|rs4428160|rs964450|rs13072044|rs1025814|rs11642944|rs581045|rs7205534|rs11125589|rs4761281|rs4905510|rs4765876|rs2047321|rs17458048|rs6495119|rs12630964|rs11126164|rs9937851|rs6958241
Malar Rash	NTHI_PATHWAY	BIOCARTA	876	42	20	1,49E-01	9,17E-01	rs266371|rs16950543|rs1543478|rs9345044|rs2820350|rs36047858|rs2811650|rs2189756|rs7173609|rs266312|rs7181907|rs6500552|rs7922090|rs696|rs2501657|rs7163945|rs3773650|rs9342261|rs7169592|rs968088
Malar Rash	HISTIDINE_METABOLISM	KEGG	654	52	20	1,50E-01	9,17E-01	rs962800|rs1357404|rs3829305|rs10973770|rs7798172|rs1873061|rs2114447|rs748465|rs8080449|rs4646770|rs10492227|rs4385902|rs7282864|rs6593011|rs7045845|rs8891|rs10521121|rs1038378|rs7280485|rs4878805
Malar Rash	INWARDLY_RECTIFYING_K_CHANNELS	REACTOME	1317	50	20	1,50E-01	9,17E-01	rs4793521|rs4517239|rs6517438|rs1396514|rs1385902|rs17703233|rs17763873|rs17763595|rs4898725|rs12147761|rs2109051|rs11621038|rs7943324|rs1167767|rs2367005|rs3129073|rs7141727|rs887030|rs6501411|rs1073572
Malar Rash	MAPK_TARGETS_NUCLEAR_EVENTS_MEDIATED_BY_MAP_KINASES	REACTOME	980	47	20	1,50E-01	9,17E-01	rs3799630|rs8029326|rs4618461|rs8004059|rs10223241|rs7183836|rs4256810|rs16899333|rs3799631|rs11105194|rs11881878|rs12436076|rs4709117|rs8030728|rs187571|rs10151713|rs12329315|rs6918384|rs4675672|rs882167
Malar Rash	INTEGRATION_OF_ENERGY_METABOLISM	REACTOME	4501	35	20	1,50E-01	9,17E-01	rs10937003|rs4972394|rs10956564|rs883541|rs8126112|rs11250154|rs16904360|rs1108656|rs7824704|rs7303990|rs10170651|rs942689|rs17703233|rs2294451|rs891700|rs878291|rs4964005|rs1688123|rs6133869|rs7809503
Malar Rash	IL22BP_PATHWAY	BIOCARTA	296	24	10	1,53E-01	9,17E-01	rs12409333|rs11570908|rs11216648|rs1571221|rs310245|rs3024912|rs310199|rs324015|rs4969168|rs7574865
Malar Rash	GLUCAGON_SIGNALING_IN_METABOLIC_REGULATION	REACTOME	1044	37	20	1,54E-01	9,17E-01	rs11720405|rs10956564|rs883541|rs16904360|rs2294451|rs4898725|rs12147761|rs11658925|rs72001|rs11621038|rs2099505|rs2239305|rs3934729|rs727432|rs13041735|rs7141727|rs887030|rs4736746|rs12680002|rs2287208
Malar Rash	SMALL_CELL_LUNG_CANCER	KEGG	3747	36	20	1,56E-01	9,17E-01	rs8092360|rs2711008|rs536173|rs4976123|rs735842|rs4410871|rs1868220|rs1992060|rs12543549|rs17752721|rs1286772|rs1057504|rs1543478|rs4987721|rs12955222|rs11561771|rs265371|rs11766675|rs572614|rs6938550
Malar Rash	METABOLISM_OF_VITAMINS_AND_COFACTORS	REACTOME	1119	33	20	1,58E-01	9,17E-01	rs6052937|rs12433426|rs241606|rs977576|rs878291|rs6586198|rs10494561|rs6038010|rs1409184|rs404678|rs7154905|rs17780226|rs1572151|rs598246|rs10231525|rs1002171|rs9399004|rs17649254|rs1776964|rs7760703
Malar Rash	CERAMIDE_PATHWAY	BIOCARTA	425	25	16	1,59E-01	9,17E-01	rs8092360|rs2711008|rs4987721|rs11561771|rs12537364|rs2721856|rs8083946|rs2155458|rs6471742|rs13402616|rs2526406|rs6920337|rs2322376|rs12458289|rs6567322|rs2236719
Malar Rash	APC_CDC20_MEDIATED_DEGRADATION_OF_NEK2A	REACTOME	396	25	9	1,60E-01	9,17E-01	rs11248424|rs7678743|rs7894765|rs9990802|rs2004845|rs11592827|rs1373293|rs17754530|rs11593744
Malar Rash	BLADDER_CANCER	KEGG	1222	34	20	1,61E-01	9,17E-01	rs5770775|rs4947491|rs4410871|rs7202333|rs12543549|rs7776830|rs6938550|rs2720662|rs4074785|rs3217992|rs11568993|rs17172446|rs12436076|rs11639281|rs3128519|rs4130120|rs3886934|rs1030102|rs13283404|rs7747931
Malar Rash	PYRIMIDINE_METABOLISM	REACTOME	792	33	20	1,61E-01	9,17E-01	rs5770775|rs37373|rs1253795|rs5760440|rs11587873|rs1152991|rs11664283|rs784600|rs1689924|rs9444331|rs9639018|rs4451581|rs1760217|rs2333874|rs4664914|rs12119882|rs13024527|rs3750187|rs2811188|rs6898640
Malar Rash	PPARA_PATHWAY	BIOCARTA	2445	35	20	1,62E-01	9,17E-01	rs3010301|rs883541|rs4976123|rs11039149|rs4410871|rs12543549|rs10009448|rs3774921|rs1543478|rs9373237|rs6007640|rs350806|rs11766675|rs12580394|rs1636808|rs2720662|rs2029401|rs2823025|rs964035|rs2286936
Malar Rash	G_BETA_GAMMA_SIGNALLING_THROUGH_PLC_BETA	REACTOME	624	27	20	1,63E-01	9,17E-01	rs10937003|rs8126112|rs2294451|rs4898725|rs12147761|rs6140629|rs13045064|rs11621038|rs6133573|rs926505|rs6077420|rs7141727|rs887030|rs2179440|rs2745772|rs2287208|rs2206423|rs12985186|rs6055562|rs6055748
Malar Rash	SYNTHESIS_OF_PIPS_AT_THE_GOLGI_MEMBRANE	REACTOME	788	29	20	1,63E-01	9,17E-01	rs11876701|rs2286489|rs4763508|rs974309|rs203150|rs2742413|rs1554513|rs1539826|rs9954372|rs13086080|rs7504866|rs4132081|rs12493491|rs1909475|rs2156481|rs16831670|rs1453037|rs10505819|rs1348293|rs898783
Malar Rash	OTHER_GLYCAN_DEGRADATION	KEGG	648	27	13	1,65E-01	9,17E-01	rs4690513|rs1038390|rs4690557|rs2178468|rs1316294|rs1993280|rs820844|rs6845027|rs10017041|rs10866222|rs3744189|rs11853141|rs12210887
Malar Rash	ASSEMBLY_OF_THE_PRE_REPLICATIVE_COMPLEX	REACTOME	911	35	20	1,67E-01	9,17E-01	rs12292911|rs13213285|rs8063430|rs3761873|rs6938550|rs577357|rs1781011|rs133416|rs7772351|rs7224730|rs4790706|rs837763|rs17213812|rs2282032|rs7747931|rs10948689|rs911361|rs6142963|rs9784758|rs6924102
Malar Rash	PYRUVATE_METABOLISM_AND_CITRIC_ACID_TCA_CYCLE	REACTOME	905	43	20	1,67E-01	9,17E-01	rs7647085|rs11203280|rs2158839|rs6791324|rs132930|rs10210248|rs1490265|rs10267797|rs17425310|rs1326466|rs7990266|rs7993336|rs7527283|rs993736|rs10247064|rs8035724|rs12652118|rs9678502|rs12611042|rs6051076
Malar Rash	ADP_SIGNALLING_THROUGH_P2RY12	REACTOME	438	23	17	1,67E-01	9,17E-01	rs11720405|rs7637803|rs2294451|rs4898725|rs12147761|rs11621038|rs7141727|rs887030|rs1466684|rs2287208|rs16863356|rs2886612|rs12985186|rs510619|rs17245731|rs508208|rs2993998
Malar Rash	SIGNALING_BY_CONSTITUTIVELY_ACTIVE_EGFR	REACTOME	503	32	13	1,69E-01	9,17E-01	rs4976123|rs4947491|rs7776830|rs11568993|rs17172446|rs706725|rs7708673|rs249479|rs7219|rs17238095|rs10488142|rs4131890|rs13244925
Malar Rash	PYRUVATE_METABOLISM	REACTOME	209	10	5	1,74E-01	9,17E-01	rs2158839|rs132930|rs10267797|rs7527283|rs12611042
Malar Rash	PROSTACYCLIN_SIGNALLING_THROUGH_PROSTACYCLIN_RECEPTOR	REACTOME	353	16	12	1,74E-01	9,17E-01	rs11720405|rs2294451|rs4898725|rs12147761|rs11621038|rs13041735|rs7141727|rs887030|rs2287208|rs12985186|rs508208|rs2993998
Malar Rash	CELL_CYCLE	KEGG	2581	32	20	1,74E-01	9,17E-01	rs11248424|rs266371|rs13213285|rs10155|rs16950543|rs4410871|rs4242594|rs12543549|rs4877110|rs12204457|rs9961435|rs883444|rs36047858|rs4734507|rs6938550|rs7173609|rs2720662|rs3108913|rs4074785|rs266312
Malar Rash	ATRBRCA_PATHWAY	BIOCARTA	238	19	10	1,75E-01	9,17E-01	rs10765682|rs12805507|rs12796488|rs499952|rs10896172|rs2244012|rs7113416|rs6926662|rs1801516|rs540723
Malar Rash	THROMBOXANE_SIGNALLING_THROUGH_TP_RECEPTOR	REACTOME	492	21	14	1,77E-01	9,17E-01	rs10937003|rs2294451|rs1688123|rs4898725|rs12147761|rs11621038|rs7141727|rs887030|rs2287208|rs4745639|rs7218921|rs12985186|rs508208|rs2993998
Malar Rash	TRIF_MEDIATED_TLR3_SIGNALING	REACTOME	2075	31	20	1,79E-01	9,17E-01	rs3799630|rs8029326|rs394558|rs4618461|rs4804833|rs1543478|rs2256331|rs8004059|rs1546745|rs9345044|rs2820350|rs2811650|rs10223241|rs2189756|rs7183836|rs4256810|rs1177614|rs16899333|rs3799631|rs1009619
Malar Rash	MAPK_SIGNALING_PATHWAY	KEGG	9833	31	20	1,80E-01	9,17E-01	rs3117915|rs9682469|rs11582551|rs4605535|rs3799630|rs4947491|rs10849275|rs1860094|rs4811174|rs13123632|rs4618461|rs926543|rs2701632|rs4410871|rs709318|rs4851541|rs4242594|rs6891913|rs12543549|rs11593080
Malar Rash	MITOCHONDRIA_PATHWAY	BIOCARTA	317	26	15	1,82E-01	9,17E-01	rs8092360|rs2711008|rs4987721|rs11561771|rs12537364|rs8083946|rs7953290|rs13402616|rs2526406|rs1052571|rs919699|rs12458289|rs6003071|rs6567322|rs2236719
Malar Rash	NGF_SIGNALLING_VIA_TRKA_FROM_THE_PLASMA_MEMBRANE	REACTOME	4847	38	20	1,82E-01	9,17E-01	rs10956564|rs3799630|rs883541|rs4976123|rs8029326|rs2721051|rs833141|rs13427229|rs9410438|rs16904360|rs4618461|rs7303990|rs714104|rs7257613|rs4964005|rs7231412|rs815808|rs6115830|rs738813|rs10223241
Malar Rash	G_ALPHA_S_SIGNALLING_EVENTS	REACTOME	5019	41	20	1,83E-01	9,17E-01	rs11720405|rs10956564|rs7445117|rs10497482|rs833141|rs575669|rs2268464|rs2573088|rs16904360|rs12196394|rs4243390|rs7713886|rs7208308|rs10185142|rs2294451|rs569647|rs221723|rs11125179|rs16941288|rs251925
Malar Rash	SULFUR_AMINO_ACID_METABOLISM	REACTOME	930	27	20	1,83E-01	9,17E-01	rs11135284|rs355396|rs13178865|rs173792|rs3825946|rs502862|rs9804151|rs4345650|rs294588|rs12903858|rs7447133|rs7723455|rs17360177|rs12188891|rs17359728|rs4868845|rs12915529|rs2298316|rs4583905|rs2027745
Malar Rash	IL_RECEPTOR_SHC_SIGNALING	REACTOME	624	46	20	1,84E-01	9,17E-01	rs12409333|rs4976123|rs11914132|rs12244380|rs7073236|rs1571221|rs310245|rs2075937|rs706725|rs1323653|rs7708673|rs791589|rs228942|rs249479|rs131849|rs2235329|rs310199|rs7219|rs4131890|rs12722561
Malar Rash	FORMATION_OF_ATP_BY_CHEMIOSMOTIC_COUPLING	REACTOME	77	7	3	1,85E-01	9,17E-01	rs2802460|rs11171911|rs1264901
Malar Rash	SIGNALING_BY_FGFR	REACTOME	4154	32	20	1,85E-01	9,17E-01	rs3117915|rs10956564|rs883541|rs4976123|rs2721051|rs833141|rs16904360|rs7303990|rs6891913|rs11593080|rs17612068|rs714104|rs7257613|rs4964005|rs815808|rs6115830|rs9578440|rs7972342|rs12619351|rs17234079
Malar Rash	NA_CL_DEPENDENT_NEUROTRANSMITTER_TRANSPORTERS	REACTOME	718	39	20	1,87E-01	9,17E-01	rs2531747|rs1400155|rs9682960|rs2286489|rs11128537|rs3900078|rs2017903|rs216250|rs6766142|rs13258461|rs999969|rs791183|rs1380412|rs10862903|rs10744557|rs4234504|rs11580716|rs6693689|rs542736|rs12491174
Malar Rash	SIGNALLING_BY_NGF	REACTOME	7869	31	20	1,87E-01	9,17E-01	rs1002420|rs10956564|rs3799630|rs883541|rs4976123|rs8029326|rs2721051|rs6789224|rs833141|rs10869741|rs13427229|rs9410438|rs9371859|rs16904360|rs4618461|rs7303990|rs16903375|rs1934127|rs1543478|rs12974044
Malar Rash	PI_METABOLISM	REACTOME	1808	27	20	1,91E-01	9,17E-01	rs4976123|rs1907108|rs11876701|rs2286489|rs4763508|rs974309|rs203150|rs2742413|rs4820944|rs11766675|rs1554513|rs7815340|rs1636808|rs195770|rs1539826|rs9954372|rs3775664|rs13086080|rs2270229|rs7048825
Malar Rash	NFKB_AND_MAP_KINASES_ACTIVATION_MEDIATED_BY_TLR4_SIGNALING_REPERTOIRE	REACTOME	2248	31	20	1,94E-01	9,17E-01	rs3799630|rs8029326|rs394558|rs4618461|rs4804833|rs1543478|rs2737191|rs2256331|rs8004059|rs1546745|rs9345044|rs2820350|rs2811650|rs10223241|rs2189756|rs7183836|rs4256810|rs1177614|rs16899333|rs3799631
Malar Rash	G_BETA_GAMMA_SIGNALLING_THROUGH_PI3KGAMMA	REACTOME	487	20	17	1,94E-01	9,17E-01	rs10937003|rs2294451|rs11766675|rs4898725|rs1636808|rs12147761|rs195770|rs11621038|rs42154|rs7141727|rs887030|rs2287208|rs726680|rs12985186|rs508208|rs2993998|rs7804859
Malar Rash	FGFR_LIGAND_BINDING_AND_ACTIVATION	REACTOME	984	26	20	1,95E-01	9,17E-01	rs3117915|rs6891913|rs11593080|rs17612068|rs9578440|rs17234079|rs3129601|rs3129596|rs13135439|rs11776272|rs4769189|rs477516|rs7989744|rs17004857|rs9634328|rs1501588|rs4866891|rs576674|rs1458038|rs4489674
Malar Rash	CHREBP2_PATHWAY	BIOCARTA	1059	37	20	1,96E-01	9,17E-01	rs10872828|rs883541|rs942689|rs4689424|rs878291|rs7809503|rs4528168|rs4734507|rs3108913|rs11881878|rs6965771|rs11658925|rs6468757|rs10889011|rs9291117|rs4425665|rs4668631|rs7893330|rs3019260|rs6678588
Malar Rash	VALINE_LEUCINE_AND_ISOLEUCINE_DEGRADATION	KEGG	1149	75	20	1,99E-01	9,17E-01	rs962800|rs9381786|rs9294177|rs10973770|rs2158839|rs4550189|rs4267943|rs9352825|rs1893441|rs2253612|rs10267797|rs277973|rs12874460|rs4646770|rs13220420|rs1273376|rs11969591|rs7045845|rs3122126|rs7763556
Malar Rash	FOCAL_ADHESION	KEGG	7733	28	20	1,99E-01	9,17E-01	rs3811159|rs8092360|rs536173|rs4976123|rs4947491|rs9283850|rs875078|rs4435197|rs2237642|rs7183535|rs16763|rs9410438|rs9333114|rs17182988|rs4284952|rs17752721|rs2684789|rs4987721|rs7257613|rs10829597
Malar Rash	GLIOMA	KEGG	2070	34	20	2,00E-01	9,17E-01	rs4976123|rs4947491|rs4435197|rs7183535|rs9410438|rs2684789|rs7257613|rs815808|rs11766675|rs7776830|rs11098195|rs2684790|rs6938550|rs1636808|rs4074785|rs3217992|rs11568993|rs17172446|rs2684802|rs1873423
Malar Rash	ACTIVATION_OF_KAINATE_RECEPTORS_UPON_GLUTAMATE_BINDING	REACTOME	1577	36	20	2,01E-01	9,17E-01	rs10937003|rs8126112|rs1984334|rs9861063|rs7760831|rs2294451|rs815808|rs4898725|rs12147761|rs6140629|rs2247218|rs2832438|rs13045064|rs7116106|rs12192481|rs11621038|rs6133573|rs926505|rs6077420|rs10282818
Malar Rash	METABOLISM_OF_NUCLEOTIDES	REACTOME	1559	29	20	2,03E-01	9,17E-01	rs5770775|rs37373|rs7875593|rs1253795|rs7027719|rs6467177|rs5760440|rs11587873|rs6691415|rs1713460|rs11980741|rs6556882|rs1152991|rs11664283|rs784600|rs1689924|rs9633478|rs9444331|rs10493606|rs1265124
Malar Rash	PEPTIDE_LIGAND_BINDING_RECEPTORS	REACTOME	5053	35	20	2,03E-01	9,17E-01	rs6579927|rs4629584|rs12215494|rs17451303|rs198311|rs4683189|rs16891982|rs13148043|rs2075355|rs237851|rs1371938|rs10197003|rs2996011|rs2256331|rs13196610|rs12873185|rs4660540|rs16941288|rs9376628|rs9389870
Malar Rash	DEATH_PATHWAY	BIOCARTA	505	34	20	2,04E-01	9,17E-01	rs8092360|rs2711008|rs1543478|rs4987721|rs11561771|rs12537364|rs7922090|rs696|rs8083946|rs2155458|rs13402616|rs7830593|rs2526406|rs6920337|rs1052571|rs7315397|rs12458289|rs6567322|rs2236719|rs10483453
Malar Rash	E2F_MEDIATED_REGULATION_OF_DNA_REPLICATION	REACTOME	452	15	11	2,06E-01	9,17E-01	rs2307389|rs3761873|rs11664283|rs6577060|rs11881878|rs7772351|rs837763|rs380691|rs8110447|rs6741290|rs9475837
Malar Rash	DAG_AND_IP3_SIGNALING	REACTOME	1644	49	20	2,06E-01	9,17E-01	rs10956564|rs883541|rs833141|rs16904360|rs7303990|rs714104|rs7257613|rs4964005|rs815808|rs7972342|rs12619351|rs12829100|rs2344498|rs11658925|rs72001|rs13432276|rs2099505|rs2239305|rs3934729|rs727432
Malar Rash	GABA_RECEPTOR_ACTIVATION	REACTOME	2705	54	20	2,09E-01	9,17E-01	rs10956564|rs4793521|rs12523641|rs4517239|rs6517438|rs16904360|rs1396514|rs7403139|rs1385902|rs17763873|rs3922855|rs9920954|rs17763595|rs4898725|rs12147761|rs12653458|rs3923156|rs2109051|rs4906896|rs72001
Malar Rash	TRANSPORT_TO_THE_GOLGI_AND_SUBSEQUENT_MODIFICATION	REACTOME	1557	41	20	2,09E-01	9,17E-01	rs718780|rs1947110|rs7156225|rs12431858|rs1295391|rs9401188|rs17108549|rs4686823|rs2406250|rs17035919|rs11692586|rs10138087|rs6571891|rs935374|rs35683|rs7761886|rs3820500|rs7741206|rs1519304|rs753897
Malar Rash	NUCLEOTIDE_BINDING_DOMAIN_LEUCINE_RICH_REPEAT_CONTAINING_RECEPTOR_NLR YS	REACTOME	1337	33	20	2,09E-01	9,17E-01	rs8092360|rs394558|rs2256331|rs4987721|rs507879|rs1546745|rs9345044|rs2820350|rs2811650|rs2189756|rs1177614|rs531486|rs2829946|rs7922090|rs467778|rs1545092|rs2852720|rs2501657|rs311636|rs8083946
Malar Rash	RAC1_PATHWAY	BIOCARTA	763	38	20	2,10E-01	9,17E-01	rs4976123|rs16903375|rs12191724|rs11766675|rs1636808|rs7631698|rs4894512|rs3019244|rs250846|rs9946939|rs706725|rs6592724|rs42154|rs1254403|rs7708673|rs820371|rs3849079|rs10415535|rs360442|rs249479
Malar Rash	TRANSPORT_OF_GLUCOSE_AND_OTHER_SUGARS_BILE_SALTS_AND_ORGANIC_ACIDS_M NE_COMPOUNDS	REACTOME	3234	37	20	2,11E-01	9,17E-01	rs1622108|rs8081130|rs2531747|rs2200086|rs7138679|rs1400155|rs9682960|rs2286489|rs10505314|rs132930|rs738803|rs1054986|rs11128537|rs10513484|rs4693788|rs3900078|rs1891092|rs1695715|rs17806653|rs12230938
Malar Rash	KERATINOCYTE_PATHWAY	BIOCARTA	1952	34	20	2,12E-01	9,17E-01	rs8092360|rs4947491|rs7111926|rs4804833|rs1543478|rs714104|rs4987721|rs7257613|rs8004059|rs7251505|rs7936180|rs7776830|rs12619351|rs2189756|rs11597105|rs11568993|rs17172446|rs7130878|rs767757|rs565536
Malar Rash	MAPK_PATHWAY	BIOCARTA	2690	29	20	2,13E-01	9,17E-01	rs3799630|rs8029326|rs13123632|rs4618461|rs4410871|rs12543549|rs4804833|rs886599|rs1543478|rs8004059|rs1612279|rs7251505|rs9345044|rs2820350|rs2811650|rs10223241|rs15614|rs2189756|rs7183836|rs2720662
Malar Rash	POTASSIUM_CHANNELS	REACTOME	4940	45	20	2,17E-01	9,17E-01	rs1864773|rs4793521|rs2471095|rs4517239|rs6517438|rs2034007|rs16910949|rs10100027|rs7530450|rs1396514|rs12131054|rs1385902|rs10170651|rs9701252|rs954785|rs6471070|rs17703233|rs17763873|rs1328384|rs2237878
Malar Rash	TCA_CYCLE_AND_RESPIRATORY_ELECTRON_TRANSPORT	REACTOME	2599	29	20	2,17E-01	9,17E-01	rs7647085|rs4854053|rs2711008|rs11203280|rs2158839|rs6791324|rs132930|rs11561771|rs7248185|rs13415895|rs2802460|rs10210248|rs1490265|rs1015196|rs11171911|rs1124729|rs4975851|rs11678404|rs10267797|rs12537364
Malar Rash	MCM_PATHWAY	BIOCARTA	234	14	8	2,21E-01	9,17E-01	rs13213285|rs1781011|rs133416|rs7772351|rs837763|rs8110447|rs10948689|rs34330
Malar Rash	LYSINE_DEGRADATION	KEGG	952	52	20	2,21E-01	9,17E-01	rs962800|rs987687|rs13099918|rs10973770|rs4550189|rs1913185|rs1919510|rs7632080|rs4526432|rs2629268|rs4646770|rs11029812|rs10513268|rs28660993|rs10247064|rs7045845|rs963660|rs2137558|rs12644042|rs3749282
Malar Rash	VIP_PATHWAY	BIOCARTA	1000	41	20	2,22E-01	9,17E-01	rs883541|rs4811174|rs4410871|rs12543549|rs1543478|rs754505|rs815808|rs2720662|rs670307|rs11658925|rs7922090|rs696|rs8090312|rs4130120|rs1030102|rs4904690|rs1294506|rs7213247|rs12547643|rs1693892
Malar Rash	P38MAPK_PATHWAY	BIOCARTA	1904	38	20	2,23E-01	9,17E-01	rs8029326|rs13123632|rs4618461|rs4410871|rs12543549|rs886599|rs1612279|rs9345044|rs2820350|rs2811650|rs10223241|rs2189756|rs7183836|rs2720662|rs4256810|rs294514|rs12436076|rs1750304|rs8030728|rs187571
Malar Rash	DRUG_METABOLISM_OTHER_ENZYMES	KEGG	1609	40	20	2,24E-01	9,17E-01	rs5770775|rs12599795|rs1253795|rs6467177|rs5760440|rs11587873|rs11980741|rs1152991|rs1841037|rs1689924|rs8192935|rs4783925|rs9639018|rs4451581|rs10936023|rs12446482|rs7017402|rs4148309|rs1760217|rs2333874
Malar Rash	SIGNALING_BY_FGFR_IN_DISEASE	REACTOME	4545	32	20	2,24E-01	9,17E-01	rs3117915|rs10956564|rs883541|rs4976123|rs2721051|rs833141|rs16904360|rs7303990|rs6891913|rs11593080|rs11837579|rs17612068|rs714104|rs7257613|rs4964005|rs815808|rs6115830|rs9578440|rs7972342|rs12619351
Malar Rash	AMINO_ACID_AND_OLIGOPEPTIDE_SLC_TRANSPORTERS	REACTOME	1707	36	20	2,24E-01	9,17E-01	rs3783260|rs2531747|rs2286489|rs7329421|rs2720546|rs17588888|rs2297320|rs4591778|rs2731897|rs3759608|rs4742012|rs10132368|rs2729384|rs7849913|rs2250474|rs1709190|rs16921457|rs216250|rs6766142|rs17244832
Malar Rash	INCRETIN_SYNTHESIS_SECRETION_AND_INACTIVATION	REACTOME	540	23	13	2,25E-01	9,17E-01	rs11250154|rs7824704|rs2294451|rs156005|rs880026|rs17834513|rs10871764|rs6599127|rs9863665|rs1377834|rs6867918|rs4840583|rs12541318
Malar Rash	KINESINS	REACTOME	566	28	15	2,26E-01	9,17E-01	rs7702812|rs947006|rs1422834|rs8076424|rs646289|rs7309966|rs2055474|rs11167725|rs1541160|rs12933548|rs861531|rs12125595|rs2552992|rs9311361|rs12272419
Malar Rash	G_PROTEIN_ACTIVATION	REACTOME	703	29	20	2,26E-01	9,17E-01	rs10937003|rs1108656|rs2294451|rs13196610|rs4898725|rs12147761|rs4626436|rs11621038|rs2269340|rs13338253|rs7141727|rs887030|rs865774|rs2287208|rs2886612|rs9479657|rs12985186|rs3790113|rs2241953|rs508208
Malar Rash	TGF_BETA_SIGNALING_PATHWAY	KEGG	3535	26	20	2,27E-01	9,17E-01	rs9283850|rs7171416|rs266371|rs16950543|rs4410871|rs2811982|rs12543549|rs1515263|rs17182988|rs6943592|rs13044457|rs6054517|rs719563|rs1489152|rs9295427|rs189727|rs9961435|rs11654392|rs11757721|rs36047858

<table>
<tr><td>Malar Rash</td><td>GLYCOSYLPHOSPHATIDYLINOSITOL_GPI_ANCHOR_BIOSYNTHESIS</td><td>KEGG</td><td>318</td><td>21</td><td>9</td><td>2,27E-01</td><td>9,17E-01</td><td>rs3761793|rs793683|rs4953402|rs12744696|rs3742875|rs7190878|rs2869665|rs1602|rs199714</td></tr>
<tr><td>Malar Rash</td><td>REGULATION_OF_WATER_BALANCE_BY_RENAL_AQUAPORINS</td><td>REACTOME</td><td>1393</td><td>35</td><td>20</td><td>2,28E-01</td><td>9,17E-01</td><td>rs11720405|rs10956564|rs883541|rs16904360|rs2294451|rs1893441|rs4898725|rs12147761|rs1435421|rs11658925|rs72001|rs10787805|rs11621038|rs2099505|rs485835|rs17612687|rs12242357|rs2239305|rs3934729|rs727432</td></tr>
<tr><td>Malar Rash</td><td>SEMA4D_INDUCED_CELL_MIGRATION_AND_GROWTH_CONE_COLLAPSE</td><td>REACTOME</td><td>433</td><td>23</td><td>12</td><td>2,28E-01</td><td>9,17E-01</td><td>rs9306310|rs788338|rs8139798|rs2276035|rs3119028|rs2239775|rs6498574|rs6498573|rs1579523|rs2213430|rs9610510|rs2413045</td></tr>
<tr><td>Malar Rash</td><td>TRAF6_MEDIATED_INDUCTION_OF_NFKB_AND_MAP_KINASES_UPON_TLR7_8_OR_9_ACTI</td><td>REACTOME</td><td>2239</td><td>31</td><td>20</td><td>2,29E-01</td><td>9,17E-01</td><td>rs3799630|rs8029326|rs394558|rs4618461|rs4804833|rs1543478|rs2256331|rs8004059|rs1546745|rs9345044|rs2820350|rs2811650|rs10223241|rs2189756|rs7183836|rs4256810|rs1177614|rs16899333|rs3799631|rs1009619</td></tr>
<tr><td>Malar Rash</td><td>G1_PATHWAY</td><td>BIOCARTA</td><td>611</td><td>37</td><td>20</td><td>2,29E-01</td><td>9,17E-01</td><td>rs266371|rs16950543|rs36047858|rs7173609|rs4074785|rs266312|rs3217992|rs6577060|rs7181907|rs7163945|rs10273030|rs1547705|rs380691|rs8110447|rs3804440|rs7169592|rs1801516|rs8109167|rs34330|rs2278545</td></tr>
<tr><td>Malar Rash</td><td>SPHINGOLIPID_DE_NOVO_BIOSYNTHESIS</td><td>REACTOME</td><td>1020</td><td>43</td><td>20</td><td>2,29E-01</td><td>9,17E-01</td><td>rs1993224|rs977122|rs17114593|rs7099450|rs9285606|rs2842085|rs11627212|rs12323862|rs10826013|rs11850756|rs10138733|rs1466447|rs7367215|rs1958987|rs6896136|rs11159273|rs12066561|rs10118605|rs2654583|rs4668089</td></tr>
<tr><td>Malar Rash</td><td>GABA_B_RECEPTOR_ACTIVATION</td><td>REACTOME</td><td>1863</td><td>52</td><td>20</td><td>2,32E-01</td><td>9,17E-01</td><td>rs10956564|rs4793521|rs4517239|rs6517438|rs16904360|rs1396514|rs1385902|rs17763873|rs17763595|rs4898725|rs12147761|rs2109051|rs72001|rs11621038|rs2099505|rs624549|rs7943324|rs2239305|rs1167767|rs3934729</td></tr>
<tr><td>Malar Rash</td><td>CELL_CELL_COMMUNICATION</td><td>REACTOME</td><td>9725</td><td>35</td><td>20</td><td>2,32E-01</td><td>9,17E-01</td><td>rs3807741|rs12960187|rs13170578|rs4976123|rs4715005|rs491198|rs6043388|rs11220608|rs7705566|rs9962572|rs6065062|rs2628204|rs7202333|rs11644732|rs3114396|rs12610011|rs12716949|rs10076681|rs17468086|rs1485781</td></tr>
<tr><td>Malar Rash</td><td>PROGESTERONE_MEDIATED_OOCYTE_MATURATION</td><td>KEGG</td><td>2535</td><td>46</td><td>20</td><td>2,33E-01</td><td>9,17E-01</td><td>rs10956564|rs3799630|rs4976123|rs10155|rs16904360|rs486992|rs601618|rs8023333|rs2684789|rs11766675|rs2684790|rs10841414|rs1636808|rs7678743|rs16899333|rs3799631|rs3191402|rs2684802|rs10505864|rs72001</td></tr>
<tr><td>Malar Rash</td><td>DEADENYLATION_OF_MRNA</td><td>REACTOME</td><td>331</td><td>27</td><td>12</td><td>2,35E-01</td><td>9,17E-01</td><td>rs968053|rs16898766|rs3108908|rs11642944|rs4761281|rs17028238|rs6958241|rs2165944|rs3741436|rs12532231|rs10876394|rs12599053</td></tr>
<tr><td>Malar Rash</td><td>SIGNALING_BY_PDGF</td><td>REACTOME</td><td>4427</td><td>37</td><td>20</td><td>2,36E-01</td><td>9,17E-01</td><td>rs3811159|rs10956564|rs883541|rs4976123|rs9283850|rs2721051|rs833141|rs16904360|rs7303990|rs17182988|rs714104|rs7257613|rs4964005|rs11757721|rs815808|rs6115830|rs6722598|rs7972342|rs12619351|rs6728818</td></tr>
<tr><td>Malar Rash</td><td>SEMA4D_IN_SEMAPHORIN_SIGNALING</td><td>REACTOME</td><td>597</td><td>30</td><td>15</td><td>2,36E-01</td><td>9,17E-01</td><td>rs9306310|rs3767742|rs788338|rs8139798|rs2276035|rs3119028|rs2239775|rs8054007|rs6498573|rs1579523|rs311384|rs2213430|rs1932435|rs9610510|rs2413045</td></tr>
<tr><td>Malar Rash</td><td>SIGNALING_BY_NOTCH1</td><td>REACTOME</td><td>3563</td><td>30</td><td>20</td><td>2,37E-01</td><td>9,17E-01</td><td>rs7690973|rs13245206|rs6856075|rs10152562|rs6444767|rs4410871|rs10513747|rs12543549|rs2920946|rs11732516|rs1877209|rs1995243|rs17182398|rs10140583|rs4310569|rs2051920|rs669669|rs12580394|rs3739809|rs12911091</td></tr>
<tr><td>Malar Rash</td><td>MEMBRANE_TRAFFICKING</td><td>REACTOME</td><td>2276</td><td>31</td><td>20</td><td>2,38E-01</td><td>9,17E-01</td><td>rs482263|rs11726508|rs7156225|rs9848910|rs6454601|rs878129|rs17108549|rs746987|rs7317958|rs7836184|rs26538|rs909626|rs17127601|rs7815340|rs9760|rs8091623|rs6884769|rs13100218|rs6571891|rs4804528</td></tr>
<tr><td>Malar Rash</td><td>FMLP_PATHWAY</td><td>BIOCARTA</td><td>1148</td><td>38</td><td>20</td><td>2,39E-01</td><td>9,17E-01</td><td>rs8126112|rs4811174|rs4763508|rs1543478|rs754505|rs1688123|rs815808|rs4801891|rs2189756|rs6140629|rs13045064|rs3019244|rs696|rs6133573|rs926505|rs6077420|rs10413815|rs6592724|rs8090312|rs2179440</td></tr>
<tr><td>Malar Rash</td><td>A_TETRASACCHARIDE_LINKER_SEQUENCE_IS_REQUIRED_FOR_GAG_SYNTHESIS</td><td>REACTOME</td><td>1731</td><td>42</td><td>20</td><td>2,40E-01</td><td>9,17E-01</td><td>rs718356|rs6455394|rs2147155|rs3820088|rs2682185|rs10955078|rs11224169|rs9584093|rs11223842|rs13010821|rs1328368|rs7966281|rs6492696|rs2292832|rs2150471|rs1280632|rs287541|rs7999055|rs7743868|rs11839320</td></tr>
<tr><td>Malar Rash</td><td>SLC_MEDIATED_TRANSMEMBRANE_TRANSPORT</td><td>REACTOME</td><td>8774</td><td>35</td><td>20</td><td>2,40E-01</td><td>9,17E-01</td><td>rs1622108|rs3783260|rs8081130|rs7145687|rs245169|rs890839|rs9310419|rs2531747|rs11248060|rs2200086|rs17025772|rs7138679|rs1400155|rs9682960|rs9289660|rs2286489|rs42792|rs11666324|rs10505314|rs7329421</td></tr>
<tr><td>Malar Rash</td><td>ASPARAGINE_N_LINKED_GLYCOSYLATION</td><td>REACTOME</td><td>2760</td><td>41</td><td>20</td><td>2,40E-01</td><td>9,17E-01</td><td>rs2410361|rs2720630|rs718780|rs1947110|rs7156225|rs12431858|rs1295391|rs9401188|rs17108549|rs4686823|rs6572331|rs2406250|rs17042585|rs17035919|rs313624|rs3121680|rs11692586|rs3742290|rs12435557|rs10138087</td></tr>
<tr><td>Malar Rash</td><td>ETHER_LIPID_METABOLISM</td><td>KEGG</td><td>1041</td><td>40</td><td>20</td><td>2,42E-01</td><td>9,17E-01</td><td>rs11582551|rs1348850|rs2701632|rs1882336|rs4820944|rs13127455|rs7631698|rs4894512|rs914832|rs800719|rs2301472|rs12066561|rs360441|rs1487492|rs1073084|rs6680812|rs2992068|rs955835|rs12564176|rs8080511</td></tr>
<tr><td>Malar Rash</td><td>SIGNALING_BY_FGFR1_MUTANTS</td><td>REACTOME</td><td>1243</td><td>35</td><td>20</td><td>2,46E-01</td><td>9,17E-01</td><td>rs3117915|rs4976123|rs6891913|rs11837579|rs17612068|rs9578440|rs3129601|rs2296078|rs3129596|rs11776272|rs4769189|rs477516|rs7989744|rs17004857|rs9634328|rs1501588|rs706725|rs3024912|rs1458038|rs1060697</td></tr>
<tr><td>Malar Rash</td><td>IL_2_SIGNALING</td><td>REACTOME</td><td>936</td><td>41</td><td>20</td><td>2,49E-01</td><td>9,17E-01</td><td>rs12409333|rs4976123|rs11914132|rs12244380|rs7073236|rs17489214|rs1571221|rs1993703|rs310245|rs2075937|rs706725|rs1760118|rs1323653|rs7708673|rs791589|rs228942|rs249479|rs7000615|rs131849|rs2235329</td></tr>
<tr><td>Malar Rash</td><td>DOWNREGULATION_OF_TGF_BETA_RECEPTOR_SIGNALING</td><td>REACTOME</td><td>521</td><td>38</td><td>18</td><td>2,49E-01</td><td>9,17E-01</td><td>rs266371|rs16950543|rs9961435|rs7173609|rs266312|rs928163|rs1855469|rs7181907|rs10896172|rs7163945|rs3773650|rs9309664|rs7169592|rs8109167|rs2278545|rs6494629|rs1564183|rs4729514</td></tr>
<tr><td>Malar Rash</td><td>HOMOLOGOUS_RECOMBINATION_REPAIR_OF_REPLICATION_INDEPENDENT_DOUBLE_ST</td><td>REACTOME</td><td>376</td><td>18</td><td>11</td><td>2,49E-01</td><td>9,17E-01</td><td>rs10765682|rs17782975|rs12796488|rs9909163|rs499952|rs2244012|rs156631|rs1801516|rs2881964|rs6463725|rs886727</td></tr>
<tr><td>Malar Rash</td><td>PGC1A_PATHWAY</td><td>BIOCARTA</td><td>959</td><td>46</td><td>20</td><td>2,50E-01</td><td>9,17E-01</td><td>rs8029326|rs4618461|rs10009448|rs3774921|rs6007640|rs815808|rs10223241|rs11098195|rs7183836|rs1750304|rs8030728|rs187571|rs4904690|rs11938486|rs7672552|rs1294506|rs1509247|rs10027997|rs1693892|rs2970848</td></tr>
<tr><td>Malar Rash</td><td>MHC_CLASS_II_ANTIGEN_PRESENTATION</td><td>REACTOME</td><td>2226</td><td>35</td><td>20</td><td>2,51E-01</td><td>9,17E-01</td><td>rs7702812|rs947006|rs10807113|rs7156225|rs275876|rs1422834|rs17108549|rs8076424|rs2857106|rs12508572|rs2292537|rs10249566|rs6571891|rs4804528|rs154973|rs35683|rs2281390|rs11083004|rs7023913|rs9277052</td></tr>
<tr><td>Malar Rash</td><td>MELANOMA</td><td>KEGG</td><td>2894</td><td>34</td><td>20</td><td>2,51E-01</td><td>9,17E-01</td><td>rs3117915|rs4976123|rs4947491|rs1430604|rs7202333|rs6891913|rs17612068|rs2684789|rs10276241|rs7648607|rs9578440|rs7995765|rs11766675|rs7776830|rs17234079|rs2684790|rs6938550|rs1636808|rs7621589|rs4074785</td></tr>
<tr><td>Malar Rash</td><td>SHC_MEDIATED_CASCADE</td><td>REACTOME</td><td>1070</td><td>26</td><td>20</td><td>2,51E-01</td><td>9,17E-01</td><td>rs3117915|rs6891913|rs11593080|rs17612068|rs9578440|rs17234079|rs3129601|rs3129596|rs13135439|rs11776272|rs4769189|rs477516|rs7989744|rs17004857|rs9634328|rs1501588|rs4866891|rs576674|rs1458038|rs4489674</td></tr>
<tr><td>Malar Rash</td><td>ASCORBATE_AND_ALDARATE_METABOLISM</td><td>KEGG</td><td>874</td><td>49</td><td>9</td><td>2,52E-01</td><td>9,17E-01</td><td>rs7216|rs10973770|rs1841037|rs4646770|rs4148309|rs7045845|rs2125312|rs2306619|rs4878805</td></tr>
<tr><td>Malar Rash</td><td>SPHINGOLIPID_METABOLISM</td><td>KEGG</td><td>1101</td><td>44</td><td>20</td><td>2,53E-01</td><td>9,17E-01</td><td>rs1037182|rs718780|rs10516601|rs1903364|rs7099450|rs2842085|rs5770953|rs10826013|rs8016092|rs11684431|rs2388996|rs914832|rs11159273|rs12066561|rs17046991|rs1555556|rs10438933|rs10118605|rs7508|rs1972988</td></tr>
<tr><td>Malar Rash</td><td>GENERATION_OF_SECOND_MESSENGER_MOLECULES</td><td>REACTOME</td><td>616</td><td>30</td><td>13</td><td>2,53E-01</td><td>9,17E-01</td><td>rs7756516|rs15614|rs3128918|rs3019244|rs9277052|rs12034829|rs30142|rs9277535|rs10045011|rs6592724|rs17465965|rs2289545|rs2769689</td></tr>
<tr><td>Malar Rash</td><td>CARDIACEGF_PATHWAY</td><td>BIOCARTA</td><td>1289</td><td>32</td><td>20</td><td>2,54E-01</td><td>9,17E-01</td><td>rs4947491|rs4410871|rs12543549|rs12762349|rs8004059|rs12873185|rs1278302|rs7776830|rs2720662|rs9544635|rs11568993|rs17172446|rs1674925|rs1873423|rs9544725|rs17675063|rs4130120|rs1030102|rs10151713|rs737063</td></tr>
<tr><td>Malar Rash</td><td>FATTY_ACYL_COA_BIOSYNTHESIS</td><td>REACTOME</td><td>404</td><td>18</td><td>10</td><td>2,55E-01</td><td>9,17E-01</td><td>rs11737840|rs4698832|rs12108641|rs10452184|rs6533493|rs13159372|rs13144344|rs10455125|rs4862417|rs7561624</td></tr>
<tr><td>Malar Rash</td><td>ACTIVATION_OF_NMDA_RECEPTOR_UPON_GLUTAMATE_BINDING_AND_POSTSYNAPTIC_</td><td>REACTOME</td><td>2117</td><td>35</td><td>20</td><td>2,58E-01</td><td>9,17E-01</td><td>rs10956564|rs3799630|rs937210|rs16904360|rs2098469|rs918168|rs4958596|rs10895854|rs815808|rs1868289|rs11098195|rs7298664|rs16899333|rs3799631|rs17830392|rs220593|rs16957102|rs17339754|rs4709117|rs2099505</td></tr>
<tr><td>Malar Rash</td><td>TRANSPORT_OF_INORGANIC_CATIONS_ANIONS_AND_AMINO_ACIDS_OLIGOPEPTIDES</td><td>REACTOME</td><td>4051</td><td>31</td><td>20</td><td>2,59E-01</td><td>9,17E-01</td><td>rs3783260|rs7145687|rs245169|rs2531747|rs11248060|rs17025772|rs9289660|rs2286489|rs42792|rs7329421|rs2720546|rs17588888|rs2297320|rs2192724|rs10135174|rs2642905|rs7857042|rs4591778|rs2731897|rs715305</td></tr>
<tr><td>Malar Rash</td><td>IL10_PATHWAY</td><td>BIOCARTA</td><td>288</td><td>23</td><td>10</td><td>2,59E-01</td><td>9,17E-01</td><td>rs12409333|rs11216648|rs133416|rs310245|rs3024912|rs3021094|rs310199|rs324015|rs7277902|rs7574865</td></tr>
<tr><td>Malar Rash</td><td>OPIOID_SIGNALLING</td><td>REACTOME</td><td>3594</td><td>37</td><td>20</td><td>2,60E-01</td><td>9,17E-01</td><td>rs11720405|rs10956564|rs883541|rs8126112|rs833141|rs16904360|rs1108656|rs7303990|rs7257613|rs2294451|rs13196610|rs4964005|rs815808|rs7972342|rs4898725|rs12147761|rs4626436|rs6140629|rs16889276|rs12829100</td></tr>
<tr><td>Malar Rash</td><td>PROCESSING_OF_CAPPED_INTRON_CONTAINING_PRE_MRNA</td><td>REACTOME</td><td>1547</td><td>28</td><td>20</td><td>2,60E-01</td><td>9,17E-01</td><td>rs9310419|rs2588882|rs2304232|rs968053|rs11081583|rs5766404|rs987607|rs10169262|rs12191724|rs9615022|rs4809388|rs2274913|rs10506328|rs4905510|rs7248208|rs17028238|rs13258200|rs723553|rs6706797|rs2282763</td></tr>
<tr><td>Malar Rash</td><td>ARF_PATHWAY</td><td>BIOCARTA</td><td>733</td><td>36</td><td>20</td><td>2,62E-01</td><td>9,17E-01</td><td>rs4976123|rs4410871|rs12543549|rs11766675|rs1636808|rs2720662|rs4074785|rs3217992|rs706725|rs42154|rs7708673|rs4130120|rs1030102|rs10153801|rs249479|rs12547643|rs2257082|rs10091329|rs4131890|rs4332094</td></tr>
<tr><td>Malar Rash</td><td>MTOR_PATHWAY</td><td>BIOCARTA</td><td>509</td><td>35</td><td>14</td><td>2,64E-01</td><td>9,17E-01</td><td>rs4976123|rs968053|rs6041884|rs17028238|rs706725|rs2165944|rs7708673|rs3741436|rs8124053|rs249479|rs1474880|rs10876394|rs4131890|rs2248293</td></tr>
<tr><td>Malar Rash</td><td>P53_SIGNALING_PATHWAY</td><td>KEGG</td><td>1897</td><td>46</td><td>20</td><td>2,64E-01</td><td>9,17E-01</td><td>rs2235437|rs2711008|rs11039149|rs4242594|rs1458451|rs4877110|rs10430828|rs11561771|rs2964356|rs4074785|rs3217992|rs3191402|rs11265889|rs12537364|rs17752673|rs1265124|rs2156223|rs4479955|rs7460047|rs3897566</td></tr>
<tr><td>Malar Rash</td><td>PLC_BETA_MEDIATED_EVENTS</td><td>REACTOME</td><td>2313</td><td>47</td><td>20</td><td>2,65E-01</td><td>9,17E-01</td><td>rs10956564|rs883541|rs8126112|rs833141|rs16904360|rs1108656|rs7303990|rs7257613|rs4964005|rs815808|rs7972342|rs6140629|rs12829100|rs13045064|rs2344498|rs11658925|rs72001|rs2099505|rs6133573|rs2269340</td></tr>
<tr><td>Malar Rash</td><td>NEUROTRANSMITTER_RELEASE_CYCLE</td><td>REACTOME</td><td>1400</td><td>38</td><td>20</td><td>2,66E-01</td><td>9,17E-01</td><td>rs3761793|rs7616006|rs1400155|rs9682960|rs13206801|rs6133869|rs11128537|rs3900078|rs4742012|rs7849913|rs2250474|rs10485738|rs1462923|rs16921457|rs2017903|rs216250|rs791183|rs9442725|rs3810950|rs807513</td></tr>
<tr><td>Malar Rash</td><td>SIGNALING_BY_NOTCH</td><td>REACTOME</td><td>4553</td><td>30</td><td>20</td><td>2,66E-01</td><td>9,17E-01</td><td>rs7690973|rs7156012|rs13245206|rs6856075|rs10152562|rs6444767|rs4410871|rs10513747|rs12543549|rs2920946|rs11732516|rs1877209|rs1995243|rs17182398|rs2267644|rs10140583|rs1075617|rs4310569|rs2051920|rs669669</td></tr>
<tr><td>Malar Rash</td><td>AKT_PATHWAY</td><td>BIOCARTA</td><td>682</td><td>38</td><td>20</td><td>2,67E-01</td><td>9,17E-01</td><td>rs4976123|rs2721051|rs1543478|rs11766675|rs1636808|rs2145811|rs7922090|rs696|rs706725|rs42154|rs7708673|rs4943768|rs17254382|rs7981045|rs249479|rs17069632|rs3020619|rs2042370|rs4131890|rs859624</td></tr>
<tr><td>Malar Rash</td><td>IL2_PATHWAY</td><td>BIOCARTA</td><td>452</td><td>32</td><td>20</td><td>2,68E-01</td><td>9,17E-01</td><td>rs12409333|rs11914132|rs12244380|rs8004059|rs7073236|rs17489214|rs1993703|rs310245|rs1760118|rs1323653|rs791589|rs10151713|rs228942|rs882167|rs8013053|rs2235329|rs310199|rs10120669|rs1760125|rs7219</td></tr>
<tr><td>Malar Rash</td><td>TRANSPORT_OF_MATURE_MRNA_DERIVED_FROM_AN_INTRONLESS_TRANSCRIPT</td><td>REACTOME</td><td>524</td><td>40</td><td>20</td><td>2,68E-01</td><td>9,17E-01</td><td>rs9310419|rs968053|rs5766404|rs987607|rs10169262|rs9615022|rs17028238|rs13258200|rs723553|rs2165944|rs4740419|rs12473402|rs11684526|rs9297042|rs7284581|rs4759082|rs1602|rs5766536|rs7982860|rs1462531</td></tr>
<tr><td>Malar Rash</td><td>TRANS_GOLGI_NETWORK_VESICLE_BUDDING</td><td>REACTOME</td><td>1106</td><td>47</td><td>20</td><td>2,69E-01</td><td>9,17E-01</td><td>rs482263|rs11726508|rs878129|rs26538|rs909626|rs17127601|rs9760|rs6884769|rs4804528|rs17185574|rs760306|rs10042243|rs14976|rs10897207|rs2272295|rs7712357|rs655246|rs17397283|rs4425006|rs220892</td></tr>
<tr><td>Malar Rash</td><td>METABOLISM_OF_AMINO_ACIDS_AND_DERIVATIVES</td><td>REACTOME</td><td>5246</td><td>26</td><td>20</td><td>2,70E-01</td><td>9,17E-01</td><td>rs2597758|rs12292911|rs3829305|rs17184300|rs987687|rs8063430|rs9678469|rs1235695|rs37373|rs17060292|rs6943592|rs11135284|rs7337312|rs9294177|rs2158839|rs355396|rs4550189|rs969111|rs11612372|rs1919510</td></tr>
<tr><td>Malar Rash</td><td>TRANSMISSION_ACROSS_CHEMICAL_SYNAPSES</td><td>REACTOME</td><td>9089</td><td>30</td><td>20</td><td>2,71E-01</td><td>9,17E-01</td><td>rs11720405|rs10956564|rs4793521|rs3799630|rs8126112|rs3761793|rs12523641|rs1984334|rs4517239|rs6517438|rs937210|rs16904360|rs926543|rs7616006|rs1396514|rs9861063|rs6558000|rs10183636|rs7403139|rs1400155</td></tr>
<tr><td>Malar Rash</td><td>SYNTHESIS_SECRETION_AND_DEACYLATION_OF_GHRELIN</td><td>REACTOME</td><td>624</td><td>43</td><td>20</td><td>2,72E-01</td><td>9,17E-01</td><td>rs2075355|rs10816540|rs649748|rs4979219|rs10513628|rs156005|rs1703007|rs1339760|rs7373873|rs13061243|rs10871764|rs10816543|rs4978629|rs7639015|rs3020619|rs1377834|rs4979515|rs16876489|rs7029391|rs6776482</td></tr>
<tr><td>Malar Rash</td><td>PI3K_CASCADE</td><td>REACTOME</td><td>2702</td><td>30</td><td>20</td><td>2,74E-01</td><td>9,17E-01</td><td>rs3117915|rs4976123|rs968053|rs6891913|rs11876701|rs11593080|rs17612068|rs942689|rs974309|rs203150|rs7809503|rs6115830|rs9578440|rs4528168|rs1554513|rs17234079|rs9588044|rs3129601|rs1539826|rs3129596</td></tr>
<tr><td>Malar Rash</td><td>CDT1_ASSOCIATION_WITH_THE_CDC6_ORC_ORIGIN_COMPLEX</td><td>REACTOME</td><td>793</td><td>32</td><td>19</td><td>2,76E-01</td><td>9,17E-01</td><td>rs12292911|rs13213285|rs8063430|rs3761873|rs577357|rs7772351|rs7224730|rs4790706|rs837763|rs17213812|rs2282032|rs6142963|rs9784758|rs6924102|rs2011713|rs473151|rs328368|rs9380326|rs13394402</td></tr>
<tr><td>Malar Rash</td><td>ORC1_REMOVAL_FROM_CHROMATIN</td><td>REACTOME</td><td>930</td><td>32</td><td>20</td><td>2,76E-01</td><td>9,17E-01</td><td>rs12292911|rs2307389|rs8063430|rs3761873|rs577357|rs1781011|rs133416|rs7772351|rs7224730|rs4790706|rs837763|rs17213812|rs2282032|rs10948689|rs34330|rs6142963|rs9784876|rs6924102|rs2011713|rs473151</td></tr>
<tr><td>Malar Rash</td><td>INSULIN_RECEPTOR_SIGNALLING_CASCADE</td><td>REACTOME</td><td>3006</td><td>30</td><td>20</td><td>2,79E-01</td><td>9,17E-01</td><td>rs3117915|rs4976123|rs9410438|rs968053|rs6891913|rs11876701|rs11593080|rs17612068|rs2477569|rs974309|rs203150|rs7809503|rs6115830|rs9578440|rs4528168|rs1554513|rs17234079|rs9588044|rs3129601|rs1539826</td></tr>
<tr><td>Malar Rash</td><td>STRIATED_MUSCLE_CONTRACTION</td><td>REACTOME</td><td>453</td><td>18</td><td>11</td><td>2,80E-01</td><td>9,17E-01</td><td>rs201603|rs178637|rs12452359|rs575144|rs907604|rs1073084|rs1243872|rs17794387|rs3750433|rs10860757|rs2071675</td></tr>
<tr><td>Malar Rash</td><td>EGF_PATHWAY</td><td>BIOCARTA</td><td>1048</td><td>43</td><td>20</td><td>2,82E-01</td><td>9,17E-01</td><td>rs12409333|rs4976123|rs4947491|rs8004059|rs7776830|rs11568993|rs17172446|rs1873423|rs310245|rs706725|rs3024912|rs2721856|rs7708673|rs10151713|rs737063|rs249479|rs432854|rs11648535|rs882167|rs8013053</td></tr>
<tr><td>Malar Rash</td><td>IL17_PATHWAY</td><td>BIOCARTA</td><td>316</td><td>6</td><td>4</td><td>2,82E-01</td><td>9,17E-01</td><td>rs2275913|rs2556|rs2767326|rs4839541</td></tr>
<tr><td>Malar Rash</td><td>AUTOIMMUNE_THYROID_DISEASE</td><td>KEGG</td><td>1646</td><td>59</td><td>20</td><td>2,82E-01</td><td>9,17E-01</td><td>rs2268464|rs7756516|rs9678469|rs885945|rs2517938|rs2857106|rs4812997|rs154973|rs3128918|rs9277052|rs2734973|rs154978|rs34111681|rs9277535|rs3758562|rs441808|rs9259650|rs17617515|rs7648642|rs9274407</td></tr>
<tr><td>Malar Rash</td><td>EARLY_PHASE_OF_HIV_LIFE_CYCLE</td><td>REACTOME</td><td>270</td><td>15</td><td>7</td><td>2,83E-01</td><td>9,17E-01</td><td>rs10197003|rs16832740|rs11687599|rs10191360|rs156631|rs9520758|rs7594034</td></tr>
<tr><td>Malar Rash</td><td>SIGNALING_BY_ERBB2</td><td>REACTOME</td><td>3847</td><td>41</td><td>20</td><td>2,85E-01</td><td>9,17E-01</td><td>rs10956564|rs883541|rs4976123|rs4947491|rs2721051|rs833141|rs16904360|rs7303990|rs4550189|rs714104|rs7257613|rs4964005|rs2888104|rs815808|rs6115830|rs7776830|rs7972342|rs12619351|rs11568993|rs17172446</td></tr>
<tr><td>Malar Rash</td><td>ECM_RECEPTOR_INTERACTION</td><td>KEGG</td><td>3602</td><td>31</td><td>20</td><td>2,85E-01</td><td>9,17E-01</td><td>rs3811159|rs536173|rs9283850|rs2237642|rs9333114|rs3820088|rs9293664|rs17182988|rs17752721|rs10955078|rs162070|rs12955222|rs362719|rs12695175|rs11757721|rs265371|rs1325914|rs2972831|rs572614|rs10901336</td></tr>
<tr><td>Malar Rash</td><td>IL_3_5_AND_GM_CSF_SIGNALING</td><td>REACTOME</td><td>1134</td><td>43</td><td>20</td><td>2,86E-01</td><td>9,17E-01</td><td>rs12409333|rs4976123|rs11914132|rs12244380|rs4734507|rs7073236|rs3108913|rs17489214|rs1571221|rs1993703|rs310245|rs2075937|rs706725|rs6468757|rs1760118|rs1323653|rs7708673|rs791589|rs10415535|rs228942</td></tr>
<tr><td>Malar Rash</td><td>PROSTATE_CANCER</td><td>KEGG</td><td>2870</td><td>34</td><td>20</td><td>2,88E-01</td><td>9,17E-01</td><td>rs8092360|rs4976123|rs4947491|rs2721051|rs4722835|rs11593080|rs2684789|rs1543478|rs4987721|rs177580|rs11766675|rs7776830|rs6719501|rs2684790|rs6938550|rs880026|rs1636808|rs11568993|rs17172446|rs2684802</td></tr>
<tr><td>Malar Rash</td><td>MYD88_MAL_CASCADE_INITIATED_ON_PLASMA_MEMBRANE</td><td>REACTOME</td><td>2527</td><td>31</td><td>20</td><td>2,88E-01</td><td>9,17E-01</td><td>rs3799630|rs8029326|rs394558|rs4618461|rs4804833|rs1543478|rs2737191|rs2256331|rs8004059|rs1546745|rs9345044|rs2820350|rs2811650|rs10223241|rs2189756|rs7183836|rs4256810|rs1177614|rs16899333|rs3799631</td></tr>
<tr><td>Malar Rash</td><td>GOLGI_ASSOCIATED_VESICLE_BIOGENESIS</td><td>REACTOME</td><td>1057</td><td>47</td><td>20</td><td>2,91E-01</td><td>9,17E-01</td><td>rs482263|rs11726508|rs878129|rs26538|rs909626|rs17127601|rs6884769|rs4804528|rs17185574|rs760306|rs10042243|rs14976|rs10897207|rs2272295|rs7712357|rs655246|rs17397283|rs4425006|rs220892|rs4504735</td></tr>
<tr><td>Malar Rash</td><td>MAL_PATHWAY</td><td>BIOCARTA</td><td>225</td><td>2</td><td>2</td><td>2,91E-01</td><td>9,17E-01</td><td>rs6580191|rs2302103</td></tr>
<tr><td>Malar Rash</td><td>GLYCOSPHINGOLIPID_BIOSYNTHESIS_LACTO_AND_NEOLACTO_SERIES</td><td>KEGG</td><td>670</td><td>40</td><td>17</td><td>2,91E-01</td><td>9,17E-01</td><td>rs6745253|rs2306969|rs12207089|rs986697|rs4935966|rs2029084|rs3772097|rs7768089|rs10497328|rs11605546|rs11823140|rs2508783|rs9804873|rs1702956|rs2230906|rs8111317|rs2111177</td></tr>
<tr><td>Malar Rash</td><td>AQUAPORIN_MEDIATED_TRANSPORT</td><td>REACTOME</td><td>1510</td><td>35</td><td>20</td><td>2,92E-01</td><td>9,17E-01</td><td>rs10937003|rs10956564|rs883541|rs16904360|rs2294451|rs1893441|rs4898725|rs12147761|rs1435421|rs11658925|rs72001|rs10787805|rs11621038|rs2099505|rs485835|rs17612687|rs12242357|rs2239305|rs7309966|rs3934729</td></tr>
<tr><td>Malar Rash</td><td>CHEMICAL_PATHWAY</td><td>BIOCARTA</td><td>585</td><td>29</td><td>20</td><td>2,93E-01</td><td>9,17E-01</td><td>rs8092360|rs2711008|rs4987721|rs11561771|rs12537364|rs1873423|rs3024912|rs8083946|rs737063|rs2526406|rs1801516|rs1243872|rs7840147|rs432854|rs11648535|rs3750433|rs4646018|rs7202459|rs9896483|rs1476813</td></tr>
<tr><td>Malar Rash</td><td>ACYL_CHAIN_REMODELLING_OF_PC</td><td>REACTOME</td><td>693</td><td>31</td><td>18</td><td>2,96E-01</td><td>9,17E-01</td><td>rs11582551|rs13427229|rs2701632|rs600415|rs4820944|rs156631|rs1487492|rs6680812|rs955835|rs650392|rs12564176|rs591393|rs4821738|rs12408941|rs1356410|rs2617605|rs818968|rs10912119</td></tr>
<tr><td>Malar Rash</td><td>AMINO_SUGAR_AND_NUCLEOTIDE_SUGAR_METABOLISM</td><td>KEGG</td><td>956</td><td>42</td><td>20</td><td>2,96E-01</td><td>9,17E-01</td><td>rs3823279|rs3800128|rs10489612|rs9392322|rs1227938|rs9503159|rs6841545|rs855351|rs1545970|rs12527698|rs17609046|rs12189843|rs820844|rs17700893|rs10823343|rs10770888|rs16924651|rs11584291|rs2282290|rs929959</td></tr>
<tr><td>Malar Rash</td><td>ENDOMETRIAL_CANCER</td><td>KEGG</td><td>3028</td><td>38</td><td>20</td><td>2,97E-01</td><td>9,17E-01</td><td>rs4976123|rs4947491|rs17018319|rs12472674|rs2135732|rs4410871|rs7202333|rs12543549|rs10762129|rs9415814|rs11766675|rs7776830|rs6719501|rs880026|rs1636808|rs2720662|rs2685159|rs11568993|rs17172446|rs7224837</td></tr>
<tr><td>Malar Rash</td><td>CARDIAC_MUSCLE_CONTRACTION</td><td>KEGG</td><td>3169</td><td>29</td><td>20</td><td>3,03E-01</td><td>9,17E-01</td><td>rs4605535|rs1860094|rs178637|rs926543|rs709318|rs1370154|rs17025772|rs533021|rs42792|rs7257613|rs886898|rs7248185|rs1023421|rs2192724|rs3774589|rs16831388|rs1015196|rs10510771|rs2284009|rs8107107</td></tr>
<tr><td>Malar Rash</td><td>BCELLSURVIVAL_PATHWAY</td><td>BIOCARTA</td><td>500</td><td>26</td><td>13</td><td>3,05E-01</td><td>9,17E-01</td><td>rs4976123|rs8004059|rs11766675|rs1636808|rs706725|rs42154|rs7708673|rs10151713|rs249479|rs8013053|rs882167|rs4131890|rs7804859</td></tr>
<tr><td>Malar Rash</td><td>SIGNALING_BY_EGFR_IN_CANCER</td><td>REACTOME</td><td>4030</td><td>39</td><td>20</td><td>3,07E-01</td><td>9,17E-01</td><td>rs10956564|rs883541|rs4976123|rs4947491|rs2721051|rs833141|rs16904360|rs7303990|rs12762349|rs714104|rs7257613|rs4964005|rs1278302|rs815808|rs6115830|rs7776830|rs7972342|rs12619351|rs10507911|rs11568993</td></tr>
<tr><td>Malar Rash</td><td>LONG_TERM_POTENTIATION</td><td>KEGG</td><td>3620</td><td>35</td><td>20</td><td>3,07E-01</td><td>9,17E-01</td><td>rs10956564|rs3799630|rs8126112|rs1860094|rs937210|rs16904360|rs7303990|rs2098469|rs918168|rs7257613|rs4958596|rs886898|rs4964005|rs815808|rs1868289|rs7972342|rs11098195|rs6140629|rs6792995|rs7298664</td></tr>
<tr><td>Malar Rash</td><td>DESTABILIZATION_OF_MRNA_BY_KSRP</td><td>REACTOME</td><td>205</td><td>16</td><td>8</td><td>3,08E-01</td><td>9,17E-01</td><td>rs1025814|rs4734507|rs11642944|rs3108913|rs6468757|rs3019260|rs12599053|rs1046909</td></tr>
<tr><td>Malar Rash</td><td>NOD1_2_SIGNALING_PATHWAY</td><td>REACTOME</td><td>881</td><td>42</td><td>20</td><td>3,08E-01</td><td>9,17E-01</td><td>rs394558|rs507879|rs1546745|rs9345044|rs2820350|rs2811650|rs2189756|rs1177614|rs531486|rs7922090|rs1545092|rs2501657|rs311636|rs9342261|rs13402616|rs12447155|rs11870477|rs9611169|rs12215452|rs4422587</td></tr>
<tr><td>Malar Rash</td><td>SYNTHESIS_SECRETION_AND_INACTIVATION_OF_GLP1</td><td>REACTOME</td><td>363</td><td>14</td><td>7</td><td>3,08E-01</td><td>9,17E-01</td><td>rs2294451|rs156005|rs880026|rs10871764|rs6599127|rs9863665|rs1377834</td></tr>
<tr><td>Malar Rash</td><td>PLATELET_CALCIUM_HOMEOSTASIS</td><td>REACTOME</td><td>1156</td><td>52</td><td>20</td><td>3,10E-01</td><td>9,17E-01</td><td>rs7303990|rs17025772|rs42792|rs4964005|rs2192724|rs715305|rs7972342|rs2540904|rs10160649|rs7567386|rs171406|rs4833241|rs2344498|rs2272295|rs227402|rs768710|rs11622923|rs2066966|rs1372804|rs2301343</td></tr>
<tr><td>Malar Rash</td><td>MITOCHONDRIAL_TRNA_AMINOACYLATION</td><td>REACTOME</td><td>500</td><td>8</td><td>6</td><td>3,11E-01</td><td>9,17E-01</td><td>rs7744190|rs9369472|rs167772|rs12132918|rs9472250|rs959086</td></tr>
<tr><td>Malar Rash</td><td>IL6_PATHWAY</td><td>BIOCARTA</td><td>401</td><td>24</td><td>12</td><td>3,12E-01</td><td>9,17E-01</td><td>rs12409333|rs8004059|rs1571221|rs13045110|rs310245|rs10151713|rs8013053|rs882167|rs310199|rs7219|rs6020379|rs11207303</td></tr>
<tr><td>Malar Rash</td><td>SYNTHESIS_OF_PIPS_AT_THE_PLASMA_MEMBRANE</td><td>REACTOME</td><td>1050</td><td>37</td><td>20</td><td>3,14E-01</td><td>9,17E-01</td><td>rs4976123|rs1907108|rs4763508|rs4820944|rs11766675|rs1636808|rs195770|rs3775664|rs2270229|rs7048825|rs1497391|rs706725|rs42154|rs7708673|rs17015594|rs17761155|rs1909475|rs249479|rs17082035|rs10505819</td></tr>
<tr><td>Malar Rash</td><td>ACTIVATION_OF_THE_MRNA_UPON_BINDING_OF_THE_CAP_BINDING_COMPLEX_AND_EI
T_BINDING_TO_43S</td><td>REACTOME</td><td>780</td><td>34</td><td>20</td><td>3,14E-01</td><td>9,17E-01</td><td>rs968053|rs2177836|rs2075751|rs16898766|rs3108908|rs10824618|rs11217128|rs17028238|rs16888536|rs226206|rs751716|rs7076791|rs3094303|rs2165944|rs12323544|rs3741436|rs5756219|rs701405|rs10876394|rs2175376</td></tr>
<tr><td>Malar Rash</td><td>INTERFERON_GAMMA_SIGNALING</td><td>REACTOME</td><td>2043</td><td>57</td><td>20</td><td>3,14E-01</td><td>9,17E-01</td><td>rs12409333|rs276984|rs10807113|rs9376268|rs885945|rs13116389|rs6091153|rs2517938|rs8061995|rs11098195|rs2396763|rs9808753|rs3128918|rs10878721|rs1571221|rs9277052|rs2734973|rs310245|rs9277535|rs441808</td></tr>
<tr><td>Malar Rash</td><td>IGF1R_PATHWAY</td><td>BIOCARTA</td><td>735</td><td>38</td><td>17</td><td>3,15E-01</td><td>9,17E-01</td><td>rs883541|rs4976123|rs2684789|rs2684790|rs2684802|rs11658925|rs7166558|rs706725|rs7708673|rs249479|rs7213247|rs11674718|rs7169544|rs4965436|rs7219|rs4131890|rs964818</td></tr>
<tr><td>Malar Rash</td><td>GLYCEROLIPID_METABOLISM</td><td>KEGG</td><td>1872</td><td>42</td><td>20</td><td>3,15E-01</td><td>9,17E-01</td><td>rs7749480|rs7216|rs1708414|rs13427229|rs11248060|rs9533044|rs10973770|rs12454833|rs17799355|rs6802450|rs9954848|rs1869138|rs549662|rs2920126|rs914832|rs13225343|rs11195813|rs3819847|rs12066561|rs4646770</td></tr>
<tr><td>Malar Rash</td><td>ENDOSOMAL_SORTING_COMPLEX_REQUIRED_FOR_TRANSPORT_ESCRT</td><td>REACTOME</td><td>318</td><td>38</td><td>16</td><td>3,17E-01</td><td>9,17E-01</td><td>rs746987|rs7836184|rs7815340|rs8091623|rs7428816|rs6769886|rs1825896|rs10091786|rs13258200|rs17687453|rs522073|rs17405840|rs1962276|rs8891|rs17689135|rs3852689</td></tr>
<tr><td>Malar Rash</td><td>METAL_ION_SLC_TRANSPORTERS</td><td>REACTOME</td><td>653</td><td>35</td><td>16</td><td>3,17E-01</td><td>9,17E-01</td><td>rs1622108|rs10505314|rs1891092|rs1695715|rs149292|rs12608998|rs13258200|rs16861637|rs10497704|rs35239|rs2771338|rs10493940|rs11685885|rs1977090|rs2233914|rs7864452</td></tr>
<tr><td>Malar Rash</td><td>AXON_GUIDANCE</td><td>KEGG</td><td>9506</td><td>30</td><td>20</td><td>3,18E-01</td><td>9,17E-01</td><td>rs12639432|rs7562441|rs265060|rs2117580|rs1487091|rs12638746|rs12695621|rs4679332|rs2410869|rs4811174|rs6596698|rs9962572|rs1384566|rs2291333|rs4309752|rs1028154|rs9519971|rs12786815|rs13061481|rs2804877</td></tr>
<tr><td>Malar Rash</td><td>TIGHT_JUNCTION_INTERACTIONS</td><td>REACTOME</td><td>715</td><td>30</td><td>17</td><td>3,19E-01</td><td>9,17E-01</td><td>rs12960187|rs12866697|rs4819808|rs16986270|rs11207864|rs17744842|rs12457111|rs6512665|rs11009506|rs10508025|rs7068747|rs1108371|rs2786551|rs2139393|rs4934677|rs6979031|rs2065949</td></tr>
</table>

Malar Rash	SIGNALING_BY_TGF_BETA_RECEPTOR_COMPLEX	REACTOME	1605	32	20	3,21E-01	9,17E-01	rs266371|rs16950543|rs4410871|rs12543549|rs6041884|rs9961435|rs36047858|rs12580394|rs7173609|rs2720662|rs266312|rs3217992|rs6577060|rs11916830|rs928163|rs1855469|rs9507713|rs7181907|rs10896172|rs944695
Malar Rash	ACTIVATION_OF_CHAPERONE_GENES_BY_XBP1S	REACTOME	761	38	20	3,22E-01	9,17E-01	rs883541|rs394558|rs17042585|rs241179|rs1515372|rs17365609|rs11217128|rs2717233|rs9311513|rs17043569|rs1464103|rs4930351|rs9311542|rs9328415|rs1243872|rs6806768|rs4461514|rs6762892|rs12495017|rs3750433
Malar Rash	LYSOSOME	KEGG	2448	41	20	3,23E-01	9,17E-01	rs4690513|rs1968930|rs2662126|rs11039149|rs11248060|rs878129|rs4690557|rs12602084|rs26538|rs12508572|rs9760|rs1061497|rs5770953|rs2292537|rs6884769|rs2178468|rs13238736|rs6681804|rs8016092|rs17770799
Malar Rash	INHIBITION_OF_INSULIN_SECRETION_BY_ADRENALINE_NORADRENALINE	REACTOME	789	30	20	3,26E-01	9,17E-01	rs10937003|rs1108656|rs2294451|rs4898725|rs12147761|rs11013044|rs11621038|rs914363|rs12219393|rs10508557|rs3934729|rs13338253|rs7141727|rs887030|rs2287208|rs2886612|rs12985186|rs3790113|rs2241953|rs17245731
Malar Rash	MCALPAIN_PATHWAY	BIOCARTA	543	30	17	3,26E-01	9,17E-01	rs883541|rs4947491|rs7776830|rs11568993|rs17172446|rs11658925|rs1254403|rs820371|rs1243872|rs7840147|rs10511418|rs7213247|rs3750433|rs7545965|rs17238095|rs10488142|rs13244925
Malar Rash	GLYCOGEN_BREAKDOWN_GLYCOGENOLYSIS	REACTOME	246	20	9	3,27E-01	9,17E-01	rs10489612|rs815808|rs6841545|rs855351|rs4904690|rs1294506|rs929959|rs1693892|rs1058903
Malar Rash	AMINE_COMPOUND_SLC_TRANSPORTERS	REACTOME	1298	31	20	3,28E-01	9,17E-01	rs2531747|rs1400155|rs9682960|rs2286489|rs1054986|rs11128537|rs3900078|rs17806653|rs601788|rs1972873|rs2017903|rs216250|rs6766142|rs13258461|rs999969|rs791183|rs1380412|rs7359232|rs10862903|rs10744557
Malar Rash	PHENYLALANINE_METABOLISM	KEGG	442	11	7	3,29E-01	9,17E-01	rs4924793|rs11612372|rs2298316|rs4385902|rs6593011|rs1539259|rs10745952
Malar Rash	DILATED_CARDIOMYOPATHY	KEGG	4636	32	20	3,31E-01	9,17E-01	rs4605535|rs10956564|rs1860094|rs178637|rs9333114|rs16904360|rs926543|rs709318|rs1370154|rs17025772|rs17752721|rs533021|rs42792|rs4272873|rs7257613|rs886898|rs2192724|rs265371|rs3774589|rs9510465
Malar Rash	INTERACTIONS_OF_VPR_WITH_HOST_CELLULAR_PROTEINS	REACTOME	480	26	14	3,31E-01	9,17E-01	rs9310419|rs5766404|rs987607|rs9615022|rs1545951|rs723553|rs4740419|rs4274569|rs9297042|rs7284581|rs4759082|rs5766536|rs7982860|rs1462531
Malar Rash	STARCH_AND_SUCROSE_METABOLISM	KEGG	2178	53	20	3,32E-01	9,17E-01	rs2618956|rs11184173|rs11761774|rs10489612|rs2055242|rs1227938|rs17021491|rs6841545|rs1409184|rs855351|rs1841037|rs10881159|rs10494011|rs7650373|rs9399004|rs10823343|rs4148309|rs17601580|rs11185393|rs439863
Malar Rash	INSULIN_SYNTHESIS_AND_PROCESSING	REACTOME	1033	32	20	3,32E-01	9,17E-01	rs845869|rs10458234|rs2493047|rs6133869|rs4532481|rs156005|rs6597054|rs11772444|rs1380355|rs10485738|rs149292|rs2206496|rs10279531|rs6131930|rs7691043|rs6044850|rs1132683|rs8042411|rs35239|rs8891
Malar Rash	PI_3K_CASCADE	REACTOME	1868	36	20	3,37E-01	9,17E-01	rs3117915|rs4976123|rs2721051|rs6891913|rs11593080|rs17612068|rs6115830|rs9578440|rs17234079|rs3129601|rs3129596|rs13135439|rs2145811|rs11776272|rs7922090|rs4769189|rs477516|rs7989744|rs17004857|rs9634328
Malar Rash	PROXIMAL_TUBULE_BICARBONATE_RECLAMATION	KEGG	458	17	10	3,38E-01	9,17E-01	rs1023421|rs16831388|rs8107107|rs1474758|rs772654|rs359535|rs10750114|rs12030460|rs4579962|rs10919049
Malar Rash	ACUTE_MYELOID_LEUKEMIA	KEGG	1892	38	20	3,40E-01	9,17E-01	rs10838702|rs4976123|rs2834874|rs4410871|rs12543549|rs573264|rs7251505|rs11766675|rs6719501|rs1636808|rs2720662|rs3781998|rs9976900|rs661374|rs2133606|rs7922090|rs11097974|rs530532|rs476529|rs12155836
Malar Rash	MISMATCH_REPAIR	KEGG	576	20	13	3,40E-01	9,17E-01	rs406334|rs2035256|rs9909163|rs156631|rs2381375|rs1765853|rs7521516|rs6753949|rs2881964|rs6463725|rs17584703|rs6151838|rs886727
Malar Rash	RESPIRATORY_ELECTRON_TRANSPORT_ATP_SYNTHESIS_BY_CHEMIOSMOTIC_COUPLINC<br>TION_BY_UNCOUPLING_PROTEINS_	REACTOME	1741	46	20	3,42E-01	9,17E-01	rs4854053|rs2711008|rs11203280|rs11561771|rs7248185|rs13415895|rs2802460|rs1015196|rs11171911|rs1124729|rs4975851|rs11678404|rs12537364|rs4804847|rs4361043|rs741448|rs11749320|rs10487881|rs2280231|rs10424438
Malar Rash	GALACTOSE_METABOLISM	KEGG	759	32	18	3,43E-01	9,17E-01	rs1708414|rs11761774|rs10489612|rs6560821|rs1227938|rs6841545|rs855351|rs13225343|rs10794989|rs10823343|rs1702956|rs929959|rs7788953|rs2388251|rs539822|rs11251481|rs2388566|rs2305198
Malar Rash	ACTIVATED_TLR4_SIGNALLING	REACTOME	2676	31	20	3,45E-01	9,17E-01	rs3799630|rs8029326|rs394558|rs4618461|rs4804833|rs1543478|rs2737191|rs2256331|rs8004059|rs1546745|rs9345044|rs2820350|rs2811650|rs10223241|rs2189756|rs7183836|rs4256810|rs1177614|rs16899333|rs3799631
Malar Rash	ALPHA_LINOLENIC_ACID_METABOLISM	KEGG	571	30	16	3,46E-01	9,17E-01	rs11582551|rs2701632|rs4820944|rs6447868|rs800719|rs2301472|rs1487492|rs6501847|rs6680812|rs955835|rs12564176|rs591393|rs4821738|rs12408941|rs818968|rs10912119
Malar Rash	N_GLYCAN_BIOSYNTHESIS	KEGG	1792	42	20	3,47E-01	9,17E-01	rs2410361|rs2720630|rs12431858|rs1295391|rs9401188|rs4686823|rs6572331|rs3121680|rs11692586|rs3742290|rs12435557|rs10138087|rs6999669|rs7761886|rs12936155|rs3820500|rs7741206|rs1519304|rs2411032|rs6444186
Malar Rash	PEROXISOMAL_LIPID_METABOLISM	REACTOME	435	28	11	3,47E-01	9,17E-01	rs1348850|rs6487792|rs6447868|rs1603382|rs26181|rs35412|rs6501847|rs12368136|rs10207932|rs2560718|rs10843287
Malar Rash	RNA_POL_III_CHAIN_ELONGATION	REACTOME	364	20	4	3,47E-01	9,17E-01	rs1383245|rs4961396|rs10101153|rs2257082
Malar Rash	FORMATION_OF_INCISION_COMPLEX_IN_GG_NER	REACTOME	545	8	6	3,48E-01	9,17E-01	rs11039149|rs2174270|rs9909163|rs2881964|rs6463725|rs886727
Malar Rash	RIBOFLAVIN_METABOLISM	KEGG	295	27	11	3,48E-01	9,17E-01	rs11039149|rs7815340|rs1409184|rs17687453|rs1557591|rs9399004|rs2501920|rs17601580|rs17082035|rs12129752|rs7011504
Malar Rash	RNA_POL_III_TRANSCRIPTION	REACTOME	861	41	20	3,49E-01	9,17E-01	rs7034140|rs10810136|rs4804833|rs2382444|rs1383245|rs12676965|rs13291088|rs3769769|rs4961396|rs303721|rs10101153|rs10756526|rs3011286|rs471088|rs1323348|rs1556032|rs1407837|rs3920568|rs501360|rs630973
Malar Rash	FRS2_MEDIATED_CASCADE	REACTOME	1192	26	20	3,50E-01	9,17E-01	rs3117915|rs6891913|rs11593080|rs17612068|rs9578440|rs17234079|rs3129601|rs3129596|rs13135439|rs11776272|rs4769189|rs477516|rs7989744|rs17004857|rs9634328|rs1501588|rs4866891|rs576674|rs1458038|rs4489674
Malar Rash	AT1R_PATHWAY	BIOCARTA	1224	47	20	3,53E-01	9,17E-01	rs4947491|rs8029326|rs4618461|rs815808|rs7776830|rs10223241|rs7183836|rs17172446|rs3019244|rs1750304|rs1873423|rs8030728|rs187571|rs2721856|rs6592724|rs4904690|rs737063|rs7000615|rs7840147|rs1294506
Malar Rash	SHC_RELATED_EVENTS	REACTOME	302	7	5	3,56E-01	9,17E-01	rs9410438|rs2241407|rs7254060|rs7219|rs11137533
Malar Rash	ANTIGEN_PRESENTATION_FOLDING_ASSEMBLY_AND_PEPTIDE_LOADING_OF_CLASS_I_N	REACTOME	830	61	18	3,57E-01	9,17E-01	rs7156225|rs885945|rs17108549|rs2517938|rs2857106|rs6571891|rs35683|rs2734973|rs17213812|rs441808|rs9259650|rs17108513|rs2853923|rs4461514|rs9784758|rs6924102|rs11730732|rs12434593
Malar Rash	SIGNALING_BY_FGFR_MUTANTS	REACTOME	1657	35	20	3,58E-01	9,17E-01	rs3117915|rs4976123|rs6891913|rs11593080|rs11837579|rs17612068|rs9578440|rs17234079|rs3129601|rs2296078|rs3129596|rs11776272|rs4769189|rs477516|rs7989744|rs17004857|rs9634328|rs1501588|rs706725|rs3024912
Malar Rash	NEURONAL_SYSTEM	REACTOME	13378	31	20	3,58E-01	9,17E-01	rs1864773|rs11720405|rs10956564|rs4793521|rs3799630|rs8126112|rs3761793|rs2471095|rs12523641|rs1984334|rs4517239|rs6517438|rs937210|rs2034007|rs16904360|rs926543|rs16910949|rs7616006|rs10100027|rs7530450
Malar Rash	RESPIRATORY_ELECTRON_TRANSPORT	REACTOME	1631	44	20	3,58E-01	9,17E-01	rs4854053|rs2711008|rs11203280|rs11561771|rs7248185|rs13415895|rs1015196|rs1124729|rs4975851|rs11678404|rs12537364|rs4804847|rs4361043|rs741448|rs11749320|rs10487881|rs2280231|rs10424438|rs1316932|rs7827095
Malar Rash	PROLONGED_ERK_ACTIVATION_EVENTS	REACTOME	429	8	5	3,58E-01	9,17E-01	rs13427229|rs472159|rs4626883|rs1041264|rs1476868
Malar Rash	CHEMOKINE_RECEPTORS_BIND_CHEMOKINES	REACTOME	726	40	17	3,59E-01	9,17E-01	rs4683189|rs10197003|rs16832740|rs2037596|rs1806786|rs1147960|rs11687599|rs10191360|rs3760386|rs1596230|rs12639224|rs4513489|rs6770096|rs6550177|rs972755|rs7594034|rs11217072
Malar Rash	ARMS_MEDIATED_ACTIVATION	REACTOME	393	8	5	3,60E-01	9,17E-01	rs13427229|rs472159|rs4626883|rs1041264|rs1476868
Malar Rash	INITIAL_TRIGGERING_OF_COMPLEMENT	REACTOME	434	17	7	3,60E-01	9,17E-01	rs2249349|rs11003300|rs2031749|rs379527|rs4935047|rs11916885|rs11002799
Malar Rash	DOUBLE_STRAND_BREAK_REPAIR	REACTOME	506	20	13	3,62E-01	9,17E-01	rs10765682|rs17782975|rs12796488|rs9909163|rs499952|rs2244012|rs156631|rs9520758|rs1801516|rs8178108|rs2881964|rs6463725|rs886727
Malar Rash	SYNTHESIS_AND_INTERCONVERSION_OF_NUCLEOTIDE_DI_AND_TRIPHOSPHATES	REACTOME	306	28	10	3,63E-01	9,17E-01	rs6691415|rs6556882|rs9633478|rs10493606|rs1265124|rs728741|rs1980412|rs12034899|rs6741290|rs3758192
Malar Rash	ACTIVATED_TAK1_MEDIATES_P38_MAPK_ACTIVATION	REACTOME	649	47	20	3,64E-01	9,17E-01	rs394558|rs1546745|rs9345044|rs2820350|rs2811650|rs2189756|rs4256810|rs1177614|rs531486|rs2501657|rs311636|rs9342261|rs11870477|rs9611169|rs12215452|rs4422587|rs6938848|rs6911411|rs1782449|rs12660904
Malar Rash	COMPLEMENT_CASCADE	REACTOME	752	37	18	3,64E-01	9,17E-01	rs2249349|rs4610335|rs4957473|rs3753394|rs761276|rs10473234|rs11003300|rs2031749|rs17611|rs6451566|rs379527|rs4935047|rs6691117|rs11916885|rs17186722|rs11002799|rs12659224|rs7711831
Malar Rash	GLEEVEC_PATHWAY	BIOCARTA	954	35	20	3,64E-01	9,17E-01	rs4976123|rs4410871|rs12543549|rs8004059|rs11766675|rs1636808|rs2720662|rs1571221|rs706725|rs3024912|rs2721856|rs42154|rs7708673|rs4130120|rs1030102|rs10151713|rs249479|rs8013053|rs882167|rs12547643
Malar Rash	AMYLOIDS	REACTOME	972	46	16	3,65E-01	9,17E-01	rs983788|rs9461204|rs2256331|rs4837820|rs2064193|rs1033440|rs728479|rs2829946|rs467778|rs2655439|rs7776065|rs2830012|rs468345|rs9358913|rs7046030|rs1935005
Malar Rash	GAP_JUNCTION_ASSEMBLY	REACTOME	330	14	7	3,65E-01	9,17E-01	rs6454601|rs7317958|rs7738656|rs1347638|rs1436908|rs12408178|rs7327522
Malar Rash	CHOLESTEROL_BIOSYNTHESIS	REACTOME	259	20	8	3,66E-01	9,17E-01	rs1056607|rs10056811|rs1891805|rs12026860|rs619895|rs2272682|rs2686184|rs1792325
Malar Rash	AXON_GUIDANCE	REACTOME	13131	31	20	3,67E-01	9,17E-01	rs7702812|rs1864773|rs3811159|rs3799630|rs265060|rs536173|rs4947491|rs875078|rs412970|rs4679332|rs9306310|rs3767742|rs9962572|rs1384566|rs788338|rs2291333|rs9861063|rs1028154|rs2059198|rs9878958
Malar Rash	POST_NMDA_RECEPTOR_ACTIVATION_EVENTS	REACTOME	1753	44	20	3,67E-01	9,17E-01	rs10956564|rs3799630|rs16904360|rs2098469|rs918168|rs815808|rs1868289|rs11098195|rs7298664|rs16899333|rs3799631|rs17830392|rs220593|rs16957102|rs17339754|rs4709117|rs2099505|rs12914792|rs7174521|rs17208905
Malar Rash	NEP_NS2_INTERACTS_WITH_THE_CELLULAR_EXPORT_MACHINERY	REACTOME	456	24	13	3,67E-01	9,17E-01	rs9310419|rs5766404|rs987607|rs9615022|rs723553|rs4740419|rs4274569|rs9297042|rs7284581|rs4759082|rs5766536|rs7982860|rs1462531
Malar Rash	REGULATION_OF_GLUCOKINASE_BY_GLUCOKINASE_REGULATORY_PROTEIN	REACTOME	459	24	13	3,70E-01	9,17E-01	rs9310419|rs5766404|rs987607|rs9615022|rs723553|rs4740419|rs11684526|rs9297042|rs7284581|rs4759082|rs5766536|rs7982860|rs1462531
Malar Rash	TRANSPORT_OF_RIBONUCLEOPROTEINS_INTO_THE_HOST_NUCLEUS	REACTOME	452	24	13	3,71E-01	9,17E-01	rs9310419|rs5766404|rs987607|rs9615022|rs723553|rs4740419|rs4274569|rs9297042|rs7284581|rs4759082|rs5766536|rs7982860|rs1462531
Malar Rash	PROTEASOME	KEGG	821	41	19	3,73E-01	9,17E-01	rs12292911|rs8063430|rs2140741|rs1474383|rs577357|rs10878721|rs7224730|rs4790706|rs17213812|rs2282032|rs7785299|rs6142963|rs9784758|rs6924102|rs2011713|rs473151|rs328368|rs9380326|rs13394402
Malar Rash	INSULIN_SIGNALING_PATHWAY	KEGG	3744	31	20	3,74E-01	9,17E-01	rs883541|rs4976123|rs4435197|rs2721051|rs7183535|rs9410438|rs968053|rs10009448|rs3774921|rs2477569|rs6091153|rs7809503|rs815808|rs4528168|rs4953402|rs11766675|rs1227938|rs9588044|rs10841414|rs1636808
Malar Rash	IGF1MTOR_PATHWAY	BIOCARTA	582	40	19	3,76E-01	9,17E-01	rs4976123|rs968053|rs2684789|rs2684790|rs17028238|rs2684802|rs703559|rs7166558|rs706725|rs2165944|rs7708673|rs249479|rs2971575|rs7978057|rs7169544|rs4965436|rs4131890|rs2248293|rs4015690
Malar Rash	CHONDROITIN_SULFATE_DERMATAN_SULFATE_METABOLISM	REACTOME	2784	36	20	3,77E-01	9,17E-01	rs718356|rs6455394|rs2147155|rs11248060|rs3820088|rs2682185|rs4965800|rs10955078|rs11224169|rs9584093|rs2975419|rs2449393|rs7926191|rs13010821|rs1328368|rs7966281|rs6492696|rs2292832|rs2150471|rs6586829
Malar Rash	SIGNALING_BY_INSULIN_RECEPTOR	REACTOME	3432	30	20	3,77E-01	9,17E-01	rs3117915|rs4976123|rs9410438|rs968053|rs6891913|rs11876701|rs11593080|rs17612068|rs2477569|rs974309|rs563201|rs203150|rs7809503|rs6115830|rs9578440|rs4528168|rs1554513|rs17234079|rs9588044|rs3129601
Malar Rash	INTERFERON_SIGNALING	REACTOME	3448	40	20	3,78E-01	9,17E-01	rs12409333|rs9310419|rs276984|rs10807113|rs968053|rs9376268|rs885945|rs4284952|rs13116389|rs5766404|rs987607|rs6091153|rs2517938|rs8061995|rs11098195|rs11246067|rs9615022|rs16827412|rs10495915|rs11071240
Malar Rash	TYROSINE_METABOLISM	KEGG	1169	41	20	3,80E-01	9,17E-01	rs4924793|rs9678469|rs2066097|rs12430699|rs7798172|rs2111699|rs8042782|rs2253612|rs2602884|rs7872903|rs2298316|rs7164048|rs1486806|rs1154454|rs4147536|rs4385902|rs6492706|rs6593011|rs1128349|rs1896429
Malar Rash	PKB_MEDIATED_EVENTS	REACTOME	583	30	16	3,80E-01	9,17E-01	rs968053|rs2477569|rs7809503|rs4528168|rs17028238|rs6965771|rs9908195|rs2165944|rs10889011|rs4425665|rs3741436|rs6678588|rs10876394|rs6490267|rs4595037|rs881109
Malar Rash	TERMINATION_OF_O_GLYCAN_BIOSYNTHESIS	REACTOME	961	30	20	3,81E-01	9,17E-01	rs2736862|rs12422396|rs17000836|rs4686823|rs6593538|rs15783|rs2114567|rs12984694|rs6667721|rs741901|rs6444186|rs7515898|rs4076950|rs13263183|rs2922494|rs17730982|rs4736675|rs881877|rs2969292|rs7014566
Malar Rash	CDMAC_PATHWAY	BIOCARTA	1018	26	20	3,83E-01	9,17E-01	rs8126112|rs4410871|rs12543549|rs1543478|rs8004059|rs2720662|rs6140629|rs13045064|rs1873423|rs696|rs6133573|rs926505|rs6077420|rs4130120|rs1030102|rs2179440|rs10151713|rs737063|rs2745772|rs432854
Malar Rash	TNFR1_PATHWAY	BIOCARTA	862	29	20	3,86E-01	9,17E-01	rs11039149|rs10047541|rs9345044|rs2820350|rs2811650|rs4761528|rs15614|rs4807269|rs3019244|rs2501657|rs2721856|rs6592724|rs2155458|rs9342261|rs13402616|rs3812062|rs12215452|rs4422587|rs8178108|rs6938848
Malar Rash	GAB1_SIGNALOSOME	REACTOME	1077	40	20	3,87E-01	9,17E-01	rs4976123|rs4947491|rs2721051|rs6115830|rs7776830|rs11568993|rs17172446|rs2145811|rs7922090|rs4404873|rs706725|rs7708673|rs4943768|rs17254382|rs7981045|rs249479|rs12329315|rs17069632|rs6018027|rs4675672
Malar Rash	TRAFFICKING_OF_AMPA_RECEPTORS	REACTOME	1132	23	20	3,87E-01	9,17E-01	rs937210|rs926543|rs9861063|rs7257613|rs4958596|rs132930|rs10895854|rs11098195|rs2284009|rs1873423|rs2284012|rs4076950|rs991910|rs737063|rs140028|rs11938486|rs1883987|rs2283984|rs1032970|rs4632855
Malar Rash	ACYL_CHAIN_REMODELLING_OF_PG	REACTOME	571	31	17	3,88E-01	9,17E-01	rs11582551|rs2701632|rs600415|rs4820944|rs1663171|rs28451400|rs1487492|rs6680812|rs955835|rs650392|rs12564176|rs591393|rs12408941|rs1356410|rs2617605|rs818968|rs10912119
Malar Rash	GAP_JUNCTION_TRAFFICKING	REACTOME	576	22	11	3,95E-01	9,17E-01	rs6454601|rs7317958|rs4804528|rs7023913|rs7738656|rs12338625|rs1347638|rs1436908|rs4425006|rs12408178|rs7327522
Malar Rash	ER_PHAGOSOME_PATHWAY	REACTOME	1416	62	20	3,95E-01	9,17E-01	rs12292911|rs8063430|rs885945|rs2517938|rs2857106|rs2416771|rs577357|rs7224730|rs2734973|rs4790706|rs17213812|rs441808|rs9259650|rs2282032|rs2853923|rs6142963|rs9784876|rs6924102|rs981446|rs2011713
Malar Rash	INTERFERON_ALPHA_BETA_SIGNALING	REACTOME	1477	57	20	3,96E-01	9,17E-01	rs12409333|rs276984|rs885945|rs13116389|rs6091153|rs2517938|rs8061995|rs11246067|rs2523809|rs310245|rs17213812|rs441808|rs16944688|rs2871398|rs9259650|rs3024912|rs729302|rs2859400|rs2853923|rs7395319
Malar Rash	DESTABILIZATION_OF_MRNA_BY_BRF1	REACTOME	204	7	4	3,96E-01	9,17E-01	rs1025814|rs4256810|rs194741|rs1046909
Malar Rash	MRNA_PROCESSING	REACTOME	1942	29	20	3,99E-01	9,17E-01	rs9310419|rs2588882|rs2304232|rs968053|rs11081583|rs5766404|rs987607|rs10169262|rs12191724|rs9976074|rs9615022|rs4809388|rs2274913|rs10506328|rs4905510|rs2174270|rs7248208|rs17028238|rs13258200|rs723553
Malar Rash	HS_GAG_DEGRADATION	REACTOME	1386	36	20	3,99E-01	9,17E-01	rs2147155|rs11248060|rs3820088|rs10955078|rs9584093|rs12602084|rs13010821|rs1328368|rs6854611|rs6492696|rs6831788|rs2292832|rs2150471|rs287541|rs7999055|rs11839320|rs4773686|rs9589601|rs2637451|rs755340
Malar Rash	AMINE_LIGAND_BINDING_RECEPTORS	REACTOME	2247	33	20	4,00E-01	9,17E-01	rs7445117|rs1235695|rs7713886|rs1213352|rs2446287|rs1739583|rs31684|rs891700|rs7937641|rs7814332|rs251925|rs9310061|rs1858045|rs6927637|rs17657156|rs4534729|rs1011427|rs543307|rs10066266|rs949318
Malar Rash	NKT_PATHWAY	BIOCARTA	638	30	20	4,01E-01	9,17E-01	rs4683189|rs9376268|rs10197003|rs16832740|rs9808753|rs10878721|rs11687599|rs10191360|rs3024685|rs6895787|rs2421049|rs2243290|rs4513489|rs6770096|rs1327474|rs8109167|rs2205181|rs6869688|rs12999517|rs6550177
Malar Rash	REGULATION_OF_IFNA_SIGNALING	REACTOME	339	20	10	4,01E-01	9,17E-01	rs12409333|rs6091153|rs310245|rs3024912|rs310199|rs2834133|rs4969168|rs2834159|rs2243600|rs2834116
Malar Rash	CDC42RAC_PATHWAY	BIOCARTA	435	32	11	4,01E-01	9,17E-01	rs4976123|rs275414|rs3019244|rs706725|rs6592724|rs7708673|rs727735|rs249479|rs7676985|rs4131890|rs13421586
Malar Rash	MPR_PATHWAY	BIOCARTA	624	47	19	4,02E-01	9,17E-01	rs883541|rs486992|rs601618|rs275414|rs6681804|rs11658925|rs3131660|rs7936649|rs13041735|rs727735|rs6018027|rs6062357|rs7213247|rs2886612|rs6090585|rs17245731|rs10501976|rs13421586|rs9635370
Malar Rash	ANTIGEN_ACTIVATES_B_CELL_RECEPTOR_LEADING_TO_GENERATION_OF_SECOND_MES	REACTOME	1299	46	20	4,02E-01	9,17E-01	rs4976123|rs2256647|rs7303990|rs2075751|rs4964005|rs815808|rs7972342|rs3785568|rs10160649|rs17489214|rs2344498|rs1993703|rs1445059|rs706725|rs12677326|rs1760118|rs7708673|rs4904690|rs10415535|rs249479
Malar Rash	JNK_C_JUN_KINASES_PHOSPHORYLATION_AND_ACTIVATION_MEDIATED_BY_ACTIVATE	REACTOME	728	37	20	4,04E-01	9,17E-01	rs394558|rs4804833|rs1546745|rs9345044|rs2820350|rs2811650|rs1177614|rs531486|rs2501657|rs311636|rs2721856|rs9342261|rs9611169|rs12215452|rs4422587|rs6938848|rs6911411|rs3745386|rs1782449|rs2043649
Malar Rash	COMP_PATHWAY	BIOCARTA	628	29	13	4,04E-01	9,17E-01	rs2249349|rs4957473|rs10473234|rs11003300|rs2031749|rs17611|rs6451566|rs379527|rs4935047|rs11916885|rs11002799|rs12659224|rs7711831
Malar Rash	DESTABILIZATION_OF_MRNA_BY_TRISTETRAPROLIN_TTP	REACTOME	179	7	4	4,04E-01	9,17E-01	rs4885036|rs4256810|rs1629174|rs1046909
Malar Rash	DOWNSTREAM_SIGNAL_TRANSDUCTION	REACTOME	3083	49	20	4,05E-01	9,17E-01	rs10956564|rs883541|rs4976123|rs2721051|rs833141|rs16904360|rs7303990|rs714104|rs7257613|rs4964005|rs815808|rs6115830|rs7972342|rs12619351|rs12829100|rs2344498|rs2145811|rs11658925|rs72001|rs13432276
Malar Rash	ATM_PATHWAY	BIOCARTA	397	19	12	4,08E-01	9,17E-01	rs10765682|rs1543478|rs12796488|rs499952|rs696|rs2244012|rs1801516|rs3765692|rs977908|rs11207303|rs540723|rs10483453
Malar Rash	HCMV_PATHWAY	BIOCARTA	547	34	14	4,11E-01	9,17E-01	rs4976123|rs11766675|rs2189756|rs1636808|rs706725|rs42154|rs7708673|rs249479|rs2364598|rs12329315|rs4675672|rs11870477|rs4131890|rs7804859
Malar Rash	INTERACTION_BETWEEN_L1_AND_ANKYRINS	REACTOME	970	35	20	4,11E-01	9,17E-01	rs1864773|rs6471070|rs13032976|rs3785568|rs11596260|rs2673580|rs11599447|rs969708|rs2920126|rs10791889|rs488758|rs4388439|rs2100644|rs2673590|rs10761461|rs7092606|rs1457783|rs2357727|rs857724|rs7560775
Malar Rash	METABOLISM_OF_CARBOHYDRATES	REACTOME	9072	28	20	4,12E-01	9,17E-01	rs756567|rs2736862|rs11184173|rs718356|rs6455394|rs9310419|rs2147155|rs4945522|rs11248060|rs718780|rs11761774|rs4871195|rs3820088|rs2682185|rs10489612|rs2055242|rs6560821|rs731096|rs2128211|rs4965800
Malar Rash	HYPERTROPHIC_CARDIOMYOPATHY_HCM	KEGG	4149	32	20	4,12E-01	9,17E-01	rs4605535|rs1860094|rs178637|rs9333114|rs926543|rs709318|rs1370154|rs17025772|rs17752721|rs533021|rs2477569|rs42792|rs4272873|rs7257613|rs886898|rs2192724|rs7809503|rs265371|rs4528168|rs3774589
Malar Rash	ACTIVATION_OF_NF_KAPPAB_IN_B_CELLS	REACTOME	1396	27	20	4,12E-01	9,17E-01	rs12292911|rs8063430|rs1543478|rs9345044|rs2820350|rs2811650|rs11762164|rs577357|rs4940757|rs7224730|rs4790706|rs1873423|rs7922090|rs17213812|rs696|rs1878806|rs2501657|rs9986945|rs2396963|rs2735587
Malar Rash	TYPE_II_DIABETES_MELLITUS	KEGG	2053	40	20	4,14E-01	9,17E-01	rs4605535|rs4976123|rs1860094|rs709318|rs17703233|rs714104|rs886898|rs11766675|rs3774589|rs1227938|rs12619351|rs9588044|rs1636808|rs11715993|rs13432276|rs12428609|rs1006385|rs198552|rs1281194|rs706725
Malar Rash	G0_AND_EARLY_G1	REACTOME	258	10	5	4,15E-01	9,17E-01	rs6577060|rs6813014|rs2871921|rs8110447|rs2835762
Malar Rash	HER2_PATHWAY	BIOCARTA	1326	41	20	4,18E-01	9,17E-01	rs4976123|rs4947491|rs7131878|rs2888104|rs11766675|rs7776830|rs1636808|rs4526844|rs17172446|rs17181630|rs1851176|rs706725|rs11155820|rs42154|rs7708673|rs191180|rs975731|rs11878377|rs12199102|rs16846140
Malar Rash	GLUCAGON_TYPE_LIGAND_RECEPTORS	REACTOME	786	29	20	4,21E-01	9,17E-01	rs10937003|rs2294451|rs4898725|rs9915294|rs12147761|rs1558477|rs670307|rs1941663|rs11621038|rs13041735|rs11682382|rs7141727|rs887030|rs1395956|rs7769547|rs12606116|rs2287208|rs4797684|rs12450747|rs6017596
Malar Rash	CELL_CYCLE_CHECKPOINTS	REACTOME	1961	34	20	4,22E-01	9,17E-01	rs12292911|rs11248424|rs13213285|rs8063430|rs883444|rs3761873|rs577357|rs1781011|rs7678743|rs133416|rs3191402|rs7894765|rs7772351|rs7224730|rs9909163|rs4790706|rs10896172|rs17213812|rs9990802|rs2004845

Malar Rash	SIGNALING_BY_FGFR1_FUSION_MUTANTS	REACTOME	639	31	13	4,25E-01	9,17E-01	rs4976123|rs11837579|rs2296078|rs706725|rs3024912|rs1060697|rs7708673|rs249479|rs202159|rs1880345|rs7219|rs4131890|rs708158
Malar Rash	CYTOSOLIC_DNA_SENSING_PATHWAY	KEGG	620	12	9	4,26E-01	9,17E-01	rs1543478|rs507879|rs12130290|rs7922090|rs2852720|rs696|rs6920337|rs2257082|rs10483453
Malar Rash	ERK_PATHWAY	BIOCARTA	1025	34	20	4,27E-01	9,17E-01	rs4947491|rs4410871|rs12543549|rs2684789|rs7776830|rs2684790|rs2720662|rs17172446|rs2684802|rs12436076|rs7166558|rs13041735|rs4130120|rs1030102|rs4626883|rs6018027|rs7676985|rs10414066|rs7169544|rs4965436
Malar Rash	INFLAMMASOMES	REACTOME	468	29	18	4,27E-01	9,17E-01	rs8092360|rs2256331|rs4987721|rs507879|rs2829946|rs467778|rs2852720|rs8083946|rs6426244|rs666462|rs7221898|rs2830012|rs3758192|rs568531|rs468345|rs12458289|rs6567322|rs2236719
Malar Rash	SIGNAL_AMPLIFICATION	REACTOME	1023	26	20	4,29E-01	9,17E-01	rs11720405|rs7637803|rs2294451|rs1688123|rs4898725|rs12147761|rs11621038|rs7141727|rs887030|rs1466684|rs1487492|rs6680812|rs955835|rs6018027|rs2287208|rs4745639|rs10935907|rs7218921|rs16863356|rs12564176
Malar Rash	SIGNALING_BY_THE_B_CELL_RECEPTOR_BCR	REACTOME	3426	33	20	4,31E-01	9,17E-01	rs12292911|rs4976123|rs2721051|rs8063430|rs2256647|rs7303990|rs1543478|rs2075751|rs4964005|rs604788|rs9345044|rs2820350|rs815808|rs2811650|rs6115830|rs7972342|rs11762164|rs577357|rs3785568|rs10160649
Malar Rash	GLYCOLYSIS_GLUCONEOGENESIS	KEGG	1391	58	20	4,31E-01	9,17E-01	rs962800|rs10489612|rs6560821|rs10973770|rs2158839|rs1227938|rs6841545|rs855351|rs2602884|rs1946741|rs6088638|rs10267797|rs1469594|rs1474758|rs4646770|rs1769259|rs10794989|rs10823343|rs1154454|rs4147536
Malar Rash	IGF1_PATHWAY	BIOCARTA	813	31	20	4,32E-01	9,17E-01	rs4976123|rs2684789|rs8004059|rs11766675|rs2684790|rs1636808|rs2684802|rs703559|rs7166558|rs706725|rs42154|rs7708673|rs10151713|rs249479|rs2971575|rs11674718|rs882167|rs8013053|rs7978057|rs7169544
Malar Rash	ALLOGRAFT_REJECTION	KEGG	1510	55	20	4,33E-01	9,17E-01	rs7756516|rs885945|rs2517938|rs2857106|rs4812997|rs154973|rs3128918|rs10878721|rs9277052|rs2734973|rs154978|rs34111681|rs9277535|rs3758562|rs441808|rs9259650|rs28365995|rs7648642|rs6895787|rs2421049
Malar Rash	RACCYCD_PATHWAY	BIOCARTA	551	31	16	4,34E-01	9,17E-01	rs4976123|rs1543478|rs6577060|rs3019244|rs7922090|rs696|rs706725|rs6592724|rs7708673|rs10273030|rs8110447|rs249479|rs34330|rs7394655|rs4131890|rs10483453
Malar Rash	ASTHMA	KEGG	770	56	16	4,34E-01	9,17E-01	rs7756516|rs2857106|rs4812997|rs154973|rs3128918|rs9277052|rs154978|rs9277535|rs28365995|rs9274407|rs2427832|rs2243290|rs3021094|rs10908702|rs9784758|rs547110
Malar Rash	ACTINY_PATHWAY	BIOCARTA	290	19	8	4,35E-01	9,17E-01	rs4389291|rs12191724|rs275414|rs727735|rs6142963|rs530991|rs13421586|rs7595898
Malar Rash	G_ALPHA1213_SIGNALLING_EVENTS	REACTOME	2855	35	20	4,36E-01	9,17E-01	rs11720405|rs4976123|rs6789224|rs9371859|rs1235695|rs16903375|rs2075751|rs31684|rs2294451|rs7814332|rs2882936|rs7611544|rs4898725|rs12147761|rs13397982|rs2304961|rs8139798|rs2276035|rs345306|rs2239775
Malar Rash	GRAFT_VERSUS_HOST_DISEASE	KEGG	1446	61	20	4,37E-01	9,17E-01	rs7756516|rs885945|rs2517938|rs2857106|rs154973|rs2281390|rs10878721|rs9277052|rs2523809|rs154978|rs34111681|rs9277535|rs3758562|rs441808|rs9259650|rs17617515|rs7648642|rs9274407|rs2853923|rs2332096
Malar Rash	RNA_POL_III_TRANSCRIPTION_INITIATION_FROM_TYPE_2_PROMOTER	REACTOME	417	21	5	4,39E-01	9,17E-01	rs1383245|rs4961396|rs10101153|rs3011286|rs2257082
Malar Rash	SPLICEOSOME	KEGG	1685	34	20	4,41E-01	9,17E-01	rs6764079|rs2588882|rs4428160|rs11081583|rs13072044|rs6067961|rs12191724|rs1130866|rs7701158|rs4809388|rs10506328|rs4747886|rs1076828|rs511278|rs481077|rs3094104|rs10016565|rs12630964|rs6706797|rs736259
Malar Rash	PENTOSE_AND_GLUCURONATE_INTERCONVERSIONS	KEGG	874	28	9	4,41E-01	9,17E-01	rs1708414|rs1841037|rs17080439|rs13225343|rs4148309|rs3738934|rs7788953|rs2125312|rs7337130
Malar Rash	YAP1_AND_WWTR1_TAZ_STIMULATED_GENE_EXPRESSION	REACTOME	1047	38	20	4,43E-01	9,17E-01	rs10513747|rs4237723|rs1995243|rs6007640|rs7316919|rs6804202|rs6794849|rs11916830|rs10948245|rs1490054|rs12637399|rs11154654|rs6500552|rs745361|rs6060034|rs1018503|rs6927367|rs4857702|rs7636407|rs2143491
Malar Rash	PKA_MEDIATED_PHOSPHORYLATION_OF_CREB	REACTOME	767	39	20	4,44E-01	9,17E-01	rs10956564|rs883541|rs16904360|rs11658925|rs72001|rs2099505|rs2239305|rs3934729|rs727432|rs4736746|rs12329315|rs12680002|rs7213247|rs4675672|rs6883259|rs8046783|rs4736520|rs7197403|rs2238439|rs273393
Malar Rash	RNA_POL_III_TRANSCRIPTION_TERMINATION	REACTOME	680	44	20	4,45E-01	9,17E-01	rs7034140|rs10810136|rs2382444|rs1383245|rs13291088|rs3769769|rs4961396|rs303721|rs10101153|rs10756526|rs471088|rs1323348|rs1556032|rs1407837|rs3920568|rs501360|rs630973|rs2257082|rs4740567|rs10117199
Malar Rash	ENERGY_DEPENDENT_REGULATION_OF_MTOR_BY_LKB1_AMPK	REACTOME	461	17	11	4,45E-01	9,17E-01	rs2477569|rs7809503|rs4528168|rs6965771|rs9908195|rs10889011|rs4425665|rs6678588|rs6490267|rs4595037|rs881109
Malar Rash	ACETYLCHOLINE_BINDING_AND_DOWNSTREAM_EVENTS	REACTOME	242	11	10	4,46E-01	9,17E-01	rs6558000|rs10183636|rs7844566|rs11556635|rs6090392|rs11071503|rs4779984|rs1879181|rs10519712|rs6494074
Malar Rash	APC_C_CDC20_MEDIATED_DEGRADATION_OF_MITOTIC_PROTEINS	REACTOME	1094	42	20	4,47E-01	9,17E-01	rs12292911|rs11248424|rs8063430|rs577357|rs7678743|rs7894765|rs7224730|rs4790706|rs17213812|rs9990802|rs2004845|rs11592827|rs2282032|rs17754530|rs6142963|rs11593744|rs9784758|rs6924102|rs2011713|rs473151
Malar Rash	ACH_PATHWAY	BIOCARTA	602	34	19	4,49E-01	9,17E-01	rs12292911|rs4976123|rs11766675|rs1636808|rs706725|rs42154|rs7708673|rs249479|rs7000615|rs7840147|rs17069632|rs6018027|rs4532674|rs7856889|rs6090585|rs13302218|rs4131890|rs859624|rs7804859
Malar Rash	SHH_PATHWAY	BIOCARTA	679	24	16	4,49E-01	9,17E-01	rs883541|rs737681|rs288746|rs7388704|rs11658925|rs2363923|rs357539|rs4849909|rs2835762|rs2286294|rs6709236|rs7213247|rs3801210|rs12972914|rs10243418|rs898695
Malar Rash	CDK_MEDIATED_PHOSPHORYLATION_AND_REMOVAL_OF_CDC6	REACTOME	698	26	15	4,50E-01	9,17E-01	rs12292911|rs8063430|rs577357|rs7224730|rs4790706|rs17213812|rs2282032|rs6142963|rs9784758|rs6924102|rs2011713|rs473151|rs328368|rs9380326|rs13394402
Malar Rash	REGULATION_OF_AUTOPHAGY	KEGG	933	29	20	4,53E-01	9,17E-01	rs11876701|rs974309|rs203150|rs26538|rs1554513|rs1539826|rs9954372|rs10878721|rs7504866|rs10889011|rs4132081|rs12493491|rs17794370|rs2156481|rs16831670|rs1453037|rs1348293|rs898783|rs1496814|rs12606897
Malar Rash	CELL_CELL_JUNCTION_ORGANIZATION	REACTOME	6211	34	20	4,56E-01	9,17E-01	rs12960187|rs13170578|rs491198|rs7705566|rs6065062|rs2628202|rs7202333|rs11644732|rs3114396|rs12716949|rs10076681|rs17468086|rs11739505|rs1593985|rs10520979|rs12866697|rs6875381|rs12925410|rs7736249|rs1541079
Malar Rash	DOWNSTREAM_TCR_SIGNALING	REACTOME	1573	41	20	4,59E-01	9,17E-01	rs4976123|rs10807113|rs1543478|rs1546745|rs9345044|rs2820350|rs2811650|rs11762164|rs11597105|rs4940757|rs2281390|rs9277052|rs531486|rs7640727|rs7922090|rs9277535|rs696|rs1878806|rs706725|rs2501657
Malar Rash	G1_S_TRANSITION	REACTOME	1941	32	20	4,59E-01	9,17E-01	rs12292911|rs2307389|rs8063430|rs4410871|rs12543549|rs3761873|rs2720662|rs577357|rs11664283|rs6577060|rs1781011|rs133416|rs11881878|rs7772351|rs7224730|rs9909163|rs4790706|rs837763|rs17213812|rs4130120
Malar Rash	RETINOL_METABOLISM	KEGG	1512	55	19	4,59E-01	9,17E-01	rs17270248|rs3125896|rs2241057|rs2602884|rs1841037|rs4846127|rs10493443|rs10515932|rs1925961|rs6722569|rs4926355|rs11188059|rs4148309|rs1154454|rs4147536|rs4646421|rs201823|rs2275620|rs11143438
Malar Rash	ACTIVATION_OF_ATR_IN_RESPONSE_TO_REPLICATION_STRESS	REACTOME	653	20	13	4,59E-01	9,17E-01	rs13213285|rs3761873|rs1781011|rs133416|rs7772351|rs9909163|rs10896172|rs1765853|rs10948689|rs2881964|rs6463725|rs540723|rs886727
Malar Rash	LYSOSOME_VESICLE_BIOGENESIS	REACTOME	420	11	7	4,62E-01	9,17E-01	rs17127601|rs9760|rs4804528|rs14976|rs17397283|rs4425006|rs4504735
Malar Rash	M_G1_TRANSITION	REACTOME	1315	34	20	4,62E-01	9,17E-01	rs12292911|rs2307389|rs8063430|rs3761873|rs6938550|rs577357|rs1781011|rs133416|rs7772351|rs7224730|rs9909163|rs4790706|rs837763|rs17213812|rs2282032|rs7747931|rs10948689|rs911361|rs5744750|rs6142963
Malar Rash	SYNTHESIS_OF_PA	REACTOME	1057	39	20	4,63E-01	9,17E-01	rs11582551|rs12542460|rs2701632|rs600415|rs9881788|rs4894512|rs2920126|rs11717683|rs11195813|rs2936517|rs10516696|rs360441|rs1487492|rs6680812|rs955835|rs6535510|rs836178|rs12564176|rs591393|rs6719376
Malar Rash	VIF_MEDIATED_DEGRADATION_OF_APOBEC3G	REACTOME	738	38	19	4,64E-01	9,17E-01	rs12292911|rs8063430|rs4550189|rs577357|rs7224730|rs4790706|rs17213812|rs6990813|rs7949655|rs2282032|rs6472810|rs6142963|rs9784876|rs6924102|rs2011713|rs473151|rs328368|rs9380326|rs13394402
Malar Rash	RAS_PATHWAY	BIOCARTA	557	43	20	4,71E-01	9,17E-01	rs4976123|rs11766675|rs1636808|rs9881788|rs4894512|rs7922090|rs9946939|rs2073925|rs706725|rs42154|rs7708673|rs7022583|rs10901235|rs360441|rs249479|rs4962095|rs12967882|rs4646018|rs4131890|rs7804859
Malar Rash	REGULATION_OF_MRNA_STABILITY_BY_PROTEINS_THAT_BIND_AU_RICH_ELEMENTS	REACTOME	1372	33	20	4,72E-01	9,17E-01	rs12292911|rs8063430|rs4885036|rs16898766|rs3108908|rs4734507|rs11642944|rs577357|rs3108913|rs4256810|rs7224730|rs4790706|rs17213812|rs6468757|rs4740419|rs3762885|rs1204494|rs737063|rs2282032|rs3019260
Malar Rash	OOCYTE_MEIOSIS	KEGG	3114	37	20	4,73E-01	9,17E-01	rs10956564|rs3799630|rs16904360|rs486992|rs601618|rs7303990|rs8023333|rs2684789|rs617621|rs6486914|rs4964005|rs815808|rs4734507|rs7972342|rs11098195|rs2684790|rs3108913|rs6792995|rs7678743|rs16899333
Malar Rash	NEUROACTIVE_LIGAND_RECEPTOR_INTERACTION	KEGG	13469	31	20	4,74E-01	9,17E-01	rs6579927|rs7445117|rs12523641|rs4629584|rs4517239|rs12215494|rs1394764|rs9809150|rs3740868|rs17451303|rs7623169|rs937210|rs9410438|rs2268464|rs13148043|rs1485149|rs6787255|rs237851|rs1235695|rs37373
Malar Rash	ACTIVATION_OF_THE_PRE_REPLICATIVE_COMPLEX	REACTOME	565	20	13	4,76E-01	9,17E-01	rs2307389|rs3761873|rs1781011|rs133416|rs7772351|rs9909163|rs837763|rs10948689|rs5744750|rs2881964|rs6463725|rs9475837|rs886727
Malar Rash	DESTABILIZATION_OF_MRNA_BY_AUF1_HNRNP_D0	REACTOME	811	32	19	4,76E-01	9,17E-01	rs12292911|rs8063430|rs16898766|rs3108908|rs577357|rs7224730|rs4790706|rs17213812|rs3762885|rs2282032|rs11968400|rs6142963|rs9784758|rs6924102|rs2011713|rs473151|rs328368|rs9380326|rs13394402
Malar Rash	THROMBIN_SIGNALLING_THROUGH_PROTEINASE_ACTIVATED_RECEPTORS_PARS	REACTOME	690	26	18	4,76E-01	9,17E-01	rs10937003|rs2294451|rs1688123|rs4898725|rs12147761|rs11621038|rs7141727|rs887030|rs6018027|rs2287208|rs4745639|rs7218921|rs3797390|rs153317|rs12985186|rs6090585|rs508208|rs2993998
Malar Rash	ANTIGEN_PROCESSING_CROSS_PRESENTATION	REACTOME	1601	53	20	4,77E-01	9,17E-01	rs12292911|rs8063430|rs885945|rs2517938|rs2857106|rs2416771|rs577357|rs7224730|rs2523809|rs4790706|rs5750326|rs17213812|rs441808|rs9259650|rs4852708|rs2282032|rs2853923|rs6142963|rs1527478|rs9784876
Malar Rash	MITOTIC_M_M_G1_PHASES	REACTOME	3176	32	20	4,78E-01	9,17E-01	rs12292911|rs11248424|rs2307389|rs8063430|rs9381786|rs617621|rs4267943|rs8076424|rs883444|rs6678961|rs3761873|rs6938550|rs577357|rs1781011|rs7678743|rs133416|rs7894765|rs35683|rs4758695|rs11071896
Malar Rash	GLUTATHIONE_CONJUGATION	REACTOME	407	19	9	4,79E-01	9,17E-01	rs6060124|rs451439|rs502862|rs9333372|rs4863683|rs673151|rs8191997|rs364932|rs4147594
Malar Rash	PACKAGING_OF_TELOMERE_ENDS	REACTOME	515	14	6	4,79E-01	9,17E-01	rs9461204|rs4096763|rs10247556|rs7776065|rs9358913|rs1316624
Malar Rash	MRNA_SPLICING	REACTOME	1057	34	20	4,80E-01	9,17E-01	rs2588882|rs2304232|rs11081583|rs10169262|rs12191724|rs4809388|rs2274913|rs10506328|rs4905510|rs7248208|rs13258200|rs6706797|rs571374|rs2027745|rs10783580|rs3762885|rs7982845|rs12473402|rs3816127|rs10859966
Malar Rash	HEPARAN_SULFATE_HEPARIN_HS_GAG_METABOLISM	REACTOME	3879	33	20	4,81E-01	9,17E-01	rs718356|rs6455394|rs2147155|rs4945522|rs11248060|rs3820088|rs2682185|rs731096|rs10955078|rs11224169|rs9584093|rs2245104|rs4787781|rs12602084|rs7984995|rs17774701|rs7926191|rs13010821|rs1328368|rs7966281
Malar Rash	REGULATION_OF_INSULIN_LIKE_GROWTH_FACTOR_IGF_ACTIVITY_BY_INSULIN_LIKE_G DING_PROTEINS_IGFBPS	REACTOME	570	12	10	4,82E-01	9,17E-01	rs7420849|rs1989775|rs4233167|rs1009728|rs11770622|rs7467853|rs3886934|rs10983127|rs2270360|rs6728171
Malar Rash	G1_S_SPECIFIC_TRANSCRIPTION	REACTOME	194	7	6	4,83E-01	9,17E-01	rs11664283|rs6577060|rs837763|rs380691|rs8110447|rs6741290
Malar Rash	PDGF_PATHWAY	BIOCARTA	1021	38	20	4,83E-01	9,17E-01	rs12409333|rs4976123|rs8004059|rs11766675|rs1636808|rs1873423|rs310245|rs706725|rs3024912|rs2721856|rs42154|rs7708673|rs10151713|rs737063|rs249479|rs7676985|rs432854|rs11648535|rs8013053|rs882167
Malar Rash	NUCLEAR_RECEPTOR_TRANSCRIPTION_PATHWAY	REACTOME	2750	42	20	4,86E-01	9,17E-01	rs1394764|rs9809150|rs11039149|rs7623169|rs6787255|rs486992|rs601618|rs9831450|rs4568061|rs6007640|rs350806|rs4774386|rs2576253|rs9844739|rs11572499|rs2029401|rs12101478|rs12471739|rs10988894|rs7936649
Malar Rash	SIGNALING_BY_RHO_GTPASES	REACTOME	4877	29	20	4,87E-01	9,17E-01	rs1882626|rs6991346|rs6789224|rs12195685|rs11101376|rs11493857|rs9371859|rs258760|rs1028154|rs10047541|rs16903375|rs7906475|rs13137847|rs258816|rs882653|rs252452|rs4953402|rs2882936|rs16990420|rs7611544
Malar Rash	CREB_PHOSPHORYLATION_THROUGH_THE_ACTIVATION_OF_RAS	REACTOME	1349	35	20	4,89E-01	9,17E-01	rs3799630|rs2098469|rs918168|rs815808|rs1868289|rs11098195|rs7298664|rs16899333|rs3799631|rs17830392|rs220593|rs16957102|rs17339754|rs4709117|rs12914792|rs7174521|rs17208905|rs571653|rs10845852|rs4904690
Malar Rash	NEGATIVE_REGULATION_OF_FGFR_SIGNALING	REACTOME	1536	25	20	4,91E-01	9,17E-01	rs3117915|rs6891913|rs11593080|rs17612068|rs9578440|rs17234079|rs10507911|rs3129601|rs3129596|rs13135439|rs11881878|rs11776272|rs1018726|rs4769189|rs477516|rs9531002|rs7989744|rs17004857|rs9634328|rs1501588
Malar Rash	BETA_ALANINE_METABOLISM	KEGG	711	54	17	4,92E-01	9,17E-01	rs7216|rs10973770|rs5760440|rs11587873|rs8049513|rs4451581|rs4646770|rs1760217|rs1273376|rs2333874|rs12119882|rs7045845|rs3750187|rs2811188|rs1038378|rs4878805|rs34961061
Malar Rash	MITOTIC_PROMETAPHASE	REACTOME	1814	36	20	4,93E-01	9,17E-01	rs11248424|rs9381786|rs617621|rs4267943|rs8076424|rs883444|rs6678961|rs7678743|rs7894765|rs35683|rs4758695|rs11071896|rs11881878|rs1520058|rs10402592|rs2099505|rs2068188|rs9990802|rs2055474|rs11837038
Malar Rash	UNFOLDED_PROTEIN_RESPONSE	REACTOME	1176	42	20	4,96E-01	9,17E-01	rs883541|rs394558|rs1025814|rs17042585|rs241179|rs11642944|rs1515372|rs17365609|rs3794648|rs11217128|rs2717233|rs9311513|rs1126700|rs17043569|rs1464103|rs4930351|rs9311542|rs9328415|rs1243872|rs6806768
Malar Rash	ARACHIDONIC_ACID_METABOLISM	KEGG	1356	37	20	4,99E-01	9,17E-01	rs11582551|rs4149244|rs2701632|rs4796573|rs4820944|rs10521140|rs1028997|rs6088638|rs12125747|rs800719|rs2301472|rs10492227|rs11188059|rs1487492|rs6680812|rs955835|rs12564176|rs3754206|rs591393|rs11593189
Malar Rash	PHOSPHATIDYLINOSITOL_SIGNALING_SYSTEM	KEGG	3813	27	20	5,01E-01	9,17E-01	rs4976123|rs8126112|rs11248060|rs1907108|rs9533044|rs7303990|rs11876701|rs9329570|rs4763508|rs974309|rs7257613|rs203150|rs6486914|rs4964005|rs4820944|rs815808|rs11766675|rs7972342|rs1636808|rs6140629
Malar Rash	REGULATION_OF_ACTIN_CYTOSKELETON	KEGG	7089	30	20	5,02E-01	9,17E-01	rs3117915|rs4976123|rs4947491|rs875078|rs16763|rs9306310|rs9333114|rs9371859|rs788338|rs4389291|rs6891913|rs11593080|rs17612068|rs1707488|rs11842475|rs5000880|rs2075751|rs2303162|rs10829597|rs891700
Malar Rash	IL1R_PATHWAY	BIOCARTA	1001	35	20	5,05E-01	9,17E-01	rs394558|rs4851541|rs1543478|rs9345044|rs2820350|rs2811650|rs2189756|rs1177614|rs7922090|rs696|rs2501657|rs887998|rs9342261|rs8109167|rs11870477|rs9611169|rs12215452|rs2205181|rs4422587|rs12632135
Malar Rash	SIGNALING_BY_ILS	REACTOME	2996	39	20	5,06E-01	9,17E-01	rs12409333|rs4976123|rs394558|rs4851541|rs11914132|rs507879|rs12244380|rs1546745|rs9345044|rs2820350|rs10276241|rs2811650|rs4734507|rs2189756|rs7073236|rs3108913|rs1177614|rs17489214|rs1152471|rs1571221
Malar Rash	TOLL_PATHWAY	BIOCARTA	1147	33	20	5,08E-01	9,17E-01	rs1543478|rs2737191|rs8004059|rs6007640|rs9345044|rs2820350|rs2811650|rs2189756|rs7843776|rs531486|rs7922090|rs12645085|rs696|rs6990813|rs2501657|rs2721856|rs2173578|rs10151713|rs9342261|rs11725282
Malar Rash	LEISHMANIA_INFECTION	KEGG	2265	30	20	5,09E-01	9,17E-01	rs12409333|rs7756516|rs9376268|rs1543478|rs2737191|rs8004059|rs9345044|rs2820350|rs2811650|rs2857106|rs9808753|rs154973|rs511278|rs2281390|rs10878721|rs1571221|rs9277052|rs531486|rs1873423|rs5750326
Malar Rash	APOPTOSIS	REACTOME	3298	34	20	5,09E-01	9,17E-01	rs12292911|rs8092360|rs2711008|rs8063430|rs9962572|rs7202333|rs4987721|rs11561771|rs4837820|rs15614|rs880026|rs577357|rs6577060|rs11597105|rs2498437|rs9506325|rs1538581|rs12537364|rs2156223|rs7224730
Malar Rash	G2_M_CHECKPOINTS	REACTOME	710	23	15	5,09E-01	9,17E-01	rs13213285|rs3761873|rs1781011|rs133416|rs3191402|rs7772351|rs9909163|rs10896172|rs1765853|rs1801516|rs10948689|rs2881964|rs6463725|rs540723|rs886727
Malar Rash	PRIMARY_IMMUNODEFICIENCY	KEGG	742	32	12	5,10E-01	9,17E-01	rs3767742|rs2075751|rs2857106|rs4812997|rs17213812|rs9603093|rs7607984|rs4480590|rs9784758|rs6924102|rs1932435|rs9612370
Malar Rash	CALCIUM_SIGNALING_PATHWAY	KEGG	9275	33	20	5,14E-01	9,17E-01	rs4605535|rs10956564|rs7445117|rs4947491|rs8126112|rs1860094|rs833141|rs12593288|rs16904360|rs709318|rs10153042|rs237851|rs7303990|rs17025772|rs1235695|rs7713886|rs7208308|rs42792|rs31684|rs7257613
Malar Rash	PANTOTHENATE_AND_COA_BIOSYNTHESIS	KEGG	611	43	17	5,15E-01	9,17E-01	rs241606|rs878291|rs6586198|rs5760440|rs11587873|rs1409184|rs4451581|rs9399004|rs1760217|rs17601580|rs2333874|rs12119882|rs3750187|rs2811188|rs3756975|rs12579934|rs34961061
Malar Rash	AGR_PATHWAY	BIOCARTA	1981	29	20	5,15E-01	9,17E-01	rs12292911|rs4947491|rs10183636|rs17752721|rs10749058|rs12480230|rs265371|rs7776830|rs15614|rs17172446|rs2327206|rs3019244|rs4947179|rs13204138|rs936216|rs4895658|rs6592724|rs17102138|rs17722300|rs1948098
Malar Rash	CA_DEPENDENT_EVENTS	REACTOME	1487	42	20	5,16E-01	9,17E-01	rs10956564|rs883541|rs833141|rs16904360|rs7257613|rs815808|rs12829100|rs11658925|rs72001|rs2099505|rs2239305|rs3934729|rs727432|rs4904690|rs11690832|rs737063|rs1487492|rs4736746|rs6680812|rs12329315
Malar Rash	FORMATION_OF_TRANSCRIPTION_COUPLED_NER_TC_NER_REPAIR_COMPLEX	REACTOME	389	3	2	5,17E-01	9,17E-01	rs2174270|rs3816127
Malar Rash	TRAF6_MEDIATED_NFKB_ACTIVATION	REACTOME	351	23	12	5,18E-01	9,17E-01	rs1543478|rs2256331|rs9506325|rs2829946|rs7922090|rs467778|rs696|rs2830012|rs2071277|rs468345|rs9550568|rs10483453
Malar Rash	AUTODEGRADATION_OF_THE_E3_UBIQUITIN_LIGASE_COP1	REACTOME	766	27	16	5,21E-01	9,17E-01	rs12292911|rs8063430|rs577357|rs7224730|rs4790706|rs17213812|rs2282032|rs1801516|rs6142963|rs9784758|rs6924102|rs2011713|rs473151|rs328368|rs9380326|rs13394402
Malar Rash	LIGAND_GATED_ION_CHANNEL_TRANSPORT	REACTOME	1051	27	20	5,25E-01	9,17E-01	rs12523641|rs7403139|rs3922855|rs897685|rs9920954|rs12653458|rs3923156|rs4906896|rs1426891|rs6879622|rs29849|rs2148174|rs7173587|rs17052610|rs4906888|rs963871|rs3098513|rs1593609|rs1863459|rs1542097
Malar Rash	NFKB_PATHWAY	BIOCARTA	870	31	20	5,27E-01	9,17E-01	rs4851541|rs1543478|rs2737191|rs9345044|rs2820350|rs2811650|rs7922090|rs1545092|rs696|rs2501657|rs887998|rs2155458|rs9342261|rs9611169|rs12215452|rs4422587|rs6938848|rs6920337|rs1418514|rs4895499
Malar Rash	GLYCINE_SERINE_AND_THREONINE_METABOLISM	KEGG	564	24	14	5,28E-01	9,17E-01	rs37373|rs2158839|rs9804151|rs10267797|rs522676|rs2027745|rs234742|rs2445887|rs2807912|rs6927188|rs6898640|rs663649|rs2282158|rs4498630
Malar Rash	CD28_DEPENDENT_PI3K_AKT_SIGNALING	REACTOME	572	30	11	5,29E-01	9,17E-01	rs4976123|rs6115830|rs706725|rs7648642|rs7708673|rs619864|rs249479|rs2332096|rs4131890|rs3792285|rs829179
Malar Rash	NICOTINATE_AND_NICOTINAMIDE_METABOLISM	KEGG	963	45	20	5,29E-01	9,17E-01	rs320654|rs10065491|rs10494561|rs1713460|rs1409184|rs2253612|rs784600|rs3213710|rs9444331|rs349587|rs1002171|rs9399004|rs10050461|rs9641363|rs17601580|rs12652118|rs249557|rs12502586|rs4703261|rs546435
Malar Rash	ARGININE_AND_PROLINE_METABOLISM	KEGG	991	47	20	5,29E-01	9,17E-01	rs7216|rs17184300|rs17060292|rs12610011|rs10973770|rs16851855|rs7911910|rs11914070|rs2298316|rs4646770|rs885063|rs389035|rs12570255|rs2608897|rs2291067|rs7045845|rs2494467|rs11188409|rs2275273|rs3782221
Malar Rash	VOLTAGE_GATED_POTASSIUM_CHANNELS	REACTOME	2491	25	20	5,30E-01	9,17E-01	rs1864773|rs2034007|rs10100027|rs12710651|rs9701252|rs6471070|rs2237878|rs1431651|rs6736615|rs4930013|rs9810320|rs7196274|rs1868750|rs12741900|rs891488|rs2283212|rs1528|rs4930128|rs151316|rs2673580
Malar Rash	RNA_POL_III_TRANSCRIPTION_INITIATION_FROM_TYPE_3_PROMOTER	REACTOME	501	25	9	5,32E-01	9,17E-01	rs4804833|rs1383245|rs12676965|rs4961396|rs10101153|rs2257082|rs2290424|rs9287078|rs3745386
Malar Rash	MTOR_SIGNALING_PATHWAY	KEGG	1290	37	20	5,34E-01	9,17E-01	rs3799630|rs4976123|rs968053|rs11766675|rs1636808|rs16899333|rs3799631|rs17028238|rs4709117|rs9908195|rs703559|rs706725|rs2165944|rs10889011|rs42154|rs7708673|rs17794370|rs3741436|rs249479|rs6918384
Malar Rash	INOSITOL_PHOSPHATE_METABOLISM	KEGG	2355	27	20	5,36E-01	9,17E-01	rs8126112|rs1907108|rs11876701|rs9329570|rs4763508|rs974309|rs203150|rs6486914|rs4820944|rs11766675|rs1636808|rs6140629|rs1539826|rs9954372|rs3775664|rs13045064|rs2270229|rs7048825|rs1497391|rs9959561
Malar Rash	GATA3_PATHWAY	BIOCARTA	898	40	20	5,38E-01	9,17E-01	rs883541|rs4811174|rs754505|rs1538512|rs11658925|rs12781427|rs1334549|rs11642615|rs2031557|rs12051063|rs2348182|rs8090312|rs2787148|rs2243290|rs17483588|rs1545157|rs7213247|rs11595087|rs4888027|rs4888034
Malar Rash	METABOLISM_OF_NON_CODING_RNA	REACTOME	611	33	19	5,41E-01	9,17E-01	rs9310419|rs5766404|rs987607|rs9322993|rs9615022|rs723553|rs4740419|rs535317|rs1264901|rs10859966|rs4274569|rs9297042|rs17574411|rs7284581|rs4759082|rs1602|rs5766536|rs7982860|rs1462531
Malar Rash	METABOLISM_OF_MRNA	REACTOME	3017	34	20	5,42E-01	9,17E-01	rs12292911|rs6764079|rs8063430|rs968053|rs2177836|rs4428160|rs13072044|rs2075751|rs4885036|rs16898766|rs3108908|rs4734507|rs10824618|rs11642944|rs581045|rs577357|rs3108913|rs4256810|rs12873548|rs4761281
Malar Rash	GCR_PATHWAY	BIOCARTA	736	40	20	5,42E-01	9,17E-01	rs4976123|rs815808|rs11766675|rs4695267|rs1636808|rs10066266|rs1042713|rs706725|rs13041735|rs42154|rs7708673|rs4904690|rs249479|rs4705280|rs1294506|rs968088|rs1693892|rs1058903|rs4131890|rs7804859
Malar Rash	P53_PATHWAY	BIOCARTA	347	15	11	5,43E-01	9,17E-01	rs8092360|rs4987721|rs8083946|rs8110447|rs1801516|rs977908|rs7394655|rs7315397|rs12458289|rs6567322|rs2236719

Malar Rash	MET_PATHWAY	BIOCARTA	1415	42	20	5,43E-01	9,17E-01	rs4976123|rs875078|rs10829597|rs8004059|rs10276241|rs12251654|rs11766675|rs1636808|rs3019244|rs472159|rs12707455|rs7909186|rs706725|rs1861932|rs6592724|rs42154|rs7708673|rs10151713|rs5745709|rs249479
Malar Rash	ARRHYTHMOGENIC_RIGHT_VENTRICULAR_CARDIOMYOPATHY_ARVC	KEGG	5871	38	20	5,43E-01	9,17E-01	rs4605535|rs12422236|rs1860094|rs17018319|rs9333114|rs12472674|rs2135732|rs926543|rs709318|rs17025772|rs17752721|rs42792|rs4272873|rs7257613|rs886898|rs10762129|rs9415814|rs2192724|rs265371|rs3774589
Malar Rash	NOS1_PATHWAY	BIOCARTA	1133	36	20	5,44E-01	9,17E-01	rs883541|rs2098469|rs918168|rs815808|rs1868289|rs7298664|rs220593|rs16957102|rs11658925|rs17339754|rs1873423|rs17208905|rs11650232|rs10845852|rs4904690|rs737063|rs1294506|rs6498758|rs432854|rs1548808
Malar Rash	MTA3_PATHWAY	BIOCARTA	333	12	9	5,46E-01	9,17E-01	rs7202333|rs10415156|rs11155820|rs13028484|rs12199102|rs12114565|rs8061932|rs9478265|rs851980
Malar Rash	TYPE_I_DIABETES_MELLITUS	KEGG	1975	49	20	5,47E-01	9,17E-01	rs10271690|rs10807113|rs885945|rs2517938|rs2857106|rs1380355|rs154973|rs2281390|rs10878721|rs2159469|rs9277052|rs2734973|rs154978|rs34111681|rs9277535|rs3758562|rs441808|rs9259650|rs28365995|rs7648642
Malar Rash	SIGNAL_TRANSDUCTION_BY_L1	REACTOME	1310	36	20	5,48E-01	9,17E-01	rs3799630|rs4947491|rs2804877|rs7776830|rs16899333|rs12452359|rs17172446|rs3799631|rs12436076|rs3019244|rs4709117|rs197770|rs4936186|rs6592724|rs1125432|rs4076950|rs12415228|rs6918384|rs1245138|rs9366043
Malar Rash	MRNA_3_END_PROCESSING	REACTOME	320	24	12	5,48E-01	9,17E-01	rs10169262|rs12191724|rs2274913|rs4905510|rs13258200|rs2027745|rs12473402|rs2000999|rs6017090|rs1602|rs2920706|rs7806
Malar Rash	IRON_UPTAKE_AND_TRANSPORT	REACTOME	733	36	19	5,49E-01	9,17E-01	rs563201|rs6709526|rs4435420|rs133416|rs13238736|rs17185574|rs760306|rs3814043|rs10897207|rs2884029|rs16861637|rs10497704|rs2542941|rs366193|rs12616710|rs3823494|rs9307048|rs2249572|rs7295552
Malar Rash	GLUCOSE_TRANSPORT	REACTOME	689	31	18	5,50E-01	9,17E-01	rs9310419|rs5766404|rs987607|rs1227938|rs9615022|rs1217128|rs723553|rs4740419|rs10823343|rs11684526|rs9297042|rs7284581|rs4759082|rs539822|rs2305198|rs5766536|rs7982860|rs1462531
Malar Rash	41BB_PATHWAY	BIOCARTA	341	15	8	5,52E-01	9,17E-01	rs1543478|rs10878721|rs7922090|rs1997703|rs696|rs2243290|rs11207303|rs10483453
Malar Rash	TRANSFERRIN_ENDOCYTOSIS_AND_RECYCLING	REACTOME	513	9	7	5,53E-01	9,17E-01	rs563201|rs13238736|rs3814043|rs366193|rs12616710|rs3823494|rs7295552
Malar Rash	ERBB_SIGNALING_PATHWAY	KEGG	4135	31	20	5,53E-01	9,17E-01	rs4976123|rs4947491|rs4435197|rs7183535|rs9410438|rs4410871|rs12543549|rs4804833|rs10749058|rs7257613|rs2888104|rs12480230|rs11766675|rs7776830|rs11098195|rs15614|rs1636808|rs2720662|rs11568993|rs17172446
Malar Rash	PEROXISOME	KEGG	2047	61	20	5,54E-01	9,17E-01	rs1348850|rs1038390|rs4149244|rs320654|rs10065491|rs969111|rs7260781|rs6487792|rs6447868|rs8049513|rs6133475|rs1603382|rs7248208|rs10493872|rs26181|rs349587|rs1891805|rs2172133|rs12741973|rs35412
Malar Rash	ANTIGEN_PROCESSING_AND_PRESENTATION	KEGG	1754	61	20	5,55E-01	9,17E-01	rs10807113|rs885945|rs2517938|rs2857106|rs154973|rs511278|rs2281390|rs9277052|rs2523809|rs154978|rs34111681|rs9277535|rs17213812|rs441808|rs9259650|rs28365995|rs9274407|rs9603093|rs12329315|rs2853923
Malar Rash	GLYCOSAMINOGLYCAN_METABOLISM	REACTOME	6096	31	20	5,56E-01	9,17E-01	rs2736862|rs718356|rs6455394|rs2147155|rs4945522|rs11248060|rs718780|rs4871195|rs3820088|rs2682185|rs731096|rs2128211|rs4965800|rs10955078|rs11224169|rs9584093|rs2975419|rs2128207|rs2245104|rs975037
Malar Rash	INTEGRIN_ALPHAIIB_BETA3_SIGNALING	REACTOME	779	25	20	5,56E-01	9,17E-01	rs4972394|rs6091153|rs7424885|rs12452359|rs17489214|rs472159|rs1993703|rs1861932|rs1760118|rs10497403|rs11063954|rs12465032|rs11015130|rs1243872|rs7840147|rs6018027|rs2113807|rs10120669|rs3750433|rs1760125
Malar Rash	TH1TH2_PATHWAY	BIOCARTA	535	31	18	5,57E-01	9,17E-01	rs9376268|rs12244380|rs7073236|rs4812997|rs9808753|rs10878721|rs3024685|rs1323653|rs6895787|rs2421049|rs791589|rs2243290|rs1327474|rs2332096|rs6869688|rs12999517|rs12722561|rs3792285
Malar Rash	ENDOCYTOSIS	KEGG	6484	32	20	5,57E-01	9,17E-01	rs12960187|rs3810367|rs11198846|rs4947491|rs2670873|rs11593080|rs885945|rs1532609|rs10197003|rs11914132|rs4833086|rs2684789|rs746987|rs12244380|rs7095121|rs10169262|rs2686749|rs7093673|rs2888104|rs16832740
Malar Rash	TCR_SIGNALING	REACTOME	2040	34	20	5,60E-01	9,17E-01	rs4976123|rs3767742|rs10807113|rs1543478|rs1546745|rs9345044|rs2820350|rs2811650|rs15614|rs1762164|rs1597105|rs4940757|rs3128918|rs3019244|rs9277052|rs531486|rs7640727|rs7922090|rs12034829|rs30142
Malar Rash	IMMUNOREGULATORY_INTERACTIONS_BETWEEN_A_LYMPHOID_AND_A_NON_LYMPHOI	REACTOME	1325	43	20	5,60E-01	9,17E-01	rs7202333|rs7643569|rs885945|rs2517938|rs11246067|rs4812997|rs2223286|rs2824164|rs2087863|rs2556|rs5011812|rs2523809|rs441808|rs9259650|rs208898|rs189947|rs7525461|rs3176860|rs2009345|rs11586136
Malar Rash	IL1_SIGNALING	REACTOME	1322	44	20	5,65E-01	9,17E-01	rs394558|rs4851541|rs1546745|rs9345044|rs2820350|rs2811650|rs2189756|rs1177614|rs1152471|rs531486|rs7922090|rs2501657|rs311636|rs2721856|rs887998|rs10483660|rs619864|rs9342261|rs387419|rs1870477
Malar Rash	ACTIVATED_NOTCH1_TRANSMITS_SIGNAL_TO_THE_NUCLEUS	REACTOME	719	38	17	5,65E-01	9,17E-01	rs17182398|rs10140583|rs7309125|rs1732803|rs8013402|rs910118|rs17590882|rs2423519|rs2871921|rs6750496|rs12473402|rs6040095|rs4275799|rs209948|rs1596518|rs12138787|rs12592197
Malar Rash	PARKINSONS_DISEASE	KEGG	2833	32	20	5,66E-01	9,17E-01	rs4854053|rs2711008|rs16863205|rs11203280|rs7793355|rs11561771|rs7248185|rs13415895|rs2802460|rs10878434|rs1015196|rs12669817|rs11171911|rs1124729|rs3121680|rs10255032|rs4975851|rs1545951|rs9606127|rs11678404
Malar Rash	TOLL_RECEPTOR_CASCADES	REACTOME	3778	29	20	5,66E-01	9,17E-01	rs3799630|rs8029326|rs394558|rs4618461|rs11876701|rs4804833|rs974309|rs1543478|rs2737191|rs2256331|rs203150|rs8004059|rs1546745|rs9345044|rs2820350|rs2811650|rs10223241|rs1554513|rs2189756|rs7183836
Malar Rash	PHASE_II_CONJUGATION	REACTOME	2059	37	20	5,67E-01	9,17E-01	rs6060124|rs11135284|rs7405412|rs13178865|rs451439|rs173792|rs502862|rs1841037|rs294588|rs7447133|rs7723455|rs9333372|rs296364|rs1354360|rs17360177|rs12188891|rs17359728|rs4868845|rs7499304|rs4583905
Malar Rash	NO1_PATHWAY	BIOCARTA	1482	38	20	5,67E-01	9,17E-01	rs3783260|rs883541|rs10183636|rs7329421|rs815808|rs10841414|rs10505864|rs5030102|rs10751211|rs9428653|rs11658925|rs3829387|rs907851|rs882953|rs1656966|rs7654599|rs10740406|rs17080484|rs4904690|rs1135029
Malar Rash	CCR3_PATHWAY	BIOCARTA	939	32	20	5,69E-01	9,17E-01	rs8126112|rs4683189|rs4763508|rs6140629|rs13045064|rs1873423|rs6133573|rs926505|rs6077420|rs13041735|rs2179440|rs737063|rs2745772|rs7840147|rs432854|rs10505819|rs2206423|rs11648535|rs6055562|rs7202459
Malar Rash	SYSTEMIC_LUPUS_ERYTHEMATOSUS	KEGG	2453	33	20	5,69E-01	9,17E-01	rs7756516|rs2098469|rs918168|rs9461204|rs4957473|rs1868289|rs2857106|rs10473234|rs313545|rs4812997|rs7298664|rs154973|rs2031749|rs511278|rs3128918|rs10878721|rs16927253|rs220593|rs16957102|rs17339754
Malar Rash	ION_TRANSPORT_BY_P_TYPE_ATPASES	REACTOME	1690	23	20	5,71E-01	9,17E-01	rs4941167|rs11149652|rs8052881|rs1023421|rs6067837|rs16831388|rs4906624|rs9551233|rs8107107|rs3742290|rs6577060|rs171406|rs1432791|rs13074509|rs1278769|rs4599383|rs7180035|rs6439260|rs1435835|rs10750114
Malar Rash	DOWNSTREAM_SIGNALING_EVENTS_OF_B_CELL_RECEPTOR_BCR	REACTOME	2127	26	20	5,72E-01	9,17E-01	rs12292911|rs2721051|rs8063430|rs1543478|rs604788|rs9345044|rs2820350|rs2811650|rs6115830|rs11762164|rs577357|rs4940757|rs7224730|rs2145811|rs4790706|rs1873423|rs7922090|rs17213812|rs696|rs1878806
Malar Rash	PYRIMIDINE_METABOLISM	KEGG	1900	31	20	5,74E-01	9,17E-01	rs5770775|rs7568378|rs1253795|rs1331191|rs5760440|rs11587873|rs1713460|rs6476904|rs1152991|rs11664283|rs784600|rs11125589|rs1689924|rs2377301|rs9444331|rs4819850|rs1265124|rs17770799|rs9639018|rs4451581
Malar Rash	RENIN_ANGIOTENSIN_SYSTEM	KEGG	467	20	12	5,74E-01	9,17E-01	rs9829238|rs878893|rs17387575|rs1718390|rs388915|rs12489516|rs6763840|rs316206|rs1456669|rs6783837|rs4459609|rs6772419
Malar Rash	CROSS_PRESENTATION_OF_SOLUBLE_EXOGENOUS_ANTIGENS_ENDOSOMES	REACTOME	713	29	17	5,75E-01	9,17E-01	rs12292911|rs8063430|rs577357|rs7224730|rs4790706|rs17213812|rs4852708|rs2282032|rs6142963|rs9784876|rs6924102|rs17006471|rs2011713|rs473151|rs328368|rs9380326|rs13394402
Malar Rash	CELL_JUNCTION_ORGANIZATION	REACTOME	6781	34	20	5,77E-01	9,17E-01	rs12960187|rs13170578|rs491198|rs7705566|rs6065062|rs2628204|rs7202333|rs1644732|rs3114396|rs12716949|rs10076681|rs17468086|rs11739505|rs1593985|rs10520979|rs12866697|rs6875381|rs12925410|rs7736249|rs1541079
Malar Rash	RAS_ACTIVATION_UOPN_CA2_INFUX_THROUGH_NMDA_RECEPTOR	REACTOME	1033	33	20	5,78E-01	9,17E-01	rs2098469|rs918168|rs815808|rs1868289|rs11098195|rs7298664|rs17830392|rs220593|rs16957102|rs17339754|rs12914792|rs174521|rs17208905|rs571653|rs10845852|rs4904690|rs11938486|rs248989|rs1294506|rs460524
Malar Rash	ETS_PATHWAY	BIOCARTA	682	33	20	5,82E-01	9,17E-01	rs7111926|rs8004059|rs7936180|rs12580394|rs756325|rs7130878|rs565536|rs11221390|rs535317|rs10151713|rs12421034|rs837493|rs7417586|rs1940363|rs6590328|rs882167|rs8013053|rs4886699|rs6940385|rs352092
Malar Rash	ACTIVATION_OF_GENES_BY_ATF4	REACTOME	316	17	8	5,83E-01	9,17E-01	rs4885036|rs11642944|rs3794648|rs1126700|rs12599053|rs17666978|rs1046909|rs17019442
Malar Rash	SCF_BETA_TRCP_MEDIATED_DEGRADATION_OF_EMI1	REACTOME	793	27	16	5,84E-01	9,17E-01	rs12292911|rs8063430|rs577357|rs7224730|rs4790706|rs17213812|rs2282032|rs621106|rs6142963|rs9784758|rs6924102|rs2011713|rs473151|rs328368|rs9380326|rs13394402
Malar Rash	MITOCHONDRIAL_PROTEIN_IMPORT	REACTOME	609	23	16	5,84E-01	9,17E-01	rs7559331|rs12647765|rs1171911|rs1545951|rs199847|rs7042509|rs8045887|rs11734660|rs2122013|rs11881243|rs2270104|rs6665064|rs9749801|rs3745386|rs6461674|rs973406
Malar Rash	PENTOSE_PHOSPHATE_PATHWAY	KEGG	616	26	14	5,85E-01	9,17E-01	rs10489612|rs6560821|rs6841545|rs855351|rs4908817|rs2987900|rs10794989|rs3738934|rs929959|rs11246311|rs335115|rs2388251|rs11251481|rs2388566
Malar Rash	INTESTINAL_IMMUNE_NETWORK_FOR_IGA_PRODUCTION	KEGG	1392	46	20	5,86E-01	9,17E-01	rs7756516|rs10197003|rs16832740|rs2857106|rs4812997|rs154973|rs2281390|rs1147960|rs9277052|rs11687599|rs10191360|rs154978|rs9277535|rs711801|rs3760386|rs12502051|rs17617515|rs7648642|rs1323653|rs9274407
Malar Rash	INTEGRIN_CELL_SURFACE_INTERACTIONS	REACTOME	2614	29	20	5,86E-01	9,17E-01	rs4972394|rs536173|rs9333114|rs7202333|rs17752721|rs1678979|rs12955222|rs6091153|rs265371|rs6722598|rs572614|rs2483675|rs7424885|rs12452359|rs8041354|rs17489214|rs472159|rs631428|rs1993703|rs10502347
Malar Rash	CARM_ER_PATHWAY	BIOCARTA	1617	49	20	5,86E-01	9,17E-01	rs13245206|rs4618461|rs10009448|rs3774921|rs713878|rs2051920|rs12580394|rs10223241|rs4526844|rs17181630|rs2174270|rs2823025|rs964035|rs10206786|rs6500552|rs187571|rs11155820|rs975731|rs1549926|rs12199102
Malar Rash	VEGF_SIGNALING_PATHWAY	KEGG	2111	37	20	5,87E-01	9,17E-01	rs11582551|rs4976123|rs4811174|rs2701632|rs754505|rs7257613|rs4820944|rs11766675|rs1636808|rs4256810|rs8139798|rs2239775|rs1873423|rs2241407|rs800719|rs2301472|rs706725|rs7654599|rs42154|rs8090312
Malar Rash	HUNTINGTONS_DISEASE	KEGG	4311	31	20	5,88E-01	9,17E-01	rs4854053|rs2711008|rs8126112|rs4722835|rs10009448|rs16863205|rs11203280|rs2098469|rs3774921|rs918168|rs177580|rs11561771|rs7248185|rs12587011|rs13415895|rs2802460|rs1015196|rs11171911|rs1124729|rs6503653
Malar Rash	REGULATION_OF_BETA_CELL_DEVELOPMENT	REACTOME	1288	34	20	5,88E-01	9,17E-01	rs2721051|rs9920517|rs6444767|rs1877209|rs2456526|rs4794756|rs17234079|rs2145811|rs6075965|rs12041297|rs4866891|rs4746864|rs4943768|rs17254382|rs6036189|rs7981045|rs7822101|rs10919814|rs4363819|rs2636914
Malar Rash	B_CELL_RECEPTOR_SIGNALING_PATHWAY	KEGG	2278	32	20	5,89E-01	9,17E-01	rs4976123|rs16763|rs4811174|rs1543478|rs2075751|rs754505|rs8004059|rs604788|rs11766675|rs2810493|rs11246067|rs11762164|rs1636808|rs3785568|rs17489214|rs8139798|rs4940757|rs345306|rs2239775|rs1993703
Malar Rash	PERK_REGULATED_GENE_EXPRESSION	REACTOME	334	17	8	5,90E-01	9,17E-01	rs1025814|rs11642944|rs3794648|rs1126700|rs12599053|rs17666978|rs1046909|rs17019442
Malar Rash	N_GLYCAN_ANTENNAE_ELONGATION_IN_THE_MEDIAL_TRANS_GOLGI	REACTOME	962	30	17	5,91E-01	9,17E-01	rs718780|rs12431858|rs4686823|rs2406250|rs11692586|rs10138087|rs1519304|rs6444186|rs10438933|rs7145108|rs2468279|rs1972988|rs1702956|rs764686|rs7233900|rs8015785|rs990729
Malar Rash	NON_SMALL_CELL_LUNG_CANCER	KEGG	2585	45	20	5,91E-01	9,17E-01	rs4976123|rs4947491|rs1868220|rs1992060|rs1286772|rs1057504|rs7257613|rs11766675|rs7776830|rs6938550|rs1636808|rs4074785|rs10510849|rs3217992|rs9856634|rs11568993|rs17172446|rs965033|rs1873423|rs731461
Malar Rash	ADHERENS_JUNCTION	KEGG	4520	30	20	5,91E-01	9,17E-01	rs4947491|rs266371|rs17018319|rs12472674|rs1695054|rs2135732|rs7202333|rs9955719|rs905476|rs2684789|rs9961435|rs6091153|rs10762129|rs9415814|rs17367018|rs12191724|rs9345044|rs2820350|rs6802243|rs36047858
Malar Rash	FAS_PATHWAY	BIOCARTA	980	35	20	5,92E-01	9,17E-01	rs10047541|rs1546745|rs9345044|rs2820350|rs2811650|rs15614|rs4807269|rs3019244|rs2501657|rs311636|rs2721856|rs6592724|rs2155458|rs9342261|rs13402616|rs3812062|rs12215452|rs4422587|rs8178108|rs6938848
Malar Rash	GLYCEROPHOSPHOLIPID_BIOSYNTHESIS	REACTOME	2483	30	20	5,92E-01	9,17E-01	rs11582551|rs7749480|rs13427229|rs12542460|rs2701632|rs600415|rs10494126|rs4820944|rs9881788|rs13039216|rs4894512|rs601788|rs549662|rs2577254|rs261926|rs642926|rs2920126|rs3847896|rs13245899|rs1663171
Malar Rash	JAK_STAT_SIGNALING_PATHWAY	KEGG	4346	32	20	5,93E-01	9,17E-01	rs12409333|rs4976123|rs735842|rs4410871|rs9678469|rs10173681|rs37373|rs12543549|rs9376268|rs11914132|rs12244380|rs1570908|rs11766675|rs17127601|rs12440889|rs10507911|rs2064193|rs1636808|rs7073236|rs2720662
Malar Rash	FATTY_ACID_METABOLISM	KEGG	776	53	19	5,94E-01	9,17E-01	rs7216|rs10973770|rs4550189|rs1893441|rs2602884|rs6447868|rs4646770|rs666105|rs1154454|rs6501847|rs4147536|rs7045845|rs13144344|rs1557502|rs2306619|rs4878805|rs4862417|rs7561624|rs2286963
Malar Rash	SEMAPHORIN_INTERACTIONS	REACTOME	2884	29	20	5,95E-01	9,17E-01	rs4679332|rs9306310|rs3767742|rs1384566|rs788338|rs2291333|rs2804877|rs418446|rs7781126|rs6695225|rs2137273|rs15614|rs10745681|rs6641888|rs8139798|rs166835|rs4457117|rs2276035|rs3119028|rs3019244
Malar Rash	PRE_NOTCH_TRANSCRIPTION_AND_TRANSLATION	REACTOME	835	34	20	5,97E-01	9,17E-01	rs1877209|rs2267644|rs1075617|rs4310569|rs669669|rs6938550|rs6577060|rs6500552|rs436388|rs1238|rs704859|rs4753191|rs7210702|rs7747931|rs4863683|rs911361|rs17287454|rs1076786|rs7394655|rs537756
Malar Rash	P53_INDEPENDENT_G1_S_DNA_DAMAGE_CHECKPOINT	REACTOME	729	28	17	5,98E-01	9,17E-01	rs12292911|rs8063430|rs577357|rs7224730|rs4790706|rs17213812|rs2282032|rs1801516|rs6142963|rs9784758|rs6924102|rs2011713|rs473151|rs540723|rs328368|rs9380326|rs13394402
Malar Rash	PAR1_PATHWAY	BIOCARTA	1941	34	20	5,98E-01	9,17E-01	rs4976123|rs8126112|rs2075751|rs9345044|rs2820350|rs4532638|rs2811650|rs7611544|rs11766675|rs6991392|rs1636808|rs6140629|rs2276035|rs13045064|rs1873423|rs6133573|rs926505|rs706725|rs2501657|rs6077420
Malar Rash	INSULIN_RECEPTOR_RECYCLING	REACTOME	492	10	8	6,00E-01	9,17E-01	rs563201|rs13238736|rs3814043|rs7254060|rs366193|rs12616710|rs3823494|rs7295552
Malar Rash	RESOLUTION_OF_AP_SITES_VIA_THE_MULTIPLE_NUCLEOTIDE_PATCH_REPLACEMENT_P	REACTOME	192	6	4	6,00E-01	9,17E-01	rs1713460|rs156631|rs10861152|rs12485319
Malar Rash	MYOSIN_PATHWAY	BIOCARTA	1253	30	20	6,01E-01	9,17E-01	rs8126112|rs7258844|rs12974044|rs4532638|rs7611544|rs6991392|rs6140629|rs2276035|rs13045064|rs1873423|rs6133573|rs926505|rs6077420|rs1254403|rs942648|rs2179440|rs820371|rs737063|rs1026892|rs2745772
Malar Rash	GLYOXYLATE_AND_DICARBOXYLATE_METABOLISM	KEGG	704	46	17	6,01E-01	9,17E-01	rs10758115|rs969111|rs7260781|rs407791|rs6133475|rs7874815|rs12524884|rs803422|rs2840246|rs2094703|rs10970969|rs1410703|rs10758063|rs1331249|rs10217534|rs1956545|rs2103866
Malar Rash	FCER1_PATHWAY	BIOCARTA	1564	38	20	6,02E-01	9,17E-01	rs4976123|rs4811174|rs4804833|rs754505|rs8004059|rs815808|rs11766675|rs15614|rs1636808|rs17489214|rs1993703|rs1873423|rs706725|rs2721856|rs1760118|rs42154|rs8090312|rs7708673|rs2427832|rs4904690
Malar Rash	ADHERENS_JUNCTIONS_INTERACTIONS	REACTOME	5496	32	20	6,04E-01	9,17E-01	rs13170578|rs491198|rs7705566|rs6065062|rs2628204|rs7202333|rs1644732|rs3114396|rs12716949|rs10076681|rs17468086|rs11739505|rs1593985|rs10520979|rs6875381|rs12925410|rs7736249|rs10062689|rs2963711|rs8046873
Malar Rash	LONG_TERM_DEPRESSION	KEGG	3926	32	20	6,05E-01	9,17E-01	rs11582551|rs8126112|rs937210|rs2701632|rs108656|rs901779|rs7303990|rs2684789|rs7257613|rs4958596|rs4964005|rs1688123|rs4820944|rs7972342|rs2684790|rs6140629|rs6792995|rs1871380|rs2684802|rs13045064
Malar Rash	PURINE_METABOLISM	KEGG	5442	35	20	6,06E-01	9,17E-01	rs10956564|rs10497482|rs833141|rs575669|rs2573088|rs16904360|rs12196394|rs4243390|rs1868220|rs901779|rs7568378|rs1057504|rs7875593|rs569647|rs221723|rs7027719|rs4804505|rs6467177|rs10882294|rs30571
Malar Rash	HOMOLOGOUS_RECOMBINATION	KEGG	730	41	19	6,07E-01	9,17E-01	rs10765682|rs2140741|rs12796488|rs1474383|rs4794136|rs9909163|rs499952|rs6464262|rs2244012|rs732466|rs7785299|rs16944894|rs2331648|rs7796764|rs4902538|rs861531|rs2881964|rs6463725|rs886727
Malar Rash	CYCLIN_E_ASSOCIATED_EVENTS_DURING_G1_S_TRANSITION_	REACTOME	1212	31	20	6,09E-01	9,17E-01	rs12292911|rs8063430|rs4410871|rs12543549|rs2720662|rs577357|rs7224730|rs4790706|rs17213812|rs1430120|rs1030102|rs8110447|rs2282032|rs3804440|rs4238844|rs34330|rs6142963|rs2547643|rs9784758|rs6924102
Malar Rash	MITOTIC_G1_G1_S_PHASES	REACTOME	2477	27	20	6,10E-01	9,17E-01	rs12292911|rs3213285|rs8063430|rs4410871|rs12543549|rs3761873|rs6938550|rs2720662|rs577357|rs4074785|rs3217992|rs11664283|rs6577060|rs1781011|rs133416|rs1881878|rs7772351|rs6813014|rs7224730|rs9909163
Malar Rash	WNT_PATHWAY	BIOCARTA	1363	28	20	6,10E-01	9,17E-01	rs4410871|rs12543549|rs9345044|rs2820350|rs36047858|rs2811650|rs9783503|rs880026|rs2720662|rs6559624|rs6500552|rs2133606|rs2163086|rs934170|rs11097974|rs2501657|rs1052015|rs4130120|rs6599127|rs1030102
Malar Rash	NGF_PATHWAY	BIOCARTA	566	31	17	6,11E-01	9,17E-01	rs4976123|rs8004059|rs11766675|rs1636808|rs706725|rs42154|rs7708673|rs10151713|rs4626883|rs249479|rs882167|rs8013053|rs7219|rs734194|rs4131890|rs11207303|rs7804859
Malar Rash	KERATAN_SULFATE_KERATIN_METABOLISM	REACTOME	737	28	20	6,12E-01	9,17E-01	rs2736862|rs718780|rs11038519|rs3772097|rs741901|rs7115683|rs820844|rs100912|rs10438933|rs10920616|rs13263183|rs11823140|rs895734|rs2922494|rs1972988|rs1702956|rs17730982|rs2666895|rs4736675|rs7233900
Malar Rash	BASE_EXCISION_REPAIR	REACTOME	209	6	4	6,13E-01	9,17E-01	rs1713460|rs156631|rs10861152|rs12485319
Malar Rash	DC_PATHWAY	BIOCARTA	573	19	11	6,14E-01	9,17E-01	rs2737191|rs4812997|rs10878721|rs2767326|rs522073|rs6895787|rs2421049|rs2243290|rs3021094|rs6869688|rs1418514
Malar Rash	SIGNALLING_TO_ERKS	REACTOME	794	31	20	6,14E-01	9,17E-01	rs13427229|rs9410438|rs7231412|rs4256810|rs10502802|rs472159|rs2241407|rs617460|rs2073925|rs7022583|rs10901235|rs4626883|rs4962095|rs6018027|rs7219|rs6090585|rs1041264|rs1476868|rs4723884|rs11137533
Malar Rash	PURINE_METABOLISM	REACTOME	581	34	20	6,14E-01	9,17E-01	rs7875593|rs7027719|rs6467177|rs1713460|rs1980741|rs784600|rs9444331|rs2269340|rs10936023|rs3123697|rs12912744|rs865774|rs10488735|rs7119498|rs3006009|rs3027238|rs2071017|rs151500|rs10932605|rs6985278
Malar Rash	CIRCADIAN_CLOCK	REACTOME	2075	34	20	6,15E-01	9,17E-01	rs12315175|rs4618461|rs1051374|rs10009448|rs3774921|rs1995243|rs6007640|rs10223241|rs4774386|rs6804202|rs6794849|rs12101478|rs1110261|rs1490054|rs1750304|rs12637399|rs1721775|rs6500552|rs187571|rs7176740
Malar Rash	3_UTR_MEDIATED_TRANSLATIONAL_REGULATION	REACTOME	1235	28	20	6,15E-01	9,17E-01	rs968053|rs2177836|rs2075751|rs16898766|rs1108908|rs10824618|rs12873548|rs11217128|rs17028238|rs12624027|rs9893674|rs11577033|rs16888536|rs226206|rs4777186|rs751716|rs7076791|rs3094303|rs2165944|rs12323544
Malar Rash	NOTCH_SIGNALING_PATHWAY	KEGG	1387	30	20	6,16E-01	9,17E-01	rs6444767|rs1152699|rs1877209|rs17182398|rs2267644|rs4310569|rs669669|rs12580394|rs1732803|rs8013402|rs910118|rs17590882|rs6500552|rs2423519|rs2871921|rs436388|rs7104859|rs2743275|rs4753191|rs837493
Malar Rash	RNA_POL_I_PROMOTER_OPENING	REACTOME	501	14	4	6,16E-01	9,17E-01	rs9461204|rs7776065|rs9358913|rs11652516
Malar Rash	ABC_FAMILY_PROTEINS_MEDIATED_TRANSPORT	REACTOME	1753	74	20	6,17E-01	9,17E-01	rs899494|rs1038390|rs12452482|rs17703233|rs4147837|rs9524864|rs4148384|rs3886119|rs10493872|rs225406|rs2172133|rs3749438|rs12869867|rs2235015|rs4148442|rs17822931|rs17031664|rs9922834|rs581244|rs1858941
Malar Rash	MYOGENESIS	REACTOME	1747	42	20	6,17E-01	9,17E-01	rs8029326|rs6065062|rs4618461|rs10934223|rs10223241|rs2189756|rs880026|rs7183836|rs1011415|rs11564394|rs1750304|rs12900903|rs7241357|rs10513883|rs8030728|rs9635539|rs187571|rs1547018|rs1115739|rs16943951
Malar Rash	SIGNALING_BY_HIPPO	REACTOME	627	22	14	6,19E-01	9,17E-01	rs11583605|rs9853621|rs11916830|rs2498437|rs1538581|rs6897282|rs16841638|rs11260657|rs7636249|rs1499395|rs17731641|rs707590|rs10895252|rs10077631
Malar Rash	TRYPTOPHAN_METABOLISM	KEGG	953	55	19	6,19E-01	9,17E-01	rs7216|rs10973770|rs4550189|rs1919510|rs2253612|rs2292537|rs1447200|rs4646770|rs149425|rs2447753|rs10247064|rs6593011|rs4646421|rs7045845|rs963660|rs1033510|rs1038378|rs6435060|rs4878805
Malar Rash	KERATAN_SULFATE_BIOSYNTHESIS	REACTOME	674	30	20	6,19E-01	9,17E-01	rs2736862|rs718780|rs11038519|rs3772097|rs741901|rs7115683|rs100912|rs10438933|rs10920616|rs13263183|rs11823140|rs895734|rs2922494|rs1972988|rs1702956|rs17730982|rs2666895|rs4736675|rs7233900|rs7014566
Malar Rash	PRE_NOTCH_PROCESSING_IN_GOLGI	REACTOME	691	33	18	6,21E-01	9,18E-01	rs7156012|rs2267644|rs17667217|rs2729107|rs436388|rs10150481|rs12432375|rs9323707|rs1162831|rs11624992|rs709918|rs962660|rs1777691|rs1702956|rs8010114|rs1024307|rs1959724|rs2039478
Malar Rash	DNA_REPLICATION	REACTOME	3548	32	20	6,25E-01	9,20E-01	rs12292911|rs11248424|rs2307389|rs8063430|rs9381786|rs617621|rs4267943|rs8076424|rs883444|rs6678961|rs3761873|rs6938550|rs577357|rs1781011|rs7678743|rs133416|rs7894765|rs35683|rs4758695|rs11071896
Malar Rash	ECM_PATHWAY	BIOCARTA	753	39	20	6,25E-01	9,20E-01	rs4976123|rs4837820|rs11766675|rs1636808|rs6580191|rs706725|rs42154|rs2302103|rs1254403|rs7708673|rs820371|rs249479|rs1243872|rs7840147|rs6018027|rs1051418|rs3750433|rs6090585|rs7046030|rs4131890
Malar Rash	HOST_INTERACTIONS_OF_HIV_FACTORS	REACTOME	2270	31	20	6,29E-01	9,22E-01	rs12292911|rs9310419|rs8063430|rs4550189|rs5766404|rs987607|rs15614|rs2523966|rs9615022|rs577357|rs1545951|rs3778709|rs2042237|rs8139591|rs7224730|rs723553|rs4790706|rs17213812|rs9259650|rs6990813
Malar Rash	LINOLEIC_ACID_METABOLISM	KEGG	716	36	20	6,29E-01	9,22E-01	rs11582551|rs2701632|rs4820944|rs12125747|rs800719|rs2301472|rs11188059|rs1487492|rs6680812|rs955835|rs12564176|rs3754206|rs591393|rs11593189|rs4821738|rs2275620|rs11677|rs4728329|rs818968|rs10912119
Malar Rash	PLATELET_ACTIVATION_SIGNALING_AND_AGGREGATION	REACTOME	6965	48	20	6,30E-01	9,22E-01	rs11720405|rs7637803|rs4972394|rs899494|rs4958487|rs4976123|rs11248060|rs9533044|rs7303990|rs714104|rs2256331|rs7257613|rs2294451|rs6091153|rs4964005|rs1688123|rs10276241|rs815808|rs11766675|rs4734507

Malar Rash	TRAFFICKING_OF_GLUR2_CONTAINING_AMPA_RECEPTORS	REACTOME	721	25	19	6,31E-01	9,22E-01	rs937210|rs7257613|rs4958596|rs132930|rs10895854|rs1873423|rs4076950|rs737063|rs4632855|rs432854|rs12187452|rs11648535|rs2926289|rs4234914|rs7202459|rs9896483|rs1476813|rs11645672|rs12522297
Malar Rash	ADP_SIGNALLING_THROUGH_P2RY1	REACTOME	863	30	20	6,33E-01	9,23E-01	rs11720405|rs2294451|rs1688123|rs4898725|rs12147761|rs11621038|rs7141727|rs887030|rs1487492|rs6680812|rs955835|rs6018027|rs2287208|rs4745639|rs10935907|rs12564176|rs591393|rs12985186|rs6090585|rs508208
Malar Rash	GLUTATHIONE_METABOLISM	KEGG	683	39	17	6,34E-01	9,23E-01	rs6060124|rs2111699|rs451439|rs10521140|rs502862|rs9333372|rs1265124|rs1980412|rs4863683|rs673151|rs7512076|rs6741290|rs8191997|rs364932|rs1405948|rs1015811|rs4147594
Malar Rash	PORPHYRIN_AND_CHLOROPHYLL_METABOLISM	KEGG	1163	37	14	6,36E-01	9,23E-01	rs473728|rs1841037|rs133416|rs17185574|rs760306|rs10897207|rs16861637|rs4148309|rs2647468|rs6502305|rs17608059|rs720712|rs173154|rs12961130
Malar Rash	BMAL1_CLOCK_NPAS2_ACTIVATES_CIRCADIAN_EXPRESSION	REACTOME	1728	33	20	6,37E-01	9,23E-01	rs12315175|rs10513747|rs10009448|rs3774921|rs1995243|rs6007640|rs4774386|rs6804202|rs6794849|rs12101478|rs1110261|rs1490054|rs12637399|rs7121775|rs6500552|rs7176740|rs555212|rs7307757|rs10778528|rs6060034
Malar Rash	SNARE_INTERACTIONS_IN_VESICULAR_TRANSPORT	KEGG	828	42	19	6,38E-01	9,23E-01	rs716169|rs4454410|rs12267930|rs6133869|rs1965155|rs10485738|rs4581170|rs10124366|rs14976|rs902829|rs2636177|rs8891|rs17808797|rs12369479|rs362560|rs17518345|rs11086652|rs362584|rs6015338
Malar Rash	NATURAL_KILLER_CELL_MEDIATED_CYTOTOXICITY	KEGG	3607	33	20	6,39E-01	9,23E-01	rs4976123|rs4435197|rs7183535|rs16763|rs4811174|rs9410438|rs9376268|rs754505|rs7257613|rs11766675|rs2810493|rs2523966|rs1636808|rs17489214|rs9808753|rs2523808|rs8139798|rs10878721|rs345306|rs3019244
Malar Rash	RNA_POL_II_TRANSCRIPTION_PRE_INITIATION_AND_PROMOTER_OPENING	REACTOME	383	4	3	6,39E-01	9,23E-01	rs2174270|rs7982845|rs3816127
Malar Rash	SYNTHESIS_OF_GLYCOSYLPHOSPHATIDYLINOSITOL_GPI	REACTOME	485	27	13	6,42E-01	9,23E-01	rs4953402|rs2137273|rs166835|rs8024211|rs1520459|rs4624112|rs17418632|rs3742875|rs7190878|rs10519132|rs10519147|rs281292|rs11070583
Malar Rash	GLUCONEOGENESIS	REACTOME	490	14	5	6,43E-01	9,23E-01	rs1474758|rs2298316|rs11084823|rs7119426|rs2987900
Malar Rash	CTNNB1_PHOSPHORYLATION_CASCADE	REACTOME	316	9	5	6,44E-01	9,23E-01	rs880026|rs11881878|rs6599127|rs9863665|rs10463643
Malar Rash	REGULATION_OF_ORNITHINE_DECARBOXYLASE_ODC	REACTOME	773	29	18	6,45E-01	9,23E-01	rs12292911|rs8063430|rs577357|rs7224730|rs4790706|rs17213812|rs7009612|rs2282032|rs1000156|rs6142963|rs9784876|rs6924102|rs1405948|rs2011713|rs473151|rs328368|rs9380326|rs13394402
Malar Rash	CHONDROITIN_SULFATE_BIOSYNTHESIS	REACTOME	846	36	17	6,47E-01	9,23E-01	rs4965800|rs2975419|rs7966281|rs6586829|rs1545970|rs12375275|rs1280632|rs4921662|rs17769571|rs11204038|rs17091198|rs4536299|rs7980528|rs17704048|rs12898809|rs8091366|rs4921664
Malar Rash	GLYCOSAMINOGLYCAN_DEGRADATION	KEGG	709	27	20	6,47E-01	9,23E-01	rs11248060|rs731096|rs12602084|rs9890916|rs6854611|rs6831788|rs9913031|rs11650346|rs12943534|rs820844|rs13240375|rs10521280|rs234687|rs6453423|rs755340|rs8070605|rs2269333|rs12451369|rs6584214|rs6878810
Malar Rash	AMI_PATHWAY	BIOCARTA	574	19	11	6,52E-01	9,26E-01	rs6722598|rs6755028|rs10187622|rs631428|rs9521642|rs555212|rs7322495|rs9555682|rs153317|rs9636346|rs1192201
Malar Rash	REGULATION_OF_MITOTIC_CELL_CYCLE	REACTOME	1318	41	20	6,53E-01	9,26E-01	rs12292911|rs11248424|rs8063430|rs577357|rs7678743|rs7894765|rs7224730|rs4790706|rs17213812|rs9990802|rs2004845|rs11592827|rs2282032|rs3804440|rs17754530|rs621106|rs6142963|rs11593744|rs9784876|rs6924102
Malar Rash	S_PHASE	REACTOME	2076	32	20	6,59E-01	9,26E-01	rs12292911|rs2307389|rs8063430|rs4410871|rs12543549|rs3761873|rs2720662|rs577357|rs1781011|rs133416|rs7772351|rs7224730|rs9909163|rs4790706|rs837763|rs17213812|rs156631|rs1765853|rs4130120|rs1030102
Malar Rash	RIP_MEDIATED_NFKB_ACTIVATION_VIA_DAI	REACTOME	328	29	16	6,61E-01	9,26E-01	rs1543478|rs2256331|rs9506325|rs2829946|rs7922090|rs467778|rs12645085|rs696|rs11725282|rs2830012|rs10027074|rs6920337|rs2071277|rs468345|rs9550568|rs10483453
Malar Rash	FC_EPSILON_RI_SIGNALING_PATHWAY	KEGG	2758	41	20	6,62E-01	9,26E-01	rs11582551|rs4976123|rs16763|rs2701632|rs4804833|rs714104|rs4820944|rs11766675|rs12619351|rs2810493|rs2189756|rs1636808|rs17489214|rs8139798|rs345306|rs2239775|rs13432276|rs1993703|rs1873423|rs800719
Malar Rash	PRE_NOTCH_EXPRESSION_AND_PROCESSING	REACTOME	1434	28	20	6,62E-01	9,26E-01	rs7156012|rs1877209|rs2267644|rs1075617|rs4310569|rs669669|rs6938550|rs17667217|rs6577060|rs6500552|rs3772097|rs436388|rs6682678|rs10150481|rs12432375|rs7104859|rs9323707|rs11628311|rs4753191|rs11624992
Malar Rash	AUTODEGRADATION_OF_CDH1_BY_CDH1_APC_C	REACTOME	857	29	17	6,64E-01	9,26E-01	rs12292911|rs8063430|rs577357|rs7224730|rs4790706|rs17213812|rs2004845|rs2282032|rs17754530|rs6142963|rs9784876|rs6924102|rs2011713|rs473151|rs328368|rs9380326|rs13394402
Malar Rash	FATTY_ACID_TRIACYLGLYCEROL_AND_KETONE_BODY_METABOLISM	REACTOME	5526	34	20	6,64E-01	9,26E-01	rs11737840|rs890839|rs17184300|rs9371859|rs12542460|rs10513747|rs10056811|rs10009448|rs4237723|rs9381786|rs3774921|rs2477569|rs1995243|rs11666324|rs4550189|rs4267943|rs6007640|rs7809503|rs6115830|rs4528168
Malar Rash	TPO_PATHWAY	BIOCARTA	814	31	20	6,65E-01	9,26E-01	rs4976123|rs8004059|rs11766675|rs1636808|rs1571221|rs1873423|rs706725|rs3024912|rs42154|rs7708673|rs10151713|rs737063|rs249479|rs432854|rs11648535|rs8013053|rs882167|rs7219|rs7202459|rs9896483
Malar Rash	RHO_PATHWAY	BIOCARTA	480	40	17	6,68E-01	9,26E-01	rs2075751|rs4837820|rs6580191|rs275414|rs7048825|rs2302103|rs1254403|rs727735|rs10838610|rs820371|rs1243872|rs6018027|rs10511418|rs3750433|rs6090585|rs7046030|rs13421586
Malar Rash	RELA_PATHWAY	BIOCARTA	208	12	9	6,71E-01	9,26E-01	rs1543478|rs6500552|rs7922090|rs696|rs2155458|rs6920337|rs11076786|rs129988|rs10483453
Malar Rash	PEPTIDE_CHAIN_ELONGATION	REACTOME	947	38	20	6,72E-01	9,26E-01	rs2177836|rs12974044|rs737204|rs10824618|rs12873548|rs11217128|rs12624027|rs9893674|rs11577033|rs226206|rs751716|rs4777186|rs7076791|rs3094303|rs12323544|rs6927367|rs701405|rs2278725|rs1720321|rs1720260
Malar Rash	PIP3_ACTIVATES_AKT_SIGNALING	REACTOME	587	21	14	6,73E-01	9,26E-01	rs2721051|rs6115830|rs2145811|rs7922090|rs4943768|rs17254382|rs7981045|rs12329315|rs17069632|rs4675672|rs34330|rs4646018|rs2248293|rs829179
Malar Rash	NKCELLS_PATHWAY	BIOCARTA	678	41	20	6,76E-01	9,26E-01	rs4976123|rs2523966|rs17489214|rs3019244|rs1993703|rs9259650|rs706725|rs1760118|rs6592724|rs7708673|rs2524005|rs10415535|rs249479|rs7000615|rs10120669|rs404046|rs1760125|rs10772294|rs4131890|rs10993754
Malar Rash	P53_DEPENDENT_G1_DNA_DAMAGE_RESPONSE	REACTOME	838	29	18	6,76E-01	9,26E-01	rs12292911|rs8063430|rs577357|rs7224730|rs4790706|rs17213812|rs8110447|rs2282032|rs1801516|rs34330|rs6142963|rs9784758|rs6924102|rs2011713|rs473151|rs328368|rs9380326|rs13394402
Malar Rash	RIG_I_MDA5_MEDIATED_INDUCTION_OF_IFN_ALPHA_BETA_PATHWAYS	REACTOME	1129	29	20	6,76E-01	9,26E-01	rs13116389|rs1543478|rs2256331|rs26538|rs2357973|rs9506325|rs12130290|rs270952|rs6500552|rs2829946|rs7922090|rs467778|rs1545092|rs696|rs2871398|rs2155458|rs10783580|rs4585264|rs197265|rs6802890
Malar Rash	VIBRIO_CHOLERAE_INFECTION	KEGG	1518	26	20	6,77E-01	9,26E-01	rs245169|rs563201|rs7257613|rs2237878|rs4930013|rs2416771|rs2283212|rs2498437|rs4930128|rs13238736|rs151316|rs1538581|rs3814043|rs2301647|rs1873423|rs2099505|rs2239305|rs13041735|rs737063|rs366193
Malar Rash	LATENT_INFECTION_OF_HOMO_SAPIENS_WITH_MYCOBACTERIUM_TUBERCULOSIS	REACTOME	589	11	9	6,78E-01	9,26E-01	rs563201|rs13238736|rs3814043|rs5750326|rs366193|rs12616710|rs3823494|rs7295552|rs3782221
Malar Rash	GLOBAL_GENOMIC_NER_GG_NER	REACTOME	823	13	9	6,79E-01	9,26E-01	rs11039149|rs2174270|rs9909163|rs156631|rs1765853|rs5744750|rs2881964|rs6463725|rs886727
Malar Rash	SYNTHESIS_OF_DNA	REACTOME	1545	30	20	6,79E-01	9,26E-01	rs12292911|rs2307389|rs8063430|rs3761873|rs577357|rs1781011|rs133416|rs7772351|rs7224730|rs9909163|rs4790706|rs837763|rs17213812|rs156631|rs1765853|rs2282032|rs10948689|rs11644122|rs5744750|rs34330
Malar Rash	CELL_ADHESION_MOLECULES_CAMS	KEGG	8813	37	20	6,79E-01	9,26E-01	rs10854450|rs7282326|rs491198|rs9984570|rs3767742|rs6065062|rs9333114|rs7756516|rs2826327|rs7202333|rs11644732|rs3114396|rs3820088|rs885945|rs725745|rs9955719|rs198095|rs10955078|rs2826409|rs12866697
Malar Rash	COMPLEMENT_AND_COAGULATION_CASCADES	KEGG	1742	34	20	6,81E-01	9,26E-01	rs2249349|rs11938564|rs4610335|rs4957473|rs3753394|rs761276|rs10473234|rs11003300|rs6755028|rs12588394|rs2031749|rs5030102|rs3782683|rs10187622|rs17611|rs1009448|rs6451566|rs555212|rs6906354|rs17824797
Malar Rash	CYTOKINE_SIGNALING_IN_IMMUNE_SYSTEM	REACTOME	6819	30	20	6,81E-01	9,26E-01	rs12409333|rs4976123|rs9310419|rs276984|rs394558|rs7756516|rs4851541|rs968053|rs37373|rs9376268|rs885945|rs4284952|rs13116389|rs11914132|rs5766404|rs507879|rs12244380|rs987607|rs6091153|rs1546745
Malar Rash	T_CELL_RECEPTOR_SIGNALING_PATHWAY	KEGG	3546	32	20	6,82E-01	9,26E-01	rs4976123|rs16763|rs3767742|rs4811174|rs9861063|rs4804833|rs1543478|rs754505|rs8004059|rs12480230|rs9345044|rs2820350|rs2811650|rs11766675|rs15614|rs2810493|rs11762164|rs1636808|rs11597105|rs2327206
Malar Rash	NEF_MEDIATES_DOWN_MODULATION_OF_CELL_SURFACE_RECEPTORS_BY_RECRUITINC<br>N_ADAPTERS	REACTOME	366	17	6	6,83E-01	9,26E-01	rs2523966|rs9259650|rs4076950|rs537497|rs2524005|rs404046
Malar Rash	ION_CHANNEL_TRANSPORT	REACTOME	2741	26	20	6,84E-01	9,26E-01	rs4941167|rs12523641|rs7403139|rs11149652|rs8052881|rs3922855|rs1023421|rs6067837|rs897685|rs9920954|rs16831388|rs4906624|rs9551233|rs8107107|rs3742290|rs6577060|rs12653458|rs3923156|rs171406|rs1432791
Malar Rash	GAP_JUNCTION	KEGG	4388	41	20	6,84E-01	9,26E-01	rs10956564|rs7445117|rs4947491|rs8126112|rs16904360|rs901779|rs7303990|rs7257613|rs4964005|rs7937641|rs1688123|rs251925|rs7776830|rs7972342|rs361557|rs6140629|rs6792995|rs11568993|rs17172446|rs4534729
Malar Rash	GLUCURONIDATION	REACTOME	656	21	4	6,85E-01	9,26E-01	rs1841037|rs4148309|rs1885277|rs2125312
Malar Rash	BCR_PATHWAY	BIOCARTA	1053	35	20	6,85E-01	9,26E-01	rs4811174|rs2075751|rs754505|rs8004059|rs815808|rs3785568|rs17489214|rs1993703|rs1873423|rs1760118|rs8090312|rs4904690|rs10151713|rs737063|rs10415535|rs1294506|rs432854|rs11648535|rs8013053|rs882167
Malar Rash	CLEAVAGE_OF_GROWING_TRANSCRIPT_IN_THE_TERMINATION_REGION_	REACTOME	435	32	17	6,87E-01	9,26E-01	rs10169262|rs12191724|rs2274913|rs4905510|rs13258200|rs2282763|rs2027745|rs17054866|rs12473402|rs11134939|rs10859966|rs17574411|rs2000999|rs6017090|rs1602|rs2920706|rs7806
Malar Rash	RIBOSOME	KEGG	1118	35	20	6,88E-01	9,26E-01	rs2177836|rs12974044|rs1669796|rs10824618|rs12873548|rs11217128|rs12624027|rs9893674|rs11577033|rs226206|rs360178|rs4777186|rs751716|rs7076791|rs3094303|rs2600814|rs17659488|rs12323544|rs8042411|rs6927367
Malar Rash	RNA_POL_I_RNA_POL_III_AND_MITOCHONDRIAL_TRANSCRIPTION	REACTOME	1830	34	20	6,88E-01	9,26E-01	rs7034140|rs10810136|rs4804833|rs9461204|rs2382444|rs1383245|rs2174270|rs4150115|rs12676965|rs13291088|rs3769769|rs4961396|rs303721|rs10101153|rs10756526|rs4533450|rs3011286|rs471088|rs1323348|rs10153801
Malar Rash	CCR5_PATHWAY	BIOCARTA	696	35	20	6,88E-01	9,26E-01	rs10197003|rs8004059|rs16832740|rs815808|rs1147960|rs11687599|rs1873423|rs10191360|rs4904690|rs10151713|rs737063|rs7000615|rs1294506|rs432854|rs11648535|rs8013053|rs882167|rs1693892|rs1058903|rs7594034
Malar Rash	INTRINSIC_PATHWAY	BIOCARTA	603	31	15	6,88E-01	9,26E-01	rs11938564|rs6722598|rs6755028|rs5030102|rs631428|rs9521642|rs7322495|rs1656966|rs9555682|rs925451|rs153317|rs822373|rs2729356|rs9636346|rs1192201
Malar Rash	WNT_SIGNALING_PATHWAY	KEGG	5611	34	20	6,90E-01	9,26E-01	rs4344329|rs8126112|rs10908780|rs266371|rs4811174|rs16950543|rs4410871|rs10513747|rs12543549|rs1152699|rs1995243|rs754505|rs7257613|rs7814396|rs9961435|rs9345044|rs2820350|rs36047858|rs2811650|rs12495039
Malar Rash	TASTE_TRANSDUCTION	KEGG	1100	41	20	6,90E-01	9,26E-01	rs10956564|rs833141|rs16904360|rs2294451|rs2495985|rs6067085|rs13041735|rs10491227|rs4577093|rs11690832|rs4736746|rs12680002|rs2963894|rs6488326|rs11261074|rs304797|rs4736520|rs4701849|rs6686865|rs3213627
Malar Rash	EFFECTS_OF_PIP2_HYDROLYSIS	REACTOME	1651	33	20	6,93E-01	9,28E-01	rs11248060|rs9533044|rs7303990|rs714104|rs4964005|rs7972342|rs12619351|rs6802450|rs11597105|rs4833241|rs767757|rs2344498|rs549662|rs13432276|rs872288|rs3819847|rs11898209|rs6803648|rs6467706|rs17168465
Malar Rash	EDG1_PATHWAY	BIOCARTA	1432	32	20	6,95E-01	9,29E-01	rs4976123|rs8126112|rs11766675|rs1636808|rs6140629|rs12452359|rs11689274|rs910632|rs13045064|rs1873423|rs6133573|rs926505|rs706725|rs6077420|rs1388452|rs42154|rs7708673|rs1491643|rs2179440|rs737063
Malar Rash	MATURITY_ONSET_DIABETES_OF_THE_YOUNG	KEGG	992	29	20	6,97E-01	9,29E-01	rs189800|rs6444767|rs13221415|rs2456526|rs4794756|rs1440971|rs6075965|rs12041297|rs4746864|rs6036189|rs7822101|rs10919814|rs4363819|rs2636914|rs922369|rs830786|rs12437920|rs12128213|rs11907005|rs7628926
Malar Rash	PLATELET_AGGREGATION_PLUG_FORMATION	REACTOME	942	25	20	6,98E-01	9,29E-01	rs4972394|rs6091153|rs7424885|rs12452359|rs17489214|rs472159|rs1993703|rs1861932|rs1760118|rs10497403|rs11063954|rs12465032|rs11015130|rs1243872|rs7840147|rs6018027|rs2113807|rs10120669|rs3750433|rs1760125
Malar Rash	VEGF_PATHWAY	BIOCARTA	883	29	20	7,00E-01	9,29E-01	rs4976123|rs11766675|rs1636808|rs6503653|rs3829387|rs1873423|rs706725|rs7654599|rs42154|rs7708673|rs17080484|rs1204494|rs737063|rs708030|rs249479|rs7840147|rs432854|rs4864949|rs11648535|rs943080
Malar Rash	FANCONI_ANEMIA_PATHWAY	REACTOME	461	20	14	7,01E-01	9,29E-01	rs12805507|rs7258303|rs2215849|rs1167998|rs7113416|rs6926662|rs1801516|rs6761469|rs7597339|rs11125778|rs6545694|rs10490113|rs10175975|rs6704661
Malar Rash	GLYCEROPHOSPHOLIPID_METABOLISM	KEGG	2549	30	20	7,02E-01	9,29E-01	rs11582551|rs7749480|rs13427229|rs11248060|rs2701632|rs9533044|rs4820944|rs6802450|rs7631698|rs4894512|rs261926|rs2920126|rs3847896|rs13245899|rs914832|rs800719|rs1663171|rs2301472|rs11717683|rs1880676
Malar Rash	CIRCADIAN_REPRESSION_OF_EXPRESSION_BY_REV_ERBA	REACTOME	1358	35	20	7,03E-01	9,29E-01	rs10513747|rs10009448|rs3774921|rs1995243|rs6007640|rs4774386|rs6804202|rs6794849|rs12101478|rs1490054|rs12637399|rs6500552|rs7176740|rs6060043|rs782926|rs1018503|rs10804867|rs7672552|rs17303530|rs1509247
Malar Rash	THYROID_CANCER	KEGG	1104	37	20	7,03E-01	9,29E-01	rs4410871|rs7202333|rs12543549|rs12261686|rs6719501|rs880026|rs2720662|rs2133606|rs11097974|rs4130120|rs6599127|rs1030102|rs1822913|rs12714137|rs8061932|rs9863665|rs2094405|rs12547643|rs17712901|rs7394655
Malar Rash	AMINOACYL_TRNA_BIOSYNTHESIS	KEGG	731	12	9	7,06E-01	9,31E-01	rs7744190|rs9369472|rs167772|rs10017010|rs12132918|rs9472250|rs2647468|rs12961130|rs12656366
Malar Rash	DIABETES_PATHWAYS	REACTOME	3395	30	20	7,09E-01	9,33E-01	rs883541|rs394558|rs10816540|rs649748|rs845869|rs10458234|rs2493047|rs4979219|rs1025814|rs6133869|rs6768086|rs7420849|rs4532481|rs17042585|rs1989775|rs241179|rs156005|rs11642944|rs6597054|rs1515372
Malar Rash	TRANSCRIPTIONAL_REGULATION_OF_WHITE_ADIPOCYTE_DIFFERENTIATION	REACTOME	2719	29	20	7,10E-01	9,33E-01	rs17184300|rs9573126|rs10513747|rs10816540|rs10009448|rs3774921|rs1983981|rs1995243|rs6007640|rs4979219|rs12580394|rs6496165|rs6804202|rs1703007|rs6794849|rs1861870|rs271399|rs9507713|rs9534965|rs13045110
Malar Rash	GROWTH_HORMONE_RECEPTOR_SIGNALING	REACTOME	773	38	19	7,14E-01	9,35E-01	rs37373|rs6091153|rs9588044|rs2064193|rs1033440|rs1571221|rs17590882|rs12428609|rs3024912|rs2655439|rs7981339|rs12473402|rs3020619|rs11674718|rs7319633|rs4969168|rs964818|rs7834615|rs1935005
Malar Rash	RORA_ACTIVATES_CIRCADIAN_EXPRESSION	REACTOME	1377	35	20	7,16E-01	9,35E-01	rs10513747|rs10009448|rs3774921|rs1995243|rs6007640|rs4774386|rs6804202|rs6794849|rs12101478|rs1490054|rs12637399|rs6500552|rs7176740|rs6060034|rs782926|rs1018503|rs10804867|rs7672552|rs17303530|rs1509247
Malar Rash	INTEGRATION_OF_PROVIRUS	REACTOME	122	1	1	7,17E-01	9,35E-01	rs9520758
Malar Rash	CYTOKINE_PATHWAY	BIOCARTA	491	13	8	7,19E-01	9,35E-01	rs2275913|rs10878721|rs12502051|rs6895787|rs2421049|rs2243290|rs3021094|rs6869688
Malar Rash	PROCESSING_OF_CAPPED_INTRONLESS_PRE_MRNA	REACTOME	274	25	12	7,20E-01	9,35E-01	rs10169262|rs4905510|rs13258200|rs2282763|rs17054866|rs12473402|rs11134939|rs10859966|rs17574411|rs1602|rs2920706|rs7806
Malar Rash	DRUG_METABOLISM_CYTOCHROME_P450	KEGG	1661	57	20	7,21E-01	9,35E-01	rs4924793|rs2111699|rs451439|rs2253612|rs2602884|rs7541160|rs1841037|rs9333372|rs11188059|rs12079736|rs4148309|rs1154454|rs4147536|rs4385902|rs4863683|rs673151|rs11593189|rs2275620|rs8191997|rs364932
Malar Rash	CREB_PATHWAY	BIOCARTA	969	36	20	7,23E-01	9,35E-01	rs883541|rs4976123|rs11098195|rs12436076|rs11658925|rs1873423|rs706725|rs13041735|rs7708673|rs737063|rs11938486|rs249479|rs12329315|rs432854|rs11648535|rs7213247|rs4675672|rs7219|rs7202459|rs9896483
Malar Rash	LEUKOCYTE_TRANSENDOTHELIAL_MIGRATION	KEGG	4793	30	20	7,24E-01	9,35E-01	rs4972394|rs4976123|rs16763|rs17018319|rs12472674|rs2135732|rs11644732|rs10197003|rs12866697|rs7257613|rs10762129|rs9415814|rs16832740|rs11766675|rs2810493|rs233557|rs880026|rs1636808|rs4819808|rs2685159
Malar Rash	DNA_REPAIR	REACTOME	2111	30	20	7,27E-01	9,35E-01	rs7068256|rs11039149|rs10765682|rs12805507|rs17782975|rs7258303|rs12796488|rs1713460|rs1990783|rs2174270|rs9909163|rs499952|rs2215849|rs1167998|rs10829620|rs2244012|rs156631|rs1765853|rs9520758|rs7113416
Malar Rash	INFLUENZA_LIFE_CYCLE	REACTOME	1671	31	20	7,27E-01	9,35E-01	rs9310419|rs7338209|rs2177836|rs12974044|rs5766404|rs987607|rs10824618|rs9615022|rs12873548|rs11217128|rs12624027|rs9893674|rs723553|rs11577033|rs226206|rs751716|rs4777186|rs7076791|rs3094303|rs4740419
Malar Rash	G_ALPHA_Z_SIGNALLING_EVENTS	REACTOME	2191	36	20	7,30E-01	9,35E-01	rs11720405|rs10956564|rs16904360|rs714104|rs7257613|rs2294451|rs12619351|rs4898725|rs12147761|rs11597105|rs767757|rs72001|rs13432276|rs1873423|rs11621038|rs2099505|rs2239305|rs3934729|rs727432|rs13041735
Malar Rash	BIOSYNTHESIS_OF_THE_N_GLYCAN_PRECURSOR_DOLICHOL_LIPID_LINKED_OLIGOSACC<br>RANSFER_TO_A_NASCENT_PROTEIN	REACTOME	355	5	5	7,30E-01	9,35E-01	rs3742290|rs1545970|rs8047337|rs4915899|rs16860002
Malar Rash	CTLA4_PATHWAY	BIOCARTA	662	31	12	7,31E-01	9,35E-01	rs4976123|rs30142|rs706725|rs7648642|rs7708673|rs249479|rs7607984|rs2332096|rs3804030|rs7219|rs4131890|rs3792285
Malar Rash	GSK3_PATHWAY	BIOCARTA	1055	35	20	7,31E-01	9,35E-01	rs4976123|rs2737191|rs880026|rs7843776|rs7738656|rs2133606|rs2163086|rs934170|rs11097974|rs6990813|rs706725|rs7708673|rs1052015|rs2173578|rs6599127|rs249479|rs9863665|rs2886612|rs1429519|rs1609566
Malar Rash	INNATE_IMMUNE_SYSTEM	REACTOME	6278	28	20	7,32E-01	9,35E-01	rs8092360|rs3799630|rs8029326|rs394558|rs2249349|rs4618461|rs11876701|rs4804833|rs13116389|rs974309|rs1543478|rs2737191|rs2256331|rs4987721|rs507879|rs203150|rs8004059|rs7107929|rs4610335|rs1546745
Malar Rash	SCFSKP2_MEDIATED_DEGRADATION_OF_P27_P21	REACTOME	813	30	19	7,33E-01	9,35E-01	rs12292911|rs8063430|rs577357|rs7224730|rs4790706|rs17213812|rs8110447|rs2282032|rs3804440|rs34330|rs6142963|rs9784758|rs6924102|rs2011713|rs473151|rs13160147|rs328368|rs9380326|rs13394402
Malar Rash	EIF4_PATHWAY	BIOCARTA	890	34	20	7,34E-01	9,35E-01	rs4976123|rs968053|rs16898766|rs3108908|rs17028238|rs1873423|rs706725|rs2165944|rs7708673|rs737063|rs249479|rs432854|rs11648535|rs11674718|rs7202459|rs9896483|rs1476813|rs11645672|rs4131890|rs2248293
Malar Rash	CXCR4_PATHWAY	BIOCARTA	1099	46	20	7,36E-01	9,35E-01	rs4976123|rs4763508|rs10197003|rs16832740|rs1147960|rs11687599|rs1873423|rs10191360|rs706725|rs7708673|rs737063|rs249479|rs7000615|rs7840147|rs432854|rs10505819|rs11648535|rs2886612|rs7594034|rs7202459
Malar Rash	GLUCOSE_METABOLISM	REACTOME	1554	46	20	7,36E-01	9,35E-01	rs756567|rs10489612|rs6560821|rs815808|rs4747994|rs6841545|rs855351|rs4747886|rs11881878|rs1474758|rs2298316|rs11084823|rs7119426|rs7650373|rs1769259|rs4904690|rs10794989|rs1294506|rs929959|rs1693892
Malar Rash	SPRY_PATHWAY	BIOCARTA	910	33	20	7,37E-01	9,35E-01	rs4947491|rs7776830|rs10507911|rs11568993|rs17172446|rs4912636|rs1018726|rs1036253|rs9531002|rs863712|rs17809737|rs6018027|rs1215447|rs7219|rs6090585|rs17238095|rs7317070|rs9318775|rs10488142|rs13244925
Malar Rash	HIV_LIFE_CYCLE	REACTOME	1529	30	20	7,38E-01	9,35E-01	rs9310419|rs10197003|rs5766404|rs987607|rs16832740|rs7815340|rs9615022|rs2174270|rs9606248|rs11687599|rs13258200|rs723553|rs17687453|rs10191360|rs9359790|rs6990813|rs522073|rs156631|rs4740419|rs2460386
Malar Rash	TRAF6_MEDIATED_IRF7_ACTIVATION	REACTOME	433	16	13	7,39E-01	9,35E-01	rs13116389|rs2357973|rs12130290|rs270952|rs6500552|rs2871398|rs197265|rs17488241|rs11076786|rs12470772|rs129988|rs10164859|rs2241500
Malar Rash	GPCR_PATHWAY	BIOCARTA	1102	36	20	7,40E-01	9,35E-01	rs883541|rs4811174|rs754505|rs8004059|rs815808|rs11658925|rs1873423|rs13041735|rs8090312|rs4904690|rs10151713|rs737063|rs12329315|rs1294506|rs432854|rs11648535|rs7213247|rs4675672|rs8013053|rs882167
Malar Rash	TNFR2_PATHWAY	BIOCARTA	414	24	14	7,40E-01	9,35E-01	rs1543478|rs2357973|rs13299328|rs270952|rs7922090|rs1545092|rs696|rs197265|rs6920337|rs12470772|rs4895499|rs10164859|rs10483453|rs2275635
Malar Rash	NO2IL12_PATHWAY	BIOCARTA	461	12	8	7,41E-01	9,35E-01	rs10878721|rs1571221|rs2767326|rs3024912|rs6895787|rs2421049|rs6869688|rs7574865
Malar Rash	BETA_DEFENSINS	REACTOME	333	15	6	7,42E-01	9,35E-01	rs7107929|rs6074578|rs4513489|rs13260325|rs1324528|rs1555679
Malar Rash	EPO_PATHWAY	BIOCARTA	255	11	7	7,45E-01	9,35E-01	rs8004059|rs1571221|rs10151713|rs882167|rs8013053|rs7219|rs11207303
Malar Rash	PTDINS_PATHWAY	BIOCARTA	1058	39	20	7,47E-01	9,35E-01	rs16763|rs6560821|rs714104|rs17181086|rs12619351|rs2810493|rs910118|rs13432276|rs2423519|rs872288|rs11898209|rs10777456|rs10794989|rs679106|rs6040095|rs11125038|rs209948|rs17033965|rs2388251|rs11251481

<table>
<tr><td></td><td></td><td></td><td></td><td></td><td></td><td></td><td></td><td></td></tr>
<tr><td>Malar Rash</td><td>PI3K_EVENTS_IN_ERBB2_SIGNALING</td><td>REACTOME</td><td>1886</td><td>40</td><td>20</td><td>7,48E-01</td><td>9,35E-01</td><td>rs4976123|rs4947491|rs2721051|rs2888104|rs6115830|rs7776830|rs11568993|rs17172446|rs1499063|rs2145811|rs7922090|rs1851176|rs706725|rs7708673|rs191180|rs4943768|rs17254382|rs7981045|rs1948098|rs16846140</td></tr>
<tr><td>Malar Rash</td><td>MEIOTIC_RECOMBINATION</td><td>REACTOME</td><td>993</td><td>37</td><td>17</td><td>7,49E-01</td><td>9,35E-01</td><td>rs10765682|rs9461204|rs12796488|rs9909163|rs499952|rs2244012|rs1366452|rs1801516|rs16944894|rs7776065|rs2055526|rs6870239|rs9358913|rs2881964|rs6463725|rs6535914|rs886727</td></tr>
<tr><td>Malar Rash</td><td>POST_TRANSLATIONAL_MODIFICATION_SYNTHESIS_OF_GPI_ANCHORED_PROTEINS</td><td>REACTOME</td><td>586</td><td>33</td><td>15</td><td>7,50E-01</td><td>9,35E-01</td><td>rs4953402|rs2137273|rs166835|rs8024211|rs1520459|rs4624112|rs17418632|rs7524360|rs3742875|rs7190878|rs10519132|rs10519147|rs281292|rs199714|rs11070583</td></tr>
<tr><td>Malar Rash</td><td>INSULIN_PATHWAY</td><td>BIOCARTA</td><td>725</td><td>32</td><td>18</td><td>7,50E-01</td><td>9,35E-01</td><td>rs4976123|rs8004059|rs11766675|rs1636808|rs706725|rs42154|rs7708673|rs10151713|rs7254060|rs249479|rs11674718|rs882167|rs8013053|rs7219|rs4131890|rs11207303|rs964818|rs7804859</td></tr>
<tr><td>Malar Rash</td><td>ACTIVATED_AMPK_STIMULATES_FATTY_ACID_OXIDATION_IN_MUSCLE</td><td>REACTOME</td><td>421</td><td>28</td><td>15</td><td>7,51E-01</td><td>9,35E-01</td><td>rs942689|rs7809503|rs4528168|rs6965771|rs10889011|rs2268403|rs4425665|rs6678588|rs6490267|rs13144344|rs1557502|rs2268389|rs4595037|rs881109|rs4862417</td></tr>
<tr><td>Malar Rash</td><td>VASOPRESSIN_REGULATED_WATER_REABSORPTION</td><td>KEGG</td><td>1100</td><td>41</td><td>20</td><td>7,51E-01</td><td>9,35E-01</td><td>rs4722835|rs10047541|rs177580|rs17181086|rs10249566|rs1435421|rs1481961|rs2099505|rs2237362|rs17612687|rs2239305|rs13041735|rs757980|rs7796539|rs1554160|rs6976396|rs12329315|rs596015|rs4675672|rs2938234</td></tr>
<tr><td>Malar Rash</td><td>CELLCYCLE_PATHWAY</td><td>BIOCARTA</td><td>459</td><td>18</td><td>10</td><td>7,55E-01</td><td>9,37E-01</td><td>rs4074785|rs3217992|rs6577060|rs10273030|rs1547705|rs8110447|rs2041731|rs34330|rs7394655|rs3217906</td></tr>
<tr><td>Malar Rash</td><td>PPARA_ACTIVATES_GENE_EXPRESSION</td><td>REACTOME</td><td>3675</td><td>32</td><td>20</td><td>7,56E-01</td><td>9,37E-01</td><td>rs890839|rs17184300|rs9371859|rs10513747|rs10056811|rs10009448|rs4237723|rs3774921|rs1995243|rs11666324|rs6007640|rs6115830|rs2882936|rs4774386|rs6804202|rs7744364|rs6794849|rs11916830|rs12101478|rs1861870</td></tr>
<tr><td>Malar Rash</td><td>TIE2_SIGNALING</td><td>REACTOME</td><td>790</td><td>32</td><td>16</td><td>7,56E-01</td><td>9,37E-01</td><td>rs4976123|rs9297397|rs17553089|rs2440445|rs524765|rs706725|rs7708673|rs249479|rs939643|rs10505096|rs7042119|rs7219|rs10497250|rs10955457|rs4131890|rs2118254</td></tr>
<tr><td>Malar Rash</td><td>TCR_PATHWAY</td><td>BIOCARTA</td><td>1517</td><td>39</td><td>20</td><td>7,62E-01</td><td>9,43E-01</td><td>rs4976123|rs4811174|rs1543478|rs754505|rs8004059|rs815808|rs11766675|rs1636808|rs1873423|rs696|rs706725|rs2721856|rs42154|rs8090312|rs7708673|rs4904690|rs10151713|rs737063|rs10415535|rs249479</td></tr>
<tr><td>Malar Rash</td><td>REGULATORY_RNA_PATHWAYS</td><td>REACTOME</td><td>345</td><td>16</td><td>10</td><td>7,64E-01</td><td>9,44E-01</td><td>rs1075617|rs8139591|rs2161006|rs9292427|rs7210702|rs3816127|rs8012576|rs8015314|rs4905265|rs2257082</td></tr>
<tr><td>Malar Rash</td><td>LATE_PHASE_OF_HIV_LIFE_CYCLE</td><td>REACTOME</td><td>1259</td><td>36</td><td>20</td><td>7,65E-01</td><td>9,44E-01</td><td>rs9310419|rs5766404|rs987607|rs7815340|rs9615022|rs2174270|rs9606248|rs13258200|rs723553|rs17687453|rs9359790|rs6990813|rs522073|rs4740419|rs2460386|rs7982845|rs7228520|rs3816127|rs2756382|rs4274569</td></tr>
<tr><td>Malar Rash</td><td>THE_ROLE_OF_NEF_IN_HIV1_REPLICATION_AND_DISEASE_PATHOGENESIS</td><td>REACTOME</td><td>936</td><td>32</td><td>11</td><td>7,74E-01</td><td>9,52E-01</td><td>rs15614|rs2523966|rs3778709|rs2042237|rs9259650|rs4076950|rs537497|rs2524005|rs17329410|rs1981739|rs404046</td></tr>
<tr><td>Malar Rash</td><td>DARPP_32_EVENTS</td><td>REACTOME</td><td>879</td><td>41</td><td>20</td><td>7,75E-01</td><td>9,52E-01</td><td>rs883541|rs815808|rs16889276|rs11881878|rs4557937|rs11658925|rs10896172|rs10514870|rs4904690|rs16890418|rs1294506|rs1961|rs7213247|rs1693892|rs1058903|rs12653662|rs17451451|rs6863462|rs2180335|rs2659504</td></tr>
<tr><td>Malar Rash</td><td>INTRINSIC_PATHWAY</td><td>REACTOME</td><td>264</td><td>18</td><td>10</td><td>7,76E-01</td><td>9,52E-01</td><td>rs11938564|rs5030102|rs1656966|rs11063954|rs4468370|rs925451|rs822373|rs917859|rs2729356|rs584337</td></tr>
<tr><td>Malar Rash</td><td>ENOS_ACTIVATION_AND_REGULATION</td><td>REACTOME</td><td>336</td><td>21</td><td>11</td><td>7,77E-01</td><td>9,52E-01</td><td>rs815808|rs4804528|rs6749871|rs380691|rs4904690|rs1294506|rs873553|rs4425006|rs1693892|rs1058903|rs2182856</td></tr>
<tr><td>Malar Rash</td><td>METABOLISM_OF_XENOBIOTICS_BY_CYTOCHROME_P450</td><td>KEGG</td><td>1674</td><td>50</td><td>20</td><td>7,80E-01</td><td>9,55E-01</td><td>rs4924793|rs2111699|rs451439|rs2602884|rs1841037|rs9333372|rs338585|rs11253048|rs11188059|rs149425|rs4148309|rs1154454|rs2447753|rs4147536|rs4385902|rs4863683|rs673151|rs4646421|rs3738042|rs11593189</td></tr>
<tr><td>Malar Rash</td><td>NITROGEN_METABOLISM</td><td>KEGG</td><td>587</td><td>24</td><td>19</td><td>7,81E-01</td><td>9,55E-01</td><td>rs3829305|rs6471849|rs9804151|rs11863558|rs359535|rs7842871|rs10492227|rs2807912|rs389035|rs1243872|rs2494467|rs663649|rs12926841|rs3750433|rs4501567|rs12708003|rs12138787|rs2071675|rs8053821</td></tr>
<tr><td>Malar Rash</td><td>REGULATION_OF_APOPTOSIS</td><td>REACTOME</td><td>1432</td><td>28</td><td>20</td><td>7,91E-01</td><td>9,58E-01</td><td>rs12292911|rs8063430|rs9962572|rs15614|rs577357|rs7224730|rs4790706|rs11639281|rs17213812|rs9955796|rs3128519|rs13283404|rs2282032|rs1442088|rs4400714|rs1373179|rs3862678|rs10426955|rs6142963|rs539822</td></tr>
<tr><td>Malar Rash</td><td>PPAR_SIGNALING_PATHWAY</td><td>KEGG</td><td>1324</td><td>25</td><td>20</td><td>7,91E-01</td><td>9,58E-01</td><td>rs890839|rs11039149|rs11190483|rs11666324|rs6007640|rs6447868|rs1474758|rs17580455|rs17808107|rs10937273|rs4879696|rs8023357|rs2102113|rs9658119|rs6501847|rs7895441|rs1980360|rs13144344|rs1527478|rs1557502</td></tr>
<tr><td>Malar Rash</td><td>FORMATION_OF_THE_TERNARY_COMPLEX_AND_SUBSEQUENTLY_THE_43S_COMPLEX</td><td>REACTOME</td><td>677</td><td>34</td><td>20</td><td>7,94E-01</td><td>9,58E-01</td><td>rs2177836|rs2075751|rs10824618|rs11217128|rs16888536|rs226206|rs751716|rs7076791|rs3094303|rs12323544|rs5756219|rs701405|rs2175376|rs1720321|rs1720260|rs10129208|rs4803512|rs13278681|rs4583476|rs2352901</td></tr>
<tr><td>Malar Rash</td><td>PROTEIN_EXPORT</td><td>KEGG</td><td>750</td><td>21</td><td>12</td><td>7,95E-01</td><td>9,58E-01</td><td>rs7140697|rs12705686|rs2416771|rs799617|rs10871764|rs7809411|rs17157477|rs1377834|rs7779264|rs981446|rs1525677|rs37642</td></tr>
<tr><td>Malar Rash</td><td>NONSENSE_MEDIATED_DECAY_ENHANCED_BY_THE_EXON_JUNCTION_COMPLEX</td><td>REACTOME</td><td>1261</td><td>35</td><td>20</td><td>7,96E-01</td><td>9,58E-01</td><td>rs2177836|rs12974044|rs16898766|rs3108908|rs10824618|rs12873548|rs11217128|rs12624027|rs11881878|rs9893674|rs11577033|rs2226099|rs226206|rs751716|rs4777186|rs7076791|rs3094303|rs12323544|rs6927367|rs701405</td></tr>
<tr><td>Malar Rash</td><td>SIGNALING_BY_ROBO_RECEPTOR</td><td>REACTOME</td><td>1900</td><td>25</td><td>20</td><td>8,01E-01</td><td>9,58E-01</td><td>rs1028154|rs13061481|rs6769992|rs12480230|rs15614|rs13010821|rs9309825|rs9875468|rs2327206|rs6681804|rs2292832|rs3019244|rs6779154|rs12130290|rs11718266|rs12034829|rs3131660|rs9477401|rs936216|rs6592724</td></tr>
<tr><td>Malar Rash</td><td>ADIPOCYTOKINE_SIGNALING_PATHWAY</td><td>KEGG</td><td>2106</td><td>33</td><td>20</td><td>8,01E-01</td><td>9,58E-01</td><td>rs198311|rs10009448|rs3774921|rs942689|rs1543478|rs6007640|rs7809503|rs4528168|rs17127601|rs9588044|rs11597105|rs1571221|rs6965771|rs16192|rs17316431|rs1474758|rs12428609|rs7922090|rs696|rs10889011</td></tr>
<tr><td>Malar Rash</td><td>FORMATION_OF_THE_HIV1_EARLY_ELONGATION_COMPLEX</td><td>REACTOME</td><td>329</td><td>9</td><td>6</td><td>8,01E-01</td><td>9,58E-01</td><td>rs2174270|rs2460386|rs7982845|rs7228520|rs3816127|rs1602</td></tr>
<tr><td>Malar Rash</td><td>OXIDATIVE_PHOSPHORYLATION</td><td>KEGG</td><td>2486</td><td>39</td><td>20</td><td>8,02E-01</td><td>9,58E-01</td><td>rs4854053|rs16863205|rs11203280|rs563201|rs7248185|rs13415895|rs2802460|rs1015196|rs11171911|rs1124729|rs6577060|rs4975851|rs13238736|rs11678404|rs4804847|rs3814043|rs2301647|rs4361043|rs741448|rs11749320</td></tr>
<tr><td>Malar Rash</td><td>PI3K_AKT_ACTIVATION</td><td>REACTOME</td><td>1161</td><td>37</td><td>20</td><td>8,02E-01</td><td>9,58E-01</td><td>rs4976123|rs2721051|rs6115830|rs9588044|rs2145811|rs12428609|rs7922090|rs706725|rs7708673|rs7981339|rs4943768|rs17254382|rs7981045|rs4626883|rs249479|rs12329315|rs17069632|rs11674718|rs4675672|rs34330</td></tr>
<tr><td>Malar Rash</td><td>MUSCLE_CONTRACTION</td><td>REACTOME</td><td>1023</td><td>34</td><td>20</td><td>8,03E-01</td><td>9,58E-01</td><td>rs201603|rs178637|rs815808|rs11202900|rs12452359|rs575144|rs907604|rs1254403|rs8054007|rs6498573|rs4904690|rs820371|rs1073084|rs1243872|rs1294506|rs10511418|rs17794387|rs7895441|rs1693892|rs3750433</td></tr>
<tr><td>Malar Rash</td><td>PTEN_PATHWAY</td><td>BIOCARTA</td><td>544</td><td>24</td><td>11</td><td>8,03E-01</td><td>9,58E-01</td><td>rs4976123|rs706725|rs7708673|rs249479|rs7840147|rs17069632|rs34330|rs7219|rs4131890|rs859624|rs2248293</td></tr>
<tr><td>Malar Rash</td><td>SIGNALING_BY_WNT</td><td>REACTOME</td><td>1071</td><td>35</td><td>20</td><td>8,03E-01</td><td>9,58E-01</td><td>rs12292911|rs8063430|rs880026|rs577357|rs11881878|rs7224730|rs4790706|rs17213812|rs6599127|rs2282032|rs9863665|rs621106|rs6142963|rs9784876|rs6924102|rs2011713|rs473151|rs10463643|rs328368|rs9380326</td></tr>
<tr><td>Malar Rash</td><td>ALANINE_ASPARTATE_AND_GLUTAMATE_METABOLISM</td><td>KEGG</td><td>841</td><td>28</td><td>17</td><td>8,04E-01</td><td>9,58E-01</td><td>rs3761793|rs37373|rs1873061|rs8080449|rs2298316|rs3123697|rs807513|rs885063|rs1273376|rs389035|rs6898640|rs2494467|rs3006009|rs10521121|rs4920566|rs923376|rs11243474</td></tr>
<tr><td>Malar Rash</td><td>CACAM_PATHWAY</td><td>BIOCARTA</td><td>401</td><td>15</td><td>10</td><td>8,04E-01</td><td>9,58E-01</td><td>rs815808|rs11098195|rs4904690|rs11938486|rs12329315|rs1294506|rs9898774|rs4675672|rs1693892|rs1058903</td></tr>
<tr><td>Malar Rash</td><td>RESPONSE_TO_ELEVATED_PLATELET_CYTOSOLIC_CA2_</td><td>REACTOME</td><td>2301</td><td>58</td><td>20</td><td>8,04E-01</td><td>9,58E-01</td><td>rs899494|rs4958487|rs2256331|rs7257613|rs10276241|rs815808|rs9524864|rs7424885|rs12588394|rs11568993|rs12452359|rs6681804|rs5030102|rs1873423|rs12707455|rs2829946|rs467778|rs10494097|rs3131660|rs569214</td></tr>
<tr><td>Malar Rash</td><td>COSTIMULATION_BY_THE_CD28_FAMILY</td><td>REACTOME</td><td>1479</td><td>46</td><td>20</td><td>8,05E-01</td><td>9,58E-01</td><td>rs4976123|rs7756516|rs6115830|rs822349|rs15614|rs2281390|rs11881878|rs3019244|rs9277052|rs7025291|rs9277535|rs706725|rs7648642|rs6592724|rs7708673|rs619864|rs10415535|rs249479|rs6018027|rs7607984</td></tr>
<tr><td>Malar Rash</td><td>SIGNALLING_TO_RAS</td><td>REACTOME</td><td>468</td><td>18</td><td>14</td><td>8,06E-01</td><td>9,58E-01</td><td>rs9410438|rs4256810|rs2241407|rs2073925|rs7022583|rs10901235|rs4626883|rs4962095|rs6018027|rs7219|rs6090585|rs4723884|rs11137533|rs6956681</td></tr>
<tr><td>Malar Rash</td><td>G1_PHASE</td><td>REACTOME</td><td>625</td><td>30</td><td>15</td><td>8,07E-01</td><td>9,58E-01</td><td>rs6938550|rs4074785|rs3217992|rs6577060|rs11881878|rs10273030|rs1547705|rs3804440|rs7747931|rs2041731|rs911361|rs34330|rs7394655|rs3217906|rs13160147</td></tr>
<tr><td>Malar Rash</td><td>TRANSLATION</td><td>REACTOME</td><td>1803</td><td>30</td><td>20</td><td>8,11E-01</td><td>9,60E-01</td><td>rs968053|rs2177836|rs12974044|rs737204|rs16898766|rs3108908|rs10824618|rs2416771|rs3121680|rs12873548|rs11217128|rs17028238|rs12624027|rs9893674|rs11577033|rs16888536|rs226206|rs4777186|rs751716|rs7076791</td></tr>
<tr><td>Malar Rash</td><td>HIV_INFECTION</td><td>REACTOME</td><td>3237</td><td>28</td><td>20</td><td>8,11E-01</td><td>9,60E-01</td><td>rs12292911|rs9310419|rs8063430|rs10197003|rs4550189|rs5766404|rs987607|rs16832740|rs15614|rs7815340|rs2523966|rs9615022|rs577357|rs1545951|rs3778709|rs2174270|rs2042237|rs8139591|rs7224730|rs11687599</td></tr>
<tr><td>Malar Rash</td><td>TRIGLYCERIDE_BIOSYNTHESIS</td><td>REACTOME</td><td>1182</td><td>31</td><td>20</td><td>8,12E-01</td><td>9,60E-01</td><td>rs11737840|rs12542460|rs4698832|rs12108641|rs2577254|rs10452184|rs642926|rs2920126|rs11717683|rs11195813|rs2936517|rs6533493|rs10516696|rs6535510|rs836178|rs13159372|rs35921314|rs13144344|rs6719376|rs10455125</td></tr>
<tr><td>Malar Rash</td><td>SIGNALING_BY_ERBB4</td><td>REACTOME</td><td>3434</td><td>35</td><td>20</td><td>8,17E-01</td><td>9,63E-01</td><td>rs4976123|rs4947491|rs2721051|rs486992|rs601618|rs37373|rs2888104|rs6115830|rs7776830|rs2064193|rs11071240|rs11568993|rs17172446|rs1499063|rs1147960|rs1033440|rs1571221|rs12446495|rs12444602|rs2145811</td></tr>
<tr><td>Malar Rash</td><td>FC_GAMMA_R_MEDIATED_PHAGOCYTOSIS</td><td>KEGG</td><td>3795</td><td>37</td><td>20</td><td>8,17E-01</td><td>9,63E-01</td><td>rs4976123|rs2670873|rs16763|rs3767742|rs600415|rs714104|rs7257613|rs10169262|rs4837820|rs12191724|rs11766675|rs12619351|rs2810493|rs1636808|rs17289507|rs17489214|rs9881788|rs8139798|rs4804528|rs511278</td></tr>
<tr><td>Malar Rash</td><td>TOLL_LIKE_RECEPTOR_SIGNALING_PATHWAY</td><td>KEGG</td><td>2339</td><td>38</td><td>20</td><td>8,20E-01</td><td>9,63E-01</td><td>rs4976123|rs4804833|rs1543478|rs2737191|rs8004059|rs9345044|rs2820350|rs2811650|rs11766675|rs2189756|rs1636808|rs4812997|rs7843776|rs12130290|rs531486|rs7922090|rs12645085|rs696|rs6990813|rs706725</td></tr>
<tr><td>Malar Rash</td><td>CD28_CO_STIMULATION</td><td>REACTOME</td><td>797</td><td>41</td><td>19</td><td>8,20E-01</td><td>9,63E-01</td><td>rs4976123|rs6115830|rs15614|rs3019244|rs706725|rs7648642|rs6592724|rs7708673|rs619864|rs10415535|rs249479|rs6018027|rs2332096|rs7219|rs6090585|rs4131890|rs3792285|rs829179|rs7834615</td></tr>
<tr><td>Malar Rash</td><td>INFLUENZA_VIRAL_RNA_TRANSCRIPTION_AND_REPLICATION</td><td>REACTOME</td><td>1153</td><td>35</td><td>20</td><td>8,20E-01</td><td>9,63E-01</td><td>rs7338209|rs2177836|rs12974044|rs10824618|rs12873548|rs11217128|rs12624027|rs9893674|rs11577033|rs226206|rs4777186|rs751716|rs7076791|rs3094303|rs12323544|rs7982845|rs6927367|rs3816127|rs701405|rs13017465</td></tr>
<tr><td>Malar Rash</td><td>RAP1_SIGNALLING</td><td>REACTOME</td><td>835</td><td>33</td><td>18</td><td>8,23E-01</td><td>9,63E-01</td><td>rs4972394|rs4734507|rs3108913|rs472159|rs6468757|rs1861932|rs10497403|rs10740406|rs12465032|rs3019260|rs12937985|rs2113807|rs17543997|rs2194717|rs11869953|rs10508938|rs1326250|rs293336</td></tr>
<tr><td>Malar Rash</td><td>EGFR_DOWNREGULATION</td><td>REACTOME</td><td>1037</td><td>35</td><td>20</td><td>8,23E-01</td><td>9,63E-01</td><td>rs4947491|rs7776830|rs10507911|rs11568993|rs17172446|rs1018726|rs1036253|rs9531002|rs863712|rs4076950|rs17809737|rs1962276|rs1215447|rs7219|rs4504735|rs17238095|rs7317070|rs9318775|rs10488142|rs13244925</td></tr>
<tr><td>Malar Rash</td><td>MRNA_SPLICING_MINOR_PATHWAY</td><td>REACTOME</td><td>383</td><td>16</td><td>11</td><td>8,24E-01</td><td>9,63E-01</td><td>rs11081583|rs4809388|rs571374|rs7982845|rs3816127|rs10859966|rs17574411|rs577935|rs6017090|rs1602|rs1134231</td></tr>
<tr><td>Malar Rash</td><td>GPVI_MEDIATED_ACTIVATION_CASCADE</td><td>REACTOME</td><td>1181</td><td>29</td><td>20</td><td>8,26E-01</td><td>9,63E-01</td><td>rs4976123|rs11766675|rs1636808|rs195770|rs17489214|rs8139798|rs345306|rs2239775|rs1993703|rs706725|rs1760118|rs42154|rs7708673|rs6583058|rs10415535|rs249479|rs3936112|rs10120669|rs726680|rs1760125</td></tr>
<tr><td>Malar Rash</td><td>ELONGATION_ARREST_AND_RECOVERY</td><td>REACTOME</td><td>246</td><td>12</td><td>6</td><td>8,26E-01</td><td>9,63E-01</td><td>rs6990813|rs2460386|rs7982845|rs7228520|rs3816127|rs6472810</td></tr>
<tr><td>Malar Rash</td><td>PHOSPHORYLATION_OF_THE_APC_C</td><td>REACTOME</td><td>219</td><td>3</td><td>2</td><td>8,35E-01</td><td>9,70E-01</td><td>rs2004845|rs17754530</td></tr>
<tr><td>Malar Rash</td><td>NOD_LIKE_RECEPTOR_SIGNALING_PATHWAY</td><td>KEGG</td><td>1406</td><td>35</td><td>20</td><td>8,35E-01</td><td>9,70E-01</td><td>rs314370|rs1543478|rs507879|rs1546745|rs9345044|rs2820350|rs2811650|rs531486|rs7922090|rs1545092|rs696|rs2501657|rs311636|rs9342261|rs13402616|rs6426244|rs666462|rs7221898|rs9611169|rs12215452</td></tr>
<tr><td>Malar Rash</td><td>REGULATION_OF_SIGNALING_BY_CBL</td><td>REACTOME</td><td>703</td><td>29</td><td>15</td><td>8,38E-01</td><td>9,71E-01</td><td>rs4976123|rs17489214|rs1993703|rs706725|rs1760118|rs7708673|rs10415535|rs249479|rs10120669|rs1760125|rs7219|rs4131890|rs10993754|rs1041264|rs7834615</td></tr>
<tr><td>Malar Rash</td><td>APC_C_CDC20_MEDIATED_DEGRADATION_OF_CYCLIN_B</td><td>REACTOME</td><td>224</td><td>3</td><td>2</td><td>8,40E-01</td><td>9,71E-01</td><td>rs2004845|rs17754530</td></tr>
<tr><td>Malar Rash</td><td>STRESS_PATHWAY</td><td>BIOCARTA</td><td>633</td><td>32</td><td>18</td><td>8,41E-01</td><td>9,71E-01</td><td>rs1543478|rs4761528|rs2189756|rs2357973|rs270952|rs7922090|rs696|rs2721856|rs197265|rs11870477|rs6920337|rs12470772|rs10164859|rs10859601|rs2322376|rs501222|rs11207303|rs10483453</td></tr>
<tr><td>Malar Rash</td><td>NUCLEOTIDE_EXCISION_REPAIR</td><td>REACTOME</td><td>956</td><td>14</td><td>10</td><td>8,44E-01</td><td>9,71E-01</td><td>rs11039149|rs2174270|rs9909163|rs156631|rs1765853|rs3816127|rs5744750|rs2881964|rs6463725|rs886727</td></tr>
<tr><td>Malar Rash</td><td>NUCLEOTIDE_EXCISION_REPAIR</td><td>KEGG</td><td>914</td><td>16</td><td>11</td><td>8,44E-01</td><td>9,71E-01</td><td>rs11039149|rs2174270|rs9909163|rs156631|rs2381375|rs1765853|rs5744750|rs2881964|rs6463725|rs17584703|rs886727</td></tr>
<tr><td>Malar Rash</td><td>HEMATOPOIETIC_CELL_LINEAGE</td><td>KEGG</td><td>1963</td><td>27</td><td>20</td><td>8,45E-01</td><td>9,71E-01</td><td>rs9829238|rs9678469|rs4851541|rs12244380|rs7073236|rs12452359|rs756325|rs17387575|rs2556|rs6715062|rs1009448|rs2767326|rs711801|rs3024685|rs522073|rs887998|rs6985210|rs1323653|rs1076594|rs791589</td></tr>
<tr><td>Malar Rash</td><td>FORMATION_OF_RNA_POL_II_ELONGATION_COMPLEX_</td><td>REACTOME</td><td>401</td><td>15</td><td>8</td><td>8,46E-01</td><td>9,71E-01</td><td>rs2174270|rs6990813|rs2460386|rs7982845|rs7228520|rs3816127|rs6472810|rs1602</td></tr>
<tr><td>Malar Rash</td><td>REGULATION_OF_GENE_EXPRESSION_IN_BETA_CELLS</td><td>REACTOME</td><td>716</td><td>22</td><td>14</td><td>8,47E-01</td><td>9,71E-01</td><td>rs2721051|rs2145811|rs6075965|rs4943768|rs17254382|rs6036189|rs7981045|rs7822101|rs830786|rs11907005|rs6849318|rs2996449|rs6036192|rs1950130</td></tr>
<tr><td>Malar Rash</td><td>CYTOKINE_CYTOKINE_RECEPTOR_INTERACTION</td><td>KEGG</td><td>6425</td><td>30</td><td>20</td><td>8,48E-01</td><td>9,71E-01</td><td>rs4947491|rs4683189|rs2364493|rs178637|rs9678469|rs4851541|rs37373|rs9376268|rs1515263|rs6943592|rs13044457|rs6054517|rs719563|rs10197003|rs11914132|rs12244380|rs11570908|rs10276241|rs16832740|rs7776830</td></tr>
<tr><td>Malar Rash</td><td>MELANOGENESIS</td><td>KEGG</td><td>4077</td><td>42</td><td>20</td><td>8,49E-01</td><td>9,71E-01</td><td>rs10956564|rs8126112|rs1430604|rs16904360|rs1108656|rs2066097|rs7257613|rs12430699|rs12873185|rs815808|rs7648607|rs6719501|rs11098195|rs880026|rs9544635|rs2744718|rs6140629|rs13045064|rs934448|rs72001</td></tr>
<tr><td>Malar Rash</td><td>OXYGEN_DEPENDENT_PROLINE_HYDROXYLATION_OF_HYPOXIA_INDUCIBLE_FACTOR_/</td><td>REACTOME</td><td>313</td><td>14</td><td>6</td><td>8,51E-01</td><td>9,71E-01</td><td>rs1625065|rs6990813|rs708030|rs3733828|rs6472810|rs1629140</td></tr>
<tr><td>Malar Rash</td><td>MICRORNA_MIRNA_BIOGENESIS</td><td>REACTOME</td><td>227</td><td>13</td><td>8</td><td>8,53E-01</td><td>9,71E-01</td><td>rs8139591|rs2161006|rs9292427|rs3816127|rs8012576|rs8015314|rs4905265|rs2257082</td></tr>
<tr><td>Malar Rash</td><td>XENOBIOTICS</td><td>REACTOME</td><td>231</td><td>9</td><td>4</td><td>8,54E-01</td><td>9,71E-01</td><td>rs11188059|rs4646421|rs11593189|rs2275620</td></tr>
<tr><td>Malar Rash</td><td>APOPTOTIC_CLEAVAGE_OF_CELLULAR_PROTEINS</td><td>REACTOME</td><td>1052</td><td>33</td><td>20</td><td>8,56E-01</td><td>9,71E-01</td><td>rs7202333|rs4837820|rs880026|rs11597105|rs2498437|rs1538581|rs9513432|rs2304762|rs1824476|rs6599127|rs13402616|rs7840147|rs4771307|rs9863665|rs8061932|rs3812062|rs688391|rs2435200|rs1470257|rs2920005</td></tr>
<tr><td>Malar Rash</td><td>BASIGIN_INTERACTIONS</td><td>REACTOME</td><td>595</td><td>19</td><td>16</td><td>8,57E-01</td><td>9,71E-01</td><td>rs132930|rs3759608|rs10132368|rs17244832|rs752503|rs12030460|rs1015260|rs2301628|rs7527283|rs3811188|rs13108353|rs17122962|rs10519463|rs12611042|rs10405246|rs10919049</td></tr>
<tr><td>Malar Rash</td><td>ABORTIVE_ELONGATION_OF_HIV1_TRANSCRIPT_IN_THE_ABSENCE_OF_TAT</td><td>REACTOME</td><td>195</td><td>7</td><td>5</td><td>8,57E-01</td><td>9,71E-01</td><td>rs2460386|rs7982845|rs7228520|rs3816127|rs1602</td></tr>
<tr><td>Malar Rash</td><td>DNA_STRAND_ELONGATION</td><td>REACTOME</td><td>672</td><td>17</td><td>12</td><td>8,58E-01</td><td>9,71E-01</td><td>rs3761873|rs1781011|rs133416|rs9909163|rs156631|rs1765853|rs10948689|rs11644122|rs2881964|rs6463725|rs9475837|rs886727</td></tr>
<tr><td>Malar Rash</td><td>BASE_EXCISION_REPAIR</td><td>KEGG</td><td>500</td><td>19</td><td>11</td><td>8,59E-01</td><td>9,71E-01</td><td>rs1713460|rs11942838|rs9506325|rs156631|rs9581000|rs5744750|rs10861152|rs12485319|rs2686184|rs9550568|rs11853141</td></tr>
<tr><td>Malar Rash</td><td>ABC_TRANSPORTERS</td><td>KEGG</td><td>2684</td><td>65</td><td>20</td><td>8,60E-01</td><td>9,71E-01</td><td>rs899494|rs12452482|rs17703233|rs4147837|rs2857106|rs9524864|rs4148384|rs3886119|rs10493872|rs1642220|rs7799912|rs6583481|rs225406|rs17213812|rs7023868|rs2172133|rs7636910|rs2472495|rs17132432|rs12869867</td></tr>
<tr><td>Malar Rash</td><td>P75_NTR_RECEPTOR_MEDIATED_SIGNALLING</td><td>REACTOME</td><td>2669</td><td>31</td><td>20</td><td>8,61E-01</td><td>9,71E-01</td><td>rs6789224|rs9371859|rs16903375|rs1543478|rs12974044|rs1546745|rs2882936|rs7611544|rs7569471|rs10509783|rs13397982|rs2304961|rs2276035|rs345306|rs17590882|rs6438837|rs4968185|rs250846|rs11113472|rs7633543</td></tr>
<tr><td>Malar Rash</td><td>MEIOTIC_SYNAPSIS</td><td>REACTOME</td><td>1031</td><td>24</td><td>15</td><td>8,62E-01</td><td>9,71E-01</td><td>rs9461204|rs214959|rs4096763|rs10247556|rs9942542|rs6557213|rs2813487|rs7776065|rs3812062|rs7145633|rs1145101|rs1744385|rs9358913|rs10431453|rs1316624</td></tr>
<tr><td>Malar Rash</td><td>APOPTOTIC_EXECUTION_PHASE</td><td>REACTOME</td><td>1211</td><td>32</td><td>20</td><td>8,65E-01</td><td>9,72E-01</td><td>rs7202333|rs4837820|rs15614|rs880026|rs11597105|rs2498437|rs9506325|rs1538581|rs9513432|rs1513763|rs1824476|rs6599127|rs13402616|rs7840147|rs4771307|rs8061932|rs9863665|rs3812062|rs688391|rs2435200</td></tr>
<tr><td>Malar Rash</td><td>NEGATIVE_REGULATORS_OF_RIG_I_MDA5_SIGNALING</td><td>REACTOME</td><td>464</td><td>20</td><td>12</td><td>8,67E-01</td><td>9,72E-01</td><td>rs26538|rs12130290|rs1545092|rs10783580|rs4585264|rs6802890|rs13247831|rs12447155|rs633724|rs7236796|rs4895499|rs2729356</td></tr>
<tr><td>Malar Rash</td><td>MEF2D_PATHWAY</td><td>BIOCARTA</td><td>684</td><td>33</td><td>20</td><td>8,69E-01</td><td>9,72E-01</td><td>rs4811174|rs754505|rs815808|rs1750304|rs1873423|rs8090312|rs4904690|rs737063|rs1294506|rs432854|rs11648535|rs1693892|rs1058903|rs6940385|rs352092|rs7202459|rs9896483|rs1476813|rs7545965|rs11645672</td></tr>
<tr><td>Malar Rash</td><td>APC_C_CDH1_MEDIATED_DEGRADATION_OF_CDC20_AND_OTHER_APC_C_CDH1_TARGET
TE_MITOSIS_EARLY_G1</td><td>REACTOME</td><td>959</td><td>32</td><td>20</td><td>8,72E-01</td><td>9,72E-01</td><td>rs12292911|rs8063430|rs577357|rs7224730|rs4790706|rs17213812|rs2004845|rs2282032|rs3804440|rs17754530|rs6142963|rs9784876|rs6924102|rs2011713|rs7806|rs473151|rs13160147|rs328368|rs9380326|rs13394402</td></tr>
<tr><td>Malar Rash</td><td>HS_GAG_BIOSYNTHESIS</td><td>REACTOME</td><td>3154</td><td>41</td><td>20</td><td>8,76E-01</td><td>9,72E-01</td><td>rs2147155|rs4945522|rs3820088|rs731096|rs10955078|rs9584093|rs2245104|rs4787781|rs7984995|rs17774701|rs13010821|rs1328368|rs9890916|rs6492696|rs1549805|rs2292832|rs2150471|rs1994958|rs9913031|rs10048789</td></tr>
<tr><td>Malar Rash</td><td>CLASS_B_2_SECRETIN_FAMILY_RECEPTORS</td><td>REACTOME</td><td>2257</td><td>26</td><td>20</td><td>8,77E-01</td><td>9,72E-01</td><td>rs11720405|rs737681|rs10185142|rs2294451|rs288746|rs6723083|rs4898725|rs9915294|rs12147761|rs7388704|rs2744718|rs1558477|rs670307|rs16979607|rs934448|rs1941663|rs11621038|rs752313|rs2163086|rs934170</td></tr>
<tr><td>Malar Rash</td><td>IL12_PATHWAY</td><td>BIOCARTA</td><td>589</td><td>25</td><td>11</td><td>8,78E-01</td><td>9,72E-01</td><td>rs2189756|rs10878721|rs1571221|rs3024912|rs6895787|rs2421049|rs11870477|rs6869688|rs12999517|rs7574865|rs11207303</td></tr>
<tr><td>Malar Rash</td><td>NRAGE_SIGNALS_DEATH_THROUGH_JNK</td><td>REACTOME</td><td>1919</td><td>33</td><td>20</td><td>8,78E-01</td><td>9,72E-01</td><td>rs6789224|rs9371859|rs16903375|rs2882936|rs7611544|rs13397982|rs2304961|rs2276035|rs345306|rs6438837|rs4968185|rs250846|rs7633543|rs8068519|rs6012419|rs9305462|rs12914792|rs7174521|rs7298165|rs13203300</td></tr>
<tr><td>Malar Rash</td><td>PATHOGENIC_ESCHERICHIA_COLI_INFECTION</td><td>KEGG</td><td>1256</td><td>31</td><td>20</td><td>8,79E-01</td><td>9,72E-01</td><td>rs7202333|rs2737191|rs4734507|rs361557|rs880026|rs3108913|rs7843776|rs6990813|rs6468757|rs2173578|rs6599127|rs17665024|rs737063|rs6929344|rs4668631|rs3019260|rs8061932|rs9863665|rs361893|rs11080571</td></tr>
<tr><td>Malar Rash</td><td>CHROMOSOME_MAINTENANCE</td><td>REACTOME</td><td>1949</td><td>35</td><td>20</td><td>8,84E-01</td><td>9,72E-01</td><td>rs2833690|rs9381786|rs9461204|rs4267943|rs9302109|rs4976741|rs214959|rs4096763|rs10247556|rs9942542|rs9909163|rs6557213|rs2099505|rs156631|rs1765853|rs2813487|rs7776065|rs3812062|rs5744750|rs7145633</td></tr>
<tr><td>Malar Rash</td><td>MEIOSIS</td><td>REACTOME</td><td>1674</td><td>31</td><td>20</td><td>8,84E-01</td><td>9,72E-01</td><td>rs10765682|rs9461204|rs12796488|rs214959|rs4096763|rs10247556|rs9942542|rs9909163|rs499952|rs6557213|rs2244012|rs1366452|rs2813487|rs1801516|rs16944894|rs7776065|rs3812062|rs2055526|rs7145633|rs1145101</td></tr>
<tr><td>Malar Rash</td><td>RNA_POL_II_PRE_TRANSCRIPTION_EVENTS</td><td>REACTOME</td><td>532</td><td>15</td><td>8</td><td>8,85E-01</td><td>9,72E-01</td><td>rs2174270|rs6990813|rs2460386|rs7982845|rs7228520|rs3816127|rs6472810|rs1602</td></tr>
<tr><td>Malar Rash</td><td>RIG_I_LIKE_RECEPTOR_SIGNALING_PATHWAY</td><td>KEGG</td><td>1361</td><td>26</td><td>20</td><td>8,86E-01</td><td>9,72E-01</td><td>rs1543478|rs9345044|rs2820350|rs2811650|rs26538|rs2357973|rs12130290|rs270952|rs7922090|rs696|rs2501657|rs2155458|rs6895787|rs2421049|rs197265|rs9342261|rs13402616|rs12447155|rs1983810|rs12215452</td></tr>
<tr><td>Malar Rash</td><td>MRNA_CAPPING</td><td>REACTOME</td><td>388</td><td>12</td><td>8</td><td>8,87E-01</td><td>9,72E-01</td><td>rs2174270|rs9359790|rs7982845|rs3816127|rs2756382|rs12208932|rs6936494|rs1602</td></tr>
<tr><td>Malar Rash</td><td>TRANSCRIPTION</td><td>REACTOME</td><td>2708</td><td>38</td><td>20</td><td>8,88E-01</td><td>9,72E-01</td><td>rs7034140|rs10810136|rs4804833|rs9461204|rs2382444|rs1383245|rs10169262|rs12191724|rs2274913|rs4905510|rs2174270|rs4150115|rs12676965|rs13258200|rs13291088|rs2282763|rs9359790|rs3769769|rs4961396|rs303721</td></tr>
<tr><td>Malar Rash</td><td>RENAL_CELL_CARCINOMA</td><td>KEGG</td><td>2164</td><td>37</td><td>20</td><td>8,89E-01</td><td>9,72E-01</td><td>rs4976123|rs7111926|rs12480230|rs10276241|rs7936180|rs11766675|rs15614|rs1636808|rs2327206|rs1625065|rs7130878|rs3019244|rs565536|rs472159|rs6500552|rs12707455|rs731461|rs936216|rs11221390|rs6990813</td></tr>
<tr><td>Malar Rash</td><td>SYNTHESIS_OF_PC</td><td>REACTOME</td><td>669</td><td>20</td><td>14</td><td>8,89E-01</td><td>9,72E-01</td><td>rs10494126|rs601788|rs2577254|rs642926|rs13245899|rs1880676|rs2282382|rs1073084|rs35921314|rs1557502|rs10431453|rs11101186|rs11690125|rs12210887</td></tr>
<tr><td>Malar Rash</td><td>TRANSPORT_OF_VITAMINS_NUCLEOSIDES_AND_RELATED_MOLECULES</td><td>REACTOME</td><td>1154</td><td>28</td><td>20</td><td>8,89E-01</td><td>9,72E-01</td><td>rs890839|rs11666324|rs7175096|rs7627477|rs9819520|rs6958502|rs2179845|rs1885277|rs11632145|rs16974632|rs2238360|rs666462|rs4400714|rs6885006|rs4778031|rs1246264|rs17185623|rs6480507|rs1542283|rs11074038</td></tr>
</table>

Malar Rash	GRB2_EVENTS_IN_ERBB2_SIGNALING	REACTOME	1202	32	20	8,89E-01	9,72E-01	rs4947491|rs2888104|rs7776830|rs11568993|rs17172446|rs1499063|rs1851176|rs191180|rs1948098|rs16846140|rs6755027|rs10432443|rs17830325|rs17186704|rs13391359|rs7423124|rs7219|rs17238095|rs11784670|rs6895139
Malar Rash	RNA_POL_II_TRANSCRIPTION	REACTOME	1050	34	20	8,89E-01	9,72E-01	rs10169262|rs12191724|rs2274913|rs4905510|rs2174270|rs13258200|rs2282763|rs9359790|rs6990813|rs2027745|rs17054866|rs2460386|rs7982845|rs7228520|rs12473402|rs11134939|rs3816127|rs10859966|rs2756382|rs6472810
Malar Rash	RNA_POL_I_TRANSCRIPTION_TERMINATION	REACTOME	335	15	9	8,89E-01	9,72E-01	rs2174270|rs4150115|rs4533450|rs10153801|rs6708081|rs3803643|rs2257082|rs11652516|rs11682963
Malar Rash	PHASE1_FUNCTIONALIZATION_OF_COMPOUNDS	REACTOME	1515	27	20	8,92E-01	9,72E-01	rs1235695|rs5297|rs2241057|rs2602884|rs6088638|rs12125747|rs338585|rs6722569|rs11844169|rs4815647|rs11188059|rs12079736|rs149425|rs1154454|rs2447753|rs4147536|rs4646421|rs3754206|rs2296241|rs7174997
Malar Rash	ALDOSTERONE_REGULATED_SODIUM_REABSORPTION	KEGG	1794	31	20	8,92E-01	9,72E-01	rs4976123|rs7257613|rs1023421|rs11766675|rs16831388|rs9588044|rs1636808|rs8107107|rs9402571|rs1873423|rs12428609|rs4583344|rs10050229|rs7943324|rs10750114|rs11724292|rs703559|rs540101|rs706725|rs42154
Malar Rash	CELL_SURFACE_INTERACTIONS_AT_THE_VASCULAR_WALL	REACTOME	2650	30	20	8,93E-01	9,72E-01	rs4976123|rs6043388|rs6034368|rs162070|rs12695175|rs132930|rs12114833|rs3759608|rs10132368|rs6755028|rs2223286|rs7424885|rs17553089|rs12452359|rs2824164|rs2087863|rs5011812|rs13043878|rs2440445|rs17244832
Malar Rash	GH_PATHWAY	BIOCARTA	963	32	20	8,94E-01	9,72E-01	rs4976123|rs11766675|rs1636808|rs1571221|rs1873423|rs706725|rs42154|rs7708673|rs7254060|rs737063|rs249479|rs432854|rs3020619|rs11648535|rs11674718|rs7219|rs7202459|rs9896483|rs1476813|rs11645672
Malar Rash	BASAL_CELL_CARCINOMA	KEGG	2166	34	20	8,94E-01	9,72E-01	rs737681|rs2035901|rs13044457|rs6054517|rs719563|rs288746|rs6719501|rs880026|rs7388704|rs2744718|rs2883154|rs7224837|rs7156353|rs934448|rs2133606|rs2163086|rs934170|rs11097974|rs2363923|rs357539
Malar Rash	LOSS_OF_NLP_FROM_MITOTIC_CENTROSOMES	REACTOME	1100	43	18	8,96E-01	9,72E-01	rs2089114|rs7726839|rs2802723|rs2296078|rs4864569|rs11881878|rs560564|rs1373293|rs437707|rs1399960|rs7960163|rs8080511|rs983679|rs7959574|rs9313132|rs2468257|rs10947089|rs208752
Malar Rash	ONE_CARBON_POOL_BY_FOLATE	KEGG	441	12	11	8,98E-01	9,72E-01	rs11664283|rs2115536|rs1572151|rs12524884|rs803422|rs380691|rs7282864|rs1801133|rs7280485|rs1956545|rs10932605
Malar Rash	CHYLOMICRON_MEDIATED_LIPID_TRANSPORT	REACTOME	435	24	9	8,99E-01	9,72E-01	rs12641768|rs1869138|rs10402592|rs2337382|rs6511720|rs4666360|rs4659356|rs693|rs12912850
Malar Rash	TRNA_AMINOACYLATION	REACTOME	795	16	13	9,00E-01	9,72E-01	rs7744190|rs9369472|rs167772|rs4401711|rs491511|rs12132918|rs2815155|rs9472250|rs2647468|rs12961130|rs959086|rs515077|rs12656366
Malar Rash	BOTULINUM_NEUROTOXICITY	REACTOME	522	18	13	9,00E-01	9,72E-01	rs12409084|rs6133869|rs10485738|rs1462923|rs8891|rs17808797|rs12369479|rs362560|rs17518345|rs925717|rs11086652|rs362584|rs6015338
Malar Rash	BIOLOGICAL_OXIDATIONS	REACTOME	3568	32	20	9,03E-01	9,74E-01	rs1235695|rs6060124|rs11135284|rs7405412|rs5297|rs13178865|rs451439|rs173792|rs2241057|rs502862|rs2602884|rs1841037|rs294588|rs7447133|rs7723455|rs9333372|rs296364|rs1354360|rs12125747|rs17360177
Malar Rash	EPITHELIAL_CELL_SIGNALING_IN_HELICOBACTER_PYLORI_INFECTION	KEGG	1535	28	20	9,06E-01	9,74E-01	rs4947491|rs1543478|rs563201|rs7776830|rs17172446|rs13238736|rs3019244|rs17590882|rs3814043|rs2301647|rs7922090|rs696|rs2721856|rs6592724|rs460707|rs12473402|rs366193|rs12616710|rs4275799|rs3823494
Malar Rash	SRP_DEPENDENT_COTRANSLATIONAL_PROTEIN_TARGETING_TO_MEMBRANE	REACTOME	1378	37	20	9,07E-01	9,74E-01	rs2177836|rs12974044|rs10824618|rs2416771|rs3121680|rs12873548|rs11217128|rs12624027|rs9893674|rs11577033|rs226206|rs4777186|rs751716|rs7076791|rs3094303|rs10871764|rs12323544|rs6927367|rs9328415|rs701405
Malar Rash	CALCINEURIN_PATHWAY	BIOCARTA	895	33	20	9,08E-01	9,74E-01	rs4811174|rs754505|rs815808|rs1873423|rs8090312|rs17789266|rs4904690|rs737063|rs16862130|rs2364598|rs1294506|rs9646736|rs432854|rs11648535|rs1693892|rs1058903|rs7202459|rs1031800|rs9896483|rs1476813
Malar Rash	DEFENSINS	REACTOME	495	19	9	9,08E-01	9,74E-01	rs7107929|rs6074578|rs10095331|rs4513489|rs13260325|rs4841831|rs2738048|rs1324528|rs1555679
Malar Rash	RNA_POL_I_TRANSCRIPTION_INITIATION	REACTOME	427	15	9	9,15E-01	9,80E-01	rs2174270|rs4150115|rs4533450|rs10153801|rs6708081|rs3803643|rs2257082|rs11652516|rs11682963
Malar Rash	NCAM1_INTERACTIONS	REACTOME	1896	32	20	9,16E-01	9,80E-01	rs3811159|rs2059198|rs10098621|rs6722598|rs6728818|rs881726|rs4703116|rs11013044|rs4496283|rs631428|rs914363|rs12219393|rs9521642|rs10508557|rs7322495|rs198552|rs4936186|rs2645782|rs2839102|rs3827848
Malar Rash	CELL_DEATH_SIGNALLING_VIA_NRAGE_NRIF_AND_NADE	REACTOME	2069	31	20	9,18E-01	9,81E-01	rs6789224|rs9371859|rs16903375|rs2882936|rs7611544|rs13397982|rs2304961|rs2276035|rs345306|rs6438837|rs4968185|rs250846|rs7633543|rs8068519|rs2871921|rs6012419|rs9305462|rs12914792|rs7174521|rs7298165
Malar Rash	RNA_POL_I_TRANSCRIPTION	REACTOME	937	29	13	9,23E-01	9,84E-01	rs9461204|rs2174270|rs4150115|rs4533450|rs10153801|rs6708081|rs7776065|rs10233641|rs3803643|rs2257082|rs9358913|rs11652516|rs11682963
Malar Rash	LAIR_PATHWAY	BIOCARTA	479	26	19	9,29E-01	9,88E-01	rs10473234|rs5030102|rs17611|rs6451566|rs711801|rs1656966|rs17524161|rs6131|rs3176860|rs11586136|rs379527|rs3783621|rs17414954|rs8060136|rs822373|rs3087439|rs1899037|rs12659224|rs7711831
Malar Rash	NDKDYNAMIN_PATHWAY	BIOCARTA	548	23	15	9,29E-01	9,88E-01	rs815808|rs7023913|rs12338625|rs11760351|rs744373|rs2043260|rs4904690|rs1294506|rs4146042|rs6733255|rs13426725|rs1693892|rs1058903|rs7942719|rs2659504
Malar Rash	PI3K_EVENTS_IN_ERBB4_SIGNALING	REACTOME	1709	37	20	9,30E-01	9,88E-01	rs4976123|rs2721051|rs2888104|rs6115830|rs1499063|rs2145811|rs7922090|rs1851176|rs706725|rs7708673|rs191180|rs4943768|rs17254382|rs7981045|rs1948098|rs16846140|rs249479|rs6755027|rs12329315|rs17069632
Malar Rash	BIOPEPTIDES_PATHWAY	BIOCARTA	1081	28	20	9,31E-01	9,88E-01	rs1688123|rs815808|rs11098195|rs1571221|rs5030102|rs1873423|rs3024912|rs1656966|rs1254403|rs4904690|rs820371|rs737063|rs11938486|rs7000615|rs1294506|rs432854|rs10511418|rs11648535|rs2435200|rs2886612
Malar Rash	NUCLEAR_SIGNALING_BY_ERBB4	REACTOME	2231	30	20	9,33E-01	9,88E-01	rs486992|rs601618|rs37373|rs2888104|rs2064193|rs1499063|rs1147960|rs1033440|rs1571221|rs12446495|rs12444602|rs17590882|rs3866638|rs531486|rs7184757|rs7936649|rs2871921|rs1851176|rs11155820|rs2655439
Malar Rash	NETRIN1_SIGNALING	REACTOME	3710	30	20	9,36E-01	9,89E-01	rs265060|rs875078|rs9962572|rs16903375|rs10829597|rs1564895|rs12251654|rs2468366|rs4270125|rs9309825|rs9642758|rs11597105|rs257258|rs17706224|rs4833241|rs17752673|rs1577876|rs2270229|rs4868388|rs1466947
Malar Rash	FORMATION_OF_FIBRIN_CLOT_CLOTTING_CASCADE	REACTOME	703	32	19	9,36E-01	9,89E-01	rs11938564|rs6755028|rs5030102|rs10187622|rs555212|rs6906354|rs1656966|rs11063954|rs2424505|rs4847313|rs4468370|rs925451|rs822373|rs917859|rs2729356|rs434602|rs2274393|rs584337|rs1993552
Malar Rash	CONVERSION_FROM_APC_C_CDC20_TO_APC_C_CDH1_IN_LATE_ANAPHASE	REACTOME	226	4	3	9,37E-01	9,89E-01	rs2004845|rs17754530|rs2270694
Malar Rash	PML_PATHWAY	BIOCARTA	384	17	8	9,39E-01	9,89E-01	rs16827412|rs6500552|rs1007119|rs1678154|rs11076786|rs129988|rs1649866|rs859624
Malar Rash	FORMATION_OF_TUBULIN_FOLDING_INTERMEDIATES_BY_CCT_TRIC	REACTOME	227	3	2	9,40E-01	9,89E-01	rs6929344|rs11080571
Malar Rash	TELOMERE_MAINTENANCE	REACTOME	1133	25	15	9,41E-01	9,89E-01	rs9461204|rs4976741|rs4096763|rs10247556|rs9909163|rs156631|rs1765853|rs7776065|rs5744750|rs9358913|rs2881964|rs6463725|rs9475837|rs1316624|rs886727
Malar Rash	PRIMARY_BILE_ACID_BIOSYNTHESIS	KEGG	439	13	8	9,42E-01	9,89E-01	rs2636968|rs11844169|rs11253048|rs35412|rs11202998|rs2560718|rs2431049|rs3732860
Malar Rash	RECRUITMENT_OF_MITOTIC_CENTROSOME_PROTEINS_AND_COMPLEXES	REACTOME	1177	45	19	9,44E-01	9,90E-01	rs4792930|rs7726839|rs2802723|rs2296078|rs4864569|rs11881878|rs560564|rs1373293|rs437707|rs1399960|rs7960163|rs8080511|rs983679|rs7959574|rs9313132|rs2468257|rs10947089|rs9550174|rs208752
Malar Rash	MITOTIC_G2_G2_M_PHASES	REACTOME	1189	42	20	9,49E-01	9,94E-01	rs4792930|rs7726839|rs2802723|rs6938550|rs2296078|rs4864569|rs3191402|rs11881878|rs560564|rs1373293|rs437707|rs1554160|rs7747931|rs4238844|rs911361|rs7960163|rs8080511|rs983679|rs7959574|rs9313132
Malar Rash	INFLAM_PATHWAY	BIOCARTA	747	21	14	9,53E-01	9,94E-01	rs756325|rs10878721|rs12502051|rs6985210|rs6895787|rs2421049|rs2243290|rs3021094|rs4739147|rs7417586|rs8109167|rs2205181|rs6869688|rs333947
Malar Rash	DEGRADATION_OF_THE_EXTRACELLULAR_MATRIX	REACTOME	580	23	12	9,56E-01	9,94E-01	rs2616483|rs17099425|rs743257|rs3886934|rs3811188|rs10090518|rs925451|rs17122962|rs8080307|rs7004633|rs656759|rs1940475
Malar Rash	TGF_BETA_RECEPTOR_SIGNALING_IN_EMT_EPITHELIAL_TO_MESENCHYMAL_TRANSITIC	REACTOME	429	16	13	9,59E-01	9,94E-01	rs6041884|rs11009506|rs3773650|rs7068747|rs8124053|rs2786551|rs8109167|rs1474880|rs4934677|rs2460002|rs4729514|rs1573129|rs2065949
Malar Rash	CYTOCHROME_P450_ARRANGED_BY_SUBSTRATE_TYPE	REACTOME	1163	29	18	9,63E-01	9,94E-01	rs5297|rs2241057|rs12125747|rs338585|rs6722569|rs11844169|rs11188059|rs149425|rs2447753|rs4646421|rs3754206|rs2296241|rs7174997|rs11593189|rs2275620|rs3732860|rs7016924|rs2616277
Malar Rash	CYTOSOLIC_TRNA_AMINOACYLATION	REACTOME	312	8	7	9,65E-01	9,94E-01	rs4401711|rs491511|rs2815155|rs2647468|rs12961130|rs515077|rs12656366
Malar Rash	PROTEOLYTIC_CLEAVAGE_OF_SNARE_COMPLEX_PROTEINS	REACTOME	382	15	10	9,65E-01	9,94E-01	rs6133869|rs10485738|rs1128349|rs17808797|rs12369479|rs362560|rs17518345|rs11086652|rs362584|rs6015338
Malar Rash	STEROID_BIOSYNTHESIS	KEGG	247	8	6	9,66E-01	9,94E-01	rs12026860|rs619895|rs2272682|rs10913728|rs2686184|rs1792325
Malar Rash	UCALPAIN_PATHWAY	BIOCARTA	426	8	7	9,66E-01	9,94E-01	rs12452359|rs1243872|rs7840147|rs6018027|rs3750433|rs6090585|rs3760372
Malar Rash	DNA_REPLICATION	KEGG	740	22	14	9,66E-01	9,94E-01	rs1781011|rs133416|rs9909163|rs156631|rs2381375|rs1765853|rs7563960|rs10948689|rs5744750|rs2881964|rs6463725|rs17584703|rs9475837|rs886727
Malar Rash	RNA_POLYMERASE	KEGG	351	7	4	9,66E-01	9,94E-01	rs10153801|rs3816127|rs259940|rs2257082
Malar Rash	COLLAGEN_FORMATION	REACTOME	3203	33	20	9,67E-01	9,94E-01	rs3811159|rs4956253|rs4701085|rs6806116|rs9293982|rs1913185|rs10998995|rs6722598|rs6728818|rs10479623|rs9832659|rs11983597|rs10019624|rs7070915|rs4736218|rs4496283|rs12547337|rs631428|rs262065|rs4655929
Malar Rash	EXTENSION_OF_TELOMERES	REACTOME	618	11	9	9,69E-01	9,94E-01	rs4976741|rs9909163|rs156631|rs1765853|rs5744750|rs2881964|rs6463725|rs9475837|rs886727
Malar Rash	SPPA_PATHWAY	BIOCARTA	1428	21	20	9,69E-01	9,94E-01	rs8126112|rs6140629|rs17489214|rs13045064|rs1993703|rs1873423|rs6133573|rs926505|rs6077420|rs1760118|rs2179440|rs737063|rs1487492|rs6680812|rs2745772|rs955835|rs7840147|rs6018027|rs432854|rs2206423
Malar Rash	P53HYPOXIA_PATHWAY	BIOCARTA	517	9	7	9,71E-01	9,94E-01	rs9909163|rs11770622|rs1801516|rs2235015|rs11968400|rs977908|rs2278501
Malar Rash	SYNTHESIS_OF_BILE_ACIDS_AND_BILE_SALTS	REACTOME	546	23	9	9,72E-01	9,94E-01	rs26181|rs11844169|rs11253048|rs35412|rs11202998|rs4148785|rs2560718|rs2431049|rs3732860
Malar Rash	LIPOPROTEIN_METABOLISM	REACTOME	1037	43	20	9,73E-01	9,94E-01	rs11076174|rs7659550|rs1801239|rs1869138|rs10402592|rs225406|rs838893|rs7023868|rs2337382|rs2472495|rs6511720|rs2740484|rs3858075|rs4666360|rs4659356|rs7922682|rs4149330|rs693|rs7678298|rs12912850
Malar Rash	PREFOLDIN_MEDIATED_TRANSFER_OF_SUBSTRATE_TO_CCT_TRIC	REACTOME	288	3	2	9,74E-01	9,94E-01	rs6929344|rs11080571
Malar Rash	CTLA4_INHIBITORY_SIGNALING	REACTOME	447	14	7	9,75E-01	9,94E-01	rs11881878|rs7648642|rs6018027|rs2332096|rs6090585|rs3792285|rs7834615
Malar Rash	LIPID_DIGESTION_MOBILIZATION_AND_TRANSPORT	REACTOME	1389	36	20	9,75E-01	9,94E-01	rs11076174|rs12641768|rs1801239|rs1869138|rs549662|rs10402592|rs10896172|rs225406|rs838893|rs7023868|rs2337382|rs2472495|rs8023357|rs13346080|rs9309664|rs2766581|rs6511720|rs2740484|rs7651814|rs3858075
Malar Rash	SMOOTH_MUSCLE_CONTRACTION	REACTOME	658	29	17	9,75E-01	9,94E-01	rs815808|rs11202900|rs1254403|rs8054007|rs6498573|rs4904690|rs820371|rs1243872|rs1294506|rs10511418|rs7895441|rs1693892|rs3750433|rs1058903|rs2071675|rs11188311|rs17456435
Malar Rash	AMINO_ACID_SYNTHESIS_AND_INTERCONVERSION_TRANSAMINATION	REACTOME	530	9	7	9,77E-01	9,94E-01	rs7915136|rs2298316|rs12570255|rs2494467|rs11188409|rs2275273|rs4498630
Malar Rash	REGULATION_OF_HYPOXIA_INDUCIBLE_FACTOR_HIF_BY_OXYGEN	REACTOME	443	30	17	9,78E-01	9,94E-01	rs1625065|rs6500552|rs7092999|rs6990813|rs708030|rs1243872|rs3733828|rs6472810|rs943080|rs6458351|rs2495712|rs9462937|rs3750433|rs11076786|rs2071675|rs129988|rs1629140
Malar Rash	POST_CHAPERONIN_TUBULIN_FOLDING_PATHWAY	REACTOME	254	4	3	9,79E-01	9,94E-01	rs6929344|rs11080571|rs354599
Malar Rash	SIGNALING_BY_SCF_KIT	REACTOME	1989	37	20	9,79E-01	9,94E-01	rs4976123|rs2721051|rs6115830|rs1440329|rs10515396|rs1571221|rs2145811|rs4957774|rs7922090|rs706725|rs3024912|rs7708673|rs4943768|rs17254382|rs2043287|rs7981045|rs737063|rs10415535|rs218237|rs249479
Malar Rash	INTEGRIN_PATHWAY	BIOCARTA	1027	23	15	9,80E-01	9,94E-01	rs7574419|rs4674147|rs6714500|rs472159|rs6435978|rs1243872|rs7840147|rs6018027|rs1430134|rs3750433|rs7219|rs6090585|rs934036|rs1041264|rs11207303
Malar Rash	NCAM_SIGNALING_FOR_NEURITE_OUT_GROWTH	REACTOME	2480	27	20	9,83E-01	9,94E-01	rs3811159|rs2059198|rs10098621|rs6722598|rs6728818|rs881726|rs4703116|rs11013044|rs12436076|rs4496283|rs631428|rs914363|rs10791889|rs12219393|rs9521642|rs10508557|rs7322495|rs198552|rs4936186|rs2645782
Malar Rash	TRANSCRIPTION_COUPLED_NER_TC_NER	REACTOME	834	12	9	9,84E-01	9,94E-01	rs2174270|rs9909163|rs156631|rs1765853|rs3816127|rs5744750|rs2881964|rs6463725|rs886727
Malar Rash	PYK2_PATHWAY	BIOCARTA	752	29	20	9,87E-01	9,94E-01	rs815808|rs3019244|rs1873423|rs2721856|rs6592724|rs4904690|rs737063|rs7000615|rs1294506|rs6018027|rs432854|rs11648535|rs1693892|rs1058903|rs7219|rs7202459|rs6090585|rs9896483|rs1476813|rs11645672
Malar Rash	EXTRACELLULAR_MATRIX_ORGANIZATION	REACTOME	3783	33	20	9,87E-01	9,94E-01	rs3811159|rs4956253|rs4701085|rs6806116|rs9293982|rs1913185|rs10998995|rs6722598|rs2616483|rs6728818|rs10479623|rs17099425|rs9832659|rs11983597|rs10019624|rs7070915|rs4736218|rs4496283|rs12547337|rs631428
Malar Rash	STEROID_HORMONE_BIOSYNTHESIS	KEGG	1491	42	20	9,88E-01	9,94E-01	rs5297|rs12602084|rs1841037|rs11253048|rs10917603|rs149425|rs4148309|rs3775774|rs2447753|rs274657|rs4646421|rs7174997|rs7546652|rs2431049|rs10426377|rs7016924|rs1977090|rs4339935|rs10904392|rs2476927
Malar Rash	LAGGING_STRAND_SYNTHESIS	REACTOME	550	8	7	9,88E-01	9,94E-01	rs9909163|rs156631|rs1765853|rs2881964|rs6463725|rs9475837|rs886727
Malar Rash	PLATELET_SENSITIZATION_BY_LDL	REACTOME	676	16	9	9,90E-01	9,95E-01	rs11881878|rs2337382|rs1487492|rs6680812|rs955835|rs12564176|rs591393|rs693|rs10912119
Malar Rash	G2_PATHWAY	BIOCARTA	354	8	7	9,94E-01	9,97E-01	rs4668631|rs1801516|rs6062357|rs977908|rs8178108|rs12611042|rs540723
Malar Rash	BILE_ACID_AND_BILE_SALT_METABOLISM	REACTOME	1176	39	16	9,96E-01	9,97E-01	rs1571513|rs1925978|rs2636968|rs11844169|rs11253048|rs35412|rs11202998|rs4148785|rs2560718|rs3918334|rs2431049|rs3732860|rs1730643|rs1888322|rs7678298|rs11618082
Malar Rash	REGULATION_OF_KIT_SIGNALING	REACTOME	587	15	11	9,96E-01	9,97E-01	rs1440329|rs737063|rs218237|rs6018027|rs7219|rs6090585|rs11723705|rs9896483|rs1476813|rs1014058|rs7834615
Malar Rash	SHC1_EVENTS_IN_ERBB4_SIGNALING	REACTOME	1072	28	18	9,97E-01	9,97E-01	rs2888104|rs1499063|rs1851176|rs191180|rs1948098|rs16846140|rs6755027|rs10432443|rs17830325|rs17186704|rs13391359|rs7423124|rs7219|rs11784670|rs6895139|rs4673677|rs6532431|rs12621602
Serositis	FORMATION_OF_TRANSCRIPTION_COUPLED_NER_TC_NER_REPAIR_COMPLEX	REACTOME	389	4	3	3,70E-03	7,50E-01	rs7188980|rs7126805|rs1794242
Serositis	GLUTATHIONE_CONJUGATION	REACTOME	407	35	13	7,90E-03	7,50E-01	rs1356553|rs7496|rs574389|rs7687822|rs9296691|rs12529923|rs17608022|rs316144|rs10874814|rs510088|rs4149202|rs12128974|rs3794953
Serositis	TRYPTOPHAN_METABOLISM	KEGG	953	44	20	8,70E-03	7,50E-01	rs2540069|rs10890819|rs1020426|rs17565061|rs2465665|rs10732359|rs6729962|rs4245824|rs762551|rs4131348|rs12577790|rs1528946|rs10279462|rs13027801|rs7221585|rs11725638|rs1432095|rs2854702|rs4878199|rs4646771
Serositis	NICOTINATE_AND_NICOTINAMIDE_METABOLISM	KEGG	963	39	20	1,10E-02	7,50E-01	rs2540069|rs2704973|rs2465665|rs6596572|rs6934819|rs10044871|rs6729962|rs1895218|rs6935458|rs9838798|rs6956300|rs2539847|rs11750377|rs10935348|rs2275007|rs10050461|rs10074159|rs17411887|rs4383836|rs2704958
Serositis	GLUTATHIONE_METABOLISM	KEGG	683	42	17	1,12E-02	7,50E-01	rs1356553|rs7496|rs574389|rs7687822|rs9296691|rs12529923|rs6588432|rs17608022|rs316144|rs10874814|rs2042045|rs3818562|rs4910907|rs510088|rs11875|rs1887546|rs12128974
Serositis	TGF_BETA_RECEPTOR_SIGNALING_ACTIVATES_SMADS	REACTOME	563	31	20	1,49E-02	7,50E-01	rs1019855|rs11071927|rs7166081|rs10518714|rs6025742|rs1345207|rs13075948|rs2852950|rs7173609|rs17702096|rs1942159|rs6025689|rs11661651|rs2635509|rs8124053|rs1790733|rs4811937|rs2917779|rs157104|rs8035972
Serositis	DOWNREGULATION_OF_TGF_BETA_RECEPTOR_SIGNALING	REACTOME	521	31	20	1,54E-02	7,50E-01	rs1019855|rs11071927|rs7166081|rs10518714|rs6025742|rs1345207|rs13075948|rs2852950|rs7173609|rs17702096|rs1942159|rs6025689|rs11661651|rs2635509|rs1790733|rs4811937|rs2917779|rs157104|rs8035972|rs2136619
Serositis	INTEGRIN_ALPHAIIB_BETA3_SIGNALING	REACTOME	779	35	20	1,68E-02	7,50E-01	rs2302456|rs12648395|rs7016497|rs6018627|rs11168228|rs2056131|rs523200|rs10878841|rs10820825|rs2292700|rs7548973|rs12810426|rs4886406|rs11695696|rs4220|rs7177101|rs3769210|rs1968510|rs702988|rs2584098
Serositis	SIGNALING_BY_NODAL	REACTOME	632	32	20	1,75E-02	7,50E-01	rs7566998|rs11071927|rs7166081|rs10518714|rs10783481|rs2852950|rs7173609|rs16827558|rs1942159|rs1048070|rs10170049|rs11661651|rs4362505|rs6598484|rs4965381|rs1520494|rs6430189|rs8035972|rs2136619|rs12490188
Serositis	TGFB_PATHWAY	BIOCARTA	817	34	20	1,88E-02	7,50E-01	rs1019855|rs11071927|rs7166081|rs10518714|rs17747965|rs1221220|rs13075948|rs2852950|rs10498970|rs7173609|rs4355511|rs4958072|rs1942159|rs11661651|rs2635509|rs1010650|rs13188321|rs2141484|rs157695|rs9362733
Serositis	PROTEIN_EXPORT	KEGG	750	89	20	2,09E-02	7,50E-01	rs4131086|rs10282292|rs37642|rs4940849|rs11153096|rs6948090|rs4270628|rs12536111|rs7874483|rs11133152|rs6553943|rs876144|rs1380100|rs4260596|rs1242744|rs1528036|rs10799322|rs6960391|rs1598100|rs10272725
Serositis	TOLL_LIKE_RECEPTOR_SIGNALING_PATHWAY	KEGG	2339	35	20	2,49E-02	7,50E-01	rs1864053|rs483808|rs7086224|rs2764938|rs13395505|rs4301703|rs17747965|rs10863430|rs4352468|rs7866830|rs1221220|rs11793751|rs17530657|rs10983903|rs1392125|rs9647337|rs11207332|rs511973|rs1366315|rs6871452
Serositis	G1_PATHWAY	BIOCARTA	611	35	20	2,50E-02	7,50E-01	rs4987876|rs11071927|rs7166081|rs10518714|rs227060|rs2282411|rs7781436|rs3759216|rs7173609|rs2188513|rs9315422|rs1642763|rs918587|rs4846488|rs8035972|rs380691|rs2298765|rs4150724|rs11855908|rs2237573
Serositis	PRE_NOTCH_TRANSCRIPTION_AND_TRANSLATION	REACTOME	835	41	20	2,72E-02	7,50E-01	rs10939104|rs11655371|rs2764938|rs10765795|rs4692114|rs522495|rs17530657|rs109771|rs11207332|rs9268005|rs2269423|rs4659248|rs1642763|rs10501835|rs10017756|rs1296720|rs2139926|rs1042317|rs2298765|rs2238419
Serositis	GLUCONEOGENESIS	REACTOME	490	28	8	2,73E-02	7,50E-01	rs10993276|rs6070157|rs764223|rs7122539|rs11189224|rs6015032|rs3861699|rs12342274
Serositis	TYPE_I_DIABETES_MELLITUS	KEGG	1975	71	20	2,89E-02	7,50E-01	rs4909140|rs1670338|rs10263846|rs1264524|rs17533090|rs6963236|rs7806066|rs9258186|rs1330581|rs2335158|rs9276711|rs11984274|rs7770216|rs9654713|rs6929796|rs17454570|rs17561|rs1075498|rs9658761|rs3135363
Serositis	BETA_DEFENSINS	REACTOME	333	14	5	2,90E-02	7,50E-01	rs12624551|rs10498781|rs16994490|rs1361121|rs1935386
Serositis	SPHINGOLIPID_METABOLISM	KEGG	1101	33	20	2,96E-02	7,50E-01	rs12133338|rs17046991|rs4903606|rs2008644|rs4074708|rs10508921|rs11794290|rs17751746|rs1930762|rs2739685|rs4758104|rs2153486|rs1072382|rs7145386|rs7665770|rs895344|rs12510440|rs2041976|rs1890911|rs10740635
Serositis	RNA_POL_III_TRANSCRIPTION_TERMINATION	REACTOME	680	42	20	3,24E-02	7,50E-01	rs17422451|rs2583680|rs717932|rs985559|rs7869160|rs13258625|rs7126805|rs14105|rs512598|rs12341611|rs870409|rs13302756|rs11782092|rs4979935|rs2288270|rs11788403|rs12350251|rs1383246|rs10810134|rs13277342
Serositis	FCER1_PATHWAY	BIOCARTA	1564	45	20	3,30E-02	7,50E-01	rs1864053|rs4699236|rs967776|rs483808|rs2764938|rs6812773|rs1487489|rs17530657|rs11207332|rs10820825|rs591393|rs1366315|rs6871452|rs660749|rs9895159|rs6067805|rs2017750|rs748938|rs4234512|rs9808216

Serositis	RNA_DEGRADATION	KEGG	1123	51	20	3,72E-02	7,50E-01	rs2967391|rs12516085|rs10203061|rs11853191|rs764223|rs4151657|rs2911407|rs17402006|rs275460|rs10479576|rs2560274|rs11879090|rs4641872|rs6550146|rs10512889|rs730424|rs8049212|rs1432295|rs17630822|rs1430778
Serositis	VALINE_LEUCINE_AND_ISOLEUCINE_DEGRADATION	KEGG	1149	42	20	3,72E-02	7,50E-01	rs2540069|rs10890819|rs9359435|rs17565061|rs2465665|rs10732359|rs6729962|rs3132810|rs12577790|rs11879090|rs9937726|rs9357584|rs11725638|rs13193950|rs4878199|rs10486549|rs11744623|rs17807445|rs976145|rs4646771
Serositis	RNA_POL_I_RNA_POL_III_AND_MITOCHONDRIAL_TRANSCRIPTION	REACTOME	1830	46	20	3,73E-02	7,50E-01	rs17422451|rs2583680|rs4541737|rs9295730|rs2283575|rs717932|rs985559|rs7869160|rs13258625|rs7126805|rs9461416|rs14105|rs512598|rs650627|rs2875859|rs129128|rs12341611|rs7568330|rs2763982|rs870409
Serositis	AMYOTROPHIC_LATERAL_SCLEROSIS_ALS	KEGG	1813	28	20	3,85E-02	7,50E-01	rs4699236|rs6812773|rs6490121|rs7686241|rs11055455|rs2927585|rs2228576|rs1434384|rs11722663|rs1421106|rs11725238|rs4780486|rs2237268|rs1362327|rs6827325|rs1862409|rs1861872|rs8049630|rs181402|rs8064140
Serositis	MET_PATHWAY	BIOCARTA	1415	37	20	3,89E-02	7,50E-01	rs11245205|rs1864053|rs2764938|rs2302456|rs7016497|rs6018627|rs11017615|rs17530657|rs11207332|rs10878841|rs7548973|rs1366315|rs6871452|rs178255|rs12251654|rs2017750|rs17822304|rs4234512|rs383542|rs4740304
Serositis	DRUG_METABOLISM_CYTOCHROME_P450	KEGG	1661	72	20	3,97E-02	7,50E-01	rs2540069|rs7496|rs2465665|rs6729962|rs7687822|rs7663410|rs9296691|rs762551|rs12529923|rs2013573|rs6738678|rs17608022|rs316144|rs7608175|rs2042045|200199|rs12978001|rs3818562|rs1920145|rs4148285
Serositis	COLORECTAL_CANCER	KEGG	2539	43	20	4,03E-02	7,50E-01	rs1019855|rs1864053|rs2221145|rs2764938|rs11071927|rs919635|rs7166081|rs10518714|rs4352468|rs2292653|rs249633|rs17530657|rs2219926|rs9647337|rs11207332|rs511973|rs1366315|rs9964756|rs6871452|rs13075948
Serositis	DEFENSINS	REACTOME	495	16	6	4,07E-02	7,50E-01	rs12624551|rs10498781|rs16994490|rs1361121|rs1935386|rs2741688
Serositis	PHASE_II_CONJUGATION	REACTOME	2059	42	20	4,26E-02	7,50E-01	rs1356553|rs7496|rs294594|rs574389|rs13178865|rs7687822|rs9296691|rs16970280|rs762551|rs6509397|rs12529923|rs4149413|rs946934|rs12153515|rs2013573|rs11135312|rs6738678|rs17608022|rs316144|rs10874814
Serositis	IL2_PATHWAY	BIOCARTA	452	32	9	4,30E-02	7,50E-01	rs2764938|rs17530657|rs11207332|rs10820825|rs2017750|rs747020|rs4234512|rs7617041|rs8004059
Serositis	PLATELET_AGGREGATION_PLUG_FORMATION	REACTOME	942	35	20	4,31E-02	7,50E-01	rs2302456|rs12648395|rs7016497|rs6018627|rs11168228|rs2056131|rs523200|rs10878841|rs10820825|rs2292700|rs7548973|rs12810426|rs4886406|rs11695696|rs6056|rs7177101|rs3769210|rs1968510|rs17128431|rs702988
Serositis	NEPHRIN_INTERACTIONS	REACTOME	1617	46	20	4,39E-02	7,50E-01	rs1864053|rs17505|rs809192|rs489725|rs17106413|rs356233|rs7590257|rs1892999|rs1108906|rs9647337|rs513444|rs10228655|rs2931462|rs1366315|rs6871452|rs4671928|rs1475034|rs356269|rs4730838|rs7131158
Serositis	TRAFFICKING_OF_GLUR2_CONTAINING_AMPA_RECEPTORS	REACTOME	721	35	20	4,47E-02	7,50E-01	rs8464|rs7686241|rs2927585|rs1434384|rs11722663|rs11725238|rs6827325|rs1862409|rs10750731|rs226434|rs7719292|rs12938937|rs1019738|rs9913908|rs509555|rs17113869|rs9324752|rs6421967|rs10155614|rs7936555
Serositis	TRANS_GOLGI_NETWORK_VESICLE_BUDDING	REACTOME	1106	26	20	4,47E-02	7,50E-01	rs909626|rs1969260|rs1160495|rs2292653|rs11811771|rs3745255|rs3019626|rs4325000|rs1805727|rs2618580|rs1805734|rs16907441|rs13198533|rs10963309|rs9296980|rs2025994|rs7450621|rs355412|rs151352|rs3755964
Serositis	ACETYLCHOLINE_BINDING_AND_DOWNSTREAM_EVENTS	REACTOME	242	19	7	4,49E-02	7,50E-01	rs6474421|rs11636101|rs1127314|rs11636753|rs2611605|rs12594247|rs11785591
Serositis	DESTABILIZATION_OF_MRNA_BY_TRISTETRAPROLIN_TTP	REACTOME	179	18	7	4,55E-02	7,50E-01	rs17014035|rs7273626|rs764223|rs11879090|rs11189224|rs3900716|rs2425650
Serositis	DOUBLE_STRAND_BREAK_REPAIR	REACTOME	506	24	12	4,56E-02	7,50E-01	rs4987876|rs867185|rs227060|rs10276762|rs16972096|rs886727|rs11614523|rs3132585|rs741048|rs17137468|rs4942486|rs2155209
Serositis	MAPK_PATHWAY	BIOCARTA	2690	46	20	4,65E-02	7,50E-01	rs17014035|rs483808|rs7086224|rs2764938|rs13395505|rs4301703|rs17747965|rs7594967|rs7756550|rs4352468|rs4965533|rs1221220|rs9366027|rs17530657|rs11100242|rs2237268|rs11207332|rs918418|rs7702020|rs11995971
Serositis	LYSINE_DEGRADATION	KEGG	952	28	20	4,67E-02	7,50E-01	rs7944449|rs10890819|rs17565061|rs10732359|rs4681238|rs1158269|rs12577790|rs1528946|rs11133439|rs2269881|rs2763982|rs12462556|rs11725638|rs4876902|rs1432095|rs2080087|rs11720981|rs2687860|rs4878199|rs4646771
Serositis	LONG_TERM_POTENTIATION	KEGG	3620	30	20	4,78E-02	7,50E-01	rs1352388|rs4699236|rs8464|rs2960288|rs6812773|rs7686241|rs17801265|rs6442887|rs6039107|rs11055455|rs3804984|rs2927585|rs11168228|rs1434384|rs11722663|rs9366027|rs1421106|rs11725238|rs7977039|rs4780486
Serositis	NITRIC_OXIDE_STIMULATES_GUANYLATE_CYCLASE	REACTOME	1895	45	20	4,81E-02	7,50E-01	rs12299821|rs4260410|rs377432|rs6490121|rs10749883|rs169290|rs1899908|rs2044316|rs1398312|rs1903999|rs728528|rs597384|rs2252621|rs4834779|rs703505|rs934803|rs10898864|rs6443558|rs7077665|rs12252380
Serositis	ATM_PATHWAY	BIOCARTA	397	43	16	4,91E-02	7,50E-01	rs4987876|rs2764938|rs867185|rs227060|rs17530657|rs11207332|rs2017750|rs1642763|rs10872893|rs2298483|rs299235|rs4648090|rs230547|rs4740363|rs7971479|rs2155209
Serositis	DESTABILIZATION_OF_MRNA_BY_BRF1	REACTOME	204	21	8	4,91E-02	7,50E-01	rs17014035|rs7273626|rs764223|rs11879090|rs17757211|rs11851414|rs11189224|rs2425650
Serositis	MEIOTIC_RECOMBINATION	REACTOME	993	58	18	5,46E-02	7,50E-01	rs4987876|rs867185|rs4541737|rs9295730|rs227060|rs10276762|rs9461416|rs129128|rs886727|rs9461204|rs299235|rs741048|rs17137468|rs10520896|rs4942486|rs9366694|rs6452168|rs2155209
Serositis	COSTIMULATION_BY_THE_CD28_FAMILY	REACTOME	1479	39	20	5,62E-02	7,50E-01	rs1864053|rs7086224|rs809192|rs2890658|rs4301703|rs35399661|rs6018627|rs6051511|rs12613960|rs1366315|rs6871452|rs10195135|rs4886406|rs12120009|rs11119881|rs4675397|rs3214047|rs747020|rs2894381|rs7354779
Serositis	RNA_POL_III_TRANSCRIPTION	REACTOME	861	46	20	5,70E-02	7,50E-01	rs17422451|rs2583680|rs717932|rs985559|rs7869160|rs13258625|rs7126805|rs14105|rs512598|rs650627|rs12341611|rs870409|rs13302756|rs11782092|rs10824587|rs2288270|rs11788403|rs12350251|rs1383246|rs10810134
Serositis	PLATELET_HOMEOSTASIS	REACTOME	4080	43	20	5,87E-02	7,50E-01	rs1352388|rs12299821|rs967776|rs4260410|rs10516614|rs377432|rs6490121|rs10749883|rs169290|rs1899908|rs9309065|rs2044316|rs1398312|rs1903999|rs12045786|rs728528|rs12553893|rs597384|rs4964002|rs591393
Serositis	SIGNALLING_TO_RAS	REACTOME	468	37	13	5,95E-02	7,50E-01	rs17014035|rs7273626|rs6018627|rs2043722|rs2254527|rs11137525|rs4234512|rs16837709|rs2519090|rs6012200|rs7290469|rs2425650|rs7617041
Serositis	EDG1_PATHWAY	BIOCARTA	1432	30	20	5,98E-02	7,50E-01	rs1864053|rs8464|rs2960288|rs6039107|rs7016497|rs6018627|rs2056131|rs13037679|rs2292700|rs2739685|rs1366315|rs6871452|rs4758104|rs12620429|rs1072382|rs1474937|rs6039104|rs1999134|rs4908127|rs12938937
Serositis	NTHI_PATHWAY	BIOCARTA	876	33	20	6,00E-02	7,50E-01	rs1019855|rs11071927|rs7166081|rs10518714|rs17747965|rs1221220|rs13075948|rs10498970|rs7173609|rs10872893|rs1010650|rs2141484|rs157695|rs9362733|rs8035972|rs1296720|rs711272|rs2238419|rs10485405|rs2818291
Serositis	CD28_CO_STIMULATION	REACTOME	797	30	20	6,19E-02	7,50E-01	rs1864053|rs7086224|rs809192|rs4301703|rs6018627|rs6051511|rs1366315|rs6871452|rs3214047|rs747020|rs10788819|rs383542|rs619864|rs2715261|rs34299|rs16897109|rs16897180|rs7245158|rs17350527|rs2867316
Serositis	GOLGI_ASSOCIATED_VESICLE_BIOGENESIS	REACTOME	1057	25	20	6,45E-02	7,50E-01	rs909626|rs1969260|rs1160495|rs2292653|rs11811771|rs3745255|rs3019626|rs4325000|rs2618580|rs16907441|rs13198533|rs10963309|rs9296980|rs2025994|rs12204417|rs355412|rs3755964|rs5763140|rs13156690|rs16877105
Serositis	CHEMICAL_PATHWAY	BIOCARTA	585	36	20	6,46E-02	7,50E-01	rs4987876|rs8464|rs13395505|rs227060|rs7016497|rs181402|rs17809603|rs2020902|rs2046136|rs1642763|rs12938937|rs2849382|rs9913908|rs907186|rs17093560|rs4381631|rs733646|rs12452826|rs11654719|rs169143
Serositis	BRANCHED_CHAIN_AMINO_ACID_CATABOLISM	REACTOME	507	28	11	6,49E-02	7,50E-01	rs10890819|rs9359435|rs3132810|rs12577790|rs11879090|rs13193950|rs10486549|rs17807445|rs976145|rs11083620|rs2444830
Serositis	NOS1_PATHWAY	BIOCARTA	1133	32	20	6,64E-02	7,50E-01	rs4699236|rs8464|rs6812773|rs6490121|rs11055455|rs1421106|rs4780486|rs1543889|rs1861872|rs8049630|rs8064140|rs1107475|rs1070474|rs12938937|rs12820037|rs11055603|rs12449282|rs9808216|rs4699228|rs2645370
Serositis	HOMOLOGOUS_RECOMBINATION_REPAIR_OF_REPLICATION_INDEPENDENT_DOUBLE_ST	REACTOME	376	23	11	6,64E-02	7,50E-01	rs4987876|rs867185|rs227060|rs10276762|rs886727|rs11614523|rs3132585|rs741048|rs17137468|rs4942486|rs2155209
Serositis	INTRINSIC_PATHWAY	BIOCARTA	603	40	19	6,82E-02	7,50E-01	rs10032695|rs2213865|rs2205895|rs627527|rs12648395|rs250724|rs9521664|rs4220|rs7646517|rs4704356|rs822373|rs11918289|rs9559710|rs7708314|rs2062632|rs6829128|rs7995158|rs11884770|rs2167370
Serositis	VEGF_SIGNALING_PATHWAY	KEGG	2111	47	20	6,95E-02	7,50E-01	rs17014035|rs1864053|rs4699236|rs967776|rs8464|rs6812773|rs7016497|rs6018627|rs6678574|rs9647337|rs591393|rs511973|rs1366315|rs6871452|rs660749|rs6067805|rs9932716|rs2020902|rs13054809|rs4240910
Serositis	PANCREATIC_CANCER	KEGG	1920	40	20	7,08E-02	7,50E-01	rs1019855|rs1864053|rs11071927|rs13395505|rs7166081|rs10518714|rs4352468|rs2043722|rs9647337|rs511973|rs2877260|rs1366315|rs7781436|rs6871452|rs13075948|rs10232489|rs2852950|rs7173609|rs2017750|rs1942159
Serositis	MAP_KINASE_ACTIVATION_IN_TLR_CASCADE	REACTOME	1697	44	20	7,11E-02	7,50E-01	rs17014035|rs483808|rs2764938|rs17747965|rs4352468|rs4965533|rs1221220|rs9366027|rs17530657|rs11207332|rs918418|rs17456901|rs7702020|rs1531846|rs1568383|rs9895159|rs10498970|rs2017750|rs6598265|rs325383
Serositis	INTEGRIN_PATHWAY	BIOCARTA	1027	44	20	7,12E-02	7,50E-01	rs2764938|rs809192|rs17106413|rs7016497|rs6018627|rs17530657|rs11207332|rs7548973|rs178255|rs4886406|rs907698|rs2017750|rs1475034|rs2156921|rs1671064|rs10932725|rs4234512|rs3791961|rs181483|rs4740304
Serositis	ANTIGEN_ACTIVATES_B_CELL_RECEPTOR_LEADING_TO_GENERATION_OF_SECOND_ME	REACTOME	1299	50	20	7,28E-02	7,50E-01	rs1352388|rs12413520|rs1864053|rs809192|rs4964002|rs10820825|rs1366315|rs6871452|rs210120|rs1503920|rs9932716|rs516321|rs4240910|rs722813|rs9808216|rs383542|rs7187863|rs6733907|rs34299|rs16897109
Serositis	AMYLOIDS	REACTOME	972	59	20	7,30E-02	7,50E-01	rs4541737|rs9295730|rs16910559|rs916950|rs2070022|rs9461416|rs468774|rs129128|rs10743413|rs4837820|rs6056|rs2234983|rs1417720|rs9461204|rs216779|rs8025462|rs13157444|rs983789|rs4244808|rs5484
Serositis	FATTY_ACYL_COA_BIOSYNTHESIS	REACTOME	404	9	7	7,49E-02	7,50E-01	rs17331746|rs9474466|rs7659090|rs4391265|rs7934642|rs9395855|rs10001138
Serositis	EPO_PATHWAY	BIOCARTA	255	27	7	7,72E-02	7,50E-01	rs2764938|rs17530657|rs11207332|rs2017750|rs4234512|rs7617041|rs8004059
Serositis	CHREBP2_PATHWAY	BIOCARTA	1059	45	20	7,84E-02	7,50E-01	rs10029948|rs17779293|rs4689410|rs7273626|rs11784209|rs6996970|rs1881638|rs4689001|rs17464037|rs2913319|rs12120009|rs11119881|rs951629|rs1480159|rs3935000|rs4585379|rs1031915|rs2302234|rs758989|rs4950385
Serositis	PYRUVATE_METABOLISM	REACTOME	209	6	2	7,87E-02	7,50E-01	rs7817292|rs2230392
Serositis	EGF_PATHWAY	BIOCARTA	1048	46	20	7,99E-02	7,50E-01	rs1864053|rs2764938|rs8464|rs13395505|rs17530657|rs11207332|rs2877260|rs1366315|rs6871452|rs10232489|rs9895159|rs2017750|rs12938937|rs4234512|rs383542|rs9913908|rs2110290|rs34299|rs16897109|rs16897180
Serositis	TCR_PATHWAY	BIOCARTA	1517	40	20	8,04E-02	7,50E-01	rs1864053|rs4699236|rs2764938|rs8464|rs809192|rs6812773|rs17530657|rs11207332|rs1366315|rs6871452|rs660749|rs9895159|rs6067805|rs2017750|rs747020|rs10872893|rs12938937|rs748938|rs4234512|rs9808216
Serositis	TPO_PATHWAY	BIOCARTA	814	45	20	8,12E-02	7,50E-01	rs1864053|rs2764938|rs8464|rs13395505|rs17530657|rs11207332|rs1366315|rs6871452|rs12938937|rs4234512|rs383542|rs9913908|rs1636808|rs34299|rs16897109|rs16897180|rs4381631|rs7617041|rs733646|rs4976028
Serositis	PATHOGENIC_ESCHERICHIA_COLI_INFECTION	KEGG	1256	37	20	8,24E-02	7,50E-01	rs10904481|rs8464|rs809192|rs6996970|rs7866830|rs12617710|rs11793751|rs10983903|rs1392125|rs17464037|rs4973401|rs1327798|rs5001582|rs151352|rs12938937|rs951629|rs13401952|rs9913908|rs3132585|rs17196637
Serositis	METABOLISM_OF_AMINO_ACIDS_AND_DERIVATIVES	REACTOME	5246	36	20	8,37E-02	7,50E-01	rs1356553|rs7944449|rs2355575|rs10890819|rs294594|rs9359435|rs1020426|rs10867272|rs13195843|rs1076153|rs7151518|rs867907|rs328349|rs934721|rs574389|rs13178865|rs11669215|rs4245824|rs2423248|rs7948797
Serositis	CD28_DEPENDENT_PI3K_AKT_SIGNALING	REACTOME	572	34	20	8,39E-02	7,50E-01	rs1864053|rs7086224|rs809192|rs4301703|rs6051511|rs1366315|rs6871452|rs747020|rs10788819|rs383542|rs619864|rs2715261|rs34299|rs16897109|rs16897180|rs17350527|rs2867316|rs4673260|rs4976028|rs7618747
Serositis	AXON_GUIDANCE	KEGG	9506	41	20	8,84E-02	7,50E-01	rs12871906|rs12514805|rs152608|rs3773195|rs10205272|rs4702617|rs4699236|rs4957709|rs919635|rs809192|rs6812773|rs11914750|rs7138694|rs2902125|rs935875|rs7016497|rs4461247|rs4233292|rs1119004|rs2636770
Serositis	GNRH_SIGNALING_PATHWAY	KEGG	3992	40	20	8,87E-02	7,50E-01	rs1352388|rs967776|rs483808|rs2764938|rs8464|rs2960288|rs17801265|rs6442887|rs6039107|rs7756550|rs3804984|rs4352468|rs1909745|rs6018627|rs12564813|rs17530657|rs7977039|rs4964002|rs11207332|rs7699538
Serositis	PROTEOLYTIC_CLEAVAGE_OF_SNARE_COMPLEX_PROTEINS	REACTOME	382	10	9	8,88E-02	7,50E-01	rs6026402|rs6026398|rs9893164|rs11870073|rs6951030|rs1487608|rs363039|rs17741348|rs2532514
Serositis	NFKB_PATHWAY	BIOCARTA	870	33	20	8,90E-02	7,50E-01	rs17747965|rs2228576|rs7866830|rs1221220|rs11793751|rs10983903|rs11123905|rs9389541|rs10498970|rs1327798|rs5001582|rs17561|rs10872893|rs1010650|rs555745|rs2141484|rs157695|rs9362733|rs711272|rs12685245
Serositis	AMINO_ACID_SYNTHESIS_AND_INTERCONVERSION_TRANSAMINATION	REACTOME	530	32	12	9,03E-02	7,50E-01	rs2355575|rs10867272|rs867907|rs1860172|rs6559415|rs1040151|rs11818084|rs17619406|rs4529531|rs2296063|rs1921915|rs3828090
Serositis	IGF1_PATHWAY	BIOCARTA	813	40	20	9,04E-02	7,50E-01	rs1864053|rs2764938|rs17530657|rs11207332|rs1366315|rs6871452|rs10498208|rs11247367|rs2017750|rs1273793|rs2684799|rs11854132|rs17822304|rs4234512|rs10933137|rs383542|rs1636808|rs34299|rs16897109|rs16897180
Serositis	IL10_PATHWAY	BIOCARTA	288	17	8	9,05E-02	7,50E-01	rs13395505|rs3024498|rs133415|rs1554286|rs17561|rs2857709|rs4849122|rs1476482
Serositis	SYSTEMIC_LUPUS_ERYTHEMATOSUS	KEGG	2453	47	20	9,05E-02	7,50E-01	rs4541737|rs9295730|rs10999147|rs11055455|rs17106413|rs17533090|rs3024498|rs920909|rs4151657|rs1421106|rs4780486|rs1543889|rs9276711|rs1532686|rs1554286|rs1861872|rs8049630|rs9461416|rs8064140|rs1107475
Serositis	AT1R_PATHWAY	BIOCARTA	1224	35	20	9,17E-02	7,50E-01	rs2764938|rs8464|rs7016497|rs4965533|rs6018627|rs17530657|rs11207332|rs2877260|rs918418|rs7702020|rs2478545|rs10232489|rs9895159|rs2017750|rs6598265|rs12938937|rs4234512|rs325383|rs9808216|rs9913908
Serositis	LYSOSOME_VESICLE_BIOGENESIS	REACTOME	420	14	9	9,22E-02	7,50E-01	rs3745255|rs4325000|rs1805727|rs1805734|rs10963309|rs151352|rs5763140|rs1887285|rs492960
Serositis	IL22BP_PATHWAY	BIOCARTA	296	21	11	9,34E-02	7,50E-01	rs13395505|rs12028945|rs11577442|rs12720253|rs4789576|rs742931|rs4527079|rs276467|rs7254474|rs280519|rs16829209
Serositis	NFKB_AND_MAP_KINASES_ACTIVATION_MEDIATED_BY_TLR4_SIGNALING_REPERTOIRE	REACTOME	2248	37	20	9,39E-02	7,50E-01	rs17014035|rs483808|rs2764938|rs17747965|rs4352468|rs4965533|rs7866830|rs1221220|rs11793751|rs9366027|rs17530657|rs10983903|rs1392125|rs11207332|rs918418|rs17456901|rs7702020|rs1531846|rs1568383|rs468774
Serositis	CGMP_EFFECTS	REACTOME	1520	38	20	9,60E-02	7,50E-01	rs12299821|rs4260410|rs377432|rs169290|rs1899908|rs2044316|rs1903999|rs728528|rs2252621|rs4834779|rs703505|rs934803|rs10898864|rs6443558|rs7077665|rs12252380|rs875590|rs12578492|rs11823971|rs787931
Serositis	MAPK_TARGETS_NUCLEAR_EVENTS_MEDIATED_BY_MAP_KINASES	REACTOME	980	44	20	9,66E-02	7,50E-01	rs17014035|rs2764938|rs4352468|rs4965533|rs9366027|rs17530657|rs11207332|rs918418|rs17456901|rs7702020|rs1568383|rs2017750|rs6598265|rs325383|rs918842|rs1864180|rs11633623|rs7438872|rs7290469|rs3799647
Serositis	RNA_POL_I_PROMOTER_OPENING	REACTOME	501	36	8	9,71E-02	7,50E-01	rs4541737|rs9295730|rs9461416|rs129128|rs7238305|rs9461204|rs9303601|rs9366694
Serositis	RAP1_SIGNALLING	REACTOME	835	27	20	9,90E-02	7,50E-01	rs7273626|rs6996970|rs11168228|rs1903999|rs11652683|rs523200|rs10878841|rs17464037|rs7548973|rs7208097|rs7077665|rs11695696|rs12252380|rs4234512|rs7177101|rs3769210|rs1904694|rs1871080|rs2584098|rs1937672
Serositis	TRAFFICKING_OF_AMPA_RECEPTORS	REACTOME	1132	30	20	1,01E-01	7,50E-01	rs8464|rs7686241|rs2927585|rs1434384|rs11722663|rs11725238|rs7699538|rs6827325|rs1862409|rs7645524|rs10750731|rs911219|rs226434|rs7719292|rs6583202|rs12938937|rs1019738|rs9843659|rs1024609|rs9913908
Serositis	SIGNALING_BY_ERBB2	REACTOME	3847	34	20	1,01E-01	7,50E-01	rs2306677|rs3924999|rs1864053|rs8464|rs2976525|rs2960288|rs809192|rs2466059|rs7273626|rs1909745|rs6018627|rs4733117|rs10503929|rs6051511|rs2711292|rs4234214|rs9856983|rs2877260|rs4569841|rs10890795
Serositis	41BB_PATHWAY	BIOCARTA	341	26	10	1,01E-01	7,50E-01	rs2764938|rs17530657|rs2237268|rs11207332|rs2017750|rs10872893|rs4648090|rs7019752|rs230547|rs10883452
Serositis	TERMINATION_OF_O_GLYCAN_BIOSYNTHESIS	REACTOME	961	29	20	1,02E-01	7,50E-01	rs1554324|rs2736868|rs1401346|rs9854005|rs2958830|rs6989475|rs7552569|rs4836593|rs2037957|rs950757|rs1868431|rs3940647|rs12025413|rs7820063|rs7000952|rs9799068|rs6767800|rs2736871|rs4316092|rs423207
Serositis	REGULATION_OF_SIGNALING_BY_CBL	REACTOME	703	44	20	1,04E-01	7,50E-01	rs1864053|rs809192|rs2302456|rs9647337|rs10820825|rs1366315|rs6871452|rs178255|rs4240910|rs3214047|rs383542|rs4740304|rs34299|rs16897109|rs1075834|rs16897180|rs7245158|rs16922298|rs4976028|rs1622208
Serositis	GLYOXYLATE_AND_DICARBOXYLATE_METABOLISM	KEGG	704	42	18	1,04E-01	7,50E-01	rs10970932|rs7037573|rs17720809|rs1331240|rs1956545|rs2423248|rs7660423|rs309455|rs7221585|rs10813645|rs2854702|rs7739248|rs17776734|rs12342878|rs9298935|rs1571231|rs7024321|rs699469
Serositis	GRB2_EVENTS_IN_ERBB2_SIGNALING	REACTOME	1202	38	20	1,04E-01	7,50E-01	rs3924999|rs2976525|rs2466059|rs7273626|rs4733117|rs10503929|rs2877260|rs13427642|rs1464443|rs10232489|rs2302984|rs12544147|rs4859540|rs10209471|rs2347505|rs4234512|rs1505376|rs2347501|rs13021324|rs2110290
Serositis	PENTOSE_PHOSPHATE_PATHWAY	KEGG	616	23	11	1,05E-01	7,50E-01	rs10993276|rs17301901|rs2236441|rs2375592|rs335124|rs17158714|rs2995965|rs2284997|rs12342274|rs2269260|rs3829910
Serositis	NA_CL_DEPENDENT_NEUROTRANSMITTER_TRANSPORTERS	REACTOME	718	35	17	1,05E-01	7,50E-01	rs11712912|rs10833366|rs747488|rs568567|rs3774128|rs7445640|rs13258461|rs13159461|rs3782370|rs17467186|rs2282810|rs2283138|rs2276433|rs3779672|rs894747|rs17298481|rs2304510
Serositis	UCALPAIN_PATHWAY	BIOCARTA	426	33	12	1,06E-01	7,50E-01	rs17106413|rs7016497|rs6018627|rs2056131|rs2292700|rs1475034|rs1671064|rs181483|rs6012200|rs9347252|rs819643|rs10940270
Serositis	PHOSPHORYLATION_OF_THE_APC_C	REACTOME	219	12	4	1,06E-01	7,50E-01	rs2942067|rs7681650|rs9590480|rs7736312
Serositis	GSK3_PATHWAY	BIOCARTA	1055	36	20	1,06E-01	7,50E-01	rs1864053|rs2221145|rs7866830|rs11793751|rs10983903|rs1392125|rs1366315|rs6871452|rs10022956|rs4355511|rs1327798|rs10489028|rs4958072|rs5001582|rs11725638|rs13188321|rs383542|rs2298765|rs2425363|rs34299
Serositis	P53_DEPENDENT_G1_DNA_DAMAGE_RESPONSE	REACTOME	838	22	20	1,07E-01	7,50E-01	rs10494494|rs4987876|rs10753110|rs227060|rs328349|rs7948797|rs3759216|rs1642763|rs12201680|rs918587|rs2917779|rs16827840|rs909473|rs11794849|rs755553|rs9380326|rs8004624|rs8005252|rs2621321|rs3859192
Serositis	LONG_TERM_DEPRESSION	KEGG	3926	35	20	1,08E-01	7,50E-01	rs1352388|rs967776|rs8464|rs6490121|rs7686241|rs10749883|rs6442887|rs6039107|rs3804984|rs2927585|rs1398312|rs1434384|rs11722663|rs2228068|rs1903999|rs12045786|rs11725238|rs597384|rs4926163|rs4964002
Serositis	G2_PATHWAY	BIOCARTA	354	15	10	1,09E-01	7,50E-01	rs4987876|rs227060|rs3745255|rs1642763|rs951629|rs2298483|rs3846484|rs6010742|rs10770043|rs7971479
Serositis	GPCR_PATHWAY	BIOCARTA	1102	37	20	1,09E-01	7,50E-01	rs4699236|rs2764938|rs8464|rs2960288|rs6812773|rs17530657|rs11207332|rs660749|rs6067805|rs4810147|rs12938937|rs748938|rs4234512|rs9808216|rs4699228|rs2645370|rs9913908|rs306872|rs918842|rs6733907
Serositis	SPHINGOLIPID_DE_NOVO_BIOSYNTHESIS	REACTOME	1020	35	20	1,09E-01	7,50E-01	rs2810043|rs12133338|rs4903606|rs2273814|rs29069|rs17751746|rs1930762|rs7145386|rs895344|rs9679935|rs10740635|rs4926703|rs12144400|rs225995|rs225864|rs2849382|rs1149580|rs12137498|rs10140406|rs13390393
Serositis	KERATAN_SULFATE_KERATIN_METABOLISM	REACTOME	737	38	20	1,09E-01	7,50E-01	rs1554324|rs2736868|rs1401346|rs4074708|rs2958830|rs6989475|rs2037957|rs950757|rs1868431|rs7820063|rs7000952|rs1466068|rs2736871|rs1667296|rs2770807|rs1503758|rs11919563|rs421801|rs6986303|rs4371486
Serositis	SMAD2_SMAD3_SMAD4_HETEROTRIMER_REGULATES_TRANSCRIPTION	REACTOME	715	35	20	1,11E-01	7,50E-01	rs9858201|rs11071927|rs7166081|rs10518714|rs13325694|rs11995971|rs2852950|rs7173609|rs1942159|rs11661651|rs2635509|rs2917779|rs8035972|rs11874742|rs2136619|rs4150724|rs11855908|rs7234567|rs7010121|rs2118612
Serositis	METABOLISM_OF_VITAMINS_AND_COFACTORS	REACTOME	1119	30	20	1,11E-01	7,50E-01	rs2704973|rs2748901|rs17184212|rs6935458|rs9838798|rs1956545|rs6956300|rs3786278|rs13245214|rs559325|rs2539847|rs10935348|rs579596|rs12218194|rs2086856|rs17182091|rs17518769|rs2236475|rs6954142|rs380691
Serositis	SHC1_EVENTS_IN_ERBB4_SIGNALING	REACTOME	1072	44	20	1,12E-01	7,50E-01	rs3924999|rs2976525|rs2466059|rs7273626|rs4733117|rs10503929|rs13427642|rs1464443|rs2302984|rs12544147|rs4859540|rs10209471|rs2347505|rs4234512|rs1505376|rs2347501|rs13021324|rs1357139|rs6860077|rs7290469
Serositis	PHOSPHATIDYLINOSITOL_SIGNALING_SYSTEM	KEGG	3813	37	20	1,12E-01	7,50E-01	rs1352388|rs1864053|rs10882406|rs9562385|rs8464|rs11146457|rs4486998|rs6442887|rs6039107|rs3804984|rs656629|rs182034|rs10241087|rs613993|rs4964002|rs9647337|rs13037679|rs6773688|rs511973|rs1029544
Serositis	RNA_POL_I_TRANSCRIPTION	REACTOME	937	47	14	1,12E-01	7,50E-01	rs4541737|rs9295730|rs9461416|rs14105|rs2875859|rs129128|rs7568330|rs2763982|rs7238305|rs9461204|rs9303601|rs9554080|rs11923928|rs9366694

Serositis	TRIF_MEDIATED_TLR3_SIGNALING	REACTOME	2075	36	20	1,13E-01	7,50E-01	rs17014035|rs483808|rs2764938|rs17747965|rs10863430|rs4352468|rs4965533|rs1221220|rs9366027|rs17530657|rs11207332|rs918418|rs17456901|rs7702020|rs1531846|rs1568383|rs17413414|rs468774|rs9895159|rs10498970
Serositis	TOB1_PATHWAY	BIOCARTA	652	47	18	1,13E-01	7,50E-01	rs1019855|rs11071927|rs7166081|rs10518714|rs2296620|rs13075948|rs7173609|rs4846488|rs8035972|rs2279455|rs12576947|rs7169633|rs7640543|rs6768081|rs4673260|rs2118612|rs10493860|rs6686571
Serositis	PDGF_PATHWAY	BIOCARTA	1021	44	20	1,14E-01	7,50E-01	rs1864053|rs2764938|rs8464|rs13395505|rs17530657|rs11207332|rs1366315|rs6871452|rs9895159|rs2017750|rs12938937|rs4234512|rs383542|rs9913908|rs1636808|rs34299|rs16897109|rs1492769|rs16897180|rs4381631
Serositis	APOPTOSIS	KEGG	1727	32	20	1,14E-01	7,50E-01	rs1864053|rs4699236|rs4987876|rs6812773|rs227060|rs2228576|rs9647337|rs11123905|rs11465990|rs511973|rs1366315|rs6871452|rs4077341|rs181402|rs17809603|rs2254527|rs2020902|rs17561|rs2046136|rs4240910
Serositis	PACKAGING_OF_TELOMERE_ENDS	REACTOME	515	31	8	1,15E-01	7,50E-01	rs4541737|rs9295730|rs9461416|rs129128|rs9461204|rs2748525|rs9366694|rs4731243
Serositis	AGR_PATHWAY	BIOCARTA	1981	31	20	1,15E-01	7,50E-01	rs3924999|rs2764938|rs2976525|rs2466059|rs7016497|rs6018627|rs4733117|rs17530657|rs10503929|rs6056665|rs11207332|rs2877260|rs221007|rs10232489|rs1764072|rs1884955|rs2017750|rs3778119|rs6569603|rs12523864
Serositis	ECM_PATHWAY	BIOCARTA	753	43	20	1,15E-01	7,50E-01	rs1864053|rs809192|rs16910559|rs7016497|rs6018627|rs1366315|rs6871452|rs4837820|rs4234512|rs8006774|rs383542|rs1636808|rs34299|rs16897109|rs16897180|rs6012200|rs7290469|rs7617041|rs4976028|rs7706821
Serositis	EFFECTS_OF_PIP2_HYDROLYSIS	REACTOME	1651	40	20	1,16E-01	7,50E-01	rs1352388|rs10516614|rs9562385|rs3783754|rs182034|rs10241087|rs523200|rs4964002|rs2711292|rs1029544|rs210120|rs1558909|rs1594485|rs1900448|rs16878220|rs2026628|rs2253650|rs971334|rs7177101|rs9870928
Serositis	FORMATION_OF_FIBRIN_CLOT_CLOTTING_CASCADE	REACTOME	703	45	20	1,17E-01	7,50E-01	rs10032695|rs2213865|rs2205895|rs12648395|rs11696134|rs12810426|rs352024|rs4220|rs7646517|rs7130900|rs13202850|rs822373|rs11918289|rs1742924|rs216856|rs7708314|rs9324220|rs2062632|rs6829128|rs4667193
Serositis	TOLL_PATHWAY	BIOCARTA	1147	35	20	1,18E-01	7,50E-01	rs2764938|rs17747965|rs7866830|rs1221220|rs11793751|rs17530657|rs10983903|rs1392125|rs11207332|rs9895159|rs10498970|rs1327798|rs2017750|rs5001582|rs10872893|rs12645085|rs1010650|rs2141484|rs157695|rs9362733
Serositis	ETS_PATHWAY	BIOCARTA	682	31	20	1,23E-01	7,50E-01	rs2764938|rs2836804|rs6913161|rs17530657|rs11207332|rs17025039|rs7948482|rs7538029|rs246396|rs2012898|rs433192|rs2129301|rs4765158|rs2410062|rs1263990|rs7937445|rs12574791|rs10893845|rs11057642|rs333968
Serositis	SULFUR_AMINO_ACID_METABOLISM	REACTOME	930	41	20	1,23E-01	7,50E-01	rs1356553|rs294594|rs574389|rs13178865|rs2780749|rs946934|rs12153515|rs11135312|rs670348|rs10874814|rs1422544|rs1366191|rs731727|rs11958300|rs1363075|rs510088|rs7723455|rs12658783|rs10515861|rs2247958
Serositis	DOWNSTREAM_SIGNAL_TRANSDUCTION	REACTOME	3083	42	20	1,24E-01	7,50E-01	rs2306677|rs1864053|rs8464|rs2960288|rs13395505|rs2302456|rs7273626|rs1909745|rs6018627|rs7590257|rs6051511|rs2711292|rs9647337|rs4234214|rs9856983|rs4569841|rs1366315|rs6871452|rs7733194|rs210120
Serositis	BCR_PATHWAY	BIOCARTA	1053	37	20	1,26E-01	7,50E-01	rs4699236|rs2764938|rs8464|rs6812773|rs17530657|rs11207332|rs10820825|rs660749|rs6067805|rs2017750|rs12938937|rs748938|rs4234512|rs9808216|rs4699228|rs2645370|rs9913908|rs306872|rs6733907|rs3811063
Serositis	SPHINGOLIPID_METABOLISM	REACTOME	1944	30	20	1,27E-01	7,50E-01	rs2810043|rs12133338|rs4903606|rs2273814|rs2008644|rs4074708|rs29069|rs10508921|rs11794290|rs17751746|rs1930762|rs2739685|rs430976|rs2560274|rs4758104|rs25413|rs2153486|rs1072382|rs7145386|rs895344
Serositis	CHRONIC_MYELOID_LEUKEMIA	KEGG	2538	37	20	1,27E-01	7,50E-01	rs1019855|rs1864053|rs11071927|rs2302456|rs7166081|rs10518714|rs6913161|rs2300401|rs6517288|rs1825887|rs9647337|rs511973|rs1366315|rs7781436|rs6871452|rs178255|rs13075948|rs11995971|rs3759216|rs1503920
Serositis	P53HYPOXIA_PATHWAY	BIOCARTA	517	22	11	1,28E-01	7,50E-01	rs4987876|rs227060|rs1486173|rs2017750|rs1642763|rs10410544|rs241280|rs11971230|rs1568261|rs881526|rs7971479
Serositis	AUTODEGRADATION_OF_THE_E3_UBIQUITIN_LIGASE_COP1	REACTOME	766	22	20	1,28E-01	7,50E-01	rs10494494|rs4987876|rs10753110|rs227060|rs328349|rs7948797|rs1642763|rs12201680|rs2917779|rs16827840|rs909473|rs11794849|rs755553|rs9380326|rs8004624|rs8005252|rs2621321|rs3859192|rs7500512|rs1424083
Serositis	O_GLYCAN_BIOSYNTHESIS	KEGG	1579	25	20	1,29E-01	7,50E-01	rs1554324|rs4719136|rs2736868|rs4952025|rs4745552|rs10497137|rs1401346|rs2958830|rs6989475|rs4719120|rs2037957|rs17166192|rs6553604|rs4696036|rs2303325|rs950757|rs1868431|rs7820063|rs7000952|rs7674455
Serositis	GLEEVEC_PATHWAY	BIOCARTA	954	48	20	1,30E-01	7,50E-01	rs1864053|rs2764938|rs13395505|rs17530657|rs11207332|rs1366315|rs6871452|rs178255|rs11995971|rs9895159|rs2017750|rs2156921|rs4234512|rs383542|rs1636808|rs34299|rs16897109|rs16897180|rs7617041|rs7010121
Serositis	RESPIRATORY_ELECTRON_TRANSPORT_ATP_SYNTHESIS_BY_CHEMIOSMOTIC_COUPLIN TION_BY_UNCOUPLING_PROTEINS_	REACTOME	1741	43	20	1,31E-01	7,50E-01	rs4923649|rs6437332|rs1345638|rs1264901|rs2252523|rs10045456|rs10777629|rs1500147|rs10777647|rs2302178|rs892492|rs12538107|rs2668424|rs4810150|rs13405880|rs4804840|rs16962149|rs300657|rs17285545|rs6070703
Serositis	SIGNALLING_TO_ERKS	REACTOME	794	47	19	1,32E-01	7,50E-01	rs17014035|rs2302456|rs7273626|rs6018627|rs2043722|rs7548973|rs11177669|rs2254527|rs11137525|rs4234512|rs4740304|rs16837709|rs2519090|rs6012200|rs7290469|rs2425650|rs7617041|rs2076590|rs1835496
Serositis	B_CELL_RECEPTOR_SIGNALING_PATHWAY	KEGG	2278	51	20	1,34E-01	7,50E-01	rs12413520|rs2555479|rs1864053|rs4699236|rs2764938|rs6812773|rs17530657|rs9647337|rs11207332|rs10820825|rs511973|rs1366315|rs6871452|rs660749|rs6067805|rs9932716|rs10465781|rs516321|rs13054809|rs4240910
Serositis	INSULIN_PATHWAY	BIOCARTA	725	45	20	1,34E-01	7,50E-01	rs1864053|rs2764938|rs17530657|rs11207332|rs1366315|rs6871452|rs10498208|rs2017750|rs1273793|rs17822304|rs4234512|rs10933137|rs383542|rs1636808|rs34299|rs16897109|rs16897180|rs7617041|rs4244808|rs4976028
Serositis	PYRUVATE_METABOLISM_AND_CITRIC_ACID_TCA_CYCLE	REACTOME	905	39	9	1,34E-01	7,50E-01	rs7817292|rs962033|rs7574229|rs7558055|rs11690708|rs2080087|rs2230392|rs2647168|rs1536002
Serositis	NEUROTRANSMITTER_RELEASE_CYCLE	REACTOME	1400	43	20	1,36E-01	7,50E-01	rs2355575|rs11712912|rs10491731|rs2781562|rs10814971|rs1330581|rs568567|rs9293878|rs3774128|rs6924133|rs9937726|rs6071173|rs16987501|rs1860172|rs7760432|rs17467186|rs130274|rs2068324|rs137326|rs2283138
Serositis	BUTANOATE_METABOLISM	KEGG	763	39	20	1,36E-01	7,50E-01	rs10890819|rs17565061|rs10732359|rs2214195|rs1330581|rs6769361|rs12577790|rs9937726|rs11725638|rs3751181|rs4878199|rs17470048|rs11744623|rs4646771|rs13083034|rs10846840|rs3798211|rs11726284|rs11058065|rs3790692
Serositis	BIOSYNTHESIS_OF_THE_N_GLYCAN_PRECURSOR_DOLICHOL_LIPID_LINKED_OLIGOSACC RANSFER_TO_A_NASCENT_PROTEIN	REACTOME	355	25	8	1,36E-01	7,50E-01	rs3739821|rs6415846|rs4898765|rs9924614|rs17122705|rs7527765|rs10454019|rs9824
Serositis	TGF_BETA_SIGNALING_PATHWAY	KEGG	3535	30	20	1,37E-01	7,50E-01	rs7495569|rs1019855|rs7566998|rs11071927|rs1458526|rs7166081|rs10518714|rs976568|rs10783481|rs2197815|rs934721|rs2414912|rs16883016|rs10490217|rs10188643|rs425491|rs12765929|rs3756990|rs9294963|rs6054590
Serositis	RHO_PATHWAY	BIOCARTA	480	22	9	1,37E-01	7,50E-01	rs16910559|rs6018627|rs907698|rs4837820|rs4969384|rs8006774|rs1174658|rs6012200|rs8072124
Serositis	PRE_NOTCH_EXPRESSION_AND_PROCESSING	REACTOME	1434	41	20	1,37E-01	7,50E-01	rs10939104|rs11655371|rs2764938|rs2150299|rs10765795|rs4692114|rs522495|rs17530657|rs1109771|rs11207332|rs9268005|rs2269423|rs4659248|rs1642763|rs10501835|rs10017756|rs17116491|rs10132156|rs4879666|rs1296720
Serositis	T_CELL_RECEPTOR_SIGNALING_PATHWAY	KEGG	3546	39	20	1,41E-01	7,50E-01	rs1864053|rs4699236|rs483808|rs7086224|rs2764938|rs809192|rs6812773|rs4301703|rs17747965|rs1221220|rs3024498|rs7590257|rs17530657|rs6056665|rs9647337|rs12613960|rs11207332|rs511973|rs1366315|rs6871452
Serositis	INTERFERON_ALPHA_BETA_SIGNALING	REACTOME	1477	55	20	1,42E-01	7,50E-01	rs2352930|rs13395505|rs398800|rs2074560|rs276978|rs1127314|rs9258186|rs12149292|rs886451|rs2236906|rs7770216|rs12720253|rs4789576|rs9882443|rs5009448|rs4527079|rs7254474|rs7761965|rs386365|rs4728142
Serositis	PROGESTERONE_MEDIATED_OOCYTE_MATURATION	KEGG	2535	34	20	1,42E-01	7,50E-01	rs1864053|rs12299821|rs2960288|rs169290|rs1899908|rs4352468|rs1909745|rs2044316|rs9366027|rs2942067|rs9647337|rs7681082|rs2282411|rs4234214|rs511973|rs9856983|rs4569841|rs1366315|rs6871452|rs7733194
Serositis	ALDOSTERONE_REGULATED_SODIUM_REABSORPTION	KEGG	1794	40	20	1,42E-01	7,50E-01	rs1787774|rs3910044|rs1864053|rs8464|rs543347|rs2228576|rs9647337|rs511973|rs1366315|rs6871452|rs10498208|rs2883917|rs2288772|rs1273793|rs4240910|rs1642763|rs7993089|rs9493929|rs12938937|rs10933137
Serositis	MYD88_MAL_CASCADE_INITIATED_ON_PLASMA_MEMBRANE	REACTOME	2527	35	20	1,43E-01	7,50E-01	rs17014035|rs483808|rs2764938|rs17747965|rs4352468|rs4965533|rs7866830|rs1221220|rs11793751|rs9366027|rs17530657|rs10983903|rs1392125|rs11207332|rs11465990|rs918418|rs17456901|rs7702020|rs1531846|rs1568383
Serositis	ATRBRCA_PATHWAY	BIOCARTA	238	23	11	1,45E-01	7,50E-01	rs4987876|rs867185|rs227060|rs337484|rs1642763|rs9311428|rs2298483|rs11756089|rs6450035|rs4942486|rs2155209
Serositis	BOTULINUM_NEUROTOXICITY	REACTOME	522	14	11	1,46E-01	7,50E-01	rs6026402|rs6026398|rs9893164|rs907698|rs11870073|rs6951030|rs1487608|rs363039|rs17741348|rs11585565|rs2532514
Serositis	ACTIVATED_TLR4_SIGNALLING	REACTOME	2676	36	20	1,47E-01	7,50E-01	rs17014035|rs483808|rs2764938|rs17747965|rs10863430|rs4352468|rs4965533|rs7866830|rs1221220|rs11793751|rs9366027|rs17530657|rs10983903|rs1392125|rs11207332|rs11465990|rs918418|rs17456901|rs7702020|rs1531846
Serositis	KERATAN_SULFATE_BIOSYNTHESIS	REACTOME	674	44	20	1,48E-01	7,50E-01	rs1554324|rs2736868|rs1401346|rs2958830|rs6989475|rs2037957|rs950757|rs1868431|rs7820063|rs7000952|rs1466068|rs2736871|rs1667296|rs4879666|rs1503758|rs11919563|rs421801|rs6986303|rs4371486|rs7814358
Serositis	NGF_PATHWAY	BIOCARTA	566	53	19	1,49E-01	7,50E-01	rs1864053|rs2764938|rs17530657|rs11207332|rs1366315|rs6871452|rs2254527|rs2017750|rs4234512|rs383542|rs1636808|rs34299|rs16897109|rs16897180|rs7617041|rs4976028|rs7706821|rs8004059|rs7379303
Serositis	REGULATION_OF_MRNA_STABILITY_BY_PROTEINS_THAT_BIND_AU_RICH_ELEMENTS	REACTOME	1372	31	20	1,49E-01	7,50E-01	rs17014035|rs8464|rs7273626|rs328349|rs764223|rs6996970|rs6822283|rs17464037|rs7948797|rs11879090|rs1345207|rs2101680|rs17005458|rs12201680|rs12938937|rs17757211|rs9913908|rs2917779|rs16827840|rs2035912
Serositis	VIRAL_MYOCARDITIS	KEGG	2750	55	20	1,50E-01	7,50E-01	rs11078839|rs2824319|rs1264524|rs3759614|rs809192|rs17533090|rs2335347|rs3744550|rs2235383|rs7153499|rs1651543|rs9276711|rs7770216|rs10779277|rs181402|rs4486969|rs17809603|rs1394606|rs3778119|rs6569603
Serositis	ACTIVATION_OF_NMDA_RECEPTOR_UPON_GLUTAMATE_BINDING_AND_POSTSYNAPTIC	REACTOME	2117	27	20	1,50E-01	7,50E-01	rs2960288|rs7686241|rs11055455|rs9968730|rs2927585|rs4704706|rs1434384|rs11722663|rs9366027|rs1421106|rs11725238|rs4780486|rs7699538|rs1362327|rs6827325|rs1862409|rs10750731|rs1861872|rs8049630|rs8064140
Serositis	RNA_POL_III_CHAIN_ELONGATION	REACTOME	364	39	18	1,50E-01	7,50E-01	rs17422451|rs2583680|rs985559|rs13258625|rs7126805|rs14105|rs512598|rs11782092|rs10824587|rs2288270|rs1383246|rs13277342|rs4246521|rs9554080|rs2410665|rs2597388|rs6586938|rs704986
Serositis	ALZHEIMERS_DISEASE	KEGG	5029	34	20	1,50E-01	7,50E-01	rs1352388|rs4699236|rs6812773|rs6437332|rs6490121|rs967232|rs1345638|rs17801265|rs6442887|rs6039107|rs1264901|rs11055455|rs2252523|rs3804984|rs2228576|rs13050861|rs10045456|rs6433541|rs1500147|rs1421106
Serositis	GLYCOSPHINGOLIPID_METABOLISM	REACTOME	924	31	20	1,51E-01	7,50E-01	rs2008644|rs4074708|rs10508921|rs11794290|rs2739685|rs430976|rs2560274|rs4758104|rs25413|rs2153486|rs1072382|rs3733895|rs2041976|rs2581952|rs1890911|rs12499919|rs412218|rs1687863|rs9616113|rs12888666
Serositis	ERK_MAPK_TARGETS	REACTOME	704	28	12	1,52E-01	7,50E-01	rs4965533|rs9366027|rs918418|rs17456901|rs7702020|rs1568383|rs6598265|rs325383|rs1864180|rs11633623|rs7290469|rs3799647
Serositis	OOCYTE_MEIOSIS	KEGG	3114	35	20	1,52E-01	7,50E-01	rs1352388|rs4699236|rs2960288|rs6812773|rs7273626|rs6442887|rs3804984|rs1909745|rs6996970|rs9366027|rs2942067|rs1022476|rs4964002|rs7699538|rs7681650|rs4234214|rs17464037|rs6773688|rs9856983|rs4569841
Serositis	CITRIC_ACID_CYCLE_TCA_CYCLE	REACTOME	675	33	7	1,52E-01	7,50E-01	rs962033|rs7574229|rs7558055|rs11690708|rs2080087|rs2647168|rs1536002
Serositis	IL_3_5_AND_GM_CSF_SIGNALING	REACTOME	1134	42	20	1,55E-01	7,58E-01	rs1864053|rs809192|rs2302456|rs6996970|rs9647337|rs10820825|rs17464037|rs1366315|rs6871452|rs178255|rs6794523|rs4240910|rs3214047|rs3792424|rs334782|rs383542|rs4740304|rs17606924|rs34299|rs16897109
Serositis	PROXIMAL_TUBULE_BICARBONATE_RECLAMATION	KEGG	458	25	11	1,58E-01	7,61E-01	rs2355575|rs6070157|rs359535|rs1642763|rs1860172|rs1203182|rs6015032|rs2296063|rs11265346|rs1921915|rs11746573
Serositis	COMPLEMENT_AND_COAGULATION_CASCADES	KEGG	1742	43	20	1,59E-01	7,61E-01	rs10032695|rs2213865|rs2205895|rs2204345|rs12648395|rs920909|rs4151657|rs11696134|rs3766404|rs12810426|rs250724|rs10779277|rs2031749|rs292001|rs4220|rs10824804|rs3776519|rs706469|rs11032363|rs7444800
Serositis	DESTABILIZATION_OF_MRNA_BY_KSRP	REACTOME	205	13	7	1,59E-01	7,61E-01	rs764223|rs6996970|rs17464037|rs11879090|rs11189224|rs6498509|rs4002957
Serositis	G2_M_CHECKPOINTS	REACTOME	710	25	20	1,60E-01	7,61E-01	rs4987876|rs227060|rs7290191|rs133415|rs10276762|rs11164955|rs4943153|rs6460075|rs886727|rs11164953|rs9311428|rs2298483|rs1928023|rs3846484|rs741048|rs2321822|rs13082666|rs17137468|rs4265124|rs12584168
Serositis	ASTHMA	KEGG	770	64	17	1,60E-01	7,61E-01	rs17533090|rs474951|rs3024498|rs917606|rs9276711|rs490358|rs1554286|rs3135363|rs2894381|rs176248|rs3135344|rs11751659|rs6032678|rs3134986|rs2857709|rs9380326|rs2621321
Serositis	RNA_POL_III_TRANSCRIPTION_INITIATION_FROM_TYPE_3_PROMOTER	REACTOME	501	40	19	1,63E-01	7,62E-01	rs17422451|rs2583680|rs985559|rs13258625|rs7126805|rs14105|rs512598|rs11782092|rs4979935|rs2288270|rs1383246|rs13277342|rs4246521|rs9554080|rs4804833|rs2410665|rs2597388|rs6586938|rs704986
Serositis	CTLA4_PATHWAY	BIOCARTA	662	41	20	1,64E-01	7,62E-01	rs1864053|rs12613960|rs1366315|rs6871452|rs28530648|rs10195135|rs714158|rs4675397|rs17822304|rs747020|rs3135363|rs7354779|rs3135344|rs383542|rs378299|rs2715261|rs34299|rs16897109|rs16897180|rs12473084
Serositis	ERBB_SIGNALING_PATHWAY	KEGG	4135	35	20	1,65E-01	7,62E-01	rs3924999|rs1864053|rs483808|rs2764938|rs8464|rs2976525|rs2302456|rs2466059|rs7016497|rs4352468|rs6018627|rs7590257|rs4733117|rs17530657|rs10503929|rs6056665|rs9647337|rs11207332|rs7699538|rs511973
Serositis	RESPIRATORY_ELECTRON_TRANSPORT	REACTOME	1631	40	20	1,67E-01	7,62E-01	rs4923649|rs6437332|rs1345638|rs2252523|rs10045456|rs10777629|rs1500147|rs10777647|rs2302178|rs892492|rs12538107|rs2668424|rs13405880|rs4804840|rs16962149|rs300662|rs17285545|rs4975854|rs2974101|rs7245
Serositis	MEF2D_PATHWAY	BIOCARTA	684	28	20	1,67E-01	7,62E-01	rs4699236|rs8464|rs6812773|rs6913161|rs660749|rs6067805|rs12938937|rs748938|rs9808216|rs4699228|rs2645370|rs9913908|rs306872|rs6733907|rs3811063|rs4381631|rs2273642|rs13430277|rs736232|rs17030758
Serositis	RNA_POL_III_TRANSCRIPTION_INITIATION_FROM_TYPE_2_PROMOTER	REACTOME	417	44	20	1,67E-01	7,62E-01	rs17422451|rs2583680|rs985559|rs13258625|rs7126805|rs14105|rs512598|rs650627|rs11782092|rs10824587|rs2288270|rs1383246|rs13277342|rs4246521|rs9554080|rs608604|rs2410665|rs2597388|rs6586938|rs704986
Serositis	APC_C_CDC20_MEDIATED_DEGRADATION_OF_CYCLIN_B	REACTOME	224	13	5	1,68E-01	7,62E-01	rs2942067|rs7681650|rs9590480|rs2917779|rs7736312
Serositis	NUCLEAR_EVENTS_KINASE_AND_TRANSCRIPTION_FACTOR_ACTIVATION	REACTOME	766	36	16	1,69E-01	7,62E-01	rs17014035|rs4965533|rs9366027|rs918418|rs17456901|rs7702020|rs1568383|rs6598265|rs325383|rs918842|rs1864180|rs11633623|rs7290469|rs3799647|rs1862884|rs10804160
Serositis	P38MAPK_PATHWAY	BIOCARTA	1904	53	20	1,71E-01	7,62E-01	rs17014035|rs967776|rs13395505|rs17747965|rs4965533|rs1221220|rs12564813|rs11100242|rs2237268|rs591393|rs918418|rs7702020|rs11995971|rs9895159|rs6671527|rs10498970|rs737523|rs6598265|rs1060478|rs325383
Serositis	ACTIVATED_AMPK_STIMULATES_FATTY_ACID_OXIDATION_IN_MUSCLE	REACTOME	421	16	9	1,72E-01	7,62E-01	rs4435442|rs1881638|rs7659090|rs4950385|rs7789699|rs2268388|rs7788961|rs9568196|rs4425665
Serositis	CONVERSION_FROM_APC_C_CDC20_TO_APC_C_CDH1_IN_LATE_ANAPHASE	REACTOME	226	15	6	1,73E-01	7,62E-01	rs2942067|rs7681650|rs491675|rs9590480|rs6577168|rs2012797
Serositis	PLC_BETA_MEDIATED_EVENTS	REACTOME	2313	39	20	1,74E-01	7,62E-01	rs1352388|rs967776|rs8464|rs2960288|rs6039107|rs1909745|rs6678574|rs4964002|rs13037679|rs4234214|rs591393|rs9856983|rs4569841|rs7733194|rs210120|rs1474937|rs6039104|rs891429|rs1550116|rs12938937
Serositis	CELL_CYCLE_CHECKPOINTS	REACTOME	1961	26	20	1,76E-01	7,62E-01	rs10494494|rs4987876|rs10753110|rs227060|rs328349|rs7290191|rs2942067|rs7681082|rs133415|rs7948797|rs6972752|rs10276762|rs11164955|rs4943153|rs3759216|rs6460075|rs886727|rs11164953|rs1642763|rs9590480
Serositis	HDAC_PATHWAY	BIOCARTA	1231	33	20	1,79E-01	7,62E-01	rs1864053|rs4699236|rs6812773|rs4965533|rs918418|rs7702020|rs1366315|rs6871452|rs660749|rs11247367|rs6067805|rs2684799|rs11854132|rs6598265|rs748938|rs2129301|rs325383|rs9808216|rs383542|rs4699228
Serositis	TNFR1_PATHWAY	BIOCARTA	862	36	20	1,80E-01	7,62E-01	rs2764938|rs17747965|rs2228576|rs1221220|rs17530657|rs11207332|rs9895159|rs10498970|rs2017750|rs2290149|rs1010650|rs555745|rs2141484|rs157695|rs9362733|rs711272|rs907186|rs10485405|rs2818291|rs2857709
Serositis	ACTIVATION_OF_THE_PRE_REPLICATIVE_COMPLEX	REACTOME	565	16	13	1,81E-01	7,62E-01	rs4540267|rs7290191|rs133415|rs10276762|rs11164955|rs886727|rs11164953|rs741048|rs13082666|rs17137468|rs4265124|rs2307373|rs941289
Serositis	REGULATION_OF_AUTOPHAGY	KEGG	933	31	14	1,81E-01	7,62E-01	rs4486998|rs656629|rs3745255|rs4764321|rs12632852|rs7240984|rs10853477|rs1865275|rs4658565|rs8087566|rs4890360|rs3851210|rs4244808|rs7037389
Serositis	DOWNREGULATION_OF_SMAD2_3_SMAD4_TRANSCRIPTIONAL_ACTIVITY	REACTOME	453	38	20	1,82E-01	7,62E-01	rs11071927|rs7166081|rs10518714|rs4448517|rs2852950|rs7173609|rs1942159|rs11661651|rs4765158|rs486755|rs1263990|rs2917779|rs8035972|rs11874742|rs2136619|rs10489667|rs907186|rs7169633|rs7234567|rs11057642
Serositis	ACTIVATION_OF_BH3_ONLY_PROTEINS	REACTOME	325	34	16	1,83E-01	7,62E-01	rs7273626|rs4286195|rs1893514|rs181402|rs17809603|rs2017750|rs724710|rs2046136|rs948769|rs1642763|rs11637681|rs2000745|rs2849382|rs7228933|rs4150724|rs2425650
Serositis	INWARDLY_RECTIFYING_K_CHANNELS	REACTOME	1317	42	20	1,84E-01	7,62E-01	rs13005001|rs2283253|rs12601653|rs16975251|rs12553893|rs1799270|rs4793331|rs726568|rs1167763|rs1999500|rs2074308|rs10841915|rs7864144|rs12601221|rs1253676|rs1992703|rs3106667|rs9914095|rs7214537|rs3785902
Serositis	SIGNALING_BY_FGFR1_FUSION_MUTANTS	REACTOME	639	46	20	1,86E-01	7,62E-01	rs1864053|rs13395505|rs2529232|rs441601|rs1366315|rs6871452|rs2156921|rs17364455|rs2529192|rs383542|rs6467778|rs2906734|rs34299|rs16897109|rs16897180|rs404523|rs131690|rs4976028|rs7706821|rs2279957
Serositis	IL1R_PATHWAY	BIOCARTA	1001	36	20	1,86E-01	7,62E-01	rs2764938|rs17747965|rs1221220|rs17530657|rs11207332|rs11123905|rs11465990|rs419598|rs10498970|rs2017750|rs17561|rs12307857|rs10872893|rs1010650|rs2141484|rs157695|rs9362733|rs4846488|rs711272|rs10485405
Serositis	TRANSCRIPTIONAL_ACTIVITY_OF_SMAD2_SMAD3_SMAD4_HETEROTRIMER	REACTOME	967	26	20	1,86E-01	7,62E-01	rs9858201|rs11071927|rs7166081|rs10518714|rs13325694|rs11995971|rs4448517|rs2852950|rs7173609|rs1942159|rs11661651|rs2635509|rs4765158|rs486755|rs1263990|rs2917779|rs8035972|rs11874742|rs2136619|rs10489667
Serositis	PROLONGED_ERK_ACTIVATION_EVENTS	REACTOME	429	33	12	1,87E-01	7,62E-01	rs2302456|rs7273626|rs7548973|rs11177669|rs2254527|rs4234512|rs4740304|rs16837709|rs7290469|rs2425650|rs7617041|rs2076590
Serositis	CELL_CYCLE	KEGG	2581	28	20	1,88E-01	7,62E-01	rs4987876|rs11071927|rs7166081|rs10518714|rs7273626|rs227060|rs6913161|rs7290191|rs6996970|rs3745255|rs2942067|rs1022476|rs7681082|rs133415|rs2282411|rs17464037|rs6972752|rs7781436|rs11164955|rs11995971
Serositis	REGULATORY_RNA_PATHWAYS	REACTOME	345	10	6	1,88E-01	7,62E-01	rs11655371|rs7126805|rs7160403|rs1110386|rs8015314|rs10848243
Serositis	GATA3_PATHWAY	BIOCARTA	898	32	20	1,89E-01	7,62E-01	rs11150179|rs8059522|rs1538242|rs979534|rs983263|rs660749|rs6067805|rs7188879|rs7904468|rs16951388|rs748938|rs946028|rs4888024|rs306872|rs2996520|rs7200600|rs7067548|rs2454062|rs1514232|rs2302234
Serositis	NON_SMALL_CELL_LUNG_CANCER	KEGG	2585	37	20	1,89E-01	7,62E-01	rs1864053|rs2594149|rs2682971|rs8464|rs12739596|rs7634073|rs10863430|rs6774513|rs1057504|rs9647337|rs511973|rs2142296|rs2877260|rs1366315|rs7781436|rs6871452|rs4679516|rs815719|rs1868220|rs10232489
Serositis	PI3K_EVENTS_IN_ERBB4_SIGNALING	REACTOME	1709	34	20	1,90E-01	7,62E-01	rs3924999|rs1864053|rs2976525|rs2466059|rs4733117|rs10503929|rs6051511|rs1366315|rs6871452|rs13427642|rs1464443|rs3759216|rs541425|rs2302984|rs2020902|rs12544147|rs4859540|rs10209471|rs2347505|rs1505376
Serositis	RAS_ACTIVATION_UOPN_CA2_INFUX_THROUGH_NMDA_RECEPTOR	REACTOME	1033	36	20	1,91E-01	7,62E-01	rs11055455|rs9968730|rs4704706|rs1421106|rs4780486|rs7699538|rs1543889|rs1861872|rs8049630|rs8064140|rs1107475|rs1070474|rs1063243|rs12820037|rs11055603|rs12449282|rs9808216|rs4958456|rs12818068|rs12369358
Serositis	PURINE_METABOLISM	KEGG	5442	31	20	1,92E-01	7,62E-01	rs2594149|rs12299821|rs6848187|rs2682971|rs2960288|rs377432|rs10749883|rs169290|rs7634073|rs11763324|rs6921041|rs1899908|rs4540267|rs26956|rs1909745|rs6934819|rs2044316|rs1398312|rs6935458|rs2190998
Serositis	PAR1_PATHWAY	BIOCARTA	1941	27	20	1,94E-01	7,62E-01	rs1864053|rs8464|rs2960288|rs17747965|rs6039107|rs1221220|rs13037679|rs221048|rs2282537|rs1366315|rs6871452|rs7611544|rs250724|rs11879985|rs907698|rs10498970|rs2291462|rs1474937|rs4681946|rs6039104
Serositis	P53_SIGNALING_PATHWAY	KEGG	1897	29	20	1,94E-01	7,62E-01	rs10494494|rs1530561|rs4987876|rs10753110|rs227060|rs4272042|rs1921765|rs4286195|rs7781436|rs1539953|rs181402|rs10497131|rs1486173|rs4035233|rs17809603|rs8053839|rs2020902|rs948769|rs2201331|rs2188513
Serositis	OPIOID_SIGNALLING	REACTOME	3594	37	20	1,95E-01	7,62E-01	rs2306677|rs4699236|rs967776|rs8464|rs2960288|rs6812773|rs6039107|rs26956|rs1909745|rs6678574|rs12553893|rs13037679|rs4234214|rs591393|rs9856983|rs4569841|rs17292684|rs7733194|rs210120|rs5010905

Serositis	SIGNALING_BY_ILS	REACTOME	2996	39	20	1,96E-01	7,62E-01	rs1864053|rs7086224|rs13395505|rs809192|rs2302456|rs4301703|rs7273626|rs17747965|rs6996970|rs1221220|rs9647337|rs11123905|rs10820825|rs17464037|rs11465990|rs1366315|rs6871452|rs1531846|rs178255|rs9895159
Serositis	SIGNALING_BY_TGF_BETA_RECEPTOR_COMPLEX	REACTOME	1605	29	20	1,96E-01	7,62E-01	rs1019855|rs9858201|rs11071927|rs7166081|rs10518714|rs13325694|rs6025742|rs1345207|rs13075948|rs11995971|rs11879985|rs4448517|rs2852950|rs7173609|rs17702096|rs1942159|rs6025689|rs11661651|rs12974783|rs2635509
Serositis	REGULATION_OF_WATER_BALANCE_BY_RENAL_AQUAPORINS	REACTOME	1393	52	20	1,96E-01	7,62E-01	rs1790786|rs2960288|rs1790799|rs1909745|rs491148|rs12185480|rs12553893|rs487455|rs4234214|rs9856983|rs4569841|rs7733194|rs4810147|rs891429|rs1550116|rs1253676|rs11070884|rs13153439|rs1925284|rs7863832
Serositis	NDKDYNAMIN_PATHWAY	BIOCARTA	548	31	17	1,97E-01	7,62E-01	rs4699236|rs6812773|rs17567|rs8104242|rs751873|rs9808216|rs4699228|rs2645370|rs518477|rs12294130|rs10263645|rs6733907|rs3811063|rs350289|rs13430277|rs17030758|rs1294506
Serositis	PGC1A_PATHWAY	BIOCARTA	959	28	20	1,98E-01	7,64E-01	rs4699236|rs6812773|rs4965533|rs2946394|rs7699538|rs918418|rs7702020|rs2970873|rs6598265|rs2129301|rs325383|rs1457115|rs9808216|rs4699228|rs2645370|rs4958456|rs1027228|rs742257|rs16874509|rs1011398
Serositis	P53_PATHWAY	BIOCARTA	347	16	11	2,00E-01	7,65E-01	rs4987876|rs227060|rs2046136|rs1642763|rs130274|rs918587|rs2849382|rs135152|rs2298765|rs7971479|rs11607976
Serositis	ELONGATION_ARREST_AND_RECOVERY	REACTOME	246	2	2	2,02E-01	7,65E-01	rs7126805|rs547005
Serositis	TRAF6_MEDIATED_INDUCTION_OF_NFKB_AND_MAP_KINASES_UPON_TLR7_8_OR_9_ACT	REACTOME	2239	39	20	2,05E-01	7,65E-01	rs17014035|rs483808|rs2764938|rs17747965|rs4352468|rs4965533|rs1221220|rs9366027|rs17530657|rs11207332|rs918418|rs17456901|rs7702020|rs1531846|rs1568383|rs468774|rs9895159|rs10498970|rs2017750|rs7973701
Serositis	PYK2_PATHWAY	BIOCARTA	752	38	20	2,05E-01	7,65E-01	rs2764938|rs8464|rs6018627|rs17530657|rs11207332|rs178255|rs9895159|rs2017750|rs12938937|rs4234512|rs9808216|rs9913908|rs6733907|rs11999145|rs4381631|rs6012200|rs7290469|rs7617041|rs13430277|rs1294506
Serositis	SYNTHESIS_OF_PIPS_AT_THE_GOLGI_MEMBRANE	REACTOME	788	24	11	2,07E-01	7,65E-01	rs4486998|rs656629|rs7240984|rs10853477|rs4473902|rs1865275|rs10505828|rs8087566|rs4890360|rs10444068|rs11044223
Serositis	DEPOSITION_OF_NEW_CENPA_CONTAINING_NUCLEOSOMES_AT_THE_CENTROMERE	REACTOME	637	41	13	2,07E-01	7,65E-01	rs4541737|rs9295730|rs9461416|rs129128|rs1550116|rs2833736|rs9461204|rs13220023|rs7736312|rs1958167|rs879665|rs9366694|rs3806929
Serositis	PI3K_EVENTS_IN_ERBB2_SIGNALING	REACTOME	1886	33	20	2,08E-01	7,65E-01	rs3924999|rs1864053|rs2976525|rs2466059|rs4733117|rs10503929|rs6051511|rs2877260|rs1366315|rs6871452|rs13427642|rs1464443|rs3759216|rs10232489|rs541425|rs2302984|rs2020902|rs12544147|rs4859540|rs10209471
Serositis	ACTINY_PATHWAY	BIOCARTA	290	13	4	2,08E-01	7,65E-01	rs4666600|rs16837709|rs1174658|rs17340684
Serositis	JNK_C_JUN_KINASES_PHOSPHORYLATION_AND_ACTIVATION_MEDIATED_BY_ACTIVATI	REACTOME	728	39	20	2,08E-01	7,65E-01	rs483808|rs17747965|rs4352468|rs1221220|rs1531846|rs9895159|rs10498970|rs2017750|rs1010650|rs2141484|rs157695|rs9362733|rs711272|rs10485405|rs2818291|rs7438872|rs10094579|rs721025|rs607071|rs9345148
Serositis	FANCONI_ANEMIA_PATHWAY	REACTOME	461	18	13	2,09E-01	7,65E-01	rs4987876|rs227060|rs8109798|rs10490114|rs848282|rs337484|rs980481|rs2917779|rs11756089|rs2160091|rs6758100|rs4942486|rs2863301
Serositis	ARMS_MEDIATED_ACTIVATION	REACTOME	393	32	11	2,09E-01	7,65E-01	rs2302456|rs7273626|rs7548973|rs2254527|rs4234512|rs4740304|rs16837709|rs7290469|rs2425650|rs7617041|rs2076590
Serositis	IL12_PATHWAY	BIOCARTA	589	27	13	2,14E-01	7,79E-01	rs2764938|rs17530657|rs11207332|rs17454570|rs2017750|rs12720253|rs1075498|rs7254474|rs280519|rs12576947|rs7721176|rs11869073|rs6686571
Serositis	TOLL_RECEPTOR_CASCADES	REACTOME	3778	37	20	2,17E-01	7,81E-01	rs17014035|rs483808|rs2764938|rs1296028|rs4486998|rs17747965|rs10863430|rs4352468|rs4965533|rs7866830|rs656629|rs1221220|rs11793751|rs9366027|rs2722195|rs17530657|rs10983903|rs7741738|rs1392125|rs11207332
Serositis	PLATELET_ACTIVATION_SIGNALING_AND_AGGREGATION	REACTOME	6965	36	20	2,18E-01	7,81E-01	rs1352388|rs1864053|rs967776|rs10516614|rs9562385|rs8464|rs2213865|rs2205895|rs809192|rs2302456|rs12648395|rs17661732|rs17106413|rs7016497|rs3783754|rs6996970|rs6018627|rs11168228|rs1034620|rs2056131
Serositis	A_TETRASACCHARIDE_LINKER_SEQUENCE_IS_REQUIRED_FOR_GAG_SYNTHESIS	REACTOME	1731	35	20	2,20E-01	7,81E-01	rs1458526|rs2228331|rs1932306|rs3734371|rs4583089|rs12819127|rs928646|rs1556466|rs7130091|rs1008953|rs2149063|rs1892984|rs736389|rs9524254|rs17205951|rs734384|rs16949539|rs12318659|rs1341660|rs6805260
Serositis	E2F_MEDIATED_REGULATION_OF_DNA_REPLICATION	REACTOME	452	14	11	2,20E-01	7,81E-01	rs4540267|rs7290191|rs2282411|rs9315422|rs918587|rs28171|rs380691|rs3846484|rs4265124|rs4150724|rs2307373
Serositis	ACH_PATHWAY	BIOCARTA	602	35	18	2,23E-01	7,81E-01	rs1864053|rs7016497|rs6018627|rs1366315|rs6871452|rs7445640|rs3001115|rs383542|rs1636808|rs34299|rs16897109|rs16897180|rs4132110|rs859722|rs6012200|rs4976028|rs7706821|rs7379303
Serositis	FAS_PATHWAY	BIOCARTA	980	37	20	2,23E-01	7,81E-01	rs2764938|rs17747965|rs1221220|rs17530657|rs11207332|rs1531846|rs9895159|rs10498970|rs2017750|rs9658761|rs7920305|rs1010650|rs555745|rs2141484|rs157695|rs9362733|rs711272|rs907186|rs10485405|rs2818291
Serositis	COMPLEMENT_CASCADE	REACTOME	752	48	20	2,24E-01	7,81E-01	rs2204345|rs920909|rs4151657|rs3766404|rs10779277|rs2031749|rs292001|rs10824804|rs3776519|rs706469|rs7444800|rs1342430|rs11118131|rs884113|rs2842754|rs630379|rs11265263|rs2808470|rs17468519|rs3750512
Serositis	SIGNALING_BY_HIPPO	REACTOME	627	32	20	2,24E-01	7,81E-01	rs9858201|rs7863144|rs7273626|rs13325694|rs333027|rs13080059|rs10501995|rs9661310|rs10038727|rs2124216|rs939980|rs10475878|rs17762297|rs10521111|rs2425650|rs17551539|rs2131431|rs6555807|rs11145477|rs3773820
Serositis	SIGNALING_BY_BMP	REACTOME	918	32	20	2,24E-01	7,81E-01	rs7495569|rs7566998|rs2414912|rs12765929|rs11713908|rs6054590|rs11680214|rs1048070|rs10170049|rs7212632|rs4362505|rs2635509|rs2091822|rs6430189|rs11931253|rs1440371|rs10926308|rs10491158|rs2381911|rs10465630
Serositis	G1_S_SPECIFIC_TRANSCRIPTION	REACTOME	194	9	7	2,26E-01	7,81E-01	rs7290191|rs2282411|rs9315422|rs918587|rs28171|rs380691|rs4150724
Serositis	METABOLISM_OF_STEROID_HORMONES_AND_VITAMINS_A_AND_D	REACTOME	745	34	16	2,27E-01	7,81E-01	rs11254339|rs11687903|rs7753322|rs1242102|rs1914172|rs476184|rs17139378|rs2161039|rs166050|rs7592152|rs12416759|rs4432229|rs9450609|rs10054105|rs2181874|rs3797179
Serositis	POTASSIUM_CHANNELS	REACTOME	4940	34	20	2,27E-01	7,81E-01	rs305691|rs1590287|rs13005001|rs4260410|rs483566|rs2283253|rs4532912|rs2283575|rs12601653|rs2283212|rs16975251|rs7945327|rs4337230|rs12553893|rs16896994|rs1799270|rs16910737|rs4793331|rs1883767|rs10863849
Serositis	APOPTOTIC_CLEAVAGE_OF_CELLULAR_PROTEINS	REACTOME	1052	44	20	2,27E-01	7,81E-01	rs7863144|rs16910559|rs2757594|rs7016497|rs1016835|rs11663972|rs16848196|rs4355511|rs4958072|rs4837820|rs2026628|rs619365|rs6939137|rs13188321|rs939980|rs17762297|rs10904989|rs7095324|rs11145477|rs1556003
Serositis	SIGNALING_BY_PDGF	REACTOME	4427	40	20	2,29E-01	7,82E-01	rs1352388|rs1864053|rs8464|rs2960288|rs13395505|rs627527|rs2302456|rs7273626|rs633762|rs1909745|rs6018627|rs12617710|rs1034620|rs6051511|rs4964002|rs2711292|rs9647337|rs425491|rs4234214|rs12476069
Serositis	TIGHT_JUNCTION	KEGG	6242	31	20	2,29E-01	7,82E-01	rs11078839|rs17505|rs10029948|rs17779293|rs4689410|rs8464|rs3759614|rs7863144|rs1430726|rs11784209|rs11686261|rs16948127|rs17106413|rs3783754|rs7290191|rs6018627|rs12495548|rs3744550|rs1734852|rs4689001
Serositis	GPVI_MEDIATED_ACTIVATION_CASCADE	REACTOME	1181	44	20	2,32E-01	7,82E-01	rs1864053|rs809192|rs1034620|rs9647337|rs10820825|rs511973|rs10487254|rs1858822|rs1366315|rs6871452|rs9932716|rs10465781|rs13054809|rs747020|rs6885261|rs383542|rs12142335|rs1636808|rs764605|rs7187863
Serositis	AMI_PATHWAY	BIOCARTA	574	21	11	2,33E-01	7,82E-01	rs627527|rs12648395|rs250724|rs9521664|rs6056|rs4704356|rs9559710|rs9324220|rs7995158|rs11884770|rs4667193
Serositis	GAP_JUNCTION	KEGG	4388	34	20	2,35E-01	7,82E-01	rs1352388|rs10904481|rs8464|rs2405871|rs2960288|rs10749883|rs6442887|rs6039107|rs3804984|rs1909745|rs6018627|rs1398312|rs1903999|rs7164838|rs597384|rs4964002|rs13037679|rs4234214|rs1388011|rs6773688
Serositis	NETRIN1_SIGNALING	REACTOME	3710	38	20	2,36E-01	7,82E-01	rs11245205|rs3773195|rs10516614|rs919635|rs809192|rs7138694|rs7016497|rs7163504|rs2545680|rs6018627|rs2636770|rs11017615|rs2219926|rs6548589|rs6857589|rs16948748|rs9876636|rs9964756|rs1483748|rs3887013
Serositis	DC_PATHWAY	BIOCARTA	573	35	16	2,38E-01	7,82E-01	rs7866830|rs3024498|rs11793751|rs10983903|rs1554286|rs17454570|rs1327798|rs5001582|rs1075498|rs12685245|rs6032678|rs1927937|rs7721176|rs11150614|rs10114983|rs17186662
Serositis	GLYCEROLIPID_METABOLISM	KEGG	1872	30	20	2,38E-01	7,82E-01	rs12133338|rs9562385|rs17007811|rs10732359|rs9366337|rs182034|rs2257140|rs10241087|rs7746765|rs1029544|rs1930762|rs1558909|rs1594485|rs4952147|rs1900448|rs895344|rs16878220|rs1921874|rs4819329|rs2269423
Serositis	APOPTOTIC_EXECUTION_PHASE	REACTOME	1211	38	20	2,39E-01	7,82E-01	rs7863144|rs16910559|rs2757594|rs7016497|rs1016835|rs11663972|rs16848196|rs9461416|rs4355511|rs4958072|rs4837820|rs2026628|rs619365|rs17266628|rs6939137|rs13188321|rs939980|rs4519192|rs17762297|rs9913503
Serositis	FORMATION_OF_INCISION_COMPLEX_IN_GG_NER	REACTOME	545	13	10	2,40E-01	7,82E-01	rs7188980|rs10276762|rs1794242|rs886727|rs12221497|rs1714473|rs741048|rs2808667|rs4443717|rs17137468
Serositis	STEROID_HORMONES	REACTOME	693	33	15	2,40E-01	7,82E-01	rs11254339|rs2284675|rs1242102|rs1914172|rs476184|rs17139378|rs2161039|rs166050|rs7592152|rs12416759|rs4432229|rs9450609|rs10054105|rs2181874|rs3797179
Serositis	TCA_CYCLE_AND_RESPIRATORY_ELECTRON_TRANSPORT	REACTOME	2599	44	20	2,42E-01	7,82E-01	rs4923649|rs6437332|rs1345638|rs1264901|rs2252523|rs7817292|rs10045456|rs962033|rs10777629|rs1500147|rs10777647|rs2302178|rs892492|rs12538107|rs7574229|rs7558055|rs2668424|rs4810150|rs13405880|rs11690708
Serositis	BASE_EXCISION_REPAIR	REACTOME	209	18	6	2,44E-01	7,82E-01	rs4135048|rs3829300|rs2275007|rs7135445|rs3781941|rs1760941
Serositis	GABA_SYNTHESIS_RELEASE_REUPTAKE_AND_DEGRADATION	REACTOME	746	30	18	2,44E-01	7,82E-01	rs11712912|rs2781562|rs1330581|rs9293878|rs3774128|rs6924133|rs9937726|rs6071173|rs16987501|rs7760432|rs17467186|rs6951030|rs363039|rs2049051|rs7751192|rs8190645|rs701492|rs2815736
Serositis	ETHER_LIPID_METABOLISM	KEGG	1041	41	20	2,45E-01	7,82E-01	rs12133338|rs967776|rs1487489|rs591393|rs1901826|rs1930762|rs895344|rs6846007|rs6865199|rs4926703|rs4871352|rs740229|rs12144400|rs1058913|rs3010644|rs12738546|rs1924736|rs360464|rs12137498|rs2070873
Serositis	INHIBITION_OF_THE_PROTEOLYTIC_ACTIVITY_OF_APC_C_REQUIRED_FOR_THE_ONSET_ TOTIC_SPINDLE_CHECKPOINT_COMPONENTS	REACTOME	365	14	6	2,45E-01	7,82E-01	rs2942067|rs7681650|rs9590480|rs10446729|rs9423256|rs11593182
Serositis	INTRINSIC_PATHWAY_FOR_APOPTOSIS	REACTOME	532	37	19	2,46E-01	7,82E-01	rs7273626|rs4286195|rs1893514|rs181402|rs17809603|rs2017750|rs2020902|rs724710|rs2046136|rs948769|rs1642763|rs11637681|rs2000745|rs2849382|rs7228933|rs17093560|rs4150724|rs2425650|rs396746
Serositis	DOWNSTREAM_TCR_SIGNALING	REACTOME	1573	44	20	2,46E-01	7,82E-01	rs6437802|rs1864053|rs17747965|rs17533090|rs1221220|rs9647337|rs1366315|rs6871452|rs1531846|rs10498970|rs7973701|rs2026628|rs12671372|rs747020|rs10872893|rs2894381|rs1010650|rs2141484|rs157695|rs9362733
Serositis	CELL_SURFACE_INTERACTIONS_AT_THE_VASCULAR_WALL	REACTOME	2650	33	20	2,47E-01	7,82E-01	rs2824319|rs1864053|rs2205895|rs809192|rs202493|rs6018627|rs1034620|rs2056131|rs2208637|rs11696134|rs1805|rs9647337|rs5367|rs2292700|rs10487254|rs1858822|rs1366315|rs6871452|rs3811632|rs12620429
Serositis	SPPA_PATHWAY	BIOCARTA	1428	33	20	2,47E-01	7,82E-01	rs967776|rs8464|rs6039107|rs7016497|rs6018627|rs1487489|rs13037679|rs10820825|rs591393|rs250724|rs1474937|rs6039104|rs12938937|rs4234512|rs4704356|rs9913908|rs3010644|rs12738546|rs10798090|rs708920
Serositis	IGF1R_PATHWAY	BIOCARTA	735	41	20	2,49E-01	7,82E-01	rs1864053|rs2960288|rs1366315|rs6871452|rs10498208|rs11247367|rs1273793|rs2684799|rs11854132|rs4234512|rs10933137|rs383542|rs34299|rs16897109|rs16897180|rs2302234|rs2471267|rs7290469|rs7617041|rs3861699
Serositis	RESOLUTION_OF_AP_SITES_VIA_THE_MULTIPLE_NUCLEOTIDE_PATCH_REPLACEMENT_I	REACTOME	192	18	6	2,49E-01	7,82E-01	rs4135048|rs3829300|rs2275007|rs7135445|rs3781941|rs1760941
Serositis	CITRATE_CYCLE_TCA_CYCLE	KEGG	1195	66	20	2,51E-01	7,82E-01	rs6070157|rs10970932|rs962033|rs7037573|rs17720809|rs1331240|rs7574229|rs7558055|rs7122539|rs11690708|rs10813645|rs2080087|rs17776734|rs12342878|rs9298935|rs17470048|rs7024321|rs6015032|rs2647168|rs1536002
Serositis	RENIN_ANGIOTENSIN_SYSTEM	KEGG	467	23	11	2,52E-01	7,82E-01	rs6896574|rs1466097|rs2478545|rs10513474|rs6866449|rs7159825|rs3915004|rs2200392|rs12494599|rs3772633|rs1532259
Serositis	TYROSINE_METABOLISM	KEGG	1169	45	20	2,53E-01	7,82E-01	rs2540069|rs1076153|rs2465665|rs6729962|rs7663410|rs791700|rs2389098|rs10993874|rs1928863|rs4371935|rs2042045|rs1486803|rs2519147|rs1789891|rs1442490|rs1154400|rs4646693|rs2715906|rs949832|rs4147778
Serositis	RETINOL_METABOLISM	KEGG	1512	75	20	2,53E-01	7,82E-01	rs1969260|rs2292653|rs7602885|rs7663410|rs2453280|rs762551|rs2013573|rs6738678|rs10889769|rs10171367|rs12978001|rs707718|rs1441826|rs12156358|rs4148285|rs1789891|rs12650761|rs12889813|rs1442490|rs12882423
Serositis	IL1_SIGNALING	REACTOME	1322	28	20	2,55E-01	7,82E-01	rs7086224|rs4301703|rs17747965|rs1221220|rs11123905|rs11465990|rs1531846|rs9895159|rs419598|rs10498970|rs7973701|rs17561|rs1881712|rs12307857|rs242413|rs1010650|rs2141484|rs157695|rs9362733|rs12816436
Serositis	CERAMIDE_PATHWAY	BIOCARTA	425	37	16	2,56E-01	7,82E-01	rs7818823|rs2228576|rs4758104|rs9895159|rs2017750|rs2046136|rs4234512|rs555745|rs6471742|rs2849382|rs2682086|rs4648090|rs7019752|rs230547|rs7290469|rs7617041
Serositis	TRANSCRIPTION	REACTOME	2708	42	20	2,57E-01	7,82E-01	rs17422451|rs2583680|rs4541737|rs9295730|rs2283575|rs717932|rs985559|rs7869160|rs13258625|rs7126805|rs8104242|rs34934568|rs9461416|rs14105|rs512598|rs650627|rs2875859|rs129128|rs12341611|rs12410049
Serositis	GLYCEROPHOSPHOLIPID_METABOLISM	KEGG	2549	31	20	2,57E-01	7,82E-01	rs12133338|rs967776|rs10505887|rs9562385|rs17007811|rs1487489|rs9366337|rs182034|rs2257140|rs10241087|rs7746765|rs591393|rs1029544|rs1930762|rs1558909|rs1594485|rs4952147|rs1900448|rs895344|rs16878220
Serositis	AQUAPORIN_MEDIATED_TRANSPORT	REACTOME	1510	52	20	2,59E-01	7,85E-01	rs1790786|rs2960288|rs1790799|rs1909745|rs491148|rs12185480|rs12553893|rs487455|rs4234214|rs9856983|rs4569841|rs7733194|rs4810147|rs891429|rs1550116|rs1253676|rs11070884|rs13153439|rs1925284|rs7863832
Serositis	DNA_STRAND_ELONGATION	REACTOME	672	31	16	2,60E-01	7,85E-01	rs4540267|rs7290191|rs133415|rs10276762|rs4943153|rs6460075|rs886727|rs6115178|rs1928023|rs741048|rs2321822|rs13082666|rs17137468|rs12584168|rs3781941|rs941289
Serositis	NEUROTROPHIN_SIGNALING_PATHWAY	KEGG	3689	36	20	2,61E-01	7,85E-01	rs17014035|rs1864053|rs483808|rs2764938|rs2302456|rs7273626|rs4352468|rs6996970|rs17309944|rs1387924|rs9366027|rs3184504|rs2160591|rs17530657|rs2237268|rs9647337|rs11207332|rs7699538|rs10878841|rs17464037
Serositis	HIVNEF_PATHWAY	BIOCARTA	1008	32	20	2,62E-01	7,85E-01	rs483808|rs16910559|rs7016497|rs2228576|rs2237268|rs181402|rs17809603|rs2017750|rs4837820|rs2020902|rs2046136|rs9658761|rs10872893|rs7920305|rs555745|rs2849382|rs2846855|rs907186|rs2857709|rs859722
Serositis	VASCULAR_SMOOTH_MUSCLE_CONTRACTION	KEGG	5845	37	20	2,63E-01	7,86E-01	rs1352388|rs192718|rs967776|rs4260410|rs8464|rs2960288|rs10749883|rs17801265|rs6442887|rs6039107|rs3804984|rs1909745|rs3783754|rs13257637|rs1398312|rs1903999|rs1865017|rs1487489|rs728528|rs11002124
Serositis	TCR_SIGNALING	REACTOME	2040	42	20	2,69E-01	8,01E-01	rs6437802|rs1864053|rs10045011|rs17747965|rs35399661|rs1221220|rs2289545|rs9647337|rs1366315|rs6871452|rs1531846|rs4886406|rs10498970|rs714158|rs7973701|rs2026628|rs12671372|rs7536180|rs747020|rs10872893
Serositis	BIOLOGICAL_OXIDATIONS	REACTOME	3568	35	20	2,71E-01	8,04E-01	rs1356553|rs7496|rs294594|rs7602885|rs574389|rs13178865|rs7687822|rs7663410|rs9296691|rs16970280|rs762551|rs6509397|rs12529923|rs4808358|rs4149413|rs946934|rs12153515|rs2013573|rs11207546|rs11135312
Serositis	FATTY_ACID_METABOLISM	KEGG	776	34	19	2,72E-01	8,05E-01	rs10890819|rs17565061|rs10732359|rs7663410|rs7659090|rs12577790|rs2631753|rs11725638|rs4878199|rs4646771|rs1789891|rs6849528|rs3798211|rs1442490|rs1154400|rs1923431|rs7216|rs3774051|rs11129780
Serositis	DARPP_32_EVENTS	REACTOME	879	23	20	2,74E-01	8,05E-01	rs4699236|rs6812773|rs26956|rs5010905|rs702524|rs1439|rs783037|rs10068809|rs4302546|rs9808216|rs4699228|rs2645370|rs1790733|rs27723|rs6733907|rs12135034|rs3811063|rs2302234|rs1961|rs2112957
Serositis	STRESS_PATHWAY	BIOCARTA	633	36	18	2,75E-01	8,05E-01	rs2764938|rs2228576|rs17530657|rs11207332|rs606458|rs9895159|rs6929796|rs2017750|rs10872893|rs2857709|rs2867316|rs4648090|rs7019752|rs230547|rs1710627|rs10883452|rs854586|rs11869073
Serositis	TELOMERE_MAINTENANCE	REACTOME	1133	56	20	2,77E-01	8,09E-01	rs4541737|rs9295730|rs4540267|rs10276762|rs7445640|rs4943153|rs9461416|rs129128|rs6460075|rs886727|rs9461204|rs2748525|rs1928023|rs741048|rs2321822|rs17137468|rs12584168|rs3781941|rs7141|rs9366694
Serositis	MEIOSIS	REACTOME	1674	56	20	2,81E-01	8,17E-01	rs4987876|rs867185|rs4541737|rs9295730|rs227060|rs1022476|rs10276762|rs9461416|rs129128|rs2813498|rs886727|rs9461204|rs6062378|rs2748525|rs2813554|rs299235|rs818442|rs741048|rs9384022|rs6010777
Serositis	VOLTAGE_GATED_POTASSIUM_CHANNELS	REACTOME	2491	41	20	2,82E-01	8,18E-01	rs305691|rs1590287|rs483566|rs4532912|rs2283212|rs7945327|rs16896994|rs1883767|rs10863849|rs7190491|rs17827624|rs10092842|rs1014756|rs7824311|rs11063441|rs4809745|rs1079714|rs4129625|rs10505589|rs11927855
Serositis	ERK5_PATHWAY	BIOCARTA	595	35	20	2,83E-01	8,18E-01	rs1864053|rs4965533|rs918418|rs7702020|rs1366315|rs6871452|rs6598265|rs325383|rs383542|rs1636808|rs16837709|rs918842|rs34299|rs16897109|rs16897180|rs1864180|rs11633623|rs7290469|rs4976028|rs1862884
Serositis	BIOSYNTHESIS_OF_UNSATURATED_FATTY_ACIDS	KEGG	463	23	11	2,84E-01	8,18E-01	rs6822001|rs9474466|rs4391265|rs2631753|rs2851682|rs6849528|rs7070601|rs7934642|rs9395855|rs10001138|rs11129780
Serositis	REGULATION_OF_IFNA_SIGNALING	REACTOME	339	14	8	2,86E-01	8,18E-01	rs13395505|rs12720253|rs4789576|rs4527079|rs7254474|rs386365|rs280519|rs7037389
Serositis	PROSTACYCLIN_SIGNALLING_THROUGH_PROSTACYCLIN_RECEPTOR	REACTOME	353	10	6	2,86E-01	8,18E-01	rs12553893|rs4810147|rs1253676|rs11070884|rs7863832|rs7618348
Serositis	FORMATION_OF_ATP_BY_CHEMIOSMOTIC_COUPLING	REACTOME	77	10	6	2,88E-01	8,19E-01	rs1264901|rs4810150|rs6070703|rs3744992|rs12274926|rs9965812
Serositis	WNT_SIGNALING_PATHWAY	KEGG	5611	34	20	2,89E-01	8,20E-01	rs2796794|rs4699236|rs2221145|rs2764938|rs8464|rs11071927|rs6812773|rs7166081|rs10467770|rs10518714|rs17747965|rs6039107|rs4352468|rs2292653|rs1221220|rs7634430|rs10089685|rs7641735|rs17530657|rs11207332
Serositis	APC_CDC20_MEDIATED_DEGRADATION_OF_NEK2A	REACTOME	396	15	7	2,91E-01	8,23E-01	rs2942067|rs7681650|rs9590480|rs10446729|rs2917779|rs9423256|rs11593182
Serositis	GABA_RECEPTOR_ACTIVATION	REACTOME	2705	31	20	2,94E-01	8,25E-01	rs13005001|rs17653240|rs2960288|rs1012549|rs12601653|rs1909745|rs11135214|rs16975251|rs12553893|rs282136|rs4472116|rs1159315|rs4234214|rs2078610|rs4793331|rs9856983|rs4569841|rs726568|rs7733194|rs1167763
Serositis	LEISHMANIA_INFECTION	KEGG	2265	49	20	2,95E-01	8,25E-01	rs2764938|rs13395505|rs17747965|rs17533090|rs7866830|rs1221220|rs3024498|rs11793751|rs17530657|rs10983903|rs11207332|rs3948793|rs1554286|rs10498970|rs17454570|rs1327798|rs5001582|rs17561|rs1075498|rs10410544
Serositis	ALK_PATHWAY	BIOCARTA	1932	33	20	2,95E-01	8,25E-01	rs7495569|rs1019855|rs17747965|rs1221220|rs2414912|rs2296620|rs918418|rs7702020|rs12765929|rs3756990|rs6054590|rs13075948|rs10498970|rs4355511|rs4958072|rs17182166|rs7212632|rs9475432|rs2336395|rs1010650
Serositis	SYNTHESIS_OF_PA	REACTOME	1057	38	20	2,96E-01	8,25E-01	rs967776|rs17007811|rs1487489|rs2257140|rs591393|rs4952147|rs1921874|rs17028984|rs4819329|rs6865199|rs2269423|rs9999681|rs10509948|rs3010644|rs12738546|rs2928596|rs10798090|rs360464|rs17007216|rs2070873
Serositis	SIGNALING_BY_SCF_KIT	REACTOME	1989	33	20	2,97E-01	8,25E-01	rs1864053|rs8464|rs13395505|rs809192|rs7273626|rs6018627|rs3184504|rs6051511|rs1366315|rs6871452|rs1005656|rs3759216|rs541425|rs2020902|rs3214047|rs11531636|rs747020|rs907092|rs10871668|rs12938937
Serositis	NEURONAL_SYSTEM	REACTOME	13378	32	20	2,98E-01	8,25E-01	rs305691|rs2355575|rs11712912|rs6026402|rs4928016|rs1590287|rs13005001|rs10491731|rs4260410|rs8464|rs17653240|rs2960288|rs483566|rs6474421|rs1012549|rs2283253|rs4532912|rs7686241|rs2283575|rs6039107
Serositis	TFF_PATHWAY	BIOCARTA	697	42	19	2,99E-01	8,26E-01	rs1864053|rs7016497|rs2877260|rs1366315|rs6871452|rs10232489|rs2020902|rs6895971|rs383542|rs1636808|rs2110290|rs34299|rs16897109|rs16897180|rs2252586|rs7290469|rs4976028|rs7706821|rs7379303
Serositis	TGF_BETA_RECEPTOR_SIGNALING_IN_EMT_EPITHELIAL_TO_MESENCHYMAL_TRANSITIO	REACTOME	429	20	12	3,01E-01	8,27E-01	rs1019855|rs13075948|rs11879985|rs12974783|rs8124053|rs10827338|rs2917779|rs2749609|rs7640543|rs6768081|rs2812672|rs10508791
Serositis	ANTIGEN_PROCESSING_AND_PRESENTATION	KEGG	1754	83	20	3,03E-01	8,30E-01	rs1264524|rs1296028|rs17533090|rs2235383|rs1485591|rs3948793|rs7770216|rs1242102|rs6929796|rs649216|rs3135363|rs2894381|rs176248|rs3135344|rs5009448|rs11751659|rs7761965|rs918842|rs3134986|rs2857709
Serositis	DAG_AND_IP3_SIGNALING	REACTOME	1644	40	20	3,04E-01	8,31E-01	rs1352388|rs8464|rs2960288|rs1909745|rs4964002|rs2711292|rs4234214|rs9856983|rs4569841|rs7733194|rs210120|rs891429|rs1550116|rs12938937|rs13153439|rs1457115|rs9808216|rs281489|rs9913908|rs918842
Serositis	AUTOIMMUNE_THYROID_DISEASE	KEGG	1646	68	20	3,09E-01	8,37E-01	rs1264524|rs17533090|rs3024498|rs733234|rs2272874|rs9276711|rs1554286|rs7770216|rs17111530|rs9658761|rs3135363|rs2894381|rs7920305|rs176248|rs3135344|rs5009448|rs11751659|rs7761965|rs2300520|rs2715261

<table>
<tr><td>Serositis</td><td>IRON_UPTAKE_AND_TRANSPORT</td><td>REACTOME</td><td>733</td><td>32</td><td>14</td><td>3,09E-01</td><td>8,37E-01</td><td>rs1530561|rs3019626|rs133415|rs1562853|rs2712353|rs6929796|rs11783925|rs9643478|rs2857709|rs11712192|rs4147778|rs570|rs4287912|rs10241449</td></tr>
<tr><td>Serositis</td><td>CK1_PATHWAY</td><td>BIOCARTA</td><td>759</td><td>25</td><td>20</td><td>3,10E-01</td><td>8,37E-01</td><td>rs4699236|rs6812773|rs6039107|rs13037679|rs1474937|rs6039104|rs4699228|rs2645370|rs1790733|rs11742698|rs708920|rs2381991|rs2440390|rs3811063|rs17065021|rs2302234|rs6086490|rs265995|rs17030758|rs3861699</td></tr>
<tr><td>Serositis</td><td>TRANSCRIPTION_COUPLED_NER_TC_NER</td><td>REACTOME</td><td>834</td><td>27</td><td>13</td><td>3,13E-01</td><td>8,41E-01</td><td>rs7188980|rs10276762|rs4943153|rs7126805|rs6460075|rs1794242|rs886727|rs1928023|rs741048|rs2321822|rs17137468|rs12584168|rs1638566</td></tr>
<tr><td>Serositis</td><td>LAGGING_STRAND_SYNTHESIS</td><td>REACTOME</td><td>550</td><td>24</td><td>11</td><td>3,13E-01</td><td>8,41E-01</td><td>rs4540267|rs10276762|rs4943153|rs6460075|rs886727|rs1928023|rs741048|rs2321822|rs17137468|rs12584168|rs3781941</td></tr>
<tr><td>Serositis</td><td>DEGRADATION_OF_THE_EXTRACELLULAR_MATRIX</td><td>REACTOME</td><td>580</td><td>29</td><td>17</td><td>3,15E-01</td><td>8,41E-01</td><td>rs10092877|rs1996637|rs7300198|rs1042704|rs7816934|rs10132585|rs1554999|rs4810482|rs7159825|rs1922314|rs655316|rs17293152|rs9926693|rs5760023|rs12272341|rs889515|rs10431961</td></tr>
<tr><td>Serositis</td><td>NGF_SIGNALLING_VIA_TRKA_FROM_THE_PLASMA_MEMBRANE</td><td>REACTOME</td><td>4847</td><td>39</td><td>20</td><td>3,17E-01</td><td>8,41E-01</td><td>rs2306677|rs17014035|rs1864053|rs8464|rs2960288|rs2302456|rs7273626|rs1909745|rs4965533|rs6018627|rs1387924|rs9366027|rs2043722|rs4325000|rs6051511|rs2711292|rs9647337|rs4234214|rs9856983|rs4569841</td></tr>
<tr><td>Serositis</td><td>CREB_PHOSPHORYLATION_THROUGH_THE_ACTIVATION_OF_RAS</td><td>REACTOME</td><td>1349</td><td>37</td><td>20</td><td>3,17E-01</td><td>8,41E-01</td><td>rs11055455|rs9968730|rs4704706|rs9366027|rs1421106|rs4780486|rs7699538|rs1362327|rs1861872|rs8049630|rs8064140|rs1107475|rs1070474|rs1063243|rs4234512|rs12820037|rs11055603|rs12449282|rs9808216|rs4958456</td></tr>
<tr><td>Serositis</td><td>KERATINOCYTE_PATHWAY</td><td>BIOCARTA</td><td>1952</td><td>31</td><td>20</td><td>3,19E-01</td><td>8,41E-01</td><td>rs483808|rs2764938|rs8464|rs2836804|rs3783754|rs2228576|rs17530657|rs2237268|rs2711292|rs11207332|rs2877260|rs7948482|rs10232489|rs9895159|rs2017750|rs2012898|rs2026628|rs2046136|rs9658761|rs10872893</td></tr>
<tr><td>Serositis</td><td>PEROXISOMAL_LIPID_METABOLISM</td><td>REACTOME</td><td>435</td><td>20</td><td>8</td><td>3,20E-01</td><td>8,41E-01</td><td>rs246966|rs1901826|rs11590871|rs2631753|rs6849528|rs495183|rs250412|rs11129780</td></tr>
<tr><td>Serositis</td><td>ENERGY_DEPENDENT_REGULATION_OF_MTOR_BY_LKB1_AMPK</td><td>REACTOME</td><td>461</td><td>19</td><td>12</td><td>3,21E-01</td><td>8,41E-01</td><td>rs4435442|rs1881638|rs4962081|rs2289766|rs1050700|rs4950385|rs999977|rs7789699|rs7788961|rs9568196|rs4425665|rs7146988</td></tr>
<tr><td>Serositis</td><td>IL6_PATHWAY</td><td>BIOCARTA</td><td>401</td><td>27</td><td>13</td><td>3,21E-01</td><td>8,41E-01</td><td>rs2764938|rs17530657|rs11207332|rs12720253|rs17822304|rs4234512|rs7254474|rs280519|rs161700|rs10045084|rs7617041|rs1476482|rs8004059</td></tr>
<tr><td>Serositis</td><td>SYNTHESIS_SECRETION_AND_DEACYLATION_OF_GHRELIN</td><td>REACTOME</td><td>624</td><td>35</td><td>15</td><td>3,22E-01</td><td>8,41E-01</td><td>rs687992|rs4940849|rs10978931|rs13098460|rs11133152|rs6553943|rs11794132|rs1380100|rs242376|rs1598100|rs2271731|rs1523287|rs11660643|rs4244808|rs13144122</td></tr>
<tr><td>Serositis</td><td>ALLOGRAFT_REJECTION</td><td>KEGG</td><td>1510</td><td>70</td><td>20</td><td>3,24E-01</td><td>8,42E-01</td><td>rs1264524|rs35399661|rs3024498|rs2272874|rs3948793|rs1554286|rs7770216|rs17454570|rs1075498|rs9658761|rs3135363|rs2894381|rs7920305|rs176248|rs3135344|rs5009448|rs11751659|rs7761965|rs2715261|rs6032678</td></tr>
<tr><td>Serositis</td><td>ADHERENS_JUNCTION</td><td>KEGG</td><td>4520</td><td>37</td><td>20</td><td>3,24E-01</td><td>8,42E-01</td><td>rs1019855|rs2221145|rs11071927|rs809192|rs7166081|rs1430726|rs10518714|rs17747965|rs11686261|rs17106413|rs2292653|rs6018627|rs1221220|rs7567671|rs966308|rs12608027|rs3755105|rs2441727|rs2877260|rs11576830</td></tr>
<tr><td>Serositis</td><td>METABOLISM_OF_XENOBIOTICS_BY_CYTOCHROME_P450</td><td>KEGG</td><td>1674</td><td>72</td><td>20</td><td>3,25E-01</td><td>8,43E-01</td><td>rs7496|rs7687822|rs7663410|rs9296691|rs762551|rs12529923|rs2013573|rs6738678|rs17608022|rs316144|rs10171367|rs2042045|rs3818562|rs4148285|rs1789891|rs184623|rs12650761|rs305968|rs1442490|rs1154400</td></tr>
<tr><td>Serositis</td><td>FC_EPSILON_RI_SIGNALING_PATHWAY</td><td>KEGG</td><td>2758</td><td>45</td><td>20</td><td>3,30E-01</td><td>8,46E-01</td><td>rs1864053|rs967776|rs483808|rs8464|rs809192|rs4352468|rs474951|rs12564813|rs2711292|rs9647337|rs10820825|rs591393|rs511973|rs1366315|rs6871452|rs9895159|rs9932716|rs2017750|rs10465781|rs13054809</td></tr>
<tr><td>Serositis</td><td>ACYL_CHAIN_REMODELLING_OF_PE</td><td>REACTOME</td><td>696</td><td>37</td><td>20</td><td>3,33E-01</td><td>8,46E-01</td><td>rs967776|rs12564813|rs9366337|rs7746765|rs17134769|rs591393|rs1679008|rs740229|rs3010644|rs12738546|rs10798090|rs2070873|rs9460465|rs2430080|rs623784|rs3123045|rs2802475|rs11231528|rs12405943|rs7535176</td></tr>
<tr><td>Serositis</td><td>AMINO_SUGAR_AND_NUCLEOTIDE_SUGAR_METABOLISM</td><td>KEGG</td><td>956</td><td>36</td><td>20</td><td>3,33E-01</td><td>8,46E-01</td><td>rs689035|rs17301901|rs6580215|rs9391934|rs4959621|rs7817301|rs4898765|rs626295|rs7043864|rs4976661|rs4343755|rs10458131|rs13144957|rs2628440|rs2995965|rs17501218|rs6928775|rs906215|rs2284997|rs2235718</td></tr>
<tr><td>Serositis</td><td>MYOSIN_PATHWAY</td><td>BIOCARTA</td><td>1253</td><td>29</td><td>20</td><td>3,34E-01</td><td>8,46E-01</td><td>rs8464|rs3760703|rs6039107|rs13037679|rs221048|rs2282537|rs7611544|rs11879985|rs907698|rs2291462|rs1474937|rs4681946|rs6039104|rs12974783|rs12938937|rs10139123|rs12119764|rs9913908|rs4876268|rs708920</td></tr>
<tr><td>Serositis</td><td>EIF4_PATHWAY</td><td>BIOCARTA</td><td>890</td><td>45</td><td>20</td><td>3,35E-01</td><td>8,46E-01</td><td>rs1864053|rs8464|rs1366315|rs6871452|rs10498208|rs6671527|rs1273793|rs12938937|rs1060478|rs10933137|rs6895971|rs383542|rs9913908|rs2230392|rs34299|rs16897109|rs16897180|rs4381631|rs7290469|rs733646</td></tr>
<tr><td>Serositis</td><td>CHONDROITIN_SULFATE_DERMATAN_SULFATE_METABOLISM</td><td>REACTOME</td><td>2784</td><td>37</td><td>20</td><td>3,36E-01</td><td>8,46E-01</td><td>rs9954485|rs1458526|rs2228331|rs1932306|rs3734371|rs4583089|rs12819127|rs928646|rs1556466|rs1807833|rs7130091|rs1482394|rs25413|rs11112047|rs1008953|rs2149063|rs1892984|rs736389|rs9524254|rs3733895</td></tr>
<tr><td>Serositis</td><td>TAK1_ACTIVATES_NFKB_BY_PHOSPHORYLATION_AND_ACTIVATION_OF_IKKS_COMPLE</td><td>REACTOME</td><td>777</td><td>32</td><td>20</td><td>3,37E-01</td><td>8,46E-01</td><td>rs17747965|rs1221220|rs1531846|rs468774|rs10498970|rs2096509|rs2269423|rs10410544|rs10872893|rs1010650|rs9978658|rs2141484|rs157695|rs9362733|rs2234983|rs4519192|rs216779|rs711272|rs10485405|rs2818291</td></tr>
<tr><td>Serositis</td><td>EXTENSION_OF_TELOMERES</td><td>REACTOME</td><td>618</td><td>27</td><td>13</td><td>3,37E-01</td><td>8,46E-01</td><td>rs4540267|rs10276762|rs7445640|rs4943153|rs6460075|rs886727|rs1928023|rs741048|rs2321822|rs17137468|rs12584168|rs3781941|rs7141</td></tr>
<tr><td>Serositis</td><td>GLYCINE_SERINE_AND_THREONINE_METABOLISM</td><td>KEGG</td><td>564</td><td>17</td><td>10</td><td>3,37E-01</td><td>8,46E-01</td><td>rs10867272|rs1541332|rs2780749|rs6559415|rs522676|rs10521022|rs4529531|rs699469|rs3828090|rs11209658</td></tr>
<tr><td>Serositis</td><td>GLYCOLYSIS</td><td>REACTOME</td><td>558</td><td>15</td><td>8</td><td>3,38E-01</td><td>8,46E-01</td><td>rs764223|rs2236441|rs6602514|rs17158714|rs2842754|rs11189224|rs2267846|rs3829910</td></tr>
<tr><td>Serositis</td><td>O_LINKED_GLYCOSYLATION_OF_MUCINS</td><td>REACTOME</td><td>2402</td><td>25</td><td>20</td><td>3,39E-01</td><td>8,46E-01</td><td>rs1554324|rs4719136|rs2736868|rs4952025|rs4745552|rs10497137|rs1401346|rs9854005|rs2958830|rs6989475|rs4719120|rs6502026|rs10493593|rs4836593|rs2037957|rs11879090|rs17166192|rs6553604|rs4696036|rs2303325</td></tr>
<tr><td>Serositis</td><td>SIGNALING_BY_CONSTITUTIVELY_ACTIVE_EGFR</td><td>REACTOME</td><td>503</td><td>28</td><td>16</td><td>3,40E-01</td><td>8,46E-01</td><td>rs1864053|rs2877260|rs1366315|rs6871452|rs10232489|rs383542|rs7254474|rs2917779|rs2110290|rs34299|rs16897109|rs16897180|rs2252586|rs4976028|rs7706821|rs7379303</td></tr>
<tr><td>Serositis</td><td>LIGAND_GATED_ION_CHANNEL_TRANSPORT</td><td>REACTOME</td><td>1051</td><td>39</td><td>20</td><td>3,40E-01</td><td>8,46E-01</td><td>rs17653240|rs2376837|rs11135214|rs282136|rs4472116|rs1159315|rs2078610|rs11161304|rs164368|rs3850733|rs6871616|rs7758893|rs4695936|rs7688285|rs6849847|rs7715214|rs6606856|rs159922|rs6890673|rs173766</td></tr>
<tr><td>Serositis</td><td>CA_DEPENDENT_EVENTS</td><td>REACTOME</td><td>1487</td><td>37</td><td>20</td><td>3,43E-01</td><td>8,50E-01</td><td>rs967776|rs8464|rs2960288|rs1909745|rs12045786|rs4234214|rs591393|rs9856983|rs4569841|rs7733194|rs891429|rs1550116|rs12938937|rs13153439|rs1457115|rs9808216|rs9913908|rs3010644|rs12738546|rs10798090</td></tr>
<tr><td>Serositis</td><td>IL_2_SIGNALING</td><td>REACTOME</td><td>936</td><td>41</td><td>20</td><td>3,45E-01</td><td>8,53E-01</td><td>rs1864053|rs7273626|rs9647337|rs10820825|rs1366315|rs6871452|rs6794523|rs4240910|rs3792424|rs747020|rs4234512|rs334782|rs383542|rs17606924|rs34299|rs16897109|rs16897180|rs7290469|rs2425650|rs7617041</td></tr>
<tr><td>Serositis</td><td>REGULATION_OF_INSULIN_SECRETION_BY_GLUCAGON_LIKE_PEPTIDE1</td><td>REACTOME</td><td>1388</td><td>37</td><td>20</td><td>3,48E-01</td><td>8,54E-01</td><td>rs2306677|rs11168228|rs12553893|rs10878841|rs4234214|rs9856983|rs7548973|rs210120|rs4810147|rs4963998|rs11695696|rs4809745|rs891429|rs1253676|rs11070884|rs499472|rs4832532|rs3769210|rs2584098|rs7863832</td></tr>
<tr><td>Serositis</td><td>ADP_SIGNALLING_THROUGH_P2RY12</td><td>REACTOME</td><td>438</td><td>8</td><td>5</td><td>3,48E-01</td><td>8,54E-01</td><td>rs12553893|rs1253676|rs11070884|rs7863832|rs7618348</td></tr>
<tr><td>Serositis</td><td>CDMAC_PATHWAY</td><td>BIOCARTA</td><td>1018</td><td>31</td><td>20</td><td>3,49E-01</td><td>8,54E-01</td><td>rs2764938|rs8464|rs6039107|rs17530657|rs11207332|rs13037679|rs11995971|rs1474937|rs6039104|rs10872893|rs12938937|rs4234512|rs9913908|rs1891111|rs708920|rs2857709|rs4381631|rs4648090|rs6086490|rs230547</td></tr>
<tr><td>Serositis</td><td>PROTEASOME_PATHWAY</td><td>BIOCARTA</td><td>280</td><td>14</td><td>9</td><td>3,50E-01</td><td>8,54E-01</td><td>rs2906041|rs7496283|rs12201680|rs909473|rs11794849|rs755553|rs8004624|rs8005252|rs9811380</td></tr>
<tr><td>Serositis</td><td>INHIBITION_OF_VOLTAGE_GATED_CA2_CHANNELS_VIA_GBETA_GAMMA_SUBUNITS</td><td>REACTOME</td><td>1082</td><td>34</td><td>20</td><td>3,52E-01</td><td>8,55E-01</td><td>rs13005001|rs12601653|rs16975251|rs12553893|rs4793331|rs726568|rs1167763|rs1999500|rs7864144|rs12601221|rs1253676|rs1992703|rs3106667|rs9914095|rs7214537|rs3785902|rs7863832|rs2486253|rs13000959|rs4817889</td></tr>
<tr><td>Serositis</td><td>SRP_DEPENDENT_COTRANSLATIONAL_PROTEIN_TARGETING_TO_MEMBRANE</td><td>REACTOME</td><td>1378</td><td>35</td><td>20</td><td>3,53E-01</td><td>8,55E-01</td><td>rs4131086|rs2252523|rs4940849|rs16935649|rs2302178|rs4270628|rs12121852|rs7126805|rs7874483|rs8017939|rs11133152|rs6553943|rs2253181|rs1953900|rs876144|rs3732680|200260|rs9502593|rs7634966|rs2687975</td></tr>
<tr><td>Serositis</td><td>NITROGEN_METABOLISM</td><td>KEGG</td><td>587</td><td>48</td><td>13</td><td>3,54E-01</td><td>8,55E-01</td><td>rs2355575|rs7830239|rs359535|rs2651236|rs1860172|rs3765966|rs8023341|rs7607205|rs12091891|rs2296063|rs1921915|rs2781087|rs11209658</td></tr>
<tr><td>Serositis</td><td>NOD_LIKE_RECEPTOR_SIGNALING_PATHWAY</td><td>KEGG</td><td>1406</td><td>33</td><td>20</td><td>3,55E-01</td><td>8,55E-01</td><td>rs4925650|rs17747965|rs4352468|rs1221220|rs2722195|rs1531846|rs9389541|rs10498970|rs2017750|rs11871409|rs2058364|rs10410544|rs10872893|rs1076420|rs1010650|rs2141484|rs157695|rs9362733|rs7123951|rs251295</td></tr>
<tr><td>Serositis</td><td>PTEN_PATHWAY</td><td>BIOCARTA</td><td>544</td><td>31</td><td>16</td><td>3,56E-01</td><td>8,56E-01</td><td>rs1864053|rs7016497|rs1366315|rs6871452|rs3759216|rs383542|rs2230392|rs34299|rs16897109|rs16897180|rs859722|rs7290469|rs4976028|rs889515|rs7706821|rs7379303</td></tr>
<tr><td>Serositis</td><td>REGULATION_OF_KIT_SIGNALING</td><td>REACTOME</td><td>587</td><td>26</td><td>20</td><td>3,57E-01</td><td>8,56E-01</td><td>rs8464|rs809192|rs6018627|rs3184504|rs1005656|rs3214047|rs747020|rs10871668|rs12938937|rs9913908|rs7245158|rs4381631|rs6012200|rs16922298|rs218273|rs733646|rs12452826|rs11654719|rs3134884|rs1409837</td></tr>
<tr><td>Serositis</td><td>MEIOTIC_SYNAPSIS</td><td>REACTOME</td><td>1031</td><td>42</td><td>17</td><td>3,60E-01</td><td>8,56E-01</td><td>rs4541737|rs9295730|rs1022476|rs9461416|rs129128|rs2813498|rs9461204|rs6062378|rs2748525|rs2813554|rs818442|rs9384022|rs6010777|rs11755392|rs4821818|rs9366694|rs4731243</td></tr>
<tr><td>Serositis</td><td>PLATELET_CALCIUM_HOMEOSTASIS</td><td>REACTOME</td><td>1156</td><td>51</td><td>19</td><td>3,62E-01</td><td>8,56E-01</td><td>rs1352388|rs10516614|rs9309065|rs4964002|rs210120|rs11914694|rs384994|rs10490046|rs697973|rs950574|rs12615150|rs6838639|rs830131|rs26310|rs1474500|rs17025029|rs12487529|rs10518290|rs4952447</td></tr>
<tr><td>Serositis</td><td>IL7_PATHWAY</td><td>BIOCARTA</td><td>699</td><td>35</td><td>19</td><td>3,62E-01</td><td>8,56E-01</td><td>rs1864053|rs809192|rs1366315|rs6871452|rs2046136|rs747020|rs383542|rs2849382|rs1423658|rs1636808|rs1296720|rs2238419|rs34299|rs16897109|rs16897180|rs4976028|rs7706821|rs1409837|rs7379303</td></tr>
<tr><td>Serositis</td><td>NCAM_SIGNALING_FOR_NEURITE_OUT_GROWTH</td><td>REACTOME</td><td>2480</td><td>34</td><td>20</td><td>3,62E-01</td><td>8,56E-01</td><td>rs4739207|rs809192|rs627527|rs7273626|rs633762|rs7016497|rs6018627|rs1563916|rs1034620|rs12476069|rs2286732|rs10487254|rs1858822|rs2931462|rs2975100|rs518347|rs1586494|rs4671928|rs9521664|rs2839102</td></tr>
<tr><td>Serositis</td><td>SNARE_INTERACTIONS_IN_VESICULAR_TRANSPORT</td><td>KEGG</td><td>828</td><td>30</td><td>20</td><td>3,64E-01</td><td>8,56E-01</td><td>rs6026402|rs4689117|rs1325172|rs6026398|rs9893164|rs11870073|rs751262|rs747898|rs2014613|rs10799451|rs6951030|rs1565623|rs1487608|rs7465378|rs11196003|rs363039|rs1159548|rs4234835|rs17741348|rs7836666</td></tr>
<tr><td>Serositis</td><td>CYTOKINE_SIGNALING_IN_IMMUNE_SYSTEM</td><td>REACTOME</td><td>6819</td><td>35</td><td>20</td><td>3,65E-01</td><td>8,56E-01</td><td>rs1864053|rs2352930|rs10169182|rs7086224|rs13395505|rs398800|rs809192|rs2074560|rs2302456|rs4301703|rs7273626|rs17747965|rs9845458|rs17533090|rs276978|rs6996970|rs1127314|rs8132|rs1221220|rs2272874</td></tr>
<tr><td>Serositis</td><td>G_ALPHA_S_SIGNALLING_EVENTS</td><td>REACTOME</td><td>5019</td><td>32</td><td>20</td><td>3,66E-01</td><td>8,56E-01</td><td>rs500817|rs12299821|rs6545061|rs2960288|rs377432|rs472418|rs169290|rs6921041|rs1899908|rs26956|rs1909745|rs981273|rs2044316|rs733234|rs888956|rs9494412|rs12553893|rs10228431|rs1539984|rs4234214</td></tr>
<tr><td>Serositis</td><td>FORMATION_OF_THE_TERNARY_COMPLEX_AND_SUBSEQUENTLY_THE_43S_COMPLEX</td><td>REACTOME</td><td>677</td><td>36</td><td>17</td><td>3,66E-01</td><td>8,56E-01</td><td>rs2252523|rs16935649|rs2302178|rs8017939|rs2253181|rs1953900|rs1074584|rs747020|rs4758037|rs4300036|rs2917779|rs5756236|rs4599669|rs3900716|rs1427827|rs4335707|rs2232997</td></tr>
<tr><td>Serositis</td><td>PD1_SIGNALING</td><td>REACTOME</td><td>398</td><td>30</td><td>13</td><td>3,69E-01</td><td>8,56E-01</td><td>rs2890658|rs17533090|rs4886406|rs747020|rs2894381|rs9270856|rs10435744|rs11751659|rs10975152|rs12576947|rs7048841|rs642093|rs6686571</td></tr>
<tr><td>Serositis</td><td>NO1_PATHWAY</td><td>BIOCARTA</td><td>1482</td><td>35</td><td>20</td><td>3,69E-01</td><td>8,56E-01</td><td>rs12299821|rs377432|rs169290|rs1899908|rs2044316|rs1903999|rs490057|rs7329421|rs10898864|rs9551705|rs7077665|rs12252380|rs12578492|rs11823971|rs7646517|rs9808216|rs822373|rs3817436|rs1904694|rs1871080</td></tr>
<tr><td>Serositis</td><td>MEMBRANE_TRAFFICKING</td><td>REACTOME</td><td>2276</td><td>25</td><td>20</td><td>3,70E-01</td><td>8,56E-01</td><td>rs909626|rs1969260|rs1160495|rs2292653|rs6018627|rs11811771|rs3745255|rs3019626|rs7164838|rs4325000|rs1805727|rs2618580|rs1805734|rs16907441|rs983694|rs13198533|rs10963309|rs9296980|rs4886077|rs17677598</td></tr>
<tr><td>Serositis</td><td>CALCIUM_SIGNALING_PATHWAY</td><td>KEGG</td><td>9275</td><td>30</td><td>20</td><td>3,71E-01</td><td>8,56E-01</td><td>rs1352388|rs192718|rs4699236|rs10882406|rs8464|rs2405871|rs6545061|rs2960288|rs6812773|rs6490121|rs967232|rs3760703|rs17801265|rs6442887|rs6039107|rs11763324|rs3804984|rs1909745|rs13257637|rs9309065</td></tr>
<tr><td>Serositis</td><td>HS_GAG_DEGRADATION</td><td>REACTOME</td><td>1386</td><td>48</td><td>20</td><td>3,75E-01</td><td>8,56E-01</td><td>rs2228331|rs1932306|rs4583089|rs928646|rs4074708|rs1556466|rs1008953|rs2149063|rs736389|rs9524254|rs16949539|rs4919276|rs1341660|rs1380332|rs1407998|rs7075080|rs1932298|rs7321299|rs7007840|rs272081</td></tr>
<tr><td>Serositis</td><td>GAB1_SIGNALOSOME</td><td>REACTOME</td><td>1077</td><td>30</td><td>20</td><td>3,76E-01</td><td>8,56E-01</td><td>rs1864053|rs6018627|rs6051511|rs2877260|rs1366315|rs6871452|rs3759216|rs10232489|rs4886406|rs541425|rs2020902|rs1782808|rs10788819|rs383542|rs1790733|rs7322396|rs918842|rs2110290|rs34299|rs16897109</td></tr>
<tr><td>Serositis</td><td>RACCYCD_PATHWAY</td><td>BIOCARTA</td><td>551</td><td>30</td><td>20</td><td>3,80E-01</td><td>8,56E-01</td><td>rs1864053|rs1366315|rs7781436|rs6871452|rs3759216|rs2188513|rs10872893|rs4234512|rs918587|rs383542|rs2298765|rs34299|rs16897109|rs16897180|rs4150724|rs4648090|rs230547|rs7290469|rs2237573|rs7617041</td></tr>
<tr><td>Serositis</td><td>CREB_PATHWAY</td><td>BIOCARTA</td><td>969</td><td>42</td><td>20</td><td>3,80E-01</td><td>8,56E-01</td><td>rs1864053|rs8464|rs2960288|rs7699538|rs1366315|rs6871452|rs4810147|rs12938937|rs383542|rs9913908|rs4958456|rs918842|rs34299|rs16897109|rs16897180|rs2302234|rs4381631|rs2471267|rs7290469|rs3861699</td></tr>
<tr><td>Serositis</td><td>IGF1MTOR_PATHWAY</td><td>BIOCARTA</td><td>582</td><td>26</td><td>15</td><td>3,82E-01</td><td>8,56E-01</td><td>rs1864053|rs1366315|rs6871452|rs11247367|rs2684799|rs11854132|rs383542|rs2230392|rs34299|rs16897109|rs16897180|rs4976028|rs7706821|rs7379303|rs2165945</td></tr>
<tr><td>Serositis</td><td>OXYGEN_DEPENDENT_PROLINE_HYDROXYLATION_OF_HYPOXIA_INDUCIBLE_FACTOR_/</td><td>REACTOME</td><td>313</td><td>9</td><td>5</td><td>3,82E-01</td><td>8,56E-01</td><td>rs10404903|rs887946|rs7141645|rs734205|rs2917779</td></tr>
<tr><td>Serositis</td><td>GABA_B_RECEPTOR_ACTIVATION</td><td>REACTOME</td><td>1863</td><td>27</td><td>20</td><td>3,83E-01</td><td>8,56E-01</td><td>rs13005001|rs2960288|rs12601653|rs1909745|rs16975251|rs12553893|rs4234214|rs4793331|rs9856983|rs4569841|rs726568|rs7733194|rs1167763|rs1999500|rs891429|rs7864144|rs1550116|rs12601221|rs1253676|rs1992703</td></tr>
<tr><td>Serositis</td><td>SIGNALING_BY_NOTCH</td><td>REACTOME</td><td>4553</td><td>41</td><td>20</td><td>3,83E-01</td><td>8,56E-01</td><td>rs11138465|rs10939104|rs11655371|rs2764938|rs669223|rs2150299|rs10765795|rs4692114|rs6913161|rs7634430|rs3898124|rs522495|rs7160413|rs11138350|rs17530657|rs6133987|rs17676727|rs1109771|rs11207332|rs9646967</td></tr>
<tr><td>Serositis</td><td>CHOLESTEROL_BIOSYNTHESIS</td><td>REACTOME</td><td>259</td><td>15</td><td>7</td><td>3,83E-01</td><td>8,56E-01</td><td>rs1296028|rs16979001|rs1063243|rs4840583|rs4840584|rs3794624|rs10098874</td></tr>
<tr><td>Serositis</td><td>MTOR_PATHWAY</td><td>BIOCARTA</td><td>509</td><td>35</td><td>17</td><td>3,86E-01</td><td>8,56E-01</td><td>rs1864053|rs1366315|rs6871452|rs6671527|rs4962081|rs1050700|rs1060478|rs8124053|rs383542|rs2230392|rs34299|rs16897109|rs16897180|rs4976028|rs7706821|rs7379303|rs2165945</td></tr>
<tr><td>Serositis</td><td>MAPK_SIGNALING_PATHWAY</td><td>KEGG</td><td>9833</td><td>28</td><td>20</td><td>3,87E-01</td><td>8,56E-01</td><td>rs1019855|rs17014035|rs4699236|rs4928016|rs967776|rs483808|rs7086224|rs2764938|rs8464|rs6812773|rs2302456|rs2283575|rs4301703|rs17801265|rs17747965|rs7594967|rs10937534|rs9968730|rs2286374|rs7756550</td></tr>
<tr><td>Serositis</td><td>NUCLEOTIDE_BINDING_DOMAIN_LEUCINE_RICH_REPEAT_CONTAINING_RECEPTOR_NLR
YS</td><td>REACTOME</td><td>1337</td><td>32</td><td>20</td><td>3,87E-01</td><td>8,56E-01</td><td>rs4925650|rs17747965|rs1221220|rs1531846|rs2357933|rs9389541|rs468774|rs10498970|rs1390762|rs7973701|rs2020902|rs11871409|rs2046136|rs2058364|rs1076420|rs1010650|rs2141484|rs157695|rs9362733|rs12816436</td></tr>
<tr><td>Serositis</td><td>DOWNSTREAM_SIGNALING_OF_ACTIVATED_FGFR</td><td>REACTOME</td><td>3636</td><td>34</td><td>20</td><td>3,88E-01</td><td>8,56E-01</td><td>rs1352388|rs1864053|rs8464|rs2960288|rs2283575|rs7273626|rs249736|rs1909745|rs6051511|rs4964002|rs2711292|rs4234214|rs9856983|rs4569841|rs1909669|rs1366315|rs6871452|rs7733194|rs210120|rs3759216</td></tr>
<tr><td>Serositis</td><td>CELL_CELL_COMMUNICATION</td><td>REACTOME</td><td>9725</td><td>34</td><td>20</td><td>3,91E-01</td><td>8,56E-01</td><td>rs4701252|rs1364298|rs1864053|rs17505|rs12924378|rs919635|rs809192|rs1547531|rs10045011|rs6089247|rs6006613|rs9812103|rs11127860|rs2960785|rs1834077|rs489725|rs17106413|rs7016497|rs422384|rs356233</td></tr>
<tr><td>Serositis</td><td>PANTOTHENATE_AND_COA_BIOSYNTHESIS</td><td>KEGG</td><td>611</td><td>23</td><td>13</td><td>3,91E-01</td><td>8,56E-01</td><td>rs3750187|rs6934819|rs6935458|rs12218194|rs3793358|rs1760217|rs7745094|rs2802530|rs4383836|rs7756163|rs2148312|rs10493107|rs7766061</td></tr>
<tr><td>Serositis</td><td>SIGNALING_BY_THE_B_CELL_RECEPTOR_BCR</td><td>REACTOME</td><td>3426</td><td>43</td><td>20</td><td>3,91E-01</td><td>8,56E-01</td><td>rs1352388|rs12413520|rs1864053|rs809192|rs17747965|rs328349|rs1221220|rs6051511|rs4964002|rs10820825|rs7948797|rs1366315|rs6871452|rs210120|rs3759216|rs541425|rs1503920|rs10498970|rs9932716|rs516321</td></tr>
<tr><td>Serositis</td><td>MTOR_SIGNALING_PATHWAY</td><td>KEGG</td><td>1290</td><td>46</td><td>20</td><td>3,92E-01</td><td>8,56E-01</td><td>rs4435442|rs1864053|rs9366027|rs9647337|rs511973|rs1366315|rs6871452|rs4962081|rs2289766|rs4240910|rs4690363|rs1050700|rs17719676|rs383542|rs1790733|rs1636808|rs34299|rs16897109|rs16897180|rs999977</td></tr>
<tr><td>Serositis</td><td>ABORTIVE_ELONGATION_OF_HIV1_TRANSCRIPT_IN_THE_ABSENCE_OF_TAT</td><td>REACTOME</td><td>195</td><td>3</td><td>3</td><td>3,92E-01</td><td>8,56E-01</td><td>rs7126805|rs547005|rs2808667</td></tr>
<tr><td>Serositis</td><td>GENERATION_OF_SECOND_MESSENGER_MOLECULES</td><td>REACTOME</td><td>616</td><td>42</td><td>16</td><td>3,93E-01</td><td>8,56E-01</td><td>rs10045011|rs17533090|rs2289545|rs714158|rs747020|rs2894381|rs6885261|rs171847|rs9270856|rs11751659|rs12655171|rs12576947|rs642093|rs2236317|rs1718414|rs6686571</td></tr>
<tr><td>Serositis</td><td>INSULIN_RECEPTOR_SIGNALLING_CASCADE</td><td>REACTOME</td><td>3006</td><td>35</td><td>20</td><td>3,93E-01</td><td>8,56E-01</td><td>rs4435442|rs1864053|rs2302456|rs2283575|rs4486998|rs7273626|rs249736|rs656629|rs1881638|rs6051511|rs9647337|rs1909669|rs1366315|rs6871452|rs7240984|rs10498208|rs11177669|rs10853477|rs4962081|rs1273793</td></tr>
<tr><td>Serositis</td><td>NOTCH_SIGNALING_PATHWAY</td><td>KEGG</td><td>1387</td><td>33</td><td>20</td><td>3,94E-01</td><td>8,56E-01</td><td>rs10939104|rs10765795|rs4692114|rs6913161|rs522495|rs6133987|rs1109771|rs736389|rs12624835|rs12487368|rs9268005|rs7159216|rs2269423|rs4659248|rs10501835|rs10017756|rs4765158|rs1263990|rs1296720|rs2139926</td></tr>
<tr><td>Serositis</td><td>AMINE_COMPOUND_SLC_TRANSPORTERS</td><td>REACTOME</td><td>1298</td><td>37</td><td>20</td><td>3,96E-01</td><td>8,56E-01</td><td>rs11712912|rs10833366|rs747488|rs568567|rs3774128|rs7445640|rs13258461|rs11659324|rs1480620|rs13159461|rs3782370|rs17467186|rs2844457|rs2282810|rs1122903|rs2283138|rs1889036|rs333217|rs2276433|rs3779672</td></tr>
<tr><td>Serositis</td><td>FORMATION_OF_THE_HIV1_EARLY_ELONGATION_COMPLEX</td><td>REACTOME</td><td>329</td><td>3</td><td>3</td><td>3,97E-01</td><td>8,56E-01</td><td>rs7126805|rs547005|rs2808667</td></tr>
<tr><td>Serositis</td><td>NOD1_2_SIGNALING_PATHWAY</td><td>REACTOME</td><td>881</td><td>32</td><td>20</td><td>3,97E-01</td><td>8,56E-01</td><td>rs17747965|rs1221220|rs1531846|rs2357933|rs9389541|rs10498970|rs1390762|rs7973701|rs2020902|rs1010650|rs2141484|rs157695|rs9362733|rs12816436|rs2846855|rs711272|rs10485405|rs2818291|rs666723|rs1049679</td></tr>
<tr><td>Serositis</td><td>FORMATION_OF_RNA_POL_II_ELONGATION_COMPLEX_</td><td>REACTOME</td><td>401</td><td>3</td><td>3</td><td>3,98E-01</td><td>8,56E-01</td><td>rs7126805|rs547005|rs2808667</td></tr>
<tr><td>Serositis</td><td>GRAFT_VERSUS_HOST_DISEASE</td><td>KEGG</td><td>1446</td><td>75</td><td>20</td><td>4,02E-01</td><td>8,56E-01</td><td>rs1264524|rs35399661|rs2235383|rs9276711|rs7770216|rs649216|rs17561|rs9658761|rs3135363|rs2894381|rs7920305|rs176248|rs3135344|rs5009448|rs11751659|rs7761965|rs2715261|rs3134986|rs2857709|rs859722</td></tr>
<tr><td>Serositis</td><td>RNA_POL_I_TRANSCRIPTION_TERMINATION</td><td>REACTOME</td><td>335</td><td>8</td><td>5</td><td>4,02E-01</td><td>8,56E-01</td><td>rs14105|rs2875859|rs7568330|rs9303601|rs9554080</td></tr>
<tr><td>Serositis</td><td>SIGNALING_BY_ROBO_RECEPTOR</td><td>REACTOME</td><td>1900</td><td>40</td><td>20</td><td>4,03E-01</td><td>8,56E-01</td><td>rs3773195|rs2228331|rs4712300|rs7590257|rs1930438|rs6056665|rs6548589|rs9876636|rs221007|rs9816491|rs9809640|rs9845867|rs7773313|rs9875468|rs6816854|rs2668361|rs2166802|rs776119|rs723765|rs2327225</td></tr>
<tr><td>Serositis</td><td>CTCF_PATHWAY</td><td>BIOCARTA</td><td>1035</td><td>35</td><td>20</td><td>4,03E-01</td><td>8,56E-01</td><td>rs1019855|rs1864053|rs2296620|rs1366315|rs6871452|rs13075948|rs11995971|rs3759216|rs1642763|rs383542|rs4846488|rs1636808|rs11931253|rs2279455|rs34299|rs16897109|rs16897180|rs7640543|rs7010121|rs6768081</td></tr>
<tr><td>Serositis</td><td>POST_NMDA_RECEPTOR_ACTIVATION_EVENTS</td><td>REACTOME</td><td>1753</td><td>39</td><td>20</td><td>4,03E-01</td><td>8,56E-01</td><td>rs2960288|rs11055455|rs9968730|rs4704706|rs9366027|rs1421106|rs4780486|rs7699538|rs1543889|rs1861872|rs8049630|rs8064140|rs1107475|rs1070474|rs891429|rs1550116|rs1063243|rs4234512|rs12820037|rs11055603</td></tr>
<tr><td>Serositis</td><td>SIGNALING_BY_FGFR_IN_DISEASE</td><td>REACTOME</td><td>4545</td><td>32</td><td>20</td><td>4,04E-01</td><td>8,56E-01</td><td>rs1352388|rs1864053|rs8464|rs2960288|rs13395505|rs2283575|rs7273626|rs249736|rs1909745|rs6018627|rs2529232|rs441601|rs6051511|rs4964002|rs2711292|rs4234214|rs9856983|rs4569841|rs1909669|rs1366315</td></tr>
<tr><td>Serositis</td><td>EPITHELIAL_CELL_SIGNALING_IN_HELICOBACTER_PYLORI_INFECTION</td><td>KEGG</td><td>1535</td><td>38</td><td>20</td><td>4,04E-01</td><td>8,56E-01</td><td>rs2764938|rs4352468|rs6018627|rs1734852|rs17530657|rs11207332|rs2877260|rs1562853|rs2712353|rs10232489|rs4886406|rs9895159|rs2071596|rs9932716|rs2276266|rs2017750|rs17822304|rs10872893|rs939980|rs7187863</td></tr>
<tr><td>Serositis</td><td>INNATE_IMMUNE_SYSTEM</td><td>REACTOME</td><td>6278</td><td>35</td><td>20</td><td>4,06E-01</td><td>8,56E-01</td><td>rs4925650|rs17014035|rs483808|rs2764938|rs2204345|rs1296028|rs4486998|rs17747965|rs12624551|rs10863430|rs4352468|rs4965533|rs7866830|rs656629|rs1221220|rs10498781|rs920909|rs11793751|rs9366027|rs4151657</td></tr>
<tr><td>Serositis</td><td>CALCINEURIN_PATHWAY</td><td>BIOCARTA</td><td>895</td><td>28</td><td>20</td><td>4,12E-01</td><td>8,62E-01</td><td>rs4699236|rs8464|rs6812773|rs660749|rs6067805|rs12938937|rs748938|rs9808216|rs4699228|rs2645370|rs9913908|rs306872|rs6733907|rs3811063|rs11999145|rs4381631|rs2273642|rs13430277|rs736232|rs17030758</td></tr>
<tr><td>Serositis</td><td>CCR3_PATHWAY</td><td>BIOCARTA</td><td>939</td><td>39</td><td>20</td><td>4,15E-01</td><td>8,62E-01</td><td>rs8464|rs6039107|rs7016497|rs13037679|rs907698|rs4810147|rs1474937|rs6039104|rs12938937|rs4234512|rs10505828|rs9913908|rs708920|rs11999145|rs4381631|rs6086490|rs7290469|rs7617041|rs11044223|rs733646</td></tr>
<tr><td>Serositis</td><td>SIGNALING_BY_FGFR_MUTANTS</td><td>REACTOME</td><td>1657</td><td>35</td><td>20</td><td>4,16E-01</td><td>8,62E-01</td><td>rs1864053|rs13395505|rs2283575|rs249736|rs2529232|rs441601|rs1909669|rs1366315|rs6871452|rs11177669|rs2156921|rs3945860|rs881301|rs1219643|rs17364455|rs3135772|rs2529192|rs7168316|rs7479018|rs630757</td></tr>
<tr><td>Serositis</td><td>CXCR4_PATHWAY</td><td>BIOCARTA</td><td>1099</td><td>35</td><td>20</td><td>4,16E-01</td><td>8,62E-01</td><td>rs1864053|rs8464|rs2302456|rs7016497|rs1366315|rs6871452|rs12938937|rs4234512|rs10505828|rs383542|rs9913908|rs34299|rs16897109|rs16897180|rs11999145|rs4381631|rs4648090|rs6430628|rs230547|rs7290469</td></tr>
<tr><td>Serositis</td><td>LYSOSOME</td><td>KEGG</td><td>2448</td><td>27</td><td>20</td><td>4,16E-01</td><td>8,62E-01</td><td>rs2702385|rs1296028|rs6811556|rs8132|rs11811771|rs3745255|rs6536158|rs4074708|rs2449545|rs1805727|rs13105690|rs1805734|rs2739685|rs1562853|rs4758104|rs25413|rs2153486|rs1072382|rs1242102|rs9620018</td></tr>
<tr><td>Serositis</td><td>METABOLISM_OF_MRNA</td><td>REACTOME</td><td>3017</td><td>31</td><td>20</td><td>4,17E-01</td><td>8,62E-01</td><td>rs17014035|rs8464|rs7273626|rs11784209|rs2252523|rs328349|rs764223|rs6996970|rs8132|rs16935649|rs6822283|rs2302178|rs17464037|rs7948797|rs10479576|rs12121852|rs7126805|rs17834271|rs11879090|rs1345207</td></tr>
</table>

<table>
<tr><td>Serositis</td><td>BAD_PATHWAY</td><td>BIOCARTA</td><td>927</td><td>35</td><td>20</td><td>4,19E-01</td><td>8,62E-01</td><td>rs1864053|rs2960288|rs1366315|rs6871452|rs1005656|rs11247367|rs2684799|rs2046136|rs11854132|rs383542|rs2849382|rs1636808|rs34299|rs16897109|rs16897180|rs2302234|rs17093560|rs2471267|rs7290469|rs218273</td></tr>
<tr><td>Serositis</td><td>REGULATION_OF_INSULIN_LIKE_GROWTH_FACTOR_IGF_ACTIVITY_BY_INSULIN_LIKE_G DING_PROTEINS_IGFBPS</td><td>REACTOME</td><td>570</td><td>23</td><td>15</td><td>4,21E-01</td><td>8,62E-01</td><td>rs6435944|rs7469847|rs12353147|rs1486173|rs7850583|rs34575650|rs11971230|rs12999653|rs240104|rs12272341|rs4244808|rs7515586|rs720683|rs1993013|rs1553009</td></tr>
<tr><td>Serositis</td><td>TRIGLYCERIDE_BIOSYNTHESIS</td><td>REACTOME</td><td>1182</td><td>29</td><td>20</td><td>4,21E-01</td><td>8,62E-01</td><td>rs17007811|rs17331746|rs9474466|rs2257140|rs7659090|rs11891008|rs4391265|rs4952147|rs1921874|rs17028984|rs4819329|rs6865199|rs2269423|rs9999681|rs966367|rs10509948|rs2928596|rs1036668|rs3745013|rs11889370</td></tr>
<tr><td>Serositis</td><td>REGULATION_OF_ACTIN_CYTOSKELETON</td><td>KEGG</td><td>7089</td><td>33</td><td>20</td><td>4,21E-01</td><td>8,62E-01</td><td>rs4886210|rs11245205|rs1864053|rs10870946|rs4666600|rs2302456|rs2283575|rs16910559|rs10937534|rs12373564|rs17106413|rs7016497|rs249736|rs1486949|rs2248656|rs11017615|rs9538396|rs2056131|rs11730652|rs6056665</td></tr>
<tr><td>Serositis</td><td>IL_RECEPTOR_SHC_SIGNALING</td><td>REACTOME</td><td>624</td><td>43</td><td>18</td><td>4,23E-01</td><td>8,62E-01</td><td>rs1864053|rs9647337|rs1366315|rs6871452|rs6794523|rs4240910|rs3792424|rs334782|rs383542|rs17606924|rs34299|rs16897109|rs16897180|rs4976028|rs1622208|rs7706821|rs7379303|rs693293</td></tr>
<tr><td>Serositis</td><td>PYRUVATE_METABOLISM</td><td>KEGG</td><td>908</td><td>36</td><td>20</td><td>4,23E-01</td><td>8,62E-01</td><td>rs10890819|rs6070157|rs10732359|rs6744662|rs309455|rs12577790|rs7122539|rs4878199|rs17470048|rs1571231|rs4646771|rs12431669|rs6015032|rs750385|rs3798211|rs618513|rs6545388|rs2268388|rs12281596|rs1923431</td></tr>
<tr><td>Serositis</td><td>CHONDROITIN_SULFATE_BIOSYNTHESIS</td><td>REACTOME</td><td>846</td><td>41</td><td>20</td><td>4,23E-01</td><td>8,62E-01</td><td>rs9954485|rs1458526|rs1807833|rs11112047|rs17205951|rs2052007|rs1350505|rs10503650|rs3765103|rs2408511|rs2505557|rs6597849|rs12427269|rs10899|rs9967331|rs12910524|rs10503653|rs173686|rs10481426|rs7357583</td></tr>
<tr><td>Serositis</td><td>PERK_REGULATED_GENE_EXPRESSION</td><td>REACTOME</td><td>334</td><td>17</td><td>11</td><td>4,24E-01</td><td>8,62E-01</td><td>rs764223|rs1485591|rs11879090|rs12739624|rs7571971|rs1126700|rs11189224|rs650229|rs6498509|rs1284605|rs1553009</td></tr>
<tr><td>Serositis</td><td>PML_PATHWAY</td><td>BIOCARTA</td><td>384</td><td>21</td><td>15</td><td>4,25E-01</td><td>8,62E-01</td><td>rs10169182|rs2228576|rs824939|rs1642763|rs9658761|rs7920305|rs1296720|rs2238419|rs2857709|rs859722|rs7550488|rs6754024|rs2059755|rs6738997|rs6436944</td></tr>
<tr><td>Serositis</td><td>METAL_ION_SLC_TRANSPORTERS</td><td>REACTOME</td><td>653</td><td>27</td><td>10</td><td>4,26E-01</td><td>8,63E-01</td><td>rs7527059|rs12153013|rs1377652|rs1453802|rs12993717|rs2434349|rs4131295|rs4976217|rs190223|rs233821</td></tr>
<tr><td>Serositis</td><td>INSULIN_SYNTHESIS_AND_PROCESSING</td><td>REACTOME</td><td>1033</td><td>34</td><td>20</td><td>4,28E-01</td><td>8,65E-01</td><td>rs1101156|rs852069|rs972736|rs12153013|rs10149366|rs1447045|rs835259|rs17135931|rs9504229|rs2804736|rs2039713|rs1933652|rs6951030|rs17687965|rs2434349|rs10274328|rs12333170|rs4131295|rs7143051|rs363039</td></tr>
<tr><td>Serositis</td><td>INTERACTION_BETWEEN_L1_AND_ANKYRINS</td><td>REACTOME</td><td>970</td><td>34</td><td>20</td><td>4,30E-01</td><td>8,65E-01</td><td>rs3793859|rs10509122|rs1904398|rs17134769|rs2931462|rs6599218|rs7824311|rs353123|rs4671928|rs10791889|rs10505589|rs1290018|rs1962131|rs4671952|rs13276777|rs10994341|rs595964|rs1757779|rs1841876|rs11219210</td></tr>
<tr><td>Serositis</td><td>ADP_SIGNALLING_THROUGH_P2RY1</td><td>REACTOME</td><td>863</td><td>40</td><td>20</td><td>4,30E-01</td><td>8,65E-01</td><td>rs967776|rs6018627|rs12564813|rs12553893|rs591393|rs308033|rs1253676|rs11070884|rs3010644|rs12738546|rs1924736|rs7863832|rs11999145|rs2430080|rs7618348|rs3123045|rs2802475|rs6012200|rs12405943|rs7535176</td></tr>
<tr><td>Serositis</td><td>FRUCTOSE_AND_MANNOSE_METABOLISM</td><td>KEGG</td><td>984</td><td>39</td><td>20</td><td>4,33E-01</td><td>8,66E-01</td><td>rs10993276|rs689035|rs9391934|rs4959621|rs2236441|rs7817301|rs6602514|rs17587917|rs4976661|rs10458131|rs17158714|rs2628440|rs2842754|rs6928775|rs906215|rs2235718|rs581720|rs2267846|rs3771783|rs3800052</td></tr>
<tr><td>Serositis</td><td>SIGNALING_BY_FGFR1_MUTANTS</td><td>REACTOME</td><td>1243</td><td>36</td><td>20</td><td>4,33E-01</td><td>8,66E-01</td><td>rs1864053|rs13395505|rs249736|rs2529232|rs441601|rs1366315|rs6871452|rs2156921|rs3945860|rs881301|rs17364455|rs2529192|rs7479018|rs630757|rs383542|rs6467778|rs2038711|rs10492454|rs2906734|rs34299</td></tr>
<tr><td>Serositis</td><td>INFLAM_PATHWAY</td><td>BIOCARTA</td><td>747</td><td>29</td><td>19</td><td>4,38E-01</td><td>8,73E-01</td><td>rs3024498|rs17025039|rs28530648|rs1554286|rs6929796|rs7538029|rs17454570|rs17561|rs433192|rs1075498|rs3135363|rs3135344|rs4846488|rs2857709|rs4849122|rs7721176|rs17701271|rs333968|rs1476482</td></tr>
<tr><td>Serositis</td><td>MITOCHONDRIAL_PROTEIN_IMPORT</td><td>REACTOME</td><td>609</td><td>21</td><td>15</td><td>4,40E-01</td><td>8,74E-01</td><td>rs2663342|rs6006613|rs2302178|rs4346637|rs16864246|rs3744992|rs10027697|rs1800651|rs2049805|rs352336|rs9938257|rs9965812|rs5764058|rs1513887|rs6586361</td></tr>
<tr><td>Serositis</td><td>MYOGENESIS</td><td>REACTOME</td><td>1747</td><td>32</td><td>20</td><td>4,42E-01</td><td>8,74E-01</td><td>rs6089247|rs7163504|rs4965533|rs7183361|rs13045227|rs17089925|rs6121828|rs918418|rs7702020|rs4925258|rs13045246|rs2298574|rs6089510|rs7271884|rs634053|rs2113445|rs1020066|rs6598265|rs9920504|rs7229244</td></tr>
<tr><td>Serositis</td><td>SYNTHESIS_OF_GLYCOSYLPHOSPHATIDYLINOSITOL_GPI</td><td>REACTOME</td><td>485</td><td>39</td><td>16</td><td>4,43E-01</td><td>8,74E-01</td><td>rs3739821|rs935875|rs6415846|rs1912976|rs1850348|rs2290344|rs281282|rs2486253|rs11702020|rs8031753|rs10519129|rs682567|rs1520460|rs1912643|rs9425618|rs12909534</td></tr>
<tr><td>Serositis</td><td>THROMBIN_SIGNALLING_THROUGH_PROTEINASE_ACTIVATED_RECEPTORS_PARS</td><td>REACTOME</td><td>690</td><td>26</td><td>14</td><td>4,44E-01</td><td>8,74E-01</td><td>rs6018627|rs12553893|rs308033|rs250724|rs1253676|rs11070884|rs4704356|rs7728450|rs7863832|rs11999145|rs7618348|rs6012200|rs7290469|rs1688123</td></tr>
<tr><td>Serositis</td><td>GLYCOSYLPHOSPHATIDYLINOSITOL_GPI_ANCHOR_BIOSYNTHESIS</td><td>KEGG</td><td>318</td><td>18</td><td>10</td><td>4,44E-01</td><td>8,74E-01</td><td>rs3739821|rs6415846|rs1028307|rs2290344|rs2486253|rs793683|rs11702020|rs2741584|rs9425618|rs199669</td></tr>
<tr><td>Serositis</td><td>INTRINSIC_PATHWAY</td><td>REACTOME</td><td>264</td><td>18</td><td>11</td><td>4,45E-01</td><td>8,74E-01</td><td>rs10032695|rs12810426|rs7646517|rs7130900|rs822373|rs11918289|rs216856|rs7708314|rs2062632|rs6829128|rs2167370</td></tr>
<tr><td>Serositis</td><td>NOTCH1_INTRACELLULAR_DOMAIN_REGULATES_TRANSCRIPTION</td><td>REACTOME</td><td>2852</td><td>33</td><td>20</td><td>4,46E-01</td><td>8,74E-01</td><td>rs11138465|rs10939104|rs669223|rs10765795|rs4692114|rs6913161|rs7634430|rs3898124|rs522495|rs11138350|rs17676727|rs9646967|rs1684274|rs11995971|rs11138447|rs10116365|rs12487368|rs17276701|rs13012239|rs305024</td></tr>
<tr><td>Serositis</td><td>HUNTINGTONS_DISEASE</td><td>KEGG</td><td>4311</td><td>31</td><td>20</td><td>4,49E-01</td><td>8,74E-01</td><td>rs6437332|rs757980|rs1345638|rs6442887|rs6039107|rs6972081|rs1264901|rs11055455|rs2252523|rs6913161|rs3804984|rs10045456|rs17309944|rs6433541|rs1500147|rs1179605|rs2946394|rs1421106|rs2302178|rs13037679</td></tr>
<tr><td>Serositis</td><td>G0_AND_EARLY_G1</td><td>REACTOME</td><td>258</td><td>7</td><td>5</td><td>4,49E-01</td><td>8,74E-01</td><td>rs2282411|rs9315422|rs918587|rs4150724|rs10033399</td></tr>
<tr><td>Serositis</td><td>BIOPEPTIDES_PATHWAY</td><td>BIOCARTA</td><td>1081</td><td>43</td><td>20</td><td>4,49E-01</td><td>8,74E-01</td><td>rs8464|rs13395505|rs809192|rs7699538|rs308033|rs2478545|rs2017750|rs7646517|rs12938937|rs4234512|rs9808216|rs9913908|rs822373|rs4958456|rs11918289|rs6733907|rs4381631|rs2062632|rs7290469|rs7617041</td></tr>
<tr><td>Serositis</td><td>FMLP_PATHWAY</td><td>BIOCARTA</td><td>1148</td><td>24</td><td>20</td><td>4,50E-01</td><td>8,74E-01</td><td>rs4699236|rs6812773|rs6039107|rs13037679|rs660749|rs6067805|rs1474937|rs6039104|rs10872893|rs748938|rs4234512|rs10505828|rs9808216|rs4699228|rs2645370|rs306872|rs708920|rs742257|rs1011398|rs6733907</td></tr>
<tr><td>Serositis</td><td>MITOCHONDRIA_PATHWAY</td><td>BIOCARTA</td><td>317</td><td>12</td><td>8</td><td>4,53E-01</td><td>8,76E-01</td><td>rs181402|rs17809603|rs2020902|rs2046136|rs2849382|rs2846855|rs17093560|rs396746</td></tr>
<tr><td>Serositis</td><td>ACTIVATED_TAK1_MEDIATES_P38_MAPK_ACTIVATION</td><td>REACTOME</td><td>649</td><td>37</td><td>20</td><td>4,53E-01</td><td>8,76E-01</td><td>rs17014035|rs17747965|rs1221220|rs1531846|rs10498970|rs1010650|rs2141484|rs157695|rs9362733|rs711272|rs10485405|rs2818291|rs10094579|rs721025|rs607071|rs9345148|rs17725371|rs2224063|rs1923076|rs11869073</td></tr>
<tr><td>Serositis</td><td>G_BETA_GAMMA_SIGNALLING_THROUGH_PI3KGAMMA</td><td>REACTOME</td><td>487</td><td>16</td><td>9</td><td>4,57E-01</td><td>8,77E-01</td><td>rs12553893|rs511973|rs1253676|rs11070884|rs1636808|rs764605|rs7863832|rs7618348|rs10491087</td></tr>
<tr><td>Serositis</td><td>SIGNALING_BY_EGFR_IN_CANCER</td><td>REACTOME</td><td>4030</td><td>37</td><td>20</td><td>4,58E-01</td><td>8,77E-01</td><td>rs2306677|rs1864053|rs8464|rs2960288|rs7273626|rs17567|rs1909745|rs6018627|rs4325000|rs6051511|rs2711292|rs4234214|rs9856983|rs2877260|rs4569841|rs12982931|rs1366315|rs6785423|rs6871452|rs7733194</td></tr>
<tr><td>Serositis</td><td>RIBOFLAVIN_METABOLISM</td><td>KEGG</td><td>295</td><td>13</td><td>9</td><td>4,59E-01</td><td>8,77E-01</td><td>rs6934819|rs6935458|rs17587917|rs12221497|rs4383836|rs7756163|rs6992452|rs10853825|rs7766061</td></tr>
<tr><td>Serositis</td><td>ACTIVATION_OF_GENES_BY_ATF4</td><td>REACTOME</td><td>316</td><td>15</td><td>10</td><td>4,60E-01</td><td>8,77E-01</td><td>rs764223|rs1485591|rs11879090|rs12739624|rs1126700|rs11189224|rs650229|rs6498509|rs1284605|rs1553009</td></tr>
<tr><td>Serositis</td><td>TRANSFERRIN_ENDOCYTOSIS_AND_RECYCLING</td><td>REACTOME</td><td>513</td><td>28</td><td>12</td><td>4,61E-01</td><td>8,77E-01</td><td>rs1530561|rs1562853|rs2712353|rs6929796|rs11783925|rs9643478|rs2857709|rs11712192|rs4147778|rs570|rs4287912|rs10241449</td></tr>
<tr><td>Serositis</td><td>SEMA4D_INDUCED_CELL_MIGRATION_AND_GROWTH_CONE_COLLAPSE</td><td>REACTOME</td><td>433</td><td>18</td><td>10</td><td>4,61E-01</td><td>8,77E-01</td><td>rs1651543|rs2282537|rs4486969|rs788330|rs13054809|rs342076|rs11265783|rs5997917|rs4801824|rs4938800</td></tr>
<tr><td>Serositis</td><td>REGULATION_OF_BETA_CELL_DEVELOPMENT</td><td>REACTOME</td><td>1288</td><td>39</td><td>20</td><td>4,62E-01</td><td>8,77E-01</td><td>rs8100056|rs10939104|rs4692114|rs2737641|rs813393|rs1156234|rs8042685|rs12487368|rs10743413|rs11934856|rs4746864|rs10017756|rs1782808|rs4812829|rs705974|rs7322396|rs745659|rs2249048|rs758989|rs2248673</td></tr>
<tr><td>Serositis</td><td>INTERFERON_SIGNALING</td><td>REACTOME</td><td>3448</td><td>52</td><td>20</td><td>4,64E-01</td><td>8,78E-01</td><td>rs2352930|rs10169182|rs13395505|rs398800|rs2074560|rs9845458|rs17533090|rs276978|rs2229857|rs8132|rs2272874|rs7699538|rs12149292|rs886451|rs2236906|rs7770216|rs12720253|rs7973701|rs4789576|rs1799969</td></tr>
<tr><td>Serositis</td><td>ARAP_PATHWAY</td><td>BIOCARTA</td><td>292</td><td>11</td><td>9</td><td>4,65E-01</td><td>8,78E-01</td><td>rs6000649|rs1799270|rs9610713|rs940243|rs12622238|rs6431986|rs793683|rs2179766|rs2024595</td></tr>
<tr><td>Serositis</td><td>POST_CHAPERONIN_TUBULIN_FOLDING_PATHWAY</td><td>REACTOME</td><td>254</td><td>3</td><td>3</td><td>4,66E-01</td><td>8,78E-01</td><td>rs6502026|rs151352|rs6490620</td></tr>
<tr><td>Serositis</td><td>GLYCOSAMINOGLYCAN_DEGRADATION</td><td>KEGG</td><td>709</td><td>40</td><td>19</td><td>4,67E-01</td><td>8,78E-01</td><td>rs2402676|rs4074708|rs25413|rs16948406|rs3733895|rs8068169|rs2996009|rs4450116|rs1013120|rs2022240|rs2874994|rs7075080|rs12601481|rs2285996|rs16875913|rs4791571|rs1541352|rs337853|rs6550191</td></tr>
<tr><td>Serositis</td><td>THROMBOXANE_SIGNALLING_THROUGH_TP_RECEPTOR</td><td>REACTOME</td><td>492</td><td>16</td><td>8</td><td>4,68E-01</td><td>8,78E-01</td><td>rs12553893|rs308033|rs1253676|rs11070884|rs7863832|rs11999145|rs7618348|rs1688123</td></tr>
<tr><td>Serositis</td><td>INTESTINAL_IMMUNE_NETWORK_FOR_IGA_PRODUCTION</td><td>KEGG</td><td>1392</td><td>56</td><td>20</td><td>4,69E-01</td><td>8,78E-01</td><td>rs12129555|rs35399661|rs3024498|rs12613960|rs3948793|rs10195135|rs1554286|rs8072293|rs4675397|rs3135363|rs2894381|rs7354779|rs176248|rs3135344|rs11751659|rs378299|rs2715261|rs6032678|rs3134986|rs12473084</td></tr>
<tr><td>Serositis</td><td>EICOSANOID_LIGAND_BINDING_RECEPTORS</td><td>REACTOME</td><td>507</td><td>16</td><td>8</td><td>4,71E-01</td><td>8,79E-01</td><td>rs3760703|rs2352039|rs966937|rs7147188|rs443383|rs6424765|rs10483285|rs990466</td></tr>
<tr><td>Serositis</td><td>ACUTE_MYELOID_LEUKEMIA</td><td>KEGG</td><td>1892</td><td>37</td><td>20</td><td>4,71E-01</td><td>8,79E-01</td><td>rs1864053|rs2221145|rs2300401|rs6517288|rs2292653|rs9647337|rs2282411|rs511973|rs1366315|rs6871452|rs1005656|rs11995971|rs10022956|rs10489028|rs11725638|rs4240910|rs9315422|rs4234512|rs383542|rs1790733</td></tr>
<tr><td>Serositis</td><td>CYTOSOLIC_TRNA_AMINOACYLATION</td><td>REACTOME</td><td>312</td><td>12</td><td>6</td><td>4,76E-01</td><td>8,85E-01</td><td>rs2949646|rs12730560|rs12112229|rs3018058|rs2530230|rs1284605</td></tr>
<tr><td>Serositis</td><td>COMP_PATHWAY</td><td>BIOCARTA</td><td>628</td><td>44</td><td>19</td><td>4,77E-01</td><td>8,85E-01</td><td>rs2204345|rs920909|rs4151657|rs2031749|rs292001|rs10824804|rs3776519|rs706469|rs7444800|rs1342430|rs630379|rs17468519|rs12412945|rs10841811|rs2384013|rs11003258|rs1343065|rs2279623|rs3908206</td></tr>
<tr><td>Serositis</td><td>CDC42RAC_PATHWAY</td><td>BIOCARTA</td><td>435</td><td>25</td><td>13</td><td>4,78E-01</td><td>8,85E-01</td><td>rs1864053|rs1366315|rs6871452|rs383542|rs34299|rs16897109|rs1492769|rs16897180|rs7660560|rs1174657|rs4976028|rs7706821|rs7379303</td></tr>
<tr><td>Serositis</td><td>INSULIN_SIGNALING_PATHWAY</td><td>KEGG</td><td>3744</td><td>51</td><td>20</td><td>4,82E-01</td><td>8,87E-01</td><td>rs1864053|rs12299821|rs10993276|rs6070157|rs2302456|rs169290|rs1899908|rs4352468|rs2044316|rs1881638|rs2946394|rs523200|rs9647337|rs511973|rs1366315|rs6871452|rs178255|rs10498208|rs6671527|rs1503920</td></tr>
<tr><td>Serositis</td><td>PI3K_AKT_ACTIVATION</td><td>REACTOME</td><td>1161</td><td>39</td><td>20</td><td>4,84E-01</td><td>8,87E-01</td><td>rs1864053|rs6051511|rs9647337|rs1366315|rs6871452|rs3759216|rs10498208|rs541425|rs2254527|rs1273793|rs2020902|rs7993089|rs10933137|rs1782808|rs10788819|rs383542|rs1790733|rs7322396|rs16837709|rs918842</td></tr>
<tr><td>Serositis</td><td>PHOSPHOLIPASE_C_MEDIATED_CASCADE</td><td>REACTOME</td><td>2628</td><td>31</td><td>20</td><td>4,87E-01</td><td>8,87E-01</td><td>rs2306677|rs8464|rs2960288|rs2283575|rs249736|rs1909745|rs2711292|rs4234214|rs9856983|rs4569841|rs1909669|rs7733194|rs210120|rs4963998|rs3945860|rs881301|rs891429|rs1219643|rs3135772|rs1550116</td></tr>
<tr><td>Serositis</td><td>DNA_REPAIR</td><td>REACTOME</td><td>2111</td><td>35</td><td>20</td><td>4,87E-01</td><td>8,87E-01</td><td>rs4987876|rs867185|rs7188980|rs4135048|rs227060|rs8109798|rs10276762|rs4943153|rs7126805|rs16972096|rs10490114|rs6460075|rs1794242|rs848279|rs886727|rs3829300|rs2275007|rs1452849|rs337484|rs9430624</td></tr>
<tr><td>Serositis</td><td>GLYCOSAMINOGLYCAN_METABOLISM</td><td>REACTOME</td><td>6096</td><td>38</td><td>20</td><td>4,88E-01</td><td>8,87E-01</td><td>rs9954485|rs7296900|rs1554324|rs2736868|rs1458526|rs9995245|rs2228331|rs1932306|rs3734371|rs4583089|rs17491203|rs12819127|rs928646|rs1424223|rs1401346|rs4074708|rs728528|rs1556466|rs1807833|rs7130091</td></tr>
<tr><td>Serositis</td><td>SHC_RELATED_EVENTS</td><td>REACTOME</td><td>302</td><td>21</td><td>7</td><td>4,90E-01</td><td>8,87E-01</td><td>rs7273626|rs11137525|rs4234512|rs7290469|rs2425650|rs7617041|rs4244808</td></tr>
<tr><td>Serositis</td><td>PI3K_CASCADE</td><td>REACTOME</td><td>2702</td><td>32</td><td>20</td><td>4,91E-01</td><td>8,87E-01</td><td>rs4435442|rs1864053|rs2283575|rs4486998|rs249736|rs656629|rs1881638|rs6051511|rs9647337|rs1909669|rs1366315|rs6871452|rs7240984|rs10498208|rs11177669|rs10853477|rs4962081|rs1273793|rs2289766|rs3945860</td></tr>
<tr><td>Serositis</td><td>AMINE_LIGAND_BINDING_RECEPTORS</td><td>REACTOME</td><td>2247</td><td>38</td><td>20</td><td>4,91E-01</td><td>8,87E-01</td><td>rs192718|rs2405871|rs9361153|rs2206819|rs13257637|rs888956|rs1158089|rs1791523|rs172470|rs10885530|rs10943413|rs2032357|rs7266945|rs1936600|rs17055767|rs659562|rs10885531|rs1509689|rs12139491|rs2082991</td></tr>
<tr><td>Serositis</td><td>STARCH_AND_SUCROSE_METABOLISM</td><td>KEGG</td><td>2178</td><td>61</td><td>20</td><td>4,92E-01</td><td>8,87E-01</td><td>rs958238|rs17301901|rs6934819|rs8132|rs4388712|rs6935458|rs523200|rs430976|rs1460421|rs2013573|rs6738678|rs2581952|rs4976661|rs7608175|rs412218|rs1841355|rs8004788|rs2995965|rs4148285|rs906215</td></tr>
<tr><td>Serositis</td><td>INTEGRIN_CELL_SURFACE_INTERACTIONS</td><td>REACTOME</td><td>2614</td><td>27</td><td>20</td><td>4,93E-01</td><td>8,87E-01</td><td>rs627527|rs2302456|rs12648395|rs7016497|rs6018627|rs11168228|rs1034620|rs2056131|rs523200|rs1805|rs10878841|rs10820825|rs2292700|rs10487254|rs1858822|rs7548973|rs12620429|rs12810426|rs4886406|rs12900942</td></tr>
<tr><td>Serositis</td><td>FACTORS_INVOLVED_IN_MEGAKARYOCYTE_DEVELOPMENT_AND_PLATELET_PRODUCT</td><td>REACTOME</td><td>3549</td><td>32</td><td>20</td><td>4,94E-01</td><td>8,87E-01</td><td>rs11245205|rs3757649|rs573851|rs6913161|rs1538242|rs3801783|rs979534|rs11017615|rs983263|rs10733789|rs3184504|rs2275593|rs10066223|rs7563440|rs9901294|rs7838094|rs17648423|rs4741821|rs2111534|rs8098869</td></tr>
<tr><td>Serositis</td><td>GH_PATHWAY</td><td>BIOCARTA</td><td>963</td><td>40</td><td>20</td><td>4,94E-01</td><td>8,87E-01</td><td>rs1864053|rs8464|rs1366315|rs6871452|rs10498208|rs1273793|rs12938937|rs4234512|rs10933137|rs6895971|rs383542|rs9913908|rs1636808|rs34299|rs16897109|rs16897180|rs4381631|rs7290469|rs7617041|rs4244808</td></tr>
<tr><td>Serositis</td><td>SIGNALING_BY_FGFR</td><td>REACTOME</td><td>4154</td><td>35</td><td>20</td><td>4,95E-01</td><td>8,87E-01</td><td>rs2306677|rs1864053|rs8464|rs2960288|rs2283575|rs7273626|rs249736|rs1909745|rs6018627|rs6051511|rs2711292|rs4234214|rs9856983|rs4569841|rs1909669|rs1366315|rs6871452|rs7733194|rs210120|rs3759216</td></tr>
<tr><td>Serositis</td><td>CTLA4_INHIBITORY_SIGNALING</td><td>REACTOME</td><td>447</td><td>21</td><td>14</td><td>4,96E-01</td><td>8,87E-01</td><td>rs809192|rs6018627|rs12120009|rs11119881|rs3214047|rs747020|rs2715261|rs7245158|rs6012200|rs16922298|rs7618747|rs2162610|rs1255771|rs1409837</td></tr>
<tr><td>Serositis</td><td>GASTRIN_CREB_SIGNALLING_PATHWAY_VIA_PKC_AND_MAPK</td><td>REACTOME</td><td>8122</td><td>33</td><td>20</td><td>4,99E-01</td><td>8,87E-01</td><td>rs1352388|rs1864053|rs192718|rs10516614|rs9562385|rs8464|rs2405871|rs3760703|rs691712|rs7273626|rs6039107|rs3783754|rs13257637|rs3771863|rs2352039|rs4907013|rs13115641|rs9366027|rs17742581|rs182034</td></tr>
<tr><td>Serositis</td><td>P53_INDEPENDENT_G1_S_DNA_DAMAGE_CHECKPOINT</td><td>REACTOME</td><td>729</td><td>27</td><td>18</td><td>5,02E-01</td><td>8,87E-01</td><td>rs4987876|rs227060|rs328349|rs7948797|rs12201680|rs2917779|rs16827840|rs2298483|rs909473|rs11794849|rs755553|rs9380326|rs8004624|rs8005252|rs2621321|rs3859192|rs7500512|rs1424083</td></tr>
<tr><td>Serositis</td><td>WNT_PATHWAY</td><td>BIOCARTA</td><td>1363</td><td>28</td><td>20</td><td>5,02E-01</td><td>8,87E-01</td><td>rs2221145|rs669223|rs17747965|rs1221220|rs11995971|rs10022956|rs10116365|rs10498970|rs4355511|rs10489028|rs4958072|rs11725638|rs1010650|rs13188321|rs2141484|rs157695|rs9362733|rs241280|rs1296720|rs2298765</td></tr>
<tr><td>Serositis</td><td>PKB_MEDIATED_EVENTS</td><td>REACTOME</td><td>583</td><td>21</td><td>14</td><td>5,02E-01</td><td>8,87E-01</td><td>rs4435442|rs1881638|rs4962081|rs2289766|rs1050700|rs4783446|rs4950385|rs999977|rs7789699|rs7788961|rs9568196|rs4425665|rs7146988|rs2165945</td></tr>
<tr><td>Serositis</td><td>SIGNALING_BY_INSULIN_RECEPTOR</td><td>REACTOME</td><td>3432</td><td>33</td><td>20</td><td>5,02E-01</td><td>8,87E-01</td><td>rs4435442|rs1864053|rs2302456|rs2283575|rs4486998|rs7273626|rs249736|rs656629|rs1881638|rs6051511|rs9647337|rs1909669|rs1366315|rs6871452|rs1562853|rs2712353|rs7240984|rs10498208|rs11177669|rs2071596</td></tr>
<tr><td>Serositis</td><td>CYTOKINE_PATHWAY</td><td>BIOCARTA</td><td>491</td><td>20</td><td>14</td><td>5,02E-01</td><td>8,87E-01</td><td>rs3024498|rs1554286|rs6929796|rs17454570|rs17561|rs1075498|rs12907134|rs9395766|rs2857709|rs4849122|rs1912124|rs7721176|rs17701271|rs1476482</td></tr>
<tr><td>Serositis</td><td>PROPANOATE_METABOLISM</td><td>KEGG</td><td>1219</td><td>59</td><td>20</td><td>5,03E-01</td><td>8,87E-01</td><td>rs10890819|rs10732359|rs962033|rs7574229|rs7558055|rs12577790|rs9937726|rs9357584|rs11690708|rs7954341|rs10506840|rs4878199|rs4646771|rs12423591|rs2516122|rs3798211|rs2268388|rs1536002|rs1923431|rs7216</td></tr>
<tr><td>Serositis</td><td>RNA_POL_I_TRANSCRIPTION_INITIATION</td><td>REACTOME</td><td>427</td><td>12</td><td>7</td><td>5,04E-01</td><td>8,88E-01</td><td>rs14105|rs2875859|rs7568330|rs2763982|rs9303601|rs9554080|rs11923928</td></tr>
<tr><td>Serositis</td><td>LIPID_DIGESTION_MOBILIZATION_AND_TRANSPORT</td><td>REACTOME</td><td>1389</td><td>31</td><td>20</td><td>5,07E-01</td><td>8,89E-01</td><td>rs11254339|rs13100991|rs2954804|rs3733156|rs7753322|rs3780542|rs1914172|rs1877059|rs17139378|rs8041525|rs964184|rs2236705|rs4073237|rs1790733|rs9637421|rs261336|rs7178362|rs2487052|rs2879094|rs8133510</td></tr>
<tr><td>Serositis</td><td>GLOBAL_GENOMIC_NER_GG_NER</td><td>REACTOME</td><td>823</td><td>31</td><td>16</td><td>5,09E-01</td><td>8,89E-01</td><td>rs7188980|rs10276762|rs4943153|rs6460075|rs1794242|rs886727|rs12221497|rs1714473|rs1928023|rs741048|rs2321822|rs2808667|rs1001440|rs17137468|rs12584168|rs1790740</td></tr>
<tr><td>Serositis</td><td>PHOSPHOLIPID_METABOLISM</td><td>REACTOME</td><td>6226</td><td>30</td><td>20</td><td>5,13E-01</td><td>8,89E-01</td><td>rs2810043|rs1864053|rs12133338|rs967776|rs10505887|rs4903606|rs17007811|rs4486998|rs2273814|rs2008644|rs656629|rs4074708|rs1487489|rs29069|rs10508921|rs9366337|rs2257140|rs11794290|rs7746765|rs9647337</td></tr>
<tr><td>Serositis</td><td>ACTIVATION_OF_ATR_IN_RESPONSE_TO_REPLICATION_STRESS</td><td>REACTOME</td><td>653</td><td>32</td><td>20</td><td>5,14E-01</td><td>8,89E-01</td><td>rs7290191|rs133415|rs10276762|rs11164955|rs4943153|rs6460075|rs886727|rs11164953|rs9311428|rs2298483|rs1928023|rs741048|rs2321822|rs13082666|rs17137468|rs4265124|rs12584168|rs6450035|rs2307373|rs941289</td></tr>
<tr><td>Serositis</td><td>ABCA_TRANSPORTERS_IN_LIPID_HOMEOSTASIS</td><td>REACTOME</td><td>464</td><td>20</td><td>12</td><td>5,15E-01</td><td>8,89E-01</td><td>rs2954804|rs12452482|rs10498030|rs2236705|rs4073237|rs2274412|rs11077922|rs161061|rs8133510|rs532345|rs3752237|rs4148202</td></tr>
<tr><td>Serositis</td><td>CTNNB1_PHOSPHORYLATION_CASCADE</td><td>REACTOME</td><td>316</td><td>12</td><td>8</td><td>5,15E-01</td><td>8,89E-01</td><td>rs12120009|rs11119881|rs4355511|rs4958072|rs13188321|rs241280|rs4919096|rs1255771</td></tr>
<tr><td>Serositis</td><td>VASOPRESSIN_REGULATED_WATER_REABSORPTION</td><td>KEGG</td><td>1100</td><td>40</td><td>20</td><td>5,18E-01</td><td>8,89E-01</td><td>rs757980|rs6972081|rs491148|rs2042898|rs487455|rs11712712|rs1637457|rs4810147|rs310361|rs1550116|rs177486|rs4073237|rs162003|rs12666636|rs918842|rs11828980|rs4800269|rs160337|rs16874653|rs41351</td></tr>
<tr><td>Serositis</td><td>INTERFERON_GAMMA_SIGNALING</td><td>REACTOME</td><td>2043</td><td>50</td><td>20</td><td>5,19E-01</td><td>8,89E-01</td><td>rs2352930|rs10169182|rs13395505|rs17533090|rs276978|rs2272874|rs7699538|rs12149292|rs2236906|rs7770216|rs4789576|rs1799969|rs12794326|rs2894381|rs5009448|rs4527079|rs9270856|rs4958456|rs6907167|rs11751659</td></tr>
<tr><td>Serositis</td><td>ANTIVIRAL_MECHANISM_BY_IFN_STIMULATED_GENES</td><td>REACTOME</td><td>1077</td><td>33</td><td>20</td><td>5,20E-01</td><td>8,89E-01</td><td>rs13395505|rs398800|rs2074560|rs9845458|rs8132|rs886451|rs7973701|rs17266628|rs3733446|rs9856128|rs1128864|rs12816436|rs2917779|rs1406334|rs830617|rs9913503|rs7196117|rs1347591|rs596283|rs10183020</td></tr>
<tr><td>Serositis</td><td>RIBOSOME</td><td>KEGG</td><td>1118</td><td>33</td><td>20</td><td>5,20E-01</td><td>8,89E-01</td><td>rs2252523|rs12885228|rs16935649|rs7812590|rs2302178|rs12121852|rs7126805|rs8017939|rs2253181|rs1953900|rs3732680|rs2687975|rs12147827|rs6743777|rs4672803|rs2917779|rs4599669|rs953978|rs137602|rs4237000</td></tr>
<tr><td>Serositis</td><td>APOPTOSIS</td><td>REACTOME</td><td>3298</td><td>29</td><td>20</td><td>5,23E-01</td><td>8,89E-01</td><td>rs919635|rs7863144|rs16910559|rs2757594|rs7273626|rs7016497|rs1016835|rs328349|rs11663972|rs2228576|rs2219926|rs7948797|rs16848196|rs4286195|rs9964756|rs1539953|rs181402|rs9461416|rs17809603|rs2928953</td></tr>
<tr><td>Serositis</td><td>RENAL_CELL_CARCINOMA</td><td>KEGG</td><td>2164</td><td>43</td><td>20</td><td>5,24E-01</td><td>8,89E-01</td><td>rs1864053|rs2764938|rs2302456|rs17530657|rs6056665|rs9647337|rs11207332|rs10878841|rs511973|rs7548973|rs1366315|rs6871452|rs2221007|rs178255|rs7948482|rs751210|rs4240910|rs4690363|rs17822304|rs7167062</td></tr>
<tr><td>Serositis</td><td>TYPE_II_DIABETES_MELLITUS</td><td>KEGG</td><td>2053</td><td>45</td><td>20</td><td>5,26E-01</td><td>8,89E-01</td><td>rs1864053|rs2283253|rs17801265|rs4352468|rs7977039|rs4926163|rs2711292|rs9647337|rs511973|rs1366315|rs6871452|rs2074308|rs10498208|rs2017750|rs1273793|rs4789576|rs4240910|rs7993089|rs4976661|rs10933137</td></tr>
<tr><td>Serositis</td><td>SMALL_CELL_LUNG_CANCER</td><td>KEGG</td><td>3747</td><td>35</td><td>20</td><td>5,26E-01</td><td>8,89E-01</td><td>rs1864053|rs2594149|rs2682971|rs627527|rs12739596|rs7634073|rs12373564|rs7016497|rs6774513|rs1057504|rs9647337|rs511973|rs2142296|rs1366315|rs7781436|rs6871452|rs4679516|rs12620429|rs815719|rs1868220</td></tr>
<tr><td>Serositis</td><td>PKA_MEDIATED_PHOSPHORYLATION_OF_CREB</td><td>REACTOME</td><td>767</td><td>36</td><td>20</td><td>5,28E-01</td><td>8,89E-01</td><td>rs2960288|rs1909745|rs4234214|rs9856983|rs4569841|rs7733194|rs891429|rs1550116|rs13153439|rs918842|rs2302234|rs2471267|rs9870651|rs4618675|rs16904362|rs3861699|rs6766238|rs8049367|rs1862884|rs868362</td></tr>
<tr><td>Serositis</td><td>KINESINS</td><td>REACTOME</td><td>566</td><td>25</td><td>12</td><td>5,30E-01</td><td>8,89E-01</td><td>rs2275593|rs10066223|rs7563440|rs17648423|rs16828222|rs8068256|rs12519348|rs4923515|rs3902665|rs101271|rs2432925|rs11172254</td></tr>
<tr><td>Serositis</td><td>PLATELET_SENSITIZATION_BY_LDL</td><td>REACTOME</td><td>676</td><td>42</td><td>20</td><td>5,30E-01</td><td>8,89E-01</td><td>rs967776|rs12564813|rs591393|rs872315|rs12120009|rs11119881|rs1877059|rs3010644|rs12738546|rs10798090|rs2879094|rs2430084|rs7585006|rs3123045|rs2802475|rs12405943|rs7535176|rs1255771|rs12137534|rs12033946</td></tr>
<tr><td>Serositis</td><td>NEF_MEDIATES_DOWN_MODULATION_OF_CELL_SURFACE_RECEPTORS_BY_RECRUITING N_ADAPTERS</td><td>REACTOME</td><td>366</td><td>26</td><td>12</td><td>5,31E-01</td><td>8,89E-01</td><td>rs3745255|rs801733|rs226434|rs747020|rs5009448|rs5763140|rs11783925|rs9643478|rs6421967|rs892666|rs4673260|rs11757750</td></tr>
<tr><td>Serositis</td><td>OXIDATIVE_PHOSPHORYLATION</td><td>KEGG</td><td>2486</td><td>33</td><td>20</td><td>5,35E-01</td><td>8,89E-01</td><td>rs6437332|rs1345638|rs1264901|rs2252523|rs10045456|rs6433541|rs1124734|rs1500147|rs2302178|rs12538107|rs1562853|rs2712353|rs2668424|rs6929796|rs4810150|rs1473092|rs13405880|rs8075653|rs4804840|rs16962149</td></tr>
<tr><td>Serositis</td><td>LINOLEIC_ACID_METABOLISM</td><td>KEGG</td><td>716</td><td>45</td><td>20</td><td>5,36E-01</td><td>8,89E-01</td><td>rs967776|rs12045786|rs591393|rs762551|rs11207546|rs11572255|rs740229|rs3010644|rs12738546|rs10798090|rs2070873|rs2430084|rs623784|rs3123045|rs2802475|rs10857733|rs12405943|rs7535176|rs12137534|rs9888067</td></tr>
<tr><td>Serositis</td><td>ACYL_CHAIN_REMODELLING_OF_PC</td><td>REACTOME</td><td>693</td><td>37</td><td>20</td><td>5,38E-01</td><td>8,89E-01</td><td>rs967776|rs12564813|rs17134769|rs591393|rs6865199|rs1679008|rs740229|rs3010644|rs12738546|rs1924736|rs2070873|rs2430080|rs623784|rs27056|rs3123045|rs2802475|rs11231528|rs2277007|rs12405943|rs7535176</td></tr>
</table>

Serositis	MHC_CLASS_II_ANTIGEN_PRESENTATION	REACTOME	2226	46	20	5,38E-01	8,89E-01	rs1296028|rs35399661|rs3745255|rs6536158|rs4325000|rs6785206|rs2275593|rs10066223|rs7563440|rs3948793|rs983694|rs17648423|rs1242102|rs226434|rs10963309|rs17677598|rs2452153|rs10791889|rs16828222|rs4917968
Serositis	EIF_PATHWAY	BIOCARTA	158	5	3	5,38E-01	8,89E-01	rs8020483|rs4783446|rs2165945
Serositis	ADHERENS_JUNCTIONS_INTERACTIONS	REACTOME	5496	38	20	5,38E-01	8,89E-01	rs4701252|rs1364298|rs12924378|rs1547531|rs6089247|rs9812103|rs11127860|rs1834077|rs422384|rs17504655|rs13045227|rs9928133|rs3852732|rs4856242|rs6451476|rs1154991|rs6121828|rs11576830|rs12921461|rs4925258
Serositis	INITIAL_TRIGGERING_OF_COMPLEMENT	REACTOME	434	24	13	5,39E-01	8,89E-01	rs2204345|rs4151657|rs2031749|rs292001|rs10824804|rs630379|rs11265263|rs12412945|rs2384013|rs11003258|rs1343065|rs2279623|rs3908206
Serositis	RNA_POL_II_TRANSCRIPTION_PRE_INITIATION_AND_PROMOTER_OPENING	REACTOME	383	8	5	5,40E-01	8,89E-01	rs7126805|rs6089608|rs378759|rs6450035|rs2261360
Serositis	INTEGRATION_OF_PROVIRUS	REACTOME	122	2	2	5,40E-01	8,89E-01	rs16972096|rs1151514
Serositis	ACTIVATION_OF_THE_MRNA_UPON_BINDING_OF_THE_CAP_BINDING_COMPLEX_AND_EI	REACTOME	780	37	18	5,40E-01	8,89E-01	rs2252523|rs16935649|rs2302178|rs8017939|rs2253181|rs1953900|rs1074584|rs12032332|rs4758037|rs4300036|rs2917779|rs5756236|rs4599669|rs3900716|rs1427827|rs4335707|rs2232997|rs2165945
	T_BINDING_TO_43S							
Serositis	STRIATED_MUSCLE_CONTRACTION	REACTOME	453	19	11	5,40E-01	8,89E-01	rs3759614|rs7153499|rs7179757|rs3738281|rs825087|rs4775607|rs17605348|rs7095324|rs819643|rs2031999|rs2041126
Serositis	ONE_CARBON_POOL_BY_FOLATE	KEGG	441	18	8	5,42E-01	8,89E-01	rs1956545|rs7660423|rs946934|rs7739248|rs380691|rs8923|rs1447656|rs1042917
Serositis	PI_METABOLISM	REACTOME	1808	41	20	5,42E-01	8,89E-01	rs1864053|rs4486998|rs656629|rs9647337|rs511973|rs1366315|rs6871452|rs2559523|rs751873|rs7240984|rs10853477|rs17587917|rs4473902|rs4240910|rs1865275|rs12170161|rs10505828|rs383542|rs1636808|rs764605
Serositis	GROWTH_HORMONE_RECEPTOR_SIGNALING	REACTOME	773	36	16	5,43E-01	8,89E-01	rs13395505|rs10498208|rs1273793|rs4789576|rs6451196|rs7993089|rs10933137|rs6895971|rs4527079|rs10521022|rs1417720|rs386365|rs1498706|rs7290469|rs37389|rs16922298
Serositis	PRION_DISEASES	KEGG	1585	41	20	5,44E-01	8,89E-01	rs809192|rs2827641|rs2827539|rs920909|rs742720|rs229477|rs2827255|rs10482915|rs2031749|rs292001|rs17561|rs3776519|rs926106|rs706469|rs2014519|rs12794326|rs7444800|rs8128017|rs1625694|rs1342430
Serositis	GLYCOLYSIS_GLUCONEOGENESIS	KEGG	1391	33	20	5,46E-01	8,89E-01	rs10993276|rs6070157|rs17301901|rs10732359|rs764223|rs7663410|rs2236441|rs6733848|rs4976661|rs2088102|rs17158714|rs4878199|rs17470048|rs2995965|rs4646771|rs1789891|rs11189224|rs906215|rs6015032|rs2284997
Serositis	NEGATIVE_REGULATORS_OF_RIG_I_MDA5_SIGNALING	REACTOME	464	31	14	5,48E-01	8,89E-01	rs10863430|rs2357933|rs9389541|rs6944913|rs1390762|rs3733446|rs2917779|rs821470|rs383020|rs8004192|rs10878182|rs871782|rs3851210|rs7259683
Serositis	THE_ROLE_OF_NEF_IN_HIV1_REPLICATION_AND_DISEASE_PATHOGENESIS	REACTOME	936	30	20	5,49E-01	8,89E-01	rs809192|rs3745255|rs801733|rs6964474|rs226434|rs7782153|rs747020|rs6962537|rs17170881|rs5009448|rs5763140|rs1345365|rs724489|rs11954030|rs11783925|rs9643478|rs6421967|rs10516068|rs892666|rs6890504
Serositis	G_ALPHA1213_SIGNALLING_EVENTS	REACTOME	2855	30	20	5,49E-01	8,89E-01	rs1864053|rs192718|rs2663342|rs9968730|rs2248656|rs13257637|rs4704706|rs12553893|rs2282537|rs1366315|rs6871452|rs172470|rs7611544|rs11879985|rs7266945|rs2291462|rs4681946|rs10465781|rs10821056|rs13054809
Serositis	PORPHYRIN_AND_CHLOROPHYLL_METABOLISM	KEGG	1163	62	16	5,49E-01	8,89E-01	rs4632823|rs3019626|rs133415|rs2013573|rs8075653|rs6738678|rs10168155|rs7734808|rs6502321|rs4148285|rs12650761|rs7438808|rs1560550|rs7913190|rs4148275|rs6832167
Serositis	TRANSLATION	REACTOME	1803	34	20	5,49E-01	8,89E-01	rs4131086|rs2252523|rs4940849|rs16935649|rs2302178|rs8020483|rs4270628|rs7817301|rs12121852|rs7126805|rs7874483|rs8017939|rs11133152|rs6553943|rs2253181|rs1953900|rs876144|rs3732680|200260|rs1074584
Serositis	XENOBIOTICS	REACTOME	231	12	5	5,52E-01	8,89E-01	rs762551|200199|rs12978001|rs10857733|rs9888067
Serositis	ABC_FAMILY_PROTEINS_MEDIATED_TRANSPORT	REACTOME	1753	52	20	5,52E-01	8,89E-01	rs2283253|rs2239322|rs2954804|rs12452482|rs2074308|rs10498030|rs10841915|rs12871476|rs4147844|rs2236705|rs4794173|rs1889547|rs4147823|rs4073237|rs4721946|rs1191234|rs2274412|rs11077922|rs950283|rs161061
Serositis	G_BETA_GAMMA_SIGNALLING_THROUGH_PLC_BETA	REACTOME	624	25	15	5,52E-01	8,89E-01	rs6039107|rs12553893|rs13037679|rs1474937|rs6039104|rs1253676|rs11070884|rs708920|rs7863832|rs6086490|rs7618348|rs2423361|rs6055922|rs11698656|rs978266
Serositis	G_ALPHA_Q_SIGNALLING_EVENTS	REACTOME	7428	32	20	5,54E-01	8,89E-01	rs2306677|rs1864053|rs192718|rs10516614|rs9562385|rs2405871|rs3760703|rs691712|rs6039107|rs3783754|rs13257637|rs3771863|rs2352039|rs4907013|rs13115641|rs17742581|rs182034|rs10241087|rs12553893|rs7517778
Serositis	BETA_ALANINE_METABOLISM	KEGG	711	28	14	5,55E-01	8,89E-01	rs3750187|rs10732359|rs1330581|rs9937726|rs12675680|rs4878199|rs1760217|rs4646771|rs2802530|rs1923431|rs7216|rs8190645|rs701492|rs3774051
Serositis	ADIPOCYTOKINE_SIGNALING_PATHWAY	KEGG	2106	33	20	5,58E-01	8,89E-01	rs2107310|rs6070157|rs12739596|rs4352468|rs2228576|rs1881638|rs2946394|rs2942067|rs7659090|rs10498208|rs2970873|rs2017750|rs1273793|rs2026628|rs751210|rs4789576|rs7993089|rs17822304|rs10410544|rs283695
Serositis	UNFOLDED_PROTEIN_RESPONSE	REACTOME	1176	29	20	5,58E-01	8,89E-01	rs764223|rs2722195|rs1485591|rs1734397|rs16856756|rs3785028|rs11879090|rs12739624|rs7571971|rs2730324|rs7627551|rs13073226|rs9502593|rs1126700|rs242376|rs2444229|rs1136882|rs2764085|rs7202704|rs10417957
Serositis	MISMATCH_REPAIR	KEGG	576	29	15	5,59E-01	8,89E-01	rs249633|rs10276762|rs4943153|rs6460075|rs886727|rs12112229|rs6703812|rs380691|rs1928023|rs741048|rs2321822|rs17137468|rs12584168|rs1638566|rs1776148
Serositis	SYNTHESIS_OF_PIPS_AT_THE_PLASMA_MEMBRANE	REACTOME	1050	43	20	5,59E-01	8,89E-01	rs1864053|rs9647337|rs511973|rs1366315|rs6871452|rs2559523|rs751873|rs4240910|rs10505828|rs383542|rs1636808|rs764605|rs34299|rs16897109|rs16897180|rs10444068|rs1500847|rs11736202|rs350289|rs7084115
Serositis	ENDOMETRIAL_CANCER	KEGG	3028	51	20	5,60E-01	8,89E-01	rs1864053|rs2221145|rs1549777|rs11686261|rs2292653|rs7567671|rs966308|rs9647337|rs3755105|rs2441727|rs511973|rs2877260|rs1366315|rs6871452|rs11995971|rs10232489|rs10022956|rs4355511|rs7570013|rs10489028
Serositis	SELENOAMINO_ACID_METABOLISM	KEGG	742	35	18	5,63E-01	8,89E-01	rs294594|rs13178865|rs2651236|rs12153515|rs11135312|rs4371935|rs1422544|rs1366191|rs11958300|rs10023658|rs1363075|rs7723455|rs12658783|rs1834|rs10515861|rs8045616|rs1284605|rs11209658
Serositis	NONSENSE_MEDIATED_DECAY_ENHANCED_BY_THE_EXON_JUNCTION_COMPLEX	REACTOME	1261	33	20	5,64E-01	8,89E-01	rs11784209|rs2252523|rs8132|rs16935649|rs2302178|rs12121852|rs7126805|rs17834271|rs2842880|rs8017939|rs2253181|rs1953900|rs3732680|rs2687975|rs6743777|rs4672803|rs2917779|rs4599669|rs2808667|rs137602
Serositis	SIGNALLING_BY_NGF	REACTOME	7869	38	20	5,66E-01	8,89E-01	rs1352388|rs17014035|rs1864053|rs2663342|rs8464|rs2960288|rs2302456|rs7273626|rs9968730|rs6913161|rs2248656|rs1909745|rs4965533|rs6018627|rs4704706|rs1387924|rs9366027|rs2043722|rs4325000|rs2160591
Serositis	GCR_PATHWAY	BIOCARTA	736	31	20	5,70E-01	8,89E-01	rs1864053|rs888956|rs1366315|rs6871452|rs4810147|rs9808216|rs383542|rs1932201|rs1636808|rs6733907|rs34299|rs16897109|rs16897180|rs877741|rs4648090|rs230547|rs13430277|rs1294506|rs4976028|rs7706821
Serositis	NFAT_PATHWAY	BIOCARTA	1626	29	20	5,72E-01	8,89E-01	rs1864053|rs4699236|rs6812773|rs918418|rs7702020|rs1366315|rs6871452|rs2478545|rs660749|rs6067805|rs2017750|rs748938|rs4234512|rs8124053|rs1457115|rs9808216|rs2071943|rs383542|rs4699228|rs2645370
Serositis	NCAM1_INTERACTIONS	REACTOME	1896	35	20	5,72E-01	8,89E-01	rs4739207|rs627527|rs633762|rs1563916|rs1034620|rs12476069|rs2286732|rs10487254|rs1858822|rs2975100|rs518347|rs1586494|rs9521664|rs2839102|rs10920106|rs10753890|rs926106|rs12794326|rs10503703|rs11704241
Serositis	MCM_PATHWAY	BIOCARTA	234	10	7	5,73E-01	8,89E-01	rs133415|rs3759216|rs918587|rs13082666|rs4265124|rs2307373|rs941289
Serositis	EXTRACELLULAR_MATRIX_ORGANIZATION	REACTOME	3783	33	20	5,74E-01	8,89E-01	rs1200562|rs3784381|rs627527|rs10092877|rs4909444|rs7713018|rs633762|rs1996637|rs7300198|rs11807560|rs1034620|rs12476069|rs2608069|rs10487254|rs1858822|rs972766|rs4681238|rs1042704|rs805722|rs1577217
Serositis	CARDIAC_MUSCLE_CONTRACTION	KEGG	3169	34	20	5,75E-01	8,89E-01	rs4928016|rs3759614|rs1345638|rs17801265|rs2286374|rs9309065|rs10045456|rs1500147|rs7153499|rs7977039|rs10437823|rs490057|rs7179757|rs9851393|rs7372541|rs13080236|rs2703029|rs4732433|rs1473092|rs384994
Serositis	INOSITOL_PHOSPHATE_METABOLISM	KEGG	2355	44	20	5,75E-01	8,89E-01	rs10882406|rs11146457|rs4486998|rs6039107|rs656629|rs613993|rs9647337|rs13037679|rs2559523|rs751873|rs7240984|rs10853477|rs9932716|rs1474937|rs10763492|rs6039104|rs4240910|rs2767419|rs1865275|rs10505828
Serositis	CELL_JUNCTION_ORGANIZATION	REACTOME	6781	34	20	5,76E-01	8,89E-01	rs4701252|rs1364298|rs12924378|rs1547531|rs6089247|rs6006613|rs9812103|rs11127860|rs1834077|rs17106413|rs422384|rs17504655|rs7290191|rs12495548|rs13045227|rs9928133|rs3852732|rs7528744|rs4856242|rs6451476
Serositis	MAL_PATHWAY	BIOCARTA	225	21	6	5,76E-01	8,89E-01	rs606458|rs2017750|rs4234512|rs10197534|rs7290469|rs7617041
Serositis	TRANSMISSION_ACROSS_CHEMICAL_SYNAPSES	REACTOME	9089	28	20	5,76E-01	8,89E-01	rs2355575|rs11712912|rs4928016|rs13005001|rs10491731|rs8464|rs17653240|rs2960288|rs6474421|rs2376837|rs7686241|rs6039107|rs12601653|rs11055455|rs9968730|rs1909745|rs2927585|rs11636101|rs1127314|rs4704706
Serositis	SYNTHESIS_AND_INTERCONVERSION_OF_NUCLEOTIDE_DI_AND_TRIPHOSPHATES	REACTOME	306	20	9	5,77E-01	8,89E-01	rs7512897|rs12565526|rs9324161|rs4964752|rs4910907|rs17100499|rs7865146|rs4135215|rs1698231
Serositis	SIGNALING_BY_NOTCH1	REACTOME	3563	33	20	5,77E-01	8,89E-01	rs11138465|rs10939104|rs669223|rs10765795|rs4692114|rs6913161|rs7634430|rs3898124|rs522495|rs7160413|rs11138350|rs6133987|rs17676727|rs9646967|rs1684274|rs11995971|rs11138447|rs10116365|rs12624835|rs12487368
Serositis	ARGININE_AND_PROLINE_METABOLISM	KEGG	991	45	20	5,77E-01	8,89E-01	rs2355575|rs6490121|rs867907|rs10732359|rs7817301|rs4920566|rs1860172|rs1040151|rs4878199|rs11818084|rs17619406|rs4646771|rs4704710|rs17509378|rs7607205|rs9605907|rs2296063|rs1923431|rs1921915|rs7216
Serositis	CELL_CELL_JUNCTION_ORGANIZATION	REACTOME	6211	36	20	5,79E-01	8,89E-01	rs4701252|rs1364298|rs12924378|rs1547531|rs6089247|rs9812103|rs11127860|rs1834077|rs422384|rs17504655|rs7290191|rs12495548|rs13045227|rs9928133|rs3852732|rs7528744|rs4856242|rs6451476|rs1154991|rs6121828
Serositis	DNA_REPLICATION	REACTOME	740	31	16	5,83E-01	8,91E-01	rs4540267|rs133415|rs10276762|rs4943153|rs6460075|rs886727|rs1928023|rs741048|rs2321822|rs4072375|rs13082666|rs17137468|rs12584168|rs1638566|rs941289|rs9568494
Serositis	NKCELLS_PATHWAY	BIOCARTA	678	32	16	5,83E-01	8,91E-01	rs1864053|rs10820825|rs1366315|rs6871452|rs5009448|rs383542|rs34299|rs16897109|rs16897180|rs892666|rs4976028|rs331679|rs7706821|rs12662611|rs6488297|rs7379303
Serositis	TIGHT_JUNCTION_INTERACTIONS	REACTOME	715	26	18	5,83E-01	8,91E-01	rs7290191|rs12495548|rs7528744|rs1425104|rs7993213|rs16986270|rs10827338|rs11721025|rs2749609|rs6809685|rs651084|rs8134233|rs3954262|rs2106136|rs12095754|rs2812672|rs7141756|rs10508791
Serositis	DEADENYLATION_DEPENDENT_MRNA_DECAY	REACTOME	622	28	16	5,84E-01	8,91E-01	rs764223|rs8132|rs10479576|rs11879090|rs4641872|rs6550146|rs17630822|rs11189224|rs7777931|rs1388794|rs11178190|rs1695739|rs7135334|rs6498509|rs10419232|rs2165945
Serositis	GLUCAGON_SIGNALING_IN_METABOLIC_REGULATION	REACTOME	1044	39	20	5,86E-01	8,91E-01	rs2960288|rs1909745|rs12553893|rs4234214|rs9856983|rs4569841|rs7733194|rs4810147|rs891429|rs1550116|rs1253676|rs13153439|rs7863832|rs2302234|rs2471267|rs9870651|rs4618675|rs16904362|rs7618348|rs3861699
Serositis	UBIQUITIN_MEDIATED_PROTEOLYSIS	KEGG	3227	36	20	5,86E-01	8,91E-01	rs1620958|rs2906041|rs10494494|rs10753110|rs543347|rs2942067|rs7681082|rs10890795|rs12577790|rs6518204|rs1503920|rs10490114|rs2288772|rs1661300|rs10945770|rs17276701|rs7973701|rs7496283|rs8053839|rs848279
Serositis	VEGF_PATHWAY	BIOCARTA	883	36	20	5,88E-01	8,91E-01	rs1864053|rs8464|rs7016497|rs1366315|rs6871452|rs12938937|rs383542|rs9913908|rs1636808|rs34299|rs16897109|rs16897180|rs4381631|rs2894536|rs733646|rs4976028|rs12452826|rs11654719|rs7706821|rs767975
Serositis	AKT_PATHWAY	BIOCARTA	682	33	20	5,89E-01	8,91E-01	rs1864053|rs1366315|rs6871452|rs2020902|rs10872893|rs6895971|rs1782808|rs383542|rs7322396|rs1636808|rs34299|rs16897109|rs16897180|rs859722|rs2324600|rs4648090|rs230547|rs10883452|rs4976028|rs7706821
Serositis	SIGNAL_AMPLIFICATION	REACTOME	1023	40	20	5,90E-01	8,91E-01	rs967776|rs6018627|rs1487489|rs12553893|rs591393|rs308033|rs1253676|rs11070884|rs3010644|rs12738546|rs10798090|rs7863832|rs11999145|rs2430084|rs7618348|rs3123045|rs2802475|rs6012200|rs12405943|rs7535176
Serositis	CCR5_PATHWAY	BIOCARTA	696	43	20	5,92E-01	8,92E-01	rs2764938|rs8464|rs17530657|rs11207332|rs2017750|rs12938937|rs9808216|rs9913908|rs6733907|rs11999145|rs4381631|rs6430628|rs13430277|rs1294506|rs733646|rs7211269|rs11654719|rs169143|rs8004059|rs9927848
Serositis	DIABETES_PATHWAYS	REACTOME	3395	36	20	5,93E-01	8,92E-01	rs6435944|rs1101156|rs852069|rs972736|rs764223|rs687992|rs7469847|rs4940849|rs2722195|rs12153013|rs8073244|rs1485591|rs10978931|rs1734397|rs16856756|rs3785028|rs12353147|rs11879090|rs12739624|rs10149366
Serositis	SPLICEOSOME	KEGG	1685	51	20	5,97E-01	8,95E-01	rs909770|rs6067104|rs8132|rs192705|rs12591523|rs8104242|rs9730519|rs34934568|rs2071596|rs4641872|rs7171890|rs12410049|rs10494363|rs12462556|rs6056|rs13111756|rs1978708|rs11740701|rs17281677|rs3171980
Serositis	PEPTIDE_LIGAND_BINDING_RECEPTORS	REACTOME	5053	30	20	5,97E-01	8,95E-01	rs8095771|rs2107310|rs500817|rs472418|rs12648009|rs691712|rs3136537|rs3771863|rs13115641|rs12956032|rs17742581|rs11696134|rs12994381|rs12704920|rs1539984|rs4790701|rs4057791|rs6089930|rs692463|rs2243057
Serositis	POST_TRANSLATIONAL_MODIFICATION_SYNTHESIS_OF_GPI_ANCHORED_PROTEINS	REACTOME	586	42	20	5,99E-01	8,97E-01	rs3739821|rs935875|rs6415846|rs1028307|rs1912976|rs1850348|rs2290344|rs281282|rs2486253|rs11702020|rs8031753|rs10519129|rs682567|rs344781|rs1520460|rs1912643|rs2741584|rs9425618|rs12909534|rs199669
Serositis	CARDIACEGF_PATHWAY	BIOCARTA	1289	44	20	6,01E-01	8,98E-01	rs2764938|rs8464|rs17530657|rs17742581|rs11207332|rs2877260|rs2478545|rs11995971|rs10232489|rs12938937|rs2071943|rs9913908|rs3915004|rs7084288|rs2110290|rs2252586|rs4381631|rs4648090|rs230547|rs12494599
Serositis	RECYCLING_PATHWAY_OF_L1	REACTOME	874	28	15	6,03E-01	8,98E-01	rs6018627|rs9366027|rs4325000|rs10066223|rs226434|rs10963309|rs12519348|rs6421967|rs6012200|rs7290469|rs492960|rs3799647|rs9347252|rs101271|rs2432925
Serositis	MRNA_SPLICING_MINOR_PATHWAY	REACTOME	383	15	8	6,03E-01	8,98E-01	rs7126805|rs34934568|rs12410049|rs10494363|rs2808667|rs2810640|rs9904900|rs4809388
Serositis	RIP_MEDIATED_NFKB_ACTIVATION_VIA_DAI	REACTOME	328	30	18	6,06E-01	9,00E-01	rs17413414|rs468774|rs4810086|rs2096509|rs2269423|rs10410544|rs10872893|rs12645085|rs9978658|rs2234983|rs11721827|rs4519192|rs216779|rs723295|rs10883452|rs10483285|rs4145277|rs380417
Serositis	NEUROTRANSMITTER_RECEPTOR_BINDING_AND_DOWNSTREAM_TRANSMISSION_IN_TH	REACTOME	6592	25	20	6,09E-01	9,00E-01	rs13005001|rs8464|rs17653240|rs2960288|rs6474421|rs1012549|rs7686241|rs6039107|rs12601653|rs11055455|rs9968730|rs1909745|rs2927585|rs11636101|rs1127314|rs4704706|rs1434384|rs11722663|rs11135214|rs9366027
	L							
Serositis	SIGNAL_TRANSDUCTION_BY_L1	REACTOME	1310	35	20	6,09E-01	9,00E-01	rs9366027|rs2056131|rs4325000|rs2506145|rs2292700|rs2877260|rs12620429|rs10232489|rs9633635|rs226434|rs10963309|rs881301|rs12794326|rs2555236|rs2513324|rs2298703|rs2110290|rs6421967|rs2252586|rs12271908
Serositis	ALPHA_LINOLENIC_ACID_METABOLISM	KEGG	571	42	20	6,10E-01	9,00E-01	rs967776|rs1487489|rs591393|rs2631753|rs2851682|rs740229|rs3010644|rs12738546|rs1924736|rs6849528|rs2070873|rs2430080|rs623784|rs3123045|rs2802475|rs12405943|rs7535176|rs12137534|rs12232304|rs12033946
Serositis	RIG_I_LIKE_RECEPTOR_SIGNALING_PATHWAY	KEGG	1361	31	20	6,11E-01	9,00E-01	rs17747965|rs10863430|rs4352468|rs1221220|rs2357933|rs10498970|rs17454570|rs1390762|rs2017750|rs1075498|rs10410544|rs10872893|rs1010650|rs555745|rs2141484|rs157695|rs9362733|rs711272|rs10485405|rs2818291
Serositis	NUCLEOTIDE_EXCISION_REPAIR	REACTOME	956	32	17	6,12E-01	9,00E-01	rs7188980|rs10276762|rs4943153|rs7126805|rs6460075|rs1794242|rs886727|rs12221497|rs1714473|rs1928023|rs741048|rs2321822|rs2808667|rs1001440|rs17137468|rs12584168|rs3781941
Serositis	SYNTHESIS_SECRETION_AND_INACTIVATION_OF_GLP1	REACTOME	363	26	13	6,12E-01	9,00E-01	rs4940849|rs11133152|rs6553943|rs10423648|rs1380100|rs4940851|rs12459138|rs1598100|rs2271731|rs8100944|rs12995983|rs11660643|rs13144122
Serositis	SIGNALING_BY_RHO_GTPASES	REACTOME	4877	24	20	6,15E-01	9,01E-01	rs3898094|rs11101342|rs10261846|rs817387|rs2663342|rs11161548|rs9968730|rs2248656|rs11893484|rs4704706|rs767573|rs1930438|rs10516826|rs10872344|rs1320213|rs2282537|rs7611544|rs11879985|rs6750487|rs10898864
Serositis	ACYL_CHAIN_REMODELLING_OF_PG	REACTOME	571	36	20	6,16E-01	9,01E-01	rs967776|rs12564813|rs591393|rs6865199|rs1679008|rs740229|rs3010644|rs12738546|rs10798090|rs2070873|rs2430084|rs623784|rs27056|rs1773353|rs3123045|rs2802475|rs2277007|rs12405943|rs7535176|rs12137534
Serositis	FOCAL_ADHESION	KEGG	7733	32	20	6,19E-01	9,01E-01	rs11245205|rs2535764|rs1864053|rs2764938|rs8464|rs809192|rs627527|rs2302456|rs6006613|rs12373564|rs17106413|rs7016497|rs9845458|rs4352468|rs6018627|rs1034620|rs11017615|rs2056131|rs11730652|rs17530657
Serositis	TRANSPORT_OF_GLUCOSE_AND_OTHER_SUGARS_BILE_SALTS_AND_ORGANIC_ACIDS_M	REACTOME	3234	41	20	6,21E-01	9,01E-01	rs11712912|rs2154505|rs10833366|rs7527059|rs11658922|rs12153013|rs747488|rs568567|rs3774128|rs17481006|rs7445640|rs13258461|rs10431557|rs11659324|rs4598727|rs1480620|rs1377652|rs4438540|rs2638270|rs751210
	NE_COMPOUNDS							
Serositis	GLUCOSE_METABOLISM	REACTOME	1554	47	20	6,21E-01	9,01E-01	rs10993276|rs958238|rs6070157|rs17301901|rs764223|rs523200|rs2236441|rs6602514|rs7122539|rs9808216|rs8004788|rs17158714|rs2995965|rs2842754|rs11189224|rs6733907|rs2229519|rs6015032|rs2284997|rs2267846
Serositis	DRUG_METABOLISM_OTHER_ENZYMES	KEGG	1609	61	20	6,22E-01	9,01E-01	rs3750187|rs4540635|rs7517175|rs7221585|rs10799655|rs2013573|rs6738678|rs8362|rs2854702|rs7608175|rs28171|rs3793358|rs12978001|rs13398821|rs1760217|rs4148285|rs2802530|rs12650761|rs11902596|rs714131
Serositis	ALANINE_ASPARTATE_AND_GLUTAMATE_METABOLISM	KEGG	841	31	14	6,25E-01	9,01E-01	rs2355575|rs1330581|rs9937726|rs4920566|rs11133439|rs1860172|rs10521022|rs7607205|rs2296063|rs1921915|rs8190645|rs701492|rs1275522|rs4930211
Serositis	EGFR_DOWNREGULATION	REACTOME	1037	34	17	6,29E-01	9,01E-01	rs6659310|rs4325000|rs2877260|rs12982931|rs8104242|rs10232489|rs226434|rs10963309|rs2917779|rs2110290|rs2184818|rs6421967|rs2252586|rs492960|rs1105073|rs7336234|rs12870493
Serositis	FC_GAMMA_R_MEDIATED_PHAGOCYTOSIS	KEGG	3795	42	20	6,29E-01	9,01E-01	rs1864053|rs12133338|rs967776|rs8464|rs2302456|rs16910559|rs1487489|rs10914127|rs2711292|rs9647337|rs10820825|rs591393|rs511973|rs1930762|rs1366315|rs6871452|rs178255|rs895344|rs9932716|rs2757500
Serositis	AMINO_ACID_AND_OLIGOPEPTIDE_SLC_TRANSPORTERS	REACTOME	1707	32	20	6,30E-01	9,01E-01	rs10491731|rs10211524|rs7874355|rs747488|rs11669215|rs4869682|rs16948748|rs7445640|rs7329421|rs3776578|rs9551705|rs6071173|rs13159461|rs16987501|rs2291350|rs3782370|rs11751616|rs4760587|rs3497|rs2209702
Serositis	ERK_PATHWAY	BIOCARTA	1025	41	20	6,31E-01	9,01E-01	rs6018627|rs10506618|rs2877260|rs11995971|rs11247367|rs10232489|rs6671527|rs4810147|rs2254527|rs2684799|rs11854132|rs7295237|rs4234512|rs1060478|rs2110290|rs1492769|rs2252586|rs2307049|rs6012200|rs7290469
Serositis	CSK_PATHWAY	BIOCARTA	554	34	18	6,32E-01	9,01E-01	rs2960288|rs28530648|rs4886406|rs4810147|rs7536180|rs747020|rs3135363|rs3135344|rs1296720|rs2238419|rs2302234|rs2471267|rs12576947|rs3861699|rs6766238|rs868362|rs10494786|rs6686571
Serositis	CYTOSOLIC_DNA_SENSING_PATHWAY	KEGG	620	39	20	6,33E-01	9,01E-01	rs10863430|rs1127314|rs14105|rs17413414|rs4810086|rs10410544|rs10872893|rs4979935|rs2288270|rs9311428|rs723295|rs4648090|rs230547|rs4246521|rs9554080|rs10878182|rs871782|rs10883452|rs10483285|rs1476482
Serositis	RESPONSE_TO_ELEVATED_PLATELET_CYTOSOLIC_CA2_	REACTOME	2301	50	20	6,34E-01	9,01E-01	rs8464|rs2213865|rs2205895|rs12648395|rs17661732|rs17106413|rs2056131|rs2292700|rs12810426|rs468774|rs352018|rs12871476|rs1475034|rs4220|rs4690363|rs7646517|rs12938937|rs17719676|rs9808216|rs13202850
Serositis	CELLCYCLE_PATHWAY	BIOCARTA	459	27	17	6,34E-01	9,01E-01	rs3745255|rs2282411|rs7781436|rs3759216|rs2188513|rs9315422|rs4130023|rs918587|rs7736312|rs2298765|rs4150724|rs2237573|rs12228898|rs6921368|rs10241695|rs445|rs11605729
Serositis	PRE_NOTCH_PROCESSING_IN_GOLGI	REACTOME	691	30	15	6,35E-01	9,01E-01	rs2150299|rs1109771|rs9268005|rs2269423|rs4659248|rs17116491|rs10132156|rs4879666|rs1269347|rs2168616|rs1888363|rs377743|rs4316682|rs4904040|rs17117189
Serositis	INTEGRATION_OF_ENERGY_METABOLISM	REACTOME	4501	36	20	6,38E-01	9,01E-01	rs2306677|rs8464|rs2960288|rs2283253|rs6039107|rs1909745|rs11168228|rs4940849|rs1881638|rs12553893|rs4926163|rs10878841|rs13037679|rs4234214|rs9856983|rs4569841|rs308033|rs7548973|rs7733194|rs210120
Serositis	NATURAL_KILLER_CELL_MEDIATED_CYTOTOXICITY	KEGG	3607	45	20	6,39E-01	9,01E-01	rs1864053|rs4699236|rs1264524|rs8464|rs809192|rs6812773|rs9647337|rs10820825|rs511973|rs1366315|rs6871452|rs7770216|rs4077341|rs181402|rs660749|rs17809603|rs6067805|rs9932716|rs10465781|rs11137525
Serositis	RNA_POLYMERASE	KEGG	351	10	7	6,40E-01	9,01E-01	rs7126805|rs14105|rs10824587|rs2288270|rs4246521|rs9554080|rs704986
Serositis	TIE2_SIGNALING	REACTOME	790	41	20	6,41E-01	9,01E-01	rs1864053|rs2208637|rs9647337|rs1366315|rs6871452|rs907092|rs383542|rs17353146|rs34299|rs16897109|rs16897180|rs11989879|rs2922861|rs899424|rs2920689|rs4976028|rs7706821|rs1293194|rs7379303|rs693293

Serositis	GLYCOGEN_BREAKDOWN_GLYCOGENOLYSIS	REACTOME	246	19	12	6,41E-01	9,01E-01	rs17301901|rs523200|rs9808216|rs8004788|rs2995965|rs6733907|rs2284997|rs13430277|rs8119343|rs1294506|rs2269260|rs13238899
Serositis	HEPARAN_SULFATE_HEPARIN_HS_GAG_METABOLISM	REACTOME	3879	39	20	6,42E-01	9,01E-01	rs1458526|rs9995245|rs2228331|rs1932306|rs3734371|rs4583089|rs17491203|rs1872444|rs928646|rs1424223|rs4074708|rs1556466|rs7130091|rs12446144|rs3924880|rs1008953|rs11562702|rs16948406|rs2149063|rs1892984
Serositis	PPARA_PATHWAY	BIOCARTA	2445	36	20	6,44E-01	9,01E-01	rs1864053|rs2764938|rs8464|rs246966|rs17530657|rs2946394|rs10009104|rs11207332|rs10057574|rs1366315|rs1158904|rs6871452|rs11995971|rs11757727|rs2970873|rs13046729|rs4895562|rs7275695|rs10872893|rs12938937
Serositis	DEADENYLATION_OF_MRNA	REACTOME	331	16	10	6,47E-01	9,01E-01	rs8132|rs10479576|rs6550146|rs17630822|rs7777931|rs1388794|rs11178190|rs7135334|rs6498509|rs2165945
Serositis	RAS_PATHWAY	BIOCARTA	557	35	20	6,48E-01	9,01E-01	rs1864053|rs1366315|rs6871452|rs2020902|rs4234512|rs383542|rs1636808|rs360464|rs2519090|rs34299|rs16897109|rs16897180|rs17093560|rs4648090|rs230547|rs7617041|rs10883452|rs4976028|rs7706821|rs7379303
Serositis	G1_PHASE	REACTOME	625	25	16	6,50E-01	9,01E-01	rs11784209|rs3745255|rs7781436|rs3759216|rs2188513|rs4130023|rs2917779|rs1042317|rs2298765|rs4150724|rs2237573|rs12228898|rs6921368|rs10241695|rs445|rs11607976
Serositis	IL2RB_PATHWAY	BIOCARTA	1296	34	20	6,52E-01	9,01E-01	rs1864053|rs10820825|rs1366315|rs6871452|rs178255|rs11995971|rs10498208|rs1273793|rs2046136|rs4789576|rs9658761|rs907092|rs7920305|rs4234512|rs10933137|rs4527079|rs383542|rs2849382|rs1636808|rs34299
Serositis	REGULATION_OF_HYPOXIA_INDUCIBLE_FACTOR_HIF_BY_OXYGEN	REACTOME	443	13	8	6,52E-01	9,01E-01	rs10404903|rs887946|rs7141645|rs734205|rs2917779|rs1296720|rs2238419|rs2894536
Serositis	METABOLISM_OF_CARBOHYDRATES	REACTOME	9072	32	20	6,54E-01	9,01E-01	rs9954485|rs7296900|rs1554324|rs10993276|rs958238|rs2736868|rs1458526|rs9995245|rs6070157|rs2228331|rs1932306|rs3734371|rs17301901|rs4583089|rs764223|rs17491203|rs4388712|rs1872444|rs928646|rs1424223
Serositis	PHOSPHORYLATION_OF_CD3_AND_TCR_ZETA_CHAINS	REACTOME	521	27	12	6,55E-01	9,01E-01	rs17533090|rs4886406|rs7536180|rs747020|rs2894381|rs9270856|rs11751659|rs12576947|rs4500045|rs642093|rs10494786|rs6686571
Serositis	TEL_PATHWAY	BIOCARTA	978	32	20	6,56E-01	9,01E-01	rs8464|rs2877260|rs7445640|rs11995971|rs11247367|rs10232489|rs2684799|rs2046136|rs11854132|rs1642763|rs12938937|rs2849382|rs9913908|rs2110290|rs2252586|rs4381631|rs7010121|rs733646|rs12452826|rs11654719
Serositis	PREFOLDIN_MEDIATED_TRANSFER_OF_SUBSTRATE_TO_CCT_TRIC	REACTOME	288	15	9	6,56E-01	9,01E-01	rs11576830|rs2249790|rs2832157|rs151352|rs11133604|rs3798211|rs1064019|rs13238899|rs6490620
Serositis	PPAR_SIGNALING_PATHWAY	KEGG	1324	43	20	6,56E-01	9,01E-01	rs6070157|rs12739596|rs6822001|rs2375592|rs7659090|rs11590871|rs2631753|rs964184|rs3003600|rs283695|rs2851682|rs4498289|rs7655458|rs12221497|rs3211828|rs10505752|rs6849528|rs4253623|rs7626560|rs1410602
Serositis	BCELLSURVIVAL_PATHWAY	BIOCARTA	500	28	16	6,57E-01	9,01E-01	rs1864053|rs1366315|rs6871452|rs7221585|rs383542|rs1636808|rs34299|rs16897109|rs16897180|rs3746186|rs4976028|rs7706821|rs10419232|rs8004059|rs7379303|rs10940270
Serositis	RNA_POL_II_PRE_TRANSCRIPTION_EVENTS	REACTOME	532	10	7	6,59E-01	9,01E-01	rs7126805|rs6089608|rs378759|rs547005|rs2808667|rs6450035|rs2261360
Serositis	FATTY_ACID_TRIACYLGLYCEROL_AND_KETONE_BODY_METABOLISM	REACTOME	5526	29	20	6,60E-01	9,01E-01	rs10890819|rs9858201|rs930105|rs2073635|rs1296028|rs17007811|rs12661042|rs17565061|rs10963994|rs17331746|rs10161338|rs7634430|rs1881638|rs9474466|rs2946394|rs2257140|rs6051511|rs2375592|rs13325694|rs1485591
Serositis	INFLAMMASOMES	REACTOME	468	30	19	6,62E-01	9,01E-01	rs4925650|rs468774|rs11871409|rs2046136|rs2058364|rs1076420|rs2234983|rs2849382|rs216779|rs17093560|rs2535584|rs4135215|rs9901001|rs224234|rs8029854|rs12048215|rs704986|rs882937|rs380417
Serositis	PYRIMIDINE_METABOLISM	REACTOME	792	36	19	6,62E-01	9,01E-01	rs3750187|rs4540635|rs7517175|rs7221585|rs10799655|rs2854702|rs28171|rs12675680|rs10521022|rs13398821|rs1760217|rs2802530|rs10017797|rs1180280|rs11902596|rs242243|rs10485305|rs10852849|rs1275522
Serositis	SYNTHESIS_OF_DNA	REACTOME	1545	24	20	6,63E-01	9,01E-01	rs4540267|rs328349|rs7290191|rs133415|rs2282411|rs7948797|rs10276762|rs4943153|rs3759216|rs6460075|rs886727|rs6115178|rs9315422|rs12201680|rs2917779|rs16827840|rs909473|rs1928023|rs11794849|rs741048
Serositis	GAP_JUNCTION_ASSEMBLY	REACTOME	330	8	5	6,63E-01	9,01E-01	rs7164838|rs11240144|rs7321901|rs811563|rs7327522
Serositis	PURINE_METABOLISM	REACTOME	581	17	11	6,63E-01	9,01E-01	rs6848187|rs2275007|rs4716059|rs7932321|rs2748525|rs1180280|rs11602469|rs7100|rs10485305|rs2299686|rs1760941
Serositis	CHROMOSOME_MAINTENANCE	REACTOME	1949	43	20	6,63E-01	9,01E-01	rs4541737|rs9295730|rs4540267|rs1022476|rs10276762|rs7445640|rs4943153|rs9461416|rs129128|rs6460075|rs2813498|rs886727|rs1550116|rs2833736|rs9461204|rs6062378|rs2748525|rs2813554|rs13220023|rs1928023
Serositis	RELA_PATHWAY	BIOCARTA	208	12	10	6,65E-01	9,01E-01	rs2228576|rs10872893|rs555745|rs1296720|rs2238419|rs2857709|rs4648090|rs230547|rs7550488|rs10883452
Serositis	COLLAGEN_FORMATION	REACTOME	3203	39	20	6,65E-01	9,01E-01	rs1200562|rs3784381|rs627527|rs4909444|rs7713018|rs633762|rs11807560|rs1034620|rs12476069|rs2608069|rs10487254|rs1858822|rs972766|rs4681238|rs805722|rs1577217|rs9521664|rs2894129|rs2839102|rs12204438
Serositis	HER2_PATHWAY	BIOCARTA	1326	39	20	6,65E-01	9,01E-01	rs1864053|rs12371626|rs2877260|rs1366315|rs6871452|rs13427642|rs1464443|rs10232489|rs10209471|rs4234512|rs1505376|rs383542|rs1038923|rs1636808|rs13021324|rs2813554|rs2110290|rs34299|rs1357139|rs16897109
Serositis	PROSTATE_CANCER	KEGG	2870	40	20	6,66E-01	9,01E-01	rs1864053|rs2221145|rs757980|rs6972081|rs2292653|rs2722195|rs9647337|rs1388011|rs511973|rs2877260|rs1909669|rs1366315|rs6871452|rs1637457|rs3759216|rs11247367|rs10232489|rs10022956|rs246396|rs2684799
Serositis	IMMUNOREGULATORY_INTERACTIONS_BETWEEN_A_LYMPHOID_AND_A_NON_LYMPHO	REACTOME	1325	41	20	6,66E-01	9,01E-01	rs2824319|rs2272874|rs1805|rs5367|rs11574599|rs7770216|rs1916040|rs649216|rs1799969|rs9479343|rs5009448|rs7761965|rs8111930|rs1790942|rs170962|rs6032678|rs2301698|rs12576947|rs892666|rs7196298
Serositis	THYROID_CANCER	KEGG	1104	28	20	6,68E-01	9,02E-01	rs2221145|rs12739596|rs2292653|rs2863242|rs11995971|rs10022956|rs10489028|rs11725638|rs1642763|rs283695|rs16837709|rs7903424|rs2506034|rs2298765|rs7626560|rs12776024|rs7290469|rs7010121|rs12350120|rs7020291
Serositis	LATENT_INFECTION_OF_HOMO_SAPIENS_WITH_MYCOBACTERIUM_TUBERCULOSIS	REACTOME	589	29	14	6,70E-01	9,03E-01	rs6490121|rs1562853|rs2712353|rs2071596|rs11783925|rs9643478|rs17509378|rs2857709|rs3754515|rs2889854|rs4147778|rs3794624|rs5995351|rs10241449
Serositis	NUCLEAR_RECEPTOR_TRANSCRIPTION_PATHWAY	REACTOME	2750	36	20	6,71E-01	9,03E-01	rs3910044|rs11807917|rs2283342|rs12739596|rs2737641|rs6787255|rs1857401|rs782931|rs8021398|rs10057574|rs10497172|rs7157192|rs2071427|rs12903220|rs2883917|rs17104397|rs2043581|rs1466122|rs9486829|rs283695
Serositis	MELANOGENESIS	KEGG	4077	25	20	6,72E-01	9,03E-01	rs2796794|rs2221145|rs8464|rs2960288|rs6039107|rs1909745|rs2292653|rs7641735|rs17742581|rs791700|rs7699538|rs3916033|rs13037679|rs4234214|rs9856983|rs4569841|rs2389098|rs7733194|rs1005656|rs10022956
Serositis	ACTIVATED_NOTCH1_TRANSMITS_SIGNAL_TO_THE_NUCLEUS	REACTOME	719	23	13	6,76E-01	9,06E-01	rs7160413|rs6133987|rs12624835|rs7159216|rs17678034|rs12427162|rs2917779|rs7302303|rs1535175|rs7584721|rs12892451|rs6104699|rs16826262
Serositis	SIGNALING_BY_ERBB4	REACTOME	3434	29	20	6,77E-01	9,06E-01	rs3924999|rs1864053|rs2976525|rs2466059|rs7273626|rs4733117|rs10503929|rs6051511|rs10492911|rs2877260|rs1366315|rs6871452|rs13427642|rs1464443|rs3759216|rs2221434|rs10232489|rs541425|rs10501995|rs2096509
Serositis	FRS2_MEDIATED_CASCADE	REACTOME	1192	28	20	6,78E-01	9,06E-01	rs2283575|rs7273626|rs249736|rs1909669|rs11177669|rs3945860|rs881301|rs1219643|rs3135772|rs9526838|rs2687975|rs4234512|rs7168316|rs7479018|rs630757|rs2038711|rs12220647|rs10492454|rs9568711|rs7323166
Serositis	N_GLYCAN_ANTENNAE_ELONGATION_IN_THE_MEDIAL_TRANS_GOLGI	REACTOME	962	24	20	6,79E-01	9,06E-01	rs901225|rs1455554|rs1586494|rs11123751|rs885036|rs12891561|rs10145560|rs9799068|rs6767800|rs2321537|rs1667296|rs2770807|rs10149555|rs17162650|rs421801|rs9290841|rs13155924|rs11897842|rs7979846|rs1502800
Serositis	MITOTIC_PROMETAPHASE	REACTOME	1814	47	20	6,81E-01	9,07E-01	rs2203751|rs4387213|rs3001080|rs6695454|rs6972752|rs17648423|rs1345207|rs17448345|rs12120009|rs11119881|rs7221585|rs1550116|rs17187301|rs10446729|rs8068256|rs1356792|rs12053340|rs17809510|rs17810229|rs13220023
Serositis	STEROID_BIOSYNTHESIS	KEGG	247	21	11	6,82E-01	9,07E-01	rs1296028|rs16979001|rs1063243|rs4840583|rs630288|rs10753193|rs4840584|rs10913728|rs17117126|rs10098874|rs2254636
Serositis	LAIR_PATHWAY	BIOCARTA	479	40	20	6,83E-01	9,07E-01	rs2205895|rs920909|rs17561|rs1799969|rs7444800|rs7646517|rs822373|rs3917709|rs11918289|rs8111930|rs170962|rs2857709|rs7196298|rs2062632|rs4849122|rs3176874|rs1476482|rs3917744|rs3087439|rs2279623
Serositis	MRNA_SPLICING	REACTOME	1057	33	20	6,84E-01	9,07E-01	rs192705|rs6822283|rs9664270|rs7126805|rs8104242|rs9730519|rs34934568|rs2101680|rs17005458|rs12410049|rs10494363|rs12462556|rs2035912|rs11907513|rs4813245|rs9660907|rs2808667|rs12460890|rs1529086|rs4965818
Serositis	G_PROTEIN_BETA_GAMMA_SIGNALLING	REACTOME	798	33	19	6,89E-01	9,08E-01	rs6039107|rs12553893|rs13037679|rs511973|rs1474937|rs6039104|rs1253676|rs11070884|rs1636808|rs764605|rs708920|rs7863832|rs6086490|rs7618348|rs2423361|rs6055922|rs10491087|rs11698656|rs978266
Serositis	PEPTIDE_CHAIN_ELONGATION	REACTOME	947	31	20	6,89E-01	9,08E-01	rs2252523|rs16935649|rs2302178|rs12121852|rs7126805|rs8017939|rs2253181|rs1953900|rs3732680|rs2687975|rs6743777|rs4672803|rs2917779|rs4599669|rs137602|rs4237000|rs11756089|rs3900716|rs1427827|rs9907408
Serositis	OTHER_GLYCAN_DEGRADATION	KEGG	648	28	18	6,90E-01	9,08E-01	rs2702385|rs6811556|rs4074708|rs2584394|rs12643657|rs7657084|rs2702450|rs2049805|rs10520399|rs161061|rs230547|rs4861798|rs1454212|rs17746001|rs11683765|rs6833962|rs6550191|rs4234868
Serositis	REGULATION_OF_INSULIN_SECRETION	REACTOME	3741	38	20	6,90E-01	9,08E-01	rs2306677|rs8464|rs2283253|rs6039107|rs11168228|rs4940849|rs12553893|rs4926163|rs10878841|rs13037679|rs4234214|rs9856983|rs308033|rs7548973|rs210120|rs2074308|rs4810147|rs12186641|rs4963998|rs1474937
Serositis	TRANSPORT_OF_VITAMINS_NUCLEOSIDES_AND_RELATED_MOLECULES	REACTOME	1154	35	20	6,93E-01	9,11E-01	rs4980345|rs7428024|rs13093879|rs2375592|rs3811976|rs11140582|rs6062771|rs10743413|rs3003600|rs12578492|rs727176|rs1246274|rs4756026|rs4331673|rs8025438|rs5484|rs2387400|rs10042048|rs703256|rs1275522
Serositis	VIBRIO_CHOLERAE_INFECTION	KEGG	1518	35	20	6,94E-01	9,11E-01	rs8464|rs7863144|rs2283212|rs7945327|rs1562853|rs2712353|rs7874483|rs6929796|rs4810147|rs9932716|rs1079714|rs1550116|rs12938937|rs940243|rs9913908|rs939980|rs7187863|rs11783925|rs9643478|rs9928191
Serositis	CYSTEINE_AND_METHIONINE_METABOLISM	KEGG	982	43	20	6,96E-01	9,12E-01	rs294594|rs13178865|rs2780749|rs946934|rs12153515|rs11135312|rs2544997|rs7354779|rs1366191|rs731727|rs11958300|rs378299|rs7084637|rs1363075|rs7723455|rs12658783|rs1834|rs10515861|rs11683424|rs10965163
Serositis	TRAF6_MEDIATED_NFKB_ACTIVATION	REACTOME	351	20	13	6,99E-01	9,13E-01	rs468774|rs2096509|rs2269423|rs10410544|rs10872893|rs9978658|rs2234983|rs4519192|rs216779|rs7019752|rs10883452|rs4145277|rs380417
Serositis	GLYCEROPHOSPHOLIPID_BIOSYNTHESIS	REACTOME	2483	33	20	7,00E-01	9,13E-01	rs967776|rs10505887|rs17007811|rs12045786|rs9366337|rs2257140|rs7746765|rs17134769|rs591393|rs11891008|rs7126805|rs4952147|rs1921874|rs17028984|rs4819329|rs6865199|rs2269423|rs1679008|rs9999681|rs12637288
Serositis	MICRORNA_MIRNA_BIOGENESIS	REACTOME	227	6	5	7,01E-01	9,13E-01	rs7126805|rs7160403|rs1110386|rs8015314|rs10848243
Serositis	VIP_PATHWAY	BIOCARTA	1000	24	20	7,02E-01	9,13E-01	rs4699236|rs6812773|rs660749|rs11995971|rs6067805|rs10872893|rs748938|rs9808216|rs4699228|rs2645370|rs306872|rs6733907|rs3811063|rs11999145|rs2302234|rs4648090|rs230547|rs2273642|rs7010121|rs13430277
Serositis	CHEMOKINE_RECEPTORS_BIND_CHEMOKINES	REACTOME	726	28	12	7,03E-01	9,13E-01	rs12994381|rs4790701|rs352027|rs11080372|rs2187490|rs16860761|rs11694155|rs871144|rs6430628|rs4490404|rs1104768|rs12504339
Serositis	GLIOMA	KEGG	2070	37	20	7,11E-01	9,20E-01	rs1864053|rs8464|rs9647337|rs7699538|rs511973|rs2877260|rs1366315|rs7781436|rs6871452|rs11247367|rs10232489|rs7474671|rs9932716|rs246396|rs2684799|rs11137525|rs4240910|rs2188513|rs11854132|rs1642763
Serositis	ION_CHANNEL_TRANSPORT	REACTOME	2741	41	20	7,14E-01	9,20E-01	rs17653240|rs2376837|rs2288581|rs11135214|rs247896|rs282136|rs4472116|rs4906640|rs1159315|rs6829033|rs2078610|rs9511813|rs17462259|rs11161304|rs164368|rs11914694|rs1609478|rs6447164|rs852219|rs1397855
Serositis	M_G1_TRANSITION	REACTOME	1315	21	20	7,14E-01	9,20E-01	rs4540267|rs328349|rs7290191|rs133415|rs7948797|rs10276762|rs11164955|rs886727|rs11164953|rs12201680|rs2917779|rs16827840|rs909473|rs1042317|rs11794849|rs741048|rs13082666|rs17137468|rs4265124|rs755553
Serositis	PARKINSONS_DISEASE	KEGG	2833	32	20	7,15E-01	9,20E-01	rs6437332|rs1345638|rs1264901|rs2252523|rs10045456|rs6433541|rs1500147|rs2302178|rs12538107|rs2638247|rs13258461|rs13057911|rs6518204|rs2668424|rs4810150|rs1473092|rs10945770|rs13405880|rs10799655|rs2020902
Serositis	MRNA_3_END_PROCESSING	REACTOME	320	9	6	7,16E-01	9,20E-01	rs8104242|rs34934568|rs2808667|rs234597|rs2474871|rs2025366
Serositis	GLYCOSAMINOGLYCAN_BIOSYNTHESIS_CHONDROITIN_SULFATE	KEGG	1372	41	20	7,16E-01	9,20E-01	rs11646300|rs3734371|rs8061749|rs1807833|rs7130091|rs1482394|rs11112047|rs1892984|rs2052007|rs11224189|rs10503650|rs3765103|rs9294881|rs4937983|rs931932|rs2115817|rs6597849|rs12797160|rs12427269|rs10899
Serositis	LEUKOCYTE_TRANSENDOTHELIAL_MIGRATION	KEGG	4793	33	20	7,16E-01	9,20E-01	rs1864053|rs8464|rs1430726|rs11686261|rs17106413|rs7016497|rs10863430|rs7290191|rs11168228|rs12495548|rs7567671|rs11730652|rs966308|rs9647337|rs10878841|rs3755105|rs1320213|rs2441727|rs511973|rs1425104
Serositis	INSULIN_RECEPTOR_RECYCLING	REACTOME	492	15	9	7,19E-01	9,22E-01	rs1562853|rs2712353|rs2071596|rs11783925|rs9643478|rs2857709|rs4147778|rs4244808|rs10241449
Serositis	NUCLEAR_SIGNALING_BY_ERBB4	REACTOME	2231	33	20	7,20E-01	9,22E-01	rs3924999|rs2976525|rs2466059|rs4733117|rs10503929|rs10492911|rs13427642|rs1464443|rs2221434|rs11225161|rs2096509|rs2302984|rs12544147|rs6451196|rs4859540|rs10209471|rs2347505|rs6895971|rs7198076|rs1505376
Serositis	ARF_PATHWAY	BIOCARTA	733	31	20	7,22E-01	9,22E-01	rs1864053|rs1366315|rs6871452|rs11995971|rs14105|rs1642763|rs383542|rs1636808|rs34299|rs16897109|rs16897180|rs2189000|rs9554080|rs7010121|rs4976028|rs10225279|rs7706821|rs4740363|rs7379303|rs7971479
Serositis	TASTE_TRANSDUCTION	KEGG	1100	33	20	7,26E-01	9,22E-01	rs10241629|rs2228576|rs4926163|rs210120|rs4920566|rs4810147|rs4809745|rs891429|rs2963899|rs11261073|rs11760312|rs1015443|rs7411058|rs4272105|rs10456425|rs2301698|rs3851578|rs4618675|rs16904362|rs503084
Serositis	ARRHYTHMOGENIC_RIGHT_VENTRICULAR_CARDIOMYOPATHY_ARVC	KEGG	5871	35	20	7,28E-01	9,22E-01	rs4928016|rs2221145|rs1430726|rs2757594|rs17801265|rs12373564|rs11686261|rs2286374|rs17106413|rs1016835|rs9309065|rs2292653|rs2335347|rs2056131|rs7567671|rs966308|rs7977039|rs10437823|rs3755105|rs2441727
Serositis	G_PROTEIN_ACTIVATION	REACTOME	703	28	13	7,28E-01	9,22E-01	rs12553893|rs17292684|rs1253676|rs11070884|rs4784650|rs875602|rs6914262|rs7863832|rs2267005|rs7618348|rs2281617|rs512053|rs17277929
Serositis	CYTOKINE_CYTOKINE_RECEPTOR_INTERACTION	KEGG	6425	25	20	7,29E-01	9,22E-01	rs1019855|rs1522747|rs7566998|rs7235828|rs3759614|rs12661042|rs12028945|rs11577442|rs10783481|rs934721|rs2228576|rs3024498|rs4492018|rs12994381|rs12145973|rs4790701|rs11123905|rs1388011|rs2877260|rs17025039
Serositis	METABOLISM_OF_NUCLEOTIDES	REACTOME	1559	33	20	7,30E-01	9,22E-01	rs11610|rs3750187|rs4540635|rs7512897|rs12565526|rs7517175|rs9324161|rs7221585|rs10799655|rs4964752|rs2275007|rs4716059|rs2854702|rs28171|rs3793358|rs7932321|rs10521022|rs13398821|rs4910907|rs2748525
Serositis	PTM_GAMMA_CARBOXYLATION_HYPUSINE_FORMATION_AND_ARYLSULFATASE_ACTIV	REACTOME	377	27	12	7,31E-01	9,22E-01	rs2560274|rs25413|rs3733895|rs722572|rs12499919|rs1687863|rs16875913|rs9324220|rs337853|rs2071421|rs7671785|rs13238899
Serositis	MCALPAIN_PATHWAY	BIOCARTA	543	22	11	7,32E-01	9,22E-01	rs7016497|rs2877260|rs10232489|rs2110290|rs2302234|rs2252586|rs7290469|rs9347252|rs3861699|rs6766238|rs10940270
Serositis	ABC_TRANSPORTERS	KEGG	2684	46	20	7,34E-01	9,22E-01	rs2283253|rs2239322|rs2954804|rs12452482|rs2125739|rs3780542|rs1568946|rs7382619|rs2074308|rs10498030|rs10841915|rs12871476|rs4147844|rs2236705|rs4794173|rs12154722|rs1889547|rs4147823|rs4073237|rs7296418
Serositis	PPARA_ACTIVATES_GENE_EXPRESSION	REACTOME	3675	25	20	7,35E-01	9,22E-01	rs9858201|rs930105|rs1296028|rs12661042|rs10963994|rs10161338|rs7634430|rs2946394|rs6051511|rs2375592|rs13325694|rs1485591|rs7659090|rs782931|rs2472299|rs3780542|rs2478545|rs2195003|rs10766077|rs9493149
Serositis	HS_GAG_BIOSYNTHESIS	REACTOME	3154	35	20	7,35E-01	9,22E-01	rs9995245|rs2228331|rs1932306|rs4583089|rs17491203|rs928646|rs1424223|rs1556466|rs12446144|rs3924880|rs1008953|rs11562702|rs16948406|rs2149063|rs736389|rs17775878|rs11696078|rs9524254|rs8056091|rs16949539
Serositis	NUCLEOTIDE_EXCISION_REPAIR	KEGG	914	33	18	7,36E-01	9,22E-01	rs7188980|rs10276762|rs4943153|rs6460075|rs1794242|rs886727|rs12221497|rs1714473|rs1928023|rs741048|rs2321822|rs2808667|rs4443717|rs17137468|rs12584168|rs1638566|rs9365947|rs11670895
Serositis	BMAL1_CLOCK_NPAS2_ACTIVATES_CIRCADIAN_EXPRESSION	REACTOME	1728	31	20	7,36E-01	9,22E-01	rs10462023|rs2704973|rs7634430|rs2946394|rs782931|rs6956300|rs10766077|rs2539847|rs2071427|rs12903220|rs2970873|rs7639315|rs934945|rs4795369|rs10832005|rs7172348|rs1027228|rs12437754|rs17237521|rs301191
Serositis	HISTIDINE_METABOLISM	KEGG	654	28	17	7,36E-01	9,22E-01	rs10174379|rs10732359|rs6756760|rs4371935|rs4878199|rs4646771|rs4646322|rs4646693|rs4147778|rs6716175|rs8045616|rs4646656|rs1522896|rs1923431|rs962800|rs1042917|rs4930211
Serositis	PI_3K_CASCADE	REACTOME	1868	28	20	7,38E-01	9,23E-01	rs1864053|rs2283575|rs249736|rs6051511|rs1909669|rs1366315|rs6871452|rs3759216|rs541425|rs11177669|rs3945860|rs881301|rs2020902|rs1219643|rs3135772|rs9526838|rs2687975|rs1782808|rs7168316|rs10788819
Serositis	N_GLYCAN_BIOSYNTHESIS	KEGG	1792	24	20	7,41E-01	9,26E-01	rs3739821|rs901225|rs6415846|rs1455554|rs13253051|rs2272550|rs11123751|rs885036|rs17657086|rs9924614|rs1495084|rs12887424|rs17657927|200260|rs1546149|rs12891561|rs4368537|rs807270|rs10145560|rs8007403
Serositis	FORMATION_OF_TUBULIN_FOLDING_INTERMEDIATES_BY_CCT_TRIC	REACTOME	227	14	8	7,45E-01	9,28E-01	rs2249790|rs2832157|rs151352|rs11133604|rs3798211|rs1064019|rs13238899|rs6490620
Serositis	GALACTOSE_METABOLISM	KEGG	759	32	20	7,46E-01	9,28E-01	rs17301901|rs8132|rs4074708|rs2236441|rs626295|rs4976661|rs4879666|rs17158714|rs2995965|rs906215|rs12703419|rs2284997|rs758989|rs581720|rs3771783|rs16973137|rs2269260|rs3829910|rs3088333|rs6550191
Serositis	ANTIGEN_PRESENTATION_FOLDING_ASSEMBLY_AND_PEPTIDE_LOADING_OF_CLASS_I_M	REACTOME	830	53	17	7,49E-01	9,31E-01	rs9258186|rs983694|rs7770216|rs7382619|rs17677598|rs5009448|rs1136882|rs7761965|rs2219893|rs4834703|rs17108576|rs892666|rs26310|rs9380326|rs12662611|rs11628065|rs4834700
Serositis	FGFR_LIGAND_BINDING_AND_ACTIVATION	REACTOME	984	27	20	7,52E-01	9,31E-01	rs2283575|rs249736|rs1909669|rs3945860|rs881301|rs1219643|rs3135772|rs9526838|rs2687975|rs7168316|rs7479018|rs630757|rs2038711|rs12220647|rs10492454|rs9568711|rs7323166|rs7169799|rs1482676|rs725600
Serositis	RAC1_PATHWAY	BIOCARTA	763	38	20	7,53E-01	9,31E-01	rs1864053|rs1366315|rs6871452|rs907698|rs383542|rs1636808|rs360464|rs769732|rs34299|rs16897109|rs1492769|rs16897180|rs252217|rs7660560|rs3754515|rs4976028|rs331679|rs7706821|rs339959|rs7379303
Serositis	CLEAVAGE_OF_GROWING_TRANSCRIPT_IN_THE_TERMINATION_REGION_	REACTOME	435	13	8	7,53E-01	9,31E-01	rs8104242|rs34934568|rs12410049|rs2808667|rs234597|rs2474871|rs2810640|rs2025366
Serositis	HEDGEHOG_SIGNALING_PATHWAY	KEGG	2151	35	20	7,55E-01	9,31E-01	rs7165405|rs11687903|rs1169848|rs7641735|rs6460003|rs3916033|rs10868482|rs1816069|rs3756990|rs6054590|rs12113716|rs17423920|rs9475432|rs2161039|rs12478509|rs6952416|rs733153|rs9894638|rs2299146|rs12711539
Serositis	HCMV_PATHWAY	BIOCARTA	547	30	18	7,56E-01	9,31E-01	rs1864053|rs1366315|rs6871452|rs383542|rs1636808|rs918842|rs34299|rs16897109|rs16897180|rs4648090|rs230547|rs7290469|rs4976028|rs1862884|rs10804160|rs7706821|rs11869073|rs7379303
Serositis	LIPOPROTEIN_METABOLISM	REACTOME	1037	30	20	7,56E-01	9,31E-01	rs11254339|rs3780542|rs1914172|rs1877059|rs17139378|rs8041525|rs964184|rs2236705|rs261336|rs7178362|rs2487052|rs2879094|rs8133510|rs407134|rs7585006|rs532345|rs261337|rs4659357|rs838853|rs11584174
Serositis	REGULATION_OF_APOPTOSIS	REACTOME	1432	36	20	7,59E-01	9,34E-01	rs919635|rs328349|rs2219926|rs7948797|rs9964756|rs2928953|rs768204|rs2020902|rs4976661|rs10868585|rs12201680|rs12605027|rs2917779|rs3793647|rs16827840|rs909473|rs11794849|rs9959180|rs16956918|rs755553
Serositis	MITOTIC_M_M_G1_PHASES	REACTOME	3176	38	20	7,62E-01	9,36E-01	rs2203751|rs4387213|rs4540267|rs328349|rs7290191|rs3001080|rs6695454|rs133415|rs7948797|rs6972752|rs10276762|rs11164955|rs17648423|rs1345207|rs17448345|rs12120009|rs11119881|rs7221585|rs886727|rs11164953
Serositis	TH1TH2_PATHWAY	BIOCARTA	535	26	12	7,64E-01	9,36E-01	rs28530648|rs17454570|rs1075498|rs3135363|rs3135344|rs6907167|rs2715261|rs6032678|rs1760084|rs7721176|rs4673260|rs7618747
Serositis	CACAM_PATHWAY	BIOCARTA	401	28	15	7,65E-01	9,36E-01	rs2942067|rs7699538|rs1457115|rs9808216|rs1718120|rs4958456|rs742257|rs918842|rs1011398|rs6733907|rs13430277|rs1294506|rs7314454|rs1862884|rs10804160
Serositis	VIF_MEDIATED_DEGRADATION_OF_APOBEC3G	REACTOME	738	28	18	7,69E-01	9,36E-01	rs328349|rs7948797|rs10890795|rs12577790|rs12201680|rs12418162|rs2917779|rs16827840|rs909473|rs11794849|rs755553|rs9380326|rs8004624|rs8005252|rs2621321|rs3859192|rs7500512|rs1424083
Serositis	MPR_PATHWAY	BIOCARTA	624	27	15	7,70E-01	9,36E-01	rs2960288|rs6018627|rs4810147|rs3846484|rs2302234|rs2471267|rs648838|rs1174658|rs6010742|rs6012200|rs4121759|rs7290469|rs3861699|rs6766238|rs868362
Serositis	MRNA_CAPPING	REACTOME	388	7	5	7,70E-01	9,36E-01	rs7126805|rs1324956|rs2808667|rs11756329|rs1325006

Serositis	3_UTR_MEDIATED_TRANSLATIONAL_REGULATION	REACTOME	1235	33	20	7,70E-01	9,36E-01	rs2252523|rs16935649|rs2302178|rs12121852|rs7126805|rs8017939|rs2253181|rs1953900|rs3732680|rs1074584|rs747020|rs4758037|rs4300036|rs2687975|rs6743777|rs4672803|rs2917779|rs5756236|rs4599669|rs137602
Serositis	PRIMARY_IMMUNODEFICIENCY	KEGG	742	46	19	7,71E-01	9,36E-01	rs12613960|rs10195135|rs7382619|rs8072293|rs4675397|rs7536180|rs747020|rs1423658|rs2219893|rs2569987|rs6032678|rs10774961|rs1075834|rs12473084|rs12576947|rs4350841|rs9380326|rs10494786|rs2299686
Serositis	GAP_JUNCTION_TRAFFICKING	REACTOME	576	11	8	7,72E-01	9,36E-01	rs7164838|rs3776519|rs2887979|rs11240144|rs7321901|rs811563|rs639441|rs7327522
Serositis	NEUROACTIVE_LIGAND_RECEPTOR_INTERACTION	KEGG	13469	27	20	7,73E-01	9,36E-01	rs163972|rs8095771|rs500817|rs192718|rs2405871|rs6545061|rs17653240|rs472418|rs12648009|rs6474421|rs2376837|rs9361153|rs7686241|rs3760703|rs2206819|rs11055455|rs2927585|rs13257637|rs981273|rs11636101
Serositis	NO2IL12_PATHWAY	BIOCARTA	461	8	8	7,74E-01	9,36E-01	rs17454570|rs12720253|rs1075498|rs7254474|rs280519|rs12576947|rs7721176|rs6686571
Serositis	TID_PATHWAY	BIOCARTA	479	25	16	7,75E-01	9,36E-01	rs2228576|rs11114668|rs1642763|rs10872893|rs7127500|rs6907167|rs222778|rs4756899|rs11031829|rs2857709|rs4648090|rs1760084|rs11031734|rs230547|rs7550488|rs878208
Serositis	ER_PHAGOSOME_PATHWAY	REACTOME	1416	46	20	7,82E-01	9,41E-01	rs328349|rs9258186|rs7948797|rs7770216|rs7382619|rs7874483|rs12201680|rs5009448|rs2917779|rs16827840|rs7761965|rs2219893|rs909473|rs11794849|rs892666|rs755553|rs9380326|rs8004624|rs8005252|rs3859192
Serositis	INCRETIN_SYNTHESIS_SECRETION_AND_INACTIVATION	REACTOME	540	32	18	7,85E-01	9,41E-01	rs4940849|rs12186641|rs11133152|rs6553943|rs10423648|rs1380100|rs4940851|rs12459138|rs1598100|rs4840583|rs2271731|rs8100944|rs12995983|rs11660643|rs4840584|rs7731484|rs13144122|rs11782574
Serositis	INHIBITION_OF_INSULIN_SECRETION_BY_ADRENALINE_NORADRENALINE	REACTOME	789	30	17	7,87E-01	9,41E-01	rs12553893|rs4234214|rs9856983|rs1253676|rs11070884|rs4784650|rs17128431|rs914363|rs7863832|rs2357929|rs7901587|rs9870651|rs2138551|rs7618348|rs7912454|rs7078741|rs7098615
Serositis	DILATED_CARDIOMYOPATHY	KEGG	4636	28	20	7,87E-01	9,41E-01	rs4928016|rs2960288|rs3759614|rs17801265|rs12373564|rs2286374|rs1909745|rs9309065|rs9510354|rs2056131|rs7153499|rs7977039|rs10437823|rs4234214|rs490057|rs2292700|rs9856983|rs4569841|rs7179757|rs9851393
Serositis	EARLY_PHASE_OF_HIV_LIFE_CYCLE	REACTOME	270	3	3	7,89E-01	9,41E-01	rs16972096|rs6430628|rs1151514
Serositis	G_ALPHA_Z_SIGNALLING_EVENTS	REACTOME	2191	26	20	7,90E-01	9,41E-01	rs8464|rs2960288|rs1909745|rs3783754|rs12553893|rs2711292|rs4234214|rs9856983|rs4569841|rs7733194|rs4810147|rs891429|rs2026628|rs1550116|rs1253676|rs12938937|rs17083981|rs11070884|rs13153439|rs6003495
Serositis	ACTIVATION_OF_KAINATE_RECEPTORS_UPON_GLUTAMATE_BINDING	REACTOME	1577	26	20	7,90E-01	9,41E-01	rs6039107|rs12553893|rs13037679|rs2252359|rs4363614|rs7645524|rs911219|rs1474937|rs6039104|rs1222955|rs16868250|rs1253676|rs2832458|rs6583202|rs4331108|rs11070884|rs9843659|rs2852577|rs9808216|rs708920
Serositis	GLUCURONIDATION	REACTOME	656	41	9	7,91E-01	9,41E-01	rs2013573|rs6738678|rs7608175|rs4148285|rs12650761|rs7438808|rs2687965|rs4148275|rs6832167
Serositis	ASCORBATE_AND_ALDARATE_METABOLISM	KEGG	874	56	14	7,91E-01	9,41E-01	rs10732359|rs2013573|rs6738678|rs10168155|rs4878199|rs4646771|rs4148285|rs12650761|rs7438808|rs1923431|rs7216|rs2687965|rs4148275|rs6832167
Serositis	ANTIGEN_PROCESSING_CROSS_PRESENTATION	REACTOME	1601	41	20	7,91E-01	9,41E-01	rs328349|rs2272874|rs7948797|rs12620429|rs7770216|rs7382619|rs7874483|rs12201680|rs5009448|rs3211828|rs2917779|rs16827840|rs7761965|rs2219893|rs909473|rs11794849|rs17683292|rs892666|rs3754515|rs755553
Serositis	AUTODEGRADATION_OF_CDH1_BY_CDH1_APC_C	REACTOME	857	32	18	7,93E-01	9,42E-01	rs328349|rs2942067|rs7681082|rs7948797|rs9590480|rs12201680|rs2917779|rs16827840|rs909473|rs11794849|rs755553|rs9380326|rs8004624|rs8005252|rs2621321|rs3859192|rs7500512|rs1424083
Serositis	DEATH_PATHWAY	BIOCARTA	505	24	17	7,94E-01	9,42E-01	rs181402|rs17809603|rs2020902|rs2046136|rs619365|rs10872893|rs555745|rs2849382|rs12186102|rs2846855|rs2867316|rs4648090|rs7019752|rs230547|rs10883452|rs3843973|rs1544570
Serositis	CARM_ER_PATHWAY	BIOCARTA	1617	26	20	7,97E-01	9,43E-01	rs6913161|rs2946394|rs12371626|rs918418|rs7702020|rs1158904|rs9646967|rs1684274|rs2970873|rs13012239|rs13046729|rs378759|rs7275695|rs519165|rs4795369|rs2129301|rs2822942|rs4765158|rs12721364|rs10177901
Serositis	CIRCADIAN_CLOCK	REACTOME	2075	29	20	7,98E-01	9,43E-01	rs10462023|rs2704973|rs7634430|rs2946394|rs782931|rs918418|rs7702020|rs6956300|rs10766077|rs2539847|rs2071427|rs12903220|rs2970873|rs7639315|rs934945|rs4795369|rs10832005|rs7172348|rs1027228|rs2917779
Serositis	SCFSKP2_MEDIATED_DEGRADATION_OF_P27_P21	REACTOME	813	28	19	8,00E-01	9,43E-01	rs328349|rs2282411|rs7948797|rs3759216|rs9315422|rs12201680|rs918587|rs2917779|rs16827840|rs909473|rs11794849|rs755553|rs9380326|rs8004624|rs8005252|rs2621321|rs3859192|rs7500512|rs1424083
Serositis	ENDOCYTOSIS	KEGG	6484	40	20	8,00E-01	9,43E-01	rs1620958|rs9364106|rs1264524|rs7607661|rs543347|rs2600330|rs17567|rs11921282|rs6018627|rs6505247|rs888956|rs4325000|rs2042898|rs9258186|rs10914127|rs2048090|rs2877260|rs1909669|rs1492286|rs223134
Serositis	PRIMARY_BILE_ACID_BIOSYNTHESIS	KEGG	439	15	7	8,02E-01	9,43E-01	rs246966|rs11808284|rs7089935|rs250412|rs11203006|rs2253200|rs2254636
Serositis	ORC1_REMOVAL_FROM_CHROMATIN	REACTOME	930	22	20	8,02E-01	9,43E-01	rs328349|rs133415|rs2282411|rs7948797|rs3759216|rs9315422|rs12201680|rs2917779|rs16827840|rs909473|rs11794849|rs13082666|rs4265124|rs755553|rs2307373|rs9380326|rs8004624|rs8005252|rs2621321|rs3859192
Serositis	RIG_I_MDA5_MEDIATED_INDUCTION_OF_IFN_ALPHA_BETA_PATHWAYS	REACTOME	1129	32	20	8,04E-01	9,43E-01	rs10863430|rs2357933|rs9389541|rs468774|rs6944913|rs1390762|rs2096509|rs2269423|rs10410544|rs10872893|rs3733446|rs9978658|rs555745|rs2234983|rs2917779|rs821470|rs1296720|rs4519192|rs216779|rs2238419
Serositis	BASAL_CELL_CARCINOMA	KEGG	2166	26	20	8,07E-01	9,43E-01	rs2796794|rs2221145|rs2292653|rs7641735|rs6460003|rs3916033|rs6054590|rs10022956|rs12113716|rs4355511|rs10508827|rs10489028|rs4958072|rs11725638|rs1642763|rs12478509|rs6952416|rs3923086|rs733153|rs1632588
Serositis	SEMA4D_IN_SEMAPHORIN_SIGNALING	REACTOME	597	24	15	8,10E-01	9,43E-01	rs1651543|rs2282537|rs4486969|rs788330|rs13054809|rs7536180|rs342076|rs11265783|rs311372|rs5997917|rs4801824|rs4938800|rs10494786|rs2237710|rs7259683
Serositis	BILE_ACID_AND_BILE_SALT_METABOLISM	REACTOME	1176	41	19	8,10E-01	9,43E-01	rs3916929|rs246966|rs9582668|rs1929081|rs11808284|rs3918333|rs10743413|rs4794173|rs4498289|rs11618565|rs7089935|rs5484|rs250412|rs4142323|rs12583004|rs11203006|rs2253200|rs2254636|rs1888322
Serositis	HYPERTROPHIC_CARDIOMYOPATHY_HCM	KEGG	4149	31	20	8,11E-01	9,43E-01	rs4928016|rs3759614|rs17801265|rs12373564|rs2286374|rs9309065|rs2335347|rs1881638|rs2056131|rs7153499|rs7977039|rs10437823|rs490057|rs2292700|rs7179757|rs9851393|rs7372541|rs12620429|rs13080236|rs2703029
Serositis	PIP3_ACTIVATES_AKT_SIGNALING	REACTOME	587	24	16	8,12E-01	9,43E-01	rs6051511|rs3759216|rs541425|rs2020902|rs1782808|rs10788819|rs1790733|rs7322396|rs918842|rs17350527|rs2324600|rs10883452|rs1638588|rs1862884|rs10804160|rs7971479
Serositis	GLYCOSAMINOGLYCAN_BIOSYNTHESIS_HEPARAN_SULFATE	KEGG	2273	27	20	8,13E-01	9,43E-01	rs9995245|rs11646300|rs3734371|rs17491203|rs8061749|rs7130091|rs11562702|rs16948406|rs1892984|rs11696078|rs2897293|rs11731956|rs8068169|rs2996009|rs1013120|rs11224189|rs10457277|rs2022240|rs2874994|rs7676999
Serositis	SHC_MEDIATED_CASCADE	REACTOME	1070	27	20	8,14E-01	9,43E-01	rs2283575|rs249736|rs1909669|rs3945860|rs881301|rs1219643|rs3135772|rs9526838|rs2687975|rs7168316|rs7479018|rs630757|rs2038711|rs12220647|rs10492454|rs9568711|rs7323166|rs7169799|rs1482676|rs725600
Serositis	P75_NTR_RECEPTOR_MEDIATED_SIGNALLING	REACTOME	2669	26	20	8,15E-01	9,43E-01	rs2663342|rs9968730|rs6913161|rs2248656|rs4704706|rs2160591|rs12356045|rs2282537|rs1531846|rs7611544|rs11879985|rs2254527|rs2291462|rs2017750|rs4681946|rs10465781|rs10821056|rs11192617|rs724710|rs2920898
Serositis	INFLUENZA_VIRAL_RNA_TRANSCRIPTION_AND_REPLICATION	REACTOME	1153	33	20	8,16E-01	9,43E-01	rs2252523|rs16935649|rs2302178|rs12121852|rs7126805|rs1771416|rs8017939|rs2253181|rs1953900|rs3732680|rs2687975|rs6743777|rs4672803|rs2917779|rs4599669|rs137602|rs4237000|rs11756089|rs3900716|rs1427827
Serositis	DORSO_VENTRAL_AXIS_FORMATION	KEGG	1149	25	20	8,16E-01	9,43E-01	rs2836804|rs1109771|rs2877260|rs7948482|rs10232489|rs9268005|rs2269423|rs2012898|rs4659248|rs11054492|rs2410062|rs7937445|rs2110290|rs2541117|rs2252586|rs10520565|rs12574791|rs10893845|rs377743|rs7290469
Serositis	DNA_REPLICATION	REACTOME	3548	36	20	8,17E-01	9,43E-01	rs2203751|rs4387213|rs4540267|rs328349|rs7290191|rs3001080|rs6695454|rs133415|rs2282411|rs7948797|rs6972752|rs10276762|rs11164955|rs4943153|rs17648423|rs1345207|rs3759216|rs17448345|rs12120009|rs11119881
Serositis	ACTIVATED_POINT_MUTANTS_OF_FGFR2	REACTOME	873	34	20	8,19E-01	9,44E-01	rs2283575|rs249736|rs1909669|rs3945860|rs1219643|rs3135772|rs7168316|rs7479018|rs630757|rs2038711|rs12220647|rs10492454|rs7323166|rs7169799|rs1482676|rs725600|rs1886859|rs3135763|rs7999069|rs4769189
Serositis	AMINO_ACID_TRANSPORT_ACROSS_THE_PLASMA_MEMBRANE	REACTOME	1025	32	20	8,22E-01	9,45E-01	rs10211524|rs747488|rs16948748|rs7445640|rs7329421|rs9551705|rs13159461|rs3782370|rs3497|rs8013529|rs2008831|rs2877489|rs448469|rs1007160|rs4299208|rs6502793|rs17612862|rs2037029|rs12833767|rs6876572
Serositis	BASE_EXCISION_REPAIR	KEGG	500	33	18	8,22E-01	9,45E-01	rs4135048|rs3829300|rs2275007|rs7135445|rs10009807|rs4690502|rs2569987|rs4519192|rs11727842|rs4840583|rs907186|rs10774961|rs1790740|rs4840584|rs4145277|rs10098874|rs1760941|rs3828486
Serositis	DESTABILIZATION_OF_MRNA_BY_AUF1_HNRNP_D0	REACTOME	811	31	20	8,27E-01	9,46E-01	rs328349|rs6822283|rs7948797|rs2101680|rs17005458|rs12201680|rs2917779|rs16827840|rs2035912|rs909473|rs11794849|rs755553|rs9380326|rs8004624|rs8005252|rs2621321|rs3859192|rs7500512|rs1424083|rs7678575
Serositis	MITOTIC_G1_G1_S_PHASES	REACTOME	2477	25	20	8,27E-01	9,46E-01	rs4540267|rs11784209|rs328349|rs7290191|rs3745255|rs133415|rs2282411|rs7948797|rs10276762|rs7781436|rs11164955|rs1995971|rs3759216|rs737523|rs886727|rs11164953|rs2188513|rs9315422|rs12201680|rs4130023
Serositis	YAP1_AND_WWTR1_TAZ_STIMULATED_GENE_EXPRESSION	REACTOME	1047	30	20	8,27E-01	9,46E-01	rs9858201|rs7634430|rs13325694|rs6940184|rs10501995|rs4795369|rs16873539|rs2124216|rs2288481|rs301193|rs1296720|rs4253623|rs2238419|rs13259607|rs16944293|rs11756089|rs7301162|rs12350120|rs11923928|rs7020291
Serositis	SYNTHESIS_OF_BILE_ACIDS_AND_BILE_SALTS	REACTOME	546	15	7	8,29E-01	9,46E-01	rs246966|rs11590871|rs7089935|rs250412|rs11203006|rs2253200|rs2254636
Serositis	L1CAM_INTERACTIONS	REACTOME	3914	25	20	8,30E-01	9,46E-01	rs3793859|rs1353825|rs10509122|rs6018627|rs9366027|rs1904398|rs2056131|rs4325000|rs13026243|rs7620137|rs11919047|rs12375332|rs2506145|rs10066223|rs17134769|rs2292700|rs2877260|rs2931462|rs6599218|rs12620429
Serositis	G1_S_TRANSITION	REACTOME	1941	22	20	8,31E-01	9,46E-01	rs4540267|rs328349|rs7290191|rs133415|rs2282411|rs7948797|rs10276762|rs11164955|rs1995971|rs3759216|rs737523|rs886727|rs11164953|rs9315422|rs12201680|rs918587|rs28171|rs7184777|rs2917779|rs16827840
Serositis	ACTIVATION_OF_NF_KAPPAB_IN_B_CELLS	REACTOME	1396	25	20	8,31E-01	9,46E-01	rs17747965|rs328349|rs1221220|rs7948797|rs10498970|rs12671372|rs10410544|rs10872893|rs12201680|rs1010650|rs13031237|rs2141484|rs157695|rs9362733|rs2917779|rs16827840|rs909473|rs11794849|rs711272|rs10485405
Serositis	HEMATOPOIETIC_CELL_LINEAGE	KEGG	1963	26	20	8,33E-01	9,46E-01	rs12373564|rs2056131|rs11123905|rs2292700|rs17025039|rs28530648|rs2275475|rs10779277|rs1005656|rs10513474|rs7538029|rs246396|rs6794523|rs17561|rs433192|rs3792424|rs880043|rs11032363|rs3135363|rs10423648
Serositis	SEMAPHORIN_INTERACTIONS	REACTOME	2884	32	20	8,35E-01	9,46E-01	rs4702617|rs809192|rs935875|rs4761579|rs1228865|rs2506145|rs1651543|rs919993|rs2282537|rs7735145|rs10068349|rs4486969|rs9633635|rs788330|rs1912976|rs8036030|rs13054809|rs1166906|rs11948826|rs7536180
Serositis	ION_TRANSPORT_BY_P_TYPE_ATPASES	REACTOME	1690	40	20	8,35E-01	9,46E-01	rs2288581|rs247896|rs4906640|rs6829033|rs9511813|rs17462259|rs11914694|rs1609478|rs6447164|rs852219|rs1397855|rs1642763|rs6861122|rs9788948|rs6021304|rs247804|rs16962994|rs4243757|rs697973|rs2061789
Serositis	MATURITY_ONSET_DIABETES_OF_THE_YOUNG	KEGG	992	33	20	8,36E-01	9,46E-01	rs2737641|rs813393|rs1156234|rs8042685|rs12487368|rs4716447|rs10743413|rs11934856|rs4746864|rs4812829|rs705974|rs745659|rs2249048|rs758989|rs2248673|rs9855959|rs4244808|rs5484|rs10259516|rs6788164
Serositis	NRAGE_SIGNALS_DEATH_THROUGH_JNK	REACTOME	1919	32	20	8,38E-01	9,46E-01	rs2663342|rs9968730|rs2248656|rs4704706|rs2282537|rs7611544|rs11879985|rs2254527|rs2291462|rs2017750|rs10465781|rs4681946|rs10821056|rs724710|rs12974783|rs2142335|rs769732|rs2833445|rs7633543|rs6803697
Serositis	PEROXISOME	KEGG	2047	37	20	8,38E-01	9,46E-01	rs6596572|rs10044871|rs246966|rs1895218|rs2423248|rs7659090|rs1901826|rs4846228|rs11808284|rs2631753|rs11750377|rs321600|rs10050461|rs10074159|rs2077560|rs1901548|rs7256750|rs17411887|rs6849528|rs7720125
Serositis	G_ALPHA_I_SIGNALLING_EVENTS	REACTOME	6453	30	20	8,42E-01	9,48E-01	rs8095771|rs2107310|rs2960288|rs12648009|rs9361153|rs2206819|rs1909745|rs3136537|rs4907013|rs6454676|rs12956032|rs11696134|rs10009104|rs11732375|rs12553893|rs7529232|rs11670254|rs12994381|rs4790701|rs4057791
Serositis	JAK_STAT_SIGNALING_PATHWAY	KEGG	4346	37	20	8,43E-01	9,48E-01	rs1864053|rs12472695|rs9309375|rs13395505|rs12028945|rs11577442|rs3024498|rs4492018|rs12145973|rs9647337|rs511973|rs1366315|rs6871452|rs1554286|rs10493076|rs1995971|rs1503920|rs6546174|rs17454570|rs12720253
Serositis	NEGATIVE_REGULATION_OF_FGFR_SIGNALING	REACTOME	1536	27	20	8,46E-01	9,49E-01	rs2283575|rs249736|rs6018627|rs1909669|rs11177669|rs6671527|rs3945860|rs881301|rs1219643|rs3135772|rs9526838|rs2687975|rs1060478|rs7168316|rs7479018|rs630757|rs2917779|rs2038711|rs12220647|rs10492454
Serositis	HOMOLOGOUS_RECOMBINATION	KEGG	730	34	18	8,46E-01	9,49E-01	rs867185|rs10276762|rs12142240|rs886727|rs1372070|rs11614523|rs741048|rs875344|rs10253827|rs17137468|rs1638566|rs1957572|rs17105585|rs6955085|rs4942486|rs3784111|rs240066|rs2155209
Serositis	ACTIVATION_OF_CHAPERONE_GENES_BY_XBP1S	REACTOME	761	30	19	8,50E-01	9,53E-01	rs1734397|rs16856756|rs2730324|rs7627551|rs13073226|rs9502593|rs242376|rs1136882|rs2444229|rs2764085|rs10417957|rs9820435|rs6778169|rs7621509|rs10413885|rs556595|rs748191|rs1529860|rs11773524
Serositis	MITOTIC_G2_G2_M_PHASES	REACTOME	1389	44	20	8,57E-01	9,60E-01	rs12371968|rs2282411|rs1672927|rs7963662|rs1345207|rs4526032|rs2471535|rs11108698|rs9315422|rs1063243|rs4917968|rs7184777|rs4924687|rs3132585|rs1537510|rs1042317|rs11632888|rs3846484|rs16987806|rs6478444
Serositis	PROTEIN_FOLDING	REACTOME	675	27	18	8,60E-01	9,61E-01	rs11576830|rs6502026|rs2249790|rs2832157|rs17276701|rs8053839|rs151352|rs884113|rs11133604|rs3798211|rs3750512|rs276278|rs6856075|rs2179766|rs1064019|rs13238899|rs2024595|rs6490620
Serositis	REGULATION_OF_MITOTIC_CELL_CYCLE	REACTOME	1318	30	20	8,64E-01	9,63E-01	rs328349|rs2942067|rs7681650|rs2282411|rs7948797|rs491675|rs9315422|rs9590480|rs12201680|rs10446729|rs2917779|rs16827840|rs909473|rs7736312|rs11794849|rs6577168|rs2012797|rs9423256|rs11593182|rs755553
Serositis	ECM_RECEPTOR_INTERACTION	KEGG	3602	26	20	8,65E-01	9,63E-01	rs2535764|rs627527|rs12373564|rs11073987|rs1034620|rs2056131|rs425491|rs12476069|rs2285625|rs2292700|rs10487254|rs1858822|rs1155911|rs8111|rs12620429|rs12810426|rs1008953|rs9294963|rs3780275|rs736389
Serositis	CASPASE_PATHWAY	BIOCARTA	327	6	5	8,68E-01	9,63E-01	rs2020902|rs2846855|rs907186|rs666723|rs1413710
Serositis	S_PHASE	REACTOME	2076	24	20	8,68E-01	9,63E-01	rs4540267|rs328349|rs7290191|rs133415|rs2282411|rs7948797|rs10276762|rs4943153|rs11995971|rs3759216|rs737523|rs6460075|rs886727|rs6115178|rs9315422|rs12201680|rs918587|rs2917779|rs16827840|rs909473
Serositis	APC_C_CDH1_MEDIATED_DEGRADATION_OF_CDC20_AND_OTHER_APC_C_CDH1_TARGETE_MITOSIS_EARLY_G1	REACTOME	959	32	18	8,70E-01	9,63E-01	rs328349|rs2942067|rs7681650|rs7948797|rs9590480|rs12201680|rs2917779|rs16827840|rs909473|rs11794849|rs755553|rs9380326|rs8004624|rs8005252|rs2621321|rs3859192|rs7500512|rs1424083
Serositis	ARACHIDONIC_ACID_METABOLISM	KEGG	1356	43	20	8,72E-01	9,63E-01	rs967776|rs12564813|rs591393|rs4808358|rs11207546|rs6588432|rs11572255|rs740229|rs6532481|rs3010644|rs12738546|rs1924736|rs10818684|rs2070873|rs2430080|rs623784|rs3823717|rs3123045|rs2802475|rs4726499
Serositis	TNFR2_PATHWAY	BIOCARTA	414	18	13	8,75E-01	9,63E-01	rs9389541|rs6929796|rs10872893|rs4978745|rs2857709|rs2867316|rs4648090|rs7019752|rs230547|rs8004192|rs1710627|rs7550488|rs10883452
Serositis	ASSOCIATION_OF_TRIC_CCT_WITH_TARGET_PROTEINS_DURING_BIOSYNTHESIS	REACTOME	359	23	14	8,75E-01	9,63E-01	rs2249790|rs2832157|rs17276701|rs8053839|rs884113|rs11133604|rs3798211|rs3750512|rs276278|rs6856075|rs2179766|rs1064019|rs13238899|rs2024595
Serositis	LOSS_OF_NLP_FROM_MITOTIC_CENTROSOMES	REACTOME	1100	48	20	8,75E-01	9,63E-01	rs12371968|rs503922|rs7963662|rs4526032|rs2471535|rs11108698|rs1063243|rs4917968|rs4924687|rs3132585|rs1537510|rs16987806|rs6478444|rs1414063|rs11108974|rs1420653|rs10521111|rs10745776|rs3810857|rs11782529
Serositis	TRANSPORT_TO_THE_GOLGI_AND_SUBSEQUENT_MODIFICATION	REACTOME	1557	24	20	8,75E-01	9,63E-01	rs901225|rs1455554|rs983694|rs2282583|rs1586494|rs11123751|rs885036|rs17677598|rs12891561|rs807270|rs10145560|rs9799068|rs6767800|rs1136882|rs2321537|rs1667296|rs4879666|rs10149555|rs382078|rs17162650
Serositis	TRNA_AMINOACYLATION	REACTOME	795	36	17	8,76E-01	9,63E-01	rs2949646|rs200215|rs12730560|rs797126|rs854208|rs12112229|rs3018058|rs1180954|rs708037|rs2530230|rs6926774|rs2844649|rs2298733|rs9405828|rs17575828|rs2018284|rs1284605
Serositis	STEROID_HORMONE_BIOSYNTHESIS	KEGG	1491	64	20	8,76E-01	9,63E-01	rs12929465|rs2472299|rs6509397|rs476184|rs2013573|rs6738678|rs10168155|rs166050|rs4148285|rs12650761|rs7934642|rs2665584|rs7438808|rs7089935|rs3862053|rs4432229|rs7289747|rs3848542|rs4148275|rs6832167
Serositis	TRANSCRIPTIONAL_REGULATION_OF_WHITE_ADIPOCYTE_DIFFERENTIATION	REACTOME	2719	32	20	8,78E-01	9,63E-01	rs930105|rs12595650|rs6070157|rs687992|rs10161338|rs7634430|rs2946394|rs10978931|rs4984485|rs2195003|rs12906287|rs1036519|rs2970873|rs2567452|rs1317758|rs6941942|rs11794132|rs4984460|rs4130023|rs4795369
Serositis	MELANOMA	KEGG	2894	33	20	8,80E-01	9,63E-01	rs1864053|rs2283575|rs10937534|rs249736|rs9647337|rs1388011|rs511973|rs2877260|rs1366315|rs7781436|rs6871452|rs11247367|rs10232489|rs246396|rs2684799|rs3945860|rs881301|rs2062832|rs4240910|rs2188513
Serositis	METABOLISM_OF_NON_CODING_RNA	REACTOME	611	22	12	8,80E-01	9,63E-01	rs1264901|rs12410049|rs1264882|rs1128864|rs2808667|rs1406334|rs7196117|rs1347591|rs2810640|rs596283|rs10183020|rs8023495
Serositis	PENTOSE_AND_GLUCURONATE_INTERCONVERSIONS	KEGG	874	56	12	8,81E-01	9,63E-01	rs2013573|rs6738678|rs9552200|rs7608175|rs4148285|rs12650761|rs4381447|rs7438808|rs9550652|rs2687965|rs4148275|rs6832167
Serositis	RECRUITMENT_OF_MITOTIC_CENTROSOME_PROTEINS_AND_COMPLEXES	REACTOME	1177	49	20	8,85E-01	9,66E-01	rs12371968|rs1672927|rs7963662|rs4526032|rs2471535|rs11108698|rs1063243|rs4917968|rs4924687|rs3132585|rs1537510|rs11632888|rs3846484|rs16987806|rs6478444|rs1414063|rs11108974|rs1420653|rs10521111|rs10745776
Serositis	CHEMOKINE_SIGNALING_PATHWAY	KEGG	5407	26	20	8,87E-01	9,66E-01	rs1864053|rs2960288|rs13395505|rs2302456|rs6039107|rs7016497|rs2248656|rs1909745|rs12553893|rs12994381|rs523200|rs9647337|rs4790701|rs10878841|rs13037679|rs4234214|rs511973|rs9856983|rs4569841|rs7548973
Serositis	NKT_PATHWAY	BIOCARTA	638	15	9	8,88E-01	9,66E-01	rs17454570|rs1075498|rs11080372|rs4846488|rs6907167|rs1760084|rs6430628|rs7721176|rs4673260
Serositis	STATHMIN_PATHWAY	BIOCARTA	456	18	9	8,91E-01	9,66E-01	rs7699538|rs1457115|rs4958456|rs7736312|rs2302234|rs12576947|rs3861699|rs6766238|rs6686571
Serositis	MITOCHONDRIAL_TRNA_AMINOACYLATION	REACTOME	500	24	12	8,91E-01	9,66E-01	rs200215|rs797126|rs854208|rs1180954|rs708037|rs2530230|rs6926774|rs2844649|rs2298733|rs9405828|rs17575828|rs2018284
Serositis	CELL_DEATH_SIGNALLING_VIA_NRAGE_NRIF_AND_NADE	REACTOME	2069	32	20	8,92E-01	9,66E-01	rs2663342|rs9968730|rs2248656|rs4704706|rs2282537|rs7611544|rs11879985|rs2254527|rs2291462|rs2017750|rs10465781|rs4681946|rs10821056|rs724710|rs12974783|rs2917779|rs2142335|rs769732|rs2833445|rs7633543
Serositis	CHYLOMICRON_MEDIATED_LIPID_TRANSPORT	REACTOME	435	16	11	8,95E-01	9,66E-01	rs1877059|rs8041525|rs964184|rs261336|rs7178362|rs2879094|rs7585006|rs261337|rs4659357|rs11584174|rs3771254
Serositis	SPRY_PATHWAY	BIOCARTA	910	26	14	8,96E-01	9,66E-01	rs6018627|rs2877260|rs10232489|rs7295237|rs4234512|rs2110290|rs2184818|rs2252586|rs6012200|rs7290469|rs7617041|rs1105073|rs17073177|rs12870493
Serositis	CDK_MEDIATED_PHOSPHORYLATION_AND_REMOVAL_OF_CDC6	REACTOME	698	22	15	8,96E-01	9,66E-01	rs328349|rs7948797|rs12201680|rs2917779|rs16827840|rs909473|rs11794849|rs755553|rs9380326|rs8004624|rs8005252|rs2621321|rs3859192|rs7500512|rs1424083
Serositis	APC_C_CDC20_MEDIATED_DEGRADATION_OF_MITOTIC_PROTEINS	REACTOME	1094	28	20	8,98E-01	9,66E-01	rs328349|rs2942067|rs7681650|rs7948797|rs9590480|rs12201680|rs10446729|rs2917779|rs16827840|rs909473|rs3846484|rs11794849|rs9423256|rs11593182|rs755553|rs9380326|rs8004624|rs8005252|rs2621321|rs3859192
Serositis	ENDOSOMAL_SORTING_COMPLEX_REQUIRED_FOR_TRANSPORT_ESCRT	REACTOME	318	10	8	8,99E-01	9,66E-01	rs4886077|rs12941604|rs2917779|rs7745188|rs10112819|rs7160883|rs16958736|rs11669345
Serositis	SIGNALING_BY_WNT	REACTOME	1071	25	20	8,99E-01	9,66E-01	rs328349|rs7948797|rs12120009|rs11119881|rs4355511|rs4958072|rs12201680|rs13188321|rs241280|rs2917779|rs16827840|rs909473|rs11794849|rs755553|rs9380326|rs8004624|rs8005252|rs2621321|rs4919096|rs3859192
Serositis	PHASE1_FUNCTIONALIZATION_OF_COMPOUNDS	REACTOME	1515	38	20	9,01E-01	9,66E-01	rs7602885|rs7663410|rs762551|rs4808358|rs11207546|rs1063243|rs11572255|rs200199|rs12978001|rs707718|rs10818684|rs1920145|rs1789891|rs184623|rs12416759|rs1442490|rs10153788|rs1795240|rs3823717|rs6139321
Serositis	PYRIMIDINE_METABOLISM	KEGG	1900	28	20	9,01E-01	9,66E-01	rs4540267|rs3750187|rs4540635|rs1585534|rs7126805|rs14105|rs7517175|rs7221585|rs10799655|rs4964752|rs2275007|rs8362|rs2854702|rs28171|rs12675680|rs4979935|rs2288270|rs13398821|rs4910907|rs7577180
Serositis	CYCLIN_E_ASSOCIATED_EVENTS_DURING_G1_S_TRANSITION_	REACTOME	1212	22	20	9,08E-01	9,71E-01	rs328349|rs2282411|rs7948797|rs11995971|rs3759216|rs737523|rs9315422|rs12201680|rs918587|rs7184777|rs2917779|rs16827840|rs909473|rs11794849|rs755553|rs10770043|rs7010121|rs9380326|rs8004624|rs8005252
Serositis	GLUCAGON_TYPE_LIGAND_RECEPTORS	REACTOME	786	16	12	9,08E-01	9,71E-01	rs12553893|rs4810147|rs1253676|rs1078008|rs11070884|rs7863832|rs2267732|rs6751578|rs7618348|rs887703|rs11687793|rs6748349

Serositis	CYTOCHROME_P450_ARRANGED_BY_SUBSTRATE_TYPE	REACTOME	1163	39	20	9,12E-01	9,71E-01	rs7602885|rs762551|rs4808358|rs11207546|rs1063243|rs12729119|200199|rs12978001|rs707718|rs184623|rs12416759|rs10153788|rs3823717|rs4432229|rs4726499|rs10857733|rs2181874|rs9888067|rs1290620|rs7271624
Serositis	TRAF6_MEDIATED_IRF7_ACTIVATION	REACTOME	433	17	10	9,12E-01	9,71E-01	rs10863430|rs1296720|rs2238419|rs17624154|rs7019752|rs1710627|rs10878182|rs871782|rs7037389|rs7259683
Serositis	AXON_GUIDANCE	REACTOME	13131	32	20	9,13E-01	9,71E-01	rs11245205|rs3773195|rs4702617|rs10516614|rs4739207|rs919635|rs809192|rs627527|rs7138694|rs2228331|rs7273626|rs3793859|rs633762|rs935875|rs7016497|rs7163504|rs1353825|rs2545680|rs10509122|rs6018627
Serositis	CROSS_PRESENTATION_OF_SOLUBLE_EXOGENOUS_ANTIGENS_ENDOSOMES	REACTOME	713	22	15	9,14E-01	9,71E-01	rs328349|rs7948797|rs12201680|rs16827840|rs909473|rs11794849|rs17683292|rs755553|rs9380326|rs8004624|rs8005252|rs2621321|rs3859192|rs7500512|rs1424083
Serositis	POST_TRANSLATIONAL_PROTEIN_MODIFICATION	REACTOME	6008	26	20	9,14E-01	9,71E-01	rs1554324|rs4719136|rs2736868|rs4952025|rs3739821|rs935875|rs901225|rs4745552|rs10497137|rs1401346|rs6415846|rs1455554|rs9854005|rs2958830|rs6989475|rs4719120|rs6502026|rs2560274|rs10493593|rs25413
Serositis	CLASS_B_2_SECRETIN_FAMILY_RECEPTORS	REACTOME	2257	23	20	9,16E-01	9,72E-01	rs2796794|rs7641735|rs12553893|rs10228431|rs6460003|rs3916033|rs10779277|rs12113716|rs4810147|rs3786657|rs10508827|rs6988640|rs10743413|rs1437416|rs6729895|rs1080519|rs1294884|rs11085905|rs1253676|rs466876
Serositis	SCF_BETA_TRCP_MEDIATED_DEGRADATION_OF_EMI1	REACTOME	793	22	15	9,23E-01	9,77E-01	rs328349|rs7948797|rs12201680|rs2917779|rs16827840|rs909473|rs11794849|rs755553|rs9380326|rs8004624|rs8005252|rs2621321|rs3859192|rs7500512|rs1424083
Serositis	AMINOACYL_TRNA_BIOSYNTHESIS	KEGG	731	29	13	9,24E-01	9,77E-01	rs200215|rs12730560|rs797126|rs854208|rs1180954|rs708037|rs2530230|rs6926774|rs2844649|rs9405828|rs17575828|rs2018284|rs1284605
Serositis	SMOOTH_MUSCLE_CONTRACTION	REACTOME	658	18	11	9,26E-01	9,77E-01	rs7179757|rs9658761|rs7920305|rs9808216|rs6733907|rs1410602|rs4775607|rs13430277|rs1294506|rs2469763|rs10940270
Serositis	PROTEASOME	KEGG	821	21	14	9,26E-01	9,77E-01	rs328349|rs7948797|rs12201680|rs16827840|rs909473|rs11794849|rs755553|rs9380326|rs8004624|rs8005252|rs2621321|rs3859192|rs7500512|rs1424083
Serositis	CIRCADIAN_REPRESSION_OF_EXPRESSION_BY_REV_ERBA	REACTOME	1358	30	20	9,27E-01	9,77E-01	rs7634430|rs2946394|rs782931|rs10766077|rs2071427|rs12903220|rs2970873|rs4795369|rs10832005|rs7172348|rs1027228|rs12437754|rs17237521|rs301191|rs1296720|rs16874509|rs4253623|rs2238419|rs13259607|rs16943318
Serositis	REGULATION_OF_GENE_EXPRESSION_IN_BETA_CELLS	REACTOME	716	24	16	9,29E-01	9,78E-01	rs813393|rs10743413|rs11934856|rs1782808|rs4812829|rs705974|rs7322396|rs745659|rs2249048|rs758989|rs2324600|rs4244808|rs5484|rs13249060|rs11725962|rs6075942
Serositis	PROCESSING_OF_CAPPED_INTRONLESS_PRE_MRNA	REACTOME	274	8	6	9,31E-01	9,78E-01	rs12410049|rs2808667|rs234597|rs2474871|rs2810640|rs2025366
Serositis	RORA_ACTIVATES_CIRCADIAN_EXPRESSION	REACTOME	1377	30	20	9,32E-01	9,78E-01	rs7634430|rs2946394|rs782931|rs10766077|rs2071427|rs12903220|rs2970873|rs4795369|rs10832005|rs7172348|rs1027228|rs12437754|rs17237521|rs301193|rs1296720|rs16874509|rs4253623|rs2238419|rs13259607|rs16943318
Serositis	SYNTHESIS_OF_PC	REACTOME	669	27	15	9,37E-01	9,83E-01	rs11891008|rs12637288|rs2844457|rs966367|rs1036668|rs3745013|rs2511437|rs11889370|rs1889036|rs1917806|rs4147778|rs934857|rs17097955|rs4341099|rs1869381
Serositis	ASSEMBLY_OF_THE_PRE_REPLICATIVE_COMPLEX	REACTOME	911	21	20	9,40E-01	9,85E-01	rs328349|rs133415|rs7948797|rs12201680|rs2917779|rs16827840|rs909473|rs1042317|rs11794849|rs13082666|rs4265124|rs755553|rs2307373|rs9380326|rs8004624|rs8005252|rs2621321|rs3859192|rs7500512|rs1424083
Serositis	CDT1_ASSOCIATION_WITH_THE_CDC6_ORC_ORIGIN_COMPLEX	REACTOME	793	24	17	9,43E-01	9,85E-01	rs328349|rs7948797|rs12201680|rs2917779|rs16827840|rs909473|rs11794849|rs4265124|rs755553|rs2307373|rs9380326|rs8004624|rs8005252|rs2621321|rs3859192|rs7500512|rs1424083
Serositis	HOST_INTERACTIONS_OF_HIV_FACTORS	REACTOME	2270	28	20	9,43E-01	9,85E-01	rs809192|rs328349|rs3745255|rs7948797|rs10890795|rs801733|rs12577790|rs1345207|rs6964474|rs226434|rs7782153|rs747020|rs12201680|rs6962537|rs12418162|rs17170881|rs17266628|rs5009448|rs5763140|rs1128864
Serositis	PROCESSING_OF_CAPPED_INTRON_CONTAINING_PRE_MRNA	REACTOME	1547	31	20	9,45E-01	9,86E-01	rs192705|rs6822283|rs9664270|rs7126805|rs8104242|rs9730519|rs34934568|rs2101680|rs17005458|rs12410049|rs10494363|rs12462556|rs1128864|rs2035912|rs11907513|rs4813245|rs9660907|rs2808667|rs12460890|rs1529086
Serositis	ENOS_ACTIVATION_AND_REGULATION	REACTOME	336	14	8	9,47E-01	9,86E-01	rs9808216|rs1574768|rs380691|rs6733907|rs17738966|rs13430277|rs1294506|rs10961558
Serositis	REGULATION_OF_ORNITHINE_DECARBOXYLASE_ODC	REACTOME	773	26	18	9,50E-01	9,87E-01	rs328349|rs7948797|rs12201680|rs2523177|rs16827840|rs11633399|rs909473|rs11794849|rs755553|rs9380326|rs16948170|rs8004624|rs8005252|rs2621321|rs3859192|rs7500512|rs651816|rs1424083
Serositis	TRANSPORT_OF_INORGANIC_CATIONS_ANIONS_AND_AMINO_ACIDS_OLIGOPEPTIDES	REACTOME	4051	28	20	9,51E-01	9,87E-01	rs10491731|rs6756097|rs9309065|rs10211524|rs10814971|rs1454542|rs747488|rs11669215|rs10222419|rs4869682|rs16948748|rs7445640|rs7329421|rs11731807|rs3776578|rs10498083|rs9551705|rs6071173|rs384994|rs13261830
Serositis	ASPARAGINE_N_LINKED_GLYCOSYLATION	REACTOME	2760	23	20	9,51E-01	9,87E-01	rs3739821|rs901225|rs6415846|rs1455554|rs983694|rs13253051|rs2282583|rs2272550|rs1586494|rs11123751|rs4898765|rs885036|rs17657086|rs2730324|rs9924614|rs17677598|rs1495084|rs7627551|rs12887424|rs13073226
Serositis	GLYCOSPHINGOLIPID_BIOSYNTHESIS_LACTO_AND_NEOLACTO_SERIES	KEGG	670	29	19	9,54E-01	9,88E-01	rs836657|rs1374277|rs1226900|rs6590202|rs9979588|rs4879666|rs527168|rs1678849|rs2263190|rs11603211|rs882779|rs1752337|rs659115|rs7578484|rs240542|rs17646831|rs10754052|rs9399799|rs3798745
Serositis	BASAL_TRANSCRIPTION_FACTORS	KEGG	386	13	9	9,56E-01	9,88E-01	rs6089608|rs378759|rs6755901|rs810364|rs17145611|rs6450035|rs3811474|rs2261360|rs7818587
Serositis	DOWNSTREAM_SIGNALING_EVENTS_OF_B_CELL_RECEPTOR_BCR	REACTOME	2127	25	20	9,56E-01	9,88E-01	rs17747965|rs328349|rs1221220|rs6051511|rs7948797|rs3759216|rs541425|rs10498970|rs2020902|rs12671372|rs10410544|rs10872893|rs12201680|rs6543716|rs7177101|rs1782808|rs1010650|rs13031237|rs10788819|rs2141484
Serositis	INFLUENZA_LIFE_CYCLE	REACTOME	1671	34	20	9,59E-01	9,89E-01	rs2252523|rs16935649|rs2302178|rs12121852|rs7126805|rs1771416|rs1345207|rs8017939|rs2253181|rs1953900|rs3732680|rs2687975|rs17266628|rs1128864|rs6743777|rs4672803|rs2917779|rs4599669|rs137602|rs1406334
Serositis	MRNA_PROCESSING	REACTOME	1942	38	20	9,62E-01	9,91E-01	rs1127314|rs192705|rs6822283|rs9664270|rs7126805|rs8104242|rs9730519|rs34934568|rs2101680|rs17005458|rs12410049|rs10494363|rs12462556|rs9353553|rs1128864|rs2035912|rs11907513|rs4813245|rs9660907|rs2808667
Serositis	MUSCLE_CONTRACTION	REACTOME	1023	34	20	9,65E-01	9,93E-01	rs3759614|rs7153499|rs7179757|rs9658761|rs7920305|rs9808216|rs3738281|rs6733907|rs1410602|rs825087|rs4775607|rs13430277|rs17605348|rs1294506|rs7095324|rs819643|rs2469763|rs2031999|rs2041126|rs10940270
Serositis	SHH_PATHWAY	BIOCARTA	679	24	18	9,67E-01	9,93E-01	rs6460003|rs12113716|rs12478509|rs6952416|rs2299146|rs12711539|rs12463908|rs2302234|rs2305944|rs13298230|rs2237428|rs3861699|rs6766238|rs757911|rs6965030|rs6955791|rs895479|rs12155027
Serositis	TRANSPORT_OF_MATURE_TRANSCRIPT_TO_CYTOPLASM	REACTOME	693	18	10	9,68E-01	9,93E-01	rs8104242|rs34934568|rs1128864|rs2808667|rs1406334|rs7196117|rs1347591|rs596283|rs10183020|rs2165945
Serositis	NEP_NS2_INTERACTS_WITH_THE_CELLULAR_EXPORT_MACHINERY	REACTOME	456	13	8	9,69E-01	9,93E-01	rs1345207|rs1128864|rs1406334|rs7196117|rs1347591|rs10848243|rs596283|rs10183020
Serositis	MTA3_PATHWAY	BIOCARTA	333	5	5	9,69E-01	9,93E-01	rs16938992|rs2813554|rs13028484|rs3020366|rs6490620
Serositis	IL17_PATHWAY	BIOCARTA	316	8	6	9,71E-01	9,93E-01	rs9395766|rs12576947|rs10924103|rs1885800|rs1476482|rs6686571
Serositis	BASIGIN_INTERACTIONS	REACTOME	595	15	10	9,75E-01	9,94E-01	rs1642763|rs880043|rs8013529|rs2877489|rs448469|rs1007160|rs12272341|rs2037029|rs731710|rs3746248
Serositis	RNA_POL_II_TRANSCRIPTION	REACTOME	1050	27	17	9,75E-01	9,94E-01	rs7126805|rs8104242|rs34934568|rs12410049|rs6089608|rs378759|rs1324956|rs547005|rs2808667|rs234597|rs6450035|rs2474871|rs11756329|rs2810640|rs1325006|rs2025366|rs2261360
Serositis	CELL_ADHESION_MOLECULES_CAMS	KEGG	8813	39	20	9,76E-01	9,94E-01	rs1264524|rs2205895|rs2890658|rs6089247|rs12373564|rs17824941|rs7163504|rs2827641|rs17533090|rs7290191|rs12495548|rs2827539|rs742720|rs13045227|rs10500175|rs7620137|rs2272874|rs9863756|rs4971785|rs12613960
Serositis	SLC_MEDIATED_TRANSMEMBRANE_TRANSPORT	REACTOME	8774	27	20	9,77E-01	9,94E-01	rs11712912|rs10491731|rs2154505|rs10833366|rs7527059|rs6756097|rs11658922|rs9309065|rs4980345|rs10211524|rs10814971|rs1454542|rs12153013|rs747488|rs11669215|rs7428024|rs13093879|rs2375592|rs10222419|rs4869682
Serositis	BLADDER_CANCER	KEGG	1222	35	20	9,83E-01	9,99E-01	rs2877260|rs11995971|rs10232489|rs4690363|rs1642763|rs10868585|rs4234512|rs4810482|rs17719676|rs3793647|rs1042317|rs2110290|rs2298765|rs2252586|rs7290469|rs2894536|rs7617041|rs7010121|rs12272341|rs2574904
Serositis	TRANSPORT_OF_RIBONUCLEOPROTEINS_INTO_THE_HOST_NUCLEUS	REACTOME	452	14	8	9,84E-01	9,99E-01	rs17266628|rs1128864|rs1406334|rs9913503|rs7196117|rs1347591|rs596283|rs10183020
Serositis	PTDINS_PATHWAY	BIOCARTA	1058	31	19	9,86E-01	1,00E+00	rs6133987|rs2236441|rs2711292|rs12624835|rs281489|rs17158714|rs4760381|rs6720958|rs1464573|rs12001180|rs11106841|rs16922298|rs2789853|rs11903923|rs11125039|rs3858101|rs3829910|rs6104699|rs483404
Serositis	NUCLEOTIDE_LIKE_PURINERGIC_RECEPTORS	REACTOME	272	5	5	9,89E-01	1,00E+00	rs3766560|rs7208308|rs12798517|rs13094696|rs1983338
Serositis	GLUCOSE_TRANSPORT	REACTOME	689	28	13	9,90E-01	1,00E+00	rs751210|rs4976661|rs1128864|rs906215|rs758989|rs581720|rs1406334|rs7196117|rs1347591|rs780094|rs3771783|rs596283|rs10183020
Serositis	INTERACTIONS_OF_VPR_WITH_HOST_CELLULAR_PROTEINS	REACTOME	480	13	8	9,92E-01	1,00E+00	rs17266628|rs1128864|rs1406334|rs7196117|rs1347591|rs596283|rs1151514|rs10183020
Serositis	ANTIGEN_PROCESSING_UBIQUITINATION_PROTEASOME_DEGRADATION	REACTOME	4788	34	20	9,93E-01	1,00E+00	rs2906041|rs12619100|rs328349|rs2942067|rs7681650|rs7948797|rs7425902|rs10204358|rs10890795|rs12632852|rs28495843|rs12577790|rs6518204|rs1503920|rs1661300|rs10945770|rs17276701|rs7973701|rs12142240|rs7496283
Serositis	HIV_INFECTION	REACTOME	3237	28	20	9,94E-01	1,00E+00	rs809192|rs328349|rs3745255|rs7948797|rs10890795|rs7126805|rs801733|rs12577790|rs1345207|rs16972096|rs6964474|rs226434|rs7782153|rs6089608|rs378759|rs747020|rs12201680|rs9353553|rs6962537|rs12418162
Serositis	REGULATION_OF_GLUCOKINASE_BY_GLUCOKINASE_REGULATORY_PROTEIN	REACTOME	459	13	8	9,95E-01	1,00E+00	rs1128864|rs758989|rs1406334|rs7196117|rs1347591|rs780094|rs596283|rs10183020
Serositis	CLASS_I_MHC_MEDIATED_ANTIGEN_PROCESSING_PRESENTATION	REACTOME	5895	33	20	9,96E-01	1,00E+00	rs2906041|rs12619100|rs328349|rs2235383|rs2942067|rs7681650|rs7948797|rs7425902|rs10204358|rs10890795|rs12632852|rs12620429|rs983694|rs7770216|rs28495843|rs7382619|rs12577790|rs6518204|rs7874483|rs1503920
Serositis	TRANSPORT_OF_MATURE_MRNA_DERIVED_FROM_AN_INTRONLESS_TRANSCRIPT	REACTOME	524	13	8	9,98E-01	1,00E+00	rs1128864|rs2808667|rs1406334|rs7196117|rs1347591|rs596283|rs10183020|rs2165945
Serositis	PHENYLALANINE_METABOLISM	KEGG	442	10	4	9,98E-01	1,00E+00	rs4646693|rs4147778|rs4646656|rs4930211
Serositis	LATE_PHASE_OF_HIV_LIFE_CYCLE	REACTOME	1259	29	20	9,99E-01	1,00E+00	rs7126805|rs1345207|rs6089608|rs378759|rs1324956|rs1128864|rs547005|rs2917779|rs2808667|rs1406334|rs6450035|rs7196117|rs1347591|rs11756329|rs10848243|rs1325006|rs596283|rs10183020|rs12250830|rs2261360
Serositis	HIV_LIFE_CYCLE	REACTOME	1529	27	20	1,00E+00	1,00E+00	rs7126805|rs1345207|rs16972096|rs6089608|rs378759|rs9353553|rs1128864|rs547005|rs2917779|rs2808667|rs1406334|rs6430628|rs6450035|rs7196117|rs1347591|rs11756329|rs10848243|rs1325006|rs596283|rs1151514
Hematologic Disorder	SPRY_PATHWAY	BIOCARTA	910	63	18	5,30E-03	9,87E-01	rs253447|rs7983710|rs4947979|rs4349043|rs6563245|rs959662|rs2110290|rs1417839|rs3853477|rs1333421|rs17006928|rs11979158|rs2888586|rs11905013|rs16967789|rs863712|rs4776783|rs13124930
Hematologic Disorder	TERMINATION_OF_O_GLYCAN_BIOSYNTHESIS	REACTOME	961	48	20	6,10E-03	9,87E-01	rs485141|rs6669101|rs2268535|rs689423|rs1609454|rs1251484|rs1895364|rs6989475|rs1571142|rs657224|rs7844620|rs7016568|rs721590|rs4337792|rs1251465|rs3928852|rs2945770|rs538977|rs10784585|rs7814358
Hematologic Disorder	O_LINKED_GLYCOSYLATION_OF_MUCINS	REACTOME	2402	45	20	8,20E-03	9,87E-01	rs485141|rs4846948|rs6870010|rs6669101|rs2268535|rs6095638|rs689423|rs1609454|rs4846904|rs7794356|rs1251484|rs1465231|rs4719095|rs1895364|rs6989475|rs1571142|rs657224|rs1241347|rs540427|rs7844620
Hematologic Disorder	FORMATION_OF_FIBRIN_CLOT_CLOTTING_CASCADE	REACTOME	703	49	16	1,46E-02	9,87E-01	rs4610017|rs2074437|rs12500151|rs9667326|rs1040172|rs4764521|rs9504738|rs3211719|rs5985|rs10203300|rs11611433|rs10016252|rs6829128|rs6796803|rs698951|rs2283333
Hematologic Disorder	FORMATION_OF_THE_TERNARY_COMPLEX_AND_SUBSEQUENTLY_THE_43S_COMPLEX	REACTOME	677	33	18	2,01E-02	9,87E-01	rs6059703|rs7902136|rs2316692|rs7814028|rs751716|rs10498413|rs979971|rs4300036|rs10895987|rs1149747|rs153107|rs4911414|rs2286204|rs11002569|rs10140464|rs2913115|rs7826090|rs4255712
Hematologic Disorder	LONG_TERM_DEPRESSION	KEGG	3926	37	20	2,30E-02	9,87E-01	rs6786487|rs198178|rs9306383|rs4958671|rs2914621|rs6056252|rs7069974|rs2278964|rs8038056|rs631134|rs4684438|rs7975710|rs8121340|rs449072|rs1572507|rs3745844|rs7829816|rs11097368|rs3806777|rs6108237
Hematologic Disorder	UNFOLDED_PROTEIN_RESPONSE	REACTOME	1176	32	20	2,54E-02	9,87E-01	rs1391962|rs1406961|rs11869535|rs10275903|rs11826421|rs6809947|rs17726874|rs1030098|rs1000174|rs1331060|rs2269337|rs2583839|rs12023776|rs2075355|rs10895987|rs2302784|rs1533079|rs8054172|rs8050801|rs398708
Hematologic Disorder	PRIMARY_IMMUNODEFICIENCY	KEGG	742	28	20	2,57E-02	9,87E-01	rs933984|401754|rs4419220|rs5751691|rs11547281|rs8065836|rs17054541|rs12120762|rs874627|rs3752008|rs131387|rs179307|rs4764622|rs1075834|rs12410250|rs12127588|rs3741920|rs4294983|rs13209654|rs8079130
Hematologic Disorder	PROCESSING_OF_CAPPED_INTRONLESS_PRE_MRNA	REACTOME	274	13	6	2,63E-02	9,87E-01	rs762286|rs665714|rs3117655|rs17605098|rs17574411|rs798741
Hematologic Disorder	CLEAVAGE_OF_GROWING_TRANSCRIPT_IN_THE_TERMINATION_REGION_	REACTOME	435	17	9	2,64E-02	9,87E-01	rs9704|rs665714|rs11578832|rs3117655|rs8072724|rs17605098|rs706130|rs17574411|rs798741
Hematologic Disorder	MRNA_3_END_PROCESSING	REACTOME	320	8	5	2,64E-02	9,87E-01	rs665714|rs11578832|rs3117655|rs8072724|rs706130
Hematologic Disorder	FORMATION_OF_ATP_BY_CHEMIOSMOTIC_COUPLING	REACTOME	77	5	2	2,71E-02	9,87E-01	rs8133991|rs2834291
Hematologic Disorder	INTERACTION_BETWEEN_L1_AND_ANKYRINS	REACTOME	970	44	18	2,97E-02	9,87E-01	rs170679|rs12593435|rs2469515|rs1358533|rs9643288|rs11216788|rs4948398|rs11219218|rs1002442|rs12355908|rs10171331|rs3787116|rs857677|rs2152833|rs2300045|rs11814752|rs10509119|rs4736413
Hematologic Disorder	ACTIVATION_OF_THE_MRNA_UPON_BINDING_OF_THE_CAP_BINDING_COMPLEX_AND_EI<br>T_BINDING_TO_43S	REACTOME	780	34	19	3,17E-02	9,87E-01	rs6059703|rs7902136|rs2316692|rs7814028|rs751716|rs10498413|rs7438406|rs979971|rs4300036|rs10895987|rs1149747|rs153107|rs4911414|rs2286204|rs11002569|rs10140464|rs2913115|rs7826090|rs4255712
Hematologic Disorder	GLYCOLYSIS	REACTOME	558	13	9	3,38E-02	9,87E-01	rs11251481|rs10903939|rs2239167|rs10105118|rs7096344|rs7093049|rs2388595|rs1574318|rs11251588
Hematologic Disorder	METABOLISM_OF_NON_CODING_RNA	REACTOME	611	22	13	3,55E-02	9,87E-01	rs3026106|rs762286|rs729405|rs132834|rs11177325|rs13219815|rs102993|rs17574411|rs7705738|rs4740419|rs1125397|rs4148761|rs1065483
Hematologic Disorder	EIF_PATHWAY	BIOCARTA	158	6	5	3,63E-02	9,87E-01	rs6059703|rs7438406|rs4911414|rs1110240|rs8059570
Hematologic Disorder	LONG_TERM_POTENTIATION	KEGG	3620	39	20	3,89E-02	9,87E-01	rs6786487|rs198178|rs4958671|rs2914621|rs1876267|rs6056252|rs3799663|rs4684438|rs7975710|rs8121340|rs7595027|rs429219|rs449072|rs6108237|rs2239093|rs2239041|rs11048455|rs7008202|rs7651990|rs4073472
Hematologic Disorder	TRAFFICKING_OF_GLUR2_CONTAINING_AMPA_RECEPTORS	REACTOME	721	43	17	4,34E-02	9,87E-01	rs198178|rs4958671|rs2914621|rs449072|rs4273649|rs9914703|rs4533948|rs10515699|rs10476798|rs226434|rs9324747|rs9925433|rs174827|rs4791032|rs2012859|rs1422884|rs11859534
Hematologic Disorder	ARACHIDONIC_ACID_METABOLISM	KEGG	1356	31	20	4,55E-02	9,87E-01	rs762286|rs13130370|rs2278964|rs631134|rs7211486|rs1572507|rs11084882|rs1937919|rs3814499|rs2284198|rs1126667|rs2540514|rs6012696|rs4820315|rs11582551|rs3754205|rs1530837|rs230632|rs330746|rs4525119
Hematologic Disorder	CALCIUM_SIGNALING_PATHWAY	KEGG	9275	44	20	4,56E-02	9,87E-01	rs6786487|rs10920115|rs175338|rs2189164|rs198178|rs3753617|rs2036068|rs11922130|rs2382070|rs10008744|rs1876267|rs10506979|rs12566751|rs2689698|rs2471005|rs6056252|rs12915265|rs1578180|rs4684438|rs7975710
Hematologic Disorder	SEMAPHORIN_INTERACTIONS	REACTOME	2884	39	20	4,56E-02	9,87E-01	rs40725|rs679423|rs999426|rs1354096|rs42192|rs6882860|rs12467258|rs6702300|rs17142743|rs4844419|rs6861460|rs6869373|rs2075589|rs1651543|rs2244639|rs10085098|rs11167990|rs12120762|rs1465061|rs6446401
Hematologic Disorder	PHOSPHATIDYLINOSITOL_SIGNALING_SYSTEM	KEGG	3813	30	20	4,74E-02	9,87E-01	rs6786487|rs198178|rs3786284|rs11922130|rs2689698|rs6056252|rs17593321|rs1992838|rs4684438|rs7975710|rs8121340|rs663591|rs7595027|rs449072|rs2278210|rs928807|rs6108237|rs11048455|rs4721330|rs7651990
Hematologic Disorder	ER_PHAGOSOME_PATHWAY	REACTOME	1416	60	20	4,95E-02	9,87E-01	rs5013310|rs1701470|rs7805129|rs2526060|401754|rs4813929|rs11845708|rs698856|rs6033024|rs7200210|rs2735007|rs8078118|rs12037569|rs2596574|rs10512267|rs2245822|rs1611350|rs7126210|rs13209654|rs4268186
Hematologic Disorder	HEPARAN_SULFATE_HEPARIN_HS_GAG_METABOLISM	REACTOME	3879	29	20	5,14E-02	9,87E-01	rs2865155|rs12596791|rs4693611|rs9584410|rs1459433|rs10492629|rs7126447|rs17565276|rs8035692|rs11223958|rs319535|rs6785758|rs2445905|rs1363761|rs8068169|rs2996009|rs11729868|rs2124368|rs1363765|rs6932261
Hematologic Disorder	IGF1MTOR_PATHWAY	BIOCARTA	582	31	17	5,33E-02	9,87E-01	rs6059703|rs8038056|rs2684806|rs6586116|rs534182|rs7438406|rs2677760|rs173704|rs4983559|rs2673825|rs11953134|rs4911414|rs12901358|rs334550|rs7162314|rs1550805|rs4966013
Hematologic Disorder	INTERACTIONS_OF_VPR_WITH_HOST_CELLULAR_PROTEINS	REACTOME	480	18	12	5,62E-02	9,87E-01	rs3026106|rs729405|rs1151514|rs4678191|rs132834|rs11177325|rs13219815|rs102993|rs4740419|rs1125397|rs4148761|rs1065483
Hematologic Disorder	INTRINSIC_PATHWAY	REACTOME	264	28	10	5,62E-02	9,87E-01	rs2074437|rs12500151|rs9667326|rs4764521|rs3211719|rs11611433|rs10016252|rs6829128|rs6796803|rs2283333
Hematologic Disorder	HS_GAG_BIOSYNTHESIS	REACTOME	3154	30	20	5,75E-02	9,87E-01	rs2865155|rs12596791|rs9584410|rs1459433|rs10492629|rs7126447|rs17565276|rs8035692|rs319535|rs2445905|rs1363761|rs8068169|rs2996009|rs11729868|rs2124368|rs1363765|rs10955841|rs9584127|rs205148|rs17621552
Hematologic Disorder	RENIN_ANGIOTENSIN_SYSTEM	KEGG	467	16	5	6,30E-02	9,87E-01	rs2478545|rs6440971|rs1027045|rs989692|rs2638363
Hematologic Disorder	FORMATION_OF_TUBULIN_FOLDING_INTERMEDIATES_BY_CCT_TRIC	REACTOME	227	3	1	6,44E-02	9,87E-01	rs9405622
Hematologic Disorder	CK1_PATHWAY	BIOCARTA	759	32	20	6,63E-02	9,87E-01	rs1876267|rs2471005|rs6056252|rs6501512|rs8121340|rs1030098|rs6086473|rs2719804|rs12364283|rs2423360|rs6133635|rs12670688|rs6086495|rs2302784|rs3801969|rs2519866|rs1471107|rs2732514|rs2327025|rs4699014
Hematologic Disorder	MRNA_PROCESSING	REACTOME	1942	40	20	6,84E-02	9,87E-01	rs3026106|rs4382847|rs9704|rs665714|rs2011786|rs729405|rs7361672|rs11084882|rs11578832|rs7438406|rs132834|rs606119|rs10431307|rs3117655|rs3744473|rs7061|rs8072724|rs6454694|rs1923416|rs2261682
Hematologic Disorder	ANTIGEN_PROCESSING_CROSS_PRESENTATION	REACTOME	1601	52	20	6,88E-02	9,87E-01	rs5013310|rs1701470|rs7805129|rs2526060|401754|rs4813929|rs11845708|rs698856|rs2274064|rs6033024|rs7200210|rs2735007|rs8078118|rs12037569|rs2596574|rs10512267|rs2245822|rs1611350|rs7126210|rs13209654
Hematologic Disorder	REGULATION_OF_GLUCOKINASE_BY_GLUCOKINASE_REGULATORY_PROTEIN	REACTOME	459	14	10	6,92E-02	9,87E-01	rs3026106|rs729405|rs132834|rs11177325|rs13219815|rs102993|rs4740419|rs1125397|rs4148761|rs1065483
Hematologic Disorder	TRANSPORT_OF_RIBONUCLEOPROTEINS_INTO_THE_HOST_NUCLEUS	REACTOME	452	15	11	7,17E-02	9,87E-01	rs3026106|rs729405|rs4678191|rs132834|rs11177325|rs13219815|rs102993|rs4740419|rs1125397|rs4148761|rs1065483
Hematologic Disorder	POST_TRANSLATIONAL_PROTEIN_MODIFICATION	REACTOME	6008	47	20	7,24E-02	9,87E-01	rs485141|rs4846948|rs1391962|rs6870010|rs6669101|rs2268535|rs6095638|rs13026308|rs689423|rs1609454|rs4846904|rs7794356|rs1251484|rs10872180|rs11713682|rs1465231|rs10431384|rs2028900|rs4719095|rs11658645
Hematologic Disorder	GNRH_SIGNALING_PATHWAY	KEGG	3992	32	20	7,64E-02	9,87E-01	rs6786487|rs10920115|rs198178|rs591676|rs6056252|rs2278964|rs631134|rs4684438|rs7975710|rs8121340|rs7595027|rs4947979|rs449072|rs1572507|rs6108237|rs2239093|rs2239041|rs11048455|rs7008202|rs7651990
Hematologic Disorder	NCAM_SIGNALING_FOR_NEURITE_OUT_GROWTH	REACTOME	2480	26	20	7,67E-02	9,87E-01	rs170679|rs10920115|rs12593435|rs17579232|rs7113799|rs10787635|rs6133053|rs2102902|rs9521585|rs12892024|rs7975385|rs9327283|rs6415964|rs1465061|rs3211098|rs4915478|rs12765367|rs2303377|rs552125|rs6664294

Hematologic Disorder	GAP_JUNCTION	KEGG	4388	29	20	7,91E-02	9,87E-01	rs6786487|rs9307949|rs198178|rs10517655|rs2471005|rs6056252|rs7069974|rs2194869|rs4684438|rs7975710|rs8121340|rs4947979|rs449072|rs11591988|rs3806777|rs6108237|rs710839|rs512756|rs907647|rs11048455
Hematologic Disorder	ACTIVATION_OF_CHAPERONE_GENES_BY_XBP1S	REACTOME	761	34	19	8,19E-02	9,87E-01	rs1391962|rs1406961|rs11826421|rs6809947|rs1030098|rs12023776|rs2075355|rs10895987|rs2302784|rs8054172|rs398708|rs6803605|rs1836586|rs12487822|rs4689388|rs3846211|rs6803009|rs16856756|rs9922624
Hematologic Disorder	JAK_STAT_SIGNALING_PATHWAY	KEGG	4346	39	20	8,38E-02	9,87E-01	rs253447|rs7983710|rs12203803|rs10512687|rs3918003|rs1561928|rs2239167|rs925246|rs13398775|rs4349043|rs7026552|rs12409877|rs9814648|rs10849024|rs6541017|rs6563245|rs2302793|rs12062136|rs959662|rs9321590
Hematologic Disorder	G_BETA_GAMMA_SIGNALLING_THROUGH_PLC_BETA	REACTOME	624	30	20	8,78E-02	9,87E-01	rs4898725|rs6056252|rs8121340|rs6807740|rs16947151|rs6086473|rs1253704|rs2719804|rs874627|rs2423360|rs6133635|rs6086495|rs4901170|rs3741920|rs675327|rs2327025|rs10907192|rs4334421|rs931647|rs12704705
Hematologic Disorder	G_PROTEIN_BETA_GAMMA_SIGNALLING	REACTOME	798	29	20	8,95E-02	9,87E-01	rs4898725|rs6056252|rs8121340|rs6807740|rs16947151|rs6086473|rs1253704|rs2719804|rs874627|rs12944446|rs2423360|rs4983559|rs6133635|rs6086495|rs4901170|rs3741920|rs675327|rs2327025|rs10907192|rs4334421
Hematologic Disorder	ALZHEIMERS_DISEASE	KEGG	5029	26	20	9,18E-02	9,87E-01	rs6786487|rs10920115|rs8133991|rs11869535|rs7910977|rs1876267|rs12654078|rs6056252|rs1054174|rs4684438|rs7975710|rs8121340|rs7595027|rs13263130|rs6108237|rs11068458|rs2239093|rs2239041|rs11048455|rs7651990
Hematologic Disorder	TRANSPORT_OF_MATURE_MRNA_DERIVED_FROM_AN_INTRONLESS_TRANSCRIPT	REACTOME	524	16	12	9,70E-02	9,87E-01	rs3026106|rs729405|rs7438406|rs132834|rs11177325|rs13219815|rs102993|rs633742|rs4740419|rs1125397|rs4148761|rs1065483
Hematologic Disorder	NEP_NS2_INTERACTS_WITH_THE_CELLULAR_EXPORT_MACHINERY	REACTOME	456	15	11	1,00E-01	9,87E-01	rs3026106|rs729405|rs132834|rs11177325|rs13219815|rs12613627|rs102993|rs4740419|rs1125397|rs4148761|rs1065483
Hematologic Disorder	METABOLISM_OF_CARBOHYDRATES	REACTOME	9072	25	20	1,02E-01	9,87E-01	rs3026106|rs2865155|rs17079451|rs12596791|rs11251481|rs4693611|rs9584410|rs1459433|rs10492629|rs6095638|rs7126447|rs17565276|rs8035692|rs11223958|rs319535|rs6785758|rs10903939|rs2445905|rs1363761|rs2239167
Hematologic Disorder	CARDIACEGF_PATHWAY	BIOCARTA	1289	38	20	1,03E-01	9,87E-01	rs198178|rs10008744|rs2478545|rs1561928|rs4947979|rs449072|rs748289|rs1870510|rs2302793|rs9471684|rs868589|rs9914703|rs2110290|rs1278302|rs6681035|rs2235360|rs6470648|rs1278396|rs2638363|rs1278300
Hematologic Disorder	TRANSLATION	REACTOME	1803	25	20	1,07E-01	9,87E-01	rs6059703|rs7902136|rs1701470|rs7805129|rs2316692|rs7814028|rs751716|rs7257982|rs10498413|rs7438406|rs4131086|rs11755449|rs979971|rs4300036|rs10895987|rs6553943|rs9928312|rs1149747|rs12620516|rs153107
Hematologic Disorder	ACETYLCHOLINE_BINDING_AND_DOWNSTREAM_EVENTS	REACTOME	242	3	2	1,09E-01	9,87E-01	rs12915265|rs1550097
Hematologic Disorder	GLYCOSAMINOGLYCAN_METABOLISM	REACTOME	6096	29	20	1,11E-01	9,87E-01	rs2865155|rs17079451|rs12596791|rs4693611|rs9584410|rs1459433|rs10492629|rs6095638|rs7126447|rs17565276|rs8035692|rs11223958|rs319535|rs6785758|rs1363761|rs2445905|rs8068169|rs9498175|rs9967331|rs2996009
Hematologic Disorder	SELENOAMINO_ACID_METABOLISM	KEGG	742	29	20	1,11E-01	9,87E-01	rs762286|rs819136|rs12142041|rs3124455|rs10077775|rs2232248|rs2028900|rs2522420|rs2915850|rs520002|rs2522425|rs1946198|rs12443981|rs2993763|rs7704440|rs10068900|rs158850|rs6088466|rs2755427|rs7905664
Hematologic Disorder	CHOLESTEROL_BIOSYNTHESIS	REACTOME	259	12	5	1,14E-01	9,87E-01	rs1340865|rs2684874|rs3750729|rs1129195|rs13358429
Hematologic Disorder	NRAGE_SIGNALS_DEATH_THROUGH_JNK	REACTOME	1919	49	20	1,16E-01	9,87E-01	rs11630971|rs12480096|rs6063285|rs2985700|rs163819|rs8034125|rs6019300|rs13405741|rs165095|rs6012542|rs9290452|rs2334915|rs1016088|rs4459929|rs1483578|rs13074616|rs2834291|rs2248656|rs8024521|rs10152417
Hematologic Disorder	SIGNALING_BY_RHO_GTPASES	REACTOME	4877	34	20	1,18E-01	9,87E-01	rs11630971|rs12480096|rs10150350|rs673377|rs1348536|rs12543681|rs6063285|rs226130|rs5751636|rs2985700|rs163819|rs8034125|rs743584|rs6019300|rs2675181|rs11096770|rs165095|rs10499162|rs11084882|rs6012542
Hematologic Disorder	VEGF_PATHWAY	BIOCARTA	883	34	20	1,23E-01	9,87E-01	rs6059703|rs198178|rs449072|rs9914703|rs2677760|rs6458350|rs173704|rs4767886|rs2235360|rs708030|rs11953134|rs4262039|rs4911414|rs12204488|rs10517342|rs10085025|rs9925433|rs174827|rs3025010|rs4791032
Hematologic Disorder	INTEGRATION_OF_PROVIRUS	REACTOME	122	7	2	1,25E-01	9,87E-01	rs1151514|rs2391626
Hematologic Disorder	SPHINGOLIPID_METABOLISM	KEGG	1101	32	20	1,28E-01	9,87E-01	rs17752182|rs11206808|rs947007|rs12081589|rs3011777|rs4935597|rs11810204|rs3892357|rs2863973|rs1362720|rs2167239|rs7069860|rs2303147|rs7018784|rs6588636|rs11938628|rs11624137|rs1234367|rs9914387|rs17469007
Hematologic Disorder	ECM_RECEPTOR_INTERACTION	KEGG	3602	37	20	1,30E-01	9,87E-01	rs1537722|rs1898722|rs17594552|rs3212588|rs7794418|rs10874639|rs12563833|rs17275376|rs12674323|rs10038|rs6861460|rs10798331|rs9521585|rs16877442|rs9765848|rs2124368|rs1271449|rs17013181|rs4764521|rs12523864
Hematologic Disorder	FMLP_PATHWAY	BIOCARTA	1148	41	20	1,34E-01	9,87E-01	rs1876267|rs6056252|rs1992838|rs8121340|rs7595027|rs7193701|rs2274064|rs6086473|rs748938|rs2055226|rs2719804|rs2238628|rs4802861|rs2423360|rs3760201|rs2947864|rs6133635|rs6086495|rs13196204|rs1555147
Hematologic Disorder	NEGATIVE_REGULATION_OF_FGFR_SIGNALING	REACTOME	1536	51	20	1,35E-01	9,87E-01	rs7983710|rs1888057|rs2174834|rs2992024|rs17072214|rs12523052|rs4349043|rs513320|rs1078363|rs6563245|rs541682|rs1054743|rs9510129|rs3806929|rs1417839|rs10105118|rs9805578|rs12220387|rs1924210|rs10477181
Hematologic Disorder	SHH_PATHWAY	BIOCARTA	679	34	20	1,36E-01	9,87E-01	rs17193211|rs6501512|rs1990135|rs4448343|rs1030098|rs776501|rs11979793|rs12670688|rs12686467|rs7777688|rs2302784|rs3801969|rs334550|rs966790|rs3801159|rs17829622|rs12530988|rs1531284|rs13243079|rs10228290
Hematologic Disorder	INTEGRIN_CELL_SURFACE_INTERACTIONS	REACTOME	2614	35	20	1,37E-01	9,87E-01	rs1537722|rs1898722|rs3212588|rs8133991|rs3769309|rs7202877|rs17275376|rs6861460|rs9521585|rs1271449|rs17013181|rs1148853|rs4764521|rs12523864|rs3211098|rs693754|rs1360290|rs4983559|rs11102314|rs552125
Hematologic Disorder	ARAP_PATHWAY	BIOCARTA	292	15	9	1,39E-01	9,87E-01	rs1406961|rs11191274|rs6501243|rs11956308|rs11574849|rs6090919|rs6472296|rs12692379|rs3758549
Hematologic Disorder	MEF2D_PATHWAY	BIOCARTA	684	36	20	1,42E-01	9,87E-01	rs198178|rs1876267|rs738809|rs7595027|rs449072|rs748938|rs2055226|rs9914703|rs1555147|rs1153942|rs2732514|rs4699014|rs7598661|rs4872495|rs9925433|rs174827|rs4791032|rs16842040|rs815804|rs2300502
Hematologic Disorder	CCR3_PATHWAY	BIOCARTA	939	42	20	1,42E-01	9,87E-01	rs198178|rs6056252|rs1992838|rs8121340|rs449072|rs6086473|rs2719804|rs9914703|rs2423360|rs6133635|rs6086495|rs17735961|rs2041190|rs17419409|rs2327025|rs12827287|rs10907192|rs9925433|rs174827|rs4791032
Hematologic Disorder	TRAFFICKING_OF_AMPA_RECEPTORS	REACTOME	1132	50	20	1,43E-01	9,87E-01	rs198178|rs4958671|rs2914621|rs449072|rs4273649|rs9914703|rs4533948|rs12535537|rs1741949|rs13189947|rs6842886|rs10476798|rs2075067|rs2238498|rs718501|rs226434|rs916891|rs9324747|rs1557809|rs9925433
Hematologic Disorder	NO1_PATHWAY	BIOCARTA	1482	43	20	1,46E-01	9,87E-01	rs3753617|rs7997827|rs6501512|rs7069974|rs7595027|rs4659495|rs710839|rs7907343|rs1030098|rs10996331|rs2454566|rs2055226|rs6673700|rs6458350|rs4983559|rs1863961|rs12670688|rs17093887|rs11045210|rs4262039
Hematologic Disorder	PROCESSING_OF_CAPPED_INTRON_CONTAINING_PRE_MRNA	REACTOME	1547	38	20	1,47E-01	9,87E-01	rs3026106|rs9704|rs665714|rs2011786|rs729405|rs7361672|rs11084882|rs11578832|rs7438406|rs132834|rs606119|rs3117655|rs3744473|rs7061|rs8072724|rs2261682|rs11177325|rs706130|rs8070091|rs1782322
Hematologic Disorder	CELL_DEATH_SIGNALLING_VIA_NRAGE_NRIF_AND_NADE	REACTOME	2069	49	20	1,47E-01	9,87E-01	rs11630971|rs12480096|rs6063285|rs2985700|rs163819|rs8034125|rs6019300|rs13405741|rs165095|rs6012542|rs9290452|rs3850839|rs2334915|rs1016088|rs4459929|rs1483578|rs13074616|rs2834291|rs2248656|rs8024521
Hematologic Disorder	ETHER_LIPID_METABOLISM	KEGG	1041	49	20	1,48E-01	9,87E-01	rs11206808|rs947007|rs12081589|rs2278964|rs631134|rs1572507|rs11810204|rs4820315|rs11582551|rs1530837|rs230632|rs330746|rs330725|rs6588636|rs3743016|rs2292023|rs776797|rs17469007|rs4431886|rs2522137
Hematologic Disorder	INTESTINAL_IMMUNE_NETWORK_FOR_IGA_PRODUCTION	KEGG	1392	31	20	1,51E-01	9,87E-01	rs933984|rs2284191|rs10508199|rs4419220|rs11591534|rs8065836|rs13410482|rs4245843|rs3752008|rs28772724|rs4722178|rs9990563|rs17212090|rs3130192|rs17488134|rs12106790|rs7139897|rs9268911|rs4294983|rs13209654
Hematologic Disorder	STRIATED_MUSCLE_CONTRACTION	REACTOME	453	26	12	1,51E-01	9,87E-01	rs9894327|rs7481661|rs2244095|rs10982444|rs11086023|rs907609|rs365990|rs1989811|rs7602715|rs10817759|rs178638|rs2035714
Hematologic Disorder	FOCAL_ADHESION	KEGG	7733	26	20	1,52E-01	9,87E-01	rs1537722|rs1898722|rs9307949|rs198178|rs11630971|rs12591920|rs3212588|rs7794418|rs707598|rs10517655|rs10874639|rs7202877|rs12563833|rs17275376|rs11826421|rs2483852|rs2194869|rs12674323|rs8038056|rs10038
Hematologic Disorder	CHYLOMICRON_MEDIATED_LIPID_TRANSPORT	REACTOME	435	13	7	1,54E-01	9,87E-01	rs7172821|rs2124368|rs1057613|rs2242064|rs4656994|rs10917070|rs875919
Hematologic Disorder	REGULATION_OF_INSULIN_SECRETION_BY_GLUCAGON_LIKE_PEPTIDE1	REACTOME	1388	37	20	1,58E-01	9,87E-01	rs3769309|rs4898725|rs6501512|rs7975710|rs6807740|rs11048455|rs7008202|rs7975385|rs1030098|rs16947151|rs953672|rs1253704|rs16974215|rs874627|rs11102314|rs12670688|rs4901170|rs10495673|rs7303557|rs2302784
Hematologic Disorder	PROTEIN_EXPORT	KEGG	750	39	20	1,59E-01	9,87E-01	rs1701470|rs3114815|rs7805129|rs12374709|rs4131086|rs38758|rs12531640|rs6553943|rs2523239|rs10512267|rs4653669|rs4268186|rs12891079|rs10799322|rs10953644|rs2168967|rs12705676|rs12327532|rs12531989|rs2966404
Hematologic Disorder	AMI_PATHWAY	BIOCARTA	574	31	14	1,60E-01	9,87E-01	rs4610017|rs9521585|rs1040172|rs3211098|rs552125|rs3211719|rs10203300|rs4889545|rs9559771|rs12476572|rs10492497|rs1556122|rs1247571|rs1652508
Hematologic Disorder	INTEGRIN_ALPHAIIB_BETA3_SIGNALING	REACTOME	779	28	20	1,62E-01	9,87E-01	rs1898722|rs3769309|rs7202877|rs1148853|rs4764521|rs4983559|rs11102314|rs2038171|rs16967035|rs3782908|rs10492302|rs11177325|rs4744514|rs2888586|rs1537741|rs10993709|rs11905013|rs290267|rs2283333|rs3820852
Hematologic Disorder	ABCA_TRANSPORTERS_IN_LIPID_HOMEOSTASIS	REACTOME	464	14	7	1,62E-01	9,87E-01	rs7419736|rs4563284|rs915843|rs1749541|rs425215|rs6744831|rs4148134
Hematologic Disorder	CTLA4_PATHWAY	BIOCARTA	662	26	14	1,63E-01	9,87E-01	rs933984|rs4419220|rs2677760|rs173704|rs858492|rs11953134|rs12106790|rs12564899|rs9268911|rs412805|rs4294983|rs3096735|rs1550805|rs16967789
Hematologic Disorder	P75_NTR_RECEPTOR_MEDIATED_SIGNALLING	REACTOME	2669	44	20	1,64E-01	9,87E-01	rs11630971|rs12480096|rs6063285|rs11072660|rs2985700|rs11638112|rs163819|rs8034125|rs6019300|rs13405741|rs165095|rs6012542|rs9290452|rs3850839|rs2334915|rs1016088|rs4459929|rs1483578|rs13074616|rs2834291
Hematologic Disorder	PHASE1_FUNCTIONALIZATION_OF_COMPOUNDS	REACTOME	1515	41	20	1,64E-01	9,87E-01	rs1741284|rs13130370|rs2276332|rs4405788|rs2284198|rs6012696|rs4466436|rs7172632|rs3754205|rs7069114|rs1826909|rs639434|rs1741318|rs10912598|rs2654846|rs12125747|rs17642708|rs1126671|rs4144915|rs1934967
Hematologic Disorder	ANTIGEN_PROCESSING_AND_PRESENTATION	KEGG	1754	54	20	1,71E-01	9,87E-01	rs5013310|rs2284191|rs10772294|401754|rs11547281|rs10158384|rs17054541|rs874627|rs28772724|rs12310310|rs2735007|rs4764622|rs2596574|rs4538747|rs3130192|rs3741920|rs2245822|rs9268911|rs4313734|rs7037389
Hematologic Disorder	NOS1_PATHWAY	BIOCARTA	1133	38	20	1,71E-01	9,87E-01	rs198178|rs1876267|rs6501512|rs7595027|rs449072|rs11068458|rs1030098|rs1650397|rs2055226|rs9914703|rs8058978|rs12670688|rs2302784|rs2284428|rs3801969|rs2268105|rs2732514|rs7202071|rs4699014|rs7598661
Hematologic Disorder	BCR_PATHWAY	BIOCARTA	1053	41	20	1,72E-01	9,87E-01	rs198178|rs1876267|rs7595027|rs449072|rs7829816|rs7193701|rs1148853|rs748938|rs2055226|rs9914703|rs6681035|rs2235360|rs13196204|rs1075834|rs17718517|rs1555147|rs4744514|rs2732514|rs2888586|rs10993709
Hematologic Disorder	GPCR_PATHWAY	BIOCARTA	1102	36	20	1,73E-01	9,87E-01	rs198178|rs1876267|rs6501512|rs7595027|rs449072|rs7193701|rs1030098|rs748938|rs2055226|rs9914703|rs6681035|rs2235360|rs12670688|rs2302784|rs3801969|rs1555147|rs2732514|rs4699014|rs7598661|rs10907192
Hematologic Disorder	GLYCOSAMINOGLYCAN_BIOSYNTHESIS_HEPARAN_SULFATE	KEGG	2273	39	20	1,73E-01	9,87E-01	rs2865155|rs9584410|rs7126447|rs17565276|rs8035692|rs11223958|rs2445905|rs8068169|rs2996009|rs11729868|rs6932261|rs10955841|rs17621552|rs2451157|rs2218348|rs2187467|rs6835462|rs11644921|rs2169860|rs923997
Hematologic Disorder	CALCINEURIN_PATHWAY	BIOCARTA	895	42	20	1,74E-01	9,87E-01	rs198178|rs1876267|rs7595027|rs449072|rs7193701|rs748938|rs2055226|rs9914703|rs12213076|rs2235360|rs9372365|rs1555147|rs2732514|rs4699014|rs7598661|rs4872495|rs9925433|rs174827|rs4791032|rs815804
Hematologic Disorder	3_UTR_MEDIATED_TRANSLATIONAL_REGULATION	REACTOME	1235	29	20	1,75E-01	9,87E-01	rs6059703|rs7902136|rs2316692|rs7814028|rs751716|rs7257982|rs10498413|rs7438406|rs979971|rs4300036|rs10895987|rs9928312|rs1149747|rs12620516|rs153107|rs4911414|rs2430949|rs2286204|rs11002569|rs10140464
Hematologic Disorder	MELANOGENESIS	KEGG	4077	39	20	1,75E-01	9,87E-01	rs6059709|rs603401|rs198178|rs680997|rs6056252|rs7430957|rs12573128|rs8121340|rs7595027|rs449072|rs1338423|rs6108237|rs7008202|rs7975385|rs4732279|rs6086473|rs953672|rs2055226|rs9471684|rs2719804
Hematologic Disorder	G_PROTEIN_ACTIVATION	REACTOME	703	27	20	1,76E-01	9,87E-01	rs9306383|rs4898725|rs6807740|rs6003495|rs16947151|rs1253704|rs874627|rs4901170|rs6941251|rs3741920|rs2617841|rs675327|rs6515016|rs10907192|rs4334421|rs931647|rs12704705|rs11157866|rs6045824|rs2058459
Hematologic Disorder	PTEN_PATHWAY	BIOCARTA	544	26	13	1,76E-01	9,87E-01	rs7202877|rs11826421|rs6586116|rs534182|rs2677760|rs173704|rs4983559|rs2673825|rs11953134|rs2888586|rs1550805|rs16967789|rs1102737
Hematologic Disorder	PYK2_PATHWAY	BIOCARTA	752	35	20	1,78E-01	9,87E-01	rs198178|rs7202877|rs7595027|rs449072|rs2055226|rs2529737|rs9914703|rs3760201|rs6681035|rs2235360|rs13196204|rs2888586|rs7598661|rs11905013|rs9925433|rs174827|rs4791032|rs815804|rs2300502|rs16967789
Hematologic Disorder	SPHINGOLIPID_DE_NOVO_BIOSYNTHESIS	REACTOME	1020	34	20	1,84E-01	9,87E-01	rs17752182|rs11206808|rs947007|rs12081589|rs3011777|rs4935597|rs11810204|rs29196|rs7069860|rs10148024|rs2303147|rs7018784|rs8093795|rs6588636|rs11844637|rs9914387|rs17469007|rs4431886|rs2333660|rs11790131
Hematologic Disorder	PLC_BETA_MEDIATED_EVENTS	REACTOME	2313	29	20	1,85E-01	9,87E-01	rs9306383|rs2036068|rs6056252|rs6501512|rs7975710|rs8121340|rs7595027|rs429219|rs1572507|rs6108237|rs11048455|rs7008202|rs7975385|rs1030098|rs6003495|rs6086473|rs953672|rs2055226|rs2719804|rs9914703
Hematologic Disorder	CDMAC_PATHWAY	BIOCARTA	1018	41	20	1,86E-01	9,87E-01	rs198178|rs6056252|rs1561928|rs8121340|rs449072|rs6086473|rs2302793|rs2719804|rs9914703|rs2423360|rs6681035|rs6133635|rs6470648|rs6086495|rs4733616|rs2327025|rs4637783|rs6995630|rs9925433|rs17103133
Hematologic Disorder	RIG_I_MDA5_MEDIATED_INDUCTION_OF_IFN_ALPHA_BETA_PATHWAYS	REACTOME	1129	31	20	1,86E-01	9,87E-01	rs2070600|rs12599479|rs744038|rs821466|rs11191274|rs13116389|rs1394839|rs621277|rs6133067|rs803523|rs12052592|rs1040411|rs10970986|rs1404358|rs12922698|rs11574849|rs11599360|rs7037389|rs2133165|rs461626
Hematologic Disorder	BIOSYNTHESIS_OF_UNSATURATED_FATTY_ACIDS	KEGG	463	25	15	1,87E-01	9,87E-01	rs1393492|rs17620632|rs7656124|rs4665830|rs6599263|rs2816373|rs11121611|rs11037685|rs1502593|rs1429146|rs17006135|rs12498468|rs174532|rs4908874|rs17041597
Hematologic Disorder	G0_AND_EARLY_G1	REACTOME	258	21	9	1,89E-01	9,87E-01	rs17193211|rs4899503|rs7015841|rs6577059|rs2448343|rs10425066|rs17068405|rs11617870|rs13706
Hematologic Disorder	TRANSPORT_OF_MATURE_TRANSCRIPT_TO_CYTOPLASM	REACTOME	693	22	16	1,89E-01	9,87E-01	rs3026106|rs729405|rs11578832|rs7438406|rs132834|rs8072724|rs11177325|rs706130|rs13219815|rs102993|rs633742|rs798741|rs4740419|rs1125397|rs4148761|rs1065483
Hematologic Disorder	CYTOKINE_CYTOKINE_RECEPTOR_INTERACTION	KEGG	6425	32	20	1,90E-01	9,87E-01	rs9307949|rs12203803|rs7662504|rs159254|rs7830593|rs10512687|rs10508199|rs10517655|rs17798043|rs4277528|rs10912560|rs3918003|rs7235828|rs7617478|rs6685520|rs11591534|rs2194869|rs948758|rs925246|rs2237432
Hematologic Disorder	MUSCLE_CONTRACTION	REACTOME	1023	43	20	1,93E-01	9,87E-01	rs9894327|rs7481661|rs7914896|rs6861460|rs7595027|rs2244095|rs2055226|rs10982444|rs11866891|rs4934433|rs16967494|rs11086023|rs907609|rs820360|rs10229310|rs7907084|rs7598661|rs7779600|rs365990|rs17169635
Hematologic Disorder	DORSO_VENTRAL_AXIS_FORMATION	KEGG	1149	34	20	1,93E-01	9,87E-01	rs2070600|rs9787850|rs12130718|rs4947979|rs922305|rs9959145|rs2110290|rs7235783|rs1648178|rs2213176|rs4759659|rs9977361|rs1107869|rs6487465|rs11979158|rs7963072|rs2888586|rs649944|rs6697258|rs2416791
Hematologic Disorder	BCELLSURVIVAL_PATHWAY	BIOCARTA	500	13	8	1,95E-01	9,87E-01	rs6861460|rs17304039|rs2677760|rs173704|rs4983559|rs11953134|rs17103133|rs1550805
Hematologic Disorder	AMYLOIDS	REACTOME	972	72	20	1,95E-01	9,87E-01	rs12047264|rs12203803|rs11177603|rs2124368|rs1394839|rs6532196|rs1009181|rs4699390|rs198853|rs1033440|rs12306121|rs461626|rs10917070|rs2830076|rs8132767|rs11932021|rs1383436|rs6939589|rs7950019|rs2255235
Hematologic Disorder	POST_CHAPERONIN_TUBULIN_FOLDING_PATHWAY	REACTOME	254	15	4	1,95E-01	9,87E-01	rs629246|rs9405622|rs8074277|rs10440685
Hematologic Disorder	CREB_PATHWAY	BIOCARTA	969	37	20	1,98E-01	9,87E-01	rs198178|rs6501512|rs449072|rs12892024|rs1030098|rs9914703|rs12535537|rs2677760|rs173704|rs4983559|rs6842886|rs12670688|rs2075067|rs11953134|rs2302784|rs13196204|rs3801969|rs2888586|rs9925433|rs174827
Hematologic Disorder	HOST_INTERACTIONS_OF_HIV_FACTORS	REACTOME	2270	30	20	2,00E-01	9,87E-01	rs3026106|rs2526060|rs6899021|401754|rs4813929|rs1053079|rs729405|rs11845708|rs698856|rs1151514|rs1420320|rs1465061|rs6033024|rs874627|rs4678191|rs3806929|rs7200210|rs132834|rs978914|rs8078118
Hematologic Disorder	VIBRIO_CHOLERAE_INFECTION	KEGG	1518	40	20	2,05E-01	9,87E-01	rs198178|rs1701470|rs7805129|rs12188497|rs449072|rs2283200|rs7469887|rs9914703|rs11974545|rs2601773|rs1080199|rs785437|rs2237875|rs2106467|rs7108478|rs2235360|rs869896|rs10082815|rs231918|rs10512267
Hematologic Disorder	ENDOCYTOSIS	KEGG	6484	50	20	2,06E-01	9,87E-01	rs3026106|rs1406961|rs5013310|rs673377|rs1348536|rs928670|rs17064520|rs17584591|rs767589|rs3768926|rs7091519|rs16869484|rs8038056|rs11096770|rs4947979|rs6801338|rs8043408|rs6566955|rs3740691|rs1025229
Hematologic Disorder	INOSITOL_PHOSPHATE_METABOLISM	KEGG	2355	29	20	2,07E-01	9,87E-01	rs3786284|rs11922130|rs2689698|rs6056252|rs17593321|rs1992838|rs663591|rs8121340|rs2278210|rs928807|rs6108237|rs11702774|rs6541017|rs6086473|rs2719804|rs6586116|rs4633807|rs534182|rs7922014|rs4121647
Hematologic Disorder	MCALPAIN_PATHWAY	BIOCARTA	543	25	15	2,07E-01	9,87E-01	rs6501512|rs6861460|rs4947979|rs1030098|rs2110290|rs4767886|rs12670688|rs2302784|rs3801969|rs1153942|rs820360|rs11979158|rs1195962|rs16842040|rs13243079
Hematologic Disorder	PENTOSE_AND_GLUCURONATE_INTERCONVERSIONS	KEGG	874	22	7	2,08E-01	9,87E-01	rs696256|rs2104712|rs4072113|rs2736486|rs7788953|rs6820734|rs9530
Hematologic Disorder	ARRHYTHMOGENIC_RIGHT_VENTRICULAR_CARDIOMYOPATHY_ARVC	KEGG	5871	35	20	2,11E-01	9,87E-01	rs10920115|rs10520308|rs3753617|rs3212588|rs6480244|rs7241357|rs9510495|rs11715062|rs17275376|rs10174961|rs10045068|rs1467913|rs12573128|rs6861460|rs2102902|rs17247184|rs4258819|rs4659495|rs6751684|rs1338423
Hematologic Disorder	ETS_PATHWAY	BIOCARTA	682	24	19	2,11E-01	9,87E-01	rs9787850|rs6689823|rs1242991|rs906304|rs1648178|rs6681035|rs9977361|rs919751|rs1107869|rs433192|rs837492|rs455328|rs17103133|rs2129301|rs7417586|rs10846681|rs565536|rs837493|rs3733678
Hematologic Disorder	PLATELET_AGGREGATION_PLUG_FORMATION	REACTOME	942	28	20	2,11E-01	9,87E-01	rs1898722|rs3769309|rs7202877|rs1148853|rs4764521|rs10885123|rs4983559|rs11102314|rs2038171|rs16967035|rs3782908|rs10492302|rs11177325|rs4744514|rs2888586|rs1537741|rs10993709|rs11905013|rs290267|rs2283333
Hematologic Disorder	VIF_MEDIATED_DEGRADATION_OF_APOBEC3G	REACTOME	738	31	19	2,12E-01	9,87E-01	rs2526060|401754|rs4813929|rs1053079|rs11845708|rs698856|rs6033024|rs7200210|rs8078118|rs12037569|rs7126210|rs13209654|rs4888688|rs963267|rs9912225|rs755553|rs8009527|rs12447239|rs241429
Hematologic Disorder	CLASS_B_2_SECRETIN_FAMILY_RECEPTORS	REACTOME	2257	35	20	2,13E-01	9,87E-01	rs467439|rs603401|rs4898725|rs680997|rs7976725|rs6807740|rs4448343|rs4527134|rs2732794|rs16947151|rs9916772|rs1253704|rs2286362|rs874627|rs351359|rs17083085|rs10492361|rs3793228|rs12686467|rs4901170
Hematologic Disorder	INHIBITION_OF_INSULIN_SECRETION_BY_ADRENALINE_NORADRENALINE	REACTOME	789	25	20	2,14E-01	9,87E-01	rs4898725|rs6807740|rs7975385|rs16947151|rs6415964|rs953672|rs1253704|rs10885123|rs874627|rs12765367|rs4901170|rs3741920|rs2617841|rs675327|rs10907192|rs4334421|rs931647|rs12704705|rs10508561|rs11157866
Hematologic Disorder	FGFR_LIGAND_BINDING_AND_ACTIVATION	REACTOME	984	40	20	2,15E-01	9,87E-01	rs1888057|rs2174834|rs2992024|rs17072214|rs12523052|rs513320|rs1078363|rs541682|rs9510129|rs3806929|rs9805578|rs12220387|rs1924210|rs10477181|rs3117972|rs8000805|rs152523|rs871346|rs11167790|rs17287322
Hematologic Disorder	BIOLOGICAL_OXIDATIONS	REACTOME	3568	35	20	2,18E-01	9,87E-01	rs1741284|rs762286|rs13130370|rs819136|rs316156|rs2276332|rs4405788|rs2284198|rs1584783|rs6497501|rs10077775|rs6012696|rs2028900|rs4466436|rs7172632|rs2522420|rs2915850|rs4072113|rs3754205|rs7069114
Hematologic Disorder	VASCULAR_SMOOTH_MUSCLE_CONTRACTION	KEGG	5845	31	20	2,25E-01	9,87E-01	rs6786487|rs10920115|rs198178|rs10008744|rs2288839|rs6056252|rs7069974|rs2278964|rs7133677|rs631134|rs4684438|rs7975710|rs8121340|rs7595027|rs449072|rs1572507|rs3806777|rs6108237|rs2239093|rs2239041
Hematologic Disorder	SPPA_PATHWAY	BIOCARTA	1428	43	20	2,26E-01	9,87E-01	rs198178|rs6056252|rs6861460|rs8121340|rs449072|rs1572507|rs2284198|rs6086473|rs1148853|rs2719804|rs9914703|rs2423360|rs6133635|rs230632|rs330746|rs6086495|rs330725|rs776797|rs4744514|rs10993709
Hematologic Disorder	GLYCEROPHOSPHOLIPID_METABOLISM	KEGG	2549	32	20	2,26E-01	9,87E-01	rs2070600|rs2278952|rs11206808|rs947007|rs12081589|rs2278964|rs631134|rs1572507|rs12572167|rs2352106|rs1975802|rs4721330|rs11810204|rs2358073|rs16947151|rs7220132|rs10508915|rs12492576|rs4820315|rs9668071
Hematologic Disorder	PENTOSE_PHOSPHATE_PATHWAY	KEGG	616	12	9	2,27E-01	9,87E-01	rs11251481|rs10903939|rs7093049|rs10034495|rs2388595|rs1574318|rs2071931|rs11251588|rs7800421
Hematologic Disorder	CCR5_PATHWAY	BIOCARTA	696	36	20	2,28E-01	9,87E-01	rs198178|rs11591534|rs7595027|rs449072|rs2055226|rs9914703|rs4245843|rs6681035|rs2235360|rs13196204|rs17488134|rs7598661|rs9925433|rs17103133|rs174827|rs4791032|rs815804|rs2300502|rs1147879|rs11859534
Hematologic Disorder	GLUTATHIONE_CONJUGATION	REACTOME	407	21	12	2,28E-01	9,87E-01	rs762286|rs316156|rs1584783|rs11807|rs2235970|rs3796633|rs10772921|rs7527279|rs7747120|rs8192100|rs1027473|rs6088635
Hematologic Disorder	LIPID_DIGESTION_MOBILIZATION_AND_TRANSPORT	REACTOME	1389	39	20	2,30E-01	9,87E-01	rs1914172|rs7172821|rs4563284|rs821466|rs2796833|rs6806526|rs4073472|rs2124368|rs1057613|rs2482430|rs7916688|rs7200210|rs915841|rs3733156|rs4708|rs2027401|rs11611433|rs2242064|rs4656994|rs425215
Hematologic Disorder	ADP_SIGNALLING_THROUGH_P2RY1	REACTOME	863	31	20	2,30E-01	9,87E-01	rs4898725|rs6807740|rs1572507|rs16947151|rs1253704|rs2296781|rs874627|rs2238628|rs230632|rs330746|rs4901170|rs330725|rs13196204|rs776797|rs3741920|rs675327|rs10907192|rs11905013|rs4334421|rs1843854

Hematologic Disorder	FCER1_PATHWAY	BIOCARTA	1564	45	20	2,32E-01	9,87E-01	rs198178|rs1876267|rs7595027|rs449072|rs1572507|rs7829816|rs7193701|rs7548864|rs1148853|rs748938|rs2055226|rs2529737|rs2677760|rs173704|rs6681035|rs230632|rs2235360|rs330746|rs330725|rs11953134
Hematologic Disorder	ANTIGEN_ACTIVATES_B_CELL_RECEPTOR_LEADING_TO_GENERATION_OF_SECOND_MES	REACTOME	1299	39	20	2,32E-01	9,87E-01	rs727152|rs750525|rs16929712|rs7975710|rs7595027|rs7829816|rs11048455|rs6541017|rs1148853|rs1465061|rs2055226|rs173704|rs6414232|rs2235360|rs11953134|rs4240896|rs7303557|rs1075834|rs4963998|rs17718517
Hematologic Disorder	TRANS_GOLGI_NETWORK_VESICLE_BUDDING	REACTOME	1106	33	20	2,32E-01	9,87E-01	rs4794873|rs7738190|rs2289599|rs3829893|rs13216265|rs11191274|rs6045109|rs1890020|rs1130861|rs6733550|rs419588|rs3731828|rs4075688|rs4961604|rs12037569|rs11574849|rs2022345|rs757718|rs2283852|rs2271050
Hematologic Disorder	VIRAL_MYOCARDITIS	KEGG	2750	51	20	2,37E-01	9,87E-01	rs9894327|rs5013310|rs2284191|rs9510495|rs2074876|rs6672395|rs10045068|rs1651543|rs4740366|rs7630352|rs1465061|rs12523864|rs2413398|rs4705000|rs11866891|rs28772724|rs2583839|rs9510702|rs2735007|rs614367
Hematologic Disorder	PEROXISOMAL_LIPID_METABOLISM	REACTOME	435	31	10	2,38E-01	9,87E-01	rs7310157|rs2555491|rs6599263|rs4931163|rs3782513|rs2349566|rs7129719|rs11605276|rs2278167|rs10500795
Hematologic Disorder	SYNTHESIS_AND_INTERCONVERSION_OF_NUCLEOTIDE_DI_AND_TRIPHOSPHATES	REACTOME	306	15	8	2,39E-01	9,87E-01	rs10040697|rs2978663|rs8190893|rs2198511|rs3817657|rs2853229|rs12031867|rs6556882
Hematologic Disorder	ENOS_ACTIVATION_AND_REGULATION	REACTOME	336	22	8	2,41E-01	9,87E-01	rs7595027|rs710839|rs2055226|rs4983559|rs12236525|rs7598661|rs815804|rs2300502
Hematologic Disorder	ACTIVATION_OF_NF_KAPPAB_IN_B_CELLS	REACTOME	1396	36	20	2,43E-01	9,87E-01	rs198178|rs2526060|401754|rs4813929|rs6461796|rs449072|rs11845708|rs698856|rs6033024|rs13217057|rs7200210|rs8078118|rs2141484|rs12037569|rs3802727|rs10485101|rs7126210|rs13209654|rs11767716|rs4888688
Hematologic Disorder	NUCLEOTIDE_LIKE_PURINERGIC_RECEPTORS	REACTOME	272	20	8	2,45E-01	9,87E-01	rs11687066|rs10857898|rs11235676|rs3394|rs2510881|rs2364813|rs12139261|rs1920397
Hematologic Disorder	HYPERTROPHIC_CARDIOMYOPATHY_HCM	KEGG	4149	29	20	2,46E-01	9,87E-01	rs10920115|rs3753617|rs3212588|rs12540943|rs9510495|rs11715062|rs17275376|rs10045068|rs1467913|rs6861460|rs2102902|rs17247184|rs4659495|rs1881628|rs2239093|rs6806680|rs2239041|rs37070|rs7975385|rs2970533
Hematologic Disorder	GLUTATHIONE_METABOLISM	KEGG	683	31	19	2,55E-01	9,87E-01	rs762286|rs316156|rs2978663|rs11084882|rs1584783|rs8190893|rs11807|rs3817657|rs2235970|rs3796633|rs2853229|rs10772921|rs7527279|rs7747120|rs6464540|rs8192100|rs1027473|rs6088635|rs1029328
Hematologic Disorder	L1CAM_INTERACTIONS	REACTOME	3914	38	20	2,57E-01	9,87E-01	rs170679|rs12593435|rs3212588|rs7113799|rs10182735|rs11970312|rs2655198|rs17275376|rs6861460|rs2469515|rs4947979|rs1358533|rs12892024|rs9310462|rs9643288|rs11216788|rs4948398|rs550840|rs6869991|rs2110290
Hematologic Disorder	LIPOPROTEIN_METABOLISM	REACTOME	1037	39	20	2,57E-01	9,87E-01	rs1914172|rs7172821|rs821466|rs2796833|rs2124368|rs1057613|rs2482430|rs7916688|rs7200210|rs915841|rs4708|rs2027401|rs11611433|rs2242064|rs4656994|rs425215|rs9665553|rs10917070|rs875919|rs2271463
Hematologic Disorder	REGULATION_OF_ORNITHINE_DECARBOXYLASE_ODC	REACTOME	773	31	20	2,58E-01	9,87E-01	rs2526060|401754|rs2436856|rs4813929|rs11845708|rs698856|rs6033024|rs7200210|rs8078118|rs12037569|rs2513897|rs7126210|rs13209654|rs4888688|rs963267|rs9912225|rs755553|rs8009527|rs12447239|rs241429
Hematologic Disorder	ACTIVATION_OF_KAINATE_RECEPTORS_UPON_GLUTAMATE_BINDING	REACTOME	1577	41	20	2,58E-01	9,87E-01	rs4898725|rs6056252|rs2832449|rs8121340|rs7595027|rs6807740|rs2832505|rs16947151|rs6086473|rs1253704|rs2055226|rs2719804|rs874627|rs1741949|rs2423360|rs7103569|rs6133635|rs6086495|rs12190843|rs4901170
Hematologic Disorder	G_ALPHA1213_SIGNALLING_EVENTS	REACTOME	2855	42	20	2,59E-01	9,87E-01	rs11630971|rs12480096|rs6063285|rs4898725|rs2985700|rs163819|rs8034125|rs6019300|rs6807740|rs165095|rs6012542|rs8100350|rs16947151|rs9290452|rs1253704|rs874627|rs1016088|rs11952170|rs4459929|rs1483578
Hematologic Disorder	GLUCOSE_TRANSPORT	REACTOME	689	17	13	2,61E-01	9,87E-01	rs3026106|rs729405|rs132834|rs11177325|rs13219815|rs102993|rs17382631|rs7897621|rs324079|rs4740419|rs1125397|rs4148761|rs1065483
Hematologic Disorder	EIF4_PATHWAY	BIOCARTA	890	34	20	2,64E-01	9,87E-01	rs198178|rs449072|rs9914703|rs1054743|rs6586116|rs534182|rs7438406|rs2677760|rs173704|rs4983559|rs2673825|rs7558386|rs11953134|rs13196204|rs1110240|rs1258033|rs9925433|rs174827|rs4791032|rs1550805
Hematologic Disorder	CXCR4_PATHWAY	BIOCARTA	1099	40	20	2,66E-01	9,87E-01	rs198178|rs7202877|rs11591534|rs1992838|rs449072|rs9914703|rs4245843|rs2677760|rs173704|rs4767886|rs2235360|rs11953134|rs17488134|rs17419409|rs12827287|rs10907192|rs9925433|rs174827|rs4791032|rs1550805
Hematologic Disorder	VIP_PATHWAY	BIOCARTA	1000	30	20	2,68E-01	9,87E-01	rs1876267|rs6501512|rs1561928|rs7595027|rs1030098|rs748938|rs2302793|rs2055226|rs17083085|rs2235360|rs6470648|rs12670688|rs3793228|rs2302784|rs3801969|rs1555147|rs4733616|rs2732514|rs4699014|rs7598661
Hematologic Disorder	SHC_MEDIATED_CASCADE	REACTOME	1070	39	20	2,70E-01	9,87E-01	rs1888057|rs2174834|rs2992024|rs17072214|rs12523052|rs513320|rs1078363|rs541682|rs17392492|rs3806929|rs9805578|rs12220387|rs1924210|rs10477181|rs3117972|rs8000805|rs152523|rs871346|rs11167790|rs6487465
Hematologic Disorder	GOLGI_ASSOCIATED_VESICLE_BIOGENESIS	REACTOME	1057	39	20	2,70E-01	9,87E-01	rs4794873|rs7738190|rs2289599|rs3829893|rs13216265|rs6045109|rs1890020|rs1130861|rs6733550|rs419588|rs3731828|rs4075688|rs4961604|rs12037569|rs2022345|rs757718|rs3788412|rs2271050|rs11102972|rs2885240
Hematologic Disorder	DESTABILIZATION_OF_MRNA_BY_AUF1_HNRNP_D0	REACTOME	811	33	20	2,71E-01	9,87E-01	rs2526060|rs2011786|401754|rs4813929|rs11845708|rs698856|rs6033024|rs7200210|rs8078118|rs12037569|rs7126210|rs13209654|rs4888688|rs4550918|rs963267|rs9912225|rs755553|rs8009527|rs12447239|rs241429
Hematologic Disorder	MTOR_PATHWAY	BIOCARTA	509	23	12	2,72E-01	9,87E-01	rs1054743|rs6586116|rs534182|rs7438406|rs2677760|rs173704|rs4983559|rs2673825|rs11953134|rs1110240|rs1258033|rs1550805
Hematologic Disorder	EGFR_DOWNREGULATION	REACTOME	1037	54	16	2,72E-01	9,87E-01	rs7983710|rs4947979|rs4349043|rs6563245|rs2110290|rs1417839|rs4961604|rs1333421|rs226434|rs17006928|rs11979158|rs10795505|rs16933086|rs16967789|rs863712|rs13124930
Hematologic Disorder	MRNA_SPLICING_MINOR_PATHWAY	REACTOME	383	20	10	2,73E-01	9,87E-01	rs3788374|rs11084882|rs9908594|rs8072724|rs2261682|rs8070091|rs633742|rs17574411|rs1613070|rs663133
Hematologic Disorder	GLYCEROLIPID_METABOLISM	KEGG	1872	35	20	2,78E-01	9,87E-01	rs2070600|rs7172821|rs11206808|rs947007|rs12081589|rs12572167|rs4721330|rs2165387|rs11810204|rs2358073|rs12492576|rs10518221|rs3043|rs10749107|rs2993263|rs3109164|rs2097055|rs6588636|rs9533042|rs1474250
Hematologic Disorder	GLUCAGON_SIGNALING_IN_METABOLIC_REGULATION	REACTOME	1044	32	20	2,79E-01	9,87E-01	rs4898725|rs6501512|rs6807740|rs7008202|rs7975385|rs1030098|rs953672|rs1253704|rs874627|rs2601773|rs902522|rs12670688|rs4901170|rs72001|rs2302784|rs3801969|rs13249746|rs3741920|rs675327|rs4565471
Hematologic Disorder	INTERFERON_ALPHA_BETA_SIGNALING	REACTOME	1477	48	20	2,80E-01	9,87E-01	rs5013310|rs744038|rs2352946|401754|rs2239167|rs11648565|rs490531|rs13116389|rs8041687|rs2735007|rs2038171|rs276947|rs2596574|rs11649515|rs10160565|rs2031913|rs12938635|rs2245822|rs7037389|rs1611350
Hematologic Disorder	SLC_MEDIATED_TRANSMEMBRANE_TRANSPORT	REACTOME	8774	38	20	2,80E-01	9,87E-01	rs3026106|rs7295598|rs12582527|rs7997827|rs1392312|rs2034755|rs12609914|rs17492143|rs2313982|rs13274956|rs12505556|rs2256453|rs3806536|rs10049581|rs7144273|rs10820785|rs729405|rs12188497|rs9820724|rs2073210
Hematologic Disorder	CA_DEPENDENT_EVENTS	REACTOME	1487	42	20	2,81E-01	9,87E-01	rs2036068|rs6501512|rs7595027|rs429219|rs1572507|rs7008202|rs7975385|rs1030098|rs953672|rs2055226|rs9914703|rs2601773|rs230632|rs902522|rs330746|rs12670688|rs17649232|rs330725|rs12468232|rs72001
Hematologic Disorder	RNA_POL_II_TRANSCRIPTION	REACTOME	1050	35	20	2,83E-01	9,87E-01	rs3788374|rs665714|rs11826421|rs1053079|rs11084882|rs11578832|rs1471420|rs3117655|rs8072724|rs6454694|rs1923416|rs2261682|rs1865094|rs17605098|rs9955264|rs706130|rs7833229|rs9344845|rs963267|rs2031449
Hematologic Disorder	FATTY_ACID_METABOLISM	KEGG	776	45	19	2,83E-01	9,87E-01	rs2276332|rs2066938|rs4665830|rs11578832|rs6599263|rs9423240|rs3043|rs1826909|rs13353058|rs2654846|rs4862439|rs3792311|rs12609870|rs7575760|rs1126671|rs7527279|rs11934173|rs1867802|rs7705738
Hematologic Disorder	O_GLYCAN_BIOSYNTHESIS	KEGG	1579	32	20	2,84E-01	9,87E-01	rs4846948|rs6870010|rs6095638|rs4846904|rs7794356|rs1465231|rs4719095|rs6989475|rs1241347|rs540427|rs7844620|rs7016568|rs10490517|rs721590|rs2945770|rs4720723|rs7814358|rs6889241|rs17199134|rs2945745
Hematologic Disorder	OXYGEN_DEPENDENT_PROLINE_HYDROXYLATION_OF_HYPOXIA_INDUCIBLE_FACTOR_/	REACTOME	313	11	8	2,86E-01	9,87E-01	rs1053079|rs7542797|rs9973653|rs708030|rs11599360|rs17034876|rs2163411|rs3736329
Hematologic Disorder	RIP_MEDIATED_NFKB_ACTIVATION_VIA_DAI	REACTOME	328	16	11	2,86E-01	9,87E-01	rs2070600|rs11191274|rs1394839|rs11574849|rs461626|rs2073145|rs2830076|rs8132767|rs204991|rs7950019|rs1056890
Hematologic Disorder	SIGNAL_AMPLIFICATION	REACTOME	1023	29	20	2,89E-01	9,87E-01	rs4898725|rs6807740|rs1572507|rs8100350|rs16947151|rs1253704|rs2296781|rs874627|rs2238628|rs230632|rs330746|rs4901170|rs330725|rs13196204|rs776797|rs3741920|rs675327|rs10907192|rs11905013|rs4334421
Hematologic Disorder	ION_CHANNEL_TRANSPORT	REACTOME	2741	29	20	2,90E-01	9,87E-01	rs6606877|rs416330|rs4921297|rs2930648|rs6289|rs10519568|rs7358822|rs2306004|rs488447|rs7623572|rs29658|rs12854617|rs422598|rs11637885|rs17116451|rs2049283|rs8052881|rs4472116|rs12518088|rs1278663
Hematologic Disorder	ACTIVATION_OF_NMDA_RECEPTOR_UPON_GLUTAMATE_BINDING_AND_POSTSYNAPTIC_	REACTOME	2117	52	20	2,91E-01	9,87E-01	rs11630971|rs4958671|rs2914621|rs3799663|rs7595027|rs429219|rs7008202|rs1650397|rs2055226|rs6889794|rs4533948|rs12535537|rs10515699|rs8024521|rs10152417|rs8058978|rs6842886|rs10476798|rs2075067|rs17591358
Hematologic Disorder	SIGNALING_BY_FGFR1_MUTANTS	REACTOME	1243	39	20	2,91E-01	9,87E-01	rs5751636|rs2174834|rs743584|rs2992024|rs17072214|rs12523052|rs1078363|rs541682|rs10878951|rs17392492|rs2677760|rs173704|rs2235360|rs9805578|rs11953134|rs1924210|rs10477181|rs140504|rs2021739|rs3117972
Hematologic Disorder	INTERFERON_SIGNALING	REACTOME	3448	38	20	2,91E-01	9,87E-01	rs3026106|rs5013310|rs7113799|rs744038|rs10502414|rs2352946|401754|rs2239167|rs729405|rs11648565|rs266549|rs13116389|rs8041687|rs9321590|rs4678191|rs12535537|rs28772724|rs1678192|rs7438406|rs659527
Hematologic Disorder	P53HYPOXIA_PATHWAY	BIOCARTA	517	35	15	2,92E-01	9,87E-01	rs17172724|rs2235067|rs4640402|rs4278081|rs12704364|rs7805659|rs13006682|rs4983559|rs675327|rs6723920|rs6750100|rs7208143|rs7790722|rs1534151|rs879965
Hematologic Disorder	OOCYTE_MEIOSIS	KEGG	3114	34	20	2,93E-01	9,87E-01	rs6786487|rs1876267|rs11970312|rs8038056|rs4684438|rs7975710|rs7595027|rs11048455|rs7008202|rs7651990|rs7975385|rs4073472|rs2684806|rs953672|rs2055226|rs6856643|rs733018|rs12535537|rs2601773|rs17057833
Hematologic Disorder	PD1_SIGNALING	REACTOME	398	22	10	2,94E-01	9,87E-01	rs2239167|rs874627|rs28772724|rs7024586|rs858492|rs4764622|rs3130192|rs3741920|rs12564899|rs2281389
Hematologic Disorder	ABC_FAMILY_PROTEINS_MEDIATED_TRANSPORT	REACTOME	1753	53	20	2,94E-01	9,87E-01	rs12700232|rs7419736|rs2235067|rs4563284|rs17675608|rs2235013|rs1889548|rs9902652|rs11974545|rs915841|rs2297633|rs4847286|rs557026|rs1628382|rs9524681|rs681187|rs425215|rs8187843|rs34972642|rs7537325
Hematologic Disorder	AUTODEGRADATION_OF_THE_E3_UBIQUITIN_LIGASE_COP1	REACTOME	766	27	18	2,95E-01	9,87E-01	rs2526060|401754|rs4813929|rs11845708|rs698856|rs6033024|rs7200210|rs8078118|rs12037569|rs7126210|rs13209654|rs4888688|rs963267|rs9912225|rs755553|rs8009527|rs12447239|rs241429
Hematologic Disorder	THROMBOXANE_SIGNALLING_THROUGH_TP_RECEPTOR	REACTOME	492	25	20	2,99E-01	9,87E-01	rs4898725|rs6807740|rs8100350|rs16947151|rs1253704|rs2296781|rs874627|rs2238628|rs4901170|rs3741920|rs675327|rs10907192|rs4334421|rs931647|rs12704705|rs11145448|rs11157866|rs2058459|rs5439|rs1253690
Hematologic Disorder	PRE_NOTCH_TRANSCRIPTION_AND_TRANSLATION	REACTOME	835	30	20	3,00E-01	9,87E-01	rs2070600|rs17825554|rs1584783|rs12103149|rs614367|rs6681035|rs679162|rs7943506|rs7930909|rs2068477|rs3796633|rs4753196|rs9907255|rs9295464|rs6577059|rs6697258|rs6808352|rs11621593|rs6831303|rs11715447
Hematologic Disorder	LINOLEIC_ACID_METABOLISM	KEGG	716	43	20	3,01E-01	9,87E-01	rs2278964|rs631134|rs1572507|rs4820315|rs11582551|rs3754205|rs1530837|rs230632|rs330746|rs330725|rs3743016|rs12125747|rs776797|rs2522137|rs776698|rs1934967|rs1843854|rs11589805|rs623784|rs4651390
Hematologic Disorder	NON_SMALL_CELL_LUNG_CANCER	KEGG	2585	36	20	3,01E-01	9,87E-01	rs7373003|rs198178|rs241669|rs2568875|rs4947979|rs449072|rs2365491|rs2121845|rs4917339|rs2362892|rs6541017|rs9914703|rs2110290|rs972795|rs2677760|rs173704|rs614367|rs4983559|rs2235360|rs679162
Hematologic Disorder	BIOPEPTIDES_PATHWAY	BIOCARTA	1081	38	20	3,04E-01	9,87E-01	rs198178|rs2478545|rs7595027|rs449072|rs1465061|rs2055226|rs9914703|rs12535537|rs6842886|rs2235360|rs841718|rs2075067|rs11792962|rs13196204|rs820360|rs2888586|rs7598661|rs10907192|rs6796803|rs9925433
Hematologic Disorder	REGULATION_OF_KIT_SIGNALING	REACTOME	587	35	15	3,04E-01	9,87E-01	rs2239167|rs7829816|rs8085725|rs1465061|rs723279|rs10745478|rs9914703|rs7245158|rs2888586|rs11905013|rs4791032|rs11151581|rs9487749|rs16967789|rs6820303
Hematologic Disorder	REGULATION_OF_MRNA_STABILITY_BY_PROTEINS_THAT_BIND_AU_RICH_ELEMENTS	REACTOME	1372	28	20	3,04E-01	9,87E-01	rs2526060|rs2011786|401754|rs4813929|rs11845708|rs698856|rs1000174|rs6033024|rs2269337|rs9914703|rs2583839|rs7200210|rs4983559|rs1629174|rs8078118|rs12037569|rs13196204|rs1533079|rs8006663|rs2135121
Hematologic Disorder	INTRINSIC_PATHWAY	BIOCARTA	603	18	12	3,05E-01	9,87E-01	rs12500151|rs9521585|rs3211098|rs552125|rs3211719|rs10016252|rs6829128|rs6796803|rs9559771|rs12476572|rs10492497|rs1556122
Hematologic Disorder	UCALPAIN_PATHWAY	BIOCARTA	426	19	8	3,06E-01	9,87E-01	rs6861460|rs540874|rs761900|rs4767886|rs2152833|rs11905013|rs1195962|rs3816492
Hematologic Disorder	P53_INDEPENDENT_G1_S_DNA_DAMAGE_CHECKPOINT	REACTOME	729	28	19	3,07E-01	9,87E-01	rs2526060|401754|rs4813929|rs11845708|rs698856|rs6033024|rs7200210|rs8078118|rs12037569|rs5752792|rs7126210|rs13209654|rs4888688|rs963267|rs9912225|rs755553|rs8009527|rs12447239|rs241429
Hematologic Disorder	ALDOSTERONE_REGULATED_SODIUM_REABSORPTION	KEGG	1794	31	20	3,09E-01	9,87E-01	rs198178|rs2758152|rs17064520|rs9493844|rs449072|rs6566955|rs10500204|rs6541017|rs2758145|rs8094453|rs9914703|rs2677760|rs173704|rs7558386|rs2200984|rs4499341|rs11953134|rs4240896|rs973608|rs7999458
Hematologic Disorder	DIABETES_PATHWAYS	REACTOME	3395	24	20	3,11E-01	9,87E-01	rs1391962|rs1406961|rs11869535|rs17172724|rs10275903|rs12106074|rs11826421|rs6809947|rs1061476|rs17726874|rs1996483|rs1030098|rs10276817|rs11187215|rs10816539|rs1000174|rs10267007|rs1331060|rs2269337|rs7875152
Hematologic Disorder	BASE_EXCISION_REPAIR	KEGG	500	17	12	3,12E-01	9,87E-01	rs7693247|rs2645400|rs2244095|rs3093926|rs2029166|rs11842915|rs16981262|rs174532|rs2303744|rs1826121|rs2268406|rs12567614
Hematologic Disorder	IL17_PATHWAY	BIOCARTA	316	20	10	3,13E-01	9,87E-01	rs11547281|rs874627|rs10777109|rs4722178|rs858492|rs4764622|rs3741920|rs12564899|rs2275913|rs5439
Hematologic Disorder	RNA_POL_III_TRANSCRIPTION_INITIATION_FROM_TYPE_2_PROMOTER	REACTOME	417	18	13	3,14E-01	9,87E-01	rs11989385|rs11084882|rs11833143|rs2926362|rs2227301|rs13277342|rs4442161|rs4922152|rs4921702|rs1372349|rs6081150|rs492553|rs510342
Hematologic Disorder	CROSS_PRESENTATION_OF_SOLUBLE_EXOGENOUS_ANTIGENS_ENDOSOMES	REACTOME	713	30	19	3,15E-01	9,87E-01	rs2526060|401754|rs4813929|rs11845708|rs698856|rs6033024|rs7200210|rs8078118|rs12037569|rs7126210|rs13209654|rs10206319|rs4888688|rs963267|rs9912225|rs755553|rs8009527|rs12447239|rs241429
Hematologic Disorder	RNA_POL_III_CHAIN_ELONGATION	REACTOME	364	18	13	3,15E-01	9,87E-01	rs11989385|rs11084882|rs11833143|rs2926362|rs2227301|rs13277342|rs4442161|rs4922152|rs4921702|rs1372349|rs6081150|rs492553|rs510342
Hematologic Disorder	NCAM1_INTERACTIONS	REACTOME	1896	25	20	3,17E-01	9,87E-01	rs10920115|rs17579232|rs7113799|rs10787635|rs6133053|rs2102902|rs9521585|rs7975385|rs9327283|rs6415964|rs3211098|rs4915478|rs12765367|rs2303377|rs552125|rs6664294|rs375007|rs6076707|rs11197510|rs10052890
Hematologic Disorder	ABC_TRANSPORTERS	KEGG	2684	63	20	3,18E-01	9,87E-01	rs12700232|rs7419736|rs10224277|rs2235067|401754|rs4563284|rs3114018|rs17675608|rs3213499|rs2235013|rs1889548|rs9902652|rs2482430|rs11974545|rs915842|rs12113960|rs2297633|rs2027401|rs4847286|rs557026
Hematologic Disorder	MYOSIN_PATHWAY	BIOCARTA	1253	36	20	3,18E-01	9,87E-01	rs198178|rs6056252|rs8121340|rs449072|rs12600950|rs6086473|rs2719804|rs9914703|rs9515380|rs2423360|rs13074616|rs6133635|rs822570|rs6086495|rs625572|rs820360|rs2327025|rs10907192|rs9925433|rs174827
Hematologic Disorder	PRION_DISEASES	KEGG	1585	37	20	3,19E-01	9,87E-01	rs1547371|rs229441|rs7113799|rs6699859|rs1450660|rs2828119|rs2826380|rs2827185|rs2183564|rs1465061|rs2828098|rs2303377|rs4722178|rs11700900|rs11088936|rs2826254|rs2826718|rs10473234|rs6076707|rs2827650
Hematologic Disorder	SYNTHESIS_OF_PA	REACTOME	1057	36	20	3,20E-01	9,87E-01	rs2070600|rs631134|rs776717|rs1572507|rs12572167|rs11582551|rs10749107|rs230632|rs330746|rs330725|rs1474250|rs2292023|rs776797|rs6756908|rs2522137|rs776698|rs1843854|rs11589805|rs623784|rs600415
Hematologic Disorder	CGMP_EFFECTS	REACTOME	1520	44	20	3,23E-01	9,87E-01	rs2036068|rs7069974|rs7133677|rs220813|rs710839|rs11729954|rs7907343|rs10996331|rs2454566|rs968757|rs221723|rs1863961|rs30887|rs17649232|rs787931|rs11045210|rs2052369|rs10497603|rs710648|rs1844396
Hematologic Disorder	CITRATE_CYCLE_TCA_CYCLE	KEGG	1195	45	20	3,23E-01	9,87E-01	rs10813415|rs10738886|rs1054174|rs17022214|rs7673954|rs2125812|rs4657015|rs2028548|rs10970986|rs9670039|rs11227623|rs2158839|rs287573|rs11732439|rs11893686|rs1040566|rs7685841|rs1411872|rs3561|rs10516988
Hematologic Disorder	SYNTHESIS_OF_PC	REACTOME	669	43	17	3,24E-01	9,87E-01	rs2278952|rs10820785|rs16947151|rs966367|rs7220132|rs10508915|rs8092807|rs859106|rs17097955|rs3906767|rs12343412|rs9469069|rs3124016|rs16985665|rs11673803|rs12471545|rs2033877
Hematologic Disorder	CDC42RAC_PATHWAY	BIOCARTA	435	9	5	3,24E-01	9,87E-01	rs11123212|rs2677760|rs173704|rs11953134|rs1550805
Hematologic Disorder	CHREBP2_PATHWAY	BIOCARTA	1059	25	20	3,25E-01	9,87E-01	rs12540943|rs6501512|rs4502283|rs1881628|rs4593210|rs1030098|rs7074811|rs1528977|rs4302525|rs10105118|rs12670688|rs278152|rs161041|rs2302784|rs3801969|rs2135121|rs7715740|rs2254230|rs1255771|rs10952328
Hematologic Disorder	ACYL_CHAIN_REMODELLING_OF_PC	REACTOME	693	46	20	3,25E-01	9,87E-01	rs631134|rs776717|rs1572507|rs4820315|rs7300327|rs9668071|rs11582551|rs230632|rs330746|rs330725|rs16981262|rs2292023|rs776797|rs2522137|rs776698|rs1843854|rs11589805|rs623784|rs600415|rs4651390
Hematologic Disorder	ION_TRANSPORT_BY_P_TYPE_ATPASES	REACTOME	1690	30	20	3,26E-01	9,87E-01	rs416330|rs4921297|rs2930648|rs7358822|rs2306004|rs7623572|rs12854617|rs422598|rs17116451|rs2049283|rs8052881|rs1278663|rs17414023|rs1403588|rs7333546|rs17593128|rs2414009|rs1609478|rs973608|rs962878
Hematologic Disorder	PIP3_ACTIVATES_AKT_SIGNALING	REACTOME	587	24	12	3,26E-01	9,87E-01	rs665821|rs3007685|rs6586116|rs534182|rs4983559|rs2673825|rs12958400|rs10507483|rs7335734|rs7321873|rs2701853|rs6721035
Hematologic Disorder	AXON_GUIDANCE	REACTOME	13131	37	20	3,27E-01	9,87E-01	rs170679|rs10920115|rs40725|rs679423|rs12593435|rs999426|rs3212588|rs1354096|rs17579232|rs42192|rs6882860|rs1442089|rs6548499|rs12467258|rs7113799|rs6702300|rs10182735|rs163819|rs3799663|rs17142743
Hematologic Disorder	PROTEASOME_PATHWAY	BIOCARTA	280	15	12	3,27E-01	9,87E-01	rs2189713|rs11845708|rs12916907|rs2340634|rs4331321|rs8078118|rs12037569|rs7126210|rs11637057|rs9912225|rs755553|rs8009527
Hematologic Disorder	DESTABILIZATION_OF_MRNA_BY_TRISTETRAPROLIN_TTP	REACTOME	179	8	4	3,27E-01	9,87E-01	rs2269337|rs2583839|rs1629174|rs882253
Hematologic Disorder	GRAFT_VERSUS_HOST_DISEASE	KEGG	1446	60	20	3,28E-01	9,87E-01	rs5013310|rs2284191|rs10772294|rs28772724|rs12310310|rs2735007|rs4722178|rs4934433|rs2596574|rs4538747|rs3130192|rs12106790|rs2245822|rs9268911|rs1611350|rs2105436|rs13209654|rs241427|rs9296068|rs11967684
Hematologic Disorder	THROMBIN_SIGNALLING_THROUGH_PROTEINASE_ACTIVATED_RECEPTORS_PARS	REACTOME	690	25	20	3,28E-01	9,87E-01	rs4898725|rs6807740|rs16947151|rs1253704|rs2296781|rs874627|rs2238628|rs4901170|rs3797418|rs2069656|rs3741920|rs675327|rs10907192|rs11905013|rs4334421|rs931647|rs12704705|rs11145448|rs11157866|rs2058459
Hematologic Disorder	GH_PATHWAY	BIOCARTA	963	33	20	3,29E-01	9,87E-01	rs198178|rs2239167|rs449072|rs10500204|rs9914703|rs2677760|rs173704|rs2235360|rs7558386|rs4499341|rs11953134|rs11792962|rs10408844|rs2288404|rs7961554|rs2888586|rs9925433|rs174827|rs4791032|rs1550805
Hematologic Disorder	PPARA_PATHWAY	BIOCARTA	2445	31	20	3,31E-01	9,87E-01	rs198178|rs1471921|rs6501512|rs328419|rs1561928|rs1242991|rs17754188|rs449072|rs6448226|rs4868204|rs4917339|rs1030098|rs12498345|rs2823035|rs9399315|rs906304|rs2302793|rs2823040|rs9914703|rs2677760
Hematologic Disorder	ANTIGEN_PRESENTATION_FOLDING_ASSEMBLY_AND_PEPTIDE_LOADING_OF_CLASS_I_M	REACTOME	830	74	18	3,33E-01	9,87E-01	rs5013310|rs11748795|401754|rs2735007|rs35684|rs28096|rs2596574|rs7152364|rs2245822|rs1611350|rs13209654|rs4834699|rs26310|rs11967684|rs1426813|rs2523554|rs2255235|rs241429
Hematologic Disorder	ASSEMBLY_OF_THE_PRE_REPLICATIVE_COMPLEX	REACTOME	911	30	20	3,33E-01	9,87E-01	rs2526060|401754|rs4813929|rs11845708|rs698856|rs9474192|rs6033024|rs7200210|rs837763|rs8078118|rs12037569|rs9295464|rs7126210|rs13209654|rs4888688|rs963267|rs9912225|rs755553|rs8009527|rs6085344
Hematologic Disorder	HIV_LIFE_CYCLE	REACTOME	1529	27	20	3,36E-01	9,87E-01	rs3026106|rs11826421|rs1053079|rs729405|rs11084882|rs1151514|rs1471420|rs874627|rs4245843|rs2391626|rs132834|rs16981262|rs6454694|rs1923416|rs4764622|rs2261682|rs1865094|rs11177325|rs3741920|rs9955264
Hematologic Disorder	RNA_POL_III_TRANSCRIPTION_INITIATION_FROM_TYPE_3_PROMOTER	REACTOME	501	21	14	3,38E-01	9,87E-01	rs11989385|rs11084882|rs11833143|rs2926362|rs2227301|rs13277342|rs7548201|rs4442161|rs4922152|rs4921702|rs1372349|rs6081150|rs492553|rs510342
Hematologic Disorder	FRUCTOSE_AND_MANNOSE_METABOLISM	KEGG	984	36	20	3,39E-01	9,87E-01	rs11251481|rs2628440|rs13026308|rs9391926|rs10903939|rs4674406|rs7096344|rs7093049|rs17772897|rs2388595|rs17587917|rs7788953|rs7768483|rs1574318|rs7897621|rs2814820|rs11251588|rs12210306|rs2761233|rs4959620
Hematologic Disorder	PHASE_II_CONJUGATION	REACTOME	2059	33	20	3,39E-01	9,87E-01	rs762286|rs819136|rs316156|rs1584783|rs6497501|rs10077775|rs2028900|rs2522420|rs2915850|rs4072113|rs520002|rs11807|rs2736486|rs4149530|rs2235970|rs2522425|rs3796633|rs1946198|rs1581680|rs8054172
Hematologic Disorder	CYSTEINE_AND_METHIONINE_METABOLISM	KEGG	982	33	20	3,40E-01	9,87E-01	rs2306787|rs819136|rs12142041|rs3124455|rs10077775|rs2028900|rs2915850|rs7916688|rs10904881|rs520002|rs1946198|rs4784991|rs2993763|rs7704440|rs10068900|rs158850|rs6088466|rs7206052|rs7946331|rs4301242
Hematologic Disorder	GLYCOSAMINOGLYCAN_DEGRADATION	KEGG	709	38	20	3,41E-01	9,87E-01	rs4693611|rs17565276|rs8068169|rs2996009|rs17621552|rs2218348|rs9915235|rs8068166|rs4693614|rs1541352|rs4792347|rs7224707|rs3756444|rs693998|rs1866430|rs4792358|rs8080163|rs12601354|rs2127998|rs12601543
Hematologic Disorder	ACTIVATED_POINT_MUTANTS_OF_FGFR2	REACTOME	873	32	20	3,41E-01	9,87E-01	rs2174834|rs2992024|rs17072214|rs12523052|rs1078363|rs541682|rs17392492|rs3806929|rs9805578|rs12220387|rs1924210|rs10477181|rs3117972|rs8000805|rs152523|rs871346|rs11167790|rs17287322|rs10737157|rs822304

<table>
<tr><td>Hematologic Disorder</td><td>HS_GAG_DEGRADATION</td><td>REACTOME</td><td>1386</td><td>31</td><td>20</td><td>3,45E-01</td><td>9,87E-01</td><td>rs4693611|rs1459433|rs10492629|rs319535|rs2124368|rs9584127|rs7577243|rs4773567|rs7329232|rs9516245|rs1980779|rs9301947|rs497836|rs4693614|rs9301898|rs9516113|rs1373863|rs10917070|rs1159752|rs12756105</td></tr>
<tr><td>Hematologic Disorder</td><td>PARKINSONS_DISEASE</td><td>KEGG</td><td>2833</td><td>42</td><td>20</td><td>3,46E-01</td><td>9,87E-01</td><td>rs10831551|rs8133991|rs10878220|rs2201144|rs875785|rs12654078|rs1054174|rs6973149|rs12205861|rs13263130|rs13140566|rs4797343|rs4310676|rs6532196|rs2834291|rs2187213|rs10268780|rs4657015|rs2292023|rs9931644</td></tr>
<tr><td>Hematologic Disorder</td><td>CLASS_I_MHC_MEDIATED_ANTIGEN_PROCESSING_PRESENTATION</td><td>REACTOME</td><td>5895</td><td>44</td><td>20</td><td>3,49E-01</td><td>9,87E-01</td><td>rs5013310|rs1701470|rs11748795|rs1575335|rs7805129|rs7716238|rs899348|rs875785|rs12436854|rs2526060|rs11253250|401754|rs4813929|rs1053079|rs3817442|rs2189713|rs1454387|rs1863703|rs12205861|rs11845708</td></tr>
<tr><td>Hematologic Disorder</td><td>CDK_MEDIATED_PHOSPHORYLATION_AND_REMOVAL_OF_CDC6</td><td>REACTOME</td><td>698</td><td>28</td><td>19</td><td>3,50E-01</td><td>9,87E-01</td><td>rs2526060|401754|rs4813929|rs11845708|rs698856|rs6033024|rs7200210|rs8078118|rs12037569|rs7126210|rs13209654|rs4888688|rs963267|rs9912225|rs755553|rs8009527|rs13706|rs12447239|rs241429</td></tr>
<tr><td>Hematologic Disorder</td><td>INTEGRIN_PATHWAY</td><td>BIOCARTA</td><td>1027</td><td>39</td><td>20</td><td>3,50E-01</td><td>9,87E-01</td><td>rs5751636|rs7202877|rs743584|rs6861460|rs540874|rs1465061|rs11243454|rs761900|rs11102314|rs6681035|rs4767886|rs10901096|rs140504|rs2888586|rs11905013|rs13426335|rs1195962|rs9487749|rs3012750|rs3816492</td></tr>
<tr><td>Hematologic Disorder</td><td>ACTINY_PATHWAY</td><td>BIOCARTA</td><td>290</td><td>11</td><td>5</td><td>3,51E-01</td><td>9,87E-01</td><td>rs1331006|rs11123212|rs560873|rs924483|rs6334</td></tr>
<tr><td>Hematologic Disorder</td><td>NFAT_PATHWAY</td><td>BIOCARTA</td><td>1626</td><td>36</td><td>20</td><td>3,54E-01</td><td>9,87E-01</td><td>rs1876267|rs3760429|rs6501512|rs2478545|rs2645400|rs7595027|rs429219|rs7193701|rs1030098|rs748938|rs2055226|rs9471684|rs2677760|rs173704|rs4983559|rs10903341|rs12670688|rs869236|rs1852730|rs11953134</td></tr>
<tr><td>Hematologic Disorder</td><td>BIOSYNTHESIS_OF_THE_N_GLYCAN_PRECURSOR_DOLICHOL_LIPID_LINKED_OLIGOSACC
RANSFER_TO_A_NASCENT_PROTEIN</td><td>REACTOME</td><td>355</td><td>13</td><td>7</td><td>3,54E-01</td><td>9,87E-01</td><td>rs13026308|rs4674406|rs481325|rs3805703|rs10479469|rs6096210|rs8047337</td></tr>
<tr><td>Hematologic Disorder</td><td>DOWNSTREAM_SIGNALING_EVENTS_OF_B_CELL_RECEPTOR_BCR</td><td>REACTOME</td><td>2127</td><td>29</td><td>20</td><td>3,57E-01</td><td>9,87E-01</td><td>rs198178|rs2526060|401754|rs4813929|rs6461796|rs449072|rs665821|rs11845708|rs698856|rs10200743|rs3007711|rs6033024|rs13217057|rs6586116|rs534182|rs7200210|rs4983559|rs2673825|rs8078118|rs12958400</td></tr>
<tr><td>Hematologic Disorder</td><td>FRS2_MEDIATED_CASCADE</td><td>REACTOME</td><td>1192</td><td>39</td><td>20</td><td>3,57E-01</td><td>9,87E-01</td><td>rs1888057|rs2174834|rs2992024|rs17072214|rs12523052|rs513320|rs1078363|rs541682|rs9510129|rs3806929|rs9805578|rs12220387|rs1924210|rs10477181|rs3117972|rs8000805|rs152523|rs871346|rs11167790|rs6487465</td></tr>
<tr><td>Hematologic Disorder</td><td>ACTIVATION_OF_GENES_BY_ATF4</td><td>REACTOME</td><td>316</td><td>17</td><td>10</td><td>3,58E-01</td><td>9,87E-01</td><td>rs10275903|rs1000174|rs1331060|rs2269337|rs2583839|rs1533079|rs10269471|rs882253|rs1613070|rs10861159</td></tr>
<tr><td>Hematologic Disorder</td><td>DEADENYLATION_DEPENDENT_MRNA_DECAY</td><td>REACTOME</td><td>622</td><td>29</td><td>15</td><td>3,58E-01</td><td>9,87E-01</td><td>rs7133677|rs11553287|rs1000174|rs6821225|rs2269337|rs2583839|rs7438406|rs10879129|rs1533079|rs2453176|rs368686|rs2429175|rs2115794|rs882253|rs10879083</td></tr>
<tr><td>Hematologic Disorder</td><td>ACYL_CHAIN_REMODELLING_OF_PE</td><td>REACTOME</td><td>696</td><td>46</td><td>20</td><td>3,58E-01</td><td>9,87E-01</td><td>rs631134|rs776717|rs1572507|rs4820315|rs7300327|rs9668071|rs11582551|rs230632|rs330746|rs330725|rs16981262|rs776797|rs2522137|rs776698|rs1843854|rs11589805|rs623784|rs600415|rs4651390|rs2386983</td></tr>
<tr><td>Hematologic Disorder</td><td>INTERFERON_GAMMA_SIGNALING</td><td>REACTOME</td><td>2043</td><td>42</td><td>20</td><td>3,59E-01</td><td>9,87E-01</td><td>rs5013310|rs7113799|rs744038|rs10502414|rs2352946|rs2239167|rs11648565|rs13116389|rs9321590|rs12535537|rs28772724|rs1678192|rs2735007|rs2303377|rs6842886|rs353645|rs2075067|rs2038171|rs276947|rs11792962</td></tr>
<tr><td>Hematologic Disorder</td><td>METABOLISM_OF_XENOBIOTICS_BY_CYTOCHROME_P450</td><td>KEGG</td><td>1674</td><td>63</td><td>20</td><td>3,59E-01</td><td>9,87E-01</td><td>rs316156|rs2276332|rs11252845|rs1937919|rs1584783|rs1826909|rs11807|rs2736486|rs2151896|rs7914666|rs4525119|rs2654846|rs3796633|rs1126673|rs1934967|rs10772921|rs7527279|rs7747120|rs4149222|rs6464540</td></tr>
<tr><td>Hematologic Disorder</td><td>SIGNALING_BY_FGFR1_FUSION_MUTANTS</td><td>REACTOME</td><td>639</td><td>32</td><td>15</td><td>3,59E-01</td><td>9,87E-01</td><td>rs5751636|rs743584|rs10878951|rs2677760|rs173704|rs2235360|rs11953134|rs140504|rs2021739|rs11048778|rs2906649|rs1550805|rs16967789|rs15556|rs13029532</td></tr>
<tr><td>Hematologic Disorder</td><td>PERK_REGULATED_GENE_EXPRESSION</td><td>REACTOME</td><td>334</td><td>17</td><td>10</td><td>3,61E-01</td><td>9,87E-01</td><td>rs10275903|rs1000174|rs1331060|rs2269337|rs2583839|rs1533079|rs10269471|rs882253|rs1613070|rs10861159</td></tr>
<tr><td>Hematologic Disorder</td><td>APC_C_CDC20_MEDIATED_DEGRADATION_OF_MITOTIC_PROTEINS</td><td>REACTOME</td><td>1094</td><td>32</td><td>20</td><td>3,61E-01</td><td>9,87E-01</td><td>rs2526060|401754|rs4813929|rs11845708|rs698856|rs6033024|rs6856643|rs17057833|rs7200210|rs1027692|rs8078118|rs12037569|rs315690|rs11599360|rs7126210|rs2448343|rs13209654|rs4888688|rs1037633|rs963267</td></tr>
<tr><td>Hematologic Disorder</td><td>NITRIC_OXIDE_STIMULATES_GUANYLATE_CYCLASE</td><td>REACTOME</td><td>1895</td><td>35</td><td>20</td><td>3,61E-01</td><td>9,87E-01</td><td>rs2036068|rs7069974|rs7133677|rs220813|rs3806777|rs710839|rs11068458|rs907647|rs11729954|rs7907343|rs10996331|rs2454566|rs968757|rs221723|rs1863961|rs30887|rs17649232|rs787931|rs11045210|rs2052369</td></tr>
<tr><td>Hematologic Disorder</td><td>DESTABILIZATION_OF_MRNA_BY_BRF1</td><td>REACTOME</td><td>204</td><td>9</td><td>5</td><td>3,62E-01</td><td>9,87E-01</td><td>rs2269337|rs2583839|rs4983559|rs8006663|rs882253</td></tr>
<tr><td>Hematologic Disorder</td><td>ADP_SIGNALLING_THROUGH_P2RY12</td><td>REACTOME</td><td>438</td><td>21</td><td>16</td><td>3,62E-01</td><td>9,87E-01</td><td>rs4898725|rs6807740|rs16947151|rs1253704|rs874627|rs4901170|rs3741920|rs675327|rs10907192|rs4334421|rs931647|rs12704705|rs11157866|rs2058459|rs5439|rs1253690</td></tr>
<tr><td>Hematologic Disorder</td><td>DAG_AND_IP3_SIGNALING</td><td>REACTOME</td><td>1644</td><td>37</td><td>20</td><td>3,63E-01</td><td>9,87E-01</td><td>rs2036068|rs6501512|rs7975710|rs7595027|rs429219|rs11048455|rs7008202|rs7975385|rs1030098|rs953672|rs2055226|rs9914703|rs2601773|rs7559522|rs2235360|rs902522|rs12670688|rs17649232|rs10497603|rs72001</td></tr>
<tr><td>Hematologic Disorder</td><td>PYRIMIDINE_METABOLISM</td><td>REACTOME</td><td>792</td><td>31</td><td>20</td><td>3,64E-01</td><td>9,87E-01</td><td>rs9704|rs7517899|rs13399758|rs10238679|rs12450758|rs4789145|rs595890|rs167508|rs1399291|rs12127542|rs9437663|rs11589238|rs4949952|rs16835912|rs290826|rs355374|rs13116598|rs10918318|rs6683842|rs10485305</td></tr>
<tr><td>Hematologic Disorder</td><td>HUNTINGTONS_DISEASE</td><td>KEGG</td><td>4311</td><td>30</td><td>20</td><td>3,65E-01</td><td>9,87E-01</td><td>rs6786487|rs8133991|rs12654078|rs6056252|rs1054174|rs4684438|rs8121340|rs794356|rs13263130|rs6108237|rs6448226|rs11084882|rs7651990|rs4732279|rs4797343|rs6086473|rs1471420|rs1179605|rs2719804|rs4968207</td></tr>
<tr><td>Hematologic Disorder</td><td>PHOSPHOLIPASE_C_MEDIATED_CASCADE</td><td>REACTOME</td><td>2628</td><td>33</td><td>20</td><td>3,65E-01</td><td>9,87E-01</td><td>rs2036068|rs1888057|rs2174834|rs6501512|rs2992024|rs7975710|rs7595027|rs17072214|rs429219|rs12523052|rs513320|rs1078363|rs11048455|rs7008202|rs7975385|rs1030098|rs953672|rs541682|rs2055226|rs9914703</td></tr>
<tr><td>Hematologic Disorder</td><td>ADHERENS_JUNCTION</td><td>KEGG</td><td>4520</td><td>30</td><td>20</td><td>3,66E-01</td><td>9,87E-01</td><td>rs10520308|rs6480244|rs3828150|rs793639|rs12467258|rs7617478|rs1822454|rs7914896|rs8038056|rs10174961|rs12573128|rs11869351|rs2239167|rs4947979|rs4258819|rs6751684|rs1338423|rs10500204|rs9971243|rs540874</td></tr>
<tr><td>Hematologic Disorder</td><td>DEFENSINS</td><td>REACTOME</td><td>495</td><td>26</td><td>16</td><td>3,68E-01</td><td>9,87E-01</td><td>rs2702938|rs13251447|rs874627|rs1339|rs6039403|rs6074947|rs2978880|rs4764622|rs11466640|rs3741920|rs3798315|rs2738050|rs6057657|rs6046492|rs17030340|rs5439</td></tr>
<tr><td>Hematologic Disorder</td><td>PTM_GAMMA_CARBOXYLATION_HYPUSINE_FORMATION_AND_ARYLSULFATASE_ACTIV</td><td>REACTOME</td><td>377</td><td>17</td><td>7</td><td>3,68E-01</td><td>9,87E-01</td><td>rs11713682|rs2028900|rs11658645|rs3211719|rs12499919|rs10126|rs918167</td></tr>
<tr><td>Hematologic Disorder</td><td>STATHMIN_PATHWAY</td><td>BIOCARTA</td><td>456</td><td>26</td><td>17</td><td>3,69E-01</td><td>9,87E-01</td><td>rs6501512|rs429219|rs1030098|rs12535537|rs6842886|rs12670688|rs858492|rs2075067|rs2302784|rs3801969|rs3797755|rs12564899|rs2448343|rs457734|rs13243079|rs2241695|rs3733619</td></tr>
<tr><td>Hematologic Disorder</td><td>PLATELET_CALCIUM_HOMEOSTASIS</td><td>REACTOME</td><td>1156</td><td>44</td><td>20</td><td>3,69E-01</td><td>9,87E-01</td><td>rs727152|rs750525|rs16929712|rs7975710|rs11048455|rs7623572|rs765555|rs7159779|rs7303557|rs12286714|rs4963998|rs35674|rs4711338|rs649775|rs884701|rs905298|rs799320|rs26310|rs10516616|rs2880875</td></tr>
<tr><td>Hematologic Disorder</td><td>PROSTACYCLIN_SIGNALLING_THROUGH_PROSTACYCLIN_RECEPTOR</td><td>REACTOME</td><td>353</td><td>22</td><td>16</td><td>3,69E-01</td><td>9,87E-01</td><td>rs4898725|rs6807740|rs16947151|rs1253704|rs874627|rs4901170|rs3741920|rs675327|rs10907192|rs4334421|rs931647|rs12704705|rs11157866|rs2058459|rs5439|rs1253690</td></tr>
<tr><td>Hematologic Disorder</td><td>DEADENYLATION_OF_MRNA</td><td>REACTOME</td><td>331</td><td>14</td><td>8</td><td>3,70E-01</td><td>9,87E-01</td><td>rs7133677|rs1000174|rs7438406|rs10879129|rs1533079|rs2453176|rs368686|rs10879083</td></tr>
<tr><td>Hematologic Disorder</td><td>TFF_PATHWAY</td><td>BIOCARTA</td><td>697</td><td>22</td><td>12</td><td>3,70E-01</td><td>9,87E-01</td><td>rs4947979|rs2110290|rs2677760|rs173704|rs4983559|rs10268780|rs11953134|rs9883073|rs11979158|rs2888586|rs1550805|rs16967789</td></tr>
<tr><td>Hematologic Disorder</td><td>ORC1_REMOVAL_FROM_CHROMATIN</td><td>REACTOME</td><td>930</td><td>30</td><td>20</td><td>3,73E-01</td><td>9,87E-01</td><td>rs2526060|401754|rs4813929|rs11845708|rs698856|rs9474192|rs6033024|rs7200210|rs837763|rs8078118|rs12037569|rs7126210|rs13209654|rs17068405|rs4888688|rs963267|rs9912225|rs755553|rs8009527|rs6085344</td></tr>
<tr><td>Hematologic Disorder</td><td>GLYCEROPHOSPHOLIPID_BIOSYNTHESIS</td><td>REACTOME</td><td>2483</td><td>30</td><td>20</td><td>3,74E-01</td><td>9,87E-01</td><td>rs2070600|rs2278952|rs7269032|rs631134|rs10820785|rs776717|rs1572507|rs12572167|rs4665830|rs16947151|rs6038219|rs966367|rs7220132|rs10508915|rs4820315|rs7300327|rs9668071|rs11582551|rs8092807|rs10749107</td></tr>
<tr><td>Hematologic Disorder</td><td>G_BETA_GAMMA_SIGNALLING_THROUGH_PI3KGAMMA</td><td>REACTOME</td><td>487</td><td>24</td><td>19</td><td>3,76E-01</td><td>9,87E-01</td><td>rs4898725|rs6807740|rs16947151|rs1253704|rs874627|rs12944446|rs4983559|rs4901170|rs3741920|rs675327|rs10907192|rs4334421|rs931647|rs12704705|rs682083|rs11157866|rs2058459|rs5439|rs1253690</td></tr>
<tr><td>Hematologic Disorder</td><td>LIGAND_GATED_ION_CHANNEL_TRANSPORT</td><td>REACTOME</td><td>1051</td><td>52</td><td>20</td><td>3,78E-01</td><td>9,87E-01</td><td>rs6606877|rs6289|rs10519568|rs488447|rs29658|rs11637885|rs4472116|rs12518088|rs1426220|rs256147|rs4263535|rs1897356|rs10051667|rs2607313|rs11135148|rs1035750|rs2715573|rs170020|rs7173054|rs2169637</td></tr>
<tr><td>Hematologic Disorder</td><td>REGULATION_OF_HYPOXIA_INDUCIBLE_FACTOR_HIF_BY_OXYGEN</td><td>REACTOME</td><td>443</td><td>16</td><td>11</td><td>3,79E-01</td><td>9,87E-01</td><td>rs1053079|rs7542797|rs9973653|rs6458350|rs708030|rs11599360|rs12204488|rs17034876|rs2163411|rs3025010|rs3736329</td></tr>
<tr><td>Hematologic Disorder</td><td>CDT1_ASSOCIATION_WITH_THE_CDC6_ORC_ORIGIN_COMPLEX</td><td>REACTOME</td><td>793</td><td>28</td><td>20</td><td>3,81E-01</td><td>9,87E-01</td><td>rs2526060|401754|rs4813929|rs11845708|rs698856|rs6033024|rs7200210|rs837763|rs8078118|rs12037569|rs7126210|rs13209654|rs4888688|rs963267|rs9912225|rs755553|rs8009527|rs13706|rs12447239|rs6085344</td></tr>
<tr><td>Hematologic Disorder</td><td>CHEMICAL_PATHWAY</td><td>BIOCARTA</td><td>585</td><td>27</td><td>15</td><td>3,81E-01</td><td>9,87E-01</td><td>rs198178|rs449072|rs9914703|rs4983559|rs4767886|rs10268780|rs12454712|rs9925433|rs174827|rs4791032|rs2850768|rs4987786|rs11859534|rs12567614|rs13029532</td></tr>
<tr><td>Hematologic Disorder</td><td>CHEMOKINE_SIGNALING_PATHWAY</td><td>KEGG</td><td>5407</td><td>34</td><td>20</td><td>3,82E-01</td><td>9,87E-01</td><td>rs198178|rs12480096|rs159254|rs928670|rs6063285|rs4898725|rs2985700|rs6899021|rs6056252|rs7202877|rs7091519|rs11591534|rs6019300|rs8121340|rs449072|rs6807740|rs7829816|rs6108237|rs7008202|rs7975385</td></tr>
<tr><td>Hematologic Disorder</td><td>ENERGY_DEPENDENT_REGULATION_OF_MTOR_BY_LKB1_AMPK</td><td>REACTOME</td><td>461</td><td>21</td><td>16</td><td>3,85E-01</td><td>9,87E-01</td><td>rs12540943|rs1881628|rs2672886|rs4969429|rs1528977|rs278152|rs11651724|rs17762352|rs10952328|rs13431868|rs4726096|rs10169231|rs12142743|rs3764640|rs4725435|rs6573304</td></tr>
<tr><td>Hematologic Disorder</td><td>OPIOID_SIGNALLING</td><td>REACTOME</td><td>3594</td><td>27</td><td>20</td><td>3,85E-01</td><td>9,87E-01</td><td>rs9306383|rs2036068|rs4898725|rs1876267|rs6056252|rs6501512|rs7975710|rs8121340|rs7595027|rs429219|rs6807740|rs1572507|rs6108237|rs11048455|rs7008202|rs7975385|rs1030098|rs6003495|rs16947151|rs6086473</td></tr>
<tr><td>Hematologic Disorder</td><td>P53_DEPENDENT_G1_DNA_DAMAGE_RESPONSE</td><td>REACTOME</td><td>838</td><td>29</td><td>20</td><td>3,86E-01</td><td>9,87E-01</td><td>rs2526060|401754|rs4813929|rs11845708|rs698856|rs6033024|rs7200210|rs8078118|rs7015841|rs12037569|rs7126210|rs13209654|rs10425066|rs4888688|rs963267|rs9912225|rs755553|rs8009527|rs12447239|rs241429</td></tr>
<tr><td>Hematologic Disorder</td><td>HDAC_PATHWAY</td><td>BIOCARTA</td><td>1231</td><td>28</td><td>20</td><td>3,86E-01</td><td>9,87E-01</td><td>rs1876267|rs738809|rs8038056|rs7595027|rs10500204|rs2684806|rs748938|rs2055226|rs2677760|rs173704|rs4983559|rs2947864|rs4499341|rs1852730|rs11953134|rs10408844|rs13196204|rs2288404|rs1555147|rs2732514</td></tr>
<tr><td>Hematologic Disorder</td><td>SIGNALING_BY_THE_B_CELL_RECEPTOR_BCR</td><td>REACTOME</td><td>3426</td><td>30</td><td>20</td><td>3,87E-01</td><td>9,87E-01</td><td>rs198178|rs727152|rs2526060|401754|rs750525|rs4813929|rs6461796|rs16929712|rs7975710|rs7595027|rs449072|rs665821|rs11845708|rs7829816|rs698856|rs10200743|rs11048455|rs6541017|rs3007711|rs1148853</td></tr>
<tr><td>Hematologic Disorder</td><td>TRIGLYCERIDE_BIOSYNTHESIS</td><td>REACTOME</td><td>1182</td><td>33</td><td>20</td><td>3,91E-01</td><td>9,87E-01</td><td>rs2070600|rs17620632|rs4502283|rs12572167|rs7656124|rs966367|rs8092807|rs10749107|rs2816373|rs11037685|rs1474250|rs2292023|rs4862439|rs3792311|rs7575760|rs1429146|rs3906767|rs6756908|rs12498468|rs807673</td></tr>
<tr><td>Hematologic Disorder</td><td>PROTEASOME</td><td>KEGG</td><td>821</td><td>30</td><td>20</td><td>3,94E-01</td><td>9,87E-01</td><td>rs2526060|401754|rs4813929|rs11845708|rs698856|rs6033024|rs7200210|rs8078118|rs12037569|rs12017236|rs7126210|rs13209654|rs4888688|rs963267|rs1404508|rs9912225|rs755553|rs8009527|rs12447239|rs241429</td></tr>
<tr><td>Hematologic Disorder</td><td>CTLA4_INHIBITORY_SIGNALING</td><td>REACTOME</td><td>447</td><td>22</td><td>9</td><td>3,94E-01</td><td>9,87E-01</td><td>rs7829816|rs1465061|rs4983559|rs10105118|rs7245158|rs12106790|rs1255771|rs11905013|rs9487749</td></tr>
<tr><td>Hematologic Disorder</td><td>MATURITY_ONSET_DIABETES_OF_THE_YOUNG</td><td>KEGG</td><td>992</td><td>30</td><td>20</td><td>3,95E-01</td><td>9,87E-01</td><td>rs1899745|rs2690035|rs1390088|rs2456525|rs10949620|rs10030773|rs7209835|rs2247328|rs830783|rs6513926|rs17138478|rs6075942|rs4238393|rs17366515|rs7961554|rs6048114|rs17659514|rs6788164|rs12306121|rs12437920</td></tr>
<tr><td>Hematologic Disorder</td><td>EDG1_PATHWAY</td><td>BIOCARTA</td><td>1432</td><td>43</td><td>20</td><td>3,96E-01</td><td>9,87E-01</td><td>rs198178|rs6056252|rs2396569|rs8121340|rs449072|rs6086473|rs2719804|rs9914703|rs2423360|rs2677760|rs173704|rs4983559|rs6133635|rs6086495|rs11953134|rs1234367|rs9914387|rs2327025|rs10907192|rs11905013</td></tr>
<tr><td>Hematologic Disorder</td><td>NEPHRIN_INTERACTIONS</td><td>REACTOME</td><td>1617</td><td>44</td><td>20</td><td>3,97E-01</td><td>9,87E-01</td><td>rs7950175|rs987493|rs2691542|rs11505717|rs549190|rs1465061|rs16974215|rs873496|rs761900|rs12420540|rs2282507|rs16972767|rs3862639|rs2677760|rs173704|rs17809941|rs10171331|rs11953134|rs11220521|rs2152833</td></tr>
<tr><td>Hematologic Disorder</td><td>NEGATIVE_REGULATORS_OF_RIG_I_MDA5_SIGNALING</td><td>REACTOME</td><td>464</td><td>33</td><td>15</td><td>3,97E-01</td><td>9,87E-01</td><td>rs12599479|rs821466|rs621277|rs6133067|rs803523|rs1040411|rs10970986|rs12922698|rs11599360|rs6084497|rs7267297|rs2683065|rs289710|rs2144825|rs8180663</td></tr>
<tr><td>Hematologic Disorder</td><td>SCF_BETA_TRCP_MEDIATED_DEGRADATION_OF_EMI1</td><td>REACTOME</td><td>793</td><td>29</td><td>20</td><td>3,98E-01</td><td>9,87E-01</td><td>rs2526060|401754|rs4813929|rs11845708|rs698856|rs6033024|rs7200210|rs8078118|rs12037569|rs3802727|rs7126210|rs13209654|rs4888688|rs963267|rs9912225|rs755553|rs8009527|rs11191017|rs12447239|rs241429</td></tr>
<tr><td>Hematologic Disorder</td><td>NEUROTRANSMITTER_RECEPTOR_BINDING_AND_DOWNSTREAM_TRANSMISSION_IN_TH
L</td><td>REACTOME</td><td>6592</td><td>40</td><td>20</td><td>3,99E-01</td><td>9,87E-01</td><td>rs198178|rs11630971|rs6606877|rs4958671|rs2914621|rs11654455|rs4898725|rs6289|rs10519568|rs6056252|rs3799663|rs12915265|rs2832449|rs8121340|rs7595027|rs429219|rs449072|rs6807740|rs488447|rs2832505</td></tr>
<tr><td>Hematologic Disorder</td><td>SYNTHESIS_OF_PIPS_AT_THE_PLASMA_MEMBRANE</td><td>REACTOME</td><td>1050</td><td>31</td><td>20</td><td>4,00E-01</td><td>9,87E-01</td><td>rs6796883|rs1992838|rs11702774|rs6541017|rs6586116|rs534182|rs7922014|rs12944446|rs2677760|rs173704|rs2673825|rs11953134|rs4240896|rs2218313|rs13296679|rs9878820|rs17419409|rs12827287|rs293777|rs6594014</td></tr>
<tr><td>Hematologic Disorder</td><td>A_TETRASACCHARIDE_LINKER_SEQUENCE_IS_REQUIRED_FOR_GAG_SYNTHESIS</td><td>REACTOME</td><td>1731</td><td>24</td><td>20</td><td>4,02E-01</td><td>9,87E-01</td><td>rs1459433|rs10492629|rs11223958|rs319535|rs6785758|rs2124368|rs6932261|rs9584127|rs7577243|rs2187467|rs4773567|rs7329232|rs9516245|rs1980779|rs2169860|rs7926027|rs256459|rs4335563|rs9301947|rs497836</td></tr>
<tr><td>Hematologic Disorder</td><td>COSTIMULATION_BY_THE_CD28_FAMILY</td><td>REACTOME</td><td>1479</td><td>31</td><td>20</td><td>4,05E-01</td><td>9,87E-01</td><td>rs933984|rs4419220|rs2239167|rs7829816|rs3007711|rs1465061|rs874627|rs28772724|rs2677760|rs173704|rs4983559|rs7024586|rs10105118|rs858492|rs11953134|rs7245158|rs4764622|rs3130192|rs17718517|rs3741920</td></tr>
<tr><td>Hematologic Disorder</td><td>MTA3_PATHWAY</td><td>BIOCARTA</td><td>333</td><td>20</td><td>8</td><td>4,07E-01</td><td>9,87E-01</td><td>rs2292963|rs980281|rs3785076|rs4953564|rs6472114|rs17081685|rs6939257|rs3020343</td></tr>
<tr><td>Hematologic Disorder</td><td>ERK_MAPK_TARGETS</td><td>REACTOME</td><td>704</td><td>25</td><td>12</td><td>4,09E-01</td><td>9,87E-01</td><td>rs11970312|rs12892024|rs7305095|rs10105118|rs1852730|rs17591358|rs13196204|rs3886478|rs2187911|rs1286086|rs10858840|rs770082</td></tr>
<tr><td>Hematologic Disorder</td><td>APC_C_CDH1_MEDIATED_DEGRADATION_OF_CDC20_AND_OTHER_APC_C_CDH1_TARGET
TE_MITOSIS_EARLY_G1</td><td>REACTOME</td><td>959</td><td>30</td><td>20</td><td>4,09E-01</td><td>9,87E-01</td><td>rs2526060|401754|rs4813929|rs11845708|rs698856|rs6033024|rs17057833|rs7200210|rs8078118|rs12037569|rs315690|rs11599360|rs7126210|rs13209654|rs3027267|rs4888688|rs1037633|rs963267|rs9912225|rs755553</td></tr>
<tr><td>Hematologic Disorder</td><td>MRNA_SPLICING</td><td>REACTOME</td><td>1057</td><td>45</td><td>20</td><td>4,10E-01</td><td>9,87E-01</td><td>rs9704|rs665714|rs2011786|rs7361672|rs11084882|rs11578832|rs606119|rs3117655|rs9910260|rs7061|rs8072724|rs2261682|rs706130|rs8070091|rs1782322|rs4550918|rs1300253|rs633742|rs17574411|rs1613070</td></tr>
<tr><td>Hematologic Disorder</td><td>HIV_INFECTION</td><td>REACTOME</td><td>3237</td><td>29</td><td>20</td><td>4,12E-01</td><td>9,87E-01</td><td>rs3026106|rs2526060|rs6899021|401754|rs11826421|rs4813929|rs1053079|rs729405|rs11845708|rs698856|rs11084882|rs1151514|rs1420320|rs1471420|rs1465061|rs6033024|rs874627|rs4678191|rs4245843|rs3806929</td></tr>
<tr><td>Hematologic Disorder</td><td>LATE_PHASE_OF_HIV_LIFE_CYCLE</td><td>REACTOME</td><td>1259</td><td>28</td><td>20</td><td>4,12E-01</td><td>9,87E-01</td><td>rs3026106|rs11826421|rs1053079|rs729405|rs11084882|rs1471420|rs132834|rs6454694|rs1923416|rs2261682|rs1865094|rs11177325|rs9955264|rs2271050|rs13219815|rs12613627|rs102993|rs7833229|rs9344845|rs963267</td></tr>
<tr><td>Hematologic Disorder</td><td>MCM_PATHWAY</td><td>BIOCARTA</td><td>234</td><td>15</td><td>5</td><td>4,13E-01</td><td>9,87E-01</td><td>rs9474192|rs10777109|rs837763|rs10425066|rs13706</td></tr>
<tr><td>Hematologic Disorder</td><td>GLYCOLYSIS_GLUCONEOGENESIS</td><td>KEGG</td><td>1391</td><td>40</td><td>20</td><td>4,13E-01</td><td>9,87E-01</td><td>rs11251481|rs2306787|rs2276332|rs10903939|rs2239167|rs7673954|rs3043|rs1826909|rs2993263|rs7093049|rs2158839|rs2654846|rs11732439|rs1040566|rs7685841|rs3561|rs2388595|rs10516988|rs1126673|rs7527279</td></tr>
<tr><td>Hematologic Disorder</td><td>CARDIAC_MUSCLE_CONTRACTION</td><td>KEGG</td><td>3169</td><td>30</td><td>20</td><td>4,14E-01</td><td>9,87E-01</td><td>rs10920115|rs3753617|rs12654078|rs11715062|rs1467913|rs2102902|rs17247184|rs4659495|rs2239093|rs6806680|rs2239041|rs37070|rs7975385|rs2970533|rs6415964|rs4915478|rs6673700|rs38553|rs12765367|rs3774425</td></tr>
<tr><td>Hematologic Disorder</td><td>G_ALPHA_Z_SIGNALLING_EVENTS</td><td>REACTOME</td><td>2191</td><td>37</td><td>20</td><td>4,15E-01</td><td>9,87E-01</td><td>rs198178|rs9306383|rs4898725|rs449072|rs6807740|rs7008202|rs7975385|rs6003495|rs16947151|rs953672|rs1253704|rs10885123|rs874627|rs9914703|rs2601773|rs1335579|rs7559522|rs902522|rs4901170|rs9397595</td></tr>
<tr><td>Hematologic Disorder</td><td>AT1R_PATHWAY</td><td>BIOCARTA</td><td>1224</td><td>42</td><td>20</td><td>4,16E-01</td><td>9,87E-01</td><td>rs198178|rs2478545|rs7595027|rs4947979|rs449072|rs2055226|rs2529737|rs9914703|rs2110290|rs6681035|rs1852730|rs2638363|rs11979158|rs2888586|rs7598661|rs11905013|rs9925433|rs174827|rs4791032|rs815804</td></tr>
<tr><td>Hematologic Disorder</td><td>ALPHA_LINOLENIC_ACID_METABOLISM</td><td>KEGG</td><td>571</td><td>54</td><td>20</td><td>4,18E-01</td><td>9,87E-01</td><td>rs2278964|rs631134|rs1572507|rs4820315|rs11582551|rs1530837|rs230632|rs330746|rs330725|rs3743016|rs776797|rs2522137|rs776698|rs1843854|rs11589805|rs623784|rs4651390|rs695117|rs11802712|rs1567102</td></tr>
<tr><td>Hematologic Disorder</td><td>REGULATION_OF_APOPTOSIS</td><td>REACTOME</td><td>1432</td><td>32</td><td>20</td><td>4,18E-01</td><td>9,87E-01</td><td>rs1442089|rs2526060|401754|rs4813929|rs10502934|rs11845708|rs698856|rs727683|rs6033024|rs7200210|rs8078118|rs4746080|rs12037569|rs12456286|rs7126210|rs4878066|rs13209654|rs893024|rs4888688|rs9966535</td></tr>
<tr><td>Hematologic Disorder</td><td>PANTOTHENATE_AND_COA_BIOSYNTHESIS</td><td>KEGG</td><td>611</td><td>28</td><td>13</td><td>4,18E-01</td><td>9,87E-01</td><td>rs9704|rs7517899|rs10771144|rs1399291|rs12127542|rs9437663|rs11589238|rs4949952|rs290826|rs355374|rs9483334|rs7267297|rs11165976</td></tr>
<tr><td>Hematologic Disorder</td><td>RESOLUTION_OF_AP_SITES_VIA_THE_MULTIPLE_NUCLEOTIDE_PATCH_REPLACEMENT_P</td><td>REACTOME</td><td>192</td><td>6</td><td>5</td><td>4,19E-01</td><td>9,87E-01</td><td>rs2244095|rs2029166|rs16981262|rs174532|rs2303744</td></tr>
<tr><td>Hematologic Disorder</td><td>BASE_EXCISION_REPAIR</td><td>REACTOME</td><td>209</td><td>6</td><td>5</td><td>4,21E-01</td><td>9,87E-01</td><td>rs2244095|rs2029166|rs16981262|rs174532|rs2303744</td></tr>
<tr><td>Hematologic Disorder</td><td>TCR_PATHWAY</td><td>BIOCARTA</td><td>1517</td><td>39</td><td>20</td><td>4,22E-01</td><td>9,87E-01</td><td>rs198178|rs1876267|rs7595027|rs449072|rs7193701|rs748938|rs1465061|rs2055226|rs2529737|rs9914703|rs2677760|rs173704|rs6681035|rs2235360|rs858492|rs252299|rs11953134|rs17718517|rs1555147|rs12564899</td></tr>
<tr><td>Hematologic Disorder</td><td>RAP1_SIGNALLING</td><td>REACTOME</td><td>835</td><td>47</td><td>20</td><td>4,22E-01</td><td>9,87E-01</td><td>rs3769309|rs7069974|rs7907343|rs10996331|rs9901827|rs2454566|rs1476462|rs11102314|rs16967035|rs3782908|rs2135121|rs10492302|rs11177325|rs10824002|rs1904006|rs1937652|rs726195|rs3820852|rs293228|rs2240079</td></tr>
<tr><td>Hematologic Disorder</td><td>COMPLEMENT_AND_COAGULATION_CASCADES</td><td>KEGG</td><td>1742</td><td>36</td><td>20</td><td>4,28E-01</td><td>9,87E-01</td><td>rs4610017|rs12500151|rs6699859|rs1450660|rs4935400|rs1040172|rs4764521|rs1571344|rs9504738|rs1143664|rs3211719|rs7083840|rs1903971|rs11003258|rs17093887|rs1389623|rs5985|rs10203300|rs10473234|rs344772</td></tr>
<tr><td>Hematologic Disorder</td><td>GLEEVEC_PATHWAY</td><td>BIOCARTA</td><td>954</td><td>34</td><td>20</td><td>4,31E-01</td><td>9,87E-01</td><td>rs5751636|rs743584|rs1561928|rs2302793|rs2529737|rs2677760|rs173704|rs4983559|rs6681035|rs6470648|rs11953134|rs11792962|rs140504|rs4733616|rs2888586|rs4637783|rs6995630|rs17103133|rs2395851|rs1550805</td></tr>
<tr><td>Hematologic Disorder</td><td>ACTIVATED_AMPK_STIMULATES_FATTY_ACID_OXIDATION_IN_MUSCLE</td><td>REACTOME</td><td>421</td><td>22</td><td>16</td><td>4,32E-01</td><td>9,87E-01</td><td>rs12540943|rs1881628|rs11578832|rs1528977|rs278152|rs4862439|rs3792311|rs17762352|rs10952328|rs13431868|rs11934173|rs4726096|rs10169231|rs12142743|rs3764640|rs4725435</td></tr>
<tr><td>Hematologic Disorder</td><td>TRAF6_MEDIATED_IRF7_ACTIVATION</td><td>REACTOME</td><td>433</td><td>42</td><td>14</td><td>4,33E-01</td><td>9,87E-01</td><td>rs744038|rs13116389|rs621277|rs6133067|rs9287799|rs10970986|rs1404358|rs7037389|rs2133165|rs6084497|rs2207701|rs2811757|rs7267297|rs12419174</td></tr>
<tr><td>Hematologic Disorder</td><td>BETA_ALANINE_METABOLISM</td><td>KEGG</td><td>711</td><td>29</td><td>16</td><td>4,34E-01</td><td>9,87E-01</td><td>rs9704|rs7517899|rs4665830|rs3043|rs1399291|rs12127542|rs9437663|rs11589238|rs4949952|rs290826|rs7527279|rs355374|rs11165976|rs7705738|rs12467996|rs10803852</td></tr>
<tr><td>Hematologic Disorder</td><td>NKCELLS_PATHWAY</td><td>BIOCARTA</td><td>678</td><td>30</td><td>17</td><td>4,37E-01</td><td>9,87E-01</td><td>rs10772294|rs2239167|rs1148853|rs12310310|rs2677760|rs173704|rs252299|rs11953134|rs17718517|rs4744514|rs10993709|rs1550805|rs8045689|rs290267|rs158688|rs4776783|rs2255235</td></tr>
<tr><td>Hematologic Disorder</td><td>RAC1_PATHWAY</td><td>BIOCARTA</td><td>763</td><td>27</td><td>13</td><td>4,38E-01</td><td>9,87E-01</td><td>rs163819|rs165095|rs11811630|rs2677760|rs173704|rs11953134|rs17718517|rs2519866|rs820360|rs2303492|rs1550805|rs10800842|rs889312</td></tr>
<tr><td>Hematologic Disorder</td><td>GLYCOGEN_BREAKDOWN_GLYCOGENOLYSIS</td><td>REACTOME</td><td>246</td><td>12</td><td>6</td><td>4,38E-01</td><td>9,87E-01</td><td>rs7595027|rs2055226|rs8052416|rs7598661|rs815804|rs2300502</td></tr>
<tr><td>Hematologic Disorder</td><td>DARPP_32_EVENTS</td><td>REACTOME</td><td>879</td><td>41</td><td>20</td><td>4,39E-01</td><td>9,87E-01</td><td>rs1876267|rs6501512|rs7595027|rs1030098|rs2055226|rs918583|rs10105118|rs4255357|rs12670688|rs2302784|rs3801969|rs2732514|rs4699014|rs7598661|rs10514862|rs815804|rs2300502|rs13243079|rs294498|rs2409741</td></tr>
<tr><td>Hematologic Disorder</td><td>PKB_MEDIATED_EVENTS</td><td>REACTOME</td><td>583</td><td>23</td><td>18</td><td>4,39E-01</td><td>9,87E-01</td><td>rs12540943|rs1881628|rs2672886|rs4969429|rs7438406|rs1528977|rs278152|rs11651724|rs17762352|rs10952328|rs13431868|rs4726096|rs10169231|rs12142743|rs8059570|rs3764640|rs4725435|rs6573304</td></tr>
<tr><td>Hematologic Disorder</td><td>ENDOMETRIAL_CANCER</td><td>KEGG</td><td>3028</td><td>30</td><td>20</td><td>4,40E-01</td><td>9,87E-01</td><td>rs10520308|rs6480244|rs11826421|rs10174961|rs1561928|rs12573128|rs4947979|rs4258819|rs6751684|rs1338423|rs9971243|rs6541017|rs17017611|rs2302793|rs2110290|rs6586116|rs534182|rs2677760|rs173704|rs614367</td></tr>
<tr><td>Hematologic Disorder</td><td>CD28_DEPENDENT_PI3K_AKT_SIGNALING</td><td>REACTOME</td><td>572</td><td>24</td><td>10</td><td>4,40E-01</td><td>9,87E-01</td><td>rs3007711|rs1465061|rs2677760|rs173704|rs4983559|rs11953134|rs12106790|rs9487749|rs1550805|rs7086224</td></tr>
<tr><td>Hematologic Disorder</td><td>AMINO_ACID_TRANSPORT_ACROSS_THE_PLASMA_MEMBRANE</td><td>REACTOME</td><td>1025</td><td>39</td><td>20</td><td>4,42E-01</td><td>9,87E-01</td><td>rs7997827|rs12609914|rs2313982|rs13144383|rs1975802|rs4640842|rs1605819|rs2955849|rs9788810|rs748680|rs1007160|rs3759608|rs12891079|rs720625|rs7685529|rs3851117|rs1107920|rs2720546|rs1003349|rs9325217</td></tr>
</table>

Hematologic Disorder	MEMBRANE_TRAFFICKING	REACTOME	2276	28	20	4,42E-01	9,87E-01	rs1406961|rs4794873|rs7738190|rs16869484|rs2289599|rs3829893|rs13216265|rs9342215|rs11191274|rs6045109|rs1890020|rs1130861|rs6733550|rs419588|rs785437|rs3731828|rs35684|rs1555772|rs7213960|rs4075688
Hematologic Disorder	PRE_NOTCH_EXPRESSION_AND_PROCESSING	REACTOME	1434	26	20	4,44E-01	9,87E-01	rs2070600|rs17825554|rs12431724|rs12274970|rs1958223|rs1584783|rs12103149|rs614367|rs6681035|rs1242551|rs679162|rs7943506|rs7930909|rs6780684|rs2068477|rs1211307|rs3796633|rs4753196|rs9907255|rs9295464
Hematologic Disorder	IL_RECEPTOR_SHC_SIGNALING	REACTOME	624	25	18	4,45E-01	9,87E-01	rs2239167|rs9814648|rs6541017|rs1887027|rs2677760|rs173704|rs11953134|rs4240896|rs11792962|rs6000495|rs163567|rs9878820|rs7910961|rs2888586|rs12409333|rs1550805|rs16967789|rs2072496
Hematologic Disorder	DESTABILIZATION_OF_MRNA_BY_KSRP	REACTOME	205	15	9	4,47E-01	9,87E-01	rs1000174|rs2269337|rs2583839|rs4983559|rs13196204|rs1533079|rs2135121|rs882253|rs1613070
Hematologic Disorder	CITRIC_ACID_CYCLE_TCA_CYCLE	REACTOME	675	30	13	4,47E-01	9,87E-01	rs1054174|rs17022214|rs2125812|rs4657015|rs2028548|rs9670039|rs2158839|rs11893686|rs1172416|rs7602538|rs3738814|rs7589648|rs7600094
Hematologic Disorder	REGULATION_OF_SIGNALING_BY_CBL	REACTOME	703	35	20	4,49E-01	9,87E-01	rs7829816|rs6541017|rs1148853|rs1465061|rs2282004|rs2677760|rs173704|rs10901096|rs11953134|rs4240896|rs7245158|rs1075834|rs17718517|rs9878820|rs4744514|rs10993709|rs9487749|rs1550805|rs3012750|rs290267
Hematologic Disorder	IGF1R_PATHWAY	BIOCARTA	735	26	20	4,49E-01	9,87E-01	rs6501512|rs8038056|rs1030098|rs2684806|rs2677760|rs173704|rs4983559|rs7558386|rs12670688|rs11953134|rs2302784|rs3801969|rs2888586|rs12901358|rs7162314|rs1550805|rs16967789|rs10243170|rs13243079|rs4966013
Hematologic Disorder	EGF_PATHWAY	BIOCARTA	1048	32	20	4,53E-01	9,87E-01	rs198178|rs4947979|rs449072|rs2529737|rs9914703|rs2110290|rs2677760|rs173704|rs6681035|rs2235360|rs841718|rs11953134|rs11979158|rs2888586|rs12409333|rs9925433|rs174827|rs17103133|rs4791032|rs1550805
Hematologic Disorder	FATTY_ACYL_COA_BIOSYNTHESIS	REACTOME	404	25	15	4,53E-01	9,87E-01	rs17620632|rs4502283|rs7656124|rs2816373|rs11037685|rs4862439|rs3792311|rs7575760|rs1429146|rs12498468|rs807673|rs11934173|rs1867802|rs3758549|rs17041597
Hematologic Disorder	GLUCAGON_TYPE_LIGAND_RECEPTORS	REACTOME	786	31	20	4,53E-01	9,87E-01	rs4898725|rs6807740|rs16947151|rs9916772|rs1253704|rs874627|rs17083085|rs3793228|rs4901170|rs2041570|rs1010004|rs3741920|rs675327|rs10907192|rs4334421|rs931647|rs12704705|rs2730260|rs8081471|rs11157866
Hematologic Disorder	CHONDROITIN_SULFATE_DERMATAN_SULFATE_METABOLISM	REACTOME	2784	23	20	4,55E-01	9,87E-01	rs17079451|rs1459433|rs10492629|rs11223958|rs319535|rs6785758|rs9498175|rs9967331|rs17728703|rs2124368|rs6932261|rs9584127|rs7577243|rs2187467|rs3798592|rs4773567|rs7329232|rs9516245|rs1980779|rs2169860
Hematologic Disorder	SCFSKP2_MEDIATED_DEGRADATION_OF_P27_P21	REACTOME	813	29	19	4,57E-01	9,87E-01	rs2526060|401754|rs4813929|rs11845708|rs6033024|rs7200210|rs8078118|rs7015841|rs12037569|rs7126210|rs13209654|rs10425066|rs17068405|rs4888688|rs9912225|rs755553|rs8009527|rs12447239|rs241429
Hematologic Disorder	MELANOMA	KEGG	2894	32	20	4,58E-01	9,87E-01	rs9307949|rs10517655|rs2174834|rs2194869|rs8038056|rs1393989|rs7430957|rs2992024|rs17072214|rs4947979|rs12523052|rs1078363|rs6541017|rs2684806|rs541682|rs2110290|rs17392492|rs6586116|rs534182|rs3806929
Hematologic Disorder	SMALL_CELL_LUNG_CANCER	KEGG	3747	37	20	4,59E-01	9,87E-01	rs7373003|rs1898722|rs241669|rs3212588|rs2568875|rs1561928|rs2365491|rs9521585|rs2121845|rs4917339|rs2362892|rs6541017|rs2302793|rs12523864|rs6586116|rs693754|rs534182|rs972795|rs2677760|rs173704
Hematologic Disorder	MYOGENESIS	REACTOME	1747	53	20	4,59E-01	9,87E-01	rs7241357|rs1499904|rs9961862|rs4500831|rs1939890|rs4740366|rs17446875|rs1970546|rs10485473|rs1894151|rs2583839|rs6028012|rs2947864|rs2427158|rs16945051|rs1852730|rs1477440|rs8099679|rs10485469|rs9883073
Hematologic Disorder	INTEGRATION_OF_ENERGY_METABOLISM	REACTOME	4501	31	20	4,59E-01	9,87E-01	rs2070600|rs12540943|rs3769309|rs244603|rs4898725|rs6056252|rs6501512|rs1578180|rs7975710|rs2645400|rs8121340|rs4502283|rs6807740|rs1881628|rs11048455|rs7008202|rs7975385|rs1030098|rs685960|rs16947151
Hematologic Disorder	SYNTHESIS_OF_BILE_ACIDS_AND_BILE_SALTS	REACTOME	546	15	8	4,61E-01	9,87E-01	rs2555491|rs4466436|rs639434|rs2151896|rs2297472|rs17642708|rs3742377|rs2278167
Hematologic Disorder	TRANSPORT_OF_VITAMINS_NUCLEOSIDES_AND_RELATED_MOLECULES	REACTOME	1154	35	20	4,64E-01	9,87E-01	rs4149126|rs2256453|rs1643033|rs11739895|rs2427372|rs2286814|rs7081|rs4854786|rs6878765|rs11045417|rs8025658|rs1246274|rs10944312|rs1676889|rs12306121|rs3743369|rs7178204|rs3740839|rs3761248|rs6480507
Hematologic Disorder	GROWTH_HORMONE_RECEPTOR_SIGNALING	REACTOME	773	27	17	4,65E-01	9,87E-01	rs12203803|rs2239167|rs7829816|rs7728464|rs7558386|rs7734558|rs2038171|rs7999458|rs11792962|rs1033440|rs6860397|rs7727306|rs6122940|rs7187776|rs13029532|rs3816997|rs2396340
Hematologic Disorder	LEUKOCYTE_TRANSENDOTHELIAL_MIGRATION	KEGG	4793	39	20	4,67E-01	9,87E-01	rs10520308|rs198178|rs6480244|rs8133991|rs3769309|rs2985700|rs7202877|rs11591534|rs10174961|rs449072|rs4258819|rs6751684|rs9971243|rs16865445|rs6541017|rs2274064|rs540874|rs17017611|rs13410482|rs9914703
Hematologic Disorder	EARLY_PHASE_OF_HIV_LIFE_CYCLE	REACTOME	270	18	10	4,67E-01	9,87E-01	rs1151514|rs874627|rs4245843|rs2391626|rs16981262|rs4764622|rs3741920|rs174532|rs2303744|rs5439
Hematologic Disorder	NATURAL_KILLER_CELL_MEDIATED_CYTOTOXICITY	KEGG	3607	51	20	4,69E-01	9,87E-01	rs198178|rs5013310|rs7830593|rs10772294|rs1876267|rs2239167|rs579589|rs449072|rs7193701|rs2596534|rs2274906|rs6922617|rs6541017|rs1148853|rs748938|rs1465061|rs16845834|rs9321590|rs9914703|rs28366099
Hematologic Disorder	M_G1_TRANSITION	REACTOME	1315	40	20	4,70E-01	9,87E-01	rs2526060|401754|rs4813929|rs11845708|rs698856|rs925192|rs10493854|rs9474192|rs6033024|rs7200210|rs837763|rs8078118|rs12037569|rs1818622|rs9295464|rs7126210|rs13209654|rs4888688|rs963267|rs9692542
Hematologic Disorder	ERK_PATHWAY	BIOCARTA	1025	30	20	4,70E-01	9,87E-01	rs8038056|rs1561928|rs4947979|rs12892024|rs2684806|rs2302793|rs2110290|rs2334915|rs1054743|rs6470648|rs2671632|rs10416531|rs4733616|rs11979158|rs2888586|rs12901358|rs10907192|rs11905013|rs4637783|rs1258033
Hematologic Disorder	CYTOCHROME_P450_ARRANGED_BY_SUBSTRATE_TYPE	REACTOME	1163	31	20	4,71E-01	9,87E-01	rs13130370|rs4405788|rs2284198|rs6012696|rs4466436|rs7172632|rs3754205|rs7069114|rs639434|rs12125747|rs17642708|rs4144915|rs1934967|rs2269429|rs3823717|rs2299893|rs11126361|rs3742377|rs2899472|rs9309462
Hematologic Disorder	RAS_ACTIVATION_UOPN_CA2_INFUX_THROUGH_NMDA_RECEPTOR	REACTOME	1033	33	20	4,75E-01	9,87E-01	rs11630971|rs7595027|rs1650397|rs2055226|rs12535537|rs8024521|rs10152417|rs8058978|rs6842886|rs2075067|rs2284428|rs2268105|rs1562008|rs11741062|rs7202071|rs7598661|rs17833967|rs815804|rs2300502|rs7174399
Hematologic Disorder	SPHINGOLIPID_METABOLISM	REACTOME	1944	30	20	4,75E-01	9,87E-01	rs11206808|rs17752182|rs947007|rs12081589|rs3011777|rs4935597|rs11810204|rs11713682|rs3892357|rs2863973|rs1362720|rs29196|rs2618956|rs11658645|rs7691016|rs2167239|rs7069860|rs10148024|rs2303147|rs7018784
Hematologic Disorder	EICOSANOID_LIGAND_BINDING_RECEPTORS	REACTOME	507	21	13	4,75E-01	9,87E-01	rs11687066|rs2260577|rs8100350|rs11851721|rs6893430|rs708491|rs348563|rs3821349|rs9535080|rs931647|rs1409166|rs1449585|rs3786989
Hematologic Disorder	SMOOTH_MUSCLE_CONTRACTION	REACTOME	658	39	18	4,76E-01	9,87E-01	rs7914896|rs6861460|rs7595027|rs2055226|rs11866891|rs4934433|rs16967494|rs11086023|rs820360|rs10229310|rs7907084|rs7598661|rs7779600|rs17169635|rs815804|rs2300502|rs2035714|rs621209
Hematologic Disorder	SIGNAL_TRANSDUCTION_BY_L1	REACTOME	1310	34	20	4,77E-01	9,87E-01	rs7113799|rs11970312|rs17275376|rs4947979|rs12892024|rs550840|rs2110290|rs3780867|rs11009311|rs2303377|rs2785262|rs4961604|rs17591358|rs226434|rs11009375|rs11979158|rs11594539|rs559928|rs2776932|rs2474741
Hematologic Disorder	SPLICEOSOME	KEGG	1685	38	20	4,79E-01	9,87E-01	rs9704|rs17825554|rs7361672|rs11845708|rs1151514|rs11578832|rs6075608|rs200177|rs622175|rs6821225|rs17198981|rs1887027|rs6733550|rs1043879|rs9908594|rs6488306|rs8072724|rs8029715|rs8070091|rs4313734
Hematologic Disorder	INCRETIN_SYNTHESIS_SECRETION_AND_INACTIVATION	REACTOME	540	43	17	4,79E-01	9,87E-01	rs244548|rs2645400|rs874627|rs7446508|rs10903341|rs6553943|rs9883073|rs7824704|rs261988|rs3741920|rs2168967|rs4840579|rs9951619|rs5439|rs7709763|rs12327532|rs681566
Hematologic Disorder	RNA_POL_III_TRANSCRIPTION_TERMINATION	REACTOME	680	24	18	4,80E-01	9,87E-01	rs11989385|rs11084882|rs13295646|rs11833143|rs2926362|rs2227301|rs13277342|rs4442161|rs630973|rs497789|rs4922152|rs4921702|rs1807429|rs1372349|rs6081150|rs10810120|rs492553|rs510342
Hematologic Disorder	GALACTOSE_METABOLISM	KEGG	759	18	14	4,81E-01	9,87E-01	rs11251481|rs10903939|rs4072113|rs869896|rs7093049|rs4726492|rs2388595|rs1362153|rs10971412|rs7788953|rs1574318|rs7897621|rs324079|rs11251588
Hematologic Disorder	HEMATOPOIETIC_CELL_LINEAGE	KEGG	1963	46	20	4,81E-01	9,87E-01	rs3212588|rs3918003|rs6861460|rs925246|rs11547281|rs9814648|rs2871444|rs6440971|rs12619383|rs13410482|rs874627|rs10777109|rs1887027|rs28772724|rs1571344|rs4722178|rs989692|rs353645|rs919751|rs4075688
Hematologic Disorder	SIGNALING_BY_FGFR_IN_DISEASE	REACTOME	4545	30	20	4,81E-01	9,87E-01	rs7983710|rs2036068|rs1888057|rs5751636|rs2174834|rs6501512|rs743584|rs2992024|rs7975710|rs7595027|rs17072214|rs429219|rs665821|rs12523052|rs4349043|rs513320|rs1078363|rs11048455|rs7008202|rs7975385
Hematologic Disorder	TASTE_TRANSDUCTION	KEGG	1100	36	20	4,83E-01	9,87E-01	rs9461937|rs7008202|rs7975385|rs953672|rs7789906|rs874627|rs2499742|rs10422148|rs2451351|rs11668370|rs17649232|rs1107660|rs4711369|rs10497603|rs3750506|rs4711338|rs13249746|rs649775|rs3741920|rs4565471
Hematologic Disorder	CSK_PATHWAY	BIOCARTA	554	29	18	4,83E-01	9,87E-01	rs6501512|rs1030098|rs12120762|rs874627|rs12670688|rs858492|rs4764622|rs2302784|rs3801969|rs12410250|rs12127588|rs3741920|rs12564899|rs9268911|rs10907192|rs10243170|rs13243079|rs5439
Hematologic Disorder	TRAF6_MEDIATED_NFKB_ACTIVATION	REACTOME	351	42	19	4,84E-01	9,87E-01	rs2070600|rs11191274|rs1394839|rs621277|rs6133067|rs10970986|rs11574849|rs2133165|rs461626|rs6084497|rs2811757|rs2830076|rs8132767|rs7267297|rs12419174|rs204991|rs7950019|rs1056890|rs889312
Hematologic Disorder	TGF_BETA_SIGNALING_PATHWAY	KEGG	3535	26	20	4,85E-01	9,87E-01	rs7662504|rs6728021|rs17798043|rs4277528|rs7617478|rs13015373|rs535343|rs1561928|rs10038|rs2237432|rs16877442|rs6140076|rs10946347|rs9765848|rs10198521|rs527929|rs17054541|rs6833281|rs2302793|rs224320
Hematologic Disorder	DRUG_METABOLISM_OTHER_ENZYMES	KEGG	1609	34	20	4,91E-01	9,87E-01	rs9704|rs11076121|rs11568314|rs7517899|rs10238679|rs2736486|rs206819|rs1399291|rs12127542|rs9437663|rs11589238|rs4949952|rs16835912|rs6499795|rs290826|rs355374|rs17323225|rs10918318|rs6683842|rs8362
Hematologic Disorder	TIGHT_JUNCTION	KEGG	6242	39	20	4,94E-01	9,87E-01	rs10520308|rs198178|rs9894327|rs6480244|rs8133991|rs2624867|rs2074876|rs2985700|rs10174961|rs987493|rs2691542|rs449072|rs4258819|rs6751684|rs1651543|rs11505717|rs9971243|rs4593210|rs16865445|rs540874
Hematologic Disorder	REGULATION_OF_WATER_BALANCE_BY_RENAL_AQUAPORINS	REACTOME	1393	32	20	4,96E-01	9,87E-01	rs4898725|rs6501512|rs6807740|rs7008202|rs7975385|rs1030098|rs16947151|rs953672|rs1253704|rs874627|rs2601773|rs902522|rs12670688|rs4901170|rs72001|rs8181443|rs2302784|rs566381|rs3801969|rs13249746
Hematologic Disorder	DILATED_CARDIOMYOPATHY	KEGG	4636	31	20	4,96E-01	9,87E-01	rs10920115|rs3753617|rs3212588|rs9510495|rs11715062|rs17275376|rs10045068|rs1467913|rs6861460|rs2102902|rs17247184|rs4659495|rs2239093|rs6806680|rs2239041|rs37070|rs7008202|rs7975385|rs2970533|rs6415964
Hematologic Disorder	GLYCINE_SERINE_AND_THREONINE_METABOLISM	KEGG	564	20	14	4,97E-01	9,87E-01	rs13299822|rs3124455|rs12449313|rs2183838|rs4623951|rs2158839|rs4538195|rs1505637|rs2047995|rs11137606|rs870541|rs694821|rs10783815|rs11137862
Hematologic Disorder	METABOLISM_OF_AMINO_ACIDS_AND_DERIVATIVES	REACTOME	5246	28	20	4,99E-01	9,87E-01	rs1741284|rs10831551|rs819136|rs4277528|rs13299822|rs10483943|rs2526060|rs1371404|401754|rs2436856|rs4813929|rs594747|rs2237432|rs11845708|rs698856|rs944605|rs1325530|rs3124455|rs10080237|rs10198521
Hematologic Disorder	REGULATORY_RNA_PATHWAYS	REACTOME	345	14	11	4,99E-01	9,87E-01	rs11084882|rs12103149|rs550353|rs2227301|rs3822360|rs2261682|rs9907255|rs669702|rs633742|rs8012576|rs663133
Hematologic Disorder	DEATH_PATHWAY	BIOCARTA	505	21	11	5,00E-01	9,87E-01	rs7830593|rs16845834|rs10268780|rs2152833|rs4872077|rs12454712|rs7367066|rs2811757|rs2850768|rs4987786|rs10489113
Hematologic Disorder	SIGNALING_BY_WNT	REACTOME	1071	32	20	5,02E-01	9,87E-01	rs2526060|401754|rs4813929|rs11845708|rs698856|rs6033024|rs7200210|rs10105118|rs8078118|rs12037569|rs3802727|rs9883073|rs7126210|rs13209654|rs1255771|rs4888688|rs963267|rs9912225|rs755553|rs8009527
Hematologic Disorder	PI_3K_CASCADE	REACTOME	1868	39	20	5,06E-01	9,87E-01	rs1888057|rs2174834|rs2992024|rs17072214|rs665821|rs12523052|rs513320|rs1078363|rs3007685|rs541682|rs17392492|rs6586116|rs534182|rs3806929|rs2677760|rs173704|rs4983559|rs2673825|rs9805578|rs12958400
Hematologic Disorder	MPR_PATHWAY	BIOCARTA	624	18	13	5,07E-01	9,87E-01	rs6501512|rs1030098|rs11123212|rs12670688|rs2302784|rs3801969|rs4809258|rs2448343|rs10907192|rs6062662|rs11905013|rs10243170|rs13243079
Hematologic Disorder	INHIBITION_OF_VOLTAGE_GATED_CA2_CHANNELS_VIA_GBETA_GAMMA_SUBUNITS	REACTOME	1082	27	20	5,08E-01	9,87E-01	rs11654455|rs4898725|rs16947151|rs1253704|rs10459889|rs3787870|rs874627|rs8079714|rs4901170|rs1073572|rs2275404|rs3741920|rs6590356|rs675327|rs10907192|rs3129073|rs4334421|rs2186344|rs931647|rs8079702
Hematologic Disorder	IL_3_5_AND_GM_CSF_SIGNALING	REACTOME	1134	30	20	5,09E-01	9,87E-01	rs2239167|rs7829816|rs9814648|rs6541017|rs1148853|rs1465061|rs11243454|rs1887027|rs2677760|rs173704|rs10901096|rs11953134|rs4240896|rs7245158|rs11792962|rs6000495|rs163567|rs1075834|rs17718517|rs9878820
Hematologic Disorder	SIGNALING_BY_FGFR	REACTOME	4154	36	20	5,09E-01	9,87E-01	rs7983710|rs2036068|rs1888057|rs2174834|rs6501512|rs2992024|rs7975710|rs7595027|rs17072214|rs429219|rs665821|rs12523052|rs4349043|rs513320|rs1078363|rs11048455|rs7008202|rs7975385|rs1030098|rs3007685
Hematologic Disorder	CYCLIN_E_ASSOCIATED_EVENTS_DURING_G1_S_TRANSITION_	REACTOME	1212	31	20	5,09E-01	9,87E-01	rs2526060|401754|rs4813929|rs1561928|rs11845708|rs2302793|rs6033024|rs7200210|rs8078118|rs6470648|rs7015841|rs12037569|rs4733616|rs7126210|rs13209654|rs9302815|rs4637783|rs10425066|rs17068405|rs6995630
Hematologic Disorder	GABA_RECEPTOR_ACTIVATION	REACTOME	2705	36	20	5,11E-01	9,87E-01	rs6606877|rs11654455|rs4898725|rs6289|rs10519568|rs488447|rs7008202|rs7975385|rs16947151|rs953672|rs1253704|rs10459889|rs11637885|rs3787870|rs874627|rs4472116|rs2601773|rs12518088|rs8079714|rs902522
Hematologic Disorder	DEGRADATION_OF_THE_EXTRACELLULAR_MATRIX	REACTOME	580	28	14	5,15E-01	9,87E-01	rs7202877|rs1061476|rs278891|rs1871050|rs11225314|rs278897|rs1683591|rs2889529|rs4085170|rs671188|rs1003349|rs8104556|rs1247571|rs1652508
Hematologic Disorder	VEGF_SIGNALING_PATHWAY	KEGG	2111	32	20	5,16E-01	9,87E-01	rs198178|rs1876267|rs2278964|rs631134|rs449072|rs1572507|rs7193701|rs6541017|rs748938|rs4820315|rs9914703|rs11582551|rs2677760|rs6458350|rs173704|rs4983559|rs1530837|rs4767886|rs230632|rs2235360
Hematologic Disorder	GLUCURONIDATION	REACTOME	656	15	3	5,16E-01	9,87E-01	rs4072113|rs2736486|rs6820734
Hematologic Disorder	INFLUENZA_LIFE_CYCLE	REACTOME	1671	28	20	5,17E-01	9,87E-01	rs3026106|rs7902136|rs729405|rs11084882|rs751716|rs7257982|rs4678191|rs10498413|rs132834|rs10895987|rs9928312|rs1149747|rs12620516|rs2261682|rs7329817|rs11177325|rs2430949|rs13219815|rs12613627|rs7981647
Hematologic Disorder	PKA_MEDIATED_PHOSPHORYLATION_OF_CREB	REACTOME	767	33	20	5,17E-01	9,87E-01	rs6501512|rs7008202|rs7975385|rs1030098|rs953672|rs2601773|rs902522|rs12670688|rs72001|rs2302784|rs3801969|rs13249746|rs4565471|rs1550116|rs12444920|rs263231|rs13407913|rs6721035|rs10243170|rs13243079
Hematologic Disorder	RETINOL_METABOLISM	KEGG	1512	62	20	5,18E-01	9,87E-01	rs2276332|rs873393|rs4405788|rs11647597|rs7069114|rs1826909|rs2736486|rs8035612|rs2654846|rs6564851|rs11865869|rs1126671|rs4144915|rs1934967|rs8026018|rs11126361|rs9309462|rs6820734|rs12978001|rs667660
Hematologic Disorder	N_GLYCAN_ANTENNAE_ELONGATION_IN_THE_MEDIAL_TRANS_GOLGI	REACTOME	962	28	14	5,19E-01	9,87E-01	rs2268535|rs6095638|rs878815|rs869896|rs16830017|rs359327|rs11813767|rs1362153|rs10858586|rs7728733|rs10971412|rs3806698|rs4141587|rs11929282
Hematologic Disorder	TEL_PATHWAY	BIOCARTA	978	27	20	5,24E-01	9,87E-01	rs8038056|rs1561928|rs4947979|rs2684806|rs2302793|rs9914703|rs2110290|rs3093926|rs4983559|rs6470648|rs4733616|rs6487465|rs11979158|rs12454712|rs12901358|rs4637783|rs6995630|rs4791032|rs2395851|rs7162314
Hematologic Disorder	LYSOSOME	KEGG	2448	30	20	5,25E-01	9,87E-01	rs2070600|rs10019475|rs7693497|rs2289599|rs11826421|rs2292963|rs13106298|rs1975802|rs11713682|rs540874|rs12528608|rs1362720|rs10798810|rs12114861|rs2297363|rs11658645|rs6849590|rs2167239|rs6854437|rs869896
Hematologic Disorder	SEMA4D_INDUCED_CELL_MIGRATION_AND_GROWTH_CONE_COLLAPSE	REACTOME	433	23	10	5,26E-01	9,87E-01	rs1651543|rs2413398|rs11866891|rs822570|rs16967494|rs9897780|rs1005570|rs1359647|rs17412099|rs10205683
Hematologic Disorder	YAP1_AND_WWTR1_TAZ_STIMULATED_GENE_EXPRESSION	REACTOME	1047	31	20	5,28E-01	9,87E-01	rs9842151|rs450110|rs4917339|rs16911828|rs11225161|rs9874964|rs12208924|rs2235734|rs997607|rs2701108|rs6940184|rs2068477|rs17787953|rs7598617|rs11022522|rs6772982|rs10774754|rs7941340|rs6808352|rs4897554
Hematologic Disorder	PLATELET_HOMEOSTASIS	REACTOME	4080	22	20	5,29E-01	9,87E-01	rs2036068|rs727152|rs4898725|rs750525|rs7069974|rs7133677|rs16929712|rs2239167|rs7975710|rs220813|rs6807740|rs1572507|rs3806777|rs710839|rs11068458|rs907647|rs11048455|rs11729954|rs7623572|rs7907343
Hematologic Disorder	NUCLEAR_EVENTS_KINASE_AND_TRANSCRIPTION_FACTOR_ACTIVATION	REACTOME	766	26	13	5,29E-01	9,87E-01	rs3799663|rs12892024|rs7305095|rs10105118|rs1852730|rs17591358|rs13196204|rs3886478|rs2187911|rs1286086|rs6721035|rs10858840|rs770082
Hematologic Disorder	BAD_PATHWAY	BIOCARTA	927	30	20	5,29E-01	9,87E-01	rs6501512|rs8038056|rs1030098|rs2684806|rs10777109|rs2677760|rs173704|rs4983559|rs12670688|rs11953134|rs2302784|rs6000495|rs3801969|rs12454712|rs12901358|rs7162314|rs1550805|rs2850768|rs10243170|rs13243079
Hematologic Disorder	NICOTINATE_AND_NICOTINAMIDE_METABOLISM	KEGG	963	42	11	5,29E-01	9,87E-01	rs11242501|rs1819628|rs16849307|rs12450758|rs4789145|rs595890|rs167508|rs7765347|rs10485305|rs10002171|rs6603811
Hematologic Disorder	PRIMARY_BILE_ACID_BIOSYNTHESIS	KEGG	439	11	6	5,29E-01	9,87E-01	rs4466436|rs639434|rs2151896|rs2297472|rs17642708|rs3742377
Hematologic Disorder	REGULATION_OF_MITOTIC_CELL_CYCLE	REACTOME	1318	28	20	5,29E-01	9,87E-01	rs2526060|401754|rs4813929|rs11845708|rs698856|rs6033024|rs6856643|rs17057833|rs7200210|rs1027692|rs8078118|rs12037569|rs315690|rs3802727|rs491675|rs11599360|rs7126210|rs2448343|rs13209654|rs3027267
Hematologic Disorder	ELONGATION_ARREST_AND_RECOVERY	REACTOME	246	11	8	5,31E-01	9,87E-01	rs1053079|rs11084882|rs2261682|rs1865094|rs633742|rs3786238|rs1613070|rs663133
Hematologic Disorder	PRE_NOTCH_PROCESSING_IN_GOLGI	REACTOME	691	26	18	5,32E-01	9,87E-01	rs2070600|rs12431724|rs12274970|rs1958223|rs2039478|rs1211307|rs10140642|rs6697258|rs10971412|rs13091236|rs4904087|rs9893372|rs828599|rs204991|rs304303|rs8192565|rs8006900|rs933209
Hematologic Disorder	SIGNALING_BY_FGFR_MUTANTS	REACTOME	1657	38	20	5,34E-01	9,87E-01	rs5751636|rs2174834|rs743584|rs2992024|rs17072214|rs12523052|rs1078363|rs541682|rs10878951|rs17392492|rs3806929|rs2677760|rs173704|rs2235360|rs9805578|rs12220387|rs11953134|rs1924210|rs10477181|rs140504
Hematologic Disorder	CELL_ADHESION_MOLECULES_CAMS	KEGG	8813	47	20	5,35E-01	9,87E-01	rs933984|rs5013310|rs17836166|rs7241357|rs8133991|rs2284191|rs3828150|rs1547371|rs229441|rs7565870|rs7113799|rs2985700|rs1822454|rs17275376|rs4419220|rs7635950|rs11547281|rs6589499|rs2828119|rs10125
Hematologic Disorder	STEROID_HORMONE_BIOSYNTHESIS	KEGG	1491	49	20	5,35E-01	9,87E-01	rs11252845|rs1937919|rs4466436|rs2684874|rs7172632|rs166050|rs11037685|rs2736486|rs2151896|rs7914666|rs4525119|rs4149530|rs1581680|rs7546652|rs2269429|rs2899472|rs433852|rs2302948|rs10923838|rs6163
Hematologic Disorder	PPAR_SIGNALING_PATHWAY	KEGG	1324	35	20	5,36E-01	9,87E-01	rs7914896|rs11826421|rs1393492|rs2555491|rs4917339|rs2165387|rs11578832|rs6599263|rs10518221|rs2286814|rs1502593|rs3109164|rs6878765|rs4862439|rs3792311|rs12609870|rs4656994|rs7575760|rs1040566|rs17006135
Hematologic Disorder	COMPLEMENT_CASCADE	REACTOME	752	30	20	5,37E-01	9,87E-01	rs6699859|rs1450660|rs4935400|rs1571344|rs1143664|rs7083840|rs1903971|rs11003258|rs1389623|rs10473234|rs700239|rs1683591|rs8264|rs9469069|rs3733001|rs2794515|rs11003138|rs11003134|rs3821801|rs930506
Hematologic Disorder	GAP_JUNCTION_ASSEMBLY	REACTOME	330	13	8	5,37E-01	9,87E-01	rs9342215|rs1555772|rs7213960|rs999095|rs12074036|rs876165|rs8064669|rs9315439
Hematologic Disorder	TRANSPORT_OF_INORGANIC_CATIONS_ANIONS_AND_AMINO_ACIDS_OLIGOPEPTIDES	REACTOME	4051	43	20	5,38E-01	9,87E-01	rs7997827|rs2034755|rs12609914|rs17492143|rs2313982|rs13274956|rs12505556|rs7144273|rs12188497|rs255049|rs13144383|rs2248465|rs4640842|rs2303715|rs12528608|rs10498084|rs7257982|rs7869301|rs1605819|rs1124329
Hematologic Disorder	TRYPTOPHAN_METABOLISM	KEGG	953	36	13	5,42E-01	9,87E-01	rs1371404|rs4665830|rs11685496|rs732215|rs3043|rs11179050|rs2126000|rs11808201|rs7527279|rs7705738|rs352887|rs1033510|rs3742384
Hematologic Disorder	GENERATION_OF_SECOND_MESSENGER_MOLECULES	REACTOME	616	34	15	5,45E-01	9,87E-01	rs1336480|rs874627|rs28772724|rs2235360|rs858492|rs252299|rs4764622|rs3130192|rs3741920|rs12564899|rs412805|rs2281389|rs8045689|rs5439|rs10045011
Hematologic Disorder	REGULATION_OF_INSULIN_LIKE_GROWTH_FACTOR_IGF_ACTIVITY_BY_INSULIN_LIKE_G DING_PROTEINS_IGFBPS	REACTOME	570	30	19	5,45E-01	9,87E-01	rs17172724|rs10275903|rs1061476|rs10276817|rs7805659|rs3770472|rs7874262|rs6478223|rs204190|rs7047400|rs7536647|rs8104556|rs11794542|rs7467853|rs1247571|rs10982912|rs1652508|rs1534150|rs879965
Hematologic Disorder	TAK1_ACTIVATES_NFKB_BY_PHOSPHORYLATION_AND_ACTIVATION_OF_IKKS_COMPLE	REACTOME	777	27	20	5,47E-01	9,87E-01	rs2070600|rs11191274|rs1394839|rs13217057|rs708030|rs2141484|rs390993|rs11574849|rs10485101|rs6570956|rs2133165|rs461626|rs4961116|rs2830076|rs8132767|rs2906766|rs1510470|rs12419174|rs9294511|rs204991
Hematologic Disorder	DOWNREGULATION_OF_TGF_BETA_RECEPTOR_SIGNALING	REACTOME	521	21	12	5,49E-01	9,87E-01	rs7617478|rs4073472|rs1539872|rs4325953|rs6099789|rs12915039|rs11687800|rs12901499|rs17702096|rs12613627|rs1005061|rs17025329
Hematologic Disorder	CTNNB1_PHOSPHORYLATION_CASCADE	REACTOME	316	8	3	5,50E-01	9,87E-01	rs10105118|rs9883073|rs1255771
Hematologic Disorder	MITOTIC_G1_G1_S_PHASES	REACTOME	2477	27	20	5,52E-01	9,87E-01	rs17193211|rs2526060|401754|rs4813929|rs1561928|rs11845708|rs4899503|rs925192|rs10849024|rs10493854|rs9474192|rs2302793|rs6033024|rs7200210|rs614367|rs7315599|rs10105118|rs837763|rs8078118|rs6470648
Hematologic Disorder	MICRORNA_MIRNA_BIOGENESIS	REACTOME	227	12	9	5,53E-01	9,87E-01	rs11084882|rs550353|rs2227301|rs3822360|rs2261682|rs669702|rs633742|rs8012576|rs663133
Hematologic Disorder	ASTHMA	KEGG	770	42	16	5,53E-01	9,87E-01	rs2284191|rs7548864|rs28772724|rs17735961|rs2041190|rs4538747|rs3130192|rs4656994|rs9268911|rs13209654|rs241427|rs9296068|rs2281389|rs3815341|rs9276711|rs181997

Hematologic Disorder	METABOLISM_OF_NUCLEOTIDES	REACTOME	1559	31	20	5,58E-01	9,87E-01	rs9704|rs10040697|rs7517899|rs13399758|rs2978663|rs10238679|rs8190893|rs2815307|rs12450758|rs10781076|rs4789145|rs595890|rs167508|rs3817657|rs206819|rs1399291|rs2853229|rs12127542|rs9437663|rs11589238
Hematologic Disorder	AUTODEGRADATION_OF_CDH1_BY_CDH1_APC_C	REACTOME	857	31	20	5,61E-01	9,87E-01	rs2526060|401754|rs4813929|rs11845708|rs6033024|rs7200210|rs8078118|rs12037569|rs315690|rs11599360|rs7126210|rs13209654|rs4888688|rs1037633|rs9912225|rs755553|rs8009527|rs6836883|rs12447239|rs241429
Hematologic Disorder	SIGNALING_BY_CONSTITUTIVELY_ACTIVE_EGFR	REACTOME	503	25	14	5,62E-01	9,87E-01	rs4947979|rs2110290|rs2677760|rs173704|rs2235360|rs11953134|rs6487465|rs11979158|rs2888586|rs926938|rs1550805|rs1994581|rs17389103|rs16967789
Hematologic Disorder	CREB_PHOSPHORYLATION_THROUGH_THE_ACTIVATION_OF_RAS	REACTOME	1349	34	20	5,63E-01	9,87E-01	rs11630971|rs11970312|rs7595027|rs1650397|rs2055226|rs12535537|rs8024521|rs10152417|rs8058978|rs6842886|rs2075067|rs17591358|rs2284428|rs2268105|rs1562008|rs11741062|rs7202071|rs7598661|rs17833967|rs2187911
Hematologic Disorder	AQUAPORIN_MEDIATED_TRANSPORT	REACTOME	1510	32	20	5,64E-01	9,87E-01	rs4898725|rs6501512|rs6807740|rs7008202|rs7975385|rs1030098|rs16947151|rs953672|rs1253704|rs874627|rs2601773|rs902522|rs12670688|rs4901170|rs4774292|rs72001|rs8181443|rs2302784|rs566381|rs3801969
Hematologic Disorder	IL_2_SIGNALING	REACTOME	936	24	20	5,65E-01	9,87E-01	rs2239167|rs9814648|rs6541017|rs1148853|rs1887027|rs2677760|rs173704|rs11953134|rs4240896|rs11792962|rs6000495|rs163567|rs9878820|rs7910961|rs4744514|rs6487465|rs2888586|rs2448343|rs10993709|rs12409333
Hematologic Disorder	LYSOSOME_VESICLE_BIOGENESIS	REACTOME	420	16	8	5,65E-01	9,87E-01	rs6733550|rs419588|rs3731828|rs4961604|rs757718|rs2283852|rs16933086|rs1254900
Hematologic Disorder	TGF_BETA_RECEPTOR_SIGNALING_ACTIVATES_SMADS	REACTOME	563	21	12	5,65E-01	9,87E-01	rs7617478|rs4073472|rs1539872|rs4325953|rs6099789|rs12915039|rs11687800|rs12901499|rs17702096|rs12613627|rs1005061|rs17025329
Hematologic Disorder	PHOSPHORYLATION_OF_CD3_AND_TCR_ZETA_CHAINS	REACTOME	521	23	12	5,66E-01	9,87E-01	rs12120762|rs874627|rs28772724|rs858492|rs4764622|rs3130192|rs12410250|rs12127588|rs3741920|rs12564899|rs2281389|rs5439
Hematologic Disorder	AMINO_SUGAR_AND_NUCLEOTIDE_SUGAR_METABOLISM	KEGG	956	37	20	5,68E-01	9,87E-01	rs2628440|rs13026308|rs9391926|rs4072113|rs4674406|rs481325|rs10857871|rs17772897|rs11102233|rs10814369|rs10841923|rs3805703|rs3756444|rs1113428|rs10019381|rs7768483|rs7897621|rs2814820|rs12210306|rs10479469
Hematologic Disorder	SRP_DEPENDENT_COTRANSLATIONAL_PROTEIN_TARGETING_TO_MEMBRANE	REACTOME	1378	32	20	5,69E-01	9,87E-01	rs7902136|rs1701470|rs7805129|rs751716|rs7257982|rs10498413|rs4131086|rs10895987|rs6553943|rs9928312|rs1149747|rs12620516|rs10512267|rs4653669|rs4268186|rs10799322|rs2430949|rs2168967|rs11002569|rs10140464
Hematologic Disorder	NOTCH_SIGNALING_PATHWAY	KEGG	1387	30	20	5,70E-01	9,87E-01	rs2070600|rs17825554|rs1242991|rs1584783|rs1359779|rs906304|rs3850839|rs7741552|rs6104695|rs7943506|rs7930909|rs1474817|rs2068477|rs3796633|rs4753196|rs1865094|rs11891208|rs1535175|rs3781408|rs837492
Hematologic Disorder	CIRCADIAN_REPRESSION_OF_EXPRESSION_BY_REV_ERBA	REACTOME	1358	33	20	5,71E-01	9,87E-01	rs341404|rs9842151|rs6448226|rs450110|rs877228|rs4917339|rs9874964|rs12909890|rs2235734|rs930358|rs4450343|rs6494219|rs3844598|rs3768984|rs4775292|rs7598617|rs2052455|rs6772982|rs17191554|rs17270571
Hematologic Disorder	BRANCHED_CHAIN_AMINO_ACID_CATABOLISM	REACTOME	507	30	12	5,74E-01	9,87E-01	rs10080237|rs11761572|rs9423240|rs13353058|rs10771144|rs2158839|rs17744890|rs534800|rs3132810|rs757141|rs4646857|rs17810414
Hematologic Disorder	PAR1_PATHWAY	BIOCARTA	1941	34	20	5,75E-01	9,87E-01	rs198178|rs6056252|rs8121340|rs449072|rs12600950|rs6086473|rs2719804|rs9914703|rs13217057|rs9515380|rs2423360|rs13074616|rs2677760|rs173704|rs6133635|rs822570|rs6086495|rs625572|rs2141484|rs11953134
Hematologic Disorder	METABOLISM_OF_STEROID_HORMONES_AND_VITAMINS_A_AND_D	REACTOME	745	40	20	5,75E-01	9,87E-01	rs1914172|rs2796833|rs11647597|rs7172632|rs166050|rs7916688|rs2268365|rs6564851|rs11865869|rs7546652|rs9665553|rs4233828|rs2269429|rs2271463|rs7788953|rs2899472|rs2302694|rs10923838|rs6163|rs10199676
Hematologic Disorder	GLYCOSAMINOGLYCAN_BIOSYNTHESIS_CHONDROITIN_SULFATE	KEGG	1372	27	20	5,76E-01	9,87E-01	rs17079451|rs11223958|rs9498175|rs17728703|rs6932261|rs2187467|rs3798592|rs2169860|rs2128333|rs12598195|rs11641613|rs7926027|rs4335563|rs13219957|rs7314687|rs11862371|rs312157|rs4255769|rs651984|rs7202082
Hematologic Disorder	PATHOGENIC_ESCHERICHIA_COLI_INFECTION	KEGG	1256	51	20	5,79E-01	9,87E-01	rs1053079|rs2806117|rs11591988|rs4740366|rs2225602|rs1465061|rs9405622|rs3909578|rs9914703|rs2583839|rs4483225|rs3785076|rs9883073|rs2135121|rs17032053|rs35580157|rs4791032|rs9487749|rs17031463|rs1131171
Hematologic Disorder	GATA3_PATHWAY	BIOCARTA	898	25	20	5,80E-01	9,87E-01	rs12261421|rs6501512|rs1030098|rs748938|rs12785018|rs3760201|rs12670688|rs2302784|rs13196204|rs7091279|rs3801969|rs1555147|rs17143686|rs7911451|rs17798093|rs1538242|rs11256133|rs1572297|rs13243079|rs7913253
Hematologic Disorder	ANTIVIRAL_MECHANISM_BY_IFN_STIMULATED_GENES	REACTOME	1077	32	20	5,82E-01	9,87E-01	rs3026106|rs729405|rs4678191|rs7438406|rs621277|rs132834|rs2235360|rs10970986|rs11177325|rs1110240|rs12409333|rs11716884|rs6787425|rs13219815|rs102993|rs1037633|rs3851366|rs6749955|rs1198329|rs4740419
Hematologic Disorder	RESPIRATORY_ELECTRON_TRANSPORT_ATP_SYNTHESIS_BY_CHEMIOSMOTIC_COUPLIN<br>TION_BY_UNCOUPLING_PROTEINS_	REACTOME	1741	49	20	5,84E-01	9,87E-01	rs8133991|rs12654078|rs1626521|rs1054174|rs10777647|rs13263130|rs4797343|rs6538582|rs2834291|rs10268780|rs4657015|rs9931644|rs10206264|rs4656994|rs10491416|rs637421|rs3738814|rs4241236|rs2161249|rs249189
Hematologic Disorder	RNA_POL_II_PRE_TRANSCRIPTION_EVENTS	REACTOME	532	23	14	5,84E-01	9,87E-01	rs11826421|rs1053079|rs11084882|rs1471420|rs2261682|rs1865094|rs7833229|rs963267|rs633742|rs3786238|rs7946331|rs1613070|rs1561734|rs663133
Hematologic Disorder	LEISHMANIA_INFECTION	KEGG	2265	37	20	5,85E-01	9,87E-01	rs198178|rs2284191|rs2239167|rs2806117|rs449072|rs2225602|rs11811630|rs13410482|rs9321590|rs13217057|rs28772724|rs4483225|rs1571344|rs6853|rs6681035|rs1339|rs1389623|rs2141484|rs11792962|rs13196204
Hematologic Disorder	PI3K_CASCADE	REACTOME	2702	34	20	5,86E-01	9,87E-01	rs12540943|rs1888057|rs2174834|rs2992024|rs17072214|rs12523052|rs1881628|rs513320|rs10500204|rs1078363|rs2672886|rs3007685|rs4969429|rs541682|rs9510129|rs4633807|rs3806929|rs7438406|rs4121647|rs2677760
Hematologic Disorder	RORA_ACTIVATES_CIRCADIAN_EXPRESSION	REACTOME	1377	33	20	5,86E-01	9,87E-01	rs341404|rs9842151|rs6448226|rs450110|rs877228|rs4917339|rs9874964|rs12909890|rs2235734|rs930358|rs4450343|rs6494219|rs3844598|rs3768984|rs4775292|rs7598617|rs2052455|rs6772982|rs17191554|rs17270571
Hematologic Disorder	STEROID_BIOSYNTHESIS	KEGG	247	6	4	5,86E-01	9,87E-01	rs2684874|rs1129195|rs2297472|rs7531307
Hematologic Disorder	CACAM_PATHWAY	BIOCARTA	401	26	15	5,86E-01	9,87E-01	rs7595027|rs429219|rs2055226|rs12535537|rs6842886|rs2075067|rs3797755|rs7598661|rs815804|rs2300502|rs6721035|rs457734|rs2241695|rs3733619|rs2236893
Hematologic Disorder	CD28_CO_STIMULATION	REACTOME	797	35	15	5,87E-01	9,87E-01	rs7829816|rs3007685|rs1465061|rs2677760|rs173704|rs4983559|rs11953134|rs7245158|rs17718517|rs12106790|rs11905013|rs9487749|rs1550805|rs16967789|rs7086224
Hematologic Disorder	SEMA4D_IN_SEMAPHORIN_SIGNALING	REACTOME	597	27	14	5,87E-01	9,87E-01	rs1651543|rs12120762|rs2413398|rs11866891|rs822570|rs16967494|rs9897780|rs12410250|rs12127588|rs12551214|rs1005570|rs1359647|rs17412099|rs10205683
Hematologic Disorder	AXON_GUIDANCE	KEGG	9506	39	20	5,87E-01	9,87E-01	rs40725|rs679423|rs7480538|rs2938818|rs1354096|rs42192|rs6882860|rs1442089|rs6548499|rs7367207|rs1876267|rs6702300|rs17142743|rs4844419|rs11591534|rs4422405|rs11936468|rs10502934|rs2675181|rs327649
Hematologic Disorder	SNARE_INTERACTIONS_IN_VESICULAR_TRANSPORT	KEGG	828	29	20	5,89E-01	9,87E-01	rs17741348|rs8073622|rs2695863|rs6818421|rs6026402|rs6733550|rs4610878|rs3731828|rs2075067|rs1591811|rs7215786|rs11087010|rs3286|rs6026373|rs255313|rs4813925|rs6583595|rs545572|rs11657426|rs4759753
Hematologic Disorder	DEPOSITION_OF_NEW_CENPA_CONTAINING_NUCLEOSOMES_AT_THE_CENTROMERE	REACTOME	637	55	13	5,94E-01	9,87E-01	rs8134439|rs2051186|rs3806929|rs762224|rs1009181|rs17690723|rs4699390|rs198853|rs1550116|rs1957173|rs6939589|rs12433547|rs12906755
Hematologic Disorder	CTCF_PATHWAY	BIOCARTA	1035	33	20	5,94E-01	9,87E-01	rs7617478|rs1561928|rs284169|rs2302793|rs6586116|rs534182|rs2677760|rs173704|rs2673825|rs6470648|rs4325953|rs11953134|rs284180|rs1887054|rs4733616|rs4637783|rs6995630|rs2395851|rs1550805|rs7526672
Hematologic Disorder	IMMUNOREGULATORY_INTERACTIONS_BETWEEN_A_LYMPHOID_AND_A_NON_LYMPHOI	REACTOME	1325	55	20	5,95E-01	9,87E-01	rs5013310|rs10772294|rs11547281|rs12310310|rs2735007|rs3785076|rs10411879|rs858492|rs1389623|rs2205292|rs2596574|rs10160565|rs208894|rs2245822|rs12564899|rs170962|rs1611350|rs202873|rs2838734|rs4818278
Hematologic Disorder	METABOLISM_OF_VITAMINS_AND_COFACTORS	REACTOME	1119	45	20	5,96E-01	9,87E-01	rs10513009|rs4502283|rs1819628|rs1525459|rs16849307|rs7760703|rs12449313|rs7568490|rs17228538|rs12947270|rs1659700|rs13328363|rs11144870|rs10275875|rs2699474|rs943415|rs4311592|rs17382631|rs3004068|rs7267297
Hematologic Disorder	METABOLISM_OF_MRNA	REACTOME	3017	29	20	5,96E-01	9,87E-01	rs7902136|rs2526060|rs2011786|401754|rs4813929|rs7133677|rs2226099|rs11553287|rs11845708|rs698856|rs11578832|rs751716|rs1000174|rs6033024|rs6821225|rs7257982|rs2269337|rs10498413|rs9914703|rs2583839
Hematologic Disorder	AMYOTROPHIC_LATERAL_SCLEROSIS_ALS	KEGG	1813	43	20	5,97E-01	9,87E-01	rs4958671|rs2914621|rs1876267|rs11068458|rs1650397|rs4273649|rs4533948|rs13189947|rs3760201|rs2947864|rs8058978|rs1061624|rs10268780|rs10476798|rs13196204|rs2284428|rs17180771|rs4656994|rs2268105|rs2732514
Hematologic Disorder	TYROSINE_METABOLISM	KEGG	1169	42	17	5,98E-01	9,87E-01	rs10831551|rs2276332|rs732215|rs2232248|rs4822458|rs1826909|rs2654846|rs2276702|rs4784991|rs1126671|rs738807|rs12238357|rs6474675|rs10183226|rs1325115|rs7206052|rs3025373
Hematologic Disorder	INSULIN_RECEPTOR_SIGNALLING_CASCADE	REACTOME	3006	35	20	5,98E-01	9,87E-01	rs12540943|rs1888057|rs11770199|rs2174834|rs2992024|rs17072214|rs12523052|rs1881628|rs513320|rs10500204|rs1078363|rs2672886|rs3007685|rs4969429|rs541682|rs17392492|rs4633807|rs3806929|rs7438406|rs4121647
Hematologic Disorder	REGULATION_OF_ACTIN_CYTOSKELETON	KEGG	7089	25	20	6,00E-01	9,87E-01	rs1898722|rs9307949|rs2036068|rs3212588|rs10517655|rs2985700|rs2174834|rs7202877|rs17275376|rs2483852|rs2194869|rs1336480|rs1393989|rs2992024|rs1072319|rs11869351|rs6861460|rs17718875|rs6875519|rs17072214
Hematologic Disorder	ARF_PATHWAY	BIOCARTA	733	38	18	6,01E-01	9,87E-01	rs1561928|rs4740366|rs2302793|rs2583839|rs2227301|rs2677760|rs173704|rs6470648|rs11953134|rs4733616|rs4637783|rs6995630|rs2395851|rs1550805|rs3218020|rs4265149|rs492553|rs510342
Hematologic Disorder	ANTIGEN_PROCESSING_UBIQUITINATION_PROTEASOME_DEGRADATION	REACTOME	4788	25	20	6,03E-01	9,87E-01	rs1575335|rs7716238|rs899348|rs875785|rs12436854|rs2526060|rs11253250|401754|rs4813929|rs1053079|rs3817442|rs2189713|rs1454387|rs1863703|rs12205861|rs11845708|rs2735202|rs698856|rs4479142|rs7601345
Hematologic Disorder	SIGNALING_BY_BMP	REACTOME	918	51	20	6,03E-01	9,87E-01	rs7662504|rs6140076|rs1560909|rs7560572|rs2700262|rs16827260|rs2225265|rs13044457|rs4662532|rs11599360|rs7263691|rs1950047|rs7546054|rs1259299|rs4454537|rs11071904|rs10511595|rs12906270|rs6726059|rs893911
Hematologic Disorder	TPO_PATHWAY	BIOCARTA	814	33	20	6,06E-01	9,87E-01	rs198178|rs449072|rs9914703|rs2677760|rs173704|rs6681035|rs2235360|rs11953134|rs11792962|rs2888586|rs9925433|rs17103133|rs174827|rs4791032|rs1550805|rs16967789|rs4776783|rs11859534|rs13029532|rs204680
Hematologic Disorder	ACUTE_MYELOID_LEUKEMIA	KEGG	1892	28	20	6,06E-01	9,87E-01	rs7279722|rs9979015|rs2248720|rs1561928|rs12573128|rs2735202|rs1338423|rs6541017|rs2302793|rs2677760|rs2715553|rs173704|rs614367|rs4983559|rs6470648|rs679162|rs11953134|rs4240896|rs13277521|rs11088302
Hematologic Disorder	RESPONSE_TO_ELEVATED_PLATELET_CYTOSOLIC_CA2_	REACTOME	2301	39	20	6,07E-01	9,87E-01	rs1898722|rs198178|rs3775478|rs7595027|rs449072|rs1394839|rs4764521|rs2055226|rs9914703|rs761900|rs16972767|rs4236049|rs6458350|rs9504738|rs10520130|rs5985|rs11611433|rs1628382|rs9524681|rs757718
Hematologic Disorder	SULFUR_AMINO_ACID_METABOLISM	REACTOME	930	31	19	6,09E-01	9,87E-01	rs819136|rs3124455|rs10077775|rs2028900|rs2915850|rs520002|rs16979344|rs676476|rs2235970|rs1946198|rs2993763|rs7704440|rs10068900|rs158850|rs6088466|rs7297662|rs4301242|rs10125400|rs13164902
Hematologic Disorder	CELL_SURFACE_INTERACTIONS_AT_THE_VASCULAR_WALL	REACTOME	2650	38	20	6,09E-01	9,87E-01	rs1898722|rs8133991|rs2313982|rs6707461|rs2239167|rs4848908|rs7829816|rs13144383|rs1975802|rs1465061|rs12988898|rs6045559|rs1605819|rs2677760|rs173704|rs2235360|rs11953134|rs11265544|rs973608|rs7124686
Hematologic Disorder	REGULATION_OF_IFNA_SIGNALING	REACTOME	339	12	6	6,10E-01	9,87E-01	rs2239167|rs2038171|rs7037389|rs12409333|rs6122940|rs13029532
Hematologic Disorder	PGC1A_PATHWAY	BIOCARTA	959	39	20	6,14E-01	9,87E-01	rs1876267|rs7595027|rs429219|rs6448226|rs2055226|rs12535537|rs6842886|rs1852730|rs2075067|rs3797755|rs2732514|rs4699014|rs7598661|rs4872495|rs2129301|rs815804|rs2300502|rs457734|rs4253662|rs2241695
Hematologic Disorder	TOB1_PATHWAY	BIOCARTA	652	19	15	6,15E-01	9,87E-01	rs7617478|rs284169|rs1887027|rs858492|rs4325953|rs284180|rs1887054|rs12564899|rs7910961|rs12915039|rs4278789|rs12901499|rs7526672|rs17025329|rs3014996
Hematologic Disorder	NUCLEAR_SIGNALING_BY_ERBB4	REACTOME	2231	34	20	6,16E-01	9,87E-01	rs12203803|rs16948096|rs2976532|rs2975498|rs11591534|rs980281|rs10501995|rs3850839|rs7728464|rs4887934|rs4673677|rs3744473|rs7734558|rs1481762|rs2439321|rs7196183|rs7562228|rs3802727|rs13252799|rs11792962
Hematologic Disorder	ACYL_CHAIN_REMODELLING_OF_PG	REACTOME	571	47	20	6,20E-01	9,87E-01	rs631134|rs776717|rs1572507|rs11582551|rs230632|rs330746|rs330725|rs2292023|rs776797|rs2522137|rs776698|rs1843854|rs11589805|rs623784|rs600415|rs4651390|rs695117|rs6085358|rs11802712|rs1567102
Hematologic Disorder	IL22BP_PATHWAY	BIOCARTA	296	13	9	6,20E-01	9,87E-01	rs12062136|rs2046068|rs2227492|rs841718|rs11792962|rs12409333|rs7418238|rs13029532|rs2072496
Hematologic Disorder	INWARDLY_RECTIFYING_K_CHANNELS	REACTOME	1317	27	20	6,25E-01	9,87E-01	rs11654455|rs4898725|rs16947151|rs1253704|rs10459889|rs3787870|rs874627|rs8079714|rs4901170|rs1073572|rs2275404|rs3741920|rs6590356|rs675327|rs10907192|rs3129073|rs4334421|rs2186344|rs931647|rs8079702
Hematologic Disorder	EPO_PATHWAY	BIOCARTA	255	10	9	6,26E-01	9,87E-01	rs2239167|rs6681035|rs2235360|rs11792962|rs2888586|rs17103133|rs16967789|rs4776783|rs204680
Hematologic Disorder	RNA_POL_III_TRANSCRIPTION	REACTOME	861	27	19	6,27E-01	9,87E-01	rs11989385|rs11084882|rs13295646|rs11833143|rs2926362|rs2227301|rs13277342|rs7548201|rs4442161|rs630973|rs497789|rs4922152|rs4921702|rs1807429|rs1372349|rs6081150|rs10810120|rs492553|rs510342
Hematologic Disorder	DC_PATHWAY	BIOCARTA	573	15	9	6,27E-01	9,87E-01	rs2806117|rs2225602|rs4483225|rs1339|rs2454909|rs17030340|rs3213094|rs12552190|rs3014996
Hematologic Disorder	P53_SIGNALING_PATHWAY	KEGG	1897	25	20	6,27E-01	9,87E-01	rs7830593|rs17172724|rs17230094|rs13159031|rs10849024|rs4278081|rs12454840|rs4150084|rs6586116|rs534182|rs7805659|rs6760597|rs614367|rs7315599|rs2673825|rs4934433|rs10268780|rs7015841|rs10520130|rs679162
Hematologic Disorder	REGULATION_OF_INSULIN_SECRETION	REACTOME	3741	31	20	6,27E-01	9,87E-01	rs3769309|rs244603|rs4898725|rs6056252|rs6501512|rs1072319|rs7975710|rs2645400|rs8121340|rs6807740|rs11048455|rs7008202|rs7975385|rs1030098|rs685960|rs16947151|rs6415964|rs6086473|rs953672|rs1253704
Hematologic Disorder	COMP_PATHWAY	BIOCARTA	628	34	20	6,28E-01	9,87E-01	rs6699859|rs1450660|rs4935400|rs1143664|rs7083840|rs1903971|rs11003258|rs1389623|rs10473234|rs700239|rs1683591|rs8264|rs9469069|rs3733001|rs11003138|rs11003134|rs3821801|rs930506|rs2840323|rs537160
Hematologic Disorder	FC_GAMMA_R_MEDIATED_PHAGOCYTOSIS	KEGG	3795	33	20	6,30E-01	9,87E-01	rs198178|rs11206808|rs1331006|rs6899021|rs947007|rs12081589|rs2278964|rs776717|rs449072|rs1572507|rs7829816|rs31568|rs1992309|rs11810204|rs6541017|rs1148853|rs12120762|rs10499602|rs4820315|rs9914703
Hematologic Disorder	PTDINS_PATHWAY	BIOCARTA	1058	34	20	6,30E-01	9,87E-01	rs11251481|rs10903939|rs7829816|rs1359779|rs6104695|rs4983559|rs7559522|rs2235360|rs1474817|rs7093049|rs17180771|rs2388595|rs334550|rs2264420|rs2254669|rs2106373|rs1574318|rs6550227|rs11251588|rs6033079
Hematologic Disorder	GLIOMA	KEGG	2070	27	20	6,31E-01	9,87E-01	rs198178|rs8038056|rs7595027|rs4947979|rs449072|rs6541017|rs2684806|rs2055226|rs9914703|rs2110290|rs12535537|rs6586116|rs534182|rs2677760|rs173704|rs614367|rs4983559|rs6842886|rs2673825|rs2235360
Hematologic Disorder	CARM_ER_PATHWAY	BIOCARTA	1617	30	20	6,31E-01	9,87E-01	rs1242991|rs12533919|rs3791500|rs6448226|rs11685345|rs2188495|rs980281|rs2823035|rs906304|rs7786745|rs12530920|rs2823040|rs614367|rs3791428|rs12579363|rs1852730|rs11168249|rs679162|rs3844598|rs3782908
Hematologic Disorder	AGR_PATHWAY	BIOCARTA	1981	28	20	6,34E-01	9,87E-01	rs2976532|rs2975498|rs6861460|rs4947979|rs6926377|rs10509470|rs17074746|rs1649947|rs12523864|rs849668|rs2110290|rs6681035|rs4767886|rs6057007|rs1856416|rs12205363|rs1481762|rs11196301|rs521803|rs2439321
Hematologic Disorder	ATM_PATHWAY	BIOCARTA	397	17	9	6,34E-01	9,87E-01	rs4740366|rs2583839|rs6681035|rs5752792|rs675327|rs12731705|rs3765705|rs8086622|rs10489113
Hematologic Disorder	NUCLEAR_RECEPTOR_TRANSCRIPTION_PATHWAY	REACTOME	2750	27	20	6,35E-01	9,87E-01	rs4478032|rs341404|rs12437118|rs7619728|rs218291|rs5002695|rs2690035|rs877228|rs4917339|rs980281|rs12909890|rs12562202|rs2247328|rs4903399|rs11622517|rs2715553|rs10995388|rs930358|rs9812637|rs830783
Hematologic Disorder	GLUCOSE_METABOLISM	REACTOME	1554	27	20	6,39E-01	9,87E-01	rs11251481|rs10903939|rs2239167|rs7595027|rs11918537|rs13072710|rs2055226|rs4072113|rs10105118|rs7096344|rs764453|rs7093049|rs11227623|rs9309906|rs8052416|rs1040566|rs3561|rs2388595|rs4784991|rs7598661
Hematologic Disorder	INHIBITION_OF_THE_PROTEOLYTIC_ACTIVITY_OF_APC_C_REQUIRED_FOR_THE_ONSET_<br>TOTIC_SPINDLE_CHECKPOINT_COMPONENTS	REACTOME	365	11	6	6,39E-01	9,87E-01	rs6856643|rs1027692|rs315690|rs11599360|rs1037633|rs6836883
Hematologic Disorder	VASOPRESSIN_REGULATED_WATER_REABSORPTION	KEGG	1100	30	20	6,43E-01	9,87E-01	rs1913462|rs7975385|rs4732279|rs953672|rs41307|rs2601773|rs754217|rs16874653|rs4807546|rs1481986|rs10488753|rs10895461|rs566381|rs17180771|rs6948572|rs177480|rs8071026|rs1550116|rs628426|rs3785089
Hematologic Disorder	NFKB_AND_MAP_KINASES_ACTIVATION_MEDIATED_BY_TLR4_SIGNALING_REPERTOIRE	REACTOME	2248	31	20	6,45E-01	9,87E-01	rs2070600|rs3799663|rs1053079|rs2806117|rs12892024|rs11191274|rs7305095|rs2225602|rs1394839|rs2529737|rs13217057|rs4483225|rs3760201|rs2947864|rs6681035|rs10105118|rs708030|rs2141484|rs1852730|rs17591358
Hematologic Disorder	AUTOIMMUNE_THYROID_DISEASE	KEGG	1646	65	20	6,45E-01	9,87E-01	rs5013310|rs2284191|rs28772724|rs2735007|rs4934433|rs2596574|rs17212090|rs3130192|rs2276702|rs12106790|rs2245822|rs9268911|rs7037389|rs1611350|rs2105436|rs13209654|rs241427|rs9296068|rs11967684|rs8022139
Hematologic Disorder	POST_NMDA_RECEPTOR_ACTIVATION_EVENTS	REACTOME	1753	42	20	6,48E-01	9,87E-01	rs11630971|rs11970312|rs7595027|rs429219|rs7008202|rs1650397|rs2055226|rs12535537|rs8024521|rs10152417|rs8058978|rs6842886|rs2075067|rs17591358|rs2284428|rs3797755|rs13249746|rs2268105|rs1562008|rs11741062
Hematologic Disorder	GLYCOSPHINGOLIPID_BIOSYNTHESIS_LACTO_AND_NEOLACTO_SERIES	KEGG	670	35	19	6,49E-01	9,87E-01	rs597697|rs567149|rs2837111|rs638338|rs7928391|rs869896|rs9295586|rs2029083|rs1362153|rs10971412|rs16854351|rs13091236|rs3806698|rs828599|rs12151513|rs2785917|rs304303|rs7601633|rs16870458
Hematologic Disorder	APC_CDC20_MEDIATED_DEGRADATION_OF_NEK2A	REACTOME	396	11	6	6,50E-01	9,87E-01	rs6856643|rs1027692|rs315690|rs11599360|rs1037633|rs6836883
Hematologic Disorder	ALLOGRAFT_REJECTION	KEGG	1510	67	20	6,52E-01	9,87E-01	rs5013310|rs2284191|rs28772724|rs2735007|rs4934433|rs2596574|rs4538747|rs3130192|rs12106790|rs2245822|rs9268911|rs1611350|rs2105436|rs13209654|rs241427|rs9296068|rs11967684|rs2281389|rs2523554|rs3948793
Hematologic Disorder	APOPTOSIS	KEGG	1727	39	20	6,52E-01	9,87E-01	rs7830593|rs1876267|rs6501512|rs1030098|rs6541017|rs622175|rs12619383|rs16845834|rs2334915|rs2677760|rs173704|rs4983559|rs6853|rs4934433|rs10268780|rs12670688|rs708030|rs11953134|rs4240896|rs2302784
Hematologic Disorder	PDGF_PATHWAY	BIOCARTA	1021	33	20	6,53E-01	9,87E-01	rs198178|rs449072|rs2529737|rs9914703|rs2677760|rs173704|rs6681035|rs2235360|rs841718|rs11953134|rs2888586|rs12409333|rs9925433|rs174827|rs17103133|rs4791032|rs1550805|rs16967789|rs4776783|rs11859534
Hematologic Disorder	TOLL_PATHWAY	BIOCARTA	1147	30	20	6,53E-01	9,87E-01	rs1053079|rs2806117|rs2225602|rs2529737|rs13217057|rs4483225|rs3760201|rs2947864|rs6853|rs6681035|rs1339|rs2141484|rs13196204|rs11466640|rs10485101|rs6570956|rs2133165|rs9916229|rs17103133|rs4253662
Hematologic Disorder	SYNTHESIS_SECRETION_AND_DEACYLATION_OF_GHRELIN	REACTOME	624	52	20	6,53E-01	9,87E-01	rs1996483|rs10816539|rs7875152|rs12338922|rs9873795|rs2075355|rs6444918|rs9854178|rs6553943|rs9812221|rs261988|rs944472|rs2168967|rs4979258|rs667052|rs4978670|rs7709763|rs12327532|rs13289749|rs1360257
Hematologic Disorder	SYNTHESIS_OF_DNA	REACTOME	1545	32	20	6,53E-01	9,87E-01	rs2526060|401754|rs4813929|rs2244095|rs11845708|rs698856|rs9474192|rs6033024|rs7200210|rs837763|rs8078118|rs12037569|rs10774976|rs16981262|rs1818622|rs7126210|rs13209654|rs17068405|rs4888688|rs1317069
Hematologic Disorder	METAL_ION_SLC_TRANSPORTERS	REACTOME	653	13	8	6,56E-01	9,87E-01	rs10049581|rs1540052|rs16861582|rs11776475|rs10940184|rs11695739|rs10038257|rs1891092
Hematologic Disorder	SIGNALING_BY_EGFR_IN_CANCER	REACTOME	4030	33	20	6,57E-01	9,87E-01	rs7983710|rs2036068|rs6501512|rs7975710|rs7595027|rs4947979|rs429219|rs665821|rs748289|rs1870510|rs4349043|rs11048455|rs7008202|rs7975385|rs1030098|rs3007711|rs6563245|rs953672|rs9845175|rs2055226
Hematologic Disorder	AMINO_ACID_AND_OLIGOPEPTIDE_SLC_TRANSPORTERS	REACTOME	1707	37	20	6,61E-01	9,87E-01	rs7997827|rs12609914|rs2313982|rs13144383|rs1975802|rs4640842|rs2303715|rs12528608|rs7257982|rs1605819|rs3887266|rs2955849|rs9788810|rs748680|rs1007160|rs1165176|rs3759608|rs12891079|rs720625|rs7685529
Hematologic Disorder	PYRUVATE_METABOLISM	KEGG	908	30	20	6,62E-01	9,87E-01	rs2306787|rs12285344|rs7673954|rs3043|rs10814535|rs12448432|rs11227623|rs2158839|rs11732439|rs1040566|rs7685841|rs3561|rs10516988|rs7527279|rs7788953|rs662361|rs7705738|rs11943890|rs6025642|rs7946331
Hematologic Disorder	DOWNSTREAM_SIGNALING_OF_ACTIVATED_FGFR	REACTOME	3636	31	20	6,62E-01	9,87E-01	rs2036068|rs1888057|rs2174834|rs6501512|rs2992024|rs7975710|rs7595027|rs17072214|rs429219|rs665821|rs12523052|rs513320|rs1078363|rs11048455|rs7008202|rs7975385|rs1030098|rs3007685|rs953672|rs541682
Hematologic Disorder	BILE_ACID_AND_BILE_SALT_METABOLISM	REACTOME	1176	28	17	6,63E-01	9,87E-01	rs12582527|rs2555491|rs4466436|rs639434|rs2151896|rs1014030|rs3916932|rs2297472|rs17642708|rs12306121|rs9519177|rs3742377|rs2278167|rs4794172|rs723603|rs4149033|rs3916947
Hematologic Disorder	LAIR_PATHWAY	BIOCARTA	479	21	13	6,63E-01	9,87E-01	rs1450660|rs13410482|rs4722178|rs1389623|rs10473234|rs3753306|rs170962|rs6796803|rs2838734|rs8264|rs3917018|rs6565191|rs4818989
Hematologic Disorder	CHRONIC_MYELOID_LEUKEMIA	KEGG	2538	34	20	6,67E-01	9,87E-01	rs7279722|rs5751636|rs9979015|rs2248720|rs7617478|rs743584|rs1561928|rs4740366|rs6541017|rs2302793|rs3863105|rs2583839|rs2677760|rs173704|rs614367|rs4983559|rs6414232|rs6470648|rs4325953|rs679162

Hematologic Disorder	BASAL_CELL_CARCINOMA	KEGG	2166	27	20	6,67E-01	9,87E-01	rs603401|rs680997|rs12573128|rs1990135|rs1863703|rs1338423|rs4448343|rs6140076|rs776501|rs11979793|rs351359|rs12892252|rs2225265|rs13044457|rs7222705|rs12686467|rs10898552|rs7135366|rs12434228|rs4765835
Hematologic Disorder	MAPK_SIGNALING_PATHWAY	KEGG	9833	26	20	6,68E-01	9,87E-01	rs10920115|rs175338|rs198178|rs11630971|rs3821709|rs1876267|rs591676|rs17521587|rs12566751|rs11715062|rs7617478|rs2174834|rs11970312|rs12304579|rs2278964|rs1393989|rs1561928|rs1467913|rs2992024|rs631134
Hematologic Disorder	NDKDYNAMIN_PATHWAY	BIOCARTA	548	32	17	6,68E-01	9,87E-01	rs1876267|rs7595027|rs11702774|rs2055226|rs10499602|rs2732514|rs4699014|rs7598661|rs4872495|rs2181190|rs815804|rs2300502|rs3003607|rs1469980|rs6851231|rs2392581|rs10501608
Hematologic Disorder	S_PHASE	REACTOME	2076	23	20	6,69E-01	9,87E-01	rs2526060|401754|rs4813929|rs1561928|rs2244095|rs11845708|rs698856|rs9474192|rs2302793|rs6033024|rs2281479|rs7200210|rs614367|rs837763|rs8078118|rs6470648|rs7015841|rs679162|rs12037569|rs10774976
Hematologic Disorder	TRANSPORT_OF_GLUCOSE_AND_OTHER_SUGARS_BILE_SALTS_AND_ORGANIC_ACIDS_M NE_COMPOUNDS	REACTOME	3234	50	20	6,71E-01	9,87E-01	rs7295598|rs1392312|rs3806536|rs10049581|rs10820785|rs9820724|rs2073210|rs3813008|rs4640842|rs4506956|rs9871974|rs218655|rs9835618|rs2283231|rs859106|rs1540052|rs2276433|rs869896|rs10747989|rs413781
Hematologic Disorder	PYRIMIDINE_METABOLISM	KEGG	1900	30	20	6,72E-01	9,87E-01	rs9704|rs2076559|rs7517899|rs13399758|rs2244095|rs11084882|rs10238679|rs11833143|rs10814571|rs2227301|rs12450758|rs4789145|rs595890|rs167508|rs3817657|rs7145427|rs2261682|rs1818622|rs1399291|rs2853229
Hematologic Disorder	IL7_PATHWAY	BIOCARTA	699	28	12	6,74E-01	9,87E-01	rs925246|rs1465061|rs2677760|rs173704|rs11953134|rs12454712|rs12409333|rs9487749|rs1550805|rs2850768|rs4987786|rs2072496
Hematologic Disorder	RNA_DEGRADATION	KEGG	1123	39	20	6,75E-01	9,87E-01	rs7133677|rs2239167|rs11553287|rs1000174|rs3750010|rs6821225|rs2269337|rs2583839|rs275499|rs10879129|rs1533079|rs4859871|rs12520016|rs2453176|rs6739788|rs1651031|rs368686|rs2429175|rs2115794|rs2037347
Hematologic Disorder	THE_ROLE_OF_NEF_IN_HIV1_REPLICATION_AND_DISEASE_PATHOGENESIS	REACTOME	936	36	20	6,78E-01	9,87E-01	rs6899021|rs1420320|rs1465061|rs874627|rs978914|rs858492|rs17071888|rs1477317|rs4764622|rs757718|rs226434|rs3788412|rs3741920|rs12656935|rs12564899|rs905155|rs259916|rs9487749|rs30084|rs5439
Hematologic Disorder	RNA_POL_II_TRANSCRIPTION_PRE_INITIATION_AND_PROMOTER_OPENING	REACTOME	383	17	11	6,78E-01	9,87E-01	rs11826421|rs11084882|rs1471420|rs2261682|rs7833229|rs963267|rs633742|rs7946331|rs1613070|rs1561734|rs663133
Hematologic Disorder	TRANSCRIPTION	REACTOME	2708	31	20	6,81E-01	9,87E-01	rs9704|rs11989385|rs665714|rs11826421|rs1053079|rs11084882|rs13295646|rs11578832|rs1471420|rs11833143|rs2926362|rs2227301|rs3117655|rs3759978|rs1009181|rs8072724|rs6454694|rs1923416|rs2068477|rs13277342
Hematologic Disorder	GAB1_SIGNALOSOME	REACTOME	1077	32	20	6,82E-01	9,87E-01	rs4947979|rs665821|rs3007685|rs2110290|rs6586116|rs534182|rs2677760|rs173704|rs4983559|rs2673825|rs12958400|rs11953134|rs10507483|rs7335734|rs11979158|rs11905013|rs7321873|rs2701853|rs1550805|rs6721035
Hematologic Disorder	SYSTEMIC_LUPUS_ERYTHEMATOSUS	KEGG	2453	40	20	6,86E-01	9,87E-01	rs9704|rs2284191|rs6699859|rs1450660|rs540874|rs1650397|rs761900|rs16972767|rs28772724|rs1143664|rs8058978|rs1389623|rs1009181|rs10473234|rs4538747|rs3130192|rs2284428|rs700239|rs1683591|rs2268105
Hematologic Disorder	INNATE_IMMUNE_SYSTEM	REACTOME	6278	32	20	6,91E-01	9,87E-01	rs6931568|rs2070600|rs12599479|rs2702938|rs744038|rs11970312|rs13251447|rs9913432|rs1053079|rs821466|rs2806117|rs6699859|rs1450660|rs12892024|rs4935400|rs11191274|rs7305095|rs13116389|rs1109225|rs2225602
Hematologic Disorder	TRANSMISSION_ACROSS_CHEMICAL_SYNAPSES	REACTOME	9089	35	20	6,93E-01	9,87E-01	rs198178|rs11630971|rs6606877|rs4958671|rs2914621|rs11654455|rs4898725|rs6289|rs11715062|rs10519568|rs6056252|rs3799663|rs12915265|rs3806536|rs2102902|rs2832449|rs8121340|rs7595027|rs2815715|rs9820724
Hematologic Disorder	PROTEOLYTIC_CLEAVAGE_OF_SNARE_COMPLEX_PROTEINS	REACTOME	382	22	16	6,94E-01	9,87E-01	rs17741348|rs8073622|rs2695863|rs6026402|rs1591811|rs7215786|rs11087010|rs6026373|rs4813925|rs11657426|rs4759753|rs12208207|rs363012|rs6951030|rs2534721|rs2695872
Hematologic Disorder	REGULATION_OF_BETA_CELL_DEVELOPMENT	REACTOME	1288	30	20	6,94E-01	9,87E-01	rs1899745|rs2690035|rs1390088|rs2456525|rs10030773|rs7209835|rs2247328|rs4983559|rs830783|rs11699172|rs17138478|rs6075942|rs4238393|rs17366515|rs7961554|rs10507483|rs7335734|rs6048114|rs17659514|rs6788164
Hematologic Disorder	ACTIVATION_OF_BH3_ONLY_PROTEINS	REACTOME	325	18	15	6,98E-01	9,87E-01	rs13405741|rs12454840|rs4983559|rs13395957|rs9966076|rs6577059|rs8071026|rs12454712|rs12957347|rs2850768|rs11123500|rs1544252|rs11617870|rs4987786|rs2333091
Hematologic Disorder	BASIGIN_INTERACTIONS	REACTOME	595	37	20	6,98E-01	9,87E-01	rs1607527|rs13144383|rs1975802|rs1605819|rs973608|rs8054172|rs9788810|rs748680|rs1200150|rs1007160|rs3759608|rs12891079|rs4239020|rs7685529|rs1107920|rs2012859|rs1003349|rs1200138|rs773433|rs7657780
Hematologic Disorder	KERATINOCYTE_PATHWAY	BIOCARTA	1952	34	20	6,99E-01	9,87E-01	rs9787850|rs198178|rs4947979|rs449072|rs2529737|rs9914703|rs2110290|rs3760201|rs1648178|rs2947864|rs6681035|rs7559522|rs1061624|rs4934433|rs9977361|rs1107869|rs4750434|rs13196204|rs11979158|rs12454712
Hematologic Disorder	HEDGEHOG_SIGNALING_PATHWAY	KEGG	2151	33	20	6,99E-01	9,87E-01	rs1863921|rs680997|rs6891344|rs1990135|rs1863703|rs4448343|rs6140076|rs1334586|rs776501|rs11979793|rs351359|rs12892252|rs2225265|rs12198986|rs13044457|rs12686467|rs12434228|rs4765835|rs4835757|rs3802727
Hematologic Disorder	SIGNALING_BY_PDGF	REACTOME	4427	27	20	7,00E-01	9,87E-01	rs9307949|rs2036068|rs10517655|rs7202877|rs6501512|rs2194869|rs7975710|rs7595027|rs429219|rs665821|rs9521585|rs16877442|rs11048455|rs7008202|rs7975385|rs9765848|rs1030098|rs3007711|rs953672|rs2055226
Hematologic Disorder	B_CELL_RECEPTOR_SIGNALING_PATHWAY	KEGG	2278	41	20	7,02E-01	9,87E-01	rs198178|rs1876267|rs6461796|rs2239167|rs449072|rs7193701|rs7829816|rs10200743|rs6541017|rs1148853|rs748938|rs2677760|rs173704|rs1571344|rs4983559|rs6681035|rs767652|rs11953134|rs4240896|rs1075834
Hematologic Disorder	GRB2_EVENTS_IN_ERBB2_SIGNALING	REACTOME	1202	34	20	7,04E-01	9,87E-01	rs2976532|rs2919384|rs4947979|rs2110290|rs4673677|rs1481762|rs2439321|rs7562228|rs13252799|rs10503907|rs17406681|rs6487465|rs11979158|rs2888586|rs3756675|rs2448343|rs926938|rs13016652|rs7693713|rs17695276
Hematologic Disorder	RNA_POLYMERASE	KEGG	351	14	11	7,05E-01	9,87E-01	rs11084882|rs11833143|rs3739574|rs2227301|rs2261682|rs633742|rs6081150|rs492553|rs1150743|rs510342|rs663133
Hematologic Disorder	ACTIVATION_OF_THE_PRE_REPLICATIVE_COMPLEX	REACTOME	565	29	10	7,06E-01	9,87E-01	rs925192|rs10493854|rs9474192|rs837763|rs1818622|rs9692542|rs13234190|rs7208143|rs13706|rs6085344
Hematologic Disorder	PROSTATE_CANCER	KEGG	2870	24	20	7,07E-01	9,87E-01	rs9307949|rs10517655|rs2194869|rs8038056|rs12573128|rs4947979|rs1338423|rs1061476|rs4732279|rs6541017|rs2684806|rs41307|rs2110290|rs6586116|rs534182|rs1565483|rs16874653|rs2677760|rs173704|rs614367
Hematologic Disorder	CYTOKINE_SIGNALING_IN_IMMUNE_SYSTEM	REACTOME	6819	39	20	7,10E-01	9,87E-01	rs3026106|rs5013310|rs12203803|rs7113799|rs744038|rs10502414|rs2352946|401754|rs2239167|rs729405|rs925246|rs11648565|rs7829816|rs266549|rs11191274|rs13116389|rs9814648|rs2871444|rs8041687|rs6541017
Hematologic Disorder	TGF_BETA_RECEPTOR_SIGNALING_IN_EMT_EPITHELIAL_TO_MESENCHYMAL_TRANSITIO	REACTOME	429	14	11	7,10E-01	9,87E-01	rs7617478|rs11009607|rs4325953|rs11265544|rs2254669|rs12449157|rs4934622|rs10827315|rs17025329|rs1359280|rs12252306
Hematologic Disorder	CELL_CYCLE_CHECKPOINTS	REACTOME	1961	30	20	7,11E-01	9,87E-01	rs2526060|401754|rs4813929|rs11845708|rs698856|rs925192|rs10493854|rs9474192|rs6033024|rs1558461|rs6856643|rs7200210|rs1027692|rs8078118|rs7015841|rs12037569|rs315690|rs10774976|rs5752792|rs11772205
Hematologic Disorder	NGF_PATHWAY	BIOCARTA	566	18	13	7,12E-01	9,87E-01	rs2334915|rs2677760|rs173704|rs6681035|rs2235360|rs2671632|rs11953134|rs2888586|rs17103133|rs1550805|rs16967789|rs4776783|rs204680
Hematologic Disorder	PHENYLALANINE_METABOLISM	KEGG	442	15	7	7,18E-01	9,87E-01	rs732215|rs4822458|rs1522307|rs4784991|rs738807|rs3817446|rs7206052
Hematologic Disorder	TRANSCRIPTIONAL_ACTIVITY_OF_SMAD2_SMAD3_SMAD4_HETEROTRIMER	REACTOME	967	26	20	7,18E-01	9,87E-01	rs1561928|rs1242991|rs906304|rs2302793|rs951538|rs6470648|rs1539872|rs2065500|rs17787953|rs757718|rs11599360|rs6577059|rs4733616|rs12915039|rs524386|rs837492|rs12901499|rs9512215|rs4637783|rs6995630
Hematologic Disorder	CYTOSOLIC_DNA_SENSING_PATHWAY	KEGG	620	38	16	7,21E-01	9,87E-01	rs2210463|rs11833143|rs621277|rs6133067|rs2227301|rs4722178|rs10970986|rs928413|rs7037389|rs6084497|rs2073145|rs7267297|rs6081150|rs492553|rs510342|rs10489113
Hematologic Disorder	MYD88_MAL_CASCADE_INITIATED_ON_PLASMA_MEMBRANE	REACTOME	2527	31	20	7,24E-01	9,87E-01	rs2070600|rs11970312|rs1053079|rs2806117|rs12892024|rs11191274|rs7305095|rs2225602|rs1394839|rs6020238|rs2529737|rs13217057|rs4483225|rs3760201|rs2947864|rs6853|rs6681035|rs10105118|rs1339|rs708030
Hematologic Disorder	PYRUVATE_METABOLISM_AND_CITRIC_ACID_TCA_CYCLE	REACTOME	905	38	20	7,26E-01	9,87E-01	rs1054174|rs17022214|rs2125812|rs4657015|rs187431|rs2028548|rs2978169|rs9670039|rs2158839|rs11893686|rs1172416|rs7602538|rs12675850|rs3738814|rs4239020|rs7589648|rs7600094|rs2012859|rs836624|rs7946331
Hematologic Disorder	G1_S_TRANSITION	REACTOME	1941	32	20	7,27E-01	9,87E-01	rs2526060|401754|rs4813929|rs1561928|rs11845708|rs925192|rs10493854|rs9474192|rs2302793|rs6033024|rs7200210|rs10105118|rs837763|rs8078118|rs6470648|rs7015841|rs12037569|rs1818622|rs6577059|rs4733616
Hematologic Disorder	TID_PATHWAY	BIOCARTA	479	8	7	7,28E-01	9,87E-01	rs9321590|rs17137037|rs1061624|rs11792962|rs4149576|rs10489113|rs3014996
Hematologic Disorder	GSK3_PATHWAY	BIOCARTA	1055	34	20	7,28E-01	9,87E-01	rs1053079|rs2806117|rs2225602|rs4483225|rs2677760|rs173704|rs614367|rs4983559|rs1555772|rs6853|rs679162|rs11953134|rs9883073|rs334550|rs1550805|rs2343115|rs12552190|rs7394655|rs10489113|rs1429519
Hematologic Disorder	SIGNALING_BY_SCF_KIT	REACTOME	1989	34	20	7,31E-01	9,87E-01	rs11770199|rs2239167|rs665821|rs7829816|rs8085725|rs3007685|rs1465061|rs723279|rs10777109|rs9914703|rs6586116|rs534182|rs2677760|rs173704|rs10515395|rs4983559|rs2673825|rs12958400|rs11953134|rs7245158
Hematologic Disorder	AMINE_COMPOUND_SLC_TRANSPORTERS	REACTOME	1298	29	20	7,31E-01	9,87E-01	rs3806536|rs10820785|rs9820724|rs4640842|rs9871974|rs9835618|rs859106|rs2276433|rs869896|rs8083023|rs2292023|rs17097955|rs4923345|rs17298481|rs1362153|rs12343412|rs872035|rs720625|rs9469069|rs3124016
Hematologic Disorder	MAPK_TARGETS_NUCLEAR_EVENTS_MEDIATED_BY_MAP_KINASES	REACTOME	980	28	15	7,31E-01	9,87E-01	rs3799663|rs12892024|rs7305095|rs6681035|rs10105118|rs1852730|rs17591358|rs13196204|rs3886478|rs17103133|rs2187911|rs1286086|rs6721035|rs10858840|rs770082
Hematologic Disorder	TYPE_II_DIABETES_MELLITUS	KEGG	2053	29	20	7,32E-01	9,87E-01	rs175338|rs12566751|rs2609485|rs2239093|rs10500204|rs2239041|rs6541017|rs10422148|rs2677760|rs3774425|rs173704|rs12408625|rs7559522|rs7558386|rs11668370|rs4499341|rs11953134|rs3750506|rs4240896|rs7999458
Hematologic Disorder	FORMATION_OF_TRANSCRIPTION_COUPLED_NER_TC_NER_REPAIR_COMPLEX	REACTOME	389	13	9	7,34E-01	9,87E-01	rs11084882|rs1029290|rs17199520|rs2261682|rs3212986|rs633742|rs7946331|rs1561734|rs663133
Hematologic Disorder	RAS_PATHWAY	BIOCARTA	557	11	7	7,35E-01	9,87E-01	rs2677760|rs173704|rs4983559|rs11953134|rs1550805|rs4776783|rs10489113
Hematologic Disorder	BETA_DEFENSINS	REACTOME	333	15	10	7,40E-01	9,87E-01	rs1339|rs6039403|rs6074947|rs2978880|rs11466640|rs3798315|rs2738050|rs6057657|rs6046492|rs17030340
Hematologic Disorder	SIGNALING_BY_INSULIN_RECEPTOR	REACTOME	3432	35	20	7,41E-01	9,87E-01	rs12540943|rs1888057|rs11770199|rs2174834|rs2992024|rs17072214|rs12523052|rs1881628|rs513320|rs10500204|rs1078363|rs2672886|rs3007711|rs4969429|rs541682|rs9510129|rs1080199|rs4633807|rs3806929|rs7438406
Hematologic Disorder	ABORTIVE_ELONGATION_OF_HIV1_TRANSCRIPT_IN_THE_ABSENCE_OF_TAT	REACTOME	195	7	7	7,41E-01	9,87E-01	rs11084882|rs2261682|rs1865094|rs633742|rs3786238|rs1613070|rs663133
Hematologic Disorder	INTRINSIC_PATHWAY_FOR_APOPTOSIS	REACTOME	532	19	16	7,43E-01	9,87E-01	rs13405741|rs12454840|rs4983559|rs13395957|rs10268780|rs9966076|rs6577059|rs8071026|rs12454712|rs12957347|rs2850768|rs11123500|rs1544252|rs11617870|rs4987786|rs2333091
Hematologic Disorder	PREFOLDIN_MEDIATED_TRANSFER_OF_SUBSTRATE_TO_CCT_TRIC	REACTOME	288	6	4	7,43E-01	9,87E-01	rs9405622|rs3892375|rs12664430|rs16999299
Hematologic Disorder	APOPTOSIS	REACTOME	3298	26	20	7,44E-01	9,87E-01	rs7830593|rs1442089|rs2526060|401754|rs4813929|rs10502934|rs13405741|rs11845708|rs698856|rs727683|rs17398435|rs11618611|rs1538583|rs6033024|rs16845834|rs12454840|rs4678191|rs17198981|rs785437|rs7200210
Hematologic Disorder	NTHI_PATHWAY	BIOCARTA	876	30	20	7,45E-01	9,87E-01	rs7617478|rs4868204|rs13217057|rs3760201|rs2947864|rs6853|rs1339|rs2141484|rs4325953|rs13196204|rs10485101|rs11746792|rs12915039|rs12901499|rs9916229|rs17025329|rs17030340|rs9294511|rs739559|rs2716227
Hematologic Disorder	MET_PATHWAY	BIOCARTA	1415	40	20	7,45E-01	9,87E-01	rs2483852|rs6861460|rs882052|rs2282004|rs7895721|rs6586116|rs534182|rs2677760|rs173704|rs11102314|rs6681035|rs4767886|rs2673825|rs10901096|rs1573822|rs11953134|rs10492302|rs11177325|rs2888586|rs11905013
Hematologic Disorder	PROTEIN_FOLDING	REACTOME	675	27	13	7,50E-01	9,87E-01	rs629246|rs9405622|rs10517563|rs2054759|rs8074277|rs9914387|rs6090919|rs3892375|rs10440685|rs12664430|rs10008975|rs16999299|rs3750512
Hematologic Disorder	ERK5_PATHWAY	BIOCARTA	595	18	11	7,52E-01	9,87E-01	rs2677760|rs173704|rs4983559|rs2235360|rs1852730|rs11953134|rs3886478|rs1550805|rs6721035|rs16967789|rs6334
Hematologic Disorder	ASSOCIATION_OF_TRIC_CCT_WITH_TARGET_PROTEINS_DURING_BIOSYNTHESIS	REACTOME	359	9	6	7,53E-01	9,87E-01	rs10517563|rs2054759|rs9914387|rs6090919|rs7685083|rs3750512
Hematologic Disorder	PLATELET_SENSITIZATION_BY_LDL	REACTOME	676	36	17	7,53E-01	9,87E-01	rs2239167|rs1572507|rs10105118|rs230632|rs330746|rs330725|rs13196204|rs776797|rs1255771|rs1843854|rs875919|rs4908343|rs4651390|rs1288516|rs695117|rs11802712|rs2076075
Hematologic Disorder	GABA_B_RECEPTOR_ACTIVATION	REACTOME	1863	36	20	7,54E-01	9,87E-01	rs11654455|rs4898725|rs7008202|rs7975385|rs16947151|rs953672|rs1253704|rs10459889|rs3787870|rs874627|rs2601773|rs8079714|rs902522|rs4901170|rs72001|rs1073572|rs13249746|rs2275404|rs3741920|rs6590356
Hematologic Disorder	NITROGEN_METABOLISM	KEGG	587	32	14	7,54E-01	9,87E-01	rs3124455|rs3850839|rs918233|rs6983167|rs187431|rs6998241|rs759688|rs10192656|rs12307364|rs17762822|rs10269471|rs13257749|rs7308827|rs4984241
Hematologic Disorder	APOPTOTIC_CLEAVAGE_OF_CELLULAR_PROTEINS	REACTOME	1052	42	18	7,54E-01	9,87E-01	rs11845708|rs17398435|rs11618611|rs7469887|rs17198981|rs785437|rs3785076|rs9392904|rs4750434|rs9883073|rs2152833|rs2757632|rs10906340|rs260530|rs661891|rs3815975|rs11839442|rs2295859
Hematologic Disorder	VALINE_LEUCINE_AND_ISOLEUCINE_DEGRADATION	KEGG	1149	41	19	7,56E-01	9,87E-01	rs2066938|rs4665830|rs10080237|rs6599263|rs11764246|rs9423240|rs3043|rs13353058|rs10771144|rs2158839|rs17744890|rs534800|rs3132810|rs7527279|rs757141|rs4646857|rs7705738|rs17810414|rs837322
Hematologic Disorder	STEROID_HORMONES	REACTOME	693	39	20	7,61E-01	9,87E-01	rs1914172|rs2796833|rs7172632|rs166050|rs7916688|rs2268365|rs7546652|rs9665553|rs4233828|rs2269429|rs2271463|rs7788953|rs2899472|rs2302694|rs10923838|rs6163|rs10199676|rs7683624|rs11254314|rs4694421
Hematologic Disorder	FORMATION_OF_RNA_POL_II_ELONGATION_COMPLEX_	REACTOME	401	15	10	7,61E-01	9,87E-01	rs1053079|rs11084882|rs2261682|rs1865094|rs633742|rs3786238|rs7946331|rs1613070|rs1561734|rs663133
Hematologic Disorder	TCA_CYCLE_AND_RESPIRATORY_ELECTRON_TRANSPORT	REACTOME	2599	46	20	7,62E-01	9,87E-01	rs8133991|rs12654078|rs1626521|rs1054174|rs10777647|rs13263130|rs4797343|rs6538582|rs17022214|rs2125812|rs2834291|rs10268780|rs4657015|rs187431|rs2028548|rs2978169|rs9670039|rs2158839|rs9931644|rs10206264
Hematologic Disorder	SIGNALING_BY_ROBO_RECEPTOR	REACTOME	1900	51	20	7,62E-01	9,87E-01	rs6548499|rs6672395|rs1336480|rs11936468|rs2675181|rs4740366|rs7650723|rs7577243|rs2583839|rs9872433|rs6057007|rs9870957|rs12054167|rs2168753|rs2670009|rs884237|rs2171958|rs17032053|rs2888586|rs2313632
Hematologic Disorder	EPITHELIAL_CELL_SIGNALING_IN_HELICOBACTER_PYLORI_INFECTION	KEGG	1535	29	20	7,63E-01	9,87E-01	rs8133991|rs10953942|rs4947979|rs7829816|rs2529737|rs2110290|rs1080199|rs785437|rs6681035|rs2235360|rs869896|rs11265544|rs13196204|rs10082815|rs11979158|rs460707|rs2837132|rs11905013|rs12111892|rs16956011
Hematologic Disorder	ASCORBATE_AND_ALDARATE_METABOLISM	KEGG	874	18	5	7,64E-01	9,87E-01	rs3043|rs2736486|rs7527279|rs7705738|rs6820734
Hematologic Disorder	GLYCOSPHINGOLIPID_METABOLISM	REACTOME	924	38	20	7,64E-01	9,87E-01	rs11713682|rs3892357|rs2863973|rs1362720|rs2618956|rs11658645|rs7691016|rs2167239|rs11624137|rs1234367|rs1638720|rs6008947|rs12499919|rs3756444|rs1420934|rs135678|rs7140737|rs918167|rs3742704|rs681566
Hematologic Disorder	FATTY_ACID_TRIACYLGLYCEROL_AND_KETONE_BODY_METABOLISM	REACTOME	5526	26	20	7,65E-01	9,87E-01	rs2070600|rs12540943|rs341404|rs2985700|rs12424322|rs6583745|rs2478545|rs17620632|rs1242991|rs12785137|rs4502283|rs9842151|rs4640402|rs1881628|rs12572167|rs716738|rs6448226|rs2352106|rs7656124|rs450110
Hematologic Disorder	SIGNALING_BY_HIPPO	REACTOME	627	22	13	7,65E-01	9,87E-01	rs10501995|rs1538583|rs6875678|rs785437|rs7521197|rs17787953|rs904534|rs6572681|rs260530|rs2010464|rs9313414|rs746145|rs685167
Hematologic Disorder	STRESS_PATHWAY	BIOCARTA	633	31	20	7,67E-01	9,87E-01	rs12817549|rs2529737|rs3760201|rs2947864|rs6681035|rs12052592|rs11614624|rs1404358|rs13196204|rs733751|rs524386|rs12818362|rs9916229|rs2957154|rs2811757|rs3858603|rs4149576|rs739559|rs10745664|rs2716227
Hematologic Disorder	DOWNSTREAM_SIGNAL_TRANSDUCTION	REACTOME	3083	31	20	7,67E-01	9,87E-01	rs2036068|rs7202877|rs6501512|rs7975710|rs7595027|rs429219|rs665821|rs11048455|rs7008202|rs7975385|rs1030098|rs3007685|rs953672|rs2055226|rs9914703|rs2282004|rs2601773|rs6586116|rs534182|rs2677760
Hematologic Disorder	NEUROACTIVE_LIGAND_RECEPTOR_INTERACTION	KEGG	13469	40	20	7,69E-01	9,87E-01	rs2189164|rs12203803|rs6606877|rs4958671|rs4478032|rs2914621|rs10934511|rs2382070|rs10008744|rs9461937|rs2300437|rs6289|rs10421380|rs2471005|rs10519568|rs7619728|rs12915265|rs16921368|rs2280011|rs1578180
Hematologic Disorder	NEF_MEDIATES_DOWN_MODULATION_OF_CELL_SURFACE_RECEPTORS_BY_RECRUITING N_ADAPTERS	REACTOME	366	13	8	7,71E-01	9,87E-01	rs874627|rs4764622|rs757718|rs226434|rs2283852|rs3741920|rs5439|rs2255235
Hematologic Disorder	POTASSIUM_CHANNELS	REACTOME	4940	31	20	7,72E-01	9,87E-01	rs11654455|rs7538655|rs4898725|rs1953248|rs36960|rs11120453|rs1566602|rs17797668|rs7133677|rs2469515|rs349358|rs2283200|rs9643288|rs4113643|rs12024924|rs16947151|rs343797|rs12436815|rs1253704|rs10459889
Hematologic Disorder	DRUG_METABOLISM_CYTOCHROME_P450	KEGG	1661	59	20	7,75E-01	9,87E-01	rs316156|rs2276332|rs1584783|rs1826909|rs11807|rs2736486|rs10912598|rs2654846|rs3796633|rs1126671|rs1934967|rs10772921|rs7527279|rs7747120|rs6464540|rs8192100|rs6820734|rs12978001|rs667660|rs1027473
Hematologic Disorder	THYROID_CANCER	KEGG	1104	30	20	7,77E-01	9,87E-01	rs1561928|rs12573128|rs1338423|rs4917339|rs2302793|rs614367|rs3785076|rs6470648|rs679162|rs9883073|rs4733616|rs6487465|rs926938|rs4637783|rs10489743|rs6995630|rs2395851|rs1994581|rs10842515|rs2343115
Hematologic Disorder	INFLAMMASOMES	REACTOME	468	38	17	7,78E-01	9,87E-01	rs9913432|rs1109225|rs1394839|rs7209554|rs12454712|rs4790801|rs461626|rs17178663|rs16955023|rs2830076|rs8132767|rs2850768|rs224205|rs6424377|rs4987786|rs6698597|rs208277
Hematologic Disorder	ACTIVATED_TLR4_SIGNALLING	REACTOME	2676	31	20	7,78E-01	9,87E-01	rs2070600|rs11970312|rs1053079|rs2806117|rs12892024|rs11191274|rs7305095|rs2225602|rs1394839|rs6020238|rs2529737|rs13217057|rs4483225|rs3760201|rs2947864|rs6853|rs6681035|rs10105118|rs1339|rs708030
Hematologic Disorder	BLADDER_CANCER	KEGG	1222	32	20	7,79E-01	9,87E-01	rs1561928|rs4947979|rs12892024|rs2302793|rs2110290|rs6458350|rs614367|rs3785076|rs6470648|rs10520130|rs679162|rs9295464|rs4733616|rs6487465|rs11979158|rs12204488|rs4878066|rs926938|rs4637783|rs6995630
Hematologic Disorder	SIGNALLING_BY_NGF	REACTOME	7869	27	20	7,80E-01	9,87E-01	rs11630971|rs12480096|rs2036068|rs6063285|rs11072660|rs2094583|rs2985700|rs11638112|rs163819|rs3799663|rs8034125|rs6501512|rs6019300|rs7975710|rs7595027|rs10869590|rs429219|rs13405741|rs665821|rs165095
Hematologic Disorder	GAP_JUNCTION_TRAFFICKING	REACTOME	576	27	17	7,80E-01	9,87E-01	rs16869484|rs9342215|rs1555772|rs7213960|rs11956308|rs999095|rs700239|rs12074036|rs876165|rs807673|rs16933086|rs8064669|rs3003607|rs6453850|rs9315439|rs10462005|rs3849768
Hematologic Disorder	SMAD2_SMAD3_SMAD4_HETEROTRIMER_REGULATES_TRANSCRIPTION	REACTOME	715	30	20	7,81E-01	9,87E-01	rs1561928|rs2302793|rs951538|rs6470648|rs1539872|rs2065500|rs17787953|rs757718|rs6577059|rs4733616|rs12915039|rs524386|rs12901499|rs9512215|rs4637783|rs6995630|rs2957154|rs2395851|rs1333050|rs3218020
Hematologic Disorder	MITOTIC_M_M_G1_PHASES	REACTOME	3176	27	20	7,82E-01	9,87E-01	rs2526060|401754|rs4813929|rs9913432|rs729405|rs11845708|rs698856|rs3781974|rs11058562|rs4073472|rs925192|rs10493854|rs9474192|rs6033024|rs1558461|rs6856643|rs7200210|rs17007652|rs35684|rs10825702
Hematologic Disorder	KERATAN_SULFATE_BIOSYNTHESIS	REACTOME	674	27	20	7,83E-01	9,87E-01	rs6095638|rs6989475|rs7844620|rs7016568|rs869896|rs721590|rs11038540|rs3743398|rs2945770|rs7814358|rs1362153|rs2945745|rs10971412|rs13091236|rs3806698|rs4141587|rs828599|rs4932438|rs304303|rs6805378
Hematologic Disorder	GLUCONEOGENESIS	REACTOME	490	14	10	7,83E-01	9,87E-01	rs2239167|rs764453|rs11227623|rs1040566|rs3561|rs4784991|rs764454|rs807673|rs7206052|rs6025642
Hematologic Disorder	NOTCH1_INTRACELLULAR_DOMAIN_REGULATES_TRANSCRIPTION	REACTOME	2852	27	20	7,83E-01	9,87E-01	rs17825554|rs1561928|rs1706710|rs1242991|rs12533919|rs9842151|rs3791500|rs10867791|rs11685345|rs1584783|rs2188495|rs17296588|rs906304|rs2302793|rs7786745|rs9874964|rs12530920|rs11790630|rs10517563|rs2054759
Hematologic Disorder	ECM_PATHWAY	BIOCARTA	753	28	15	7,84E-01	9,87E-01	rs6861460|rs1465061|rs2677760|rs173704|rs4767886|rs11953134|rs3844598|rs820360|rs11905013|rs9487749|rs1550805|rs4776783|rs2547547|rs11741808|rs8009215
Hematologic Disorder	PI_METABOLISM	REACTOME	1808	28	20	7,85E-01	9,87E-01	rs6796883|rs1992838|rs11702774|rs6541017|rs6586116|rs4633807|rs534182|rs7922014|rs12944446|rs4121647|rs2677760|rs173704|rs989885|rs2673825|rs11953134|rs4240896|rs2218313|rs13296679|rs9878820|rs17419409
Hematologic Disorder	KERATAN_SULFATE_KERATIN_METABOLISM	REACTOME	737	27	20	7,85E-01	9,87E-01	rs6095638|rs6989475|rs7844620|rs7016568|rs869896|rs721590|rs11038540|rs3743398|rs2945770|rs7814358|rs3756444|rs1362153|rs2945745|rs10971412|rs13091236|rs3806698|rs4141587|rs828599|rs4932438|rs304303

Hematologic Disorder	RACCYCD_PATHWAY	BIOCARTA	551	18	12	7,87E-01	9,87E-01	rs2677760|rs173704|rs614367|rs4983559|rs679162|rs11953134|rs6577059|rs10425066|rs1550805|rs11617870|rs7394655|rs10489113
Hematologic Disorder	SHC1_EVENTS_IN_ERBB4_SIGNALING	REACTOME	1072	33	20	7,89E-01	9,87E-01	rs2976532|rs2975498|rs4673677|rs1481762|rs2439321|rs7562228|rs13252799|rs10503907|rs17406681|rs6487465|rs2888586|rs3756675|rs2448343|rs926938|rs13016652|rs7693713|rs17695276|rs10932383|rs1994581|rs17389103
Hematologic Disorder	PI3K_EVENTS_IN_ERBB2_SIGNALING	REACTOME	1886	32	20	7,89E-01	9,87E-01	rs2976532|rs2919384|rs4947979|rs665821|rs3007711|rs2110290|rs6586116|rs534182|rs2677760|rs173704|rs4983559|rs4673677|rs2673825|rs1481762|rs12958400|rs2439321|rs11953134|rs7562228|rs13252799|rs10503907
Hematologic Disorder	VOLTAGE_GATED_POTASSIUM_CHANNELS	REACTOME	2491	31	20	7,89E-01	9,87E-01	rs1953248|rs1566602|rs2469515|rs349358|rs2283200|rs9643288|rs4113643|rs12024924|rs343797|rs12436815|rs1387671|rs1980561|rs529446|rs3757105|rs2237875|rs2106467|rs17727131|rs1501560|rs7108478|rs346613
Hematologic Disorder	HISTIDINE_METABOLISM	KEGG	654	16	11	7,90E-01	9,87E-01	rs732215|rs2232248|rs3043|rs11656519|rs12923|rs12307364|rs748465|rs7527279|rs7705738|rs16840472|rs7308827
Hematologic Disorder	DOWNREGULATION_OF_SMAD2_3_SMAD4_TRANSCRIPTIONAL_ACTIVITY	REACTOME	453	16	11	7,92E-01	9,87E-01	rs1242991|rs906304|rs1539872|rs11599360|rs12915039|rs837492|rs12901499|rs10846681|rs12567614|rs837493|rs6573304
Hematologic Disorder	FC_EPSILON_RI_SIGNALING_PATHWAY	KEGG	2758	29	20	7,92E-01	9,87E-01	rs198178|rs2278964|rs631134|rs449072|rs1572507|rs7829816|rs7548864|rs6541017|rs1148853|rs1465061|rs4820315|rs2529737|rs9914703|rs11582551|rs2677760|rs3760201|rs173704|rs4983559|rs2947864|rs1530837
Hematologic Disorder	PROLONGED_ERK_ACTIVATION_EVENTS	REACTOME	429	23	14	7,93E-01	9,87E-01	rs2282004|rs2334915|rs11102314|rs2235360|rs10901096|rs6487465|rs2448343|rs926938|rs3012750|rs1994581|rs10842515|rs10793894|rs4776783|rs6334
Hematologic Disorder	TH1TH2_PATHWAY	BIOCARTA	535	14	7	7,93E-01	9,87E-01	rs9321590|rs1887027|rs12106790|rs7910961|rs9268911|rs3213094|rs3014996
Hematologic Disorder	INFLAM_PATHWAY	BIOCARTA	747	28	13	7,94E-01	9,87E-01	rs925246|rs874627|rs4722178|rs9990563|rs4764622|rs433192|rs3741920|rs9268911|rs7417586|rs7526672|rs5439|rs3213094|rs3014996
Hematologic Disorder	MITOTIC_PROMETAPHASE	REACTOME	1814	40	20	7,95E-01	9,87E-01	rs9913432|rs729405|rs3781974|rs11058562|rs4073472|rs1558461|rs6856643|rs17007652|rs35684|rs10825702|rs10105118|rs1027692|rs6550401|rs6997684|rs2044107|rs11772205|rs11177325|rs3027267|rs1255771|rs1550116
Hematologic Disorder	SIGNALING_BY_ERBB4	REACTOME	3434	33	20	7,97E-01	9,87E-01	rs12203803|rs16948096|rs2976532|rs2975498|rs11591534|rs4947979|rs665821|rs3007685|rs980281|rs11225161|rs3850839|rs7728464|rs2110290|rs6586116|rs534182|rs4887934|rs2677760|rs173704|rs4983559|rs4673677
Hematologic Disorder	G2_PATHWAY	BIOCARTA	354	8	8	7,97E-01	9,87E-01	rs2281479|rs5752792|rs4809258|rs675327|rs2448343|rs6062662|rs8178179|rs1010608
Hematologic Disorder	RENAL_CELL_CARCINOMA	KEGG	2164	27	20	7,98E-01	9,87E-01	rs9787850|rs1053079|rs11072905|rs6541017|rs12591047|rs7542797|rs12440290|rs9973653|rs2282004|rs2677760|rs6458350|rs173704|rs1648178|rs4983559|rs11102314|rs6681035|rs6057007|rs708030|rs10901096|rs1107869
Hematologic Disorder	NA_CL_DEPENDENT_NEUROTRANSMITTER_TRANSPORTERS	REACTOME	718	27	18	7,99E-01	9,87E-01	rs9820724|rs4640842|rs9871974|rs9835618|rs2276433|rs869896|rs2292023|rs4923345|rs17298481|rs1362153|rs720625|rs4922130|rs2270650|rs2171003|rs10088489|rs2134253|rs2241695|rs555044
Hematologic Disorder	TIE2_SIGNALING	REACTOME	790	42	20	8,00E-01	9,87E-01	rs6707461|rs12988898|rs2677760|rs173704|rs11953134|rs3923113|rs9878820|rs6487465|rs2888586|rs1654701|rs2959777|rs926938|rs581724|rs3737187|rs1550805|rs1994581|rs10842515|rs1433195|rs16967789|rs13274462
Hematologic Disorder	ATRBRCA_PATHWAY	BIOCARTA	238	3	3	8,01E-01	9,87E-01	rs9534093|rs5752792|rs153012
Hematologic Disorder	IL2RB_PATHWAY	BIOCARTA	1296	25	20	8,01E-01	9,87E-01	rs1561928|rs2239167|rs1148853|rs2302793|rs1887027|rs2677760|rs173704|rs4983559|rs4934433|rs6470648|rs7558386|rs2060941|rs11953134|rs4733616|rs7910961|rs4744514|rs12454712|rs2888586|rs2105436|rs10993709
Hematologic Disorder	BMAL1_CLOCK_NPAS2_ACTIVATES_CIRCADIAN_EXPRESSION	REACTOME	1728	30	20	8,02E-01	9,87E-01	rs341404|rs9842151|rs6448226|rs450110|rs877228|rs4917339|rs9874964|rs12909890|rs2235734|rs930358|rs3211719|rs4450343|rs2303147|rs6494219|rs3844598|rs3768984|rs4775292|rs7598617|rs757718|rs2052455
Hematologic Disorder	G_ALPHA_S_SIGNALLING_EVENTS	REACTOME	5019	30	20	8,05E-01	9,87E-01	rs9306383|rs2036068|rs2382070|rs4898725|rs2300437|rs2471005|rs7976725|rs220813|rs6807740|rs7008202|rs7975385|rs6003495|rs16947151|rs9916772|rs953672|rs1253704|rs874627|rs17076377|rs2601773|rs9947403
Hematologic Disorder	PI3K_EVENTS_IN_ERBB4_SIGNALING	REACTOME	1709	34	20	8,06E-01	9,87E-01	rs2976532|rs2975498|rs665821|rs3007711|rs6586116|rs534182|rs2677760|rs173704|rs4983559|rs4673677|rs2673825|rs1481762|rs12958400|rs2439321|rs11953134|rs7562228|rs13252799|rs10503907|rs17406681|rs10507483
Hematologic Disorder	SHC_RELATED_EVENTS	REACTOME	302	21	14	8,06E-01	9,87E-01	rs10500204|rs4499341|rs10408844|rs2288404|rs6487465|rs2888586|rs2448343|rs926938|rs1994581|rs10842515|rs4804366|rs16967789|rs12460755|rs4776783
Hematologic Disorder	PEPTIDE_CHAIN_ELONGATION	REACTOME	947	35	19	8,07E-01	9,87E-01	rs7902136|rs751716|rs7257982|rs10498413|rs11755449|rs10895987|rs9928312|rs1149747|rs12620516|rs2430949|rs11002569|rs10140464|rs433852|rs9907408|rs2913115|rs6605321|rs4255712|rs1003644|rs1452402
Hematologic Disorder	BASAL_TRANSCRIPTION_FACTORS	KEGG	386	14	9	8,08E-01	9,87E-01	rs11826421|rs1471420|rs7833229|rs7004630|rs963267|rs633742|rs7946331|rs1613070|rs6587341
Hematologic Disorder	TRAF6_MEDIATED_INDUCTION_OF_NFKB_AND_MAP_KINASES_UPON_TLR7_8_OR_9_ACTI	REACTOME	2239	27	20	8,09E-01	9,87E-01	rs2070600|rs11970312|rs12892024|rs11191274|rs7305095|rs1394839|rs6020238|rs2529737|rs13217057|rs3760201|rs2947864|rs6853|rs6681035|rs10105118|rs708030|rs242400|rs2141484|rs1852730|rs17591358|rs390993
Hematologic Disorder	PI3K_AKT_ACTIVATION	REACTOME	1161	32	20	8,09E-01	9,87E-01	rs665821|rs3007685|rs2334915|rs6586116|rs534182|rs2677760|rs173704|rs4983559|rs2673825|rs7558386|rs12958400|rs11953134|rs7999458|rs9878820|rs10507483|rs7335734|rs7321873|rs2701853|rs1550805|rs6721035
Hematologic Disorder	MTOR_SIGNALING_PATHWAY	KEGG	1290	27	20	8,10E-01	9,87E-01	rs11970312|rs2672886|rs6541017|rs4969429|rs7438406|rs2677760|rs6458350|rs173704|rs4983559|rs17591358|rs11953134|rs4240896|rs11651724|rs9878820|rs17762352|rs12204488|rs13431868|rs3025010|rs1550805|rs309770
Hematologic Disorder	REGULATION_OF_GENE_EXPRESSION_IN_BETA_CELLS	REACTOME	716	32	18	8,13E-01	9,87E-01	rs1390088|rs10030773|rs4983559|rs830783|rs11699172|rs6075942|rs17366515|rs7961554|rs10507483|rs7335734|rs6048114|rs12306121|rs7321873|rs2701853|rs1462436|rs1949109|rs1885088|rs2424455
Hematologic Disorder	OTHER_GLYCAN_DEGRADATION	KEGG	648	23	15	8,13E-01	9,87E-01	rs10019475|rs13106298|rs3892357|rs227284|rs3756444|rs2584396|rs10520399|rs7691749|rs6552275|rs8072347|rs4862816|rs17801853|rs7691205|rs10489113|rs681566
Hematologic Disorder	RECYCLING_PATHWAY_OF_L1	REACTOME	874	43	20	8,15E-01	9,87E-01	rs11970312|rs12892024|rs550840|rs6869991|rs4961604|rs17591358|rs10066223|rs1579282|rs226434|rs559928|rs11905013|rs2187911|rs16933086|rs1286086|rs7829347|rs2083709|rs961548|rs3003607|rs1867042|rs17182398
Hematologic Disorder	ARMS_MEDIATED_ACTIVATION	REACTOME	393	21	13	8,18E-01	9,87E-01	rs2282004|rs2334915|rs11102314|rs10901096|rs6487465|rs2448343|rs926938|rs3012750|rs1994581|rs10842515|rs10793894|rs4776783|rs6334
Hematologic Disorder	EFFECTS_OF_PIP2_HYDROLYSIS	REACTOME	1651	31	20	8,18E-01	9,87E-01	rs2852786|rs7975710|rs11048455|rs4721330|rs2358073|rs12492576|rs7559522|rs4750434|rs9533042|rs7303557|rs6461149|rs347413|rs2148656|rs16967035|rs12286714|rs17764589|rs4963998|rs4711338|rs649775|rs951974
Hematologic Disorder	RNA_POL_I_PROMOTER_OPENING	REACTOME	501	49	5	8,19E-01	9,87E-01	rs1009181|rs4699390|rs198853|rs9303601|rs6939589
Hematologic Disorder	PHOSPHOLIPID_METABOLISM	REACTOME	6226	30	20	8,19E-01	9,87E-01	rs2070600|rs2278952|rs17752182|rs11206808|rs7269032|rs6796883|rs947007|rs12081589|rs1992838|rs631134|rs10820785|rs3011777|rs776717|rs4935597|rs1572507|rs12572167|rs4665830|rs11810204|rs11713682|rs3892357
Hematologic Disorder	PROPANOATE_METABOLISM	KEGG	1219	33	17	8,19E-01	9,87E-01	rs2306787|rs4665830|rs17022214|rs2125812|rs3043|rs9670039|rs11893686|rs1172416|rs7602538|rs7589648|rs7600094|rs7527279|rs4646857|rs7705738|rs7946331|rs6088635|rs837322
Hematologic Disorder	IRON_UPTAKE_AND_TRANSPORT	REACTOME	733	30	14	8,19E-01	9,87E-01	rs3114018|rs1080199|rs12947270|rs4075688|rs869896|rs16861582|rs10082815|rs9784454|rs16843078|rs11230882|rs17721027|rs4732330|rs5747285|rs9922624
Hematologic Disorder	PPARA_ACTIVATES_GENE_EXPRESSION	REACTOME	3675	32	20	8,20E-01	9,87E-01	rs341404|rs2985700|rs12424322|rs6583745|rs2478545|rs12785137|rs9842151|rs4640402|rs716738|rs6448226|rs450110|rs877228|rs4917339|rs16911828|rs11578832|rs11225161|rs9874964|rs12909890|rs7220132|rs2482430
Hematologic Disorder	APOPTOTIC_EXECUTION_PHASE	REACTOME	1211	40	20	8,22E-01	9,87E-01	rs11845708|rs17398435|rs11618611|rs1538583|rs4678191|rs17198981|rs785437|rs3785076|rs1009181|rs9392904|rs4750434|rs9883073|rs2152833|rs2757632|rs10906340|rs7367066|rs260530|rs9295684|rs661891|rs3815975
Hematologic Disorder	ALANINE_ASPARTATE_AND_GLUTAMATE_METABOLISM	KEGG	841	28	18	8,22E-01	9,87E-01	rs13399758|rs2073210|rs944605|rs918233|rs6938431|rs4538195|rs759688|rs11656519|rs10192656|rs4784991|rs3805703|rs9384741|rs1215988|rs10269471|rs7206052|rs12467996|rs10479469|rs10803852
Hematologic Disorder	TRIF_MEDIATED_TLR3_SIGNALING	REACTOME	2075	28	20	8,22E-01	9,87E-01	rs2070600|rs3799663|rs12892024|rs11191274|rs7305095|rs1394839|rs2529737|rs13217057|rs3760201|rs2947864|rs6681035|rs10105118|rs708030|rs2141484|rs1852730|rs17591358|rs390993|rs13196204|rs11574849|rs10485101
Hematologic Disorder	TOLL_RECEPTOR_CASCADES	REACTOME	3778	33	20	8,23E-01	9,87E-01	rs6931568|rs2070600|rs3799663|rs1053079|rs2806117|rs12892024|rs11191274|rs7305095|rs2225602|rs1394839|rs6020238|rs2529737|rs13217057|rs4633807|rs4483225|rs4121647|rs3760201|rs2947864|rs6853|rs6681035
Hematologic Disorder	WNT_SIGNALING_PATHWAY	KEGG	5611	32	20	8,23E-01	9,87E-01	rs603401|rs198178|rs680997|rs1876267|rs853275|rs6056252|rs1561928|rs12573128|rs8121340|rs449072|rs1456391|rs7193701|rs9842151|rs1338423|rs494660|rs6108237|rs4956081|rs10849024|rs10864439|rs9812599
Hematologic Disorder	FACTORS_INVOLVED_IN_MEGAKARYOCYTE_DEVELOPMENT_AND_PLATELET_PRODUCT	REACTOME	3549	32	20	8,24E-01	9,87E-01	rs744038|rs6899021|rs12261421|rs6501512|rs2483852|rs1410285|rs2645400|rs11130267|rs13116389|rs4740366|rs10837628|rs1030098|rs8100350|rs882052|rs12785018|rs6717108|rs10488675|rs6869991|rs7895721|rs2583839
Hematologic Disorder	INITIAL_TRIGGERING_OF_COMPLEMENT	REACTOME	434	28	17	8,24E-01	9,87E-01	rs4935400|rs1143664|rs7083840|rs1903971|rs11003258|rs1389623|rs1683591|rs9469069|rs3733001|rs2794515|rs11003138|rs11003134|rs3821801|rs930506|rs2840323|rs537160|rs12220324
Hematologic Disorder	FANCONI_ANEMIA_PATHWAY	REACTOME	461	9	6	8,25E-01	9,87E-01	rs4325734|rs9534093|rs10493324|rs10175975|rs249869|rs1230
Hematologic Disorder	P38MAPK_PATHWAY	BIOCARTA	1904	52	20	8,26E-01	9,87E-01	rs11625206|rs1561928|rs1572507|rs12892024|rs2302793|rs2529737|rs1054743|rs13217057|rs2947864|rs230632|rs330746|rs6470648|rs2141484|rs1852730|rs330725|rs13196204|rs776797|rs10485101|rs4733616|rs4637783
Hematologic Disorder	LATENT_INFECTION_OF_HOMO_SAPIENS_WITH_MYCOBACTERIUM_TUBERCULOSIS	REACTOME	589	20	12	8,26E-01	9,87E-01	rs11068458|rs11811630|rs1080199|rs869896|rs10082815|rs11913594|rs16843078|rs17721027|rs4732330|rs5747285|rs9922624|rs741998
Hematologic Disorder	CONVERSION_FROM_APC_C_CDC20_TO_APC_C_CDH1_IN_LATE_ANAPHASE	REACTOME	226	11	5	8,27E-01	9,87E-01	rs315690|rs491675|rs11599360|rs1037633|rs6836883
Hematologic Disorder	ASPARAGINE_N_LINKED_GLYCOSYLATION	REACTOME	2760	35	20	8,27E-01	9,87E-01	rs1391962|rs2268535|rs6095638|rs13026308|rs10872180|rs10431384|rs1495085|rs4674406|rs35684|rs481325|rs878815|rs751687|rs869896|rs16830017|rs7593939|rs7152364|rs1146298|rs359327|rs9387640|rs11813767
Hematologic Disorder	PURINE_METABOLISM	KEGG	5442	32	20	8,28E-01	9,87E-01	rs7373003|rs241669|rs2036068|rs2076559|rs220813|rs2244095|rs2365491|rs3806777|rs11084882|rs907647|rs11729954|rs2121845|rs7008202|rs10125|rs7975385|rs2362892|rs953672|rs968757|rs2522420|rs11833143
Hematologic Disorder	GCR_PATHWAY	BIOCARTA	736	34	18	8,34E-01	9,87E-01	rs7595027|rs2055226|rs2677760|rs173704|rs4983559|rs11934749|rs11953134|rs7598661|rs10907192|rs2125391|rs1058793|rs1550805|rs6840205|rs815804|rs2300502|rs4388509|rs10489113|rs156768
Hematologic Disorder	INSULIN_RECEPTOR_RECYCLING	REACTOME	492	21	14	8,34E-01	9,87E-01	rs10500204|rs1080199|rs4499341|rs869896|rs10408844|rs2288404|rs10082815|rs4804366|rs16843078|rs17721027|rs12460755|rs4732330|rs5747285|rs9922624
Hematologic Disorder	ACH_PATHWAY	BIOCARTA	602	25	15	8,34E-01	9,87E-01	rs2677760|rs173704|rs4983559|rs521803|rs11953134|rs10759469|rs524408|rs3001115|rs7126210|rs11905013|rs4978958|rs1550805|rs1550097|rs1102737|rs2853676
Hematologic Disorder	RIBOSOME	KEGG	1118	44	20	8,35E-01	9,87E-01	rs7902136|rs751716|rs7257982|rs10498413|rs12594729|rs10895987|rs17289605|rs9928312|rs1149747|rs12620516|rs1528474|rs2430949|rs11002569|rs10140464|rs7177131|rs433852|rs9907408|rs2913115|rs6605321|rs4255712
Hematologic Disorder	IGF1_PATHWAY	BIOCARTA	813	23	17	8,36E-01	9,87E-01	rs8038056|rs2684806|rs2677760|rs173704|rs6681035|rs7558386|rs11953134|rs2888586|rs12901358|rs17103133|rs7162314|rs1550805|rs16967789|rs4966013|rs4776783|rs204680|rs2396340
Hematologic Disorder	NONSENSE_MEDIATED_DECAY_ENHANCED_BY_THE_EXON_JUNCTION_COMPLEX	REACTOME	1261	46	20	8,36E-01	9,87E-01	rs7902136|rs2226099|rs11578832|rs751716|rs7257982|rs10498413|rs9898819|rs10895987|rs9928312|rs1149747|rs12620516|rs11257408|rs2430949|rs11002569|rs10140464|rs433852|rs9907408|rs2913115|rs7908725|rs2062983
Hematologic Disorder	BOTULINUM_NEUROTOXICITY	REACTOME	522	27	19	8,37E-01	9,87E-01	rs17741348|rs8073622|rs2695863|rs6026402|rs1591811|rs7215786|rs11087010|rs6026373|rs17046030|rs4813925|rs11657426|rs12121078|rs4759753|rs12208207|rs363012|rs6951030|rs2534721|rs10800842|rs2695872
Hematologic Disorder	NKT_PATHWAY	BIOCARTA	638	14	12	8,38E-01	9,87E-01	rs9321590|rs874627|rs4245843|rs6770096|rs4764622|rs3741920|rs11080372|rs7526672|rs5439|rs3213094|rs7645716|rs3014996
Hematologic Disorder	DOWNSTREAM_TCR_SIGNALING	REACTOME	1573	27	20	8,39E-01	9,87E-01	rs6461796|rs6020238|rs874627|rs13217057|rs6586116|rs534182|rs28772724|rs2677760|rs173704|rs2673825|rs858492|rs2141484|rs4750434|rs390993|rs11953134|rs4764622|rs3130192|rs10485101|rs9878820|rs3741920
Hematologic Disorder	GLYOXYLATE_AND_DICARBOXYLATE_METABOLISM	KEGG	704	56	20	8,42E-01	9,87E-01	rs10813415|rs10738886|rs563440|rs10814535|rs10970986|rs372957|rs1411872|rs2150637|rs3765953|rs6077242|rs10970374|rs7863381|rs17289402|rs16918170|rs10970083|rs867469|rs745686|rs6140362|rs780758|rs1331249
Hematologic Disorder	RHO_PATHWAY	BIOCARTA	480	17	14	8,43E-01	9,87E-01	rs11869351|rs11123212|rs822570|rs3844598|rs4969254|rs13296679|rs820360|rs11905013|rs1359647|rs10800842|rs17412099|rs11741808|rs2547547|rs8009215
Hematologic Disorder	PYRUVATE_METABOLISM	REACTOME	209	11	9	8,44E-01	9,87E-01	rs149156|rs2978169|rs2158839|rs4239020|rs2012859|rs836624|rs7946331|rs7190459|rs773433
Hematologic Disorder	SIGNALING_BY_ERBB2	REACTOME	3847	28	20	8,44E-01	9,87E-01	rs2036068|rs2976532|rs2975498|rs6501512|rs7975710|rs7595027|rs4947979|rs429219|rs665821|rs11048455|rs7008202|rs7975385|rs1030098|rs3007685|rs953672|rs1465061|rs2055226|rs9914703|rs2110290|rs2601773
Hematologic Disorder	G_ALPHA_Q_SIGNALLING_EVENTS	REACTOME	7428	32	20	8,45E-01	9,87E-01	rs13131815|rs2852786|rs928670|rs4898725|rs10008744|rs6056252|rs163819|rs7091519|rs2478545|rs11687066|rs1072319|rs13212082|rs7975710|rs8121340|rs13107347|rs6807740|rs165095|rs17221044|rs6108237|rs7610820
Hematologic Disorder	DNA_REPLICATION	REACTOME	3548	27	20	8,46E-01	9,87E-01	rs2526060|401754|rs4813929|rs9913432|rs729405|rs2244095|rs11845708|rs698856|rs3781974|rs11058562|rs4073472|rs925192|rs10493854|rs9474192|rs6033024|rs1558461|rs6856643|rs7200210|rs17007652|rs35684
Hematologic Disorder	MEIOTIC_SYNAPSIS	REACTOME	1031	62	20	8,47E-01	9,87E-01	rs2813487|rs2623981|rs9383976|rs7740449|rs1009181|rs11913200|rs6997684|rs4887840|rs2093045|rs4699390|rs4313734|rs198853|rs9322366|rs6010770|rs12449157|rs1256061|rs2813498|rs9397084|rs3784929|rs6939589
Hematologic Disorder	PHOSPHORYLATION_OF_THE_APC_C	REACTOME	219	8	5	8,48E-01	9,87E-01	rs315690|rs11599360|rs2448343|rs1037633|rs6836883
Hematologic Disorder	IL1_SIGNALING	REACTOME	1322	45	20	8,51E-01	9,87E-01	rs2871444|rs12619383|rs6020238|rs2529737|rs13217057|rs2947864|rs6853|rs12504990|rs708030|rs242400|rs2141484|rs390993|rs3802727|rs10485101|rs17090708|rs6570956|rs2133165|rs9916229|rs4961116|rs1541435
Hematologic Disorder	APC_C_CDC20_MEDIATED_DEGRADATION_OF_CYCLIN_B	REACTOME	224	8	5	8,54E-01	9,87E-01	rs315690|rs11599360|rs2448343|rs1037633|rs6836883
Hematologic Disorder	INFLUENZA_VIRAL_RNA_TRANSCRIPTION_AND_REPLICATION	REACTOME	1153	31	20	8,55E-01	9,87E-01	rs7902136|rs11084882|rs751716|rs7257982|rs10498413|rs10895987|rs9928312|rs1149747|rs12620516|rs2261682|rs7329817|rs2430949|rs7981647|rs1337792|rs11002569|rs10140464|rs433852|rs9907408|rs633742|rs2913115
Hematologic Disorder	DNA_REPLICATION	KEGG	740	24	12	8,57E-01	9,87E-01	rs2244095|rs9474192|rs10774976|rs16981262|rs1818622|rs1317069|rs4943153|rs174532|rs2303744|rs9692542|rs13234190|rs7208143
Hematologic Disorder	INSULIN_SIGNALING_PATHWAY	KEGG	3744	29	20	8,57E-01	9,87E-01	rs12540943|rs3828150|rs7914896|rs6501512|rs7595027|rs4502283|rs4730626|rs1881628|rs6448226|rs10500204|rs4073472|rs1030098|rs2672886|rs6541017|rs4969429|rs2055226|rs11243454|rs1054743|rs7438406|rs2677760
Hematologic Disorder	NUCLEOTIDE_BINDING_DOMAIN_LEUCINE_RICH_REPEAT_CONTAINING_RECEPTOR_NLR YS	REACTOME	1337	35	20	8,57E-01	9,87E-01	rs9913432|rs1109225|rs1394839|rs7209554|rs6020238|rs13217057|rs2947864|rs708030|rs2141484|rs390993|rs12922698|rs13196204|rs10485101|rs12454712|rs4790801|rs6570956|rs2133165|rs461626|rs9916229|rs17178663
Hematologic Disorder	NEURONAL_SYSTEM	REACTOME	13378	35	20	8,63E-01	9,87E-01	rs198178|rs11630971|rs6606877|rs4958671|rs2914621|rs11654455|rs7538655|rs4898725|rs1953248|rs36960|rs6289|rs11120453|rs11715062|rs10519568|rs6056252|rs3799663|rs12915265|rs1566602|rs3806536|rs17797668
Hematologic Disorder	TRANSCRIPTIONAL_REGULATION_OF_WHITE_ADIPOCYTE_DIFFERENTIATION	REACTOME	2719	29	20	8,63E-01	9,87E-01	rs2440684|rs12424322|rs1242991|rs9842151|rs1036519|rs716738|rs6448226|rs450110|rs4917339|rs10816539|rs906304|rs9874964|rs7220132|rs218655|rs7875152|rs2235734|rs12338922|rs10520802|rs1629174|rs951538
Hematologic Disorder	HCMV_PATHWAY	BIOCARTA	547	21	15	8,64E-01	9,87E-01	rs2677760|rs3760201|rs173704|rs4983559|rs2947864|rs11953134|rs13196204|rs9916229|rs1550805|rs6721035|rs739559|rs4776783|rs2716227|rs10489113|rs889312
Hematologic Disorder	CHROMOSOME_MAINTENANCE	REACTOME	1949	32	20	8,65E-01	9,87E-01	rs2244095|rs8134439|rs2051186|rs2813487|rs4968207|rs3806929|rs2833736|rs2623981|rs9383976|rs7740449|rs1009181|rs17690723|rs10774976|rs11913200|rs16981262|rs6997684|rs1818622|rs4887840|rs2093045|rs4699390
Hematologic Disorder	AMINO_ACID_SYNTHESIS_AND_INTERCONVERSION_TRANSAMINATION	REACTOME	530	24	12	8,65E-01	9,87E-01	rs13299822|rs2183838|rs1505637|rs4784991|rs7919469|rs2047995|rs11137606|rs870541|rs10269471|rs7206052|rs2293968|rs11137862
Hematologic Disorder	TYPE_I_DIABETES_MELLITUS	KEGG	1975	58	20	8,65E-01	9,87E-01	rs5013310|rs2284191|rs6952172|rs28772724|rs2735007|rs4934433|rs2596574|rs17212090|rs3130192|rs12106790|rs2245822|rs1638006|rs9268911|rs2058522|rs1611350|rs2105436|rs13209654|rs241427|rs9296068|rs4716858
Hematologic Disorder	INSULIN_PATHWAY	BIOCARTA	725	25	18	8,66E-01	9,87E-01	rs10500204|rs2677760|rs173704|rs6681035|rs7558386|rs4499341|rs11953134|rs10408844|rs2288404|rs2888586|rs17103133|rs1550805|rs4804366|rs16967789|rs12460755|rs4776783|rs204680|rs2396340
Hematologic Disorder	PLATELET_ACTIVATION_SIGNALING_AND_AGGREGATION	REACTOME	6965	28	20	8,67E-01	9,87E-01	rs1898722|rs198178|rs2852786|rs3769309|rs4898725|rs7202877|rs3775478|rs7975710|rs7595027|rs449072|rs6807740|rs1572507|rs7829816|rs11048455|rs4721330|rs2358073|rs8100350|rs16947151|rs1394839|rs1148853
Hematologic Disorder	PML_PATHWAY	BIOCARTA	384	14	12	8,68E-01	9,87E-01	rs1678192|rs2715553|rs1061624|rs4934433|rs890669|rs2105436|rs12664430|rs4149576|rs3130014|rs16825445|rs1102737|rs13706
Hematologic Disorder	AKT_PATHWAY	BIOCARTA	682	16	11	8,69E-01	9,87E-01	rs2677760|rs173704|rs4983559|rs11953134|rs10507483|rs7335734|rs7321873|rs2701853|rs1550805|rs10489113|rs1102737
Hematologic Disorder	SIGNALING_BY_TGF_BETA_RECEPTOR_COMPLEX	REACTOME	1605	27	20	8,69E-01	9,87E-01	rs7617478|rs1561928|rs1242991|rs4073472|rs906304|rs2302793|rs11009607|rs951538|rs6470648|rs1539872|rs2065500|rs4325953|rs11265544|rs17787953|rs757718|rs6099789|rs11599360|rs6577059|rs4733616|rs12915039
Hematologic Disorder	G1_PHASE	REACTOME	625	19	15	8,72E-01	9,87E-01	rs10849024|rs614367|rs7315599|rs10105118|rs2065500|rs679162|rs9295464|rs6577059|rs1333050|rs3218020|rs741245|rs11617870|rs7394655|rs1561734|rs3217810
Hematologic Disorder	BUTANOATE_METABOLISM	KEGG	763	28	18	8,73E-01	9,87E-01	rs2066938|rs4665830|rs7673954|rs3043|rs163234|rs11732439|rs7685841|rs10516988|rs11646919|rs9838874|rs7527279|rs12447998|rs11915185|rs7705738|rs11943890|rs12467996|rs10803852|rs11097485
Hematologic Disorder	MITOCHONDRIAL_PROTEIN_IMPORT	REACTOME	609	17	16	8,74E-01	9,87E-01	rs1863703|rs4689604|rs764453|rs13021051|rs4823185|rs1865094|rs2611512|rs5752792|rs1651031|rs4143897|rs764454|rs12563749|rs10758442|rs2969344|rs1130635|rs7716228
Hematologic Disorder	G1_PATHWAY	BIOCARTA	611	28	17	8,74E-01	9,87E-01	rs4740366|rs2583839|rs614367|rs2065500|rs679162|rs6577059|rs12915039|rs2448343|rs334550|rs12901499|rs10425066|rs17068405|rs7526672|rs1333050|rs3218020|rs11617870|rs7394655
Hematologic Disorder	IL10_PATHWAY	BIOCARTA	288	10	6	8,74E-01	9,87E-01	rs4722178|rs841718|rs12409333|rs268691|rs13029532|rs999261
Hematologic Disorder	PORPHYRIN_AND_CHLOROPHYLL_METABOLISM	KEGG	1163	43	17	8,77E-01	9,87E-01	rs7857932|rs9872119|rs4895324|rs2736486|rs8066253|rs16861582|rs11078205|rs12123041|rs4895332|rs1736442|rs268691|rs11230882|rs1029659|rs6820734|rs10794023|rs9530|rs17148024
Hematologic Disorder	MAPK_PATHWAY	BIOCARTA	2690	44	20	8,80E-01	9,87E-01	rs11625206|rs591676|rs3799663|rs1561928|rs12892024|rs2302793|rs938875|rs11687570|rs550840|rs2529737|rs1054743|rs13217057|rs3760201|rs2947864|rs1509498|rs6681035|rs6470648|rs2141484|rs1852730|rs17591358

Hematologic Disorder	G1_S_SPECIFIC_TRANSCRIPTION	REACTOME	194	10	8	8,81E-01	9,87E-01	rs837763|rs6577059|rs2448343|rs10425066|rs17068405|rs11617870|rs13706|rs7193473
Hematologic Disorder	ALK_PATHWAY	BIOCARTA	1932	24	20	8,82E-01	9,87E-01	rs17798043|rs7617478|rs2645400|rs6140076|rs284169|rs13217057|rs12892252|rs2225265|rs10903341|rs13044457|rs869236|rs2141484|rs1852730|rs4438436|rs4325953|rs12434228|rs9883073|rs1276430|rs284180|rs1887054
Hematologic Disorder	GASTRIN_CREB_SIGNALLING_PATHWAY_VIA_PKC_AND_MAPK	REACTOME	8122	32	20	8,82E-01	9,87E-01	rs2189164|rs2852786|rs928670|rs4898725|rs10008744|rs6056252|rs163819|rs11970312|rs7091519|rs2478545|rs11687066|rs1072319|rs13212082|rs7975710|rs8121340|rs13107347|rs4947979|rs6807740|rs165095|rs17221044
Hematologic Disorder	COLORECTAL_CANCER	KEGG	2539	26	20	8,83E-01	9,87E-01	rs1442089|rs7617478|rs1561928|rs12573128|rs10502934|rs1338423|rs727683|rs6541017|rs2302793|rs2677760|rs173704|rs614367|rs4983559|rs6681035|rs10268780|rs6470648|rs7222705|rs1539872|rs4325953|rs679162
Hematologic Disorder	NO2IL12_PATHWAY	BIOCARTA	461	11	10	8,85E-01	9,87E-01	rs874627|rs858492|rs11792962|rs4764622|rs3741920|rs12564899|rs5439|rs3213094|rs13029532|rs3014996
Hematologic Disorder	CELLCYCLE_PATHWAY	BIOCARTA	459	20	16	8,86E-01	9,87E-01	rs10849024|rs614367|rs7315599|rs2065500|rs679162|rs6577059|rs2448343|rs10425066|rs17068405|rs1333050|rs3218020|rs741245|rs11617870|rs7394655|rs1561734|rs3217810
Hematologic Disorder	SYNTHESIS_SECRETION_AND_INACTIVATION_OF_GLP1	REACTOME	363	20	11	8,87E-01	9,87E-01	rs874627|rs6553943|rs9883073|rs261988|rs3741920|rs2168967|rs9951619|rs5439|rs7709763|rs12327532|rs681566
Hematologic Disorder	ACTIVATION_OF_ATR_IN_RESPONSE_TO_REPLICATION_STRESS	REACTOME	653	26	12	8,87E-01	9,87E-01	rs925192|rs10493854|rs9474192|rs10774976|rs1317069|rs4943153|rs9692542|rs153012|rs13234190|rs7208143|rs6085344|rs13706
Hematologic Disorder	DNA_STRAND_ELONGATION	REACTOME	672	25	13	8,88E-01	9,87E-01	rs2244095|rs9474192|rs10774976|rs16981262|rs1818622|rs1317069|rs4943153|rs174532|rs2303744|rs9692542|rs13234190|rs7208143|rs6085344
Hematologic Disorder	NFKB_PATHWAY	BIOCARTA	870	46	20	8,88E-01	9,87E-01	rs2806117|rs2225602|rs12619383|rs13217057|rs4483225|rs6853|rs1061624|rs2141484|rs10485101|rs2133165|rs4149576|rs12419174|rs9294511|rs2683065|rs12552190|rs713155|rs8180657|rs10489113|rs889312|rs4513756
Hematologic Disorder	RESPIRATORY_ELECTRON_TRANSPORT	REACTOME	1631	39	20	8,88E-01	9,87E-01	rs12654078|rs1054174|rs10777647|rs13263130|rs4797343|rs6538582|rs10268780|rs4657015|rs9931644|rs10206264|rs4656994|rs10491416|rs637421|rs3738814|rs4241236|rs2161249|rs249189|rs1500258|rs12188123|rs11991704
Hematologic Disorder	EXTENSION_OF_TELOMERES	REACTOME	618	24	13	8,90E-01	9,87E-01	rs2244095|rs4968207|rs10774976|rs16981262|rs1818622|rs1317069|rs4943153|rs174532|rs2303744|rs9692542|rs13234190|rs7208143|rs2853676
Hematologic Disorder	TNFR2_PATHWAY	BIOCARTA	414	20	11	8,92E-01	9,87E-01	rs4868204|rs1061624|rs9287799|rs1404358|rs11746792|rs2811757|rs2683065|rs2144825|rs8180663|rs10489113|rs889312
Hematologic Disorder	PACKAGING_OF_TELOMERE_ENDS	REACTOME	515	40	8	8,95E-01	9,87E-01	rs1009181|rs4887840|rs4699390|rs198853|rs12449157|rs3784929|rs6939589|rs7781740
Hematologic Disorder	LAGGING_STRAND_SYNTHESIS	REACTOME	550	21	11	8,95E-01	9,87E-01	rs2244095|rs10774976|rs16981262|rs1818622|rs1317069|rs4943153|rs174532|rs2303744|rs9692542|rs13234190|rs7208143
Hematologic Disorder	GABA_SYNTHESIS_RELEASE_REUPTAKE_AND_DEGRADATION	REACTOME	746	29	18	8,96E-01	9,87E-01	rs2815715|rs9820724|rs9871974|rs9835618|rs2200528|rs11087010|rs1416546|rs17046030|rs4813925|rs817365|rs363012|rs13215369|rs2171003|rs9341358|rs12467996|rs6951030|rs555044|rs10803852
Hematologic Disorder	P53_PATHWAY	BIOCARTA	347	12	9	8,97E-01	9,87E-01	rs614367|rs679162|rs675327|rs12454712|rs10425066|rs5754289|rs2850768|rs4987786|rs7394655
Hematologic Disorder	CHEMOKINE_RECEPTORS_BIND_CHEMOKINES	REACTOME	726	30	20	8,98E-01	9,87E-01	rs11591534|rs4245843|rs6770096|rs17735961|rs2063979|rs9288627|rs2041190|rs17488134|rs11080372|rs3798315|rs2236938|rs3815341|rs1875266|rs4790701|rs1147879|rs950817|rs1129763|rs13062158|rs4796144|rs7645716
Hematologic Disorder	XENOBIOTICS	REACTOME	231	6	3	8,98E-01	9,87E-01	rs1934967|rs12978001|rs305946
Hematologic Disorder	WNT_PATHWAY	BIOCARTA	1363	30	20	8,99E-01	9,87E-01	rs1561928|rs10867791|rs17296588|rs2302793|rs13217057|rs614367|rs6470648|rs2141484|rs679162|rs3802727|rs9883073|rs7038882|rs3739581|rs10485101|rs4733616|rs334550|rs4637783|rs11139170|rs6995630|rs2395851
Hematologic Disorder	GPVI_MEDIATED_ACTIVATION_CASCADE	REACTOME	1181	30	20	9,00E-01	9,87E-01	rs7829816|rs1148853|rs1465061|rs12944446|rs2677760|rs173704|rs4983559|rs252299|rs11953134|rs17718517|rs4656994|rs9878820|rs4744514|rs10993709|rs2254669|rs16956011|rs9487749|rs1550805|rs682083|rs8045689
Hematologic Disorder	SIGNALING_BY_NOTCH	REACTOME	4553	23	20	9,00E-01	9,87E-01	rs2070600|rs17825554|rs12431724|rs1561928|rs1706710|rs1242991|rs12533919|rs9842151|rs3791500|rs10867791|rs11685345|rs12274970|rs1958223|rs1584783|rs2188495|rs1359779|rs17296588|rs906304|rs2302793|rs7786745
Hematologic Disorder	ADHERENS_JUNCTIONS_INTERACTIONS	REACTOME	5496	30	20	9,02E-01	9,87E-01	rs7730479|rs7241357|rs10520938|rs1350214|rs17359062|rs6589499|rs11845708|rs2330539|rs7706649|rs7717247|rs10511074|rs1392152|rs1510165|rs7189335|rs17446875|rs8057899|rs1366497|rs1970546|rs2937524|rs10485473
Hematologic Disorder	TRANSPORT_TO_THE_GOLGI_AND_SUBSEQUENT_MODIFICATION	REACTOME	1557	31	20	9,03E-01	9,87E-01	rs2268535|rs6095638|rs10872180|rs35684|rs878815|rs869896|rs16830017|rs7593939|rs7152364|rs1009549|rs359327|rs9387640|rs11813767|rs12144850|rs1362153|rs10858586|rs7728733|rs4834699|rs26310|rs10971412
Hematologic Disorder	RNA_POL_I_RNA_POL_III_AND_MITOCHONDRIAL_TRANSCRIPTION	REACTOME	1830	30	20	9,06E-01	9,87E-01	rs11989385|rs11084882|rs13295646|rs11833143|rs2926362|rs2227301|rs3759978|rs1009181|rs2068477|rs13277342|rs7548201|rs4699390|rs4442161|rs630973|rs497789|rs198853|rs891502|rs4922152|rs6808352|rs4921702
Hematologic Disorder	T_CELL_RECEPTOR_SIGNALING_PATHWAY	KEGG	3546	26	20	9,07E-01	9,87E-01	rs933984|rs1876267|rs4419220|rs6461796|rs2239167|rs11547281|rs7193701|rs6541017|rs748938|rs12120762|rs1465061|rs874627|rs13217057|rs1741949|rs2677760|rs173704|rs4983559|rs6681035|rs6414232|rs2235360
Hematologic Disorder	G2_M_CHECKPOINTS	REACTOME	710	28	14	9,07E-01	9,87E-01	rs925192|rs10493854|rs9474192|rs10774976|rs5752792|rs2448343|rs1317069|rs4943153|rs9692542|rs153012|rs13234190|rs7208143|rs6085344|rs13706
Hematologic Disorder	TCR_SIGNALING	REACTOME	2040	26	20	9,10E-01	9,87E-01	rs1336480|rs6461796|rs12120762|rs6020238|rs874627|rs13217057|rs6586116|rs534182|rs28772724|rs2677760|rs173704|rs2673825|rs2235360|rs858492|rs252299|rs2141484|rs4750434|rs390993|rs11953134|rs4764622
Hematologic Disorder	EXTRACELLULAR_MATRIX_ORGANIZATION	REACTOME	3783	25	20	9,11E-01	9,87E-01	rs7645967|rs9447453|rs10874639|rs7202877|rs9521585|rs13094190|rs1061476|rs278891|rs12788428|rs10740358|rs7070979|rs2688768|rs874627|rs1871050|rs3211098|rs11788110|rs3113725|rs16927852|rs9296821|rs2279723
Hematologic Disorder	IL2_PATHWAY	BIOCARTA	452	18	15	9,12E-01	9,87E-01	rs1148853|rs1887027|rs6681035|rs4744514|rs7910961|rs2888586|rs10993709|rs12409333|rs17103133|rs290267|rs16967789|rs158688|rs4776783|rs2072496|rs204680
Hematologic Disorder	MRNA_CAPPING	REACTOME	388	20	14	9,12E-01	9,87E-01	rs11084882|rs6454694|rs1923416|rs2261682|rs1865094|rs9955264|rs9344845|rs2031449|rs633742|rs749420|rs7946331|rs1613070|rs1561734|rs663133
Hematologic Disorder	SIGNALING_BY_NOTCH1	REACTOME	3563	26	20	9,12E-01	9,87E-01	rs17825554|rs1561928|rs1706710|rs1242991|rs12533919|rs9842151|rs3791500|rs10867791|rs11685345|rs1584783|rs2188495|rs1359779|rs17296588|rs906304|rs2302793|rs7786745|rs3850839|rs9874964|rs12530920|rs11790630
Hematologic Disorder	TRANSFERRIN_ENDOCYTOSIS_AND_RECYCLING	REACTOME	513	13	9	9,13E-01	9,87E-01	rs1080199|rs4075688|rs869896|rs10082815|rs16843078|rs17721027|rs4732330|rs5747285|rs9922624
Hematologic Disorder	RELA_PATHWAY	BIOCARTA	208	11	9	9,13E-01	9,87E-01	rs1061624|rs3844598|rs2052455|rs2133165|rs4149576|rs12419174|rs10489113|rs2547547|rs11741808
Hematologic Disorder	HER2_PATHWAY	BIOCARTA	1326	34	20	9,14E-01	9,87E-01	rs4947979|rs980281|rs2110290|rs2677760|rs173704|rs4673677|rs4722178|rs12579363|rs11953134|rs7562228|rs17406681|rs7971370|rs11979158|rs2888586|rs13016652|rs1001408|rs1550805|rs17081685|rs10932383|rs16846917
Hematologic Disorder	CYTOKINE_PATHWAY	BIOCARTA	491	8	5	9,15E-01	9,87E-01	rs4722178|rs9990563|rs2275913|rs3213094|rs3014996
Hematologic Disorder	CIRCADIAN_CLOCK	REACTOME	2075	29	20	9,15E-01	9,87E-01	rs341404|rs9842151|rs6448226|rs450110|rs877228|rs4917339|rs9874964|rs12909890|rs2235734|rs930358|rs3211719|rs4450343|rs1852730|rs2303147|rs6494219|rs3802727|rs3844598|rs3768984|rs4775292|rs7598617
Hematologic Disorder	FORMATION_OF_THE_HIV1_EARLY_ELONGATION_COMPLEX	REACTOME	329	11	9	9,16E-01	9,87E-01	rs11084882|rs2261682|rs1865094|rs633742|rs3786238|rs7946331|rs1613070|rs1561734|rs663133
Hematologic Disorder	MHC_CLASS_II_ANTIGEN_PRESENTATION	REACTOME	2226	31	20	9,18E-01	9,87E-01	rs2284191|rs1913462|rs2292963|rs540874|rs6869991|rs754217|rs28772724|rs35684|rs7142555|rs1481986|rs1992300|rs4961604|rs10488753|rs10066223|rs3130192|rs757718|rs4243971|rs1579282|rs10895461|rs226434
Hematologic Disorder	RIBOFLAVIN_METABOLISM	KEGG	295	11	7	9,18E-01	9,87E-01	rs3820127|rs17587917|rs11144870|rs7120118|rs7765347|rs1395006|rs6776932
Hematologic Disorder	MITOCHONDRIAL_TRNA_AMINOACYLATION	REACTOME	500	31	16	9,19E-01	9,87E-01	rs959086|rs17140468|rs2432762|rs2003879|rs797134|rs941987|rs2713834|rs797126|rs9405834|rs3784929|rs12525112|rs17753334|rs3131921|rs10485196|rs2432793|rs2270003
Hematologic Disorder	TRANSCRIPTION_COUPLED_NER_TC_NER	REACTOME	834	26	18	9,19E-01	9,87E-01	rs2244095|rs11084882|rs1029290|rs17199520|rs10774976|rs16981262|rs2261682|rs3212986|rs1317069|rs4943153|rs633742|rs2303744|rs9692542|rs13234190|rs7946331|rs7208143|rs1561734|rs663133
Hematologic Disorder	TGFB_PATHWAY	BIOCARTA	817	29	17	9,20E-01	9,87E-01	rs7617478|rs13217057|rs3785076|rs1539872|rs2141484|rs4325953|rs10485101|rs12915039|rs12901499|rs7526672|rs17025329|rs9294511|rs4776783|rs713155|rs4513756|rs4707681|rs17555739
Hematologic Disorder	COLLAGEN_FORMATION	REACTOME	3203	25	20	9,20E-01	9,87E-01	rs7645967|rs9447453|rs10874639|rs9521585|rs13094190|rs12788428|rs10740358|rs7070979|rs2688768|rs874627|rs3211098|rs11788110|rs3113725|rs16927852|rs9296821|rs2279723|rs552125|rs3776817|rs1297941|rs6664294
Hematologic Disorder	TNFR1_PATHWAY	BIOCARTA	862	33	20	9,23E-01	9,87E-01	rs12817549|rs2529737|rs13217057|rs6681035|rs2141484|rs11614624|rs2152833|rs733751|rs10485101|rs12818362|rs7367066|rs7120118|rs2811757|rs3858603|rs8178179|rs4149576|rs921|rs9294511|rs10745664|rs2697920
Hematologic Disorder	IL6_PATHWAY	BIOCARTA	401	13	10	9,24E-01	9,87E-01	rs6681035|rs4722178|rs11792962|rs2888586|rs12409333|rs17103133|rs16967789|rs4776783|rs2072496|rs204680
Hematologic Disorder	GLYCOSYLPHOSPHATIDYLINOSITOL_GPI_ANCHOR_BIOSYNTHESIS	KEGG	318	4	4	9,24E-01	9,87E-01	rs2275404|rs6088565|rs2273595|rs17164861
Hematologic Disorder	MISMATCH_REPAIR	KEGG	576	17	10	9,26E-01	9,87E-01	rs2244095|rs10774976|rs16981262|rs1317069|rs4943153|rs2303744|rs2897298|rs9692542|rs13234190|rs7208143
Hematologic Disorder	IL1R_PATHWAY	BIOCARTA	1001	42	20	9,26E-01	9,87E-01	rs12619383|rs13217057|rs3760201|rs2947864|rs6853|rs6681035|rs4722178|rs708030|rs2141484|rs13196204|rs10485101|rs2133165|rs9916229|rs7526672|rs10937439|rs12419174|rs9294511|rs739559|rs2716227|rs713155
Hematologic Disorder	TIGHT_JUNCTION_INTERACTIONS	REACTOME	715	33	19	9,26E-01	9,87E-01	rs2985700|rs16865445|rs11009607|rs481781|rs11265544|rs12529366|rs12040893|rs12449157|rs4934622|rs10827315|rs377239|rs4598521|rs13051127|rs6849819|rs1359280|rs1054185|rs16865352|rs12252306|rs7516332
Hematologic Disorder	PEROXISOME	KEGG	2047	47	20	9,28E-01	9,87E-01	rs7310157|rs11242501|rs2073210|rs2555491|rs6599263|rs3213499|rs4931162|rs3782513|rs2349566|rs4623951|rs10842054|rs6938431|rs206819|rs10789465|rs4538195|rs681187|rs4862439|rs3792311|rs7129719|rs7575760
Hematologic Disorder	ACTIVATED_TAK1_MEDIATES_P38_MAPK_ACTIVATION	REACTOME	649	29	20	9,29E-01	9,87E-01	rs13217057|rs3760201|rs2947864|rs708030|rs2141484|rs390993|rs13196204|rs10485101|rs6570956|rs2133165|rs9916229|rs4961116|rs2906766|rs1510470|rs12419174|rs9294511|rs739559|rs2716227|rs713155|rs4513756
Hematologic Disorder	SIGNALLING_TO_RAS	REACTOME	468	17	13	9,29E-01	9,87E-01	rs2334915|rs10496561|rs13196204|rs6487465|rs2888586|rs2448343|rs926938|rs11905013|rs1994581|rs17389103|rs16967789|rs4776783|rs6334
Hematologic Disorder	MAP_KINASE_ACTIVATION_IN_TLR_CASCADE	REACTOME	1697	34	20	9,30E-01	9,87E-01	rs11970312|rs12892024|rs7305095|rs2529737|rs13217057|rs3760201|rs2947864|rs6681035|rs10105118|rs708030|rs2141484|rs1852730|rs17591358|rs390993|rs13196204|rs10485101|rs2448343|rs6570956|rs2133165|rs9916229
Hematologic Disorder	MITOCHONDRIA_PATHWAY	BIOCARTA	317	9	6	9,31E-01	9,87E-01	rs10268780|rs12454712|rs7367066|rs2850768|rs4987786|rs2293968
Hematologic Disorder	NOD1_2_SIGNALING_PATHWAY	REACTOME	881	28	20	9,32E-01	9,87E-01	rs6020238|rs13217057|rs2947864|rs708030|rs2141484|rs390993|rs12922698|rs13196204|rs10485101|rs6570956|rs2133165|rs9916229|rs4961116|rs2906766|rs1510470|rs12419174|rs9294511|rs739559|rs2683065|rs2716227
Hematologic Disorder	INSULIN_SYNTHESIS_AND_PROCESSING	REACTOME	1033	41	20	9,33E-01	9,87E-01	rs12106074|rs11187215|rs10267007|rs4901761|rs6597267|rs11087010|rs11907226|rs261988|rs4814615|rs10940184|rs4813925|rs4901750|rs11749327|rs10038257|rs10517365|rs363012|rs7143894|rs16999102|rs7709763|rs6951030
Hematologic Disorder	GLOBAL_GENOMIC_NER_GG_NER	REACTOME	823	25	15	9,33E-01	9,87E-01	rs2244095|rs1029290|rs17199520|rs10774976|rs16981262|rs784673|rs3212986|rs1317069|rs4943153|rs2303744|rs9692542|rs13234190|rs7946331|rs7208143|rs1561734
Hematologic Disorder	ENDOSOMAL_SORTING_COMPLEX_REQUIRED_FOR_TRANSPORT_ESCRT	REACTOME	318	6	4	9,34E-01	9,87E-01	rs2271050|rs10795505|rs17405840|rs4581063
Hematologic Disorder	ERBB_SIGNALING_PATHWAY	KEGG	4135	34	20	9,34E-01	9,87E-01	rs198178|rs2976532|rs2919384|rs6672395|rs1561928|rs4947979|rs449072|rs10509470|rs4740366|rs6541017|rs2302793|rs1649947|rs2529737|rs9914703|rs2110290|rs12535537|rs2583839|rs2677760|rs173704|rs4983559
Hematologic Disorder	CYTOSOLIC_TRNA_AMINOACYLATION	REACTOME	312	11	7	9,34E-01	9,87E-01	rs7072230|rs12123041|rs7097067|rs3784929|rs4401711|rs12327342|rs3742384
Hematologic Disorder	KINESINS	REACTOME	566	24	15	9,34E-01	9,87E-01	rs6869991|rs7142555|rs1992300|rs10066223|rs4243971|rs1579282|rs1890981|rs2957407|rs994626|rs961548|rs1464898|rs12451373|rs775251|rs11622651|rs9903429
Hematologic Disorder	MEIOSIS	REACTOME	1674	43	20	9,35E-01	9,87E-01	rs2813487|rs12449313|rs2623981|rs9383976|rs7740449|rs1009181|rs11575845|rs11913200|rs6997684|rs9534093|rs4887840|rs2093045|rs805293|rs17379153|rs4699390|rs4313734|rs198853|rs9322366|rs17312807|rs6010770
Hematologic Disorder	CELL_CELL_JUNCTION_ORGANIZATION	REACTOME	6211	31	20	9,36E-01	9,87E-01	rs7730479|rs7241357|rs10520938|rs2985700|rs1350214|rs17359062|rs6589499|rs11845708|rs4485945|rs7706649|rs7717247|rs10511074|rs1392152|rs16865445|rs1510165|rs7189335|rs17446875|rs8057899|rs1366497|rs1970546
Hematologic Disorder	SIGNALLING_TO_ERKS	REACTOME	794	29	20	9,37E-01	9,87E-01	rs2282004|rs2334915|rs11102314|rs2235360|rs10901096|rs10496561|rs13196204|rs6487465|rs2888586|rs2448343|rs926938|rs11905013|rs3012750|rs1994581|rs10842515|rs16967789|rs10793894|rs8090510|rs4776783|rs6334
Hematologic Disorder	RNA_POL_I_TRANSCRIPTION_TERMINATION	REACTOME	335	11	8	9,37E-01	9,87E-01	rs2227301|rs3759978|rs891502|rs9303601|rs492553|rs7946331|rs510342|rs1561734
Hematologic Disorder	PEPTIDE_LIGAND_BINDING_RECEPTORS	REACTOME	5053	35	20	9,38E-01	9,87E-01	rs13131815|rs10008744|rs10421380|rs11591534|rs2478545|rs13212082|rs13107347|rs17304749|rs17221044|rs2664175|rs7610820|rs17557166|rs4396295|rs1394839|rs7775745|rs9471684|rs12050331|rs1016492|rs4245843|rs4802861
Hematologic Disorder	SIGNALING_BY_NODAL	REACTOME	632	30	17	9,38E-01	9,87E-01	rs7560572|rs16827260|rs1539872|rs4662532|rs1950047|rs12915039|rs10804386|rs12901499|rs3784485|rs6598477|rs3934681|rs1037118|rs6598483|rs886853|rs1000914|rs10511595|rs6726059
Hematologic Disorder	RIG_I_LIKE_RECEPTOR_SIGNALING_PATHWAY	KEGG	1361	40	20	9,39E-01	9,87E-01	rs7026552|rs13217057|rs621277|rs6133067|rs803523|rs12052592|rs10812605|rs2141484|rs1040411|rs10970986|rs1404358|rs12922698|rs13196204|rs10485101|rs7037389|rs2133165|rs6084497|rs2207701|rs2811757|rs7262903
Hematologic Disorder	NETRIN1_SIGNALING	REACTOME	3710	34	20	9,40E-01	9,87E-01	rs1442089|rs163819|rs2483852|rs11936468|rs10502934|rs165095|rs31568|rs727683|rs11632854|rs12584914|rs882052|rs1465061|rs7650723|rs10866630|rs7895721|rs6890724|rs2560861|rs992852|rs2235360|rs17732939
Hematologic Disorder	SYNTHESIS_OF_PIPS_AT_THE_GOLGI_MEMBRANE	REACTOME	788	31	18	9,41E-01	9,87E-01	rs1992838|rs4633807|rs4121647|rs479198|rs17419409|rs12827287|rs307123|rs13209604|rs9946713|rs1954852|rs621042|rs11876701|rs17697518|rs1130635|rs9950453|rs325363|rs2305689|rs8089360
Hematologic Disorder	SYNTHESIS_OF_GLYCOSYLPHOSPHATIDYLINOSITOL_GPI	REACTOME	485	6	5	9,41E-01	9,87E-01	rs281215|rs2275404|rs2273595|rs17164861|rs2033735
Hematologic Disorder	IL12_PATHWAY	BIOCARTA	589	13	12	9,42E-01	9,87E-01	rs2947864|rs6681035|rs858492|rs11792962|rs13196204|rs12564899|rs9916229|rs739559|rs3213094|rs2716227|rs13029532|rs3014996
Hematologic Disorder	E2F_MEDIATED_REGULATION_OF_DNA_REPLICATION	REACTOME	452	20	11	9,42E-01	9,87E-01	rs10105118|rs837763|rs1818622|rs6577059|rs2448343|rs10425066|rs17068405|rs11617870|rs7193473|rs13706|rs6085344
Hematologic Disorder	MAL_PATHWAY	BIOCARTA	225	9	8	9,44E-01	9,87E-01	rs3844598|rs7961554|rs524386|rs2957154|rs4776783|rs889312|rs11741808|rs2547547
Hematologic Disorder	ONE_CARBON_POOL_BY_FOLATE	KEGG	441	11	8	9,44E-01	9,87E-01	rs12449313|rs563440|rs3772431|rs7604425|rs10783815|rs6495449|rs745686|rs3772428
Hematologic Disorder	HOMOLOGOUS_RECOMBINATION	KEGG	730	17	12	9,44E-01	9,87E-01	rs2244095|rs12449313|rs181565|rs9534093|rs1404508|rs2256224|rs7141945|rs9692542|rs13234190|rs8009944|rs7208143|rs2930961
Hematologic Disorder	MEIOTIC_RECOMBINATION	REACTOME	993	67	14	9,46E-01	9,87E-01	rs12449313|rs1009181|rs11575845|rs9534093|rs805293|rs17379153|rs4699390|rs198853|rs17312807|rs8086622|rs9692542|rs13234190|rs6939589|rs7208143
Hematologic Disorder	OXIDATIVE_PHOSPHORYLATION	KEGG	2486	35	20	9,46E-01	9,87E-01	rs8133991|rs12654078|rs1054174|rs13263130|rs4797343|rs7072230|rs959086|rs1080199|rs2834291|rs4657015|rs869896|rs8066253|rs9931644|rs10206264|rs10082815|rs11078205|rs8056122|rs4656994|rs6577059|rs10491416
Hematologic Disorder	NEUROTRANSMITTER_RELEASE_CYCLE	REACTOME	1400	31	20	9,47E-01	9,87E-01	rs3806536|rs2815715|rs9820724|rs1016742|rs10508915|rs9871974|rs9835618|rs2200528|rs11087010|rs1416546|rs17046030|rs4813925|rs5754289|rs2097312|rs817365|rs363012|rs13215369|rs2171003|rs9341358|rs12467996
Hematologic Disorder	NUCLEOTIDE_EXCISION_REPAIR	REACTOME	956	29	19	9,48E-01	9,87E-01	rs2244095|rs11084882|rs1029290|rs17199520|rs10774976|rs16981262|rs2261682|rs784673|rs3212986|rs1317069|rs4943153|rs633742|rs2303744|rs9692542|rs13234190|rs7946331|rs7208143|rs1561734|rs663133
Hematologic Disorder	ARGININE_AND_PROLINE_METABOLISM	KEGG	991	28	18	9,50E-01	9,87E-01	rs944605|rs11068458|rs3043|rs918233|rs4623951|rs759688|rs2078265|rs2608905|rs10192656|rs4784991|rs7919469|rs7527279|rs1215988|rs500608|rs7705738|rs7206052|rs1814288|rs12610011
Hematologic Disorder	ACTIVATED_NOTCH1_TRANSMITS_SIGNAL_TO_THE_NUCLEUS	REACTOME	719	24	17	9,51E-01	9,87E-01	rs1359779|rs3850839|rs1838343|rs6104695|rs1474817|rs1535175|rs3017036|rs17762822|rs1033583|rs4768307|rs11177623|rs6033079|rs1869135|rs6750496|rs7270364|rs17182398|rs7763013
Hematologic Disorder	NUCLEOTIDE_EXCISION_REPAIR	KEGG	914	25	15	9,51E-01	9,87E-01	rs2244095|rs1029290|rs17199520|rs10774976|rs16981262|rs784673|rs3212986|rs1317069|rs4943153|rs2303744|rs9692542|rs13234190|rs7946331|rs7208143|rs1561734
Hematologic Disorder	PROGESTERONE_MEDIATED_OOCYTE_MATURATION	KEGG	2535	36	20	9,52E-01	9,87E-01	rs11970312|rs8038056|rs7008202|rs7975385|rs6541017|rs2684806|rs953672|rs2281479|rs6856643|rs2601773|rs2677760|rs173704|rs4983559|rs1863961|rs1027692|rs902522|rs11045210|rs17591358|rs315690|rs11953134
Hematologic Disorder	UBIQUITIN_MEDIATED_PROTEOLYSIS	KEGG	3227	31	20	9,53E-01	9,87E-01	rs17064520|rs875785|rs1053079|rs2189713|rs12205861|rs4325734|rs6566955|rs12916907|rs2340634|rs8094453|rs10517563|rs2054759|rs2806532|rs4331321|rs2187213|rs6414232|rs10895987|rs708030|rs315690|rs3802727
Hematologic Disorder	HOMOLOGOUS_RECOMBINATION_REPAIR_OF_REPLICATION_INDEPENDENT_DOUBLE_STR	REACTOME	376	18	9	9,53E-01	9,87E-01	rs16981262|rs9534093|rs2048718|rs2106664|rs9969042|rs2303744|rs9692542|rs13234190|rs7208143
Hematologic Disorder	RNA_POL_I_TRANSCRIPTION_INITIATION	REACTOME	427	20	12	9,54E-01	9,87E-01	rs2227301|rs3759978|rs2068477|rs891502|rs6808352|rs9469069|rs11715447|rs9303601|rs492553|rs7946331|rs510342|rs1561734
Hematologic Disorder	DOUBLE_STRAND_BREAK_REPAIR	REACTOME	506	23	11	9,54E-01	9,87E-01	rs2391626|rs16981262|rs9534093|rs2048718|rs2106664|rs3909184|rs8178179|rs2303744|rs9692542|rs13234190|rs7208143
Hematologic Disorder	LYSINE_DEGRADATION	KEGG	952	23	20	9,55E-01	9,87E-01	rs4665830|rs2592984|rs3043|rs10501080|rs7689415|rs9863274|rs6800639|rs11228150|rs894013|rs9883003|rs9469069|rs7527279|rs706327|rs1206381|rs4923429|rs7907802|rs6675934|rs7705738|rs2602248|rs2269881
Hematologic Disorder	NOD_LIKE_RECEPTOR_SIGNALING_PATHWAY	KEGG	1406	43	20	9,57E-01	9,87E-01	rs9913432|rs1109225|rs7209554|rs13217057|rs4722178|rs17735961|rs2141484|rs390993|rs2041190|rs13196204|rs10485101|rs4790801|rs6570956|rs2133165|rs17178663|rs16955023|rs4961116|rs3815341|rs2906766|rs1510470
Hematologic Disorder	TRNA_AMINOACYLATION	REACTOME	795	39	20	9,57E-01	9,87E-01	rs10509321|rs959086|rs17140468|rs2432762|rs2003879|rs797134|rs12123041|rs941987|rs2713834|rs797126|rs9405834|rs7097067|rs3784929|rs12525112|rs17753334|rs3131921|rs4401711|rs10485196|rs2432793|rs3742384

Hematologic Disorder	POST_TRANSLATIONAL_MODIFICATION_SYNTHESIS_OF_GPI_ANCHORED_PROTEINS	REACTOME	586	10	8	9,58E-01	9,87E-01	rs281215|rs344772|rs2275404|rs6088565|rs2273595|rs17164861|rs6096210|rs2033735
Hematologic Disorder	SIGNALING_BY_ILS	REACTOME	2996	34	20	9,60E-01	9,87E-01	rs2239167|rs925246|rs7829816|rs11191274|rs9814648|rs2871444|rs6541017|rs1148853|rs1465061|rs12619383|rs6020238|rs2529737|rs11243454|rs13217057|rs1887027|rs2677760|rs173704|rs2947864|rs6853|rs4722178
Hematologic Disorder	CELL_JUNCTION_ORGANIZATION	REACTOME	6781	31	20	9,60E-01	9,87E-01	rs7730479|rs7241357|rs10520938|rs2985700|rs1350214|rs11826421|rs17359062|rs6589499|rs11845708|rs4485945|rs7706649|rs7717247|rs10511074|rs1392152|rs16865445|rs1510165|rs7189335|rs17446875|rs8057899|rs1366497
Hematologic Disorder	AMINE_LIGAND_BINDING_RECEPTORS	REACTOME	2247	32	20	9,61E-01	9,87E-01	rs2382070|rs2471005|rs1072319|rs11706283|rs685960|rs12364283|rs10885123|rs7531091|rs11952170|rs8091476|rs10095546|rs2802402|rs472151|rs9686886|rs1471107|rs1364041|rs2581831|rs6906147|rs12748947|rs9351108
Hematologic Disorder	FORMATION_OF_INCISION_COMPLEX_IN_GG_NER	REACTOME	545	16	9	9,61E-01	9,87E-01	rs1029290|rs17199520|rs784673|rs3212986|rs9692542|rs13234190|rs7946331|rs7208143|rs1561734
Hematologic Disorder	ADIPOCYTOKINE_SIGNALING_PATHWAY	KEGG	2106	27	20	9,61E-01	9,87E-01	rs12540943|rs1881628|rs6448226|rs12409877|rs4917339|rs272711|rs1528977|rs4983559|rs1061624|rs7558386|rs278152|rs4750434|rs7999458|rs11792962|rs4862439|rs3792311|rs12609870|rs7575760|rs1040566|rs10848550
Hematologic Disorder	REGULATION_OF_AUTOPHAGY	KEGG	933	28	15	9,63E-01	9,87E-01	rs4633807|rs4121647|rs803523|rs989885|rs1040411|rs7037389|rs307123|rs9946713|rs1954852|rs11876701|rs17697518|rs9950453|rs325363|rs8089360|rs3014996
Hematologic Disorder	TELOMERE_MAINTENANCE	REACTOME	1133	60	20	9,64E-01	9,87E-01	rs2244095|rs4968207|rs1009181|rs10774976|rs16981262|rs1818622|rs4887840|rs4699390|rs198853|rs12449157|rs1317069|rs4943153|rs174532|rs2303744|rs9692542|rs3784929|rs13234190|rs6939589|rs7208143|rs7781740
Hematologic Disorder	CELL_CELL_COMMUNICATION	REACTOME	9725	31	20	9,65E-01	9,87E-01	rs7950175|rs7730479|rs7241357|rs1442089|rs10520938|rs2985700|rs1350214|rs11826421|rs17359062|rs10502934|rs2239167|rs987493|rs2691542|rs6589499|rs11845708|rs4485945|rs7706649|rs7717247|rs727683|rs10511074
Hematologic Disorder	TOLL_LIKE_RECEPTOR_SIGNALING_PATHWAY	KEGG	2339	28	20	9,65E-01	9,87E-01	rs1053079|rs2806117|rs2225602|rs6541017|rs2529737|rs13217057|rs4483225|rs2677760|rs3760201|rs173704|rs4983559|rs2947864|rs6853|rs6681035|rs4722178|rs1339|rs2141484|rs11953134|rs4240896|rs13196204
Hematologic Disorder	CASPASE_PATHWAY	BIOCARTA	327	5	5	9,68E-01	9,87E-01	rs10268780|rs7367066|rs921|rs12567614|rs2272256
Hematologic Disorder	PURINE_METABOLISM	REACTOME	581	27	14	9,69E-01	9,87E-01	rs10781076|rs4789145|rs595890|rs167508|rs206819|rs926938|rs17323225|rs10485305|rs6848187|rs819851|rs7604425|rs7206953|rs1033510|rs860762
Hematologic Disorder	CELL_CYCLE	KEGG	2581	32	20	9,69E-01	9,87E-01	rs1561928|rs4740366|rs925192|rs10849024|rs10493854|rs9474192|rs2302793|rs1558461|rs2281479|rs6856643|rs17057833|rs2583839|rs2283231|rs614367|rs7315599|rs1027692|rs6470648|rs7015841|rs1539872|rs2065500
Hematologic Disorder	PANCREATIC_CANCER	KEGG	1920	26	20	9,69E-01	9,87E-01	rs7617478|rs4947979|rs6541017|rs2110290|rs2677760|rs6458350|rs173704|rs614367|rs4983559|rs1539872|rs4325953|rs10496561|rs679162|rs11953134|rs4240896|rs9534093|rs399819|rs9295464|rs9878820|rs6487465
Hematologic Disorder	PROXIMAL_TUBULE_BICARBONATE_RECLAMATION	KEGG	458	14	11	9,71E-01	9,87E-01	rs973608|rs4839534|rs1040566|rs1200150|rs3561|rs11805919|rs11749327|rs1200138|rs4957044|rs6025642|rs4839066
Hematologic Disorder	AMINOACYL_TRNA_BIOSYNTHESIS	KEGG	731	30	20	9,71E-01	9,87E-01	rs9423240|rs17140468|rs2432762|rs2003879|rs797134|rs13353058|rs12123041|rs941987|rs797126|rs9405834|rs8038261|rs3784929|rs12525112|rs17753334|rs3131921|rs10485196|rs2432793|rs12327342|rs3742384|rs2270003
Hematologic Disorder	DNA_REPAIR	REACTOME	2111	29	20	9,73E-01	9,87E-01	rs12917|rs2244095|rs737094|rs4325734|rs11084882|rs1029290|rs2391626|rs1978759|rs17199520|rs2029166|rs10774976|rs16981262|rs9534093|rs992574|rs2261682|rs784673|rs7764543|rs2048718|rs2106664|rs3212986
Hematologic Disorder	RNA_POL_I_TRANSCRIPTION	REACTOME	937	68	16	9,74E-01	9,87E-01	rs2227301|rs3759978|rs1009181|rs2068477|rs4699390|rs198853|rs891502|rs6808352|rs9469069|rs11715447|rs9303601|rs492553|rs6939589|rs7946331|rs510342|rs1561734
Hematologic Disorder	G_ALPHA_I_SIGNALLING_EVENTS	REACTOME	6453	25	20	9,74E-01	9,87E-01	rs9306383|rs4898725|rs10421380|rs13116176|rs11591534|rs2478545|rs11687066|rs6807740|rs17304749|rs2260577|rs2664175|rs512756|rs17557166|rs10857898|rs7008202|rs11706283|rs7975385|rs2500236|rs6003495|rs4396295
Hematologic Disorder	41BB_PATHWAY	BIOCARTA	341	12	8	9,76E-01	9,87E-01	rs6681035|rs13196204|rs10139934|rs2811757|rs10489113|rs889312|rs8005104|rs3014996
Hematologic Disorder	JNK_C_JUN_KINASES_PHOSPHORYLATION_AND_ACTIVATION_MEDIATED_BY_ACTIVATE	REACTOME	728	29	18	9,77E-01	9,87E-01	rs2529737|rs13217057|rs708030|rs2141484|rs390993|rs10485101|rs6570956|rs2133165|rs4961116|rs2906766|rs1510470|rs12419174|rs9294511|rs713155|rs4513756|rs10944547|rs17555739|rs8082104
Hematologic Disorder	CERAMIDE_PATHWAY	BIOCARTA	425	18	11	9,77E-01	9,87E-01	rs2529737|rs10268780|rs12454712|rs2811757|rs4149576|rs2850768|rs4987786|rs4776783|rs10489113|rs889312|rs8082104
Hematologic Disorder	NGF_SIGNALLING_VIA_TRKA_FROM_THE_PLASMA_MEMBRANE	REACTOME	4847	31	20	9,77E-01	9,87E-01	rs2036068|rs3799663|rs6501512|rs7975710|rs7595027|rs429219|rs665821|rs12892024|rs7305095|rs11048455|rs7008202|rs7975385|rs1030098|rs3007685|rs953672|rs938875|rs2055226|rs9914703|rs11243454|rs2334915
Hematologic Disorder	FAS_PATHWAY	BIOCARTA	980	29	20	9,79E-01	9,87E-01	rs2529737|rs13217057|rs6681035|rs4934433|rs2141484|rs390993|rs2152833|rs10485101|rs2105436|rs7367066|rs4961116|rs12664430|rs8178179|rs1510470|rs921|rs3130014|rs9294511|rs12567614|rs713155|rs1102737
Hematologic Disorder	RECRUITMENT_OF_MITOTIC_CENTROSOME_PROTEINS_AND_COMPLEXES	REACTOME	1177	42	18	9,79E-01	9,87E-01	rs4907726|rs11933720|rs11108793|rs12670688|rs741339|rs1562203|rs3780674|rs3801969|rs2448343|rs12111310|rs4762388|rs10790180|rs11108995|rs13243079|rs12832163|rs8258|rs746145|rs10507088
Hematologic Disorder	CHONDROITIN_SULFATE_BIOSYNTHESIS	REACTOME	846	30	17	9,80E-01	9,87E-01	rs9967331|rs256459|rs2591454|rs1056695|rs309568|rs4661210|rs7314687|rs312157|rs11112143|rs4535751|rs219233|rs1704907|rs861158|rs17035996|rs1476614|rs6597849|rs12579969
Hematologic Disorder	MITOTIC_G2_G2_M_PHASES	REACTOME	1389	39	20	9,83E-01	9,88E-01	rs8381|rs2281479|rs11933720|rs11108793|rs12670688|rs741339|rs1562203|rs3780674|rs3801969|rs9295464|rs2448343|rs9302815|rs12111310|rs17068405|rs12613627|rs4762388|rs10790180|rs1010608|rs11108995|rs13243079
Hematologic Disorder	HIVNEF_PATHWAY	BIOCARTA	1008	29	20	9,83E-01	9,88E-01	rs12817549|rs1061624|rs4934433|rs10268780|rs11614624|rs2152833|rs733751|rs12454712|rs2105436|rs12818362|rs7367066|rs2811757|rs3858603|rs8178179|rs12664430|rs4149576|rs2850768|rs921|rs3130014|rs4987786
Hematologic Disorder	NEUROTROPHIN_SIGNALING_PATHWAY	KEGG	3689	33	20	9,84E-01	9,88E-01	rs16941424|rs11970312|rs12304579|rs7595027|rs429219|rs12892024|rs4740366|rs6541017|rs938875|rs2055226|rs550840|rs2282004|rs2334915|rs12535537|rs2583839|rs2677760|rs173704|rs4983559|rs11102314|rs6681035
Hematologic Disorder	STARCH_AND_SUCROSE_METABOLISM	KEGG	2178	48	20	9,88E-01	9,90E-01	rs12741933|rs11918537|rs13072710|rs2618956|rs4072113|rs7691016|rs2736486|rs2050592|rs9309906|rs4726492|rs642497|rs710862|rs9483334|rs7897621|rs324079|rs11184173|rs6820734|rs9530|rs13066021|rs2578300
Hematologic Disorder	LOSS_OF_NLP_FROM_MITOTIC_CENTROSOMES	REACTOME	1100	33	16	9,89E-01	9,91E-01	rs11933720|rs11108793|rs12670688|rs741339|rs3780674|rs3801969|rs2448343|rs12111310|rs4762388|rs10790180|rs11108995|rs13243079|rs12832163|rs8258|rs746145|rs10507088
Hematologic Disorder	N_GLYCAN_BIOSYNTHESIS	KEGG	1792	42	19	9,94E-01	9,94E-01	rs2268535|rs10872180|rs1495085|rs878815|rs751687|rs869896|rs16830017|rs1009549|rs9387640|rs12144850|rs1362153|rs7728733|rs10971412|rs11783448|rs9320730|rs11929282|rs3811244|rs6096210|rs8047337
Immunologic Disorder	CHEMOKINE_RECEPTORS_BIND_CHEMOKINES	REACTOME	726	35	20	6,10E-03	9,95E-01	rs7604823|rs7677281|rs17414505|rs3138112|rs4674259|rs1933107|rs6744322|rs12691883|rs2011946|rs1904836|rs2056296|rs4972960|rs10191360|rs1933089|rs9868689|rs6761387|rs3733236|rs12409982|rs875969|rs6841674
Immunologic Disorder	NATURAL_KILLER_CELL_MEDIATED_CYTOTOXICITY	KEGG	3607	41	20	7,20E-03	9,95E-01	rs895437|rs649216|rs5865|rs16897466|rs2510257|rs775859|rs1641661|rs9975358|rs3115612|rs766389|rs2251396|rs11066320|rs4278155|rs4963867|rs7910825|rs12765241|rs4896251|rs11135693|rs9299346|rs6063664
Immunologic Disorder	ALDOSTERONE_REGULATED_SODIUM_REABSORPTION	KEGG	1794	46	20	1,13E-02	9,95E-01	rs4365706|rs588407|rs16897466|rs17201100|rs7749856|rs11964762|rs9493891|rs10023128|rs868840|rs4963867|rs8088164|rs2396303|rs9493883|rs1634079|rs11657742|rs7943324|rs6849903|rs10050229|rs13320527|rs1879827
Immunologic Disorder	CHEMOKINE_SIGNALING_PATHWAY	KEGG	5407	31	20	1,14E-02	9,95E-01	rs7604823|rs7677281|rs8115510|rs16897466|rs2510257|rs10485723|rs17414505|rs227130|rs3138112|rs2423360|rs3819579|rs7239317|rs1882070|rs12545028|rs4855077|rs4963867|rs304737|rs2409416|rs7140817|rs13052373
Immunologic Disorder	RAS_ACTIVATION_UOPN_CA2_INFUX_THROUGH_NMDA_RECEPTOR	REACTOME	1033	46	20	1,30E-02	9,95E-01	rs11055651|rs4408387|rs10514211|rs10772692|rs4703825|rs7974275|rs1861787|rs12921725|rs10845809|rs765688|rs10505778|rs9645754|rs766831|rs1379270|rs34116|rs4704699|rs4332745|rs722008|rs11125132|rs11055603
Immunologic Disorder	EIF_PATHWAY	BIOCARTA	158	16	2	1,57E-02	9,95E-01	rs3767248|rs12930464
Immunologic Disorder	NEUROTRANSMITTER_RECEPTOR_BINDING_AND_DOWNSTREAM_TRANSMISSION_IN_TH<br>L	REACTOME	6592	38	20	1,66E-02	9,95E-01	rs4514648|rs1051774|rs11055651|rs2836285|rs4408387|rs1034841|rs9303513|rs10485723|rs10514211|rs6290|rs227130|rs2423360|rs10772692|rs2832535|rs3755768|rs4703825|rs12545028|rs7974275|rs1073572|rs4855077
Immunologic Disorder	ATRBRCA_PATHWAY	BIOCARTA	238	4	1	1,69E-02	9,95E-01	rs4657
Immunologic Disorder	PANTOTHENATE_AND_COA_BIOSYNTHESIS	KEGG	611	49	20	2,19E-02	9,95E-01	rs9493120|rs6934819|rs12023630|rs17117613|rs12039696|rs12061124|rs6917833|rs12041436|rs6681958|rs2294757|rs2352745|rs7731228|rs10493895|rs1471841|rs7756163|rs6935458|rs492734|rs290821|rs11047702|rs9493099
Immunologic Disorder	MYOSIN_PATHWAY	BIOCARTA	1253	37	20	2,31E-02	9,95E-01	rs11929318|rs10485723|rs227130|rs2423360|rs208354|rs17202361|rs503473|rs11923190|rs1952966|rs12119764|rs2062154|rs820363|rs8115925|rs798524|rs10229817|rs4938800|rs7039739|rs17786782|rs1015092|rs2745755
Immunologic Disorder	SYNTHESIS_SECRETION_AND_DEACYLATION_OF_GHRELIN	REACTOME	624	47	20	2,48E-02	9,95E-01	rs7038715|rs634454|rs17233136|rs4542003|rs10490815|rs7659414|rs8027776|rs3792747|rs2123861|rs17062878|rs1026483|rs1411355|rs7811892|rs1004446|rs3761656|rs4529218|rs1851741|rs11135469|rs10978943|rs791600
Immunologic Disorder	INTRINSIC_PATHWAY	REACTOME	264	7	4	3,03E-02	9,95E-01	rs723188|rs12602483|rs17491334|rs2283332
Immunologic Disorder	PEPTIDE_LIGAND_BINDING_RECEPTORS	REACTOME	5053	25	20	3,11E-02	9,95E-01	rs7604823|rs7677281|rs2306718|rs10506460|rs2281617|rs17414505|rs7146347|rs37244|rs3138112|rs4895594|rs466609|rs2286469|rs13410673|rs10490815|rs4396295|rs6941251|rs92|rs400819|rs4674257|rs6537662
Immunologic Disorder	POST_CHAPERONIN_TUBULIN_FOLDING_PATHWAY	REACTOME	254	20	4	3,42E-02	9,95E-01	rs9747279|rs652265|rs6699315|rs11080567
Immunologic Disorder	LIGAND_GATED_ION_CHANNEL_TRANSPORT	REACTOME	1051	50	20	3,46E-02	9,95E-01	rs4514648|rs6290|rs10083549|rs11852692|rs4354903|rs11606194|rs2044081|rs17561473|rs12493550|rs17738971|rs1484363|rs4485963|rs6606856|rs6576565|rs11740084|rs4695935|rs4887546|rs8023912|rs7762714|rs3816596
Immunologic Disorder	NO1_PATHWAY	BIOCARTA	1482	29	20	3,49E-02	9,95E-01	rs12058655|rs7146347|rs17540215|rs7907343|rs12797628|rs7117518|rs10925415|rs1937701|rs16835237|rs10058772|rs2392695|rs13168072|rs2127155|rs9579422|rs1926212|rs7303397|rs17631340|rs1427988|rs3794405|rs17073762
Immunologic Disorder	CREB_PHOSPHORYLATION_THROUGH_THE_ACTIVATION_OF_RAS	REACTOME	1349	47	20	3,54E-02	9,95E-01	rs11055651|rs4408387|rs10514211|rs10772692|rs4703825|rs7974275|rs1861787|rs12921725|rs3817828|rs10845809|rs765688|rs10505778|rs9645754|rs766831|rs1379270|rs34116|rs4704699|rs4332745|rs722008|rs11125132
Immunologic Disorder	PAR1_PATHWAY	BIOCARTA	1941	35	20	3,73E-02	9,95E-01	rs16897466|rs11929318|rs10485723|rs37244|rs227130|rs2423360|rs208354|rs17202361|rs304737|rs503473|rs11923190|rs12119764|rs2062154|rs8115925|rs798524|rs10229817|rs4938800|rs9359935|rs2886612|rs13320527
Immunologic Disorder	INOSITOL_PHOSPHATE_METABOLISM	KEGG	2355	38	20	3,87E-02	9,95E-01	rs8115510|rs10485723|rs227130|rs2305030|rs350292|rs2423360|rs2212963|rs3819579|rs11082159|rs10509534|rs2636638|rs1997696|rs1130635|rs2151414|rs7899961|rs4297784|rs8115925|rs6481369|rs7197601|rs1865275
Immunologic Disorder	GABA_RECEPTOR_ACTIVATION	REACTOME	2705	38	20	4,15E-02	9,95E-01	rs4514648|rs2836285|rs1034841|rs6290|rs12545028|rs1073572|rs10083549|rs304737|rs11852692|rs7140817|rs4354903|rs2532019|rs948215|rs2044081|rs17561473|rs236504|rs13168690|rs17230421|rs17738971|rs1484363
Immunologic Disorder	CALCIUM_SIGNALING_PATHWAY	KEGG	9275	31	20	4,23E-02	9,95E-01	rs4779632|rs8115510|rs2102996|rs1034258|rs12058655|rs17347606|rs10506460|rs2104483|rs1730146|rs10485723|rs7146347|rs37244|rs227130|rs2305030|rs675498|rs2423360|rs12018744|rs3819579|rs12328595|rs13393014
Immunologic Disorder	G_ALPHA_I_SIGNALLING_EVENTS	REACTOME	6453	26	20	4,38E-02	9,95E-01	rs7604823|rs2325102|rs7677281|rs2281617|rs1730146|rs17414505|rs7146347|rs3138112|rs12603232|rs466609|rs12545028|rs954385|rs4855077|rs2286469|rs304737|rs7140817|rs12213790|rs4396295|rs6941251|rs2532019
Immunologic Disorder	INITIAL_TRIGGERING_OF_COMPLEMENT	REACTOME	434	44	13	4,62E-02	9,95E-01	rs11003448|rs698090|rs10824895|rs6467|rs1917186|rs2592887|rs16861812|rs11064498|rs7474987|rs4935411|rs7474747|rs1917190|rs16842484
Immunologic Disorder	TYPE_I_DIABETES_MELLITUS	KEGG	1975	44	20	4,82E-02	9,95E-01	rs10227495|rs4680551|rs1641661|rs3115612|rs10486212|rs987870|rs7910825|rs1670340|rs12765241|rs10262207|rs1611493|rs2243131|rs9640065|rs881468|rs1982126|rs28367832|rs11984274|rs4315909|rs17500510|rs9276933
Immunologic Disorder	POST_NMDA_RECEPTOR_ACTIVATION_EVENTS	REACTOME	1753	49	20	4,82E-02	9,95E-01	rs11055651|rs4408387|rs10514211|rs10772692|rs4703825|rs12545028|rs7974275|rs1861787|rs12921725|rs7214864|rs3817828|rs10845809|rs2193151|rs11651131|rs10505778|rs9645754|rs766831|rs1379270|rs34116|rs4704699
Immunologic Disorder	ALZHEIMERS_DISEASE	KEGG	5029	37	20	5,59E-02	9,95E-01	rs4779632|rs8115510|rs11055651|rs4408387|rs10485723|rs227130|rs11745467|rs3794176|rs2423360|rs10772692|rs3819579|rs466609|rs7974275|rs7910825|rs1861787|rs12765241|rs12921725|rs11077773|rs1800649|rs400819
Immunologic Disorder	RIBOFLAVIN_METABOLISM	KEGG	295	26	11	5,73E-02	9,95E-01	rs9493120|rs6934819|rs4802742|rs756607|rs12459891|rs7756163|rs6935458|rs11994010|rs9493099|rs2411335|rs10503595
Immunologic Disorder	NEUROACTIVE_LIGAND_RECEPTOR_INTERACTION	KEGG	13469	36	20	5,88E-02	9,95E-01	rs4514648|rs2325102|rs1034258|rs11055651|rs2306718|rs10506460|rs4408387|rs2281617|rs2104483|rs1730146|rs7629975|rs7146347|rs37244|rs6290|rs4895594|rs712786|rs10772692|rs12018744|rs2832535|rs6445959
Immunologic Disorder	TRANSMISSION_ACROSS_CHEMICAL_SYNAPSES	REACTOME	9089	40	20	6,16E-02	9,95E-01	rs4514648|rs1051774|rs11055651|rs2836285|rs1482574|rs4408387|rs1034841|rs9303513|rs10485723|rs10514211|rs6290|rs227130|rs2423360|rs10772692|rs2832535|rs3755768|rs2074615|rs4703825|rs12545028|rs7974275
Immunologic Disorder	SHC1_EVENTS_IN_ERBB4_SIGNALING	REACTOME	1072	25	20	6,46E-02	9,95E-01	rs2102996|rs17347606|rs12328595|rs13393014|rs4963867|rs776385|rs2919390|rs16846161|rs4672649|rs10503904|rs6580353|rs10497961|rs7826312|rs4150196|rs1481756|rs718607|rs7557116|rs9792249|rs4886755|rs16879142
Immunologic Disorder	NEURONAL_SYSTEM	REACTOME	13378	40	20	6,48E-02	9,95E-01	rs4514648|rs337683|rs1051774|rs588407|rs11055651|rs2836285|rs1482574|rs9688792|rs4408387|rs1034841|rs9303513|rs10485723|rs11102357|rs10514211|rs6290|rs227130|rs4809563|rs2423360|rs10772692|rs2832535
Immunologic Disorder	PROLONGED_ERK_ACTIVATION_EVENTS	REACTOME	429	19	9	6,50E-02	9,95E-01	rs10858075|rs4963867|rs28628360|rs11102314|rs2296950|rs7523086|rs17388587|rs2334915|rs2868161
Immunologic Disorder	COMPLEMENT_CASCADE	REACTOME	752	57	20	6,80E-02	9,95E-01	rs11003448|rs698090|rs2842704|rs10824895|rs923109|rs6467|rs2019724|rs1917186|rs2592887|rs16861812|rs11064498|rs12021671|rs399443|rs7474987|rs4935411|rs7474747|rs3805226|rs1917190|rs391781|rs647962
Immunologic Disorder	MTOR_PATHWAY	BIOCARTA	509	33	11	6,84E-02	9,95E-01	rs3767248|rs16897466|rs10509534|rs13320527|rs13173003|rs706725|rs6449947|rs2161529|rs3782148|rs10515074|rs6871452
Immunologic Disorder	NOS1_PATHWAY	BIOCARTA	1133	47	20	6,90E-02	9,95E-01	rs11055651|rs4408387|rs10772692|rs7974275|rs1861787|rs12921725|rs2469749|rs10845809|rs2193151|rs10505778|rs9645754|rs766831|rs4332745|rs722008|rs11125132|rs11055603|rs2302234|rs3804357|rs10781329|rs316369
Immunologic Disorder	ARMS_MEDIATED_ACTIVATION	REACTOME	393	19	9	7,25E-02	9,95E-01	rs10858075|rs4963867|rs28628360|rs11102314|rs2296950|rs7523086|rs17388587|rs2334915|rs2868161
Immunologic Disorder	GENERATION_OF_SECOND_MESSENGER_MOLECULES	REACTOME	616	56	17	7,49E-02	9,95E-01	rs895437|rs5865|rs987870|rs8045689|rs17500510|rs704856|rs315734|rs9276291|rs695161|rs2236317|rs11956872|rs2995094|rs1362811|rs107140|rs527589|rs858490|rs1004420
Immunologic Disorder	SMOOTH_MUSCLE_CONTRACTION	REACTOME	658	29	20	7,50E-02	9,95E-01	rs2119685|rs7910825|rs12765241|rs1934158|rs501603|rs10519327|rs8060975|rs215573|rs820363|rs1363950|rs13163497|rs11125132|rs13240924|rs10824069|rs1050162|rs752364|rs10188058|rs1441734|rs1409711|rs975885
Immunologic Disorder	NETRIN1_SIGNALING	REACTOME	3710	27	20	7,58E-02	9,95E-01	rs12375339|rs11924816|rs7435615|rs4750517|rs10502934|rs1592818|rs551357|rs17023932|rs8033109|rs2163783|rs10521156|rs10511131|rs10823677|rs1499772|rs253324|rs2077753|rs1483319|rs12324320|rs587198|rs9418677
Immunologic Disorder	OPIOID_SIGNALLING	REACTOME	3594	40	20	7,60E-02	9,95E-01	rs12046844|rs8115510|rs2281617|rs10485723|rs227130|rs2423360|rs3819579|rs12545028|rs4855077|rs298026|rs304737|rs7140817|rs17649232|rs6941251|rs2532019|rs13168690|rs1997696|rs4599070|rs6081597|rs8115925
Immunologic Disorder	GRB2_EVENTS_IN_ERBB2_SIGNALING	REACTOME	1202	25	20	8,21E-02	9,95E-01	rs2102996|rs17347606|rs12328595|rs13393014|rs4963867|rs776385|rs2919390|rs16846161|rs6974790|rs4672649|rs10503904|rs6580353|rs10497961|rs7826312|rs4150196|rs1481756|rs718607|rs7557116|rs1140475|rs9792249
Immunologic Disorder	GLYCOSAMINOGLYCAN_BIOSYNTHESIS_HEPARAN_SULFATE	KEGG	2273	30	20	8,48E-02	9,95E-01	rs12445976|rs9890008|rs7938320|rs11696078|rs9999101|rs3785690|rs2865155|rs1859906|rs1111994|rs3845669|rs517231|rs12615148|rs11223864|rs1944866|rs11231168|rs722769|rs16948248|rs2635351|rs7765174|rs4131119
Immunologic Disorder	G_ALPHA1213_SIGNALLING_EVENTS	REACTOME	2855	32	20	8,53E-02	9,95E-01	rs16897466|rs11929318|rs10514211|rs6578414|rs208354|rs17202361|rs894628|rs4703825|rs4855077|rs7266945|rs503473|rs13098182|rs2409416|rs7140817|rs11923190|rs13052373|rs6019300|rs2062154|rs1886010|rs10109963
Immunologic Disorder	CYTOKINE_CYTOKINE_RECEPTOR_INTERACTION	KEGG	6425	30	20	8,68E-02	9,95E-01	rs7604823|rs7677281|rs4680551|rs17414505|rs6706968|rs3138112|rs3773650|rs9975358|rs991694|rs260693|rs10493377|rs4278155|rs7628250|rs7910825|rs11208677|rs604045|rs12666352|rs1980499|rs12765241|rs4655690
Immunologic Disorder	LONG_TERM_POTENTIATION	KEGG	3620	44	20	8,75E-02	9,95E-01	rs8115510|rs11055651|rs4408387|rs10485723|rs227130|rs2423360|rs10772692|rs3819579|rs12545028|rs7974275|rs4963867|rs1861787|rs12921725|rs9299346|rs3817828|rs1997696|rs2469749|rs10845809|rs2193151|rs11102314
Immunologic Disorder	METABOLISM_OF_NON_CODING_RNA	REACTOME	611	33	17	8,78E-02	9,95E-01	rs2368443|rs2740349|rs85276|rs1484931|rs6770415|rs4684917|rs7596050|rs4951031|rs6434036|rs773546|rs5766533|rs17757841|rs580438|rs11634402|rs7202364|rs12465639|rs11097233
Immunologic Disorder	SEMA4D_IN_SEMAPHORIN_SIGNALING	REACTOME	597	32	20	8,85E-02	9,95E-01	rs6578414|rs7239317|rs17054953|rs503473|rs8060975|rs215573|rs2012928|rs4938800|rs1476002|rs12551214|rs343144|rs4367942|rs962199|rs1050162|rs752364|rs11030106|rs7259209|rs4669700|rs12474095|rs713753
Immunologic Disorder	ACTIVATION_OF_NMDA_RECEPTOR_UPON_GLUTAMATE_BINDING_AND_POSTSYNAPTIC_	REACTOME	2117	48	20	8,90E-02	9,95E-01	rs11055651|rs4408387|rs10514211|rs10772692|rs4703825|rs12545028|rs7974275|rs1861787|rs12921725|rs7214864|rs3817828|rs10845809|rs2193151|rs11651131|rs10505778|rs9645754|rs766831|rs1493383|rs1461236|rs1379270
Immunologic Disorder	AMYOTROPHIC_LATERAL_SCLEROSIS_ALS	KEGG	1813	53	20	9,01E-02	9,95E-01	rs11055651|rs4408387|rs10772692|rs7974275|rs1981725|rs2272722|rs1861787|rs12921725|rs9299346|rs2469749|rs10845809|rs2193151|rs10505778|rs4817415|rs9645754|rs766831|rs1493383|rs1461236|rs4332745|rs1016860
Immunologic Disorder	PHOSPHATIDYLINOSITOL_SIGNALING_SYSTEM	KEGG	3813	38	20	9,52E-02	9,95E-01	rs8115510|rs16897466|rs10485723|rs227130|rs2305030|rs11868917|rs350292|rs2423360|rs1943595|rs3819579|rs11082159|rs10509534|rs3890760|rs706578|rs2636638|rs1880553|rs1997696|rs1130635|rs11657742|rs2151414
Immunologic Disorder	BETA_ALANINE_METABOLISM	KEGG	711	48	17	9,73E-02	9,95E-01	rs7374394|rs12023630|rs959707|rs10918239|rs12061124|rs4578194|rs12041436|rs10918228|rs6681958|rs10493895|rs12964208|rs12554375|rs6566816|rs406303|rs290821|rs738814|rs11165924
Immunologic Disorder	ANTIVIRAL_MECHANISM_BY_IFN_STIMULATED_GENES	REACTOME	1077	45	20	1,06E-01	9,95E-01	rs3767248|rs11868917|rs2368443|rs1484931|rs10511409|rs1920650|rs6770415|rs4684917|rs11632652|rs7596050|rs6434036|rs9876561|rs7593878|rs3851167|rs773546|rs3024908|rs5766533|rs4648202|rs1551319|rs17505069
Immunologic Disorder	PI3K_EVENTS_IN_ERBB4_SIGNALING	REACTOME	1709	24	20	1,07E-01	9,95E-01	rs2102996|rs16897466|rs17347606|rs12328595|rs13393014|rs10509534|rs7322396|rs776385|rs10876227|rs2919390|rs16846161|rs4672649|rs10503904|rs13320527|rs6580353|rs7991808|rs10497961|rs7826312|rs13173003|rs4150196
Immunologic Disorder	CGMP_EFFECTS	REACTOME	1520	37	20	1,08E-01	9,95E-01	rs17540215|rs7907343|rs12797628|rs7117518|rs634651|rs16897963|rs1937701|rs17649232|rs537557|rs12191997|rs1926212|rs4599070|rs7303397|rs17631340|rs1427988|rs9295312|rs13320527|rs221723|rs12789511|rs3781913
Immunologic Disorder	P75_NTR_RECEPTOR_MEDIATED_SIGNALLING	REACTOME	2669	34	20	1,11E-01	9,95E-01	rs7162113|rs11929318|rs10514211|rs10858075|rs894628|rs4703825|rs2137110|rs28628360|rs503473|rs13098182|rs2409416|rs11923190|rs13052373|rs6019300|rs2926449|rs2062154|rs737329|rs7022396|rs4938800|rs1379270
Immunologic Disorder	SYNTHESIS_SECRETION_AND_INACTIVATION_OF_GLP1	REACTOME	363	24	11	1,14E-01	9,95E-01	rs7659414|rs8027776|rs3792747|rs2123861|rs17062878|rs1026483|rs7811892|rs3761656|rs4529218|rs11135469|rs791600
Immunologic Disorder	INWARDLY_RECTIFYING_K_CHANNELS	REACTOME	1317	53	20	1,14E-01	9,95E-01	rs588407|rs2836285|rs1034841|rs1073572|rs7140817|rs236504|rs17230421|rs7943324|rs1283798|rs2186344|rs2691775|rs4816566|rs17763873|rs4817889|rs1482313|rs1023545|rs2652452|rs2509585|rs642771|rs236525
Immunologic Disorder	HUNTINGTONS_DISEASE	KEGG	4311	47	20	1,15E-01	9,95E-01	rs8115510|rs11055651|rs4408387|rs10485723|rs227130|rs11745467|rs3794176|rs2423360|rs10772692|rs3819579|rs1509241|rs7974275|rs1861787|rs11077773|rs1800649|rs1997696|rs6902440|rs10845809|rs765688|rs9309485
Immunologic Disorder	SEMA4D_INDUCED_CELL_MIGRATION_AND_GROWTH_CONE_COLLAPSE	REACTOME	433	33	20	1,16E-01	9,95E-01	rs6578414|rs17202361|rs17054953|rs503473|rs8060975|rs215573|rs2012928|rs4938800|rs1476002|rs343144|rs4367942|rs962199|rs1050162|rs752364|rs11030106|rs4669700|rs12474095|rs713753|rs5997919|rs7045789

Immunologic Disorder	COMPLEMENT_AND_COAGULATION_CASCADES	KEGG	1742	53	20	1,17E-01	9,95E-01	rs11003448|rs698090|rs7146347|rs37244|rs723188|rs2842704|rs10824895|rs923109|rs13388743|rs6467|rs2019724|rs1917186|rs16861812|rs11850274|rs11064498|rs922866|rs17491334|rs3851513|rs12437294|rs3024459
Immunologic Disorder	SIGNALLING_TO_RAS	REACTOME	468	23	13	1,19E-01	9,95E-01	rs10858075|rs4963867|rs28628360|rs7022409|rs1125467|rs2043722|rs10486801|rs7523086|rs10486802|rs944485|rs17388587|rs2334915|rs2868161
Immunologic Disorder	CSK_PATHWAY	BIOCARTA	554	36	17	1,19E-01	9,95E-01	rs895437|rs5865|rs17136507|rs1378942|rs2395174|rs704856|rs2302234|rs2395182|rs10781329|rs1404909|rs695161|rs1296720|rs2995094|rs3135363|rs858490|rs12726043|rs1004420
Immunologic Disorder	SRP_DEPENDENT_COTRANSLATIONAL_PROTEIN_TARGETING_TO_MEMBRANE	REACTOME	1378	30	20	1,21E-01	9,95E-01	rs4704|rs3365|rs4599669|rs10824609|rs7659414|rs8027776|rs17510142|rs7219604|rs2305345|rs2070658|rs1561|rs2123861|rs17062878|rs1026483|rs1848466|rs1007482|rs457372|rs478309|rs11902670|rs9817549
Immunologic Disorder	PRIMARY_IMMUNODEFICIENCY	KEGG	742	41	18	1,22E-01	9,95E-01	rs895437|rs5865|rs2028373|rs11593133|rs11567697|rs1399602|rs2071482|rs695161|rs4808100|rs9937676|rs4675391|rs12572872|rs117821|rs8074984|rs12726043|rs6753271|rs1004420|rs10483213
Immunologic Disorder	TRANSPORT_OF_RIBONUCLEOPROTEINS_INTO_THE_HOST_NUCLEUS	REACTOME	452	25	14	1,22E-01	9,95E-01	rs2368443|rs1484931|rs10511409|rs6770415|rs4684917|rs7596050|rs6434036|rs773546|rs5766533|rs17757841|rs580438|rs7202364|rs12465639|rs11097233
Immunologic Disorder	DORSO_VENTRAL_AXIS_FORMATION	KEGG	1149	32	20	1,26E-01	9,95E-01	rs1797648|rs1793327|rs12577895|rs4963867|rs10845433|rs2723807|rs7126782|rs204995|rs6974790|rs2239172|rs7927013|rs693797|rs2045349|rs621922|rs6690616|rs762718|rs2836776|rs1140475|rs2836781|rs10790921
Immunologic Disorder	ABORTIVE_ELONGATION_OF_HIV1_TRANSCRIPT_IN_THE_ABSENCE_OF_TAT	REACTOME	195	10	2	1,35E-01	9,95E-01	rs503347|rs4487686
Immunologic Disorder	SPRY_PATHWAY	BIOCARTA	910	46	17	1,36E-01	9,95E-01	rs6563245|rs6563216|rs10879175|rs10506596|rs4105088|rs6974790|rs1333404|rs1930911|rs9601627|rs2624189|rs9601679|rs1140475|rs3113392|rs4145077|rs11977660|rs9565596|rs1382559
Immunologic Disorder	FORMATION_OF_ATP_BY_CHEMIOSMOTIC_COUPLING	REACTOME	77	4	2	1,37E-01	9,95E-01	rs11077773|rs3745004
Immunologic Disorder	PI3K_EVENTS_IN_ERBB2_SIGNALING	REACTOME	1886	24	20	1,37E-01	9,95E-01	rs2102996|rs16897466|rs17347606|rs12328595|rs13393014|rs10509534|rs7322396|rs776385|rs10876227|rs2919390|rs16846161|rs6974790|rs4672649|rs10503904|rs13320527|rs6580353|rs7991808|rs10497961|rs7826312|rs13173003
Immunologic Disorder	GAP_JUNCTION	KEGG	4388	40	20	1,37E-01	9,95E-01	rs8115510|rs1034258|rs10485723|rs227130|rs2423360|rs1719361|rs12018744|rs3819579|rs17540215|rs7907343|rs12545028|rs4963867|rs17405661|rs304737|rs1937701|rs2532019|rs13168690|rs1997696|rs1926212|rs7764196
Immunologic Disorder	RNA_DEGRADATION	KEGG	1123	33	20	1,39E-01	9,95E-01	rs8111911|rs1864571|rs240542|rs10853869|rs3740526|rs16969675|rs6792874|rs7024667|rs1893352|rs1011128|rs13293339|rs10415765|rs11638991|rs3809425|rs1353498|rs1921589|rs1545229|rs2241790|rs435584|rs10469954
Immunologic Disorder	AMINE_LIGAND_BINDING_RECEPTORS	REACTOME	2247	26	20	1,40E-01	9,95E-01	rs1034258|rs2104483|rs1730146|rs12018744|rs954385|rs10925763|rs7266945|rs11195623|rs16838192|rs6597459|rs6864183|rs17310690|rs10109963|rs3739216|rs9534591|rs2171543|rs6888329|rs472585|rs10068298|rs2011827
Immunologic Disorder	INTRINSIC_PATHWAY	BIOCARTA	603	21	9	1,41E-01	9,95E-01	rs37244|rs6436654|rs9515201|rs4675141|rs9521699|rs153317|rs7991229|rs4447275|rs7318486
Immunologic Disorder	NRAGE_SIGNALS_DEATH_THROUGH_JNK	REACTOME	1919	30	20	1,41E-01	9,95E-01	rs11929318|rs10514211|rs10858075|rs894628|rs4703825|rs28628360|rs503473|rs13098182|rs2409416|rs11923190|rs13052373|rs6019300|rs2062154|rs737329|rs7022396|rs4938800|rs1379270|rs1808563|rs34116|rs9984499
Immunologic Disorder	GRAFT_VERSUS_HOST_DISEASE	KEGG	1446	68	20	1,43E-01	9,95E-01	rs649216|rs1641661|rs1610751|rs987870|rs7910825|rs12765241|rs1611493|rs28367832|rs17500510|rs9276933|rs3135342|rs9275312|rs9264967|rs9276291|rs2681417|rs2395182|rs2048874|rs117821|rs241398|rs982764
Immunologic Disorder	MELANOGENESIS	KEGG	4077	42	20	1,44E-01	9,95E-01	rs8115510|rs7833751|rs10485723|rs6558063|rs227130|rs2423360|rs4655036|rs3819579|rs973330|rs12545028|rs4963867|rs7623610|rs304737|rs2094405|rs2532019|rs13168690|rs1997696|rs2710952|rs2807362|rs8115925
Immunologic Disorder	ALLOGRAFT_REJECTION	KEGG	1510	69	20	1,46E-01	9,95E-01	rs4680551|rs1641661|rs3115612|rs987870|rs7910825|rs12765241|rs1611493|rs2243131|rs28367832|rs4315909|rs17500510|rs9276933|rs4988821|rs9275312|rs9264967|rs9276291|rs2681417|rs2395182|rs2421057|rs117821
Immunologic Disorder	N_GLYCAN_ANTENNAE_ELONGATION_IN_THE_MEDIAL_TRANS_GOLGI	REACTOME	962	40	20	1,47E-01	9,95E-01	rs6444190|rs1352323|rs6095638|rs7617523|rs1147445|rs17103248|rs10513809|rs547177|rs7176079|rs12311449|rs4429522|rs9808181|rs1111961|rs12246530|rs12894020|rs11720551|rs11074059|rs745775|rs8015785|rs3931230
Immunologic Disorder	SYNTHESIS_OF_PC	REACTOME	669	40	15	1,53E-01	9,95E-01	rs627538|rs600734|rs1124605|rs3934427|rs396106|rs1164|rs10493879|rs6703265|rs13232524|rs13394440|rs7980436|rs915012|rs10197467|rs7545324|rs13288985
Immunologic Disorder	INTERACTIONS_OF_VPR_WITH_HOST_CELLULAR_PROTEINS	REACTOME	480	26	15	1,54E-01	9,95E-01	rs2368443|rs1484931|rs10511409|rs6770415|rs4684917|rs7596050|rs6434036|rs773546|rs5766533|rs17757841|rs7046713|rs580438|rs7202364|rs12465639|rs11097233
Immunologic Disorder	G_PROTEIN_BETA_GAMMA_SIGNALLING	REACTOME	798	35	20	1,55E-01	9,95E-01	rs10485723|rs227130|rs2423360|rs4855077|rs7140817|rs11657742|rs8115925|rs17153654|rs12488540|rs1015092|rs2745755|rs12704705|rs10498440|rs874269|rs11078772|rs12886319|rs1253676|rs12536419|rs2876140|rs291353
Immunologic Disorder	INCRETIN_SYNTHESIS_SECRETION_AND_INACTIVATION	REACTOME	540	25	12	1,56E-01	9,95E-01	rs7659414|rs8027776|rs3792747|rs2123861|rs17062878|rs1026483|rs7811892|rs3761656|rs4529218|rs11135469|rs2409809|rs791600
Immunologic Disorder	TGF_BETA_RECEPTOR_SIGNALING_IN_EMT_EPITHELIAL_TO_MESENCHYMAL_TRANSITI(	REACTOME	429	21	9	1,59E-01	9,95E-01	rs3773650|rs991694|rs1160769|rs1002386|rs2476997|rs6792117|rs1985313|rs1019855|rs6979
Immunologic Disorder	MUSCLE_CONTRACTION	REACTOME	1023	27	20	1,60E-01	9,95E-01	rs2119685|rs7910825|rs12765241|rs1124605|rs1934158|rs501603|rs10519327|rs8060975|rs215573|rs820363|rs1363950|rs8003073|rs13163497|rs7248997|rs11125132|rs13240924|rs10824069|rs1050162|rs201615|rs1263784
Immunologic Disorder	TRIGLYCERIDE_BIOSYNTHESIS	REACTOME	1182	35	20	1,65E-01	9,95E-01	rs627538|rs12199314|rs6870653|rs4795194|rs10452184|rs10182865|rs204995|rs3934427|rs2046731|rs1164|rs558491|rs9463908|rs7561624|rs7680893|rs6455717|rs11195662|rs3733796|rs2862996|rs4106571|rs904729
Immunologic Disorder	DC_PATHWAY	BIOCARTA	573	32	16	1,65E-01	9,95E-01	rs9847023|rs4932145|rs2243131|rs1930714|rs1410201|rs1329049|rs1928295|rs7046061|rs4315909|rs10984022|rs1996215|rs2421057|rs2806112|rs2028299|rs11177048|rs6895787
Immunologic Disorder	PLATELET_CALCIUM_HOMEOSTASIS	REACTOME	1156	49	20	1,66E-01	9,95E-01	rs7126870|rs6727414|rs1499772|rs6502751|rs11651131|rs709641|rs210120|rs13414508|rs10191613|rs11890040|rs10743591|rs3782309|rs427632|rs4367942|rs7561383|rs715252|rs2373901|rs6486795|rs10835270|rs830145
Immunologic Disorder	DRUG_METABOLISM_OTHER_ENZYMES	KEGG	1609	42	20	1,66E-01	9,95E-01	rs1689924|rs12023630|rs2572023|rs11165950|rs6830865|rs12061124|rs10031344|rs12120077|rs6681958|rs131820|rs1253795|rs17323225|rs207444|rs10493895|rs34778440|rs6437133|rs10755053|rs6997340|rs11902596|rs8047963
Immunologic Disorder	PROTEIN_EXPORT	KEGG	750	53	19	1,67E-01	9,95E-01	rs12673482|rs7659414|rs6948090|rs8027776|rs2305345|rs2123861|rs17062878|rs1026483|rs478309|rs9817549|rs2254990|rs10953644|rs1528035|rs4260596|rs1648628|rs214455|rs799617|rs1569256|rs6960391
Immunologic Disorder	HER2_PATHWAY	BIOCARTA	1326	28	20	1,69E-01	9,95E-01	rs2102996|rs16897466|rs17347606|rs12328595|rs13393014|rs16846161|rs6974790|rs4672649|rs13320527|rs10784600|rs161700|rs17153654|rs10497961|rs13173003|rs10506503|rs706725|rs9383939|rs7557116|rs1140475|rs3020418
Immunologic Disorder	COMP_PATHWAY	BIOCARTA	628	51	17	1,71E-01	9,95E-01	rs11003448|rs698090|rs10824895|rs6467|rs1917186|rs16861812|rs11064498|rs399443|rs3742089|rs7474987|rs4935411|rs7474747|rs3805226|rs3742088|rs1917190|rs391781|rs647962
Immunologic Disorder	LEUKOCYTE_TRANSENDOTHELIAL_MIGRATION	KEGG	4793	32	20	1,71E-01	9,95E-01	rs3802901|rs482533|rs4601648|rs16897466|rs2510257|rs17202361|rs11639525|rs11066320|rs6557449|rs10216570|rs304737|rs4378885|rs10175610|rs12598326|rs1952966|rs17235168|rs10997482|rs233551|rs11102314|rs11657742
Immunologic Disorder	RIG_I_MDA5_MEDIATED_INDUCTION_OF_IFN_ALPHA_BETA_PATHWAYS	REACTOME	1129	30	20	1,72E-01	9,95E-01	rs11868917|rs17826255|rs12446199|rs466609|rs9938543|rs400819|rs204995|rs7670291|rs2076756|rs7046061|rs17136507|rs10878177|rs3769823|rs457581|rs3006475|rs710092|rs1710627|rs2829946|rs9550565|rs1296720
Immunologic Disorder	METABOLISM_OF_NUCLEOTIDES	REACTOME	1559	58	20	1,72E-01	9,95E-01	rs1689924|rs1265122|rs12023630|rs7086415|rs959707|rs2041296|rs7100550|rs12061124|rs6420125|rs10861171|rs12041436|rs6681958|rs131820|rs1565850|rs1253795|rs2551698|rs17323225|rs207444|rs10493895|rs7217560
Immunologic Disorder	LYSINE_DEGRADATION	KEGG	952	48	20	1,75E-01	9,95E-01	rs7374394|rs3735477|rs7643465|rs7621828|rs10918239|rs899875|rs2864419|rs2459139|rs4578194|rs10918228|rs13103186|rs1047636|rs11132836|rs4342076|rs267738|rs578641|rs12554375|rs1913185|rs12572872|rs1022004
Immunologic Disorder	REGULATION_OF_GLUCOKINASE_BY_GLUCOKINASE_REGULATORY_PROTEIN	REACTOME	459	24	13	1,79E-01	9,95E-01	rs2368443|rs1484931|rs6770415|rs4684917|rs7596050|rs6434036|rs773546|rs5766533|rs17757841|rs580438|rs7202364|rs12465639|rs11097233
Immunologic Disorder	NEP_NS2_INTERACTS_WITH_THE_CELLULAR_EXPORT_MACHINERY	REACTOME	456	24	13	1,79E-01	9,95E-01	rs2368443|rs1484931|rs6770415|rs4684917|rs7596050|rs6434036|rs773546|rs5766533|rs17757841|rs580438|rs7202364|rs12465639|rs11097233
Immunologic Disorder	TRANSPORT_OF_MATURE_MRNA_DERIVED_FROM_AN_INTRONLESS_TRANSCRIPT	REACTOME	524	24	13	1,80E-01	9,95E-01	rs2368443|rs1484931|rs6770415|rs4684917|rs7596050|rs6434036|rs773546|rs5766533|rs17757841|rs580438|rs7202364|rs12465639|rs11097233
Immunologic Disorder	TCR_SIGNALING	REACTOME	2040	41	20	1,81E-01	9,95E-01	rs895437|rs5865|rs16897466|rs4750517|rs987870|rs10509534|rs13238172|rs587198|rs8045689|rs9359935|rs13320527|rs7815195|rs7824819|rs17500510|rs10806443|rs6125883|rs1378942|rs704856|rs6924218|rs4357599
Immunologic Disorder	G_ALPHA_Q_SIGNALLING_EVENTS	REACTOME	7428	29	20	1,81E-01	9,95E-01	rs8115510|rs2306718|rs16897466|rs10506460|rs10485723|rs7146347|rs4750517|rs37244|rs227130|rs4895594|rs11868917|rs2423360|rs12018744|rs3819579|rs466609|rs10925763|rs4855077|rs7266945|rs13098182|rs13410673
Immunologic Disorder	GLYCINE_SERINE_AND_THREONINE_METABOLISM	KEGG	564	25	14	1,85E-01	9,95E-01	rs2510257|rs12449313|rs12683330|rs7875845|rs10975640|rs129901|rs3821869|rs4077632|rs234792|rs7035933|rs40443|rs7031908|rs13294797|rs921986
Immunologic Disorder	GOLGI_ASSOCIATED_VESICLE_BIOGENESIS	REACTOME	1057	37	20	1,85E-01	9,95E-01	rs2279186|rs10187424|rs10493377|rs6473202|rs12192757|rs998731|rs17745273|rs941022|rs3812162|rs10069818|rs8078165|rs677909|rs2618604|rs10889546|rs7536700|rs11881315|rs2283857|rs2231754|rs16933086|rs2072824
Immunologic Disorder	CTCF_PATHWAY	BIOCARTA	1035	31	20	1,87E-01	9,95E-01	rs16897466|rs3773650|rs991694|rs2029356|rs604045|rs10509534|rs9642880|rs3009935|rs13320527|rs7812998|rs17153654|rs13173003|rs2033098|rs706725|rs12029576|rs947712|rs2296620|rs6792117|rs1019855|rs1041159
Immunologic Disorder	RHO_PATHWAY	BIOCARTA	480	24	11	1,87E-01	9,95E-01	rs17202361|rs1952966|rs736730|rs2151414|rs820363|rs10824069|rs10781329|rs3829078|rs11144133|rs4766522|rs3771099
Immunologic Disorder	AMI_PATHWAY	BIOCARTA	574	30	12	1,91E-01	9,95E-01	rs37244|rs6436654|rs13388743|rs9515201|rs4675141|rs4889673|rs9521699|rs153317|rs7991229|rs783166|rs4447275|rs7318486
Immunologic Disorder	CELL_DEATH_SIGNALLING_VIA_NRAGE_NRIF_AND_NADE	REACTOME	2069	30	20	1,92E-01	9,95E-01	rs11929318|rs10514211|rs10858075|rs894628|rs4703825|rs28628360|rs503473|rs13098182|rs2409416|rs11923190|rs13052373|rs6019300|rs2062154|rs1886010|rs7022396|rs4938800|rs1379270|rs1808563|rs34116|rs9984499
Immunologic Disorder	VASCULAR_SMOOTH_MUSCLE_CONTRACTION	KEGG	5845	34	20	1,93E-01	9,95E-01	rs8115510|rs10506460|rs1865017|rs10485723|rs4750517|rs227130|rs2423360|rs2119685|rs208354|rs3819579|rs17540215|rs7907343|rs7239317|rs12545028|rs7266945|rs7910825|rs503473|rs1937701|rs12765241|rs12904722
Immunologic Disorder	TID_PATHWAY	BIOCARTA	479	28	14	1,94E-01	9,95E-01	rs2108333|rs16934382|rs4896251|rs2279664|rs11823078|rs8904|rs1006099|rs12210503|rs10140922|rs3914898|rs1012919|rs816060|rs11177048|rs932503
Immunologic Disorder	NEGATIVE_REGULATORS_OF_RIG_I_MDA5_SIGNALING	REACTOME	464	26	17	1,95E-01	9,95E-01	rs11868917|rs17826255|rs12446199|rs9938543|rs7670291|rs2076756|rs10878177|rs710092|rs12206804|rs1994174|rs10485352|rs640353|rs2717532|rs6133067|rs7699006|rs627346|rs6802890
Immunologic Disorder	TRANSPORT_TO_THE_GOLGI_AND_SUBSEQUENT_MODIFICATION	REACTOME	1557	35	20	1,96E-01	9,95E-01	rs6444190|rs1352323|rs6095638|rs12198857|rs7617523|rs10490815|rs1147445|rs4940866|rs17103248|rs10513809|rs547177|rs709641|rs7176079|rs12311449|rs4429522|rs9808181|rs11730732|rs1111961|rs12525680|rs12246530
Immunologic Disorder	PLC_BETA_MEDIATED_EVENTS	REACTOME	2313	44	20	1,97E-01	9,95E-01	rs8115510|rs10485723|rs227130|rs2423360|rs3819579|rs12545028|rs304737|rs17649232|rs2532019|rs13168690|rs1997696|rs4599070|rs8115925|rs210120|rs9922362|rs10743591|rs2886612|rs3782309|rs7615501|rs4629562
Immunologic Disorder	PML_PATHWAY	BIOCARTA	384	29	12	1,97E-01	9,95E-01	rs4886844|rs7910825|rs12765241|rs6728176|rs17136507|rs1370923|rs1612907|rs1296720|rs7588554|rs982764|rs13411586|rs816060
Immunologic Disorder	APOPTOSIS	KEGG	1727	38	20	1,98E-01	9,95E-01	rs16897466|rs10858075|rs4278155|rs7628250|rs7910825|rs28628360|rs12765241|rs11135693|rs9299346|rs2469749|rs1047266|rs11657742|rs547243|rs1030021|rs11883987|rs13320527|rs17196143|rs3769823|rs653118|rs17153654
Immunologic Disorder	REGULATION_OF_IFNA_SIGNALING	REACTOME	339	14	9	1,99E-01	9,95E-01	rs9975358|rs4811068|rs2834112|rs7046061|rs3024908|rs10811518|rs2284550|rs8074003|rs13038695
Immunologic Disorder	EICOSANOID_LIGAND_BINDING_RECEPTORS	REACTOME	507	16	6	2,00E-01	9,95E-01	rs1776503|rs8005977|rs10462010|rs930420|rs1449587|rs4340954
Immunologic Disorder	INHIBITION_OF_VOLTAGE_GATED_CA2_CHANNELS_VIA_GBETA_GAMMA_SUBUNITS	REACTOME	1082	43	20	2,01E-01	9,95E-01	rs2836285|rs1034841|rs1073572|rs7140817|rs948215|rs236504|rs17230421|rs2186344|rs2691775|rs4816566|rs17763873|rs4817889|rs1482313|rs1023545|rs2652452|rs642771|rs236525|rs1930409|rs10498440|rs8129867
Immunologic Disorder	PHOSPHOLIPID_METABOLISM	REACTOME	6226	46	20	2,06E-01	9,95E-01	rs1619030|rs627538|rs6781371|rs16897466|rs12199314|rs350292|rs1943595|rs267733|rs9839984|rs6870653|rs611899|rs14003|rs2467458|rs2074625|rs11082159|rs10509534|rs756607|rs2636638|rs1124605|rs2868932
Immunologic Disorder	N_GLYCAN_BIOSYNTHESIS	KEGG	1792	39	20	2,07E-01	9,95E-01	rs6444190|rs4704|rs1869676|rs9832807|rs12198857|rs7617523|rs2070658|rs1147445|rs17103248|rs11231168|rs10513809|rs547177|rs8081232|rs6707475|rs4429522|rs7615501|rs9808181|rs1111961|rs9831111|rs2811491
Immunologic Disorder	ADIPOCYTOKINE_SIGNALING_PATHWAY	KEGG	2106	43	20	2,09E-01	9,95E-01	rs17201100|rs4750517|rs1509241|rs10493377|rs11066320|rs11208677|rs6952398|rs92|rs7214864|rs2396303|rs1555949|rs10182865|rs7858838|rs587198|rs11651131|rs7314454|rs9805049|rs10889011|rs7533324|rs1107376
Immunologic Disorder	SYSTEMIC_LUPUS_ERYTHEMATOSUS	KEGG	2453	58	20	2,09E-01	9,95E-01	rs11055651|rs4408387|rs10772692|rs987870|rs7974275|rs1861787|rs12921725|rs6467|rs10845809|rs765688|rs10505778|rs9645754|rs766831|rs2268969|rs17500510|rs9276933|rs4332745|rs11064498|rs3135342|rs722008
Immunologic Disorder	PYRIMIDINE_METABOLISM	REACTOME	792	42	20	2,09E-01	9,95E-01	rs1689924|rs12023630|rs959707|rs12061124|rs12041436|rs6681958|rs131820|rs1253795|rs10493895|rs7217560|rs4241759|rs867744|rs2298582|rs167508|rs6437133|rs10755053|rs11902596|rs40443|rs16851444|rs290821
Immunologic Disorder	TRANS_GOLGI_NETWORK_VESICLE_BUDDING	REACTOME	1106	36	20	2,10E-01	9,95E-01	rs2279186|rs10187424|rs10493377|rs6473202|rs12192757|rs998731|rs17745273|rs941022|rs3812162|rs10069818|rs8078165|rs677909|rs2618604|rs2279597|rs10889546|rs7536700|rs11881315|rs2283857|rs2231754|rs16933086
Immunologic Disorder	G_BETA_GAMMA_SIGNALLING_THROUGH_PLC_BETA	REACTOME	624	31	20	2,12E-01	9,95E-01	rs10485723|rs227130|rs2423360|rs4855077|rs7140817|rs8115925|rs12488540|rs1015092|rs2745755|rs12704705|rs10498440|rs12886319|rs1253676|rs2876140|rs291353|rs12595160|rs4659946|rs961090|rs6086495|rs8023017
Immunologic Disorder	FORMATION_OF_FIBRIN_CLOT_CLOTTING_CASCADE	REACTOME	703	24	10	2,13E-01	9,95E-01	rs723188|rs12602483|rs13388743|rs922866|rs17491334|rs3851513|rs3024459|rs2283332|rs11757860|rs407447
Immunologic Disorder	ASCORBATE_AND_ALDARATE_METABOLISM	KEGG	874	33	12	2,16E-01	9,95E-01	rs2608836|rs6830865|rs10918239|rs10031344|rs4578194|rs10918228|rs34778440|rs12554375|rs8138406|rs6600793|rs6717546|rs1347047
Immunologic Disorder	GLYCEROPHOSPHOLIPID_BIOSYNTHESIS	REACTOME	2483	39	20	2,16E-01	9,95E-01	rs1619030|rs627538|rs12199314|rs6870653|rs611899|rs14003|rs2467458|rs1124605|rs204995|rs3934427|rs531592|rs396106|rs28688444|rs2046731|rs1164|rs558491|rs4803329|rs6672057|rs10493879|rs6455717
Immunologic Disorder	INNATE_IMMUNE_SYSTEM	REACTOME	6278	36	20	2,17E-01	9,95E-01	rs1679802|rs11003448|rs698090|rs11105194|rs11868917|rs3808969|rs4704|rs2212963|rs17826255|rs12446199|rs466609|rs716097|rs9938543|rs10244937|rs2842704|rs11082159|rs317985|rs10824895|rs2066843|rs400819
Immunologic Disorder	G_PROTEIN_ACTIVATION	REACTOME	703	33	20	2,20E-01	9,95E-01	rs2281617|rs4855077|rs304737|rs7140817|rs6941251|rs6081597|rs2886612|rs9371781|rs12488540|rs2272381|rs12704705|rs10498440|rs12886319|rs1253676|rs10223804|rs2065139|rs12626044|rs291353|rs12595160|rs518596
Immunologic Disorder	ACTIVATION_OF_KAINATE_RECEPTORS_UPON_GLUTAMATE_BINDING	REACTOME	1577	32	20	2,21E-01	9,95E-01	rs10485723|rs227130|rs2423360|rs2832535|rs3755768|rs4855077|rs7140817|rs6656317|rs12577638|rs8115925|rs3767087|rs2253438|rs1984334|rs7116106|rs3802912|rs11125132|rs12488540|rs1015092|rs2745755|rs12704705
Immunologic Disorder	NKT_PATHWAY	BIOCARTA	638	28	17	2,21E-01	9,95E-01	rs9847023|rs604045|rs4896251|rs2243131|rs12691883|rs3009935|rs4315909|rs2011946|rs2056296|rs12029576|rs10191360|rs947712|rs2421057|rs12210503|rs3914898|rs11177048|rs6895787
Immunologic Disorder	GLYCOSAMINOGLYCAN_DEGRADATION	KEGG	709	35	20	2,22E-01	9,95E-01	rs9890008|rs3785690|rs1859906|rs722769|rs16948248|rs4505759|rs4889841|rs726799|rs12451382|rs3826365|rs2279597|rs10474567|rs12219903|rs820846|rs8081226|rs17818103|rs9900703|rs11651136|rs2108702|rs12947875
Immunologic Disorder	GSK3_PATHWAY	BIOCARTA	1055	34	20	2,22E-01	9,95E-01	rs16897466|rs304737|rs2710952|rs7764196|rs1930714|rs1392125|rs1410201|rs1329049|rs1893352|rs931771|rs637462|rs1928295|rs2886612|rs13320527|rs778584|rs10984022|rs1996215|rs13173003|rs706725|rs2806112
Immunologic Disorder	AUTOIMMUNE_THYROID_DISEASE	KEGG	1646	68	20	2,22E-01	9,95E-01	rs1641661|rs3115612|rs987870|rs9362387|rs7910825|rs12765241|rs1611493|rs180219|rs28367832|rs17500510|rs9276933|rs2395174|rs9275312|rs9264967|rs9276291|rs2681417|rs2395182|rs733234|rs10811518|rs117821
Immunologic Disorder	TRANSCRIPTIONAL_REGULATION_OF_WHITE_ADIPOCYTE_DIFFERENTIATION	REACTOME	2719	37	20	2,23E-01	9,95E-01	rs6496143|rs2306718|rs7038715|rs1509241|rs680548|rs634454|rs4542003|rs4445045|rs10520792|rs17089943|rs1555949|rs184946|rs7858838|rs12308345|rs7713987|rs4857735|rs2035590|rs3782267|rs12907625|rs1411355
Immunologic Disorder	ACYL_CHAIN_REMODELLING_OF_PG	REACTOME	571	41	17	2,24E-01	9,95E-01	rs1619030|rs6870653|rs28688444|rs6672057|rs3733796|rs2261612|rs10798106|rs1093733|rs17544558|rs904729|rs2292023|rs12086010|rs6665890|rs10494603|rs2962033|rs28451400|rs645953
Immunologic Disorder	METABOLISM_OF_XENOBIOTICS_BY_CYTOCHROME_P450	KEGG	1674	38	20	2,28E-01	9,95E-01	rs3794176|rs2572023|rs6830865|rs10918239|rs10031344|rs1042389|rs4336933|rs2274536|rs10918228|rs6904935|rs1571858|rs915908|rs2279345|rs34778440|rs2470890|rs2270941|rs11252845|rs1238741|rs1465936|rs1937881
Immunologic Disorder	TOB1_PATHWAY	BIOCARTA	652	28	17	2,29E-01	9,95E-01	rs3773650|rs991694|rs2029356|rs604045|rs3009935|rs266335|rs704856|rs12029576|rs947712|rs2296620|rs6792117|rs1019855|rs1041159|rs2995094|rs920293|rs858490|rs11177048
Immunologic Disorder	DILATED_CARDIOMYOPATHY	KEGG	4636	32	20	2,30E-01	9,95E-01	rs1051774|rs1034258|rs754298|rs12058655|rs4894044|rs9303513|rs12620429|rs12545028|rs604045|rs6727414|rs1375526|rs10925415|rs2532019|rs16835237|rs1934158|rs2392695|rs13168690|rs12203956|rs9849746|rs2127155
Immunologic Disorder	DRUG_METABOLISM_CYTOCHROME_P450	KEGG	1661	40	20	2,30E-01	9,95E-01	rs3794176|rs2572023|rs6830865|rs10918239|rs10031344|rs1042389|rs2274536|rs10918228|rs6904935|rs7886938|rs1571858|rs915908|rs6608458|rs2279345|rs34778440|rs2470890|rs1238741|rs1465936|rs4646785|rs2654846
Immunologic Disorder	PLATELET_AGGREGATION_PLUG_FORMATION	REACTOME	942	27	20	2,31E-01	9,95E-01	rs2152305|rs723188|rs4811068|rs954385|rs11195623|rs11102314|rs1537745|rs787033|rs1378942|rs10492301|rs4561129|rs17491334|rs3107340|rs10506562|rs8036582|rs945296|rs2678289|rs2283332|rs6581827|rs3829078
Immunologic Disorder	STARCH_AND_SUCROSE_METABOLISM	KEGG	2178	57	20	2,33E-01	9,95E-01	rs7183546|rs7767111|rs6934819|rs2608836|rs9290242|rs6830865|rs10031344|rs10998736|rs4856401|rs7797360|rs439863|rs7756163|rs34778440|rs6935458|rs3800993|rs9875791|rs6531592|rs12500105|rs11185098|rs11722340
Immunologic Disorder	SPHINGOLIPID_DE_NOVO_BIOSYNTHESIS	REACTOME	1020	57	20	2,39E-01	9,95E-01	rs9883862|rs267733|rs1580254|rs2074625|rs2868932|rs892987|rs1341347|rs10497340|rs1372050|rs16831830|rs11675308|rs12336689|rs10483381|rs17785945|rs13353121|rs7856222|rs11810204|rs4912230|rs723060|rs2236317
Immunologic Disorder	CARDIAC_MUSCLE_CONTRACTION	KEGG	3169	27	20	2,39E-01	9,95E-01	rs1051774|rs754298|rs12058655|rs9303513|rs11745467|rs6727414|rs1375526|rs10925415|rs1800649|rs1934158|rs16835237|rs2392695|rs9849746|rs2127155|rs6942458|rs13414508|rs2898543|rs7428777|rs10191613|rs2286383
Immunologic Disorder	ARRHYTHMOGENIC_RIGHT_VENTRICULAR_CARDIOMYOPATHY_ARVC	KEGG	5871	31	20	2,41E-01	9,95E-01	rs482533|rs1051774|rs11564334|rs4601648|rs754298|rs12058655|rs9303513|rs12620429|rs6727414|rs10175610|rs1375526|rs10925415|rs2094405|rs16835237|rs17235168|rs2392695|rs12203956|rs9849746|rs2127155|rs6942458
Immunologic Disorder	GASTRIN_CREB_SIGNALLING_PATHWAY_VIA_PKC_AND_MAPK	REACTOME	8122	29	20	2,41E-01	9,95E-01	rs8115510|rs2306718|rs16897466|rs10506460|rs10485723|rs7146347|rs4750517|rs37244|rs227130|rs4895594|rs11868917|rs2423360|rs12018744|rs3819579|rs466609|rs10925763|rs4855077|rs7266945|rs4963867|rs13098182
Immunologic Disorder	MITOTIC_PROMETAPHASE	REACTOME	1814	39	20	2,41E-01	9,95E-01	rs2090500|rs7074757|rs11582225|rs7244453|rs10490815|rs1678002|rs7683090|rs1801376|rs12355683|rs709641|rs1506048|rs2045418|rs9907283|rs7309390|rs7211218|rs12571653|rs1052053|rs12911848|rs11082819|rs1553367
Immunologic Disorder	MEMBRANE_TRAFFICKING	REACTOME	2276	36	20	2,43E-01	9,95E-01	rs2279186|rs1719361|rs10187424|rs10493377|rs6473202|rs12192757|rs6453850|rs998731|rs10490815|rs17745273|rs1280054|rs17687453|rs10795505|rs941022|rs3812162|rs7764196|rs17089022|rs709641|rs10737381|rs10069818
Immunologic Disorder	IGF1MTOR_PATHWAY	BIOCARTA	582	16	11	2,45E-01	9,95E-01	rs16897466|rs10509534|rs13320527|rs10794486|rs2684796|rs13173003|rs706725|rs6449947|rs2161529|rs10515074|rs6871452
Immunologic Disorder	PTM_GAMMA_CARBOXYLATION_HYPUSINE_FORMATION_AND_ARYLSULFATASE_ACTIV	REACTOME	377	12	7	2,46E-01	9,95E-01	rs10187424|rs956274|rs3744307|rs10474567|rs891943|rs17818103|rs2194051
Immunologic Disorder	O_LINKED_GLYCOSYLATION_OF_MUCINS	REACTOME	2402	31	20	2,46E-01	9,95E-01	rs1791534|rs11672574|rs6444190|rs17143909|rs6095638|rs7405583|rs7617523|rs10183136|rs1474925|rs10513809|rs11950904|rs6968731|rs707085|rs10932055|rs6976906|rs9901757|rs10931759|rs16834313|rs1364419|rs12658598

Immunologic Disorder	NITRIC_OXIDE_STIMULATES_GUANYLATE_CYCLASE	REACTOME	1895	36	20	2,50E-01	9,95E-01	rs17540215|rs7907343|rs12797628|rs7117518|rs634651|rs16897963|rs1937701|rs17649232|rs537557|rs12191997|rs1926212|rs4599070|rs7303397|rs17631340|rs1427988|rs9295312|rs13320527|rs221725|rs11945064|rs12789511
Immunologic Disorder	T_CELL_RECEPTOR_SIGNALING_PATHWAY	KEGG	3546	33	20	2,51E-01	9,95E-01	rs895437|rs5865|rs16897466|rs2510257|rs4750517|rs3755768|rs9870951|rs4963867|rs13238172|rs7648548|rs9299346|rs6063664|rs2469749|rs587198|rs11657742|rs6118746|rs11632939|rs8045689|rs9359935|rs13218316
Immunologic Disorder	G0_AND_EARLY_G1	REACTOME	258	5	3	2,51E-01	9,95E-01	rs2835741|rs11909066|rs695161
Immunologic Disorder	ANTIGEN_PROCESSING_AND_PRESENTATION	KEGG	1754	74	20	2,52E-01	9,95E-01	rs649216|rs3115612|rs987870|rs1242119|rs6897881|rs1611493|rs1299525|rs28367832|rs4839|rs17500510|rs9276933|rs2071482|rs3135342|rs9275312|rs9264967|rs9276291|rs2395182|rs10811518|rs117821|rs241404
Immunologic Disorder	PI_METABOLISM	REACTOME	1808	42	20	2,54E-01	9,95E-01	rs16897466|rs350292|rs2212963|rs11082159|rs10509534|rs756607|rs2636638|rs1130635|rs11657742|rs2151414|rs4297784|rs2492977|rs1865275|rs13320527|rs4553709|rs307118|rs11013118|rs1109303|rs17153654|rs4919128
Immunologic Disorder	ACYL_CHAIN_REMODELLING_OF_PC	REACTOME	693	40	17	2,59E-01	9,95E-01	rs1619030|rs6870653|rs6672057|rs16981247|rs3733796|rs2261612|rs17340109|rs10798106|rs1093733|rs17544558|rs904729|rs2292023|rs12086010|rs10798080|rs10494603|rs2962033|rs645953
Immunologic Disorder	EDG1_PATHWAY	BIOCARTA	1432	37	20	2,60E-01	9,95E-01	rs16897466|rs10485723|rs227130|rs2423360|rs12620429|rs304737|rs12041998|rs8115925|rs12041851|rs2886612|rs13320527|rs17153654|rs1015092|rs2745755|rs13173003|rs12756662|rs706725|rs1404909|rs1507964|rs4381631
Immunologic Disorder	NDKDYNAMIN_PATHWAY	BIOCARTA	548	24	14	2,60E-01	9,95E-01	rs350292|rs2041296|rs11761049|rs17745273|rs2469749|rs677909|rs11125132|rs3804357|rs7854612|rs13002829|rs669336|rs1848116|rs815802|rs2732514
Immunologic Disorder	GAP_JUNCTION_TRAFFICKING	REACTOME	576	33	11	2,60E-01	9,95E-01	rs6453850|rs1280054|rs7764196|rs10737381|rs8078165|rs6454601|rs12119977|rs7854612|rs11881315|rs16933086|rs7899248
Immunologic Disorder	ERK_PATHWAY	BIOCARTA	1025	34	20	2,61E-01	9,95E-01	rs4595639|rs10879175|rs10858075|rs28628360|rs6510636|rs6974790|rs9642880|rs2488320|rs7812998|rs10794486|rs4587680|rs7523086|rs2684796|rs2033098|rs11009195|rs1507964|rs2671642|rs1140475|rs7156757|rs6581958
Immunologic Disorder	CARM_ER_PATHWAY	BIOCARTA	1617	39	20	2,62E-01	9,95E-01	rs2526640|rs6543560|rs11685345|rs1509241|rs2285431|rs12308345|rs9636384|rs10247238|rs3782267|rs12054920|rs2655215|rs908263|rs843458|rs6860708|rs701078|rs17136507|rs4235306|rs11764843|rs7726306|rs2214850
Immunologic Disorder	PROTEIN_FOLDING	REACTOME	675	55	16	2,63E-01	9,95E-01	rs9747279|rs4712311|rs652265|rs6699315|rs4723235|rs6865121|rs2292267|rs4810909|rs10824092|rs9992175|rs2024595|rs11080567|rs7749814|rs10516289|rs1393015|rs2272313
Immunologic Disorder	POTASSIUM_CHANNELS	REACTOME	4940	41	20	2,63E-01	9,95E-01	rs337683|rs588407|rs2836285|rs9688792|rs1034841|rs11102357|rs4809563|rs337701|rs1073572|rs85276|rs17811161|rs7140817|rs12134651|rs639622|rs236504|rs13264567|rs4832505|rs17230421|rs7552646|rs1883767
Immunologic Disorder	NUCLEAR_RECEPTOR_TRANSCRIPTION_PATHWAY	REACTOME	2750	35	20	2,65E-01	9,95E-01	rs4365706|rs745011|rs2868094|rs10023128|rs868840|rs10495039|rs7114704|rs1393385|rs1555949|rs10193997|rs10876227|rs4237855|rs7858838|rs518162|rs755292|rs830308|rs1634079|rs7555255|rs12723457|rs6849903
Immunologic Disorder	TGFB_PATHWAY	BIOCARTA	817	28	20	2,66E-01	9,95E-01	rs3773650|rs991694|rs604045|rs3009935|rs9359935|rs266335|rs17136507|rs10806443|rs2063327|rs11661651|rs6924218|rs284513|rs17236851|rs3859312|rs12029576|rs7746039|rs947712|rs6792117|rs1296720|rs2103994
Immunologic Disorder	THROMBIN_SIGNALLING_THROUGH_PROTEINASE_ACTIVATED_RECEPTORS_PARS	REACTOME	690	39	20	2,68E-01	9,95E-01	rs37244|rs208354|rs4855077|rs7140817|rs798524|rs10229817|rs7039739|rs17786782|rs12488540|rs12704705|rs153317|rs1162211|rs10498440|rs12886319|rs1253676|rs291353|rs12595160|rs33932857|rs4659946|rs8023017
Immunologic Disorder	PI3K_AKT_ACTIVATION	REACTOME	1161	35	20	2,72E-01	9,95E-01	rs16897466|rs17201100|rs10858075|rs28628360|rs10509534|rs7322396|rs2396303|rs10876227|rs13320527|rs7523086|rs7991808|rs9521472|rs13173003|rs706725|rs12454900|rs2396306|rs874269|rs13398058|rs6449947|rs9480865
Immunologic Disorder	APOPTOSIS	REACTOME	3298	48	20	2,74E-01	9,95E-01	rs500804|rs4750517|rs10502934|rs10268808|rs1719361|rs551357|rs17202361|rs7910825|rs2892190|rs10511409|rs12765241|rs10823677|rs6457689|rs11135693|rs1047266|rs587198|rs4129439|rs1957107|rs490332|rs727514
Immunologic Disorder	TRANSLATION	REACTOME	1803	30	20	2,78E-01	9,95E-01	rs4704|rs3365|rs4599669|rs10824609|rs7659414|rs8027776|rs17510142|rs7219604|rs2305345|rs2070658|rs1561|rs2123861|rs17062878|rs1026483|rs4606077|rs1848466|rs1007482|rs457372|rs478309|rs11902670
Immunologic Disorder	ECM_PATHWAY	BIOCARTA	753	38	20	2,78E-01	9,95E-01	rs16897466|rs7239317|rs1952966|rs820363|rs2488320|rs13163497|rs13218316|rs13320527|rs17153654|rs4587680|rs13173003|rs11009195|rs706725|rs3765141|rs6449947|rs12536419|rs3829078|rs2161529|rs11153318|rs4766522
Immunologic Disorder	BASE_EXCISION_REPAIR	REACTOME	209	10	6	2,80E-01	9,95E-01	rs2261316|rs2029166|rs16981247|rs4135054|rs2682587|rs2723875
Immunologic Disorder	EIF4_PATHWAY	BIOCARTA	890	52	20	2,80E-01	9,95E-01	rs3767248|rs16897466|rs17201100|rs10509534|rs2396303|rs13320527|rs6872878|rs13173003|rs706725|rs2396306|rs4381631|rs8081614|rs4791036|rs13398058|rs6449947|rs2161529|rs169143|rs6451620|rs10515074|rs7219495
Immunologic Disorder	SIGNALLING_TO_ERKS	REACTOME	794	34	20	2,81E-01	9,95E-01	rs10858075|rs4963867|rs28628360|rs7022409|rs4890408|rs11102314|rs2296950|rs1125467|rs2043722|rs10486801|rs7523086|rs6507478|rs6507515|rs10486802|rs944485|rs17388587|rs10163682|rs2334915|rs2868161|rs7239883
Immunologic Disorder	FATTY_ACID_METABOLISM	KEGG	776	36	20	2,81E-01	9,95E-01	rs7374394|rs10918239|rs666105|rs10182865|rs9392576|rs11934603|rs4578194|rs10918228|rs9947207|rs7561624|rs679596|rs7218656|rs12554375|rs1238741|rs2654846|rs17194178|rs11129780|rs1335277|rs2305508|rs7249356
Immunologic Disorder	FORMATION_OF_TUBULIN_FOLDING_INTERMEDIATES_BY_CCT_TRIC	REACTOME	227	10	3	2,85E-01	9,95E-01	rs6865121|rs2292267|rs11080567
Immunologic Disorder	GABA_B_RECEPTOR_ACTIVATION	REACTOME	1863	41	20	2,89E-01	9,95E-01	rs2836285|rs1034841|rs12545028|rs1073572|rs304737|rs7140817|rs2532019|rs948215|rs236504|rs13168690|rs17230421|rs2186344|rs2691775|rs9922362|rs4816566|rs17763873|rs2886612|rs4629562|rs13190668|rs4817889
Immunologic Disorder	GLUCONEOGENESIS	REACTOME	490	31	14	2,91E-01	9,95E-01	rs3793932|rs12768505|rs7944999|rs10258885|rs9649679|rs764223|rs13380644|rs11983987|rs4511520|rs10781329|rs6025645|rs9934713|rs8049025|rs4544229
Immunologic Disorder	ACYL_CHAIN_REMODELLING_OF_PE	REACTOME	696	27	13	2,92E-01	9,95E-01	rs1619030|rs6672057|rs16981247|rs2261612|rs17340109|rs10798106|rs1093733|rs17544558|rs12086010|rs10798080|rs7528851|rs645953|rs499899
Immunologic Disorder	MITOCHONDRIAL_PROTEIN_IMPORT	REACTOME	609	21	10	2,94E-01	9,95E-01	rs2286469|rs10257369|rs1130635|rs10258885|rs12693099|rs3783694|rs3745004|rs4407280|rs730168|rs938275
Immunologic Disorder	ION_CHANNEL_TRANSPORT	REACTOME	2741	42	20	2,94E-01	9,95E-01	rs4514648|rs6290|rs10083549|rs2575484|rs11852692|rs4782977|rs12595426|rs4354903|rs11606194|rs2044081|rs17561473|rs12493550|rs12448765|rs17738971|rs709641|rs1484363|rs4485963|rs11161255|rs1933199|rs6606856
Immunologic Disorder	CHOLESTEROL_BIOSYNTHESIS	REACTOME	259	5	3	2,95E-01	9,95E-01	rs6699315|rs4839|rs12676995
Immunologic Disorder	GALACTOSE_METABOLISM	KEGG	759	38	15	2,97E-01	9,95E-01	rs7183546|rs11251480|rs10998736|rs7797360|rs9419445|rs4433480|rs3800993|rs10903880|rs2322659|rs11251555|rs6531592|rs2409044|rs10903900|rs1872837|rs2812365
Immunologic Disorder	IGF1_PATHWAY	BIOCARTA	813	33	20	2,97E-01	9,95E-01	rs16897466|rs17201100|rs11066320|rs2396303|rs13320527|rs4767879|rs10794486|rs17153654|rs2684796|rs13173003|rs2624189|rs706725|rs741847|rs2396306|rs13398058|rs6449947|rs12536419|rs2161529|rs10515074|rs6871452
Immunologic Disorder	NGF_PATHWAY	BIOCARTA	566	30	16	2,98E-01	9,95E-01	rs16897466|rs10858075|rs28628360|rs13320527|rs17153654|rs7523086|rs13173003|rs706725|rs2671642|rs741847|rs6449947|rs12536419|rs2161529|rs10515074|rs2334915|rs6871452
Immunologic Disorder	ACTINY_PATHWAY	BIOCARTA	290	16	6	2,98E-01	9,95E-01	rs9581718|rs736730|rs6718462|rs4731121|rs2286831|rs3771099
Immunologic Disorder	CHRONIC_MYELOID_LEUKEMIA	KEGG	2538	29	20	2,99E-01	9,95E-01	rs16897466|rs3773650|rs991694|rs9870951|rs11066320|rs4963867|rs10936575|rs604045|rs744451|rs730830|rs7648548|rs17152433|rs11657742|rs5996534|rs9642880|rs3009935|rs1125467|rs2834906|rs266335|rs13320527
Immunologic Disorder	LYSOSOME_VESICLE_BIOGENESIS	REACTOME	420	25	13	2,99E-01	9,95E-01	rs10187424|rs10493377|rs941022|rs3812162|rs8078165|rs2279597|rs10889546|rs11881315|rs2283857|rs16933086|rs1749969|rs11075904|rs1805775
Immunologic Disorder	SYNTHESIS_OF_PIPS_AT_THE_PLASMA_MEMBRANE	REACTOME	1050	32	20	3,00E-01	9,95E-01	rs16897466|rs350292|rs10509534|rs2636638|rs11657742|rs2151414|rs13320527|rs11013118|rs1109303|rs17153654|rs4919128|rs13173003|rs764190|rs706725|rs10781329|rs10000770|rs11078772|rs228271|rs2667096|rs3756121
Immunologic Disorder	PENTOSE_AND_GLUCURONATE_INTERCONVERSIONS	KEGG	874	24	8	3,01E-01	9,95E-01	rs2608836|rs6830865|rs10031344|rs34778440|rs2270941|rs6600793|rs6717546|rs1347047
Immunologic Disorder	INSULIN_PATHWAY	BIOCARTA	725	33	20	3,01E-01	9,95E-01	rs16897466|rs17201100|rs11066320|rs2396303|rs13320527|rs4767879|rs17153654|rs13173003|rs2624189|rs706725|rs741847|rs1004446|rs2396306|rs13398058|rs6449947|rs12536419|rs2161529|rs34883124|rs10515074|rs6871452
Immunologic Disorder	RENAL_CELL_CARCINOMA	KEGG	2164	28	20	3,06E-01	9,95E-01	rs16897466|rs1793327|rs11066320|rs12577895|rs4963867|rs14003|rs604045|rs12666352|rs1465645|rs11102314|rs11657742|rs3858961|rs382649|rs6118746|rs11632939|rs2296950|rs3009935|rs17697359|rs13320527|rs17136507
Immunologic Disorder	INTERFERON_SIGNALING	REACTOME	3448	57	20	3,06E-01	9,95E-01	rs3767248|rs9975358|rs3115612|rs11868917|rs4886844|rs2368443|rs987870|rs4811068|rs1484931|rs10511409|rs1920650|rs4896251|rs6770415|rs4684917|rs1611493|rs1532113|rs11632652|rs7596050|rs2834112|rs28367832
Immunologic Disorder	NKCELLS_PATHWAY	BIOCARTA	678	32	18	3,07E-01	9,95E-01	rs16897466|rs1610751|rs1611493|rs8045689|rs2488320|rs13320527|rs1808563|rs4587680|rs13173003|rs11009195|rs706725|rs6449947|rs2161529|rs1148844|rs527589|rs10515074|rs6871452|rs2475193
Immunologic Disorder	GABA_SYNTHESIS_RELEASE_REUPTAKE_AND_DEGRADATION	REACTOME	746	43	15	3,11E-01	9,95E-01	rs1482574|rs10510403|rs16987501|rs9342910|rs6460052|rs10861034|rs17466478|rs2596852|rs362560|rs1569579|rs12190935|rs6951030|rs2944371|rs9826914|rs3774121
Immunologic Disorder	HYPERTROPHIC_CARDIOMYOPATHY_HCM	KEGG	4149	34	20	3,11E-01	9,95E-01	rs1051774|rs754298|rs12058655|rs4894044|rs9303513|rs12620429|rs604045|rs6727414|rs1375526|rs10925415|rs6952398|rs1934158|rs16835237|rs2392695|rs12203956|rs9849746|rs2127155|rs6942458|rs941022|rs13080492
Immunologic Disorder	RAP1_SIGNALLING	REACTOME	835	28	20	3,12E-01	9,95E-01	rs17540215|rs7907343|rs829420|rs1937701|rs1926212|rs11102314|rs17631340|rs6480687|rs10740303|rs10492301|rs10781329|rs10506562|rs8036582|rs1904024|rs4935027|rs1954624|rs293310|rs6581827|rs10520094|rs12766318
Immunologic Disorder	AMINO_ACID_SYNTHESIS_AND_INTERCONVERSION_TRANSAMINATION	REACTOME	530	23	13	3,18E-01	9,95E-01	rs3793932|rs7875845|rs12768505|rs4934297|rs13380644|rs4077632|rs4511520|rs234792|rs1475332|rs9934713|rs8049025|rs13294797|rs4544229
Immunologic Disorder	AXON_GUIDANCE	KEGG	9506	22	20	3,20E-01	9,95E-01	rs12375339|rs11924816|rs9942475|rs9445063|rs7435615|rs10502934|rs12914000|rs1592818|rs951556|rs551357|rs597332|rs17023932|rs12477147|rs17202361|rs9452527|rs1559677|rs4761558|rs1032491|rs2163783|rs17054953
Immunologic Disorder	CHYLOMICRON_MEDIATED_LIPID_TRANSPORT	REACTOME	435	32	16	3,20E-01	9,95E-01	rs4775073|rs10516451|rs9426785|rs97458|rs11581077|rs1607267|rs261337|rs881980|rs754524|rs9644636|rs312046|rs4666291|rs7568769|rs417344|rs936960|rs12474685
Immunologic Disorder	APC_C_CDC20_MEDIATED_DEGRADATION_OF_MITOTIC_PROTEINS	REACTOME	1094	40	20	3,20E-01	9,95E-01	rs10268808|rs7074757|rs2892190|rs6457689|rs7683090|rs1801376|rs4921283|rs9876561|rs1957107|rs807293|rs6736093|rs764190|rs6575122|rs2293361|rs2716601|rs12436216|rs16972457|rs228271|rs8075668|rs7105122
Immunologic Disorder	ASTHMA	KEGG	770	53	15	3,21E-01	9,95E-01	rs17254450|rs987870|rs17500510|rs9276933|rs3135342|rs9275312|rs740002|rs9276291|rs2395182|rs11741137|rs16942771|rs117821|rs241398|rs3135363|rs9469310
Immunologic Disorder	METAL_ION_SLC_TRANSPORTERS	REACTOME	653	41	15	3,22E-01	9,95E-01	rs2043128|rs2224|rs948419|rs10208012|rs2304704|rs2771338|rs1891092|rs801289|rs11743274|rs9438392|rs12352784|rs6734593|rs16861582|rs1243441|rs9277937
Immunologic Disorder	GLUCURONIDATION	REACTOME	656	21	7	3,26E-01	9,95E-01	rs2608836|rs6830865|rs10031344|rs17497947|rs6600793|rs6717546|rs1347047
Immunologic Disorder	TRAF6_MEDIATED_NFKB_ACTIVATION	REACTOME	351	29	19	3,27E-01	9,95E-01	rs11868917|rs17826255|rs466609|rs400819|rs204995|rs457581|rs3006488|rs2829946|rs9550565|rs8904|rs9551925|rs10140922|rs331455|rs12655019|rs1012919|rs11747611|rs6133067|rs3014875|rs12431309
Immunologic Disorder	GLYCOSPHINGOLIPID_BIOSYNTHESIS_LACTO_AND_NEOLACTO_SERIES	KEGG	670	33	18	3,28E-01	9,95E-01	rs8111317|rs505922|rs240542|rs17751811|rs838133|rs1379868|rs2279861|rs673086|rs836709|rs9295586|rs4808100|rs9689151|rs7855713|rs7025162|rs7582020|rs12294732|rs10754052|rs582037
Immunologic Disorder	AMYLOIDS	REACTOME	972	34	16	3,28E-01	9,95E-01	rs466609|rs400819|rs17078876|rs9426785|rs11581077|rs457581|rs4534818|rs983788|rs7760713|rs2829946|rs1004446|rs2592900|rs869894|rs12562819|rs11966463|rs9461416
Immunologic Disorder	TRAFFICKING_OF_AMPA_RECEPTORS	REACTOME	1132	30	20	3,29E-01	9,95E-01	rs1051774|rs9303513|rs3755768|rs6453850|rs1280054|rs2076370|rs1493383|rs1461236|rs372332|rs740804|rs2267339|rs2189290|rs1945968|rs891943|rs4381631|rs8081614|rs4791036|rs2344819|rs11710744|rs384842
Immunologic Disorder	NOTCH_SIGNALING_PATHWAY	KEGG	1387	34	20	3,34E-01	9,95E-01	rs2094347|rs744451|rs2423539|rs730830|rs10005500|rs17152433|rs204995|rs12308345|rs3782267|rs17288919|rs5756685|rs7647261|rs6131146|rs701078|rs693797|rs17136507|rs1472075|rs7893862|rs12417347|rs10846681
Immunologic Disorder	TRAF6_MEDIATED_IRF7_ACTIVATION	REACTOME	433	15	13	3,38E-01	9,95E-01	rs11868917|rs17826255|rs7046061|rs17136507|rs10878177|rs1710627|rs1296720|rs12504466|rs1425552|rs10811518|rs17624154|rs331455|rs6133067
Immunologic Disorder	GLUCOSE_TRANSPORT	REACTOME	689	32	16	3,42E-01	9,95E-01	rs2368443|rs1484931|rs6770415|rs4684917|rs10998736|rs7596050|rs6434036|rs773546|rs5766533|rs17757841|rs580438|rs3738516|rs7202364|rs12465639|rs1872837|rs11097233
Immunologic Disorder	SIGNALING_BY_CONSTITUTIVELY_ACTIVE_EGFR	REACTOME	503	19	13	3,46E-01	9,95E-01	rs16897466|rs4963867|rs6974790|rs13320527|rs13173003|rs706725|rs1140475|rs6449947|rs2161529|rs17388587|rs10515074|rs11977660|rs6871452
Immunologic Disorder	REGULATION_OF_AUTOPHAGY	KEGG	933	49	20	3,47E-01	9,95E-01	rs1943595|rs11082159|rs1888176|rs4297784|rs2780883|rs10889011|rs1865275|rs2796498|rs4553709|rs307118|rs2077507|rs710092|rs881536|rs1004446|rs2156480|rs8089360|rs10811518|rs3804332|rs520522|rs11177048
Immunologic Disorder	DOWNREGULATION_OF_TGF_BETA_RECEPTOR_SIGNALING	REACTOME	521	26	14	3,47E-01	9,95E-01	rs3773650|rs991694|rs3752496|rs266335|rs2063327|rs11661651|rs3859312|rs6792117|rs6025689|rs1019855|rs920293|rs6099823|rs7351039|rs4464148
Immunologic Disorder	ONE_CARBON_POOL_BY_FOLATE	KEGG	441	18	9	3,48E-01	9,95E-01	rs12449313|rs12899781|rs7630090|rs6745054|rs2298582|rs1252109|rs7739248|rs921986|rs685487
Immunologic Disorder	GAP_JUNCTION_ASSEMBLY	REACTOME	330	17	5	3,48E-01	9,95E-01	rs7764196|rs10737381|rs6454601|rs12119977|rs7899248
Immunologic Disorder	INFLUENZA_LIFE_CYCLE	REACTOME	1671	33	20	3,49E-01	9,95E-01	rs3365|rs2368443|rs4599669|rs10824609|rs1484931|rs10511409|rs17510142|rs7219604|rs1561|rs6770415|rs4684917|rs7596050|rs1848466|rs1007482|rs6434036|rs457372|rs8078165|rs11902670|rs773546|rs1115882
Immunologic Disorder	ACTIVATED_AMPK_STIMULATES_FATTY_ACID_OXIDATION_IN_MUSCLE	REACTOME	421	18	12	3,49E-01	9,95E-01	rs6952398|rs17762352|rs10889011|rs2796498|rs10849926|rs9568196|rs6950343|rs679596|rs9591258|rs4425665|rs7805942|rs2305508
Immunologic Disorder	SIGNALING_BY_FGFR1_FUSION_MUTANTS	REACTOME	639	29	20	3,52E-01	9,95E-01	rs16897466|rs4769946|rs404284|rs12537526|rs5996534|rs13320527|rs10784767|rs13173003|rs3024908|rs706725|rs1981405|rs5751614|rs9900213|rs6449947|rs13232623|rs2161529|rs12668172|rs3789311|rs10515074|rs6871452
Immunologic Disorder	RETINOL_METABOLISM	KEGG	1512	80	20	3,52E-01	9,95E-01	rs10889769|rs2572023|rs6830865|rs10031344|rs941022|rs1042389|rs12501328|rs17270248|rs4646570|rs2279345|rs34778440|rs2470890|rs725957|rs4143225|rs1238741|rs12062307|rs2654846|rs6600793|rs6717546|rs1347047
Immunologic Disorder	LONG_TERM_DEPRESSION	KEGG	3926	34	20	3,54E-01	9,95E-01	rs8115510|rs10485723|rs227130|rs2423360|rs208354|rs3819579|rs17540215|rs7907343|rs4963867|rs304737|rs1937701|rs12904722|rs1997696|rs1926212|rs2169202|rs17631340|rs8115925|rs210120|rs8113506|rs798524
Immunologic Disorder	NICOTINATE_AND_NICOTINAMIDE_METABOLISM	KEGG	963	36	20	3,56E-01	9,95E-01	rs9493119|rs6934819|rs179883|rs17155849|rs6596572|rs4122274|rs1153987|rs7217560|rs7756163|rs6935458|rs867744|rs167508|rs17356935|rs1217461|rs10001565|rs11744767|rs1760930|rs12136213|rs13236748|rs9493099
Immunologic Disorder	ETS_PATHWAY	BIOCARTA	682	28	20	3,57E-01	9,95E-01	rs1793327|rs12577895|rs197414|rs12308345|rs3782267|rs7555325|rs701078|rs10846681|rs333947|rs2836776|rs2836781|rs741847|rs2014340|rs10790921|rs7972214|rs12318072|rs6537883|rs10846670|rs1110904|rs870844
Immunologic Disorder	PURINE_METABOLISM	KEGG	5442	41	20	3,58E-01	9,95E-01	rs11208751|rs9493120|rs6934819|rs1265122|rs10510816|rs2395097|rs12797628|rs12545028|rs7117518|rs634651|rs298026|rs16897963|rs17649232|rs2755427|rs2041296|rs2532019|rs7100550|rs13168690|rs6081160|rs537557
Immunologic Disorder	ASPARAGINE_N_LINKED_GLYCOSYLATION	REACTOME	2760	30	20	3,59E-01	9,95E-01	rs6444190|rs4704|rs1869676|rs1352323|rs6095638|rs12198857|rs7617523|rs10490815|rs2070658|rs1147445|rs1869241|rs4940866|rs17103248|rs11231168|rs10513809|rs547177|rs709641|rs6707475|rs7176079|rs7633722
Immunologic Disorder	TGF_BETA_RECEPTOR_SIGNALING_ACTIVATES_SMADS	REACTOME	563	26	14	3,60E-01	9,95E-01	rs3773650|rs991694|rs3752496|rs266335|rs2063327|rs11661651|rs3859312|rs6792117|rs6025689|rs1019855|rs920293|rs6099823|rs7351039|rs4464148
Immunologic Disorder	BUTANOATE_METABOLISM	KEGG	763	47	19	3,60E-01	9,95E-01	rs7374394|rs7616534|rs2686084|rs10918239|rs4578194|rs10918228|rs2301770|rs4129215|rs9923280|rs1569579|rs11647477|rs11710823|rs12554375|rs954241|rs1155876|rs7307700|rs12503593|rs316738|rs2107232
Immunologic Disorder	PHENYLALANINE_METABOLISM	KEGG	442	25	16	3,67E-01	9,95E-01	rs3793932|rs3794176|rs7312593|rs12768505|rs4788547|rs13380644|rs4511520|rs1465936|rs4646785|rs9934713|rs7977571|rs8049025|rs6539049|rs4544229|rs7980567|rs11612372
Immunologic Disorder	WNT_SIGNALING_PATHWAY	KEGG	5611	41	20	3,68E-01	9,95E-01	rs8115510|rs7833751|rs10485723|rs6558063|rs227130|rs2423360|rs4655036|rs3819579|rs17202361|rs2719373|rs744451|rs11181782|rs4445045|rs1678002|rs730830|rs2094405|rs10506210|rs17152433|rs9299346|rs1997696
Immunologic Disorder	IGF1R_PATHWAY	BIOCARTA	735	34	18	3,68E-01	9,95E-01	rs16897466|rs17201100|rs2396303|rs13320527|rs10794486|rs2302234|rs2684796|rs13173003|rs706725|rs10781329|rs1404909|rs2396306|rs13398058|rs6449947|rs2161529|rs10515074|rs6871452|rs16867044
Immunologic Disorder	ERK_MAPK_TARGETS	REACTOME	704	31	20	3,69E-01	9,95E-01	rs11105194|rs3817828|rs1519959|rs12054920|rs1688545|rs6860708|rs8026614|rs7726306|rs7727198|rs1019206|rs17555326|rs3799577|rs3886478|rs7156757|rs796038|rs6558135|rs17353559|rs12199759|rs2345258|rs667656
Immunologic Disorder	ENERGY_DEPENDENT_REGULATION_OF_MTOR_BY_LKB1_AMPK	REACTOME	461	18	11	3,70E-01	9,95E-01	rs6952398|rs17762352|rs10889011|rs2796498|rs9568196|rs6950343|rs2589142|rs9591258|rs4425665|rs7805942|rs6565498
Immunologic Disorder	ADHERENS_JUNCTION	KEGG	4520	40	20	3,71E-01	9,95E-01	rs482533|rs4601648|rs4752894|rs3773650|rs991694|rs10879175|rs1719361|rs4811068|rs9581718|rs4852507|rs10506596|rs2094405|rs17235168|rs523223|rs501603|rs10997482|rs4105088|rs6974790|rs1368965|rs4938724
Immunologic Disorder	HS_GAG_BIOSYNTHESIS	KEGG	3154	28	20	3,75E-01	9,95E-01	rs9890008|rs7938320|rs11696078|rs9999101|rs3785690|rs2865155|rs9924390|rs2284277|rs1859906|rs3845669|rs7986556|rs517231|rs12615148|rs12855016|rs1581826|rs9426785|rs722769|rs16948248|rs7325810|rs2635351
Immunologic Disorder	MAPK_SIGNALING_PATHWAY	KEGG	9833	25	20	3,78E-01	9,95E-01	rs1051774|rs754298|rs17322289|rs6491679|rs11105194|rs9303513|rs8111317|rs10514211|rs3773650|rs4595639|rs991694|rs10908915|rs675498|rs10879175|rs208354|rs10858075|rs12743521|rs6444054|rs4703825|rs4963867
Immunologic Disorder	IL6_PATHWAY	BIOCARTA	401	13	8	3,79E-01	9,95E-01	rs11066320|rs4811006|rs4767879|rs161700|rs4808100|rs741847|rs16884711|rs2904254
Immunologic Disorder	REGULATION_OF_HYPOXIA_INDUCIBLE_FACTOR_HIF_BY_OXYGEN	REACTOME	443	18	11	3,81E-01	9,95E-01	rs2495726|rs1934158|rs1465645|rs17136507|rs2790860|rs10827475|rs1296720|rs2035423|rs7145164|rs1994174|rs10504553
Immunologic Disorder	INFLAM_PATHWAY	BIOCARTA	747	34	20	3,85E-01	9,95E-01	rs9847023|rs604045|rs2243131|rs7555325|rs3009935|rs7046061|rs4315909|rs4988821|rs333947|rs16906594|rs2395182|rs12029576|rs947712|rs2421057|rs9916158|rs2048874|rs1227631|rs3135363|rs6537883|rs2322180
Immunologic Disorder	ACTIVATION_OF_CHAPERONE_GENES_BY_XBP1S	REACTOME	761	44	20	3,87E-01	9,95E-01	rs13331733|rs10490815|rs3846211|rs1869241|rs9309485|rs970952|rs7633722|rs2437215|rs2218972|rs478309|rs9817549|rs8047140|rs4939627|rs3829078|rs902130|rs370476|rs4312645|rs10215237|rs6793950|rs1568082
Immunologic Disorder	RNA_POLYMERASE	KEGG	351	22	7	3,87E-01	9,95E-01	rs6081160|rs3132128|rs6539267|rs510342|rs4487686|rs9261258|rs2247746
Immunologic Disorder	G_ALPHA_S_SIGNALLING_EVENTS	REACTOME	5019	32	20	3,88E-01	9,95E-01	rs11208751|rs1034258|rs2104483|rs9362387|rs12797628|rs12545028|rs7117518|rs4855077|rs634651|rs298026|rs304737|rs7140817|rs16897963|rs17649232|rs12674049|rs255112|rs2532019|rs736730|rs13168690|rs6864183
Immunologic Disorder	FATTY_ACYL_COA_BIOSYNTHESIS	REACTOME	404	21	12	3,88E-01	9,95E-01	rs4795194|rs10452184|rs10182865|rs9463908|rs7561624|rs7680893|rs2862996|rs11726880|rs6838244|rs11098070|rs2817090|rs7690709
Immunologic Disorder	CYTOKINE_SIGNALING_IN_IMMUNE_SYSTEM	REACTOME	6819	46	20	3,89E-01	9,95E-01	rs1679802|rs3767248|rs16897466|rs17201100|rs9975358|rs3115612|rs11868917|rs4886844|rs2368443|rs987870|rs4811068|rs7628250|rs4963867|rs12666352|rs1484931|rs10511409|rs1920650|rs4896251|rs2066843|rs2396303

Immunologic Disorder	GLUTATHIONE_METABOLISM	KEGG	683	40	18	3,89E-01	9,95E-01	rs1265122|rs10918239|rs4932145|rs2274536|rs10918228|rs6904935|rs3760194|rs1571858|rs2551698|rs1265143|rs1265139|rs818169|rs574389|rs795602|rs2028299|rs6955931|rs2853228|rs7896809
Immunologic Disorder	SIGNALING_BY_FGFR1_MUTANTS	REACTOME	1243	32	20	3,91E-01	9,95E-01	rs16897466|rs4769946|rs404284|rs2517037|rs823600|rs12537526|rs5996534|rs13262310|rs666028|rs2038712|rs10870911|rs13320527|rs3129596|rs10784767|rs13173003|rs3024908|rs13249548|rs706725|rs1981405|rs9509959
Immunologic Disorder	IRON_UPTAKE_AND_TRANSPORT	REACTOME	733	48	20	3,91E-01	9,95E-01	rs3794176|rs661821|rs6532059|rs1512348|rs896189|rs2304704|rs2454016|rs7562668|rs6473885|rs3093988|rs6713990|rs6759240|rs1532222|rs16861582|rs4804662|rs902130|rs4668394|rs721950|rs496380|rs4960948
Immunologic Disorder	CLASS_B_2_SECRETIN_FAMILY_RECEPTORS	REACTOME	2257	39	20	3,93E-01	9,95E-01	rs7833751|rs6558063|rs7258925|rs4655036|rs4855077|rs7140817|rs12674049|rs255112|rs6952416|rs1006470|rs1410489|rs2710956|rs2807362|rs17356059|rs7349332|rs7797477|rs4383344|rs2797921|rs6768050|rs7624718
Immunologic Disorder	TCR_PATHWAY	BIOCARTA	1517	30	20	3,93E-01	9,95E-01	rs895437|rs5865|rs16897466|rs6063664|rs2469749|rs8045689|rs13218316|rs13320527|rs1808563|rs17153654|rs6021266|rs704856|rs11125132|rs13173003|rs2624189|rs706725|rs3804357|rs6063634|rs695161|rs1997815
Immunologic Disorder	RNA_POL_III_TRANSCRIPTION	REACTOME	861	42	20	3,97E-01	9,95E-01	rs9407553|rs10961344|rs6081160|rs4077515|rs10961490|rs6539267|rs907076|rs393543|rs10810132|rs510342|rs4961396|rs16858688|rs11145922|rs7145164|rs7857772|rs2995094|rs4922157|rs9406469|rs12930464|rs1075
Immunologic Disorder	ANTIGEN_PROCESSING_CROSS_PRESENTATION	REACTOME	1601	53	20	3,97E-01	9,95E-01	rs3115612|rs10268808|rs12620429|rs2892190|rs10046115|rs1611493|rs7714122|rs28367832|rs1957107|rs807293|rs764190|rs2303138|rs9264967|rs6575122|rs2293361|rs2716601|rs5995351|rs12436216|rs16972457|rs228271
Immunologic Disorder	PYRUVATE_METABOLISM	REACTOME	209	13	7	4,00E-01	9,95E-01	rs693163|rs2076370|rs11032981|rs1539624|rs11710823|rs3133978|rs1808417
Immunologic Disorder	INTEGRIN_ALPHAIIB_BETA3_SIGNALING	REACTOME	779	24	20	4,01E-01	9,95E-01	rs2152305|rs723188|rs4811068|rs11102314|rs1537745|rs787033|rs1378942|rs10492301|rs17491334|rs10506562|rs8036582|rs945296|rs2678289|rs2283332|rs6581827|rs3829078|rs10520094|rs1148844|rs9989778|rs6753702
Immunologic Disorder	PTEN_PATHWAY	BIOCARTA	544	25	14	4,02E-01	9,95E-01	rs16897466|rs10509534|rs2488320|rs13320527|rs4587680|rs13173003|rs11009195|rs706725|rs6449947|rs9480865|rs2161529|rs10515074|rs6871452|rs2475193
Immunologic Disorder	DEADENYLATION_DEPENDENT_MRNA_DECAY	REACTOME	622	31	16	4,08E-01	9,95E-01	rs240542|rs10853869|rs3740526|rs6792874|rs7024667|rs1893352|rs10415765|rs1545229|rs2241790|rs435584|rs7975725|rs11642944|rs10973537|rs1239915|rs582037|rs4291799
Immunologic Disorder	PYRIMIDINE_METABOLISM	KEGG	1900	46	20	4,08E-01	9,95E-01	rs1689924|rs1265122|rs12023630|rs11165950|rs2041296|rs12061124|rs6081160|rs10861171|rs7575062|rs9261212|rs12120077|rs6539267|rs6681958|rs131820|rs2039300|rs4669069|rs1253795|rs10493895|rs7217560|rs510342
Immunologic Disorder	PEPTIDE_CHAIN_ELONGATION	REACTOME	947	29	20	4,09E-01	9,95E-01	rs3365|rs4599669|rs10824609|rs17510142|rs7219604|rs1561|rs1848466|rs1007482|rs457372|rs11902670|rs1115882|rs1249123|rs2851387|rs2436526|rs4979816|rs13325754|rs12879285|rs3745624|rs4149455|rs10824618
Immunologic Disorder	ANTIGEN_ACTIVATES_B_CELL_RECEPTOR_LEADING_TO_GENERATION_OF_SECOND_ME	REACTOME	1299	29	20	4,10E-01	9,95E-01	rs7126870|rs16897466|rs9870951|rs7648548|rs210120|rs7197601|rs10743591|rs13218316|rs3782309|rs1808563|rs4367942|rs11125132|rs11644646|rs2618455|rs13173003|rs17793122|rs706725|rs6486795|rs4254322|rs10835270
Immunologic Disorder	TAK1_ACTIVATES_NFKB_BY_PHOSPHORYLATION_AND_ACTIVATION_OF_IKKS_COMPLE	REACTOME	777	33	20	4,10E-01	9,95E-01	rs466609|rs2066843|rs400819|rs204995|rs9359935|rs10806443|rs457581|rs3006475|rs6961625|rs6924218|rs284513|rs2829946|rs9550565|rs7746039|rs8904|rs2616052|rs9551925|rs10140922|rs331455|rs10257280
Immunologic Disorder	TERMINATION_OF_O_GLYCAN_BIOSYNTHESIS	REACTOME	961	29	20	4,11E-01	9,95E-01	rs11672574|rs6444190|rs7617523|rs10513809|rs6968731|rs741901|rs2403981|rs3873342|rs1975605|rs12422396|rs7260214|rs2306851|rs1868430|rs11720551|rs11825977|rs4985526|rs7820063|rs3780670|rs16904967|rs6981912
Immunologic Disorder	SEMAPHORIN_INTERACTIONS	REACTOME	2884	43	20	4,11E-01	9,95E-01	rs12914000|rs6578414|rs17202361|rs1559677|rs4761558|rs1032491|rs17054953|rs7710998|rs503473|rs4761585|rs2657031|rs8060975|rs215573|rs12189367|rs3847794|rs1781030|rs2012928|rs10763940|rs984576|rs2488320
Immunologic Disorder	TRYPTOPHAN_METABOLISM	KEGG	953	43	20	4,15E-01	9,95E-01	rs7374394|rs3735477|rs10918239|rs899875|rs4578194|rs1347929|rs10918228|rs12138459|rs930420|rs1053230|rs2470890|rs4617138|rs11132836|rs12554375|rs17110690|rs12231356|rs172424|rs16888662|rs1022004|rs12155014
Immunologic Disorder	SYNTHESIS_AND_INTERCONVERSION_OF_NUCLEOTIDE_DI_AND_TRIPHOSPHATES	REACTOME	306	34	16	4,16E-01	9,95E-01	rs1265122|rs2041296|rs6420125|rs10861171|rs1565850|rs2551698|rs1265143|rs6680677|rs1874819|rs1265139|rs6556889|rs3780670|rs10518441|rs7537574|rs2853228|rs6539138
Immunologic Disorder	NUCLEAR_EVENTS_KINASE_AND_TRANSCRIPTION_FACTOR_ACTIVATION	REACTOME	766	31	20	4,18E-01	9,95E-01	rs1009619|rs3817828|rs1519959|rs12054920|rs1688545|rs6860708|rs8026614|rs7726306|rs7727198|rs1019206|rs17555326|rs3799577|rs3886478|rs7156757|rs796038|rs6558135|rs17353559|rs12199759|rs2345258|rs667656
Immunologic Disorder	JAK_STAT_SIGNALING_PATHWAY	KEGG	4346	36	20	4,20E-01	9,95E-01	rs6563245|rs16897466|rs9847023|rs6563216|rs9975358|rs9870951|rs10493377|rs11066320|rs12028951|rs4445045|rs4655690|rs4896251|rs7648548|rs741347|rs1373635|rs10795505|rs3856848|rs11657742|rs1532113|rs2834112
Immunologic Disorder	REGULATORY_RNA_PATHWAYS	REACTOME	345	6	5	4,24E-01	9,95E-01	rs12103149|rs4487686|rs11958935|rs7405515|rs442277
Immunologic Disorder	HOST_INTERACTIONS_OF_HIV_FACTORS	REACTOME	2270	36	20	4,24E-01	9,95E-01	rs1610751|rs10268808|rs2368443|rs1882070|rs2892190|rs1484931|rs10511409|rs6457689|rs17071888|rs6770415|rs4684917|rs1611493|rs7596050|rs6434036|rs13218316|rs873491|rs1957107|rs12654453|rs12532031|rs372332
Immunologic Disorder	ASSOCIATION_OF_TRIC_CCT_WITH_TARGET_PROTEINS_DURING_BIOSYNTHESIS	REACTOME	359	34	11	4,25E-01	9,95E-01	rs4712311|rs4723235|rs6865121|rs2292267|rs4810909|rs10824092|rs9992175|rs2024595|rs7749814|rs10516289|rs1393015
Immunologic Disorder	TRANSPORT_OF_MATURE_TRANSCRIPT_TO_CYTOPLASM	REACTOME	693	32	17	4,26E-01	9,95E-01	rs2368443|rs1484931|rs6770415|rs4684917|rs7596050|rs3737984|rs6434036|rs773546|rs2282763|rs5766533|rs17757841|rs580438|rs7202364|rs12465639|rs2334321|rs6093835|rs11097233
Immunologic Disorder	METABOLISM_OF_STEROID_HORMONES_AND_VITAMINS_A_AND_D	REACTOME	745	23	14	4,26E-01	9,95E-01	rs9362387|rs716097|rs6467|rs12501328|rs17270248|rs7099855|rs31309|rs4736349|rs10923819|rs4667591|rs12907723|rs10883782|rs12522757|rs10500804
Immunologic Disorder	ACTIVATION_OF_BH3_ONLY_PROTEINS	REACTOME	325	14	8	4,26E-01	9,95E-01	rs500804|rs1016860|rs956572|rs1439287|rs719450|rs2868161|rs12454650|rs4940927
Immunologic Disorder	DESTABILIZATION_OF_MRNA_BY_TRISTETRAPROLIN_TTP	REACTOME	179	14	7	4,28E-01	9,95E-01	rs3740526|rs9628|rs7024667|rs1545229|rs10973537|rs2868161|rs4291799
Immunologic Disorder	CITRIC_ACID_CYCLE_TCA_CYCLE	REACTOME	675	17	10	4,29E-01	9,95E-01	rs3735477|rs4832076|rs9649679|rs11983987|rs10496307|rs2647209|rs13428219|rs7647085|rs12155014|rs10193241
Immunologic Disorder	STEROID_HORMONES	REACTOME	693	21	13	4,29E-01	9,95E-01	rs9362387|rs716097|rs6467|rs17270248|rs7099855|rs31309|rs4736349|rs10923819|rs4667591|rs12907723|rs10883782|rs12522757|rs10500804
Immunologic Disorder	PROPANOATE_METABOLISM	KEGG	1219	34	18	4,30E-01	9,95E-01	rs7374394|rs4795194|rs4832076|rs10918239|rs4578194|rs9554684|rs10918228|rs1921038|rs10849926|rs10496307|rs11631023|rs6546670|rs12554375|rs13428219|rs406303|rs7647085|rs1435969|rs10193241
Immunologic Disorder	O_GLYCAN_BIOSYNTHESIS	KEGG	1579	31	20	4,30E-01	9,95E-01	rs1791534|rs17143909|rs6095638|rs10183136|rs1474925|rs11950904|rs707085|rs10932055|rs6976906|rs10931759|rs16834313|rs1364419|rs12658598|rs741901|rs2403981|rs12216907|rs7807431|rs1975605|rs621293|rs16917989
Immunologic Disorder	GPVI_MEDIATED_ACTIVATION_CASCADE	REACTOME	1181	31	20	4,31E-01	9,95E-01	rs16897466|rs6578414|rs2521206|rs11657742|rs7197601|rs8045689|rs1476002|rs13218316|rs13320527|rs1808563|rs343144|rs4367942|rs17153654|rs10487254|rs11644646|rs13173003|rs315734|rs962199|rs17793122|rs706725
Immunologic Disorder	NEF_MEDIATES_DOWN_MODULATION_OF_CELL_SURFACE_RECEPTORS_BY_RECRUITIN N_ADAPTERS	REACTOME	366	21	11	4,32E-01	9,95E-01	rs1610751|rs1611493|rs873491|rs372332|rs13259308|rs695161|rs2283857|rs384842|rs11075904|rs6753271|rs1004420
Immunologic Disorder	TRANSFERRIN_ENDOCYTOSIS_AND_RECYCLING	REACTOME	513	42	17	4,33E-01	9,95E-01	rs3794176|rs661821|rs1512348|rs896189|rs2454016|rs7562668|rs6473885|rs3093988|rs6713990|rs1532222|rs4804662|rs902130|rs721950|rs496380|rs4960948|rs7579208|rs13236345
Immunologic Disorder	GLYCEROLIPID_METABOLISM	KEGG	1872	35	20	4,34E-01	9,95E-01	rs4775073|rs12199314|rs11868917|rs706578|rs1880553|rs10918239|rs204995|rs4578194|rs10918228|rs1341347|rs2046731|rs7422272|rs558491|rs12963082|rs12246792|rs6455717|rs4823168|rs11195662|rs7230869|rs217600
Immunologic Disorder	THE_ROLE_OF_NEF_IN_HIV1_REPLICATION_AND_DISEASE_PATHOGENESIS	REACTOME	936	36	20	4,35E-01	9,95E-01	rs1610751|rs1882070|rs17071888|rs1611493|rs13218316|rs873491|rs12654453|rs12532031|rs372332|rs6473885|rs1986619|rs704856|rs373777|rs695161|rs2283857|rs2717947|rs384842|rs2995094|rs11153318|rs4723582
Immunologic Disorder	REGULATION_OF_GENE_EXPRESSION_IN_BETA_CELLS	REACTOME	716	36	17	4,36E-01	9,95E-01	rs2868094|rs7322396|rs6047982|rs830786|rs7991808|rs1515013|rs11725962|rs6073418|rs11065374|rs1004446|rs6036219|rs2060604|rs874269|rs717248|rs2920914|rs2757072|rs559080
Immunologic Disorder	IMMUNOREGULATORY_INTERACTIONS_BETWEEN_A_LYMPHOID_AND_A_NON_LYMPHO	REACTOME	1325	41	20	4,38E-01	9,95E-01	rs649216|rs3115612|rs595872|rs1784873|rs2824321|rs1611493|rs2488320|rs28367832|rs1749313|rs4587680|rs3107634|rs704856|rs12419365|rs9264967|rs11009195|rs2824115|rs9937676|rs2824278|rs2995094|rs2253907
Immunologic Disorder	ABC_FAMILY_PROTEINS_MEDIATED_TRANSPORT	REACTOME	1753	57	20	4,43E-01	9,95E-01	rs7330196|rs8081909|rs12921748|rs10521168|rs12493550|rs11654057|rs4147967|rs1678388|rs1283798|rs323073|rs4781699|rs4148407|rs4147819|rs17205859|rs9302064|rs8071366|rs10877173|rs4672731|rs12443685|rs8001940
Immunologic Disorder	NO2IL12_PATHWAY	BIOCARTA	461	16	11	4,44E-01	9,95E-01	rs9847023|rs2243131|rs4315909|rs704856|rs3024908|rs7560255|rs2421057|rs2995094|rs858490|rs11177048|rs6895787
Immunologic Disorder	ENDOMETRIAL_CANCER	KEGG	3028	31	20	4,45E-01	9,95E-01	rs482533|rs4601648|rs16897466|rs4963867|rs10509534|rs4852507|rs2094405|rs17235168|rs10997482|rs11657742|rs6974790|rs9642880|rs1368965|rs6721643|rs1904608|rs10997565|rs931771|rs13320527|rs17020744|rs7812998
Immunologic Disorder	SPPA_PATHWAY	BIOCARTA	1428	30	20	4,50E-01	9,95E-01	rs10485723|rs37244|rs227130|rs2423360|rs304737|rs8115925|rs2488320|rs13163497|rs2886612|rs4587680|rs1015092|rs2745755|rs10798106|rs11009195|rs2058265|rs153317|rs17544558|rs4381631|rs8081614|rs4791036
Immunologic Disorder	MPR_PATHWAY	BIOCARTA	624	27	15	4,51E-01	9,95E-01	rs304737|rs736730|rs518162|rs4809258|rs6062357|rs2886612|rs2302234|rs1404909|rs10781329|rs2012987|rs1535211|rs1942836|rs6062662|rs660541|rs3771099
Immunologic Disorder	INTERACTION_BETWEEN_L1_AND_ANKYRINS	REACTOME	970	34	20	4,53E-01	9,95E-01	rs4809563|rs1362890|rs7831492|rs7003385|rs7645358|rs6675810|rs2673576|rs4902311|rs2445884|rs4935840|rs13258868|rs17155687|rs10509122|rs2153522|rs4737021|rs2038442|rs4233808|rs7428779|rs4968681|rs1757779
Immunologic Disorder	XENOBIOTICS	REACTOME	231	7	5	4,53E-01	9,95E-01	rs1042389|rs915908|rs2279345|rs2470890|rs1536827
Immunologic Disorder	MELANOMA	KEGG	2894	30	20	4,53E-01	9,95E-01	rs16897466|rs6491679|rs8111317|rs4963867|rs17405661|rs7623610|rs12666352|rs10509534|rs2517037|rs12419388|rs823600|rs9518498|rs11657742|rs6974790|rs13262310|rs666028|rs838133|rs2038712|rs10870911|rs17506327
Immunologic Disorder	SIGNALING_BY_ERBB2	REACTOME	3847	24	20	4,55E-01	9,95E-01	rs2102996|rs16897466|rs17347606|rs12328595|rs13393014|rs12545028|rs4963867|rs10509534|rs17649232|rs7322396|rs2532019|rs776385|rs10876227|rs13168690|rs2919390|rs16846161|rs4599070|rs6974790|rs3752496|rs4672649
Immunologic Disorder	P53_SIGNALING_PATHWAY	KEGG	1897	31	20	4,55E-01	9,95E-01	rs1265122|rs10908915|rs10497131|rs7910825|rs12995726|rs10509534|rs4445045|rs12765241|rs11135693|rs10163809|rs1047266|rs11762916|rs3769823|rs1478604|rs6977956|rs11063059|rs2881873|rs300193|rs1265143|rs1486137
Immunologic Disorder	ANTIGEN_PRESENTATION_FOLDING_ASSEMBLY_AND_PEPTIDE_LOADING_OF_CLASS_I_M	REACTOME	830	45	15	4,56E-01	9,95E-01	rs3115612|rs10490815|rs1611493|rs709641|rs28367832|rs11730732|rs2071482|rs9264967|rs151823|rs117821|rs2253907|rs7676892|rs1632949|rs12433553|rs7156225
Immunologic Disorder	BASE_EXCISION_REPAIR	KEGG	500	20	13	4,57E-01	9,95E-01	rs2261316|rs2678681|rs6846031|rs2135615|rs16981247|rs2029166|rs9550565|rs4135054|rs9551925|rs2682587|rs3730463|rs2723875|rs12431309
Immunologic Disorder	SYNTHESIS_OF_GLYCOSYLPHOSPHATIDYLINOSITOL_GPI	REACTOME	485	35	15	4,58E-01	9,95E-01	rs12914000|rs1559677|rs9673061|rs12323943|rs11633288|rs2269615|rs12902710|rs16958772|rs3742872|rs2273595|rs514874|rs766132|rs17271669|rs647488|rs1912640
Immunologic Disorder	PANCREATIC_CANCER	KEGG	1920	28	20	4,60E-01	9,95E-01	rs16897466|rs3773650|rs991694|rs4963867|rs604045|rs7022409|rs11657742|rs6974790|rs382649|rs3009935|rs1476002|rs10520358|rs266335|rs13320527|rs2043722|rs2869433|rs17153654|rs6780561|rs10486801|rs13110230
Immunologic Disorder	REGULATION_OF_APOPTOSIS	REACTOME	1432	40	20	4,61E-01	9,95E-01	rs10502934|rs10268808|rs551357|rs2892190|rs10823677|rs6457689|rs4129439|rs1957107|rs490332|rs727514|rs1105384|rs807293|rs764190|rs6575122|rs2293361|rs2716601|rs2277257|rs2277259|rs8099038|rs12436216
Immunologic Disorder	ELONGATION_ARREST_AND_RECOVERY	REACTOME	246	11	3	4,62E-01	9,95E-01	rs503347|rs4487686|rs10504553
Immunologic Disorder	RNA_POL_III_TRANSCRIPTION_TERMINATION	REACTOME	680	29	17	4,62E-01	9,95E-01	rs9407553|rs10961344|rs6081160|rs10961490|rs6539267|rs10810132|rs510342|rs4961396|rs7857772|rs4922157|rs9406469|rs12930464|rs1075|rs4921702|rs13296301|rs2247746|rs8192986
Immunologic Disorder	RIP_MEDIATED_NFKB_ACTIVATION_VIA_DAI	REACTOME	328	27	17	4,63E-01	9,95E-01	rs466609|rs400819|rs204995|rs7691384|rs457581|rs3006475|rs10415336|rs2829946|rs9550565|rs956239|rs8904|rs9551925|rs10140922|rs13038139|rs1012919|rs3014875|rs12431309
Immunologic Disorder	TH1TH2_PATHWAY	BIOCARTA	535	24	14	4,66E-01	9,95E-01	rs9847023|rs4896251|rs2243131|rs4315909|rs3135342|rs2681417|rs2395182|rs2421057|rs12210503|rs3914898|rs3135363|rs2332096|rs11177048|rs6895787
Immunologic Disorder	INHIBITION_OF_INSULIN_SECRETION_BY_ADRENALINE_NORADRENALINE	REACTOME	789	34	20	4,66E-01	9,95E-01	rs954385|rs4855077|rs304737|rs7140817|rs11195623|rs2886612|rs4561129|rs12488540|rs3107340|rs12704705|rs10498440|rs12886319|rs7975385|rs1253676|rs12267833|rs7096359|rs291353|rs12595160|rs4659946|rs8023017
Immunologic Disorder	SPLICEOSOME	KEGG	1685	40	20	4,69E-01	9,95E-01	rs8111911|rs4763206|rs10061188|rs10853869|rs1870194|rs12121067|rs9970867|rs3737984|rs4356184|rs6135844|rs7523056|rs2339648|rs10415765|rs12441204|rs514524|rs3093988|rs435584|rs10259241|rs17825554|rs6853501
Immunologic Disorder	SIGNALING_BY_HIPPO	REACTOME	627	24	15	4,70E-01	9,95E-01	rs17322289|rs1719361|rs500755|rs13094395|rs9439587|rs193466|rs13065308|rs770701|rs722420|rs1499395|rs1422422|rs2868161|rs7522344|rs7652697|rs916374
Immunologic Disorder	CTNNB1_PHOSPHORYLATION_CASCADE	REACTOME	316	5	4	4,72E-01	9,95E-01	rs1678002|rs7145618|rs4919096|rs4355511
Immunologic Disorder	SELENOAMINO_ACID_METABOLISM	KEGG	742	21	10	4,72E-01	9,95E-01	rs2755427|rs2028898|rs10058433|rs3760194|rs4130719|rs12414133|rs2522420|rs12411782|rs7446290|rs10515868
Immunologic Disorder	ER_PHAGOSOME_PATHWAY	REACTOME	1416	50	20	4,74E-01	9,95E-01	rs3115612|rs10268808|rs2892190|rs6457689|rs1611493|rs28367832|rs1957107|rs807293|rs764190|rs9264967|rs6575122|rs2293361|rs2716601|rs12436216|rs16972457|rs228271|rs8075668|rs1648628|rs7105122|rs117821
Immunologic Disorder	NEUROTRANSMITTER_RELEASE_CYCLE	REACTOME	1400	39	20	4,74E-01	9,95E-01	rs1482574|rs2074615|rs2858347|rs5998715|rs10510403|rs9609608|rs737088|rs16987501|rs9342910|rs6460052|rs4401921|rs10861034|rs17466478|rs2596852|rs362560|rs1679933|rs1569579|rs12190935|rs6951030|rs2944371
Immunologic Disorder	ARACHIDONIC_ACID_METABOLISM	KEGG	1356	37	20	4,75E-01	9,95E-01	rs759391|rs4147584|rs12904722|rs1290622|rs1558139|rs759998|rs1042389|rs884115|rs4338847|rs3760194|rs6672057|rs915908|rs2409758|rs2279345|rs2261612|rs10798106|rs1093733|rs2058265|rs17544558|rs4808358
Immunologic Disorder	MET_PATHWAY	BIOCARTA	1415	23	20	4,78E-01	9,95E-01	rs16897466|rs11066320|rs12666352|rs10509534|rs9418677|rs11102314|rs2296950|rs2488320|rs13163497|rs13320527|rs975360|rs4767879|rs17153654|rs4587680|rs10492301|rs11017780|rs13173003|rs2624189|rs11009195|rs706725
Immunologic Disorder	PATHOGENIC_ESCHERICHIA_COLI_INFECTION	KEGG	1256	41	20	4,80E-01	9,95E-01	rs7239317|rs6580921|rs736730|rs1930714|rs1392125|rs1410201|rs1329049|rs2488320|rs13218316|rs1928295|rs1436649|rs778584|rs10984022|rs1996215|rs4587680|rs7590257|rs2177640|rs11009195|rs2806112|rs3769504
Immunologic Disorder	ENDOCYTOSIS	KEGG	6484	35	20	4,81E-01	9,95E-01	rs2102996|rs1034258|rs17347606|rs37244|rs3115612|rs10211191|rs6685064|rs12328595|rs9870951|rs13393014|rs10493377|rs11203986|rs925037|rs6506685|rs8088164|rs13102063|rs7648548|rs4674259|rs10519327|rs17687453
Immunologic Disorder	REGULATION_OF_BETA_CELL_DEVELOPMENT	REACTOME	1288	30	20	4,82E-01	9,95E-01	rs2868094|rs7322396|rs1360127|rs6047982|rs10005500|rs4411624|rs830786|rs7647261|rs2737621|rs10919803|rs3861927|rs1472075|rs9892543|rs1474054|rs7991808|rs1515013|rs11725962|rs6073418|rs11065374|rs12069085
Immunologic Disorder	METABOLISM_OF_VITAMINS_AND_COFACTORS	REACTOME	1119	40	20	4,83E-01	9,95E-01	rs9493120|rs12449313|rs179883|rs12039696|rs1500367|rs254489|rs592261|rs17339942|rs10513009|rs7731228|rs1047636|rs7756163|rs6897965|rs6935458|rs598246|rs17356935|rs2187250|rs228578|rs3738516|rs3133803
Immunologic Disorder	FORMATION_OF_THE_HIV1_EARLY_ELONGATION_COMPLEX	REACTOME	329	11	3	4,86E-01	9,95E-01	rs503347|rs4487686|rs886420
Immunologic Disorder	SYNTHESIS_OF_PIPS_AT_THE_GOLGI_MEMBRANE	REACTOME	788	52	19	4,87E-01	9,95E-01	rs1943595|rs11082159|rs1130635|rs4297784|rs2492977|rs1865275|rs4553709|rs307118|rs4919128|rs2992950|rs2156480|rs8089360|rs11044171|rs17339449|rs7185187|rs520522|rs6568602|rs3764459|rs9885672
Immunologic Disorder	MATURITY_ONSET_DIABETES_OF_THE_YOUNG	KEGG	992	34	20	4,89E-01	9,95E-01	rs2868094|rs6047982|rs11768251|rs830786|rs7647261|rs2737621|rs10919803|rs3861927|rs1472075|rs9892543|rs1474054|rs1515013|rs11725962|rs6073418|rs11065374|rs12069085|rs990394|rs1004446|rs6036219|rs2060604
Immunologic Disorder	NEGATIVE_REGULATION_OF_FGFR_SIGNALING	REACTOME	1536	37	20	4,90E-01	9,95E-01	rs6563245|rs6563216|rs17510142|rs2517037|rs12419388|rs823600|rs13262310|rs1333404|rs666028|rs2038712|rs10870911|rs10908200|rs3787011|rs1930911|rs9601627|rs3129596|rs9601679|rs13249548|rs9509959|rs11745375
Immunologic Disorder	PROTEASOME	KEGG	821	47	20	4,91E-01	9,95E-01	rs10268808|rs2892190|rs6457689|rs1957107|rs1464807|rs9508068|rs807293|rs764190|rs6575122|rs2293361|rs2716601|rs7785299|rs12436216|rs16972457|rs228271|rs8075668|rs7105122|rs117821|rs16930278|rs8066176
Immunologic Disorder	ACTIVATED_NOTCH1_TRANSMITS_SIGNAL_TO_THE_NUCLEUS	REACTOME	719	25	12	4,91E-01	9,95E-01	rs2094347|rs2423539|rs6131146|rs2396663|rs911336|rs12138787|rs2181219|rs4973190|rs11177540|rs1018534|rs1674098|rs2423516
Immunologic Disorder	EGFR_DOWNREGULATION	REACTOME	1037	39	19	4,92E-01	9,95E-01	rs6563245|rs6563216|rs10795505|rs6974790|rs1333404|rs8078165|rs372332|rs1930911|rs9601627|rs9601679|rs1140475|rs3113392|rs4145077|rs384842|rs16933086|rs2473322|rs11977660|rs9565596|rs1382559
Immunologic Disorder	RIBOSOME	KEGG	1118	28	20	4,95E-01	9,95E-01	rs3365|rs4599669|rs10824609|rs17510142|rs7219604|rs1561|rs1848466|rs1007482|rs457372|rs9845497|rs11902670|rs1115882|rs1249123|rs2851387|rs2436526|rs858893|rs4979816|rs13325754|rs12879285|rs3745624
Immunologic Disorder	SIGNALING_BY_NODAL	REACTOME	632	29	20	4,96E-01	9,95E-01	rs4965900|rs10876227|rs4965897|rs7566998|rs2641530|rs266335|rs12476865|rs7002959|rs2063327|rs11661651|rs7169026|rs3859312|rs6598456|rs10514714|rs28469650|rs9480865|rs4377290|rs7047712|rs1866411|rs920293
Immunologic Disorder	NONSENSE_MEDIATED_DECAY_ENHANCED_BY_THE_EXON_JUNCTION_COMPLEX	REACTOME	1261	31	20	4,98E-01	9,95E-01	rs3365|rs4750154|rs4599669|rs10824609|rs17510142|rs7219604|rs1561|rs1848466|rs3737984|rs1007482|rs457372|rs1052053|rs11902670|rs1115882|rs1249123|rs2851387|rs2436526|rs4979816|rs13325754|rs942537
Immunologic Disorder	SIGNALING_BY_RHO_GTPASES	REACTOME	4877	27	20	5,00E-01	9,95E-01	rs11929318|rs10514211|rs2175766|rs6578414|rs9904572|rs894628|rs4703825|rs17281570|rs1044011|rs503473|rs13098182|rs1116757|rs2409416|rs7670874|rs11923190|rs13052373|rs9965330|rs13102063|rs6019300|rs1817149
Immunologic Disorder	DOWNSTREAM_TCR_SIGNALING	REACTOME	1573	40	20	5,04E-01	9,95E-01	rs16897466|rs4750517|rs987870|rs10509534|rs13238172|rs587198|rs9359935|rs13320527|rs17500510|rs10806443|rs6125883|rs704856|rs6924218|rs4357599|rs284513|rs13173003|rs9276291|rs706725|rs695161|rs7746039
Immunologic Disorder	EARLY_PHASE_OF_HIV_LIFE_CYCLE	REACTOME	270	10	7	5,05E-01	9,95E-01	rs12691883|rs2011946|rs16981247|rs2056296|rs10191360|rs1872066|rs7046713
Immunologic Disorder	NON_SMALL_CELL_LUNG_CANCER	KEGG	2585	29	20	5,08E-01	9,95E-01	rs16897466|rs17322289|rs10510816|rs1580816|rs4963867|rs6550967|rs4858703|rs1555949|rs7858838|rs9311770|rs11657742|rs7638130|rs6974790|rs12108196|rs7197601|rs4681063|rs13320527|rs4681044|rs7533324|rs17153654
Immunologic Disorder	BASAL_CELL_CARCINOMA	KEGG	2166	36	20	5,14E-01	9,95E-01	rs7833751|rs6558063|rs4655036|rs1980499|rs2094405|rs6952416|rs811153|rs2710956|rs2810061|rs2807362|rs17356059|rs7155858|rs4766398|rs7796149|rs7349332|rs931771|rs6085802|rs2575580|rs2797921|rs6768050
Immunologic Disorder	ENDOSOMAL_SORTING_COMPLEX_REQUIRED_FOR_TRANSPORT_ESCRT	REACTOME	318	16	8	5,15E-01	9,95E-01	rs17687453|rs10795505|rs17089022|rs1825896|rs13042412|rs2782528|rs3213601|rs2378026
Immunologic Disorder	METABOLISM_OF_AMINO_ACIDS_AND_DERIVATIVES	REACTOME	5246	34	20	5,18E-01	9,95E-01	rs2261316|rs3793932|rs6706968|rs7145370|rs10268808|rs10483947|rs9362387|rs1209157|rs3735477|rs827294|rs2892190|rs12449313|rs7312593|rs11698712|rs6457689|rs7875845|rs10918239|rs2415361|rs1491503|rs2028898
Immunologic Disorder	POST_TRANSLATIONAL_PROTEIN_MODIFICATION	REACTOME	6008	37	20	5,20E-01	9,95E-01	rs1791534|rs11672574|rs6444190|rs12914000|rs4704|rs10187424|rs1869676|rs1559677|rs17143909|rs1352323|rs6095638|rs12198857|rs7405583|rs7617523|rs10490815|rs10183136|rs2070658|rs1147445|rs1869241|rs4940866
Immunologic Disorder	SYNTHESIS_OF_PA	REACTOME	1057	34	20	5,21E-01	9,95E-01	rs12199314|rs6870653|rs204995|rs531592|rs2046731|rs558491|rs6672057|rs6455717|rs6780561|rs11195662|rs3733796|rs2261612|rs10798106|rs1093733|rs4106571|rs17544558|rs904729|rs3913795|rs2292023|rs12106865
Immunologic Disorder	DESTABILIZATION_OF_MRNA_BY_BRF1	REACTOME	204	16	8	5,22E-01	9,95E-01	rs3740526|rs9628|rs7024667|rs1545229|rs10973537|rs8019383|rs2868161|rs4291799

Immunologic Disorder	POST_TRANSLATIONAL_MODIFICATION_SYNTHESIS_OF_GPI_ANCHORED_PROTEINS	REACTOME	586	38	17	5,23E-01	9,95E-01	rs12914000|rs1559677|rs9673061|rs12323943|rs11633288|rs2049363|rs2269615|rs12902710|rs16958772|rs3742872|rs2273595|rs4949652|rs514874|rs766132|rs17271669|rs647488|rs1912640
Immunologic Disorder	B_CELL_RECEPTOR_SIGNALING_PATHWAY	KEGG	2278	29	20	5,24E-01	9,95E-01	rs16897466|rs2510257|rs595872|rs4963867|rs3890760|rs767652|rs13238172|rs11685205|rs9299346|rs6063664|rs2469749|rs11657742|rs7197601|rs1365487|rs1476002|rs13320527|rs4468807|rs12551214|rs1808563|rs17153654
Immunologic Disorder	RAC1_PATHWAY	BIOCARTA	763	32	20	5,25E-01	9,95E-01	rs16897466|rs2555159|rs820363|rs13320527|rs1808563|rs17153654|rs6780561|rs13343175|rs13173003|rs706725|rs2723663|rs1507964|rs32815|rs8096628|rs6449947|rs12536419|rs2161529|rs527589|rs12655019|rs4509829
Immunologic Disorder	ALK_PATHWAY	BIOCARTA	1932	31	20	5,26E-01	9,95E-01	rs3773650|rs991694|rs2029356|rs604045|rs1980499|rs7769728|rs811153|rs2710956|rs2810061|rs12054920|rs7155858|rs3009935|rs6085802|rs6860708|rs7273197|rs9359935|rs10806443|rs7726306|rs4811808|rs7727198
Immunologic Disorder	CYTOSOLIC_TRNA_AMINOACYLATION	REACTOME	312	16	6	5,26E-01	9,95E-01	rs309137|rs2039521|rs556155|rs925210|rs193466|rs7443214
Immunologic Disorder	NOTCH1_INTRACELLULAR_DOMAIN_REGULATES_TRANSCRIPTION	REACTOME	2852	34	20	5,27E-01	9,95E-01	rs2526640|rs6543560|rs435806|rs11685345|rs10512091|rs2285431|rs10005500|rs2378377|rs12553514|rs12308345|rs9636384|rs11794108|rs4857735|rs10247238|rs10780408|rs3782267|rs17288919|rs7856583|rs9642880|rs2655215
Immunologic Disorder	REGULATION_OF_ORNITHINE_DECARBOXYLASE_ODC	REACTOME	773	42	20	5,27E-01	9,95E-01	rs10268808|rs2892190|rs10046115|rs1957107|rs757233|rs807293|rs764190|rs1064825|rs6575122|rs2293361|rs2716601|rs12436216|rs16972457|rs228271|rs8075668|rs7105122|rs818169|rs117821|rs16930278|rs8066176
Immunologic Disorder	CYTOKINE_PATHWAY	BIOCARTA	491	28	17	5,28E-01	9,95E-01	rs9847023|rs7179134|rs2243131|rs7046061|rs4315909|rs740002|rs8193036|rs413917|rs2421057|rs1741137|rs2048874|rs12907723|rs2322180|rs10030060|rs11177048|rs6895787|rs8193040
Immunologic Disorder	PREFOLDIN_MEDIATED_TRANSFER_OF_SUBSTRATE_TO_CCT_TRIC	REACTOME	288	11	4	5,29E-01	9,95E-01	rs6865121|rs2292267|rs11080567|rs2272313
Immunologic Disorder	REGULATION_OF_MITOTIC_CELL_CYCLE	REACTOME	1318	40	20	5,30E-01	9,95E-01	rs10268808|rs7074757|rs2892190|rs6457689|rs7683090|rs1801376|rs4921283|rs9876561|rs1957107|rs807293|rs6736093|rs764190|rs1063302|rs6575122|rs2293361|rs2716601|rs8058610|rs12436216|rs16972457|rs228271
Immunologic Disorder	SIGNALING_BY_ERBB4	REACTOME	3434	24	20	5,30E-01	9,95E-01	rs2102996|rs16897466|rs17347606|rs12328595|rs13393014|rs4963867|rs10509534|rs7322396|rs776385|rs10876227|rs2919390|rs58162|rs16846161|rs6974790|rs4672649|rs10503904|rs9937072|rs13320527|rs736918|rs6580353
Immunologic Disorder	GLYCOSAMINOGLYCAN_BIOSYNTHESIS_CHONDROITIN_SULFATE	KEGG	1372	33	20	5,30E-01	9,95E-01	rs12445976|rs6783962|rs483981|rs1111994|rs11223864|rs1944866|rs11231168|rs1123373|rs7765174|rs2640882|rs1994788|rs4921664|rs6983139|rs2029737|rs4308750|rs2282608|rs6539155|rs12280153|rs1342761|rs9377154
Immunologic Disorder	EFFECTS_OF_PIP2_HYDROLYSIS	REACTOME	1651	36	20	5,31E-01	9,95E-01	rs4750517|rs11868917|rs706578|rs1499772|rs1880553|rs587198|rs1033910|rs210120|rs10495929|rs7422272|rs10743591|rs3782309|rs1838793|rs7615501|rs4264601|rs7558342|rs4952779|rs4357599|rs798790|rs6751933
Immunologic Disorder	PLATELET_HOMEOSTASIS	REACTOME	4080	24	20	5,32E-01	9,95E-01	rs7126870|rs17540215|rs7907343|rs12797628|rs7117518|rs4855077|rs634651|rs6727414|rs7140817|rs16897963|rs1937701|rs1678002|rs17649232|rs1499772|rs6502751|rs537557|rs12191997|rs1926212|rs11651131|rs4599070
Immunologic Disorder	ADP_SIGNALLING_THROUGH_P2RY12	REACTOME	438	22	15	5,34E-01	9,95E-01	rs4855077|rs304737|rs7140817|rs2886612|rs12488540|rs12704705|rs10498440|rs12886319|rs1253676|rs6770918|rs291353|rs12595160|rs4659946|rs8023017|rs10802797
Immunologic Disorder	MAPK_TARGETS_NUCLEAR_EVENTS_MEDIATED_BY_MAP_KINASES	REACTOME	980	30	20	5,36E-01	9,95E-01	rs11105194|rs3817828|rs1519959|rs12054920|rs1688545|rs6860708|rs8026614|rs2869433|rs7726306|rs7727198|rs1019206|rs17555326|rs3799577|rs3886478|rs7156757|rs741847|rs796038|rs6558135|rs17353559|rs12199759
Immunologic Disorder	SULFUR_AMINO_ACID_METABOLISM	REACTOME	930	32	20	5,38E-01	9,95E-01	rs2682578|rs3793932|rs2028898|rs10058433|rs12768505|rs2599442|rs11083711|rs2160907|rs4130719|rs1030588|rs12964208|rs8141597|rs1252109|rs12148892|rs574389|rs12411782|rs7446290|rs13300268|rs10515868|rs11750906
Immunologic Disorder	INTEGRIN_CELL_SURFACE_INTERACTIONS	REACTOME	2614	31	20	5,41E-01	9,95E-01	rs2976230|rs1757096|rs2152305|rs12620429|rs723188|rs4811068|rs170209|rs6436654|rs12203956|rs2521206|rs11102314|rs9515201|rs13080492|rs1537745|rs3212588|rs787033|rs2488320|rs4675141|rs13163497|rs17442186
Immunologic Disorder	GLYCOLYSIS	REACTOME	558	16	10	5,42E-01	9,95E-01	rs11251480|rs923109|rs764223|rs9419445|rs4433480|rs10903880|rs11251555|rs10903900|rs1341928|rs1535694
Immunologic Disorder	DNA_REPLICATION	KEGG	740	33	17	5,43E-01	9,95E-01	rs7322765|rs9540340|rs2073032|rs17482047|rs9570962|rs16981247|rs12602230|rs4943189|rs3786136|rs1832265|rs9535573|rs9539881|rs2164210|rs13082666|rs10258834|rs309126|rs7357092
Immunologic Disorder	GCR_PATHWAY	BIOCARTA	736	32	20	5,44E-01	9,95E-01	rs16897466|rs11934749|rs7671780|rs6888329|rs13320527|rs17153654|rs6580285|rs1125132|rs12650894|rs13173003|rs706725|rs17100410|rs12562819|rs6449947|rs12536419|rs2161529|rs13002829|rs10515074|rs6871452|rs815802
Immunologic Disorder	PD1_SIGNALING	REACTOME	398	37	12	5,44E-01	9,95E-01	rs987870|rs4143813|rs17500510|rs1378942|rs704856|rs9276291|rs695161|rs2995094|rs16923198|rs858490|rs1004420|rs7865645
Immunologic Disorder	GLUCAGON_TYPE_LIGAND_RECEPTORS	REACTOME	786	30	20	5,45E-01	9,95E-01	rs4855077|rs7140817|rs12674049|rs1410489|rs4383344|rs2756117|rs1124425|rs12488540|rs7767039|rs1988035|rs12704705|rs399867|rs1981701|rs10498440|rs3761656|rs16940526|rs7803859|rs12886319|rs1253676|rs1007783
Immunologic Disorder	NEUROTROPHIN_SIGNALING_PATHWAY	KEGG	3689	35	20	5,46E-01	9,95E-01	rs16897466|rs17201100|rs10858075|rs8033590|rs171853|rs11066320|rs4963867|rs11638486|rs11612899|rs28628360|rs10897495|rs2396303|rs8030107|rs3817828|rs10849265|rs11102314|rs11657742|rs2296950|rs7197601|rs1125467
Immunologic Disorder	RIG_I_LIKE_RECEPTOR_SIGNALING_PATHWAY	KEGG	1361	31	20	5,47E-01	9,95E-01	rs4680551|rs11868917|rs12446199|rs2243131|rs2076756|rs9359935|rs7046061|rs4315909|rs10806443|rs2869433|rs10878177|rs3769823|rs710092|rs6924218|rs284513|rs1710627|rs7746039|rs2421057|rs8904|rs2103994
Immunologic Disorder	MHC_CLASS_II_ANTIGEN_PRESENTATION	REACTOME	2226	36	20	5,48E-01	9,95E-01	rs987870|rs716097|rs2588568|rs10490815|rs1800649|rs6785206|rs7235655|rs9309485|rs709641|rs6966540|rs1057452|rs7935469|rs2299268|rs1299525|rs277311|rs2045418|rs4839|rs9907283|rs8078165|rs17500510
Immunologic Disorder	IL7_PATHWAY	BIOCARTA	699	26	20	5,48E-01	9,95E-01	rs16897466|rs11567697|rs13218316|rs13320527|rs17136507|rs17153654|rs1016860|rs13173003|rs956572|rs16906594|rs706725|rs695161|rs4808100|rs1296720|rs9900213|rs6449947|rs12536419|rs1227631|rs2161529|rs11153318
Immunologic Disorder	HEPARAN_SULFATE_HEPARIN_HS_GAG_METABOLISM	REACTOME	3879	26	20	5,49E-01	9,95E-01	rs9890008|rs7938320|rs11696078|rs9999101|rs3785690|rs2865155|rs9924390|rs2284277|rs1859906|rs3845669|rs1111994|rs7986931|rs517231|rs12615148|rs11223864|rs12855016|rs1581826|rs1944866|rs11231168|rs9426785
Immunologic Disorder	CELL_ADHESION_MOLECULES_CAMS	KEGG	8813	41	20	5,51E-01	9,95E-01	rs3802901|rs11564334|rs12621581|rs2446886|rs10277969|rs3115612|rs11215593|rs17749762|rs12620429|rs987870|rs11639525|rs10216570|rs7809670|rs886972|rs4378885|rs2284277|rs12598326|rs2827029|rs1917950|rs6790921
Immunologic Disorder	MITOTIC_M_M_G1_PHASES	REACTOME	3176	44	20	5,51E-01	9,95E-01	rs2090500|rs10268808|rs7074757|rs1582225|rs2892190|rs7244453|rs10490815|rs1678002|rs6457689|rs7683090|rs1801376|rs12355683|rs709641|rs2073032|rs1506048|rs2045418|rs9907283|rs7309390|rs1957107|rs7211218
Immunologic Disorder	STRIATED_MUSCLE_CONTRACTION	REACTOME	453	20	13	5,51E-01	9,95E-01	rs1124605|rs1934158|rs1363950|rs8003073|rs7248997|rs201615|rs1263784|rs707210|rs178638|rs742698|rs12147570|rs4509829|rs16922319
Immunologic Disorder	REGULATION_OF_ACTIN_CYTOSKELETON	KEGG	7089	31	20	5,53E-01	9,95E-01	rs16897466|rs6491679|rs2510257|rs746347|rs8111317|rs37244|rs2976230|rs208354|rs17202361|rs12620429|rs1711782|rs170209|rs10925763|rs4963867|rs831207|rs17405661|rs503473|rs2409416|rs13052373|rs16838192
Immunologic Disorder	PHOSPHORYLATION_OF_CD3_AND_TCR_ZETA_CHAINS	REACTOME	521	42	14	5,54E-01	9,95E-01	rs987870|rs7815195|rs7824819|rs17500510|rs1378942|rs704856|rs2705497|rs9276291|rs695161|rs4445178|rs2995094|rs858490|rs12726043|rs1004420
Immunologic Disorder	NUCLEOTIDE_BINDING_DOMAIN_LEUCINE_RICH_REPEAT_CONTAINING_RECEPTOR_NLR<br>YS	REACTOME	1337	30	20	5,58E-01	9,95E-01	rs12446199|rs466609|rs2066843|rs400819|rs4077515|rs4925654|rs9359935|rs10806443|rs3769823|rs457581|rs6125883|rs6961625|rs10925019|rs1016860|rs6924218|rs284513|rs956572|rs2829946|rs1613367|rs7746039
Immunologic Disorder	SIGNALING_BY_FGFR_MUTANTS	REACTOME	1657	32	20	5,58E-01	9,95E-01	rs16897466|rs4769946|rs4963867|rs404284|rs2517037|rs823600|rs12537526|rs5996534|rs13262310|rs666028|rs2038712|rs10870911|rs13320527|rs3787011|rs3129596|rs10784767|rs13173003|rs13249548|rs3024908|rs706725
Immunologic Disorder	REGULATION_OF_INSULIN_SECRETION	REACTOME	3741	28	20	5,59E-01	9,95E-01	rs10485723|rs227130|rs2423360|rs12545028|rs954385|rs10925763|rs4855077|rs304737|rs7140817|rs7659414|rs8027776|rs11195623|rs16838192|rs3792747|rs4832505|rs2123861|rs17062878|rs11102314|rs1026483|rs8115925
Immunologic Disorder	GAB1_SIGNALOSOME	REACTOME	1077	27	20	5,60E-01	9,95E-01	rs16897466|rs10509534|rs7322396|rs10876227|rs6974790|rs13320527|rs7815195|rs7824819|rs1378942|rs7991808|rs4489281|rs13173003|rs706725|rs7241455|rs1140475|rs874269|rs4445178|rs6449947|rs9480865|rs683590
Immunologic Disorder	PTDINS_PATHWAY	BIOCARTA	1058	38	20	5,61E-01	9,95E-01	rs2094347|rs2510257|rs2423539|rs11251480|rs6131146|rs10495929|rs1838793|rs9419445|rs4264601|rs7558342|rs4952779|rs6751933|rs4433480|rs10903880|rs11251555|rs2181219|rs4760516|rs10903900|rs7585985|rs1562653
Immunologic Disorder	DARPP_32_EVENTS	REACTOME	879	54	20	5,61E-01	9,95E-01	rs12046844|rs298026|rs271831|rs35305|rs28044|rs11125132|rs2302234|rs1867724|rs3804357|rs10781329|rs6869149|rs10064439|rs13002829|rs13176438|rs7732249|rs1208797|rs6696880|rs2238646|rs2303929|rs1848116
Immunologic Disorder	BCELLSURVIVAL_PATHWAY	BIOCARTA	500	35	19	5,61E-01	9,95E-01	rs16897466|rs2488320|rs13163497|rs13320527|rs7211218|rs17153654|rs4587680|rs13173003|rs11009195|rs706725|rs741847|rs2277922|rs3765141|rs6449947|rs12536419|rs2161529|rs10515074|rs6871452|rs2475193
Immunologic Disorder	VIP_PATHWAY	BIOCARTA	1000	26	20	5,62E-01	9,95E-01	rs12674049|rs6063664|rs2469749|rs1410489|rs9642880|rs7812998|rs7039739|rs17786782|rs2756117|rs6021266|rs1124425|rs1125132|rs2302234|rs2033098|rs7767039|rs399867|rs3804357|rs10781329|rs6063634|rs1997815
Immunologic Disorder	ETHER_LIPID_METABOLISM	KEGG	1041	43	20	5,66E-01	9,95E-01	rs6870653|rs12904722|rs1124605|rs10930785|rs1341347|rs3826706|rs11689608|rs6672057|rs6780561|rs3733796|rs2261612|rs10497508|rs10798106|rs1093733|rs11810204|rs17544558|rs904729|rs4912230|rs2292023|rs7980436
Immunologic Disorder	LIPOPROTEIN_METABOLISM	REACTOME	1037	30	20	5,67E-01	9,95E-01	rs4775073|rs10516451|rs2487052|rs9426785|rs97458|rs1581077|rs7099855|rs1607267|rs261337|rs3857979|rs881980|rs701089|rs4765600|rs10991522|rs4783961|rs754524|rs9644636|rs312046|rs4666291|rs4715
Immunologic Disorder	EPITHELIAL_CELL_SIGNALING_IN_HELICOBACTER_PYLORI_INFECTION	KEGG	1535	35	20	5,69E-01	9,95E-01	rs3794176|rs1719361|rs11066320|rs4674257|rs1512348|rs6974790|rs896189|rs7197601|rs2454016|rs7562668|rs4767879|rs2869433|rs1378942|rs6473885|rs1259158|rs6961625|rs11644646|rs17793122|rs3093988|rs4150196
Immunologic Disorder	SIGNALING_BY_INSULIN_RECEPTOR	REACTOME	3432	33	20	5,69E-01	9,95E-01	rs16897466|rs17201100|rs3794176|rs2212963|rs10248619|rs4963867|rs11082159|rs6952398|rs17510142|rs2396303|rs2517037|rs12419388|rs1512348|rs823600|rs896189|rs13262310|rs4297784|rs666028|rs2038712|rs17762352
Immunologic Disorder	IL1_SIGNALING	REACTOME	1322	40	20	5,71E-01	9,95E-01	rs1679802|rs7628250|rs2066843|rs1030021|rs4901623|rs11883987|rs9359935|rs17196143|rs10806443|rs6125883|rs6961625|rs6924218|rs284513|rs7746039|rs10808338|rs2440134|rs17090892|rs2103994|rs2334659|rs4097
Immunologic Disorder	ASSEMBLY_OF_THE_PRE_REPLICATIVE_COMPLEX	REACTOME	911	36	20	5,71E-01	9,95E-01	rs10268808|rs2892190|rs6457689|rs2073032|rs1957107|rs807293|rs764190|rs9348428|rs6575122|rs4727590|rs2293361|rs2716601|rs12436216|rs16972457|rs228271|rs8075668|rs2164210|rs13082666|rs7105122|rs117821
Immunologic Disorder	APC_C_CDH1_MEDIATED_DEGRADATION_OF_CDC20_AND_OTHER_APC_C_CDH1_TARGET<br>TE_MITOSIS_EARLY_G1	REACTOME	959	35	20	5,72E-01	9,95E-01	rs10268808|rs2892190|rs10046115|rs4921283|rs9876561|rs1957107|rs807293|rs6736093|rs764190|rs6575122|rs2293361|rs2716601|rs8058610|rs12436216|rs16972457|rs228271|rs8075668|rs7105122|rs1994174|rs12352784
Immunologic Disorder	DIABETES_PATHWAYS	REACTOME	3395	45	20	5,72E-01	9,95E-01	rs7038715|rs12133739|rs13331733|rs634454|rs17233136|rs374560|rs4542003|rs10490815|rs7659414|rs3740526|rs8027776|rs3846211|rs1869241|rs3792747|rs2123861|rs17062878|rs9309485|rs1026483|rs2035567|rs7024667
Immunologic Disorder	ERBB_SIGNALING_PATHWAY	KEGG	4135	27	20	5,72E-01	9,95E-01	rs2102996|rs16897466|rs17347606|rs17099627|rs12328595|rs9870951|rs13393014|rs4963867|rs7648548|rs776385|rs2919390|rs16846161|rs11657742|rs6974790|rs4076319|rs4672649|rs6118746|rs10503904|rs9642880|rs11632939
Immunologic Disorder	PRE_NOTCH_TRANSCRIPTION_AND_TRANSLATION	REACTOME	835	32	15	5,73E-01	9,95E-01	rs12103149|rs10005500|rs204995|rs17288919|rs693797|rs17136507|rs12417347|rs9348428|rs17825554|rs1296720|rs2948091|rs677598|rs7405515|rs795981|rs795987
Immunologic Disorder	CCR3_PATHWAY	BIOCARTA	939	30	19	5,74E-01	9,95E-01	rs10485723|rs227130|rs2423360|rs8115925|rs7039739|rs17786782|rs1015092|rs2745755|rs4381631|rs8081614|rs4791036|rs4669700|rs11044171|rs2876140|rs169143|rs4509829|rs6086495|rs7219495|rs6055912
Immunologic Disorder	INSULIN_RECEPTOR_SIGNALLING_CASCADE	REACTOME	3006	30	20	5,74E-01	9,95E-01	rs16897466|rs17201100|rs2212963|rs10248619|rs4963867|rs11082159|rs6952398|rs17510142|rs2396303|rs2517037|rs12419388|rs823601|rs13262310|rs4297784|rs666028|rs2038712|rs17762352|rs10870911|rs10908200|rs10889011
Immunologic Disorder	COLORECTAL_CANCER	KEGG	2539	27	20	5,76E-01	9,95E-01	rs16897466|rs10502934|rs3773650|rs991694|rs4963867|rs604045|rs7022409|rs2094405|rs11657742|rs9642880|rs3009935|rs931771|rs4129439|rs1476002|rs266335|rs13320527|rs7812998|rs727514|rs2869433|rs7211218
Immunologic Disorder	LAGGING_STRAND_SYNTHESIS	REACTOME	550	22	11	5,76E-01	9,95E-01	rs9540340|rs17482047|rs9570962|rs16981247|rs12602230|rs4943189|rs3786136|rs1832265|rs9539881|rs10258834|rs7357092
Immunologic Disorder	TGF_BETA_SIGNALING_PATHWAY	KEGG	3535	37	20	5,76E-01	9,95E-01	rs11693395|rs6706968|rs3773650|rs991694|rs7239317|rs535343|rs218225|rs604045|rs1980499|rs983072|rs7769728|rs17642074|rs811153|rs2810061|rs9368070|rs9642880|rs2630720|rs7155858|rs3009935|rs6085802
Immunologic Disorder	IL2RB_PATHWAY	BIOCARTA	1296	33	20	5,79E-01	9,95E-01	rs16897466|rs17201100|rs7910825|rs12765241|rs2396303|rs9642880|rs13320527|rs7812998|rs17153654|rs1016860|rs13173003|rs2033098|rs956572|rs706725|rs4808100|rs741847|rs2396306|rs9900213|rs13398058|rs6449947
Immunologic Disorder	RENIN_ANGIOTENSIN_SYSTEM	KEGG	467	12	9	5,79E-01	9,95E-01	rs4932145|rs7714122|rs252628|rs1466097|rs2303138|rs3772622|rs9289775|rs2028299|rs418757
Immunologic Disorder	INSULIN_RECEPTOR_RECYCLING	REACTOME	492	39	17	5,80E-01	9,95E-01	rs3794176|rs1512348|rs896189|rs2454016|rs7562668|rs6473885|rs3093988|rs6713990|rs1004446|rs1532222|rs902130|rs721950|rs34883124|rs4960948|rs7579208|rs13236345|rs4247374
Immunologic Disorder	TEL_PATHWAY	BIOCARTA	978	24	20	5,82E-01	9,95E-01	rs4963867|rs2678681|rs9644708|rs11776114|rs6974790|rs2184282|rs9642880|rs7812998|rs10794486|rs1016860|rs2684796|rs2033098|rs956572|rs1140475|rs4381631|rs8081614|rs4791036|rs17388587|rs4552863|rs16902328
Immunologic Disorder	PYRUVATE_METABOLISM_AND_CITRIC_ACID_TCA_CYCLE	REACTOME	905	31	18	5,86E-01	9,95E-01	rs3735477|rs4832076|rs693163|rs2076370|rs11032981|rs9649679|rs11983987|rs10496307|rs2647209|rs1539624|rs11710823|rs3133978|rs13428219|rs1808417|rs7647085|rs7001334|rs12155014|rs10193241
Immunologic Disorder	HS_GAG_DEGRADATION	REACTOME	1386	33	20	5,87E-01	9,95E-01	rs2284277|rs7986556|rs12855016|rs1581826|rs9426785|rs7325810|rs4773614|rs4505759|rs4889841|rs1581077|rs12855484|rs959390|rs9584214|rs7329232|rs7992498|rs497836|rs1070073|rs12219903|rs1316929|rs1373838
Immunologic Disorder	CYTOCHROME_P450_ARRANGED_BY_SUBSTRATE_TYPE	REACTOME	1163	58	20	5,87E-01	9,95E-01	rs2572023|rs1290622|rs1558139|rs6467|rs759998|rs3764561|rs1042389|rs915908|rs2279345|rs2470890|rs2058265|rs4808358|rs4736349|rs695149|rs11572285|rs10883782|rs7271624|rs12192544|rs11085969|rs10500804
Immunologic Disorder	UNFOLDED_PROTEIN_RESPONSE	REACTOME	1176	43	20	5,88E-01	9,95E-01	rs13331733|rs10490815|rs3740526|rs3846211|rs1869241|rs9309485|rs7024667|rs970952|rs7633722|rs2437215|rs2218972|rs478309|rs9817549|rs1545229|rs8047140|rs12467314|rs4939627|rs4783961|rs7571971|rs3829078
Immunologic Disorder	PEROXISOMAL_LIPID_METABOLISM	REACTOME	435	26	13	5,89E-01	9,95E-01	rs11934603|rs10930785|rs531592|rs11689608|rs1050146|rs4148828|rs10497508|rs7218656|rs7194178|rs2560718|rs11129780|rs12369827|rs4681689
Immunologic Disorder	SIGNALING_BY_NOTCH1	REACTOME	3563	31	20	5,90E-01	9,95E-01	rs2526640|rs6543560|rs2094347|rs435806|rs11685345|rs10512091|rs2423539|rs2285431|rs10005500|rs2378377|rs12553514|rs12308345|rs9636384|rs11794108|rs4857735|rs10247238|rs10780408|rs3782267|rs17288919|rs7856583
Immunologic Disorder	SIGNALLING_BY_NGF	REACTOME	7869	25	20	5,91E-01	9,95E-01	rs7162113|rs16897466|rs17201100|rs1009619|rs11929318|rs10514211|rs10858075|rs894628|rs4703825|rs4965900|rs12545028|rs2137110|rs4963867|rs28628360|rs10509534|rs7022409|rs503473|rs13098182|rs2409416|rs4890408
Immunologic Disorder	CK1_PATHWAY	BIOCARTA	759	27	19	5,91E-01	9,95E-01	rs10485723|rs227130|rs2423360|rs8115925|rs2302234|rs1015092|rs2745755|rs3804357|rs10781329|rs2876140|rs265976|rs3186454|rs4896857|rs6086495|rs6864341|rs2303929|rs1848116|rs6055912|rs2732514
Immunologic Disorder	TOLL_RECEPTOR_CASCADES	REACTOME	3778	37	20	5,93E-01	9,95E-01	rs1679802|rs1009619|rs3808969|rs1943595|rs466609|rs1242119|rs11082159|rs317985|rs2066843|rs400819|rs3817828|rs204995|rs4297784|rs1519959|rs12054920|rs1930714|rs1392125|rs7691384|rs7197601|rs1410201
Immunologic Disorder	NOD1_2_SIGNALING_PATHWAY	REACTOME	881	33	20	5,94E-01	9,95E-01	rs12446199|rs2066843|rs4077515|rs9359935|rs10806443|rs3769823|rs6125883|rs6961625|rs6924218|rs284513|rs1613367|rs7746039|rs2440134|rs2103994|rs1145922|rs12206804|rs640353|rs1862710|rs11070318|rs331455
Immunologic Disorder	A_TETRASACCHARIDE_LINKER_SEQUENCE_IS_REQUIRED_FOR_GAG_SYNTHESIS	REACTOME	1731	29	20	5,97E-01	9,95E-01	rs2284277|rs1111994|rs7986931|rs11223864|rs12855016|rs1581826|rs1944866|rs11231168|rs9426785|rs7325810|rs380331|rs11581077|rs7765174|rs12855484|rs959390|rs17422291|rs6785758|rs9584214|rs7329232|rs7315891
Immunologic Disorder	TRAF6_MEDIATED_INDUCTION_OF_NFKB_AND_MAP_KINASES_UPON_TLR7_8_OR_9_ACT	REACTOME	2239	31	20	5,98E-01	9,95E-01	rs1679802|rs11105194|rs466609|rs2066843|rs400819|rs3817828|rs204995|rs1519959|rs12054920|rs1688545|rs4901623|rs6860708|rs8026614|rs9359935|rs10806443|rs2869433|rs457581|rs6125883|rs7726306|rs3006475
Immunologic Disorder	ABC_TRANSPORTERS	KEGG	2684	47	20	5,98E-01	9,95E-01	rs7330196|rs2487052|rs8081909|rs12921748|rs6532059|rs10521168|rs12493550|rs10236459|rs11654057|rs4147967|rs1678388|rs1283798|rs323073|rs4781699|rs519236|rs4148407|rs4147819|rs2071482|rs17205859|rs4148828
Immunologic Disorder	NUCLEAR_SIGNALING_BY_ERBB4	REACTOME	2231	25	20	6,01E-01	9,95E-01	rs2102996|rs17347606|rs12328595|rs13393014|rs776385|rs2919390|rs518162|rs16846161|rs4672649|rs9937072|rs736918|rs6580353|rs6872878|rs10497961|rs1904836|rs7184686|rs7826312|rs4150196|rs1481756
Immunologic Disorder	VIRAL_MYOCARDITIS	KEGG	2750	52	20	6,02E-01	9,95E-01	rs3767248|rs1641661|rs1610751|rs987870|rs1784873|rs12203956|rs2824321|rs8060975|rs1611493|rs215573|rs2012928|rs8003073|rs28367832|rs7732608|rs1476002|rs13218316|rs17500510|rs3769823|rs9276933|rs3135342
Immunologic Disorder	TIGHT_JUNCTION_INTERACTIONS	REACTOME	715	31	12	6,03E-01	9,95E-01	rs4378885|rs9856741|rs11764572|rs6700677|rs1160769|rs1002386|rs2476997|rs1985313|rs532413|rs12748525|rs6979|rs9676180
Immunologic Disorder	INFLAMMASOMES	REACTOME	468	23	17	6,04E-01	9,95E-01	rs466609|rs400819|rs4925654|rs457581|rs10925019|rs1016860|rs956572|rs2829946|rs1613367|rs1076420|rs880888|rs1653583|rs9988572|rs3806265|rs17178663|rs12454650|rs524533
Immunologic Disorder	LATENT_INFECTION_OF_HOMO_SAPIENS_WITH_MYCOBACTERIUM_TUBERCULOSIS	REACTOME	589	42	19	6,06E-01	9,95E-01	rs3794176|rs1512348|rs17078876|rs896189|rs2454016|rs7562668|rs13259308|rs3093988|rs6713990|rs5995351|rs1532222|rs902130|rs721950|rs4960948|rs7579208|rs2889854|rs13236345|rs4492907|rs1004356
Immunologic Disorder	VOLTAGE_GATED_POTASSIUM_CHANNELS	REACTOME	2491	27	20	6,07E-01	9,95E-01	rs9688792|rs11102357|rs4809563|rs85276|rs17811161|rs12134651|rs639622|rs13264567|rs4832505|rs7552646|rs1883767|rs1496328|rs9860501|rs2784140|rs12741034|rs674163|rs7156354|rs2673576|rs7548910|rs7528073
Immunologic Disorder	CTLA4_PATHWAY	BIOCARTA	662	29	20	6,08E-01	9,95E-01	rs16897466|rs11066320|rs13320527|rs4767879|rs3135342|rs704856|rs13173003|rs2681417|rs2395182|rs706725|rs695161|rs4675391|rs6449947|rs2995094|rs2161529|rs3135363|rs858490|rs1004420|rs10515074|rs2332096
Immunologic Disorder	INFLUENZA_VIRAL_RNA_TRANSCRIPTION_AND_REPLICATION	REACTOME	1153	27	20	6,09E-01	9,95E-01	rs3365|rs4599669|rs10824609|rs17510142|rs7219604|rs1561|rs1848466|rs1007482|rs457372|rs11902670|rs1115882|rs4487686|rs1249123|rs2851387|rs2436526|rs4979816|rs13112870|rs13325754|rs12879285|rs3745624
Immunologic Disorder	FMLP_PATHWAY	BIOCARTA	1148	27	20	6,10E-01	9,95E-01	rs10485723|rs227130|rs2423360|rs6063664|rs2469749|rs8115925|rs6021266|rs11125132|rs1015092|rs2745755|rs3804357|rs6063634|rs1997815|rs2440134|rs8904|rs17695032|rs11044171|rs2876140|rs10140922|rs13002829
Immunologic Disorder	ERK5_PATHWAY	BIOCARTA	595	32	19	6,12E-01	9,95E-01	rs16897466|rs12054920|rs6860708|rs8026614|rs13320527|rs7726306|rs17153654|rs7727198|rs13173003|rs3886478|rs706725|rs6449947|rs12536419|rs2161529|rs10515074|rs6871452|rs4534823|rs7168482|rs16903232
Immunologic Disorder	INHIBITION_OF_THE_PROTEOLYTIC_ACTIVITY_OF_APC_C_REQUIRED_FOR_THE_ONSET_<br>TOTIC_SPINDLE_CHECKPOINT_COMPONENTS	REACTOME	365	32	15	6,14E-01	9,95E-01	rs7074757|rs7683090|rs1801376|rs9876561|rs6736093|rs12644766|rs1994174|rs12352784|rs10794599|rs1027692|rs7588325|rs436862|rs9590483|rs17663924|rs1801389
Immunologic Disorder	CYSTEINE_AND_METHIONINE_METABOLISM	KEGG	982	36	20	6,14E-01	9,95E-01	rs3793932|rs2038574|rs2028898|rs10058433|rs12768505|rs4788547|rs2160907|rs13380644|rs4130719|rs11631023|rs4511520|rs1465825|rs8141597|rs1252109|rs11254413|rs12411782|rs9934713|rs8049025|rs2016089|rs4544229
Immunologic Disorder	HISTIDINE_METABOLISM	KEGG	654	38	18	6,14E-01	9,95E-01	rs3794176|rs10918239|rs4578194|rs10918228|rs1799388|rs16837531|rs12964208|rs12554375|rs6738271|rs1465936|rs7487781|rs4954961|rs4646785|rs1455996|rs6566816|rs2071048|rs2241136|rs12301817
Immunologic Disorder	AUTODEGRADATION_OF_CDH1_BY_CDH1_APC_C	REACTOME	857	44	20	6,14E-01	9,95E-01	rs10268808|rs2892190|rs10046115|rs9876561|rs1957107|rs807293|rs6736093|rs764190|rs6575122|rs2716601|rs12436216|rs16972457|rs228271|rs8075668|rs7105122|rs1994174|rs12352784|rs117821|rs16930278|rs4369862
Immunologic Disorder	PYRUVATE_METABOLISM	KEGG	908	36	20	6,16E-01	9,95E-01	rs4795194|rs10918239|rs4578194|rs10918228|rs4235653|rs7107498|rs9649679|rs10849926|rs11983987|rs11631023|rs4129215|rs507211|rs6025645|rs10163789|rs11710823|rs12554375|rs954241|rs12503593|rs582282|rs10501622
Immunologic Disorder	IL22BP_PATHWAY	BIOCARTA	296	11	9	6,17E-01	9,95E-01	rs741347|rs12100005|rs3024908|rs7560255|rs4808100|rs2598483|rs9900213|rs5029846|rs8074003

Immunologic Disorder	PKB_MEDIATED_EVENTS	REACTOME	583	30	15	6,19E-01	9,95E-01	rs6952398|rs17762352|rs10889011|rs2796498|rs9568196|rs6950343|rs2589142|rs9591258|rs11023350|rs874269|rs4425665|rs7805942|rs7975725|rs6565498|rs12930464
Immunologic Disorder	MYD88_MAL_CASCADE_INITIATED_ON_PLASMA_MEMBRANE	REACTOME	2527	30	20	6,22E-01	9,95E-01	rs1679802|rs11105194|rs466609|rs2066843|rs400819|rs3817828|rs204995|rs1519959|rs12054920|rs1930714|rs1392125|rs1410201|rs1329049|rs1893352|rs637462|rs1688545|rs4901623|rs6860708|rs8026614|rs9359935
Immunologic Disorder	PI3K_CASCADE	REACTOME	2702	31	20	6,22E-01	9,95E-01	rs16897466|rs17201100|rs1943595|rs11082159|rs6952398|rs17510142|rs2396303|rs2517037|rs12419388|rs823600|rs13262310|rs4297784|rs666028|rs2038712|rs17762352|rs10870911|rs10908200|rs10889011|rs1865275|rs13320527
Immunologic Disorder	NTHI_PATHWAY	BIOCARTA	876	33	20	6,23E-01	9,95E-01	rs3773650|rs991694|rs9359935|rs266335|rs17136507|rs10806443|rs6580285|rs6924218|rs284513|rs17100410|rs7746039|rs2440134|rs6792117|rs1296720|rs8904|rs2616052|rs1019855|rs10140922|rs11129780|rs920293
Immunologic Disorder	TYROSINE_METABOLISM	KEGG	1169	36	20	6,24E-01	9,95E-01	rs3793932|rs3794176|rs973330|rs12768505|rs12292212|rs4788547|rs13380644|rs7048594|rs4511520|rs1370275|rs1004446|rs1238741|rs1465936|rs4646785|rs2654846|rs9934713|rs2073835|rs2075508|rs8049025|rs4297126
Immunologic Disorder	INSULIN_SIGNALING_PATHWAY	KEGG	3744	28	20	6,24E-01	9,95E-01	rs16897466|rs17201100|rs1509241|rs4811068|rs9870951|rs4963867|rs4795194|rs6510636|rs3890760|rs6952398|rs7322396|rs7648548|rs2396303|rs501603|rs11657742|rs10998736|rs7303397|rs1427988|rs2296950|rs4933211
Immunologic Disorder	G_ALPHA_Z_SIGNALLING_EVENTS	REACTOME	2191	32	20	6,25E-01	9,95E-01	rs4750517|rs12545028|rs954385|rs4855077|rs304737|rs7140817|rs2532019|rs11195623|rs13168690|rs587198|rs1033910|rs10495929|rs9922362|rs2886612|rs1838793|rs7615501|rs4629562|rs2790090|rs4264601|rs7558342
Immunologic Disorder	LINOLEIC_ACID_METABOLISM	KEGG	716	31	14	6,25E-01	9,95E-01	rs2572023|rs12904722|rs6672057|rs915908|rs2261612|rs2470890|rs10798106|rs1093733|rs17544558|rs12086010|rs11572285|rs10798080|rs10494603|rs1536827
Immunologic Disorder	SIGNALING_BY_THE_B_CELL_RECEPTOR_BCR	REACTOME	3426	32	20	6,26E-01	9,95E-01	rs7126870|rs16897466|rs10268808|rs9870951|rs4963867|rs2892190|rs10509534|rs7322396|rs6457689|rs13238172|rs7648548|rs11685205|rs10876227|rs210120|rs7197601|rs10743591|rs9359935|rs13218316|rs3782309|rs1957107
Immunologic Disorder	NFKB_PATHWAY	BIOCARTA	870	45	20	6,26E-01	9,95E-01	rs1930714|rs1030021|rs1410201|rs1329049|rs11883987|rs9359935|rs1928295|rs10806443|rs10984022|rs1996215|rs6924218|rs284513|rs7746039|rs2806112|rs8904|rs2616052|rs12206804|rs2048874|rs10140922|rs640353
Immunologic Disorder	GH_PATHWAY	BIOCARTA	963	36	20	6,26E-01	9,95E-01	rs16897466|rs17201100|rs2396303|rs13320527|rs6872878|rs17153654|rs13173003|rs706725|rs11065374|rs1004446|rs2396306|rs4381631|rs8081614|rs4791036|rs9900213|rs13398058|rs6449947|rs12536419|rs2161529|rs169143
Immunologic Disorder	SHC_MEDIATED_CASCADE	REACTOME	1070	38	20	6,27E-01	9,95E-01	rs4963867|rs17510142|rs2517037|rs12419388|rs823600|rs13262310|rs666028|rs2038712|rs10870911|rs10908200|rs3787011|rs3129596|rs13249548|rs9509959|rs11745375|rs451643|rs249923|rs17388587|rs475738|rs9509982
Immunologic Disorder	ALPHA_LINOLENIC_ACID_METABOLISM	KEGG	571	30	13	6,27E-01	9,95E-01	rs12904722|rs11934603|rs6672057|rs2261612|rs10798106|rs7482316|rs1093733|rs7218656|rs17544558|rs17194178|rs12086010|rs6665890|rs7528851
Immunologic Disorder	ORC1_REMOVAL_FROM_CHROMATIN	REACTOME	930	40	20	6,29E-01	9,95E-01	rs10268808|rs2892190|rs10046115|rs2073032|rs1957107|rs807293|rs764190|rs6575122|rs4727590|rs2293361|rs2716601|rs12436216|rs16972457|rs228271|rs8075668|rs2164210|rs13082666|rs7105122|rs117821|rs16930278
Immunologic Disorder	CELL_SURFACE_INTERACTIONS_AT_THE_VASCULAR_WALL	REACTOME	2650	35	20	6,32E-01	9,95E-01	rs3802901|rs16897466|rs12620429|rs4963867|rs1784873|rs4848901|rs2521206|rs2824321|rs2130007|rs2076370|rs97458|rs603085|rs1433176|rs17575616|rs2488320|rs10145863|rs202490|rs13218316|rs13320527|rs1259158
Immunologic Disorder	MTOR_SIGNALING_PATHWAY	KEGG	1290	30	20	6,35E-01	9,95E-01	rs16897466|rs3817828|rs11657742|rs382649|rs17762352|rs10889011|rs10520358|rs13320527|rs13184620|rs2796498|rs17153654|rs9568196|rs13110230|rs13173003|rs3799577|rs2589142|rs881536|rs706725|rs9591258|rs3775198
Immunologic Disorder	CDT1_ASSOCIATION_WITH_THE_CDC6_ORC_ORIGIN_COMPLEX	REACTOME	793	44	20	6,35E-01	9,95E-01	rs10268808|rs2892190|rs10046115|rs1957107|rs807293|rs764190|rs6575122|rs4727590|rs2293361|rs2716601|rs12436216|rs16972457|rs228271|rs8075668|rs7105122|rs117821|rs4074236|rs16930278|rs8066176|rs3782115
Immunologic Disorder	DEADENYLATION_OF_MRNA	REACTOME	331	11	5	6,36E-01	9,95E-01	rs10853869|rs2241790|rs7975725|rs11642944|rs1239915
Immunologic Disorder	PHASE_II_CONJUGATION	REACTOME	2059	39	20	6,36E-01	9,95E-01	rs13331733|rs2608836|rs2755427|rs6830865|rs10918239|rs2028898|rs10031344|rs10058433|rs10918228|rs6904935|rs1571858|rs4130719|rs8047140|rs6497501|rs2470890|rs17497947|rs7498905|rs12964208|rs6997340|rs2522420
Immunologic Disorder	PENTOSE_PHOSPHATE_PATHWAY	KEGG	616	32	16	6,37E-01	9,95E-01	rs11251480|rs12995771|rs4691924|rs9419445|rs3782545|rs12642830|rs7633966|rs10794337|rs6963707|rs4433480|rs10903880|rs11251555|rs6531592|rs10903900|rs4908817|rs4687717
Immunologic Disorder	GLUCAGON_SIGNALING_IN_METABOLIC_REGULATION	REACTOME	1044	32	20	6,38E-01	9,95E-01	rs12545028|rs4855077|rs7140817|rs2532019|rs13168690|rs9922362|rs4629562|rs13190668|rs2302234|rs12488540|rs12704705|rs1404909|rs10781329|rs263241|rs10498440|rs3761656|rs12680002|rs12886319|rs7016570|rs7975385
Immunologic Disorder	DESTABILIZATION_OF_MRNA_BY_KSRP	REACTOME	205	12	7	6,40E-01	9,95E-01	rs3740526|rs7024667|rs1545229|rs11642944|rs10973537|rs7814838|rs4291799
Immunologic Disorder	BAD_PATHWAY	BIOCARTA	927	34	20	6,40E-01	9,95E-01	rs16897466|rs3020827|rs1507955|rs13320527|rs10794486|rs17153654|rs1016860|rs2302234|rs2684796|rs13173003|rs956572|rs2270941|rs706725|rs10781329|rs1404909|rs131849|rs2166019|rs6449947|rs12536419|rs2161529
Immunologic Disorder	TASTE_TRANSDUCTION	KEGG	1100	38	20	6,43E-01	9,95E-01	rs11745136|rs12545028|rs17649232|rs4599070|rs210120|rs8113506|rs9461937|rs1370666|rs10409463|rs10781329|rs7139183|rs6944302|rs4701849|rs263241|rs12680002|rs7016570|rs7975385|rs1873246|rs7765294|rs6467133
Immunologic Disorder	GLUTATHIONE_CONJUGATION	REACTOME	407	18	10	6,44E-01	9,95E-01	rs10918239|rs10918228|rs6904935|rs1571858|rs12964208|rs574389|rs795602|rs11807|rs6955931|rs7896809
Immunologic Disorder	MYOGENESIS	REACTOME	1747	27	20	6,44E-01	9,95E-01	rs11564334|rs17749762|rs1437467|rs6089291|rs7963905|rs8031866|rs12054920|rs13381800|rs6860708|rs8026614|rs999367|rs7726306|rs6065062|rs6089510|rs7727198|rs1305277|rs2012125|rs9304511|rs1944343|rs2440134
Immunologic Disorder	3_UTR_MEDIATED_TRANSLATIONAL_REGULATION	REACTOME	1235	27	20	6,45E-01	9,95E-01	rs3365|rs4599669|rs10824609|rs17510142|rs7219604|rs1561|rs1848466|rs1007482|rs457372|rs11902670|rs1115882|rs10955775|rs1249123|rs2851387|rs7825662|rs2436526|rs16888522|rs4979816|rs13325754|rs12879285
Immunologic Disorder	PPAR_SIGNALING_PATHWAY	KEGG	1324	26	20	6,46E-01	9,95E-01	rs7374394|rs7114704|rs1555949|rs501603|rs10182865|rs7858838|rs11934603|rs2130007|rs7533324|rs7561624|rs17351947|rs7125320|rs679596|rs4823529|rs6855734|rs7482316|rs7218656|rs6025645|rs4917339|rs11794310
Immunologic Disorder	APC_CDC20_MEDIATED_DEGRADATION_OF_NEK2A	REACTOME	396	34	17	6,46E-01	9,95E-01	rs7074757|rs7683090|rs1801376|rs9876561|rs6736093|rs10494940|rs12096414|rs12644766|rs1994174|rs12352784|rs10794599|rs1027692|rs17588325|rs4369862|rs9590483|rs17663924|rs1801389
Immunologic Disorder	ADHERENS_JUNCTIONS_INTERACTIONS	REACTOME	5496	35	20	6,46E-01	9,95E-01	rs11564334|rs2446886|rs11215593|rs17749762|rs4782717|rs3101011|rs11639525|rs7709151|rs12444964|rs4353494|rs4518142|rs12598326|rs8055843|rs10472700|rs4782749|rs523223|rs4577701|rs6089291|rs233551|rs1703043
Immunologic Disorder	DNA_REPLICATION	REACTOME	3548	42	20	6,49E-01	9,95E-01	rs2090500|rs10268808|rs7074757|rs11582225|rs2892190|rs7244453|rs10490815|rs1678002|rs10046115|rs7683090|rs1801376|rs12355683|rs9540340|rs709641|rs2073032|rs1506048|rs2045418|rs3815795|rs9907283|rs7309390
Immunologic Disorder	VIF_MEDIATED_DEGRADATION_OF_APOBEC3G	REACTOME	738	42	20	6,50E-01	9,95E-01	rs10268808|rs2892190|rs6457689|rs1957107|rs807293|rs764190|rs6575122|rs2293361|rs2716601|rs12436216|rs16972457|rs228271|rs8075668|rs7105122|rs117821|rs16930278|rs139272|rs8066176|rs3782115|rs12292911
Immunologic Disorder	IL1R_PATHWAY	BIOCARTA	1001	38	20	6,51E-01	9,95E-01	rs7628250|rs604045|rs1030021|rs3009935|rs11883987|rs9359935|rs17196143|rs7046061|rs10806443|rs6924218|rs284513|rs12029576|rs7746039|rs947712|rs2440134|rs8904|rs2616052|rs2334659|rs1152918|rs2048874
Immunologic Disorder	CDK_MEDIATED_PHOSPHORYLATION_AND_REMOVAL_OF_CDC6	REACTOME	698	44	20	6,52E-01	9,95E-01	rs10268808|rs2892190|rs10046115|rs1957107|rs807293|rs764190|rs6575122|rs2293361|rs2716601|rs12436216|rs16972457|rs228271|rs8075668|rs7105122|rs117821|rs16930278|rs8066176|rs3782115|rs12292911|rs4498476
Immunologic Disorder	METABOLISM_OF_MRNA	REACTOME	3017	35	20	6,52E-01	9,95E-01	rs10268808|rs3365|rs2892190|rs4750154|rs4599669|rs10824609|rs240542|rs10853869|rs3740526|rs10046115|rs17510142|rs7219604|rs1561|rs1848466|rs9628|rs7024667|rs1893352|rs3737984|rs1007482|rs457372
Immunologic Disorder	PLATELET_ACTIVATION_SIGNALING_AND_AGGREGATION	REACTOME	6965	24	20	6,52E-01	9,95E-01	rs16897466|rs4750517|rs37244|rs7330196|rs11868917|rs6578414|rs208354|rs2152305|rs466609|rs723188|rs4811068|rs954385|rs4855077|rs604045|rs12666352|rs304737|rs4246027|rs7140817|rs706578|rs1499772
Immunologic Disorder	PROCESSING_OF_CAPPED_INTRONLESS_PRE_MRNA	REACTOME	274	8	6	6,53E-01	9,95E-01	rs9970867|rs3809425|rs2282763|rs9937484|rs11749500|rs4653184
Immunologic Disorder	CROSS_PRESENTATION_OF_SOLUBLE_EXOGENOUS_ANTIGENS_ENDOSOMES	REACTOME	713	44	20	6,54E-01	9,95E-01	rs10268808|rs2892190|rs10046115|rs1957107|rs807293|rs764190|rs6575122|rs2293361|rs2716601|rs12436216|rs16972457|rs228271|rs8075668|rs7105122|rs117821|rs16930278|rs8066176|rs3782115|rs12292911|rs4498476
Immunologic Disorder	FC_GAMMA_R_MEDIATED_PHAGOCYTOSIS	KEGG	3795	25	20	6,54E-01	9,95E-01	rs1619030|rs16897466|rs2510257|rs9581718|rs3890760|rs12904722|rs253324|rs11761049|rs736730|rs17071888|rs11657742|rs2151414|rs11745637|rs7817803|rs7722385|rs1341347|rs7197601|rs9297810|rs8045689|rs17289507
Immunologic Disorder	SIGNAL_TRANSDUCTION_BY_L1	REACTOME	1310	40	20	6,57E-01	9,95E-01	rs12620429|rs10897495|rs3817828|rs6974790|rs13080492|rs10763940|rs2488320|rs8078165|rs3781878|rs10827185|rs372332|rs2083709|rs7947910|rs3799577|rs11009195|rs11641695|rs1140475|rs7156757|rs2555236|rs384842
Immunologic Disorder	TRANSPORT_OF_VITAMINS_NUCLEOSIDES_AND_RELATED_MOLECULES	REACTOME	1154	38	20	6,58E-01	9,95E-01	rs11853372|rs2236553|rs4149028|rs11038673|rs17596814|rs6480513|rs7027670|rs4149057|rs2249998|rs2279861|rs12685438|rs3787538|rs17497947|rs12439582|rs12917346|rs17695287|rs586046|rs7045243|rs709370|rs11038643
Immunologic Disorder	UCALPAIN_PATHWAY	BIOCARTA	426	16	10	6,59E-01	9,95E-01	rs2268969|rs2488320|rs13163497|rs4587680|rs11009195|rs707210|rs975885|rs11227147|rs3829078|rs2475193
Immunologic Disorder	FGFR_LIGAND_BINDING_AND_ACTIVATION	REACTOME	984	37	19	6,59E-01	9,95E-01	rs17510142|rs2517037|rs12419388|rs823601|rs13262310|rs666028|rs2038712|rs10870911|rs10908200|rs3787011|rs3129596|rs13249548|rs9509959|rs11745375|rs451643|rs249923|rs475738|rs9509982|rs630757
Immunologic Disorder	THROMBOXANE_SIGNALLING_THROUGH_TP_RECEPTOR	REACTOME	492	22	15	6,59E-01	9,95E-01	rs4855077|rs7140817|rs7039739|rs17786782|rs12488540|rs12704705|rs1162211|rs10498440|rs12886319|rs1253676|rs291353|rs12595160|rs4659946|rs8023017|rs10802797
Immunologic Disorder	NUCLEOTIDE_LIKE_PURINERGIC_RECEPTORS	REACTOME	272	11	6	6,61E-01	9,95E-01	rs12028677|rs12139261|rs10920570|rs9855770|rs3766566|rs6770918
Immunologic Disorder	PGC1A_PATHWAY	BIOCARTA	959	38	20	6,61E-01	9,95E-01	rs1509241|rs2469749|rs12054920|rs6860708|rs8026614|rs4235306|rs7726306|rs1159937|rs11125132|rs7727198|rs1388331|rs4823529|rs31309|rs3804357|rs7709420|rs891943|rs1491380|rs17613848|rs13002829|rs9800032
Immunologic Disorder	SIGNALING_BY_FGFR_IN_DISEASE	REACTOME	4545	27	20	6,61E-01	9,95E-01	rs6563245|rs16897466|rs6563216|rs4283545|rs4769946|rs12545028|rs4963867|rs10509534|rs404284|rs17649232|rs7322396|rs2532019|rs17510142|rs2517037|rs12419388|rs10876227|rs13168690|rs823601|rs12537526|rs4599070
Immunologic Disorder	INTEGRATION_OF_ENERGY_METABOLISM	REACTOME	4501	27	20	6,61E-01	9,95E-01	rs10485723|rs227130|rs2423360|rs12545028|rs954385|rs4855077|rs10925763|rs4795194|rs304737|rs6952398|rs7140817|rs7659414|rs8027776|rs2532019|rs11195623|rs16838192|rs13168690|rs3792747|rs204995|rs4832505
Immunologic Disorder	SCF_BETA_TRCP_MEDIATED_DEGRADATION_OF_EMI1	REACTOME	793	42	20	6,63E-01	9,95E-01	rs10268808|rs2892190|rs10046115|rs1957107|rs807293|rs764190|rs6575122|rs2293361|rs2716601|rs12436216|rs16972457|rs228271|rs8075668|rs7105122|rs17760556|rs117821|rs734423|rs16930278|rs8066176|rs3782115
Immunologic Disorder	P53_INDEPENDENT_G1_S_DNA_DAMAGE_CHECKPOINT	REACTOME	729	44	20	6,63E-01	9,95E-01	rs10268808|rs2892190|rs6457689|rs1957107|rs807293|rs764190|rs6575122|rs2293361|rs2716601|rs12436216|rs16972457|rs228271|rs8075668|rs7105122|rs117821|rs16930278|rs8066176|rs3782115|rs12292911|rs4498476
Immunologic Disorder	AUTODEGRADATION_OF_THE_E3_UBIQUITIN_LIGASE_COP1	REACTOME	766	44	20	6,64E-01	9,95E-01	rs10268808|rs2892190|rs6457689|rs1957107|rs807293|rs764190|rs6575122|rs2293361|rs2716601|rs12436216|rs16972457|rs228271|rs8075668|rs7105122|rs117821|rs16930278|rs8066176|rs3782115|rs12292911|rs4498476
Immunologic Disorder	CDMAC_PATHWAY	BIOCARTA	1018	29	20	6,65E-01	9,95E-01	rs10485723|rs227130|rs2423360|rs9642880|rs8115925|rs7812998|rs1015092|rs2745755|rs2033098|rs741847|rs8904|rs4381631|rs8081614|rs4791036|rs2876140|rs10140922|rs4552863|rs169143|rs16902328|rs1012919
Immunologic Disorder	RESPONSE_TO_ELEVATED_PLATELET_CYTOSOLIC_CA2_	REACTOME	2301	30	20	6,67E-01	9,95E-01	rs7330196|rs466609|rs723188|rs604045|rs12666352|rs4246027|rs400819|rs941022|rs4817415|rs382649|rs1678388|rs2268969|rs17661732|rs3009935|rs10520358|rs975360|rs457581|rs1478604|rs922866|rs17491334
Immunologic Disorder	ACTIVATED_POINT_MUTANTS_OF_FGFR2	REACTOME	873	29	15	6,67E-01	9,95E-01	rs2517037|rs823601|rs13262310|rs666028|rs2038712|rs10870911|rs3787011|rs3129596|rs13249548|rs9509959|rs11745375|rs249923|rs475738|rs9509982|rs630757
Immunologic Disorder	BIOSYNTHESIS_OF_UNSATURATED_FATTY_ACIDS	KEGG	463	25	17	6,67E-01	9,95E-01	rs923109|rs10452184|rs11934603|rs9463908|rs7680893|rs17351947|rs7482316|rs2862996|rs7218656|rs11726880|rs17194178|rs6838244|rs11098070|rs11129780|rs2817090|rs6832679|rs7690709
Immunologic Disorder	COLLAGEN_FORMATION	REACTOME	3203	29	20	6,69E-01	9,95E-01	rs10759690|rs2002284|rs11687617|rs2126642|rs7643465|rs751181|rs7621828|rs6436654|rs881821|rs13109282|rs2521206|rs1507270|rs2459139|rs2318345|rs9515201|rs11787950|rs4841920|rs4736183|rs1413294|rs4675141
Immunologic Disorder	KINESINS	REACTOME	566	29	14	6,70E-01	9,95E-01	rs1057452|rs277311|rs2045418|rs9907283|rs861528|rs7142555|rs1251370|rs1553367|rs8013773|rs6734278|rs17554407|rs6706373|rs11160756|rs3824735
Immunologic Disorder	MAL_PATHWAY	BIOCARTA	225	12	6	6,72E-01	9,95E-01	rs17202361|rs11065374|rs12655019|rs11747611|rs10197534|rs2473322
Immunologic Disorder	PURINE_METABOLISM	REACTOME	581	41	13	6,74E-01	9,95E-01	rs7086415|rs7100550|rs17323225|rs207444|rs10869134|rs867744|rs167508|rs2748531|rs1760930|rs831525|rs8075078|rs16885|rs313163
Immunologic Disorder	ACTIVATED_TLR4_SIGNALLING	REACTOME	2676	30	20	6,75E-01	9,95E-01	rs1679802|rs11105194|rs466609|rs2066843|rs400819|rs3817828|rs204995|rs1519959|rs12054920|rs1930714|rs1392125|rs7691384|rs1410201|rs1329049|rs1893352|rs637462|rs1688545|rs4901623|rs6860708|rs8026614
Immunologic Disorder	TIGHT_JUNCTION	KEGG	6242	32	20	6,79E-01	9,95E-01	rs482533|rs4601648|rs4750517|rs948311|rs1719361|rs2290505|rs1383067|rs4963867|rs10216570|rs304737|rs4378885|rs10509534|rs4852507|rs17235168|rs1480151|rs2471577|rs2868932|rs10997482|rs587198|rs9829259
Immunologic Disorder	DOWNREGULATION_OF_SMAD2_3_SMAD4_TRANSCRIPTIONAL_ACTIVITY	REACTOME	453	27	20	6,82E-01	9,95E-01	rs12308345|rs3782267|rs727929|rs266335|rs701078|rs2063327|rs11661651|rs10846681|rs11661340|rs17236851|rs3859312|rs695161|rs2014340|rs1994174|rs7972214|rs12318072|rs10846670|rs1110904|rs870844|rs10910050
Immunologic Disorder	TFF_PATHWAY	BIOCARTA	697	41	20	6,82E-01	9,95E-01	rs16897466|rs6974790|rs2488320|rs13320527|rs6872878|rs17153654|rs4587680|rs13173003|rs11009195|rs706725|rs1140475|rs6449947|rs12536419|rs2161529|rs1862710|rs12700583|rs6451620|rs7315397|rs10515074|rs11977660
Immunologic Disorder	RAS_PATHWAY	BIOCARTA	557	29	19	6,83E-01	9,95E-01	rs16897466|rs7022409|rs13320527|rs17153654|rs6780561|rs10486801|rs13173003|rs706725|rs10486802|rs36064377|rs6449947|rs12536419|rs2161529|rs1862710|rs328994|rs10515074|rs2473322|rs6871452|rs9848505
Immunologic Disorder	MCM_PATHWAY	BIOCARTA	234	8	5	6,88E-01	9,95E-01	rs2073032|rs4727590|rs2164210|rs13082666|rs309126
Immunologic Disorder	REGULATION_OF_INSULIN_LIKE_GROWTH_FACTOR_IGF_ACTIVITY_BY_INSULIN_LIKE_G DING_PROTEINS_IGFBPS	REACTOME	570	38	17	6,91E-01	9,95E-01	rs12133739|rs374560|rs11762916|rs6977956|rs7125320|rs6478233|rs1486137|rs717963|rs1004446|rs6946252|rs7420849|rs783166|rs17513961|rs470168|rs7536647|rs198977|rs2294649
Immunologic Disorder	HDAC_PATHWAY	BIOCARTA	1231	23	20	6,92E-01	9,95E-01	rs16897466|rs6063664|rs2469749|rs12054920|rs6860708|rs8026614|rs13320527|rs10794486|rs7726306|rs17153654|rs6021266|rs11125132|rs7727198|rs2684796|rs13173003|rs3886478|rs706725|rs3804357|rs6063634|rs1997815
Immunologic Disorder	SNARE_INTERACTIONS_IN_VESICULAR_TRANSPORT	KEGG	828	43	20	6,92E-01	9,95E-01	rs10187424|rs12259064|rs12766378|rs486409|rs2695934|rs6460052|rs12022368|rs228729|rs362560|rs9313618|rs10460623|rs6951030|rs2315141|rs12938765|rs11086650|rs12740112|rs2842888|rs3782115|rs10902671|rs29784
Immunologic Disorder	PROSTACYCLIN_SIGNALLING_THROUGH_PROSTACYCLIN_RECEPTOR	REACTOME	353	17	12	6,92E-01	9,95E-01	rs4855077|rs7140817|rs12488540|rs12704705|rs10498440|rs12886319|rs1253676|rs291353|rs12595160|rs4659946|rs8023017|rs10802797
Immunologic Disorder	CXCR4_PATHWAY	BIOCARTA	1099	28	20	6,95E-01	9,95E-01	rs16897466|rs304737|rs12691883|rs2886612|rs13320527|rs2011946|rs7039739|rs17786782|rs1904836|rs13173003|rs2056296|rs706725|rs10191360|rs4381631|rs8081614|rs4791036|rs6449947|rs11044171|rs2161529|rs169143
Immunologic Disorder	GNRH_SIGNALING_PATHWAY	KEGG	3992	35	20	6,95E-01	9,95E-01	rs8115510|rs10485723|rs227130|rs2423360|rs3819579|rs9362387|rs12545028|rs4963867|rs12904722|rs2532019|rs13168690|rs1997696|rs6974790|rs8115925|rs210120|rs9922362|rs10743591|rs3782309|rs2869433|rs7615501
Immunologic Disorder	EXTENSION_OF_TELOMERES	REACTOME	618	23	12	6,98E-01	9,95E-01	rs9540340|rs17482047|rs9570962|rs16981247|rs12602230|rs4943189|rs3786136|rs1832265|rs9539881|rs10258834|rs8104760|rs7357092
Immunologic Disorder	SIGNALING_BY_FGFR	REACTOME	4154	27	20	6,98E-01	9,95E-01	rs6563245|rs16897466|rs6563216|rs4283545|rs12545028|rs4963867|rs10509534|rs17649232|rs7322396|rs2532019|rs17510142|rs2517037|rs12419388|rs10876227|rs13168690|rs823600|rs4599070|rs13262310|rs1333404|rs666028
Immunologic Disorder	TOLL_PATHWAY	BIOCARTA	1147	31	20	6,99E-01	9,95E-01	rs1930714|rs1392125|rs7691384|rs1410201|rs1329049|rs1893352|rs637462|rs9359935|rs1928295|rs10806443|rs778584|rs10984022|rs1996215|rs6924218|rs284513|rs4823529|rs7661887|rs7746039|rs741847|rs2806112
Immunologic Disorder	SIGNALING_BY_WNT	REACTOME	1071	35	20	7,03E-01	9,95E-01	rs10268808|rs2892190|rs1678002|rs10046115|rs1957107|rs807293|rs764190|rs6575122|rs2293361|rs7145618|rs2716601|rs4919096|rs12436216|rs16972457|rs228271|rs8075668|rs7105122|rs17760556|rs117821|rs734423
Immunologic Disorder	G2_PATHWAY	BIOCARTA	354	11	10	7,04E-01	9,95E-01	rs4809258|rs6062357|rs2177640|rs2282128|rs8178179|rs8058610|rs2012987|rs1535211|rs6062662|rs2281479
Immunologic Disorder	MRNA_CAPPING	REACTOME	388	5	3	7,04E-01	9,95E-01	rs10944384|rs4487686|rs886420
Immunologic Disorder	ACH_PATHWAY	BIOCARTA	602	33	17	7,04E-01	9,95E-01	rs16897466|rs10980549|rs13320527|rs4332201|rs17153654|rs13173003|rs706725|rs3824867|rs6449947|rs10980573|rs12536419|rs9480865|rs2161529|rs10515074|rs12292911|rs6871452|rs6477778
Immunologic Disorder	PI_3K_CASCADE	REACTOME	1868	31	20	7,05E-01	9,95E-01	rs16897466|rs10509534|rs7322396|rs17510142|rs2517037|rs12419388|rs10876227|rs823600|rs13262310|rs666028|rs2038712|rs10870911|rs10908200|rs13320527|rs3787011|rs3129596|rs7991808|rs13173003|rs13249548|rs706725
Immunologic Disorder	MISMATCH_REPAIR	KEGG	576	26	14	7,07E-01	9,95E-01	rs9540340|rs406334|rs17482047|rs9570962|rs16981247|rs12602230|rs4943189|rs3786136|rs1832265|rs9539881|rs10258834|rs3797887|rs10490302|rs7357092
Immunologic Disorder	CDC42RAC_PATHWAY	BIOCARTA	435	27	16	7,07E-01	9,95E-01	rs16897466|rs736730|rs13320527|rs13173003|rs706725|rs1507964|rs4731121|rs6449947|rs2161529|rs2286831|rs527589|rs10515074|rs3771099|rs2473322|rs11133330|rs6871452
Immunologic Disorder	DESTABILIZATION_OF_MRNA_BY_AUF1_HNRNP_D0	REACTOME	811	44	20	7,08E-01	9,95E-01	rs10268808|rs2892190|rs10046115|rs1957107|rs807293|rs764190|rs6575122|rs2293361|rs2716601|rs12436216|rs16972457|rs228271|rs8075668|rs7105122|rs117821|rs16930278|rs8066176|rs3782115|rs12292911|rs4498476
Immunologic Disorder	INTERFERON_GAMMA_SIGNALING	REACTOME	2043	41	20	7,09E-01	9,95E-01	rs1610751|rs4886844|rs987870|rs4811068|rs4896251|rs1611493|rs1532113|rs28367832|rs4616352|rs3781878|rs17500510|rs7615501|rs353590|rs7947910|rs391023|rs2025258|rs1612907|rs9264967|rs9276291|rs3024908
Immunologic Disorder	ACTIVATION_OF_GENES_BY_ATF4	REACTOME	316	14	8	7,11E-01	9,95E-01	rs3740526|rs7024667|rs1545229|rs4783961|rs2499854|rs11642944|rs10973537|rs4291799
Immunologic Disorder	BASAL_TRANSCRIPTION_FACTORS	KEGG	386	20	14	7,12E-01	9,95E-01	rs7004630|rs6902440|rs3811474|rs13238568|rs9858561|rs3779641|rs7813680|rs519336|rs3811473|rs13237829|rs13082232|rs11682325|rs17750066|rs886420
Immunologic Disorder	ARF_PATHWAY	BIOCARTA	733	28	18	7,12E-01	9,95E-01	rs16897466|rs9642880|rs13320527|rs7812998|rs17153654|rs510342|rs13173003|rs2033098|rs706725|rs6449947|rs12536419|rs2161529|rs7853654|rs4552863|rs2269337|rs16902328|rs10515074|rs6871452
Immunologic Disorder	PEROXISOME	KEGG	2047	29	20	7,14E-01	9,95E-01	rs7374394|rs11698712|rs666105|rs10182865|rs9392576|rs11934603|rs10930785|rs17155849|rs4817415|rs531592|rs6596572|rs9374174|rs4122274|rs1153987|rs11689608|rs9618193|rs7561624|rs17323225|rs207444|rs11050146
Immunologic Disorder	ARAP_PATHWAY	BIOCARTA	292	12	5	7,15E-01	9,95E-01	rs4810909|rs796985|rs12950719|rs2024595|rs276719
Immunologic Disorder	G1_S_SPECIFIC_TRANSCRIPTION	REACTOME	194	1	1	7,17E-01	9,95E-01	rs2298582
Immunologic Disorder	APOPTOTIC_EXECUTION_PHASE	REACTOME	1211	46	20	7,17E-01	9,95E-01	rs4750517|rs1719361|rs7239317|rs10511409|rs587198|rs3769823|rs7615501|rs7760713|rs6597269|rs4357599|rs7220988|rs9550565|rs2271354|rs706494|rs9551925|rs12738235|rs11663972|rs242557|rs1409876|rs17374045
Immunologic Disorder	GLYCEROPHOSPHOLIPID_METABOLISM	KEGG	2549	35	20	7,19E-01	9,95E-01	rs12199314|rs11868917|rs6870653|rs2467458|rs706578|rs12904722|rs1880553|rs1124605|rs204995|rs531592|rs28688444|rs1341347|rs2046731|rs2006771|rs7422272|rs558491|rs6672057|rs6455717|rs177088|rs6780561

Immunologic Disorder	RESPIRATORY_ELECTRON_TRANSPORT_ATP_SYNTHESIS_BY_CHEMIOSMOTIC_COUPLIN(TION_BY_UNCOUPLING_PROTEINS_	REACTOME	1741	33	20	7,19E-01	9,95E-01	rs11745467|rs3794176|rs11077773|rs1800649|rs2255408|rs16899656|rs2898543|rs4720913|rs7180462|rs778584|rs4149553|rs17759300|rs17701871|rs2647209|rs6867920|rs3745004|rs12985735|rs10269406|rs11670086|rs2971028
Immunologic Disorder	AKT_PATHWAY	BIOCARTA	682	40	19	7,20E-01	9,95E-01	rs16897466|rs7322396|rs13320527|rs6872878|rs17153654|rs7991808|rs13173003|rs706725|rs8904|rs6449947|rs12536419|rs9480865|rs10140922|rs2161529|rs1862710|rs6451620|rs1012919|rs10515074|rs6871452
Immunologic Disorder	SCFSKP2_MEDIATED_DEGRADATION_OF_P27_P21	REACTOME	813	43	19	7,21E-01	9,95E-01	rs10268808|rs2892190|rs6457689|rs1957107|rs807293|rs764190|rs6575122|rs2716601|rs12436216|rs16972457|rs228271|rs8075668|rs7105122|rs117821|rs16930278|rs8066176|rs3782115|rs12292911|rs4498476
Immunologic Disorder	IL_RECEPTOR_SHC_SIGNALING	REACTOME	624	21	15	7,21E-01	9,95E-01	rs16897466|rs3856848|rs13320527|rs13173003|rs706725|rs1981405|rs131849|rs4808100|rs6449947|rs2161529|rs4054727|rs10515074|rs6871452|rs2075726|rs3804795
Immunologic Disorder	INTERFERON_ALPHA_BETA_SIGNALING	REACTOME	1477	41	20	7,22E-01	9,95E-01	rs9975358|rs1610751|rs4811068|rs1611493|rs2834112|rs28367832|rs4497664|rs7046061|rs1983177|rs2071482|rs391023|rs2025258|rs9264967|rs3024908|rs12504466|rs1425552|rs10811518|rs17624154|rs2240193|rs303211
Immunologic Disorder	P53_DEPENDENT_G1_DNA_DAMAGE_RESPONSE	REACTOME	838	44	20	7,22E-01	9,95E-01	rs10268808|rs2892190|rs6457689|rs1957107|rs807293|rs764190|rs6575122|rs2293361|rs2716601|rs12436216|rs16972457|rs228271|rs8075668|rs7105122|rs117821|rs16930278|rs8066176|rs3782115|rs12292911|rs4498476
Immunologic Disorder	HIV_INFECTION	REACTOME	3237	41	20	7,23E-01	9,95E-01	rs1610751|rs10268808|rs2368443|rs1882070|rs2892190|rs1484931|rs10511409|rs6457689|rs17071888|rs17687453|rs6770415|rs503347|rs4684917|rs1611493|rs7596050|rs12691883|rs10944384|rs6434036|rs13218316|rs873491
Immunologic Disorder	RESOLUTION_OF_AP_SITES_VIA_THE_MULTIPLE_NUCLEOTIDE_PATCH_REPLACEMENT_I	REACTOME	192	7	4	7,24E-01	9,95E-01	rs16981247|rs2029166|rs4135054|rs2723875
Immunologic Disorder	ACTIVATION_OF_ATR_IN_RESPONSE_TO_REPLICATION_STRESS	REACTOME	653	29	15	7,25E-01	9,95E-01	rs9540340|rs2073032|rs17482047|rs9570962|rs12602230|rs4943189|rs3786136|rs1832265|rs9539881|rs4727590|rs2164210|rs13082666|rs10258834|rs309126|rs7357092
Immunologic Disorder	NGF_SIGNALLING_VIA_TRKA_FROM_THE_PLASMA_MEMBRANE	REACTOME	4847	24	20	7,26E-01	9,95E-01	rs16897466|rs17201100|rs1009619|rs10858075|rs12545028|rs4963867|rs28628360|rs10509534|rs7022409|rs4890408|rs17649232|rs7322396|rs2532019|rs2396303|rs3817828|rs10876227|rs13168690|rs11102314|rs4599070|rs1519959
Immunologic Disorder	RESPIRATORY_ELECTRON_TRANSPORT	REACTOME	1631	31	20	7,28E-01	9,95E-01	rs11745467|rs3794176|rs1800649|rs2255408|rs16899656|rs2898543|rs4720913|rs7180462|rs778584|rs4149553|rs17759300|rs17701871|rs2647209|rs6867920|rs12985735|rs10269406|rs11670086|rs2971028|rs12548235|rs993248
Immunologic Disorder	BIOLOGICAL_OXIDATIONS	REACTOME	3568	45	20	7,29E-01	9,95E-01	rs13331733|rs2608836|rs2572023|rs2755427|rs6830865|rs10918239|rs1290622|rs2028898|rs1558139|rs6467|rs759998|rs3764561|rs10031344|rs1042389|rs10058433|rs10918228|rs6904935|rs7886938|rs1571858|rs915908
Immunologic Disorder	SIGNALING_BY_ROBO_RECEPTOR	REACTOME	1900	32	20	7,31E-01	9,95E-01	rs11924816|rs7435615|rs10511131|rs2077753|rs7432274|rs6118746|rs17016015|rs12487085|rs4539916|rs11632939|rs7590257|rs1379659|rs6775462|rs2648512|rs2600164|rs1316929|rs3769504|rs2708987|rs1478049|rs4686340
Immunologic Disorder	TCA_CYCLE_AND_RESPIRATORY_ELECTRON_TRANSPORT	REACTOME	2599	29	20	7,32E-01	9,95E-01	rs11745467|rs3794176|rs3735477|rs4832076|rs11077773|rs1800649|rs2255408|rs693163|rs16899656|rs2898543|rs2076370|rs4720913|rs11032981|rs7180462|rs9649679|rs778584|rs4149553|rs17759300|rs11983987|rs10496307
Immunologic Disorder	PHASE1_FUNCTIONALIZATION_OF_COMPOUNDS	REACTOME	1515	59	20	7,33E-01	9,95E-01	rs2572023|rs1290622|rs1558139|rs6467|rs759998|rs3764561|rs1042389|rs7886938|rs915908|rs6608458|rs2279345|rs2470890|rs2058265|rs4808358|rs4736349|rs2654846|rs695149|rs11572285|rs10883782|rs7271624
Immunologic Disorder	TOLL_LIKE_RECEPTOR_SIGNALING_PATHWAY	KEGG	2339	29	20	7,33E-01	9,95E-01	rs16897466|rs9847023|rs9975358|rs11657742|rs2834112|rs2243131|rs1930714|rs1392125|rs7691384|rs1410201|rs1329049|rs1893352|rs637462|rs9359935|rs1928295|rs13320527|rs7046061|rs4315909|rs10806443|rs2869433
Immunologic Disorder	FRS2_MEDIATED_CASCADE	REACTOME	1192	38	20	7,35E-01	9,95E-01	rs4963867|rs17510142|rs2517037|rs12419388|rs823601|rs13262310|rs666028|rs2038712|rs10870911|rs10908200|rs3787011|rs3129596|rs13249548|rs9509959|rs11745375|rs451643|rs249923|rs17388587|rs475738|rs9509982
Immunologic Disorder	INTEGRATION_OF_PROVIRUS	REACTOME	122	2	2	7,36E-01	9,95E-01	rs1872066|rs7046713
Immunologic Disorder	SHC_RELATED_EVENTS	REACTOME	302	11	8	7,37E-01	9,95E-01	rs4963867|rs1125467|rs1004446|rs944485|rs17388587|rs34883124|rs2868161|rs4247374
Immunologic Disorder	OXYGEN_DEPENDENT_PROLINE_HYDROXYLATION_OF_HYPOXIA_INDUCIBLE_FACTOR_/	REACTOME	313	12	7	7,37E-01	9,95E-01	rs1465645|rs2790860|rs10827475|rs2035423|rs7145164|rs1994174|rs10504553
Immunologic Disorder	CASPASE_PATHWAY	BIOCARTA	327	16	10	7,40E-01	9,95E-01	rs1641661|rs3769823|rs1613367|rs2271354|rs12738235|rs1862710|rs12700583|rs7315397|rs10999439|rs921
Immunologic Disorder	FRUCTOSE_AND_MANNOSE_METABOLISM	KEGG	984	35	18	7,40E-01	9,95E-01	rs761147|rs756607|rs11251480|rs3800083|rs923109|rs10998736|rs9419445|rs4433480|rs10903880|rs11251555|rs11994010|rs10903900|rs1341928|rs3800128|rs1872837|rs1535694|rs10503595|rs2318089
Immunologic Disorder	CHREBP2_PATHWAY	BIOCARTA	1059	46	20	7,41E-01	9,95E-01	rs4795194|rs6952398|rs1678002|rs1480151|rs1447598|rs10889011|rs2796498|rs13434196|rs2177640|rs6950343|rs2302234|rs10781329|rs7145618|rs4425665|rs10782370|rs9419596|rs7805942|rs17105330|rs2868161|rs7814838
Immunologic Disorder	REGULATION_OF_INSULIN_SECRETION_BY_GLUCAGON_LIKE_PEPTIDE1	REACTOME	1388	25	20	7,42E-01	9,95E-01	rs12545028|rs4855077|rs7140817|rs4832505|rs11102314|rs210120|rs10743591|rs3782309|rs12479002|rs1870822|rs12614549|rs10492301|rs2302234|rs12488540|rs12704705|rs10781329|rs10506562|rs263241|rs10498440|rs3761656
Immunologic Disorder	INTEGRIN_PATHWAY	BIOCARTA	1027	39	20	7,43E-01	9,95E-01	rs7239317|rs11102314|rs5996534|rs2268969|rs2296950|rs2488320|rs13163497|rs13218316|rs1378942|rs4587680|rs10824069|rs11009195|rs5751614|rs707210|rs975885|rs11227147|rs3829078|rs11153318|rs6714412|rs2475193
Immunologic Disorder	NFKB_AND_MAP_KINASES_ACTIVATION_MEDIATED_BY_TLR4_SIGNALING_REPERTOIRE	REACTOME	2248	26	20	7,43E-01	9,95E-01	rs1009619|rs466609|rs2066843|rs400819|rs3817828|rs204995|rs1519959|rs12054920|rs1930714|rs1392125|rs7691384|rs1410201|rs1329049|rs1688545|rs6860708|rs8026614|rs9359935|rs1928295|rs10806443|rs2869433
Immunologic Disorder	G_BETA_GAMMA_SIGNALLING_THROUGH_PI3KGAMMA	REACTOME	487	30	18	7,43E-01	9,95E-01	rs4855077|rs7140817|rs11657742|rs17153654|rs12488540|rs12704705|rs10498440|rs874269|rs11078772|rs12886319|rs1253676|rs12536419|rs291353|rs12595160|rs4659946|rs559080|rs8023017|rs10802797
Immunologic Disorder	VEGF_PATHWAY	BIOCARTA	883	31	20	7,44E-01	9,95E-01	rs16897466|rs10058772|rs13168072|rs3794405|rs13320527|rs17153654|rs13173003|rs9554325|rs706725|rs4381631|rs8081614|rs7145164|rs4791036|rs2125489|rs6449947|rs12536419|rs2161529|rs169143|rs10515074|rs7219495
Immunologic Disorder	LATE_PHASE_OF_HIV_LIFE_CYCLE	REACTOME	1259	42	20	7,45E-01	9,95E-01	rs2368443|rs1484931|rs17687453|rs6770415|rs503347|rs4684917|rs7596050|rs10944384|rs6434036|rs773546|rs2269744|rs4487686|rs5766533|rs9858561|rs3779641|rs519336|rs17757841|rs580438|rs13082232|rs7202364
Immunologic Disorder	KERATAN_SULFATE_BIOSYNTHESIS	REACTOME	674	25	15	7,46E-01	9,95E-01	rs6095638|rs2012019|rs741901|rs2403981|rs7115683|rs1975605|rs2279861|rs4808100|rs1868430|rs4985526|rs7820063|rs16904967|rs1539144|rs6981912|rs756379
Immunologic Disorder	CELL_CELL_JUNCTION_ORGANIZATION	REACTOME	6211	34	20	7,46E-01	9,95E-01	rs11564334|rs2446886|rs11215593|rs17749762|rs4782717|rs3101011|rs11639525|rs7709151|rs4378885|rs12444964|rs7203355|rs4518142|rs12598326|rs8055843|rs10472700|rs4782749|rs523223|rs4577701|rs6089291|rs233551
Immunologic Disorder	ARGININE_AND_PROLINE_METABOLISM	KEGG	991	29	20	7,47E-01	9,95E-01	rs3793932|rs10918239|rs12768505|rs4934297|rs4578194|rs10918228|rs11622835|rs13380644|rs556258|rs4511520|rs12554375|rs1547033|rs84314|rs1475332|rs818169|rs914971|rs344807|rs9934713|rs12554609|rs10932334
Immunologic Disorder	REGULATION_OF_SIGNALING_BY_CBL	REACTOME	703	27	16	7,48E-01	9,95E-01	rs16897466|rs2296950|rs13218316|rs13320527|rs1808563|rs13173003|rs706725|rs6449947|rs3214047|rs2161529|rs11153318|rs1148844|rs10515074|rs6871452|rs12674523|rs178255
Immunologic Disorder	GROWTH_HORMONE_RECEPTOR_SIGNALING	REACTOME	773	34	18	7,49E-01	9,95E-01	rs17201100|rs4811068|rs2396303|rs6872878|rs9521472|rs3024908|rs2396306|rs9900213|rs13398058|rs40443|rs996969|rs11966463|rs6451620|rs1330543|rs8074003|rs13038695|rs12674523|rs16867044
Immunologic Disorder	DNA_STRAND_ELONGATION	REACTOME	672	31	18	7,49E-01	9,95E-01	rs9540340|rs2073032|rs3815795|rs17482047|rs9570962|rs16981247|rs12602230|rs4943189|rs3786136|rs1832265|rs9539881|rs2164210|rs13082666|rs10258834|rs11783920|rs309126|rs11644122|rs7357092
Immunologic Disorder	SIGNALING_BY_NOTCH	REACTOME	4553	31	20	7,50E-01	9,95E-01	rs2526640|rs6543560|rs2094347|rs435806|rs12103149|rs11685345|rs10512091|rs2423539|rs2285431|rs10005500|rs2378377|rs12553514|rs204995|rs12308345|rs9636384|rs11794108|rs4857735|rs10247238|rs10780408|rs3782267
Immunologic Disorder	RECYCLING_PATHWAY_OF_L1	REACTOME	874	29	16	7,50E-01	9,95E-01	rs10897495|rs3817828|rs277311|rs8078165|rs372332|rs2083709|rs3799577|rs7156757|rs7109133|rs7854612|rs11881315|rs384842|rs16933086|rs12199759|rs7831864|rs2345258
Immunologic Disorder	OXIDATIVE_PHOSPHORYLATION	KEGG	2486	34	20	7,51E-01	9,95E-01	rs2001515|rs11745467|rs3794176|rs11077773|rs958556|rs1800649|rs1512348|rs16899656|rs896189|rs2898543|rs4720913|rs2454016|rs7562668|rs778584|rs6473885|rs4149553|rs17759300|rs17701871|rs2647209|rs3093988
Immunologic Disorder	JNK_C_JUN_KINASES_PHOSPHORYLATION_AND_ACTIVATION_MEDIATED_BY_ACTIVATI	REACTOME	728	28	14	7,51E-01	9,95E-01	rs2066843|rs9359935|rs10806443|rs2869433|rs6961625|rs6924218|rs284513|rs7746039|rs2616052|rs5006575|rs17627593|rs331455|rs6955931|rs7699978
Immunologic Disorder	CACAM_PATHWAY	BIOCARTA	401	16	14	7,54E-01	9,95E-01	rs7214864|rs11651131|rs7314454|rs11125132|rs31309|rs10774589|rs7709420|rs891943|rs2288684|rs13002829|rs9800032|rs3797617|rs815802|rs12750212
Immunologic Disorder	PIP3_ACTIVATES_AKT_SIGNALING	REACTOME	587	24	14	7,57E-01	9,95E-01	rs10509534|rs7322396|rs10876227|rs7991808|rs7241455|rs874269|rs9480865|rs683590|rs6084242|rs1862710|rs865961|rs559080|rs879107|rs7234377
Immunologic Disorder	G1_PATHWAY	BIOCARTA	611	15	11	7,58E-01	9,95E-01	rs604045|rs3009935|rs266335|rs12029576|rs695161|rs947712|rs7853654|rs7793983|rs2269337|rs920293|rs7781436
Immunologic Disorder	EXTRACELLULAR_MATRIX_ORGANIZATION	REACTOME	3783	29	20	7,60E-01	9,95E-01	rs10759690|rs2002284|rs11687617|rs2126642|rs4726572|rs7643465|rs751181|rs7621828|rs6436654|rs881821|rs10517867|rs2521206|rs1507270|rs2459139|rs6469604|rs2318345|rs9515201|rs11787950|rs4841920|rs4736183
Immunologic Disorder	TYPE_II_DIABETES_MELLITUS	KEGG	2053	32	20	7,62E-01	9,95E-01	rs16897466|rs17201100|rs675498|rs2396303|rs11657742|rs10998736|rs556321|rs8113506|rs10495929|rs13320527|rs1838793|rs2869433|rs7615501|rs4765929|rs4148407|rs4264601|rs17153654|rs7558342|rs4952779|rs10409463
Immunologic Disorder	PROTEASOME_PATHWAY	BIOCARTA	280	18	13	7,62E-01	9,95E-01	rs10268808|rs1957107|rs764190|rs2811491|rs12436216|rs228271|rs7105122|rs16930278|rs7181164|rs1557874|rs8066176|rs12292911|rs4498476
Immunologic Disorder	GLYOXYLATE_AND_DICARBOXYLATE_METABOLISM	KEGG	704	30	15	7,63E-01	9,95E-01	rs11698712|rs17191070|rs6745054|rs9649679|rs11983987|rs7857257|rs288877|rs1115506|rs2298028|rs7049218|rs6140342|rs7739248|rs838530|rs2205824|rs1556395
Immunologic Disorder	RNA_POL_III_TRANSCRIPTION_INITIATION_FROM_TYPE_3_PROMOTER	REACTOME	501	34	15	7,64E-01	9,95E-01	rs6081160|rs4077515|rs6539267|rs907076|rs510342|rs4961396|rs16858688|rs11145922|rs7145164|rs2995094|rs4922157|rs12930464|rs1075|rs4921702|rs2247746
Immunologic Disorder	G2_M_CHECKPOINTS	REACTOME	710	29	15	7,64E-01	9,95E-01	rs9540340|rs2073032|rs17482047|rs9570962|rs12602230|rs4943189|rs3786136|rs1832265|rs9539881|rs4727590|rs2164210|rs13082666|rs10258834|rs309126|rs7357092
Immunologic Disorder	SIGNALING_BY_SCF_KIT	REACTOME	1989	27	20	7,64E-01	9,95E-01	rs16897466|rs10248619|rs4963867|rs10509534|rs7322396|rs10876227|rs3184504|rs3020827|rs9944770|rs13218316|rs1507955|rs13320527|rs1808563|rs4957806|rs7991808|rs13173003|rs3024908|rs706725|rs1981405|rs6861689
Immunologic Disorder	NEPHRIN_INTERACTIONS	REACTOME	1617	27	20	7,65E-01	9,95E-01	rs16897466|rs3802819|rs2471577|rs11604082|rs1784230|rs2268969|rs4937179|rs13218316|rs13320527|rs12668317|rs2364913|rs12617710|rs12154832|rs13173003|rs7931141|rs706725|rs10254114|rs707210|rs12978914|rs3769504
Immunologic Disorder	LOSS_OF_NLP_FROM_MITOTIC_CENTROSOMES	REACTOME	1100	40	18	7,66E-01	9,95E-01	rs34541|rs870777|rs9309485|rs4837708|rs10777859|rs11794773|rs10494940|rs2158138|rs4924687|rs1333924|rs8058610|rs12096414|rs11104736|rs12422443|rs1894923|rs3789311|rs1993067|rs719450
Immunologic Disorder	MCALPAIN_PATHWAY	BIOCARTA	543	28	16	7,66E-01	9,95E-01	rs6974790|rs820363|rs2488320|rs13163497|rs4587680|rs2302234|rs11009195|rs10781329|rs1140475|rs975885|rs11227147|rs3829078|rs4509829|rs11977660|rs6674492|rs2475193
Immunologic Disorder	PERK_REGULATED_GENE_EXPRESSION	REACTOME	334	16	10	7,66E-01	9,95E-01	rs3740526|rs7024667|rs1545229|rs12467314|rs4783961|rs7571971|rs2499854|rs11642944|rs10973537|rs4291799
Immunologic Disorder	VALINE_LEUCINE_AND_ISOLEUCINE_DEGRADATION	KEGG	1149	36	20	7,68E-01	9,95E-01	rs7374394|rs10918239|rs4578194|rs9554684|rs10918228|rs9947207|rs11752339|rs2352745|rs1471841|rs10498923|rs6546670|rs2075624|rs12554375|rs2125894|rs723587|rs492734|rs11129780|rs1751796|rs316738|rs11047702
Immunologic Disorder	CITRATE_CYCLE_TCA_CYCLE	KEGG	1195	39	20	7,68E-01	9,95E-01	rs3735477|rs4832076|rs17191070|rs7944999|rs9649679|rs11983987|rs10496307|rs2647209|rs4129215|rs7857257|rs288877|rs1115506|rs6025645|rs11710823|rs954241|rs13428219|rs12503593|rs7049218|rs7647085|rs1556395
Immunologic Disorder	PROGESTERONE_MEDIATED_OOCYTE_MATURATION	KEGG	2535	31	20	7,70E-01	9,95E-01	rs16897466|rs12545028|rs4963867|rs304737|rs2532019|rs3817828|rs7683090|rs13168690|rs518162|rs11657742|rs7303397|rs1427988|rs9922362|rs2886612|rs13320527|rs2869433|rs10794486|rs4629562|rs17153654|rs13190668
Immunologic Disorder	PROTEOLYTIC_CLEAVAGE_OF_SNARE_COMPLEX_PROTEINS	REACTOME	382	18	13	7,70E-01	9,95E-01	rs486409|rs2695934|rs6460052|rs12022368|rs362560|rs10460623|rs6951030|rs2315141|rs8076161|rs11086650|rs12740112|rs2842888|rs10902671
Immunologic Disorder	TRIF_MEDIATED_TLR3_SIGNALING	REACTOME	2075	25	20	7,72E-01	9,95E-01	rs11105194|rs466609|rs2066843|rs400819|rs3817828|rs204995|rs1519959|rs12054920|rs7691384|rs1688545|rs6860708|rs8026614|rs9359935|rs10806443|rs2869433|rs10878177|rs457581|rs7726306|rs3006475|rs6961625
Immunologic Disorder	RNA_POL_I_RNA_POL_III_AND_MITOCHONDRIAL_TRANSCRIPTION	REACTOME	1830	36	20	7,72E-01	9,95E-01	rs9407553|rs10961344|rs6081160|rs4077515|rs10961490|rs6539267|rs907076|rs12245545|rs393543|rs10810132|rs510342|rs7760713|rs803269|rs2188148|rs4961396|rs16858688|rs10208|rs871820|rs1995152|rs2948091
Immunologic Disorder	LIPID_DIGESTION_MOBILIZATION_AND_TRANSPORT	REACTOME	1389	29	20	7,73E-01	9,95E-01	rs4775073|rs10516451|rs2487052|rs9426785|rs97458|rs11581077|rs7099855|rs1607267|rs4682910|rs10781329|rs261337|rs3857979|rs881980|rs701089|rs4765600|rs10991522|rs4783961|rs754524|rs1110060|rs9644636
Immunologic Disorder	RNA_POL_III_TRANSCRIPTION_INITIATION_FROM_TYPE_2_PROMOTER	REACTOME	417	18	10	7,73E-01	9,95E-01	rs6081160|rs6539267|rs393543|rs510342|rs4961396|rs4922157|rs12930464|rs1075|rs4921702|rs2247746
Immunologic Disorder	SHH_PATHWAY	BIOCARTA	679	23	13	7,75E-01	9,95E-01	rs6952416|rs2835741|rs7796149|rs11909066|rs6463057|rs2302234|rs10781329|rs10122178|rs1869026|rs6961071|rs12616114|rs7801545|rs17172023
Immunologic Disorder	REGULATION_OF_MRNA_STABILITY_BY_PROTEINS_THAT_BIND_AU_RICH_ELEMENTS	REACTOME	1372	38	20	7,77E-01	9,95E-01	rs10268808|rs2892190|rs3740526|rs6457689|rs9628|rs7024667|rs1957107|rs7615501|rs807293|rs1545229|rs764190|rs6575122|rs2293361|rs2716601|rs2958405|rs12436216|rs16972457|rs228271|rs8075668|rs4381631
Immunologic Disorder	IL12_PATHWAY	BIOCARTA	589	20	15	7,77E-01	9,95E-01	rs4680551|rs2243131|rs4315909|rs704856|rs3024908|rs7560255|rs2421057|rs2440134|rs2995094|rs858490|rs11177048|rs11656130|rs6895787|rs9895446|rs2715815
Immunologic Disorder	TRANSCRIPTIONAL_ACTIVITY_OF_SMAD2_SMAD3_SMAD4_HETEROTRIMER	REACTOME	967	26	20	7,77E-01	9,95E-01	rs12308345|rs3782267|rs9642880|rs727929|rs266335|rs701078|rs7812998|rs2063327|rs11661651|rs10846681|rs2033098|rs11661340|rs17236851|rs3859312|rs13065308|rs695161|rs2014340|rs722420|rs1994174|rs7972214
Immunologic Disorder	INTRINSIC_PATHWAY_FOR_APOPTOSIS	REACTOME	532	25	15	7,79E-01	9,95E-01	rs500804|rs3769823|rs1016860|rs2269744|rs956572|rs2270941|rs1439287|rs1862710|rs12700583|rs7315397|rs719450|rs8104760|rs2868161|rs12454650|rs4940927
Immunologic Disorder	SIGNALING_BY_EGFR_IN_CANCER	REACTOME	4030	35	20	7,79E-01	9,95E-01	rs6563245|rs16897466|rs6563216|rs12545028|rs4963867|rs10509534|rs17649232|rs7322396|rs2532019|rs2366698|rs10876227|rs13168690|rs10795505|rs6974790|rs4599070|rs1333404|rs210120|rs9422812|rs11244948|rs10495929
Immunologic Disorder	LEISHMANIA_INFECTION	KEGG	2265	36	20	7,80E-01	9,95E-01	rs9847023|rs987870|rs604045|rs4896251|rs2243131|rs1930714|rs1410201|rs1329049|rs2488320|rs3009935|rs9359935|rs1928295|rs4315909|rs17500510|rs10806443|rs9276933|rs10984022|rs1996215|rs4587680|rs4988821
Immunologic Disorder	TIE2_SIGNALING	REACTOME	790	26	19	7,81E-01	9,95E-01	rs16897466|rs4963867|rs603085|rs1433176|rs13320527|rs13173003|rs706725|rs6707461|rs6449947|rs2161529|rs17388587|rs12998964|rs2442592|rs2922861|rs1283681|rs10515074|rs617333|rs6871452|rs903489
Immunologic Disorder	FACTORS_INVOLVED_IN_MEGAKARYOCYTE_DEVELOPMENT_AND_PLATELET_PRODUCT	REACTOME	3549	53	20	7,83E-01	9,95E-01	rs1360963|rs376047|rs661356|rs10244937|rs1480353|rs12491673|rs7235524|rs4607530|rs17071888|rs3184504|rs4747801|rs9418677|rs1357159|rs1057452|rs6466389|rs277311|rs2045418|rs9907283|rs2039300|rs7046061
Immunologic Disorder	SPHINGOLIPID_METABOLISM	REACTOME	1944	47	20	7,86E-01	9,95E-01	rs9883862|rs267733|rs1580254|rs2074625|rs2868932|rs892987|rs1341347|rs956274|rs3744307|rs10497340|rs1372050|rs16831830|rs11675308|rs439863|rs12336689|rs10483381|rs17785945|rs13353121|rs2293764|rs10474567
Immunologic Disorder	KERATINOCYTE_PATHWAY	BIOCARTA	1952	31	20	7,86E-01	9,95E-01	rs1793327|rs4750517|rs12577895|rs7910825|rs12765241|rs587198|rs1033910|rs6974790|rs10495929|rs1838793|rs7615501|rs4264601|rs7558342|rs1016860|rs4952779|rs4357599|rs798790|rs6751933|rs956572|rs2836776
Immunologic Disorder	FORMATION_OF_RNA_POL_II_ELONGATION_COMPLEX_	REACTOME	401	12	4	7,87E-01	9,95E-01	rs503347|rs4487686|rs10504553|rs886420
Immunologic Disorder	GATA3_PATHWAY	BIOCARTA	898	34	19	7,87E-01	9,95E-01	rs10514476|rs6063664|rs4747801|rs4747795|rs6021266|rs9319543|rs2302234|rs16950922|rs4747803|rs11648191|rs17686166|rs10781329|rs6063634|rs1997815|rs11255728|rs11255764|rs1559341|rs998139|rs1419294
Immunologic Disorder	HIV_LIFE_CYCLE	REACTOME	1529	37	20	7,89E-01	9,95E-01	rs2368443|rs1484931|rs17687453|rs6770415|rs503347|rs4684917|rs7596050|rs12691883|rs10944384|rs6434036|rs2011946|rs16981247|rs773546|rs2269744|rs2056296|rs4487686|rs10191360|rs5766533|rs9858561|rs1872066
Immunologic Disorder	CELLCYCLE_PATHWAY	BIOCARTA	459	6	6	7,89E-01	9,95E-01	rs4445045|rs11063059|rs11611593|rs7793983|rs2075146|rs7781436
Immunologic Disorder	BOTULINUM_NEUROTOXICITY	REACTOME	522	19	14	7,89E-01	9,95E-01	rs486409|rs2695934|rs6460052|rs12022368|rs10861034|rs362560|rs10460623|rs6951030|rs2315141|rs12938765|rs11086650|rs12740112|rs2842888|rs10902671
Immunologic Disorder	PKA_MEDIATED_PHOSPHORYLATION_OF_CREB	REACTOME	767	44	20	7,90E-01	9,95E-01	rs12545028|rs2532019|rs13168690|rs9922362|rs4629562|rs13190668|rs2302234|rs1404909|rs10781329|rs263241|rs12680002|rs7016570|rs7975385|rs10098764|rs1329795|rs12681426|rs10103835|rs7713650|rs1369450|rs7818937
Immunologic Disorder	TRANSPORT_OF_INORGANIC_CATIONS_ANIONS_AND_AMINO_ACIDS_OLIGOPEPTIDES	REACTOME	4051	34	20	7,92E-01	9,95E-01	rs2051134|rs12625137|rs10169443|rs2074615|rs2328409|rs6727414|rs10485438|rs2290046|rs9579422|rs10964216|rs16987501|rs3497|rs2383113|rs13414508|rs10191613|rs4505759|rs1554672|rs11890040|rs10145863|rs4339600
Immunologic Disorder	REGULATION_OF_KIT_SIGNALING	REACTOME	587	29	16	7,93E-01	9,95E-01	rs3184504|rs3020827|rs9944770|rs13218316|rs1507955|rs695161|rs2166019|rs12454733|rs4381631|rs8081614|rs4791036|rs3214047|rs11153318|rs1004420|rs7219495|rs12674523
Immunologic Disorder	KERATAN_SULFATE_KERATIN_METABOLISM	REACTOME	737	28	17	7,93E-01	9,95E-01	rs6095638|rs2012019|rs741901|rs2403981|rs7115683|rs1975605|rs2279861|rs2279597|rs820846|rs4808100|rs1868430|rs4985526|rs7820063|rs16904967|rs1539144|rs6981912|rs756379
Immunologic Disorder	MEIOTIC_SYNAPSIS	REACTOME	1031	27	18	7,95E-01	9,95E-01	rs7738912|rs9384022|rs8006145|rs9371582|rs6467022|rs7760713|rs2025258|rs9397501|rs4984803|rs2289938|rs2271354|rs360622|rs2748531|rs6979|rs1890100|rs9461416|rs4764812|rs1339729
Immunologic Disorder	SIGNAL_AMPLIFICATION	REACTOME	1023	30	20	7,95E-01	9,95E-01	rs4855077|rs304737|rs7140817|rs2886612|rs7039739|rs17786782|rs12488540|rs10798106|rs12704705|rs17544558|rs1162211|rs9855770|rs10498440|rs12886319|rs1253676|rs6770918|rs12086010|rs10798080|rs10494603|rs291353
Immunologic Disorder	MRNA_SPLICING_MINOR_PATHWAY	REACTOME	383	6	4	7,96E-01	9,95E-01	rs4951031|rs4487686|rs788008|rs6093835
Immunologic Disorder	PRE_NOTCH_PROCESSING_IN_GOLGI	REACTOME	691	25	10	7,97E-01	9,95E-01	rs204995|rs5756685|rs693797|rs11846825|rs1242449|rs17117532|rs3026445|rs4899801|rs1263459|rs12433856
Immunologic Disorder	AMINE_COMPOUND_SLC_TRANSPORTERS	REACTOME	1298	41	20	7,99E-01	9,95E-01	rs11659324|rs600734|rs10510403|rs396106|rs2240783|rs6763180|rs10493879|rs6499775|rs877788|rs17466478|rs2596852|rs4394904|rs460700|rs6703265|rs1471239|rs11710744|rs2944371|rs4932227|rs9826914|rs721950
Immunologic Disorder	NCAM1_INTERACTIONS	REACTOME	1896	34	20	8,01E-01	9,95E-01	rs11687617|rs1352323|rs6133126|rs6436654|rs2521206|rs9327283|rs9515201|rs12679408|rs4841920|rs7176079|rs4675141|rs11676186|rs3781878|rs2413599|rs4148407|rs7947910|rs10487254|rs1379868|rs592121|rs9521699
Immunologic Disorder	TRANSPORT_OF_GLUCOSE_AND_OTHER_SUGARS_BILE_SALTS_AND_ORGANIC_ACIDS_M NE_COMPOUNDS	REACTOME	3234	31	20	8,03E-01	9,95E-01	rs738803|rs2043128|rs11659324|rs600734|rs10510403|rs2224|rs11153238|rs948419|rs1327257|rs8129503|rs396106|rs2076370|rs7758229|rs10208012|rs2304704|rs2771338|rs11174735|rs9374174|rs1891092|rs2240783
Immunologic Disorder	APOPTOTIC_CLEAVAGE_OF_CELLULAR_PROTEINS	REACTOME	1052	48	20	8,04E-01	9,95E-01	rs4750517|rs1719361|rs17202361|rs587198|rs3769823|rs7615501|rs6597269|rs4357599|rs7220988|rs2271354|rs706494|rs11663972|rs242557|rs1409876|rs17374045|rs4355511|rs11253614|rs337475|rs10906429|rs7915166
Immunologic Disorder	DAG_AND_IP3_SIGNALING	REACTOME	1644	36	20	8,05E-01	9,95E-01	rs12545028|rs17649232|rs2532019|rs13168690|rs4599070|rs210120|rs10495929|rs9922362|rs10743591|rs3782309|rs1838793|rs7615501|rs4629562|rs4264601|rs1370666|rs7558342|rs4952779|rs13190668|rs11125132|rs2302234

Immunologic Disorder	RECRUITMENT_OF_MITOTIC_CENTROSOME_PROTEINS_AND_COMPLEXES	REACTOME	1177	47	20	8,05E-01	9,95E-01	rs34530|rs870777|rs4907532|rs9309485|rs4837708|rs10777859|rs11794773|rs10494940|rs2158138|rs4924687|rs1333924|rs8058610|rs12096414|rs11104736|rs5006363|rs12422443|rs1894923|rs3789311|rs1993067|rs719450
Immunologic Disorder	CELL_CYCLE_CHECKPOINTS	REACTOME	1961	42	20	8,06E-01	9,95E-01	rs10268808|rs7074757|rs2892190|rs6457689|rs7683090|rs1801376|rs9540340|rs2073032|rs9876561|rs1957107|rs17482047|rs807293|rs9570962|rs6736093|rs12602230|rs4943189|rs3786136|rs764190|rs1832265|rs9539881
Immunologic Disorder	RNA_POL_III_CHAIN_ELONGATION	REACTOME	364	17	9	8,06E-01	9,95E-01	rs6081160|rs6539267|rs510342|rs4961396|rs4922157|rs12930464|rs1075|rs4921702|rs2247746
Immunologic Disorder	DOWNSTREAM_SIGNALING_EVENTS_OF_B_CELL_RECEPTOR_BCR	REACTOME	2127	34	20	8,07E-01	9,95E-01	rs10268808|rs4963867|rs2892190|rs10509534|rs7322396|rs10046115|rs13238172|rs11685205|rs10876227|rs9359935|rs1957107|rs10806443|rs807293|rs7991808|rs6924218|rs284513|rs764190|rs6575122|rs8036582|rs11066
Immunologic Disorder	MITOCHONDRIAL_TRNA_AMINOACYLATION	REACTOME	500	28	13	8,08E-01	9,95E-01	rs1977058|rs9660185|rs727238|rs10807297|rs10899545|rs2372948|rs12132918|rs17576051|rs9472250|rs1535298|rs1815271|rs2293341|rs886420
Immunologic Disorder	AXON_GUIDANCE	REACTOME	13131	23	20	8,09E-01	9,95E-01	rs12375339|rs11924816|rs7435615|rs4750517|rs10502934|rs12914000|rs1592818|rs4809563|rs6578414|rs551357|rs17023932|rs8033109|rs17202361|rs12620429|rs1559677|rs4761558|rs3755768|rs1032491|rs2163783|rs17054953
Immunologic Disorder	FANCONI_ANEMIA_PATHWAY	REACTOME	461	29	12	8,12E-01	9,95E-01	rs4657|rs10889328|rs13399274|rs4439380|rs42840|rs13029039|rs10490113|rs4325734|rs12713380|rs6731791|rs929641|rs449499
Immunologic Disorder	RNA_POL_I_PROMOTER_OPENING	REACTOME	501	9	4	8,13E-01	9,95E-01	rs7760713|rs10208|rs1630732|rs9461416
Immunologic Disorder	OTHER_GLYCAN_DEGRADATION	KEGG	648	22	14	8,14E-01	9,95E-01	rs17064802|rs2122136|rs892445|rs2716805|rs17716609|rs2293764|rs820846|rs1464292|rs9790604|rs13151036|rs4861798|rs6552352|rs13140529|rs12233819
Immunologic Disorder	VIBRIO_CHOLERAE_INFECTION	KEGG	1518	40	20	8,14E-01	9,95E-01	rs3794176|rs1719361|rs2532019|rs1512348|rs896189|rs7197601|rs2454016|rs7562668|rs1676904|rs6473885|rs7108037|rs11644646|rs17793122|rs3093988|rs6713990|rs4930006|rs10781329|rs4254322|rs4381631|rs11825977
Immunologic Disorder	ACUTE_MYELOID_LEUKEMIA	KEGG	1892	34	20	8,15E-01	9,95E-01	rs16897466|rs4886844|rs4963867|rs2094405|rs11657742|rs9642880|rs3020827|rs931771|rs2834906|rs1507955|rs13320527|rs7812998|rs10891629|rs17153654|rs4255473|rs11701104|rs13173003|rs2033098|rs706725|rs2166019
Immunologic Disorder	GLEEVEC_PATHWAY	BIOCARTA	954	29	20	8,15E-01	9,95E-01	rs16897466|rs5996534|rs9642880|rs13320527|rs7812998|rs17153654|rs13173003|rs2033098|rs3024908|rs706725|rs5751614|rs741847|rs9900213|rs6449947|rs12536419|rs2161529|rs4552863|rs16902328|rs12655019|rs11747611
Immunologic Disorder	E2F_MEDIATED_REGULATION_OF_DNA_REPLICATION	REACTOME	452	4	2	8,17E-01	9,95E-01	rs4727590|rs2298582
Immunologic Disorder	HOMOLOGOUS_RECOMBINATION_REPAIR_OF_REPLICATION_INDEPENDENT_DOUBLE_ST	REACTOME	376	12	8	8,17E-01	9,95E-01	rs17482047|rs16981247|rs10849582|rs12602230|rs3786136|rs10258834|rs7357092|rs11571468
Immunologic Disorder	SYNTHESIS_OF_DNA	REACTOME	1545	35	20	8,17E-01	9,95E-01	rs10268808|rs2892190|rs10046115|rs9540340|rs2073032|rs3815795|rs1957107|rs17482047|rs807293|rs9570962|rs16981247|rs12602230|rs4943189|rs3786136|rs764190|rs1832265|rs9539881|rs6575122|rs4727590|rs2293361
Immunologic Disorder	HEDGEHOG_SIGNALING_PATHWAY	KEGG	2151	29	20	8,22E-01	9,95E-01	rs4655036|rs2074625|rs1980499|rs6952416|rs7769728|rs811153|rs2810061|rs2807362|rs17356059|rs7155858|rs4766398|rs7796149|rs7349332|rs6085802|rs17270248|rs7273197|rs2575580|rs6768050|rs7624718|rs4811808
Immunologic Disorder	CLEAVAGE_OF_GROWING_TRANSCRIPT_IN_THE_TERMINATION_REGION_	REACTOME	435	15	9	8,22E-01	9,95E-01	rs4951031|rs3737984|rs3809425|rs2282763|rs9937484|rs11749500|rs4653184|rs2334321|rs6093835
Immunologic Disorder	DEPOSITION_OF_NEW_CENPA_CONTAINING_NUCLEOSOMES_AT_THE_CENTROMERE	REACTOME	637	27	13	8,22E-01	9,95E-01	rs406387|rs7606893|rs8134439|rs7760713|rs2173746|rs11562980|rs12124096|rs585513|rs460262|rs7697867|rs9461416|rs10483587|rs7171260
Immunologic Disorder	YAP1_AND_WWTR1_TAZ_STIMULATED_GENE_EXPRESSION	REACTOME	1047	28	20	8,23E-01	9,95E-01	rs12310617|rs1555949|rs7858838|rs4857735|rs17136507|rs16911712|rs6772982|rs2047564|rs2236018|rs4823529|rs13065308|rs16828012|rs722420|rs1296720|rs4917339|rs2948091|rs11794310|rs12562819|rs4539982|rs2095252
Immunologic Disorder	CLASS_I_MHC_MEDIATED_ANTIGEN_PROCESSING_PRESENTATION	REACTOME	5895	48	20	8,24E-01	9,95E-01	rs12179534|rs705551|rs3115612|rs10268808|rs6685064|rs12620429|rs9870951|rs4667005|rs2892190|rs10490815|rs10046115|rs7648548|rs9323205|rs6942330|rs4687569|rs1611493|rs7714122|rs709641|rs3752496|rs7670291
Immunologic Disorder	MAP_KINASE_ACTIVATION_IN_TLR_CASCADE	REACTOME	1697	28	20	8,24E-01	9,95E-01	rs1009619|rs2066843|rs3817828|rs1519959|rs12054920|rs1688545|rs6860708|rs8026614|rs9359935|rs10806443|rs2869433|rs7726306|rs6961625|rs7727198|rs6924218|rs1019206|rs284513|rs17555326|rs3799577|rs3886478
Immunologic Disorder	TNFR2_PATHWAY	BIOCARTA	414	15	10	8,26E-01	9,95E-01	rs4978374|rs1710627|rs8904|rs12206804|rs10140922|rs640353|rs12655019|rs1012919|rs11747611|rs816060
Immunologic Disorder	FATTY_ACID_TRIACYLGLYCEROL_AND_KETONE_BODY_METABOLISM	REACTOME	5526	33	20	8,27E-01	9,95E-01	rs627538|rs2306718|rs12199314|rs1509241|rs6870653|rs680548|rs4795194|rs7616534|rs2487052|rs6952398|rs2686084|rs12310617|rs1555949|rs10452184|rs10182865|rs7858838|rs204995|rs12308345|rs7713987|rs10861171
Immunologic Disorder	WNT_PATHWAY	BIOCARTA	1363	28	20	8,28E-01	9,95E-01	rs730830|rs2710952|rs11794108|rs7856583|rs9642880|rs7028704|rs931771|rs9359935|rs17136507|rs10806443|rs7812998|rs6924218|rs284513|rs1186145|rs2033098|rs11139346|rs695161|rs7746039|rs7218768|rs1296720
Immunologic Disorder	ABCA_TRANSPORTERS_IN_LIPID_HOMEOSTASIS	REACTOME	464	6	5	8,28E-01	9,95E-01	rs323073|rs10877173|rs4672731|rs6501919|rs7419736
Immunologic Disorder	SIGNALING_BY_ILS	REACTOME	2996	33	20	8,30E-01	9,95E-01	rs1679802|rs16897466|rs7628250|rs4963867|rs12666352|rs2066843|rs3856848|rs1030021|rs2296950|rs4901623|rs11883987|rs9359935|rs11567697|rs13218316|rs13320527|rs17196143|rs975360|rs10806443|rs1808563|rs6125883
Immunologic Disorder	INTESTINAL_IMMUNE_NETWORK_FOR_IGA_PRODUCTION	KEGG	1392	54	20	8,31E-01	9,95E-01	rs987870|rs2028373|rs12691883|rs2011946|rs17500510|rs9276933|rs4988821|rs9275312|rs1904836|rs9559300|rs2056296|rs9276291|rs2681417|rs2395182|rs10191360|rs3850997|rs4675391|rs3770136|rs117821|rs241398
Immunologic Disorder	M_G1_TRANSITION	REACTOME	1315	35	20	8,31E-01	9,95E-01	rs10268808|rs2892190|rs6457689|rs2073032|rs1957107|rs17482047|rs807293|rs12602230|rs3786136|rs764190|rs9348428|rs6575122|rs4727590|rs2293361|rs2716601|rs12436216|rs16972457|rs228271|rs8075668|rs2164210
Immunologic Disorder	AMINO_SUGAR_AND_NUCLEOTIDE_SUGAR_METABOLISM	KEGG	956	36	20	8,32E-01	9,95E-01	rs10776724|rs761147|rs2608836|rs3800083|rs10814369|rs10998736|rs820846|rs10938371|rs6531592|rs2486072|rs1559113|rs4878646|rs3800128|rs1872837|rs7680159|rs7707082|rs12047673|rs2909075|rs2318089|rs2812365
Immunologic Disorder	CD28_DEPENDENT_PI3K_AKT_SIGNALING	REACTOME	572	26	18	8,32E-01	9,95E-01	rs16897466|rs13218316|rs13320527|rs13173003|rs2681417|rs706725|rs695161|rs874269|rs6449947|rs683590|rs6084242|rs2161529|rs11153318|rs1004420|rs10515074|rs2332096|rs559080|rs6871452
Immunologic Disorder	PHOSPHORYLATION_OF_THE_APC_C	REACTOME	219	11	7	8,33E-01	9,95E-01	rs9876561|rs6736093|rs8058610|rs1994174|rs12352784|rs4369862|rs9590483
Immunologic Disorder	ACTIVATED_TAK1_MEDIATES_P38_MAPK_ACTIVATION	REACTOME	649	22	14	8,33E-01	9,95E-01	rs2066843|rs9359935|rs10806443|rs6961625|rs6924218|rs284513|rs7746039|rs2440134|rs2103994|rs331455|rs6955931|rs11656130|rs9895446|rs2715815
Immunologic Disorder	G1_PHASE	REACTOME	625	7	7	8,34E-01	9,95E-01	rs4445045|rs11063059|rs9348428|rs11611593|rs7793983|rs2075146|rs7781436
Immunologic Disorder	ACTIVATION_OF_NF_KAPPAB_IN_B_CELLS	REACTOME	1396	35	20	8,34E-01	9,95E-01	rs10268808|rs2892190|rs6457689|rs13238172|rs9359935|rs1957107|rs10806443|rs807293|rs6924218|rs284513|rs764190|rs6575122|rs2293361|rs2716601|rs7746039|rs6726160|rs12436216|rs16972457|rs228271|rs8904
Immunologic Disorder	SIGNALING_BY_TGF_BETA_RECEPTOR_COMPLEX	REACTOME	1605	23	20	8,34E-01	9,95E-01	rs3773650|rs991694|rs12308345|rs3752496|rs3782267|rs9642880|rs727929|rs266335|rs701078|rs7812998|rs1160769|rs1002386|rs2063327|rs11661651|rs10846681|rs2033098|rs11661340|rs17236851|rs3859312|rs13065308
Immunologic Disorder	STEROID_BIOSYNTHESIS	KEGG	247	9	4	8,35E-01	9,95E-01	rs4839|rs2492778|rs12676995|rs12358054
Immunologic Disorder	AQUAPORIN_MEDIATED_TRANSPORT	REACTOME	1510	38	20	8,35E-01	9,95E-01	rs12545028|rs4855077|rs7140817|rs2532019|rs13168690|rs8032239|rs9922362|rs6672010|rs4629562|rs9920375|rs13190668|rs1760798|rs2302234|rs12488540|rs12704705|rs17716413|rs1404909|rs10781329|rs263241|rs10498440
Immunologic Disorder	CELL_JUNCTION_ORGANIZATION	REACTOME	6781	34	20	8,39E-01	9,95E-01	rs11564334|rs2446886|rs11215593|rs17749762|rs4782717|rs3101011|rs11639525|rs7709151|rs4378885|rs12444964|rs4353494|rs4518142|rs12598326|rs8055843|rs10472700|rs4782749|rs523223|rs4577701|rs6089291|rs233551
Immunologic Disorder	THYROID_CANCER	KEGG	1104	34	20	8,39E-01	9,95E-01	rs4963867|rs2094405|rs1555949|rs7858838|rs9642880|rs931771|rs7533324|rs7812998|rs12620738|rs4255473|rs2033098|rs4917339|rs11794310|rs7896811|rs17388587|rs7079711|rs4552863|rs16902328|rs17567325|rs9277937
Immunologic Disorder	TNFR1_PATHWAY	BIOCARTA	862	33	18	8,41E-01	9,95E-01	rs2362419|rs7114704|rs9359935|rs10806443|rs3769823|rs6924218|rs284513|rs8178179|rs7746039|rs2616052|rs2271354|rs12738235|rs527589|rs12655019|rs7957946|rs11747611|rs1005890|rs921
Immunologic Disorder	APC_C_CDC20_MEDIATED_DEGRADATION_OF_CYCLIN_B	REACTOME	224	10	6	8,42E-01	9,95E-01	rs9876561|rs6736093|rs1994174|rs12352784|rs4369862|rs9590483
Immunologic Disorder	IL17_PATHWAY	BIOCARTA	316	12	9	8,43E-01	9,95E-01	rs17575616|rs704856|rs8193036|rs1413917|rs9916158|rs2995094|rs858490|rs6753271|rs8193040
Immunologic Disorder	TRNA_AMINOACYLATION	REACTOME	795	44	19	8,43E-01	9,95E-01	rs1977058|rs9660185|rs309137|rs2039521|rs727238|rs10807297|rs10899545|rs556155|rs925210|rs193466|rs2372948|rs12132918|rs17576051|rs7443214|rs9472250|rs1535298|rs1815271|rs2293341|rs886420
Immunologic Disorder	COSTIMULATION_BY_THE_CD28_FAMILY	REACTOME	1479	47	20	8,43E-01	9,95E-01	rs16897466|rs987870|rs1678002|rs4143813|rs13218316|rs13320527|rs17500510|rs1808563|rs1378942|rs704856|rs13173003|rs9276291|rs2681417|rs706725|rs7145618|rs695161|rs874269|rs4675391|rs6449947|rs2995094
Immunologic Disorder	AMINOACYL_TRNA_BIOSYNTHESIS	KEGG	731	42	18	8,44E-01	9,95E-01	rs1977058|rs9660185|rs309137|rs727238|rs10807297|rs10899545|rs556155|rs925210|rs193466|rs2372948|rs12132918|rs17576051|rs7443214|rs9472250|rs1535298|rs1815271|rs2293341|rs886420
Immunologic Disorder	P53HYPOXIA_PATHWAY	BIOCARTA	517	22	13	8,44E-01	9,95E-01	rs2035567|rs6977956|rs12602230|rs3786136|rs1486137|rs1064825|rs2270941|rs11870150|rs7145164|rs6946252|rs17513961|rs7604966|rs8104760
Immunologic Disorder	AMINO_ACID_TRANSPORT_ACROSS_THE_PLASMA_MEMBRANE	REACTOME	1025	27	20	8,45E-01	9,95E-01	rs9579422|rs3497|rs10145863|rs6763180|rs17073762|rs1571784|rs1822743|rs737039|rs752503|rs1105168|rs3770705|rs17740674|rs4394904|rs1345091|rs2588156|rs10519465|rs2729384|rs1471239|rs11710744|rs9933029
Immunologic Disorder	ACTIVATION_OF_THE_PRE_REPLICATIVE_COMPLEX	REACTOME	565	17	10	8,45E-01	9,95E-01	rs2073032|rs17482047|rs12602230|rs3786136|rs4727590|rs2164210|rs13082666|rs10258834|rs309126|rs7357092
Immunologic Disorder	SMALL_CELL_LUNG_CANCER	KEGG	3747	32	20	8,46E-01	9,95E-01	rs16897466|rs10510816|rs1580816|rs12620429|rs10509534|rs6550967|rs6436654|rs4858703|rs1555949|rs12203956|rs7858838|rs9311770|rs11657742|rs7638130|rs1532113|rs9515201|rs9642880|rs3212588|rs12108196|rs2488320
Immunologic Disorder	AGR_PATHWAY	BIOCARTA	1981	27	20	8,46E-01	9,95E-01	rs17099627|rs776385|rs12203956|rs2919390|rs6974790|rs4076319|rs6118746|rs10503904|rs11632939|rs2488320|rs10980549|rs13163497|rs4332201|rs6580353|rs4587680|rs7826312|rs11009195|rs1481756|rs3824867|rs1140475
Immunologic Disorder	IL_2_SIGNALING	REACTOME	936	25	20	8,47E-01	9,95E-01	rs16897466|rs4963867|rs3856848|rs13320527|rs13173003|rs706725|rs1981405|rs131849|rs695161|rs4808100|rs9900213|rs6449947|rs2161529|rs17388587|rs1148844|rs4054727|rs1004420|rs10515074|rs2868161|rs6871452
Immunologic Disorder	RACCYCD_PATHWAY	BIOCARTA	551	21	14	8,49E-01	9,95E-01	rs16897466|rs13320527|rs13173003|rs706725|rs8904|rs6449947|rs10140922|rs2161529|rs7793983|rs527589|rs1012919|rs10515074|rs7781436|rs6871452
Immunologic Disorder	CYTOSOLIC_DNA_SENSING_PATHWAY	KEGG	620	25	14	8,49E-01	9,95E-01	rs6081160|rs6539267|rs7046061|rs10878177|rs510342|rs1613367|rs8904|rs3733236|rs10811518|rs10140922|rs13038139|rs1012919|rs6133067|rs2247746
Immunologic Disorder	GLYCOSYLPHOSPHATIDYLINOSITOL_GPI_ANCHOR_BIOSYNTHESIS	KEGG	318	13	9	8,52E-01	9,95E-01	rs2049363|rs2269615|rs796985|rs12902710|rs3742872|rs2273595|rs4949652|rs514874|rs2717532
Immunologic Disorder	CA_DEPENDENT_EVENTS	REACTOME	1487	40	20	8,53E-01	9,95E-01	rs12545028|rs17649232|rs2532019|rs13168690|rs4599070|rs9922362|rs7615501|rs4629562|rs1370666|rs13190668|rs11125132|rs2302234|rs31309|rs10798106|rs10781329|rs1404909|rs7709420|rs17544558|rs263241|rs12680002
Immunologic Disorder	ECM_RECEPTOR_INTERACTION	KEGG	3602	34	20	8,54E-01	9,95E-01	rs1757096|rs12620429|rs723188|rs11687617|rs2126642|rs1018829|rs2284277|rs1553311|rs6436654|rs12203956|rs2521206|rs6467|rs941022|rs9426785|rs9515201|rs13080492|rs4841920|rs3212588|rs11581077|rs2488320
Immunologic Disorder	DOUBLE_STRAND_BREAK_REPAIR	REACTOME	506	14	10	8,54E-01	9,95E-01	rs17482047|rs16981247|rs10849582|rs12602230|rs3786136|rs8178179|rs1872066|rs10258834|rs7357092|rs11571468
Immunologic Disorder	FAS_PATHWAY	BIOCARTA	980	35	19	8,56E-01	9,95E-01	rs7910825|rs12765241|rs9359935|rs10806443|rs3769823|rs6924218|rs284513|rs8178179|rs2287147|rs7746039|rs2616052|rs7690165|rs2271354|rs982764|rs12738235|rs527589|rs12655019|rs11747611|rs921
Immunologic Disorder	MRNA_3_END_PROCESSING	REACTOME	320	10	5	8,58E-01	9,95E-01	rs3737984|rs3809425|rs9937484|rs2334321|rs6093835
Immunologic Disorder	EPO_PATHWAY	BIOCARTA	255	3	2	8,60E-01	9,95E-01	rs741847|rs9900213
Immunologic Disorder	SLC_MEDIATED_TRANSMEMBRANE_TRANSPORT	REACTOME	8774	32	20	8,61E-01	9,95E-01	rs2051134|rs12625137|rs738803|rs2043128|rs11853372|rs10169443|rs2368443|rs2074615|rs2328409|rs11659324|rs600734|rs6727414|rs1484931|rs10485438|rs2236553|rs2290046|rs10510403|rs6770415|rs4149028|rs4684917
Immunologic Disorder	PARKINSONS_DISEASE	KEGG	2833	31	20	8,63E-01	9,95E-01	rs10799655|rs11745467|rs3794176|rs6685064|rs11077773|rs1800649|rs5993761|rs16899656|rs7291584|rs12292212|rs2898543|rs4720913|rs4239838|rs778584|rs623817|rs4149553|rs17759300|rs9458499|rs17701871|rs2647209
Immunologic Disorder	GLUCOSE_METABOLISM	REACTOME	1554	39	20	8,63E-01	9,95E-01	rs3793932|rs11251480|rs923109|rs12768505|rs7944999|rs10258885|rs4856401|rs9649679|rs764223|rs9419445|rs13380644|rs11983987|rs11125132|rs4511520|rs4433480|rs10781329|rs6025645|rs9875791|rs10903880|rs11251555
Immunologic Disorder	CONVERSION_FROM_APC_C_CDC20_TO_APC_C_CDH1_IN_LATE_ANAPHASE	REACTOME	226	13	8	8,65E-01	9,95E-01	rs9876561|rs6736093|rs1063302|rs1994174|rs12352784|rs4369862|rs9590483|rs2809823
Immunologic Disorder	CYCLIN_E_ASSOCIATED_EVENTS_DURING_G1_S_TRANSITION_	REACTOME	1212	41	20	8,68E-01	9,95E-01	rs10268808|rs2892190|rs10046115|rs9642880|rs1957107|rs7812998|rs807293|rs2033098|rs764190|rs6575122|rs2716601|rs12436216|rs16972457|rs228271|rs8075668|rs7105122|rs117821|rs4552863|rs16930278|rs16902328
Immunologic Disorder	RORA_ACTIVATES_CIRCADIAN_EXPRESSION	REACTOME	1377	34	20	8,69E-01	9,95E-01	rs1509241|rs1555949|rs7858838|rs4857735|rs2290430|rs17136507|rs4235306|rs1159937|rs6772982|rs12989454|rs1388331|rs2047564|rs679596|rs4823529|rs11634318|rs1589703|rs4074920|rs456998|rs11894671|rs16828012
Immunologic Disorder	MEIOTIC_RECOMBINATION	REACTOME	993	22	12	8,69E-01	9,95E-01	rs12449313|rs17482047|rs12602230|rs7760713|rs3786136|rs5750616|rs4821792|rs10258834|rs9461416|rs921986|rs10520896|rs7357092
Immunologic Disorder	NITROGEN_METABOLISM	KEGG	587	27	14	8,70E-01	9,95E-01	rs1934158|rs4934297|rs289806|rs2688655|rs359535|rs12138787|rs7961807|rs1475332|rs2241136|rs3765965|rs10932334|rs12301817|rs290558|rs186471
Immunologic Disorder	CIRCADIAN_REPRESSION_OF_EXPRESSION_BY_REV_ERBA	REACTOME	1358	34	20	8,71E-01	9,95E-01	rs1509241|rs1555949|rs7858838|rs4857735|rs2290430|rs17136507|rs4235306|rs1159937|rs6772982|rs12989454|rs1388331|rs2047564|rs4823529|rs11634318|rs1589703|rs4074920|rs456998|rs11894671|rs16828012|rs1296720
Immunologic Disorder	MTA3_PATHWAY	BIOCARTA	333	12	6	8,71E-01	9,95E-01	rs930420|rs9383939|rs695161|rs3020418|rs6557164|rs9671696
Immunologic Disorder	UBIQUITIN_MEDIATED_PROTEOLYSIS	KEGG	3227	32	20	8,72E-01	9,95E-01	rs705551|rs4886844|rs6685064|rs9870951|rs4667005|rs8088164|rs7648548|rs1532113|rs3752496|rs7670291|rs2910411|rs2754513|rs6869437|rs12534197|rs9876561|rs4239838|rs9477599|rs13287325|rs900791|rs6566946
Immunologic Disorder	CHONDROITIN_SULFATE_DERMATAN_SULFATE_METABOLISM	REACTOME	2784	28	20	8,72E-01	9,95E-01	rs6783962|rs2284277|rs483981|rs1111994|rs7986556|rs11223864|rs12855016|rs1581826|rs1944866|rs11231168|rs9426785|rs7325810|rs1380331|rs4505759|rs11581077|rs1123373|rs7765174|rs2640882|rs12855484|rs1994788
Immunologic Disorder	CCR5_PATHWAY	BIOCARTA	696	24	16	8,72E-01	9,95E-01	rs12691883|rs2011946|rs7039739|rs17786782|rs1904836|rs11125132|rs2056296|rs10191360|rs741847|rs4381631|rs8081614|rs4791036|rs169143|rs13002829|rs7219495|rs815802
Immunologic Disorder	GLYCOLYSIS_GLUCONEOGENESIS	KEGG	1391	34	20	8,75E-01	9,95E-01	rs3794176|rs11251480|rs10918239|rs10998736|rs4578194|rs10918228|rs764223|rs9419445|rs11631023|rs4129215|rs4433480|rs6025645|rs11710823|rs12554375|rs10903880|rs1238741|rs1465936|rs954241|rs11251555|rs6531592
Immunologic Disorder	HOMOLOGOUS_RECOMBINATION	KEGG	730	26	16	8,75E-01	9,95E-01	rs12449313|rs1464807|rs861528|rs17482047|rs10849582|rs12602230|rs3786136|rs7785299|rs927221|rs2256224|rs10258834|rs2253168|rs921986|rs7357092|rs11571468|rs757369
Immunologic Disorder	STEROID_HORMONE_BIOSYNTHESIS	KEGG	1491	41	20	8,75E-01	9,95E-01	rs2572023|rs6830865|rs6467|rs10031344|rs4336933|rs34778440|rs2862996|rs4736349|rs11252845|rs1937881|rs10923819|rs4149455|rs10883782|rs12522757|rs6600793|rs6717546|rs12056107|rs1347047|rs9277937|rs10795227
Immunologic Disorder	BLADDER_CANCER	KEGG	1222	31	20	8,76E-01	9,95E-01	rs4963867|rs6974790|rs382649|rs9642880|rs131820|rs10520358|rs7812998|rs1105384|rs1478604|rs13110230|rs7125320|rs2033098|rs9348428|rs3775198|rs1140475|rs7156757|rs3118846|rs4282116|rs17388587|rs4552863
Immunologic Disorder	HCMV_PATHWAY	BIOCARTA	547	23	16	8,77E-01	9,95E-01	rs16897466|rs13320527|rs17153654|rs13173003|rs706725|rs2440134|rs6449947|rs12536419|rs2161529|rs12655019|rs11747611|rs10515074|rs6871452|rs11656130|rs9895446|rs2715815
Immunologic Disorder	CELL_CELL_COMMUNICATION	REACTOME	9725	29	20	8,78E-01	9,95E-01	rs11564334|rs16897466|rs2446886|rs10502934|rs11215593|rs17749762|rs4782717|rs3101011|rs11639525|rs2837867|rs10521156|rs7709151|rs3802819|rs4378885|rs12444964|rs7203355|rs2837801|rs4518142|rs12598326|rs2837361
Immunologic Disorder	PPARA_PATHWAY	BIOCARTA	2445	32	20	8,78E-01	9,95E-01	rs16897466|rs7374394|rs1509241|rs7114704|rs1555949|rs7858838|rs12308345|rs3782267|rs9642880|rs978180|rs13320527|rs701078|rs17136507|rs4235306|rs7812998|rs1159937|rs17153654|rs2302234|rs1388331|rs10846681
Immunologic Disorder	ADP_SIGNALLING_THROUGH_P2RY1	REACTOME	863	34	20	8,78E-01	9,95E-01	rs4855077|rs7140817|rs7039739|rs17786782|rs12488540|rs10798106|rs12704705|rs17544558|rs1162211|rs9855770|rs10498440|rs12886319|rs1253676|rs12086010|rs10798080|rs10494603|rs291353|rs12595160|rs4659946|rs8023017
Immunologic Disorder	PORPHYRIN_AND_CHLOROPHYLL_METABOLISM	KEGG	1163	40	18	8,78E-01	9,95E-01	rs3853397|rs6830865|rs10031344|rs727238|rs34778440|rs1043836|rs9900346|rs8071450|rs2269221|rs16861582|rs12188286|rs3088235|rs10951743|rs6600793|rs6717546|rs10521248|rs1347047|rs11078200
Immunologic Disorder	DEATH_PATHWAY	BIOCARTA	505	21	15	8,79E-01	9,95E-01	rs11135693|rs1047266|rs3769823|rs1016860|rs956572|rs8904|rs4268128|rs10140922|rs12738235|rs1862710|rs12700583|rs1012919|rs7315397|rs12454650|rs337475
Immunologic Disorder	ACETYLCHOLINE_BINDING_AND_DOWNSTREAM_EVENTS	REACTOME	242	15	9	8,79E-01	9,95E-01	rs1567883|rs12442954|rs6495309|rs16970006|rs6062894|rs6090392|rs7167632|rs2337980|rs9672615
Immunologic Disorder	NCAM_SIGNALING_FOR_NEURITE_OUT_GROWTH	REACTOME	2480	27	20	8,80E-01	9,95E-01	rs11687617|rs1352323|rs4963867|rs6133126|rs6436654|rs2521206|rs9327283|rs9515201|rs12679408|rs4841920|rs7176079|rs4675141|rs6675810|rs13218316|rs11676186|rs3781878|rs4902311|rs2445884|rs2413599|rs4148407
Immunologic Disorder	INSULIN_SYNTHESIS_AND_PROCESSING	REACTOME	1033	29	20	8,80E-01	9,95E-01	rs3792747|rs1955161|rs10518808|rs6460052|rs2611249|rs1211554|rs362560|rs1251370|rs4360172|rs2250704|rs4691213|rs1004446|rs11743274|rs6951030|rs2269044|rs4529218|rs6136050|rs1007912|rs12509278|rs11135469
Immunologic Disorder	P53_PATHWAY	BIOCARTA	347	11	8	8,81E-01	9,95E-01	rs737088|rs1016860|rs956572|rs2270941|rs7287772|rs919699|rs8104760|rs12454650
Immunologic Disorder	BMAL1_CLOCK_NPAS2_ACTIVATES_CIRCADIAN_EXPRESSION	REACTOME	1728	33	20	8,81E-01	9,95E-01	rs7121775|rs1509241|rs179883|rs1555949|rs7858838|rs4857735|rs2290430|rs17136507|rs4235306|rs1159937|rs11038695|rs6580285|rs6772982|rs12989454|rs1388331|rs2047564|rs4823529|rs11634318|rs1589703|rs4074920
Immunologic Disorder	REGULATION_OF_WATER_BALANCE_BY_RENAL_AQUAPORINS	REACTOME	1393	32	20	8,82E-01	9,95E-01	rs12545028|rs4855077|rs7140817|rs2532019|rs13168690|rs9922362|rs4629562|rs13190668|rs2302234|rs12488540|rs12704705|rs17716413|rs10781329|rs1404909|rs263241|rs10498440|rs12680002|rs1786648|rs12886319|rs7016570
Immunologic Disorder	GPCR_PATHWAY	BIOCARTA	1102	26	20	8,83E-01	9,95E-01	rs304737|rs6063664|rs2469749|rs2886612|rs7039739|rs17786782|rs6021266|rs11125132|rs2302234|rs3804357|rs1404909|rs10781329|rs6063634|rs1997815|rs741847|rs4381631|rs8081614|rs4791036|rs169143|rs13002829
Immunologic Disorder	LYSOSOME	KEGG	2448	25	20	8,83E-01	9,95E-01	rs3794176|rs1242119|rs17064802|rs2122136|rs661821|rs4932145|rs941022|rs896189|rs10069818|rs4505759|rs4889841|rs3744307|rs1299525|rs892445|rs4839|rs2716805|rs8078165|rs13259308|rs17716609|rs727238
Immunologic Disorder	FORMATION_OF_THE_TERNARY_COMPLEX_AND_SUBSEQUENTLY_THE_43S_COMPLEX	REACTOME	677	34	16	8,86E-01	9,95E-01	rs4599669|rs10824609|rs11902670|rs10955775|rs1249123|rs7825662|rs16888522|rs4979816|rs12879285|rs10824618|rs4803512|rs8460|rs10505278|rs2277841|rs1959573|rs12786251

Immunologic Disorder	SMAD2_SMAD3_SMAD4_HETEROTRIMER_REGULATES_TRANSCRIPTION	REACTOME	715	29	18	8,86E-01	9,95E-01	rs9642880|rs727929|rs266335|rs7812998|rs2063327|rs11661651|rs2033098|rs11661340|rs3859312|rs13065308|rs695161|rs722420|rs4552863|rs16902328|rs920293|rs7351039|rs4464148|rs7652697
Immunologic Disorder	MITOTIC_G2_G2_M_PHASES	REACTOME	1389	43	20	8,88E-01	9,95E-01	rs34541|rs870777|rs4907532|rs9309485|rs4837708|rs10777859|rs11794773|rs2282128|rs10494940|rs2158138|rs4924687|rs9348428|rs1333924|rs8058610|rs12096414|rs11104736|rs5006363|rs12422443|rs1894923|rs2281479
Immunologic Disorder	PRION_DISEASES	KEGG	1585	30	20	8,88E-01	9,95E-01	rs6133126|rs2827029|rs2825756|rs4817415|rs2828035|rs232405|rs13218316|rs3781878|rs1783396|rs8128017|rs7947910|rs1041752|rs2270941|rs10781329|rs11701201|rs2828074|rs399443|rs6139434|rs12625444|rs11910207
Immunologic Disorder	TPO_PATHWAY	BIOCARTA	814	27	19	8,88E-01	9,95E-01	rs16897466|rs13320527|rs17153654|rs13173003|rs2624189|rs3024908|rs706725|rs741847|rs4381631|rs8081614|rs4791036|rs9900213|rs6449947|rs12536419|rs2161529|rs169143|rs10515074|rs7219495|rs6871452
Immunologic Disorder	ALANINE_ASPARTATE_AND_GLUTAMATE_METABOLISM	KEGG	841	32	19	8,90E-01	9,95E-01	rs3793932|rs12768505|rs4934297|rs9374174|rs13380644|rs4511520|rs1569579|rs1475332|rs914971|rs40443|rs9934713|rs12554609|rs10932334|rs11966294|rs1559113|rs8049025|rs186471|rs7707082|rs4544229
Immunologic Disorder	AMINO_ACID_AND_OLIGOPEPTIDE_SLC_TRANSPORTERS	REACTOME	1707	25	20	8,90E-01	9,95E-01	rs2074615|rs2290046|rs9579422|rs16987501|rs3497|rs10145863|rs6763180|rs491600|rs17073762|rs1571784|rs1822743|rs737039|rs3823151|rs4401921|rs752503|rs1105168|rs7311469|rs3770705|rs1679933|rs17740674
Immunologic Disorder	CTLA4_INHIBITORY_SIGNALING	REACTOME	447	15	12	8,92E-01	9,95E-01	rs1678002|rs13218316|rs2681417|rs7145618|rs695161|rs874269|rs3214047|rs11153318|rs1004420|rs2332096|rs559080|rs12674523
Immunologic Disorder	MEIOSIS	REACTOME	1674	28	20	8,93E-01	9,95E-01	rs7738912|rs9384022|rs12449313|rs17482047|rs8006145|rs9371582|rs12602230|rs6467022|rs7760713|rs2025258|rs3786136|rs9397501|rs4984803|rs5750616|rs2289938|rs2271354|rs4821792|rs360622|rs10258834|rs2748531
Immunologic Disorder	DOWNSTREAM_SIGNALING_OF_ACTIVATED_FGFR	REACTOME	3636	28	20	8,94E-01	9,95E-01	rs16897466|rs12545028|rs4963867|rs10509534|rs17649232|rs7322396|rs2532019|rs17510142|rs2517037|rs12419388|rs10876227|rs13168690|rs823600|rs4599070|rs13262310|rs666028|rs210120|rs2038712|rs10870911|rs10908200
Immunologic Disorder	PACKAGING_OF_TELOMERE_ENDS	REACTOME	515	7	5	8,94E-01	9,95E-01	rs6467022|rs7760713|rs2748531|rs6979|rs9461416
Immunologic Disorder	RELA_PATHWAY	BIOCARTA	208	10	8	8,95E-01	9,95E-01	rs17136507|rs456998|rs1296720|rs8904|rs10140922|rs331455|rs1012919|rs816060
Immunologic Disorder	BRANCHED_CHAIN_AMINO_ACID_CATABOLISM	REACTOME	507	27	11	8,96E-01	9,95E-01	rs11752339|rs2352745|rs1471841|rs10498923|rs2075624|rs2125894|rs723587|rs492734|rs1751796|rs11047702|rs17356884
Immunologic Disorder	CD28_CO_STIMULATION	REACTOME	797	27	20	8,97E-01	9,95E-01	rs16897466|rs13218316|rs13320527|rs1808563|rs13173003|rs2681417|rs706725|rs695161|rs874269|rs6449947|rs683590|rs6084242|rs3214047|rs2161529|rs11153318|rs107140|rs527589|rs1004420|rs10515074|rs2473322
Immunologic Disorder	GLYCOSAMINOGLYCAN_METABOLISM	REACTOME	6096	26	20	8,98E-01	9,95E-01	rs9890008|rs7938320|rs11696078|rs9999101|rs3785690|rs2865155|rs6095638|rs6783962|rs9924390|rs2284277|rs1859906|rs483981|rs3845669|rs1111994|rs7986931|rs517231|rs12493550|rs12615148|rs11223864|rs12855016
Immunologic Disorder	PLATELET_SENSITIZATION_BY_LDL	REACTOME	676	35	18	8,99E-01	9,95E-01	rs1678002|rs97458|rs3737984|rs1607267|rs10798106|rs2185077|rs7145618|rs17544558|rs10493173|rs754524|rs12086010|rs10798080|rs10494603|rs312046|rs12726763|rs4666291|rs7568769|rs12474685
Immunologic Disorder	ANTIGEN_PROCESSING_UBIQUITINATION_PROTEASOME_DEGRADATION	REACTOME	4788	40	20	9,02E-01	9,95E-01	rs12179534|rs705551|rs10268808|rs6685064|rs9870951|rs4667005|rs2892190|rs6457689|rs7648548|rs9323205|rs6942330|rs4687569|rs7714122|rs3752496|rs7670291|rs2910411|rs195579|rs12534197|rs9876561|rs4239838
Immunologic Disorder	MICRORNA_MIRNA_BIOGENESIS	REACTOME	227	4	3	9,03E-01	9,95E-01	rs4487686|rs11958935|rs442277
Immunologic Disorder	DOWNSTREAM_SIGNAL_TRANSDUCTION	REACTOME	3083	34	20	9,04E-01	9,95E-01	rs16897466|rs12545028|rs4963867|rs10509534|rs17649232|rs7322396|rs2532019|rs10876227|rs13168690|rs4599070|rs210120|rs2296950|rs10495929|rs9922362|rs10743591|rs13320527|rs3782309|rs1838793|rs7615501|rs4629562
Immunologic Disorder	BASIGIN_INTERACTIONS	REACTOME	595	31	20	9,07E-01	9,95E-01	rs2076370|rs2488320|rs10145863|rs4587680|rs7125320|rs752503|rs7215959|rs6440052|rs11009195|rs1539624|rs10519465|rs16852004|rs1322487|rs9933029|rs11670126|rs981257|rs1376091|rs470168|rs17782215|rs2475193
Immunologic Disorder	NA_CL_DEPENDENT_NEUROTRANSMITTER_TRANSPORTERS	REACTOME	718	28	17	9,08E-01	9,95E-01	rs10510403|rs2240783|rs6763180|rs6499775|rs17466478|rs2596852|rs4394904|rs460700|rs1471239|rs11710744|rs2944371|rs9826914|rs721950|rs9800032|rs2242446|rs3774121|rs2531747
Immunologic Disorder	CHROMOSOME_MAINTENANCE	REACTOME	1949	27	20	9,13E-01	9,95E-01	rs7738912|rs9384022|rs9540340|rs406387|rs7606893|rs8134439|rs17482047|rs8006145|rs9371582|rs9570962|rs16981247|rs12602230|rs6467022|rs7760713|rs579327|rs2025258|rs4943189|rs3786136|rs9397501|rs4984803
Immunologic Disorder	HIVNEF_PATHWAY	BIOCARTA	1008	33	20	9,16E-01	9,95E-01	rs2362419|rs7910825|rs12765241|rs3769823|rs7615501|rs1016860|rs8178179|rs956572|rs2624189|rs8904|rs2271354|rs10140922|rs982764|rs12738235|rs1862710|rs12655019|rs12700583|rs1012919|rs7957946|rs11747611
Immunologic Disorder	TRANSCRIPTION_COUPLED_NER_TC_NER	REACTOME	834	28	16	9,17E-01	9,95E-01	rs9540340|rs17482047|rs9570962|rs16981247|rs12602230|rs4943189|rs3786136|rs4487686|rs1832265|rs9539881|rs10258834|rs2025448|rs4134815|rs2051547|rs7357092|rs886420
Immunologic Disorder	DEGRADATION_OF_THE_EXTRACELLULAR_MATRIX	REACTOME	580	30	17	9,17E-01	9,95E-01	rs4726572|rs6469604|rs1382104|rs7125320|rs7004633|rs11781588|rs12222856|rs9634312|rs12334533|rs783166|rs655316|rs470168|rs198977|rs17099425|rs11225394|rs198979|rs2003241
Immunologic Disorder	TELOMERE_MAINTENANCE	REACTOME	1133	30	17	9,18E-01	9,95E-01	rs9540340|rs17482047|rs9570962|rs16981247|rs12602230|rs6467022|rs7760713|rs4943189|rs3786136|rs1832265|rs9539881|rs10258834|rs2748531|rs6979|rs9461416|rs8104760|rs7357092
Immunologic Disorder	TRAFFICKING_OF_GLUR2_CONTAINING_AMPA_RECEPTORS	REACTOME	721	32	15	9,18E-01	9,95E-01	rs2076370|rs1493383|rs1461236|rs372332|rs1945968|rs4381631|rs8081614|rs4791036|rs2344819|rs11710744|rs384842|rs169143|rs10515690|rs7116745|rs7219495
Immunologic Disorder	FC_EPSILON_RI_SIGNALING_PATHWAY	KEGG	2758	26	20	9,19E-01	9,95E-01	rs16897466|rs2510257|rs4963867|rs3890760|rs12904722|rs11657742|rs7197601|rs8045689|rs10495929|rs1476002|rs13218316|rs13320527|rs4468807|rs1838793|rs2869433|rs1808563|rs7615501|rs6672057|rs4264601|rs17153654
Immunologic Disorder	GLOBAL_GENOMIC_NER_GG_NER	REACTOME	823	28	17	9,19E-01	9,95E-01	rs9540340|rs17482047|rs9570962|rs16981247|rs12602230|rs4943189|rs3786136|rs1832265|rs9539881|rs13292544|rs12336762|rs2291120|rs10258834|rs2051547|rs7357092|rs886420|rs17195683
Immunologic Disorder	VEGF_SIGNALING_PATHWAY	KEGG	2111	28	20	9,20E-01	9,95E-01	rs16897466|rs4963867|rs12904722|rs9299346|rs6063664|rs2469749|rs11657742|rs7197601|rs1476002|rs13320527|rs6672057|rs17153654|rs6021266|rs11644646|rs13173003|rs17793122|rs2261612|rs1064825|rs10798106|rs706725
Immunologic Disorder	PROCESSING_OF_CAPPED_INTRON_CONTAINING_PRE_MRNA	REACTOME	1547	35	20	9,22E-01	9,95E-01	rs2368443|rs1484931|rs6770415|rs4684917|rs12121067|rs7596050|rs9970867|rs3737984|rs6434036|rs6135844|rs3809425|rs773546|rs4487686|rs2282763|rs5766533|rs2022747|rs6571850|rs9937484|rs17757841|rs12352784
Immunologic Disorder	PHOSPHOLIPASE_C_MEDIATED_CASCADE	REACTOME	2628	35	20	9,24E-01	9,95E-01	rs12545028|rs17649232|rs2532019|rs17510142|rs2517037|rs12419388|rs13168690|rs823601|rs4599070|rs13262310|rs666028|rs210120|rs2038712|rs10870911|rs10908200|rs10495929|rs9922362|rs10743591|rs3782309|rs3787011
Immunologic Disorder	CHONDROITIN_SULFATE_BIOSYNTHESIS	REACTOME	846	29	19	9,24E-01	9,95E-01	rs6783962|rs1123373|rs1994788|rs4921664|rs6983139|rs4470745|rs2029737|rs12229646|rs4308750|rs12653308|rs6539155|rs16943623|rs4399617|rs17128441|rs2012387|rs3889350|rs4921659|rs2969078|rs2559639
Immunologic Disorder	STATHMIN_PATHWAY	BIOCARTA	456	14	10	9,26E-01	9,95E-01	rs704856|rs2302234|rs31309|rs10781329|rs7709420|rs891943|rs2995094|rs887346|rs858490|rs3797617
Immunologic Disorder	PDGF_PATHWAY	BIOCARTA	1021	28	20	9,26E-01	9,95E-01	rs16897466|rs13320527|rs17153654|rs13173003|rs2624189|rs3024908|rs706725|rs1507964|rs7560255|rs741847|rs2598483|rs4381631|rs8081614|rs4791036|rs9900213|rs6449947|rs12536419|rs2161529|rs169143|rs12655019
Immunologic Disorder	NFAT_PATHWAY	BIOCARTA	1626	23	20	9,29E-01	9,95E-01	rs16897466|rs6063664|rs2469749|rs12054920|rs6860708|rs13320527|rs17136507|rs7726306|rs17153654|rs6021266|rs11125132|rs7727198|rs2302234|rs13173003|rs31309|rs4150196|rs11731252|rs706725|rs3804357|rs10781329
Immunologic Disorder	CIRCADIAN_CLOCK	REACTOME	2075	33	20	9,30E-01	9,95E-01	rs7121775|rs1509241|rs179883|rs1555949|rs7858838|rs4857735|rs12054920|rs6860708|rs2290430|rs17136507|rs4235306|rs7726306|rs1159937|rs11038695|rs6580285|rs7727198|rs6772982|rs12989454|rs1388331|rs2047564
Immunologic Disorder	IL_3_5_AND_GM_CSF_SIGNALING	REACTOME	1134	30	20	9,30E-01	9,95E-01	rs16897466|rs3856848|rs2296950|rs13218316|rs13320527|rs1808563|rs13173003|rs706725|rs1981405|rs131849|rs4808100|rs9900213|rs6449947|rs3214047|rs2161529|rs11153318|rs1148844|rs4054727|rs10515074|rs6871452
Immunologic Disorder	EGF_PATHWAY	BIOCARTA	1048	26	20	9,31E-01	9,95E-01	rs16897466|rs6974790|rs13320527|rs13173003|rs2624189|rs3024908|rs706725|rs7560255|rs1140475|rs741847|rs2598483|rs4381631|rs8081614|rs4791036|rs9900213|rs6449947|rs2161529|rs169143|rs12655019|rs11747611
Immunologic Disorder	RNA_POL_I_TRANSCRIPTION_TERMINATION	REACTOME	335	8	7	9,32E-01	9,95E-01	rs510342|rs10208|rs871820|rs1995152|rs1056616|rs4150115|rs886420
Immunologic Disorder	MITOCHONDRIA_PATHWAY	BIOCARTA	317	13	10	9,33E-01	9,95E-01	rs3769823|rs1016860|rs956572|rs2270941|rs12738235|rs1862710|rs12700583|rs919699|rs8104760|rs12454650
Immunologic Disorder	SIGNALING_BY_BMP	REACTOME	918	26	20	9,34E-01	9,95E-01	rs1980499|rs6085802|rs7566998|rs6816834|rs12476865|rs3796433|rs12765929|rs6842938|rs8024414|rs235721|rs1994174|rs568752|rs7047712|rs11071920|rs1866411|rs2439368|rs2209982|rs10910050|rs10485705|rs7351039
Immunologic Disorder	IL10_PATHWAY	BIOCARTA	288	9	7	9,34E-01	9,95E-01	rs3024908|rs7560255|rs2598483|rs9900213|rs2048874|rs10951743|rs2834165
Immunologic Disorder	FOCAL_ADHESION	KEGG	7733	31	20	9,35E-01	9,95E-01	rs10519186|rs16897466|rs2510257|rs7239317|rs1757096|rs12620429|rs723188|rs11687617|rs2126642|rs17405661|rs12666352|rs10509534|rs1018829|rs4445045|rs1952966|rs6436654|rs10058772|rs13168072|rs12203956|rs2521206
Immunologic Disorder	NOD_LIKE_RECEPTOR_SIGNALING_PATHWAY	KEGG	1406	34	20	9,35E-01	9,95E-01	rs2066843|rs4077515|rs4925654|rs9359935|rs10806443|rs2869433|rs3769823|rs6961625|rs10925019|rs6924218|rs284513|rs44267|rs1613367|rs7746039|rs1076420|rs8904|rs2616052|rs11145922|rs1449046|rs12206804
Immunologic Disorder	ACTIVATION_OF_THE_MRNA_UPON_BINDING_OF_THE_CAP_BINDING_COMPLEX_AND_EI<br>T_BINDING_TO_43S	REACTOME	780	38	17	9,35E-01	9,95E-01	rs4599669|rs10824609|rs11902670|rs10955775|rs1249123|rs7825662|rs16888522|rs4979816|rs12879285|rs10824618|rs4803512|rs7975725|rs8460|rs10505278|rs2277841|rs12101212|rs12786251
Immunologic Disorder	CALCINEURIN_PATHWAY	BIOCARTA	895	23	20	9,36E-01	9,95E-01	rs6063664|rs2469749|rs7039739|rs17786782|rs6021266|rs11125132|rs6931833|rs3804357|rs6063634|rs1997815|rs17233751|rs4381631|rs8081614|rs4791036|rs169143|rs13002829|rs7219495|rs11153415|rs1848116|rs815802
Immunologic Disorder	SIGNALING_BY_PDGF	REACTOME	4427	29	20	9,38E-01	9,95E-01	rs16897466|rs11687617|rs12545028|rs4963867|rs17405661|rs10509534|rs17649232|rs7322396|rs2532019|rs6436654|rs10876227|rs13168690|rs2521206|rs4599070|rs9515201|rs4841920|rs210120|rs2296950|rs4675141|rs10495929
Immunologic Disorder	DEFENSINS	REACTOME	495	13	7	9,41E-01	9,95E-01	rs16925377|rs2738108|rs1837790|rs2702842|rs6937697|rs2196239|rs2738165
Immunologic Disorder	PROXIMAL_TUBULE_BICARBONATE_RECLAMATION	KEGG	458	20	13	9,42E-01	9,95E-01	rs4934297|rs6440052|rs16846575|rs359535|rs6025645|rs16831388|rs2019655|rs16852004|rs1322487|rs981257|rs11608153|rs1377523|rs9428340
Immunologic Disorder	TRANSCRIPTION	REACTOME	2708	40	20	9,45E-01	9,95E-01	rs9407553|rs10961344|rs6081160|rs503347|rs4077515|rs10961490|rs4951031|rs6539267|rs3737984|rs10944384|rs907076|rs12245545|rs393543|rs10810132|rs510342|rs3809425|rs7760713|rs803269|rs2188148|rs4487686
Immunologic Disorder	GLYCOGEN_BREAKDOWN_GLYCOGENOLYSIS	REACTOME	246	7	6	9,47E-01	9,95E-01	rs11125132|rs6531592|rs12149656|rs13002829|rs7144098|rs815802
Immunologic Disorder	CELL_CYCLE	KEGG	2581	30	20	9,49E-01	9,95E-01	rs7074757|rs10908915|rs604045|rs4445045|rs7683090|rs1801376|rs4921283|rs9642880|rs2073032|rs3009935|rs266335|rs17136507|rs7812998|rs11063059|rs6736093|rs2177640|rs2282128|rs10766424|rs2063327|rs11661651
Immunologic Disorder	LAIR_PATHWAY	BIOCARTA	479	21	11	9,52E-01	9,95E-01	rs2488320|rs4587680|rs11009195|rs399443|rs2048874|rs3770136|rs3805226|rs7551819|rs3917016|rs391781|rs2475193
Immunologic Disorder	RNA_POL_II_PRE_TRANSCRIPTION_EVENTS	REACTOME	532	20	9	9,54E-01	9,95E-01	rs503347|rs4487686|rs9858561|rs3779641|rs519336|rs13082232|rs17750066|rs10504553|rs886420
Immunologic Disorder	S_PHASE	REACTOME	2076	35	20	9,55E-01	9,95E-01	rs10268808|rs2892190|rs10046115|rs9540340|rs9642880|rs2073032|rs3815795|rs1957107|rs7812998|rs17482047|rs807293|rs9570962|rs16981247|rs2282128|rs12602230|rs4943189|rs3786136|rs2033098|rs764190|rs1832265
Immunologic Disorder	BIOSYNTHESIS_OF_THE_N_GLYCAN_PRECURSOR_DOLICHOL_LIPID_LINKED_OLIGOSACC<br>RANSFER_TO_A_NASCENT_PROTEIN	REACTOME	355	13	8	9,56E-01	9,95E-01	rs7615501|rs11833948|rs3790867|rs9824|rs2766005|rs1559113|rs7707082|rs11147621
Immunologic Disorder	MEF2D_PATHWAY	BIOCARTA	684	21	19	9,56E-01	9,95E-01	rs6063664|rs2469749|rs6021266|rs11125132|rs3804357|rs6063634|rs695161|rs1997815|rs4381631|rs8081614|rs4791036|rs5760189|rs169143|rs13002829|rs7219495|rs1848116|rs6674492|rs815802|rs2732514
Immunologic Disorder	CERAMIDE_PATHWAY	BIOCARTA	425	11	9	9,58E-01	9,95E-01	rs3769823|rs1016860|rs956572|rs2270941|rs12655019|rs12700583|rs11747611|rs8104760|rs12454650
Immunologic Disorder	BETA_DEFENSINS	REACTOME	333	8	5	9,58E-01	9,95E-01	rs1837790|rs2702842|rs6937697|rs2196239|rs2738165
Immunologic Disorder	RNA_POL_I_TRANSCRIPTION_INITIATION	REACTOME	427	10	9	9,59E-01	9,95E-01	rs510342|rs10208|rs871820|rs1995152|rs2948091|rs1056616|rs2025448|rs4150115|rs886420
Immunologic Disorder	CREB_PATHWAY	BIOCARTA	969	30	20	9,60E-01	9,95E-01	rs16897466|rs13320527|rs2302234|rs13173003|rs706725|rs1404909|rs10781329|rs7156757|rs891943|rs4381631|rs8081614|rs4791036|rs6449947|rs2161529|rs169143|rs887346|rs3797617|rs10515074|rs7219495|rs6871452
Immunologic Disorder	BILE_ACID_AND_BILE_SALT_METABOLISM	REACTOME	1176	38	20	9,64E-01	9,95E-01	rs4149028|rs7140017|rs3916933|rs4148407|rs7997220|rs4149057|rs1571513|rs7318504|rs7338828|rs12261436|rs9300812|rs16961268|rs2560718|rs16963394|rs4148412|rs12358054|rs12192544|rs10508135|rs12056107|rs9514099
Immunologic Disorder	L1CAM_INTERACTIONS	REACTOME	3914	31	20	9,65E-01	9,95E-01	rs4809563|rs12620429|rs3755768|rs10897495|rs6790921|rs1362890|rs3817828|rs7831492|rs6974790|rs13080492|rs1564585|rs7003385|rs7645358|rs10763940|rs3212588|rs2488320|rs13163497|rs10511228|rs277311|rs17442186
Immunologic Disorder	IL2_PATHWAY	BIOCARTA	452	7	6	9,67E-01	9,95E-01	rs695161|rs4808100|rs741847|rs9900213|rs1148844|rs1004420
Immunologic Disorder	GLIOMA	KEGG	2070	30	20	9,67E-01	9,95E-01	rs16897466|rs4963867|rs10509534|rs11657742|rs6974790|rs7197601|rs1125467|rs13320527|rs10794486|rs17153654|rs11125132|rs11644646|rs2684796|rs13173003|rs17793122|rs9348428|rs706725|rs1507964|rs1140475|rs874269
Immunologic Disorder	ION_TRANSPORT_BY_P_TYPE_ATPASES	REACTOME	1690	33	20	9,67E-01	9,95E-01	rs2575484|rs4782977|rs12595426|rs12448765|rs709641|rs11161255|rs1933199|rs12650894|rs487586|rs247888|rs12902281|rs6440052|rs12485872|rs7174452|rs1968274|rs2914819|rs10519505|rs1460352|rs16831388|rs2049283
Immunologic Disorder	PRE_NOTCH_EXPRESSION_AND_PROCESSING	REACTOME	1434	39	20	9,67E-01	9,95E-01	rs12103149|rs10005500|rs204995|rs17288919|rs5756685|rs693797|rs17136507|rs12417347|rs11846825|rs9348428|rs1242449|rs17825554|rs17117532|rs1296720|rs2948091|rs677598|rs3026445|rs4899801|rs7405515|rs1263459
Immunologic Disorder	CARDIACEGF_PATHWAY	BIOCARTA	1289	26	20	9,68E-01	9,95E-01	rs2366698|rs6974790|rs9642880|rs9422812|rs11244948|rs7812998|rs2763404|rs2033098|rs1140475|rs741847|rs4381631|rs8081614|rs4791036|rs13140736|rs1330876|rs17023204|rs3772622|rs9462724|rs4552863|rs169143
Immunologic Disorder	NUCLEOTIDE_EXCISION_REPAIR	KEGG	914	30	19	9,68E-01	9,95E-01	rs9540340|rs17482047|rs9570962|rs16981247|rs12602230|rs4943189|rs3786136|rs1832265|rs9539881|rs13292544|rs12336762|rs2291120|rs10258834|rs2025448|rs1124275|rs2051547|rs7357092|rs886420|rs17195683
Immunologic Disorder	BIOPEPTIDES_PATHWAY	BIOCARTA	1081	26	20	9,70E-01	9,95E-01	rs304737|rs820363|rs13218316|rs2886612|rs11125132|rs3024908|rs7220988|rs7560255|rs891943|rs2598483|rs4381631|rs8081614|rs4791036|rs9900213|rs11153318|rs169143|rs13002829|rs242557|rs9800032|rs3797617
Immunologic Disorder	MRNA_PROCESSING	REACTOME	1942	35	20	9,70E-01	9,95E-01	rs2368443|rs1484931|rs6770415|rs4684917|rs12121067|rs7596050|rs4951031|rs3737984|rs10944384|rs6434036|rs6135844|rs3809425|rs773546|rs4487686|rs2282763|rs5766533|rs2377084|rs2022747|rs6571850|rs9937484
Immunologic Disorder	NUCLEOTIDE_EXCISION_REPAIR	REACTOME	956	32	20	9,71E-01	9,95E-01	rs9540340|rs17482047|rs9570962|rs16981247|rs12602230|rs4943189|rs3786136|rs4487686|rs1832265|rs9539881|rs13292544|rs12336762|rs2291120|rs10258834|rs2025448|rs4134815|rs2051547|rs7357092|rs886420|rs17195683
Immunologic Disorder	RNA_POL_II_TRANSCRIPTION_PRE_INITIATION_AND_PROMOTER_OPENING	REACTOME	383	11	7	9,72E-01	9,95E-01	rs4487686|rs9858561|rs3779641|rs519336|rs13082232|rs17750066|rs886420
Immunologic Disorder	FCER1_PATHWAY	BIOCARTA	1564	28	20	9,74E-01	9,95E-01	rs16897466|rs6063664|rs2469749|rs13320527|rs1808563|rs17153654|rs6021266|rs11125132|rs13173003|rs10798106|rs706725|rs3804357|rs6063634|rs1997815|rs17544558|rs741847|rs6449947|rs12536419|rs2161529|rs12086010
Immunologic Disorder	FORMATION_OF_INCISION_COMPLEX_IN_GG_NER	REACTOME	545	15	11	9,75E-01	9,95E-01	rs17482047|rs12602230|rs3786136|rs13292544|rs12336762|rs2291120|rs10258834|rs2051547|rs7357092|rs886420|rs17195683
Immunologic Disorder	MAPK_PATHWAY	BIOCARTA	2690	29	20	9,75E-01	9,95E-01	rs6444054|rs604045|rs6510636|rs10897495|rs3817828|rs12054920|rs9642880|rs3009935|rs6860708|rs8026614|rs9359935|rs10806443|rs7812998|rs2869433|rs7726306|rs2083709|rs7629245|rs7727198|rs6924218|rs284513
Immunologic Disorder	PROSTATE_CANCER	KEGG	2870	24	20	9,75E-01	9,95E-01	rs16897466|rs4963867|rs17405661|rs10509534|rs7322396|rs2094405|rs11657742|rs6974790|rs931771|rs13320527|rs17136507|rs10008978|rs10794486|rs17153654|rs1016860|rs7991808|rs4255473|rs260808|rs2684796|rs13173003
Immunologic Disorder	HEMATOPOIETIC_CELL_LINEAGE	KEGG	1963	42	20	9,76E-01	9,95E-01	rs4932145|rs3856848|rs1030021|rs7555325|rs3212588|rs3020827|rs13163497|rs11883987|rs11567697|rs1507955|rs778584|rs353590|rs4972828|rs4988821|rs333947|rs7215959|rs16906594|rs2395182|rs12021671|rs1108338
Immunologic Disorder	METABOLISM_OF_CARBOHYDRATES	REACTOME	9072	25	20	9,77E-01	9,95E-01	rs9890008|rs3793932|rs7938320|rs11696078|rs9999101|rs3785690|rs2368443|rs2865155|rs6095638|rs6783962|rs9924390|rs1484931|rs2284277|rs11251480|rs1859906|rs483981|rs3845669|rs1111994|rs9290242|rs7986931
Immunologic Disorder	P38MAPK_PATHWAY	BIOCARTA	1904	25	20	9,78E-01	9,95E-01	rs604045|rs12054920|rs9642880|rs3009935|rs6860708|rs8026614|rs9359935|rs10806443|rs7812998|rs7726306|rs7727198|rs6924218|rs284513|rs2033098|rs10798106|rs3024908|rs12029576|rs7746039|rs7156757|rs17544558
Immunologic Disorder	AT1R_PATHWAY	BIOCARTA	1224	27	20	9,78E-01	9,95E-01	rs6974790|rs12054920|rs6860708|rs8026614|rs7039739|rs7726306|rs17786782|rs11125132|rs7727198|rs1140475|rs4381631|rs8081614|rs4791036|rs3772622|rs169143|rs13002829|rs527589|rs12655019|rs11747611|rs7219495
Immunologic Disorder	DNA_REPAIR	REACTOME	2111	26	20	9,81E-01	9,95E-01	rs2261316|rs4657|rs1891008|rs9540340|rs12253191|rs10889328|rs13399274|rs17482047|rs9570962|rs4439380|rs16981247|rs2029166|rs10849582|rs12602230|rs7087821|rs4943189|rs3786136|rs4487686|rs8178179|rs1832265
Immunologic Disorder	ENOS_ACTIVATION_AND_REGULATION	REACTOME	336	10	8	9,82E-01	9,95E-01	rs11125132|rs4731121|rs11881315|rs2286831|rs13002829|rs4142081|rs815802|rs10961558
Immunologic Disorder	G1_S_TRANSITION	REACTOME	1941	34	20	9,82E-01	9,95E-01	rs10268808|rs2892190|rs6457689|rs9642880|rs2073032|rs1957107|rs7812998|rs17482047|rs807293|rs12602230|rs3786136|rs2033098|rs764190|rs6575122|rs4727590|rs2716601|rs2298582|rs12436216|rs16972457|rs228271
Immunologic Disorder	GLYCOSPHINGOLIPID_METABOLISM	REACTOME	924	20	16	9,84E-01	9,95E-01	rs956274|rs3744307|rs439863|rs2293764|rs10474567|rs820846|rs2805478|rs891943|rs1476387|rs17818103|rs12500105|rs2194051|rs11722340|rs1507710|rs8009654|rs7825369
Immunologic Disorder	STRESS_PATHWAY	BIOCARTA	633	23	13	9,85E-01	9,95E-01	rs2362419|rs1710627|rs2440134|rs8904|rs10140922|rs12655019|rs1012919|rs7957946|rs11747611|rs1005890|rs11656130|rs9895446|rs2715815
Immunologic Disorder	PPARA_ACTIVATES_GENE_EXPRESSION	REACTOME	3675	32	20	9,85E-01	9,95E-01	rs2306718|rs1509241|rs680548|rs2487052|rs12310617|rs1555949|rs7858838|rs7713987|rs10861171|rs4857735|rs2035590|rs4839|rs2290430|rs9381536|rs17136507|rs4235306|rs1159937|rs16911712|rs4148828|rs6772982
Immunologic Disorder	RNA_POL_I_TRANSCRIPTION	REACTOME	937	18	12	9,85E-01	9,95E-01	rs510342|rs7760713|rs10208|rs871820|rs1995152|rs2948091|rs1630732|rs1056616|rs2025448|rs4150115|rs9461416|rs886420
Immunologic Disorder	OOCYTE_MEIOSIS	KEGG	3114	31	20	9,86E-01	9,95E-01	rs12545028|rs1678002|rs2532019|rs9299346|rs3817828|rs7683090|rs13168690|rs2469749|rs518162|rs4921283|rs210120|rs1506048|rs9922362|rs10743591|rs3782309|rs10794486|rs4629562|rs13190668|rs6736093|rs2177640
Immunologic Disorder	ATM_PATHWAY	BIOCARTA	397	8	6	9,86E-01	9,95E-01	rs3765692|rs8904|rs10140922|rs7853654|rs2269337|rs1012919

Immunologic Disorder	RNA_POL_II_TRANSCRIPTION	REACTOME	1050	37	19	9,88E-01	9,95E-01	rs503347|rs4951031|rs3737984|rs10944384|rs3809425|rs4487686|rs2282763|rs9858561|rs3779641|rs519336|rs9937484|rs11749500|rs4653184|rs13082232|rs2334321|rs17750066|rs10504553|rs6093835|rs886420
Immunologic Disorder	SPHINGOLIPID_METABOLISM	KEGG	1101	35	20	9,90E-01	9,95E-01	rs892987|rs1341347|rs6842323|rs12336689|rs17785945|rs2293764|rs7856222|rs11810204|rs4912230|rs2805478|rs1460784|rs1476387|rs2236317|rs4912189|rs4269197|rs1507710|rs10220397|rs6450317|rs2404985|rs8009654
Immunologic Disorder	CHEMICAL_PATHWAY	BIOCARTA	585	16	15	9,90E-01	9,95E-01	rs1016860|rs956572|rs3024908|rs2270941|rs4381631|rs8081614|rs4791036|rs3829078|rs169143|rs1862710|rs12700583|rs919699|rs7219495|rs8104760|rs12454650
Immunologic Disorder	MRNA_SPLICING	REACTOME	1057	26	15	9,90E-01	9,95E-01	rs12121067|rs9970867|rs3737984|rs6135844|rs3809425|rs4487686|rs9937484|rs12352784|rs11688232|rs296885|rs788008|rs1398968|rs2334321|rs602186|rs6093835
Immunologic Disorder	MITOTIC_G1_G1_S_PHASES	REACTOME	2477	34	20	9,90E-01	9,95E-01	rs10268808|rs2892190|rs4445045|rs10046115|rs2835741|rs9642880|rs2073032|rs1957107|rs7812998|rs11909066|rs17482047|rs807293|rs11063059|rs12602230|rs3786136|rs2033098|rs764190|rs9348428|rs6575122|rs4727590
Immunologic Disorder	BCR_PATHWAY	BIOCARTA	1053	21	20	9,90E-01	9,95E-01	rs6063664|rs2469749|rs1808563|rs6021266|rs11125132|rs3804357|rs6063634|rs1997815|rs741847|rs4381631|rs8081614|rs4791036|rs169143|rs1148844|rs13002829|rs12655019|rs11747611|rs7219495|rs1848116|rs815802
Immunologic Disorder	FORMATION_OF_TRANSCRIPTION_COUPLED_NER_TC_NER_REPAIR_COMPLEX	REACTOME	389	6	5	9,90E-01	9,95E-01	rs4487686|rs2025448|rs4134815|rs2051547|rs886420
Immunologic Disorder	VASOPRESSIN_REGULATED_WATER_REABSORPTION	KEGG	1100	48	20	9,90E-01	9,95E-01	rs2532019|rs9309485|rs6966540|rs7935469|rs2299268|rs10781329|rs7931713|rs1024551|rs1786648|rs7975385|rs2299278|rs177505|rs6953524|rs12419850|rs6948022|rs9882323|rs487455|rs467323|rs719450|rs921
Immunologic Disorder	41BB_PATHWAY	BIOCARTA	341	8	6	9,94E-01	9,98E-01	rs8904|rs10140922|rs12655019|rs1012919|rs11747611|rs11177048
Immunologic Disorder	PYK2_PATHWAY	BIOCARTA	752	16	14	9,96E-01	9,98E-01	rs7039739|rs17786782|rs11125132|rs4381631|rs8081614|rs4791036|rs169143|rs13002829|rs527589|rs12655019|rs11747611|rs7219495|rs815802|rs178255
Immunologic Disorder	PRIMARY_BILE_ACID_BIOSYNTHESIS	KEGG	439	12	6	9,97E-01	9,98E-01	rs12261436|rs2560718|rs12358054|rs12192544|rs12056107|rs4681689
Immunologic Disorder	SYNTHESIS_OF_BILE_ACIDS_AND_BILE_SALTS	REACTOME	546	12	6	9,99E-01	9,99E-01	rs12261436|rs2560718|rs12358054|rs12192544|rs12056107|rs4681689
Neurologic Disorder	TNFR2_PATHWAY	BIOCARTA	414	17	9	1,00E-03	7,45E-01	rs730503|rs4698855|rs472391|rs10958713|rs8016947|rs1516096|rs11154954|rs1267070|rs17112705
Neurologic Disorder	PANCREATIC_CANCER	KEGG	1920	42	20	3,50E-03	7,45E-01	rs3911080|rs4698855|rs930655|rs454305|rs6692647|rs4963857|rs465531|rs17041245|rs6872249|rs2761367|rs759169|rs1962515|rs3741403|rs266359|rs6783860|rs660442|rs2374594|rs12666347|rs1423236|rs17005702
Neurologic Disorder	NOD1_2_SIGNALING_PATHWAY	REACTOME	881	36	20	4,50E-03	7,45E-01	rs730503|rs7810486|rs1884257|rs40383|rs11869073|rs2440134|rs472391|rs3730327|rs7754397|rs1129880|rs661838|rs6980760|rs997284|rs6001585|rs6454860|rs1483382|rs474246|rs2715815|rs11867183|rs743923
Neurologic Disorder	AMINE_COMPOUND_SLC_TRANSPORTERS	REACTOME	1298	53	20	4,90E-03	7,45E-01	rs258097|rs10153360|rs12758601|rs1123617|rs2924149|rs1362832|rs6499775|rs2241694|rs10862814|rs2518100|rs10775478|rs3785152|rs4922132|rs4975646|rs1039122|rs4567513|rs10446439|rs363397|rs2270637|rs877788
Neurologic Disorder	ACTINY_PATHWAY	BIOCARTA	290	13	2	5,30E-03	7,45E-01	rs1078631|rs7595941
Neurologic Disorder	NUCLEOTIDE_BINDING_DOMAIN_LEUCINE_RICH_REPEAT_CONTAINING_RECEPTOR_NLRYS	REACTOME	1337	31	20	5,60E-03	7,45E-01	rs730503|rs2829949|rs7810486|rs1884257|rs9901001|rs40383|rs11869073|rs2440134|rs472391|rs3730327|rs7754397|rs2829929|rs1129880|rs661838|rs4941185|rs10759403|rs6980760|rs997284|rs6001585|rs224205
Neurologic Disorder	TID_PATHWAY	BIOCARTA	479	24	9	7,60E-03	7,85E-01	rs4698855|rs2237956|rs5030311|rs3758947|rs161353|rs11031716|rs10958713|rs8016947|rs3850209
Neurologic Disorder	HCMV_PATHWAY	BIOCARTA	547	30	15	8,70E-03	7,85E-01	rs4698855|rs465531|rs2360969|rs11869073|rs2440134|rs6872249|rs3730327|rs1423236|rs997284|rs4470720|rs4776783|rs474246|rs2715815|rs11867183|rs602932
Neurologic Disorder	GLIOMA	KEGG	2070	47	20	9,00E-03	7,85E-01	rs3911080|rs930655|rs454305|rs11938486|rs4963857|rs465531|rs1457596|rs17041245|rs919751|rs2241694|rs12948922|rs4381535|rs6872437|rs759169|rs1962515|rs12462766|rs10413199|rs2374594|rs11736538|rs12666347
Neurologic Disorder	CARDIACEGF_PATHWAY	BIOCARTA	1289	40	20	1,01E-02	7,85E-01	rs4698855|rs9296345|rs6772419|rs6925906|rs6597745|rs17041245|rs12948922|rs4332094|rs2142200|rs759169|rs4129666|rs2395851|rs7835350|rs12548864|rs12666347|rs3729908|rs11813508|rs1324768|rs11159135|rs884904
Neurologic Disorder	NEGATIVE_REGULATORS_OF_RIG_I_MDA5_SIGNALING	REACTOME	464	29	20	1,11E-02	7,85E-01	rs730503|rs8056241|rs710092|rs472391|rs223366|rs26532|rs11662928|rs17212649|rs683456|rs7865082|rs4435493|rs11118087|rs11639875|rs532726|rs3761034|rs11154954|rs12529626|rs9900686|rs336279|rs3851210
Neurologic Disorder	RAS_PATHWAY	BIOCARTA	557	28	11	1,18E-02	7,85E-01	rs4698855|rs465531|rs6872249|rs1962515|rs660442|rs1423236|rs4470720|rs4776783|rs2124147|rs3850186|rs17112705
Neurologic Disorder	TRANSPORT_OF_GLUCOSE_AND_OTHER_SUGARS_BILE_SALTS_AND_ORGANIC_ACIDS_MNE_COMPOUNDS	REACTOME	3234	55	20	1,29E-02	7,92E-01	rs273805|rs258097|rs7604163|rs10153360|rs12758601|rs1123617|rs2924149|rs1362832|rs3810915|rs6499775|rs2241694|rs10862814|rs823082|rs4820571|rs2518100|rs10775478|rs3785152|rs10987639|rs10931679|rs4922132
Neurologic Disorder	PROSTATE_CANCER	KEGG	2870	42	20	1,58E-02	8,99E-01	rs3911080|rs4698855|rs930655|rs454305|rs4963857|rs465531|rs260852|rs1457596|rs17041245|rs919751|rs2360969|rs6872437|rs2568218|rs759169|rs11226342|rs1962515|rs176632|rs660442|rs16830730|rs11736538
Neurologic Disorder	NFKB_PATHWAY	BIOCARTA	870	39	20	1,69E-02	8,99E-01	rs730503|rs4698855|rs1884257|rs4851543|rs10983712|rs472391|rs7744|rs7754397|rs6001585|rs6454860|rs9362744|rs10958713|rs8016947|rs2239621|rs11154954|rs2160227|rs12623383|rs9362878|rs1410202|rs17112705
Neurologic Disorder	NON_SMALL_CELL_LUNG_CANCER	KEGG	2585	42	20	1,96E-02	9,62E-01	rs3911080|rs930655|rs454305|rs13064561|rs4963857|rs465531|rs4679614|rs17632419|rs17041245|rs745830|rs7340603|rs12948922|rs643629|rs6872437|rs759169|rs1962515|rs13325331|rs660442|rs17325940|rs2374594
Neurologic Disorder	G_ALPHA_Q_SIGNALLING_EVENTS	REACTOME	7428	41	20	2,11E-02	9,62E-01	rs2803545|rs1046248|rs6794593|rs2829949|rs13098182|rs7356986|rs2474265|rs13402503|rs10802789|rs6772419|rs6925906|rs4557033|rs10907192|rs465531|rs7250851|rs10280384|rs3821525|rs2423356|rs12251540|rs3755677
Neurologic Disorder	RIG_I_MDA5_MEDIATED_INDUCTION_OF_IFN_ALPHA_BETA_PATHWAYS	REACTOME	1129	28	20	2,17E-02	9,62E-01	rs730503|rs8056241|rs2829949|rs710092|rs472391|rs2829929|rs223366|rs26532|rs11662928|rs17212649|rs683456|rs7865082|rs4435493|rs11118087|rs11639875|rs4978113|rs532726|rs1077767|rs3761034|rs10958713
Neurologic Disorder	NOD_LIKE_RECEPTOR_SIGNALING_PATHWAY	KEGG	1406	39	20	2,62E-02	9,80E-01	rs730503|rs4698855|rs1884257|rs9901001|rs40383|rs472391|rs159293|rs3730327|rs7754397|rs1129880|rs661838|rs6980760|rs6001585|rs224205|rs6454860|rs10229203|rs1483382|rs743923|rs237004|rs2716932
Neurologic Disorder	GASTRIN_CREB_SIGNALLING_PATHWAY_VIA_PKC_AND_MAPK	REACTOME	8122	41	20	2,99E-02	9,80E-01	rs2803545|rs1046248|rs6794593|rs2829949|rs13098182|rs7356986|rs2474265|rs13402503|rs10802789|rs6772419|rs6925906|rs4963857|rs4557033|rs10907192|rs465531|rs7250851|rs10280384|rs3821525|rs2423356|rs3755677
Neurologic Disorder	NA_CL_DEPENDENT_NEUROTRANSMITTER_TRANSPORTERS	REACTOME	718	40	20	3,03E-02	9,80E-01	rs258097|rs2924149|rs6499775|rs2241694|rs10862814|rs3785152|rs4922132|rs4975646|rs1039122|rs4567513|rs10446439|rs363397|rs2270637|rs12316387|rs10779127|rs12488144|rs1682821|rs933555|rs617217|rs12638941
Neurologic Disorder	HS_GAG_DEGRADATION	REACTOME	1386	35	20	3,06E-02	9,80E-01	rs9556369|rs1445270|rs2575738|rs9561545|rs34719083|rs11729350|rs1007973|rs1929035|rs3771254|rs9523030|rs7997908|rs535828|rs7562354|rs10492621|rs1333674|rs7321645|rs7992010|rs2491142|rs17189103|rs9522718
Neurologic Disorder	RACCYCD_PATHWAY	BIOCARTA	551	29	13	3,10E-02	9,80E-01	rs4698855|rs465531|rs6872437|rs1962515|rs6592724|rs2374594|rs1423236|rs4470720|rs9368950|rs10958713|rs8016947|rs9380590|rs17112705
Neurologic Disorder	FMLP_PATHWAY	BIOCARTA	1148	25	20	3,33E-02	9,80E-01	rs4698855|rs1569736|rs10907192|rs7250851|rs2423356|rs11869073|rs2440134|rs3730327|rs771941|rs1962515|rs8090560|rs6592724|rs7271063|rs7259810|rs997284|rs10505811|rs6077411|rs4776783|rs6055685|rs6086714
Neurologic Disorder	NCAM1_INTERACTIONS	REACTOME	1896	36	20	3,95E-02	9,80E-01	rs518558|rs4751955|rs4747345|rs4842157|rs16946687|rs1459590|rs16975294|rs7477742|rs2548278|rs10891524|rs4663708|rs7917174|rs10858253|rs1295881|rs4145072|rs2059198|rs10828803|rs4773209|rs6998973|rs587409
Neurologic Disorder	RNA_POL_III_TRANSCRIPTION_INITIATION_FROM_TYPE_3_PROMOTER	REACTOME	501	29	13	3,97E-02	9,80E-01	rs4922220|rs11779820|rs17093344|rs6132068|rs12581936|rs6081172|rs2062348|rs4119055|rs4961408|rs10095297|rs11204109|rs17093001|rs10503682
Neurologic Disorder	REGULATION_OF_APOPTOSIS	REACTOME	1432	57	20	4,32E-02	9,80E-01	rs8033658|rs2219556|rs704375|rs11922435|rs1816793|rs400223|rs6583176|rs13338214|rs12598046|rs1316489|rs4316851|rs2962783|rs755859|rs17031765|rs1329600|rs11642698|rs4795311|rs417940|rs17475756|rs12447239
Neurologic Disorder	CERAMIDE_PATHWAY	BIOCARTA	425	18	12	4,48E-02	9,80E-01	rs4698855|rs973807|rs4737509|rs1962515|rs660442|rs4941185|rs4776783|rs12962644|rs1026825|rs1016860|rs8083946|rs12693932
Neurologic Disorder	RNA_POL_III_TRANSCRIPTION_INITIATION_FROM_TYPE_2_PROMOTER	REACTOME	417	29	13	4,58E-02	9,80E-01	rs4922220|rs11779820|rs17093344|rs6132068|rs12581936|rs6081172|rs2062348|rs4119055|rs4961408|rs10095297|rs11204109|rs17093001|rs10503682
Neurologic Disorder	GAP_JUNCTION_TRAFFICKING	REACTOME	576	33	14	5,17E-02	9,80E-01	rs9320821|rs716289|rs2025880|rs9450659|rs843346|rs11757506|rs11919795|rs11240144|rs6665029|rs3805787|rs1342475|rs644339|rs9315439|rs292246
Neurologic Disorder	ANTIGEN_PROCESSING_CROSS_PRESENTATION	REACTOME	1601	43	20	5,50E-02	9,80E-01	rs11974777|rs704375|rs3211834|rs1265646|rs11922435|rs13191519|rs400223|rs7755852|rs13338214|rs12598046|rs9258895|rs755859|rs7287350|rs17031765|rs11642698|rs3772832|rs4795311|rs417940|rs28367729|rs12447239
Neurologic Disorder	RNA_POL_III_CHAIN_ELONGATION	REACTOME	364	29	13	5,58E-02	9,80E-01	rs4922220|rs11779820|rs17093344|rs6132068|rs12581936|rs6081172|rs2062348|rs4119055|rs4961408|rs10095297|rs11204109|rs17093001|rs10503682
Neurologic Disorder	ATM_PATHWAY	BIOCARTA	397	21	8	5,62E-02	9,80E-01	rs4698855|rs16942|rs2697675|rs6470522|rs9368950|rs8016947|rs9380590|rs1295686
Neurologic Disorder	TIE2_SIGNALING	REACTOME	790	30	12	5,63E-02	9,80E-01	rs4963857|rs465531|rs615532|rs6872437|rs1319745|rs2515466|rs1423236|rs4470720|rs2208637|rs12626011|rs7303282|rs1283658
Neurologic Disorder	A_TETRASACCHARIDE_LINKER_SEQUENCE_IS_REQUIRED_FOR_GAG_SYNTHESIS	REACTOME	1731	32	20	5,78E-02	9,80E-01	rs9556369|rs1445270|rs2575738|rs309556|rs9561545|rs12750012|rs1007973|rs1929035|rs3771254|rs9523030|rs7997908|rs6762394|rs535828|rs9364126|rs7562354|rs10492621|rs1333674|rs12280153|rs7321645|rs7992010
Neurologic Disorder	G0_AND_EARLY_G1	REACTOME	258	9	5	6,00E-02	9,80E-01	rs2070235|rs3091249|rs9315420|rs6073285|rs3897903
Neurologic Disorder	BLADDER_CANCER	KEGG	1222	58	20	6,10E-02	9,80E-01	rs8033658|rs4963857|rs17041245|rs4332094|rs2142200|rs11070217|rs759169|rs1962515|rs1316489|rs3741403|rs4129666|rs2395851|rs7835350|rs12548864|rs12666347|rs1329600|rs884904|rs4776783|rs17475756|rs2183938
Neurologic Disorder	NICOTINATE_AND_NICOTINAMIDE_METABOLISM	KEGG	963	32	20	6,25E-02	9,80E-01	rs17153071|rs10041164|rs7771841|rs9362210|rs17156226|rs784600|rs6603811|rs6939185|rs11096544|rs10069201|rs586364|rs1180275|rs17341747|rs12635210|rs784619|rs11742538|rs11828|rs16834034|rs6881783|rs9940962
Neurologic Disorder	GLYCOSPHINGOLIPID_BIOSYNTHESIS_LACTO_AND_NEOLACTO_SERIES	KEGG	670	47	20	6,37E-02	9,80E-01	rs10426709|rs11220473|rs6913109|rs9377313|rs11754559|rs948360|rs588774|rs2785910|rs4937131|rs11156122|rs6571057|rs7855713|rs500498|rs240542|rs12294732|rs750592|rs6570428|rs304303|rs7584792|rs10930282
Neurologic Disorder	BETA_DEFENSINS	REACTOME	333	7	5	6,56E-02	9,80E-01	rs6039403|rs1418258|rs4513489|rs4710191|rs7757618
Neurologic Disorder	NCAM_SIGNALING_FOR_NEURITE_OUT_GROWTH	REACTOME	2480	34	20	6,61E-02	9,80E-01	rs518558|rs4751955|rs4747345|rs4842157|rs4963857|rs16946687|rs1459590|rs16975294|rs7477742|rs2548278|rs2360969|rs10891524|rs4663707|rs7917174|rs10858253|rs1295881|rs4145072|rs1962515|rs2059198|rs10828803
Neurologic Disorder	CYTOSOLIC_DNA_SENSING_PATHWAY	KEGG	620	38	20	6,87E-02	9,80E-01	rs4698855|rs17498196|rs6132068|rs12581936|rs6081172|rs12943654|rs996029|rs1330383|rs17212649|rs10229203|rs4119055|rs7865082|rs16924159|rs11118087|rs4978113|rs11743030|rs10958713|rs8016947|rs10125010|rs17112705
Neurologic Disorder	ACTIVATION_OF_THE_PRE_REPLICATIVE_COMPLEX	REACTOME	565	26	13	7,16E-02	9,80E-01	rs487989|rs9450763|rs7980317|rs2985689|rs11814554|rs11764107|rs7916990|rs8077346|rs2307373|rs133422|rs3735461|rs2891652|rs677740
Neurologic Disorder	PRE_NOTCH_PROCESSING_IN_GOLGI	REACTOME	691	31	16	7,18E-02	9,80E-01	rs3761441|rs532385|rs17576984|rs3759929|rs17115605|rs8006798|rs6502762|rs1114312|rs17653970|rs8016995|rs17642827|rs10498559|rs1959012|rs1777697|rs750592|rs304303
Neurologic Disorder	PROTEASOME	KEGG	821	40	20	7,19E-02	9,80E-01	rs912521|rs704375|rs2140741|rs11922435|rs400223|rs13338214|rs12598046|rs755859|rs17031765|rs11642698|rs4795311|rs417940|rs12447239|rs7212835|rs9508068|rs40610|rs2665833|rs4813030|rs16972410|rs12867747
Neurologic Disorder	AKT_PATHWAY	BIOCARTA	682	36	15	7,33E-02	9,80E-01	rs4698855|rs465531|rs6872249|rs13354167|rs660442|rs1423236|rs3776841|rs4470720|rs3800230|rs2721047|rs3812889|rs10958713|rs8016947|rs7322396|rs17112705
Neurologic Disorder	INTRINSIC_PATHWAY	REACTOME	264	12	5	7,33E-02	9,80E-01	rs4253419|rs4253236|rs5030102|rs925453|rs3211719
Neurologic Disorder	IL1R_PATHWAY	BIOCARTA	1001	32	20	7,48E-02	9,80E-01	rs4698855|rs6692647|rs1884257|rs4851543|rs11869073|rs2440134|rs7744|rs3730327|rs7754397|rs997284|rs6001585|rs6454860|rs10229203|rs474246|rs2715815|rs11867183|rs743923|rs9362744|rs10958713|rs8016947
Neurologic Disorder	INHIBITION_OF_INSULIN_SECRETION_BY_ADRENALINE_NORADRENALINE	REACTOME	789	42	20	7,65E-02	9,80E-01	rs4747345|rs10907192|rs7477742|rs7098615|rs7916268|rs1295881|rs11195534|rs10828803|rs4898712|rs9822061|rs12706724|rs739431|rs9630890|rs10885138|rs1108656|rs6674438|rs2024497|rs1878247|rs6510696|rs2272274
Neurologic Disorder	CELL_ADHESION_MOLECULES_CAMS	KEGG	8813	48	20	7,68E-02	9,80E-01	rs6930052|rs2032728|rs10490170|rs2826516|rs2575738|rs2880989|rs2011815|rs2710091|rs1941136|rs656068|rs1269621|rs2826334|rs309556|rs13332162|rs12587703|rs2707575|rs10184153|rs12611635|rs10129666|rs11777908
Neurologic Disorder	CDC42RAC_PATHWAY	BIOCARTA	435	24	9	7,82E-02	9,80E-01	rs465531|rs6872249|rs7595941|rs6592724|rs11736538|rs1423236|rs4470720|rs17691810|rs1492769
Neurologic Disorder	EXTENSION_OF_TELOMERES	REACTOME	618	31	20	7,91E-02	9,80E-01	rs487989|rs7980317|rs1062708|rs9592217|rs12877281|rs11147544|rs10951757|rs2985689|rs1925089|rs1359389|rs7324308|rs7996386|rs8077346|rs7994717|rs690705|rs2922666|rs509360|rs2304136|rs3735461|rs174556
Neurologic Disorder	PEPTIDE_LIGAND_BINDING_RECEPTORS	REACTOME	5053	33	20	8,14E-02	9,80E-01	rs566416|rs1046248|rs2829949|rs9296345|rs2474265|rs6431547|rs6772419|rs6925906|rs10115467|rs7250851|rs10280384|rs2961739|rs3110052|rs10178143|rs4585434|rs1012178|rs4513489|rs6089784|rs2271933|rs1557399
Neurologic Disorder	41BB_PATHWAY	BIOCARTA	341	17	10	8,18E-02	9,80E-01	rs4698855|rs10139934|rs3730327|rs7566401|rs602932|rs9402839|rs10958713|rs8016947|rs1295686|rs17112705
Neurologic Disorder	RENAL_CELL_CARCINOMA	KEGG	2164	43	20	8,23E-02	9,80E-01	rs3911080|rs930655|rs454305|rs7552931|rs4963857|rs465531|rs797377|rs6872437|rs6583176|rs1962515|rs3741403|rs3738483|rs6592724|rs12563264|rs6118687|rs1423236|rs17005702|rs4470720|rs4776783|rs1940359
Neurologic Disorder	SYNTHESIS_OF_DNA	REACTOME	1545	32	20	8,39E-02	9,80E-01	rs487989|rs9450763|rs704375|rs7980317|rs11922435|rs9592217|rs400223|rs12877281|rs11147544|rs10951757|rs13338214|rs12598046|rs2985689|rs1925089|rs9315420|rs755859|rs17031765|rs1359389|rs11642698|rs7324308
Neurologic Disorder	MPR_PATHWAY	BIOCARTA	624	11	6	8,75E-02	9,80E-01	rs10907192|rs7595941|rs12706724|rs13038037|rs6513412|rs6092708
Neurologic Disorder	DNA_REPLICATION	KEGG	740	29	20	8,99E-02	9,80E-01	rs487989|rs7980317|rs9592217|rs12877281|rs11147544|rs10951757|rs537786|rs2985689|rs1925089|rs17651654|rs1359389|rs7324308|rs7996386|rs8077346|rs7994717|rs690705|rs2922666|rs509360|rs2304136|rs133422
Neurologic Disorder	NKCELLS_PATHWAY	BIOCARTA	678	30	12	9,01E-02	9,80E-01	rs461970|rs465531|rs6872437|rs9259916|rs6592724|rs1423236|rs4470720|rs4776783|rs7846568|rs10993754|rs308195|rs2524005
Neurologic Disorder	ERBB_SIGNALING_PATHWAY	KEGG	4135	33	20	9,13E-02	9,80E-01	rs3911080|rs930655|rs454305|rs11938486|rs4963857|rs465531|rs10490697|rs17041245|rs6704686|rs2241694|rs7605126|rs12948922|rs4332094|rs2142200|rs4381535|rs4894936|rs2163349|rs6872249|rs6583176|rs759169
Neurologic Disorder	NTHI_PATHWAY	BIOCARTA	876	30	20	9,29E-02	9,80E-01	rs4698855|rs1884257|rs11869073|rs2440134|rs7744|rs3730327|rs7754397|rs266359|rs1129880|rs6783860|rs997284|rs6454860|rs8032542|rs12521436|rs474246|rs2715815|rs11867183|rs602932|rs9362744|rs10958713
Neurologic Disorder	HDAC_PATHWAY	BIOCARTA	1231	47	20	9,60E-02	9,80E-01	rs1569736|rs465531|rs738809|rs1457596|rs11953993|rs11746572|rs11869073|rs2440134|rs6872249|rs8041078|rs3730327|rs8090560|rs1423236|rs7259810|rs997284|rs4470720|rs6219|rs10514306|rs3827321|rs474246
Neurologic Disorder	E2F_MEDIATED_REGULATION_OF_DNA_REPLICATION	REACTOME	452	21	11	9,73E-02	9,80E-01	rs487989|rs9450763|rs7980317|rs9315420|rs3776841|rs2307373|rs1650647|rs2891652|rs11666474|rs7585544|rs677740
Neurologic Disorder	MAPK_PATHWAY	BIOCARTA	2690	39	20	9,76E-02	9,80E-01	rs4698855|rs7552931|rs2011940|rs11953993|rs1884257|rs10139934|rs7116712|rs11746572|rs4332094|rs2360969|rs2142200|rs10897495|rs11869073|rs2440134|rs8041078|rs6583176|rs3730327|rs7754397|rs1962515|rs10050099
Neurologic Disorder	METABOLISM_OF_VITAMINS_AND_COFACTORS	REACTOME	1119	37	20	9,82E-02	9,80E-01	rs17153071|rs7771841|rs12218194|rs6603811|rs13025803|rs3794948|rs8129445|rs10274162|rs6939185|rs17280674|rs4726739|rs631108|rs2987981|rs7781216|rs17212649|rs41684|rs10487629|rs743463|rs12635210|rs2477703
Neurologic Disorder	CDMAC_PATHWAY	BIOCARTA	1018	29	20	1,00E-01	9,80E-01	rs4698855|rs2423356|rs12948922|rs4332094|rs2142200|rs771941|rs1962515|rs4129666|rs2395851|rs7835350|rs12548864|rs7271063|rs3729908|rs11159135|rs6077411|rs4776783|rs6055685|rs6086714|rs3785383|rs16959227
Neurologic Disorder	RAS_ACTIVATION_UOPN_CA2_INFUX_THROUGH_NMDA_RECEPTOR	REACTOME	1033	38	20	1,01E-01	9,80E-01	rs11612284|rs170290|rs11938486|rs2300267|rs2241694|rs11055587|rs316369|rs7206547|rs17833967|rs11055612|rs7259810|rs2314978|rs7979850|rs2160173|rs7301754|rs34117|rs10492846|rs4286653|rs734446|rs11741062
Neurologic Disorder	RESPONSE_TO_ELEVATED_PLATELET_CYTOSOLIC_CA2_	REACTOME	2301	42	20	1,01E-01	9,80E-01	rs11974777|rs2829949|rs7552931|rs4148483|rs3211834|rs17041245|rs2292867|rs12948922|rs9590125|rs11070217|rs17557564|rs2363880|rs1115219|rs3741403|rs3748316|rs2829929|rs4902681|rs7760746|rs7259810|rs3729908
Neurologic Disorder	INTERACTION_BETWEEN_L1_AND_ANKYRINS	REACTOME	970	40	20	1,04E-01	9,80E-01	rs2075728|rs1269621|rs7374540|rs13255835|rs7807090|rs12549656|rs10101217|rs11779500|rs1006955|rs7895653|rs1695777|rs521184|rs10184275|rs870379|rs10994443|rs2290559|rs7002144|rs10791889|rs1269637|rs12355908
Neurologic Disorder	TELOMERE_MAINTENANCE	REACTOME	1133	37	20	1,07E-01	9,80E-01	rs487989|rs7980317|rs1062708|rs9592217|rs12877281|rs11147544|rs10951757|rs10088657|rs2985689|rs1925089|rs1359389|rs7324308|rs198838|rs7996386|rs8077346|rs7994717|rs690705|rs2922666|rs509360|rs2304136
Neurologic Disorder	ERK5_PATHWAY	BIOCARTA	595	40	14	1,07E-01	9,80E-01	rs465531|rs11953993|rs11746572|rs2360969|rs6872249|rs8041078|rs1423236|rs4470720|rs10514306|rs1833895|rs4483828|rs6860708|rs11247123|rs153685
Neurologic Disorder	STRESS_PATHWAY	BIOCARTA	633	32	17	1,09E-01	9,80E-01	rs4698855|rs7810486|rs11869073|rs2440134|rs3730327|rs997284|rs3742064|rs474246|rs2715815|rs11867183|rs602932|rs10958713|rs8016947|rs1516096|rs1267070|rs974189|rs17112705
Neurologic Disorder	MCM_PATHWAY	BIOCARTA	234	9	6	1,13E-01	9,80E-01	rs9450763|rs764833|rs17227367|rs2307373|rs133422|rs2891652
Neurologic Disorder	MITOCHONDRIAL_PROTEIN_IMPORT	REACTOME	609	31	17	1,13E-01	9,80E-01	rs6006615|rs730168|rs2950390|rs11760823|rs7252142|rs9873033|rs2802944|rs11588577|rs2802962|rs13245850|rs11654885|rs2674000|rs2663342|rs2235779|rs12704840|rs3770459|rs3793451
Neurologic Disorder	SIGNALING_BY_CONSTITUTIVELY_ACTIVE_EGFR	REACTOME	503	41	16	1,14E-01	9,80E-01	rs4963857|rs465531|rs17041245|rs6872437|rs759169|rs12666347|rs1423236|rs4470720|rs884904|rs4435493|rs6837153|rs10488142|rs3761034|rs1525642|rs7303282|rs17586344
Neurologic Disorder	DEGRADATION_OF_THE_EXTRACELLULAR_MATRIX	REACTOME	580	46	16	1,21E-01	9,80E-01	rs4253239|rs1428814|rs2454331|rs3759929|rs2664352|rs2616500|rs2681300|rs10504870|rs598693|rs925453|rs4789939|rs1247571|rs3761008|rs2250205|rs1922314|rs2274756
Neurologic Disorder	STATHMIN_PATHWAY	BIOCARTA	456	23	8	1,22E-01	9,80E-01	rs11938486|rs2241694|rs3782046|rs10918708|rs6698995|rs4286653|rs2071863|rs25923
Neurologic Disorder	CYSTEINE_AND_METHIONINE_METABOLISM	KEGG	982	45	20	1,23E-01	9,80E-01	rs9429768|rs3794492|rs10836312|rs2059098|rs3972|rs158845|rs152395|rs8062660|rs283525|rs10811629|rs11076318|rs6895949|rs2915200|rs10068900|rs442756|rs1363072|rs1472623|rs11958300|rs29861|rs4868753

Neurologic Disorder	CREB_PHOSPHORYLATION_THROUGH_THE_ACTIVATION_OF_RAS	REACTOME	1349	35	20	1,24E-01	9,80E-01	rs11612284|rs170290|rs11938486|rs2300267|rs2241694|rs2360969|rs1962515|rs11055587|rs316369|rs6903559|rs7206547|rs17833967|rs11055612|rs7259810|rs2314978|rs7979850|rs2160173|rs7301754|rs34117|rs10492846
Neurologic Disorder	GAP_JUNCTION_ASSEMBLY	REACTOME	330	27	11	1,26E-01	9,80E-01	rs9320821|rs716289|rs9450659|rs11757506|rs11240144|rs6665029|rs3805787|rs1342475|rs644339|rs9315439|rs292246
Neurologic Disorder	ASCORBATE_AND_ALDARATE_METABOLISM	KEGG	874	10	7	1,30E-01	9,80E-01	rs11249454|rs4578194|rs7551594|rs4878784|rs11937734|rs2608822|rs10027817
Neurologic Disorder	GATA3_PATHWAY	BIOCARTA	898	27	20	1,31E-01	9,80E-01	rs1569736|rs11255531|rs7094402|rs2787148|rs4243170|rs3730327|rs521331|rs4888024|rs477461|rs8090560|rs10905311|rs4385796|rs30387|rs386687|rs10508374|rs2348182|rs602932|rs2388896|rs6096477|rs6126251
Neurologic Disorder	NFAT_PATHWAY	BIOCARTA	1626	48	20	1,31E-01	9,80E-01	rs7356986|rs6925906|rs1569736|rs465531|rs1457596|rs11953993|rs11746572|rs6872249|rs17472986|rs3730327|rs1962515|rs8090560|rs16830730|rs6553727|rs1423236|rs7259810|rs3804158|rs4470720|rs4510815|rs6219
Neurologic Disorder	TEL_PATHWAY	BIOCARTA	978	31	20	1,32E-01	9,80E-01	rs4963857|rs12948922|rs4332094|rs2142200|rs759169|rs2409571|rs4841167|rs4129666|rs9644708|rs2395851|rs10088657|rs7835350|rs12548864|rs4941185|rs12666347|rs3776841|rs7217258|rs884904|rs16959227|rs2684788
Neurologic Disorder	M_G1_TRANSITION	REACTOME	1315	41	20	1,33E-01	9,80E-01	rs487989|rs9450763|rs704375|rs7980317|rs11922435|rs400223|rs13338214|rs12598046|rs2985689|rs11814554|rs755859|rs11764107|rs17031765|rs11642698|rs4795311|rs417940|rs7916990|rs8077346|rs12447239|rs4435493
Neurologic Disorder	ARF_PATHWAY	BIOCARTA	733	43	15	1,34E-01	9,80E-01	rs465531|rs4332094|rs2142200|rs6872437|rs4129666|rs2395851|rs7835350|rs12548864|rs1423236|rs4470720|rs12543298|rs2189000|rs4410871|rs16903124|rs4733593
Neurologic Disorder	TOLL_PATHWAY	BIOCARTA	1147	31	20	1,34E-01	9,80E-01	rs4698855|rs1884257|rs10983712|rs11869073|rs2440134|rs7744|rs3730327|rs7754397|rs12330015|rs6007640|rs661838|rs997284|rs6001585|rs11159135|rs6454860|rs474246|rs2715815|rs11867183|rs743923|rs237004
Neurologic Disorder	RAC1_PATHWAY	BIOCARTA	763	45	17	1,39E-01	9,80E-01	rs461970|rs465531|rs4766526|rs17545756|rs6872249|rs6592724|rs11736538|rs1423236|rs4470720|rs2124147|rs7212835|rs17691810|rs820335|rs3911406|rs1492769|rs308195|rs3850186
Neurologic Disorder	SLC_MEDIATED_TRANSMEMBRANE_TRANSPORT	REACTOME	8774	47	20	1,41E-01	9,80E-01	rs273805|rs258097|rs720680|rs7604163|rs10153360|rs3818057|rs12758601|rs1123617|rs780662|rs838626|rs13431075|rs2924149|rs1362832|rs3810915|rs9508477|rs6499775|rs2241694|rs10862814|rs1000940|rs823082
Neurologic Disorder	REGULATION_OF_SIGNALING_BY_CBL	REACTOME	703	23	12	1,43E-01	9,80E-01	rs461970|rs465531|rs6872249|rs7905645|rs1423236|rs4470720|rs11188666|rs12547683|rs7245158|rs10993754|rs308195|rs6916861
Neurologic Disorder	CHROMOSOME_MAINTENANCE	REACTOME	1949	41	20	1,49E-01	9,80E-01	rs487989|rs10185631|rs7980317|rs2833690|rs1062708|rs9592217|rs1500480|rs12877281|rs16942|rs11147544|rs10951757|rs7700605|rs10088657|rs2985689|rs1925089|rs1359389|rs7324308|rs1266294|rs2459111|rs8134439
Neurologic Disorder	GLYCOSYLPHOSPHATIDYLINOSITOL_GPI_ANCHOR_BIOSYNTHESIS	KEGG	318	19	11	1,51E-01	9,80E-01	rs6776064|rs2689672|rs2741538|rs1934080|rs4916595|rs199714|rs13149933|rs4916596|rs2070139|rs9807301|rs4791665
Neurologic Disorder	PYRUVATE_METABOLISM	REACTOME	209	21	4	1,52E-01	9,80E-01	rs12693008|rs2767038|rs132930|rs1216539
Neurologic Disorder	LAGGING_STRAND_SYNTHESIS	REACTOME	550	32	19	1,53E-01	9,80E-01	rs487989|rs7980317|rs9592217|rs12877281|rs11147544|rs10951757|rs1925089|rs1359389|rs7324308|rs7996386|rs8077346|rs7994717|rs690705|rs2922666|rs509360|rs2304136|rs3735461|rs174556|rs677740
Neurologic Disorder	STEROID_HORMONE_BIOSYNTHESIS	KEGG	1491	39	20	1,54E-01	9,80E-01	rs7980317|rs11249454|rs2518824|rs1458928|rs10876933|rs232542|rs2414099|rs4555110|rs6518591|rs12555788|rs1937849|rs1906889|rs166050|rs7048593|rs17361616|500437|rs819271|rs4864976|rs2470176|rs11253042
Neurologic Disorder	ACTIVATION_OF_KAINATE_RECEPTORS_UPON_GLUTAMATE_BINDING	REACTOME	1577	30	20	1,57E-01	9,80E-01	rs4839797|rs363512|rs10907192|rs218378|rs2423356|rs2782901|rs2399927|rs771941|rs989467|rs3741403|rs11777589|rs660442|rs4898712|rs7271063|rs7259810|rs12660459|rs6077411|rs652008|rs9630890|rs10892600
Neurologic Disorder	S_PHASE	REACTOME	2076	36	20	1,60E-01	9,80E-01	rs487989|rs9450763|rs704375|rs7980317|rs11922435|rs4332094|rs9592217|rs2142200|rs400223|rs12877281|rs11147544|rs10951757|rs13338214|rs12598046|rs4129666|rs2395851|rs7835350|rs2985689|rs12548864|rs1925089
Neurologic Disorder	HEMATOPOIETIC_CELL_LINEAGE	KEGG	1963	45	20	1,63E-01	9,80E-01	rs11974777|rs10184153|rs3211834|rs764833|rs919751|rs2292867|rs4851543|rs575330|rs216123|rs16926995|rs2363880|rs3782046|rs12461927|rs917846|rs1541435|rs831630|rs11688011|rs1425802|rs10229203|rs2239512
Neurologic Disorder	GCR_PATHWAY	BIOCARTA	736	36	18	1,64E-01	9,80E-01	rs4698855|rs10907192|rs465531|rs6872249|rs1423236|rs7259810|rs4470720|rs12521436|rs3857420|rs2400707|rs17462424|rs6513412|rs13135920|rs12650894|rs6733907|rs9314809|rs10512016|rs6092708
Neurologic Disorder	IGF1R_PATHWAY	BIOCARTA	735	29	12	1,65E-01	9,80E-01	rs465531|rs6872249|rs1962515|rs660442|rs1423236|rs4470720|rs11687971|rs4776783|rs2972153|rs12467492|rs6730045|rs2684788
Neurologic Disorder	MTOR_PATHWAY	BIOCARTA	509	24	13	1,67E-01	9,80E-01	rs465531|rs6872249|rs1050700|rs8063461|rs1423236|rs3776841|rs4470720|rs7190284|rs1074078|rs1474879|rs945559|rs6134733|rs1294690
Neurologic Disorder	VIP_PATHWAY	BIOCARTA	1000	28	20	1,67E-01	9,80E-01	rs4698855|rs1569736|rs4332094|rs2142200|rs9651304|rs4129666|rs2395851|rs8090560|rs7835350|rs12548864|rs9371268|rs7259810|rs17083085|rs12543298|rs4410871|rs6096477|rs6126251|rs6021247|rs17828764|rs1052653
Neurologic Disorder	IL17_PATHWAY	BIOCARTA	316	15	6	1,68E-01	9,80E-01	rs764833|rs3782046|rs10229203|rs17227367|rs10918708|rs6698995
Neurologic Disorder	CDT1_ASSOCIATION_WITH_THE_CDC6_ORC_ORIGIN_COMPLEX	REACTOME	793	38	20	1,68E-01	9,80E-01	rs9450763|rs704375|rs11922435|rs400223|rs13338214|rs12598046|rs755859|rs17031765|rs11642698|rs4795311|rs417940|rs12447239|rs4435493|rs7212835|rs40610|rs2307373|rs3761034|rs2584624|rs4813030|rs2891652
Neurologic Disorder	MYOSIN_PATHWAY	BIOCARTA	1253	33	20	1,68E-01	9,80E-01	rs272107|rs10907192|rs3919447|rs2423356|rs17288929|rs12948922|rs11765386|rs771941|rs10951757|rs1208204|rs7271063|rs3729908|rs1035395|rs17830000|rs2908297|rs6077411|rs6055685|rs6086714|rs3785383|rs16959227
Neurologic Disorder	ACTIVATED_TAK1_MEDIATES_P38_MAPK_ACTIVATION	REACTOME	649	35	20	1,69E-01	9,80E-01	rs1884257|rs40383|rs11869073|rs2440134|rs3730327|rs7754397|rs1129880|rs661838|rs6980760|rs997284|rs6001585|rs6454860|rs1483382|rs474246|rs2715815|rs11867183|rs743923|rs237004|rs9362744|rs6908296
Neurologic Disorder	NOS1_PATHWAY	BIOCARTA	1133	29	20	1,69E-01	9,80E-01	rs11612284|rs170290|rs2300267|rs12948922|rs2293055|rs11055587|rs316369|rs7206547|rs17833967|rs11055612|rs7259810|rs2314978|rs3729908|rs7979850|rs2160173|rs7301754|rs1879417|rs10492846|rs3785383|rs16959227
Neurologic Disorder	P38MAPK_PATHWAY	BIOCARTA	1904	48	20	1,69E-01	9,80E-01	rs6692647|rs11953993|rs1884257|rs12749748|rs11746572|rs4332094|rs2360969|rs2142200|rs10912053|rs11869073|rs2440134|rs8041078|rs3730327|rs7754397|rs10050099|rs4129666|rs2395851|rs7835350|rs12548864|rs7566401
Neurologic Disorder	IGF1MTOR_PATHWAY	BIOCARTA	582	34	17	1,69E-01	9,80E-01	rs465531|rs1457596|rs6806377|rs6872249|rs16830730|rs1423236|rs3776841|rs4470720|rs843358|rs6219|rs1074078|rs2684788|rs6214|rs945559|rs9815292|rs2125111|rs651933
Neurologic Disorder	RNA_POL_III_TRANSCRIPTION_TERMINATION	REACTOME	680	41	19	1,69E-01	9,80E-01	rs4922220|rs11779820|rs17093344|rs6132068|rs12581936|rs1327510|rs6081172|rs2062348|rs683474|rs4119055|rs4961408|rs10810136|rs10961271|rs17709614|rs10095297|rs7018762|rs11204109|rs17093001|rs10503682
Neurologic Disorder	RIBOFLAVIN_METABOLISM	KEGG	295	22	8	1,70E-01	9,80E-01	rs7771841|rs3820127|rs6939185|rs1942492|rs11994010|rs9553572|rs6587041|rs13278771
Neurologic Disorder	AUTODEGRADATION_OF_CDH1_BY_CDH1_APC_C	REACTOME	857	34	20	1,71E-01	9,80E-01	rs704375|rs16930959|rs11922435|rs400223|rs13338214|rs12598046|rs6742309|rs755859|rs17031765|rs11642698|rs4795311|rs6550808|rs417940|rs17842119|rs12447239|rs4435493|rs7212835|rs40610|rs28653722|rs3761034
Neurologic Disorder	LEISHMANIA_INFECTION	KEGG	2265	43	20	1,74E-01	9,80E-01	rs6930052|rs4698855|rs6692647|rs10184153|rs1884257|rs10983712|rs7744|rs3730327|rs12944039|rs7754397|rs1129880|rs661838|rs7287350|rs3729908|rs6001585|rs11159135|rs6454860|rs3785383|rs540722|rs3135021
Neurologic Disorder	PTDINS_PATHWAY	BIOCARTA	1058	33	20	1,74E-01	9,80E-01	rs1232587|rs13402503|rs4557033|rs3753242|rs13023787|rs843346|rs660442|rs16830730|rs17158495|rs17158795|rs6134162|rs11919795|rs2127956|rs506134|rs17020967|rs2075876|rs183984|rs12547683|rs9409864|rs585156
Neurologic Disorder	GSK3_PATHWAY	BIOCARTA	1055	41	20	1,75E-01	9,80E-01	rs4698855|rs9320821|rs465531|rs10983712|rs6872249|rs7744|rs16830730|rs11757506|rs1423236|rs3776841|rs12706724|rs4470720|rs2431238|rs3805787|rs3750144|rs2239621|rs637462|rs6802872|rs9810950|rs1410202
Neurologic Disorder	SIGNALING_BY_SCF_KIT	REACTOME	1989	28	20	1,75E-01	9,80E-01	rs461970|rs1843195|rs4963857|rs465531|rs764833|rs12948922|rs3759929|rs2360969|rs6872249|rs6503691|rs1962515|rs2245693|rs7226990|rs917846|rs11204910|rs660442|rs8063461|rs1423236|rs6594334|rs1019000
Neurologic Disorder	INNATE_IMMUNE_SYSTEM	REACTOME	6278	33	20	1,75E-01	9,80E-01	rs730503|rs8056241|rs2829949|rs710092|rs7810486|rs6039403|rs11953993|rs1884257|rs2587616|rs1418258|rs10983712|rs9901001|rs11746572|rs2360969|rs40383|rs11869073|rs2218810|rs2440134|rs10473234|rs8041078
Neurologic Disorder	MITOCHONDRIAL_TRNA_AMINOACYLATION	REACTOME	500	24	13	1,75E-01	9,80E-01	rs3818057|rs1983800|rs6597165|rs11962015|rs12153956|rs2295085|rs17140654|rs2240396|rs6924018|rs9369476|rs13294|rs765878|rs2068871
Neurologic Disorder	TRANSFERRIN_ENDOCYTOSIS_AND_RECYCLING	REACTOME	513	29	15	1,76E-01	9,80E-01	rs1296826|rs577866|rs1115219|rs6994201|rs10954211|rs2460221|rs2401158|rs2075609|rs2270637|rs7464428|rs10124511|rs745554|rs7853552|rs4927870|rs7511769
Neurologic Disorder	SYNTHESIS_OF_GLYCOSYLPHOSPHATIDYLINOSITOL_GPI	REACTOME	485	20	10	1,76E-01	9,80E-01	rs6776064|rs1918638|rs11577096|rs13149933|rs504729|rs1849295|rs1972118|rs7178329|rs9807301|rs4791665
Neurologic Disorder	ECM_RECEPTOR_INTERACTION	KEGG	3602	30	20	1,77E-01	9,80E-01	rs11974777|rs2575738|rs4842157|rs2437095|rs10184153|rs3211834|rs34719083|rs1265646|rs2292867|rs16877483|rs16975294|rs7807090|rs4663708|rs3771254|rs10858253|rs11070217|rs4145072|rs9505962|rs7544531|rs16926995
Neurologic Disorder	DORSO_VENTRAL_AXIS_FORMATION	KEGG	1149	35	20	1,77E-01	9,80E-01	rs532385|rs4963857|rs2855709|rs17576984|rs17818396|rs759169|rs6692418|rs16840117|rs1381528|rs1114312|rs12666347|rs6674139|rs884904|rs2710293|rs4776783|rs1940359|rs6672784|rs2156932|rs6457903|rs1001445
Neurologic Disorder	PYRIMIDINE_METABOLISM	KEGG	1900	34	20	1,77E-01	9,80E-01	rs487989|rs7980317|rs10918295|rs9362210|rs784600|rs6132068|rs2215134|rs12581936|rs3807034|rs10951757|rs6081172|rs11697114|rs6518591|rs3821996|rs6683957|rs11096544|rs4672066|rs2985689|rs7217258|rs17745314
Neurologic Disorder	PROTEIN_FOLDING	REACTOME	675	33	14	1,79E-01	9,80E-01	rs10942832|rs1418927|rs10824092|rs11727661|rs6023019|rs3844450|rs6856075|rs710750|rs9477550|rs11893547|rs4270584|rs1937746|rs12977802|rs151358
Neurologic Disorder	CXCR4_PATHWAY	BIOCARTA	1099	34	20	1,80E-01	9,80E-01	rs4698855|rs10907192|rs465531|rs12948922|rs6872249|rs1962515|rs1423236|rs12706724|rs3729908|rs10505811|rs4470720|rs4776783|rs3785383|rs16959227|rs11890854|rs7371043|rs1147963|rs7016497|rs120909|rs12691883
Neurologic Disorder	ASTHMA	KEGG	770	16	7	1,82E-01	9,80E-01	rs6930052|rs3135021|rs17351243|rs11569333|rs4813003|rs12435849|rs1295686
Neurologic Disorder	MUSCLE_CONTRACTION	REACTOME	1023	42	20	1,84E-01	9,80E-01	rs6509933|rs4766526|rs1265646|rs6509931|rs8039242|rs11647019|rs3811178|rs7481661|rs1029513|rs4917690|rs7259810|rs13411881|rs1926196|rs12595658|rs3772832|rs2805787|rs1441734|rs2097657|rs7907756|rs913912
Neurologic Disorder	DNA_STRAND_ELONGATION	REACTOME	672	33	20	1,88E-01	9,80E-01	rs487989|rs7980317|rs9592217|rs12877281|rs11147544|rs10951757|rs1925089|rs1359389|rs7324308|rs7996386|rs8077346|rs7994717|rs690705|rs2922666|rs509360|rs2304136|rs133422|rs3735461|rs174556|rs677740
Neurologic Disorder	SMALL_CELL_LUNG_CANCER	KEGG	3747	28	20	1,91E-01	9,80E-01	rs4698855|rs512868|rs13064561|rs2437095|rs465531|rs4679614|rs17632419|rs745830|rs16975294|rs7340603|rs7807090|rs4332094|rs643629|rs2142200|rs6872437|rs4145072|rs12944039|rs13325331|rs4129666|rs4285344
Neurologic Disorder	ARMS_MEDIATED_ACTIVATION	REACTOME	393	15	7	1,92E-01	9,80E-01	rs4963857|rs1962515|rs13008971|rs4776783|rs17623382|rs11102933|rs7303282
Neurologic Disorder	ENOS_ACTIVATION_AND_REGULATION	REACTOME	336	13	8	1,93E-01	9,80E-01	rs2878172|rs10756597|rs7688672|rs2191502|rs7259810|rs12685826|rs1650647|rs6733907
Neurologic Disorder	RELA_PATHWAY	BIOCARTA	208	8	6	1,94E-01	9,80E-01	rs4698855|rs10958713|rs8016947|rs2238419|rs17112705|rs11076785
Neurologic Disorder	CTCF_PATHWAY	BIOCARTA	1035	37	20	1,97E-01	9,80E-01	rs7552931|rs465531|rs4332094|rs2142200|rs6872437|rs4129666|rs2296620|rs2395851|rs6783860|rs7835350|rs12548864|rs1874572|rs1423236|rs3776841|rs4470720|rs1074078|rs1805110|rs12543298|rs945559|rs4410871
Neurologic Disorder	POST_TRANSLATIONAL_MODIFICATION_SYNTHESIS_OF_GPI_ANCHORED_PROTEINS	REACTOME	586	28	15	1,97E-01	9,80E-01	rs6776064|rs1918638|rs2689672|rs2741538|rs11577096|rs199714|rs13149933|rs504729|rs6126126|rs1849295|rs1972118|rs2070139|rs7178329|rs9807301|rs4791665
Neurologic Disorder	ORC1_REMOVAL_FROM_CHROMATIN	REACTOME	930	37	20	2,03E-01	9,80E-01	rs9450763|rs704375|rs11922435|rs400223|rs13338214|rs12598046|rs9315420|rs755859|rs17031765|rs11642698|rs4795311|rs417940|rs12447239|rs4435493|rs7212835|rs9368950|rs40610|rs2307373|rs133422|rs3761034
Neurologic Disorder	EXTRACELLULAR_MATRIX_ORGANIZATION	REACTOME	3783	24	20	2,03E-01	9,80E-01	rs518558|rs13111857|rs4253239|rs12234520|rs4691230|rs4842157|rs2714819|rs1428814|rs2454331|rs16975294|rs1355685|rs3759929|rs4663708|rs4949455|rs10858253|rs17775314|rs12652833|rs4145072|rs2541232|rs2664352
Neurologic Disorder	RENIN_ANGIOTENSIN_SYSTEM	KEGG	467	11	6	2,04E-01	9,80E-01	rs6772419|rs1466097|rs2182574|rs2033078|rs12107440|rs16894413
Neurologic Disorder	CACAM_PATHWAY	BIOCARTA	401	16	9	2,06E-01	9,80E-01	rs11938486|rs2241694|rs2360969|rs7259810|rs4286653|rs6733907|rs6703522|rs25923|rs11615578
Neurologic Disorder	DEPOSITION_OF_NEW_CENPA_CONTAINING_NUCLEOSOMES_AT_THE_CENTROMERE	REACTOME	637	42	12	2,07E-01	9,80E-01	rs10185631|rs2833690|rs1500480|rs7700605|rs1266294|rs8134439|rs198838|rs2549854|rs213548|rs2249418|rs10144117|rs9860614
Neurologic Disorder	IL_RECEPTOR_SHC_SIGNALING	REACTOME	624	16	8	2,11E-01	9,80E-01	rs465531|rs6872437|rs1423236|rs4470720|rs6776572|rs525563|rs228935|rs651933
Neurologic Disorder	DEFENSINS	REACTOME	495	11	7	2,12E-01	9,80E-01	rs6039403|rs1418258|rs4513489|rs4710191|rs7461956|rs4300027|rs7757618
Neurologic Disorder	MYOGENESIS	REACTOME	1747	52	20	2,13E-01	9,80E-01	rs2032728|rs11953993|rs16985602|rs11746572|rs4925249|rs11869073|rs2440134|rs8041078|rs3730327|rs6071519|rs9949107|rs1129880|rs16984422|rs817689|rs6065046|rs997284|rs751722|rs17207681|rs10514306|rs474246
Neurologic Disorder	AUTODEGRADATION_OF_THE_E3_UBIQUITIN_LIGASE_COP1	REACTOME	766	40	20	2,16E-01	9,80E-01	rs704375|rs12736942|rs11922435|rs400223|rs13338214|rs12598046|rs755859|rs17031765|rs11642698|rs4795311|rs417940|rs12447239|rs4435493|rs7212835|rs40610|rs3761034|rs2584624|rs4813030|rs16972410|rs12446725
Neurologic Disorder	CCR3_PATHWAY	BIOCARTA	939	32	20	2,16E-01	9,80E-01	rs10907192|rs3919447|rs2423356|rs12948922|rs771941|rs1962515|rs4669725|rs7271063|rs3729908|rs10505811|rs6077411|rs4776783|rs6055685|rs6086714|rs3785383|rs16959227|rs6055853|rs6513412|rs7016497|rs120909
Neurologic Disorder	LATENT_INFECTION_OF_HOMO_SAPIENS_WITH_MYCOBACTERIUM_TUBERCULOSIS	REACTOME	589	26	17	2,17E-01	9,80E-01	rs1296826|rs2293055|rs12944039|rs6994201|rs10954211|rs2460221|rs2401158|rs7287350|rs2075609|rs2270637|rs1879417|rs7464428|rs10124511|rs4683235|rs7853552|rs17078876|rs7511769
Neurologic Disorder	GLEEVEC_PATHWAY	BIOCARTA	954	48	20	2,17E-01	9,80E-01	rs465531|rs4332094|rs2142200|rs6872249|rs6503691|rs1962515|rs6003709|rs4129666|rs2395851|rs660442|rs7835350|rs12548864|rs1423236|rs11159135|rs4470720|rs4776783|rs12543298|rs4410871|rs16903124|rs1882832
Neurologic Disorder	TCR_PATHWAY	BIOCARTA	1517	33	20	2,18E-01	9,80E-01	rs4698855|rs461970|rs1569736|rs465531|rs12948922|rs6872437|rs1962515|rs3782046|rs8090560|rs1423236|rs7259810|rs3729908|rs11953892|rs11159135|rs4470720|rs4776783|rs7735400|rs3785383|rs16959227|rs10918708
Neurologic Disorder	PTEN_PATHWAY	BIOCARTA	544	20	8	2,18E-01	9,80E-01	rs465531|rs6872437|rs1423236|rs4470720|rs3800230|rs945559|rs7016497|rs11785444
Neurologic Disorder	RNA_POLYMERASE	KEGG	351	39	11	2,18E-01	9,80E-01	rs6132068|rs12581936|rs3807035|rs6081172|rs3821996|rs7217258|rs3134879|rs4119055|rs11743030|rs1107674|rs12977802
Neurologic Disorder	ACYL_CHAIN_REMODELLING_OF_PG	REACTOME	571	40	16	2,19E-01	9,80E-01	rs10058172|rs12749748|rs10912053|rs1076205|rs600415|rs1800627|rs776686|rs1938506|rs6425147|rs17411112|rs1548796|rs10753000|rs1487492|rs7554410|rs3761431|rs10912174
Neurologic Disorder	GLOBAL_GENOMIC_NER_GG_NER	REACTOME	823	35	20	2,20E-01	9,80E-01	rs817827|rs9592217|rs12877281|rs11147544|rs10951757|rs8054783|rs1573638|rs2985689|rs1925089|rs1359389|rs7324308|rs7996386|rs12336793|rs8077346|rs7994717|rs690705|rs2922666|rs2304136|rs9925917|rs1124303
Neurologic Disorder	PURINE_METABOLISM	REACTOME	581	33	20	2,20E-01	9,80E-01	rs9362210|rs784600|rs4812848|rs1908335|rs899011|rs11096544|rs2198009|rs2071017|rs3127468|rs13411881|rs586364|rs1180275|rs7103258|rs784619|rs12912744|rs1126432|rs7118388|rs11076720|rs10179712|rs714510
Neurologic Disorder	NOTCH_SIGNALING_PATHWAY	KEGG	1387	29	20	2,21E-01	9,80E-01	rs1232587|rs3761441|rs532385|rs17576984|rs7893862|rs4525961|rs10501844|rs843346|rs6779720|rs12365545|rs1114312|rs1693663|rs6134162|rs11919795|rs12580394|rs4686655|rs6777021|rs3757016|rs11021461|rs2072793
Neurologic Disorder	HYPERTROPHIC_CARDIOMYOPATHY_HCM	KEGG	4149	43	20	2,21E-01	9,80E-01	rs6692647|rs4747345|rs2239048|rs2437095|rs4726096|rs1457596|rs6509933|rs10184153|rs3919447|rs3912834|rs7198466|rs1265646|rs2292867|rs7477742|rs6509931|rs886940|rs2042995|rs8039242|rs1295881|rs886898
Neurologic Disorder	EDG1_PATHWAY	BIOCARTA	1432	33	20	2,23E-01	9,80E-01	rs10907192|rs465531|rs2423356|rs2292867|rs12948922|rs12407294|rs6872249|rs771941|rs4261706|rs9434153|rs13263637|rs6436755|rs11736538|rs1423236|rs7271063|rs12706724|rs3729908|rs4470720|rs6077411|rs6055685
Neurologic Disorder	ACTIVATION_OF_ATR_IN_RESPONSE_TO_REPLICATION_STRESS	REACTOME	653	31	18	2,26E-01	9,80E-01	rs9450763|rs9592217|rs12877281|rs11147544|rs1925089|rs11814554|rs11764107|rs1359389|rs7324308|rs7916990|rs7996386|rs8077346|rs7994717|rs690705|rs2307373|rs133422|rs3735461|rs2891652
Neurologic Disorder	CHRONIC_MYELOID_LEUKEMIA	KEGG	2538	30	20	2,27E-01	9,80E-01	rs4698855|rs7552931|rs4963857|rs465531|rs7893862|rs4332094|rs2142200|rs4381535|rs4894936|rs6872249|rs6503691|rs1962515|rs6003709|rs4129666|rs266359|rs2395851|rs6783860|rs660442|rs2834698|rs7835350
Neurologic Disorder	NUCLEAR_EVENTS_KINASE_AND_TRANSCRIPTION_FACTOR_ACTIVATION	REACTOME	766	55	20	2,28E-01	9,80E-01	rs11953993|rs11746572|rs2360969|rs8041078|rs3730327|rs6903559|rs9873175|rs963921|rs1129880|rs12540995|rs3776841|rs3742064|rs10514306|rs602932|rs9459785|rs9347129|rs9295348|rs1833895|rs845647|rs4483828
Neurologic Disorder	METABOLISM_OF_NUCLEOTIDES	REACTOME	1559	36	20	2,32E-01	9,80E-01	rs10782645|rs10918295|rs9362210|rs10493604|rs784600|rs4812848|rs10782647|rs1908335|rs899011|rs6683957|rs11096544|rs2198009|rs2071017|rs10759403|rs3127468|rs13411881|rs2291702|rs6741915|rs586364|rs1180275
Neurologic Disorder	ACH_PATHWAY	BIOCARTA	602	27	13	2,34E-01	9,80E-01	rs465531|rs6872437|rs4978952|rs3791729|rs660442|rs1423236|rs4470720|rs3800230|rs7016497|rs7846568|rs1550097|rs2846451|rs11785444
Neurologic Disorder	REGULATION_OF_ACTIN_CYTOSKELETON	KEGG	7089	36	20	2,36E-01	9,80E-01	rs1046248|rs11978556|rs461970|rs4704350|rs10802789|rs272107|rs4963857|rs465531|rs3911489|rs260852|rs12914045|rs2184778|rs10184153|rs4766526|rs17041245|rs919751|rs1265646|rs3011577|rs2292867|rs1851710
Neurologic Disorder	ACUTE_MYELOID_LEUKEMIA	KEGG	1892	31	20	2,37E-01	9,80E-01	rs4698855|rs4963857|rs465531|rs4332094|rs2142200|rs6872249|rs2568218|rs6503691|rs1962515|rs4129666|rs917846|rs176632|rs2395851|rs660442|rs2834698|rs7835350|rs12548864|rs10488697|rs9315420|rs12681007
Neurologic Disorder	ASSEMBLY_OF_THE_PRE_REPLICATIVE_COMPLEX	REACTOME	911	38	20	2,37E-01	9,80E-01	rs9450763|rs704375|rs11922435|rs400223|rs13338214|rs12598046|rs755859|rs17031765|rs11642698|rs4795311|rs417940|rs12447239|rs4435493|rs7212835|rs40610|rs2307373|rs133422|rs3761034|rs2665833|rs4813030
Neurologic Disorder	P53_DEPENDENT_G1_DNA_DAMAGE_RESPONSE	REACTOME	838	39	20	2,37E-01	9,80E-01	rs704375|rs12736942|rs11922435|rs400223|rs13338214|rs12598046|rs755859|rs17031765|rs11642698|rs4795311|rs417940|rs12447239|rs4435493|rs7212835|rs9368950|rs40610|rs3761034|rs2584624|rs4813030|rs9380590
Neurologic Disorder	ECM_PATHWAY	BIOCARTA	753	32	12	2,38E-01	9,80E-01	rs465531|rs3919447|rs6872437|rs1962515|rs1423236|rs4470720|rs4776783|rs7016497|rs7641248|rs3911406|rs6916861|rs11785444
Neurologic Disorder	DESTABILIZATION_OF_MRNA_BY_AUF1_HNRNP_D0	REACTOME	811	38	20	2,40E-01	9,80E-01	rs704375|rs11922435|rs400223|rs13338214|rs12598046|rs2868355|rs755859|rs17031765|rs11642698|rs12510329|rs4795311|rs417940|rs17517994|rs12447239|rs4435493|rs7212835|rs40610|rs3761034|rs2665833|rs4813030
Neurologic Disorder	RIP_MEDIATED_NFKB_ACTIVATION_VIA_DAI	REACTOME	328	10	7	2,40E-01	9,80E-01	rs2829949|rs2829929|rs4807650|rs10958713|rs8016947|rs6516727|rs17112705
Neurologic Disorder	G1_S_TRANSITION	REACTOME	1941	37	20	2,41E-01	9,80E-01	rs487989|rs9450763|rs704375|rs7980317|rs11922435|rs4332094|rs2142200|rs400223|rs13338214|rs12598046|rs4129666|rs2395851|rs7835350|rs2985689|rs12548864|rs11814554|rs9315420|rs755859|rs3776841|rs11764107
Neurologic Disorder	PURINE_METABOLISM	KEGG	5442	30	20	2,41E-01	9,80E-01	rs37576|rs487989|rs13064561|rs7771841|rs10782645|rs7980317|rs7733296|rs9362210|rs4679614|rs10493604|rs17632419|rs705789|rs745830|rs6570053|rs784600|rs6132068|rs4812848|rs2215134|rs7340603|rs2269142
Neurologic Disorder	SIGNALLING_TO_RAS	REACTOME	468	23	13	2,42E-01	9,80E-01	rs4963857|rs3730327|rs1962515|rs1129880|rs12462766|rs10413199|rs4776783|rs2241407|rs4877062|rs602932|rs11102933|rs7021743|rs7303282

Neurologic Disorder	SIGNALING_BY_FGFR1_FUSION_MUTANTS	REACTOME	639	30	17	2,44E-01	9,80E-01	rs465531|rs1874326|rs6872437|rs6503691|rs6003709|rs11048776|rs1423236|rs4470720|rs4073362|rs6973312|rs6581852|rs7812254|rs1880345|rs201492|rs11977362|rs1725604|rs435359
Neurologic Disorder	G2_M_CHECKPOINTS	REACTOME	710	31	18	2,48E-01	9,80E-01	rs9450763|rs9592217|rs12877281|rs11147544|rs1925089|rs11814554|rs11764107|rs1359389|rs7324308|rs7916990|rs7996386|rs8077346|rs7994717|rs690705|rs2307373|rs133422|rs3735461|rs2891652
Neurologic Disorder	SYSTEMIC_LUPUS_ERYTHEMATOSUS	KEGG	2453	37	20	2,48E-01	9,80E-01	rs6930052|rs11612284|rs170290|rs2300267|rs2218810|rs10473234|rs11055587|rs316369|rs16918961|rs4902681|rs7206547|rs17833967|rs11055612|rs2314978|rs7736582|rs7979850|rs2160173|rs7301754|rs9831894|rs3776206
Neurologic Disorder	GLUTATHIONE_METABOLISM	KEGG	683	28	17	2,49E-01	9,80E-01	rs6898463|rs3818562|rs5996675|rs7551594|rs2297763|rs6464540|rs13114510|rs510088|rs4712036|rs9296695|rs2602218|rs9474314|rs12580698|rs2287396|rs7585544|rs2911670|rs11807
Neurologic Disorder	STRIATED_MUSCLE_CONTRACTION	REACTOME	453	38	18	2,50E-01	9,80E-01	rs6509933|rs4766526|rs6509931|rs8039242|rs3811178|rs7481661|rs1029513|rs12595658|rs2805787|rs1989811|rs913912|rs825080|rs4664057|rs7225575|rs2149981|rs17031746|rs3811454|rs359281
Neurologic Disorder	SIGNALING_BY_HIPPO	REACTOME	627	15	9	2,52E-01	9,80E-01	rs1775395|rs11260671|rs2309428|rs950601|rs9322206|rs6991043|rs5006364|rs4753145|rs12555245
Neurologic Disorder	POST_CHAPERONIN_TUBULIN_FOLDING_PATHWAY	REACTOME	254	14	5	2,52E-01	9,80E-01	rs10942832|rs3844450|rs1937746|rs12977802|rs151358
Neurologic Disorder	REGULATION_OF_KIT_SIGNALING	REACTOME	587	26	16	2,53E-01	9,80E-01	rs1843195|rs764833|rs12948922|rs2245693|rs7226990|rs917846|rs17227367|rs16959227|rs12547683|rs218268|rs9963024|rs7245158|rs17634425|rs1725604|rs6916861|rs12450534
Neurologic Disorder	ONE_CARBON_POOL_BY_FOLATE	KEGG	441	17	11	2,54E-01	9,80E-01	rs6905272|rs538017|rs1771845|rs2987981|rs6745054|rs2839128|rs9478918|rs11757561|rs803447|rs1650647|rs17357592
Neurologic Disorder	AMINOACYL_TRNA_BIOSYNTHESIS	KEGG	731	31	18	2,55E-01	9,80E-01	rs3818057|rs1983800|rs6597165|rs11745439|rs11962015|rs12153956|rs2053701|rs2295085|rs611060|rs17140654|rs2240396|rs6924018|rs9369476|rs3733977|rs13294|rs925210|rs765878|rs2068871
Neurologic Disorder	CTLA4_PATHWAY	BIOCARTA	662	32	13	2,55E-01	9,80E-01	rs465531|rs6872249|rs3782046|rs2838515|rs1423236|rs4470720|rs13420393|rs9831894|rs3115961|rs10918708|rs6698995|rs2278385|rs4673268
Neurologic Disorder	CYCLIN_E_ASSOCIATED_EVENTS_DURING_G1_S_TRANSITION_	REACTOME	1212	38	20	2,59E-01	9,80E-01	rs704375|rs11922435|rs4332094|rs2142200|rs400223|rs13338214|rs12598046|rs4129666|rs2395851|rs7835350|rs12548864|rs9315420|rs755859|rs3776841|rs17031765|rs11642698|rs4795311|rs417940|rs12447239|rs4435493
Neurologic Disorder	RETINOL_METABOLISM	KEGG	1512	35	12	2,62E-01	9,80E-01	rs4072868|rs2068888|rs11249454|rs6736570|rs8022613|rs1400681|rs678040|rs10027817|rs4852846|rs4618985|rs1958297|rs7602885
Neurologic Disorder	NUCLEOTIDE_EXCISION_REPAIR	REACTOME	956	34	20	2,62E-01	9,80E-01	rs817827|rs9592217|rs12877281|rs11147544|rs10951757|rs8054783|rs3821996|rs1573638|rs2985689|rs1925089|rs1359389|rs7324308|rs7217258|rs7996386|rs12336793|rs8077346|rs7994717|rs690705|rs2922666|rs2304136
Neurologic Disorder	BIOPEPTIDES_PATHWAY	BIOCARTA	1081	37	20	2,63E-01	9,80E-01	rs11938486|rs10907192|rs2241694|rs12948922|rs17545756|rs3730327|rs6503691|rs1962515|rs7259810|rs12706724|rs3729908|rs4776783|rs2182574|rs3785383|rs16959227|rs602932|rs1467967|rs4286653|rs120909|rs5030102
Neurologic Disorder	APC_C_CDH1_MEDIATED_DEGRADATION_OF_CDC20_AND_OTHER_APC_C_CDH1_TARGET<br>TE_MITOSIS_EARLY_G1	REACTOME	959	34	20	2,65E-01	9,80E-01	rs704375|rs16930959|rs11922435|rs400223|rs13338214|rs12598046|rs6742309|rs755859|rs17031765|rs11642698|rs4795311|rs6550808|rs417940|rs17842119|rs12447239|rs4435493|rs7212835|rs40610|rs28653722|rs3761034
Neurologic Disorder	RNA_POL_III_TRANSCRIPTION	REACTOME	861	41	19	2,66E-01	9,80E-01	rs4922220|rs11779820|rs17093344|rs6132068|rs12581936|rs1327510|rs6081172|rs2062348|rs683474|rs4119055|rs4961408|rs10810136|rs10961271|rs17709614|rs10095297|rs7018762|rs11204109|rs17093001|rs10503682
Neurologic Disorder	MITOTIC_G1_G1_S_PHASES	REACTOME	2477	34	20	2,69E-01	9,80E-01	rs2070235|rs487989|rs9450763|rs704375|rs7980317|rs11922435|rs4332094|rs2142200|rs400223|rs13338214|rs12598046|rs3091249|rs4129666|rs2395851|rs12370927|rs7835350|rs2985689|rs2374594|rs12548864|rs9369325
Neurologic Disorder	PROTEASOME_PATHWAY	BIOCARTA	280	27	13	2,69E-01	9,80E-01	rs704375|rs11922435|rs2714747|rs7496283|rs17031765|rs4795311|rs2526025|rs7212835|rs40610|rs6017596|rs6031882|rs8003074|rs2811491
Neurologic Disorder	ATRBRCA_PATHWAY	BIOCARTA	238	22	6	2,69E-01	9,80E-01	rs2761367|rs16942|rs2697675|rs6470522|rs9634672|rs1295686
Neurologic Disorder	G_BETA_GAMMA_SIGNALLING_THROUGH_PI3KGAMMA	REACTOME	487	10	7	2,71E-01	9,80E-01	rs10907192|rs4898712|rs9630890|rs6674438|rs2024497|rs6510696|rs6972935
Neurologic Disorder	LEUKOCYTE_TRANSENDOTHELIAL_MIGRATION	KEGG	4793	46	20	2,73E-01	9,80E-01	rs9397270|rs11978556|rs6757169|rs461970|rs2880989|rs465531|rs13332162|rs482533|rs10184153|rs4766526|rs11777908|rs9322526|rs471398|rs12948922|rs6872437|rs3730327|rs10951757|rs12643103|rs4616804|rs1368909
Neurologic Disorder	IMMUNOREGULATORY_INTERACTIONS_BETWEEN_A_LYMPHOID_AND_A_NON_LYMPHO	REACTOME	1325	51	20	2,74E-01	9,80E-01	rs2824327|rs11155703|rs7259347|rs1501813|rs13191519|rs7755852|rs4936757|rs3782046|rs9258895|rs1617582|rs3107638|rs2073917|rs28367729|rs2010259|rs10918708|rs6698995|rs9266440|rs3094691|rs2900420|rs1801552
Neurologic Disorder	NGF_PATHWAY	BIOCARTA	566	23	11	2,75E-01	9,80E-01	rs465531|rs6872249|rs1962515|rs1423236|rs11159135|rs4470720|rs4776783|rs6037986|rs11102933|rs1882832|rs8013053
Neurologic Disorder	NEP_NS2_INTERACTS_WITH_THE_CELLULAR_EXPORT_MACHINERY	REACTOME	456	35	16	2,76E-01	9,80E-01	rs13431075|rs9679654|rs1000940|rs905639|rs283525|rs2350623|rs132813|rs12693351|rs1406334|rs7697711|rs9985182|rs357111|rs103612|rs1462529|rs3809335|rs2882359
Neurologic Disorder	ERK_PATHWAY	BIOCARTA	1025	31	20	2,77E-01	9,80E-01	rs10907192|rs7965899|rs4332094|rs2142200|rs759169|rs1962515|rs4129666|rs2395851|rs7835350|rs12548864|rs11736538|rs12666347|rs4760932|rs3776841|rs884904|rs4776783|rs2684788|rs12543298|rs17691810|rs6513412
Neurologic Disorder	ALDOSTERONE_REGULATED_SODIUM_REABSORPTION	KEGG	1794	33	20	2,77E-01	9,80E-01	rs10434131|rs2048546|rs4963857|rs465531|rs1457596|rs17256769|rs12948922|rs13112423|rs6872249|rs9493844|rs6694434|rs12323112|rs1423236|rs3729908|rs10520008|rs4470720|rs2073636|rs11687971|rs6219|rs1543661
Neurologic Disorder	ACTIVATED_NOTCH1_TRANSMITS_SIGNAL_TO_THE_NUCLEUS	REACTOME	719	31	18	2,78E-01	9,80E-01	rs1232587|rs6750496|rs6436879|rs4973205|rs611908|rs6134162|rs6736713|rs9905578|rs7155375|rs4435493|rs10752637|rs6033079|rs12968949|rs3761034|rs7146712|rs6033014|rs10784789|rs2193218
Neurologic Disorder	SCFSKP2_MEDIATED_DEGRADATION_OF_P27_P21	REACTOME	813	35	20	2,79E-01	9,80E-01	rs704375|rs11922435|rs400223|rs13338214|rs12598046|rs9315420|rs755859|rs3776841|rs17031765|rs11642698|rs4795311|rs417940|rs12447239|rs4435493|rs7212835|rs9368950|rs40610|rs3761034|rs2584624|rs2041074
Neurologic Disorder	ER_PHAGOSOME_PATHWAY	REACTOME	1416	38	20	2,85E-01	9,80E-01	rs704375|rs11922435|rs13191519|rs400223|rs7755852|rs13338214|rs12598046|rs9258895|rs755859|rs17031765|rs11642698|rs4795311|rs417940|rs28367729|rs12447239|rs4435493|rs7212835|rs9266440|rs40610|rs3094691
Neurologic Disorder	CONVERSION_FROM_APC_C_CDC20_TO_APC_C_CDH1_IN_LATE_ANAPHASE	REACTOME	226	13	8	2,90E-01	9,80E-01	rs16930959|rs478859|rs6742309|rs6550808|rs17842119|rs28653722|rs1464095|rs4848611
Neurologic Disorder	REGULATORY_RNA_PATHWAYS	REACTOME	345	17	7	2,92E-01	9,80E-01	rs175169|rs3821996|rs2033719|rs8014347|rs7217258|rs1107674|rs12977802
Neurologic Disorder	TRAFFICKING_OF_AMPA_RECEPTORS	REACTOME	1132	41	20	2,92E-01	9,80E-01	rs11938486|rs7198466|rs2241694|rs12948922|rs1557809|rs843346|rs2107118|rs637569|rs12507865|rs7936555|rs3729908|rs11919795|rs132930|rs10420331|rs3785383|rs16959227|rs4958671|rs12596171|rs4286653|rs120909
Neurologic Disorder	COLLAGEN_FORMATION	REACTOME	3203	28	20	2,93E-01	9,80E-01	rs518558|rs13111857|rs12234520|rs4691230|rs4842157|rs2714819|rs16975294|rs1355685|rs4663708|rs4949455|rs10858253|rs17775314|rs12652833|rs4145072|rs2541232|rs1249710|rs10943218|rs17819585|rs7747377|rs17415692
Neurologic Disorder	STEROID_HORMONES	REACTOME	693	32	20	2,93E-01	9,80E-01	rs1062708|rs1458928|rs2414099|rs4555110|rs1276720|rs12555788|rs12504112|rs17139378|rs6975650|rs166050|rs932742|rs830958|rs7048593|rs3012499|rs11857713|rs3787557|rs819271|rs4864976|rs2470176|rs2247506
Neurologic Disorder	ARACHIDONIC_ACID_METABOLISM	KEGG	1356	39	20	2,96E-01	9,80E-01	rs6898463|rs41723|rs12749748|rs5996675|rs10912053|rs17764723|rs1076205|rs1800627|rs6503078|rs776686|rs1937849|rs2733750|rs4650729|rs800719|rs4924592|rs10818684|rs2212922|rs6425147|rs17411112|rs7806848
Neurologic Disorder	NUCLEOTIDE_EXCISION_REPAIR	KEGG	914	35	20	2,97E-01	9,80E-01	rs817827|rs9592217|rs12877281|rs11147544|rs10951757|rs8054783|rs12446097|rs2985689|rs1925089|rs1359389|rs7324308|rs7996386|rs12336793|rs8077346|rs7994717|rs690705|rs2922666|rs2304136|rs9925917|rs1124303
Neurologic Disorder	ANTIGEN_PROCESSING_AND_PRESENTATION	KEGG	1754	51	20	3,01E-01	9,80E-01	rs6930052|rs6918969|rs7259347|rs13191519|rs2360969|rs7755852|rs9258895|rs28367729|rs3135021|rs1952015|rs4978113|rs9266440|rs3094691|rs10125010|rs2361651|rs2517938|rs2517861|rs1325158|rs4072864|rs13202464
Neurologic Disorder	LYSINE_DEGRADATION	KEGG	952	42	19	3,02E-01	9,80E-01	rs13108615|rs1355685|rs7689415|rs4578194|rs7551594|rs4878784|rs2661067|rs7521445|rs1258191|rs7666900|rs9535241|rs3774051|rs1913185|rs6816|rs1348657|rs11725821|rs12629663|rs2141473|rs11716171
Neurologic Disorder	EGF_PATHWAY	BIOCARTA	1048	39	20	3,03E-01	9,80E-01	rs465531|rs17041245|rs12948922|rs6872249|rs759169|rs6503691|rs1962515|rs12666347|rs1423236|rs3729908|rs11953892|rs11159135|rs4470720|rs884904|rs4776783|rs7735400|rs3785383|rs16959227|rs10488142|rs6037986
Neurologic Disorder	KERATINOCYTE_PATHWAY	BIOCARTA	1952	39	20	3,03E-01	9,80E-01	rs4698855|rs13402503|rs4557033|rs17041245|rs12948922|rs11869073|rs2440134|rs759169|rs3730327|rs13023787|rs1962515|rs4941185|rs12666347|rs3776841|rs3729908|rs997284|rs1926196|rs11159135|rs506134|rs183984
Neurologic Disorder	TRNA_AMINOACYLATION	REACTOME	795	31	18	3,04E-01	9,80E-01	rs3818057|rs1983800|rs6597165|rs11745439|rs11962015|rs12153956|rs7905657|rs2295085|rs611060|rs17140654|rs2240396|rs6924018|rs9369476|rs3733977|rs13294|rs925210|rs765878|rs2068871
Neurologic Disorder	REGULATION_OF_MRNA_STABILITY_BY_PROTEINS_THAT_BIND_AU_RICH_ELEMENTS	REACTOME	1372	35	20	3,04E-01	9,80E-01	rs704375|rs11922435|rs9679654|rs12948922|rs400223|rs3730327|rs13338214|rs12598046|rs7252142|rs2868355|rs1129880|rs755859|rs17031765|rs12675103|rs11642698|rs12510329|rs4795311|rs417940|rs17517994|rs12447239
Neurologic Disorder	POST_NMDA_RECEPTOR_ACTIVATION_EVENTS	REACTOME	1753	34	20	3,05E-01	9,80E-01	rs11612284|rs170290|rs11938486|rs2300267|rs2241694|rs2360969|rs1962515|rs11055587|rs316369|rs6903559|rs7206547|rs17833967|rs11055612|rs7259810|rs2314978|rs7979850|rs2160173|rs7301754|rs34117|rs12548296
Neurologic Disorder	SELENOAMINO_ACID_METABOLISM	KEGG	742	50	20	3,05E-01	9,80E-01	rs9429768|rs5996675|rs2059098|rs3972|rs158845|rs10225988|rs6895949|rs10068900|rs17487394|rs1363072|rs2726165|rs6935937|rs11958300|rs4868753|rs1399406|rs10070913|rs10057578|rs1330674|rs9934579|rs13112642
Neurologic Disorder	SIGNALING_BY_PDGF	REACTOME	4427	24	20	3,07E-01	9,80E-01	rs518558|rs13402503|rs4842157|rs4963857|rs4557033|rs465531|rs260852|rs10490697|rs919751|rs16877483|rs16975294|rs7605126|rs12948922|rs3759929|rs2360969|rs4663707|rs10858253|rs2163349|rs11070217|rs6872437
Neurologic Disorder	METAL_ION_SLC_TRANSPORTERS	REACTOME	653	37	17	3,09E-01	9,80E-01	rs7604163|rs16930959|rs823082|rs10931679|rs1397744|rs4505549|rs2352267|rs3737580|rs13436110|rs10505292|rs951776|rs10282940|rs7608598|rs457490|rs6693456|rs1155301|rs1472487
Neurologic Disorder	TRANSPORT_OF_RIBONUCLEOPROTEINS_INTO_THE_HOST_NUCLEUS	REACTOME	452	38	17	3,10E-01	9,80E-01	rs13431075|rs4678192|rs1000940|rs905639|rs283525|rs2350623|rs132813|rs12693351|rs1406334|rs7697711|rs9985182|rs357111|rs103612|rs9879508|rs1462529|rs3809335|rs2882359
Neurologic Disorder	PAR1_PATHWAY	BIOCARTA	1941	33	20	3,11E-01	9,80E-01	rs272107|rs10907192|rs465531|rs2423356|rs1884257|rs17288929|rs12948922|rs6872437|rs11765386|rs771941|rs7754397|rs1208204|rs1423236|rs7271063|rs12706724|rs3729908|rs17830000|rs4470720|rs6454860|rs6077411
Neurologic Disorder	AMYOTROPHIC_LATERAL_SCLEROSIS_ALS	KEGG	1813	28	20	3,11E-01	9,80E-01	rs11612284|rs170290|rs2300267|rs11869073|rs2440134|rs3730327|rs2293055|rs11055587|rs316369|rs16927467|rs1129880|rs660442|rs12507865|rs4941185|rs7206547|rs11033098|rs17833967|rs11055612|rs2314978|rs997284
Neurologic Disorder	TRAF6_MEDIATED_NFKB_ACTIVATION	REACTOME	351	12	9	3,12E-01	9,80E-01	rs2829949|rs2829929|rs17212649|rs7865082|rs10958713|rs8016947|rs9900686|rs6516727|rs17112705
Neurologic Disorder	FC_EPSILON_RI_SIGNALING_PATHWAY	KEGG	2758	40	20	3,13E-01	9,80E-01	rs461970|rs13402503|rs4963857|rs4557033|rs465531|rs12749748|rs12948922|rs12046362|rs11869073|rs2440134|rs12693008|rs6872437|rs3730327|rs1076205|rs13023787|rs1962515|rs1129880|rs1800627|rs776686|rs1423236
Neurologic Disorder	G2_PATHWAY	BIOCARTA	354	16	5	3,14E-01	9,80E-01	rs16942|rs2213178|rs13038037|rs9368950|rs9380590
Neurologic Disorder	PROLONGED_ERK_ACTIVATION_EVENTS	REACTOME	429	17	9	3,16E-01	9,80E-01	rs4963857|rs1962515|rs13008971|rs516391|rs4776783|rs17623382|rs11102933|rs7303282|rs7979636
Neurologic Disorder	PROTEOLYTIC_CLEAVAGE_OF_SNARE_COMPLEX_PROTEINS	REACTOME	382	14	7	3,20E-01	9,80E-01	rs7965988|rs7138800|rs4812024|rs2277672|rs3744656|rs6077718|rs10848194
Neurologic Disorder	TRANSCRIPTION_COUPLED_NER_TC_NER	REACTOME	834	33	20	3,20E-01	9,80E-01	rs9592217|rs12877281|rs11147544|rs10951757|rs8054783|rs3821996|rs1573638|rs2985689|rs1925089|rs1359389|rs7324308|rs7217258|rs7996386|rs8077346|rs7994717|rs690705|rs2922666|rs2304136|rs9925917|rs3745364
Neurologic Disorder	MAPK_TARGETS_NUCLEAR_EVENTS_MEDIATED_BY_MAP_KINASES	REACTOME	980	52	20	3,21E-01	9,80E-01	rs11953993|rs11746572|rs2360969|rs8041078|rs3730327|rs6903559|rs9873175|rs963921|rs1129880|rs12540995|rs3776841|rs7566401|rs11159135|rs3742064|rs10514306|rs743923|rs9459785|rs9347129|rs9295348|rs1833895
Neurologic Disorder	METABOLISM_OF_STEROID_HORMONES_AND_VITAMINS_A_AND_D	REACTOME	745	32	20	3,21E-01	9,80E-01	rs1062708|rs1458928|rs2414099|rs4555110|rs1276720|rs12555788|rs12504112|rs17139378|rs6975650|rs166050|rs932742|rs830958|rs7048593|rs3012499|rs11857713|rs3787557|rs819271|rs4864976|rs2470176|rs2247506
Neurologic Disorder	TAK1_ACTIVATES_NFKB_BY_PHOSPHORYLATION_AND_ACTIVATION_OF_IKKS_COMPLE	REACTOME	777	45	19	3,21E-01	9,80E-01	rs2829949|rs1884257|rs40383|rs7754397|rs2829929|rs661838|rs6980760|rs6001585|rs6454860|rs1483382|rs237004|rs9362744|rs6908296|rs10958713|rs8016947|rs9362878|rs6516727|rs17112705|rs10087480
Neurologic Disorder	HOMOLOGOUS_RECOMBINATION	KEGG	730	36	20	3,21E-01	9,80E-01	rs2140741|rs3102854|rs17102087|rs2761367|rs10951757|rs2697675|rs17102086|rs8012659|rs928901|rs365783|rs8077346|rs6470522|rs9634672|rs2922666|rs7141573|rs17835218|rs401549|rs986645|rs3735461|rs4899243
Neurologic Disorder	SCF_BETA_TRCP_MEDIATED_DEGRADATION_OF_EMI1	REACTOME	793	36	20	3,21E-01	9,80E-01	rs704375|rs11922435|rs400223|rs13338214|rs12598046|rs755859|rs3776841|rs17031765|rs11642698|rs4795311|rs417940|rs12447239|rs4435493|rs7212835|rs40610|rs3761034|rs2665833|rs2041074|rs4813030|rs16972410
Neurologic Disorder	INSULIN_RECEPTOR_RECYCLING	REACTOME	492	22	14	3,22E-01	9,80E-01	rs1296826|rs6994201|rs10954211|rs2460221|rs2401158|rs2075609|rs2270637|rs4804377|rs7464428|rs10124511|rs2229431|rs7853552|rs11668751|rs7511769
Neurologic Disorder	G_BETA_GAMMA_SIGNALLING_THROUGH_PLC_BETA	REACTOME	624	27	19	3,22E-01	9,80E-01	rs10907192|rs2423356|rs771941|rs3741403|rs660442|rs4898712|rs7271063|rs6077411|rs9630890|rs6055685|rs6086714|rs6674438|rs2024497|rs6055853|rs6510696|rs2719774|rs6055926|rs2719807|rs6972935
Neurologic Disorder	CHONDROITIN_SULFATE_DERMATAN_SULFATE_METABOLISM	REACTOME	2784	31	20	3,24E-01	9,80E-01	rs9556369|rs1445270|rs2575738|rs309556|rs9561545|rs34719083|rs1007973|rs1929035|rs3771254|rs9523030|rs7997908|rs6762394|rs17035996|rs535828|rs9364126|rs7562354|rs10492621|rs1333674|rs3798592|rs12280153
Neurologic Disorder	PHENYLALANINE_METABOLISM	KEGG	442	27	15	3,25E-01	9,80E-01	rs4405161|rs4820571|rs8062660|rs283525|rs11076318|rs4965732|rs7306243|rs10912626|rs29861|rs6425233|rs7497607|rs1418191|rs2514051|rs11575387|rs11644079
Neurologic Disorder	APC_C_CDC20_MEDIATED_DEGRADATION_OF_MITOTIC_PROTEINS	REACTOME	1094	33	20	3,26E-01	9,80E-01	rs704375|rs16930959|rs11922435|rs400223|rs13338214|rs12598046|rs6742309|rs755859|rs17031765|rs11642698|rs4795311|rs6550808|rs417940|rs17842119|rs12447239|rs10902882|rs4435493|rs7212835|rs40610|rs28653722
Neurologic Disorder	APOPTOSIS	REACTOME	3298	47	20	3,26E-01	9,80E-01	rs8033658|rs2219556|rs704375|rs11714395|rs4678192|rs11922435|rs1816793|rs400223|rs2466178|rs6583176|rs13338214|rs2309428|rs12598046|rs1316489|rs3862706|rs660442|rs4316851|rs1800627|rs2962783|rs4941185
Neurologic Disorder	CD28_CO_STIMULATION	REACTOME	797	32	20	3,26E-01	9,80E-01	rs461970|rs465531|rs6872437|rs6583176|rs11204910|rs6592724|rs1423236|rs4470720|rs9831894|rs1074078|rs9292729|rs3115961|rs728975|rs12547683|rs2049233|rs7245158|rs6583177|rs7086224|rs6916861|rs308195
Neurologic Disorder	CREB_PATHWAY	BIOCARTA	969	41	20	3,27E-01	9,80E-01	rs11938486|rs465531|rs2241694|rs12948922|rs2360969|rs6872249|rs3730327|rs1423236|rs3729908|rs4470720|rs3785383|rs16959227|rs743923|rs4286653|rs6513412|rs120909|rs7196303|rs17634425|rs11074594|rs12450534
Neurologic Disorder	CELL_CYCLE_CHECKPOINTS	REACTOME	1961	30	20	3,31E-01	9,80E-01	rs9450763|rs704375|rs12736942|rs16930959|rs11922435|rs9592217|rs400223|rs12877281|rs11147544|rs13338214|rs12598046|rs6742309|rs1925089|rs11814554|rs755859|rs11764107|rs17031765|rs1359389|rs11642698|rs7324308
Neurologic Disorder	T_CELL_RECEPTOR_SIGNALING_PATHWAY	KEGG	3546	34	20	3,32E-01	9,80E-01	rs4698855|rs461970|rs1569736|rs4963857|rs465531|rs10490697|rs1884257|rs7605126|rs4894936|rs2163349|rs12693008|rs6872437|rs6583176|rs3730327|rs7754397|rs1962515|rs3782046|rs1129880|rs8090560|rs16830730
Neurologic Disorder	ETHER_LIPID_METABOLISM	KEGG	1041	47	20	3,32E-01	9,80E-01	rs10058172|rs11810204|rs12749748|rs884277|rs12046362|rs1076205|rs1510486|rs1800627|rs776686|rs4650729|rs800719|rs4924592|rs4912291|rs6425147|rs2124147|rs17411112|rs1548796|rs10753000|rs1487492|rs12139100
Neurologic Disorder	PRE_NOTCH_TRANSCRIPTION_AND_TRANSLATION	REACTOME	835	23	15	3,33E-01	9,80E-01	rs532385|rs17576984|rs4525961|rs10501844|rs2033719|rs12365545|rs1114312|rs11021461|rs470141|rs7697053|rs7930909|rs687782|rs2238419|rs11076785|rs9295464
Neurologic Disorder	TYROSINE_METABOLISM	KEGG	1169	50	20	3,33E-01	9,80E-01	rs1407998|rs2518824|rs1446255|rs4820571|rs10120066|rs1325154|rs6518591|rs8062660|rs283525|rs10225988|rs11076318|rs4965732|rs1942492|rs2519154|rs6935937|500437|rs10960466|rs29861|rs2150696|rs7497607
Neurologic Disorder	AMYLOIDS	REACTOME	972	39	18	3,34E-01	9,80E-01	rs2829949|rs12750012|rs710794|rs4932450|rs2829929|rs1047953|rs2736994|rs2595361|rs198838|rs2744119|rs4220|rs6850389|rs4683235|rs6695377|rs17078876|rs6516727|rs2808658|rs7544500
Neurologic Disorder	PATHOGENIC_ESCHERICHIA_COLI_INFECTION	KEGG	1256	35	20	3,38E-01	9,80E-01	rs11695567|rs727266|rs10490697|rs10983712|rs7605126|rs12948922|rs2163349|rs4669725|rs10496402|rs12675103|rs3844450|rs16959227|rs4851076|rs2055036|rs6743358|rs7609295|rs17634425|rs1937746|rs4973417|rs1465641
Neurologic Disorder	SYNTHESIS_AND_INTERCONVERSION_OF_NUCLEOTIDE_DI_AND_TRIPHOSPHATES	REACTOME	306	32	11	3,38E-01	9,80E-01	rs10782645|rs10493604|rs10782647|rs10759403|rs17100499|rs4561|rs5028961|rs2054020|rs11162331|rs7585544|rs2911670
Neurologic Disorder	SULFUR_AMINO_ACID_METABOLISM	REACTOME	930	44	20	3,39E-01	9,80E-01	rs10836312|rs2059098|rs3972|rs158845|rs152395|rs1915161|rs10811629|rs6895949|rs2985403|rs10068900|rs442756|rs1363072|rs3213403|rs510088|rs1472623|rs4712036|rs442573|rs11958300|rs4868753|rs10070913
Neurologic Disorder	ADP_SIGNALLING_THROUGH_P2RY12	REACTOME	438	15	10	3,39E-01	9,80E-01	rs10907192|rs4898712|rs12706724|rs739431|rs9630890|rs6674438|rs2024497|rs7637803|rs6510696|rs6972935
Neurologic Disorder	CD28_DEPENDENT_PI3K_AKT_SIGNALING	REACTOME	572	21	13	3,39E-01	9,80E-01	rs465531|rs6872249|rs11204910|rs1423236|rs4470720|rs9831894|rs1074078|rs9292729|rs3115961|rs728975|rs7086224|rs6916861|rs4673268
Neurologic Disorder	COLORECTAL_CANCER	KEGG	2539	35	20	3,41E-01	9,80E-01	rs2219556|rs6692647|rs4963857|rs465531|rs4332094|rs1816793|rs2142200|rs6872249|rs2568218|rs1962515|rs4129666|rs266359|rs176632|rs2395851|rs6783860|rs660442|rs16830730|rs7835350|rs4316851|rs12548864
Neurologic Disorder	G_ALPHA1213_SIGNALLING_EVENTS	REACTOME	2855	34	20	3,41E-01	9,80E-01	rs13098182|rs461970|rs272107|rs10907192|rs465531|rs3821525|rs3755677|rs17288929|rs12319134|rs6872249|rs11765386|rs715779|rs11751128|rs4669725|rs333332|rs4898712|rs332414|rs13275416|rs1423236|rs4470720
Neurologic Disorder	EGFR_DOWNREGULATION	REACTOME	1037	39	20	3,43E-01	9,80E-01	rs2025880|rs17041245|rs9531286|rs7321702|rs759169|rs843346|rs1947372|rs1519238|rs12666347|rs11919795|rs1519242|rs884904|rs9318729|rs4435493|rs11792652|rs10488142|rs7997421|rs1582191|rs3761034|rs6845023
Neurologic Disorder	IRON_UPTAKE_AND_TRANSPORT	REACTOME	733	34	18	3,45E-01	9,80E-01	rs1296826|rs577866|rs1115219|rs6994201|rs10954211|rs2460221|rs2401158|rs2075609|rs2270637|rs2352267|rs7464428|rs10124511|rs745554|rs951776|rs7853552|rs4927870|rs4148155|rs7511769
Neurologic Disorder	P75_NTR_RECEPTOR_MEDIATED_SIGNALLING	REACTOME	2669	36	20	3,45E-01	9,80E-01	rs13098182|rs461970|rs272107|rs7810486|rs3821525|rs3755677|rs17288929|rs12319134|rs40383|rs7744|rs715779|rs11751128|rs12461927|rs660442|rs333332|rs6980760|rs332414|rs2926457|rs34117|rs1483382
Neurologic Disorder	ERK_MAPK_TARGETS	REACTOME	704	58	20	3,46E-01	9,80E-01	rs11953993|rs11746572|rs8041078|rs3730327|rs6903559|rs9873175|rs963921|rs1129880|rs12540995|rs3776841|rs10514306|rs602932|rs9459785|rs9347129|rs9295348|rs1833895|rs1927432|rs4483828|rs6860708|rs11666474
Neurologic Disorder	GLYOXYLATE_AND_DICARBOXYLATE_METABOLISM	KEGG	704	36	17	3,46E-01	9,80E-01	rs6905272|rs538017|rs4442231|rs1771845|rs1418026|rs2987981|rs6745054|rs2888998|rs1556138|rs9649679|rs9478918|rs2207717|rs11757561|rs803447|rs2375107|rs10511887|rs6055103
Neurologic Disorder	CELL_DEATH_SIGNALLING_VIA_NRAGE_NRIF_AND_NADE	REACTOME	2069	33	20	3,47E-01	9,80E-01	rs13098182|rs461970|rs272107|rs7810486|rs3821525|rs3755677|rs17288929|rs12319134|rs715779|rs11751128|rs660442|rs333332|rs332414|rs34117|rs7745332|rs9322494|rs2833456|rs735098|rs10425594|rs9290452
Neurologic Disorder	FORMATION_OF_ATP_BY_CHEMIOSMOTIC_COUPLING	REACTOME	77	9	5	3,51E-01	9,80E-01	rs2950390|rs3746106|rs7108613|rs1264897|rs151358
Neurologic Disorder	MAP_KINASE_ACTIVATION_IN_TLR_CASCADE	REACTOME	1697	37	20	3,52E-01	9,80E-01	rs11953993|rs1884257|rs11746572|rs2360969|rs40383|rs11869073|rs2440134|rs8041078|rs3730327|rs7754397|rs6903559|rs9873175|rs963921|rs1129880|rs12540995|rs661838|rs6980760|rs3776841|rs7566401|rs997284
Neurologic Disorder	MICRORNA_MIRNA_BIOGENESIS	REACTOME	227	16	6	3,52E-01	9,80E-01	rs175169|rs3821996|rs8014347|rs7217258|rs1107674|rs12977802

Neurologic Disorder	AT1R_PATHWAY	BIOCARTA	1224	44	20	3,53E-01	9,80E-01	rs6772419|rs11953993|rs12948922|rs11746572|rs8041078|rs759169|rs1962515|rs6592724|rs12666347|rs7259810|rs7566401|rs3729908|rs884904|rs4776783|rs10514306|rs2182574|rs3785383|rs16959227|rs7016497|rs10488142
Neurologic Disorder	TFF_PATHWAY	BIOCARTA	697	36	16	3,54E-01	9,80E-01	rs465531|rs6872249|rs759169|rs13354167|rs660442|rs12666347|rs1423236|rs4470720|rs884904|rs7016497|rs10488142|rs1525642|rs6802872|rs9810950|rs11785444|rs17586344
Neurologic Disorder	ARRHYTHMOGENIC_RIGHT_VENTRICULAR_CARDIOMYOPATHY_ARVC	KEGG	5871	44	20	3,58E-01	9,80E-01	rs2032728|rs9320821|rs6757169|rs4747345|rs2239048|rs2437095|rs482533|rs10184153|rs3912834|rs7198466|rs1265646|rs2292867|rs7477742|rs886940|rs1295881|rs2568218|rs886898|rs1557809|rs10737816|rs2107118
Neurologic Disorder	INTERACTIONS_OF_VPR_WITH_HOST_CELLULAR_PROTEINS	REACTOME	480	39	18	3,58E-01	9,80E-01	rs13431075|rs4678192|rs1000940|rs905639|rs283525|rs2350623|rs132813|rs12693351|rs1406334|rs7697711|rs9985182|rs357111|rs103612|rs9879508|rs1776897|rs1462529|rs3809335|rs2882359
Neurologic Disorder	INTRINSIC_PATHWAY	BIOCARTA	603	38	20	3,59E-01	9,80E-01	rs4253419|rs16975294|rs4145072|rs4773209|rs587409|rs1192201|rs6492270|rs4253236|rs1470428|rs4220|rs1951626|rs9559718|rs10220027|rs5030102|rs925453|rs3742207|rs17514517|rs3211719|rs6718820|rs12868126
Neurologic Disorder	PPARA_ACTIVATES_GENE_EXPRESSION	REACTOME	3675	40	20	3,61E-01	9,80E-01	rs11974777|rs6918969|rs2914757|rs3012755|rs4149313|rs3211834|rs10850626|rs1783978|rs715779|rs11751128|rs1929841|rs7667067|rs754313|rs12330015|rs7636407|rs6007640|rs3816358|rs10785976|rs11746690|rs150596
Neurologic Disorder	NFKB_AND_MAP_KINASES_ACTIVATION_MEDIATED_BY_TLR4_SIGNALING_REPERTOIRE	REACTOME	2248	36	20	3,61E-01	9,80E-01	rs2829949|rs11953993|rs1884257|rs10983712|rs11746572|rs2360969|rs40383|rs11869073|rs2440134|rs8041078|rs3730327|rs7754397|rs6903559|rs9873175|rs963921|rs2829929|rs1129880|rs12540995|rs661838|rs6980760
Neurologic Disorder	RHO_PATHWAY	BIOCARTA	480	25	9	3,63E-01	9,80E-01	rs3919447|rs4744669|rs7595941|rs1208204|rs820335|rs12338099|rs7502321|rs3911406|rs3180427
Neurologic Disorder	SPRY_PATHWAY	BIOCARTA	910	38	20	3,63E-01	9,80E-01	rs17041245|rs9531286|rs7321702|rs759169|rs1962515|rs1947372|rs1519238|rs12666347|rs11953892|rs1519242|rs884904|rs4776783|rs7735400|rs9318729|rs10488142|rs7997421|rs1582191|rs7443175|rs6845023|rs2329188
Neurologic Disorder	VIF_MEDIATED_DEGRADATION_OF_APOBEC3G	REACTOME	738	39	20	3,64E-01	9,80E-01	rs704375|rs11922435|rs400223|rs13338214|rs12598046|rs755859|rs17031765|rs11642698|rs4795311|rs417940|rs12447239|rs4435493|rs7212835|rs40610|rs3761034|rs10890795|rs2584624|rs4813030|rs16972410|rs12446725
Neurologic Disorder	GLUCURONIDATION	REACTOME	656	4	4	3,65E-01	9,80E-01	rs11249454|rs11937734|rs2608822|rs10027817
Neurologic Disorder	CDK_MEDIATED_PHOSPHORYLATION_AND_REMOVAL_OF_CDC6	REACTOME	698	37	20	3,67E-01	9,80E-01	rs704375|rs11922435|rs400223|rs13338214|rs12598046|rs755859|rs17031765|rs11642698|rs4795311|rs417940|rs12447239|rs4435493|rs7212835|rs40610|rs3761034|rs2584624|rs4813030|rs16972410|rs12446725|rs16827840
Neurologic Disorder	REGULATION_OF_GLUCOKINASE_BY_GLUCOKINASE_REGULATORY_PROTEIN	REACTOME	459	41	18	3,68E-01	9,80E-01	rs13431075|rs1000940|rs10951757|rs905639|rs283525|rs2350623|rs2908297|rs132813|rs12693351|rs1406334|rs7697711|rs9985182|rs357111|rs2922666|rs103612|rs1462529|rs3809335|rs2882359
Neurologic Disorder	LYSOSOME_VESICLE_BIOGENESIS	REACTOME	420	9	6	3,69E-01	9,80E-01	rs2025880|rs611908|rs10789179|rs11792652|rs15958|rs10810812
Neurologic Disorder	PPARA_PATHWAY	BIOCARTA	2445	34	20	3,69E-01	9,80E-01	rs11974777|rs2823165|rs465531|rs3211834|rs12948922|rs4332094|rs2142200|rs6872437|rs6503691|rs12944039|rs12330015|rs4129666|rs6007640|rs1363292|rs4308418|rs2395851|rs7835350|rs12548864|rs1423236|rs3729908
Neurologic Disorder	BAD_PATHWAY	BIOCARTA	927	39	19	3,69E-01	9,80E-01	rs465531|rs1457596|rs764833|rs6872437|rs917846|rs660442|rs4941185|rs1423236|rs4470720|rs6219|rs17227367|rs2684788|rs6214|rs218268|rs12962644|rs1026825|rs1016860|rs2125111|rs8083946
Neurologic Disorder	PLATELET_ACTIVATION_SIGNALING_AND_AGGREGATION	REACTOME	6965	36	20	3,69E-01	9,80E-01	rs11974777|rs2829949|rs7552931|rs461970|rs13402503|rs4557033|rs10907192|rs465531|rs1678383|rs3211834|rs17041245|rs2292867|rs12749748|rs7098615|rs3753242|rs12948922|rs7805162|rs7916268|rs12046362|rs1558909
Neurologic Disorder	SIGNALING_BY_ROBO_RECEPTOR	REACTOME	1900	35	20	3,69E-01	9,80E-01	rs3911489|rs2184778|rs10490697|rs7605126|rs10222369|rs10015865|rs11175211|rs2163349|rs4686339|rs6583176|rs7562354|rs6592724|rs9309828|rs6118687|rs2198200|rs1154398|rs1349008|rs4624600|rs675465|rs11715608
Neurologic Disorder	P53_INDEPENDENT_G1_S_DNA_DAMAGE_CHECKPOINT	REACTOME	729	37	20	3,71E-01	9,80E-01	rs704375|rs11922435|rs400223|rs13338214|rs12598046|rs755859|rs17031765|rs11642698|rs4795311|rs417940|rs12447239|rs4435493|rs7212835|rs40610|rs3761034|rs2584624|rs4813030|rs16972410|rs12446725|rs16827840
Neurologic Disorder	THROMBIN_SIGNALLING_THROUGH_PROTEINASE_ACTIVATED_RECEPTORS_PARS	REACTOME	690	17	13	3,71E-01	9,80E-01	rs10907192|rs11765386|rs4898712|rs611908|rs10869955|rs9630890|rs9905578|rs6674438|rs1801258|rs2024497|rs6510696|rs990000|rs6972935
Neurologic Disorder	TRIF_MEDIATED_TLR3_SIGNALING	REACTOME	2075	37	20	3,74E-01	9,80E-01	rs2829949|rs11953993|rs1884257|rs11746572|rs2360969|rs40383|rs11869073|rs2440134|rs8041078|rs3730327|rs7754397|rs6903559|rs9873175|rs963921|rs2829929|rs1129880|rs12540995|rs661838|rs6980760|rs3776841
Neurologic Disorder	IL_2_SIGNALING	REACTOME	936	28	15	3,75E-01	9,80E-01	rs4963857|rs465531|rs6872249|rs6503691|rs1962515|rs1423236|rs4470720|rs4776783|rs6776572|rs525563|rs7846568|rs228935|rs10993754|rs7303282|rs651933
Neurologic Disorder	CYTOCHROME_P450_ARRANGED_BY_SUBSTRATE_TYPE	REACTOME	1163	65	20	3,76E-01	9,80E-01	rs2068888|rs41723|rs1458928|rs232542|rs2414099|rs4555110|rs6736570|rs4646522|rs1957516|rs3787557|rs2470176|rs1400681|rs7806848|rs927068|rs952884|rs678040|rs2296241|rs10409750|rs11090076|rs1438921
Neurologic Disorder	BASAL_TRANSCRIPTION_FACTORS	KEGG	386	25	11	3,76E-01	9,80E-01	rs2267824|rs7524919|rs6469845|rs2304196|rs7012791|rs6089583|rs1613070|rs7833229|rs3811474|rs2158797|rs6755901
Neurologic Disorder	ACYL_CHAIN_REMODELLING_OF_PC	REACTOME	693	45	20	3,81E-01	9,80E-01	rs10058172|rs12749748|rs12046362|rs1076205|rs600415|rs13008971|rs1800627|rs776686|rs1938506|rs6425147|rs17411112|rs2111937|rs1548796|rs10753000|rs1487492|rs7554410|rs3761431|rs4821738|rs10912174|rs747527
Neurologic Disorder	ACTIVATION_OF_NMDA_RECEPTOR_UPON_GLUTAMATE_BINDING_AND_POSTSYNAPTIC_	REACTOME	2117	36	20	3,82E-01	9,80E-01	rs11612284|rs170290|rs11938486|rs2300267|rs2241694|rs2360969|rs1962515|rs11055587|rs316369|rs6903559|rs637569|rs12507865|rs7206547|rs7936555|rs17833967|rs11055612|rs7259810|rs2314978|rs7979850|rs2160173
Neurologic Disorder	TERMINATION_OF_O_GLYCAN_BIOSYNTHESIS	REACTOME	961	30	20	3,83E-01	9,80E-01	rs1119017|rs7550651|rs7018169|rs209256|rs12793968|rs7430813|rs10767555|rs7644047|rs1503766|rs2945746|rs1886689|rs3920772|rs2292897|rs7806980|rs11162133|rs1436085|rs6968731|rs8111161|rs11775725|rs2764654
Neurologic Disorder	ASSOCIATION_OF_TRIC_CCT_WITH_TARGET_PROTEINS_DURING_BIOSYNTHESIS	REACTOME	359	14	7	3,83E-01	9,80E-01	rs10824092|rs11727661|rs6856075|rs710750|rs9477550|rs11893547|rs4270584
Neurologic Disorder	SYNTHESIS_OF_PA	REACTOME	1057	41	20	3,83E-01	9,80E-01	rs10058172|rs12749748|rs10912053|rs1076205|rs2609920|rs600415|rs4573621|rs1800627|rs776686|rs1337988|rs4650729|rs11195813|rs12546427|rs6425147|rs1332793|rs2124147|rs17411112|rs1548796|rs10753000|rs6535510
Neurologic Disorder	IGF1_PATHWAY	BIOCARTA	813	46	20	3,84E-01	9,80E-01	rs465531|rs1457596|rs6872437|rs1962515|rs1423236|rs11953892|rs11159135|rs4470720|rs11687971|rs6219|rs4776783|rs7735400|rs2972153|rs12467492|rs6730045|rs2684788|rs6214|rs6037986|rs2125111|rs2624171
Neurologic Disorder	B_CELL_RECEPTOR_SIGNALING_PATHWAY	KEGG	2278	27	20	3,85E-01	9,80E-01	rs4698855|rs461970|rs1569736|rs4963857|rs465531|rs6872437|rs4917749|rs1962515|rs12461927|rs6705101|rs8090560|rs16830730|rs7905645|rs17238159|rs4670209|rs1423236|rs3729908|rs11159135|rs4470720|rs4405546
Neurologic Disorder	SHC_RELATED_EVENTS	REACTOME	302	17	12	3,87E-01	9,80E-01	rs4963857|rs1962515|rs12462766|rs10413199|rs4776783|rs2241407|rs4804377|rs4877062|rs2229431|rs11668751|rs7021743|rs7303282
Neurologic Disorder	ACTIVATED_AMPK_STIMULATES_FATTY_ACID_OXIDATION_IN_MUSCLE	REACTOME	421	20	13	3,88E-01	9,80E-01	rs4726096|rs10168275|rs7667067|rs10278273|rs13431868|rs7776530|rs7778356|rs9535241|rs9316447|rs12476589|rs4726099|rs11764602|rs4766587
Neurologic Disorder	HOMOLOGOUS_RECOMBINATION_REPAIR_OF_REPLICATION_INDEPENDENT_DOUBLE_ST	REACTOME	376	38	12	3,88E-01	9,80E-01	rs7222799|rs2761367|rs16942|rs2697675|rs8077346|rs6470522|rs9634672|rs2304136|rs12906017|rs4986765|rs3735461|rs1295686
Neurologic Disorder	PHOSPHORYLATION_OF_THE_APC_C	REACTOME	219	10	7	3,89E-01	9,80E-01	rs16930959|rs6742309|rs6550808|rs17842119|rs28653722|rs1464095|rs4848611
Neurologic Disorder	MAL_PATHWAY	BIOCARTA	225	11	4	3,91E-01	9,80E-01	rs1962515|rs7953249|rs4776783|rs3105099
Neurologic Disorder	GH_PATHWAY	BIOCARTA	963	38	20	3,93E-01	9,80E-01	rs465531|rs12948922|rs6872249|rs13354167|rs6503691|rs1962515|rs1423236|rs3729908|rs4470720|rs7953249|rs11687971|rs4776783|rs2972153|rs3785383|rs16959227|rs12467492|rs6730045|rs4804377|rs120909|rs2229431
Neurologic Disorder	DILATED_CARDIOMYOPATHY	KEGG	4636	43	20	3,94E-01	9,80E-01	rs7552931|rs4747345|rs2239048|rs2437095|rs1457596|rs6509933|rs10184153|rs4766526|rs3912834|rs7198466|rs1265646|rs2292867|rs7477742|rs6509931|rs886940|rs2042995|rs8039242|rs1295881|rs886898|rs1557809
Neurologic Disorder	REGULATION_OF_AUTOPHAGY	KEGG	933	46	20	3,94E-01	9,80E-01	rs710092|rs2587616|rs17124275|rs17596234|rs970238|rs26532|rs2315174|rs683456|rs17767371|rs1124625|rs4978113|rs1391812|rs1496814|rs532726|rs12959903|rs1539826|rs12134779|rs3845563|rs9304251|rs10125010
Neurologic Disorder	TRAF6_MEDIATED_INDUCTION_OF_NFKB_AND_MAP_KINASES_UPON_TLR7_8_OR_9_ACTI	REACTOME	2239	36	20	3,94E-01	9,80E-01	rs2829949|rs11953993|rs1884257|rs11746572|rs2360969|rs40383|rs11869073|rs2440134|rs8041078|rs7744|rs3730327|rs7754397|rs6903559|rs9873175|rs963921|rs2829929|rs1129880|rs12540995|rs661838|rs6980760
Neurologic Disorder	REGULATION_OF_MITOTIC_CELL_CYCLE	REACTOME	1318	35	20	3,95E-01	9,80E-01	rs704375|rs16930959|rs11922435|rs400223|rs478859|rs13338214|rs12598046|rs6742309|rs9315420|rs755859|rs3776841|rs17031765|rs11642698|rs4795311|rs9883834|rs417940|rs17842119|rs12447239|rs10902882|rs4435493
Neurologic Disorder	SIGNAL_TRANSDUCTION_BY_L1	REACTOME	1310	33	20	3,98E-01	9,80E-01	rs2025880|rs2292867|rs7916174|rs10891524|rs10897495|rs759169|rs843346|rs6903559|rs1902409|rs6592724|rs6481838|rs12666347|rs11919795|rs2785284|rs884904|rs4776783|rs1436109|rs2250706|rs2785252|rs11792652
Neurologic Disorder	FORMATION_OF_FIBRIN_CLOT_CLOTTING_CASCADE	REACTOME	703	27	11	3,99E-01	9,80E-01	rs4253419|rs7760746|rs4253236|rs1058242|rs4220|rs7767612|rs1951626|rs5030102|rs755656|rs925453|rs3211719
Neurologic Disorder	SIGNALING_BY_FGFR_MUTANTS	REACTOME	1657	32	20	4,01E-01	9,80E-01	rs4963857|rs465531|rs1874326|rs1851710|rs6872249|rs17472986|rs1545033|rs6503691|rs6003709|rs11048776|rs6859263|rs2320607|rs823616|rs9510005|rs1423236|rs3804158|rs9506807|rs516391|rs4470720|rs1368406
Neurologic Disorder	PYRUVATE_METABOLISM	KEGG	908	35	17	4,02E-01	9,80E-01	rs730168|rs3794492|rs4578194|rs7551594|rs7122539|rs2207632|rs4878784|rs6975650|rs11864516|rs9649679|rs971524|rs6083725|rs7788953|rs1945146|rs4930187|rs6592301|rs4766587
Neurologic Disorder	INFLAMMASOMES	REACTOME	468	22	15	4,03E-01	9,80E-01	rs2829949|rs9901001|rs2829929|rs4941185|rs10759403|rs224205|rs2716932|rs12962644|rs1026825|rs1008588|rs1016860|rs8083946|rs396146|rs6516727|rs4790793
Neurologic Disorder	FATTY_ACID_METABOLISM	KEGG	776	22	12	4,04E-01	9,80E-01	rs7744|rs4578194|rs9392560|rs7551594|rs7667067|rs4878784|rs11195905|rs3774051|rs2239621|rs638329|rs8095238|rs12825376
Neurologic Disorder	TRANSCRIPTIONAL_REGULATION_OF_WHITE_ADIPOCYTE_DIFFERENTIATION	REACTOME	2719	29	20	4,05E-01	9,80E-01	rs11974777|rs2914757|rs3012755|rs4979619|rs3211834|rs10850626|rs1783978|rs2964513|rs2577001|rs12330015|rs9651304|rs7636407|rs6007640|rs150596|rs16937001|rs9369325|rs10520809|rs11067684|rs7030021|rs12580394
Neurologic Disorder	TRANSPORT_OF_VITAMINS_NUCLEOSIDES_AND_RELATED_MOLECULES	REACTOME	1154	82	20	4,08E-01	9,80E-01	rs720680|rs3818057|rs780662|rs7024751|rs3825875|rs2895719|rs4247079|rs16974600|rs2295085|rs4980345|rs7182304|rs2417940|rs1015951|rs8033892|rs1871332|rs11140525|rs1165|rs9790150|rs4854789|rs4290453
Neurologic Disorder	PRE_NOTCH_EXPRESSION_AND_PROCESSING	REACTOME	1434	29	20	4,08E-01	9,80E-01	rs3761441|rs532385|rs17576984|rs4525961|rs3759929|rs17115605|rs10501844|rs8006798|rs2033719|rs6502762|rs12365545|rs1114312|rs17653970|rs8016995|rs17642827|rs11021461|rs470141|rs7697053|rs10498559|rs7930909
Neurologic Disorder	NRAGE_SIGNALS_DEATH_THROUGH_JNK	REACTOME	1919	33	20	4,08E-01	9,80E-01	rs13098182|rs461970|rs272107|rs3821525|rs3755677|rs17288929|rs12319134|rs715779|rs11751128|rs660442|rs333332|rs332414|rs34117|rs7745332|rs9322494|rs2833456|rs735098|rs10425594|rs9290452|rs17057445
Neurologic Disorder	BCR_PATHWAY	BIOCARTA	1053	26	20	4,10E-01	9,80E-01	rs461970|rs1569736|rs12948922|rs3730327|rs1962515|rs8090560|rs7905645|rs7259810|rs3729908|rs11159135|rs4776783|rs3785383|rs16959227|rs11188666|rs12547683|rs602932|rs6096477|rs6126251|rs6021247|rs17828764
Neurologic Disorder	GAB1_SIGNALOSOME	REACTOME	1077	36	20	4,11E-01	9,80E-01	rs465531|rs17041245|rs2360969|rs6872249|rs759169|rs11204910|rs660442|rs8063461|rs12666347|rs1423236|rs4470720|rs7190284|rs884904|rs1074078|rs9292729|rs6837153|rs9368950|rs3800230|rs4436084|rs945559
Neurologic Disorder	PREFOLDIN_MEDIATED_TRANSFER_OF_SUBSTRATE_TO_CCT_TRIC	REACTOME	288	10	7	4,11E-01	9,80E-01	rs1418927|rs6023019|rs3844450|rs710750|rs11893547|rs1937746|rs151358
Neurologic Disorder	GRAFT_VERSUS_HOST_DISEASE	KEGG	1446	44	19	4,12E-01	9,80E-01	rs6930052|rs7259347|rs13191519|rs7755852|rs9258895|rs1926196|rs10229203|rs9831894|rs28367729|rs3115961|rs3135021|rs9266440|rs3094691|rs2517938|rs2517861|rs4673268|rs2894125|rs7085850|rs13202464
Neurologic Disorder	AMINE_LIGAND_BINDING_RECEPTORS	REACTOME	2247	45	20	4,13E-01	9,80E-01	rs6794593|rs10802789|rs1857586|rs3011577|rs7098615|rs7765655|rs7916268|rs11195534|rs659562|rs266004|rs1886333|rs1358781|rs2710734|rs2840837|rs13275416|rs1125489|rs9448054|rs9787221|rs12197997|rs6899577
Neurologic Disorder	IL1_SIGNALING	REACTOME	1322	30	20	4,14E-01	9,80E-01	rs1884257|rs4851543|rs40383|rs11869073|rs2440134|rs7744|rs7754397|rs1541435|rs661838|rs6980760|rs10483660|rs3776841|rs997284|rs6001585|rs6454860|rs1483382|rs4776783|rs474246|rs728975|rs2715815
Neurologic Disorder	CROSS_PRESENTATION_OF_SOLUBLE_EXOGENOUS_ANTIGENS_ENDOSOMES	REACTOME	713	35	20	4,15E-01	9,80E-01	rs704375|rs11922435|rs400223|rs13338214|rs12598046|rs755859|rs17031765|rs11642698|rs4795311|rs417940|rs12447239|rs7212835|rs40610|rs13421115|rs2665833|rs4813030|rs16972410|rs12446725|rs16827840|rs7192167
Neurologic Disorder	TPO_PATHWAY	BIOCARTA	814	38	20	4,17E-01	9,80E-01	rs465531|rs12948922|rs6872249|rs6503691|rs1962515|rs1423236|rs3729908|rs11953892|rs11159135|rs4470720|rs4776783|rs7735400|rs3785383|rs16959227|rs6037986|rs120909|rs7196303|rs17634425|rs11074594|rs12450534
Neurologic Disorder	KINESINS	REACTOME	566	22	7	4,17E-01	9,80E-01	rs6731972|rs2910469|rs4074324|rs10742181|rs994626|rs211315|rs6890862
Neurologic Disorder	RIG_I_LIKE_RECEPTOR_SIGNALING_PATHWAY	KEGG	1361	32	20	4,17E-01	9,80E-01	rs4698855|rs710092|rs1884257|rs3730327|rs7754397|rs1129880|rs26532|rs11662928|rs6454860|rs17212649|rs683456|rs7865082|rs540722|rs11118087|rs4978113|rs743923|rs532726|rs4493656|rs10967976|rs9362744
Neurologic Disorder	HOST_INTERACTIONS_OF_HIV_FACTORS	REACTOME	2270	37	20	4,20E-01	9,80E-01	rs704375|rs13431075|rs4678192|rs11922435|rs9679654|rs175169|rs400223|rs1000940|rs6583176|rs13338214|rs905639|rs12598046|rs843346|rs7700605|rs6994201|rs9259806|rs283525|rs10265823|rs12656935|rs755859
Neurologic Disorder	MTOR_SIGNALING_PATHWAY	KEGG	1290	34	20	4,20E-01	9,80E-01	rs465531|rs1457596|rs10168275|rs6872249|rs1050700|rs3791729|rs3741403|rs6903559|rs8063461|rs1423236|rs2672886|rs9915378|rs13431868|rs4470720|rs7190284|rs6219|rs1074078|rs9292729|rs6214|rs3829572
Neurologic Disorder	JNK_C_JUN_KINASES_PHOSPHORYLATION_AND_ACTIVATION_MEDIATED_BY_ACTIVATE	REACTOME	728	37	14	4,21E-01	9,80E-01	rs1884257|rs40383|rs7754397|rs661838|rs6980760|rs6001585|rs6454860|rs1483382|rs237004|rs9362744|rs6908296|rs11249687|rs9362878|rs10087480
Neurologic Disorder	EIF4_PATHWAY	BIOCARTA	890	42	20	4,22E-01	9,80E-01	rs465531|rs12948922|rs6872249|rs3730327|rs13354167|rs1423236|rs3729908|rs4470720|rs11687971|rs1074078|rs2972153|rs3785383|rs16959227|rs12467492|rs6730045|rs743923|rs945559|rs120909|rs7196303|rs17634425
Neurologic Disorder	INFLUENZA_LIFE_CYCLE	REACTOME	1671	32	20	4,23E-01	9,80E-01	rs13431075|rs4678192|rs2025880|rs9679654|rs7219464|rs3826322|rs1000940|rs905639|rs1149747|rs2976044|rs8082649|rs3821996|rs1811384|rs283525|rs36631|rs4789626|rs2350623|rs6791696|rs137653|rs7217258
Neurologic Disorder	OXYGEN_DEPENDENT_PROLINE_HYDROXYLATION_OF_HYPOXIA_INDUCIBLE_FACTOR_/	REACTOME	313	12	6	4,24E-01	9,80E-01	rs797377|rs223366|rs8102595|rs4435493|rs12435848|rs3761034
Neurologic Disorder	G1_PATHWAY	BIOCARTA	611	22	12	4,25E-01	9,80E-01	rs7552931|rs266359|rs16830730|rs2374594|rs9315420|rs8032542|rs9368950|rs1650647|rs7156293|rs9380590|rs446304|rs920293
Neurologic Disorder	PENTOSE_AND_GLUCURONATE_INTERCONVERSIONS	KEGG	874	18	12	4,26E-01	9,80E-01	rs11249454|rs169045|rs12018394|rs2070487|rs6975650|rs11937734|rs1105267|rs2608822|rs1928371|rs7788953|rs7321765|rs10027817
Neurologic Disorder	SPPA_PATHWAY	BIOCARTA	1428	33	20	4,26E-01	9,80E-01	rs10907192|rs41723|rs2423356|rs12749748|rs12948922|rs12046362|rs771941|rs1962515|rs7271063|rs12706724|rs3729908|rs1938506|rs6077411|rs4776783|rs6055685|rs10818684|rs6086714|rs3785383|rs16959227|rs6425147
Neurologic Disorder	ANTIVIRAL_MECHANISM_BY_IFN_STIMULATED_GENES	REACTOME	1077	34	20	4,26E-01	9,80E-01	rs13431075|rs4678192|rs10492591|rs1000940|rs905639|rs441437|rs3791729|rs283525|rs2350623|rs6550808|rs132813|rs12693351|rs7865082|rs1406334|rs9481657|rs4435493|rs7697711|rs9985182|rs357111|rs17008958
Neurologic Disorder	LINOLEIC_ACID_METABOLISM	KEGG	716	40	20	4,27E-01	9,80E-01	rs12749748|rs10912053|rs17764723|rs1076205|rs4728329|rs1800627|rs776686|rs1938506|rs800719|rs4924592|rs6425147|rs17411112|rs11078523|rs1548796|rs10753000|rs678040|rs1487492|rs12139100|rs7554410|rs3761431
Neurologic Disorder	FRS2_MEDIATED_CASCADE	REACTOME	1192	33	20	4,27E-01	9,80E-01	rs4963857|rs581971|rs1851710|rs1545033|rs17472986|rs1962515|rs445775|rs6859263|rs2320607|rs823616|rs9510005|rs3804158|rs9506807|rs516391|rs1368406|rs4776783|rs13312770|rs10796853|rs755698|rs17585055
Neurologic Disorder	FCER1_PATHWAY	BIOCARTA	1564	36	20	4,28E-01	9,80E-01	rs461970|rs1569736|rs465531|rs12749748|rs10912053|rs6872249|rs6583176|rs1962515|rs8090560|rs1423236|rs7259810|rs3729908|rs11159135|rs4470720|rs1938506|rs4776783|rs3785383|rs6425147|rs12547683|rs6096477
Neurologic Disorder	VIRAL_MYOCARDITIS	KEGG	2750	38	20	4,30E-01	9,80E-01	rs6930052|rs2824327|rs2437095|rs1501813|rs13191519|rs7630352|rs7755852|rs11647019|rs3811178|rs9578539|rs4285344|rs9258895|rs9893651|rs1651543|rs2191502|rs9550895|rs2073917|rs9831894|rs28367729|rs1989811
Neurologic Disorder	PANTOTHENATE_AND_COA_BIOSYNTHESIS	KEGG	611	39	18	4,32E-01	9,80E-01	rs7771841|rs12218194|rs4140553|rs6939185|rs6683957|rs17212649|rs3002273|rs11165823|rs2477703|rs492734|rs16928163|rs10493889|rs290826|rs2669429|rs12041436|rs12917120|rs12974796|rs4976562
Neurologic Disorder	PHASE_II_CONJUGATION	REACTOME	2059	35	20	4,33E-01	9,80E-01	rs12990757|rs11249454|rs2518824|rs5996675|rs2059098|rs158845|rs7551594|rs1801280|rs6518591|rs739230|rs2297763|rs6895949|rs10068900|rs17487394|rs1363072|rs13114510|rs2726165|rs11937734|rs510088|rs2547231
Neurologic Disorder	GENERATION_OF_SECOND_MESSENGER_MOLECULES	REACTOME	616	35	15	4,34E-01	9,80E-01	rs2184778|rs6583176|rs3782046|rs10045011|rs6592724|rs10915846|rs10918708|rs1466020|rs3135021|rs6698995|rs2049233|rs6583177|rs2278385|rs6736735|rs878078
Neurologic Disorder	REGULATION_OF_ORNITHINE_DECARBOXYLASE_ODC	REACTOME	773	35	20	4,35E-01	9,80E-01	rs704375|rs11922435|rs400223|rs13338214|rs12598046|rs755859|rs17031765|rs11642698|rs4795311|rs417940|rs12447239|rs4734668|rs7212835|rs40610|rs2584624|rs4813030|rs16972410|rs12446725|rs16827840|rs7192167
Neurologic Disorder	NEUROTRANSMITTER_RECEPTOR_BINDING_AND_DOWNSTREAM_TRANSMISSION_IN_TH<br>L	REACTOME	6592	32	20	4,35E-01	9,80E-01	rs4839797|rs11612284|rs363512|rs170290|rs8066734|rs11938486|rs10907192|rs11650230|rs218378|rs2300267|rs209348|rs7198466|rs2423356|rs2241694|rs2782901|rs12948922|rs7690580|rs2360969|rs1551901|rs2399927
Neurologic Disorder	G_PROTEIN_BETA_GAMMA_SIGNALLING	REACTOME	798	27	19	4,36E-01	9,80E-01	rs10907192|rs2423356|rs771941|rs3741403|rs660442|rs4898712|rs7271063|rs6077411|rs9630890|rs6055685|rs6086714|rs6674438|rs2024497|rs6055853|rs6510696|rs2719774|rs6055926|rs2719807|rs6972935
Neurologic Disorder	NATURAL_KILLER_CELL_MEDIATED_CYTOTOXICITY	KEGG	3607	31	20	4,37E-01	9,80E-01	rs461970|rs1569736|rs11155703|rs509749|rs4963857|rs465531|rs7259347|rs13191519|rs12948922|rs4381535|rs6872249|rs2466178|rs7755852|rs1962515|rs9258895|rs1126644|rs9383633|rs8090560|rs6592724|rs12462766
Neurologic Disorder	SIGNALING_BY_NODAL	REACTOME	632	36	20	4,37E-01	9,80E-01	rs3806702|rs3759929|rs266359|rs7166898|rs4664888|rs7560572|rs11630183|rs9635893|rs8032542|rs2238655|rs6598483|rs2706055|rs4965898|rs2061039|rs13429725|rs3800230|rs7181923|rs4662505|rs17512976|rs1792684
Neurologic Disorder	ABCA_TRANSPORTERS_IN_LIPID_HOMEOSTASIS	REACTOME	464	9	6	4,38E-01	9,80E-01	rs3752237|rs2372479|rs4245794|rs16853324|rs4672731|rs4245786
Neurologic Disorder	SIGNALING_BY_FGFR1_MUTANTS	REACTOME	1243	35	20	4,38E-01	9,80E-01	rs465531|rs1874326|rs1851710|rs6872249|rs17472986|rs6503691|rs6003709|rs11048776|rs2320607|rs823616|rs9510005|rs1423236|rs3804158|rs9506807|rs4470720|rs13312770|rs4073362|rs6973312|rs6581852|rs7812254
Neurologic Disorder	SMAD2_SMAD3_SMAD4_HETEROTRIMER_REGULATES_TRANSCRIPTION	REACTOME	715	31	20	4,39E-01	9,80E-01	rs4332094|rs2142200|rs4129666|rs266359|rs2395851|rs7835350|rs12548864|rs1662820|rs9635893|rs9957562|rs8032542|rs12151827|rs12605548|rs4435493|rs12543298|rs4410871|rs6073285|rs3761034|rs16903124|rs3897903
Neurologic Disorder	PKB_MEDIATED_EVENTS	REACTOME	583	28	20	4,39E-01	9,80E-01	rs4726096|rs10168275|rs1050700|rs8063461|rs2672886|rs9915378|rs10278273|rs13431868|rs7190284|rs7776530|rs1074078|rs7778356|rs3829572|rs9535241|rs9316447|rs12476589|rs4726099|rs2299965|rs2589142|rs12948054
Neurologic Disorder	LIPOPROTEIN_METABOLISM	REACTOME	1037	64	20	4,41E-01	9,80E-01	rs4149313|rs34719083|rs3771254|rs12934552|rs1929841|rs10495717|rs1276720|rs838878|rs2020927|rs17139378|rs2472508|rs7043894|rs3012499|rs312049|rs12886225|rs7087360|rs10820743|rs3847300|rs1373656|rs1561166
Neurologic Disorder	INHIBITION_OF_VOLTAGE_GATED_CA2_CHANNELS_VIA_GBETA_GAMMA_SUBUNITS	REACTOME	1082	34	20	4,41E-01	9,80E-01	rs8066734|rs10907192|rs11650230|rs10512574|rs10180851|rs4898712|rs1934080|rs1940455|rs9903628|rs9630890|rs3025643|rs16837945|rs9906150|rs6674438|rs7221237|rs1999498|rs9983351|rs978708|rs6510696|rs13422673
Neurologic Disorder	POTASSIUM_CHANNELS	REACTOME	4940	35	20	4,43E-01	9,80E-01	rs780235|rs2075728|rs8066734|rs780231|rs10907192|rs11650230|rs7820230|rs526654|rs10092842|rs7116712|rs9856750|rs10505713|rs1891716|rs10832305|rs2077982|rs10512574|rs1605128|rs1874952|rs282880|rs4899951
Neurologic Disorder	FAS_PATHWAY	BIOCARTA	980	44	20	4,44E-01	9,80E-01	rs1884257|rs40383|rs6583176|rs7754397|rs3219110|rs2213178|rs3827730|rs6592724|rs1800627|rs6980760|rs1926196|rs4353229|rs6454860|rs1483382|rs2049233|rs9362744|rs6583177|rs17773680|rs6670527|rs9362878

Neurologic Disorder	BASE_EXCISION_REPAIR	KEGG	500	40	18	4,44E-01	9,80E-01	rs9318648|rs10951757|rs3219110|rs1050112|rs8063461|rs2985689|rs7190284|rs3213403|rs12863638|rs2922666|rs509360|rs2304136|rs10958713|rs2583194|rs965078|rs174556|rs2293464|rs2686187
Neurologic Disorder	DEATH_PATHWAY	BIOCARTA	505	28	17	4,45E-01	9,80E-01	rs4698855|rs2466178|rs1800627|rs4941185|rs9902027|rs4353229|rs12962644|rs231989|rs8016947|rs1026825|rs3138158|rs17773680|rs1016860|rs6670527|rs8083946|rs7582581|rs17112705
Neurologic Disorder	MEF2D_PATHWAY	BIOCARTA	684	24	20	4,47E-01	9,80E-01	rs1569736|rs738809|rs12948922|rs8090560|rs7259810|rs3729908|rs3827321|rs3757016|rs3785383|rs16959227|rs6096477|rs6126251|rs6021247|rs17828764|rs1052653|rs120909|rs4811174|rs6067742|rs645860|rs7196303
Neurologic Disorder	CARDIAC_MUSCLE_CONTRACTION	KEGG	3169	38	20	4,47E-01	9,80E-01	rs4747345|rs2239048|rs6509933|rs4766526|rs3912834|rs7198466|rs7477742|rs6509931|rs886940|rs8039242|rs1295881|rs886898|rs1557809|rs3811178|rs10737816|rs2107118|rs3753617|rs2370413|rs258675|rs10828803
Neurologic Disorder	MYD88_MAL_CASCADE_INITIATED_ON_PLASMA_MEMBRANE	REACTOME	2527	32	20	4,49E-01	9,80E-01	rs2829949|rs11953993|rs1884257|rs10983712|rs11746572|rs2360969|rs40383|rs11869073|rs2440134|rs8041078|rs7744|rs3730327|rs7754397|rs6903559|rs9873175|rs963921|rs2829929|rs1129880|rs12540995|rs661838
Neurologic Disorder	ABC_TRANSPORTERS	KEGG	2684	52	20	4,49E-01	9,80E-01	rs4149313|rs1662694|rs4148483|rs17545756|rs9590125|rs3752237|rs9918728|rs2372479|rs1929841|rs246240|rs4557646|rs4148382|rs2020927|rs1149222|rs1470291|rs4245794|rs2472508|rs3758947|rs2067043|rs945067
Neurologic Disorder	ARGININE_AND_PROLINE_METABOLISM	KEGG	991	33	20	4,49E-01	9,80E-01	rs6539460|rs10797768|rs4578194|rs2293055|rs7551594|rs12944039|rs8062660|rs4878784|rs11076318|rs3770685|rs9618419|rs1879417|rs10901213|rs11577155|rs29861|rs11790847|rs804429|rs12632423|rs556258|rs7031579
Neurologic Disorder	MRNA_3_END_PROCESSING	REACTOME	320	10	8	4,51E-01	9,80E-01	rs3972|rs1783983|rs575274|rs4916595|rs1076691|rs547976|rs8006358|rs12479650
Neurologic Disorder	THROMBOXANE_SIGNALLING_THROUGH_TP_RECEPTOR	REACTOME	492	13	10	4,51E-01	9,80E-01	rs10907192|rs4898712|rs10869955|rs9630890|rs6674438|rs1801258|rs2024497|rs6510696|rs990000|rs6972935
Neurologic Disorder	EPITHELIAL_CELL_SIGNALING_IN_HELICOBACTER_PYLORI_INFECTION	KEGG	1535	29	20	4,51E-01	9,80E-01	rs4698855|rs1296826|rs759169|rs3730327|rs6994201|rs1129880|rs10954211|rs2460221|rs2401158|rs6592724|rs11265544|rs12666347|rs2075609|rs2270637|rs4405546|rs884904|rs7197601|rs1147491|rs12547683|rs7464428
Neurologic Disorder	TRANSPORT_OF_MATURE_TRANSCRIPT_TO_CYTOPLASM	REACTOME	693	35	20	4,52E-01	9,80E-01	rs13431075|rs1000940|rs3972|rs905639|rs283525|rs575274|rs2350623|rs4916595|rs132813|rs1076691|rs12693351|rs1406334|rs7697711|rs9985182|rs357111|rs103612|rs1462529|rs547976|rs3809335|rs8006358
Neurologic Disorder	ENERGY_DEPENDENT_REGULATION_OF_MTOR_BY_LKB1_AMPK	REACTOME	461	29	20	4,57E-01	9,80E-01	rs4726096|rs10168275|rs1050700|rs8063461|rs2672886|rs9915378|rs10278273|rs13431868|rs7190284|rs7776530|rs7778356|rs3829572|rs9535241|rs9316447|rs12476589|rs4726099|rs2299965|rs2589142|rs12948054|rs11764602
Neurologic Disorder	DNA_REPLICATION	REACTOME	3548	38	20	4,58E-01	9,80E-01	rs487989|rs9450763|rs704375|rs7980317|rs10917799|rs449397|rs11922435|rs9679654|rs9592217|rs400223|rs12877281|rs11147544|rs10951757|rs2910469|rs13338214|rs12598046|rs35674|rs2985689|rs6769686|rs1925089
Neurologic Disorder	SHC_MEDIATED_CASCADE	REACTOME	1070	34	20	4,58E-01	9,80E-01	rs4963857|rs581971|rs1851710|rs1545033|rs17472986|rs445775|rs6859263|rs2320607|rs823616|rs9510005|rs3804158|rs9506807|rs1368406|rs13312770|rs10796853|rs755698|rs17585055|rs9509983|rs9506810|rs7730111
Neurologic Disorder	TIGHT_JUNCTION	KEGG	6242	33	20	4,60E-01	9,80E-01	rs17416597|rs11978556|rs6757169|rs13402503|rs9877901|rs17466502|rs4963857|rs4557033|rs482533|rs4766526|rs11777908|rs3753242|rs12948922|rs7630352|rs6685516|rs11647019|rs13023787|rs10951757|rs4807508|rs2309428
Neurologic Disorder	DOUBLE_STRAND_BREAK_REPAIR	REACTOME	506	42	14	4,62E-01	9,80E-01	rs7222799|rs2761367|rs16942|rs2697675|rs2213178|rs8077346|rs6470522|rs9634672|rs2304136|rs12906017|rs4986765|rs3735461|rs1295686|rs3777018
Neurologic Disorder	MELANOMA	KEGG	2894	38	20	4,63E-01	9,80E-01	rs4963857|rs465531|rs260852|rs1457596|rs17041245|rs919751|rs1851710|rs7322500|rs1927381|rs6872249|rs17472986|rs1545033|rs759169|rs11226342|rs1962515|rs12488465|rs6859263|rs7334198|rs660442|rs2320607
Neurologic Disorder	RNA_DEGRADATION	KEGG	1123	30	20	4,64E-01	9,80E-01	rs11656715|rs275447|rs10511937|rs9798936|rs2047321|rs4671871|rs1503242|rs13174522|rs4672066|rs7047479|rs1503229|rs1863000|rs11130040|rs36643|rs12656353|rs8062746|rs240542|rs934797|rs715412|rs9628
Neurologic Disorder	COMPLEMENT_AND_COAGULATION_CASCADES	KEGG	1742	40	20	4,64E-01	9,80E-01	rs4253419|rs1046248|rs1001420|rs10473234|rs2222547|rs1800450|rs3748316|rs12489890|rs831630|rs4581040|rs7760746|rs7736582|rs10033900|rs4253236|rs10824650|rs2070862|rs1058242|rs6056|rs1901167|rs7527218
Neurologic Disorder	MATURITY_ONSET_DIABETES_OF_THE_YOUNG	KEGG	992	25	20	4,64E-01	9,80E-01	rs2414223|rs4746864|rs874511|rs10951757|rs6779720|rs4612653|rs12029406|rs2908297|rs4686655|rs2158254|rs6777021|rs7953249|rs2737621|rs922369|rs2246072|rs35976751|rs2922666|rs4687500|rs2737665|rs6493576
Neurologic Disorder	HIVNEF_PATHWAY	BIOCARTA	1008	36	20	4,65E-01	9,80E-01	rs4698855|rs7810486|rs6583176|rs3219110|rs2213178|rs1800627|rs4941185|rs11953892|rs1926196|rs4353229|rs7735400|rs9402839|rs7016497|rs2049233|rs12962644|rs8016947|rs1026825|rs6583177|rs17773680|rs974189
Neurologic Disorder	TRANSPORT_OF_MATURE_MRNA_DERIVED_FROM_AN_INTRONLESS_TRANSCRIPT	REACTOME	524	36	17	4,65E-01	9,80E-01	rs13431075|rs1000940|rs905639|rs283525|rs2350623|rs4916595|rs132813|rs12693351|rs1406334|rs7697711|rs9985182|rs357111|rs103612|rs1462529|rs3809335|rs8006358|rs2882359
Neurologic Disorder	AUTOIMMUNE_THYROID_DISEASE	KEGG	1646	45	20	4,65E-01	9,80E-01	rs6930052|rs13191519|rs7755852|rs9258895|rs4736618|rs1926196|rs2687809|rs932742|rs9831894|rs28367729|rs3115961|rs7844210|rs1810396|rs13263087|rs3135021|rs4978113|rs9266440|rs3094691|rs10125010|rs17351243
Neurologic Disorder	ACTIVATION_OF_NF_KAPPAB_IN_B_CELLS	REACTOME	1396	34	20	4,66E-01	9,80E-01	rs704375|rs1884257|rs11922435|rs400223|rs7754397|rs13338214|rs12598046|rs6555976|rs17238159|rs755859|rs3776841|rs3729908|rs17031765|rs11642698|rs4795311|rs417940|rs6454860|rs6953804|rs12447239|rs3785383
Neurologic Disorder	PROSTACYCLIN_SIGNALLING_THROUGH_PROSTACYCLIN_RECEPTOR	REACTOME	353	12	9	4,67E-01	9,80E-01	rs10907192|rs4898712|rs9630890|rs6674438|rs2024497|rs6513412|rs6510696|rs6972935|rs6092708
Neurologic Disorder	REGULATION_OF_INSULIN_SECRETION	REACTOME	3741	36	20	4,67E-01	9,80E-01	rs2288419|rs10802789|rs4747345|rs10907192|rs2423356|rs3011577|rs7477742|rs7098615|rs6509931|rs12948922|rs2288519|rs7916268|rs1295881|rs771941|rs11195534|rs3741403|rs16878921|rs2471637|rs10828803|rs4690696
Neurologic Disorder	VEGF_PATHWAY	BIOCARTA	883	35	20	4,68E-01	9,80E-01	rs465531|rs12948922|rs6806377|rs6872249|rs7252142|rs3738483|rs1423236|rs3729908|rs4470720|rs843358|rs3785383|rs16959227|rs1903066|rs7016497|rs12435848|rs9462937|rs120909|rs7525733|rs9815292|rs7196303
Neurologic Disorder	PGC1A_PATHWAY	BIOCARTA	959	47	20	4,68E-01	9,80E-01	rs11938486|rs11953993|rs2241694|rs11746572|rs8041078|rs12330015|rs6007640|rs9787810|rs7259810|rs10514306|rs11730701|rs4286653|rs17828764|rs2946399|rs1833895|rs4483828|rs6733907|rs6860708|rs4823529|rs2732510
Neurologic Disorder	ALANINE_ASPARTATE_AND_GLUTAMATE_METABOLISM	KEGG	841	33	20	4,69E-01	9,80E-01	rs9372252|rs10797768|rs9873033|rs8062660|rs283525|rs11076318|rs3770685|rs3127468|rs2291702|rs2313427|rs10901213|rs12473304|rs11577155|rs29861|rs11790847|rs6932938|rs12940665|rs7031579|rs11243665|rs9937726
Neurologic Disorder	IL2RB_PATHWAY	BIOCARTA	1296	30	20	4,70E-01	9,80E-01	rs465531|rs4332094|rs2142200|rs6872249|rs6503691|rs1962515|rs4129666|rs2395851|rs660442|rs7835350|rs12548864|rs4941185|rs1423236|rs1926196|rs11159135|rs4470720|rs11687971|rs2972153|rs12467492|rs6730045
Neurologic Disorder	HS_GAG_BIOSYNTHESIS	REACTOME	3154	30	20	4,70E-01	9,80E-01	rs9556369|rs1445270|rs2575738|rs9561545|rs34719083|rs1948234|rs1007973|rs1929035|rs3771254|rs9523030|rs7997908|rs535828|rs7014925|rs12508213|rs7562354|rs10492621|rs2218348|rs1333674|rs1390102|rs11633040
Neurologic Disorder	SYNTHESIS_OF_PC	REACTOME	669	29	14	4,71E-01	9,80E-01	rs12758601|rs7903496|rs1971516|rs2075609|rs10182021|rs10820780|rs12992291|rs10493879|rs4326315|rs6741502|rs12741793|rs639210|rs1453496|rs7858499
Neurologic Disorder	DARPP_32_EVENTS	REACTOME	879	39	20	4,75E-01	9,84E-01	rs37576|rs7733296|rs17545756|rs6863462|rs13362124|rs3776841|rs7259810|rs7728286|rs35731903|rs783037|rs17828764|rs1556805|rs26950|rs6733907|rs2732510|rs11666474|rs2277921|rs599381|rs17780860|rs783060
Neurologic Disorder	PHASE1_FUNCTIONALIZATION_OF_COMPOUNDS	REACTOME	1515	46	20	4,79E-01	9,85E-01	rs2068888|rs41723|rs1458928|rs232542|rs2414099|rs4555110|rs2207632|rs6736570|rs6052369|rs4646522|rs1957516|rs10818684|rs3787557|rs2470176|rs1400681|rs7806848|rs1741295|rs927068|rs952884|rs678040
Neurologic Disorder	GLUCAGON_TYPE_LIGAND_RECEPTORS	REACTOME	786	24	20	4,82E-01	9,85E-01	rs10907192|rs10255835|rs1605504|rs4898712|rs4794013|rs1332010|rs9371268|rs1507727|rs17083085|rs1558227|rs9630890|rs1941663|rs6674438|rs9470963|rs4988498|rs2024497|rs6513412|rs6017596|rs6510696|rs10305522
Neurologic Disorder	EPO_PATHWAY	BIOCARTA	255	13	8	4,85E-01	9,85E-01	rs6503691|rs1962515|rs11159135|rs4776783|rs2291516|rs6037986|rs1882832|rs8013053
Neurologic Disorder	LONG_TERM_POTENTIATION	KEGG	3620	34	20	4,85E-01	9,85E-01	rs11612284|rs170290|rs11938486|rs2239048|rs4963857|rs2300267|rs2423356|rs2241694|rs12948922|rs771941|rs886898|rs1962515|rs11055587|rs316369|rs2370413|rs3741403|rs6903559|rs3805018|rs660442|rs12507865
Neurologic Disorder	INWARDLY_RECTIFYING_K_CHANNELS	REACTOME	1317	38	20	4,85E-01	9,85E-01	rs8066734|rs10907192|rs11650230|rs10512574|rs10180851|rs4898712|rs1934080|rs3758947|rs2067043|rs1940455|rs9903628|rs9630890|rs3025643|rs16837945|rs9906150|rs6674438|rs7221237|rs1999498|rs9983351|rs978708
Neurologic Disorder	SIGNALING_BY_NOTCH	REACTOME	4553	25	20	4,86E-01	9,85E-01	rs1232587|rs3761441|rs6750496|rs532385|rs273472|rs6436879|rs17576984|rs2769957|rs11138631|rs4525961|rs11630420|rs3759929|rs4332094|rs2142200|rs4895799|rs17115605|rs10501844|rs11727661|rs8027532|rs10746589
Neurologic Disorder	APC_C_CDC20_MEDIATED_DEGRADATION_OF_CYCLIN_B	REACTOME	224	14	9	4,89E-01	9,85E-01	rs16930959|rs6742309|rs6550808|rs17842119|rs4435493|rs28653722|rs3761034|rs1464095|rs4848611
Neurologic Disorder	TRANSCRIPTIONAL_ACTIVITY_OF_SMAD2_SMAD3_SMAD4_HETEROTRIMER	REACTOME	967	29	20	4,91E-01	9,85E-01	rs4332094|rs2142200|rs4129666|rs266359|rs3219110|rs2395851|rs7835350|rs12548864|rs223366|rs12580394|rs1662820|rs9635893|rs9957562|rs8032542|rs12119470|rs6664266|rs12151827|rs12605548|rs4435493|rs12543298
Neurologic Disorder	LAIR_PATHWAY	BIOCARTA	479	28	13	4,93E-01	9,85E-01	rs10184153|rs1001420|rs10473234|rs7736582|rs10229203|rs2073917|rs1901167|rs5030102|rs831864|rs8060136|rs373469|rs9934579|rs1179766
Neurologic Disorder	MISMATCH_REPAIR	KEGG	576	31	19	4,94E-01	9,85E-01	rs9592217|rs12877281|rs11147544|rs10951757|rs1925089|rs1359389|rs7324308|rs2348238|rs7996386|rs8077346|rs7994717|rs690705|rs2922666|rs2304136|rs11949801|rs836817|rs2066789|rs4149963|rs3735461
Neurologic Disorder	ALPHA_LINOLENIC_ACID_METABOLISM	KEGG	571	41	19	4,94E-01	9,85E-01	rs12749748|rs12046362|rs1076205|rs174449|rs1800627|rs776686|rs4650729|rs800719|rs4924592|rs6425147|rs17411112|rs1548796|rs10753000|rs1487492|rs12139100|rs7554410|rs3761431|rs4821738|rs10912174
Neurologic Disorder	VEGF_SIGNALING_PATHWAY	KEGG	2111	35	20	4,94E-01	9,85E-01	rs1569736|rs4963857|rs465531|rs12749748|rs12948922|rs10912053|rs6872437|rs3730327|rs1076205|rs1962515|rs1129880|rs660442|rs8090560|rs12462766|rs10413199|rs1800627|rs776686|rs1423236|rs3729908|rs4470720
Neurologic Disorder	CYTOSOLIC_TRNA_AMINOACYLATION	REACTOME	312	7	5	4,96E-01	9,85E-01	rs11745439|rs7905657|rs611060|rs3733977|rs925210
Neurologic Disorder	NO1_PATHWAY	BIOCARTA	1482	34	20	4,97E-01	9,85E-01	rs1046248|rs9508477|rs2038916|rs998133|rs7688672|rs17549153|rs10737816|rs3753617|rs1937690|rs2191502|rs7259810|rs17633019|rs9551699|rs7303830|rs12117452|rs10505867|rs10925402|rs1903066|rs7112131|rs9462937
Neurologic Disorder	HEPARAN_SULFATE_HEPARIN_HS_GAG_METABOLISM	REACTOME	3879	28	20	4,98E-01	9,85E-01	rs9556369|rs1445270|rs2575738|rs309556|rs9561545|rs12750012|rs1948234|rs11729350|rs1007973|rs1929035|rs3771254|rs9523030|rs7997908|rs6762394|rs535828|rs7014925|rs9364126|rs12508213|rs7562354|rs10492621
Neurologic Disorder	MCALPAIN_PATHWAY	BIOCARTA	543	35	13	5,04E-01	9,85E-01	rs3919447|rs17041245|rs759169|rs12666347|rs884904|rs7016497|rs10488142|rs1525642|rs820335|rs3911406|rs12977802|rs11785444|rs17586344
Neurologic Disorder	BCELLSURVIVAL_PATHWAY	BIOCARTA	500	24	13	5,05E-01	9,85E-01	rs465531|rs6872249|rs1423236|rs11159135|rs4470720|rs4353229|rs1074078|rs10402864|rs17773680|rs2277921|rs12608504|rs1882832|rs8013053
Neurologic Disorder	FOCAL_ADHESION	KEGG	7733	34	20	5,05E-01	9,85E-01	rs6006615|rs11978556|rs461970|rs4077746|rs4842157|rs2437095|rs465531|rs260852|rs1457596|rs10184153|rs3919447|rs17041245|rs919751|rs1265646|rs16877483|rs2292867|rs16975294|rs7807090|rs12948922|rs4663708
Neurologic Disorder	SIGNALING_BY_WNT	REACTOME	1071	34	20	5,08E-01	9,85E-01	rs704375|rs11922435|rs400223|rs13338214|rs12598046|rs755859|rs3776841|rs17031765|rs11642698|rs4795311|rs417940|rs673995|rs12447239|rs4435493|rs7212835|rs726558|rs40610|rs2431238|rs3761034|rs2665833
Neurologic Disorder	TYPE_II_DIABETES_MELLITUS	KEGG	2053	37	20	5,08E-01	9,85E-01	rs13402503|rs2239048|rs4557033|rs465531|rs3753242|rs6872437|rs886898|rs13023787|rs10951757|rs2370413|rs3750510|rs2239028|rs12323112|rs1423236|rs16927947|rs3758947|rs4765951|rs16042|rs2067043|rs2908297
Neurologic Disorder	TRYPTOPHAN_METABOLISM	KEGG	953	63	20	5,08E-01	9,85E-01	rs4662299|rs2166480|rs4578194|rs7551594|rs232542|rs7837357|rs283525|rs4878784|rs2661067|rs1258191|rs10506645|rs2302340|rs3774051|rs12129636|rs6816|rs354693|rs7118388|rs16834034|rs11575387|rs11124907
Neurologic Disorder	G_ALPHA_I_SIGNALLING_EVENTS	REACTOME	6453	31	20	5,08E-01	9,85E-01	rs566416|rs1046248|rs2829949|rs2474265|rs10907192|rs7250851|rs10027926|rs2238256|rs10857887|rs2961739|rs7098615|rs10801043|rs7916268|rs12407294|rs4585434|rs1012178|rs1548687|rs4513489|rs1341458|rs1557399
Neurologic Disorder	GLUCOSE_TRANSPORT	REACTOME	689	38	20	5,10E-01	9,85E-01	rs13431075|rs1000940|rs10951757|rs905639|rs283525|rs2350623|rs2908297|rs132813|rs12693351|rs1406334|rs7697711|rs9985182|rs357111|rs2922666|rs161626|rs7897621|rs103612|rs35046440|rs1462529|rs3809335
Neurologic Disorder	TOLL_LIKE_RECEPTOR_SIGNALING_PATHWAY	KEGG	2339	31	20	5,11E-01	9,85E-01	rs4698855|rs465531|rs1884257|rs10983712|rs11869073|rs2440134|rs6872249|rs7744|rs3730327|rs7754397|rs1129880|rs12943654|rs661838|rs1423236|rs997284|rs6001585|rs2284552|rs11159135|rs4470720|rs6454860
Neurologic Disorder	MITOTIC_M_M_G1_PHASES	REACTOME	3176	37	20	5,11E-01	9,85E-01	rs487989|rs9450763|rs704375|rs7980317|rs10917799|rs449397|rs11922435|rs9679654|rs400223|rs2910469|rs13338214|rs12598046|rs35674|rs6769686|rs2985689|rs11814554|rs755859|rs3776841|rs11764107|rs17031765
Neurologic Disorder	PDGF_PATHWAY	BIOCARTA	1021	39	20	5,11E-01	9,85E-01	rs465531|rs12948922|rs6872437|rs6503691|rs1962515|rs11736538|rs1423236|rs3729908|rs11953892|rs11159135|rs4470720|rs4776783|rs7735400|rs3785383|rs16959227|rs17691810|rs6037986|rs120909|rs525563|rs7196303
Neurologic Disorder	THYROID_CANCER	KEGG	1104	32	20	5,12E-01	9,85E-01	rs4963857|rs4332094|rs2565190|rs2142200|rs2568218|rs4129666|rs176632|rs2395851|rs7835350|rs12548864|rs10900290|rs6547602|rs4776783|rs12543298|rs17762406|rs12629751|rs17623382|rs4410871|rs16903124|rs10761415
Neurologic Disorder	PYRIMIDINE_METABOLISM	REACTOME	792	29	20	5,14E-01	9,85E-01	rs10918295|rs9362210|rs784600|rs6683957|rs2291702|rs6741915|rs586364|rs1180275|rs3002273|rs3821536|rs11165823|rs1454371|rs784619|rs10493889|rs4775749|rs290826|rs1253795|rs517483|rs1934272|rs2669429
Neurologic Disorder	LATE_PHASE_OF_HIV_LIFE_CYCLE	REACTOME	1259	33	20	5,18E-01	9,85E-01	rs13431075|rs9679654|rs7524919|rs175169|rs1000940|rs905639|rs3821996|rs283525|rs2350623|rs4916595|rs7217258|rs132813|rs2304196|rs12693351|rs12151827|rs1406334|rs11994010|rs4435493|rs7697711|rs9985182
Neurologic Disorder	ACTIVATED_TLR4_SIGNALLING	REACTOME	2676	32	20	5,19E-01	9,85E-01	rs2829949|rs11953993|rs1884257|rs10983712|rs11746572|rs2360969|rs40383|rs11869073|rs2440134|rs8041078|rs7744|rs3730327|rs7754397|rs6903559|rs9873175|rs963921|rs2829929|rs1129880|rs12540995|rs661838
Neurologic Disorder	DOWNSTREAM_SIGNAL_TRANSDUCTION	REACTOME	3083	32	20	5,20E-01	9,85E-01	rs13402503|rs4963857|rs4557033|rs465531|rs10490697|rs919751|rs7605126|rs12948922|rs2360969|rs2163349|rs6872437|rs6503691|rs13023787|rs1962515|rs11204910|rs7204987|rs660442|rs8063461|rs11736538|rs2198200
Neurologic Disorder	COSTIMULATION_BY_THE_CD28_FAMILY	REACTOME	1479	33	20	5,20E-01	9,85E-01	rs461970|rs465531|rs721363|rs6872437|rs6583176|rs3782046|rs11204910|rs6592724|rs2838515|rs1423236|rs3776841|rs4470720|rs13420393|rs9831894|rs673995|rs1074078|rs9292729|rs3115961|rs10918708|rs3135021
Neurologic Disorder	GROWTH_HORMONE_RECEPTOR_SIGNALING	REACTOME	773	37	13	5,21E-01	9,85E-01	rs13354167|rs6503691|rs1047953|rs12323112|rs2291702|rs11687971|rs1543661|rs2972153|rs12467492|rs6730045|rs2744119|rs12547683|rs1763036
Neurologic Disorder	STEROID_BIOSYNTHESIS	KEGG	247	8	5	5,22E-01	9,85E-01	rs12122753|rs2839128|rs7068816|rs1792326|rs2686187
Neurologic Disorder	GPCR_PATHWAY	BIOCARTA	1102	24	20	5,24E-01	9,85E-01	rs1569736|rs10907192|rs12948922|rs2360969|rs1962515|rs8090560|rs7259810|rs12706724|rs3729908|rs11159135|rs4776783|rs3785383|rs16959227|rs6513412|rs6096477|rs6126251|rs6021247|rs17828764|rs1052653|rs120909
Neurologic Disorder	CALCINEURIN_PATHWAY	BIOCARTA	895	29	20	5,25E-01	9,85E-01	rs10166760|rs1569736|rs12948922|rs8090560|rs7259810|rs3729908|rs3785383|rs16959227|rs12193394|rs9368950|rs9488123|rs6096477|rs6126251|rs6021247|rs17828764|rs1052653|rs120909|rs4811174|rs6067742|rs645860
Neurologic Disorder	ENDOMETRIAL_CANCER	KEGG	3028	36	20	5,28E-01	9,85E-01	rs6757169|rs4963857|rs465531|rs482533|rs17041245|rs4332094|rs2142200|rs6872437|rs2568218|rs759169|rs1962515|rs4129666|rs176632|rs1368909|rs2395851|rs660442|rs17017249|rs16830730|rs7835350|rs6755695
Neurologic Disorder	LIPID_DIGESTION_MOBILIZATION_AND_TRANSPORT	REACTOME	1389	60	20	5,29E-01	9,85E-01	rs4149313|rs12750012|rs3771254|rs12934552|rs1929841|rs10495717|rs1276720|rs838878|rs2020927|rs2191502|rs17139378|rs4245794|rs2472508|rs7975139|rs7043894|rs758726|rs3012499|rs312049|rs12886225|rs7087360
Neurologic Disorder	ADP_SIGNALLING_THROUGH_P2RY1	REACTOME	863	40	20	5,30E-01	9,85E-01	rs10907192|rs12749748|rs12046362|rs3730327|rs4898712|rs4650729|rs10869955|rs9630890|rs6674438|rs1801258|rs6425147|rs743923|rs2024497|rs1548796|rs10753000|rs6510696|rs1487492|rs1403501|rs7554410|rs990000
Neurologic Disorder	BIOLOGICAL_OXIDATIONS	REACTOME	3568	34	20	5,32E-01	9,85E-01	rs2068888|rs12990757|rs41723|rs11249454|rs2518824|rs5996675|rs2059098|rs1458928|rs158845|rs7551594|rs232542|rs2414099|rs4555110|rs1801280|rs6518591|rs2207632|rs739230|rs6736570|rs6052369|rs2297763
Neurologic Disorder	SIGNALLING_TO_ERKS	REACTOME	794	34	20	5,32E-01	9,85E-01	rs4963857|rs3730327|rs1962515|rs1129880|rs13008971|rs12462766|rs10413199|rs4525577|rs516391|rs4776783|rs2241407|rs4877062|rs743923|rs17623382|rs7235528|rs11102933|rs16977124|rs7021743|rs7303282|rs7979636
Neurologic Disorder	SHH_PATHWAY	BIOCARTA	679	14	10	5,34E-01	9,85E-01	rs2195894|rs6459975|rs1458806|rs16830730|rs4849909|rs1932075|rs10249283|rs10274882|rs721347|rs10486731
Neurologic Disorder	NEUROTROPHIN_SIGNALING_PATHWAY	KEGG	3689	35	20	5,35E-01	9,85E-01	rs4698855|rs11938486|rs4963857|rs465531|rs2013444|rs2241694|rs40383|rs4074967|rs10897495|rs4381535|rs12693008|rs6872249|rs3730327|rs1962515|rs7164531|rs6903559|rs2606160|rs1129880|rs13008971|rs660442
Neurologic Disorder	GLUTATHIONE_CONJUGATION	REACTOME	407	16	11	5,36E-01	9,85E-01	rs5996675|rs7551594|rs2297763|rs13114510|rs510088|rs4712036|rs9296695|rs2602218|rs9474314|rs12580698|rs11807
Neurologic Disorder	METABOLISM_OF_XENOBIOTICS_BY_CYTOCHROME_P450	KEGG	1674	48	20	5,36E-01	9,85E-01	rs3818562|rs11249454|rs1011382|rs7551594|rs232542|rs2297763|rs1937849|rs4965732|rs6464540|rs13114510|rs11253042|rs9296695|rs678040|rs2602218|rs3738043|rs9474314|rs7497607|rs12580698|rs2514051|rs2287396
Neurologic Disorder	GLYCINE_SERINE_AND_THREONINE_METABOLISM	KEGG	564	27	14	5,37E-01	9,85E-01	rs6539460|rs3972|rs10780262|rs7031908|rs2291702|rs10735532|rs2519154|rs1547258|rs1129187|rs2118653|rs12349155|rs7848869|rs592762|rs3010681
Neurologic Disorder	FC_GAMMA_R_MEDIATED_PHAGOCYTOSIS	KEGG	3795	35	20	5,39E-01	9,85E-01	rs461970|rs13402503|rs4557033|rs465531|rs1078631|rs11810204|rs12749748|rs12948922|rs4459703|rs884277|rs10912053|rs2959319|rs4744669|rs6872437|rs13023787|rs1962515|rs847915|rs16869106|rs2060984|rs600415
Neurologic Disorder	PPAR_SIGNALING_PATHWAY	KEGG	1324	27	20	5,39E-01	9,85E-01	rs11974777|rs3211834|rs7744|rs174449|rs1346291|rs6838184|rs7667067|rs12330015|rs6007640|rs4917690|rs7907756|rs11195905|rs7707569|rs12629751|rs3821799|rs3774051|rs11670764|rs943347|rs4498289|rs2239621
Neurologic Disorder	O_GLYCAN_BIOSYNTHESIS	KEGG	1579	34	20	5,39E-01	9,85E-01	rs1119017|rs16841278|rs7018169|rs10819377|rs10006633|rs799770|rs7953158|rs707094|rs1030197|rs6436807|rs16967040|rs7515331|rs2108790|rs1503766|rs6435200|rs7698210|rs2945746|rs10260052|rs2292897|rs4726119
Neurologic Disorder	IL10_PATHWAY	BIOCARTA	288	9	6	5,41E-01	9,85E-01	rs6503691|rs2284552|rs10229203|rs2834168|rs525563|rs17351243
Neurologic Disorder	CELL_CELL_COMMUNICATION	REACTOME	9725	34	20	5,44E-01	9,85E-01	rs2032728|rs2219556|rs6006615|rs10076681|rs2880989|rs7193606|rs465531|rs13332162|rs1387370|rs10490697|rs2318044|rs12602314|rs16985602|rs7605126|rs471398|rs2514351|rs10805805|rs9873710|rs1816793|rs4925249
Neurologic Disorder	TOB1_PATHWAY	BIOCARTA	652	30	16	5,44E-01	9,85E-01	rs6692647|rs3782046|rs2296620|rs266359|rs6783860|rs8032542|rs3115961|rs10918708|rs1805110|rs6698995|rs7156293|rs1295686|rs4673268|rs7536947|rs446304|rs920293
Neurologic Disorder	PEROXISOME	KEGG	2047	28	20	5,47E-01	9,85E-01	rs9372252|rs10041164|rs6539460|rs17156226|rs7744|rs9392560|rs12944039|rs10106087|rs7667067|rs710130|rs9787810|rs7526517|rs11598878|rs12197995|rs6464540|rs9605514|rs7808249|rs153000|rs2207717|rs8022613
Neurologic Disorder	NUCLEOTIDE_LIKE_PURINERGIC_RECEPTORS	REACTOME	272	18	5	5,47E-01	9,85E-01	rs10857887|rs7637803|rs578065|rs1403501|rs2510881
Neurologic Disorder	ADHERENS_JUNCTIONS_INTERACTIONS	REACTOME	5496	41	20	5,50E-01	9,85E-01	rs2032728|rs10076681|rs2880989|rs7193606|rs13332162|rs1387370|rs16985602|rs471398|rs2514351|rs10805805|rs9873710|rs4925249|rs4235537|rs7206005|rs6071519|rs4452532|rs7732246|rs570521|rs977628|rs7188209
Neurologic Disorder	PROTEIN_EXPORT	KEGG	750	50	18	5,52E-01	9,85E-01	rs6933329|rs10253118|rs1011382|rs1115219|rs4690696|rs2257148|rs1093677|rs13247393|rs937213|rs1528034|rs1534166|rs12705710|rs11031384|rs10280436|rs12331128|rs10499751|rs12508008|rs9860614
Neurologic Disorder	TASTE_TRANSDUCTION	KEGG	1100	31	20	5,53E-01	9,85E-01	rs4112274|rs10907192|rs17256769|rs3846591|rs1016898|rs3750510|rs6961082|rs16042|rs10256011|rs41483|rs12548296|rs6513412|rs237484|rs7009064|rs1865550|rs4595035|rs4736520|rs238551|rs2387174|rs3887555
Neurologic Disorder	SMOOTH_MUSCLE_CONTRACTION	REACTOME	658	39	20	5,53E-01	9,85E-01	rs1265646|rs8039242|rs11647019|rs1029513|rs4917690|rs7259810|rs13411881|rs1926196|rs12595658|rs3772832|rs1441734|rs7907756|rs7800283|rs943347|rs7800113|rs7641248|rs7777356|rs3911406|rs6733907|rs3180427
Neurologic Disorder	REGULATION_OF_BETA_CELL_DEVELOPMENT	REACTOME	1288	27	20	5,55E-01	9,85E-01	rs2414223|rs4746864|rs4525961|rs874511|rs10951757|rs6859263|rs6779720|rs4612653|rs12029406|rs2908297|rs4686655|rs2158254|rs6777021|rs7953249|rs2737621|rs922369|rs2246072|rs35976751|rs7697053|rs2922666

Neurologic Disorder	NITRIC_OXIDE_STIMULATES_GUANYLATE_CYCLASE	REACTOME	1895	35	20	5,56E-01	9,85E-01	rs705789|rs9856750|rs2038916|rs2269142|rs998133|rs7688672|rs17549153|rs2293055|rs12944039|rs282880|rs2128650|rs3432|rs1937690|rs7281786|rs6443552|rs7303830|rs1879417|rs10505867|rs3819902|rs1143960
Neurologic Disorder	ALK_PATHWAY	BIOCARTA	1932	48	20	5,57E-01	9,85E-01	rs6692647|rs3919447|rs11953993|rs1884257|rs11746572|rs6763683|rs733664|rs7754397|rs2296620|rs9367669|rs6783860|rs16830730|rs9844738|rs1874572|rs7566401|rs7953249|rs1374547|rs6454860|rs4510815|rs4841578
Neurologic Disorder	METABOLISM_OF_NON_CODING_RNA	REACTOME	611	36	20	5,60E-01	9,85E-01	rs13431075|rs1000940|rs905639|rs283525|rs2350623|rs4916595|rs132813|rs7306887|rs12693351|rs1406334|rs762283|rs7697711|rs9985182|rs357111|rs7726729|rs103612|rs1462529|rs3809335|rs11240609|rs2882359
Neurologic Disorder	HUNTINGTONS_DISEASE	KEGG	4311	28	20	5,60E-01	9,85E-01	rs7794510|rs11612284|rs1870990|rs2300267|rs2423356|rs2025880|rs4854069|rs2950390|rs2360969|rs771941|rs843346|rs11055587|rs3741403|rs13024804|rs3821996|rs3805018|rs660442|rs480211|rs3746106|rs2863720
Neurologic Disorder	MELANOGENESIS	KEGG	4077	35	20	5,62E-01	9,85E-01	rs9296345|rs6925906|rs11938486|rs4963857|rs764833|rs2423356|rs997782|rs2241694|rs1407998|rs12948922|rs2360969|rs1446255|rs2568218|rs771941|rs1962515|rs843346|rs10120066|rs3741403|rs3757552|rs917846
Neurologic Disorder	KERATAN_SULFATE_BIOSYNTHESIS	REACTOME	674	25	19	5,63E-01	9,85E-01	rs1119017|rs1539143|rs948360|rs7018169|rs1503766|rs2945746|rs2595361|rs2292897|rs4141587|rs10777288|rs6801461|rs11775725|rs1488682|rs11647932|rs750592|rs6981912|rs304303|rs7584792|rs1554324
Neurologic Disorder	TNFR1_PATHWAY	BIOCARTA	862	34	17	5,63E-01	9,85E-01	rs7810486|rs1884257|rs6583176|rs7754397|rs3219110|rs2213178|rs6592724|rs6454860|rs2049233|rs9362744|rs6583177|rs974189|rs6670527|rs9362878|rs12693932|rs2293464|rs17165293
Neurologic Disorder	DNA_REPAIR	REACTOME	2111	38	20	5,64E-01	9,85E-01	rs817827|rs7100805|rs7222799|rs9592217|rs12877281|rs2761367|rs16942|rs11147544|rs10951757|rs8054783|rs7091108|rs2697675|rs3821996|rs2213178|rs1573638|rs8063461|rs2985689|rs1925089|rs1359389|rs7324308
Neurologic Disorder	LIGAND_GATED_ION_CHANNEL_TRANSPORT	REACTOME	1051	34	20	5,65E-01	9,85E-01	rs209348|rs7690580|rs7183534|rs387661|rs7658410|rs1363518|rs12708616|rs6854637|rs1352062|rs5022631|rs3922855|rs6892782|rs4695955|rs1365591|rs10517664|rs248368|rs7167848|rs10155173|rs282129|rs11161293
Neurologic Disorder	SIGNAL_AMPLIFICATION	REACTOME	1023	36	20	5,65E-01	9,85E-01	rs10907192|rs12749748|rs10912053|rs3730327|rs4898712|rs12706724|rs739431|rs1938506|rs10869955|rs9630890|rs6674438|rs1801258|rs6425147|rs602932|rs2024497|rs7637803|rs1548796|rs10753000|rs6510696|rs1487492
Neurologic Disorder	TGF_BETA_RECEPTOR_SIGNALING_ACTIVATES_SMADS	REACTOME	563	27	17	5,66E-01	9,85E-01	rs9679654|rs3759929|rs266359|rs6783860|rs7975139|rs9635893|rs9957562|rs8032542|rs4435493|rs1474879|rs6134733|rs3761034|rs1294690|rs169409|rs1792684|rs446304|rs920293
Neurologic Disorder	GRB2_EVENTS_IN_ERBB2_SIGNALING	REACTOME	1202	43	20	5,67E-01	9,85E-01	rs4963857|rs17041245|rs6704686|rs759169|rs1962515|rs16845944|rs12666347|rs17646307|rs884904|rs17669781|rs4733122|rs900102|rs4776783|rs4281084|rs10503913|rs10488142|rs2466066|rs1525642|rs6758369|rs13260061
Neurologic Disorder	DESTABILIZATION_OF_MRNA_BY_KSRP	REACTOME	205	11	8	5,70E-01	9,85E-01	rs3730327|rs1129880|rs12675103|rs11130040|rs743923|rs4382556|rs1613070|rs3740526
Neurologic Disorder	RESOLUTION_OF_AP_SITES_VIA_THE_MULTIPLE_NUCLEOTIDE_PATCH_REPLACEMENT_P	REACTOME	192	10	8	5,70E-01	9,85E-01	rs10951757|rs8063461|rs7190284|rs2922666|rs509360|rs2304136|rs10958713|rs174556
Neurologic Disorder	GAP_JUNCTION	KEGG	4388	31	20	5,71E-01	9,85E-01	rs11695567|rs9320821|rs4963857|rs260852|rs2423356|rs17041245|rs919751|rs2038916|rs12948922|rs998133|rs759169|rs7688672|rs17549153|rs11226342|rs771941|rs2128650|rs1962515|rs3741403|rs266004|rs7204987
Neurologic Disorder	CGMP_EFFECTS	REACTOME	1520	39	20	5,72E-01	9,85E-01	rs705789|rs9856750|rs2038916|rs2269142|rs998133|rs7688672|rs17549153|rs282880|rs3432|rs1937690|rs7281786|rs6443552|rs7303830|rs10505867|rs3819902|rs1143960|rs7112131|rs17582581|rs7916242|rs1922128
Neurologic Disorder	FGFR_LIGAND_BINDING_AND_ACTIVATION	REACTOME	984	33	20	5,72E-01	9,85E-01	rs581971|rs1851710|rs17472986|rs1545033|rs445775|rs6859263|rs2320607|rs823616|rs9510005|rs3804158|rs9506807|rs1368406|rs13312770|rs10796853|rs755698|rs17585055|rs9509983|rs9506810|rs7730111|rs6556301
Neurologic Disorder	CELL_JUNCTION_ORGANIZATION	REACTOME	6781	38	20	5,75E-01	9,85E-01	rs2032728|rs6006615|rs10076681|rs2880989|rs7193606|rs13332162|rs1387370|rs16985602|rs471398|rs2514351|rs10805805|rs9873710|rs4925249|rs4235537|rs6685516|rs7206005|rs6071519|rs4452532|rs7732246|rs570521
Neurologic Disorder	BASE_EXCISION_REPAIR	REACTOME	209	11	9	5,75E-01	9,85E-01	rs10951757|rs8063461|rs7190284|rs3213403|rs2922666|rs509360|rs2304136|rs10958713|rs174556
Neurologic Disorder	MRNA_SPLICING	REACTOME	1057	31	20	5,77E-01	9,85E-01	rs17462632|rs3972|rs7544531|rs1783983|rs11089445|rs3821996|rs2868355|rs575274|rs4916595|rs12510329|rs7217258|rs17206784|rs1076691|rs2304196|rs7306887|rs17517994|rs762283|rs10817476|rs2232003|rs2607426
Neurologic Disorder	RNA_POL_I_PROMOTER_OPENING	REACTOME	501	17	2	5,78E-01	9,85E-01	rs198838|rs527544
Neurologic Disorder	GLYCOSAMINOGLYCAN_METABOLISM	REACTOME	6096	24	20	5,78E-01	9,85E-01	rs9556369|rs1445270|rs2575738|rs1119017|rs7250851|rs309556|rs9561545|rs12750012|rs1948234|rs11729350|rs1007973|rs1539143|rs948360|rs4802841|rs1929035|rs7018169|rs4981034|rs3771254|rs9523030|rs7997908
Neurologic Disorder	ION_TRANSPORT_BY_P_TYPE_ATPASES	REACTOME	1690	43	20	5,80E-01	9,85E-01	rs9507379|rs9998984|rs671127|rs8025743|rs8025577|rs4907561|rs9511399|rs35674|rs10507340|rs6502762|rs4298115|rs1397857|rs6694434|rs4941167|rs7337905|rs4906745|rs16831388|rs7334443|rs12650894|rs2248985
Neurologic Disorder	FACTORS_INVOLVED_IN_MEGAKARYOCYTE_DEVELOPMENT_AND_PLATELET_PRODUCT	REACTOME	3549	29	20	5,83E-01	9,85E-01	rs1612612|rs11255531|rs2957457|rs4741823|rs7094402|rs3801796|rs2787148|rs2914441|rs6731972|rs17545756|rs2729549|rs4742037|rs10262821|rs2910469|rs9584969|rs521331|rs477461|rs7905277|rs4074324|rs8012659
Neurologic Disorder	PROCESSING_OF_CAPPED_INTRONLESS_PRE_MRNA	REACTOME	274	13	8	5,84E-01	9,85E-01	rs1783983|rs4916595|rs1039322|rs17311679|rs7306887|rs762283|rs8006358|rs11240609
Neurologic Disorder	DOWNREGULATION_OF_TGF_BETA_RECEPTOR_SIGNALING	REACTOME	521	19	13	5,84E-01	9,85E-01	rs9679654|rs266359|rs6783860|rs7975139|rs9635893|rs9957562|rs8032542|rs4435493|rs3761034|rs169409|rs1792684|rs446304|rs920293
Neurologic Disorder	MHC_CLASS_II_ANTIGEN_PRESENTATION	REACTOME	2226	54	20	5,86E-01	9,85E-01	rs6930052|rs2025880|rs2457684|rs6731972|rs12577323|rs17108729|rs2910469|rs843346|rs35674|rs4074324|rs12421587|rs10516556|rs2588568|rs11919795|rs10742181|rs754217|rs4800180|rs3135021|rs490998|rs11792652
Neurologic Disorder	DOWNSTREAM_SIGNALING_EVENTS_OF_B_CELL_RECEPTOR_BCR	REACTOME	2127	29	20	5,87E-01	9,85E-01	rs4963857|rs704375|rs1884257|rs11922435|rs2360969|rs400223|rs13338214|rs7754397|rs12598046|rs11204910|rs6705101|rs660442|rs8063461|rs6555976|rs17238159|rs4670209|rs755859|rs3776841|rs3729908|rs17031765
Neurologic Disorder	FATTY_ACID_TRIACYLGLYCEROL_AND_KETONE_BODY_METABOLISM	REACTOME	5526	38	20	5,87E-01	9,85E-01	rs11974777|rs6918969|rs2914757|rs3012755|rs4149313|rs10058172|rs4726096|rs3211834|rs10850626|rs1783978|rs715779|rs1971516|rs11751128|rs2609920|rs1929841|rs7667067|rs754313|rs12330015|rs7636407|rs6007640
Neurologic Disorder	NEF_MEDIATES_DOWN_MODULATION_OF_CELL_SURFACE_RECEPTORS_BY_RECRUITIN N_ADAPTERS	REACTOME	366	26	9	5,87E-01	9,85E-01	rs843346|rs6994201|rs9259916|rs11919795|rs3115961|rs7464428|rs2524005|rs4673268|rs17370523
Neurologic Disorder	SIGNALING_BY_RHO_GTPASES	REACTOME	4877	26	20	5,87E-01	9,85E-01	rs13098182|rs461970|rs272107|rs3785732|rs17543981|rs3821525|rs3755677|rs2107622|rs17288929|rs10222369|rs12319134|rs3793252|rs11175211|rs10517355|rs6817930|rs17094556|rs2466178|rs4686339|rs3752237|rs715779
Neurologic Disorder	TGFB_PATHWAY	BIOCARTA	817	32	20	5,87E-01	9,85E-01	rs7552931|rs1884257|rs7754397|rs266359|rs6783860|rs6001585|rs9635893|rs6454860|rs9957562|rs8032542|rs4776783|rs2431238|rs9362744|rs1792684|rs1801552|rs9362878|rs7156293|rs4783676|rs2238419|rs446304
Neurologic Disorder	KERATAN_SULFATE_KERATIN_METABOLISM	REACTOME	737	25	20	5,95E-01	9,90E-01	rs1119017|rs1539143|rs948360|rs7018169|rs1503766|rs2945746|rs2595361|rs2292897|rs820846|rs4141587|rs10777288|rs6801461|rs11775725|rs15958|rs1488682|rs11647932|rs750592|rs6981912|rs304303|rs7584792
Neurologic Disorder	PROCESSING_OF_CAPPED_INTRON_CONTAINING_PRE_MRNA	REACTOME	1547	35	20	5,97E-01	9,90E-01	rs17462632|rs13431075|rs1000940|rs3972|rs7544531|rs1783983|rs905639|rs11089445|rs3821996|rs2868355|rs283525|rs575274|rs2350623|rs4916595|rs12510329|rs7217258|rs17206784|rs132813|rs1076691|rs2304196
Neurologic Disorder	CHYLOMICRON_MEDIATED_LIPID_TRANSPORT	REACTOME	435	14	9	5,98E-01	9,90E-01	rs34719083|rs3771254|rs10495717|rs312049|rs1373656|rs7605009|rs2242062|rs7544500|rs2899632
Neurologic Disorder	TRANSMISSION_ACROSS_CHEMICAL_SYNAPSES	REACTOME	9089	33	20	5,98E-01	9,90E-01	rs4839797|rs11612284|rs363512|rs170290|rs8066734|rs11938486|rs4747345|rs10907192|rs11650230|rs218378|rs2300267|rs2924149|rs209348|rs10797768|rs3912834|rs7198466|rs2423356|rs2241694|rs2782901|rs2518824
Neurologic Disorder	SIGNALING_BY_NOTCH1	REACTOME	3563	30	20	5,99E-01	9,90E-01	rs1232587|rs6750496|rs273472|rs6436879|rs2769957|rs11138631|rs4525961|rs11630420|rs4332094|rs2142200|rs4895799|rs10501844|rs11727661|rs8027532|rs10746589|rs4877600|rs4973205|rs7636407|rs4129666|rs2525055
Neurologic Disorder	INSULIN_PATHWAY	BIOCARTA	725	40	20	6,00E-01	9,90E-01	rs465531|rs6872249|rs1962515|rs1423236|rs11953892|rs11159135|rs4470720|rs11687971|rs4776783|rs7735400|rs2972153|rs12467492|rs6730045|rs4804377|rs6037986|rs2229431|rs11668751|rs2624171|rs1882832|rs8013053
Neurologic Disorder	IL7_PATHWAY	BIOCARTA	699	32	17	6,03E-01	9,90E-01	rs465531|rs575330|rs6872249|rs6503691|rs4941185|rs1423236|rs4470720|rs525563|rs12962644|rs1026825|rs7846568|rs1016860|rs8083946|rs2010128|rs6916861|rs2238419|rs11076785
Neurologic Disorder	CASPASE_PATHWAY	BIOCARTA	327	21	10	6,04E-01	9,90E-01	rs7810486|rs3219110|rs1800627|rs4353229|rs17773680|rs6670527|rs12693932|rs2293464|rs2894125|rs17165293
Neurologic Disorder	NEUROACTIVE_LIGAND_RECEPTOR_INTERACTION	KEGG	13469	31	20	6,05E-01	9,90E-01	rs1046248|rs6794593|rs4839797|rs11612284|rs2474265|rs363512|rs170290|rs10802789|rs6772419|rs17824615|rs1857586|rs7250851|rs218378|rs326416|rs2300267|rs209348|rs2124811|rs10857887|rs3011577|rs1599766
Neurologic Disorder	SPHINGOLIPID_DE_NOVO_BIOSYNTHESIS	REACTOME	1020	39	20	6,07E-01	9,90E-01	rs8041714|rs11810204|rs884277|rs6100061|rs10483379|rs10133136|rs735678|rs587316|rs7521445|rs1191571|rs16855727|rs4912291|rs964987|rs12358176|rs1953725|rs983833|rs2967625|rs10811186|rs12589478|rs17826464
Neurologic Disorder	PROPANOATE_METABOLISM	KEGG	1219	38	20	6,07E-01	9,90E-01	rs3794492|rs4553987|rs4578194|rs7551594|rs12431036|rs2207632|rs4878784|rs10948479|rs2313427|rs1890139|rs7992993|rs3949482|rs9381755|rs1340343|rs4331688|rs3774051|rs7611269|rs6083725|rs17022214|rs4489814
Neurologic Disorder	ADIPOCYTOKINE_SIGNALING_PATHWAY	KEGG	2106	31	20	6,07E-01	9,90E-01	rs11974777|rs4698855|rs4726096|rs3211834|rs7667067|rs12330015|rs6007640|rs2006643|rs12323112|rs10278273|rs11687971|rs1543661|rs16207|rs7776530|rs1074078|rs4750565|rs661891|rs2972153|rs7778356|rs12467492
Neurologic Disorder	INTEGRATION_OF_ENERGY_METABOLISM	REACTOME	4501	36	20	6,08E-01	9,90E-01	rs2288419|rs10802789|rs4747345|rs10907192|rs4726096|rs2423356|rs3011577|rs7477742|rs7098615|rs6509931|rs12948922|rs2288519|rs7916268|rs1295881|rs771941|rs11195534|rs3741403|rs16878921|rs7204987|rs2471637
Neurologic Disorder	CLEAVAGE_OF_GROWING_TRANSCRIPT_IN_THE_TERMINATION_REGION_	REACTOME	435	19	13	6,09E-01	9,90E-01	rs3972|rs1783983|rs575274|rs4916595|rs1039322|rs1076691|rs17311679|rs7306887|rs762283|rs547976|rs8006358|rs11240609|rs12479650
Neurologic Disorder	UBIQUITIN_MEDIATED_PROTEOLYSIS	KEGG	3227	42	20	6,11E-01	9,90E-01	rs512868|rs12736942|rs16930959|rs10200577|rs13191310|rs9346906|rs1947240|rs9355357|rs4894936|rs2466178|rs11727661|rs16942|rs2714747|rs12524235|rs877226|rs3782046|rs3798964|rs7558428|rs7496283|rs4290619
Neurologic Disorder	INHIBITION_OF_THE_PROTEOLYTIC_ACTIVITY_OF_APC_C_REQUIRED_FOR_THE_ONSET_ TOTIC_SPINDLE_CHECKPOINT_COMPONENTS	REACTOME	365	13	10	6,11E-01	9,90E-01	rs16930959|rs6742309|rs6550808|rs17842119|rs10902882|rs28653722|rs2495774|rs1464095|rs7654241|rs4848611
Neurologic Disorder	IL12_PATHWAY	BIOCARTA	589	37	20	6,12E-01	9,90E-01	rs11869073|rs2440134|rs3730327|rs1799988|rs3782046|rs997284|rs540722|rs10918708|rs474246|rs2715815|rs11867183|rs602932|rs6698995|rs4493656|rs17129782|rs10515794|rs7258015|rs662959|rs7636840|rs17810546
Neurologic Disorder	ACTIVATION_OF_GENES_BY_ATF4	REACTOME	316	13	9	6,13E-01	9,90E-01	rs6918969|rs11130040|rs650229|rs4382556|rs1613070|rs3740526|rs3890801|rs1229568|rs12086247
Neurologic Disorder	CHEMOKINE_RECEPTORS_BIND_CHEMOKINES	REACTOME	726	32	19	6,15E-01	9,90E-01	rs566416|rs4513489|rs4972960|rs12943654|rs7685130|rs1491961|rs11890854|rs7371043|rs1147963|rs223848|rs12691883|rs9868689|rs4710191|rs17416271|rs543721|rs7757618|rs1027241|rs1737168|rs9993375
Neurologic Disorder	CHEMOKINE_SIGNALING_PATHWAY	KEGG	5407	31	20	6,19E-01	9,90E-01	rs4698855|rs566416|rs461970|rs4963857|rs10907192|rs465531|rs2423356|rs3753242|rs4381535|rs6872437|rs159293|rs4513489|rs715779|rs771941|rs6503691|rs1962515|rs11751128|rs3741403|rs4972960|rs7204987
Neurologic Disorder	PERK_REGULATED_GENE_EXPRESSION	REACTOME	334	13	9	6,23E-01	9,90E-01	rs6918969|rs11130040|rs650229|rs4382556|rs1613070|rs3740526|rs3890801|rs1229568|rs12086247
Neurologic Disorder	FANCONI_ANEMIA_PATHWAY	REACTOME	461	24	9	6,27E-01	9,90E-01	rs2761367|rs16942|rs4435493|rs9634672|rs11157432|rs3934711|rs17049508|rs3761034|rs8107282
Neurologic Disorder	SIGNALING_BY_ILS	REACTOME	2996	32	20	6,29E-01	9,90E-01	rs461970|rs4963857|rs465531|rs1884257|rs4851543|rs40383|rs575330|rs11869073|rs2440134|rs6872249|rs7744|rs6503691|rs7754397|rs1962515|rs1541435|rs661838|rs7905645|rs6980760|rs10483660|rs1423236
Neurologic Disorder	BOTULINUM_NEUROTOXICITY	REACTOME	522	17	10	6,33E-01	9,90E-01	rs7965988|rs7138800|rs4812024|rs1845181|rs2277672|rs3744656|rs3923371|rs6077718|rs10848194|rs634986
Neurologic Disorder	SIGNALING_BY_EGFR_IN_CANCER	REACTOME	4030	37	20	6,33E-01	9,90E-01	rs13402503|rs6597745|rs4963857|rs4557033|rs465531|rs2025880|rs17041245|rs9531286|rs12948922|rs7321702|rs2360969|rs6872249|rs759169|rs13023787|rs1962515|rs843346|rs11204910|rs7204987|rs660442|rs1947372
Neurologic Disorder	INTEGRIN_PATHWAY	BIOCARTA	1027	27	17	6,37E-01	9,90E-01	rs1583109|rs1962515|rs6464546|rs6003709|rs4902681|rs2191502|rs4776783|rs6714500|rs3791975|rs490998|rs7016497|rs3867132|rs6916861|rs3180427|rs12977802|rs729725|rs11785444
Neurologic Disorder	FORMATION_OF_INCISION_COMPLEX_IN_GG_NER	REACTOME	545	27	11	6,39E-01	9,90E-01	rs817827|rs8054783|rs1573638|rs12336793|rs8077346|rs9925917|rs1124303|rs3735461|rs10817938|rs16962919|rs6872466
Neurologic Disorder	VIBRIO_CHOLERAE_INFECTION	KEGG	1518	33	20	6,40E-01	9,90E-01	rs1296826|rs12948922|rs10832305|rs12523625|rs2309428|rs6994201|rs7204987|rs10954211|rs17607283|rs2460221|rs2401158|rs3729908|rs2075609|rs2270637|rs4405546|rs7197601|rs2531982|rs7945327|rs3785383|rs16959227
Neurologic Disorder	SNARE_INTERACTIONS_IN_VESICULAR_TRANSPORT	KEGG	828	29	14	6,41E-01	9,90E-01	rs7965988|rs7138800|rs4812024|rs216478|rs6446665|rs41542|rs10100935|rs2280544|rs12162703|rs2277672|rs4610878|rs3744656|rs6077718|rs10848194
Neurologic Disorder	ACYL_CHAIN_REMODELLING_OF_PE	REACTOME	696	44	20	6,41E-01	9,90E-01	rs12749748|rs12046362|rs1076205|rs600415|rs13008971|rs1800627|rs776686|rs4650729|rs6425147|rs17411112|rs2111937|rs1548796|rs10753000|rs1487492|rs7554410|rs3761431|rs4821738|rs2457334|rs10912174|rs747527
Neurologic Disorder	PEPTIDE_CHAIN_ELONGATION	REACTOME	947	32	20	6,42E-01	9,90E-01	rs7219464|rs3826322|rs1149747|rs2976044|rs8082649|rs1811384|rs11755449|rs36631|rs4789626|rs6791696|rs137653|rs2140386|rs4435493|rs16995300|rs1570026|rs1302|rs219446|rs3829570|rs7688462|rs12632423
Neurologic Disorder	PI_3K_CASCADE	REACTOME	1868	29	20	6,44E-01	9,90E-01	rs465531|rs581971|rs1851710|rs2360969|rs6872437|rs17472986|rs1545033|rs445775|rs6859263|rs11204910|rs660442|rs2320607|rs823616|rs8063461|rs9510005|rs1423236|rs3804158|rs9506807|rs516391|rs4470720
Neurologic Disorder	PACKAGING_OF_TELOMERE_ENDS	REACTOME	515	15	4	6,46E-01	9,90E-01	rs10088657|rs198838|rs3863241|rs1126432
Neurologic Disorder	G_PROTEIN_ACTIVATION	REACTOME	703	24	14	6,47E-01	9,90E-01	rs10907192|rs4898712|rs12706724|rs739431|rs9630890|rs1108656|rs6674438|rs9384159|rs9478476|rs2024497|rs6510696|rs4820537|rs6972935|rs563649
Neurologic Disorder	SIGNALING_BY_TGF_BETA_RECEPTOR_COMPLEX	REACTOME	1605	25	20	6,49E-01	9,90E-01	rs9679654|rs3753242|rs3759929|rs4332094|rs2142200|rs4129666|rs266359|rs3219110|rs2395851|rs6783860|rs7835350|rs12548864|rs223366|rs11265544|rs7975139|rs12580394|rs1662820|rs9635893|rs9957562|rs8032542
Neurologic Disorder	RAP1_SIGNALLING	REACTOME	835	29	20	6,50E-01	9,90E-01	rs2038916|rs998133|rs17549153|rs1962515|rs1937690|rs2060773|rs12675103|rs9909561|rs829391|rs10203199|rs2004738|rs17653133|rs11869953|rs3769234|rs7916242|rs2275364|rs1922128|rs2238142|rs2242420|rs11815205
Neurologic Disorder	ALLOGRAFT_REJECTION	KEGG	1510	40	20	6,51E-01	9,90E-01	rs6930052|rs13191519|rs7755852|rs9258895|rs1926196|rs9831894|rs28367729|rs3115961|rs540722|rs3135021|rs9266440|rs3094691|rs4493656|rs17351243|rs10515794|rs11569333|rs662959|rs7636840|rs4813003|rs2517938
Neurologic Disorder	AMI_PATHWAY	BIOCARTA	574	32	18	6,58E-01	9,90E-01	rs16975294|rs4145072|rs4773209|rs587409|rs1192201|rs4581040|rs6492270|rs1470428|rs6056|rs1951626|rs9559718|rs10220027|rs3742207|rs1247571|rs17514517|rs3211719|rs6718820|rs12868126
Neurologic Disorder	STARCH_AND_SUCROSE_METABOLISM	KEGG	2178	47	20	6,62E-01	9,90E-01	rs7771841|rs11249454|rs721976|rs1062708|rs10951757|rs6939185|rs7623391|rs1351067|rs6664173|rs2908297|rs9290221|rs11937734|rs1448398|rs6548335|rs6844466|rs7692977|rs11185098|rs9863717|rs2922666|rs9325372
Neurologic Disorder	AXON_GUIDANCE	REACTOME	13131	38	20	6,66E-01	9,90E-01	rs2938769|rs2219556|rs3779972|rs518558|rs4751955|rs4747345|rs4842157|rs4963857|rs1269621|rs3911489|rs7374540|rs16946687|rs7675169|rs2184778|rs1459590|rs10490697|rs2025880|rs2292867|rs16975294|rs12602314
Neurologic Disorder	NEGATIVE_REGULATION_OF_FGFR_SIGNALING	REACTOME	1536	30	20	6,70E-01	9,90E-01	rs581971|rs9531286|rs1851710|rs7321702|rs17472986|rs1545033|rs445775|rs6859263|rs2320607|rs823616|rs9510005|rs3776841|rs3804158|rs9506807|rs516391|rs1368406|rs13312770|rs9318729|rs4435493|rs10796853
Neurologic Disorder	SIGNALING_BY_THE_B_CELL_RECEPTOR_BCR	REACTOME	3426	30	20	6,71E-01	9,90E-01	rs461970|rs4963857|rs465531|rs704375|rs1884257|rs11922435|rs2360969|rs400223|rs4894936|rs6872437|rs4917749|rs7754397|rs13338214|rs12598046|rs11204910|rs6705101|rs660442|rs7905645|rs8063461|rs6555976
Neurologic Disorder	PD1_SIGNALING	REACTOME	398	17	5	6,71E-01	9,90E-01	rs3782046|rs10918708|rs3135021|rs6698995|rs10481593
Neurologic Disorder	SIGNALING_BY_INSULIN_RECEPTOR	REACTOME	3432	38	20	6,73E-01	9,90E-01	rs1296826|rs4963857|rs465531|rs4726096|rs581971|rs2587616|rs1851710|rs10168275|rs6872249|rs1050700|rs1545033|rs17472986|rs1962515|rs445775|rs6994201|rs6859263|rs11204910|rs17596234|rs10954211|rs2320607
Neurologic Disorder	APC_CDC20_MEDIATED_DEGRADATION_OF_NEK2A	REACTOME	396	17	12	6,74E-01	9,90E-01	rs16930959|rs6742309|rs6550808|rs17842119|rs10902882|rs4435493|rs28653722|rs2495774|rs3761034|rs1464095|rs7654241|rs4848611
Neurologic Disorder	ANTIGEN_PRESENTATION_FOLDING_ASSEMBLY_AND_PEPTIDE_LOADING_OF_CLASS_I_M	REACTOME	830	39	16	6,75E-01	9,90E-01	rs13191519|rs17108729|rs7755852|rs35674|rs9258895|rs10516556|rs28367729|rs9266440|rs3094691|rs7147299|rs2517938|rs10490815|rs17108576|rs2517861|rs3775835|rs13202464
Neurologic Disorder	WNT_PATHWAY	BIOCARTA	1363	35	20	6,75E-01	9,90E-01	rs1884257|rs4332094|rs2142200|rs9902833|rs7754397|rs4129666|rs2395851|rs16830730|rs7835350|rs12548864|rs2779625|rs3776841|rs6001585|rs6454860|rs10867778|rs10122814|rs12543298|rs12057062|rs4410871|rs11654191
Neurologic Disorder	CELL_CELL_JUNCTION_ORGANIZATION	REACTOME	6211	38	20	6,78E-01	9,90E-01	rs2032728|rs10076681|rs2880989|rs7193606|rs13332162|rs1387370|rs16985602|rs471398|rs2514351|rs10805805|rs9873710|rs4925249|rs4235537|rs6685516|rs7206005|rs6071519|rs4452532|rs7732246|rs570521|rs977628
Neurologic Disorder	CLASS_I_MHC_MEDIATED_ANTIGEN_PROCESSING_PRESENTATION	REACTOME	5895	28	20	6,79E-01	9,90E-01	rs11974777|rs704375|rs7259473|rs3444|rs16930959|rs3211834|rs1265646|rs11922435|rs10200577|rs13191519|rs13191310|rs9346906|rs17011494|rs400223|rs9355357|rs4894936|rs17102087|rs11727661|rs10511272|rs17108729
Neurologic Disorder	GLYCOGEN_BREAKDOWN_GLYCOGENOLYSIS	REACTOME	246	14	8	6,81E-01	9,90E-01	rs7259810|rs6664173|rs3809676|rs6844466|rs7692977|rs1890706|rs4832960|rs6733907
Neurologic Disorder	PI3K_AKT_ACTIVATION	REACTOME	1161	29	20	6,81E-01	9,90E-01	rs465531|rs2360969|rs6872249|rs11204910|rs660442|rs8063461|rs12323112|rs1423236|rs4470720|rs7190284|rs11687971|rs1543661|rs1074078|rs9292729|rs2972153|rs12467492|rs6730045|rs9368950|rs3800230|rs945559
Neurologic Disorder	MEMBRANE_TRAFFICKING	REACTOME	2276	30	20	6,82E-01	9,90E-01	rs9320821|rs716289|rs2025880|rs9450659|rs2041374|rs17108729|rs843346|rs17091328|rs35674|rs6988243|rs11757506|rs6059504|rs611908|rs10516556|rs26532|rs3799765|rs7181201|rs11919795|rs11240144|rs1844358
Neurologic Disorder	MEIOTIC_RECOMBINATION	REACTOME	993	44	13	6,83E-01	9,90E-01	rs2761367|rs16942|rs2697675|rs13175928|rs198838|rs8077346|rs6470522|rs9634672|rs401549|rs6452136|rs3735461|rs1295686|rs1521009
Neurologic Disorder	HISTIDINE_METABOLISM	KEGG	654	27	19	6,85E-01	9,90E-01	rs16840311|rs4578194|rs7551594|rs4878784|rs10225988|rs1357404|rs4965732|rs2839128|rs6935937|rs4954961|rs6738271|rs7497607|rs2514051|rs12940665|rs4954746|rs1330674|rs11575387|rs17357592|rs1025607
Neurologic Disorder	INSULIN_RECEPTOR_SIGNALLING_CASCADE	REACTOME	3006	37	20	6,87E-01	9,90E-01	rs4963857|rs465531|rs4726096|rs581971|rs2587616|rs1851710|rs10168275|rs6872249|rs1050700|rs17472986|rs1545033|rs1962515|rs445775|rs6859263|rs11204910|rs17596234|rs2320607|rs823616|rs8063461|rs970238
Neurologic Disorder	HIV_LIFE_CYCLE	REACTOME	1529	33	20	6,89E-01	9,90E-01	rs13431075|rs9679654|rs7524919|rs175169|rs1000940|rs1799988|rs905639|rs3821996|rs283525|rs2350623|rs4916595|rs7217258|rs132813|rs2304196|rs12693351|rs12151827|rs1406334|rs11994010|rs11890854|rs4435493
Neurologic Disorder	NONSENSE_MEDIATED_DECAY_ENHANCED_BY_THE_EXON_JUNCTION_COMPLEX	REACTOME	1261	32	20	6,91E-01	9,90E-01	rs7079388|rs7219464|rs3826322|rs1149747|rs2976044|rs8082649|rs1811384|rs3776841|rs36631|rs4789626|rs4916595|rs6791696|rs137653|rs2019872|rs2140386|rs4435493|rs16995300|rs1570026|rs1302|rs219446
Neurologic Disorder	DRUG_METABOLISM_OTHER_ENZYMES	KEGG	1609	36	20	6,93E-01	9,90E-01	rs10918295|rs11249454|rs11697114|rs1801280|rs8047963|rs6683957|rs6741915|rs12446482|rs7199731|rs3002273|rs3821536|rs11165823|rs678040|rs2410561|rs10493889|rs2739737|rs290826|rs1253795|rs10179712|rs714510

Neurologic Disorder	IL_3_5_AND_GM_CSF_SIGNALING	REACTOME	1134	30	18	6,94E-01	9,90E-01	rs461970|rs465531|rs6872249|rs6503691|rs7905645|rs1423236|rs12675103|rs4470720|rs6776572|rs11188666|rs12547683|rs525563|rs7245158|rs228935|rs10993754|rs651933|rs308195|rs6916861
Neurologic Disorder	CCR5_PATHWAY	BIOCARTA	696	29	20	6,95E-01	9,90E-01	rs12948922|rs3730327|rs1799988|rs12943654|rs7259810|rs3729908|rs11159135|rs3785383|rs16959227|rs11890854|rs7371043|rs602932|rs1147963|rs120909|rs12691883|rs7846568|rs7196303|rs17634425|rs543721|rs6733907
Neurologic Disorder	ARAP_PATHWAY	BIOCARTA	292	19	9	6,97E-01	9,90E-01	rs4459703|rs4796821|rs3802680|rs4919626|rs10752637|rs907165|rs16866947|rs5758941|rs1050671
Neurologic Disorder	RIBOSOME	KEGG	1118	34	20	6,97E-01	9,90E-01	rs7219464|rs3826322|rs1149747|rs2976044|rs8082649|rs1811384|rs2985689|rs36631|rs4789626|rs6791696|rs137653|rs4955755|rs2140386|rs10139381|rs4435493|rs16995300|rs1570026|rs1302|rs219446|rs3829570
Neurologic Disorder	SIGNALING_BY_ERBB4	REACTOME	3434	36	20	7,00E-01	9,90E-01	rs11642715|rs4963857|rs465531|rs17041245|rs6704686|rs2360969|rs270434|rs3853362|rs6872249|rs759169|rs8045027|rs13354167|rs6503691|rs9924268|rs1962515|rs11204910|rs3798577|rs660442|rs661838|rs9929802
Neurologic Disorder	OXIDATIVE_PHOSPHORYLATION	KEGG	2486	32	20	7,01E-01	9,90E-01	rs9507379|rs1296826|rs4854069|rs2950390|rs8065212|rs9511399|rs6994201|rs13024804|rs10507340|rs10954211|rs2460221|rs2401158|rs480211|rs12767413|rs3746106|rs3753362|rs7249519|rs12538107|rs2075609|rs2270637
Neurologic Disorder	TRANSPORT_TO_THE_GOLGI_AND_SUBSEQUENT_MODIFICATION	REACTOME	1557	44	20	7,01E-01	9,90E-01	rs16946687|rs7572142|rs7586009|rs7769107|rs17108729|rs35674|rs2881586|rs2985689|rs3784722|rs10516556|rs2321546|rs2595361|rs4659050|rs4141587|rs1683542|rs11886886|rs7147299|rs11629679|rs729169|rs11104005
Neurologic Disorder	GLUCONEOGENESIS	REACTOME	490	27	15	7,03E-01	9,90E-01	rs11760823|rs7122539|rs8062660|rs11076318|rs9649679|rs10989500|rs4582459|rs29861|rs13245850|rs578597|rs13912|rs4930187|rs12704840|rs3770459|rs11644079
Neurologic Disorder	SIGNALLING_BY_NGF	REACTOME	7869	29	20	7,06E-01	9,90E-01	rs13098182|rs461970|rs13402503|rs272107|rs4963857|rs7810486|rs4557033|rs465531|rs2013444|rs3821525|rs2025880|rs11953993|rs3755677|rs17288929|rs12948922|rs3759929|rs11746572|rs12319134|rs2360969|rs40383
Neurologic Disorder	PLATELET_SENSITIZATION_BY_LDL	REACTOME	676	44	20	7,08E-01	9,90E-01	rs12749748|rs10912053|rs3730327|rs10495717|rs3776841|rs4650729|rs673995|rs6425147|rs743923|rs726558|rs6588474|rs1548796|rs10753000|rs312049|rs1487492|rs7554410|rs736449|rs7605009|rs11666474|rs10912174
Neurologic Disorder	GLYCEROLIPID_METABOLISM	KEGG	1872	38	20	7,09E-01	9,90E-01	rs11810204|rs7805162|rs884277|rs1558909|rs4578194|rs7551594|rs2609920|rs6838184|rs814620|rs13008971|rs2268833|rs4573621|rs4878784|rs10486056|rs3810622|rs1337988|rs6975650|rs4634526|rs11195813|rs2239624
Neurologic Disorder	RECYCLING_PATHWAY_OF_L1	REACTOME	874	34	16	7,10E-01	9,90E-01	rs2025880|rs10897495|rs843346|rs6903559|rs1637572|rs11919795|rs11792652|rs9459785|rs9347129|rs9295348|rs845647|rs9644117|rs10810812|rs538147|rs17370523|rs6890862
Neurologic Disorder	SHC1_EVENTS_IN_ERBB4_SIGNALING	REACTOME	1072	33	17	7,11E-01	9,90E-01	rs4963857|rs6704686|rs1962515|rs16845944|rs17646307|rs17669781|rs4733122|rs900102|rs4776783|rs4281084|rs10503913|rs2466066|rs6758369|rs13260061|rs7303282|rs6468085|rs4531002
Neurologic Disorder	DOWNSTREAM_TCR_SIGNALING	REACTOME	1573	43	20	7,11E-01	9,90E-01	rs465531|rs1884257|rs40383|rs6872249|rs7754397|rs3782046|rs661838|rs17238159|rs6980760|rs1423236|rs4470720|rs6454860|rs1483382|rs4750565|rs661891|rs6953804|rs10918708|rs3135021|rs6698995|rs945559
Neurologic Disorder	PYK2_PATHWAY	BIOCARTA	752	27	17	7,12E-01	9,90E-01	rs12948922|rs3730327|rs1962515|rs6592724|rs7259810|rs3729908|rs4776783|rs3785383|rs16959227|rs743923|rs120909|rs7846568|rs7196303|rs17634425|rs6733907|rs11074594|rs12450534
Neurologic Disorder	SPLICEOSOME	KEGG	1685	28	20	7,12E-01	9,90E-01	rs11656715|rs472029|rs12640588|rs704787|rs6444089|rs3972|rs12736858|rs7544531|rs11089445|rs575274|rs4351004|rs4916595|rs897880|rs4622984|rs17206784|rs1076691|rs7306887|rs762283|rs36643|rs254262
Neurologic Disorder	MRNA_PROCESSING	REACTOME	1942	35	20	7,12E-01	9,90E-01	rs17462632|rs13431075|rs11088978|rs1000940|rs3972|rs7544531|rs1783983|rs905639|rs11089445|rs3821996|rs2868355|rs283525|rs575274|rs6488567|rs2350623|rs4916595|rs12510329|rs7217258|rs11738432|rs17206784
Neurologic Disorder	ABORTIVE_ELONGATION_OF_HIV1_TRANSCRIPT_IN_THE_ABSENCE_OF_TAT	REACTOME	195	11	8	7,12E-01	9,90E-01	rs3821996|rs4916595|rs7217258|rs2304196|rs1107674|rs1613070|rs2363127|rs12977802
Neurologic Disorder	ANTIGEN_ACTIVATES_B_CELL_RECEPTOR_LEADING_TO_GENERATION_OF_SECOND_MES	REACTOME	1299	35	20	7,13E-01	9,90E-01	rs461970|rs465531|rs4894936|rs6872437|rs4917749|rs7905645|rs1423236|rs7259810|rs4470720|rs4405546|rs2618450|rs7197601|rs11188666|rs12547683|rs12761929|rs4243218|rs10993754|rs1436187|rs6733907|rs6916861
Neurologic Disorder	CELL_SURFACE_INTERACTIONS_AT_THE_VASCULAR_WALL	REACTOME	2650	32	20	7,13E-01	9,90E-01	rs2824327|rs4963857|rs465531|rs2292867|rs1501813|rs615532|rs2318044|rs6872437|rs10495717|rs1319745|rs12461927|rs2515466|rs1126644|rs2191502|rs11265544|rs10239535|rs1423236|rs4329925|rs132930|rs4470720
Neurologic Disorder	SPHINGOLIPID_METABOLISM	KEGG	1101	50	20	7,14E-01	9,90E-01	rs11810204|rs884277|rs7151140|rs13108249|rs2008644|rs1436222|rs13263637|rs735678|rs1426790|rs2595361|rs11159798|rs4912291|rs12358176|rs10811186|rs12589478|rs768185|rs2139155|rs10740635|rs878078|rs11624137
Neurologic Disorder	ACTIVATED_POINT_MUTANTS_OF_FGFR2	REACTOME	873	30	20	7,14E-01	9,90E-01	rs1851710|rs1545033|rs17472986|rs6859263|rs2320607|rs823616|rs9510005|rs3804158|rs9506807|rs1368406|rs13312770|rs10796853|rs755698|rs17585055|rs9509983|rs9506810|rs7730111|rs7999201|rs10870911|rs10908244
Neurologic Disorder	TCR_SIGNALING	REACTOME	2040	42	20	7,16E-01	9,90E-01	rs465531|rs2184778|rs1884257|rs40383|rs6872437|rs6583176|rs7754397|rs3782046|rs10045011|rs661838|rs6592724|rs17238159|rs6980760|rs1423236|rs4470720|rs6454860|rs1483382|rs4750565|rs661891|rs6953804
Neurologic Disorder	L1CAM_INTERACTIONS	REACTOME	3914	34	20	7,18E-01	9,90E-01	rs2075728|rs1269621|rs7374540|rs2025880|rs2292867|rs13255835|rs7807090|rs7916174|rs10891524|rs10897495|rs12549656|rs759169|rs10101217|rs843346|rs6903559|rs1902409|rs11779500|rs6782048|rs6592724|rs334411
Neurologic Disorder	ENDOSOMAL_SORTING_COMPLEX_REQUIRED_FOR_TRANSPORT_ESCRT	REACTOME	318	16	10	7,18E-01	9,90E-01	rs17091328|rs6059504|rs1844358|rs11994010|rs4435493|rs13015620|rs3761034|rs13912|rs873084|rs7502321
Neurologic Disorder	G_ALPHA_S_SIGNALLING_EVENTS	REACTOME	5019	36	20	7,19E-01	9,90E-01	rs37576|rs10907192|rs1857586|rs10115467|rs7733296|rs705789|rs10228431|rs6570053|rs1062708|rs7765655|rs11609312|rs10255835|rs17248872|rs6916194|rs12515498|rs2331226|rs11773493|rs6863462|rs13362124|rs659562
Neurologic Disorder	HIV_INFECTION	REACTOME	3237	28	20	7,20E-01	9,90E-01	rs704375|rs13431075|rs4678192|rs11922435|rs9679654|rs7524919|rs175169|rs400223|rs1000940|rs6583176|rs1799988|rs13338214|rs905639|rs12598046|rs843346|rs7700605|rs6994201|rs3821996|rs9259830|rs283525
Neurologic Disorder	RNA_POL_II_TRANSCRIPTION_PRE_INITIATION_AND_PROMOTER_OPENING	REACTOME	383	15	10	7,20E-01	9,90E-01	rs7524919|rs3821996|rs7217258|rs2304196|rs6089583|rs1107674|rs1613070|rs7833229|rs12977802|rs6872466
Neurologic Disorder	GABA_RECEPTOR_ACTIVATION	REACTOME	2705	35	20	7,22E-01	9,90E-01	rs8066734|rs10907192|rs11650230|rs209348|rs7690580|rs1551901|rs7183534|rs10512574|rs3786306|rs387661|rs7204987|rs7658410|rs1363518|rs10180851|rs4898712|rs16950272|rs1934080|rs9822061|rs12706724|rs3775536
Neurologic Disorder	PI3K_EVENTS_IN_ERBB2_SIGNALING	REACTOME	1886	35	20	7,22E-01	9,90E-01	rs465531|rs17041245|rs6704686|rs2360969|rs6872249|rs759169|rs11204910|rs660442|rs8063461|rs16845944|rs12666347|rs1423236|rs4470720|rs7190284|rs17646307|rs884904|rs17669781|rs4733122|rs900102|rs1074078
Neurologic Disorder	PORPHYRIN_AND_CHLOROPHYLL_METABOLISM	KEGG	1163	20	11	7,22E-01	9,90E-01	rs11249454|rs4481375|rs10900751|rs7913190|rs6882369|rs951776|rs7525733|rs4979243|rs10027817|rs17669311|rs16948995
Neurologic Disorder	NEPHRIN_INTERACTIONS	REACTOME	1617	50	20	7,24E-01	9,90E-01	rs465531|rs10490697|rs7605126|rs2163349|rs6872249|rs10790878|rs4937131|rs6590203|rs16885938|rs4902681|rs1393001|rs1423236|rs4470720|rs12420540|rs4851076|rs514463|rs6743358|rs7609295|rs3807763|rs1465641
Neurologic Disorder	PI3K_CASCADE	REACTOME	2702	41	20	7,25E-01	9,90E-01	rs465531|rs4726096|rs581971|rs2587616|rs1851710|rs10168275|rs6872249|rs1050700|rs1545033|rs17472986|rs445775|rs6859263|rs11204910|rs17596234|rs2320607|rs823616|rs8063461|rs970238|rs9510005|rs12323112
Neurologic Disorder	NOTCH1_INTRACELLULAR_DOMAIN_REGULATES_TRANSCRIPTION	REACTOME	2852	30	20	7,26E-01	9,90E-01	rs273472|rs2769957|rs11138631|rs4525961|rs11630420|rs4332094|rs2142200|rs4895799|rs10501844|rs11727661|rs8027532|rs10746589|rs4877600|rs9818162|rs4129666|rs2525055|rs6779720|rs12365545|rs2395851|rs1129880
Neurologic Disorder	AGR_PATHWAY	BIOCARTA	1981	38	20	7,26E-01	9,90E-01	rs11068958|rs2437095|rs4978952|rs6583176|rs759169|rs10509455|rs4285344|rs2152745|rs6592724|rs6118687|rs1414857|rs12666347|rs9948714|rs7098143|rs17646307|rs884904|rs17669781|rs4733122|rs900102|rs4281084
Neurologic Disorder	OPIOID_SIGNALLING	REACTOME	3594	32	20	7,27E-01	9,90E-01	rs37576|rs10907192|rs7733296|rs2423356|rs12749748|rs12948922|rs2360969|rs1551901|rs12046362|rs17545756|rs771941|rs3786306|rs6863462|rs13362124|rs3741403|rs7204987|rs660442|rs4898712|rs9822061|rs7271063
Neurologic Disorder	GABA_B_RECEPTOR_ACTIVATION	REACTOME	1863	31	20	7,27E-01	9,90E-01	rs8066734|rs10907192|rs11650230|rs1551901|rs10512574|rs3786306|rs7204987|rs10180851|rs4898712|rs1934080|rs9822061|rs12706724|rs739431|rs1940455|rs9903628|rs9630890|rs3025643|rs2531982|rs16837945|rs12548296
Neurologic Disorder	ETS_PATHWAY	BIOCARTA	682	33	20	7,27E-01	9,90E-01	rs919751|rs1176542|rs216123|rs1176541|rs12749198|rs12580394|rs11159135|rs1940359|rs3757016|rs3733678|rs6073285|rs7540934|rs7304356|rs3897903|rs11605437|rs7529675|rs11610586|rs538681|rs10499080|rs12049869
Neurologic Disorder	TGF_BETA_RECEPTOR_SIGNALING_IN_EMT_EPITHELIAL_TO_MESENCHYMAL_TRANSITI	REACTOME	429	16	12	7,28E-01	9,90E-01	rs3753242|rs6783860|rs11265544|rs2057227|rs4435493|rs1474879|rs6134733|rs3761034|rs1294690|rs12036736|rs17462455|rs8110645
Neurologic Disorder	NEURONAL_SYSTEM	REACTOME	13378	32	20	7,30E-01	9,90E-01	rs780235|rs3779972|rs4839797|rs11612284|rs363512|rs170290|rs8066734|rs11938486|rs4747345|rs780231|rs10907192|rs11650230|rs218378|rs2300267|rs2924149|rs209348|rs7820230|rs10797768|rs7965988|rs3912834
Neurologic Disorder	SYNTHESIS_OF_PIPS_AT_THE_GOLGI_MEMBRANE	REACTOME	788	44	20	7,30E-01	9,90E-01	rs2587616|rs17596234|rs4428539|rs970238|rs10505811|rs2315174|rs5751684|rs1866046|rs17767371|rs7671799|rs1496814|rs12959903|rs1336628|rs1539826|rs2742413|rs9304251|rs10483104|rs833831|rs203180|rs1941590
Neurologic Disorder	OTHER_GLYCAN_DEGRADATION	KEGG	648	15	11	7,31E-01	9,90E-01	rs4690513|rs1903568|rs10520381|rs10520394|rs820846|rs11727636|rs12501924|rs10520382|rs12643657|rs569945|rs9376755
Neurologic Disorder	COMP_PATHWAY	BIOCARTA	628	33	18	7,33E-01	9,90E-01	rs2218810|rs10473234|rs2222547|rs1800450|rs12489890|rs7736582|rs10824650|rs1901167|rs12762596|rs10824829|rs10824854|rs3761008|rs373469|rs10218945|rs1992528|rs1179766|rs710475|rs631090
Neurologic Disorder	CK1_PATHWAY	BIOCARTA	759	28	20	7,37E-01	9,90E-01	rs2423356|rs17545756|rs771941|rs266004|rs7271063|rs3776841|rs6077411|rs6055685|rs6086714|rs7212835|rs6055853|rs17828764|rs2719774|rs10891556|rs6055926|rs4130345|rs2719807|rs2732510|rs6913797|rs2201677
Neurologic Disorder	HER2_PATHWAY	BIOCARTA	1326	45	20	7,38E-01	9,90E-01	rs465531|rs6704686|rs6872437|rs759169|rs1962515|rs3798577|rs16845944|rs12666347|rs1423236|rs4470720|rs10229203|rs884904|rs4776783|rs985192|rs10488142|rs10878506|rs1525642|rs6758369|rs2504067|rs17586344
Neurologic Disorder	PRION_DISEASES	KEGG	1585	42	20	7,40E-01	9,90E-01	rs2826516|rs2826334|rs10891524|rs1001420|rs10473234|rs2827255|rs9982844|rs2855412|rs7736582|rs10229203|rs4776783|rs1436109|rs6052751|rs1901167|rs420778|rs2827563|rs2826979|rs8130625|rs1041740|rs2218618
Neurologic Disorder	ION_CHANNEL_TRANSPORT	REACTOME	2741	33	20	7,40E-01	9,90E-01	rs9507379|rs9998984|rs209348|rs671127|rs8025743|rs8025577|rs7183534|rs4907561|rs9511399|rs387661|rs35674|rs7658410|rs10507340|rs6502762|rs1363518|rs4298115|rs1397857|rs16950272|rs6694434|rs4941167
Neurologic Disorder	O_LINKED_GLYCOSYLATION_OF_MUCINS	REACTOME	2402	34	20	7,41E-01	9,90E-01	rs1119017|rs16841278|rs7550651|rs7018169|rs10819377|rs10006633|rs209256|rs799770|rs7953158|rs12793968|rs7430813|rs707094|rs1030197|rs6436807|rs16967040|rs7515331|rs10767555|rs7644047|rs2108790|rs1503766
Neurologic Disorder	G_ALPHA_Z_SIGNALLING_EVENTS	REACTOME	2191	36	20	7,42E-01	9,90E-01	rs13402503|rs4557033|rs10907192|rs7098615|rs12948922|rs7916268|rs13023787|rs11195534|rs7204987|rs4898712|rs9478395|rs9822061|rs12706724|rs3729908|rs739431|rs506134|rs183984|rs1038555|rs9630890|rs10885138
Neurologic Disorder	CSK_PATHWAY	BIOCARTA	554	21	11	7,43E-01	9,90E-01	rs10907192|rs3782046|rs10918708|rs1466020|rs6698995|rs6513412|rs670197|rs6736735|rs2238419|rs6092708|rs11076785
Neurologic Disorder	VALINE_LEUCINE_AND_ISOLEUCINE_DEGRADATION	KEGG	1149	34	20	7,46E-01	9,90E-01	rs7744|rs4578194|rs7551594|rs12431036|rs283525|rs4878784|rs4305722|rs3122126|rs2313427|rs1890139|rs3949482|rs9381755|rs42099|rs6443842|rs492734|rs3774051|rs16928163|rs9443804|rs17454070|rs2239621
Neurologic Disorder	GNRH_SIGNALING_PATHWAY	KEGG	3992	28	20	7,48E-01	9,90E-01	rs11938486|rs4963857|rs2239048|rs2423356|rs2241694|rs1062708|rs12749748|rs12948922|rs10912053|rs11869073|rs2440134|rs759169|rs3730327|rs771941|rs1076205|rs886898|rs1962515|rs2370413|rs3741403|rs7204987
Neurologic Disorder	METABOLISM_OF_MRNA	REACTOME	3017	31	20	7,49E-01	9,90E-01	rs704375|rs1701476|rs11922435|rs9679654|rs7219464|rs12948922|rs3826322|rs400223|rs9798936|rs3730327|rs13338214|rs12598046|rs1149747|rs2976044|rs7252142|rs8082649|rs2868355|rs1811384|rs1129880|rs755859
Neurologic Disorder	APOPTOSIS	KEGG	1727	33	20	7,49E-01	9,90E-01	rs4698855|rs465531|rs4851543|rs6872249|rs2466178|rs7744|rs660442|rs1800627|rs4941185|rs1423236|rs1926196|rs4470720|rs4353229|rs9314261|rs17828764|rs10958713|rs12962644|rs231989|rs8016947|rs2239621
Neurologic Disorder	TRAFFICKING_OF_GLUR2_CONTAINING_AMPA_RECEPTORS	REACTOME	721	45	20	7,51E-01	9,90E-01	rs12948922|rs843346|rs637569|rs12507865|rs7936555|rs3729908|rs11919795|rs132930|rs3785383|rs16959227|rs4958671|rs120909|rs7196303|rs4958596|rs11741511|rs17634425|rs2216649|rs11074594|rs12450534|rs17370523
Neurologic Disorder	PRIMARY_IMMUNODEFICIENCY	KEGG	742	17	13	7,51E-01	9,90E-01	rs4812848|rs3782046|rs7905645|rs13420393|rs2075876|rs131387|rs11188666|rs1466020|rs670197|rs6736735|rs11569333|rs4813003|rs4072864
Neurologic Disorder	ACTIVATION_OF_CHAPERONE_GENES_BY_XBP1S	REACTOME	761	35	19	7,53E-01	9,90E-01	rs836852|rs560577|rs1115219|rs6806768|rs2730324|rs2555141|rs848194|rs673995|rs9957722|rs1568082|rs9815624|rs5762839|rs8043811|rs3846211|rs17043569|rs2488809|rs1534166|rs9561932|rs10490815
Neurologic Disorder	NETRIN1_SIGNALING	REACTOME	3710	32	20	7,53E-01	9,90E-01	rs2938769|rs2219556|rs12602314|rs10015865|rs1816793|rs972031|rs12515863|rs12592710|rs12268044|rs16869106|rs2865384|rs1014194|rs7905277|rs17165991|rs1535386|rs11745138|rs883241|rs4316851|rs9309828|rs1865737
Neurologic Disorder	CARM_ER_PATHWAY	BIOCARTA	1617	58	20	7,59E-01	9,90E-01	rs2823165|rs11953993|rs11746572|rs16942|rs3798577|rs2525055|rs1129880|rs7789412|rs13429807|rs1178122|rs7789571|rs12580394|rs1684268|rs7217258|rs848194|rs9905578|rs2304196|rs10514306|rs3757016|rs7802855
Neurologic Disorder	IL22BP_PATHWAY	BIOCARTA	296	8	4	7,60E-01	9,90E-01	rs6503691|rs525563|rs7258015|rs3795300
Neurologic Disorder	AMINO_ACID_SYNTHESIS_AND_INTERCONVERSION_TRANSAMINATION	REACTOME	530	24	13	7,61E-01	9,90E-01	rs10797768|rs8062660|rs11076318|rs10780262|rs10735532|rs11577155|rs29861|rs12349155|rs7848869|rs592762|rs3010681|rs11644079|rs1229568
Neurologic Disorder	UCALPAIN_PATHWAY	BIOCARTA	426	15	8	7,65E-01	9,90E-01	rs2292867|rs4902681|rs490998|rs7016497|rs3867132|rs12977802|rs729725|rs11785444
Neurologic Disorder	NUCLEAR_SIGNALING_BY_ERBB4	REACTOME	2231	37	20	7,66E-01	9,90E-01	rs11642715|rs6704686|rs270434|rs3853362|rs8045027|rs13354167|rs6503691|rs9924268|rs3798577|rs661838|rs9929802|rs1047953|rs16845944|rs1882958|rs11150150|rs3776841|rs2291702|rs4888831|rs17646307|rs17669781
Neurologic Disorder	SRP_DEPENDENT_COTRANSLATIONAL_PROTEIN_TARGETING_TO_MEMBRANE	REACTOME	1378	31	20	7,66E-01	9,90E-01	rs7219464|rs1011382|rs3826322|rs1149747|rs2976044|rs1115219|rs8082649|rs1811384|rs4690696|rs36631|rs1093677|rs4789626|rs6791696|rs137653|rs2140386|rs4435493|rs16995300|rs1570026|rs937213|rs6017596
Neurologic Disorder	PROXIMAL_TUBULE_BICARBONATE_RECLAMATION	KEGG	458	21	12	7,69E-01	9,90E-01	rs10755174|rs2602049|rs6694434|rs739431|rs16831388|rs422914|rs2247114|rs2579309|rs13912|rs1200131|rs13222180|rs10737544
Neurologic Disorder	SYNTHESIS_SECRETION_AND_DEACYLATION_OF_GHRELIN	REACTOME	624	23	13	7,70E-01	9,90E-01	rs4979619|rs4690696|rs7030021|rs7037931|rs7341756|rs7734417|rs2039803|rs6976221|rs4731437|rs10490815|rs12331128|rs12491503|rs40515
Neurologic Disorder	DEADENYLATION_OF_MRNA	REACTOME	331	7	6	7,71E-01	9,90E-01	rs9798936|rs1863000|rs36643|rs789327|rs6550155|rs13255392
Neurologic Disorder	GLYCOSPHINGOLIPID_METABOLISM	REACTOME	924	43	19	7,71E-01	9,90E-01	rs7151140|rs168700|rs2008644|rs1436222|rs13263637|rs12054837|rs4834370|rs1426790|rs569523|rs11159798|rs820846|rs4286653|rs793396|rs234910|rs3846677|rs11714594|rs4697350|rs11624137|rs10445209
Neurologic Disorder	DOWNSTREAM_SIGNALING_OF_ACTIVATED_FGFR	REACTOME	3636	34	20	7,72E-01	9,90E-01	rs13402503|rs4963857|rs4557033|rs465531|rs581971|rs1851710|rs12948922|rs2360969|rs6872249|rs17472986|rs1545033|rs13023787|rs1962515|rs445775|rs6859263|rs11204910|rs7204987|rs660442|rs2320607|rs823616
Neurologic Disorder	PLATELET_AGGREGATION_PLUG_FORMATION	REACTOME	942	35	20	7,72E-01	9,90E-01	rs2292867|rs7098615|rs7916268|rs11195534|rs2060773|rs10741114|rs12265119|rs10885138|rs10203199|rs17653133|rs6056|rs7016497|rs1878247|rs3769234|rs7908674|rs10993754|rs2238142|rs11815280|rs3006835|rs11785444
Neurologic Disorder	CELL_CYCLE	KEGG	2581	30	20	7,74E-01	9,90E-01	rs6692647|rs9450763|rs16930959|rs4332094|rs2142200|rs478859|rs4129666|rs266359|rs2213178|rs2395851|rs12370927|rs16830730|rs6742309|rs7835350|rs2374594|rs12548864|rs9369325|rs9315420|rs3776841|rs11764107
Neurologic Disorder	IL2_PATHWAY	BIOCARTA	452	15	10	7,74E-01	9,90E-01	rs6503691|rs1962515|rs11159135|rs4776783|rs6037986|rs525563|rs228935|rs10993754|rs1882832|rs8013053
Neurologic Disorder	MITOCHONDRIA_PATHWAY	BIOCARTA	317	20	12	7,74E-01	9,90E-01	rs4988366|rs1800627|rs4941185|rs4353229|rs4559081|rs12962644|rs1026825|rs17773680|rs1016860|rs6670527|rs8083946|rs12693932
Neurologic Disorder	TOLL_RECEPTOR_CASCADES	REACTOME	3778	39	20	7,74E-01	9,90E-01	rs2829949|rs11953993|rs1884257|rs2587616|rs10983712|rs11746572|rs2360969|rs40383|rs11869073|rs2440134|rs8041078|rs7744|rs3730327|rs201061|rs7754397|rs2326809|rs6903559|rs9873175|rs17596234|rs963921
Neurologic Disorder	ACTIVATION_OF_BH3_ONLY_PROTEINS	REACTOME	325	10	9	7,75E-01	9,90E-01	rs3862706|rs660442|rs4941185|rs2032809|rs12962644|rs1026825|rs1016860|rs8083946|rs2585838
Neurologic Disorder	CHEMICAL_PATHWAY	BIOCARTA	585	29	20	7,77E-01	9,90E-01	rs12948922|rs3219110|rs660442|rs1800627|rs4941185|rs3729908|rs4353229|rs3785383|rs16959227|rs7016497|rs120909|rs12962644|rs1026825|rs7196303|rs17634425|rs17773680|rs1016860|rs8083946|rs11074594|rs12450534
Neurologic Disorder	UNFOLDED_PROTEIN_RESPONSE	REACTOME	1176	34	20	7,77E-01	9,90E-01	rs6918969|rs836852|rs560577|rs1115219|rs6806768|rs2730324|rs2555141|rs848194|rs673995|rs9957722|rs1568082|rs11130040|rs9815624|rs5762839|rs8043811|rs3846211|rs17043569|rs650229|rs4964375|rs4382556
Neurologic Disorder	COMPLEMENT_CASCADE	REACTOME	752	33	20	7,78E-01	9,90E-01	rs2218810|rs10473234|rs2222547|rs1800450|rs12489890|rs7736582|rs10033900|rs10824650|rs1901167|rs12762596|rs10824829|rs1323722|rs10824854|rs3761008|rs2842754|rs373469|rs582317|rs10218945|rs1992528|rs1179766
Neurologic Disorder	INFLUENZA_VIRAL_RNA_TRANSCRIPTION_AND_REPLICATION	REACTOME	1153	34	20	7,78E-01	9,90E-01	rs7219464|rs3826322|rs1149747|rs2976044|rs8082649|rs3821996|rs1811384|rs36631|rs4789626|rs6791696|rs137653|rs7217258|rs2140386|rs2304196|rs4435493|rs16995300|rs1570026|rs1302|rs219446|rs3829570
Neurologic Disorder	P53_SIGNALING_PATHWAY	KEGG	1897	38	20	7,79E-01	9,90E-01	rs4121859|rs1457596|rs12736942|rs11070217|rs2466178|rs7229801|rs3862706|rs12370927|rs8063461|rs2374594|rs9369325|rs1926196|rs7935166|rs7190284|rs1940455|rs6219|rs17584066|rs2453839|rs4470909|rs7102184
Neurologic Disorder	TRANSCRIPTION	REACTOME	2708	31	20	7,81E-01	9,90E-01	rs4922220|rs11779820|rs17093344|rs7524919|rs6132068|rs3972|rs12581936|rs1327510|rs1783983|rs6081172|rs3821996|rs575274|rs4916595|rs7217258|rs11738432|rs2062348|rs1076691|rs683474|rs2304196|rs17311679
Neurologic Disorder	RNA_POL_I_RNA_POL_III_AND_MITOCHONDRIAL_TRANSCRIPTION	REACTOME	1830	50	20	7,82E-01	9,90E-01	rs4922220|rs11779820|rs17093344|rs6132068|rs12581936|rs1327510|rs6081172|rs2062348|rs683474|rs198838|rs7255568|rs4119055|rs527544|rs4961408|rs10810136|rs10961271|rs17709614|rs10095297|rs7018762|rs11204109
Neurologic Disorder	FATTY_ACYL_COA_BIOSYNTHESIS	REACTOME	404	9	6	7,83E-01	9,90E-01	rs7667067|rs11737840|rs11195905|rs149293|rs13159372|rs10012953
Neurologic Disorder	PENTOSE_PHOSPHATE_PATHWAY	KEGG	616	27	14	7,84E-01	9,90E-01	rs11937784|rs9435168|rs17158495|rs17158795|rs2075876|rs10989500|rs6844466|rs7692977|rs578597|rs735940|rs4832960|rs11251371|rs7633966|rs2388288
Neurologic Disorder	CELLCYCLE_PATHWAY	BIOCARTA	459	18	10	7,84E-01	9,90E-01	rs12370927|rs2374594|rs9369325|rs9315420|rs9368950|rs6073285|rs3897903|rs10849006|rs9380590|rs6872466
Neurologic Disorder	GLYCEROPHOSPHOLIPID_METABOLISM	KEGG	2549	36	20	7,85E-01	9,90E-01	rs10058172|rs11810204|rs12749748|rs7805162|rs884277|rs10912053|rs1558909|rs1076205|rs7903496|rs2609920|rs11899029|rs13008971|rs2268833|rs4573621|rs1800627|rs10486056|rs776686|rs1337988|rs36631|rs11046483
Neurologic Disorder	INTESTINAL_IMMUNE_NETWORK_FOR_IGA_PRODUCTION	KEGG	1392	47	20	7,85E-01	9,90E-01	rs6930052|rs10184153|rs16972387|rs2838515|rs13420393|rs10229203|rs9831894|rs3115961|rs11890854|rs3135021|rs7371043|rs1147963|rs12691883|rs17351243|rs543721|rs831864|rs11569333|rs4813003|rs1295686|rs4673268
Neurologic Disorder	ALZHEIMERS_DISEASE	KEGG	5029	30	20	7,85E-01	9,90E-01	rs2829949|rs11612284|rs170290|rs2239048|rs2300267|rs2423356|rs4854069|rs2950390|rs2076954|rs17545756|rs771941|rs2293055|rs886898|rs11055587|rs316369|rs2370413|rs3741403|rs13024804|rs3805018|rs1571729
Neurologic Disorder	SPHINGOLIPID_METABOLISM	REACTOME	1944	28	20	7,86E-01	9,90E-01	rs8041714|rs11810204|rs884277|rs7151140|rs6100061|rs168700|rs2008644|rs1436222|rs10483379|rs13263637|rs10133136|rs735678|rs587316|rs12054837|rs7521445|rs4834370|rs1426790|rs1191571|rs569523|rs12882426
Neurologic Disorder	AMINO_SUGAR_AND_NUCLEOTIDE_SUGAR_METABOLISM	KEGG	956	33	20	7,87E-01	9,90E-01	rs163918|rs476594|rs10951757|rs3780354|rs492620|rs2908297|rs11937734|rs12473304|rs4959620|rs6844466|rs7692977|rs820846|rs3823276|rs3800070|rs2922666|rs2608822|rs10046843|rs7897621|rs4985037|rs348500
Neurologic Disorder	SIGNALING_BY_FGFR_IN_DISEASE	REACTOME	4545	30	20	7,88E-01	9,90E-01	rs13402503|rs4963857|rs4557033|rs465531|rs1874326|rs581971|rs9531286|rs1851710|rs12948922|rs7321702|rs2360969|rs6872249|rs17472986|rs1545033|rs6503691|rs13023787|rs1962515|rs6003709|rs445775|rs11048776
Neurologic Disorder	MRNA_SPLICING_MINOR_PATHWAY	REACTOME	383	21	15	7,89E-01	9,90E-01	rs7544531|rs3821996|rs4916595|rs7217258|rs1076691|rs2304196|rs7306887|rs762283|rs626785|rs1107674|rs3171927|rs1613070|rs11240609|rs12977802|rs12479650

Neurologic Disorder	ACETYLCHOLINE_BINDING_AND_DOWNSTREAM_EVENTS	REACTOME	242	8	6	7,90E-01	9,90E-01	rs3791729|rs17360319|rs1550097|rs2063722|rs12442954|rs11735437
Neurologic Disorder	SIGNALING_BY_FGFR	REACTOME	4154	34	20	7,97E-01	9,90E-01	rs13402503|rs4963857|rs4557033|rs465531|rs581971|rs9531286|rs1851710|rs12948922|rs7321702|rs2360969|rs6872437|rs1545033|rs17472986|rs13023787|rs1962515|rs445775|rs6859263|rs11204910|rs7204987|rs660442
Neurologic Disorder	RNA_POL_II_PRE_TRANSCRIPTION_EVENTS	REACTOME	532	21	14	7,99E-01	9,90E-01	rs7524919|rs3821996|rs4916595|rs7217258|rs2304196|rs12151827|rs6089583|rs731945|rs1107674|rs1613070|rs7833229|rs2363127|rs12977802|rs6872466
Neurologic Disorder	PHOSPHOLIPASE_C_MEDIATED_CASCADE	REACTOME	2628	35	20	7,99E-01	9,90E-01	rs13402503|rs4557033|rs581971|rs1851710|rs12948922|rs2360969|rs1545033|rs17472986|rs13023787|rs445775|rs6859263|rs7204987|rs2320607|rs823616|rs9510005|rs9822061|rs3804158|rs7259810|rs9506807|rs506134
Neurologic Disorder	PIP3_ACTIVATES_AKT_SIGNALING	REACTOME	587	22	17	8,00E-01	9,90E-01	rs2360969|rs11204910|rs660442|rs8063461|rs7190284|rs1074078|rs9292729|rs9368950|rs3800230|rs945559|rs2721047|rs3812889|rs7322396|rs2302264|rs9380590|rs517665|rs17112705
Neurologic Disorder	PYRUVATE_METABOLISM_AND_CITRIC_ACID_TCA_CYCLE	REACTOME	905	56	20	8,04E-01	9,90E-01	rs4553987|rs12693008|rs2767038|rs12563264|rs10069201|rs132930|rs9649679|rs7992993|rs2295059|rs1216539|rs1340343|rs4331688|rs7611269|rs17022214|rs2555586|rs4638986|rs4657015|rs10184915|rs750595|rs6547449
Neurologic Disorder	WNT_SIGNALING_PATHWAY	KEGG	5611	35	20	8,05E-01	9,90E-01	rs1253035|rs1569736|rs11938486|rs1564115|rs2423356|rs997782|rs1884257|rs7893862|rs2241694|rs6573248|rs12948922|rs2866911|rs4332094|rs2142200|rs6573249|rs2568218|rs929701|rs771941|rs9902833|rs10494343
Neurologic Disorder	INSULIN_SIGNALING_PATHWAY	KEGG	3744	25	20	8,05E-01	9,90E-01	rs17703591|rs4963857|rs465531|rs4726096|rs17565737|rs1062708|rs3753242|rs4381535|rs4894936|rs6872249|rs1050700|rs10951757|rs1962515|rs3791729|rs660442|rs16830730|rs8063461|rs12462766|rs10413199|rs4917690
Neurologic Disorder	ELONGATION_ARREST_AND_RECOVERY	REACTOME	246	14	9	8,05E-01	9,90E-01	rs3821996|rs7217258|rs2304196|rs12151827|rs731945|rs1107674|rs1613070|rs2363127|rs12977802
Neurologic Disorder	CALCIUM_SIGNALING_PATHWAY	KEGG	9275	39	20	8,11E-01	9,90E-01	rs1046248|rs6794593|rs170290|rs10802789|rs6772419|rs11938486|rs2239048|rs1857586|rs2423356|rs919751|rs3011577|rs6704686|rs2241694|rs3110052|rs12948922|rs1551901|rs2076954|rs2079373|rs759169|rs771941
Neurologic Disorder	FORMATION_OF_TRANSCRIPTION_COUPLED_NER_TC_NER_REPAIR_COMPLEX	REACTOME	389	22	11	8,14E-01	9,90E-01	rs8054783|rs3821996|rs1573638|rs7217258|rs9925917|rs3745364|rs1107674|rs794078|rs12977802|rs16962919|rs6872466
Neurologic Disorder	INITIAL_TRIGGERING_OF_COMPLEMENT	REACTOME	434	22	12	8,16E-01	9,90E-01	rs2222547|rs1800450|rs12489890|rs10824650|rs12762596|rs10824829|rs10824854|rs3761008|rs10218945|rs1992528|rs710475|rs631090
Neurologic Disorder	SEMA4D_INDUCED_CELL_MIGRATION_AND_GROWTH_CONE_COLLAPSE	REACTOME	433	14	9	8,17E-01	9,90E-01	rs11647019|rs4669725|rs1651543|rs788332|rs2413459|rs4820273|rs8143159|rs8075851|rs17286488
Neurologic Disorder	SIGNALING_BY_ERBB2	REACTOME	3847	42	20	8,20E-01	9,90E-01	rs13402503|rs4963857|rs4557033|rs465531|rs17041245|rs6704686|rs12948922|rs2360969|rs6872249|rs759169|rs13023787|rs4807508|rs1962515|rs11204910|rs7204987|rs660442|rs8063461|rs16845944|rs12666347|rs9822061
Neurologic Disorder	THE_ROLE_OF_NEF_IN_HIV1_REPLICATION_AND_DISEASE_PATHOGENESIS	REACTOME	936	41	20	8,21E-01	9,90E-01	rs6583176|rs843346|rs6994201|rs9259806|rs10265823|rs12656935|rs11919795|rs2893542|rs3115961|rs10918708|rs7464428|rs6698995|rs6872596|rs2049233|rs6583177|rs10249209|rs12532146|rs7811413|rs6916861|rs2524005
Neurologic Disorder	PHOSPHORYLATION_OF_CD3_AND_TCR_ZETA_CHAINS	REACTOME	521	19	7	8,24E-01	9,90E-01	rs3782046|rs10918708|rs3135021|rs6698995|rs4436084|rs670197|rs2705499
Neurologic Disorder	SYNTHESIS_OF_PIPS_AT_THE_PLASMA_MEMBRANE	REACTOME	1050	33	18	8,24E-01	9,90E-01	rs465531|rs4744669|rs6872249|rs10000770|rs1423236|rs10505811|rs4470720|rs7671799|rs13125853|rs945559|rs9553572|rs3761431|rs12338099|rs651933|rs15362|rs648648|rs2055212|rs9356200
Neurologic Disorder	ABC_FAMILY_PROTEINS_MEDIATED_TRANSPORT	REACTOME	1753	45	20	8,25E-01	9,90E-01	rs4148483|rs9590125|rs3752237|rs2372479|rs246240|rs4148382|rs1470291|rs4245794|rs3758947|rs2067043|rs486879|rs13233308|rs16853324|rs7984157|rs903880|rs1008822|rs4148493|rs4672731|rs4245786|rs2238472
Neurologic Disorder	PML_PATHWAY	BIOCARTA	384	21	10	8,26E-01	9,90E-01	rs12471618|rs3754940|rs1926196|rs12694866|rs11885648|rs824918|rs930140|rs2238419|rs7085850|rs11076785
Neurologic Disorder	INOSITOL_PHOSPHATE_METABOLISM	KEGG	2355	42	20	8,27E-01	9,90E-01	rs1612612|rs2423356|rs2587616|rs4744669|rs771941|rs3741403|rs17596234|rs2955005|rs660442|rs10000770|rs970238|rs7271063|rs6108249|rs7915483|rs10841085|rs10505811|rs4519018|rs2315174|rs4405546|rs6077411
Neurologic Disorder	DOWNREGULATION_OF_SMAD2_3_SMAD4_TRANSCRIPTIONAL_ACTIVITY	REACTOME	453	27	19	8,27E-01	9,90E-01	rs266359|rs3219110|rs223366|rs12580394|rs1662820|rs9635893|rs8032542|rs12119470|rs6664266|rs12605548|rs4435493|rs3761034|rs7304356|rs1792684|rs11610586|rs10858047|rs2293464|rs446304|rs920293
Neurologic Disorder	PI3K_EVENTS_IN_ERBB4_SIGNALING	REACTOME	1709	32	20	8,27E-01	9,90E-01	rs465531|rs6704686|rs2360969|rs6872437|rs11204910|rs660442|rs8063461|rs16845944|rs1423236|rs4470720|rs7190284|rs17646307|rs17669781|rs4733122|rs900102|rs1074078|rs9292729|rs4281084|rs10503913|rs9368950
Neurologic Disorder	INSULIN_SYNTHESIS_AND_PROCESSING	REACTOME	1033	29	20	8,28E-01	9,90E-01	rs10483681|rs1033807|rs1150824|rs3737580|rs7734417|rs2247114|rs13436110|rs908024|rs13242614|rs7081888|rs1203336|rs4275150|rs835259|rs4960355|rs4691212|rs4865041|rs2145915|rs2476842|rs4959352|rs6693456
Neurologic Disorder	TRANSLATION	REACTOME	1803	29	20	8,28E-01	9,90E-01	rs7219464|rs6806377|rs1011382|rs3826322|rs1149747|rs2976044|rs1115219|rs8082649|rs1811384|rs4690696|rs11755449|rs12541647|rs36631|rs1093677|rs4789626|rs6791696|rs137653|rs843358|rs11775744|rs2140386
Neurologic Disorder	INTERFERON_SIGNALING	REACTOME	3448	38	20	8,30E-01	9,90E-01	rs11938486|rs13431075|rs4678192|rs2241694|rs13191519|rs1211304|rs10891524|rs1000940|rs7755852|rs7970893|rs905639|rs441437|rs16926995|rs3791729|rs9258895|rs283525|rs3754940|rs13331155|rs2350623|rs2284552
Neurologic Disorder	GLYCEROPHOSPHOLIPID_BIOSYNTHESIS	REACTOME	2483	35	20	8,30E-01	9,90E-01	rs12758601|rs10058172|rs12749748|rs10912053|rs1076205|rs7903496|rs1971516|rs2609920|rs600415|rs13008971|rs4573621|rs1800627|rs776686|rs3810622|rs1337988|rs36631|rs11046483|rs2075609|rs4650729|rs10182021
Neurologic Disorder	AMINO_ACID_TRANSPORT_ACROSS_THE_PLASMA_MEMBRANE	REACTOME	1025	37	19	8,31E-01	9,90E-01	rs9508477|rs10862814|rs4567513|rs10446439|rs4283235|rs739431|rs9551699|rs313853|rs1450453|rs12316387|rs10490211|rs2601011|rs12488144|rs7214236|rs12638941|rs299133|rs6706514|rs3904922|rs1215964
Neurologic Disorder	GPVI_MEDIATED_ACTIVATION_CASCADE	REACTOME	1181	34	20	8,32E-01	9,90E-01	rs461970|rs465531|rs3753242|rs6872437|rs10239535|rs1423236|rs4470720|rs4405546|rs7197601|rs10235102|rs12547683|rs73143|rs4243218|rs10993754|rs2413459|rs4820273|rs12036736|rs6916861|rs308195|rs42530
Neurologic Disorder	NDKDYNAMIN_PATHWAY	BIOCARTA	548	30	20	8,32E-01	9,90E-01	rs11887389|rs843346|rs3768857|rs2080284|rs7259810|rs11919795|rs11761049|rs11760351|rs17828764|rs2028211|rs934826|rs6947865|rs659023|rs6733907|rs310441|rs2732510|rs648648|rs9356200|rs2201677|rs17370523
Neurologic Disorder	APOPTOTIC_EXECUTION_PHASE	REACTOME	1211	30	20	8,33E-01	9,90E-01	rs11714395|rs4678192|rs6583176|rs2309428|rs1800627|rs4353229|rs4582459|rs9513432|rs6550461|rs4750565|rs661891|rs1467967|rs2431238|rs7016497|rs10904914|rs2049233|rs5006364|rs336611|rs9879508|rs12868939
Neurologic Disorder	AXON_GUIDANCE	KEGG	9506	30	20	8,33E-01	9,90E-01	rs2938769|rs2219556|rs1437642|rs1569736|rs7613488|rs4963857|rs10490697|rs17187720|rs7674285|rs12602314|rs7605126|rs3759929|rs728637|rs13144749|rs10222369|rs10015865|rs7916174|rs4696666|rs1816793|rs1918638
Neurologic Disorder	DRUG_METABOLISM_CYTOCHROME_P450	KEGG	1661	31	20	8,34E-01	9,90E-01	rs3818562|rs11249454|rs7551594|rs2297763|rs4965732|rs6464540|rs13114510|rs9296695|rs678040|rs2602218|rs9474314|rs7497607|rs4916273|rs11090076|rs12580698|rs2514051|rs10912598|rs2287396|rs10027817|rs16834034
Neurologic Disorder	VOLTAGE_GATED_POTASSIUM_CHANNELS	REACTOME	2491	37	20	8,36E-01	9,90E-01	rs3779972|rs526654|rs10092842|rs1891716|rs10832305|rs1605128|rs1874952|rs10504537|rs11779500|rs991436|rs2471637|rs17803126|rs12036302|rs757511|rs735636|rs4902186|rs7825542|rs12470356|rs12735839|rs13087932
Neurologic Disorder	EFFECTS_OF_PIP2_HYDROLYSIS	REACTOME	1651	42	20	8,36E-01	9,90E-01	rs13402503|rs4557033|rs7805162|rs1558909|rs13023787|rs2268833|rs10486056|rs4634526|rs506134|rs183984|rs2239624|rs4750565|rs1989820|rs661891|rs17653133|rs10904914|rs17834568|rs585156|rs1868388|rs1541612
Neurologic Disorder	RNA_POL_II_TRANSCRIPTION	REACTOME	1050	29	20	8,37E-01	9,90E-01	rs7524919|rs3972|rs1783983|rs3821996|rs575274|rs4916595|rs7217258|rs1039322|rs1076691|rs2304196|rs17311679|rs7306887|rs12151827|rs762283|rs6089591|rs731945|rs1107674|rs17492709|rs1613070|rs7833229
Neurologic Disorder	LONG_TERM_DEPRESSION	KEGG	3926	25	20	8,38E-01	9,90E-01	rs4963857|rs1457596|rs2423356|rs12749748|rs2038916|rs12948922|rs998133|rs10912053|rs11765386|rs7688672|rs17549153|rs771941|rs1076205|rs2293055|rs2128650|rs1962515|rs3741403|rs1937690|rs3805018|rs660442
Neurologic Disorder	INTERFERON_GAMMA_SIGNALING	REACTOME	2043	37	20	8,39E-01	9,90E-01	rs11938486|rs2241694|rs13191519|rs10891524|rs7755852|rs7970893|rs16926995|rs9258895|rs3754940|rs13331155|rs1425802|rs12694866|rs11885648|rs28367729|rs1436109|rs1467558|rs3135021|rs1489595|rs9430018|rs9266440
Neurologic Disorder	REGULATION_OF_HYPOXIA_INDUCIBLE_FACTOR_HIF_BY_OXYGEN	REACTOME	443	19	12	8,40E-01	9,90E-01	rs797377|rs3738483|rs223366|rs8102595|rs4435493|rs12435848|rs9462937|rs3761034|rs2238419|rs1358980|rs11076785|rs2495718
Neurologic Disorder	G1_S_SPECIFIC_TRANSCRIPTION	REACTOME	194	5	3	8,40E-01	9,90E-01	rs9315420|rs1650647|rs7585544
Neurologic Disorder	MITOTIC_G2_G2_M_PHASES	REACTOME	1389	53	20	8,40E-01	9,90E-01	rs12594262|rs720336|rs9679654|rs7969578|rs1129880|rs9550130|rs9315420|rs11109097|rs3844450|rs2289815|rs3828569|rs989264|rs8258|rs17751083|rs1582341|rs10046843|rs11038993|rs2277663|rs4790353|rs3017756
Neurologic Disorder	GLYCOLYSIS_GLUCONEOGENESIS	KEGG	1391	28	20	8,41E-01	9,90E-01	rs3794492|rs4578194|rs7551594|rs10951757|rs2207632|rs4878784|rs17158495|rs17158795|rs4965732|rs2908297|rs2075876|rs10989500|rs4582459|rs6844466|rs7692977|rs971524|rs2922666|rs161626|rs7897621|rs578597
Neurologic Disorder	RECRUITMENT_OF_MITOTIC_CENTROSOME_PROTEINS_AND_COMPLEXES	REACTOME	1177	53	20	8,41E-01	9,90E-01	rs12594262|rs720336|rs7969578|rs1129880|rs9550130|rs11109097|rs3844450|rs2289815|rs3828569|rs989264|rs8258|rs17751083|rs1582341|rs10972175|rs11038993|rs2277663|rs4790353|rs3017756|rs11666474|rs435359
Neurologic Disorder	PI_METABOLISM	REACTOME	1808	44	20	8,42E-01	9,90E-01	rs465531|rs2587616|rs4744669|rs6872249|rs17596234|rs4428539|rs10020322|rs970238|rs1423236|rs10505811|rs4470720|rs2315174|rs5751684|rs1866046|rs17767371|rs11994010|rs7671799|rs13125853|rs1496814|rs945559
Neurologic Disorder	FORMATION_OF_THE_HIV1_EARLY_ELONGATION_COMPLEX	REACTOME	329	12	9	8,43E-01	9,90E-01	rs3821996|rs4916595|rs7217258|rs2304196|rs1107674|rs1613070|rs2363127|rs12977802|rs6872466
Neurologic Disorder	BUTANOATE_METABOLISM	KEGG	763	33	20	8,43E-01	9,90E-01	rs4578194|rs7551594|rs2686110|rs4878784|rs4728329|rs11058085|rs2313427|rs6798107|rs4765026|rs971524|rs3775972|rs3774051|rs6497490|rs2107232|rs4857595|rs11047649|rs9937726|rs1629679|rs1641002|rs12825376
Neurologic Disorder	NO2IL12_PATHWAY	BIOCARTA	461	25	14	8,44E-01	9,90E-01	rs12944039|rs1799988|rs3782046|rs540722|rs10918708|rs6698995|rs4493656|rs17129782|rs10515794|rs7258015|rs662959|rs7636840|rs17810546|rs7250425
Neurologic Disorder	FORMATION_OF_TUBULIN_FOLDING_INTERMEDIATES_BY_CCT_TRIC	REACTOME	227	5	5	8,45E-01	9,90E-01	rs3844450|rs710750|rs11893547|rs1937746|rs151358
Neurologic Disorder	TGF_BETA_SIGNALING_PATHWAY	KEGG	3535	33	20	8,45E-01	9,90E-01	rs13105878|rs7552931|rs16877483|rs4332094|rs2142200|rs11070217|rs6763683|rs733664|rs9505962|rs4455423|rs13271702|rs2247450|rs4129666|rs4952316|rs7568633|rs9367669|rs6431907|rs2395851|rs6783860|rs4664888
Neurologic Disorder	BIOSYNTHESIS_OF_UNSATURATED_FATTY_ACIDS	KEGG	463	15	12	8,46E-01	9,90E-01	rs7744|rs174449|rs11737840|rs11254654|rs2357221|rs509360|rs2054780|rs2239621|rs1929492|rs10012953|rs4693052|rs2229594
Neurologic Disorder	TYPE_I_DIABETES_MELLITUS	KEGG	1975	42	20	8,48E-01	9,90E-01	rs6930052|rs13191519|rs7755852|rs9258895|rs11765127|rs1926196|rs9831894|rs28367729|rs3115961|rs2366831|rs540722|rs3135021|rs10256092|rs9266440|rs3094691|rs4493656|rs4691212|rs6972776|rs9654699|rs10515794
Neurologic Disorder	TIGHT_JUNCTION_INTERACTIONS	REACTOME	715	28	16	8,49E-01	9,90E-01	rs6685516|rs4616804|rs11265544|rs9865021|rs3844450|rs9981696|rs2057227|rs2788616|rs11207865|rs5748285|rs7999180|rs17462455|rs16865412|rs3011901|rs4506761|rs10804925
Neurologic Disorder	PROGESTERONE_MEDIATED_OOCYTE_MATURATION	KEGG	2535	33	20	8,50E-01	9,90E-01	rs4963857|rs465531|rs1457596|rs16930959|rs6872249|rs3730327|rs1962515|rs6903559|rs7204987|rs1129880|rs6742309|rs9315420|rs9822061|rs1423236|rs12706724|rs739431|rs4470720|rs6219|rs7303830|rs17842119
Neurologic Disorder	NITROGEN_METABOLISM	KEGG	587	15	10	8,52E-01	9,90E-01	rs10797768|rs3770685|rs289806|rs11577155|rs422914|rs2611339|rs11646115|rs1229568|rs541902|rs1025607
Neurologic Disorder	ADHERENS_JUNCTION	KEGG	4520	38	20	8,52E-01	9,90E-01	rs6757169|rs1941136|rs656068|rs482533|rs1078631|rs1884257|rs471398|rs9873710|rs2568218|rs759169|rs10769317|rs9902833|rs7754397|rs570521|rs266359|rs176632|rs1368909|rs6783860|rs17017249|rs4902681
Neurologic Disorder	TRANS_GOLGI_NETWORK_VESICLE_BUDDING	REACTOME	1106	36	20	8,53E-01	9,90E-01	rs2025880|rs2041374|rs6988243|rs611908|rs26532|rs3799765|rs7181201|rs10805919|rs17271418|rs10789179|rs3802680|rs4919626|rs597619|rs11792652|rs4245633|rs745554|rs7755435|rs15958|rs10810812|rs6080886
Neurologic Disorder	CITRATE_CYCLE_TCA_CYCLE	KEGG	1195	48	20	8,54E-01	9,90E-01	rs4553987|rs4442231|rs7122539|rs12563264|rs2888998|rs1556138|rs1258191|rs9649679|rs7992993|rs971524|rs2295059|rs1340343|rs4331688|rs7611269|rs17022214|rs4638986|rs6816|rs2375107|rs4657015|rs10184915
Neurologic Disorder	PEROXISOMAL_LIPID_METABOLISM	REACTOME	435	15	10	8,55E-01	9,90E-01	rs7744|rs11598878|rs7808249|rs11050116|rs591670|rs2239621|rs13434020|rs12368136|rs10219485|rs7092925
Neurologic Disorder	BASIGIN_INTERACTIONS	REACTOME	595	18	11	8,55E-01	9,90E-01	rs12461927|rs2191502|rs4329925|rs132930|rs1450453|rs1216539|rs299133|rs1200131|rs7251432|rs5749696|rs10737544
Neurologic Disorder	GLYCOSAMINOGLYCAN_DEGRADATION	KEGG	709	21	15	8,55E-01	9,90E-01	rs11729350|rs2218348|rs3785690|rs12054837|rs569523|rs820846|rs12672990|rs234910|rs15958|rs3846677|rs11076720|rs1029682|rs4919234|rs2323178|rs11078168
Neurologic Disorder	CIRCADIAN_CLOCK	REACTOME	2075	46	20	8,57E-01	9,90E-01	rs17153071|rs11953993|rs11746572|rs2360969|rs12330015|rs9818162|rs6007640|rs3816358|rs7659060|rs16937001|rs11694387|rs17237486|rs3776841|rs7566401|rs12442490|rs13025524|rs10514306|rs12521436|rs4435493|rs17024869
Neurologic Disorder	MAPK_SIGNALING_PATHWAY	KEGG	9833	33	20	8,58E-01	9,90E-01	rs4698855|rs6692647|rs1569736|rs4747345|rs2239048|rs4963857|rs12749729|rs2013444|rs3912834|rs7198466|rs11953993|rs17041245|rs919751|rs1884257|rs7116712|rs4851543|rs12749748|rs7477742|rs1979050|rs1851710
Neurologic Disorder	NKT_PATHWAY	BIOCARTA	638	29	20	8,60E-01	9,90E-01	rs6692647|rs4513489|rs12943654|rs3115961|rs1491961|rs540722|rs11890854|rs7371043|rs4493656|rs17129782|rs12691883|rs10515794|rs543721|rs3850209|rs7156293|rs662959|rs7636840|rs1295686|rs17810546|rs4673268
Neurologic Disorder	PTM_GAMMA_CARBOXYLATION_HYPUSINE_FORMATION_AND_ARYLSULFATASE_ACTIV	REACTOME	377	21	12	8,61E-01	9,90E-01	rs3759929|rs12054837|rs4834370|rs569523|rs4286653|rs793396|rs234910|rs3846677|rs11714594|rs3211719|rs722572|rs10445209
Neurologic Disorder	EICOSANOID_LIGAND_BINDING_RECEPTORS	REACTOME	507	11	6	8,63E-01	9,90E-01	rs12651829|rs803012|rs8005977|rs6701594|rs2241362|rs4603079
Neurologic Disorder	NGF_SIGNALLING_VIA_TRKA_FROM_THE_PLASMA_MEMBRANE	REACTOME	4847	38	20	8,64E-01	9,90E-01	rs13402503|rs4963857|rs4557033|rs465531|rs2013444|rs2025880|rs11953993|rs12948922|rs11746572|rs2360969|rs6872437|rs8041078|rs3730327|rs13023787|rs1962515|rs843346|rs6903559|rs11204910|rs7204987|rs1605504
Neurologic Disorder	METABOLISM_OF_CARBOHYDRATES	REACTOME	9072	23	20	8,65E-01	9,90E-01	rs9556369|rs1445270|rs2575738|rs1119017|rs7250851|rs309556|rs13431075|rs9561545|rs12750012|rs1948234|rs11729350|rs1007973|rs1539143|rs721976|rs1062708|rs948360|rs4802841|rs1929035|rs7018169|rs4981034
Neurologic Disorder	CITRIC_ACID_CYCLE_TCA_CYCLE	REACTOME	675	39	15	8,65E-01	9,90E-01	rs4553987|rs12563264|rs10069201|rs9649679|rs7992993|rs2295059|rs1340343|rs4331688|rs7611269|rs17022214|rs4638986|rs4657015|rs10184915|rs750595|rs6547449
Neurologic Disorder	PARKINSONS_DISEASE	KEGG	2833	33	20	8,65E-01	9,90E-01	rs2190117|rs4854069|rs2950390|rs13191310|rs9346906|rs9355357|rs12524235|rs3798964|rs4922132|rs11089296|rs13024804|rs4975646|rs480211|rs3746106|rs1039122|rs3753362|rs7249519|rs12538107|rs363397|rs2736994
Neurologic Disorder	INFLAM_PATHWAY	BIOCARTA	747	27	15	8,68E-01	9,90E-01	rs7552931|rs575330|rs10229203|rs540722|rs4493656|rs7540934|rs17351243|rs10515794|rs7529675|rs2010128|rs7156293|rs662959|rs7636840|rs1295686|rs17810546
Neurologic Disorder	FORMATION_OF_RNA_POL_II_ELONGATION_COMPLEX_	REACTOME	401	16	11	8,68E-01	9,90E-01	rs3821996|rs4916595|rs7217258|rs2304196|rs12151827|rs731945|rs1107674|rs1613070|rs2363127|rs12977802|rs6872466
Neurologic Disorder	N_GLYCAN_ANTENNAE_ELONGATION_IN_THE_MEDIAL_TRANS_GOLGI	REACTOME	962	30	15	8,71E-01	9,90E-01	rs16946687|rs2985689|rs3784722|rs2321546|rs2595361|rs4141587|rs1683542|rs11886886|rs11629679|rs729169|rs11104005|rs11692586|rs31614|rs750592|rs6444192
Neurologic Disorder	GOLGI_ASSOCIATED_VESICLE_BIOGENESIS	REACTOME	1057	38	20	8,74E-01	9,90E-01	rs2025880|rs2041374|rs6988243|rs611908|rs26532|rs3799765|rs7181201|rs10805919|rs17271418|rs10789179|rs597619|rs11792652|rs4245633|rs745554|rs7755435|rs10810812|rs6080886|rs4927870|rs659023|rs742208
Neurologic Disorder	POST_TRANSLATIONAL_PROTEIN_MODIFICATION	REACTOME	6008	26	20	8,74E-01	9,90E-01	rs6776064|rs1119017|rs16946687|rs7572142|rs7586009|rs7769107|rs16841278|rs3759929|rs7550651|rs7018169|rs1918638|rs7000884|rs10819377|rs10006633|rs209256|rs799770|rs17108729|rs7953158|rs12793968|rs34088
Neurologic Disorder	DESTABILIZATION_OF_MRNA_BY_TRISTETRAPROLIN_TTP	REACTOME	179	7	5	8,76E-01	9,90E-01	rs10425594|rs11130040|rs9628|rs4382556|rs3740526
Neurologic Disorder	ANTIGEN_PROCESSING_UBIQUITINATION_PROTEASOME_DEGRADATION	REACTOME	4788	30	20	8,77E-01	9,90E-01	rs704375|rs7259473|rs3444|rs16930959|rs11922435|rs10200577|rs13191310|rs9346906|rs1947240|rs400223|rs9355357|rs4894936|rs17102087|rs11727661|rs10511272|rs2714747|rs12524235|rs13338214|rs1559631|rs12598046
Neurologic Disorder	CHREBP2_PATHWAY	BIOCARTA	1059	30	20	8,78E-01	9,90E-01	rs4726096|rs10951757|rs11156546|rs3776841|rs12675103|rs10278273|rs2908297|rs673995|rs7776530|rs7778356|rs17145817|rs11727636|rs7069785|rs726558|rs2922666|rs9697030|rs4726099|rs9650417|rs736449|rs11666474
Neurologic Disorder	REGULATION_OF_IFNA_SIGNALING	REACTOME	339	14	6	8,78E-01	9,90E-01	rs2284552|rs4978113|rs2834168|rs525563|rs10125010|rs7258015
Neurologic Disorder	SEMA4D_IN_SEMAPHORIN_SIGNALING	REACTOME	597	19	11	8,79E-01	9,90E-01	rs11647019|rs4669725|rs1651543|rs10972530|rs788332|rs670197|rs2413459|rs4820273|rs5997917|rs8075851|rs17286488
Neurologic Disorder	AMINO_ACID_AND_OLIGOPEPTIDE_SLC_TRANSPORTERS	REACTOME	1707	32	20	8,80E-01	9,90E-01	rs9508477|rs10862814|rs7022772|rs9634511|rs16927467|rs11033098|rs4567513|rs10446439|rs11110371|rs16987501|rs927352|rs739431|rs4283235|rs9551699|rs313853|rs4742014|rs1450453|rs301434|rs1289393|rs7958082
Neurologic Disorder	IL6_PATHWAY	BIOCARTA	401	18	10	8,82E-01	9,90E-01	rs1962515|rs11159135|rs10229203|rs4776783|rs6037986|rs525563|rs7258015|rs4811006|rs1882832|rs8013053
Neurologic Disorder	EARLY_PHASE_OF_HIV_LIFE_CYCLE	REACTOME	270	11	10	8,84E-01	9,90E-01	rs1799988|rs11890854|rs7371043|rs509360|rs2304136|rs12691883|rs543721|rs1776897|rs174556|rs3777018
Neurologic Disorder	MET_PATHWAY	BIOCARTA	1415	29	20	8,84E-01	9,90E-01	rs465531|rs6872437|rs1962515|rs7905277|rs6592724|rs7906619|rs1423236|rs11953892|rs11159135|rs4470720|rs11017910|rs4776783|rs12771596|rs7735400|rs4464906|rs6837153|rs945559|rs868567|rs7016497|rs8181424
Neurologic Disorder	CLASS_B_2_SECRETIN_FAMILY_RECEPTORS	REACTOME	2257	31	20	8,84E-01	9,90E-01	rs10907192|rs10228431|rs997782|rs12300399|rs10255835|rs2331226|rs3757552|rs1605504|rs6459975|rs1458806|rs2923088|rs4898712|rs4794013|rs1332010|rs9371268|rs1507727|rs9305046|rs557077|rs17212187|rs1480524
Neurologic Disorder	NEUROTRANSMITTER_RELEASE_CYCLE	REACTOME	1400	42	20	8,85E-01	9,90E-01	rs2924149|rs7022772|rs7903496|rs363397|rs16987501|rs1845181|rs7616006|rs1361310|rs13020302|rs2313427|rs4742014|rs7036061|rs6927917|rs301434|rs301431|rs1682821|rs10491734|rs617217|rs6753886|rs16921457
Neurologic Disorder	TRIGLYCERIDE_BIOSYNTHESIS	REACTOME	1182	30	20	8,85E-01	9,90E-01	rs10058172|rs1971516|rs2609920|rs7667067|rs4573621|rs1337988|rs10182021|rs11737840|rs11195813|rs12546427|rs1332793|rs11195905|rs12992291|rs6535510|rs149293|rs9307828|rs6741502|rs2293286|rs13159372|rs1453496
Neurologic Disorder	CHONDROITIN_SULFATE_BIOSYNTHESIS	REACTOME	846	32	19	8,86E-01	9,90E-01	rs309556|rs17035996|rs11768384|rs1046650|rs11985507|rs309599|rs4661210|rs3802330|rs924584|rs4929810|rs17481063|rs2958595|rs12456123|rs835489|rs1485803|rs2975419|rs12582801|rs7919939|rs3026785
Neurologic Disorder	INCRETIN_SYNTHESIS_SECRETION_AND_INACTIVATION	REACTOME	540	32	18	8,89E-01	9,90E-01	rs16878921|rs4690696|rs4794013|rs7231030|rs4510815|rs4841578|rs2239512|rs7734417|rs4865694|rs6976221|rs4731437|rs6802872|rs2898292|rs4940851|rs936431|rs9810950|rs12331128|rs1062219
Neurologic Disorder	PKA_MEDIATED_PHOSPHORYLATION_OF_CREB	REACTOME	767	23	12	8,90E-01	9,90E-01	rs2360969|rs7204987|rs9822061|rs2531982|rs12548296|rs7009064|rs4630884|rs2238455|rs1540620|rs1865550|rs4736520|rs1344840
Neurologic Disorder	MRNA_CAPPING	REACTOME	388	14	10	8,90E-01	9,90E-01	rs3821996|rs4916595|rs7217258|rs2304196|rs1107674|rs17492709|rs1613070|rs2748178|rs12977802|rs6872466
Neurologic Disorder	XENOBIOTICS	REACTOME	231	2	1	8,91E-01	9,90E-01	rs11090076
Neurologic Disorder	PLATELET_HOMEOSTASIS	REACTOME	4080	25	20	8,91E-01	9,90E-01	rs10907192|rs705789|rs9856750|rs12749748|rs2038916|rs2269142|rs998133|rs10912053|rs7688672|rs3730327|rs17549153|rs2293055|rs12944039|rs282880|rs2128650|rs10495717|rs3432|rs35674|rs1937690|rs7281786
Neurologic Disorder	RESPIRATORY_ELECTRON_TRANSPORT_ATP_SYNTHESIS_BY_CHEMIOSMOTIC_COUPLING	REACTOME	1741	29	20	8,93E-01	9,90E-01	rs4854069|rs2950390|rs13024804|rs12461410|rs480211|rs3746106|rs3753362|rs7249519|rs12538107|rs2075609|rs7108613|rs1031609|rs266805|rs10514609|rs4805324|rs4808206|rs10486095|rs2159077|rs2295059|rs254262

	TION_BY_UNCOUPLING_PROTEINS_							
Neurologic Disorder	LYSOSOME	KEGG	2448	29	20	8,97E-01	9,90E-01	rs2025880|rs2457684|rs577866|rs3803533|rs7151140|rs4690513|rs10824092|rs168700|rs6994201|rs1903568|rs13263637|rs12508223|rs4581040|rs10520381|rs26532|rs12054837|rs7181201|rs2075609|rs10805919|rs161353
Neurologic Disorder	INTEGRIN_ALPHAIIB_BETA3_SIGNALING	REACTOME	779	22	14	8,97E-01	9,90E-01	rs2292867|rs2060773|rs10741114|rs12265119|rs10203199|rs17653133|rs6056|rs7016497|rs3769234|rs10993754|rs2238142|rs11815280|rs3006835|rs11785444
Neurologic Disorder	ENDOCYTOSIS	KEGG	6484	22	20	8,98E-01	9,90E-01	rs11068958|rs167564|rs360856|rs360891|rs2025880|rs17041245|rs919751|rs10102187|rs6704686|rs10520579|rs13191519|rs11714011|rs3753242|rs2914441|rs4459703|rs2565190|rs10517355|rs2959319|rs4744669|rs4894936
Neurologic Disorder	DAG_AND_IP3_SIGNALING	REACTOME	1644	36	20	8,98E-01	9,90E-01	rs13402503|rs4557033|rs12948922|rs2360969|rs13023787|rs7204987|rs9822061|rs7259810|rs506134|rs183984|rs2531982|rs12548296|rs16959227|rs1143960|rs585156|rs7009064|rs1868388|rs4630884|rs2238455|rs1540620
Neurologic Disorder	VASCULAR_SMOOTH_MUSCLE_CONTRACTION	KEGG	5845	27	20	8,99E-01	9,90E-01	rs13402503|rs6772419|rs2239048|rs4557033|rs2423356|rs10228431|rs9856750|rs12749748|rs2038916|rs12948922|rs998133|rs12046362|rs11765386|rs17549153|rs771941|rs1076205|rs11647019|rs886898|rs13023787|rs2331226
Neurologic Disorder	3_UTR_MEDIATED_TRANSLATIONAL_REGULATION	REACTOME	1235	32	20	9,01E-01	9,90E-01	rs7219464|rs3826322|rs1149747|rs2976044|rs8082649|rs1811384|rs36631|rs4789626|rs6791696|rs137653|rs2140386|rs4435493|rs16995300|rs1570026|rs1302|rs219446|rs3829570|rs7688462|rs12632423|rs3761034
Neurologic Disorder	BETA_ALANINE_METABOLISM	KEGG	711	32	17	9,03E-01	9,90E-01	rs4578194|rs7551594|rs6683957|rs4878784|rs2313427|rs3002273|rs11165823|rs3774051|rs10493889|rs290826|rs2669429|rs12120077|rs12464840|rs9937726|rs1629679|rs1641002|rs1641021
Neurologic Disorder	PRIMARY_BILE_ACID_BIOSYNTHESIS	KEGG	439	20	10	9,03E-01	9,90E-01	rs1906889|rs1957516|rs7906894|rs17361616|rs952884|rs11670764|rs13434020|rs6969282|rs1929492|rs2229594
Neurologic Disorder	P53HYPOXIA_PATHWAY	BIOCARTA	517	23	12	9,04E-01	9,90E-01	rs13426275|rs17584066|rs10193943|rs13233308|rs8077346|rs2453839|rs4470909|rs9368950|rs12435848|rs788735|rs9380590|rs1462282
Neurologic Disorder	GLYCOLYSIS	REACTOME	558	22	13	9,06E-01	9,90E-01	rs17158495|rs17158795|rs3776841|rs2075876|rs10989500|rs4582459|rs2387015|rs578597|rs2054780|rs11251371|rs11666474|rs602395|rs2388288
Neurologic Disorder	DIABETES_PATHWAYS	REACTOME	3395	28	20	9,07E-01	9,90E-01	rs6918969|rs10483681|rs9674544|rs4979619|rs1033807|rs836852|rs560577|rs1115219|rs4690696|rs6806768|rs7515586|rs909941|rs2730324|rs7030021|rs2555141|rs848194|rs673995|rs10119888|rs17584066|rs9957722
Neurologic Disorder	MITOTIC_PROMETAPHASE	REACTOME	1814	36	20	9,07E-01	9,90E-01	rs10917799|rs449397|rs9679654|rs2910469|rs35674|rs6769686|rs3776841|rs7975139|rs10742181|rs11636566|rs673995|rs10902882|rs1466962|rs11764590|rs12911848|rs726558|rs2549854|rs2495774|rs994626|rs11038993
Neurologic Disorder	CYTOKINE_SIGNALING_IN_IMMUNE_SYSTEM	REACTOME	6819	27	20	9,08E-01	9,90E-01	rs461970|rs11938486|rs4963857|rs465531|rs13431075|rs4678192|rs1884257|rs2241694|rs4851543|rs13191519|rs1211304|rs40383|rs10891524|rs575330|rs11869073|rs2440134|rs1000940|rs6872437|rs7744|rs7755852
Neurologic Disorder	GLUCOSE_METABOLISM	REACTOME	1554	26	20	9,09E-01	9,90E-01	rs1062708|rs11760823|rs7122539|rs8062660|rs17158495|rs17158795|rs11076318|rs3776841|rs7259810|rs6664173|rs3809676|rs2075876|rs9649679|rs10989500|rs4582459|rs6844466|rs7692977|rs29861|rs13245850|rs2387015
Neurologic Disorder	INTRINSIC_PATHWAY_FOR_APOPTOSIS	REACTOME	532	18	13	9,10E-01	9,90E-01	rs3862706|rs660442|rs4941185|rs4353229|rs2032809|rs4559081|rs12962644|rs1026825|rs17773680|rs1016860|rs8083946|rs12693932|rs2585838
Neurologic Disorder	REGULATION_OF_GENE_EXPRESSION_IN_BETA_CELLS	REACTOME	716	16	9	9,14E-01	9,92E-01	rs10951757|rs2908297|rs7953249|rs2922666|rs2721047|rs3812889|rs7322396|rs6065725|rs16939058
Neurologic Disorder	ASPARAGINE_N_LINKED_GLYCOSYLATION	REACTOME	2760	38	20	9,15E-01	9,92E-01	rs16946687|rs7572142|rs7586009|rs7769107|rs7000884|rs17108729|rs34088|rs10872983|rs35674|rs2881586|rs2985689|rs3784722|rs2730324|rs10516556|rs2321546|rs4982619|rs2595361|rs4659050|rs12092678|rs4141587
Neurologic Disorder	APOPTOTIC_CLEAVAGE_OF_CELLULAR_PROTEINS	REACTOME	1052	28	20	9,19E-01	9,92E-01	rs11714395|rs2309428|rs1800627|rs4353229|rs4582459|rs9513432|rs6550461|rs4750565|rs661891|rs1467967|rs2431238|rs7016497|rs10904914|rs5006364|rs336611|rs12868939|rs2488809|rs17773680|rs1801552|rs12555245
Neurologic Disorder	FORMATION_OF_THE_TERNARY_COMPLEX_AND_SUBSEQUENTLY_THE_43S_COMPLEX	REACTOME	677	23	15	9,19E-01	9,92E-01	rs1149747|rs2976044|rs1811384|rs36631|rs2140386|rs4435493|rs1570026|rs4900928|rs7254299|rs803686|rs2175376|rs11747683|rs11847329|rs16888536|rs2352907
Neurologic Disorder	PLC_BETA_MEDIATED_EVENTS	REACTOME	2313	29	20	9,21E-01	9,92E-01	rs2423356|rs12749748|rs12948922|rs2360969|rs10912053|rs771941|rs3741403|rs7204987|rs660442|rs9822061|rs6108249|rs7271063|rs7259810|rs12706724|rs739431|rs1938506|rs6077411|rs1108656|rs6108237|rs2531982
Neurologic Disorder	CTLA4_INHIBITORY_SIGNALING	REACTOME	447	12	9	9,21E-01	9,92E-01	rs3776841|rs9831894|rs673995|rs12547683|rs726558|rs7245158|rs736449|rs6916861|rs11666474
Neurologic Disorder	PHOSPHATIDYLINOSITOL_SIGNALING_SYSTEM	KEGG	3813	46	20	9,22E-01	9,92E-01	rs1612612|rs465531|rs2423356|rs2587616|rs12948922|rs7805162|rs1558909|rs4744669|rs6872437|rs771941|rs3741403|rs3805018|rs17596234|rs2955005|rs660442|rs2268833|rs10000770|rs970238|rs10486056|rs1423236
Neurologic Disorder	INTEGRIN_CELL_SURFACE_INTERACTIONS	REACTOME	2614	31	20	9,22E-01	9,92E-01	rs2437095|rs1265646|rs2292867|rs16975294|rs11070217|rs4145072|rs4285344|rs4773209|rs12700171|rs587409|rs2060773|rs1192201|rs334411|rs10904603|rs11265544|rs1934940|rs10239535|rs6492270|rs2069176|rs3772832
Neurologic Disorder	REGULATION_OF_INSULIN_SECRETION_BY_GLUCAGON_LIKE_PEPTIDE1	REACTOME	1388	29	20	9,23E-01	9,92E-01	rs10907192|rs2471637|rs2060773|rs4898712|rs17803126|rs1332010|rs9822061|rs735636|rs12470356|rs9630890|rs12614549|rs10203199|rs12548296|rs6674438|rs10180853|rs9470963|rs2024497|rs6513412|rs6510696|rs237484
Neurologic Disorder	SYNTHESIS_SECRETION_AND_INACTIVATION_OF_GLP1	REACTOME	363	18	11	9,24E-01	9,92E-01	rs4690696|rs7231030|rs2239512|rs7734417|rs6976221|rs4731437|rs6802872|rs4940851|rs936431|rs9810950|rs12331128
Neurologic Disorder	SYNTHESIS_OF_BILE_ACIDS_AND_BILE_SALTS	REACTOME	546	20	10	9,25E-01	9,92E-01	rs1906889|rs1957516|rs7906894|rs17361616|rs952884|rs11670764|rs13434020|rs6969282|rs1929492|rs2229594
Neurologic Disorder	METABOLISM_OF_AMINO_ACIDS_AND_DERIVATIVES	REACTOME	5246	39	20	9,28E-01	9,93E-01	rs704375|rs6539460|rs4662299|rs848080|rs10797768|rs11922435|rs1062708|rs2166480|rs400223|rs10836312|rs2059098|rs3972|rs4578194|rs158845|rs152395|rs2059693|rs7551594|rs13338214|rs12598046|rs7837357
Neurologic Disorder	REGULATION_OF_INSULIN_LIKE_GROWTH_FACTOR_IGF_ACTIVITY_BY_INSULIN_LIKE_G<br>DING_PROTEINS_IGFBPS	REACTOME	570	24	14	9,28E-01	9,93E-01	rs7515586|rs909941|rs10119888|rs17584066|rs2453839|rs4470909|rs12115454|rs788735|rs2206509|rs12137641|rs1247571|rs6478223|rs1462282|rs7855710
Neurologic Disorder	NUCLEAR_RECEPTOR_TRANSCRIPTION_PATHWAY	REACTOME	2750	28	20	9,29E-01	9,93E-01	rs10434131|rs2048546|rs13112423|rs4746781|rs17668665|rs12330015|rs6007640|rs1363292|rs3798577|rs1980211|rs9849581|rs4612653|rs12029406|rs1676303|rs17237486|rs9787810|rs10520008|rs4808611|rs12436385|rs16830505
Neurologic Disorder	CHOLESTEROL_BIOSYNTHESIS	REACTOME	259	4	3	9,32E-01	9,93E-01	rs2839128|rs1792326|rs2686187
Neurologic Disorder	SIGNALING_BY_BMP	REACTOME	918	37	18	9,33E-01	9,93E-01	rs733664|rs4455423|rs7560572|rs223366|rs1874572|rs9957562|rs12119470|rs2706055|rs13429725|rs2423161|rs4662505|rs12685826|rs1147700|rs7171416|rs1147695|rs1515263|rs9968396|rs4390543
Neurologic Disorder	JAK_STAT_SIGNALING_PATHWAY	KEGG	4346	37	20	9,34E-01	9,93E-01	rs512868|rs465531|rs9531286|rs10512692|rs4332094|rs7321702|rs2142200|rs575330|rs4894936|rs6872249|rs13354167|rs6503691|rs4129666|rs2395851|rs12370927|rs1947372|rs1047953|rs7835350|rs2244238|rs12548864
Neurologic Disorder	RESPIRATORY_ELECTRON_TRANSPORT	REACTOME	1631	33	20	9,35E-01	9,93E-01	rs4854069|rs13024804|rs12461410|rs480211|rs3753362|rs7249519|rs12538107|rs2075609|rs1031609|rs266805|rs10514609|rs4805324|rs4808206|rs10486095|rs2159077|rs2295059|rs254262|rs218969|rs2276986|rs17716170
Neurologic Disorder	CYTOKINE_CYTOKINE_RECEPTOR_INTERACTION	KEGG	6425	39	20	9,37E-01	9,93E-01	rs566416|rs6692647|rs887796|rs764833|rs17041245|rs919751|rs10512692|rs4851543|rs575330|rs2466178|rs159293|rs759169|rs4513489|rs216123|rs13354167|rs4455423|rs3811178|rs16972387|rs3741403|rs917846
Neurologic Disorder	CA_DEPENDENT_EVENTS	REACTOME	1487	40	20	9,39E-01	9,93E-01	rs12749748|rs12948922|rs2360969|rs12046362|rs7204987|rs9822061|rs7259810|rs1938506|rs2531982|rs12548296|rs16959227|rs6425147|rs1143960|rs1548796|rs10753000|rs1487492|rs7009064|rs4630884|rs7554410|rs2238455
Neurologic Disorder	CTNNB1_PHOSPHORYLATION_CASCADE	REACTOME	316	9	8	9,41E-01	9,93E-01	rs3776841|rs673995|rs726558|rs2431238|rs6802872|rs736449|rs11666474|rs9810950
Neurologic Disorder	HEDGEHOG_SIGNALING_PATHWAY	KEGG	2151	27	20	9,41E-01	9,93E-01	rs2195894|rs997782|rs3909324|rs13170938|rs3757552|rs9367669|rs6459975|rs17821630|rs1458806|rs16830730|rs6555976|rs557077|rs1374547|rs11609521|rs3734444|rs6092437|rs830958|rs17356059|rs584632|rs4849909
Neurologic Disorder	DESTABILIZATION_OF_MRNA_BY_BRF1	REACTOME	204	7	5	9,42E-01	9,93E-01	rs11130040|rs9628|rs4382556|rs12894045|rs3740526
Neurologic Disorder	FRUCTOSE_AND_MANNOSE_METABOLISM	KEGG	984	41	20	9,42E-01	9,93E-01	rs476594|rs4728329|rs17158495|rs17158795|rs6975650|rs2075876|rs10989500|rs4959620|rs11994010|rs3823276|rs3800070|rs2387015|rs9553572|rs7897621|rs578597|rs2054780|rs4985037|rs7788953|rs3823271|rs12197419
Neurologic Disorder	VASOPRESSIN_REGULATED_WATER_REABSORPTION	KEGG	1100	29	20	9,44E-01	9,94E-01	rs2360969|rs17374415|rs7204987|rs12421587|rs2863720|rs2127956|rs6499096|rs849314|rs2531982|rs4878538|rs6513412|rs7794304|rs10486581|rs2214097|rs2299269|rs2238455|rs10459005|rs1860758|rs7944909|rs10486588
Neurologic Disorder	INTERFERON_ALPHA_BETA_SIGNALING	REACTOME	1477	44	20	9,50E-01	9,97E-01	rs13191519|rs7755852|rs7970893|rs441437|rs9258895|rs13331155|rs2284552|rs28367729|rs4978113|rs9430018|rs9266440|rs2834168|rs3094691|rs525563|rs1077767|rs11073795|rs10125010|rs7190274|rs305059|rs12920485
Neurologic Disorder	BIOSYNTHESIS_OF_THE_N_GLYCAN_PRECURSOR_DOLICHOL_LIPID_LINKED_OLIGOSACC<br>RANSFER_TO_A_NASCENT_PROTEIN	REACTOME	355	13	7	9,51E-01	9,97E-01	rs12092678|rs6126126|rs3890845|rs4985037|rs12021563|rs628629|rs10041598
Neurologic Disorder	GALACTOSE_METABOLISM	KEGG	759	37	20	9,52E-01	9,97E-01	rs10951757|rs17158495|rs17158795|rs6975650|rs2908297|rs2075876|rs6844466|rs7692977|rs2922666|rs161626|rs12903690|rs10046843|rs7897621|rs4832960|rs12535134|rs7788953|rs11251371|rs10998700|rs750592|rs2388288
Neurologic Disorder	INTEGRATION_OF_PROVIRUS	REACTOME	122	2	2	9,53E-01	9,97E-01	rs1776897|rs3777018
Neurologic Disorder	TCA_CYCLE_AND_RESPIRATORY_ELECTRON_TRANSPORT	REACTOME	2599	33	20	9,54E-01	9,97E-01	rs4854069|rs2950390|rs4553987|rs12693008|rs2767038|rs13024804|rs12461410|rs480211|rs3746106|rs12563264|rs3753362|rs7249519|rs12538107|rs10069201|rs2075609|rs132930|rs7108613|rs9649679|rs1031609|rs266805
Neurologic Disorder	SEMAPHORIN_INTERACTIONS	REACTOME	2884	30	20	9,55E-01	9,97E-01	rs7675169|rs3759929|rs7916174|rs1918638|rs17545756|rs406898|rs6583176|rs11647019|rs1902409|rs1499582|rs6592724|rs4669725|rs3755847|rs1651543|rs6481838|rs1340113|rs2785284|rs10972530|rs32974|rs2463506
Neurologic Disorder	BMAL1_CLOCK_NPAS2_ACTIVATES_CIRCADIAN_EXPRESSION	REACTOME	1728	31	20	9,57E-01	9,97E-01	rs17153071|rs12330015|rs7636407|rs6007640|rs3816358|rs7659060|rs16937001|rs11694387|rs17237486|rs16943758|rs13025524|rs12521436|rs17024869|rs6811520|rs11730701|rs1302|rs4774388|rs2946399|rs7693492|rs1657800
Neurologic Disorder	GABA_SYNTHESIS_RELEASE_REUPTAKE_AND_DEGRADATION	REACTOME	746	22	16	9,59E-01	9,97E-01	rs2924149|rs16987501|rs1845181|rs1361310|rs2313427|rs6927917|rs1682821|rs892601|rs2950798|rs9937726|rs6077718|rs1629679|rs1641002|rs2744604|rs1641021|rs634986
Neurologic Disorder	G1_PHASE	REACTOME	625	25	16	9,61E-01	9,97E-01	rs12370927|rs2374594|rs9369325|rs3776841|rs4435493|rs9368950|rs6073285|rs3761034|rs2041074|rs3897903|rs10849006|rs9650417|rs11666474|rs9380590|rs6872466|rs9295464
Neurologic Disorder	EIF_PATHWAY	BIOCARTA	158	2	1	9,62E-01	9,97E-01	rs2250205
Neurologic Disorder	MTA3_PATHWAY	BIOCARTA	333	12	8	9,62E-01	9,97E-01	rs3798577|rs985192|rs1937746|rs1801552|rs6020218|rs4783676|rs2504067|rs758944
Neurologic Disorder	BRANCHED_CHAIN_AMINO_ACID_CATABOLISM	REACTOME	507	19	10	9,63E-01	9,97E-01	rs3122126|rs42099|rs6443842|rs492734|rs16928163|rs9443804|rs17454070|rs3805914|rs12464840|rs12974796
Neurologic Disorder	ACTIVATION_OF_THE_MRNA_UPON_BINDING_OF_THE_CAP_BINDING_COMPLEX_AND_EI<br>T_BINDING_TO_43S	REACTOME	780	24	16	9,63E-01	9,97E-01	rs1149747|rs2976044|rs1811384|rs36631|rs2140386|rs4435493|rs1570026|rs4900928|rs7254299|rs803686|rs2175376|rs13255392|rs11747683|rs11847329|rs16888536|rs2352907
Neurologic Disorder	BASAL_CELL_CARCINOMA	KEGG	2166	28	20	9,66E-01	9,99E-01	rs2195894|rs997782|rs2568218|rs843346|rs3757552|rs176632|rs6459975|rs1458806|rs16830730|rs12452196|rs11919795|rs7215016|rs4563070|rs557077|rs17212187|rs1480524|rs791464|rs11609521|rs6547602|rs17356059
Neurologic Disorder	GLUCAGON_SIGNALING_IN_METABOLIC_REGULATION	REACTOME	1044	31	20	9,69E-01	9,99E-01	rs10907192|rs7204987|rs4898712|rs9822061|rs9630890|rs2531982|rs12548296|rs6674438|rs2024497|rs6513412|rs6510696|rs7009064|rs4630884|rs2238455|rs1540620|rs1865550|rs4736520|rs1344840|rs6972935|rs6092708
Neurologic Disorder	PHOSPHOLIPID_METABOLISM	REACTOME	6226	34	20	9,69E-01	9,99E-01	rs12758601|rs10058172|rs465531|rs8041714|rs2587616|rs11810204|rs12749748|rs884277|rs12046362|rs7151140|rs4744669|rs6872437|rs6100061|rs168700|rs1076205|rs2008644|rs7903496|rs1971516|rs1436222|rs2609920
Neurologic Disorder	TRAF6_MEDIATED_IRF7_ACTIVATION	REACTOME	433	19	11	9,73E-01	1,00E+00	rs17212649|rs7865082|rs11118087|rs4978113|rs1077767|rs10125010|rs1516096|rs1267070|rs9900686|rs2238419|rs11076785
Neurologic Disorder	P53_PATHWAY	BIOCARTA	347	9	8	9,74E-01	1,00E+00	rs4941185|rs9368950|rs12962644|rs1026825|rs1016860|rs8083946|rs9380590|rs7287772
Neurologic Disorder	CIRCADIAN_REPRESSION_OF_EXPRESSION_BY_REV_ERBA	REACTOME	1358	31	20	9,74E-01	1,00E+00	rs12330015|rs9818162|rs6007640|rs3816358|rs16937001|rs11694387|rs17237486|rs12442490|rs13025524|rs17024869|rs6811520|rs11730701|rs1302|rs4774388|rs2946399|rs1657800|rs6012188|rs13043637|rs16828012|rs10851691
Neurologic Disorder	MEIOSIS	REACTOME	1674	46	20	9,76E-01	1,00E+00	rs2761367|rs16942|rs2697675|rs13175928|rs10088657|rs2459111|rs198838|rs8077346|rs6470522|rs9634672|rs6090153|rs9383626|rs6010789|rs3863241|rs1126432|rs401549|rs1890100|rs10513002|rs2763022|rs6452136
Neurologic Disorder	BILE_ACID_AND_BILE_SALT_METABOLISM	REACTOME	1176	58	20	9,77E-01	1,00E+00	rs12430125|rs10459302|rs1906889|rs1957516|rs7906894|rs17361616|rs7707569|rs2417940|rs1333286|rs7999223|rs952884|rs11670764|rs4772604|rs4498289|rs2575139|rs885520|rs13434020|rs6969282|rs1929492|rs1274749
Neurologic Disorder	RORA_ACTIVATES_CIRCADIAN_EXPRESSION	REACTOME	1377	31	20	9,77E-01	1,00E+00	rs12330015|rs9818162|rs6007640|rs3816358|rs16937001|rs11694387|rs17237486|rs1025676|rs13025524|rs17024869|rs6811520|rs11730701|rs1302|rs4774388|rs2946399|rs1657800|rs6012188|rs13043637|rs16828012|rs10851691
Neurologic Disorder	PLATELET_CALCIUM_HOMEOSTASIS	REACTOME	1156	23	14	9,79E-01	1,00E+00	rs35674|rs6502762|rs2042016|rs17271418|rs373857|rs1989820|rs380082|rs7623572|rs12615150|rs11679585|rs10771283|rs7139251|rs12650123|rs12485425
Neurologic Disorder	LOSS_OF_NLP_FROM_MITOTIC_CENTROSOMES	REACTOME	1100	36	19	9,80E-01	1,00E+00	rs720336|rs7969578|rs11109097|rs3844450|rs3828569|rs989264|rs8258|rs17751083|rs1582341|rs10972175|rs11038993|rs2277663|rs4790353|rs3017756|rs11666474|rs435359|rs758944|rs2281212|rs2138349
Neurologic Disorder	DEADENYLATION_DEPENDENT_MRNA_DECAY	REACTOME	622	17	13	9,82E-01	1,00E+00	rs9798936|rs1863000|rs11130040|rs36643|rs240542|rs715412|rs9628|rs4382556|rs789327|rs9855053|rs6550155|rs13255392|rs3740526
Neurologic Disorder	OOCYTE_MEIOSIS	KEGG	3114	32	20	9,86E-01	1,00E+00	rs11938486|rs1457596|rs16930959|rs2241694|rs6903559|rs7204987|rs3805018|rs1129880|rs6742309|rs6555976|rs6769686|rs9822061|rs3776841|rs7259810|rs12675103|rs7975139|rs10841085|rs9852752|rs6219|rs673995
Neurologic Disorder	MEIOTIC_SYNAPSIS	REACTOME	1031	40	18	9,89E-01	1,00E+00	rs16942|rs10088657|rs2459111|rs198838|rs6090153|rs9383626|rs6010789|rs6472716|rs1126432|rs1890100|rs10513002|rs2763022|rs2256191|rs1416388|rs884587|rs696516|rs9383986|rs17165293
Neurologic Disorder	TRANSPORT_OF_INORGANIC_CATIONS_ANIONS_AND_AMINO_ACIDS_OLIGOPEPTIDES	REACTOME	4051	33	20	9,90E-01	1,00E+00	rs838626|rs9508477|rs10862814|rs7022772|rs12523625|rs9634511|rs7150406|rs2051134|rs12912107|rs10755174|rs4839620|rs16927467|rs2385635|rs17607283|rs1182652|rs2602049|rs11033098|rs4672969|rs4567513|rs10446439
Neurologic Disorder	CYTOKINE_PATHWAY	BIOCARTA	491	12	10	9,91E-01	1,00E+00	rs10229203|rs11857713|rs540722|rs4493656|rs17351243|rs10515794|rs662959|rs7636840|rs1295686|rs17810546
Neurologic Disorder	DC_PATHWAY	BIOCARTA	573	15	12	9,92E-01	1,00E+00	rs10983712|rs540722|rs4493656|rs17351243|rs10515794|rs11569333|rs662959|rs7636840|rs4813003|rs1295686|rs17810546|rs913365
Neurologic Disorder	GLYCOSAMINOGLYCAN_BIOSYNTHESIS_HEPARAN_SULFATE	KEGG	2273	27	20	9,93E-01	1,00E+00	rs1948234|rs7014925|rs9364126|rs12508213|rs8063678|rs2218348|rs1390102|rs11633040|rs3785690|rs12280153|rs4946052|rs12641904|rs17862059|rs1948223|rs224463|rs7830231|rs3737580|rs10447360|rs4937917|rs11683275
Neurologic Disorder	GLYCOSAMINOGLYCAN_BIOSYNTHESIS_CHONDROITIN_SULFATE	KEGG	1372	26	20	9,93E-01	1,00E+00	rs17035996|rs9364126|rs8063678|rs3798592|rs12280153|rs11768384|rs11985507|rs3802330|rs10447360|rs4937917|rs4929810|rs17481063|rs12201109|rs9351798|rs2958595|rs6940223|rs835489|rs4937902|rs2975419|rs12445976
Neurologic Disorder	YAP1_AND_WWTR1_TAZ_STIMULATED_GENE_EXPRESSION	REACTOME	1047	36	18	9,95E-01	1,00E+00	rs12330015|rs9818162|rs6007640|rs16937001|rs11830449|rs17410829|rs6012188|rs13043637|rs16828012|rs9815008|rs2862632|rs1004448|rs4823529|rs13089230|rs486054|rs2238419|rs3921860|rs11076785
Neurologic Disorder	REGULATION_OF_WATER_BALANCE_BY_RENAL_AQUAPORINS	REACTOME	1393	28	20	9,96E-01	1,00E+00	rs10907192|rs7204987|rs4898712|rs9822061|rs9630890|rs2531982|rs12548296|rs6674438|rs4878538|rs2024497|rs6513412|rs6510696|rs7009064|rs4630884|rs2238455|rs1540620|rs1865550|rs4736520|rs12456750|rs1344840
Neurologic Disorder	N_GLYCAN_BIOSYNTHESIS	KEGG	1792	52	20	9,96E-01	1,00E+00	rs7769107|rs7000884|rs10872983|rs2985689|rs2321546|rs4982619|rs4659050|rs12092678|rs1683542|rs6126126|rs3890845|rs11886886|rs6017596|rs10483269|rs6031882|rs12681567|rs4789394|rs12021563|rs11692586|rs31614
Neurologic Disorder	TH1TH2_PATHWAY	BIOCARTA	535	16	16	9,98E-01	1,00E+00	rs9831894|rs3115961|rs540722|rs4493656|rs17129782|rs10515794|rs11569333|rs3850209|rs662959|rs7636840|rs4813003|rs1295686|rs17810546|rs4673268|rs3024672|rs7250425
Neurologic Disorder	RNA_POL_I_TRANSCRIPTION	REACTOME	937	20	5	9,98E-01	1,00E+00	rs198838|rs527544|rs2941256|rs6872466|rs16867179
Neurologic Disorder	AQUAPORIN_MEDIATED_TRANSPORT	REACTOME	1510	27	20	9,98E-01	1,00E+00	rs10907192|rs7204987|rs4898712|rs9822061|rs9630890|rs2531982|rs12548296|rs6674438|rs4878538|rs2024497|rs6513412|rs6510696|rs7009064|rs10518966|rs4630884|rs2238455|rs1540620|rs1865550|rs4736520|rs12456750
Neurologic Disorder	RNA_POL_I_TRANSCRIPTION_TERMINATION	REACTOME	335	2	2	9,99E-01	1,00E+00	rs6872466|rs16867179
Neurologic Disorder	RNA_POL_I_TRANSCRIPTION_INITIATION	REACTOME	427	3	3	1,00E+00	1,00E+00	rs2941256|rs6872466|rs16867179
Renal Disorder	MRNA_SPLICING_MINOR_PATHWAY	REACTOME	383	44	14	7,00E-04	4,33E-01	rs503156|rs1109009|rs3796531|rs6761|rs1212343|rs6132649|rs4916595|rs4942391|rs9916811|rs7982845|rs6734|rs3821996|rs6114527|rs2254883
Renal Disorder	VALINE_LEUCINE_AND_ISOLEUCINE_DEGRADATION	KEGG	1149	35	20	1,90E-03	4,33E-01	rs1006087|rs4657468|rs4646864|rs2444827|rs4578194|rs757141|rs837322|rs6784193|rs757140|rs4379190|rs12337623|rs3828090|rs1348335|rs1641002|rs10492559|rs7757271|rs9810603|rs3924411|rs16970653|rs10918228
Renal Disorder	BUTANOATE_METABOLISM	KEGG	763	44	20	2,90E-03	4,33E-01	rs1006087|rs4657468|rs6416611|rs4578194|rs6784193|rs1330581|rs2686067|rs4379190|rs12337623|rs3828090|rs8190612|rs6784865|rs1348335|rs1641002|rs9810603|rs10918228|rs6830624|rs6772127|rs12744386|rs876848
Renal Disorder	PYRUVATE_METABOLISM	KEGG	908	31	20	3,20E-03	4,33E-01	rs1006087|rs4657468|rs625566|rs4578194|rs12448432|rs12285344|rs6723088|rs654136|rs4379190|rs12337623|rs973639|rs1348335|rs949349|rs10918228|rs1937782|rs989925|rs262498|rs4732025|rs1016331|rs6025639
Renal Disorder	RNA_POL_II_TRANSCRIPTION	REACTOME	1050	48	20	3,40E-03	4,33E-01	rs1109009|rs3796531|rs1676996|rs6761|rs6508433|rs8009789|rs1212343|rs6132649|rs731945|rs4916595|rs234605|rs10917420|rs3748269|rs234587|rs17311679|rs4942391|rs6142711|rs9916811|rs8060037|rs7982845
Renal Disorder	PROPANOATE_METABOLISM	KEGG	1219	50	20	3,50E-03	4,33E-01	rs1006087|rs4657468|rs4646864|rs4578194|rs11715476|rs12232909|rs17070878|rs837322|rs6784193|rs4379190|rs12337623|rs2147502|rs1348335|rs1641002|rs10492559|rs9810603|rs1921032|rs10918228|rs7646423|rs752409
Renal Disorder	NEUROTRANSMITTER_RELEASE_CYCLE	REACTOME	1400	42	20	3,80E-03	4,33E-01	rs4980806|rs241905|rs12494778|rs2789588|rs713996|rs2051569|rs241707|rs1322183|rs130274|rs2228622|rs1330581|rs2697152|rs8190612|rs363222|rs6905900|rs1641002|rs911719|rs867687|rs7855993|rs1917806
Renal Disorder	GLYCOLYSIS_GLUCONEOGENESIS	KEGG	1391	36	20	4,90E-03	4,89E-01	rs1006087|rs9423466|rs2638449|rs4657468|rs1556632|rs4578194|rs7924157|rs4379190|rs12337623|rs10762277|rs973639|rs1348335|rs324079|rs3829185|rs2995948|rs217463|rs6671606|rs10918228|rs2388268|rs6025639

Renal Disorder	GALACTOSE_METABOLISM	KEGG	759	41	20	6,50E-03	5,51E-01	rs9423466|rs2638449|rs8024732|rs1556632|rs7924157|rs6963697|rs10762277|rs324079|rs3829185|rs2995948|rs217463|rs6671606|rs6954522|rs3770387|rs2183124|rs4732025|rs4485425|rs2388268|rs12257705|rs7089499
Renal Disorder	GLYCOSAMINOGLYCAN_BIOSYNTHESIS_HEPARAN_SULFATE	KEGG	2273	84	20	7,00E-03	5,51E-01	rs17862070|rs12509776|rs11776828|rs12927816|rs4782044|rs11223864|rs240920|rs7840309|rs7697986|rs1289500|rs7202082|rs530664|rs9999101|rs6699536|rs17123397|rs12509327|rs1506792|rs3845669|rs7932707|rs11224189
Renal Disorder	BETA_ALANINE_METABOLISM	KEGG	711	53	20	7,60E-03	5,51E-01	rs1006087|rs4657468|rs4578194|rs6784193|rs1330581|rs4379190|rs12337623|rs8190612|rs1348335|rs1641002|rs4415625|rs9810603|rs10875076|rs2786507|rs10918228|rs876848|rs17593512|rs12119882|rs10973770|rs12961730
Renal Disorder	FORMATION_OF_RNA_POL_II_ELONGATION_COMPLEX_	REACTOME	401	49	14	1,10E-02	6,67E-01	rs3796531|rs6761|rs8009789|rs1212343|rs731945|rs4916595|rs10917420|rs3748269|rs4942391|rs7982845|rs6734|rs3821996|rs2285877|rs2254883
Renal Disorder	RNA_POL_II_PRE_TRANSCRIPTION_EVENTS	REACTOME	532	59	20	1,12E-02	6,67E-01	rs3796531|rs1676996|rs6761|rs6508433|rs8009789|rs1212343|rs731945|rs4916595|rs10917420|rs3748269|rs4942391|rs6142711|rs7982845|rs6734|rs3821996|rs2285877|rs2254883|rs13242458|rs11191666|rs11040933
Renal Disorder	MRNA_SPLICING	REACTOME	1057	39	20	1,17E-02	6,67E-01	rs503156|rs1109009|rs3796531|rs6761|rs1212343|rs6132649|rs4916595|rs12046563|rs234605|rs234587|rs4942391|rs10124515|rs12459507|rs9916811|rs4982437|rs8060037|rs7982845|rs6734|rs17102539|rs3821996
Renal Disorder	FATTY_ACID_METABOLISM	KEGG	776	31	20	1,33E-02	6,67E-01	rs1006087|rs4657468|rs4578194|rs6835696|rs1135889|rs6784193|rs4379190|rs12337623|rs1348335|rs9810603|rs4142082|rs899491|rs10918228|rs7070744|rs17593512|rs12577790|rs1229976|rs9378878|rs6692958|rs10973770
Renal Disorder	P53_PATHWAY	BIOCARTA	347	25	12	1,40E-02	6,67E-01	rs241905|rs713996|rs7315397|rs12962644|rs130274|rs135029|rs11547635|rs2288726|rs10860361|rs137500|rs399413|rs17318844
Renal Disorder	AGR_PATHWAY	BIOCARTA	1981	41	20	1,42E-02	6,67E-01	rs17664708|rs2760494|rs7742931|rs9492123|rs2423401|rs7274475|rs11167020|rs2364598|rs12905386|rs13204138|rs1714424|rs17722300|rs17749288|rs1160798|rs265454|rs10748834|rs2439321|rs306962|rs12611955|rs9390184
Renal Disorder	TRANSCRIPTION	REACTOME	2708	45	20	1,76E-02	7,80E-01	rs1109009|rs3796531|rs303721|rs1676996|rs1554669|rs6761|rs487674|rs6508433|rs2616211|rs7004724|rs2798716|rs8009789|rs2031195|rs1212343|rs6132649|rs731945|rs4916595|rs2239896|rs234605|rs10917420
Renal Disorder	FORMATION_OF_THE_HIV1_EARLY_ELONGATION_COMPLEX	REACTOME	329	39	10	2,02E-02	8,07E-01	rs3796531|rs6761|rs1212343|rs4916595|rs3748269|rs4942391|rs7982845|rs6734|rs3821996|rs2254883
Renal Disorder	NEF_MEDIATES_DOWN_MODULATION_OF_CELL_SURFACE_RECEPTORS_BY_RECRUITIN N_ADAPTERS	REACTOME	366	22	9	2,28E-02	8,07E-01	rs503156|rs11861296|rs9643478|rs1181390|rs4675367|rs7465143|rs11919795|rs7946917|rs2231216
Renal Disorder	POST_TRANSLATIONAL_PROTEIN_MODIFICATION	REACTOME	6008	43	20	2,38E-02	8,07E-01	rs7550651|rs1436086|rs1037138|rs1242923|rs4686838|rs6803605|rs7739078|rs6415846|rs3811244|rs9688182|rs625149|rs652856|rs13136852|rs12246530|rs2305042|rs7529530|rs12304768|rs3780668|rs11564099|rs2239896
Renal Disorder	N_GLYCAN_BIOSYNTHESIS	KEGG	1792	55	20	2,60E-02	8,07E-01	rs1037138|rs1242923|rs4686838|rs7739078|rs6415846|rs3811244|rs625149|rs12304768|rs11564099|rs2239896|rs2207067|rs16830230|rs4821901|rs1707113|rs10801963|rs11684754|rs6594405|rs2256556|rs3004318|rs3739821
Renal Disorder	PROCESSING_OF_CAPPED_INTRON_CONTAINING_PRE_MRNA	REACTOME	1547	37	20	2,65E-02	8,07E-01	rs503156|rs1109009|rs3796531|rs3852030|rs6761|rs1212343|rs6132649|rs4916595|rs12046563|rs4837334|rs234605|rs9551193|rs234587|rs4942391|rs2022747|rs10124515|rs444319|rs12459507|rs9916811|rs4982437
Renal Disorder	ELONGATION_ARREST_AND_RECOVERY	REACTOME	246	31	10	2,67E-02	8,07E-01	rs3796531|rs6761|rs8009789|rs731945|rs10917420|rs4942391|rs7982845|rs3821996|rs2285877|rs2254883
Renal Disorder	HISTIDINE_METABOLISM	KEGG	654	41	20	2,69E-02	8,07E-01	rs1006087|rs4657468|rs11074678|rs4578194|rs4379190|rs12337623|rs1348335|rs1873061|rs2288742|rs10918228|rs1973076|rs2509712|rs2279124|rs10973770|rs12961730|rs1038378|rs3794950|rs1885490|rs486764|rs2839127
Renal Disorder	METABOLISM_OF_NON_CODING_RNA	REACTOME	611	40	18	2,72E-02	8,07E-01	rs1109009|rs3852030|rs1212343|rs6132649|rs4916595|rs3744741|rs4837334|rs9551193|rs444319|rs630814|rs6734|rs584096|rs9678612|rs4338396|rs103612|rs6114527|rs2644714|rs11770879
Renal Disorder	LATE_PHASE_OF_HIV_LIFE_CYCLE	REACTOME	1259	47	20	2,73E-02	8,07E-01	rs3796531|rs1676996|rs3852030|rs6761|rs6508433|rs8009789|rs1212343|rs731945|rs4916595|rs4837334|rs443678|rs10917420|rs9551193|rs3748269|rs4942391|rs6142711|rs444319|rs7982845|rs630814|rs6734
Renal Disorder	ASCORBATE_AND_ALDARATE_METABOLISM	KEGG	874	110	18	2,92E-02	8,32E-01	rs1006087|rs4657468|rs4578194|rs2018985|rs11892031|rs4379190|rs12337623|rs1348335|rs1018124|rs10918228|rs4593203|rs6822259|rs10973770|rs1038378|rs1885490|rs486764|rs4148307|rs1555480
Renal Disorder	NOD_LIKE_RECEPTOR_SIGNALING_PATHWAY	KEGG	1406	35	20	3,05E-02	8,39E-01	rs2291782|rs11653896|rs11072638|rs2869441|rs7768419|rs17103274|rs1613367|rs9494850|rs1860180|rs10499196|rs10455523|rs3769823|rs360712|rs1460769|rs7793163|rs9988572|rs2548468|rs9362732|rs7658485|rs9362744
Renal Disorder	HEDGEHOG_SIGNALING_PATHWAY	KEGG	2151	34	20	3,18E-02	8,46E-01	rs1402704|rs11773897|rs10079778|rs3926693|rs6459961|rs6038619|rs277534|rs721347|rs235710|rs1529702|rs1927087|rs6054501|rs6827794|rs9789747|rs996544|rs210363|rs6107891|rs4944092|rs776504|rs13231026
Renal Disorder	TERMINATION_OF_O_GLYCAN_BIOSYNTHESIS	REACTOME	961	39	20	3,36E-02	8,61E-01	rs7550651|rs1436086|rs4686838|rs7529530|rs4836593|rs3780668|rs427589|rs7960018|rs1436085|rs4628548|rs6660107|rs10782620|rs1360878|rs544858|rs17116558|rs2304921|rs387831|rs2147791|rs17730982|rs2251330
Renal Disorder	ASPARAGINE_N_LINKED_GLYCOSYLATION	REACTOME	2760	35	20	3,51E-02	8,61E-01	rs1037138|rs1242923|rs4686838|rs6803605|rs7739078|rs6415846|rs3811244|rs625149|rs12246530|rs2305042|rs12304768|rs11564099|rs2239896|rs2207067|rs1430004|rs10510302|rs16830230|rs4821901|rs1707113|rs3784730
Renal Disorder	ABORTIVE_ELONGATION_OF_HIV1_TRANSCRIPT_IN_THE_ABSENCE_OF_TAT	REACTOME	195	28	9	3,56E-02	8,61E-01	rs3796531|rs6761|rs1212343|rs4916595|rs4942391|rs7982845|rs6734|rs3821996|rs2254883
Renal Disorder	VOLTAGE_GATED_POTASSIUM_CHANNELS	REACTOME	2491	42	20	3,67E-02	8,61E-01	rs9874417|rs10082810|rs7813186|rs2640492|rs12193344|rs2283214|rs6063604|rs3904621|rs1891716|rs231915|rs4628571|rs9297840|rs9881034|rs16995777|rs2403773|rs11102354|rs2283174|rs7555432|rs12282937|rs727233
Renal Disorder	REGULATORY_RNA_PATHWAYS	REACTOME	345	30	10	4,21E-02	9,22E-01	rs3796531|rs6761|rs4789523|rs443678|rs470113|rs4867349|rs3821996|rs2254883|rs7958457|rs3804043
Renal Disorder	TRYPTOPHAN_METABOLISM	KEGG	953	35	20	4,36E-02	9,22E-01	rs1006087|rs4657468|rs10204598|rs4578194|rs6784193|rs4379190|rs12337623|rs1348335|rs9810603|rs12231341|rs1609380|rs10918228|rs10020753|rs1042541|rs3760138|rs17593512|rs12577790|rs10973770|rs354690|rs7100934
Renal Disorder	IL6_PATHWAY	BIOCARTA	401	14	8	4,37E-02	9,22E-01	rs2760494|rs11582424|rs7793163|rs12409333|rs17822304|rs11066320|rs3742768|rs4537545
Renal Disorder	HIV_LIFE_CYCLE	REACTOME	1529	45	20	4,39E-02	9,22E-01	rs3796531|rs1676996|rs3852030|rs6761|rs4954410|rs6508433|rs8009789|rs1212343|rs731945|rs4916595|rs4837334|rs443678|rs10917420|rs9551193|rs3748269|rs4942391|rs6142711|rs444319|rs2303744|rs7982845
Renal Disorder	SYNTHESIS_SECRETION_AND_DEACYLATION_OF_GHRELIN	REACTOME	624	36	20	4,59E-02	9,39E-01	rs17768133|rs1832741|rs1004446|rs9948204|rs10520349|rs10978854|rs2094919|rs4272449|rs1880147|rs1819730|rs560104|rs4731437|rs965174|rs2075355|rs7341756|rs2936427|rs715827|rs2292462|rs11660643|rs12528807
Renal Disorder	PTDINS_PATHWAY	BIOCARTA	1058	40	20	4,75E-02	9,48E-01	rs9423466|rs2638449|rs1556632|rs1179486|rs10510496|rs7924157|rs6705717|rs6074178|rs2486335|rs2668020|rs6108746|rs6755308|rs17189227|rs281501|rs6078029|rs2244621|rs7971059|rs3731949|rs4953334|rs2388268
Renal Disorder	LYSINE_DEGRADATION	KEGG	952	29	20	4,98E-02	9,69E-01	rs1006087|rs4657468|rs4578194|rs6784193|rs4379190|rs12337623|rs1348335|rs9568221|rs9810603|rs12459507|rs1609380|rs12487322|rs10918228|rs9568203|rs2602248|rs16857208|rs17593512|rs12577790|rs10973770|rs7100934
Renal Disorder	FOCAL_ADHESION	KEGG	7733	27	20	5,26E-02	9,93E-01	rs4478405|rs10205184|rs3813668|rs2760494|rs1319475|rs6727015|rs7742931|rs1179486|rs9492123|rs4966009|rs441250|rs2869441|rs2423401|rs2282735|rs958766|rs525007|rs7274475|rs6958081|rs12962644|rs11167020
Renal Disorder	STEROID_HORMONE_BIOSYNTHESIS	KEGG	1491	110	20	5,35E-02	9,93E-01	rs10204598|rs1884722|rs2018985|rs11892031|rs1902586|rs7081411|rs1220709|rs2572023|rs476184|rs7546652|rs3753519|rs1018124|rs2955162|rs4593203|rs11992601|rs6822259|rs3756426|rs17133974|rs4646312|rs1119864
Renal Disorder	MRNA_CAPPING	REACTOME	388	48	13	5,73E-02	1,00E+00	rs3796531|rs6761|rs1212343|rs4916595|rs3748269|rs4942391|rs7982845|rs6734|rs3821996|rs10502424|rs2254883|rs9294406|rs749420
Renal Disorder	CLEAVAGE_OF_GROWING_TRANSCRIPT_IN_THE_TERMINATION_REGION_	REACTOME	435	32	17	5,86E-02	1,00E+00	rs1109009|rs1212343|rs6132649|rs4916595|rs234605|rs234587|rs17311679|rs9916811|rs8060037|rs7714390|rs6734|rs1241713|rs8021276|rs6114527|rs4981468|rs11749500|rs17108140
Renal Disorder	IL2_PATHWAY	BIOCARTA	452	31	15	6,03E-02	1,00E+00	rs2760494|rs1760125|rs2991215|rs2386841|rs9695993|rs228979|rs12409333|rs192703|rs1881735|rs4749882|rs291765|rs3742768|rs228975|rs10993698|rs7090530
Renal Disorder	MRNA_PROCESSING	REACTOME	1942	36	20	6,04E-02	1,00E+00	rs503156|rs1109009|rs3796531|rs3852030|rs6761|rs1212343|rs6132649|rs4916595|rs12046563|rs4837334|rs234605|rs9551193|rs3748269|rs2838824|rs234587|rs17311679|rs4942391|rs2022747|rs10124515|rs444319
Renal Disorder	PENTOSE_PHOSPHATE_PATHWAY	KEGG	616	36	20	6,06E-02	1,00E+00	rs9423466|rs2638449|rs1556632|rs7924157|rs10794337|rs2995948|rs217463|rs6671606|rs7980712|rs2388268|rs12257705|rs7089499|rs2239560|rs11861842|rs2065682|rs4465231|rs7899347|rs9423540|rs2838551|rs422732
Renal Disorder	41BB_PATHWAY	BIOCARTA	341	25	9	6,19E-02	1,00E+00	rs2760494|rs17103274|rs1153681|rs10784678|rs10748094|rs10878729|rs1881735|rs2306365|rs10883452
Renal Disorder	GLUCOSE_METABOLISM	REACTOME	1554	50	20	6,28E-02	1,00E+00	rs9423466|rs2638449|rs1556632|rs9857268|rs2578349|rs6723088|rs7924157|rs2490286|rs973639|rs6487241|rs2995948|rs217463|rs17425778|rs2239942|rs6671606|rs3770387|rs3763609|rs1457604|rs1878583|rs2388268
Renal Disorder	RNA_POL_II_TRANSCRIPTION_PRE_INITIATION_AND_PROMOTER_OPENING	REACTOME	383	50	14	6,54E-02	1,00E+00	rs3796531|rs1676996|rs6761|rs6508433|rs3748269|rs4942391|rs6142711|rs7982845|rs3821996|rs2254883|rs13242458|rs11191666|rs11040933|rs9291949
Renal Disorder	RECRUITMENT_OF_MITOTIC_CENTROSOME_PROTEINS_AND_COMPLEXES	REACTOME	1177	43	20	6,66E-02	1,00E+00	rs4837764|rs17692377|rs9550158|rs876992|rs10984956|rs3780674|rs6120015|rs11104736|rs2249060|rs2497600|rs7736312|rs17120218|rs2702332|rs7040576|rs9295910|rs7296755|rs2293079|rs1421411|rs2283113|rs9550174
Renal Disorder	PROCESSING_OF_CAPPED_INTRONLESS_PRE_MRNA	REACTOME	274	30	15	6,97E-02	1,00E+00	rs1109009|rs1212343|rs6132649|rs4916595|rs234605|rs234587|rs17311679|rs8060037|rs7714390|rs6734|rs1241713|rs8021276|rs6114527|rs4981468|rs11749500
Renal Disorder	THE_ROLE_OF_NEF_IN_HIV1_REPLICATION_AND_DISEASE_PATHOGENESIS	REACTOME	936	34	20	7,10E-02	1,00E+00	rs503156|rs1554669|rs11861296|rs1723015|rs9643478|rs7794173|rs4385221|rs1181390|rs4675366|rs7465143|rs10226593|rs1559463|rs13242914|rs1718414|rs2084385|rs17073074|rs10951511|rs11919795|rs1986619|rs17534481
Renal Disorder	MTOR_SIGNALING_PATHWAY	KEGG	1290	35	20	7,37E-02	1,00E+00	rs3813668|rs3805492|rs2087210|rs441250|rs1004446|rs3761840|rs2301106|rs874269|rs11739413|rs7559811|rs2048753|rs2293338|rs7698057|rs594942|rs11876|rs2244621|rs17153616|rs3828246|rs9568203|rs2231410
Renal Disorder	RELA_PATHWAY	BIOCARTA	208	10	5	7,49E-02	1,00E+00	rs6489711|rs17103274|rs10498658|rs2306365|rs10883452
Renal Disorder	ARGININE_AND_PROLINE_METABOLISM	KEGG	991	28	20	7,55E-02	1,00E+00	rs1006087|rs4657468|rs1405948|rs4578194|rs9658265|rs4379190|rs12337623|rs2490286|rs1348335|rs914976|rs4920566|rs10918228|rs186471|rs10901826|rs885815|rs10973770|rs156055|rs2306619|rs10911044|rs3755259
Renal Disorder	GABA_SYNTHESIS_RELEASE_REUPTAKE_AND_DEGRADATION	REACTOME	746	44	18	7,61E-02	1,00E+00	rs4980806|rs2789588|rs1322183|rs1330581|rs2697152|rs8190612|rs6905900|rs1641002|rs911719|rs883382|rs6104567|rs4610207|rs876848|rs2304911|rs874628|rs961072|rs9293878|rs10455248
Renal Disorder	BASE_EXCISION_REPAIR	KEGG	500	33	17	7,74E-02	1,00E+00	rs7327256|rs9318648|rs12485319|rs4135063|rs874583|rs2005618|rs4769346|rs2303744|rs11876|rs2244095|rs2323973|rs2048075|rs818707|rs3213403|rs2629768|rs159146|rs2862909
Renal Disorder	TRANSLATION	REACTOME	1803	36	20	7,78E-02	1,00E+00	rs2291782|rs17768133|rs1074584|rs625149|rs9948204|rs6469646|rs6469170|rs12587507|rs10520349|rs7006843|rs6501697|rs3748269|rs16892766|rs1133099|rs1435961|rs1649990|rs1043149|rs8026641|rs2958522|rs12942418
Renal Disorder	HIV_INFECTION	REACTOME	3237	39	20	8,40E-02	1,00E+00	rs503156|rs3796531|rs1676996|rs1554669|rs3852030|rs6761|rs4954410|rs11861296|rs6508433|rs8009789|rs4576294|rs1212343|rs1723015|rs731945|rs4916595|rs241437|rs4837334|rs443678|rs10917420|rs9643478
Renal Disorder	SHH_PATHWAY	BIOCARTA	679	34	20	8,70E-02	1,00E+00	rs11773897|rs6459961|rs277534|rs721347|rs2835809|rs776504|rs13231026|rs10491176|rs898109|rs817184|rs2286562|rs1991769|rs11763188|rs3763609|rs2835810|rs316689|rs17709725|rs10786696|rs846401|rs2298330
Renal Disorder	RAP1_SIGNALLING	REACTOME	835	28	20	8,74E-02	1,00E+00	rs1326250|rs4734497|rs1937652|rs12801636|rs9895126|rs829374|rs1904006|rs4935225|rs756819|rs9907411|rs10997716|rs1937701|rs2339794|rs1530378|rs3763609|rs17601717|rs10508951|rs13019093|rs1890548|rs17464037
Renal Disorder	LEUKOCYTE_TRANSENDOTHELIAL_MIGRATION	KEGG	4793	33	20	8,88E-02	1,00E+00	rs8000535|rs3813668|rs1825428|rs1179486|rs1094972|rs868032|rs7605424|rs2862431|rs525007|rs4954410|rs11167020|rs7099767|rs25800|rs6716817|rs2383289|rs12801636|rs11812710|rs6788984|rs12761828|rs10865451
Renal Disorder	TRANSPORT_TO_THE_GOLGI_AND_SUBSEQUENT_MODIFICATION	REACTOME	1557	41	20	8,94E-02	1,00E+00	rs1037138|rs4686838|rs7739078|rs12246530|rs2305042|rs2207067|rs16830230|rs4821901|rs1707113|rs3784730|rs10801963|rs7142869|rs13205436|rs4777987|rs11684754|rs1947110|rs6594405|rs439182|rs11852344|rs2183124
Renal Disorder	BASAL_CELL_CARCINOMA	KEGG	2166	24	20	9,19E-02	1,00E+00	rs1402704|rs11773897|rs6459961|rs6038619|rs277534|rs721347|rs235710|rs1413611|rs6054501|rs11196203|rs7904519|rs6788984|rs6827794|rs996544|rs210363|rs4944092|rs6107891|rs776504|rs13231026|rs4765835
Renal Disorder	ACYL_CHAIN_REMODELLING_OF_PC	REACTOME	693	34	20	9,26E-02	1,00E+00	rs622442|rs1148757|rs2701632|rs12733186|rs4551123|rs2307278|rs13160153|rs10454443|rs2303744|rs1668547|rs1668589|rs645953|rs7554573|rs2250227|rs3815252|rs11589805|rs2144438|rs3825786|rs7530853|rs622450
Renal Disorder	ATM_PATHWAY	BIOCARTA	397	28	9	9,40E-02	1,00E+00	rs2760494|rs17103274|rs6985793|rs7853654|rs1881735|rs4648545|rs2181484|rs2306365|rs11792889
Renal Disorder	BRANCHED_CHAIN_AMINO_ACID_CATABOLISM	REACTOME	507	60	14	9,49E-02	1,00E+00	rs4646864|rs2444827|rs757141|rs757140|rs7757271|rs3924411|rs16970653|rs277952|rs2072293|rs12577790|rs1259022|rs3853607|rs42101|rs11764246
Renal Disorder	METABOLISM_OF_AMINO_ACIDS_AND_DERIVATIVES	REACTOME	5246	32	20	9,50E-02	1,00E+00	rs12194954|rs11637483|rs4657468|rs4646864|rs1405948|rs1217356|rs2444827|rs4578194|rs1004446|rs757141|rs11626687|rs11679258|rs4576294|rs6116189|rs568520|rs652800|rs241437|rs1368421|rs757140|rs17105367
Renal Disorder	CGMP_EFFECTS	REACTOME	1520	34	20	9,54E-02	1,00E+00	rs11042893|rs1326250|rs1135029|rs1937652|rs4235953|rs4583026|rs6565616|rs9365906|rs483832|rs2162734|rs1904006|rs4935225|rs10516652|rs341078|rs11045301|rs220785|rs17193|rs10997716|rs1937701|rs6928737
Renal Disorder	CITRIC_ACID_CYCLE_TCA_CYCLE	REACTOME	675	61	15	9,65E-02	1,00E+00	rs3934860|rs11715476|rs12232909|rs17070878|rs2147502|rs773652|rs7646423|rs2325899|rs752409|rs9526420|rs2503449|rs1991542|rs17479135|rs2923861|rs2361508
Renal Disorder	RIG_I_MDA5_MEDIATED_INDUCTION_OF_IFN_ALPHA_BETA_PATHWAYS	REACTOME	1129	28	20	9,69E-02	1,00E+00	rs7327256|rs1549669|rs3756103|rs7768419|rs17103274|rs11006121|rs9494850|rs10499196|rs34129552|rs7143468|rs1342852|rs6978998|rs3769823|rs1994174|rs3787620|rs461626|rs10498658|rs261537|rs17686080|rs8126493
Renal Disorder	METABOLISM_OF_CARBOHYDRATES	REACTOME	9072	38	20	9,83E-02	1,00E+00	rs9423466|rs17862070|rs12509776|rs2638449|rs11776828|rs2147155|rs1556632|rs11223864|rs9857268|rs2637426|rs2578349|rs7840309|rs7697986|rs3852030|rs7356549|rs2288236|rs6723088|rs7924157|rs1289500|rs4309396
Renal Disorder	REGULATION_OF_BETA_CELL_DEVELOPMENT	REACTOME	1288	37	20	9,84E-02	1,00E+00	rs4687504|rs3006246|rs1004446|rs6656594|rs2755217|rs677874|rs10919806|rs874269|rs3110649|rs16878154|rs6694219|rs1877207|rs2435234|rs4687488|rs7983897|rs3094509|rs1116123|rs662702|rs16939060|rs2821320
Renal Disorder	ACTIVATION_OF_CHAPERONE_GENES_BY_XBP1S	REACTOME	761	36	20	1,01E-01	1,00E+00	rs240920|rs6803605|rs7793585|rs1198873|rs1430004|rs10510302|rs7639294|rs6802161|rs7621754|rs2295797|rs343419|rs2884289|rs4031375|rs12544882|rs9880553|rs2075355|rs9561932|rs10480450|rs7621509|rs6774516
Renal Disorder	MICRORNA_MIRNA_BIOGENESIS	REACTOME	227	24	8	1,02E-01	1,00E+00	rs3796531|rs6761|rs443678|rs4867349|rs3821996|rs2254883|rs7958457|rs3804043
Renal Disorder	TEL_PATHWAY	BIOCARTA	978	40	20	1,05E-01	1,00E+00	rs9642880|rs4966009|rs6761|rs12962644|rs6601306|rs3924649|rs2975842|rs4381631|rs2684792|rs12440962|rs3889392|rs16960228|rs10095762|rs4635969|rs7168213|rs11871468|rs17746482|rs4245566|rs10435712|rs1993596
Renal Disorder	ACYL_CHAIN_REMODELLING_OF_PE	REACTOME	696	33	20	1,06E-01	1,00E+00	rs622442|rs1148757|rs2701632|rs12733186|rs2307278|rs17560881|rs10454443|rs2303744|rs1668547|rs6907076|rs1668589|rs645953|rs7554573|rs2250227|rs3815252|rs11589805|rs2144438|rs3825786|rs7530853|rs622450
Renal Disorder	GPVI_MEDIATED_ACTIVATION_CASCADE	REACTOME	1181	34	20	1,09E-01	1,00E+00	rs3813668|rs1760125|rs525007|rs2991215|rs11646140|rs195771|rs874269|rs9695993|rs2075555|rs2128833|rs656709|rs2668020|rs4666415|rs17020088|rs252353|rs192703|rs12039210|rs4794117|rs17073074|rs17153616
Renal Disorder	REGULATION_OF_ACTIN_CYTOSKELETON	KEGG	7089	25	20	1,10E-01	1,00E+00	rs10205184|rs3813668|rs1319475|rs6727015|rs1112903|rs1179486|rs263931|rs7987595|rs2976230|rs10488597|rs2423401|rs1716|rs7318731|rs1004446|rs525007|rs7274475|rs8001712|rs11167020|rs25800|rs6716817
Renal Disorder	FORMATION_OF_THE_TERNARY_COMPLEX_AND_SUBSEQUENTLY_THE_43S_COMPLEX	REACTOME	677	40	18	1,11E-01	1,00E+00	rs1074584|rs6469646|rs6469170|rs12587507|rs7006843|rs16892766|rs1133099|rs1435961|rs1649990|rs7826090|rs4075144|rs4145041|rs10140464|rs1720321|rs1881129|rs11902670|rs1790157|rs10407035
Renal Disorder	CITRATE_CYCLE_TCA_CYCLE	KEGG	1195	53	20	1,13E-01	1,00E+00	rs3934860|rs6723088|rs11715476|rs12232909|rs17070878|rs2094703|rs2304497|rs2147502|rs773652|rs973639|rs7875673|rs7646423|rs2325899|rs262498|rs10970923|rs752409|rs9526420|rs6025639|rs2888998|rs2503449
Renal Disorder	EPO_PATHWAY	BIOCARTA	255	11	4	1,15E-01	1,00E+00	rs2760494|rs1881740|rs3742768|rs8108707
Renal Disorder	IMMUNOREGULATORY_INTERACTIONS_BETWEEN_A_LYMPHOID_AND_A_NON_LYMPHO	REACTOME	1325	37	20	1,15E-01	1,00E+00	rs202892|rs169374|rs1554669|rs2032288|rs1723015|rs9863580|rs9865917|rs12419365|rs11799952|rs2073917|rs1298954|rs8131116|rs405509|rs1009897|rs13047336|rs12111032|rs11586136|rs3107634|rs13078891|rs281423
Renal Disorder	PORPHYRIN_AND_CHLOROPHYLL_METABOLISM	KEGG	1163	100	20	1,17E-01	1,00E+00	rs2018985|rs11892031|rs11871705|rs7922546|rs2239896|rs2078340|rs17752881|rs1018124|rs907638|rs7705728|rs1461161|rs4593203|rs999212|rs6822259|rs643788|rs818704|rs1366485|rs7624706|rs1181574|rs10486752
Renal Disorder	INTEGRIN_CELL_SURFACE_INTERACTIONS	REACTOME	2614	49	20	1,18E-01	1,00E+00	rs10205184|rs1319475|rs9492123|rs2976230|rs1716|rs1760125|rs2991215|rs11167020|rs10911203|rs12006094|rs1330351|rs4366552|rs178323|rs11606866|rs9695993|rs2075555|rs306962|rs12419365|rs1318662|rs2073917
Renal Disorder	MATURITY_ONSET_DIABETES_OF_THE_YOUNG	KEGG	992	38	20	1,19E-01	1,00E+00	rs4687504|rs3006246|rs1004446|rs6656594|rs677874|rs10919806|rs3110649|rs6694219|rs2435234|rs4687488|rs7983897|rs3094509|rs1116123|rs662702|rs16939060|rs2821320|rs1203951|rs2270604|rs190925|rs12625315
Renal Disorder	ACYL_CHAIN_REMODELLING_OF_PG	REACTOME	571	41	19	1,21E-01	1,00E+00	rs622442|rs1148757|rs2701632|rs12733186|rs4551123|rs13160153|rs10454443|rs1668547|rs1668589|rs645953|rs7554573|rs2250227|rs11589805|rs2144438|rs3825786|rs7530853|rs622450|rs9729852|rs6426616
Renal Disorder	SIGNALING_BY_BMP	REACTOME	918	34	20	1,24E-01	1,00E+00	rs12123578|rs6038619|rs235710|rs6054501|rs11006121|rs1837291|rs996544|rs6107891|rs1994174|rs6038621|rs3858284|rs9293817|rs2145274|rs6085775|rs2225265|rs6504246|rs4303700|rs173107|rs4233580|rs12129753
Renal Disorder	PROXIMAL_TUBULE_BICARBONATE_RECLAMATION	KEGG	458	42	20	1,27E-01	1,00E+00	rs6864158|rs6723088|rs9329911|rs1405376|rs12695746|rs860294|rs973639|rs17159722|rs7841820|rs1000597|rs2672027|rs262498|rs4839061|rs6025639|rs16852004|rs4811873|rs4803520|rs11748410|rs1901709|rs17028097
Renal Disorder	TOB1_PATHWAY	BIOCARTA	652	34	20	1,31E-01	1,00E+00	rs1554669|rs6660484|rs2386841|rs1723015|rs1805113|rs1181390|rs4675367|rs412705|rs10784678|rs10748094|rs10878729|rs11071939|rs4749882|rs4372735|rs6662137|rs17131536|rs17534481|rs6809777|rs4938507|rs7090530
Renal Disorder	ADP_SIGNALLING_THROUGH_P2RY1	REACTOME	863	35	20	1,33E-01	1,00E+00	rs1148757|rs308039|rs12878420|rs12746334|rs12733186|rs12985186|rs12352752|rs3786947|rs10454443|rs6681938|rs8113423|rs17474654|rs7554573|rs2250227|rs16947151|rs887030|rs12881038|rs3746070|rs1253676|rs11083841
Renal Disorder	RENAL_CELL_CARCINOMA	KEGG	2164	38	20	1,33E-01	1,00E+00	rs3813668|rs2760494|rs441250|rs2423401|rs958766|rs12147685|rs7274475|rs12905386|rs183642|rs17722300|rs2301106|rs3924649|rs874269|rs712285|rs10793894|rs914596|rs8042361|rs17822304|rs1718414|rs2084385
Renal Disorder	GLYCOSPHINGOLIPID_BIOSYNTHESIS_LACTO_AND_NEOLACTO_SERIES	KEGG	670	20	10	1,34E-01	1,00E+00	rs7764197|rs7755748|rs597152|rs1225999|rs1004369|rs2183124|rs10865331|rs7256058|rs4173|rs6713082
Renal Disorder	STRESS_PATHWAY	BIOCARTA	633	33	14	1,37E-01	1,00E+00	rs2760494|rs6489711|rs17103274|rs10859602|rs2715825|rs10498658|rs1485888|rs1881735|rs1267034|rs2306365|rs10883452|rs10783388|rs7585907|rs7810486
Renal Disorder	RNA_POL_III_TRANSCRIPTION	REACTOME	861	41	20	1,37E-01	1,00E+00	rs303721|rs1554669|rs487674|rs2616211|rs7004724|rs2798716|rs2031195|rs7021022|rs4871975|rs17221107|rs7875762|rs12930464|rs11204135|rs1319462|rs9644645|rs9512817|rs2254883|rs2616241|rs2273933|rs10810057
Renal Disorder	POTASSIUM_CHANNELS	REACTOME	4940	38	20	1,38E-01	1,00E+00	rs9874417|rs10082810|rs17344557|rs7813186|rs2640492|rs12193344|rs2283214|rs6063604|rs3904621|rs1891716|rs1167763|rs12878420|rs231915|rs12746334|rs2961957|rs4628571|rs199214|rs16838062|rs9297840|rs9881034
Renal Disorder	TRANSCRIPTIONAL_REGULATION_OF_WHITE_ADIPOCYTE_DIFFERENTIATION	REACTOME	2719	29	20	1,39E-01	1,00E+00	rs4478405|rs1832741|rs6496143|rs10850606|rs1555208|rs1147289|rs2577001|rs7168486|rs9850957|rs10055872|rs9298360|rs2987392|rs4430696|rs1110462|rs6866285|rs934782|rs1410117|rs10978854|rs2094919|rs6857002

<table>
<tr><td></td><td></td><td></td><td></td><td></td><td></td><td></td><td></td><td></td></tr>
<tr><td>Renal Disorder</td><td>INFLAMMASOMES</td><td>REACTOME</td><td>468</td><td>37</td><td>17</td><td>1,40E-01</td><td>1,00E+00</td><td>rs11653896|rs11072638|rs12962644|rs1613367|rs3787620|rs9988572|rs461626|rs8126493|rs400819|rs1076420|rs925599|rs7219822|rs2716858|rs891781|rs503720|rs741810|rs7283136</td></tr>
<tr><td>Renal Disorder</td><td>NATURAL_KILLER_CELL_MEDIATED_CYTOTOXICITY</td><td>KEGG</td><td>3607</td><td>28</td><td>20</td><td>1,41E-01</td><td>1,00E+00</td><td>rs1394320|rs3813668|rs1179486|rs1554669|rs1760125|rs525007|rs1133782|rs2991215|rs3924519|rs11646140|rs33063|rs1723015|rs3924649|rs3093998|rs503832|rs10509323|rs4381631|rs9695993|rs231998|rs2486335</td></tr>
<tr><td>Renal Disorder</td><td>TRANSPORT_OF_MATURE_MRNA_DERIVED_FROM_AN_INTRONLESS_TRANSCRIPT</td><td>REACTOME</td><td>524</td><td>29</td><td>16</td><td>1,42E-01</td><td>1,00E+00</td><td>rs3852030|rs1212343|rs4916595|rs4837334|rs9551193|rs444319|rs630814|rs6734|rs7698057|rs1241713|rs9678612|rs4338396|rs3763851|rs103612|rs8021276|rs11770879</td></tr>
<tr><td>Renal Disorder</td><td>STEROID_HORMONES</td><td>REACTOME</td><td>693</td><td>47</td><td>19</td><td>1,43E-01</td><td>1,00E+00</td><td>rs3926693|rs1884722|rs1902586|rs16846797|rs9789747|rs476184|rs7546652|rs2181874|rs10222695|rs3753519|rs2302694|rs4732025|rs152883|rs3756426|rs1119864|rs2259458|rs2899469|rs10923838|rs2356825</td></tr>
<tr><td>Renal Disorder</td><td>G_ALPHA_I_SIGNALLING_EVENTS</td><td>REACTOME</td><td>6453</td><td>27</td><td>20</td><td>1,43E-01</td><td>1,00E+00</td><td>rs7757020|rs563587|rs2531995|rs12127134|rs2332807|rs628208|rs10488597|rs10776728|rs11077666|rs4954410|rs9323567|rs1876527|rs12568515|rs4678029|rs1167763|rs12878420|rs2373154|rs12746334|rs2531982|rs2341942</td></tr>
<tr><td>Renal Disorder</td><td>REGULATION_OF_GLUCOKINASE_BY_GLUCOKINASE_REGULATORY_PROTEIN</td><td>REACTOME</td><td>459</td><td>16</td><td>9</td><td>1,45E-01</td><td>1,00E+00</td><td>rs3852030|rs4837334|rs9551193|rs444319|rs630814|rs9678612|rs4338396|rs103612|rs11770879</td></tr>
<tr><td>Renal Disorder</td><td>MITOTIC_G2_G2_M_PHASES</td><td>REACTOME</td><td>1389</td><td>42</td><td>20</td><td>1,46E-01</td><td>1,00E+00</td><td>rs4837764|rs17692377|rs9550158|rs876992|rs10984956|rs3780674|rs6120015|rs11104736|rs2249060|rs2497600|rs7736312|rs17120218|rs2702332|rs7040576|rs942042|rs9295910|rs746720|rs7296755|rs2293079|rs1421411</td></tr>
<tr><td>Renal Disorder</td><td>NEP_NS2_INTERACTS_WITH_THE_CELLULAR_EXPORT_MACHINERY</td><td>REACTOME</td><td>456</td><td>16</td><td>9</td><td>1,47E-01</td><td>1,00E+00</td><td>rs3852030|rs4837334|rs9551193|rs444319|rs630814|rs9678612|rs4338396|rs103612|rs11770879</td></tr>
<tr><td>Renal Disorder</td><td>STARCH_AND_SUCROSE_METABOLISM</td><td>KEGG</td><td>2178</td><td>85</td><td>20</td><td>1,50E-01</td><td>1,00E+00</td><td>rs11070370|rs9857268|rs2578349|rs2018985|rs11892031|rs6963697|rs10762277|rs324079|rs1391212|rs3829185|rs6487241|rs2995948|rs217463|rs17425778|rs1018124|rs6671606|rs6954522|rs3770387|rs11185369|rs4514289</td></tr>
<tr><td>Renal Disorder</td><td>TRANSPORT_OF_RIBONUCLEOPROTEINS_INTO_THE_HOST_NUCLEUS</td><td>REACTOME</td><td>452</td><td>16</td><td>9</td><td>1,50E-01</td><td>1,00E+00</td><td>rs3852030|rs4837334|rs9551193|rs444319|rs630814|rs9678612|rs4338396|rs103612|rs11770879</td></tr>
<tr><td>Renal Disorder</td><td>TRAF6_MEDIATED_NFKB_ACTIVATION</td><td>REACTOME</td><td>351</td><td>26</td><td>10</td><td>1,50E-01</td><td>1,00E+00</td><td>rs7327256|rs17103274|rs34129552|rs3787620|rs461626|rs8126493|rs400819|rs2306365|rs10883452|rs7283136</td></tr>
<tr><td>Renal Disorder</td><td>LOSS_OF_NLP_FROM_MITOTIC_CENTROSOMES</td><td>REACTOME</td><td>1100</td><td>37</td><td>20</td><td>1,51E-01</td><td>1,00E+00</td><td>rs10760097|rs17692377|rs876992|rs10984956|rs3780674|rs6120015|rs11104736|rs2249060|rs2497600|rs2702332|rs7040576|rs9295910|rs7296755|rs2293079|rs1421411|rs2283113|rs10777848|rs7314573|rs9405048|rs4076557</td></tr>
<tr><td>Renal Disorder</td><td>WNT_SIGNALING_PATHWAY</td><td>KEGG</td><td>5611</td><td>25</td><td>20</td><td>1,55E-01</td><td>1,00E+00</td><td>rs4478405|rs1402704|rs2760494|rs12243975|rs9642880|rs11866594|rs2869441|rs11715237|rs978486|rs9688182|rs6716817|rs12905386|rs33063|rs1529702|rs9850957|rs1990623|rs8009789|rs1413611|rs2034614|rs730830</td></tr>
<tr><td>Renal Disorder</td><td>CERAMIDE_PATHWAY</td><td>BIOCARTA</td><td>425</td><td>20</td><td>9</td><td>1,55E-01</td><td>1,00E+00</td><td>rs6489711|rs12962644|rs3769823|rs10498658|rs2244621|rs1881740|rs2306365|rs4758415|rs477895</td></tr>
<tr><td>Renal Disorder</td><td>PEROXISOME</td><td>KEGG</td><td>2047</td><td>42</td><td>20</td><td>1,58E-01</td><td>1,00E+00</td><td>rs3934860|rs10463469|rs11679258|rs4531246|rs3736330|rs444369|rs34236|rs4233453|rs6835696|rs1135889|rs2545618|rs6784193|rs3822464|rs9810603|rs12582802|rs4142082|rs899491|rs1990745|rs11242540|rs1573736</td></tr>
<tr><td>Renal Disorder</td><td>NITRIC_OXIDE_STIMULATES_GUANYLATE_CYCLASE</td><td>REACTOME</td><td>1895</td><td>34</td><td>20</td><td>1,58E-01</td><td>1,00E+00</td><td>rs11042893|rs1326250|rs1135029|rs9658265|rs1937652|rs4235953|rs4583026|rs6565616|rs9365906|rs483832|rs2162734|rs1904006|rs4935225|rs10516652|rs11722434|rs341078|rs11045301|rs220785|rs17193|rs10997716</td></tr>
<tr><td>Renal Disorder</td><td>OXYGEN_DEPENDENT_PROLINE_HYDROXYLATION_OF_HYPOXIA_INDUCIBLE_FACTOR_</td><td>REACTOME</td><td>313</td><td>19</td><td>11</td><td>1,62E-01</td><td>1,00E+00</td><td>rs12147685|rs11006121|rs2301106|rs712285|rs1994174|rs261537|rs8003133|rs2285877|rs6742517|rs8102595|rs16912145</td></tr>
<tr><td>Renal Disorder</td><td>PPARA_PATHWAY</td><td>BIOCARTA</td><td>2445</td><td>31</td><td>20</td><td>1,64E-01</td><td>1,00E+00</td><td>rs3813668|rs2760494|rs9642880|rs1004446|rs10463469|rs1555208|rs1147289|rs2364598|rs17103274|rs17605851|rs1135889|rs6784193|rs4381631|rs10482869|rs6857002|rs1988145|rs1805352|rs10491176|rs1111348|rs9884852</td></tr>
<tr><td>Renal Disorder</td><td>STATHMIN_PATHWAY</td><td>BIOCARTA</td><td>456</td><td>22</td><td>14</td><td>1,64E-01</td><td>1,00E+00</td><td>rs1554669|rs9688182|rs1723015|rs7736312|rs10491176|rs4724298|rs2286562|rs3763609|rs316689|rs17534481|rs4938507|rs2450246|rs10892234|rs10923137</td></tr>
<tr><td>Renal Disorder</td><td>T_CELL_RECEPTOR_SIGNALING_PATHWAY</td><td>KEGG</td><td>3546</td><td>30</td><td>20</td><td>1,68E-01</td><td>1,00E+00</td><td>rs3813668|rs2760494|rs1179486|rs1554669|rs2423401|rs525007|rs7274475|rs11646140|rs25800|rs12905386|rs33063|rs17103274|rs1723015|rs17722300|rs7605392|rs3924649|rs874269|rs596866|rs10455523|rs7914</td></tr>
<tr><td>Renal Disorder</td><td>IL_3_5_AND_GM_CSF_SIGNALING</td><td>REACTOME</td><td>1134</td><td>36</td><td>20</td><td>1,68E-01</td><td>1,00E+00</td><td>rs3813668|rs1760125|rs2991215|rs2386841|rs4734497|rs7914|rs9695993|rs10793894|rs743562|rs2668020|rs228979|rs5756391|rs12409333|rs192703|rs17073074|rs17464037|rs1123551|rs13173003|rs853812|rs13259960</td></tr>
<tr><td>Renal Disorder</td><td>METABOLISM_OF_NUCLEOTIDES</td><td>REACTOME</td><td>1559</td><td>49</td><td>20</td><td>1,68E-01</td><td>1,00E+00</td><td>rs4910146|rs1689924|rs6539145|rs11042855|rs17001868|rs12036993|rs944664|rs1934272|rs17664900|rs3780663|rs4415625|rs2298582|rs10017797|rs4290308|rs10875076|rs2786507|rs6922|rs7030035|rs1998393|rs1573736</td></tr>
<tr><td>Renal Disorder</td><td>RESOLUTION_OF_AP_SITES_VIA_THE_MULTIPLE_NUCLEOTIDE_PATCH_REPLACEMENT_</td><td>REACTOME</td><td>192</td><td>15</td><td>8</td><td>1,69E-01</td><td>1,00E+00</td><td>rs12485319|rs4135063|rs2005618|rs2303744|rs11876|rs2244095|rs2629768|rs159146</td></tr>
<tr><td>Renal Disorder</td><td>IL12_PATHWAY</td><td>BIOCARTA</td><td>589</td><td>36</td><td>18</td><td>1,70E-01</td><td>1,00E+00</td><td>rs2760494|rs1554669|rs1723015|rs360712|rs10515797|rs10784678|rs2715825|rs1869624|rs3790565|rs10748094|rs10878729|rs1881740|rs17534481|rs4938507|rs11924144|rs6444106|rs10892234|rs11465597</td></tr>
<tr><td>Renal Disorder</td><td>GLYCEROLIPID_METABOLISM</td><td>KEGG</td><td>1872</td><td>23</td><td>20</td><td>1,71E-01</td><td>1,00E+00</td><td>rs1006087|rs4657468|rs1815326|rs4578194|rs2357960|rs711237|rs9566906|rs4379190|rs12337623|rs12988612|rs1348335|rs34129552|rs17560881|rs2076213|rs1528098|rs10486048|rs2920123|rs928389|rs6907076|rs10918228</td></tr>
<tr><td>Renal Disorder</td><td>METABOLISM_OF_STEROID_HORMONES_AND_VITAMINS_A_AND_D</td><td>REACTOME</td><td>745</td><td>47</td><td>19</td><td>1,71E-01</td><td>1,00E+00</td><td>rs3926693|rs1884722|rs1902586|rs16846797|rs9789747|rs476184|rs7546652|rs2181874|rs10222695|rs3753519|rs2302694|rs4732025|rs152883|rs3756426|rs1119864|rs2259458|rs2899469|rs10923838|rs2356827</td></tr>
<tr><td>Renal Disorder</td><td>CELLCYCLE_PATHWAY</td><td>BIOCARTA</td><td>459</td><td>25</td><td>12</td><td>1,74E-01</td><td>1,00E+00</td><td>rs4478405|rs17772958|rs7736312|rs1861178|rs2811709|rs9381100|rs4943383|rs9576040|rs7133323|rs2450246|rs399413|rs17318844</td></tr>
<tr><td>Renal Disorder</td><td>NKCELLS_PATHWAY</td><td>BIOCARTA</td><td>678</td><td>39</td><td>18</td><td>1,75E-01</td><td>1,00E+00</td><td>rs3813668|rs1760125|rs2991215|rs11646140|rs9695993|rs360712|rs252353|rs192703|rs13173003|rs853812|rs291765|rs3019245|rs7641889|rs6449947|rs3019244|rs10993698|rs830474|rs10054692</td></tr>
<tr><td>Renal Disorder</td><td>MRNA_3_END_PROCESSING</td><td>REACTOME</td><td>320</td><td>16</td><td>11</td><td>1,75E-01</td><td>1,00E+00</td><td>rs1212343|rs4916595|rs234605|rs234587|rs9916811|rs8060037|rs6734|rs1241713|rs8021276|rs4981468|rs17108140</td></tr>
<tr><td>Renal Disorder</td><td>AQUAPORIN_MEDIATED_TRANSPORT</td><td>REACTOME</td><td>1510</td><td>34</td><td>20</td><td>1,76E-01</td><td>1,00E+00</td><td>rs11578377|rs2531995|rs12878420|rs12746334|rs2531982|rs1891842|rs1865550|rs12985186|rs7229075|rs853600|rs2899624|rs10491176|rs17159722|rs2457971|rs2286562|rs6681938|rs1000597|rs8113423|rs17474654|rs3763609</td></tr>
<tr><td>Renal Disorder</td><td>GLYCOLYSIS</td><td>REACTOME</td><td>558</td><td>41</td><td>19</td><td>1,79E-01</td><td>1,00E+00</td><td>rs9423466|rs2638449|rs1556632|rs7924157|rs2239942|rs2388268|rs12257705|rs7089499|rs11861842|rs2065682|rs7899347|rs9423540|rs2838551|rs10411335|rs422732|rs13293668|rs12304476|rs2106446|rs633045</td></tr>
<tr><td>Renal Disorder</td><td>PYRIMIDINE_METABOLISM</td><td>KEGG</td><td>1900</td><td>44</td><td>20</td><td>1,80E-01</td><td>1,00E+00</td><td>rs1689924|rs3796531|rs6761|rs6539145|rs3742810|rs6713599|rs12036993|rs13413734|rs944664|rs1934272|rs17664900|rs308003|rs4415625|rs2298582|rs10017797|rs4290308|rs10875076|rs4871975|rs2786507|rs6922</td></tr>
<tr><td>Renal Disorder</td><td>GLYCOSAMINOGLYCAN_BIOSYNTHESIS_CHONDROITIN_SULFATE</td><td>KEGG</td><td>1372</td><td>45</td><td>20</td><td>1,81E-01</td><td>1,00E+00</td><td>rs12927816|rs4782044|rs11223864|rs7356549|rs1289500|rs10861257|rs7202082|rs12544497|rs9422277|rs2254827|rs7932707|rs11224189|rs12935114|rs4319439|rs9488914|rs7816924|rs2125193|rs4238653|rs2975420|rs476135</td></tr>
<tr><td>Renal Disorder</td><td>RNA_POLYMERASE</td><td>KEGG</td><td>351</td><td>24</td><td>9</td><td>1,81E-01</td><td>1,00E+00</td><td>rs3796531|rs6761|rs4871975|rs3821996|rs17221107|rs9512817|rs2254883|rs730283|rs10153801</td></tr>
<tr><td>Renal Disorder</td><td>HIVNEF_PATHWAY</td><td>BIOCARTA</td><td>1008</td><td>33</td><td>20</td><td>1,84E-01</td><td>1,00E+00</td><td>rs6489711|rs919699|rs12962644|rs11167020|rs17103274|rs10859602|rs874583|rs306962|rs3769823|rs443627|rs10498658|rs1718414|rs2084385|rs7840147|rs2288726|rs7615501|rs10860361|rs1485888|rs1881740|rs2306365</td></tr>
<tr><td>Renal Disorder</td><td>N_GLYCAN_ANTENNAE_ELONGATION_IN_THE_MEDIAL_TRANS_GOLGI</td><td>REACTOME</td><td>962</td><td>50</td><td>20</td><td>1,86E-01</td><td>1,00E+00</td><td>rs1037138|rs4686838|rs12246530|rs16830230|rs4821901|rs3784730|rs4777987|rs11684754|rs6594405|rs439182|rs11852344|rs2183124|rs7494064|rs11629679|rs2900041|rs12147958|rs10483781|rs2290049|rs3797680|rs12964617</td></tr>
<tr><td>Renal Disorder</td><td>GLUCURONIDATION</td><td>REACTOME</td><td>656</td><td>82</td><td>8</td><td>1,87E-01</td><td>1,00E+00</td><td>rs2018985|rs11892031|rs1018124|rs4072113|rs4593203|rs6822259|rs17620012|rs4148307</td></tr>
<tr><td>Renal Disorder</td><td>CHEMICAL_PATHWAY</td><td>BIOCARTA</td><td>585</td><td>37</td><td>20</td><td>1,91E-01</td><td>1,00E+00</td><td>rs7315397|rs12962644|rs11167020|rs874583|rs4381631|rs306962|rs443627|rs3889392|rs2295797|rs16960228|rs7840147|rs2288726|rs2244621|rs10860361|rs11871468|rs3124743|rs1003599|rs16959569|rs4788426|rs477895</td></tr>
<tr><td>Renal Disorder</td><td>NEGATIVE_REGULATORS_OF_RIG_I_MDA5_SIGNALING</td><td>REACTOME</td><td>464</td><td>30</td><td>17</td><td>1,94E-01</td><td>1,00E+00</td><td>rs1549669|rs7768419|rs11006121|rs9494850|rs10499196|rs7143468|rs6978998|rs1994174|rs261537|rs10214930|rs657597|rs4148171|rs626776|rs1496642|rs7958457|rs17718931|rs16912145</td></tr>
<tr><td>Renal Disorder</td><td>SYNTHESIS_SECRETION_AND_INACTIVATION_OF_GLP1</td><td>REACTOME</td><td>363</td><td>29</td><td>16</td><td>1,95E-01</td><td>1,00E+00</td><td>rs17768133|rs9948204|rs677874|rs6788984|rs10520349|rs662702|rs4731437|rs965174|rs2936427|rs8100944|rs429988|rs2292462|rs741529|rs11660643|rs9551421|rs8098067</td></tr>
<tr><td>Renal Disorder</td><td>TNFR2_PATHWAY</td><td>BIOCARTA</td><td>414</td><td>24</td><td>14</td><td>2,00E-01</td><td>1,00E+00</td><td>rs7768419|rs17103274|rs9494850|rs10499196|rs7874280|rs7143468|rs12517663|rs10498658|rs657597|rs1267034|rs2306365|rs10883452|rs7585907|rs749864</td></tr>
<tr><td>Renal Disorder</td><td>HS_GAG_BIOSYNTHESIS</td><td>REACTOME</td><td>3154</td><td>74</td><td>20</td><td>2,01E-01</td><td>1,00E+00</td><td>rs17862070|rs12509776|rs11776828|rs2147155|rs2637426|rs7840309|rs7697986|rs4309396|rs530664|rs9999101|rs12509327|rs1506792|rs3845669|rs9524299|rs3848064|rs4400920|rs9523342|rs2651458|rs9589601|rs7981707</td></tr>
<tr><td>Renal Disorder</td><td>3_UTR_MEDIATED_TRANSLATIONAL_REGULATION</td><td>REACTOME</td><td>1235</td><td>41</td><td>20</td><td>2,01E-01</td><td>1,00E+00</td><td>rs2291782|rs1074584|rs6469646|rs6469170|rs12587507|rs7006843|rs6501697|rs16892766|rs1133099|rs1435961|rs1649990|rs2958522|rs12942418|rs7826090|rs7698057|rs4075144|rs6800812|rs9907408|rs4145041|rs10140464</td></tr>
<tr><td>Renal Disorder</td><td>INSULIN_SIGNALING_PATHWAY</td><td>KEGG</td><td>3744</td><td>31</td><td>20</td><td>2,01E-01</td><td>1,00E+00</td><td>rs1548395|rs3813668|rs3805492|rs2087210|rs2869441|rs1004446|rs7822664|rs7984646|rs3761840|rs4078293|rs4583026|rs2755217|rs2940341|rs3924649|rs874269|rs11739413|rs2162734|rs10762277|rs7914|rs973639</td></tr>
<tr><td>Renal Disorder</td><td>ENDOMETRIAL_CANCER</td><td>KEGG</td><td>3028</td><td>45</td><td>20</td><td>2,02E-01</td><td>1,00E+00</td><td>rs3813668|rs1825428|rs868032|rs9642880|rs7605424|rs2862431|rs7099767|rs11196203|rs11812710|rs7904519|rs6788984|rs3924649|rs12761828|rs10865451|rs874269|rs4074716|rs946199|rs10496234|rs219463|rs6741085</td></tr>
<tr><td>Renal Disorder</td><td>ARRHYTHMOGENIC_RIGHT_VENTRICULAR_CARDIOMYOPATHY_ARVC</td><td>KEGG</td><td>5871</td><td>40</td><td>20</td><td>2,02E-01</td><td>1,00E+00</td><td>rs10205184|rs7984956|rs1319475|rs6727015|rs1825428|rs9492123|rs868032|rs7605424|rs2862431|rs1674535|rs1229487|rs7099767|rs10420331|rs1356733|rs11196203|rs10996472|rs7904519|rs6788984|rs4245919|rs9947576</td></tr>
<tr><td>Renal Disorder</td><td>SELENOAMINO_ACID_METABOLISM</td><td>KEGG</td><td>742</td><td>59</td><td>18</td><td>2,03E-01</td><td>1,00E+00</td><td>rs11074678|rs2726670|rs1368421|rs11746351|rs2807907|rs17062465|rs4649923|rs2546324|rs6892131|rs2288742|rs1562337|rs1359277|rs12410281|rs4489081|rs10023658|rs11745553|rs203217|rs12142041</td></tr>
<tr><td>Renal Disorder</td><td>REGULATION_OF_AUTOPHAGY</td><td>KEGG</td><td>933</td><td>52</td><td>20</td><td>2,04E-01</td><td>1,00E+00</td><td>rs3805492|rs1004446|rs7238810|rs9959057|rs10502786|rs1943600|rs8089834|rs10784678|rs10748094|rs12458976|rs3764459|rs12327116|rs4146444|rs10878729|rs12606897|rs28674674|rs12957072|rs12960377|rs9304251|rs8090817</td></tr>
<tr><td>Renal Disorder</td><td>BAD_PATHWAY</td><td>BIOCARTA</td><td>927</td><td>30</td><td>20</td><td>2,05E-01</td><td>1,00E+00</td><td>rs3813668|rs4966009|rs12962644|rs13114243|rs2684792|rs10491176|rs5756391|rs12440962|rs1014058|rs2286562|rs3763609|rs11632057|rs2726626|rs2244621|rs17153616|rs1589880|rs849430|rs13173003|rs853812|rs1004717</td></tr>
<tr><td>Renal Disorder</td><td>INTERACTIONS_OF_VPR_WITH_HOST_CELLULAR_PROTEINS</td><td>REACTOME</td><td>480</td><td>19</td><td>11</td><td>2,05E-01</td><td>1,00E+00</td><td>rs3852030|rs4837334|rs9551193|rs7667259|rs444319|rs630814|rs9678612|rs4338396|rs103612|rs2665515|rs11770879</td></tr>
<tr><td>Renal Disorder</td><td>HEPARAN_SULFATE_HEPARIN_HS_GAG_METABOLISM</td><td>REACTOME</td><td>3879</td><td>72</td><td>20</td><td>2,07E-01</td><td>1,00E+00</td><td>rs17862070|rs12509776|rs11776828|rs2147155|rs11223864|rs2637426|rs7840309|rs7697986|rs1289500|rs4309396|rs530664|rs9999101|rs12509327|rs1506792|rs3845669|rs7323264|rs3848064|rs4400920|rs9523342|rs2651458</td></tr>
<tr><td>Renal Disorder</td><td>SMALL_CELL_LUNG_CANCER</td><td>KEGG</td><td>3747</td><td>32</td><td>20</td><td>2,11E-01</td><td>1,00E+00</td><td>rs6807877|rs3813668|rs6727015|rs7742931|rs9492123|rs9642880|rs2282735|rs919699|rs12962644|rs11167020|rs10911203|rs17103274|rs1160798|rs874269|rs6446081|rs2121794|rs7143468|rs306962|rs1805352|rs3762013</td></tr>
<tr><td>Renal Disorder</td><td>G_PROTEIN_BETA_GAMMA_SIGNALLING</td><td>REACTOME</td><td>798</td><td>28</td><td>20</td><td>2,12E-01</td><td>1,00E+00</td><td>rs12905386|rs12878420|rs12746334|rs195771|rs874269|rs12985186|rs6055957|rs6516421|rs6681938|rs8113423|rs17474654|rs16947151|rs594942|rs887030|rs2244621|rs12881038|rs17153616|rs849430|rs1253676|rs1004717</td></tr>
<tr><td>Renal Disorder</td><td>PURINE_METABOLISM</td><td>REACTOME</td><td>581</td><td>43</td><td>14</td><td>2,12E-01</td><td>1,00E+00</td><td>rs4910146|rs11042855|rs17001868|rs944664|rs6922|rs7030035|rs1998393|rs12039225|rs313229|rs207432|rs951409|rs2198009|rs7939845|rs11001053</td></tr>
<tr><td>Renal Disorder</td><td>DIABETES_PATHWAYS</td><td>REACTOME</td><td>3395</td><td>42</td><td>20</td><td>2,17E-01</td><td>1,00E+00</td><td>rs17768133|rs1832741|rs6136050|rs240920|rs6803605|rs1004446|rs12324775|rs9948204|rs7793585|rs1198873|rs10520349|rs1430004|rs10510302|rs9922943|rs7639294|rs10978854|rs6044732|rs2094919|rs9911085|rs7214466</td></tr>
<tr><td>Renal Disorder</td><td>REGULATION_OF_HYPOXIA_INDUCIBLE_FACTOR_HIF_BY_OXYGEN</td><td>REACTOME</td><td>443</td><td>21</td><td>13</td><td>2,18E-01</td><td>1,00E+00</td><td>rs12147685|rs11006121|rs2301106|rs712285|rs1994174|rs261537|rs2495727|rs8003133|rs2285877|rs6742517|rs4507587|rs8102595|rs16912145</td></tr>
<tr><td>Renal Disorder</td><td>PROTEIN_EXPORT</td><td>KEGG</td><td>750</td><td>40</td><td>18</td><td>2,21E-01</td><td>1,00E+00</td><td>rs17768133|rs1525500|rs9948204|rs799617|rs10520349|rs1043149|rs8026641|rs7795082|rs965174|rs1558078|rs10499749|rs2292462|rs11660643|rs6971934|rs1569256|rs7803359|rs2412473|rs398704</td></tr>
<tr><td>Renal Disorder</td><td>NO2IL12_PATHWAY</td><td>BIOCARTA</td><td>461</td><td>22</td><td>12</td><td>2,21E-01</td><td>1,00E+00</td><td>rs1554669|rs1723015|rs10515797|rs10784678|rs1869624|rs3790565|rs10748094|rs10878729|rs17534481|rs4938507|rs10892234|rs10923137</td></tr>
<tr><td>Renal Disorder</td><td>B_CELL_RECEPTOR_SIGNALING_PATHWAY</td><td>KEGG</td><td>2278</td><td>29</td><td>20</td><td>2,22E-01</td><td>1,00E+00</td><td>rs3813668|rs2760494|rs1179486|rs1760125|rs525007|rs2991215|rs33063|rs17103274|rs12551214|rs3924649|rs874269|rs9695993|rs2486335|rs6123013|rs656709|rs13031194|rs1878808|rs11799952|rs2668020|rs7193701</td></tr>
<tr><td>Renal Disorder</td><td>GATA3_PATHWAY</td><td>BIOCARTA</td><td>898</td><td>28</td><td>20</td><td>2,23E-01</td><td>1,00E+00</td><td>rs1243438|rs12444808|rs30405|rs11648597|rs10490944|rs250157|rs10491176|rs6123013|rs827618|rs743562|rs2286562|rs3763609|rs3813583|rs10508351|rs10795608|rs1149986|rs10905548|rs4239343|rs316689|rs10905399</td></tr>
<tr><td>Renal Disorder</td><td>NEURONAL_SYSTEM</td><td>REACTOME</td><td>13378</td><td>29</td><td>20</td><td>2,24E-01</td><td>1,00E+00</td><td>rs9874417|rs10082810|rs2531995|rs4980806|rs7716847|rs17344557|rs7813186|rs241905|rs2640492|rs12494778|rs2789588|rs2051248|rs2075716|rs713996|rs2051569|rs241707|rs1674535|rs12193344|rs2283214|rs6063604</td></tr>
<tr><td>Renal Disorder</td><td>BASE_EXCISION_REPAIR</td><td>REACTOME</td><td>209</td><td>16</td><td>9</td><td>2,24E-01</td><td>1,00E+00</td><td>rs12485319|rs4135063|rs2005618|rs2303744|rs11876|rs2244095|rs3213403|rs2629768|rs159146</td></tr>
<tr><td>Renal Disorder</td><td>SIGNALING_BY_ROBO_RECEPTOR</td><td>REACTOME</td><td>1900</td><td>29</td><td>20</td><td>2,25E-01</td><td>1,00E+00</td><td>rs519813|rs6797462|rs2423401|rs7274475|rs12905386|rs6803548|rs1163758|rs17722300|rs7605392|rs17049971|rs7853654|rs7666974|rs2700331|rs914596|rs17379883|rs13320269|rs1718414|rs2084385|rs17613956|rs1162704</td></tr>
<tr><td>Renal Disorder</td><td>IL_2_SIGNALING</td><td>REACTOME</td><td>936</td><td>31</td><td>20</td><td>2,28E-01</td><td>1,00E+00</td><td>rs3813668|rs1760125|rs2991215|rs2386841|rs3924649|rs9695993|rs743562|rs228979|rs5756391|rs12409333|rs192703|rs12459940|rs13173003|rs853812|rs4749882|rs1755994|rs228975|rs7641889|rs25887|rs6449947</td></tr>
<tr><td>Renal Disorder</td><td>DEATH_PATHWAY</td><td>BIOCARTA</td><td>505</td><td>32</td><td>16</td><td>2,30E-01</td><td>1,00E+00</td><td>rs7315397|rs12962644|rs17103274|rs231998|rs3769823|rs443627|rs10498658|rs2288726|rs10860361|rs16910106|rs2306365|rs3124743|rs12082157|rs10883452|rs12226338|rs2604532</td></tr>
<tr><td>Renal Disorder</td><td>SPLICEOSOME</td><td>KEGG</td><td>1685</td><td>31</td><td>20</td><td>2,30E-01</td><td>1,00E+00</td><td>rs503156|rs1109009|rs1212343|rs6132649|rs4916595|rs3093998|rs12046563|rs3857113|rs36664|rs10517052|rs10124515|rs12459507|rs9916811|rs4982437|rs2123463|rs4312745|rs2297334|rs7089227|rs2232739|rs6734</td></tr>
<tr><td>Renal Disorder</td><td>NUCLEOTIDE_BINDING_DOMAIN_LEUCINE_RICH_REPEAT_CONTAINING_RECEPTOR_NLR
YS</td><td>REACTOME</td><td>1337</td><td>28</td><td>20</td><td>2,31E-01</td><td>1,00E+00</td><td>rs11653896|rs11072638|rs12962644|rs7768419|rs1613367|rs9494850|rs10499196|rs10455523|rs3769823|rs3787620|rs9988572|rs2715825|rs461626|rs9362732|rs9362744|rs8126493|rs9351265|rs400819|rs1076420|rs2046316</td></tr>
<tr><td>Renal Disorder</td><td>APOPTOSIS</td><td>KEGG</td><td>1727</td><td>29</td><td>20</td><td>2,32E-01</td><td>1,00E+00</td><td>rs3813668|rs6489711|rs1133782|rs919699|rs12962644|rs3924519|rs17103274|rs874269|rs1152918|rs231998|rs10491176|rs3769823|rs6582484|rs5756391|rs443627|rs2286562|rs10498658|rs7873986|rs3763609|rs17733355</td></tr>
<tr><td>Renal Disorder</td><td>ARAP_PATHWAY</td><td>BIOCARTA</td><td>292</td><td>12</td><td>7</td><td>2,33E-01</td><td>1,00E+00</td><td>rs5758941|rs6743450|rs16866947|rs1890060|rs17198191|rs940243|rs2267364</td></tr>
<tr><td>Renal Disorder</td><td>TID_PATHWAY</td><td>BIOCARTA</td><td>479</td><td>21</td><td>12</td><td>2,34E-01</td><td>1,00E+00</td><td>rs6489711|rs17103274|rs11613504|rs10784678|rs10748094|rs10878729|rs9376268|rs8127569|rs2306365|rs2284553|rs399413|rs2270024</td></tr>
<tr><td>Renal Disorder</td><td>ENERGY_DEPENDENT_REGULATION_OF_MTOR_BY_LKB1_AMPK</td><td>REACTOME</td><td>461</td><td>25</td><td>14</td><td>2,36E-01</td><td>1,00E+00</td><td>rs3805492|rs3761840|rs7559811|rs2048753|rs1881643|rs11876|rs9568203|rs2231410|rs953221|rs2727564|rs2536075|rs7789674|rs4962081|rs10257529</td></tr>
<tr><td>Renal Disorder</td><td>SIGNAL_AMPLIFICATION</td><td>REACTOME</td><td>1023</td><td>35</td><td>20</td><td>2,37E-01</td><td>1,00E+00</td><td>rs1148757|rs308039|rs12878420|rs12746334|rs12733186|rs12985186|rs12352752|rs3786947|rs10454443|rs6681938|rs8113423|rs17474654|rs7554573|rs2250227|rs16947151|rs887030|rs12881038|rs3746070|rs1253676|rs11083841</td></tr>
<tr><td>Renal Disorder</td><td>ACTIVATION_OF_THE_MRNA_UPON_BINDING_OF_THE_CAP_BINDING_COMPLEX_AND_EI
T_BINDING_TO_43S</td><td>REACTOME</td><td>780</td><td>44</td><td>20</td><td>2,39E-01</td><td>1,00E+00</td><td>rs1074584|rs6469646|rs6469170|rs12587507|rs7006843|rs16892766|rs1133099|rs1435961|rs1649990|rs7826090|rs7698057|rs4075144|rs4145041|rs10140464|rs1720321|rs1881129|rs11902670|rs1790157|rs2230571|rs10407035</td></tr>
<tr><td>Renal Disorder</td><td>ACTIVATED_AMPK_STIMULATES_FATTY_ACID_OXIDATION_IN_MUSCLE</td><td>REACTOME</td><td>421</td><td>26</td><td>12</td><td>2,39E-01</td><td>1,00E+00</td><td>rs3805492|rs6835696|rs7559811|rs1881643|rs9568203|rs1016331|rs953221|rs2727564|rs2536075|rs7789674|rs10257529|rs17108140</td></tr>
<tr><td>Renal Disorder</td><td>METABOLISM_OF_XENOBIOTICS_BY_CYTOCHROME_P450</td><td>KEGG</td><td>1674</td><td>91</td><td>20</td><td>2,40E-01</td><td>1,00E+00</td><td>rs10204598|rs2018985|rs11892031|rs7081411|rs2572023|rs1695|rs4149195|rs1018124|rs10918228|rs4593203|rs10786172|rs13114510|rs12646059|rs6822259|rs34511626|rs1934975|rs7355084|rs4147599|rs396216|rs2132847</td></tr>
<tr><td>Renal Disorder</td><td>FRUCTOSE_AND_MANNOSE_METABOLISM</td><td>KEGG</td><td>984</td><td>38</td><td>20</td><td>2,41E-01</td><td>1,00E+00</td><td>rs9423466|rs2638449|rs1556632|rs7924157|rs10762277|rs13197291|rs3829185|rs7015243|rs4732025|rs2388268|rs12257705|rs7089499|rs17587917|rs11861842|rs2065682|rs7899347|rs693830|rs4769414|rs9423540|rs3800143</td></tr>
<tr><td>Renal Disorder</td><td>AMINO_SUGAR_AND_NUCLEOTIDE_SUGAR_METABOLISM</td><td>KEGG</td><td>956</td><td>30</td><td>20</td><td>2,49E-01</td><td>1,00E+00</td><td>rs1113428|rs888352|rs1043313|rs10762277|rs16872173|rs6851930|rs13197291|rs6835058|rs3829185|rs2995948|rs217463|rs6671606|rs4072113|rs16850799|rs2486072|rs4485425|rs1059273|rs7733582|rs11944003|rs918481</td></tr>
<tr><td>Renal Disorder</td><td>ERBB_SIGNALING_PATHWAY</td><td>KEGG</td><td>4135</td><td>33</td><td>20</td><td>2,53E-01</td><td>1,00E+00</td><td>rs3813668|rs17664708|rs2760494|rs9642880|rs2423401|rs2869441|rs7274475|rs9688182|rs11167020|rs12905386|rs1714424|rs17722300|rs17749288|rs7605392|rs3924649|rs874269|rs265454|rs10748834|rs4381631|rs7914</td></tr>
<tr><td>Renal Disorder</td><td>TRANSPORT_OF_MATURE_TRANSCRIPT_TO_CYTOPLASM</td><td>REACTOME</td><td>693</td><td>31</td><td>18</td><td>2,53E-01</td><td>1,00E+00</td><td>rs3852030|rs1212343|rs4916595|rs4837334|rs9551193|rs444319|rs9916811|rs630814|rs6734|rs7698057|rs1241713|rs9678612|rs4338396|rs3763851|rs103612|rs8021276|rs17108140|rs11770879</td></tr>
<tr><td>Renal Disorder</td><td>INFLUENZA_LIFE_CYCLE</td><td>REACTOME</td><td>1671</td><td>40</td><td>20</td><td>2,53E-01</td><td>1,00E+00</td><td>rs2291782|rs3796531|rs3852030|rs6761|rs1043313|rs12587507|rs4837334|rs6501697|rs9551193|rs4942391|rs1133099|rs444319|rs585871|rs1435961|rs1649990|rs2958522|rs7982845|rs12942418|rs630814|rs4075144</td></tr>
<tr><td>Renal Disorder</td><td>THROMBIN_SIGNALLING_THROUGH_PROTEINASE_ACTIVATED_RECEPTORS_PARS</td><td>REACTOME</td><td>690</td><td>36</td><td>20</td><td>2,54E-01</td><td>1,00E+00</td><td>rs308039|rs12878420|rs12746334|rs12985186|rs12352752|rs3786947|rs7728560|rs6681938|rs8113423|rs17474654|rs16947151|rs887030|rs12881038|rs3746070|rs1253676|rs4752932|rs11083841|rs10493754|rs17124637|rs1276309</td></tr>
<tr><td>Renal Disorder</td><td>INTRINSIC_PATHWAY_FOR_APOPTOSIS</td><td>REACTOME</td><td>532</td><td>29</td><td>15</td><td>2,54E-01</td><td>1,00E+00</td><td>rs7315397|rs12962644|rs3769823|rs652196|rs210179|rs443627|rs9308742|rs2288726|rs2244621|rs10860361|rs1881735|rs1107480|rs3124743|rs396746|rs477895</td></tr>
<tr><td>Renal Disorder</td><td>CTLA4_PATHWAY</td><td>BIOCARTA</td><td>662</td><td>38</td><td>20</td><td>2,55E-01</td><td>1,00E+00</td><td>rs3813668|rs1554669|rs25800|rs1723015|rs1181390|rs4675366|rs28605404|rs17822304|rs10932047|rs12616245|rs13173003|rs853812|rs11066320|rs17534481|rs7641889|rs4938507|rs6449947|rs2858870|rs7283760|rs10892234</td></tr>
<tr><td>Renal Disorder</td><td>THROMBOXANE_SIGNALLING_THROUGH_TP_RECEPTOR</td><td>REACTOME</td><td>492</td><td>35</td><td>19</td><td>2,56E-01</td><td>1,00E+00</td><td>rs308039|rs12878420|rs12746334|rs12985186|rs12352752|rs3786947|rs6681938|rs8113423|rs17474654|rs16947151|rs887030|rs12881038|rs3746070|rs1253676|rs11083841|rs12075532|rs17124637|rs1276309|rs6700907</td></tr>
<tr><td>Renal Disorder</td><td>AMINE_LIGAND_BINDING_RECEPTORS</td><td>REACTOME</td><td>2247</td><td>31</td><td>20</td><td>2,58E-01</td><td>1,00E+00</td><td>rs7757020|rs1112903|rs10488597|rs1876527|rs2039860|rs636293|rs10495431|rs946637|rs4568821|rs11956891|rs4075571|rs7727161|rs9353427|rs2039801|rs2150043|rs16832154|rs9450430|rs942472|rs6076665|rs12778878</td></tr>
<tr><td>Renal Disorder</td><td>FC_EPSILON_RI_SIGNALING_PATHWAY</td><td>KEGG</td><td>2758</td><td>30</td><td>20</td><td>2,58E-01</td><td>1,00E+00</td><td>rs3813668|rs1179486|rs2869441|rs1148757|rs1760125|rs525007|rs2991215|rs11646140|rs2701632|rs3924649|rs874269|rs12733186|rs6705717|rs4381631|rs9695993|rs2486335|rs656709|rs13031194|rs743562|rs2668020</td></tr>
<tr><td>Renal Disorder</td><td>SULFUR_AMINO_ACID_METABOLISM</td><td>REACTOME</td><td>930</td><td>52</td><td>18</td><td>2,61E-01</td><td>1,00E+00</td><td>rs11637483|rs1368421|rs2490286|rs6493166|rs11746351|rs568529|rs2807907|rs17062465|rs4649923|rs2546324|rs6892131|rs13300268|rs1915190|rs12410281|rs4489081|rs11745553|rs3794950|rs3213403</td></tr>
<tr><td>Renal Disorder</td><td>UNFOLDED_PROTEIN_RESPONSE</td><td>REACTOME</td><td>1176</td><td>37</td><td>20</td><td>2,61E-01</td><td>1,00E+00</td><td>rs240920|rs6803605|rs7793585|rs1198873|rs1430004|rs10510302|rs9922943|rs9872641|rs9911085|rs6802161|rs3091237|rs7621754|rs2295797|rs9784647|rs738288|rs10861147|rs343419|rs11244096|rs13429247|rs4031375</td></tr>
<tr><td>Renal Disorder</td><td>MITOCHONDRIA_PATHWAY</td><td>BIOCARTA</td><td>317</td><td>22</td><td>12</td><td>2,61E-01</td><td>1,00E+00</td><td>rs7315397|rs12962644|rs3769823|rs210179|rs443627|rs2288726|rs10860361|rs3124743|rs12082157|rs2293964|rs396746|rs2604532</td></tr>
<tr><td>Renal Disorder</td><td>CASPASE_PATHWAY</td><td>BIOCARTA</td><td>327</td><td>20</td><td>12</td><td>2,62E-01</td><td>1,00E+00</td><td>rs7315397|rs874583|rs1613367|rs10509323|rs3769823|rs2288726|rs10860361|rs3124743|rs12082157|rs566275|rs7810486|rs2604532</td></tr>
</table>

Renal Disorder	REGULATION_OF_SIGNALING_BY_CBL	REACTOME	703	41	20	2,65E-01	1,00E+00	rs3813668|rs1760125|rs2991215|rs7914|rs9695993|rs10793894|rs2668020|rs192703|rs17073074|rs1123551|rs13173003|rs853812|rs1755994|rs10901077|rs7641889|rs6449947|rs1027990|rs10457242|rs10993698|rs12967001
Renal Disorder	EARLY_PHASE_OF_HIV_LIFE_CYCLE	REACTOME	270	8	4	2,69E-01	1,00E+00	rs4954410|rs2303744|rs7987249|rs2665515
Renal Disorder	NUCLEAR_RECEPTOR_TRANSCRIPTION_PATHWAY	REACTOME	2750	38	20	2,69E-01	1,00E+00	rs17668665|rs3006246|rs2361287|rs1555208|rs6656594|rs6494232|rs935344|rs11917719|rs2813703|rs17605851|rs2818747|rs10919806|rs834834|rs2461796|rs6694219|rs1988145|rs3020317|rs1805352|rs3762013|rs9658119
Renal Disorder	GLYCOSYLPHOSPHATIDYLINOSITOL_GPI_ANCHOR_BIOSYNTHESIS	KEGG	318	23	12	2,70E-01	1,00E+00	rs6415846|rs1212343|rs4916595|rs8007199|rs6734|rs3739821|rs13102358|rs17069203|rs10518440|rs7763|rs527365|rs2835569
Renal Disorder	PYRUVATE_METABOLISM_AND_CITRIC_ACID_TCA_CYCLE	REACTOME	905	68	20	2,71E-01	1,00E+00	rs3934860|rs11715476|rs12232909|rs17070878|rs2147502|rs773652|rs2421763|rs7646423|rs2325899|rs2732553|rs752409|rs9526420|rs474908|rs2503449|rs1991542|rs17479135|rs2923861|rs2361508|rs724714|rs756847
Renal Disorder	PI3K_CASCADE	REACTOME	2702	32	20	2,72E-01	1,00E+00	rs3813668|rs3805492|rs263931|rs7318731|rs1004446|rs7984646|rs7238810|rs3761840|rs2517037|rs249736|rs874269|rs7559811|rs9959057|rs10502786|rs4770252|rs2048753|rs1379707|rs1536433|rs1943600|rs1881643
Renal Disorder	BASAL_TRANSCRIPTION_FACTORS	KEGG	386	42	16	2,73E-01	1,00E+00	rs1676996|rs6508433|rs6755901|rs3748269|rs4942391|rs6142711|rs757575|rs7982845|rs876970|rs3763851|rs2301908|rs13242458|rs11191666|rs4879592|rs11040933|rs9291949
Renal Disorder	TFF_PATHWAY	BIOCARTA	697	31	20	2,73E-01	1,00E+00	rs3813668|rs7315397|rs11167020|rs6788984|rs306962|rs7840147|rs2288726|rs2244621|rs17153616|rs10860361|rs849430|rs13173003|rs853812|rs17746482|rs4245566|rs1004717|rs429988|rs7641889|rs6449947|rs2877260
Renal Disorder	REGULATION_OF_INSULIN_SECRETION	REACTOME	3741	25	20	2,74E-01	1,00E+00	rs10082810|rs17768133|rs1112903|rs1004446|rs308039|rs12905386|rs9948204|rs12878420|rs1422259|rs12746334|rs677874|rs12832680|rs6788984|rs10495431|rs946637|rs10520349|rs4568821|rs1865550|rs1994474|rs12985186
Renal Disorder	SIGNALING_BY_ILS	REACTOME	2996	36	20	2,76E-01	1,00E+00	rs3813668|rs11582424|rs10198990|rs958766|rs1760125|rs2991215|rs2386841|rs1382973|rs4734497|rs3924649|rs1613367|rs1679804|rs1152918|rs10455523|rs7914|rs9695993|rs10793894|rs12434440|rs743562|rs360712
Renal Disorder	REGULATION_OF_INSULIN_LIKE_GROWTH_FACTOR_IGF_ACTIVITY_BY_INSULIN_LIKE_G DING_PROTEINS_IGFBPS	REACTOME	570	16	9	2,76E-01	1,00E+00	rs1004446|rs7214466|rs6478230|rs2206510|rs7540375|rs11981109|rs4752932|rs2280699|rs2294649
Renal Disorder	DRUG_METABOLISM_OTHER_ENZYMES	KEGG	1609	120	20	2,77E-01	1,00E+00	rs1689924|rs2018985|rs11892031|rs12036993|rs1934272|rs2572023|rs4415625|rs1415683|rs1420566|rs1018124|rs2786507|rs7817770|rs4593203|rs6822259|rs13333135|rs12119882|rs1353035|rs4304627|rs207432|rs4734782
Renal Disorder	HOST_INTERACTIONS_OF_HIV_FACTORS	REACTOME	2270	35	20	2,78E-01	1,00E+00	rs503156|rs1554669|rs3852030|rs11861296|rs4576294|rs1723015|rs241437|rs4837334|rs443678|rs9643478|rs9551193|rs7794173|rs4385221|rs1181390|rs7667259|rs4675367|rs7465143|rs444319|rs10226593|rs1559463
Renal Disorder	RNA_POL_III_TRANSCRIPTION_TERMINATION	REACTOME	680	46	20	2,79E-01	1,00E+00	rs303721|rs487674|rs2616211|rs7004724|rs2031195|rs7021022|rs4871975|rs17221107|rs7875762|rs12930464|rs11204135|rs9644645|rs9512817|rs2254883|rs2616241|rs2273933|rs10810057|rs2208919|rs10810134|rs4668163
Renal Disorder	IL2RB_PATHWAY	BIOCARTA	1296	28	20	2,81E-01	1,00E+00	rs3813668|rs9642880|rs1760125|rs2991215|rs12962644|rs2386841|rs7914|rs9695993|rs228979|rs10095762|rs12409333|rs1317063|rs192703|rs2244621|rs17153616|rs849430|rs13173003|rs853812|rs4749882|rs1004717
Renal Disorder	ECM_RECEPTOR_INTERACTION	KEGG	3602	50	20	2,82E-01	1,00E+00	rs10205184|rs1319475|rs6727015|rs7742931|rs9492123|rs2282735|rs6958081|rs10911203|rs9393162|rs12006094|rs1330351|rs4366552|rs2645772|rs1160798|rs3794125|rs2075555|rs7169775|rs7551235|rs3762013|rs2651458
Renal Disorder	INSULIN_RECEPTOR_RECYCLING	REACTOME	492	34	16	2,83E-01	1,00E+00	rs1004446|rs1198873|rs9643478|rs296913|rs7465143|rs7966867|rs3852876|rs1390939|rs12468286|rs896210|rs13238831|rs2229431|rs2460222|rs7258382|rs12659817|rs3856465
Renal Disorder	CLASS_B_2_SECRETIN_FAMILY_RECEPTORS	REACTOME	2257	24	20	2,84E-01	1,00E+00	rs1402704|rs11773897|rs6459961|rs8113491|rs721347|rs12878420|rs12746334|rs1413611|rs10421327|rs1429890|rs12985186|rs4944092|rs1867935|rs1294857|rs358817|rs4798777|rs315580|rs557077|rs7596898|rs1124425
Renal Disorder	SRP_DEPENDENT_COTRANSLATIONAL_PROTEIN_TARGETING_TO_MEMBRANE	REACTOME	1378	46	20	2,86E-01	1,00E+00	rs2291782|rs17768133|rs625149|rs9948204|rs12587507|rs10520349|rs6501697|rs1133099|rs1435961|rs1649990|rs1043149|rs8026641|rs2958522|rs12942418|rs4075144|rs6800812|rs9907408|rs4145041|rs965174|rs10499749
Renal Disorder	SYNTHESIS_AND_INTERCONVERSION_OF_NUCLEOTIDE_DI_AND_TRIPHOSPHATES	REACTOME	306	12	7	2,88E-01	1,00E+00	rs6539145|rs3780663|rs4290308|rs1573736|rs6556882|rs9324161|rs10987759
Renal Disorder	FORMATION_OF_TRANSCRIPTION_COUPLED_NER_TC_NER_REPAIR_COMPLEX	REACTOME	389	48	15	2,89E-01	1,00E+00	rs3796531|rs6761|rs7722373|rs3748269|rs2157072|rs3821996|rs1646314|rs7190295|rs6537537|rs7195205|rs794084|rs2254883|rs12930200|rs4150282|rs6498466
Renal Disorder	PYRIMIDINE_METABOLISM	REACTOME	792	57	20	2,90E-01	1,00E+00	rs1689924|rs12036993|rs944664|rs1934272|rs17664900|rs4415625|rs2298582|rs10017797|rs1890138|rs2786507|rs6922|rs1998393|rs12039225|rs12119882|rs313229|rs13375516|rs4734782|rs11132155|rs11783979|rs12567432
Renal Disorder	RIP_MEDIATED_NFKB_ACTIVATION_VIA_DAI	REACTOME	328	32	14	2,91E-01	1,00E+00	rs7327256|rs17103274|rs34129552|rs3787620|rs461626|rs10498658|rs7668666|rs8126493|rs400819|rs2306365|rs4811887|rs10883452|rs9996894|rs7283136
Renal Disorder	ADHERENS_JUNCTION	KEGG	4520	46	20	2,96E-01	1,00E+00	rs7991448|rs1825428|rs868032|rs4966009|rs7605424|rs2862431|rs7099767|rs4938726|rs183642|rs11196203|rs10996472|rs7904519|rs6788984|rs2113001|rs12761828|rs10865451|rs4074716|rs6506561|rs10455523|rs2684792
Renal Disorder	INFLUENZA_VIRAL_RNA_TRANSCRIPTION_AND_REPLICATION	REACTOME	1153	42	20	2,96E-01	1,00E+00	rs2291782|rs3796531|rs6761|rs12587507|rs6501697|rs4942391|rs1133099|rs585871|rs1435961|rs1649990|rs2958522|rs7982845|rs12942418|rs4075144|rs3821996|rs6800812|rs9907408|rs4145041|rs7983524|rs9561932
Renal Disorder	INTEGRATION_OF_ENERGY_METABOLISM	REACTOME	4501	24	20	2,97E-01	1,00E+00	rs10082810|rs2531995|rs17768133|rs1112903|rs1004446|rs308039|rs12905386|rs9948204|rs12878420|rs1422259|rs12746334|rs2531982|rs677874|rs12832680|rs2304497|rs6788984|rs10495431|rs946637|rs10520349|rs4568821
Renal Disorder	G_BETA_GAMMA_SIGNALLING_THROUGH_PLC_BETA	REACTOME	624	29	20	2,97E-01	1,00E+00	rs12905386|rs12878420|rs12746334|rs12985186|rs6055957|rs6516421|rs6681938|rs8113423|rs17474654|rs16947151|rs594942|rs887030|rs2244621|rs12881038|rs1253676|rs11083841|rs1534897|rs6055927|rs10493754|rs17124637
Renal Disorder	PKB_MEDIATED_EVENTS	REACTOME	583	31	18	2,99E-01	1,00E+00	rs3805492|rs3761840|rs874269|rs7559811|rs2048753|rs1881643|rs7698057|rs11876|rs9568203|rs2231410|rs953221|rs12930464|rs2727564|rs2536075|rs7789674|rs4962081|rs10257529|rs2230571
Renal Disorder	INCRETIN_SYNTHESIS_SECRETION_AND_INACTIVATION	REACTOME	540	28	20	2,99E-01	1,00E+00	rs17768133|rs9948204|rs677874|rs6788984|rs10520349|rs662702|rs10112596|rs4731437|rs965174|rs4865694|rs17153698|rs2936427|rs8100944|rs429988|rs10069886|rs2292462|rs741529|rs6862321|rs11660643|rs9551421
Renal Disorder	RNA_POL_I_RNA_POL_III_AND_MITOCHONDRIAL_TRANSCRIPTION	REACTOME	1830	47	20	3,05E-01	1,00E+00	rs303721|rs1554669|rs487674|rs2616211|rs7004724|rs2798716|rs2031195|rs2239896|rs7021022|rs3748269|rs1994174|rs10826175|rs4871975|rs3786254|rs17221107|rs7875762|rs4485425|rs6537537|rs12930464|rs643788
Renal Disorder	NFKB_PATHWAY	BIOCARTA	870	30	20	3,07E-01	1,00E+00	rs6489711|rs7768419|rs17103274|rs9494850|rs7858743|rs10499196|rs10455523|rs1329057|rs9362732|rs10498658|rs9362744|rs9351265|rs9362786|rs657597|rs2306365|rs2818291|rs10883452|rs12001550|rs2144363|rs10983899
Renal Disorder	VIRAL_MYOCARDITIS	KEGG	2750	44	20	3,09E-01	1,00E+00	rs202892|rs7984956|rs169374|rs9492123|rs2032288|rs2857106|rs2012928|rs10509323|rs7853654|rs1181390|rs3769823|rs4675366|rs9285248|rs241436|rs13204672|rs7721069|rs429916|rs443627|rs2073917|rs8131116
Renal Disorder	NGF_PATHWAY	BIOCARTA	566	32	14	3,10E-01	1,00E+00	rs3813668|rs2760494|rs657770|rs17153616|rs849430|rs1881735|rs13173003|rs853812|rs1004717|rs3742768|rs7641889|rs6449947|rs830474|rs10054692
Renal Disorder	PENTOSE_AND_GLUCURONATE_INTERCONVERSIONS	KEGG	874	99	11	3,10E-01	1,00E+00	rs2018985|rs11892031|rs1018124|rs3770387|rs4593203|rs6822259|rs4732025|rs169045|rs17620012|rs2259458|rs4148307
Renal Disorder	VIBRIO_CHOLERAE_INFECTION	KEGG	1518	35	20	3,12E-01	1,00E+00	rs2531995|rs2283214|rs231915|rs1198873|rs2531982|rs10055964|rs9643478|rs4381631|rs2283174|rs296913|rs1977232|rs12282937|rs7465143|rs7966867|rs2237892|rs3889392|rs16960228|rs1390939|rs12468286|rs3763609
Renal Disorder	BIOSYNTHESIS_OF_THE_N_GLYCAN_PRECURSOR_DOLICHOL_LIPID_LINKED_OLIGOSACC RANSFER_TO_A_NASCENT_PROTEIN	REACTOME	355	35	15	3,12E-01	1,00E+00	rs6415846|rs12304768|rs11564099|rs2239896|rs3004318|rs3739821|rs7615501|rs643788|rs4430364|rs12423422|rs7866897|rs7733582|rs918481|rs633045|rs11789383
Renal Disorder	ACTIVATION_OF_BH3_ONLY_PROTEINS	REACTOME	325	17	8	3,15E-01	1,00E+00	rs12962644|rs652196|rs443627|rs9308742|rs2244621|rs1881735|rs1107480|rs477895
Renal Disorder	VEGF_PATHWAY	BIOCARTA	883	42	20	3,16E-01	1,00E+00	rs3813668|rs12453963|rs11167020|rs2301106|rs4381631|rs3748269|rs306962|rs3889392|rs16960228|rs7840147|rs17153616|rs11871468|rs849430|rs13173003|rs853812|rs1004717|rs13188129|rs7641889|rs6449947|rs1003599
Renal Disorder	LATENT_INFECTION_OF_HOMO_SAPIENS_WITH_MYCOBACTERIUM_TUBERCULOSIS	REACTOME	589	32	14	3,21E-01	1,00E+00	rs9658265|rs1198873|rs9643478|rs296913|rs7465143|rs7966867|rs1390939|rs12468286|rs896210|rs13238831|rs2460222|rs4868237|rs6490121|rs3856465
Renal Disorder	DOWNREGULATION_OF_SMAD2_3_SMAD4_TRANSCRIPTIONAL_ACTIVITY	REACTOME	453	26	17	3,21E-01	1,00E+00	rs1147289|rs621231|rs11006121|rs874583|rs1994174|rs412705|rs556765|rs1989138|rs6504246|rs85440|rs11071939|rs3782262|rs3782267|rs701078|rs10773097|rs893473|rs16912145
Renal Disorder	IGF1_PATHWAY	BIOCARTA	813	33	20	3,23E-01	1,00E+00	rs3813668|rs2760494|rs4966009|rs2684792|rs12440962|rs1317063|rs7168213|rs17822304|rs17153616|rs849430|rs1881735|rs13173003|rs853812|rs11066320|rs1004717|rs3742768|rs6725556|rs7641889|rs6449947|rs7168671
Renal Disorder	INOSITOL_PHOSPHATE_METABOLISM	KEGG	2355	31	20	3,27E-01	1,00E+00	rs1547643|rs4646864|rs1223577|rs12905386|rs16976931|rs7238810|rs731945|rs11044232|rs9959057|rs10502786|rs2928464|rs9365723|rs6055957|rs3740360|rs12686355|rs1943600|rs6516421|rs8089834|rs10747068|rs17761155
Renal Disorder	INSULIN_RECEPTOR_SIGNALLING_CASCADE	REACTOME	3006	32	20	3,27E-01	1,00E+00	rs3813668|rs3805492|rs263931|rs7318731|rs1004446|rs7984646|rs7238810|rs3761840|rs2517037|rs249736|rs3924649|rs874269|rs7559811|rs9959057|rs10502786|rs4770252|rs2048753|rs1379707|rs1536433|rs1943600
Renal Disorder	CYTOSOLIC_DNA_SENSING_PATHWAY	KEGG	620	24	14	3,30E-01	1,00E+00	rs17103274|rs9972960|rs1613367|rs360712|rs7793163|rs4871975|rs10498658|rs17221107|rs387149|rs2306365|rs9512817|rs4811887|rs10883452|rs741810
Renal Disorder	GENERATION_OF_SECOND_MESSENGER_MOLECULES	REACTOME	616	50	20	3,30E-01	1,00E+00	rs1554669|rs11646140|rs25800|rs1723015|rs13204672|rs252353|rs35571839|rs1718414|rs2084385|rs10995|rs12166465|rs9276516|rs9611234|rs3019245|rs17534481|rs4938507|rs3019244|rs10892234|rs2298887|rs2179915
Renal Disorder	PPARA_ACTIVATES_GENE_EXPRESSION	REACTOME	3675	31	20	3,33E-01	1,00E+00	rs10235960|rs2018985|rs11892031|rs16912392|rs6539145|rs10850606|rs1555208|rs6494232|rs6835696|rs9850957|rs10055872|rs7865126|rs1135889|rs7941340|rs2987392|rs1110462|rs11969866|rs3828090|rs6866285|rs1410117
Renal Disorder	FORMATION_OF_ATP_BY_CHEMIOSMOTIC_COUPLING	REACTOME	77	1	1	3,35E-01	1,00E+00	rs3745004
Renal Disorder	SYNTHESIS_OF_PA	REACTOME	1057	30	20	3,36E-01	1,00E+00	rs1148757|rs2701632|rs12733186|rs711237|rs12988612|rs34129552|rs4551123|rs13160153|rs10454443|rs2920123|rs1668547|rs1668589|rs645953|rs7554573|rs2250227|rs10504041|rs11720070|rs836178|rs12989107|rs11589805
Renal Disorder	TIGHT_JUNCTION_INTERACTIONS	REACTOME	715	27	13	3,36E-01	1,00E+00	rs8000535|rs9676180|rs12866697|rs1705589|rs7551399|rs1286831|rs4497528|rs2063490|rs11009554|rs2835416|rs2057227|rs6122972|rs790055
Renal Disorder	TRANSFERRIN_ENDOCYTOSIS_AND_RECYCLING	REACTOME	513	34	13	3,37E-01	1,00E+00	rs1198873|rs9643478|rs296913|rs865108|rs7465143|rs7966867|rs1390939|rs12468286|rs896210|rs13238831|rs2460222|rs4868237|rs3856465
Renal Disorder	SPPA_PATHWAY	BIOCARTA	1428	36	20	3,37E-01	1,00E+00	rs1148757|rs1760125|rs2991215|rs11167020|rs12733186|rs4381631|rs9695993|rs306962|rs6055957|rs6516421|rs10454443|rs7728560|rs3889392|rs16960228|rs6681938|rs192703|rs7554573|rs2250227|rs7840147|rs11871468
Renal Disorder	DEFENSINS	REACTOME	495	3	1	3,38E-01	1,00E+00	rs2741692
Renal Disorder	ADIPOCYTOKINE_SIGNALING_PATHWAY	KEGG	2106	29	20	3,41E-01	1,00E+00	rs3805492|rs2869441|rs6489711|rs1555208|rs7984646|rs6835696|rs17103274|rs874269|rs596866|rs973639|rs324079|rs6857002|rs1805352|rs3762013|rs9884852|rs10021542|rs1881643|rs759869|rs1409875|rs1460769
Renal Disorder	RETINOL_METABOLISM	KEGG	1512	97	20	3,43E-01	1,00E+00	rs348472|rs2018985|rs11892031|rs17820763|rs2572023|rs747574|rs1018124|rs4593203|rs10786172|rs6822259|rs6735281|rs1934975|rs2275875|rs1229976|rs6692958|rs9888067|rs889125|rs1010570|rs4918317|rs1154454
Renal Disorder	O_LINKED_GLYCOSYLATION_OF_MUCINS	REACTOME	2402	35	20	3,45E-01	1,00E+00	rs7550651|rs1436086|rs4686838|rs13136852|rs7529530|rs4836593|rs3780668|rs427589|rs10052189|rs10849124|rs7690646|rs7801523|rs7960018|rs1436085|rs2241430|rs683969|rs17594632|rs4628548|rs6660107|rs10865331
Renal Disorder	CELL_SURFACE_INTERACTIONS_AT_THE_VASCULAR_WALL	REACTOME	2650	34	20	3,45E-01	1,00E+00	rs202892|rs3813668|rs2922860|rs169374|rs10505105|rs2032288|rs3924649|rs503832|rs12695746|rs11606866|rs2075555|rs925864|rs619145|rs4601425|rs12419365|rs2668020|rs12406751|rs3117638|rs2073917|rs557856
Renal Disorder	BIOLOGICAL_OXIDATIONS	REACTOME	3568	77	20	3,50E-01	1,00E+00	rs10204598|rs2726670|rs348472|rs1217356|rs2018985|rs11892031|rs1902586|rs6116189|rs863915|rs1368421|rs1220709|rs11746351|rs2572023|rs1695|rs2181874|rs17062465|rs2604299|rs4149195|rs1018124|rs2546324
Renal Disorder	CHEMOKINE_RECEPTORS_BIND_CHEMOKINES	REACTOME	726	44	16	3,51E-01	1,00E+00	rs4954410|rs2373154|rs9972960|rs1860180|rs9993375|rs7677281|rs871144|rs9868689|rs223848|rs1513253|rs2064298|rs4423707|rs3780891|rs8073784|rs11080372|rs6693141
Renal Disorder	TNFR1_PATHWAY	BIOCARTA	862	31	20	3,53E-01	1,00E+00	rs2760494|rs6489711|rs10859602|rs874583|rs10455523|rs3769823|rs9362732|rs10498658|rs1718414|rs2084385|rs9362744|rs9351265|rs1485888|rs9362786|rs1881735|rs3019245|rs12082157|rs2818291|rs3019244|rs2144363
Renal Disorder	INHIBITION_OF_VOLTAGE_GATED_CA2_CHANNELS_VIA_GBETA_GAMMA_SUBUNITS	REACTOME	1082	30	20	3,54E-01	1,00E+00	rs1167763|rs12878420|rs12746334|rs2961957|rs16838062|rs7209800|rs12985186|rs2079514|rs6681938|rs872091|rs8113423|rs1787337|rs17474654|rs2836048|rs16947151|rs7226144|rs887030|rs12881038|rs196074|rs2779572
Renal Disorder	FC_GAMMA_R_MEDIATED_PHAGOCYTOSIS	KEGG	3795	31	20	3,55E-01	1,00E+00	rs622442|rs3813668|rs1179486|rs1148757|rs1760125|rs525007|rs2991215|rs11646140|rs4601326|rs1440014|rs6743450|rs4639572|rs874269|rs12733186|rs6705717|rs4381631|rs9695993|rs4129967|rs4385221|rs2486335
Renal Disorder	CCR3_PATHWAY	BIOCARTA	939	40	20	3,58E-01	1,00E+00	rs11167020|rs6716817|rs1860180|rs11044232|rs4381631|rs306962|rs6055957|rs753770|rs6516421|rs3889392|rs16960228|rs6681938|rs7840147|rs11871468|rs1534897|rs13041735|rs6055927|rs17347805|rs8073784|rs1003599
Renal Disorder	PURINE_METABOLISM	KEGG	5442	37	20	3,61E-01	1,00E+00	rs2531995|rs4910146|rs6807877|rs2726670|rs3796531|rs1342236|rs6761|rs1135029|rs11042855|rs289383|rs3742810|rs4235953|rs1700186|rs4583026|rs2531982|rs6565616|rs9365906|rs483832|rs944664|rs6446081
Renal Disorder	GLUTATHIONE_METABOLISM	KEGG	683	32	20	3,61E-01	1,00E+00	rs3934860|rs1405948|rs1695|rs4149195|rs10918228|rs13114510|rs12646059|rs34511626|rs2028299|rs7355084|rs2007084|rs4147599|rs396216|rs6688451|rs2132847|rs9333372|rs885815|rs6004011|rs4932145|rs10735234
Renal Disorder	SEMA4D_IN_SEMAPHORIN_SIGNALING	REACTOME	597	35	20	3,66E-01	1,00E+00	rs6716817|rs183642|rs12551214|rs2012928|rs753770|rs2128833|rs4666415|rs220076|rs1579523|rs8075851|rs2278902|rs342076|rs1002397|rs17054953|rs872313|rs714180|rs686912|rs17138948|rs136206|rs713753
Renal Disorder	G_BETA_GAMMA_SIGNALLING_THROUGH_PI3KGAMMA	REACTOME	487	36	20	3,68E-01	1,00E+00	rs12878420|rs12746334|rs195771|rs874269|rs12985186|rs6681938|rs8113423|rs17474654|rs16947151|rs887030|rs12881038|rs17153616|rs849430|rs1253676|rs1004717|rs11083841|rs10493754|rs17124637|rs1276309|rs6700907
Renal Disorder	P53_SIGNALING_PATHWAY	KEGG	1897	33	20	3,70E-01	1,00E+00	rs4478405|rs12544597|rs7315397|rs17230094|rs7935663|rs7120821|rs17772958|rs726825|rs3846484|rs1861178|rs3769823|rs11824683|rs865108|rs657899|rs714679|rs643571|rs652913|rs732292|rs2303076|rs443627
Renal Disorder	PHASE1_FUNCTIONALIZATION_OF_COMPOUNDS	REACTOME	1515	49	20	3,71E-01	1,00E+00	rs10204598|rs348472|rs1217356|rs1902586|rs6116189|rs863915|rs2572023|rs2181874|rs2065996|rs10786172|rs12459140|rs11992601|rs6735281|rs1934975|rs527643|rs1229976|rs9888067|rs4556118|rs162556|rs1010570
Renal Disorder	TRANSCRIPTION_COUPLED_NER_TC_NER	REACTOME	834	47	20	3,73E-01	1,00E+00	rs3796531|rs6761|rs7722373|rs12602230|rs3748269|rs4790838|rs2303744|rs2157072|rs3821996|rs1646314|rs7190295|rs6537537|rs2244095|rs2323973|rs7195205|rs7332652|rs7208143|rs794084|rs2254883|rs8181839
Renal Disorder	ALPHA_LINOLENIC_ACID_METABOLISM	KEGG	571	40	18	3,77E-01	1,00E+00	rs1148757|rs1135889|rs2701632|rs12733186|rs10454443|rs1668547|rs899491|rs7554573|rs2250227|rs1712435|rs11589805|rs2144438|rs7530853|rs8025614|rs622450|rs9729852|rs6426616|rs1678305
Renal Disorder	ACTIVATED_POINT_MUTANTS_OF_FGFR2	REACTOME	873	26	15	3,77E-01	1,00E+00	rs263931|rs7318731|rs2517037|rs249736|rs4770252|rs1379707|rs1536433|rs17099156|rs4770192|rs822248|rs6859263|rs7914068|rs1860228|rs10788184|rs4424770
Renal Disorder	FCER1_PATHWAY	BIOCARTA	1564	37	20	3,78E-01	1,00E+00	rs3813668|rs2760494|rs1148757|rs1760125|rs2991215|rs12733186|rs9695993|rs6123013|rs2668020|rs10454443|rs7193701|rs1718414|rs192703|rs7554573|rs2084385|rs2250227|rs17733355|rs17153616|rs849430|rs1881740
Renal Disorder	ENDOSOMAL_SORTING_COMPLEX_REQUIRED_FOR_TRANSPORT_ESCRT	REACTOME	318	21	5	3,79E-01	1,00E+00	rs6769886|rs873104|rs734484|rs1002765|rs7160883
Renal Disorder	TRIGLYCERIDE_BIOSYNTHESIS	REACTOME	1182	33	20	3,79E-01	1,00E+00	rs11695702|rs6835696|rs1887571|rs2304497|rs711237|rs12988612|rs11740632|rs34129552|rs4551123|rs13160153|rs2920123|rs10484663|rs7070744|rs10504041|rs2577264|rs11720070|rs836178|rs12989107|rs17007461|rs989950
Renal Disorder	EIF_PATHWAY	BIOCARTA	158	13	6	3,79E-01	1,00E+00	rs12453963|rs7698057|rs12930464|rs7154496|rs2403370|rs2230571
Renal Disorder	ENDOCYTOSIS	KEGG	6484	32	20	3,80E-01	1,00E+00	rs12647577|rs3740694|rs4980806|rs6586773|rs16963744|rs4966009|rs5758941|rs2386841|rs4954410|rs1043313|rs3957526|rs10510496|rs6769886|rs4601326|rs183642|rs2316426|rs10194786|rs4639572|rs1891842|rs4837334
Renal Disorder	RNA_POL_III_TRANSCRIPTION_INITIATION_FROM_TYPE_3_PROMOTER	REACTOME	501	40	17	3,81E-01	1,00E+00	rs1554669|rs2616211|rs7004724|rs2798716|rs4871975|rs17221107|rs12930464|rs11204135|rs1319462|rs9644645|rs9512817|rs2254883|rs2616241|rs12676965|rs10101364|rs6486361|rs11785348
Renal Disorder	MELANOMA	KEGG	2894	40	20	3,81E-01	1,00E+00	rs3813668|rs263931|rs4966009|rs7987595|rs7318731|rs958766|rs183642|rs2517037|rs249736|rs9307949|rs3924649|rs874269|rs939688|rs2684792|rs4770252|rs1379707|rs1536433|rs17405661|rs12440962|rs942042
Renal Disorder	NKT_PATHWAY	BIOCARTA	638	34	20	3,82E-01	1,00E+00	rs4954410|rs2373154|rs9972960|rs1181390|rs4675367|rs743562|rs10515797|rs10784678|rs3790565|rs10748094|rs10878729|rs9376268|rs8127569|rs6662137|rs25887|rs2284553|rs11080372|rs6498016|rs11465597|rs4252340
Renal Disorder	CD28_DEPENDENT_PI3K_AKT_SIGNALING	REACTOME	572	22	12	3,83E-01	1,00E+00	rs3813668|rs874269|rs1181390|rs4675367|rs17073074|rs13173003|rs853812|rs7641889|rs6449947|rs10457242|rs830474|rs10054692
Renal Disorder	INTEGRIN_ALPHAIIB_BETA3_SIGNALING	REACTOME	779	35	20	3,83E-01	1,00E+00	rs1760125|rs2991215|rs11167020|rs9695993|rs306962|rs1318662|rs2295797|rs192703|rs7840147|rs17516906|rs13019093|rs787068|rs1890548|rs291765|rs12426178|rs1250079|rs4913455|rs6726101|rs1986155|rs10492301
Renal Disorder	REGULATION_OF_WATER_BALANCE_BY_RENAL_AQUAPORINS	REACTOME	1393	32	20	3,83E-01	1,00E+00	rs2531995|rs12878420|rs12746334|rs2531982|rs1891842|rs1865550|rs12985186|rs7229075|rs853600|rs10491176|rs17159722|rs2457971|rs2286562|rs6681938|rs1000597|rs8113423|rs17474654|rs3763609|rs16947151|rs887030
Renal Disorder	NOTCH_SIGNALING_PATHWAY	KEGG	1387	27	20	3,83E-01	1,00E+00	rs4687504|rs12243975|rs9999030|rs1147289|rs1250129|rs6074178|rs16878154|rs1877207|rs4678200|rs17659152|rs3761441|rs3124607|rs4687488|rs6108746|rs7951485|rs11245515|rs17189227|rs12646059|rs6078029|rs10870070
Renal Disorder	PEROXISOMAL_LIPID_METABOLISM	REACTOME	435	22	10	3,84E-01	1,00E+00	rs10463469|rs1135889|rs3822464|rs899491|rs92631|rs7866897|rs531592|rs11050103|rs11789383|rs1678305
Renal Disorder	SIGNALING_BY_INSULIN_RECEPTOR	REACTOME	3432	30	20	3,88E-01	1,00E+00	rs3813668|rs3805492|rs263931|rs7318731|rs1004446|rs7984646|rs7238810|rs1198873|rs3761840|rs2517037|rs249736|rs3924649|rs874269|rs7559811|rs9959057|rs10502786|rs9643478|rs4770252|rs2048753|rs1379707
Renal Disorder	NO1_PATHWAY	BIOCARTA	1482	27	20	3,89E-01	1,00E+00	rs1326250|rs1135029|rs1937652|rs4583026|rs8016905|rs2162734|rs1904006|rs4935225|rs10516652|rs341078|rs12611955|rs11045301|rs10491176|rs10997716|rs1937701|rs2339794|rs341065|rs2286562|rs11823971|rs3763609
Renal Disorder	GLYCOSAMINOGLYCAN_METABOLISM	REACTOME	6096	55	20	3,89E-01	1,00E+00	rs17862070|rs12509776|rs11776828|rs2147155|rs11223864|rs2637426|rs7840309|rs7697986|rs7356549|rs2288236|rs1289500|rs4309396|rs10861257|rs530664|rs10853672|rs6508463|rs9999101|rs3794125|rs4148559|rs16872173
Renal Disorder	NITROGEN_METABOLISM	KEGG	587	43	15	3,92E-01	1,00E+00	rs9329911|rs1464327|rs2807907|rs7841820|rs4649923|rs17762822|rs289810|rs186471|rs11634841|rs4501567|rs10494266|rs12410598|rs10911044|rs562271|rs2254327
Renal Disorder	G0_AND_EARLY_G1	REACTOME	258	29	10	3,92E-01	1,00E+00	rs17096208|rs2835809|rs7189819|rs887594|rs2072293|rs4943383|rs9576040|rs2835810|rs4140876|rs2298330
Renal Disorder	PPAR_SIGNALING_PATHWAY	KEGG	1324	34	20	3,94E-01	1,00E+00	rs1555208|rs6835696|rs1135889|rs9298360|rs6784193|rs10846765|rs3828090|rs973639|rs1805352|rs3762013|rs9658119|rs17456435|rs9810603|rs4917696|rs899491|rs997942|rs11090911|rs11742783|rs7070744|rs2120825

Renal Disorder	INSULIN_PATHWAY	BIOCARTA	725	29	20	3,95E-01	1,00E+00	rs3813668|rs2760494|rs1004446|rs3852876|rs1317063|rs17822304|rs17153616|rs849430|rs1881740|rs13173003|rs853812|rs11066320|rs1004717|rs2229431|rs3742768|rs6725556|rs7258382|rs7641889|rs6449947|rs10052248
Renal Disorder	PROGESTERONE_MEDIATED_OOCYTE_MATURATION	KEGG	2535	31	20	3,96E-01	1,00E+00	rs2531995|rs3813668|rs4966009|rs2869441|rs1004446|rs4583026|rs2531982|rs3924649|rs874269|rs2162734|rs1865550|rs2684792|rs3846484|rs11045301|rs1460769|rs12440962|rs13409926|rs2324716|rs3763609|rs11632057
Renal Disorder	PROSTATE_CANCER	KEGG	2870	28	20	3,98E-01	1,00E+00	rs3813668|rs4966009|rs1884722|rs1004446|rs12962644|rs17103274|rs2755217|rs11196203|rs7904519|rs6788984|rs9307949|rs3924649|rs874269|rs939688|rs7777049|rs2684792|rs1695|rs946199|rs17405661|rs12440962
Renal Disorder	GLUCOSE_TRANSPORT	REACTOME	689	27	17	3,98E-01	1,00E+00	rs3852030|rs4837334|rs10762277|rs9551193|rs324079|rs3829185|rs444319|rs630814|rs9678612|rs4338396|rs1754154|rs7976243|rs6699129|rs103612|rs691057|rs8067341|rs11770879
Renal Disorder	GABA_RECEPTOR_ACTIVATION	REACTOME	2705	32	20	4,02E-01	1,00E+00	rs2531995|rs7716847|rs2075716|rs1167763|rs12878420|rs12746334|rs2961957|rs2531982|rs16838062|rs1372491|rs3922855|rs12050748|rs1865550|rs7209800|rs12985186|rs641010|rs2079514|rs12198870|rs6681938|rs872091
Renal Disorder	P75_NTR_RECEPTOR_MEDIATED_SIGNALLING	REACTOME	2669	38	20	4,02E-01	1,00E+00	rs1045739|rs1826785|rs1362991|rs525007|rs12356045|rs17103274|rs6019409|rs4681888|rs12767512|rs333327|rs657770|rs4147184|rs11698214|rs7774757|rs656709|rs4681908|rs4918129|rs12480425|rs17020088|rs6095298
Renal Disorder	ACTINY_PATHWAY	BIOCARTA	290	8	5	4,04E-01	1,00E+00	rs6724135|rs465316|rs9553868|rs4338396|rs9581718
Renal Disorder	TIE2_SIGNALING	REACTOME	790	37	20	4,06E-01	1,00E+00	rs3813668|rs2922860|rs10505105|rs3924649|rs4601425|rs3117638|rs1013779|rs9789645|rs13173003|rs853812|rs11788154|rs7641889|rs6449947|rs8453|rs574628|rs1954720|rs10167913|rs1556170|rs17389103|rs2922861
Renal Disorder	INNATE_IMMUNE_SYSTEM	REACTOME	6278	35	20	4,07E-01	1,00E+00	rs11653896|rs2760494|rs7327256|rs11072638|rs2869441|rs10198990|rs12962644|rs1549669|rs1382973|rs625149|rs9848727|rs3756103|rs186037|rs2794515|rs7768419|rs7238810|rs17103274|rs11006121|rs1613367|rs9494850
Renal Disorder	TRANSCRIPTIONAL_ACTIVITY_OF_SMAD2_SMAD3_SMAD4_HETEROTRIMER	REACTOME	967	28	20	4,10E-01	1,00E+00	rs9642880|rs1147289|rs2364598|rs621231|rs11006121|rs874583|rs1994174|rs412705|rs10095762|rs556765|rs1989138|rs6504246|rs85440|rs11071939|rs2811712|rs3782262|rs3782267|rs701078|rs10091329|rs4939832
Renal Disorder	GLUCAGON_SIGNALING_IN_METABOLIC_REGULATION	REACTOME	1044	30	20	4,10E-01	1,00E+00	rs2531995|rs12878420|rs12746334|rs2531982|rs1865550|rs12985186|rs10491176|rs2286562|rs6681938|rs8113423|rs17474654|rs3763609|rs887030|rs6555491|rs12881038|rs1589880|rs1253676|rs11083841|rs316689|rs13041735
Renal Disorder	CYTOKINE_CYTOKINE_RECEPTOR_INTERACTION	KEGG	6425	31	20	4,12E-01	1,00E+00	rs6478106|rs11582424|rs441250|rs13436213|rs6489711|rs958766|rs1133782|rs2386841|rs4954410|rs3924519|rs6038619|rs8065836|rs2205960|rs235710|rs2373154|rs183642|rs568520|rs6054501|rs9972960|rs10501372
Renal Disorder	EPITHELIAL_CELL_SIGNALING_IN_HELICOBACTER_PYLORI_INFECTION	KEGG	1535	50	20	4,13E-01	1,00E+00	rs2760494|rs2869441|rs460707|rs17103274|rs1198873|rs183642|rs9643478|rs11606866|rs296913|rs7465143|rs2668020|rs1460769|rs7966867|rs1390939|rs12468286|rs17822304|rs7658485|rs896210|rs12640395|rs1881735
Renal Disorder	TRANSMISSION_ACROSS_CHEMICAL_SYNAPSES	REACTOME	9089	24	20	4,14E-01	1,00E+00	rs2531995|rs4980806|rs7716847|rs241905|rs12494778|rs2789588|rs2051248|rs2075716|rs713996|rs2051569|rs241707|rs1674535|rs1322183|rs9688182|rs130274|rs12905386|rs3133861|rs1167763|rs12878420|rs10420331
Renal Disorder	CHONDROITIN_SULFATE_BIOSYNTHESIS	REACTOME	846	31	20	4,15E-01	1,00E+00	rs7356549|rs10861257|rs10853672|rs6508463|rs12544497|rs9422277|rs17715721|rs2254827|rs1420950|rs4319439|rs11106114|rs7816924|rs13180395|rs2975420|rs1352033|rs12318001|rs2029737|rs11245310|rs1907800|rs13265751
Renal Disorder	SIGNALLING_BY_NGF	REACTOME	7869	33	20	4,16E-01	1,00E+00	rs2531995|rs903547|rs1045739|rs3813668|rs4745416|rs917199|rs1826785|rs8026441|rs1362991|rs525007|rs12356045|rs1043313|rs7984646|rs9848727|rs17103274|rs6019409|rs2755217|rs2531982|rs4681888|rs12767512
Renal Disorder	TRANSPORT_OF_INORGANIC_CATIONS_ANIONS_AND_AMINO_ACIDS_OLIGOPEPTIDES	REACTOME	4051	31	20	4,18E-01	1,00E+00	rs17298185|rs4980806|rs6864158|rs2361204|rs1322183|rs12047830|rs2588233|rs2228622|rs7036497|rs1405376|rs9792282|rs10055964|rs12568779|rs7645841|rs12635345|rs11727046|rs11712783|rs7730843|rs368161|rs11709751
Renal Disorder	OTHER_GLYCAN_DEGRADATION	KEGG	648	34	15	4,22E-01	1,00E+00	rs7681905|rs1947202|rs6552287|rs16872173|rs880240|rs12233819|rs12744386|rs10434287|rs17067582|rs1395479|rs1455661|rs13140529|rs10520433|rs7673686|rs2383278
Renal Disorder	GOLGI_ASSOCIATED_VESICLE_BIOGENESIS	REACTOME	1057	46	20	4,24E-01	1,00E+00	rs7725100|rs1043313|rs11861296|rs4519603|rs2025994|rs6863273|rs13165062|rs2232739|rs907638|rs2072824|rs11726508|rs17340112|rs742106|rs2038521|rs10485575|rs909626|rs12000388|rs6111721|rs2304911|rs3780247
Renal Disorder	P53HYPOXIA_PATHWAY	BIOCARTA	517	15	9	4,25E-01	1,00E+00	rs2301106|rs12602230|rs4790838|rs353241|rs11981109|rs1881740|rs6750100|rs7208143|rs6543296
Renal Disorder	TRAF6_MEDIATED_INDUCTION_OF_NFKB_AND_MAP_KINASES_UPON_TLR7_8_OR_9_ACT	REACTOME	2239	41	20	4,25E-01	1,00E+00	rs2760494|rs7327256|rs2869441|rs10198990|rs1382973|rs9848727|rs17103274|rs1153681|rs1679804|rs10455523|rs34129552|rs12434440|rs3787620|rs6582484|rs1460769|rs17555326|rs2715825|rs461626|rs9362732|rs7658485
Renal Disorder	PATHOGENIC_ESCHERICHIA_COLI_INFECTION	KEGG	1256	43	20	4,26E-01	1,00E+00	rs4734497|rs6716817|rs6788984|rs7605392|rs9810952|rs7858743|rs4381631|rs7853654|rs753770|rs1329057|rs3889392|rs16960228|rs4973410|rs10458071|rs17073074|rs17464037|rs9295910|rs7843776|rs11871468|rs2293079
Renal Disorder	CYTOKINE_PATHWAY	BIOCARTA	491	23	12	4,27E-01	1,00E+00	rs743562|rs360712|rs10515797|rs10784678|rs7793163|rs10748094|rs10878729|rs7717673|rs12907134|rs9990563|rs13117878|rs25887
Renal Disorder	LYSOSOME	KEGG	2448	33	20	4,27E-01	1,00E+00	rs217111|rs7725100|rs1043313|rs7681905|rs1947202|rs11861296|rs12476858|rs6552287|rs1756673|rs6845262|rs9643478|rs16872173|rs34129552|rs880240|rs1075845|rs296913|rs12233819|rs7465143|rs12601802|rs7966867
Renal Disorder	ANTIGEN_ACTIVATES_B_CELL_RECEPTOR_LEADING_TO_GENERATION_OF_SECOND_ME	REACTOME	1299	37	20	4,30E-01	1,00E+00	rs3813668|rs1760125|rs2991215|rs12832680|rs1994474|rs7914|rs9695993|rs2220168|rs2668020|rs9668898|rs11048716|rs6584088|rs2035440|rs192703|rs17073074|rs4889384|rs13173003|rs853812|rs563654|rs11083841
Renal Disorder	DEPOSITION_OF_NEW_CENPA_CONTAINING_NUCLEOSOMES_AT_THE_CENTROMERE	REACTOME	637	42	16	4,30E-01	1,00E+00	rs1500480|rs11563256|rs2239896|rs10492892|rs7736312|rs3004318|rs7665370|rs6657480|rs7606893|rs643788|rs12590125|rs10946808|rs1341758|rs4665273|rs2302154|rs7741445
Renal Disorder	PRIMARY_BILE_ACID_BIOSYNTHESIS	KEGG	439	24	9	4,31E-01	1,00E+00	rs10463469|rs3822464|rs6586175|rs92631|rs11992601|rs882610|rs4556118|rs10788637|rs7002672
Renal Disorder	ALDOSTERONE_REGULATED_SODIUM_REABSORPTION	KEGG	1794	28	20	4,33E-01	1,00E+00	rs3813668|rs6489711|rs1004446|rs7984646|rs3924649|rs12695746|rs2461796|rs6566946|rs860294|rs4381631|rs12446463|rs228433|rs985122|rs11724772|rs727981|rs3753519|rs3852876|rs3889392|rs529445|rs16960228
Renal Disorder	MYOGENESIS	REACTOME	1747	29	20	4,35E-01	1,00E+00	rs6121823|rs13040003|rs6089510|rs8097498|rs2207542|rs16941815|rs6788984|rs9947576|rs2426987|rs7853654|rs6129005|rs6061373|rs4801145|rs2715825|rs6071385|rs525205|rs4925207|rs8090143|rs2426913|rs1644047
Renal Disorder	MYD88_MAL_CASCADE_INITIATED_ON_PLASMA_MEMBRANE	REACTOME	2527	35	20	4,36E-01	1,00E+00	rs2760494|rs7327256|rs2869441|rs10198990|rs1382973|rs9848727|rs17103274|rs1153681|rs7858743|rs1679804|rs1152918|rs10455523|rs34129552|rs12434440|rs1329057|rs3787620|rs6582484|rs1460769|rs17555326|rs2715825
Renal Disorder	PHOSPHORYLATION_OF_CD3_AND_TCR_ZETA_CHAINS	REACTOME	521	33	10	4,38E-01	1,00E+00	rs1554669|rs1723015|rs13204672|rs35571839|rs9276487|rs17534481|rs4938507|rs10892234|rs7009399|rs2179915
Renal Disorder	DILATED_CARDIOMYOPATHY	KEGG	4636	46	20	4,40E-01	1,00E+00	rs10205184|rs2531995|rs7984956|rs1319475|rs6727015|rs9492123|rs1674535|rs1229487|rs10420331|rs1356733|rs2531982|rs4245919|rs1865550|rs11130462|rs17759800|rs368161|rs2150043|rs11086023|rs886940|rs9285248
Renal Disorder	SYNTHESIS_OF_BILE_ACIDS_AND_BILE_SALTS	REACTOME	546	24	9	4,42E-01	1,00E+00	rs10463469|rs3822464|rs6586175|rs92631|rs11992601|rs882610|rs4556118|rs10788637|rs7002672
Renal Disorder	LYSOSOME_VESICLE_BIOGENESIS	REACTOME	420	22	10	4,45E-01	1,00E+00	rs1043313|rs11861296|rs2232739|rs17340112|rs2038521|rs12000388|rs2304911|rs3780247|rs3744382|rs12714147
Renal Disorder	REGULATION_OF_INSULIN_SECRETION_BY_GLUCAGON_LIKE_PEPTIDE1	REACTOME	1388	32	20	4,46E-01	1,00E+00	rs10082810|rs12878420|rs12746334|rs12832680|rs1865550|rs1994474|rs12985186|rs10491176|rs2220168|rs7300093|rs2286562|rs6681938|rs11048716|rs8113423|rs10771297|rs17474654|rs3763609|rs16947151|rs7600722|rs887030
Renal Disorder	MITOCHONDRIAL_TRNA_AMINOACYLATION	REACTOME	500	16	7	4,47E-01	1,00E+00	rs4970972|rs1853861|rs6938112|rs6764923|rs730594|rs6793847|rs9328293
Renal Disorder	IGF1R_PATHWAY	BIOCARTA	735	37	20	4,48E-01	1,00E+00	rs3813668|rs4966009|rs2684792|rs10491176|rs12440962|rs2286562|rs1317063|rs3763609|rs11632057|rs2244621|rs1589880|rs13173003|rs853812|rs316689|rs6725556|rs7641889|rs6449947|rs7168671|rs11770994|rs830474
Renal Disorder	APOPTOTIC_EXECUTION_PHASE	REACTOME	1211	30	20	4,49E-01	1,00E+00	rs7327256|rs11167020|rs6788984|rs596866|rs7831595|rs306962|rs3769823|rs16848195|rs1409875|rs9310549|rs4750351|rs10904914|rs1718414|rs2084385|rs7840147|rs943451|rs336629|rs7615501|rs604663|rs16910106
Renal Disorder	HYPERTROPHIC_CARDIOMYOPATHY_HCM	KEGG	4149	46	20	4,50E-01	1,00E+00	rs10205184|rs7984956|rs1319475|rs3805492|rs6727015|rs9492123|rs1674535|rs1229487|rs10420331|rs1356733|rs4245919|rs11130462|rs17759800|rs368161|rs11086023|rs886940|rs9285248|rs1881643|rs12813682|rs7793163
Renal Disorder	INTESTINAL_IMMUNE_NETWORK_FOR_IGA_PRODUCTION	KEGG	1392	35	20	4,53E-01	1,00E+00	rs6489711|rs4954410|rs8065836|rs2857106|rs1181390|rs9922891|rs4675367|rs743562|rs241436|rs13204672|rs7793163|rs429916|rs2580874|rs10932047|rs12616245|rs9990563|rs4273077|rs13117878|rs9276472|rs4749882
Renal Disorder	PHASE_II_CONJUGATION	REACTOME	2059	80	20	4,54E-01	1,00E+00	rs2726670|rs2018985|rs11892031|rs1368421|rs1220709|rs11746351|rs1695|rs17062465|rs2604299|rs4149195|rs1018124|rs2546324|rs6892131|rs3770387|rs10918228|rs7817770|rs4593203|rs13114510|rs6709978|rs12646059
Renal Disorder	ACTIVATION_OF_KAINATE_RECEPTORS_UPON_GLUTAMATE_BINDING	REACTOME	1577	27	20	4,54E-01	1,00E+00	rs2051248|rs12905386|rs3133861|rs12878420|rs12746334|rs16823912|rs9390939|rs12985186|rs3995732|rs6055957|rs6516421|rs10892629|rs6681938|rs8113423|rs17474654|rs768858|rs1465164|rs16947151|rs594942|rs1269711
Renal Disorder	PLATELET_AGGREGATION_PLUG_FORMATION	REACTOME	942	34	20	4,55E-01	1,00E+00	rs1760125|rs2991215|rs11167020|rs9695993|rs306962|rs1318662|rs12778878|rs2295797|rs192703|rs7840147|rs17516906|rs13019093|rs787068|rs1890548|rs4076557|rs291765|rs12426178|rs1250079|rs4913455|rs4917596
Renal Disorder	SIGNALING_BY_FGFR1_MUTANTS	REACTOME	1243	28	20	4,58E-01	1,00E+00	rs3813668|rs263931|rs7318731|rs2517037|rs249736|rs4770252|rs2906725|rs1379707|rs430435|rs1536433|rs407366|rs11974028|rs7825208|rs17099156|rs13173003|rs853812|rs4770192|rs822248|rs7641889|rs6449947
Renal Disorder	ALANINE_ASPARTATE_AND_GLUTAMATE_METABOLISM	KEGG	841	40	19	4,60E-01	1,00E+00	rs11679258|rs17001868|rs1330581|rs8190612|rs2490286|rs1641002|rs914976|rs4920566|rs1873061|rs2509712|rs186471|rs876848|rs7355583|rs7733582|rs918481|rs10911044|rs10736660|rs2254327|rs970169
Renal Disorder	PRION_DISEASES	KEGG	1585	35	20	4,61E-01	1,00E+00	rs186037|rs2826875|rs967173|rs10911203|rs2827765|rs3851570|rs9976114|rs2826895|rs2004385|rs7793163|rs3124607|rs17201103|rs4936186|rs978815|rs1245138|rs1011838|rs3763609|rs11603384|rs17073074|rs873803
Renal Disorder	TRANS_GOLGI_NETWORK_VESICLE_BUDDING	REACTOME	1106	46	20	4,64E-01	1,00E+00	rs7725100|rs1043313|rs11861296|rs4519603|rs2025994|rs6863273|rs13165062|rs2232739|rs907638|rs2072824|rs11726508|rs17340112|rs742106|rs1890060|rs2038521|rs10485575|rs909626|rs12000388|rs6111721|rs2304911
Renal Disorder	CSK_PATHWAY	BIOCARTA	554	25	15	4,65E-01	1,00E+00	rs1554669|rs1723015|rs10491176|rs2286562|rs6681938|rs28605404|rs3763609|rs1589880|rs316689|rs13041735|rs17534481|rs4938507|rs2858870|rs11770994|rs10892234
Renal Disorder	HOMOLOGOUS_RECOMBINATION	KEGG	730	35	19	4,65E-01	1,00E+00	rs419893|rs1016548|rs11211262|rs6985793|rs12602230|rs4790838|rs5756807|rs4902562|rs2244095|rs7402445|rs7208143|rs206111|rs4145409|rs861528|rs3092989|rs10258735|rs4899246|rs17482047|rs1023529
Renal Disorder	LIGAND_GATED_ION_CHANNEL_TRANSPORT	REACTOME	1051	27	20	4,66E-01	1,00E+00	rs7716847|rs2075716|rs1372491|rs3922855|rs12050748|rs4695935|rs12198870|rs2200457|rs10022789|rs751994|rs9362636|rs209348|rs1035751|rs10517664|rs1863455|rs255487|rs10519552|rs4707529|rs748464|rs13193513
Renal Disorder	MITOCHONDRIAL_PROTEIN_IMPORT	REACTOME	609	26	17	4,67E-01	1,00E+00	rs11731396|rs7559331|rs773652|rs7667259|rs1133099|rs405509|rs10266523|rs3745004|rs989925|rs3763609|rs3821096|rs3212276|rs16849483|rs7531062|rs11938388|rs7190048|rs11726245
Renal Disorder	SHC_RELATED_EVENTS	REACTOME	302	13	9	4,68E-01	1,00E+00	rs1004446|rs3924649|rs3852876|rs12459940|rs2229431|rs7258382|rs8453|rs10842515|rs350825
Renal Disorder	G1_PHASE	REACTOME	625	37	17	4,69E-01	1,00E+00	rs4478405|rs17772958|rs1861178|rs942042|rs11135929|rs7189819|rs746720|rs2811709|rs9381100|rs12173146|rs7133323|rs3735219|rs10411335|rs4531067|rs399413|rs633045|rs17318844
Renal Disorder	TCR_PATHWAY	BIOCARTA	1517	37	20	4,69E-01	1,00E+00	rs3813668|rs2760494|rs1554669|rs11646140|rs17103274|rs1723015|rs4381631|rs6123013|rs7193701|rs252353|rs3889392|rs16960228|rs17733355|rs17073074|rs17153616|rs11871468|rs849430|rs1881740|rs13173003|rs853812
Renal Disorder	INWARDLY_RECTIFYING_K_CHANNELS	REACTOME	1317	29	20	4,71E-01	1,00E+00	rs1167763|rs12878420|rs12746334|rs2961957|rs16838062|rs7209800|rs12985186|rs2079514|rs6681938|rs872091|rs8113423|rs1787337|rs17474654|rs2836048|rs16947151|rs7226144|rs887030|rs12881038|rs196074|rs722341
Renal Disorder	A_TETRASACCHARIDE_LINKER_SEQUENCE_IS_REQUIRED_FOR_GAG_SYNTHESIS	REACTOME	1731	42	20	4,71E-01	1,00E+00	rs2147155|rs11223864|rs2637426|rs1289500|rs9524299|rs3848064|rs4400920|rs9523342|rs2651458|rs9589601|rs7981707|rs7932707|rs11224189|rs3848044|rs12867304|rs11106114|rs6492589|rs878949|rs1391005|rs13180395
Renal Disorder	DRUG_METABOLISM_CYTOCHROME_P450	KEGG	1661	83	20	4,72E-01	1,00E+00	rs2018985|rs11892031|rs2572023|rs1695|rs4149195|rs1018124|rs10918228|rs2354432|rs4593203|rs1409655|rs13114510|rs12646059|rs6822259|rs34511626|rs1934975|rs7355084|rs4147599|rs396216|rs2132847|rs9333372
Renal Disorder	IRON_UPTAKE_AND_TRANSPORT	REACTOME	733	39	18	4,75E-01	1,00E+00	rs2728125|rs1198873|rs9643478|rs296913|rs865108|rs7465143|rs7966867|rs907638|rs1390939|rs12468286|rs896210|rs13238831|rs7624706|rs2460222|rs1284864|rs1109748|rs4868237|rs3856465
Renal Disorder	INTERACTION_BETWEEN_L1_AND_ANKYRINS	REACTOME	970	41	20	4,76E-01	1,00E+00	rs1406275|rs1992294|rs9297840|rs2403773|rs2052313|rs9856587|rs11599447|rs2300045|rs17333297|rs1457784|rs4667911|rs1805124|rs13385399|rs353123|rs12273127|rs12154880|rs1447218|rs8025614|rs10101217|rs13411795
Renal Disorder	G_PROTEIN_ACTIVATION	REACTOME	703	37	20	4,76E-01	1,00E+00	rs12878420|rs12746334|rs1319339|rs12985186|rs1799917|rs3790112|rs6681938|rs8113423|rs17474654|rs16947151|rs887030|rs12881038|rs1253676|rs2235751|rs11083841|rs9479657|rs10493754|rs17124637|rs1276309|rs598832
Renal Disorder	AMINOACYL_TRNA_BIOSYNTHESIS	KEGG	731	22	11	4,77E-01	1,00E+00	rs4970972|rs17034466|rs1853861|rs6894208|rs6938112|rs6764923|rs730594|rs156319|rs6793847|rs9328293|rs1003000
Renal Disorder	INITIAL_TRIGGERING_OF_COMPLEMENT	REACTOME	434	17	9	4,77E-01	1,00E+00	rs186037|rs2794515|rs2072633|rs292007|rs2204757|rs2794520|rs10824842|rs237554|rs11003231
Renal Disorder	IL17_PATHWAY	BIOCARTA	316	17	12	4,79E-01	1,00E+00	rs1554669|rs1723015|rs12406751|rs7793163|rs1009897|rs17534481|rs25887|rs4938507|rs656801|rs10892234|rs10923137|rs938486
Renal Disorder	PROSTACYCLIN_SIGNALLING_THROUGH_PROSTACYCLIN_RECEPTOR	REACTOME	353	30	16	4,79E-01	1,00E+00	rs12878420|rs12746334|rs12985186|rs6681938|rs8113423|rs17474654|rs16947151|rs887030|rs12881038|rs1253676|rs11083841|rs13041735|rs10493754|rs17124637|rs1276309|rs6700907
Renal Disorder	SMAD2_SMAD3_SMAD4_HETEROTRIMER_REGULATES_TRANSCRIPTION	REACTOME	715	15	11	4,79E-01	1,00E+00	rs9642880|rs2364598|rs412705|rs10095762|rs85440|rs11071939|rs2811712|rs10091329|rs4939832|rs893473|rs12869996
Renal Disorder	FATTY_ACID_TRIACYLGLYCEROL_AND_KETONE_BODY_METABOLISM	REACTOME	5526	31	20	4,80E-01	1,00E+00	rs10235960|rs11695702|rs2018985|rs11892031|rs16912392|rs6539145|rs10850606|rs1555208|rs1147289|rs6494232|rs1543932|rs6835696|rs9850957|rs10055872|rs1887571|rs7865126|rs837322|rs1135889|rs2304497|rs7941340
Renal Disorder	BCR_PATHWAY	BIOCARTA	1053	42	20	4,81E-01	1,00E+00	rs2760494|rs1760125|rs2991215|rs4381631|rs9695993|rs6123013|rs2668020|rs7193701|rs3889392|rs16960228|rs192703|rs17733355|rs11871468|rs1881740|rs4239343|rs1755994|rs11083841|rs3742768|rs1027990|rs10993698
Renal Disorder	ACTIVATED_TLR4_SIGNALLING	REACTOME	2676	35	20	4,82E-01	1,00E+00	rs2760494|rs7327256|rs2869441|rs10198990|rs1382973|rs9848727|rs17103274|rs1153681|rs7858743|rs1679804|rs1152918|rs10455523|rs34129552|rs7143468|rs12434440|rs1329057|rs3787620|rs6582484|rs1460769|rs17555326
Renal Disorder	DEADENYLATION_OF_MRNA	REACTOME	331	29	9	4,82E-01	1,00E+00	rs7138763|rs36664|rs4761134|rs1271211|rs7698057|rs871683|rs6948145|rs2230571|rs12922485
Renal Disorder	TH1TH2_PATHWAY	BIOCARTA	535	31	18	4,84E-01	1,00E+00	rs2386841|rs1181390|rs4675366|rs360712|rs10515797|rs10784678|rs3790565|rs10748094|rs28605404|rs10878729|rs9376268|rs4749882|rs8127569|rs2858870|rs2284553|rs7090530|rs6498016|rs11465597
Renal Disorder	SIGNALING_BY_RHO_GTPASES	REACTOME	4877	23	20	4,84E-01	1,00E+00	rs1409084|rs9597687|rs12647577|rs6797462|rs525007|rs9398918|rs4838605|rs6019409|rs6803548|rs12218054|rs4681888|rs1569707|rs333327|rs10491035|rs7596229|rs9315226|rs2410038|rs220531|rs11732086|rs6750487
Renal Disorder	ACH_PATHWAY	BIOCARTA	602	34	20	4,85E-01	1,00E+00	rs3813668|rs11167020|rs4796421|rs306962|rs4635969|rs7840147|rs2244621|rs17153616|rs3828246|rs849430|rs484754|rs13173003|rs853812|rs1004717|rs7641889|rs6449947|rs2021840|rs1190452|rs859637|rs6018257
Renal Disorder	ABC_TRANSPORTERS	KEGG	2684	65	20	4,85E-01	1,00E+00	rs10231745|rs212082|rs4148426|rs2728125|rs2857106|rs4148434|rs11772384|rs12054261|rs717707|rs241436|rs12582802|rs1975194|rs17132823|rs3752237|rs38780|rs4148006|rs9524681|rs2277122|rs12718322|rs3213473
Renal Disorder	NUCLEOTIDE_EXCISION_REPAIR	REACTOME	956	47	20	4,85E-01	1,00E+00	rs3796531|rs6761|rs7722373|rs12602230|rs3748269|rs4790838|rs2303744|rs2157072|rs3821996|rs1646314|rs7190295|rs6537537|rs2244095|rs2323973|rs7195205|rs7332652|rs7038659|rs7208143|rs794084|rs2254883
Renal Disorder	LINOLEIC_ACID_METABOLISM	KEGG	716	36	20	4,86E-01	1,00E+00	rs1148757|rs2701632|rs12733186|rs2572023|rs10454443|rs1668547|rs1409655|rs7554573|rs2250227|rs1934975|rs1712435|rs11589805|rs2144438|rs9888067|rs7530853|rs8025614|rs1010570|rs622450|rs9729852|rs6426616
Renal Disorder	NEUROTROPHIN_SIGNALING_PATHWAY	KEGG	3689	32	20	4,87E-01	1,00E+00	rs1045739|rs3813668|rs2760494|rs2869441|rs12962644|rs9688182|rs7984646|rs4734497|rs17103274|rs4078293|rs3924649|rs874269|rs1152918|rs10793894|rs657770|rs7853654|rs4724298|rs299847|rs11632476|rs6582484
Renal Disorder	CHYLOMICRON_MEDIATED_LIPID_TRANSPORT	REACTOME	435	17	9	4,89E-01	1,00E+00	rs8111157|rs11668477|rs925864|rs405509|rs8039477|rs878949|rs7172930|rs7601441|rs388065
Renal Disorder	SEMAPHORIN_INTERACTIONS	REACTOME	2884	43	20	4,92E-01	1,00E+00	rs6882497|rs2722987|rs652856|rs6716817|rs659632|rs183642|rs10863725|rs12551214|rs2776937|rs11945109|rs2012928|rs12659273|rs6950591|rs477077|rs11009311|rs2713616|rs200094|rs17299268|rs753770|rs2128833
Renal Disorder	NA_CL_DEPENDENT_NEUROTRANSMITTER_TRANSPORTERS	REACTOME	718	30	18	4,95E-01	1,00E+00	rs4980806|rs2697152|rs7523468|rs363222|rs3779672|rs3922763|rs12490575|rs4610207|rs7970932|rs9844272|rs12230144|rs7959178|rs9429225|rs11580716|rs10060827|rs3785152|rs2270650|rs2428965
Renal Disorder	METABOLISM_OF_VITAMINS_AND_COFACTORS	REACTOME	1119	32	20	4,96E-01	1,00E+00	rs8096713|rs9948558|rs1523955|rs7789134|rs3096|rs11621555|rs9369152|rs9847705|rs1754154|rs1881847|rs7976243|rs6699129|rs11151933|rs16849220|rs6979644|rs17339921|rs17649254|rs1776967|rs7585481|rs675220
Renal Disorder	CYTOSOLIC_TRNA_AMINOACYLATION	REACTOME	312	8	5	5,02E-01	1,00E+00	rs17034466|rs6894208|rs2039521|rs156319|rs1003000
Renal Disorder	GROWTH_HORMONE_RECEPTOR_SIGNALING	REACTOME	773	32	13	5,03E-01	1,00E+00	rs13436213|rs7984646|rs12521272|rs2668020|rs1317063|rs9466269|rs6725556|rs2962101|rs9904782|rs6933317|rs1027990|rs6903568|rs4444401
Renal Disorder	SYNTHESIS_OF_PIPS_AT_THE_GOLGI_MEMBRANE	REACTOME	788	47	19	5,05E-01	1,00E+00	rs7238810|rs11044232|rs9959057|rs10502786|rs1943600|rs8089834|rs12457578|rs12490575|rs3764459|rs12327116|rs4146444|rs12606897|rs2305688|rs12957072|rs7771000|rs12960377|rs9304251|rs8090817|rs10499054
Renal Disorder	TYPE_I_DIABETES_MELLITUS	KEGG	1975	42	20	5,08E-01	1,00E+00	rs1983298|rs1004446|rs2857106|rs1330581|rs10509323|rs8190612|rs2335832|rs1181390|rs4675367|rs241436|rs10515797|rs13204672|rs10784678|rs429916|rs10748094|rs221219|rs10878729|rs12111032|rs6944306|rs876848
Renal Disorder	INSULIN_SYNTHESIS_AND_PROCESSING	REACTOME	1033	41	20	5,09E-01	1,00E+00	rs6136050|rs1004446|rs12324775|rs6044732|rs1977232|rs8019012|rs883382|rs6104567|rs2788236|rs835264|rs7768444|rs2936427|rs7703932|rs1286371|rs2317079|rs2304911|rs17134857|rs13175238|rs11857829|rs6950383
Renal Disorder	GAP_JUNCTION_ASSEMBLY	REACTOME	330	11	5	5,10E-01	1,00E+00	rs1886748|rs6929363|rs952477|rs6919417|rs9342215
Renal Disorder	SYNTHESIS_OF_GLYCOSYLPHOSPHATIDYLINOSITOL_GPI	REACTOME	485	47	18	5,13E-01	1,00E+00	rs6415846|rs652856|rs477077|rs8007199|rs7174373|rs3739821|rs8034783|rs12437829|rs13102358|rs17069203|rs1912629|rs7763|rs166835|rs527365|rs4261469|rs2835569|rs10519150|rs2178099
Renal Disorder	G1_S_SPECIFIC_TRANSCRIPTION	REACTOME	194	4	3	5,16E-01	1,00E+00	rs2298582|rs4943383|rs9576040
Renal Disorder	CCR5_PATHWAY	BIOCARTA	696	36	19	5,16E-01	1,00E+00	rs2760494|rs4954410|rs9972960|rs4381631|rs3091237|rs3889392|rs16960228|rs11871468|rs1881735|rs1024611|rs11083841|rs3742768|rs3780891|rs1003599|rs11626241|rs16959569|rs4788426|rs10137869|rs9897002
Renal Disorder	APOPTOTIC_CLEAVAGE_OF_CELLULAR_PROTEINS	REACTOME	1052	32	20	5,19E-01	1,00E+00	rs11167020|rs6788984|rs596866|rs7831595|rs306962|rs3769823|rs16848195|rs1409875|rs9310549|rs4750351|rs10904914|rs7840147|rs943451|rs336629|rs7615501|rs604663|rs16910106|rs832179|rs429988|rs17154230
Renal Disorder	GLYCEROPHOSPHOLIPID_BIOSYNTHESIS	REACTOME	2483	30	20	5,19E-01	1,00E+00	rs622442|rs11695702|rs1148757|rs6053183|rs2701632|rs12733186|rs711237|rs12988612|rs34129552|rs4551123|rs2307278|rs17560881|rs13160153|rs2076213|rs867687|rs6133248|rs10454443|rs1917806|rs2920123|rs2303744
Renal Disorder	TRAF6_MEDIATED_IRF7_ACTIVATION	REACTOME	433	13	8	5,22E-01	1,00E+00	rs3756103|rs1342852|rs17686080|rs7655371|rs1267034|rs1077767|rs7585907|rs1059492

Renal Disorder	MEIOTIC_RECOMBINATION	REACTOME	993	62	20	5,25E-01	1,00E+00	rs10043677|rs2239896|rs6985793|rs13162827|rs12602230|rs17312807|rs4790838|rs975072|rs1980455|rs4485425|rs7402445|rs643788|rs7208143|rs206111|rs10946808|rs9686666|rs1341758|rs3092989|rs10258735|rs17482047
Renal Disorder	RNA_DEGRADATION	KEGG	1123	48	20	5,28E-01	1,00E+00	rs10199432|rs7138763|rs234605|rs36664|rs234587|rs1844710|rs4761134|rs1271211|rs2072633|rs9784647|rs11553287|rs13032405|rs11244096|rs7602687|rs7187181|rs10814620|rs871683|rs275490|rs12153548|rs11707321
Renal Disorder	DESTABILIZATION_OF_MRNA_BY_KSRP	REACTOME	205	15	8	5,29E-01	1,00E+00	rs4734497|rs9784647|rs17464037|rs11244096|rs13259960|rs10814620|rs11707321|rs12922485
Renal Disorder	ECM_PATHWAY	BIOCARTA	753	38	20	5,29E-01	1,00E+00	rs3813668|rs11167020|rs2383289|rs306962|rs2295797|rs7840147|rs17125|rs17073074|rs17153616|rs1275659|rs849430|rs13173003|rs853812|rs1004717|rs7641889|rs6449947|rs10457242|rs12340264|rs1499280|rs6018257
Renal Disorder	LONG_TERM_DEPRESSION	KEGG	3926	26	20	5,29E-01	1,00E+00	rs4966009|rs1148757|rs12669290|rs1326250|rs308039|rs9658265|rs12905386|rs1937652|rs1422259|rs362848|rs12832680|rs2701632|rs3924649|rs11933263|rs12733186|rs1994474|rs9324756|rs4381631|rs2684792|rs1904006
Renal Disorder	COLORECTAL_CANCER	KEGG	2539	34	20	5,31E-01	1,00E+00	rs3813668|rs2760494|rs9642880|rs2869441|rs12962644|rs9966514|rs11196203|rs7904519|rs6788984|rs3924649|rs874269|rs946199|rs412705|rs1460769|rs219463|rs10095762|rs4940144|rs7658485|rs4401133|rs2244621
Renal Disorder	PD1_SIGNALING	REACTOME	398	37	12	5,34E-01	1,00E+00	rs1554669|rs1723015|rs13204672|rs35571839|rs9276472|rs17534481|rs4938507|rs1556188|rs7869190|rs10892234|rs16923198|rs6914616
Renal Disorder	NUCLEOTIDE_LIKE_PURINERGIC_RECEPTORS	REACTOME	272	18	6	5,35E-01	1,00E+00	rs10776728|rs4944831|rs1565573|rs7652832|rs7625688|rs557451
Renal Disorder	CIRCADIAN_REPRESSION_OF_EXPRESSION_BY_REV_ERBA	REACTOME	1358	29	20	5,35E-01	1,00E+00	rs16912392|rs1555208|rs6494232|rs9850957|rs6486099|rs6857002|rs1805352|rs9884852|rs10021542|rs754499|rs2899664|rs16873865|rs6494217|rs17204910|rs11090911|rs12630639|rs1234805|rs6803450|rs6810541|rs13295631
Renal Disorder	PRE_NOTCH_TRANSCRIPTION_AND_TRANSLATION	REACTOME	835	29	19	5,36E-01	1,00E+00	rs2760494|rs9999030|rs4789523|rs16878154|rs1877207|rs470113|rs942042|rs7951485|rs12646059|rs746720|rs9291482|rs1238|rs16878186|rs6816542|rs6771941|rs4598633|rs10939100|rs17318844|rs736349
Renal Disorder	GABA_B_RECEPTOR_ACTIVATION	REACTOME	1863	30	20	5,38E-01	1,00E+00	rs2531995|rs1167763|rs12878420|rs12746334|rs2961957|rs2531982|rs16838062|rs1865550|rs7209800|rs12985186|rs641010|rs2079514|rs6681938|rs872091|rs8113423|rs1787337|rs17474654|rs2836048|rs16947151|rs7226144
Renal Disorder	IL22BP_PATHWAY	BIOCARTA	296	18	11	5,43E-01	1,00E+00	rs11570908|rs2643626|rs1869624|rs12409333|rs11216667|rs1026791|rs4330872|rs276567|rs9904782|rs10892204|rs4444401
Renal Disorder	REGULATION_OF_GENE_EXPRESSION_IN_BETA_CELLS	REACTOME	716	26	15	5,46E-01	1,00E+00	rs1004446|rs2755217|rs677874|rs874269|rs7983897|rs662702|rs16939060|rs1203951|rs12625315|rs6132536|rs17008250|rs10957761|rs169226|rs1169307|rs1397734
Renal Disorder	CHOLESTEROL_BIOSYNTHESIS	REACTOME	259	5	4	5,47E-01	1,00E+00	rs2075906|rs11076696|rs1891805|rs10161401
Renal Disorder	RORA_ACTIVATES_CIRCADIAN_EXPRESSION	REACTOME	1377	29	20	5,48E-01	1,00E+00	rs16912392|rs1555208|rs6494232|rs9850957|rs6486099|rs6857002|rs1805352|rs9884852|rs10021542|rs754499|rs2899664|rs16873865|rs6494217|rs17204910|rs11090911|rs12630639|rs1234805|rs6803450|rs6810541|rs13295631
Renal Disorder	FAS_PATHWAY	BIOCARTA	980	28	20	5,50E-01	1,00E+00	rs2760494|rs7660964|rs874583|rs10455523|rs3769823|rs9362732|rs1718414|rs2084385|rs9362744|rs9351265|rs11097115|rs2046316|rs9362786|rs1881740|rs3124743|rs3019245|rs12082157|rs2818291|rs40383|rs2021840
Renal Disorder	DNA_REPAIR	REACTOME	2111	45	20	5,51E-01	1,00E+00	rs3796531|rs12485319|rs6761|rs4135063|rs1990783|rs934419|rs2239896|rs6985793|rs7722373|rs12602230|rs3748269|rs2005618|rs2027136|rs4790838|rs2303744|rs2157072|rs420259|rs8107282|rs3821996|rs1646314
Renal Disorder	SIGNALING_BY_FGFR1_FUSION_MUTANTS	REACTOME	639	24	13	5,51E-01	1,00E+00	rs3813668|rs2906725|rs430435|rs407366|rs11974028|rs13173003|rs853812|rs7641889|rs6449947|rs6003709|rs1060697|rs830474|rs10054692
Renal Disorder	TRANSPORT_OF_GLUCOSE_AND_OTHER_SUGARS_BILE_SALTS_AND_ORGANIC_ACIDS_M NE_COMPOUNDS	REACTOME	3234	33	20	5,54E-01	1,00E+00	rs11173212|rs4895396|rs4980806|rs6569926|rs7603445|rs7544111|rs11778200|rs2023705|rs2697152|rs7523468|rs363222|rs2811689|rs13266634|rs3779672|rs2360101|rs4810539|rs3922763|rs7017745|rs3199966|rs12490575
Renal Disorder	FATTY_ACYL_COA_BIOSYNTHESIS	REACTOME	404	25	14	5,54E-01	1,00E+00	rs6835696|rs1887571|rs2304497|rs11740632|rs10484663|rs7070744|rs2817110|rs16891209|rs7744440|rs7097428|rs7694475|rs2285655|rs7211875|rs710251
Renal Disorder	TAK1_ACTIVATES_NFKB_BY_PHOSPHORYLATION_AND_ACTIVATION_OF_IKKS_COMPLE	REACTOME	777	38	20	5,56E-01	1,00E+00	rs7327256|rs17103274|rs10455523|rs34129552|rs3787620|rs461626|rs9362732|rs9362744|rs8126493|rs9351265|rs400819|rs2046316|rs9362786|rs2306365|rs2818291|rs40383|rs10883452|rs2144363|rs12679902|rs7283136
Renal Disorder	CDC42RAC_PATHWAY	BIOCARTA	435	20	11	5,62E-01	1,00E+00	rs3813668|rs6724135|rs465316|rs13173003|rs853812|rs3019245|rs7641889|rs6449947|rs3019244|rs830474|rs10054692
Renal Disorder	MET_PATHWAY	BIOCARTA	1415	27	20	5,63E-01	1,00E+00	rs3813668|rs2760494|rs958766|rs11167020|rs183642|rs10793894|rs306962|rs17822304|rs10830079|rs7840147|rs1890548|rs17153616|rs2887070|rs1275659|rs1123551|rs4338396|rs849430|rs1881740|rs12459940|rs13173003
Renal Disorder	IGF1MTOR_PATHWAY	BIOCARTA	582	34	17	5,65E-01	1,00E+00	rs3813668|rs4966009|rs2684792|rs12440962|rs7698057|rs7168213|rs13173003|rs853812|rs7641889|rs6449947|rs7168671|rs703592|rs756847|rs1709642|rs4603211|rs830474|rs10054692
Renal Disorder	CYTOKINE_SIGNALING_IN_IMMUNE_SYSTEM	REACTOME	6819	31	20	5,67E-01	1,00E+00	rs3813668|rs11582424|rs1532472|rs13436213|rs10198990|rs958766|rs1760125|rs2991215|rs3852030|rs2386841|rs1382973|rs9688182|rs7984646|rs3756103|rs4734497|rs4576294|rs3851570|rs3924649|rs241437|rs1613367
Renal Disorder	CHEMOKINE_SIGNALING_PATHWAY	KEGG	5407	27	20	5,69E-01	1,00E+00	rs2531995|rs3813668|rs1179486|rs525007|rs4954410|rs11167020|rs25800|rs6716817|rs12905386|rs12878420|rs2373154|rs12746334|rs17103274|rs6019409|rs9972960|rs2531982|rs3924649|rs874269|rs1860180|rs1865550
Renal Disorder	TRANSPORT_OF_VITAMINS_NUCLEOSIDES_AND_RELATED_MOLECULES	REACTOME	1154	47	20	5,73E-01	1,00E+00	rs6496875|rs10501421|rs6062689|rs7175096|rs7182304|rs207963|rs9369465|rs2487068|rs16946175|rs12422149|rs2061714|rs11635769|rs997942|rs11742783|rs4149056|rs709370|rs6062840|rs1399936|rs6478827|rs6559770
Renal Disorder	CXCR4_PATHWAY	BIOCARTA	1099	38	20	5,76E-01	1,00E+00	rs3813668|rs4954410|rs11167020|rs11044232|rs4381631|rs306962|rs3889392|rs16960228|rs6681938|rs7840147|rs11871468|rs13173003|rs853812|rs2306365|rs3780891|rs7641889|rs6449947|rs1003599|rs16959569|rs4788426
Renal Disorder	THYROID_CANCER	KEGG	1104	30	20	5,76E-01	1,00E+00	rs9642880|rs11196203|rs7904519|rs6788984|rs3924649|rs1805352|rs946199|rs3762013|rs219463|rs10095762|rs2066394|rs2120825|rs13295631|rs6719501|rs12459940|rs2053025|rs11819326|rs10205484|rs3763745|rs429988
Renal Disorder	SEMA4D_INDUCED_CELL_MIGRATION_AND_GROWTH_CONE_COLLAPSE	REACTOME	433	32	18	5,76E-01	1,00E+00	rs6716817|rs2012928|rs753770|rs2128833|rs4666415|rs220076|rs1579523|rs8075851|rs2278902|rs342076|rs17054953|rs872313|rs686912|rs136206|rs713753|rs4405007|rs7572838|rs7251148
Renal Disorder	GLUCAGON_TYPE_LIGAND_RECEPTORS	REACTOME	786	28	20	5,77E-01	1,00E+00	rs12878420|rs12746334|rs12985186|rs4798777|rs1124425|rs6681938|rs8113423|rs680314|rs17474654|rs16947151|rs887030|rs12881038|rs11123523|rs2068278|rs1253676|rs4870142|rs11083841|rs13041735|rs342541|rs3823082
Renal Disorder	AMYOTROPHIC_LATERAL_SCLEROSIS_ALS	KEGG	1813	30	20	5,79E-01	1,00E+00	rs6489711|rs7315397|rs12962644|rs10510496|rs9658265|rs1613367|rs9324756|rs492014|rs12054636|rs2715825|rs443627|rs10025251|rs1972578|rs4246043|rs405509|rs7873986|rs17733355|rs2288726|rs2502807|rs2244621
Renal Disorder	DOWNSTREAM_TCR_SIGNALING	REACTOME	1573	33	20	5,81E-01	1,00E+00	rs3813668|rs1554669|rs17103274|rs1723015|rs596866|rs10455523|rs1878808|rs1409875|rs13204672|rs35571839|rs9362732|rs4750351|rs10904914|rs13080704|rs9362744|rs943451|rs9351265|rs604663|rs2046316|rs9362786
Renal Disorder	SYSTEMIC_LUPUS_ERYTHEMATOSUS	KEGG	2453	38	20	5,82E-01	1,00E+00	rs1109009|rs186037|rs2857106|rs6132649|rs2239896|rs2004385|rs1181390|rs4675366|rs11799952|rs2300875|rs241436|rs13204672|rs10784678|rs17201103|rs429916|rs10748094|rs1972578|rs1884634|rs2072633|rs10878729
Renal Disorder	CHONDROITIN_SULFATE_DERMATAN_SULFATE_METABOLISM	REACTOME	2784	28	20	5,82E-01	1,00E+00	rs2147155|rs11223864|rs2637426|rs7356549|rs1289500|rs10861257|rs10853672|rs6508463|rs16872173|rs12544497|rs9422277|rs7323264|rs3848064|rs4400920|rs9523342|rs2651458|rs17715721|rs9589601|rs2254827|rs7981707
Renal Disorder	ASTHMA	KEGG	770	65	15	5,84E-01	1,00E+00	rs2857106|rs1860180|rs743562|rs241436|rs13204672|rs429916|rs7717673|rs983671|rs9276472|rs6930052|rs25887|rs8073784|rs917606|rs1015166|rs9275582
Renal Disorder	RNA_POL_III_CHAIN_ELONGATION	REACTOME	364	30	12	5,84E-01	1,00E+00	rs2616211|rs7004724|rs4871975|rs17221107|rs12930464|rs11204135|rs9644645|rs9512817|rs2254883|rs2616241|rs10101364|rs11785348
Renal Disorder	IL10_PATHWAY	BIOCARTA	288	15	9	5,85E-01	1,00E+00	rs2643626|rs7793163|rs1869624|rs12409333|rs11216667|rs2236758|rs1181574|rs10486752|rs10892204
Renal Disorder	TYROSINE_METABOLISM	KEGG	1169	51	20	5,88E-01	1,00E+00	rs11074678|rs1004446|rs2490286|rs11787985|rs1339266|rs13293727|rs7188406|rs10809590|rs2288742|rs996697|rs2279124|rs2389098|rs4646312|rs1229976|rs2519155|rs2162549|rs1154454|rs9678469|rs1540053|rs11018541
Renal Disorder	MITOTIC_PROMETAPHASE	REACTOME	1814	39	20	5,88E-01	1,00E+00	rs1878553|rs4938050|rs2957385|rs4822075|rs6120015|rs10492892|rs17001200|rs387865|rs3846484|rs6667880|rs3004318|rs9898519|rs1995785|rs12220494|rs6657480|rs2760217|rs11119881|rs12068067|rs6981928|rs7546833
Renal Disorder	GAP_JUNCTION_TRAFFICKING	REACTOME	576	26	12	5,88E-01	1,00E+00	rs1043313|rs1886748|rs17473058|rs6929363|rs276698|rs11919795|rs952477|rs6919417|rs9342215|rs2231216|rs3744382|rs9352247
Renal Disorder	CELL_CELL_COMMUNICATION	REACTOME	9725	27	20	5,89E-01	1,00E+00	rs8000535|rs3813668|rs2837630|rs3909729|rs1094972|rs10061989|rs2282735|rs1471804|rs3848788|rs6121823|rs11167020|rs4818176|rs7194177|rs12523586|rs9818995|rs13040003|rs4938726|rs12925685|rs6089510|rs2468449
Renal Disorder	ANTIGEN_PRESENTATION_FOLDING_ASSEMBLY_AND_PEPTIDE_LOADING_OF_CLASS_I_M	REACTOME	830	47	9	5,91E-01	1,00E+00	rs2857106|rs7142869|rs241436|rs12111032|rs11730728|rs8182000|rs1015166|rs7676892|rs11718213
Renal Disorder	JAK_STAT_SIGNALING_PATHWAY	KEGG	4346	30	20	5,93E-01	1,00E+00	rs4478405|rs3813668|rs11582424|rs9642880|rs13436213|rs2386841|rs8074124|rs10501372|rs9601816|rs874269|rs11570908|rs9545368|rs12910308|rs12521272|rs2643626|rs7914|rs17772958|rs10219897|rs1861178|rs743562
Renal Disorder	INHIBITION_OF_INSULIN_SECRETION_BY_ADRENALINE_NORADRENALINE	REACTOME	789	37	20	5,93E-01	1,00E+00	rs12878420|rs12746334|rs12985186|rs1799917|rs3790112|rs12778878|rs6681938|rs8113423|rs17474654|rs16947151|rs887030|rs12881038|rs1253676|rs1277765|rs11083841|rs4917596|rs10493754|rs17124637|rs1277738|rs11013137
Renal Disorder	POST_TRANSLATIONAL_MODIFICATION_SYNTHESIS_OF_GPI_ANCHORED_PROTEINS	REACTOME	586	50	20	5,93E-01	1,00E+00	rs6415846|rs652856|rs477077|rs8007199|rs7174373|rs3739821|rs8034783|rs12437829|rs13102358|rs1912629|rs17069203|rs10518440|rs7763|rs344772|rs166835|rs527365|rs4261469|rs2835569|rs12917114|rs2178099
Renal Disorder	TIGHT_JUNCTION	KEGG	6242	43	20	5,98E-01	1,00E+00	rs8000535|rs7765757|rs1825428|rs868032|rs1147782|rs7605424|rs2862431|rs460707|rs7099767|rs10996472|rs2012928|rs6788984|rs3924649|rs12761828|rs17441629|rs10865451|rs874269|rs9810952|rs596866|rs6705717
Renal Disorder	CELL_CELL_JUNCTION_ORGANIZATION	REACTOME	6211	30	20	6,00E-01	1,00E+00	rs8000535|rs1094972|rs10061989|rs1471804|rs6121823|rs7194177|rs12523586|rs9818995|rs13040003|rs4938726|rs12925685|rs6089510|rs2468449|rs2207542|rs7204173|rs17423077|rs7719157|rs6788984|rs9947576|rs2426987
Renal Disorder	GLYCINE_SERINE_AND_THREONINE_METABOLISM	KEGG	564	28	17	6,03E-01	1,00E+00	rs11679258|rs3828090|rs4877550|rs2807907|rs4649923|rs12815112|rs11137677|rs10521484|rs7355583|rs10975629|rs12410598|rs10514151|rs9835128|rs1611123|rs77905|rs129934|rs3009696
Renal Disorder	TRAFFICKING_OF_AMPA_RECEPTORS	REACTOME	1132	32	20	6,06E-01	1,00E+00	rs9688182|rs10420331|rs9324756|rs4381631|rs4724298|rs492014|rs12054636|rs10025251|rs3889392|rs16960228|rs4246043|rs768858|rs158196|rs7198466|rs276698|rs11871468|rs6792258|rs11919795|rs578772|rs1438949
Renal Disorder	NOD1_2_SIGNALING_PATHWAY	REACTOME	881	28	20	6,07E-01	1,00E+00	rs7768419|rs1613367|rs9494850|rs10499196|rs10455523|rs3769823|rs2715825|rs9362732|rs9362744|rs9351265|rs2046316|rs9362786|rs657597|rs2818291|rs40383|rs10883452|rs1496642|rs2144363|rs566275|rs7810486
Renal Disorder	GNRH_SIGNALING_PATHWAY	KEGG	3992	27	20	6,07E-01	1,00E+00	rs2531995|rs2760494|rs2869441|rs1148757|rs308039|rs9688182|rs12905386|rs4078293|rs2531982|rs12832680|rs2701632|rs3924649|rs12733186|rs1865550|rs1994474|rs4381631|rs6055957|rs3786947|rs4724298|rs2220168
Renal Disorder	IL_RECEPTOR_SHC_SIGNALING	REACTOME	624	32	18	6,07E-01	1,00E+00	rs3813668|rs2386841|rs743562|rs228979|rs5756391|rs12409333|rs13173003|rs853812|rs4749882|rs228975|rs7641889|rs25887|rs6449947|rs7090530|rs334807|rs2075937|rs830474|rs10054692
Renal Disorder	TOLL_PATHWAY	BIOCARTA	1147	27	20	6,08E-01	1,00E+00	rs2760494|rs1555208|rs17103274|rs7858743|rs10455523|rs1329057|rs2715825|rs9362732|rs11090911|rs7668666|rs9362744|rs9351265|rs7843776|rs9362786|rs1881735|rs2306365|rs3742768|rs2818291|rs10883452|rs5743942
Renal Disorder	PHOSPHORYLATION_OF_THE_APC_C	REACTOME	219	14	10	6,08E-01	1,00E+00	rs11006121|rs3846484|rs1994174|rs2324716|rs2311552|rs12352784|rs11718625|rs2450246|rs16912145|rs12647998
Renal Disorder	TRNA_AMINOACYLATION	REACTOME	795	24	12	6,08E-01	1,00E+00	rs4970972|rs17034466|rs1853861|rs6894208|rs6938112|rs6764923|rs730594|rs2039521|rs156319|rs6793847|rs9328293|rs1003000
Renal Disorder	PACKAGING_OF_TELOMERE_ENDS	REACTOME	515	23	7	6,08E-01	1,00E+00	rs2239896|rs2975842|rs643788|rs4096763|rs10946808|rs1341758|rs7741445
Renal Disorder	HOMOLOGOUS_RECOMBINATION_REPAIR_OF_REPLICATION_INDEPENDENT_DOUBLE_ST	REACTOME	376	26	14	6,11E-01	1,00E+00	rs2239896|rs6985793|rs12602230|rs4790838|rs2303744|rs9295910|rs643788|rs9405048|rs7208143|rs206111|rs3092989|rs10258735|rs17482047|rs4986765
Renal Disorder	DESTABILIZATION_OF_MRNA_BY_BRF1	REACTOME	204	8	5	6,12E-01	1,00E+00	rs8003773|rs9784647|rs11244096|rs10814620|rs11707321
Renal Disorder	REGULATION_OF_IFNA_SIGNALING	REACTOME	339	12	6	6,12E-01	1,00E+00	rs2066808|rs2834139|rs12409333|rs2236758|rs9904782|rs4444401
Renal Disorder	EDG1_PATHWAY	BIOCARTA	1432	27	20	6,16E-01	1,00E+00	rs12127134|rs3813668|rs11167020|rs4381631|rs306962|rs6055957|rs6516421|rs3889392|rs16960228|rs6681938|rs11782529|rs7541500|rs7387252|rs7840147|rs17153616|rs11871468|rs1589880|rs849430|rs13173003|rs853812
Renal Disorder	PLATELET_HOMEOSTASIS	REACTOME	4080	30	20	6,16E-01	1,00E+00	rs1148757|rs11042893|rs1326250|rs1135029|rs9658265|rs1937652|rs4235953|rs12878420|rs12746334|rs4583026|rs6565616|rs12832680|rs9365906|rs483832|rs12733186|rs2162734|rs7672750|rs1994474|rs12985186|rs1904006
Renal Disorder	ETHER_LIPID_METABOLISM	KEGG	1041	34	20	6,17E-01	1,00E+00	rs1148757|rs2701632|rs12733186|rs4551123|rs13160153|rs6552744|rs10454443|rs928389|rs1668547|rs7554573|rs2250227|rs1712435|rs11206718|rs1953116|rs11589805|rs2144438|rs7530853|rs8025614|rs6819977|rs622450
Renal Disorder	CACAM_PATHWAY	BIOCARTA	401	12	9	6,18E-01	1,00E+00	rs9688182|rs4724298|rs11615578|rs2288684|rs7213777|rs11083841|rs918842|rs11626241|rs10137869
Renal Disorder	TASTE_TRANSDUCTION	KEGG	1100	30	20	6,19E-01	1,00E+00	rs6489711|rs12905386|rs1422259|rs1865550|rs12446463|rs42372|rs4920566|rs6963381|rs6681938|rs9380409|rs3763609|rs3887555|rs3759253|rs4711369|rs2966704|rs316689|rs13041735|rs937035|rs10270183|rs11085835
Renal Disorder	INTEGRIN_PATHWAY	BIOCARTA	1027	30	20	6,20E-01	1,00E+00	rs2760494|rs11167020|rs10793894|rs306962|rs2300875|rs2295797|rs1884634|rs7840147|rs17073074|rs723113|rs1890548|rs1275659|rs1123551|rs4338396|rs17106421|rs1881735|rs12459940|rs7255180|rs4726612|rs10901077
Renal Disorder	TCR_SIGNALING	REACTOME	2040	36	20	6,21E-01	1,00E+00	rs3813668|rs1554669|rs11646140|rs25800|rs17103274|rs1723015|rs596866|rs10455523|rs1878808|rs1409875|rs13204672|rs252353|rs35571839|rs9362732|rs4750351|rs10904914|rs1718414|rs2084385|rs13080704|rs9362744
Renal Disorder	SHC1_EVENTS_IN_ERBB4_SIGNALING	REACTOME	1072	40	20	6,22E-01	1,00E+00	rs17664708|rs1714424|rs17749288|rs3924649|rs2439321|rs1505374|rs16846161|rs1503486|rs13008370|rs6742399|rs12459940|rs7822564|rs10954845|rs4584111|rs7574462|rs1357136|rs8453|rs987320|rs11097054|rs2466059
Renal Disorder	PDGF_PATHWAY	BIOCARTA	1021	37	20	6,22E-01	1,00E+00	rs3813668|rs2760494|rs4381631|rs2066808|rs1869624|rs3889392|rs16960228|rs12409333|rs17153616|rs11871468|rs849430|rs1881735|rs13173003|rs853812|rs1004717|rs3742768|rs7641889|rs6449947|rs1003599|rs10052248
Renal Disorder	WNT_PATHWAY	BIOCARTA	1363	26	20	6,25E-01	1,00E+00	rs9642880|rs1250129|rs10867605|rs6788984|rs10455523|rs11794108|rs9658119|rs12948818|rs2627013|rs353241|rs1429529|rs219463|rs9362732|rs10095762|rs9362744|rs7791011|rs9351265|rs11860497|rs11190921|rs9362786
Renal Disorder	IL7_PATHWAY	BIOCARTA	699	31	15	6,26E-01	1,00E+00	rs3813668|rs12962644|rs12409333|rs17073074|rs17153616|rs849430|rs13173003|rs853812|rs1004717|rs7641889|rs6449947|rs10457242|rs830474|rs11551174|rs10054692
Renal Disorder	AMINO_ACID_AND_OLIGOPEPTIDE_SLC_TRANSPORTERS	REACTOME	1707	29	20	6,26E-01	1,00E+00	rs4980806|rs1322183|rs2588233|rs2228622|rs9792282|rs7730843|rs2301539|rs9910870|rs911719|rs7855993|rs11059909|rs2455258|rs3922763|rs1136995|rs12490575|rs2562571|rs17503950|rs10404771|rs10401807|rs759664
Renal Disorder	PML_PATHWAY	BIOCARTA	384	26	14	6,27E-01	1,00E+00	rs6489711|rs2218064|rs4085400|rs866270|rs13411586|rs2042300|rs6436952|rs2021840|rs1599871|rs1367415|rs859637|rs399413|rs2396763|rs3130014
Renal Disorder	SIGNALLING_TO_RAS	REACTOME	468	15	9	6,27E-01	1,00E+00	rs917199|rs3924649|rs12459940|rs13224312|rs8453|rs4723910|rs10842515|rs350825|rs6018257
Renal Disorder	GLYOXYLATE_AND_DICARBOXYLATE_METABOLISM	KEGG	704	28	18	6,33E-01	1,00E+00	rs6723088|rs4233453|rs2094703|rs773652|rs7875673|rs262498|rs10970923|rs1042541|rs1022632|rs2888998|rs6055076|rs12346019|rs942382|rs2223534|rs2005390|rs2281603|rs17028097|rs6695488
Renal Disorder	CYSTEINE_AND_METHIONINE_METABOLISM	KEGG	982	51	18	6,35E-01	1,00E+00	rs1368421|rs2490286|rs11746351|rs568529|rs2807907|rs17062465|rs7188406|rs4649923|rs12815112|rs2546324|rs6892131|rs13300268|rs12410598|rs4489081|rs11745553|rs802682|rs12142041|rs970169
Renal Disorder	AMINO_ACID_TRANSPORT_ACROSS_THE_PLASMA_MEMBRANE	REACTOME	1025	28	20	6,35E-01	1,00E+00	rs4980806|rs2588233|rs9792282|rs2301539|rs9910870|rs3922763|rs12490575|rs17503950|rs10404771|rs759664|rs3742057|rs745961|rs7970932|rs2070870|rs1975802|rs9844272|rs2250202|rs12230144|rs7959178|rs1105168
Renal Disorder	SYNTHESIS_OF_PC	REACTOME	669	24	16	6,36E-01	1,00E+00	rs11695702|rs867687|rs1917806|rs3199966|rs16947151|rs1917804|rs1979021|rs2577264|rs7861505|rs989950|rs610261|rs4949673|rs2716651|rs7587168|rs4646371|rs12340765
Renal Disorder	NEGATIVE_REGULATION_OF_FGFR_SIGNALING	REACTOME	1536	32	20	6,37E-01	1,00E+00	rs263931|rs7318731|rs9601816|rs2517037|rs249736|rs9545368|rs7914|rs4770252|rs1379707|rs10219897|rs1536433|rs6671527|rs9593627|rs4761243|rs1936544|rs7825208|rs17099156|rs17431894|rs12429667|rs4770192
Renal Disorder	HDAC_PATHWAY	BIOCARTA	1231	31	20	6,37E-01	1,00E+00	rs3813668|rs4966009|rs1004446|rs2684792|rs6123013|rs12440962|rs2715825|rs3852876|rs8141797|rs11632057|rs1644047|rs17733355|rs17153616|rs849430|rs13173003|rs853812|rs4239343|rs1004717|rs2229431|rs11083841
Renal Disorder	CYTOCHROME_P450_ARRANGED_BY_SUBSTRATE_TYPE	REACTOME	1163	52	20	6,38E-01	1,00E+00	rs10204598|rs1902586|rs863915|rs2572023|rs2181874|rs2065996|rs1409655|rs12459140|rs11992601|rs6735281|rs1934975|rs527643|rs9888067|rs4556118|rs162556|rs1010570|rs4918317|rs7002672|rs10256972|rs2899469
Renal Disorder	CA_DEPENDENT_EVENTS	REACTOME	1487	36	20	6,39E-01	1,00E+00	rs2531995|rs1148757|rs2531982|rs12733186|rs1865550|rs4381631|rs10491176|rs10454443|rs3889392|rs16960228|rs2286562|rs3763609|rs7554573|rs2250227|rs6555491|rs7615501|rs11871468|rs1589880|rs11083841|rs2144438
Renal Disorder	BMAL1_CLOCK_NPAS2_ACTIVATES_CIRCADIAN_EXPRESSION	REACTOME	1728	26	20	6,42E-01	1,00E+00	rs16912392|rs1555208|rs6494232|rs9850957|rs7689448|rs7300833|rs6486099|rs6857002|rs1805352|rs9884852|rs10021542|rs754499|rs2899664|rs6442925|rs16873865|rs6494217|rs17204910|rs11090911|rs12630639|rs1234805
Renal Disorder	PLC_BETA_MEDIATED_EVENTS	REACTOME	2313	28	20	6,42E-01	1,00E+00	rs2531995|rs1148757|rs12905386|rs2531982|rs12832680|rs12733186|rs1865550|rs1994474|rs4381631|rs6055957|rs10491176|rs2220168|rs6516421|rs10454443|rs1799917|rs7300093|rs3790112|rs3889392|rs16960228|rs2286562
Renal Disorder	CELL_JUNCTION_ORGANIZATION	REACTOME	6781	29	20	6,43E-01	1,00E+00	rs8000535|rs1094972|rs10061989|rs2282735|rs1471804|rs6121823|rs7194177|rs12523586|rs9818995|rs13040003|rs4938726|rs12925685|rs6089510|rs2468449|rs2207542|rs7204173|rs17423077|rs7719157|rs6788984|rs9947576
Renal Disorder	SIGNALING_BY_FGFR_MUTANTS	REACTOME	1657	27	20	6,44E-01	1,00E+00	rs3813668|rs263931|rs7318731|rs2517037|rs249736|rs3924649|rs4770252|rs2906725|rs1379707|rs430435|rs1536433|rs407366|rs11974028|rs4761243|rs7825208|rs17099156|rs13173003|rs853812|rs4770192|rs822248
Renal Disorder	HUNTINGTONS_DISEASE	KEGG	4311	44	20	6,44E-01	1,00E+00	rs10235960|rs3796531|rs1676996|rs7315397|rs6761|rs1043313|rs2364598|rs444369|rs12905386|rs6508433|rs254738|rs7777049|rs17163678|rs6857002|rs6055957|rs7667259|rs12521803|rs3769823|rs1133099|rs1994174
Renal Disorder	SIGNALING_BY_NODAL	REACTOME	632	35	16	6,46E-01	1,00E+00	rs8026441|rs4664888|rs1837291|rs2277589|rs412705|rs6598465|rs11071939|rs484754|rs4965900|rs4233580|rs886853|rs1438885|rs6715004|rs2643358|rs893473|rs7607970
Renal Disorder	RNA_POL_I_PROMOTER_OPENING	REACTOME	501	32	7	6,46E-01	1,00E+00	rs2239896|rs3786254|rs4485425|rs643788|rs10946808|rs1341758|rs7741445
Renal Disorder	NONSENSE_MEDIATED_DECAY_ENHANCED_BY_THE_EXON_JUNCTION_COMPLEX	REACTOME	1261	34	20	6,47E-01	1,00E+00	rs2291782|rs1212343|rs12587507|rs4916595|rs6501697|rs1133099|rs1435961|rs1649990|rs2958522|rs12942418|rs6734|rs11135929|rs4075144|rs6800812|rs9907408|rs4145041|rs10140464|rs2842880|rs10425596|rs1720321
Renal Disorder	PTM_GAMMA_CARBOXYLATION_HYPUSINE_FORMATION_AND_ARYLSULFATASE_ACTIV	REACTOME	377	19	10	6,51E-01	1,00E+00	rs9688182|rs12601802|rs11672955|rs6816578|rs12503095|rs4076557|rs7211107|rs13164947|rs11949551|rs16972915

Renal Disorder	PHOSPHATIDYLINOSITOL_SIGNALING_SYSTEM	KEGG	3813	29	20	6,52E-01	1,00E+00	rs1547643|rs3813668|rs1223577|rs6053183|rs12905386|rs16976931|rs7238810|rs4078293|rs12832680|rs2357960|rs11044232|rs9566906|rs1994474|rs9959057|rs10502786|rs4381631|rs9365723|rs6055957|rs2220168|rs3740360
Renal Disorder	TOLL_LIKE_RECEPTOR_SIGNALING_PATHWAY	KEGG	2339	31	20	6,52E-01	1,00E+00	rs3813668|rs2760494|rs2869441|rs17103274|rs9972960|rs874269|rs7858743|rs10455523|rs7143468|rs3769823|rs1329057|rs6582484|rs10515797|rs1460769|rs2834139|rs7793163|rs2715825|rs9362732|rs10498658|rs7658485
Renal Disorder	STEROID_BIOSYNTHESIS	KEGG	247	7	5	6,53E-01	1,00E+00	rs2075906|rs6586175|rs7094601|rs2280699|rs586977
Renal Disorder	BILE_ACID_AND_BILE_SALT_METABOLISM	REACTOME	1176	39	20	6,54E-01	1,00E+00	rs10463469|rs1361576|rs1996083|rs1336608|rs3822464|rs6586175|rs12021074|rs4149056|rs4497522|rs92631|rs11992601|rs9519177|rs882610|rs4556118|rs4148418|rs3916933|rs1399936|rs10788637|rs4772597|rs7002672
Renal Disorder	VEGF_SIGNALING_PATHWAY	KEGG	2111	34	20	6,55E-01	1,00E+00	rs3813668|rs1148757|rs11167020|rs33063|rs2701632|rs3924649|rs874269|rs12733186|rs4381631|rs306962|rs6123013|rs10454443|rs7193701|rs3889392|rs1668547|rs16960228|rs7873986|rs7554573|rs244418|rs2250227
Renal Disorder	MAPK_TARGETS_NUCLEAR_EVENTS_MEDIATED_BY_MAP_KINASES	REACTOME	980	46	20	6,58E-01	1,00E+00	rs2760494|rs2869441|rs9848727|rs1153681|rs1460769|rs17555326|rs7658485|rs1644047|rs17555250|rs12640395|rs1881740|rs3742768|rs2341674|rs10783388|rs731288|rs325392|rs10411335|rs16903232|rs918842|rs633045
Renal Disorder	RAS_PATHWAY	BIOCARTA	557	35	17	6,59E-01	1,00E+00	rs3813668|rs917199|rs2244621|rs17153616|rs849430|rs13173003|rs853812|rs1004717|rs2306365|rs13224312|rs10883452|rs7641889|rs6449947|rs4723910|rs830474|rs477895|rs10054692
Renal Disorder	PI_METABOLISM	REACTOME	1808	31	20	6,65E-01	1,00E+00	rs3813668|rs7238810|rs195771|rs11044232|rs9959057|rs10502786|rs9365723|rs12686355|rs7015243|rs1943600|rs8089834|rs17761155|rs12457578|rs3764459|rs12490575|rs12327116|rs1556751|rs4146444|rs1750043|rs17153616
Renal Disorder	CELL_ADHESION_MOLECULES_CAMS	KEGG	8813	30	20	6,65E-01	1,00E+00	rs8000535|rs1319475|rs1382421|rs16854920|rs6727015|rs1094972|rs6121823|rs2826875|rs13040003|rs10220329|rs4938726|rs2857106|rs967173|rs6089510|rs2207542|rs2827765|rs3851570|rs4725745|rs1015931|rs9976114
Renal Disorder	UCALPAIN_PATHWAY	BIOCARTA	426	27	16	6,69E-01	1,00E+00	rs11167020|rs306962|rs2300875|rs2295797|rs1884634|rs7840147|rs723113|rs1275659|rs4338396|rs17106421|rs7255180|rs10802557|rs1418071|rs1499280|rs17106672|rs6018257
Renal Disorder	NOTCH1_INTRACELLULAR_DOMAIN_REGULATES_TRANSCRIPTION	REACTOME	2852	31	20	6,72E-01	1,00E+00	rs4877187|rs4687504|rs9642880|rs9999030|rs1147289|rs9850957|rs2301106|rs10780347|rs16878154|rs11794108|rs2655215|rs1877207|rs6756261|rs13430446|rs1347370|rs6543557|rs3791446|rs2627013|rs12908056|rs2467781
Renal Disorder	ADP_SIGNALLING_THROUGH_P2RY12	REACTOME	438	30	18	6,73E-01	1,00E+00	rs12878420|rs12746334|rs12985186|rs6681938|rs8113423|rs17474654|rs16947151|rs887030|rs12881038|rs1253676|rs11083841|rs12075532|rs17124637|rs1276309|rs17204376|rs6700907|rs10486925|rs9875152
Renal Disorder	SLC_MEDIATED_TRANSMEMBRANE_TRANSPORT	REACTOME	8774	29	20	6,74E-01	1,00E+00	rs17298185|rs11173212|rs9385677|rs4980806|rs6864158|rs2361204|rs6569926|rs3852030|rs1322183|rs12047830|rs7603445|rs2588233|rs7544111|rs2228622|rs7036497|rs1405376|rs11778200|rs2023705|rs6496875|rs4837334
Renal Disorder	LAIR_PATHWAY	BIOCARTA	479	16	9	6,75E-01	1,00E+00	rs2004385|rs7793163|rs17201103|rs2073917|rs11586136|rs11166507|rs281437|rs7035682|rs237554
Renal Disorder	COMP_PATHWAY	BIOCARTA	628	22	10	6,76E-01	1,00E+00	rs186037|rs2004385|rs17201103|rs2072633|rs292007|rs2204757|rs10824842|rs7035682|rs237554|rs11003231
Renal Disorder	ATRBRCA_PATHWAY	BIOCARTA	238	22	7	6,77E-01	1,00E+00	rs6985793|rs2686830|rs206111|rs9462085|rs527365|rs3092989|rs9291949
Renal Disorder	CREB_PATHWAY	BIOCARTA	969	30	20	6,78E-01	1,00E+00	rs3813668|rs9688182|rs4381631|rs10491176|rs4724298|rs3889392|rs16960228|rs2286562|rs3763609|rs11871468|rs1589880|rs13173003|rs853812|rs316689|rs13041735|rs7641889|rs6449947|rs918842|rs1003599|rs11770994
Renal Disorder	ALK_PATHWAY	BIOCARTA	1932	27	20	6,78E-01	1,00E+00	rs6660484|rs6038619|rs235710|rs6054501|rs6788984|rs1153681|rs996544|rs1805113|rs10455523|rs210363|rs6107891|rs6038621|rs3858284|rs1429529|rs2145274|rs9362732|rs6085775|rs1644047|rs10497191|rs9362744
Renal Disorder	RNA_POL_III_TRANSCRIPTION_INITIATION_FROM_TYPE_2_PROMOTER	REACTOME	417	31	13	6,78E-01	1,00E+00	rs2616211|rs7004724|rs4871975|rs17221107|rs12930464|rs11204135|rs9644645|rs9512817|rs2254883|rs2616241|rs586977|rs10101364|rs11785348
Renal Disorder	ADHERENS_JUNCTIONS_INTERACTIONS	REACTOME	5496	29	20	6,79E-01	1,00E+00	rs1094972|rs10061989|rs1471804|rs6121823|rs7194177|rs12523586|rs9818995|rs13040003|rs4938726|rs12925685|rs6089510|rs2468449|rs2207542|rs7204173|rs17423077|rs7719157|rs6788984|rs9947576|rs2426987|rs2549160
Renal Disorder	RHO_PATHWAY	BIOCARTA	480	25	15	6,79E-01	1,00E+00	rs2383289|rs6724135|rs12686355|rs2295797|rs465316|rs1556751|rs17125|rs10735625|rs10125301|rs10458657|rs11038977|rs12340264|rs4969349|rs4969367|rs6018257
Renal Disorder	CDMAC_PATHWAY	BIOCARTA	1018	32	20	6,81E-01	1,00E+00	rs2760494|rs9642880|rs17103274|rs4381631|rs6055957|rs6516421|rs3889392|rs16960228|rs10095762|rs11871468|rs2306365|rs1534897|rs3742768|rs6055927|rs17347805|rs10091329|rs1003599|rs708916|rs16959569|rs4788426
Renal Disorder	G_ALPHA_Z_SIGNALLING_EVENTS	REACTOME	2191	34	20	6,81E-01	1,00E+00	rs2531995|rs12878420|rs12746334|rs2531982|rs2790090|rs476003|rs596866|rs6705717|rs1865550|rs1674729|rs12985186|rs4381631|rs11621184|rs1409875|rs3889392|rs12778878|rs16960228|rs6681938|rs8113423|rs4750351
Renal Disorder	POST_CHAPERONIN_TUBULIN_FOLDING_PATHWAY	REACTOME	254	11	8	6,82E-01	1,00E+00	rs7734027|rs10484911|rs10458071|rs254412|rs2293079|rs4986129|rs1542891|rs9501935
Renal Disorder	SIGNALING_BY_ERBB2	REACTOME	3847	35	20	6,82E-01	1,00E+00	rs2531995|rs3813668|rs17664708|rs16963744|rs1714424|rs2755217|rs2531982|rs12832680|rs17749288|rs3924649|rs874269|rs6705717|rs1865550|rs1994474|rs4381631|rs2439321|rs10491176|rs2220168|rs1505374|rs7300093
Renal Disorder	PLATELET_CALCIUM_HOMEOSTASIS	REACTOME	1156	45	20	6,83E-01	1,00E+00	rs12832680|rs7672750|rs1994474|rs368161|rs2220168|rs759691|rs7300093|rs1861303|rs11719939|rs11048716|rs10771297|rs13079701|rs417614|rs17032939|rs7213777|rs1049376|rs2278556|rs4964001|rs1380429|rs2430890
Renal Disorder	EICOSANOID_LIGAND_BINDING_RECEPTORS	REACTOME	507	32	13	6,83E-01	1,00E+00	rs1020360|rs7147188|rs12748050|rs919208|rs1980839|rs4546432|rs8004654|rs11083841|rs3760703|rs1899982|rs2357460|rs520171|rs11851721
Renal Disorder	MHC_CLASS_II_ANTIGEN_PRESENTATION	REACTOME	2226	36	20	6,84E-01	1,00E+00	rs217111|rs1043313|rs11861296|rs2857106|rs2957385|rs319345|rs6845262|rs7142869|rs7156395|rs10038958|rs241436|rs13204672|rs429916|rs1692793|rs420259|rs2748220|rs2384319|rs11730728|rs10020753|rs6706373
Renal Disorder	MYOSIN_PATHWAY	BIOCARTA	1253	30	20	6,84E-01	1,00E+00	rs8001712|rs2383289|rs4681888|rs11619224|rs4381631|rs6055957|rs4681908|rs6516421|rs3889392|rs16960228|rs6681938|rs12740413|rs17756915|rs17125|rs11871468|rs13070800|rs13280012|rs1534897|rs3760703|rs9522182
Renal Disorder	BIOPEPTIDES_PATHWAY	BIOCARTA	1081	36	20	6,87E-01	1,00E+00	rs308039|rs9688182|rs4381631|rs2066808|rs3786947|rs4724298|rs1869624|rs3889392|rs16960228|rs6681938|rs17125|rs17073074|rs11871468|rs1881735|rs12459940|rs4076557|rs11083841|rs10457242|rs1003599|rs11626241
Renal Disorder	OOCYTE_MEIOSIS	KEGG	3114	35	20	6,87E-01	1,00E+00	rs2531995|rs4966009|rs1004446|rs9688182|rs4734497|rs1529702|rs4078293|rs2531982|rs12832680|rs1865550|rs1994474|rs2684792|rs7736312|rs4724298|rs2961920|rs2220168|rs12440962|rs13409926|rs7300093|rs11048716
Renal Disorder	APC_C_CDC20_MEDIATED_DEGRADATION_OF_CYCLIN_B	REACTOME	224	15	11	6,87E-01	1,00E+00	rs11006121|rs3846484|rs1994174|rs2324716|rs2311552|rs12352784|rs11718625|rs2450246|rs16912145|rs12647998|rs710251
Renal Disorder	PRE_NOTCH_EXPRESSION_AND_PROCESSING	REACTOME	1434	33	20	6,88E-01	1,00E+00	rs2760494|rs9999030|rs4789523|rs16878154|rs3748269|rs1877207|rs17616659|rs470113|rs229833|rs10898931|rs3761441|rs942042|rs7951485|rs2183124|rs12646059|rs746720|rs9291482|rs2225382|rs1238|rs16878186
Renal Disorder	ALLOGRAFT_REJECTION	KEGG	1510	68	19	6,89E-01	1,00E+00	rs2857106|rs10509323|rs1181390|rs4675367|rs743562|rs241436|rs10515797|rs13204672|rs10784678|rs429916|rs10748094|rs10878729|rs12111032|rs9276516|rs6930052|rs2021840|rs859637|rs1015166|rs9275582
Renal Disorder	JNK_C_JUN_KINASES_PHOSPHORYLATION_AND_ACTIVATION_MEDIATED_BY_ACTIVATI	REACTOME	728	34	16	6,89E-01	1,00E+00	rs2869441|rs10455523|rs1460769|rs9362732|rs7658485|rs9362744|rs9351265|rs2046316|rs12640395|rs9362786|rs1881740|rs2818291|rs40383|rs2144363|rs12679902|rs1504281
Renal Disorder	COMPLEMENT_CASCADE	REACTOME	752	28	14	6,90E-01	1,00E+00	rs186037|rs2794515|rs2004385|rs17201103|rs2072633|rs292007|rs2204757|rs1571344|rs2794520|rs10824842|rs7035682|rs2808470|rs237554|rs11003231
Renal Disorder	GRAFT_VERSUS_HOST_DISEASE	KEGG	1446	69	19	6,91E-01	1,00E+00	rs2857106|rs10509323|rs1181390|rs4675366|rs241436|rs13204672|rs10784678|rs7793163|rs429916|rs10748094|rs10878729|rs12111032|rs9276472|rs6930052|rs17771967|rs2021840|rs859637|rs1015166|rs9275595
Renal Disorder	GLEEVEC_PATHWAY	BIOCARTA	954	37	19	6,92E-01	1,00E+00	rs3813668|rs2760494|rs9642880|rs10095762|rs2244621|rs17153616|rs849430|rs1881740|rs13173003|rs853812|rs1004717|rs3742768|rs7641889|rs6449947|rs6003709|rs10091329|rs830474|rs477895|rs10054692
Renal Disorder	PYRUVATE_METABOLISM	REACTOME	209	11	5	6,94E-01	1,00E+00	rs2421763|rs2732553|rs474908|rs2031686|rs756847
Renal Disorder	PEPTIDE_CHAIN_ELONGATION	REACTOME	947	36	20	6,94E-01	1,00E+00	rs2291782|rs12587507|rs6501697|rs1133099|rs1435961|rs1649990|rs2958522|rs12942418|rs4075144|rs6800812|rs9907408|rs4145041|rs10140464|rs10425596|rs1720321|rs2395626|rs11902670|rs16953470|rs1790157|rs4777189
Renal Disorder	EGF_PATHWAY	BIOCARTA	1048	34	20	6,96E-01	1,00E+00	rs3813668|rs2760494|rs4381631|rs2066808|rs1869624|rs3889392|rs16960228|rs12409333|rs11871468|rs1881735|rs13173003|rs853812|rs17746482|rs4245566|rs3742768|rs7641889|rs6449947|rs1003599|rs2877260|rs10052248
Renal Disorder	XENOBIOTICS	REACTOME	231	7	4	6,97E-01	1,00E+00	rs10786172|rs1934975|rs9888067|rs1010570
Renal Disorder	SYNTHESIS_OF_PIPS_AT_THE_PLASMA_MEMBRANE	REACTOME	1050	27	20	6,97E-01	1,00E+00	rs3813668|rs195771|rs11044232|rs9365723|rs12686355|rs17761155|rs1556751|rs1750043|rs17153616|rs10735625|rs849430|rs13173003|rs853812|rs10125301|rs1004717|rs11930849|rs7641889|rs1476592|rs6449947|rs4769414
Renal Disorder	PTEN_PATHWAY	BIOCARTA	544	27	16	6,98E-01	1,00E+00	rs3813668|rs11167020|rs306962|rs7840147|rs484754|rs13173003|rs853812|rs7641889|rs6449947|rs2021840|rs859637|rs756847|rs11040933|rs4603211|rs830474|rs10054692
Renal Disorder	PHOSPHOLIPASE_C_MEDIATED_CASCADE	REACTOME	2628	28	20	6,98E-01	1,00E+00	rs2531995|rs263931|rs7318731|rs2531982|rs2517037|rs249736|rs12832680|rs6705717|rs1865550|rs1994474|rs4381631|rs4770252|rs1379707|rs10491176|rs2220168|rs1536433|rs9668898|rs3889392|rs16960228|rs2286562
Renal Disorder	PHENYLALANINE_METABOLISM	KEGG	442	9	5	6,99E-01	1,00E+00	rs2490286|rs7188406|rs2279124|rs7309105|rs970169
Renal Disorder	GRB2_EVENTS_IN_ERBB2_SIGNALING	REACTOME	1202	39	20	7,01E-01	1,00E+00	rs17664708|rs1714424|rs17749288|rs3924649|rs2439321|rs1505374|rs16846161|rs1503486|rs13008370|rs6742399|rs12459940|rs7822564|rs17746482|rs4245566|rs10954845|rs4584111|rs7574462|rs1357136|rs8453|rs987320
Renal Disorder	RIBOSOME	KEGG	1118	36	20	7,02E-01	1,00E+00	rs2291782|rs12587507|rs7177131|rs6501697|rs1133099|rs1435961|rs1649990|rs2958522|rs12942418|rs4075144|rs6800812|rs9907408|rs4145041|rs10140464|rs10425596|rs1720321|rs2395626|rs11902670|rs16953470|rs1790157
Renal Disorder	AMINO_ACID_SYNTHESIS_AND_INTERCONVERSION_TRANSAMINATION	REACTOME	530	21	11	7,02E-01	1,00E+00	rs3828090|rs2490286|rs4877550|rs11137677|rs10521484|rs10901826|rs2293964|rs10911044|rs2254327|rs3009696|rs970169
Renal Disorder	DOWNSTREAM_SIGNALING_OF_ACTIVATED_FGFR	REACTOME	3636	26	20	7,02E-01	1,00E+00	rs2531995|rs3813668|rs263931|rs7318731|rs2755217|rs2531982|rs2517037|rs249736|rs12832680|rs3924649|rs874269|rs6705717|rs1865550|rs1994474|rs4381631|rs4770252|rs1379707|rs10491176|rs2220168|rs1536433
Renal Disorder	CK1_PATHWAY	BIOCARTA	759	23	17	7,03E-01	1,00E+00	rs362848|rs11956891|rs6055957|rs10491176|rs6516421|rs2286562|rs3763609|rs6556186|rs265993|rs1534897|rs316689|rs6055927|rs11214654|rs17347805|rs708916|rs1033398|rs703745
Renal Disorder	ASSOCIATION_OF_TRIC_CCT_WITH_TARGET_PROTEINS_DURING_BIOSYNTHESIS	REACTOME	359	14	7	7,03E-01	1,00E+00	rs12199169|rs2842863|rs2325899|rs1014146|rs4394301|rs9297054|rs1474298
Renal Disorder	VASOPRESSIN_REGULATED_WATER_REABSORPTION	KEGG	1100	28	20	7,04E-01	1,00E+00	rs2531995|rs10510496|rs2531982|rs319345|rs7777049|rs2295797|rs420259|rs3763609|rs10951201|rs41351|rs7926726|rs1435421|rs886816|rs316689|rs13041735|rs17180771|rs2292814|rs2304911|rs2072341|rs2040215
Renal Disorder	DAG_AND_IP3_SIGNALING	REACTOME	1644	34	20	7,04E-01	1,00E+00	rs2531995|rs2531982|rs12832680|rs6705717|rs1865550|rs1994474|rs4381631|rs10491176|rs2220168|rs7300093|rs3889392|rs16960228|rs2286562|rs11048716|rs2035440|rs3763609|rs6755308|rs281501|rs6555491|rs7615501
Renal Disorder	NFKB_AND_MAP_KINASES_ACTIVATION_MEDIATED_BY_TLR4_SIGNALING_REPERTOIRE	REACTOME	2248	36	20	7,06E-01	1,00E+00	rs2760494|rs7327256|rs2869441|rs9848727|rs17103274|rs1153681|rs7858743|rs10455523|rs34129552|rs1329057|rs3787620|rs1460769|rs17555326|rs2715825|rs461626|rs9362732|rs10498658|rs7658485|rs1644047|rs7668666
Renal Disorder	TPO_PATHWAY	BIOCARTA	814	40	20	7,08E-01	1,00E+00	rs3813668|rs2760494|rs4381631|rs3889392|rs16960228|rs17153616|rs11871468|rs849430|rs13173003|rs853812|rs1004717|rs3742768|rs7641889|rs6449947|rs1003599|rs10052248|rs16959569|rs4788426|rs830474|rs710251
Renal Disorder	HCMV_PATHWAY	BIOCARTA	547	33	17	7,08E-01	1,00E+00	rs3813668|rs2364598|rs2715825|rs17153616|rs849430|rs12459940|rs13173003|rs853812|rs1004717|rs2306365|rs7641889|rs6449947|rs918842|rs399413|rs350825|rs830474|rs10054692
Renal Disorder	CIRCADIAN_CLOCK	REACTOME	2075	29	20	7,09E-01	1,00E+00	rs16912392|rs1555208|rs6494232|rs9850957|rs7689448|rs2301106|rs7300833|rs1153681|rs6486099|rs6857002|rs1805352|rs9884852|rs10021542|rs754499|rs2899664|rs6442925|rs16873865|rs6494217|rs17204910|rs11090911
Renal Disorder	PROLONGED_ERK_ACTIVATION_EVENTS	REACTOME	429	18	10	7,09E-01	1,00E+00	rs3924649|rs10793894|rs4761243|rs1890548|rs1123551|rs12459940|rs10901077|rs8453|rs17389103|rs350825
Renal Disorder	HS_GAG_DEGRADATION	REACTOME	1386	44	20	7,11E-01	1,00E+00	rs2147155|rs2637426|rs7323264|rs3848064|rs4400920|rs9523342|rs2651458|rs9589601|rs7981707|rs3848044|rs12867304|rs6492589|rs878949|rs9583786|rs1407998|rs7323523|rs7601441|rs1445267|rs9560673|rs12875742
Renal Disorder	ARF_PATHWAY	BIOCARTA	733	35	20	7,15E-01	1,00E+00	rs3813668|rs9642880|rs7853654|rs2189000|rs10095762|rs2811709|rs17153616|rs849430|rs13173003|rs853812|rs1004717|rs9512817|rs7641889|rs6449947|rs10091329|rs399413|rs10153801|rs11792889|rs830474|rs10054692
Renal Disorder	ACETYLCHOLINE_BINDING_AND_DOWNSTREAM_EVENTS	REACTOME	242	18	10	7,16E-01	1,00E+00	rs2337980|rs12611955|rs11556635|rs3828246|rs10497426|rs17360319|rs1190452|rs6494223|rs1955259|rs4779939
Renal Disorder	DOUBLE_STRAND_BREAK_REPAIR	REACTOME	506	28	15	7,17E-01	1,00E+00	rs2239896|rs6985793|rs12602230|rs4790838|rs2303744|rs9295910|rs643788|rs9405048|rs7208143|rs206111|rs3092989|rs7987249|rs10258735|rs17482047|rs4986765
Renal Disorder	TOLL_RECEPTOR_CASCADES	REACTOME	3778	33	20	7,17E-01	1,00E+00	rs2760494|rs7327256|rs2869441|rs10198990|rs1382973|rs9848727|rs7238810|rs17103274|rs9959057|rs1153681|rs7858743|rs10502786|rs1679804|rs1152918|rs10455523|rs34129552|rs7143468|rs12434440|rs1329057|rs1943600
Renal Disorder	KERATINOCYTE_PATHWAY	BIOCARTA	1952	38	20	7,18E-01	1,00E+00	rs2760494|rs6489711|rs12962644|rs2364598|rs17103274|rs596866|rs6705717|rs4381631|rs11621184|rs1409875|rs2715825|rs3889392|rs16960228|rs10498658|rs4750351|rs10904914|rs6755308|rs943451|rs281501|rs7615501
Renal Disorder	ION_CHANNEL_TRANSPORT	REACTOME	2741	30	20	7,18E-01	1,00E+00	rs7716847|rs6490959|rs2075716|rs4941086|rs1372491|rs3922855|rs12695746|rs12050748|rs9817842|rs860294|rs4769434|rs4695935|rs1511493|rs8040030|rs17643649|rs4505988|rs759691|rs898500|rs968332|rs12198870
Renal Disorder	MTOR_PATHWAY	BIOCARTA	509	33	17	7,18E-01	1,00E+00	rs3813668|rs3761840|rs6671527|rs7698057|rs11876|rs2231410|rs13173003|rs853812|rs2403370|rs7641889|rs6449947|rs4962081|rs756847|rs4603211|rs2230571|rs830474|rs10054692
Renal Disorder	GH_PATHWAY	BIOCARTA	963	37	20	7,18E-01	1,00E+00	rs3813668|rs1004446|rs4381631|rs3852876|rs3889392|rs16960228|rs1317063|rs17153616|rs11871468|rs849430|rs13173003|rs853812|rs1004717|rs2229431|rs6725556|rs7258382|rs7641889|rs6449947|rs1003599|rs16959569
Renal Disorder	BLADDER_CANCER	KEGG	1222	30	20	7,18E-01	1,00E+00	rs9642880|rs441250|rs3924649|rs1930056|rs4878104|rs10746816|rs1953040|rs942042|rs10095762|rs10512188|rs4462564|rs594942|rs2244621|rs746720|rs2811709|rs332236|rs4690458|rs12459940|rs17746482|rs4245566
Renal Disorder	AKT_PATHWAY	BIOCARTA	682	41	19	7,19E-01	1,00E+00	rs3813668|rs17103274|rs2755217|rs2244621|rs17153616|rs849430|rs484754|rs13173003|rs853812|rs1004717|rs2306365|rs10883452|rs7641889|rs6449947|rs2021840|rs859637|rs830474|rs477895|rs10054692
Renal Disorder	ARMS_MEDIATED_ACTIVATION	REACTOME	393	15	9	7,19E-01	1,00E+00	rs3924649|rs10793894|rs1890548|rs1123551|rs12459940|rs10901077|rs8453|rs10842515|rs350825
Renal Disorder	CD28_CO_STIMULATION	REACTOME	797	36	20	7,20E-01	1,00E+00	rs3813668|rs874269|rs1181390|rs4675366|rs2668020|rs1718414|rs2084385|rs17073074|rs12166465|rs13173003|rs853812|rs9611234|rs3019245|rs7641889|rs6449947|rs3019244|rs1027990|rs10457242|rs12967001|rs6018257
Renal Disorder	APOPTOSIS	REACTOME	3298	29	20	7,24E-01	1,00E+00	rs7327256|rs6489711|rs919699|rs12962644|rs11167020|rs4576294|rs9966514|rs6788984|rs241437|rs596866|rs7831595|rs11732086|rs231998|rs306962|rs3769823|rs652196|rs210179|rs16848195|rs1930056|rs1409875
Renal Disorder	PIP3_ACTIVATES_AKT_SIGNALING	REACTOME	587	17	9	7,24E-01	1,00E+00	rs2755217|rs874269|rs11876|rs2244621|rs484754|rs10883452|rs918842|rs4603211|rs477895
Renal Disorder	G_ALPHA1213_SIGNALLING_EVENTS	REACTOME	2855	32	20	7,24E-01	1,00E+00	rs3813668|rs525007|rs6716817|rs12878420|rs12746334|rs6019409|rs636293|rs4681888|rs333327|rs12985186|rs11907586|rs7774757|rs753770|rs2128833|rs656709|rs4681908|rs12480425|rs4666415|rs17020088|rs6095298
Renal Disorder	SHC_MEDIATED_CASCADE	REACTOME	1070	29	20	7,27E-01	1,00E+00	rs263931|rs7318731|rs2517037|rs249736|rs3924649|rs4770252|rs1379707|rs1536433|rs7825208|rs17099156|rs17431894|rs4770192|rs822248|rs6859263|rs7914068|rs8453|rs743682|rs1860228|rs10788184|rs4424770
Renal Disorder	EFFECTS_OF_PIP2_HYDROLYSIS	REACTOME	1651	37	20	7,30E-01	1,00E+00	rs12832680|rs2357960|rs9566906|rs596866|rs6705717|rs7672750|rs1994474|rs11621184|rs2220168|rs1528098|rs1409875|rs10486048|rs7300093|rs11048716|rs4750351|rs2035440|rs10904914|rs7777178|rs6755308|rs943451
Renal Disorder	CONVERSION_FROM_APC_C_CDC20_TO_APC_C_CDH1_IN_LATE_ANAPHASE	REACTOME	226	12	9	7,31E-01	1,00E+00	rs11006121|rs1994174|rs2324716|rs2311552|rs12352784|rs11718625|rs16912145|rs12647998|rs710251
Renal Disorder	NEPHRIN_INTERACTIONS	REACTOME	1617	37	20	7,33E-01	1,00E+00	rs3813668|rs3909729|rs7605392|rs12537793|rs7783680|rs2300875|rs4112714|rs2513427|rs7125248|rs1884634|rs611849|rs12807269|rs10893608|rs17073074|rs723113|rs10953565|rs7928765|rs17106421|rs13173003|rs853812
Renal Disorder	COSTIMULATION_BY_THE_CD28_FAMILY	REACTOME	1479	31	20	7,34E-01	1,00E+00	rs3813668|rs1554669|rs1723015|rs874269|rs1181390|rs4675367|rs2668020|rs13204672|rs35571839|rs1718414|rs2084385|rs11119881|rs17073074|rs12166465|rs7546833|rs10932047|rs12616245|rs13173003|rs853812|rs9276472
Renal Disorder	TRIF_MEDIATED_TLR3_SIGNALING	REACTOME	2075	35	20	7,34E-01	1,00E+00	rs2760494|rs7327256|rs2869441|rs9848727|rs17103274|rs1153681|rs10455523|rs34129552|rs3787620|rs1460769|rs17555326|rs2715825|rs461626|rs9362732|rs10498658|rs7658485|rs1644047|rs7668666|rs9362744|rs8126493
Renal Disorder	INTERFERON_GAMMA_SIGNALING	REACTOME	2043	31	20	7,35E-01	1,00E+00	rs1532472|rs9688182|rs3756103|rs3851570|rs3794125|rs1342852|rs4724298|rs2161737|rs10128562|rs13204672|rs10784678|rs866270|rs4936186|rs10748094|rs35571839|rs1245138|rs1915033|rs12409333|rs13411586|rs10878729
Renal Disorder	MITOTIC_G1_G1_S_PHASES	REACTOME	2477	29	20	7,37E-01	1,00E+00	rs4478405|rs17096208|rs9642880|rs4576294|rs241437|rs12602230|rs2835809|rs17772958|rs7736312|rs1861178|rs2298582|rs10948689|rs4790838|rs236106|rs942042|rs10095762|rs10268808|rs11135929|rs7189819|rs887594
Renal Disorder	MEIOSIS	REACTOME	1674	35	20	7,37E-01	1,00E+00	rs10043677|rs2239896|rs2975842|rs6985793|rs13162827|rs12602230|rs17312807|rs360712|rs2123463|rs4790838|rs975072|rs1980455|rs4485425|rs6981928|rs7402445|rs643788|rs12213919|rs2635443|rs584336|rs7208143
Renal Disorder	PLATELET_SENSITIZATION_BY_LDL	REACTOME	676	37	16	7,38E-01	1,00E+00	rs1148757|rs12733186|rs925864|rs10454443|rs7554573|rs2250227|rs11119881|rs7546833|rs2144438|rs10411335|rs9729852|rs9892152|rs633045|rs6588478|rs388065|rs10493173
Renal Disorder	MCALPAIN_PATHWAY	BIOCARTA	543	28	19	7,40E-01	1,00E+00	rs11167020|rs306962|rs10491176|rs2295797|rs2286562|rs3763609|rs7840147|rs17125|rs1275659|rs4338396|rs17746482|rs4245566|rs7255180|rs316689|rs1499280|rs2877260|rs12080565|rs11760524|rs721412
Renal Disorder	FRS2_MEDIATED_CASCADE	REACTOME	1192	28	20	7,41E-01	1,00E+00	rs263931|rs7318731|rs2517037|rs249736|rs3924649|rs4770252|rs1379707|rs1536433|rs4761243|rs7825208|rs17099156|rs17431894|rs2395788|rs12459940|rs4770192|rs822248|rs6859263|rs7914068|rs8453|rs743682
Renal Disorder	PROTEOLYTIC_CLEAVAGE_OF_SNARE_COMPLEX_PROTEINS	REACTOME	382	23	10	7,43E-01	1,00E+00	rs2532520|rs883382|rs6104567|rs8071640|rs11086651|rs3763851|rs7209802|rs4286803|rs2304911|rs1591811
Renal Disorder	NETRIN1_SIGNALING	REACTOME	3710	31	20	7,47E-01	1,00E+00	rs519813|rs2483548|rs17056621|rs11167020|rs11949924|rs9966514|rs9888659|rs187384|rs16947893|rs596866|rs7672750|rs7170984|rs4129967|rs306962|rs9910870|rs900617|rs7666974|rs17542080|rs9548050|rs1409875
Renal Disorder	PI3K_EVENTS_IN_ERBB4_SIGNALING	REACTOME	1709	35	20	7,49E-01	1,00E+00	rs3813668|rs17664708|rs1714424|rs2755217|rs17749288|rs874269|rs2439321|rs1505374|rs16846161|rs1503486|rs11876|rs2244621|rs13008370|rs6742399|rs484754|rs13173003|rs853812|rs7822564|rs10954845|rs4584111
Renal Disorder	IL1_SIGNALING	REACTOME	1322	40	20	7,50E-01	1,00E+00	rs10198990|rs1382973|rs1679804|rs1152918|rs10455523|rs12434440|rs6582484|rs12940055|rs2715825|rs9362732|rs9362744|rs9351265|rs11190921|rs2046316|rs9362786|rs2053025|rs3213448|rs2818291|rs40383|rs10883452
Renal Disorder	CHREBP2_PATHWAY	BIOCARTA	1059	31	20	7,51E-01	1,00E+00	rs3805492|rs4734497|rs10491176|rs3935000|rs1881643|rs2286562|rs17721635|rs11135929|rs3763609|rs11119881|rs17464037|rs7546833|rs953221|rs4327561|rs13259960|rs2727564|rs7866897|rs316689|rs2536075|rs7789674

Renal Disorder	MELANOGENESIS	KEGG	4077	24	20	7,52E-01	1,00E+00	rs1402704|rs2531995|rs9688182|rs12905386|rs1413611|rs4078293|rs2531982|rs11196203|rs7904519|rs6788984|rs3924649|rs1865550|rs13114243|rs4381631|rs4944092|rs11787985|rs4765835|rs1339266|rs6055957|rs4724298
Renal Disorder	GLYCOGEN_BREAKDOWN_GLYCOGENOLYSIS	REACTOME	246	11	10	7,53E-01	1,00E+00	rs2995948|rs217463|rs6671606|rs1457604|rs7144098|rs12889552|rs11083841|rs8004788|rs11626241|rs10137869
Renal Disorder	NUCLEOTIDE_EXCISION_REPAIR	KEGG	914	40	20	7,53E-01	1,00E+00	rs7769220|rs7722373|rs12602230|rs3748269|rs4790838|rs2303744|rs2157072|rs1646314|rs7190295|rs6537537|rs2244095|rs2323973|rs7195205|rs7332652|rs7038659|rs7208143|rs8181839|rs751511|rs818707|rs12930200
Renal Disorder	SIGNALING_BY_FGFR	REACTOME	4154	25	20	7,55E-01	1,00E+00	rs2531995|rs3813668|rs263931|rs7318731|rs2755217|rs2531982|rs9601816|rs2517037|rs249736|rs12832680|rs3924649|rs874269|rs6705717|rs1865550|rs1994474|rs9545368|rs4381631|rs7914|rs4770252|rs1379707
Renal Disorder	NUCLEAR_SIGNALING_BY_ERBB4	REACTOME	2231	33	20	7,55E-01	1,00E+00	rs17664708|rs13436213|rs1714424|rs17749288|rs9930132|rs7198400|rs12521272|rs2738627|rs2439321|rs3020317|rs1505374|rs17762822|rs11863844|rs16846161|rs7199110|rs1503486|rs7108698|rs11642715|rs12444602|rs9466269
Renal Disorder	LIPOPROTEIN_METABOLISM	REACTOME	1037	31	19	7,55E-01	1,00E+00	rs10846760|rs8111157|rs11668477|rs925864|rs4715|rs4871975|rs7499892|rs405509|rs11057761|rs8039477|rs878949|rs7172930|rs10820726|rs701090|rs7601441|rs225436|rs4810479|rs388065|rs2356827
Renal Disorder	GLUTATHIONE_CONJUGATION	REACTOME	407	28	14	7,57E-01	1,00E+00	rs1695|rs4149195|rs10918228|rs13114510|rs12646059|rs34511626|rs7355084|rs4147599|rs396216|rs2132847|rs9333372|rs3794950|rs1024834|rs2297763
Renal Disorder	PGC1A_PATHWAY	BIOCARTA	959	30	20	7,60E-01	1,00E+00	rs9688182|rs1555208|rs6857002|rs4724298|rs9884852|rs10021542|rs16873865|rs11090911|rs1644047|rs17733355|rs6810541|rs10517023|rs11083841|rs325392|rs16903232|rs17733133|rs11600990|rs4823529|rs11626241|rs10009448
Renal Disorder	ABCA_TRANSPORTERS_IN_LIPID_HOMEOSTASIS	REACTOME	464	12	6	7,61E-01	1,00E+00	rs12582802|rs3752237|rs3809046|rs4245794|rs626776|rs225436
Renal Disorder	AMYLOIDS	REACTOME	972	56	20	7,61E-01	1,00E+00	rs1004446|rs2239896|rs3787620|rs1961839|rs461626|rs17015738|rs9466269|rs878949|rs8126493|rs4485425|rs400819|rs643788|rs6933317|rs10946808|rs1341758|rs6903568|rs12340264|rs8025462|rs3764478|rs7741445
Renal Disorder	MAPK_PATHWAY	BIOCARTA	2690	27	20	7,61E-01	1,00E+00	rs2760494|rs9642880|rs2869441|rs2364598|rs17103274|rs12801636|rs874269|rs1153681|rs10455523|rs1950167|rs12961853|rs9810603|rs6671527|rs1460769|rs12940055|rs2715825|rs11100237|rs9362732|rs10498658|rs10095762
Renal Disorder	PKA_MEDIATED_PHOSPHORYLATION_OF_CREB	REACTOME	767	27	19	7,61E-01	1,00E+00	rs2531995|rs2531982|rs1865550|rs10491176|rs2286562|rs3763609|rs6555491|rs1589880|rs316689|rs2072341|rs17828481|rs918842|rs4665273|rs10451896|rs11770994|rs11649508|rs4785953|rs2290910|rs11543317
Renal Disorder	CTCF_PATHWAY	BIOCARTA	1035	39	20	7,62E-01	1,00E+00	rs3813668|rs9642880|rs6660484|rs1805113|rs10095762|rs2811709|rs17153616|rs849430|rs13173003|rs853812|rs1004717|rs6662137|rs17131536|rs7641889|rs6809777|rs6449947|rs10091329|rs1461084|rs4603211|rs830474
Renal Disorder	IL1R_PATHWAY	BIOCARTA	1001	34	20	7,63E-01	1,00E+00	rs2760494|rs17103274|rs1152918|rs10455523|rs7793163|rs2715825|rs9362732|rs9362744|rs9351265|rs9362786|rs1881735|rs6662137|rs2306365|rs3213448|rs2818291|rs10883452|rs5743942|rs289067|rs2144363|rs4252340
Renal Disorder	DC_PATHWAY	BIOCARTA	573	24	16	7,64E-01	1,00E+00	rs7858743|rs743562|rs1329057|rs10515797|rs10784678|rs10748094|rs10878729|rs2028299|rs2007084|rs4932145|rs25887|rs4522435|rs12001550|rs10983899|rs10923137|rs4836716
Renal Disorder	VIP_PATHWAY	BIOCARTA	1000	26	20	7,65E-01	1,00E+00	rs9642880|rs17103274|rs10491176|rs6123013|rs1124425|rs2286562|rs10095762|rs680314|rs3763609|rs17733355|rs4746697|rs4870142|rs4239343|rs2306365|rs11083841|rs316689|rs3823082|rs10883452|rs192846|rs10091329
Renal Disorder	SIGNALING_BY_HIPPO	REACTOME	627	16	11	7,65E-01	1,00E+00	rs4976592|rs6894208|rs10157819|rs770713|rs7108698|rs950601|rs6555807|rs10737109|rs17322289|rs2124216|rs17608107
Renal Disorder	ACUTE_MYELOID_LEUKEMIA	KEGG	1892	25	20	7,66E-01	1,00E+00	rs3813668|rs9642880|rs11196203|rs7904519|rs3924649|rs874269|rs2268284|rs13114243|rs946199|rs9658119|rs1014058|rs219463|rs10095762|rs17116453|rs238928|rs2726626|rs2244621|rs17153616|rs4435678|rs10488695
Renal Disorder	CARM_ER_PATHWAY	BIOCARTA	1617	35	20	7,70E-01	1,00E+00	rs6761|rs1147289|rs10482869|rs2655215|rs6857002|rs3020317|rs6756261|rs13430446|rs1111348|rs9884852|rs10021542|rs6543557|rs3791446|rs16873865|rs2717344|rs1644047|rs1168292|rs996688|rs10499515|rs6810541
Renal Disorder	FANCONI_ANEMIA_PATHWAY	REACTOME	461	16	10	7,71E-01	1,00E+00	rs934419|rs420259|rs8107282|rs42830|rs2561883|rs206111|rs9462085|rs527365|rs3092989|rs3934711
Renal Disorder	GLUCONEOGENESIS	REACTOME	490	25	17	7,74E-01	1,00E+00	rs6723088|rs2490286|rs973639|rs2239942|rs3763609|rs262498|rs3821096|rs6025639|rs316689|rs11861842|rs4811873|rs422732|rs13293668|rs2106446|rs17028097|rs1040566|rs970169
Renal Disorder	FGFR_LIGAND_BINDING_AND_ACTIVATION	REACTOME	984	31	20	7,76E-01	1,00E+00	rs263931|rs7318731|rs2517037|rs249736|rs4770252|rs1379707|rs1536433|rs7825208|rs17099156|rs17431894|rs4770192|rs822248|rs6859263|rs7914068|rs743682|rs1860228|rs10788184|rs4424770|rs9591501|rs731089
Renal Disorder	ION_TRANSPORT_BY_P_TYPE_ATPASES	REACTOME	1690	33	20	7,80E-01	1,00E+00	rs6490959|rs4941086|rs12695746|rs9817842|rs860294|rs4769434|rs1511493|rs8040030|rs17643649|rs4505988|rs759691|rs898500|rs968332|rs2239942|rs11719939|rs13079701|rs9634423|rs6013258|rs4839061|rs1553890
Renal Disorder	ETS_PATHWAY	BIOCARTA	682	26	16	7,82E-01	1,00E+00	rs2760494|rs1147289|rs12025765|rs7189819|rs584096|rs7516187|rs3782262|rs3782267|rs3742768|rs701078|rs333947|rs2228440|rs7115037|rs10790952|rs10773097|rs2836776
Renal Disorder	LIPID_DIGESTION_MOBILIZATION_AND_TRANSPORT	REACTOME	1389	26	20	7,82E-01	1,00E+00	rs10846760|rs9298360|rs8111157|rs11668477|rs925864|rs4715|rs13409926|rs4871975|rs7499892|rs405509|rs11057761|rs8039477|rs3763609|rs878949|rs4245794|rs7172930|rs10820726|rs701090|rs567384|rs316689
Renal Disorder	SIGNALLING_TO_ERKS	REACTOME	794	29	17	7,89E-01	1,00E+00	rs917199|rs3924649|rs10793894|rs4761243|rs1442655|rs1890548|rs1123551|rs12459940|rs673709|rs615258|rs10901077|rs13224312|rs8453|rs4723910|rs10842515|rs350825|rs6018257
Renal Disorder	RACCYCD_PATHWAY	BIOCARTA	551	26	14	7,89E-01	1,00E+00	rs3813668|rs17103274|rs13173003|rs853812|rs2306365|rs3019245|rs10883452|rs7641889|rs6449947|rs3019244|rs399413|rs17318844|rs830474|rs10054692
Renal Disorder	AXON_GUIDANCE	KEGG	9506	31	20	7,90E-01	1,00E+00	rs519813|rs2483548|rs6797462|rs6950509|rs2423401|rs6882497|rs2722987|rs7274475|rs4954410|rs652856|rs10917268|rs11167020|rs6716817|rs12905386|rs9294570|rs33063|rs16862708|rs11035640|rs9966514|rs659632
Renal Disorder	SIGNALING_BY_THE_B_CELL_RECEPTOR_BCR	REACTOME	3426	32	20	7,91E-01	1,00E+00	rs3813668|rs1760125|rs2991215|rs1529702|rs4576294|rs17103274|rs2755217|rs12832680|rs3924649|rs241437|rs874269|rs1994474|rs10455523|rs7914|rs9695993|rs2220168|rs1878808|rs2668020|rs7300093|rs11048716
Renal Disorder	NICOTINATE_AND_NICOTINAMIDE_METABOLISM	KEGG	963	53	20	7,93E-01	1,00E+00	rs34236|rs2545618|rs944664|rs2604299|rs9847705|rs6922|rs1998393|rs1990745|rs11242540|rs10479267|rs6596573|rs12039225|rs16849220|rs11214959|rs313229|rs675220|rs2271170|rs1348223|rs2540052|rs2241080
Renal Disorder	CTNNB1_PHOSPHORYLATION_CASCADE	REACTOME	316	16	10	7,94E-01	1,00E+00	rs6788984|rs353241|rs11119881|rs7546833|rs11860497|rs429988|rs10411335|rs4355511|rs633045|rs7200589
Renal Disorder	ERK_PATHWAY	BIOCARTA	1025	32	20	7,95E-01	1,00E+00	rs9642880|rs4966009|rs2684792|rs657770|rs6671527|rs12440962|rs6681938|rs10095762|rs7168213|rs10506601|rs12459940|rs17746482|rs4245566|rs13041735|rs10091329|rs7168671|rs2877260|rs350825|rs11760524|rs6018257
Renal Disorder	INTRINSIC_PATHWAY	REACTOME	264	10	4	7,95E-01	1,00E+00	rs1008728|rs11132389|rs4752932|rs2510280
Renal Disorder	MTA3_PATHWAY	BIOCARTA	333	15	8	7,97E-01	1,00E+00	rs3020317|rs6020178|rs1298954|rs2293079|rs11861842|rs1284300|rs7186053|rs9383939
Renal Disorder	GLYCEROPHOSPHOLIPID_METABOLISM	KEGG	2549	29	20	7,97E-01	1,00E+00	rs1148757|rs6053183|rs1543932|rs2701632|rs2357960|rs12733186|rs711237|rs9566906|rs12988612|rs34129552|rs4551123|rs17560881|rs13160153|rs867687|rs10454443|rs1917806|rs1528098|rs10486048|rs2920123|rs928389
Renal Disorder	AT1R_PATHWAY	BIOCARTA	1224	32	20	7,99E-01	1,00E+00	rs2760494|rs11167020|rs1153681|rs4381631|rs306962|rs3889392|rs16960228|rs1644047|rs7840147|rs11871468|rs1881735|rs12459940|rs17746482|rs4245566|rs389566|rs11083841|rs3019245|rs325392|rs3019244|rs12721331
Renal Disorder	FORMATION_OF_TUBULIN_FOLDING_INTERMEDIATES_BY_CCT_TRIC	REACTOME	227	7	5	7,99E-01	1,00E+00	rs2842863|rs10458071|rs2293079|rs1474298|rs9501935
Renal Disorder	TRAFFICKING_OF_GLUR2_CONTAINING_AMPA_RECEPTORS	REACTOME	721	35	20	8,00E-01	1,00E+00	rs9324756|rs4381631|rs492014|rs12054636|rs10025251|rs3889392|rs16960228|rs4246043|rs11871468|rs11919795|rs578772|rs1438949|rs1862319|rs11167606|rs648762|rs1003599|rs2914621|rs2231216|rs1942971|rs16959569
Renal Disorder	G2_PATHWAY	BIOCARTA	354	4	3	8,02E-01	1,00E+00	rs7736312|rs2450246|rs4669407
Renal Disorder	DESTABILIZATION_OF_MRNA_BY_TRISTETRAPROLIN_TTP	REACTOME	179	6	5	8,05E-01	1,00E+00	rs9784647|rs11244096|rs10814620|rs11707321|rs10407035
Renal Disorder	PI_3K_CASCADE	REACTOME	1868	26	20	8,08E-01	1,00E+00	rs3813668|rs263931|rs7318731|rs2755217|rs2517037|rs249736|rs874269|rs4770252|rs1379707|rs1536433|rs4761243|rs11876|rs2244621|rs7825208|rs17099156|rs17431894|rs484754|rs13173003|rs853812|rs4770192
Renal Disorder	PLATELET_ACTIVATION_SIGNALING_AND_AGGREGATION	REACTOME	6965	28	20	8,09E-01	1,00E+00	rs3813668|rs441250|rs1148757|rs958766|rs1760125|rs525007|rs2991215|rs4148426|rs308039|rs11167020|rs11646140|rs4734497|rs12878420|rs12746334|rs12832680|rs195771|rs874269|rs2357960|rs10770|rs12733186
Renal Disorder	INTERFERON_ALPHA_BETA_SIGNALING	REACTOME	1477	40	20	8,10E-01	1,00E+00	rs1532472|rs3756103|rs4576294|rs241437|rs2066808|rs1342852|rs2834139|rs1915033|rs12409333|rs17686080|rs12111032|rs4239266|rs7655371|rs9933359|rs1077767|rs2236758|rs729302|rs4556628|rs9904782|rs12149292
Renal Disorder	SPHINGOLIPID_DE_NOVO_BIOSYNTHESIS	REACTOME	1020	47	20	8,11E-01	1,00E+00	rs11169282|rs7179325|rs7145669|rs29145|rs2886905|rs7183379|rs9679935|rs928389|rs7087539|rs4797414|rs11206718|rs1953116|rs4965316|rs7148794|rs659259|rs11621931|rs11676002|rs1957463|rs3845727|rs10513906
Renal Disorder	PANCREATIC_CANCER	KEGG	1920	35	20	8,13E-01	1,00E+00	rs3813668|rs917199|rs441250|rs2869441|rs3924649|rs874269|rs412705|rs1460769|rs942042|rs12409333|rs7658485|rs594942|rs2244621|rs746720|rs2811709|rs17153616|rs12640395|rs849430|rs11071939|rs4690458
Renal Disorder	MCM_PATHWAY	BIOCARTA	234	9	6	8,14E-01	1,00E+00	rs10948689|rs6950715|rs133431|rs1994189|rs13242458|rs782428
Renal Disorder	REGULATION_OF_ORNITHINE_DECARBOXYLASE_ODC	REACTOME	773	39	20	8,15E-01	1,00E+00	rs1405948|rs4576294|rs241437|rs1533182|rs10257369|rs2513892|rs1064576|rs885815|rs963267|rs2526057|rs4069666|rs4904662|rs1052748|rs3755259|rs11764195|rs2357804|rs1015166|rs11877878|rs16972457|rs16960774
Renal Disorder	SIGNALING_BY_CONSTITUTIVELY_ACTIVE_EGFR	REACTOME	503	31	17	8,16E-01	1,00E+00	rs3813668|rs3924649|rs7914|rs13173003|rs853812|rs17746482|rs4245566|rs7641889|rs6449947|rs8453|rs2877260|rs17389103|rs11760524|rs10023724|rs830474|rs721412|rs10054692
Renal Disorder	GSK3_PATHWAY	BIOCARTA	1055	24	20	8,16E-01	1,00E+00	rs3813668|rs6788984|rs7858743|rs1329057|rs1429529|rs219463|rs7791011|rs7843776|rs11860497|rs13173003|rs853812|rs2465153|rs6919417|rs2306365|rs429988|rs7641889|rs6449947|rs931771|rs5743942|rs12001550
Renal Disorder	GAP_JUNCTION	KEGG	4388	22	20	8,17E-01	1,00E+00	rs2531995|rs1326250|rs308039|rs12905386|rs1937652|rs362848|rs2039860|rs2531982|rs12832680|rs9307949|rs3924649|rs939688|rs11956891|rs1865550|rs1994474|rs4381631|rs1904006|rs4935225|rs10516652|rs11722434
Renal Disorder	AMINE_COMPOUND_SLC_TRANSPORTERS	REACTOME	1298	30	20	8,18E-01	1,00E+00	rs4980806|rs2697152|rs7523468|rs363222|rs3779672|rs3922763|rs12490575|rs3199966|rs4610207|rs1979021|rs13020302|rs7970932|rs9844272|rs12230144|rs7861505|rs7959178|rs9429225|rs4949673|rs11580716|rs10060827
Renal Disorder	YAP1_AND_WWTR1_TAZ_STIMULATED_GENE_EXPRESSION	REACTOME	1047	31	20	8,20E-01	1,00E+00	rs1555208|rs9850957|rs7941340|rs1321080|rs1805352|rs7108698|rs11090911|rs12630639|rs11022522|rs6803450|rs16944288|rs13295631|rs11603665|rs7429325|rs2395626|rs1209035|rs2113431|rs2772395|rs4823529|rs7619263
Renal Disorder	KINESINS	REACTOME	566	21	12	8,21E-01	1,00E+00	rs2957385|rs7156395|rs10038958|rs2384319|rs6706373|rs351294|rs861528|rs1508947|rs9916504|rs7142555|rs6427229|rs1457354
Renal Disorder	EGFR_DOWNREGULATION	REACTOME	1037	40	20	8,22E-01	1,00E+00	rs1043313|rs9601816|rs9545368|rs7914|rs10219897|rs9593627|rs1936544|rs2038521|rs12429667|rs11919795|rs12000388|rs17746482|rs4245566|rs1349683|rs7989244|rs3780247|rs2877260|rs2231216|rs3744382|rs1176270
Renal Disorder	ABC_FAMILY_PROTEINS_MEDIATED_TRANSPORT	REACTOME	1753	56	20	8,22E-01	1,00E+00	rs212082|rs4148426|rs4148434|rs4148559|rs12582802|rs3752237|rs4148006|rs9524681|rs3213473|rs722341|rs3809046|rs4245794|rs2293079|rs943288|rs4148418|rs2766482|rs9561797|rs626776|rs225436|rs4147975
Renal Disorder	E2F_MEDIATED_REGULATION_OF_DNA_REPLICATION	REACTOME	452	21	12	8,26E-01	1,00E+00	rs3846484|rs2298582|rs236106|rs6950715|rs4943383|rs9377723|rs9576040|rs1994189|rs2450246|rs10411335|rs399413|rs633045
Renal Disorder	BCELLSURVIVAL_PATHWAY	BIOCARTA	500	28	15	8,26E-01	1,00E+00	rs3813668|rs17153616|rs1042541|rs1275659|rs849430|rs13173003|rs853812|rs1004717|rs3742768|rs3124743|rs7641889|rs6449947|rs975886|rs830474|rs10054692
Renal Disorder	INFLAM_PATHWAY	BIOCARTA	747	27	15	8,26E-01	1,00E+00	rs743562|rs10515797|rs10784678|rs7793163|rs10748094|rs28605404|rs10878729|rs9990563|rs13117878|rs6662137|rs10425596|rs25887|rs2858870|rs333947|rs4252340
Renal Disorder	SIGNALING_BY_NOTCH	REACTOME	4553	31	20	8,27E-01	1,00E+00	rs4877187|rs4687504|rs2760494|rs9642880|rs9999030|rs1147289|rs4789523|rs9850957|rs2301106|rs10780347|rs6074178|rs16878154|rs11794108|rs3748269|rs2655215|rs1877207|rs17616659|rs6756261|rs13430446|rs470113
Renal Disorder	PAR1_PATHWAY	BIOCARTA	1941	29	20	8,29E-01	1,00E+00	rs3813668|rs8001712|rs4681888|rs11619224|rs10455523|rs4381631|rs6055957|rs4681908|rs6516421|rs7728560|rs3889392|rs16960228|rs6681938|rs9362732|rs12740413|rs17756915|rs9362744|rs9351265|rs17153616|rs11871468
Renal Disorder	PI3K_EVENTS_IN_ERBB2_SIGNALING	REACTOME	1886	35	20	8,29E-01	1,00E+00	rs3813668|rs17664708|rs1714424|rs2755217|rs17749288|rs874269|rs2439321|rs1505374|rs16846161|rs1503486|rs11876|rs2244621|rs13008370|rs6742399|rs484754|rs13173003|rs853812|rs7822564|rs17746482|rs4245566
Renal Disorder	PROTEIN_FOLDING	REACTOME	675	25	15	8,29E-01	1,00E+00	rs12199169|rs7734027|rs2842863|rs10484911|rs2325899|rs10458071|rs254412|rs2293079|rs1014146|rs4986129|rs1542891|rs4394301|rs9297054|rs1474298|rs9501935
Renal Disorder	ACTIVATED_NOTCH1_TRANSMITS_SIGNAL_TO_THE_NUCLEUS	REACTOME	719	28	19	8,30E-01	1,00E+00	rs6074178|rs17659152|rs6732014|rs17762822|rs12892451|rs6108746|rs958110|rs17189227|rs6078029|rs10139403|rs10494266|rs3887640|rs4972905|rs1596518|rs1674098|rs7300813|rs17182398|rs903630|rs12192239
Renal Disorder	INHIBITION_OF_THE_PROTEOLYTIC_ACTIVITY_OF_APC_C_REQUIRED_FOR_THE_ONSET_OF_ANAPHASE_BY_MITOTIC_SPINDLE_CHECKPOINT_COMPONENTS	REACTOME	365	13	10	8,30E-01	1,00E+00	rs11006121|rs1994174|rs2324716|rs2311552|rs12352784|rs11718625|rs2058765|rs16912145|rs12647998|rs710251
Renal Disorder	SIGNALING_BY_FGFR_IN_DISEASE	REACTOME	4545	24	20	8,31E-01	1,00E+00	rs2531995|rs3813668|rs263931|rs7318731|rs2755217|rs2531982|rs9601816|rs2517037|rs249736|rs12832680|rs3924649|rs874269|rs6705717|rs1865550|rs1994474|rs9545368|rs4381631|rs7914|rs4770252|rs2906725
Renal Disorder	TYPE_II_DIABETES_MELLITUS	KEGG	2053	33	20	8,31E-01	1,00E+00	rs3813668|rs2869441|rs1004446|rs7984646|rs1422259|rs6705717|rs10762277|rs3829185|rs1460769|rs3852876|rs7983897|rs1317063|rs7658485|rs6755308|rs281501|rs17153616|rs7615501|rs3731949|rs722341|rs4953334
Renal Disorder	GPCR_PATHWAY	BIOCARTA	1102	31	20	8,34E-01	1,00E+00	rs2760494|rs4381631|rs10491176|rs6123013|rs7193701|rs3889392|rs16960228|rs2286562|rs6681938|rs3763609|rs17733355|rs11871468|rs1589880|rs4239343|rs11083841|rs316689|rs13041735|rs3742768|rs918842|rs1003599
Renal Disorder	TGF_BETA_SIGNALING_PATHWAY	KEGG	3535	28	20	8,35E-01	1,00E+00	rs9642880|rs6038619|rs2364598|rs6716817|rs235710|rs4664888|rs9393162|rs568520|rs6054501|rs1837291|rs996544|rs17704722|rs210363|rs6107891|rs10033464|rs17637848|rs7670455|rs753770|rs6038621|rs412705
Renal Disorder	RIG_I_LIKE_RECEPTOR_SIGNALING_PATHWAY	KEGG	1361	32	20	8,35E-01	1,00E+00	rs2869441|rs17103274|rs10455523|rs7143468|rs3769823|rs10515797|rs1460769|rs9362732|rs10498658|rs12115747|rs7658485|rs9362744|rs9351265|rs12640395|rs9362786|rs11792285|rs1881735|rs1267034|rs2306365|rs4148171
Renal Disorder	METAL_ION_SLC_TRANSPORTERS	REACTOME	653	31	19	8,36E-01	1,00E+00	rs7603445|rs11778200|rs13266634|rs2360101|rs7017745|rs759072|rs4617186|rs7703932|rs1286371|rs7624706|rs12352784|rs7605057|rs2033899|rs13175238|rs992385|rs439205|rs12727395|rs11809572|rs11688042
Renal Disorder	DOWNSTREAM_SIGNAL_TRANSDUCTION	REACTOME	3083	29	20	8,37E-01	1,00E+00	rs2531995|rs3813668|rs2755217|rs2531982|rs12832680|rs7605392|rs3924649|rs874269|rs6705717|rs1865550|rs1994474|rs4381631|rs10793894|rs10491176|rs2220168|rs9668898|rs3889392|rs16960228|rs2286562|rs11048716
Renal Disorder	OPIOID_SIGNALLING	REACTOME	3594	28	20	8,38E-01	1,00E+00	rs2531995|rs1148757|rs12905386|rs12878420|rs12746334|rs2531982|rs12832680|rs12733186|rs1319339|rs1865550|rs1994474|rs12985186|rs4381631|rs641010|rs6055957|rs10491176|rs2220168|rs6516421|rs10454443|rs1799917
Renal Disorder	NEUROACTIVE_LIGAND_RECEPTOR_INTERACTION	KEGG	13469	27	20	8,39E-01	1,00E+00	rs7757020|rs7716847|rs12127134|rs1112903|rs10490893|rs989506|rs7375509|rs2075716|rs10488597|rs13436213|rs10776728|rs11077666|rs884700|rs1876527|rs11917719|rs3133861|rs1167763|rs362848|rs2039860|rs4676410
Renal Disorder	SIGNALING_BY_NOTCH1	REACTOME	3563	31	20	8,40E-01	1,00E+00	rs4877187|rs4687504|rs9642880|rs9999030|rs1147289|rs9850957|rs2301106|rs10780347|rs6074178|rs16878154|rs11794108|rs2655215|rs1877207|rs6756261|rs13430446|rs17659152|rs1347370|rs6543557|rs6732014|rs3791446
Renal Disorder	DNA_REPLICATION	KEGG	740	29	18	8,41E-01	1,00E+00	rs9535573|rs12602230|rs10948689|rs4790838|rs2303744|rs2244095|rs2323973|rs9377723|rs133431|rs7332652|rs7208143|rs8181839|rs751511|rs818707|rs13242458|rs10258735|rs17482047|rs782428
Renal Disorder	REGULATION_OF_KIT_SIGNALING	REACTOME	587	34	20	8,42E-01	1,00E+00	rs13114243|rs4381631|rs7914|rs2668020|rs1014058|rs3889392|rs16960228|rs17073074|rs2726626|rs11871468|rs3184504|rs1027990|rs10513985|rs10457242|rs1003599|rs12967001|rs16959569|rs6018257|rs2711390|rs9897002
Renal Disorder	APC_CDC20_MEDIATED_DEGRADATION_OF_NEK2A	REACTOME	396	13	10	8,42E-01	1,00E+00	rs11006121|rs1994174|rs2324716|rs2311552|rs12352784|rs11718625|rs2058765|rs16912145|rs12647998|rs710251
Renal Disorder	TCA_CYCLE_AND_RESPIRATORY_ELECTRON_TRANSPORT	REACTOME	2599	48	20	8,42E-01	1,00E+00	rs3934860|rs11715476|rs12232909|rs17070878|rs254738|rs17163678|rs2147502|rs773652|rs2421763|rs12521803|rs1133099|rs10206058|rs6874335|rs218968|rs4147661|rs3745004|rs7646423|rs41426|rs2325899|rs17163764
Renal Disorder	CHRONIC_MYELOID_LEUKEMIA	KEGG	2538	26	20	8,44E-01	1,00E+00	rs3813668|rs12243975|rs9642880|rs17103274|rs730830|rs3924649|rs874269|rs2268284|rs7914|rs1362770|rs7853654|rs412705|rs942042|rs10095762|rs7635759|rs17822304|rs11245515|rs7648140|rs2244621|rs746720
Renal Disorder	MEMBRANE_TRAFFICKING	REACTOME	2276	44	20	8,45E-01	1,00E+00	rs7725100|rs1043313|rs11861296|rs4519603|rs6769886|rs2025994|rs6863273|rs13165062|rs7142869|rs1886748|rs17473058|rs6929363|rs2232739|rs907638|rs873104|rs734484|rs2072824|rs11726508|rs11730728|rs17340112
Renal Disorder	SIGNALING_BY_TGF_BETA_RECEPTOR_COMPLEX	REACTOME	1605	28	20	8,47E-01	1,00E+00	rs9642880|rs1147289|rs2364598|rs621231|rs11006121|rs874583|rs1994174|rs412705|rs13409926|rs10095762|rs556765|rs1989138|rs6504246|rs85440|rs11071939|rs2811712|rs4811914|rs3782262|rs3782267|rs169409
Renal Disorder	PREFOLDIN_MEDIATED_TRANSFER_OF_SUBSTRATE_TO_CCT_TRIC	REACTOME	288	7	5	8,47E-01	1,00E+00	rs2842863|rs10458071|rs2293079|rs1474298|rs9501935
Renal Disorder	PHOSPHOLIPID_METABOLISM	REACTOME	6226	31	20	8,48E-01	1,00E+00	rs622442|rs11695702|rs3813668|rs1148757|rs9688182|rs11169282|rs6053183|rs7238810|rs7179325|rs7145669|rs2701632|rs195771|rs12733186|rs711237|rs1756673|rs11044232|rs9959057|rs12988612|rs10502786|rs16872173
Renal Disorder	DEGRADATION_OF_THE_EXTRACELLULAR_MATRIX	REACTOME	580	9	8	8,48E-01	1,00E+00	rs13277637|rs527832|rs3875143|rs10504868|rs2616506|rs3851539|rs4406450|rs6499933
Renal Disorder	GLIOMA	KEGG	2070	33	20	8,49E-01	1,00E+00	rs3813668|rs4966009|rs9688182|rs4078293|rs3924649|rs874269|rs4381631|rs2684792|rs4724298|rs12440962|rs3889392|rs16960228|rs942042|rs7168213|rs746720|rs2811709|rs17153616|rs11871468|rs849430|rs12459940
Renal Disorder	REGULATION_OF_APOPTOSIS	REACTOME	1432	39	20	8,52E-01	1,00E+00	rs4576294|rs9966514|rs241437|rs11732086|rs1930056|rs4878104|rs10746816|rs1953040|rs10512188|rs10266523|rs4940144|rs1718414|rs2084385|rs4401133|rs10027921|rs332236|rs1064576|rs4347699|rs963267|rs2526057
Renal Disorder	HER2_PATHWAY	BIOCARTA	1326	28	20	8,52E-01	1,00E+00	rs3813668|rs11582424|rs3020317|rs1505374|rs7793163|rs16846161|rs1168292|rs13008370|rs17153616|rs6742399|rs849430|rs13173003|rs853812|rs17746482|rs4245566|rs6581709|rs1004717|rs10784600|rs7574462|rs7641889
Renal Disorder	LAGGING_STRAND_SYNTHESIS	REACTOME	550	19	11	8,53E-01	1,00E+00	rs12602230|rs4790838|rs2303744|rs2244095|rs9377723|rs7330677|rs7208143|rs8181839|rs751511|rs10258735|rs17482047
Renal Disorder	SPRY_PATHWAY	BIOCARTA	910	30	20	8,53E-01	1,00E+00	rs9601816|rs9545368|rs7914|rs10219897|rs4105088|rs253447|rs9593627|rs1936544|rs10506601|rs12429667|rs17746482|rs4245566|rs1349683|rs7989244|rs2278345|rs2877260|rs10052248|rs1176270|rs11760524|rs6018257
Renal Disorder	MPR_PATHWAY	BIOCARTA	624	25	18	8,54E-01	1,00E+00	rs6724135|rs3846484|rs10491176|rs2286562|rs6681938|rs465316|rs3763609|rs1589880|rs4338396|rs516693|rs316689|rs13041735|rs2450246|rs8025182|rs11770994|rs600677|rs6018257|rs10486925
Renal Disorder	ERK_MAPK_TARGETS	REACTOME	704	22	10	8,54E-01	1,00E+00	rs9848727|rs17555326|rs1644047|rs17555250|rs2341674|rs731288|rs325392|rs10411335|rs16903232|rs633045

Renal Disorder	P38MAPK_PATHWAY	BIOCARTA	1904	29	20	8,57E-01	1,00E+00	rs9642880|rs1148757|rs12733186|rs1153681|rs10455523|rs1950167|rs6671527|rs10454443|rs2715825|rs11100237|rs9362732|rs10498658|rs10095762|rs7554573|rs1644047|rs2023955|rs2250227|rs9362744|rs10143368|rs9351265
Renal Disorder	ERK5_PATHWAY	BIOCARTA	595	29	14	8,58E-01	1,00E+00	rs3813668|rs1644047|rs17153616|rs849430|rs13173003|rs853812|rs1004717|rs7641889|rs6449947|rs325392|rs16903232|rs918842|rs830474|rs10054692
Renal Disorder	SNARE_INTERACTIONS_IN_VESICULAR_TRANSPORT	KEGG	828	44	20	8,59E-01	1,00E+00	rs7794610|rs11196125|rs7014552|rs2532520|rs883382|rs2232739|rs6104567|rs8071640|rs1492366|rs7070744|rs11086651|rs3763851|rs7209802|rs8543|rs4286803|rs2304911|rs1591811|rs11730104|rs6583640|rs12714147
Renal Disorder	FORMATION_OF_INCISION_COMPLEX_IN_GG_NER	REACTOME	545	38	15	8,59E-01	1,00E+00	rs12602230|rs3748269|rs4790838|rs2157072|rs1646314|rs7190295|rs7195205|rs7038659|rs7208143|rs12930200|rs10258735|rs1323804|rs17482047|rs4150282|rs6498466
Renal Disorder	NRAGE_SIGNALS_DEATH_THROUGH_JNK	REACTOME	1919	27	20	8,60E-01	1,00E+00	rs525007|rs6019409|rs4681888|rs333327|rs657770|rs11698214|rs7774757|rs656709|rs4681908|rs12480425|rs17020088|rs6095298|rs4810774|rs12627433|rs4778857|rs11086250|rs13052373|rs12039210|rs9308742|rs17214843
Renal Disorder	SIGNALING_BY_ERBB4	REACTOME	3434	34	20	8,61E-01	1,00E+00	rs3813668|rs17664708|rs13436213|rs1714424|rs2755217|rs17749288|rs3924649|rs9930132|rs874269|rs7198400|rs12521272|rs2738627|rs2439321|rs3020317|rs1505374|rs17762822|rs11863844|rs16846161|rs7199110|rs1503486
Renal Disorder	EIF4_PATHWAY	BIOCARTA	890	39	20	8,63E-01	1,00E+00	rs3813668|rs4381631|rs6671527|rs3889392|rs16960228|rs1317063|rs7698057|rs11871468|rs13173003|rs853812|rs2403370|rs6725556|rs7641889|rs6449947|rs1003599|rs756847|rs16959569|rs4603211|rs4788426|rs2230571
Renal Disorder	DEADENYLATION_DEPENDENT_MRNA_DECAY	REACTOME	622	41	18	8,64E-01	1,00E+00	rs7138763|rs36664|rs4761134|rs1271211|rs7698057|rs9784647|rs11553287|rs11244096|rs10814620|rs871683|rs11707321|rs2429135|rs2007366|rs6948145|rs10892286|rs2128162|rs2230571|rs12922485
Renal Disorder	SIGNAL_TRANSDUCTION_BY_L1	REACTOME	1310	35	20	8,64E-01	1,00E+00	rs1043313|rs3851570|rs2776937|rs11009311|rs4936186|rs1245138|rs16959812|rs730172|rs17296436|rs7825208|rs873803|rs2038521|rs10827244|rs12459940|rs928814|rs11919795|rs12000388|rs17746482|rs4245566|rs3019245
Renal Disorder	EXTENSION_OF_TELOMERES	REACTOME	618	22	13	8,65E-01	1,00E+00	rs12602230|rs4790838|rs2303744|rs4635969|rs2244095|rs2323973|rs9377723|rs7330677|rs7208143|rs8181839|rs751511|rs10258735|rs17482047
Renal Disorder	RAC1_PATHWAY	BIOCARTA	763	26	14	8,65E-01	1,00E+00	rs3813668|rs17125|rs17153616|rs849430|rs13173003|rs853812|rs1004717|rs7712871|rs3019245|rs7641889|rs6449947|rs3019244|rs830474|rs10054692
Renal Disorder	OXIDATIVE_PHOSPHORYLATION	KEGG	2486	34	20	8,68E-01	1,00E+00	rs6490959|rs11871705|rs1198873|rs254738|rs17163678|rs9643478|rs2078340|rs12521803|rs296913|rs1133099|rs7465143|rs374954|rs10206058|rs7966867|rs6874335|rs2239942|rs218968|rs4147661|rs1390939|rs12468286
Renal Disorder	CHROMOSOME_MAINTENANCE	REACTOME	1949	26	20	8,70E-01	1,00E+00	rs1500480|rs11563256|rs2239896|rs2975842|rs12602230|rs10492892|rs3846484|rs360712|rs2123463|rs4790838|rs2303744|rs3004318|rs4635969|rs7665370|rs6657480|rs7606893|rs6981928|rs2244095|rs643788|rs2323973
Renal Disorder	NEUROTRANSMITTER_RECEPTOR_BINDING_AND_DOWNSTREAM_TRANSMISSION_IN_TH<br>L	REACTOME	6592	25	20	8,71E-01	1,00E+00	rs2531995|rs7716847|rs2051248|rs2075716|rs9688182|rs12905386|rs3133861|rs1167763|rs12878420|rs10420331|rs12746334|rs2961957|rs2531982|rs16838062|rs1372491|rs3922855|rs2337980|rs16823912|rs9390939|rs12050748
Renal Disorder	APC_C_CDC20_MEDIATED_DEGRADATION_OF_MITOTIC_PROTEINS	REACTOME	1094	38	20	8,71E-01	1,00E+00	rs4576294|rs11006121|rs241437|rs7736312|rs2961920|rs1994174|rs10257369|rs2324716|rs2311552|rs1064576|rs963267|rs2526057|rs12352784|rs11718625|rs2058765|rs4069666|rs2450246|rs11159958|rs1052748|rs11764195
Renal Disorder	AUTOIMMUNE_THYROID_DISEASE	KEGG	1646	60	20	8,72E-01	1,00E+00	rs2857106|rs10509323|rs1181390|rs4675367|rs743562|rs241436|rs13204672|rs10108988|rs429916|rs12111032|rs9276472|rs10197319|rs6930052|rs917986|rs2021840|rs9678469|rs859637|rs1015166|rs2071399|rs9275595
Renal Disorder	GLYCOSPHINGOLIPID_METABOLISM	REACTOME	924	38	20	8,72E-01	1,00E+00	rs9688182|rs1756673|rs16872173|rs12601802|rs2295797|rs11782529|rs7387252|rs1991798|rs2185456|rs6816578|rs1547308|rs12503095|rs7211107|rs4747209|rs13164947|rs11949551|rs4758415|rs16972915|rs17126237|rs4810479
Renal Disorder	METABOLISM_OF_MRNA	REACTOME	3017	38	20	8,72E-01	1,00E+00	rs2291782|rs7138763|rs4734497|rs4576294|rs1212343|rs12587507|rs4916595|rs241437|rs6501697|rs4381631|rs36664|rs1133099|rs1435961|rs1649990|rs8003773|rs3889392|rs2958522|rs16960228|rs12942418|rs4761134
Renal Disorder	MAP_KINASE_ACTIVATION_IN_TLR_CASCADE	REACTOME	1697	40	20	8,75E-01	1,00E+00	rs2760494|rs2869441|rs9848727|rs1153681|rs10455523|rs1460769|rs17555326|rs2715825|rs9362732|rs7658485|rs1644047|rs9362744|rs9351265|rs17555250|rs2046316|rs12640395|rs9362786|rs1881735|rs12459940|rs3742768
Renal Disorder	SPHINGOLIPID_METABOLISM	REACTOME	1944	41	20	8,75E-01	1,00E+00	rs9688182|rs11169282|rs7179325|rs7145669|rs1756673|rs16872173|rs29145|rs2886905|rs7183379|rs9679935|rs12601802|rs928389|rs2295797|rs11782529|rs7087539|rs7387252|rs4797414|rs1991798|rs2185456|rs6816578
Renal Disorder	ARACHIDONIC_ACID_METABOLISM	KEGG	1356	31	20	8,77E-01	1,00E+00	rs1148757|rs2701632|rs12733186|rs10454443|rs1668547|rs7215288|rs1409655|rs7554573|rs12459140|rs2250227|rs1934975|rs1712435|rs527643|rs6688451|rs17133974|rs11589805|rs2835270|rs6692958|rs2144438|rs9888067
Renal Disorder	CARDIACEGF_PATHWAY	BIOCARTA	1289	34	20	8,81E-01	1,00E+00	rs2760494|rs9642880|rs4381631|rs11244777|rs3889392|rs16960228|rs2859338|rs10095762|rs10507880|rs11871468|rs17746482|rs4245566|rs4714351|rs2306365|rs389566|rs3742768|rs12098279|rs4714469|rs12721331|rs10091329
Renal Disorder	PRIMARY_IMMUNODEFICIENCY	KEGG	742	55	17	8,82E-01	1,00E+00	rs8065836|rs2857106|rs241436|rs9612385|rs2580874|rs10932047|rs12616245|rs4273077|rs4938507|rs6002551|rs1015166|rs12169718|rs10892234|rs11188666|rs10208393|rs938486|rs2800953
Renal Disorder	APC_C_CDH1_MEDIATED_DEGRADATION_OF_CDC20_AND_OTHER_APC_C_CDH1_TARGET<br>TE_MITOSIS_EARLY_G1	REACTOME	959	40	20	8,84E-01	1,00E+00	rs4576294|rs11006121|rs241437|rs2961920|rs1994174|rs10268808|rs2324716|rs2311552|rs1064576|rs963267|rs2526057|rs12352784|rs11718625|rs4069666|rs11159958|rs1052748|rs11764195|rs2357804|rs1015166|rs11877878
Renal Disorder	PROTEASOME_PATHWAY	BIOCARTA	280	14	9	8,85E-01	1,00E+00	rs1977036|rs10268808|rs1064576|rs1052748|rs11764195|rs4331321|rs2357804|rs16960774|rs2719889
Renal Disorder	G2_M_CHECKPOINTS	REACTOME	710	29	19	8,85E-01	1,00E+00	rs12602230|rs2686830|rs3846484|rs10948689|rs4790838|rs236106|rs6950715|rs133431|rs7332652|rs1994189|rs7208143|rs8181839|rs751511|rs2450246|rs13242458|rs10258735|rs17482047|rs11764107|rs782428
Renal Disorder	ACTIVATED_TAK1_MEDIATES_P38_MAPK_ACTIVATION	REACTOME	649	16	12	8,86E-01	1,00E+00	rs10455523|rs2715825|rs9362732|rs9362744|rs9351265|rs2046316|rs9362786|rs2818291|rs40383|rs2144363|rs12679902|rs1504281
Renal Disorder	G_ALPHA_S_SIGNALLING_EVENTS	REACTOME	5019	29	20	8,86E-01	1,00E+00	rs2531995|rs1342236|rs1135029|rs289383|rs4235953|rs12878420|rs12746334|rs4583026|rs2531982|rs9365906|rs6755901|rs483832|rs1429890|rs11956891|rs2162734|rs1865550|rs12985186|rs1867935|rs7727161|rs1294857
Renal Disorder	CTLA4_INHIBITORY_SIGNALING	REACTOME	447	22	12	8,88E-01	1,00E+00	rs874269|rs2668020|rs11119881|rs17073074|rs7546833|rs10197319|rs10411335|rs1027990|rs10457242|rs12967001|rs633045|rs6018257
Renal Disorder	SMOOTH_MUSCLE_CONTRACTION	REACTOME	658	30	20	8,88E-01	1,00E+00	rs17432675|rs11086023|rs17456435|rs220076|rs4917696|rs2295797|rs4775571|rs16946166|rs17125|rs1275659|rs11083841|rs10458657|rs13415017|rs16981095|rs7586415|rs975886|rs1623605|rs11626241|rs4405494|rs10137869
Renal Disorder	RNA_POL_I_TRANSCRIPTION_TERMINATION	REACTOME	335	16	5	8,89E-01	1,00E+00	rs3748269|rs9512817|rs2254883|rs17011628|rs10153801
Renal Disorder	MEIOTIC_SYNAPSIS	REACTOME	1031	38	17	8,89E-01	1,00E+00	rs2239896|rs2975842|rs360712|rs2123463|rs6981928|rs643788|rs12213919|rs2635443|rs584336|rs9478325|rs4096763|rs2419572|rs10946808|rs2235229|rs1341758|rs8006145|rs7741445
Renal Disorder	NGF_SIGNALLING_VIA_TRKA_FROM_THE_PLASMA_MEMBRANE	REACTOME	4847	32	20	8,89E-01	1,00E+00	rs2531995|rs3813668|rs917199|rs1043313|rs7984646|rs9848727|rs2755217|rs2531982|rs12832680|rs3924649|rs874269|rs6705717|rs1865550|rs1994474|rs4381631|rs10793894|rs10491176|rs2220168|rs4798777|rs7300093
Renal Disorder	DNA_STRAND_ELONGATION	REACTOME	672	25	16	8,90E-01	1,00E+00	rs12602230|rs10948689|rs4790838|rs236106|rs2303744|rs2244095|rs9377723|rs133431|rs7330677|rs7208143|rs8181839|rs751511|rs13242458|rs10258735|rs17482047|rs782428
Renal Disorder	FACTORS_INVOLVED_IN_MEGAKARYOCYTE_DEVELOPMENT_AND_PLATELET_PRODUCT	REACTOME	3549	36	20	8,91E-01	1,00E+00	rs1243438|rs10510496|rs3756103|rs7757054|rs2713604|rs2957385|rs7248924|rs10837628|rs10490944|rs1460578|rs7853654|rs4385221|rs1342852|rs10491176|rs7156395|rs827618|rs2709624|rs10038958|rs3209441|rs8081915
Renal Disorder	SIGNALING_BY_PDGF	REACTOME	4427	26	20	8,92E-01	1,00E+00	rs2531995|rs3813668|rs9393162|rs2755217|rs2531982|rs4366552|rs2645772|rs12832680|rs9307949|rs7605392|rs3924649|rs874269|rs939688|rs6705717|rs1865550|rs1994474|rs4381631|rs2075555|rs10793894|rs10491176
Renal Disorder	ACTIVATION_OF_THE_PRE_REPLICATIVE_COMPLEX	REACTOME	565	24	15	8,92E-01	1,00E+00	rs12602230|rs10948689|rs4790838|rs236106|rs6950715|rs2323973|rs9377723|rs133431|rs1994189|rs7208143|rs13242458|rs10258735|rs17482047|rs11764107|rs782428
Renal Disorder	INTERFERON_SIGNALING	REACTOME	3448	30	20	8,94E-01	1,00E+00	rs1532472|rs3852030|rs9688182|rs3756103|rs4576294|rs3851570|rs241437|rs4837334|rs3794125|rs9551193|rs2643626|rs1342852|rs4724298|rs2161737|rs444319|rs10128562|rs2293338|rs13204672|rs10784678|rs2834139
Renal Disorder	CELL_DEATH_SIGNALLING_VIA_NRAGE_NRIF_AND_NADE	REACTOME	2069	26	20	8,95E-01	1,00E+00	rs525007|rs6019409|rs4681888|rs333327|rs657770|rs11907586|rs7774757|rs656709|rs4681908|rs12480425|rs17020088|rs6095298|rs4810774|rs3004318|rs17762822|rs12627433|rs4778857|rs11086250|rs13052373|rs12039210
Renal Disorder	ASSEMBLY_OF_THE_PRE_REPLICATIVE_COMPLEX	REACTOME	911	42	20	8,95E-01	1,00E+00	rs4576294|rs241437|rs10948689|rs236106|rs942042|rs10268808|rs6950715|rs746720|rs1064576|rs133431|rs1994189|rs963267|rs2526057|rs4069666|rs11159958|rs1052748|rs11764195|rs13242458|rs2357804|rs1015166
Renal Disorder	SIGNALING_BY_EGFR_IN_CANCER	REACTOME	4030	30	20	8,95E-01	1,00E+00	rs2531995|rs3813668|rs1043313|rs2755217|rs2531982|rs9601816|rs12832680|rs3924649|rs874269|rs6705717|rs1865550|rs1994474|rs9545368|rs4381631|rs7914|rs10219897|rs10491176|rs2220168|rs7300093|rs11244777
Renal Disorder	PRE_NOTCH_PROCESSING_IN_GOLGI	REACTOME	691	26	14	8,97E-01	1,00E+00	rs3748269|rs17616659|rs229833|rs10898931|rs3761441|rs2183124|rs2225382|rs17588820|rs1571377|rs162663|rs1769392|rs7148568|rs17116019|rs986055
Renal Disorder	VASCULAR_SMOOTH_MUSCLE_CONTRACTION	KEGG	5845	26	20	8,97E-01	1,00E+00	rs2531995|rs1148757|rs11042893|rs1326250|rs308039|rs6716817|rs12905386|rs1937652|rs4078293|rs2531982|rs636293|rs12832680|rs2701632|rs12733186|rs596866|rs6705717|rs1865550|rs1994474|rs4381631|rs1904006
Renal Disorder	SIGNALING_BY_SCF_KIT	REACTOME	1989	36	20	9,02E-01	1,00E+00	rs3813668|rs2755217|rs2113001|rs3924649|rs874269|rs13114243|rs4381631|rs7914|rs2668020|rs1014058|rs3889392|rs16960228|rs17073074|rs12166465|rs11876|rs2726626|rs2244621|rs11871468|rs484754|rs12459940
Renal Disorder	AUTODEGRADATION_OF_CDH1_BY_CDH1_APC_C	REACTOME	857	40	20	9,05E-01	1,00E+00	rs4576294|rs11006121|rs241437|rs1994174|rs10268808|rs2324716|rs2311552|rs1064576|rs2526057|rs12352784|rs11718625|rs4069666|rs4904662|rs1052748|rs11764195|rs2357804|rs1015166|rs16972457|rs16960774|rs2716605
Renal Disorder	MITOTIC_M_M_G1_PHASES	REACTOME	3176	34	20	9,06E-01	1,00E+00	rs1878553|rs4576294|rs4938050|rs2957385|rs4822075|rs241437|rs6120015|rs12602230|rs10492892|rs17001200|rs387865|rs3846484|rs10948689|rs6667880|rs4790838|rs236106|rs3004318|rs9898519|rs1995785|rs942042
Renal Disorder	LEISHMANIA_INFECTION	KEGG	2265	40	20	9,08E-01	1,00E+00	rs2760494|rs2857106|rs17103274|rs7858743|rs10455523|rs1329057|rs6582484|rs241436|rs10515797|rs13204672|rs10784678|rs429916|rs10748094|rs9362732|rs12409333|rs10878729|rs9376268|rs9362744|rs9351265|rs9362786
Renal Disorder	CELL_CYCLE	KEGG	2581	34	20	9,08E-01	1,00E+00	rs4478405|rs9642880|rs4734497|rs12695746|rs17772958|rs7853654|rs7736312|rs1861178|rs2961920|rs657899|rs10948689|rs412705|rs652913|rs12004815|rs942042|rs10095762|rs2324716|rs7189819|rs11672955|rs17464037
Renal Disorder	ACTIVATION_OF_NF_KAPPAB_IN_B_CELLS	REACTOME	1396	39	20	9,09E-01	1,00E+00	rs1529702|rs4576294|rs17103274|rs241437|rs10455523|rs1878808|rs9362732|rs10257369|rs9362744|rs9351265|rs1064576|rs11190921|rs9362786|rs2053025|rs2306365|rs963267|rs2526057|rs2818291|rs10883452|rs3735219
Renal Disorder	REGULATION_OF_MRNA_STABILITY_BY_PROTEINS_THAT_BIND_AU_RICH_ELEMENTS	REACTOME	1372	41	20	9,11E-01	1,00E+00	rs4734497|rs4576294|rs241437|rs4381631|rs8003773|rs3889392|rs16960228|rs10268808|rs9784647|rs17464037|rs11244096|rs7615501|rs11871468|rs1064576|rs13259960|rs10814620|rs103612|rs963267|rs2526057|rs11707321
Renal Disorder	BASIGIN_INTERACTIONS	REACTOME	595	14	9	9,12E-01	1,00E+00	rs12695746|rs10404771|rs745961|rs16852004|rs2070870|rs1975802|rs890397|rs756847|rs3783436
Renal Disorder	MISMATCH_REPAIR	KEGG	576	19	15	9,12E-01	1,00E+00	rs12602230|rs4658535|rs4790838|rs2303744|rs2244095|rs7332652|rs7208143|rs9789343|rs8181839|rs751511|rs7584256|rs7603494|rs10258735|rs17482047|rs175057
Renal Disorder	TELOMERE_MAINTENANCE	REACTOME	1133	45	20	9,13E-01	1,00E+00	rs2239896|rs2975842|rs12602230|rs4790838|rs2303744|rs4635969|rs2244095|rs643788|rs2323973|rs9377723|rs7330677|rs7208143|rs4096763|rs8181839|rs751511|rs10946808|rs1341758|rs10258735|rs17482047|rs7741445
Renal Disorder	BOTULINUM_NEUROTOXICITY	REACTOME	522	28	14	9,14E-01	1,00E+00	rs2532520|rs883382|rs6104567|rs8071640|rs11086651|rs3763851|rs7209802|rs4348778|rs11590011|rs4286803|rs2304911|rs1591811|rs961072|rs4418670
Renal Disorder	NCAM1_INTERACTIONS	REACTOME	1896	28	20	9,17E-01	1,00E+00	rs1356733|rs3851570|rs4366552|rs2645772|rs3784730|rs17159534|rs2075555|rs4777987|rs3106796|rs518558|rs1481003|rs4936186|rs1245138|rs11852344|rs17498886|rs4794117|rs564031|rs10933164|rs434222|rs873803
Renal Disorder	PROTEASOME	KEGG	821	45	20	9,17E-01	1,00E+00	rs4576294|rs241437|rs12017236|rs10784678|rs10748094|rs10268808|rs10878729|rs1064576|rs963267|rs2526057|rs4904662|rs1052748|rs11764195|rs2357804|rs1015166|rs11877878|rs16972457|rs16960774|rs2716605|rs2230571
Renal Disorder	HEMATOPOIETIC_CELL_LINEAGE	KEGG	1963	25	20	9,17E-01	1,00E+00	rs6727015|rs11582424|rs2386841|rs3794125|rs13114243|rs743562|rs10128562|rs7793163|rs1014058|rs35571839|rs1009897|rs353644|rs2726626|rs2028299|rs1275659|rs2007084|rs4749882|rs8100944|rs2553828|rs1571344
Renal Disorder	ACTIVATION_OF_ATR_IN_RESPONSE_TO_REPLICATION_STRESS	REACTOME	653	26	18	9,18E-01	1,00E+00	rs12602230|rs2686830|rs10948689|rs4790838|rs236106|rs6950715|rs133431|rs7332652|rs1994189|rs7208143|rs8181839|rs751511|rs13242458|rs10258735|rs17482047|rs11764107|rs9291949|rs782428
Renal Disorder	SIGNALING_BY_WNT	REACTOME	1071	39	20	9,21E-01	1,00E+00	rs4576294|rs6788984|rs241437|rs353241|rs10266523|rs11119881|rs7546833|rs11860497|rs1064576|rs11190921|rs12173146|rs429988|rs963267|rs2526057|rs3735219|rs4069666|rs10411335|rs11159958|rs1052748|rs11764195
Renal Disorder	ENOS_ACTIVATION_AND_REGULATION	REACTOME	336	8	8	9,22E-01	1,00E+00	rs10516652|rs10961558|rs1343567|rs11083841|rs11626241|rs3845727|rs2182856|rs10137869
Renal Disorder	NON_SMALL_CELL_LUNG_CANCER	KEGG	2585	32	20	9,23E-01	1,00E+00	rs6807877|rs3813668|rs3924649|rs874269|rs6446081|rs2121794|rs4381631|rs1805352|rs3762013|rs11721188|rs12631825|rs241706|rs3889392|rs17061789|rs1385713|rs16960228|rs942042|rs6446152|rs2244621|rs746720
Renal Disorder	GLOBAL_GENOMIC_NER_GG_NER	REACTOME	823	43	20	9,23E-01	1,00E+00	rs12602230|rs3748269|rs4790838|rs2303744|rs2157072|rs1646314|rs7190295|rs2244095|rs2323973|rs7195205|rs7330677|rs7038659|rs7208143|rs8181839|rs751511|rs12930200|rs10258735|rs1323804|rs17482047|rs4150282
Renal Disorder	FMLP_PATHWAY	BIOCARTA	1148	26	20	9,25E-01	1,00E+00	rs17103274|rs11044232|rs6055957|rs6123013|rs6516421|rs7193701|rs2715825|rs6681938|rs17733355|rs3746070|rs12459940|rs4239343|rs2306365|rs11083841|rs1534897|rs6055927|rs3019245|rs17347805|rs3019244|rs17733133
Renal Disorder	PI3K_AKT_ACTIVATION	REACTOME	1161	42	19	9,25E-01	1,00E+00	rs3813668|rs7984646|rs2755217|rs874269|rs1317063|rs11876|rs2244621|rs484754|rs13173003|rs853812|rs10883452|rs6725556|rs7641889|rs6449947|rs918842|rs4603211|rs830474|rs477895|rs10054692
Renal Disorder	PANTOTHENATE_AND_COA_BIOSYNTHESIS	KEGG	611	34	10	9,25E-01	1,00E+00	rs4415625|rs10875076|rs2786507|rs9321367|rs12119882|rs4304627|rs4734782|rs12211125|rs11783979|rs12567432
Renal Disorder	BIOSYNTHESIS_OF_UNSATURATED_FATTY_ACIDS	KEGG	463	24	15	9,26E-01	1,00E+00	rs1135889|rs899491|rs10484663|rs11625677|rs16855357|rs4748380|rs17593512|rs17351947|rs2817110|rs7744440|rs7694475|rs4908874|rs1678305|rs7529205|rs2303842
Renal Disorder	RNA_POL_I_TRANSCRIPTION	REACTOME	937	50	14	9,28E-01	1,00E+00	rs2239896|rs3748269|rs3786254|rs4485425|rs6537537|rs643788|rs9512817|rs2254883|rs10946808|rs1341758|rs6771941|rs17011628|rs10153801|rs7741445
Renal Disorder	NCAM_SIGNALING_FOR_NEURITE_OUT_GROWTH	REACTOME	2480	30	20	9,31E-01	1,00E+00	rs11167020|rs1356733|rs3851570|rs4366552|rs2645772|rs3924649|rs3784730|rs17159534|rs2075555|rs306962|rs4777987|rs3106796|rs518558|rs1481003|rs4936186|rs1245138|rs11852344|rs17498886|rs4794117|rs7840147
Renal Disorder	ORC1_REMOVAL_FROM_CHROMATIN	REACTOME	930	41	20	9,31E-01	1,00E+00	rs4576294|rs241437|rs10948689|rs236106|rs10257369|rs6950715|rs1064576|rs4943383|rs133431|rs9576040|rs1994189|rs963267|rs2526057|rs4069666|rs11159958|rs1052748|rs11764195|rs13242458|rs2357804|rs1015166
Renal Disorder	PYK2_PATHWAY	BIOCARTA	752	29	18	9,31E-01	1,00E+00	rs2760494|rs4381631|rs3889392|rs16960228|rs11871468|rs1881740|rs12459940|rs11083841|rs3019245|rs3019244|rs1003599|rs11626241|rs16959569|rs350825|rs6018257|rs4788426|rs10137869|rs9897002
Renal Disorder	MAL_PATHWAY	BIOCARTA	225	9	5	9,35E-01	1,00E+00	rs4338396|rs1881735|rs12459940|rs350825|rs1169307
Renal Disorder	NUCLEAR_EVENTS_KINASE_AND_TRANSCRIPTION_FACTOR_ACTIVATION	REACTOME	766	26	12	9,36E-01	1,00E+00	rs9848727|rs17555326|rs1644047|rs17555250|rs2341674|rs10783388|rs731288|rs325392|rs10411335|rs16903232|rs918842|rs633045
Renal Disorder	CARDIAC_MUSCLE_CONTRACTION	KEGG	3169	30	20	9,36E-01	1,00E+00	rs1674535|rs1229487|rs10420331|rs1356733|rs4245919|rs254738|rs12695746|rs11130462|rs17759800|rs860294|rs368161|rs12521803|rs11086023|rs886940|rs10206058|rs12813682|rs6874335|rs1861303|rs10280428|rs716540
Renal Disorder	RESPONSE_TO_ELEVATED_PLATELET_CYTOSOLIC_CA2_	REACTOME	2301	36	20	9,36E-01	1,00E+00	rs441250|rs958766|rs4148426|rs10770|rs4148434|rs4381631|rs8077638|rs2300875|rs3787620|rs4713936|rs3889392|rs2295797|rs16960228|rs461626|rs1884634|rs9524681|rs4462564|rs17516906|rs594942|rs8126493
Renal Disorder	CALCINEURIN_PATHWAY	BIOCARTA	895	28	19	9,36E-01	1,00E+00	rs2364598|rs4381631|rs6123013|rs7193701|rs3889392|rs16960228|rs17733355|rs11871468|rs4239343|rs11083841|rs2348305|rs1003599|rs17733133|rs11626241|rs16959569|rs4788426|rs12963519|rs10137869|rs9897002
Renal Disorder	NTHI_PATHWAY	BIOCARTA	876	29	20	9,36E-01	1,00E+00	rs17103274|rs10455523|rs412705|rs2715825|rs9362732|rs12517663|rs9362744|rs9351265|rs11071939|rs9362786|rs2306365|rs2818291|rs10883452|rs6809777|rs2144363|rs10515523|rs1461084|rs893473|rs749864|rs1504281
Renal Disorder	ACTIVATION_OF_GENES_BY_ATF4	REACTOME	316	17	13	9,38E-01	1,00E+00	rs3091237|rs9784647|rs738288|rs11244096|rs10814620|rs1024611|rs11707321|rs3125289|rs4131751|rs6539125|rs12922485|rs6918969|rs4257050
Renal Disorder	REGULATION_OF_MITOTIC_CELL_CYCLE	REACTOME	1318	40	20	9,38E-01	1,00E+00	rs4576294|rs11006121|rs241437|rs3846484|rs2961920|rs1994174|rs10268808|rs2324716|rs2311552|rs1064576|rs11190921|rs4943383|rs12173146|rs9576040|rs963267|rs2526057|rs12352784|rs11718625|rs3735219|rs2058765
Renal Disorder	RECYCLING_PATHWAY_OF_L1	REACTOME	874	30	12	9,39E-01	1,00E+00	rs1043313|rs10038958|rs2038521|rs11919795|rs12000388|rs351294|rs327234|rs3780247|rs17182398|rs2231216|rs3744382|rs6018257
Renal Disorder	RNA_POL_I_TRANSCRIPTION_INITIATION	REACTOME	427	18	7	9,39E-01	1,00E+00	rs3748269|rs6537537|rs9512817|rs2254883|rs6771941|rs17011628|rs10153801
Renal Disorder	PARKINSONS_DISEASE	KEGG	2833	33	20	9,40E-01	1,00E+00	rs1004446|rs919699|rs625149|rs254738|rs17163678|rs12208181|rs363222|rs7667259|rs12521803|rs3779672|rs1133099|rs374954|rs10206058|rs9458439|rs6874335|rs768150|rs218968|rs4147661|rs3811909|rs3745004
Renal Disorder	INTEGRATION_OF_PROVIRUS	REACTOME	122	3	2	9,41E-01	1,00E+00	rs7987249|rs2665515
Renal Disorder	RIBOFLAVIN_METABOLISM	KEGG	295	14	8	9,42E-01	1,00E+00	rs7015243|rs17587917|rs3849413|rs693830|rs4769414|rs4921773|rs11018541|rs2269692
Renal Disorder	NOS1_PATHWAY	BIOCARTA	1133	28	20	9,44E-01	1,00E+00	rs9658265|rs4381631|rs10491176|rs3889392|rs1972578|rs16960228|rs2286562|rs3763609|rs17733355|rs11871468|rs837697|rs11083841|rs316689|rs1543889|rs4617674|rs1003599|rs17733133|rs2540165|rs10744035|rs11626241
Renal Disorder	RENIN_ANGIOTENSIN_SYSTEM	KEGG	467	22	12	9,44E-01	1,00E+00	rs669262|rs2028299|rs16861156|rs2007084|rs12109329|rs389566|rs4932145|rs9968047|rs12721331|rs984920|rs4810479|rs2033078
Renal Disorder	M_G1_TRANSITION	REACTOME	1315	42	20	9,45E-01	1,00E+00	rs4576294|rs241437|rs12602230|rs10948689|rs4790838|rs236106|rs942042|rs10268808|rs6950715|rs746720|rs1064576|rs2323973|rs9377723|rs133431|rs1994189|rs7208143|rs963267|rs2526057|rs4069666|rs11159958
Renal Disorder	ANTIVIRAL_MECHANISM_BY_IFN_STIMULATED_GENES	REACTOME	1077	35	20	9,46E-01	1,00E+00	rs3852030|rs4837334|rs9551193|rs444319|rs2293338|rs630814|rs12409333|rs7698057|rs9678612|rs3828246|rs4338396|rs2403370|rs103612|rs11718625|rs35191309|rs12148733|rs2230571|rs11632652|rs1658347|rs11770879
Renal Disorder	MAPK_SIGNALING_PATHWAY	KEGG	9833	29	20	9,53E-01	1,00E+00	rs2760494|rs9642880|rs263931|rs7987595|rs2869441|rs1148757|rs7318731|rs6489711|rs1674535|rs1229487|rs9848727|rs10420331|rs1422259|rs1356733|rs12801636|rs2517037|rs249736|rs4245919|rs2701632|rs3924649
Renal Disorder	CALCIUM_SIGNALING_PATHWAY	KEGG	9275	23	20	9,54E-01	1,00E+00	rs2531995|rs1547643|rs1112903|rs10488597|rs1223577|rs308039|rs9688182|rs9658265|rs12905386|rs1422259|rs362848|rs2039860|rs4078293|rs2531982|rs636293|rs12832680|rs2291730|rs6755901|rs10495431|rs946637
Renal Disorder	PERK_REGULATED_GENE_EXPRESSION	REACTOME	334	18	14	9,56E-01	1,00E+00	rs3091237|rs9784647|rs738288|rs11244096|rs10814620|rs1024611|rs11707321|rs3125289|rs4131751|rs6539125|rs2365106|rs12922485|rs6918969|rs4257050

<table>
<tr><td>Renal Disorder</td><td>DNA_REPLICATION</td><td>REACTOME</td><td>3548</td><td>33</td><td>20</td><td>9,57E-01</td><td>1,00E+00</td><td>rs1878553|rs4576294|rs4938050|rs2957385|rs4822075|rs241437|rs6120015|rs12602230|rs10492892|rs17001200|rs387865|rs7736312|rs10948689|rs6667880|rs4790838|rs236106|rs2303744|rs3004318|rs9898519|rs1995785</td></tr>
<tr><td>Renal Disorder</td><td>INTRINSIC_PATHWAY</td><td>BIOCARTA</td><td>603</td><td>15</td><td>10</td><td>9,59E-01</td><td>1,00E+00</td><td>rs7728560|rs1008728|rs11132389|rs10933164|rs4076557|rs10933175|rs9521650|rs419244|rs7317390|rs4771685</td></tr>
<tr><td>Renal Disorder</td><td>GCR_PATHWAY</td><td>BIOCARTA</td><td>736</td><td>35</td><td>20</td><td>9,63E-01</td><td>1,00E+00</td><td>rs3813668|rs6681938|rs17153616|rs849430|rs13173003|rs853812|rs1004717|rs2306365|rs11083841|rs7875996|rs13041735|rs7641889|rs6449947|rs10515523|rs12555078|rs2125391|rs2033897|rs11626241|rs830474|rs10137869</td></tr>
<tr><td>Renal Disorder</td><td>DOWNSTREAM_SIGNALING_EVENTS_OF_B_CELL_RECEPTOR_BCR</td><td>REACTOME</td><td>2127</td><td>30</td><td>20</td><td>9,63E-01</td><td>1,00E+00</td><td>rs1529702|rs4576294|rs17103274|rs2755217|rs3924649|rs241437|rs874269|rs10455523|rs1878808|rs9362732|rs10268808|rs9362744|rs11876|rs9351265|rs2244621|rs1064576|rs11190921|rs9362786|rs484754|rs12173146</td></tr>
<tr><td>Renal Disorder</td><td>RESPIRATORY_ELECTRON_TRANSPORT</td><td>REACTOME</td><td>1631</td><td>33</td><td>20</td><td>9,64E-01</td><td>1,00E+00</td><td>rs254738|rs17163678|rs12521803|rs1133099|rs10206058|rs6874335|rs218968|rs4147661|rs41426|rs17163764|rs1500147|rs17771284|rs4537274|rs1840816|rs12421329|rs1806990|rs4254432|rs10181407|rs4725269|rs7240172</td></tr>
<tr><td>Renal Disorder</td><td>MUSCLE_CONTRACTION</td><td>REACTOME</td><td>1023</td><td>30</td><td>20</td><td>9,64E-01</td><td>1,00E+00</td><td>rs17432675|rs11086023|rs17456435|rs220076|rs4917696|rs2295797|rs4775571|rs16946166|rs17125|rs1275659|rs2244095|rs11083841|rs10458657|rs13415017|rs10802557|rs16981095|rs7586415|rs4683304|rs1418071|rs1499280</td></tr>
<tr><td>Renal Disorder</td><td>G_ALPHA_Q_SIGNALLING_EVENTS</td><td>REACTOME</td><td>7428</td><td>21</td><td>20</td><td>9,66E-01</td><td>1,00E+00</td><td>rs3813668|rs1112903|rs308039|rs228648|rs12568515|rs12905386|rs4678029|rs12878420|rs12746334|rs2039860|rs6772051|rs636293|rs333327|rs12832680|rs10495431|rs946637|rs6731427|rs8016905|rs2357960|rs4568821</td></tr>
<tr><td>Renal Disorder</td><td>CDT1_ASSOCIATION_WITH_THE_CDC6_ORC_ORIGIN_COMPLEX</td><td>REACTOME</td><td>793</td><td>42</td><td>20</td><td>9,69E-01</td><td>1,00E+00</td><td>rs4576294|rs241437|rs236106|rs10257369|rs6950715|rs1064576|rs1994189|rs963267|rs2526057|rs4069666|rs11159958|rs1052748|rs11764195|rs2357804|rs1015166|rs11877878|rs16972457|rs16960774|rs2716605|rs2230571</td></tr>
<tr><td>Renal Disorder</td><td>CYCLIN_E_ASSOCIATED_EVENTS_DURING_G1_S_TRANSITION_</td><td>REACTOME</td><td>1212</td><td>41</td><td>20</td><td>9,69E-01</td><td>1,00E+00</td><td>rs9642880|rs4576294|rs241437|rs10095762|rs10268808|rs10143368|rs1064576|rs4943383|rs2053025|rs9576040|rs2526057|rs3735219|rs4069666|rs11159958|rs1052748|rs11764195|rs8020793|rs10091329|rs2357804|rs10129544</td></tr>
<tr><td>Renal Disorder</td><td>FORMATION_OF_FIBRIN_CLOT_CLOTTING_CASCADE</td><td>REACTOME</td><td>703</td><td>20</td><td>11</td><td>9,70E-01</td><td>1,00E+00</td><td>rs1008728|rs10874870|rs4667193|rs11132389|rs2424505|rs4076557|rs12613071|rs9405911|rs3863223|rs3024422|rs2510280</td></tr>
<tr><td>Renal Disorder</td><td>ONE_CARBON_POOL_BY_FOLATE</td><td>KEGG</td><td>441</td><td>7</td><td>7</td><td>9,71E-01</td><td>1,00E+00</td><td>rs2298582|rs4646703|rs4679103|rs942382|rs2166766|rs2281603|rs2839127</td></tr>
<tr><td>Renal Disorder</td><td>GAB1_SIGNALOSOME</td><td>REACTOME</td><td>1077</td><td>36</td><td>20</td><td>9,71E-01</td><td>1,00E+00</td><td>rs3813668|rs2755217|rs874269|rs11876|rs2244621|rs484754|rs13173003|rs853812|rs17746482|rs4245566|rs10883452|rs7641889|rs6449947|rs918842|rs2877260|rs6018257|rs11760524|rs7009399|rs4603211|rs10023724</td></tr>
<tr><td>Renal Disorder</td><td>DOWNREGULATION_OF_TGF_BETA_RECEPTOR_SIGNALING</td><td>REACTOME</td><td>521</td><td>18</td><td>13</td><td>9,72E-01</td><td>1,00E+00</td><td>rs412705|rs13409926|rs6504246|rs11071939|rs4811914|rs157091|rs6070233|rs6809777|rs4233728|rs4939832|rs1461084|rs17702096|rs893473</td></tr>
<tr><td>Renal Disorder</td><td>CELL_CYCLE_CHECKPOINTS</td><td>REACTOME</td><td>1961</td><td>33</td><td>20</td><td>9,72E-01</td><td>1,00E+00</td><td>rs4576294|rs11006121|rs241437|rs12602230|rs2686830|rs7736312|rs1994174|rs10948689|rs732292|rs4790838|rs236106|rs10266523|rs2324716|rs6950715|rs2311552|rs1064576|rs1002170|rs133431|rs7332652|rs1994189</td></tr>
<tr><td>Renal Disorder</td><td>AUTODEGRADATION_OF_THE_E3_UBIQUITIN_LIGASE_COP1</td><td>REACTOME</td><td>766</td><td>40</td><td>19</td><td>9,74E-01</td><td>1,00E+00</td><td>rs4576294|rs241437|rs732292|rs10268808|rs1064576|rs963267|rs2526057|rs4069666|rs11159958|rs1052748|rs11764195|rs2357804|rs1015166|rs11877878|rs16972457|rs16960774|rs2716605|rs2230571|rs425594</td></tr>
<tr><td>Renal Disorder</td><td>L1CAM_INTERACTIONS</td><td>REACTOME</td><td>3914</td><td>36</td><td>20</td><td>9,74E-01</td><td>1,00E+00</td><td>rs1043313|rs1564585|rs1406275|rs10911203|rs1992294|rs3851570|rs2776937|rs9297840|rs11009311|rs2403773|rs2052313|rs1504056|rs12497579|rs9856587|rs10038958|rs11599447|rs2300045|rs4936186|rs1245138|rs17333297</td></tr>
<tr><td>Renal Disorder</td><td>RESPIRATORY_ELECTRON_TRANSPORT_ATP_SYNTHESIS_BY_CHEMIOSMOTIC_COUPLIN(
TION_BY_UNCOUPLING_PROTEINS_</td><td>REACTOME</td><td>1741</td><td>33</td><td>20</td><td>9,74E-01</td><td>1,00E+00</td><td>rs254738|rs17163678|rs12521803|rs1133099|rs10206058|rs6874335|rs218968|rs4147661|rs3745004|rs41426|rs17163764|rs1500147|rs17771284|rs4537274|rs1840816|rs12421329|rs1806990|rs4254432|rs10181407|rs4725269</td></tr>
<tr><td>Renal Disorder</td><td>AMI_PATHWAY</td><td>BIOCARTA</td><td>574</td><td>14</td><td>10</td><td>9,75E-01</td><td>1,00E+00</td><td>rs7728560|rs4667193|rs10933164|rs4076557|rs10933175|rs12613071|rs9521650|rs419244|rs7317390|rs4771685</td></tr>
<tr><td>Renal Disorder</td><td>G1_PATHWAY</td><td>BIOCARTA</td><td>611</td><td>22</td><td>12</td><td>9,76E-01</td><td>1,00E+00</td><td>rs7853654|rs412705|rs2811709|rs11071939|rs4943383|rs9576040|rs6662137|rs399413|rs17318844|rs11792889|rs893473|rs4252340</td></tr>
<tr><td>Renal Disorder</td><td>CROSS_PRESENTATION_OF_SOLUBLE_EXOGENOUS_ANTIGENS_ENDOSOMES</td><td>REACTOME</td><td>713</td><td>41</td><td>19</td><td>9,76E-01</td><td>1,00E+00</td><td>rs4576294|rs241437|rs10268808|rs9891713|rs1064576|rs963267|rs2526057|rs4069666|rs4904662|rs1052748|rs11764195|rs2357804|rs1015166|rs11877878|rs16972457|rs16960774|rs2716605|rs2230571|rs425594</td></tr>
<tr><td>Renal Disorder</td><td>G1_S_TRANSITION</td><td>REACTOME</td><td>1941</td><td>30</td><td>20</td><td>9,77E-01</td><td>1,00E+00</td><td>rs9642880|rs4576294|rs241437|rs12602230|rs3846484|rs2298582|rs10948689|rs4790838|rs236106|rs10095762|rs10268808|rs10143368|rs6950715|rs1064576|rs4943383|rs2323973|rs9377723|rs12173146|rs133431|rs9576040</td></tr>
<tr><td>Renal Disorder</td><td>ANTIGEN_PROCESSING_AND_PRESENTATION</td><td>KEGG</td><td>1754</td><td>101</td><td>20</td><td>9,78E-01</td><td>1,00E+00</td><td>rs2857106|rs241436|rs13204672|rs2123463|rs429916|rs1692793|rs2748220|rs12111032|rs9276516|rs6930052|rs17771967|rs4131751|rs874628|rs918842|rs6539125|rs1015166|rs9275582|rs938486|rs2800953|rs6918969</td></tr>
<tr><td>Renal Disorder</td><td>TGF_BETA_RECEPTOR_SIGNALING_ACTIVATES_SMADS</td><td>REACTOME</td><td>563</td><td>18</td><td>13</td><td>9,78E-01</td><td>1,00E+00</td><td>rs412705|rs13409926|rs6504246|rs11071939|rs4811914|rs157091|rs6070233|rs6809777|rs4233728|rs4939832|rs1461084|rs17702096|rs893473</td></tr>
<tr><td>Renal Disorder</td><td>SYNTHESIS_OF_DNA</td><td>REACTOME</td><td>1545</td><td>35</td><td>20</td><td>9,78E-01</td><td>1,00E+00</td><td>rs4576294|rs241437|rs12602230|rs10948689|rs4790838|rs236106|rs2303744|rs10257369|rs6950715|rs2244095|rs1064576|rs4943383|rs2323973|rs9377723|rs133431|rs9576040|rs7332652|rs1994189|rs7208143|rs963267</td></tr>
<tr><td>Renal Disorder</td><td>MEF2D_PATHWAY</td><td>BIOCARTA</td><td>684</td><td>28</td><td>20</td><td>9,80E-01</td><td>1,00E+00</td><td>rs4381631|rs6123013|rs3889392|rs4820581|rs16960228|rs17733355|rs11871468|rs4239343|rs7255180|rs11083841|rs1003599|rs17733133|rs11626241|rs16959569|rs12080565|rs4788426|rs2282476|rs12963519|rs10137869|rs9897002</td></tr>
<tr><td>Renal Disorder</td><td>GLYCOSAMINOGLYCAN_DEGRADATION</td><td>KEGG</td><td>709</td><td>28</td><td>19</td><td>9,80E-01</td><td>1,00E+00</td><td>rs530664|rs16872173|rs12945447|rs9906816|rs11656638|rs10260580|rs1013124|rs6502246|rs7219146|rs8082242|rs8082497|rs8082455|rs7210358|rs2022247|rs13164947|rs3785690|rs17666787|rs11248060|rs11726245</td></tr>
<tr><td>Renal Disorder</td><td>SCF_BETA_TRCP_MEDIATED_DEGRADATION_OF_EMI1</td><td>REACTOME</td><td>793</td><td>43</td><td>20</td><td>9,81E-01</td><td>1,00E+00</td><td>rs4576294|rs241437|rs10257369|rs1064576|rs11190921|rs2053025|rs963267|rs2526057|rs3735219|rs4069666|rs4904662|rs1052748|rs11764195|rs2357804|rs1015166|rs11877878|rs16972457|rs16960774|rs2716605|rs2230571</td></tr>
<tr><td>Renal Disorder</td><td>COMPLEMENT_AND_COAGULATION_CASCADES</td><td>KEGG</td><td>1742</td><td>32</td><td>20</td><td>9,81E-01</td><td>1,00E+00</td><td>rs186037|rs8016905|rs8077638|rs2004385|rs7728560|rs17201103|rs1008728|rs10874870|rs2072633|rs4667193|rs11132389|rs1799722|rs2424505|rs17090790|rs292007|rs9316180|rs1243164|rs4752932|rs2204757|rs1571344</td></tr>
<tr><td>Renal Disorder</td><td>CDK_MEDIATED_PHOSPHORYLATION_AND_REMOVAL_OF_CDC6</td><td>REACTOME</td><td>698</td><td>39</td><td>18</td><td>9,81E-01</td><td>1,00E+00</td><td>rs4576294|rs241437|rs10268808|rs1064576|rs963267|rs2526057|rs4069666|rs11159958|rs1052748|rs11764195|rs2357804|rs1015166|rs11877878|rs16972457|rs16960774|rs2716605|rs2230571|rs425594</td></tr>
<tr><td>Renal Disorder</td><td>LONG_TERM_POTENTIATION</td><td>KEGG</td><td>3620</td><td>25</td><td>20</td><td>9,82E-01</td><td>1,00E+00</td><td>rs9688182|rs12905386|rs362848|rs4078293|rs12832680|rs3924649|rs1865550|rs1994474|rs9324756|rs4381631|rs6055957|rs4724298|rs2220168|rs492014|rs6516421|rs12054636|rs13409926|rs7300093|rs10025251|rs3889392</td></tr>
<tr><td>Renal Disorder</td><td>KERATAN_SULFATE_KERATIN_METABOLISM</td><td>REACTOME</td><td>737</td><td>24</td><td>15</td><td>9,82E-01</td><td>1,00E+00</td><td>rs16872173|rs13070425|rs6801461|rs2183124|rs4540819|rs10865331|rs4932435|rs7256058|rs10800925|rs17730982|rs11073813|rs12964617|rs9643302|rs8039131|rs6713082</td></tr>
<tr><td>Renal Disorder</td><td>AXON_GUIDANCE</td><td>REACTOME</td><td>13131</td><td>29</td><td>20</td><td>9,82E-01</td><td>1,00E+00</td><td>rs519813|rs2483548|rs6797462|rs2423401|rs6882497|rs17056621|rs2722987|rs7274475|rs1043313|rs1564585|rs652856|rs1406275|rs11167020|rs6716817|rs11949924|rs12905386|rs10911203|rs1356733|rs9966514|rs659632</td></tr>
<tr><td>Renal Disorder</td><td>VIF_MEDIATED_DEGRADATION_OF_APOBEC3G</td><td>REACTOME</td><td>738</td><td>41</td><td>20</td><td>9,82E-01</td><td>1,00E+00</td><td>rs4576294|rs241437|rs10257369|rs2285877|rs1064576|rs12577790|rs963267|rs2526057|rs4069666|rs11159958|rs1052748|rs11764195|rs2357804|rs1015166|rs11877878|rs16972457|rs16960774|rs2716605|rs2230571|rs425594</td></tr>
<tr><td>Renal Disorder</td><td>P53_DEPENDENT_G1_DNA_DAMAGE_RESPONSE</td><td>REACTOME</td><td>838</td><td>40</td><td>19</td><td>9,83E-01</td><td>1,00E+00</td><td>rs4576294|rs241437|rs732292|rs10257369|rs1064576|rs963267|rs2526057|rs4069666|rs4904662|rs1052748|rs11764195|rs2357804|rs1015166|rs11877878|rs16972457|rs16960774|rs2716605|rs2230571|rs425594</td></tr>
<tr><td>Renal Disorder</td><td>NFAT_PATHWAY</td><td>BIOCARTA</td><td>1626</td><td>24</td><td>20</td><td>9,84E-01</td><td>1,00E+00</td><td>rs3813668|rs10491176|rs6123013|rs7193701|rs353241|rs2286562|rs16982097|rs2859338|rs3763609|rs1644047|rs17733355|rs17153616|rs10112596|rs4338396|rs849430|rs17153698|rs1881735|rs13173003|rs853812|rs4239343</td></tr>
<tr><td>Renal Disorder</td><td>P53_INDEPENDENT_G1_S_DNA_DAMAGE_CHECKPOINT</td><td>REACTOME</td><td>729</td><td>39</td><td>18</td><td>9,84E-01</td><td>1,00E+00</td><td>rs4576294|rs241437|rs10257369|rs1064576|rs963267|rs2526057|rs4069666|rs11159958|rs1052748|rs11764195|rs2357804|rs1015166|rs11877878|rs16972457|rs16960774|rs2716605|rs2230571|rs425594</td></tr>
<tr><td>Renal Disorder</td><td>DARPP_32_EVENTS</td><td>REACTOME</td><td>879</td><td>26</td><td>17</td><td>9,84E-01</td><td>1,00E+00</td><td>rs10491176|rs10440630|rs2286562|rs3763609|rs7733296|rs6859121|rs13355401|rs11083841|rs316689|rs10052657|rs10411335|rs874628|rs11626241|rs633045|rs6877743|rs11959349|rs10137869</td></tr>
<tr><td>Renal Disorder</td><td>NDKDYNAMIN_PATHWAY</td><td>BIOCARTA</td><td>548</td><td>17</td><td>12</td><td>9,84E-01</td><td>1,00E+00</td><td>rs9365723|rs1750043|rs17733355|rs11919795|rs11083841|rs4723777|rs17733133|rs2231216|rs11626241|rs6588412|rs4373430|rs10137869</td></tr>
<tr><td>Renal Disorder</td><td>GASTRIN_CREB_SIGNALLING_PATHWAY_VIA_PKC_AND_MAPK</td><td>REACTOME</td><td>8122</td><td>21</td><td>20</td><td>9,85E-01</td><td>1,00E+00</td><td>rs3813668|rs1112903|rs308039|rs228648|rs12568515|rs12905386|rs4678029|rs12878420|rs12746334|rs2039860|rs6772051|rs636293|rs333327|rs12832680|rs10495431|rs946637|rs6731427|rs3924649|rs8016905|rs2357960</td></tr>
<tr><td>Renal Disorder</td><td>STRIATED_MUSCLE_CONTRACTION</td><td>REACTOME</td><td>453</td><td>21</td><td>13</td><td>9,87E-01</td><td>1,00E+00</td><td>rs11086023|rs4775571|rs16946166|rs2244095|rs10802557|rs16981095|rs12107311|rs1418071|rs13091025|rs4405494|rs11110856|rs907609|rs17031542</td></tr>
<tr><td>Renal Disorder</td><td>SCFSKP2_MEDIATED_DEGRADATION_OF_P27_P21</td><td>REACTOME</td><td>813</td><td>45</td><td>20</td><td>9,87E-01</td><td>1,00E+00</td><td>rs4576294|rs241437|rs10257369|rs1064576|rs4943383|rs12173146|rs9576040|rs2526057|rs3735219|rs4069666|rs11159958|rs1052748|rs11764195|rs2357804|rs1015166|rs16972457|rs16960774|rs2716605|rs2230571|rs425594</td></tr>
<tr><td>Renal Disorder</td><td>COLLAGEN_FORMATION</td><td>REACTOME</td><td>3203</td><td>24</td><td>20</td><td>9,89E-01</td><td>1,00E+00</td><td>rs12642840|rs4366552|rs2910111|rs2645772|rs1469598|rs949794|rs2075555|rs1425353|rs3762013|rs4715|rs11161747|rs2485071|rs3106796|rs518558|rs10739406|rs11788110|rs747574|rs7624656|rs710521|rs4871975</td></tr>
<tr><td>Renal Disorder</td><td>ANTIGEN_PROCESSING_UBIQUITINATION_PROTEASOME_DEGRADATION</td><td>REACTOME</td><td>4788</td><td>25</td><td>20</td><td>9,89E-01</td><td>1,00E+00</td><td>rs7754366|rs1529702|rs17658803|rs4331462|rs4576294|rs11006121|rs9368020|rs1977036|rs241437|rs6459644|rs11210996|rs1446438|rs246105|rs304103|rs2120823|rs12208181|rs4678200|rs10490578|rs1994174|rs2552346</td></tr>
<tr><td>Renal Disorder</td><td>ALZHEIMERS_DISEASE</td><td>KEGG</td><td>5029</td><td>25</td><td>20</td><td>9,90E-01</td><td>1,00E+00</td><td>rs6489711|rs7315397|rs9658265|rs12905386|rs1799986|rs4078293|rs12832680|rs2291730|rs254738|rs682639|rs17163678|rs1994474|rs6055957|rs12521803|rs2220168|rs3769823|rs9911085|rs1133099|rs374954|rs6516421</td></tr>
<tr><td>Renal Disorder</td><td>RAS_ACTIVATION_UOPN_CA2_INFUX_THROUGH_NMDA_RECEPTOR</td><td>REACTOME</td><td>1033</td><td>24</td><td>20</td><td>9,90E-01</td><td>1,00E+00</td><td>rs9688182|rs4724298|rs1972578|rs4778857|rs17214843|rs2979676|rs837697|rs11083841|rs1543889|rs10802557|rs4617674|rs734446|rs12153097|rs2540165|rs1418071|rs10744035|rs11626241|rs10045942|rs26900|rs11644511</td></tr>
<tr><td>Renal Disorder</td><td>DESTABILIZATION_OF_MRNA_BY_AUF1_HNRNP_D0</td><td>REACTOME</td><td>811</td><td>41</td><td>19</td><td>9,91E-01</td><td>1,00E+00</td><td>rs4576294|rs241437|rs10268808|rs1064576|rs963267|rs2526057|rs4550918|rs4069666|rs11159958|rs1052748|rs11764195|rs2357804|rs1015166|rs11877878|rs16972457|rs16960774|rs2716605|rs2230571|rs425594</td></tr>
<tr><td>Renal Disorder</td><td>KERATAN_SULFATE_BIOSYNTHESIS</td><td>REACTOME</td><td>674</td><td>22</td><td>14</td><td>9,91E-01</td><td>1,00E+00</td><td>rs13070425|rs6801461|rs2183124|rs4540819|rs10865331|rs4932435|rs7256058|rs10800925|rs17730982|rs11073813|rs12964617|rs9643302|rs8039131|rs6713082</td></tr>
<tr><td>Renal Disorder</td><td>TGFB_PATHWAY</td><td>BIOCARTA</td><td>817</td><td>23</td><td>18</td><td>9,92E-01</td><td>1,00E+00</td><td>rs10455523|rs412705|rs9362732|rs9362744|rs9351265|rs11071939|rs9362786|rs6662137|rs2818291|rs6809777|rs2144363|rs4939832|rs1461084|rs4355511|rs893473|rs7186053|rs4252340|rs1504281</td></tr>
<tr><td>Renal Disorder</td><td>SPHINGOLIPID_METABOLISM</td><td>KEGG</td><td>1101</td><td>36</td><td>19</td><td>9,92E-01</td><td>1,00E+00</td><td>rs7145669|rs1756673|rs928389|rs11782529|rs7087539|rs7387252|rs2185456|rs11206718|rs1547308|rs1953116|rs11722311|rs4758415|rs11621931|rs11676002|rs17126237|rs12964617|rs2994565|rs12444616|rs3011777</td></tr>
<tr><td>Renal Disorder</td><td>S_PHASE</td><td>REACTOME</td><td>2076</td><td>29</td><td>20</td><td>9,93E-01</td><td>1,00E+00</td><td>rs9642880|rs4576294|rs241437|rs12602230|rs10948689|rs4790838|rs236106|rs2303744|rs10095762|rs10266523|rs10143368|rs6950715|rs2244095|rs1064576|rs4943383|rs2323973|rs9377723|rs2053025|rs133431|rs9576040</td></tr>
<tr><td>Renal Disorder</td><td>PEPTIDE_LIGAND_BINDING_RECEPTORS</td><td>REACTOME</td><td>5053</td><td>29</td><td>20</td><td>9,95E-01</td><td>1,00E+00</td><td>rs11077666|rs4954410|rs228648|rs2373154|rs9972960|rs6731427|rs8016905|rs1860180|rs1319339|rs12215494|rs3821318|rs1042048|rs10830139|rs12203515|rs7853654|rs3829185|rs918242|rs1284077|rs759869|rs3787620</td></tr>
<tr><td>Renal Disorder</td><td>DORSO_VENTRAL_AXIS_FORMATION</td><td>KEGG</td><td>1149</td><td>30</td><td>20</td><td>9,95E-01</td><td>1,00E+00</td><td>rs4872471|rs3924649|rs10732562|rs3124607|rs12096679|rs10870070|rs17746482|rs4245566|rs2008864|rs12129753|rs7525061|rs12967204|rs2877260|rs7115037|rs10790952|rs10842515|rs10848080|rs11760524|rs1940640|rs2836776</td></tr>
<tr><td>Renal Disorder</td><td>TGF_BETA_RECEPTOR_SIGNALING_IN_EMT_EPITHELIAL_TO_MESENCHYMAL_TRANSITI(</td><td>REACTOME</td><td>429</td><td>6</td><td>5</td><td>9,96E-01</td><td>1,00E+00</td><td>rs11009554|rs2057227|rs6809777|rs1461084|rs790055</td></tr>
<tr><td>Renal Disorder</td><td>ACTIVATION_OF_NMDA_RECEPTOR_UPON_GLUTAMATE_BINDING_AND_POSTSYNAPTIC_</td><td>REACTOME</td><td>2117</td><td>28</td><td>20</td><td>9,96E-01</td><td>1,00E+00</td><td>rs9688182|rs1865550|rs9324756|rs4724298|rs492014|rs12054636|rs10025251|rs1972578|rs4246043|rs4778857|rs17214843|rs2979676|rs1589880|rs2288684|rs578772|rs837697|rs1438949|rs1862319|rs7213777|rs11167606</td></tr>
<tr><td>Renal Disorder</td><td>EXTRACELLULAR_MATRIX_ORGANIZATION</td><td>REACTOME</td><td>3783</td><td>23</td><td>20</td><td>9,97E-01</td><td>1,00E+00</td><td>rs12642840|rs13277637|rs4366552|rs2910111|rs2645772|rs1469598|rs949794|rs2075555|rs1425353|rs3762013|rs4715|rs11161747|rs2485071|rs3106796|rs518558|rs10739406|rs527832|rs11788110|rs3875143|rs747574</td></tr>
<tr><td>Renal Disorder</td><td>ER_PHAGOSOME_PATHWAY</td><td>REACTOME</td><td>1416</td><td>72</td><td>20</td><td>9,97E-01</td><td>1,00E+00</td><td>rs2857106|rs241436|rs10257369|rs12111032|rs1064576|rs10499749|rs963267|rs2526057|rs4069666|rs11159958|rs1052748|rs6971934|rs11764195|rs2357804|rs1569256|rs1015166|rs11877878|rs16972457|rs16960774|rs2716605</td></tr>
<tr><td>Renal Disorder</td><td>O_GLYCAN_BIOSYNTHESIS</td><td>KEGG</td><td>1579</td><td>29</td><td>20</td><td>9,98E-01</td><td>1,00E+00</td><td>rs13136852|rs10052189|rs10849124|rs7690646|rs2079778|rs7801523|rs2241430|rs683969|rs17594632|rs7223597|rs7574842|rs1558002|rs11767193|rs12621256|rs7666914|rs17637385|rs462515|rs2046482|rs17730982|rs11769145</td></tr>
<tr><td>Renal Disorder</td><td>UBIQUITIN_MEDIATED_PROTEOLYSIS</td><td>KEGG</td><td>3227</td><td>25</td><td>20</td><td>9,98E-01</td><td>1,00E+00</td><td>rs1529702|rs4331462|rs11006121|rs1977036|rs6566946|rs7722373|rs7914|rs12208181|rs1994174|rs732292|rs9458439|rs768150|rs529445|rs6084276|rs261537|rs559046|rs2324716|rs873104|rs1051738|rs11672955</td></tr>
<tr><td>Renal Disorder</td><td>ANTIGEN_PROCESSING_CROSS_PRESENTATION</td><td>REACTOME</td><td>1601</td><td>73</td><td>20</td><td>9,98E-01</td><td>1,00E+00</td><td>rs2857106|rs241436|rs10268808|rs12111032|rs9891713|rs1064576|rs10499749|rs963267|rs2526057|rs4069666|rs4904662|rs1052748|rs6971934|rs11764195|rs2357804|rs1569256|rs1015166|rs11877878|rs16972457|rs16960774</td></tr>
<tr><td>Renal Disorder</td><td>CREB_PHOSPHORYLATION_THROUGH_THE_ACTIVATION_OF_RAS</td><td>REACTOME</td><td>1349</td><td>25</td><td>20</td><td>9,99E-01</td><td>1,00E+00</td><td>rs9688182|rs4724298|rs1972578|rs4778857|rs17214843|rs2979676|rs837697|rs11083841|rs1362327|rs10802557|rs4617674|rs734446|rs918842|rs12153097|rs2540165|rs1418071|rs10744035|rs11626241|rs10045942|rs26900</td></tr>
<tr><td>Renal Disorder</td><td>CLASS_I_MHC_MEDIATED_ANTIGEN_PROCESSING_PRESENTATION</td><td>REACTOME</td><td>5895</td><td>35</td><td>20</td><td>9,99E-01</td><td>1,00E+00</td><td>rs7754366|rs1529702|rs17658803|rs2857106|rs4331462|rs11006121|rs9368020|rs1977036|rs6459644|rs11210996|rs1446438|rs246105|rs304103|rs7142869|rs2120823|rs12208181|rs4678200|rs10490578|rs1994174|rs2552346</td></tr>
<tr><td>Renal Disorder</td><td>POST_NMDA_RECEPTOR_ACTIVATION_EVENTS</td><td>REACTOME</td><td>1753</td><td>24</td><td>20</td><td>1,00E+00</td><td>1,00E+00</td><td>rs9688182|rs1865550|rs4724298|rs1972578|rs4778857|rs17214843|rs2979676|rs1589880|rs2288684|rs837697|rs7213777|rs11083841|rs316689|rs1543889|rs10802557|rs4617674|rs734446|rs918842|rs12153097|rs2540165</td></tr>
<tr><td>Renal Disorder</td><td>BETA_DEFENSINS</td><td>REACTOME</td><td>333</td><td>0</td><td>0</td><td>1,00E+00</td><td>1,00E+00</td><td>NA</td></tr>
</table>

Abbreviations: Database, database from the Molecular Signatures Databases (MSigDB) containing the indicated genetic pathway; FDR, False Discovery Rate; P, empirical set-based P-value; Pathway, genetic pathways tested for association with the 11 SLE clinical phenotypes of systemic lupus erythematosus; $SNPS_{PATH}$, number of SNPs mapping to a particular pathway; SLE phenotype, clinical phenotypes of systemic lupus erythematosus tested for association with each pathway; SNPs, list of SNPs passing the pathway analysis thresholds; $SNPS_1$, total number of SNPs below the P-value threshold; $SNPS_2$, number of significant SNPs also passing the LD criteria.

2.3 Table S3. Association results of the SNPs from the VEGF pathway associated with oral ulcers in SLE.

POPULATION	SNP	CHR	POSITION	A1	A2	OR	P	GENE
Discovery	rs2373929	7	150714812	A	G	1.91	2.64E-5	*NOS3*
Discovery	rs17709898	4	55957723	G	A	0.53	1.50E-4	*KDR*
Discovery	rs743507	7	150707488	G	A	0.53	2.61E-4	*NOS3*
Discovery	rs10940145	5	67290972	G	A	0.55	3.26E-4	*PIK3R1*
Discovery	rs7396875	11	551405	G	A	2.01	7.16E-4	*HRAS*
Discovery	rs7726943	5	67818581	G	A	1.68	7.80E-4	*PIK3R1*
Discovery	rs10461535	5	67218216	G	A	2.17	1.30E-3	*PIK3R1*
Discovery	rs227913	17	64607394	C	A	0.62	2.67E-3	*PRKCA*
Discovery	rs2372183	5	67197955	A	G	2.06	2.98E-3	*PIK3R1*
Discovery	rs4976028	5	67290733	G	A	1.70	3.46E-3	*PIK3R1*
Discovery	rs904132	4	55802108	G	A	0.40	5.78E-3	*KDR*
Discovery	rs11133360	4	55982752	G	A	0.67	7.40E-3	*KDR*
Discovery	rs9892886	17	64382588	G	A	1.73	8.06E-3	*PRKCA*
Discovery	rs11860367	16	24011167	G	A	0.66	8.25E-3	*PRKCB*
Discovery	rs10515068	5	67450499	C	A	1.61	1.02E-2	*PIK3R1*
Discovery	rs7719763	5	67681425	A	G	1.47	1.38E-2	*PIK3R1*
Discovery	rs8051034	16	23925134	C	A	0.40	1.53E-2	*PRKCB*
Discovery	rs863818	5	67554023	G	A	0.69	1.65E-2	*PIK3R1*
Discovery	rs10515059	5	67241677	A	C	0.65	2.22E-2	*PIK3R1*
Discovery	rs6504424	17	64387328	G	A	1.49	2.27E-2	*PRKCA*
Replication	rs17626553	13	28968770	G	A	0.43	2.35E-4	*FLT1*
Replication	rs1324057	13	28925435	A	G	0.52	2.56E-4	*FLT1*
Replication	rs11959164	5	180096972	G	A	1.61	1.44E-3	*FLT4*
Replication	rs10515077	5	67613801	C	A	2.26	2.86E-3	*PIK3R1*
Replication	rs6129760	20	39746403	G	A	1.61	3.29E-3	*PLCG1*
Replication	rs11744801	5	67301383	C	A	0.56	3.56E-3	*PIK3R1*
Replication	rs7202459	16	24039013	G	A	0.64	4.25E-3	*PRKCB*
Replication	rs6842830	4	55873926	G	A	0.65	4.31E-3	*KDR*
Replication	rs3844278	3	10211918	A	G	0.52	5.15E-3	*VHL*
Replication	rs9550377	13	29101681	A	G	2.03	8.17E-3	*FLT1*
Replication	rs2228246	20	39792063	G	A	0.59	8.75E-3	*PLCG1*
Replication	rs17086609	13	28929711	G	A	1.50	1.02E-2	*FLT1*
Replication	rs10067755	5	67307462	G	A	1.46	1.15E-2	*PIK3R1*
Replication	rs6947821	7	150729212	G	A	1.48	1.21E-2	*NOS3*
Replication	rs4290512	17	64395039	A	C	0.43	1.39E-2	*PRKCA*
Replication	rs2283540	16	24130770	A	G	0.68	1.53E-2	*PRKCB*
Replication	rs708030	3	10212494	G	A	1.43	1.83E-2	*VHL*
Replication	rs10283368	8	141679817	A	G	0.70	2.00E-2	*PTK2*
Replication	rs4791036	17	64797135	G	A	0.62	2.29E-2	*PRKCA*
Replication	rs2125489	4	55953483	A	G	0.55	2.39E-2	*KDR*

Abbreviations: A1, minor allele; A2, major allele; Chr, chromosome; Position, SNP base pair in build GRCh37/hg19; OR, Odds Ratio; P, P-value obtained from the single-marker association analysis; SNP, Single-Nucleotide Polymorphism.

2.4 Table S4. Statistical significance of the differential expression analysis performed on the VEGF pathway genes after immunotherapy.

GENE	$P_{BETAMETHASONE\ VALERATE}$	$P_{IMIQUIMOD}$	$P_{DIPHENCYPRONE}$	$P_{PIMECROLIMUS}$
ARNT	1,10E-02	5,26E-03	1,22E-01	2,63E-02
EIF1	4,93E-02	2,65E-06	6,68E-04	3,31E-01
EIF1AX	6,93E-02	2,88E-03	1,33E-01	5,56E-01
EIF2B1	6,82E-01	6,49E-03	9,26E-01	7,20E-01
EIF2B2	1,87E-02	2,99E-01	4,66E-01	1,86E-01
EIF2B3	3,57E-01	5,91E-01	4,15E-02	8,83E-01
EIF2B4	8,87E-03	7,67E-01	1,25E-01	3,33E-01
EIF2B5	8,20E-01	4,92E-01	3,61E-01	8,41E-01
EIF2S1	2,37E-03	7,54E-03	1,20E-01	1,16E-02
EIF2S2	1,20E-01	7,64E-06	2,18E-03	2,63E-01
EIF2S3	2,57E-01	1,69E-02	1,64E-01	5,93E-01
ELAVL1	5,34E-03	6,83E-03	4,95E-02	1,61E-01
FLT1	4,86E-03	1,70E-02	6,32E-02	5,89E-02
FLT4	3,05E-01	2,73E-01	2,11E-01	2,91E-01
HIF1A	1,76E-01	2,46E-02	9,17E-02	6,53E-01
HRAS	1,04E-03	1,21E-08	5,19E-01	2,78E-02
KDR	1,80E-01	6,39E-02	3,55E-01	2,94E-01
NOS3	6,31E-01	6,32E-01	3,96E-01	3,21E-01
PIK3CA	9,54E-02	1,42E-08	1,09E-01	1,62E-01
PIK3CG	2,08E-01	1,95E-01	1,85E-01	5,18E-01
PIK3R1	1,12E-04	4,70E-06	2,76E-04	1,66E-01
PLCG1	2,44E-01	2,06E-01	1,74E-02	1,91E-01
PRKCA	1,84E-01	2,50E-01	1,75E-01	1,82E-01
PRKCB	3,19E-01	5,34E-02	1,56E-01	3,53E-01
PTK2	3,01E-04	6,72E-09	7,51E-02	1,44E-01
PXN	2,18E-01	8,53E-02	4,97E-01	1,08E-01
SHC1	4,17E-04	9,64E-03	2,94E-01	9,82E-02
VEGFA	2,24E-04	2,20E-11	3,34E-02	2,88E-02
VHL	6,66E-02	7,61E-02	2,64E-01	5,51E-01

Abbreviations: Gene, gene from the VEGF genetic pathway (only the most significant probe was considered for those genes mapped by more than one probe); $P_{BETAMETHASONE\ VALERATE}$, P-value obtained from the differential expression analysis comparing samples before (N=10) and after (N=10) betamethasone valerate treatment; $P_{DIPHENCYPRONE}$, P-value obtained from the differential expression analysis comparing samples before (N=22) and after (N=22) diphencyprone treatment; $P_{IMIQUIMOD}$, P-value obtained from the differential expression analysis comparing samples before (N=6) and after (N=54) imiquimod treatment; $P_{PIMECROLIMUS}$, P-value obtained from the differential expression analysis comparing samples before (N=10) and after (N=10) pimecrolimus treatment.

2.5 Table S5. Statistical significance of the VEGF genetic pathway perturbation after topical immunotherapy.

VEGF PATHWAY	BETAMETHASONE VALERATE	IMIQUIMOD	DIPHENCYPRONE	PIMECROLIMUS
**DEG (n)**	12	16	7	4
**NDEG (n)**	17	13	22	25
**Total Genes (n)**	29	29	29	29
**P-value**	5.69E-9	5.38E-14	4.59E-4	5.50E-2

Abbreviations: DEG, number of VEGF pathway genes that were differentially expressed comparing untreated and treated samples ($P<0.05$); NDEG, number of VEGF pathway genes that were not differentially expressed comparing untreated and treated samples ($P>0.05$); P-value, statistical significance of the VEGF genetic pathway perturbation (Binomial test); Total Genes, total number of genes mapping to the VEGF genetic pathway; VEGF pathway, features of the VEGF genetic pathway related with the differential gene expression analysis.

2.6 Table S6. Established genetic variants for SLE risk.

SNP	CHR	POSITION	LOCUS	RISK ALLELE
rs2476601	1	114377568	*PTPN22*	A
rs1801274	1	161479745	*FCGR2A*	C
rs704840	1	173226195	*TNFSF4*	G
rs17849501	1	183542323	*SMG7-NCF2*	T
rs3024505	1	206939904	*IL10*	T
rs9782955	1	236039877	*LYST*	C
rs2111485	2	163110536	*IFIH1*	G
rs3768792	2	213871709	*IKZF2*	C
rs6740462	2	65667272	*SPRED2*	A
rs11889341	2	191943742	*STAT4*	T
rs9311676	3	58470351	*ABHD6-PXK*	C
rs564799	3	159728987	*IL12A*	C
rs10028805	4	102737250	*BANK1*	G
rs7726414	5	133431834	*TCF7-SKP1*	T
rs10036748	5	150458146	*TNIP1*	T
rs2431697	5	159879978	*MIR146A*	T
rs9462027	6	34797241	*UHRF1BP1*	A
rs6568431	6	106588806	*PRDM1-ATG5*	A
rs6932056	6	138242437	*TNFAIP3*	C
rs1270942	6	31918860	*MHC class III*	C
rs849142	7	28185891	*JAZF1*	A
rs4917014	7	50305863	*IKZF1*	T
rs10488631	7	128594183	*IRF5*	C
rs2736340	8	11343973	*BLK*	T
rs2663052	10	50069395	*WDFY4*	C
rs4948496	10	63805617	*ARID5B*	C
rs3794060	11	71187679	*DHCR7-NADSYN1*	C
rs2732549	11	35088399	*CD44*	T
rs7941765	11	128499000	*ETS1-FLI1*	C
rs12802200	11	566936	*IRF7*	C
rs10774625	12	111910219	*SH2B3*	A
rs1059312	12	129278864	*SLC15A4*	C
rs4902562	14	68731458	*RAD51B*	A
rs2289583	15	75311036	*CSK*	A
rs9652601	16	11174365	*CIITA-SOCS1*	G
rs34572943	16	31272353	*ITGAM*	A
rs11644034	16	85972612	*IRF8*	G
rs2286672	17	4712617	*PLD2*	T
rs2941509	17	37921194	*IKZF3*	A
rs2304256	19	10475652	*TYK2*	C
rs7444	22	21976934	*UBE2L3*	C
rs887369	X	30577846	*CXorf21*	C
rs1734787	X	153325446	*IRAK1-MECP2*	C

Abbreviations: Chr, chromosome; Position, SNP base pair in build GRCh37/hg19; SNP, Single-Nucleotide Polymorphism.

2.7 Table S7. Reference biological pathways selected for the present study.

PATHWAY	DATABASE	GENES
RELA_PATHWAY	BIOCARTA	16
BCELLSURVIVAL_PATHWAY	BIOCARTA	16
CACAM_PATHWAY	BIOCARTA	16
CDMAC_PATHWAY	BIOCARTA	16
EIF_PATHWAY	BIOCARTA	16
GATA3_PATHWAY	BIOCARTA	16
IL22BP_PATHWAY	BIOCARTA	16
P53_PATHWAY	BIOCARTA	16
ACH_PATHWAY	BIOCARTA	16
CDC42RAC_PATHWAY	BIOCARTA	16
SHH_PATHWAY	BIOCARTA	16
LAIR_PATHWAY	BIOCARTA	17
HCMV_PATHWAY	BIOCARTA	17
IL17_PATHWAY	BIOCARTA	17
IL10_PATHWAY	BIOCARTA	17
IL7_PATHWAY	BIOCARTA	17
NO2IL12_PATHWAY	BIOCARTA	17
CK1_PATHWAY	BIOCARTA	17
PML_PATHWAY	BIOCARTA	17
41BB_PATHWAY	BIOCARTA	17
ARF_PATHWAY	BIOCARTA	17
ARAP_PATHWAY	BIOCARTA	18
MCM_PATHWAY	BIOCARTA	18
ETS_PATHWAY	BIOCARTA	18
NGF_PATHWAY	BIOCARTA	18
PTEN_PATHWAY	BIOCARTA	18
CARDIACEGF_PATHWAY	BIOCARTA	18
ERK5_PATHWAY	BIOCARTA	18
SPRY_PATHWAY	BIOCARTA	18
TEL_PATHWAY	BIOCARTA	18
TNFR2_PATHWAY	BIOCARTA	18
UCALPAIN_PATHWAY	BIOCARTA	18
TID_PATHWAY	BIOCARTA	19
COMP_PATHWAY	BIOCARTA	19
MTA3_PATHWAY	BIOCARTA	19
EPO_PATHWAY	BIOCARTA	19
MAL_PATHWAY	BIOCARTA	19
STATHMIN_PATHWAY	BIOCARTA	19
TGFB_PATHWAY	BIOCARTA	19
TH1TH2_PATHWAY	BIOCARTA	19
AMI_PATHWAY	BIOCARTA	20
ATM_PATHWAY	BIOCARTA	20
CCR5_PATHWAY	BIOCARTA	20
NKCELLS_PATHWAY	BIOCARTA	20
IGF1MTOR_PATHWAY	BIOCARTA	20
ACTINY_PATHWAY	BIOCARTA	20
CALCINEURIN_PATHWAY	BIOCARTA	21
NDKDYNAMIN_PATHWAY	BIOCARTA	21
IGF1_PATHWAY	BIOCARTA	21
ATRBRCA_PATHWAY	BIOCARTA	21
MITOCHONDRIA_PATHWAY	BIOCARTA	21
TOB1_PATHWAY	BIOCARTA	21
CTLA4_PATHWAY	BIOCARTA	21
TFF_PATHWAY	BIOCARTA	21
AKT_PATHWAY	BIOCARTA	22
CHEMICAL_PATHWAY	BIOCARTA	22
SPPA_PATHWAY	BIOCARTA	22
CERAMIDE_PATHWAY	BIOCARTA	22
GCR_PATHWAY	BIOCARTA	22
CYTOKINE_PATHWAY	BIOCARTA	22
DC_PATHWAY	BIOCARTA	22
IL2_PATHWAY	BIOCARTA	22
IL6_PATHWAY	BIOCARTA	22
INSULIN_PATHWAY	BIOCARTA	22
HER2_PATHWAY	BIOCARTA	22
CASPASE_PATHWAY	BIOCARTA	23
CCR3_PATHWAY	BIOCARTA	23
CTCF_PATHWAY	BIOCARTA	23
CELLCYCLE_PATHWAY	BIOCARTA	23
P53HYPOXIA_PATHWAY	BIOCARTA	23
IL12_PATHWAY	BIOCARTA	23
GLEEVEC_PATHWAY	BIOCARTA	23
INTRINSIC_PATHWAY	BIOCARTA	23
MTOR_PATHWAY	BIOCARTA	23
IGF1R_PATHWAY	BIOCARTA	23
NFKB_PATHWAY	BIOCARTA	23
PTDINS_PATHWAY	BIOCARTA	23
RAC1_PATHWAY	BIOCARTA	23
RAS_PATHWAY	BIOCARTA	23
MEF2D_PATHWAY	BIOCARTA	23
CSK_PATHWAY	BIOCARTA	24
G2_PATHWAY	BIOCARTA	24
CXCR4_PATHWAY	BIOCARTA	24
ECM_PATHWAY	BIOCARTA	24
NTHI_PATHWAY	BIOCARTA	24
NOS1_PATHWAY	BIOCARTA	24
EIF4_PATHWAY	BIOCARTA	24
TPO_PATHWAY	BIOCARTA	24
MCALPAIN_PATHWAY	BIOCARTA	25
STRESS_PATHWAY	BIOCARTA	25
RACCYCD_PATHWAY	BIOCARTA	26
BAD_PATHWAY	BIOCARTA	26
PGC1A_PATHWAY	BIOCARTA	26
WNT_PATHWAY	BIOCARTA	26

GSK3_PATHWAY	BIOCARTA	27
EDG1_PATHWAY	BIOCARTA	27
CREB_PATHWAY	BIOCARTA	27
G1_PATHWAY	BIOCARTA	28
ERK_PATHWAY	BIOCARTA	28
GH_PATHWAY	BIOCARTA	28
PROTEASOME_PATHWAY	BIOCARTA	28
INFLAM_PATHWAY	BIOCARTA	29
VIP_PATHWAY	BIOCARTA	29
NKT_PATHWAY	BIOCARTA	29
TNFR1_PATHWAY	BIOCARTA	29
VEGF_PATHWAY	BIOCARTA	29
FAS_PATHWAY	BIOCARTA	30
EGF_PATHWAY	BIOCARTA	31
PYK2_PATHWAY	BIOCARTA	31
MYOSIN_PATHWAY	BIOCARTA	31
HDAC_PATHWAY	BIOCARTA	32
PDGF_PATHWAY	BIOCARTA	32
RHO_PATHWAY	BIOCARTA	32
NO1_PATHWAY	BIOCARTA	33
DEATH_PATHWAY	BIOCARTA	33
IL1R_PATHWAY	BIOCARTA	33
MPR_PATHWAY	BIOCARTA	34
CARM_ER_PATHWAY	BIOCARTA	35
AGR_PATHWAY	BIOCARTA	36
AT1R_PATHWAY	BIOCARTA	36
ALK_PATHWAY	BIOCARTA	37
BCR_PATHWAY	BIOCARTA	37
MET_PATHWAY	BIOCARTA	37
GPCR_PATHWAY	BIOCARTA	37
PAR1_PATHWAY	BIOCARTA	37
TOLL_PATHWAY	BIOCARTA	37
IL2RB_PATHWAY	BIOCARTA	38
INTEGRIN_PATHWAY	BIOCARTA	38
FMLP_PATHWAY	BIOCARTA	39
P38MAPK_PATHWAY	BIOCARTA	40
FCER1_PATHWAY	BIOCARTA	41
CHREBP2_PATHWAY	BIOCARTA	42
BIOPEPTIDES_PATHWAY	BIOCARTA	45
KERATINOCYTE_PATHWAY	BIOCARTA	46
TCR_PATHWAY	BIOCARTA	49
NFAT_PATHWAY	BIOCARTA	56
HIVNEF_PATHWAY	BIOCARTA	58
PPARA_PATHWAY	BIOCARTA	58
MAPK_PATHWAY	BIOCARTA	87
PRIMARY_BILE_ACID_BIOSYNTHESIS	KEGG	16
OTHER_GLYCAN_DEGRADATION	KEGG	16
GLYOXYLATE_AND_DICARBOXYLATE_METABOLISM	KEGG	16
RIBOFLAVIN_METABOLISM	KEGG	16
PANTOTHENATE_AND_COA_BIOSYNTHESIS	KEGG	16
STEROID_BIOSYNTHESIS	KEGG	17
ONE_CARBON_POOL_BY_FOLATE	KEGG	17
RENIN_ANGIOTENSIN_SYSTEM	KEGG	17
PHENYLALANINE_METABOLISM	KEGG	18
ALPHA_LINOLENIC_ACID_METABOLISM	KEGG	19
GLYCOSAMINOGLYCAN_DEGRADATION	KEGG	21
BETA_ALANINE_METABOLISM	KEGG	22
GLYCOSAMINOGLYCAN_BIOSYNTHESIS_CHONDROITIN_SULFATE	KEGG	22
BIOSYNTHESIS_OF_UNSATURATED_FATTY_ACIDS	KEGG	22
NITROGEN_METABOLISM	KEGG	23
MISMATCH_REPAIR	KEGG	23
PROXIMAL_TUBULE_BICARBONATE_RECLAMATION	KEGG	23
NICOTINATE_AND_NICOTINAMIDE_METABOLISM	KEGG	24
PROTEIN_EXPORT	KEGG	24
ASCORBATE_AND_ALDARATE_METABOLISM	KEGG	25
GLYCOSYLPHOSPHATIDYLINOSITOL_GPI_ANCHOR_BIOSYNTHESIS	KEGG	25
DORSO_VENTRAL_AXIS_FORMATION	KEGG	25
MATURITY_ONSET_DIABETES_OF_THE_YOUNG	KEGG	25
GALACTOSE_METABOLISM	KEGG	26
SELENOAMINO_ACID_METABOLISM	KEGG	26
GLYCOSAMINOGLYCAN_BIOSYNTHESIS_HEPARAN_SULFATE	KEGG	26
GLYCOSPHINGOLIPID_BIOSYNTHESIS_LACTO_AND_NEOLACTO_SERIES	KEGG	26
PENTOSE_PHOSPHATE_PATHWAY	KEGG	27
PENTOSE_AND_GLUCURONATE_INTERCONVERSIONS	KEGG	28
HOMOLOGOUS_RECOMBINATION	KEGG	28
HISTIDINE_METABOLISM	KEGG	29
LINOLEIC_ACID_METABOLISM	KEGG	29
RNA_POLYMERASE	KEGG	29
THYROID_CANCER	KEGG	29
O_GLYCAN_BIOSYNTHESIS	KEGG	30
ASTHMA	KEGG	30
GLYCINE_SERINE_AND_THREONINE_METABOLISM	KEGG	31
CITRATE_CYCLE_TCA_CYCLE	KEGG	32
ALANINE_ASPARTATE_AND_GLUTAMATE_METABOLISM	KEGG	32
ETHER_LIPID_METABOLISM	KEGG	33
PROPANOATE_METABOLISM	KEGG	33
FRUCTOSE_AND_MANNOSE_METABOLISM	KEGG	34
CYSTEINE_AND_METHIONINE_METABOLISM	KEGG	34
BUTANOATE_METABOLISM	KEGG	34
BASE_EXCISION_REPAIR	KEGG	35
REGULATION_OF_AUTOPHAGY	KEGG	35
PRION_DISEASES	KEGG	35
PRIMARY_IMMUNODEFICIENCY	KEGG	35
BASAL_TRANSCRIPTION_FACTORS	KEGG	36
DNA_REPLICATION	KEGG	36
SNARE_INTERACTIONS_IN_VESICULAR_TRANSPORT	KEGG	38
ALLOGRAFT_REJECTION	KEGG	38
TRYPTOPHAN_METABOLISM	KEGG	40
SPHINGOLIPID_METABOLISM	KEGG	40
PYRUVATE_METABOLISM	KEGG	40
PORPHYRIN_AND_CHLOROPHYLL_METABOLISM	KEGG	41

AMINOACYL_TRNA_BIOSYNTHESIS	KEGG	41
FATTY_ACID_METABOLISM	KEGG	42
TYROSINE_METABOLISM	KEGG	42
ALDOSTERONE_REGULATED_SODIUM_REABSORPTION	KEGG	42
BLADDER_CANCER	KEGG	42
GRAFT_VERSUS_HOST_DISEASE	KEGG	42
VALINE_LEUCINE_AND_ISOLEUCINE_DEGRADATION	KEGG	44
LYSINE_DEGRADATION	KEGG	44
AMINO_SUGAR_AND_NUCLEOTIDE_SUGAR_METABOLISM	KEGG	44
ABC_TRANSPORTERS	KEGG	44
NUCLEOTIDE_EXCISION_REPAIR	KEGG	44
TYPE_I_DIABETES_MELLITUS	KEGG	44
VASOPRESSIN_REGULATED_WATER_REABSORPTION	KEGG	44
N_GLYCAN_BIOSYNTHESIS	KEGG	46
NOTCH_SIGNALING_PATHWAY	KEGG	47
TYPE_II_DIABETES_MELLITUS	KEGG	47
PROTEASOME	KEGG	48
INTESTINAL_IMMUNE_NETWORK_FOR_IGA_PRODUCTION	KEGG	48
GLYCEROLIPID_METABOLISM	KEGG	49
GLUTATHIONE_METABOLISM	KEGG	50
DRUG_METABOLISM_OTHER_ENZYMES	KEGG	51
STARCH_AND_SUCROSE_METABOLISM	KEGG	52
MTOR_SIGNALING_PATHWAY	KEGG	52
TASTE_TRANSDUCTION	KEGG	52
ENDOMETRIAL_CANCER	KEGG	52
AMYOTROPHIC_LATERAL_SCLEROSIS_ALS	KEGG	53
AUTOIMMUNE_THYROID_DISEASE	KEGG	53
ARGININE_AND_PROLINE_METABOLISM	KEGG	54
INOSITOL_PHOSPHATE_METABOLISM	KEGG	54
NON_SMALL_CELL_LUNG_CANCER	KEGG	54
STEROID_HORMONE_BIOSYNTHESIS	KEGG	55
BASAL_CELL_CARCINOMA	KEGG	55
HEDGEHOG_SIGNALING_PATHWAY	KEGG	56
CYTOSOLIC_DNA_SENSING_PATHWAY	KEGG	56
VIBRIO_CHOLERAE_INFECTION	KEGG	56
ARACHIDONIC_ACID_METABOLISM	KEGG	58
RNA_DEGRADATION	KEGG	59
PATHOGENIC_ESCHERICHIA_COLI_INFECTION	KEGG	59
ACUTE_MYELOID_LEUKEMIA	KEGG	60
GLYCOLYSIS_GLUCONEOGENESIS	KEGG	62
NOD_LIKE_RECEPTOR_SIGNALING_PATHWAY	KEGG	62
COLORECTAL_CANCER	KEGG	62
RETINOL_METABOLISM	KEGG	64
GLIOMA	KEGG	65
ADIPOCYTOKINE_SIGNALING_PATHWAY	KEGG	67
EPITHELIAL_CELL_SIGNALING_IN_HELICOBACTER_PYLORI_INFECTION	KEGG	68
PPAR_SIGNALING_PATHWAY	KEGG	69
P53_SIGNALING_PATHWAY	KEGG	69
COMPLEMENT_AND_COAGULATION_CASCADES	KEGG	69
METABOLISM_OF_XENOBIOTICS_BY_CYTOCHROME_P450	KEGG	70
LONG_TERM_POTENTIATION	KEGG	70
LONG_TERM_DEPRESSION	KEGG	70
RENAL_CELL_CARCINOMA	KEGG	70
PANCREATIC_CANCER	KEGG	70
RIG_I_LIKE_RECEPTOR_SIGNALING_PATHWAY	KEGG	71
MELANOMA	KEGG	71
DRUG_METABOLISM_CYTOCHROME_P450	KEGG	72
LEISHMANIA_INFECTION	KEGG	72
CHRONIC_MYELOID_LEUKEMIA	KEGG	73
VIRAL_MYOCARDITIS	KEGG	73
ADHERENS_JUNCTION	KEGG	75
B_CELL_RECEPTOR_SIGNALING_PATHWAY	KEGG	75
PHOSPHATIDYLINOSITOL_SIGNALING_SYSTEM	KEGG	76
VEGF_SIGNALING_PATHWAY	KEGG	76
ARRHYTHMOGENIC_RIGHT_VENTRICULAR_CARDIOMYOPATHY_ARVC	KEGG	76
GLYCEROPHOSPHOLIPID_METABOLISM	KEGG	77
PEROXISOME	KEGG	78
FC_EPSILON_RI_SIGNALING_PATHWAY	KEGG	79
CARDIAC_MUSCLE_CONTRACTION	KEGG	80
ECM_RECEPTOR_INTERACTION	KEGG	84
SMALL_CELL_LUNG_CANCER	KEGG	84
HYPERTROPHIC_CARDIOMYOPATHY_HCM	KEGG	85
TGF_BETA_SIGNALING_PATHWAY	KEGG	86
PROGESTERONE_MEDIATED_OOCYTE_MATURATION	KEGG	86
ERBB_SIGNALING_PATHWAY	KEGG	87
RIBOSOME	KEGG	88
APOPTOSIS	KEGG	88
HEMATOPOIETIC_CELL_LINEAGE	KEGG	88
ANTIGEN_PROCESSING_AND_PRESENTATION	KEGG	89
PROSTATE_CANCER	KEGG	89
GAP_JUNCTION	KEGG	90
DILATED_CARDIOMYOPATHY	KEGG	92
FC_GAMMA_R_MEDIATED_PHAGOCYTOSIS	KEGG	97
PYRIMIDINE_METABOLISM	KEGG	98
GNRH_SIGNALING_PATHWAY	KEGG	101
TOLL_LIKE_RECEPTOR_SIGNALING_PATHWAY	KEGG	102
MELANOGENESIS	KEGG	102
T_CELL_RECEPTOR_SIGNALING_PATHWAY	KEGG	108
OOCYTE_MEIOSIS	KEGG	114
VASCULAR_SMOOTH_MUSCLE_CONTRACTION	KEGG	115
LEUKOCYTE_TRANSENDOTHELIAL_MIGRATION	KEGG	118
LYSOSOME	KEGG	121
NEUROTROPHIN_SIGNALING_PATHWAY	KEGG	126
SPLICEOSOME	KEGG	128
CELL_CYCLE	KEGG	128
AXON_GUIDANCE	KEGG	129
PARKINSONS_DISEASE	KEGG	133
CELL_ADHESION_MOLECULES_CAMS	KEGG	134
TIGHT_JUNCTION	KEGG	134
OXIDATIVE_PHOSPHORYLATION	KEGG	135
NATURAL_KILLER_CELL_MEDIATED_CYTOTOXICITY	KEGG	137

INSULIN_SIGNALING_PATHWAY	KEGG	137
UBIQUITIN_MEDIATED_PROTEOLYSIS	KEGG	138
SYSTEMIC_LUPUS_ERYTHEMATOSUS	KEGG	140
WNT_SIGNALING_PATHWAY	KEGG	151
JAK_STAT_SIGNALING_PATHWAY	KEGG	155
PURINE_METABOLISM	KEGG	159
ALZHEIMERS_DISEASE	KEGG	169
CALCIUM_SIGNALING_PATHWAY	KEGG	178
ENDOCYTOSIS	KEGG	183
HUNTINGTONS_DISEASE	KEGG	185
CHEMOKINE_SIGNALING_PATHWAY	KEGG	190
FOCAL_ADHESION	KEGG	201
REGULATION_OF_ACTIN_CYTOSKELETON	KEGG	216
MAPK_SIGNALING_PATHWAY	KEGG	267
CYTOKINE_CYTOKINE_RECEPTOR_INTERACTION	KEGG	267
NEUROACTIVE_LIGAND_RECEPTOR_INTERACTION	KEGG	272
CTNNB1_PHOSPHORYLATION_CASCADE	REACTOME	16
PRE_NOTCH_PROCESSING_IN_GOLGI	REACTOME	16
TGF_BETA_RECEPTOR_SIGNALING_IN_EMT_EPITHELIAL_TO_MESENCHYMAL_TRANSITION	REACTOME	16
ACTIVATED_POINT_MUTANTS_OF_FGFR2	REACTOME	16
ACYL_CHAIN_REMODELLING_OF_PG	REACTOME	16
PHOSPHORYLATION_OF_CD3_AND_TCR_ZETA_CHAINS	REACTOME	16
XENOBIOTICS	REACTOME	16
REGULATION_OF_INSULIN_LIKE_GROWTH_FACTOR_IGF_ACTIVITY_BY_INSULIN_LIKE_GROWTH_FACTOR_BINDING_PROTEINS_IGFBPS	REACTOME	16
ACETYLCHOLINE_BINDING_AND_DOWNSTREAM_EVENTS	REACTOME	16
NUCLEOTIDE_LIKE_PURINERGIC_RECEPTORS	REACTOME	16
EICOSANOID_LIGAND_BINDING_RECEPTORS	REACTOME	16
TRAFFICKING_OF_GLUR2_CONTAINING_AMPA_RECEPTORS	REACTOME	16
SYNTHESIS_SECRETION_AND_DEACYLATION_OF_GHRELIN	REACTOME	16
JNK_C_JUN_KINASES_PHOSPHORYLATION_AND_ACTIVATION_MEDIATED_BY_ACTIVATED_HUMAN_TAK1	REACTOME	16
PLATELET_SENSITIZATION_BY_LDL	REACTOME	16
FORMATION_OF_ATP_BY_CHEMIOSMOTIC_COUPLING	REACTOME	16
CHYLOMICRON_MEDIATED_LIPID_TRANSPORT	REACTOME	16
INTEGRATION_OF_PROVIRUS	REACTOME	16
INITIAL_TRIGGERING_OF_COMPLEMENT	REACTOME	16
REGULATION_OF_KIT_SIGNALING	REACTOME	17
PROTEOLYTIC_CLEAVAGE_OF_SNARE_COMPLEX_PROTEINS	REACTOME	17
RESOLUTION_OF_AP_SITES_VIA_THE_MULTIPLE_NUCLEOTIDE_PATCH_REPLACEMENT_PATHWAY	REACTOME	17
ARMS_MEDIATED_ACTIVATION	REACTOME	17
SYNTHESIS_OF_PIPS_AT_THE_GOLGI_MEMBRANE	REACTOME	17
TIE2_SIGNALING	REACTOME	17
HOMOLOGOUS_RECOMBINATION_REPAIR_OF_REPLICATION_INDEPENDENT_DOUBLE_STRAND_BREAKS	REACTOME	17
BRANCHED_CHAIN_AMINO_ACID_CATABOLISM	REACTOME	17
NA_CL_DEPENDENT_NEUROTRANSMITTER_TRANSPORTERS	REACTOME	17
RAS_ACTIVATION_UOPN_CA2_INFUX_THROUGH_NMDA_RECEPTOR	REACTOME	17
AMINO_ACID_SYNTHESIS_AND_INTERCONVERSION_TRANSAMINATION	REACTOME	17
RAP1_SIGNALLING	REACTOME	17
GABA_SYNTHESIS_RELEASE_REUPTAKE_AND_DEGRADATION	REACTOME	17
DESTABILIZATION_OF_MRNA_BY_BRF1	REACTOME	17
DESTABILIZATION_OF_MRNA_BY_KSRP	REACTOME	17
DESTABILIZATION_OF_MRNA_BY_TRISTETRAPROLIN_TTP	REACTOME	17
INTRINSIC_PATHWAY	REACTOME	17
ACTIVATION_OF_BH3_ONLY_PROTEINS	REACTOME	17
RNA_POL_III_CHAIN_ELONGATION	REACTOME	17
INFLAMMASOMES	REACTOME	17
SYNTHESIS_OF_GLYCOSYLPHOSPHATIDYLINOSITOL_GPI	REACTOME	17
SHC_RELATED_EVENTS	REACTOME	17
GLYCOGEN_BREAKDOWN_GLYCOGENOLYSIS	REACTOME	18
SIGNALING_BY_NODAL	REACTOME	18
ABCA_TRANSPORTERS_IN_LIPID_HOMEOSTASIS	REACTOME	18
SIGNALING_BY_CONSTITUTIVELY_ACTIVE_EGFR	REACTOME	18
RIP_MEDIATED_NFKB_ACTIVATION_VIA_DAI	REACTOME	18
OXYGEN_DEPENDENT_PROLINE_HYDROXYLATION_OF_HYPOXIA_INDUCIBLE_FACTOR_ALPHA	REACTOME	18
SYNTHESIS_OF_PC	REACTOME	18
FATTY_ACYL_COA_BIOSYNTHESIS	REACTOME	18
PKA_MEDIATED_PHOSPHORYLATION_OF_CREB	REACTOME	18
PD1_SIGNALING	REACTOME	18
ENERGY_DEPENDENT_REGULATION_OF_MTOR_BY_LKB1_AMPK	REACTOME	18
ACTIVATED_TAK1_MEDIATES_P38_MAPK_ACTIVATION	REACTOME	18
REGULATION_OF_SIGNALING_BY_CBL	REACTOME	18
PLATELET_CALCIUM_HOMEOSTASIS	REACTOME	18
N_GLYCAN_ANTENNAE_ELONGATION_IN_THE_MEDIAL_TRANS_GOLGI	REACTOME	18
GLUCURONIDATION	REACTOME	18
GAP_JUNCTION_ASSEMBLY	REACTOME	18
BASE_EXCISION_REPAIR	REACTOME	19
SYNTHESIS_OF_BILE_ACIDS_AND_BILE_SALTS	REACTOME	19
ACTIVATED_AMPK_STIMULATES_FATTY_ACID_OXIDATION_IN_MUSCLE	REACTOME	19
BOTULINUM_NEUROTOXICITY	REACTOME	19
PROLONGED_ERK_ACTIVATION_EVENTS	REACTOME	19
SIGNALING_BY_FGFR1_FUSION_MUTANTS	REACTOME	19
POST_CHAPERONIN_TUBULIN_FOLDING_PATHWAY	REACTOME	19
PYRUVATE_METABOLISM	REACTOME	19
SYNTHESIS_AND_INTERCONVERSION_OF_NUCLEOTIDE_DI_AND_TRIPHOSPHATES	REACTOME	19
CGMP_EFFECTS	REACTOME	19
PROSTACYCLIN_SIGNALLING_THROUGH_PROSTACYCLIN_RECEPTOR	REACTOME	19
SYNTHESIS_SECRETION_AND_INACTIVATION_OF_GLP1	REACTOME	19
LAGGING_STRAND_SYNTHESIS	REACTOME	19
RNA_POL_III_TRANSCRIPTION_TERMINATION	REACTOME	19
G1_S_SPECIFIC_TRANSCRIPTION	REACTOME	19
SHC1_EVENTS_IN_ERBB4_SIGNALING	REACTOME	20
HS_GAG_DEGRADATION	REACTOME	20
DOWNREGULATION_OF_SMAD2_3_SMAD4_TRANSCRIPTIONAL_ACTIVITY	REACTOME	20
ENOS_ACTIVATION_AND_REGULATION	REACTOME	20
REGULATION_OF_GENE_EXPRESSION_IN_BETA_CELLS	REACTOME	20
G_BETA_GAMMA_SIGNALLING_THROUGH_PLC_BETA	REACTOME	20
NEPHRIN_INTERACTIONS	REACTOME	20
NEF_MEDIATES_DOWN_MODULATION_OF_CELL_SURFACE_RECEPTORS_BY_RECRUITING_THEM_TO_CLATHRIN_ADAPTERS	REACTOME	21
CHONDROITIN_SULFATE_BIOSYNTHESIS	REACTOME	21
ACYL_CHAIN_REMODELLING_OF_PE	REACTOME	21
ERK_MAPK_TARGETS	REACTOME	21
MITOCHONDRIAL_TRNA_AMINOACYLATION	REACTOME	21

INSULIN_SYNTHESIS_AND_PROCESSING	REACTOME	21
PEROXISOMAL_LIPID_METABOLISM	REACTOME	21
CTLA4_INHIBITORY_SIGNALING	REACTOME	21
ADP_SIGNALLING_THROUGH_P2RY12	REACTOME	21
TRAF6_MEDIATED_NFKB_ACTIVATION	REACTOME	21
LIGAND_GATED_ION_CHANNEL_TRANSPORT	REACTOME	21
EARLY_PHASE_OF_HIV_LIFE_CYCLE	REACTOME	21
ANTIGEN_PRESENTATION_FOLDING_ASSEMBLY_AND_PEPTIDE_LOADING_OF_CLASS_I_MHC	REACTOME	21
RNA_POL_I_TRANSCRIPTION_TERMINATION	REACTOME	22
GRB2_EVENTS_IN_ERBB2_SIGNALING	REACTOME	22
SIGNALING_BY_HIPPO	REACTOME	22
ACYL_CHAIN_REMODELLING_OF_PC	REACTOME	22
FORMATION_OF_TUBULIN_FOLDING_INTERMEDIATES_BY_CCT_TRIC	REACTOME	22
CD28_DEPENDENT_PI3K_AKT_SIGNALING	REACTOME	22
DEADENYLATION_OF_MRNA	REACTOME	22
METAL_ION_SLC_TRANSPORTERS	REACTOME	22
INCRETIN_SYNTHESIS_SECRETION_AND_INACTIVATION	REACTOME	22
CONVERSION_FROM_APC_C_CDC20_TO_APC_C_CDH1_IN_LATE_ANAPHASE	REACTOME	22
FGFR_LIGAND_BINDING_AND_ACTIVATION	REACTOME	22
RNA_POL_III_TRANSCRIPTION_INITIATION_FROM_TYPE_2_PROMOTER	REACTOME	23
INSULIN_RECEPTOR_RECYCLING	REACTOME	23
CIRCADIAN_REPRESSION_OF_EXPRESSION_BY_REV_ERBA	REACTOME	23
SIGNALING_BY_BMP	REACTOME	23
DOWNREGULATION_OF_TGF_BETA_RECEPTOR_SIGNALING	REACTOME	23
MICRORNA_MIRNA_BIOGENESIS	REACTOME	23
PROCESSING_OF_CAPPED_INTRONLESS_PRE_MRNA	REACTOME	23
LYSOSOME_VESICLE_BIOGENESIS	REACTOME	23
THROMBOXANE_SIGNALLING_THROUGH_TP_RECEPTOR	REACTOME	23
TAK1_ACTIVATES_NFKB_BY_PHOSPHORYLATION_AND_ACTIVATION_OF_IKKS_COMPLEX	REACTOME	23
INTERACTION_BETWEEN_L1_AND_ANKYRINS	REACTOME	23
FORMATION_OF_INCISION_COMPLEX_IN_GG_NER	REACTOME	23
ABORTIVE_ELONGATION_OF_HIV1_TRANSCRIPT_IN_THE_ABSENCE_OF_TAT	REACTOME	23
PHOSPHORYLATION_OF_THE_APC_C	REACTOME	23
GLUTATHIONE_CONJUGATION	REACTOME	23
INHIBITION_OF_THE_PROTEOLYTIC_ACTIVITY_OF_APC_C_REQUIRED_FOR_THE_ONSET_OF_ANAPHASE_BY_MITOTIC_SPINDLE_CHECKPOINT_COMPONENTS	REACTOME	24
GROWTH_HORMONE_RECEPTOR_SIGNALING	REACTOME	24
SULFUR_AMINO_ACID_METABOLISM	REACTOME	24
TERMINATION_OF_O_GLYCAN_BIOSYNTHESIS	REACTOME	24
RORA_ACTIVATES_CIRCADIAN_EXPRESSION	REACTOME	24
YAP1_AND_WWTR1_TAZ_STIMULATED_GENE_EXPRESSION	REACTOME	24
NUCLEAR_EVENTS_KINASE_AND_TRANSCRIPTION_FACTOR_ACTIVATION	REACTOME	24
CYTOSOLIC_TRNA_AMINOACYLATION	REACTOME	24
DOUBLE_STRAND_BREAK_REPAIR	REACTOME	24
KINESINS	REACTOME	24
REGULATION_OF_IFNA_SIGNALING	REACTOME	24
CHOLESTEROL_BIOSYNTHESIS	REACTOME	24
PYRIMIDINE_METABOLISM	REACTOME	24
G0_AND_EARLY_G1	REACTOME	25
REGULATION_OF_HYPOXIA_INDUCIBLE_FACTOR_HIF_BY_OXYGEN	REACTOME	25
A_TETRASACCHARIDE_LINKER_SEQUENCE_IS_REQUIRED_FOR_GAG_SYNTHESIS	REACTOME	25
EGFR_DOWNREGULATION	REACTOME	25
DARPP_32_EVENTS	REACTOME	25
INHIBITION_OF_INSULIN_SECRETION_BY_ADRENALINE_NORADRENALINE	REACTOME	25
FANCONI_ANEMIA_PATHWAY	REACTOME	25
ADP_SIGNALLING_THROUGH_P2RY1	REACTOME	25
G_BETA_GAMMA_SIGNALLING_THROUGH_PI3KGAMMA	REACTOME	25
SMOOTH_MUSCLE_CONTRACTION	REACTOME	25
EFFECTS_OF_PIP2_HYDROLYSIS	REACTOME	25
NITRIC_OXIDE_STIMULATES_GUANYLATE_CYCLASE	REACTOME	25
INHIBITION_OF_VOLTAGE_GATED_CA2_CHANNELS_VIA_GBETA_GAMMA_SUBUNITS	REACTOME	25
TRANSFERRIN_ENDOCYTOSIS_AND_RECYCLING	REACTOME	25
RNA_POL_I_TRANSCRIPTION_INITIATION	REACTOME	25
TGF_BETA_RECEPTOR_SIGNALING_ACTIVATES_SMADS	REACTOME	26
KERATAN_SULFATE_BIOSYNTHESIS	REACTOME	26
REGULATORY_RNA_PATHWAYS	REACTOME	26
CITRIC_ACID_CYCLE_TCA_CYCLE	REACTOME	26
ACTIVATION_OF_GENES_BY_ATF4	REACTOME	26
POST_TRANSLATIONAL_MODIFICATION_SYNTHESIS_OF_GPI_ANCHORED_PROTEINS	REACTOME	26
RNA_POL_III_TRANSCRIPTION_INITIATION_FROM_TYPE_3_PROMOTER	REACTOME	26
APC_C_CDC20_MEDIATED_DEGRADATION_OF_CYCLIN_B	REACTOME	26
PTM_GAMMA_CARBOXYLATION_HYPUSINE_FORMATION_AND_ARYLSULFATASE_ACTIVATION	REACTOME	27
BILE_ACID_AND_BILE_SALT_METABOLISM	REACTOME	27
ACTIVATED_NOTCH1_TRANSMITS_SIGNAL_TO_THE_NUCLEUS	REACTOME	27
SIGNALLING_TO_RAS	REACTOME	27
SMAD2_SMAD3_SMAD4_HETEROTRIMER_REGULATES_TRANSCRIPTION	REACTOME	27
SYNTHESIS_OF_PA	REACTOME	27
GENERATION_OF_SECOND_MESSENGER_MOLECULES	REACTOME	27
G_PROTEIN_ACTIVATION	REACTOME	27
INTEGRIN_ALPHAIIB_BETA3_SIGNALING	REACTOME	27
ASSOCIATION_OF_TRIC_CCT_WITH_TARGET_PROTEINS_DURING_BIOSYNTHESIS	REACTOME	27
STRIATED_MUSCLE_CONTRACTION	REACTOME	27
ADHERENS_JUNCTIONS_INTERACTIONS	REACTOME	27
SEMA4D_INDUCED_CELL_MIGRATION_AND_GROWTH_CONE_COLLAPSE	REACTOME	27
CREB_PHOSPHORYLATION_THROUGH_THE_ACTIVATION_OF_RAS	REACTOME	27
AMINE_COMPOUND_SLC_TRANSPORTERS	REACTOME	27
RECYCLING_PATHWAY_OF_L1	REACTOME	27
IL_RECEPTOR_SHC_SIGNALING	REACTOME	27
ENDOSOMAL_SORTING_COMPLEX_REQUIRED_FOR_TRANSPORT_ESCRT	REACTOME	27
NEP_NS2_INTERACTS_WITH_THE_CELLULAR_EXPORT_MACHINERY	REACTOME	27
TRANSPORT_OF_RIBONUCLEOPROTEINS_INTO_THE_HOST_NUCLEUS	REACTOME	27
REGULATION_OF_GLUCOKINASE_BY_GLUCOKINASE_REGULATORY_PROTEIN	REACTOME	27
EXTENSION_OF_TELOMERES	REACTOME	27
GAP_JUNCTION_TRAFFICKING	REACTOME	27
PREFOLDIN_MEDIATED_TRANSFER_OF_SUBSTRATE_TO_CCT_TRIC	REACTOME	28
TRAFFICKING_OF_AMPA_RECEPTORS	REACTOME	28
G_PROTEIN_BETA_GAMMA_SIGNALLING	REACTOME	28
MYOGENESIS	REACTOME	28
SHC_MEDIATED_CASCADE	REACTOME	28
LIPOPROTEIN_METABOLISM	REACTOME	28
THE_ROLE_OF_NEF_IN_HIV1_REPLICATION_AND_DISEASE_PATHOGENESIS	REACTOME	28
APC_CDC20_MEDIATED_DEGRADATION_OF_NEK2A	REACTOME	28

PRE_NOTCH_TRANSCRIPTION_AND_TRANSLATION	REACTOME	29
DEGRADATION_OF_THE_EXTRACELLULAR_MATRIX	REACTOME	29
ANTIGEN_ACTIVATES_B_CELL_RECEPTOR_LEADING_TO_GENERATION_OF_SECOND_MESSENGERS	REACTOME	29
GLYCOLYSIS	REACTOME	29
STEROID_HORMONES	REACTOME	29
PERK_REGULATED_GENE_EXPRESSION	REACTOME	29
TIGHT_JUNCTION_INTERACTIONS	REACTOME	29
BIOSYNTHESIS_OF_THE_N_GLYCAN_PRECURSOR_DOLICHOL_LIPID_LINKED_OLIGOSACCHARIDE_LLO_AND_TRANSFER_TO_A_NASCENT_PROTEIN	REACTOME	29
PKB_MEDIATED_EVENTS	REACTOME	29
PIP3_ACTIVATES_AKT_SIGNALING	REACTOME	29
SIGNALING_BY_FGFR1_MUTANTS	REACTOME	30
KERATAN_SULFATE_KERATIN_METABOLISM	REACTOME	30
BASIGIN_INTERACTIONS	REACTOME	30
REGULATION_OF_BETA_CELL_DEVELOPMENT	REACTOME	30
MRNA_CAPPING	REACTOME	30
CA_DEPENDENT_EVENTS	REACTOME	30
SIGNALING_BY_ROBO_RECEPTOR	REACTOME	30
FORMATION_OF_TRANSCRIPTION_COUPLED_NER_TC_NER_REPAIR_COMPLEX	REACTOME	30
MAPK_TARGETS_NUCLEAR_EVENTS_MEDIATED_BY_MAP_KINASES	REACTOME	30
TRAF6_MEDIATED_IRF7_ACTIVATION	REACTOME	30
NOD1_2_SIGNALING_PATHWAY	REACTOME	30
DNA_STRAND_ELONGATION	REACTOME	30
INTRINSIC_PATHWAY_FOR_APOPTOSIS	REACTOME	30
ACTIVATION_OF_THE_PRE_REPLICATIVE_COMPLEX	REACTOME	31
SPHINGOLIPID_DE_NOVO_BIOSYNTHESIS	REACTOME	31
SYNTHESIS_OF_PIPS_AT_THE_PLASMA_MEMBRANE	REACTOME	31
HS_GAG_BIOSYNTHESIS	REACTOME	31
AMINO_ACID_TRANSPORT_ACROSS_THE_PLASMA_MEMBRANE	REACTOME	31
GPVI_MEDIATED_ACTIVATION_CASCADE	REACTOME	31
SIGNAL_AMPLIFICATION	REACTOME	31
ACTIVATION_OF_KAINATE_RECEPTORS_UPON_GLUTAMATE_BINDING	REACTOME	31
TRANSPORT_OF_VITAMINS_NUCLEOSIDES_AND_RELATED_MOLECULES	REACTOME	31
NEGATIVE_REGULATORS_OF_RIG_I_MDA5_SIGNALING	REACTOME	31
INWARDLY_RECTIFYING_K_CHANNELS	REACTOME	31
DAG_AND_IP3_SIGNALING	REACTOME	32
ELONGATION_ARREST_AND_RECOVERY	REACTOME	32
CD28_CO_STIMULATION	REACTOME	32
SEMA4D_IN_SEMAPHORIN_SIGNALING	REACTOME	32
FORMATION_OF_FIBRIN_CLOT_CLOTTING_CASCADE	REACTOME	32
THROMBIN_SIGNALLING_THROUGH_PROTEINASE_ACTIVATED_RECEPTORS_PARS	REACTOME	32
COMPLEMENT_CASCADE	REACTOME	32
LATENT_INFECTION_OF_HOMO_SAPIENS_WITH_MYCOBACTERIUM_TUBERCULOSIS	REACTOME	33
RNA_POL_III_TRANSCRIPTION	REACTOME	33
TRANSPORT_OF_MATURE_MRNA_DERIVED_FROM_AN_INTRONLESS_TRANSCRIPT	REACTOME	33
GLUCAGON_TYPE_LIGAND_RECEPTORS	REACTOME	33
POST_NMDA_RECEPTOR_ACTIVATION_EVENTS	REACTOME	33
TRANSPORT_TO_THE_GOLGI_AND_SUBSEQUENT_MODIFICATION	REACTOME	33
PURINE_METABOLISM	REACTOME	33
INTERACTIONS_OF_VPR_WITH_HOST_CELLULAR_PROTEINS	REACTOME	33
NEUROTRANSMITTER_RELEASE_CYCLE	REACTOME	34
GLUCONEOGENESIS	REACTOME	34
ABC_FAMILY_PROTEINS_MEDIATED_TRANSPORT	REACTOME	34
GLUCAGON_SIGNALING_IN_METABOLIC_REGULATION	REACTOME	34
SIGNAL_TRANSDUCTION_BY_L1	REACTOME	34
ION_TRANSPORT_BY_P_TYPE_ATPASES	REACTOME	34
FORMATION_OF_THE_HIV1_EARLY_ELONGATION_COMPLEX	REACTOME	34
METABOLISM_OF_STEROID_HORMONES_AND_VITAMINS_A_AND_D	REACTOME	35
MRNA_3_END_PROCESSING	REACTOME	35
GLOBAL_GENOMIC_NER_GG_NER	REACTOME	35
E2F_MEDIATED_REGULATION_OF_DNA_REPLICATION	REACTOME	35
BMAL1_CLOCK_NPAS2_ACTIVATES_CIRCADIAN_EXPRESSION	REACTOME	36
SIGNALLING_TO_ERKS	REACTOME	36
FRS2_MEDIATED_CASCADE	REACTOME	36
IRON_UPTAKE_AND_TRANSPORT	REACTOME	36
PLATELET_AGGREGATION_PLUG_FORMATION	REACTOME	36
NEGATIVE_REGULATION_OF_FGFR_SIGNALING	REACTOME	37
DOWNSTREAM_TCR_SIGNALING	REACTOME	37
ACTIVATION_OF_NMDA_RECEPTOR_UPON_GLUTAMATE_BINDING_AND_POSTSYNAPTIC_EVENTS	REACTOME	37
PI3K_EVENTS_IN_ERBB4_SIGNALING	REACTOME	38
NUCLEAR_SIGNALING_BY_ERBB4	REACTOME	38
GLYCOSPHINGOLIPID_METABOLISM	REACTOME	38
TRIGLYCERIDE_BIOSYNTHESIS	REACTOME	38
TRANSCRIPTIONAL_ACTIVITY_OF_SMAD2_SMAD3_SMAD4_HETEROTRIMER	REACTOME	38
PI3K_AKT_ACTIVATION	REACTOME	38
GAB1_SIGNALOSOME	REACTOME	38
G1_PHASE	REACTOME	38
AMINE_LIGAND_BINDING_RECEPTORS	REACTOME	38
GLUCOSE_TRANSPORT	REACTOME	38
GABA_B_RECEPTOR_ACTIVATION	REACTOME	38
ACTIVATION_OF_ATR_IN_RESPONSE_TO_REPLICATION_STRESS	REACTOME	38
NCAM1_INTERACTIONS	REACTOME	39
IL1_SIGNALING	REACTOME	39
APOPTOTIC_CLEAVAGE_OF_CELLULAR_PROTEINS	REACTOME	40
RNA_POL_II_TRANSCRIPTION_PRE_INITIATION_AND_PROMOTER_OPENING	REACTOME	41
NETRIN1_SIGNALING	REACTOME	41
IL_2_SIGNALING	REACTOME	41
BETA_DEFENSINS	REACTOME	42
TRNA_AMINOACYLATION	REACTOME	42
NRAGE_SIGNALS_DEATH_THROUGH_JNK	REACTOME	43
PLC_BETA_MEDIATED_EVENTS	REACTOME	43
REGULATION_OF_INSULIN_SECRETION_BY_GLUCAGON_LIKE_PEPTIDE1	REACTOME	43
IL_3_5_AND_GM_CSF_SIGNALING	REACTOME	43
VOLTAGE_GATED_POTASSIUM_CHANNELS	REACTOME	43
PI3K_EVENTS_IN_ERBB2_SIGNALING	REACTOME	44
PRE_NOTCH_EXPRESSION_AND_PROCESSING	REACTOME	44
SIGNALING_BY_FGFR_MUTANTS	REACTOME	44
G_ALPHA_Z_SIGNALLING_EVENTS	REACTOME	44
REGULATION_OF_WATER_BALANCE_BY_RENAL_AQUAPORINS	REACTOME	44
CLEAVAGE_OF_GROWING_TRANSCRIPT_IN_THE_TERMINATION_REGION_	REACTOME	44
TRANSCRIPTION_COUPLED_NER_TC_NER	REACTOME	45
MRNA_SPLICING_MINOR_PATHWAY	REACTOME	45

FORMATION_OF_RNA_POL_II_ELONGATION_COMPLEX_	REACTOME	45
G2_M_CHECKPOINTS	REACTOME	45
NOTCH1_INTRACELLULAR_DOMAIN_REGULATES_TRANSCRIPTION	REACTOME	46
ACTIVATION_OF_CHAPERONE_GENES_BY_XBP1S	REACTOME	46
LIPID_DIGESTION_MOBILIZATION_AND_TRANSPORT	REACTOME	46
NUCLEOTIDE_BINDING_DOMAIN_LEUCINE_RICH_REPEAT_CONTAINING_RECEPTOR_NLR_SIGNALING_PATHWAYS	REACTOME	46
PYRUVATE_METABOLISM_AND_CITRIC_ACID_TCA_CYCLE	REACTOME	48
CROSS_PRESENTATION_OF_SOLUBLE_EXOGENOUS_ANTIGENS_ENDOSOMES	REACTOME	48
PI_METABOLISM	REACTOME	48
CDK_MEDIATED_PHOSPHORYLATION_AND_REMOVAL_OF_CDC6	REACTOME	48
MUSCLE_CONTRACTION	REACTOME	48
DEADENYLATION_DEPENDENT_MRNA_DECAY	REACTOME	48
PACKAGING_OF_TELOMERE_ENDS	REACTOME	48
METABOLISM_OF_NON_CODING_RNA	REACTOME	49
CHONDROITIN_SULFATE_DERMATAN_SULFATE_METABOLISM	REACTOME	49
REGULATION_OF_ORNITHINE_DECARBOXYLASE_ODC	REACTOME	49
NUCLEAR_RECEPTOR_TRANSCRIPTION_PATHWAY	REACTOME	49
AMINO_ACID_AND_OLIGOPEPTIDE_SLC_TRANSPORTERS	REACTOME	49
MAP_KINASE_ACTIVATION_IN_TLR_CASCADE	REACTOME	50
METABOLISM_OF_VITAMINS_AND_COFACTORS	REACTOME	51
DEFENSINS	REACTOME	51
P53_INDEPENDENT_G1_S_DNA_DAMAGE_CHECKPOINT	REACTOME	51
CYTOCHROME_P450_ARRANGED_BY_SUBSTRATE_TYPE	REACTOME	51
NUCLEOTIDE_EXCISION_REPAIR	REACTOME	51
AUTODEGRADATION_OF_THE_E3_UBIQUITIN_LIGASE_COP1	REACTOME	51
AQUAPORIN_MEDIATED_TRANSPORT	REACTOME	51
SCF_BETA_TRCP_MEDIATED_DEGRADATION_OF_EMI1	REACTOME	51
HEPARAN_SULFATE_HEPARIN_HS_GAG_METABOLISM	REACTOME	52
GABA_RECEPTOR_ACTIVATION	REACTOME	52
VIF_MEDIATED_DEGRADATION_OF_APOBEC3G	REACTOME	52
PROTEIN_FOLDING	REACTOME	53
GOLGI_ASSOCIATED_VESICLE_BIOGENESIS	REACTOME	53
CIRCADIAN_CLOCK	REACTOME	53
DESTABILIZATION_OF_MRNA_BY_AUF1_HNRNP_D0	REACTOME	53
TCR_SIGNALING	REACTOME	54
TRANSPORT_OF_MATURE_TRANSCRIPT_TO_CYTOPLASM	REACTOME	54
PHOSPHOLIPASE_C_MEDIATED_CASCADE	REACTOME	54
APOPTOTIC_EXECUTION_PHASE	REACTOME	54
ION_CHANNEL_TRANSPORT	REACTOME	55
CELL_CELL_JUNCTION_ORGANIZATION	REACTOME	56
CDT1_ASSOCIATION_WITH_THE_CDC6_ORC_ORIGIN_COMPLEX	REACTOME	56
PI_3K_CASCADE	REACTOME	56
SCFSKP2_MEDIATED_DEGRADATION_OF_P27_P21	REACTOME	56
CHEMOKINE_RECEPTORS_BIND_CHEMOKINES	REACTOME	57
P53_DEPENDENT_G1_DNA_DAMAGE_RESPONSE	REACTOME	57
MITOCHONDRIAL_PROTEIN_IMPORT	REACTOME	58
COLLAGEN_FORMATION	REACTOME	58
REGULATION_OF_APOPTOSIS	REACTOME	58
O_LINKED_GLYCOSYLATION_OF_MUCINS	REACTOME	59
LOSS_OF_NLP_FROM_MITOTIC_CENTROSOMES	REACTOME	59
TRANS_GOLGI_NETWORK_VESICLE_BUDDING	REACTOME	60
CELL_DEATH_SIGNALLING_VIA_NRAGE_NRIF_AND_NADE	REACTOME	60
ER_PHAGOSOME_PATHWAY	REACTOME	61
RNA_POL_II_PRE_TRANSCRIPTION_EVENTS	REACTOME	61
RNA_POL_I_PROMOTER_OPENING	REACTOME	62
COSTIMULATION_BY_THE_CD28_FAMILY	REACTOME	63
INTERFERON_GAMMA_SIGNALING	REACTOME	63
SIGNALING_BY_TGF_BETA_RECEPTOR_COMPLEX	REACTOME	63
ACTIVATION_OF_NF_KAPPAB_IN_B_CELLS	REACTOME	64
NCAM_SIGNALING_FOR_NEURITE_OUT_GROWTH	REACTOME	64
DEPOSITION_OF_NEW_CENPA_CONTAINING_NUCLEOSOMES_AT_THE_CENTROMERE	REACTOME	64
INTERFERON_ALPHA_BETA_SIGNALING	REACTOME	64
AUTODEGRADATION_OF_CDH1_BY_CDH1_APC_C	REACTOME	64
SIGNALING_BY_WNT	REACTOME	65
CYCLIN_E_ASSOCIATED_EVENTS_DURING_G1_S_TRANSITION_	REACTOME	65
ASSEMBLY_OF_THE_PRE_REPLICATIVE_COMPLEX	REACTOME	65
ANTIVIRAL_MECHANISM_BY_IFN_STIMULATED_GENES	REACTOME	66
RECRUITMENT_OF_MITOTIC_CENTROSOME_PROTEINS_AND_COMPLEXES	REACTOME	66
ORC1_REMOVAL_FROM_CHROMATIN	REACTOME	67
SEMAPHORIN_INTERACTIONS	REACTOME	68
SPHINGOLIPID_METABOLISM	REACTOME	69
GLUCOSE_METABOLISM	REACTOME	69
IMMUNOREGULATORY_INTERACTIONS_BETWEEN_A_LYMPHOID_AND_A_NON_LYMPHOID_CELL	REACTOME	70
SIGNALING_BY_NOTCH1	REACTOME	70
PHASE1_FUNCTIONALIZATION_OF_COMPOUNDS	REACTOME	70
PHASE_II_CONJUGATION	REACTOME	70
PI3K_CASCADE	REACTOME	71
METABOLISM_OF_NUCLEOTIDES	REACTOME	72
NFKB_AND_MAP_KINASES_ACTIVATION_MEDIATED_BY_TLR4_SIGNALING_REPERTOIRE	REACTOME	72
TRANSCRIPTIONAL_REGULATION_OF_WHITE_ADIPOCYTE_DIFFERENTIATION	REACTOME	72
APC_C_CDH1_MEDIATED_DEGRADATION_OF_CDC20_AND_OTHER_APC_C_CDH1_TARGETED_PROTEINS_IN_LATE_MITOSIS_EARLY_G1	REACTOME	72
RIG_I_MDA5_MEDIATED_INDUCTION_OF_IFN_ALPHA_BETA_PATHWAYS	REACTOME	73
APC_C_CDC20_MEDIATED_DEGRADATION_OF_MITOTIC_PROTEINS	REACTOME	73
MEIOTIC_SYNAPSIS	REACTOME	73
FORMATION_OF_THE_TERNARY_COMPLEX_AND_SUBSEQUENTLY_THE_43S_COMPLEX	REACTOME	74
TRIF_MEDIATED_TLR3_SIGNALING	REACTOME	74
G_ALPHA1213_SIGNALLING_EVENTS	REACTOME	74
TELOMERE_MAINTENANCE	REACTOME	75
ANTIGEN_PROCESSING_CROSS_PRESENTATION	REACTOME	76
TRAF6_MEDIATED_INDUCTION_OF_NFKB_AND_MAP_KINASES_UPON_TLR7_8_OR_9_ACTIVATION	REACTOME	77
SIGNALING_BY_SCF_KIT	REACTOME	78
OPIOID_SIGNALLING	REACTOME	78
CELL_JUNCTION_ORGANIZATION	REACTOME	78
PLATELET_HOMEOSTASIS	REACTOME	78
INTEGRIN_CELL_SURFACE_INTERACTIONS	REACTOME	79
RESPIRATORY_ELECTRON_TRANSPORT	REACTOME	79
UNFOLDED_PROTEIN_RESPONSE	REACTOME	80
P75_NTR_RECEPTOR_MEDIATED_SIGNALLING	REACTOME	81
M_G1_TRANSITION	REACTOME	81
MITOTIC_G2_G2_M_PHASES	REACTOME	81
ASPARAGINE_N_LINKED_GLYCOSYLATION	REACTOME	81

GLYCEROPHOSPHOLIPID_BIOSYNTHESIS	REACTOME	82
MYD88_MAL_CASCADE_INITIATED_ON_PLASMA_MEMBRANE	REACTOME	83
AMYLOIDS	REACTOME	83
ACTIVATION_OF_THE_MRNA_UPON_BINDING_OF_THE_CAP_BINDING_COMPLEX_AND_EIFS_AND_SUBSEQUENT_BINDING_TO_43S	REACTOME	84
REGULATION_OF_MRNA_STABILITY_BY_PROTEINS_THAT_BIND_AU_RICH_ELEMENTS	REACTOME	84
REGULATION_OF_MITOTIC_CELL_CYCLE	REACTOME	85
L1CAM_INTERACTIONS	REACTOME	86
MEIOTIC_RECOMBINATION	REACTOME	86
EXTRACELLULAR_MATRIX_ORGANIZATION	REACTOME	87
INSULIN_RECEPTOR_SIGNALLING_CASCADE	REACTOME	87
MITOTIC_PROMETAPHASE	REACTOME	87
CLASS_B_2_SECRETIN_FAMILY_RECEPTORS	REACTOME	88
RESPONSE_TO_ELEVATED_PLATELET_CYTOSOLIC_CA2_	REACTOME	89
RNA_POL_I_TRANSCRIPTION	REACTOME	89
TRANSPORT_OF_GLUCOSE_AND_OTHER_SUGARS_BILE_SALTS_AND_ORGANIC_ACIDS_METAL_IONS_AND_AMINE_COMPOUNDS	REACTOME	89
SIGNALING_BY_ERBB4	REACTOME	90
CELL_SURFACE_INTERACTIONS_AT_THE_VASCULAR_WALL	REACTOME	91
MHC_CLASS_II_ANTIGEN_PRESENTATION	REACTOME	91
SYNTHESIS_OF_DNA	REACTOME	92
REGULATION_OF_INSULIN_SECRETION	REACTOME	93
ACTIVATED_TLR4_SIGNALLING	REACTOME	93
TRANSPORT_OF_INORGANIC_CATIONS_ANIONS_AND_AMINO_ACIDS_OLIGOPEPTIDES	REACTOME	94
DOWNSTREAM_SIGNAL_TRANSDUCTION	REACTOME	95
DOWNSTREAM_SIGNALING_EVENTS_OF_B_CELL_RECEPTOR_BCR	REACTOME	97
RESPIRATORY_ELECTRON_TRANSPORT_ATP_SYNTHESIS_BY_CHEMIOSMOTIC_COUPLING_AND_HEAT_PRODUCTION_BY_UNCOUPLING_PROTEINS_	REACTOME	98
POTASSIUM_CHANNELS	REACTOME	98
DOWNSTREAM_SIGNALING_OF_ACTIVATED_FGFR	REACTOME	100
SIGNALING_BY_ERBB2	REACTOME	101
SIGNALING_BY_NOTCH	REACTOME	103
PPARA_ACTIVATES_GENE_EXPRESSION	REACTOME	104
LATE_PHASE_OF_HIV_LIFE_CYCLE	REACTOME	104
RNA_POL_II_TRANSCRIPTION	REACTOME	105
SIGNALING_BY_ILS	REACTOME	107
SIGNALING_BY_INSULIN_RECEPTOR	REACTOME	108
SIGNALING_BY_EGFR_IN_CANCER	REACTOME	109
S_PHASE	REACTOME	109
GLYCOSAMINOGLYCAN_METABOLISM	REACTOME	111
MRNA_SPLICING	REACTOME	111
G1_S_TRANSITION	REACTOME	112
DNA_REPAIR	REACTOME	112
SIGNALING_BY_FGFR	REACTOME	112
SIGNALING_BY_RHO_GTPASES	REACTOME	113
MEIOSIS	REACTOME	116
TOLL_RECEPTOR_CASCADES	REACTOME	118
CELL_CELL_COMMUNICATION	REACTOME	120
INTEGRATION_OF_ENERGY_METABOLISM	REACTOME	120
G_ALPHA_S_SIGNALLING_EVENTS	REACTOME	121
SIGNALING_BY_PDGF	REACTOME	122
RNA_POL_I_RNA_POL_III_AND_MITOCHONDRIAL_TRANSCRIPTION	REACTOME	122
CHROMOSOME_MAINTENANCE	REACTOME	122
CELL_CYCLE_CHECKPOINTS	REACTOME	124
HIV_LIFE_CYCLE	REACTOME	125
SIGNALING_BY_THE_B_CELL_RECEPTOR_BCR	REACTOME	126
SIGNALING_BY_FGFR_IN_DISEASE	REACTOME	127
MEMBRANE_TRAFFICKING	REACTOME	129
FACTORS_INVOLVED_IN_MEGAKARYOCYTE_DEVELOPMENT_AND_PLATELET_PRODUCTION	REACTOME	132
HOST_INTERACTIONS_OF_HIV_FACTORS	REACTOME	132
DIABETES_PATHWAYS	REACTOME	133
NGF_SIGNALLING_VIA_TRKA_FROM_THE_PLASMA_MEMBRANE	REACTOME	137
NEUROTRANSMITTER_RECEPTOR_BINDING_AND_DOWNSTREAM_TRANSMISSION_IN_THE_POSTSYNAPTIC_CELL	REACTOME	137
MITOTIC_G1_G1_S_PHASES	REACTOME	137
BIOLOGICAL_OXIDATIONS	REACTOME	139
PROCESSING_OF_CAPPED_INTRON_CONTAINING_PRE_MRNA	REACTOME	140
TCA_CYCLE_AND_RESPIRATORY_ELECTRON_TRANSPORT	REACTOME	141
APOPTOSIS	REACTOME	148
PEPTIDE_CHAIN_ELONGATION	REACTOME	153
INTERFERON_SIGNALING	REACTOME	159
MRNA_PROCESSING	REACTOME	161
FATTY_ACID_TRIACYLGLYCEROL_AND_KETONE_BODY_METABOLISM	REACTOME	168
INFLUENZA_VIRAL_RNA_TRANSCRIPTION_AND_REPLICATION	REACTOME	169
MITOTIC_M_M_G1_PHASES	REACTOME	172
3_UTR_MEDIATED_TRANSLATIONAL_REGULATION	REACTOME	176
NONSENSE_MEDIATED_DECAY_ENHANCED_BY_THE_EXON_JUNCTION_COMPLEX	REACTOME	176
SRP_DEPENDENT_COTRANSLATIONAL_PROTEIN_TARGETING_TO_MEMBRANE	REACTOME	179
G_ALPHA_Q_SIGNALLING_EVENTS	REACTOME	184
TRANSMISSION_ACROSS_CHEMICAL_SYNAPSES	REACTOME	186
PEPTIDE_LIGAND_BINDING_RECEPTORS	REACTOME	188
POST_TRANSLATIONAL_PROTEIN_MODIFICATION	REACTOME	188
DNA_REPLICATION	REACTOME	192
G_ALPHA_I_SIGNALLING_EVENTS	REACTOME	195
PHOSPHOLIPID_METABOLISM	REACTOME	198
METABOLISM_OF_AMINO_ACIDS_AND_DERIVATIVES	REACTOME	200
INFLUENZA_LIFE_CYCLE	REACTOME	203
GASTRIN_CREB_SIGNALLING_PATHWAY_VIA_PKC_AND_MAPK	REACTOME	205
HIV_INFECTION	REACTOME	207
PLATELET_ACTIVATION_SIGNALING_AND_AGGREGATION	REACTOME	208
TRANSCRIPTION	REACTOME	210
ANTIGEN_PROCESSING_UBIQUITINATION_PROTEASOME_DEGRADATION	REACTOME	212
SIGNALLING_BY_NGF	REACTOME	217
TRANSLATION	REACTOME	222
SLC_MEDIATED_TRANSMEMBRANE_TRANSPORT	REACTOME	241
METABOLISM_OF_CARBOHYDRATES	REACTOME	247
AXON_GUIDANCE	REACTOME	251
CLASS_I_MHC_MEDIATED_ANTIGEN_PROCESSING_PRESENTATION	REACTOME	251
CYTOKINE_SIGNALING_IN_IMMUNE_SYSTEM	REACTOME	270
NEURONAL_SYSTEM	REACTOME	279
INNATE_IMMUNE_SYSTEM	REACTOME	279
METABOLISM_OF_MRNA	REACTOME	284

Abbreviations: Genes, number of genes contained in each particular pathway.

3. Supplementary Figures

3.1 Figure S1. Principal components of the SLE discovery cohort.

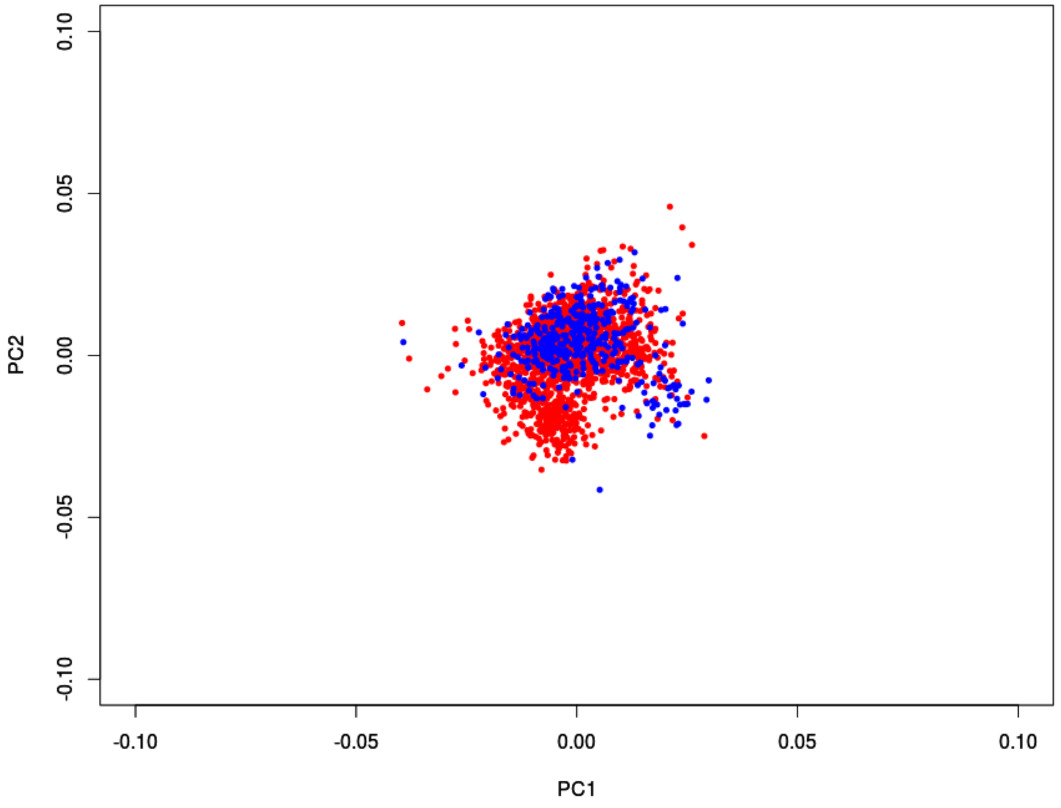

Figure S1. Principal components of the SLE discovery cohort. The SLE patients (blue dots) and control patients (red dots) are plotted according to their first (PC1) and second (PC2) principal components estimated in EIGENSTRAT using the genome-wide data. In the present figure, samples showing an outlier genetic background (i.e. samples with >6 standard deviations in any of the 10 principal components of variation) were excluded.

4. References

1. Julia A, Tortosa R, Hernanz JM, Canete JD, Fonseca E, Ferrandiz C, Unamuno P, Puig L, Fernandez-Sueiro JL, Sanmarti R *et al*: Risk variants for psoriasis vulgaris in a large case-control collection and association with clinical subphenotypes. *Hum Mol Genet* 2012, 21(20):4549-4557. Epub 2012 Jul 4519.

2. Tan EM, Cohen AS, Fries JF, Masi AT, McShane DJ, Rothfield NF, Schaller JG, Talal N, Winchester RJ: The 1982 revised criteria for the classification of systemic lupus erythematosus. *Arthritis Rheum* 1982, 25(11):1271-1277.

3. Purcell S, Neale B, Todd-Brown K, Thomas L, Ferreira MA, Bender D, Maller J, Sklar P, de Bakker PI, Daly MJ *et al*: PLINK: a tool set for whole-genome association and population-based linkage analyses. *Am J Hum Genet* 2007, 81(3):559-575. Epub 2007 Jul 2025.

4. Aterido A, Julia A, Ferrandiz C, Puig L, Fonseca E, Fernandez-Lopez E, Dauden E, Sanchez-Carazo JL, Lopez-Estebaranz JL, Moreno-Ramirez D *et al*: Genome-Wide Pathway Analysis Identifies Genetic Pathways Associated with Psoriasis. *J Invest Dermatol* 2016, 136(3):593-602. doi: 510.1016/j.jid.2015.1011.1026. Epub 2015 Dec 1029.

5. Price AL, Patterson NJ, Plenge RM, Weinblatt ME, Shadick NA, Reich D: Principal components analysis corrects for stratification in genome-wide association studies. *Nat Genet* 2006, 38(8):904-909. Epub 2006 Jul 2023.

6. Delaneau O, Zagury JF, Marchini J: Improved whole-chromosome phasing for disease and population genetic studies. *Nature methods* 2013, 10(1):5-6.

7. Howie B, Marchini J, Stephens M: Genotype imputation with thousands of genomes. *G3 (Bethesda, Md)* 2011, 1(6):457-470.

8. Abecasis GR, Auton A, Brooks LD, DePristo MA, Durbin RM, Handsaker RE, Kang HM, Marth GT, McVean GA: An integrated map of genetic variation from 1,092 human genomes. *Nature* 2012, 491(7422):56-65.

9. Edgar R, Domrachev M, Lash AE: Gene Expression Omnibus: NCBI gene expression and hybridization array data repository. *Nucleic Acids Res* 2002, 30(1):207-210.

10. Chang AY, Werth VP: Treatment of cutaneous lupus. *Curr Rheumatol Rep* 2011, 13(4):300-307. doi: 310.1007/s11926-11011-10180-z.

11. Khandpur S, Sharma VK, Sumanth K: Topical immunomodulators in dermatology. *J Postgrad Med* 2004, 50(2):131-139.

12. Allison DB, Cui X, Page GP, Sabripour M: Microarray data analysis: from disarray to consolidation and consensus. *Nat Rev Genet* 2006, 7(1):55-65.

13. Bolstad BM, Irizarry RA, Astrand M, Speed TP: A comparison of normalization methods for high density oligonucleotide array data based on variance and bias. *Bioinformatics* 2003, 19(2):185-193.

14. Ihaka R, Gentleman G: R: A language for data analysis and graphics. *Journal of Computational and Graphical Statistics* 1996, 5(5):299-314.
